# Direct stroke unit access versus a hub-and-spoke model with telemedicine-assisted CT in Germany: a cross-sectional geospatial analysis

**DOI:** 10.1016/j.lanepe.2026.101604

**Published:** 2026-02-05

**Authors:** Lars Masanneck, Marius Vach, Julian Caspers, Christian Rubbert, David von der Lieth, Sven G. Meuth, Marc Pawlitzki, Michael Gliem

**Affiliations:** a Department of Neurology, University Hospital and Medical Faculty University Hospital Düsseldorf, Düsseldorf, Germany; b Department of Diagnostic and Interventional Radiology, University Hospital and Medical Faculty University Hospital Düsseldorf, Düsseldorf, Germany; c Fire and Rescue Services, City of Düsseldorf, Düsseldorf, Germany

**Keywords:** Stroke, Models of care, Outcome, Telemedicine

## Abstract

**Background:**

Timely reperfusion offers the greatest benefit in acute ischaemic stroke within the first hour after onset. However, geographic disparities in stroke care access persist across Germany. Despite the potential of telemedicine and mobile stroke units, nationwide data that quantify existing care gaps or systematically investigate the benefit of early imaging with subsequent thrombolysis in locally accessible, CT-equipped hospitals with telemedicine are lacking. This study modelled nationwide access and compared direct transfer to specialised hospitals with a hub-and-spoke strategy (nearest CT plus telemedicine) for early thrombolysis.

**Methods:**

We performed a cross-sectional geospatial analysis combining national facility registries and 2023 hospital quality reports (data collected February 1st–July 23rd, 2025). We mapped German CT-equipped hospitals (n = 1475), stroke-ready hospitals (≥100 annual cases of “complex neurological treatment of acute stroke”, n = 463) and certified stroke units (n = 349). For these facilities we modelled driving-time access up to 60 min in 5-min intervals using a local installation of openrouteservice, overlaying these on population and settlement grids. Additional scenarios simulated variations in ambulance speed (default: standard openrouteservice vehicle speed) or additional in-hospital delays when using a hub-and-spoke scenario. We then compared hub-and-spoke versus direct transfer to specialised hospitals on a national, state- and county-level.

**Findings:**

Within 30 min, nearly all residents (82,484,915/83,420,000; 98.9%) could reach a CT-equipped hospital, 90.0% (75,051,793) a stroke-ready hospital, but only 85.0% (70,875,055) a certified stroke unit. Compared with direct transfer to a stroke unit, a hub-and-spoke pathway would let 36.4% of inhabitants start imaging ≥10 min sooner (assuming normal speed, other scenarios varying from 4.5% to 40.0%) and for 14.2% it could save ≥20 min (varying from 1.1% to 18.2% across scenarios). Estimated benefits of a hub-and-spoke model depended on assumed driving speed and declined with simulated CT delays. Rural regions had particularly pronounced access gaps to stroke care, as evidenced by lower levels of urbanisation in regions with a higher hub-and-spoke benefit potential. State-level analysis illustrated heterogeneity, with 48.6% of inhabitants potentially benefitting from a hub-and-spoke model in Saxony-Anhalt, but <5% in city-states (assuming normal driving speed and 10-min delay).

**Interpretation:**

Marked intra-national inequities in rapid accessibility to stroke care persist. Leveraging CT-equipped hospitals with telemedicine could enable timelier thrombolysis within a hub-and-spoke model. Open questions regarding implementation and economics should be investigated.

**Funding:**

University Hospital Düsseldorf, the B. Braun Foundation, and the Ministry of Economic Affairs, Innovation, Digitalization and Energy of the State of North Rhine-Westphalia (funding number 005–2008-0055).

**Research in context**

**Evidence before this study**

Timely access to reperfusion therapy is critical in acute ischaemic stroke, with intravenous thrombolysis within the first 60 min (“golden hour”) yielding the greatest clinical benefit. However, geographical disparities in access to stroke units persist across Germany.

We searched PubMed for studies from 1st January 2000 to 30th June 2025 using the search terms “stroke” OR “stroke care” AND “isochrone” OR “telemedicine”. No filters were applied with regard to the study design.

Previous research has highlighted the potential of telemedicine, mobile stroke units, and prehospital triage to improve treatment rates, but no prior nationwide study has systematically quantified population-level coverage gaps or evaluated the impact of early in-hospital imaging at the nearest CT-equipped facility combined with teleconsultation on golden-hour access.

**Added value of this study**

This study presents a nationwide analysis modelling driving-time access to the nearest CT-equipped hospitals, hospitals frequently treating stroke patients, certified stroke units, and thrombectomy centres in Germany. Simulating a hub-and-spoke model that exploits those CT scanners plus teleconsultation shows 30.3 million people (36.4% of the German population) could begin imaging ≥10 min sooner and 9.5% would save ≥20 min compared to direct-transfer to stroke units, with 26.9% of residents still benefiting after a universal 10-min in-hospital delay. Benefits are visualised on local levels and cluster in rural and eastern regions, underscoring persistent inequity. Coupled with growing evidence for telestroke networks, mobile stroke units and single-bolus tenecteplase, these findings suggest that repurposing ubiquitous CT capacity might be a rapid, scalable and cost-conscious route to equitable golden-hour thrombolysis. By providing open-access geospatial data across different assumptions and scenarios and an interactive visualisation tool, this study offers a scalable framework for data-driven optimisation of stroke systems of care and enables local authorities to adapt strategies tailored to the region. The framework and underlying code can further easily be used for other healthcare research.

**Implications of all the available evidence**

Our findings underscore substantial intra-national disparities in stroke unit access, particularly in rural regions, and demonstrate that pragmatic adaptations to current care pathways could significantly improve equity and timeliness of stroke treatment in Germany as well as further countries in Europe. These insights support the further implementation and evaluation of early in-hospital imaging at the nearest CT-equipped facility and teleconsultation as a complement to existing stroke systems. More broadly, our approach can inform strategic planning nationally or in other countries seeking to optimise access to time-critical interventions.

## Introduction

Acute ischaemic stroke remains the foremost cause of adult disability and the second leading cause of death in high-income countries.^1^^,^^2^ Beyond population growth and ageing, the rising global stroke burden is further fuelled by the limited effectiveness of current primary prevention strategies for stroke and cardiovascular disease. Disparities and major gaps in access to acute stroke care persist, especially in rural areas.^3^ These gaps are particularly critical given that timely recanalisation of the occluded vessel—whether through intravenous thrombolysis (IVT) or endovascular thrombectomy–is essential for preserving penumbral tissue and minimising long-term disability.^4^ Early-treated patients have a markedly higher probability to benefit from treatment.^5^^,^^6^ Addressing persistent disparities and closing major gaps in access to acute stroke care has been recognised as a key research priority by the European Stroke Organisation (ESO).^7^

Currently, less than 15% of patients across Europe receive acute reperfusion therapies,^8^ largely due to delayed presentation and limited access to timely stroke care.^9^ For every 10-min interval delay in 100 patients, 1.2 had worse ambulation and 0.8 showed higher dependence at discharge.^10^ This treatment gap is increasingly being understood through data-driven mapping approaches, which have proven essential for identifying geographic disparities in stroke service provision–particularly in rural or underserved regions,^8^ but even in urban areas, substantial prehospital delays persist. In the Berlin STEMO trial, only 5% of thrombolysed patients receiving conventional care were treated within the golden hour (≤60 min from symptom onset), highlighting the limitations of standard stroke workflows even within a highly developed metropolitan system.^11^^,^^12^ To improve access to time-critical stroke interventions, multiple strategies have proven effective—including prehospital triage systems with teleconsultation, centralised ambulance coordination, and structured training of stroke unit teams.^9^ In particular, regional disparities in stroke unit availability and transportation times highlight the potential of teleneurology concepts,^13^ mobile stroke units^14^ and dynamic, map-based routing systems^15^ to further reduce delays and guide patients more efficiently to appropriate care. We therefore set out to compare the current status quo of direct transfer to a certified stroke unit with an extended model: initiating imaging and IVT under telemedical supervision at the nearest CT-equipped facility, even if not a certified stroke unit.^16^ This could significantly reduce treatment times, particularly in periurban and rural areas, by leveraging existing CT infrastructure and enabling parallel workflows in stroke care.^16^^,^^17^

To evaluate the impact and scalability of this approach, its potential should be systematically assessed across the national healthcare landscape. In this context, we conducted a nationwide German analysis modelling driving-time access to (1) CT-equipped hospitals, (2) stroke-ready hospitals with frequent stroke treatments, and (3) certified stroke units at 5–60 min thresholds. We then directly compared the direct transfer to a specialised hospital with a telemedicine-enabled hub-and-spoke paradigm—initiating thrombolysis at the nearest CT hospital followed by transfer—to quantify potential gains in timely stroke care accessibility.

## Methods

### Data collection

To obtain a list of all hospitals with CT scanner, all hospitals listed in the German Hospital Directory (https://www.deutsches-krankenhaus-verzeichnis.de) that reported having performed native CT examinations (operation and procedure (OPS)-code 3–20∗) in the most recent 2023 reporting year were identified. Facilities were deduplicated and those documenting fewer than three CT procedures in 2023 were excluded. (Tele-)Stroke unit data were obtained from the German Stroke Society's publicly available database on February 7, 2025 (https://www.dsg-info.de/stroke-units-neurovaskulaere-netzwerke/).^18^ For thrombectomy centres, data from the German Stroke Society were manually cross-referenced and matched with the list provided on the DeGIR website (https://degir.de/degir-dgnr-zentren/, module E and EF)^19^—also on February 7, 2025. To include hospitals that treat substantial numbers of stroke patients but are not (yet) certified as stroke units, we obtained access to the federally mandated German hospital quality reports and downloaded the 2023 edition on 23 July 2025. We then selected hospitals that had billed OPS 8–981 (“complex neurological treatment of acute stroke”) or OPS 8–98b (“other acute neurological stroke treatment”, without or with tele-consultation) at least 100 times in 2023. Hospitals meeting this OPS threshold but not classified as certified stroke units as well as all stroke units were assigned to a new category, labelled “stroke-ready hospitals”.

### Isochrone calculation

The updated calculation builds on previously published work.^20^^,^^21^ Accessibility was quantified via driving-time isochrones, which delineate areas reachable within specific travel durations. Using a local Docker installation of openrouteservice (version 9.1.2)^22^^,^^23^ with the “driving-car” profile, isochrones were computed with a maximum travel time of 1 h (3600 s) and at 5-min intervals (300 s). For each centre, isochrones corresponding to 5 up to 60 min were generated and processed as polygon geometries using the Shapely library. A thus-defined isochrone is a geographic area showing how far one can travel from a specific point within a certain time by car. To account for potential variations in emergency response conditions, additional isochrones were generated assuming increased (1.2×) and decreased (0.8×) driving speeds, representing scenarios of expedited and congested travel, respectively.

### Geospatial aggregation and population analysis

Stroke unit classifications were extracted according to the official classes of German stroke unit certification. Isochrone polygons for centres within each investigated category (e.g., all hospitals with documented CT use, all stroke units, regional stroke units, supra-regional stroke units–with the latter providing advanced care and serving populations beyond a single administrative region–and thrombectomy centres) were aggregated via a union operation to create a composite coverage area for each travel time threshold. We thus merged the areas reachable by car within a certain time to show overall coverage for that type of care. These unioned geometries were then used as masks for spatial population analysis. Population estimates were derived by overlaying the aggregated isochrone polygons onto the most recent release of the Global Human Settlement Population Grid with population estimates for 2025 (GHS-POP E2025)^24^^,^^25^ using Rasterio for raster masking. The total population within each coverage area was computed to obtain both absolute and percentage estimates relative to the national population according to the used dataset. A population of 83,420,000 residents for Germany was assumed.

### Time benefit analysis and scenario comparison

To quantify the potential advantages of accessing CT-equipped hospitals versus stroke units, we performed a grid-based comparative analysis across Germany using a 1-km resolution spatial grid. For each grid point, minimum travel times to the nearest CT hospital and nearest stroke unit were calculated using the respective precomputed isochrone datasets. Time benefits were defined as the difference in travel time (stroke unit time minus CT hospital time), with a 10-min threshold applied to identify clinically significant advantages. To account for varying real-world conditions, six scenarios were analysed: three representing different driving speeds (normal conditions according to openrouteservice, emergency response with +20% speed, and congested traffic with −20% speed), and three incorporating time penalties of 10, 20, or 30 min added to CT hospital access to simulate potential delays from reduced institutional experience or teleneurology consultation requirements. We chose these different driving speed and procedural scenarios as bounding conditions in the absence of harmonised emergency service data and empirical procedural data across the nation. Areas were classified into benefit categories compared with direct-shipment-to-centre ranging from high (≥30 min saved), medium (20–30 min), and low (10–20 min) time savings, to regions where only CT hospitals were accessible within 60 min, areas where neither service was reachable within 60 min, and areas with minimal differences (<10 min) considered clinically irrelevant. Population coverage for each scenario was calculated by overlaying these classified benefit areas with the GHS-POP dataset.

To analyse the urban-rural characteristics of exemplary isochrones and their potential benefit areas, we utilised the GHS-Settlement Model dataset.^25^ Areas were classified as urban (codes 30, 23, 22, 21; encompassing urban centres, towns, and suburban areas), rural (codes 13, 12, 11; rural areas of varying densities), or ‘other’ (water bodies [code 10] and all remaining categories).

For further fine-grained local analyses on state and county level, openly available geographical datasets^26^^,^^27^ based on official government data were used to repeat the respective analyses. Road network data were sourced from OpenStreetMap (https://www.openstreetmap.org) on February 7th, 2025.^28^ A schematic high-level overview of the analyses can be found in Supplement S1.

### Data analysis and visualisation

All analyses were implemented in Python (version 3.12.9) with all source code and used packages available in the respective GitHub repository: https://github.com/MasanneckLab/GeoStroke-Analyses.

### Interactive web application for data exploration

To further enhance the opportunities to explore the numerous resulting datasets, we developed an interactive web-based visualiser (https://masannecklab.github.io/GeoStroke-Visualizer/), allowing users to intuitively explore access to CT or stroke care by state or county, visualise benefits under different scenarios, explore absolute and relative population coverage and download the respective results. This web application is a static, client–side interface built with vanilla HTML, CSS, and JavaScript, hosted via GitHub Pages, which dynamically loads region-specific metadata and visualisation assets from a structured directory of pre-rendered images and data files. Its full source code is also available in the repository.

### Ethical consideration

An ethical approval was not required, since exclusively publicly available datasets were used. As this study involved no people and relied solely on geospatial analyses, informed consent was not required.

## Results

### Access to CT-equipped hospitals

**Fig. 1:**
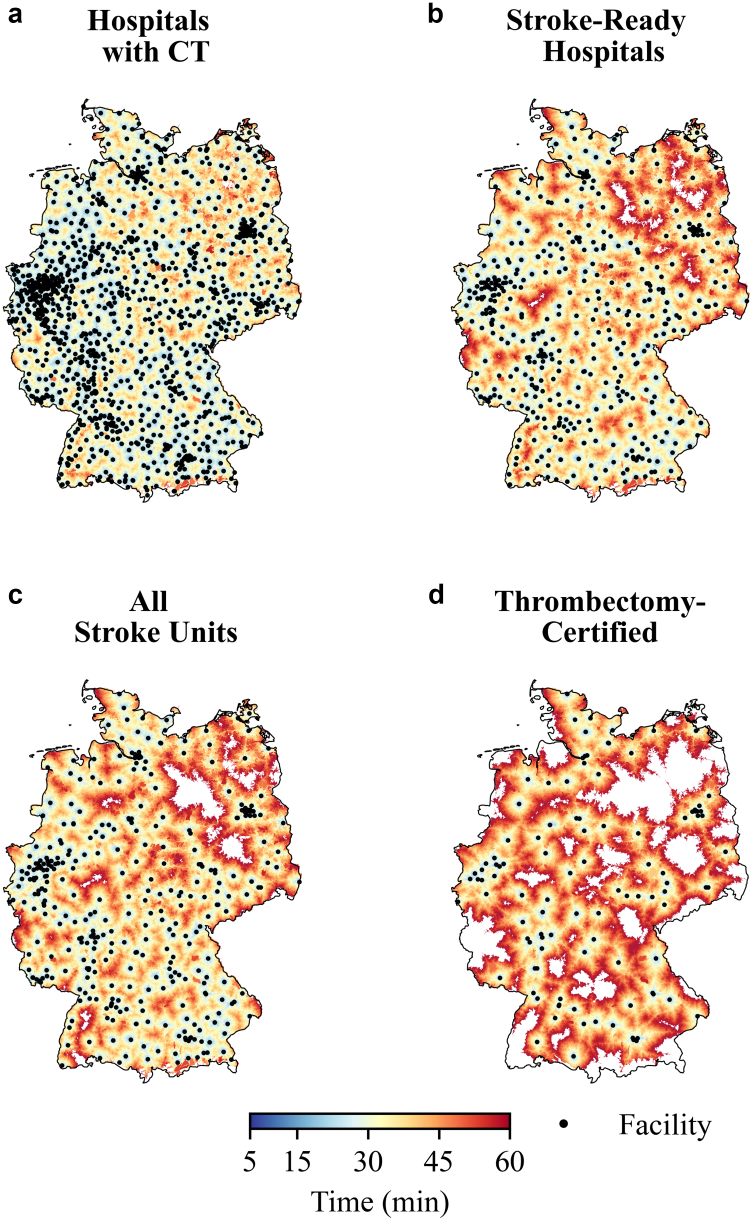
**Geographic access to CT imaging and stroke care facilities in Germany.** Maps of Germany showing 5–60-min drive-time isochrones (light blue to red) around a) all hospitals with on-site CT scanners, b) hospitals that frequently treat stroke patients or are certified stroke units, c) only certified stroke units, and d) thrombectomy-capable stroke units. Black dots mark the respective facilities. Areas not reached within 60 min are left white. For population coverage see Table 1. For more detailed state or county analyses see Supplements S3–S6 or the interactive web application https://masannecklab.github.io/GeoStroke-Visualizer/.

Within 15 min, 63,660,670 people (76.3%) could reach at least one of 1475 hospitals with on-site CT. Coverage rose sharply to 82,484,915 (98.9%) by 30 min, 83,342,930 (99.9%) by 45 min, and 83,366,218 (99.9%) by 60 min (Table 1, Fig. 1A). Thus, nearly the entire population could access initial imaging for suspected stroke within half an hour. While there appeared to be regions in north-eastern, central and south-western Germany with difficulties to access, the number of regions without access within 45 min was very small.

**Table 1:** Population coverage statistics of isochrones.

Type	Time (min)	Covered population	Percentage (%)
CT-equipped hospitals	15	63,660,670	76.3
	30	82,484,915	98.9
	45	83,342,930	99.9
	60	83,366,218	99.9
Stroke-Ready hospitals	15	42,673,811	51.2
	30	75,051,793	90.0
	45	82,019,212	98.3
	60	83,183,701	99.7
All certified stroke units	15	38,387,496	46.0
	30	70,875,055	85.0
	45	80,767,201	96.8
	60	82,764,286	99.2
Only Supraregional stroke units	15	25,172,663	30.2
	30	54,634,780	65.5
	45	73,448,888	88.1
	60	80,924,726	97.0
Only Regional/Telemedicine supported stroke units	15	20,818,316	25.0
	30	55,953,360	67.1
	45	75,255,717	90.2
	60	81,427,645	97.6
Only Thrombectomy-Certified Stroke Units	15	19,726,605	23.7
	30	47,994,024	57.5
	45	67,453,075	80.9
	60	77,172,441	92.5
Only telemedicine supported stroke units (Subset of Regional)	15	1,383,768	1.7
	30	8,534,560	10.2
	45	19,694,013	23.6
	60	33,312,753	39.9

The table reports the number and percentage of residents within 15-, 30-, 45-, and 60-min driving time (“isochrones”) of different categories of hospitals. Coverage is shown for all hospitals with on-site CT scanners, *stroke-ready hospitals* (defined as hospitals performing ≥100 OPS-coded acute stroke treatments annually), all certified stroke units, supraregional stroke units, regional/telemedicine-supported stroke units, thrombectomy-certified stroke units, and the subset of only telemedicine-supported stroke units. Population coverage estimates are based on the 2025 Estimate Global Human Settlement Population Grid and national isochrone modelling using openrouteservice.

### Access to stroke-ready hospitals

Already at the 15-min threshold, 42,673,811 people (51.2%) could reach at least one of 463 stroke-ready hospitals. Coverage increased further to 75,051,793 (90.0%) by 30 min, 82,019,212 (98.3%), by 45 min, and 83,183,701 (99.7%) by 60 min (Table 1, Fig. 1B).

### Access to certified stroke units

**Fig. 2:**
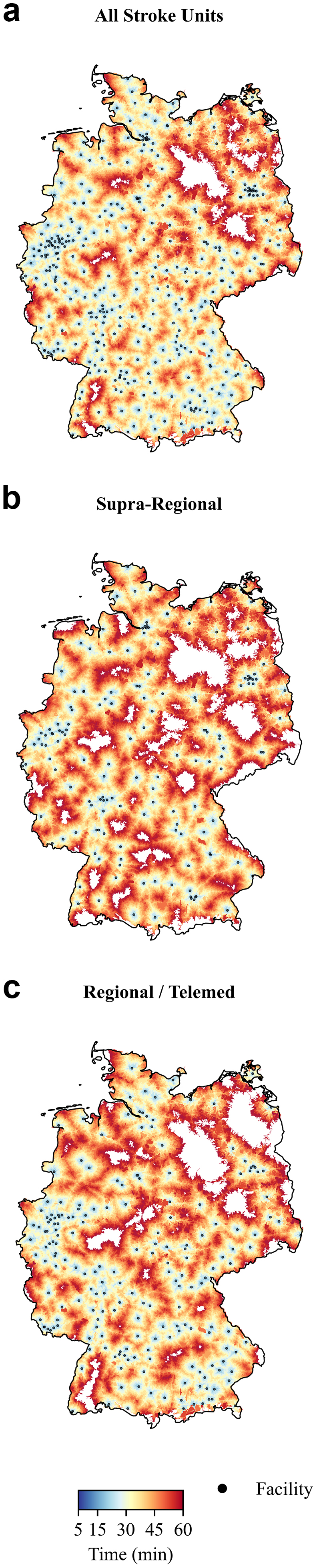
**National coverage by stroke unit tier.** Drive-time isochrones (5–60-min) around a) all certified stroke units, b) supra-regional stroke units, and c) regional/telemedical stroke units in Germany. Black dots mark each stroke unit. Areas not reached within 60 min are left white. For population coverage see Table 1. For a map of coverage of only telemedicine-supported regional stroke units see Supplement S2.

Overall certified stroke-unit coverage was substantially lower than CT-hospital coverage at shorter intervals (Table 1, Fig. 1C). At 15 min, only 38,387,496 residents (46.0%) lived within reach of at least one of 349 certified stroke units. By 30 min, coverage improved to 70,875,055 (85.0%), increasing further to 80,767,201 (96.8%) at 45 min and 82,764,286 (99.2%) at 60 min. A further stratification by supra-regional and regional/telemedicine-supported stroke unit can be found in Fig. 2. These analyses already revealed substantial local differences with incomplete coverage across parts of northern, eastern and south-western Germany. We further compared stroke unit access to thrombectomy-certified neurovascular centres, which was the most geographically constrained. Only 19,726,605 residents (23.7%) were within 15 min of a DeGIR-thrombectomy-certified centre; 47,994,024 (57.5%) within 30 min; 67,453,075 (80.9%) within 45 min; and 77,172,441 (92.5%) within 60 min (Table 1, Fig. 1D). Regional stroke units supported by telemedicine were particularly common in southern Germany, especially Bavaria, with a notable example in northern Germany being the only stroke unit on an island on Rügen (see Supplement S2). Reflecting their location in less densely populated areas, these units covered only 1,383,768 (1.7%) residents within 15 min and 8,534,560 (10.2%) within 30 min (see Table 1). Detailed state- or county-wise analyses with higher temporal resolution can also be accessed in the web application and Supplement S3–S6.

When analysing the degree of urbanisation covered by these isochrones, there was a consistent decrease in the proportion of urbanised areas, from 36.5% within 15-min stroke unit isochrones down to just 9.9% within the 60-min isochrones for all CT-equipped hospitals. In line with this, the 15-min stroke-unit isochrones covered 43.0% (15,088 km^2^) of all German urban areas, but only 11.6% (26,280 km^2^) of all rural areas. And while rural coverage increased to 54.6% (164,643 km^2^) for 30-min stroke unit isochrones, all hospitals with CT already covered 89.5% (284,712 km^2^) at 30 min (see Supplement S7 for detailed area measurements and urbanisation data).

### Benefit analyses—when and where does the hub-and-spoke paradigm yield greatest impact?

**Fig. 3:**
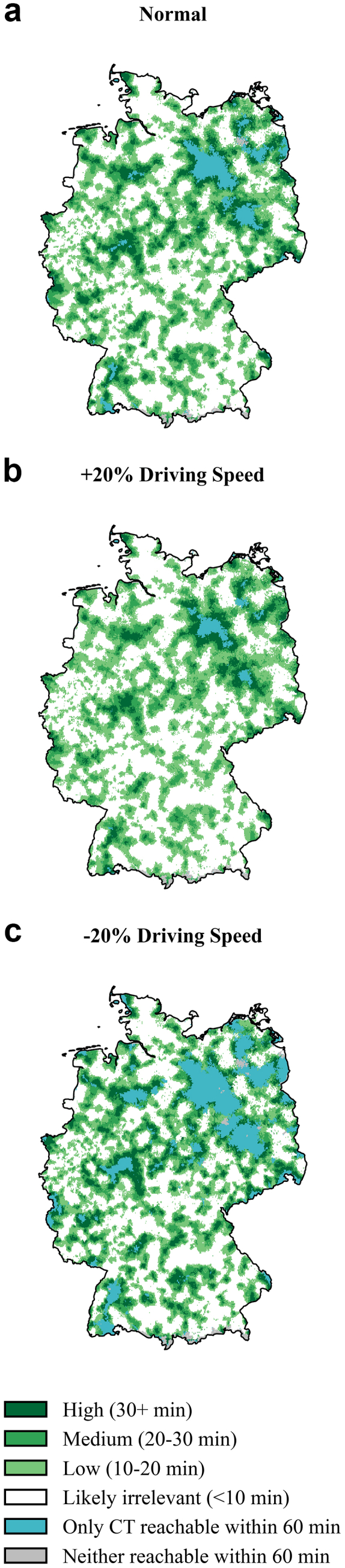
**Time benefit estimation of hospital with CT versus certified stroke unit across Germany.** Maps of Germany showing estimated time benefit of reaching the nearest hospital with a CT scanner versus a certified stroke unit under three driving speed scenarios: a) normal driving speed, b) increased speed (+20%), and c) reduced speed (−20%). Time benefit categories reflect estimated additional drive-time required to reach a stroke unit compared to a CT-equipped hospital: high (30+ min), medium (20–30 min), low (10–20 min), or likely irrelevant (<10 min). Areas where only CT access is available within 60 min are shown in light blue, and regions without access to any analysed facility within 60 min are shown in grey. For quantitative analyses of these scenarios, see Table 2. For the same analysis focussing on stroke-ready hospitals versus all CT-equipped hospitals, refer to Supplement S9. County or state-wise representations and statistics can be found in Supplements S11 and S12 or on the interactive web application https://masannecklab.github.io/GeoStroke-Visualizer/.

Our analysis revealed varying degrees of benefit across different regions in Germany (see Fig. 3A). Assuming normal vehicle speed, the majority of the population (52,133,844; 62.7%) experienced a difference of less than 10 min. However, slightly more than one-third of the German population (36.4%; 30.3 million inhabitants) would reach a CT-capable hospital at least 10 min faster than a certified stroke unit. When stratified by benefit, 22.2% of the population would save 10–20 min, 9.4% 20–30 min, and 4.8% more than half an hour. A marginal 0.7% of residents even live in areas where a stroke unit is out of 60-min reach, yet a CT facility is accessible within 60 min. Of note, 0.7% live in areas where neither facility could be reached within 60 min, yielding the comparison impossible (see Table 2). While areas with likely no benefit showed higher shares of urban areas (13.5%), these declined in areas with 10-min benefit (8.3%), 20–30-min benefit (5.9%) and >30-min benefit (6.4%). Areas with only CT-access had an especially low degree of urbanisation (1.2%), whereas areas that could not be reached from either facility within 60 min showed low urbanisation (2.9%) while also having high shares of water bodies or uninhabited areas (12.7%) (see Supplement S8).

**Table 2:** Benefit calculations for different drip-and-ship scenarios.

Benefit compared to	Benefit category	Scenario: normal	Scenario: +20% driving speed	Scenario: −20% driving speed	Scenario: +10 min penalty	Scenario: +20 min penalty	Scenario: +30 min penalty
Certified stroke units	Neither reachable within 60 min	213,885 (0.3%)	206,279 (0.2%)	229,184 (0.3%)	213,885 (0.3%)	213,885 (0.3%)	213,885 (0.3%)
	Only CT reachable within 60 min	574,085 (0.7%)	186,189 (0.2%)	1,880,509 (2.3%)	574,085 (0.7%)	574,085 (0.7%)	574,085 (0.7%)
	Likely irrelevant (<10 min)	52,133,844 (62.7%)	56,222,940 (67.6%)	47,760,782 (57.4%)	59,954,572 (72.1%)	71,307,861 (85.7%)	78,600,079 (94.5%)
	Low (10–20 min)	18,441,260 (22.2%)	17,913,479 (21.5%)	18,140,731 (21.8%)	14,158,787 (17.0%)	7,641,977 (9.2%)	2,850,726 (3.4%)
	Medium (20–30 min)	7,813,066 (9.4%)	6,413,206 (7.7%)	9,007,366 (10.8%)	5,855,786 (7.0%)	2,625,470 (3.2%)	955,902 (1.1%)
	High (>30 min)	4,018,524 (4.8%)	2,252,573 (2.7%)	6,176,094 (7.4%)	2,437,551 (2.9%)	831,379 (1.0%)	0 (0.0%)
Stroke-Ready hospitals	Neither reachable within 60 min	213,678 (0.3%)	205,650 (0.2%)	227,921 (0.3%)	213,678 (0.3%)	213,678 (0.3%)	213,678 (0.3%)
	Only CT reachable within 60 min	171,991 (0.2%)	59,442 (0.1%)	882,526 (1.1%)	171,991 (0.2%)	171,991 (0.2%)	171,991 (0.2%)
	Likely irrelevant (<10 min)	58,380,216 (70.2%)	62,302,259 (74.9%)	54,076,203 (65.0%)	65,882,828 (79.2%)	75,544,675 (90.8%)	80,549,666 (96.8%)
	Low (10–20 min)	16,312,764 (19.6%)	15,179,861 (18.2%)	16,873,686 (20.3%)	11,777,980 (14.2%)	5,246,886 (6.3%)	1,763,872 (2.1%)
	Medium (20–30 min)	5,784,046 (7.0%)	4,268,748 (5.1%)	7,082,466 (8.5%)	3,774,794 (4.5%)	1,599,329 (1.9%)	495,460 (0.6%)
	High (>30 min)	2,331,971 (2.8%)	1,178,706 (1.4%)	4,051,864 (4.9%)	1,373,396 (1.7%)	418,105 (0.5%)	0 (0.0%)

The table presents the number and percentage of German residents who would experience a clinically relevant time benefit (≥10 min earlier access) by being transported to the nearest CT-equipped hospital compared with the nearest certified stroke unit (*upper half*) or compared with the nearest stroke-ready hospital (*lower half*). Benefit categories are defined as low (10–20 min faster), medium (20–30 min), and high (>30 min). “Likely irrelevant” indicates differences <10 min “Only CT reachable” indicates areas where stroke units were outside the 60-min isochrone but CT hospitals were still reachable, while “Neither reachable” denotes populations outside 60-min reach of both facilities. Results are shown under six scenarios: baseline driving speed, increased speed (+20%), decreased speed (−20%), and baseline speed with added 10-, 20-, or 30-min in-hospital penalty times to simulate potential delays at CT hospitals due to lack of stroke specialisation.

Altering pre-hospital conditions shifted the magnitude of benefit but not its general geographic distribution pattern. Faster transfer (+20% driving speed) trimmed the share of population with a benefit of more than 10 min to 31.9%, whereas slower transfer (−20% driving speed) enlarged it to 40.0% (see Fig. 3 B and C; see Table 2).

**Fig. 4:**
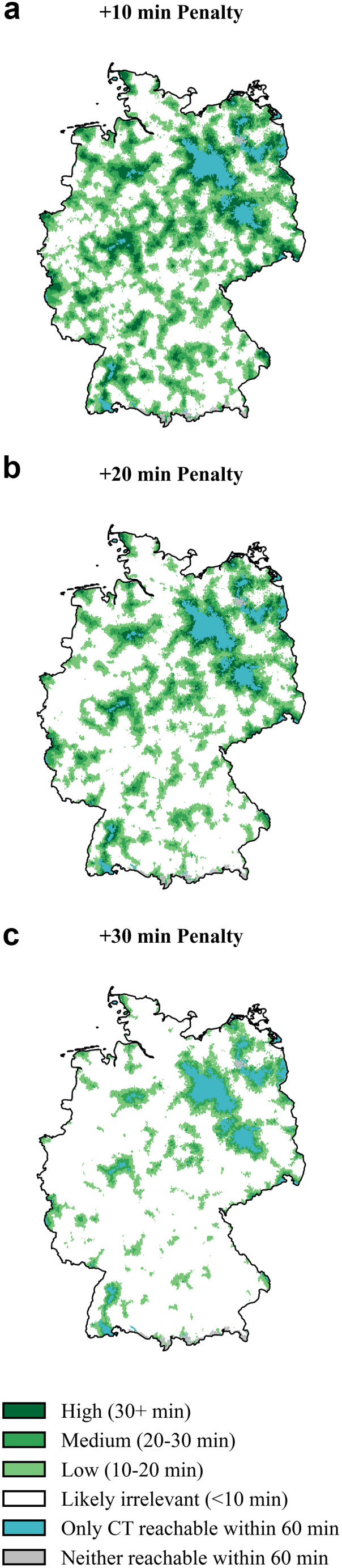
**Simulated impact of delays when accessing CT-equipped hospitals instead of stroke units.** Maps of Germany showing estimated time benefit of reaching the nearest hospital with a CT scanner versus a certified stroke unit under three hypothetical penalty scenarios applied to CT-equipped hospitals to simulate delays due to lack of specialised stroke care (e.g., less experienced staff, teleconsulting etc.): a) +10 min penalty, b) +20 min penalty, and c) +30 min penalty. Time benefit categories reflect additional drive-time to reach a stroke unit compared to a CT-equipped hospital adjusted by the respective penalty: high (30+ min), medium (20–30 min), low (10–20 min), or likely irrelevant (<10 min). Areas where only CT access remains within 60 min are shown in light blue, and regions without access to either facility type within 60 min are shown in grey. For corresponding quantitative analyses, see Table 2. For analogous analyses comparing stroke-ready hospitals with all CT-equipped hospitals, refer to Supplement S10. Further population statistics are available in Supplements S11 and S12 or via the interactive tool at https://masannecklab.github.io/GeoStroke-Visualizer/.

As primarily navigating to a CT-equipped hospital with less stroke experience and the potential setup of teleneurological support might incur additional delays, we calculated scenarios assuming normal driving speed with fixed penalty times added. Introducing a moderate penalty of 10 or 20 min markedly reduced potential beneficiaries (from 36.4% down to 26.9% and 13.4%, respectively). Imposing a universal 30-min delay almost abolished the advantage (4.6%) (see Fig. 4; see Table 2).

When calculating potential benefits from all CT-equipped hospitals to stroke-ready hospitals (n = 463) instead of certified stroke units (n = 349), the share of the population that might benefit from a direct-to-CT strategy fell as expected (e.g., to 29.4% in the baseline scenario down from 36.4% for only certified stroke units) (see Table 2 and Supplements S9 and S10 for details). Exact state- or county-wise calculations can be found in the web application and Supplements S11 and S12.

### Regional disparities and potential for localised approaches

As seen in the wider analysis above, differences between access to specialised stroke care and hospital-based CT imaging more generally differ immensely locally. A hub-and-spoke model with care at the next-best CT would thus provide the greatest value in areas with a large discrepancy between stroke unit access and access to local hospitals with CTs. Although no substantial differences were observed in highly urbanised states such as Berlin, Hamburg, and Bremen (likely no benefit for >95% of the population for CT versus Certified stroke units in normal driving scenario with 10 min teleneurology penalty), notable disparities emerged in certain other federal states. This is especially the case for north-eastern states such as Mecklenburg-Vorpommern (39.2% of population with potential benefit in same scenario, 3.7% can only reach CT within 60 min), Brandenburg (45.6% with potential benefit in same scenario; 7.2% can only reach CT within 60 min) or Saxony-Anhalt (48.6% of population with potential benefit in same scenario, 6.6% can only reach CT within 60 min). However, even larger states with comparably good overall coverage, such as the Western German North Rhine-Westphalia (78.4% of population likely no benefit, with potential (mostly low) benefit for 21.6% in same scenario) show relevant local variability on a county level, with for example the county “Hochsauerlandkreis” having large discrepancies (35.0% likely no benefit versus 63.0% with potential benefit). For all local representations on state and county-level see Supplements S3–S6 and S10–S11, or the interactive web application, which enables users to explore state- and county-level scenarios, provides intuitive visualisations along with detailed area and population estimates (https://masannecklab.github.io/GeoStroke-Visualizer/). Illustrative web application screenshot examples of the state North Rhine-Westphalia and the county “Hochsauerlandkreis” in the mentioned scenario can be found in Supplements S13 and S14.

## Discussion

The safety and effectiveness of ischaemic stroke treatment are highly dependent on fast prehospital and intra-hospital processing times.^10^ Using isochrone analysis, our study shows that although certified stroke units are widely distributed across Germany, their geographical distribution does not consistently match the time-critical requirements of acute stroke care. By modelling travel times, we identified significant regional differences in accessibility. While urban and densely populated regions tend to have a high level of stroke care within acceptable time windows, accessibility is significantly worse in some rural and structurally underserved areas.^29^^,^^30^ In these regions, patients are often faced with long transfer times that can exceed the recommended treatment windows for IVT or endovascular therapy. Given a German registry's median door-to-needle (thrombolysis) time of 30 min,^31^ a maximum 30-min transfer interval is required to be able to achieve thrombolysis within 60 min of symptom onset in an ideal setting.^16^ Under the current distribution of certified stroke units, only 85% of the German population meets this criterion. These findings highlight a mismatch between infrastructure availability and clinical urgency and emphasise the need for targeted health system planning to address geographic disparities in stroke care. As a potential response to this gap, leveraging the existing CT infrastructure across all hospitals could allow a substantial proportion of patients to receive essential imaging—and thus essential treatment—much earlier. Thus, utilising any CT-equipped hospital for initial imaging and teleassessment elevates 30-min coverage to 98.9%. A 15-min transfer interval is currently achievable for 46.0% and could be increased to 76.3% of the German population by the proposed model, indicating that a hub-and-spoke model could dramatically improve equitable access to thrombolysis during the golden hour. In addition, this approach may enable earlier contact between stroke specialists and patients via telemedicine, as well as provide preliminary insights into CT findings. This could support more informed early decision-making, including directing ambulances to thrombectomy-capable centres.

Such a strategy should only be implemented in settings where it offers a substantial benefit compared to direct transfer to a certified stroke centre. Our results provide the necessary granularity to inform the development of region-specific strategies, accounting for local disparities in healthcare infrastructure. While utilising the nearest CT-equipped facility may not yield significant benefits in densely populated city-states such as Hamburg or Berlin, regions in states like Saxony-Anhalt or Mecklenburg-Western Pomerania could substantially benefit, particularly with the expansion of telemedical stroke services. This approach should not be considered in isolation; rather, it should be complemented by additional strategies evaluated in our analyses, such as enhancing telemedicine-supported certified stroke units—a concept successfully implemented in Bavaria, Germany's largest state, to achieve comparably high coverage despite its size.

Our analysis highlights the disadvantage rural areas face: only 54.6% of rural areas have access to a stroke unit within 30 min, compared to 89.5% with access to a CT-equipped hospital (urban areas: 87.1% versus 99.4%). Regionally tailored strategies, as proposed here, could help close this gap. Recent evidence suggests telemedicine may be particularly effective in rural settings and populations,^32^ highlighting the need to support especially the parts of a country with weaker infrastructure. Thus, pronounced differences between counties and states made visible through the here presented work underscore the need for tailored, data-driven interventions.

To better assess the real-world applicability of our approach, we incorporated variability into a detailed time benefit and scenario analysis. Using a nationwide, grid-based comparison of travel times to CT-equipped hospitals versus certified stroke units or just hospitals that treat many stroke patients, we modelled multiple driving scenarios and accounted for potential institutional delays. These analyses not only revealed regional disparities in access but also demonstrated the potential impact of our proposed hub-and-spoke approach under different assumptions. As such, it may serve as a blueprint for evaluating hub-and-spoke stroke care models in other geographic settings and health systems. The aim of reducing the processing times in the prehospital chain of care led to the establishment of mobile stroke units, which, by including the possibility to perform CT-imaging within the ambulance, allow fast identification of patients eligible for thrombolysis.^14^ A recent study showed that thrombolysis within the first 60 min of symptom onset—enabled primarily through mobile stroke unit care–was associated with nearly twice the odds of an excellent clinical outcome compared to later treatment, without an increased risk of haemorrhage.^33^^,^^34^ Nevertheless, introducing mobile stroke units on a larger scale is hampered by immense costs of purchasing and operating them, raising uncertainties about cost-effectiveness.^35^

The previously proposed approach of in-ambulance video-consulting, including video-guided NIHSS assessment to evaluate eligibility for thrombolysis therapy, could be used to improve the rescue chain without the constraints of mobile stroke units.^16^ Notably, in-transit clinical assessment has been shown to reduce treatment times without compromising safety.^36^, ^37^, ^38^ Remote evaluation of the NIHSS via telemedicine has proven reliable,^39^ and thrombolysis administered within telestroke networks has demonstrated both safety and efficacy.^37^^,^^40^ However, these approaches have not yet proven to be cost-effective and resource-efficient, warranting further precisely designed implementation studies.

The feasibility of administering IVT in hospitals with CT scanners but lacking structured stroke workflows is a valid concern. Telestroke systems have demonstrated to effectively achieve treatment times comparable to those of dedicated stroke centres^34^^,^^41^^,^^42^ relying on trained personnel, rapid assessment, and streamlined protocols. Evidence from telestroke programs further indicates that improvements in IVT rates depend on integrated care structures, interprofessional team training, and robust quality management. We propose a stepwise exploratory implementation, beginning with teleassessment prehospitally, including NIHSS evaluation in the ambulance. Patients would then be transported to the stroke centre and treated by the prior teleconsultant and neurological team. In a subsequent phase—guided by insights from the initial rollout—imaging could be performed at a CT-equipped facility, followed by the administration of thrombolysis. A mobile device–based approach would ensure continuous expert stroke supervision during thrombolysis, even outside a certified stroke centre. To ensure prompt secondary transportation to a dedicated stroke centre, part of the thrombolysis supervision and risk management would be performed by emergency medical services (EMS) personnel in the ambulance, given fast CT processing times. Emergency physicians, already skilled in managing stroke-related issues like blood pressure and agitation, could help bridge gaps in hospital workflows when supported by telemedicine and clear protocols. Successful implementation should be accompanied by substantial training of EMS personnel and by establishment of a safety board supervising processing time and outcomes.^43^ Moreover, EMS personnel are already accustomed to managing drip-and-ship patients needing bridging-thrombolysis in the ambulance, as well as stroke patients initially presenting to non-stroke centre emergency departments which are subsequently transported to a stroke unit. In our experience, complications tend to arise later in the care pathway, when the patient has been admitted to a stroke unit. Finally, the single-bolus agent tenecteplase—due to its ease of administration and favourable pharmacokinetics—may enhance the practicality of IVT in non-specialised settings.^44^

Conceptually, the pathway we describe extends existing hospital-to-hospital telestroke networks into the prehospital setting. Existing organisational recommendations of the German Stroke Society^45^ and earlier European frameworks^46^ should be adapted to make EMS personnel the first point of contact for teleneurologists. Exemplary regulatory and organisational requirements adapted from these recommendations and extended to the hub-and-spoke setting are summarised in Panel 1. We admit that depending on country-specific regulation, different legal constraints may exist.

Panel 1 **Minimum requirements for commissioning the described hub-and-spoke model.**

• **24/7 infrastructure**: Continuous CT availability, laboratory availability; capacity to initiate intravenous thrombolysis or adequate EMS resources.
• **Specialist access**: Immediate round-the-clock teleneurology by stroke specialists, with defined response-time targets and reliable backup.
• **Technology and data protection**: Secure real-time audio-video and rapid image transfer, GDPR-compliant with audit logs and tested fallbacks.
• **Protocols and EMS integration**: SOPs for prehospital triage and video-assisted scoring, processing and monitoring. Local protocols for EMS and hospital cooperation.
• **Overcoming governance and liability barriers**: Formal hub–spoke agreements specifying roles, documentation standards, escalation pathways and medico-legal cover.
• **Quality assurance**: Registry-based monitoring of door-to-CT/needle, thrombolysis and mimic rates, and outcomes, with scheduled audit and feedback.
• **Stepwise rollout:** Gathering data and improving processes gradually, from prehospital teleneurological setting to integration into clinical pathways.
• **Training and competency**: Initial and recurrent joint training and simulation for EMS and ED teams.

Panel: Essential operational, technical, and governance prerequisites for commissioning a prehospital, telemedicine-enabled hub-and-spoke thrombolysis pathway, integrating EMS as the first point of contact. EMS, emergency medical services; GDPR, General Data Protection Regulation; SOP, standard operating procedure.

Further challenges are stroke mimics, which make up a relevant proportion of patients transported and treated under stroke codes. Decreasing door-to-needle times may even increase stroke mimics treated with thrombolysis.^47^, ^48^, ^49^ Analyses of teleneurological settings have indicated a high proportion of stroke mimics among those receiving thrombolysis, with a recent monocentre study reporting 24%^50^ and potentially even higher rates in less controlled settings such as the one proposed here. Nevertheless, thrombolysis in stroke mimics appears to be safer than in actual stroke patients^51^ and tools such as the Telestroke Mimic Score^52^ along with extensive EMS education may help mitigate this issue.

A key limitation of our analysis is the lack of reliable data on the 24/7 availability of CT imaging across all hospitals, which is essential for consistent and timely stroke care and might alter analyses (especially at night). It may also be discussed, whether CT angiography imaging should also be a minimum requirement, which might alter analyses to a small degree. Moreover, while all data sources were carefully selected and cross-validated where possible, they may still contain inaccuracies or omissions that could influence the results. Although, while we tested multiple sensitivity scenarios, these rely on uniform speed multipliers and fixed delay penalties specified as pragmatic bounding conditions. Importantly, they may not capture structural or locally varying features that shape real-world intervals—e.g., time-of-day congestion, urban–rural differences in dispatch and on-scene times, or loading/unloading and door-to-CT processes–and could widen or narrow absolute gains. In the absence of harmonised, nationwide EMS data, empirical calibration and validation were not possible, however, future work should couple this framework to such datasets. Similarly, the 10, 20 and 30-min delays were chosen as rough estimates and should be revised with better empirical data from real-world data objectifying delays through non-specialised and telemedical settings. Furthermore, the implementation of video-based thrombolysis outside certified stroke units faces unresolved legal, financial, and organisational challenges that vary between healthcare systems. Licencing restrictions, liability concerns, and the need for robust quality assurance frameworks represent significant barriers. Safe and effective implementation will require clear regulatory guidance, structured oversight, and well-defined responsibilities. Moreover, we acknowledge that the economic implications of implementing a hub-and-spoke model—considering the required infrastructure, training, and protocol development—remain uncertain. To translate this concept into clinical practice, prospective studies and implementation science approaches will be crucial to evaluate its feasibility, safety, real-world effectiveness, and cost-efficiency. In addition, it is important to emphasise that there is no one-size-fits-all approach to improving access to timely stroke recanalisation therapies; instead, strategies must be tailored to the specific regional context and healthcare infrastructure.^53^

In conclusion, our findings underscore substantial regional disparities in timely stroke care and highlight the potential of integrating prehospital teleconsultation and early imaging at CT-equipped facilities as a scalable, pragmatic complement to existing stroke systems. The here introduced approach is adaptable to other geographic regions and may serve as a transferable blueprint for other time-sensitive emergency care systems. Tailored adaptations of the here proposed hub-and-spoke pathway may help optimise equity and treatment access in Germany and beyond, particularly in underserved regions.

## Contributors

LM and MV contributed to data analysis and verification. LM, MP, and MG developed the study concept. LM, MV, and CR were responsible for data collection. JC, SGM, DvdL, MG, and MP contributed to interpretation of results. LM, MP, and MG prepared the initial draft. All authors contributed to writing, review, and editing of the final manuscript. All authors had full access to the data and were responsible for the decision to submit the manuscript.

## Data sharing statement

The data underlying this study is publicly available. Underlying code is available in the respective GitHub repository https://github.com/MasanneckLab/GeoStroke-Analyses. An interactive web application, which lets users explore the datasets on a local level and across different scenarios is available here: https://masannecklab.github.io/GeoStroke-Visualizer/. This web application also offers downloads of large parts of the results.

## Declaration of generative AI and AI-assisted technologies in the writing process

During the preparation of this work the authors used Grammarly, DeepL and ChatGPT in order to refine written language. Large language model-based technology was used to help write and refactor code underlying the analysis. While using these tools, the authors reviewed and edited the content as needed and take full responsibility for the content of the publication.

## Editor note

The Lancet Group takes a neutral position with respect to territorial claims in published maps and institutional affiliations.

## Declaration of interests

**LM** reports no conflicts of interest related to this study. He has received honoraria for lecturing, consulting, and travel expenses for attending meetings from Biogen, Merck, Sanofi, argenX, Roche, Alexion, and Novartis, all outside the scope of this work. His research is funded by the German Multiple Sclerosis Foundation (DMSG) and the Deutsche Forschungsgemeinschaft (DFG, German Research Foundation)—493659010. **MV** reports no conflicts of interest. He received funding from the Medical Faculty of the Heinrich-Heine-University Duesseldorf, Germany through a Clinician Scientist program. **JC** reports no conflicts of interest related to this study. His research has been funded by Ministry of Economic Affairs, Innovation, Digitalization and Energy of the State of North Rhine-Westphalia (MWIDE NRW, 005–2008-0055), the German Federal Ministry of Education and Research (BMBF)–01GP2113C. He has received honoraria for lecturing and travel expenses from Allergan and Pfizer, all outside the scope of this work. **CR and DR** report no conflicts of interest. **SGM** reports no conflicts of interest related to this study. He has received grants from the German Ministry for Education and Research (BMBF), the Bundesinstitut für Risikobewertung (BfR), the Deutsche Forschungsgemeinschaft (DFG), the Else Kröner Fresenius Foundation, the Gemeinsame Bundesausschuss (G-BA), the German Academic Exchange Service, the Hertie Foundation, the Interdisciplinary Center for Clinical Studies (IZKF) Muenster, and the German Foundation Neurology. He has received payment or honoraria for lectures, presentations or educational events from Almirall, Amicus Therapeutics Germany, ArgenX, Bayer Health Care, Biogen, Celgene, Diamed, Genzyme, MedDay Pharmaceuticals, Merck Serono, Novartis, Neuraxpharm, Novo Nordisk, ONO Pharma, Roche, Sanofi-Aventis, Chugai Pharma, QuintilesIMS, and Teva. He has received support for attending meetings and/or travel from Almirall, Amicus Therapeutics Germany, ArgenX, Bayer Health Care, Biogen, Celgene, Diamed, Genzyme, MedDay Pharmaceuticals, Merck Serono, Novartis, Neuraxpharm, Novo Nordisk, ONO Pharma, Roche, Sanofi-Aventis, Chugai Pharma, QuintilesIMS, and Teva. He reports other financial or non-financial interests with Alexion, Almirall, Amicus Therapeutics Germany, Biogen, Diamed, Fresenius Medical Care, Genzyme, HERZ Burgdorf, Merck Serono, Novartis, ONO Pharma, Roche, and Teva.

**MP** reports no conflicts of interest related to this study. He has received payment or honoraria for lectures, presentations, or educational events from Argenx, Alexion, Biogen, Bayer, Demecan, Hexal, Merck, Sanofi, Takeda, Teva, Janssen, Roche, and Novartis. He has received support for attending meetings and/or travel from Alexion, Biogen, Merck, Sanofi, and Novartis.

He has received honoraria for lecturing and travel expenses for attending meetings from Alexion, ArgenX, Bayer Health Care, Biogen, Demecan, Hexal, Merck Serono, Neuraxpharm, Novartis, Roche, Sanofi-Aventis, Takeda, and Teva. His research is funded by ArgenX, Biogen, Demecan, Hexal, Novartis, Neuraxpharm and Roche, all outside the scope of this study. **MG** reports no conflicts of interest related to this study. He has received honoraria for lecturing and travel expenses from Roche, Novartis, Pfizer, Bristol-Myers Squibb all outside the scope of this work.

## Appendix A Supplementary data

Supplementary File

Translated Abstract
