# Supplementary material for: Direct stroke unit access versus a hub-and-spoke model with telemedicine-assisted CT in Germany: a cross-sectional geospatial analysis
Source: Lancet Reg Health Eur. 2026 Feb 5;63:101604. doi: 10.1016/j.lanepe.2026.101604 (PMC12906117; doi:10.1016/j.lanepe.2026.101604)
Supplement: Translated Abstract [file mmc2.pdf]

This translation in German was submitted by the authors and we reproduce it as supplied. It has not been peer reviewed. Our editorial processes have only been applied to the original abstract in English, which should serve as reference for this manuscript.

Translated abstract (German)

Hintergrund: Eine frühzeitige Reperfusion innerhalb der ersten Stunde nach Symptombeginn bietet den größten therapeutischen Nutzen beim akuten ischämischen Schlaganfall. In Deutschland bestehen jedoch weiterhin ausgeprägte regionale Unterschiede im Zugang zur Schlaganfallversorgung. Obwohl Telemedizin und mobile Stroke Units vielversprechende Ansätze darstellen, fehlen bislang bundesweite Daten, die bestehende Versorgungslücken quantifizieren oder den Nutzen einer frühen Bildgebung mit anschließender Thrombolysebehandlung in lokal erreichbaren, CT-ausgestatteten Kliniken mit telemedizinischer Anbindung systematisch untersuchen. Diese Studie modelliert den deutschlandweiten Zugang zur Schlaganfallversorgung und vergleicht den direkten Transport in ein Zentrum mit einer Hub-and-Spoke-Strategie (nächstgelegenes CT plus Telemedizin) für eine frühe Thrombolyse.

Methoden: Wir führten eine geospatiale Querschnittsanalyse durch, in die nationale Versorgungsregister und die Qualitätsberichte der Krankenhäuser von 2023 einflossen (Datenerhebung 1. Februar bis 14. Juli 2025). Erfasst wurden CT-ausgestattete Krankenhäuser (n = 1 475), „stroke-ready" Krankenhäuser (≥100 jährliche Fälle „Komplexe neurologische Behandlung des akuten Schlaganfalls", n = 463) und zertifizierte Stroke Units (n = 349). Für diese Einrichtungen wurden Fahrzeitisochronen bis 60 Minuten in 5-Minuten-Intervallen mithilfe einer lokalen Installation von openrouteservice berechnet und mit Bevölkerungs- sowie Siedlungsrastern überlagert. Zusätzliche Szenarien simulierten unterschiedliche Rettungswagengeschwindigkeiten (Standardannahme: die in openrouteservice hinterlegten Fahrzeuggeschwindigkeiten) sowie zusätzliche innerklinische Verzögerungen beim Einsatz des Hub-and-Spoke-Ansatzes. Anschließend wurden Hub-and-Spoke-Strategie und direkter Transport in spezialisierte Krankenhäuser auf Bundes-, Länder- und Kreisebene verglichen.

Ergebnisse: Innerhalb von 30 Minuten erreichten 82 484 915/83 420 000 Einwohner (98,9 %) ein CT-Krankenhaus, 90,0 % (75 051 793) ein stroke-ready Krankenhaus, jedoch nur 85,0 % (70 875 055) eine zertifizierte Stroke Unit. Im Vergleich zum direkten Stroke-Unit-Transport würde ein Hub-and-Spoke-Ansatz 36,4 % der Bevölkerung eine Bildgebung ≥10 Minuten früher ermöglichen (bei angenommener normaler Fahrgeschwindigkeit; in alternativen Szenarien zwischen 4,5 % und 40,0 %) und bei 14,2 % eine Zeitersparnis von ≥20 Minuten (1,1 % bis 18,2 % in verschiedenen Szenarien). Der geschätzte Nutzen des Hub-and-Spoke-Modells hing von den angenommenen Fahrgeschwindigkeiten ab und nahm bei simulierten CT-bedingten Verzögerungen ab. In ländlichen Regionen zeigten

sich besonders ausgeprägte Versorgungslücken, was sich in niedrigeren Urbanisierungsgraden der Regionen mit höherem Nutzenpotenzial für das Hub-and-Spoke-Modell widerspiegelte. Die Analyse auf Bundeslandebene verdeutlichte Heterogenität, mit einem potenziellen Nutzen für 48,6 % der Bevölkerung in Sachsen-Anhalt, jedoch <5 % in Stadtstaaten (angenommene normale Fahrgeschwindigkeit und 10-minütige Verzögerung).

Interpretation: Deutliche innerdeutsche Ungleichheiten im schnellen Zugang zur Schlaganfallversorgung bestehen fort. Die Nutzung vorhandener CT-Kapazitäten unter telemedizinischer Aufsicht könnte im Rahmen eines Hub-and-Spoke-Modells eine frühere Thrombolyse ermöglichen. Offene Fragen hinsichtlich Umsetzung und Wirtschaftlichkeit sollten weiter untersucht werden.

Funding: Die Studie wurde durch Mittel des Universitätsklinikums Düsseldorf, der B. Braun-Stiftung sowie des Ministeriums für Wirtschaft, Innovation, Digitalisierung und Energie des Landes Nordrhein-Westfalen (Förderkennzeichen 005–2008-0055) unterstützt.
