# Supplementary material for: Direct stroke unit access versus a hub-and-spoke model with telemedicine-assisted CT in Germany: a cross-sectional geospatial analysis
Source: Lancet Reg Health Eur. 2026 Feb 5;63:101604. doi: 10.1016/j.lanepe.2026.101604 (PMC12906117; doi:10.1016/j.lanepe.2026.101604)
Supplement: Supplementary File [file mmc1.pdf]

Table of Contents

Supplement S1 - Methods Depiction 2
Supplement S2 - Telemed Single Panel 3
Supplement S3 - State Isochrones Analysis Tables 4
Supplement S4 - State Isochrones Analysis Figures 20
Supplement S5 - County Isochrones Analysis Tables 36
Supplement S6 - County Isochrones Analysis Figures 436
Supplement S7 - Rurality-Urbanity Analyses of Isochrones 836
Supplement S8 - Rurality-Urbanity Analyses of Benefits 837
Supplement S9 - Stroke-Ready Speed Scenarios 838
Supplement S10 - Stroke-Ready Penalty Scenarios 839
Supplement S11 - State Benefit Scenario Tables 840
Supplement S12 - County Benefit Scenario Tables 1032
Supplement S13 - NRW WebApp Example 5832
Supplement S14 - HSK WebApp Example 5833

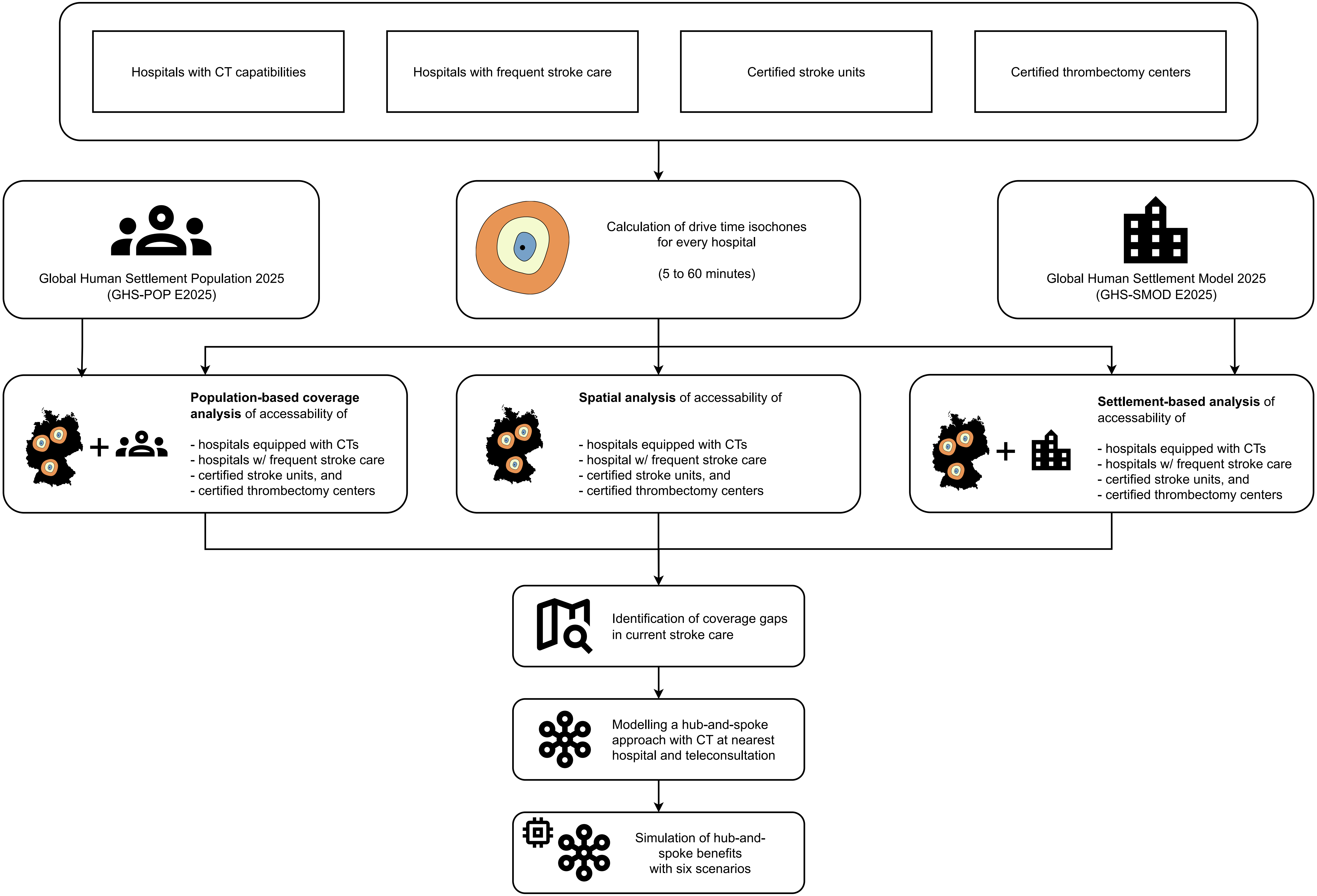
Hospitals with CT capabilities
Hospitals with frequent stroke care
Certified stroke units
Certified thrombectomy centers
Global Human Settlement Population 2025 (GHS-POP E2025)
Calculation of drive time isochones for every hospital
(5 to 60 minutes)
Global Human Settlement Model 2025 (GHS-SMOD E2025)
Population-based coverage analysis of accessability of
- hospitals equipped with CTs
- hospitals w/ frequent stroke care
- certified stroke units, and
- certified thrombectomy centers
Spatial analysis of accessability of
- hospitals equipped with CTs
- hospital w/ frequent stroke care
- certified stroke units, and
- certified thrombectomy centers
Settlement-based analysis of accessability of
- hospitals equipped with CTs
- hospitals w/ frequent stroke care
- certified stroke units, and
- certified thrombectomy centers
Identification of coverage gaps in current stroke care
Modelling a hub-and-spoke approach with CT at nearest hospital and teleconsultation
Simulation of hub-and-spoke benefits with six scenarios

Telemedical Stroke Units

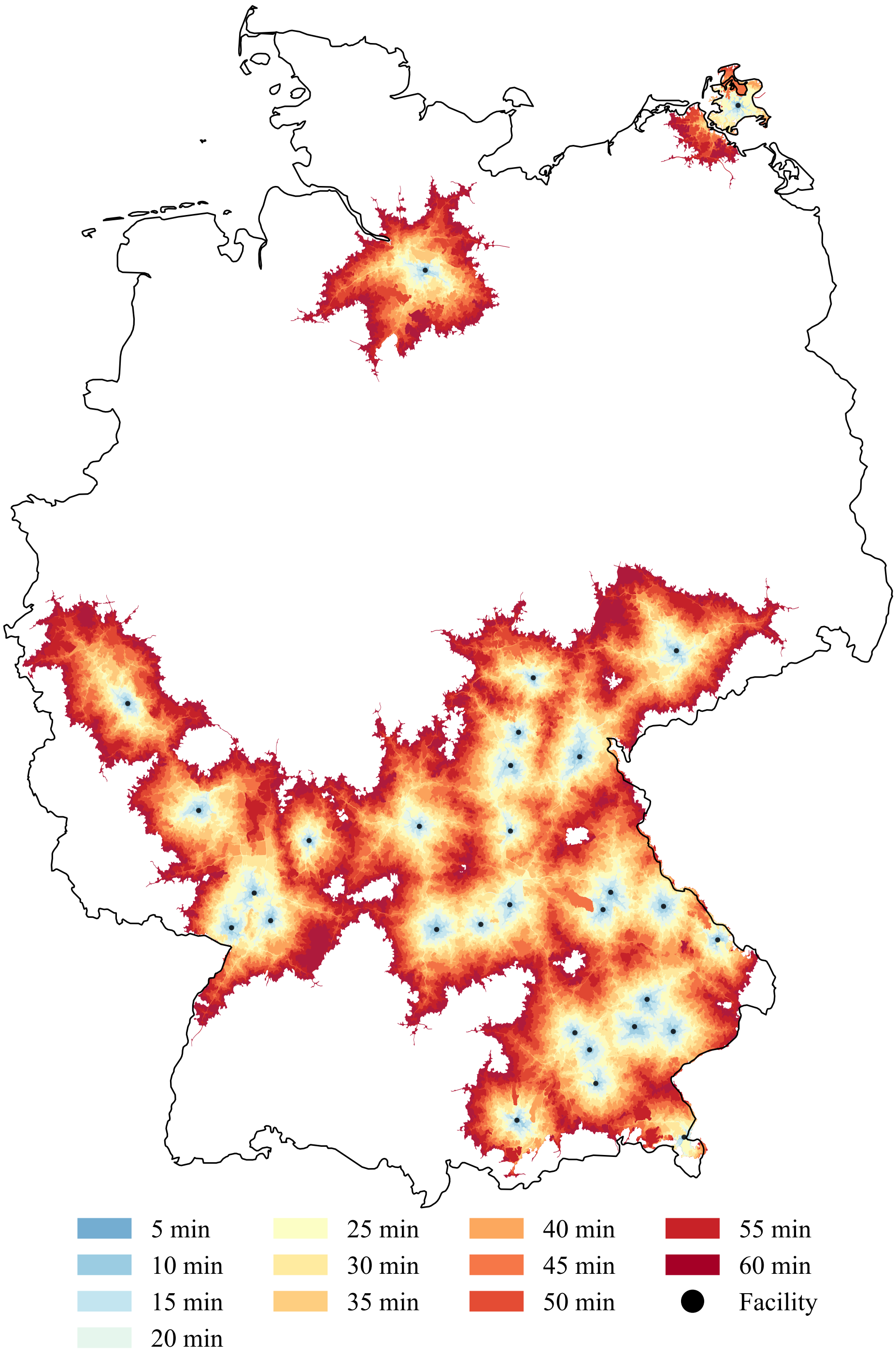

Population Coverage in Baden-Württemberg

Condition	Drive Time (min)	Covered Population	Coverage (%)
Hospitals with CT	5	1582790	14.52
Hospitals with CT	10	4326811	39.69
Hospitals with CT	15	7376076	67.67
Hospitals with CT	20	9317244	85.47
Hospitals with CT	25	10340575	94.86
Hospitals with CT	30	10761361	98.72
Hospitals with CT	35	10857033	99.6
Hospitals with CT	40	10883991	99.85
Hospitals with CT	45	10891930	99.92
Hospitals with CT	50	10895685	99.95
Hospitals with CT	55	10895182	99.95
Hospitals with CT	60	10896021	99.96
Stroke-Ready Hospitals	5	745034	6.83
Stroke-Ready Hospitals	10	2447749	22.46
Stroke-Ready Hospitals	15	4797836	44.01
Stroke-Ready Hospitals	20	7161283	65.7
Stroke-Ready Hospitals	25	9017287	82.72
Stroke-Ready Hospitals	30	10073997	92.42
Stroke-Ready Hospitals	35	10619941	97.43
Stroke-Ready Hospitals	40	10809034	99.16
Stroke-Ready Hospitals	45	10875583	99.77
Stroke-Ready Hospitals	50	10892137	99.92
Stroke-Ready Hospitals	55	10894114	99.94
Stroke-Ready Hospitals	60	10894459	99.94
Stroke Units	5	619763	5.69
Stroke Units	10	2077841	19.06
Stroke Units	15	4060078	37.25
Stroke Units	20	6074668	55.73
Stroke Units	25	7790547	71.47
Stroke Units	30	8975669	82.34
Stroke Units	35	9786078	89.78
Stroke Units	40	10259642	94.12
Stroke Units	45	10523847	96.54
Stroke Units	50	10656128	97.76
Stroke Units	55	10731989	98.45
Stroke Units	60	10785669	98.95

Population Coverage in Bayern

Condition	Drive Time (min)	Covered Population	Coverage (%)
Hospitals with CT	5	2863575	21.52
Hospitals with CT	10	6529581	49.06
Hospitals with CT	15	9705965	72.93
Hospitals with CT	20	11946493	89.77
Hospitals with CT	25	12917419	97.06
Hospitals with CT	30	13228410	99.4
Hospitals with CT	35	13275426	99.75
Hospitals with CT	40	13288158	99.85
Hospitals with CT	45	13294302	99.89
Hospitals with CT	50	13295550	99.9
Hospitals with CT	55	13294650	99.9
Hospitals with CT	60	13295885	99.9
Stroke-Ready Hospitals	5	1044109	7.85
Stroke-Ready Hospitals	10	3382596	25.42
Stroke-Ready Hospitals	15	6239022	46.88
Stroke-Ready Hospitals	20	8957793	67.31
Stroke-Ready Hospitals	25	10825767	81.34
Stroke-Ready Hospitals	30	12122323	91.09
Stroke-Ready Hospitals	35	12809720	96.25
Stroke-Ready Hospitals	40	13105623	98.48
Stroke-Ready Hospitals	45	13247065	99.54
Stroke-Ready Hospitals	50	13288657	99.85
Stroke-Ready Hospitals	55	13291021	99.87
Stroke-Ready Hospitals	60	13293980	99.89
Stroke Units	5	998426	7.5
Stroke Units	10	3271972	24.59
Stroke Units	15	6010881	45.17
Stroke Units	20	8592642	64.56
Stroke Units	25	10461265	78.61
Stroke Units	30	11865885	89.16
Stroke Units	35	12676645	95.25
Stroke Units	40	13048757	98.05
Stroke Units	45	13224604	99.37
Stroke Units	50	13277853	99.77
Stroke Units	55	13286897	99.84
Stroke Units	60	13292794	99.88

Population Coverage in Berlin

Condition	Drive Time (min)	Covered Population	Coverage (%)
Hospitals with CT	5	1421892	39.35
Hospitals with CT	10	3201327	88.6
Hospitals with CT	15	3583044	99.16
Hospitals with CT	20	3611117	99.94
Hospitals with CT	25	3612930	99.99
Hospitals with CT	30	3613074	99.99
Hospitals with CT	35	3613426	100.0
Hospitals with CT	40	3613426	100.0
Hospitals with CT	45	3613426	100.0
Hospitals with CT	50	3613426	100.0
Hospitals with CT	55	3613426	100.0
Hospitals with CT	60	3613426	100.0
Stroke-Ready Hospitals	5	637493	17.64
Stroke-Ready Hospitals	10	2120358	58.68
Stroke-Ready Hospitals	15	3269306	90.48
Stroke-Ready Hospitals	20	3586166	99.25
Stroke-Ready Hospitals	25	3607985	99.85
Stroke-Ready Hospitals	30	3612501	99.97
Stroke-Ready Hospitals	35	3613426	100.0
Stroke-Ready Hospitals	40	3613426	100.0
Stroke-Ready Hospitals	45	3613426	100.0
Stroke-Ready Hospitals	50	3613426	100.0
Stroke-Ready Hospitals	55	3613426	100.0
Stroke-Ready Hospitals	60	3613426	100.0
Stroke Units	5	598179	16.55
Stroke Units	10	2103243	58.21
Stroke Units	15	3259071	90.19
Stroke Units	20	3586133	99.24
Stroke Units	25	3607985	99.85
Stroke Units	30	3612331	99.97
Stroke Units	35	3613426	100.0
Stroke Units	40	3613426	100.0
Stroke Units	45	3613426	100.0
Stroke Units	50	3613426	100.0
Stroke Units	55	3613426	100.0
Stroke Units	60	3613426	100.0

Population Coverage in Brandenburg

Condition	Drive Time (min)	Covered Population	Coverage (%)
Hospitals with CT	5	454063	18.52
Hospitals with CT	10	1091250	44.51
Hospitals with CT	15	1599104	65.23
Hospitals with CT	20	2006161	81.83
Hospitals with CT	25	2255690	92.01
Hospitals with CT	30	2369759	96.66
Hospitals with CT	35	2432487	99.22
Hospitals with CT	40	2443004	99.65
Hospitals with CT	45	2447867	99.85
Hospitals with CT	50	2448326	99.86
Hospitals with CT	55	2447312	99.82
Hospitals with CT	60	2447743	99.84
Stroke-Ready Hospitals	5	141724	5.78
Stroke-Ready Hospitals	10	396886	16.19
Stroke-Ready Hospitals	15	737165	30.07
Stroke-Ready Hospitals	20	1156233	47.16
Stroke-Ready Hospitals	25	1540462	62.83
Stroke-Ready Hospitals	30	1818622	74.18
Stroke-Ready Hospitals	35	1998635	81.52
Stroke-Ready Hospitals	40	2116842	86.34
Stroke-Ready Hospitals	45	2231667	91.03
Stroke-Ready Hospitals	50	2261081	92.23
Stroke-Ready Hospitals	55	2370218	96.68
Stroke-Ready Hospitals	60	2407280	98.19
Stroke Units	5	111185	4.54
Stroke Units	10	327367	13.35
Stroke Units	15	612112	24.97
Stroke Units	20	935496	38.16
Stroke Units	25	1252474	51.09
Stroke Units	30	1534309	62.58
Stroke Units	35	1721374	70.21
Stroke Units	40	1853646	75.61
Stroke Units	45	1971630	80.42
Stroke Units	50	2076050	84.68
Stroke Units	55	2176589	88.78
Stroke Units	60	2277059	92.88

Population Coverage in Bremen

Condition	Drive Time (min)	Covered Population	Coverage (%)
Hospitals with CT	5	209504	39.99
Hospitals with CT	10	449080	85.72
Hospitals with CT	15	497066	94.88
Hospitals with CT	20	506964	96.77
Hospitals with CT	25	508350	97.03
Hospitals with CT	30	508548	97.07
Hospitals with CT	35	523903	100.0
Hospitals with CT	40	523903	100.0
Hospitals with CT	45	523903	100.0
Hospitals with CT	50	523903	100.0
Hospitals with CT	55	523903	100.0
Hospitals with CT	60	523903	100.0
Stroke-Ready Hospitals	5	79718	15.22
Stroke-Ready Hospitals	10	242766	46.34
Stroke-Ready Hospitals	15	392303	74.88
Stroke-Ready Hospitals	20	479302	91.49
Stroke-Ready Hospitals	25	504635	96.32
Stroke-Ready Hospitals	30	506866	96.75
Stroke-Ready Hospitals	35	523903	100.0
Stroke-Ready Hospitals	40	523903	100.0
Stroke-Ready Hospitals	45	523903	100.0
Stroke-Ready Hospitals	50	523903	100.0
Stroke-Ready Hospitals	55	523903	100.0
Stroke-Ready Hospitals	60	523903	100.0
Stroke Units	5	55732	10.64
Stroke Units	10	167851	32.04
Stroke Units	15	338087	64.53
Stroke Units	20	478088	91.26
Stroke Units	25	504407	96.28
Stroke Units	30	506692	96.71
Stroke Units	35	523903	100.0
Stroke Units	40	523903	100.0
Stroke Units	45	523903	100.0
Stroke Units	50	523903	100.0
Stroke Units	55	523903	100.0
Stroke Units	60	523903	100.0

Population Coverage in Hamburg

Condition	Drive Time (min)	Covered Population	Coverage (%)
Hospitals with CT	5	663784	35.88
Hospitals with CT	10	1397324	75.54
Hospitals with CT	15	1749657	94.58
Hospitals with CT	20	1813839	98.05
Hospitals with CT	25	1840826	99.51
Hospitals with CT	30	1841330	99.54
Hospitals with CT	35	1841406	99.54
Hospitals with CT	40	1841408	99.54
Hospitals with CT	45	1841408	99.54
Hospitals with CT	50	1841417	99.54
Hospitals with CT	55	1841315	99.54
Hospitals with CT	60	1841336	99.54
Stroke-Ready Hospitals	5	280910	15.19
Stroke-Ready Hospitals	10	1001353	54.13
Stroke-Ready Hospitals	15	1463335	79.1
Stroke-Ready Hospitals	20	1713787	92.64
Stroke-Ready Hospitals	25	1824769	98.64
Stroke-Ready Hospitals	30	1839591	99.44
Stroke-Ready Hospitals	35	1841313	99.54
Stroke-Ready Hospitals	40	1841356	99.54
Stroke-Ready Hospitals	45	1841397	99.54
Stroke-Ready Hospitals	50	1841399	99.54
Stroke-Ready Hospitals	55	1841261	99.53
Stroke-Ready Hospitals	60	1841354	99.54
Stroke Units	5	280910	15.19
Stroke Units	10	1001353	54.13
Stroke Units	15	1463335	79.1
Stroke Units	20	1713787	92.64
Stroke Units	25	1824769	98.64
Stroke Units	30	1839591	99.44
Stroke Units	35	1841312	99.54
Stroke Units	40	1841356	99.54
Stroke Units	45	1841383	99.54
Stroke Units	50	1841385	99.54
Stroke Units	55	1841261	99.53
Stroke Units	60	1841354	99.54

Population Coverage in Hessen

Condition	Drive Time (min)	Covered Population	Coverage (%)
Hospitals with CT	5	1406560	22.49
Hospitals with CT	10	3315715	53.01
Hospitals with CT	15	5012136	80.14
Hospitals with CT	20	5872902	93.9
Hospitals with CT	25	6143166	98.22
Hospitals with CT	30	6228032	99.58
Hospitals with CT	35	6250949	99.94
Hospitals with CT	40	6254316	100.0
Hospitals with CT	45	6254410	100.0
Hospitals with CT	50	6254410	100.0
Hospitals with CT	55	6254410	100.0
Hospitals with CT	60	6254410	100.0
Stroke-Ready Hospitals	5	581349	9.3
Stroke-Ready Hospitals	10	2021417	32.32
Stroke-Ready Hospitals	15	3593327	57.45
Stroke-Ready Hospitals	20	4603230	73.6
Stroke-Ready Hospitals	25	5315737	84.99
Stroke-Ready Hospitals	30	5747792	91.9
Stroke-Ready Hospitals	35	6013571	96.15
Stroke-Ready Hospitals	40	6138335	98.14
Stroke-Ready Hospitals	45	6191870	99.0
Stroke-Ready Hospitals	50	6239892	99.77
Stroke-Ready Hospitals	55	6250347	99.94
Stroke-Ready Hospitals	60	6253805	99.99
Stroke Units	5	497445	7.95
Stroke Units	10	1764865	28.22
Stroke Units	15	3291490	52.63
Stroke Units	20	4301341	68.77
Stroke Units	25	5046304	80.68
Stroke Units	30	5483872	87.68
Stroke Units	35	5803746	92.79
Stroke Units	40	6014653	96.17
Stroke Units	45	6142142	98.2
Stroke Units	50	6204165	99.2
Stroke Units	55	6222191	99.48
Stroke Units	60	6248955	99.91

Population Coverage in Mecklenburg-Vorpommern

Condition	Drive Time (min)	Covered Population	Coverage (%)
Hospitals with CT	5	257718	17.49
Hospitals with CT	10	553587	37.57
Hospitals with CT	15	799728	54.28
Hospitals with CT	20	1033787	70.17
Hospitals with CT	25	1227505	83.31
Hospitals with CT	30	1347342	91.45
Hospitals with CT	35	1418554	96.28
Hospitals with CT	40	1440208	97.75
Hospitals with CT	45	1453198	98.63
Hospitals with CT	50	1463160	99.31
Hospitals with CT	55	1463881	99.36
Hospitals with CT	60	1465811	99.49
Stroke-Ready Hospitals	5	115365	7.83
Stroke-Ready Hospitals	10	290905	19.74
Stroke-Ready Hospitals	15	440679	29.91
Stroke-Ready Hospitals	20	580334	39.39
Stroke-Ready Hospitals	25	756866	51.37
Stroke-Ready Hospitals	30	889394	60.37
Stroke-Ready Hospitals	35	1028105	69.78
Stroke-Ready Hospitals	40	1149647	78.03
Stroke-Ready Hospitals	45	1245758	84.55
Stroke-Ready Hospitals	50	1311215	89.0
Stroke-Ready Hospitals	55	1376007	93.39
Stroke-Ready Hospitals	60	1423547	96.62
Stroke Units	5	103417	7.02
Stroke Units	10	280018	19.01
Stroke Units	15	437635	29.7
Stroke Units	20	579160	39.31
Stroke Units	25	756401	51.34
Stroke Units	30	887540	60.24
Stroke Units	35	1013707	68.8
Stroke Units	40	1128797	76.61
Stroke Units	45	1233272	83.71
Stroke Units	50	1303352	88.46
Stroke Units	55	1367889	92.84
Stroke Units	60	1416498	96.14

Population Coverage in Niedersachsen

Condition	Drive Time (min)	Covered Population	Coverage (%)
Hospitals with CT	5	1409630	17.48
Hospitals with CT	10	3447696	42.75
Hospitals with CT	15	5406337	67.03
Hospitals with CT	20	6863503	85.1
Hospitals with CT	25	7612628	94.39
Hospitals with CT	30	7938078	98.42
Hospitals with CT	35	8023939	99.49
Hospitals with CT	40	8035556	99.63
Hospitals with CT	45	8038749	99.67
Hospitals with CT	50	8042575	99.72
Hospitals with CT	55	8043191	99.73
Hospitals with CT	60	8044362	99.74
Stroke-Ready Hospitals	5	569540	7.06
Stroke-Ready Hospitals	10	1726515	21.41
Stroke-Ready Hospitals	15	3058069	37.92
Stroke-Ready Hospitals	20	4505790	55.87
Stroke-Ready Hospitals	25	5794260	71.84
Stroke-Ready Hospitals	30	6765254	83.88
Stroke-Ready Hospitals	35	7321085	90.77
Stroke-Ready Hospitals	40	7675269	95.17
Stroke-Ready Hospitals	45	7886546	97.79
Stroke-Ready Hospitals	50	7998258	99.17
Stroke-Ready Hospitals	55	8025171	99.5
Stroke-Ready Hospitals	60	8034863	99.62
Stroke Units	5	472583	5.86
Stroke Units	10	1391315	17.25
Stroke Units	15	2468047	30.6
Stroke Units	20	3691578	45.77
Stroke Units	25	4956410	61.45
Stroke Units	30	6126865	75.97
Stroke Units	35	6857849	85.03
Stroke Units	40	7385222	91.57
Stroke Units	45	7725692	95.79
Stroke Units	50	7914191	98.13
Stroke Units	55	7986750	99.03
Stroke Units	60	8020805	99.45

Population Coverage in Nordrhein-Westfalen

Condition	Drive Time (min)	Covered Population	Coverage (%)
Hospitals with CT	5	5117981	27.84
Hospitals with CT	10	12109418	65.88
Hospitals with CT	15	16244371	88.37
Hospitals with CT	20	17839583	97.05
Hospitals with CT	25	18277111	99.43
Hospitals with CT	30	18350234	99.83
Hospitals with CT	35	18358016	99.87
Hospitals with CT	40	18360540	99.89
Hospitals with CT	45	18361316	99.89
Hospitals with CT	50	18361491	99.89
Hospitals with CT	55	18360724	99.89
Hospitals with CT	60	18361483	99.89
Stroke-Ready Hospitals	5	1820387	9.9
Stroke-Ready Hospitals	10	6464798	35.17
Stroke-Ready Hospitals	15	11394645	61.99
Stroke-Ready Hospitals	20	14870859	80.9
Stroke-Ready Hospitals	25	16851721	91.68
Stroke-Ready Hospitals	30	17617364	95.84
Stroke-Ready Hospitals	35	17958886	97.7
Stroke-Ready Hospitals	40	18113086	98.54
Stroke-Ready Hospitals	45	18234611	99.2
Stroke-Ready Hospitals	50	18295760	99.53
Stroke-Ready Hospitals	55	18337035	99.76
Stroke-Ready Hospitals	60	18358489	99.88
Stroke Units	5	1542785	8.39
Stroke Units	10	5535245	30.11
Stroke Units	15	10198365	55.48
Stroke Units	20	13696917	74.52
Stroke Units	25	15867383	86.32
Stroke Units	30	16980605	92.38
Stroke Units	35	17714742	96.37
Stroke Units	40	18020143	98.04
Stroke Units	45	18207282	99.05
Stroke Units	50	18288064	99.49
Stroke Units	55	18330601	99.72
Stroke Units	60	18355223	99.86

Population Coverage in Rheinland-Pfalz

Condition	Drive Time (min)	Covered Population	Coverage (%)
Hospitals with CT	5	756667	18.17
Hospitals with CT	10	1907648	45.8
Hospitals with CT	15	3044556	73.09
Hospitals with CT	20	3749633	90.02
Hospitals with CT	25	4066393	97.63
Hospitals with CT	30	4132862	99.22
Hospitals with CT	35	4151461	99.67
Hospitals with CT	40	4160067	99.87
Hospitals with CT	45	4163858	99.97
Hospitals with CT	50	4164193	99.97
Hospitals with CT	55	4163782	99.96
Hospitals with CT	60	4164036	99.97
Stroke-Ready Hospitals	5	338818	8.13
Stroke-Ready Hospitals	10	1036142	24.88
Stroke-Ready Hospitals	15	1892300	45.43
Stroke-Ready Hospitals	20	2752173	66.07
Stroke-Ready Hospitals	25	3280216	78.75
Stroke-Ready Hospitals	30	3661129	87.9
Stroke-Ready Hospitals	35	3880141	93.15
Stroke-Ready Hospitals	40	4008201	96.23
Stroke-Ready Hospitals	45	4092961	98.26
Stroke-Ready Hospitals	50	4139506	99.38
Stroke-Ready Hospitals	55	4157810	99.82
Stroke-Ready Hospitals	60	4162278	99.93
Stroke Units	5	245870	5.9
Stroke Units	10	805951	19.35
Stroke Units	15	1487709	35.72
Stroke Units	20	2293624	55.07
Stroke Units	25	2975385	71.43
Stroke Units	30	3455408	82.96
Stroke Units	35	3765466	90.4
Stroke Units	40	3952953	94.9
Stroke Units	45	4069405	97.7
Stroke Units	50	4131057	99.18
Stroke Units	55	4154697	99.75
Stroke Units	60	4161922	99.92

Population Coverage in Saarland

Condition	Drive Time (min)	Covered Population	Coverage (%)
Hospitals with CT	5	162530	16.68
Hospitals with CT	10	477145	48.96
Hospitals with CT	15	765351	78.53
Hospitals with CT	20	925694	94.98
Hospitals with CT	25	964879	99.0
Hospitals with CT	30	972452	99.77
Hospitals with CT	35	972896	99.82
Hospitals with CT	40	972937	99.82
Hospitals with CT	45	972959	99.83
Hospitals with CT	50	973042	99.83
Hospitals with CT	55	972893	99.82
Hospitals with CT	60	973022	99.83
Stroke-Ready Hospitals	5	96010	9.85
Stroke-Ready Hospitals	10	345634	35.46
Stroke-Ready Hospitals	15	663054	68.03
Stroke-Ready Hospitals	20	817775	83.9
Stroke-Ready Hospitals	25	892588	91.58
Stroke-Ready Hospitals	30	937085	96.15
Stroke-Ready Hospitals	35	964959	99.01
Stroke-Ready Hospitals	40	972761	99.81
Stroke-Ready Hospitals	45	972948	99.83
Stroke-Ready Hospitals	50	972948	99.83
Stroke-Ready Hospitals	55	972969	99.83
Stroke-Ready Hospitals	60	973026	99.83
Stroke Units	5	96010	9.85
Stroke Units	10	345634	35.46
Stroke Units	15	663054	68.03
Stroke Units	20	817775	83.9
Stroke Units	25	892588	91.58
Stroke Units	30	937059	96.14
Stroke Units	35	964959	99.01
Stroke Units	40	972752	99.81
Stroke Units	45	972948	99.83
Stroke Units	50	972948	99.83
Stroke Units	55	972966	99.83
Stroke Units	60	973025	99.83

Population Coverage in Sachsen-Anhalt

Condition	Drive Time (min)	Covered Population	Coverage (%)
Hospitals with CT	5	488294	23.22
Hospitals with CT	10	1045827	49.73
Hospitals with CT	15	1435574	68.26
Hospitals with CT	20	1751174	83.26
Hospitals with CT	25	1950847	92.76
Hospitals with CT	30	2043968	97.18
Hospitals with CT	35	2080073	98.9
Hospitals with CT	40	2099903	99.84
Hospitals with CT	45	2102288	99.96
Hospitals with CT	50	2102544	99.97
Hospitals with CT	55	2103214	100.0
Hospitals with CT	60	2103214	100.0
Stroke-Ready Hospitals	5	194111	9.23
Stroke-Ready Hospitals	10	592874	28.19
Stroke-Ready Hospitals	15	867645	41.25
Stroke-Ready Hospitals	20	1107708	52.67
Stroke-Ready Hospitals	25	1353373	64.35
Stroke-Ready Hospitals	30	1601866	76.16
Stroke-Ready Hospitals	35	1796595	85.42
Stroke-Ready Hospitals	40	1905710	90.61
Stroke-Ready Hospitals	45	1957085	93.05
Stroke-Ready Hospitals	50	1997711	94.98
Stroke-Ready Hospitals	55	2061690	98.03
Stroke-Ready Hospitals	60	2086884	99.22
Stroke Units	5	130431	6.2
Stroke Units	10	419719	19.96
Stroke Units	15	600710	28.56
Stroke Units	20	755683	35.93
Stroke Units	25	970137	46.13
Stroke Units	30	1204483	57.27
Stroke Units	35	1415138	67.28
Stroke Units	40	1609150	76.51
Stroke Units	45	1739917	82.73
Stroke Units	50	1828917	86.96
Stroke Units	55	1914523	91.03
Stroke Units	60	1967960	93.57

Population Coverage in Sachsen

Condition	Drive Time (min)	Covered Population	Coverage (%)
Hospitals with CT	5	708201	17.2
Hospitals with CT	10	1939386	47.09
Hospitals with CT	15	3113673	75.6
Hospitals with CT	20	3797134	92.2
Hospitals with CT	25	4017391	97.54
Hospitals with CT	30	4020523	97.62
Hospitals with CT	35	4107283	99.73
Hospitals with CT	40	4115622	99.93
Hospitals with CT	45	4117589	99.98
Hospitals with CT	50	4117629	99.98
Hospitals with CT	55	4117174	99.97
Hospitals with CT	60	4117391	99.97
Stroke-Ready Hospitals	5	294506	7.15
Stroke-Ready Hospitals	10	1021366	24.8
Stroke-Ready Hospitals	15	1819784	44.19
Stroke-Ready Hospitals	20	2576840	62.57
Stroke-Ready Hospitals	25	3096419	75.18
Stroke-Ready Hospitals	30	3427441	83.22
Stroke-Ready Hospitals	35	3690031	89.6
Stroke-Ready Hospitals	40	3856803	93.65
Stroke-Ready Hospitals	45	3961437	96.19
Stroke-Ready Hospitals	50	4050330	98.34
Stroke-Ready Hospitals	55	4095184	99.43
Stroke-Ready Hospitals	60	4112355	99.85
Stroke Units	5	247330	6.01
Stroke Units	10	876436	21.28
Stroke Units	15	1522012	36.96
Stroke Units	20	2222827	53.97
Stroke Units	25	2812403	68.29
Stroke Units	30	3197733	77.64
Stroke Units	35	3501038	85.01
Stroke Units	40	3739363	90.79
Stroke Units	45	3888125	94.41
Stroke Units	50	4000047	97.12
Stroke Units	55	4066955	98.75
Stroke Units	60	4098687	99.52

Population Coverage in Schleswig-Holstein

Condition	Drive Time (min)	Covered Population	Coverage (%)
Hospitals with CT	5	514897	18.58
Hospitals with CT	10	1195495	43.14
Hospitals with CT	15	1842149	66.48
Hospitals with CT	20	2316764	83.6
Hospitals with CT	25	2606390	94.06
Hospitals with CT	30	2720891	98.19
Hospitals with CT	35	2750092	99.24
Hospitals with CT	40	2755071	99.42
Hospitals with CT	45	2758417	99.54
Hospitals with CT	50	2760847	99.63
Hospitals with CT	55	2759383	99.58
Hospitals with CT	60	2760875	99.63
Stroke-Ready Hospitals	5	240705	8.69
Stroke-Ready Hospitals	10	633150	22.85
Stroke-Ready Hospitals	15	1060314	38.26
Stroke-Ready Hospitals	20	1589598	57.36
Stroke-Ready Hospitals	25	2100979	75.82
Stroke-Ready Hospitals	30	2403947	86.75
Stroke-Ready Hospitals	35	2551496	92.07
Stroke-Ready Hospitals	40	2650304	95.64
Stroke-Ready Hospitals	45	2702717	97.53
Stroke-Ready Hospitals	50	2723620	98.29
Stroke-Ready Hospitals	55	2727131	98.41
Stroke-Ready Hospitals	60	2732026	98.59
Stroke Units	5	234447	8.46
Stroke Units	10	621469	22.43
Stroke Units	15	1055585	38.09
Stroke Units	20	1581955	57.09
Stroke Units	25	2095120	75.61
Stroke Units	30	2399777	86.6
Stroke Units	35	2549245	91.99
Stroke Units	40	2648179	95.56
Stroke Units	45	2701543	97.49
Stroke Units	50	2723277	98.27
Stroke Units	55	2727018	98.41
Stroke Units	60	2731942	98.59

Population Coverage in Thüringen

Condition	Drive Time (min)	Covered Population	Coverage (%)
Hospitals with CT	5	314113	15.47
Hospitals with CT	10	831903	40.98
Hospitals with CT	15	1274415	62.77
Hospitals with CT	20	1646637	81.11
Hospitals with CT	25	1855586	91.4
Hospitals with CT	30	1965451	96.81
Hospitals with CT	35	2012408	99.12
Hospitals with CT	40	2026921	99.84
Hospitals with CT	45	2030127	100.0
Hospitals with CT	50	2030174	100.0
Hospitals with CT	55	2030174	100.0
Hospitals with CT	60	2030174	100.0
Stroke-Ready Hospitals	5	135963	6.7
Stroke-Ready Hospitals	10	471325	23.22
Stroke-Ready Hospitals	15	847157	41.73
Stroke-Ready Hospitals	20	1188479	58.54
Stroke-Ready Hospitals	25	1469945	72.4
Stroke-Ready Hospitals	30	1705910	84.03
Stroke-Ready Hospitals	35	1878980	92.55
Stroke-Ready Hospitals	40	1991633	98.1
Stroke-Ready Hospitals	45	2025778	99.78
Stroke-Ready Hospitals	50	2030012	99.99
Stroke-Ready Hospitals	55	2030174	100.0
Stroke-Ready Hospitals	60	2030174	100.0
Stroke Units	5	125448	6.18
Stroke Units	10	442670	21.8
Stroke Units	15	790736	38.95
Stroke Units	20	1097126	54.04
Stroke Units	25	1347406	66.37
Stroke Units	30	1568633	77.27
Stroke Units	35	1780042	87.68
Stroke Units	40	1938942	95.51
Stroke Units	45	1997018	98.37
Stroke Units	50	2025471	99.77
Stroke Units	55	2029832	99.98
Stroke Units	60	2030160	100.0

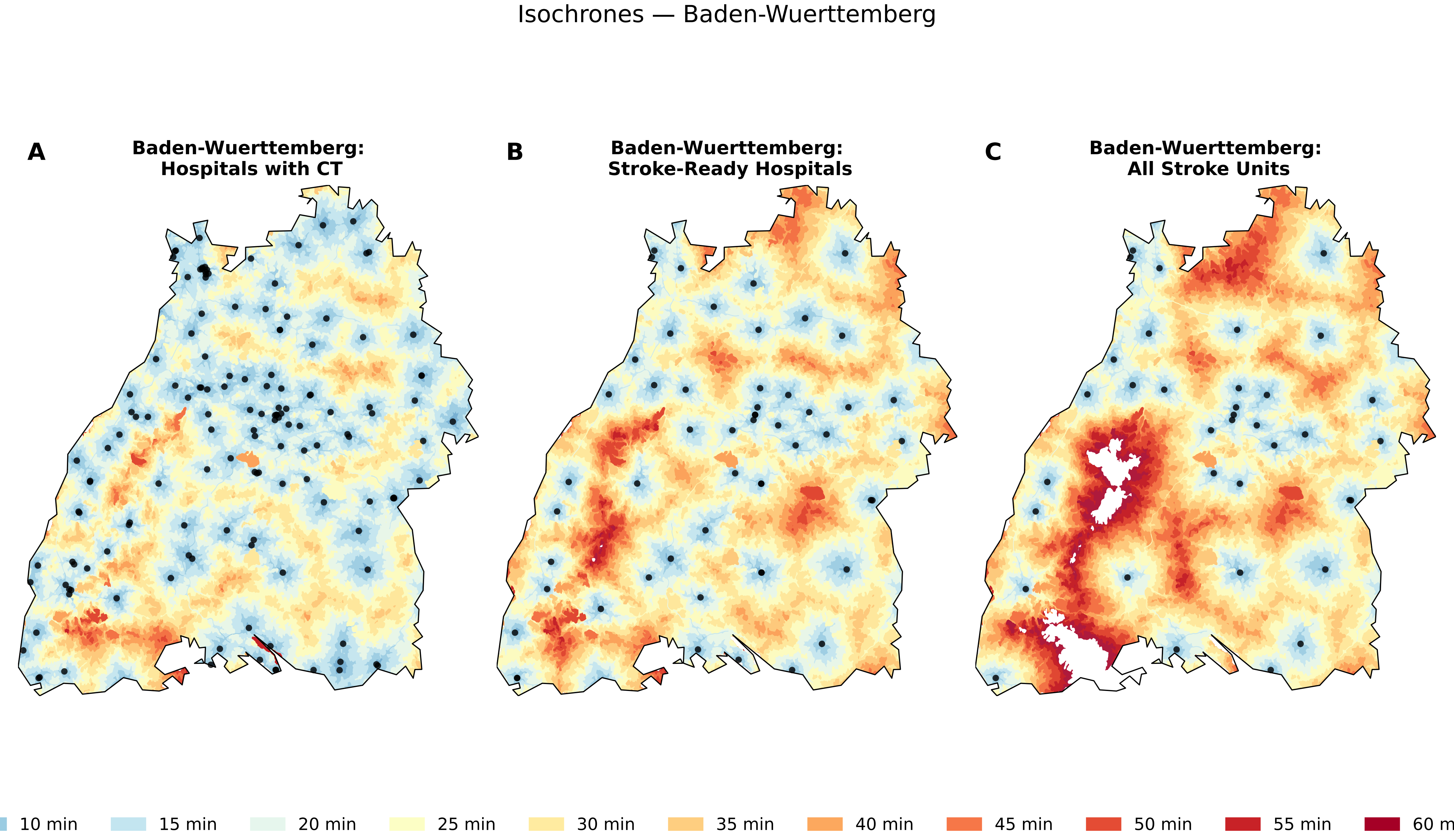
Isochrones — Baden-Wuerttemberg
A
Baden-Wuerttemberg:
Hospitals with CT
B
Baden-Wuerttemberg:
Stroke-Ready Hospitals
C
Baden-Wuerttemberg:
All Stroke Units
10 min
15 min
20 min
25 min
30 min
35 min
40 min
45 min
50 min
55 min
60 m

Isochrones — Bavaria

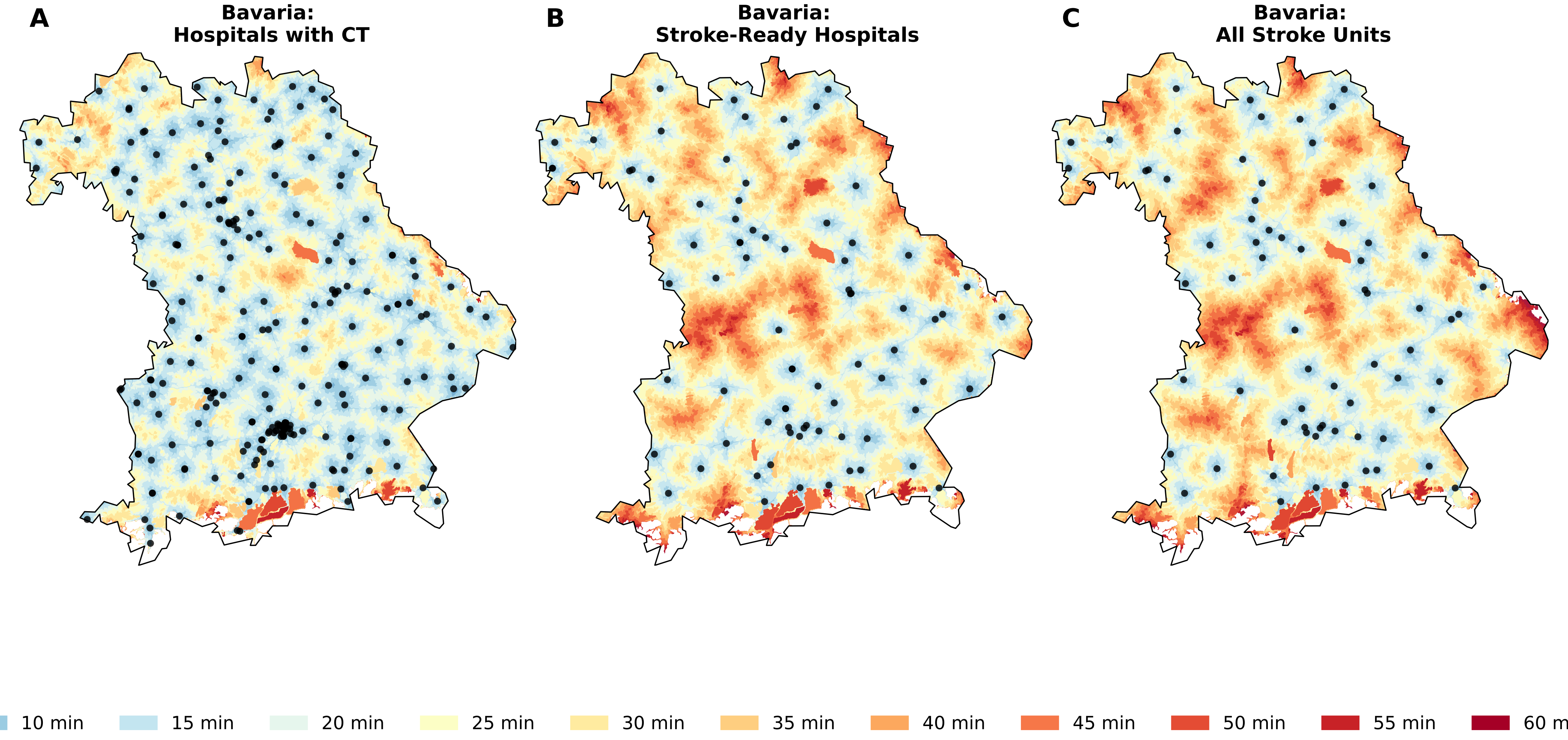

Isochrones — Berlin

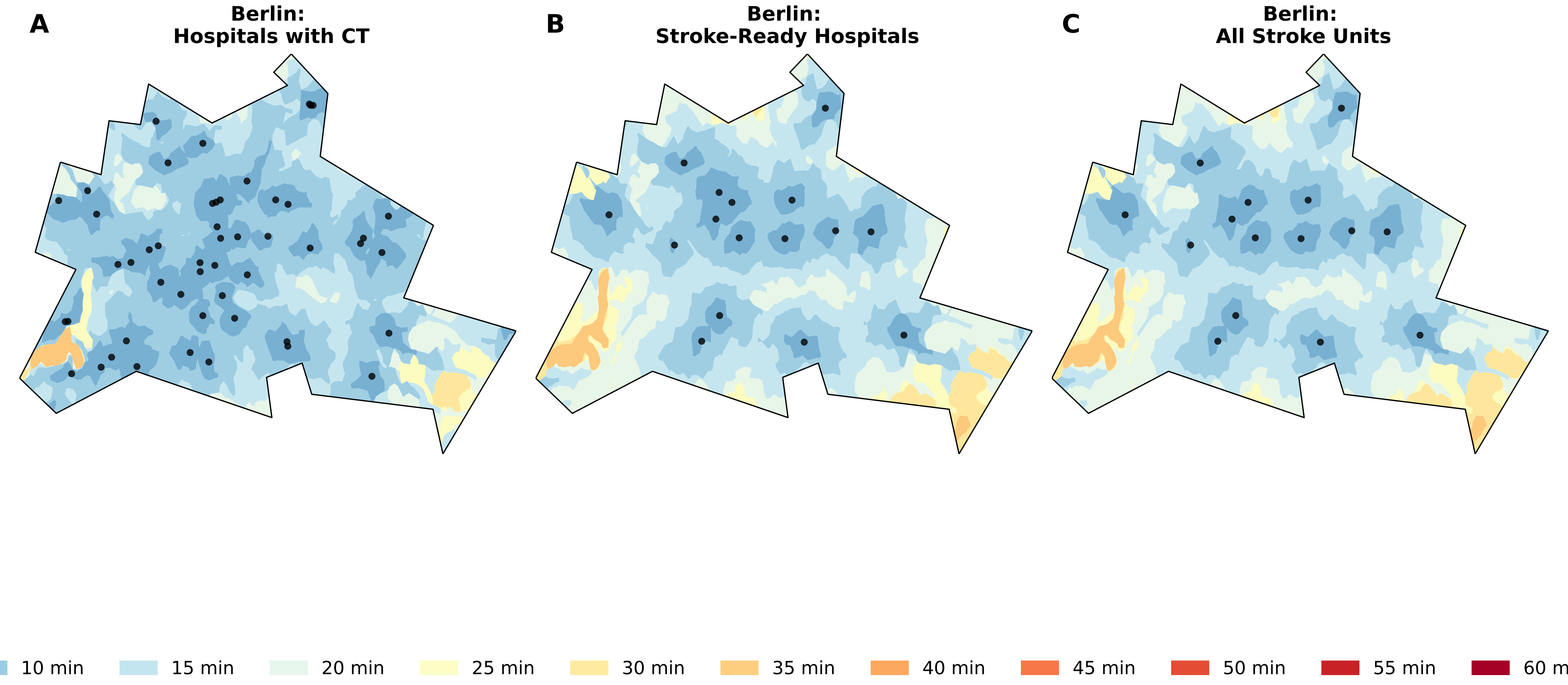

Isochrones — Brandenburg

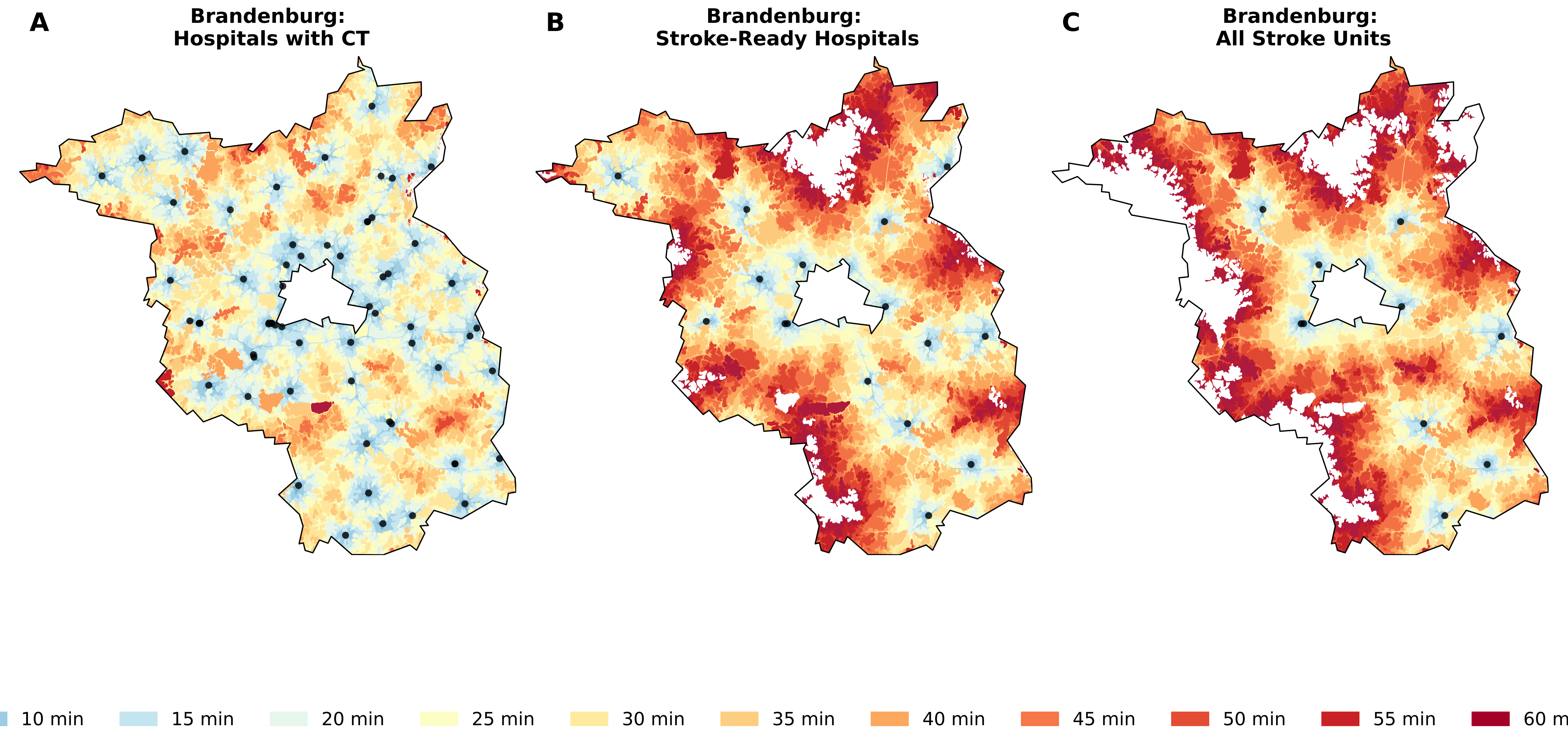

Isochrones — Bremen

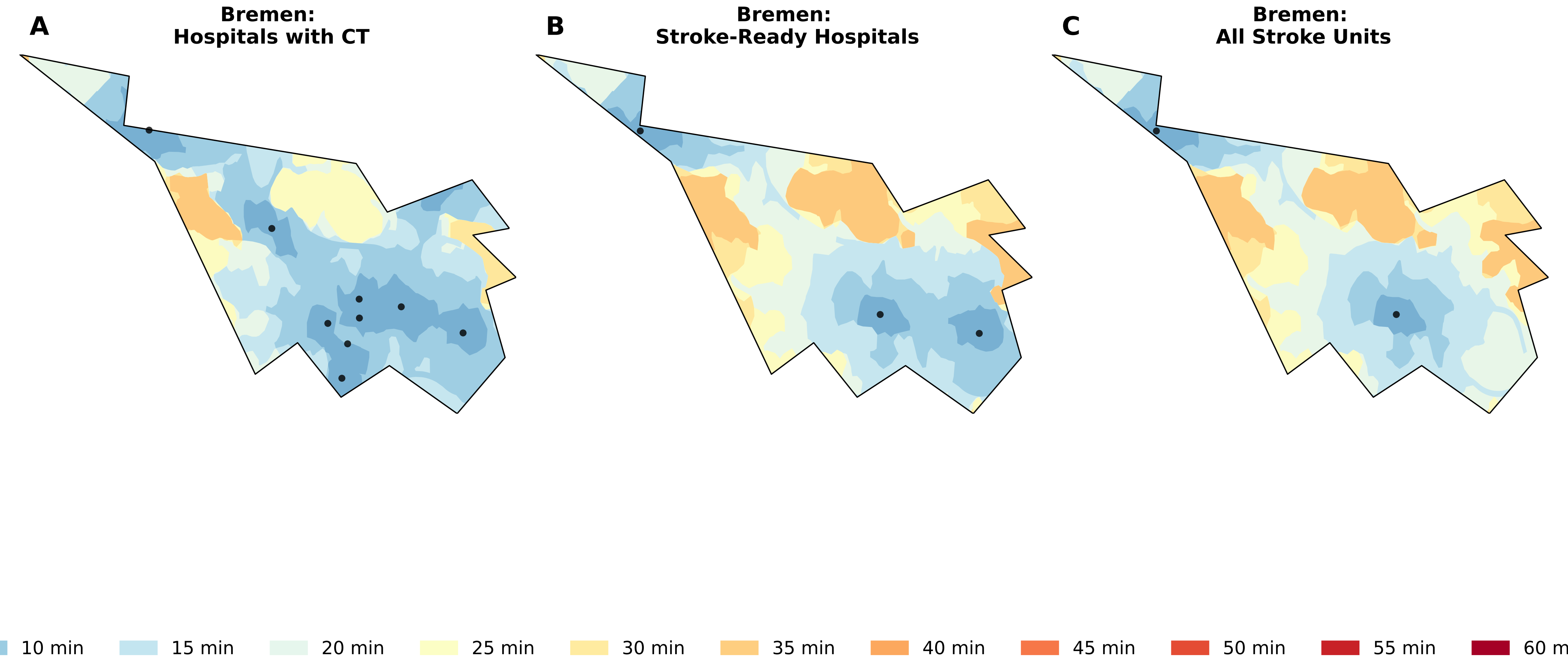

Isochrones — Hamburg

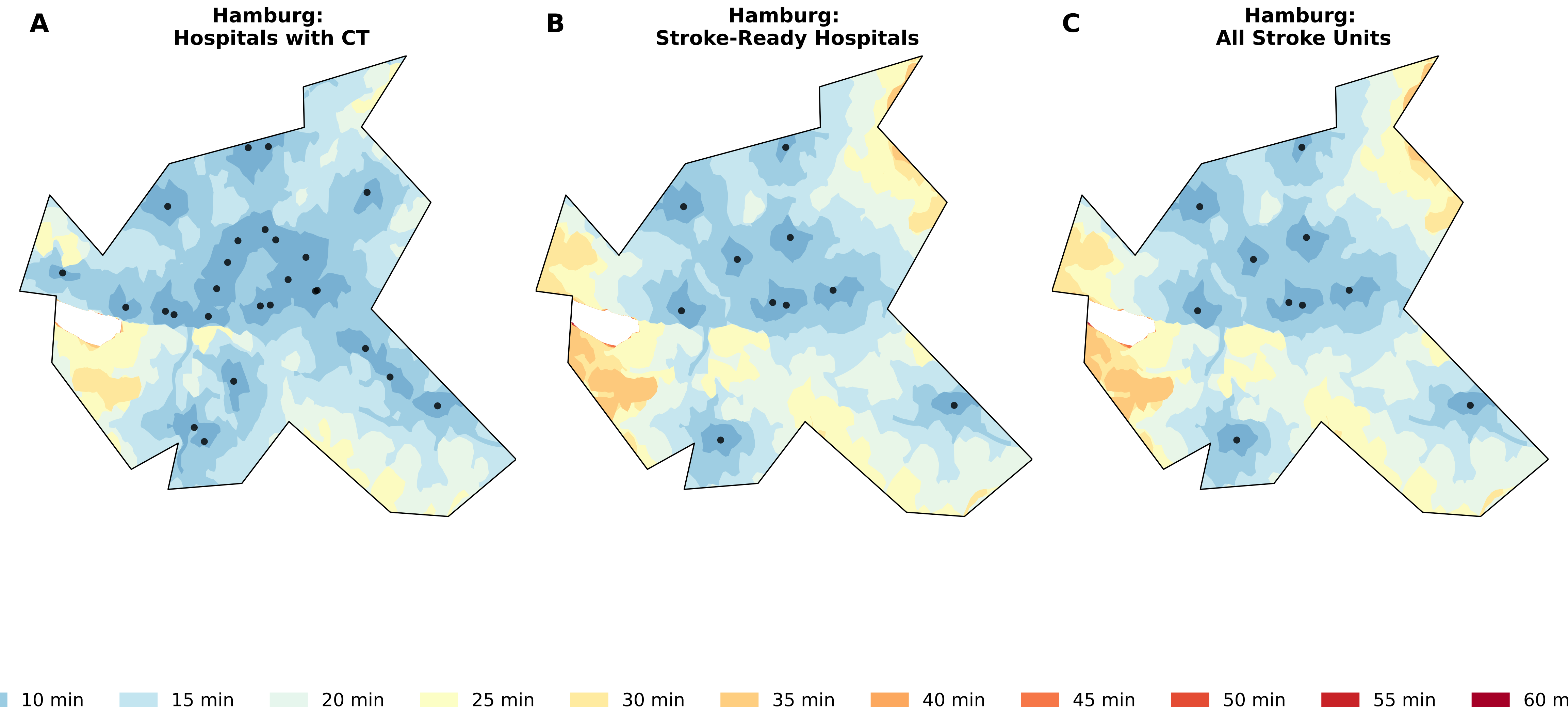

Isochrones — Hesse

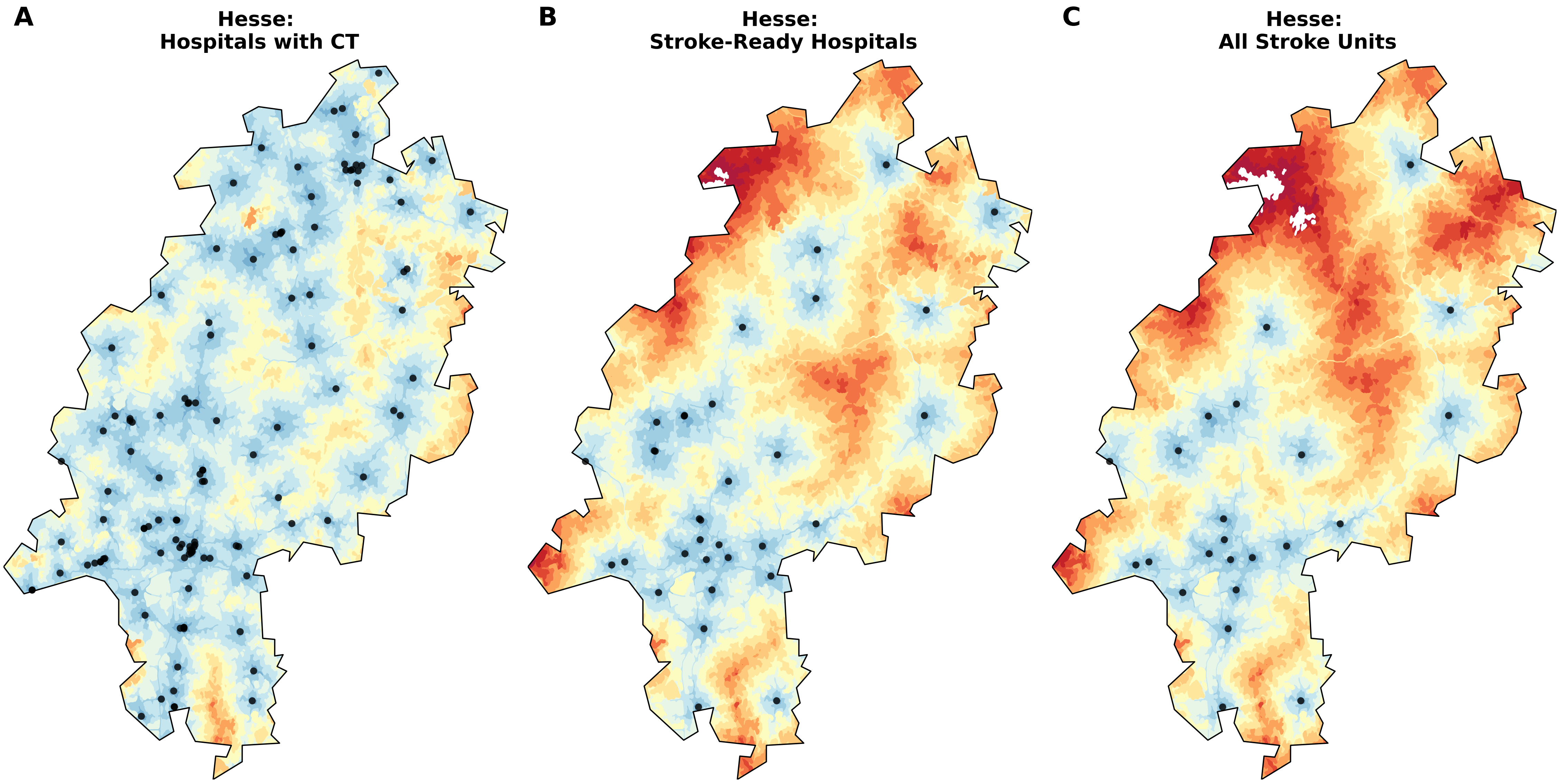

10 min | 15 min | 20 min | 25 min | 30 min | 35 min | 40 min | 45 min | 50 min | 55 min

Isochrones — Mecklenburg-Western Pomerania

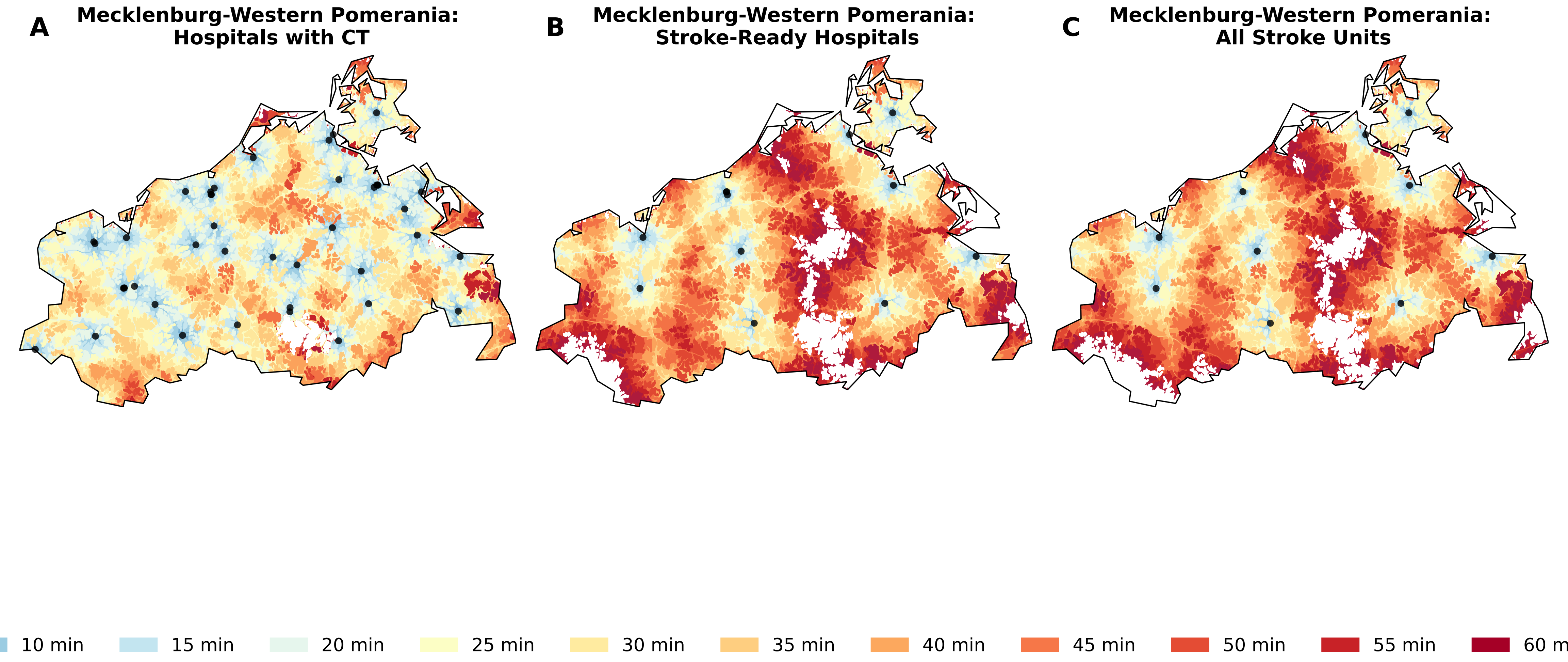

Isochrones — Lower Saxony

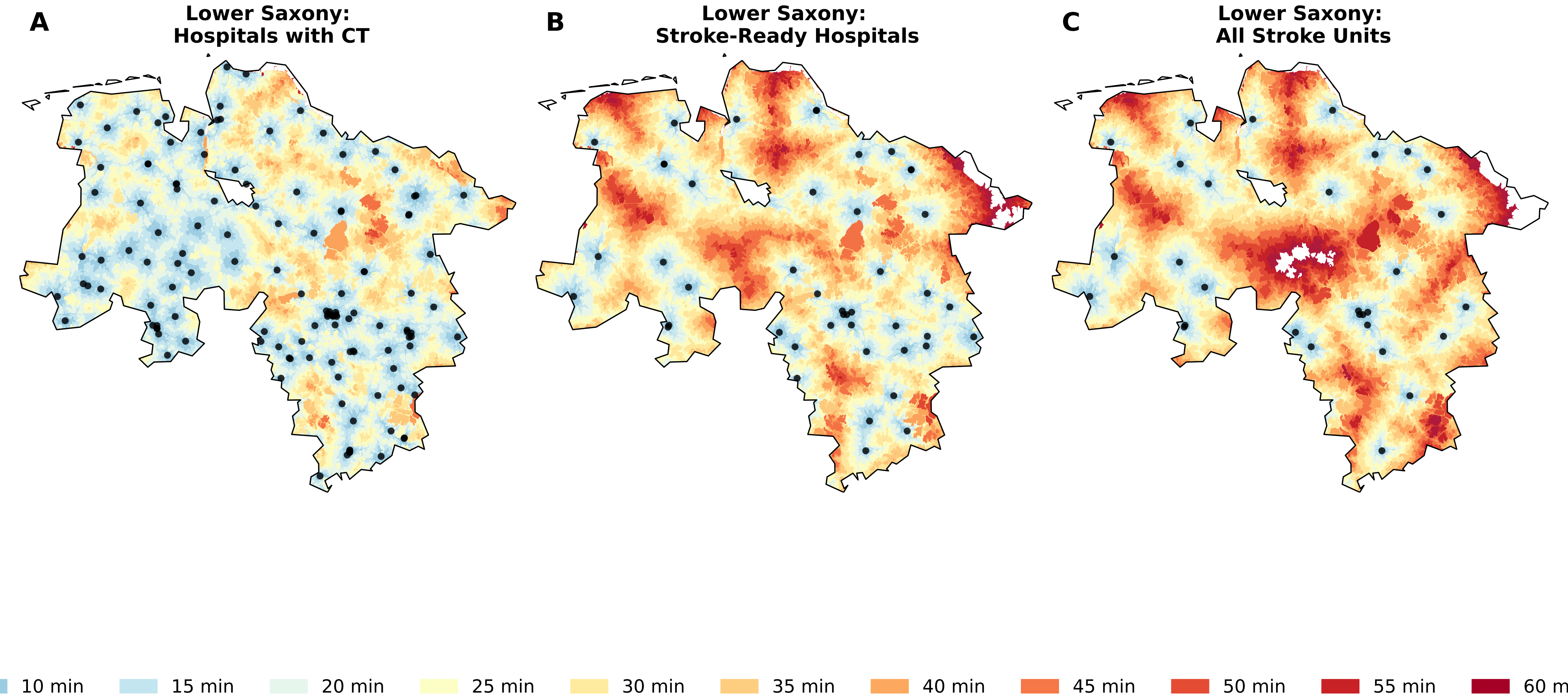

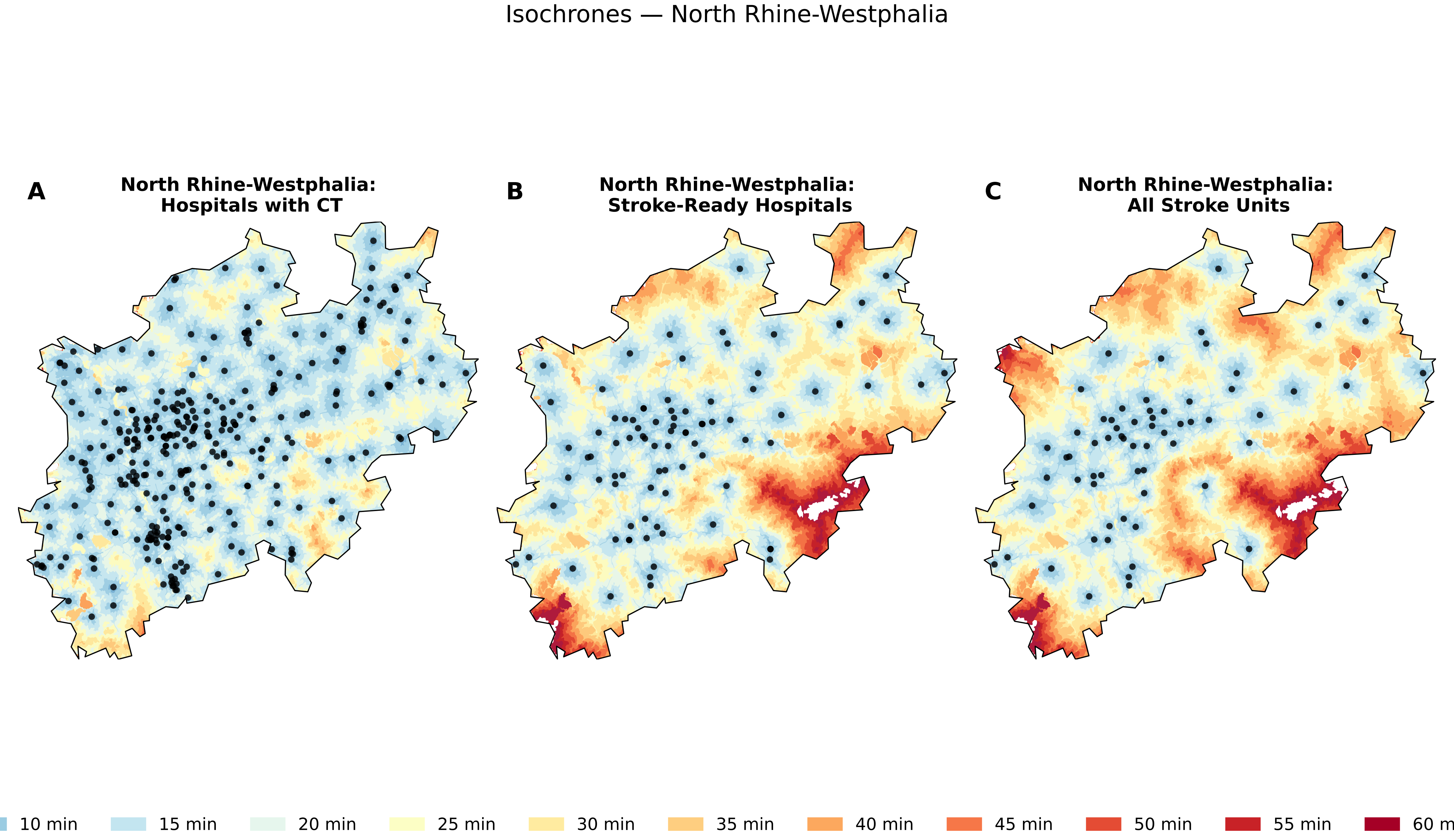
Isochrones — North Rhine-Westphalia
A
North Rhine-Westphalia:
Hospitals with CT
B
North Rhine-Westphalia:
Stroke-Ready Hospitals
C
North Rhine-Westphalia:
All Stroke Units
10 min
15 min
20 min
25 min
30 min
35 min
40 min
45 min
50 min
55 min
60 m

Isochrones — Rhineland-Palatinate

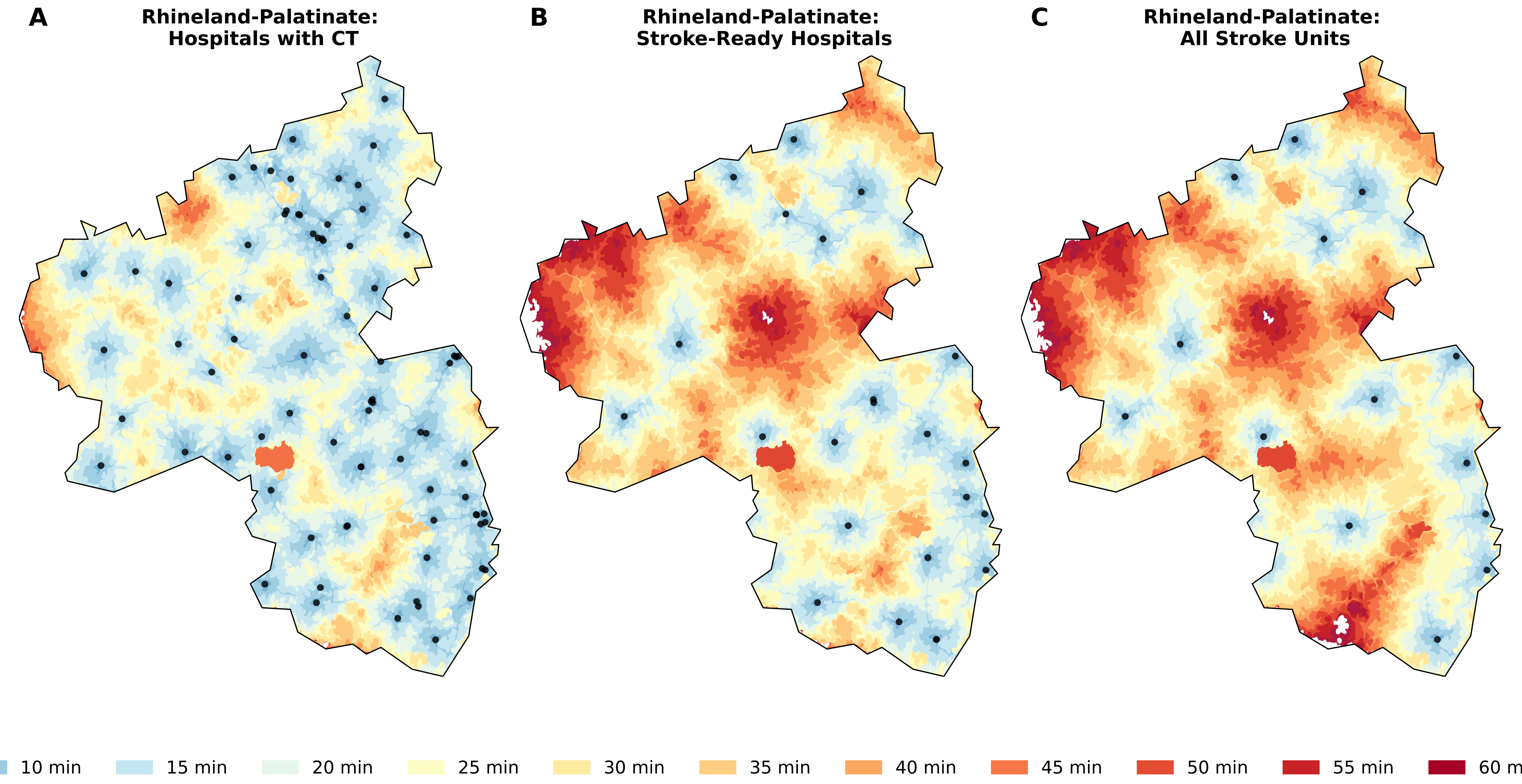

Isochrones — Saarland

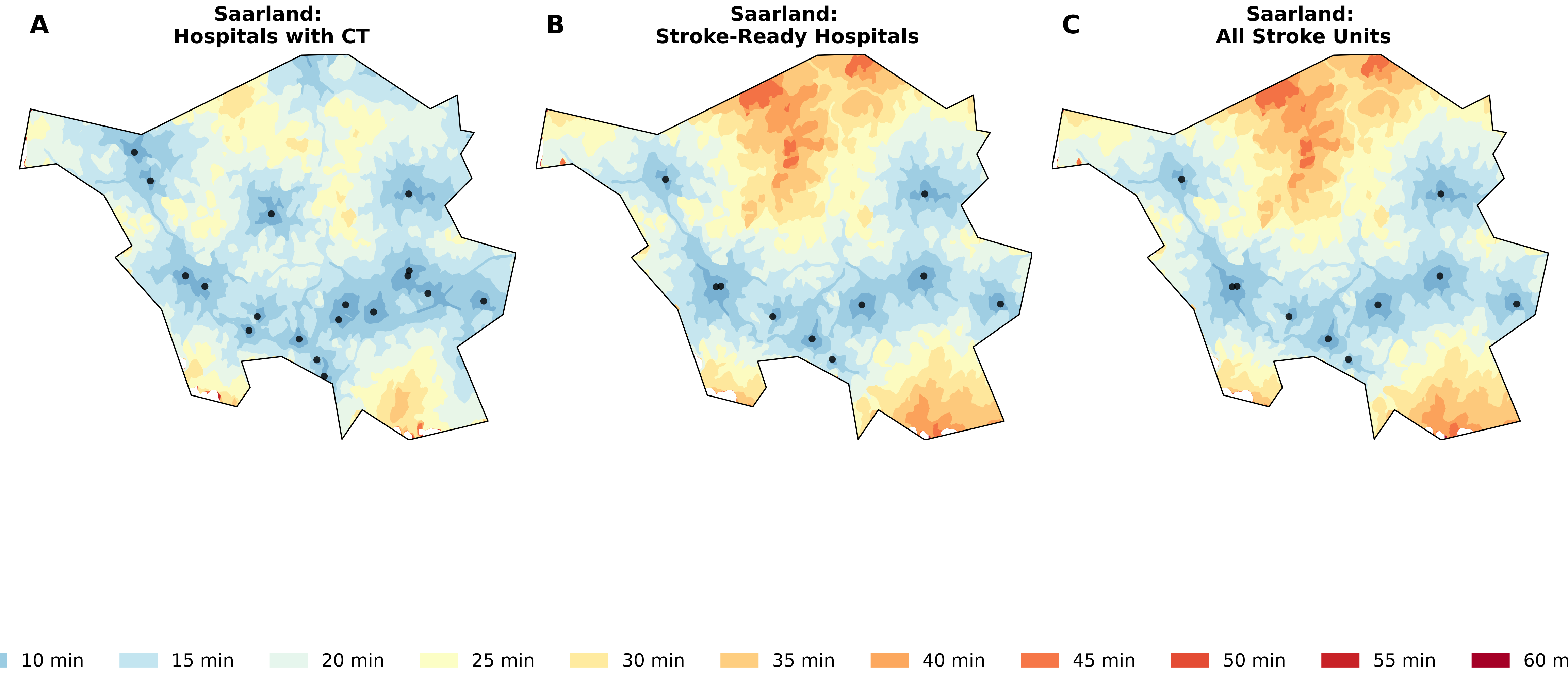

Isochrones — Saxony-Anhalt

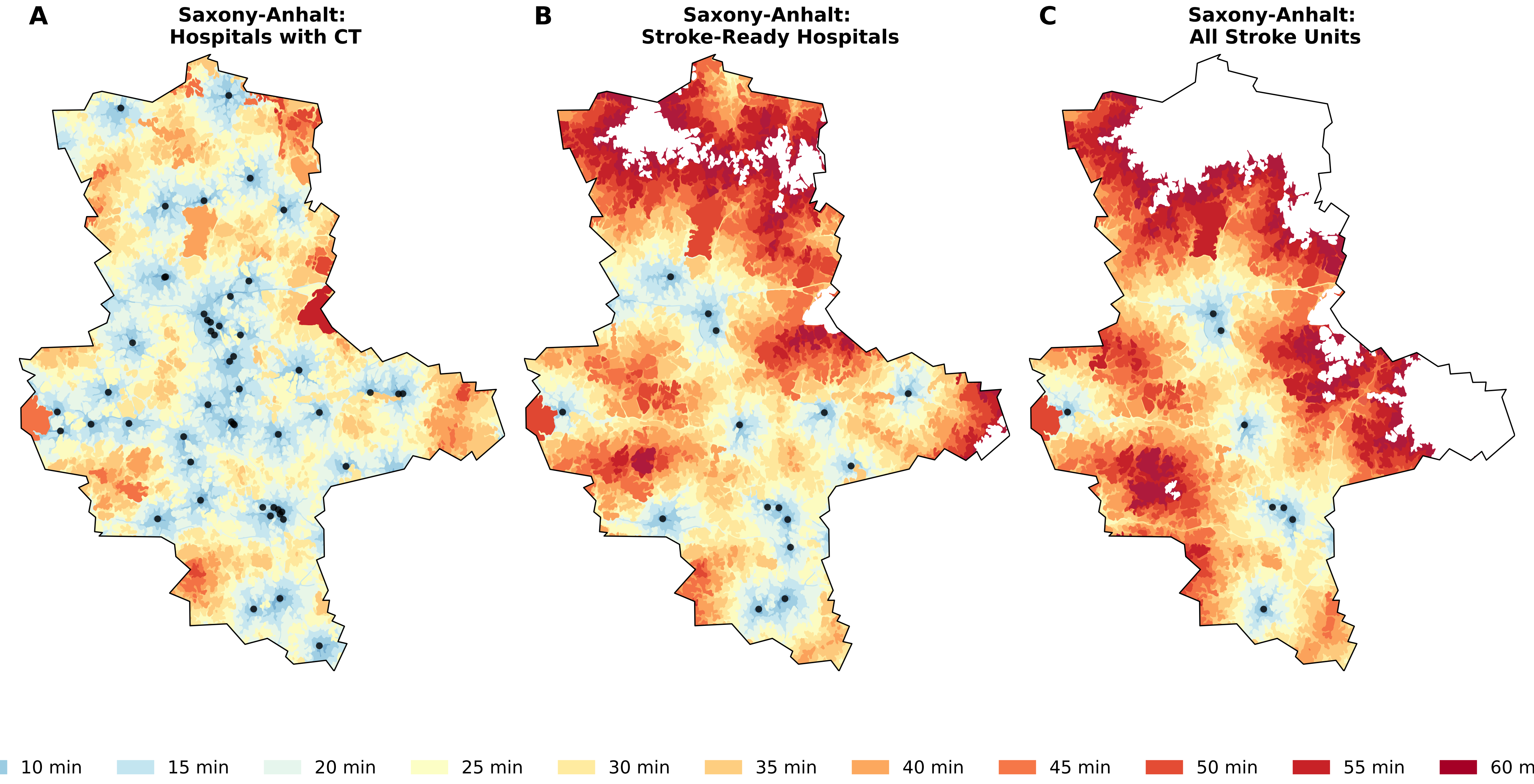

Isochrones — Saxony

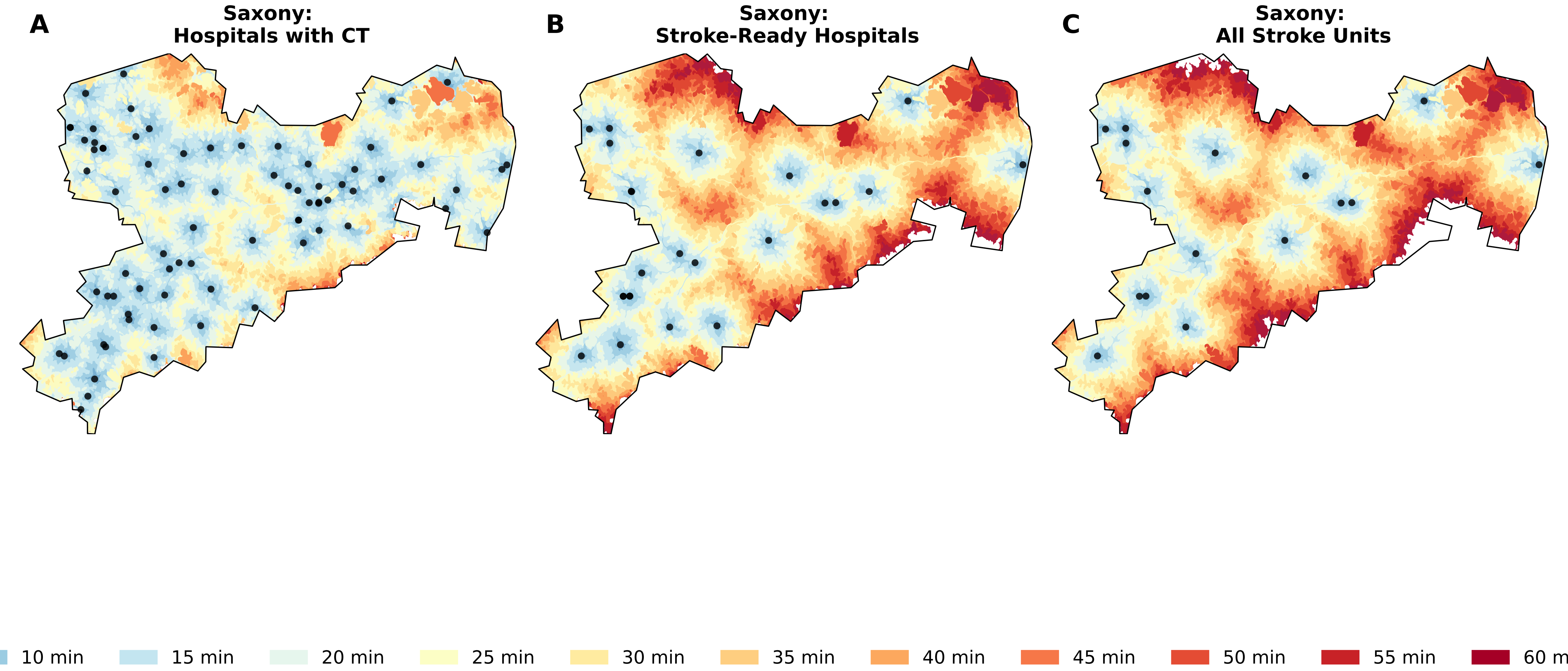

Isochrones — Schleswig-Holstein

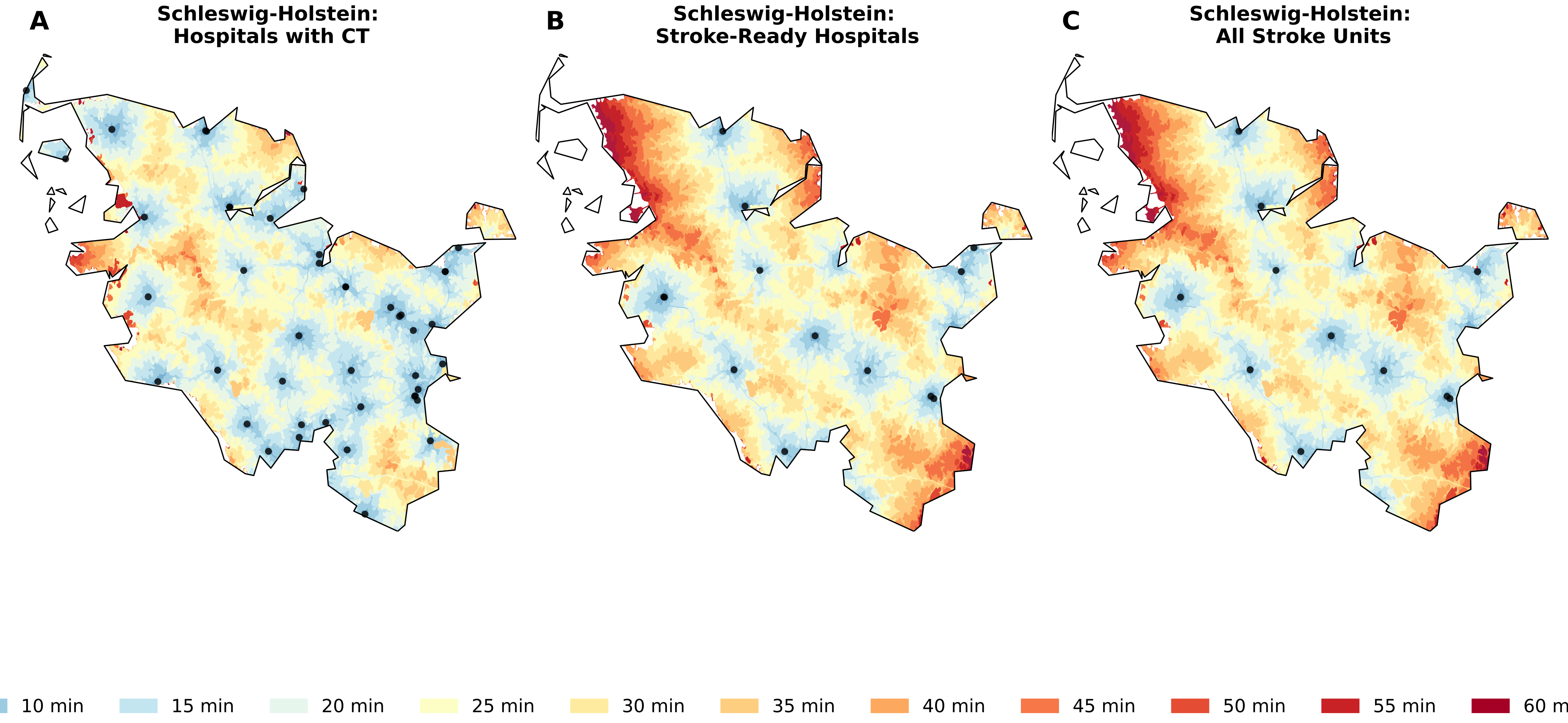

Isochrones — Thuringia

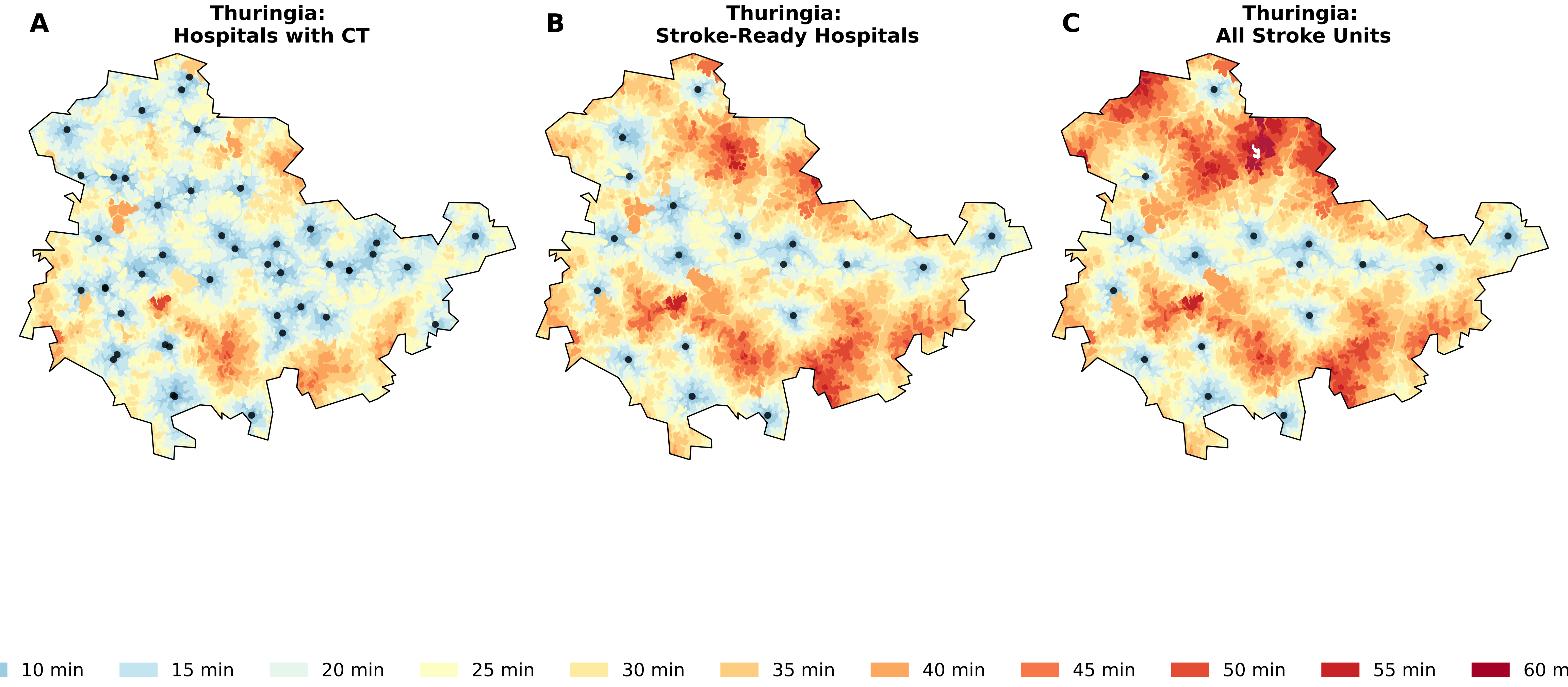

Population Coverage - Kreisfreie Stadt Neumünster (Schleswig-Holstein)

Condition	Drive Time (min)	Covered Population	Coverage (%)
Hospitals with CT	5	33068	38.49
Hospitals with CT	10	73598	85.66
Hospitals with CT	15	85514	99.52
Hospitals with CT	20	85904	99.98
Hospitals with CT	25	85923	100.0
Hospitals with CT	30	85923	100.0
Hospitals with CT	35	85923	100.0
Hospitals with CT	40	85923	100.0
Hospitals with CT	45	85923	100.0
Hospitals with CT	50	85923	100.0
Hospitals with CT	55	85923	100.0
Hospitals with CT	60	85923	100.0
Stroke-Ready Hospitals	5	34862	40.57
Stroke-Ready Hospitals	10	72095	83.91
Stroke-Ready Hospitals	15	85380	99.37
Stroke-Ready Hospitals	20	85901	99.98
Stroke-Ready Hospitals	25	85923	100.0
Stroke-Ready Hospitals	30	85923	100.0
Stroke-Ready Hospitals	35	85923	100.0
Stroke-Ready Hospitals	40	85923	100.0
Stroke-Ready Hospitals	45	85923	100.0
Stroke-Ready Hospitals	50	85923	100.0
Stroke-Ready Hospitals	55	85923	100.0
Stroke-Ready Hospitals	60	85923	100.0
Stroke Units	5	34862	40.57
Stroke Units	10	72095	83.91
Stroke Units	15	85380	99.37
Stroke Units	20	85901	99.98
Stroke Units	25	85923	100.0
Stroke Units	30	85923	100.0
Stroke Units	35	85923	100.0
Stroke Units	40	85923	100.0
Stroke Units	45	85923	100.0
Stroke Units	50	85923	100.0
Stroke Units	55	85923	100.0
Stroke Units	60	85923	100.0

Population Coverage - Kreisfreie Stadt Osnabrück (Niedersachsen)

Condition	Drive Time (min)	Covered Population	Coverage (%)
Hospitals with CT	5	62386	40.39
Hospitals with CT	10	142098	92.01
Hospitals with CT	15	153984	99.7
Hospitals with CT	20	154441	100.0
Hospitals with CT	25	154441	100.0
Hospitals with CT	30	154441	100.0
Hospitals with CT	35	154441	100.0
Hospitals with CT	40	154441	100.0
Hospitals with CT	45	154441	100.0
Hospitals with CT	50	154441	100.0
Hospitals with CT	55	154441	100.0
Hospitals with CT	60	154441	100.0
Stroke-Ready Hospitals	5	14495	9.39
Stroke-Ready Hospitals	10	73650	47.69
Stroke-Ready Hospitals	15	136044	88.09
Stroke-Ready Hospitals	20	153228	99.21
Stroke-Ready Hospitals	25	154432	99.99
Stroke-Ready Hospitals	30	154441	100.0
Stroke-Ready Hospitals	35	154441	100.0
Stroke-Ready Hospitals	40	154441	100.0
Stroke-Ready Hospitals	45	154441	100.0
Stroke-Ready Hospitals	50	154441	100.0
Stroke-Ready Hospitals	55	154441	100.0
Stroke-Ready Hospitals	60	154441	100.0
Stroke Units	5	14495	9.39
Stroke Units	10	73650	47.69
Stroke Units	15	136044	88.09
Stroke Units	20	153228	99.21
Stroke Units	25	154432	99.99
Stroke Units	30	154441	100.0
Stroke Units	35	154441	100.0
Stroke Units	40	154441	100.0
Stroke Units	45	154441	100.0
Stroke Units	50	154441	100.0
Stroke Units	55	154441	100.0
Stroke Units	60	154441	100.0

Population Coverage - Kreisfreie Stadt Mönchengladbach (Nordrhein-Westfalen)

Condition	Drive Time (min)	Covered Population	Coverage (%)
Hospitals with CT	5	90958	33.18
Hospitals with CT	10	207902	75.84
Hospitals with CT	15	271794	99.15
Hospitals with CT	20	274121	100.0
Hospitals with CT	25	274121	100.0
Hospitals with CT	30	274121	100.0
Hospitals with CT	35	274121	100.0
Hospitals with CT	40	274121	100.0
Hospitals with CT	45	274121	100.0
Hospitals with CT	50	274121	100.0
Hospitals with CT	55	274121	100.0
Hospitals with CT	60	274121	100.0
Stroke-Ready Hospitals	5	25182	9.19
Stroke-Ready Hospitals	10	112181	40.92
Stroke-Ready Hospitals	15	222671	81.23
Stroke-Ready Hospitals	20	264038	96.32
Stroke-Ready Hospitals	25	274121	100.0
Stroke-Ready Hospitals	30	274121	100.0
Stroke-Ready Hospitals	35	274121	100.0
Stroke-Ready Hospitals	40	274121	100.0
Stroke-Ready Hospitals	45	274121	100.0
Stroke-Ready Hospitals	50	274121	100.0
Stroke-Ready Hospitals	55	274121	100.0
Stroke-Ready Hospitals	60	274121	100.0
Stroke Units	5	25182	9.19
Stroke Units	10	112181	40.92
Stroke Units	15	222671	81.23
Stroke Units	20	264038	96.32
Stroke Units	25	274121	100.0
Stroke Units	30	274121	100.0
Stroke Units	35	274121	100.0
Stroke Units	40	274121	100.0
Stroke Units	45	274121	100.0
Stroke Units	50	274121	100.0
Stroke Units	55	274121	100.0
Stroke Units	60	274121	100.0

Population Coverage - Kreis Coesfeld (Nordrhein-Westfalen)

Condition	Drive Time (min)	Covered Population	Coverage (%)
Hospitals with CT	5	54472	25.23
Hospitals with CT	10	104385	48.34
Hospitals with CT	15	186723	86.47
Hospitals with CT	20	215270	99.69
Hospitals with CT	25	215927	100.0
Hospitals with CT	30	215936	100.0
Hospitals with CT	35	215936	100.0
Hospitals with CT	40	215936	100.0
Hospitals with CT	45	215936	100.0
Hospitals with CT	50	215936	100.0
Hospitals with CT	55	215936	100.0
Hospitals with CT	60	215936	100.0
Stroke-Ready Hospitals	5	34669	16.06
Stroke-Ready Hospitals	10	64990	30.1
Stroke-Ready Hospitals	15	100290	46.44
Stroke-Ready Hospitals	20	149091	69.04
Stroke-Ready Hospitals	25	209012	96.79
Stroke-Ready Hospitals	30	215864	99.97
Stroke-Ready Hospitals	35	215936	100.0
Stroke-Ready Hospitals	40	215936	100.0
Stroke-Ready Hospitals	45	215936	100.0
Stroke-Ready Hospitals	50	215936	100.0
Stroke-Ready Hospitals	55	215936	100.0
Stroke-Ready Hospitals	60	215936	100.0
Stroke Units	5	18929	8.77
Stroke Units	10	33449	15.49
Stroke Units	15	47127	21.82
Stroke Units	20	90932	42.11
Stroke Units	25	181303	83.96
Stroke Units	30	208546	96.58
Stroke Units	35	215576	99.83
Stroke Units	40	215936	100.0
Stroke Units	45	215936	100.0
Stroke Units	50	215936	100.0
Stroke Units	55	215936	100.0
Stroke Units	60	215936	100.0

Population Coverage - Kreis Paderborn (Nordrhein-Westfalen)

Condition	Drive Time (min)	Covered Population	Coverage (%)
Hospitals with CT	5	82971	25.1
Hospitals with CT	10	169029	51.14
Hospitals with CT	15	244295	73.91
Hospitals with CT	20	304277	92.06
Hospitals with CT	25	329929	99.82
Hospitals with CT	30	330521	100.0
Hospitals with CT	35	330521	100.0
Hospitals with CT	40	330521	100.0
Hospitals with CT	45	330521	100.0
Hospitals with CT	50	330521	100.0
Hospitals with CT	55	330521	100.0
Hospitals with CT	60	330521	100.0
Stroke-Ready Hospitals	5	33172	10.04
Stroke-Ready Hospitals	10	99849	30.21
Stroke-Ready Hospitals	15	141523	42.82
Stroke-Ready Hospitals	20	214087	64.77
Stroke-Ready Hospitals	25	289585	87.61
Stroke-Ready Hospitals	30	323927	98.01
Stroke-Ready Hospitals	35	330214	99.91
Stroke-Ready Hospitals	40	330479	99.99
Stroke-Ready Hospitals	45	330494	99.99
Stroke-Ready Hospitals	50	330521	100.0
Stroke-Ready Hospitals	55	330521	100.0
Stroke-Ready Hospitals	60	330521	100.0
Stroke Units	5	33172	10.04
Stroke Units	10	99849	30.21
Stroke Units	15	141523	42.82
Stroke Units	20	214056	64.76
Stroke Units	25	285317	86.32
Stroke Units	30	323654	97.92
Stroke Units	35	330214	99.91
Stroke Units	40	330479	99.99
Stroke Units	45	330494	99.99
Stroke Units	50	330521	100.0
Stroke Units	55	330521	100.0
Stroke Units	60	330521	100.0

Population Coverage - Kreisfreie Stadt Kassel (Hessen)

Condition	Drive Time (min)	Covered Population	Coverage (%)
Hospitals with CT	5	117344	57.04
Hospitals with CT	10	189841	92.28
Hospitals with CT	15	205719	100.0
Hospitals with CT	20	205719	100.0
Hospitals with CT	25	205719	100.0
Hospitals with CT	30	205719	100.0
Hospitals with CT	35	205719	100.0
Hospitals with CT	40	205719	100.0
Hospitals with CT	45	205719	100.0
Hospitals with CT	50	205719	100.0
Hospitals with CT	55	205719	100.0
Hospitals with CT	60	205719	100.0
Stroke-Ready Hospitals	5	31687	15.4
Stroke-Ready Hospitals	10	102784	49.96
Stroke-Ready Hospitals	15	182959	88.94
Stroke-Ready Hospitals	20	205467	99.88
Stroke-Ready Hospitals	25	205692	99.99
Stroke-Ready Hospitals	30	205719	100.0
Stroke-Ready Hospitals	35	205719	100.0
Stroke-Ready Hospitals	40	205719	100.0
Stroke-Ready Hospitals	45	205719	100.0
Stroke-Ready Hospitals	50	205719	100.0
Stroke-Ready Hospitals	55	205719	100.0
Stroke-Ready Hospitals	60	205719	100.0
Stroke Units	5	31687	15.4
Stroke Units	10	102784	49.96
Stroke Units	15	182959	88.94
Stroke Units	20	205467	99.88
Stroke Units	25	205692	99.99
Stroke Units	30	205719	100.0
Stroke Units	35	205719	100.0
Stroke Units	40	205719	100.0
Stroke Units	45	205719	100.0
Stroke Units	50	205719	100.0
Stroke Units	55	205719	100.0
Stroke Units	60	205719	100.0

Population Coverage - Landkreis Hersfeld-Rotenburg (Hessen)

Condition	Drive Time (min)	Covered Population	Coverage (%)
Hospitals with CT	5	17350	14.35
Hospitals with CT	10	36968	30.58
Hospitals with CT	15	62075	51.35
Hospitals with CT	20	79824	66.03
Hospitals with CT	25	103212	85.38
Hospitals with CT	30	113779	94.12
Hospitals with CT	35	120710	99.85
Hospitals with CT	40	120856	99.97
Hospitals with CT	45	120889	100.0
Hospitals with CT	50	120889	100.0
Hospitals with CT	55	120889	100.0
Hospitals with CT	60	120889	100.0
Stroke-Ready Hospitals	5	10486	8.67
Stroke-Ready Hospitals	10	25124	20.78
Stroke-Ready Hospitals	15	34934	28.9
Stroke-Ready Hospitals	20	48894	40.45
Stroke-Ready Hospitals	25	74362	61.51
Stroke-Ready Hospitals	30	101396	83.88
Stroke-Ready Hospitals	35	116864	96.67
Stroke-Ready Hospitals	40	120550	99.72
Stroke-Ready Hospitals	45	120889	100.0
Stroke-Ready Hospitals	50	120889	100.0
Stroke-Ready Hospitals	55	120889	100.0
Stroke-Ready Hospitals	60	120889	100.0
Stroke Units	5	10486	8.67
Stroke Units	10	25124	20.78
Stroke Units	15	34934	28.9
Stroke Units	20	48894	40.45
Stroke Units	25	74362	61.51
Stroke Units	30	100198	82.88
Stroke Units	35	115495	95.54
Stroke Units	40	120232	99.46
Stroke Units	45	120889	100.0
Stroke Units	50	120889	100.0
Stroke Units	55	120889	100.0
Stroke Units	60	120889	100.0

Population Coverage - Landkreis Schwalm-Eder-Kreis (Hessen)

Condition	Drive Time (min)	Covered Population	Coverage (%)
Hospitals with CT	5	20131	11.07
Hospitals with CT	10	51496	28.32
Hospitals with CT	15	97243	53.48
Hospitals with CT	20	137497	75.62
Hospitals with CT	25	166217	91.41
Hospitals with CT	30	181787	99.98
Hospitals with CT	35	181827	100.0
Hospitals with CT	40	181827	100.0
Hospitals with CT	45	181827	100.0
Hospitals with CT	50	181827	100.0
Hospitals with CT	55	181827	100.0
Hospitals with CT	60	181827	100.0
Stroke-Ready Hospitals	5	6048	3.33
Stroke-Ready Hospitals	10	16145	8.88
Stroke-Ready Hospitals	15	34244	18.83
Stroke-Ready Hospitals	20	69180	38.05
Stroke-Ready Hospitals	25	114236	62.83
Stroke-Ready Hospitals	30	150836	82.96
Stroke-Ready Hospitals	35	172108	94.66
Stroke-Ready Hospitals	40	178359	98.09
Stroke-Ready Hospitals	45	181749	99.96
Stroke-Ready Hospitals	50	181827	100.0
Stroke-Ready Hospitals	55	181827	100.0
Stroke-Ready Hospitals	60	181827	100.0
Stroke Units	5	0	0.0
Stroke Units	10	0	0.0
Stroke Units	15	0	0.0
Stroke Units	20	1388	0.76
Stroke Units	25	20817	11.45
Stroke Units	30	55161	30.34
Stroke Units	35	102476	56.36
Stroke Units	40	134260	73.84
Stroke Units	45	171095	94.1
Stroke Units	50	181752	99.96
Stroke Units	55	181827	100.0
Stroke Units	60	181827	100.0

Population Coverage - Landkreis Westerwaldkreis (Rheinland-Pfalz)

Condition	Drive Time (min)	Covered Population	Coverage (%)
Hospitals with CT	5	18763	9.12
Hospitals with CT	10	85229	41.41
Hospitals with CT	15	148555	72.18
Hospitals with CT	20	186026	90.38
Hospitals with CT	25	202171	98.22
Hospitals with CT	30	205820	100.0
Hospitals with CT	35	205824	100.0
Hospitals with CT	40	205824	100.0
Hospitals with CT	45	205824	100.0
Hospitals with CT	50	205824	100.0
Hospitals with CT	55	205824	100.0
Hospitals with CT	60	205824	100.0
Stroke-Ready Hospitals	5	3388	1.65
Stroke-Ready Hospitals	10	28826	14.01
Stroke-Ready Hospitals	15	87829	42.67
Stroke-Ready Hospitals	20	128481	62.42
Stroke-Ready Hospitals	25	151104	73.41
Stroke-Ready Hospitals	30	177158	86.07
Stroke-Ready Hospitals	35	205016	99.61
Stroke-Ready Hospitals	40	205824	100.0
Stroke-Ready Hospitals	45	205824	100.0
Stroke-Ready Hospitals	50	205824	100.0
Stroke-Ready Hospitals	55	205824	100.0
Stroke-Ready Hospitals	60	205824	100.0
Stroke Units	5	3388	1.65
Stroke Units	10	28826	14.01
Stroke Units	15	87829	42.67
Stroke Units	20	128481	62.42
Stroke Units	25	151104	73.41
Stroke Units	30	173371	84.23
Stroke Units	35	199318	96.84
Stroke Units	40	205614	99.9
Stroke Units	45	205824	100.0
Stroke Units	50	205824	100.0
Stroke Units	55	205824	100.0
Stroke Units	60	205824	100.0

Population Coverage - Kreisfreie Stadt Frankenthal (Pfalz) (Rheinland-Pfalz)

Condition	Drive Time (min)	Covered Population	Coverage (%)
Hospitals with CT	5	10503	23.12
Hospitals with CT	10	42433	93.4
Hospitals with CT	15	45406	99.94
Hospitals with CT	20	45433	100.0
Hospitals with CT	25	45433	100.0
Hospitals with CT	30	45433	100.0
Hospitals with CT	35	45433	100.0
Hospitals with CT	40	45433	100.0
Hospitals with CT	45	45433	100.0
Hospitals with CT	50	45433	100.0
Hospitals with CT	55	45433	100.0
Hospitals with CT	60	45433	100.0
Stroke-Ready Hospitals	5	9333	20.54
Stroke-Ready Hospitals	10	39994	88.03
Stroke-Ready Hospitals	15	45390	99.91
Stroke-Ready Hospitals	20	45433	100.0
Stroke-Ready Hospitals	25	45433	100.0
Stroke-Ready Hospitals	30	45433	100.0
Stroke-Ready Hospitals	35	45433	100.0
Stroke-Ready Hospitals	40	45433	100.0
Stroke-Ready Hospitals	45	45433	100.0
Stroke-Ready Hospitals	50	45433	100.0
Stroke-Ready Hospitals	55	45433	100.0
Stroke-Ready Hospitals	60	45433	100.0
Stroke Units	5	0	0.0
Stroke Units	10	0	0.0
Stroke Units	15	25382	55.87
Stroke Units	20	45403	99.93
Stroke Units	25	45433	100.0
Stroke Units	30	45433	100.0
Stroke Units	35	45433	100.0
Stroke Units	40	45433	100.0
Stroke Units	45	45433	100.0
Stroke Units	50	45433	100.0
Stroke Units	55	45433	100.0
Stroke Units	60	45433	100.0

Population Coverage - Landkreis Böblingen (Baden-Württemberg)

Condition	Drive Time (min)	Covered Population	Coverage (%)
Hospitals with CT	5	45924	12.33
Hospitals with CT	10	166272	44.66
Hospitals with CT	15	294006	78.96
Hospitals with CT	20	357871	96.11
Hospitals with CT	25	372128	99.94
Hospitals with CT	30	372228	99.97
Hospitals with CT	35	372240	99.97
Hospitals with CT	40	372349	100.0
Hospitals with CT	45	372349	100.0
Hospitals with CT	50	372349	100.0
Hospitals with CT	55	372349	100.0
Hospitals with CT	60	372349	100.0
Stroke-Ready Hospitals	5	5705	1.53
Stroke-Ready Hospitals	10	33796	9.08
Stroke-Ready Hospitals	15	85067	22.85
Stroke-Ready Hospitals	20	183002	49.15
Stroke-Ready Hospitals	25	309197	83.04
Stroke-Ready Hospitals	30	363142	97.53
Stroke-Ready Hospitals	35	372198	99.96
Stroke-Ready Hospitals	40	372349	100.0
Stroke-Ready Hospitals	45	372349	100.0
Stroke-Ready Hospitals	50	372349	100.0
Stroke-Ready Hospitals	55	372349	100.0
Stroke-Ready Hospitals	60	372349	100.0
Stroke Units	5	5705	1.53
Stroke Units	10	33796	9.08
Stroke Units	15	82965	22.28
Stroke Units	20	161882	43.48
Stroke Units	25	287181	77.13
Stroke Units	30	352795	94.75
Stroke Units	35	371417	99.75
Stroke Units	40	372349	100.0
Stroke Units	45	372349	100.0
Stroke Units	50	372349	100.0
Stroke Units	55	372349	100.0
Stroke Units	60	372349	100.0

Population Coverage - Landkreis Cham (Bayern)

Condition	Drive Time (min)	Covered Population	Coverage (%)
Hospitals with CT	5	9773	7.56
Hospitals with CT	10	30854	23.86
Hospitals with CT	15	58391	45.15
Hospitals with CT	20	95793	74.06
Hospitals with CT	25	118695	91.77
Hospitals with CT	30	126665	97.93
Hospitals with CT	35	128982	99.72
Hospitals with CT	40	129265	99.94
Hospitals with CT	45	129300	99.97
Hospitals with CT	50	129304	99.97
Hospitals with CT	55	129292	99.96
Hospitals with CT	60	129302	99.97
Stroke-Ready Hospitals	5	6109	4.72
Stroke-Ready Hospitals	10	18363	14.2
Stroke-Ready Hospitals	15	31679	24.49
Stroke-Ready Hospitals	20	60816	47.02
Stroke-Ready Hospitals	25	89468	69.17
Stroke-Ready Hospitals	30	109048	84.31
Stroke-Ready Hospitals	35	119649	92.51
Stroke-Ready Hospitals	40	125177	96.78
Stroke-Ready Hospitals	45	129134	99.84
Stroke-Ready Hospitals	50	129238	99.92
Stroke-Ready Hospitals	55	129261	99.94
Stroke-Ready Hospitals	60	129287	99.96
Stroke Units	5	6109	4.72
Stroke Units	10	18363	14.2
Stroke Units	15	31679	24.49
Stroke Units	20	60816	47.02
Stroke Units	25	89415	69.13
Stroke Units	30	108991	84.27
Stroke Units	35	119634	92.5
Stroke Units	40	125177	96.78
Stroke Units	45	129134	99.84
Stroke Units	50	129238	99.92
Stroke Units	55	129261	99.94
Stroke Units	60	129287	99.96

Population Coverage - Landkreis Saarlouis (Saarland)

Condition	Drive Time (min)	Covered Population	Coverage (%)
Hospitals with CT	5	20729	11.07
Hospitals with CT	10	83976	44.86
Hospitals with CT	15	159630	85.27
Hospitals with CT	20	186432	99.59
Hospitals with CT	25	187117	99.95
Hospitals with CT	30	187163	99.98
Hospitals with CT	35	187182	99.99
Hospitals with CT	40	187182	99.99
Hospitals with CT	45	187182	99.99
Hospitals with CT	50	187182	99.99
Hospitals with CT	55	187127	99.96
Hospitals with CT	60	187128	99.96
Stroke-Ready Hospitals	5	14680	7.84
Stroke-Ready Hospitals	10	68828	36.77
Stroke-Ready Hospitals	15	128557	68.67
Stroke-Ready Hospitals	20	152984	81.72
Stroke-Ready Hospitals	25	167648	89.55
Stroke-Ready Hospitals	30	181786	97.1
Stroke-Ready Hospitals	35	186965	99.87
Stroke-Ready Hospitals	40	187168	99.98
Stroke-Ready Hospitals	45	187172	99.98
Stroke-Ready Hospitals	50	187172	99.98
Stroke-Ready Hospitals	55	187138	99.96
Stroke-Ready Hospitals	60	187138	99.96
Stroke Units	5	14680	7.84
Stroke Units	10	68828	36.77
Stroke Units	15	128557	68.67
Stroke Units	20	152984	81.72
Stroke Units	25	167648	89.55
Stroke Units	30	181786	97.1
Stroke Units	35	186965	99.87
Stroke Units	40	187168	99.98
Stroke Units	45	187172	99.98
Stroke Units	50	187172	99.98
Stroke Units	55	187138	99.96
Stroke Units	60	187138	99.96

Population Coverage - Landkreis Anhalt-Bitterfeld (Sachsen-Anhalt)

Condition	Drive Time (min)	Covered Population	Coverage (%)
Hospitals with CT	5	33056	22.41
Hospitals with CT	10	58710	39.8
Hospitals with CT	15	99556	67.5
Hospitals with CT	20	134864	91.44
Hospitals with CT	25	146585	99.38
Hospitals with CT	30	147479	99.99
Hospitals with CT	35	147490	100.0
Hospitals with CT	40	147496	100.0
Hospitals with CT	45	147496	100.0
Hospitals with CT	50	147496	100.0
Hospitals with CT	55	147497	100.0
Hospitals with CT	60	147497	100.0
Stroke-Ready Hospitals	5	5448	3.69
Stroke-Ready Hospitals	10	19445	13.18
Stroke-Ready Hospitals	15	39561	26.82
Stroke-Ready Hospitals	20	70715	47.94
Stroke-Ready Hospitals	25	98901	67.05
Stroke-Ready Hospitals	30	124660	84.52
Stroke-Ready Hospitals	35	135360	91.77
Stroke-Ready Hospitals	40	141433	95.89
Stroke-Ready Hospitals	45	143891	97.56
Stroke-Ready Hospitals	50	147348	99.9
Stroke-Ready Hospitals	55	147470	99.98
Stroke-Ready Hospitals	60	147495	100.0
Stroke Units	5	0	0.0
Stroke Units	10	0	0.0
Stroke Units	15	0	0.0
Stroke Units	20	830	0.56
Stroke Units	25	10726	7.27
Stroke Units	30	49278	33.41
Stroke Units	35	87843	59.56
Stroke Units	40	112473	76.25
Stroke Units	45	124698	84.54
Stroke Units	50	140486	95.25
Stroke Units	55	143650	97.39
Stroke Units	60	146609	99.4

Population Coverage - Landkreis Jerichower Land (Sachsen-Anhalt)

Condition	Drive Time (min)	Covered Population	Coverage (%)
Hospitals with CT	5	9706	12.06
Hospitals with CT	10	29348	36.46
Hospitals with CT	15	43544	54.09
Hospitals with CT	20	57014	70.83
Hospitals with CT	25	71337	88.62
Hospitals with CT	30	78089	97.01
Hospitals with CT	35	79713	99.02
Hospitals with CT	40	79784	99.11
Hospitals with CT	45	79816	99.15
Hospitals with CT	50	79845	99.19
Hospitals with CT	55	80499	100.0
Hospitals with CT	60	80499	100.0
Stroke-Ready Hospitals	5	0	0.0
Stroke-Ready Hospitals	10	0	0.0
Stroke-Ready Hospitals	15	272	0.34
Stroke-Ready Hospitals	20	3738	4.64
Stroke-Ready Hospitals	25	15086	18.74
Stroke-Ready Hospitals	30	34662	43.06
Stroke-Ready Hospitals	35	49593	61.61
Stroke-Ready Hospitals	40	63655	79.08
Stroke-Ready Hospitals	45	71787	89.18
Stroke-Ready Hospitals	50	78148	97.08
Stroke-Ready Hospitals	55	79603	98.89
Stroke-Ready Hospitals	60	79836	99.18
Stroke Units	5	0	0.0
Stroke Units	10	0	0.0
Stroke Units	15	272	0.34
Stroke Units	20	3738	4.64
Stroke Units	25	15046	18.69
Stroke Units	30	33129	41.16
Stroke Units	35	40122	49.84
Stroke Units	40	49326	61.28
Stroke Units	45	55194	68.57
Stroke Units	50	59858	74.36
Stroke Units	55	66616	82.75
Stroke Units	60	74676	92.77

Population Coverage - Landkreis Kyffhäuserkreis (Thüringen)

Condition	Drive Time (min)	Covered Population	Coverage (%)
Hospitals with CT	5	9206	13.21
Hospitals with CT	10	15286	21.93
Hospitals with CT	15	18014	25.85
Hospitals with CT	20	34164	49.02
Hospitals with CT	25	51346	73.68
Hospitals with CT	30	63390	90.96
Hospitals with CT	35	68505	98.3
Hospitals with CT	40	69615	99.89
Hospitals with CT	45	69692	100.0
Hospitals with CT	50	69692	100.0
Hospitals with CT	55	69692	100.0
Hospitals with CT	60	69692	100.0
Stroke-Ready Hospitals	5	0	0.0
Stroke-Ready Hospitals	10	0	0.0
Stroke-Ready Hospitals	15	412	0.59
Stroke-Ready Hospitals	20	9263	13.29
Stroke-Ready Hospitals	25	16462	23.62
Stroke-Ready Hospitals	30	32938	47.26
Stroke-Ready Hospitals	35	52588	75.46
Stroke-Ready Hospitals	40	64736	92.89
Stroke-Ready Hospitals	45	69181	99.27
Stroke-Ready Hospitals	50	69675	99.98
Stroke-Ready Hospitals	55	69692	100.0
Stroke-Ready Hospitals	60	69692	100.0
Stroke Units	5	0	0.0
Stroke Units	10	0	0.0
Stroke Units	15	0	0.0
Stroke Units	20	0	0.0
Stroke Units	25	89	0.13
Stroke Units	30	3182	4.57
Stroke Units	35	13821	19.83
Stroke Units	40	34655	49.73
Stroke Units	45	48923	70.2
Stroke Units	50	66168	94.94
Stroke Units	55	69380	99.55
Stroke Units	60	69678	99.98

Population Coverage - Landkreis Peine (Niedersachsen)

Condition	Drive Time (min)	Covered Population	Coverage (%)
Hospitals with CT	5	8229	6.3
Hospitals with CT	10	45155	34.54
Hospitals with CT	15	93274	71.36
Hospitals with CT	20	122455	93.68
Hospitals with CT	25	128284	98.14
Hospitals with CT	30	130717	100.0
Hospitals with CT	35	130717	100.0
Hospitals with CT	40	130717	100.0
Hospitals with CT	45	130717	100.0
Hospitals with CT	50	130717	100.0
Hospitals with CT	55	130717	100.0
Hospitals with CT	60	130717	100.0
Stroke-Ready Hospitals	5	5104	3.9
Stroke-Ready Hospitals	10	40006	30.61
Stroke-Ready Hospitals	15	82864	63.39
Stroke-Ready Hospitals	20	112335	85.94
Stroke-Ready Hospitals	25	125641	96.12
Stroke-Ready Hospitals	30	130712	100.0
Stroke-Ready Hospitals	35	130717	100.0
Stroke-Ready Hospitals	40	130717	100.0
Stroke-Ready Hospitals	45	130717	100.0
Stroke-Ready Hospitals	50	130717	100.0
Stroke-Ready Hospitals	55	130717	100.0
Stroke-Ready Hospitals	60	130717	100.0
Stroke Units	5	0	0.0
Stroke Units	10	0	0.0
Stroke Units	15	0	0.0
Stroke Units	20	637	0.49
Stroke Units	25	25509	19.51
Stroke Units	30	81487	62.34
Stroke Units	35	115023	87.99
Stroke Units	40	130337	99.71
Stroke Units	45	130717	100.0
Stroke Units	50	130717	100.0
Stroke Units	55	130717	100.0
Stroke Units	60	130717	100.0

Population Coverage - Kreisfreie Stadt Leverkusen (Nordrhein-Westfalen)

Condition	Drive Time (min)	Covered Population	Coverage (%)
Hospitals with CT	5	32201	20.04
Hospitals with CT	10	107335	66.8
Hospitals with CT	15	145808	90.75
Hospitals with CT	20	160614	99.97
Hospitals with CT	25	160670	100.0
Hospitals with CT	30	160670	100.0
Hospitals with CT	35	160670	100.0
Hospitals with CT	40	160670	100.0
Hospitals with CT	45	160670	100.0
Hospitals with CT	50	160670	100.0
Hospitals with CT	55	160670	100.0
Hospitals with CT	60	160670	100.0
Stroke-Ready Hospitals	5	18954	11.8
Stroke-Ready Hospitals	10	71175	44.3
Stroke-Ready Hospitals	15	127799	79.54
Stroke-Ready Hospitals	20	148549	92.46
Stroke-Ready Hospitals	25	160670	100.0
Stroke-Ready Hospitals	30	160670	100.0
Stroke-Ready Hospitals	35	160670	100.0
Stroke-Ready Hospitals	40	160670	100.0
Stroke-Ready Hospitals	45	160670	100.0
Stroke-Ready Hospitals	50	160670	100.0
Stroke-Ready Hospitals	55	160670	100.0
Stroke-Ready Hospitals	60	160670	100.0
Stroke Units	5	18954	11.8
Stroke Units	10	71175	44.3
Stroke Units	15	127799	79.54
Stroke Units	20	148549	92.46
Stroke Units	25	160670	100.0
Stroke Units	30	160670	100.0
Stroke Units	35	160670	100.0
Stroke Units	40	160670	100.0
Stroke Units	45	160670	100.0
Stroke Units	50	160670	100.0
Stroke Units	55	160670	100.0
Stroke Units	60	160670	100.0

Population Coverage - Kreis Ennepe-Ruhr-Kreis (Nordrhein-Westfalen)

Condition	Drive Time (min)	Covered Population	Coverage (%)
Hospitals with CT	5	75378	22.66
Hospitals with CT	10	205803	61.87
Hospitals with CT	15	298388	89.7
Hospitals with CT	20	326319	98.09
Hospitals with CT	25	332629	99.99
Hospitals with CT	30	332661	100.0
Hospitals with CT	35	332661	100.0
Hospitals with CT	40	332661	100.0
Hospitals with CT	45	332661	100.0
Hospitals with CT	50	332661	100.0
Hospitals with CT	55	332661	100.0
Hospitals with CT	60	332661	100.0
Stroke-Ready Hospitals	5	28087	8.44
Stroke-Ready Hospitals	10	150888	45.36
Stroke-Ready Hospitals	15	272939	82.05
Stroke-Ready Hospitals	20	321367	96.6
Stroke-Ready Hospitals	25	324935	97.68
Stroke-Ready Hospitals	30	330178	99.25
Stroke-Ready Hospitals	35	332630	99.99
Stroke-Ready Hospitals	40	332661	100.0
Stroke-Ready Hospitals	45	332661	100.0
Stroke-Ready Hospitals	50	332661	100.0
Stroke-Ready Hospitals	55	332661	100.0
Stroke-Ready Hospitals	60	332661	100.0
Stroke Units	5	19619	5.9
Stroke Units	10	111906	33.64
Stroke Units	15	209399	62.95
Stroke Units	20	275081	82.69
Stroke Units	25	297665	89.48
Stroke Units	30	312101	93.82
Stroke Units	35	329705	99.11
Stroke Units	40	332439	99.93
Stroke Units	45	332661	100.0
Stroke Units	50	332661	100.0
Stroke Units	55	332661	100.0
Stroke Units	60	332661	100.0

Population Coverage - Kreis Märkischer Kreis (Nordrhein-Westfalen)

Condition	Drive Time (min)	Covered Population	Coverage (%)
Hospitals with CT	5	72126	17.36
Hospitals with CT	10	232426	55.95
Hospitals with CT	15	333199	80.21
Hospitals with CT	20	396832	95.53
Hospitals with CT	25	413557	99.56
Hospitals with CT	30	415394	100.0
Hospitals with CT	35	415394	100.0
Hospitals with CT	40	415394	100.0
Hospitals with CT	45	415394	100.0
Hospitals with CT	50	415394	100.0
Hospitals with CT	55	415394	100.0
Hospitals with CT	60	415394	100.0
Stroke-Ready Hospitals	5	31227	7.52
Stroke-Ready Hospitals	10	92703	22.32
Stroke-Ready Hospitals	15	148843	35.83
Stroke-Ready Hospitals	20	257195	61.92
Stroke-Ready Hospitals	25	346155	83.33
Stroke-Ready Hospitals	30	387693	93.33
Stroke-Ready Hospitals	35	410828	98.9
Stroke-Ready Hospitals	40	414862	99.87
Stroke-Ready Hospitals	45	415255	99.97
Stroke-Ready Hospitals	50	415392	100.0
Stroke-Ready Hospitals	55	415394	100.0
Stroke-Ready Hospitals	60	415394	100.0
Stroke Units	5	14522	3.5
Stroke Units	10	43619	10.5
Stroke Units	15	68190	16.42
Stroke Units	20	118911	28.63
Stroke Units	25	218038	52.49
Stroke Units	30	328941	79.19
Stroke Units	35	405742	97.68
Stroke Units	40	414840	99.87
Stroke Units	45	415255	99.97
Stroke Units	50	415392	100.0
Stroke Units	55	415394	100.0
Stroke Units	60	415394	100.0

Population Coverage - Landkreis Ahrweiler (Rheinland-Pfalz)

Condition	Drive Time (min)	Covered Population	Coverage (%)
Hospitals with CT	5	23375	18.14
Hospitals with CT	10	60160	46.68
Hospitals with CT	15	84168	65.3
Hospitals with CT	20	97274	75.47
Hospitals with CT	25	109635	85.06
Hospitals with CT	30	115219	89.4
Hospitals with CT	35	121243	94.07
Hospitals with CT	40	127161	98.66
Hospitals with CT	45	128886	100.0
Hospitals with CT	50	128887	100.0
Hospitals with CT	55	128887	100.0
Hospitals with CT	60	128887	100.0
Stroke-Ready Hospitals	5	14539	11.28
Stroke-Ready Hospitals	10	27922	21.66
Stroke-Ready Hospitals	15	44006	34.14
Stroke-Ready Hospitals	20	73043	56.67
Stroke-Ready Hospitals	25	104888	81.38
Stroke-Ready Hospitals	30	112192	87.05
Stroke-Ready Hospitals	35	115768	89.82
Stroke-Ready Hospitals	40	120985	93.87
Stroke-Ready Hospitals	45	127523	98.94
Stroke-Ready Hospitals	50	128883	100.0
Stroke-Ready Hospitals	55	128887	100.0
Stroke-Ready Hospitals	60	128887	100.0
Stroke Units	5	14539	11.28
Stroke Units	10	27903	21.65
Stroke Units	15	42820	33.22
Stroke Units	20	63586	49.33
Stroke Units	25	98842	76.69
Stroke Units	30	112039	86.93
Stroke Units	35	115768	89.82
Stroke Units	40	120985	93.87
Stroke Units	45	127256	98.73
Stroke Units	50	128882	100.0
Stroke Units	55	128887	100.0
Stroke Units	60	128887	100.0

Population Coverage - Stadtkreis Baden-Baden (Baden-Württemberg)

Condition	Drive Time (min)	Covered Population	Coverage (%)
Hospitals with CT	5	13992	22.23
Hospitals with CT	10	44657	70.95
Hospitals with CT	15	59882	95.14
Hospitals with CT	20	62917	99.96
Hospitals with CT	25	62927	99.98
Hospitals with CT	30	62940	100.0
Hospitals with CT	35	62941	100.0
Hospitals with CT	40	62941	100.0
Hospitals with CT	45	62941	100.0
Hospitals with CT	50	62941	100.0
Hospitals with CT	55	62941	100.0
Hospitals with CT	60	62941	100.0
Stroke-Ready Hospitals	5	0	0.0
Stroke-Ready Hospitals	10	10	0.02
Stroke-Ready Hospitals	15	6412	10.19
Stroke-Ready Hospitals	20	20186	32.07
Stroke-Ready Hospitals	25	33886	53.84
Stroke-Ready Hospitals	30	55131	87.59
Stroke-Ready Hospitals	35	62178	98.79
Stroke-Ready Hospitals	40	62913	99.96
Stroke-Ready Hospitals	45	62921	99.97
Stroke-Ready Hospitals	50	62939	100.0
Stroke-Ready Hospitals	55	62941	100.0
Stroke-Ready Hospitals	60	62941	100.0
Stroke Units	5	0	0.0
Stroke Units	10	10	0.02
Stroke Units	15	6412	10.19
Stroke Units	20	20186	32.07
Stroke Units	25	33886	53.84
Stroke Units	30	55131	87.59
Stroke Units	35	62178	98.79
Stroke Units	40	62913	99.96
Stroke Units	45	62921	99.97
Stroke Units	50	62938	100.0
Stroke Units	55	62941	100.0
Stroke Units	60	62941	100.0

Population Coverage - Landkreis Waldshut (Baden-Württemberg)

Condition	Drive Time (min)	Covered Population	Coverage (%)
Hospitals with CT	5	4980	2.81
Hospitals with CT	10	14831	8.37
Hospitals with CT	15	35769	20.19
Hospitals with CT	20	68244	38.51
Hospitals with CT	25	103436	58.37
Hospitals with CT	30	141989	80.13
Hospitals with CT	35	153824	86.81
Hospitals with CT	40	167086	94.29
Hospitals with CT	45	173146	97.71
Hospitals with CT	50	176501	99.6
Hospitals with CT	55	176287	99.48
Hospitals with CT	60	176359	99.52
Stroke-Ready Hospitals	5	4952	2.79
Stroke-Ready Hospitals	10	14942	8.43
Stroke-Ready Hospitals	15	30769	17.36
Stroke-Ready Hospitals	20	57891	32.67
Stroke-Ready Hospitals	25	86876	49.03
Stroke-Ready Hospitals	30	119319	67.33
Stroke-Ready Hospitals	35	155122	87.54
Stroke-Ready Hospitals	40	168506	95.09
Stroke-Ready Hospitals	45	173349	97.82
Stroke-Ready Hospitals	50	176532	99.62
Stroke-Ready Hospitals	55	176192	99.43
Stroke-Ready Hospitals	60	176237	99.45
Stroke Units	5	0	0.0
Stroke Units	10	0	0.0
Stroke Units	15	0	0.0
Stroke Units	20	0	0.0
Stroke Units	25	0	0.0
Stroke Units	30	3480	1.96
Stroke Units	35	15144	8.55
Stroke Units	40	29750	16.79
Stroke Units	45	39522	22.3
Stroke Units	50	55372	31.25
Stroke Units	55	73328	41.38
Stroke Units	60	90700	51.18

Population Coverage - Landkreis Bad Tölz-Wolfratshausen (Bayern)

Condition	Drive Time (min)	Covered Population	Coverage (%)
Hospitals with CT	5	28861	22.87
Hospitals with CT	10	68520	54.31
Hospitals with CT	15	111654	88.49
Hospitals with CT	20	121158	96.03
Hospitals with CT	25	123350	97.76
Hospitals with CT	30	124165	98.41
Hospitals with CT	35	124956	99.04
Hospitals with CT	40	125078	99.13
Hospitals with CT	45	125454	99.43
Hospitals with CT	50	125881	99.77
Hospitals with CT	55	125986	99.85
Hospitals with CT	60	125990	99.86
Stroke-Ready Hospitals	5	11592	9.19
Stroke-Ready Hospitals	10	28981	22.97
Stroke-Ready Hospitals	15	46349	36.74
Stroke-Ready Hospitals	20	69124	54.79
Stroke-Ready Hospitals	25	97041	76.91
Stroke-Ready Hospitals	30	123272	97.7
Stroke-Ready Hospitals	35	124137	98.39
Stroke-Ready Hospitals	40	124619	98.77
Stroke-Ready Hospitals	45	125145	99.19
Stroke-Ready Hospitals	50	125847	99.74
Stroke-Ready Hospitals	55	126003	99.87
Stroke-Ready Hospitals	60	126003	99.87
Stroke Units	5	11592	9.19
Stroke Units	10	28981	22.97
Stroke Units	15	46349	36.74
Stroke Units	20	69124	54.79
Stroke Units	25	97041	76.91
Stroke Units	30	123272	97.7
Stroke Units	35	124066	98.33
Stroke Units	40	124616	98.77
Stroke Units	45	125145	99.19
Stroke Units	50	125847	99.74
Stroke Units	55	126003	99.87
Stroke Units	60	126003	99.87

Population Coverage - Landkreis Passau (Bayern)

Condition	Drive Time (min)	Covered Population	Coverage (%)
Hospitals with CT	5	15617	8.04
Hospitals with CT	10	42457	21.86
Hospitals with CT	15	84575	43.54
Hospitals with CT	20	146832	75.6
Hospitals with CT	25	183435	94.44
Hospitals with CT	30	194022	99.89
Hospitals with CT	35	194105	99.93
Hospitals with CT	40	194112	99.94
Hospitals with CT	45	194112	99.94
Hospitals with CT	50	194113	99.94
Hospitals with CT	55	194079	99.92
Hospitals with CT	60	194118	99.94
Stroke-Ready Hospitals	5	4333	2.23
Stroke-Ready Hospitals	10	11399	5.87
Stroke-Ready Hospitals	15	36716	18.9
Stroke-Ready Hospitals	20	77094	39.69
Stroke-Ready Hospitals	25	118327	60.92
Stroke-Ready Hospitals	30	159838	82.29
Stroke-Ready Hospitals	35	181853	93.63
Stroke-Ready Hospitals	40	190101	97.87
Stroke-Ready Hospitals	45	193854	99.8
Stroke-Ready Hospitals	50	194104	99.93
Stroke-Ready Hospitals	55	194057	99.91
Stroke-Ready Hospitals	60	194091	99.93
Stroke Units	5	0	0.0
Stroke Units	10	3	0.0
Stroke Units	15	2714	1.4
Stroke Units	20	24288	12.5
Stroke Units	25	75093	38.66
Stroke Units	30	122822	63.23
Stroke Units	35	166276	85.61
Stroke Units	40	184116	94.79
Stroke Units	45	189713	97.67
Stroke Units	50	191839	98.77
Stroke Units	55	193925	99.84
Stroke Units	60	194089	99.92

Population Coverage - Landkreis Lichtenfels (Bayern)

Condition	Drive Time (min)	Covered Population	Coverage (%)
Hospitals with CT	5	6918	10.0
Hospitals with CT	10	20302	29.34
Hospitals with CT	15	36078	52.14
Hospitals with CT	20	47875	69.19
Hospitals with CT	25	64499	93.21
Hospitals with CT	30	69197	100.0
Hospitals with CT	35	69197	100.0
Hospitals with CT	40	69197	100.0
Hospitals with CT	45	69197	100.0
Hospitals with CT	50	69197	100.0
Hospitals with CT	55	69197	100.0
Hospitals with CT	60	69197	100.0
Stroke-Ready Hospitals	5	3145	4.55
Stroke-Ready Hospitals	10	17714	25.6
Stroke-Ready Hospitals	15	40299	58.24
Stroke-Ready Hospitals	20	52608	76.03
Stroke-Ready Hospitals	25	65787	95.07
Stroke-Ready Hospitals	30	69197	100.0
Stroke-Ready Hospitals	35	69197	100.0
Stroke-Ready Hospitals	40	69197	100.0
Stroke-Ready Hospitals	45	69197	100.0
Stroke-Ready Hospitals	50	69197	100.0
Stroke-Ready Hospitals	55	69197	100.0
Stroke-Ready Hospitals	60	69197	100.0
Stroke Units	5	3145	4.55
Stroke Units	10	17714	25.6
Stroke Units	15	40299	58.24
Stroke Units	20	52608	76.03
Stroke Units	25	65787	95.07
Stroke Units	30	69197	100.0
Stroke Units	35	69197	100.0
Stroke Units	40	69197	100.0
Stroke Units	45	69197	100.0
Stroke Units	50	69197	100.0
Stroke Units	55	69197	100.0
Stroke Units	60	69197	100.0

Population Coverage - Landkreis Wunsiedel i.Fichtelgebirge (Bayern)

Condition	Drive Time (min)	Covered Population	Coverage (%)
Hospitals with CT	5	15436	20.32
Hospitals with CT	10	34705	45.67
Hospitals with CT	15	56663	74.57
Hospitals with CT	20	69659	91.67
Hospitals with CT	25	75243	99.02
Hospitals with CT	30	75956	99.96
Hospitals with CT	35	75960	99.97
Hospitals with CT	40	75961	99.97
Hospitals with CT	45	75967	99.98
Hospitals with CT	50	75967	99.98
Hospitals with CT	55	75961	99.97
Hospitals with CT	60	75961	99.97
Stroke-Ready Hospitals	5	0	0.0
Stroke-Ready Hospitals	10	0	0.0
Stroke-Ready Hospitals	15	1082	1.43
Stroke-Ready Hospitals	20	13807	18.17
Stroke-Ready Hospitals	25	26770	35.23
Stroke-Ready Hospitals	30	41159	54.17
Stroke-Ready Hospitals	35	67685	89.08
Stroke-Ready Hospitals	40	74587	98.16
Stroke-Ready Hospitals	45	75927	99.92
Stroke-Ready Hospitals	50	75959	99.96
Stroke-Ready Hospitals	55	75943	99.94
Stroke-Ready Hospitals	60	75948	99.95
Stroke Units	5	0	0.0
Stroke Units	10	0	0.0
Stroke Units	15	1082	1.43
Stroke Units	20	13807	18.17
Stroke Units	25	26770	35.23
Stroke Units	30	41159	54.17
Stroke Units	35	67685	89.08
Stroke Units	40	74587	98.16
Stroke Units	45	75927	99.92
Stroke Units	50	75959	99.96
Stroke Units	55	75943	99.94
Stroke Units	60	75948	99.95

Population Coverage - Landkreis Ansbach (Bayern)

Condition	Drive Time (min)	Covered Population	Coverage (%)
Hospitals with CT	5	10426	5.64
Hospitals with CT	10	33321	18.02
Hospitals with CT	15	68446	37.02
Hospitals with CT	20	129526	70.06
Hospitals with CT	25	177457	95.99
Hospitals with CT	30	184876	100.0
Hospitals with CT	35	184876	100.0
Hospitals with CT	40	184876	100.0
Hospitals with CT	45	184876	100.0
Hospitals with CT	50	184876	100.0
Hospitals with CT	55	184876	100.0
Hospitals with CT	60	184876	100.0
Stroke-Ready Hospitals	5	3047	1.65
Stroke-Ready Hospitals	10	17725	9.59
Stroke-Ready Hospitals	15	38865	21.02
Stroke-Ready Hospitals	20	89015	48.15
Stroke-Ready Hospitals	25	144017	77.9
Stroke-Ready Hospitals	30	165348	89.44
Stroke-Ready Hospitals	35	178159	96.37
Stroke-Ready Hospitals	40	184098	99.58
Stroke-Ready Hospitals	45	184876	100.0
Stroke-Ready Hospitals	50	184876	100.0
Stroke-Ready Hospitals	55	184876	100.0
Stroke-Ready Hospitals	60	184876	100.0
Stroke Units	5	3047	1.65
Stroke Units	10	17725	9.59
Stroke Units	15	38830	21.0
Stroke Units	20	87813	47.5
Stroke Units	25	143602	77.67
Stroke Units	30	165348	89.44
Stroke Units	35	178159	96.37
Stroke Units	40	184098	99.58
Stroke Units	45	184876	100.0
Stroke Units	50	184876	100.0
Stroke Units	55	184876	100.0
Stroke Units	60	184876	100.0

Population Coverage - Kreisfreie Stadt Würzburg (Bayern)

Condition	Drive Time (min)	Covered Population	Coverage (%)
Hospitals with CT	5	64670	50.48
Hospitals with CT	10	109799	85.71
Hospitals with CT	15	124526	97.2
Hospitals with CT	20	128110	100.0
Hospitals with CT	25	128110	100.0
Hospitals with CT	30	128110	100.0
Hospitals with CT	35	128110	100.0
Hospitals with CT	40	128110	100.0
Hospitals with CT	45	128110	100.0
Hospitals with CT	50	128110	100.0
Hospitals with CT	55	128110	100.0
Hospitals with CT	60	128110	100.0
Stroke-Ready Hospitals	5	30047	23.45
Stroke-Ready Hospitals	10	99866	77.95
Stroke-Ready Hospitals	15	117186	91.47
Stroke-Ready Hospitals	20	125240	97.76
Stroke-Ready Hospitals	25	128110	100.0
Stroke-Ready Hospitals	30	128110	100.0
Stroke-Ready Hospitals	35	128110	100.0
Stroke-Ready Hospitals	40	128110	100.0
Stroke-Ready Hospitals	45	128110	100.0
Stroke-Ready Hospitals	50	128110	100.0
Stroke-Ready Hospitals	55	128110	100.0
Stroke-Ready Hospitals	60	128110	100.0
Stroke Units	5	30047	23.45
Stroke Units	10	99866	77.95
Stroke Units	15	117186	91.47
Stroke Units	20	125240	97.76
Stroke Units	25	128110	100.0
Stroke Units	30	128110	100.0
Stroke Units	35	128110	100.0
Stroke Units	40	128110	100.0
Stroke Units	45	128110	100.0
Stroke Units	50	128110	100.0
Stroke Units	55	128110	100.0
Stroke Units	60	128110	100.0

Population Coverage - Landkreis Oder-Spree (Brandenburg)

Condition	Drive Time (min)	Covered Population	Coverage (%)
Hospitals with CT	5	29702	17.25
Hospitals with CT	10	95457	55.43
Hospitals with CT	15	131666	76.45
Hospitals with CT	20	143637	83.41
Hospitals with CT	25	155507	90.3
Hospitals with CT	30	170094	98.77
Hospitals with CT	35	171988	99.87
Hospitals with CT	40	172173	99.98
Hospitals with CT	45	172179	99.98
Hospitals with CT	50	172179	99.98
Hospitals with CT	55	172166	99.97
Hospitals with CT	60	172188	99.98
Stroke-Ready Hospitals	5	2817	1.64
Stroke-Ready Hospitals	10	14028	8.15
Stroke-Ready Hospitals	15	48582	28.21
Stroke-Ready Hospitals	20	87569	50.85
Stroke-Ready Hospitals	25	119635	69.47
Stroke-Ready Hospitals	30	151752	88.12
Stroke-Ready Hospitals	35	163032	94.67
Stroke-Ready Hospitals	40	167406	97.21
Stroke-Ready Hospitals	45	169043	98.16
Stroke-Ready Hospitals	50	171136	99.37
Stroke-Ready Hospitals	55	172079	99.92
Stroke-Ready Hospitals	60	172105	99.94
Stroke Units	5	310	0.18
Stroke Units	10	9411	5.46
Stroke Units	15	31686	18.4
Stroke Units	20	52126	30.27
Stroke Units	25	93802	54.47
Stroke Units	30	135144	78.47
Stroke Units	35	152907	88.79
Stroke Units	40	164189	95.34
Stroke Units	45	168223	97.68
Stroke Units	50	170952	99.27
Stroke Units	55	172067	99.91
Stroke Units	60	172096	99.93

Population Coverage - Landkreis Potsdam-Mittelmark (Brandenburg)

Condition	Drive Time (min)	Covered Population	Coverage (%)
Hospitals with CT	5	13774	6.79
Hospitals with CT	10	65046	32.04
Hospitals with CT	15	117710	57.99
Hospitals with CT	20	164777	81.17
Hospitals with CT	25	191453	94.31
Hospitals with CT	30	199963	98.5
Hospitals with CT	35	201390	99.21
Hospitals with CT	40	202798	99.9
Hospitals with CT	45	202943	99.97
Hospitals with CT	50	202952	99.98
Hospitals with CT	55	202998	100.0
Hospitals with CT	60	202998	100.0
Stroke-Ready Hospitals	5	0	0.0
Stroke-Ready Hospitals	10	8	0.0
Stroke-Ready Hospitals	15	13986	6.89
Stroke-Ready Hospitals	20	81806	40.3
Stroke-Ready Hospitals	25	116124	57.2
Stroke-Ready Hospitals	30	137666	67.82
Stroke-Ready Hospitals	35	161271	79.44
Stroke-Ready Hospitals	40	177574	87.48
Stroke-Ready Hospitals	45	189816	93.51
Stroke-Ready Hospitals	50	198712	97.89
Stroke-Ready Hospitals	55	201745	99.38
Stroke-Ready Hospitals	60	202720	99.86
Stroke Units	5	0	0.0
Stroke Units	10	8	0.0
Stroke Units	15	13094	6.45
Stroke Units	20	74873	36.88
Stroke Units	25	101499	50.0
Stroke Units	30	118258	58.26
Stroke Units	35	134749	66.38
Stroke Units	40	149178	73.49
Stroke Units	45	159561	78.6
Stroke Units	50	173169	85.31
Stroke Units	55	185400	91.33
Stroke Units	60	193726	95.43

Population Coverage - Landkreis Uckermark (Brandenburg)

Condition	Drive Time (min)	Covered Population	Coverage (%)
Hospitals with CT	5	25957	22.36
Hospitals with CT	10	50153	43.21
Hospitals with CT	15	63001	54.27
Hospitals with CT	20	74308	64.01
Hospitals with CT	25	97013	83.57
Hospitals with CT	30	107955	93.0
Hospitals with CT	35	112268	96.71
Hospitals with CT	40	113824	98.06
Hospitals with CT	45	114263	98.43
Hospitals with CT	50	114324	98.49
Hospitals with CT	55	113516	97.79
Hospitals with CT	60	113779	98.02
Stroke-Ready Hospitals	5	8540	7.36
Stroke-Ready Hospitals	10	16097	13.87
Stroke-Ready Hospitals	15	19521	16.82
Stroke-Ready Hospitals	20	22736	19.59
Stroke-Ready Hospitals	25	39181	33.75
Stroke-Ready Hospitals	30	47405	40.84
Stroke-Ready Hospitals	35	52019	44.81
Stroke-Ready Hospitals	40	58412	50.32
Stroke-Ready Hospitals	45	72972	62.86
Stroke-Ready Hospitals	50	84998	73.22
Stroke-Ready Hospitals	55	92699	79.86
Stroke-Ready Hospitals	60	97772	84.23
Stroke Units	5	0	0.0
Stroke Units	10	0	0.0
Stroke Units	15	0	0.0
Stroke Units	20	0	0.0
Stroke Units	25	0	0.0
Stroke Units	30	41	0.04
Stroke Units	35	1857	1.6
Stroke Units	40	10540	9.08
Stroke Units	45	20093	17.31
Stroke Units	50	36870	31.76
Stroke Units	55	56490	48.66
Stroke Units	60	78580	67.69

Population Coverage - Landkreis Sächsische Schweiz-Osterzgebirge (Sachsen)

Condition	Drive Time (min)	Covered Population	Coverage (%)
Hospitals with CT	5	20327	8.83
Hospitals with CT	10	65180	28.3
Hospitals with CT	15	136197	59.14
Hospitals with CT	20	189711	82.37
Hospitals with CT	25	214397	93.09
Hospitals with CT	30	223066	96.85
Hospitals with CT	35	228050	99.02
Hospitals with CT	40	229874	99.81
Hospitals with CT	45	230044	99.88
Hospitals with CT	50	230051	99.89
Hospitals with CT	55	229893	99.82
Hospitals with CT	60	229972	99.85
Stroke-Ready Hospitals	5	0	0.0
Stroke-Ready Hospitals	10	30	0.01
Stroke-Ready Hospitals	15	5705	2.48
Stroke-Ready Hospitals	20	30945	13.44
Stroke-Ready Hospitals	25	66302	28.79
Stroke-Ready Hospitals	30	133518	57.97
Stroke-Ready Hospitals	35	169257	73.49
Stroke-Ready Hospitals	40	196515	85.33
Stroke-Ready Hospitals	45	211732	91.93
Stroke-Ready Hospitals	50	222889	96.78
Stroke-Ready Hospitals	55	227346	98.71
Stroke-Ready Hospitals	60	229620	99.7
Stroke Units	5	0	0.0
Stroke Units	10	0	0.0
Stroke Units	15	2730	1.19
Stroke Units	20	23260	10.1
Stroke Units	25	52740	22.9
Stroke Units	30	99775	43.32
Stroke Units	35	142653	61.94
Stroke Units	40	173320	75.26
Stroke Units	45	185782	80.67
Stroke Units	50	199747	86.73
Stroke Units	55	210200	91.27
Stroke Units	60	216886	94.17

Population Coverage - Kreisfreie Stadt Leipzig (Sachsen)

Condition	Drive Time (min)	Covered Population	Coverage (%)
Hospitals with CT	5	105470	17.03
Hospitals with CT	10	367693	59.37
Hospitals with CT	15	535199	86.42
Hospitals with CT	20	615969	99.46
Hospitals with CT	25	619326	100.0
Hospitals with CT	30	619326	100.0
Hospitals with CT	35	619326	100.0
Hospitals with CT	40	619326	100.0
Hospitals with CT	45	619326	100.0
Hospitals with CT	50	619326	100.0
Hospitals with CT	55	619326	100.0
Hospitals with CT	60	619326	100.0
Stroke-Ready Hospitals	5	76166	12.3
Stroke-Ready Hospitals	10	271378	43.82
Stroke-Ready Hospitals	15	441632	71.31
Stroke-Ready Hospitals	20	589160	95.13
Stroke-Ready Hospitals	25	619127	99.97
Stroke-Ready Hospitals	30	619326	100.0
Stroke-Ready Hospitals	35	619326	100.0
Stroke-Ready Hospitals	40	619326	100.0
Stroke-Ready Hospitals	45	619326	100.0
Stroke-Ready Hospitals	50	619326	100.0
Stroke-Ready Hospitals	55	619326	100.0
Stroke-Ready Hospitals	60	619326	100.0
Stroke Units	5	76166	12.3
Stroke Units	10	271378	43.82
Stroke Units	15	441632	71.31
Stroke Units	20	589160	95.13
Stroke Units	25	619127	99.97
Stroke Units	30	619326	100.0
Stroke Units	35	619326	100.0
Stroke Units	40	619326	100.0
Stroke Units	45	619326	100.0
Stroke Units	50	619326	100.0
Stroke Units	55	619326	100.0
Stroke Units	60	619326	100.0

Population Coverage - Kreisfreie Stadt Jena (Thüringen)

Condition	Drive Time (min)	Covered Population	Coverage (%)
Hospitals with CT	5	13384	13.35
Hospitals with CT	10	32752	32.67
Hospitals with CT	15	80789	80.59
Hospitals with CT	20	98304	98.06
Hospitals with CT	25	100246	100.0
Hospitals with CT	30	100246	100.0
Hospitals with CT	35	100246	100.0
Hospitals with CT	40	100246	100.0
Hospitals with CT	45	100246	100.0
Hospitals with CT	50	100246	100.0
Hospitals with CT	55	100246	100.0
Hospitals with CT	60	100246	100.0
Stroke-Ready Hospitals	5	12099	12.07
Stroke-Ready Hospitals	10	23945	23.89
Stroke-Ready Hospitals	15	69716	69.54
Stroke-Ready Hospitals	20	93756	93.53
Stroke-Ready Hospitals	25	99839	99.59
Stroke-Ready Hospitals	30	100239	99.99
Stroke-Ready Hospitals	35	100246	100.0
Stroke-Ready Hospitals	40	100246	100.0
Stroke-Ready Hospitals	45	100246	100.0
Stroke-Ready Hospitals	50	100246	100.0
Stroke-Ready Hospitals	55	100246	100.0
Stroke-Ready Hospitals	60	100246	100.0
Stroke Units	5	12099	12.07
Stroke Units	10	23945	23.89
Stroke Units	15	69716	69.54
Stroke Units	20	93756	93.53
Stroke Units	25	99839	99.59
Stroke Units	30	100239	99.99
Stroke Units	35	100246	100.0
Stroke Units	40	100246	100.0
Stroke Units	45	100246	100.0
Stroke Units	50	100246	100.0
Stroke Units	55	100246	100.0
Stroke Units	60	100246	100.0

Population Coverage - Landkreis Gotha (Thüringen)

Condition	Drive Time (min)	Covered Population	Coverage (%)
Hospitals with CT	5	11482	9.39
Hospitals with CT	10	53315	43.6
Hospitals with CT	15	84813	69.36
Hospitals with CT	20	108964	89.11
Hospitals with CT	25	121466	99.34
Hospitals with CT	30	122178	99.92
Hospitals with CT	35	122219	99.95
Hospitals with CT	40	122229	99.96
Hospitals with CT	45	122229	99.96
Hospitals with CT	50	122276	100.0
Hospitals with CT	55	122276	100.0
Hospitals with CT	60	122276	100.0
Stroke-Ready Hospitals	5	8210	6.71
Stroke-Ready Hospitals	10	34286	28.04
Stroke-Ready Hospitals	15	65776	53.79
Stroke-Ready Hospitals	20	97761	79.95
Stroke-Ready Hospitals	25	116509	95.28
Stroke-Ready Hospitals	30	121825	99.63
Stroke-Ready Hospitals	35	121937	99.72
Stroke-Ready Hospitals	40	122173	99.92
Stroke-Ready Hospitals	45	122183	99.92
Stroke-Ready Hospitals	50	122196	99.93
Stroke-Ready Hospitals	55	122276	100.0
Stroke-Ready Hospitals	60	122276	100.0
Stroke Units	5	8210	6.71
Stroke Units	10	32629	26.69
Stroke Units	15	61047	49.93
Stroke Units	20	91179	74.57
Stroke Units	25	113648	92.94
Stroke Units	30	121733	99.56
Stroke Units	35	121937	99.72
Stroke Units	40	122173	99.92
Stroke Units	45	122183	99.92
Stroke Units	50	122196	99.93
Stroke Units	55	122276	100.0
Stroke Units	60	122276	100.0

Population Coverage - Kreis Pinneberg (Schleswig-Holstein)

Condition	Drive Time (min)	Covered Population	Coverage (%)
Hospitals with CT	5	36693	11.85
Hospitals with CT	10	137897	44.54
Hospitals with CT	15	242025	78.17
Hospitals with CT	20	292351	94.42
Hospitals with CT	25	304250	98.26
Hospitals with CT	30	307093	99.18
Hospitals with CT	35	307588	99.34
Hospitals with CT	40	307694	99.38
Hospitals with CT	45	307807	99.41
Hospitals with CT	50	307969	99.47
Hospitals with CT	55	307894	99.44
Hospitals with CT	60	307923	99.45
Stroke-Ready Hospitals	5	17362	5.61
Stroke-Ready Hospitals	10	77320	24.97
Stroke-Ready Hospitals	15	132792	42.89
Stroke-Ready Hospitals	20	217945	70.39
Stroke-Ready Hospitals	25	288956	93.32
Stroke-Ready Hospitals	30	305296	98.6
Stroke-Ready Hospitals	35	307310	99.25
Stroke-Ready Hospitals	40	307371	99.27
Stroke-Ready Hospitals	45	307393	99.28
Stroke-Ready Hospitals	50	307877	99.44
Stroke-Ready Hospitals	55	307380	99.28
Stroke-Ready Hospitals	60	307755	99.4
Stroke Units	5	17362	5.61
Stroke Units	10	77320	24.97
Stroke Units	15	132792	42.89
Stroke Units	20	217945	70.39
Stroke Units	25	288956	93.32
Stroke Units	30	305296	98.6
Stroke Units	35	307310	99.25
Stroke Units	40	307371	99.27
Stroke Units	45	307393	99.28
Stroke Units	50	307877	99.44
Stroke Units	55	307380	99.28
Stroke Units	60	307755	99.4

Population Coverage - Kreis Schleswig-Flensburg (Schleswig-Holstein)

Condition	Drive Time (min)	Covered Population	Coverage (%)
Hospitals with CT	5	19521	9.72
Hospitals with CT	10	39839	19.83
Hospitals with CT	15	69885	34.79
Hospitals with CT	20	122288	60.88
Hospitals with CT	25	168729	84.01
Hospitals with CT	30	190807	95.0
Hospitals with CT	35	199152	99.15
Hospitals with CT	40	200320	99.73
Hospitals with CT	45	200402	99.77
Hospitals with CT	50	200448	99.8
Hospitals with CT	55	199914	99.53
Hospitals with CT	60	200043	99.6
Stroke-Ready Hospitals	5	20420	10.17
Stroke-Ready Hospitals	10	40609	20.22
Stroke-Ready Hospitals	15	71918	35.81
Stroke-Ready Hospitals	20	115074	57.29
Stroke-Ready Hospitals	25	157330	78.33
Stroke-Ready Hospitals	30	176531	87.89
Stroke-Ready Hospitals	35	189136	94.17
Stroke-Ready Hospitals	40	196575	97.87
Stroke-Ready Hospitals	45	199765	99.46
Stroke-Ready Hospitals	50	200121	99.63
Stroke-Ready Hospitals	55	199765	99.46
Stroke-Ready Hospitals	60	199775	99.46
Stroke Units	5	20420	10.17
Stroke Units	10	40609	20.22
Stroke Units	15	71918	35.81
Stroke Units	20	115074	57.29
Stroke Units	25	157330	78.33
Stroke Units	30	176531	87.89
Stroke Units	35	189136	94.17
Stroke Units	40	196543	97.85
Stroke Units	45	199765	99.46
Stroke Units	50	200121	99.63
Stroke Units	55	199765	99.46
Stroke Units	60	199775	99.46

Population Coverage - Kreis Steinburg (Schleswig-Holstein)

Condition	Drive Time (min)	Covered Population	Coverage (%)
Hospitals with CT	5	12855	9.65
Hospitals with CT	10	31465	23.62
Hospitals with CT	15	59565	44.72
Hospitals with CT	20	94135	70.68
Hospitals with CT	25	114725	86.14
Hospitals with CT	30	126835	95.23
Hospitals with CT	35	132805	99.71
Hospitals with CT	40	132963	99.83
Hospitals with CT	45	132978	99.84
Hospitals with CT	50	133030	99.88
Hospitals with CT	55	133054	99.9
Hospitals with CT	60	133084	99.92
Stroke-Ready Hospitals	5	13500	10.14
Stroke-Ready Hospitals	10	29355	22.04
Stroke-Ready Hospitals	15	52064	39.09
Stroke-Ready Hospitals	20	82749	62.13
Stroke-Ready Hospitals	25	104874	78.74
Stroke-Ready Hospitals	30	117355	88.11
Stroke-Ready Hospitals	35	131227	98.53
Stroke-Ready Hospitals	40	132896	99.78
Stroke-Ready Hospitals	45	132955	99.82
Stroke-Ready Hospitals	50	132999	99.86
Stroke-Ready Hospitals	55	132915	99.79
Stroke-Ready Hospitals	60	132969	99.83
Stroke Units	5	13500	10.14
Stroke Units	10	29355	22.04
Stroke Units	15	52064	39.09
Stroke Units	20	82749	62.13
Stroke Units	25	104873	78.74
Stroke Units	30	117355	88.11
Stroke Units	35	131227	98.53
Stroke Units	40	132895	99.78
Stroke Units	45	132955	99.82
Stroke Units	50	132999	99.86
Stroke Units	55	132891	99.77
Stroke Units	60	132960	99.83

Population Coverage - Landkreis Verden (Niedersachsen)

Condition	Drive Time (min)	Covered Population	Coverage (%)
Hospitals with CT	5	22588	17.01
Hospitals with CT	10	55135	41.51
Hospitals with CT	15	88638	66.73
Hospitals with CT	20	120104	90.42
Hospitals with CT	25	129979	97.86
Hospitals with CT	30	132827	100.0
Hospitals with CT	35	132828	100.0
Hospitals with CT	40	132828	100.0
Hospitals with CT	45	132828	100.0
Hospitals with CT	50	132828	100.0
Hospitals with CT	55	132828	100.0
Hospitals with CT	60	132828	100.0
Stroke-Ready Hospitals	5	91	0.07
Stroke-Ready Hospitals	10	3591	2.7
Stroke-Ready Hospitals	15	31753	23.91
Stroke-Ready Hospitals	20	54502	41.03
Stroke-Ready Hospitals	25	91777	69.09
Stroke-Ready Hospitals	30	117412	88.39
Stroke-Ready Hospitals	35	124458	93.7
Stroke-Ready Hospitals	40	131849	99.26
Stroke-Ready Hospitals	45	132828	100.0
Stroke-Ready Hospitals	50	132828	100.0
Stroke-Ready Hospitals	55	132828	100.0
Stroke-Ready Hospitals	60	132828	100.0
Stroke Units	5	0	0.0
Stroke Units	10	0	0.0
Stroke Units	15	307	0.23
Stroke Units	20	12540	9.44
Stroke Units	25	42961	32.34
Stroke Units	30	76258	57.41
Stroke Units	35	112425	84.64
Stroke Units	40	122618	92.31
Stroke Units	45	127619	96.08
Stroke Units	50	130364	98.14
Stroke Units	55	131738	99.18
Stroke Units	60	132828	100.0

Population Coverage - Kreisfreie Stadt Delmenhorst (Niedersachsen)

Condition	Drive Time (min)	Covered Population	Coverage (%)
Hospitals with CT	5	1943	2.65
Hospitals with CT	10	23891	32.55
Hospitals with CT	15	69057	94.09
Hospitals with CT	20	73353	99.94
Hospitals with CT	25	73395	100.0
Hospitals with CT	30	73395	100.0
Hospitals with CT	35	73395	100.0
Hospitals with CT	40	73395	100.0
Hospitals with CT	45	73395	100.0
Hospitals with CT	50	73395	100.0
Hospitals with CT	55	73395	100.0
Hospitals with CT	60	73395	100.0
Stroke-Ready Hospitals	5	0	0.0
Stroke-Ready Hospitals	10	0	0.0
Stroke-Ready Hospitals	15	0	0.0
Stroke-Ready Hospitals	20	51	0.07
Stroke-Ready Hospitals	25	12462	16.98
Stroke-Ready Hospitals	30	62597	85.29
Stroke-Ready Hospitals	35	73395	100.0
Stroke-Ready Hospitals	40	73395	100.0
Stroke-Ready Hospitals	45	73395	100.0
Stroke-Ready Hospitals	50	73395	100.0
Stroke-Ready Hospitals	55	73395	100.0
Stroke-Ready Hospitals	60	73395	100.0
Stroke Units	5	0	0.0
Stroke Units	10	0	0.0
Stroke Units	15	0	0.0
Stroke Units	20	51	0.07
Stroke Units	25	12276	16.73
Stroke Units	30	62553	85.23
Stroke Units	35	73395	100.0
Stroke Units	40	73395	100.0
Stroke Units	45	73395	100.0
Stroke Units	50	73395	100.0
Stroke Units	55	73395	100.0
Stroke Units	60	73395	100.0

Population Coverage - Kreis Kleve (Nordrhein-Westfalen)

Condition	Drive Time (min)	Covered Population	Coverage (%)
Hospitals with CT	5	79124	25.99
Hospitals with CT	10	179338	58.9
Hospitals with CT	15	264970	87.02
Hospitals with CT	20	299859	98.48
Hospitals with CT	25	303557	99.69
Hospitals with CT	30	303837	99.78
Hospitals with CT	35	303940	99.82
Hospitals with CT	40	303960	99.82
Hospitals with CT	45	304003	99.84
Hospitals with CT	50	304066	99.86
Hospitals with CT	55	303953	99.82
Hospitals with CT	60	304084	99.87
Stroke-Ready Hospitals	5	20327	6.68
Stroke-Ready Hospitals	10	69321	22.77
Stroke-Ready Hospitals	15	157939	51.87
Stroke-Ready Hospitals	20	232419	76.33
Stroke-Ready Hospitals	25	276373	90.76
Stroke-Ready Hospitals	30	299636	98.4
Stroke-Ready Hospitals	35	303496	99.67
Stroke-Ready Hospitals	40	303642	99.72
Stroke-Ready Hospitals	45	303720	99.75
Stroke-Ready Hospitals	50	303925	99.81
Stroke-Ready Hospitals	55	303786	99.77
Stroke-Ready Hospitals	60	303923	99.81
Stroke Units	5	0	0.0
Stroke Units	10	667	0.22
Stroke Units	15	14851	4.88
Stroke Units	20	34890	11.46
Stroke Units	25	74605	24.5
Stroke Units	30	122828	40.34
Stroke Units	35	199675	65.58
Stroke Units	40	226763	74.47
Stroke Units	45	277511	91.14
Stroke Units	50	297288	97.63
Stroke Units	55	302731	99.42
Stroke Units	60	303828	99.78

Population Coverage - Kreis Herford (Nordrhein-Westfalen)

Condition	Drive Time (min)	Covered Population	Coverage (%)
Hospitals with CT	5	69863	27.91
Hospitals with CT	10	178778	71.43
Hospitals with CT	15	238462	95.27
Hospitals with CT	20	250230	99.97
Hospitals with CT	25	250295	100.0
Hospitals with CT	30	250295	100.0
Hospitals with CT	35	250295	100.0
Hospitals with CT	40	250295	100.0
Hospitals with CT	45	250295	100.0
Hospitals with CT	50	250295	100.0
Hospitals with CT	55	250295	100.0
Hospitals with CT	60	250295	100.0
Stroke-Ready Hospitals	5	11192	4.47
Stroke-Ready Hospitals	10	38767	15.49
Stroke-Ready Hospitals	15	78890	31.52
Stroke-Ready Hospitals	20	136885	54.69
Stroke-Ready Hospitals	25	198547	79.33
Stroke-Ready Hospitals	30	243447	97.26
Stroke-Ready Hospitals	35	250272	99.99
Stroke-Ready Hospitals	40	250295	100.0
Stroke-Ready Hospitals	45	250295	100.0
Stroke-Ready Hospitals	50	250295	100.0
Stroke-Ready Hospitals	55	250295	100.0
Stroke-Ready Hospitals	60	250295	100.0
Stroke Units	5	11192	4.47
Stroke Units	10	38767	15.49
Stroke Units	15	78890	31.52
Stroke Units	20	136689	54.61
Stroke Units	25	195977	78.3
Stroke Units	30	240584	96.12
Stroke Units	35	250265	99.99
Stroke Units	40	250295	100.0
Stroke Units	45	250295	100.0
Stroke Units	50	250295	100.0
Stroke Units	55	250295	100.0
Stroke Units	60	250295	100.0

Population Coverage - Kreisfreie Stadt Dortmund (Nordrhein-Westfalen)

Condition	Drive Time (min)	Covered Population	Coverage (%)
Hospitals with CT	5	222137	36.33
Hospitals with CT	10	543874	88.94
Hospitals with CT	15	611480	100.0
Hospitals with CT	20	611483	100.0
Hospitals with CT	25	611483	100.0
Hospitals with CT	30	611483	100.0
Hospitals with CT	35	611483	100.0
Hospitals with CT	40	611483	100.0
Hospitals with CT	45	611483	100.0
Hospitals with CT	50	611483	100.0
Hospitals with CT	55	611483	100.0
Hospitals with CT	60	611483	100.0
Stroke-Ready Hospitals	5	88248	14.43
Stroke-Ready Hospitals	10	312879	51.17
Stroke-Ready Hospitals	15	580994	95.01
Stroke-Ready Hospitals	20	611483	100.0
Stroke-Ready Hospitals	25	611483	100.0
Stroke-Ready Hospitals	30	611483	100.0
Stroke-Ready Hospitals	35	611483	100.0
Stroke-Ready Hospitals	40	611483	100.0
Stroke-Ready Hospitals	45	611483	100.0
Stroke-Ready Hospitals	50	611483	100.0
Stroke-Ready Hospitals	55	611483	100.0
Stroke-Ready Hospitals	60	611483	100.0
Stroke Units	5	83993	13.74
Stroke Units	10	310524	50.78
Stroke Units	15	580185	94.88
Stroke Units	20	611483	100.0
Stroke Units	25	611483	100.0
Stroke Units	30	611483	100.0
Stroke Units	35	611483	100.0
Stroke Units	40	611483	100.0
Stroke Units	45	611483	100.0
Stroke Units	50	611483	100.0
Stroke Units	55	611483	100.0
Stroke Units	60	611483	100.0

Population Coverage - Kreisfreie Stadt Wiesbaden (Hessen)

Condition	Drive Time (min)	Covered Population	Coverage (%)
Hospitals with CT	5	101386	36.54
Hospitals with CT	10	217452	78.36
Hospitals with CT	15	277475	99.99
Hospitals with CT	20	277497	100.0
Hospitals with CT	25	277503	100.0
Hospitals with CT	30	277503	100.0
Hospitals with CT	35	277503	100.0
Hospitals with CT	40	277503	100.0
Hospitals with CT	45	277503	100.0
Hospitals with CT	50	277503	100.0
Hospitals with CT	55	277503	100.0
Hospitals with CT	60	277503	100.0
Stroke-Ready Hospitals	5	41532	14.97
Stroke-Ready Hospitals	10	177632	64.01
Stroke-Ready Hospitals	15	265563	95.7
Stroke-Ready Hospitals	20	277488	99.99
Stroke-Ready Hospitals	25	277503	100.0
Stroke-Ready Hospitals	30	277503	100.0
Stroke-Ready Hospitals	35	277503	100.0
Stroke-Ready Hospitals	40	277503	100.0
Stroke-Ready Hospitals	45	277503	100.0
Stroke-Ready Hospitals	50	277503	100.0
Stroke-Ready Hospitals	55	277503	100.0
Stroke-Ready Hospitals	60	277503	100.0
Stroke Units	5	41532	14.97
Stroke Units	10	177632	64.01
Stroke Units	15	265563	95.7
Stroke Units	20	277488	99.99
Stroke Units	25	277503	100.0
Stroke Units	30	277503	100.0
Stroke Units	35	277503	100.0
Stroke Units	40	277503	100.0
Stroke Units	45	277503	100.0
Stroke Units	50	277503	100.0
Stroke Units	55	277503	100.0
Stroke Units	60	277503	100.0

Population Coverage - Landkreis Hochtaunuskreis (Hessen)

Condition	Drive Time (min)	Covered Population	Coverage (%)
Hospitals with CT	5	32746	14.08
Hospitals with CT	10	142306	61.2
Hospitals with CT	15	219765	94.51
Hospitals with CT	20	232369	99.93
Hospitals with CT	25	232520	100.0
Hospitals with CT	30	232520	100.0
Hospitals with CT	35	232520	100.0
Hospitals with CT	40	232520	100.0
Hospitals with CT	45	232520	100.0
Hospitals with CT	50	232520	100.0
Hospitals with CT	55	232520	100.0
Hospitals with CT	60	232520	100.0
Stroke-Ready Hospitals	5	15747	6.77
Stroke-Ready Hospitals	10	85573	36.8
Stroke-Ready Hospitals	15	155682	66.95
Stroke-Ready Hospitals	20	201346	86.59
Stroke-Ready Hospitals	25	227411	97.8
Stroke-Ready Hospitals	30	232499	99.99
Stroke-Ready Hospitals	35	232520	100.0
Stroke-Ready Hospitals	40	232520	100.0
Stroke-Ready Hospitals	45	232520	100.0
Stroke-Ready Hospitals	50	232520	100.0
Stroke-Ready Hospitals	55	232520	100.0
Stroke-Ready Hospitals	60	232520	100.0
Stroke Units	5	15602	6.71
Stroke Units	10	84022	36.14
Stroke Units	15	154912	66.62
Stroke Units	20	198000	85.15
Stroke Units	25	227120	97.68
Stroke Units	30	232497	99.99
Stroke Units	35	232520	100.0
Stroke Units	40	232520	100.0
Stroke Units	45	232520	100.0
Stroke Units	50	232520	100.0
Stroke Units	55	232520	100.0
Stroke Units	60	232520	100.0

Population Coverage - Landkreis Kassel (Hessen)

Condition	Drive Time (min)	Covered Population	Coverage (%)
Hospitals with CT	5	27325	11.53
Hospitals with CT	10	116222	49.04
Hospitals with CT	15	206973	87.33
Hospitals with CT	20	235580	99.4
Hospitals with CT	25	237000	100.0
Hospitals with CT	30	237000	100.0
Hospitals with CT	35	237000	100.0
Hospitals with CT	40	237000	100.0
Hospitals with CT	45	237000	100.0
Hospitals with CT	50	237000	100.0
Hospitals with CT	55	237000	100.0
Hospitals with CT	60	237000	100.0
Stroke-Ready Hospitals	5	1632	0.69
Stroke-Ready Hospitals	10	21784	9.19
Stroke-Ready Hospitals	15	60143	25.38
Stroke-Ready Hospitals	20	111501	47.05
Stroke-Ready Hospitals	25	157692	66.54
Stroke-Ready Hospitals	30	191044	80.61
Stroke-Ready Hospitals	35	222749	93.99
Stroke-Ready Hospitals	40	233860	98.68
Stroke-Ready Hospitals	45	237000	100.0
Stroke-Ready Hospitals	50	237000	100.0
Stroke-Ready Hospitals	55	237000	100.0
Stroke-Ready Hospitals	60	237000	100.0
Stroke Units	5	1632	0.69
Stroke Units	10	21784	9.19
Stroke Units	15	60143	25.38
Stroke Units	20	111501	47.05
Stroke Units	25	157065	66.27
Stroke Units	30	188020	79.33
Stroke Units	35	214108	90.34
Stroke Units	40	233602	98.57
Stroke Units	45	236998	100.0
Stroke Units	50	237000	100.0
Stroke Units	55	237000	100.0
Stroke Units	60	237000	100.0

Population Coverage - Landkreis Cochem-Zell (Rheinland-Pfalz)

Condition	Drive Time (min)	Covered Population	Coverage (%)
Hospitals with CT	5	4798	7.46
Hospitals with CT	10	11454	17.8
Hospitals with CT	15	23460	36.45
Hospitals with CT	20	46321	71.98
Hospitals with CT	25	62911	97.76
Hospitals with CT	30	64313	99.93
Hospitals with CT	35	64356	100.0
Hospitals with CT	40	64356	100.0
Hospitals with CT	45	64356	100.0
Hospitals with CT	50	64356	100.0
Hospitals with CT	55	64356	100.0
Hospitals with CT	60	64356	100.0
Stroke-Ready Hospitals	5	0	0.0
Stroke-Ready Hospitals	10	0	0.0
Stroke-Ready Hospitals	15	0	0.0
Stroke-Ready Hospitals	20	966	1.5
Stroke-Ready Hospitals	25	6739	10.47
Stroke-Ready Hospitals	30	19731	30.66
Stroke-Ready Hospitals	35	33808	52.53
Stroke-Ready Hospitals	40	45302	70.39
Stroke-Ready Hospitals	45	53873	83.71
Stroke-Ready Hospitals	50	61645	95.79
Stroke-Ready Hospitals	55	63743	99.05
Stroke-Ready Hospitals	60	64351	99.99
Stroke Units	5	0	0.0
Stroke Units	10	0	0.0
Stroke Units	15	0	0.0
Stroke Units	20	966	1.5
Stroke Units	25	6739	10.47
Stroke Units	30	19731	30.66
Stroke Units	35	33808	52.53
Stroke Units	40	45302	70.39
Stroke Units	45	53873	83.71
Stroke Units	50	61645	95.79
Stroke Units	55	63743	99.05
Stroke Units	60	64351	99.99

Population Coverage - Landkreis Rhein-Lahn-Kreis (Rheinland-Pfalz)

Condition	Drive Time (min)	Covered Population	Coverage (%)
Hospitals with CT	5	14703	11.79
Hospitals with CT	10	53867	43.21
Hospitals with CT	15	86951	69.74
Hospitals with CT	20	114109	91.53
Hospitals with CT	25	124608	99.95
Hospitals with CT	30	124674	100.0
Hospitals with CT	35	124674	100.0
Hospitals with CT	40	124674	100.0
Hospitals with CT	45	124674	100.0
Hospitals with CT	50	124674	100.0
Hospitals with CT	55	124674	100.0
Hospitals with CT	60	124674	100.0
Stroke-Ready Hospitals	5	2291	1.84
Stroke-Ready Hospitals	10	22250	17.85
Stroke-Ready Hospitals	15	42159	33.82
Stroke-Ready Hospitals	20	64059	51.38
Stroke-Ready Hospitals	25	77291	61.99
Stroke-Ready Hospitals	30	94175	75.54
Stroke-Ready Hospitals	35	104452	83.78
Stroke-Ready Hospitals	40	115585	92.71
Stroke-Ready Hospitals	45	121500	97.45
Stroke-Ready Hospitals	50	124082	99.53
Stroke-Ready Hospitals	55	124644	99.98
Stroke-Ready Hospitals	60	124674	100.0
Stroke Units	5	2291	1.84
Stroke Units	10	22250	17.85
Stroke Units	15	42159	33.82
Stroke Units	20	64059	51.38
Stroke Units	25	77291	61.99
Stroke Units	30	94175	75.54
Stroke Units	35	104452	83.78
Stroke Units	40	115585	92.71
Stroke Units	45	121499	97.45
Stroke Units	50	124081	99.52
Stroke Units	55	124644	99.98
Stroke Units	60	124674	100.0

Population Coverage - Stadtkreis Heilbronn (Baden-Württemberg)

Condition	Drive Time (min)	Covered Population	Coverage (%)
Hospitals with CT	5	13837	11.24
Hospitals with CT	10	59169	48.07
Hospitals with CT	15	120764	98.11
Hospitals with CT	20	122968	99.9
Hospitals with CT	25	123086	100.0
Hospitals with CT	30	123086	100.0
Hospitals with CT	35	123086	100.0
Hospitals with CT	40	123086	100.0
Hospitals with CT	45	123086	100.0
Hospitals with CT	50	123086	100.0
Hospitals with CT	55	123086	100.0
Hospitals with CT	60	123086	100.0
Stroke-Ready Hospitals	5	15156	12.31
Stroke-Ready Hospitals	10	60332	49.02
Stroke-Ready Hospitals	15	121047	98.34
Stroke-Ready Hospitals	20	122974	99.91
Stroke-Ready Hospitals	25	123086	100.0
Stroke-Ready Hospitals	30	123086	100.0
Stroke-Ready Hospitals	35	123086	100.0
Stroke-Ready Hospitals	40	123086	100.0
Stroke-Ready Hospitals	45	123086	100.0
Stroke-Ready Hospitals	50	123086	100.0
Stroke-Ready Hospitals	55	123086	100.0
Stroke-Ready Hospitals	60	123086	100.0
Stroke Units	5	15156	12.31
Stroke Units	10	60332	49.02
Stroke Units	15	121047	98.34
Stroke Units	20	122974	99.91
Stroke Units	25	123086	100.0
Stroke Units	30	123086	100.0
Stroke Units	35	123086	100.0
Stroke Units	40	123086	100.0
Stroke Units	45	123086	100.0
Stroke Units	50	123086	100.0
Stroke Units	55	123086	100.0
Stroke Units	60	123086	100.0

Population Coverage - Landkreis Schwandorf (Bayern)

Condition	Drive Time (min)	Covered Population	Coverage (%)
Hospitals with CT	5	32148	21.6
Hospitals with CT	10	74524	50.07
Hospitals with CT	15	120386	80.88
Hospitals with CT	20	144931	97.37
Hospitals with CT	25	148744	99.94
Hospitals with CT	30	148830	99.99
Hospitals with CT	35	148832	100.0
Hospitals with CT	40	148833	100.0
Hospitals with CT	45	148835	100.0
Hospitals with CT	50	148835	100.0
Hospitals with CT	55	148833	100.0
Hospitals with CT	60	148833	100.0
Stroke-Ready Hospitals	5	19140	12.86
Stroke-Ready Hospitals	10	46199	31.04
Stroke-Ready Hospitals	15	71187	47.83
Stroke-Ready Hospitals	20	104790	70.41
Stroke-Ready Hospitals	25	120480	80.95
Stroke-Ready Hospitals	30	134177	90.15
Stroke-Ready Hospitals	35	141816	95.28
Stroke-Ready Hospitals	40	146267	98.27
Stroke-Ready Hospitals	45	148768	99.95
Stroke-Ready Hospitals	50	148833	100.0
Stroke-Ready Hospitals	55	148833	100.0
Stroke-Ready Hospitals	60	148836	100.0
Stroke Units	5	19140	12.86
Stroke Units	10	46199	31.04
Stroke Units	15	71187	47.83
Stroke Units	20	104790	70.41
Stroke Units	25	120480	80.95
Stroke Units	30	134177	90.15
Stroke Units	35	141816	95.28
Stroke Units	40	146267	98.27
Stroke Units	45	148768	99.95
Stroke Units	50	148833	100.0
Stroke Units	55	148833	100.0
Stroke Units	60	148836	100.0

Population Coverage - Landkreis Unterallgäu (Bayern)

Condition	Drive Time (min)	Covered Population	Coverage (%)
Hospitals with CT	5	18194	12.79
Hospitals with CT	10	42282	29.72
Hospitals with CT	15	92986	65.36
Hospitals with CT	20	129249	90.85
Hospitals with CT	25	140893	99.03
Hospitals with CT	30	142272	100.0
Hospitals with CT	35	142272	100.0
Hospitals with CT	40	142272	100.0
Hospitals with CT	45	142272	100.0
Hospitals with CT	50	142272	100.0
Hospitals with CT	55	142272	100.0
Hospitals with CT	60	142272	100.0
Stroke-Ready Hospitals	5	126	0.09
Stroke-Ready Hospitals	10	9421	6.62
Stroke-Ready Hospitals	15	30050	21.12
Stroke-Ready Hospitals	20	79729	56.04
Stroke-Ready Hospitals	25	111413	78.31
Stroke-Ready Hospitals	30	127644	89.72
Stroke-Ready Hospitals	35	136748	96.12
Stroke-Ready Hospitals	40	141238	99.27
Stroke-Ready Hospitals	45	142272	100.0
Stroke-Ready Hospitals	50	142272	100.0
Stroke-Ready Hospitals	55	142272	100.0
Stroke-Ready Hospitals	60	142272	100.0
Stroke Units	5	126	0.09
Stroke Units	10	9421	6.62
Stroke Units	15	28139	19.78
Stroke Units	20	67191	47.23
Stroke Units	25	100721	70.8
Stroke Units	30	123795	87.01
Stroke Units	35	134580	94.59
Stroke Units	40	140172	98.52
Stroke Units	45	142203	99.95
Stroke Units	50	142272	100.0
Stroke Units	55	142272	100.0
Stroke Units	60	142272	100.0

Population Coverage - Kreisfreie Stadt Frankfurt (Oder) (Brandenburg)

Condition	Drive Time (min)	Covered Population	Coverage (%)
Hospitals with CT	5	30237	53.88
Hospitals with CT	10	51981	92.63
Hospitals with CT	15	55417	98.76
Hospitals with CT	20	55761	99.37
Hospitals with CT	25	55799	99.44
Hospitals with CT	30	55799	99.44
Hospitals with CT	35	55896	99.61
Hospitals with CT	40	55944	99.69
Hospitals with CT	45	55944	99.69
Hospitals with CT	50	55945	99.7
Hospitals with CT	55	55846	99.52
Hospitals with CT	60	55859	99.54
Stroke-Ready Hospitals	5	2961	5.28
Stroke-Ready Hospitals	10	13673	24.37
Stroke-Ready Hospitals	15	47720	85.04
Stroke-Ready Hospitals	20	55289	98.53
Stroke-Ready Hospitals	25	55694	99.25
Stroke-Ready Hospitals	30	55694	99.25
Stroke-Ready Hospitals	35	55695	99.25
Stroke-Ready Hospitals	40	55715	99.29
Stroke-Ready Hospitals	45	55715	99.29
Stroke-Ready Hospitals	50	55715	99.29
Stroke-Ready Hospitals	55	55678	99.22
Stroke-Ready Hospitals	60	55693	99.25
Stroke Units	5	2961	5.28
Stroke Units	10	13673	24.37
Stroke Units	15	47720	85.04
Stroke Units	20	55289	98.53
Stroke Units	25	55694	99.25
Stroke Units	30	55694	99.25
Stroke Units	35	55695	99.25
Stroke Units	40	55695	99.25
Stroke Units	45	55695	99.25
Stroke Units	50	55695	99.25
Stroke Units	55	55673	99.21
Stroke Units	60	55688	99.24

Population Coverage - Landkreis Dahme-Spreewald (Brandenburg)

Condition	Drive Time (min)	Covered Population	Coverage (%)
Hospitals with CT	5	21827	13.77
Hospitals with CT	10	59839	37.76
Hospitals with CT	15	105395	66.51
Hospitals with CT	20	134682	84.99
Hospitals with CT	25	147093	92.82
Hospitals with CT	30	154001	97.18
Hospitals with CT	35	157496	99.39
Hospitals with CT	40	158160	99.81
Hospitals with CT	45	158379	99.94
Hospitals with CT	50	158469	100.0
Hospitals with CT	55	158469	100.0
Hospitals with CT	60	158469	100.0
Stroke-Ready Hospitals	5	7962	5.02
Stroke-Ready Hospitals	10	15057	9.5
Stroke-Ready Hospitals	15	25742	16.24
Stroke-Ready Hospitals	20	46390	29.27
Stroke-Ready Hospitals	25	95004	59.95
Stroke-Ready Hospitals	30	141805	89.48
Stroke-Ready Hospitals	35	153793	97.05
Stroke-Ready Hospitals	40	156094	98.5
Stroke-Ready Hospitals	45	157644	99.48
Stroke-Ready Hospitals	50	158339	99.92
Stroke-Ready Hospitals	55	158469	100.0
Stroke-Ready Hospitals	60	158469	100.0
Stroke Units	5	7230	4.56
Stroke Units	10	12672	8.0
Stroke Units	15	19804	12.5
Stroke Units	20	29376	18.54
Stroke Units	25	51620	32.57
Stroke Units	30	113285	71.49
Stroke Units	35	143258	90.4
Stroke Units	40	154242	97.33
Stroke Units	45	157002	99.07
Stroke Units	50	158057	99.74
Stroke Units	55	158161	99.81
Stroke Units	60	158469	100.0

Population Coverage - Landkreis Leipzig (Sachsen)

Condition	Drive Time (min)	Covered Population	Coverage (%)
Hospitals with CT	5	38572	15.1
Hospitals with CT	10	90869	35.58
Hospitals with CT	15	173655	67.99
Hospitals with CT	20	239153	93.63
Hospitals with CT	25	255097	99.87
Hospitals with CT	30	255305	99.95
Hospitals with CT	35	255425	100.0
Hospitals with CT	40	255425	100.0
Hospitals with CT	45	255425	100.0
Hospitals with CT	50	255425	100.0
Hospitals with CT	55	255425	100.0
Hospitals with CT	60	255425	100.0
Stroke-Ready Hospitals	5	7340	2.87
Stroke-Ready Hospitals	10	18572	7.27
Stroke-Ready Hospitals	15	55031	21.54
Stroke-Ready Hospitals	20	126452	49.51
Stroke-Ready Hospitals	25	197197	77.2
Stroke-Ready Hospitals	30	235699	92.28
Stroke-Ready Hospitals	35	253483	99.24
Stroke-Ready Hospitals	40	255391	99.99
Stroke-Ready Hospitals	45	255425	100.0
Stroke-Ready Hospitals	50	255425	100.0
Stroke-Ready Hospitals	55	255425	100.0
Stroke-Ready Hospitals	60	255425	100.0
Stroke Units	5	6088	2.38
Stroke Units	10	18045	7.07
Stroke Units	15	53780	21.06
Stroke Units	20	126231	49.42
Stroke Units	25	196634	76.98
Stroke Units	30	234653	91.87
Stroke Units	35	252817	98.98
Stroke Units	40	255186	99.91
Stroke Units	45	255425	100.0
Stroke Units	50	255425	100.0
Stroke Units	55	255425	100.0
Stroke Units	60	255425	100.0

Population Coverage - Kreisfreie Stadt Magdeburg (Sachsen-Anhalt)

Condition	Drive Time (min)	Covered Population	Coverage (%)
Hospitals with CT	5	76403	30.65
Hospitals with CT	10	201730	80.92
Hospitals with CT	15	247285	99.19
Hospitals with CT	20	248500	99.68
Hospitals with CT	25	249300	100.0
Hospitals with CT	30	249300	100.0
Hospitals with CT	35	249300	100.0
Hospitals with CT	40	249300	100.0
Hospitals with CT	45	249300	100.0
Hospitals with CT	50	249300	100.0
Hospitals with CT	55	249300	100.0
Hospitals with CT	60	249300	100.0
Stroke-Ready Hospitals	5	22074	8.85
Stroke-Ready Hospitals	10	130237	52.24
Stroke-Ready Hospitals	15	213769	85.75
Stroke-Ready Hospitals	20	242076	97.1
Stroke-Ready Hospitals	25	246564	98.9
Stroke-Ready Hospitals	30	248425	99.65
Stroke-Ready Hospitals	35	249285	99.99
Stroke-Ready Hospitals	40	249300	100.0
Stroke-Ready Hospitals	45	249300	100.0
Stroke-Ready Hospitals	50	249300	100.0
Stroke-Ready Hospitals	55	249300	100.0
Stroke-Ready Hospitals	60	249300	100.0
Stroke Units	5	22074	8.85
Stroke Units	10	130237	52.24
Stroke Units	15	213769	85.75
Stroke Units	20	242076	97.1
Stroke Units	25	246564	98.9
Stroke Units	30	248425	99.65
Stroke Units	35	249285	99.99
Stroke Units	40	249300	100.0
Stroke Units	45	249300	100.0
Stroke Units	50	249300	100.0
Stroke Units	55	249300	100.0
Stroke Units	60	249300	100.0

Population Coverage - Landkreis Wartburgkreis (Thüringen)

Condition	Drive Time (min)	Covered Population	Coverage (%)
Hospitals with CT	5	21347	15.46
Hospitals with CT	10	55326	40.07
Hospitals with CT	15	78382	56.77
Hospitals with CT	20	102971	74.58
Hospitals with CT	25	123069	89.13
Hospitals with CT	30	134743	97.59
Hospitals with CT	35	138054	99.99
Hospitals with CT	40	138067	99.99
Hospitals with CT	45	138075	100.0
Hospitals with CT	50	138075	100.0
Hospitals with CT	55	138075	100.0
Hospitals with CT	60	138075	100.0
Stroke-Ready Hospitals	5	22939	16.61
Stroke-Ready Hospitals	10	49013	35.5
Stroke-Ready Hospitals	15	67302	48.74
Stroke-Ready Hospitals	20	92353	66.89
Stroke-Ready Hospitals	25	117493	85.09
Stroke-Ready Hospitals	30	129501	93.79
Stroke-Ready Hospitals	35	135761	98.32
Stroke-Ready Hospitals	40	138066	99.99
Stroke-Ready Hospitals	45	138075	100.0
Stroke-Ready Hospitals	50	138075	100.0
Stroke-Ready Hospitals	55	138075	100.0
Stroke-Ready Hospitals	60	138075	100.0
Stroke Units	5	22939	16.61
Stroke Units	10	49013	35.5
Stroke Units	15	66697	48.31
Stroke Units	20	90615	65.63
Stroke Units	25	114229	82.73
Stroke Units	30	129284	93.63
Stroke Units	35	135727	98.3
Stroke Units	40	138066	99.99
Stroke Units	45	138075	100.0
Stroke Units	50	138075	100.0
Stroke Units	55	138075	100.0
Stroke Units	60	138075	100.0

Population Coverage - Landkreis Unstrut-Hainich-Kreis (Thüringen)

Condition	Drive Time (min)	Covered Population	Coverage (%)
Hospitals with CT	5	30863	34.19
Hospitals with CT	10	58267	64.55
Hospitals with CT	15	73391	81.3
Hospitals with CT	20	85401	94.61
Hospitals with CT	25	90136	99.85
Hospitals with CT	30	90216	99.94
Hospitals with CT	35	90240	99.97
Hospitals with CT	40	90269	100.0
Hospitals with CT	45	90269	100.0
Hospitals with CT	50	90269	100.0
Hospitals with CT	55	90269	100.0
Hospitals with CT	60	90269	100.0
Stroke-Ready Hospitals	5	9195	10.19
Stroke-Ready Hospitals	10	20047	22.21
Stroke-Ready Hospitals	15	45177	50.05
Stroke-Ready Hospitals	20	70884	78.53
Stroke-Ready Hospitals	25	82256	91.12
Stroke-Ready Hospitals	30	89333	98.96
Stroke-Ready Hospitals	35	90160	99.88
Stroke-Ready Hospitals	40	90211	99.94
Stroke-Ready Hospitals	45	90269	100.0
Stroke-Ready Hospitals	50	90269	100.0
Stroke-Ready Hospitals	55	90269	100.0
Stroke-Ready Hospitals	60	90269	100.0
Stroke Units	5	346	0.38
Stroke Units	10	4846	5.37
Stroke Units	15	23894	26.47
Stroke Units	20	41002	45.42
Stroke Units	25	49020	54.3
Stroke Units	30	61119	67.71
Stroke Units	35	81322	90.09
Stroke Units	40	87159	96.55
Stroke Units	45	90124	99.84
Stroke Units	50	90269	100.0
Stroke Units	55	90269	100.0
Stroke Units	60	90269	100.0

Population Coverage - Kreisfreie Stadt Bielefeld (Nordrhein-Westfalen)

Condition	Drive Time (min)	Covered Population	Coverage (%)
Hospitals with CT	5	112513	32.18
Hospitals with CT	10	238409	68.19
Hospitals with CT	15	342330	97.91
Hospitals with CT	20	349622	100.0
Hospitals with CT	25	349622	100.0
Hospitals with CT	30	349622	100.0
Hospitals with CT	35	349622	100.0
Hospitals with CT	40	349622	100.0
Hospitals with CT	45	349622	100.0
Hospitals with CT	50	349622	100.0
Hospitals with CT	55	349622	100.0
Hospitals with CT	60	349622	100.0
Stroke-Ready Hospitals	5	40690	11.64
Stroke-Ready Hospitals	10	156319	44.71
Stroke-Ready Hospitals	15	254151	72.69
Stroke-Ready Hospitals	20	332229	95.02
Stroke-Ready Hospitals	25	349622	100.0
Stroke-Ready Hospitals	30	349622	100.0
Stroke-Ready Hospitals	35	349622	100.0
Stroke-Ready Hospitals	40	349622	100.0
Stroke-Ready Hospitals	45	349622	100.0
Stroke-Ready Hospitals	50	349622	100.0
Stroke-Ready Hospitals	55	349622	100.0
Stroke-Ready Hospitals	60	349622	100.0
Stroke Units	5	10628	3.04
Stroke Units	10	77862	22.27
Stroke Units	15	198420	56.75
Stroke Units	20	301390	86.2
Stroke Units	25	349238	99.89
Stroke Units	30	349622	100.0
Stroke Units	35	349622	100.0
Stroke Units	40	349622	100.0
Stroke Units	45	349622	100.0
Stroke Units	50	349622	100.0
Stroke Units	55	349622	100.0
Stroke Units	60	349622	100.0

Population Coverage - Kreisfreie Stadt Bochum (Nordrhein-Westfalen)

Condition	Drive Time (min)	Covered Population	Coverage (%)
Hospitals with CT	5	131617	34.04
Hospitals with CT	10	355140	91.84
Hospitals with CT	15	386703	100.0
Hospitals with CT	20	386703	100.0
Hospitals with CT	25	386703	100.0
Hospitals with CT	30	386703	100.0
Hospitals with CT	35	386703	100.0
Hospitals with CT	40	386703	100.0
Hospitals with CT	45	386703	100.0
Hospitals with CT	50	386703	100.0
Hospitals with CT	55	386703	100.0
Hospitals with CT	60	386703	100.0
Stroke-Ready Hospitals	5	81308	21.03
Stroke-Ready Hospitals	10	298841	77.28
Stroke-Ready Hospitals	15	380153	98.31
Stroke-Ready Hospitals	20	386703	100.0
Stroke-Ready Hospitals	25	386703	100.0
Stroke-Ready Hospitals	30	386703	100.0
Stroke-Ready Hospitals	35	386703	100.0
Stroke-Ready Hospitals	40	386703	100.0
Stroke-Ready Hospitals	45	386703	100.0
Stroke-Ready Hospitals	50	386703	100.0
Stroke-Ready Hospitals	55	386703	100.0
Stroke-Ready Hospitals	60	386703	100.0
Stroke Units	5	51451	13.31
Stroke Units	10	248861	64.35
Stroke Units	15	363121	93.9
Stroke Units	20	386684	99.99
Stroke Units	25	386703	100.0
Stroke Units	30	386703	100.0
Stroke Units	35	386703	100.0
Stroke Units	40	386703	100.0
Stroke Units	45	386703	100.0
Stroke Units	50	386703	100.0
Stroke Units	55	386703	100.0
Stroke Units	60	386703	100.0

Population Coverage - Landkreis Fulda (Hessen)

Condition	Drive Time (min)	Covered Population	Coverage (%)
Hospitals with CT	5	49315	22.39
Hospitals with CT	10	107956	49.01
Hospitals with CT	15	155830	70.75
Hospitals with CT	20	190301	86.4
Hospitals with CT	25	205327	93.22
Hospitals with CT	30	217006	98.52
Hospitals with CT	35	220204	99.97
Hospitals with CT	40	220259	100.0
Hospitals with CT	45	220259	100.0
Hospitals with CT	50	220259	100.0
Hospitals with CT	55	220259	100.0
Hospitals with CT	60	220259	100.0
Stroke-Ready Hospitals	5	21780	9.89
Stroke-Ready Hospitals	10	65365	29.68
Stroke-Ready Hospitals	15	105896	48.08
Stroke-Ready Hospitals	20	145035	65.85
Stroke-Ready Hospitals	25	178361	80.98
Stroke-Ready Hospitals	30	207884	94.38
Stroke-Ready Hospitals	35	218771	99.32
Stroke-Ready Hospitals	40	220259	100.0
Stroke-Ready Hospitals	45	220259	100.0
Stroke-Ready Hospitals	50	220259	100.0
Stroke-Ready Hospitals	55	220259	100.0
Stroke-Ready Hospitals	60	220259	100.0
Stroke Units	5	21780	9.89
Stroke Units	10	65365	29.68
Stroke Units	15	105896	48.08
Stroke Units	20	145035	65.85
Stroke Units	25	178361	80.98
Stroke Units	30	207884	94.38
Stroke Units	35	218771	99.32
Stroke Units	40	220259	100.0
Stroke Units	45	220259	100.0
Stroke Units	50	220259	100.0
Stroke Units	55	220259	100.0
Stroke Units	60	220259	100.0

Population Coverage - Landkreis Rastatt (Baden-Württemberg)

Condition	Drive Time (min)	Covered Population	Coverage (%)
Hospitals with CT	5	37168	16.36
Hospitals with CT	10	96649	42.54
Hospitals with CT	15	169721	74.71
Hospitals with CT	20	204479	90.0
Hospitals with CT	25	223891	98.55
Hospitals with CT	30	226742	99.8
Hospitals with CT	35	227044	99.94
Hospitals with CT	40	227160	99.99
Hospitals with CT	45	227168	99.99
Hospitals with CT	50	227168	99.99
Hospitals with CT	55	227145	99.98
Hospitals with CT	60	227169	99.99
Stroke-Ready Hospitals	5	17891	7.88
Stroke-Ready Hospitals	10	41747	18.38
Stroke-Ready Hospitals	15	75334	33.16
Stroke-Ready Hospitals	20	107250	47.21
Stroke-Ready Hospitals	25	150826	66.39
Stroke-Ready Hospitals	30	185072	81.46
Stroke-Ready Hospitals	35	212855	93.69
Stroke-Ready Hospitals	40	223624	98.43
Stroke-Ready Hospitals	45	226729	99.8
Stroke-Ready Hospitals	50	227102	99.96
Stroke-Ready Hospitals	55	227125	99.97
Stroke-Ready Hospitals	60	227133	99.98
Stroke Units	5	17891	7.88
Stroke Units	10	41747	18.38
Stroke Units	15	75334	33.16
Stroke Units	20	107250	47.21
Stroke Units	25	150826	66.39
Stroke Units	30	184953	81.41
Stroke Units	35	212617	93.59
Stroke Units	40	221045	97.3
Stroke Units	45	223118	98.21
Stroke Units	50	226377	99.64
Stroke Units	55	226711	99.79
Stroke Units	60	227012	99.92

Population Coverage - Landkreis Breisgau-Hochschwarzwald (Baden-Württemberg)

Condition	Drive Time (min)	Covered Population	Coverage (%)
Hospitals with CT	5	17544	6.86
Hospitals with CT	10	55383	21.65
Hospitals with CT	15	120949	47.28
Hospitals with CT	20	189100	73.93
Hospitals with CT	25	236681	92.53
Hospitals with CT	30	249998	97.73
Hospitals with CT	35	254526	99.5
Hospitals with CT	40	255472	99.87
Hospitals with CT	45	255609	99.93
Hospitals with CT	50	255798	100.0
Hospitals with CT	55	255773	99.99
Hospitals with CT	60	255797	100.0
Stroke-Ready Hospitals	5	10332	4.04
Stroke-Ready Hospitals	10	38390	15.01
Stroke-Ready Hospitals	15	78130	30.54
Stroke-Ready Hospitals	20	139758	54.64
Stroke-Ready Hospitals	25	201235	78.67
Stroke-Ready Hospitals	30	232675	90.96
Stroke-Ready Hospitals	35	250522	97.94
Stroke-Ready Hospitals	40	254965	99.67
Stroke-Ready Hospitals	45	255442	99.86
Stroke-Ready Hospitals	50	255786	99.99
Stroke-Ready Hospitals	55	255767	99.99
Stroke-Ready Hospitals	60	255777	99.99
Stroke Units	5	0	0.0
Stroke Units	10	276	0.11
Stroke Units	15	16903	6.61
Stroke Units	20	54007	21.11
Stroke Units	25	95817	37.46
Stroke Units	30	139838	54.67
Stroke Units	35	188403	73.65
Stroke Units	40	218466	85.41
Stroke Units	45	240848	94.15
Stroke Units	50	248765	97.25
Stroke Units	55	252694	98.79
Stroke Units	60	254857	99.63

Population Coverage - Landkreis Ebersberg (Bayern)

Condition	Drive Time (min)	Covered Population	Coverage (%)
Hospitals with CT	5	8425	6.11
Hospitals with CT	10	32805	23.77
Hospitals with CT	15	84754	61.42
Hospitals with CT	20	128048	92.8
Hospitals with CT	25	136328	98.8
Hospitals with CT	30	137985	100.0
Hospitals with CT	35	137985	100.0
Hospitals with CT	40	137985	100.0
Hospitals with CT	45	137985	100.0
Hospitals with CT	50	137985	100.0
Hospitals with CT	55	137985	100.0
Hospitals with CT	60	137985	100.0
Stroke-Ready Hospitals	5	7796	5.65
Stroke-Ready Hospitals	10	27424	19.88
Stroke-Ready Hospitals	15	81170	58.83
Stroke-Ready Hospitals	20	125439	90.91
Stroke-Ready Hospitals	25	135123	97.93
Stroke-Ready Hospitals	30	137981	100.0
Stroke-Ready Hospitals	35	137985	100.0
Stroke-Ready Hospitals	40	137985	100.0
Stroke-Ready Hospitals	45	137985	100.0
Stroke-Ready Hospitals	50	137985	100.0
Stroke-Ready Hospitals	55	137985	100.0
Stroke-Ready Hospitals	60	137985	100.0
Stroke Units	5	7796	5.65
Stroke Units	10	27424	19.88
Stroke Units	15	81170	58.83
Stroke Units	20	125439	90.91
Stroke Units	25	135123	97.93
Stroke Units	30	137981	100.0
Stroke Units	35	137985	100.0
Stroke Units	40	137985	100.0
Stroke Units	45	137985	100.0
Stroke Units	50	137985	100.0
Stroke Units	55	137985	100.0
Stroke Units	60	137985	100.0

Population Coverage - Landkreis Rottal-Inn (Bayern)

Condition	Drive Time (min)	Covered Population	Coverage (%)
Hospitals with CT	5	15760	12.92
Hospitals with CT	10	33616	27.57
Hospitals with CT	15	63982	52.47
Hospitals with CT	20	99472	81.57
Hospitals with CT	25	119679	98.14
Hospitals with CT	30	121936	99.99
Hospitals with CT	35	121941	99.99
Hospitals with CT	40	121941	99.99
Hospitals with CT	45	121941	99.99
Hospitals with CT	50	121941	99.99
Hospitals with CT	55	121930	99.98
Hospitals with CT	60	121941	99.99
Stroke-Ready Hospitals	5	7372	6.05
Stroke-Ready Hospitals	10	16548	13.57
Stroke-Ready Hospitals	15	35413	29.04
Stroke-Ready Hospitals	20	73216	60.04
Stroke-Ready Hospitals	25	106261	87.14
Stroke-Ready Hospitals	30	116142	95.24
Stroke-Ready Hospitals	35	120796	99.05
Stroke-Ready Hospitals	40	121937	99.99
Stroke-Ready Hospitals	45	121939	99.99
Stroke-Ready Hospitals	50	121939	99.99
Stroke-Ready Hospitals	55	121927	99.98
Stroke-Ready Hospitals	60	121936	99.99
Stroke Units	5	7372	6.05
Stroke Units	10	15527	12.73
Stroke Units	15	30387	24.92
Stroke Units	20	51322	42.08
Stroke Units	25	77781	63.78
Stroke Units	30	101905	83.56
Stroke Units	35	115853	95.0
Stroke Units	40	121688	99.79
Stroke Units	45	121939	99.99
Stroke Units	50	121939	99.99
Stroke Units	55	121926	99.98
Stroke Units	60	121936	99.99

Population Coverage - Landkreis Bamberg (Bayern)

Condition	Drive Time (min)	Covered Population	Coverage (%)
Hospitals with CT	5	6077	4.03
Hospitals with CT	10	27381	18.16
Hospitals with CT	15	87384	57.95
Hospitals with CT	20	138797	92.04
Hospitals with CT	25	149936	99.43
Hospitals with CT	30	150798	100.0
Hospitals with CT	35	150798	100.0
Hospitals with CT	40	150798	100.0
Hospitals with CT	45	150798	100.0
Hospitals with CT	50	150798	100.0
Hospitals with CT	55	150798	100.0
Hospitals with CT	60	150798	100.0
Stroke-Ready Hospitals	5	0	0.0
Stroke-Ready Hospitals	10	2999	1.99
Stroke-Ready Hospitals	15	12058	8.0
Stroke-Ready Hospitals	20	50700	33.62
Stroke-Ready Hospitals	25	105303	69.83
Stroke-Ready Hospitals	30	135536	89.88
Stroke-Ready Hospitals	35	145105	96.23
Stroke-Ready Hospitals	40	150685	99.93
Stroke-Ready Hospitals	45	150798	100.0
Stroke-Ready Hospitals	50	150798	100.0
Stroke-Ready Hospitals	55	150798	100.0
Stroke-Ready Hospitals	60	150798	100.0
Stroke Units	5	0	0.0
Stroke Units	10	2999	1.99
Stroke Units	15	12058	8.0
Stroke Units	20	50700	33.62
Stroke Units	25	105303	69.83
Stroke Units	30	135536	89.88
Stroke Units	35	144180	95.61
Stroke Units	40	150684	99.92
Stroke Units	45	150798	100.0
Stroke Units	50	150798	100.0
Stroke Units	55	150798	100.0
Stroke Units	60	150798	100.0

Population Coverage - Landkreis Schweinfurt (Bayern)

Condition	Drive Time (min)	Covered Population	Coverage (%)
Hospitals with CT	5	11384	9.73
Hospitals with CT	10	46626	39.87
Hospitals with CT	15	92748	79.31
Hospitals with CT	20	111249	95.13
Hospitals with CT	25	114190	97.64
Hospitals with CT	30	116911	99.97
Hospitals with CT	35	116947	100.0
Hospitals with CT	40	116949	100.0
Hospitals with CT	45	116949	100.0
Hospitals with CT	50	116949	100.0
Hospitals with CT	55	116949	100.0
Hospitals with CT	60	116949	100.0
Stroke-Ready Hospitals	5	0	0.0
Stroke-Ready Hospitals	10	6340	5.42
Stroke-Ready Hospitals	15	31523	26.95
Stroke-Ready Hospitals	20	73523	62.87
Stroke-Ready Hospitals	25	106373	90.96
Stroke-Ready Hospitals	30	116178	99.34
Stroke-Ready Hospitals	35	116947	100.0
Stroke-Ready Hospitals	40	116948	100.0
Stroke-Ready Hospitals	45	116949	100.0
Stroke-Ready Hospitals	50	116949	100.0
Stroke-Ready Hospitals	55	116949	100.0
Stroke-Ready Hospitals	60	116949	100.0
Stroke Units	5	0	0.0
Stroke Units	10	6340	5.42
Stroke Units	15	31523	26.95
Stroke Units	20	73523	62.87
Stroke Units	25	106373	90.96
Stroke Units	30	116178	99.34
Stroke Units	35	116947	100.0
Stroke Units	40	116948	100.0
Stroke Units	45	116949	100.0
Stroke Units	50	116949	100.0
Stroke Units	55	116949	100.0
Stroke Units	60	116949	100.0

Population Coverage - Landkreis St. Wendel (Saarland)

Condition	Drive Time (min)	Covered Population	Coverage (%)
Hospitals with CT	5	7087	8.31
Hospitals with CT	10	21032	24.66
Hospitals with CT	15	47351	55.52
Hospitals with CT	20	80966	94.93
Hospitals with CT	25	85260	99.97
Hospitals with CT	30	85286	100.0
Hospitals with CT	35	85286	100.0
Hospitals with CT	40	85286	100.0
Hospitals with CT	45	85286	100.0
Hospitals with CT	50	85286	100.0
Hospitals with CT	55	85286	100.0
Hospitals with CT	60	85286	100.0
Stroke-Ready Hospitals	5	6164	7.23
Stroke-Ready Hospitals	10	19220	22.54
Stroke-Ready Hospitals	15	33180	38.9
Stroke-Ready Hospitals	20	56034	65.7
Stroke-Ready Hospitals	25	69602	81.61
Stroke-Ready Hospitals	30	81082	95.07
Stroke-Ready Hospitals	35	85070	99.75
Stroke-Ready Hospitals	40	85273	99.98
Stroke-Ready Hospitals	45	85286	100.0
Stroke-Ready Hospitals	50	85286	100.0
Stroke-Ready Hospitals	55	85286	100.0
Stroke-Ready Hospitals	60	85286	100.0
Stroke Units	5	6164	7.23
Stroke Units	10	19220	22.54
Stroke Units	15	33180	38.9
Stroke Units	20	56034	65.7
Stroke Units	25	69602	81.61
Stroke Units	30	81082	95.07
Stroke Units	35	85070	99.75
Stroke Units	40	85273	99.98
Stroke Units	45	85286	100.0
Stroke Units	50	85286	100.0
Stroke Units	55	85286	100.0
Stroke Units	60	85286	100.0

Population Coverage - Landkreis Harz (Sachsen-Anhalt)

Condition	Drive Time (min)	Covered Population	Coverage (%)
Hospitals with CT	5	49035	24.5
Hospitals with CT	10	97156	48.55
Hospitals with CT	15	136808	68.37
Hospitals with CT	20	172115	86.01
Hospitals with CT	25	186466	93.19
Hospitals with CT	30	194869	97.38
Hospitals with CT	35	199226	99.56
Hospitals with CT	40	199940	99.92
Hospitals with CT	45	200102	100.0
Hospitals with CT	50	200102	100.0
Hospitals with CT	55	200102	100.0
Hospitals with CT	60	200102	100.0
Stroke-Ready Hospitals	5	15396	7.69
Stroke-Ready Hospitals	10	28835	14.41
Stroke-Ready Hospitals	15	33933	16.96
Stroke-Ready Hospitals	20	53393	26.68
Stroke-Ready Hospitals	25	77346	38.65
Stroke-Ready Hospitals	30	112294	56.12
Stroke-Ready Hospitals	35	160602	80.26
Stroke-Ready Hospitals	40	186776	93.34
Stroke-Ready Hospitals	45	198644	99.27
Stroke-Ready Hospitals	50	199896	99.9
Stroke-Ready Hospitals	55	200000	99.95
Stroke-Ready Hospitals	60	200102	100.0
Stroke Units	5	15396	7.69
Stroke Units	10	28835	14.41
Stroke Units	15	33933	16.96
Stroke Units	20	53393	26.68
Stroke Units	25	77346	38.65
Stroke Units	30	112285	56.11
Stroke Units	35	159061	79.49
Stroke Units	40	182724	91.32
Stroke Units	45	192335	96.12
Stroke Units	50	196867	98.38
Stroke Units	55	199622	99.76
Stroke Units	60	200102	100.0

Population Coverage - Kreisfreie Stadt Gera (Thüringen)

Condition	Drive Time (min)	Covered Population	Coverage (%)
Hospitals with CT	5	8218	7.49
Hospitals with CT	10	53517	48.8
Hospitals with CT	15	92025	83.92
Hospitals with CT	20	107376	97.92
Hospitals with CT	25	109643	99.99
Hospitals with CT	30	109657	100.0
Hospitals with CT	35	109657	100.0
Hospitals with CT	40	109657	100.0
Hospitals with CT	45	109657	100.0
Hospitals with CT	50	109657	100.0
Hospitals with CT	55	109657	100.0
Hospitals with CT	60	109657	100.0
Stroke-Ready Hospitals	5	4472	4.08
Stroke-Ready Hospitals	10	40370	36.81
Stroke-Ready Hospitals	15	86234	78.64
Stroke-Ready Hospitals	20	99654	90.88
Stroke-Ready Hospitals	25	105574	96.28
Stroke-Ready Hospitals	30	109079	99.47
Stroke-Ready Hospitals	35	109599	99.95
Stroke-Ready Hospitals	40	109657	100.0
Stroke-Ready Hospitals	45	109657	100.0
Stroke-Ready Hospitals	50	109657	100.0
Stroke-Ready Hospitals	55	109657	100.0
Stroke-Ready Hospitals	60	109657	100.0
Stroke Units	5	4472	4.08
Stroke Units	10	40370	36.81
Stroke Units	15	86234	78.64
Stroke Units	20	99654	90.88
Stroke Units	25	105574	96.28
Stroke Units	30	109079	99.47
Stroke Units	35	109543	99.9
Stroke Units	40	109657	100.0
Stroke Units	45	109657	100.0
Stroke Units	50	109657	100.0
Stroke Units	55	109657	100.0
Stroke Units	60	109657	100.0

Population Coverage - Kreisfreie Stadt Weimar (Thüringen)

Condition	Drive Time (min)	Covered Population	Coverage (%)
Hospitals with CT	5	10084	17.48
Hospitals with CT	10	36997	64.12
Hospitals with CT	15	53173	92.16
Hospitals with CT	20	57345	99.39
Hospitals with CT	25	57698	100.0
Hospitals with CT	30	57698	100.0
Hospitals with CT	35	57698	100.0
Hospitals with CT	40	57698	100.0
Hospitals with CT	45	57698	100.0
Hospitals with CT	50	57698	100.0
Hospitals with CT	55	57698	100.0
Hospitals with CT	60	57698	100.0
Stroke-Ready Hospitals	5	10137	17.57
Stroke-Ready Hospitals	10	37189	64.46
Stroke-Ready Hospitals	15	53142	92.1
Stroke-Ready Hospitals	20	57345	99.39
Stroke-Ready Hospitals	25	57694	99.99
Stroke-Ready Hospitals	30	57698	100.0
Stroke-Ready Hospitals	35	57698	100.0
Stroke-Ready Hospitals	40	57698	100.0
Stroke-Ready Hospitals	45	57698	100.0
Stroke-Ready Hospitals	50	57698	100.0
Stroke-Ready Hospitals	55	57698	100.0
Stroke-Ready Hospitals	60	57698	100.0
Stroke Units	5	10137	17.57
Stroke Units	10	37189	64.46
Stroke Units	15	53142	92.1
Stroke Units	20	57345	99.39
Stroke Units	25	57694	99.99
Stroke Units	30	57698	100.0
Stroke Units	35	57698	100.0
Stroke Units	40	57698	100.0
Stroke Units	45	57698	100.0
Stroke Units	50	57698	100.0
Stroke Units	55	57698	100.0
Stroke Units	60	57698	100.0

Population Coverage - Landkreis Holzminden (Niedersachsen)

Condition	Drive Time (min)	Covered Population	Coverage (%)
Hospitals with CT	5	0	0.0
Hospitals with CT	10	711	0.99
Hospitals with CT	15	15613	21.75
Hospitals with CT	20	36792	51.25
Hospitals with CT	25	51209	71.33
Hospitals with CT	30	69366	96.63
Hospitals with CT	35	71660	99.82
Hospitals with CT	40	71788	100.0
Hospitals with CT	45	71788	100.0
Hospitals with CT	50	71788	100.0
Hospitals with CT	55	71788	100.0
Hospitals with CT	60	71788	100.0
Stroke-Ready Hospitals	5	0	0.0
Stroke-Ready Hospitals	10	707	0.98
Stroke-Ready Hospitals	15	15130	21.08
Stroke-Ready Hospitals	20	28871	40.22
Stroke-Ready Hospitals	25	38669	53.87
Stroke-Ready Hospitals	30	53587	74.65
Stroke-Ready Hospitals	35	62938	87.67
Stroke-Ready Hospitals	40	68573	95.52
Stroke-Ready Hospitals	45	71664	99.83
Stroke-Ready Hospitals	50	71788	100.0
Stroke-Ready Hospitals	55	71788	100.0
Stroke-Ready Hospitals	60	71788	100.0
Stroke Units	5	0	0.0
Stroke Units	10	707	0.98
Stroke Units	15	14992	20.88
Stroke Units	20	27388	38.15
Stroke Units	25	35507	49.46
Stroke Units	30	45082	62.8
Stroke Units	35	55832	77.77
Stroke Units	40	61782	86.06
Stroke Units	45	66673	92.87
Stroke Units	50	71578	99.71
Stroke Units	55	71785	99.99
Stroke Units	60	71788	100.0

Population Coverage - Landkreis Grafschaft Bentheim (Niedersachsen)

Condition	Drive Time (min)	Covered Population	Coverage (%)
Hospitals with CT	5	26901	19.5
Hospitals with CT	10	75148	54.47
Hospitals with CT	15	111089	80.52
Hospitals with CT	20	121284	87.91
Hospitals with CT	25	129553	93.9
Hospitals with CT	30	135590	98.27
Hospitals with CT	35	137293	99.51
Hospitals with CT	40	137364	99.56
Hospitals with CT	45	137474	99.64
Hospitals with CT	50	137572	99.71
Hospitals with CT	55	137487	99.65
Hospitals with CT	60	137593	99.73
Stroke-Ready Hospitals	5	17596	12.75
Stroke-Ready Hospitals	10	54847	39.75
Stroke-Ready Hospitals	15	73992	53.63
Stroke-Ready Hospitals	20	95667	69.34
Stroke-Ready Hospitals	25	127331	92.29
Stroke-Ready Hospitals	30	134828	97.72
Stroke-Ready Hospitals	35	137210	99.45
Stroke-Ready Hospitals	40	137336	99.54
Stroke-Ready Hospitals	45	137415	99.6
Stroke-Ready Hospitals	50	137492	99.65
Stroke-Ready Hospitals	55	137251	99.48
Stroke-Ready Hospitals	60	137387	99.58
Stroke Units	5	17596	12.75
Stroke Units	10	54847	39.75
Stroke Units	15	73992	53.63
Stroke Units	20	95667	69.34
Stroke Units	25	127331	92.29
Stroke Units	30	134828	97.72
Stroke Units	35	137210	99.45
Stroke Units	40	137336	99.54
Stroke Units	45	137412	99.6
Stroke Units	50	137480	99.64
Stroke Units	55	137251	99.48
Stroke Units	60	137386	99.58

Population Coverage - Kreis Höxter (Nordrhein-Westfalen)

Condition	Drive Time (min)	Covered Population	Coverage (%)
Hospitals with CT	5	32460	22.73
Hospitals with CT	10	77528	54.3
Hospitals with CT	15	114742	80.36
Hospitals with CT	20	135427	94.84
Hospitals with CT	25	142452	99.76
Hospitals with CT	30	142791	100.0
Hospitals with CT	35	142791	100.0
Hospitals with CT	40	142791	100.0
Hospitals with CT	45	142791	100.0
Hospitals with CT	50	142791	100.0
Hospitals with CT	55	142791	100.0
Hospitals with CT	60	142791	100.0
Stroke-Ready Hospitals	5	13352	9.35
Stroke-Ready Hospitals	10	32307	22.63
Stroke-Ready Hospitals	15	48039	33.64
Stroke-Ready Hospitals	20	68837	48.21
Stroke-Ready Hospitals	25	98527	69.0
Stroke-Ready Hospitals	30	116257	81.42
Stroke-Ready Hospitals	35	135644	94.99
Stroke-Ready Hospitals	40	142780	99.99
Stroke-Ready Hospitals	45	142791	100.0
Stroke-Ready Hospitals	50	142791	100.0
Stroke-Ready Hospitals	55	142791	100.0
Stroke-Ready Hospitals	60	142791	100.0
Stroke Units	5	11323	7.93
Stroke Units	10	21969	15.39
Stroke Units	15	32622	22.85
Stroke Units	20	48157	33.73
Stroke Units	25	70741	49.54
Stroke Units	30	100267	70.22
Stroke Units	35	124134	86.93
Stroke Units	40	142186	99.58
Stroke Units	45	142791	100.0
Stroke Units	50	142791	100.0
Stroke Units	55	142791	100.0
Stroke Units	60	142791	100.0

Population Coverage - Landkreis Marburg-Biedenkopf (Hessen)

Condition	Drive Time (min)	Covered Population	Coverage (%)
Hospitals with CT	5	36032	14.81
Hospitals with CT	10	87241	35.85
Hospitals with CT	15	139787	57.44
Hospitals with CT	20	194528	79.93
Hospitals with CT	25	237954	97.78
Hospitals with CT	30	243362	100.0
Hospitals with CT	35	243362	100.0
Hospitals with CT	40	243362	100.0
Hospitals with CT	45	243362	100.0
Hospitals with CT	50	243362	100.0
Hospitals with CT	55	243362	100.0
Hospitals with CT	60	243362	100.0
Stroke-Ready Hospitals	5	1606	0.66
Stroke-Ready Hospitals	10	17167	7.05
Stroke-Ready Hospitals	15	79159	32.53
Stroke-Ready Hospitals	20	129276	53.12
Stroke-Ready Hospitals	25	175020	71.92
Stroke-Ready Hospitals	30	190533	78.29
Stroke-Ready Hospitals	35	197948	81.34
Stroke-Ready Hospitals	40	213123	87.57
Stroke-Ready Hospitals	45	233080	95.77
Stroke-Ready Hospitals	50	242454	99.63
Stroke-Ready Hospitals	55	243362	100.0
Stroke-Ready Hospitals	60	243362	100.0
Stroke Units	5	1606	0.66
Stroke Units	10	14872	6.11
Stroke Units	15	71178	29.25
Stroke Units	20	118547	48.71
Stroke Units	25	153112	62.92
Stroke Units	30	181486	74.57
Stroke Units	35	194576	79.95
Stroke Units	40	211914	87.08
Stroke Units	45	230237	94.61
Stroke Units	50	241237	99.13
Stroke Units	55	243362	100.0
Stroke Units	60	243362	100.0

Population Coverage - Landkreis Rhein-Hunsrück-Kreis (Rheinland-Pfalz)

Condition	Drive Time (min)	Covered Population	Coverage (%)
Hospitals with CT	5	17827	16.98
Hospitals with CT	10	42227	40.23
Hospitals with CT	15	69739	66.44
Hospitals with CT	20	94221	89.77
Hospitals with CT	25	102386	97.55
Hospitals with CT	30	104906	99.95
Hospitals with CT	35	104959	100.0
Hospitals with CT	40	104962	100.0
Hospitals with CT	45	104962	100.0
Hospitals with CT	50	104962	100.0
Hospitals with CT	55	104962	100.0
Hospitals with CT	60	104962	100.0
Stroke-Ready Hospitals	5	0	0.0
Stroke-Ready Hospitals	10	0	0.0
Stroke-Ready Hospitals	15	0	0.0
Stroke-Ready Hospitals	20	260	0.25
Stroke-Ready Hospitals	25	3875	3.69
Stroke-Ready Hospitals	30	13998	13.34
Stroke-Ready Hospitals	35	37878	36.09
Stroke-Ready Hospitals	40	68749	65.5
Stroke-Ready Hospitals	45	88910	84.71
Stroke-Ready Hospitals	50	100813	96.05
Stroke-Ready Hospitals	55	104401	99.47
Stroke-Ready Hospitals	60	104961	100.0
Stroke Units	5	0	0.0
Stroke Units	10	0	0.0
Stroke Units	15	0	0.0
Stroke Units	20	260	0.25
Stroke Units	25	3875	3.69
Stroke Units	30	13998	13.34
Stroke Units	35	37878	36.09
Stroke Units	40	68749	65.5
Stroke Units	45	88888	84.69
Stroke Units	50	100813	96.05
Stroke Units	55	104401	99.47
Stroke Units	60	104961	100.0

Population Coverage - Landkreis Eifelkreis Bitburg-Prüm (Rheinland-Pfalz)

Condition	Drive Time (min)	Covered Population	Coverage (%)
Hospitals with CT	5	10707	10.66
Hospitals with CT	10	22170	22.06
Hospitals with CT	15	40471	40.27
Hospitals with CT	20	61360	61.06
Hospitals with CT	25	83202	82.79
Hospitals with CT	30	89824	89.38
Hospitals with CT	35	97926	97.44
Hospitals with CT	40	100192	99.7
Hospitals with CT	45	100444	99.95
Hospitals with CT	50	100470	99.98
Hospitals with CT	55	100459	99.96
Hospitals with CT	60	100476	99.98
Stroke-Ready Hospitals	5	0	0.0
Stroke-Ready Hospitals	10	0	0.0
Stroke-Ready Hospitals	15	0	0.0
Stroke-Ready Hospitals	20	8	0.01
Stroke-Ready Hospitals	25	3520	3.5
Stroke-Ready Hospitals	30	23760	23.64
Stroke-Ready Hospitals	35	46600	46.37
Stroke-Ready Hospitals	40	61632	61.33
Stroke-Ready Hospitals	45	76663	76.29
Stroke-Ready Hospitals	50	88419	87.98
Stroke-Ready Hospitals	55	96102	95.63
Stroke-Ready Hospitals	60	99243	98.75
Stroke Units	5	0	0.0
Stroke Units	10	0	0.0
Stroke Units	15	0	0.0
Stroke Units	20	8	0.01
Stroke Units	25	3520	3.5
Stroke Units	30	23760	23.64
Stroke Units	35	46600	46.37
Stroke Units	40	61632	61.33
Stroke Units	45	76663	76.29
Stroke Units	50	88419	87.98
Stroke Units	55	96102	95.63
Stroke Units	60	99243	98.75

Population Coverage - Landkreis Erding (Bayern)

Condition	Drive Time (min)	Covered Population	Coverage (%)
Hospitals with CT	5	32003	23.58
Hospitals with CT	10	71187	52.45
Hospitals with CT	15	108473	79.92
Hospitals with CT	20	133315	98.22
Hospitals with CT	25	135714	99.99
Hospitals with CT	30	135731	100.0
Hospitals with CT	35	135731	100.0
Hospitals with CT	40	135731	100.0
Hospitals with CT	45	135731	100.0
Hospitals with CT	50	135731	100.0
Hospitals with CT	55	135731	100.0
Hospitals with CT	60	135731	100.0
Stroke-Ready Hospitals	5	14280	10.52
Stroke-Ready Hospitals	10	32388	23.86
Stroke-Ready Hospitals	15	54044	39.82
Stroke-Ready Hospitals	20	85188	62.76
Stroke-Ready Hospitals	25	116467	85.81
Stroke-Ready Hospitals	30	135708	99.98
Stroke-Ready Hospitals	35	135731	100.0
Stroke-Ready Hospitals	40	135731	100.0
Stroke-Ready Hospitals	45	135731	100.0
Stroke-Ready Hospitals	50	135731	100.0
Stroke-Ready Hospitals	55	135731	100.0
Stroke-Ready Hospitals	60	135731	100.0
Stroke Units	5	14280	10.52
Stroke Units	10	32388	23.86
Stroke Units	15	54044	39.82
Stroke Units	20	85188	62.76
Stroke Units	25	116467	85.81
Stroke Units	30	135708	99.98
Stroke Units	35	135731	100.0
Stroke Units	40	135731	100.0
Stroke Units	45	135731	100.0
Stroke Units	50	135731	100.0
Stroke Units	55	135731	100.0
Stroke Units	60	135731	100.0

Population Coverage - Landkreis Deggendorf (Bayern)

Condition	Drive Time (min)	Covered Population	Coverage (%)
Hospitals with CT	5	15677	13.12
Hospitals with CT	10	53789	45.03
Hospitals with CT	15	82134	68.76
Hospitals with CT	20	109407	91.59
Hospitals with CT	25	117635	98.48
Hospitals with CT	30	119443	99.99
Hospitals with CT	35	119450	100.0
Hospitals with CT	40	119450	100.0
Hospitals with CT	45	119450	100.0
Hospitals with CT	50	119450	100.0
Hospitals with CT	55	119450	100.0
Hospitals with CT	60	119450	100.0
Stroke-Ready Hospitals	5	16130	13.5
Stroke-Ready Hospitals	10	43068	36.06
Stroke-Ready Hospitals	15	65653	54.96
Stroke-Ready Hospitals	20	85629	71.69
Stroke-Ready Hospitals	25	107956	90.38
Stroke-Ready Hospitals	30	117077	98.01
Stroke-Ready Hospitals	35	119366	99.93
Stroke-Ready Hospitals	40	119450	100.0
Stroke-Ready Hospitals	45	119450	100.0
Stroke-Ready Hospitals	50	119450	100.0
Stroke-Ready Hospitals	55	119450	100.0
Stroke-Ready Hospitals	60	119450	100.0
Stroke Units	5	16130	13.5
Stroke Units	10	43068	36.06
Stroke Units	15	65653	54.96
Stroke Units	20	85629	71.69
Stroke Units	25	107956	90.38
Stroke Units	30	117077	98.01
Stroke Units	35	119366	99.93
Stroke Units	40	119450	100.0
Stroke Units	45	119450	100.0
Stroke Units	50	119450	100.0
Stroke Units	55	119450	100.0
Stroke Units	60	119450	100.0

Population Coverage - Landkreis Weißenburg-Gunzenhausen (Bayern)

Condition	Drive Time (min)	Covered Population	Coverage (%)
Hospitals with CT	5	9982	10.46
Hospitals with CT	10	31109	32.61
Hospitals with CT	15	59468	62.33
Hospitals with CT	20	83209	87.21
Hospitals with CT	25	93574	98.07
Hospitals with CT	30	95405	99.99
Hospitals with CT	35	95411	100.0
Hospitals with CT	40	95411	100.0
Hospitals with CT	45	95411	100.0
Hospitals with CT	50	95411	100.0
Hospitals with CT	55	95411	100.0
Hospitals with CT	60	95411	100.0
Stroke-Ready Hospitals	5	2225	2.33
Stroke-Ready Hospitals	10	11001	11.53
Stroke-Ready Hospitals	15	20533	21.52
Stroke-Ready Hospitals	20	38258	40.1
Stroke-Ready Hospitals	25	58314	61.12
Stroke-Ready Hospitals	30	73497	77.03
Stroke-Ready Hospitals	35	84023	88.06
Stroke-Ready Hospitals	40	91610	96.02
Stroke-Ready Hospitals	45	95228	99.81
Stroke-Ready Hospitals	50	95411	100.0
Stroke-Ready Hospitals	55	95411	100.0
Stroke-Ready Hospitals	60	95411	100.0
Stroke Units	5	2225	2.33
Stroke Units	10	11001	11.53
Stroke Units	15	20533	21.52
Stroke Units	20	38258	40.1
Stroke Units	25	58314	61.12
Stroke Units	30	73497	77.03
Stroke Units	35	84023	88.06
Stroke Units	40	91610	96.02
Stroke Units	45	95228	99.81
Stroke Units	50	95411	100.0
Stroke Units	55	95411	100.0
Stroke Units	60	95411	100.0

Population Coverage - Landkreis Lindau (Bodensee) (Bayern)

Condition	Drive Time (min)	Covered Population	Coverage (%)
Hospitals with CT	5	7771	9.31
Hospitals with CT	10	29434	35.27
Hospitals with CT	15	43836	52.52
Hospitals with CT	20	52187	62.53
Hospitals with CT	25	70757	84.78
Hospitals with CT	30	81411	97.54
Hospitals with CT	35	83185	99.67
Hospitals with CT	40	83347	99.86
Hospitals with CT	45	83366	99.89
Hospitals with CT	50	83404	99.93
Hospitals with CT	55	83156	99.63
Hospitals with CT	60	83293	99.8
Stroke-Ready Hospitals	5	0	0.0
Stroke-Ready Hospitals	10	0	0.0
Stroke-Ready Hospitals	15	0	0.0
Stroke-Ready Hospitals	20	0	0.0
Stroke-Ready Hospitals	25	7	0.01
Stroke-Ready Hospitals	30	7331	8.78
Stroke-Ready Hospitals	35	36275	43.46
Stroke-Ready Hospitals	40	53862	64.54
Stroke-Ready Hospitals	45	74652	89.45
Stroke-Ready Hospitals	50	82281	98.59
Stroke-Ready Hospitals	55	83060	99.52
Stroke-Ready Hospitals	60	83147	99.62
Stroke Units	5	0	0.0
Stroke Units	10	0	0.0
Stroke Units	15	0	0.0
Stroke Units	20	0	0.0
Stroke Units	25	7	0.01
Stroke Units	30	7331	8.78
Stroke Units	35	36275	43.46
Stroke Units	40	53862	64.54
Stroke Units	45	74652	89.45
Stroke Units	50	82281	98.59
Stroke Units	55	83060	99.52
Stroke Units	60	83147	99.62

Population Coverage - Kreisfreie Stadt Rostock (Mecklenburg-Vorpommern)

Condition	Drive Time (min)	Covered Population	Coverage (%)
Hospitals with CT	5	39328	20.77
Hospitals with CT	10	95395	50.38
Hospitals with CT	15	127466	67.32
Hospitals with CT	20	170270	89.93
Hospitals with CT	25	184119	97.25
Hospitals with CT	30	186153	98.32
Hospitals with CT	35	187513	99.04
Hospitals with CT	40	189069	99.86
Hospitals with CT	45	189154	99.9
Hospitals with CT	50	189195	99.93
Hospitals with CT	55	189093	99.87
Hospitals with CT	60	189170	99.91
Stroke-Ready Hospitals	5	33733	17.82
Stroke-Ready Hospitals	10	75777	40.02
Stroke-Ready Hospitals	15	99485	52.55
Stroke-Ready Hospitals	20	141642	74.81
Stroke-Ready Hospitals	25	174912	92.38
Stroke-Ready Hospitals	30	185011	97.72
Stroke-Ready Hospitals	35	186518	98.51
Stroke-Ready Hospitals	40	189031	99.84
Stroke-Ready Hospitals	45	189167	99.91
Stroke-Ready Hospitals	50	189209	99.93
Stroke-Ready Hospitals	55	189195	99.93
Stroke-Ready Hospitals	60	189199	99.93
Stroke Units	5	21784	11.51
Stroke Units	10	65664	34.68
Stroke Units	15	98569	52.06
Stroke Units	20	141224	74.59
Stroke Units	25	174606	92.22
Stroke Units	30	184588	97.49
Stroke Units	35	185662	98.06
Stroke Units	40	188743	99.69
Stroke Units	45	189035	99.84
Stroke Units	50	189080	99.87
Stroke Units	55	189195	99.93
Stroke Units	60	189199	99.93

Population Coverage - Landkreis Rostock (Mecklenburg-Vorpommern)

Condition	Drive Time (min)	Covered Population	Coverage (%)
Hospitals with CT	5	19624	9.84
Hospitals with CT	10	58021	29.1
Hospitals with CT	15	92108	46.19
Hospitals with CT	20	126800	63.59
Hospitals with CT	25	162275	81.38
Hospitals with CT	30	184464	92.51
Hospitals with CT	35	196637	98.62
Hospitals with CT	40	198446	99.52
Hospitals with CT	45	198743	99.67
Hospitals with CT	50	198937	99.77
Hospitals with CT	55	198872	99.74
Hospitals with CT	60	199000	99.8
Stroke-Ready Hospitals	5	3623	1.82
Stroke-Ready Hospitals	10	22325	11.2
Stroke-Ready Hospitals	15	38443	19.28
Stroke-Ready Hospitals	20	51994	26.08
Stroke-Ready Hospitals	25	83319	41.79
Stroke-Ready Hospitals	30	121191	60.78
Stroke-Ready Hospitals	35	151138	75.8
Stroke-Ready Hospitals	40	167677	84.09
Stroke-Ready Hospitals	45	181929	91.24
Stroke-Ready Hospitals	50	194629	97.61
Stroke-Ready Hospitals	55	197930	99.26
Stroke-Ready Hospitals	60	198726	99.66
Stroke Units	5	3623	1.82
Stroke Units	10	21584	10.82
Stroke Units	15	36369	18.24
Stroke Units	20	51271	25.71
Stroke Units	25	83093	41.67
Stroke Units	30	120781	60.57
Stroke Units	35	150706	75.58
Stroke Units	40	167369	83.94
Stroke Units	45	181634	91.09
Stroke Units	50	194319	97.45
Stroke Units	55	197845	99.22
Stroke Units	60	198624	99.61

Population Coverage - Landkreis Vorpommern-Greifswald (Mecklenburg-Vorpommern)

Condition	Drive Time (min)	Covered Population	Coverage (%)
Hospitals with CT	5	72449	31.94
Hospitals with CT	10	100360	44.25
Hospitals with CT	15	126484	55.76
Hospitals with CT	20	157054	69.24
Hospitals with CT	25	191075	84.24
Hospitals with CT	30	201931	89.03
Hospitals with CT	35	207461	91.47
Hospitals with CT	40	212745	93.8
Hospitals with CT	45	217883	96.06
Hospitals with CT	50	223214	98.41
Hospitals with CT	55	224186	98.84
Hospitals with CT	60	224964	99.18
Stroke-Ready Hospitals	5	35607	15.7
Stroke-Ready Hospitals	10	55491	24.47
Stroke-Ready Hospitals	15	68907	30.38
Stroke-Ready Hospitals	20	81868	36.09
Stroke-Ready Hospitals	25	101043	44.55
Stroke-Ready Hospitals	30	112685	49.68
Stroke-Ready Hospitals	35	137996	60.84
Stroke-Ready Hospitals	40	163893	72.26
Stroke-Ready Hospitals	45	181639	80.08
Stroke-Ready Hospitals	50	189348	83.48
Stroke-Ready Hospitals	55	197868	87.24
Stroke-Ready Hospitals	60	207195	91.35
Stroke Units	5	35607	15.7
Stroke Units	10	55491	24.47
Stroke Units	15	68907	30.38
Stroke Units	20	81868	36.09
Stroke Units	25	101043	44.55
Stroke Units	30	112685	49.68
Stroke Units	35	137996	60.84
Stroke Units	40	163293	71.99
Stroke Units	45	179472	79.13
Stroke Units	50	186836	82.37
Stroke Units	55	196183	86.49
Stroke Units	60	205705	90.69

Population Coverage - Landkreis Mittelsachsen (Sachsen)

Condition	Drive Time (min)	Covered Population	Coverage (%)
Hospitals with CT	5	39497	13.41
Hospitals with CT	10	94603	32.13
Hospitals with CT	15	157316	53.42
Hospitals with CT	20	239710	81.41
Hospitals with CT	25	283679	96.34
Hospitals with CT	30	231289	78.55
Hospitals with CT	35	294106	99.88
Hospitals with CT	40	294459	100.0
Hospitals with CT	45	294464	100.0
Hospitals with CT	50	294464	100.0
Hospitals with CT	55	294464	100.0
Hospitals with CT	60	294464	100.0
Stroke-Ready Hospitals	5	14987	5.09
Stroke-Ready Hospitals	10	49655	16.86
Stroke-Ready Hospitals	15	78401	26.63
Stroke-Ready Hospitals	20	108931	36.99
Stroke-Ready Hospitals	25	157011	53.32
Stroke-Ready Hospitals	30	210707	71.56
Stroke-Ready Hospitals	35	257271	87.37
Stroke-Ready Hospitals	40	282516	95.94
Stroke-Ready Hospitals	45	294061	99.86
Stroke-Ready Hospitals	50	294444	99.99
Stroke-Ready Hospitals	55	294464	100.0
Stroke-Ready Hospitals	60	294464	100.0
Stroke Units	5	14987	5.09
Stroke Units	10	49655	16.86
Stroke Units	15	78401	26.63
Stroke Units	20	107884	36.64
Stroke Units	25	152324	51.73
Stroke Units	30	201779	68.52
Stroke Units	35	252307	85.68
Stroke Units	40	281695	95.66
Stroke Units	45	294061	99.86
Stroke Units	50	294444	99.99
Stroke Units	55	294464	100.0
Stroke Units	60	294464	100.0

Population Coverage - Landkreis Schmalkalden-Meiningen (Thüringen)

Condition	Drive Time (min)	Covered Population	Coverage (%)
Hospitals with CT	5	19821	17.12
Hospitals with CT	10	37261	32.18
Hospitals with CT	15	69931	60.39
Hospitals with CT	20	96999	83.77
Hospitals with CT	25	107584	92.91
Hospitals with CT	30	113431	97.96
Hospitals with CT	35	115691	99.91
Hospitals with CT	40	115787	99.99
Hospitals with CT	45	115799	100.0
Hospitals with CT	50	115799	100.0
Hospitals with CT	55	115799	100.0
Hospitals with CT	60	115799	100.0
Stroke-Ready Hospitals	5	2376	2.05
Stroke-Ready Hospitals	10	8583	7.41
Stroke-Ready Hospitals	15	24935	21.53
Stroke-Ready Hospitals	20	38606	33.34
Stroke-Ready Hospitals	25	59627	51.49
Stroke-Ready Hospitals	30	77356	66.8
Stroke-Ready Hospitals	35	94675	81.76
Stroke-Ready Hospitals	40	113315	97.86
Stroke-Ready Hospitals	45	115783	99.99
Stroke-Ready Hospitals	50	115797	100.0
Stroke-Ready Hospitals	55	115799	100.0
Stroke-Ready Hospitals	60	115799	100.0
Stroke Units	5	2376	2.05
Stroke Units	10	8583	7.41
Stroke Units	15	24935	21.53
Stroke Units	20	38606	33.34
Stroke Units	25	59627	51.49
Stroke Units	30	77356	66.8
Stroke Units	35	94675	81.76
Stroke Units	40	113315	97.86
Stroke Units	45	115783	99.99
Stroke Units	50	115797	100.0
Stroke Units	55	115799	100.0
Stroke Units	60	115799	100.0

Population Coverage - Kreisfreie Stadt Lübeck (Schleswig-Holstein)

Condition	Drive Time (min)	Covered Population	Coverage (%)
Hospitals with CT	5	75914	34.83
Hospitals with CT	10	169222	77.65
Hospitals with CT	15	214534	98.44
Hospitals with CT	20	216109	99.16
Hospitals with CT	25	216272	99.24
Hospitals with CT	30	217803	99.94
Hospitals with CT	35	217834	99.96
Hospitals with CT	40	217838	99.96
Hospitals with CT	45	217841	99.96
Hospitals with CT	50	217866	99.97
Hospitals with CT	55	217850	99.96
Hospitals with CT	60	217890	99.98
Stroke-Ready Hospitals	5	40695	18.67
Stroke-Ready Hospitals	10	104828	48.1
Stroke-Ready Hospitals	15	160033	73.43
Stroke-Ready Hospitals	20	188060	86.29
Stroke-Ready Hospitals	25	200591	92.04
Stroke-Ready Hospitals	30	212831	97.66
Stroke-Ready Hospitals	35	214493	98.42
Stroke-Ready Hospitals	40	217683	99.89
Stroke-Ready Hospitals	45	217687	99.89
Stroke-Ready Hospitals	50	217805	99.94
Stroke-Ready Hospitals	55	217766	99.92
Stroke-Ready Hospitals	60	217772	99.93
Stroke Units	5	40695	18.67
Stroke Units	10	104828	48.1
Stroke Units	15	160033	73.43
Stroke Units	20	188060	86.29
Stroke Units	25	200591	92.04
Stroke Units	30	212831	97.66
Stroke Units	35	214493	98.42
Stroke Units	40	217683	99.89
Stroke Units	45	217687	99.89
Stroke Units	50	217805	99.94
Stroke Units	55	217730	99.91
Stroke Units	60	217772	99.93

Population Coverage - Kreis Nordfriesland (Schleswig-Holstein)

Condition	Drive Time (min)	Covered Population	Coverage (%)
Hospitals with CT	5	28566	17.02
Hospitals with CT	10	62532	37.25
Hospitals with CT	15	90089	53.66
Hospitals with CT	20	115302	68.68
Hospitals with CT	25	142547	84.91
Hospitals with CT	30	151725	90.38
Hospitals with CT	35	155154	92.42
Hospitals with CT	40	157715	93.94
Hospitals with CT	45	160582	95.65
Hospitals with CT	50	162527	96.81
Hospitals with CT	55	162266	96.66
Hospitals with CT	60	162411	96.74
Stroke-Ready Hospitals	5	0	0.0
Stroke-Ready Hospitals	10	0	0.0
Stroke-Ready Hospitals	15	0	0.0
Stroke-Ready Hospitals	20	1212	0.72
Stroke-Ready Hospitals	25	4967	2.96
Stroke-Ready Hospitals	30	14187	8.45
Stroke-Ready Hospitals	35	32760	19.51
Stroke-Ready Hospitals	40	71948	42.86
Stroke-Ready Hospitals	45	108019	64.34
Stroke-Ready Hospitals	50	126775	75.52
Stroke-Ready Hospitals	55	131386	78.26
Stroke-Ready Hospitals	60	134621	80.19
Stroke Units	5	0	0.0
Stroke Units	10	0	0.0
Stroke Units	15	0	0.0
Stroke Units	20	431	0.26
Stroke Units	25	4501	2.68
Stroke Units	30	13048	7.77
Stroke Units	35	32086	19.11
Stroke Units	40	70714	42.12
Stroke Units	45	107282	63.9
Stroke Units	50	126723	75.48
Stroke Units	55	131342	78.23
Stroke Units	60	134580	80.16

Population Coverage - Landkreis Northeim (Niedersachsen)

Condition	Drive Time (min)	Covered Population	Coverage (%)
Hospitals with CT	5	10084	7.52
Hospitals with CT	10	43971	32.79
Hospitals with CT	15	80529	60.05
Hospitals with CT	20	110597	82.47
Hospitals with CT	25	128688	95.96
Hospitals with CT	30	134023	99.93
Hospitals with CT	35	134107	100.0
Hospitals with CT	40	134111	100.0
Hospitals with CT	45	134112	100.0
Hospitals with CT	50	134112	100.0
Hospitals with CT	55	134112	100.0
Hospitals with CT	60	134112	100.0
Stroke-Ready Hospitals	5	3375	2.52
Stroke-Ready Hospitals	10	25104	18.72
Stroke-Ready Hospitals	15	51747	38.59
Stroke-Ready Hospitals	20	84966	63.35
Stroke-Ready Hospitals	25	105642	78.77
Stroke-Ready Hospitals	30	114887	85.66
Stroke-Ready Hospitals	35	120539	89.88
Stroke-Ready Hospitals	40	133108	99.25
Stroke-Ready Hospitals	45	134112	100.0
Stroke-Ready Hospitals	50	134112	100.0
Stroke-Ready Hospitals	55	134112	100.0
Stroke-Ready Hospitals	60	134112	100.0
Stroke Units	5	0	0.0
Stroke Units	10	0	0.0
Stroke Units	15	2174	1.62
Stroke Units	20	9576	7.14
Stroke Units	25	35685	26.61
Stroke Units	30	70303	52.42
Stroke Units	35	88703	66.14
Stroke Units	40	125699	93.73
Stroke Units	45	134103	99.99
Stroke Units	50	134111	100.0
Stroke Units	55	134112	100.0
Stroke Units	60	134112	100.0

Population Coverage - Kreisfreie Stadt Wilhelmshaven (Niedersachsen)

Condition	Drive Time (min)	Covered Population	Coverage (%)
Hospitals with CT	5	8239	10.97
Hospitals with CT	10	54589	72.68
Hospitals with CT	15	68930	91.77
Hospitals with CT	20	70715	94.14
Hospitals with CT	25	71040	94.58
Hospitals with CT	30	73357	97.66
Hospitals with CT	35	73357	97.66
Hospitals with CT	40	73362	97.67
Hospitals with CT	45	73366	97.67
Hospitals with CT	50	73366	97.67
Hospitals with CT	55	73366	97.67
Hospitals with CT	60	73436	97.77
Stroke-Ready Hospitals	5	0	0.0
Stroke-Ready Hospitals	10	404	0.54
Stroke-Ready Hospitals	15	25147	33.48
Stroke-Ready Hospitals	20	64824	86.3
Stroke-Ready Hospitals	25	70477	93.83
Stroke-Ready Hospitals	30	73262	97.54
Stroke-Ready Hospitals	35	73262	97.54
Stroke-Ready Hospitals	40	73307	97.6
Stroke-Ready Hospitals	45	73308	97.6
Stroke-Ready Hospitals	50	73340	97.64
Stroke-Ready Hospitals	55	73309	97.6
Stroke-Ready Hospitals	60	73310	97.6
Stroke Units	5	0	0.0
Stroke Units	10	404	0.54
Stroke Units	15	25147	33.48
Stroke Units	20	64824	86.3
Stroke Units	25	70477	93.83
Stroke Units	30	73262	97.54
Stroke Units	35	73262	97.54
Stroke Units	40	73307	97.6
Stroke Units	45	73308	97.6
Stroke Units	50	73340	97.64
Stroke Units	55	73309	97.6
Stroke Units	60	73310	97.6

Population Coverage - Kreisfreie Stadt Essen (Nordrhein-Westfalen)

Condition	Drive Time (min)	Covered Population	Coverage (%)
Hospitals with CT	5	214316	35.92
Hospitals with CT	10	511161	85.67
Hospitals with CT	15	596672	100.0
Hospitals with CT	20	596676	100.0
Hospitals with CT	25	596676	100.0
Hospitals with CT	30	596676	100.0
Hospitals with CT	35	596676	100.0
Hospitals with CT	40	596676	100.0
Hospitals with CT	45	596676	100.0
Hospitals with CT	50	596676	100.0
Hospitals with CT	55	596676	100.0
Hospitals with CT	60	596676	100.0
Stroke-Ready Hospitals	5	133736	22.41
Stroke-Ready Hospitals	10	412340	69.11
Stroke-Ready Hospitals	15	558015	93.52
Stroke-Ready Hospitals	20	595807	99.85
Stroke-Ready Hospitals	25	596661	100.0
Stroke-Ready Hospitals	30	596676	100.0
Stroke-Ready Hospitals	35	596676	100.0
Stroke-Ready Hospitals	40	596676	100.0
Stroke-Ready Hospitals	45	596676	100.0
Stroke-Ready Hospitals	50	596676	100.0
Stroke-Ready Hospitals	55	596676	100.0
Stroke-Ready Hospitals	60	596676	100.0
Stroke Units	5	133736	22.41
Stroke Units	10	412340	69.11
Stroke Units	15	558015	93.52
Stroke Units	20	595774	99.85
Stroke Units	25	596661	100.0
Stroke Units	30	596676	100.0
Stroke Units	35	596676	100.0
Stroke Units	40	596676	100.0
Stroke Units	45	596676	100.0
Stroke Units	50	596676	100.0
Stroke Units	55	596676	100.0
Stroke Units	60	596676	100.0

Population Coverage - Kreisfreie Stadt Münster (Nordrhein-Westfalen)

Condition	Drive Time (min)	Covered Population	Coverage (%)
Hospitals with CT	5	152653	41.84
Hospitals with CT	10	286859	78.63
Hospitals with CT	15	357935	98.11
Hospitals with CT	20	364740	99.97
Hospitals with CT	25	364831	100.0
Hospitals with CT	30	364831	100.0
Hospitals with CT	35	364831	100.0
Hospitals with CT	40	364831	100.0
Hospitals with CT	45	364831	100.0
Hospitals with CT	50	364831	100.0
Hospitals with CT	55	364831	100.0
Hospitals with CT	60	364831	100.0
Stroke-Ready Hospitals	5	53695	14.72
Stroke-Ready Hospitals	10	194590	53.34
Stroke-Ready Hospitals	15	323093	88.56
Stroke-Ready Hospitals	20	352220	96.54
Stroke-Ready Hospitals	25	363885	99.74
Stroke-Ready Hospitals	30	364831	100.0
Stroke-Ready Hospitals	35	364831	100.0
Stroke-Ready Hospitals	40	364831	100.0
Stroke-Ready Hospitals	45	364831	100.0
Stroke-Ready Hospitals	50	364831	100.0
Stroke-Ready Hospitals	55	364831	100.0
Stroke-Ready Hospitals	60	364831	100.0
Stroke Units	5	53695	14.72
Stroke Units	10	194590	53.34
Stroke Units	15	323093	88.56
Stroke Units	20	352220	96.54
Stroke Units	25	363885	99.74
Stroke Units	30	364831	100.0
Stroke Units	35	364831	100.0
Stroke Units	40	364831	100.0
Stroke Units	45	364831	100.0
Stroke Units	50	364831	100.0
Stroke Units	55	364831	100.0
Stroke Units	60	364831	100.0

Population Coverage - Kreis Recklinghausen (Nordrhein-Westfalen)

Condition	Drive Time (min)	Covered Population	Coverage (%)
Hospitals with CT	5	199674	32.39
Hospitals with CT	10	486488	78.92
Hospitals with CT	15	579014	93.93
Hospitals with CT	20	615352	99.83
Hospitals with CT	25	616387	100.0
Hospitals with CT	30	616413	100.0
Hospitals with CT	35	616413	100.0
Hospitals with CT	40	616413	100.0
Hospitals with CT	45	616413	100.0
Hospitals with CT	50	616413	100.0
Hospitals with CT	55	616413	100.0
Hospitals with CT	60	616413	100.0
Stroke-Ready Hospitals	5	78223	12.69
Stroke-Ready Hospitals	10	276179	44.8
Stroke-Ready Hospitals	15	445480	72.27
Stroke-Ready Hospitals	20	550224	89.26
Stroke-Ready Hospitals	25	615388	99.83
Stroke-Ready Hospitals	30	616391	100.0
Stroke-Ready Hospitals	35	616413	100.0
Stroke-Ready Hospitals	40	616413	100.0
Stroke-Ready Hospitals	45	616413	100.0
Stroke-Ready Hospitals	50	616413	100.0
Stroke-Ready Hospitals	55	616413	100.0
Stroke-Ready Hospitals	60	616413	100.0
Stroke Units	5	78183	12.68
Stroke Units	10	276179	44.8
Stroke Units	15	445451	72.27
Stroke Units	20	550011	89.23
Stroke Units	25	615388	99.83
Stroke Units	30	616391	100.0
Stroke Units	35	616413	100.0
Stroke Units	40	616413	100.0
Stroke Units	45	616413	100.0
Stroke Units	50	616413	100.0
Stroke Units	55	616413	100.0
Stroke Units	60	616413	100.0

Population Coverage - Landkreis Offenbach (Hessen)

Condition	Drive Time (min)	Covered Population	Coverage (%)
Hospitals with CT	5	32146	9.42
Hospitals with CT	10	128293	37.59
Hospitals with CT	15	272331	79.79
Hospitals with CT	20	339924	99.59
Hospitals with CT	25	341311	100.0
Hospitals with CT	30	341311	100.0
Hospitals with CT	35	341311	100.0
Hospitals with CT	40	341311	100.0
Hospitals with CT	45	341311	100.0
Hospitals with CT	50	341311	100.0
Hospitals with CT	55	341311	100.0
Hospitals with CT	60	341311	100.0
Stroke-Ready Hospitals	5	43484	12.74
Stroke-Ready Hospitals	10	132684	38.87
Stroke-Ready Hospitals	15	273280	80.07
Stroke-Ready Hospitals	20	340938	99.89
Stroke-Ready Hospitals	25	341311	100.0
Stroke-Ready Hospitals	30	341311	100.0
Stroke-Ready Hospitals	35	341311	100.0
Stroke-Ready Hospitals	40	341311	100.0
Stroke-Ready Hospitals	45	341311	100.0
Stroke-Ready Hospitals	50	341311	100.0
Stroke-Ready Hospitals	55	341311	100.0
Stroke-Ready Hospitals	60	341311	100.0
Stroke Units	5	29117	8.53
Stroke Units	10	93128	27.29
Stroke Units	15	214793	62.93
Stroke Units	20	328797	96.33
Stroke Units	25	341308	100.0
Stroke Units	30	341311	100.0
Stroke Units	35	341311	100.0
Stroke Units	40	341311	100.0
Stroke Units	45	341311	100.0
Stroke Units	50	341311	100.0
Stroke Units	55	341311	100.0
Stroke Units	60	341311	100.0

Population Coverage - Kreisfreie Stadt Trier (Rheinland-Pfalz)

Condition	Drive Time (min)	Covered Population	Coverage (%)
Hospitals with CT	5	13768	10.71
Hospitals with CT	10	66728	51.92
Hospitals with CT	15	107916	83.96
Hospitals with CT	20	127916	99.53
Hospitals with CT	25	128523	100.0
Hospitals with CT	30	128525	100.0
Hospitals with CT	35	128525	100.0
Hospitals with CT	40	128525	100.0
Hospitals with CT	45	128525	100.0
Hospitals with CT	50	128525	100.0
Hospitals with CT	55	128525	100.0
Hospitals with CT	60	128525	100.0
Stroke-Ready Hospitals	5	27200	21.16
Stroke-Ready Hospitals	10	75930	59.08
Stroke-Ready Hospitals	15	117174	91.17
Stroke-Ready Hospitals	20	128506	99.99
Stroke-Ready Hospitals	25	128525	100.0
Stroke-Ready Hospitals	30	128525	100.0
Stroke-Ready Hospitals	35	128525	100.0
Stroke-Ready Hospitals	40	128525	100.0
Stroke-Ready Hospitals	45	128525	100.0
Stroke-Ready Hospitals	50	128525	100.0
Stroke-Ready Hospitals	55	128525	100.0
Stroke-Ready Hospitals	60	128525	100.0
Stroke Units	5	27200	21.16
Stroke Units	10	75930	59.08
Stroke Units	15	117174	91.17
Stroke Units	20	128506	99.99
Stroke Units	25	128525	100.0
Stroke Units	30	128525	100.0
Stroke Units	35	128525	100.0
Stroke Units	40	128525	100.0
Stroke Units	45	128525	100.0
Stroke Units	50	128525	100.0
Stroke Units	55	128525	100.0
Stroke Units	60	128525	100.0

Population Coverage - Landkreis Bernkastel-Wittlich (Rheinland-Pfalz)

Condition	Drive Time (min)	Covered Population	Coverage (%)
Hospitals with CT	5	4030	3.51
Hospitals with CT	10	18600	16.22
Hospitals with CT	15	42916	37.43
Hospitals with CT	20	71238	62.12
Hospitals with CT	25	109218	95.24
Hospitals with CT	30	114501	99.85
Hospitals with CT	35	114671	100.0
Hospitals with CT	40	114671	100.0
Hospitals with CT	45	114671	100.0
Hospitals with CT	50	114671	100.0
Hospitals with CT	55	114671	100.0
Hospitals with CT	60	114671	100.0
Stroke-Ready Hospitals	5	6700	5.84
Stroke-Ready Hospitals	10	17146	14.95
Stroke-Ready Hospitals	15	29707	25.91
Stroke-Ready Hospitals	20	48500	42.29
Stroke-Ready Hospitals	25	65432	57.06
Stroke-Ready Hospitals	30	92843	80.96
Stroke-Ready Hospitals	35	111724	97.43
Stroke-Ready Hospitals	40	114515	99.86
Stroke-Ready Hospitals	45	114671	100.0
Stroke-Ready Hospitals	50	114671	100.0
Stroke-Ready Hospitals	55	114671	100.0
Stroke-Ready Hospitals	60	114671	100.0
Stroke Units	5	6700	5.84
Stroke Units	10	17146	14.95
Stroke Units	15	29707	25.91
Stroke Units	20	48500	42.29
Stroke Units	25	65432	57.06
Stroke Units	30	92843	80.96
Stroke Units	35	111724	97.43
Stroke Units	40	114515	99.86
Stroke Units	45	114671	100.0
Stroke Units	50	114671	100.0
Stroke Units	55	114671	100.0
Stroke Units	60	114671	100.0

Population Coverage - Kreisfreie Stadt Worms (Rheinland-Pfalz)

Condition	Drive Time (min)	Covered Population	Coverage (%)
Hospitals with CT	5	9106	11.12
Hospitals with CT	10	49385	60.32
Hospitals with CT	15	78389	95.74
Hospitals with CT	20	81412	99.44
Hospitals with CT	25	81674	99.76
Hospitals with CT	30	81872	100.0
Hospitals with CT	35	81874	100.0
Hospitals with CT	40	81874	100.0
Hospitals with CT	45	81874	100.0
Hospitals with CT	50	81874	100.0
Hospitals with CT	55	81874	100.0
Hospitals with CT	60	81874	100.0
Stroke-Ready Hospitals	5	9596	11.72
Stroke-Ready Hospitals	10	52385	63.98
Stroke-Ready Hospitals	15	79164	96.69
Stroke-Ready Hospitals	20	81560	99.62
Stroke-Ready Hospitals	25	81699	99.79
Stroke-Ready Hospitals	30	81868	99.99
Stroke-Ready Hospitals	35	81874	100.0
Stroke-Ready Hospitals	40	81874	100.0
Stroke-Ready Hospitals	45	81874	100.0
Stroke-Ready Hospitals	50	81874	100.0
Stroke-Ready Hospitals	55	81874	100.0
Stroke-Ready Hospitals	60	81874	100.0
Stroke Units	5	9596	11.72
Stroke Units	10	52385	63.98
Stroke Units	15	78927	96.4
Stroke Units	20	81560	99.62
Stroke Units	25	81699	99.79
Stroke Units	30	81868	99.99
Stroke Units	35	81874	100.0
Stroke Units	40	81874	100.0
Stroke Units	45	81874	100.0
Stroke Units	50	81874	100.0
Stroke Units	55	81874	100.0
Stroke Units	60	81874	100.0

Population Coverage - Landkreis Heilbronn (Baden-Württemberg)

Condition	Drive Time (min)	Covered Population	Coverage (%)
Hospitals with CT	5	12728	3.84
Hospitals with CT	10	59129	17.84
Hospitals with CT	15	134431	40.56
Hospitals with CT	20	229150	69.15
Hospitals with CT	25	292777	88.34
Hospitals with CT	30	329491	99.42
Hospitals with CT	35	331404	100.0
Hospitals with CT	40	331404	100.0
Hospitals with CT	45	331404	100.0
Hospitals with CT	50	331404	100.0
Hospitals with CT	55	331404	100.0
Hospitals with CT	60	331404	100.0
Stroke-Ready Hospitals	5	0	0.0
Stroke-Ready Hospitals	10	2603	0.79
Stroke-Ready Hospitals	15	39682	11.97
Stroke-Ready Hospitals	20	147852	44.61
Stroke-Ready Hospitals	25	264614	79.85
Stroke-Ready Hospitals	30	310867	93.8
Stroke-Ready Hospitals	35	325236	98.14
Stroke-Ready Hospitals	40	331187	99.93
Stroke-Ready Hospitals	45	331400	100.0
Stroke-Ready Hospitals	50	331404	100.0
Stroke-Ready Hospitals	55	331404	100.0
Stroke-Ready Hospitals	60	331404	100.0
Stroke Units	5	0	0.0
Stroke Units	10	2529	0.76
Stroke Units	15	34130	10.3
Stroke Units	20	106364	32.1
Stroke Units	25	203745	61.48
Stroke Units	30	275634	83.17
Stroke Units	35	312943	94.43
Stroke Units	40	329784	99.51
Stroke Units	45	331398	100.0
Stroke Units	50	331404	100.0
Stroke Units	55	331404	100.0
Stroke Units	60	331404	100.0

Population Coverage - Kreisfreie Stadt Ingolstadt (Bayern)

Condition	Drive Time (min)	Covered Population	Coverage (%)
Hospitals with CT	5	50596	37.42
Hospitals with CT	10	101178	74.83
Hospitals with CT	15	126219	93.34
Hospitals with CT	20	135197	99.98
Hospitals with CT	25	135219	100.0
Hospitals with CT	30	135219	100.0
Hospitals with CT	35	135219	100.0
Hospitals with CT	40	135219	100.0
Hospitals with CT	45	135219	100.0
Hospitals with CT	50	135219	100.0
Hospitals with CT	55	135219	100.0
Hospitals with CT	60	135219	100.0
Stroke-Ready Hospitals	5	14705	10.88
Stroke-Ready Hospitals	10	46967	34.73
Stroke-Ready Hospitals	15	104970	77.63
Stroke-Ready Hospitals	20	128657	95.15
Stroke-Ready Hospitals	25	135194	99.98
Stroke-Ready Hospitals	30	135218	100.0
Stroke-Ready Hospitals	35	135219	100.0
Stroke-Ready Hospitals	40	135219	100.0
Stroke-Ready Hospitals	45	135219	100.0
Stroke-Ready Hospitals	50	135219	100.0
Stroke-Ready Hospitals	55	135219	100.0
Stroke-Ready Hospitals	60	135219	100.0
Stroke Units	5	14705	10.88
Stroke Units	10	46967	34.73
Stroke Units	15	104970	77.63
Stroke Units	20	128657	95.15
Stroke Units	25	135194	99.98
Stroke Units	30	135218	100.0
Stroke Units	35	135219	100.0
Stroke Units	40	135219	100.0
Stroke Units	45	135219	100.0
Stroke Units	50	135219	100.0
Stroke Units	55	135219	100.0
Stroke Units	60	135219	100.0

Population Coverage - Landkreis Forchheim (Bayern)

Condition	Drive Time (min)	Covered Population	Coverage (%)
Hospitals with CT	5	10223	8.63
Hospitals with CT	10	37602	31.76
Hospitals with CT	15	66061	55.79
Hospitals with CT	20	100556	84.92
Hospitals with CT	25	114158	96.41
Hospitals with CT	30	118411	100.0
Hospitals with CT	35	118411	100.0
Hospitals with CT	40	118411	100.0
Hospitals with CT	45	118411	100.0
Hospitals with CT	50	118411	100.0
Hospitals with CT	55	118411	100.0
Hospitals with CT	60	118411	100.0
Stroke-Ready Hospitals	5	5683	4.8
Stroke-Ready Hospitals	10	25571	21.6
Stroke-Ready Hospitals	15	43454	36.7
Stroke-Ready Hospitals	20	68067	57.48
Stroke-Ready Hospitals	25	88321	74.59
Stroke-Ready Hospitals	30	102452	86.52
Stroke-Ready Hospitals	35	115969	97.94
Stroke-Ready Hospitals	40	118409	100.0
Stroke-Ready Hospitals	45	118411	100.0
Stroke-Ready Hospitals	50	118411	100.0
Stroke-Ready Hospitals	55	118411	100.0
Stroke-Ready Hospitals	60	118411	100.0
Stroke Units	5	5683	4.8
Stroke Units	10	25571	21.6
Stroke Units	15	43454	36.7
Stroke Units	20	68067	57.48
Stroke Units	25	88321	74.59
Stroke Units	30	102452	86.52
Stroke Units	35	115662	97.68
Stroke Units	40	118409	100.0
Stroke Units	45	118411	100.0
Stroke Units	50	118411	100.0
Stroke Units	55	118411	100.0
Stroke Units	60	118411	100.0

Population Coverage - Landkreis Erlangen-Höchstadt (Bayern)

Condition	Drive Time (min)	Covered Population	Coverage (%)
Hospitals with CT	5	18342	13.35
Hospitals with CT	10	58756	42.76
Hospitals with CT	15	112268	81.71
Hospitals with CT	20	135204	98.4
Hospitals with CT	25	137371	99.98
Hospitals with CT	30	137399	100.0
Hospitals with CT	35	137399	100.0
Hospitals with CT	40	137399	100.0
Hospitals with CT	45	137399	100.0
Hospitals with CT	50	137399	100.0
Hospitals with CT	55	137399	100.0
Hospitals with CT	60	137399	100.0
Stroke-Ready Hospitals	5	0	0.0
Stroke-Ready Hospitals	10	8628	6.28
Stroke-Ready Hospitals	15	28303	20.6
Stroke-Ready Hospitals	20	44999	32.75
Stroke-Ready Hospitals	25	92683	67.46
Stroke-Ready Hospitals	30	127018	92.44
Stroke-Ready Hospitals	35	137228	99.88
Stroke-Ready Hospitals	40	137399	100.0
Stroke-Ready Hospitals	45	137399	100.0
Stroke-Ready Hospitals	50	137399	100.0
Stroke-Ready Hospitals	55	137399	100.0
Stroke-Ready Hospitals	60	137399	100.0
Stroke Units	5	0	0.0
Stroke Units	10	8628	6.28
Stroke Units	15	27325	19.89
Stroke Units	20	40764	29.67
Stroke Units	25	83931	61.09
Stroke Units	30	122133	88.89
Stroke Units	35	133032	96.82
Stroke Units	40	136138	99.08
Stroke Units	45	137358	99.97
Stroke Units	50	137399	100.0
Stroke Units	55	137399	100.0
Stroke Units	60	137399	100.0

Population Coverage - Landkreis Mansfeld-Südharz (Sachsen-Anhalt)

Condition	Drive Time (min)	Covered Population	Coverage (%)
Hospitals with CT	5	25746	20.12
Hospitals with CT	10	53607	41.9
Hospitals with CT	15	93998	73.47
Hospitals with CT	20	113547	88.75
Hospitals with CT	25	123048	96.18
Hospitals with CT	30	126175	98.62
Hospitals with CT	35	127761	99.86
Hospitals with CT	40	127859	99.94
Hospitals with CT	45	127937	100.0
Hospitals with CT	50	127937	100.0
Hospitals with CT	55	127937	100.0
Hospitals with CT	60	127937	100.0
Stroke-Ready Hospitals	5	11577	9.05
Stroke-Ready Hospitals	10	21657	16.93
Stroke-Ready Hospitals	15	31894	24.93
Stroke-Ready Hospitals	20	43281	33.83
Stroke-Ready Hospitals	25	73037	57.09
Stroke-Ready Hospitals	30	101698	79.49
Stroke-Ready Hospitals	35	122317	95.61
Stroke-Ready Hospitals	40	127568	99.71
Stroke-Ready Hospitals	45	127924	99.99
Stroke-Ready Hospitals	50	127937	100.0
Stroke-Ready Hospitals	55	127937	100.0
Stroke-Ready Hospitals	60	127937	100.0
Stroke Units	5	0	0.0
Stroke Units	10	0	0.0
Stroke Units	15	0	0.0
Stroke Units	20	591	0.46
Stroke Units	25	6077	4.75
Stroke Units	30	23030	18.0
Stroke Units	35	45463	35.54
Stroke Units	40	96849	75.7
Stroke Units	45	119113	93.1
Stroke Units	50	124691	97.46
Stroke Units	55	125978	98.47
Stroke Units	60	127924	99.99

Population Coverage - Kreis Minden-Lübbecke (Nordrhein-Westfalen)

Condition	Drive Time (min)	Covered Population	Coverage (%)
Hospitals with CT	5	36634	11.84
Hospitals with CT	10	151187	48.85
Hospitals with CT	15	235369	76.05
Hospitals with CT	20	282391	91.25
Hospitals with CT	25	302414	97.72
Hospitals with CT	30	306997	99.2
Hospitals with CT	35	309312	99.95
Hospitals with CT	40	309473	100.0
Hospitals with CT	45	309473	100.0
Hospitals with CT	50	309473	100.0
Hospitals with CT	55	309473	100.0
Hospitals with CT	60	309473	100.0
Stroke-Ready Hospitals	5	10048	3.25
Stroke-Ready Hospitals	10	57439	18.56
Stroke-Ready Hospitals	15	110338	35.65
Stroke-Ready Hospitals	20	177093	57.22
Stroke-Ready Hospitals	25	204507	66.08
Stroke-Ready Hospitals	30	224103	72.41
Stroke-Ready Hospitals	35	253333	81.86
Stroke-Ready Hospitals	40	283557	91.63
Stroke-Ready Hospitals	45	308443	99.67
Stroke-Ready Hospitals	50	309473	100.0
Stroke-Ready Hospitals	55	309473	100.0
Stroke-Ready Hospitals	60	309473	100.0
Stroke Units	5	10048	3.25
Stroke Units	10	57439	18.56
Stroke Units	15	110338	35.65
Stroke Units	20	177093	57.22
Stroke Units	25	204507	66.08
Stroke Units	30	223961	72.37
Stroke Units	35	252882	81.71
Stroke Units	40	283556	91.63
Stroke Units	45	308443	99.67
Stroke Units	50	309473	100.0
Stroke Units	55	309473	100.0
Stroke Units	60	309473	100.0

Population Coverage - Kreisfreie Stadt Hamm (Nordrhein-Westfalen)

Condition	Drive Time (min)	Covered Population	Coverage (%)
Hospitals with CT	5	50583	28.84
Hospitals with CT	10	105601	60.21
Hospitals with CT	15	159407	90.88
Hospitals with CT	20	175400	100.0
Hospitals with CT	25	175400	100.0
Hospitals with CT	30	175400	100.0
Hospitals with CT	35	175400	100.0
Hospitals with CT	40	175400	100.0
Hospitals with CT	45	175400	100.0
Hospitals with CT	50	175400	100.0
Hospitals with CT	55	175400	100.0
Hospitals with CT	60	175400	100.0
Stroke-Ready Hospitals	5	13430	7.66
Stroke-Ready Hospitals	10	61390	35.0
Stroke-Ready Hospitals	15	115475	65.84
Stroke-Ready Hospitals	20	171117	97.56
Stroke-Ready Hospitals	25	175400	100.0
Stroke-Ready Hospitals	30	175400	100.0
Stroke-Ready Hospitals	35	175400	100.0
Stroke-Ready Hospitals	40	175400	100.0
Stroke-Ready Hospitals	45	175400	100.0
Stroke-Ready Hospitals	50	175400	100.0
Stroke-Ready Hospitals	55	175400	100.0
Stroke-Ready Hospitals	60	175400	100.0
Stroke Units	5	13430	7.66
Stroke Units	10	61390	35.0
Stroke Units	15	115475	65.84
Stroke Units	20	171117	97.56
Stroke Units	25	175400	100.0
Stroke Units	30	175400	100.0
Stroke Units	35	175400	100.0
Stroke Units	40	175400	100.0
Stroke Units	45	175400	100.0
Stroke Units	50	175400	100.0
Stroke Units	55	175400	100.0
Stroke Units	60	175400	100.0

Population Coverage - Landkreis Main-Taunus-Kreis (Hessen)

Condition	Drive Time (min)	Covered Population	Coverage (%)
Hospitals with CT	5	1021	0.44
Hospitals with CT	10	57237	24.73
Hospitals with CT	15	203409	87.89
Hospitals with CT	20	230751	99.71
Hospitals with CT	25	231433	100.0
Hospitals with CT	30	231433	100.0
Hospitals with CT	35	231433	100.0
Hospitals with CT	40	231433	100.0
Hospitals with CT	45	231433	100.0
Hospitals with CT	50	231433	100.0
Hospitals with CT	55	231433	100.0
Hospitals with CT	60	231433	100.0
Stroke-Ready Hospitals	5	737	0.32
Stroke-Ready Hospitals	10	54374	23.49
Stroke-Ready Hospitals	15	188292	81.36
Stroke-Ready Hospitals	20	214548	92.7
Stroke-Ready Hospitals	25	227661	98.37
Stroke-Ready Hospitals	30	231433	100.0
Stroke-Ready Hospitals	35	231433	100.0
Stroke-Ready Hospitals	40	231433	100.0
Stroke-Ready Hospitals	45	231433	100.0
Stroke-Ready Hospitals	50	231433	100.0
Stroke-Ready Hospitals	55	231433	100.0
Stroke-Ready Hospitals	60	231433	100.0
Stroke Units	5	737	0.32
Stroke Units	10	54374	23.49
Stroke Units	15	188292	81.36
Stroke Units	20	214548	92.7
Stroke Units	25	227661	98.37
Stroke Units	30	231433	100.0
Stroke Units	35	231433	100.0
Stroke Units	40	231433	100.0
Stroke Units	45	231433	100.0
Stroke Units	50	231433	100.0
Stroke Units	55	231433	100.0
Stroke Units	60	231433	100.0

Population Coverage - Kreisfreie Stadt Ludwigshafen am Rhein (Rheinland-Pfalz)

Condition	Drive Time (min)	Covered Population	Coverage (%)
Hospitals with CT	5	78635	48.18
Hospitals with CT	10	121658	74.55
Hospitals with CT	15	134506	82.42
Hospitals with CT	20	163201	100.0
Hospitals with CT	25	163201	100.0
Hospitals with CT	30	163201	100.0
Hospitals with CT	35	163201	100.0
Hospitals with CT	40	163201	100.0
Hospitals with CT	45	163201	100.0
Hospitals with CT	50	163201	100.0
Hospitals with CT	55	163201	100.0
Hospitals with CT	60	163201	100.0
Stroke-Ready Hospitals	5	30864	18.91
Stroke-Ready Hospitals	10	92820	56.88
Stroke-Ready Hospitals	15	133556	81.84
Stroke-Ready Hospitals	20	161223	98.79
Stroke-Ready Hospitals	25	163201	100.0
Stroke-Ready Hospitals	30	163201	100.0
Stroke-Ready Hospitals	35	163201	100.0
Stroke-Ready Hospitals	40	163201	100.0
Stroke-Ready Hospitals	45	163201	100.0
Stroke-Ready Hospitals	50	163201	100.0
Stroke-Ready Hospitals	55	163201	100.0
Stroke-Ready Hospitals	60	163201	100.0
Stroke Units	5	30864	18.91
Stroke Units	10	91335	55.97
Stroke Units	15	133134	81.58
Stroke Units	20	161223	98.79
Stroke Units	25	163201	100.0
Stroke Units	30	163201	100.0
Stroke Units	35	163201	100.0
Stroke Units	40	163201	100.0
Stroke Units	45	163201	100.0
Stroke Units	50	163201	100.0
Stroke Units	55	163201	100.0
Stroke Units	60	163201	100.0

Population Coverage - Landkreis Esslingen (Baden-Württemberg)

Condition	Drive Time (min)	Covered Population	Coverage (%)
Hospitals with CT	5	75585	14.62
Hospitals with CT	10	212289	41.05
Hospitals with CT	15	402618	77.85
Hospitals with CT	20	505236	97.69
Hospitals with CT	25	516786	99.93
Hospitals with CT	30	517115	99.99
Hospitals with CT	35	517159	100.0
Hospitals with CT	40	517159	100.0
Hospitals with CT	45	517159	100.0
Hospitals with CT	50	517159	100.0
Hospitals with CT	55	517159	100.0
Hospitals with CT	60	517159	100.0
Stroke-Ready Hospitals	5	48149	9.31
Stroke-Ready Hospitals	10	113251	21.9
Stroke-Ready Hospitals	15	248662	48.08
Stroke-Ready Hospitals	20	396550	76.68
Stroke-Ready Hospitals	25	499015	96.49
Stroke-Ready Hospitals	30	516710	99.91
Stroke-Ready Hospitals	35	517159	100.0
Stroke-Ready Hospitals	40	517159	100.0
Stroke-Ready Hospitals	45	517159	100.0
Stroke-Ready Hospitals	50	517159	100.0
Stroke-Ready Hospitals	55	517159	100.0
Stroke-Ready Hospitals	60	517159	100.0
Stroke Units	5	48149	9.31
Stroke Units	10	112750	21.8
Stroke Units	15	243719	47.13
Stroke Units	20	395311	76.44
Stroke Units	25	499015	96.49
Stroke Units	30	516710	99.91
Stroke Units	35	517159	100.0
Stroke Units	40	517159	100.0
Stroke Units	45	517159	100.0
Stroke Units	50	517159	100.0
Stroke Units	55	517159	100.0
Stroke Units	60	517159	100.0

Population Coverage - Landkreis Dachau (Bayern)

Condition	Drive Time (min)	Covered Population	Coverage (%)
Hospitals with CT	5	18460	12.3
Hospitals with CT	10	70714	47.1
Hospitals with CT	15	124215	82.74
Hospitals with CT	20	147222	98.06
Hospitals with CT	25	150131	100.0
Hospitals with CT	30	150131	100.0
Hospitals with CT	35	150131	100.0
Hospitals with CT	40	150131	100.0
Hospitals with CT	45	150131	100.0
Hospitals with CT	50	150131	100.0
Hospitals with CT	55	150131	100.0
Hospitals with CT	60	150131	100.0
Stroke-Ready Hospitals	5	18986	12.65
Stroke-Ready Hospitals	10	57228	38.12
Stroke-Ready Hospitals	15	91677	61.06
Stroke-Ready Hospitals	20	121040	80.62
Stroke-Ready Hospitals	25	142026	94.6
Stroke-Ready Hospitals	30	149993	99.91
Stroke-Ready Hospitals	35	150131	100.0
Stroke-Ready Hospitals	40	150131	100.0
Stroke-Ready Hospitals	45	150131	100.0
Stroke-Ready Hospitals	50	150131	100.0
Stroke-Ready Hospitals	55	150131	100.0
Stroke-Ready Hospitals	60	150131	100.0
Stroke Units	5	18882	12.58
Stroke Units	10	54977	36.62
Stroke Units	15	90039	59.97
Stroke Units	20	117433	78.22
Stroke Units	25	140913	93.86
Stroke Units	30	149027	99.26
Stroke Units	35	150131	100.0
Stroke Units	40	150131	100.0
Stroke Units	45	150131	100.0
Stroke Units	50	150131	100.0
Stroke Units	55	150131	100.0
Stroke Units	60	150131	100.0

Population Coverage - Landkreis Bad Kissingen (Bayern)

Condition	Drive Time (min)	Covered Population	Coverage (%)
Hospitals with CT	5	16788	15.75
Hospitals with CT	10	33770	31.67
Hospitals with CT	15	55822	52.35
Hospitals with CT	20	84671	79.41
Hospitals with CT	25	99347	93.17
Hospitals with CT	30	104515	98.02
Hospitals with CT	35	105779	99.21
Hospitals with CT	40	106625	100.0
Hospitals with CT	45	106625	100.0
Hospitals with CT	50	106625	100.0
Hospitals with CT	55	106625	100.0
Hospitals with CT	60	106625	100.0
Stroke-Ready Hospitals	5	0	0.0
Stroke-Ready Hospitals	10	0	0.0
Stroke-Ready Hospitals	15	191	0.18
Stroke-Ready Hospitals	20	6698	6.28
Stroke-Ready Hospitals	25	24540	23.02
Stroke-Ready Hospitals	30	49400	46.33
Stroke-Ready Hospitals	35	83196	78.03
Stroke-Ready Hospitals	40	103533	97.1
Stroke-Ready Hospitals	45	106457	99.84
Stroke-Ready Hospitals	50	106625	100.0
Stroke-Ready Hospitals	55	106625	100.0
Stroke-Ready Hospitals	60	106625	100.0
Stroke Units	5	0	0.0
Stroke Units	10	0	0.0
Stroke Units	15	191	0.18
Stroke Units	20	6698	6.28
Stroke Units	25	24540	23.02
Stroke Units	30	49400	46.33
Stroke Units	35	83196	78.03
Stroke Units	40	103533	97.1
Stroke Units	45	106457	99.84
Stroke Units	50	106625	100.0
Stroke Units	55	106625	100.0
Stroke Units	60	106625	100.0

Population Coverage - Kreisfreie Stadt Erfurt (Thüringen)

Condition	Drive Time (min)	Covered Population	Coverage (%)
Hospitals with CT	5	36751	14.87
Hospitals with CT	10	170868	69.12
Hospitals with CT	15	226086	91.45
Hospitals with CT	20	245741	99.4
Hospitals with CT	25	247222	100.0
Hospitals with CT	30	247222	100.0
Hospitals with CT	35	247222	100.0
Hospitals with CT	40	247222	100.0
Hospitals with CT	45	247222	100.0
Hospitals with CT	50	247222	100.0
Hospitals with CT	55	247222	100.0
Hospitals with CT	60	247222	100.0
Stroke-Ready Hospitals	5	24044	9.73
Stroke-Ready Hospitals	10	124029	50.17
Stroke-Ready Hospitals	15	194832	78.81
Stroke-Ready Hospitals	20	237900	96.23
Stroke-Ready Hospitals	25	246872	99.86
Stroke-Ready Hospitals	30	247216	100.0
Stroke-Ready Hospitals	35	247222	100.0
Stroke-Ready Hospitals	40	247222	100.0
Stroke-Ready Hospitals	45	247222	100.0
Stroke-Ready Hospitals	50	247222	100.0
Stroke-Ready Hospitals	55	247222	100.0
Stroke-Ready Hospitals	60	247222	100.0
Stroke Units	5	24044	9.73
Stroke Units	10	124029	50.17
Stroke Units	15	194832	78.81
Stroke Units	20	237900	96.23
Stroke Units	25	246872	99.86
Stroke Units	30	247216	100.0
Stroke Units	35	247222	100.0
Stroke Units	40	247222	100.0
Stroke Units	45	247222	100.0
Stroke Units	50	247222	100.0
Stroke Units	55	247222	100.0
Stroke Units	60	247222	100.0

Population Coverage - Kreisfreie Stadt Flensburg (Schleswig-Holstein)

Condition	Drive Time (min)	Covered Population	Coverage (%)
Hospitals with CT	5	36532	43.19
Hospitals with CT	10	75760	89.56
Hospitals with CT	15	83589	98.82
Hospitals with CT	20	83590	98.82
Hospitals with CT	25	83590	98.82
Hospitals with CT	30	83590	98.82
Hospitals with CT	35	83590	98.82
Hospitals with CT	40	83597	98.82
Hospitals with CT	45	83597	98.82
Hospitals with CT	50	83597	98.82
Hospitals with CT	55	84272	99.62
Hospitals with CT	60	84334	99.7
Stroke-Ready Hospitals	5	37509	44.34
Stroke-Ready Hospitals	10	75818	89.63
Stroke-Ready Hospitals	15	83544	98.76
Stroke-Ready Hospitals	20	83560	98.78
Stroke-Ready Hospitals	25	83560	98.78
Stroke-Ready Hospitals	30	83560	98.78
Stroke-Ready Hospitals	35	83560	98.78
Stroke-Ready Hospitals	40	83582	98.81
Stroke-Ready Hospitals	45	83582	98.81
Stroke-Ready Hospitals	50	83582	98.81
Stroke-Ready Hospitals	55	83542	98.76
Stroke-Ready Hospitals	60	83579	98.8
Stroke Units	5	37509	44.34
Stroke Units	10	75818	89.63
Stroke Units	15	83544	98.76
Stroke Units	20	83560	98.78
Stroke Units	25	83560	98.78
Stroke Units	30	83560	98.78
Stroke Units	35	83560	98.78
Stroke Units	40	83582	98.81
Stroke Units	45	83582	98.81
Stroke Units	50	83582	98.81
Stroke Units	55	83542	98.76
Stroke Units	60	83579	98.8

Population Coverage - Kreis Dithmarschen (Schleswig-Holstein)

Condition	Drive Time (min)	Covered Population	Coverage (%)
Hospitals with CT	5	15574	11.37
Hospitals with CT	10	40907	29.86
Hospitals with CT	15	57442	41.93
Hospitals with CT	20	87988	64.23
Hospitals with CT	25	125313	91.47
Hospitals with CT	30	134532	98.2
Hospitals with CT	35	135687	99.04
Hospitals with CT	40	136008	99.28
Hospitals with CT	45	136188	99.41
Hospitals with CT	50	136301	99.49
Hospitals with CT	55	135828	99.15
Hospitals with CT	60	136225	99.44
Stroke-Ready Hospitals	5	17265	12.6
Stroke-Ready Hospitals	10	31767	23.19
Stroke-Ready Hospitals	15	44926	32.79
Stroke-Ready Hospitals	20	74162	54.13
Stroke-Ready Hospitals	25	92475	67.5
Stroke-Ready Hospitals	30	116108	84.75
Stroke-Ready Hospitals	35	132511	96.73
Stroke-Ready Hospitals	40	134760	98.37
Stroke-Ready Hospitals	45	135309	98.77
Stroke-Ready Hospitals	50	135383	98.82
Stroke-Ready Hospitals	55	134844	98.43
Stroke-Ready Hospitals	60	135019	98.56
Stroke Units	5	17165	12.53
Stroke Units	10	29053	21.21
Stroke Units	15	41352	30.18
Stroke Units	20	67646	49.38
Stroke Units	25	91481	66.78
Stroke Units	30	115025	83.96
Stroke Units	35	131767	96.18
Stroke Units	40	134225	97.98
Stroke Units	45	135108	98.62
Stroke Units	50	135210	98.7
Stroke Units	55	134844	98.43
Stroke Units	60	135019	98.56

Population Coverage - Landkreis Hameln-Pyrmont (Niedersachsen)

Condition	Drive Time (min)	Covered Population	Coverage (%)
Hospitals with CT	5	51712	35.08
Hospitals with CT	10	101250	68.69
Hospitals with CT	15	132877	90.15
Hospitals with CT	20	145770	98.89
Hospitals with CT	25	147388	99.99
Hospitals with CT	30	147400	100.0
Hospitals with CT	35	147401	100.0
Hospitals with CT	40	147401	100.0
Hospitals with CT	45	147401	100.0
Hospitals with CT	50	147401	100.0
Hospitals with CT	55	147401	100.0
Hospitals with CT	60	147401	100.0
Stroke-Ready Hospitals	5	14120	9.58
Stroke-Ready Hospitals	10	28519	19.35
Stroke-Ready Hospitals	15	41636	28.25
Stroke-Ready Hospitals	20	71040	48.2
Stroke-Ready Hospitals	25	105190	71.36
Stroke-Ready Hospitals	30	130743	88.7
Stroke-Ready Hospitals	35	139919	94.92
Stroke-Ready Hospitals	40	145093	98.43
Stroke-Ready Hospitals	45	147250	99.9
Stroke-Ready Hospitals	50	147401	100.0
Stroke-Ready Hospitals	55	147401	100.0
Stroke-Ready Hospitals	60	147401	100.0
Stroke Units	5	5661	3.84
Stroke Units	10	11476	7.79
Stroke Units	15	21974	14.91
Stroke Units	20	42966	29.15
Stroke Units	25	73729	50.02
Stroke Units	30	98612	66.9
Stroke Units	35	121100	82.16
Stroke Units	40	142121	96.42
Stroke Units	45	145328	98.59
Stroke Units	50	147381	99.99
Stroke Units	55	147388	99.99
Stroke Units	60	147401	100.0

Population Coverage - Landkreis Celle (Niedersachsen)

Condition	Drive Time (min)	Covered Population	Coverage (%)
Hospitals with CT	5	17658	10.02
Hospitals with CT	10	47891	27.17
Hospitals with CT	15	68955	39.11
Hospitals with CT	20	101235	57.42
Hospitals with CT	25	133407	75.67
Hospitals with CT	30	156725	88.9
Hospitals with CT	35	172185	97.67
Hospitals with CT	40	174868	99.19
Hospitals with CT	45	176229	99.96
Hospitals with CT	50	176292	100.0
Hospitals with CT	55	176292	100.0
Hospitals with CT	60	176292	100.0
Stroke-Ready Hospitals	5	15746	8.93
Stroke-Ready Hospitals	10	46197	26.21
Stroke-Ready Hospitals	15	67602	38.35
Stroke-Ready Hospitals	20	98660	55.96
Stroke-Ready Hospitals	25	131947	74.85
Stroke-Ready Hospitals	30	154817	87.82
Stroke-Ready Hospitals	35	171190	97.11
Stroke-Ready Hospitals	40	174112	98.76
Stroke-Ready Hospitals	45	176220	99.96
Stroke-Ready Hospitals	50	176292	100.0
Stroke-Ready Hospitals	55	176292	100.0
Stroke-Ready Hospitals	60	176292	100.0
Stroke Units	5	15746	8.93
Stroke Units	10	46197	26.21
Stroke Units	15	67602	38.35
Stroke Units	20	98348	55.79
Stroke Units	25	129203	73.29
Stroke Units	30	146015	82.83
Stroke Units	35	160817	91.22
Stroke Units	40	169434	96.11
Stroke Units	45	175517	99.56
Stroke Units	50	175805	99.72
Stroke Units	55	176292	100.0
Stroke Units	60	176292	100.0

Population Coverage - Landkreis Harburg (Niedersachsen)

Condition	Drive Time (min)	Covered Population	Coverage (%)
Hospitals with CT	5	23011	9.46
Hospitals with CT	10	63881	26.25
Hospitals with CT	15	150173	61.71
Hospitals with CT	20	198691	81.64
Hospitals with CT	25	235822	96.9
Hospitals with CT	30	243010	99.85
Hospitals with CT	35	243195	99.93
Hospitals with CT	40	243364	100.0
Hospitals with CT	45	243364	100.0
Hospitals with CT	50	243364	100.0
Hospitals with CT	55	243364	100.0
Hospitals with CT	60	243364	100.0
Stroke-Ready Hospitals	5	20692	8.5
Stroke-Ready Hospitals	10	57512	23.63
Stroke-Ready Hospitals	15	110746	45.51
Stroke-Ready Hospitals	20	166464	68.4
Stroke-Ready Hospitals	25	218334	89.71
Stroke-Ready Hospitals	30	241923	99.41
Stroke-Ready Hospitals	35	243197	99.93
Stroke-Ready Hospitals	40	243364	100.0
Stroke-Ready Hospitals	45	243364	100.0
Stroke-Ready Hospitals	50	243364	100.0
Stroke-Ready Hospitals	55	243364	100.0
Stroke-Ready Hospitals	60	243364	100.0
Stroke Units	5	20692	8.5
Stroke Units	10	57512	23.63
Stroke Units	15	110746	45.51
Stroke Units	20	166464	68.4
Stroke Units	25	217869	89.52
Stroke Units	30	241895	99.4
Stroke Units	35	243192	99.93
Stroke Units	40	243364	100.0
Stroke Units	45	243364	100.0
Stroke Units	50	243364	100.0
Stroke Units	55	243364	100.0
Stroke Units	60	243364	100.0

Population Coverage - Kreisfreie Stadt Düsseldorf (Nordrhein-Westfalen)

Condition	Drive Time (min)	Covered Population	Coverage (%)
Hospitals with CT	5	284796	42.1
Hospitals with CT	10	556890	82.33
Hospitals with CT	15	675764	99.9
Hospitals with CT	20	676319	99.98
Hospitals with CT	25	676424	100.0
Hospitals with CT	30	676424	100.0
Hospitals with CT	35	676424	100.0
Hospitals with CT	40	676424	100.0
Hospitals with CT	45	676424	100.0
Hospitals with CT	50	676424	100.0
Hospitals with CT	55	676424	100.0
Hospitals with CT	60	676424	100.0
Stroke-Ready Hospitals	5	140557	20.78
Stroke-Ready Hospitals	10	428131	63.29
Stroke-Ready Hospitals	15	631351	93.34
Stroke-Ready Hospitals	20	665825	98.43
Stroke-Ready Hospitals	25	675943	99.93
Stroke-Ready Hospitals	30	676424	100.0
Stroke-Ready Hospitals	35	676424	100.0
Stroke-Ready Hospitals	40	676424	100.0
Stroke-Ready Hospitals	45	676424	100.0
Stroke-Ready Hospitals	50	676424	100.0
Stroke-Ready Hospitals	55	676424	100.0
Stroke-Ready Hospitals	60	676424	100.0
Stroke Units	5	140557	20.78
Stroke Units	10	428131	63.29
Stroke Units	15	631351	93.34
Stroke Units	20	665825	98.43
Stroke Units	25	675943	99.93
Stroke Units	30	676424	100.0
Stroke Units	35	676424	100.0
Stroke Units	40	676424	100.0
Stroke Units	45	676424	100.0
Stroke Units	50	676424	100.0
Stroke Units	55	676424	100.0
Stroke Units	60	676424	100.0

Population Coverage - Kreis Düren (Nordrhein-Westfalen)

Condition	Drive Time (min)	Covered Population	Coverage (%)
Hospitals with CT	5	32991	12.87
Hospitals with CT	10	127044	49.58
Hospitals with CT	15	209292	81.67
Hospitals with CT	20	242928	94.8
Hospitals with CT	25	254993	99.51
Hospitals with CT	30	256161	99.96
Hospitals with CT	35	256182	99.97
Hospitals with CT	40	256259	100.0
Hospitals with CT	45	256259	100.0
Hospitals with CT	50	256259	100.0
Hospitals with CT	55	256259	100.0
Hospitals with CT	60	256259	100.0
Stroke-Ready Hospitals	5	9092	3.55
Stroke-Ready Hospitals	10	59470	23.21
Stroke-Ready Hospitals	15	99093	38.67
Stroke-Ready Hospitals	20	174136	67.95
Stroke-Ready Hospitals	25	232622	90.78
Stroke-Ready Hospitals	30	253691	99.0
Stroke-Ready Hospitals	35	256062	99.92
Stroke-Ready Hospitals	40	256225	99.99
Stroke-Ready Hospitals	45	256226	99.99
Stroke-Ready Hospitals	50	256230	99.99
Stroke-Ready Hospitals	55	256234	99.99
Stroke-Ready Hospitals	60	256259	100.0
Stroke Units	5	9092	3.55
Stroke Units	10	59470	23.21
Stroke Units	15	99093	38.67
Stroke Units	20	174136	67.95
Stroke Units	25	232622	90.78
Stroke Units	30	253691	99.0
Stroke Units	35	256062	99.92
Stroke Units	40	256225	99.99
Stroke Units	45	256226	99.99
Stroke Units	50	256230	99.99
Stroke Units	55	256234	99.99
Stroke Units	60	256259	100.0

Population Coverage - Kreis Soest (Nordrhein-Westfalen)

Condition	Drive Time (min)	Covered Population	Coverage (%)
Hospitals with CT	5	79230	26.84
Hospitals with CT	10	158377	53.66
Hospitals with CT	15	234042	79.3
Hospitals with CT	20	274274	92.93
Hospitals with CT	25	294135	99.66
Hospitals with CT	30	295120	99.99
Hospitals with CT	35	295145	100.0
Hospitals with CT	40	295145	100.0
Hospitals with CT	45	295145	100.0
Hospitals with CT	50	295145	100.0
Hospitals with CT	55	295145	100.0
Hospitals with CT	60	295145	100.0
Stroke-Ready Hospitals	5	31809	10.78
Stroke-Ready Hospitals	10	100219	33.96
Stroke-Ready Hospitals	15	136886	46.38
Stroke-Ready Hospitals	20	236391	80.09
Stroke-Ready Hospitals	25	265056	89.81
Stroke-Ready Hospitals	30	278604	94.4
Stroke-Ready Hospitals	35	294045	99.63
Stroke-Ready Hospitals	40	295138	100.0
Stroke-Ready Hospitals	45	295139	100.0
Stroke-Ready Hospitals	50	295145	100.0
Stroke-Ready Hospitals	55	295145	100.0
Stroke-Ready Hospitals	60	295145	100.0
Stroke Units	5	31809	10.78
Stroke Units	10	100219	33.96
Stroke Units	15	135245	45.82
Stroke Units	20	233787	79.21
Stroke Units	25	265055	89.81
Stroke Units	30	278604	94.4
Stroke Units	35	294045	99.63
Stroke Units	40	295138	100.0
Stroke Units	45	295139	100.0
Stroke Units	50	295145	100.0
Stroke Units	55	295145	100.0
Stroke Units	60	295145	100.0

Population Coverage - Kreisfreie Stadt Darmstadt (Hessen)

Condition	Drive Time (min)	Covered Population	Coverage (%)
Hospitals with CT	5	71186	46.96
Hospitals with CT	10	123536	81.49
Hospitals with CT	15	150671	99.39
Hospitals with CT	20	151216	99.75
Hospitals with CT	25	151599	100.0
Hospitals with CT	30	151599	100.0
Hospitals with CT	35	151599	100.0
Hospitals with CT	40	151599	100.0
Hospitals with CT	45	151599	100.0
Hospitals with CT	50	151599	100.0
Hospitals with CT	55	151599	100.0
Hospitals with CT	60	151599	100.0
Stroke-Ready Hospitals	5	47118	31.08
Stroke-Ready Hospitals	10	113683	74.99
Stroke-Ready Hospitals	15	149772	98.79
Stroke-Ready Hospitals	20	151599	100.0
Stroke-Ready Hospitals	25	151599	100.0
Stroke-Ready Hospitals	30	151599	100.0
Stroke-Ready Hospitals	35	151599	100.0
Stroke-Ready Hospitals	40	151599	100.0
Stroke-Ready Hospitals	45	151599	100.0
Stroke-Ready Hospitals	50	151599	100.0
Stroke-Ready Hospitals	55	151599	100.0
Stroke-Ready Hospitals	60	151599	100.0
Stroke Units	5	47118	31.08
Stroke Units	10	113683	74.99
Stroke Units	15	149772	98.79
Stroke Units	20	151599	100.0
Stroke Units	25	151599	100.0
Stroke Units	30	151599	100.0
Stroke Units	35	151599	100.0
Stroke Units	40	151599	100.0
Stroke Units	45	151599	100.0
Stroke Units	50	151599	100.0
Stroke Units	55	151599	100.0
Stroke Units	60	151599	100.0

Population Coverage - Kreisfreie Stadt Offenbach am Main (Hessen)

Condition	Drive Time (min)	Covered Population	Coverage (%)
Hospitals with CT	5	76789	62.37
Hospitals with CT	10	118628	96.35
Hospitals with CT	15	123120	100.0
Hospitals with CT	20	123120	100.0
Hospitals with CT	25	123120	100.0
Hospitals with CT	30	123120	100.0
Hospitals with CT	35	123120	100.0
Hospitals with CT	40	123120	100.0
Hospitals with CT	45	123120	100.0
Hospitals with CT	50	123120	100.0
Hospitals with CT	55	123120	100.0
Hospitals with CT	60	123120	100.0
Stroke-Ready Hospitals	5	70704	57.43
Stroke-Ready Hospitals	10	101721	82.62
Stroke-Ready Hospitals	15	123106	99.99
Stroke-Ready Hospitals	20	123120	100.0
Stroke-Ready Hospitals	25	123120	100.0
Stroke-Ready Hospitals	30	123120	100.0
Stroke-Ready Hospitals	35	123120	100.0
Stroke-Ready Hospitals	40	123120	100.0
Stroke-Ready Hospitals	45	123120	100.0
Stroke-Ready Hospitals	50	123120	100.0
Stroke-Ready Hospitals	55	123120	100.0
Stroke-Ready Hospitals	60	123120	100.0
Stroke Units	5	70704	57.43
Stroke Units	10	101721	82.62
Stroke Units	15	123106	99.99
Stroke Units	20	123120	100.0
Stroke Units	25	123120	100.0
Stroke Units	30	123120	100.0
Stroke Units	35	123120	100.0
Stroke Units	40	123120	100.0
Stroke Units	45	123120	100.0
Stroke Units	50	123120	100.0
Stroke Units	55	123120	100.0
Stroke Units	60	123120	100.0

Population Coverage - Landkreis Werra-Meißner-Kreis (Hessen)

Condition	Drive Time (min)	Covered Population	Coverage (%)
Hospitals with CT	5	22914	22.72
Hospitals with CT	10	49287	48.87
Hospitals with CT	15	65896	65.34
Hospitals with CT	20	85306	84.59
Hospitals with CT	25	98563	97.73
Hospitals with CT	30	100647	99.8
Hospitals with CT	35	100748	99.9
Hospitals with CT	40	100848	100.0
Hospitals with CT	45	100848	100.0
Hospitals with CT	50	100848	100.0
Hospitals with CT	55	100848	100.0
Hospitals with CT	60	100848	100.0
Stroke-Ready Hospitals	5	11741	11.64
Stroke-Ready Hospitals	10	25252	25.04
Stroke-Ready Hospitals	15	33485	33.2
Stroke-Ready Hospitals	20	45708	45.32
Stroke-Ready Hospitals	25	62144	61.62
Stroke-Ready Hospitals	30	77667	77.01
Stroke-Ready Hospitals	35	96917	96.1
Stroke-Ready Hospitals	40	100808	99.96
Stroke-Ready Hospitals	45	100848	100.0
Stroke-Ready Hospitals	50	100848	100.0
Stroke-Ready Hospitals	55	100848	100.0
Stroke-Ready Hospitals	60	100848	100.0
Stroke Units	5	0	0.0
Stroke Units	10	0	0.0
Stroke Units	15	1168	1.16
Stroke Units	20	2644	2.62
Stroke Units	25	6990	6.93
Stroke Units	30	13832	13.72
Stroke Units	35	38908	38.58
Stroke Units	40	73084	72.47
Stroke Units	45	89352	88.6
Stroke Units	50	99398	98.56
Stroke Units	55	100848	100.0
Stroke Units	60	100848	100.0

Population Coverage - Landkreis Mayen-Koblenz (Rheinland-Pfalz)

Condition	Drive Time (min)	Covered Population	Coverage (%)
Hospitals with CT	5	45635	21.03
Hospitals with CT	10	107155	49.37
Hospitals with CT	15	165504	76.26
Hospitals with CT	20	196336	90.46
Hospitals with CT	25	211039	97.23
Hospitals with CT	30	216429	99.72
Hospitals with CT	35	217040	100.0
Hospitals with CT	40	217040	100.0
Hospitals with CT	45	217040	100.0
Hospitals with CT	50	217040	100.0
Hospitals with CT	55	217040	100.0
Hospitals with CT	60	217040	100.0
Stroke-Ready Hospitals	5	7949	3.66
Stroke-Ready Hospitals	10	32697	15.07
Stroke-Ready Hospitals	15	94590	43.58
Stroke-Ready Hospitals	20	143699	66.21
Stroke-Ready Hospitals	25	167105	76.99
Stroke-Ready Hospitals	30	197173	90.85
Stroke-Ready Hospitals	35	210513	96.99
Stroke-Ready Hospitals	40	215608	99.34
Stroke-Ready Hospitals	45	216689	99.84
Stroke-Ready Hospitals	50	217040	100.0
Stroke-Ready Hospitals	55	217040	100.0
Stroke-Ready Hospitals	60	217040	100.0
Stroke Units	5	0	0.0
Stroke Units	10	1994	0.92
Stroke Units	15	53995	24.88
Stroke Units	20	117497	54.14
Stroke Units	25	156835	72.26
Stroke Units	30	188560	86.88
Stroke Units	35	209682	96.61
Stroke Units	40	215004	99.06
Stroke Units	45	215929	99.49
Stroke Units	50	217040	100.0
Stroke Units	55	217040	100.0
Stroke Units	60	217040	100.0

Population Coverage - Landkreis Bad Dürkheim (Rheinland-Pfalz)

Condition	Drive Time (min)	Covered Population	Coverage (%)
Hospitals with CT	5	21824	16.23
Hospitals with CT	10	52460	39.02
Hospitals with CT	15	108905	81.01
Hospitals with CT	20	126703	94.25
Hospitals with CT	25	128744	95.76
Hospitals with CT	30	132111	98.27
Hospitals with CT	35	134211	99.83
Hospitals with CT	40	134438	100.0
Hospitals with CT	45	134438	100.0
Hospitals with CT	50	134438	100.0
Hospitals with CT	55	134438	100.0
Hospitals with CT	60	134438	100.0
Stroke-Ready Hospitals	5	0	0.0
Stroke-Ready Hospitals	10	1818	1.35
Stroke-Ready Hospitals	15	34283	25.5
Stroke-Ready Hospitals	20	85991	63.96
Stroke-Ready Hospitals	25	119308	88.75
Stroke-Ready Hospitals	30	131117	97.53
Stroke-Ready Hospitals	35	134098	99.75
Stroke-Ready Hospitals	40	134438	100.0
Stroke-Ready Hospitals	45	134438	100.0
Stroke-Ready Hospitals	50	134438	100.0
Stroke-Ready Hospitals	55	134438	100.0
Stroke-Ready Hospitals	60	134438	100.0
Stroke Units	5	0	0.0
Stroke Units	10	0	0.0
Stroke Units	15	172	0.13
Stroke Units	20	16452	12.24
Stroke Units	25	91659	68.18
Stroke Units	30	120676	89.76
Stroke Units	35	125408	93.28
Stroke Units	40	130775	97.28
Stroke Units	45	134413	99.98
Stroke Units	50	134438	100.0
Stroke Units	55	134438	100.0
Stroke Units	60	134438	100.0

Population Coverage - Landkreis Landsberg am Lech (Bayern)

Condition	Drive Time (min)	Covered Population	Coverage (%)
Hospitals with CT	5	12426	10.28
Hospitals with CT	10	39990	33.07
Hospitals with CT	15	64802	53.59
Hospitals with CT	20	97901	80.96
Hospitals with CT	25	118038	97.61
Hospitals with CT	30	120927	100.0
Hospitals with CT	35	120930	100.0
Hospitals with CT	40	120930	100.0
Hospitals with CT	45	120930	100.0
Hospitals with CT	50	120930	100.0
Hospitals with CT	55	120930	100.0
Hospitals with CT	60	120930	100.0
Stroke-Ready Hospitals	5	9170	7.58
Stroke-Ready Hospitals	10	32613	26.97
Stroke-Ready Hospitals	15	57014	47.15
Stroke-Ready Hospitals	20	83221	68.82
Stroke-Ready Hospitals	25	109561	90.6
Stroke-Ready Hospitals	30	120383	99.55
Stroke-Ready Hospitals	35	120827	99.91
Stroke-Ready Hospitals	40	120863	99.94
Stroke-Ready Hospitals	45	120930	100.0
Stroke-Ready Hospitals	50	120930	100.0
Stroke-Ready Hospitals	55	120930	100.0
Stroke-Ready Hospitals	60	120930	100.0
Stroke Units	5	0	0.0
Stroke Units	10	0	0.0
Stroke Units	15	0	0.0
Stroke Units	20	21	0.02
Stroke Units	25	8022	6.63
Stroke Units	30	63840	52.79
Stroke Units	35	111595	92.28
Stroke Units	40	120840	99.93
Stroke Units	45	120861	99.94
Stroke Units	50	120930	100.0
Stroke Units	55	120930	100.0
Stroke Units	60	120930	100.0

Population Coverage - Landkreis Donau-Ries (Bayern)

Condition	Drive Time (min)	Covered Population	Coverage (%)
Hospitals with CT	5	20859	15.65
Hospitals with CT	10	51057	38.3
Hospitals with CT	15	86559	64.92
Hospitals with CT	20	109447	82.09
Hospitals with CT	25	127027	95.28
Hospitals with CT	30	133104	99.84
Hospitals with CT	35	133324	100.0
Hospitals with CT	40	133324	100.0
Hospitals with CT	45	133324	100.0
Hospitals with CT	50	133324	100.0
Hospitals with CT	55	133324	100.0
Hospitals with CT	60	133324	100.0
Stroke-Ready Hospitals	5	0	0.0
Stroke-Ready Hospitals	10	0	0.0
Stroke-Ready Hospitals	15	0	0.0
Stroke-Ready Hospitals	20	11	0.01
Stroke-Ready Hospitals	25	890	0.67
Stroke-Ready Hospitals	30	13229	9.92
Stroke-Ready Hospitals	35	41633	31.23
Stroke-Ready Hospitals	40	90898	68.18
Stroke-Ready Hospitals	45	121857	91.4
Stroke-Ready Hospitals	50	132504	99.39
Stroke-Ready Hospitals	55	133324	100.0
Stroke-Ready Hospitals	60	133324	100.0
Stroke Units	5	0	0.0
Stroke Units	10	0	0.0
Stroke Units	15	0	0.0
Stroke Units	20	11	0.01
Stroke Units	25	890	0.67
Stroke Units	30	13229	9.92
Stroke Units	35	41633	31.23
Stroke Units	40	90898	68.18
Stroke Units	45	121857	91.4
Stroke Units	50	132504	99.39
Stroke Units	55	133324	100.0
Stroke Units	60	133324	100.0

Population Coverage - Landkreis Märkisch-Oderland (Brandenburg)

Condition	Drive Time (min)	Covered Population	Coverage (%)
Hospitals with CT	5	25727	13.97
Hospitals with CT	10	57275	31.11
Hospitals with CT	15	117183	63.65
Hospitals with CT	20	164672	89.44
Hospitals with CT	25	179937	97.73
Hospitals with CT	30	183492	99.66
Hospitals with CT	35	183813	99.84
Hospitals with CT	40	183908	99.89
Hospitals with CT	45	183932	99.9
Hospitals with CT	50	183976	99.93
Hospitals with CT	55	183875	99.87
Hospitals with CT	60	183926	99.9
Stroke-Ready Hospitals	5	1879	1.02
Stroke-Ready Hospitals	10	6265	3.4
Stroke-Ready Hospitals	15	18614	10.11
Stroke-Ready Hospitals	20	71123	38.63
Stroke-Ready Hospitals	25	95893	52.08
Stroke-Ready Hospitals	30	115000	62.46
Stroke-Ready Hospitals	35	137448	74.65
Stroke-Ready Hospitals	40	158965	86.34
Stroke-Ready Hospitals	45	170132	92.41
Stroke-Ready Hospitals	50	176509	95.87
Stroke-Ready Hospitals	55	180878	98.24
Stroke-Ready Hospitals	60	183280	99.55
Stroke Units	5	1879	1.02
Stroke Units	10	6265	3.4
Stroke Units	15	18614	10.11
Stroke Units	20	71123	38.63
Stroke Units	25	95893	52.08
Stroke Units	30	115000	62.46
Stroke Units	35	137434	74.65
Stroke Units	40	158536	86.11
Stroke Units	45	169961	92.31
Stroke Units	50	176374	95.8
Stroke Units	55	180869	98.24
Stroke Units	60	183266	99.54

Population Coverage - Landkreis Prignitz (Brandenburg)

Condition	Drive Time (min)	Covered Population	Coverage (%)
Hospitals with CT	5	14278	19.2
Hospitals with CT	10	21052	28.31
Hospitals with CT	15	31260	42.03
Hospitals with CT	20	54739	73.61
Hospitals with CT	25	65042	87.46
Hospitals with CT	30	70564	94.88
Hospitals with CT	35	71890	96.67
Hospitals with CT	40	73342	98.62
Hospitals with CT	45	74336	99.96
Hospitals with CT	50	74367	100.0
Hospitals with CT	55	74368	100.0
Hospitals with CT	60	74368	100.0
Stroke-Ready Hospitals	5	6348	8.54
Stroke-Ready Hospitals	10	10801	14.52
Stroke-Ready Hospitals	15	16641	22.38
Stroke-Ready Hospitals	20	35560	47.82
Stroke-Ready Hospitals	25	50779	68.28
Stroke-Ready Hospitals	30	61452	82.63
Stroke-Ready Hospitals	35	68325	91.87
Stroke-Ready Hospitals	40	72236	97.13
Stroke-Ready Hospitals	45	73662	99.05
Stroke-Ready Hospitals	50	22645	30.45
Stroke-Ready Hospitals	55	74134	99.69
Stroke-Ready Hospitals	60	74204	99.78
Stroke Units	5	0	0.0
Stroke Units	10	0	0.0
Stroke Units	15	0	0.0
Stroke Units	20	0	0.0
Stroke Units	25	675	0.91
Stroke Units	30	1900	2.56
Stroke Units	35	3225	4.34
Stroke Units	40	5431	7.3
Stroke Units	45	11333	15.24
Stroke Units	50	20365	27.38
Stroke Units	55	25001	33.62
Stroke Units	60	31386	42.2

Population Coverage - Landkreis Burgenlandkreis (Sachsen-Anhalt)

Condition	Drive Time (min)	Covered Population	Coverage (%)
Hospitals with CT	5	32798	19.86
Hospitals with CT	10	73185	44.32
Hospitals with CT	15	104405	63.23
Hospitals with CT	20	139270	84.34
Hospitals with CT	25	150390	91.08
Hospitals with CT	30	155363	94.09
Hospitals with CT	35	160020	96.91
Hospitals with CT	40	163901	99.26
Hospitals with CT	45	165104	99.99
Hospitals with CT	50	165120	100.0
Hospitals with CT	55	165120	100.0
Hospitals with CT	60	165120	100.0
Stroke-Ready Hospitals	5	28736	17.4
Stroke-Ready Hospitals	10	52638	31.88
Stroke-Ready Hospitals	15	71126	43.08
Stroke-Ready Hospitals	20	98139	59.43
Stroke-Ready Hospitals	25	110353	66.83
Stroke-Ready Hospitals	30	132177	80.05
Stroke-Ready Hospitals	35	156079	94.52
Stroke-Ready Hospitals	40	162413	98.36
Stroke-Ready Hospitals	45	165088	99.98
Stroke-Ready Hospitals	50	165115	100.0
Stroke-Ready Hospitals	55	165120	100.0
Stroke-Ready Hospitals	60	165120	100.0
Stroke Units	5	13596	8.23
Stroke Units	10	22370	13.55
Stroke Units	15	31438	19.04
Stroke Units	20	44513	26.96
Stroke Units	25	81100	49.12
Stroke Units	30	102890	62.31
Stroke Units	35	125581	76.05
Stroke Units	40	159178	96.4
Stroke Units	45	164902	99.87
Stroke Units	50	165115	100.0
Stroke Units	55	165120	100.0
Stroke Units	60	165120	100.0

Population Coverage - Landkreis Stendal (Sachsen-Anhalt)

Condition	Drive Time (min)	Covered Population	Coverage (%)
Hospitals with CT	5	20286	19.6
Hospitals with CT	10	34936	33.75
Hospitals with CT	15	50952	49.23
Hospitals with CT	20	70521	68.13
Hospitals with CT	25	83120	80.3
Hospitals with CT	30	92191	89.07
Hospitals with CT	35	97638	94.33
Hospitals with CT	40	102906	99.42
Hospitals with CT	45	103334	99.83
Hospitals with CT	50	103507	100.0
Hospitals with CT	55	103507	100.0
Hospitals with CT	60	103507	100.0
Stroke-Ready Hospitals	5	0	0.0
Stroke-Ready Hospitals	10	0	0.0
Stroke-Ready Hospitals	15	0	0.0
Stroke-Ready Hospitals	20	37	0.04
Stroke-Ready Hospitals	25	475	0.46
Stroke-Ready Hospitals	30	3070	2.97
Stroke-Ready Hospitals	35	7941	7.67
Stroke-Ready Hospitals	40	18100	17.49
Stroke-Ready Hospitals	45	31995	30.91
Stroke-Ready Hospitals	50	44775	43.26
Stroke-Ready Hospitals	55	87783	84.81
Stroke-Ready Hospitals	60	99421	96.05
Stroke Units	5	0	0.0
Stroke Units	10	0	0.0
Stroke Units	15	0	0.0
Stroke Units	20	0	0.0
Stroke Units	25	0	0.0
Stroke Units	30	3	0.0
Stroke Units	35	666	0.64
Stroke Units	40	2902	2.8
Stroke Units	45	10651	10.29
Stroke Units	50	31456	30.39
Stroke Units	55	53866	52.04
Stroke Units	60	61126	59.06

Population Coverage - Landkreis Weimarer Land (Thüringen)

Condition	Drive Time (min)	Covered Population	Coverage (%)
Hospitals with CT	5	16101	21.82
Hospitals with CT	10	34656	46.98
Hospitals with CT	15	50923	69.03
Hospitals with CT	20	64276	87.13
Hospitals with CT	25	70731	95.87
Hospitals with CT	30	73754	99.97
Hospitals with CT	35	73775	100.0
Hospitals with CT	40	73775	100.0
Hospitals with CT	45	73775	100.0
Hospitals with CT	50	73775	100.0
Hospitals with CT	55	73775	100.0
Hospitals with CT	60	73775	100.0
Stroke-Ready Hospitals	5	763	1.03
Stroke-Ready Hospitals	10	6415	8.7
Stroke-Ready Hospitals	15	18316	24.83
Stroke-Ready Hospitals	20	30294	41.06
Stroke-Ready Hospitals	25	39579	53.65
Stroke-Ready Hospitals	30	54677	74.11
Stroke-Ready Hospitals	35	71250	96.58
Stroke-Ready Hospitals	40	73631	99.81
Stroke-Ready Hospitals	45	73775	100.0
Stroke-Ready Hospitals	50	73775	100.0
Stroke-Ready Hospitals	55	73775	100.0
Stroke-Ready Hospitals	60	73775	100.0
Stroke Units	5	763	1.03
Stroke Units	10	6415	8.7
Stroke Units	15	18316	24.83
Stroke Units	20	30294	41.06
Stroke Units	25	39579	53.65
Stroke Units	30	54677	74.11
Stroke Units	35	71250	96.58
Stroke Units	40	73631	99.81
Stroke Units	45	73775	100.0
Stroke Units	50	73775	100.0
Stroke Units	55	73775	100.0
Stroke Units	60	73775	100.0

Population Coverage - Kreisfreie Stadt Kiel (Schleswig-Holstein)

Condition	Drive Time (min)	Covered Population	Coverage (%)
Hospitals with CT	5	95578	36.88
Hospitals with CT	10	152093	58.68
Hospitals with CT	15	217110	83.77
Hospitals with CT	20	253681	97.88
Hospitals with CT	25	258500	99.74
Hospitals with CT	30	258501	99.74
Hospitals with CT	35	258556	99.76
Hospitals with CT	40	258597	99.78
Hospitals with CT	45	258688	99.81
Hospitals with CT	50	258703	99.82
Hospitals with CT	55	258555	99.76
Hospitals with CT	60	258761	99.84
Stroke-Ready Hospitals	5	39172	15.11
Stroke-Ready Hospitals	10	121263	46.79
Stroke-Ready Hospitals	15	191518	73.9
Stroke-Ready Hospitals	20	245242	94.63
Stroke-Ready Hospitals	25	257166	99.23
Stroke-Ready Hospitals	30	257242	99.26
Stroke-Ready Hospitals	35	257423	99.33
Stroke-Ready Hospitals	40	257503	99.36
Stroke-Ready Hospitals	45	257503	99.36
Stroke-Ready Hospitals	50	257968	99.54
Stroke-Ready Hospitals	55	258308	99.67
Stroke-Ready Hospitals	60	258761	99.84
Stroke Units	5	39172	15.11
Stroke Units	10	121263	46.79
Stroke Units	15	191518	73.9
Stroke Units	20	245242	94.63
Stroke Units	25	257166	99.23
Stroke Units	30	257242	99.26
Stroke Units	35	257423	99.33
Stroke Units	40	257503	99.36
Stroke Units	45	257503	99.36
Stroke Units	50	257968	99.54
Stroke Units	55	258308	99.67
Stroke Units	60	258761	99.84

Population Coverage - Landkreis Gifhorn (Niedersachsen)

Condition	Drive Time (min)	Covered Population	Coverage (%)
Hospitals with CT	5	8503	4.94
Hospitals with CT	10	26221	15.23
Hospitals with CT	15	72728	42.23
Hospitals with CT	20	129345	75.11
Hospitals with CT	25	163102	94.72
Hospitals with CT	30	172026	99.9
Hospitals with CT	35	172195	100.0
Hospitals with CT	40	172195	100.0
Hospitals with CT	45	172202	100.0
Hospitals with CT	50	172202	100.0
Hospitals with CT	55	172202	100.0
Hospitals with CT	60	172202	100.0
Stroke-Ready Hospitals	5	5620	3.26
Stroke-Ready Hospitals	10	39636	23.02
Stroke-Ready Hospitals	15	68901	40.01
Stroke-Ready Hospitals	20	107417	62.38
Stroke-Ready Hospitals	25	141733	82.31
Stroke-Ready Hospitals	30	153016	88.86
Stroke-Ready Hospitals	35	162490	94.36
Stroke-Ready Hospitals	40	169756	98.58
Stroke-Ready Hospitals	45	172191	99.99
Stroke-Ready Hospitals	50	172201	100.0
Stroke-Ready Hospitals	55	172202	100.0
Stroke-Ready Hospitals	60	172202	100.0
Stroke Units	5	0	0.0
Stroke Units	10	18	0.01
Stroke Units	15	3871	2.25
Stroke Units	20	11391	6.62
Stroke Units	25	34704	20.15
Stroke Units	30	83673	48.59
Stroke Units	35	111989	65.03
Stroke Units	40	146597	85.13
Stroke Units	45	168133	97.64
Stroke Units	50	172155	99.97
Stroke Units	55	172202	100.0
Stroke Units	60	172202	100.0

Population Coverage - Landkreis Helmstedt (Niedersachsen)

Condition	Drive Time (min)	Covered Population	Coverage (%)
Hospitals with CT	5	18699	20.91
Hospitals with CT	10	28202	31.54
Hospitals with CT	15	50100	56.03
Hospitals with CT	20	77229	86.37
Hospitals with CT	25	87047	97.35
Hospitals with CT	30	89233	99.8
Hospitals with CT	35	89401	99.99
Hospitals with CT	40	89413	100.0
Hospitals with CT	45	89413	100.0
Hospitals with CT	50	89413	100.0
Hospitals with CT	55	89413	100.0
Hospitals with CT	60	89413	100.0
Stroke-Ready Hospitals	5	15670	17.53
Stroke-Ready Hospitals	10	26155	29.25
Stroke-Ready Hospitals	15	44825	50.13
Stroke-Ready Hospitals	20	75466	84.4
Stroke-Ready Hospitals	25	86829	97.11
Stroke-Ready Hospitals	30	88738	99.24
Stroke-Ready Hospitals	35	89403	99.99
Stroke-Ready Hospitals	40	89413	100.0
Stroke-Ready Hospitals	45	89413	100.0
Stroke-Ready Hospitals	50	89413	100.0
Stroke-Ready Hospitals	55	89413	100.0
Stroke-Ready Hospitals	60	89413	100.0
Stroke Units	5	0	0.0
Stroke Units	10	0	0.0
Stroke Units	15	3322	3.72
Stroke Units	20	16439	18.39
Stroke Units	25	33971	37.99
Stroke Units	30	65512	73.27
Stroke Units	35	70308	78.63
Stroke Units	40	78585	87.89
Stroke Units	45	89234	99.8
Stroke Units	50	89413	100.0
Stroke Units	55	89413	100.0
Stroke Units	60	89413	100.0

Population Coverage - Landkreis Lüchow-Dannenberg (Niedersachsen)

Condition	Drive Time (min)	Covered Population	Coverage (%)
Hospitals with CT	5	3972	8.13
Hospitals with CT	10	8413	17.23
Hospitals with CT	15	16939	34.68
Hospitals with CT	20	32015	65.55
Hospitals with CT	25	42591	87.2
Hospitals with CT	30	46130	94.45
Hospitals with CT	35	48267	98.82
Hospitals with CT	40	48790	99.9
Hospitals with CT	45	48841	100.0
Hospitals with CT	50	48841	100.0
Hospitals with CT	55	48841	100.0
Hospitals with CT	60	48841	100.0
Stroke-Ready Hospitals	5	0	0.0
Stroke-Ready Hospitals	10	0	0.0
Stroke-Ready Hospitals	15	0	0.0
Stroke-Ready Hospitals	20	0	0.0
Stroke-Ready Hospitals	25	0	0.0
Stroke-Ready Hospitals	30	0	0.0
Stroke-Ready Hospitals	35	1478	3.03
Stroke-Ready Hospitals	40	6521	13.35
Stroke-Ready Hospitals	45	11288	23.11
Stroke-Ready Hospitals	50	24607	50.38
Stroke-Ready Hospitals	55	39576	81.03
Stroke-Ready Hospitals	60	45001	92.14
Stroke Units	5	0	0.0
Stroke Units	10	0	0.0
Stroke Units	15	0	0.0
Stroke Units	20	0	0.0
Stroke Units	25	0	0.0
Stroke Units	30	0	0.0
Stroke Units	35	1477	3.03
Stroke Units	40	6396	13.1
Stroke Units	45	10892	22.3
Stroke Units	50	23699	48.52
Stroke Units	55	36969	75.69
Stroke Units	60	40874	83.69

Population Coverage - Landkreis Rotenburg (Wümme) (Niedersachsen)

Condition	Drive Time (min)	Covered Population	Coverage (%)
Hospitals with CT	5	14489	8.84
Hospitals with CT	10	30757	18.76
Hospitals with CT	15	56506	34.46
Hospitals with CT	20	99127	60.45
Hospitals with CT	25	132519	80.82
Hospitals with CT	30	160295	97.76
Hospitals with CT	35	163751	99.86
Hospitals with CT	40	163974	100.0
Hospitals with CT	45	163974	100.0
Hospitals with CT	50	163974	100.0
Hospitals with CT	55	163974	100.0
Hospitals with CT	60	163974	100.0
Stroke-Ready Hospitals	5	8073	4.92
Stroke-Ready Hospitals	10	16092	9.81
Stroke-Ready Hospitals	15	29016	17.7
Stroke-Ready Hospitals	20	49880	30.42
Stroke-Ready Hospitals	25	74437	45.4
Stroke-Ready Hospitals	30	96533	58.87
Stroke-Ready Hospitals	35	120419	73.44
Stroke-Ready Hospitals	40	140306	85.57
Stroke-Ready Hospitals	45	155279	94.7
Stroke-Ready Hospitals	50	162973	99.39
Stroke-Ready Hospitals	55	163930	99.97
Stroke-Ready Hospitals	60	163974	100.0
Stroke Units	5	8073	4.92
Stroke Units	10	16092	9.81
Stroke Units	15	28920	17.64
Stroke Units	20	45314	27.64
Stroke Units	25	68984	42.07
Stroke Units	30	95887	58.48
Stroke Units	35	117824	71.86
Stroke Units	40	136959	83.52
Stroke Units	45	154459	94.2
Stroke Units	50	162229	98.94
Stroke Units	55	163930	99.97
Stroke Units	60	163974	100.0

Population Coverage - Kreis Euskirchen (Nordrhein-Westfalen)

Condition	Drive Time (min)	Covered Population	Coverage (%)
Hospitals with CT	5	15442	8.2
Hospitals with CT	10	41357	21.97
Hospitals with CT	15	71151	37.8
Hospitals with CT	20	122419	65.03
Hospitals with CT	25	159181	84.56
Hospitals with CT	30	183082	97.26
Hospitals with CT	35	186511	99.08
Hospitals with CT	40	188223	99.99
Hospitals with CT	45	188236	100.0
Hospitals with CT	50	188237	100.0
Hospitals with CT	55	188236	100.0
Hospitals with CT	60	188237	100.0
Stroke-Ready Hospitals	5	10806	5.74
Stroke-Ready Hospitals	10	36785	19.54
Stroke-Ready Hospitals	15	64591	34.31
Stroke-Ready Hospitals	20	96752	51.4
Stroke-Ready Hospitals	25	126379	67.14
Stroke-Ready Hospitals	30	140811	74.81
Stroke-Ready Hospitals	35	157878	83.87
Stroke-Ready Hospitals	40	167866	89.18
Stroke-Ready Hospitals	45	176992	94.03
Stroke-Ready Hospitals	50	183249	97.35
Stroke-Ready Hospitals	55	186142	98.89
Stroke-Ready Hospitals	60	188210	99.99
Stroke Units	5	10806	5.74
Stroke Units	10	36785	19.54
Stroke Units	15	64591	34.31
Stroke Units	20	96752	51.4
Stroke Units	25	126379	67.14
Stroke Units	30	140811	74.81
Stroke Units	35	157878	83.87
Stroke Units	40	167866	89.18
Stroke Units	45	176992	94.03
Stroke Units	50	183249	97.35
Stroke Units	55	186142	98.89
Stroke Units	60	188210	99.99

Population Coverage - Kreis Olpe (Nordrhein-Westfalen)

Condition	Drive Time (min)	Covered Population	Coverage (%)
Hospitals with CT	5	32258	23.82
Hospitals with CT	10	60167	44.43
Hospitals with CT	15	98859	73.0
Hospitals with CT	20	128738	95.07
Hospitals with CT	25	132833	98.09
Hospitals with CT	30	135075	99.75
Hospitals with CT	35	135418	100.0
Hospitals with CT	40	135418	100.0
Hospitals with CT	45	135418	100.0
Hospitals with CT	50	135418	100.0
Hospitals with CT	55	135418	100.0
Hospitals with CT	60	135418	100.0
Stroke-Ready Hospitals	5	0	0.0
Stroke-Ready Hospitals	10	0	0.0
Stroke-Ready Hospitals	15	1142	0.84
Stroke-Ready Hospitals	20	22005	16.25
Stroke-Ready Hospitals	25	53542	39.54
Stroke-Ready Hospitals	30	59129	43.66
Stroke-Ready Hospitals	35	64083	47.32
Stroke-Ready Hospitals	40	81415	60.12
Stroke-Ready Hospitals	45	109169	80.62
Stroke-Ready Hospitals	50	128640	94.99
Stroke-Ready Hospitals	55	135291	99.91
Stroke-Ready Hospitals	60	135418	100.0
Stroke Units	5	0	0.0
Stroke Units	10	0	0.0
Stroke Units	15	973	0.72
Stroke Units	20	21193	15.65
Stroke Units	25	52172	38.53
Stroke Units	30	59129	43.66
Stroke Units	35	64081	47.32
Stroke Units	40	81414	60.12
Stroke Units	45	109127	80.59
Stroke Units	50	128619	94.98
Stroke Units	55	135291	99.91
Stroke Units	60	135418	100.0

Population Coverage - Landkreis Lahn-Dill-Kreis (Hessen)

Condition	Drive Time (min)	Covered Population	Coverage (%)
Hospitals with CT	5	28606	11.19
Hospitals with CT	10	102795	40.2
Hospitals with CT	15	191987	75.08
Hospitals with CT	20	240607	94.09
Hospitals with CT	25	255091	99.75
Hospitals with CT	30	255712	100.0
Hospitals with CT	35	255722	100.0
Hospitals with CT	40	255722	100.0
Hospitals with CT	45	255722	100.0
Hospitals with CT	50	255722	100.0
Hospitals with CT	55	255722	100.0
Hospitals with CT	60	255722	100.0
Stroke-Ready Hospitals	5	18706	7.31
Stroke-Ready Hospitals	10	64586	25.26
Stroke-Ready Hospitals	15	113245	44.28
Stroke-Ready Hospitals	20	135113	52.84
Stroke-Ready Hospitals	25	172705	67.54
Stroke-Ready Hospitals	30	215027	84.09
Stroke-Ready Hospitals	35	240934	94.22
Stroke-Ready Hospitals	40	253919	99.29
Stroke-Ready Hospitals	45	255722	100.0
Stroke-Ready Hospitals	50	255722	100.0
Stroke-Ready Hospitals	55	255722	100.0
Stroke-Ready Hospitals	60	255722	100.0
Stroke Units	5	8842	3.46
Stroke Units	10	37219	14.55
Stroke Units	15	87467	34.2
Stroke Units	20	119446	46.71
Stroke Units	25	143608	56.16
Stroke Units	30	182072	71.2
Stroke Units	35	227331	88.9
Stroke Units	40	243091	95.06
Stroke Units	45	254556	99.54
Stroke Units	50	255722	100.0
Stroke Units	55	255722	100.0
Stroke Units	60	255722	100.0

Population Coverage - Landkreis Ravensburg (Baden-Württemberg)

Condition	Drive Time (min)	Covered Population	Coverage (%)
Hospitals with CT	5	35654	12.88
Hospitals with CT	10	97296	35.15
Hospitals with CT	15	149973	54.19
Hospitals with CT	20	195464	70.62
Hospitals with CT	25	251553	90.89
Hospitals with CT	30	275990	99.72
Hospitals with CT	35	276742	99.99
Hospitals with CT	40	276768	100.0
Hospitals with CT	45	276768	100.0
Hospitals with CT	50	276768	100.0
Hospitals with CT	55	276768	100.0
Hospitals with CT	60	276768	100.0
Stroke-Ready Hospitals	5	19528	7.06
Stroke-Ready Hospitals	10	61733	22.31
Stroke-Ready Hospitals	15	102424	37.01
Stroke-Ready Hospitals	20	128447	46.41
Stroke-Ready Hospitals	25	180038	65.05
Stroke-Ready Hospitals	30	242268	87.53
Stroke-Ready Hospitals	35	272994	98.64
Stroke-Ready Hospitals	40	276212	99.8
Stroke-Ready Hospitals	45	276768	100.0
Stroke-Ready Hospitals	50	276768	100.0
Stroke-Ready Hospitals	55	276768	100.0
Stroke-Ready Hospitals	60	276768	100.0
Stroke Units	5	19528	7.06
Stroke Units	10	61733	22.31
Stroke Units	15	102424	37.01
Stroke Units	20	128447	46.41
Stroke Units	25	180038	65.05
Stroke Units	30	242248	87.53
Stroke Units	35	272994	98.64
Stroke Units	40	276212	99.8
Stroke Units	45	276768	100.0
Stroke Units	50	276768	100.0
Stroke Units	55	276768	100.0
Stroke Units	60	276768	100.0

Population Coverage - Kreisfreie Stadt Rosenheim (Bayern)

Condition	Drive Time (min)	Covered Population	Coverage (%)
Hospitals with CT	5	29337	46.85
Hospitals with CT	10	55855	89.2
Hospitals with CT	15	62621	100.0
Hospitals with CT	20	62621	100.0
Hospitals with CT	25	62621	100.0
Hospitals with CT	30	62621	100.0
Hospitals with CT	35	62621	100.0
Hospitals with CT	40	62621	100.0
Hospitals with CT	45	62621	100.0
Hospitals with CT	50	62621	100.0
Hospitals with CT	55	62621	100.0
Hospitals with CT	60	62621	100.0
Stroke-Ready Hospitals	5	28408	45.36
Stroke-Ready Hospitals	10	55440	88.53
Stroke-Ready Hospitals	15	62515	99.83
Stroke-Ready Hospitals	20	62621	100.0
Stroke-Ready Hospitals	25	62621	100.0
Stroke-Ready Hospitals	30	62621	100.0
Stroke-Ready Hospitals	35	62621	100.0
Stroke-Ready Hospitals	40	62621	100.0
Stroke-Ready Hospitals	45	62621	100.0
Stroke-Ready Hospitals	50	62621	100.0
Stroke-Ready Hospitals	55	62621	100.0
Stroke-Ready Hospitals	60	62621	100.0
Stroke Units	5	28408	45.36
Stroke Units	10	55440	88.53
Stroke Units	15	62515	99.83
Stroke Units	20	62621	100.0
Stroke Units	25	62621	100.0
Stroke Units	30	62621	100.0
Stroke Units	35	62621	100.0
Stroke Units	40	62621	100.0
Stroke Units	45	62621	100.0
Stroke Units	50	62621	100.0
Stroke Units	55	62621	100.0
Stroke Units	60	62621	100.0

Population Coverage - Landkreis Freising (Bayern)

Condition	Drive Time (min)	Covered Population	Coverage (%)
Hospitals with CT	5	14018	8.01
Hospitals with CT	10	37398	21.38
Hospitals with CT	15	56252	32.16
Hospitals with CT	20	121663	69.55
Hospitals with CT	25	169313	96.79
Hospitals with CT	30	174924	100.0
Hospitals with CT	35	174926	100.0
Hospitals with CT	40	174926	100.0
Hospitals with CT	45	174926	100.0
Hospitals with CT	50	174926	100.0
Hospitals with CT	55	174926	100.0
Hospitals with CT	60	174926	100.0
Stroke-Ready Hospitals	5	14018	8.01
Stroke-Ready Hospitals	10	37017	21.16
Stroke-Ready Hospitals	15	48873	27.94
Stroke-Ready Hospitals	20	86653	49.54
Stroke-Ready Hospitals	25	153254	87.61
Stroke-Ready Hospitals	30	169771	97.05
Stroke-Ready Hospitals	35	174306	99.65
Stroke-Ready Hospitals	40	174926	100.0
Stroke-Ready Hospitals	45	174926	100.0
Stroke-Ready Hospitals	50	174926	100.0
Stroke-Ready Hospitals	55	174926	100.0
Stroke-Ready Hospitals	60	174926	100.0
Stroke Units	5	14018	8.01
Stroke Units	10	37017	21.16
Stroke Units	15	48873	27.94
Stroke Units	20	86653	49.54
Stroke Units	25	153254	87.61
Stroke Units	30	169771	97.05
Stroke Units	35	174306	99.65
Stroke Units	40	174926	100.0
Stroke Units	45	174926	100.0
Stroke Units	50	174926	100.0
Stroke Units	55	174926	100.0
Stroke Units	60	174926	100.0

Population Coverage - Landkreis Garmisch-Partenkirchen (Bayern)

Condition	Drive Time (min)	Covered Population	Coverage (%)
Hospitals with CT	5	20326	23.37
Hospitals with CT	10	43289	49.77
Hospitals with CT	15	58892	67.71
Hospitals with CT	20	68268	78.49
Hospitals with CT	25	76168	87.57
Hospitals with CT	30	83782	96.33
Hospitals with CT	35	85068	97.8
Hospitals with CT	40	85358	98.14
Hospitals with CT	45	85961	98.83
Hospitals with CT	50	85986	98.86
Hospitals with CT	55	85949	98.82
Hospitals with CT	60	86035	98.92
Stroke-Ready Hospitals	5	9402	10.81
Stroke-Ready Hospitals	10	19299	22.19
Stroke-Ready Hospitals	15	25679	29.52
Stroke-Ready Hospitals	20	33510	38.53
Stroke-Ready Hospitals	25	41887	48.16
Stroke-Ready Hospitals	30	68207	78.42
Stroke-Ready Hospitals	35	71615	82.34
Stroke-Ready Hospitals	40	74862	86.07
Stroke-Ready Hospitals	45	81376	93.56
Stroke-Ready Hospitals	50	85484	98.28
Stroke-Ready Hospitals	55	85727	98.56
Stroke-Ready Hospitals	60	85762	98.6
Stroke Units	5	9402	10.81
Stroke Units	10	19299	22.19
Stroke Units	15	25679	29.52
Stroke Units	20	33510	38.53
Stroke Units	25	41887	48.16
Stroke Units	30	68207	78.42
Stroke Units	35	71615	82.34
Stroke Units	40	74862	86.07
Stroke Units	45	81372	93.56
Stroke Units	50	85437	98.23
Stroke Units	55	85727	98.56
Stroke Units	60	85759	98.6

Population Coverage - Landkreis Regensburg (Bayern)

Condition	Drive Time (min)	Covered Population	Coverage (%)
Hospitals with CT	5	6705	3.46
Hospitals with CT	10	32122	16.58
Hospitals with CT	15	101939	52.62
Hospitals with CT	20	167651	86.55
Hospitals with CT	25	186449	96.25
Hospitals with CT	30	192995	99.63
Hospitals with CT	35	193711	100.0
Hospitals with CT	40	193711	100.0
Hospitals with CT	45	193711	100.0
Hospitals with CT	50	193711	100.0
Hospitals with CT	55	193711	100.0
Hospitals with CT	60	193711	100.0
Stroke-Ready Hospitals	5	2170	1.12
Stroke-Ready Hospitals	10	18522	9.56
Stroke-Ready Hospitals	15	70106	36.19
Stroke-Ready Hospitals	20	141429	73.01
Stroke-Ready Hospitals	25	170627	88.08
Stroke-Ready Hospitals	30	187621	96.86
Stroke-Ready Hospitals	35	192548	99.4
Stroke-Ready Hospitals	40	193459	99.87
Stroke-Ready Hospitals	45	193711	100.0
Stroke-Ready Hospitals	50	193711	100.0
Stroke-Ready Hospitals	55	193711	100.0
Stroke-Ready Hospitals	60	193711	100.0
Stroke Units	5	461	0.24
Stroke Units	10	16807	8.68
Stroke Units	15	69466	35.86
Stroke Units	20	139815	72.18
Stroke Units	25	170147	87.84
Stroke Units	30	186543	96.3
Stroke Units	35	192548	99.4
Stroke Units	40	193459	99.87
Stroke Units	45	193711	100.0
Stroke Units	50	193711	100.0
Stroke Units	55	193711	100.0
Stroke Units	60	193711	100.0

Population Coverage - Landkreis Tirschenreuth (Bayern)

Condition	Drive Time (min)	Covered Population	Coverage (%)
Hospitals with CT	5	11373	14.89
Hospitals with CT	10	25340	33.17
Hospitals with CT	15	50606	66.24
Hospitals with CT	20	74079	96.96
Hospitals with CT	25	76215	99.76
Hospitals with CT	30	76391	99.99
Hospitals with CT	35	76398	100.0
Hospitals with CT	40	76401	100.0
Hospitals with CT	45	76401	100.0
Hospitals with CT	50	76401	100.0
Hospitals with CT	55	76390	99.98
Hospitals with CT	60	76401	100.0
Stroke-Ready Hospitals	5	0	0.0
Stroke-Ready Hospitals	10	0	0.0
Stroke-Ready Hospitals	15	0	0.0
Stroke-Ready Hospitals	20	1353	1.77
Stroke-Ready Hospitals	25	16029	20.98
Stroke-Ready Hospitals	30	38120	49.89
Stroke-Ready Hospitals	35	61978	81.12
Stroke-Ready Hospitals	40	69373	90.8
Stroke-Ready Hospitals	45	76337	99.92
Stroke-Ready Hospitals	50	76383	99.98
Stroke-Ready Hospitals	55	76385	99.98
Stroke-Ready Hospitals	60	76398	100.0
Stroke Units	5	0	0.0
Stroke Units	10	0	0.0
Stroke Units	15	0	0.0
Stroke Units	20	1353	1.77
Stroke Units	25	16029	20.98
Stroke Units	30	38120	49.89
Stroke Units	35	61978	81.12
Stroke Units	40	69373	90.8
Stroke Units	45	76337	99.92
Stroke Units	50	76383	99.98
Stroke Units	55	76385	99.98
Stroke Units	60	76398	100.0

Population Coverage - Kreisfreie Stadt Kaufbeuren (Bayern)

Condition	Drive Time (min)	Covered Population	Coverage (%)
Hospitals with CT	5	16399	38.19
Hospitals with CT	10	33316	77.59
Hospitals with CT	15	42932	99.98
Hospitals with CT	20	42940	100.0
Hospitals with CT	25	42940	100.0
Hospitals with CT	30	42940	100.0
Hospitals with CT	35	42940	100.0
Hospitals with CT	40	42940	100.0
Hospitals with CT	45	42940	100.0
Hospitals with CT	50	42940	100.0
Hospitals with CT	55	42940	100.0
Hospitals with CT	60	42940	100.0
Stroke-Ready Hospitals	5	8550	19.91
Stroke-Ready Hospitals	10	32340	75.31
Stroke-Ready Hospitals	15	42929	99.97
Stroke-Ready Hospitals	20	42940	100.0
Stroke-Ready Hospitals	25	42940	100.0
Stroke-Ready Hospitals	30	42940	100.0
Stroke-Ready Hospitals	35	42940	100.0
Stroke-Ready Hospitals	40	42940	100.0
Stroke-Ready Hospitals	45	42940	100.0
Stroke-Ready Hospitals	50	42940	100.0
Stroke-Ready Hospitals	55	42940	100.0
Stroke-Ready Hospitals	60	42940	100.0
Stroke Units	5	8550	19.91
Stroke Units	10	32340	75.31
Stroke Units	15	42929	99.97
Stroke Units	20	42940	100.0
Stroke Units	25	42940	100.0
Stroke Units	30	42940	100.0
Stroke Units	35	42940	100.0
Stroke Units	40	42940	100.0
Stroke Units	45	42940	100.0
Stroke Units	50	42940	100.0
Stroke Units	55	42940	100.0
Stroke Units	60	42940	100.0

Population Coverage - Landkreis Oberallgäu (Bayern)

Condition	Drive Time (min)	Covered Population	Coverage (%)
Hospitals with CT	5	28522	18.31
Hospitals with CT	10	55194	35.42
Hospitals with CT	15	106029	68.05
Hospitals with CT	20	135322	86.85
Hospitals with CT	25	148751	95.47
Hospitals with CT	30	152916	98.14
Hospitals with CT	35	153299	98.39
Hospitals with CT	40	153390	98.44
Hospitals with CT	45	153434	98.47
Hospitals with CT	50	153442	98.48
Hospitals with CT	55	153228	98.34
Hospitals with CT	60	153288	98.38
Stroke-Ready Hospitals	5	0	0.0
Stroke-Ready Hospitals	10	826	0.53
Stroke-Ready Hospitals	15	35310	22.66
Stroke-Ready Hospitals	20	59672	38.3
Stroke-Ready Hospitals	25	77417	49.69
Stroke-Ready Hospitals	30	116162	74.55
Stroke-Ready Hospitals	35	127126	81.59
Stroke-Ready Hospitals	40	137058	87.96
Stroke-Ready Hospitals	45	145638	93.47
Stroke-Ready Hospitals	50	151565	97.27
Stroke-Ready Hospitals	55	152745	98.03
Stroke-Ready Hospitals	60	153118	98.27
Stroke Units	5	0	0.0
Stroke Units	10	826	0.53
Stroke Units	15	35310	22.66
Stroke Units	20	59672	38.3
Stroke Units	25	77417	49.69
Stroke Units	30	116162	74.55
Stroke Units	35	127126	81.59
Stroke Units	40	137058	87.96
Stroke Units	45	145638	93.47
Stroke Units	50	151565	97.27
Stroke Units	55	152745	98.03
Stroke Units	60	153118	98.27

Population Coverage - Landkreis Regionalverband Saarbrücken (Saarland)

Condition	Drive Time (min)	Covered Population	Coverage (%)
Hospitals with CT	5	73295	21.75
Hospitals with CT	10	206732	61.34
Hospitals with CT	15	301851	89.56
Hospitals with CT	20	330479	98.05
Hospitals with CT	25	335940	99.67
Hospitals with CT	30	336860	99.95
Hospitals with CT	35	336902	99.96
Hospitals with CT	40	336902	99.96
Hospitals with CT	45	336902	99.96
Hospitals with CT	50	336917	99.96
Hospitals with CT	55	336894	99.96
Hospitals with CT	60	336894	99.96
Stroke-Ready Hospitals	5	45922	13.63
Stroke-Ready Hospitals	10	153536	45.55
Stroke-Ready Hospitals	15	282477	83.81
Stroke-Ready Hospitals	20	316961	94.04
Stroke-Ready Hospitals	25	328870	97.58
Stroke-Ready Hospitals	30	336387	99.81
Stroke-Ready Hospitals	35	336894	99.96
Stroke-Ready Hospitals	40	336915	99.96
Stroke-Ready Hospitals	45	336915	99.96
Stroke-Ready Hospitals	50	336915	99.96
Stroke-Ready Hospitals	55	336861	99.95
Stroke-Ready Hospitals	60	336880	99.95
Stroke Units	5	45922	13.63
Stroke Units	10	153536	45.55
Stroke Units	15	282477	83.81
Stroke Units	20	316961	94.04
Stroke Units	25	328870	97.58
Stroke Units	30	336387	99.81
Stroke Units	35	336894	99.96
Stroke Units	40	336915	99.96
Stroke Units	45	336915	99.96
Stroke Units	50	336915	99.96
Stroke Units	55	336861	99.95
Stroke Units	60	336880	99.95

Population Coverage - Landkreis Altenburger Land (Thüringen)

Condition	Drive Time (min)	Covered Population	Coverage (%)
Hospitals with CT	5	4939	5.87
Hospitals with CT	10	23266	27.67
Hospitals with CT	15	43888	52.2
Hospitals with CT	20	72375	86.08
Hospitals with CT	25	83768	99.63
Hospitals with CT	30	84080	100.0
Hospitals with CT	35	84080	100.0
Hospitals with CT	40	84080	100.0
Hospitals with CT	45	84080	100.0
Hospitals with CT	50	84080	100.0
Hospitals with CT	55	84080	100.0
Hospitals with CT	60	84080	100.0
Stroke-Ready Hospitals	5	7744	9.21
Stroke-Ready Hospitals	10	25130	29.89
Stroke-Ready Hospitals	15	38031	45.23
Stroke-Ready Hospitals	20	58794	69.93
Stroke-Ready Hospitals	25	74340	88.42
Stroke-Ready Hospitals	30	83276	99.04
Stroke-Ready Hospitals	35	84079	100.0
Stroke-Ready Hospitals	40	84080	100.0
Stroke-Ready Hospitals	45	84080	100.0
Stroke-Ready Hospitals	50	84080	100.0
Stroke-Ready Hospitals	55	84080	100.0
Stroke-Ready Hospitals	60	84080	100.0
Stroke Units	5	7744	9.21
Stroke Units	10	25130	29.89
Stroke Units	15	38030	45.23
Stroke Units	20	57881	68.84
Stroke Units	25	72001	85.63
Stroke Units	30	82827	98.51
Stroke Units	35	84079	100.0
Stroke Units	40	84080	100.0
Stroke Units	45	84080	100.0
Stroke Units	50	84080	100.0
Stroke Units	55	84080	100.0
Stroke Units	60	84080	100.0

Population Coverage - Landkreis Diepholz (Niedersachsen)

Condition	Drive Time (min)	Covered Population	Coverage (%)
Hospitals with CT	5	22832	10.81
Hospitals with CT	10	51742	24.5
Hospitals with CT	15	110520	52.34
Hospitals with CT	20	182637	86.49
Hospitals with CT	25	207643	98.33
Hospitals with CT	30	211169	100.0
Hospitals with CT	35	211170	100.0
Hospitals with CT	40	211170	100.0
Hospitals with CT	45	211170	100.0
Hospitals with CT	50	211170	100.0
Hospitals with CT	55	211170	100.0
Hospitals with CT	60	211170	100.0
Stroke-Ready Hospitals	5	0	0.0
Stroke-Ready Hospitals	10	0	0.0
Stroke-Ready Hospitals	15	1471	0.7
Stroke-Ready Hospitals	20	19947	9.45
Stroke-Ready Hospitals	25	71094	33.67
Stroke-Ready Hospitals	30	97561	46.2
Stroke-Ready Hospitals	35	125756	59.55
Stroke-Ready Hospitals	40	171553	81.24
Stroke-Ready Hospitals	45	204216	96.71
Stroke-Ready Hospitals	50	211060	99.95
Stroke-Ready Hospitals	55	211170	100.0
Stroke-Ready Hospitals	60	211170	100.0
Stroke Units	5	0	0.0
Stroke Units	10	0	0.0
Stroke Units	15	1126	0.53
Stroke Units	20	19078	9.03
Stroke Units	25	67653	32.04
Stroke Units	30	92504	43.81
Stroke Units	35	112253	53.16
Stroke Units	40	140312	66.45
Stroke Units	45	166975	79.07
Stroke Units	50	184983	87.6
Stroke Units	55	200296	94.85
Stroke Units	60	208035	98.52

Population Coverage - Kreisfreie Stadt Emden (Niedersachsen)

Condition	Drive Time (min)	Covered Population	Coverage (%)
Hospitals with CT	5	10627	21.26
Hospitals with CT	10	27830	55.68
Hospitals with CT	15	39952	79.93
Hospitals with CT	20	43445	86.92
Hospitals with CT	25	43825	87.68
Hospitals with CT	30	43936	87.9
Hospitals with CT	35	44787	89.6
Hospitals with CT	40	45104	90.24
Hospitals with CT	45	45109	90.25
Hospitals with CT	50	48242	96.52
Hospitals with CT	55	49912	99.86
Hospitals with CT	60	49912	99.86
Stroke-Ready Hospitals	5	11067	22.14
Stroke-Ready Hospitals	10	27895	55.81
Stroke-Ready Hospitals	15	39911	79.85
Stroke-Ready Hospitals	20	43437	86.9
Stroke-Ready Hospitals	25	43823	87.67
Stroke-Ready Hospitals	30	43933	87.9
Stroke-Ready Hospitals	35	44055	88.14
Stroke-Ready Hospitals	40	44369	88.77
Stroke-Ready Hospitals	45	45098	90.23
Stroke-Ready Hospitals	50	49880	99.79
Stroke-Ready Hospitals	55	49912	99.86
Stroke-Ready Hospitals	60	49912	99.86
Stroke Units	5	11067	22.14
Stroke Units	10	27895	55.81
Stroke Units	15	39911	79.85
Stroke Units	20	43437	86.9
Stroke Units	25	43823	87.67
Stroke Units	30	43933	87.9
Stroke Units	35	44055	88.14
Stroke Units	40	44369	88.77
Stroke Units	45	45098	90.23
Stroke Units	50	49880	99.79
Stroke Units	55	49912	99.86
Stroke Units	60	49912	99.86

Population Coverage - Kreisfreie Stadt Gelsenkirchen (Nordrhein-Westfalen)

Condition	Drive Time (min)	Covered Population	Coverage (%)
Hospitals with CT	5	137140	49.02
Hospitals with CT	10	260163	92.99
Hospitals with CT	15	279785	100.0
Hospitals with CT	20	279785	100.0
Hospitals with CT	25	279785	100.0
Hospitals with CT	30	279785	100.0
Hospitals with CT	35	279785	100.0
Hospitals with CT	40	279785	100.0
Hospitals with CT	45	279785	100.0
Hospitals with CT	50	279785	100.0
Hospitals with CT	55	279785	100.0
Hospitals with CT	60	279785	100.0
Stroke-Ready Hospitals	5	49370	17.65
Stroke-Ready Hospitals	10	164528	58.81
Stroke-Ready Hospitals	15	267380	95.57
Stroke-Ready Hospitals	20	279785	100.0
Stroke-Ready Hospitals	25	279785	100.0
Stroke-Ready Hospitals	30	279785	100.0
Stroke-Ready Hospitals	35	279785	100.0
Stroke-Ready Hospitals	40	279785	100.0
Stroke-Ready Hospitals	45	279785	100.0
Stroke-Ready Hospitals	50	279785	100.0
Stroke-Ready Hospitals	55	279785	100.0
Stroke-Ready Hospitals	60	279785	100.0
Stroke Units	5	49370	17.65
Stroke Units	10	164467	58.78
Stroke Units	15	267317	95.54
Stroke Units	20	279785	100.0
Stroke Units	25	279785	100.0
Stroke Units	30	279785	100.0
Stroke Units	35	279785	100.0
Stroke Units	40	279785	100.0
Stroke Units	45	279785	100.0
Stroke Units	50	279785	100.0
Stroke Units	55	279785	100.0
Stroke Units	60	279785	100.0

Population Coverage - Kreis Warendorf (Nordrhein-Westfalen)

Condition	Drive Time (min)	Covered Population	Coverage (%)
Hospitals with CT	5	67057	24.65
Hospitals with CT	10	149663	55.01
Hospitals with CT	15	237058	87.13
Hospitals with CT	20	265439	97.56
Hospitals with CT	25	272058	100.0
Hospitals with CT	30	272070	100.0
Hospitals with CT	35	272070	100.0
Hospitals with CT	40	272070	100.0
Hospitals with CT	45	272070	100.0
Hospitals with CT	50	272070	100.0
Hospitals with CT	55	272070	100.0
Hospitals with CT	60	272070	100.0
Stroke-Ready Hospitals	5	26764	9.84
Stroke-Ready Hospitals	10	79565	29.24
Stroke-Ready Hospitals	15	129654	47.65
Stroke-Ready Hospitals	20	194811	71.6
Stroke-Ready Hospitals	25	244092	89.72
Stroke-Ready Hospitals	30	272042	99.99
Stroke-Ready Hospitals	35	272070	100.0
Stroke-Ready Hospitals	40	272070	100.0
Stroke-Ready Hospitals	45	272070	100.0
Stroke-Ready Hospitals	50	272070	100.0
Stroke-Ready Hospitals	55	272070	100.0
Stroke-Ready Hospitals	60	272070	100.0
Stroke Units	5	18540	6.81
Stroke Units	10	46235	16.99
Stroke Units	15	76250	28.03
Stroke Units	20	122486	45.02
Stroke Units	25	166454	61.18
Stroke Units	30	214800	78.95
Stroke Units	35	247662	91.03
Stroke Units	40	269145	98.92
Stroke Units	45	272070	100.0
Stroke Units	50	272070	100.0
Stroke Units	55	272070	100.0
Stroke Units	60	272070	100.0

Population Coverage - Stadtkreis Ulm (Baden-Württemberg)

Condition	Drive Time (min)	Covered Population	Coverage (%)
Hospitals with CT	5	29974	24.95
Hospitals with CT	10	80424	66.96
Hospitals with CT	15	111121	92.51
Hospitals with CT	20	120028	99.93
Hospitals with CT	25	120114	100.0
Hospitals with CT	30	120114	100.0
Hospitals with CT	35	120114	100.0
Hospitals with CT	40	120114	100.0
Hospitals with CT	45	120114	100.0
Hospitals with CT	50	120114	100.0
Hospitals with CT	55	120114	100.0
Hospitals with CT	60	120114	100.0
Stroke-Ready Hospitals	5	14410	12.0
Stroke-Ready Hospitals	10	45595	37.96
Stroke-Ready Hospitals	15	93977	78.24
Stroke-Ready Hospitals	20	118238	98.44
Stroke-Ready Hospitals	25	120114	100.0
Stroke-Ready Hospitals	30	120114	100.0
Stroke-Ready Hospitals	35	120114	100.0
Stroke-Ready Hospitals	40	120114	100.0
Stroke-Ready Hospitals	45	120114	100.0
Stroke-Ready Hospitals	50	120114	100.0
Stroke-Ready Hospitals	55	120114	100.0
Stroke-Ready Hospitals	60	120114	100.0
Stroke Units	5	14410	12.0
Stroke Units	10	45595	37.96
Stroke Units	15	93977	78.24
Stroke Units	20	118238	98.44
Stroke Units	25	120114	100.0
Stroke Units	30	120114	100.0
Stroke Units	35	120114	100.0
Stroke Units	40	120114	100.0
Stroke Units	45	120114	100.0
Stroke Units	50	120114	100.0
Stroke Units	55	120114	100.0
Stroke Units	60	120114	100.0

Population Coverage - Landkreis Traunstein (Bayern)

Condition	Drive Time (min)	Covered Population	Coverage (%)
Hospitals with CT	5	16132	9.14
Hospitals with CT	10	41162	23.32
Hospitals with CT	15	84008	47.6
Hospitals with CT	20	128742	72.94
Hospitals with CT	25	155788	88.26
Hospitals with CT	30	169708	96.15
Hospitals with CT	35	172682	97.83
Hospitals with CT	40	173454	98.27
Hospitals with CT	45	175211	99.27
Hospitals with CT	50	176050	99.74
Hospitals with CT	55	176069	99.75
Hospitals with CT	60	176123	99.78
Stroke-Ready Hospitals	5	12844	7.28
Stroke-Ready Hospitals	10	25169	14.26
Stroke-Ready Hospitals	15	51659	29.27
Stroke-Ready Hospitals	20	94107	53.32
Stroke-Ready Hospitals	25	131031	74.24
Stroke-Ready Hospitals	30	159993	90.65
Stroke-Ready Hospitals	35	172478	97.72
Stroke-Ready Hospitals	40	173410	98.25
Stroke-Ready Hospitals	45	175127	99.22
Stroke-Ready Hospitals	50	175808	99.61
Stroke-Ready Hospitals	55	176038	99.74
Stroke-Ready Hospitals	60	176098	99.77
Stroke Units	5	12844	7.28
Stroke Units	10	25169	14.26
Stroke Units	15	51659	29.27
Stroke Units	20	94107	53.32
Stroke Units	25	131031	74.24
Stroke Units	30	159993	90.65
Stroke Units	35	172478	97.72
Stroke Units	40	173410	98.25
Stroke Units	45	175127	99.22
Stroke Units	50	175808	99.61
Stroke Units	55	176038	99.74
Stroke Units	60	176098	99.77

Population Coverage - Kreisfreie Stadt Regensburg (Bayern)

Condition	Drive Time (min)	Covered Population	Coverage (%)
Hospitals with CT	5	76054	44.72
Hospitals with CT	10	140947	82.88
Hospitals with CT	15	168911	99.32
Hospitals with CT	20	170062	100.0
Hospitals with CT	25	170062	100.0
Hospitals with CT	30	170062	100.0
Hospitals with CT	35	170062	100.0
Hospitals with CT	40	170062	100.0
Hospitals with CT	45	170062	100.0
Hospitals with CT	50	170062	100.0
Hospitals with CT	55	170062	100.0
Hospitals with CT	60	170062	100.0
Stroke-Ready Hospitals	5	58409	34.35
Stroke-Ready Hospitals	10	130944	77.0
Stroke-Ready Hospitals	15	167555	98.53
Stroke-Ready Hospitals	20	170062	100.0
Stroke-Ready Hospitals	25	170062	100.0
Stroke-Ready Hospitals	30	170062	100.0
Stroke-Ready Hospitals	35	170062	100.0
Stroke-Ready Hospitals	40	170062	100.0
Stroke-Ready Hospitals	45	170062	100.0
Stroke-Ready Hospitals	50	170062	100.0
Stroke-Ready Hospitals	55	170062	100.0
Stroke-Ready Hospitals	60	170062	100.0
Stroke Units	5	57149	33.61
Stroke Units	10	129492	76.14
Stroke Units	15	167521	98.51
Stroke Units	20	170062	100.0
Stroke Units	25	170062	100.0
Stroke Units	30	170062	100.0
Stroke Units	35	170062	100.0
Stroke Units	40	170062	100.0
Stroke Units	45	170062	100.0
Stroke Units	50	170062	100.0
Stroke Units	55	170062	100.0
Stroke Units	60	170062	100.0

Population Coverage - Kreisfreie Stadt Weiden i.d.OPf. (Bayern)

Condition	Drive Time (min)	Covered Population	Coverage (%)
Hospitals with CT	5	11737	27.1
Hospitals with CT	10	34843	80.44
Hospitals with CT	15	42617	98.39
Hospitals with CT	20	43316	100.0
Hospitals with CT	25	43316	100.0
Hospitals with CT	30	43316	100.0
Hospitals with CT	35	43316	100.0
Hospitals with CT	40	43316	100.0
Hospitals with CT	45	43316	100.0
Hospitals with CT	50	43316	100.0
Hospitals with CT	55	43316	100.0
Hospitals with CT	60	43316	100.0
Stroke-Ready Hospitals	5	16160	37.31
Stroke-Ready Hospitals	10	36715	84.76
Stroke-Ready Hospitals	15	43067	99.43
Stroke-Ready Hospitals	20	43316	100.0
Stroke-Ready Hospitals	25	43316	100.0
Stroke-Ready Hospitals	30	43316	100.0
Stroke-Ready Hospitals	35	43316	100.0
Stroke-Ready Hospitals	40	43316	100.0
Stroke-Ready Hospitals	45	43316	100.0
Stroke-Ready Hospitals	50	43316	100.0
Stroke-Ready Hospitals	55	43316	100.0
Stroke-Ready Hospitals	60	43316	100.0
Stroke Units	5	16160	37.31
Stroke Units	10	36715	84.76
Stroke Units	15	43067	99.43
Stroke Units	20	43316	100.0
Stroke Units	25	43316	100.0
Stroke Units	30	43316	100.0
Stroke Units	35	43316	100.0
Stroke Units	40	43316	100.0
Stroke Units	45	43316	100.0
Stroke Units	50	43316	100.0
Stroke Units	55	43316	100.0
Stroke Units	60	43316	100.0

Population Coverage - Kreisfreie Stadt Ansbach (Bayern)

Condition	Drive Time (min)	Covered Population	Coverage (%)
Hospitals with CT	5	7677	18.63
Hospitals with CT	10	29803	72.34
Hospitals with CT	15	37411	90.8
Hospitals with CT	20	41148	99.87
Hospitals with CT	25	41201	100.0
Hospitals with CT	30	41201	100.0
Hospitals with CT	35	41201	100.0
Hospitals with CT	40	41201	100.0
Hospitals with CT	45	41201	100.0
Hospitals with CT	50	41201	100.0
Hospitals with CT	55	41201	100.0
Hospitals with CT	60	41201	100.0
Stroke-Ready Hospitals	5	7502	18.21
Stroke-Ready Hospitals	10	29655	71.98
Stroke-Ready Hospitals	15	37405	90.79
Stroke-Ready Hospitals	20	41148	99.87
Stroke-Ready Hospitals	25	41201	100.0
Stroke-Ready Hospitals	30	41201	100.0
Stroke-Ready Hospitals	35	41201	100.0
Stroke-Ready Hospitals	40	41201	100.0
Stroke-Ready Hospitals	45	41201	100.0
Stroke-Ready Hospitals	50	41201	100.0
Stroke-Ready Hospitals	55	41201	100.0
Stroke-Ready Hospitals	60	41201	100.0
Stroke Units	5	7502	18.21
Stroke Units	10	29655	71.98
Stroke Units	15	37405	90.79
Stroke Units	20	41148	99.87
Stroke Units	25	41201	100.0
Stroke Units	30	41201	100.0
Stroke Units	35	41201	100.0
Stroke Units	40	41201	100.0
Stroke Units	45	41201	100.0
Stroke Units	50	41201	100.0
Stroke Units	55	41201	100.0
Stroke Units	60	41201	100.0

Population Coverage - Landkreis Rhön-Grabfeld (Bayern)

Condition	Drive Time (min)	Covered Population	Coverage (%)
Hospitals with CT	5	2258	2.75
Hospitals with CT	10	11791	14.35
Hospitals with CT	15	33419	40.67
Hospitals with CT	20	53661	65.3
Hospitals with CT	25	74960	91.22
Hospitals with CT	30	81793	99.53
Hospitals with CT	35	82178	100.0
Hospitals with CT	40	82178	100.0
Hospitals with CT	45	82178	100.0
Hospitals with CT	50	82178	100.0
Hospitals with CT	55	82178	100.0
Hospitals with CT	60	82178	100.0
Stroke-Ready Hospitals	5	650	0.79
Stroke-Ready Hospitals	10	6263	7.62
Stroke-Ready Hospitals	15	22728	27.66
Stroke-Ready Hospitals	20	45033	54.8
Stroke-Ready Hospitals	25	67861	82.58
Stroke-Ready Hospitals	30	78523	95.55
Stroke-Ready Hospitals	35	80808	98.33
Stroke-Ready Hospitals	40	82170	99.99
Stroke-Ready Hospitals	45	82178	100.0
Stroke-Ready Hospitals	50	82178	100.0
Stroke-Ready Hospitals	55	82178	100.0
Stroke-Ready Hospitals	60	82178	100.0
Stroke Units	5	650	0.79
Stroke Units	10	6263	7.62
Stroke Units	15	22728	27.66
Stroke Units	20	45033	54.8
Stroke Units	25	67861	82.58
Stroke Units	30	78523	95.55
Stroke Units	35	80808	98.33
Stroke Units	40	82170	99.99
Stroke Units	45	82178	100.0
Stroke Units	50	82178	100.0
Stroke Units	55	82178	100.0
Stroke Units	60	82178	100.0

Population Coverage - Landkreis Ostprignitz-Ruppin (Brandenburg)

Condition	Drive Time (min)	Covered Population	Coverage (%)
Hospitals with CT	5	19364	20.45
Hospitals with CT	10	36573	38.62
Hospitals with CT	15	49602	52.37
Hospitals with CT	20	66375	70.08
Hospitals with CT	25	80217	84.7
Hospitals with CT	30	67328	71.09
Hospitals with CT	35	92193	97.34
Hospitals with CT	40	92358	97.52
Hospitals with CT	45	94519	99.8
Hospitals with CT	50	94678	99.97
Hospitals with CT	55	94710	100.0
Hospitals with CT	60	94710	100.0
Stroke-Ready Hospitals	5	9277	9.8
Stroke-Ready Hospitals	10	20020	21.14
Stroke-Ready Hospitals	15	26493	27.97
Stroke-Ready Hospitals	20	33784	35.67
Stroke-Ready Hospitals	25	41119	43.42
Stroke-Ready Hospitals	30	48196	50.89
Stroke-Ready Hospitals	35	62593	66.09
Stroke-Ready Hospitals	40	80175	84.65
Stroke-Ready Hospitals	45	89558	94.56
Stroke-Ready Hospitals	50	91935	97.07
Stroke-Ready Hospitals	55	94312	99.58
Stroke-Ready Hospitals	60	94558	99.84
Stroke Units	5	9277	9.8
Stroke Units	10	20020	21.14
Stroke Units	15	26493	27.97
Stroke Units	20	33738	35.62
Stroke Units	25	40656	42.93
Stroke Units	30	47895	50.57
Stroke Units	35	62191	65.66
Stroke Units	40	75239	79.44
Stroke Units	45	87442	92.33
Stroke Units	50	90847	95.92
Stroke Units	55	92636	97.81
Stroke Units	60	93777	99.02

Population Coverage - Kreis Plön (Schleswig-Holstein)

Condition	Drive Time (min)	Covered Population	Coverage (%)
Hospitals with CT	5	7394	5.74
Hospitals with CT	10	25623	19.88
Hospitals with CT	15	47893	37.15
Hospitals with CT	20	72557	56.29
Hospitals with CT	25	104430	81.01
Hospitals with CT	30	122177	94.78
Hospitals with CT	35	127300	98.75
Hospitals with CT	40	127904	99.22
Hospitals with CT	45	127934	99.24
Hospitals with CT	50	127956	99.26
Hospitals with CT	55	128034	99.32
Hospitals with CT	60	128131	99.4
Stroke-Ready Hospitals	5	0	0.0
Stroke-Ready Hospitals	10	2111	1.64
Stroke-Ready Hospitals	15	6642	5.15
Stroke-Ready Hospitals	20	26329	20.42
Stroke-Ready Hospitals	25	60423	46.87
Stroke-Ready Hospitals	30	97309	75.49
Stroke-Ready Hospitals	35	115743	89.79
Stroke-Ready Hospitals	40	126938	98.47
Stroke-Ready Hospitals	45	127493	98.9
Stroke-Ready Hospitals	50	127552	98.95
Stroke-Ready Hospitals	55	127869	99.19
Stroke-Ready Hospitals	60	127990	99.29
Stroke Units	5	0	0.0
Stroke Units	10	2111	1.64
Stroke Units	15	6642	5.15
Stroke Units	20	26329	20.42
Stroke Units	25	60423	46.87
Stroke Units	30	97280	75.46
Stroke Units	35	115715	89.76
Stroke Units	40	126912	98.45
Stroke Units	45	127469	98.88
Stroke Units	50	127547	98.94
Stroke Units	55	127869	99.19
Stroke Units	60	127990	99.29

Population Coverage - Landkreis Nienburg (Weser) (Niedersachsen)

Condition	Drive Time (min)	Covered Population	Coverage (%)
Hospitals with CT	5	9234	7.61
Hospitals with CT	10	25700	21.17
Hospitals with CT	15	39573	32.59
Hospitals with CT	20	53538	44.09
Hospitals with CT	25	76332	62.87
Hospitals with CT	30	105240	86.68
Hospitals with CT	35	120138	98.95
Hospitals with CT	40	121419	100.0
Hospitals with CT	45	121419	100.0
Hospitals with CT	50	121419	100.0
Hospitals with CT	55	121419	100.0
Hospitals with CT	60	121419	100.0
Stroke-Ready Hospitals	5	9234	7.61
Stroke-Ready Hospitals	10	25700	21.17
Stroke-Ready Hospitals	15	39058	32.17
Stroke-Ready Hospitals	20	50460	41.56
Stroke-Ready Hospitals	25	64009	52.72
Stroke-Ready Hospitals	30	81794	67.37
Stroke-Ready Hospitals	35	102812	84.68
Stroke-Ready Hospitals	40	117388	96.68
Stroke-Ready Hospitals	45	121024	99.67
Stroke-Ready Hospitals	50	121419	100.0
Stroke-Ready Hospitals	55	121419	100.0
Stroke-Ready Hospitals	60	121419	100.0
Stroke Units	5	0	0.0
Stroke Units	10	0	0.0
Stroke Units	15	0	0.0
Stroke Units	20	0	0.0
Stroke Units	25	0	0.0
Stroke Units	30	527	0.43
Stroke Units	35	7339	6.04
Stroke Units	40	20009	16.48
Stroke Units	45	36931	30.42
Stroke Units	50	73671	60.68
Stroke Units	55	98380	81.03
Stroke Units	60	114741	94.5

Population Coverage - Landkreis Emsland (Niedersachsen)

Condition	Drive Time (min)	Covered Population	Coverage (%)
Hospitals with CT	5	68233	21.16
Hospitals with CT	10	131415	40.76
Hospitals with CT	15	186932	57.97
Hospitals with CT	20	255440	79.22
Hospitals with CT	25	306580	95.08
Hospitals with CT	30	321364	99.67
Hospitals with CT	35	322185	99.92
Hospitals with CT	40	322246	99.94
Hospitals with CT	45	322255	99.94
Hospitals with CT	50	322289	99.95
Hospitals with CT	55	322226	99.93
Hospitals with CT	60	322251	99.94
Stroke-Ready Hospitals	5	13578	4.21
Stroke-Ready Hospitals	10	29076	9.02
Stroke-Ready Hospitals	15	50189	15.57
Stroke-Ready Hospitals	20	117815	36.54
Stroke-Ready Hospitals	25	180596	56.01
Stroke-Ready Hospitals	30	222481	69.0
Stroke-Ready Hospitals	35	250094	77.56
Stroke-Ready Hospitals	40	268443	83.25
Stroke-Ready Hospitals	45	289496	89.78
Stroke-Ready Hospitals	50	319641	99.13
Stroke-Ready Hospitals	55	322022	99.87
Stroke-Ready Hospitals	60	322145	99.91
Stroke Units	5	13578	4.21
Stroke Units	10	29076	9.02
Stroke Units	15	50189	15.57
Stroke Units	20	117815	36.54
Stroke Units	25	180596	56.01
Stroke Units	30	222481	69.0
Stroke Units	35	250094	77.56
Stroke Units	40	268443	83.25
Stroke Units	45	289496	89.78
Stroke Units	50	319641	99.13
Stroke Units	55	322022	99.87
Stroke Units	60	322145	99.91

Population Coverage - Landkreis Wittmund (Niedersachsen)

Condition	Drive Time (min)	Covered Population	Coverage (%)
Hospitals with CT	5	6823	11.98
Hospitals with CT	10	11712	20.57
Hospitals with CT	15	17307	30.39
Hospitals with CT	20	30319	53.24
Hospitals with CT	25	49097	86.22
Hospitals with CT	30	54163	95.11
Hospitals with CT	35	54254	95.27
Hospitals with CT	40	54299	95.35
Hospitals with CT	45	54316	95.38
Hospitals with CT	50	54324	95.4
Hospitals with CT	55	54194	95.17
Hospitals with CT	60	54241	95.25
Stroke-Ready Hospitals	5	0	0.0
Stroke-Ready Hospitals	10	16	0.03
Stroke-Ready Hospitals	15	886	1.56
Stroke-Ready Hospitals	20	3724	6.54
Stroke-Ready Hospitals	25	16311	28.64
Stroke-Ready Hospitals	30	24134	42.38
Stroke-Ready Hospitals	35	31004	54.45
Stroke-Ready Hospitals	40	40765	71.59
Stroke-Ready Hospitals	45	47185	82.86
Stroke-Ready Hospitals	50	53647	94.21
Stroke-Ready Hospitals	55	54185	95.15
Stroke-Ready Hospitals	60	54202	95.18
Stroke Units	5	0	0.0
Stroke Units	10	16	0.03
Stroke Units	15	886	1.56
Stroke Units	20	3724	6.54
Stroke Units	25	16311	28.64
Stroke Units	30	24134	42.38
Stroke Units	35	31004	54.45
Stroke Units	40	40765	71.59
Stroke Units	45	47185	82.86
Stroke Units	50	53647	94.21
Stroke Units	55	54185	95.15
Stroke Units	60	54202	95.18

Population Coverage - Kreis Hochsauerlandkreis (Nordrhein-Westfalen)

Condition	Drive Time (min)	Covered Population	Coverage (%)
Hospitals with CT	5	58838	22.43
Hospitals with CT	10	129844	49.49
Hospitals with CT	15	187747	71.56
Hospitals with CT	20	235379	89.71
Hospitals with CT	25	258360	98.47
Hospitals with CT	30	262315	99.98
Hospitals with CT	35	262366	100.0
Hospitals with CT	40	262369	100.0
Hospitals with CT	45	262369	100.0
Hospitals with CT	50	262369	100.0
Hospitals with CT	55	262369	100.0
Hospitals with CT	60	262369	100.0
Stroke-Ready Hospitals	5	6961	2.65
Stroke-Ready Hospitals	10	34881	13.29
Stroke-Ready Hospitals	15	58959	22.47
Stroke-Ready Hospitals	20	84992	32.39
Stroke-Ready Hospitals	25	106051	40.42
Stroke-Ready Hospitals	30	136764	52.13
Stroke-Ready Hospitals	35	159746	60.89
Stroke-Ready Hospitals	40	185953	70.87
Stroke-Ready Hospitals	45	220755	84.14
Stroke-Ready Hospitals	50	238502	90.9
Stroke-Ready Hospitals	55	255471	97.37
Stroke-Ready Hospitals	60	260999	99.48
Stroke Units	5	6961	2.65
Stroke Units	10	34881	13.29
Stroke Units	15	58947	22.47
Stroke Units	20	84961	32.38
Stroke Units	25	106051	40.42
Stroke Units	30	136764	52.13
Stroke Units	35	159746	60.89
Stroke Units	40	185953	70.87
Stroke Units	45	220307	83.97
Stroke Units	50	237477	90.51
Stroke Units	55	250168	95.35
Stroke Units	60	258223	98.42

Population Coverage - Landkreis Rheingau-Taunus-Kreis (Hessen)

Condition	Drive Time (min)	Covered Population	Coverage (%)
Hospitals with CT	5	26038	14.21
Hospitals with CT	10	69700	38.03
Hospitals with CT	15	137354	74.95
Hospitals with CT	20	175044	95.52
Hospitals with CT	25	180222	98.35
Hospitals with CT	30	183251	100.0
Hospitals with CT	35	183255	100.0
Hospitals with CT	40	183255	100.0
Hospitals with CT	45	183255	100.0
Hospitals with CT	50	183255	100.0
Hospitals with CT	55	183255	100.0
Hospitals with CT	60	183255	100.0
Stroke-Ready Hospitals	5	0	0.0
Stroke-Ready Hospitals	10	415	0.23
Stroke-Ready Hospitals	15	15971	8.72
Stroke-Ready Hospitals	20	43492	23.73
Stroke-Ready Hospitals	25	103867	56.68
Stroke-Ready Hospitals	30	144935	79.09
Stroke-Ready Hospitals	35	172434	94.1
Stroke-Ready Hospitals	40	178260	97.27
Stroke-Ready Hospitals	45	180943	98.74
Stroke-Ready Hospitals	50	182799	99.75
Stroke-Ready Hospitals	55	183221	99.98
Stroke-Ready Hospitals	60	183255	100.0
Stroke Units	5	0	0.0
Stroke Units	10	415	0.23
Stroke Units	15	15971	8.72
Stroke Units	20	43492	23.73
Stroke Units	25	103772	56.63
Stroke Units	30	144365	78.78
Stroke Units	35	172428	94.09
Stroke Units	40	178260	97.27
Stroke Units	45	180943	98.74
Stroke Units	50	182799	99.75
Stroke Units	55	183219	99.98
Stroke Units	60	183255	100.0

Population Coverage - Landkreis Göppingen (Baden-Württemberg)

Condition	Drive Time (min)	Covered Population	Coverage (%)
Hospitals with CT	5	26506	10.6
Hospitals with CT	10	79742	31.9
Hospitals with CT	15	169756	67.9
Hospitals with CT	20	210271	84.11
Hospitals with CT	25	240676	96.27
Hospitals with CT	30	249862	99.95
Hospitals with CT	35	249996	100.0
Hospitals with CT	40	249996	100.0
Hospitals with CT	45	249996	100.0
Hospitals with CT	50	249996	100.0
Hospitals with CT	55	249996	100.0
Hospitals with CT	60	249996	100.0
Stroke-Ready Hospitals	5	29254	11.7
Stroke-Ready Hospitals	10	74130	29.65
Stroke-Ready Hospitals	15	159818	63.93
Stroke-Ready Hospitals	20	199314	79.73
Stroke-Ready Hospitals	25	234808	93.92
Stroke-Ready Hospitals	30	247846	99.14
Stroke-Ready Hospitals	35	249996	100.0
Stroke-Ready Hospitals	40	249996	100.0
Stroke-Ready Hospitals	45	249996	100.0
Stroke-Ready Hospitals	50	249996	100.0
Stroke-Ready Hospitals	55	249996	100.0
Stroke-Ready Hospitals	60	249996	100.0
Stroke Units	5	29254	11.7
Stroke Units	10	73914	29.57
Stroke Units	15	157100	62.84
Stroke Units	20	199294	79.72
Stroke Units	25	234806	93.92
Stroke Units	30	247846	99.14
Stroke Units	35	249992	100.0
Stroke Units	40	249996	100.0
Stroke Units	45	249996	100.0
Stroke Units	50	249996	100.0
Stroke Units	55	249996	100.0
Stroke Units	60	249996	100.0

Population Coverage - Stadtkreis Freiburg im Breisgau (Baden-Württemberg)

Condition	Drive Time (min)	Covered Population	Coverage (%)
Hospitals with CT	5	87500	33.86
Hospitals with CT	10	205711	79.6
Hospitals with CT	15	242439	93.81
Hospitals with CT	20	258147	99.89
Hospitals with CT	25	258335	99.96
Hospitals with CT	30	258373	99.97
Hospitals with CT	35	258439	100.0
Hospitals with CT	40	258439	100.0
Hospitals with CT	45	258439	100.0
Hospitals with CT	50	258439	100.0
Hospitals with CT	55	258439	100.0
Hospitals with CT	60	258439	100.0
Stroke-Ready Hospitals	5	24358	9.43
Stroke-Ready Hospitals	10	105461	40.81
Stroke-Ready Hospitals	15	211879	81.98
Stroke-Ready Hospitals	20	250141	96.79
Stroke-Ready Hospitals	25	257759	99.74
Stroke-Ready Hospitals	30	258043	99.85
Stroke-Ready Hospitals	35	258306	99.95
Stroke-Ready Hospitals	40	258439	100.0
Stroke-Ready Hospitals	45	258439	100.0
Stroke-Ready Hospitals	50	258439	100.0
Stroke-Ready Hospitals	55	258439	100.0
Stroke-Ready Hospitals	60	258439	100.0
Stroke Units	5	24358	9.43
Stroke Units	10	105461	40.81
Stroke Units	15	211879	81.98
Stroke Units	20	250141	96.79
Stroke Units	25	257759	99.74
Stroke Units	30	258043	99.85
Stroke Units	35	258295	99.94
Stroke Units	40	258439	100.0
Stroke Units	45	258439	100.0
Stroke Units	50	258439	100.0
Stroke Units	55	258439	100.0
Stroke Units	60	258439	100.0

Population Coverage - Landkreis Landshut (Bayern)

Condition	Drive Time (min)	Covered Population	Coverage (%)
Hospitals with CT	5	7376	4.74
Hospitals with CT	10	40394	25.95
Hospitals with CT	15	92090	59.15
Hospitals with CT	20	132181	84.9
Hospitals with CT	25	147805	94.94
Hospitals with CT	30	155478	99.86
Hospitals with CT	35	155690	100.0
Hospitals with CT	40	155690	100.0
Hospitals with CT	45	155690	100.0
Hospitals with CT	50	155690	100.0
Hospitals with CT	55	155690	100.0
Hospitals with CT	60	155690	100.0
Stroke-Ready Hospitals	5	7118	4.57
Stroke-Ready Hospitals	10	25011	16.06
Stroke-Ready Hospitals	15	65719	42.21
Stroke-Ready Hospitals	20	102977	66.14
Stroke-Ready Hospitals	25	133893	86.0
Stroke-Ready Hospitals	30	146638	94.19
Stroke-Ready Hospitals	35	155529	99.9
Stroke-Ready Hospitals	40	155690	100.0
Stroke-Ready Hospitals	45	155690	100.0
Stroke-Ready Hospitals	50	155690	100.0
Stroke-Ready Hospitals	55	155690	100.0
Stroke-Ready Hospitals	60	155690	100.0
Stroke Units	5	7118	4.57
Stroke Units	10	25011	16.06
Stroke Units	15	65719	42.21
Stroke Units	20	102952	66.13
Stroke Units	25	133806	85.94
Stroke Units	30	146208	93.91
Stroke Units	35	155387	99.81
Stroke Units	40	155690	100.0
Stroke Units	45	155690	100.0
Stroke Units	50	155690	100.0
Stroke Units	55	155690	100.0
Stroke Units	60	155690	100.0

Population Coverage - Landkreis Regen (Bayern)

Condition	Drive Time (min)	Covered Population	Coverage (%)
Hospitals with CT	5	6359	8.14
Hospitals with CT	10	20151	25.81
Hospitals with CT	15	34344	43.99
Hospitals with CT	20	54429	69.72
Hospitals with CT	25	73499	94.14
Hospitals with CT	30	77829	99.69
Hospitals with CT	35	77968	99.87
Hospitals with CT	40	77968	99.87
Hospitals with CT	45	78003	99.91
Hospitals with CT	50	78004	99.91
Hospitals with CT	55	78017	99.93
Hospitals with CT	60	78017	99.93
Stroke-Ready Hospitals	5	3803	4.87
Stroke-Ready Hospitals	10	11064	14.17
Stroke-Ready Hospitals	15	20317	26.02
Stroke-Ready Hospitals	20	33932	43.46
Stroke-Ready Hospitals	25	50018	64.07
Stroke-Ready Hospitals	30	65523	83.93
Stroke-Ready Hospitals	35	75674	96.93
Stroke-Ready Hospitals	40	77972	99.87
Stroke-Ready Hospitals	45	78010	99.92
Stroke-Ready Hospitals	50	78010	99.92
Stroke-Ready Hospitals	55	78014	99.93
Stroke-Ready Hospitals	60	78017	99.93
Stroke Units	5	3803	4.87
Stroke Units	10	11064	14.17
Stroke Units	15	20317	26.02
Stroke Units	20	33932	43.46
Stroke Units	25	50018	64.07
Stroke Units	30	65523	83.93
Stroke Units	35	75674	96.93
Stroke Units	40	77972	99.87
Stroke Units	45	78010	99.92
Stroke Units	50	78010	99.92
Stroke Units	55	78008	99.92
Stroke Units	60	78011	99.92

Population Coverage - Kreisfreie Stadt Coburg (Bayern)

Condition	Drive Time (min)	Covered Population	Coverage (%)
Hospitals with CT	5	12712	30.72
Hospitals with CT	10	36315	87.75
Hospitals with CT	15	41288	99.77
Hospitals with CT	20	41384	100.0
Hospitals with CT	25	41384	100.0
Hospitals with CT	30	41384	100.0
Hospitals with CT	35	41384	100.0
Hospitals with CT	40	41384	100.0
Hospitals with CT	45	41384	100.0
Hospitals with CT	50	41384	100.0
Hospitals with CT	55	41384	100.0
Hospitals with CT	60	41384	100.0
Stroke-Ready Hospitals	5	12708	30.71
Stroke-Ready Hospitals	10	36301	87.72
Stroke-Ready Hospitals	15	41288	99.77
Stroke-Ready Hospitals	20	41384	100.0
Stroke-Ready Hospitals	25	41384	100.0
Stroke-Ready Hospitals	30	41384	100.0
Stroke-Ready Hospitals	35	41384	100.0
Stroke-Ready Hospitals	40	41384	100.0
Stroke-Ready Hospitals	45	41384	100.0
Stroke-Ready Hospitals	50	41384	100.0
Stroke-Ready Hospitals	55	41384	100.0
Stroke-Ready Hospitals	60	41384	100.0
Stroke Units	5	12708	30.71
Stroke Units	10	36301	87.72
Stroke Units	15	41288	99.77
Stroke Units	20	41384	100.0
Stroke Units	25	41384	100.0
Stroke Units	30	41384	100.0
Stroke Units	35	41384	100.0
Stroke Units	40	41384	100.0
Stroke Units	45	41384	100.0
Stroke Units	50	41384	100.0
Stroke Units	55	41384	100.0
Stroke Units	60	41384	100.0

Population Coverage - Landkreis Neunkirchen (Saarland)

Condition	Drive Time (min)	Covered Population	Coverage (%)
Hospitals with CT	5	28982	22.76
Hospitals with CT	10	59725	46.9
Hospitals with CT	15	99288	77.97
Hospitals with CT	20	124361	97.66
Hospitals with CT	25	127327	99.99
Hospitals with CT	30	127343	100.0
Hospitals with CT	35	127343	100.0
Hospitals with CT	40	127343	100.0
Hospitals with CT	45	127343	100.0
Hospitals with CT	50	127343	100.0
Hospitals with CT	55	127343	100.0
Hospitals with CT	60	127343	100.0
Stroke-Ready Hospitals	5	18260	14.34
Stroke-Ready Hospitals	10	51249	40.24
Stroke-Ready Hospitals	15	87923	69.04
Stroke-Ready Hospitals	20	118739	93.24
Stroke-Ready Hospitals	25	126877	99.63
Stroke-Ready Hospitals	30	127343	100.0
Stroke-Ready Hospitals	35	127343	100.0
Stroke-Ready Hospitals	40	127343	100.0
Stroke-Ready Hospitals	45	127343	100.0
Stroke-Ready Hospitals	50	127343	100.0
Stroke-Ready Hospitals	55	127343	100.0
Stroke-Ready Hospitals	60	127343	100.0
Stroke Units	5	18260	14.34
Stroke Units	10	51249	40.24
Stroke Units	15	87923	69.04
Stroke Units	20	118739	93.24
Stroke Units	25	126877	99.63
Stroke Units	30	127343	100.0
Stroke Units	35	127343	100.0
Stroke Units	40	127343	100.0
Stroke Units	45	127343	100.0
Stroke Units	50	127343	100.0
Stroke Units	55	127343	100.0
Stroke Units	60	127343	100.0

Population Coverage - Landkreis Nordwestmecklenburg (Mecklenburg-Vorpommern)

Condition	Drive Time (min)	Covered Population	Coverage (%)
Hospitals with CT	5	23586	16.23
Hospitals with CT	10	50403	34.68
Hospitals with CT	15	74059	50.95
Hospitals with CT	20	101008	69.49
Hospitals with CT	25	128287	88.26
Hospitals with CT	30	141704	97.49
Hospitals with CT	35	143897	99.0
Hospitals with CT	40	144294	99.27
Hospitals with CT	45	144440	99.37
Hospitals with CT	50	144645	99.51
Hospitals with CT	55	143994	99.07
Hospitals with CT	60	144602	99.48
Stroke-Ready Hospitals	5	15273	10.51
Stroke-Ready Hospitals	10	35252	24.25
Stroke-Ready Hospitals	15	50638	34.84
Stroke-Ready Hospitals	20	63776	43.88
Stroke-Ready Hospitals	25	93733	64.49
Stroke-Ready Hospitals	30	116269	79.99
Stroke-Ready Hospitals	35	132012	90.82
Stroke-Ready Hospitals	40	141144	97.1
Stroke-Ready Hospitals	45	143811	98.94
Stroke-Ready Hospitals	50	144112	99.15
Stroke-Ready Hospitals	55	143469	98.7
Stroke-Ready Hospitals	60	144121	99.15
Stroke Units	5	15273	10.51
Stroke Units	10	35252	24.25
Stroke Units	15	50638	34.84
Stroke Units	20	63776	43.88
Stroke Units	25	93731	64.49
Stroke Units	30	116268	79.99
Stroke Units	35	132012	90.82
Stroke Units	40	141144	97.1
Stroke Units	45	143778	98.92
Stroke Units	50	144055	99.11
Stroke Units	55	143358	98.63
Stroke Units	60	143983	99.06

Population Coverage - Kreisfreie Stadt Herne (Nordrhein-Westfalen)

Condition	Drive Time (min)	Covered Population	Coverage (%)
Hospitals with CT	5	90113	54.53
Hospitals with CT	10	165021	99.87
Hospitals with CT	15	165244	100.0
Hospitals with CT	20	165244	100.0
Hospitals with CT	25	165244	100.0
Hospitals with CT	30	165244	100.0
Hospitals with CT	35	165244	100.0
Hospitals with CT	40	165244	100.0
Hospitals with CT	45	165244	100.0
Hospitals with CT	50	165244	100.0
Hospitals with CT	55	165244	100.0
Hospitals with CT	60	165244	100.0
Stroke-Ready Hospitals	5	33683	20.38
Stroke-Ready Hospitals	10	112120	67.85
Stroke-Ready Hospitals	15	165232	99.99
Stroke-Ready Hospitals	20	165244	100.0
Stroke-Ready Hospitals	25	165244	100.0
Stroke-Ready Hospitals	30	165244	100.0
Stroke-Ready Hospitals	35	165244	100.0
Stroke-Ready Hospitals	40	165244	100.0
Stroke-Ready Hospitals	45	165244	100.0
Stroke-Ready Hospitals	50	165244	100.0
Stroke-Ready Hospitals	55	165244	100.0
Stroke-Ready Hospitals	60	165244	100.0
Stroke Units	5	33683	20.38
Stroke Units	10	112120	67.85
Stroke Units	15	165232	99.99
Stroke Units	20	165244	100.0
Stroke Units	25	165244	100.0
Stroke Units	30	165244	100.0
Stroke Units	35	165244	100.0
Stroke Units	40	165244	100.0
Stroke Units	45	165244	100.0
Stroke Units	50	165244	100.0
Stroke Units	55	165244	100.0
Stroke Units	60	165244	100.0

Population Coverage - Landkreis Altenkirchen (Westerwald) (Rheinland-Pfalz)

Condition	Drive Time (min)	Covered Population	Coverage (%)
Hospitals with CT	5	6927	5.19
Hospitals with CT	10	19791	14.84
Hospitals with CT	15	58140	43.58
Hospitals with CT	20	97956	73.43
Hospitals with CT	25	130634	97.92
Hospitals with CT	30	133409	100.0
Hospitals with CT	35	133409	100.0
Hospitals with CT	40	133409	100.0
Hospitals with CT	45	133409	100.0
Hospitals with CT	50	133409	100.0
Hospitals with CT	55	133409	100.0
Hospitals with CT	60	133409	100.0
Stroke-Ready Hospitals	5	0	0.0
Stroke-Ready Hospitals	10	220	0.17
Stroke-Ready Hospitals	15	8212	6.16
Stroke-Ready Hospitals	20	23937	17.94
Stroke-Ready Hospitals	25	36179	27.12
Stroke-Ready Hospitals	30	62860	47.12
Stroke-Ready Hospitals	35	86975	65.19
Stroke-Ready Hospitals	40	116468	87.3
Stroke-Ready Hospitals	45	132729	99.49
Stroke-Ready Hospitals	50	133409	100.0
Stroke-Ready Hospitals	55	133409	100.0
Stroke-Ready Hospitals	60	133409	100.0
Stroke Units	5	0	0.0
Stroke Units	10	220	0.17
Stroke Units	15	6880	5.16
Stroke Units	20	23210	17.4
Stroke Units	25	34999	26.24
Stroke Units	30	54983	41.21
Stroke Units	35	76359	57.24
Stroke Units	40	110899	83.13
Stroke Units	45	130066	97.49
Stroke Units	50	133409	100.0
Stroke Units	55	133409	100.0
Stroke Units	60	133409	100.0

Population Coverage - Kreisfreie Stadt Kaiserslautern (Rheinland-Pfalz)

Condition	Drive Time (min)	Covered Population	Coverage (%)
Hospitals with CT	5	35756	32.27
Hospitals with CT	10	83966	75.77
Hospitals with CT	15	109224	98.57
Hospitals with CT	20	110718	99.91
Hospitals with CT	25	110813	100.0
Hospitals with CT	30	110813	100.0
Hospitals with CT	35	110813	100.0
Hospitals with CT	40	110813	100.0
Hospitals with CT	45	110813	100.0
Hospitals with CT	50	110813	100.0
Hospitals with CT	55	110813	100.0
Hospitals with CT	60	110813	100.0
Stroke-Ready Hospitals	5	28469	25.69
Stroke-Ready Hospitals	10	81283	73.35
Stroke-Ready Hospitals	15	109003	98.37
Stroke-Ready Hospitals	20	110262	99.5
Stroke-Ready Hospitals	25	110813	100.0
Stroke-Ready Hospitals	30	110813	100.0
Stroke-Ready Hospitals	35	110813	100.0
Stroke-Ready Hospitals	40	110813	100.0
Stroke-Ready Hospitals	45	110813	100.0
Stroke-Ready Hospitals	50	110813	100.0
Stroke-Ready Hospitals	55	110813	100.0
Stroke-Ready Hospitals	60	110813	100.0
Stroke Units	5	28469	25.69
Stroke Units	10	81283	73.35
Stroke Units	15	109003	98.37
Stroke Units	20	110262	99.5
Stroke Units	25	110813	100.0
Stroke Units	30	110813	100.0
Stroke Units	35	110813	100.0
Stroke Units	40	110813	100.0
Stroke Units	45	110813	100.0
Stroke Units	50	110813	100.0
Stroke Units	55	110813	100.0
Stroke Units	60	110813	100.0

Population Coverage - Landkreis Mainz-Bingen (Rheinland-Pfalz)

Condition	Drive Time (min)	Covered Population	Coverage (%)
Hospitals with CT	5	10262	4.88
Hospitals with CT	10	36480	17.35
Hospitals with CT	15	96078	45.7
Hospitals with CT	20	173194	82.39
Hospitals with CT	25	206756	98.35
Hospitals with CT	30	210177	99.98
Hospitals with CT	35	210187	99.98
Hospitals with CT	40	210219	100.0
Hospitals with CT	45	210219	100.0
Hospitals with CT	50	210219	100.0
Hospitals with CT	55	210219	100.0
Hospitals with CT	60	210219	100.0
Stroke-Ready Hospitals	5	0	0.0
Stroke-Ready Hospitals	10	603	0.29
Stroke-Ready Hospitals	15	20877	9.93
Stroke-Ready Hospitals	20	100919	48.01
Stroke-Ready Hospitals	25	181818	86.49
Stroke-Ready Hospitals	30	205277	97.65
Stroke-Ready Hospitals	35	207036	98.49
Stroke-Ready Hospitals	40	209844	99.82
Stroke-Ready Hospitals	45	210218	100.0
Stroke-Ready Hospitals	50	210219	100.0
Stroke-Ready Hospitals	55	210219	100.0
Stroke-Ready Hospitals	60	210219	100.0
Stroke Units	5	0	0.0
Stroke Units	10	603	0.29
Stroke Units	15	20274	9.64
Stroke Units	20	97543	46.4
Stroke Units	25	176917	84.16
Stroke Units	30	205083	97.56
Stroke Units	35	207033	98.48
Stroke Units	40	209844	99.82
Stroke Units	45	210218	100.0
Stroke Units	50	210219	100.0
Stroke Units	55	210219	100.0
Stroke Units	60	210219	100.0

Population Coverage - Landkreis Heidenheim (Baden-Württemberg)

Condition	Drive Time (min)	Covered Population	Coverage (%)
Hospitals with CT	5	9915	7.75
Hospitals with CT	10	34601	27.03
Hospitals with CT	15	75134	58.7
Hospitals with CT	20	110126	86.04
Hospitals with CT	25	127615	99.71
Hospitals with CT	30	127989	100.0
Hospitals with CT	35	127989	100.0
Hospitals with CT	40	127989	100.0
Hospitals with CT	45	127989	100.0
Hospitals with CT	50	127989	100.0
Hospitals with CT	55	127989	100.0
Hospitals with CT	60	127989	100.0
Stroke-Ready Hospitals	5	9678	7.56
Stroke-Ready Hospitals	10	32134	25.11
Stroke-Ready Hospitals	15	59146	46.21
Stroke-Ready Hospitals	20	92932	72.61
Stroke-Ready Hospitals	25	119710	93.53
Stroke-Ready Hospitals	30	124536	97.3
Stroke-Ready Hospitals	35	126270	98.66
Stroke-Ready Hospitals	40	127642	99.73
Stroke-Ready Hospitals	45	127989	100.0
Stroke-Ready Hospitals	50	127989	100.0
Stroke-Ready Hospitals	55	127989	100.0
Stroke-Ready Hospitals	60	127989	100.0
Stroke Units	5	9678	7.56
Stroke Units	10	32134	25.11
Stroke Units	15	59146	46.21
Stroke Units	20	92932	72.61
Stroke Units	25	119710	93.53
Stroke Units	30	124536	97.3
Stroke Units	35	126270	98.66
Stroke Units	40	127642	99.73
Stroke Units	45	127989	100.0
Stroke Units	50	127989	100.0
Stroke Units	55	127989	100.0
Stroke Units	60	127989	100.0

Population Coverage - Stadtkreis Pforzheim (Baden-Württemberg)

Condition	Drive Time (min)	Covered Population	Coverage (%)
Hospitals with CT	5	63804	51.06
Hospitals with CT	10	107922	86.36
Hospitals with CT	15	123136	98.54
Hospitals with CT	20	124962	100.0
Hospitals with CT	25	124962	100.0
Hospitals with CT	30	124962	100.0
Hospitals with CT	35	124962	100.0
Hospitals with CT	40	124962	100.0
Hospitals with CT	45	124962	100.0
Hospitals with CT	50	124962	100.0
Hospitals with CT	55	124962	100.0
Hospitals with CT	60	124962	100.0
Stroke-Ready Hospitals	5	20275	16.23
Stroke-Ready Hospitals	10	82751	66.22
Stroke-Ready Hospitals	15	115069	92.08
Stroke-Ready Hospitals	20	124893	99.94
Stroke-Ready Hospitals	25	124962	100.0
Stroke-Ready Hospitals	30	124962	100.0
Stroke-Ready Hospitals	35	124962	100.0
Stroke-Ready Hospitals	40	124962	100.0
Stroke-Ready Hospitals	45	124962	100.0
Stroke-Ready Hospitals	50	124962	100.0
Stroke-Ready Hospitals	55	124962	100.0
Stroke-Ready Hospitals	60	124962	100.0
Stroke Units	5	20275	16.23
Stroke Units	10	82751	66.22
Stroke Units	15	115069	92.08
Stroke Units	20	124893	99.94
Stroke Units	25	124962	100.0
Stroke Units	30	124962	100.0
Stroke Units	35	124962	100.0
Stroke Units	40	124962	100.0
Stroke Units	45	124962	100.0
Stroke Units	50	124962	100.0
Stroke Units	55	124962	100.0
Stroke Units	60	124962	100.0

Population Coverage - Landkreis Tübingen (Baden-Württemberg)

Condition	Drive Time (min)	Covered Population	Coverage (%)
Hospitals with CT	5	16610	7.6
Hospitals with CT	10	46029	21.06
Hospitals with CT	15	90559	41.44
Hospitals with CT	20	160371	73.38
Hospitals with CT	25	209606	95.91
Hospitals with CT	30	218469	99.97
Hospitals with CT	35	218527	100.0
Hospitals with CT	40	218538	100.0
Hospitals with CT	45	218538	100.0
Hospitals with CT	50	218538	100.0
Hospitals with CT	55	218538	100.0
Hospitals with CT	60	218538	100.0
Stroke-Ready Hospitals	5	16898	7.73
Stroke-Ready Hospitals	10	53581	24.52
Stroke-Ready Hospitals	15	88921	40.69
Stroke-Ready Hospitals	20	150414	68.83
Stroke-Ready Hospitals	25	198860	91.0
Stroke-Ready Hospitals	30	211804	96.92
Stroke-Ready Hospitals	35	218527	99.99
Stroke-Ready Hospitals	40	218538	100.0
Stroke-Ready Hospitals	45	218538	100.0
Stroke-Ready Hospitals	50	218538	100.0
Stroke-Ready Hospitals	55	218538	100.0
Stroke-Ready Hospitals	60	218538	100.0
Stroke Units	5	16898	7.73
Stroke Units	10	53581	24.52
Stroke Units	15	88241	40.38
Stroke Units	20	137314	62.83
Stroke Units	25	173996	79.62
Stroke Units	30	202759	92.78
Stroke Units	35	217157	99.37
Stroke Units	40	218538	100.0
Stroke Units	45	218538	100.0
Stroke Units	50	218538	100.0
Stroke Units	55	218538	100.0
Stroke Units	60	218538	100.0

Population Coverage - Landkreis Bodenseekreis (Baden-Württemberg)

Condition	Drive Time (min)	Covered Population	Coverage (%)
Hospitals with CT	5	29528	13.36
Hospitals with CT	10	77762	35.18
Hospitals with CT	15	169368	76.61
Hospitals with CT	20	212068	95.93
Hospitals with CT	25	218710	98.93
Hospitals with CT	30	220603	99.79
Hospitals with CT	35	220812	99.88
Hospitals with CT	40	220860	99.9
Hospitals with CT	45	220920	99.93
Hospitals with CT	50	220929	99.93
Hospitals with CT	55	220472	99.73
Hospitals with CT	60	220770	99.86
Stroke-Ready Hospitals	5	3764	1.7
Stroke-Ready Hospitals	10	24791	11.21
Stroke-Ready Hospitals	15	67143	30.37
Stroke-Ready Hospitals	20	110836	50.14
Stroke-Ready Hospitals	25	165678	74.94
Stroke-Ready Hospitals	30	210690	95.3
Stroke-Ready Hospitals	35	219054	99.09
Stroke-Ready Hospitals	40	220657	99.81
Stroke-Ready Hospitals	45	220696	99.83
Stroke-Ready Hospitals	50	220698	99.83
Stroke-Ready Hospitals	55	220327	99.66
Stroke-Ready Hospitals	60	220486	99.73
Stroke Units	5	3764	1.7
Stroke Units	10	24791	11.21
Stroke Units	15	67143	30.37
Stroke Units	20	110836	50.14
Stroke Units	25	165678	74.94
Stroke Units	30	210690	95.3
Stroke Units	35	219054	99.09
Stroke Units	40	220657	99.81
Stroke Units	45	220696	99.83
Stroke Units	50	220698	99.83
Stroke Units	55	220315	99.66
Stroke Units	60	220474	99.73

Population Coverage - Landkreis Miesbach (Bayern)

Condition	Drive Time (min)	Covered Population	Coverage (%)
Hospitals with CT	5	3690	3.74
Hospitals with CT	10	21110	21.38
Hospitals with CT	15	32080	32.49
Hospitals with CT	20	49782	50.42
Hospitals with CT	25	72498	73.43
Hospitals with CT	30	89903	91.05
Hospitals with CT	35	95917	97.15
Hospitals with CT	40	97277	98.52
Hospitals with CT	45	97778	99.03
Hospitals with CT	50	97889	99.14
Hospitals with CT	55	97907	99.16
Hospitals with CT	60	98009	99.26
Stroke-Ready Hospitals	5	4153	4.21
Stroke-Ready Hospitals	10	21598	21.87
Stroke-Ready Hospitals	15	32069	32.48
Stroke-Ready Hospitals	20	49713	50.35
Stroke-Ready Hospitals	25	71650	72.57
Stroke-Ready Hospitals	30	89726	90.88
Stroke-Ready Hospitals	35	95766	96.99
Stroke-Ready Hospitals	40	97124	98.37
Stroke-Ready Hospitals	45	97713	98.96
Stroke-Ready Hospitals	50	97849	99.1
Stroke-Ready Hospitals	55	97841	99.09
Stroke-Ready Hospitals	60	97911	99.17
Stroke Units	5	4153	4.21
Stroke Units	10	21598	21.87
Stroke Units	15	32069	32.48
Stroke Units	20	49713	50.35
Stroke Units	25	71650	72.57
Stroke Units	30	89726	90.88
Stroke Units	35	95766	96.99
Stroke Units	40	97124	98.37
Stroke Units	45	97713	98.96
Stroke Units	50	97849	99.1
Stroke Units	55	97841	99.09
Stroke Units	60	97911	99.17

Population Coverage - Landkreis Mühldorf a.Inn (Bayern)

Condition	Drive Time (min)	Covered Population	Coverage (%)
Hospitals with CT	5	7684	6.95
Hospitals with CT	10	24658	22.29
Hospitals with CT	15	57650	52.11
Hospitals with CT	20	103781	93.8
Hospitals with CT	25	110636	100.0
Hospitals with CT	30	110636	100.0
Hospitals with CT	35	110636	100.0
Hospitals with CT	40	110636	100.0
Hospitals with CT	45	110636	100.0
Hospitals with CT	50	110636	100.0
Hospitals with CT	55	110636	100.0
Hospitals with CT	60	110636	100.0
Stroke-Ready Hospitals	5	0	0.0
Stroke-Ready Hospitals	10	1074	0.97
Stroke-Ready Hospitals	15	11451	10.35
Stroke-Ready Hospitals	20	51418	46.48
Stroke-Ready Hospitals	25	85343	77.14
Stroke-Ready Hospitals	30	110636	100.0
Stroke-Ready Hospitals	35	110636	100.0
Stroke-Ready Hospitals	40	110636	100.0
Stroke-Ready Hospitals	45	110636	100.0
Stroke-Ready Hospitals	50	110636	100.0
Stroke-Ready Hospitals	55	110636	100.0
Stroke-Ready Hospitals	60	110636	100.0
Stroke Units	5	0	0.0
Stroke Units	10	1074	0.97
Stroke Units	15	11451	10.35
Stroke Units	20	51418	46.48
Stroke Units	25	85343	77.14
Stroke Units	30	110636	100.0
Stroke Units	35	110636	100.0
Stroke Units	40	110636	100.0
Stroke Units	45	110636	100.0
Stroke Units	50	110636	100.0
Stroke Units	55	110636	100.0
Stroke Units	60	110636	100.0

Population Coverage - Landkreis Neuburg-Schrobenhausen (Bayern)

Condition	Drive Time (min)	Covered Population	Coverage (%)
Hospitals with CT	5	26004	27.23
Hospitals with CT	10	52416	54.9
Hospitals with CT	15	77093	80.74
Hospitals with CT	20	92386	96.76
Hospitals with CT	25	95480	100.0
Hospitals with CT	30	95483	100.0
Hospitals with CT	35	95483	100.0
Hospitals with CT	40	95483	100.0
Hospitals with CT	45	95483	100.0
Hospitals with CT	50	95483	100.0
Hospitals with CT	55	95483	100.0
Hospitals with CT	60	95483	100.0
Stroke-Ready Hospitals	5	0	0.0
Stroke-Ready Hospitals	10	0	0.0
Stroke-Ready Hospitals	15	162	0.17
Stroke-Ready Hospitals	20	9100	9.53
Stroke-Ready Hospitals	25	38233	40.04
Stroke-Ready Hospitals	30	68204	71.43
Stroke-Ready Hospitals	35	80195	83.99
Stroke-Ready Hospitals	40	89867	94.12
Stroke-Ready Hospitals	45	95462	99.98
Stroke-Ready Hospitals	50	95483	100.0
Stroke-Ready Hospitals	55	95483	100.0
Stroke-Ready Hospitals	60	95483	100.0
Stroke Units	5	0	0.0
Stroke Units	10	0	0.0
Stroke Units	15	162	0.17
Stroke Units	20	9100	9.53
Stroke Units	25	38233	40.04
Stroke Units	30	68204	71.43
Stroke Units	35	80195	83.99
Stroke Units	40	89867	94.12
Stroke Units	45	95462	99.98
Stroke Units	50	95483	100.0
Stroke Units	55	95483	100.0
Stroke Units	60	95483	100.0

Population Coverage - Landkreis Starnberg (Bayern)

Condition	Drive Time (min)	Covered Population	Coverage (%)
Hospitals with CT	5	47859	35.57
Hospitals with CT	10	106116	78.88
Hospitals with CT	15	133649	99.34
Hospitals with CT	20	134302	99.83
Hospitals with CT	25	134450	99.94
Hospitals with CT	30	134537	100.0
Hospitals with CT	35	134537	100.0
Hospitals with CT	40	134537	100.0
Hospitals with CT	45	134537	100.0
Hospitals with CT	50	134537	100.0
Hospitals with CT	55	134537	100.0
Hospitals with CT	60	134537	100.0
Stroke-Ready Hospitals	5	5966	4.43
Stroke-Ready Hospitals	10	12344	9.18
Stroke-Ready Hospitals	15	27821	20.68
Stroke-Ready Hospitals	20	90022	66.91
Stroke-Ready Hospitals	25	120829	89.81
Stroke-Ready Hospitals	30	134083	99.66
Stroke-Ready Hospitals	35	134469	99.95
Stroke-Ready Hospitals	40	134469	99.95
Stroke-Ready Hospitals	45	134537	100.0
Stroke-Ready Hospitals	50	134537	100.0
Stroke-Ready Hospitals	55	134537	100.0
Stroke-Ready Hospitals	60	134537	100.0
Stroke Units	5	0	0.0
Stroke Units	10	0	0.0
Stroke Units	15	8211	6.1
Stroke Units	20	57367	42.64
Stroke Units	25	96890	72.02
Stroke Units	30	129637	96.36
Stroke Units	35	134253	99.79
Stroke Units	40	134426	99.92
Stroke Units	45	134448	99.93
Stroke Units	50	134537	100.0
Stroke Units	55	134537	100.0
Stroke Units	60	134537	100.0

Population Coverage - Landkreis Fürth (Bayern)

Condition	Drive Time (min)	Covered Population	Coverage (%)
Hospitals with CT	5	0	0.0
Hospitals with CT	10	501	0.42
Hospitals with CT	15	44399	37.4
Hospitals with CT	20	106851	90.01
Hospitals with CT	25	116903	98.48
Hospitals with CT	30	118705	100.0
Hospitals with CT	35	118705	100.0
Hospitals with CT	40	118705	100.0
Hospitals with CT	45	118705	100.0
Hospitals with CT	50	118705	100.0
Hospitals with CT	55	118705	100.0
Hospitals with CT	60	118705	100.0
Stroke-Ready Hospitals	5	0	0.0
Stroke-Ready Hospitals	10	1157	0.97
Stroke-Ready Hospitals	15	61900	52.15
Stroke-Ready Hospitals	20	107801	90.81
Stroke-Ready Hospitals	25	117513	99.0
Stroke-Ready Hospitals	30	118705	100.0
Stroke-Ready Hospitals	35	118705	100.0
Stroke-Ready Hospitals	40	118705	100.0
Stroke-Ready Hospitals	45	118705	100.0
Stroke-Ready Hospitals	50	118705	100.0
Stroke-Ready Hospitals	55	118705	100.0
Stroke-Ready Hospitals	60	118705	100.0
Stroke Units	5	0	0.0
Stroke Units	10	1157	0.97
Stroke Units	15	61841	52.1
Stroke Units	20	107786	90.8
Stroke Units	25	117511	98.99
Stroke Units	30	118705	100.0
Stroke Units	35	118705	100.0
Stroke Units	40	118705	100.0
Stroke Units	45	118705	100.0
Stroke Units	50	118705	100.0
Stroke Units	55	118705	100.0
Stroke Units	60	118705	100.0

Population Coverage - Landkreis Kitzingen (Bayern)

Condition	Drive Time (min)	Covered Population	Coverage (%)
Hospitals with CT	5	3087	3.38
Hospitals with CT	10	21474	23.49
Hospitals with CT	15	51150	55.94
Hospitals with CT	20	80156	87.67
Hospitals with CT	25	90313	98.78
Hospitals with CT	30	91414	99.98
Hospitals with CT	35	91429	100.0
Hospitals with CT	40	91429	100.0
Hospitals with CT	45	91429	100.0
Hospitals with CT	50	91429	100.0
Hospitals with CT	55	91429	100.0
Hospitals with CT	60	91429	100.0
Stroke-Ready Hospitals	5	2328	2.55
Stroke-Ready Hospitals	10	16264	17.79
Stroke-Ready Hospitals	15	33030	36.13
Stroke-Ready Hospitals	20	50754	55.51
Stroke-Ready Hospitals	25	69146	75.63
Stroke-Ready Hospitals	30	89291	97.66
Stroke-Ready Hospitals	35	91424	99.99
Stroke-Ready Hospitals	40	91429	100.0
Stroke-Ready Hospitals	45	91429	100.0
Stroke-Ready Hospitals	50	91429	100.0
Stroke-Ready Hospitals	55	91429	100.0
Stroke-Ready Hospitals	60	91429	100.0
Stroke Units	5	2328	2.55
Stroke Units	10	16264	17.79
Stroke Units	15	33030	36.13
Stroke Units	20	50754	55.51
Stroke Units	25	69146	75.63
Stroke Units	30	89279	97.65
Stroke Units	35	91423	99.99
Stroke Units	40	91429	100.0
Stroke Units	45	91429	100.0
Stroke Units	50	91429	100.0
Stroke Units	55	91429	100.0
Stroke Units	60	91429	100.0

Population Coverage - Landkreis Günzburg (Bayern)

Condition	Drive Time (min)	Covered Population	Coverage (%)
Hospitals with CT	5	32079	25.64
Hospitals with CT	10	73295	58.58
Hospitals with CT	15	110333	88.18
Hospitals with CT	20	122773	98.12
Hospitals with CT	25	125060	99.95
Hospitals with CT	30	125098	99.98
Hospitals with CT	35	125119	100.0
Hospitals with CT	40	125119	100.0
Hospitals with CT	45	125119	100.0
Hospitals with CT	50	125119	100.0
Hospitals with CT	55	125119	100.0
Hospitals with CT	60	125119	100.0
Stroke-Ready Hospitals	5	3352	2.68
Stroke-Ready Hospitals	10	20762	16.59
Stroke-Ready Hospitals	15	46329	37.03
Stroke-Ready Hospitals	20	71846	57.42
Stroke-Ready Hospitals	25	85124	68.03
Stroke-Ready Hospitals	30	92985	74.32
Stroke-Ready Hospitals	35	107114	85.61
Stroke-Ready Hospitals	40	121975	97.49
Stroke-Ready Hospitals	45	125119	100.0
Stroke-Ready Hospitals	50	125119	100.0
Stroke-Ready Hospitals	55	125119	100.0
Stroke-Ready Hospitals	60	125119	100.0
Stroke Units	5	3352	2.68
Stroke Units	10	20762	16.59
Stroke Units	15	46329	37.03
Stroke Units	20	71846	57.42
Stroke Units	25	85124	68.03
Stroke Units	30	92985	74.32
Stroke Units	35	107114	85.61
Stroke Units	40	121684	97.25
Stroke Units	45	125118	100.0
Stroke Units	50	125119	100.0
Stroke Units	55	125119	100.0
Stroke Units	60	125119	100.0

Population Coverage - Landkreis Havelland (Brandenburg)

Condition	Drive Time (min)	Covered Population	Coverage (%)
Hospitals with CT	5	20819	13.75
Hospitals with CT	10	45499	30.05
Hospitals with CT	15	90404	59.71
Hospitals with CT	20	130141	85.95
Hospitals with CT	25	142036	93.81
Hospitals with CT	30	149092	98.47
Hospitals with CT	35	150734	99.55
Hospitals with CT	40	151294	99.92
Hospitals with CT	45	151408	100.0
Hospitals with CT	50	151413	100.0
Hospitals with CT	55	151413	100.0
Hospitals with CT	60	151413	100.0
Stroke-Ready Hospitals	5	7765	5.13
Stroke-Ready Hospitals	10	14033	9.27
Stroke-Ready Hospitals	15	31993	21.13
Stroke-Ready Hospitals	20	81652	53.93
Stroke-Ready Hospitals	25	100935	66.66
Stroke-Ready Hospitals	30	109800	72.52
Stroke-Ready Hospitals	35	116849	77.17
Stroke-Ready Hospitals	40	121565	80.29
Stroke-Ready Hospitals	45	135332	89.38
Stroke-Ready Hospitals	50	144918	95.71
Stroke-Ready Hospitals	55	148634	98.16
Stroke-Ready Hospitals	60	150541	99.42
Stroke Units	5	0	0.0
Stroke Units	10	880	0.58
Stroke Units	15	9677	6.39
Stroke Units	20	39838	26.31
Stroke Units	25	58579	38.69
Stroke Units	30	77586	51.24
Stroke Units	35	92202	60.89
Stroke Units	40	99160	65.49
Stroke Units	45	103032	68.05
Stroke Units	50	105242	69.51
Stroke Units	55	107189	70.79
Stroke Units	60	111267	73.49

Population Coverage - Landkreis Teltow-Fläming (Brandenburg)

Condition	Drive Time (min)	Covered Population	Coverage (%)
Hospitals with CT	5	16115	10.26
Hospitals with CT	10	39595	25.21
Hospitals with CT	15	54515	34.71
Hospitals with CT	20	88692	56.47
Hospitals with CT	25	128960	82.1
Hospitals with CT	30	147435	93.87
Hospitals with CT	35	155620	99.08
Hospitals with CT	40	156845	99.86
Hospitals with CT	45	156957	99.93
Hospitals with CT	50	156976	99.94
Hospitals with CT	55	156986	99.95
Hospitals with CT	60	157070	100.0
Stroke-Ready Hospitals	5	0	0.0
Stroke-Ready Hospitals	10	0	0.0
Stroke-Ready Hospitals	15	254	0.16
Stroke-Ready Hospitals	20	8923	5.68
Stroke-Ready Hospitals	25	47848	30.46
Stroke-Ready Hospitals	30	77735	49.49
Stroke-Ready Hospitals	35	97462	62.05
Stroke-Ready Hospitals	40	110960	70.64
Stroke-Ready Hospitals	45	133038	84.7
Stroke-Ready Hospitals	50	148138	94.31
Stroke-Ready Hospitals	55	155006	98.69
Stroke-Ready Hospitals	60	156774	99.81
Stroke Units	5	0	0.0
Stroke Units	10	0	0.0
Stroke Units	15	121	0.08
Stroke Units	20	7157	4.56
Stroke Units	25	40405	25.72
Stroke Units	30	63093	40.17
Stroke Units	35	78495	49.97
Stroke Units	40	93847	59.75
Stroke Units	45	121873	77.59
Stroke Units	50	134902	85.89
Stroke Units	55	142250	90.57
Stroke Units	60	149761	95.35

Population Coverage - Landkreis Börde (Sachsen-Anhalt)

Condition	Drive Time (min)	Covered Population	Coverage (%)
Hospitals with CT	5	4872	3.14
Hospitals with CT	10	36908	23.75
Hospitals with CT	15	84196	54.19
Hospitals with CT	20	124475	80.11
Hospitals with CT	25	143836	92.57
Hospitals with CT	30	153751	98.95
Hospitals with CT	35	155043	99.78
Hospitals with CT	40	155375	99.99
Hospitals with CT	45	155383	100.0
Hospitals with CT	50	155383	100.0
Hospitals with CT	55	155383	100.0
Hospitals with CT	60	155383	100.0
Stroke-Ready Hospitals	5	3781	2.43
Stroke-Ready Hospitals	10	18002	11.59
Stroke-Ready Hospitals	15	48385	31.14
Stroke-Ready Hospitals	20	86850	55.89
Stroke-Ready Hospitals	25	108827	70.04
Stroke-Ready Hospitals	30	124717	80.26
Stroke-Ready Hospitals	35	137442	88.45
Stroke-Ready Hospitals	40	152935	98.42
Stroke-Ready Hospitals	45	155023	99.77
Stroke-Ready Hospitals	50	155383	100.0
Stroke-Ready Hospitals	55	155383	100.0
Stroke-Ready Hospitals	60	155383	100.0
Stroke Units	5	14	0.01
Stroke Units	10	3333	2.15
Stroke Units	15	19279	12.41
Stroke Units	20	46881	30.17
Stroke Units	25	69836	44.94
Stroke Units	30	96789	62.29
Stroke Units	35	117500	75.62
Stroke Units	40	135789	87.39
Stroke Units	45	150624	96.94
Stroke Units	50	154953	99.72
Stroke Units	55	155382	100.0
Stroke Units	60	155383	100.0

Population Coverage - Landkreis Stade (Niedersachsen)

Condition	Drive Time (min)	Covered Population	Coverage (%)
Hospitals with CT	5	23286	11.71
Hospitals with CT	10	71144	35.78
Hospitals with CT	15	101303	50.94
Hospitals with CT	20	147078	73.96
Hospitals with CT	25	178940	89.99
Hospitals with CT	30	186779	93.93
Hospitals with CT	35	192793	96.95
Hospitals with CT	40	194085	97.6
Hospitals with CT	45	194166	97.64
Hospitals with CT	50	194394	97.76
Hospitals with CT	55	194373	97.75
Hospitals with CT	60	194434	97.78
Stroke-Ready Hospitals	5	10938	5.5
Stroke-Ready Hospitals	10	32587	16.39
Stroke-Ready Hospitals	15	54016	27.16
Stroke-Ready Hospitals	20	86149	43.32
Stroke-Ready Hospitals	25	129478	65.11
Stroke-Ready Hospitals	30	176884	88.95
Stroke-Ready Hospitals	35	186481	93.78
Stroke-Ready Hospitals	40	189975	95.54
Stroke-Ready Hospitals	45	192448	96.78
Stroke-Ready Hospitals	50	193401	97.26
Stroke-Ready Hospitals	55	194167	97.65
Stroke-Ready Hospitals	60	194337	97.73
Stroke Units	5	10859	5.46
Stroke Units	10	32561	16.37
Stroke Units	15	53998	27.16
Stroke Units	20	86003	43.25
Stroke Units	25	129415	65.08
Stroke Units	30	176844	88.93
Stroke Units	35	186435	93.76
Stroke Units	40	189942	95.52
Stroke Units	45	192436	96.77
Stroke Units	50	193382	97.25
Stroke Units	55	194167	97.65
Stroke Units	60	194337	97.73

Population Coverage - Kreisfreie Stadt Oldenburg (Oldb) (Niedersachsen)

Condition	Drive Time (min)	Covered Population	Coverage (%)
Hospitals with CT	5	40500	25.32
Hospitals with CT	10	121495	75.96
Hospitals with CT	15	155247	97.06
Hospitals with CT	20	159948	100.0
Hospitals with CT	25	159948	100.0
Hospitals with CT	30	159948	100.0
Hospitals with CT	35	159948	100.0
Hospitals with CT	40	159948	100.0
Hospitals with CT	45	159948	100.0
Hospitals with CT	50	159948	100.0
Hospitals with CT	55	159948	100.0
Hospitals with CT	60	159948	100.0
Stroke-Ready Hospitals	5	25748	16.1
Stroke-Ready Hospitals	10	98465	61.56
Stroke-Ready Hospitals	15	144062	90.07
Stroke-Ready Hospitals	20	159936	99.99
Stroke-Ready Hospitals	25	159948	100.0
Stroke-Ready Hospitals	30	159948	100.0
Stroke-Ready Hospitals	35	159948	100.0
Stroke-Ready Hospitals	40	159948	100.0
Stroke-Ready Hospitals	45	159948	100.0
Stroke-Ready Hospitals	50	159948	100.0
Stroke-Ready Hospitals	55	159948	100.0
Stroke-Ready Hospitals	60	159948	100.0
Stroke Units	5	25748	16.1
Stroke Units	10	98465	61.56
Stroke Units	15	144062	90.07
Stroke Units	20	159936	99.99
Stroke Units	25	159948	100.0
Stroke Units	30	159948	100.0
Stroke Units	35	159948	100.0
Stroke Units	40	159948	100.0
Stroke Units	45	159948	100.0
Stroke Units	50	159948	100.0
Stroke Units	55	159948	100.0
Stroke Units	60	159948	100.0

Population Coverage - Landkreis Cloppenburg (Niedersachsen)

Condition	Drive Time (min)	Covered Population	Coverage (%)
Hospitals with CT	5	32681	19.92
Hospitals with CT	10	68842	41.96
Hospitals with CT	15	128569	78.36
Hospitals with CT	20	153042	93.27
Hospitals with CT	25	163605	99.71
Hospitals with CT	30	164076	100.0
Hospitals with CT	35	164076	100.0
Hospitals with CT	40	164076	100.0
Hospitals with CT	45	164076	100.0
Hospitals with CT	50	164076	100.0
Hospitals with CT	55	164076	100.0
Hospitals with CT	60	164076	100.0
Stroke-Ready Hospitals	5	0	0.0
Stroke-Ready Hospitals	10	153	0.09
Stroke-Ready Hospitals	15	4603	2.81
Stroke-Ready Hospitals	20	11380	6.94
Stroke-Ready Hospitals	25	34999	21.33
Stroke-Ready Hospitals	30	96831	59.02
Stroke-Ready Hospitals	35	140214	85.46
Stroke-Ready Hospitals	40	157396	95.93
Stroke-Ready Hospitals	45	161716	98.56
Stroke-Ready Hospitals	50	163939	99.92
Stroke-Ready Hospitals	55	164076	100.0
Stroke-Ready Hospitals	60	164076	100.0
Stroke Units	5	0	0.0
Stroke Units	10	153	0.09
Stroke Units	15	4603	2.81
Stroke Units	20	11380	6.94
Stroke Units	25	34999	21.33
Stroke Units	30	96831	59.02
Stroke Units	35	140214	85.46
Stroke Units	40	157396	95.93
Stroke Units	45	161716	98.56
Stroke Units	50	163939	99.92
Stroke Units	55	164076	100.0
Stroke Units	60	164076	100.0

Population Coverage - Kreisfreie Stadt Wuppertal (Nordrhein-Westfalen)

Condition	Drive Time (min)	Covered Population	Coverage (%)
Hospitals with CT	5	121672	33.89
Hospitals with CT	10	278001	77.42
Hospitals with CT	15	353670	98.5
Hospitals with CT	20	359067	100.0
Hospitals with CT	25	359073	100.0
Hospitals with CT	30	359073	100.0
Hospitals with CT	35	359073	100.0
Hospitals with CT	40	359073	100.0
Hospitals with CT	45	359073	100.0
Hospitals with CT	50	359073	100.0
Hospitals with CT	55	359073	100.0
Hospitals with CT	60	359073	100.0
Stroke-Ready Hospitals	5	76776	21.38
Stroke-Ready Hospitals	10	225784	62.88
Stroke-Ready Hospitals	15	322906	89.93
Stroke-Ready Hospitals	20	358062	99.72
Stroke-Ready Hospitals	25	359070	100.0
Stroke-Ready Hospitals	30	359073	100.0
Stroke-Ready Hospitals	35	359073	100.0
Stroke-Ready Hospitals	40	359073	100.0
Stroke-Ready Hospitals	45	359073	100.0
Stroke-Ready Hospitals	50	359073	100.0
Stroke-Ready Hospitals	55	359073	100.0
Stroke-Ready Hospitals	60	359073	100.0
Stroke Units	5	76776	21.38
Stroke Units	10	224272	62.46
Stroke Units	15	321347	89.49
Stroke Units	20	355470	99.0
Stroke Units	25	356967	99.41
Stroke Units	30	359073	100.0
Stroke Units	35	359073	100.0
Stroke Units	40	359073	100.0
Stroke Units	45	359073	100.0
Stroke Units	50	359073	100.0
Stroke Units	55	359073	100.0
Stroke Units	60	359073	100.0

Population Coverage - Landkreis Odenwaldkreis (Hessen)

Condition	Drive Time (min)	Covered Population	Coverage (%)
Hospitals with CT	5	15081	15.41
Hospitals with CT	10	35767	36.54
Hospitals with CT	15	60005	61.3
Hospitals with CT	20	80550	82.29
Hospitals with CT	25	89628	91.56
Hospitals with CT	30	97376	99.47
Hospitals with CT	35	97884	99.99
Hospitals with CT	40	97890	100.0
Hospitals with CT	45	97890	100.0
Hospitals with CT	50	97890	100.0
Hospitals with CT	55	97890	100.0
Hospitals with CT	60	97890	100.0
Stroke-Ready Hospitals	5	8669	8.86
Stroke-Ready Hospitals	10	22862	23.36
Stroke-Ready Hospitals	15	30580	31.24
Stroke-Ready Hospitals	20	47024	48.04
Stroke-Ready Hospitals	25	63054	64.41
Stroke-Ready Hospitals	30	87733	89.62
Stroke-Ready Hospitals	35	97566	99.67
Stroke-Ready Hospitals	40	97890	100.0
Stroke-Ready Hospitals	45	97890	100.0
Stroke-Ready Hospitals	50	97890	100.0
Stroke-Ready Hospitals	55	97890	100.0
Stroke-Ready Hospitals	60	97890	100.0
Stroke Units	5	8669	8.86
Stroke Units	10	22862	23.36
Stroke Units	15	30580	31.24
Stroke Units	20	47024	48.04
Stroke Units	25	63054	64.41
Stroke Units	30	87702	89.59
Stroke Units	35	97565	99.67
Stroke Units	40	97890	100.0
Stroke Units	45	97890	100.0
Stroke Units	50	97890	100.0
Stroke Units	55	97890	100.0
Stroke Units	60	97890	100.0

Population Coverage - Landkreis Karlsruhe (Baden-Württemberg)

Condition	Drive Time (min)	Covered Population	Coverage (%)
Hospitals with CT	5	29045	6.66
Hospitals with CT	10	110200	25.28
Hospitals with CT	15	230761	52.95
Hospitals with CT	20	376717	86.44
Hospitals with CT	25	423536	97.18
Hospitals with CT	30	435719	99.97
Hospitals with CT	35	435835	100.0
Hospitals with CT	40	435835	100.0
Hospitals with CT	45	435835	100.0
Hospitals with CT	50	435835	100.0
Hospitals with CT	55	435835	100.0
Hospitals with CT	60	435835	100.0
Stroke-Ready Hospitals	5	17352	3.98
Stroke-Ready Hospitals	10	66955	15.36
Stroke-Ready Hospitals	15	148556	34.09
Stroke-Ready Hospitals	20	292783	67.18
Stroke-Ready Hospitals	25	386736	88.73
Stroke-Ready Hospitals	30	422276	96.89
Stroke-Ready Hospitals	35	435570	99.94
Stroke-Ready Hospitals	40	435827	100.0
Stroke-Ready Hospitals	45	435835	100.0
Stroke-Ready Hospitals	50	435835	100.0
Stroke-Ready Hospitals	55	435835	100.0
Stroke-Ready Hospitals	60	435835	100.0
Stroke Units	5	17352	3.98
Stroke Units	10	66955	15.36
Stroke Units	15	148556	34.09
Stroke Units	20	292783	67.18
Stroke Units	25	384847	88.3
Stroke Units	30	421646	96.74
Stroke Units	35	432646	99.27
Stroke Units	40	435827	100.0
Stroke Units	45	435835	100.0
Stroke Units	50	435835	100.0
Stroke Units	55	435835	100.0
Stroke Units	60	435835	100.0

Population Coverage - Kreisfreie Stadt Landshut (Bayern)

Condition	Drive Time (min)	Covered Population	Coverage (%)
Hospitals with CT	5	39461	57.14
Hospitals with CT	10	64828	93.87
Hospitals with CT	15	67596	97.88
Hospitals with CT	20	69058	100.0
Hospitals with CT	25	69058	100.0
Hospitals with CT	30	69058	100.0
Hospitals with CT	35	69058	100.0
Hospitals with CT	40	69058	100.0
Hospitals with CT	45	69058	100.0
Hospitals with CT	50	69058	100.0
Hospitals with CT	55	69058	100.0
Hospitals with CT	60	69058	100.0
Stroke-Ready Hospitals	5	13996	20.27
Stroke-Ready Hospitals	10	51770	74.97
Stroke-Ready Hospitals	15	66093	95.71
Stroke-Ready Hospitals	20	68543	99.25
Stroke-Ready Hospitals	25	69058	100.0
Stroke-Ready Hospitals	30	69058	100.0
Stroke-Ready Hospitals	35	69058	100.0
Stroke-Ready Hospitals	40	69058	100.0
Stroke-Ready Hospitals	45	69058	100.0
Stroke-Ready Hospitals	50	69058	100.0
Stroke-Ready Hospitals	55	69058	100.0
Stroke-Ready Hospitals	60	69058	100.0
Stroke Units	5	13996	20.27
Stroke Units	10	51770	74.97
Stroke Units	15	66093	95.71
Stroke Units	20	68543	99.25
Stroke Units	25	69058	100.0
Stroke Units	30	69058	100.0
Stroke Units	35	69058	100.0
Stroke Units	40	69058	100.0
Stroke Units	45	69058	100.0
Stroke Units	50	69058	100.0
Stroke Units	55	69058	100.0
Stroke Units	60	69058	100.0

Population Coverage - Kreisfreie Stadt Erlangen (Bayern)

Condition	Drive Time (min)	Covered Population	Coverage (%)
Hospitals with CT	5	40670	32.12
Hospitals with CT	10	94183	74.38
Hospitals with CT	15	126344	99.78
Hospitals with CT	20	126620	99.99
Hospitals with CT	25	126629	100.0
Hospitals with CT	30	126629	100.0
Hospitals with CT	35	126629	100.0
Hospitals with CT	40	126629	100.0
Hospitals with CT	45	126629	100.0
Hospitals with CT	50	126629	100.0
Hospitals with CT	55	126629	100.0
Hospitals with CT	60	126629	100.0
Stroke-Ready Hospitals	5	14622	11.55
Stroke-Ready Hospitals	10	63227	49.93
Stroke-Ready Hospitals	15	120260	94.97
Stroke-Ready Hospitals	20	126602	99.98
Stroke-Ready Hospitals	25	126618	99.99
Stroke-Ready Hospitals	30	126629	100.0
Stroke-Ready Hospitals	35	126629	100.0
Stroke-Ready Hospitals	40	126629	100.0
Stroke-Ready Hospitals	45	126629	100.0
Stroke-Ready Hospitals	50	126629	100.0
Stroke-Ready Hospitals	55	126629	100.0
Stroke-Ready Hospitals	60	126629	100.0
Stroke Units	5	14622	11.55
Stroke Units	10	63227	49.93
Stroke Units	15	120260	94.97
Stroke Units	20	126602	99.98
Stroke Units	25	126618	99.99
Stroke Units	30	126629	100.0
Stroke Units	35	126629	100.0
Stroke Units	40	126629	100.0
Stroke Units	45	126629	100.0
Stroke Units	50	126629	100.0
Stroke Units	55	126629	100.0
Stroke Units	60	126629	100.0

Population Coverage - Landkreis Haßberge (Bayern)

Condition	Drive Time (min)	Covered Population	Coverage (%)
Hospitals with CT	5	15165	17.4
Hospitals with CT	10	33627	38.58
Hospitals with CT	15	60365	69.25
Hospitals with CT	20	76905	88.23
Hospitals with CT	25	85752	98.38
Hospitals with CT	30	87164	100.0
Hospitals with CT	35	87166	100.0
Hospitals with CT	40	87166	100.0
Hospitals with CT	45	87166	100.0
Hospitals with CT	50	87166	100.0
Hospitals with CT	55	87166	100.0
Hospitals with CT	60	87166	100.0
Stroke-Ready Hospitals	5	0	0.0
Stroke-Ready Hospitals	10	0	0.0
Stroke-Ready Hospitals	15	380	0.44
Stroke-Ready Hospitals	20	3762	4.32
Stroke-Ready Hospitals	25	14211	16.3
Stroke-Ready Hospitals	30	56255	64.54
Stroke-Ready Hospitals	35	81077	93.02
Stroke-Ready Hospitals	40	87155	99.99
Stroke-Ready Hospitals	45	87166	100.0
Stroke-Ready Hospitals	50	87166	100.0
Stroke-Ready Hospitals	55	87166	100.0
Stroke-Ready Hospitals	60	87166	100.0
Stroke Units	5	0	0.0
Stroke Units	10	0	0.0
Stroke Units	15	380	0.44
Stroke Units	20	3762	4.32
Stroke Units	25	14211	16.3
Stroke Units	30	56255	64.54
Stroke Units	35	81077	93.02
Stroke Units	40	87155	99.99
Stroke Units	45	87166	100.0
Stroke Units	50	87166	100.0
Stroke Units	55	87166	100.0
Stroke Units	60	87166	100.0

Population Coverage - Landkreis Aichach-Friedberg (Bayern)

Condition	Drive Time (min)	Covered Population	Coverage (%)
Hospitals with CT	5	27157	20.36
Hospitals with CT	10	54506	40.86
Hospitals with CT	15	86578	64.9
Hospitals with CT	20	112039	83.98
Hospitals with CT	25	131519	98.58
Hospitals with CT	30	133411	100.0
Hospitals with CT	35	133411	100.0
Hospitals with CT	40	133411	100.0
Hospitals with CT	45	133411	100.0
Hospitals with CT	50	133411	100.0
Hospitals with CT	55	133411	100.0
Hospitals with CT	60	133411	100.0
Stroke-Ready Hospitals	5	0	0.0
Stroke-Ready Hospitals	10	4	0.0
Stroke-Ready Hospitals	15	5294	3.97
Stroke-Ready Hospitals	20	33511	25.12
Stroke-Ready Hospitals	25	76954	57.68
Stroke-Ready Hospitals	30	110312	82.69
Stroke-Ready Hospitals	35	127627	95.66
Stroke-Ready Hospitals	40	133124	99.78
Stroke-Ready Hospitals	45	133411	100.0
Stroke-Ready Hospitals	50	133411	100.0
Stroke-Ready Hospitals	55	133411	100.0
Stroke-Ready Hospitals	60	133411	100.0
Stroke Units	5	0	0.0
Stroke Units	10	4	0.0
Stroke Units	15	5294	3.97
Stroke Units	20	33503	25.11
Stroke Units	25	76943	57.67
Stroke Units	30	109314	81.94
Stroke Units	35	127569	95.62
Stroke Units	40	133124	99.78
Stroke Units	45	133411	100.0
Stroke Units	50	133411	100.0
Stroke Units	55	133411	100.0
Stroke Units	60	133411	100.0

Population Coverage - Landkreis Dillingen a.d.Donau (Bayern)

Condition	Drive Time (min)	Covered Population	Coverage (%)
Hospitals with CT	5	9877	10.17
Hospitals with CT	10	35045	36.09
Hospitals with CT	15	64942	66.88
Hospitals with CT	20	90380	93.08
Hospitals with CT	25	97052	99.95
Hospitals with CT	30	97098	100.0
Hospitals with CT	35	97098	100.0
Hospitals with CT	40	97098	100.0
Hospitals with CT	45	97098	100.0
Hospitals with CT	50	97098	100.0
Hospitals with CT	55	97098	100.0
Hospitals with CT	60	97098	100.0
Stroke-Ready Hospitals	5	0	0.0
Stroke-Ready Hospitals	10	2	0.0
Stroke-Ready Hospitals	15	496	0.51
Stroke-Ready Hospitals	20	13109	13.5
Stroke-Ready Hospitals	25	31371	32.31
Stroke-Ready Hospitals	30	64718	66.65
Stroke-Ready Hospitals	35	86496	89.08
Stroke-Ready Hospitals	40	92086	94.84
Stroke-Ready Hospitals	45	94137	96.95
Stroke-Ready Hospitals	50	96991	99.89
Stroke-Ready Hospitals	55	97098	100.0
Stroke-Ready Hospitals	60	97098	100.0
Stroke Units	5	0	0.0
Stroke Units	10	2	0.0
Stroke Units	15	496	0.51
Stroke Units	20	13109	13.5
Stroke Units	25	31371	32.31
Stroke Units	30	64718	66.65
Stroke Units	35	86496	89.08
Stroke Units	40	92086	94.84
Stroke Units	45	94137	96.95
Stroke Units	50	96991	99.89
Stroke Units	55	97098	100.0
Stroke Units	60	97098	100.0

Population Coverage - Landkreis Oberspreewald-Lausitz (Brandenburg)

Condition	Drive Time (min)	Covered Population	Coverage (%)
Hospitals with CT	5	12850	11.85
Hospitals with CT	10	33968	31.32
Hospitals with CT	15	47799	44.07
Hospitals with CT	20	76502	70.54
Hospitals with CT	25	95049	87.64
Hospitals with CT	30	106430	98.13
Hospitals with CT	35	107861	99.45
Hospitals with CT	40	108458	100.0
Hospitals with CT	45	108458	100.0
Hospitals with CT	50	108458	100.0
Hospitals with CT	55	108458	100.0
Hospitals with CT	60	108458	100.0
Stroke-Ready Hospitals	5	5542	5.11
Stroke-Ready Hospitals	10	18015	16.61
Stroke-Ready Hospitals	15	27599	25.45
Stroke-Ready Hospitals	20	55809	51.46
Stroke-Ready Hospitals	25	75290	69.42
Stroke-Ready Hospitals	30	101433	93.52
Stroke-Ready Hospitals	35	107486	99.1
Stroke-Ready Hospitals	40	108294	99.85
Stroke-Ready Hospitals	45	108458	100.0
Stroke-Ready Hospitals	50	108458	100.0
Stroke-Ready Hospitals	55	108458	100.0
Stroke-Ready Hospitals	60	108458	100.0
Stroke Units	5	5542	5.11
Stroke Units	10	18015	16.61
Stroke Units	15	27599	25.45
Stroke Units	20	55809	51.46
Stroke Units	25	75290	69.42
Stroke Units	30	101429	93.52
Stroke Units	35	107421	99.04
Stroke Units	40	108294	99.85
Stroke Units	45	108458	100.0
Stroke Units	50	108458	100.0
Stroke Units	55	108458	100.0
Stroke Units	60	108458	100.0

Population Coverage - Landkreis Bautzen (Sachsen)

Condition	Drive Time (min)	Covered Population	Coverage (%)
Hospitals with CT	5	62408	21.66
Hospitals with CT	10	136657	47.44
Hospitals with CT	15	195374	67.82
Hospitals with CT	20	243501	84.52
Hospitals with CT	25	279640	97.07
Hospitals with CT	30	287105	99.66
Hospitals with CT	35	287967	99.96
Hospitals with CT	40	287972	99.96
Hospitals with CT	45	288081	100.0
Hospitals with CT	50	288081	100.0
Hospitals with CT	55	288077	100.0
Hospitals with CT	60	288080	100.0
Stroke-Ready Hospitals	5	15821	5.49
Stroke-Ready Hospitals	10	33816	11.74
Stroke-Ready Hospitals	15	66377	23.04
Stroke-Ready Hospitals	20	94266	32.72
Stroke-Ready Hospitals	25	131291	45.57
Stroke-Ready Hospitals	30	156321	54.26
Stroke-Ready Hospitals	35	212663	73.82
Stroke-Ready Hospitals	40	252656	87.7
Stroke-Ready Hospitals	45	267506	92.86
Stroke-Ready Hospitals	50	280359	97.32
Stroke-Ready Hospitals	55	288018	99.98
Stroke-Ready Hospitals	60	288065	99.99
Stroke Units	5	13438	4.66
Stroke Units	10	28849	10.01
Stroke Units	15	39261	13.63
Stroke Units	20	52849	18.34
Stroke Units	25	83338	28.93
Stroke Units	30	109401	37.98
Stroke Units	35	172461	59.86
Stroke Units	40	225012	78.11
Stroke Units	45	253717	88.07
Stroke Units	50	267424	92.83
Stroke Units	55	282692	98.13
Stroke Units	60	287842	99.91

Population Coverage - Kreisfreie Stadt Dessau-Roßlau (Sachsen-Anhalt)

Condition	Drive Time (min)	Covered Population	Coverage (%)
Hospitals with CT	5	8014	9.53
Hospitals with CT	10	43094	51.23
Hospitals with CT	15	63545	75.55
Hospitals with CT	20	78421	93.23
Hospitals with CT	25	83145	98.85
Hospitals with CT	30	84103	99.99
Hospitals with CT	35	84113	100.0
Hospitals with CT	40	84113	100.0
Hospitals with CT	45	84113	100.0
Hospitals with CT	50	84113	100.0
Hospitals with CT	55	84113	100.0
Hospitals with CT	60	84113	100.0
Stroke-Ready Hospitals	5	7668	9.12
Stroke-Ready Hospitals	10	41567	49.42
Stroke-Ready Hospitals	15	62077	73.8
Stroke-Ready Hospitals	20	70742	84.1
Stroke-Ready Hospitals	25	79838	94.92
Stroke-Ready Hospitals	30	83732	99.55
Stroke-Ready Hospitals	35	84005	99.87
Stroke-Ready Hospitals	40	84005	99.87
Stroke-Ready Hospitals	45	84113	100.0
Stroke-Ready Hospitals	50	84113	100.0
Stroke-Ready Hospitals	55	84113	100.0
Stroke-Ready Hospitals	60	84113	100.0
Stroke Units	5	0	0.0
Stroke Units	10	0	0.0
Stroke Units	15	0	0.0
Stroke Units	20	0	0.0
Stroke Units	25	0	0.0
Stroke Units	30	7	0.01
Stroke Units	35	762	0.91
Stroke Units	40	18472	21.96
Stroke Units	45	53328	63.4
Stroke Units	50	69746	82.92
Stroke Units	55	79734	94.79
Stroke Units	60	83386	99.14

Population Coverage - Landkreis Lüneburg (Niedersachsen)

Condition	Drive Time (min)	Covered Population	Coverage (%)
Hospitals with CT	5	18365	10.3
Hospitals with CT	10	54066	30.33
Hospitals with CT	15	90159	50.58
Hospitals with CT	20	127640	71.6
Hospitals with CT	25	150994	84.7
Hospitals with CT	30	171881	96.42
Hospitals with CT	35	177197	99.4
Hospitals with CT	40	177979	99.84
Hospitals with CT	45	178268	100.0
Hospitals with CT	50	178268	100.0
Hospitals with CT	55	178268	100.0
Hospitals with CT	60	178268	100.0
Stroke-Ready Hospitals	5	20385	11.44
Stroke-Ready Hospitals	10	57314	32.15
Stroke-Ready Hospitals	15	93033	52.19
Stroke-Ready Hospitals	20	123059	69.03
Stroke-Ready Hospitals	25	136845	76.76
Stroke-Ready Hospitals	30	149251	83.72
Stroke-Ready Hospitals	35	157132	88.14
Stroke-Ready Hospitals	40	167633	94.03
Stroke-Ready Hospitals	45	172123	96.55
Stroke-Ready Hospitals	50	173195	97.15
Stroke-Ready Hospitals	55	173518	97.34
Stroke-Ready Hospitals	60	174596	97.94
Stroke Units	5	20366	11.42
Stroke Units	10	57197	32.09
Stroke Units	15	93018	52.18
Stroke Units	20	122908	68.95
Stroke Units	25	136563	76.61
Stroke Units	30	148563	83.34
Stroke Units	35	157014	88.08
Stroke Units	40	167612	94.02
Stroke Units	45	171909	96.43
Stroke Units	50	173182	97.15
Stroke Units	55	173518	97.34
Stroke Units	60	174596	97.94

Population Coverage - Kreisfreie Stadt Bremerhaven (Bremen)

Condition	Drive Time (min)	Covered Population	Coverage (%)
Hospitals with CT	5	18909	16.37
Hospitals with CT	10	76296	66.04
Hospitals with CT	15	110143	95.33
Hospitals with CT	20	114590	99.18
Hospitals with CT	25	114628	99.21
Hospitals with CT	30	114639	99.22
Hospitals with CT	35	114640	99.22
Hospitals with CT	40	114640	99.22
Hospitals with CT	45	114640	99.22
Hospitals with CT	50	114662	99.24
Hospitals with CT	55	114585	99.18
Hospitals with CT	60	114625	99.21
Stroke-Ready Hospitals	5	2498	2.16
Stroke-Ready Hospitals	10	15258	13.21
Stroke-Ready Hospitals	15	72010	62.33
Stroke-Ready Hospitals	20	112559	97.42
Stroke-Ready Hospitals	25	114114	98.77
Stroke-Ready Hospitals	30	114310	98.94
Stroke-Ready Hospitals	35	114310	98.94
Stroke-Ready Hospitals	40	114483	99.09
Stroke-Ready Hospitals	45	114545	99.14
Stroke-Ready Hospitals	50	114594	99.18
Stroke-Ready Hospitals	55	114460	99.07
Stroke-Ready Hospitals	60	114508	99.11
Stroke Units	5	2498	2.16
Stroke Units	10	15258	13.21
Stroke Units	15	72010	62.33
Stroke Units	20	112559	97.42
Stroke Units	25	114114	98.77
Stroke Units	30	114310	98.94
Stroke Units	35	114310	98.94
Stroke Units	40	114483	99.09
Stroke Units	45	114545	99.14
Stroke Units	50	114594	99.18
Stroke Units	55	114460	99.07
Stroke Units	60	114483	99.09

Population Coverage - Kreisfreie Stadt Koblenz (Rheinland-Pfalz)

Condition	Drive Time (min)	Covered Population	Coverage (%)
Hospitals with CT	5	63232	50.24
Hospitals with CT	10	113205	89.95
Hospitals with CT	15	125853	100.0
Hospitals with CT	20	125857	100.0
Hospitals with CT	25	125857	100.0
Hospitals with CT	30	125857	100.0
Hospitals with CT	35	125857	100.0
Hospitals with CT	40	125857	100.0
Hospitals with CT	45	125857	100.0
Hospitals with CT	50	125857	100.0
Hospitals with CT	55	125857	100.0
Hospitals with CT	60	125857	100.0
Stroke-Ready Hospitals	5	31658	25.15
Stroke-Ready Hospitals	10	91466	72.67
Stroke-Ready Hospitals	15	123967	98.5
Stroke-Ready Hospitals	20	125857	100.0
Stroke-Ready Hospitals	25	125857	100.0
Stroke-Ready Hospitals	30	125857	100.0
Stroke-Ready Hospitals	35	125857	100.0
Stroke-Ready Hospitals	40	125857	100.0
Stroke-Ready Hospitals	45	125857	100.0
Stroke-Ready Hospitals	50	125857	100.0
Stroke-Ready Hospitals	55	125857	100.0
Stroke-Ready Hospitals	60	125857	100.0
Stroke Units	5	31658	25.15
Stroke Units	10	91466	72.67
Stroke Units	15	123967	98.5
Stroke Units	20	125857	100.0
Stroke Units	25	125857	100.0
Stroke Units	30	125857	100.0
Stroke Units	35	125857	100.0
Stroke Units	40	125857	100.0
Stroke Units	45	125857	100.0
Stroke Units	50	125857	100.0
Stroke Units	55	125857	100.0
Stroke Units	60	125857	100.0

Population Coverage - Kreisfreie Stadt Pirmasens (Rheinland-Pfalz)

Condition	Drive Time (min)	Covered Population	Coverage (%)
Hospitals with CT	5	18107	48.59
Hospitals with CT	10	35281	94.69
Hospitals with CT	15	37261	100.0
Hospitals with CT	20	37262	100.0
Hospitals with CT	25	37262	100.0
Hospitals with CT	30	37262	100.0
Hospitals with CT	35	37262	100.0
Hospitals with CT	40	37262	100.0
Hospitals with CT	45	37262	100.0
Hospitals with CT	50	37262	100.0
Hospitals with CT	55	37262	100.0
Hospitals with CT	60	37262	100.0
Stroke-Ready Hospitals	5	18107	48.59
Stroke-Ready Hospitals	10	35185	94.43
Stroke-Ready Hospitals	15	37261	100.0
Stroke-Ready Hospitals	20	37262	100.0
Stroke-Ready Hospitals	25	37262	100.0
Stroke-Ready Hospitals	30	37262	100.0
Stroke-Ready Hospitals	35	37262	100.0
Stroke-Ready Hospitals	40	37262	100.0
Stroke-Ready Hospitals	45	37262	100.0
Stroke-Ready Hospitals	50	37262	100.0
Stroke-Ready Hospitals	55	37262	100.0
Stroke-Ready Hospitals	60	37262	100.0
Stroke Units	5	0	0.0
Stroke Units	10	0	0.0
Stroke Units	15	0	0.0
Stroke Units	20	0	0.0
Stroke Units	25	0	0.0
Stroke Units	30	1575	4.23
Stroke Units	35	31742	85.19
Stroke Units	40	37200	99.83
Stroke Units	45	37260	100.0
Stroke Units	50	37262	100.0
Stroke Units	55	37262	100.0
Stroke Units	60	37262	100.0

Population Coverage - Landkreis Donnersbergkreis (Rheinland-Pfalz)

Condition	Drive Time (min)	Covered Population	Coverage (%)
Hospitals with CT	5	10715	13.82
Hospitals with CT	10	25045	32.31
Hospitals with CT	15	55927	72.15
Hospitals with CT	20	75883	97.89
Hospitals with CT	25	77511	99.99
Hospitals with CT	30	77517	100.0
Hospitals with CT	35	77517	100.0
Hospitals with CT	40	77517	100.0
Hospitals with CT	45	77517	100.0
Hospitals with CT	50	77517	100.0
Hospitals with CT	55	77517	100.0
Hospitals with CT	60	77517	100.0
Stroke-Ready Hospitals	5	0	0.0
Stroke-Ready Hospitals	10	0	0.0
Stroke-Ready Hospitals	15	7034	9.07
Stroke-Ready Hospitals	20	27141	35.01
Stroke-Ready Hospitals	25	57554	74.25
Stroke-Ready Hospitals	30	77342	99.77
Stroke-Ready Hospitals	35	77517	100.0
Stroke-Ready Hospitals	40	77517	100.0
Stroke-Ready Hospitals	45	77517	100.0
Stroke-Ready Hospitals	50	77517	100.0
Stroke-Ready Hospitals	55	77517	100.0
Stroke-Ready Hospitals	60	77517	100.0
Stroke Units	5	0	0.0
Stroke Units	10	0	0.0
Stroke Units	15	0	0.0
Stroke Units	20	1588	2.05
Stroke Units	25	18528	23.9
Stroke Units	30	61643	79.52
Stroke Units	35	71858	92.7
Stroke Units	40	77515	100.0
Stroke Units	45	77517	100.0
Stroke Units	50	77517	100.0
Stroke Units	55	77517	100.0
Stroke Units	60	77517	100.0

Population Coverage - Stadtkreis Heidelberg (Baden-Württemberg)

Condition	Drive Time (min)	Covered Population	Coverage (%)
Hospitals with CT	5	91229	59.1
Hospitals with CT	10	133307	86.36
Hospitals with CT	15	153271	99.29
Hospitals with CT	20	154354	99.99
Hospitals with CT	25	154366	100.0
Hospitals with CT	30	154366	100.0
Hospitals with CT	35	154366	100.0
Hospitals with CT	40	154366	100.0
Hospitals with CT	45	154366	100.0
Hospitals with CT	50	154366	100.0
Hospitals with CT	55	154366	100.0
Hospitals with CT	60	154366	100.0
Stroke-Ready Hospitals	5	21821	14.14
Stroke-Ready Hospitals	10	71208	46.13
Stroke-Ready Hospitals	15	124821	80.86
Stroke-Ready Hospitals	20	150217	97.31
Stroke-Ready Hospitals	25	154328	99.98
Stroke-Ready Hospitals	30	154366	100.0
Stroke-Ready Hospitals	35	154366	100.0
Stroke-Ready Hospitals	40	154366	100.0
Stroke-Ready Hospitals	45	154366	100.0
Stroke-Ready Hospitals	50	154366	100.0
Stroke-Ready Hospitals	55	154366	100.0
Stroke-Ready Hospitals	60	154366	100.0
Stroke Units	5	21821	14.14
Stroke Units	10	71208	46.13
Stroke Units	15	124821	80.86
Stroke Units	20	150217	97.31
Stroke Units	25	154328	99.98
Stroke Units	30	154366	100.0
Stroke Units	35	154366	100.0
Stroke Units	40	154366	100.0
Stroke Units	45	154366	100.0
Stroke Units	50	154366	100.0
Stroke Units	55	154366	100.0
Stroke Units	60	154366	100.0

Population Coverage - Kreisfreie Stadt Straubing (Bayern)

Condition	Drive Time (min)	Covered Population	Coverage (%)
Hospitals with CT	5	20025	42.66
Hospitals with CT	10	43561	92.8
Hospitals with CT	15	46888	99.88
Hospitals with CT	20	46942	100.0
Hospitals with CT	25	46942	100.0
Hospitals with CT	30	46942	100.0
Hospitals with CT	35	46942	100.0
Hospitals with CT	40	46942	100.0
Hospitals with CT	45	46942	100.0
Hospitals with CT	50	46942	100.0
Hospitals with CT	55	46942	100.0
Hospitals with CT	60	46942	100.0
Stroke-Ready Hospitals	5	20092	42.8
Stroke-Ready Hospitals	10	43455	92.57
Stroke-Ready Hospitals	15	46888	99.88
Stroke-Ready Hospitals	20	46942	100.0
Stroke-Ready Hospitals	25	46942	100.0
Stroke-Ready Hospitals	30	46942	100.0
Stroke-Ready Hospitals	35	46942	100.0
Stroke-Ready Hospitals	40	46942	100.0
Stroke-Ready Hospitals	45	46942	100.0
Stroke-Ready Hospitals	50	46942	100.0
Stroke-Ready Hospitals	55	46942	100.0
Stroke-Ready Hospitals	60	46942	100.0
Stroke Units	5	20092	42.8
Stroke Units	10	43455	92.57
Stroke Units	15	46888	99.88
Stroke Units	20	46942	100.0
Stroke Units	25	46942	100.0
Stroke Units	30	46942	100.0
Stroke Units	35	46942	100.0
Stroke Units	40	46942	100.0
Stroke Units	45	46942	100.0
Stroke Units	50	46942	100.0
Stroke Units	55	46942	100.0
Stroke Units	60	46942	100.0

Population Coverage - Landkreis Straubing-Bogen (Bayern)

Condition	Drive Time (min)	Covered Population	Coverage (%)
Hospitals with CT	5	11811	11.75
Hospitals with CT	10	33678	33.5
Hospitals with CT	15	61938	61.62
Hospitals with CT	20	87642	87.19
Hospitals with CT	25	98718	98.2
Hospitals with CT	30	100485	99.96
Hospitals with CT	35	100522	100.0
Hospitals with CT	40	100522	100.0
Hospitals with CT	45	100522	100.0
Hospitals with CT	50	100522	100.0
Hospitals with CT	55	100522	100.0
Hospitals with CT	60	100522	100.0
Stroke-Ready Hospitals	5	0	0.0
Stroke-Ready Hospitals	10	3096	3.08
Stroke-Ready Hospitals	15	23709	23.59
Stroke-Ready Hospitals	20	56807	56.51
Stroke-Ready Hospitals	25	78549	78.14
Stroke-Ready Hospitals	30	91804	91.33
Stroke-Ready Hospitals	35	99838	99.32
Stroke-Ready Hospitals	40	100522	100.0
Stroke-Ready Hospitals	45	100522	100.0
Stroke-Ready Hospitals	50	100522	100.0
Stroke-Ready Hospitals	55	100522	100.0
Stroke-Ready Hospitals	60	100522	100.0
Stroke Units	5	0	0.0
Stroke Units	10	3096	3.08
Stroke Units	15	23709	23.59
Stroke Units	20	56807	56.51
Stroke Units	25	78417	78.01
Stroke Units	30	90917	90.45
Stroke Units	35	99782	99.26
Stroke Units	40	100522	100.0
Stroke Units	45	100522	100.0
Stroke Units	50	100522	100.0
Stroke Units	55	100522	100.0
Stroke Units	60	100522	100.0

Population Coverage - Landkreis Neumarkt i.d.OPf. (Bayern)

Condition	Drive Time (min)	Covered Population	Coverage (%)
Hospitals with CT	5	7936	5.99
Hospitals with CT	10	34700	26.2
Hospitals with CT	15	57457	43.38
Hospitals with CT	20	70455	53.19
Hospitals with CT	25	95474	72.07
Hospitals with CT	30	120016	90.6
Hospitals with CT	35	127692	96.4
Hospitals with CT	40	131190	99.04
Hospitals with CT	45	132467	100.0
Hospitals with CT	50	132467	100.0
Hospitals with CT	55	132467	100.0
Hospitals with CT	60	132467	100.0
Stroke-Ready Hospitals	5	8097	6.11
Stroke-Ready Hospitals	10	34391	25.96
Stroke-Ready Hospitals	15	54685	41.28
Stroke-Ready Hospitals	20	68380	51.62
Stroke-Ready Hospitals	25	91616	69.16
Stroke-Ready Hospitals	30	112065	84.6
Stroke-Ready Hospitals	35	119677	90.34
Stroke-Ready Hospitals	40	124396	93.91
Stroke-Ready Hospitals	45	132002	99.65
Stroke-Ready Hospitals	50	132467	100.0
Stroke-Ready Hospitals	55	132467	100.0
Stroke-Ready Hospitals	60	132467	100.0
Stroke Units	5	8097	6.11
Stroke Units	10	34391	25.96
Stroke Units	15	54685	41.28
Stroke Units	20	68380	51.62
Stroke Units	25	91616	69.16
Stroke Units	30	112065	84.6
Stroke Units	35	119675	90.34
Stroke Units	40	124394	93.91
Stroke Units	45	132002	99.65
Stroke Units	50	132467	100.0
Stroke Units	55	132467	100.0
Stroke Units	60	132467	100.0

Population Coverage - Kreisfreie Stadt Schwabach (Bayern)

Condition	Drive Time (min)	Covered Population	Coverage (%)
Hospitals with CT	5	20936	49.69
Hospitals with CT	10	41648	98.85
Hospitals with CT	15	42132	100.0
Hospitals with CT	20	42132	100.0
Hospitals with CT	25	42132	100.0
Hospitals with CT	30	42132	100.0
Hospitals with CT	35	42132	100.0
Hospitals with CT	40	42132	100.0
Hospitals with CT	45	42132	100.0
Hospitals with CT	50	42132	100.0
Hospitals with CT	55	42132	100.0
Hospitals with CT	60	42132	100.0
Stroke-Ready Hospitals	5	24442	58.01
Stroke-Ready Hospitals	10	41814	99.25
Stroke-Ready Hospitals	15	42132	100.0
Stroke-Ready Hospitals	20	42132	100.0
Stroke-Ready Hospitals	25	42132	100.0
Stroke-Ready Hospitals	30	42132	100.0
Stroke-Ready Hospitals	35	42132	100.0
Stroke-Ready Hospitals	40	42132	100.0
Stroke-Ready Hospitals	45	42132	100.0
Stroke-Ready Hospitals	50	42132	100.0
Stroke-Ready Hospitals	55	42132	100.0
Stroke-Ready Hospitals	60	42132	100.0
Stroke Units	5	20387	48.39
Stroke Units	10	41672	98.91
Stroke Units	15	42132	100.0
Stroke Units	20	42132	100.0
Stroke Units	25	42132	100.0
Stroke Units	30	42132	100.0
Stroke Units	35	42132	100.0
Stroke Units	40	42132	100.0
Stroke Units	45	42132	100.0
Stroke Units	50	42132	100.0
Stroke Units	55	42132	100.0
Stroke Units	60	42132	100.0

Population Coverage - Kreisfreie Stadt Brandenburg an der Havel (Brandenburg)

Condition	Drive Time (min)	Covered Population	Coverage (%)
Hospitals with CT	5	15412	22.31
Hospitals with CT	10	47911	69.34
Hospitals with CT	15	58658	84.9
Hospitals with CT	20	64292	93.05
Hospitals with CT	25	65957	95.46
Hospitals with CT	30	66174	95.78
Hospitals with CT	35	69067	99.96
Hospitals with CT	40	69067	99.96
Hospitals with CT	45	69092	100.0
Hospitals with CT	50	69092	100.0
Hospitals with CT	55	69092	100.0
Hospitals with CT	60	69092	100.0
Stroke-Ready Hospitals	5	4646	6.72
Stroke-Ready Hospitals	10	22472	32.52
Stroke-Ready Hospitals	15	42563	61.6
Stroke-Ready Hospitals	20	58371	84.48
Stroke-Ready Hospitals	25	64346	93.13
Stroke-Ready Hospitals	30	65574	94.91
Stroke-Ready Hospitals	35	68990	99.85
Stroke-Ready Hospitals	40	69002	99.87
Stroke-Ready Hospitals	45	69092	100.0
Stroke-Ready Hospitals	50	69092	100.0
Stroke-Ready Hospitals	55	69092	100.0
Stroke-Ready Hospitals	60	69092	100.0
Stroke Units	5	0	0.0
Stroke Units	10	0	0.0
Stroke Units	15	0	0.0
Stroke Units	20	0	0.0
Stroke Units	25	0	0.0
Stroke Units	30	0	0.0
Stroke Units	35	0	0.0
Stroke Units	40	439	0.64
Stroke Units	45	2401	3.48
Stroke Units	50	14539	21.04
Stroke Units	55	33907	49.08
Stroke Units	60	57878	83.77

Population Coverage - Landkreis Ludwigslust-Parchim (Mecklenburg-Vorpommern)

Condition	Drive Time (min)	Covered Population	Coverage (%)
Hospitals with CT	5	29051	14.82
Hospitals with CT	10	49903	25.46
Hospitals with CT	15	74219	37.86
Hospitals with CT	20	103424	52.76
Hospitals with CT	25	137227	70.01
Hospitals with CT	30	168654	86.04
Hospitals with CT	35	192047	97.98
Hospitals with CT	40	195080	99.52
Hospitals with CT	45	195641	99.81
Hospitals with CT	50	196015	100.0
Hospitals with CT	55	196016	100.0
Hospitals with CT	60	196016	100.0
Stroke-Ready Hospitals	5	564	0.29
Stroke-Ready Hospitals	10	4460	2.28
Stroke-Ready Hospitals	15	6055	3.09
Stroke-Ready Hospitals	20	11924	6.08
Stroke-Ready Hospitals	25	29577	15.09
Stroke-Ready Hospitals	30	46010	23.47
Stroke-Ready Hospitals	35	79133	40.37
Stroke-Ready Hospitals	40	115279	58.81
Stroke-Ready Hospitals	45	147722	75.36
Stroke-Ready Hospitals	50	160818	82.04
Stroke-Ready Hospitals	55	176395	89.99
Stroke-Ready Hospitals	60	186496	95.14
Stroke Units	5	564	0.29
Stroke Units	10	4460	2.28
Stroke Units	15	6055	3.09
Stroke Units	20	11924	6.08
Stroke Units	25	29561	15.08
Stroke Units	30	45005	22.96
Stroke Units	35	65981	33.66
Stroke Units	40	95874	48.91
Stroke Units	45	138168	70.49
Stroke Units	50	155962	79.57
Stroke Units	55	170508	86.99
Stroke Units	60	181489	92.59

Population Coverage - Landkreis Saalfeld-Rudolstadt (Thüringen)

Condition	Drive Time (min)	Covered Population	Coverage (%)
Hospitals with CT	5	17530	18.11
Hospitals with CT	10	38282	39.54
Hospitals with CT	15	57999	59.9
Hospitals with CT	20	71141	73.48
Hospitals with CT	25	77817	80.37
Hospitals with CT	30	82494	85.2
Hospitals with CT	35	90825	93.81
Hospitals with CT	40	96498	99.67
Hospitals with CT	45	96820	100.0
Hospitals with CT	50	96820	100.0
Hospitals with CT	55	96820	100.0
Hospitals with CT	60	96820	100.0
Stroke-Ready Hospitals	5	6364	6.57
Stroke-Ready Hospitals	10	15744	16.26
Stroke-Ready Hospitals	15	30468	31.47
Stroke-Ready Hospitals	20	52194	53.91
Stroke-Ready Hospitals	25	64356	66.47
Stroke-Ready Hospitals	30	73990	76.42
Stroke-Ready Hospitals	35	83375	86.11
Stroke-Ready Hospitals	40	90225	93.19
Stroke-Ready Hospitals	45	94947	98.07
Stroke-Ready Hospitals	50	96775	99.95
Stroke-Ready Hospitals	55	96820	100.0
Stroke-Ready Hospitals	60	96820	100.0
Stroke Units	5	6364	6.57
Stroke Units	10	15744	16.26
Stroke Units	15	30468	31.47
Stroke Units	20	52194	53.91
Stroke Units	25	64356	66.47
Stroke Units	30	73990	76.42
Stroke Units	35	83375	86.11
Stroke Units	40	90225	93.19
Stroke Units	45	94947	98.07
Stroke Units	50	96775	99.95
Stroke Units	55	96820	100.0
Stroke Units	60	96820	100.0

Population Coverage - Kreis Ostholstein (Schleswig-Holstein)

Condition	Drive Time (min)	Covered Population	Coverage (%)
Hospitals with CT	5	65094	32.23
Hospitals with CT	10	112222	55.56
Hospitals with CT	15	157946	78.2
Hospitals with CT	20	181991	90.11
Hospitals with CT	25	193495	95.8
Hospitals with CT	30	197938	98.0
Hospitals with CT	35	199518	98.79
Hospitals with CT	40	199972	99.01
Hospitals with CT	45	200049	99.05
Hospitals with CT	50	200287	99.17
Hospitals with CT	55	199215	98.64
Hospitals with CT	60	199867	98.96
Stroke-Ready Hospitals	5	23250	11.51
Stroke-Ready Hospitals	10	37145	18.39
Stroke-Ready Hospitals	15	54849	27.16
Stroke-Ready Hospitals	20	112609	55.76
Stroke-Ready Hospitals	25	159293	78.87
Stroke-Ready Hospitals	30	184845	91.52
Stroke-Ready Hospitals	35	197621	97.85
Stroke-Ready Hospitals	40	199488	98.77
Stroke-Ready Hospitals	45	199666	98.86
Stroke-Ready Hospitals	50	199703	98.88
Stroke-Ready Hospitals	55	198222	98.14
Stroke-Ready Hospitals	60	199001	98.53
Stroke Units	5	17225	8.53
Stroke Units	10	28360	14.04
Stroke Units	15	53442	26.46
Stroke Units	20	111910	55.41
Stroke Units	25	154918	76.7
Stroke Units	30	182881	90.55
Stroke Units	35	196739	97.41
Stroke Units	40	198906	98.48
Stroke Units	45	199377	98.72
Stroke Units	50	199552	98.8
Stroke Units	55	198177	98.12
Stroke Units	60	198968	98.51

Population Coverage - Kreis Rendsburg-Eckernförde (Schleswig-Holstein)

Condition	Drive Time (min)	Covered Population	Coverage (%)
Hospitals with CT	5	13482	4.86
Hospitals with CT	10	68429	24.68
Hospitals with CT	15	153518	55.36
Hospitals with CT	20	207560	74.85
Hospitals with CT	25	260541	93.95
Hospitals with CT	30	274953	99.15
Hospitals with CT	35	275803	99.46
Hospitals with CT	40	275837	99.47
Hospitals with CT	45	275861	99.48
Hospitals with CT	50	275933	99.5
Hospitals with CT	55	275544	99.36
Hospitals with CT	60	275811	99.46
Stroke-Ready Hospitals	5	4094	1.48
Stroke-Ready Hospitals	10	32222	11.62
Stroke-Ready Hospitals	15	82239	29.66
Stroke-Ready Hospitals	20	135172	48.74
Stroke-Ready Hospitals	25	215966	77.88
Stroke-Ready Hospitals	30	261003	94.12
Stroke-Ready Hospitals	35	269167	97.06
Stroke-Ready Hospitals	40	271977	98.08
Stroke-Ready Hospitals	45	274874	99.12
Stroke-Ready Hospitals	50	275507	99.35
Stroke-Ready Hospitals	55	275434	99.32
Stroke-Ready Hospitals	60	275622	99.39
Stroke Units	5	4094	1.48
Stroke Units	10	32222	11.62
Stroke Units	15	82239	29.66
Stroke Units	20	135155	48.74
Stroke Units	25	215832	77.83
Stroke Units	30	260967	94.11
Stroke Units	35	269150	97.06
Stroke Units	40	271977	98.08
Stroke Units	45	274874	99.12
Stroke Units	50	275507	99.35
Stroke Units	55	275434	99.32
Stroke Units	60	275622	99.39

Population Coverage - Landkreis Ostalbkreis (Baden-Württemberg)

Condition	Drive Time (min)	Covered Population	Coverage (%)
Hospitals with CT	5	46310	14.88
Hospitals with CT	10	134110	43.08
Hospitals with CT	15	220111	70.7
Hospitals with CT	20	286316	91.97
Hospitals with CT	25	307868	98.89
Hospitals with CT	30	311081	99.92
Hospitals with CT	35	311330	100.0
Hospitals with CT	40	311331	100.0
Hospitals with CT	45	311331	100.0
Hospitals with CT	50	311331	100.0
Hospitals with CT	55	311331	100.0
Hospitals with CT	60	311331	100.0
Stroke-Ready Hospitals	5	22403	7.2
Stroke-Ready Hospitals	10	70956	22.79
Stroke-Ready Hospitals	15	142894	45.9
Stroke-Ready Hospitals	20	221531	71.16
Stroke-Ready Hospitals	25	269171	86.46
Stroke-Ready Hospitals	30	294973	94.75
Stroke-Ready Hospitals	35	308522	99.1
Stroke-Ready Hospitals	40	311331	100.0
Stroke-Ready Hospitals	45	311331	100.0
Stroke-Ready Hospitals	50	311331	100.0
Stroke-Ready Hospitals	55	311331	100.0
Stroke-Ready Hospitals	60	311331	100.0
Stroke Units	5	14191	4.56
Stroke Units	10	47954	15.4
Stroke Units	15	78975	25.37
Stroke Units	20	123019	39.51
Stroke Units	25	181770	58.39
Stroke Units	30	256543	82.4
Stroke Units	35	298054	95.74
Stroke Units	40	305939	98.27
Stroke Units	45	311331	100.0
Stroke Units	50	311331	100.0
Stroke Units	55	311331	100.0
Stroke Units	60	311331	100.0

Population Coverage - Landkreis Emmendingen (Baden-Württemberg)

Condition	Drive Time (min)	Covered Population	Coverage (%)
Hospitals with CT	5	14789	9.14
Hospitals with CT	10	48884	30.19
Hospitals with CT	15	101054	62.42
Hospitals with CT	20	129850	80.21
Hospitals with CT	25	151165	93.37
Hospitals with CT	30	160533	99.16
Hospitals with CT	35	161790	99.94
Hospitals with CT	40	161885	99.99
Hospitals with CT	45	161895	100.0
Hospitals with CT	50	161895	100.0
Hospitals with CT	55	161893	100.0
Hospitals with CT	60	161893	100.0
Stroke-Ready Hospitals	5	7021	4.34
Stroke-Ready Hospitals	10	22027	13.61
Stroke-Ready Hospitals	15	39940	24.67
Stroke-Ready Hospitals	20	76422	47.2
Stroke-Ready Hospitals	25	113368	70.03
Stroke-Ready Hospitals	30	137174	84.73
Stroke-Ready Hospitals	35	153656	94.91
Stroke-Ready Hospitals	40	159898	98.77
Stroke-Ready Hospitals	45	161270	99.61
Stroke-Ready Hospitals	50	161822	99.95
Stroke-Ready Hospitals	55	161859	99.98
Stroke-Ready Hospitals	60	161854	99.97
Stroke Units	5	0	0.0
Stroke Units	10	0	0.0
Stroke Units	15	869	0.54
Stroke Units	20	30737	18.99
Stroke Units	25	84497	52.19
Stroke Units	30	127564	78.79
Stroke Units	35	145286	89.74
Stroke Units	40	153574	94.86
Stroke Units	45	159427	98.48
Stroke Units	50	161084	99.5
Stroke Units	55	161715	99.89
Stroke Units	60	161836	99.96

Population Coverage - Kreisfreie Stadt München (Bayern)

Condition	Drive Time (min)	Covered Population	Coverage (%)
Hospitals with CT	5	857341	51.3
Hospitals with CT	10	1516693	90.76
Hospitals with CT	15	1663204	99.52
Hospitals with CT	20	1671153	100.0
Hospitals with CT	25	1671155	100.0
Hospitals with CT	30	1671155	100.0
Hospitals with CT	35	1671155	100.0
Hospitals with CT	40	1671155	100.0
Hospitals with CT	45	1671155	100.0
Hospitals with CT	50	1671155	100.0
Hospitals with CT	55	1671155	100.0
Hospitals with CT	60	1671155	100.0
Stroke-Ready Hospitals	5	190725	11.41
Stroke-Ready Hospitals	10	809060	48.41
Stroke-Ready Hospitals	15	1397645	83.63
Stroke-Ready Hospitals	20	1641833	98.25
Stroke-Ready Hospitals	25	1671142	100.0
Stroke-Ready Hospitals	30	1671155	100.0
Stroke-Ready Hospitals	35	1671155	100.0
Stroke-Ready Hospitals	40	1671155	100.0
Stroke-Ready Hospitals	45	1671155	100.0
Stroke-Ready Hospitals	50	1671155	100.0
Stroke-Ready Hospitals	55	1671155	100.0
Stroke-Ready Hospitals	60	1671155	100.0
Stroke Units	5	190725	11.41
Stroke Units	10	809060	48.41
Stroke Units	15	1397645	83.63
Stroke Units	20	1641833	98.25
Stroke Units	25	1671142	100.0
Stroke Units	30	1671155	100.0
Stroke Units	35	1671155	100.0
Stroke Units	40	1671155	100.0
Stroke Units	45	1671155	100.0
Stroke Units	50	1671155	100.0
Stroke Units	55	1671155	100.0
Stroke Units	60	1671155	100.0

Population Coverage - Landkreis Bayreuth (Bayern)

Condition	Drive Time (min)	Covered Population	Coverage (%)
Hospitals with CT	5	7139	6.56
Hospitals with CT	10	24271	22.29
Hospitals with CT	15	49327	45.29
Hospitals with CT	20	87507	80.35
Hospitals with CT	25	102265	93.9
Hospitals with CT	30	108620	99.73
Hospitals with CT	35	108910	100.0
Hospitals with CT	40	108910	100.0
Hospitals with CT	45	108910	100.0
Hospitals with CT	50	108910	100.0
Hospitals with CT	55	108910	100.0
Hospitals with CT	60	108910	100.0
Stroke-Ready Hospitals	5	459	0.42
Stroke-Ready Hospitals	10	11102	10.19
Stroke-Ready Hospitals	15	26002	23.88
Stroke-Ready Hospitals	20	46747	42.92
Stroke-Ready Hospitals	25	67019	61.54
Stroke-Ready Hospitals	30	95990	88.14
Stroke-Ready Hospitals	35	106093	97.41
Stroke-Ready Hospitals	40	108090	99.25
Stroke-Ready Hospitals	45	108910	100.0
Stroke-Ready Hospitals	50	108910	100.0
Stroke-Ready Hospitals	55	108910	100.0
Stroke-Ready Hospitals	60	108910	100.0
Stroke Units	5	459	0.42
Stroke Units	10	6003	5.51
Stroke Units	15	16378	15.04
Stroke Units	20	38212	35.09
Stroke Units	25	62561	57.44
Stroke Units	30	89236	81.94
Stroke Units	35	101693	93.37
Stroke Units	40	107498	98.7
Stroke Units	45	108910	100.0
Stroke Units	50	108910	100.0
Stroke Units	55	108910	100.0
Stroke Units	60	108910	100.0

Population Coverage - Landkreis Kronach (Bayern)

Condition	Drive Time (min)	Covered Population	Coverage (%)
Hospitals with CT	5	9002	12.67
Hospitals with CT	10	16056	22.59
Hospitals with CT	15	33179	46.68
Hospitals with CT	20	48774	68.62
Hospitals with CT	25	56686	79.75
Hospitals with CT	30	62751	88.28
Hospitals with CT	35	67092	94.39
Hospitals with CT	40	70986	99.87
Hospitals with CT	45	71079	100.0
Hospitals with CT	50	71079	100.0
Hospitals with CT	55	71079	100.0
Hospitals with CT	60	71079	100.0
Stroke-Ready Hospitals	5	0	0.0
Stroke-Ready Hospitals	10	0	0.0
Stroke-Ready Hospitals	15	0	0.0
Stroke-Ready Hospitals	20	1416	1.99
Stroke-Ready Hospitals	25	13017	18.31
Stroke-Ready Hospitals	30	34883	49.08
Stroke-Ready Hospitals	35	55044	77.44
Stroke-Ready Hospitals	40	63927	89.94
Stroke-Ready Hospitals	45	70651	99.4
Stroke-Ready Hospitals	50	71077	100.0
Stroke-Ready Hospitals	55	71079	100.0
Stroke-Ready Hospitals	60	71079	100.0
Stroke Units	5	0	0.0
Stroke Units	10	0	0.0
Stroke Units	15	0	0.0
Stroke Units	20	1416	1.99
Stroke Units	25	13017	18.31
Stroke Units	30	34883	49.08
Stroke Units	35	55044	77.44
Stroke Units	40	63927	89.94
Stroke Units	45	70651	99.4
Stroke Units	50	71077	100.0
Stroke Units	55	71079	100.0
Stroke Units	60	71079	100.0

Population Coverage - Landkreis Aschaffenburg (Bayern)

Condition	Drive Time (min)	Covered Population	Coverage (%)
Hospitals with CT	5	2675	1.49
Hospitals with CT	10	13988	7.78
Hospitals with CT	15	64033	35.6
Hospitals with CT	20	126861	70.54
Hospitals with CT	25	163095	90.68
Hospitals with CT	30	179581	99.85
Hospitals with CT	35	179846	100.0
Hospitals with CT	40	179849	100.0
Hospitals with CT	45	179849	100.0
Hospitals with CT	50	179849	100.0
Hospitals with CT	55	179849	100.0
Hospitals with CT	60	179849	100.0
Stroke-Ready Hospitals	5	2859	1.59
Stroke-Ready Hospitals	10	14619	8.13
Stroke-Ready Hospitals	15	66194	36.81
Stroke-Ready Hospitals	20	125706	69.9
Stroke-Ready Hospitals	25	163772	91.06
Stroke-Ready Hospitals	30	179717	99.93
Stroke-Ready Hospitals	35	179844	100.0
Stroke-Ready Hospitals	40	179849	100.0
Stroke-Ready Hospitals	45	179849	100.0
Stroke-Ready Hospitals	50	179849	100.0
Stroke-Ready Hospitals	55	179849	100.0
Stroke-Ready Hospitals	60	179849	100.0
Stroke Units	5	2847	1.58
Stroke Units	10	10206	5.68
Stroke Units	15	41201	22.91
Stroke Units	20	115042	63.97
Stroke Units	25	163290	90.79
Stroke Units	30	179708	99.92
Stroke Units	35	179844	100.0
Stroke Units	40	179849	100.0
Stroke Units	45	179849	100.0
Stroke Units	50	179849	100.0
Stroke Units	55	179849	100.0
Stroke Units	60	179849	100.0

Population Coverage - Landkreis Saalekreis (Sachsen-Anhalt)

Condition	Drive Time (min)	Covered Population	Coverage (%)
Hospitals with CT	5	789	0.47
Hospitals with CT	10	8049	4.76
Hospitals with CT	15	28492	16.87
Hospitals with CT	20	84046	49.75
Hospitals with CT	25	143722	85.07
Hospitals with CT	30	164818	97.56
Hospitals with CT	35	167753	99.3
Hospitals with CT	40	168765	99.9
Hospitals with CT	45	168927	100.0
Hospitals with CT	50	168935	100.0
Hospitals with CT	55	168935	100.0
Hospitals with CT	60	168935	100.0
Stroke-Ready Hospitals	5	8501	5.03
Stroke-Ready Hospitals	10	27284	16.15
Stroke-Ready Hospitals	15	48284	28.58
Stroke-Ready Hospitals	20	93563	55.38
Stroke-Ready Hospitals	25	142856	84.56
Stroke-Ready Hospitals	30	163237	96.63
Stroke-Ready Hospitals	35	167838	99.35
Stroke-Ready Hospitals	40	168802	99.92
Stroke-Ready Hospitals	45	168927	100.0
Stroke-Ready Hospitals	50	168935	100.0
Stroke-Ready Hospitals	55	168935	100.0
Stroke-Ready Hospitals	60	168935	100.0
Stroke Units	5	1016	0.6
Stroke Units	10	3211	1.9
Stroke Units	15	21776	12.89
Stroke Units	20	64497	38.18
Stroke Units	25	126134	74.66
Stroke Units	30	152808	90.45
Stroke Units	35	165063	97.71
Stroke Units	40	167923	99.4
Stroke Units	45	168473	99.73
Stroke Units	50	168827	99.94
Stroke Units	55	168935	100.0
Stroke Units	60	168935	100.0

Population Coverage - Kreisfreie Stadt Wolfsburg (Niedersachsen)

Condition	Drive Time (min)	Covered Population	Coverage (%)
Hospitals with CT	5	10260	8.41
Hospitals with CT	10	48380	39.63
Hospitals with CT	15	98143	80.4
Hospitals with CT	20	121455	99.5
Hospitals with CT	25	121976	99.93
Hospitals with CT	30	122066	100.0
Hospitals with CT	35	122066	100.0
Hospitals with CT	40	122066	100.0
Hospitals with CT	45	122066	100.0
Hospitals with CT	50	122066	100.0
Hospitals with CT	55	122066	100.0
Hospitals with CT	60	122066	100.0
Stroke-Ready Hospitals	5	10073	8.25
Stroke-Ready Hospitals	10	47397	38.83
Stroke-Ready Hospitals	15	97832	80.15
Stroke-Ready Hospitals	20	121382	99.44
Stroke-Ready Hospitals	25	121976	99.93
Stroke-Ready Hospitals	30	122066	100.0
Stroke-Ready Hospitals	35	122066	100.0
Stroke-Ready Hospitals	40	122066	100.0
Stroke-Ready Hospitals	45	122066	100.0
Stroke-Ready Hospitals	50	122066	100.0
Stroke-Ready Hospitals	55	122066	100.0
Stroke-Ready Hospitals	60	122066	100.0
Stroke Units	5	10073	8.25
Stroke Units	10	47397	38.83
Stroke Units	15	97621	79.97
Stroke Units	20	121296	99.37
Stroke Units	25	121837	99.81
Stroke Units	30	122066	100.0
Stroke Units	35	122066	100.0
Stroke Units	40	122066	100.0
Stroke Units	45	122066	100.0
Stroke Units	50	122066	100.0
Stroke Units	55	122066	100.0
Stroke Units	60	122066	100.0

Population Coverage - Landkreis Wolfenbüttel (Niedersachsen)

Condition	Drive Time (min)	Covered Population	Coverage (%)
Hospitals with CT	5	9025	7.53
Hospitals with CT	10	43671	36.45
Hospitals with CT	15	76949	64.23
Hospitals with CT	20	102483	85.55
Hospitals with CT	25	116541	97.28
Hospitals with CT	30	119403	99.67
Hospitals with CT	35	119788	99.99
Hospitals with CT	40	119794	100.0
Hospitals with CT	45	119794	100.0
Hospitals with CT	50	119794	100.0
Hospitals with CT	55	119794	100.0
Hospitals with CT	60	119794	100.0
Stroke-Ready Hospitals	5	8746	7.3
Stroke-Ready Hospitals	10	41432	34.59
Stroke-Ready Hospitals	15	61885	51.66
Stroke-Ready Hospitals	20	89512	74.72
Stroke-Ready Hospitals	25	112712	94.09
Stroke-Ready Hospitals	30	119333	99.61
Stroke-Ready Hospitals	35	119788	99.99
Stroke-Ready Hospitals	40	119794	100.0
Stroke-Ready Hospitals	45	119794	100.0
Stroke-Ready Hospitals	50	119794	100.0
Stroke-Ready Hospitals	55	119794	100.0
Stroke-Ready Hospitals	60	119794	100.0
Stroke Units	5	0	0.0
Stroke Units	10	0	0.0
Stroke Units	15	1834	1.53
Stroke Units	20	42106	35.15
Stroke Units	25	78007	65.12
Stroke Units	30	103345	86.27
Stroke Units	35	115284	96.24
Stroke Units	40	119675	99.9
Stroke Units	45	119794	100.0
Stroke Units	50	119794	100.0
Stroke Units	55	119794	100.0
Stroke Units	60	119794	100.0

Population Coverage - Landkreis Ammerland (Niedersachsen)

Condition	Drive Time (min)	Covered Population	Coverage (%)
Hospitals with CT	5	8615	7.09
Hospitals with CT	10	16679	13.73
Hospitals with CT	15	46285	38.11
Hospitals with CT	20	103345	85.08
Hospitals with CT	25	120687	99.36
Hospitals with CT	30	121462	100.0
Hospitals with CT	35	121462	100.0
Hospitals with CT	40	121462	100.0
Hospitals with CT	45	121462	100.0
Hospitals with CT	50	121462	100.0
Hospitals with CT	55	121462	100.0
Hospitals with CT	60	121462	100.0
Stroke-Ready Hospitals	5	7713	6.35
Stroke-Ready Hospitals	10	16179	13.32
Stroke-Ready Hospitals	15	40496	33.34
Stroke-Ready Hospitals	20	90448	74.47
Stroke-Ready Hospitals	25	112616	92.72
Stroke-Ready Hospitals	30	121252	99.83
Stroke-Ready Hospitals	35	121462	100.0
Stroke-Ready Hospitals	40	121462	100.0
Stroke-Ready Hospitals	45	121462	100.0
Stroke-Ready Hospitals	50	121462	100.0
Stroke-Ready Hospitals	55	121462	100.0
Stroke-Ready Hospitals	60	121462	100.0
Stroke Units	5	7713	6.35
Stroke Units	10	16179	13.32
Stroke Units	15	40496	33.34
Stroke Units	20	90448	74.47
Stroke Units	25	112616	92.72
Stroke Units	30	121252	99.83
Stroke Units	35	121462	100.0
Stroke Units	40	121462	100.0
Stroke Units	45	121462	100.0
Stroke Units	50	121462	100.0
Stroke Units	55	121462	100.0
Stroke Units	60	121462	100.0

Population Coverage - Kreis Rhein-Kreis Neuss (Nordrhein-Westfalen)

Condition	Drive Time (min)	Covered Population	Coverage (%)
Hospitals with CT	5	73978	15.71
Hospitals with CT	10	247731	52.61
Hospitals with CT	15	416615	88.48
Hospitals with CT	20	470535	99.93
Hospitals with CT	25	470844	100.0
Hospitals with CT	30	470844	100.0
Hospitals with CT	35	470844	100.0
Hospitals with CT	40	470844	100.0
Hospitals with CT	45	470844	100.0
Hospitals with CT	50	470844	100.0
Hospitals with CT	55	470844	100.0
Hospitals with CT	60	470844	100.0
Stroke-Ready Hospitals	5	19929	4.23
Stroke-Ready Hospitals	10	83564	17.75
Stroke-Ready Hospitals	15	249039	52.89
Stroke-Ready Hospitals	20	400044	84.96
Stroke-Ready Hospitals	25	457615	97.19
Stroke-Ready Hospitals	30	470621	99.95
Stroke-Ready Hospitals	35	470844	100.0
Stroke-Ready Hospitals	40	470844	100.0
Stroke-Ready Hospitals	45	470844	100.0
Stroke-Ready Hospitals	50	470844	100.0
Stroke-Ready Hospitals	55	470844	100.0
Stroke-Ready Hospitals	60	470844	100.0
Stroke Units	5	19929	4.23
Stroke Units	10	83564	17.75
Stroke Units	15	249039	52.89
Stroke Units	20	400044	84.96
Stroke Units	25	457615	97.19
Stroke Units	30	470621	99.95
Stroke Units	35	470844	100.0
Stroke Units	40	470844	100.0
Stroke Units	45	470844	100.0
Stroke Units	50	470844	100.0
Stroke Units	55	470844	100.0
Stroke Units	60	470844	100.0

Population Coverage - Kreis Lippe (Nordrhein-Westfalen)

Condition	Drive Time (min)	Covered Population	Coverage (%)
Hospitals with CT	5	47479	13.76
Hospitals with CT	10	138949	40.26
Hospitals with CT	15	214178	62.05
Hospitals with CT	20	300054	86.93
Hospitals with CT	25	343658	99.57
Hospitals with CT	30	345090	99.98
Hospitals with CT	35	345159	100.0
Hospitals with CT	40	345159	100.0
Hospitals with CT	45	345159	100.0
Hospitals with CT	50	345159	100.0
Hospitals with CT	55	345159	100.0
Hospitals with CT	60	345159	100.0
Stroke-Ready Hospitals	5	15106	4.38
Stroke-Ready Hospitals	10	38945	11.28
Stroke-Ready Hospitals	15	71623	20.75
Stroke-Ready Hospitals	20	163364	47.33
Stroke-Ready Hospitals	25	266706	77.27
Stroke-Ready Hospitals	30	324824	94.11
Stroke-Ready Hospitals	35	344778	99.89
Stroke-Ready Hospitals	40	345086	99.98
Stroke-Ready Hospitals	45	345159	100.0
Stroke-Ready Hospitals	50	345159	100.0
Stroke-Ready Hospitals	55	345159	100.0
Stroke-Ready Hospitals	60	345159	100.0
Stroke Units	5	15021	4.35
Stroke Units	10	34723	10.06
Stroke Units	15	65194	18.89
Stroke Units	20	148464	43.01
Stroke Units	25	234265	67.87
Stroke Units	30	312331	90.49
Stroke Units	35	344644	99.85
Stroke Units	40	345086	99.98
Stroke Units	45	345159	100.0
Stroke Units	50	345159	100.0
Stroke Units	55	345159	100.0
Stroke Units	60	345159	100.0

Population Coverage - Kreisfreie Stadt Hagen (Nordrhein-Westfalen)

Condition	Drive Time (min)	Covered Population	Coverage (%)
Hospitals with CT	5	55934	28.02
Hospitals with CT	10	141302	70.77
Hospitals with CT	15	190512	95.42
Hospitals with CT	20	198985	99.66
Hospitals with CT	25	199638	99.99
Hospitals with CT	30	199659	100.0
Hospitals with CT	35	199659	100.0
Hospitals with CT	40	199659	100.0
Hospitals with CT	45	199659	100.0
Hospitals with CT	50	199659	100.0
Hospitals with CT	55	199659	100.0
Hospitals with CT	60	199659	100.0
Stroke-Ready Hospitals	5	24257	12.15
Stroke-Ready Hospitals	10	97216	48.69
Stroke-Ready Hospitals	15	162054	81.17
Stroke-Ready Hospitals	20	194205	97.27
Stroke-Ready Hospitals	25	198436	99.39
Stroke-Ready Hospitals	30	199659	100.0
Stroke-Ready Hospitals	35	199659	100.0
Stroke-Ready Hospitals	40	199659	100.0
Stroke-Ready Hospitals	45	199659	100.0
Stroke-Ready Hospitals	50	199659	100.0
Stroke-Ready Hospitals	55	199659	100.0
Stroke-Ready Hospitals	60	199659	100.0
Stroke Units	5	0	0.0
Stroke Units	10	325	0.16
Stroke Units	15	12745	6.38
Stroke Units	20	58318	29.21
Stroke Units	25	165483	82.88
Stroke Units	30	194296	97.31
Stroke Units	35	199278	99.81
Stroke Units	40	199659	100.0
Stroke Units	45	199659	100.0
Stroke Units	50	199659	100.0
Stroke Units	55	199659	100.0
Stroke Units	60	199659	100.0

Population Coverage - Landkreis Alzey-Worms (Rheinland-Pfalz)

Condition	Drive Time (min)	Covered Population	Coverage (%)
Hospitals with CT	5	16345	12.63
Hospitals with CT	10	38864	30.04
Hospitals with CT	15	100621	77.76
Hospitals with CT	20	120841	93.39
Hospitals with CT	25	128493	99.3
Hospitals with CT	30	129241	99.88
Hospitals with CT	35	129390	100.0
Hospitals with CT	40	129394	100.0
Hospitals with CT	45	129394	100.0
Hospitals with CT	50	129394	100.0
Hospitals with CT	55	129394	100.0
Hospitals with CT	60	129394	100.0
Stroke-Ready Hospitals	5	9085	7.02
Stroke-Ready Hospitals	10	26408	20.41
Stroke-Ready Hospitals	15	71128	54.97
Stroke-Ready Hospitals	20	115333	89.13
Stroke-Ready Hospitals	25	128876	99.6
Stroke-Ready Hospitals	30	129197	99.85
Stroke-Ready Hospitals	35	129390	100.0
Stroke-Ready Hospitals	40	129390	100.0
Stroke-Ready Hospitals	45	129394	100.0
Stroke-Ready Hospitals	50	129394	100.0
Stroke-Ready Hospitals	55	129394	100.0
Stroke-Ready Hospitals	60	129394	100.0
Stroke Units	5	0	0.0
Stroke Units	10	2940	2.27
Stroke Units	15	24882	19.23
Stroke Units	20	68798	53.17
Stroke Units	25	121753	94.09
Stroke Units	30	129163	99.82
Stroke Units	35	129386	99.99
Stroke Units	40	129390	100.0
Stroke Units	45	129394	100.0
Stroke Units	50	129394	100.0
Stroke Units	55	129394	100.0
Stroke Units	60	129394	100.0

Population Coverage - Landkreis Germersheim (Rheinland-Pfalz)

Condition	Drive Time (min)	Covered Population	Coverage (%)
Hospitals with CT	5	24035	18.67
Hospitals with CT	10	73889	57.4
Hospitals with CT	15	115183	89.48
Hospitals with CT	20	128340	99.7
Hospitals with CT	25	128667	99.96
Hospitals with CT	30	128715	99.99
Hospitals with CT	35	128715	99.99
Hospitals with CT	40	128715	99.99
Hospitals with CT	45	128715	99.99
Hospitals with CT	50	128715	99.99
Hospitals with CT	55	128719	100.0
Hospitals with CT	60	128719	100.0
Stroke-Ready Hospitals	5	8253	6.41
Stroke-Ready Hospitals	10	21190	16.46
Stroke-Ready Hospitals	15	66701	51.82
Stroke-Ready Hospitals	20	117231	91.07
Stroke-Ready Hospitals	25	128595	99.9
Stroke-Ready Hospitals	30	128688	99.97
Stroke-Ready Hospitals	35	128688	99.97
Stroke-Ready Hospitals	40	128689	99.97
Stroke-Ready Hospitals	45	128690	99.97
Stroke-Ready Hospitals	50	128715	99.99
Stroke-Ready Hospitals	55	128715	99.99
Stroke-Ready Hospitals	60	128717	100.0
Stroke Units	5	8253	6.41
Stroke Units	10	21190	16.46
Stroke Units	15	66692	51.81
Stroke Units	20	117186	91.04
Stroke Units	25	128591	99.9
Stroke Units	30	128688	99.97
Stroke Units	35	128688	99.97
Stroke Units	40	128689	99.97
Stroke Units	45	128690	99.97
Stroke Units	50	128715	99.99
Stroke Units	55	128688	99.97
Stroke Units	60	128717	100.0

Population Coverage - Landkreis Neckar-Odenwald-Kreis (Baden-Württemberg)

Condition	Drive Time (min)	Covered Population	Coverage (%)
Hospitals with CT	5	20911	14.59
Hospitals with CT	10	55593	38.78
Hospitals with CT	15	98840	68.94
Hospitals with CT	20	127766	89.12
Hospitals with CT	25	140875	98.26
Hospitals with CT	30	143342	99.98
Hospitals with CT	35	143370	100.0
Hospitals with CT	40	143370	100.0
Hospitals with CT	45	143370	100.0
Hospitals with CT	50	143370	100.0
Hospitals with CT	55	143370	100.0
Hospitals with CT	60	143370	100.0
Stroke-Ready Hospitals	5	7826	5.46
Stroke-Ready Hospitals	10	28428	19.83
Stroke-Ready Hospitals	15	46654	32.54
Stroke-Ready Hospitals	20	69099	48.2
Stroke-Ready Hospitals	25	82996	57.89
Stroke-Ready Hospitals	30	98425	68.65
Stroke-Ready Hospitals	35	119331	83.23
Stroke-Ready Hospitals	40	135474	94.49
Stroke-Ready Hospitals	45	143370	100.0
Stroke-Ready Hospitals	50	143370	100.0
Stroke-Ready Hospitals	55	143370	100.0
Stroke-Ready Hospitals	60	143370	100.0
Stroke Units	5	0	0.0
Stroke Units	10	0	0.0
Stroke Units	15	0	0.0
Stroke Units	20	0	0.0
Stroke Units	25	158	0.11
Stroke Units	30	4288	2.99
Stroke Units	35	20377	14.21
Stroke Units	40	49607	34.6
Stroke Units	45	99230	69.21
Stroke Units	50	140463	97.97
Stroke Units	55	143370	100.0
Stroke Units	60	143370	100.0

Population Coverage - Landkreis Enzkreis (Baden-Württemberg)

Condition	Drive Time (min)	Covered Population	Coverage (%)
Hospitals with CT	5	17194	8.78
Hospitals with CT	10	81375	41.53
Hospitals with CT	15	163579	83.49
Hospitals with CT	20	191546	97.76
Hospitals with CT	25	195919	99.99
Hospitals with CT	30	195931	100.0
Hospitals with CT	35	195931	100.0
Hospitals with CT	40	195931	100.0
Hospitals with CT	45	195936	100.0
Hospitals with CT	50	195936	100.0
Hospitals with CT	55	195936	100.0
Hospitals with CT	60	195936	100.0
Stroke-Ready Hospitals	5	0	0.0
Stroke-Ready Hospitals	10	1971	1.01
Stroke-Ready Hospitals	15	40871	20.86
Stroke-Ready Hospitals	20	106638	54.42
Stroke-Ready Hospitals	25	156362	79.8
Stroke-Ready Hospitals	30	181743	92.76
Stroke-Ready Hospitals	35	192919	98.46
Stroke-Ready Hospitals	40	195837	99.95
Stroke-Ready Hospitals	45	195920	99.99
Stroke-Ready Hospitals	50	195936	100.0
Stroke-Ready Hospitals	55	195936	100.0
Stroke-Ready Hospitals	60	195936	100.0
Stroke Units	5	0	0.0
Stroke Units	10	1971	1.01
Stroke Units	15	40871	20.86
Stroke Units	20	106623	54.42
Stroke Units	25	155086	79.15
Stroke Units	30	181741	92.76
Stroke Units	35	192919	98.46
Stroke Units	40	195837	99.95
Stroke Units	45	195920	99.99
Stroke Units	50	195936	100.0
Stroke Units	55	195936	100.0
Stroke Units	60	195936	100.0

Population Coverage - Landkreis Berchtesgadener Land (Bayern)

Condition	Drive Time (min)	Covered Population	Coverage (%)
Hospitals with CT	5	30736	29.06
Hospitals with CT	10	58719	55.52
Hospitals with CT	15	84521	79.92
Hospitals with CT	20	99592	94.17
Hospitals with CT	25	103880	98.22
Hospitals with CT	30	104413	98.73
Hospitals with CT	35	104602	98.91
Hospitals with CT	40	104643	98.95
Hospitals with CT	45	104664	98.97
Hospitals with CT	50	104706	99.01
Hospitals with CT	55	104597	98.9
Hospitals with CT	60	104636	98.94
Stroke-Ready Hospitals	5	13284	12.56
Stroke-Ready Hospitals	10	18998	17.96
Stroke-Ready Hospitals	15	25277	23.9
Stroke-Ready Hospitals	20	35604	33.67
Stroke-Ready Hospitals	25	51076	48.3
Stroke-Ready Hospitals	30	75278	71.18
Stroke-Ready Hospitals	35	92557	87.52
Stroke-Ready Hospitals	40	103240	97.62
Stroke-Ready Hospitals	45	104229	98.56
Stroke-Ready Hospitals	50	104387	98.7
Stroke-Ready Hospitals	55	104415	98.73
Stroke-Ready Hospitals	60	104528	98.84
Stroke Units	5	13284	12.56
Stroke Units	10	18998	17.96
Stroke Units	15	25277	23.9
Stroke Units	20	35604	33.67
Stroke Units	25	51076	48.3
Stroke Units	30	75278	71.18
Stroke Units	35	92557	87.52
Stroke Units	40	103240	97.62
Stroke Units	45	104229	98.56
Stroke Units	50	104387	98.7
Stroke Units	55	104415	98.73
Stroke Units	60	104528	98.84

Population Coverage - Kreisfreie Stadt Hof (Bayern)

Condition	Drive Time (min)	Covered Population	Coverage (%)
Hospitals with CT	5	22743	50.82
Hospitals with CT	10	42523	95.03
Hospitals with CT	15	44723	99.94
Hospitals with CT	20	44749	100.0
Hospitals with CT	25	44749	100.0
Hospitals with CT	30	44749	100.0
Hospitals with CT	35	44749	100.0
Hospitals with CT	40	44749	100.0
Hospitals with CT	45	44749	100.0
Hospitals with CT	50	44749	100.0
Hospitals with CT	55	44749	100.0
Hospitals with CT	60	44749	100.0
Stroke-Ready Hospitals	5	22001	49.17
Stroke-Ready Hospitals	10	42318	94.57
Stroke-Ready Hospitals	15	44723	99.94
Stroke-Ready Hospitals	20	44749	100.0
Stroke-Ready Hospitals	25	44749	100.0
Stroke-Ready Hospitals	30	44749	100.0
Stroke-Ready Hospitals	35	44749	100.0
Stroke-Ready Hospitals	40	44749	100.0
Stroke-Ready Hospitals	45	44749	100.0
Stroke-Ready Hospitals	50	44749	100.0
Stroke-Ready Hospitals	55	44749	100.0
Stroke-Ready Hospitals	60	44749	100.0
Stroke Units	5	22001	49.17
Stroke Units	10	42318	94.57
Stroke Units	15	44723	99.94
Stroke Units	20	44749	100.0
Stroke Units	25	44749	100.0
Stroke Units	30	44749	100.0
Stroke Units	35	44749	100.0
Stroke Units	40	44749	100.0
Stroke Units	45	44749	100.0
Stroke Units	50	44749	100.0
Stroke Units	55	44749	100.0
Stroke Units	60	44749	100.0

Population Coverage - Landkreis Kulmbach (Bayern)

Condition	Drive Time (min)	Covered Population	Coverage (%)
Hospitals with CT	5	5743	7.59
Hospitals with CT	10	26689	35.27
Hospitals with CT	15	53708	70.97
Hospitals with CT	20	73709	97.4
Hospitals with CT	25	75677	100.0
Hospitals with CT	30	75678	100.0
Hospitals with CT	35	75678	100.0
Hospitals with CT	40	75678	100.0
Hospitals with CT	45	75678	100.0
Hospitals with CT	50	75678	100.0
Hospitals with CT	55	75678	100.0
Hospitals with CT	60	75678	100.0
Stroke-Ready Hospitals	5	2695	3.56
Stroke-Ready Hospitals	10	17843	23.58
Stroke-Ready Hospitals	15	41192	54.43
Stroke-Ready Hospitals	20	63676	84.14
Stroke-Ready Hospitals	25	73450	97.06
Stroke-Ready Hospitals	30	75639	99.95
Stroke-Ready Hospitals	35	75678	100.0
Stroke-Ready Hospitals	40	75678	100.0
Stroke-Ready Hospitals	45	75678	100.0
Stroke-Ready Hospitals	50	75678	100.0
Stroke-Ready Hospitals	55	75678	100.0
Stroke-Ready Hospitals	60	75678	100.0
Stroke Units	5	2695	3.56
Stroke Units	10	17843	23.58
Stroke Units	15	40991	54.17
Stroke Units	20	63635	84.09
Stroke Units	25	72791	96.19
Stroke Units	30	75613	99.91
Stroke Units	35	75678	100.0
Stroke Units	40	75678	100.0
Stroke Units	45	75678	100.0
Stroke Units	50	75678	100.0
Stroke Units	55	75678	100.0
Stroke Units	60	75678	100.0

Population Coverage - Kreisfreie Stadt Memmingen (Bayern)

Condition	Drive Time (min)	Covered Population	Coverage (%)
Hospitals with CT	5	28533	65.89
Hospitals with CT	10	42142	97.32
Hospitals with CT	15	43264	99.91
Hospitals with CT	20	43300	100.0
Hospitals with CT	25	43302	100.0
Hospitals with CT	30	43302	100.0
Hospitals with CT	35	43302	100.0
Hospitals with CT	40	43302	100.0
Hospitals with CT	45	43302	100.0
Hospitals with CT	50	43302	100.0
Hospitals with CT	55	43302	100.0
Hospitals with CT	60	43302	100.0
Stroke-Ready Hospitals	5	28486	65.78
Stroke-Ready Hospitals	10	42149	97.34
Stroke-Ready Hospitals	15	43264	99.91
Stroke-Ready Hospitals	20	43300	100.0
Stroke-Ready Hospitals	25	43302	100.0
Stroke-Ready Hospitals	30	43302	100.0
Stroke-Ready Hospitals	35	43302	100.0
Stroke-Ready Hospitals	40	43302	100.0
Stroke-Ready Hospitals	45	43302	100.0
Stroke-Ready Hospitals	50	43302	100.0
Stroke-Ready Hospitals	55	43302	100.0
Stroke-Ready Hospitals	60	43302	100.0
Stroke Units	5	28486	65.78
Stroke Units	10	42149	97.34
Stroke Units	15	43264	99.91
Stroke Units	20	43300	100.0
Stroke Units	25	43302	100.0
Stroke Units	30	43302	100.0
Stroke Units	35	43302	100.0
Stroke Units	40	43302	100.0
Stroke Units	45	43302	100.0
Stroke Units	50	43302	100.0
Stroke Units	55	43302	100.0
Stroke Units	60	43302	100.0

Population Coverage - Landkreis Augsburg (Bayern)

Condition	Drive Time (min)	Covered Population	Coverage (%)
Hospitals with CT	5	36102	14.54
Hospitals with CT	10	114648	46.18
Hospitals with CT	15	170609	68.72
Hospitals with CT	20	219565	88.44
Hospitals with CT	25	243626	98.13
Hospitals with CT	30	248209	99.98
Hospitals with CT	35	248265	100.0
Hospitals with CT	40	248265	100.0
Hospitals with CT	45	248265	100.0
Hospitals with CT	50	248265	100.0
Hospitals with CT	55	248265	100.0
Hospitals with CT	60	248265	100.0
Stroke-Ready Hospitals	5	12791	5.15
Stroke-Ready Hospitals	10	42289	17.03
Stroke-Ready Hospitals	15	87744	35.34
Stroke-Ready Hospitals	20	154477	62.22
Stroke-Ready Hospitals	25	220055	88.64
Stroke-Ready Hospitals	30	237743	95.76
Stroke-Ready Hospitals	35	244711	98.57
Stroke-Ready Hospitals	40	248083	99.93
Stroke-Ready Hospitals	45	248265	100.0
Stroke-Ready Hospitals	50	248265	100.0
Stroke-Ready Hospitals	55	248265	100.0
Stroke-Ready Hospitals	60	248265	100.0
Stroke Units	5	12791	5.15
Stroke Units	10	42276	17.03
Stroke Units	15	78558	31.64
Stroke Units	20	126038	50.77
Stroke Units	25	191122	76.98
Stroke Units	30	229935	92.62
Stroke Units	35	240424	96.84
Stroke Units	40	245840	99.02
Stroke Units	45	248264	100.0
Stroke Units	50	248265	100.0
Stroke Units	55	248265	100.0
Stroke Units	60	248265	100.0

Population Coverage - Landkreis Vorpommern-Rügen (Mecklenburg-Vorpommern)

Condition	Drive Time (min)	Covered Population	Coverage (%)
Hospitals with CT	5	36566	17.83
Hospitals with CT	10	88100	42.96
Hospitals with CT	15	109489	53.39
Hospitals with CT	20	131439	64.09
Hospitals with CT	25	155889	76.01
Hospitals with CT	30	174171	84.93
Hospitals with CT	35	187650	91.5
Hospitals with CT	40	191269	93.27
Hospitals with CT	45	196040	95.59
Hospitals with CT	50	200003	97.53
Hospitals with CT	55	200661	97.85
Hospitals with CT	60	201251	98.13
Stroke-Ready Hospitals	5	14862	7.25
Stroke-Ready Hospitals	10	41896	20.43
Stroke-Ready Hospitals	15	67697	33.01
Stroke-Ready Hospitals	20	83035	40.49
Stroke-Ready Hospitals	25	103218	50.33
Stroke-Ready Hospitals	30	122252	59.61
Stroke-Ready Hospitals	35	137895	67.24
Stroke-Ready Hospitals	40	153031	74.62
Stroke-Ready Hospitals	45	163715	79.83
Stroke-Ready Hospitals	50	175330	85.49
Stroke-Ready Hospitals	55	186843	91.11
Stroke-Ready Hospitals	60	195350	95.26
Stroke Units	5	14862	7.25
Stroke Units	10	41896	20.43
Stroke Units	15	67697	33.01
Stroke Units	20	83035	40.49
Stroke Units	25	103218	50.33
Stroke Units	30	122252	59.61
Stroke Units	35	137895	67.24
Stroke Units	40	153013	74.61
Stroke Units	45	163630	79.79
Stroke Units	50	175236	85.45
Stroke Units	55	186614	91.0
Stroke Units	60	195181	95.17

Population Coverage - Landkreis Zwickau (Sachsen)

Condition	Drive Time (min)	Covered Population	Coverage (%)
Hospitals with CT	5	67772	21.36
Hospitals with CT	10	174182	54.91
Hospitals with CT	15	282113	88.93
Hospitals with CT	20	314934	99.28
Hospitals with CT	25	317217	100.0
Hospitals with CT	30	317217	100.0
Hospitals with CT	35	317217	100.0
Hospitals with CT	40	317217	100.0
Hospitals with CT	45	317217	100.0
Hospitals with CT	50	317217	100.0
Hospitals with CT	55	317217	100.0
Hospitals with CT	60	317217	100.0
Stroke-Ready Hospitals	5	37234	11.74
Stroke-Ready Hospitals	10	107327	33.83
Stroke-Ready Hospitals	15	185937	58.62
Stroke-Ready Hospitals	20	275214	86.76
Stroke-Ready Hospitals	25	313344	98.78
Stroke-Ready Hospitals	30	317199	99.99
Stroke-Ready Hospitals	35	317217	100.0
Stroke-Ready Hospitals	40	317217	100.0
Stroke-Ready Hospitals	45	317217	100.0
Stroke-Ready Hospitals	50	317217	100.0
Stroke-Ready Hospitals	55	317217	100.0
Stroke-Ready Hospitals	60	317217	100.0
Stroke Units	5	26204	8.26
Stroke Units	10	85964	27.1
Stroke Units	15	149541	47.14
Stroke Units	20	215426	67.91
Stroke Units	25	299777	94.5
Stroke Units	30	316853	99.89
Stroke Units	35	317217	100.0
Stroke Units	40	317217	100.0
Stroke Units	45	317217	100.0
Stroke Units	50	317217	100.0
Stroke Units	55	317217	100.0
Stroke Units	60	317217	100.0

Population Coverage - Landkreis Görlitz (Sachsen)

Condition	Drive Time (min)	Covered Population	Coverage (%)
Hospitals with CT	5	53895	21.55
Hospitals with CT	10	114831	45.91
Hospitals with CT	15	165317	66.1
Hospitals with CT	20	205117	82.01
Hospitals with CT	25	225558	90.18
Hospitals with CT	30	238600	95.39
Hospitals with CT	35	245888	98.31
Hospitals with CT	40	248274	99.26
Hospitals with CT	45	249752	99.85
Hospitals with CT	50	249771	99.86
Hospitals with CT	55	249587	99.79
Hospitals with CT	60	249704	99.83
Stroke-Ready Hospitals	5	18887	7.55
Stroke-Ready Hospitals	10	48607	19.43
Stroke-Ready Hospitals	15	58605	23.43
Stroke-Ready Hospitals	20	67263	26.89
Stroke-Ready Hospitals	25	80304	32.11
Stroke-Ready Hospitals	30	105492	42.18
Stroke-Ready Hospitals	35	119950	47.96
Stroke-Ready Hospitals	40	140643	56.23
Stroke-Ready Hospitals	45	167660	67.03
Stroke-Ready Hospitals	50	210513	84.17
Stroke-Ready Hospitals	55	232123	92.81
Stroke-Ready Hospitals	60	245034	97.97
Stroke Units	5	18887	7.55
Stroke Units	10	48607	19.43
Stroke Units	15	58605	23.43
Stroke Units	20	67263	26.89
Stroke Units	25	80304	32.11
Stroke Units	30	105492	42.18
Stroke Units	35	119950	47.96
Stroke Units	40	140643	56.23
Stroke Units	45	167660	67.03
Stroke Units	50	210513	84.17
Stroke Units	55	232109	92.8
Stroke Units	60	245029	97.97

Population Coverage - Landkreis Sonneberg (Thüringen)

Condition	Drive Time (min)	Covered Population	Coverage (%)
Hospitals with CT	5	11603	21.62
Hospitals with CT	10	21337	39.76
Hospitals with CT	15	28187	52.52
Hospitals with CT	20	36047	67.17
Hospitals with CT	25	42227	78.68
Hospitals with CT	30	48231	89.87
Hospitals with CT	35	53192	99.11
Hospitals with CT	40	53535	99.75
Hospitals with CT	45	53668	100.0
Hospitals with CT	50	53668	100.0
Hospitals with CT	55	53668	100.0
Hospitals with CT	60	53668	100.0
Stroke-Ready Hospitals	5	10822	20.17
Stroke-Ready Hospitals	10	20989	39.11
Stroke-Ready Hospitals	15	27341	50.95
Stroke-Ready Hospitals	20	34523	64.33
Stroke-Ready Hospitals	25	40799	76.02
Stroke-Ready Hospitals	30	44063	82.1
Stroke-Ready Hospitals	35	50233	93.6
Stroke-Ready Hospitals	40	53068	98.88
Stroke-Ready Hospitals	45	53668	100.0
Stroke-Ready Hospitals	50	53668	100.0
Stroke-Ready Hospitals	55	53668	100.0
Stroke-Ready Hospitals	60	53668	100.0
Stroke Units	5	10822	20.17
Stroke Units	10	20989	39.11
Stroke Units	15	27341	50.95
Stroke Units	20	34523	64.33
Stroke Units	25	40799	76.02
Stroke Units	30	44063	82.1
Stroke Units	35	50233	93.6
Stroke Units	40	53068	98.88
Stroke Units	45	53668	100.0
Stroke Units	50	53668	100.0
Stroke Units	55	53668	100.0
Stroke Units	60	53668	100.0

Population Coverage - Kreisfreie Stadt Köln (Nordrhein-Westfalen)

Condition	Drive Time (min)	Covered Population	Coverage (%)
Hospitals with CT	5	463857	38.17
Hospitals with CT	10	962558	79.21
Hospitals with CT	15	1192036	98.1
Hospitals with CT	20	1215070	99.99
Hospitals with CT	25	1215184	100.0
Hospitals with CT	30	1215184	100.0
Hospitals with CT	35	1215184	100.0
Hospitals with CT	40	1215184	100.0
Hospitals with CT	45	1215184	100.0
Hospitals with CT	50	1215184	100.0
Hospitals with CT	55	1215184	100.0
Hospitals with CT	60	1215184	100.0
Stroke-Ready Hospitals	5	68367	5.63
Stroke-Ready Hospitals	10	417408	34.35
Stroke-Ready Hospitals	15	969408	79.77
Stroke-Ready Hospitals	20	1187048	97.68
Stroke-Ready Hospitals	25	1215163	100.0
Stroke-Ready Hospitals	30	1215184	100.0
Stroke-Ready Hospitals	35	1215184	100.0
Stroke-Ready Hospitals	40	1215184	100.0
Stroke-Ready Hospitals	45	1215184	100.0
Stroke-Ready Hospitals	50	1215184	100.0
Stroke-Ready Hospitals	55	1215184	100.0
Stroke-Ready Hospitals	60	1215184	100.0
Stroke Units	5	60021	4.94
Stroke Units	10	346306	28.5
Stroke Units	15	873718	71.9
Stroke Units	20	1186970	97.68
Stroke Units	25	1215163	100.0
Stroke Units	30	1215184	100.0
Stroke Units	35	1215184	100.0
Stroke Units	40	1215184	100.0
Stroke Units	45	1215184	100.0
Stroke Units	50	1215184	100.0
Stroke Units	55	1215184	100.0
Stroke Units	60	1215184	100.0

Population Coverage - Kreis Heinsberg (Nordrhein-Westfalen)

Condition	Drive Time (min)	Covered Population	Coverage (%)
Hospitals with CT	5	38392	15.34
Hospitals with CT	10	117696	47.02
Hospitals with CT	15	209142	83.55
Hospitals with CT	20	238612	95.32
Hospitals with CT	25	248489	99.26
Hospitals with CT	30	250115	99.91
Hospitals with CT	35	250135	99.92
Hospitals with CT	40	250162	99.93
Hospitals with CT	45	250231	99.96
Hospitals with CT	50	250255	99.97
Hospitals with CT	55	250219	99.96
Hospitals with CT	60	250278	99.98
Stroke-Ready Hospitals	5	19806	7.91
Stroke-Ready Hospitals	10	51836	20.71
Stroke-Ready Hospitals	15	105644	42.2
Stroke-Ready Hospitals	20	157995	63.11
Stroke-Ready Hospitals	25	218265	87.19
Stroke-Ready Hospitals	30	246819	98.6
Stroke-Ready Hospitals	35	250030	99.88
Stroke-Ready Hospitals	40	250045	99.89
Stroke-Ready Hospitals	45	250105	99.91
Stroke-Ready Hospitals	50	250123	99.92
Stroke-Ready Hospitals	55	250147	99.93
Stroke-Ready Hospitals	60	250214	99.95
Stroke Units	5	19806	7.91
Stroke Units	10	51836	20.71
Stroke Units	15	105644	42.2
Stroke Units	20	157995	63.11
Stroke Units	25	218265	87.19
Stroke Units	30	246819	98.6
Stroke Units	35	250030	99.88
Stroke Units	40	250045	99.89
Stroke Units	45	250105	99.91
Stroke Units	50	250123	99.92
Stroke Units	55	250147	99.93
Stroke Units	60	250214	99.95

Population Coverage - Landkreis Bergstraße (Hessen)

Condition	Drive Time (min)	Covered Population	Coverage (%)
Hospitals with CT	5	62799	23.6
Hospitals with CT	10	146601	55.1
Hospitals with CT	15	180807	67.96
Hospitals with CT	20	211693	79.57
Hospitals with CT	25	227237	85.41
Hospitals with CT	30	251056	94.36
Hospitals with CT	35	262928	98.82
Hospitals with CT	40	265995	99.98
Hospitals with CT	45	266057	100.0
Hospitals with CT	50	266057	100.0
Hospitals with CT	55	266057	100.0
Hospitals with CT	60	266057	100.0
Stroke-Ready Hospitals	5	9302	3.5
Stroke-Ready Hospitals	10	26243	9.86
Stroke-Ready Hospitals	15	75139	28.24
Stroke-Ready Hospitals	20	155097	58.29
Stroke-Ready Hospitals	25	191116	71.83
Stroke-Ready Hospitals	30	226625	85.18
Stroke-Ready Hospitals	35	251042	94.36
Stroke-Ready Hospitals	40	265428	99.76
Stroke-Ready Hospitals	45	265964	99.97
Stroke-Ready Hospitals	50	266057	100.0
Stroke-Ready Hospitals	55	266057	100.0
Stroke-Ready Hospitals	60	266057	100.0
Stroke Units	5	9302	3.5
Stroke Units	10	26243	9.86
Stroke Units	15	75090	28.22
Stroke Units	20	151717	57.02
Stroke Units	25	190931	71.76
Stroke Units	30	226623	85.18
Stroke Units	35	251023	94.35
Stroke Units	40	265428	99.76
Stroke Units	45	265964	99.97
Stroke Units	50	266057	100.0
Stroke Units	55	266057	100.0
Stroke Units	60	266057	100.0

Population Coverage - Landkreis Wetteraukreis (Hessen)

Condition	Drive Time (min)	Covered Population	Coverage (%)
Hospitals with CT	5	50504	16.9
Hospitals with CT	10	124240	41.57
Hospitals with CT	15	209267	70.02
Hospitals with CT	20	282226	94.43
Hospitals with CT	25	298769	99.97
Hospitals with CT	30	298863	100.0
Hospitals with CT	35	298863	100.0
Hospitals with CT	40	298863	100.0
Hospitals with CT	45	298863	100.0
Hospitals with CT	50	298863	100.0
Hospitals with CT	55	298863	100.0
Hospitals with CT	60	298863	100.0
Stroke-Ready Hospitals	5	19076	6.38
Stroke-Ready Hospitals	10	64174	21.47
Stroke-Ready Hospitals	15	137833	46.12
Stroke-Ready Hospitals	20	229889	76.92
Stroke-Ready Hospitals	25	288706	96.6
Stroke-Ready Hospitals	30	296944	99.36
Stroke-Ready Hospitals	35	298863	100.0
Stroke-Ready Hospitals	40	298863	100.0
Stroke-Ready Hospitals	45	298863	100.0
Stroke-Ready Hospitals	50	298863	100.0
Stroke-Ready Hospitals	55	298863	100.0
Stroke-Ready Hospitals	60	298863	100.0
Stroke Units	5	1934	0.65
Stroke Units	10	12570	4.21
Stroke Units	15	44720	14.96
Stroke Units	20	143885	48.14
Stroke Units	25	272450	91.16
Stroke Units	30	296747	99.29
Stroke Units	35	298863	100.0
Stroke Units	40	298863	100.0
Stroke Units	45	298863	100.0
Stroke Units	50	298863	100.0
Stroke Units	55	298863	100.0
Stroke Units	60	298863	100.0

Population Coverage - Kreisfreie Stadt Landau in der Pfalz (Rheinland-Pfalz)

Condition	Drive Time (min)	Covered Population	Coverage (%)
Hospitals with CT	5	28868	54.89
Hospitals with CT	10	49406	93.93
Hospitals with CT	15	52520	99.86
Hospitals with CT	20	52520	99.86
Hospitals with CT	25	52520	99.86
Hospitals with CT	30	52560	99.93
Hospitals with CT	35	52594	100.0
Hospitals with CT	40	52596	100.0
Hospitals with CT	45	52596	100.0
Hospitals with CT	50	52596	100.0
Hospitals with CT	55	52596	100.0
Hospitals with CT	60	52596	100.0
Stroke-Ready Hospitals	5	0	0.0
Stroke-Ready Hospitals	10	1254	2.38
Stroke-Ready Hospitals	15	9494	18.05
Stroke-Ready Hospitals	20	51943	98.76
Stroke-Ready Hospitals	25	52520	99.86
Stroke-Ready Hospitals	30	52520	99.86
Stroke-Ready Hospitals	35	52544	99.9
Stroke-Ready Hospitals	40	52588	99.99
Stroke-Ready Hospitals	45	52596	100.0
Stroke-Ready Hospitals	50	52596	100.0
Stroke-Ready Hospitals	55	52596	100.0
Stroke-Ready Hospitals	60	52596	100.0
Stroke Units	5	0	0.0
Stroke Units	10	0	0.0
Stroke Units	15	2329	4.43
Stroke Units	20	45570	86.64
Stroke Units	25	52415	99.66
Stroke Units	30	52520	99.86
Stroke Units	35	52544	99.9
Stroke Units	40	52552	99.92
Stroke Units	45	52594	100.0
Stroke Units	50	52596	100.0
Stroke Units	55	52596	100.0
Stroke Units	60	52596	100.0

Population Coverage - Stadtkreis Stuttgart (Baden-Württemberg)

Condition	Drive Time (min)	Covered Population	Coverage (%)
Hospitals with CT	5	223259	34.89
Hospitals with CT	10	445508	69.62
Hospitals with CT	15	609479	95.24
Hospitals with CT	20	639874	99.99
Hospitals with CT	25	639946	100.0
Hospitals with CT	30	639946	100.0
Hospitals with CT	35	639946	100.0
Hospitals with CT	40	639946	100.0
Hospitals with CT	45	639946	100.0
Hospitals with CT	50	639946	100.0
Hospitals with CT	55	639946	100.0
Hospitals with CT	60	639946	100.0
Stroke-Ready Hospitals	5	114927	17.96
Stroke-Ready Hospitals	10	326119	50.96
Stroke-Ready Hospitals	15	519197	81.13
Stroke-Ready Hospitals	20	632495	98.84
Stroke-Ready Hospitals	25	639946	100.0
Stroke-Ready Hospitals	30	639946	100.0
Stroke-Ready Hospitals	35	639946	100.0
Stroke-Ready Hospitals	40	639946	100.0
Stroke-Ready Hospitals	45	639946	100.0
Stroke-Ready Hospitals	50	639946	100.0
Stroke-Ready Hospitals	55	639946	100.0
Stroke-Ready Hospitals	60	639946	100.0
Stroke Units	5	114927	17.96
Stroke Units	10	326119	50.96
Stroke Units	15	519197	81.13
Stroke Units	20	632495	98.84
Stroke Units	25	639946	100.0
Stroke Units	30	639946	100.0
Stroke Units	35	639946	100.0
Stroke Units	40	639946	100.0
Stroke Units	45	639946	100.0
Stroke Units	50	639946	100.0
Stroke Units	55	639946	100.0
Stroke Units	60	639946	100.0

Population Coverage - Landkreis Konstanz (Baden-Württemberg)

Condition	Drive Time (min)	Covered Population	Coverage (%)
Hospitals with CT	5	57681	20.73
Hospitals with CT	10	150406	54.05
Hospitals with CT	15	224675	80.74
Hospitals with CT	20	265023	95.24
Hospitals with CT	25	272720	98.01
Hospitals with CT	30	277576	99.75
Hospitals with CT	35	277661	99.78
Hospitals with CT	40	277662	99.78
Hospitals with CT	45	277671	99.79
Hospitals with CT	50	277714	99.8
Hospitals with CT	55	277251	99.64
Hospitals with CT	60	277693	99.8
Stroke-Ready Hospitals	5	7561	2.72
Stroke-Ready Hospitals	10	63793	22.93
Stroke-Ready Hospitals	15	135725	48.78
Stroke-Ready Hospitals	20	225484	81.03
Stroke-Ready Hospitals	25	266460	95.76
Stroke-Ready Hospitals	30	274902	98.79
Stroke-Ready Hospitals	35	277230	99.63
Stroke-Ready Hospitals	40	277411	99.69
Stroke-Ready Hospitals	45	277441	99.7
Stroke-Ready Hospitals	50	277464	99.71
Stroke-Ready Hospitals	55	276903	99.51
Stroke-Ready Hospitals	60	277011	99.55
Stroke Units	5	4443	1.6
Stroke Units	10	51410	18.48
Stroke Units	15	82042	29.48
Stroke Units	20	140115	50.35
Stroke Units	25	175974	63.24
Stroke Units	30	195720	70.34
Stroke Units	35	227342	81.7
Stroke Units	40	264366	95.01
Stroke Units	45	276892	99.51
Stroke Units	50	277212	99.62
Stroke Units	55	276713	99.44
Stroke Units	60	276846	99.49

Population Coverage - Landkreis Sigmaringen (Baden-Württemberg)

Condition	Drive Time (min)	Covered Population	Coverage (%)
Hospitals with CT	5	7037	5.47
Hospitals with CT	10	22038	17.13
Hospitals with CT	15	31040	24.13
Hospitals with CT	20	59527	46.27
Hospitals with CT	25	90606	70.42
Hospitals with CT	30	124540	96.8
Hospitals with CT	35	128657	100.0
Hospitals with CT	40	128658	100.0
Hospitals with CT	45	128658	100.0
Hospitals with CT	50	128658	100.0
Hospitals with CT	55	128658	100.0
Hospitals with CT	60	128658	100.0
Stroke-Ready Hospitals	5	7038	5.47
Stroke-Ready Hospitals	10	21851	16.98
Stroke-Ready Hospitals	15	28867	22.44
Stroke-Ready Hospitals	20	48850	37.97
Stroke-Ready Hospitals	25	71012	55.19
Stroke-Ready Hospitals	30	114532	89.02
Stroke-Ready Hospitals	35	127508	99.11
Stroke-Ready Hospitals	40	128658	100.0
Stroke-Ready Hospitals	45	128658	100.0
Stroke-Ready Hospitals	50	128658	100.0
Stroke-Ready Hospitals	55	128658	100.0
Stroke-Ready Hospitals	60	128658	100.0
Stroke Units	5	7037	5.47
Stroke Units	10	21829	16.97
Stroke Units	15	28812	22.39
Stroke Units	20	48815	37.94
Stroke Units	25	70586	54.86
Stroke Units	30	113854	88.49
Stroke Units	35	127389	99.01
Stroke Units	40	128637	99.98
Stroke Units	45	128658	100.0
Stroke Units	50	128658	100.0
Stroke Units	55	128658	100.0
Stroke Units	60	128658	100.0

Population Coverage - Landkreis Kelheim (Bayern)

Condition	Drive Time (min)	Covered Population	Coverage (%)
Hospitals with CT	5	24546	20.6
Hospitals with CT	10	59241	49.72
Hospitals with CT	15	85947	72.14
Hospitals with CT	20	107513	90.24
Hospitals with CT	25	117852	98.91
Hospitals with CT	30	119147	100.0
Hospitals with CT	35	119147	100.0
Hospitals with CT	40	119147	100.0
Hospitals with CT	45	119147	100.0
Hospitals with CT	50	119147	100.0
Hospitals with CT	55	119147	100.0
Hospitals with CT	60	119147	100.0
Stroke-Ready Hospitals	5	0	0.0
Stroke-Ready Hospitals	10	0	0.0
Stroke-Ready Hospitals	15	7667	6.44
Stroke-Ready Hospitals	20	19027	15.97
Stroke-Ready Hospitals	25	44311	37.19
Stroke-Ready Hospitals	30	77042	64.66
Stroke-Ready Hospitals	35	106876	89.7
Stroke-Ready Hospitals	40	115445	96.89
Stroke-Ready Hospitals	45	119135	99.99
Stroke-Ready Hospitals	50	119147	100.0
Stroke-Ready Hospitals	55	119147	100.0
Stroke-Ready Hospitals	60	119147	100.0
Stroke Units	5	0	0.0
Stroke Units	10	0	0.0
Stroke Units	15	6788	5.7
Stroke Units	20	15576	13.07
Stroke Units	25	37321	31.32
Stroke Units	30	74114	62.2
Stroke Units	35	103867	87.18
Stroke Units	40	113800	95.51
Stroke Units	45	119093	99.95
Stroke Units	50	119147	100.0
Stroke Units	55	119147	100.0
Stroke Units	60	119147	100.0

Population Coverage - Kreisfreie Stadt Schweinfurt (Bayern)

Condition	Drive Time (min)	Covered Population	Coverage (%)
Hospitals with CT	5	28551	52.97
Hospitals with CT	10	53478	99.21
Hospitals with CT	15	53817	99.84
Hospitals with CT	20	53903	100.0
Hospitals with CT	25	53903	100.0
Hospitals with CT	30	53903	100.0
Hospitals with CT	35	53903	100.0
Hospitals with CT	40	53903	100.0
Hospitals with CT	45	53903	100.0
Hospitals with CT	50	53903	100.0
Hospitals with CT	55	53903	100.0
Hospitals with CT	60	53903	100.0
Stroke-Ready Hospitals	5	10532	19.54
Stroke-Ready Hospitals	10	40562	75.25
Stroke-Ready Hospitals	15	53480	99.21
Stroke-Ready Hospitals	20	53903	100.0
Stroke-Ready Hospitals	25	53903	100.0
Stroke-Ready Hospitals	30	53903	100.0
Stroke-Ready Hospitals	35	53903	100.0
Stroke-Ready Hospitals	40	53903	100.0
Stroke-Ready Hospitals	45	53903	100.0
Stroke-Ready Hospitals	50	53903	100.0
Stroke-Ready Hospitals	55	53903	100.0
Stroke-Ready Hospitals	60	53903	100.0
Stroke Units	5	10532	19.54
Stroke Units	10	40562	75.25
Stroke Units	15	53480	99.21
Stroke Units	20	53903	100.0
Stroke Units	25	53903	100.0
Stroke Units	30	53903	100.0
Stroke Units	35	53903	100.0
Stroke Units	40	53903	100.0
Stroke Units	45	53903	100.0
Stroke Units	50	53903	100.0
Stroke Units	55	53903	100.0
Stroke Units	60	53903	100.0

Population Coverage - Landkreis Neu-Ulm (Bayern)

Condition	Drive Time (min)	Covered Population	Coverage (%)
Hospitals with CT	5	20331	11.74
Hospitals with CT	10	66597	38.45
Hospitals with CT	15	138482	79.94
Hospitals with CT	20	170484	98.42
Hospitals with CT	25	172993	99.86
Hospitals with CT	30	173227	100.0
Hospitals with CT	35	173227	100.0
Hospitals with CT	40	173227	100.0
Hospitals with CT	45	173227	100.0
Hospitals with CT	50	173227	100.0
Hospitals with CT	55	173227	100.0
Hospitals with CT	60	173227	100.0
Stroke-Ready Hospitals	5	0	0.0
Stroke-Ready Hospitals	10	0	0.0
Stroke-Ready Hospitals	15	6761	3.9
Stroke-Ready Hospitals	20	43854	25.32
Stroke-Ready Hospitals	25	120358	69.48
Stroke-Ready Hospitals	30	168502	97.27
Stroke-Ready Hospitals	35	173224	100.0
Stroke-Ready Hospitals	40	173227	100.0
Stroke-Ready Hospitals	45	173227	100.0
Stroke-Ready Hospitals	50	173227	100.0
Stroke-Ready Hospitals	55	173227	100.0
Stroke-Ready Hospitals	60	173227	100.0
Stroke Units	5	0	0.0
Stroke Units	10	0	0.0
Stroke Units	15	6761	3.9
Stroke Units	20	43854	25.32
Stroke Units	25	120358	69.48
Stroke Units	30	168502	97.27
Stroke Units	35	173224	100.0
Stroke Units	40	173227	100.0
Stroke Units	45	173227	100.0
Stroke Units	50	173227	100.0
Stroke Units	55	173227	100.0
Stroke Units	60	173227	100.0

Population Coverage - Landkreis Oberhavel (Brandenburg)

Condition	Drive Time (min)	Covered Population	Coverage (%)
Hospitals with CT	5	36396	18.14
Hospitals with CT	10	114469	57.06
Hospitals with CT	15	152324	75.93
Hospitals with CT	20	170111	84.79
Hospitals with CT	25	185779	92.6
Hospitals with CT	30	196760	98.08
Hospitals with CT	35	199052	99.22
Hospitals with CT	40	200370	99.88
Hospitals with CT	45	200599	99.99
Hospitals with CT	50	200613	100.0
Hospitals with CT	55	200616	100.0
Hospitals with CT	60	200616	100.0
Stroke-Ready Hospitals	5	7688	3.83
Stroke-Ready Hospitals	10	31775	15.84
Stroke-Ready Hospitals	15	49894	24.87
Stroke-Ready Hospitals	20	91369	45.54
Stroke-Ready Hospitals	25	136530	68.06
Stroke-Ready Hospitals	30	154292	76.91
Stroke-Ready Hospitals	35	161850	80.68
Stroke-Ready Hospitals	40	166474	82.98
Stroke-Ready Hospitals	45	174592	87.03
Stroke-Ready Hospitals	50	179085	89.27
Stroke-Ready Hospitals	55	184269	91.85
Stroke-Ready Hospitals	60	193485	96.45
Stroke Units	5	7688	3.83
Stroke Units	10	31775	15.84
Stroke Units	15	49894	24.87
Stroke Units	20	91369	45.54
Stroke Units	25	136501	68.04
Stroke Units	30	154277	76.9
Stroke Units	35	161848	80.68
Stroke Units	40	166474	82.98
Stroke Units	45	174592	87.03
Stroke Units	50	179085	89.27
Stroke Units	55	184269	91.85
Stroke Units	60	193485	96.45

Population Coverage - Landkreis Spree-Neiße (Brandenburg)

Condition	Drive Time (min)	Covered Population	Coverage (%)
Hospitals with CT	5	22806	19.11
Hospitals with CT	10	48760	40.87
Hospitals with CT	15	68373	57.31
Hospitals with CT	20	83442	69.94
Hospitals with CT	25	98522	82.58
Hospitals with CT	30	111898	93.79
Hospitals with CT	35	117781	98.72
Hospitals with CT	40	118827	99.59
Hospitals with CT	45	119139	99.86
Hospitals with CT	50	119261	99.96
Hospitals with CT	55	119151	99.87
Hospitals with CT	60	119200	99.91
Stroke-Ready Hospitals	5	0	0.0
Stroke-Ready Hospitals	10	696	0.58
Stroke-Ready Hospitals	15	4513	3.78
Stroke-Ready Hospitals	20	13900	11.65
Stroke-Ready Hospitals	25	46899	39.31
Stroke-Ready Hospitals	30	72380	60.67
Stroke-Ready Hospitals	35	84844	71.11
Stroke-Ready Hospitals	40	90291	75.68
Stroke-Ready Hospitals	45	92324	77.38
Stroke-Ready Hospitals	50	102500	85.91
Stroke-Ready Hospitals	55	118221	99.09
Stroke-Ready Hospitals	60	119036	99.77
Stroke Units	5	0	0.0
Stroke Units	10	696	0.58
Stroke Units	15	4513	3.78
Stroke Units	20	13900	11.65
Stroke Units	25	46899	39.31
Stroke Units	30	72380	60.67
Stroke Units	35	84844	71.11
Stroke Units	40	90291	75.68
Stroke Units	45	92324	77.38
Stroke Units	50	102500	85.91
Stroke Units	55	118221	99.09
Stroke Units	60	118972	99.72

Population Coverage - Landkreis Ilm-Kreis (Thüringen)

Condition	Drive Time (min)	Covered Population	Coverage (%)
Hospitals with CT	5	11888	12.32
Hospitals with CT	10	22233	23.04
Hospitals with CT	15	30259	31.36
Hospitals with CT	20	38078	39.46
Hospitals with CT	25	55342	57.35
Hospitals with CT	30	84317	87.37
Hospitals with CT	35	90333	93.61
Hospitals with CT	40	94001	97.41
Hospitals with CT	45	96501	100.0
Hospitals with CT	50	96501	100.0
Hospitals with CT	55	96501	100.0
Hospitals with CT	60	96501	100.0
Stroke-Ready Hospitals	5	0	0.0
Stroke-Ready Hospitals	10	0	0.0
Stroke-Ready Hospitals	15	108	0.11
Stroke-Ready Hospitals	20	3878	4.02
Stroke-Ready Hospitals	25	19486	20.19
Stroke-Ready Hospitals	30	43176	44.74
Stroke-Ready Hospitals	35	64597	66.94
Stroke-Ready Hospitals	40	91547	94.87
Stroke-Ready Hospitals	45	96364	99.86
Stroke-Ready Hospitals	50	96500	100.0
Stroke-Ready Hospitals	55	96501	100.0
Stroke-Ready Hospitals	60	96501	100.0
Stroke Units	5	0	0.0
Stroke Units	10	0	0.0
Stroke Units	15	108	0.11
Stroke Units	20	3878	4.02
Stroke Units	25	19486	20.19
Stroke Units	30	43176	44.74
Stroke Units	35	64597	66.94
Stroke Units	40	91547	94.87
Stroke Units	45	96364	99.86
Stroke Units	50	96500	100.0
Stroke Units	55	96501	100.0
Stroke Units	60	96501	100.0

Population Coverage - Landkreis Greiz (Thüringen)

Condition	Drive Time (min)	Covered Population	Coverage (%)
Hospitals with CT	5	7419	8.08
Hospitals with CT	10	16219	17.67
Hospitals with CT	15	30984	33.75
Hospitals with CT	20	54486	59.35
Hospitals with CT	25	71801	78.21
Hospitals with CT	30	84923	92.5
Hospitals with CT	35	91729	99.92
Hospitals with CT	40	91806	100.0
Hospitals with CT	45	91806	100.0
Hospitals with CT	50	91806	100.0
Hospitals with CT	55	91806	100.0
Hospitals with CT	60	91806	100.0
Stroke-Ready Hospitals	5	0	0.0
Stroke-Ready Hospitals	10	737	0.8
Stroke-Ready Hospitals	15	3573	3.89
Stroke-Ready Hospitals	20	13381	14.58
Stroke-Ready Hospitals	25	34159	37.21
Stroke-Ready Hospitals	30	50014	54.48
Stroke-Ready Hospitals	35	72729	79.22
Stroke-Ready Hospitals	40	85430	93.05
Stroke-Ready Hospitals	45	91786	99.98
Stroke-Ready Hospitals	50	91806	100.0
Stroke-Ready Hospitals	55	91806	100.0
Stroke-Ready Hospitals	60	91806	100.0
Stroke Units	5	0	0.0
Stroke Units	10	737	0.8
Stroke Units	15	3573	3.89
Stroke Units	20	13381	14.58
Stroke Units	25	34128	37.17
Stroke Units	30	49525	53.95
Stroke Units	35	72707	79.2
Stroke Units	40	85419	93.04
Stroke Units	45	91786	99.98
Stroke Units	50	91806	100.0
Stroke Units	55	91806	100.0
Stroke Units	60	91806	100.0

Population Coverage - Kreisfreie Stadt Bottrop (Nordrhein-Westfalen)

Condition	Drive Time (min)	Covered Population	Coverage (%)
Hospitals with CT	5	41907	35.83
Hospitals with CT	10	105170	89.91
Hospitals with CT	15	116967	100.0
Hospitals with CT	20	116967	100.0
Hospitals with CT	25	116967	100.0
Hospitals with CT	30	116967	100.0
Hospitals with CT	35	116967	100.0
Hospitals with CT	40	116967	100.0
Hospitals with CT	45	116967	100.0
Hospitals with CT	50	116967	100.0
Hospitals with CT	55	116967	100.0
Hospitals with CT	60	116967	100.0
Stroke-Ready Hospitals	5	21678	18.53
Stroke-Ready Hospitals	10	78508	67.12
Stroke-Ready Hospitals	15	116300	99.43
Stroke-Ready Hospitals	20	116967	100.0
Stroke-Ready Hospitals	25	116967	100.0
Stroke-Ready Hospitals	30	116967	100.0
Stroke-Ready Hospitals	35	116967	100.0
Stroke-Ready Hospitals	40	116967	100.0
Stroke-Ready Hospitals	45	116967	100.0
Stroke-Ready Hospitals	50	116967	100.0
Stroke-Ready Hospitals	55	116967	100.0
Stroke-Ready Hospitals	60	116967	100.0
Stroke Units	5	21678	18.53
Stroke Units	10	78494	67.11
Stroke Units	15	116298	99.43
Stroke Units	20	116967	100.0
Stroke Units	25	116967	100.0
Stroke Units	30	116967	100.0
Stroke Units	35	116967	100.0
Stroke Units	40	116967	100.0
Stroke Units	45	116967	100.0
Stroke Units	50	116967	100.0
Stroke Units	55	116967	100.0
Stroke Units	60	116967	100.0

Population Coverage - Kreisfreie Stadt Speyer (Rheinland-Pfalz)

Condition	Drive Time (min)	Covered Population	Coverage (%)
Hospitals with CT	5	29098	56.64
Hospitals with CT	10	50605	98.51
Hospitals with CT	15	51249	99.76
Hospitals with CT	20	51358	99.97
Hospitals with CT	25	51373	100.0
Hospitals with CT	30	51373	100.0
Hospitals with CT	35	51373	100.0
Hospitals with CT	40	51373	100.0
Hospitals with CT	45	51373	100.0
Hospitals with CT	50	51373	100.0
Hospitals with CT	55	51373	100.0
Hospitals with CT	60	51373	100.0
Stroke-Ready Hospitals	5	21524	41.9
Stroke-Ready Hospitals	10	50563	98.42
Stroke-Ready Hospitals	15	51255	99.77
Stroke-Ready Hospitals	20	51356	99.97
Stroke-Ready Hospitals	25	51373	100.0
Stroke-Ready Hospitals	30	51373	100.0
Stroke-Ready Hospitals	35	51373	100.0
Stroke-Ready Hospitals	40	51373	100.0
Stroke-Ready Hospitals	45	51373	100.0
Stroke-Ready Hospitals	50	51373	100.0
Stroke-Ready Hospitals	55	51373	100.0
Stroke-Ready Hospitals	60	51373	100.0
Stroke Units	5	21524	41.9
Stroke Units	10	50563	98.42
Stroke Units	15	51255	99.77
Stroke Units	20	51356	99.97
Stroke Units	25	51373	100.0
Stroke Units	30	51373	100.0
Stroke Units	35	51373	100.0
Stroke Units	40	51373	100.0
Stroke Units	45	51373	100.0
Stroke Units	50	51373	100.0
Stroke Units	55	51373	100.0
Stroke Units	60	51373	100.0

Population Coverage - Stadtkreis Mannheim (Baden-Württemberg)

Condition	Drive Time (min)	Covered Population	Coverage (%)
Hospitals with CT	5	79455	22.9
Hospitals with CT	10	207505	59.81
Hospitals with CT	15	335848	96.8
Hospitals with CT	20	346922	99.99
Hospitals with CT	25	346949	100.0
Hospitals with CT	30	346949	100.0
Hospitals with CT	35	346949	100.0
Hospitals with CT	40	346949	100.0
Hospitals with CT	45	346949	100.0
Hospitals with CT	50	346949	100.0
Hospitals with CT	55	346949	100.0
Hospitals with CT	60	346949	100.0
Stroke-Ready Hospitals	5	39970	11.52
Stroke-Ready Hospitals	10	173017	49.87
Stroke-Ready Hospitals	15	293790	84.68
Stroke-Ready Hospitals	20	346651	99.91
Stroke-Ready Hospitals	25	346949	100.0
Stroke-Ready Hospitals	30	346949	100.0
Stroke-Ready Hospitals	35	346949	100.0
Stroke-Ready Hospitals	40	346949	100.0
Stroke-Ready Hospitals	45	346949	100.0
Stroke-Ready Hospitals	50	346949	100.0
Stroke-Ready Hospitals	55	346949	100.0
Stroke-Ready Hospitals	60	346949	100.0
Stroke Units	5	39970	11.52
Stroke Units	10	173009	49.87
Stroke Units	15	281919	81.26
Stroke Units	20	343794	99.09
Stroke Units	25	346949	100.0
Stroke Units	30	346949	100.0
Stroke Units	35	346949	100.0
Stroke Units	40	346949	100.0
Stroke Units	45	346949	100.0
Stroke Units	50	346949	100.0
Stroke Units	55	346949	100.0
Stroke Units	60	346949	100.0

Population Coverage - Landkreis Tuttlingen (Baden-Württemberg)

Condition	Drive Time (min)	Covered Population	Coverage (%)
Hospitals with CT	5	0	0.0
Hospitals with CT	10	1937	1.43
Hospitals with CT	15	25087	18.57
Hospitals with CT	20	53708	39.76
Hospitals with CT	25	77817	57.61
Hospitals with CT	30	122708	90.84
Hospitals with CT	35	134893	99.86
Hospitals with CT	40	135083	100.0
Hospitals with CT	45	135084	100.0
Hospitals with CT	50	135084	100.0
Hospitals with CT	55	135084	100.0
Hospitals with CT	60	135084	100.0
Stroke-Ready Hospitals	5	7524	5.57
Stroke-Ready Hospitals	10	26293	19.46
Stroke-Ready Hospitals	15	61273	45.36
Stroke-Ready Hospitals	20	102798	76.1
Stroke-Ready Hospitals	25	127503	94.39
Stroke-Ready Hospitals	30	134237	99.37
Stroke-Ready Hospitals	35	135084	100.0
Stroke-Ready Hospitals	40	135084	100.0
Stroke-Ready Hospitals	45	135084	100.0
Stroke-Ready Hospitals	50	135084	100.0
Stroke-Ready Hospitals	55	135084	100.0
Stroke-Ready Hospitals	60	135084	100.0
Stroke Units	5	0	0.0
Stroke Units	10	0	0.0
Stroke Units	15	6	0.0
Stroke Units	20	11579	8.57
Stroke Units	25	24016	17.78
Stroke Units	30	44619	33.03
Stroke Units	35	82249	60.89
Stroke Units	40	114505	84.77
Stroke Units	45	128486	95.12
Stroke Units	50	134721	99.73
Stroke Units	55	135084	100.0
Stroke Units	60	135084	100.0

Population Coverage - Landkreis Zollernalbkreis (Baden-Württemberg)

Condition	Drive Time (min)	Covered Population	Coverage (%)
Hospitals with CT	5	33219	17.74
Hospitals with CT	10	82110	43.84
Hospitals with CT	15	138035	73.71
Hospitals with CT	20	173657	92.73
Hospitals with CT	25	184384	98.46
Hospitals with CT	30	187226	99.97
Hospitals with CT	35	187275	100.0
Hospitals with CT	40	187275	100.0
Hospitals with CT	45	187275	100.0
Hospitals with CT	50	187275	100.0
Hospitals with CT	55	187275	100.0
Hospitals with CT	60	187275	100.0
Stroke-Ready Hospitals	5	14038	7.5
Stroke-Ready Hospitals	10	41467	22.14
Stroke-Ready Hospitals	15	77529	41.4
Stroke-Ready Hospitals	20	113604	60.66
Stroke-Ready Hospitals	25	161184	86.07
Stroke-Ready Hospitals	30	182912	97.67
Stroke-Ready Hospitals	35	186999	99.85
Stroke-Ready Hospitals	40	187275	100.0
Stroke-Ready Hospitals	45	187275	100.0
Stroke-Ready Hospitals	50	187275	100.0
Stroke-Ready Hospitals	55	187275	100.0
Stroke-Ready Hospitals	60	187275	100.0
Stroke Units	5	0	0.0
Stroke Units	10	6	0.0
Stroke Units	15	4275	2.28
Stroke Units	20	8920	4.76
Stroke Units	25	21753	11.62
Stroke Units	30	36670	19.58
Stroke Units	35	85780	45.8
Stroke Units	40	151150	80.71
Stroke Units	45	184499	98.52
Stroke Units	50	187267	100.0
Stroke Units	55	187275	100.0
Stroke Units	60	187275	100.0

Population Coverage - Landkreis Eichstätt (Bayern)

Condition	Drive Time (min)	Covered Population	Coverage (%)
Hospitals with CT	5	17041	13.05
Hospitals with CT	10	48207	36.91
Hospitals with CT	15	93634	71.69
Hospitals with CT	20	118520	90.74
Hospitals with CT	25	128189	98.14
Hospitals with CT	30	130481	99.9
Hospitals with CT	35	130617	100.0
Hospitals with CT	40	130617	100.0
Hospitals with CT	45	130617	100.0
Hospitals with CT	50	130617	100.0
Hospitals with CT	55	130617	100.0
Hospitals with CT	60	130617	100.0
Stroke-Ready Hospitals	5	429	0.33
Stroke-Ready Hospitals	10	5389	4.13
Stroke-Ready Hospitals	15	18107	13.86
Stroke-Ready Hospitals	20	39258	30.06
Stroke-Ready Hospitals	25	66472	50.89
Stroke-Ready Hospitals	30	85881	65.75
Stroke-Ready Hospitals	35	102661	78.6
Stroke-Ready Hospitals	40	120917	92.57
Stroke-Ready Hospitals	45	127287	97.45
Stroke-Ready Hospitals	50	130612	100.0
Stroke-Ready Hospitals	55	130617	100.0
Stroke-Ready Hospitals	60	130617	100.0
Stroke Units	5	429	0.33
Stroke Units	10	5389	4.13
Stroke Units	15	18107	13.86
Stroke Units	20	39258	30.06
Stroke Units	25	66472	50.89
Stroke Units	30	85881	65.75
Stroke Units	35	102661	78.6
Stroke Units	40	120908	92.57
Stroke Units	45	127171	97.36
Stroke Units	50	130612	100.0
Stroke Units	55	130617	100.0
Stroke Units	60	130617	100.0

Population Coverage - Landkreis Freyung-Grafenau (Bayern)

Condition	Drive Time (min)	Covered Population	Coverage (%)
Hospitals with CT	5	8077	10.03
Hospitals with CT	10	18959	23.55
Hospitals with CT	15	44746	55.58
Hospitals with CT	20	61828	76.8
Hospitals with CT	25	75109	93.3
Hospitals with CT	30	80204	99.62
Hospitals with CT	35	80437	99.91
Hospitals with CT	40	80438	99.91
Hospitals with CT	45	80459	99.94
Hospitals with CT	50	80462	99.94
Hospitals with CT	55	80474	99.96
Hospitals with CT	60	80480	99.97
Stroke-Ready Hospitals	5	4236	5.26
Stroke-Ready Hospitals	10	11194	13.9
Stroke-Ready Hospitals	15	27182	33.76
Stroke-Ready Hospitals	20	39706	49.32
Stroke-Ready Hospitals	25	56979	70.78
Stroke-Ready Hospitals	30	73471	91.26
Stroke-Ready Hospitals	35	78803	97.88
Stroke-Ready Hospitals	40	80438	99.91
Stroke-Ready Hospitals	45	80438	99.91
Stroke-Ready Hospitals	50	80438	99.91
Stroke-Ready Hospitals	55	80448	99.93
Stroke-Ready Hospitals	60	80449	99.93
Stroke Units	5	0	0.0
Stroke Units	10	0	0.0
Stroke Units	15	0	0.0
Stroke Units	20	433	0.54
Stroke Units	25	5080	6.31
Stroke Units	30	13360	16.6
Stroke Units	35	27339	33.96
Stroke Units	40	44933	55.81
Stroke Units	45	61850	76.83
Stroke Units	50	71026	88.22
Stroke Units	55	76131	94.56
Stroke Units	60	79315	98.52

Population Coverage - Landkreis Main-Spessart (Bayern)

Condition	Drive Time (min)	Covered Population	Coverage (%)
Hospitals with CT	5	8596	6.54
Hospitals with CT	10	18782	14.29
Hospitals with CT	15	32449	24.68
Hospitals with CT	20	51349	39.06
Hospitals with CT	25	87458	66.53
Hospitals with CT	30	125217	95.25
Hospitals with CT	35	131354	99.92
Hospitals with CT	40	131455	100.0
Hospitals with CT	45	131455	100.0
Hospitals with CT	50	131455	100.0
Hospitals with CT	55	131455	100.0
Hospitals with CT	60	131455	100.0
Stroke-Ready Hospitals	5	10711	8.15
Stroke-Ready Hospitals	10	19088	14.52
Stroke-Ready Hospitals	15	29647	22.55
Stroke-Ready Hospitals	20	47504	36.14
Stroke-Ready Hospitals	25	81931	62.33
Stroke-Ready Hospitals	30	112380	85.49
Stroke-Ready Hospitals	35	126514	96.24
Stroke-Ready Hospitals	40	131098	99.73
Stroke-Ready Hospitals	45	131452	100.0
Stroke-Ready Hospitals	50	131455	100.0
Stroke-Ready Hospitals	55	131455	100.0
Stroke-Ready Hospitals	60	131455	100.0
Stroke Units	5	10711	8.15
Stroke Units	10	19088	14.52
Stroke Units	15	29647	22.55
Stroke Units	20	47504	36.14
Stroke Units	25	81931	62.33
Stroke Units	30	112380	85.49
Stroke Units	35	126514	96.24
Stroke Units	40	131098	99.73
Stroke Units	45	131452	100.0
Stroke Units	50	131455	100.0
Stroke Units	55	131455	100.0
Stroke Units	60	131455	100.0

Population Coverage - Kreisfreie Stadt Potsdam (Brandenburg)

Condition	Drive Time (min)	Covered Population	Coverage (%)
Hospitals with CT	5	92966	45.28
Hospitals with CT	10	166041	80.87
Hospitals with CT	15	194569	94.77
Hospitals with CT	20	204247	99.48
Hospitals with CT	25	205119	99.9
Hospitals with CT	30	205315	100.0
Hospitals with CT	35	205315	100.0
Hospitals with CT	40	205315	100.0
Hospitals with CT	45	205315	100.0
Hospitals with CT	50	205315	100.0
Hospitals with CT	55	205315	100.0
Hospitals with CT	60	205315	100.0
Stroke-Ready Hospitals	5	48162	23.46
Stroke-Ready Hospitals	10	112340	54.72
Stroke-Ready Hospitals	15	183439	89.35
Stroke-Ready Hospitals	20	200277	97.55
Stroke-Ready Hospitals	25	204749	99.72
Stroke-Ready Hospitals	30	205299	99.99
Stroke-Ready Hospitals	35	205315	100.0
Stroke-Ready Hospitals	40	205315	100.0
Stroke-Ready Hospitals	45	205315	100.0
Stroke-Ready Hospitals	50	205315	100.0
Stroke-Ready Hospitals	55	205315	100.0
Stroke-Ready Hospitals	60	205315	100.0
Stroke Units	5	48162	23.46
Stroke Units	10	112340	54.72
Stroke Units	15	183280	89.27
Stroke Units	20	199262	97.05
Stroke Units	25	204713	99.71
Stroke Units	30	205274	99.98
Stroke Units	35	205315	100.0
Stroke Units	40	205315	100.0
Stroke Units	45	205315	100.0
Stroke Units	50	205315	100.0
Stroke Units	55	205315	100.0
Stroke Units	60	205315	100.0

Population Coverage - Landkreis Nordsachsen (Sachsen)

Condition	Drive Time (min)	Covered Population	Coverage (%)
Hospitals with CT	5	44851	24.37
Hospitals with CT	10	70527	38.32
Hospitals with CT	15	109465	59.47
Hospitals with CT	20	141010	76.61
Hospitals with CT	25	155698	84.58
Hospitals with CT	30	171088	92.95
Hospitals with CT	35	180684	98.16
Hospitals with CT	40	183986	99.95
Hospitals with CT	45	184073	100.0
Hospitals with CT	50	184073	100.0
Hospitals with CT	55	184073	100.0
Hospitals with CT	60	184073	100.0
Stroke-Ready Hospitals	5	5796	3.15
Stroke-Ready Hospitals	10	13045	7.09
Stroke-Ready Hospitals	15	28293	15.37
Stroke-Ready Hospitals	20	58401	31.73
Stroke-Ready Hospitals	25	86962	47.24
Stroke-Ready Hospitals	30	113676	61.76
Stroke-Ready Hospitals	35	134176	72.89
Stroke-Ready Hospitals	40	147146	79.94
Stroke-Ready Hospitals	45	165804	90.08
Stroke-Ready Hospitals	50	176868	96.09
Stroke-Ready Hospitals	55	182902	99.36
Stroke-Ready Hospitals	60	184033	99.98
Stroke Units	5	5796	3.15
Stroke Units	10	13045	7.09
Stroke Units	15	28293	15.37
Stroke Units	20	57357	31.16
Stroke Units	25	84428	45.87
Stroke Units	30	105807	57.48
Stroke Units	35	124724	67.76
Stroke Units	40	141211	76.71
Stroke Units	45	160491	87.19
Stroke Units	50	172292	93.6
Stroke Units	55	178772	97.12
Stroke Units	60	183121	99.48

Population Coverage - Landkreis Cuxhaven (Niedersachsen)

Condition	Drive Time (min)	Covered Population	Coverage (%)
Hospitals with CT	5	22917	11.51
Hospitals with CT	10	60537	30.41
Hospitals with CT	15	103081	51.78
Hospitals with CT	20	134671	67.65
Hospitals with CT	25	162745	81.75
Hospitals with CT	30	186679	93.78
Hospitals with CT	35	197063	98.99
Hospitals with CT	40	197893	99.41
Hospitals with CT	45	197925	99.43
Hospitals with CT	50	197944	99.44
Hospitals with CT	55	197572	99.25
Hospitals with CT	60	197858	99.39
Stroke-Ready Hospitals	5	1536	0.77
Stroke-Ready Hospitals	10	3207	1.61
Stroke-Ready Hospitals	15	14117	7.09
Stroke-Ready Hospitals	20	42641	21.42
Stroke-Ready Hospitals	25	65112	32.71
Stroke-Ready Hospitals	30	98313	49.39
Stroke-Ready Hospitals	35	142612	71.64
Stroke-Ready Hospitals	40	169330	85.06
Stroke-Ready Hospitals	45	185264	93.07
Stroke-Ready Hospitals	50	193420	97.16
Stroke-Ready Hospitals	55	196726	98.82
Stroke-Ready Hospitals	60	197400	99.16
Stroke Units	5	1536	0.77
Stroke Units	10	3207	1.61
Stroke Units	15	14117	7.09
Stroke Units	20	42641	21.42
Stroke Units	25	65112	32.71
Stroke Units	30	98313	49.39
Stroke Units	35	142612	71.64
Stroke Units	40	169330	85.06
Stroke Units	45	185259	93.06
Stroke Units	50	193413	97.16
Stroke Units	55	196726	98.82
Stroke Units	60	197400	99.16

Population Coverage - Kreisfreie Stadt Bremen (Bremen)

Condition	Drive Time (min)	Covered Population	Coverage (%)
Hospitals with CT	5	216456	36.41
Hospitals with CT	10	477408	80.3
Hospitals with CT	15	541239	91.04
Hospitals with CT	20	564310	94.92
Hospitals with CT	25	566011	95.21
Hospitals with CT	30	566277	95.25
Hospitals with CT	35	581700	97.85
Hospitals with CT	40	581700	97.85
Hospitals with CT	45	581784	97.86
Hospitals with CT	50	581967	97.89
Hospitals with CT	55	581664	97.84
Hospitals with CT	60	581963	97.89
Stroke-Ready Hospitals	5	92473	15.55
Stroke-Ready Hospitals	10	268889	45.23
Stroke-Ready Hospitals	15	434828	73.14
Stroke-Ready Hospitals	20	530514	89.24
Stroke-Ready Hospitals	25	562853	94.68
Stroke-Ready Hospitals	30	564451	94.94
Stroke-Ready Hospitals	35	581594	97.83
Stroke-Ready Hospitals	40	581594	97.83
Stroke-Ready Hospitals	45	581628	97.83
Stroke-Ready Hospitals	50	581766	97.86
Stroke-Ready Hospitals	55	581377	97.79
Stroke-Ready Hospitals	60	581739	97.85
Stroke Units	5	68579	11.54
Stroke Units	10	194594	32.73
Stroke Units	15	381087	64.1
Stroke Units	20	529414	89.05
Stroke Units	25	562631	94.64
Stroke Units	30	564283	94.92
Stroke Units	35	581594	97.83
Stroke Units	40	581594	97.83
Stroke Units	45	581628	97.83
Stroke Units	50	581766	97.86
Stroke Units	55	581356	97.79
Stroke Units	60	581715	97.85

Population Coverage - Landkreis Groß-Gerau (Hessen)

Condition	Drive Time (min)	Covered Population	Coverage (%)
Hospitals with CT	5	35365	13.62
Hospitals with CT	10	117292	45.18
Hospitals with CT	15	190738	73.48
Hospitals with CT	20	251309	96.81
Hospitals with CT	25	259379	99.92
Hospitals with CT	30	259573	99.99
Hospitals with CT	35	259585	100.0
Hospitals with CT	40	259593	100.0
Hospitals with CT	45	259593	100.0
Hospitals with CT	50	259593	100.0
Hospitals with CT	55	259593	100.0
Hospitals with CT	60	259593	100.0
Stroke-Ready Hospitals	5	19999	7.7
Stroke-Ready Hospitals	10	72178	27.8
Stroke-Ready Hospitals	15	154883	59.66
Stroke-Ready Hospitals	20	211607	81.52
Stroke-Ready Hospitals	25	252840	97.4
Stroke-Ready Hospitals	30	259470	99.95
Stroke-Ready Hospitals	35	259583	100.0
Stroke-Ready Hospitals	40	259589	100.0
Stroke-Ready Hospitals	45	259593	100.0
Stroke-Ready Hospitals	50	259593	100.0
Stroke-Ready Hospitals	55	259593	100.0
Stroke-Ready Hospitals	60	259593	100.0
Stroke Units	5	19999	7.7
Stroke Units	10	72178	27.8
Stroke Units	15	154883	59.66
Stroke Units	20	211607	81.52
Stroke Units	25	252840	97.4
Stroke Units	30	259470	99.95
Stroke Units	35	259583	100.0
Stroke Units	40	259589	100.0
Stroke Units	45	259593	100.0
Stroke Units	50	259593	100.0
Stroke Units	55	259593	100.0
Stroke Units	60	259593	100.0

Population Coverage - Landkreis Limburg-Weilburg (Hessen)

Condition	Drive Time (min)	Covered Population	Coverage (%)
Hospitals with CT	5	22995	13.3
Hospitals with CT	10	70576	40.83
Hospitals with CT	15	123310	71.34
Hospitals with CT	20	159653	92.37
Hospitals with CT	25	172824	99.99
Hospitals with CT	30	172837	100.0
Hospitals with CT	35	172837	100.0
Hospitals with CT	40	172837	100.0
Hospitals with CT	45	172837	100.0
Hospitals with CT	50	172837	100.0
Hospitals with CT	55	172837	100.0
Hospitals with CT	60	172837	100.0
Stroke-Ready Hospitals	5	11981	6.93
Stroke-Ready Hospitals	10	39267	22.72
Stroke-Ready Hospitals	15	76880	44.48
Stroke-Ready Hospitals	20	129952	75.19
Stroke-Ready Hospitals	25	170584	98.7
Stroke-Ready Hospitals	30	172834	100.0
Stroke-Ready Hospitals	35	172837	100.0
Stroke-Ready Hospitals	40	172837	100.0
Stroke-Ready Hospitals	45	172837	100.0
Stroke-Ready Hospitals	50	172837	100.0
Stroke-Ready Hospitals	55	172837	100.0
Stroke-Ready Hospitals	60	172837	100.0
Stroke Units	5	11755	6.8
Stroke Units	10	37246	21.55
Stroke Units	15	72977	42.22
Stroke Units	20	122227	70.72
Stroke Units	25	166734	96.47
Stroke Units	30	172207	99.64
Stroke Units	35	172830	100.0
Stroke Units	40	172837	100.0
Stroke Units	45	172837	100.0
Stroke Units	50	172837	100.0
Stroke Units	55	172837	100.0
Stroke Units	60	172837	100.0

Population Coverage - Landkreis Waldeck-Frankenberg (Hessen)

Condition	Drive Time (min)	Covered Population	Coverage (%)
Hospitals with CT	5	45099	28.54
Hospitals with CT	10	84927	53.75
Hospitals with CT	15	130998	82.91
Hospitals with CT	20	152992	96.83
Hospitals with CT	25	157602	99.75
Hospitals with CT	30	157966	99.98
Hospitals with CT	35	157989	99.99
Hospitals with CT	40	158003	100.0
Hospitals with CT	45	158003	100.0
Hospitals with CT	50	158003	100.0
Hospitals with CT	55	158003	100.0
Hospitals with CT	60	158003	100.0
Stroke-Ready Hospitals	5	0	0.0
Stroke-Ready Hospitals	10	1372	0.87
Stroke-Ready Hospitals	15	15749	9.97
Stroke-Ready Hospitals	20	25116	15.9
Stroke-Ready Hospitals	25	32908	20.83
Stroke-Ready Hospitals	30	41649	26.36
Stroke-Ready Hospitals	35	69571	44.03
Stroke-Ready Hospitals	40	92238	58.38
Stroke-Ready Hospitals	45	106167	67.19
Stroke-Ready Hospitals	50	144697	91.58
Stroke-Ready Hospitals	55	154362	97.7
Stroke-Ready Hospitals	60	157933	99.96
Stroke Units	5	0	0.0
Stroke Units	10	0	0.0
Stroke Units	15	0	0.0
Stroke Units	20	0	0.0
Stroke Units	25	1193	0.76
Stroke Units	30	3304	2.09
Stroke Units	35	25150	15.92
Stroke Units	40	57872	36.63
Stroke Units	45	82524	52.23
Stroke Units	50	111675	70.68
Stroke Units	55	125829	79.64
Stroke Units	60	152582	96.57

Population Coverage - Landkreis Trier-Saarburg (Rheinland-Pfalz)

Condition	Drive Time (min)	Covered Population	Coverage (%)
Hospitals with CT	5	9382	6.24
Hospitals with CT	10	24395	16.23
Hospitals with CT	15	53291	35.45
Hospitals with CT	20	108998	72.51
Hospitals with CT	25	142372	94.71
Hospitals with CT	30	150223	99.93
Hospitals with CT	35	150288	99.98
Hospitals with CT	40	150303	99.99
Hospitals with CT	45	150306	99.99
Hospitals with CT	50	150306	99.99
Hospitals with CT	55	150299	99.98
Hospitals with CT	60	150307	99.99
Stroke-Ready Hospitals	5	0	0.0
Stroke-Ready Hospitals	10	1447	0.96
Stroke-Ready Hospitals	15	19225	12.79
Stroke-Ready Hospitals	20	57227	38.07
Stroke-Ready Hospitals	25	93276	62.05
Stroke-Ready Hospitals	30	125869	83.73
Stroke-Ready Hospitals	35	147788	98.31
Stroke-Ready Hospitals	40	150302	99.99
Stroke-Ready Hospitals	45	150311	99.99
Stroke-Ready Hospitals	50	150313	99.99
Stroke-Ready Hospitals	55	150300	99.98
Stroke-Ready Hospitals	60	150306	99.99
Stroke Units	5	0	0.0
Stroke Units	10	1447	0.96
Stroke Units	15	19225	12.79
Stroke Units	20	57227	38.07
Stroke Units	25	93276	62.05
Stroke Units	30	125869	83.73
Stroke Units	35	147788	98.31
Stroke Units	40	150302	99.99
Stroke Units	45	150311	99.99
Stroke Units	50	150313	99.99
Stroke Units	55	150300	99.98
Stroke Units	60	150306	99.99

Population Coverage - Landkreis Reutlingen (Baden-Württemberg)

Condition	Drive Time (min)	Covered Population	Coverage (%)
Hospitals with CT	5	31006	10.41
Hospitals with CT	10	104205	35.0
Hospitals with CT	15	195131	65.54
Hospitals with CT	20	264451	88.83
Hospitals with CT	25	290028	97.42
Hospitals with CT	30	297424	99.9
Hospitals with CT	35	297711	100.0
Hospitals with CT	40	297711	100.0
Hospitals with CT	45	297711	100.0
Hospitals with CT	50	297711	100.0
Hospitals with CT	55	297711	100.0
Hospitals with CT	60	297711	100.0
Stroke-Ready Hospitals	5	21017	7.06
Stroke-Ready Hospitals	10	75942	25.51
Stroke-Ready Hospitals	15	138944	46.67
Stroke-Ready Hospitals	20	197839	66.45
Stroke-Ready Hospitals	25	234842	78.88
Stroke-Ready Hospitals	30	254681	85.55
Stroke-Ready Hospitals	35	276794	92.97
Stroke-Ready Hospitals	40	285813	96.0
Stroke-Ready Hospitals	45	296629	99.64
Stroke-Ready Hospitals	50	297711	100.0
Stroke-Ready Hospitals	55	297711	100.0
Stroke-Ready Hospitals	60	297711	100.0
Stroke Units	5	21017	7.06
Stroke Units	10	75942	25.51
Stroke Units	15	138944	46.67
Stroke Units	20	197839	66.45
Stroke Units	25	234842	78.88
Stroke Units	30	254677	85.55
Stroke Units	35	276793	92.97
Stroke Units	40	285813	96.0
Stroke Units	45	296629	99.64
Stroke Units	50	297711	100.0
Stroke Units	55	297711	100.0
Stroke Units	60	297711	100.0

Population Coverage - Kreisfreie Stadt Schwerin (Mecklenburg-Vorpommern)

Condition	Drive Time (min)	Covered Population	Coverage (%)
Hospitals with CT	5	12848	13.28
Hospitals with CT	10	49306	50.95
Hospitals with CT	15	77688	80.28
Hospitals with CT	20	91960	95.03
Hospitals with CT	25	96683	99.91
Hospitals with CT	30	96772	100.0
Hospitals with CT	35	96772	100.0
Hospitals with CT	40	96772	100.0
Hospitals with CT	45	96772	100.0
Hospitals with CT	50	96772	100.0
Hospitals with CT	55	96772	100.0
Hospitals with CT	60	96772	100.0
Stroke-Ready Hospitals	5	2585	2.67
Stroke-Ready Hospitals	10	30301	31.31
Stroke-Ready Hospitals	15	61211	63.25
Stroke-Ready Hospitals	20	81103	83.81
Stroke-Ready Hospitals	25	95733	98.93
Stroke-Ready Hospitals	30	96684	99.91
Stroke-Ready Hospitals	35	96772	100.0
Stroke-Ready Hospitals	40	96772	100.0
Stroke-Ready Hospitals	45	96772	100.0
Stroke-Ready Hospitals	50	96772	100.0
Stroke-Ready Hospitals	55	96772	100.0
Stroke-Ready Hospitals	60	96772	100.0
Stroke Units	5	2585	2.67
Stroke Units	10	30301	31.31
Stroke Units	15	61211	63.25
Stroke Units	20	81103	83.81
Stroke Units	25	95733	98.93
Stroke Units	30	96684	99.91
Stroke Units	35	96772	100.0
Stroke Units	40	96772	100.0
Stroke Units	45	96772	100.0
Stroke Units	50	96772	100.0
Stroke Units	55	96772	100.0
Stroke Units	60	96772	100.0

Population Coverage - Kreisfreie Stadt Hamburg (Hamburg)

Condition	Drive Time (min)	Covered Population	Coverage (%)
Hospitals with CT	5	650582	36.13
Hospitals with CT	10	1359788	75.52
Hospitals with CT	15	1690592	93.89
Hospitals with CT	20	1757960	97.63
Hospitals with CT	25	1791276	99.48
Hospitals with CT	30	1791826	99.51
Hospitals with CT	35	1791919	99.52
Hospitals with CT	40	1791932	99.52
Hospitals with CT	45	1791932	99.52
Hospitals with CT	50	1791960	99.52
Hospitals with CT	55	1791798	99.51
Hospitals with CT	60	1791895	99.52
Stroke-Ready Hospitals	5	269819	14.99
Stroke-Ready Hospitals	10	978185	54.33
Stroke-Ready Hospitals	15	1414573	78.56
Stroke-Ready Hospitals	20	1660275	92.21
Stroke-Ready Hospitals	25	1778034	98.75
Stroke-Ready Hospitals	30	1790013	99.41
Stroke-Ready Hospitals	35	1791805	99.51
Stroke-Ready Hospitals	40	1791847	99.52
Stroke-Ready Hospitals	45	1791889	99.52
Stroke-Ready Hospitals	50	1791901	99.52
Stroke-Ready Hospitals	55	1791758	99.51
Stroke-Ready Hospitals	60	1791857	99.52
Stroke Units	5	269819	14.99
Stroke Units	10	978185	54.33
Stroke Units	15	1414573	78.56
Stroke Units	20	1660275	92.21
Stroke Units	25	1778034	98.75
Stroke Units	30	1790013	99.41
Stroke Units	35	1791803	99.51
Stroke Units	40	1791847	99.52
Stroke Units	45	1791875	99.52
Stroke Units	50	1791887	99.52
Stroke Units	55	1791758	99.51
Stroke Units	60	1791857	99.52

Population Coverage - Kreisfreie Stadt Braunschweig (Niedersachsen)

Condition	Drive Time (min)	Covered Population	Coverage (%)
Hospitals with CT	5	95291	38.63
Hospitals with CT	10	193353	78.38
Hospitals with CT	15	241257	97.8
Hospitals with CT	20	246619	99.98
Hospitals with CT	25	246678	100.0
Hospitals with CT	30	246678	100.0
Hospitals with CT	35	246678	100.0
Hospitals with CT	40	246678	100.0
Hospitals with CT	45	246678	100.0
Hospitals with CT	50	246678	100.0
Hospitals with CT	55	246678	100.0
Hospitals with CT	60	246678	100.0
Stroke-Ready Hospitals	5	1723	0.7
Stroke-Ready Hospitals	10	27746	11.25
Stroke-Ready Hospitals	15	92648	37.56
Stroke-Ready Hospitals	20	176056	71.37
Stroke-Ready Hospitals	25	239295	97.01
Stroke-Ready Hospitals	30	246678	100.0
Stroke-Ready Hospitals	35	246678	100.0
Stroke-Ready Hospitals	40	246678	100.0
Stroke-Ready Hospitals	45	246678	100.0
Stroke-Ready Hospitals	50	246678	100.0
Stroke-Ready Hospitals	55	246678	100.0
Stroke-Ready Hospitals	60	246678	100.0
Stroke Units	5	1705	0.69
Stroke Units	10	15585	6.32
Stroke Units	15	90799	36.81
Stroke Units	20	169486	68.71
Stroke Units	25	220945	89.57
Stroke Units	30	246126	99.78
Stroke Units	35	246678	100.0
Stroke Units	40	246678	100.0
Stroke Units	45	246678	100.0
Stroke Units	50	246678	100.0
Stroke Units	55	246678	100.0
Stroke Units	60	246678	100.0

Population Coverage - Landkreis Leer (Niedersachsen)

Condition	Drive Time (min)	Covered Population	Coverage (%)
Hospitals with CT	5	9592	5.63
Hospitals with CT	10	33040	19.38
Hospitals with CT	15	64865	38.04
Hospitals with CT	20	120794	70.84
Hospitals with CT	25	157137	92.15
Hospitals with CT	30	164142	96.26
Hospitals with CT	35	165286	96.93
Hospitals with CT	40	165299	96.94
Hospitals with CT	45	165299	96.94
Hospitals with CT	50	165301	96.94
Hospitals with CT	55	165300	96.94
Hospitals with CT	60	165306	96.94
Stroke-Ready Hospitals	5	0	0.0
Stroke-Ready Hospitals	10	0	0.0
Stroke-Ready Hospitals	15	1367	0.8
Stroke-Ready Hospitals	20	13901	8.15
Stroke-Ready Hospitals	25	45701	26.8
Stroke-Ready Hospitals	30	78158	45.83
Stroke-Ready Hospitals	35	108912	63.87
Stroke-Ready Hospitals	40	138444	81.19
Stroke-Ready Hospitals	45	155561	91.23
Stroke-Ready Hospitals	50	165250	96.91
Stroke-Ready Hospitals	55	165297	96.93
Stroke-Ready Hospitals	60	165301	96.94
Stroke Units	5	0	0.0
Stroke Units	10	0	0.0
Stroke Units	15	1367	0.8
Stroke Units	20	13901	8.15
Stroke Units	25	45701	26.8
Stroke Units	30	78158	45.83
Stroke Units	35	108912	63.87
Stroke Units	40	138444	81.19
Stroke Units	45	155561	91.23
Stroke Units	50	165250	96.91
Stroke Units	55	165297	96.93
Stroke Units	60	165301	96.94

Population Coverage - Kreisfreie Stadt Duisburg (Nordrhein-Westfalen)

Condition	Drive Time (min)	Covered Population	Coverage (%)
Hospitals with CT	5	263458	47.65
Hospitals with CT	10	509328	92.12
Hospitals with CT	15	528319	95.55
Hospitals with CT	20	552894	100.0
Hospitals with CT	25	552897	100.0
Hospitals with CT	30	552903	100.0
Hospitals with CT	35	552903	100.0
Hospitals with CT	40	552903	100.0
Hospitals with CT	45	552903	100.0
Hospitals with CT	50	552903	100.0
Hospitals with CT	55	552903	100.0
Hospitals with CT	60	552903	100.0
Stroke-Ready Hospitals	5	55732	10.08
Stroke-Ready Hospitals	10	275020	49.74
Stroke-Ready Hospitals	15	475442	85.99
Stroke-Ready Hospitals	20	542580	98.13
Stroke-Ready Hospitals	25	552897	100.0
Stroke-Ready Hospitals	30	552903	100.0
Stroke-Ready Hospitals	35	552903	100.0
Stroke-Ready Hospitals	40	552903	100.0
Stroke-Ready Hospitals	45	552903	100.0
Stroke-Ready Hospitals	50	552903	100.0
Stroke-Ready Hospitals	55	552903	100.0
Stroke-Ready Hospitals	60	552903	100.0
Stroke Units	5	15328	2.77
Stroke Units	10	146863	26.56
Stroke Units	15	385102	69.65
Stroke Units	20	511159	92.45
Stroke Units	25	537657	97.24
Stroke Units	30	552903	100.0
Stroke Units	35	552903	100.0
Stroke Units	40	552903	100.0
Stroke Units	45	552903	100.0
Stroke Units	50	552903	100.0
Stroke Units	55	552903	100.0
Stroke Units	60	552903	100.0

Population Coverage - Kreis Siegen-Wittgenstein (Nordrhein-Westfalen)

Condition	Drive Time (min)	Covered Population	Coverage (%)
Hospitals with CT	5	62297	21.24
Hospitals with CT	10	144753	49.35
Hospitals with CT	15	206820	70.51
Hospitals with CT	20	262084	89.35
Hospitals with CT	25	285564	97.36
Hospitals with CT	30	292563	99.74
Hospitals with CT	35	293293	99.99
Hospitals with CT	40	293319	100.0
Hospitals with CT	45	293319	100.0
Hospitals with CT	50	293319	100.0
Hospitals with CT	55	293319	100.0
Hospitals with CT	60	293319	100.0
Stroke-Ready Hospitals	5	33960	11.58
Stroke-Ready Hospitals	10	112166	38.24
Stroke-Ready Hospitals	15	164789	56.18
Stroke-Ready Hospitals	20	203957	69.53
Stroke-Ready Hospitals	25	242339	82.62
Stroke-Ready Hospitals	30	252196	85.98
Stroke-Ready Hospitals	35	252897	86.22
Stroke-Ready Hospitals	40	256338	87.39
Stroke-Ready Hospitals	45	261406	89.12
Stroke-Ready Hospitals	50	270965	92.38
Stroke-Ready Hospitals	55	283726	96.73
Stroke-Ready Hospitals	60	292761	99.81
Stroke Units	5	26299	8.97
Stroke Units	10	97536	33.25
Stroke Units	15	151055	51.5
Stroke Units	20	184501	62.9
Stroke Units	25	214887	73.26
Stroke Units	30	241756	82.42
Stroke Units	35	252804	86.19
Stroke Units	40	256333	87.39
Stroke Units	45	261406	89.12
Stroke Units	50	270511	92.22
Stroke Units	55	283719	96.73
Stroke Units	60	292761	99.81

Population Coverage - Landkreis Schwarzwald-Baar-Kreis (Baden-Württemberg)

Condition	Drive Time (min)	Covered Population	Coverage (%)
Hospitals with CT	5	4812	2.32
Hospitals with CT	10	52615	25.42
Hospitals with CT	15	94173	45.49
Hospitals with CT	20	124845	60.31
Hospitals with CT	25	166450	80.41
Hospitals with CT	30	187893	90.77
Hospitals with CT	35	203882	98.49
Hospitals with CT	40	206928	99.96
Hospitals with CT	45	207005	100.0
Hospitals with CT	50	207005	100.0
Hospitals with CT	55	207005	100.0
Hospitals with CT	60	207005	100.0
Stroke-Ready Hospitals	5	772	0.37
Stroke-Ready Hospitals	10	22870	11.05
Stroke-Ready Hospitals	15	77258	37.32
Stroke-Ready Hospitals	20	111577	53.9
Stroke-Ready Hospitals	25	149612	72.27
Stroke-Ready Hospitals	30	177842	85.91
Stroke-Ready Hospitals	35	190693	92.12
Stroke-Ready Hospitals	40	199633	96.44
Stroke-Ready Hospitals	45	203282	98.2
Stroke-Ready Hospitals	50	206494	99.75
Stroke-Ready Hospitals	55	206952	99.97
Stroke-Ready Hospitals	60	206993	99.99
Stroke Units	5	772	0.37
Stroke Units	10	22870	11.05
Stroke Units	15	74768	36.12
Stroke Units	20	110264	53.27
Stroke Units	25	145758	70.41
Stroke Units	30	170186	82.21
Stroke Units	35	180248	87.07
Stroke Units	40	189846	91.71
Stroke Units	45	197773	95.54
Stroke Units	50	204549	98.81
Stroke Units	55	206821	99.91
Stroke Units	60	206981	99.99

Population Coverage - Kreisfreie Stadt Amberg (Bayern)

Condition	Drive Time (min)	Covered Population	Coverage (%)
Hospitals with CT	5	19824	46.26
Hospitals with CT	10	40693	94.96
Hospitals with CT	15	42852	100.0
Hospitals with CT	20	42852	100.0
Hospitals with CT	25	42852	100.0
Hospitals with CT	30	42852	100.0
Hospitals with CT	35	42852	100.0
Hospitals with CT	40	42852	100.0
Hospitals with CT	45	42852	100.0
Hospitals with CT	50	42852	100.0
Hospitals with CT	55	42852	100.0
Hospitals with CT	60	42852	100.0
Stroke-Ready Hospitals	5	19352	45.16
Stroke-Ready Hospitals	10	40260	93.95
Stroke-Ready Hospitals	15	42852	100.0
Stroke-Ready Hospitals	20	42852	100.0
Stroke-Ready Hospitals	25	42852	100.0
Stroke-Ready Hospitals	30	42852	100.0
Stroke-Ready Hospitals	35	42852	100.0
Stroke-Ready Hospitals	40	42852	100.0
Stroke-Ready Hospitals	45	42852	100.0
Stroke-Ready Hospitals	50	42852	100.0
Stroke-Ready Hospitals	55	42852	100.0
Stroke-Ready Hospitals	60	42852	100.0
Stroke Units	5	19352	45.16
Stroke Units	10	40260	93.95
Stroke Units	15	42852	100.0
Stroke Units	20	42852	100.0
Stroke Units	25	42852	100.0
Stroke Units	30	42852	100.0
Stroke Units	35	42852	100.0
Stroke Units	40	42852	100.0
Stroke Units	45	42852	100.0
Stroke Units	50	42852	100.0
Stroke Units	55	42852	100.0
Stroke Units	60	42852	100.0

Population Coverage - Kreisfreie Stadt Bayreuth (Bayern)

Condition	Drive Time (min)	Covered Population	Coverage (%)
Hospitals with CT	5	34658	47.18
Hospitals with CT	10	67488	91.87
Hospitals with CT	15	72929	99.27
Hospitals with CT	20	73464	100.0
Hospitals with CT	25	73464	100.0
Hospitals with CT	30	73464	100.0
Hospitals with CT	35	73464	100.0
Hospitals with CT	40	73464	100.0
Hospitals with CT	45	73464	100.0
Hospitals with CT	50	73464	100.0
Hospitals with CT	55	73464	100.0
Hospitals with CT	60	73464	100.0
Stroke-Ready Hospitals	5	25755	35.06
Stroke-Ready Hospitals	10	65666	89.38
Stroke-Ready Hospitals	15	73274	99.74
Stroke-Ready Hospitals	20	73464	100.0
Stroke-Ready Hospitals	25	73464	100.0
Stroke-Ready Hospitals	30	73464	100.0
Stroke-Ready Hospitals	35	73464	100.0
Stroke-Ready Hospitals	40	73464	100.0
Stroke-Ready Hospitals	45	73464	100.0
Stroke-Ready Hospitals	50	73464	100.0
Stroke-Ready Hospitals	55	73464	100.0
Stroke-Ready Hospitals	60	73464	100.0
Stroke Units	5	15364	20.91
Stroke Units	10	54187	73.76
Stroke Units	15	73087	99.49
Stroke Units	20	73456	99.99
Stroke Units	25	73464	100.0
Stroke Units	30	73464	100.0
Stroke Units	35	73464	100.0
Stroke Units	40	73464	100.0
Stroke Units	45	73464	100.0
Stroke Units	50	73464	100.0
Stroke Units	55	73464	100.0
Stroke Units	60	73464	100.0

Population Coverage - Landkreis Nürnberger Land (Bayern)

Condition	Drive Time (min)	Covered Population	Coverage (%)
Hospitals with CT	5	31677	18.63
Hospitals with CT	10	82952	48.77
Hospitals with CT	15	131947	77.58
Hospitals with CT	20	157507	92.61
Hospitals with CT	25	167874	98.7
Hospitals with CT	30	170062	99.99
Hospitals with CT	35	170077	100.0
Hospitals with CT	40	170077	100.0
Hospitals with CT	45	170077	100.0
Hospitals with CT	50	170077	100.0
Hospitals with CT	55	170077	100.0
Hospitals with CT	60	170077	100.0
Stroke-Ready Hospitals	5	3401	2.0
Stroke-Ready Hospitals	10	28076	16.51
Stroke-Ready Hospitals	15	53384	31.39
Stroke-Ready Hospitals	20	107667	63.3
Stroke-Ready Hospitals	25	134149	78.88
Stroke-Ready Hospitals	30	157271	92.47
Stroke-Ready Hospitals	35	166037	97.62
Stroke-Ready Hospitals	40	170013	99.96
Stroke-Ready Hospitals	45	170077	100.0
Stroke-Ready Hospitals	50	170077	100.0
Stroke-Ready Hospitals	55	170077	100.0
Stroke-Ready Hospitals	60	170077	100.0
Stroke Units	5	3401	2.0
Stroke Units	10	28076	16.51
Stroke Units	15	53384	31.39
Stroke Units	20	107667	63.3
Stroke Units	25	134149	78.88
Stroke Units	30	157271	92.47
Stroke Units	35	166037	97.62
Stroke Units	40	170013	99.96
Stroke Units	45	170077	100.0
Stroke Units	50	170077	100.0
Stroke Units	55	170077	100.0
Stroke Units	60	170077	100.0

Population Coverage - Landkreis Roth (Bayern)

Condition	Drive Time (min)	Covered Population	Coverage (%)
Hospitals with CT	5	5389	4.18
Hospitals with CT	10	23810	18.49
Hospitals with CT	15	64133	49.8
Hospitals with CT	20	103190	80.13
Hospitals with CT	25	124309	96.53
Hospitals with CT	30	128777	100.0
Hospitals with CT	35	128777	100.0
Hospitals with CT	40	128777	100.0
Hospitals with CT	45	128777	100.0
Hospitals with CT	50	128777	100.0
Hospitals with CT	55	128777	100.0
Hospitals with CT	60	128777	100.0
Stroke-Ready Hospitals	5	7228	5.61
Stroke-Ready Hospitals	10	26579	20.64
Stroke-Ready Hospitals	15	69406	53.9
Stroke-Ready Hospitals	20	100106	77.74
Stroke-Ready Hospitals	25	115246	89.49
Stroke-Ready Hospitals	30	121258	94.16
Stroke-Ready Hospitals	35	127560	99.06
Stroke-Ready Hospitals	40	128777	100.0
Stroke-Ready Hospitals	45	128777	100.0
Stroke-Ready Hospitals	50	128777	100.0
Stroke-Ready Hospitals	55	128777	100.0
Stroke-Ready Hospitals	60	128777	100.0
Stroke Units	5	7228	5.61
Stroke Units	10	25586	19.87
Stroke Units	15	68492	53.19
Stroke Units	20	100088	77.72
Stroke Units	25	115246	89.49
Stroke Units	30	121258	94.16
Stroke Units	35	127560	99.06
Stroke Units	40	128777	100.0
Stroke Units	45	128777	100.0
Stroke Units	50	128777	100.0
Stroke Units	55	128777	100.0
Stroke Units	60	128777	100.0

Population Coverage - Kreisfreie Stadt Aschaffenburg (Bayern)

Condition	Drive Time (min)	Covered Population	Coverage (%)
Hospitals with CT	5	2767	3.94
Hospitals with CT	10	37196	52.92
Hospitals with CT	15	61687	87.76
Hospitals with CT	20	70200	99.87
Hospitals with CT	25	70292	100.0
Hospitals with CT	30	70292	100.0
Hospitals with CT	35	70292	100.0
Hospitals with CT	40	70292	100.0
Hospitals with CT	45	70292	100.0
Hospitals with CT	50	70292	100.0
Hospitals with CT	55	70292	100.0
Hospitals with CT	60	70292	100.0
Stroke-Ready Hospitals	5	2843	4.05
Stroke-Ready Hospitals	10	38371	54.59
Stroke-Ready Hospitals	15	62643	89.12
Stroke-Ready Hospitals	20	70203	99.87
Stroke-Ready Hospitals	25	70292	100.0
Stroke-Ready Hospitals	30	70292	100.0
Stroke-Ready Hospitals	35	70292	100.0
Stroke-Ready Hospitals	40	70292	100.0
Stroke-Ready Hospitals	45	70292	100.0
Stroke-Ready Hospitals	50	70292	100.0
Stroke-Ready Hospitals	55	70292	100.0
Stroke-Ready Hospitals	60	70292	100.0
Stroke Units	5	2843	4.05
Stroke Units	10	38371	54.59
Stroke Units	15	62636	89.11
Stroke Units	20	70200	99.87
Stroke Units	25	70292	100.0
Stroke Units	30	70292	100.0
Stroke Units	35	70292	100.0
Stroke Units	40	70292	100.0
Stroke Units	45	70292	100.0
Stroke Units	50	70292	100.0
Stroke Units	55	70292	100.0
Stroke Units	60	70292	100.0

Population Coverage - Landkreis Merzig-Wadern (Saarland)

Condition	Drive Time (min)	Covered Population	Coverage (%)
Hospitals with CT	5	8290	8.29
Hospitals with CT	10	29387	29.38
Hospitals with CT	15	52726	52.72
Hospitals with CT	20	83456	83.44
Hospitals with CT	25	99030	99.01
Hospitals with CT	30	100006	99.99
Hospitals with CT	35	100006	99.99
Hospitals with CT	40	100006	99.99
Hospitals with CT	45	100011	99.99
Hospitals with CT	50	100018	100.0
Hospitals with CT	55	99998	99.98
Hospitals with CT	60	100015	100.0
Stroke-Ready Hospitals	5	3713	3.71
Stroke-Ready Hospitals	10	20034	20.03
Stroke-Ready Hospitals	15	39810	39.8
Stroke-Ready Hospitals	20	62543	62.53
Stroke-Ready Hospitals	25	76883	76.87
Stroke-Ready Hospitals	30	82795	82.78
Stroke-Ready Hospitals	35	94307	94.29
Stroke-Ready Hospitals	40	99902	99.88
Stroke-Ready Hospitals	45	100009	99.99
Stroke-Ready Hospitals	50	100011	99.99
Stroke-Ready Hospitals	55	100002	99.98
Stroke-Ready Hospitals	60	100012	99.99
Stroke Units	5	3713	3.71
Stroke Units	10	20034	20.03
Stroke Units	15	39810	39.8
Stroke Units	20	62543	62.53
Stroke Units	25	76883	76.87
Stroke Units	30	82795	82.78
Stroke Units	35	94307	94.29
Stroke Units	40	99902	99.88
Stroke Units	45	100009	99.99
Stroke Units	50	100011	99.99
Stroke Units	55	100002	99.98
Stroke Units	60	100012	99.99

Population Coverage - Landkreis Vogtlandkreis (Sachsen)

Condition	Drive Time (min)	Covered Population	Coverage (%)
Hospitals with CT	5	43609	19.86
Hospitals with CT	10	98188	44.72
Hospitals with CT	15	150273	68.45
Hospitals with CT	20	203895	92.87
Hospitals with CT	25	214417	97.67
Hospitals with CT	30	219083	99.79
Hospitals with CT	35	219411	99.94
Hospitals with CT	40	219423	99.95
Hospitals with CT	45	219473	99.97
Hospitals with CT	50	219474	99.97
Hospitals with CT	55	219463	99.96
Hospitals with CT	60	219475	99.97
Stroke-Ready Hospitals	5	20740	9.45
Stroke-Ready Hospitals	10	73332	33.4
Stroke-Ready Hospitals	15	114419	52.12
Stroke-Ready Hospitals	20	143220	65.24
Stroke-Ready Hospitals	25	179992	81.99
Stroke-Ready Hospitals	30	190105	86.59
Stroke-Ready Hospitals	35	205771	93.73
Stroke-Ready Hospitals	40	213313	97.16
Stroke-Ready Hospitals	45	216825	98.76
Stroke-Ready Hospitals	50	218112	99.35
Stroke-Ready Hospitals	55	219136	99.82
Stroke-Ready Hospitals	60	219448	99.96
Stroke Units	5	12043	5.49
Stroke Units	10	42828	19.51
Stroke Units	15	64344	29.31
Stroke Units	20	91184	41.53
Stroke Units	25	140063	63.8
Stroke Units	30	177091	80.66
Stroke Units	35	195205	88.91
Stroke Units	40	207460	94.5
Stroke Units	45	216500	98.61
Stroke Units	50	218110	99.35
Stroke Units	55	219121	99.81
Stroke Units	60	219426	99.95

Population Coverage - Landkreis Altmarkkreis Salzwedel (Sachsen-Anhalt)

Condition	Drive Time (min)	Covered Population	Coverage (%)
Hospitals with CT	5	13086	16.94
Hospitals with CT	10	27335	35.39
Hospitals with CT	15	36679	47.49
Hospitals with CT	20	49142	63.63
Hospitals with CT	25	59666	77.26
Hospitals with CT	30	71753	92.91
Hospitals with CT	35	76269	98.75
Hospitals with CT	40	77148	99.89
Hospitals with CT	45	77231	100.0
Hospitals with CT	50	77231	100.0
Hospitals with CT	55	77231	100.0
Hospitals with CT	60	77231	100.0
Stroke-Ready Hospitals	5	0	0.0
Stroke-Ready Hospitals	10	0	0.0
Stroke-Ready Hospitals	15	0	0.0
Stroke-Ready Hospitals	20	2	0.0
Stroke-Ready Hospitals	25	584	0.76
Stroke-Ready Hospitals	30	2359	3.06
Stroke-Ready Hospitals	35	5142	6.66
Stroke-Ready Hospitals	40	18359	23.77
Stroke-Ready Hospitals	45	25369	32.85
Stroke-Ready Hospitals	50	35626	46.13
Stroke-Ready Hospitals	55	53031	68.67
Stroke-Ready Hospitals	60	65915	85.35
Stroke Units	5	0	0.0
Stroke Units	10	0	0.0
Stroke Units	15	0	0.0
Stroke Units	20	0	0.0
Stroke Units	25	0	0.0
Stroke Units	30	0	0.0
Stroke Units	35	116	0.15
Stroke Units	40	2966	3.84
Stroke Units	45	10041	13.0
Stroke Units	50	19691	25.5
Stroke Units	55	42977	55.65
Stroke Units	60	55391	71.72

Population Coverage - Kreis Segeberg (Schleswig-Holstein)

Condition	Drive Time (min)	Covered Population	Coverage (%)
Hospitals with CT	5	56063	20.47
Hospitals with CT	10	132553	48.39
Hospitals with CT	15	196345	71.68
Hospitals with CT	20	265703	97.0
Hospitals with CT	25	273861	99.98
Hospitals with CT	30	273911	100.0
Hospitals with CT	35	273911	100.0
Hospitals with CT	40	273911	100.0
Hospitals with CT	45	273911	100.0
Hospitals with CT	50	273911	100.0
Hospitals with CT	55	273911	100.0
Hospitals with CT	60	273911	100.0
Stroke-Ready Hospitals	5	15966	5.83
Stroke-Ready Hospitals	10	47456	17.33
Stroke-Ready Hospitals	15	118718	43.34
Stroke-Ready Hospitals	20	177288	64.72
Stroke-Ready Hospitals	25	256263	93.56
Stroke-Ready Hospitals	30	273209	99.74
Stroke-Ready Hospitals	35	273911	100.0
Stroke-Ready Hospitals	40	273911	100.0
Stroke-Ready Hospitals	45	273911	100.0
Stroke-Ready Hospitals	50	273911	100.0
Stroke-Ready Hospitals	55	273911	100.0
Stroke-Ready Hospitals	60	273911	100.0
Stroke Units	5	15966	5.83
Stroke Units	10	47456	17.33
Stroke Units	15	118718	43.34
Stroke Units	20	177288	64.72
Stroke Units	25	256263	93.56
Stroke Units	30	273209	99.74
Stroke Units	35	273911	100.0
Stroke Units	40	273911	100.0
Stroke Units	45	273911	100.0
Stroke Units	50	273911	100.0
Stroke Units	55	273911	100.0
Stroke Units	60	273911	100.0

Population Coverage - Landkreis Osnabrück (Niedersachsen)

Condition	Drive Time (min)	Covered Population	Coverage (%)
Hospitals with CT	5	42421	12.01
Hospitals with CT	10	136313	38.6
Hospitals with CT	15	253583	71.8
Hospitals with CT	20	323394	91.57
Hospitals with CT	25	348594	98.71
Hospitals with CT	30	353117	99.99
Hospitals with CT	35	353157	100.0
Hospitals with CT	40	353157	100.0
Hospitals with CT	45	353157	100.0
Hospitals with CT	50	353157	100.0
Hospitals with CT	55	353157	100.0
Hospitals with CT	60	353157	100.0
Stroke-Ready Hospitals	5	3570	1.01
Stroke-Ready Hospitals	10	16530	4.68
Stroke-Ready Hospitals	15	60394	17.1
Stroke-Ready Hospitals	20	151531	42.91
Stroke-Ready Hospitals	25	257609	72.94
Stroke-Ready Hospitals	30	317365	89.87
Stroke-Ready Hospitals	35	337155	95.47
Stroke-Ready Hospitals	40	347971	98.53
Stroke-Ready Hospitals	45	352914	99.93
Stroke-Ready Hospitals	50	353157	100.0
Stroke-Ready Hospitals	55	353157	100.0
Stroke-Ready Hospitals	60	353157	100.0
Stroke Units	5	3570	1.01
Stroke Units	10	16530	4.68
Stroke Units	15	60381	17.1
Stroke Units	20	146820	41.57
Stroke Units	25	241950	68.51
Stroke Units	30	304378	86.19
Stroke Units	35	332910	94.27
Stroke Units	40	347772	98.48
Stroke Units	45	352914	99.93
Stroke Units	50	353157	100.0
Stroke Units	55	353157	100.0
Stroke Units	60	353157	100.0

Population Coverage - Kreisfreie Stadt Oberhausen (Nordrhein-Westfalen)

Condition	Drive Time (min)	Covered Population	Coverage (%)
Hospitals with CT	5	101202	47.03
Hospitals with CT	10	211980	98.52
Hospitals with CT	15	215167	100.0
Hospitals with CT	20	215167	100.0
Hospitals with CT	25	215167	100.0
Hospitals with CT	30	215167	100.0
Hospitals with CT	35	215167	100.0
Hospitals with CT	40	215167	100.0
Hospitals with CT	45	215167	100.0
Hospitals with CT	50	215167	100.0
Hospitals with CT	55	215167	100.0
Hospitals with CT	60	215167	100.0
Stroke-Ready Hospitals	5	32406	15.06
Stroke-Ready Hospitals	10	128755	59.84
Stroke-Ready Hospitals	15	194663	90.47
Stroke-Ready Hospitals	20	215167	100.0
Stroke-Ready Hospitals	25	215167	100.0
Stroke-Ready Hospitals	30	215167	100.0
Stroke-Ready Hospitals	35	215167	100.0
Stroke-Ready Hospitals	40	215167	100.0
Stroke-Ready Hospitals	45	215167	100.0
Stroke-Ready Hospitals	50	215167	100.0
Stroke-Ready Hospitals	55	215167	100.0
Stroke-Ready Hospitals	60	215167	100.0
Stroke Units	5	29509	13.71
Stroke Units	10	121052	56.26
Stroke Units	15	193303	89.84
Stroke Units	20	215167	100.0
Stroke Units	25	215167	100.0
Stroke Units	30	215167	100.0
Stroke Units	35	215167	100.0
Stroke Units	40	215167	100.0
Stroke Units	45	215167	100.0
Stroke Units	50	215167	100.0
Stroke Units	55	215167	100.0
Stroke Units	60	215167	100.0

Population Coverage - Kreisfreie Stadt Remscheid (Nordrhein-Westfalen)

Condition	Drive Time (min)	Covered Population	Coverage (%)
Hospitals with CT	5	39262	36.19
Hospitals with CT	10	85638	78.94
Hospitals with CT	15	106284	97.97
Hospitals with CT	20	108490	100.0
Hospitals with CT	25	108490	100.0
Hospitals with CT	30	108490	100.0
Hospitals with CT	35	108490	100.0
Hospitals with CT	40	108490	100.0
Hospitals with CT	45	108490	100.0
Hospitals with CT	50	108490	100.0
Hospitals with CT	55	108490	100.0
Hospitals with CT	60	108490	100.0
Stroke-Ready Hospitals	5	8059	7.43
Stroke-Ready Hospitals	10	28689	26.44
Stroke-Ready Hospitals	15	68062	62.74
Stroke-Ready Hospitals	20	100230	92.39
Stroke-Ready Hospitals	25	108409	99.92
Stroke-Ready Hospitals	30	108490	100.0
Stroke-Ready Hospitals	35	108490	100.0
Stroke-Ready Hospitals	40	108490	100.0
Stroke-Ready Hospitals	45	108490	100.0
Stroke-Ready Hospitals	50	108490	100.0
Stroke-Ready Hospitals	55	108490	100.0
Stroke-Ready Hospitals	60	108490	100.0
Stroke Units	5	8059	7.43
Stroke Units	10	28689	26.44
Stroke Units	15	68044	62.72
Stroke Units	20	100223	92.38
Stroke Units	25	108409	99.92
Stroke Units	30	108490	100.0
Stroke Units	35	108490	100.0
Stroke Units	40	108490	100.0
Stroke Units	45	108490	100.0
Stroke Units	50	108490	100.0
Stroke Units	55	108490	100.0
Stroke Units	60	108490	100.0

Population Coverage - Kreis Mettmann (Nordrhein-Westfalen)

Condition	Drive Time (min)	Covered Population	Coverage (%)
Hospitals with CT	5	77909	16.45
Hospitals with CT	10	240849	50.84
Hospitals with CT	15	444170	93.76
Hospitals with CT	20	473526	99.96
Hospitals with CT	25	473726	100.0
Hospitals with CT	30	473726	100.0
Hospitals with CT	35	473726	100.0
Hospitals with CT	40	473726	100.0
Hospitals with CT	45	473726	100.0
Hospitals with CT	50	473726	100.0
Hospitals with CT	55	473726	100.0
Hospitals with CT	60	473726	100.0
Stroke-Ready Hospitals	5	0	0.0
Stroke-Ready Hospitals	10	17349	3.66
Stroke-Ready Hospitals	15	198775	41.96
Stroke-Ready Hospitals	20	410580	86.67
Stroke-Ready Hospitals	25	473420	99.94
Stroke-Ready Hospitals	30	473726	100.0
Stroke-Ready Hospitals	35	473726	100.0
Stroke-Ready Hospitals	40	473726	100.0
Stroke-Ready Hospitals	45	473726	100.0
Stroke-Ready Hospitals	50	473726	100.0
Stroke-Ready Hospitals	55	473726	100.0
Stroke-Ready Hospitals	60	473726	100.0
Stroke Units	5	0	0.0
Stroke Units	10	17018	3.59
Stroke Units	15	187051	39.49
Stroke Units	20	394561	83.29
Stroke Units	25	473420	99.94
Stroke Units	30	473726	100.0
Stroke Units	35	473726	100.0
Stroke Units	40	473726	100.0
Stroke Units	45	473726	100.0
Stroke Units	50	473726	100.0
Stroke Units	55	473726	100.0
Stroke Units	60	473726	100.0

Population Coverage - Kreis Rhein-Sieg-Kreis (Nordrhein-Westfalen)

Condition	Drive Time (min)	Covered Population	Coverage (%)
Hospitals with CT	5	97433	16.7
Hospitals with CT	10	249497	42.78
Hospitals with CT	15	400197	68.61
Hospitals with CT	20	523330	89.72
Hospitals with CT	25	574200	98.44
Hospitals with CT	30	583274	100.0
Hospitals with CT	35	583276	100.0
Hospitals with CT	40	583276	100.0
Hospitals with CT	45	583276	100.0
Hospitals with CT	50	583276	100.0
Hospitals with CT	55	583276	100.0
Hospitals with CT	60	583276	100.0
Stroke-Ready Hospitals	5	14061	2.41
Stroke-Ready Hospitals	10	75237	12.9
Stroke-Ready Hospitals	15	235241	40.33
Stroke-Ready Hospitals	20	411514	70.55
Stroke-Ready Hospitals	25	531058	91.05
Stroke-Ready Hospitals	30	555875	95.3
Stroke-Ready Hospitals	35	569480	97.63
Stroke-Ready Hospitals	40	579347	99.33
Stroke-Ready Hospitals	45	583276	100.0
Stroke-Ready Hospitals	50	583276	100.0
Stroke-Ready Hospitals	55	583276	100.0
Stroke-Ready Hospitals	60	583276	100.0
Stroke Units	5	14061	2.41
Stroke Units	10	75237	12.9
Stroke Units	15	235241	40.33
Stroke Units	20	407630	69.89
Stroke Units	25	507790	87.06
Stroke Units	30	539490	92.49
Stroke Units	35	557269	95.54
Stroke Units	40	575973	98.75
Stroke Units	45	583142	99.98
Stroke Units	50	583276	100.0
Stroke Units	55	583276	100.0
Stroke Units	60	583276	100.0

Population Coverage - Kreis Borken (Nordrhein-Westfalen)

Condition	Drive Time (min)	Covered Population	Coverage (%)
Hospitals with CT	5	57089	15.43
Hospitals with CT	10	161117	43.54
Hospitals with CT	15	270870	73.2
Hospitals with CT	20	346684	93.69
Hospitals with CT	25	368551	99.6
Hospitals with CT	30	369557	99.87
Hospitals with CT	35	369642	99.9
Hospitals with CT	40	369730	99.92
Hospitals with CT	45	369851	99.95
Hospitals with CT	50	369868	99.96
Hospitals with CT	55	369731	99.92
Hospitals with CT	60	369792	99.94
Stroke-Ready Hospitals	5	17100	4.62
Stroke-Ready Hospitals	10	37392	10.11
Stroke-Ready Hospitals	15	70686	19.1
Stroke-Ready Hospitals	20	164130	44.36
Stroke-Ready Hospitals	25	242715	65.59
Stroke-Ready Hospitals	30	299535	80.95
Stroke-Ready Hospitals	35	346843	93.74
Stroke-Ready Hospitals	40	368410	99.56
Stroke-Ready Hospitals	45	369603	99.89
Stroke-Ready Hospitals	50	369658	99.9
Stroke-Ready Hospitals	55	369607	99.89
Stroke-Ready Hospitals	60	369708	99.92
Stroke Units	5	17100	4.62
Stroke Units	10	37392	10.11
Stroke Units	15	69421	18.76
Stroke Units	20	142596	38.54
Stroke Units	25	219134	59.22
Stroke Units	30	267200	72.21
Stroke Units	35	330379	89.29
Stroke Units	40	366943	99.17
Stroke Units	45	369446	99.84
Stroke Units	50	369596	99.89
Stroke Units	55	369599	99.89
Stroke Units	60	369705	99.91

Population Coverage - Landkreis Vulkaneifel (Rheinland-Pfalz)

Condition	Drive Time (min)	Covered Population	Coverage (%)
Hospitals with CT	5	5790	9.21
Hospitals with CT	10	18422	29.31
Hospitals with CT	15	35234	56.06
Hospitals with CT	20	50781	80.79
Hospitals with CT	25	60951	96.97
Hospitals with CT	30	62826	99.95
Hospitals with CT	35	62855	100.0
Hospitals with CT	40	62855	100.0
Hospitals with CT	45	62855	100.0
Hospitals with CT	50	62855	100.0
Hospitals with CT	55	62855	100.0
Hospitals with CT	60	62855	100.0
Stroke-Ready Hospitals	5	0	0.0
Stroke-Ready Hospitals	10	0	0.0
Stroke-Ready Hospitals	15	99	0.16
Stroke-Ready Hospitals	20	5391	8.58
Stroke-Ready Hospitals	25	13000	20.68
Stroke-Ready Hospitals	30	24183	38.48
Stroke-Ready Hospitals	35	32373	51.5
Stroke-Ready Hospitals	40	38955	61.98
Stroke-Ready Hospitals	45	50406	80.19
Stroke-Ready Hospitals	50	57807	91.97
Stroke-Ready Hospitals	55	62692	99.74
Stroke-Ready Hospitals	60	62855	100.0
Stroke Units	5	0	0.0
Stroke Units	10	0	0.0
Stroke Units	15	99	0.16
Stroke Units	20	5391	8.58
Stroke Units	25	13000	20.68
Stroke Units	30	24183	38.48
Stroke Units	35	32373	51.5
Stroke Units	40	38955	61.98
Stroke Units	45	50406	80.19
Stroke Units	50	57807	91.97
Stroke Units	55	62692	99.74
Stroke Units	60	62855	100.0

Population Coverage - Landkreis Kaiserslautern (Rheinland-Pfalz)

Condition	Drive Time (min)	Covered Population	Coverage (%)
Hospitals with CT	5	4392	4.11
Hospitals with CT	10	15061	14.09
Hospitals with CT	15	43239	40.45
Hospitals with CT	20	89220	83.46
Hospitals with CT	25	105706	98.88
Hospitals with CT	30	106907	100.0
Hospitals with CT	35	106907	100.0
Hospitals with CT	40	106907	100.0
Hospitals with CT	45	106907	100.0
Hospitals with CT	50	106907	100.0
Hospitals with CT	55	106907	100.0
Hospitals with CT	60	106907	100.0
Stroke-Ready Hospitals	5	0	0.0
Stroke-Ready Hospitals	10	2	0.0
Stroke-Ready Hospitals	15	5010	4.69
Stroke-Ready Hospitals	20	46645	43.63
Stroke-Ready Hospitals	25	96244	90.03
Stroke-Ready Hospitals	30	106695	99.8
Stroke-Ready Hospitals	35	106907	100.0
Stroke-Ready Hospitals	40	106907	100.0
Stroke-Ready Hospitals	45	106907	100.0
Stroke-Ready Hospitals	50	106907	100.0
Stroke-Ready Hospitals	55	106907	100.0
Stroke-Ready Hospitals	60	106907	100.0
Stroke Units	5	0	0.0
Stroke Units	10	2	0.0
Stroke Units	15	5010	4.69
Stroke Units	20	46645	43.63
Stroke Units	25	95959	89.76
Stroke Units	30	105578	98.76
Stroke Units	35	106894	99.99
Stroke Units	40	106907	100.0
Stroke Units	45	106907	100.0
Stroke Units	50	106907	100.0
Stroke Units	55	106907	100.0
Stroke Units	60	106907	100.0

Population Coverage - Landkreis Calw (Baden-Württemberg)

Condition	Drive Time (min)	Covered Population	Coverage (%)
Hospitals with CT	5	9234	6.09
Hospitals with CT	10	40417	26.66
Hospitals with CT	15	84049	55.43
Hospitals with CT	20	122466	80.77
Hospitals with CT	25	140934	92.95
Hospitals with CT	30	149722	98.74
Hospitals with CT	35	151381	99.84
Hospitals with CT	40	151448	99.88
Hospitals with CT	45	151629	100.0
Hospitals with CT	50	151629	100.0
Hospitals with CT	55	151629	100.0
Hospitals with CT	60	151629	100.0
Stroke-Ready Hospitals	5	5811	3.83
Stroke-Ready Hospitals	10	21547	14.21
Stroke-Ready Hospitals	15	40755	26.88
Stroke-Ready Hospitals	20	65267	43.04
Stroke-Ready Hospitals	25	88757	58.54
Stroke-Ready Hospitals	30	111728	73.69
Stroke-Ready Hospitals	35	144607	95.37
Stroke-Ready Hospitals	40	151334	99.81
Stroke-Ready Hospitals	45	151422	99.86
Stroke-Ready Hospitals	50	151594	99.98
Stroke-Ready Hospitals	55	151629	100.0
Stroke-Ready Hospitals	60	151629	100.0
Stroke Units	5	0	0.0
Stroke Units	10	0	0.0
Stroke Units	15	0	0.0
Stroke Units	20	4415	2.91
Stroke Units	25	10455	6.9
Stroke Units	30	31060	20.48
Stroke Units	35	65604	43.27
Stroke Units	40	98479	64.95
Stroke Units	45	121409	80.07
Stroke Units	50	138733	91.5
Stroke Units	55	146654	96.72
Stroke Units	60	151495	99.91

Population Coverage - Kreisfreie Stadt Berlin (Berlin)

Condition	Drive Time (min)	Covered Population	Coverage (%)
Hospitals with CT	5	1421007	39.16
Hospitals with CT	10	3195363	88.06
Hospitals with CT	15	3591907	98.99
Hospitals with CT	20	3626479	99.94
Hospitals with CT	25	3628199	99.99
Hospitals with CT	30	3628346	99.99
Hospitals with CT	35	3628698	100.0
Hospitals with CT	40	3628698	100.0
Hospitals with CT	45	3628698	100.0
Hospitals with CT	50	3628698	100.0
Hospitals with CT	55	3628698	100.0
Hospitals with CT	60	3628698	100.0
Stroke-Ready Hospitals	5	635988	17.53
Stroke-Ready Hospitals	10	2119364	58.41
Stroke-Ready Hospitals	15	3280906	90.42
Stroke-Ready Hospitals	20	3590073	98.94
Stroke-Ready Hospitals	25	3620475	99.77
Stroke-Ready Hospitals	30	3627325	99.96
Stroke-Ready Hospitals	35	3628698	100.0
Stroke-Ready Hospitals	40	3628698	100.0
Stroke-Ready Hospitals	45	3628698	100.0
Stroke-Ready Hospitals	50	3628698	100.0
Stroke-Ready Hospitals	55	3628698	100.0
Stroke-Ready Hospitals	60	3628698	100.0
Stroke Units	5	596674	16.44
Stroke Units	10	2102248	57.93
Stroke Units	15	3270671	90.13
Stroke Units	20	3590040	98.93
Stroke Units	25	3620475	99.77
Stroke Units	30	3627251	99.96
Stroke Units	35	3628698	100.0
Stroke Units	40	3628698	100.0
Stroke Units	45	3628698	100.0
Stroke Units	50	3628698	100.0
Stroke Units	55	3628698	100.0
Stroke Units	60	3628698	100.0

Population Coverage - Kreis Stormarn (Schleswig-Holstein)

Condition	Drive Time (min)	Covered Population	Coverage (%)
Hospitals with CT	5	27489	11.27
Hospitals with CT	10	85787	35.16
Hospitals with CT	15	186929	76.62
Hospitals with CT	20	233069	95.53
Hospitals with CT	25	243017	99.61
Hospitals with CT	30	243553	99.83
Hospitals with CT	35	243968	100.0
Hospitals with CT	40	243968	100.0
Hospitals with CT	45	243968	100.0
Hospitals with CT	50	243968	100.0
Hospitals with CT	55	243968	100.0
Hospitals with CT	60	243968	100.0
Stroke-Ready Hospitals	5	0	0.0
Stroke-Ready Hospitals	10	1951	0.8
Stroke-Ready Hospitals	15	33461	13.72
Stroke-Ready Hospitals	20	95738	39.24
Stroke-Ready Hospitals	25	165128	67.68
Stroke-Ready Hospitals	30	232034	95.11
Stroke-Ready Hospitals	35	243742	99.91
Stroke-Ready Hospitals	40	243968	100.0
Stroke-Ready Hospitals	45	243968	100.0
Stroke-Ready Hospitals	50	243968	100.0
Stroke-Ready Hospitals	55	243968	100.0
Stroke-Ready Hospitals	60	243968	100.0
Stroke Units	5	0	0.0
Stroke Units	10	1951	0.8
Stroke Units	15	33461	13.72
Stroke Units	20	95738	39.24
Stroke Units	25	165128	67.68
Stroke Units	30	232034	95.11
Stroke Units	35	243742	99.91
Stroke Units	40	243968	100.0
Stroke Units	45	243968	100.0
Stroke Units	50	243968	100.0
Stroke Units	55	243968	100.0
Stroke Units	60	243968	100.0

Population Coverage - Landkreis Hildesheim (Niedersachsen)

Condition	Drive Time (min)	Covered Population	Coverage (%)
Hospitals with CT	5	44791	15.25
Hospitals with CT	10	130977	44.59
Hospitals with CT	15	208625	71.03
Hospitals with CT	20	266757	90.82
Hospitals with CT	25	289243	98.48
Hospitals with CT	30	293642	99.98
Hospitals with CT	35	293715	100.0
Hospitals with CT	40	293715	100.0
Hospitals with CT	45	293715	100.0
Hospitals with CT	50	293715	100.0
Hospitals with CT	55	293715	100.0
Hospitals with CT	60	293715	100.0
Stroke-Ready Hospitals	5	20834	7.09
Stroke-Ready Hospitals	10	81843	27.86
Stroke-Ready Hospitals	15	147830	50.33
Stroke-Ready Hospitals	20	193384	65.84
Stroke-Ready Hospitals	25	224201	76.33
Stroke-Ready Hospitals	30	248191	84.5
Stroke-Ready Hospitals	35	265590	90.42
Stroke-Ready Hospitals	40	272516	92.78
Stroke-Ready Hospitals	45	290287	98.83
Stroke-Ready Hospitals	50	293673	99.99
Stroke-Ready Hospitals	55	293715	100.0
Stroke-Ready Hospitals	60	293715	100.0
Stroke Units	5	20834	7.09
Stroke Units	10	81843	27.86
Stroke Units	15	143686	48.92
Stroke Units	20	182331	62.08
Stroke Units	25	215724	73.45
Stroke Units	30	246567	83.95
Stroke Units	35	262619	89.41
Stroke Units	40	271475	92.43
Stroke Units	45	288567	98.25
Stroke Units	50	293663	99.98
Stroke Units	55	293713	100.0
Stroke Units	60	293715	100.0

Population Coverage - Kreis Viersen (Nordrhein-Westfalen)

Condition	Drive Time (min)	Covered Population	Coverage (%)
Hospitals with CT	5	77297	26.11
Hospitals with CT	10	153934	51.99
Hospitals with CT	15	269446	91.01
Hospitals with CT	20	291608	98.5
Hospitals with CT	25	294144	99.35
Hospitals with CT	30	294215	99.38
Hospitals with CT	35	294216	99.38
Hospitals with CT	40	294236	99.38
Hospitals with CT	45	294242	99.39
Hospitals with CT	50	294243	99.39
Hospitals with CT	55	294236	99.38
Hospitals with CT	60	294241	99.39
Stroke-Ready Hospitals	5	18861	6.37
Stroke-Ready Hospitals	10	77581	26.2
Stroke-Ready Hospitals	15	209162	70.65
Stroke-Ready Hospitals	20	269830	91.14
Stroke-Ready Hospitals	25	293855	99.26
Stroke-Ready Hospitals	30	294134	99.35
Stroke-Ready Hospitals	35	294209	99.38
Stroke-Ready Hospitals	40	294218	99.38
Stroke-Ready Hospitals	45	294219	99.38
Stroke-Ready Hospitals	50	294236	99.38
Stroke-Ready Hospitals	55	294222	99.38
Stroke-Ready Hospitals	60	294237	99.38
Stroke Units	5	18861	6.37
Stroke Units	10	77581	26.2
Stroke Units	15	209162	70.65
Stroke Units	20	269830	91.14
Stroke Units	25	293855	99.26
Stroke Units	30	294134	99.35
Stroke Units	35	294209	99.38
Stroke Units	40	294218	99.38
Stroke Units	45	294219	99.38
Stroke Units	50	294231	99.38
Stroke Units	55	294222	99.38
Stroke Units	60	294237	99.38

Population Coverage - Kreis Rheinisch-Bergischer Kreis (Nordrhein-Westfalen)

Condition	Drive Time (min)	Covered Population	Coverage (%)
Hospitals with CT	5	65425	21.71
Hospitals with CT	10	183584	60.92
Hospitals with CT	15	252430	83.76
Hospitals with CT	20	300854	99.83
Hospitals with CT	25	301279	99.97
Hospitals with CT	30	301372	100.0
Hospitals with CT	35	301372	100.0
Hospitals with CT	40	301372	100.0
Hospitals with CT	45	301372	100.0
Hospitals with CT	50	301372	100.0
Hospitals with CT	55	301372	100.0
Hospitals with CT	60	301372	100.0
Stroke-Ready Hospitals	5	34266	11.37
Stroke-Ready Hospitals	10	135766	45.05
Stroke-Ready Hospitals	15	206087	68.38
Stroke-Ready Hospitals	20	281010	93.24
Stroke-Ready Hospitals	25	298967	99.2
Stroke-Ready Hospitals	30	301282	99.97
Stroke-Ready Hospitals	35	301372	100.0
Stroke-Ready Hospitals	40	301372	100.0
Stroke-Ready Hospitals	45	301372	100.0
Stroke-Ready Hospitals	50	301372	100.0
Stroke-Ready Hospitals	55	301372	100.0
Stroke-Ready Hospitals	60	301372	100.0
Stroke Units	5	27337	9.07
Stroke Units	10	86589	28.73
Stroke Units	15	172016	57.08
Stroke Units	20	233463	77.47
Stroke Units	25	282245	93.65
Stroke Units	30	299629	99.42
Stroke Units	35	301258	99.96
Stroke Units	40	301372	100.0
Stroke Units	45	301372	100.0
Stroke Units	50	301372	100.0
Stroke Units	55	301372	100.0
Stroke Units	60	301372	100.0

Population Coverage - Kreisfreie Stadt Zweibrücken (Rheinland-Pfalz)

Condition	Drive Time (min)	Covered Population	Coverage (%)
Hospitals with CT	5	22837	70.04
Hospitals with CT	10	31663	97.1
Hospitals with CT	15	32607	100.0
Hospitals with CT	20	32607	100.0
Hospitals with CT	25	32607	100.0
Hospitals with CT	30	32607	100.0
Hospitals with CT	35	32607	100.0
Hospitals with CT	40	32607	100.0
Hospitals with CT	45	32607	100.0
Hospitals with CT	50	32607	100.0
Hospitals with CT	55	32607	100.0
Hospitals with CT	60	32607	100.0
Stroke-Ready Hospitals	5	0	0.0
Stroke-Ready Hospitals	10	363	1.11
Stroke-Ready Hospitals	15	9587	29.4
Stroke-Ready Hospitals	20	30339	93.04
Stroke-Ready Hospitals	25	32569	99.88
Stroke-Ready Hospitals	30	32607	100.0
Stroke-Ready Hospitals	35	32607	100.0
Stroke-Ready Hospitals	40	32607	100.0
Stroke-Ready Hospitals	45	32607	100.0
Stroke-Ready Hospitals	50	32607	100.0
Stroke-Ready Hospitals	55	32607	100.0
Stroke-Ready Hospitals	60	32607	100.0
Stroke Units	5	0	0.0
Stroke Units	10	363	1.11
Stroke Units	15	9587	29.4
Stroke Units	20	29573	90.69
Stroke Units	25	32534	99.77
Stroke Units	30	32607	100.0
Stroke Units	35	32607	100.0
Stroke Units	40	32607	100.0
Stroke Units	45	32607	100.0
Stroke Units	50	32607	100.0
Stroke Units	55	32607	100.0
Stroke Units	60	32607	100.0

Population Coverage - Landkreis Rhein-Pfalz-Kreis (Rheinland-Pfalz)

Condition	Drive Time (min)	Covered Population	Coverage (%)
Hospitals with CT	5	2860	1.88
Hospitals with CT	10	50142	32.88
Hospitals with CT	15	139684	91.6
Hospitals with CT	20	152270	99.85
Hospitals with CT	25	152495	100.0
Hospitals with CT	30	152495	100.0
Hospitals with CT	35	152495	100.0
Hospitals with CT	40	152495	100.0
Hospitals with CT	45	152495	100.0
Hospitals with CT	50	152495	100.0
Hospitals with CT	55	152495	100.0
Hospitals with CT	60	152495	100.0
Stroke-Ready Hospitals	5	2444	1.6
Stroke-Ready Hospitals	10	21532	14.12
Stroke-Ready Hospitals	15	86360	56.63
Stroke-Ready Hospitals	20	150863	98.93
Stroke-Ready Hospitals	25	152495	100.0
Stroke-Ready Hospitals	30	152495	100.0
Stroke-Ready Hospitals	35	152495	100.0
Stroke-Ready Hospitals	40	152495	100.0
Stroke-Ready Hospitals	45	152495	100.0
Stroke-Ready Hospitals	50	152495	100.0
Stroke-Ready Hospitals	55	152495	100.0
Stroke-Ready Hospitals	60	152495	100.0
Stroke Units	5	0	0.0
Stroke Units	10	11635	7.63
Stroke Units	15	60477	39.66
Stroke Units	20	139899	91.74
Stroke Units	25	152489	100.0
Stroke Units	30	152495	100.0
Stroke Units	35	152495	100.0
Stroke Units	40	152495	100.0
Stroke Units	45	152495	100.0
Stroke Units	50	152495	100.0
Stroke Units	55	152495	100.0
Stroke Units	60	152495	100.0

Population Coverage - Landkreis Altötting (Bayern)

Condition	Drive Time (min)	Covered Population	Coverage (%)
Hospitals with CT	5	14293	12.82
Hospitals with CT	10	29241	26.23
Hospitals with CT	15	61037	54.76
Hospitals with CT	20	94099	84.41
Hospitals with CT	25	106500	95.54
Hospitals with CT	30	106804	95.81
Hospitals with CT	35	106909	95.91
Hospitals with CT	40	107268	96.23
Hospitals with CT	45	108646	97.46
Hospitals with CT	50	108775	97.58
Hospitals with CT	55	108695	97.51
Hospitals with CT	60	108786	97.59
Stroke-Ready Hospitals	5	15448	13.86
Stroke-Ready Hospitals	10	27697	24.85
Stroke-Ready Hospitals	15	60957	54.68
Stroke-Ready Hospitals	20	91573	82.15
Stroke-Ready Hospitals	25	104284	93.55
Stroke-Ready Hospitals	30	106670	95.69
Stroke-Ready Hospitals	35	106768	95.78
Stroke-Ready Hospitals	40	106802	95.81
Stroke-Ready Hospitals	45	106882	95.88
Stroke-Ready Hospitals	50	106884	95.88
Stroke-Ready Hospitals	55	106781	95.79
Stroke-Ready Hospitals	60	108468	97.3
Stroke Units	5	15448	13.86
Stroke Units	10	27697	24.85
Stroke Units	15	60957	54.68
Stroke Units	20	91573	82.15
Stroke Units	25	104284	93.55
Stroke Units	30	106670	95.69
Stroke Units	35	106768	95.78
Stroke Units	40	106802	95.81
Stroke Units	45	106820	95.83
Stroke Units	50	106826	95.83
Stroke Units	55	106781	95.79
Stroke Units	60	108468	97.3

Population Coverage - Landkreis Pfaffenhofen a.d.Ilm (Bayern)

Condition	Drive Time (min)	Covered Population	Coverage (%)
Hospitals with CT	5	10327	8.34
Hospitals with CT	10	31681	25.58
Hospitals with CT	15	67983	54.88
Hospitals with CT	20	119836	96.74
Hospitals with CT	25	123875	100.0
Hospitals with CT	30	123875	100.0
Hospitals with CT	35	123875	100.0
Hospitals with CT	40	123875	100.0
Hospitals with CT	45	123875	100.0
Hospitals with CT	50	123875	100.0
Hospitals with CT	55	123875	100.0
Hospitals with CT	60	123875	100.0
Stroke-Ready Hospitals	5	9605	7.75
Stroke-Ready Hospitals	10	30221	24.4
Stroke-Ready Hospitals	15	43448	35.07
Stroke-Ready Hospitals	20	70361	56.8
Stroke-Ready Hospitals	25	91395	73.78
Stroke-Ready Hospitals	30	111677	90.15
Stroke-Ready Hospitals	35	121837	98.36
Stroke-Ready Hospitals	40	123875	100.0
Stroke-Ready Hospitals	45	123875	100.0
Stroke-Ready Hospitals	50	123875	100.0
Stroke-Ready Hospitals	55	123875	100.0
Stroke-Ready Hospitals	60	123875	100.0
Stroke Units	5	9605	7.75
Stroke Units	10	30221	24.4
Stroke Units	15	43448	35.07
Stroke Units	20	70361	56.8
Stroke Units	25	91395	73.78
Stroke Units	30	111677	90.15
Stroke Units	35	121751	98.29
Stroke Units	40	123875	100.0
Stroke Units	45	123875	100.0
Stroke Units	50	123875	100.0
Stroke Units	55	123875	100.0
Stroke Units	60	123875	100.0

Population Coverage - Kreisfreie Stadt Bamberg (Bayern)

Condition	Drive Time (min)	Covered Population	Coverage (%)
Hospitals with CT	5	6117	7.86
Hospitals with CT	10	35888	46.12
Hospitals with CT	15	76830	98.72
Hospitals with CT	20	77784	99.95
Hospitals with CT	25	77824	100.0
Hospitals with CT	30	77824	100.0
Hospitals with CT	35	77824	100.0
Hospitals with CT	40	77824	100.0
Hospitals with CT	45	77824	100.0
Hospitals with CT	50	77824	100.0
Hospitals with CT	55	77824	100.0
Hospitals with CT	60	77824	100.0
Stroke-Ready Hospitals	5	2985	3.84
Stroke-Ready Hospitals	10	23397	30.06
Stroke-Ready Hospitals	15	53888	69.24
Stroke-Ready Hospitals	20	72177	92.74
Stroke-Ready Hospitals	25	77719	99.87
Stroke-Ready Hospitals	30	77824	100.0
Stroke-Ready Hospitals	35	77824	100.0
Stroke-Ready Hospitals	40	77824	100.0
Stroke-Ready Hospitals	45	77824	100.0
Stroke-Ready Hospitals	50	77824	100.0
Stroke-Ready Hospitals	55	77824	100.0
Stroke-Ready Hospitals	60	77824	100.0
Stroke Units	5	2985	3.84
Stroke Units	10	23397	30.06
Stroke Units	15	53888	69.24
Stroke Units	20	72177	92.74
Stroke Units	25	77719	99.87
Stroke Units	30	77824	100.0
Stroke Units	35	77824	100.0
Stroke Units	40	77824	100.0
Stroke Units	45	77824	100.0
Stroke Units	50	77824	100.0
Stroke Units	55	77824	100.0
Stroke Units	60	77824	100.0

Population Coverage - Landkreis Hof (Bayern)

Condition	Drive Time (min)	Covered Population	Coverage (%)
Hospitals with CT	5	22678	22.59
Hospitals with CT	10	50542	50.34
Hospitals with CT	15	88140	87.79
Hospitals with CT	20	98764	98.37
Hospitals with CT	25	100353	99.95
Hospitals with CT	30	100363	99.96
Hospitals with CT	35	100363	99.96
Hospitals with CT	40	100386	99.98
Hospitals with CT	45	100386	99.98
Hospitals with CT	50	100387	99.98
Hospitals with CT	55	100363	99.96
Hospitals with CT	60	100386	99.98
Stroke-Ready Hospitals	5	8952	8.92
Stroke-Ready Hospitals	10	26897	26.79
Stroke-Ready Hospitals	15	60908	60.66
Stroke-Ready Hospitals	20	80351	80.03
Stroke-Ready Hospitals	25	89194	88.84
Stroke-Ready Hospitals	30	96090	95.7
Stroke-Ready Hospitals	35	99736	99.34
Stroke-Ready Hospitals	40	100363	99.96
Stroke-Ready Hospitals	45	100363	99.96
Stroke-Ready Hospitals	50	100363	99.96
Stroke-Ready Hospitals	55	100357	99.95
Stroke-Ready Hospitals	60	100381	99.98
Stroke Units	5	8952	8.92
Stroke Units	10	26897	26.79
Stroke Units	15	60908	60.66
Stroke Units	20	80351	80.03
Stroke Units	25	89194	88.84
Stroke Units	30	96090	95.7
Stroke Units	35	99736	99.34
Stroke Units	40	100363	99.96
Stroke Units	45	100363	99.96
Stroke Units	50	100363	99.96
Stroke Units	55	100357	99.95
Stroke Units	60	100381	99.98

Population Coverage - Landkreis Miltenberg (Bayern)

Condition	Drive Time (min)	Covered Population	Coverage (%)
Hospitals with CT	5	7549	5.67
Hospitals with CT	10	22822	17.14
Hospitals with CT	15	59157	44.43
Hospitals with CT	20	97099	72.93
Hospitals with CT	25	125489	94.25
Hospitals with CT	30	130973	98.37
Hospitals with CT	35	133126	99.99
Hospitals with CT	40	133146	100.0
Hospitals with CT	45	133146	100.0
Hospitals with CT	50	133146	100.0
Hospitals with CT	55	133146	100.0
Hospitals with CT	60	133146	100.0
Stroke-Ready Hospitals	5	7549	5.67
Stroke-Ready Hospitals	10	22591	16.97
Stroke-Ready Hospitals	15	55525	41.7
Stroke-Ready Hospitals	20	87335	65.59
Stroke-Ready Hospitals	25	103342	77.62
Stroke-Ready Hospitals	30	121594	91.32
Stroke-Ready Hospitals	35	129450	97.22
Stroke-Ready Hospitals	40	132801	99.74
Stroke-Ready Hospitals	45	133146	100.0
Stroke-Ready Hospitals	50	133146	100.0
Stroke-Ready Hospitals	55	133146	100.0
Stroke-Ready Hospitals	60	133146	100.0
Stroke Units	5	7549	5.67
Stroke Units	10	22591	16.97
Stroke Units	15	55525	41.7
Stroke Units	20	87335	65.59
Stroke Units	25	103342	77.62
Stroke Units	30	121594	91.32
Stroke Units	35	129248	97.07
Stroke Units	40	132801	99.74
Stroke Units	45	133146	100.0
Stroke Units	50	133146	100.0
Stroke Units	55	133146	100.0
Stroke Units	60	133146	100.0

Population Coverage - Kreisfreie Stadt Augsburg (Bayern)

Condition	Drive Time (min)	Covered Population	Coverage (%)
Hospitals with CT	5	115312	40.22
Hospitals with CT	10	277537	96.81
Hospitals with CT	15	285905	99.73
Hospitals with CT	20	286595	99.97
Hospitals with CT	25	286672	99.99
Hospitals with CT	30	286672	99.99
Hospitals with CT	35	286689	100.0
Hospitals with CT	40	286689	100.0
Hospitals with CT	45	286689	100.0
Hospitals with CT	50	286689	100.0
Hospitals with CT	55	286689	100.0
Hospitals with CT	60	286689	100.0
Stroke-Ready Hospitals	5	14415	5.03
Stroke-Ready Hospitals	10	68025	23.73
Stroke-Ready Hospitals	15	183285	63.93
Stroke-Ready Hospitals	20	277275	96.72
Stroke-Ready Hospitals	25	286550	99.95
Stroke-Ready Hospitals	30	286647	99.99
Stroke-Ready Hospitals	35	286689	100.0
Stroke-Ready Hospitals	40	286689	100.0
Stroke-Ready Hospitals	45	286689	100.0
Stroke-Ready Hospitals	50	286689	100.0
Stroke-Ready Hospitals	55	286689	100.0
Stroke-Ready Hospitals	60	286689	100.0
Stroke Units	5	14415	5.03
Stroke Units	10	68025	23.73
Stroke Units	15	183285	63.93
Stroke Units	20	277275	96.72
Stroke Units	25	286549	99.95
Stroke Units	30	286647	99.99
Stroke Units	35	286689	100.0
Stroke Units	40	286689	100.0
Stroke Units	45	286689	100.0
Stroke Units	50	286689	100.0
Stroke Units	55	286689	100.0
Stroke Units	60	286689	100.0

Population Coverage - Landkreis Barnim (Brandenburg)

Condition	Drive Time (min)	Covered Population	Coverage (%)
Hospitals with CT	5	27565	16.17
Hospitals with CT	10	81018	47.53
Hospitals with CT	15	120193	70.52
Hospitals with CT	20	150214	88.13
Hospitals with CT	25	163890	96.16
Hospitals with CT	30	168656	98.95
Hospitals with CT	35	170134	99.82
Hospitals with CT	40	170322	99.93
Hospitals with CT	45	170428	99.99
Hospitals with CT	50	170429	99.99
Hospitals with CT	55	170427	99.99
Hospitals with CT	60	170428	99.99
Stroke-Ready Hospitals	5	9898	5.81
Stroke-Ready Hospitals	10	38312	22.48
Stroke-Ready Hospitals	15	74253	43.56
Stroke-Ready Hospitals	20	110718	64.96
Stroke-Ready Hospitals	25	132411	77.69
Stroke-Ready Hospitals	30	150585	88.35
Stroke-Ready Hospitals	35	165327	97.0
Stroke-Ready Hospitals	40	168565	98.9
Stroke-Ready Hospitals	45	169879	99.67
Stroke-Ready Hospitals	50	170158	99.83
Stroke-Ready Hospitals	55	170327	99.93
Stroke-Ready Hospitals	60	170335	99.94
Stroke Units	5	9898	5.81
Stroke Units	10	38312	22.48
Stroke Units	15	74253	43.56
Stroke Units	20	110718	64.96
Stroke Units	25	132411	77.69
Stroke Units	30	150488	88.29
Stroke Units	35	163739	96.07
Stroke Units	40	167147	98.07
Stroke Units	45	169400	99.39
Stroke Units	50	170125	99.81
Stroke Units	55	170299	99.92
Stroke Units	60	170314	99.92

Population Coverage - Landkreis Meißen (Sachsen)

Condition	Drive Time (min)	Covered Population	Coverage (%)
Hospitals with CT	5	49518	21.62
Hospitals with CT	10	121122	52.88
Hospitals with CT	15	179425	78.33
Hospitals with CT	20	214773	93.76
Hospitals with CT	25	226958	99.08
Hospitals with CT	30	228752	99.86
Hospitals with CT	35	229065	100.0
Hospitals with CT	40	229065	100.0
Hospitals with CT	45	229070	100.0
Hospitals with CT	50	229070	100.0
Hospitals with CT	55	229070	100.0
Hospitals with CT	60	229070	100.0
Stroke-Ready Hospitals	5	5519	2.41
Stroke-Ready Hospitals	10	25587	11.17
Stroke-Ready Hospitals	15	52007	22.7
Stroke-Ready Hospitals	20	93255	40.71
Stroke-Ready Hospitals	25	139554	60.92
Stroke-Ready Hospitals	30	166859	72.84
Stroke-Ready Hospitals	35	196427	85.75
Stroke-Ready Hospitals	40	218812	95.52
Stroke-Ready Hospitals	45	227346	99.25
Stroke-Ready Hospitals	50	228556	99.78
Stroke-Ready Hospitals	55	229070	100.0
Stroke-Ready Hospitals	60	229070	100.0
Stroke Units	5	5519	2.41
Stroke Units	10	25587	11.17
Stroke Units	15	52007	22.7
Stroke Units	20	93255	40.71
Stroke Units	25	139554	60.92
Stroke Units	30	166859	72.84
Stroke Units	35	196427	85.75
Stroke Units	40	218812	95.52
Stroke Units	45	227346	99.25
Stroke Units	50	228556	99.78
Stroke Units	55	229070	100.0
Stroke Units	60	229070	100.0

Population Coverage - Kreisfreie Stadt Suhl (Thüringen)

Condition	Drive Time (min)	Covered Population	Coverage (%)
Hospitals with CT	5	17607	52.86
Hospitals with CT	10	27962	83.95
Hospitals with CT	15	30666	92.07
Hospitals with CT	20	31818	95.53
Hospitals with CT	25	32803	98.48
Hospitals with CT	30	33206	99.69
Hospitals with CT	35	33308	100.0
Hospitals with CT	40	33308	100.0
Hospitals with CT	45	33308	100.0
Hospitals with CT	50	33308	100.0
Hospitals with CT	55	33308	100.0
Hospitals with CT	60	33308	100.0
Stroke-Ready Hospitals	5	2673	8.03
Stroke-Ready Hospitals	10	15743	47.27
Stroke-Ready Hospitals	15	27195	81.65
Stroke-Ready Hospitals	20	30625	91.95
Stroke-Ready Hospitals	25	32733	98.27
Stroke-Ready Hospitals	30	32815	98.52
Stroke-Ready Hospitals	35	33191	99.65
Stroke-Ready Hospitals	40	33308	100.0
Stroke-Ready Hospitals	45	33308	100.0
Stroke-Ready Hospitals	50	33308	100.0
Stroke-Ready Hospitals	55	33308	100.0
Stroke-Ready Hospitals	60	33308	100.0
Stroke Units	5	2673	8.03
Stroke Units	10	15743	47.27
Stroke Units	15	27195	81.65
Stroke Units	20	30625	91.95
Stroke Units	25	32733	98.27
Stroke Units	30	32815	98.52
Stroke Units	35	33191	99.65
Stroke Units	40	33308	100.0
Stroke Units	45	33308	100.0
Stroke Units	50	33308	100.0
Stroke Units	55	33308	100.0
Stroke Units	60	33308	100.0

Population Coverage - Landkreis Eichsfeld (Thüringen)

Condition	Drive Time (min)	Covered Population	Coverage (%)
Hospitals with CT	5	10666	11.27
Hospitals with CT	10	18797	19.85
Hospitals with CT	15	40167	42.42
Hospitals with CT	20	70048	73.98
Hospitals with CT	25	94028	99.31
Hospitals with CT	30	94679	100.0
Hospitals with CT	35	94683	100.0
Hospitals with CT	40	94683	100.0
Hospitals with CT	45	94683	100.0
Hospitals with CT	50	94683	100.0
Hospitals with CT	55	94683	100.0
Hospitals with CT	60	94683	100.0
Stroke-Ready Hospitals	5	1665	1.76
Stroke-Ready Hospitals	10	11798	12.46
Stroke-Ready Hospitals	15	30812	32.54
Stroke-Ready Hospitals	20	44168	46.65
Stroke-Ready Hospitals	25	67507	71.3
Stroke-Ready Hospitals	30	89087	94.09
Stroke-Ready Hospitals	35	94566	99.88
Stroke-Ready Hospitals	40	94683	100.0
Stroke-Ready Hospitals	45	94683	100.0
Stroke-Ready Hospitals	50	94683	100.0
Stroke-Ready Hospitals	55	94683	100.0
Stroke-Ready Hospitals	60	94683	100.0
Stroke Units	5	0	0.0
Stroke Units	10	0	0.0
Stroke Units	15	1449	1.53
Stroke Units	20	4041	4.27
Stroke Units	25	10631	11.23
Stroke Units	30	19092	20.17
Stroke Units	35	48437	51.16
Stroke Units	40	78276	82.67
Stroke Units	45	87488	92.4
Stroke Units	50	93733	99.0
Stroke Units	55	94674	99.99
Stroke Units	60	94683	100.0

Population Coverage - Landkreis Göttingen (Niedersachsen)

Condition	Drive Time (min)	Covered Population	Coverage (%)
Hospitals with CT	5	84391	25.22
Hospitals with CT	10	194717	58.2
Hospitals with CT	15	263220	78.67
Hospitals with CT	20	309218	92.42
Hospitals with CT	25	332032	99.24
Hospitals with CT	30	334359	99.94
Hospitals with CT	35	334571	100.0
Hospitals with CT	40	334571	100.0
Hospitals with CT	45	334571	100.0
Hospitals with CT	50	334571	100.0
Hospitals with CT	55	334571	100.0
Hospitals with CT	60	334571	100.0
Stroke-Ready Hospitals	5	18759	5.61
Stroke-Ready Hospitals	10	82120	24.54
Stroke-Ready Hospitals	15	164601	49.2
Stroke-Ready Hospitals	20	216100	64.59
Stroke-Ready Hospitals	25	260333	77.81
Stroke-Ready Hospitals	30	306802	91.7
Stroke-Ready Hospitals	35	331937	99.21
Stroke-Ready Hospitals	40	334426	99.96
Stroke-Ready Hospitals	45	334569	100.0
Stroke-Ready Hospitals	50	334571	100.0
Stroke-Ready Hospitals	55	334571	100.0
Stroke-Ready Hospitals	60	334571	100.0
Stroke Units	5	16591	4.96
Stroke Units	10	64495	19.28
Stroke Units	15	131077	39.18
Stroke Units	20	161986	48.42
Stroke Units	25	200835	60.03
Stroke Units	30	249580	74.6
Stroke Units	35	278757	83.32
Stroke Units	40	310699	92.86
Stroke Units	45	332517	99.39
Stroke Units	50	334440	99.96
Stroke Units	55	334543	99.99
Stroke Units	60	334571	100.0

Population Coverage - Landkreis Schaumburg (Niedersachsen)

Condition	Drive Time (min)	Covered Population	Coverage (%)
Hospitals with CT	5	10166	6.52
Hospitals with CT	10	44709	28.69
Hospitals with CT	15	80526	51.67
Hospitals with CT	20	103848	66.64
Hospitals with CT	25	128888	82.7
Hospitals with CT	30	149228	95.76
Hospitals with CT	35	155445	99.75
Hospitals with CT	40	155842	100.0
Hospitals with CT	45	155842	100.0
Hospitals with CT	50	155842	100.0
Hospitals with CT	55	155842	100.0
Hospitals with CT	60	155842	100.0
Stroke-Ready Hospitals	5	6985	4.48
Stroke-Ready Hospitals	10	35765	22.95
Stroke-Ready Hospitals	15	58100	37.28
Stroke-Ready Hospitals	20	98159	62.99
Stroke-Ready Hospitals	25	125253	80.37
Stroke-Ready Hospitals	30	149496	95.93
Stroke-Ready Hospitals	35	155804	99.98
Stroke-Ready Hospitals	40	155842	100.0
Stroke-Ready Hospitals	45	155842	100.0
Stroke-Ready Hospitals	50	155842	100.0
Stroke-Ready Hospitals	55	155842	100.0
Stroke-Ready Hospitals	60	155842	100.0
Stroke Units	5	6985	4.48
Stroke Units	10	35765	22.95
Stroke Units	15	58100	37.28
Stroke Units	20	98159	62.99
Stroke Units	25	123753	79.41
Stroke Units	30	144850	92.95
Stroke Units	35	151185	97.01
Stroke Units	40	155728	99.93
Stroke Units	45	155802	99.97
Stroke Units	50	155842	100.0
Stroke Units	55	155842	100.0
Stroke Units	60	155842	100.0

Population Coverage - Landkreis Aurich (Niedersachsen)

Condition	Drive Time (min)	Covered Population	Coverage (%)
Hospitals with CT	5	23956	12.72
Hospitals with CT	10	56389	29.94
Hospitals with CT	15	93935	49.88
Hospitals with CT	20	136284	72.37
Hospitals with CT	25	162132	86.1
Hospitals with CT	30	177987	94.51
Hospitals with CT	35	180213	95.7
Hospitals with CT	40	180266	95.72
Hospitals with CT	45	180282	95.73
Hospitals with CT	50	180342	95.76
Hospitals with CT	55	180252	95.72
Hospitals with CT	60	180298	95.74
Stroke-Ready Hospitals	5	0	0.0
Stroke-Ready Hospitals	10	1747	0.93
Stroke-Ready Hospitals	15	7235	3.84
Stroke-Ready Hospitals	20	16005	8.5
Stroke-Ready Hospitals	25	27411	14.56
Stroke-Ready Hospitals	30	53193	28.25
Stroke-Ready Hospitals	35	96324	51.15
Stroke-Ready Hospitals	40	130990	69.56
Stroke-Ready Hospitals	45	164762	87.49
Stroke-Ready Hospitals	50	175255	93.06
Stroke-Ready Hospitals	55	179324	95.22
Stroke-Ready Hospitals	60	180129	95.65
Stroke Units	5	0	0.0
Stroke Units	10	1747	0.93
Stroke Units	15	7235	3.84
Stroke Units	20	16005	8.5
Stroke Units	25	27411	14.56
Stroke Units	30	53193	28.25
Stroke Units	35	96324	51.15
Stroke Units	40	130990	69.56
Stroke Units	45	164762	87.49
Stroke Units	50	175255	93.06
Stroke Units	55	179324	95.22
Stroke Units	60	180129	95.65

Population Coverage - Landkreis Friesland (Niedersachsen)

Condition	Drive Time (min)	Covered Population	Coverage (%)
Hospitals with CT	5	13375	13.68
Hospitals with CT	10	29518	30.19
Hospitals with CT	15	61784	63.18
Hospitals with CT	20	85393	87.32
Hospitals with CT	25	93880	96.0
Hospitals with CT	30	95800	97.96
Hospitals with CT	35	96262	98.44
Hospitals with CT	40	96306	98.48
Hospitals with CT	45	96307	98.48
Hospitals with CT	50	96313	98.49
Hospitals with CT	55	96169	98.34
Hospitals with CT	60	96244	98.42
Stroke-Ready Hospitals	5	5242	5.36
Stroke-Ready Hospitals	10	13327	13.63
Stroke-Ready Hospitals	15	29457	30.12
Stroke-Ready Hospitals	20	67538	69.06
Stroke-Ready Hospitals	25	90584	92.63
Stroke-Ready Hospitals	30	93909	96.03
Stroke-Ready Hospitals	35	95780	97.94
Stroke-Ready Hospitals	40	96087	98.26
Stroke-Ready Hospitals	45	96095	98.27
Stroke-Ready Hospitals	50	96095	98.27
Stroke-Ready Hospitals	55	96093	98.26
Stroke-Ready Hospitals	60	96137	98.31
Stroke Units	5	5242	5.36
Stroke Units	10	13327	13.63
Stroke Units	15	29457	30.12
Stroke Units	20	67538	69.06
Stroke Units	25	90584	92.63
Stroke Units	30	93909	96.03
Stroke Units	35	95780	97.94
Stroke Units	40	96087	98.26
Stroke Units	45	96095	98.27
Stroke Units	50	96095	98.27
Stroke Units	55	96093	98.26
Stroke Units	60	96137	98.31

Population Coverage - Landkreis Wesermarsch (Niedersachsen)

Condition	Drive Time (min)	Covered Population	Coverage (%)
Hospitals with CT	5	12600	14.15
Hospitals with CT	10	38260	42.96
Hospitals with CT	15	67205	75.46
Hospitals with CT	20	76693	86.11
Hospitals with CT	25	84059	94.38
Hospitals with CT	30	86582	97.21
Hospitals with CT	35	87256	97.97
Hospitals with CT	40	88389	99.24
Hospitals with CT	45	88418	99.27
Hospitals with CT	50	88471	99.33
Hospitals with CT	55	88346	99.19
Hospitals with CT	60	88613	99.49
Stroke-Ready Hospitals	5	0	0.0
Stroke-Ready Hospitals	10	64	0.07
Stroke-Ready Hospitals	15	3782	4.25
Stroke-Ready Hospitals	20	10835	12.17
Stroke-Ready Hospitals	25	20953	23.53
Stroke-Ready Hospitals	30	48185	54.1
Stroke-Ready Hospitals	35	77615	87.14
Stroke-Ready Hospitals	40	83291	93.52
Stroke-Ready Hospitals	45	85703	96.23
Stroke-Ready Hospitals	50	87793	98.57
Stroke-Ready Hospitals	55	88051	98.86
Stroke-Ready Hospitals	60	88280	99.12
Stroke Units	5	0	0.0
Stroke Units	10	64	0.07
Stroke Units	15	3782	4.25
Stroke Units	20	10835	12.17
Stroke Units	25	20953	23.53
Stroke Units	30	48185	54.1
Stroke Units	35	77615	87.14
Stroke Units	40	83291	93.52
Stroke Units	45	85703	96.23
Stroke Units	50	87793	98.57
Stroke Units	55	88051	98.86
Stroke Units	60	88235	99.07

Population Coverage - Landkreis Main-Kinzig-Kreis (Hessen)

Condition	Drive Time (min)	Covered Population	Coverage (%)
Hospitals with CT	5	65341	15.97
Hospitals with CT	10	158439	38.72
Hospitals with CT	15	300579	73.46
Hospitals with CT	20	386106	94.36
Hospitals with CT	25	408865	99.92
Hospitals with CT	30	409184	100.0
Hospitals with CT	35	409184	100.0
Hospitals with CT	40	409184	100.0
Hospitals with CT	45	409184	100.0
Hospitals with CT	50	409184	100.0
Hospitals with CT	55	409184	100.0
Hospitals with CT	60	409184	100.0
Stroke-Ready Hospitals	5	43874	10.72
Stroke-Ready Hospitals	10	117558	28.73
Stroke-Ready Hospitals	15	245842	60.08
Stroke-Ready Hospitals	20	335662	82.03
Stroke-Ready Hospitals	25	378395	92.48
Stroke-Ready Hospitals	30	394254	96.35
Stroke-Ready Hospitals	35	406100	99.25
Stroke-Ready Hospitals	40	408057	99.72
Stroke-Ready Hospitals	45	409182	100.0
Stroke-Ready Hospitals	50	409184	100.0
Stroke-Ready Hospitals	55	409184	100.0
Stroke-Ready Hospitals	60	409184	100.0
Stroke Units	5	43874	10.72
Stroke Units	10	117557	28.73
Stroke Units	15	245541	60.01
Stroke Units	20	335286	81.94
Stroke Units	25	377678	92.3
Stroke Units	30	394254	96.35
Stroke Units	35	406100	99.25
Stroke Units	40	408057	99.72
Stroke Units	45	409182	100.0
Stroke Units	50	409184	100.0
Stroke Units	55	409184	100.0
Stroke Units	60	409184	100.0

Population Coverage - Landkreis Vogelsbergkreis (Hessen)

Condition	Drive Time (min)	Covered Population	Coverage (%)
Hospitals with CT	5	15582	14.52
Hospitals with CT	10	35783	33.34
Hospitals with CT	15	56931	53.05
Hospitals with CT	20	81799	76.22
Hospitals with CT	25	104962	97.8
Hospitals with CT	30	107315	99.99
Hospitals with CT	35	107324	100.0
Hospitals with CT	40	107324	100.0
Hospitals with CT	45	107324	100.0
Hospitals with CT	50	107324	100.0
Hospitals with CT	55	107324	100.0
Hospitals with CT	60	107324	100.0
Stroke-Ready Hospitals	5	0	0.0
Stroke-Ready Hospitals	10	0	0.0
Stroke-Ready Hospitals	15	917	0.86
Stroke-Ready Hospitals	20	5952	5.55
Stroke-Ready Hospitals	25	17048	15.88
Stroke-Ready Hospitals	30	49778	46.38
Stroke-Ready Hospitals	35	75134	70.01
Stroke-Ready Hospitals	40	97933	91.25
Stroke-Ready Hospitals	45	107316	99.99
Stroke-Ready Hospitals	50	107324	100.0
Stroke-Ready Hospitals	55	107324	100.0
Stroke-Ready Hospitals	60	107324	100.0
Stroke Units	5	0	0.0
Stroke Units	10	0	0.0
Stroke Units	15	917	0.86
Stroke Units	20	4746	4.42
Stroke Units	25	12274	11.44
Stroke Units	30	32679	30.45
Stroke Units	35	63820	59.46
Stroke Units	40	93310	86.94
Stroke Units	45	107206	99.89
Stroke Units	50	107324	100.0
Stroke Units	55	107324	100.0
Stroke Units	60	107324	100.0

Population Coverage - Landkreis Neuwied (Rheinland-Pfalz)

Condition	Drive Time (min)	Covered Population	Coverage (%)
Hospitals with CT	5	37389	20.18
Hospitals with CT	10	108663	58.64
Hospitals with CT	15	172723	93.2
Hospitals with CT	20	184855	99.75
Hospitals with CT	25	185227	99.95
Hospitals with CT	30	185317	100.0
Hospitals with CT	35	185317	100.0
Hospitals with CT	40	185317	100.0
Hospitals with CT	45	185317	100.0
Hospitals with CT	50	185317	100.0
Hospitals with CT	55	185317	100.0
Hospitals with CT	60	185317	100.0
Stroke-Ready Hospitals	5	5045	2.72
Stroke-Ready Hospitals	10	12424	6.7
Stroke-Ready Hospitals	15	61307	33.08
Stroke-Ready Hospitals	20	121160	65.38
Stroke-Ready Hospitals	25	159378	86.0
Stroke-Ready Hospitals	30	183745	99.15
Stroke-Ready Hospitals	35	185317	100.0
Stroke-Ready Hospitals	40	185317	100.0
Stroke-Ready Hospitals	45	185317	100.0
Stroke-Ready Hospitals	50	185317	100.0
Stroke-Ready Hospitals	55	185317	100.0
Stroke-Ready Hospitals	60	185317	100.0
Stroke Units	5	5045	2.72
Stroke Units	10	10329	5.57
Stroke Units	15	30049	16.22
Stroke Units	20	95361	51.46
Stroke Units	25	145918	78.74
Stroke Units	30	172178	92.91
Stroke Units	35	184979	99.82
Stroke Units	40	185317	100.0
Stroke Units	45	185317	100.0
Stroke Units	50	185317	100.0
Stroke Units	55	185317	100.0
Stroke Units	60	185317	100.0

Population Coverage - Kreisfreie Stadt Mainz (Rheinland-Pfalz)

Condition	Drive Time (min)	Covered Population	Coverage (%)
Hospitals with CT	5	62180	29.05
Hospitals with CT	10	167001	78.01
Hospitals with CT	15	210713	98.43
Hospitals with CT	20	214068	100.0
Hospitals with CT	25	214068	100.0
Hospitals with CT	30	214068	100.0
Hospitals with CT	35	214068	100.0
Hospitals with CT	40	214068	100.0
Hospitals with CT	45	214068	100.0
Hospitals with CT	50	214068	100.0
Hospitals with CT	55	214068	100.0
Hospitals with CT	60	214068	100.0
Stroke-Ready Hospitals	5	34687	16.2
Stroke-Ready Hospitals	10	134314	62.74
Stroke-Ready Hospitals	15	206105	96.28
Stroke-Ready Hospitals	20	213779	99.87
Stroke-Ready Hospitals	25	214068	100.0
Stroke-Ready Hospitals	30	214068	100.0
Stroke-Ready Hospitals	35	214068	100.0
Stroke-Ready Hospitals	40	214068	100.0
Stroke-Ready Hospitals	45	214068	100.0
Stroke-Ready Hospitals	50	214068	100.0
Stroke-Ready Hospitals	55	214068	100.0
Stroke-Ready Hospitals	60	214068	100.0
Stroke Units	5	34687	16.2
Stroke Units	10	134314	62.74
Stroke Units	15	206105	96.28
Stroke Units	20	213779	99.87
Stroke Units	25	214068	100.0
Stroke Units	30	214068	100.0
Stroke Units	35	214068	100.0
Stroke Units	40	214068	100.0
Stroke Units	45	214068	100.0
Stroke Units	50	214068	100.0
Stroke Units	55	214068	100.0
Stroke Units	60	214068	100.0

Population Coverage - Landkreis Hohenlohekreis (Baden-Württemberg)

Condition	Drive Time (min)	Covered Population	Coverage (%)
Hospitals with CT	5	10199	9.33
Hospitals with CT	10	30372	27.78
Hospitals with CT	15	50202	45.92
Hospitals with CT	20	63674	58.24
Hospitals with CT	25	86145	78.8
Hospitals with CT	30	102732	93.97
Hospitals with CT	35	108249	99.01
Hospitals with CT	40	109326	100.0
Hospitals with CT	45	109326	100.0
Hospitals with CT	50	109326	100.0
Hospitals with CT	55	109326	100.0
Hospitals with CT	60	109326	100.0
Stroke-Ready Hospitals	5	12112	11.08
Stroke-Ready Hospitals	10	31974	29.25
Stroke-Ready Hospitals	15	52335	47.87
Stroke-Ready Hospitals	20	65244	59.68
Stroke-Ready Hospitals	25	87016	79.59
Stroke-Ready Hospitals	30	103925	95.06
Stroke-Ready Hospitals	35	109128	99.82
Stroke-Ready Hospitals	40	109326	100.0
Stroke-Ready Hospitals	45	109326	100.0
Stroke-Ready Hospitals	50	109326	100.0
Stroke-Ready Hospitals	55	109326	100.0
Stroke-Ready Hospitals	60	109326	100.0
Stroke Units	5	0	0.0
Stroke Units	10	0	0.0
Stroke Units	15	860	0.79
Stroke Units	20	8324	7.61
Stroke Units	25	40572	37.11
Stroke Units	30	77056	70.48
Stroke Units	35	102309	93.58
Stroke Units	40	108401	99.15
Stroke Units	45	109326	100.0
Stroke Units	50	109326	100.0
Stroke Units	55	109326	100.0
Stroke Units	60	109326	100.0

Population Coverage - Landkreis Ortenaukreis (Baden-Württemberg)

Condition	Drive Time (min)	Covered Population	Coverage (%)
Hospitals with CT	5	52823	12.55
Hospitals with CT	10	160025	38.02
Hospitals with CT	15	251723	59.81
Hospitals with CT	20	334780	79.55
Hospitals with CT	25	393105	93.41
Hospitals with CT	30	414246	98.43
Hospitals with CT	35	419776	99.75
Hospitals with CT	40	420615	99.95
Hospitals with CT	45	420805	99.99
Hospitals with CT	50	420825	100.0
Hospitals with CT	55	420789	99.99
Hospitals with CT	60	420811	99.99
Stroke-Ready Hospitals	5	30597	7.27
Stroke-Ready Hospitals	10	83376	19.81
Stroke-Ready Hospitals	15	135604	32.22
Stroke-Ready Hospitals	20	196145	46.61
Stroke-Ready Hospitals	25	280977	66.77
Stroke-Ready Hospitals	30	346732	82.39
Stroke-Ready Hospitals	35	379880	90.27
Stroke-Ready Hospitals	40	398205	94.62
Stroke-Ready Hospitals	45	412699	98.06
Stroke-Ready Hospitals	50	418853	99.53
Stroke-Ready Hospitals	55	420737	99.97
Stroke-Ready Hospitals	60	420801	99.99
Stroke Units	5	30597	7.27
Stroke Units	10	83376	19.81
Stroke Units	15	135604	32.22
Stroke Units	20	196139	46.61
Stroke Units	25	280715	66.7
Stroke Units	30	345861	82.18
Stroke Units	35	377182	89.62
Stroke Units	40	393717	93.55
Stroke Units	45	406454	96.58
Stroke Units	50	414160	98.41
Stroke Units	55	419433	99.66
Stroke Units	60	420516	99.92

Population Coverage - Landkreis Neustadt a.d.Aisch-Bad Windsheim (Bayern)

Condition	Drive Time (min)	Covered Population	Coverage (%)
Hospitals with CT	5	13591	13.39
Hospitals with CT	10	28447	28.03
Hospitals with CT	15	56666	55.83
Hospitals with CT	20	86813	85.53
Hospitals with CT	25	99424	97.96
Hospitals with CT	30	101488	99.99
Hospitals with CT	35	101500	100.0
Hospitals with CT	40	101500	100.0
Hospitals with CT	45	101500	100.0
Hospitals with CT	50	101500	100.0
Hospitals with CT	55	101500	100.0
Hospitals with CT	60	101500	100.0
Stroke-Ready Hospitals	5	4459	4.39
Stroke-Ready Hospitals	10	14391	14.18
Stroke-Ready Hospitals	15	25578	25.2
Stroke-Ready Hospitals	20	44586	43.93
Stroke-Ready Hospitals	25	64023	63.08
Stroke-Ready Hospitals	30	82681	81.46
Stroke-Ready Hospitals	35	100909	99.42
Stroke-Ready Hospitals	40	101498	100.0
Stroke-Ready Hospitals	45	101500	100.0
Stroke-Ready Hospitals	50	101500	100.0
Stroke-Ready Hospitals	55	101500	100.0
Stroke-Ready Hospitals	60	101500	100.0
Stroke Units	5	0	0.0
Stroke Units	10	0	0.0
Stroke Units	15	2	0.0
Stroke Units	20	2488	2.45
Stroke Units	25	18984	18.7
Stroke Units	30	39497	38.91
Stroke Units	35	72985	71.91
Stroke Units	40	92785	91.41
Stroke Units	45	101315	99.82
Stroke Units	50	101500	100.0
Stroke Units	55	101500	100.0
Stroke Units	60	101500	100.0

Population Coverage - Landkreis Goslar (Niedersachsen)

Condition	Drive Time (min)	Covered Population	Coverage (%)
Hospitals with CT	5	23128	16.91
Hospitals with CT	10	67343	49.23
Hospitals with CT	15	107346	78.47
Hospitals with CT	20	119461	87.33
Hospitals with CT	25	128826	94.17
Hospitals with CT	30	135758	99.24
Hospitals with CT	35	136667	99.9
Hospitals with CT	40	136674	99.91
Hospitals with CT	45	136797	100.0
Hospitals with CT	50	136797	100.0
Hospitals with CT	55	136797	100.0
Hospitals with CT	60	136797	100.0
Stroke-Ready Hospitals	5	2359	1.72
Stroke-Ready Hospitals	10	11780	8.61
Stroke-Ready Hospitals	15	20684	15.12
Stroke-Ready Hospitals	20	35436	25.9
Stroke-Ready Hospitals	25	78436	57.34
Stroke-Ready Hospitals	30	128510	93.94
Stroke-Ready Hospitals	35	135704	99.2
Stroke-Ready Hospitals	40	136488	99.77
Stroke-Ready Hospitals	45	136547	99.82
Stroke-Ready Hospitals	50	136797	100.0
Stroke-Ready Hospitals	55	136797	100.0
Stroke-Ready Hospitals	60	136797	100.0
Stroke Units	5	2359	1.72
Stroke Units	10	11780	8.61
Stroke Units	15	20684	15.12
Stroke Units	20	34519	25.23
Stroke Units	25	68717	50.23
Stroke Units	30	121671	88.94
Stroke Units	35	132613	96.94
Stroke Units	40	134581	98.38
Stroke Units	45	135817	99.28
Stroke Units	50	136749	99.96
Stroke Units	55	136770	99.98
Stroke Units	60	136797	100.0

Population Coverage - Landkreis Region Hannover (Niedersachsen)

Condition	Drive Time (min)	Covered Population	Coverage (%)
Hospitals with CT	5	309139	26.86
Hospitals with CT	10	662136	57.54
Hospitals with CT	15	884110	76.83
Hospitals with CT	20	1062865	92.36
Hospitals with CT	25	1120896	97.41
Hospitals with CT	30	1142568	99.29
Hospitals with CT	35	1150065	99.94
Hospitals with CT	40	1150754	100.0
Hospitals with CT	45	1150754	100.0
Hospitals with CT	50	1150754	100.0
Hospitals with CT	55	1150754	100.0
Hospitals with CT	60	1150754	100.0
Stroke-Ready Hospitals	5	218045	18.95
Stroke-Ready Hospitals	10	541990	47.1
Stroke-Ready Hospitals	15	760642	66.1
Stroke-Ready Hospitals	20	960778	83.49
Stroke-Ready Hospitals	25	1089911	94.71
Stroke-Ready Hospitals	30	1138132	98.9
Stroke-Ready Hospitals	35	1149794	99.92
Stroke-Ready Hospitals	40	1150754	100.0
Stroke-Ready Hospitals	45	1150754	100.0
Stroke-Ready Hospitals	50	1150754	100.0
Stroke-Ready Hospitals	55	1150754	100.0
Stroke-Ready Hospitals	60	1150754	100.0
Stroke Units	5	209974	18.25
Stroke Units	10	511812	44.48
Stroke Units	15	701250	60.94
Stroke Units	20	853802	74.2
Stroke Units	25	984804	85.58
Stroke Units	30	1075074	93.42
Stroke Units	35	1124990	97.76
Stroke Units	40	1140443	99.1
Stroke Units	45	1146673	99.65
Stroke Units	50	1150584	99.99
Stroke Units	55	1150754	100.0
Stroke Units	60	1150754	100.0

Population Coverage - Landkreis Vechta (Niedersachsen)

Condition	Drive Time (min)	Covered Population	Coverage (%)
Hospitals with CT	5	41585	30.63
Hospitals with CT	10	70690	52.06
Hospitals with CT	15	126057	92.84
Hospitals with CT	20	135500	99.79
Hospitals with CT	25	135779	100.0
Hospitals with CT	30	135779	100.0
Hospitals with CT	35	135779	100.0
Hospitals with CT	40	135779	100.0
Hospitals with CT	45	135779	100.0
Hospitals with CT	50	135779	100.0
Hospitals with CT	55	135779	100.0
Hospitals with CT	60	135779	100.0
Stroke-Ready Hospitals	5	9086	6.69
Stroke-Ready Hospitals	10	17303	12.74
Stroke-Ready Hospitals	15	36835	27.13
Stroke-Ready Hospitals	20	49537	36.48
Stroke-Ready Hospitals	25	67232	49.52
Stroke-Ready Hospitals	30	86755	63.89
Stroke-Ready Hospitals	35	110351	81.27
Stroke-Ready Hospitals	40	127750	94.09
Stroke-Ready Hospitals	45	133809	98.55
Stroke-Ready Hospitals	50	135779	100.0
Stroke-Ready Hospitals	55	135779	100.0
Stroke-Ready Hospitals	60	135779	100.0
Stroke Units	5	9086	6.69
Stroke Units	10	17303	12.74
Stroke Units	15	36835	27.13
Stroke Units	20	49537	36.48
Stroke Units	25	67232	49.52
Stroke Units	30	86755	63.89
Stroke Units	35	110351	81.27
Stroke Units	40	127719	94.06
Stroke Units	45	133808	98.55
Stroke Units	50	135779	100.0
Stroke Units	55	135779	100.0
Stroke Units	60	135779	100.0

Population Coverage - Kreis Städteregion Aachen (Nordrhein-Westfalen)

Condition	Drive Time (min)	Covered Population	Coverage (%)
Hospitals with CT	5	124719	22.45
Hospitals with CT	10	298301	53.69
Hospitals with CT	15	467566	84.15
Hospitals with CT	20	545819	98.24
Hospitals with CT	25	554428	99.79
Hospitals with CT	30	555103	99.91
Hospitals with CT	35	555251	99.93
Hospitals with CT	40	555326	99.95
Hospitals with CT	45	555334	99.95
Hospitals with CT	50	555338	99.95
Hospitals with CT	55	555178	99.92
Hospitals with CT	60	555333	99.95
Stroke-Ready Hospitals	5	16583	2.98
Stroke-Ready Hospitals	10	79120	14.24
Stroke-Ready Hospitals	15	256655	46.19
Stroke-Ready Hospitals	20	422034	75.96
Stroke-Ready Hospitals	25	501245	90.21
Stroke-Ready Hospitals	30	523012	94.13
Stroke-Ready Hospitals	35	529024	95.21
Stroke-Ready Hospitals	40	535760	96.43
Stroke-Ready Hospitals	45	545004	98.09
Stroke-Ready Hospitals	50	549688	98.93
Stroke-Ready Hospitals	55	552055	99.36
Stroke-Ready Hospitals	60	555025	99.89
Stroke Units	5	16583	2.98
Stroke Units	10	79120	14.24
Stroke Units	15	256655	46.19
Stroke Units	20	422034	75.96
Stroke Units	25	501245	90.21
Stroke Units	30	523012	94.13
Stroke Units	35	529024	95.21
Stroke Units	40	535760	96.43
Stroke Units	45	545004	98.09
Stroke Units	50	549688	98.93
Stroke Units	55	552055	99.36
Stroke Units	60	555025	99.89

Population Coverage - Kreis Steinfurt (Nordrhein-Westfalen)

Condition	Drive Time (min)	Covered Population	Coverage (%)
Hospitals with CT	5	53145	12.19
Hospitals with CT	10	145875	33.46
Hospitals with CT	15	245911	56.41
Hospitals with CT	20	359222	82.41
Hospitals with CT	25	424490	97.38
Hospitals with CT	30	435907	100.0
Hospitals with CT	35	435907	100.0
Hospitals with CT	40	435907	100.0
Hospitals with CT	45	435907	100.0
Hospitals with CT	50	435907	100.0
Hospitals with CT	55	435907	100.0
Hospitals with CT	60	435907	100.0
Stroke-Ready Hospitals	5	17400	3.99
Stroke-Ready Hospitals	10	47992	11.01
Stroke-Ready Hospitals	15	81455	18.69
Stroke-Ready Hospitals	20	158024	36.25
Stroke-Ready Hospitals	25	258153	59.22
Stroke-Ready Hospitals	30	359976	82.58
Stroke-Ready Hospitals	35	426015	97.73
Stroke-Ready Hospitals	40	435907	100.0
Stroke-Ready Hospitals	45	435907	100.0
Stroke-Ready Hospitals	50	435907	100.0
Stroke-Ready Hospitals	55	435907	100.0
Stroke-Ready Hospitals	60	435907	100.0
Stroke Units	5	17400	3.99
Stroke Units	10	47992	11.01
Stroke Units	15	81455	18.69
Stroke Units	20	157807	36.2
Stroke Units	25	251296	57.65
Stroke Units	30	340636	78.14
Stroke Units	35	418692	96.05
Stroke Units	40	435907	100.0
Stroke Units	45	435907	100.0
Stroke Units	50	435907	100.0
Stroke Units	55	435907	100.0
Stroke Units	60	435907	100.0

Population Coverage - Kreis Gütersloh (Nordrhein-Westfalen)

Condition	Drive Time (min)	Covered Population	Coverage (%)
Hospitals with CT	5	64781	18.26
Hospitals with CT	10	176845	49.85
Hospitals with CT	15	290061	81.76
Hospitals with CT	20	342423	96.51
Hospitals with CT	25	354706	99.98
Hospitals with CT	30	354789	100.0
Hospitals with CT	35	354789	100.0
Hospitals with CT	40	354789	100.0
Hospitals with CT	45	354789	100.0
Hospitals with CT	50	354789	100.0
Hospitals with CT	55	354789	100.0
Hospitals with CT	60	354789	100.0
Stroke-Ready Hospitals	5	0	0.0
Stroke-Ready Hospitals	10	566	0.16
Stroke-Ready Hospitals	15	23870	6.73
Stroke-Ready Hospitals	20	103478	29.17
Stroke-Ready Hospitals	25	266888	75.22
Stroke-Ready Hospitals	30	353280	99.57
Stroke-Ready Hospitals	35	354777	100.0
Stroke-Ready Hospitals	40	354789	100.0
Stroke-Ready Hospitals	45	354789	100.0
Stroke-Ready Hospitals	50	354789	100.0
Stroke-Ready Hospitals	55	354789	100.0
Stroke-Ready Hospitals	60	354789	100.0
Stroke Units	5	0	0.0
Stroke Units	10	396	0.11
Stroke Units	15	13022	3.67
Stroke Units	20	47608	13.42
Stroke Units	25	162418	45.78
Stroke Units	30	292518	82.45
Stroke Units	35	339354	95.65
Stroke Units	40	353968	99.77
Stroke Units	45	354789	100.0
Stroke Units	50	354789	100.0
Stroke Units	55	354789	100.0
Stroke Units	60	354789	100.0

Population Coverage - Kreis Unna (Nordrhein-Westfalen)

Condition	Drive Time (min)	Covered Population	Coverage (%)
Hospitals with CT	5	95387	24.63
Hospitals with CT	10	251334	64.91
Hospitals with CT	15	363760	93.94
Hospitals with CT	20	387151	99.98
Hospitals with CT	25	387212	100.0
Hospitals with CT	30	387212	100.0
Hospitals with CT	35	387212	100.0
Hospitals with CT	40	387212	100.0
Hospitals with CT	45	387212	100.0
Hospitals with CT	50	387212	100.0
Hospitals with CT	55	387212	100.0
Hospitals with CT	60	387212	100.0
Stroke-Ready Hospitals	5	44309	11.44
Stroke-Ready Hospitals	10	104729	27.05
Stroke-Ready Hospitals	15	229576	59.29
Stroke-Ready Hospitals	20	362649	93.66
Stroke-Ready Hospitals	25	386942	99.93
Stroke-Ready Hospitals	30	387212	100.0
Stroke-Ready Hospitals	35	387212	100.0
Stroke-Ready Hospitals	40	387212	100.0
Stroke-Ready Hospitals	45	387212	100.0
Stroke-Ready Hospitals	50	387212	100.0
Stroke-Ready Hospitals	55	387212	100.0
Stroke-Ready Hospitals	60	387212	100.0
Stroke Units	5	44309	11.44
Stroke Units	10	104225	26.92
Stroke Units	15	219949	56.8
Stroke Units	20	356388	92.04
Stroke Units	25	386668	99.86
Stroke Units	30	387212	100.0
Stroke Units	35	387212	100.0
Stroke Units	40	387212	100.0
Stroke Units	45	387212	100.0
Stroke Units	50	387212	100.0
Stroke Units	55	387212	100.0
Stroke Units	60	387212	100.0

Population Coverage - Landkreis Darmstadt-Dieburg (Hessen)

Condition	Drive Time (min)	Covered Population	Coverage (%)
Hospitals with CT	5	22787	7.89
Hospitals with CT	10	62783	21.73
Hospitals with CT	15	211630	73.24
Hospitals with CT	20	276646	95.74
Hospitals with CT	25	286528	99.16
Hospitals with CT	30	288900	99.98
Hospitals with CT	35	288966	100.0
Hospitals with CT	40	288966	100.0
Hospitals with CT	45	288966	100.0
Hospitals with CT	50	288966	100.0
Hospitals with CT	55	288966	100.0
Hospitals with CT	60	288966	100.0
Stroke-Ready Hospitals	5	0	0.0
Stroke-Ready Hospitals	10	6042	2.09
Stroke-Ready Hospitals	15	63495	21.97
Stroke-Ready Hospitals	20	184339	63.79
Stroke-Ready Hospitals	25	243379	84.22
Stroke-Ready Hospitals	30	277131	95.9
Stroke-Ready Hospitals	35	287382	99.45
Stroke-Ready Hospitals	40	288917	99.98
Stroke-Ready Hospitals	45	288966	100.0
Stroke-Ready Hospitals	50	288966	100.0
Stroke-Ready Hospitals	55	288966	100.0
Stroke-Ready Hospitals	60	288966	100.0
Stroke Units	5	0	0.0
Stroke Units	10	6042	2.09
Stroke Units	15	60834	21.05
Stroke Units	20	161196	55.78
Stroke Units	25	231040	79.95
Stroke Units	30	275662	95.4
Stroke Units	35	287382	99.45
Stroke Units	40	288917	99.98
Stroke Units	45	288966	100.0
Stroke Units	50	288966	100.0
Stroke Units	55	288966	100.0
Stroke Units	60	288966	100.0

Population Coverage - Landkreis Gießen (Hessen)

Condition	Drive Time (min)	Covered Population	Coverage (%)
Hospitals with CT	5	53463	20.72
Hospitals with CT	10	156326	60.59
Hospitals with CT	15	224585	87.05
Hospitals with CT	20	252627	97.92
Hospitals with CT	25	257986	100.0
Hospitals with CT	30	257998	100.0
Hospitals with CT	35	257998	100.0
Hospitals with CT	40	257998	100.0
Hospitals with CT	45	257998	100.0
Hospitals with CT	50	257998	100.0
Hospitals with CT	55	257998	100.0
Hospitals with CT	60	257998	100.0
Stroke-Ready Hospitals	5	13678	5.3
Stroke-Ready Hospitals	10	56379	21.85
Stroke-Ready Hospitals	15	157549	61.07
Stroke-Ready Hospitals	20	211719	82.06
Stroke-Ready Hospitals	25	240900	93.37
Stroke-Ready Hospitals	30	257807	99.93
Stroke-Ready Hospitals	35	257998	100.0
Stroke-Ready Hospitals	40	257998	100.0
Stroke-Ready Hospitals	45	257998	100.0
Stroke-Ready Hospitals	50	257998	100.0
Stroke-Ready Hospitals	55	257998	100.0
Stroke-Ready Hospitals	60	257998	100.0
Stroke Units	5	13678	5.3
Stroke Units	10	56375	21.85
Stroke Units	15	157549	61.07
Stroke Units	20	211719	82.06
Stroke Units	25	240900	93.37
Stroke Units	30	257807	99.93
Stroke Units	35	257998	100.0
Stroke Units	40	257998	100.0
Stroke Units	45	257998	100.0
Stroke Units	50	257998	100.0
Stroke Units	55	257998	100.0
Stroke Units	60	257998	100.0

Population Coverage - Kreisfreie Stadt Neustadt an der Weinstraße (Rheinland-Pfalz)

Condition	Drive Time (min)	Covered Population	Coverage (%)
Hospitals with CT	5	27503	51.2
Hospitals with CT	10	48089	89.52
Hospitals with CT	15	53573	99.73
Hospitals with CT	20	53654	99.88
Hospitals with CT	25	53696	99.96
Hospitals with CT	30	53717	100.0
Hospitals with CT	35	53717	100.0
Hospitals with CT	40	53717	100.0
Hospitals with CT	45	53717	100.0
Hospitals with CT	50	53717	100.0
Hospitals with CT	55	53717	100.0
Hospitals with CT	60	53717	100.0
Stroke-Ready Hospitals	5	27503	51.2
Stroke-Ready Hospitals	10	48089	89.52
Stroke-Ready Hospitals	15	53560	99.71
Stroke-Ready Hospitals	20	53636	99.85
Stroke-Ready Hospitals	25	53696	99.96
Stroke-Ready Hospitals	30	53717	100.0
Stroke-Ready Hospitals	35	53717	100.0
Stroke-Ready Hospitals	40	53717	100.0
Stroke-Ready Hospitals	45	53717	100.0
Stroke-Ready Hospitals	50	53717	100.0
Stroke-Ready Hospitals	55	53717	100.0
Stroke-Ready Hospitals	60	53717	100.0
Stroke Units	5	0	0.0
Stroke Units	10	0	0.0
Stroke Units	15	0	0.0
Stroke Units	20	332	0.62
Stroke Units	25	32127	59.81
Stroke Units	30	52649	98.01
Stroke Units	35	53563	99.71
Stroke Units	40	53712	99.99
Stroke Units	45	53717	100.0
Stroke Units	50	53717	100.0
Stroke Units	55	53717	100.0
Stroke Units	60	53717	100.0

Population Coverage - Landkreis Main-Tauber-Kreis (Baden-Württemberg)

Condition	Drive Time (min)	Covered Population	Coverage (%)
Hospitals with CT	5	17830	13.55
Hospitals with CT	10	42624	32.38
Hospitals with CT	15	77236	58.68
Hospitals with CT	20	99410	75.53
Hospitals with CT	25	117558	89.32
Hospitals with CT	30	131597	99.98
Hospitals with CT	35	131619	100.0
Hospitals with CT	40	131619	100.0
Hospitals with CT	45	131619	100.0
Hospitals with CT	50	131619	100.0
Hospitals with CT	55	131619	100.0
Hospitals with CT	60	131619	100.0
Stroke-Ready Hospitals	5	9769	7.42
Stroke-Ready Hospitals	10	21693	16.48
Stroke-Ready Hospitals	15	42760	32.49
Stroke-Ready Hospitals	20	56584	42.99
Stroke-Ready Hospitals	25	76322	57.99
Stroke-Ready Hospitals	30	96363	73.21
Stroke-Ready Hospitals	35	108950	82.78
Stroke-Ready Hospitals	40	124302	94.44
Stroke-Ready Hospitals	45	131619	100.0
Stroke-Ready Hospitals	50	131619	100.0
Stroke-Ready Hospitals	55	131619	100.0
Stroke-Ready Hospitals	60	131619	100.0
Stroke Units	5	9769	7.42
Stroke Units	10	21693	16.48
Stroke Units	15	42760	32.49
Stroke Units	20	56584	42.99
Stroke Units	25	76322	57.99
Stroke Units	30	96363	73.21
Stroke Units	35	108950	82.78
Stroke Units	40	124302	94.44
Stroke Units	45	131619	100.0
Stroke Units	50	131619	100.0
Stroke Units	55	131619	100.0
Stroke Units	60	131619	100.0

Population Coverage - Landkreis Freudenstadt (Baden-Württemberg)

Condition	Drive Time (min)	Covered Population	Coverage (%)
Hospitals with CT	5	9069	7.82
Hospitals with CT	10	18196	15.7
Hospitals with CT	15	33922	29.27
Hospitals with CT	20	68029	58.69
Hospitals with CT	25	105543	91.06
Hospitals with CT	30	115388	99.55
Hospitals with CT	35	115821	99.93
Hospitals with CT	40	115862	99.96
Hospitals with CT	45	115895	99.99
Hospitals with CT	50	115907	100.0
Hospitals with CT	55	115907	100.0
Hospitals with CT	60	115907	100.0
Stroke-Ready Hospitals	5	9188	7.93
Stroke-Ready Hospitals	10	18177	15.68
Stroke-Ready Hospitals	15	33376	28.8
Stroke-Ready Hospitals	20	50317	43.41
Stroke-Ready Hospitals	25	71311	61.52
Stroke-Ready Hospitals	30	101725	87.76
Stroke-Ready Hospitals	35	115088	99.29
Stroke-Ready Hospitals	40	115862	99.96
Stroke-Ready Hospitals	45	115893	99.99
Stroke-Ready Hospitals	50	115907	100.0
Stroke-Ready Hospitals	55	115907	100.0
Stroke-Ready Hospitals	60	115907	100.0
Stroke Units	5	0	0.0
Stroke Units	10	0	0.0
Stroke Units	15	0	0.0
Stroke Units	20	0	0.0
Stroke Units	25	0	0.0
Stroke Units	30	50	0.04
Stroke Units	35	11602	10.01
Stroke Units	40	22280	19.22
Stroke Units	45	29303	25.28
Stroke Units	50	37594	32.43
Stroke Units	55	61536	53.09
Stroke Units	60	87097	75.14

Population Coverage - Landkreis Rosenheim (Bayern)

Condition	Drive Time (min)	Covered Population	Coverage (%)
Hospitals with CT	5	38207	14.9
Hospitals with CT	10	122805	47.89
Hospitals with CT	15	207020	80.73
Hospitals with CT	20	245205	95.63
Hospitals with CT	25	253745	98.96
Hospitals with CT	30	255148	99.5
Hospitals with CT	35	255692	99.72
Hospitals with CT	40	255737	99.73
Hospitals with CT	45	255763	99.74
Hospitals with CT	50	255787	99.75
Hospitals with CT	55	255750	99.74
Hospitals with CT	60	255798	99.76
Stroke-Ready Hospitals	5	14637	5.71
Stroke-Ready Hospitals	10	66218	25.82
Stroke-Ready Hospitals	15	113908	44.42
Stroke-Ready Hospitals	20	166819	65.06
Stroke-Ready Hospitals	25	215864	84.18
Stroke-Ready Hospitals	30	247716	96.61
Stroke-Ready Hospitals	35	254365	99.2
Stroke-Ready Hospitals	40	255317	99.57
Stroke-Ready Hospitals	45	255689	99.71
Stroke-Ready Hospitals	50	255741	99.74
Stroke-Ready Hospitals	55	255659	99.7
Stroke-Ready Hospitals	60	255689	99.71
Stroke Units	5	14637	5.71
Stroke Units	10	66218	25.82
Stroke Units	15	113908	44.42
Stroke Units	20	166819	65.06
Stroke Units	25	215864	84.18
Stroke Units	30	247716	96.61
Stroke Units	35	254365	99.2
Stroke Units	40	255317	99.57
Stroke Units	45	255689	99.71
Stroke Units	50	255741	99.74
Stroke Units	55	255659	99.7
Stroke Units	60	255689	99.71

Population Coverage - Kreisfreie Stadt Passau (Bayern)

Condition	Drive Time (min)	Covered Population	Coverage (%)
Hospitals with CT	5	11178	21.65
Hospitals with CT	10	29195	56.55
Hospitals with CT	15	50441	97.7
Hospitals with CT	20	51617	99.98
Hospitals with CT	25	51629	100.0
Hospitals with CT	30	51629	100.0
Hospitals with CT	35	51629	100.0
Hospitals with CT	40	51629	100.0
Hospitals with CT	45	51630	100.0
Hospitals with CT	50	51630	100.0
Hospitals with CT	55	51630	100.0
Hospitals with CT	60	51630	100.0
Stroke-Ready Hospitals	5	8571	16.6
Stroke-Ready Hospitals	10	27595	53.45
Stroke-Ready Hospitals	15	49358	95.6
Stroke-Ready Hospitals	20	51617	99.98
Stroke-Ready Hospitals	25	51629	100.0
Stroke-Ready Hospitals	30	51629	100.0
Stroke-Ready Hospitals	35	51629	100.0
Stroke-Ready Hospitals	40	51629	100.0
Stroke-Ready Hospitals	45	51630	100.0
Stroke-Ready Hospitals	50	51630	100.0
Stroke-Ready Hospitals	55	51629	100.0
Stroke-Ready Hospitals	60	51629	100.0
Stroke Units	5	8571	16.6
Stroke Units	10	27595	53.45
Stroke Units	15	49358	95.6
Stroke Units	20	51617	99.98
Stroke Units	25	51629	100.0
Stroke Units	30	51629	100.0
Stroke Units	35	51629	100.0
Stroke Units	40	51629	100.0
Stroke Units	45	51629	100.0
Stroke Units	50	51630	100.0
Stroke Units	55	51629	100.0
Stroke Units	60	51629	100.0

Population Coverage - Landkreis Coburg (Bayern)

Condition	Drive Time (min)	Covered Population	Coverage (%)
Hospitals with CT	5	3343	3.72
Hospitals with CT	10	20961	23.32
Hospitals with CT	15	55663	61.93
Hospitals with CT	20	81430	90.59
Hospitals with CT	25	89876	99.99
Hospitals with CT	30	89886	100.0
Hospitals with CT	35	89886	100.0
Hospitals with CT	40	89886	100.0
Hospitals with CT	45	89886	100.0
Hospitals with CT	50	89886	100.0
Hospitals with CT	55	89886	100.0
Hospitals with CT	60	89886	100.0
Stroke-Ready Hospitals	5	1100	1.22
Stroke-Ready Hospitals	10	14306	15.92
Stroke-Ready Hospitals	15	46019	51.2
Stroke-Ready Hospitals	20	77215	85.9
Stroke-Ready Hospitals	25	89265	99.31
Stroke-Ready Hospitals	30	89885	100.0
Stroke-Ready Hospitals	35	89886	100.0
Stroke-Ready Hospitals	40	89886	100.0
Stroke-Ready Hospitals	45	89886	100.0
Stroke-Ready Hospitals	50	89886	100.0
Stroke-Ready Hospitals	55	89886	100.0
Stroke-Ready Hospitals	60	89886	100.0
Stroke Units	5	1100	1.22
Stroke Units	10	14306	15.92
Stroke Units	15	46019	51.2
Stroke Units	20	77215	85.9
Stroke Units	25	89265	99.31
Stroke Units	30	89885	100.0
Stroke Units	35	89886	100.0
Stroke Units	40	89886	100.0
Stroke Units	45	89886	100.0
Stroke Units	50	89886	100.0
Stroke Units	55	89886	100.0
Stroke Units	60	89886	100.0

Population Coverage - Landkreis Ostallgäu (Bayern)

Condition	Drive Time (min)	Covered Population	Coverage (%)
Hospitals with CT	5	6811	4.85
Hospitals with CT	10	26700	19.02
Hospitals with CT	15	57586	41.02
Hospitals with CT	20	104231	74.24
Hospitals with CT	25	125528	89.41
Hospitals with CT	30	135403	96.44
Hospitals with CT	35	139061	99.05
Hospitals with CT	40	139832	99.6
Hospitals with CT	45	139891	99.64
Hospitals with CT	50	139917	99.66
Hospitals with CT	55	139830	99.6
Hospitals with CT	60	139898	99.64
Stroke-Ready Hospitals	5	0	0.0
Stroke-Ready Hospitals	10	3737	2.66
Stroke-Ready Hospitals	15	31874	22.7
Stroke-Ready Hospitals	20	55538	39.56
Stroke-Ready Hospitals	25	85517	60.91
Stroke-Ready Hospitals	30	106252	75.68
Stroke-Ready Hospitals	35	127857	91.07
Stroke-Ready Hospitals	40	134150	95.55
Stroke-Ready Hospitals	45	137046	97.61
Stroke-Ready Hospitals	50	139681	99.49
Stroke-Ready Hospitals	55	139615	99.44
Stroke-Ready Hospitals	60	139778	99.56
Stroke Units	5	0	0.0
Stroke Units	10	2819	2.01
Stroke Units	15	16471	11.73
Stroke Units	20	36549	26.03
Stroke Units	25	79667	56.74
Stroke Units	30	104718	74.59
Stroke Units	35	127857	91.07
Stroke Units	40	134150	95.55
Stroke Units	45	137046	97.61
Stroke Units	50	139681	99.49
Stroke Units	55	139615	99.44
Stroke Units	60	139750	99.54

Population Coverage - Landkreis Saarpfalz-Kreis (Saarland)

Condition	Drive Time (min)	Covered Population	Coverage (%)
Hospitals with CT	5	24273	17.69
Hospitals with CT	10	77502	56.48
Hospitals with CT	15	104312	76.02
Hospitals with CT	20	120658	87.93
Hospitals with CT	25	131792	96.04
Hospitals with CT	30	137112	99.92
Hospitals with CT	35	137211	99.99
Hospitals with CT	40	137215	100.0
Hospitals with CT	45	137218	100.0
Hospitals with CT	50	137218	100.0
Hospitals with CT	55	137196	99.98
Hospitals with CT	60	137212	100.0
Stroke-Ready Hospitals	5	7270	5.3
Stroke-Ready Hospitals	10	32753	23.87
Stroke-Ready Hospitals	15	91528	66.7
Stroke-Ready Hospitals	20	108868	79.34
Stroke-Ready Hospitals	25	122782	89.48
Stroke-Ready Hospitals	30	129691	94.51
Stroke-Ready Hospitals	35	135734	98.92
Stroke-Ready Hospitals	40	137214	100.0
Stroke-Ready Hospitals	45	137218	100.0
Stroke-Ready Hospitals	50	137218	100.0
Stroke-Ready Hospitals	55	137206	99.99
Stroke-Ready Hospitals	60	137212	100.0
Stroke Units	5	7270	5.3
Stroke Units	10	32753	23.87
Stroke Units	15	91528	66.7
Stroke Units	20	108868	79.34
Stroke Units	25	122782	89.48
Stroke Units	30	129620	94.46
Stroke Units	35	135734	98.92
Stroke Units	40	137205	99.99
Stroke Units	45	137218	100.0
Stroke Units	50	137218	100.0
Stroke Units	55	137203	99.99
Stroke Units	60	137211	99.99

Population Coverage - Landkreis Nordhausen (Thüringen)

Condition	Drive Time (min)	Covered Population	Coverage (%)
Hospitals with CT	5	12596	16.34
Hospitals with CT	10	35974	46.66
Hospitals with CT	15	56083	72.74
Hospitals with CT	20	67457	87.49
Hospitals with CT	25	76857	99.68
Hospitals with CT	30	76983	99.84
Hospitals with CT	35	77103	100.0
Hospitals with CT	40	77103	100.0
Hospitals with CT	45	77103	100.0
Hospitals with CT	50	77103	100.0
Hospitals with CT	55	77103	100.0
Hospitals with CT	60	77103	100.0
Stroke-Ready Hospitals	5	4971	6.45
Stroke-Ready Hospitals	10	23650	30.67
Stroke-Ready Hospitals	15	37016	48.01
Stroke-Ready Hospitals	20	47555	61.68
Stroke-Ready Hospitals	25	59244	76.84
Stroke-Ready Hospitals	30	74057	96.05
Stroke-Ready Hospitals	35	76791	99.6
Stroke-Ready Hospitals	40	77023	99.9
Stroke-Ready Hospitals	45	77103	100.0
Stroke-Ready Hospitals	50	77103	100.0
Stroke-Ready Hospitals	55	77103	100.0
Stroke-Ready Hospitals	60	77103	100.0
Stroke Units	5	4971	6.45
Stroke Units	10	23650	30.67
Stroke Units	15	37015	48.01
Stroke Units	20	45764	59.35
Stroke Units	25	54114	70.18
Stroke Units	30	67205	87.16
Stroke Units	35	73667	95.54
Stroke Units	40	76703	99.48
Stroke Units	45	77095	99.99
Stroke Units	50	77103	100.0
Stroke Units	55	77103	100.0
Stroke Units	60	77103	100.0

Population Coverage - Landkreis Uelzen (Niedersachsen)

Condition	Drive Time (min)	Covered Population	Coverage (%)
Hospitals with CT	5	12691	13.63
Hospitals with CT	10	36863	39.59
Hospitals with CT	15	61225	65.75
Hospitals with CT	20	80062	85.98
Hospitals with CT	25	89430	96.04
Hospitals with CT	30	92723	99.58
Hospitals with CT	35	92870	99.74
Hospitals with CT	40	92871	99.74
Hospitals with CT	45	93113	100.0
Hospitals with CT	50	93113	100.0
Hospitals with CT	55	93113	100.0
Hospitals with CT	60	93113	100.0
Stroke-Ready Hospitals	5	4305	4.62
Stroke-Ready Hospitals	10	18833	20.23
Stroke-Ready Hospitals	15	36589	39.3
Stroke-Ready Hospitals	20	48759	52.37
Stroke-Ready Hospitals	25	72156	77.49
Stroke-Ready Hospitals	30	88405	94.94
Stroke-Ready Hospitals	35	92090	98.9
Stroke-Ready Hospitals	40	92818	99.68
Stroke-Ready Hospitals	45	93113	100.0
Stroke-Ready Hospitals	50	93113	100.0
Stroke-Ready Hospitals	55	93113	100.0
Stroke-Ready Hospitals	60	93113	100.0
Stroke Units	5	4305	4.62
Stroke Units	10	18833	20.23
Stroke Units	15	36589	39.3
Stroke Units	20	48759	52.37
Stroke Units	25	72103	77.44
Stroke Units	30	88405	94.94
Stroke Units	35	92090	98.9
Stroke Units	40	92818	99.68
Stroke Units	45	92973	99.85
Stroke Units	50	93113	100.0
Stroke Units	55	93113	100.0
Stroke Units	60	93113	100.0

Population Coverage - Kreisfreie Stadt Mülheim an der Ruhr (Nordrhein-Westfalen)

Condition	Drive Time (min)	Covered Population	Coverage (%)
Hospitals with CT	5	38502	21.37
Hospitals with CT	10	161570	89.66
Hospitals with CT	15	179942	99.85
Hospitals with CT	20	180205	100.0
Hospitals with CT	25	180205	100.0
Hospitals with CT	30	180205	100.0
Hospitals with CT	35	180205	100.0
Hospitals with CT	40	180205	100.0
Hospitals with CT	45	180205	100.0
Hospitals with CT	50	180205	100.0
Hospitals with CT	55	180205	100.0
Hospitals with CT	60	180205	100.0
Stroke-Ready Hospitals	5	28253	15.68
Stroke-Ready Hospitals	10	119342	66.23
Stroke-Ready Hospitals	15	179156	99.42
Stroke-Ready Hospitals	20	180205	100.0
Stroke-Ready Hospitals	25	180205	100.0
Stroke-Ready Hospitals	30	180205	100.0
Stroke-Ready Hospitals	35	180205	100.0
Stroke-Ready Hospitals	40	180205	100.0
Stroke-Ready Hospitals	45	180205	100.0
Stroke-Ready Hospitals	50	180205	100.0
Stroke-Ready Hospitals	55	180205	100.0
Stroke-Ready Hospitals	60	180205	100.0
Stroke Units	5	28253	15.68
Stroke Units	10	119342	66.23
Stroke Units	15	179156	99.42
Stroke Units	20	180205	100.0
Stroke Units	25	180205	100.0
Stroke Units	30	180205	100.0
Stroke Units	35	180205	100.0
Stroke Units	40	180205	100.0
Stroke Units	45	180205	100.0
Stroke Units	50	180205	100.0
Stroke Units	55	180205	100.0
Stroke Units	60	180205	100.0

Population Coverage - Kreisfreie Stadt Bonn (Nordrhein-Westfalen)

Condition	Drive Time (min)	Covered Population	Coverage (%)
Hospitals with CT	5	141537	43.46
Hospitals with CT	10	295630	90.76
Hospitals with CT	15	325463	99.92
Hospitals with CT	20	325710	100.0
Hospitals with CT	25	325710	100.0
Hospitals with CT	30	325710	100.0
Hospitals with CT	35	325710	100.0
Hospitals with CT	40	325710	100.0
Hospitals with CT	45	325710	100.0
Hospitals with CT	50	325710	100.0
Hospitals with CT	55	325710	100.0
Hospitals with CT	60	325710	100.0
Stroke-Ready Hospitals	5	47919	14.71
Stroke-Ready Hospitals	10	165702	50.87
Stroke-Ready Hospitals	15	261182	80.19
Stroke-Ready Hospitals	20	304401	93.46
Stroke-Ready Hospitals	25	325618	99.97
Stroke-Ready Hospitals	30	325710	100.0
Stroke-Ready Hospitals	35	325710	100.0
Stroke-Ready Hospitals	40	325710	100.0
Stroke-Ready Hospitals	45	325710	100.0
Stroke-Ready Hospitals	50	325710	100.0
Stroke-Ready Hospitals	55	325710	100.0
Stroke-Ready Hospitals	60	325710	100.0
Stroke Units	5	47919	14.71
Stroke Units	10	165702	50.87
Stroke Units	15	261182	80.19
Stroke Units	20	304401	93.46
Stroke Units	25	325618	99.97
Stroke Units	30	325710	100.0
Stroke Units	35	325710	100.0
Stroke Units	40	325710	100.0
Stroke Units	45	325710	100.0
Stroke Units	50	325710	100.0
Stroke Units	55	325710	100.0
Stroke Units	60	325710	100.0

Population Coverage - Landkreis Birkenfeld (Rheinland-Pfalz)

Condition	Drive Time (min)	Covered Population	Coverage (%)
Hospitals with CT	5	12061	14.58
Hospitals with CT	10	34677	41.91
Hospitals with CT	15	57861	69.92
Hospitals with CT	20	74733	90.31
Hospitals with CT	25	80915	97.78
Hospitals with CT	30	81077	97.98
Hospitals with CT	35	81106	98.01
Hospitals with CT	40	81107	98.01
Hospitals with CT	45	82749	100.0
Hospitals with CT	50	82749	100.0
Hospitals with CT	55	82749	100.0
Hospitals with CT	60	82749	100.0
Stroke-Ready Hospitals	5	4223	5.1
Stroke-Ready Hospitals	10	18830	22.76
Stroke-Ready Hospitals	15	30640	37.03
Stroke-Ready Hospitals	20	43400	52.45
Stroke-Ready Hospitals	25	56879	68.74
Stroke-Ready Hospitals	30	76049	91.9
Stroke-Ready Hospitals	35	80582	97.38
Stroke-Ready Hospitals	40	80954	97.83
Stroke-Ready Hospitals	45	81123	98.03
Stroke-Ready Hospitals	50	82749	100.0
Stroke-Ready Hospitals	55	82749	100.0
Stroke-Ready Hospitals	60	82749	100.0
Stroke Units	5	4223	5.1
Stroke Units	10	18830	22.76
Stroke Units	15	30640	37.03
Stroke Units	20	43400	52.45
Stroke Units	25	56879	68.74
Stroke Units	30	76048	91.9
Stroke Units	35	80582	97.38
Stroke Units	40	80954	97.83
Stroke Units	45	81123	98.03
Stroke Units	50	82749	100.0
Stroke Units	55	82749	100.0
Stroke Units	60	82749	100.0

Population Coverage - Landkreis Rems-Murr-Kreis (Baden-Württemberg)

Condition	Drive Time (min)	Covered Population	Coverage (%)
Hospitals with CT	5	41205	9.86
Hospitals with CT	10	125796	30.11
Hospitals with CT	15	288084	68.95
Hospitals with CT	20	347709	83.22
Hospitals with CT	25	389801	93.29
Hospitals with CT	30	407104	97.44
Hospitals with CT	35	416202	99.61
Hospitals with CT	40	417819	100.0
Hospitals with CT	45	417819	100.0
Hospitals with CT	50	417819	100.0
Hospitals with CT	55	417819	100.0
Hospitals with CT	60	417819	100.0
Stroke-Ready Hospitals	5	38563	9.23
Stroke-Ready Hospitals	10	105301	25.2
Stroke-Ready Hospitals	15	257615	61.66
Stroke-Ready Hospitals	20	339934	81.36
Stroke-Ready Hospitals	25	377402	90.33
Stroke-Ready Hospitals	30	392915	94.04
Stroke-Ready Hospitals	35	403197	96.5
Stroke-Ready Hospitals	40	417151	99.84
Stroke-Ready Hospitals	45	417819	100.0
Stroke-Ready Hospitals	50	417819	100.0
Stroke-Ready Hospitals	55	417819	100.0
Stroke-Ready Hospitals	60	417819	100.0
Stroke Units	5	20056	4.8
Stroke Units	10	64783	15.51
Stroke Units	15	185422	44.38
Stroke Units	20	279772	66.96
Stroke Units	25	355039	84.97
Stroke Units	30	373370	89.36
Stroke Units	35	395837	94.74
Stroke Units	40	415652	99.48
Stroke Units	45	417819	100.0
Stroke Units	50	417819	100.0
Stroke Units	55	417819	100.0
Stroke Units	60	417819	100.0

Population Coverage - Landkreis Schwäbisch Hall (Baden-Württemberg)

Condition	Drive Time (min)	Covered Population	Coverage (%)
Hospitals with CT	5	17241	9.03
Hospitals with CT	10	60921	31.92
Hospitals with CT	15	94729	49.63
Hospitals with CT	20	131632	68.96
Hospitals with CT	25	160502	84.08
Hospitals with CT	30	180448	94.53
Hospitals with CT	35	190390	99.74
Hospitals with CT	40	190887	100.0
Hospitals with CT	45	190887	100.0
Hospitals with CT	50	190887	100.0
Hospitals with CT	55	190887	100.0
Hospitals with CT	60	190887	100.0
Stroke-Ready Hospitals	5	1938	1.02
Stroke-Ready Hospitals	10	19539	10.24
Stroke-Ready Hospitals	15	50595	26.51
Stroke-Ready Hospitals	20	67815	35.53
Stroke-Ready Hospitals	25	98575	51.64
Stroke-Ready Hospitals	30	147965	77.51
Stroke-Ready Hospitals	35	182274	95.49
Stroke-Ready Hospitals	40	190688	99.9
Stroke-Ready Hospitals	45	190887	100.0
Stroke-Ready Hospitals	50	190887	100.0
Stroke-Ready Hospitals	55	190887	100.0
Stroke-Ready Hospitals	60	190887	100.0
Stroke Units	5	1938	1.02
Stroke Units	10	19539	10.24
Stroke Units	15	50593	26.5
Stroke Units	20	67222	35.22
Stroke Units	25	95898	50.24
Stroke Units	30	146671	76.84
Stroke Units	35	180145	94.37
Stroke Units	40	190644	99.87
Stroke Units	45	190887	100.0
Stroke Units	50	190887	100.0
Stroke Units	55	190887	100.0
Stroke Units	60	190887	100.0

Population Coverage - Landkreis Rottweil (Baden-Württemberg)

Condition	Drive Time (min)	Covered Population	Coverage (%)
Hospitals with CT	5	28095	20.42
Hospitals with CT	10	54295	39.47
Hospitals with CT	15	82946	60.3
Hospitals with CT	20	106228	77.23
Hospitals with CT	25	126682	92.09
Hospitals with CT	30	135064	98.19
Hospitals with CT	35	137532	99.98
Hospitals with CT	40	137556	100.0
Hospitals with CT	45	137556	100.0
Hospitals with CT	50	137556	100.0
Hospitals with CT	55	137556	100.0
Hospitals with CT	60	137556	100.0
Stroke-Ready Hospitals	5	10420	7.58
Stroke-Ready Hospitals	10	35172	25.57
Stroke-Ready Hospitals	15	46217	33.6
Stroke-Ready Hospitals	20	58399	42.45
Stroke-Ready Hospitals	25	92301	67.1
Stroke-Ready Hospitals	30	116551	84.73
Stroke-Ready Hospitals	35	129643	94.25
Stroke-Ready Hospitals	40	135701	98.65
Stroke-Ready Hospitals	45	137450	99.92
Stroke-Ready Hospitals	50	137556	100.0
Stroke-Ready Hospitals	55	137556	100.0
Stroke-Ready Hospitals	60	137556	100.0
Stroke Units	5	0	0.0
Stroke Units	10	0	0.0
Stroke Units	15	307	0.22
Stroke Units	20	4959	3.61
Stroke Units	25	26949	19.59
Stroke Units	30	46985	34.16
Stroke Units	35	76454	55.58
Stroke Units	40	106944	77.75
Stroke Units	45	122862	89.32
Stroke Units	50	128838	93.66
Stroke Units	55	136167	98.99
Stroke Units	60	137233	99.77

Population Coverage - Landkreis Amberg-Sulzbach (Bayern)

Condition	Drive Time (min)	Covered Population	Coverage (%)
Hospitals with CT	5	15240	14.28
Hospitals with CT	10	44070	41.3
Hospitals with CT	15	69475	65.11
Hospitals with CT	20	87490	81.99
Hospitals with CT	25	101881	95.48
Hospitals with CT	30	105722	99.08
Hospitals with CT	35	106691	99.99
Hospitals with CT	40	106692	99.99
Hospitals with CT	45	106705	100.0
Hospitals with CT	50	106705	100.0
Hospitals with CT	55	106705	100.0
Hospitals with CT	60	106705	100.0
Stroke-Ready Hospitals	5	0	0.0
Stroke-Ready Hospitals	10	9305	8.72
Stroke-Ready Hospitals	15	27472	25.75
Stroke-Ready Hospitals	20	60505	56.7
Stroke-Ready Hospitals	25	80827	75.75
Stroke-Ready Hospitals	30	93827	87.93
Stroke-Ready Hospitals	35	103541	97.04
Stroke-Ready Hospitals	40	105861	99.21
Stroke-Ready Hospitals	45	106446	99.76
Stroke-Ready Hospitals	50	106705	100.0
Stroke-Ready Hospitals	55	106705	100.0
Stroke-Ready Hospitals	60	106705	100.0
Stroke Units	5	0	0.0
Stroke Units	10	9305	8.72
Stroke Units	15	27472	25.75
Stroke Units	20	60505	56.7
Stroke Units	25	80827	75.75
Stroke Units	30	93827	87.93
Stroke Units	35	103541	97.04
Stroke Units	40	105861	99.21
Stroke Units	45	106446	99.76
Stroke Units	50	106705	100.0
Stroke Units	55	106705	100.0
Stroke Units	60	106705	100.0

Population Coverage - Kreisfreie Stadt Kempten (Allgäu) (Bayern)

Condition	Drive Time (min)	Covered Population	Coverage (%)
Hospitals with CT	5	30620	44.71
Hospitals with CT	10	64213	93.76
Hospitals with CT	15	68488	100.0
Hospitals with CT	20	68488	100.0
Hospitals with CT	25	68488	100.0
Hospitals with CT	30	68488	100.0
Hospitals with CT	35	68488	100.0
Hospitals with CT	40	68488	100.0
Hospitals with CT	45	68488	100.0
Hospitals with CT	50	68488	100.0
Hospitals with CT	55	68488	100.0
Hospitals with CT	60	68488	100.0
Stroke-Ready Hospitals	5	30190	44.08
Stroke-Ready Hospitals	10	64137	93.65
Stroke-Ready Hospitals	15	68488	100.0
Stroke-Ready Hospitals	20	68488	100.0
Stroke-Ready Hospitals	25	68488	100.0
Stroke-Ready Hospitals	30	68488	100.0
Stroke-Ready Hospitals	35	68488	100.0
Stroke-Ready Hospitals	40	68488	100.0
Stroke-Ready Hospitals	45	68488	100.0
Stroke-Ready Hospitals	50	68488	100.0
Stroke-Ready Hospitals	55	68488	100.0
Stroke-Ready Hospitals	60	68488	100.0
Stroke Units	5	30190	44.08
Stroke Units	10	64137	93.65
Stroke Units	15	68488	100.0
Stroke Units	20	68488	100.0
Stroke Units	25	68488	100.0
Stroke Units	30	68488	100.0
Stroke Units	35	68488	100.0
Stroke Units	40	68488	100.0
Stroke Units	45	68488	100.0
Stroke Units	50	68488	100.0
Stroke Units	55	68488	100.0
Stroke Units	60	68488	100.0

Population Coverage - Landkreis Mecklenburgische Seenplatte (Mecklenburg-Vorpommern)

Condition	Drive Time (min)	Covered Population	Coverage (%)
Hospitals with CT	5	29733	12.44
Hospitals with CT	10	74014	30.98
Hospitals with CT	15	126352	52.88
Hospitals with CT	20	162820	68.14
Hospitals with CT	25	188393	78.84
Hospitals with CT	30	214713	89.86
Hospitals with CT	35	228145	95.48
Hospitals with CT	40	235418	98.53
Hospitals with CT	45	237610	99.44
Hospitals with CT	50	238052	99.63
Hospitals with CT	55	237949	99.58
Hospitals with CT	60	238131	99.66
Stroke-Ready Hospitals	5	9134	3.82
Stroke-Ready Hospitals	10	29197	12.22
Stroke-Ready Hospitals	15	56269	23.55
Stroke-Ready Hospitals	20	67695	28.33
Stroke-Ready Hospitals	25	81181	33.98
Stroke-Ready Hospitals	30	98461	41.21
Stroke-Ready Hospitals	35	116970	48.95
Stroke-Ready Hospitals	40	138687	58.04
Stroke-Ready Hospitals	45	157997	66.12
Stroke-Ready Hospitals	50	182399	76.34
Stroke-Ready Hospitals	55	208809	87.39
Stroke-Ready Hospitals	60	228115	95.47
Stroke Units	5	9134	3.82
Stroke Units	10	29197	12.22
Stroke Units	15	56269	23.55
Stroke Units	20	67695	28.33
Stroke Units	25	81181	33.98
Stroke Units	30	98461	41.21
Stroke Units	35	116970	48.95
Stroke Units	40	138687	58.04
Stroke Units	45	157997	66.12
Stroke Units	50	182399	76.34
Stroke Units	55	208795	87.38
Stroke Units	60	228102	95.46

Population Coverage - Kreisfreie Stadt Chemnitz (Sachsen)

Condition	Drive Time (min)	Covered Population	Coverage (%)
Hospitals with CT	5	52794	17.83
Hospitals with CT	10	183345	61.91
Hospitals with CT	15	268010	90.49
Hospitals with CT	20	293036	98.94
Hospitals with CT	25	296171	100.0
Hospitals with CT	30	296171	100.0
Hospitals with CT	35	296171	100.0
Hospitals with CT	40	296171	100.0
Hospitals with CT	45	296171	100.0
Hospitals with CT	50	296171	100.0
Hospitals with CT	55	296171	100.0
Hospitals with CT	60	296171	100.0
Stroke-Ready Hospitals	5	12891	4.35
Stroke-Ready Hospitals	10	67304	22.72
Stroke-Ready Hospitals	15	191525	64.67
Stroke-Ready Hospitals	20	262423	88.61
Stroke-Ready Hospitals	25	288416	97.38
Stroke-Ready Hospitals	30	295366	99.73
Stroke-Ready Hospitals	35	296171	100.0
Stroke-Ready Hospitals	40	296171	100.0
Stroke-Ready Hospitals	45	296171	100.0
Stroke-Ready Hospitals	50	296171	100.0
Stroke-Ready Hospitals	55	296171	100.0
Stroke-Ready Hospitals	60	296171	100.0
Stroke Units	5	0	0.0
Stroke Units	10	2910	0.98
Stroke Units	15	56500	19.08
Stroke Units	20	147192	49.7
Stroke Units	25	230962	77.98
Stroke Units	30	287300	97.0
Stroke Units	35	295942	99.92
Stroke Units	40	296171	100.0
Stroke Units	45	296171	100.0
Stroke Units	50	296171	100.0
Stroke Units	55	296171	100.0
Stroke Units	60	296171	100.0

Population Coverage - Landkreis Erzgebirgskreis (Sachsen)

Condition	Drive Time (min)	Covered Population	Coverage (%)
Hospitals with CT	5	34247	10.43
Hospitals with CT	10	104117	31.7
Hospitals with CT	15	215160	65.5
Hospitals with CT	20	296560	90.29
Hospitals with CT	25	323572	98.51
Hospitals with CT	30	327943	99.84
Hospitals with CT	35	328397	99.98
Hospitals with CT	40	328446	99.99
Hospitals with CT	45	328446	99.99
Hospitals with CT	50	328446	99.99
Hospitals with CT	55	328451	100.0
Hospitals with CT	60	328452	100.0
Stroke-Ready Hospitals	5	16921	5.15
Stroke-Ready Hospitals	10	48149	14.66
Stroke-Ready Hospitals	15	93246	28.39
Stroke-Ready Hospitals	20	163852	49.88
Stroke-Ready Hospitals	25	229535	69.88
Stroke-Ready Hospitals	30	274875	83.68
Stroke-Ready Hospitals	35	300323	91.43
Stroke-Ready Hospitals	40	312083	95.01
Stroke-Ready Hospitals	45	318288	96.9
Stroke-Ready Hospitals	50	326557	99.42
Stroke-Ready Hospitals	55	328361	99.97
Stroke-Ready Hospitals	60	328435	99.99
Stroke Units	5	5998	1.83
Stroke Units	10	25004	7.61
Stroke Units	15	48708	14.83
Stroke Units	20	92049	28.02
Stroke Units	25	130967	39.87
Stroke Units	30	167796	51.09
Stroke Units	35	205568	62.58
Stroke Units	40	258610	78.73
Stroke Units	45	289753	88.21
Stroke Units	50	315250	95.98
Stroke Units	55	325255	99.02
Stroke Units	60	327374	99.67

Population Coverage - Kreisfreie Stadt Dresden (Sachsen)

Condition	Drive Time (min)	Covered Population	Coverage (%)
Hospitals with CT	5	96263	15.64
Hospitals with CT	10	321247	52.2
Hospitals with CT	15	548711	89.16
Hospitals with CT	20	607946	98.79
Hospitals with CT	25	615406	100.0
Hospitals with CT	30	615406	100.0
Hospitals with CT	35	615406	100.0
Hospitals with CT	40	615406	100.0
Hospitals with CT	45	615406	100.0
Hospitals with CT	50	615406	100.0
Hospitals with CT	55	615406	100.0
Hospitals with CT	60	615406	100.0
Stroke-Ready Hospitals	5	62200	10.11
Stroke-Ready Hospitals	10	265498	43.14
Stroke-Ready Hospitals	15	448304	72.85
Stroke-Ready Hospitals	20	567202	92.17
Stroke-Ready Hospitals	25	613812	99.74
Stroke-Ready Hospitals	30	615406	100.0
Stroke-Ready Hospitals	35	615406	100.0
Stroke-Ready Hospitals	40	615406	100.0
Stroke-Ready Hospitals	45	615406	100.0
Stroke-Ready Hospitals	50	615406	100.0
Stroke-Ready Hospitals	55	615406	100.0
Stroke-Ready Hospitals	60	615406	100.0
Stroke Units	5	62200	10.11
Stroke Units	10	265498	43.14
Stroke Units	15	447881	72.78
Stroke Units	20	563951	91.64
Stroke Units	25	610132	99.14
Stroke Units	30	614356	99.83
Stroke Units	35	615406	100.0
Stroke Units	40	615406	100.0
Stroke Units	45	615406	100.0
Stroke Units	50	615406	100.0
Stroke Units	55	615406	100.0
Stroke Units	60	615406	100.0

Population Coverage - Landkreis Hildburghausen (Thüringen)

Condition	Drive Time (min)	Covered Population	Coverage (%)
Hospitals with CT	5	9452	16.05
Hospitals with CT	10	14105	23.95
Hospitals with CT	15	26381	44.79
Hospitals with CT	20	45773	77.72
Hospitals with CT	25	55023	93.42
Hospitals with CT	30	57610	97.81
Hospitals with CT	35	58733	99.72
Hospitals with CT	40	58848	99.92
Hospitals with CT	45	58897	100.0
Hospitals with CT	50	58897	100.0
Hospitals with CT	55	58897	100.0
Hospitals with CT	60	58897	100.0
Stroke-Ready Hospitals	5	7385	12.54
Stroke-Ready Hospitals	10	12485	21.2
Stroke-Ready Hospitals	15	18593	31.57
Stroke-Ready Hospitals	20	37543	63.74
Stroke-Ready Hospitals	25	49892	84.71
Stroke-Ready Hospitals	30	56820	96.47
Stroke-Ready Hospitals	35	58656	99.59
Stroke-Ready Hospitals	40	58848	99.92
Stroke-Ready Hospitals	45	58897	100.0
Stroke-Ready Hospitals	50	58897	100.0
Stroke-Ready Hospitals	55	58897	100.0
Stroke-Ready Hospitals	60	58897	100.0
Stroke Units	5	7385	12.54
Stroke Units	10	12485	21.2
Stroke Units	15	18593	31.57
Stroke Units	20	37543	63.74
Stroke Units	25	49892	84.71
Stroke Units	30	56820	96.47
Stroke Units	35	58656	99.59
Stroke Units	40	58848	99.92
Stroke Units	45	58897	100.0
Stroke Units	50	58897	100.0
Stroke Units	55	58897	100.0
Stroke Units	60	58897	100.0

Population Coverage - Kreis Herzogtum Lauenburg (Schleswig-Holstein)

Condition	Drive Time (min)	Covered Population	Coverage (%)
Hospitals with CT	5	27439	14.05
Hospitals with CT	10	62487	31.99
Hospitals with CT	15	97264	49.79
Hospitals with CT	20	137518	70.39
Hospitals with CT	25	169786	86.91
Hospitals with CT	30	191903	98.23
Hospitals with CT	35	195341	99.99
Hospitals with CT	40	195353	100.0
Hospitals with CT	45	195353	100.0
Hospitals with CT	50	195353	100.0
Hospitals with CT	55	195353	100.0
Hospitals with CT	60	195353	100.0
Stroke-Ready Hospitals	5	6227	3.19
Stroke-Ready Hospitals	10	18231	9.33
Stroke-Ready Hospitals	15	32761	16.77
Stroke-Ready Hospitals	20	59002	30.2
Stroke-Ready Hospitals	25	87966	45.03
Stroke-Ready Hospitals	30	113549	58.13
Stroke-Ready Hospitals	35	146491	74.99
Stroke-Ready Hospitals	40	183829	94.1
Stroke-Ready Hospitals	45	194498	99.56
Stroke-Ready Hospitals	50	195260	99.95
Stroke-Ready Hospitals	55	195287	99.97
Stroke-Ready Hospitals	60	195353	100.0
Stroke Units	5	6227	3.19
Stroke Units	10	18231	9.33
Stroke Units	15	32761	16.77
Stroke Units	20	59002	30.2
Stroke Units	25	87966	45.03
Stroke Units	30	113549	58.13
Stroke Units	35	146491	74.99
Stroke Units	40	183807	94.09
Stroke Units	45	194493	99.56
Stroke Units	50	195260	99.95
Stroke Units	55	195287	99.97
Stroke Units	60	195353	100.0

Population Coverage - Landkreis Oldenburg (Niedersachsen)

Condition	Drive Time (min)	Covered Population	Coverage (%)
Hospitals with CT	5	14831	11.62
Hospitals with CT	10	27095	21.23
Hospitals with CT	15	72600	56.89
Hospitals with CT	20	121767	95.41
Hospitals with CT	25	127616	100.0
Hospitals with CT	30	127619	100.0
Hospitals with CT	35	127619	100.0
Hospitals with CT	40	127619	100.0
Hospitals with CT	45	127619	100.0
Hospitals with CT	50	127619	100.0
Hospitals with CT	55	127619	100.0
Hospitals with CT	60	127619	100.0
Stroke-Ready Hospitals	5	0	0.0
Stroke-Ready Hospitals	10	60	0.05
Stroke-Ready Hospitals	15	5396	4.23
Stroke-Ready Hospitals	20	34145	26.76
Stroke-Ready Hospitals	25	70357	55.13
Stroke-Ready Hospitals	30	98217	76.96
Stroke-Ready Hospitals	35	120781	94.64
Stroke-Ready Hospitals	40	126501	99.12
Stroke-Ready Hospitals	45	127619	100.0
Stroke-Ready Hospitals	50	127619	100.0
Stroke-Ready Hospitals	55	127619	100.0
Stroke-Ready Hospitals	60	127619	100.0
Stroke Units	5	0	0.0
Stroke Units	10	60	0.05
Stroke Units	15	5396	4.23
Stroke Units	20	34049	26.68
Stroke Units	25	70316	55.1
Stroke Units	30	97256	76.21
Stroke Units	35	119987	94.02
Stroke Units	40	126431	99.07
Stroke Units	45	127618	100.0
Stroke Units	50	127619	100.0
Stroke Units	55	127619	100.0
Stroke Units	60	127619	100.0

Population Coverage - Kreisfreie Stadt Krefeld (Nordrhein-Westfalen)

Condition	Drive Time (min)	Covered Population	Coverage (%)
Hospitals with CT	5	75409	31.16
Hospitals with CT	10	220427	91.09
Hospitals with CT	15	241943	99.98
Hospitals with CT	20	241981	100.0
Hospitals with CT	25	241982	100.0
Hospitals with CT	30	241982	100.0
Hospitals with CT	35	241982	100.0
Hospitals with CT	40	241982	100.0
Hospitals with CT	45	241982	100.0
Hospitals with CT	50	241982	100.0
Hospitals with CT	55	241982	100.0
Hospitals with CT	60	241982	100.0
Stroke-Ready Hospitals	5	64699	26.74
Stroke-Ready Hospitals	10	173832	71.84
Stroke-Ready Hospitals	15	229959	95.03
Stroke-Ready Hospitals	20	241973	100.0
Stroke-Ready Hospitals	25	241982	100.0
Stroke-Ready Hospitals	30	241982	100.0
Stroke-Ready Hospitals	35	241982	100.0
Stroke-Ready Hospitals	40	241982	100.0
Stroke-Ready Hospitals	45	241982	100.0
Stroke-Ready Hospitals	50	241982	100.0
Stroke-Ready Hospitals	55	241982	100.0
Stroke-Ready Hospitals	60	241982	100.0
Stroke Units	5	64699	26.74
Stroke Units	10	173832	71.84
Stroke Units	15	229959	95.03
Stroke Units	20	241973	100.0
Stroke Units	25	241982	100.0
Stroke Units	30	241982	100.0
Stroke Units	35	241982	100.0
Stroke Units	40	241982	100.0
Stroke Units	45	241982	100.0
Stroke Units	50	241982	100.0
Stroke Units	55	241982	100.0
Stroke Units	60	241982	100.0

Population Coverage - Kreis Oberbergischer Kreis (Nordrhein-Westfalen)

Condition	Drive Time (min)	Covered Population	Coverage (%)
Hospitals with CT	5	61393	22.83
Hospitals with CT	10	146019	54.31
Hospitals with CT	15	234586	87.25
Hospitals with CT	20	264992	98.56
Hospitals with CT	25	268868	100.0
Hospitals with CT	30	268868	100.0
Hospitals with CT	35	268868	100.0
Hospitals with CT	40	268868	100.0
Hospitals with CT	45	268868	100.0
Hospitals with CT	50	268868	100.0
Hospitals with CT	55	268868	100.0
Hospitals with CT	60	268868	100.0
Stroke-Ready Hospitals	5	14762	5.49
Stroke-Ready Hospitals	10	43195	16.07
Stroke-Ready Hospitals	15	83700	31.13
Stroke-Ready Hospitals	20	138799	51.62
Stroke-Ready Hospitals	25	195112	72.57
Stroke-Ready Hospitals	30	236192	87.85
Stroke-Ready Hospitals	35	263848	98.13
Stroke-Ready Hospitals	40	268728	99.95
Stroke-Ready Hospitals	45	268868	100.0
Stroke-Ready Hospitals	50	268868	100.0
Stroke-Ready Hospitals	55	268868	100.0
Stroke-Ready Hospitals	60	268868	100.0
Stroke Units	5	0	0.0
Stroke Units	10	0	0.0
Stroke Units	15	140	0.05
Stroke Units	20	849	0.32
Stroke Units	25	17141	6.38
Stroke Units	30	100825	37.5
Stroke Units	35	220117	81.87
Stroke Units	40	261372	97.21
Stroke Units	45	268455	99.85
Stroke Units	50	268868	100.0
Stroke Units	55	268868	100.0
Stroke Units	60	268868	100.0

Population Coverage - Landkreis Kusel (Rheinland-Pfalz)

Condition	Drive Time (min)	Covered Population	Coverage (%)
Hospitals with CT	5	2140	2.92
Hospitals with CT	10	12080	16.51
Hospitals with CT	15	35178	48.07
Hospitals with CT	20	58823	80.37
Hospitals with CT	25	69365	94.78
Hospitals with CT	30	73096	99.88
Hospitals with CT	35	73105	99.89
Hospitals with CT	40	73106	99.89
Hospitals with CT	45	73186	100.0
Hospitals with CT	50	73186	100.0
Hospitals with CT	55	73186	100.0
Hospitals with CT	60	73186	100.0
Stroke-Ready Hospitals	5	0	0.0
Stroke-Ready Hospitals	10	482	0.66
Stroke-Ready Hospitals	15	5377	7.35
Stroke-Ready Hospitals	20	22195	30.33
Stroke-Ready Hospitals	25	41398	56.57
Stroke-Ready Hospitals	30	57959	79.19
Stroke-Ready Hospitals	35	68838	94.06
Stroke-Ready Hospitals	40	73105	99.89
Stroke-Ready Hospitals	45	73105	99.89
Stroke-Ready Hospitals	50	73186	100.0
Stroke-Ready Hospitals	55	73186	100.0
Stroke-Ready Hospitals	60	73186	100.0
Stroke Units	5	0	0.0
Stroke Units	10	10	0.01
Stroke Units	15	3561	4.87
Stroke Units	20	17339	23.69
Stroke Units	25	31766	43.4
Stroke Units	30	45076	61.59
Stroke Units	35	56010	76.53
Stroke Units	40	70098	95.78
Stroke Units	45	73105	99.89
Stroke Units	50	73186	100.0
Stroke Units	55	73186	100.0
Stroke Units	60	73186	100.0

Population Coverage - Landkreis Fürstenfeldbruck (Bayern)

Condition	Drive Time (min)	Covered Population	Coverage (%)
Hospitals with CT	5	26075	12.13
Hospitals with CT	10	79054	36.78
Hospitals with CT	15	169447	78.84
Hospitals with CT	20	205768	95.73
Hospitals with CT	25	214845	99.96
Hospitals with CT	30	214938	100.0
Hospitals with CT	35	214938	100.0
Hospitals with CT	40	214938	100.0
Hospitals with CT	45	214938	100.0
Hospitals with CT	50	214938	100.0
Hospitals with CT	55	214938	100.0
Hospitals with CT	60	214938	100.0
Stroke-Ready Hospitals	5	24024	11.18
Stroke-Ready Hospitals	10	58767	27.34
Stroke-Ready Hospitals	15	147581	68.66
Stroke-Ready Hospitals	20	203367	94.62
Stroke-Ready Hospitals	25	214795	99.93
Stroke-Ready Hospitals	30	214938	100.0
Stroke-Ready Hospitals	35	214938	100.0
Stroke-Ready Hospitals	40	214938	100.0
Stroke-Ready Hospitals	45	214938	100.0
Stroke-Ready Hospitals	50	214938	100.0
Stroke-Ready Hospitals	55	214938	100.0
Stroke-Ready Hospitals	60	214938	100.0
Stroke Units	5	24024	11.18
Stroke Units	10	58767	27.34
Stroke Units	15	147580	68.66
Stroke Units	20	203367	94.62
Stroke Units	25	214760	99.92
Stroke Units	30	214938	100.0
Stroke Units	35	214938	100.0
Stroke Units	40	214938	100.0
Stroke Units	45	214938	100.0
Stroke Units	50	214938	100.0
Stroke Units	55	214938	100.0
Stroke Units	60	214938	100.0

Population Coverage - Landkreis Weilheim-Schongau (Bayern)

Condition	Drive Time (min)	Covered Population	Coverage (%)
Hospitals with CT	5	33535	24.83
Hospitals with CT	10	63008	46.65
Hospitals with CT	15	96622	71.53
Hospitals with CT	20	118672	87.86
Hospitals with CT	25	129966	96.22
Hospitals with CT	30	133736	99.01
Hospitals with CT	35	135007	99.95
Hospitals with CT	40	135035	99.97
Hospitals with CT	45	135057	99.99
Hospitals with CT	50	135059	99.99
Hospitals with CT	55	135059	99.99
Hospitals with CT	60	135059	99.99
Stroke-Ready Hospitals	5	16782	12.42
Stroke-Ready Hospitals	10	27397	20.28
Stroke-Ready Hospitals	15	44930	33.26
Stroke-Ready Hospitals	20	80814	59.83
Stroke-Ready Hospitals	25	91836	67.99
Stroke-Ready Hospitals	30	110995	82.17
Stroke-Ready Hospitals	35	129594	95.94
Stroke-Ready Hospitals	40	132747	98.28
Stroke-Ready Hospitals	45	134600	99.65
Stroke-Ready Hospitals	50	135059	99.99
Stroke-Ready Hospitals	55	135059	99.99
Stroke-Ready Hospitals	60	135059	99.99
Stroke Units	5	16782	12.42
Stroke Units	10	25921	19.19
Stroke Units	15	41849	30.98
Stroke Units	20	78669	58.24
Stroke Units	25	90186	66.77
Stroke Units	30	102166	75.64
Stroke Units	35	125182	92.68
Stroke Units	40	132621	98.18
Stroke Units	45	134574	99.63
Stroke Units	50	135059	99.99
Stroke Units	55	135059	99.99
Stroke Units	60	135059	99.99

Population Coverage - Landkreis Neustadt a.d.Waldnaab (Bayern)

Condition	Drive Time (min)	Covered Population	Coverage (%)
Hospitals with CT	5	5962	6.03
Hospitals with CT	10	22821	23.08
Hospitals with CT	15	43551	44.05
Hospitals with CT	20	79679	80.58
Hospitals with CT	25	92787	93.84
Hospitals with CT	30	97379	98.49
Hospitals with CT	35	98789	99.91
Hospitals with CT	40	98861	99.98
Hospitals with CT	45	98867	99.99
Hospitals with CT	50	98869	99.99
Hospitals with CT	55	98864	99.99
Hospitals with CT	60	98867	99.99
Stroke-Ready Hospitals	5	0	0.0
Stroke-Ready Hospitals	10	8207	8.3
Stroke-Ready Hospitals	15	24819	25.1
Stroke-Ready Hospitals	20	55953	56.59
Stroke-Ready Hospitals	25	73294	74.13
Stroke-Ready Hospitals	30	86412	87.39
Stroke-Ready Hospitals	35	96824	97.92
Stroke-Ready Hospitals	40	98064	99.18
Stroke-Ready Hospitals	45	98316	99.43
Stroke-Ready Hospitals	50	98835	99.96
Stroke-Ready Hospitals	55	98836	99.96
Stroke-Ready Hospitals	60	98853	99.98
Stroke Units	5	0	0.0
Stroke Units	10	8207	8.3
Stroke Units	15	24819	25.1
Stroke Units	20	55953	56.59
Stroke Units	25	73294	74.13
Stroke Units	30	86412	87.39
Stroke Units	35	96824	97.92
Stroke Units	40	98064	99.18
Stroke Units	45	98315	99.43
Stroke Units	50	98835	99.96
Stroke Units	55	98836	99.96
Stroke Units	60	98845	99.97

Population Coverage - Landkreis Würzburg (Bayern)

Condition	Drive Time (min)	Covered Population	Coverage (%)
Hospitals with CT	5	1721	1.04
Hospitals with CT	10	33699	20.42
Hospitals with CT	15	94281	57.12
Hospitals with CT	20	137936	83.57
Hospitals with CT	25	159665	96.73
Hospitals with CT	30	164946	99.93
Hospitals with CT	35	165060	100.0
Hospitals with CT	40	165062	100.0
Hospitals with CT	45	165062	100.0
Hospitals with CT	50	165062	100.0
Hospitals with CT	55	165062	100.0
Hospitals with CT	60	165062	100.0
Stroke-Ready Hospitals	5	0	0.0
Stroke-Ready Hospitals	10	10748	6.51
Stroke-Ready Hospitals	15	63682	38.58
Stroke-Ready Hospitals	20	108568	65.77
Stroke-Ready Hospitals	25	137038	83.02
Stroke-Ready Hospitals	30	155779	94.38
Stroke-Ready Hospitals	35	164569	99.7
Stroke-Ready Hospitals	40	165061	100.0
Stroke-Ready Hospitals	45	165062	100.0
Stroke-Ready Hospitals	50	165062	100.0
Stroke-Ready Hospitals	55	165062	100.0
Stroke-Ready Hospitals	60	165062	100.0
Stroke Units	5	0	0.0
Stroke Units	10	10748	6.51
Stroke Units	15	63682	38.58
Stroke Units	20	108568	65.77
Stroke Units	25	137038	83.02
Stroke Units	30	155779	94.38
Stroke Units	35	164569	99.7
Stroke Units	40	165061	100.0
Stroke Units	45	165062	100.0
Stroke Units	50	165062	100.0
Stroke Units	55	165062	100.0
Stroke Units	60	165062	100.0

Population Coverage - Landkreis Elbe-Elster (Brandenburg)

Condition	Drive Time (min)	Covered Population	Coverage (%)
Hospitals with CT	5	18895	18.54
Hospitals with CT	10	35414	34.74
Hospitals with CT	15	51306	50.33
Hospitals with CT	20	72791	71.41
Hospitals with CT	25	89803	88.09
Hospitals with CT	30	100234	98.33
Hospitals with CT	35	101785	99.85
Hospitals with CT	40	101936	100.0
Hospitals with CT	45	101939	100.0
Hospitals with CT	50	101939	100.0
Hospitals with CT	55	101939	100.0
Hospitals with CT	60	101939	100.0
Stroke-Ready Hospitals	5	0	0.0
Stroke-Ready Hospitals	10	0	0.0
Stroke-Ready Hospitals	15	0	0.0
Stroke-Ready Hospitals	20	144	0.14
Stroke-Ready Hospitals	25	1419	1.39
Stroke-Ready Hospitals	30	2748	2.7
Stroke-Ready Hospitals	35	16457	16.14
Stroke-Ready Hospitals	40	30060	29.49
Stroke-Ready Hospitals	45	46067	45.19
Stroke-Ready Hospitals	50	61967	60.79
Stroke-Ready Hospitals	55	72168	70.8
Stroke-Ready Hospitals	60	86972	85.32
Stroke Units	5	0	0.0
Stroke Units	10	0	0.0
Stroke Units	15	0	0.0
Stroke Units	20	144	0.14
Stroke Units	25	1419	1.39
Stroke Units	30	2748	2.7
Stroke Units	35	16457	16.14
Stroke Units	40	30060	29.49
Stroke Units	45	46067	45.19
Stroke Units	50	61967	60.79
Stroke Units	55	72017	70.65
Stroke Units	60	86259	84.62

Population Coverage - Landkreis Osterholz (Niedersachsen)

Condition	Drive Time (min)	Covered Population	Coverage (%)
Hospitals with CT	5	32064	28.59
Hospitals with CT	10	55627	49.6
Hospitals with CT	15	88549	78.95
Hospitals with CT	20	105135	93.74
Hospitals with CT	25	111385	99.32
Hospitals with CT	30	111753	99.64
Hospitals with CT	35	111863	99.74
Hospitals with CT	40	112152	100.0
Hospitals with CT	45	112152	100.0
Hospitals with CT	50	112152	100.0
Hospitals with CT	55	112152	100.0
Hospitals with CT	60	112152	100.0
Stroke-Ready Hospitals	5	896	0.8
Stroke-Ready Hospitals	10	5612	5.0
Stroke-Ready Hospitals	15	17637	15.73
Stroke-Ready Hospitals	20	34113	30.42
Stroke-Ready Hospitals	25	50119	44.69
Stroke-Ready Hospitals	30	77567	69.16
Stroke-Ready Hospitals	35	94303	84.08
Stroke-Ready Hospitals	40	106679	95.12
Stroke-Ready Hospitals	45	111019	98.99
Stroke-Ready Hospitals	50	112107	99.96
Stroke-Ready Hospitals	55	112152	100.0
Stroke-Ready Hospitals	60	112152	100.0
Stroke Units	5	896	0.8
Stroke Units	10	5612	5.0
Stroke Units	15	17637	15.73
Stroke Units	20	34113	30.42
Stroke Units	25	50116	44.69
Stroke Units	30	77236	68.87
Stroke Units	35	92953	82.88
Stroke Units	40	106629	95.08
Stroke Units	45	111019	98.99
Stroke Units	50	112107	99.96
Stroke Units	55	112152	100.0
Stroke Units	60	112152	100.0

Population Coverage - Landkreis Heidekreis (Niedersachsen)

Condition	Drive Time (min)	Covered Population	Coverage (%)
Hospitals with CT	5	19000	13.99
Hospitals with CT	10	35228	25.93
Hospitals with CT	15	58852	43.32
Hospitals with CT	20	88978	65.5
Hospitals with CT	25	115774	85.23
Hospitals with CT	30	131982	97.16
Hospitals with CT	35	134527	99.03
Hospitals with CT	40	135224	99.54
Hospitals with CT	45	135844	100.0
Hospitals with CT	50	135844	100.0
Hospitals with CT	55	135844	100.0
Hospitals with CT	60	135844	100.0
Stroke-Ready Hospitals	5	8317	6.12
Stroke-Ready Hospitals	10	16987	12.51
Stroke-Ready Hospitals	15	24163	17.79
Stroke-Ready Hospitals	20	42593	31.35
Stroke-Ready Hospitals	25	72124	53.09
Stroke-Ready Hospitals	30	104529	76.95
Stroke-Ready Hospitals	35	121535	89.47
Stroke-Ready Hospitals	40	134263	98.84
Stroke-Ready Hospitals	45	135844	100.0
Stroke-Ready Hospitals	50	135844	100.0
Stroke-Ready Hospitals	55	135844	100.0
Stroke-Ready Hospitals	60	135844	100.0
Stroke Units	5	0	0.0
Stroke Units	10	0	0.0
Stroke Units	15	0	0.0
Stroke Units	20	27	0.02
Stroke Units	25	1482	1.09
Stroke Units	30	9455	6.96
Stroke Units	35	36234	26.67
Stroke Units	40	79634	58.62
Stroke Units	45	123660	91.03
Stroke Units	50	130878	96.34
Stroke Units	55	135128	99.47
Stroke Units	60	135841	100.0

Population Coverage - Kreisfreie Stadt Solingen (Nordrhein-Westfalen)

Condition	Drive Time (min)	Covered Population	Coverage (%)
Hospitals with CT	5	47338	29.88
Hospitals with CT	10	149113	94.11
Hospitals with CT	15	158278	99.9
Hospitals with CT	20	158407	99.98
Hospitals with CT	25	158438	100.0
Hospitals with CT	30	158438	100.0
Hospitals with CT	35	158438	100.0
Hospitals with CT	40	158438	100.0
Hospitals with CT	45	158438	100.0
Hospitals with CT	50	158438	100.0
Hospitals with CT	55	158438	100.0
Hospitals with CT	60	158438	100.0
Stroke-Ready Hospitals	5	36010	22.73
Stroke-Ready Hospitals	10	131938	83.27
Stroke-Ready Hospitals	15	157662	99.51
Stroke-Ready Hospitals	20	158348	99.94
Stroke-Ready Hospitals	25	158438	100.0
Stroke-Ready Hospitals	30	158438	100.0
Stroke-Ready Hospitals	35	158438	100.0
Stroke-Ready Hospitals	40	158438	100.0
Stroke-Ready Hospitals	45	158438	100.0
Stroke-Ready Hospitals	50	158438	100.0
Stroke-Ready Hospitals	55	158438	100.0
Stroke-Ready Hospitals	60	158438	100.0
Stroke Units	5	0	0.0
Stroke Units	10	480	0.3
Stroke Units	15	6288	3.97
Stroke Units	20	86111	54.35
Stroke Units	25	157136	99.18
Stroke Units	30	158438	100.0
Stroke Units	35	158438	100.0
Stroke Units	40	158438	100.0
Stroke Units	45	158438	100.0
Stroke Units	50	158438	100.0
Stroke Units	55	158438	100.0
Stroke Units	60	158438	100.0

Population Coverage - Landkreis Bad Kreuznach (Rheinland-Pfalz)

Condition	Drive Time (min)	Covered Population	Coverage (%)
Hospitals with CT	5	42417	26.48
Hospitals with CT	10	81211	50.7
Hospitals with CT	15	125219	78.18
Hospitals with CT	20	152544	95.24
Hospitals with CT	25	160152	99.99
Hospitals with CT	30	160169	100.0
Hospitals with CT	35	160169	100.0
Hospitals with CT	40	160169	100.0
Hospitals with CT	45	160169	100.0
Hospitals with CT	50	160169	100.0
Hospitals with CT	55	160169	100.0
Hospitals with CT	60	160169	100.0
Stroke-Ready Hospitals	5	30147	18.82
Stroke-Ready Hospitals	10	59637	37.23
Stroke-Ready Hospitals	15	97368	60.79
Stroke-Ready Hospitals	20	122638	76.57
Stroke-Ready Hospitals	25	142683	89.08
Stroke-Ready Hospitals	30	155306	96.96
Stroke-Ready Hospitals	35	159959	99.87
Stroke-Ready Hospitals	40	160169	100.0
Stroke-Ready Hospitals	45	160169	100.0
Stroke-Ready Hospitals	50	160169	100.0
Stroke-Ready Hospitals	55	160169	100.0
Stroke-Ready Hospitals	60	160169	100.0
Stroke Units	5	15353	9.59
Stroke Units	10	50766	31.7
Stroke Units	15	81221	50.71
Stroke Units	20	104335	65.14
Stroke Units	25	129527	80.87
Stroke Units	30	147191	91.9
Stroke Units	35	157595	98.39
Stroke Units	40	159528	99.6
Stroke Units	45	160169	100.0
Stroke Units	50	160169	100.0
Stroke Units	55	160169	100.0
Stroke Units	60	160169	100.0

Population Coverage - Landkreis Südwestpfalz (Rheinland-Pfalz)

Condition	Drive Time (min)	Covered Population	Coverage (%)
Hospitals with CT	5	4842	4.32
Hospitals with CT	10	19814	17.67
Hospitals with CT	15	62309	55.56
Hospitals with CT	20	89214	79.56
Hospitals with CT	25	100799	89.89
Hospitals with CT	30	110329	98.38
Hospitals with CT	35	112051	99.92
Hospitals with CT	40	112070	99.94
Hospitals with CT	45	112099	99.96
Hospitals with CT	50	112099	99.96
Hospitals with CT	55	112126	99.99
Hospitals with CT	60	112127	99.99
Stroke-Ready Hospitals	5	0	0.0
Stroke-Ready Hospitals	10	4549	4.06
Stroke-Ready Hospitals	15	31313	27.92
Stroke-Ready Hospitals	20	66041	58.89
Stroke-Ready Hospitals	25	93702	83.56
Stroke-Ready Hospitals	30	110527	98.56
Stroke-Ready Hospitals	35	112051	99.92
Stroke-Ready Hospitals	40	112069	99.94
Stroke-Ready Hospitals	45	112085	99.95
Stroke-Ready Hospitals	50	112085	99.95
Stroke-Ready Hospitals	55	112117	99.98
Stroke-Ready Hospitals	60	112117	99.98
Stroke Units	5	0	0.0
Stroke Units	10	0	0.0
Stroke Units	15	2223	1.98
Stroke Units	20	9705	8.65
Stroke Units	25	16599	14.8
Stroke Units	30	38397	34.24
Stroke Units	35	55665	49.64
Stroke Units	40	70595	62.95
Stroke Units	45	91732	81.8
Stroke Units	50	103519	92.31
Stroke Units	55	108888	97.1
Stroke Units	60	111739	99.64

Population Coverage - Landkreis Ludwigsburg (Baden-Württemberg)

Condition	Drive Time (min)	Covered Population	Coverage (%)
Hospitals with CT	5	79168	15.01
Hospitals with CT	10	243806	46.22
Hospitals with CT	15	417934	79.22
Hospitals with CT	20	495651	93.95
Hospitals with CT	25	526715	99.84
Hospitals with CT	30	527543	100.0
Hospitals with CT	35	527543	100.0
Hospitals with CT	40	527543	100.0
Hospitals with CT	45	527543	100.0
Hospitals with CT	50	527543	100.0
Hospitals with CT	55	527543	100.0
Hospitals with CT	60	527543	100.0
Stroke-Ready Hospitals	5	24505	4.65
Stroke-Ready Hospitals	10	78145	14.81
Stroke-Ready Hospitals	15	191719	36.34
Stroke-Ready Hospitals	20	296397	56.18
Stroke-Ready Hospitals	25	391444	74.2
Stroke-Ready Hospitals	30	452627	85.8
Stroke-Ready Hospitals	35	502151	95.19
Stroke-Ready Hospitals	40	525918	99.69
Stroke-Ready Hospitals	45	527542	100.0
Stroke-Ready Hospitals	50	527543	100.0
Stroke-Ready Hospitals	55	527543	100.0
Stroke-Ready Hospitals	60	527543	100.0
Stroke Units	5	24505	4.65
Stroke Units	10	78145	14.81
Stroke Units	15	191719	36.34
Stroke Units	20	296397	56.18
Stroke Units	25	391444	74.2
Stroke Units	30	452626	85.8
Stroke Units	35	502151	95.19
Stroke Units	40	525914	99.69
Stroke Units	45	527542	100.0
Stroke Units	50	527543	100.0
Stroke Units	55	527543	100.0
Stroke Units	60	527543	100.0

Population Coverage - Landkreis Lörrach (Baden-Württemberg)

Condition	Drive Time (min)	Covered Population	Coverage (%)
Hospitals with CT	5	26589	11.02
Hospitals with CT	10	76395	31.67
Hospitals with CT	15	144165	59.77
Hospitals with CT	20	209641	86.92
Hospitals with CT	25	226446	93.88
Hospitals with CT	30	230142	95.42
Hospitals with CT	35	234418	97.19
Hospitals with CT	40	239502	99.3
Hospitals with CT	45	240692	99.79
Hospitals with CT	50	240771	99.82
Hospitals with CT	55	240632	99.77
Hospitals with CT	60	240857	99.86
Stroke-Ready Hospitals	5	15717	6.52
Stroke-Ready Hospitals	10	44360	18.39
Stroke-Ready Hospitals	15	101334	42.01
Stroke-Ready Hospitals	20	165707	68.7
Stroke-Ready Hospitals	25	210967	87.47
Stroke-Ready Hospitals	30	222590	92.29
Stroke-Ready Hospitals	35	230442	95.54
Stroke-Ready Hospitals	40	233466	96.79
Stroke-Ready Hospitals	45	239263	99.2
Stroke-Ready Hospitals	50	240544	99.73
Stroke-Ready Hospitals	55	240488	99.71
Stroke-Ready Hospitals	60	240757	99.82
Stroke Units	5	15717	6.52
Stroke Units	10	44360	18.39
Stroke Units	15	100193	41.54
Stroke Units	20	159846	66.27
Stroke Units	25	200324	83.05
Stroke Units	30	216552	89.78
Stroke Units	35	226468	93.89
Stroke Units	40	228600	94.78
Stroke Units	45	231158	95.84
Stroke Units	50	235717	97.73
Stroke Units	55	239562	99.32
Stroke Units	60	240457	99.69

Population Coverage - Landkreis Alb-Donau-Kreis (Baden-Württemberg)

Condition	Drive Time (min)	Covered Population	Coverage (%)
Hospitals with CT	5	29713	15.55
Hospitals with CT	10	64250	33.62
Hospitals with CT	15	115063	60.21
Hospitals with CT	20	161588	84.55
Hospitals with CT	25	186101	97.38
Hospitals with CT	30	191082	99.99
Hospitals with CT	35	191107	100.0
Hospitals with CT	40	191107	100.0
Hospitals with CT	45	191107	100.0
Hospitals with CT	50	191107	100.0
Hospitals with CT	55	191107	100.0
Hospitals with CT	60	191107	100.0
Stroke-Ready Hospitals	5	4370	2.29
Stroke-Ready Hospitals	10	9328	4.88
Stroke-Ready Hospitals	15	20772	10.87
Stroke-Ready Hospitals	20	45229	23.67
Stroke-Ready Hospitals	25	109257	57.17
Stroke-Ready Hospitals	30	163724	85.67
Stroke-Ready Hospitals	35	187485	98.1
Stroke-Ready Hospitals	40	190691	99.78
Stroke-Ready Hospitals	45	191105	100.0
Stroke-Ready Hospitals	50	191107	100.0
Stroke-Ready Hospitals	55	191107	100.0
Stroke-Ready Hospitals	60	191107	100.0
Stroke Units	5	4370	2.29
Stroke Units	10	9328	4.88
Stroke Units	15	20772	10.87
Stroke Units	20	45229	23.67
Stroke Units	25	109257	57.17
Stroke Units	30	163724	85.67
Stroke Units	35	187485	98.1
Stroke Units	40	190691	99.78
Stroke Units	45	191105	100.0
Stroke Units	50	191107	100.0
Stroke Units	55	191107	100.0
Stroke Units	60	191107	100.0

Population Coverage - Landkreis Biberach (Baden-Württemberg)

Condition	Drive Time (min)	Covered Population	Coverage (%)
Hospitals with CT	5	13118	6.79
Hospitals with CT	10	40856	21.14
Hospitals with CT	15	69815	36.13
Hospitals with CT	20	125120	64.74
Hospitals with CT	25	176438	91.3
Hospitals with CT	30	193194	99.97
Hospitals with CT	35	193257	100.0
Hospitals with CT	40	193257	100.0
Hospitals with CT	45	193257	100.0
Hospitals with CT	50	193257	100.0
Hospitals with CT	55	193257	100.0
Hospitals with CT	60	193257	100.0
Stroke-Ready Hospitals	5	10990	5.69
Stroke-Ready Hospitals	10	38447	19.89
Stroke-Ready Hospitals	15	63678	32.95
Stroke-Ready Hospitals	20	114234	59.11
Stroke-Ready Hospitals	25	170655	88.3
Stroke-Ready Hospitals	30	192629	99.68
Stroke-Ready Hospitals	35	193257	100.0
Stroke-Ready Hospitals	40	193257	100.0
Stroke-Ready Hospitals	45	193257	100.0
Stroke-Ready Hospitals	50	193257	100.0
Stroke-Ready Hospitals	55	193257	100.0
Stroke-Ready Hospitals	60	193257	100.0
Stroke Units	5	10990	5.69
Stroke Units	10	38447	19.89
Stroke Units	15	63659	32.94
Stroke Units	20	114225	59.11
Stroke Units	25	170620	88.29
Stroke Units	30	192629	99.68
Stroke Units	35	193257	100.0
Stroke Units	40	193257	100.0
Stroke Units	45	193257	100.0
Stroke Units	50	193257	100.0
Stroke Units	55	193257	100.0
Stroke Units	60	193257	100.0

Population Coverage - Kreisfreie Stadt Fürth (Bayern)

Condition	Drive Time (min)	Covered Population	Coverage (%)
Hospitals with CT	5	22505	14.97
Hospitals with CT	10	93770	62.39
Hospitals with CT	15	150284	99.99
Hospitals with CT	20	150301	100.0
Hospitals with CT	25	150301	100.0
Hospitals with CT	30	150301	100.0
Hospitals with CT	35	150301	100.0
Hospitals with CT	40	150301	100.0
Hospitals with CT	45	150301	100.0
Hospitals with CT	50	150301	100.0
Hospitals with CT	55	150301	100.0
Hospitals with CT	60	150301	100.0
Stroke-Ready Hospitals	5	33336	22.18
Stroke-Ready Hospitals	10	121926	81.12
Stroke-Ready Hospitals	15	150285	99.99
Stroke-Ready Hospitals	20	150301	100.0
Stroke-Ready Hospitals	25	150301	100.0
Stroke-Ready Hospitals	30	150301	100.0
Stroke-Ready Hospitals	35	150301	100.0
Stroke-Ready Hospitals	40	150301	100.0
Stroke-Ready Hospitals	45	150301	100.0
Stroke-Ready Hospitals	50	150301	100.0
Stroke-Ready Hospitals	55	150301	100.0
Stroke-Ready Hospitals	60	150301	100.0
Stroke Units	5	33336	22.18
Stroke Units	10	121926	81.12
Stroke Units	15	150285	99.99
Stroke Units	20	150301	100.0
Stroke Units	25	150301	100.0
Stroke Units	30	150301	100.0
Stroke Units	35	150301	100.0
Stroke Units	40	150301	100.0
Stroke Units	45	150301	100.0
Stroke Units	50	150301	100.0
Stroke Units	55	150301	100.0
Stroke Units	60	150301	100.0

Population Coverage - Kreisfreie Stadt Halle (Saale) (Sachsen-Anhalt)

Condition	Drive Time (min)	Covered Population	Coverage (%)
Hospitals with CT	5	138447	55.34
Hospitals with CT	10	227620	90.99
Hospitals with CT	15	248844	99.47
Hospitals with CT	20	250050	99.95
Hospitals with CT	25	250054	99.95
Hospitals with CT	30	250172	100.0
Hospitals with CT	35	250172	100.0
Hospitals with CT	40	250172	100.0
Hospitals with CT	45	250172	100.0
Hospitals with CT	50	250172	100.0
Hospitals with CT	55	250172	100.0
Hospitals with CT	60	250172	100.0
Stroke-Ready Hospitals	5	64122	25.63
Stroke-Ready Hospitals	10	208987	83.54
Stroke-Ready Hospitals	15	246960	98.72
Stroke-Ready Hospitals	20	249678	99.8
Stroke-Ready Hospitals	25	250172	100.0
Stroke-Ready Hospitals	30	250172	100.0
Stroke-Ready Hospitals	35	250172	100.0
Stroke-Ready Hospitals	40	250172	100.0
Stroke-Ready Hospitals	45	250172	100.0
Stroke-Ready Hospitals	50	250172	100.0
Stroke-Ready Hospitals	55	250172	100.0
Stroke-Ready Hospitals	60	250172	100.0
Stroke Units	5	64122	25.63
Stroke Units	10	208987	83.54
Stroke Units	15	246959	98.72
Stroke Units	20	249550	99.75
Stroke Units	25	250172	100.0
Stroke Units	30	250172	100.0
Stroke Units	35	250172	100.0
Stroke Units	40	250172	100.0
Stroke Units	45	250172	100.0
Stroke Units	50	250172	100.0
Stroke Units	55	250172	100.0
Stroke Units	60	250172	100.0

Population Coverage - Landkreis Wittenberg (Sachsen-Anhalt)

Condition	Drive Time (min)	Covered Population	Coverage (%)
Hospitals with CT	5	19210	16.84
Hospitals with CT	10	41388	36.28
Hospitals with CT	15	49397	43.3
Hospitals with CT	20	60473	53.01
Hospitals with CT	25	83314	73.03
Hospitals with CT	30	97149	85.16
Hospitals with CT	35	106215	93.1
Hospitals with CT	40	113807	99.76
Hospitals with CT	45	114056	99.98
Hospitals with CT	50	114083	100.0
Hospitals with CT	55	114083	100.0
Hospitals with CT	60	114083	100.0
Stroke-Ready Hospitals	5	12592	11.04
Stroke-Ready Hospitals	10	21531	18.87
Stroke-Ready Hospitals	15	37596	32.96
Stroke-Ready Hospitals	20	45229	39.65
Stroke-Ready Hospitals	25	61558	53.96
Stroke-Ready Hospitals	30	83761	73.42
Stroke-Ready Hospitals	35	96584	84.66
Stroke-Ready Hospitals	40	103565	90.78
Stroke-Ready Hospitals	45	106053	92.96
Stroke-Ready Hospitals	50	111788	97.99
Stroke-Ready Hospitals	55	113810	99.76
Stroke-Ready Hospitals	60	113999	99.93
Stroke Units	5	0	0.0
Stroke Units	10	0	0.0
Stroke Units	15	0	0.0
Stroke Units	20	0	0.0
Stroke Units	25	0	0.0
Stroke Units	30	0	0.0
Stroke Units	35	39	0.03
Stroke Units	40	2979	2.61
Stroke Units	45	12194	10.69
Stroke Units	50	18658	16.36
Stroke Units	55	34105	29.9
Stroke Units	60	50719	44.46

Population Coverage - Landkreis Saale-Holzland-Kreis (Thüringen)

Condition	Drive Time (min)	Covered Population	Coverage (%)
Hospitals with CT	5	15530	20.52
Hospitals with CT	10	34198	45.17
Hospitals with CT	15	52342	69.14
Hospitals with CT	20	71338	94.24
Hospitals with CT	25	75683	99.97
Hospitals with CT	30	75702	100.0
Hospitals with CT	35	75702	100.0
Hospitals with CT	40	75702	100.0
Hospitals with CT	45	75702	100.0
Hospitals with CT	50	75702	100.0
Hospitals with CT	55	75702	100.0
Hospitals with CT	60	75702	100.0
Stroke-Ready Hospitals	5	91	0.12
Stroke-Ready Hospitals	10	1365	1.8
Stroke-Ready Hospitals	15	5463	7.22
Stroke-Ready Hospitals	20	24804	32.77
Stroke-Ready Hospitals	25	52026	68.73
Stroke-Ready Hospitals	30	72287	95.49
Stroke-Ready Hospitals	35	75638	99.92
Stroke-Ready Hospitals	40	75702	100.0
Stroke-Ready Hospitals	45	75702	100.0
Stroke-Ready Hospitals	50	75702	100.0
Stroke-Ready Hospitals	55	75702	100.0
Stroke-Ready Hospitals	60	75702	100.0
Stroke Units	5	91	0.12
Stroke Units	10	1365	1.8
Stroke Units	15	5463	7.22
Stroke Units	20	24703	32.63
Stroke Units	25	50463	66.66
Stroke Units	30	71435	94.36
Stroke Units	35	75619	99.89
Stroke Units	40	75702	100.0
Stroke Units	45	75702	100.0
Stroke Units	50	75702	100.0
Stroke Units	55	75702	100.0
Stroke Units	60	75702	100.0

Population Coverage - Kreisfreie Stadt Salzgitter (Niedersachsen)

Condition	Drive Time (min)	Covered Population	Coverage (%)
Hospitals with CT	5	33014	33.0
Hospitals with CT	10	59797	59.77
Hospitals with CT	15	99918	99.87
Hospitals with CT	20	100052	100.0
Hospitals with CT	25	100052	100.0
Hospitals with CT	30	100052	100.0
Hospitals with CT	35	100052	100.0
Hospitals with CT	40	100052	100.0
Hospitals with CT	45	100052	100.0
Hospitals with CT	50	100052	100.0
Hospitals with CT	55	100052	100.0
Hospitals with CT	60	100052	100.0
Stroke-Ready Hospitals	5	21994	21.98
Stroke-Ready Hospitals	10	39918	39.9
Stroke-Ready Hospitals	15	78316	78.27
Stroke-Ready Hospitals	20	87455	87.41
Stroke-Ready Hospitals	25	97759	97.71
Stroke-Ready Hospitals	30	100013	99.96
Stroke-Ready Hospitals	35	100052	100.0
Stroke-Ready Hospitals	40	100052	100.0
Stroke-Ready Hospitals	45	100052	100.0
Stroke-Ready Hospitals	50	100052	100.0
Stroke-Ready Hospitals	55	100052	100.0
Stroke-Ready Hospitals	60	100052	100.0
Stroke Units	5	0	0.0
Stroke Units	10	0	0.0
Stroke Units	15	128	0.13
Stroke Units	20	16833	16.82
Stroke Units	25	72305	72.27
Stroke Units	30	84594	84.55
Stroke Units	35	98740	98.69
Stroke Units	40	100052	100.0
Stroke Units	45	100052	100.0
Stroke Units	50	100052	100.0
Stroke Units	55	100052	100.0
Stroke Units	60	100052	100.0

Population Coverage - Kreis Wesel (Nordrhein-Westfalen)

Condition	Drive Time (min)	Covered Population	Coverage (%)
Hospitals with CT	5	127404	26.82
Hospitals with CT	10	315838	66.48
Hospitals with CT	15	447341	94.16
Hospitals with CT	20	473837	99.74
Hospitals with CT	25	474724	99.92
Hospitals with CT	30	475083	100.0
Hospitals with CT	35	475084	100.0
Hospitals with CT	40	475084	100.0
Hospitals with CT	45	475084	100.0
Hospitals with CT	50	475084	100.0
Hospitals with CT	55	475084	100.0
Hospitals with CT	60	475084	100.0
Stroke-Ready Hospitals	5	42421	8.93
Stroke-Ready Hospitals	10	145525	30.63
Stroke-Ready Hospitals	15	282988	59.57
Stroke-Ready Hospitals	20	410555	86.42
Stroke-Ready Hospitals	25	468614	98.64
Stroke-Ready Hospitals	30	474706	99.92
Stroke-Ready Hospitals	35	474901	99.96
Stroke-Ready Hospitals	40	475084	100.0
Stroke-Ready Hospitals	45	475084	100.0
Stroke-Ready Hospitals	50	475084	100.0
Stroke-Ready Hospitals	55	475084	100.0
Stroke-Ready Hospitals	60	475084	100.0
Stroke Units	5	41800	8.8
Stroke Units	10	127721	26.88
Stroke Units	15	232647	48.97
Stroke Units	20	370678	78.02
Stroke Units	25	446954	94.08
Stroke Units	30	468290	98.57
Stroke Units	35	474100	99.79
Stroke Units	40	475084	100.0
Stroke Units	45	475084	100.0
Stroke Units	50	475084	100.0
Stroke Units	55	475084	100.0
Stroke Units	60	475084	100.0

Population Coverage - Kreis Rhein-Erft-Kreis (Nordrhein-Westfalen)

Condition	Drive Time (min)	Covered Population	Coverage (%)
Hospitals with CT	5	95188	20.94
Hospitals with CT	10	223888	49.26
Hospitals with CT	15	370795	81.58
Hospitals with CT	20	447327	98.42
Hospitals with CT	25	454112	99.91
Hospitals with CT	30	454529	100.0
Hospitals with CT	35	454529	100.0
Hospitals with CT	40	454529	100.0
Hospitals with CT	45	454529	100.0
Hospitals with CT	50	454529	100.0
Hospitals with CT	55	454529	100.0
Hospitals with CT	60	454529	100.0
Stroke-Ready Hospitals	5	22577	4.97
Stroke-Ready Hospitals	10	55234	12.15
Stroke-Ready Hospitals	15	167574	36.87
Stroke-Ready Hospitals	20	322572	70.97
Stroke-Ready Hospitals	25	446887	98.32
Stroke-Ready Hospitals	30	454295	99.95
Stroke-Ready Hospitals	35	454529	100.0
Stroke-Ready Hospitals	40	454529	100.0
Stroke-Ready Hospitals	45	454529	100.0
Stroke-Ready Hospitals	50	454529	100.0
Stroke-Ready Hospitals	55	454529	100.0
Stroke-Ready Hospitals	60	454529	100.0
Stroke Units	5	22577	4.97
Stroke Units	10	55234	12.15
Stroke Units	15	167574	36.87
Stroke Units	20	322572	70.97
Stroke Units	25	446887	98.32
Stroke Units	30	454295	99.95
Stroke Units	35	454529	100.0
Stroke Units	40	454529	100.0
Stroke Units	45	454529	100.0
Stroke Units	50	454529	100.0
Stroke Units	55	454529	100.0
Stroke Units	60	454529	100.0

Population Coverage - Kreisfreie Stadt Frankfurt am Main (Hessen)

Condition	Drive Time (min)	Covered Population	Coverage (%)
Hospitals with CT	5	360725	43.3
Hospitals with CT	10	740166	88.84
Hospitals with CT	15	812465	97.52
Hospitals with CT	20	833128	100.0
Hospitals with CT	25	833128	100.0
Hospitals with CT	30	833128	100.0
Hospitals with CT	35	833128	100.0
Hospitals with CT	40	833128	100.0
Hospitals with CT	45	833128	100.0
Hospitals with CT	50	833128	100.0
Hospitals with CT	55	833128	100.0
Hospitals with CT	60	833128	100.0
Stroke-Ready Hospitals	5	133622	16.04
Stroke-Ready Hospitals	10	616237	73.97
Stroke-Ready Hospitals	15	813006	97.58
Stroke-Ready Hospitals	20	814310	97.74
Stroke-Ready Hospitals	25	833128	100.0
Stroke-Ready Hospitals	30	833128	100.0
Stroke-Ready Hospitals	35	833128	100.0
Stroke-Ready Hospitals	40	833128	100.0
Stroke-Ready Hospitals	45	833128	100.0
Stroke-Ready Hospitals	50	833128	100.0
Stroke-Ready Hospitals	55	833128	100.0
Stroke-Ready Hospitals	60	833128	100.0
Stroke Units	5	109263	13.11
Stroke Units	10	528705	63.46
Stroke Units	15	796261	95.57
Stroke Units	20	814306	97.74
Stroke Units	25	833128	100.0
Stroke Units	30	833128	100.0
Stroke Units	35	833128	100.0
Stroke Units	40	833128	100.0
Stroke Units	45	833128	100.0
Stroke Units	50	833128	100.0
Stroke Units	55	833128	100.0
Stroke Units	60	833128	100.0

Population Coverage - Landkreis Südliche Weinstraße (Rheinland-Pfalz)

Condition	Drive Time (min)	Covered Population	Coverage (%)
Hospitals with CT	5	3598	3.22
Hospitals with CT	10	22871	20.45
Hospitals with CT	15	82913	74.12
Hospitals with CT	20	108722	97.19
Hospitals with CT	25	111776	99.92
Hospitals with CT	30	111823	99.97
Hospitals with CT	35	111860	100.0
Hospitals with CT	40	111860	100.0
Hospitals with CT	45	111860	100.0
Hospitals with CT	50	111860	100.0
Hospitals with CT	55	111860	100.0
Hospitals with CT	60	111860	100.0
Stroke-Ready Hospitals	5	3730	3.33
Stroke-Ready Hospitals	10	23657	21.15
Stroke-Ready Hospitals	15	63377	56.66
Stroke-Ready Hospitals	20	100521	89.86
Stroke-Ready Hospitals	25	109342	97.75
Stroke-Ready Hospitals	30	111770	99.92
Stroke-Ready Hospitals	35	111852	99.99
Stroke-Ready Hospitals	40	111860	100.0
Stroke-Ready Hospitals	45	111860	100.0
Stroke-Ready Hospitals	50	111860	100.0
Stroke-Ready Hospitals	55	111860	100.0
Stroke-Ready Hospitals	60	111860	100.0
Stroke Units	5	0	0.0
Stroke Units	10	1371	1.23
Stroke Units	15	12771	11.42
Stroke Units	20	36925	33.01
Stroke Units	25	74273	66.4
Stroke Units	30	95862	85.7
Stroke Units	35	106225	94.96
Stroke Units	40	111397	99.59
Stroke Units	45	111836	99.98
Stroke Units	50	111860	100.0
Stroke Units	55	111860	100.0
Stroke Units	60	111860	100.0

Population Coverage - Stadtkreis Karlsruhe (Baden-Württemberg)

Condition	Drive Time (min)	Covered Population	Coverage (%)
Hospitals with CT	5	48020	14.28
Hospitals with CT	10	154371	45.89
Hospitals with CT	15	239185	71.1
Hospitals with CT	20	311730	92.67
Hospitals with CT	25	336107	99.92
Hospitals with CT	30	336385	100.0
Hospitals with CT	35	336385	100.0
Hospitals with CT	40	336385	100.0
Hospitals with CT	45	336385	100.0
Hospitals with CT	50	336385	100.0
Hospitals with CT	55	336385	100.0
Hospitals with CT	60	336385	100.0
Stroke-Ready Hospitals	5	35392	10.52
Stroke-Ready Hospitals	10	126414	37.58
Stroke-Ready Hospitals	15	225783	67.12
Stroke-Ready Hospitals	20	321801	95.66
Stroke-Ready Hospitals	25	336098	99.91
Stroke-Ready Hospitals	30	336385	100.0
Stroke-Ready Hospitals	35	336385	100.0
Stroke-Ready Hospitals	40	336385	100.0
Stroke-Ready Hospitals	45	336385	100.0
Stroke-Ready Hospitals	50	336385	100.0
Stroke-Ready Hospitals	55	336385	100.0
Stroke-Ready Hospitals	60	336385	100.0
Stroke Units	5	35392	10.52
Stroke Units	10	126414	37.58
Stroke Units	15	225783	67.12
Stroke Units	20	321801	95.66
Stroke Units	25	336098	99.91
Stroke Units	30	336385	100.0
Stroke Units	35	336385	100.0
Stroke Units	40	336385	100.0
Stroke Units	45	336385	100.0
Stroke Units	50	336385	100.0
Stroke Units	55	336385	100.0
Stroke Units	60	336385	100.0

Population Coverage - Landkreis Rhein-Neckar-Kreis (Baden-Württemberg)

Condition	Drive Time (min)	Covered Population	Coverage (%)
Hospitals with CT	5	34335	6.35
Hospitals with CT	10	165601	30.61
Hospitals with CT	15	373151	68.97
Hospitals with CT	20	490543	90.67
Hospitals with CT	25	529849	97.94
Hospitals with CT	30	538483	99.53
Hospitals with CT	35	540963	99.99
Hospitals with CT	40	541020	100.0
Hospitals with CT	45	541020	100.0
Hospitals with CT	50	541020	100.0
Hospitals with CT	55	541020	100.0
Hospitals with CT	60	541020	100.0
Stroke-Ready Hospitals	5	8451	1.56
Stroke-Ready Hospitals	10	27317	5.05
Stroke-Ready Hospitals	15	107950	19.95
Stroke-Ready Hospitals	20	332999	61.55
Stroke-Ready Hospitals	25	479522	88.63
Stroke-Ready Hospitals	30	518840	95.9
Stroke-Ready Hospitals	35	536630	99.19
Stroke-Ready Hospitals	40	540304	99.87
Stroke-Ready Hospitals	45	541020	100.0
Stroke-Ready Hospitals	50	541020	100.0
Stroke-Ready Hospitals	55	541020	100.0
Stroke-Ready Hospitals	60	541020	100.0
Stroke Units	5	31	0.01
Stroke Units	10	7666	1.42
Stroke Units	15	70465	13.02
Stroke Units	20	247424	45.73
Stroke Units	25	382747	70.75
Stroke Units	30	448106	82.83
Stroke Units	35	502431	92.87
Stroke Units	40	525125	97.06
Stroke Units	45	539030	99.63
Stroke Units	50	541020	100.0
Stroke Units	55	541020	100.0
Stroke Units	60	541020	100.0

Population Coverage - Landkreis München (Bayern)

Condition	Drive Time (min)	Covered Population	Coverage (%)
Hospitals with CT	5	23664	6.87
Hospitals with CT	10	103940	30.19
Hospitals with CT	15	238573	69.29
Hospitals with CT	20	333081	96.73
Hospitals with CT	25	340154	98.79
Hospitals with CT	30	344318	100.0
Hospitals with CT	35	344325	100.0
Hospitals with CT	40	344325	100.0
Hospitals with CT	45	344325	100.0
Hospitals with CT	50	344325	100.0
Hospitals with CT	55	344325	100.0
Hospitals with CT	60	344325	100.0
Stroke-Ready Hospitals	5	10830	3.15
Stroke-Ready Hospitals	10	66007	19.17
Stroke-Ready Hospitals	15	144431	41.95
Stroke-Ready Hospitals	20	274844	79.82
Stroke-Ready Hospitals	25	335485	97.43
Stroke-Ready Hospitals	30	343093	99.64
Stroke-Ready Hospitals	35	344325	100.0
Stroke-Ready Hospitals	40	344325	100.0
Stroke-Ready Hospitals	45	344325	100.0
Stroke-Ready Hospitals	50	344325	100.0
Stroke-Ready Hospitals	55	344325	100.0
Stroke-Ready Hospitals	60	344325	100.0
Stroke Units	5	10830	3.15
Stroke Units	10	66007	19.17
Stroke Units	15	144431	41.95
Stroke Units	20	274844	79.82
Stroke Units	25	335485	97.43
Stroke Units	30	343093	99.64
Stroke Units	35	344325	100.0
Stroke Units	40	344325	100.0
Stroke Units	45	344325	100.0
Stroke Units	50	344325	100.0
Stroke Units	55	344325	100.0
Stroke Units	60	344325	100.0

Population Coverage - Landkreis Dingolfing-Landau (Bayern)

Condition	Drive Time (min)	Covered Population	Coverage (%)
Hospitals with CT	5	9475	10.03
Hospitals with CT	10	30222	31.99
Hospitals with CT	15	69019	73.06
Hospitals with CT	20	85687	90.71
Hospitals with CT	25	93069	98.52
Hospitals with CT	30	94464	100.0
Hospitals with CT	35	94464	100.0
Hospitals with CT	40	94464	100.0
Hospitals with CT	45	94464	100.0
Hospitals with CT	50	94464	100.0
Hospitals with CT	55	94464	100.0
Hospitals with CT	60	94464	100.0
Stroke-Ready Hospitals	5	4701	4.98
Stroke-Ready Hospitals	10	17427	18.45
Stroke-Ready Hospitals	15	43469	46.02
Stroke-Ready Hospitals	20	65756	69.61
Stroke-Ready Hospitals	25	84372	89.32
Stroke-Ready Hospitals	30	89821	95.08
Stroke-Ready Hospitals	35	94349	99.88
Stroke-Ready Hospitals	40	94464	100.0
Stroke-Ready Hospitals	45	94464	100.0
Stroke-Ready Hospitals	50	94464	100.0
Stroke-Ready Hospitals	55	94464	100.0
Stroke-Ready Hospitals	60	94464	100.0
Stroke Units	5	4701	4.98
Stroke Units	10	17427	18.45
Stroke Units	15	43469	46.02
Stroke Units	20	65756	69.61
Stroke Units	25	84372	89.32
Stroke Units	30	89821	95.08
Stroke Units	35	94349	99.88
Stroke Units	40	94464	100.0
Stroke Units	45	94464	100.0
Stroke Units	50	94464	100.0
Stroke Units	55	94464	100.0
Stroke Units	60	94464	100.0

Population Coverage - Kreisfreie Stadt Nürnberg (Bayern)

Condition	Drive Time (min)	Covered Population	Coverage (%)
Hospitals with CT	5	202411	39.06
Hospitals with CT	10	402094	77.6
Hospitals with CT	15	489704	94.5
Hospitals with CT	20	518192	100.0
Hospitals with CT	25	518192	100.0
Hospitals with CT	30	518192	100.0
Hospitals with CT	35	518192	100.0
Hospitals with CT	40	518192	100.0
Hospitals with CT	45	518192	100.0
Hospitals with CT	50	518192	100.0
Hospitals with CT	55	518192	100.0
Hospitals with CT	60	518192	100.0
Stroke-Ready Hospitals	5	28606	5.52
Stroke-Ready Hospitals	10	75084	14.49
Stroke-Ready Hospitals	15	293868	56.71
Stroke-Ready Hospitals	20	500059	96.5
Stroke-Ready Hospitals	25	517039	99.78
Stroke-Ready Hospitals	30	518192	100.0
Stroke-Ready Hospitals	35	518192	100.0
Stroke-Ready Hospitals	40	518192	100.0
Stroke-Ready Hospitals	45	518192	100.0
Stroke-Ready Hospitals	50	518192	100.0
Stroke-Ready Hospitals	55	518192	100.0
Stroke-Ready Hospitals	60	518192	100.0
Stroke Units	5	28606	5.52
Stroke Units	10	74626	14.4
Stroke Units	15	292072	56.36
Stroke Units	20	500059	96.5
Stroke Units	25	517039	99.78
Stroke Units	30	518192	100.0
Stroke Units	35	518192	100.0
Stroke Units	40	518192	100.0
Stroke Units	45	518192	100.0
Stroke Units	50	518192	100.0
Stroke Units	55	518192	100.0
Stroke Units	60	518192	100.0

Population Coverage - Kreisfreie Stadt Cottbus (Brandenburg)

Condition	Drive Time (min)	Covered Population	Coverage (%)
Hospitals with CT	5	20089	19.22
Hospitals with CT	10	62607	59.91
Hospitals with CT	15	93060	89.05
Hospitals with CT	20	102108	97.71
Hospitals with CT	25	104085	99.6
Hospitals with CT	30	104494	99.99
Hospitals with CT	35	104504	100.0
Hospitals with CT	40	104504	100.0
Hospitals with CT	45	104504	100.0
Hospitals with CT	50	104504	100.0
Hospitals with CT	55	104504	100.0
Hospitals with CT	60	104504	100.0
Stroke-Ready Hospitals	5	19807	18.95
Stroke-Ready Hospitals	10	61915	59.25
Stroke-Ready Hospitals	15	91330	87.39
Stroke-Ready Hospitals	20	101902	97.51
Stroke-Ready Hospitals	25	104062	99.58
Stroke-Ready Hospitals	30	104354	99.86
Stroke-Ready Hospitals	35	104364	99.87
Stroke-Ready Hospitals	40	104364	99.87
Stroke-Ready Hospitals	45	104504	100.0
Stroke-Ready Hospitals	50	104504	100.0
Stroke-Ready Hospitals	55	104504	100.0
Stroke-Ready Hospitals	60	104504	100.0
Stroke Units	5	19807	18.95
Stroke Units	10	61915	59.25
Stroke Units	15	91330	87.39
Stroke Units	20	101902	97.51
Stroke Units	25	104062	99.58
Stroke Units	30	104354	99.86
Stroke Units	35	104364	99.87
Stroke Units	40	104364	99.87
Stroke Units	45	104504	100.0
Stroke Units	50	104504	100.0
Stroke Units	55	104504	100.0
Stroke Units	60	104504	100.0

Population Coverage - Landkreis Salzlandkreis (Sachsen-Anhalt)

Condition	Drive Time (min)	Covered Population	Coverage (%)
Hospitals with CT	5	56525	32.06
Hospitals with CT	10	111482	63.23
Hospitals with CT	15	146207	82.92
Hospitals with CT	20	166353	94.35
Hospitals with CT	25	173726	98.53
Hospitals with CT	30	175954	99.79
Hospitals with CT	35	176317	100.0
Hospitals with CT	40	176317	100.0
Hospitals with CT	45	176317	100.0
Hospitals with CT	50	176317	100.0
Hospitals with CT	55	176317	100.0
Hospitals with CT	60	176317	100.0
Stroke-Ready Hospitals	5	14209	8.06
Stroke-Ready Hospitals	10	21617	12.26
Stroke-Ready Hospitals	15	31793	18.03
Stroke-Ready Hospitals	20	48495	27.5
Stroke-Ready Hospitals	25	85650	48.58
Stroke-Ready Hospitals	30	134969	76.55
Stroke-Ready Hospitals	35	171456	97.24
Stroke-Ready Hospitals	40	175937	99.78
Stroke-Ready Hospitals	45	176151	99.91
Stroke-Ready Hospitals	50	176317	100.0
Stroke-Ready Hospitals	55	176317	100.0
Stroke-Ready Hospitals	60	176317	100.0
Stroke Units	5	14209	8.06
Stroke Units	10	21617	12.26
Stroke Units	15	31793	18.03
Stroke Units	20	48495	27.5
Stroke Units	25	85650	48.58
Stroke Units	30	134959	76.54
Stroke Units	35	171450	97.24
Stroke Units	40	175930	99.78
Stroke Units	45	176144	99.9
Stroke Units	50	176310	100.0
Stroke Units	55	176317	100.0
Stroke Units	60	176317	100.0

Population Coverage - Landkreis Sömmerda (Thüringen)

Condition	Drive Time (min)	Covered Population	Coverage (%)
Hospitals with CT	5	11090	17.42
Hospitals with CT	10	16743	26.29
Hospitals with CT	15	29898	46.95
Hospitals with CT	20	48154	75.62
Hospitals with CT	25	59036	92.71
Hospitals with CT	30	61803	97.06
Hospitals with CT	35	63640	99.94
Hospitals with CT	40	63676	100.0
Hospitals with CT	45	63676	100.0
Hospitals with CT	50	63676	100.0
Hospitals with CT	55	63676	100.0
Hospitals with CT	60	63676	100.0
Stroke-Ready Hospitals	5	0	0.0
Stroke-Ready Hospitals	10	0	0.0
Stroke-Ready Hospitals	15	2539	3.99
Stroke-Ready Hospitals	20	8212	12.9
Stroke-Ready Hospitals	25	20351	31.96
Stroke-Ready Hospitals	30	42901	67.37
Stroke-Ready Hospitals	35	54512	85.61
Stroke-Ready Hospitals	40	61176	96.07
Stroke-Ready Hospitals	45	63642	99.95
Stroke-Ready Hospitals	50	63664	99.98
Stroke-Ready Hospitals	55	63676	100.0
Stroke-Ready Hospitals	60	63676	100.0
Stroke Units	5	0	0.0
Stroke Units	10	0	0.0
Stroke Units	15	2539	3.99
Stroke Units	20	8212	12.9
Stroke Units	25	20346	31.95
Stroke Units	30	42164	66.22
Stroke Units	35	51886	81.48
Stroke Units	40	57649	90.53
Stroke Units	45	62485	98.13
Stroke Units	50	63588	99.86
Stroke Units	55	63654	99.97
Stroke Units	60	63676	100.0

Population Coverage - Landkreis Saale-Orla-Kreis (Thüringen)

Condition	Drive Time (min)	Covered Population	Coverage (%)
Hospitals with CT	5	6527	8.67
Hospitals with CT	10	15347	20.38
Hospitals with CT	15	20179	26.8
Hospitals with CT	20	34990	46.46
Hospitals with CT	25	48579	64.51
Hospitals with CT	30	65056	86.39
Hospitals with CT	35	71917	95.5
Hospitals with CT	40	75226	99.89
Hospitals with CT	45	75306	100.0
Hospitals with CT	50	75306	100.0
Hospitals with CT	55	75306	100.0
Hospitals with CT	60	75306	100.0
Stroke-Ready Hospitals	5	0	0.0
Stroke-Ready Hospitals	10	0	0.0
Stroke-Ready Hospitals	15	0	0.0
Stroke-Ready Hospitals	20	429	0.57
Stroke-Ready Hospitals	25	7014	9.31
Stroke-Ready Hospitals	30	18772	24.93
Stroke-Ready Hospitals	35	45717	60.71
Stroke-Ready Hospitals	40	65756	87.32
Stroke-Ready Hospitals	45	73632	97.78
Stroke-Ready Hospitals	50	75303	100.0
Stroke-Ready Hospitals	55	75306	100.0
Stroke-Ready Hospitals	60	75306	100.0
Stroke Units	5	0	0.0
Stroke Units	10	0	0.0
Stroke Units	15	0	0.0
Stroke Units	20	429	0.57
Stroke Units	25	7014	9.31
Stroke Units	30	18772	24.93
Stroke Units	35	45717	60.71
Stroke Units	40	65756	87.32
Stroke Units	45	73632	97.78
Stroke Units	50	75303	100.0
Stroke Units	55	75306	100.0
Stroke Units	60	75306	100.0

Isochrones — Kreisfreie Stadt Neumünster (Schleswig-Holstein)

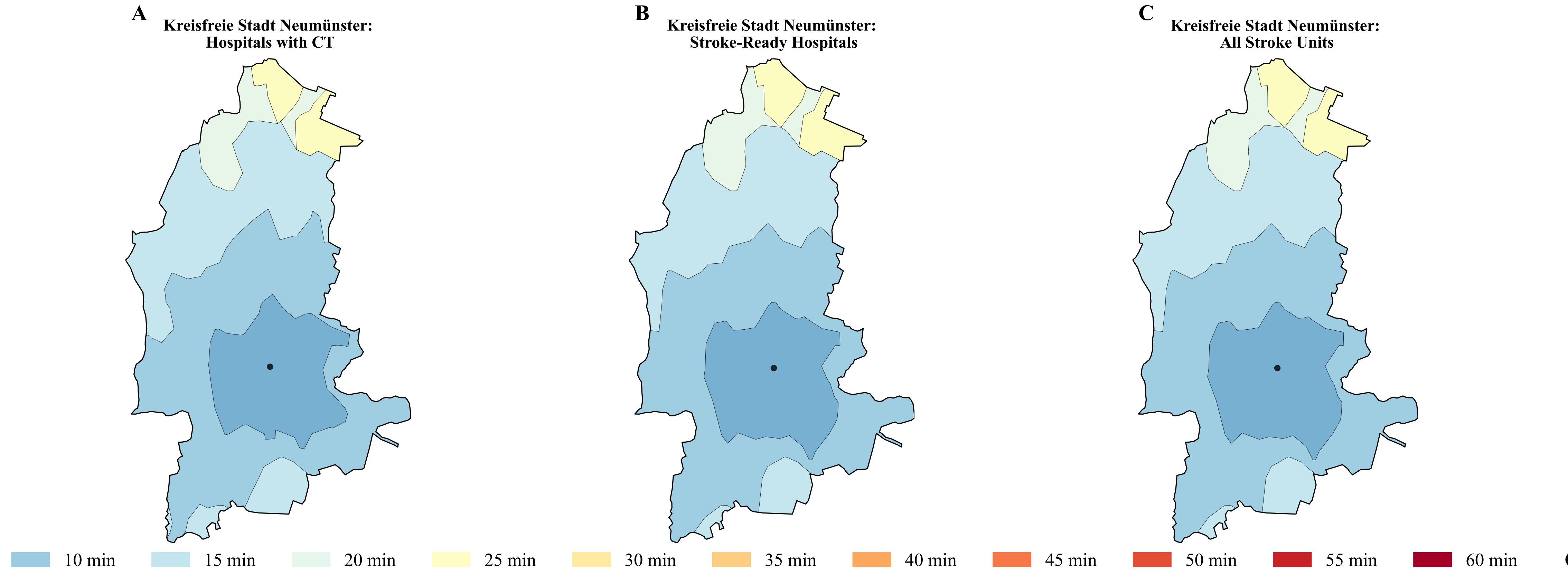

Isochrones — Kreisfreie Stadt Osnabrück (Niedersachsen)

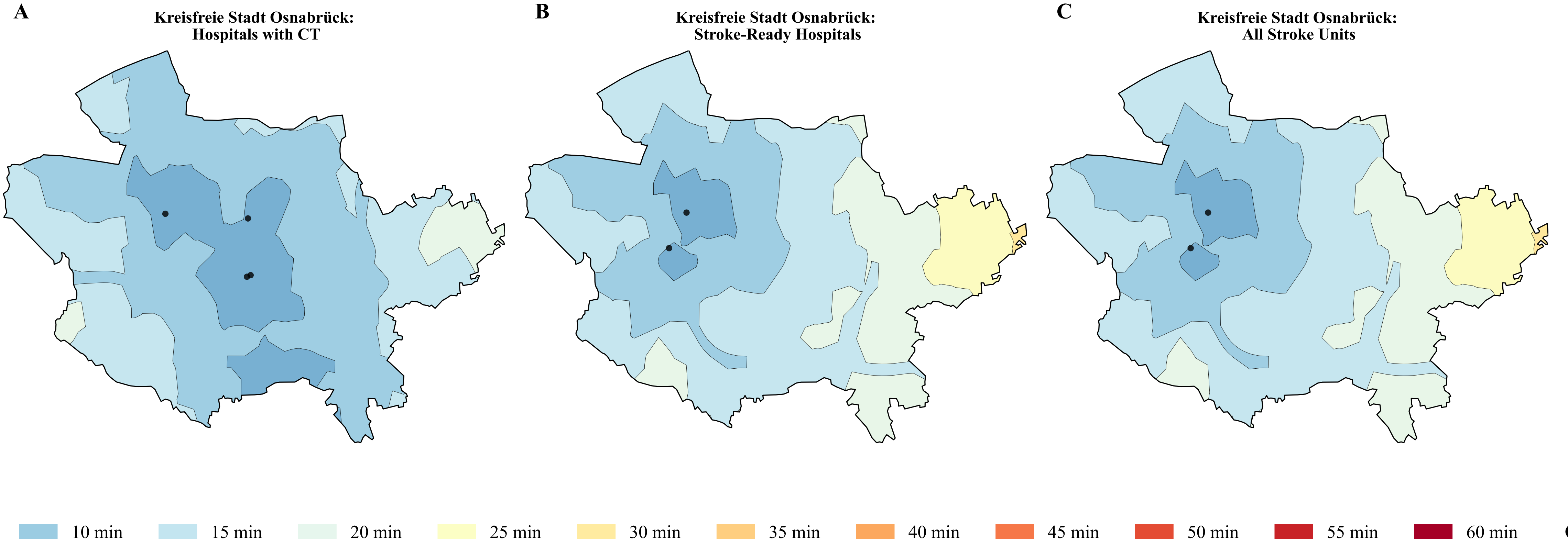

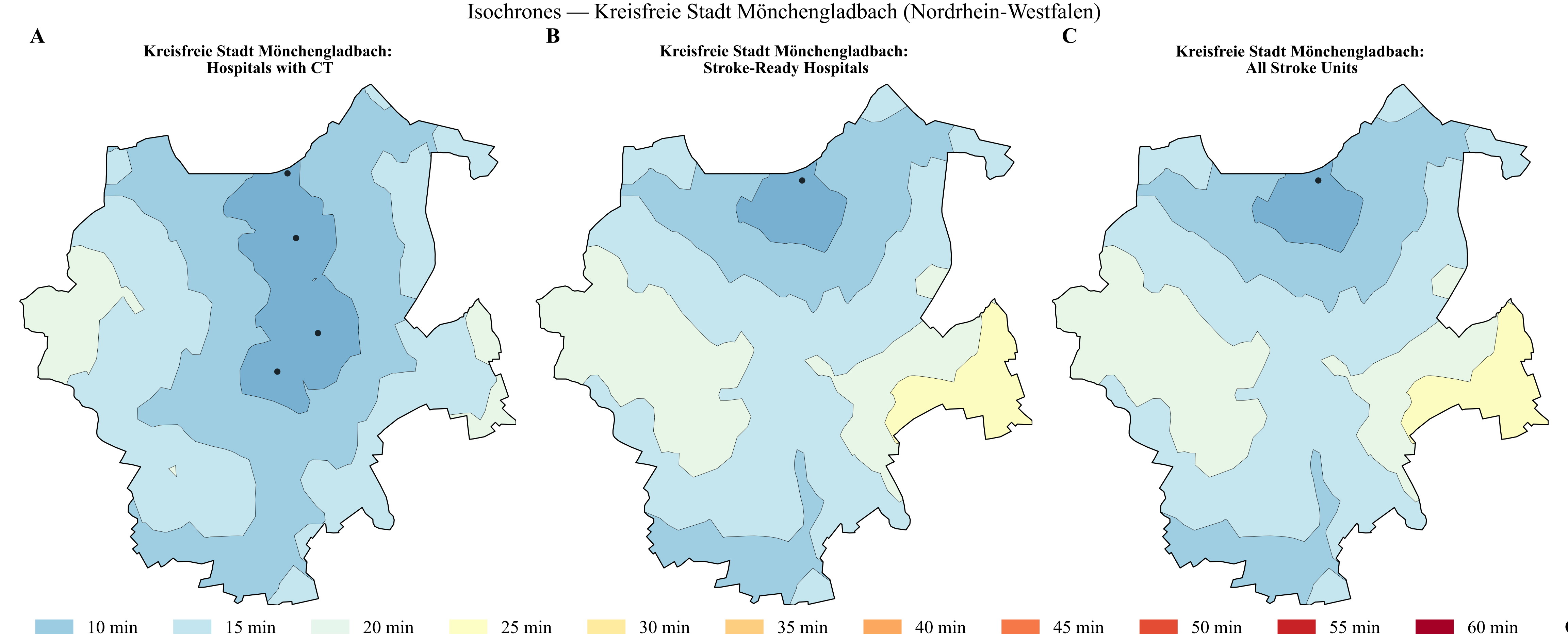
Isochrones — Kreisfreie Stadt Mönchengladbach (Nordrhein-Westfalen)
A
Kreisfreie Stadt Mönchengladbach:
Hospitals with CT
B
Kreisfreie Stadt Mönchengladbach:
Stroke-Ready Hospitals
C
Kreisfreie Stadt Mönchengladbach:
All Stroke Units
10 min
15 min
20 min
25 min
30 min
35 min
40 min
45 min
50 min
55 min
60 min

Isochrones — Kreis Coesfeld (Nordrhein-Westfalen)

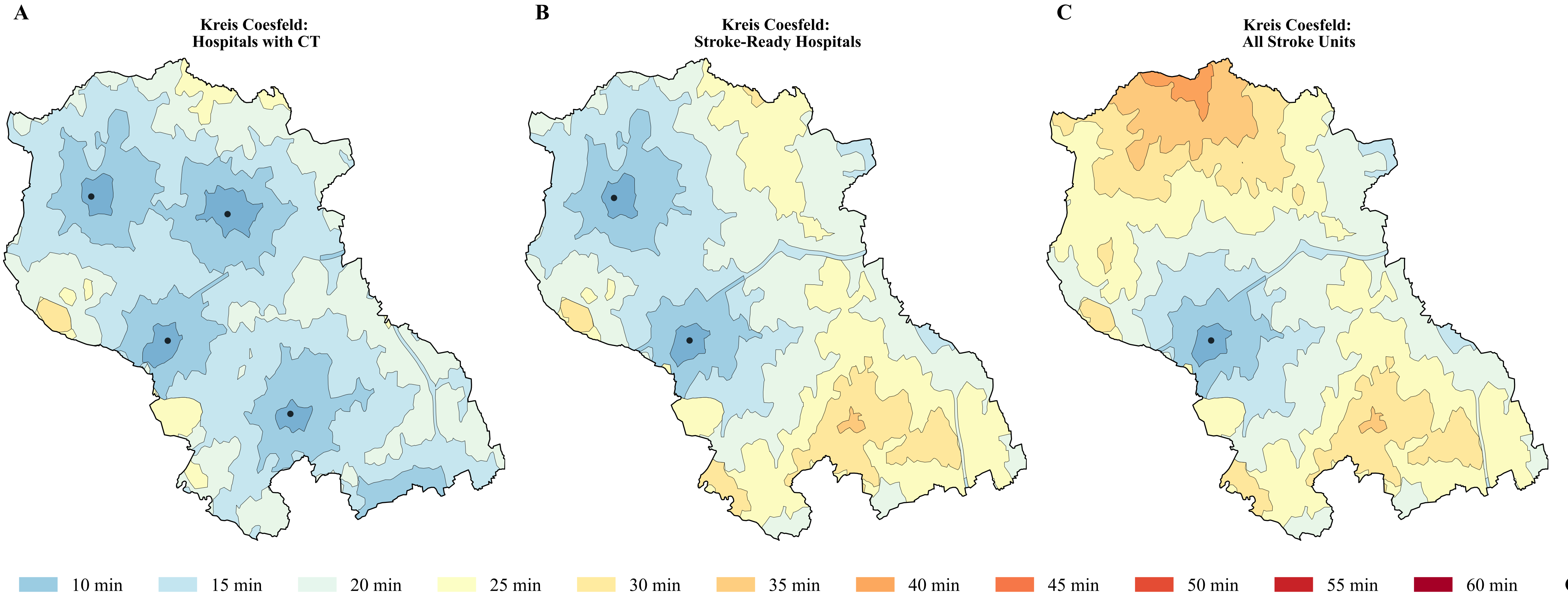

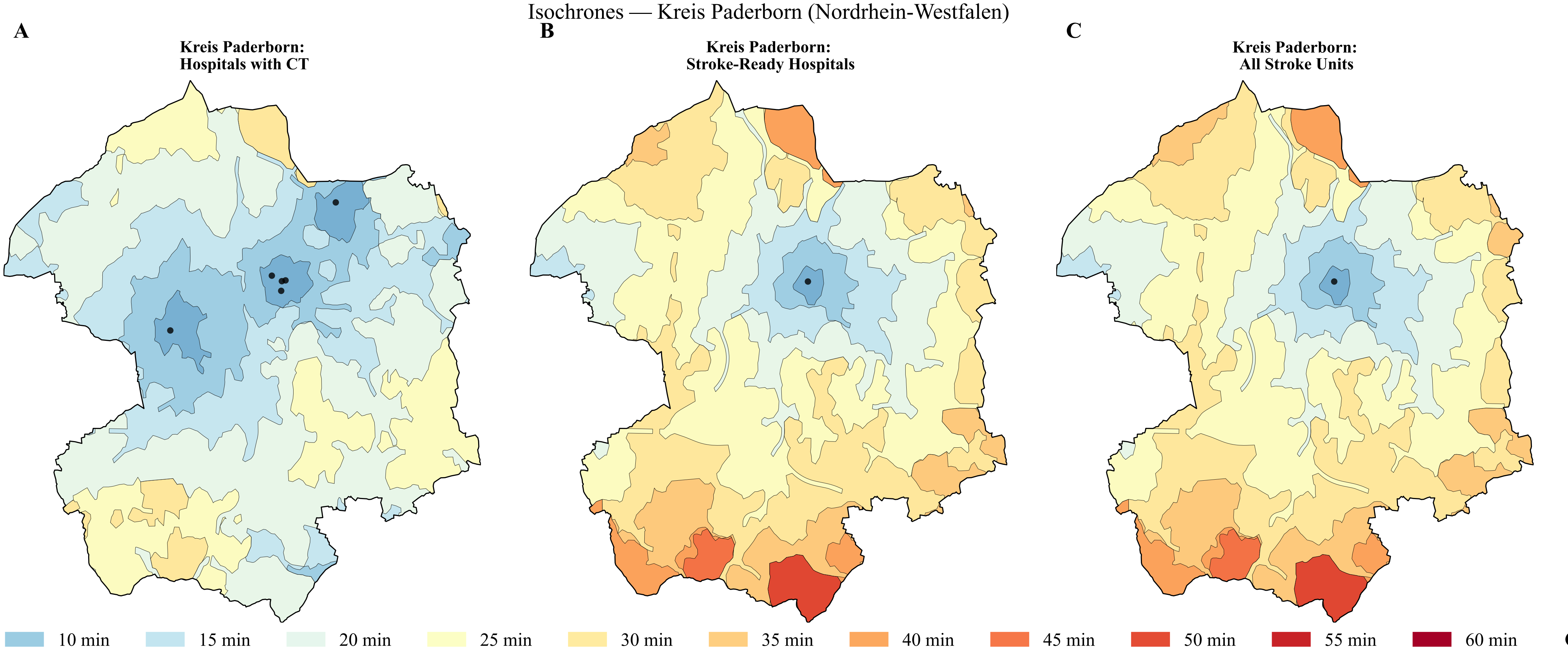

Isochrones — Kreis Paderborn (Nordrhein-Westfalen)
A
Kreis Paderborn:
Hospitals with CT
B
Kreis Paderborn:
Stroke-Ready Hospitals
C
Kreis Paderborn:
All Stroke Units
10 min
15 min
20 min
25 min
30 min
35 min
40 min
45 min
50 min
55 min
60 min

Isochrones — Kreisfreie Stadt Kassel (Hessen)

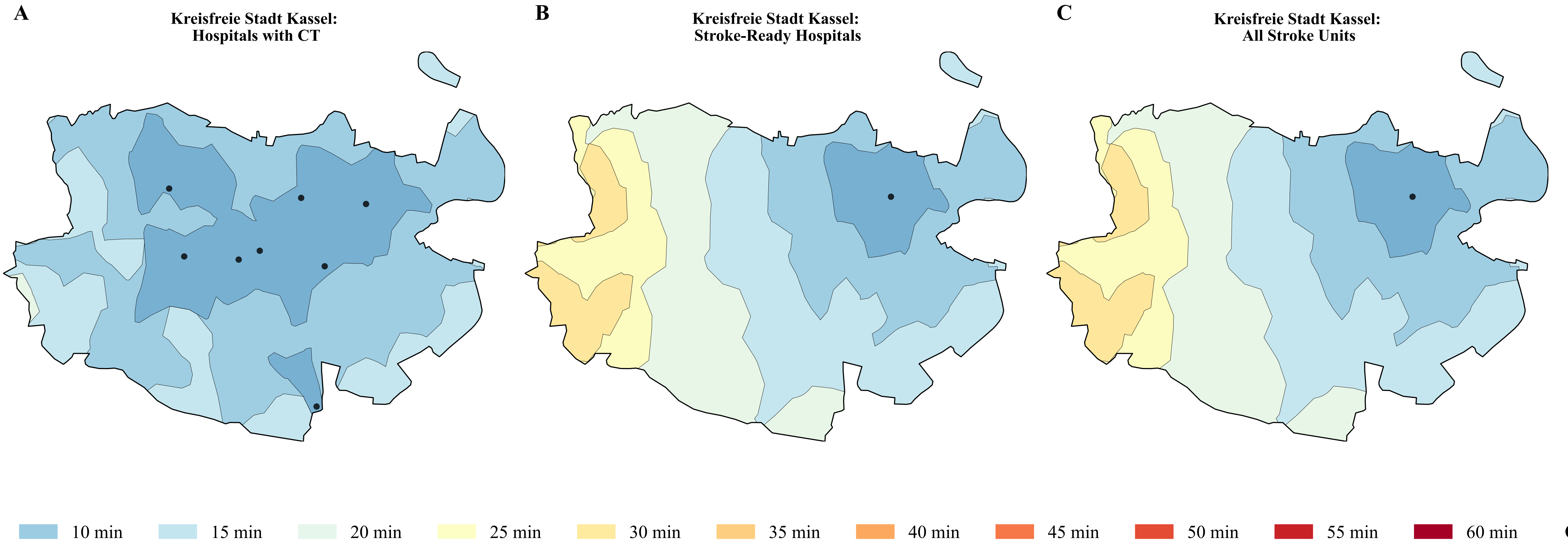

Isochrones — Landkreis Hersfeld-Rotenburg (Hessen)

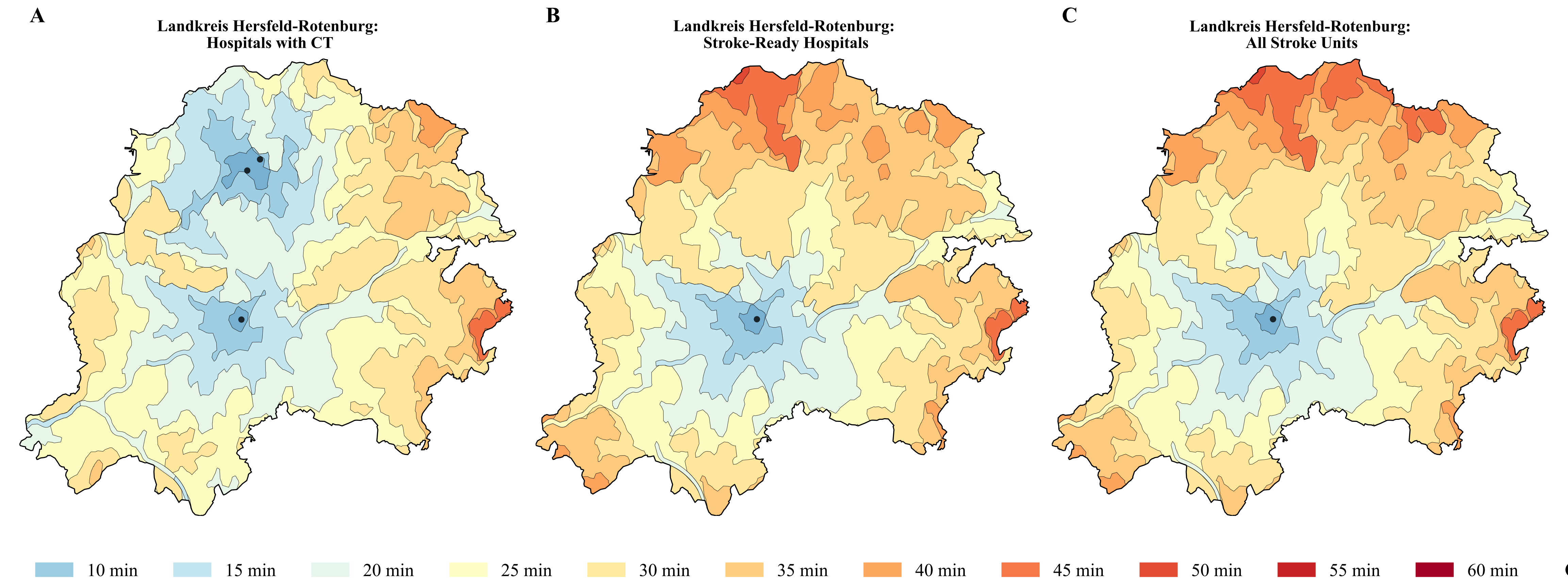

Isochrones — Landkreis Schwalm-Eder-Kreis (Hessen)

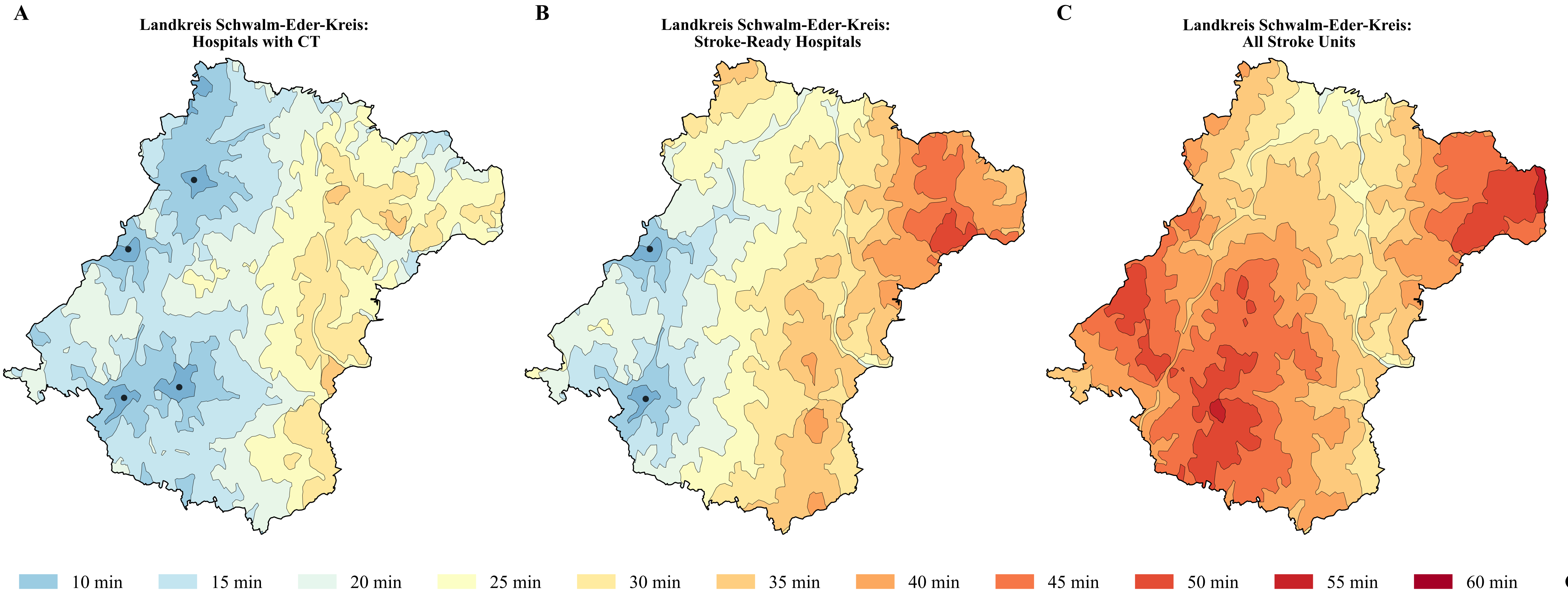

Isochrones — Landkreis Westerwaldkreis (Rheinland-Pfalz)

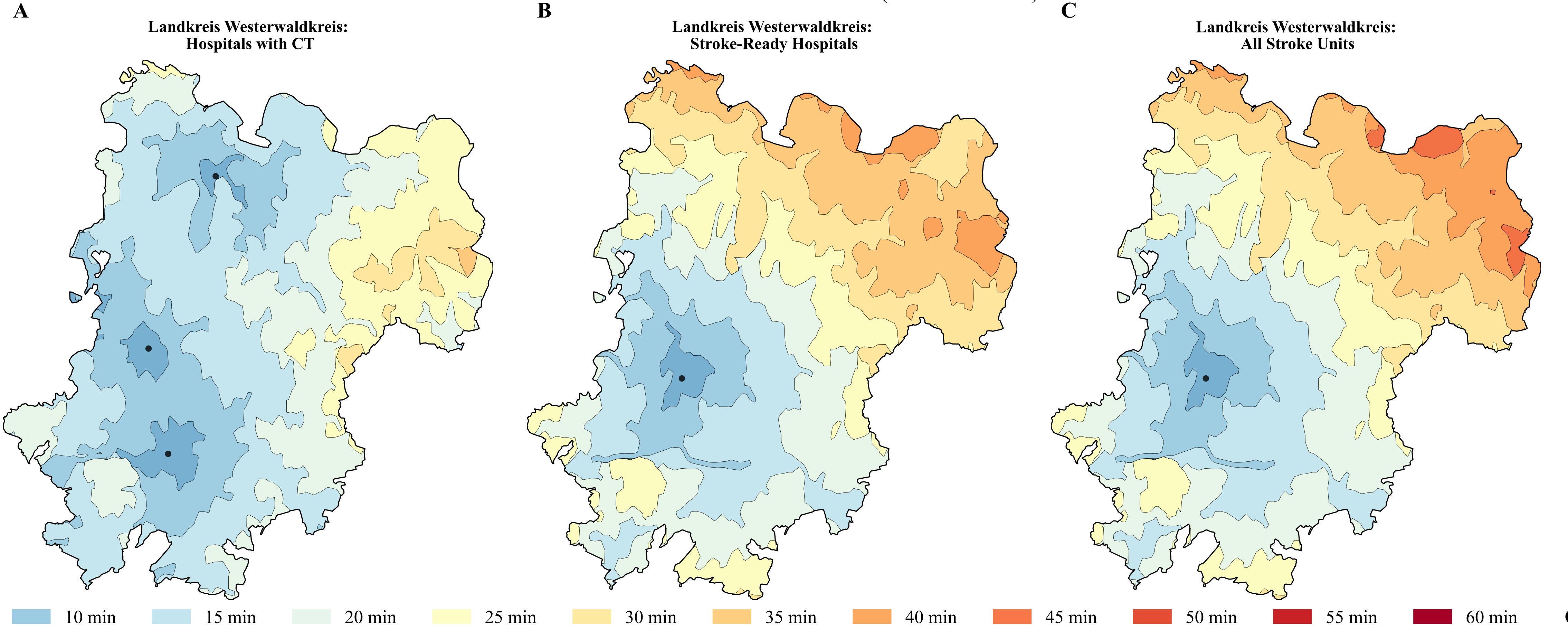

Isochrones — Kreisfreie Stadt Frankenthal (Pfalz) (Rheinland-Pfalz)

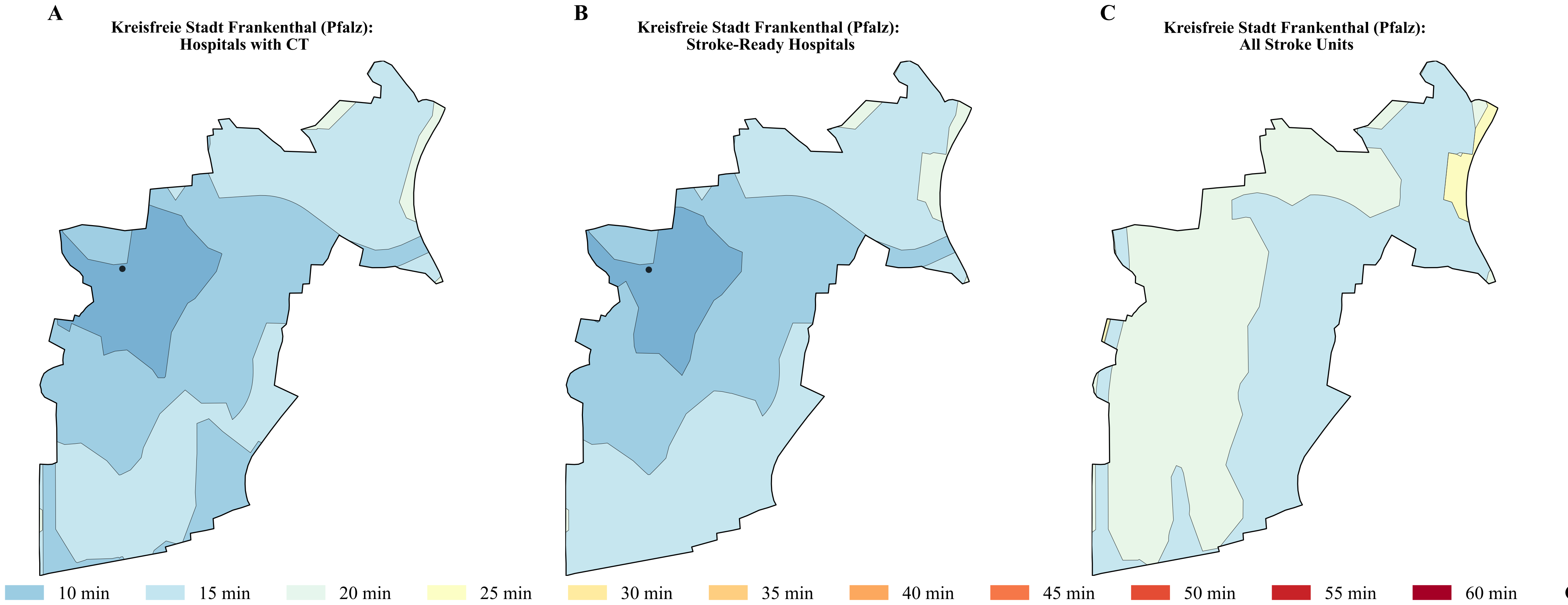

Isochrones — Landkreis Böblingen (Baden-Württemberg)

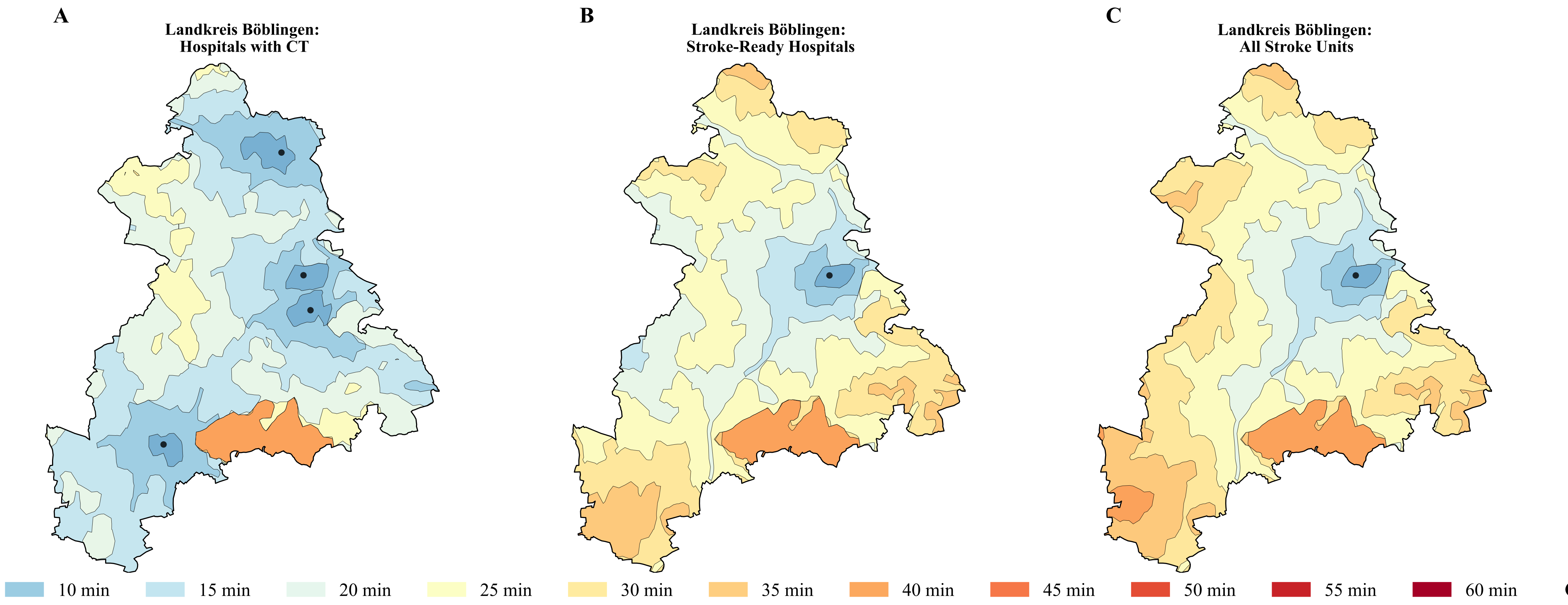

Isochrones — Landkreis Cham (Bayern)

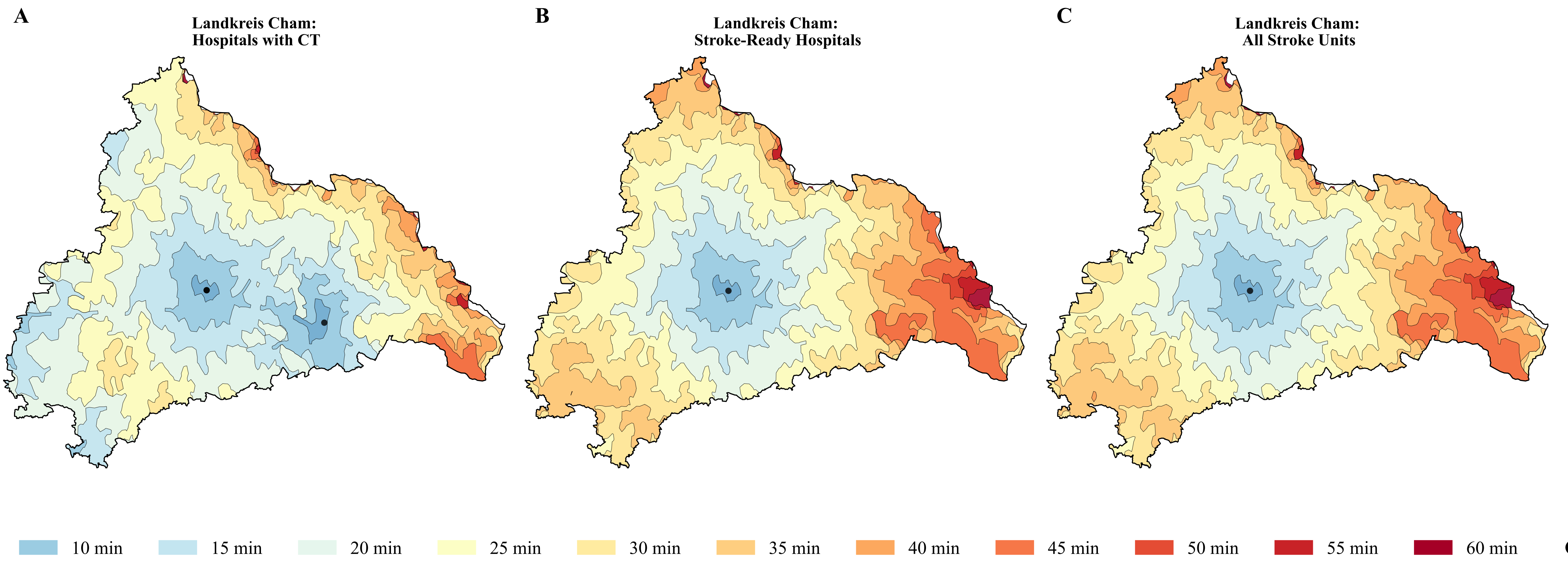

Isochrones — Landkreis Saarlouis (Saarland)

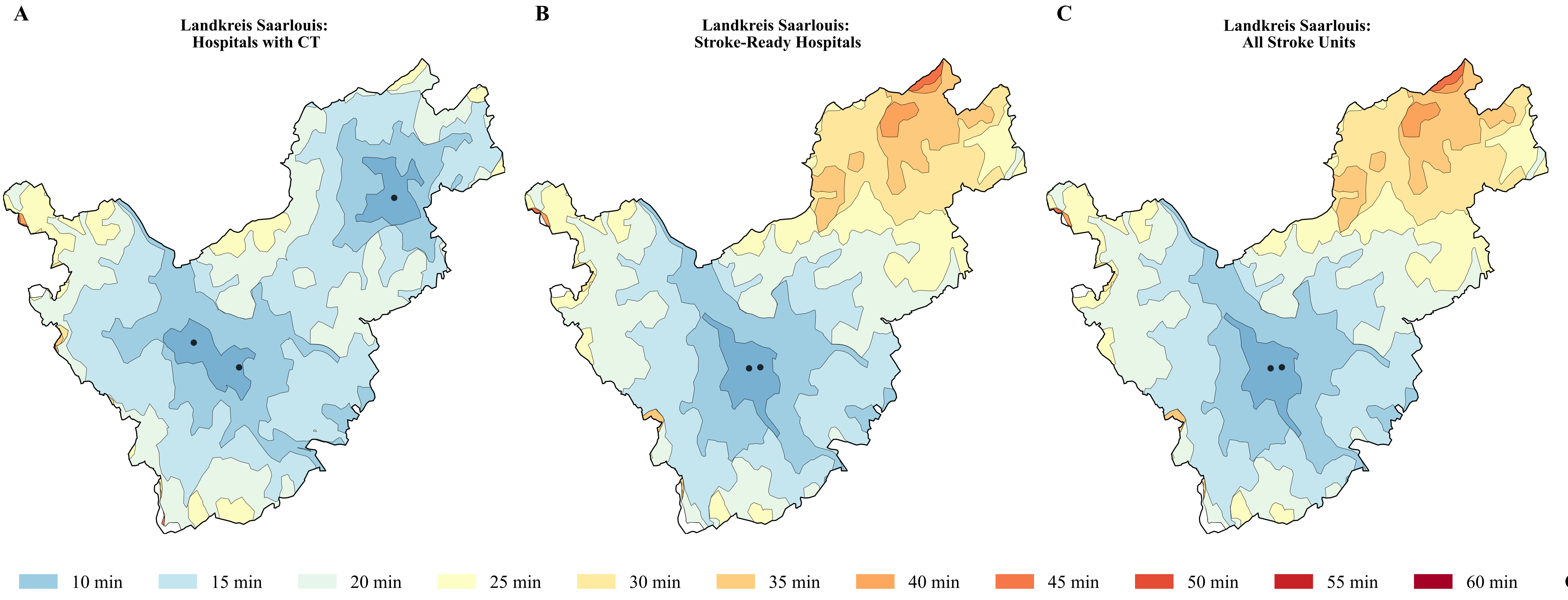

Isochrones — Landkreis Anhalt-Bitterfeld (Sachsen-Anhalt)

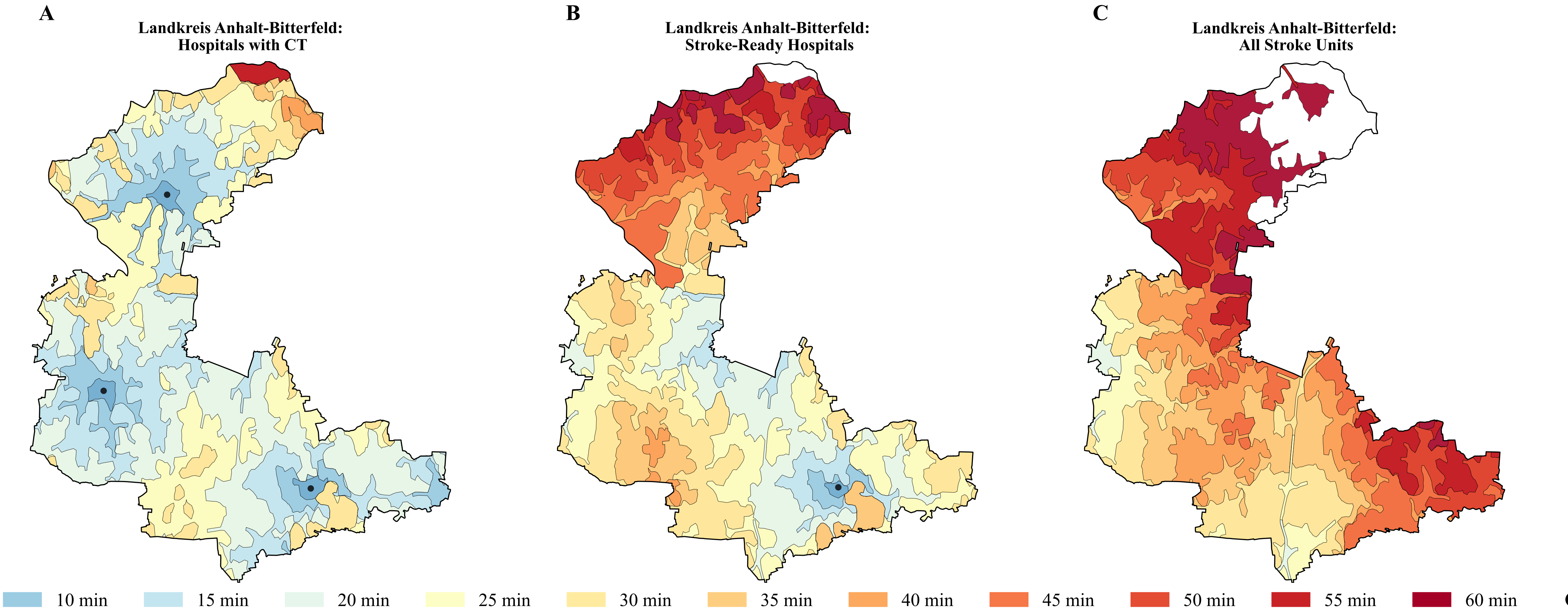

Isochrones — Landkreis Jerichower Land (Sachsen-Anhalt)

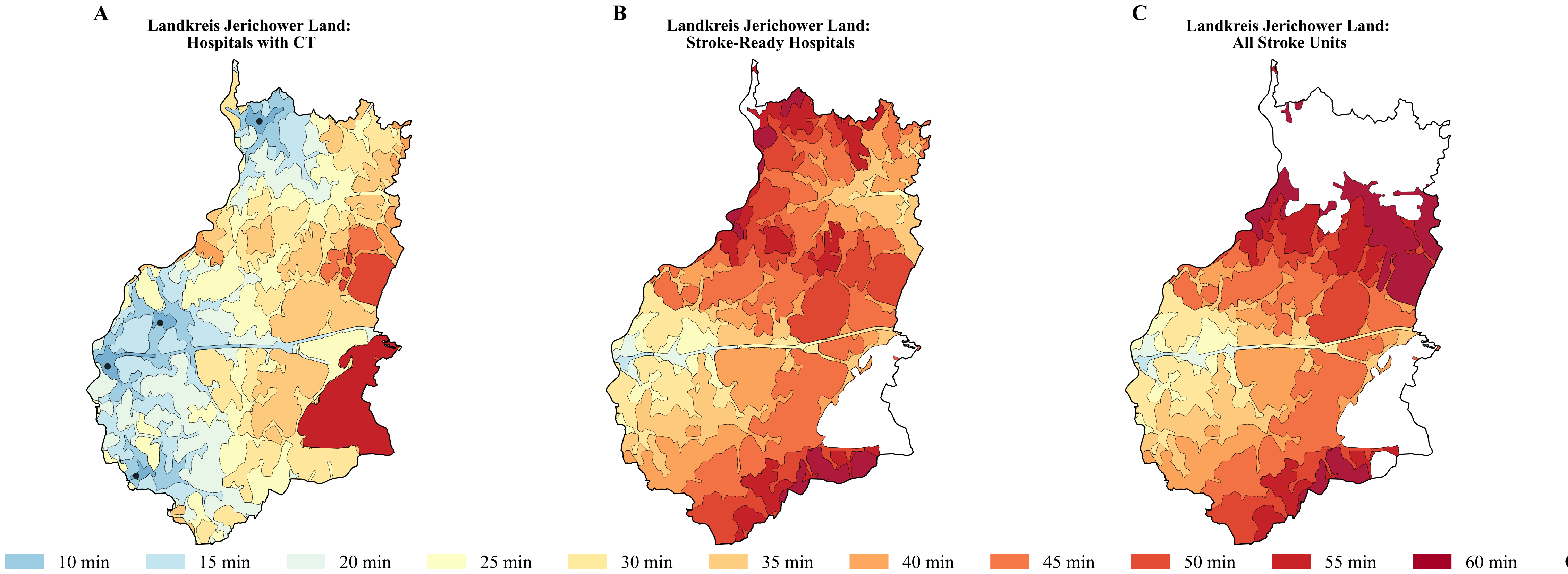

Isochrones — Landkreis Kyffhäuserkreis (Thüringen)

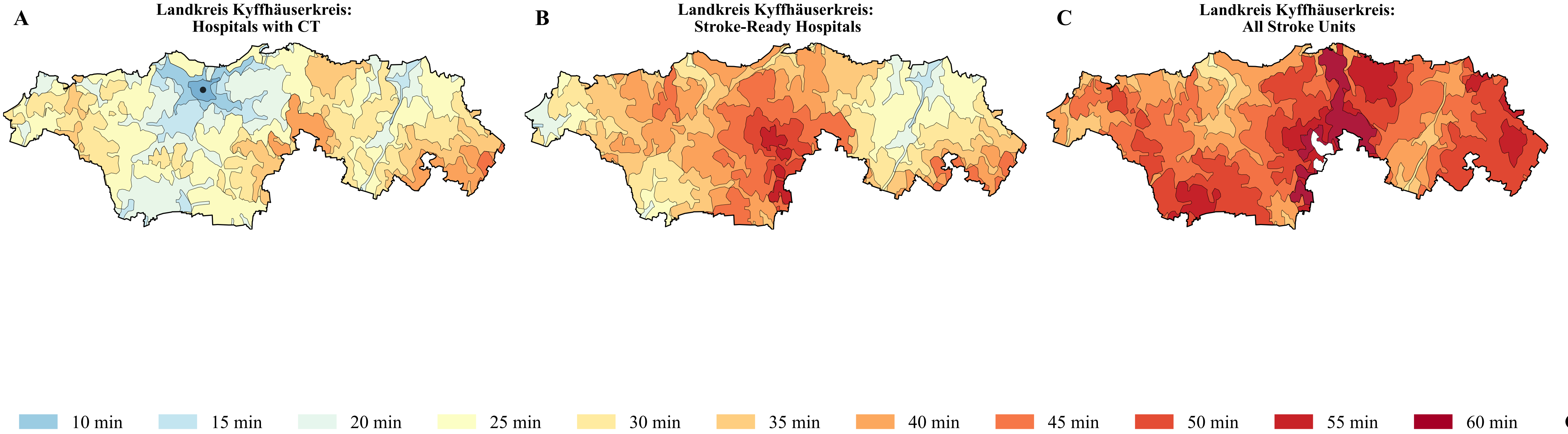

Isochrones — Landkreis Peine (Niedersachsen)

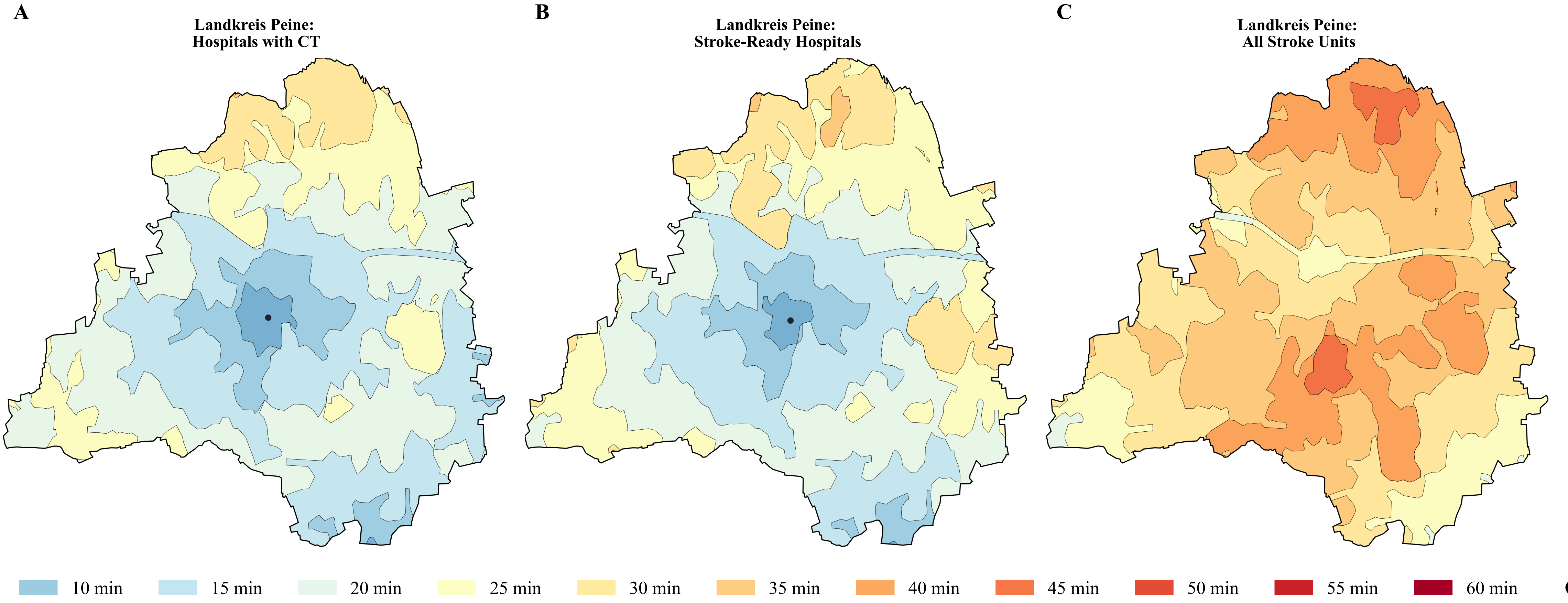

Isochrones — Kreisfreie Stadt Leverkusen (Nordrhein-Westfalen)

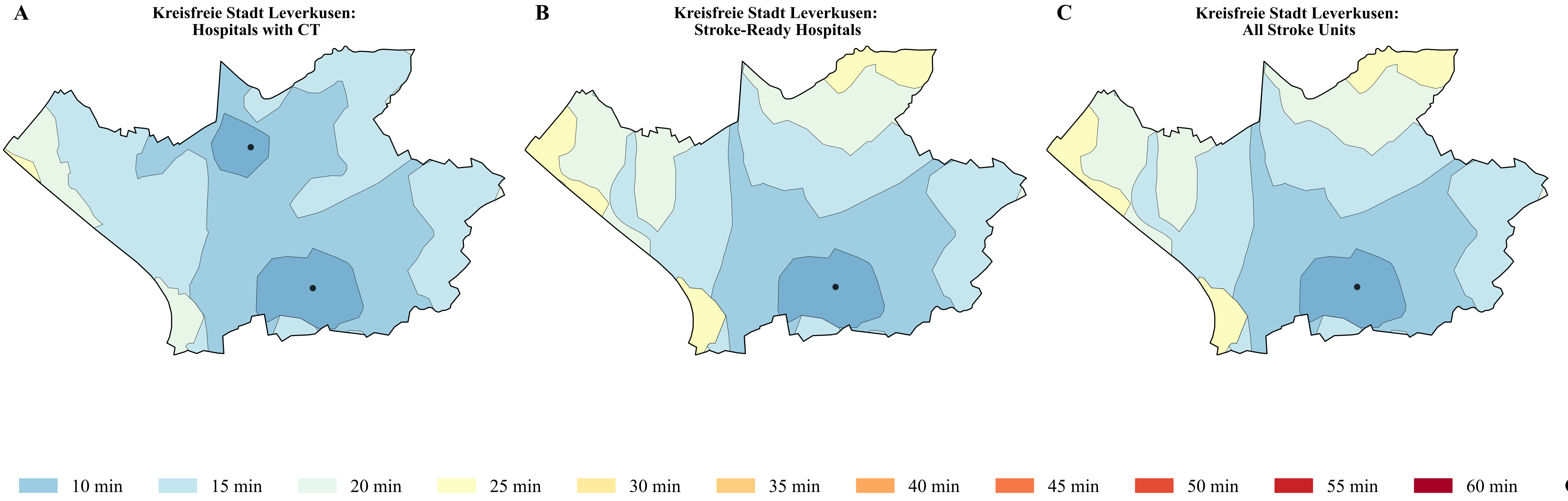

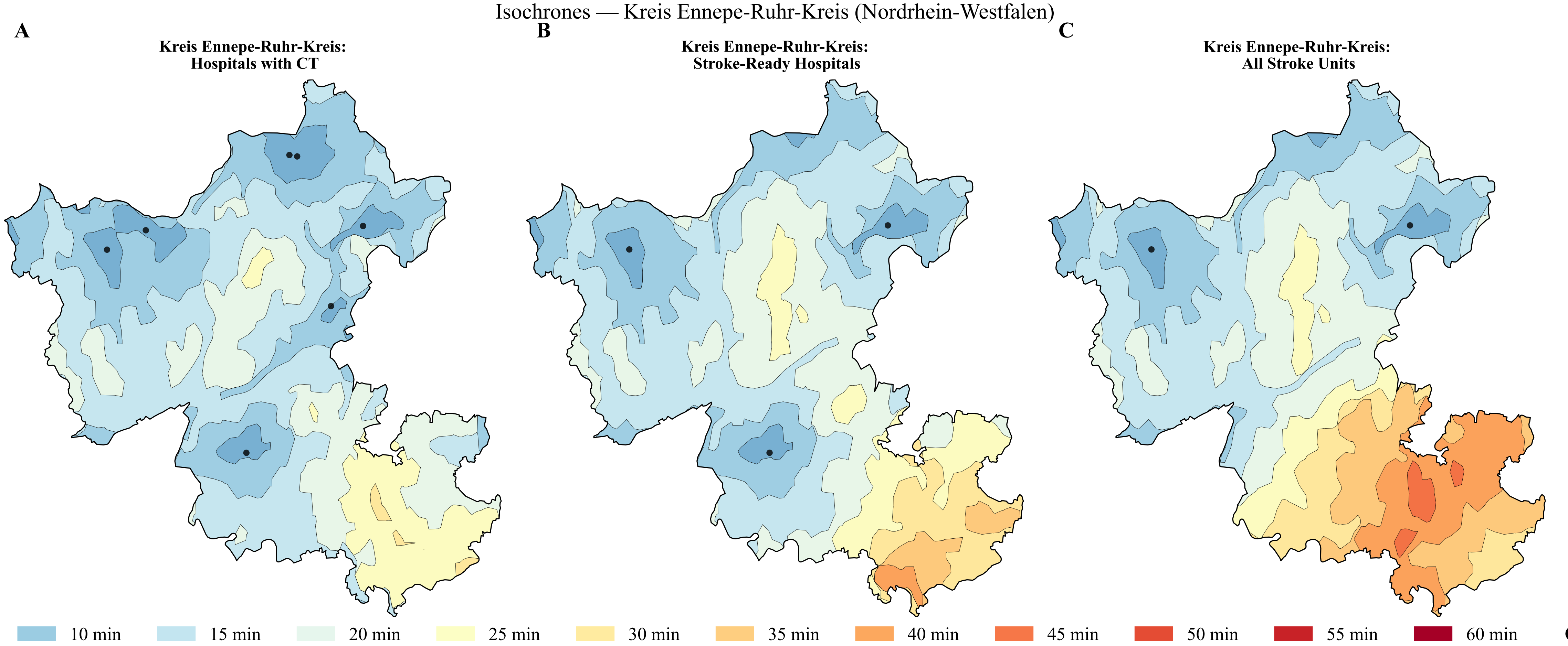

Isochrones — Kreis Ennepe-Ruhr-Kreis (Nordrhein-Westfalen)
A
Kreis Ennepe-Ruhr-Kreis:
Hospitals with CT
B
Kreis Ennepe-Ruhr-Kreis:
Stroke-Ready Hospitals
C
Kreis Ennepe-Ruhr-Kreis:
All Stroke Units
10 min
15 min
20 min
25 min
30 min
35 min
40 min
45 min
50 min
55 min
60 min

Isochrones — Kreis Märkischer Kreis (Nordrhein-Westfalen)

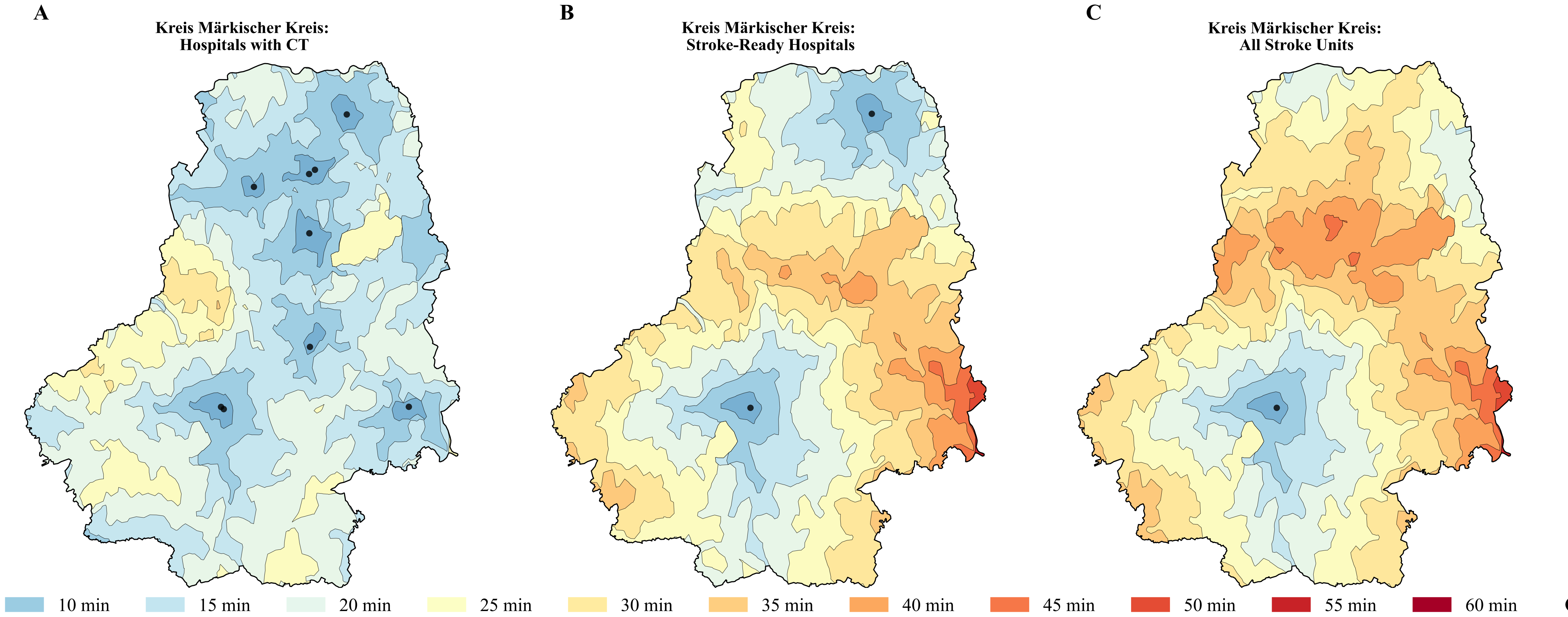

Isochrones — Landkreis Ahrweiler (Rheinland-Pfalz)

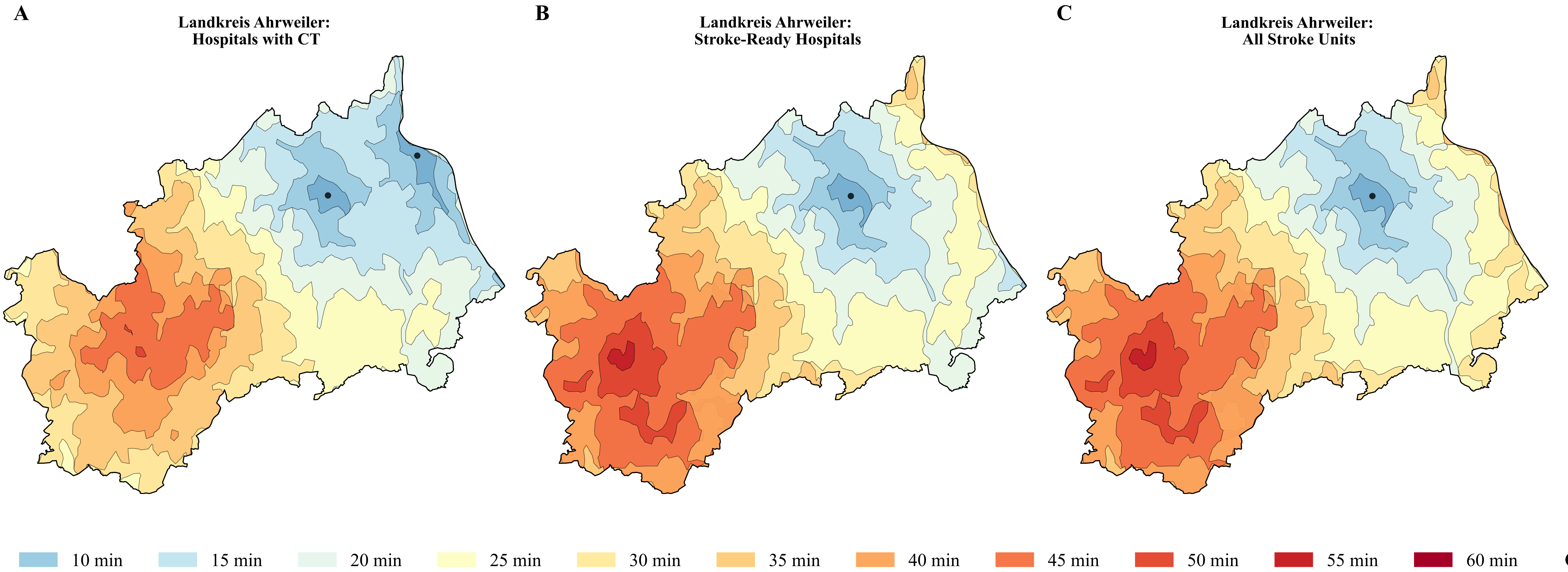

Isochrones — Stadtkreis Baden-Baden (Baden-Württemberg)

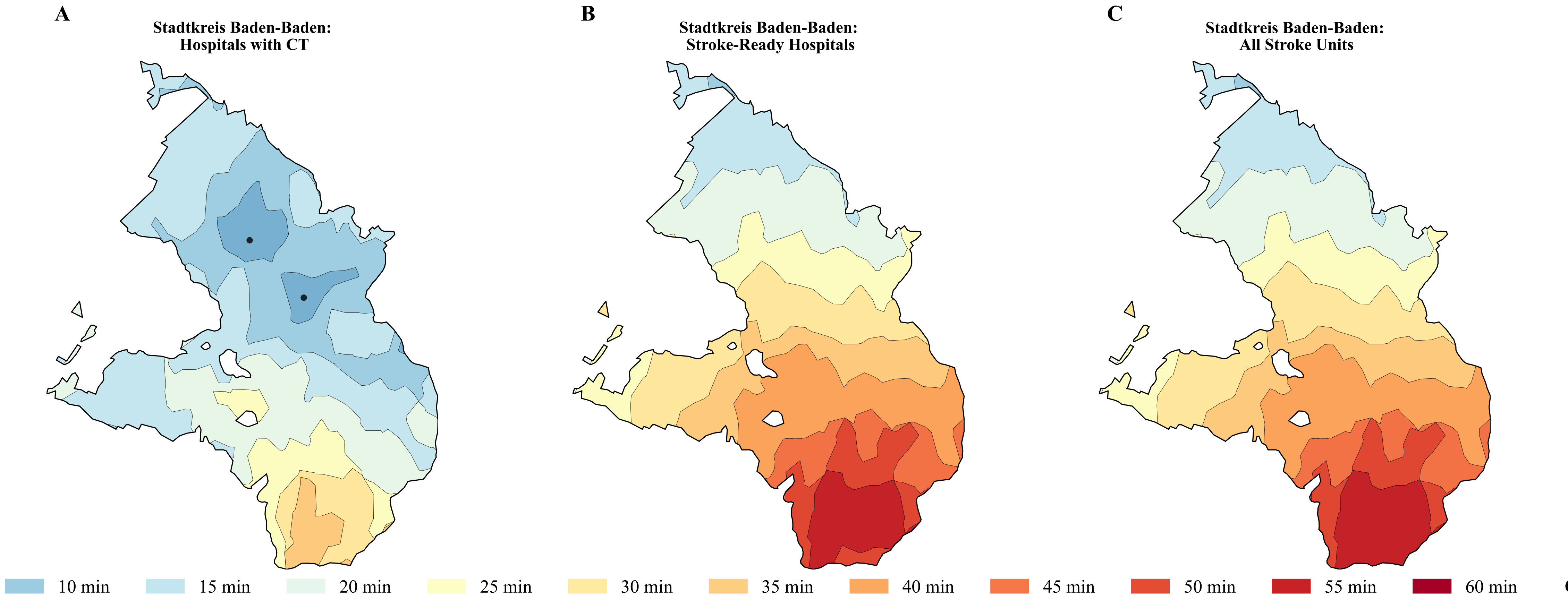

Isochrones — Landkreis Waldshut (Baden-Württemberg)

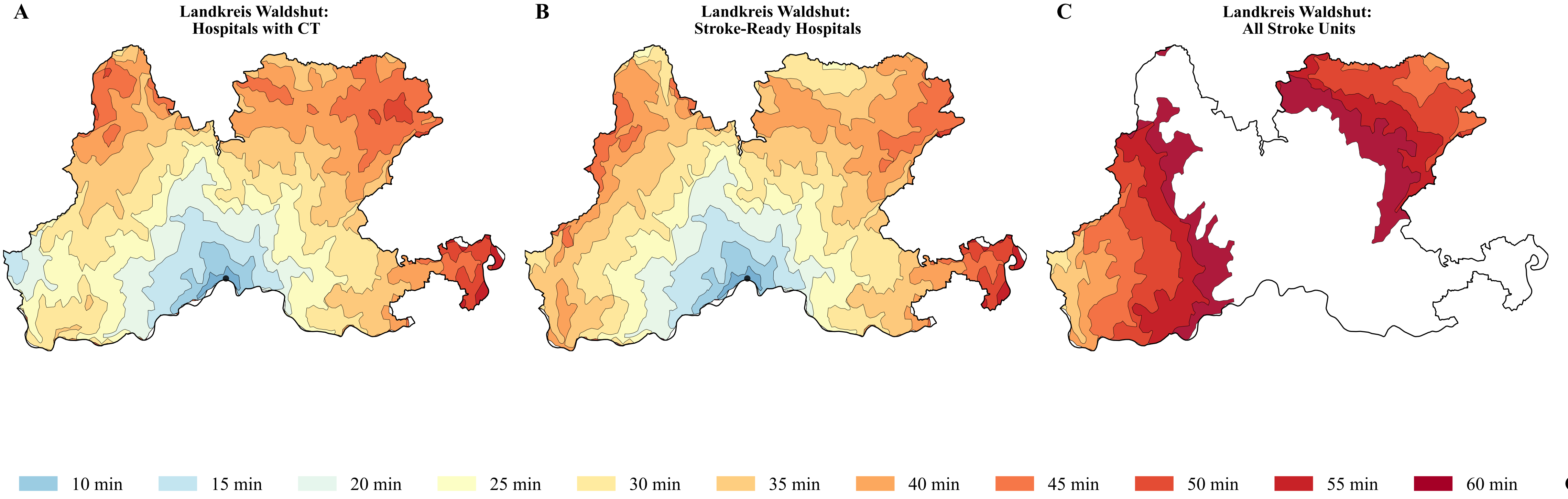

Isochrones — Landkreis Bad Tölz-Wolfratshausen (Bayern)

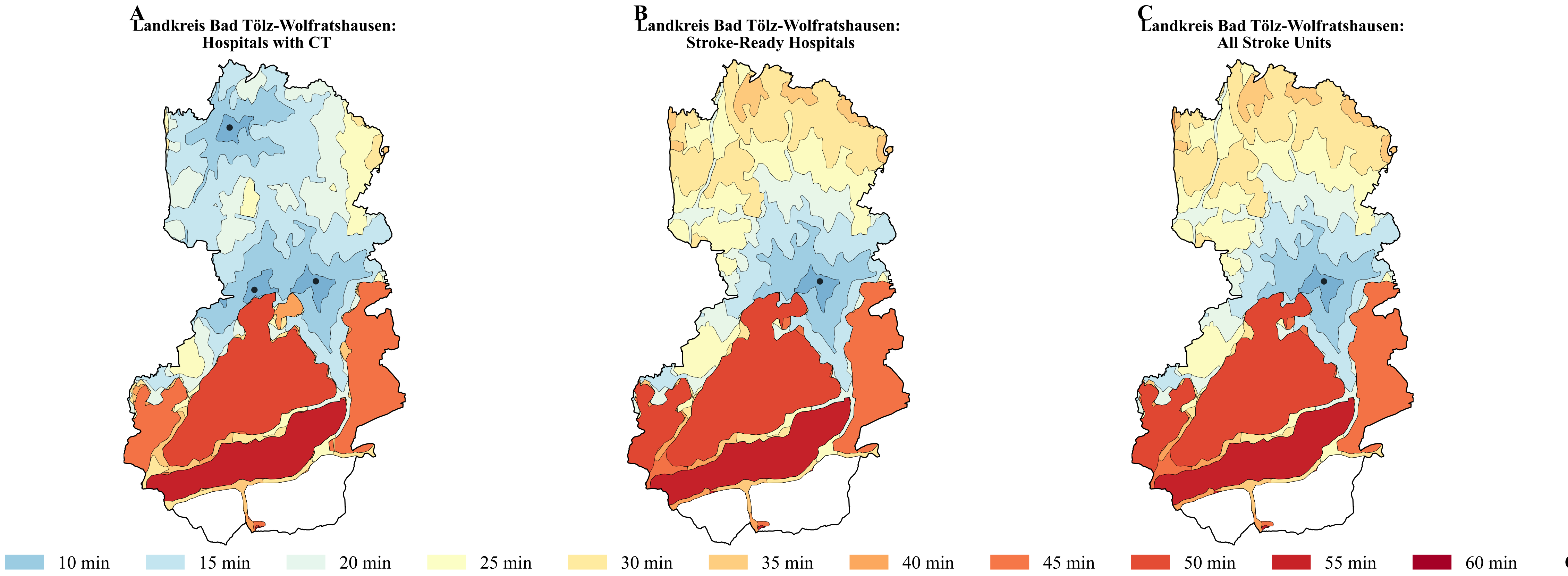

Isochrones — Landkreis Passau (Bayern)

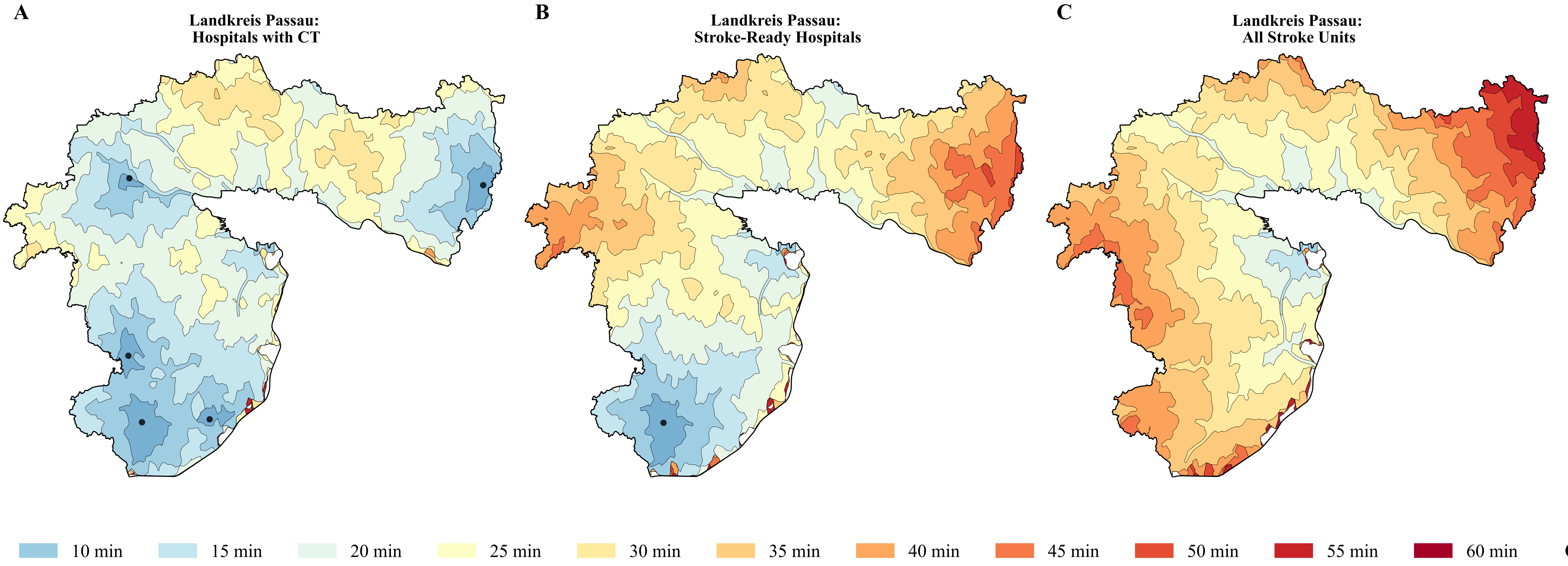

Isochrones — Landkreis Lichtenfels (Bayern)

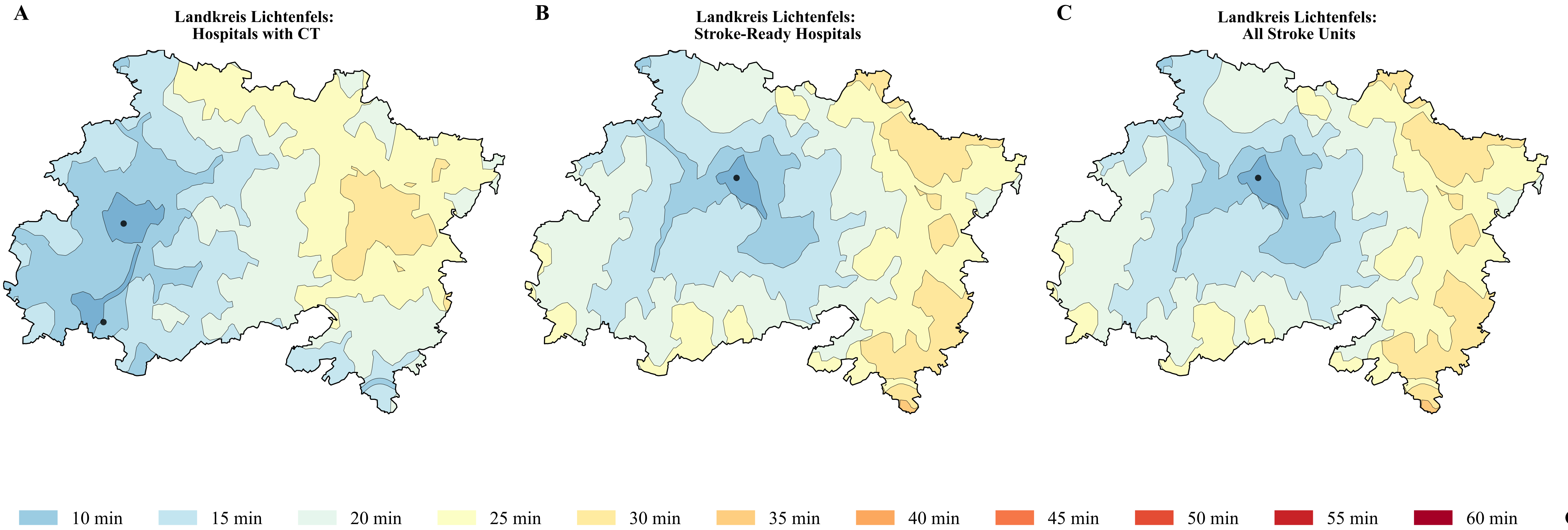

Isochrones — Landkreis Wunsiedel i.Fichtelgebirge (Bayern)

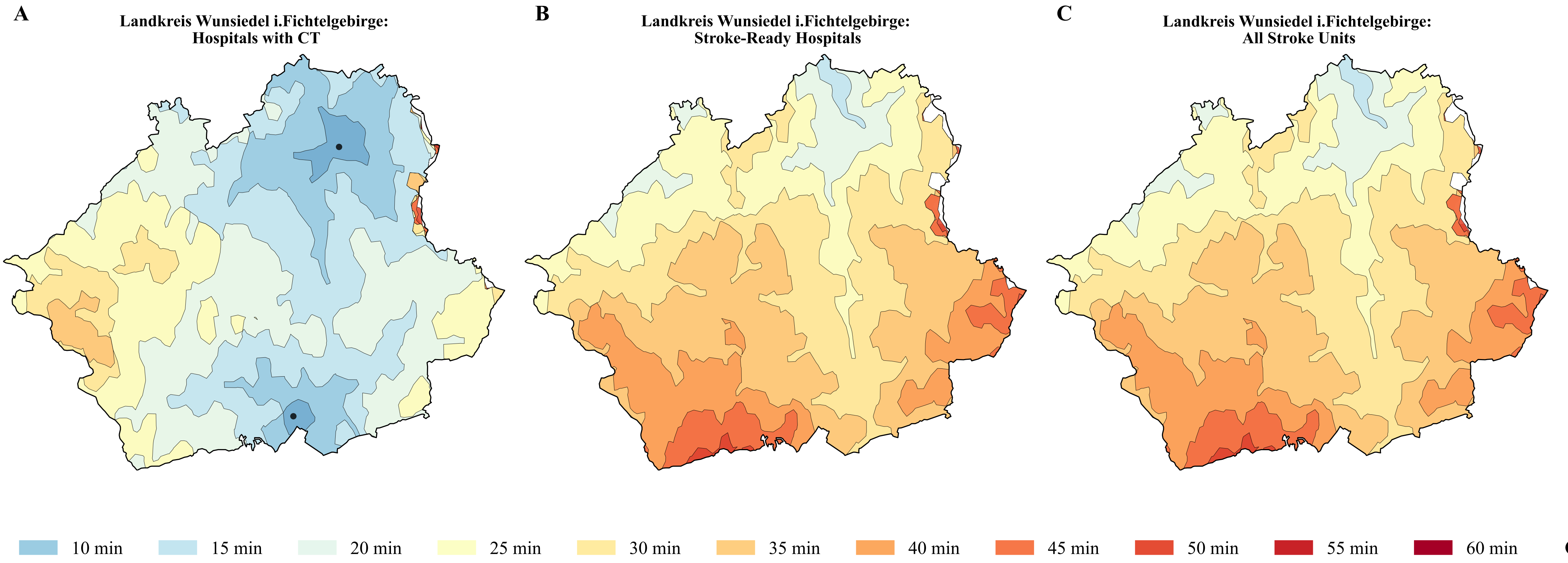

Isochrones — Landkreis Ansbach (Bayern)

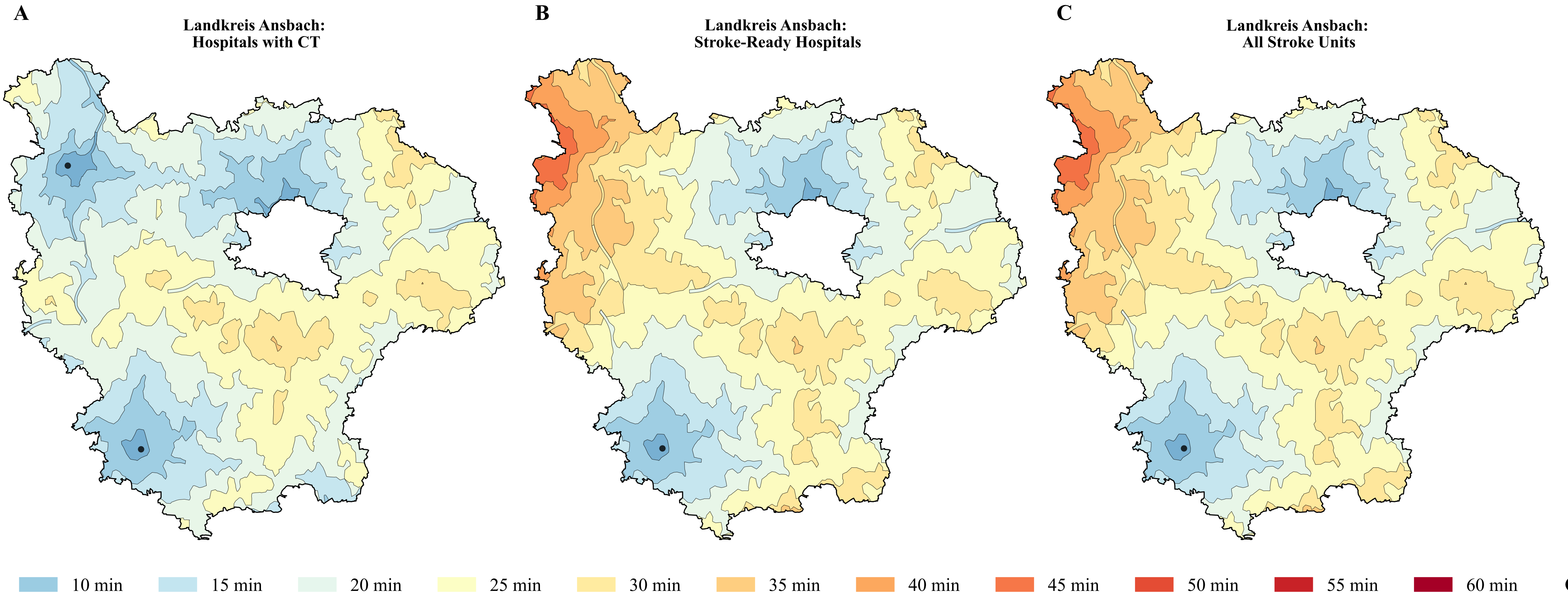

Isochrones — Kreisfreie Stadt Würzburg (Bayern)

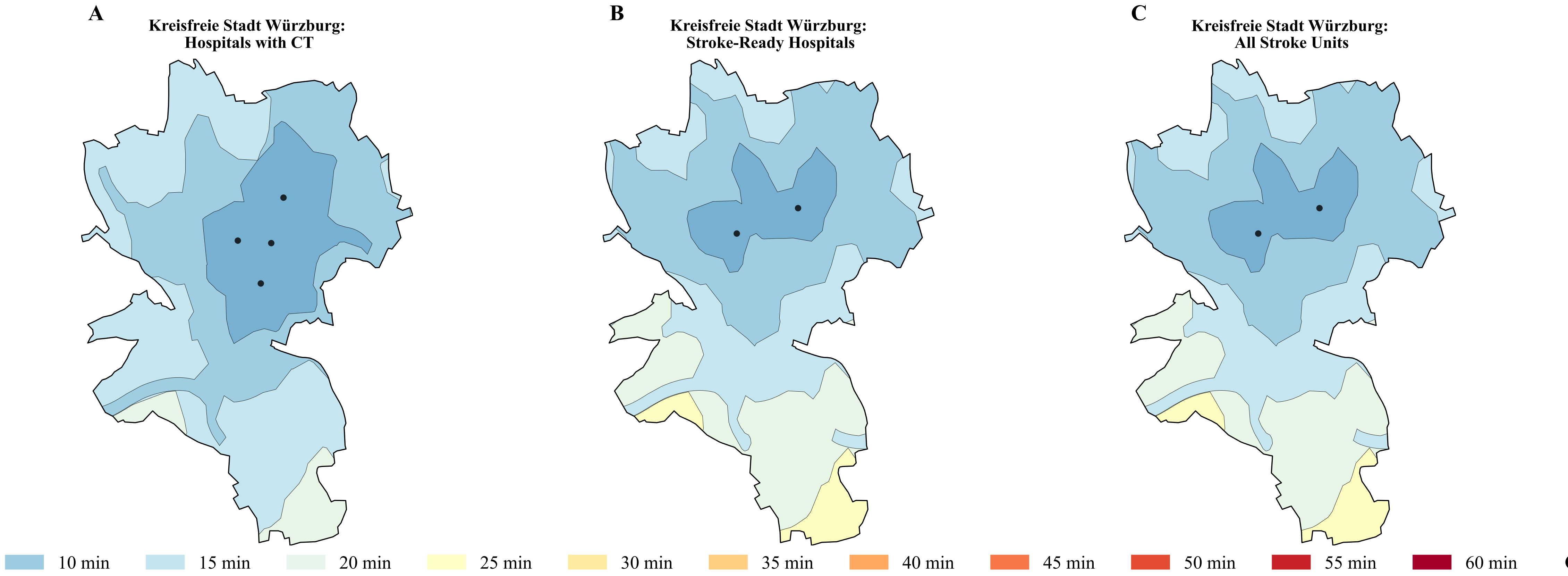

Isochrones — Landkreis Oder-Spree (Brandenburg)

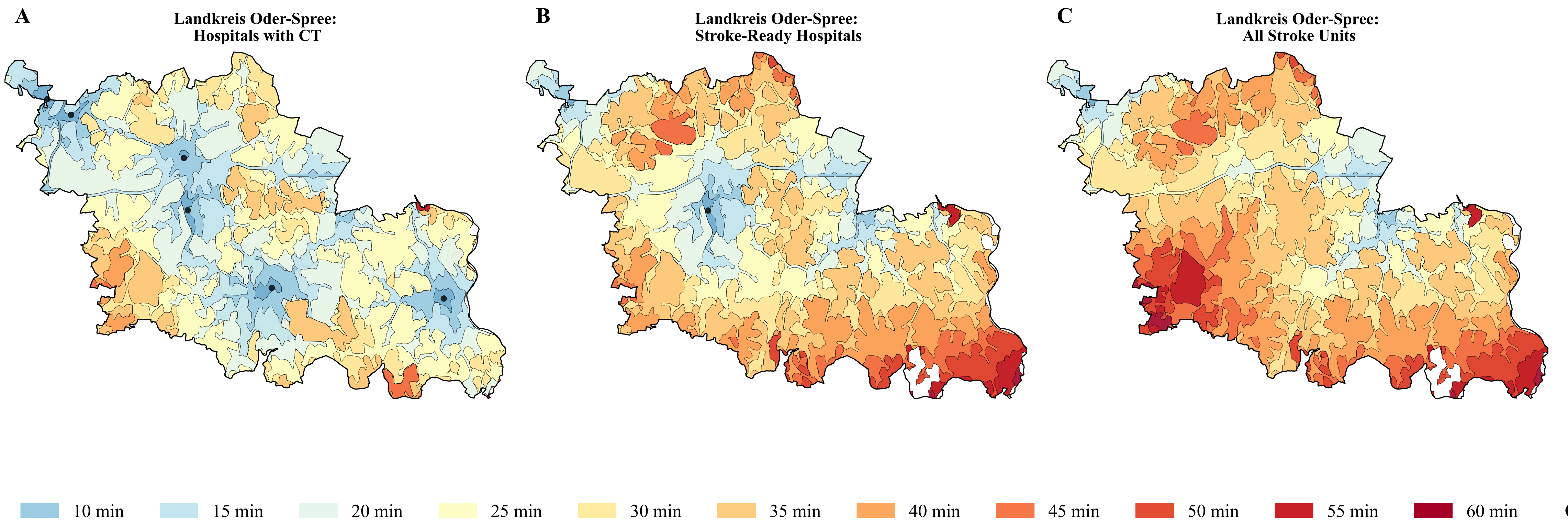

Isochrones — Landkreis Potsdam-Mittelmark (Brandenburg)

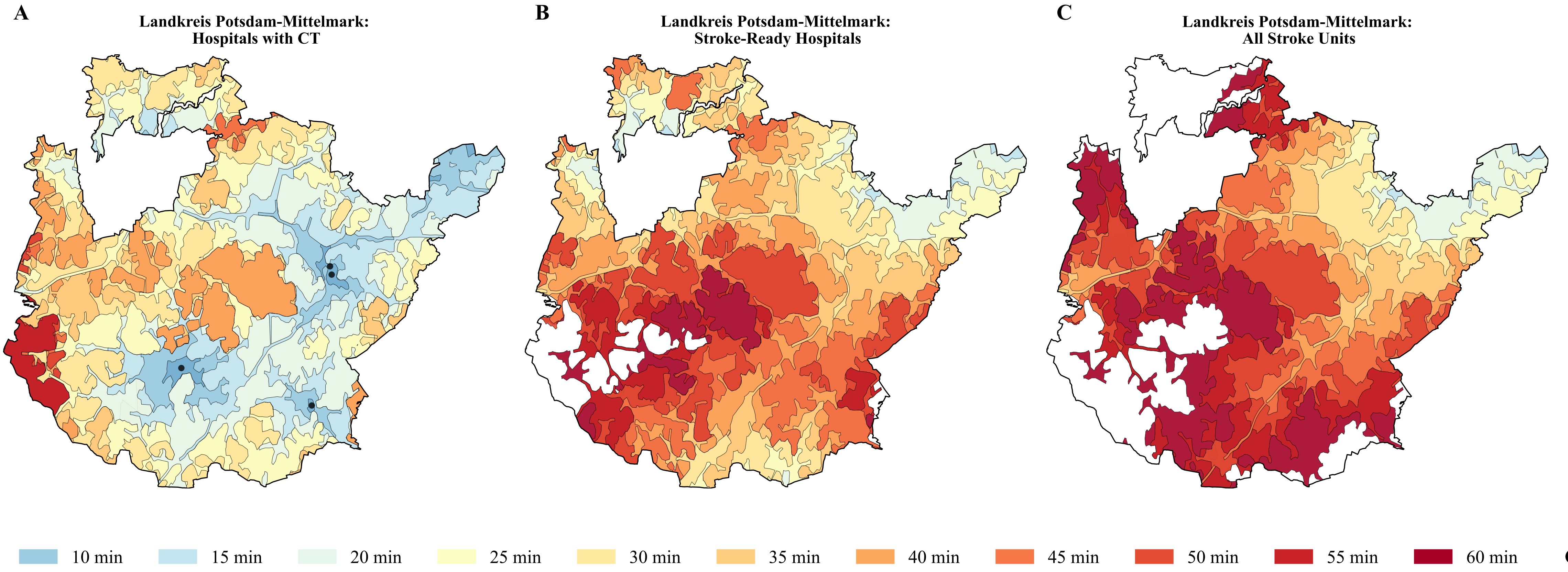

Isochrones — Landkreis Uckermark (Brandenburg)

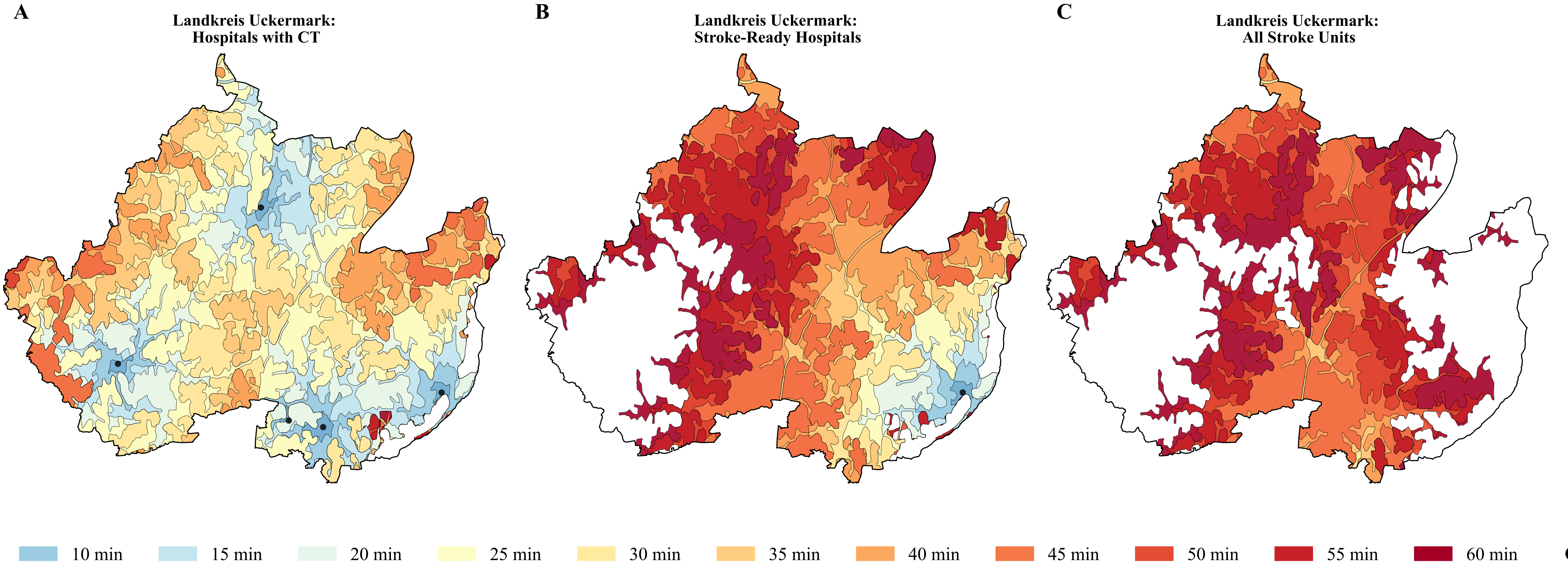

Isochrones — Landkreis Sächsische Schweiz-Osterzgebirge (Sachsen)

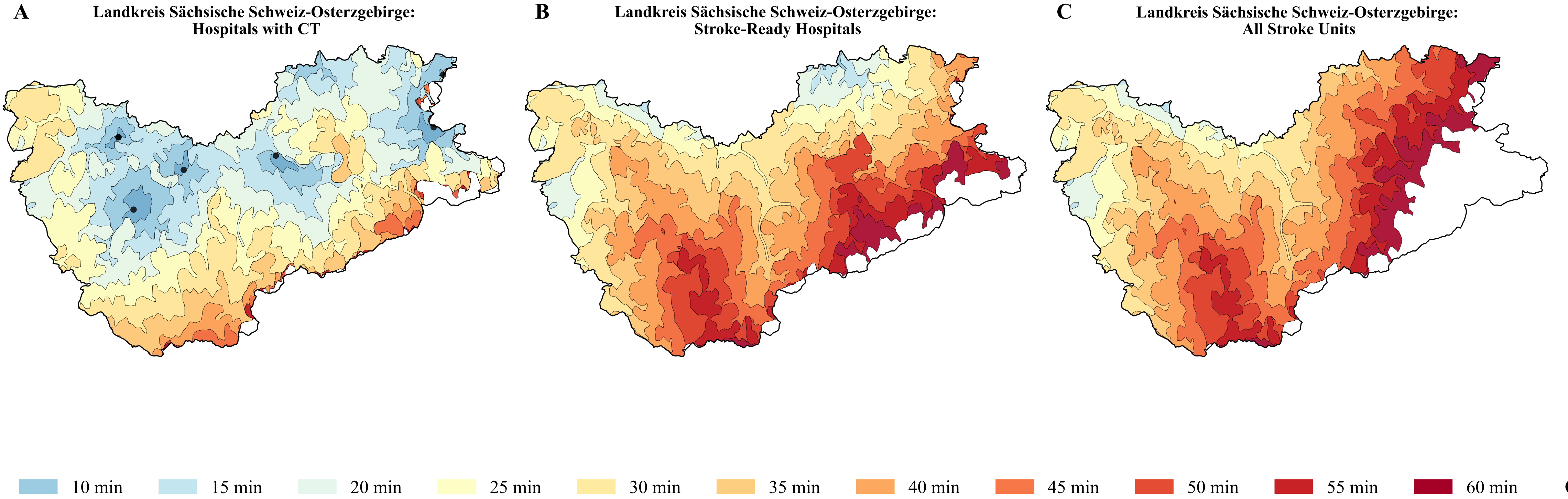

Isochrones — Kreisfreie Stadt Leipzig (Sachsen)

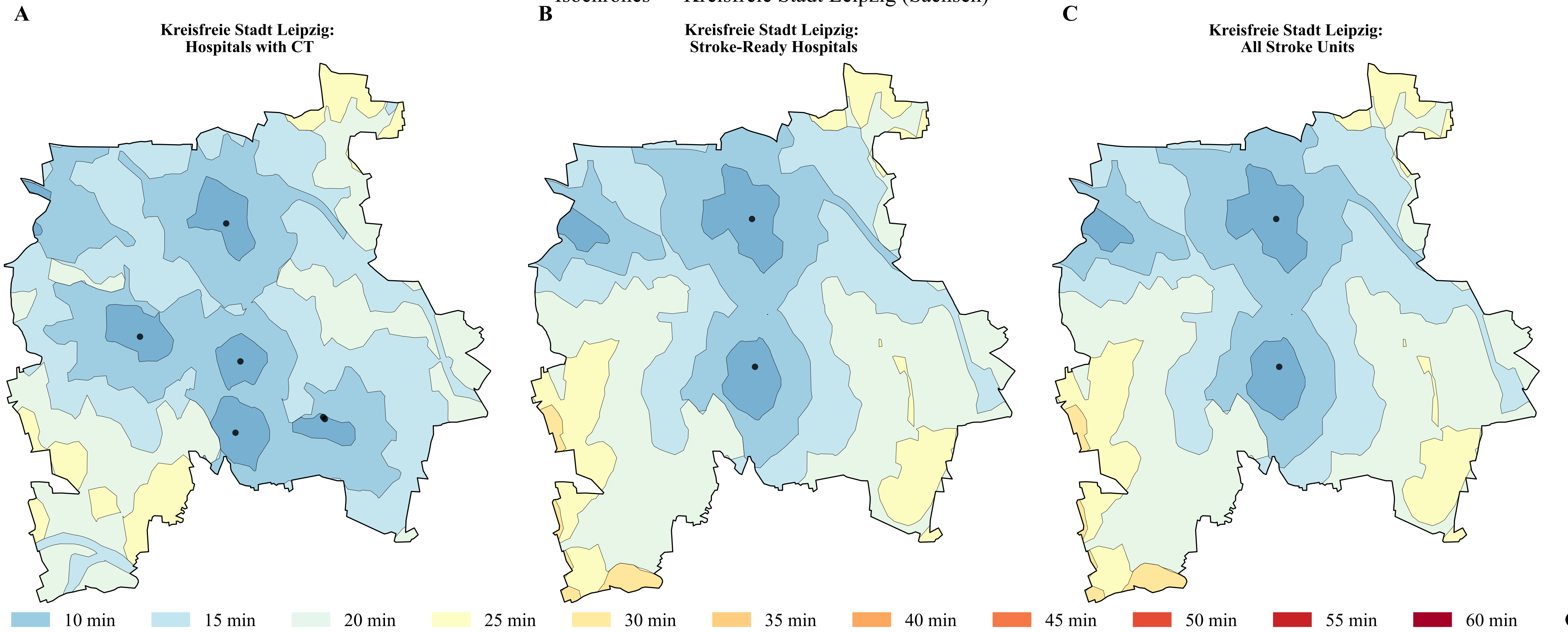

Isochrones — Kreisfreie Stadt Jena (Thüringen)

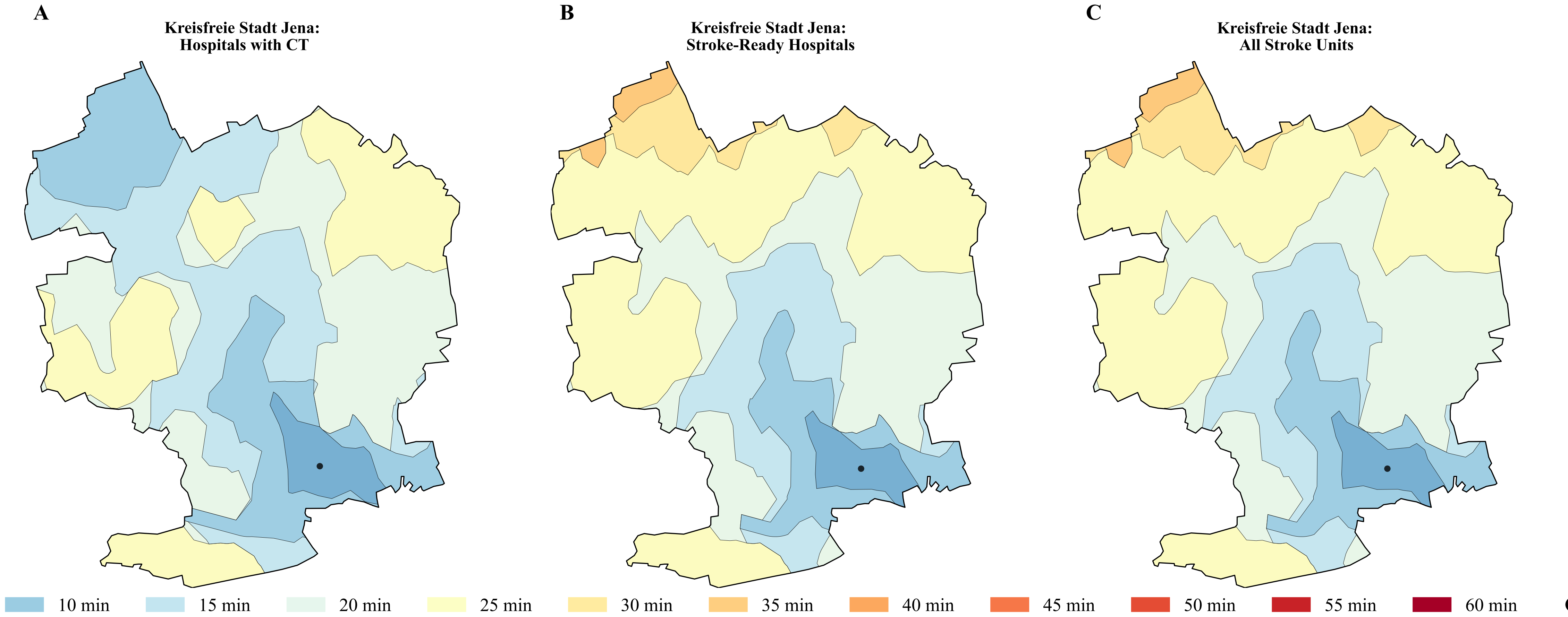

Isochrones — Landkreis Gotha (Thüringen)

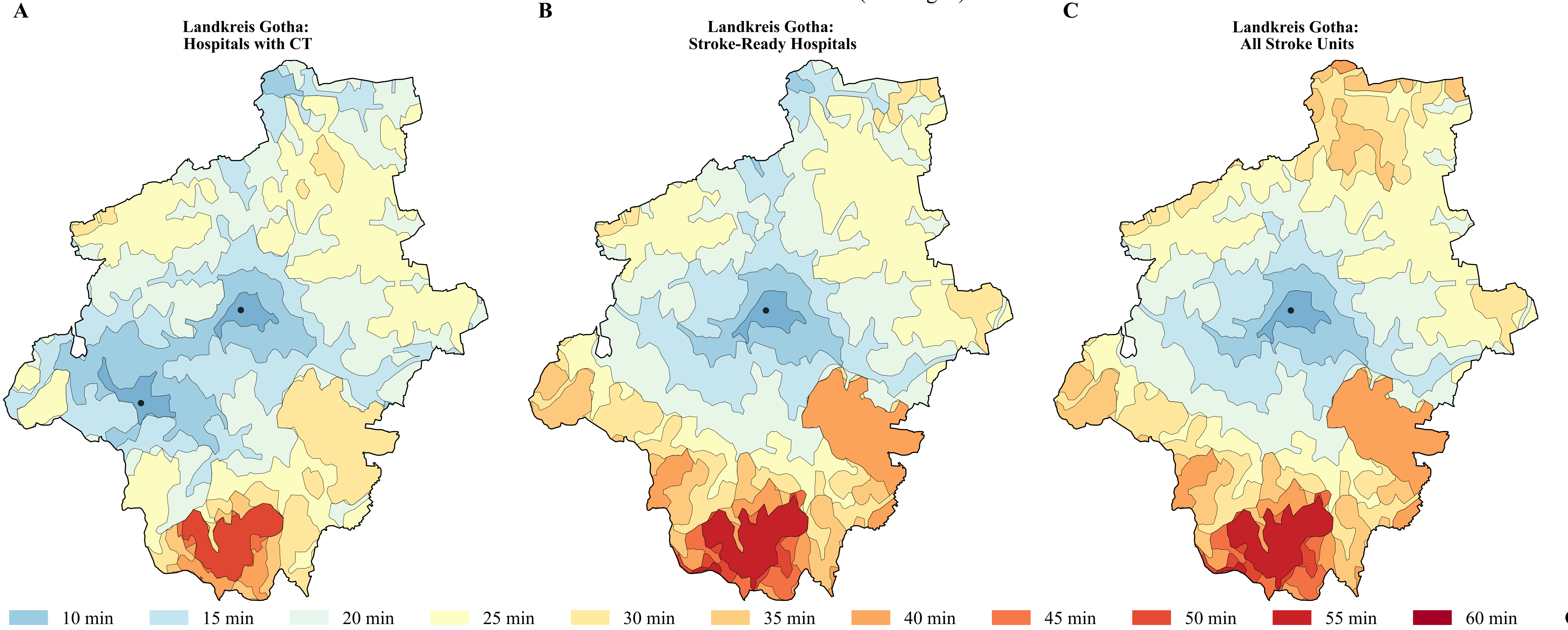

Isochrones — Kreis Pinneberg (Schleswig-Holstein)

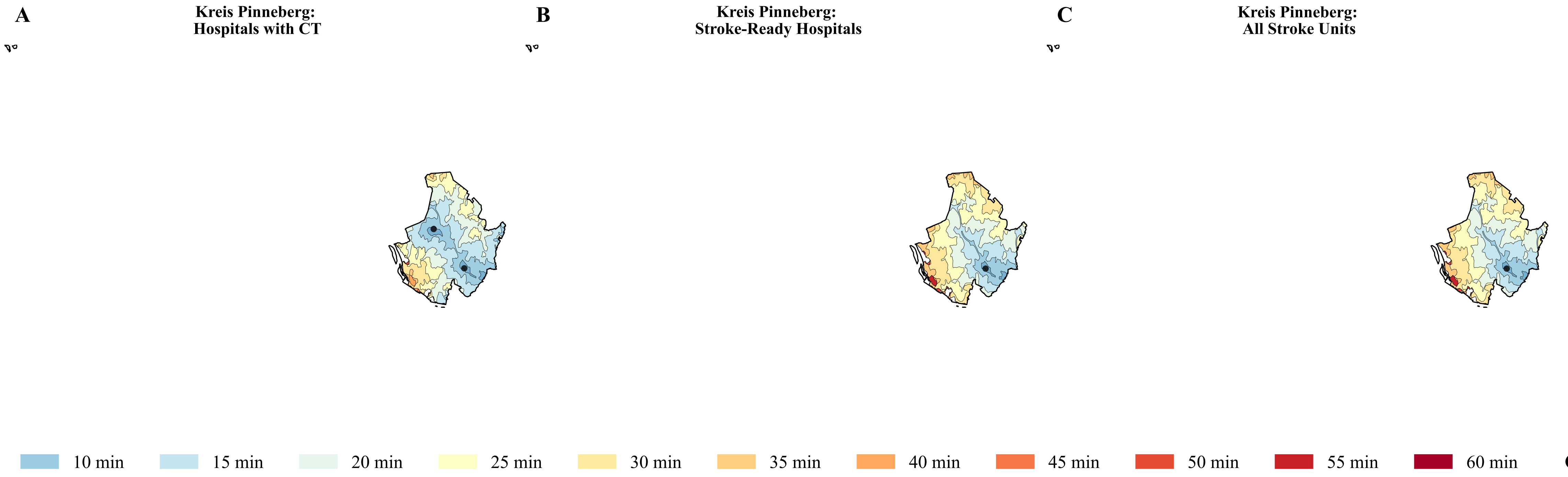

Isochrones — Kreis Schleswig-Flensburg (Schleswig-Holstein)

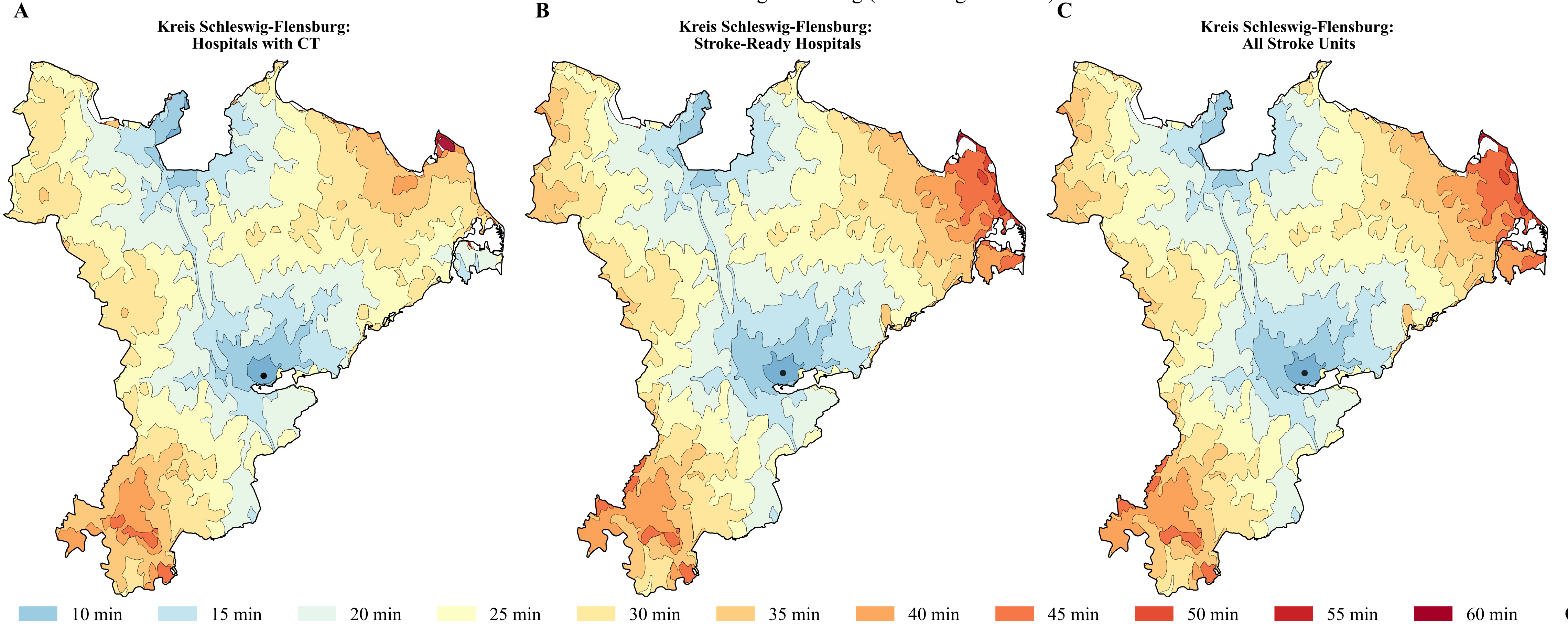

Isochrones — Kreis Steinburg (Schleswig-Holstein)

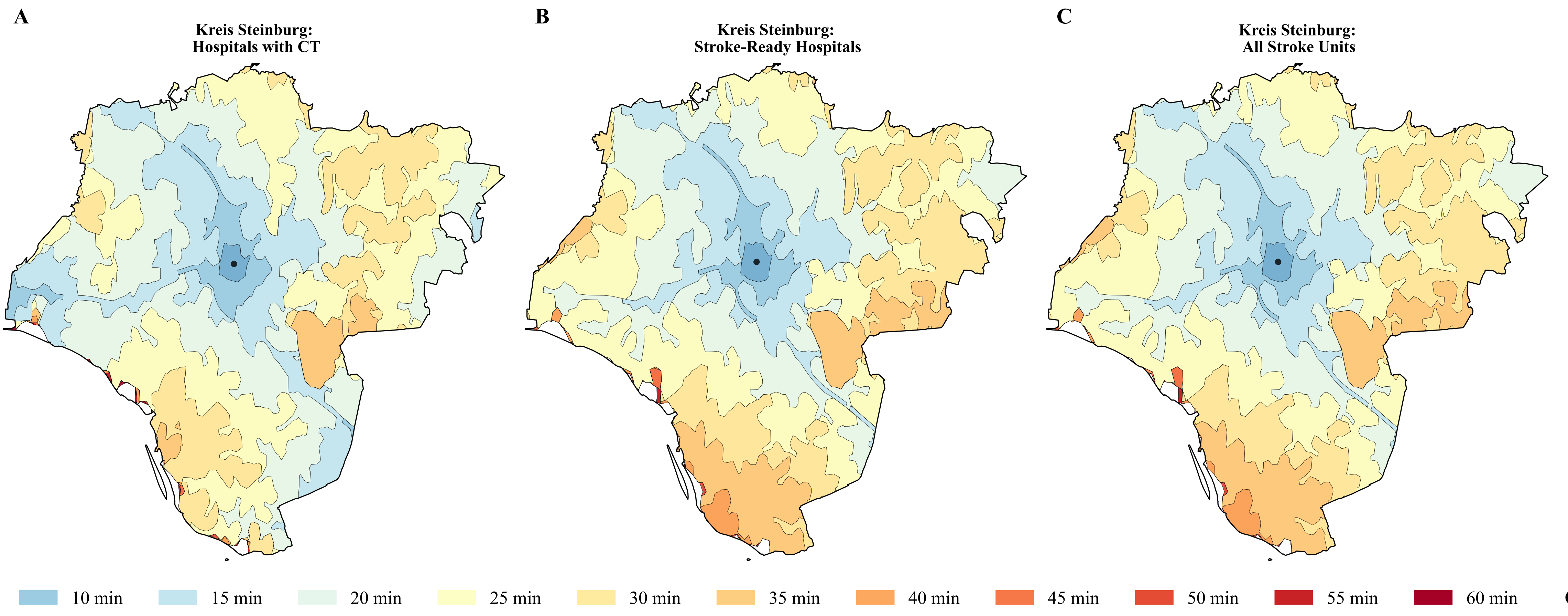

Isochrones — Landkreis Verden (Niedersachsen)

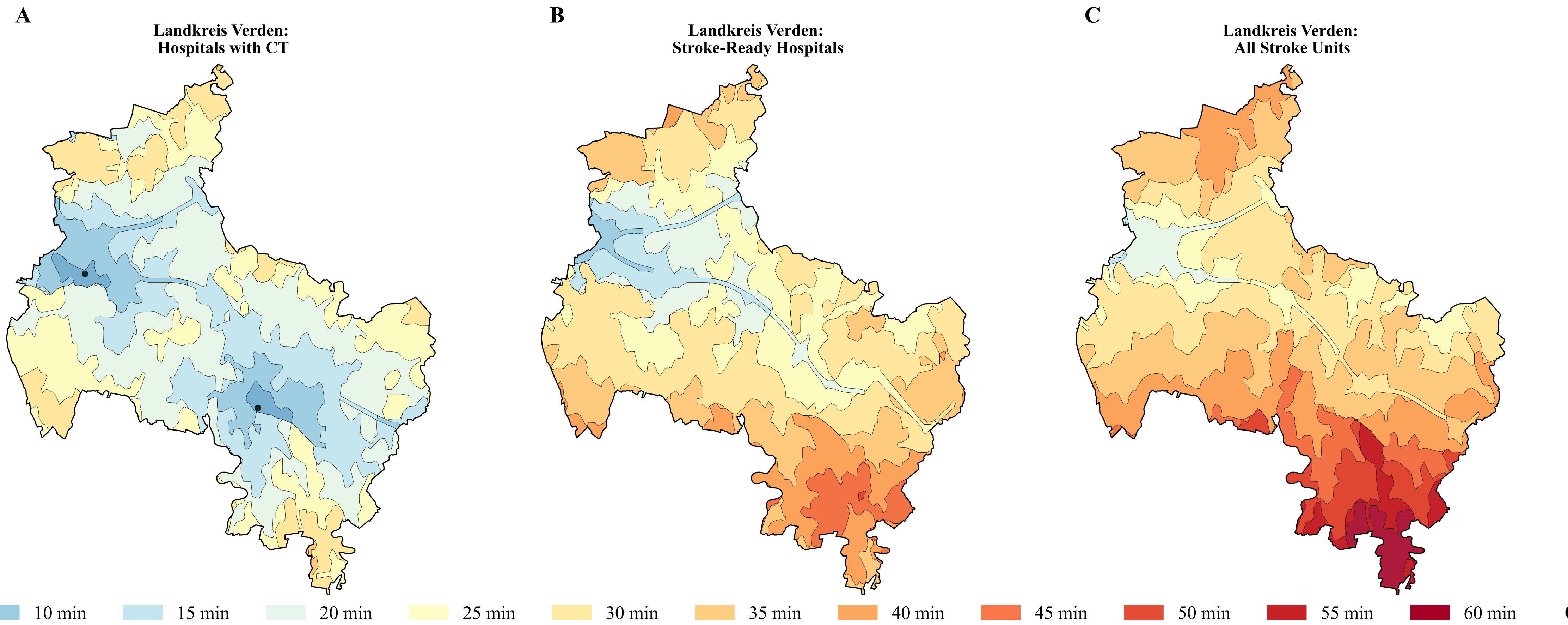

Isochrones — Kreisfreie Stadt Delmenhorst (Niedersachsen)

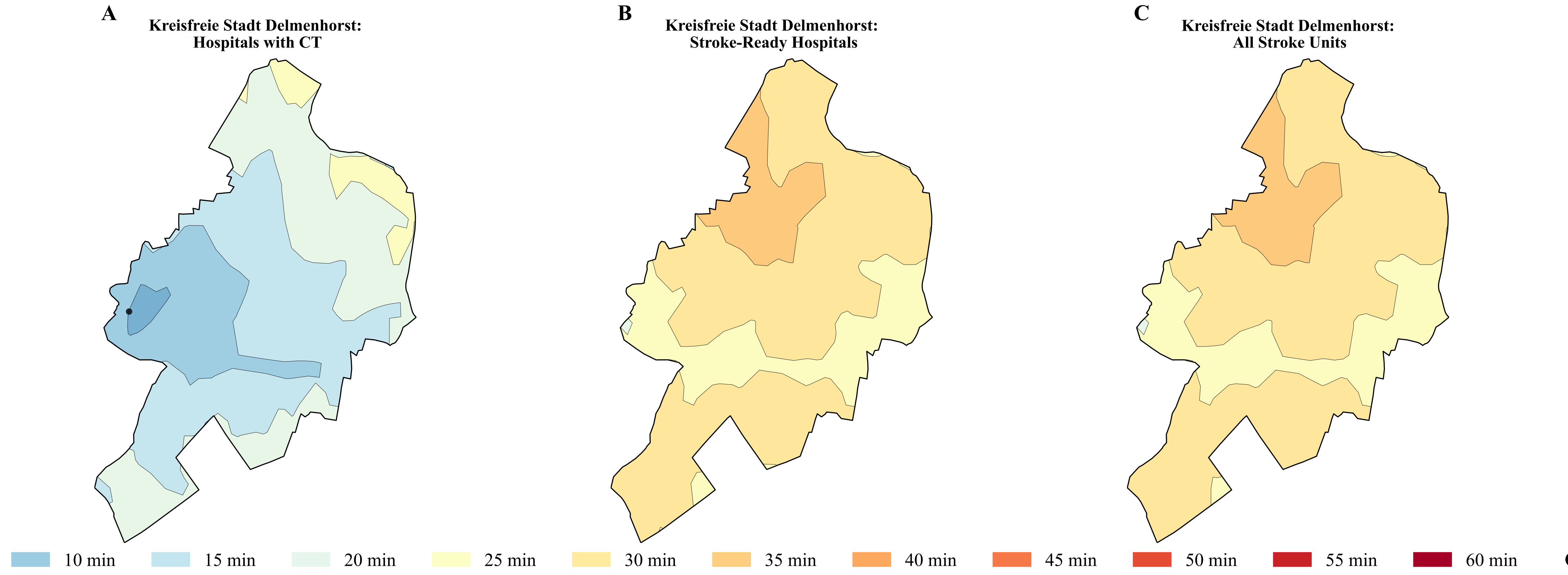

Isochrones — Kreis Kleve (Nordrhein-Westfalen)

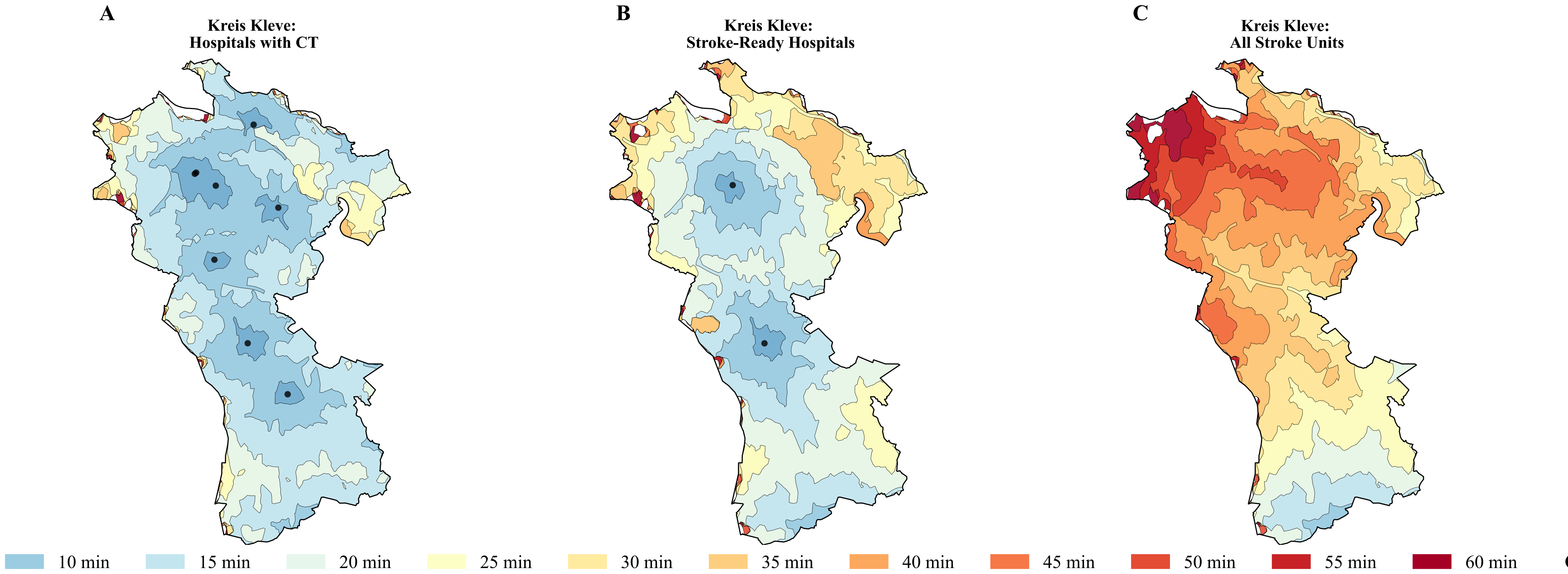

Isochrones — Kreis Herford (Nordrhein-Westfalen)

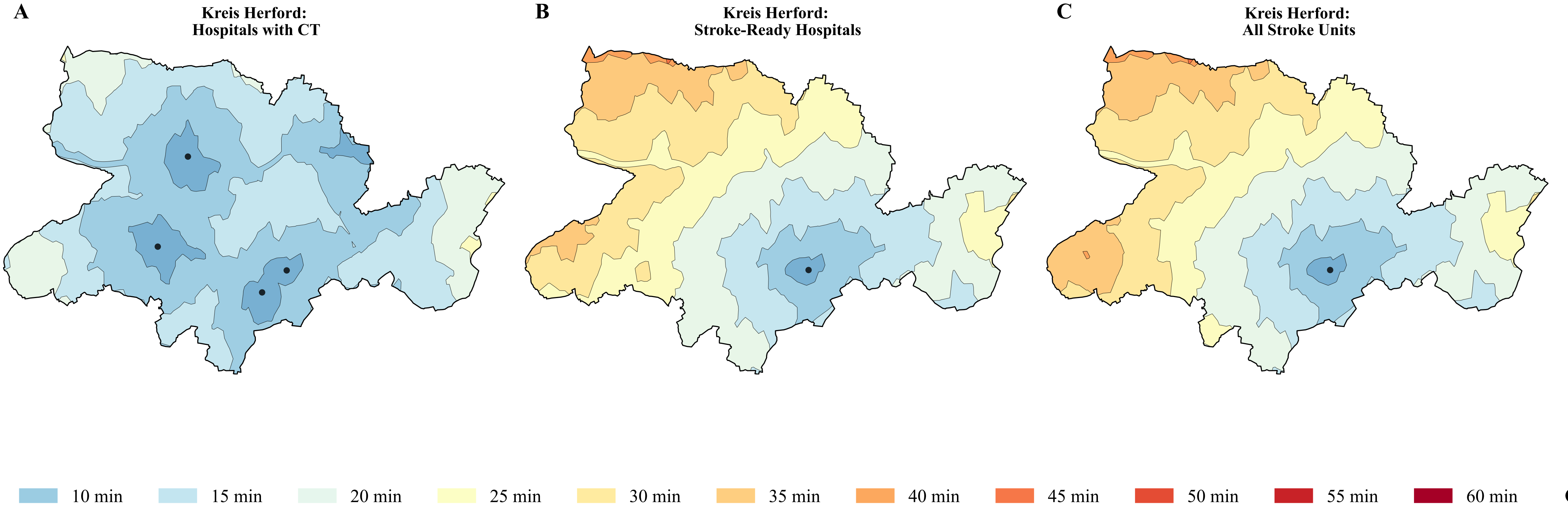

Isochrones — Kreisfreie Stadt Dortmund (Nordrhein-Westfalen)

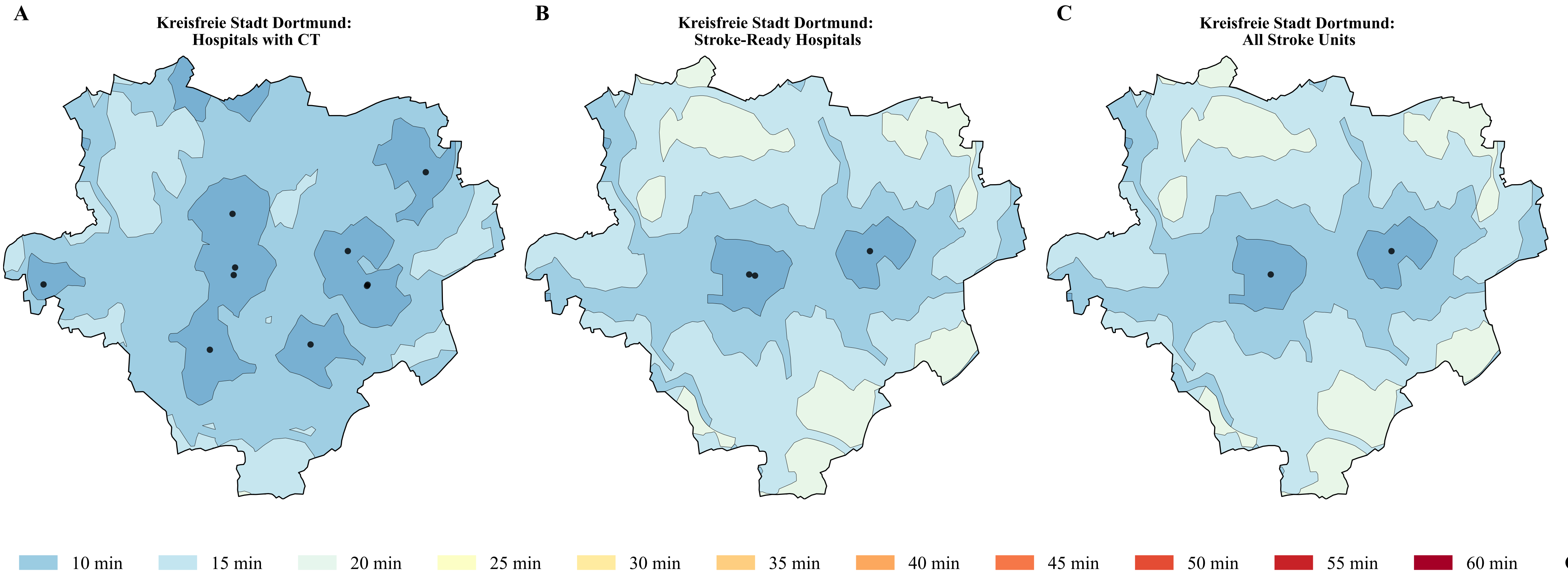

Isochrones — Kreisfreie Stadt Wiesbaden (Hessen)

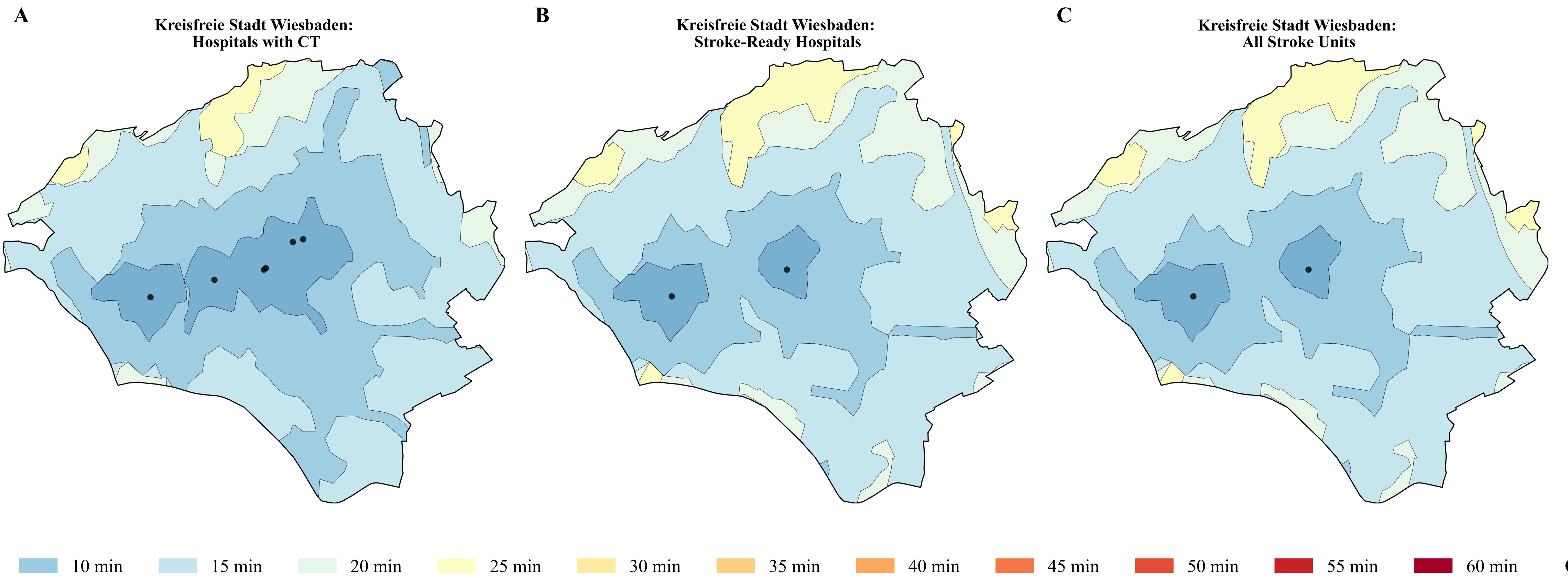

Isochrones — Landkreis Hochtaunuskreis (Hessen)

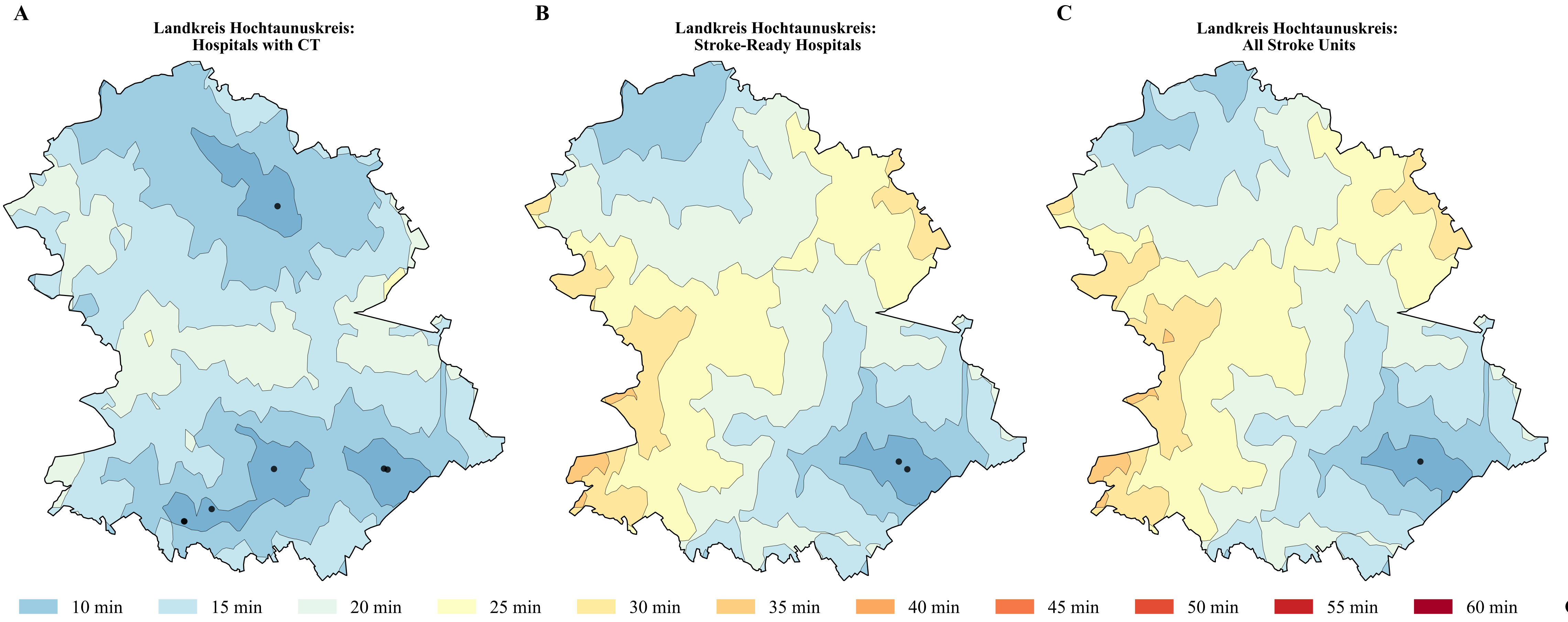

Isochrones — Landkreis Kassel (Hessen)

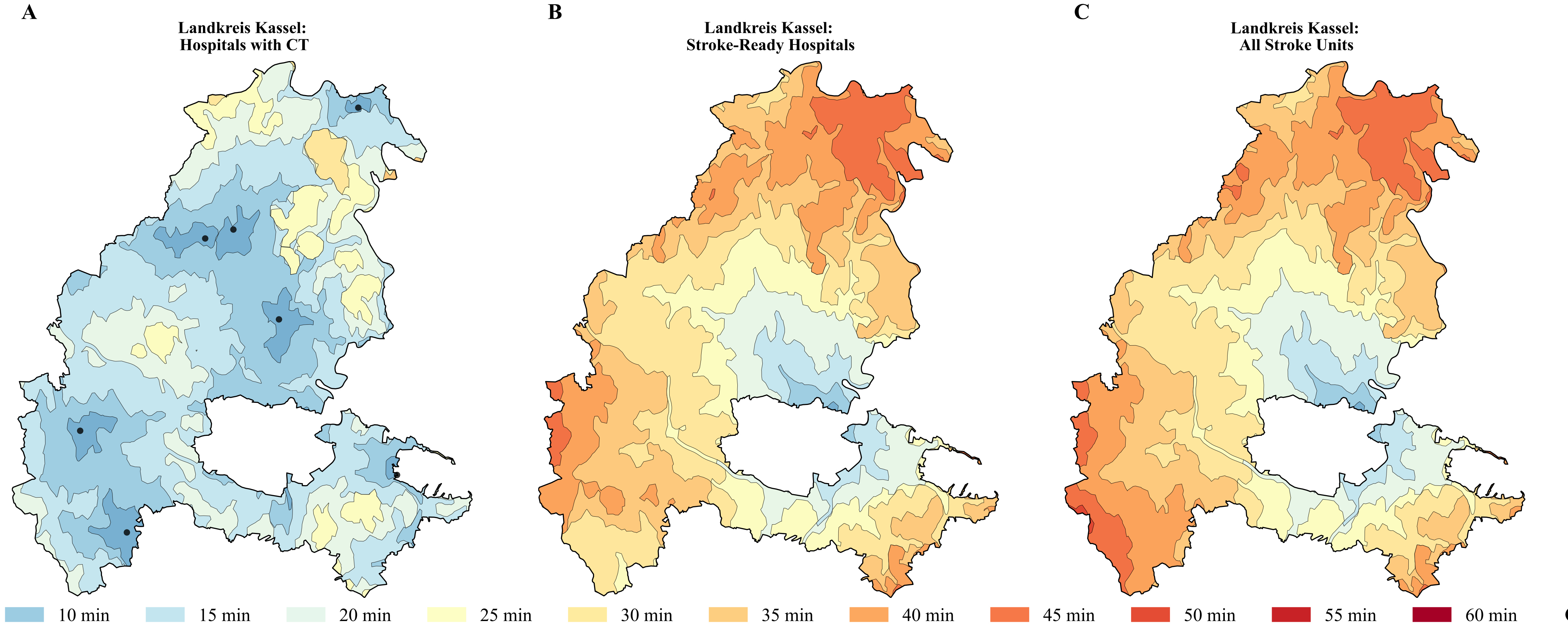

Isochrones — Landkreis Cochem-Zell (Rheinland-Pfalz)

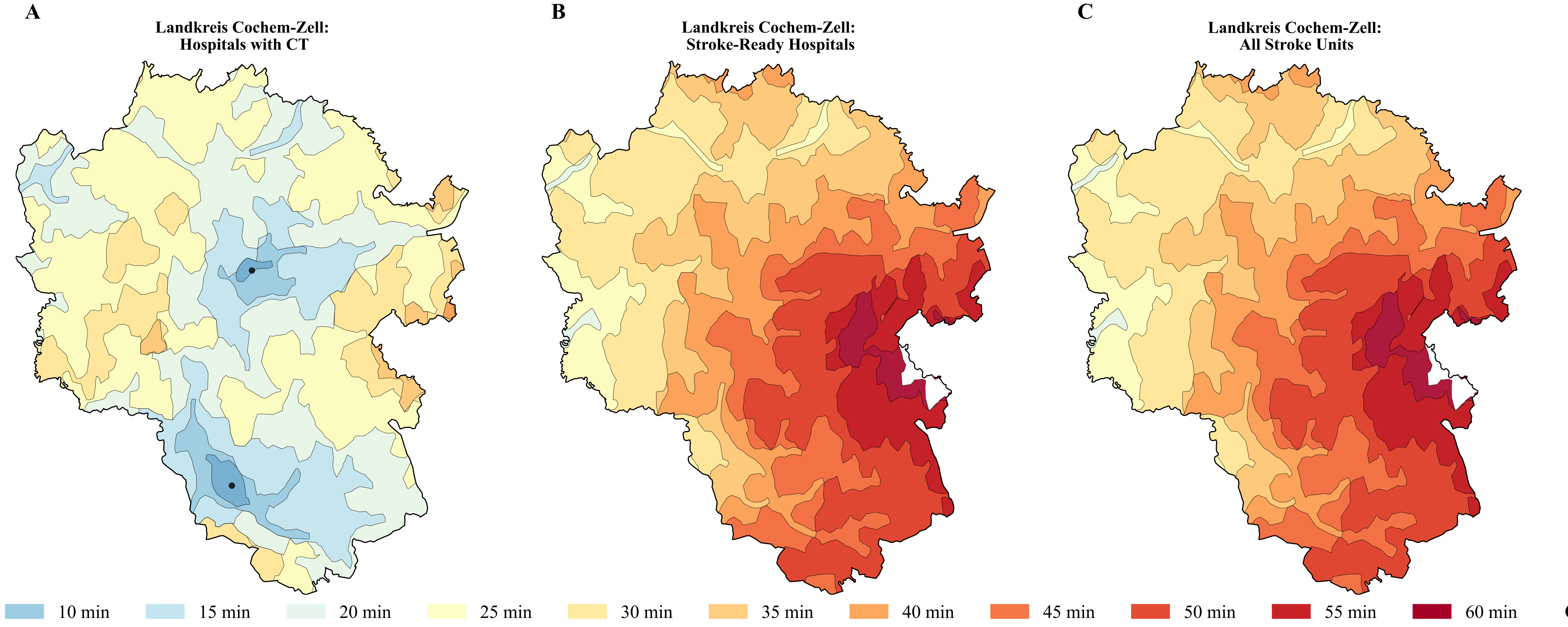

Isochrones — Landkreis Rhein-Lahn-Kreis (Rheinland-Pfalz)

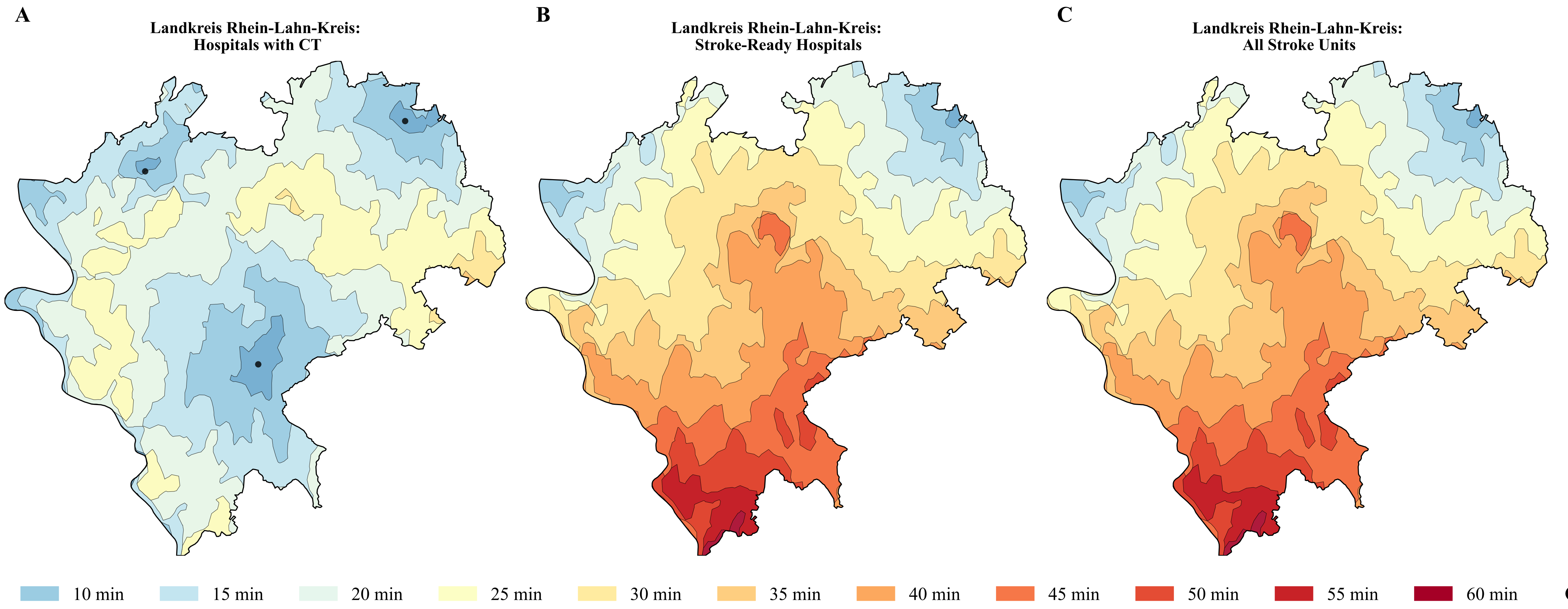

Isochrones — Stadtkreis Heilbronn (Baden-Württemberg)

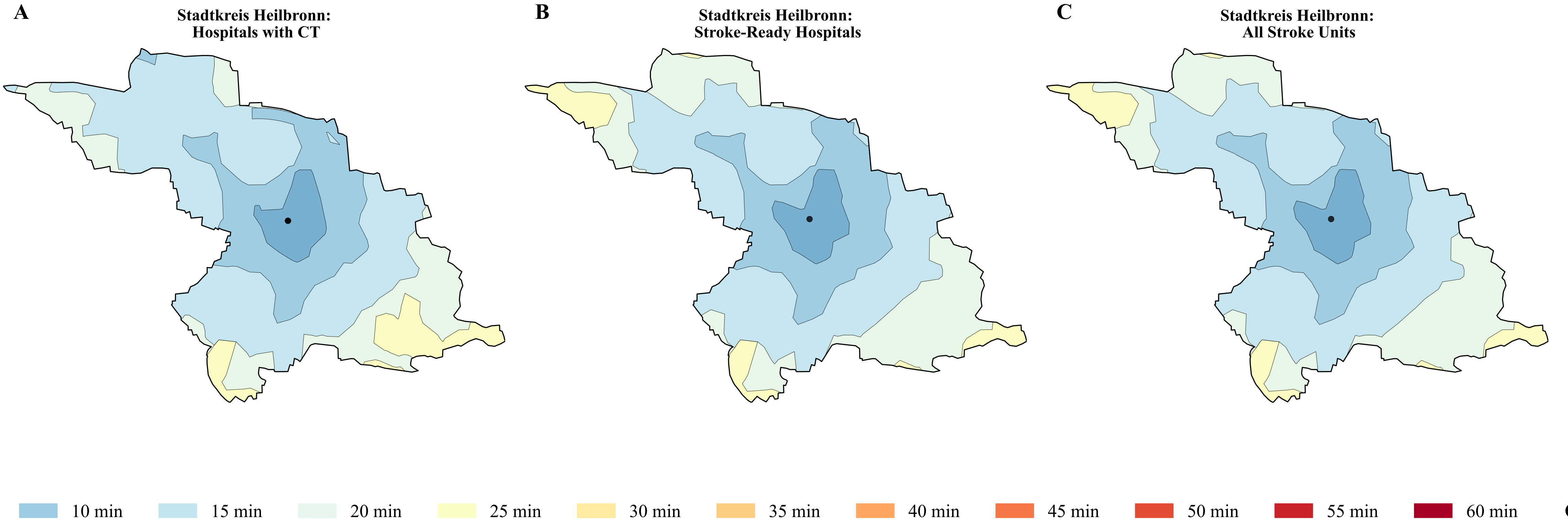

Isochrones — Landkreis Schwandorf (Bayern)

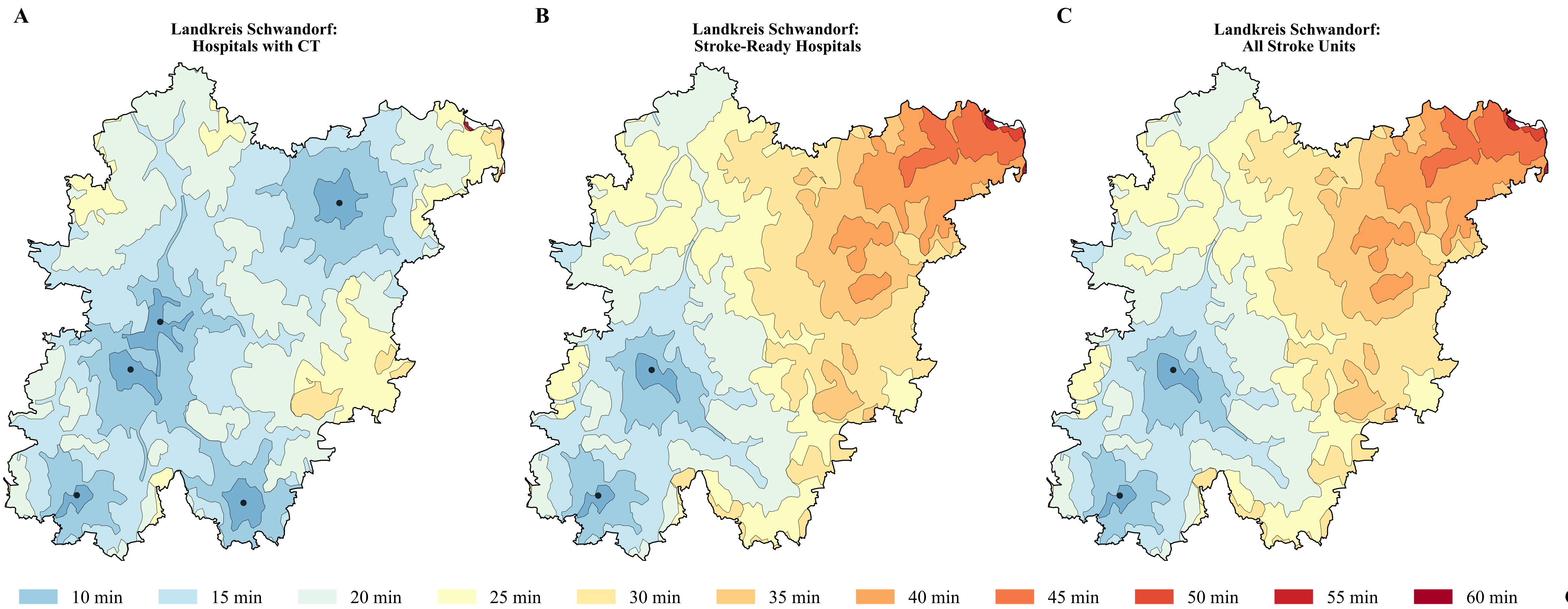

Isochrones — Landkreis Unterallgäu (Bayern)

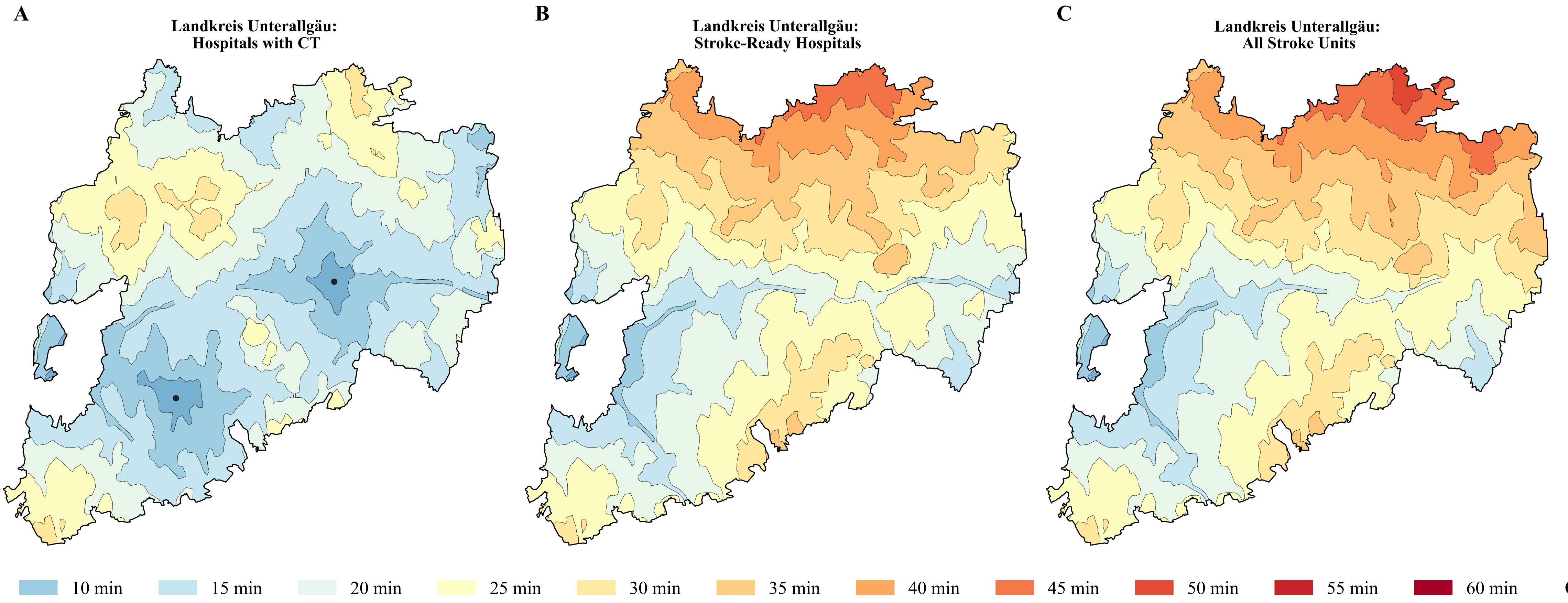

Isochrones — Kreisfreie Stadt Frankfurt (Oder) (Brandenburg)

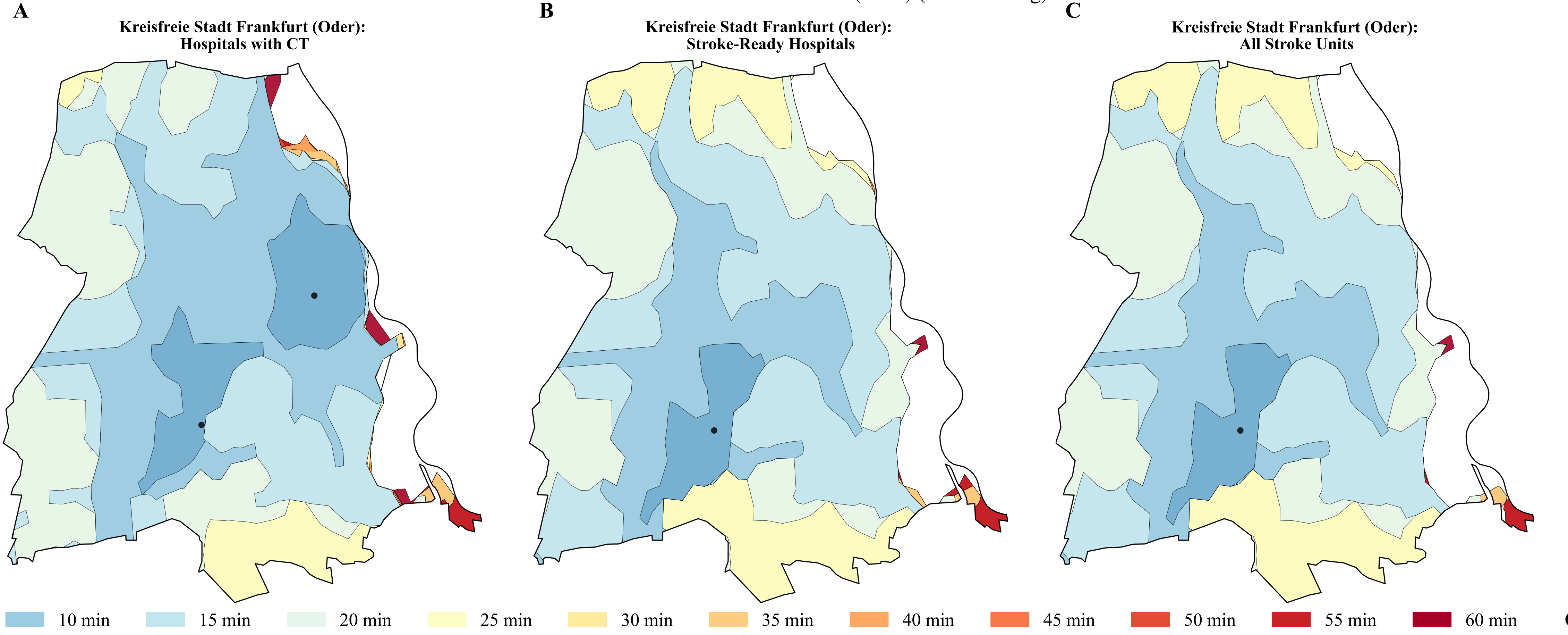

Isochrones — Landkreis Dahme-Spreewald (Brandenburg)

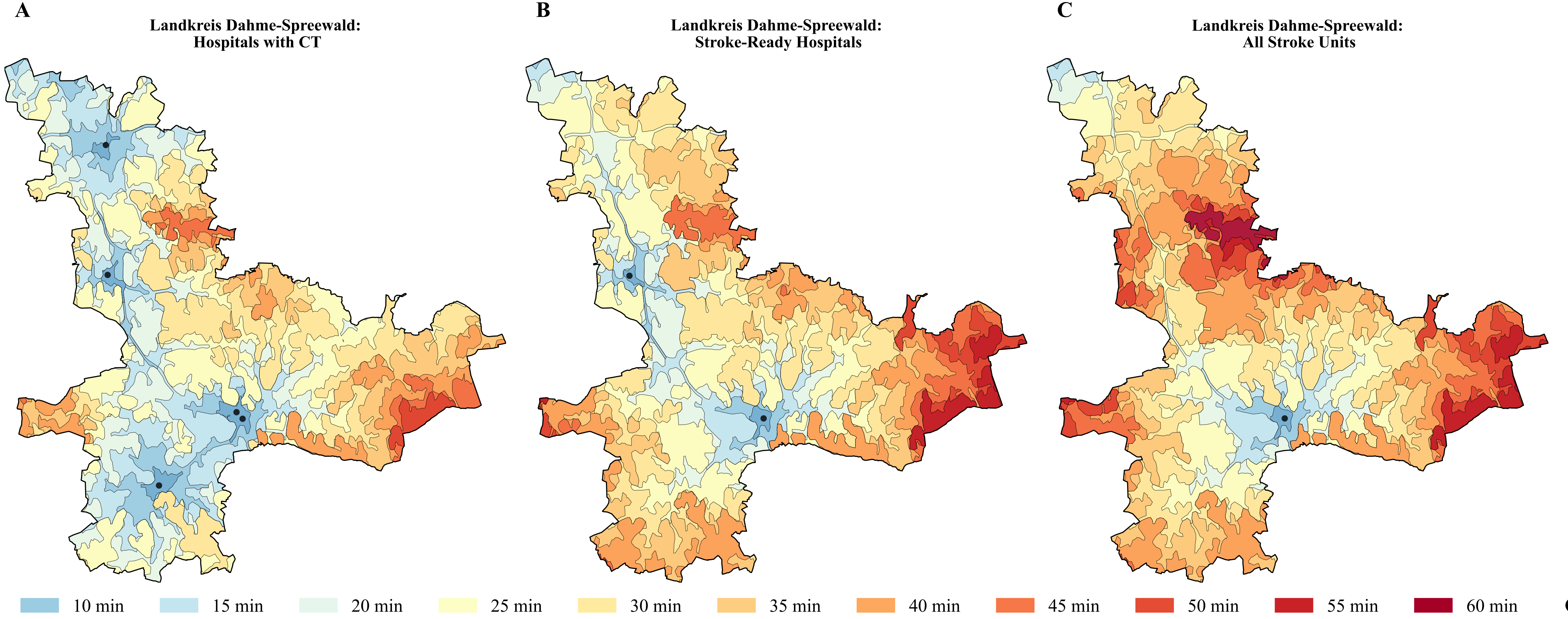

Isochrones — Landkreis Leipzig (Sachsen)

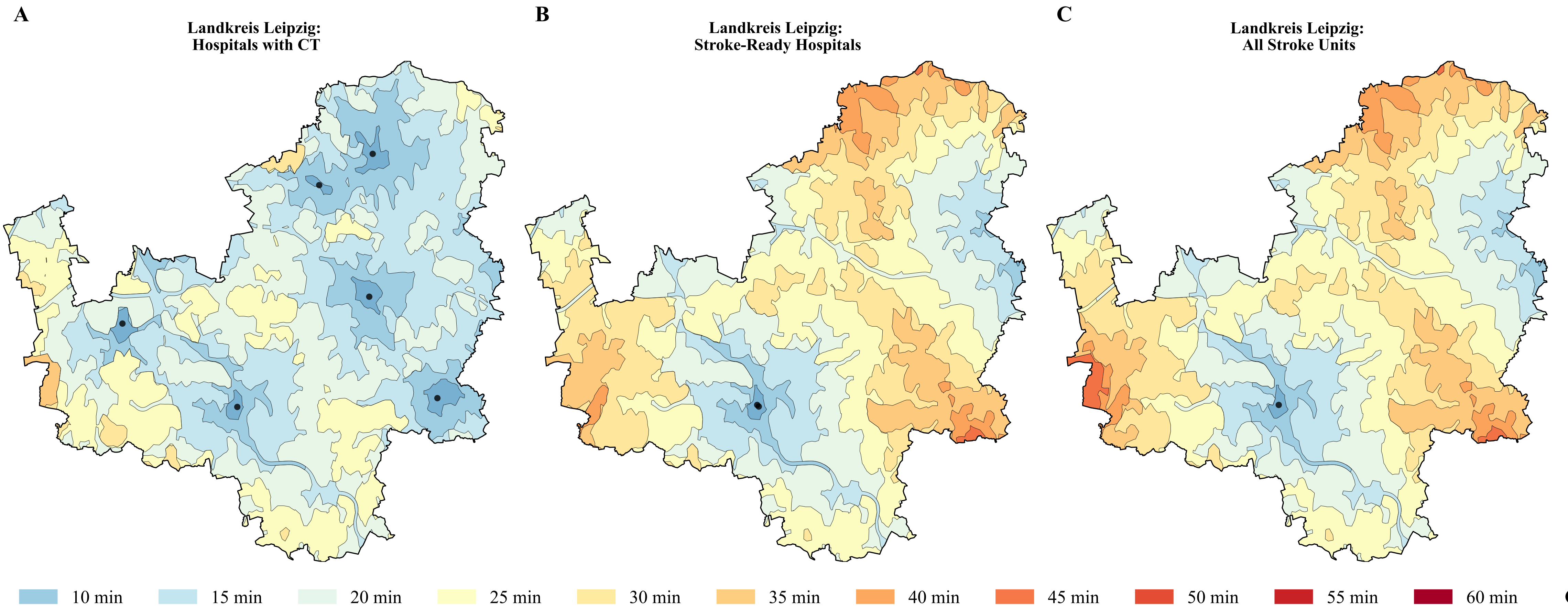

Isochrones — Kreisfreie Stadt Magdeburg (Sachsen-Anhalt)

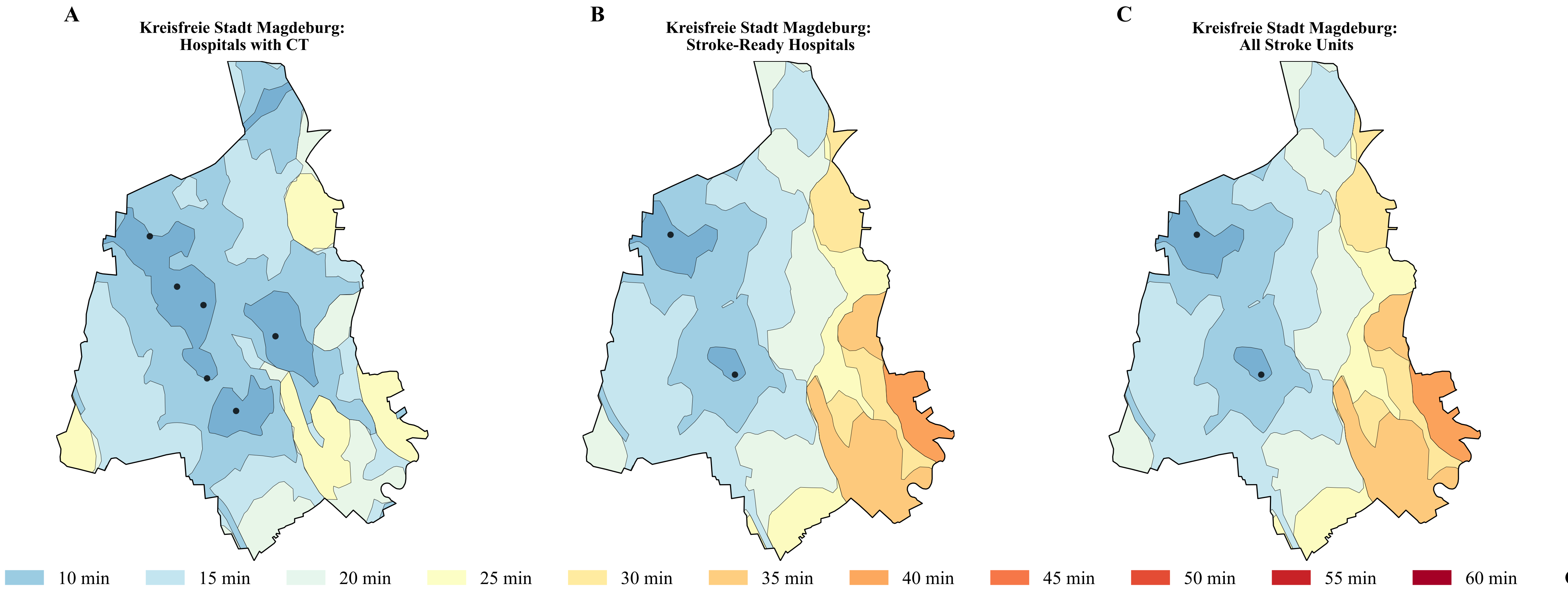

Isochrones — Landkreis Wartburgkreis (Thüringen)

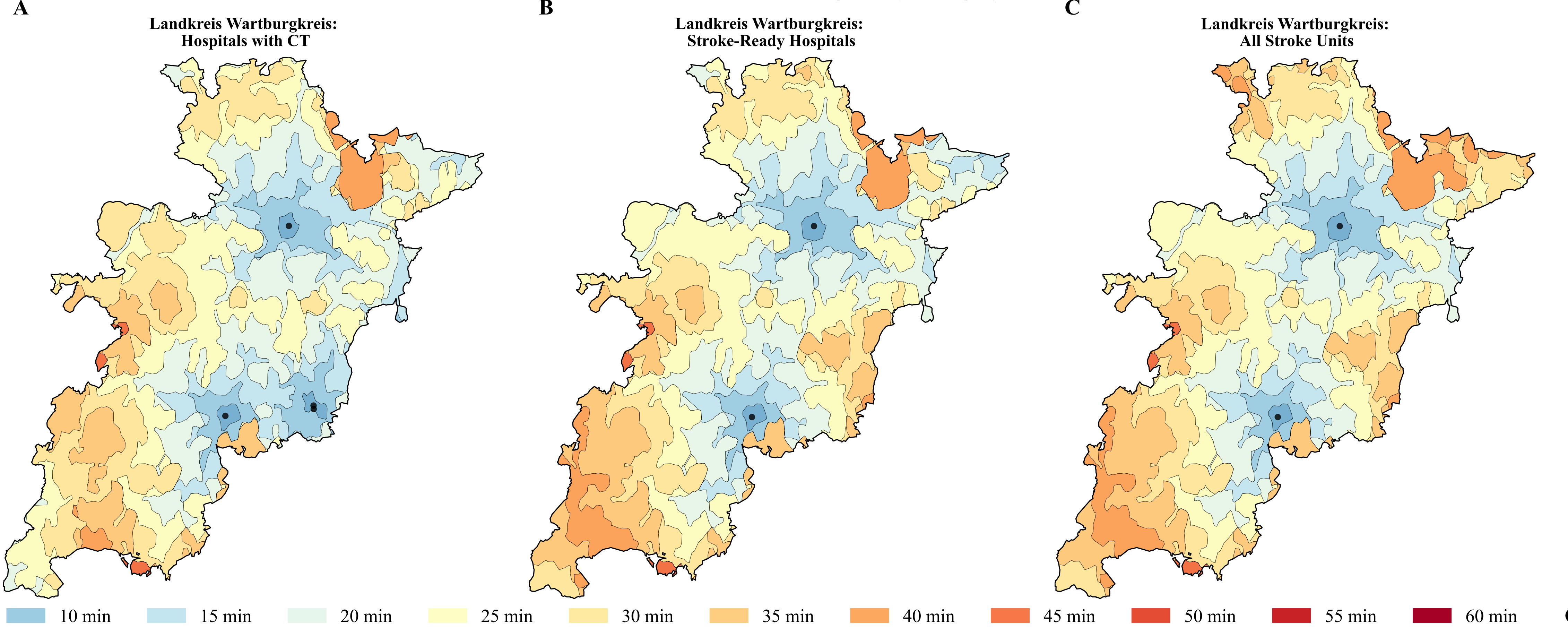

Isochrones — Landkreis Unstrut-Hainich-Kreis (Thüringen)

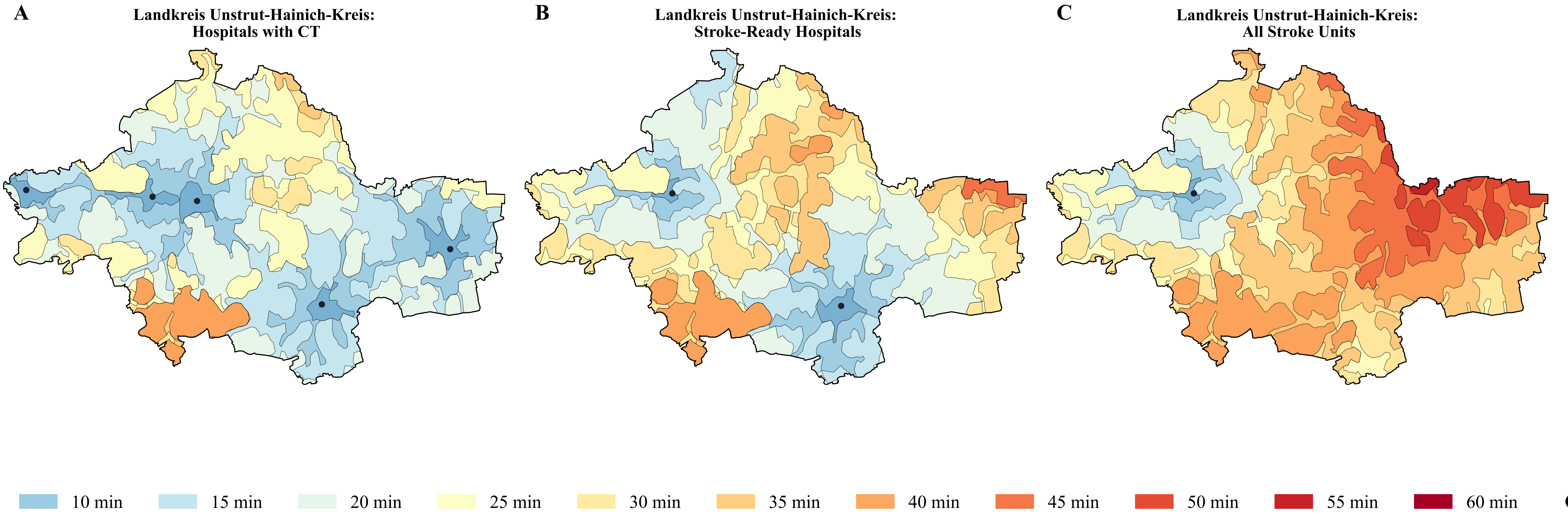

Isochrones — Kreisfreie Stadt Bielefeld (Nordrhein-Westfalen)

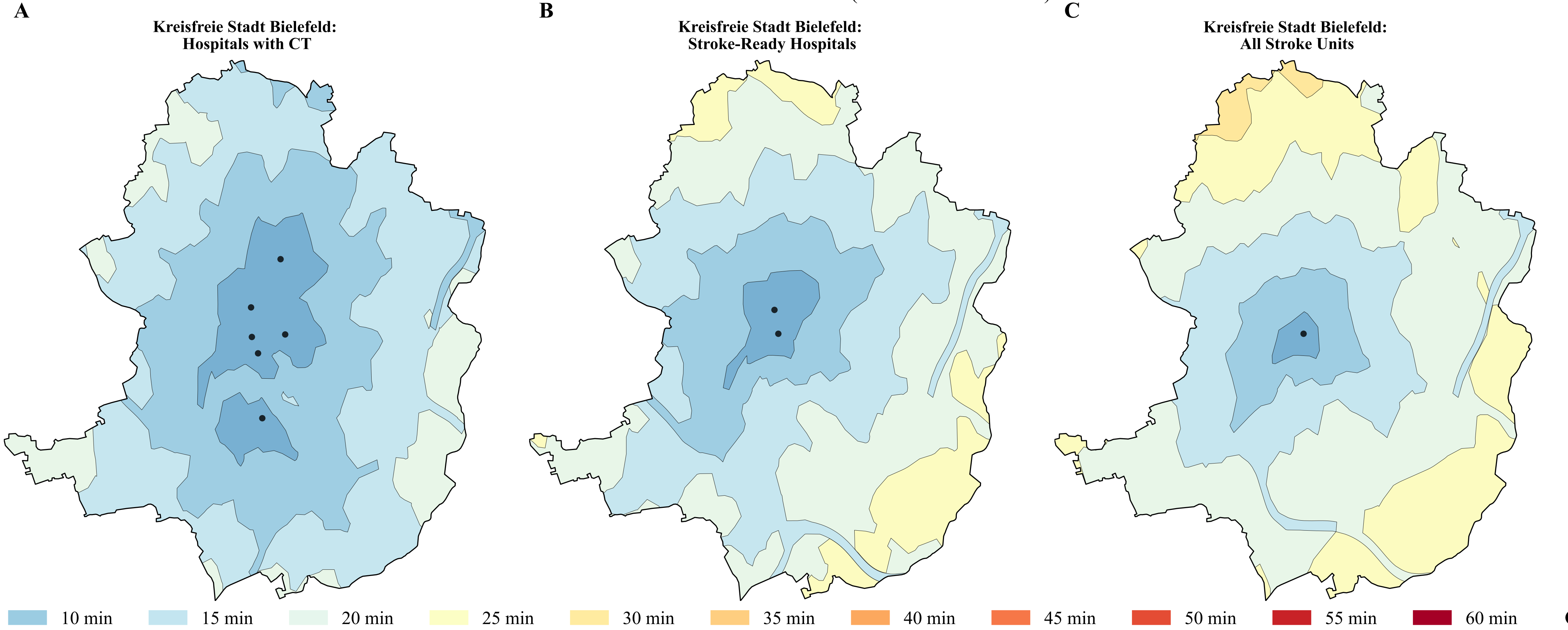

Isochrones — Kreisfreie Stadt Bochum (Nordrhein-Westfalen)

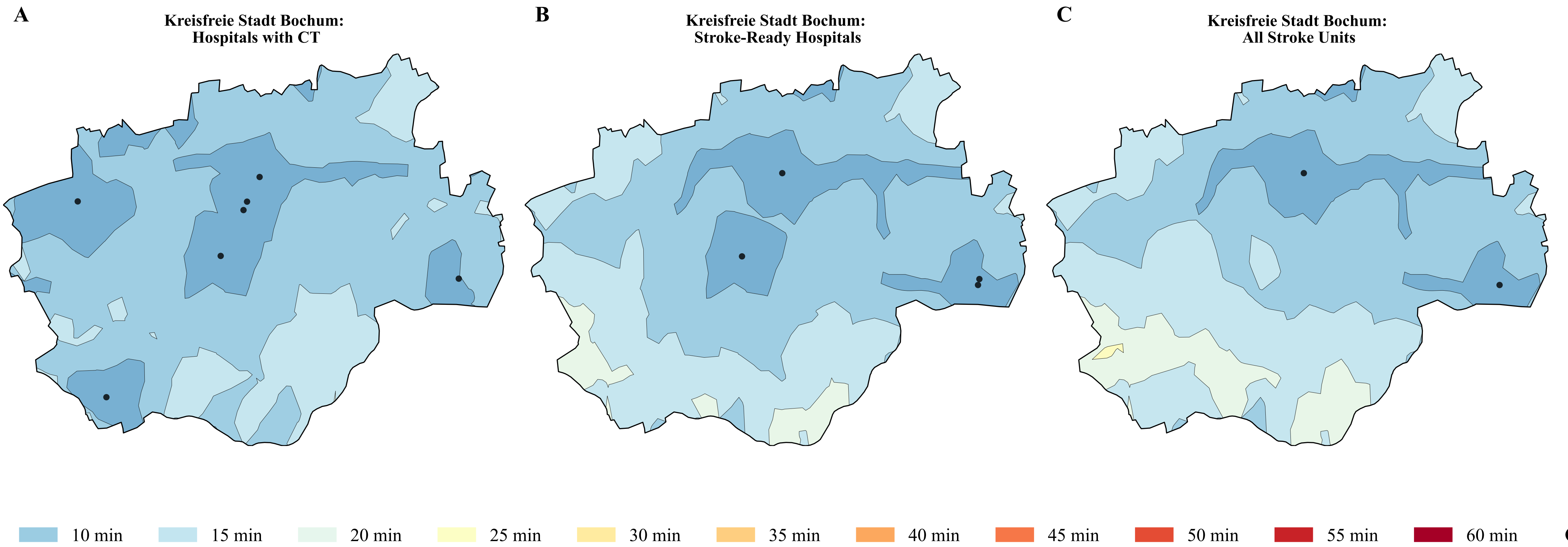

Isochrones — Landkreis Fulda (Hessen)

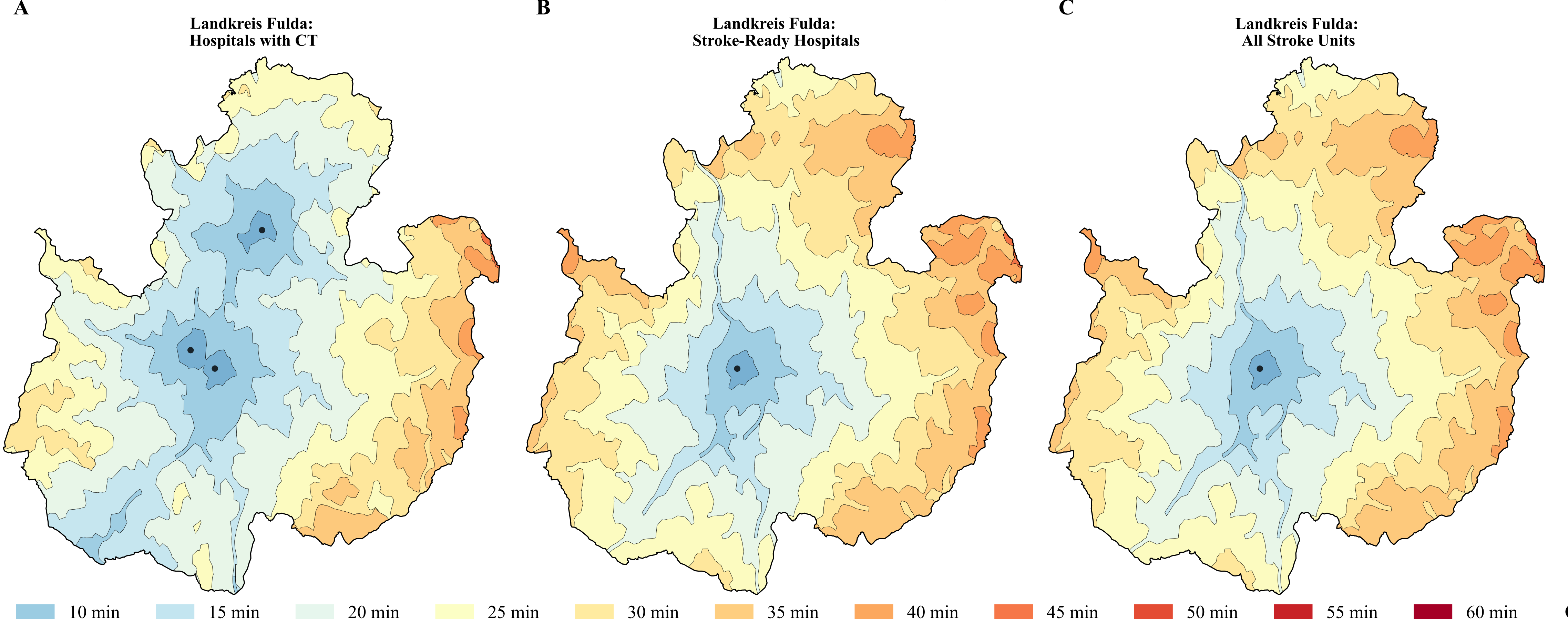

Isochrones — Landkreis Rastatt (Baden-Württemberg)

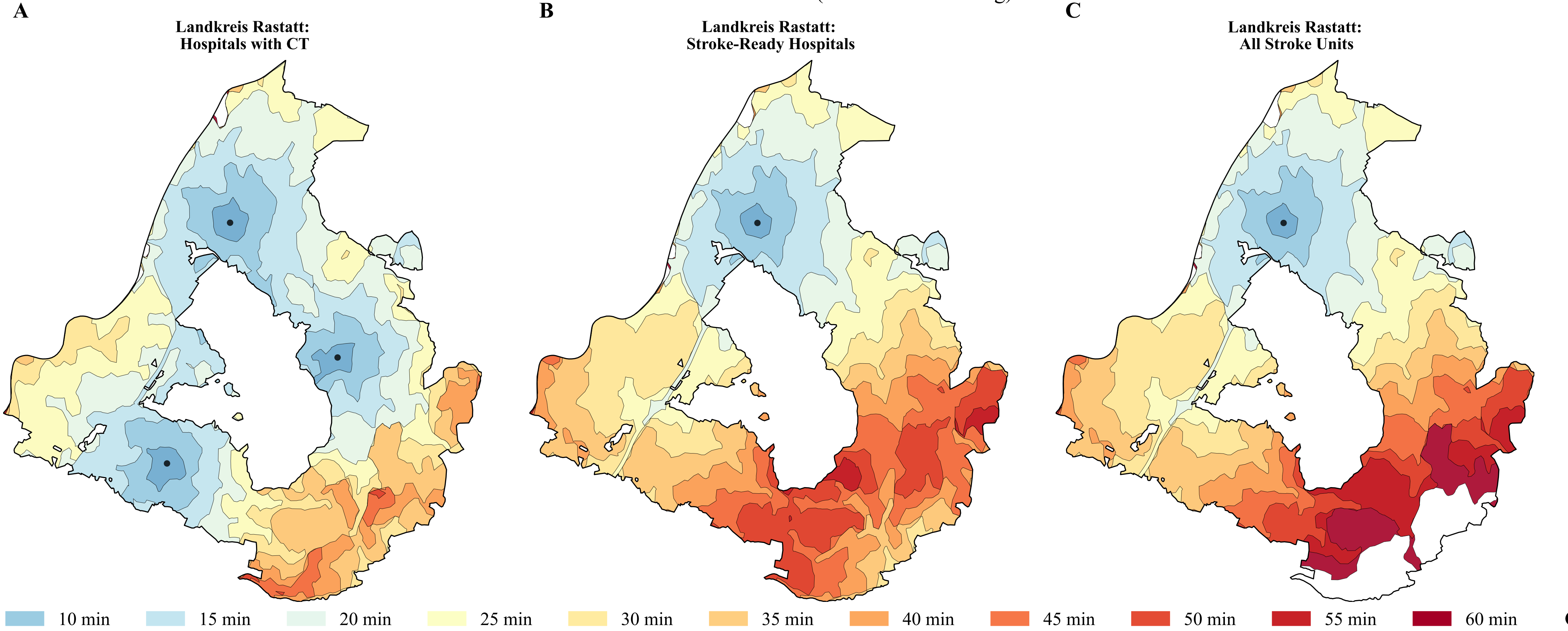

Isochrones — Landkreis Breisgau-Hochschwarzwald (Baden-Württemberg)

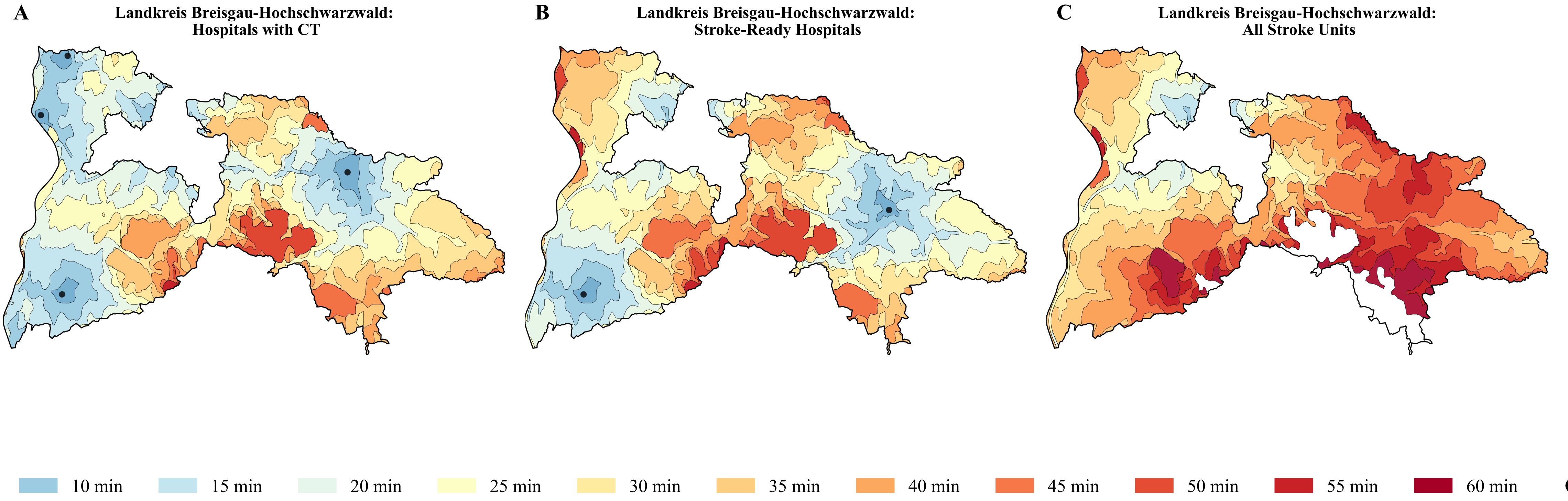

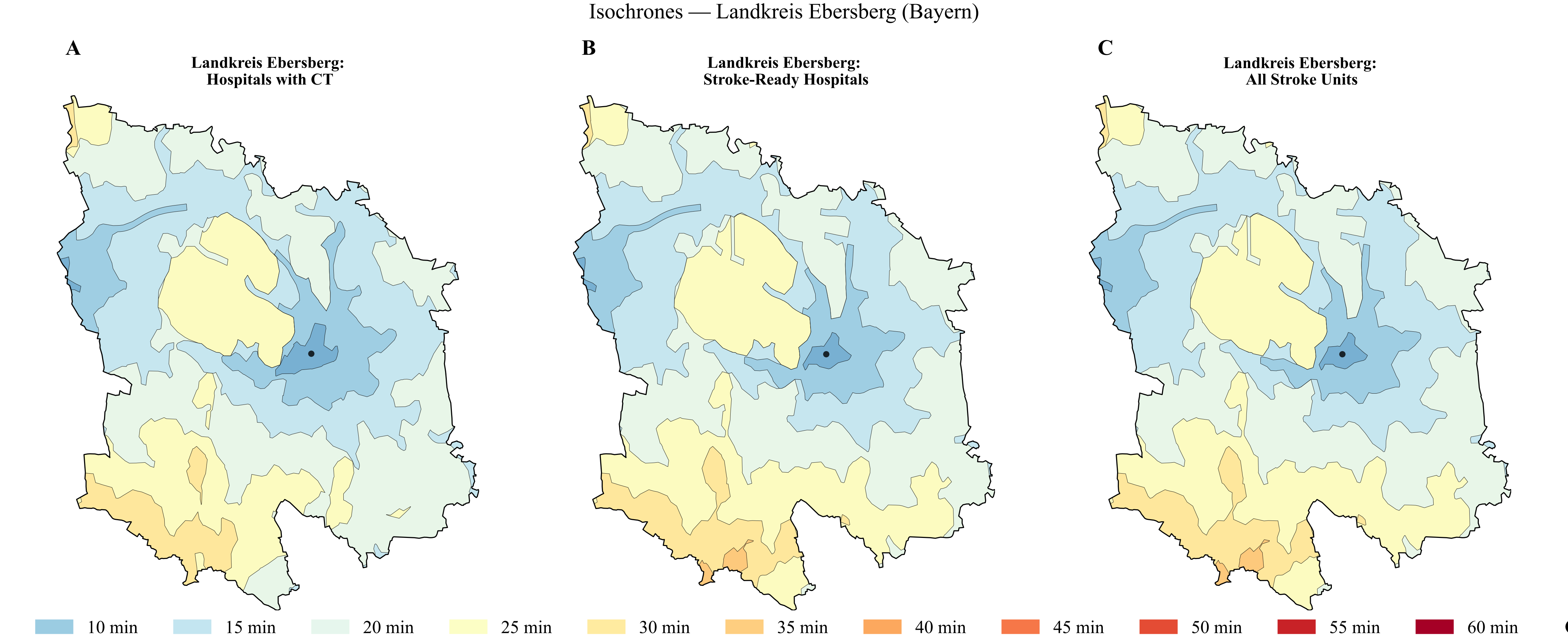
Isochrones — Landkreis Ebersberg (Bayern)
A
Landkreis Ebersberg:
Hospitals with CT
B
Landkreis Ebersberg:
Stroke-Ready Hospitals
C
Landkreis Ebersberg:
All Stroke Units
10 min
15 min
20 min
25 min
30 min
35 min
40 min
45 min
50 min
55 min
60 min

Isochrones — Landkreis Rottal-Inn (Bayern)

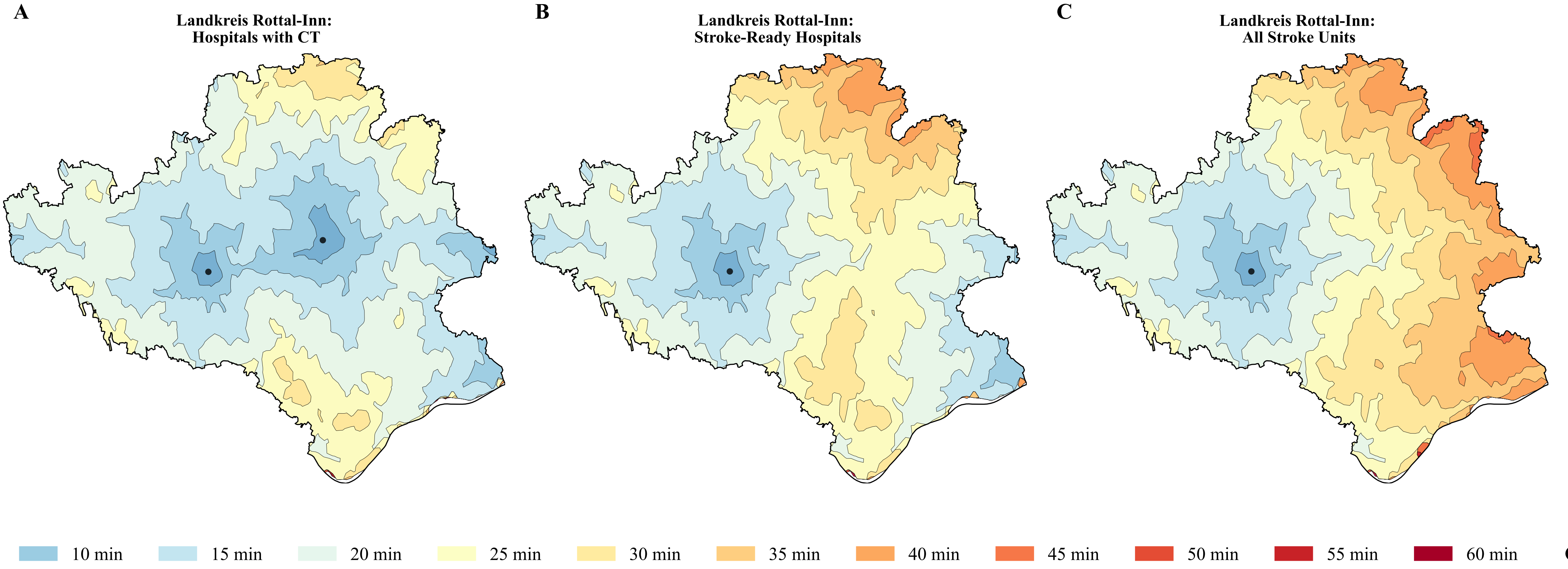

Isochrones — Landkreis Bamberg (Bayern)

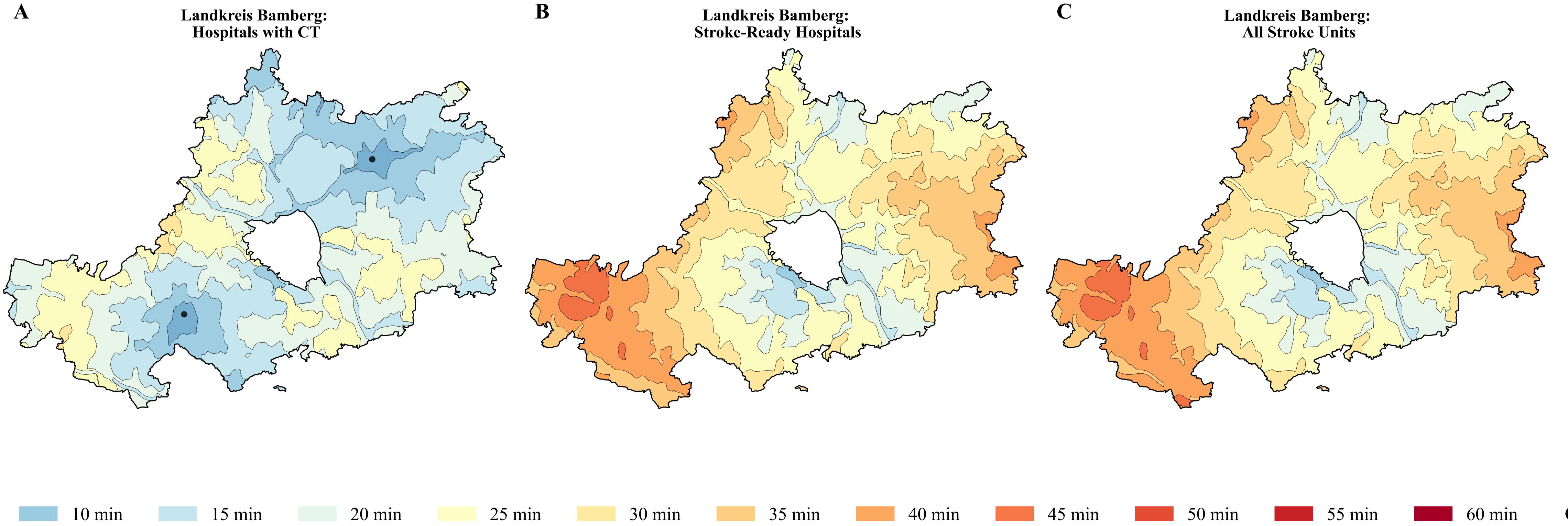

Isochrones — Landkreis Schweinfurt (Bayern)

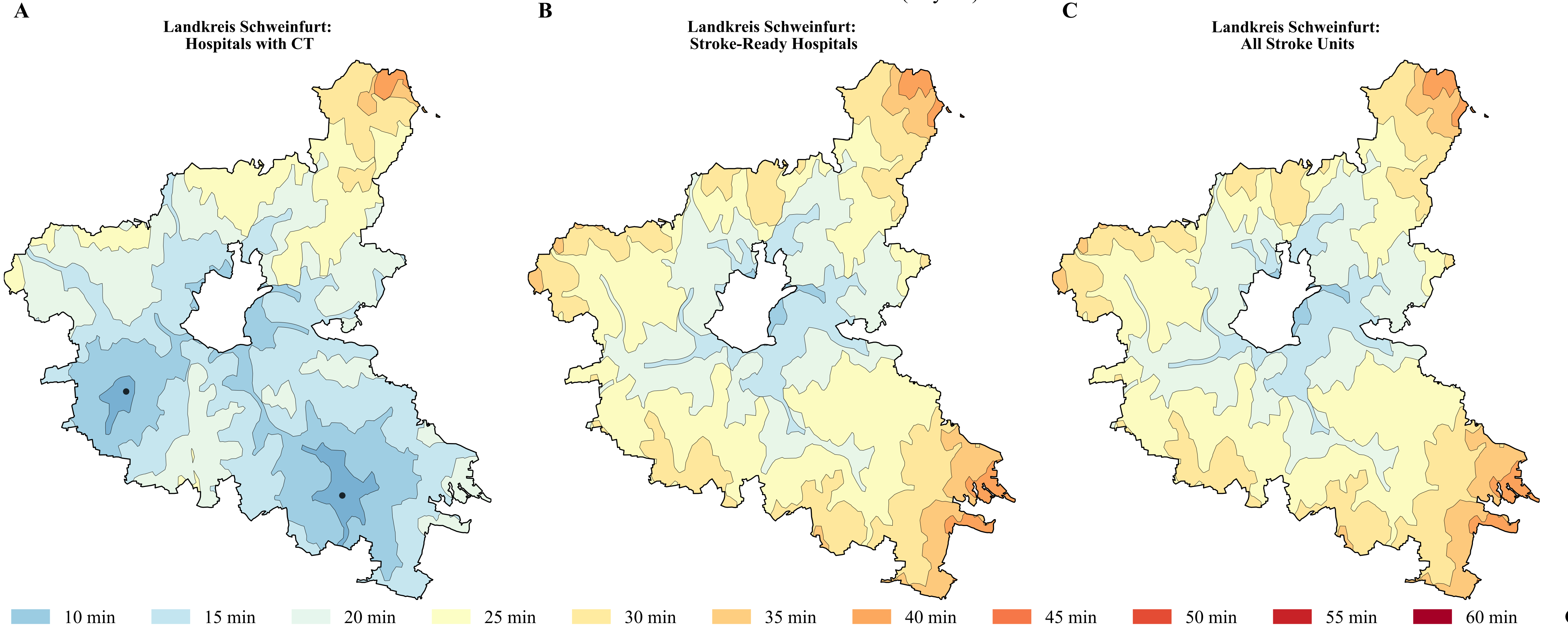

Isochrones — Landkreis St. Wendel (Saarland)

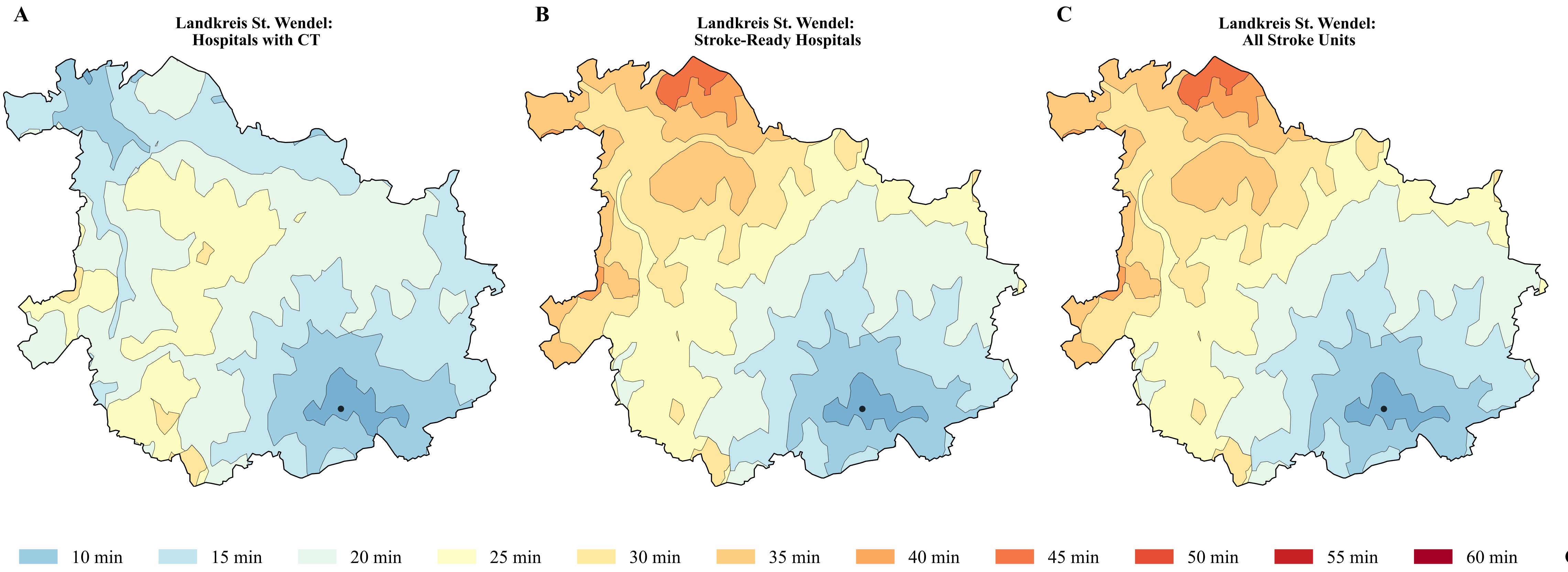

Isochrones — Landkreis Harz (Sachsen-Anhalt)

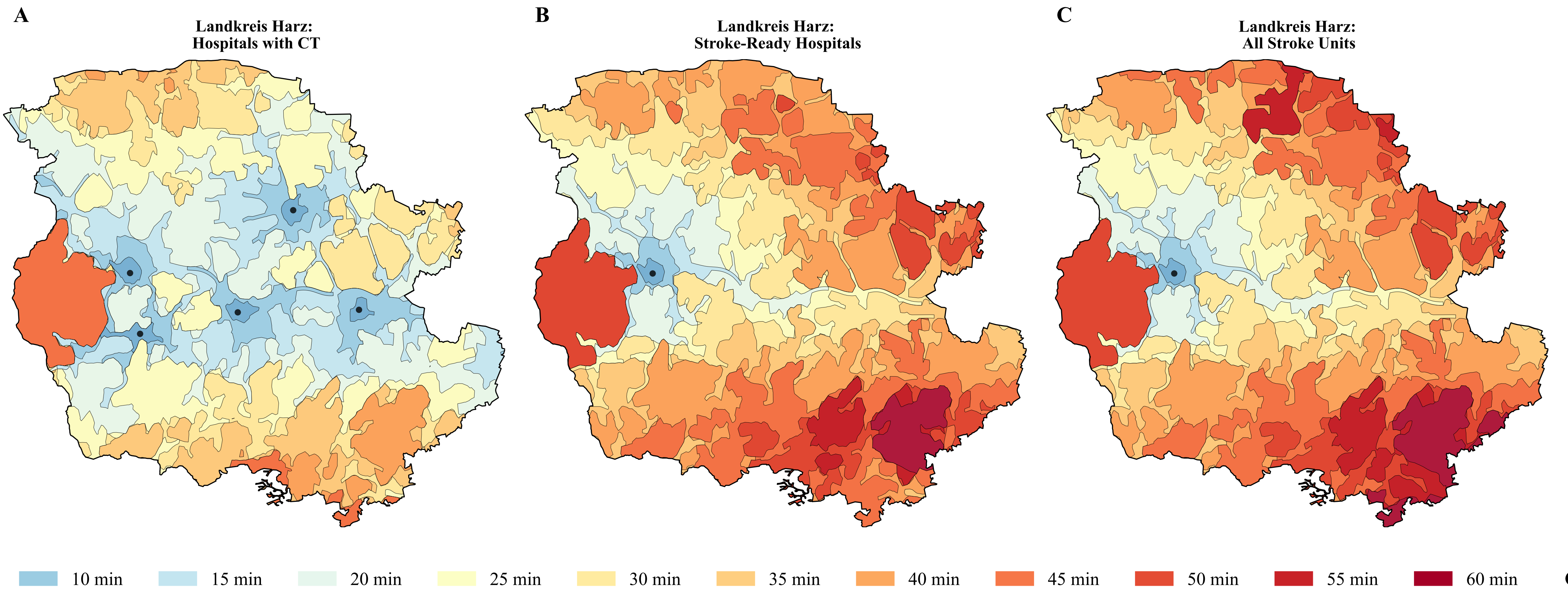

Isochrones — Kreisfreie Stadt Gera (Thüringen)

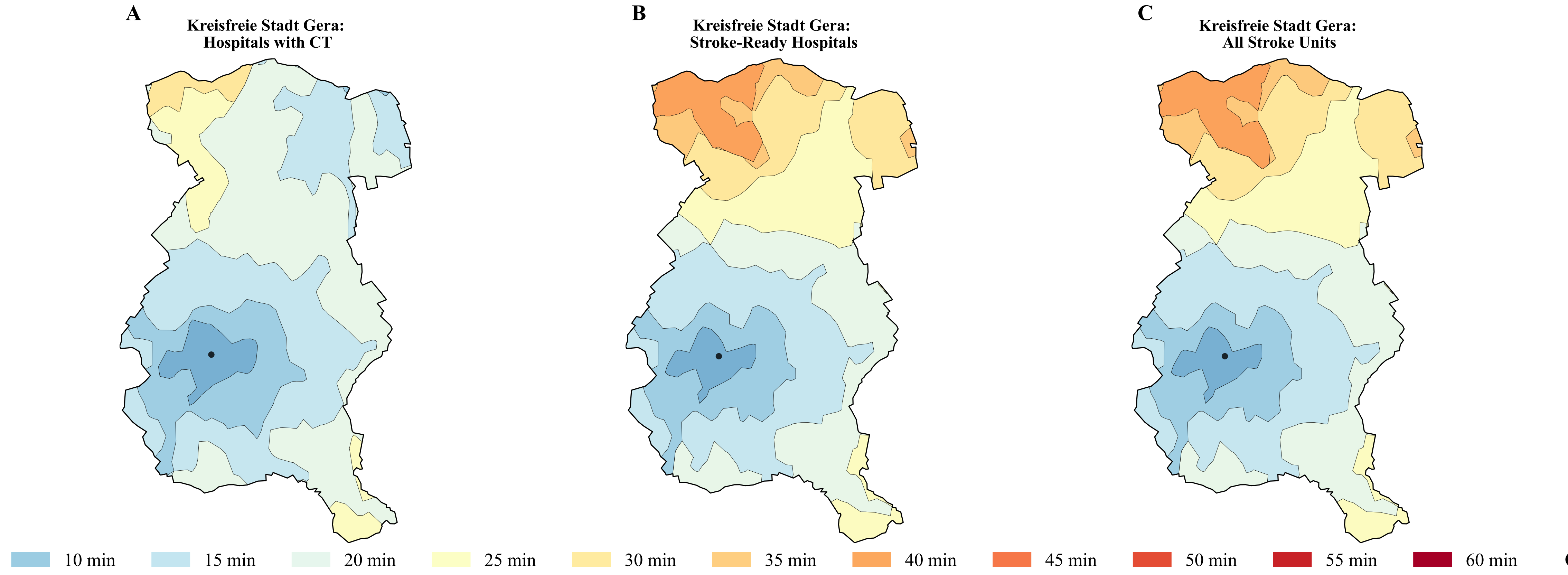

Isochrones — Kreisfreie Stadt Weimar (Thüringen)

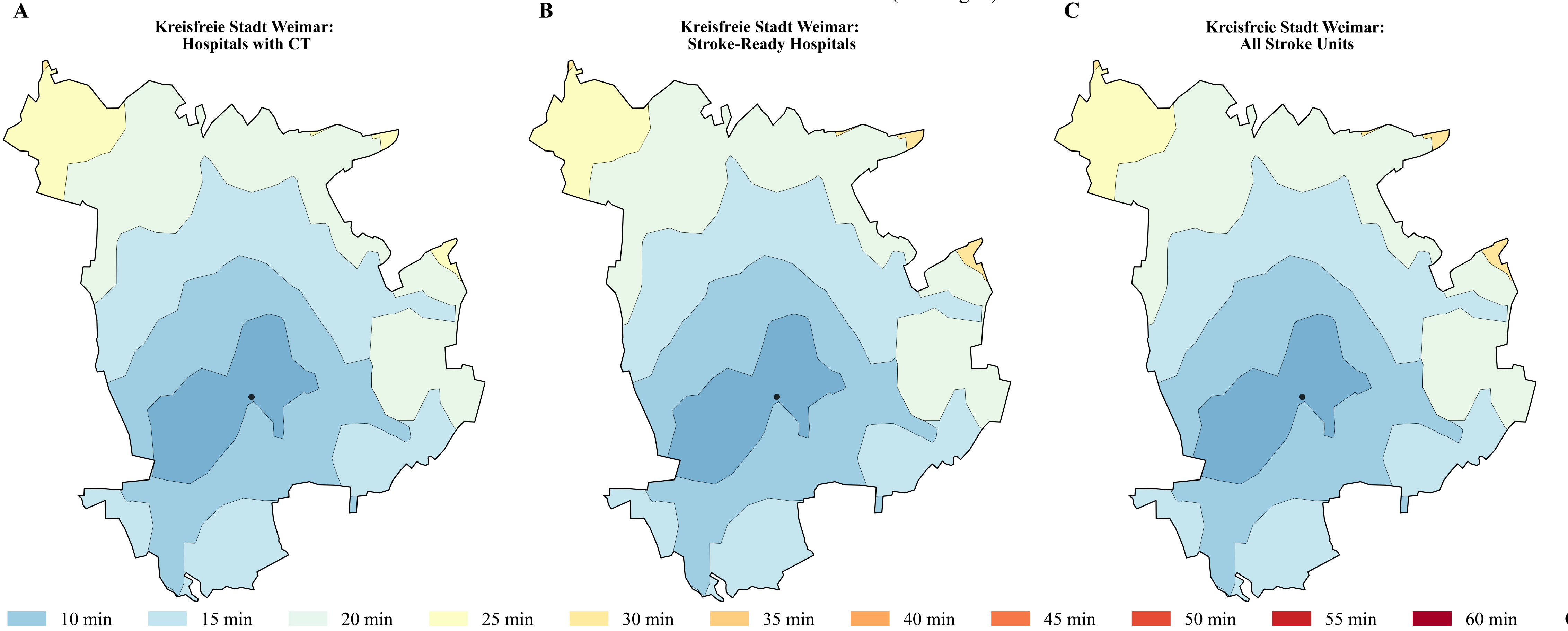

Isochrones — Landkreis Holzminden (Niedersachsen)

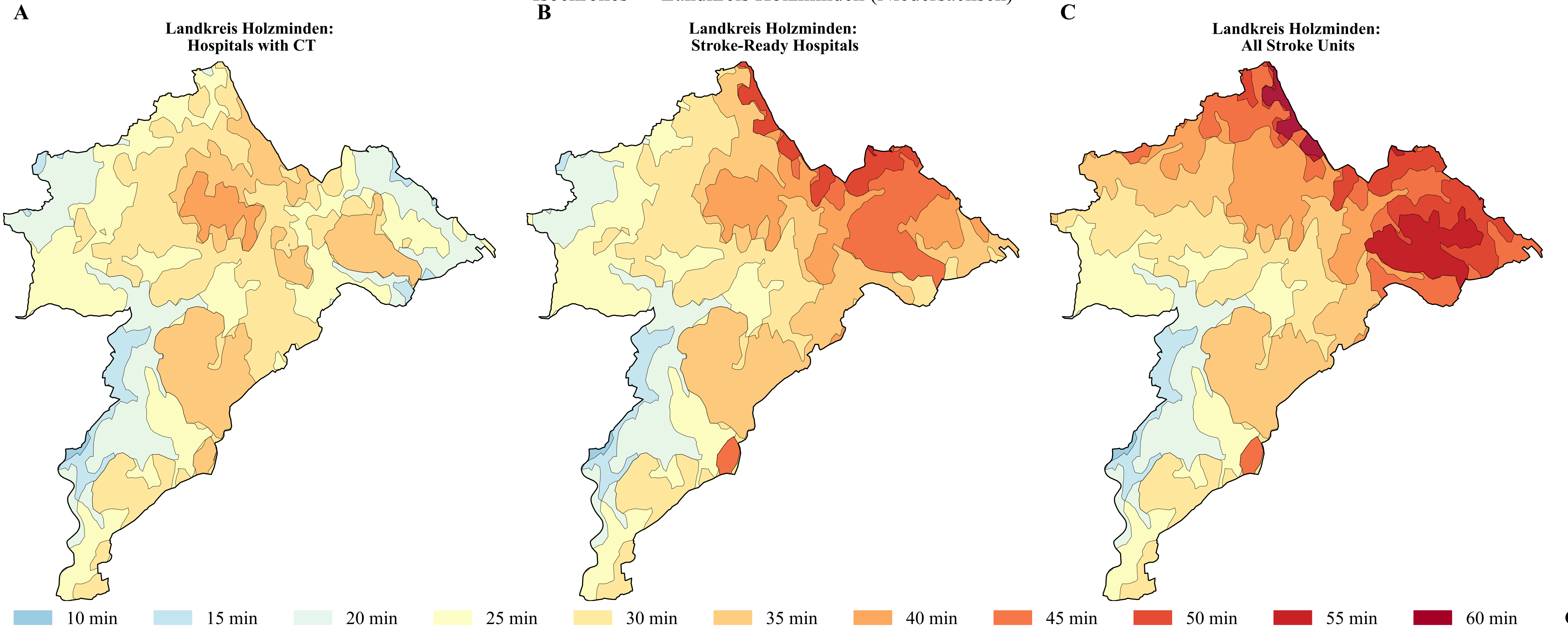

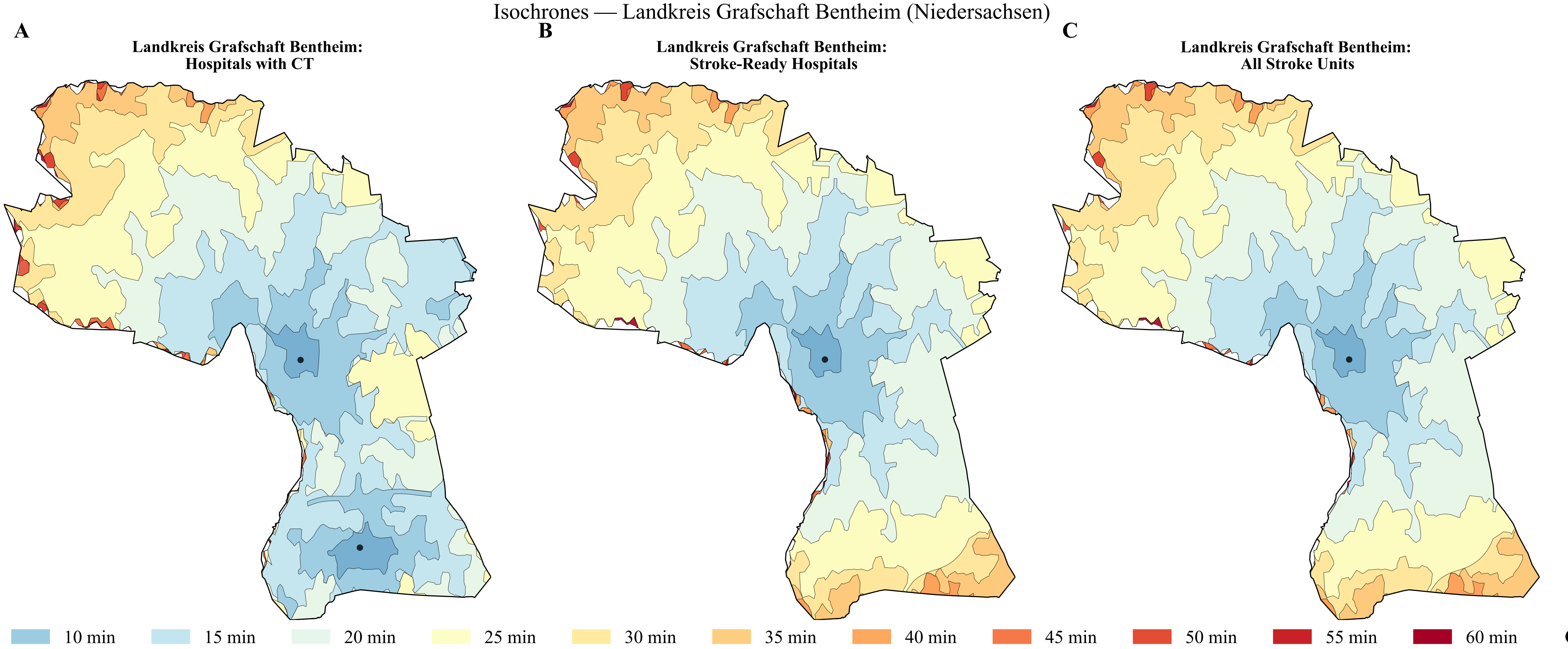

Isochrones — Landkreis Grafschaft Bentheim (Niedersachsen)
A
Landkreis Grafschaft Bentheim:
Hospitals with CT
B
Landkreis Grafschaft Bentheim:
Stroke-Ready Hospitals
C
Landkreis Grafschaft Bentheim:
All Stroke Units
10 min
15 min
20 min
25 min
30 min
35 min
40 min
45 min
50 min
55 min
60 min

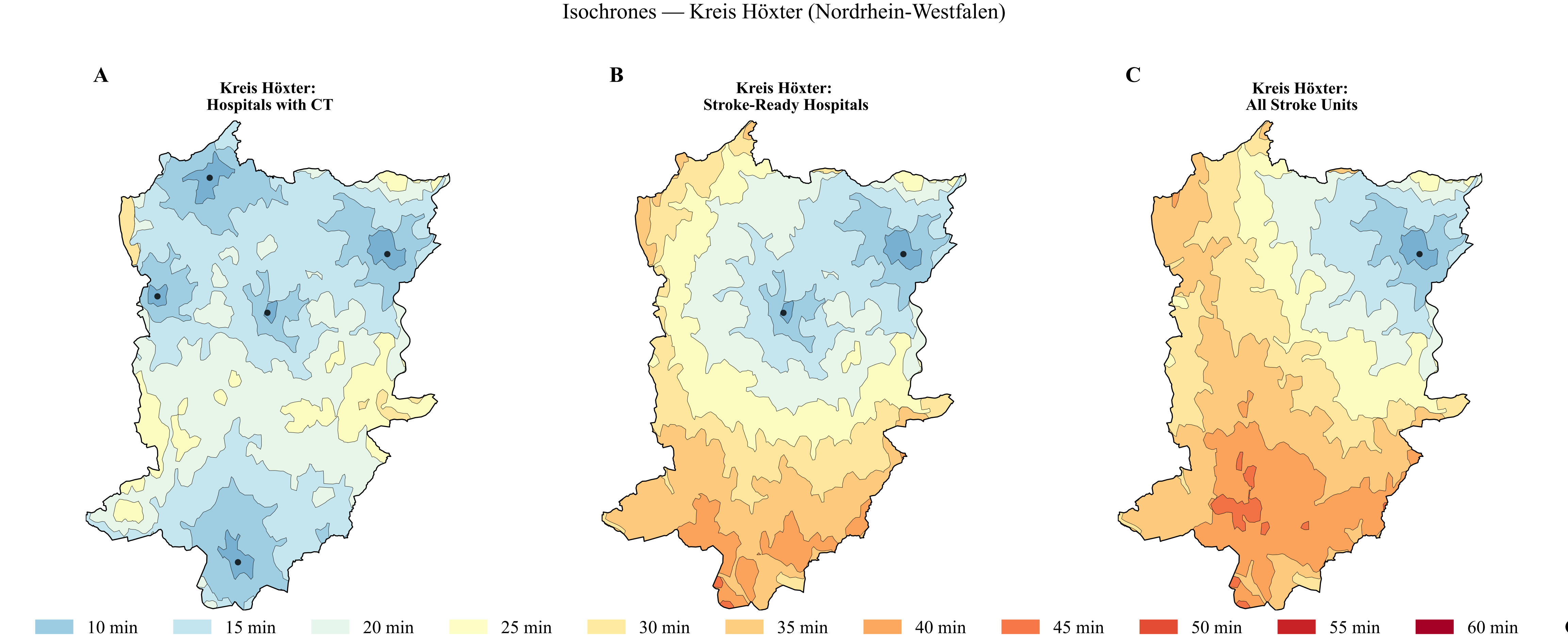
Isochrones — Kreis Höxter (Nordrhein-Westfalen)
A
Kreis Höxter:
Hospitals with CT
B
Kreis Höxter:
Stroke-Ready Hospitals
C
Kreis Höxter:
All Stroke Units
10 min
15 min
20 min
25 min
30 min
35 min
40 min
45 min
50 min
55 min
60 min

Isochrones — Landkreis Marburg-Biedenkopf (Hessen)

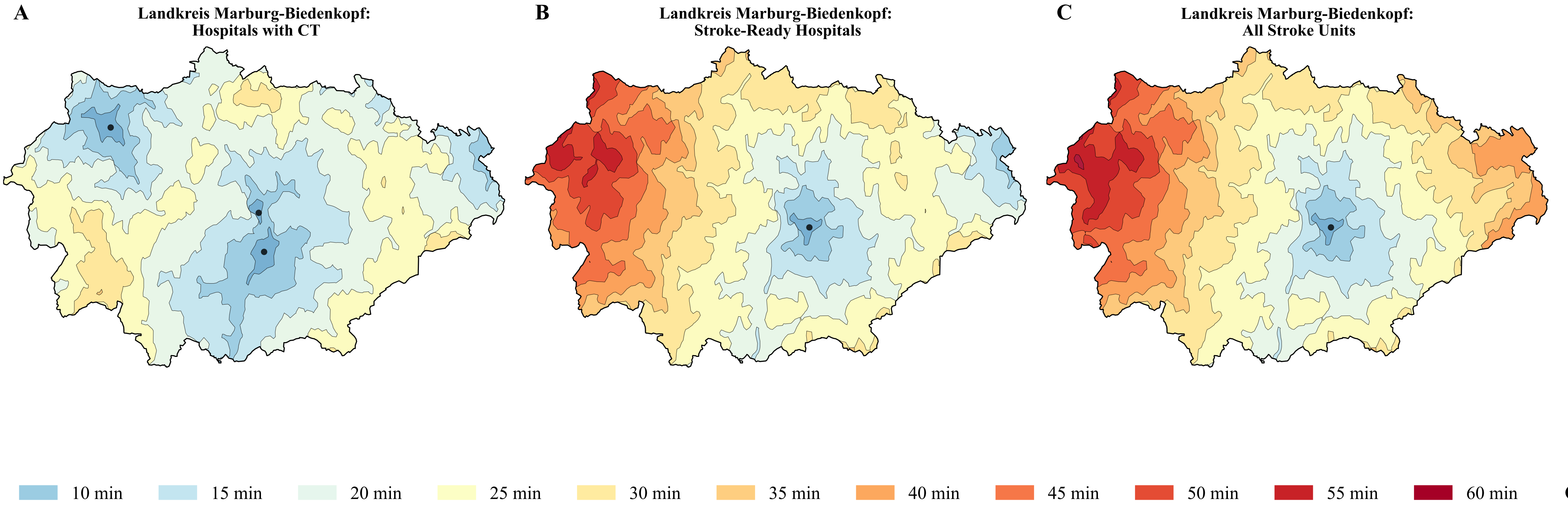

Isochrones — Landkreis Rhein-Hunsrück-Kreis (Rheinland-Pfalz)

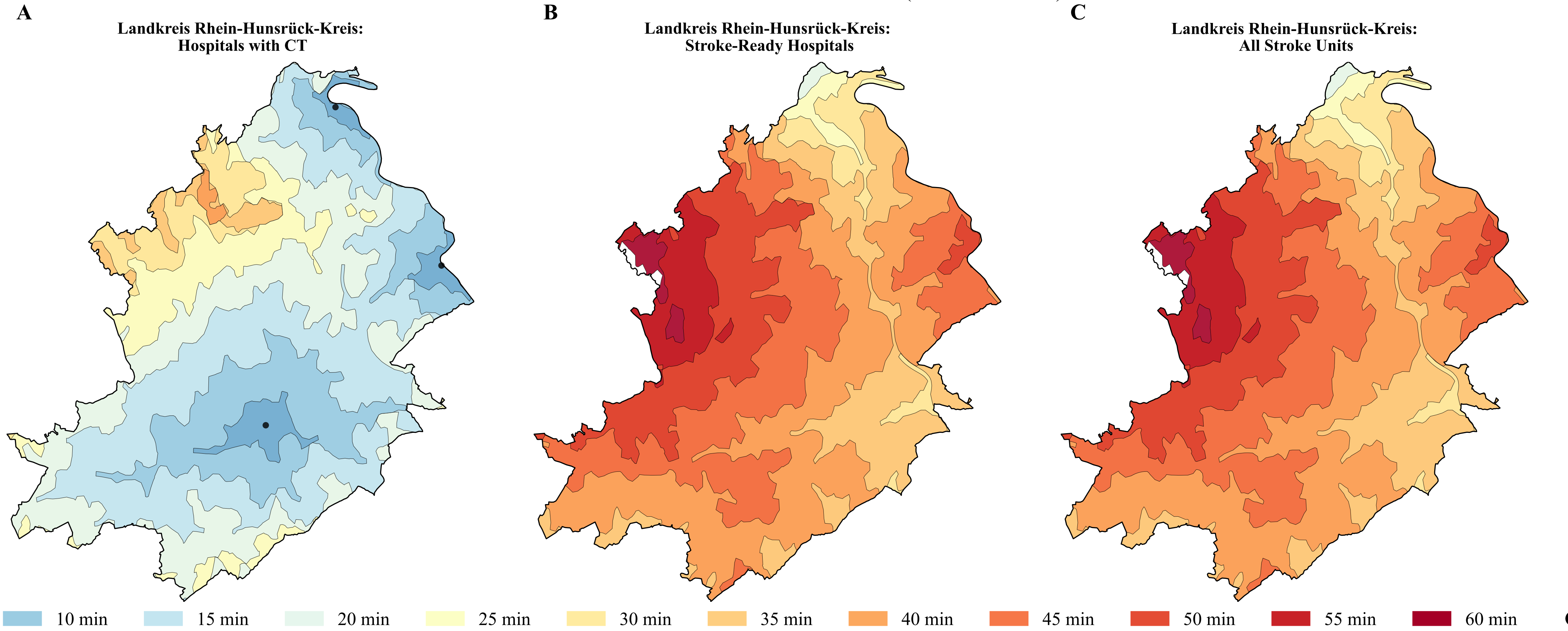

Isochrones — Landkreis Eifelkreis Bitburg-Prüm (Rheinland-Pfalz)

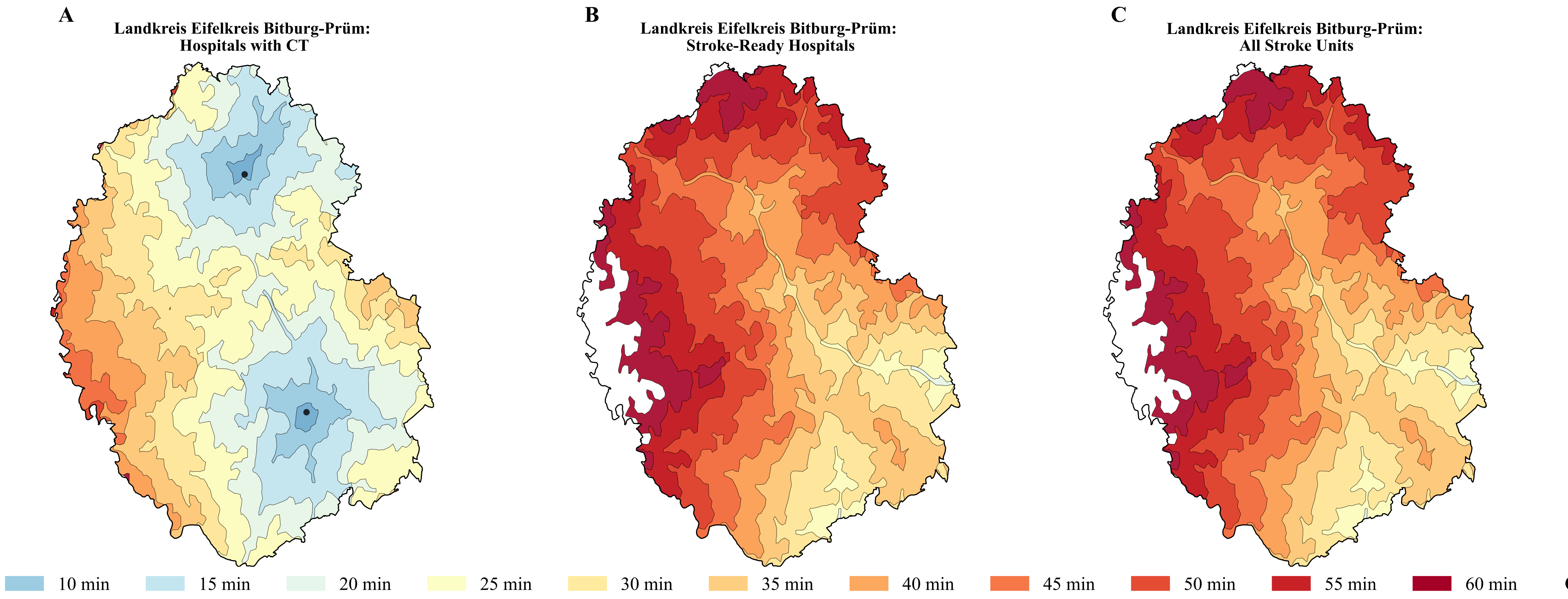

Isochrones — Landkreis Erding (Bayern)

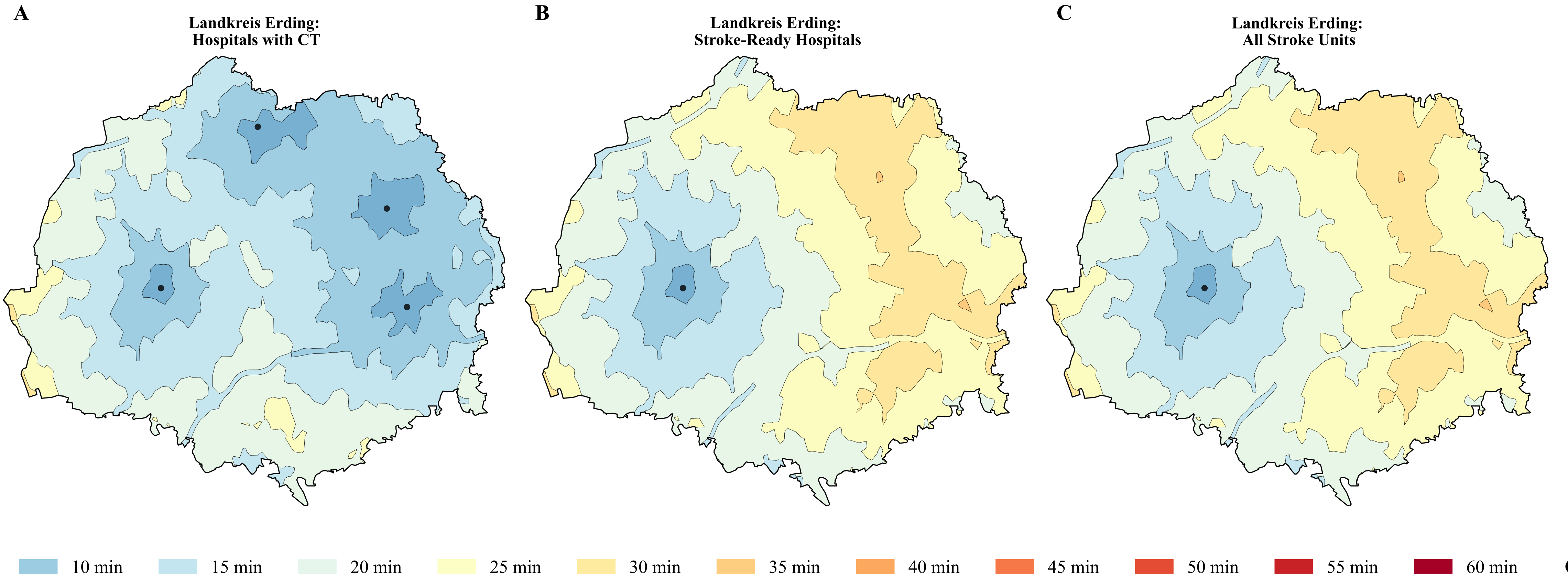

Isochrones — Landkreis Deggendorf (Bayern)

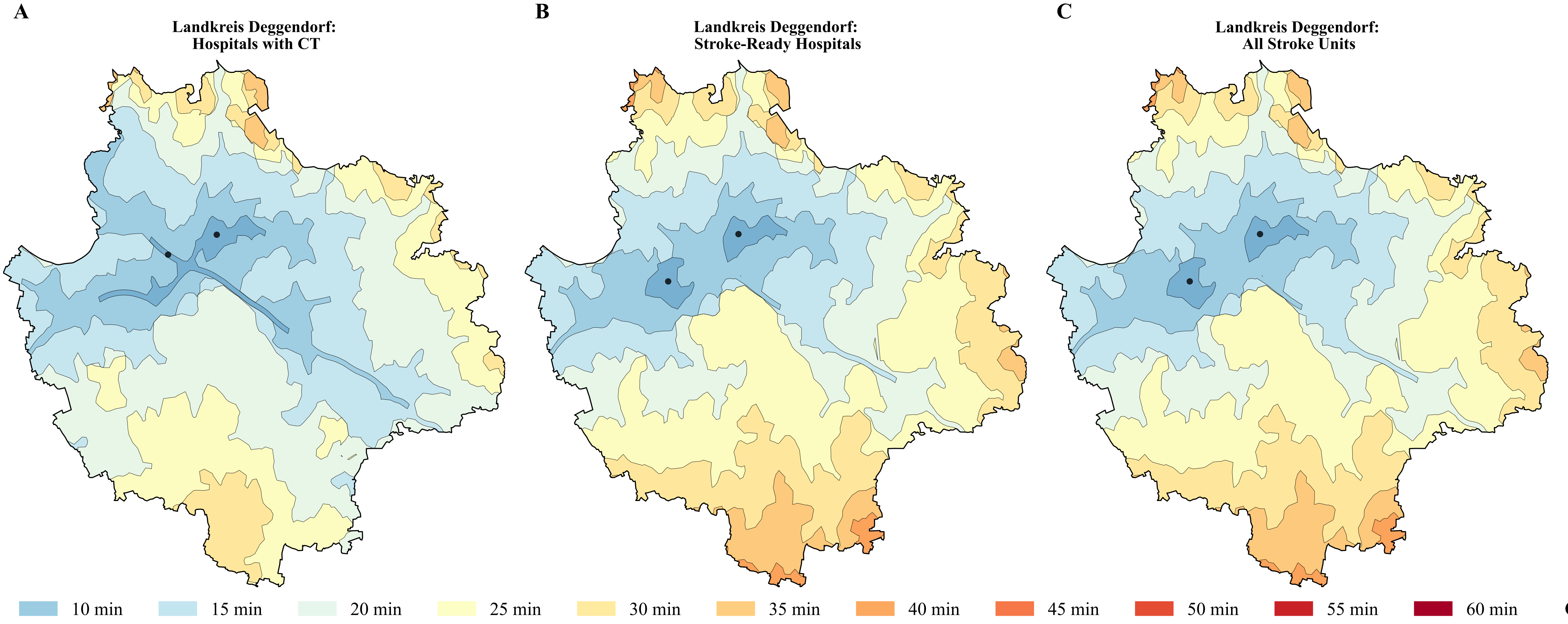

Isochrones — Landkreis Weißenburg-Gunzenhausen (Bayern)

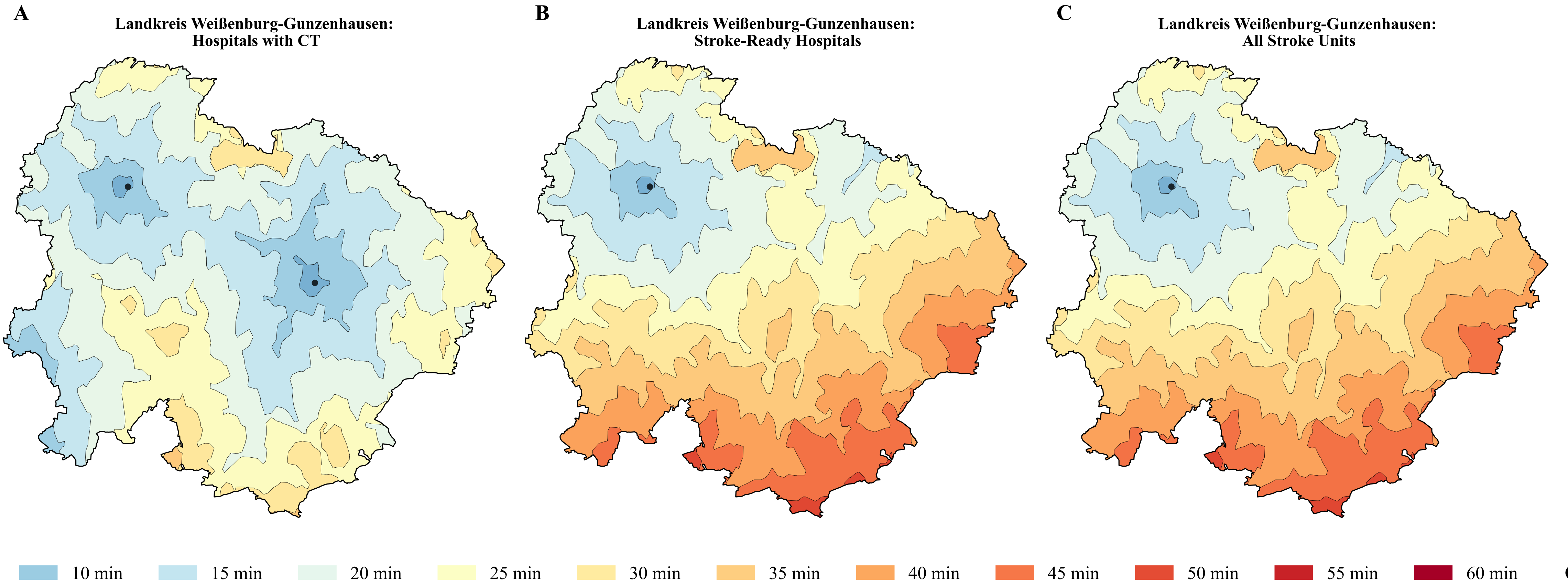

Isochrones — Landkreis Lindau (Bodensee) (Bayern)

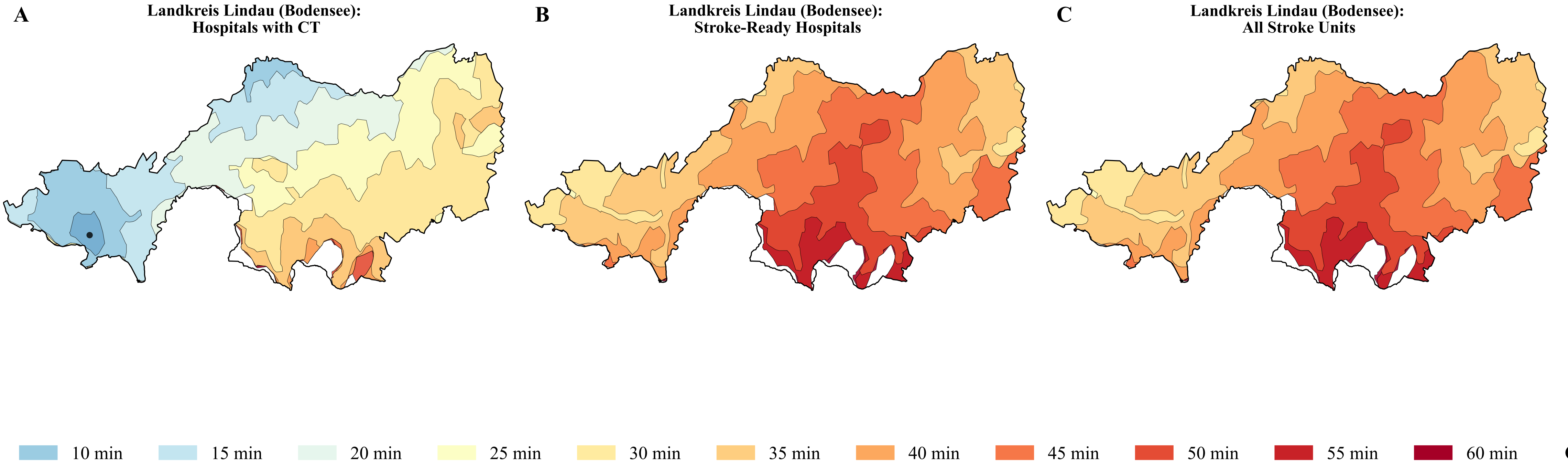

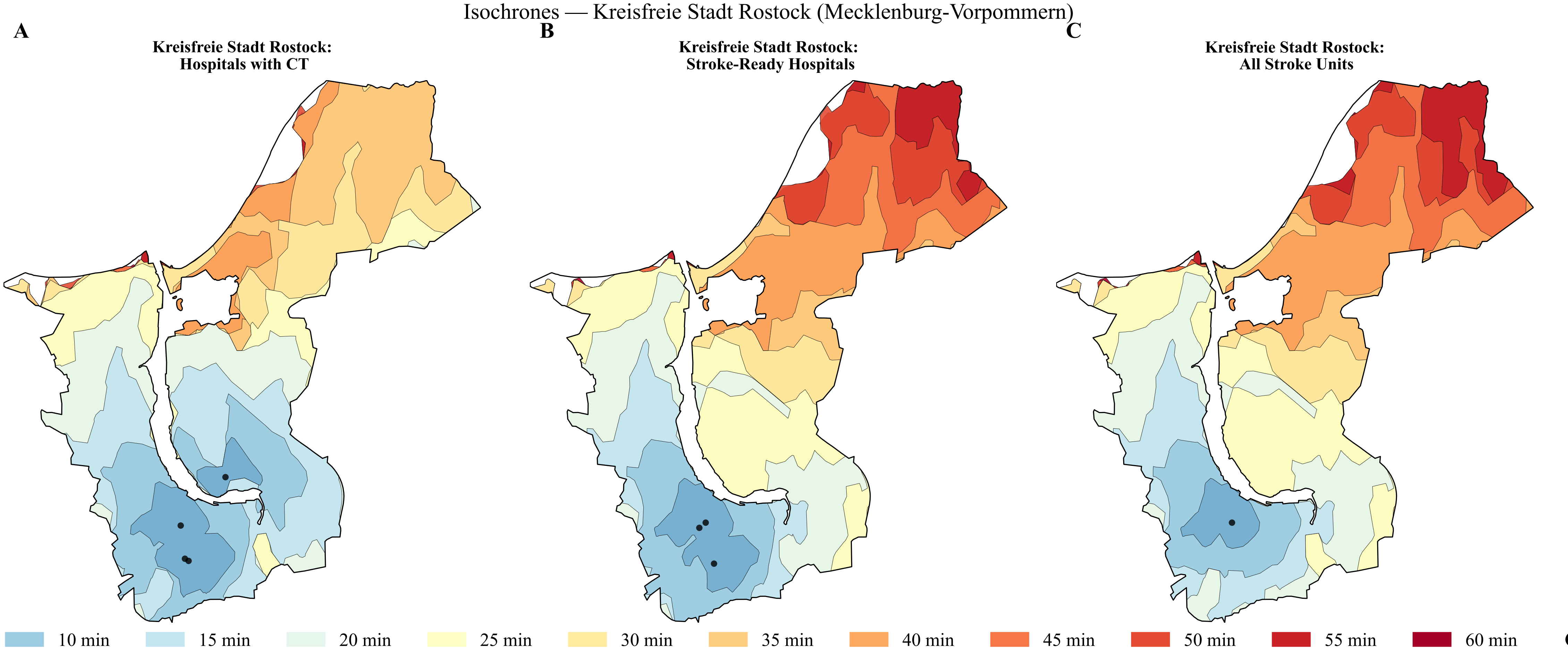
Isochrones — Kreisfreie Stadt Rostock (Mecklenburg-Vorpommern)
A
Kreisfreie Stadt Rostock:
Hospitals with CT
B
Kreisfreie Stadt Rostock:
Stroke-Ready Hospitals
C
Kreisfreie Stadt Rostock:
All Stroke Units
10 min
15 min
20 min
25 min
30 min
35 min
40 min
45 min
50 min
55 min
60 min

Isochrones — Landkreis Rostock (Mecklenburg-Vorpommern)

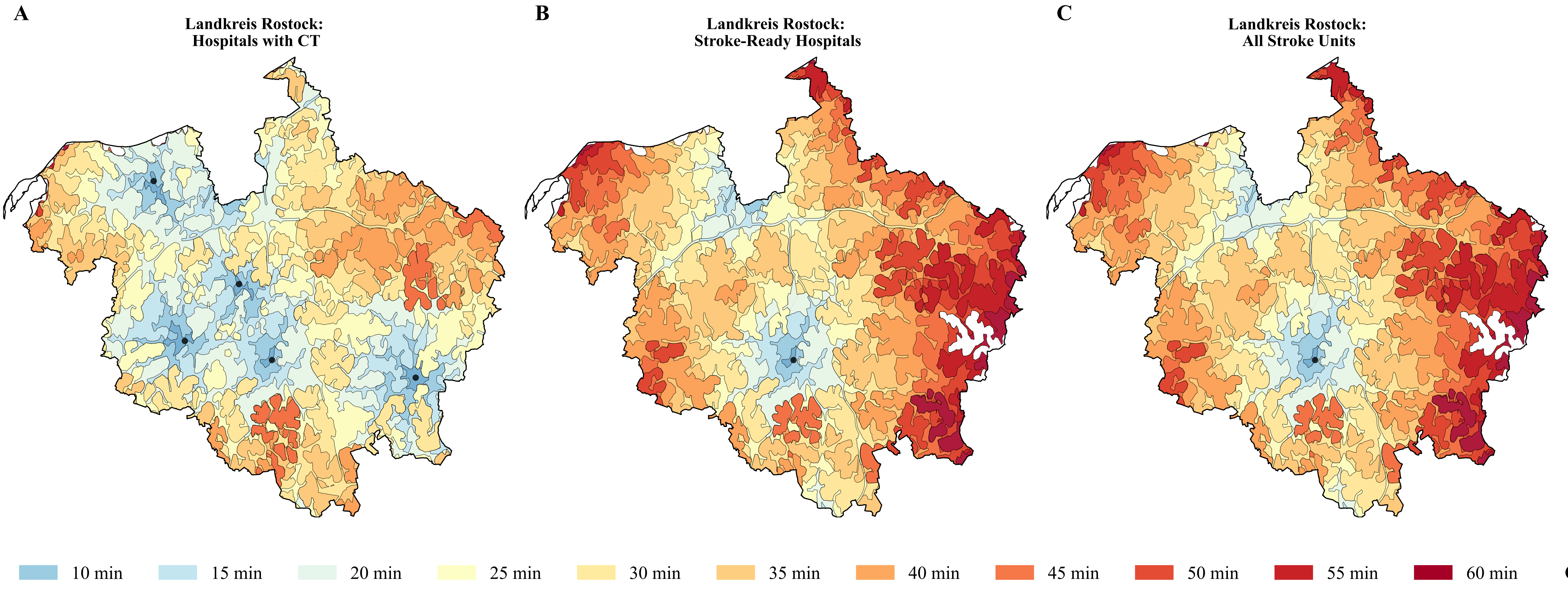

Isochrones — Landkreis Vorpommern-Greifswald (Mecklenburg-Vorpommern)

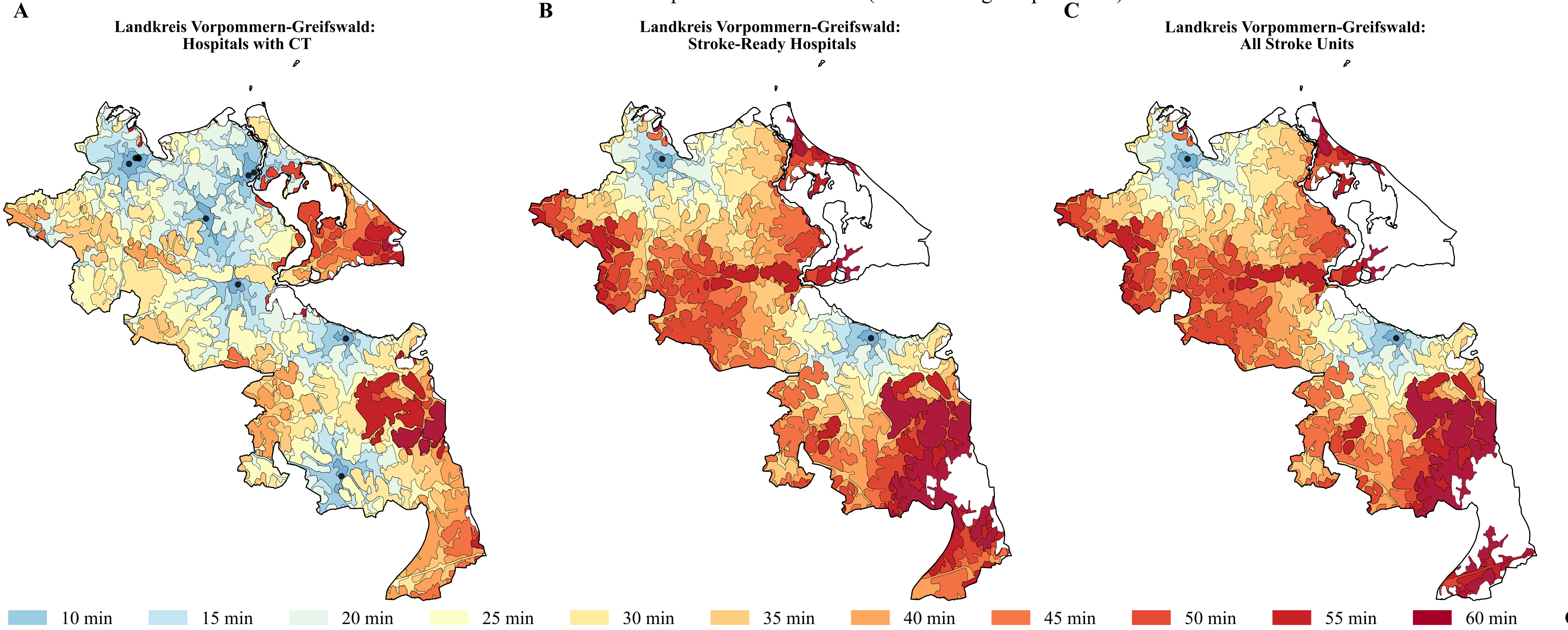

Isochrones — Landkreis Mittelsachsen (Sachsen)

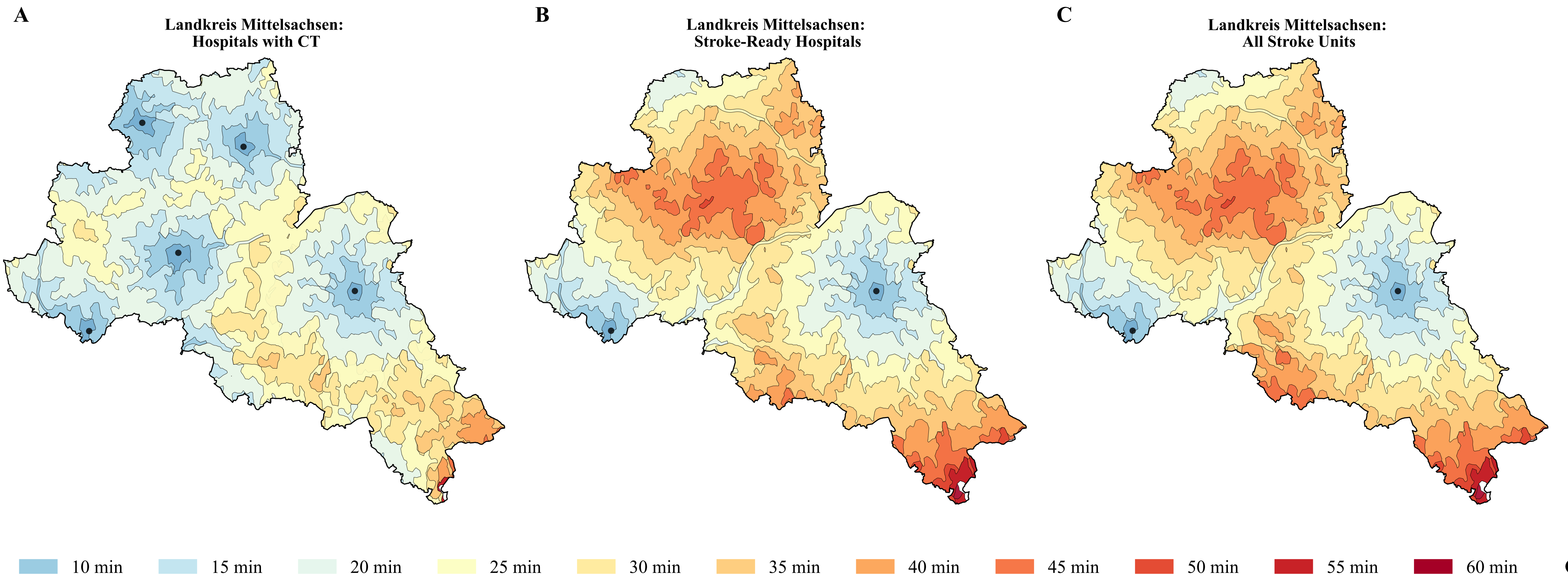

Isochrones — Landkreis Schmalkalden-Meiningen (Thüringen)

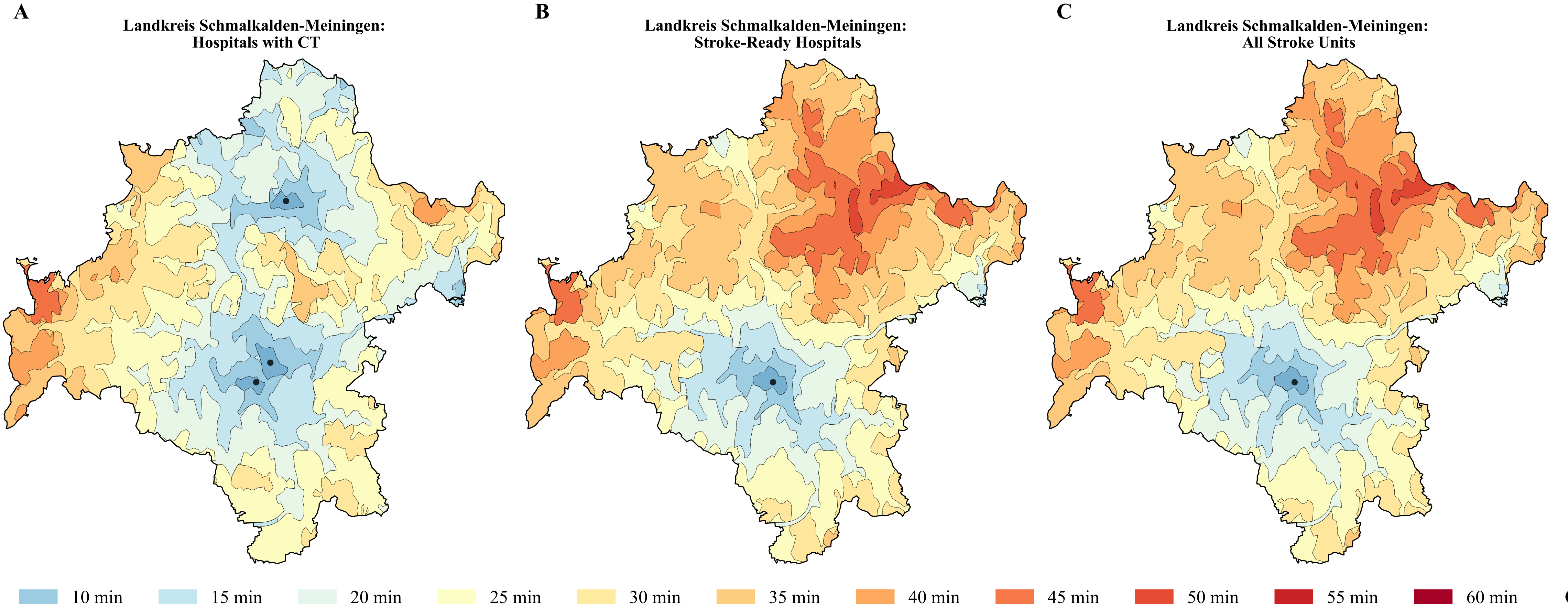

Isochrones — Kreisfreie Stadt Lübeck (Schleswig-Holstein)

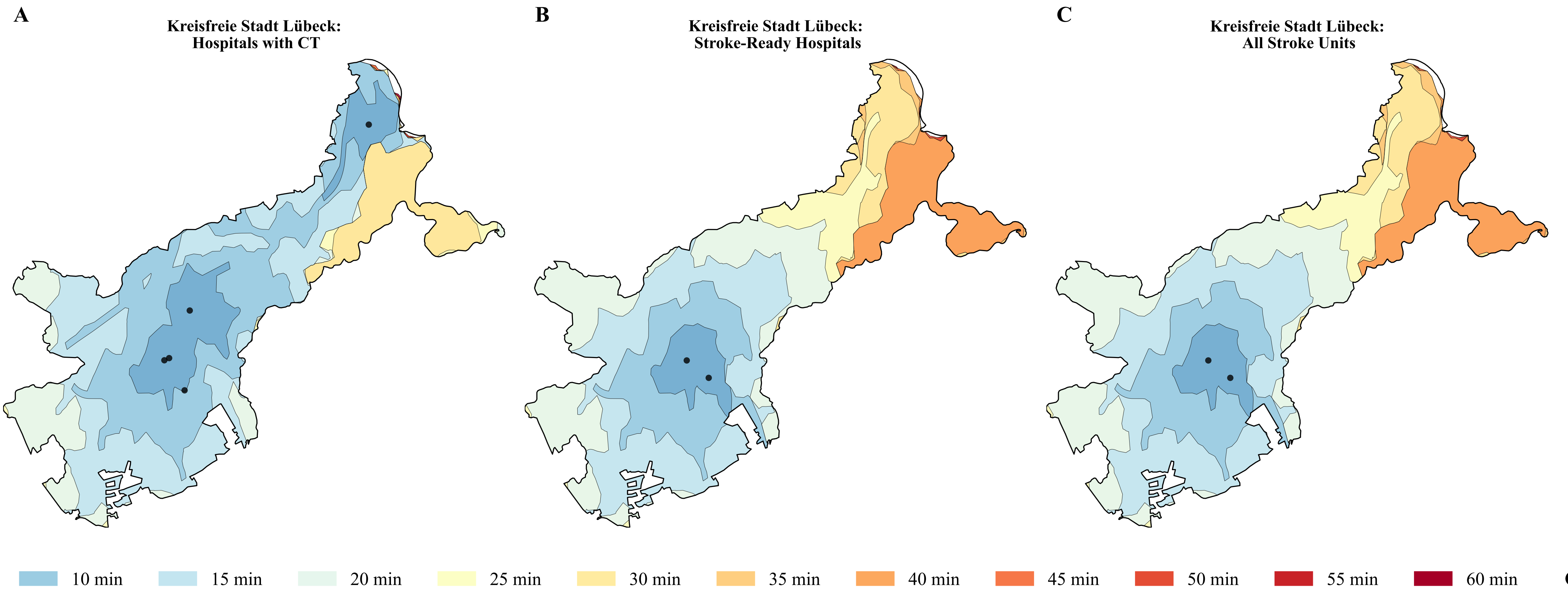

Isochrones — Kreis Nordfriesland (Schleswig-Holstein)

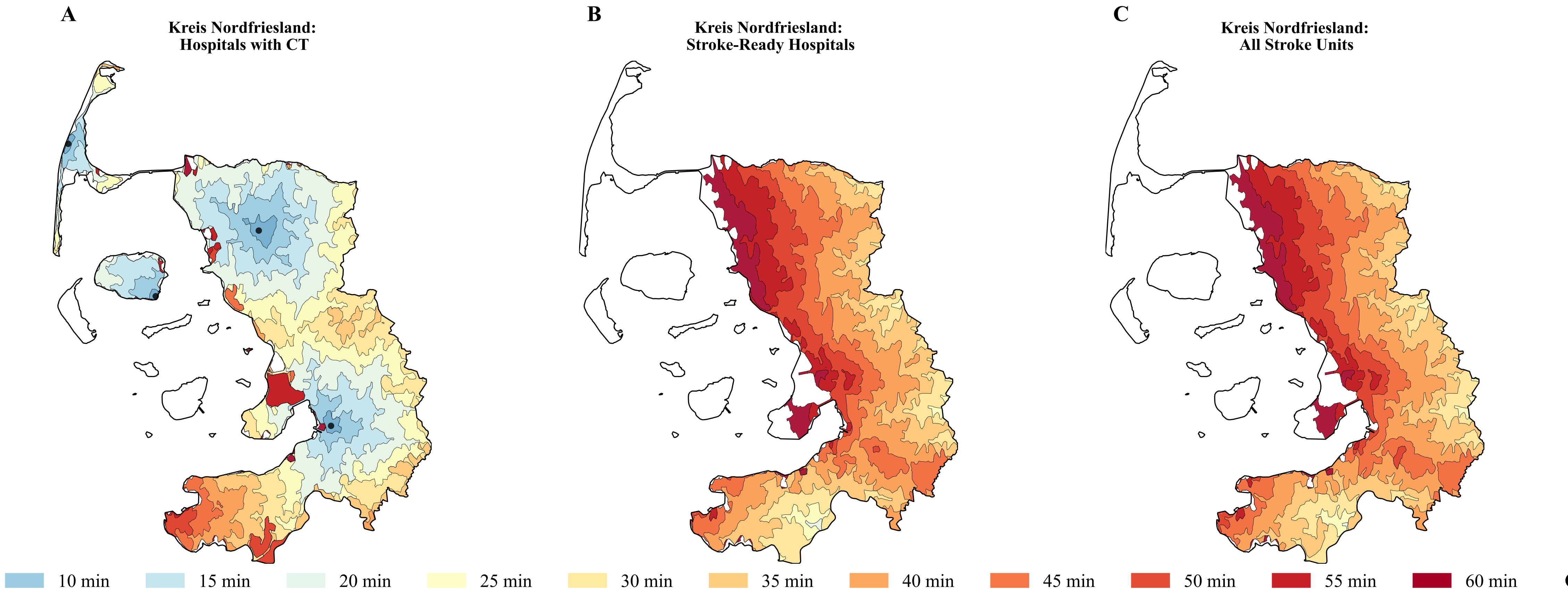

Isochrones — Landkreis Northeim (Niedersachsen)

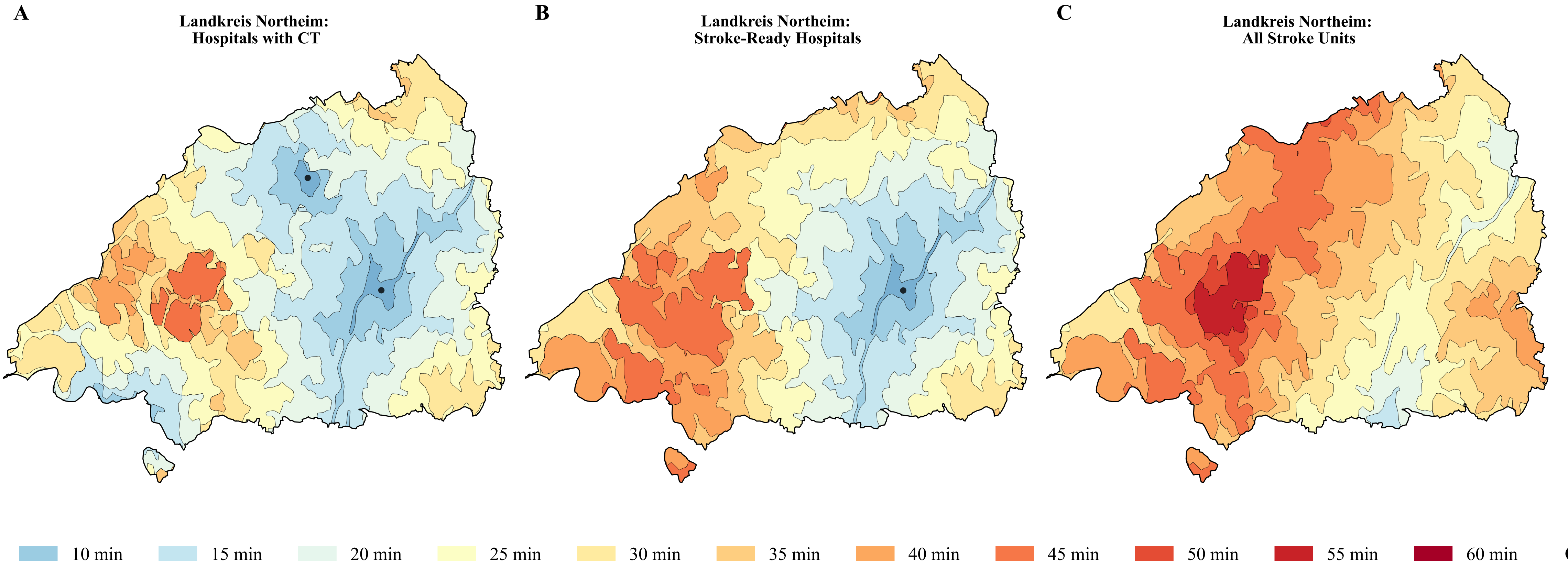

Isochrones — Kreisfreie Stadt Wilhelmshaven (Niedersachsen)

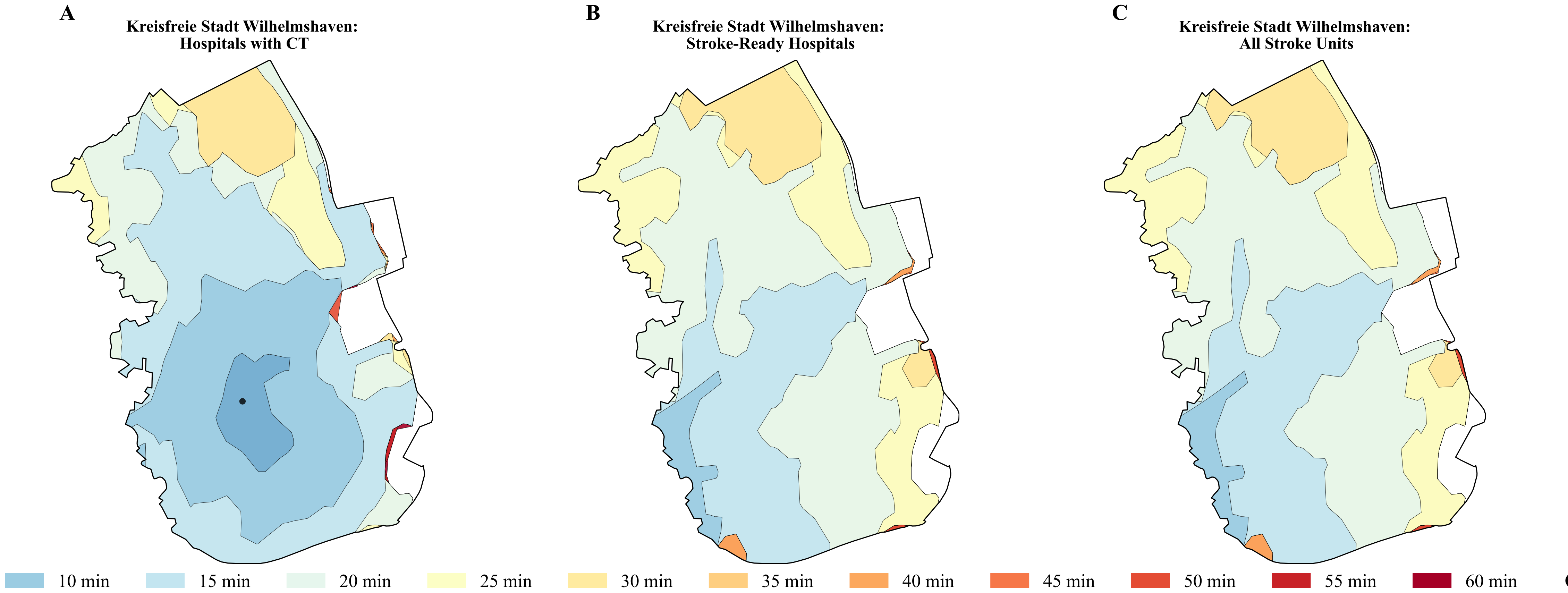

Isochrones — Kreisfreie Stadt Essen (Nordrhein-Westfalen)

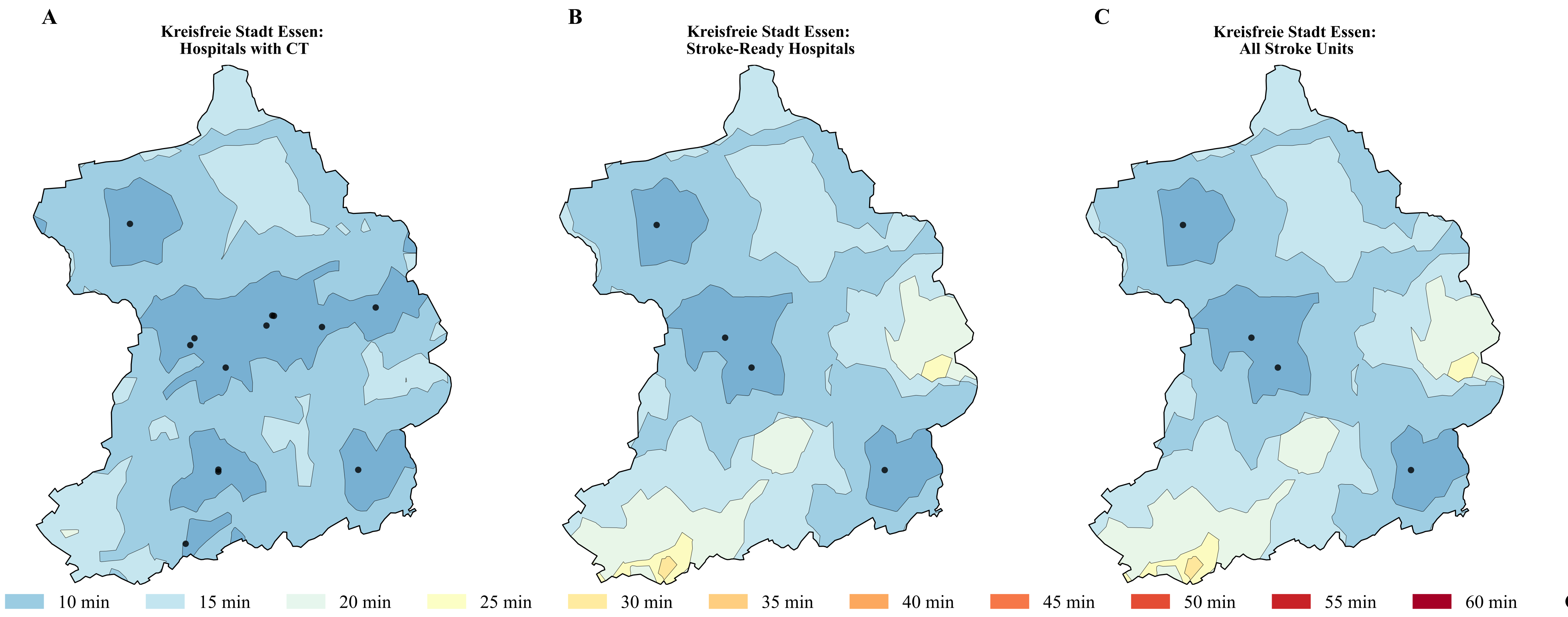

Isochrones — Kreisfreie Stadt Münster (Nordrhein-Westfalen)

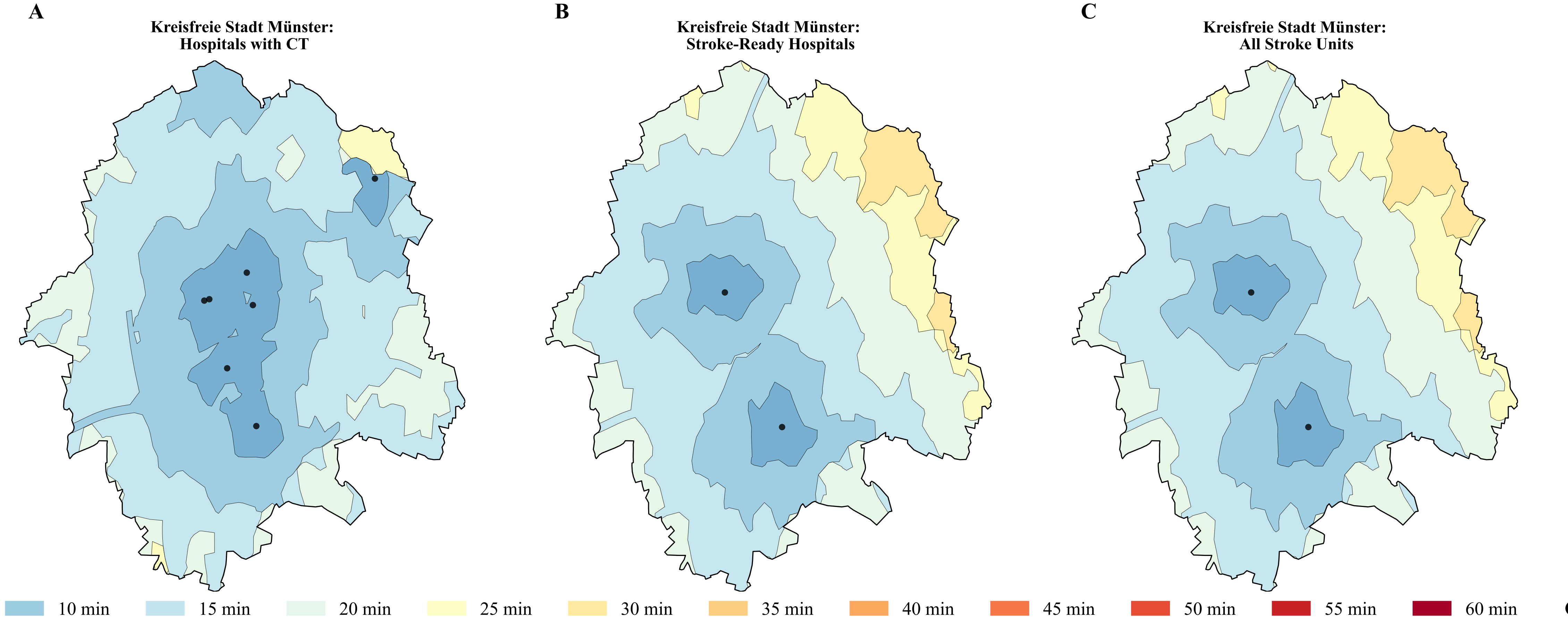

527 / 5833

Isochrones — Kreis Recklinghausen (Nordrhein-Westfalen)

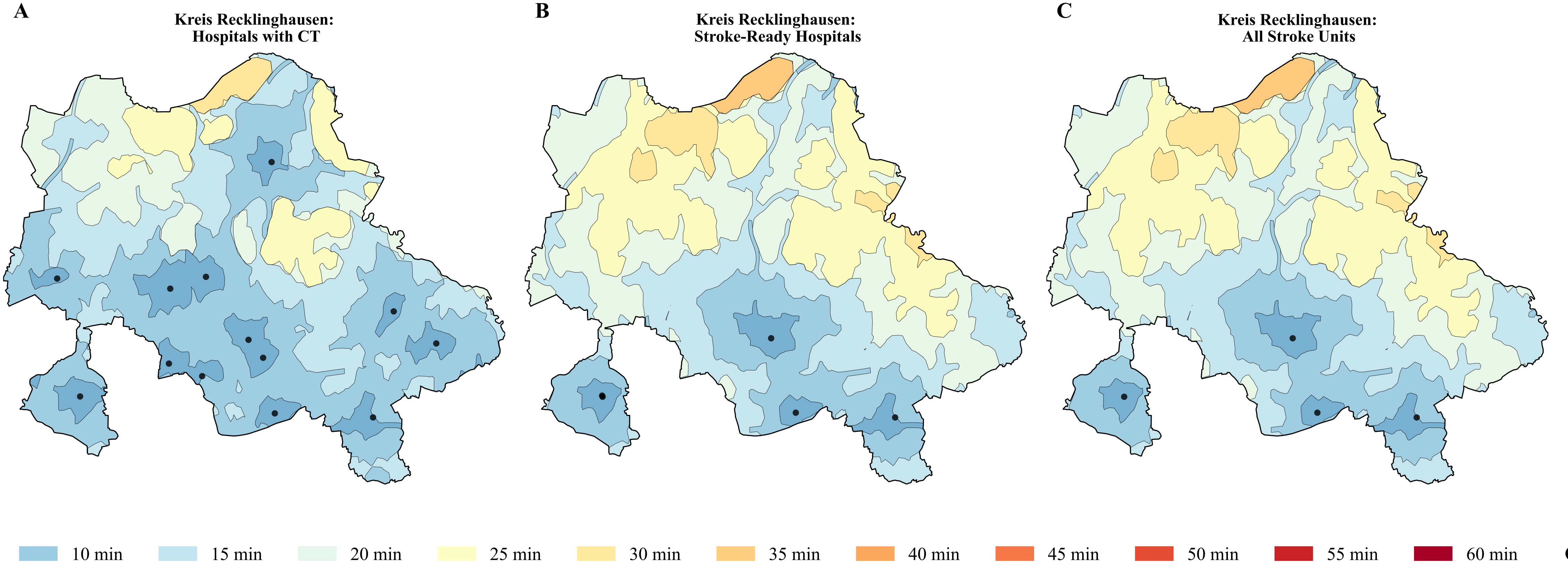

Isochrones — Landkreis Offenbach (Hessen)

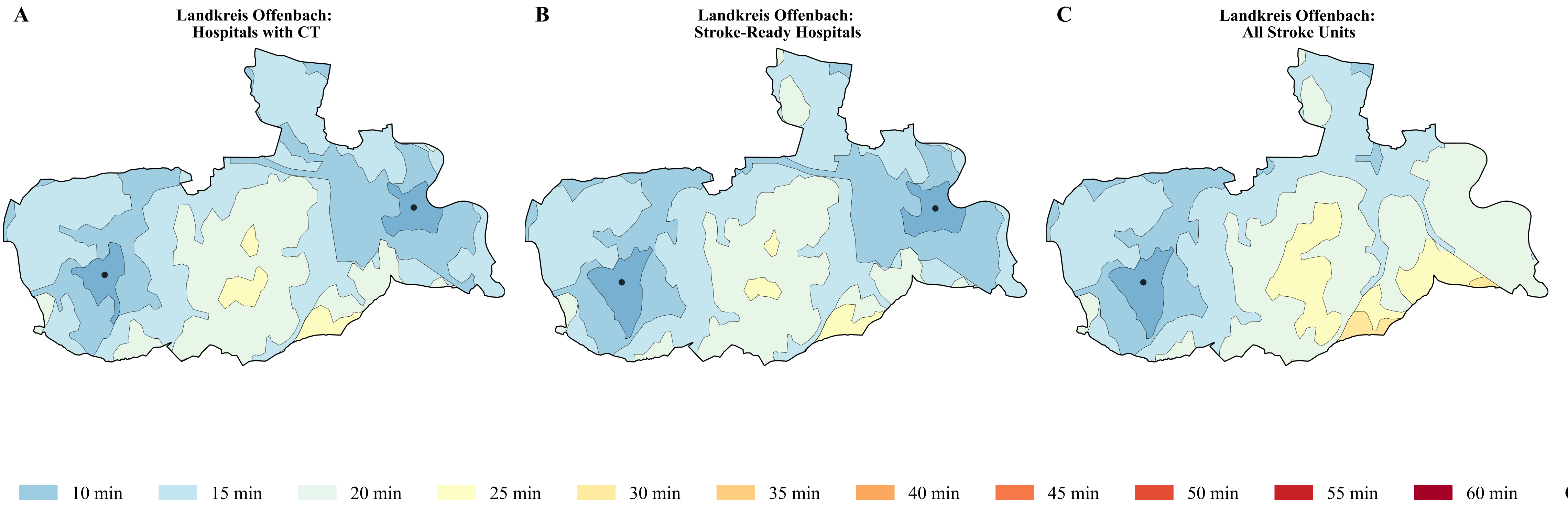

Isochrones — Kreisfreie Stadt Trier (Rheinland-Pfalz)

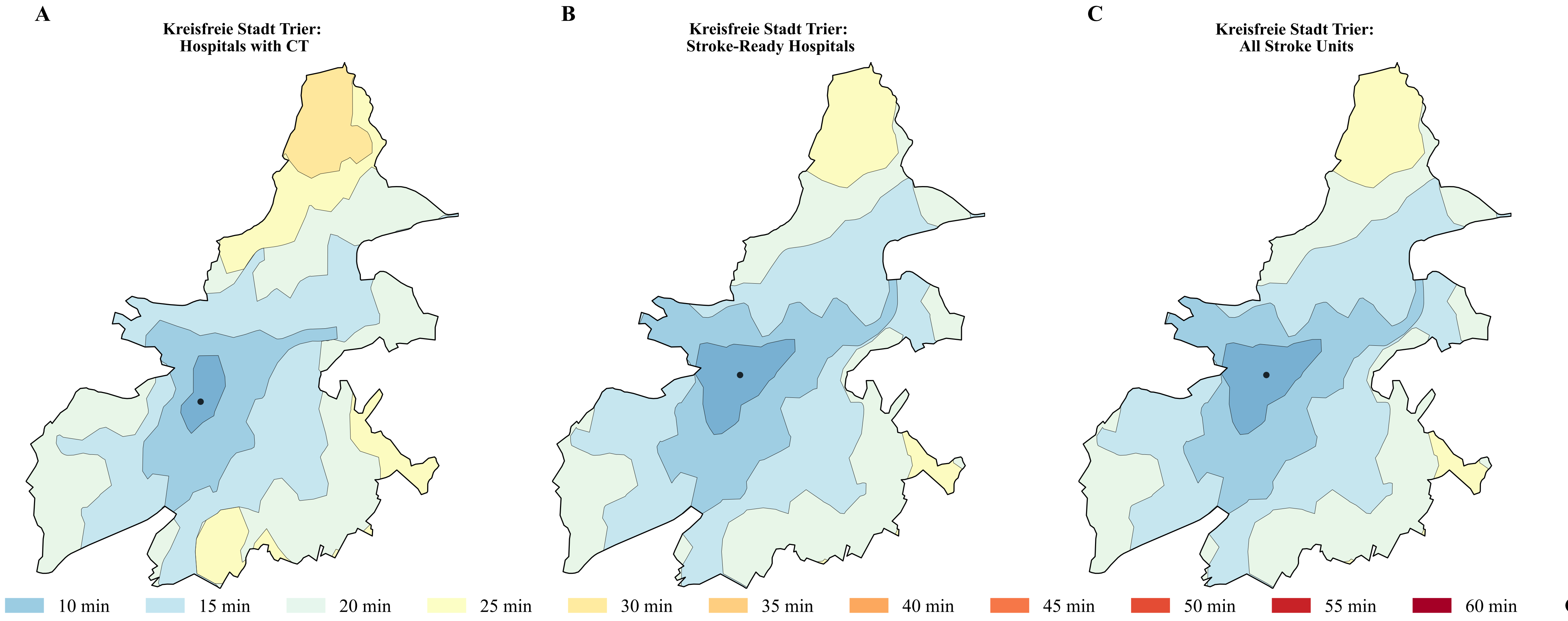

Isochrones — Landkreis Bernkastel-Wittlich (Rheinland-Pfalz)

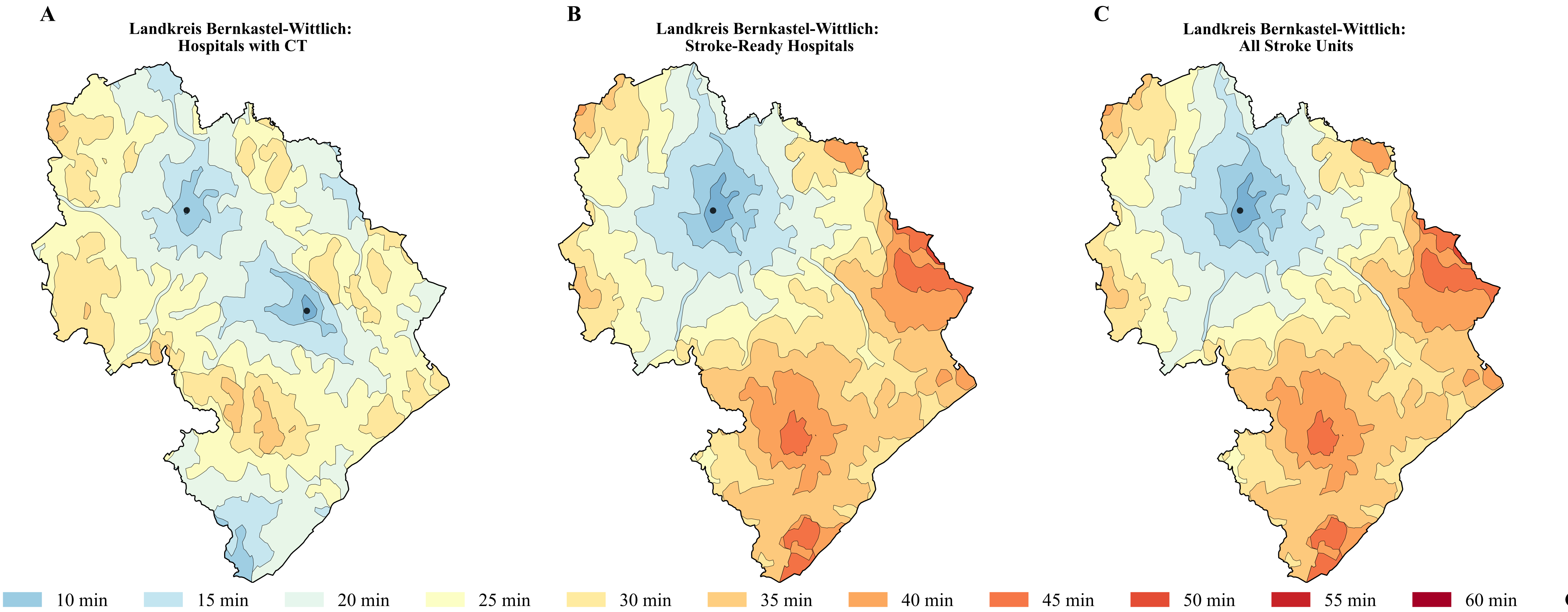

Isochrones — Kreisfreie Stadt Worms (Rheinland-Pfalz)

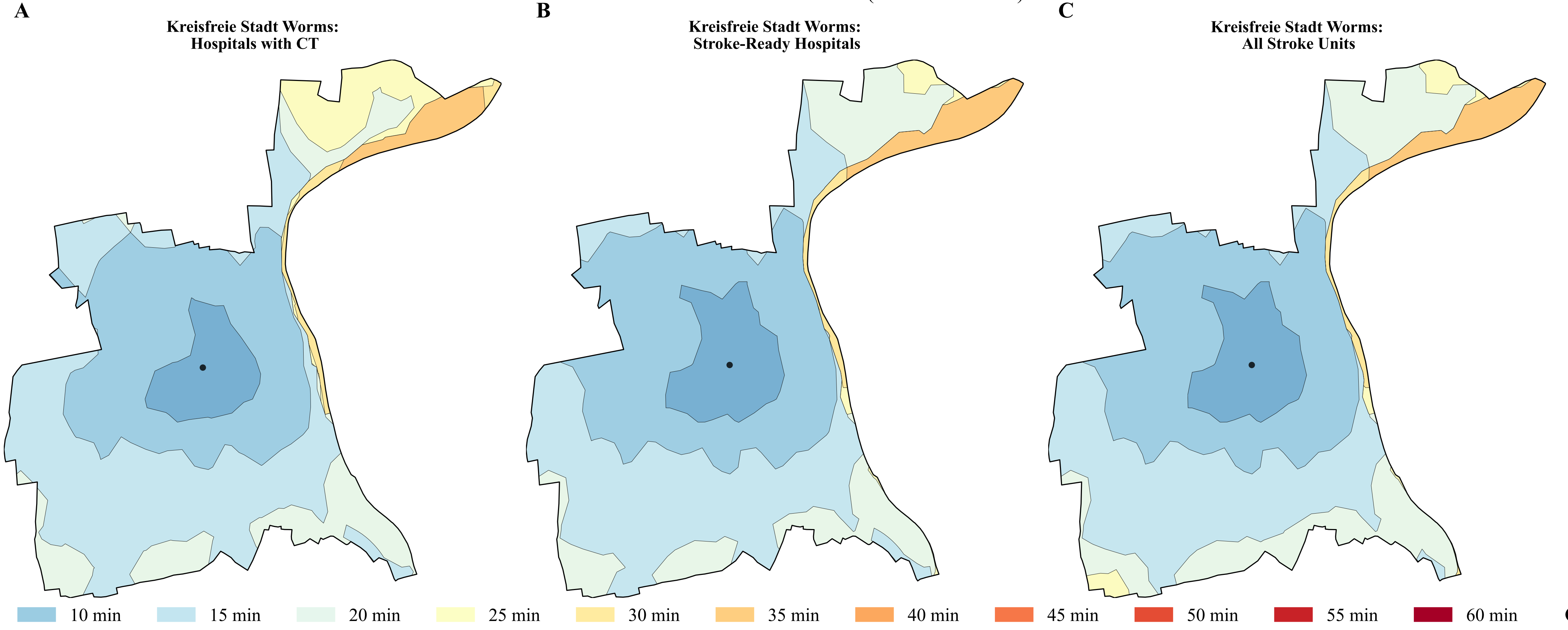

Isochrones — Landkreis Heilbronn (Baden-Württemberg)

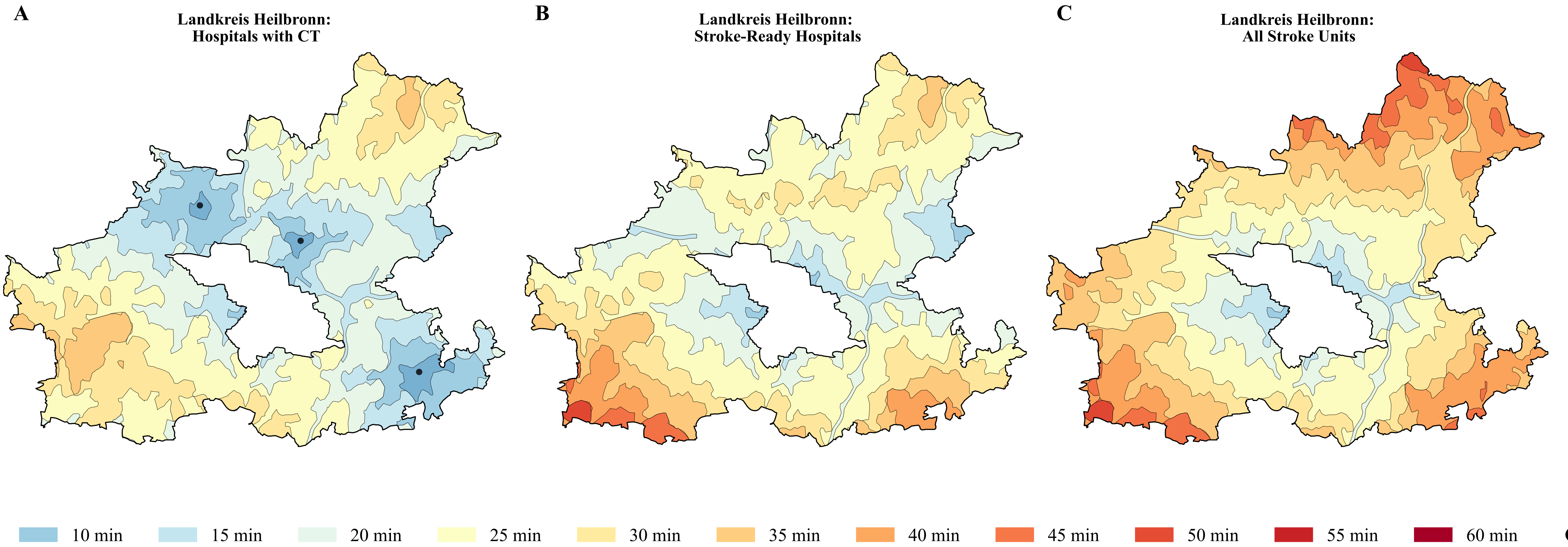

Isochrones — Kreisfreie Stadt Ingolstadt (Bayern)

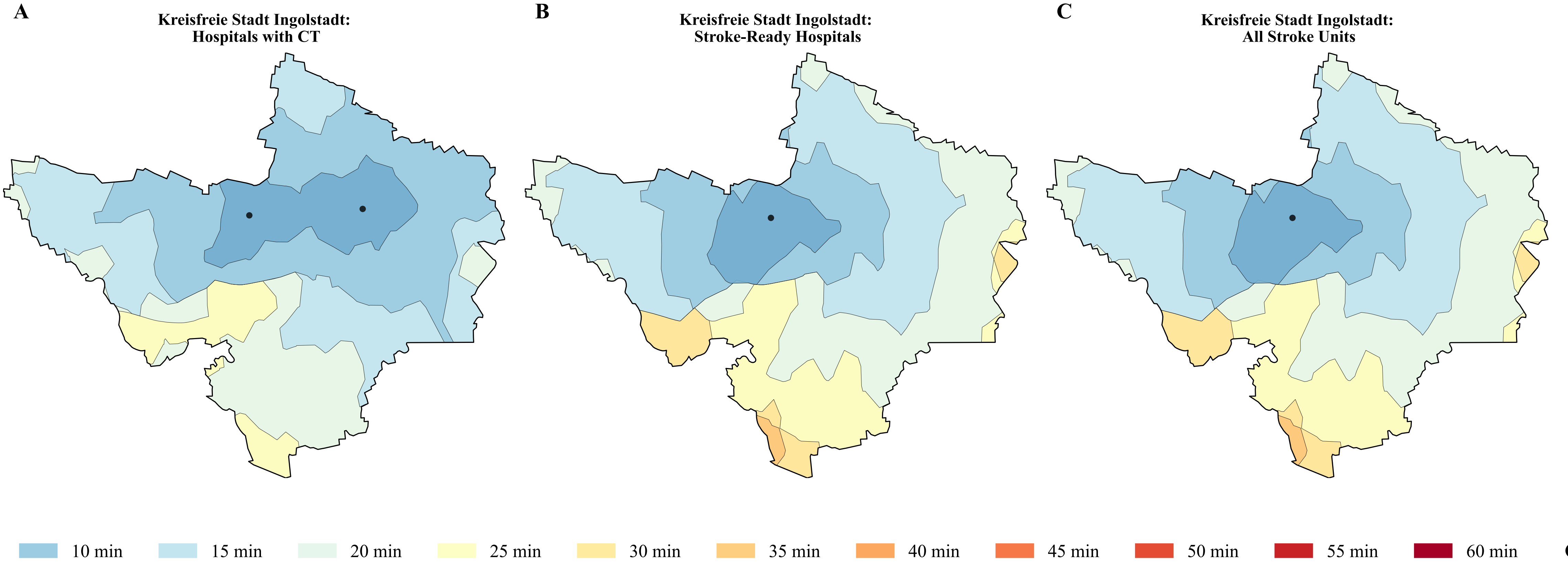

Isochrones — Landkreis Forchheim (Bayern)

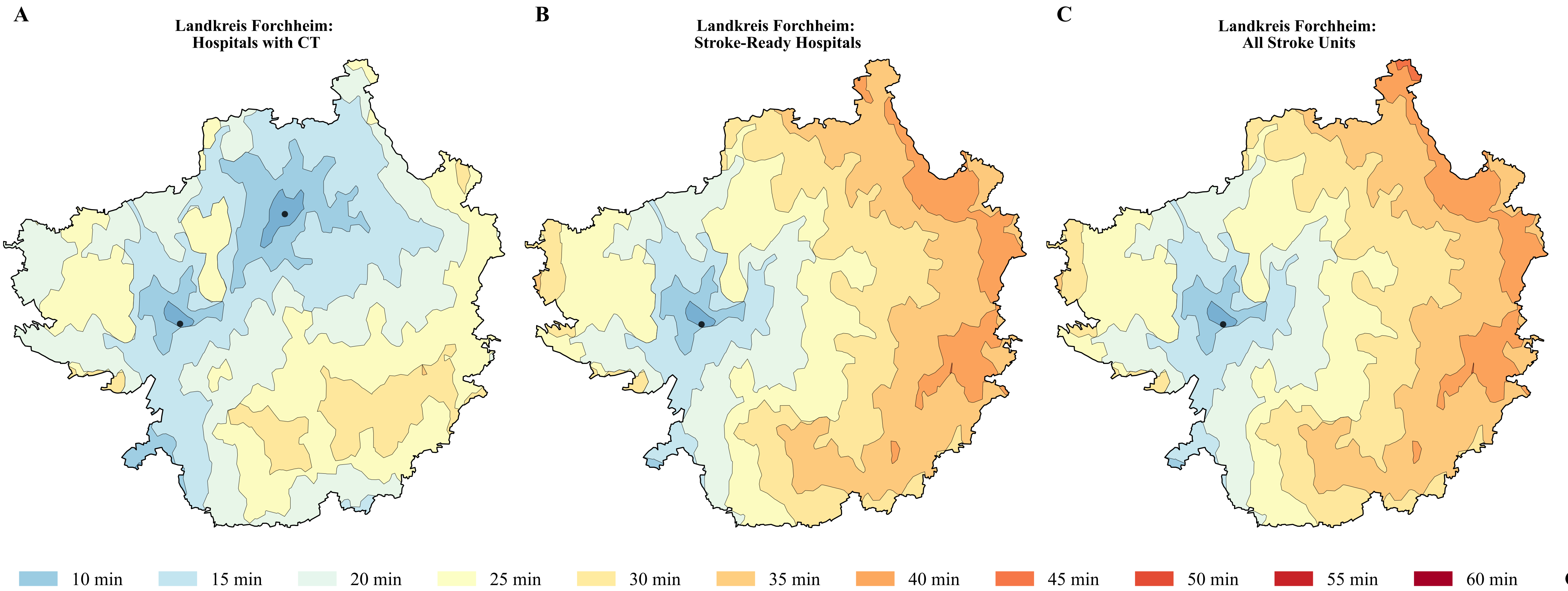

Isochrones — Landkreis Erlangen-Höchstadt (Bayern)

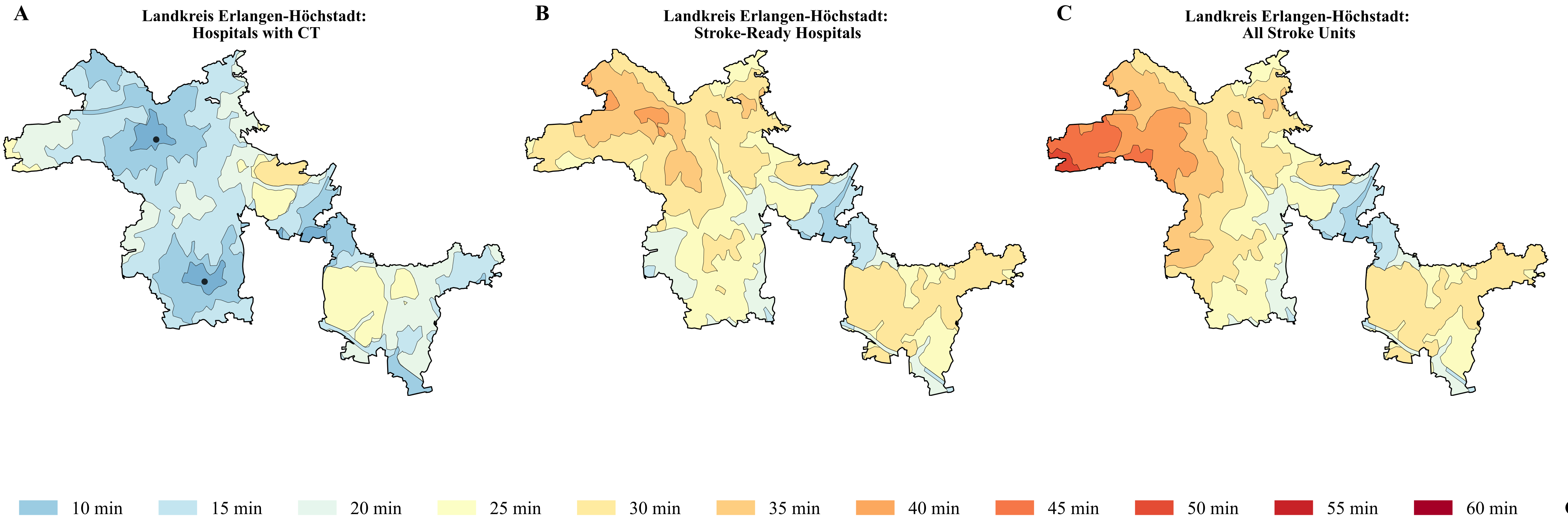

Isochrones — Landkreis Mansfeld-Südharz (Sachsen-Anhalt)

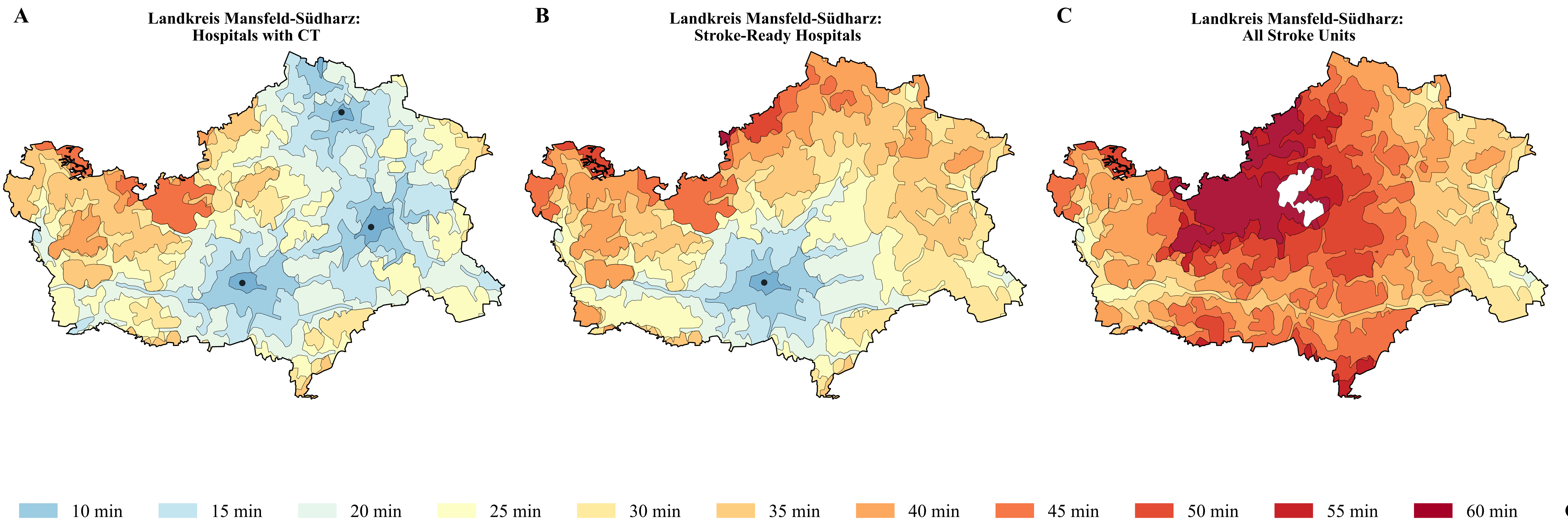

Isochrones — Kreis Minden-Lübbecke (Nordrhein-Westfalen)

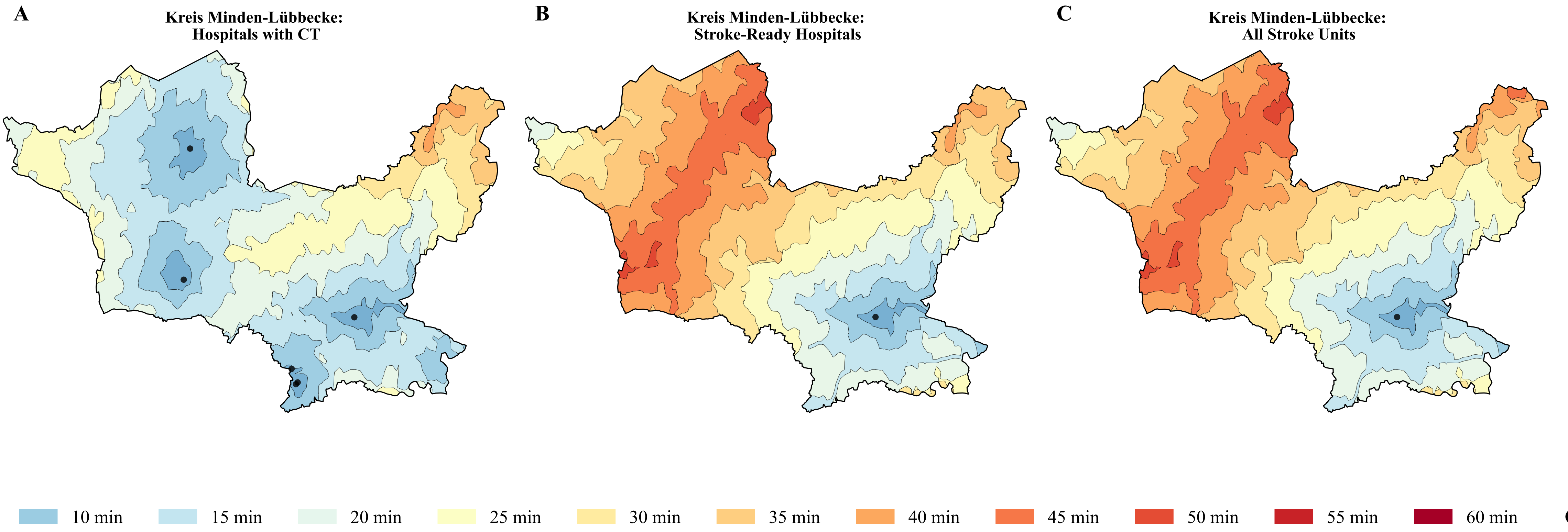

Isochrones — Kreisfreie Stadt Hamm (Nordrhein-Westfalen)

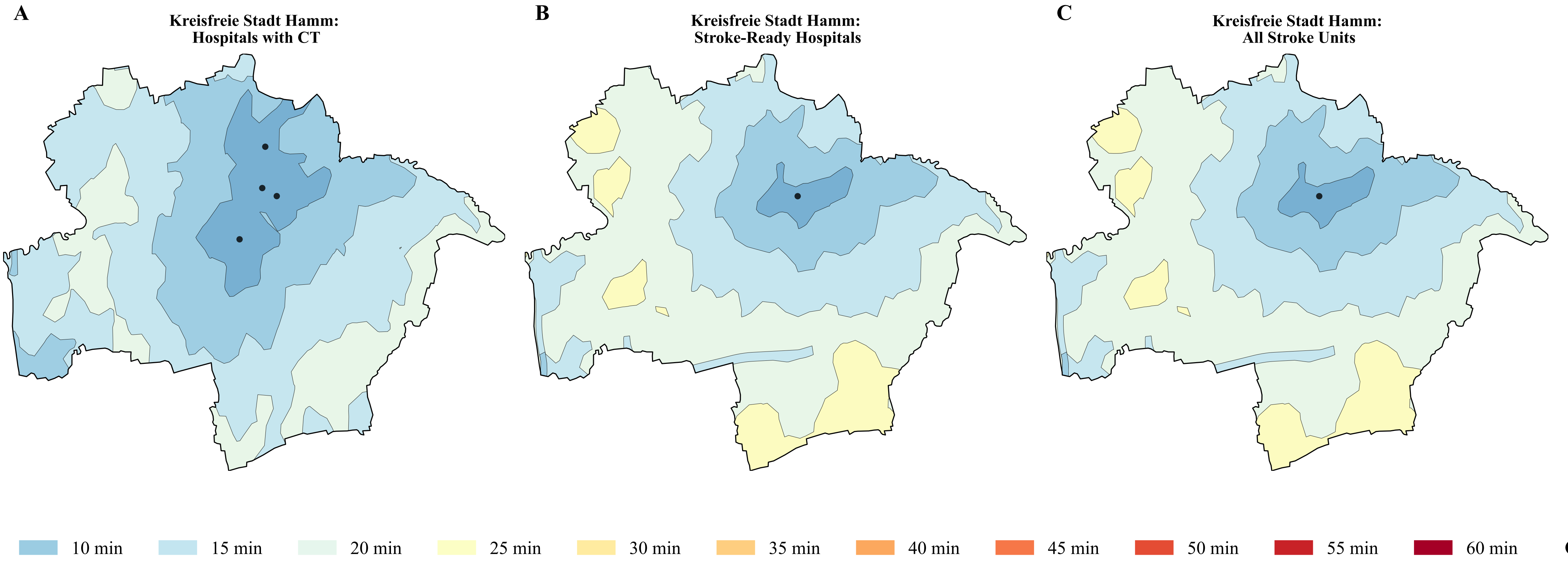

Isochrones — Landkreis Main-Taunus-Kreis (Hessen)

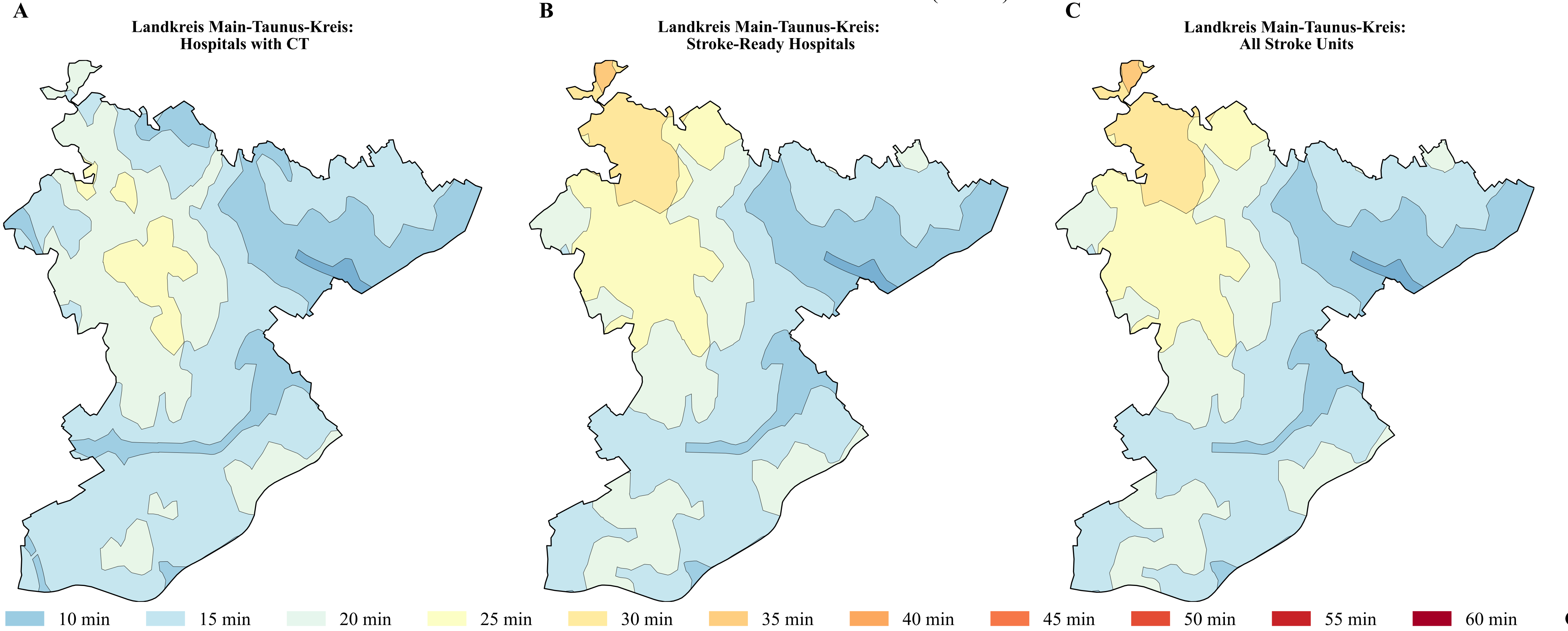

Isochrones — Kreisfreie Stadt Ludwigshafen am Rhein (Rheinland-Pfalz)

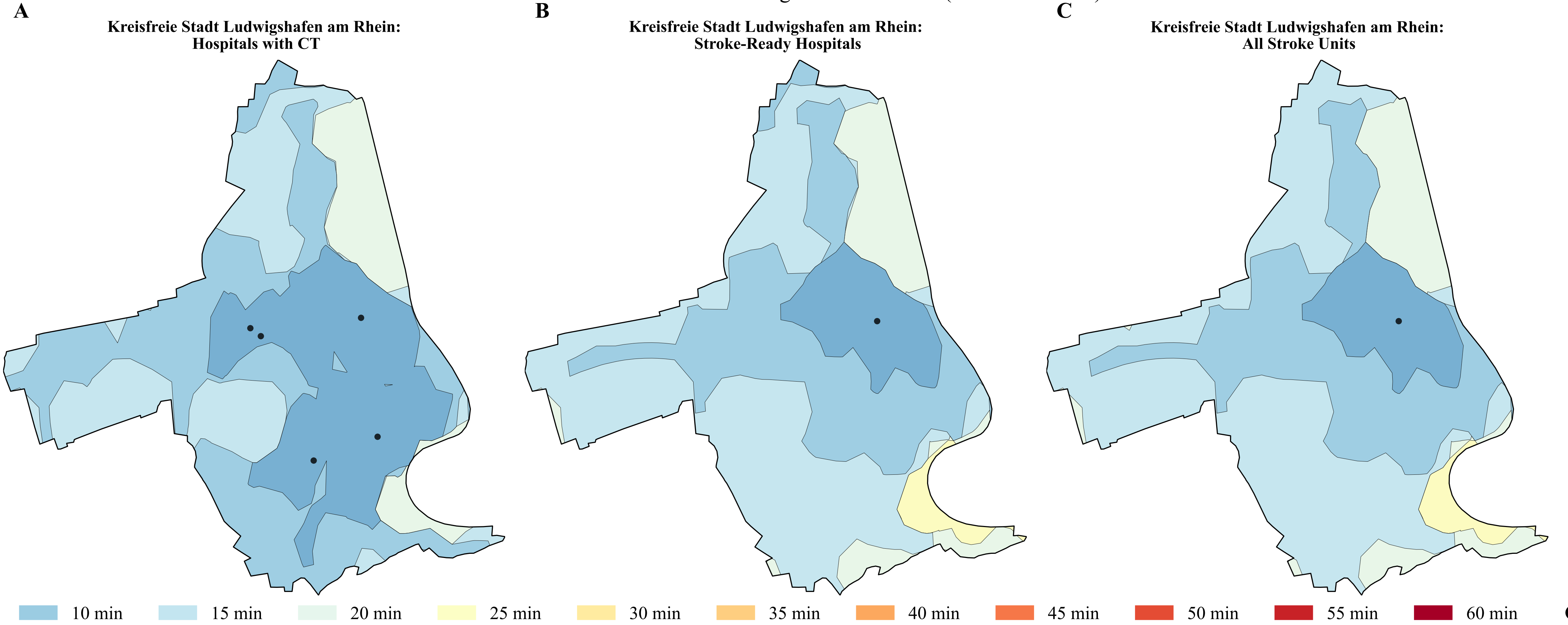

Isochrones — Landkreis Esslingen (Baden-Württemberg)

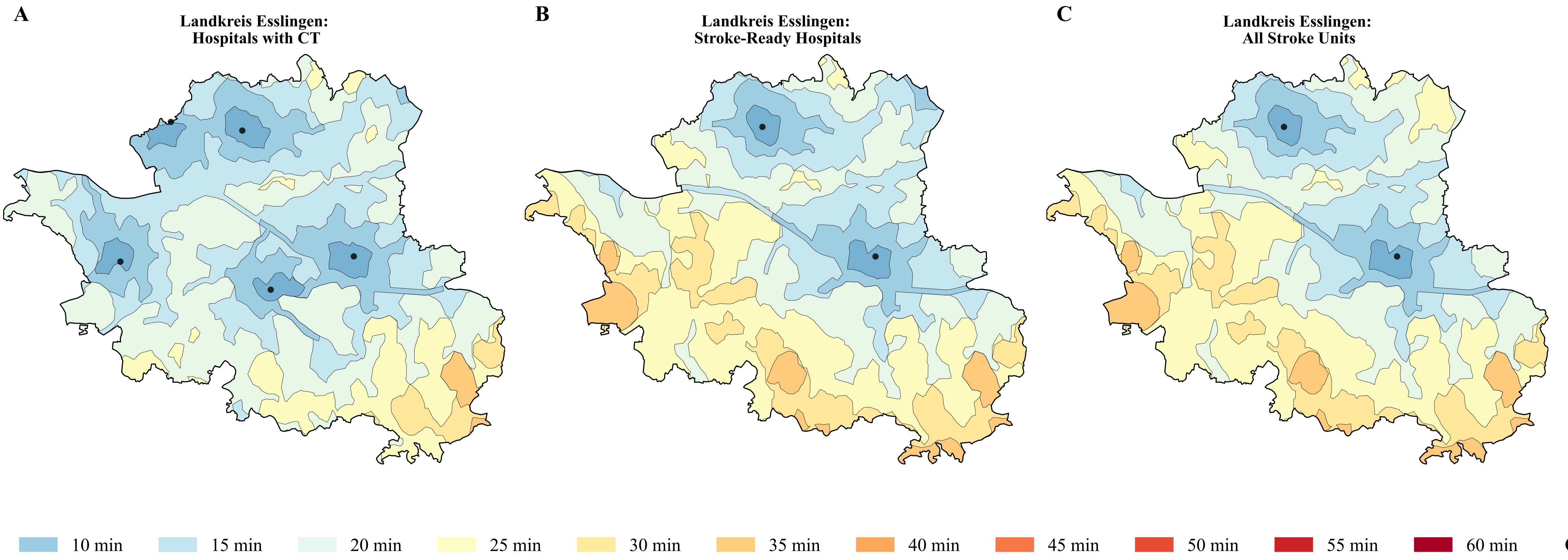

Isochrones — Landkreis Dachau (Bayern)

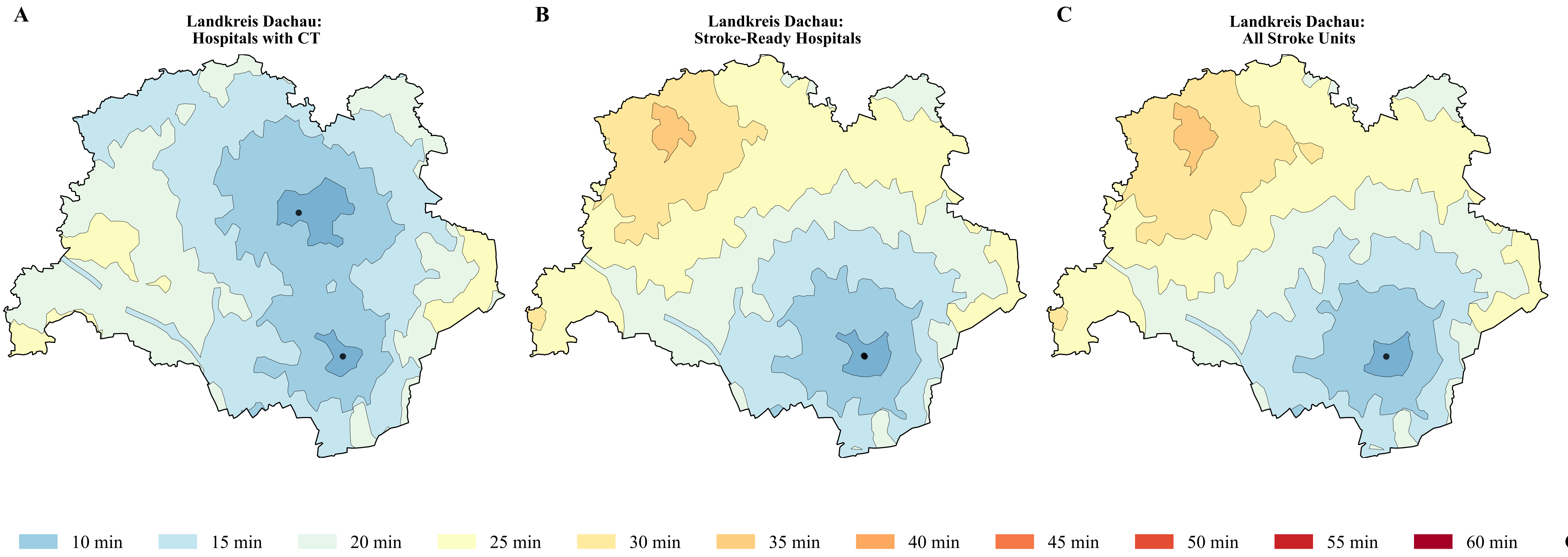

Isochrones — Landkreis Bad Kissingen (Bayern)

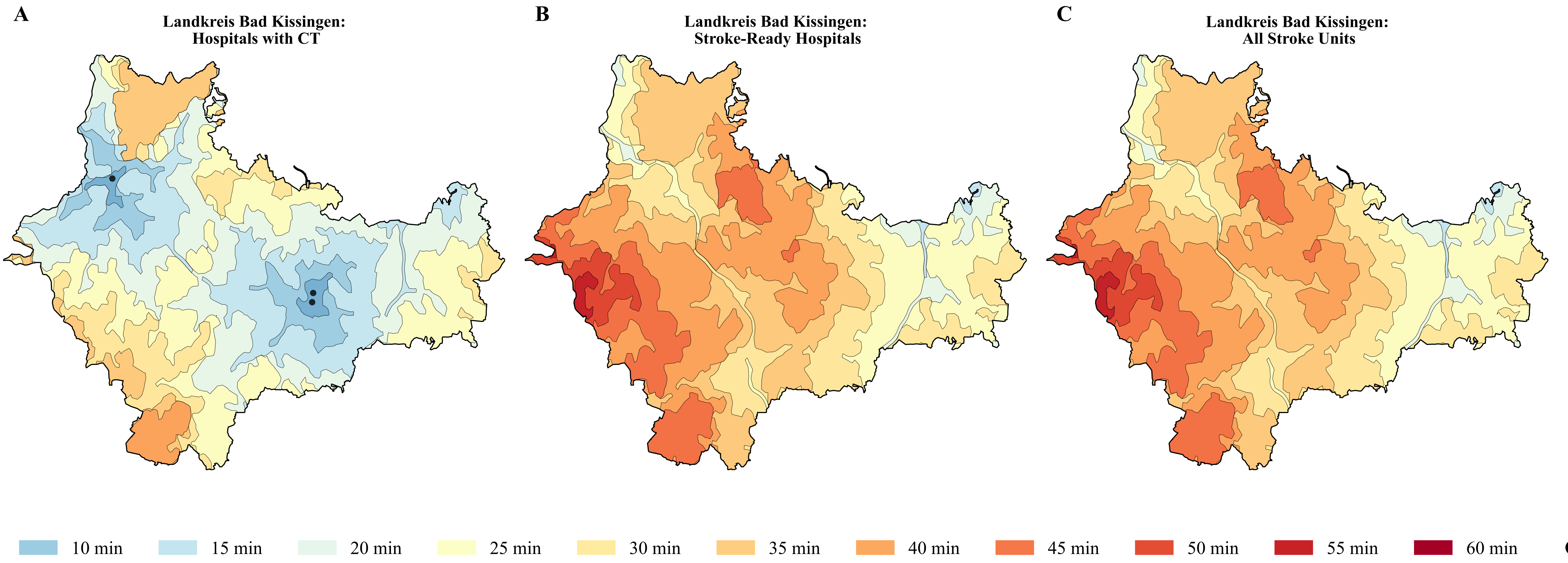

Isochrones — Kreisfreie Stadt Erfurt (Thüringen)

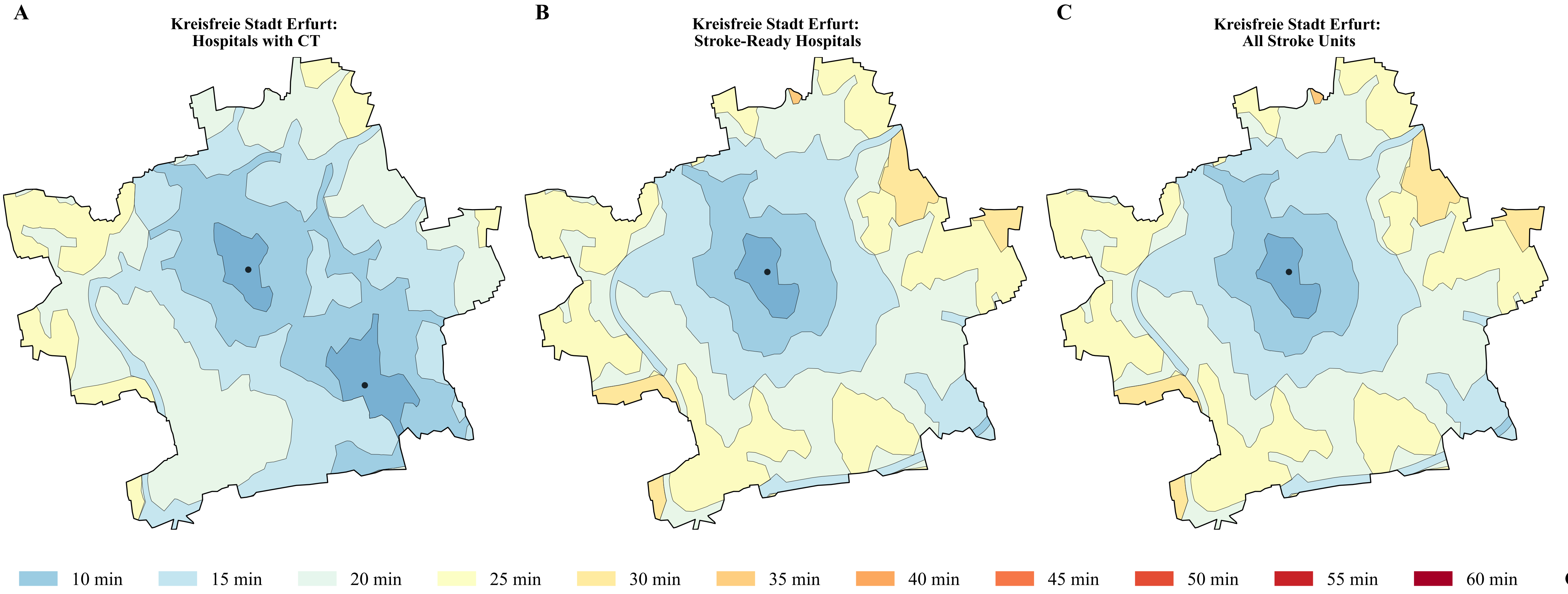

Isochrones — Kreisfreie Stadt Flensburg (Schleswig-Holstein)

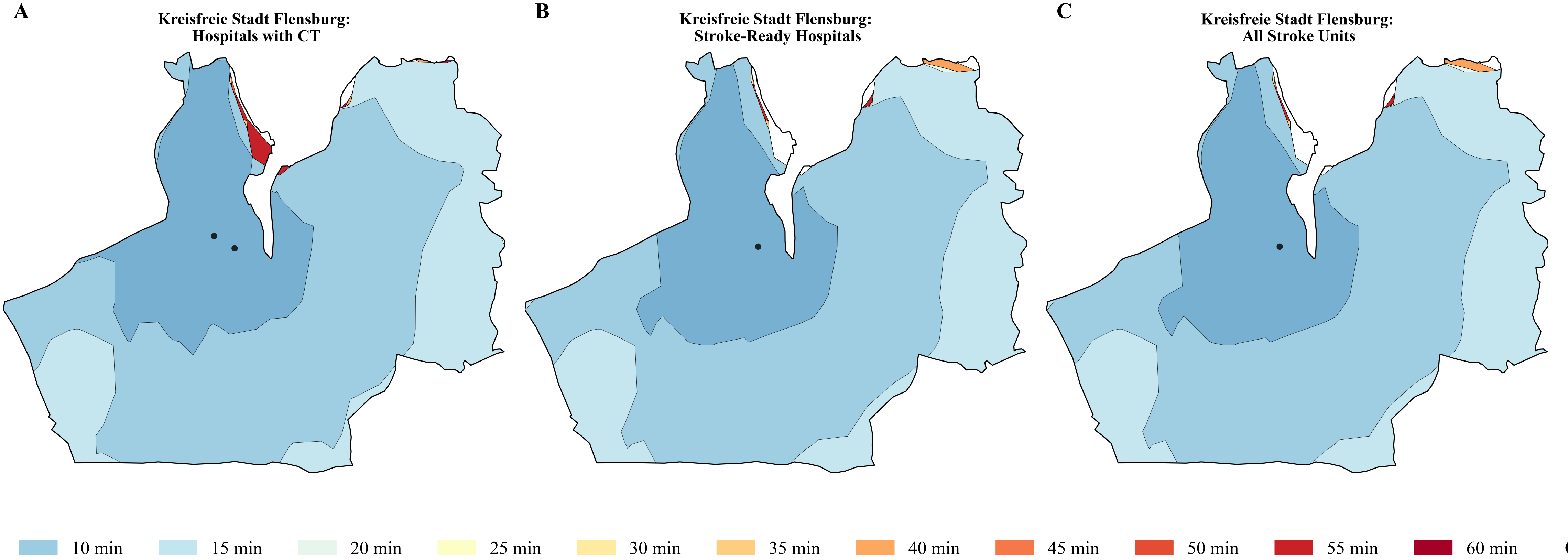

Isochrones — Kreis Dithmarschen (Schleswig-Holstein)

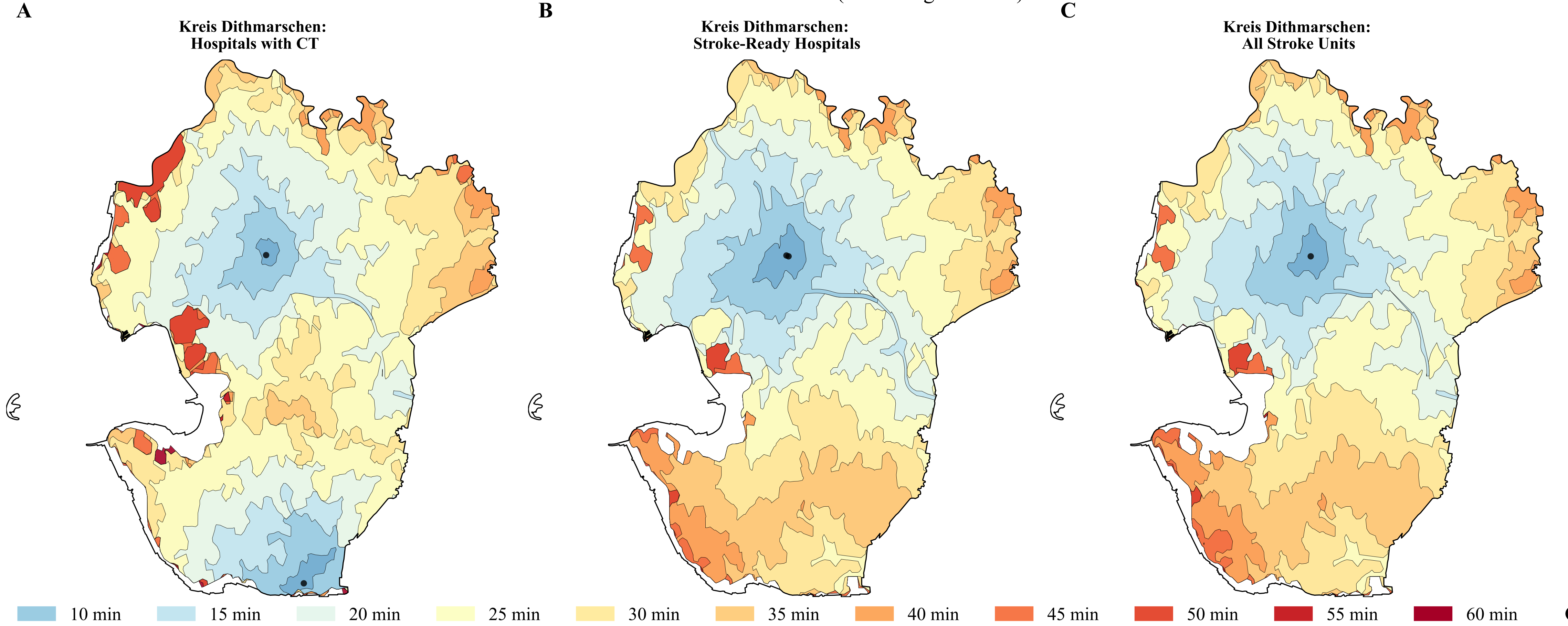

Isochrones — Landkreis Hameln-Pyrmont (Niedersachsen)

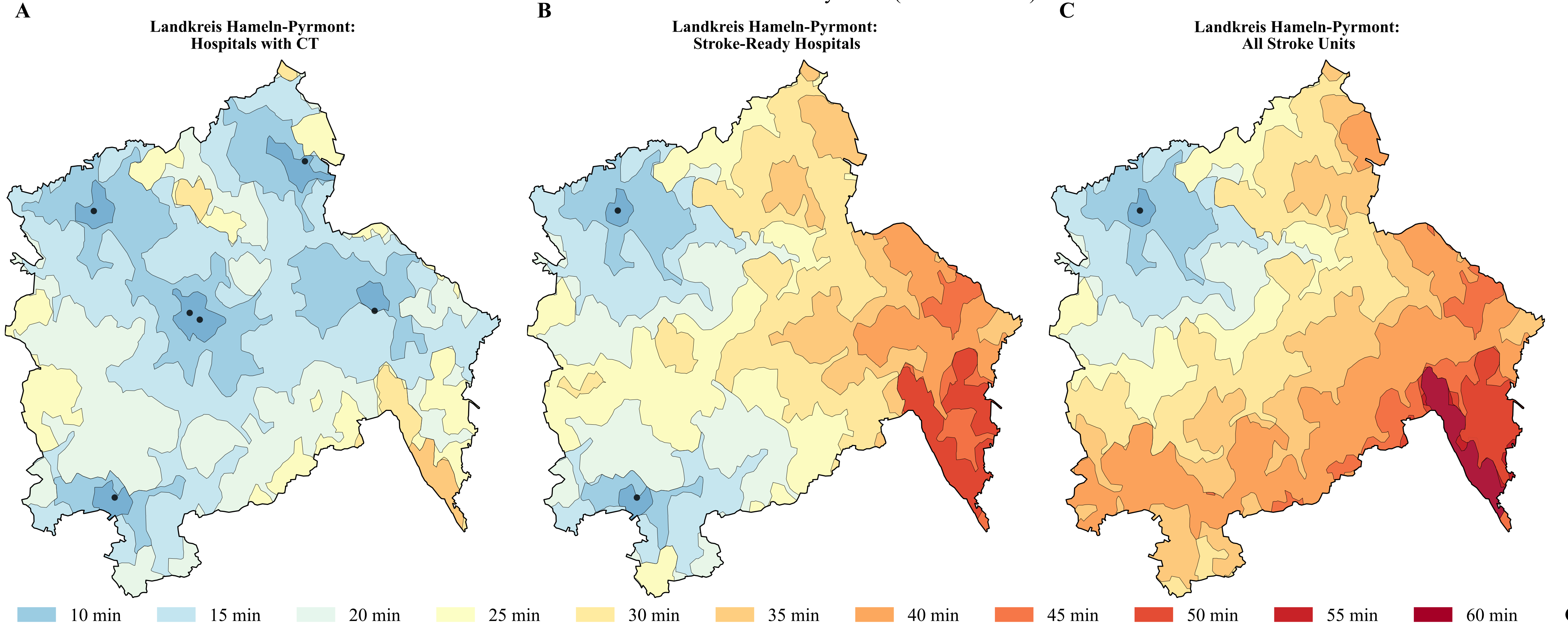

Isochrones — Landkreis Celle (Niedersachsen)

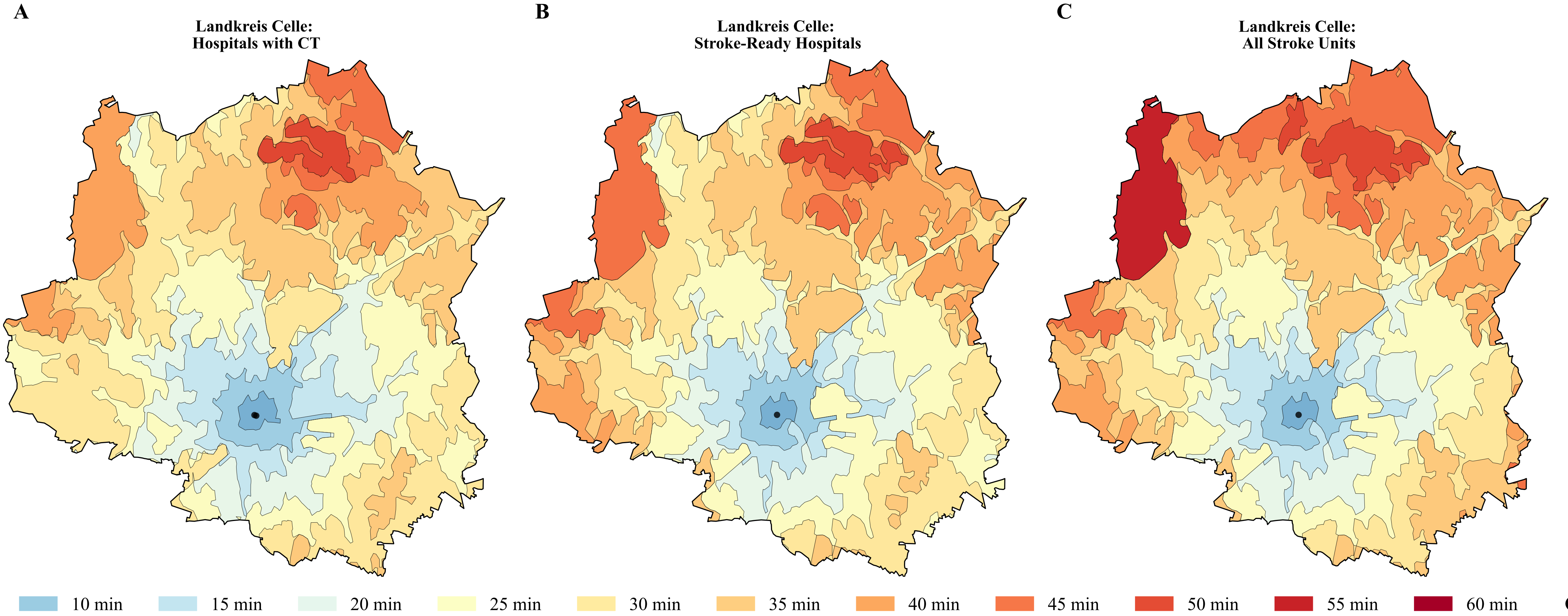

Isochrones — Landkreis Harburg (Niedersachsen)

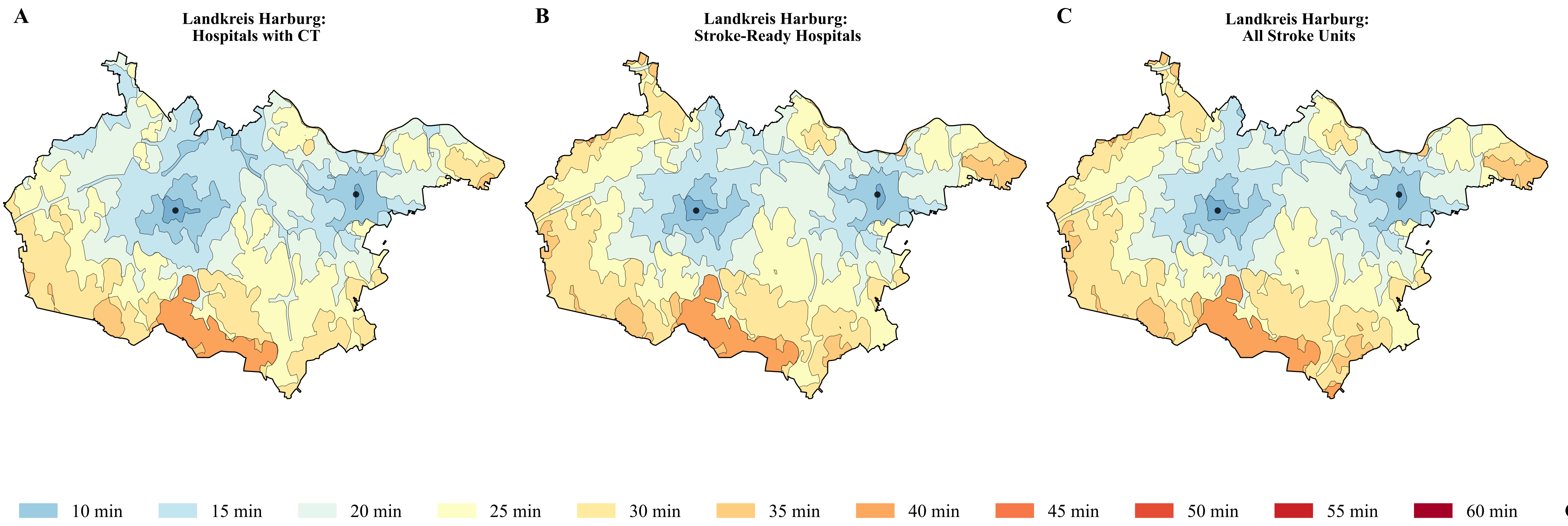

Isochrones — Kreisfreie Stadt Düsseldorf (Nordrhein-Westfalen)

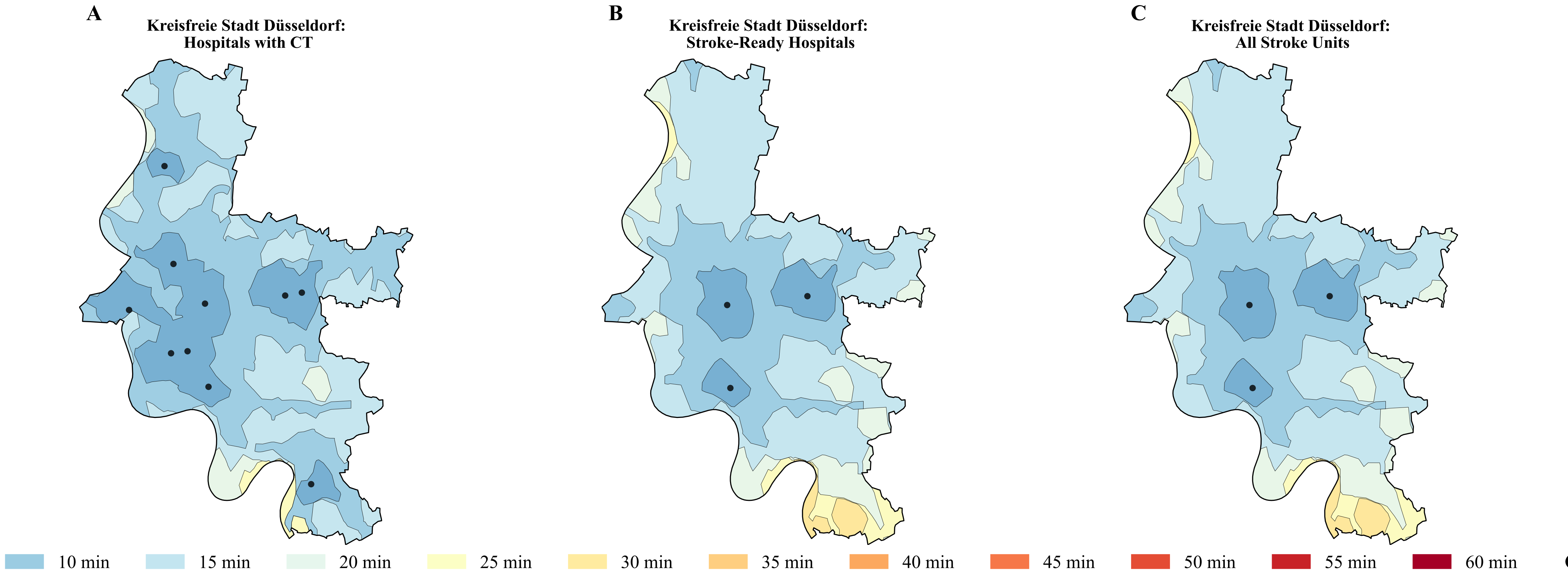

Isochrones — Kreis Düren (Nordrhein-Westfalen)

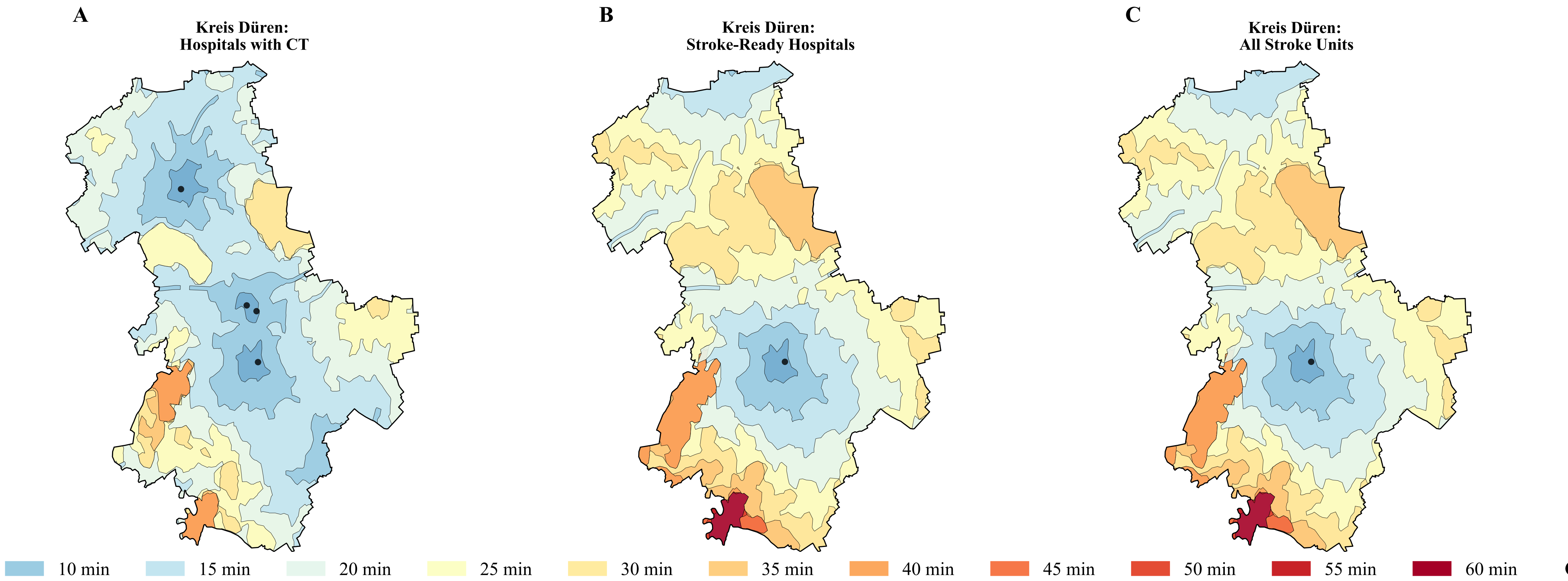

Isochrones — Kreis Soest (Nordrhein-Westfalen)

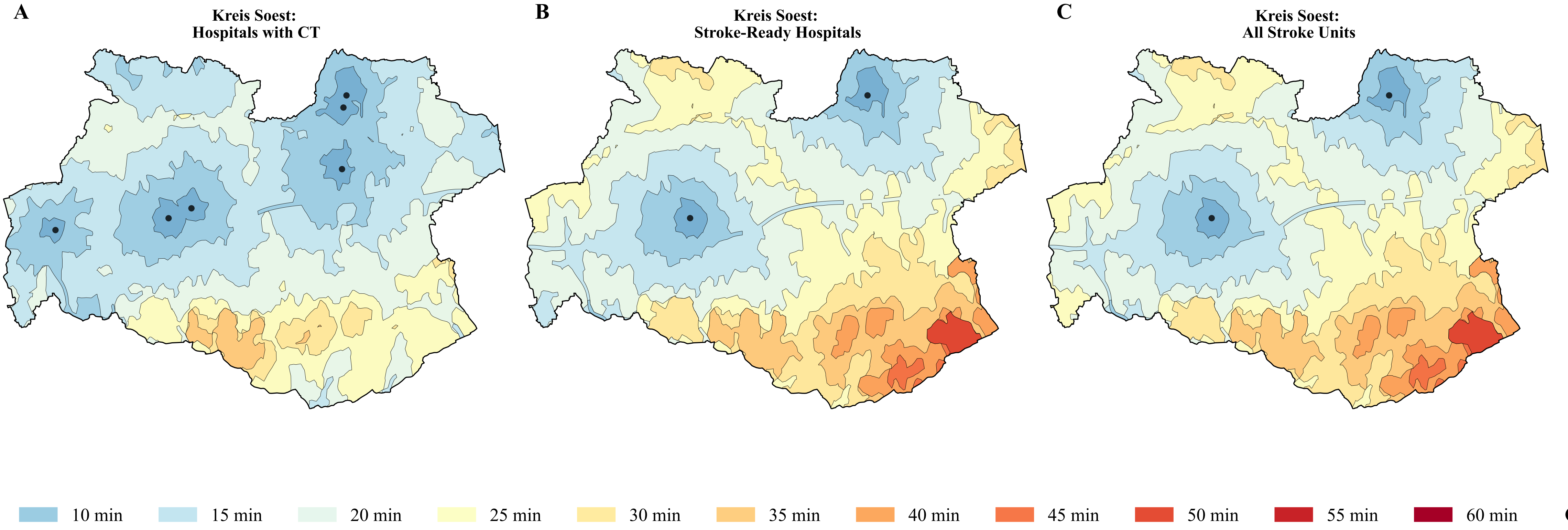

Isochrones — Kreisfreie Stadt Darmstadt (Hessen)

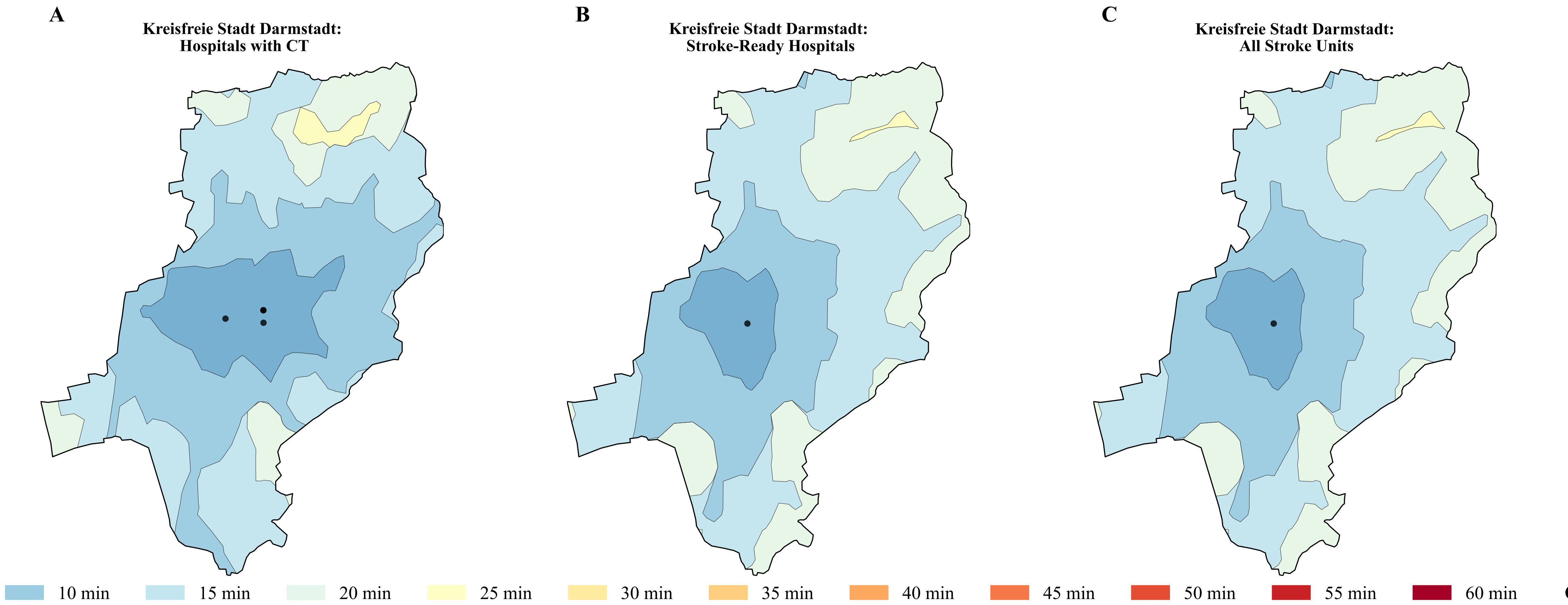

Isochrones — Kreisfreie Stadt Offenbach am Main (Hessen)

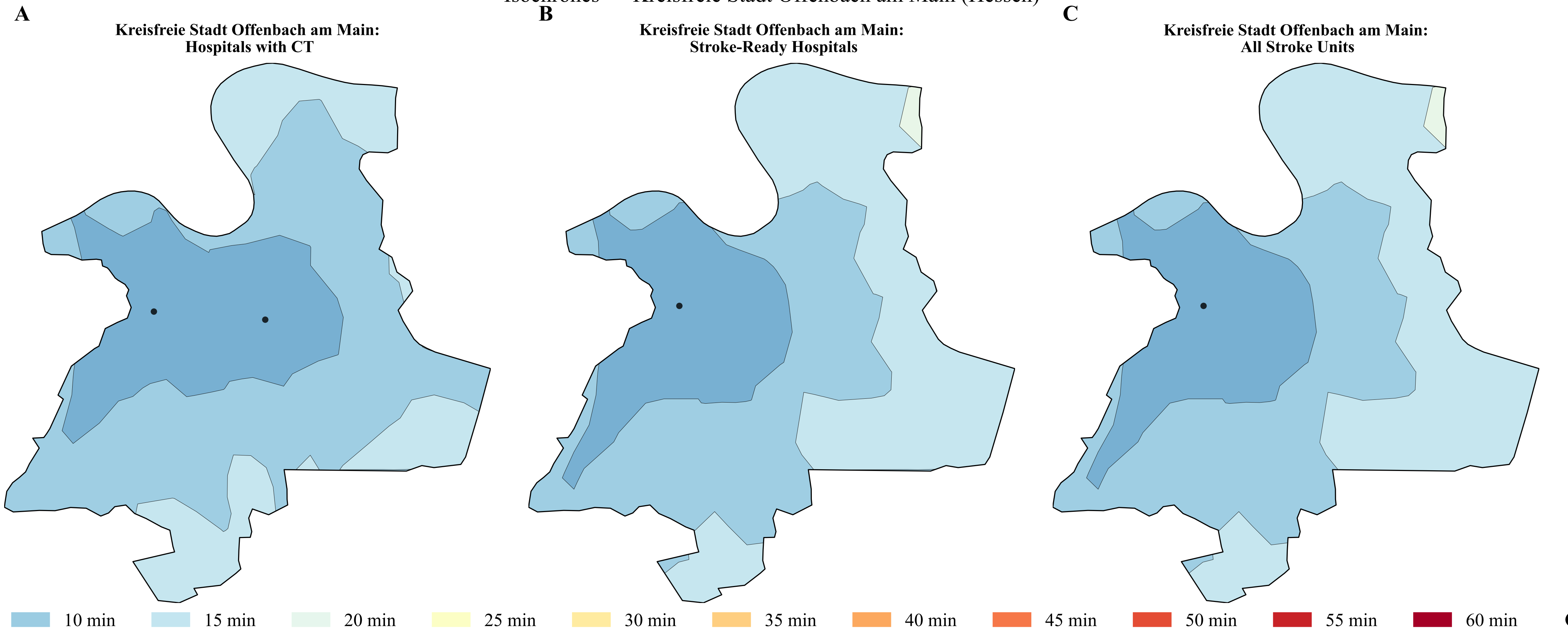

Isochrones — Landkreis Werra-Meißner-Kreis (Hessen)

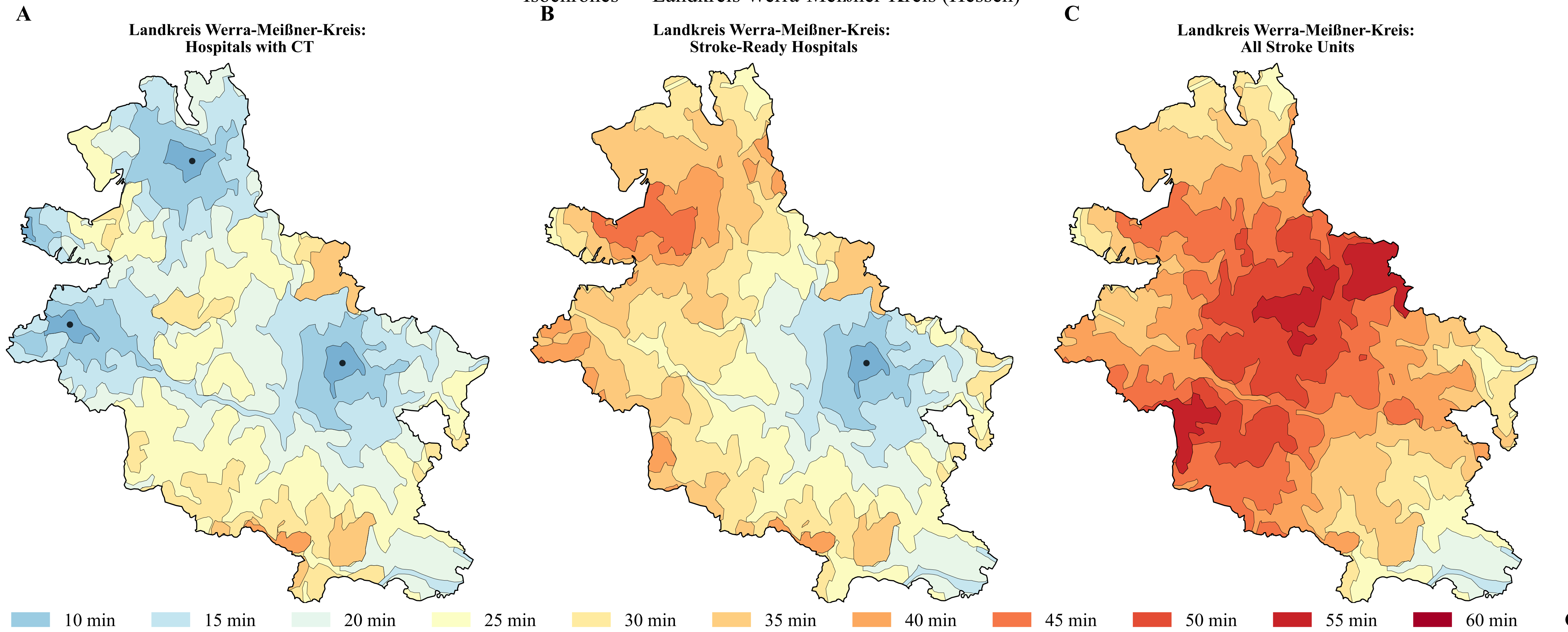

Isochrones — Landkreis Mayen-Koblenz (Rheinland-Pfalz)

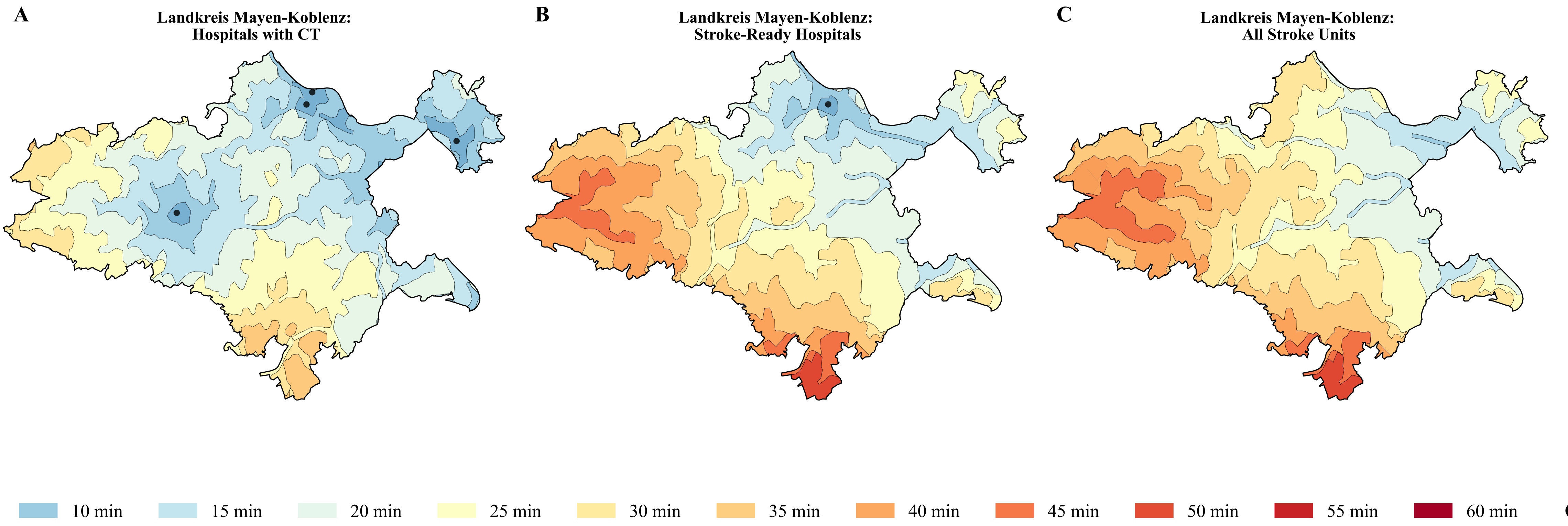

Isochrones — Landkreis Bad Dürkheim (Rheinland-Pfalz)

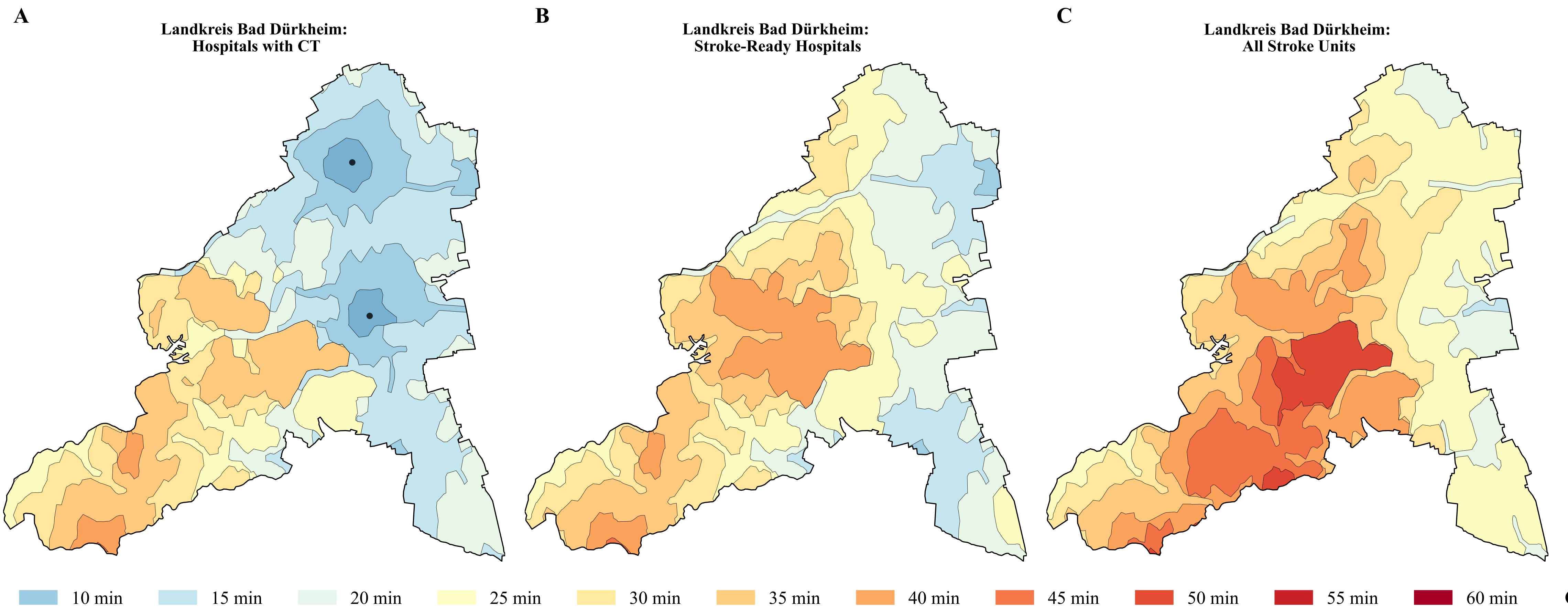

Isochrones — Landkreis Landsberg am Lech (Bayern)

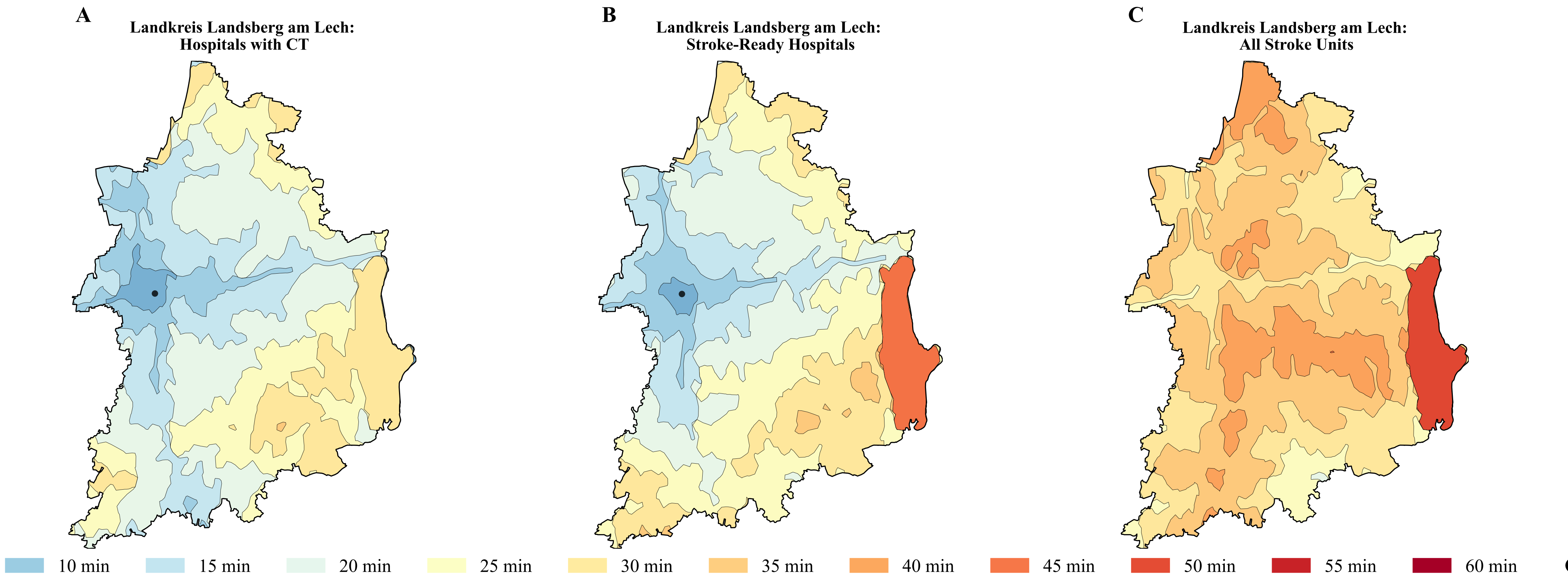

Isochrones — Landkreis Donau-Ries (Bayern)

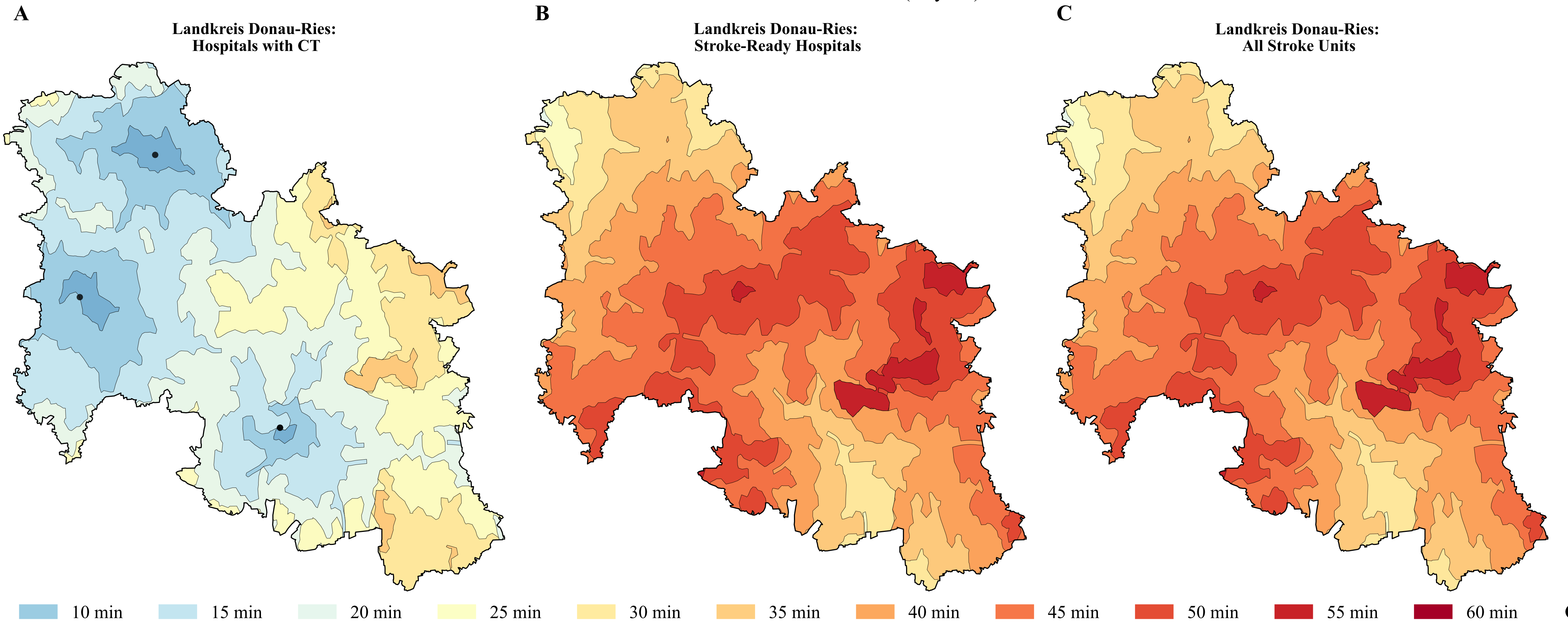

Isochrones — Landkreis Märkisch-Oderland (Brandenburg)

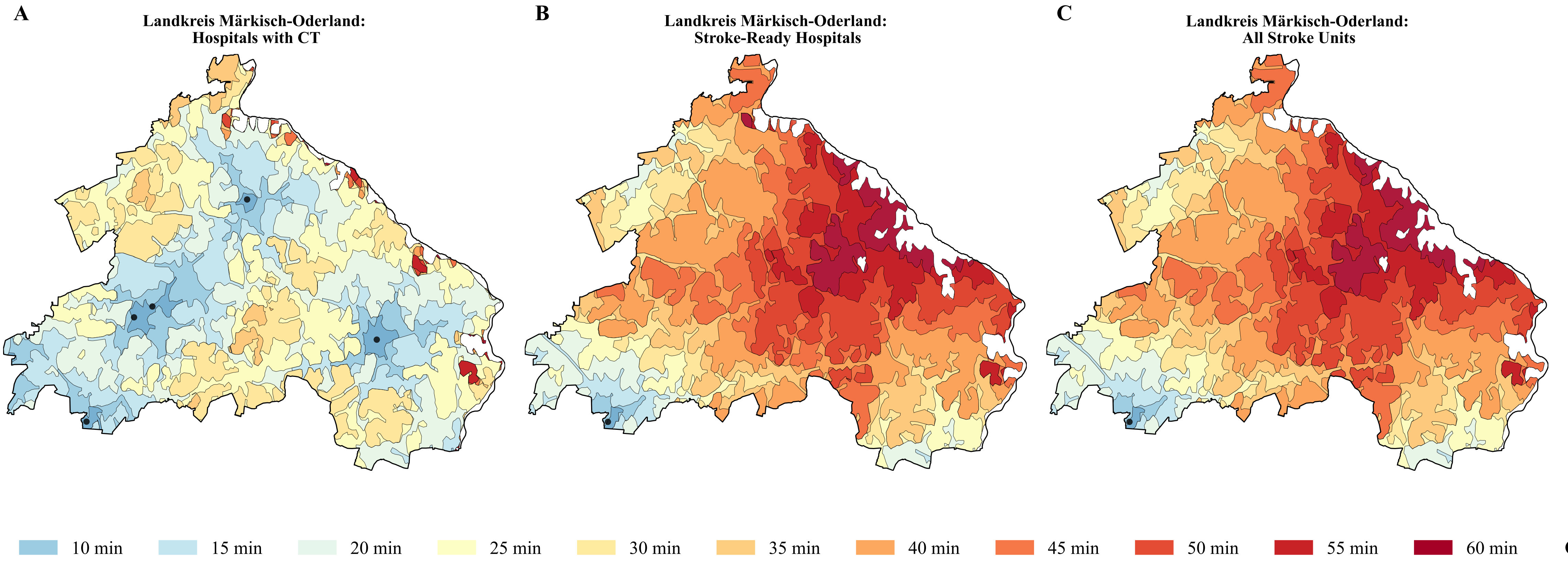

Isochrones — Landkreis Prignitz (Brandenburg)

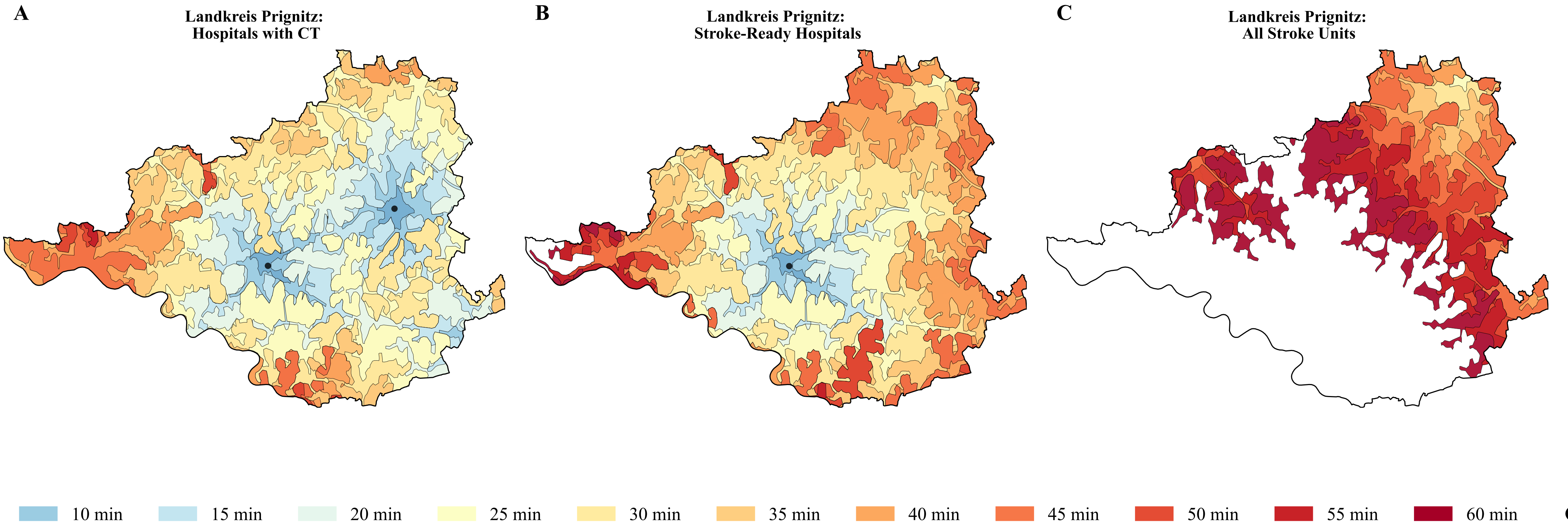

Isochrones — Landkreis Burgenlandkreis (Sachsen-Anhalt)

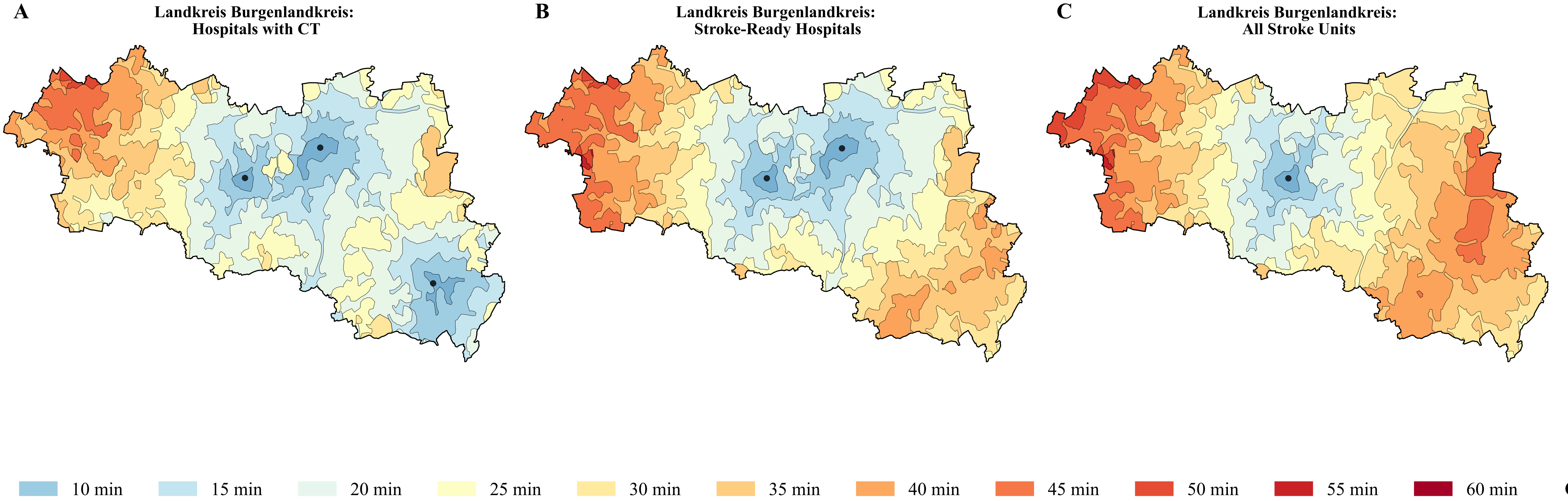

Isochrones — Landkreis Stendal (Sachsen-Anhalt)

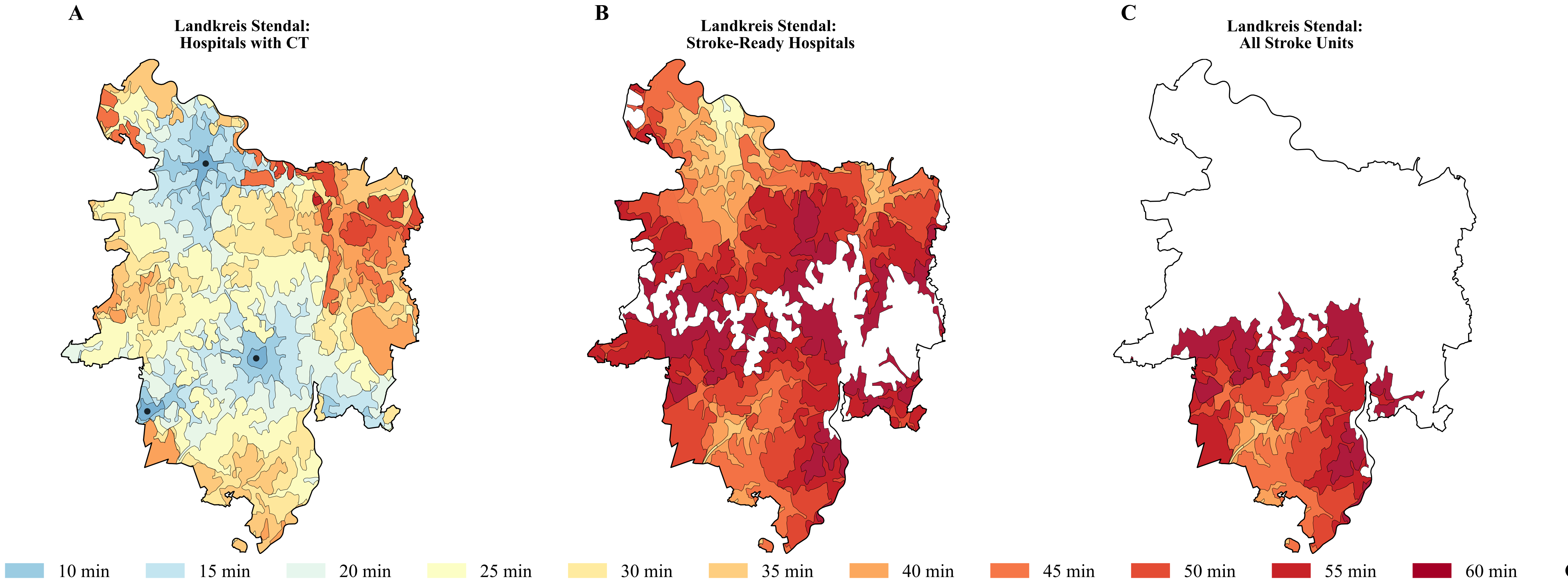

Isochrones — Landkreis Weimarer Land (Thüringen)

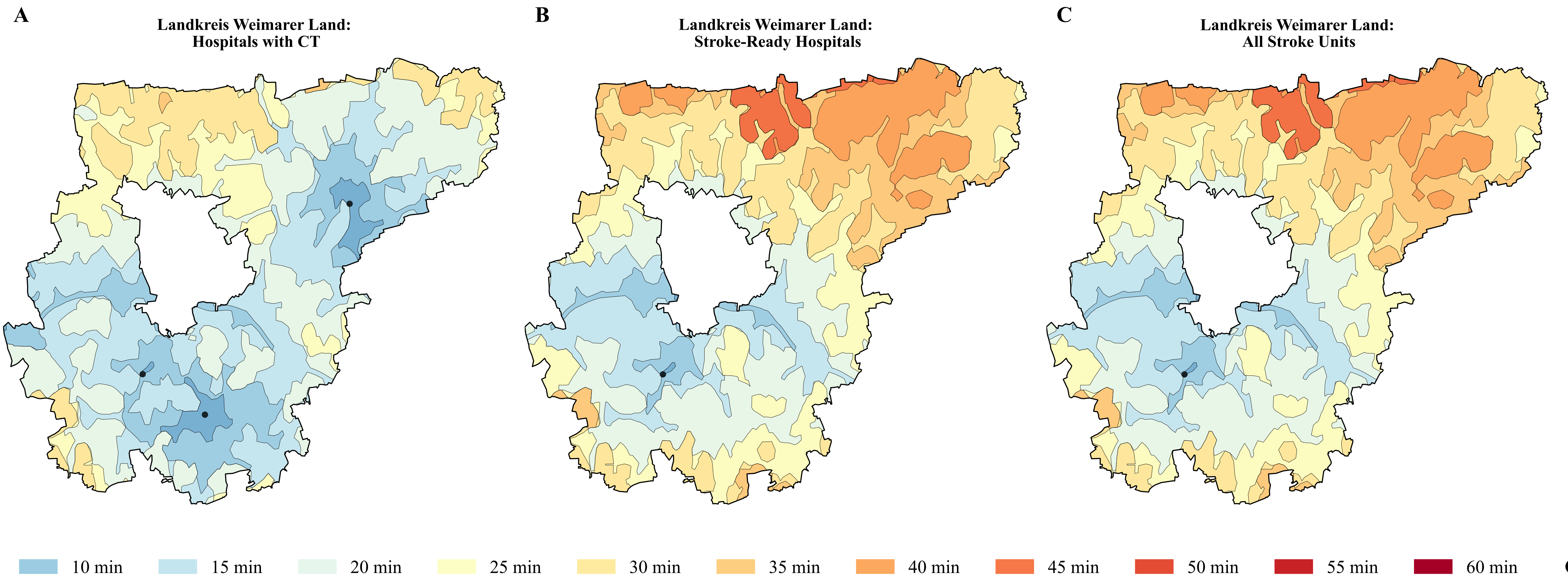

Isochrones — Kreisfreie Stadt Kiel (Schleswig-Holstein)

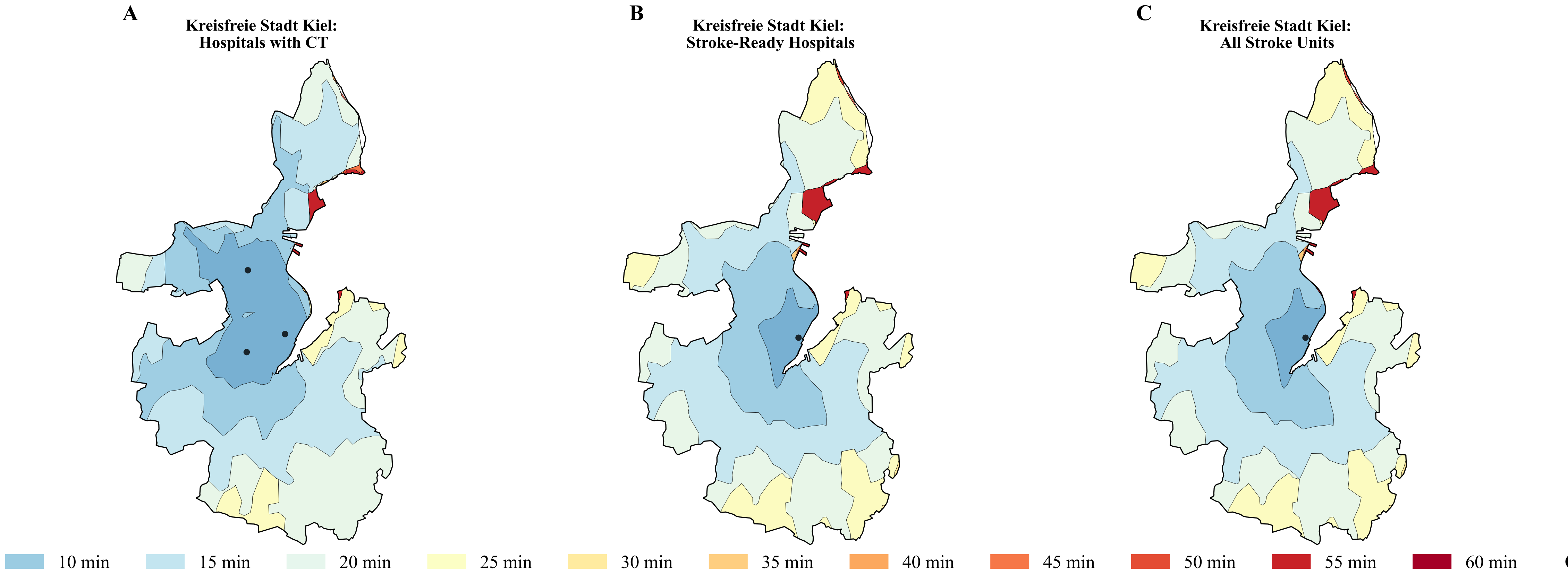

Isochrones — Landkreis Gifhorn (Niedersachsen)

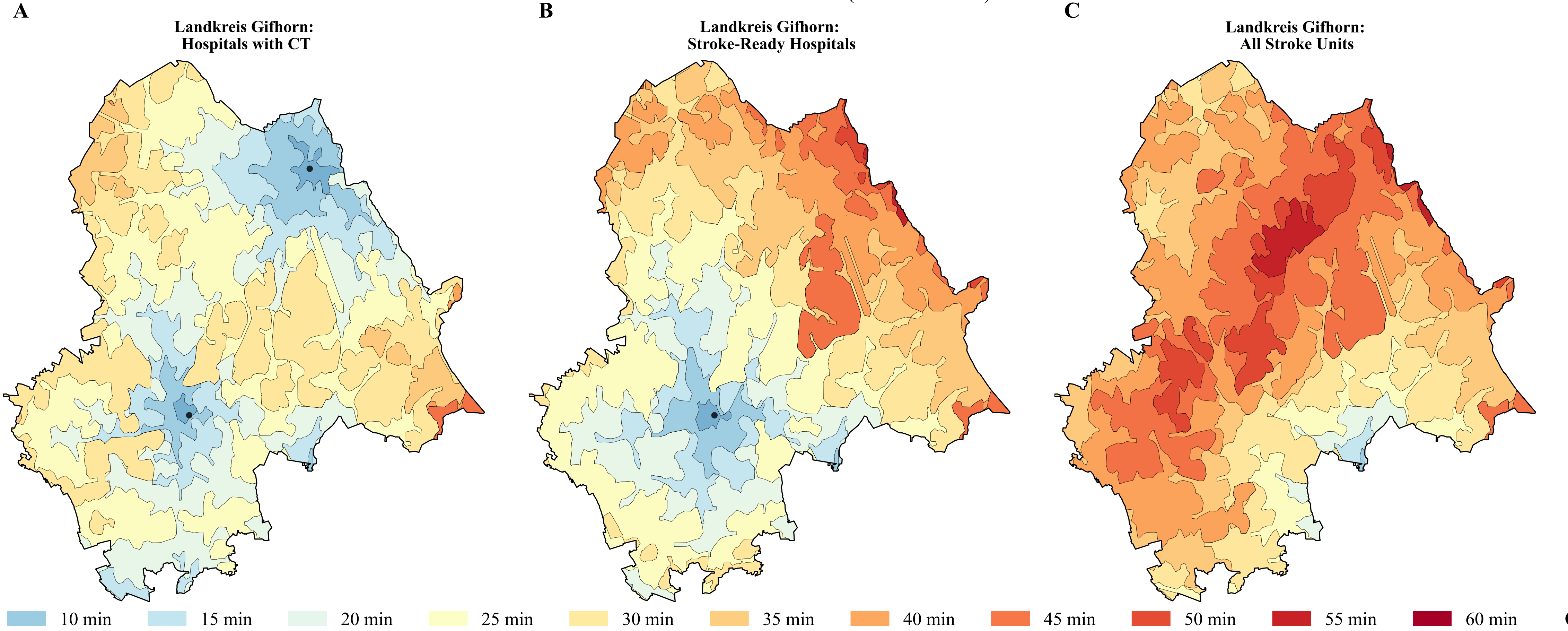

Isochrones — Landkreis Helmstedt (Niedersachsen)

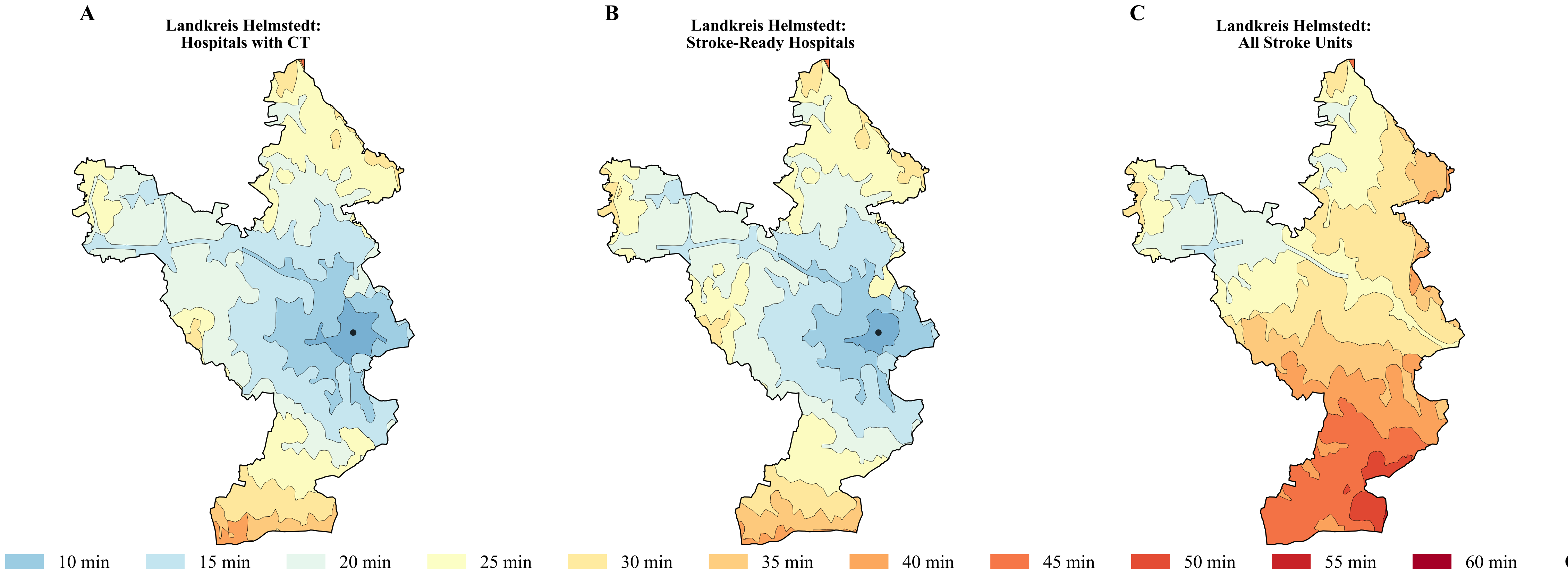

Isochrones — Landkreis Lüchow-Dannenberg (Niedersachsen)

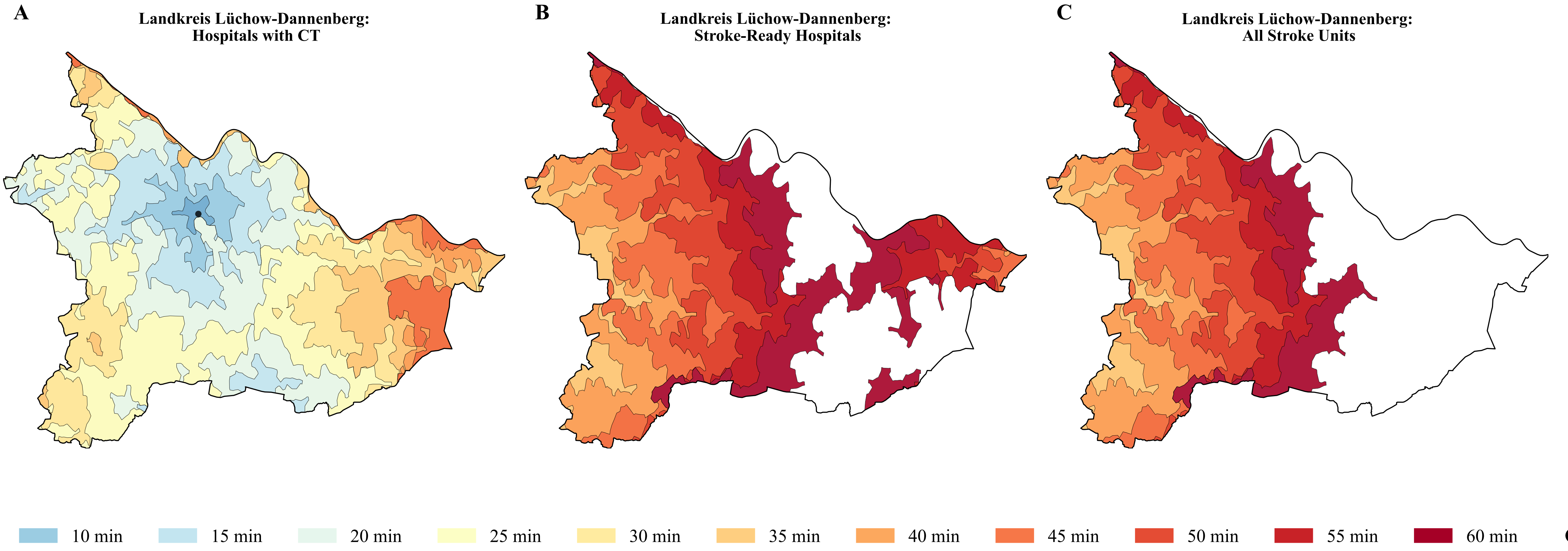

Isochrones — Landkreis Rotenburg (Wümme) (Niedersachsen)

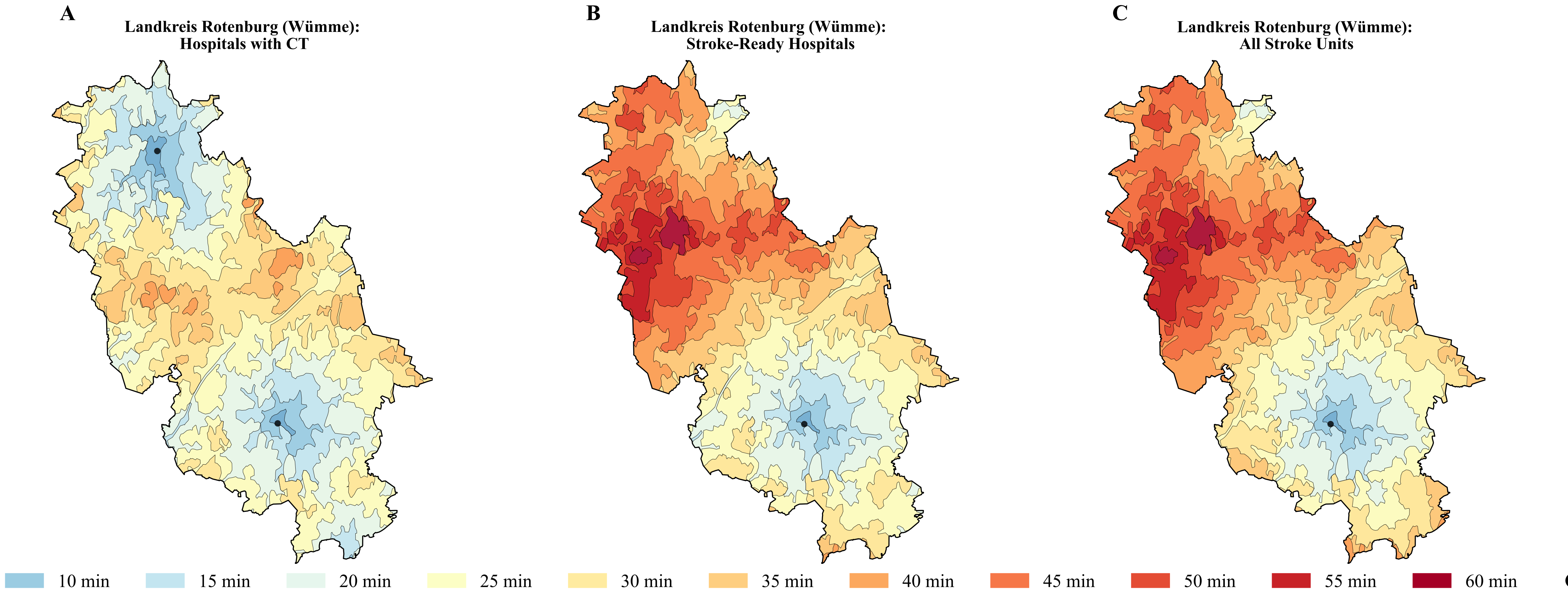

Isochrones — Kreis Euskirchen (Nordrhein-Westfalen)

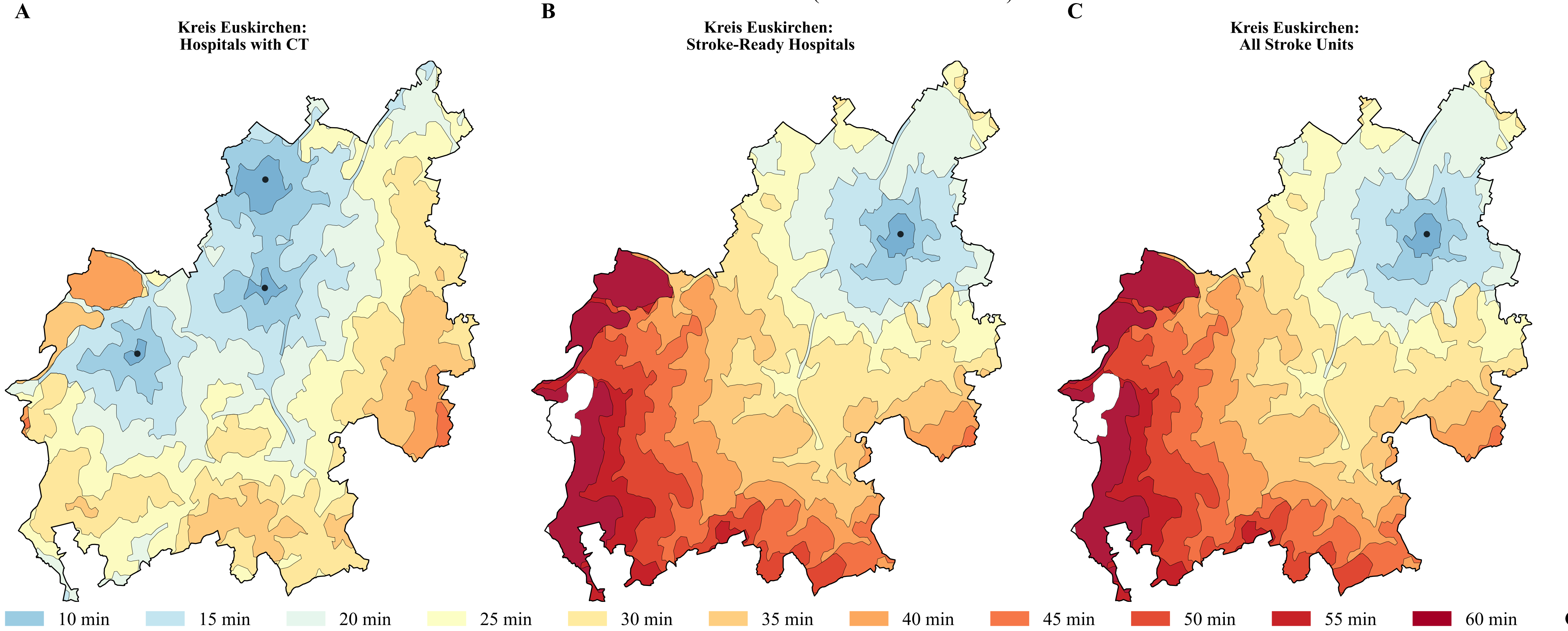

Isochrones — Kreis Olpe (Nordrhein-Westfalen)

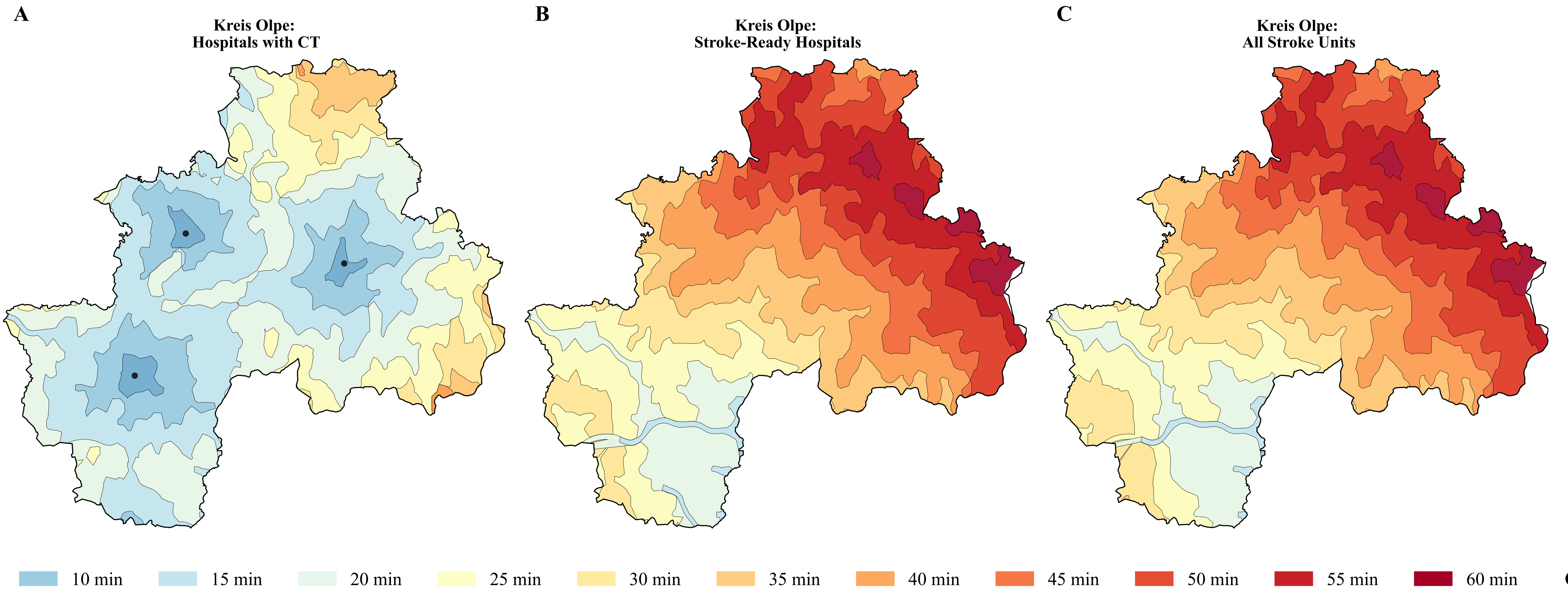

Isochrones — Landkreis Lahn-Dill-Kreis (Hessen)

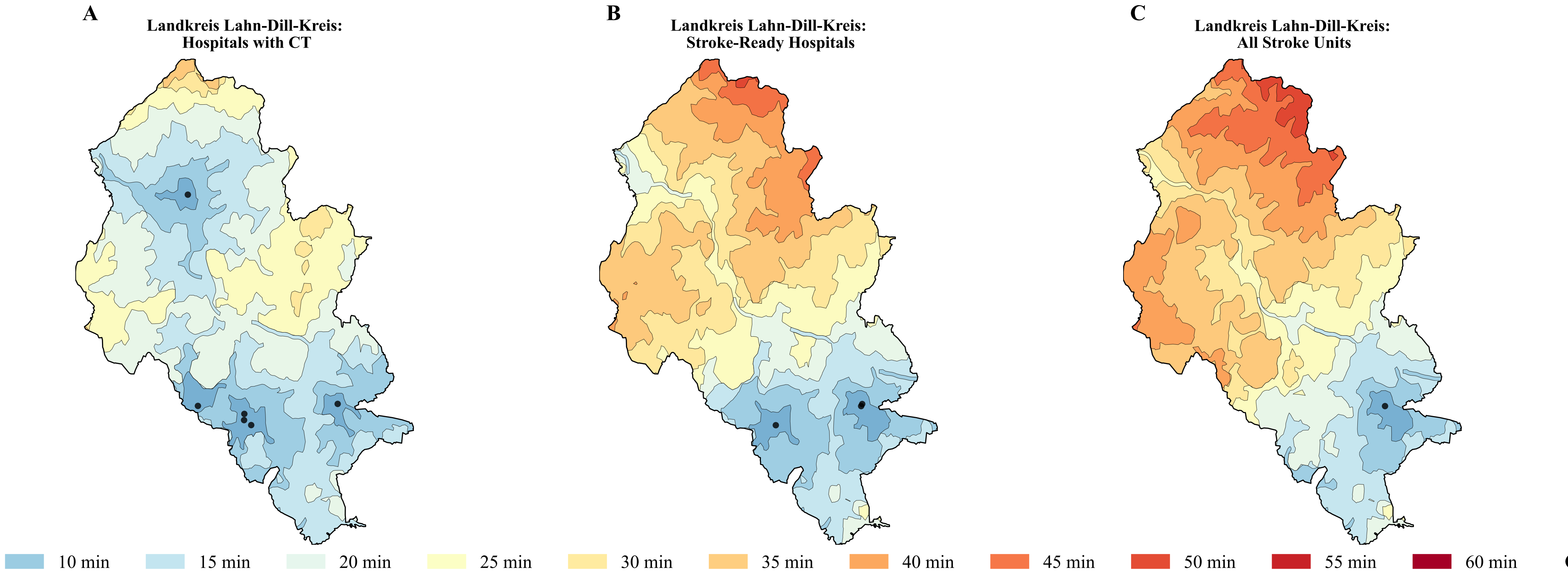

Isochrones — Landkreis Ravensburg (Baden-Württemberg)

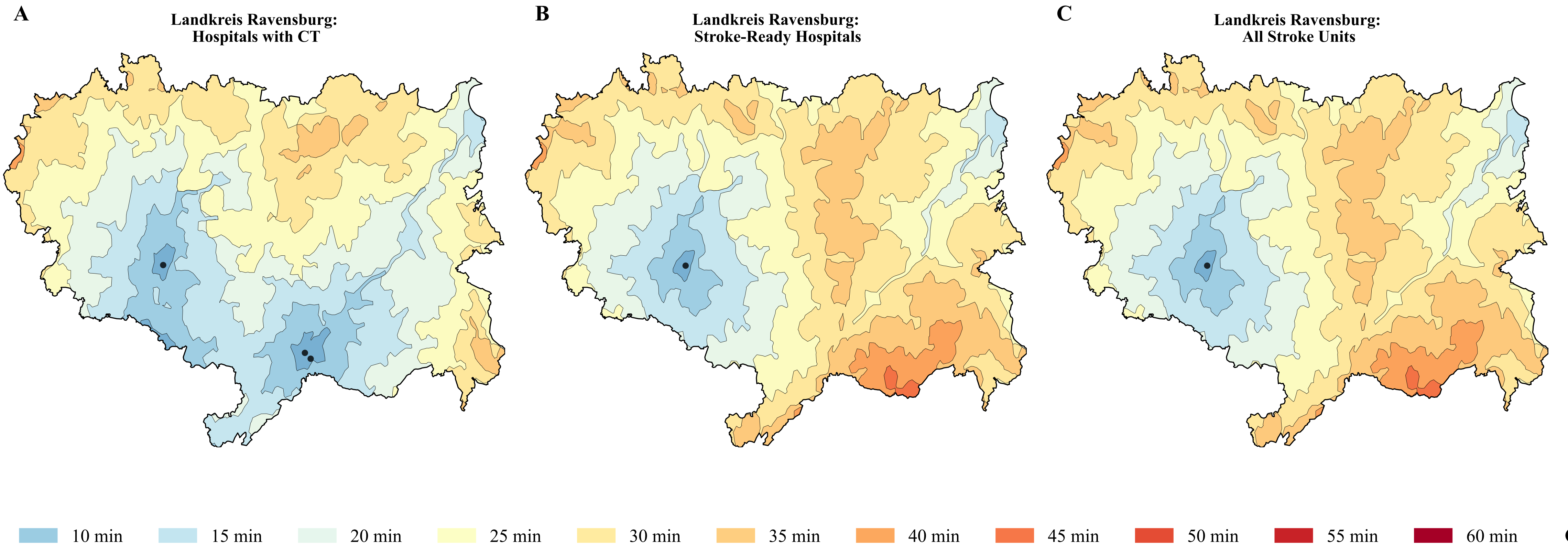

Isochrones — Kreisfreie Stadt Rosenheim (Bayern)

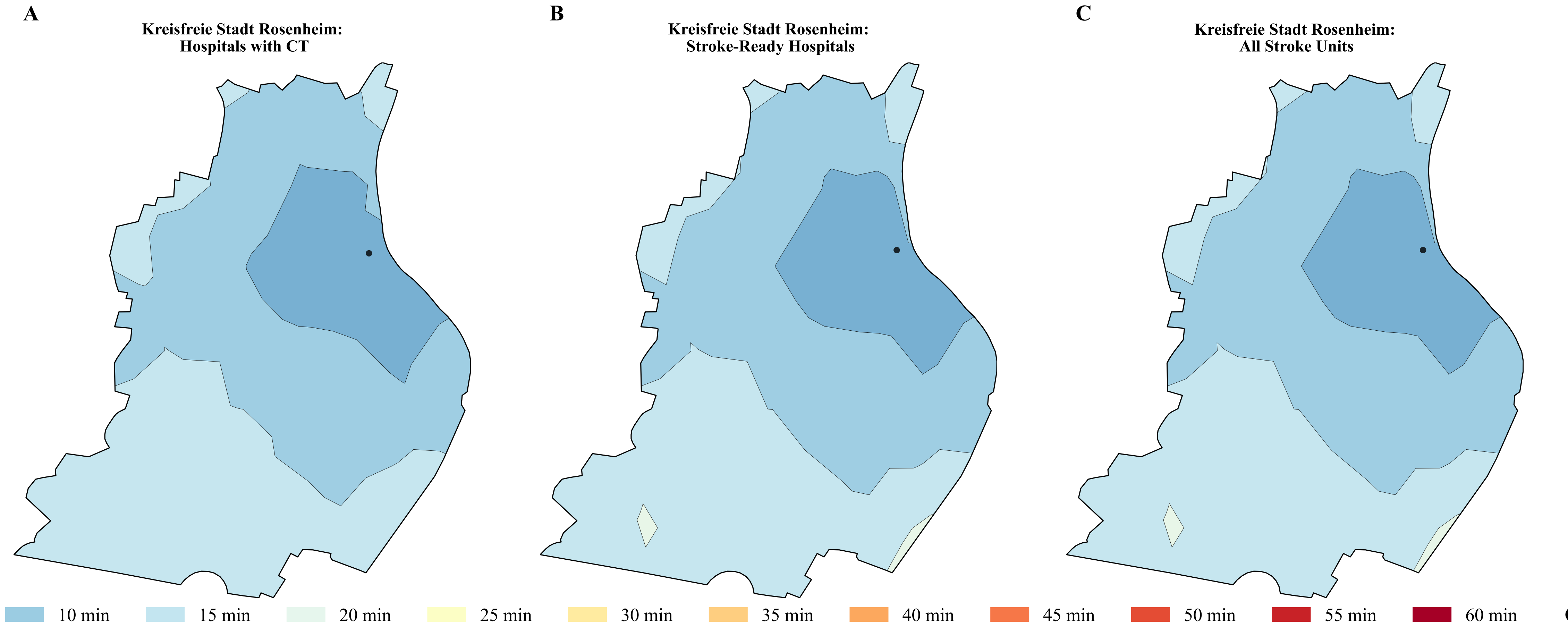

Isochrones — Landkreis Freising (Bayern)

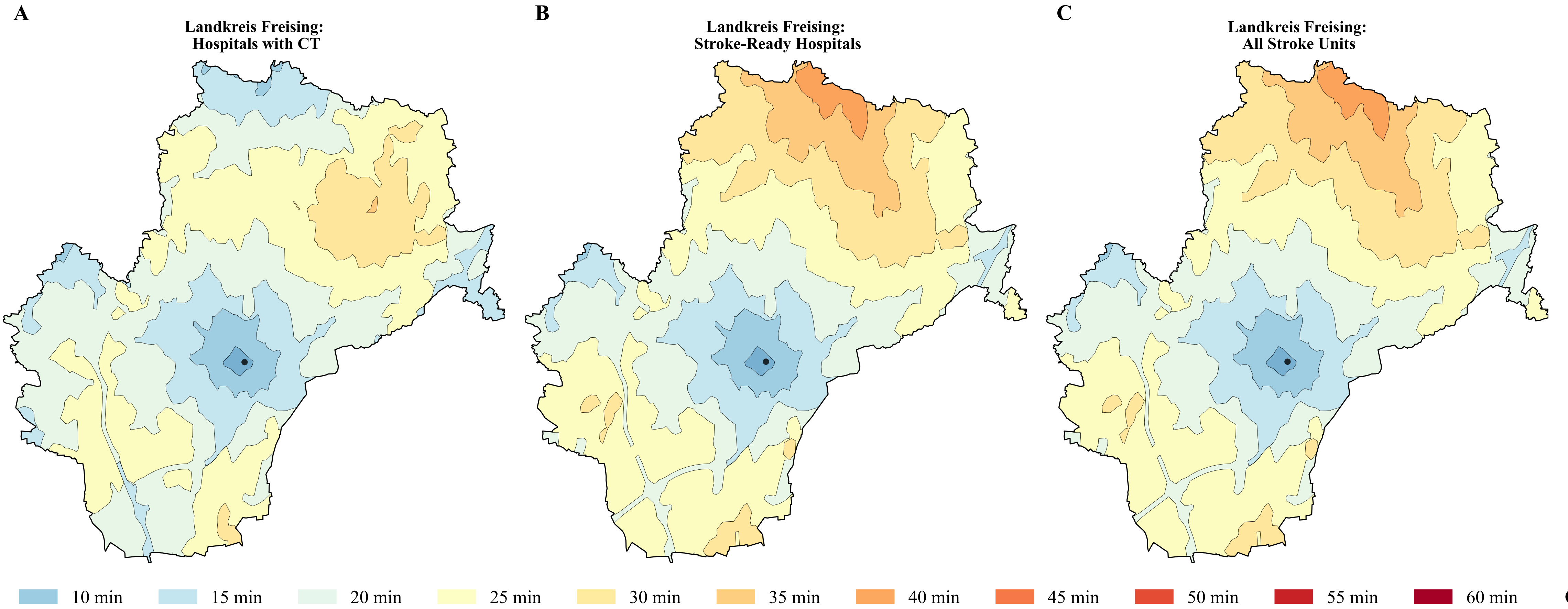

Isochrones — Landkreis Garmisch-Partenkirchen (Bayern)

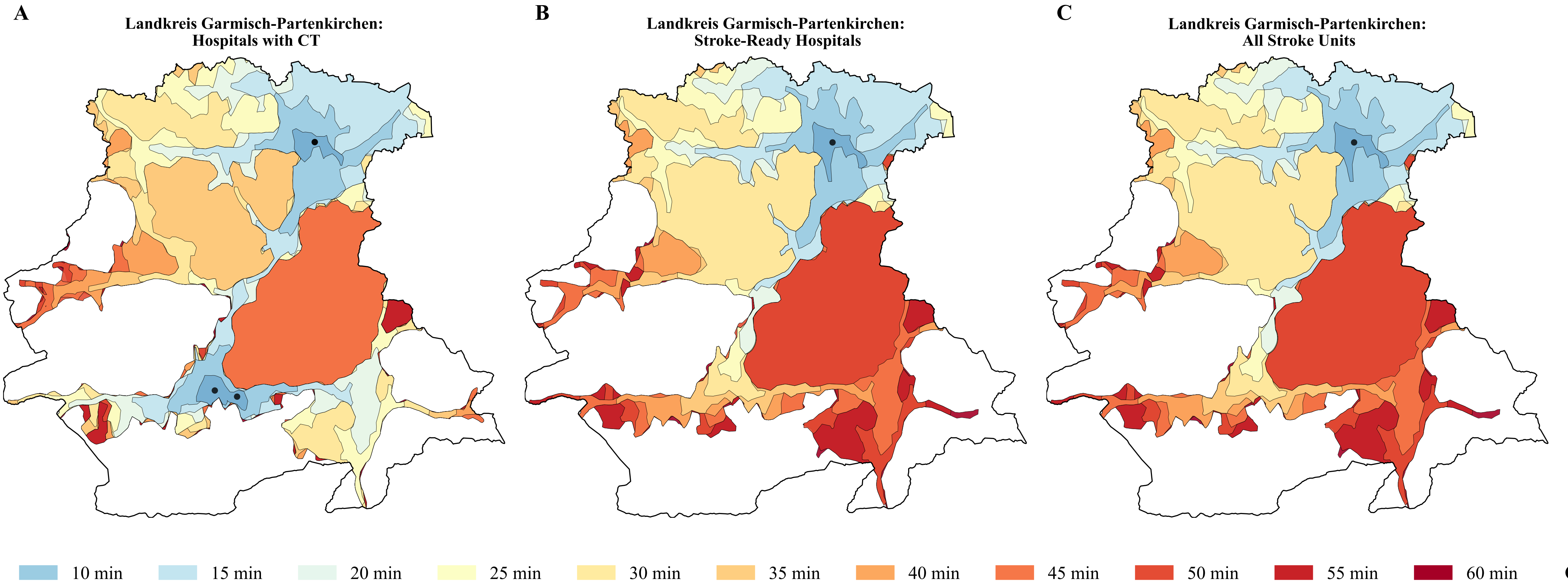

Isochrones — Landkreis Regensburg (Bayern)

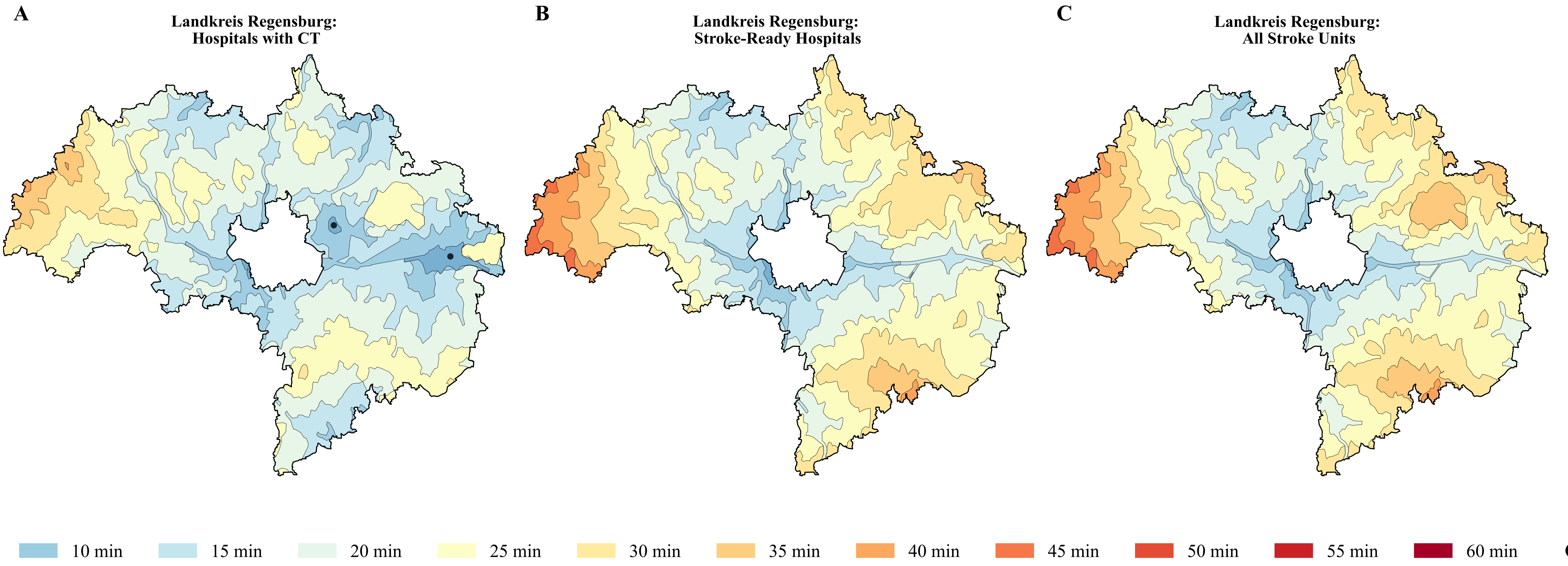

Isochrones — Landkreis Tirschenreuth (Bayern)

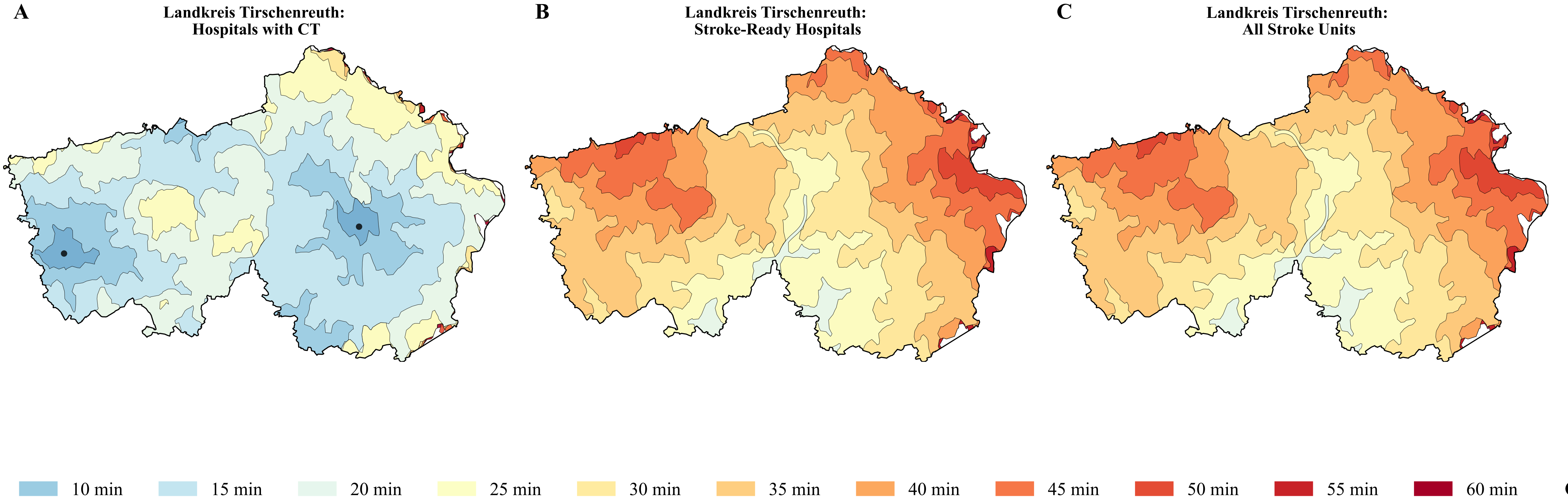

Isochrones — Kreisfreie Stadt Kaufbeuren (Bayern)

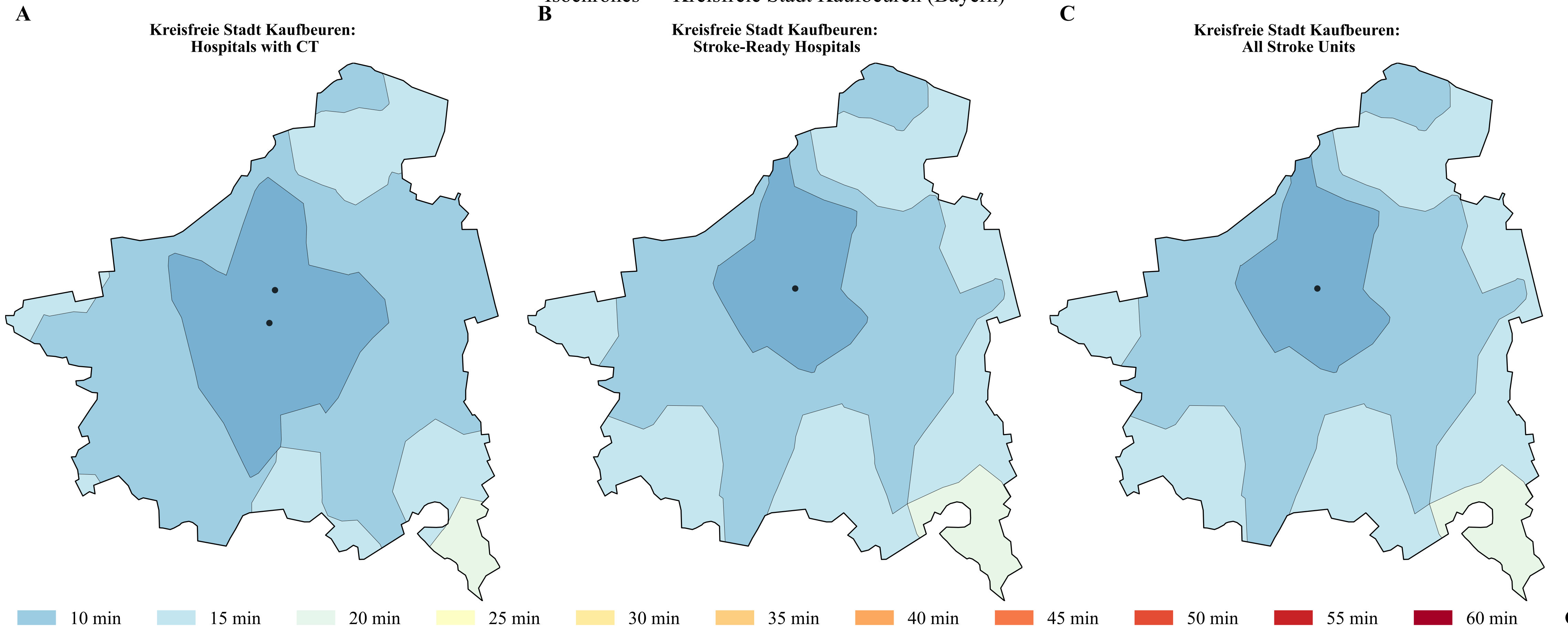

Isochrones — Landkreis Oberallgäu (Bayern)

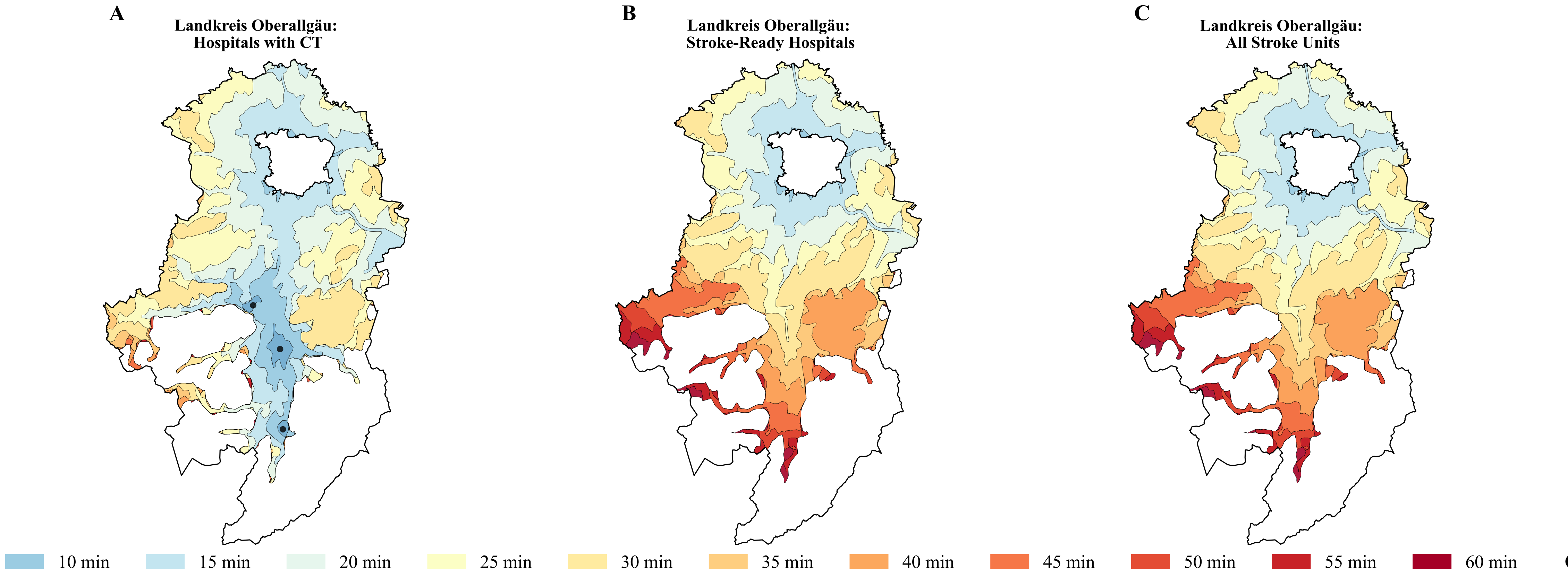

Isochrones — Landkreis Regionalverband Saarbrücken (Saarland)

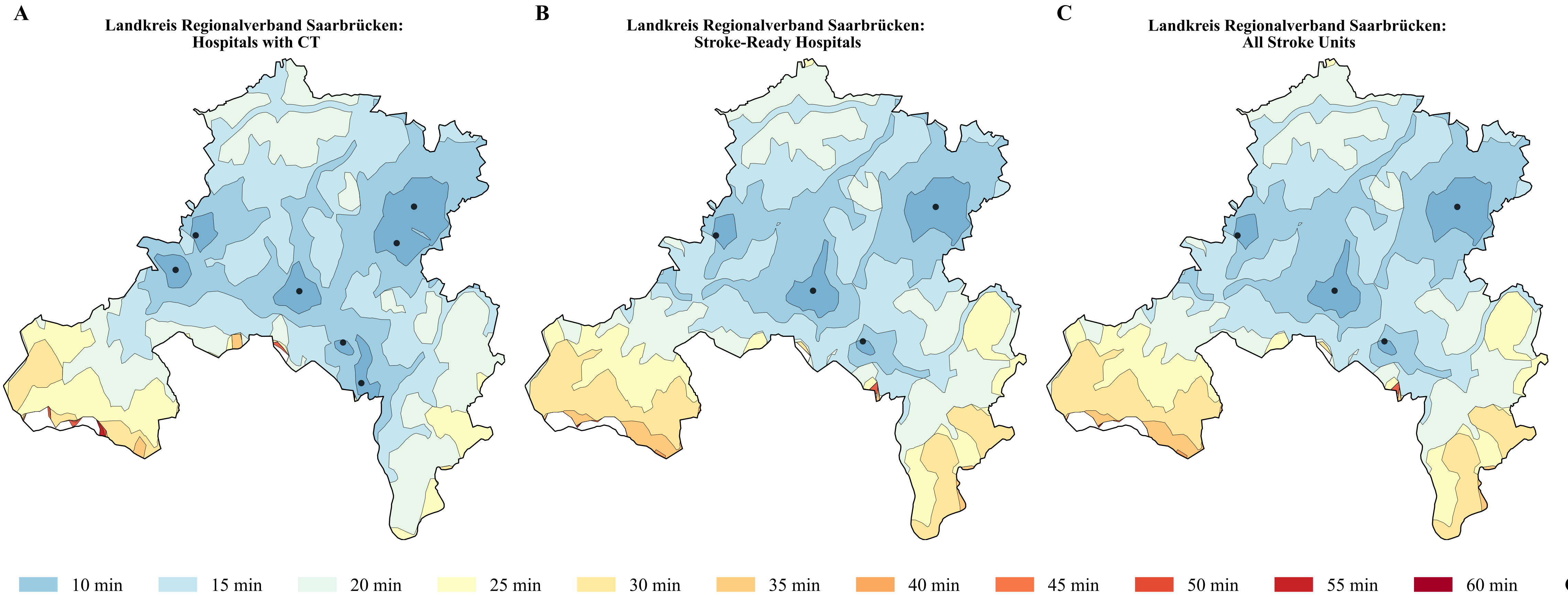

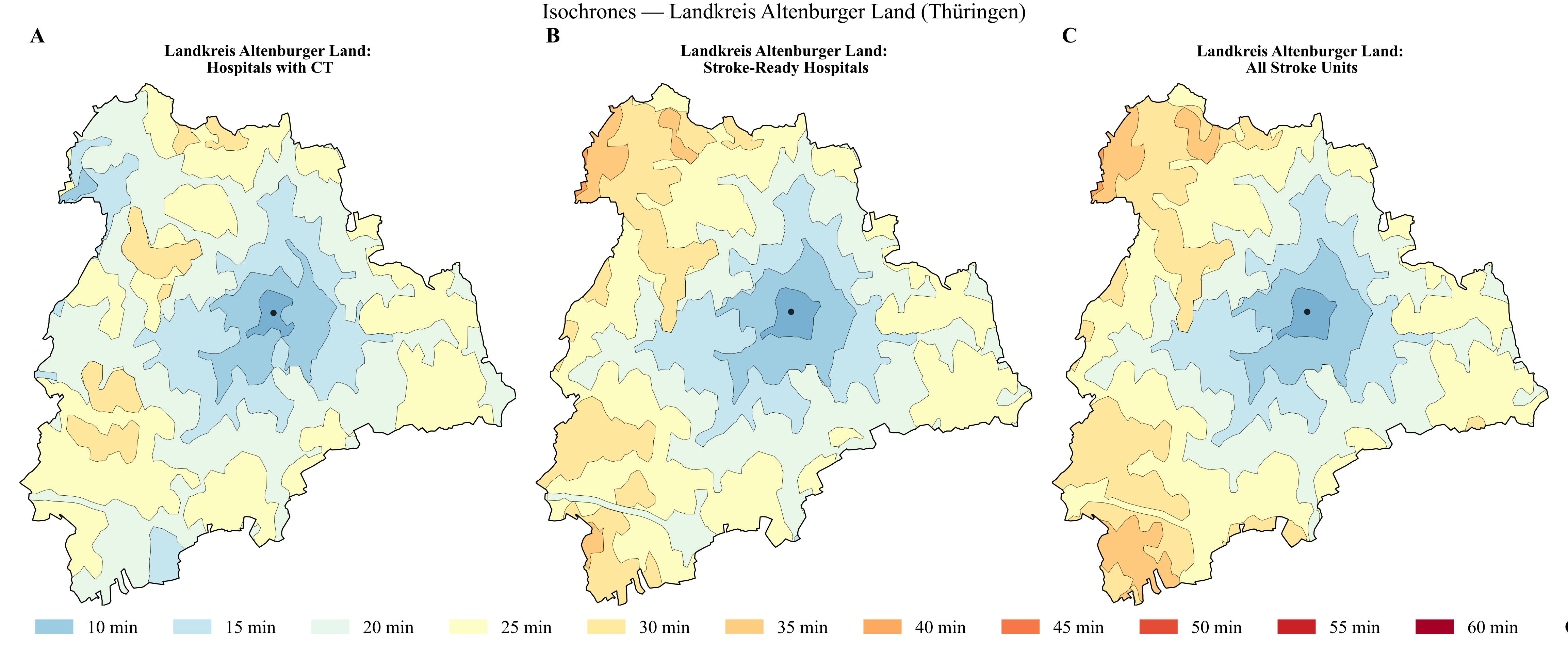

Isochrones — Landkreis Altenburger Land (Thüringen)
A
Landkreis Altenburger Land:
Hospitals with CT
B
Landkreis Altenburger Land:
Stroke-Ready Hospitals
C
Landkreis Altenburger Land:
All Stroke Units
10 min
15 min
20 min
25 min
30 min
35 min
40 min
45 min
50 min
55 min
60 min

Isochrones — Landkreis Diepholz (Niedersachsen)

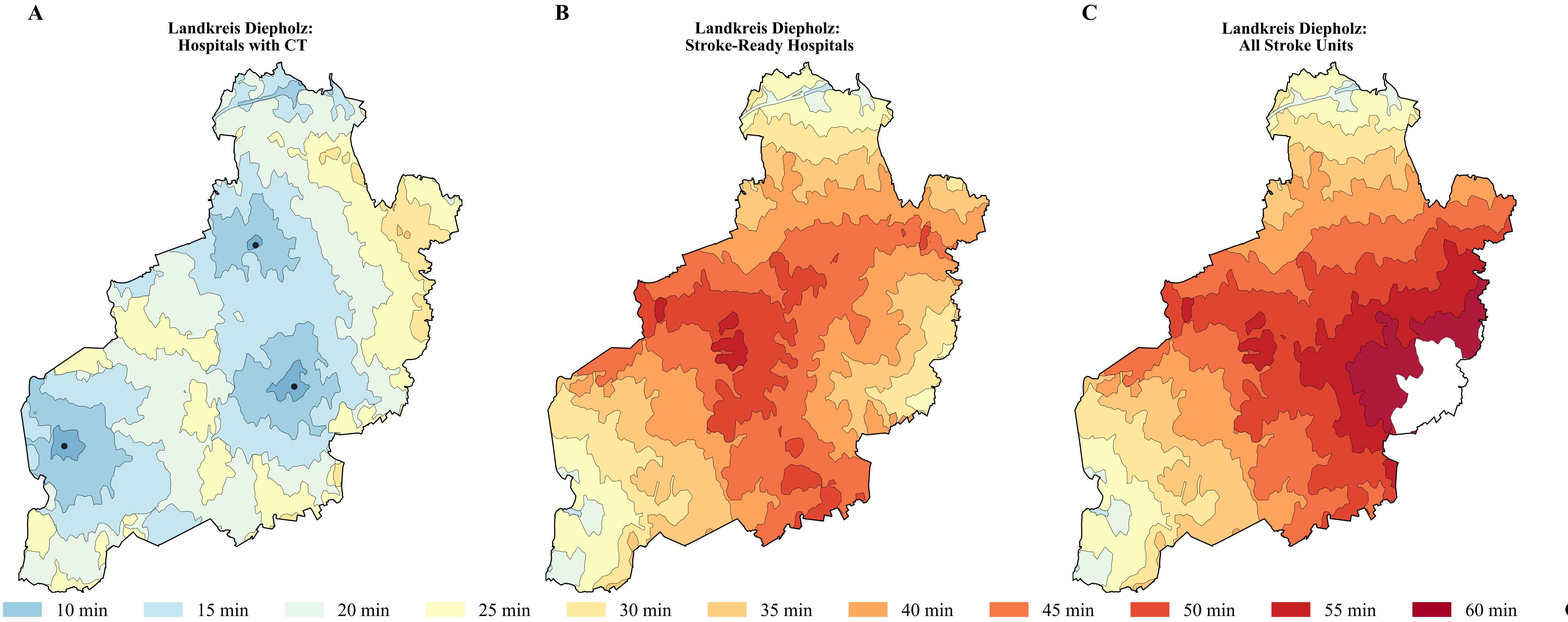

584 / 5833

Isochrones — Kreisfreie Stadt Emden (Niedersachsen)

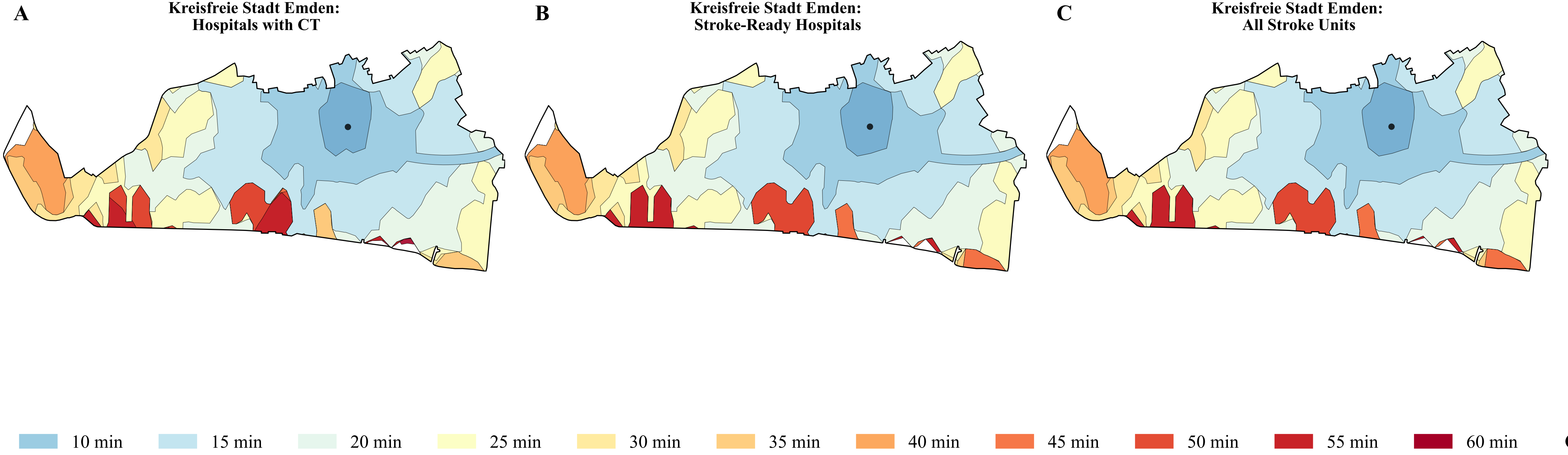

Isochrones — Kreisfreie Stadt Gelsenkirchen (Nordrhein-Westfalen)

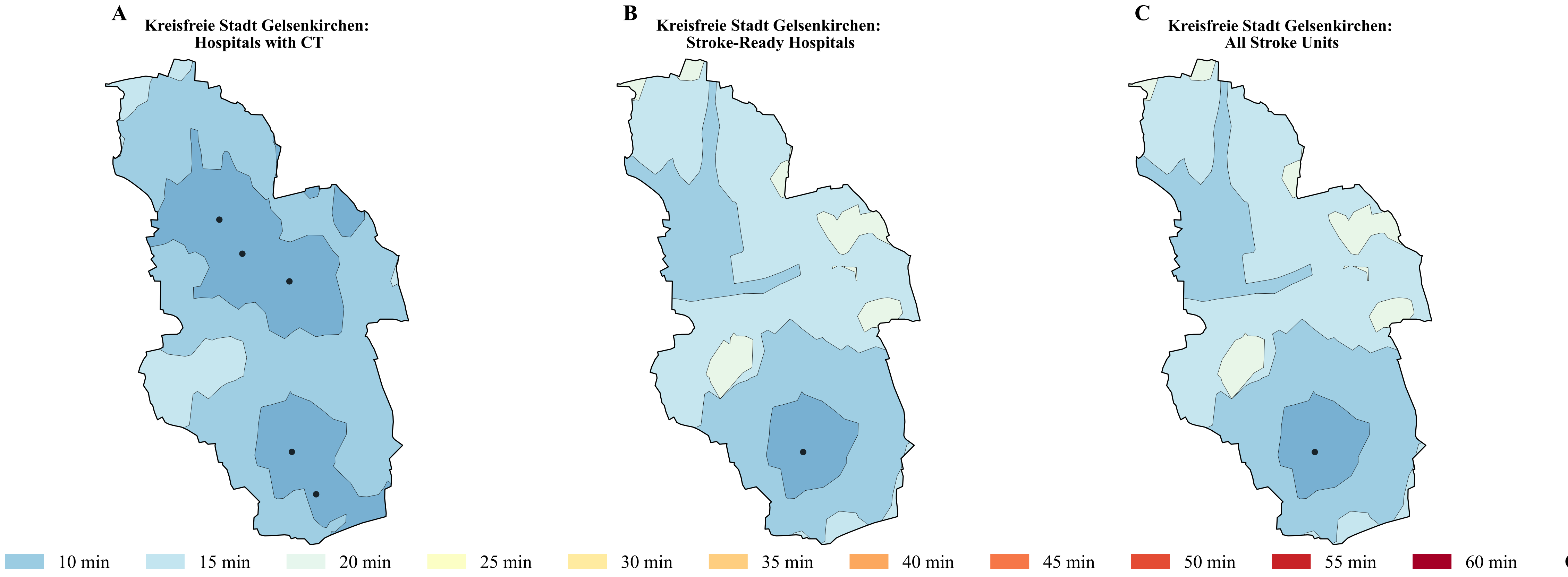

Isochrones — Kreis Warendorf (Nordrhein-Westfalen)

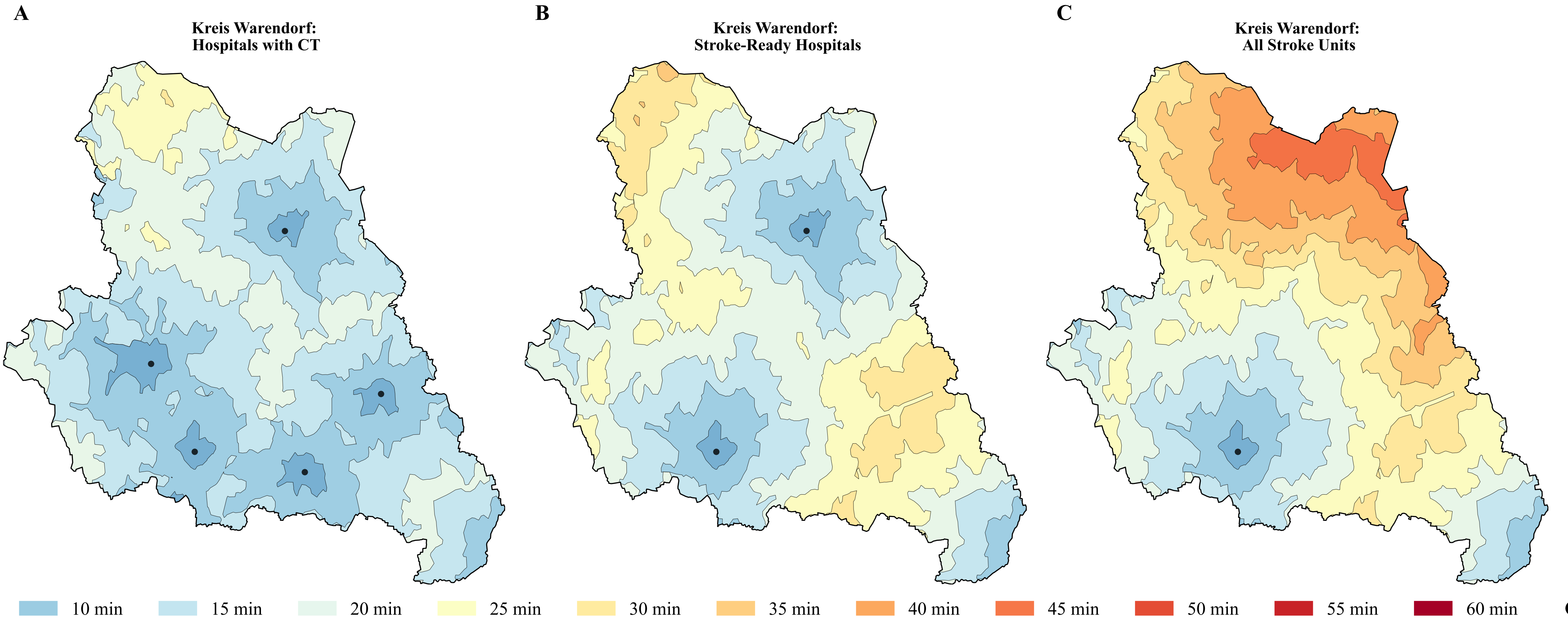

Isochrones — Stadtkreis Ulm (Baden-Württemberg)

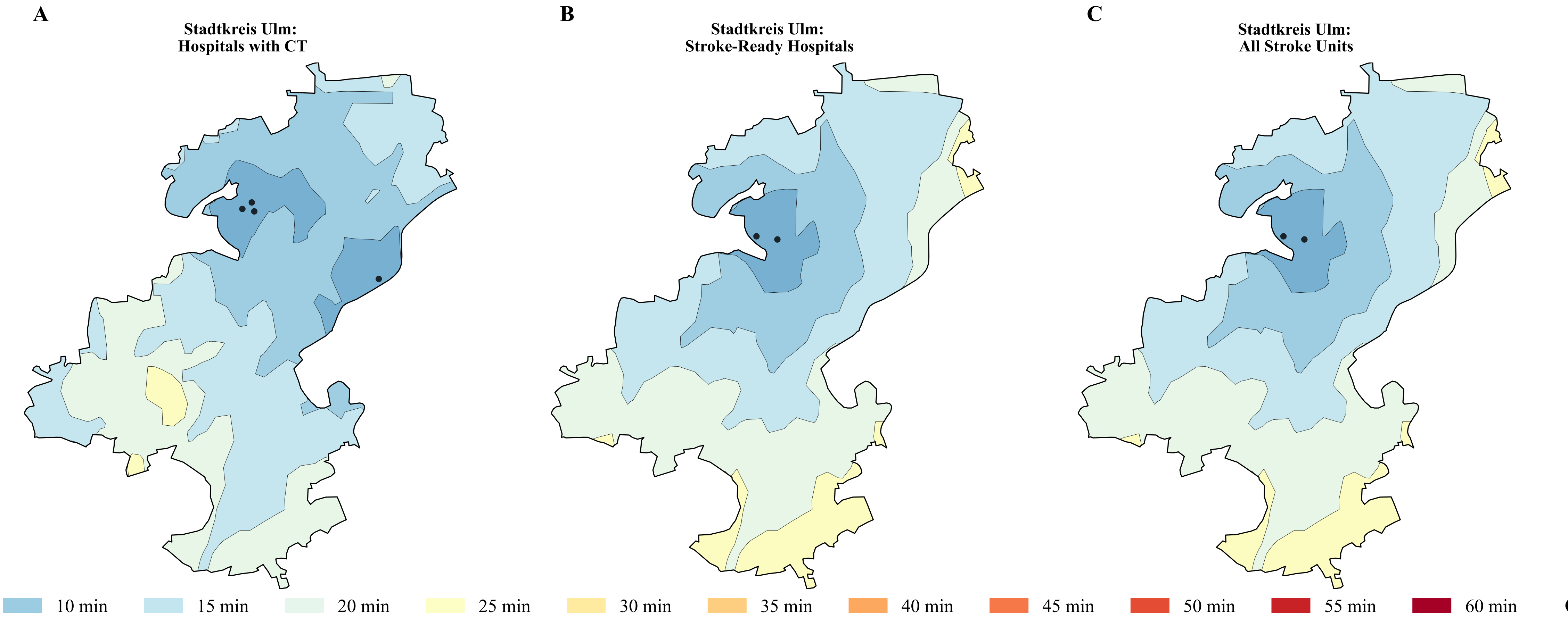

Isochrones — Landkreis Traunstein (Bayern)

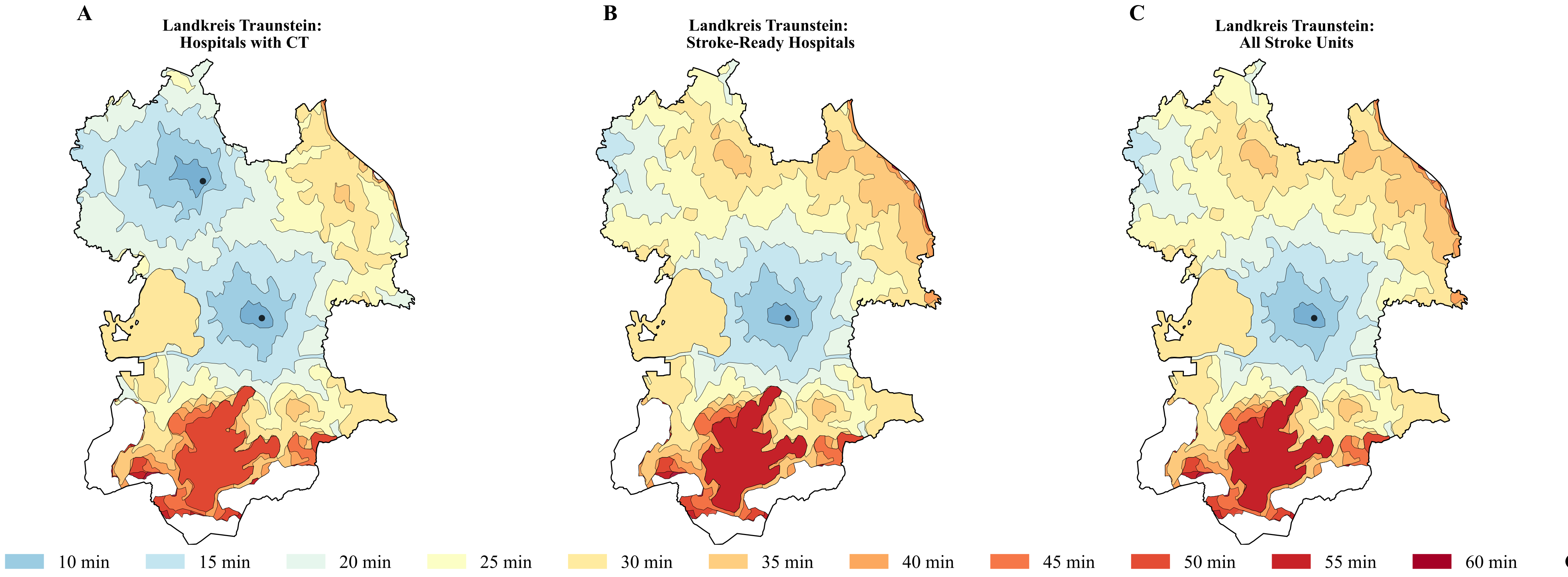

Isochrones — Kreisfreie Stadt Regensburg (Bayern)

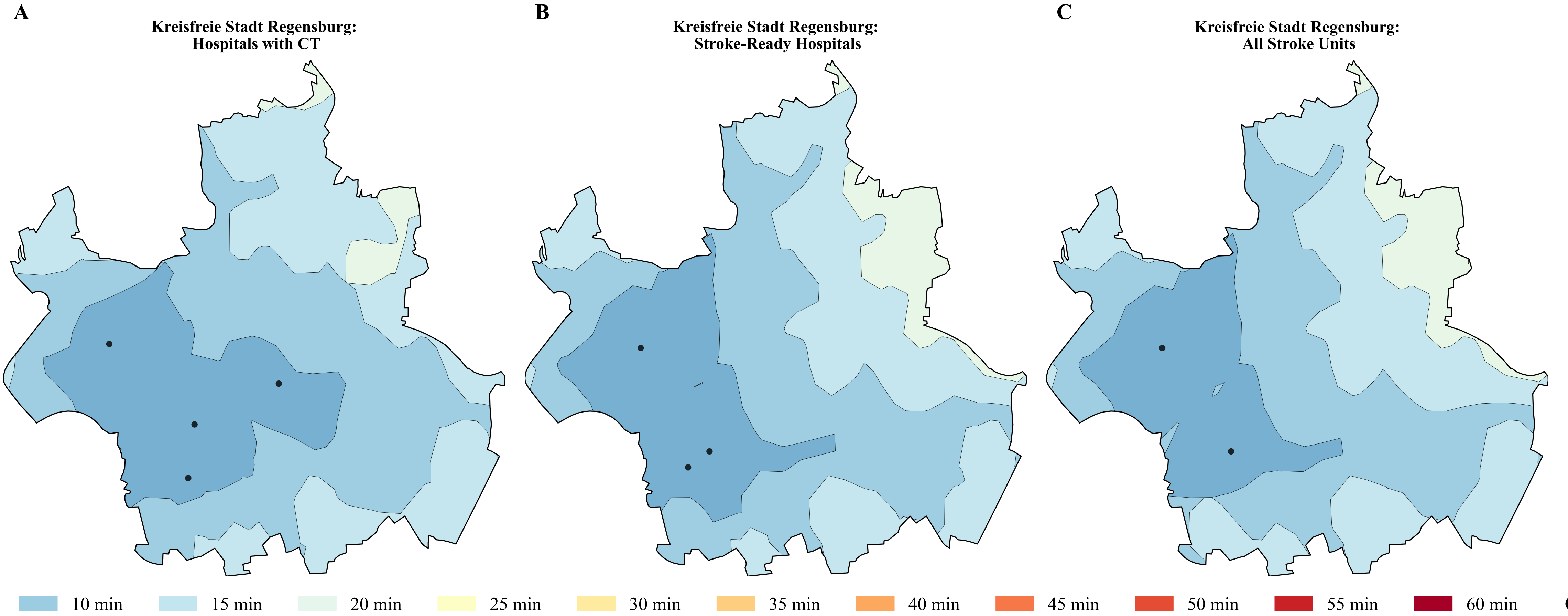

Isochrones — Kreisfreie Stadt Weiden i.d.OPf. (Bayern)

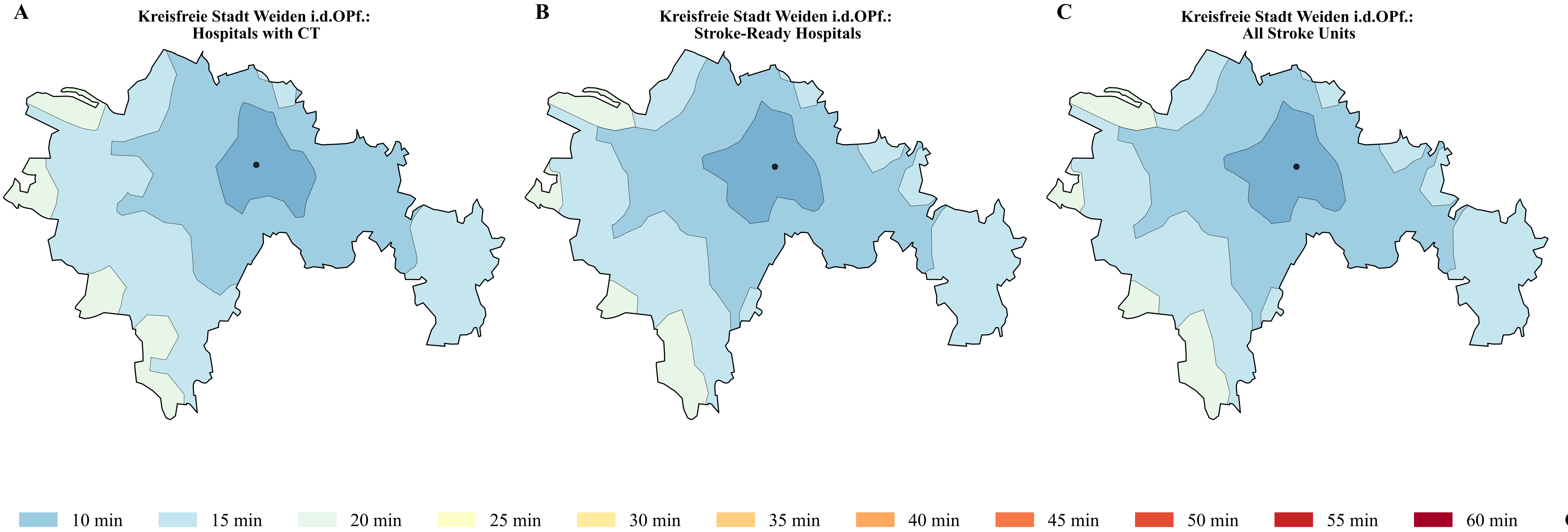

Isochrones — Kreisfreie Stadt Ansbach (Bayern)

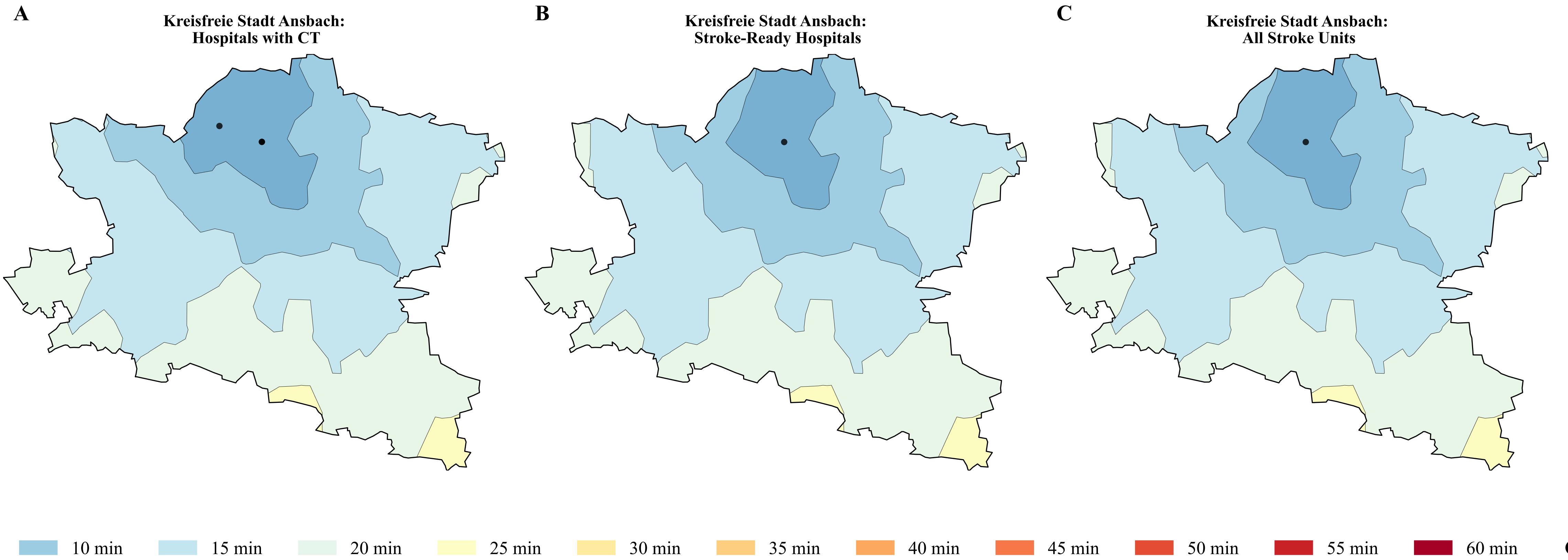

Isochrones — Landkreis Rhön-Grabfeld (Bayern)

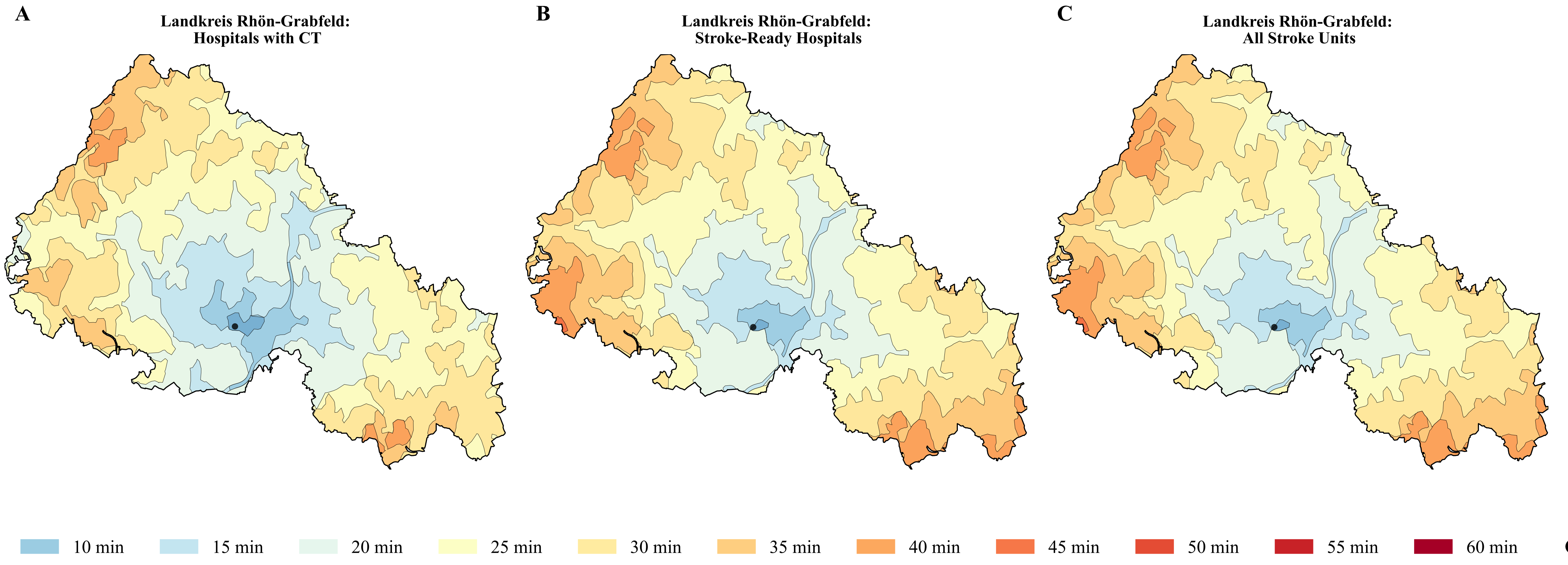

Isochrones — Landkreis Ostprignitz-Ruppin (Brandenburg)

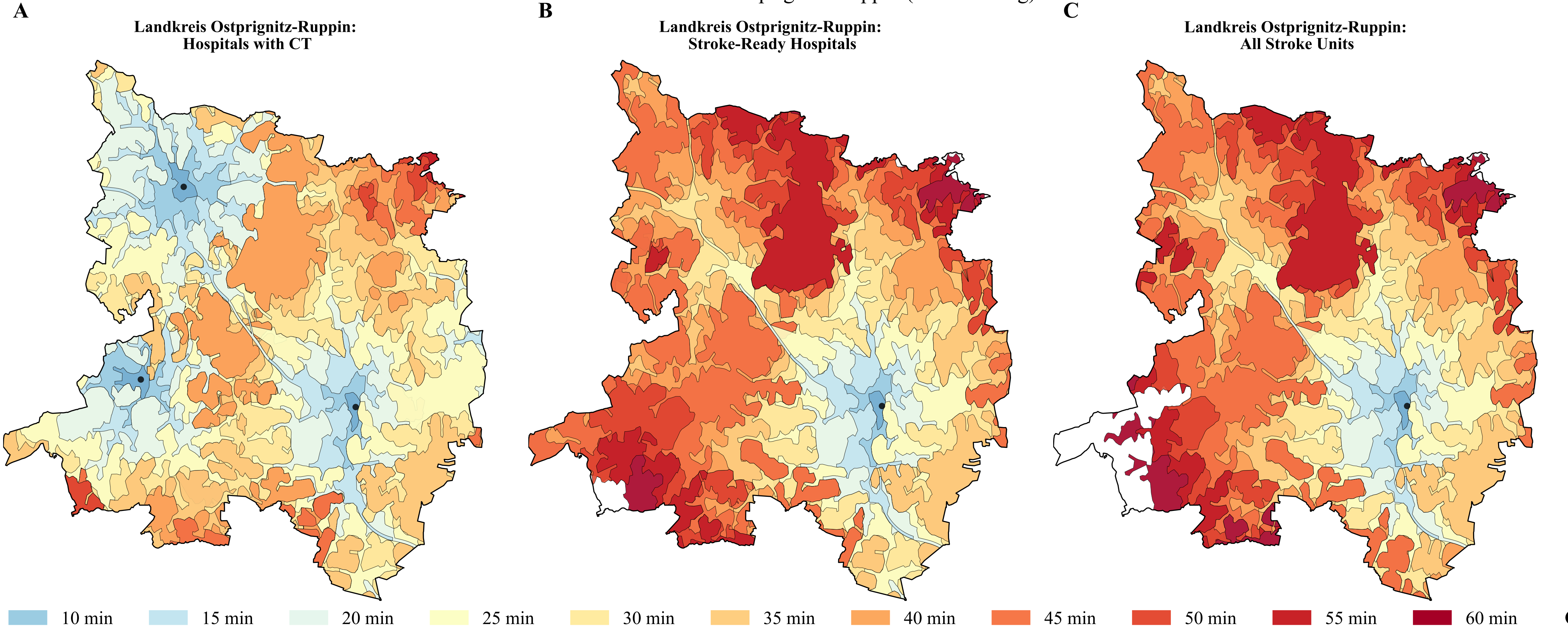

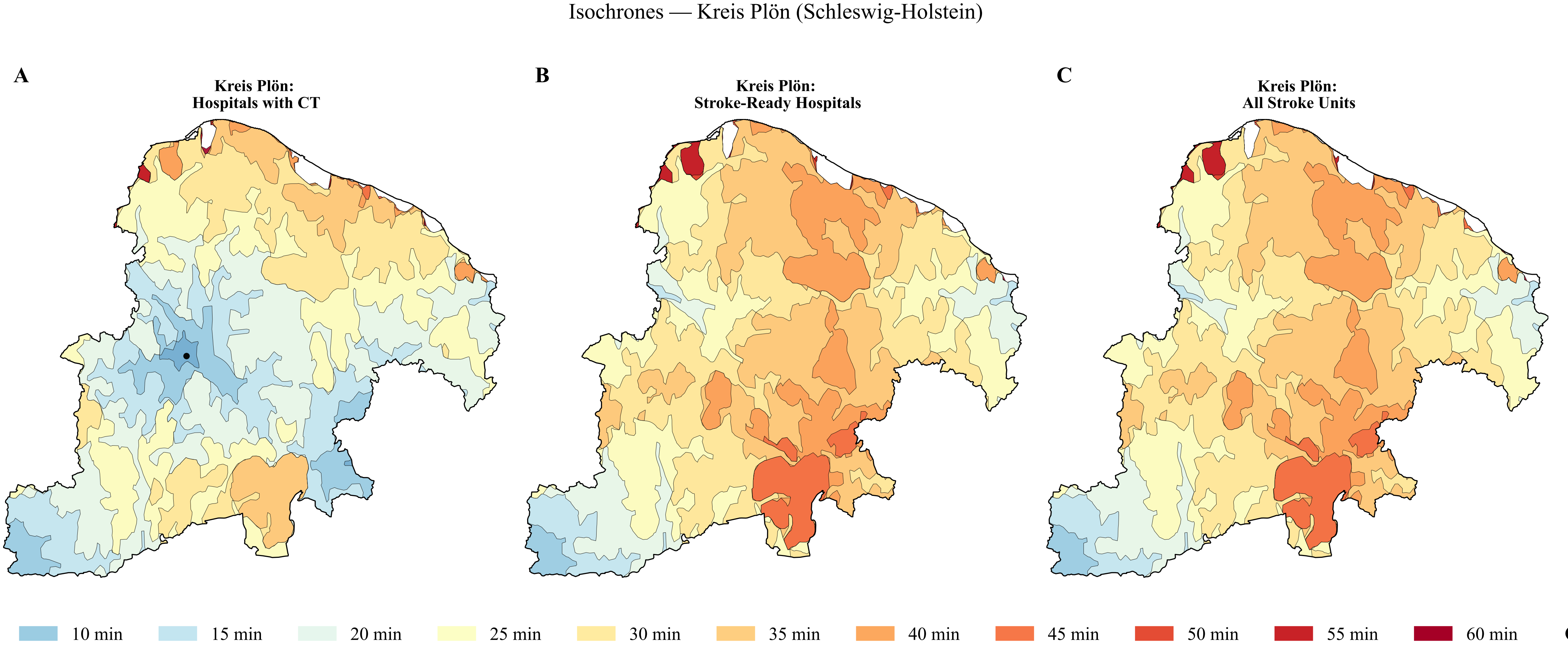

Isochrones — Kreis Plön (Schleswig-Holstein)
A
Kreis Plön:
Hospitals with CT
B
Kreis Plön:
Stroke-Ready Hospitals
C
Kreis Plön:
All Stroke Units
10 min
15 min
20 min
25 min
30 min
35 min
40 min
45 min
50 min
55 min
60 min

Isochrones — Landkreis Nienburg (Weser) (Niedersachsen)

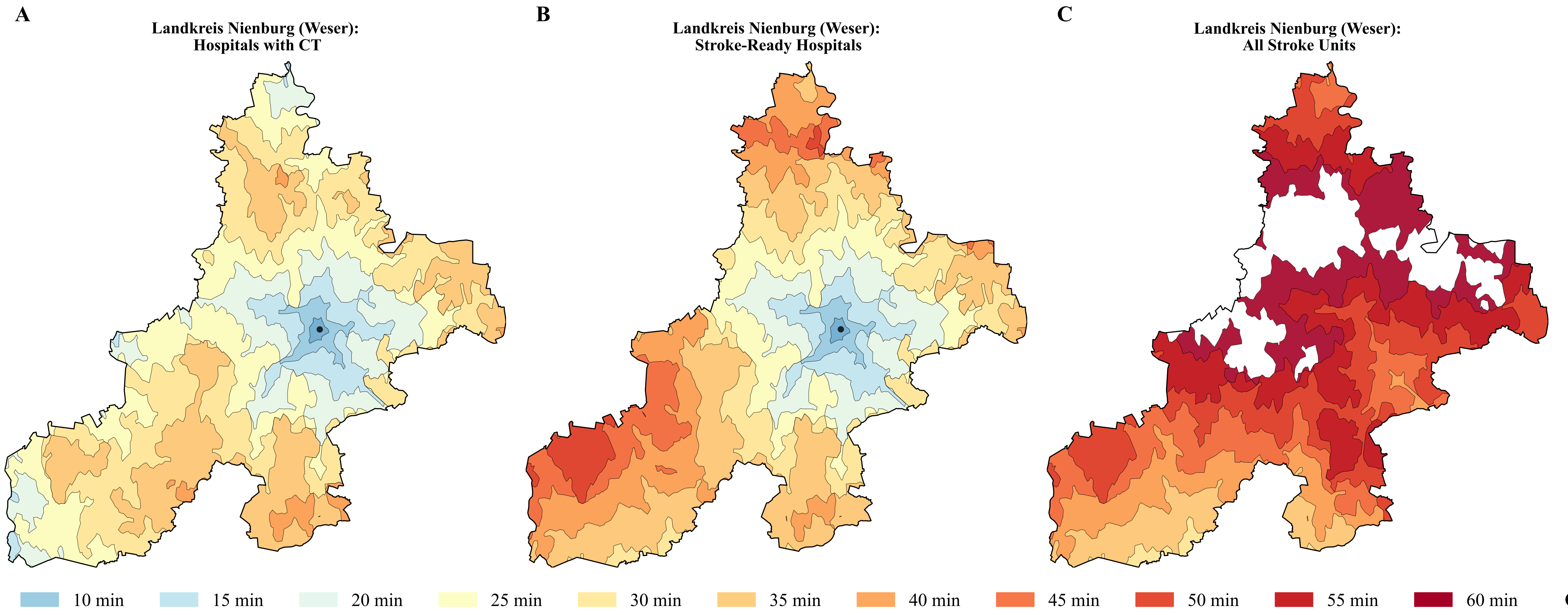

Isochrones — Landkreis Emsland (Niedersachsen)

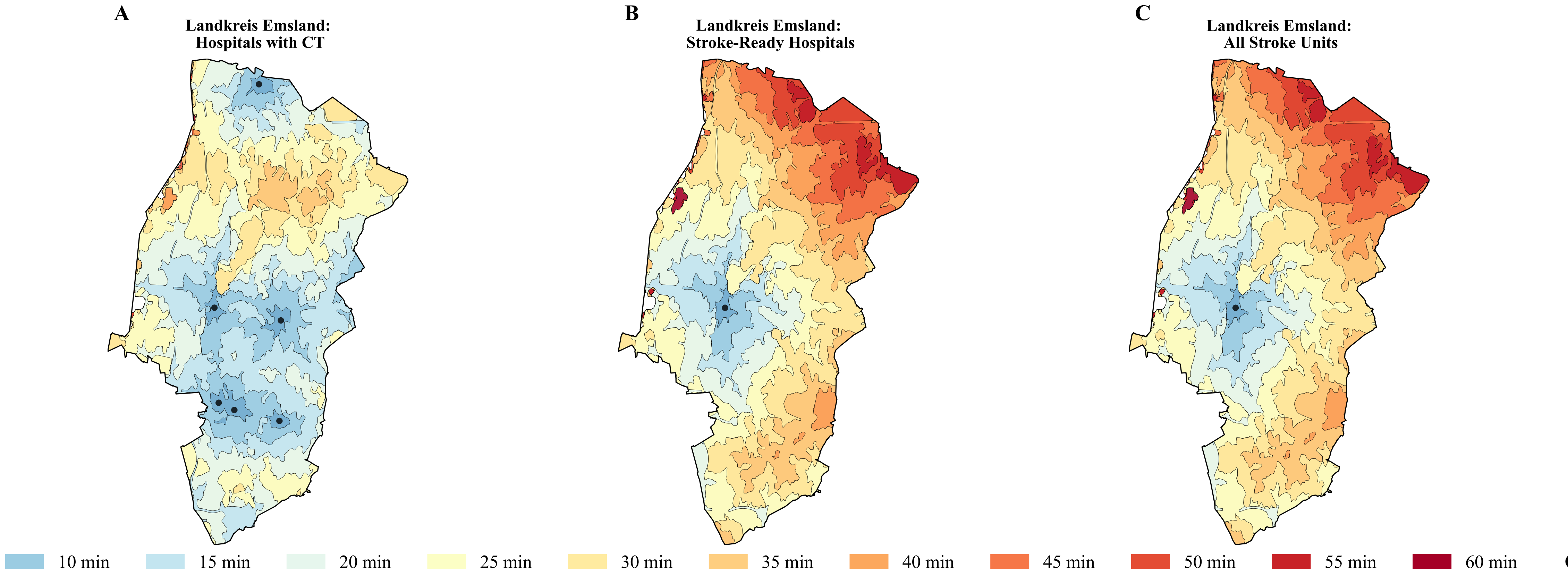

Isochrones — Landkreis Wittmund (Niedersachsen)

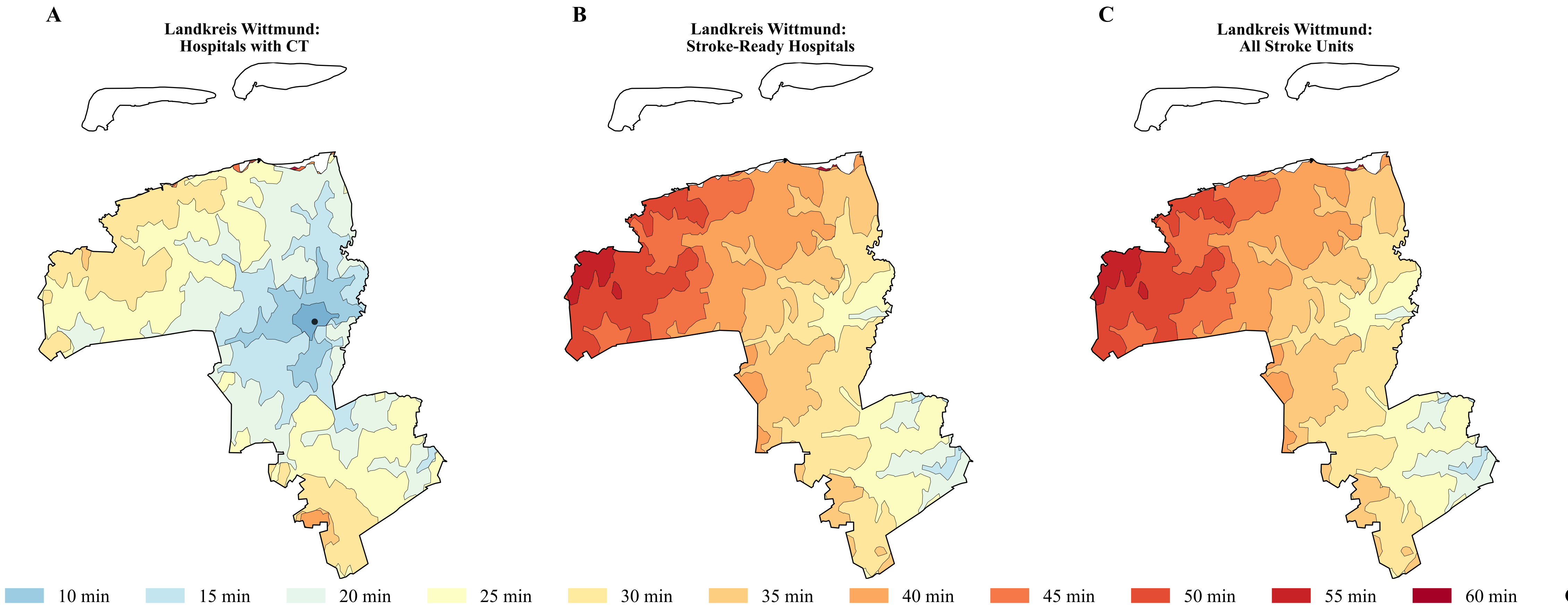

Isochrones — Kreis Hochsauerlandkreis (Nordrhein-Westfalen)

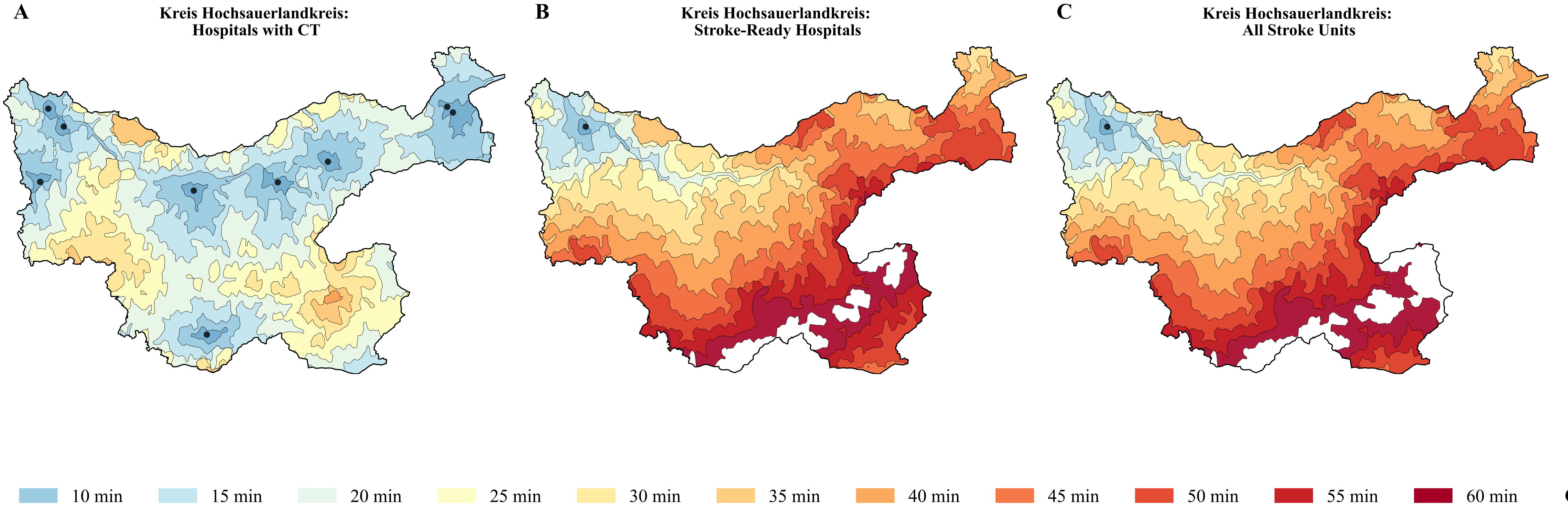

Isochrones — Landkreis Rheingau-Taunus-Kreis (Hessen)

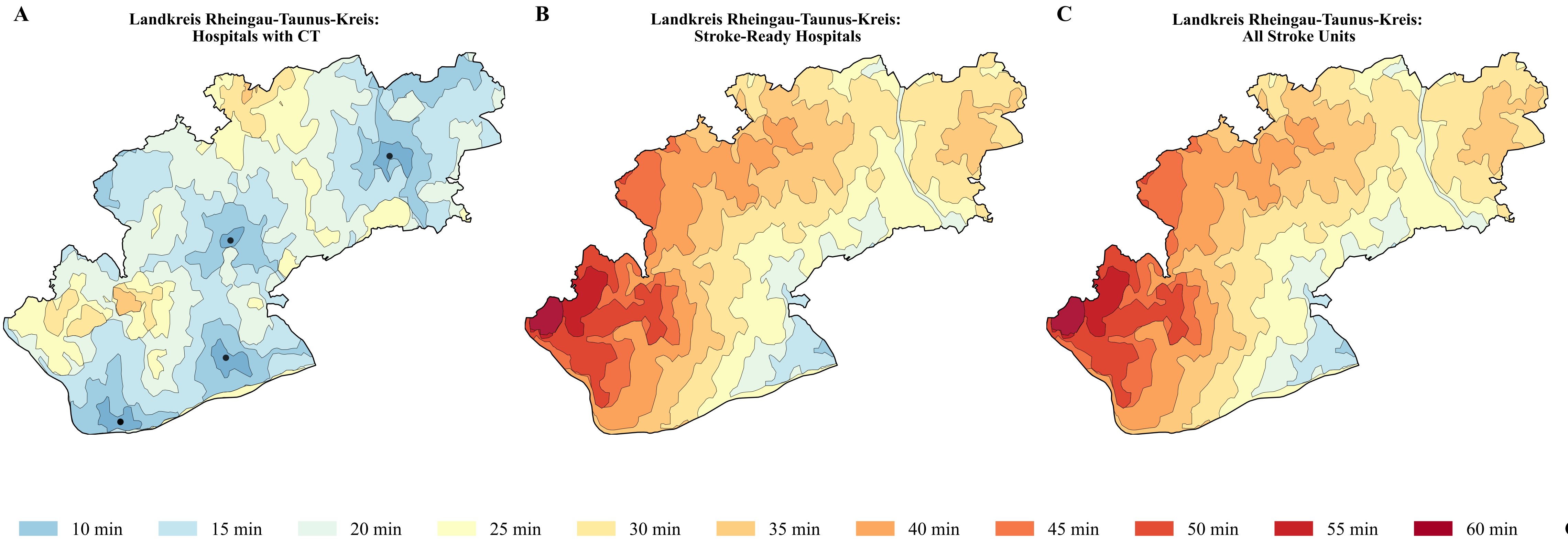

Isochrones — Landkreis Göppingen (Baden-Württemberg)

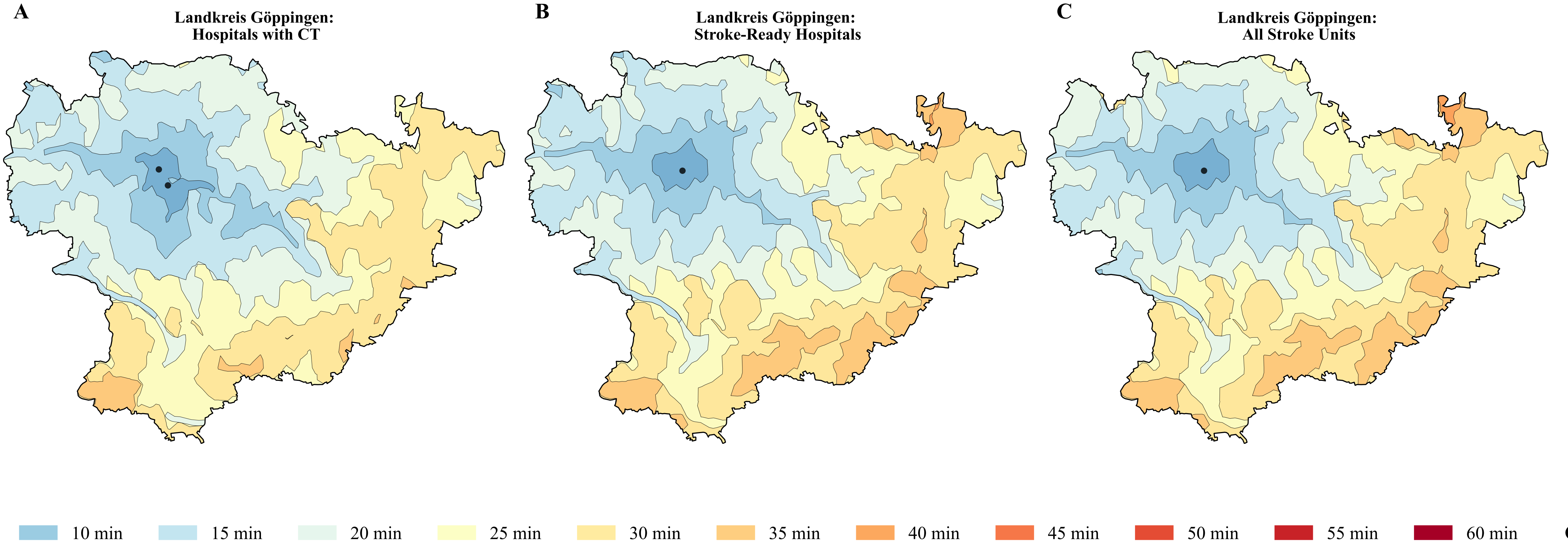

Isochrones — Stadtkreis Freiburg im Breisgau (Baden-Württemberg)

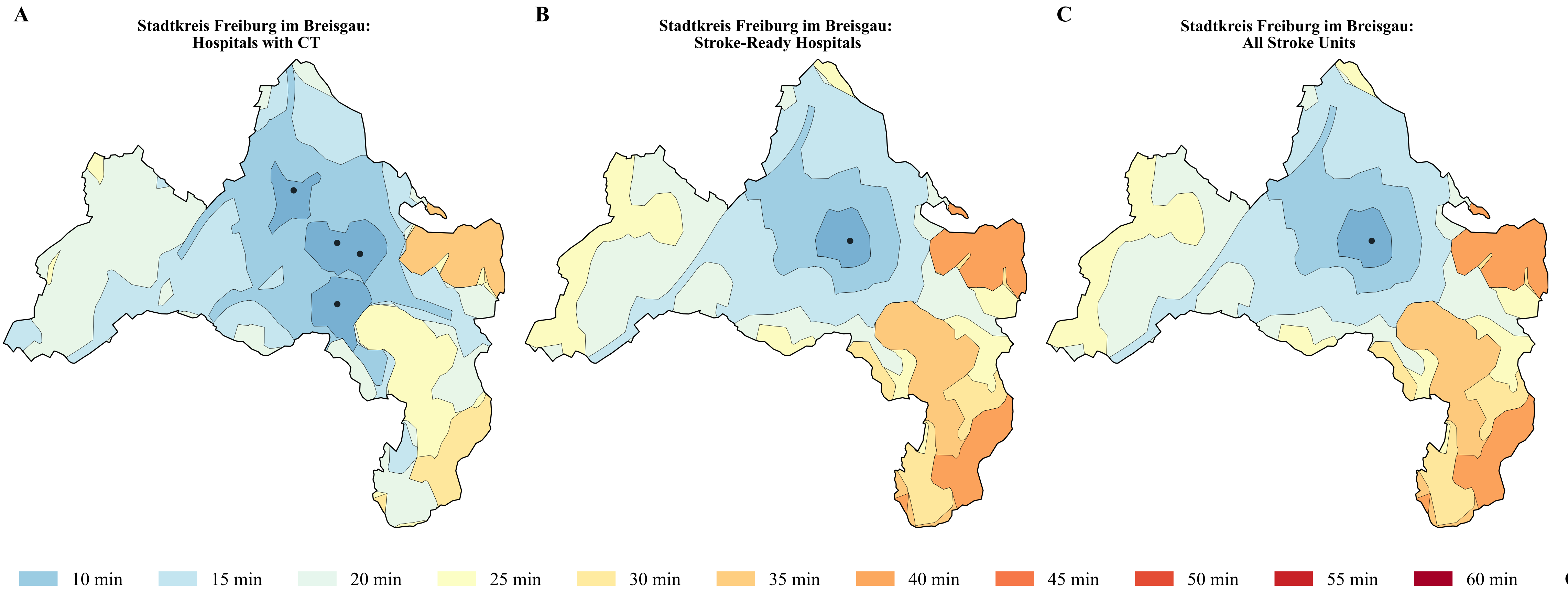

602 / 5833

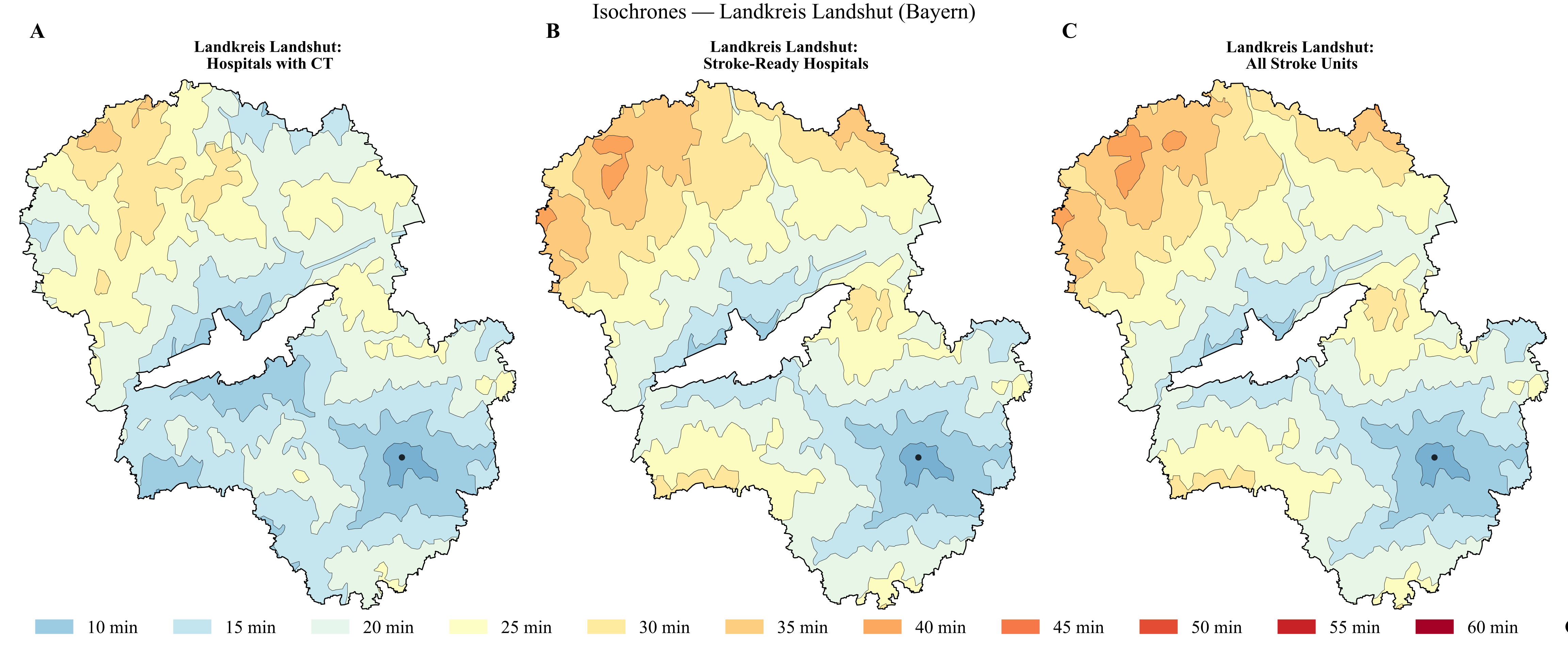
Isochrones — Landkreis Landshut (Bayern)
A
Landkreis Landshut:
Hospitals with CT
B
Landkreis Landshut:
Stroke-Ready Hospitals
C
Landkreis Landshut:
All Stroke Units
10 min
15 min
20 min
25 min
30 min
35 min
40 min
45 min
50 min
55 min
60 min

Isochrones — Landkreis Regen (Bayern)

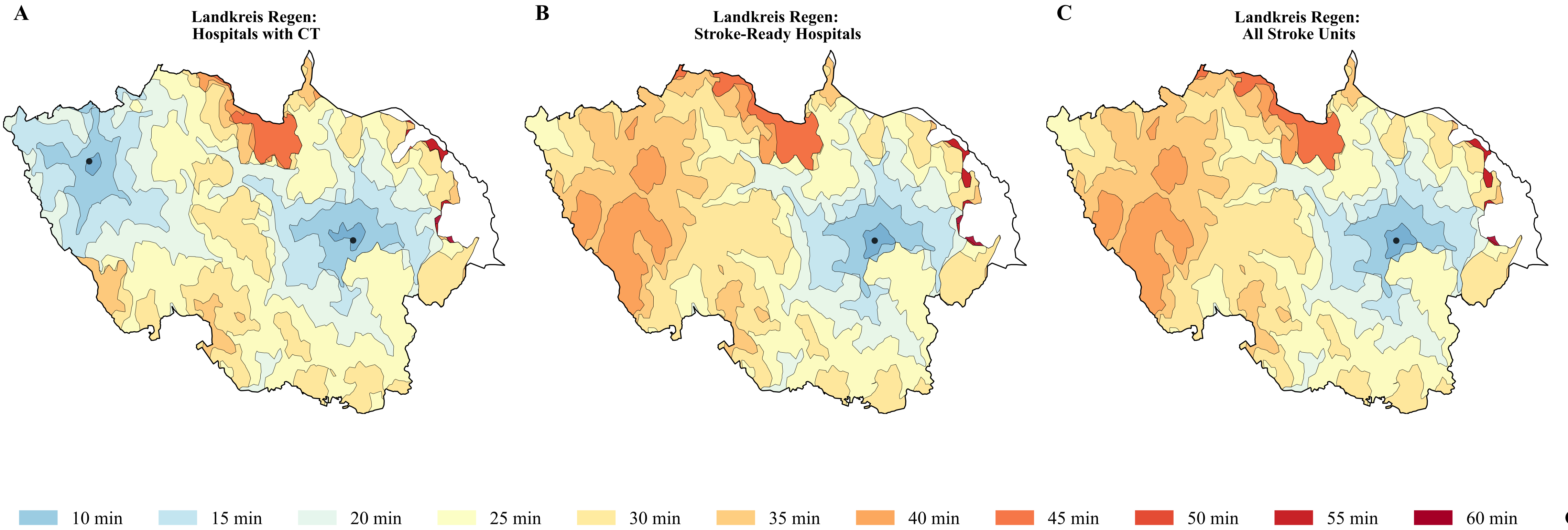

Isochrones — Kreisfreie Stadt Coburg (Bayern)

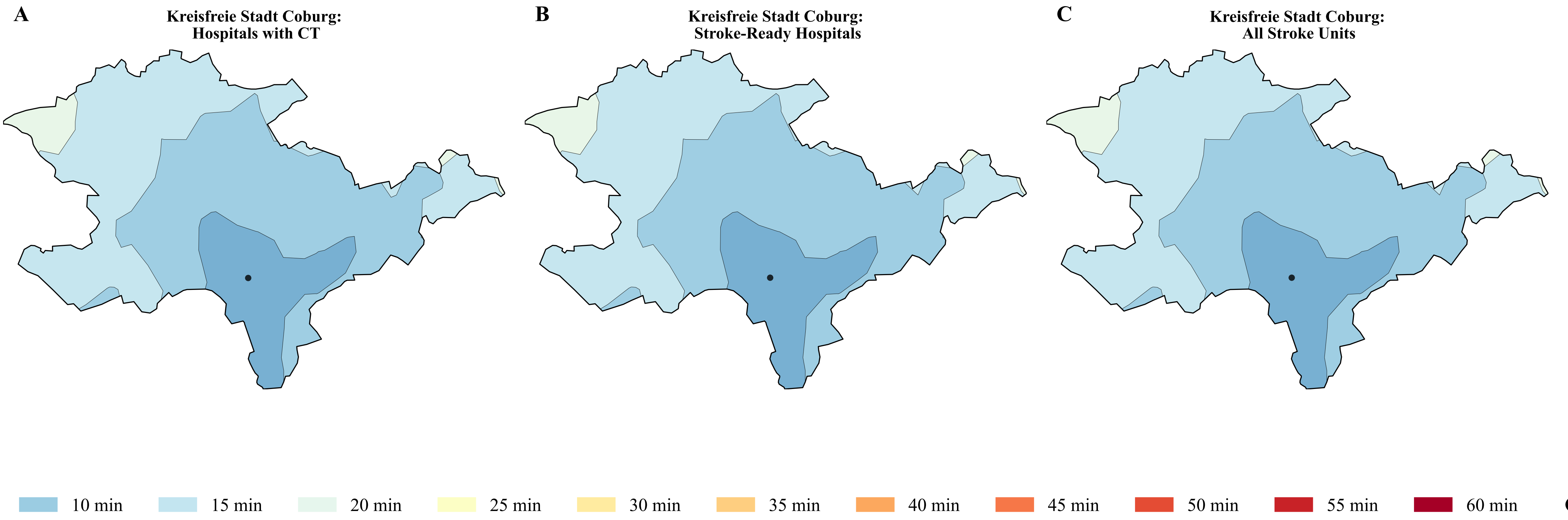

Isochrones — Landkreis Neunkirchen (Saarland)

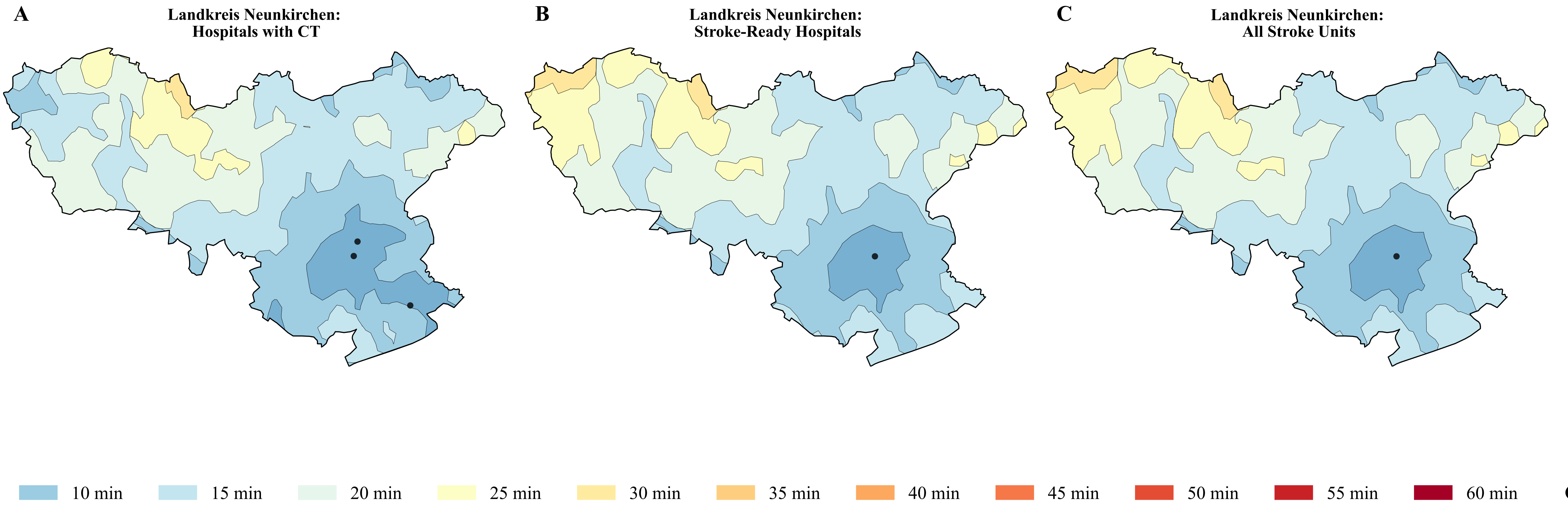

Isochrones — Landkreis Nordwestmecklenburg (Mecklenburg-Vorpommern)

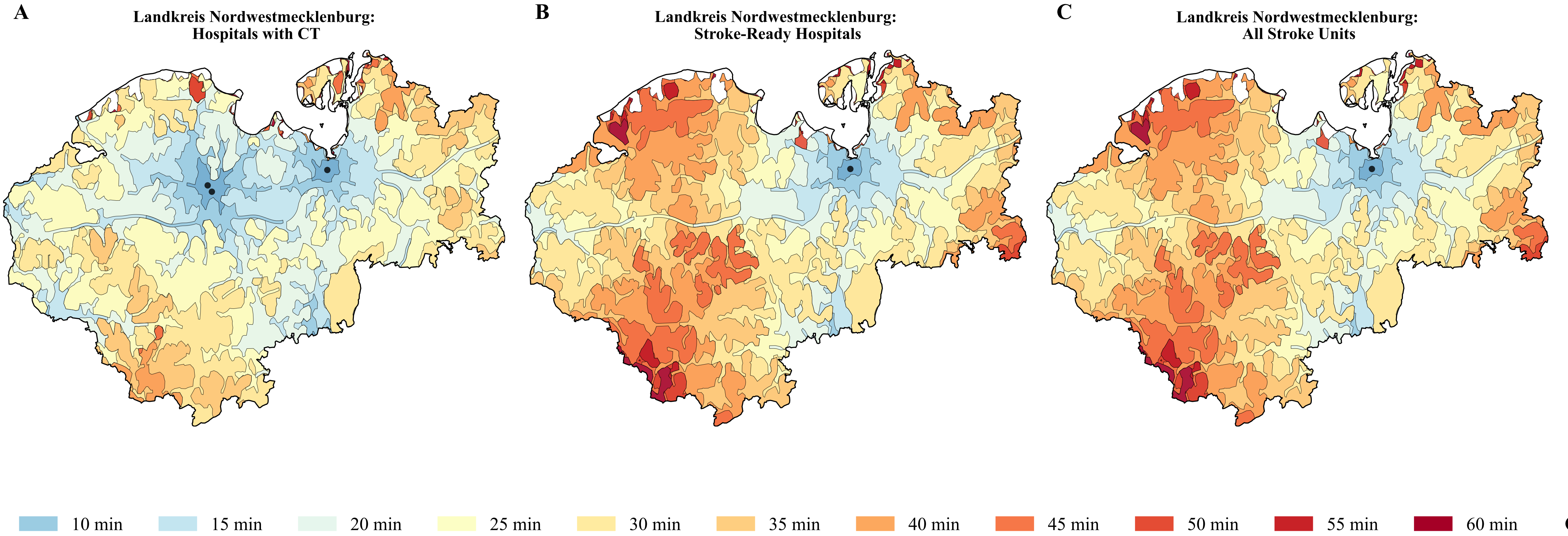

Isochrones — Kreisfreie Stadt Herne (Nordrhein-Westfalen)

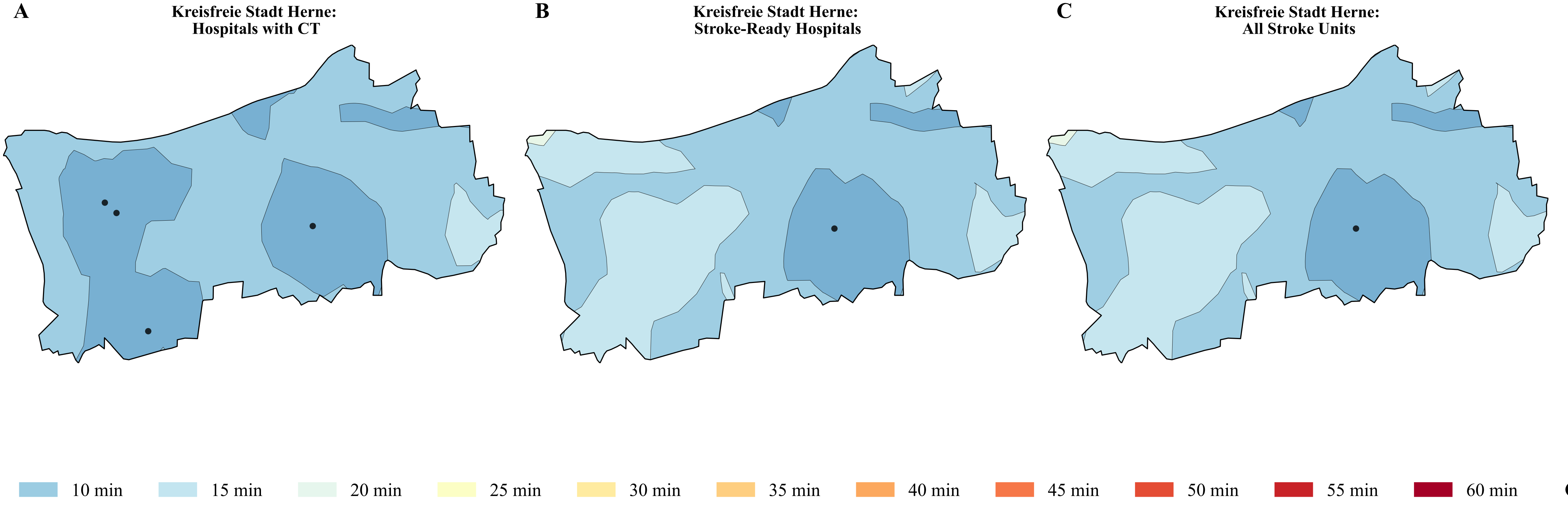

Isochrones — Landkreis Altenkirchen (Westerwald) (Rheinland-Pfalz)

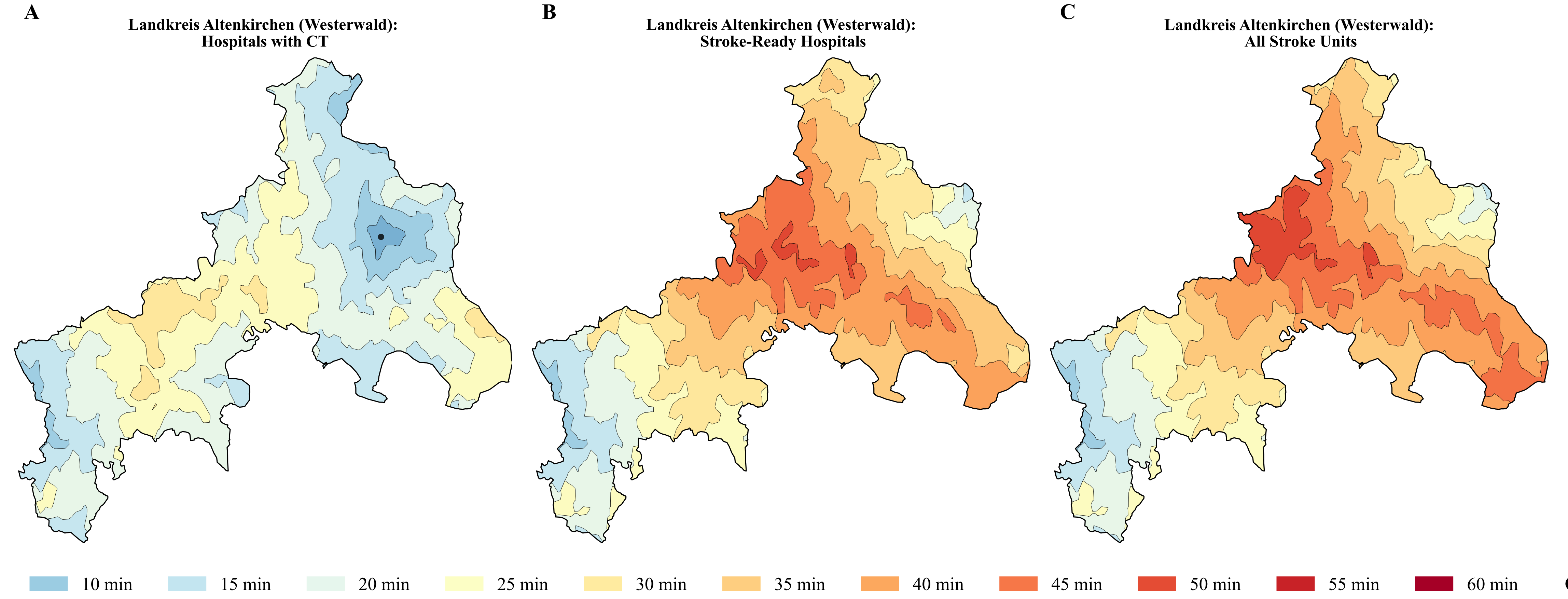

Isochrones — Kreisfreie Stadt Kaiserslautern (Rheinland-Pfalz)

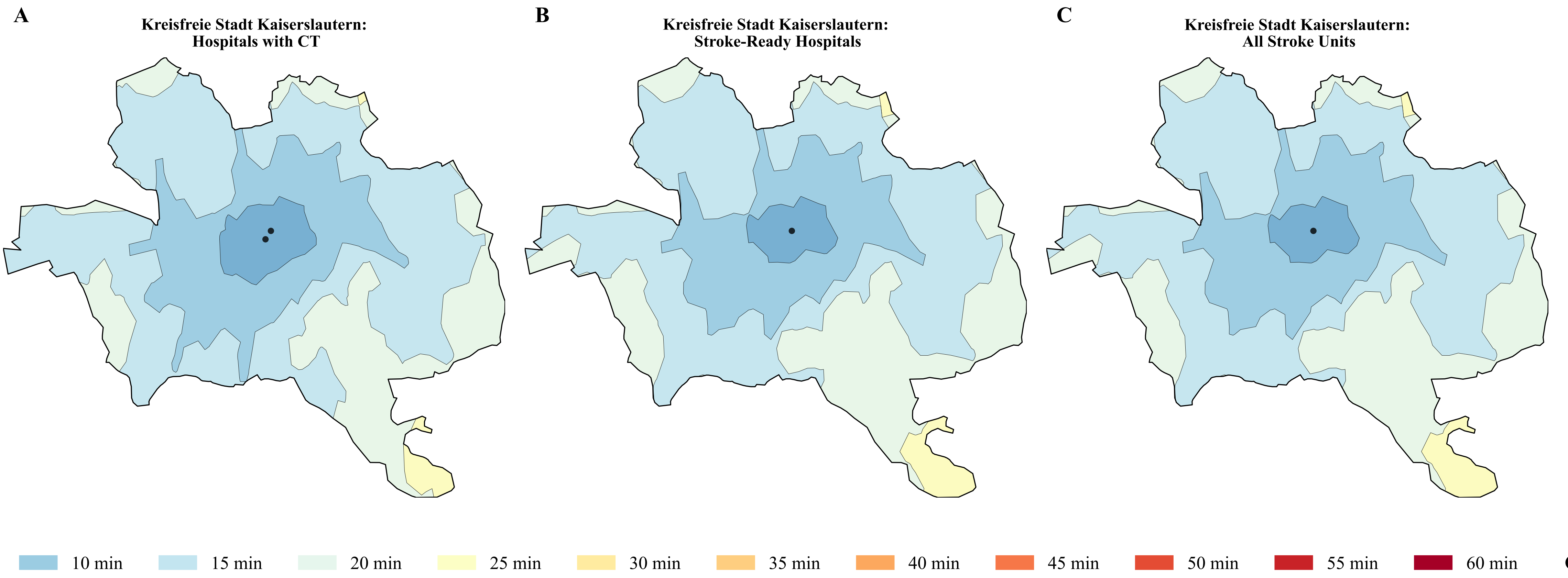

Isochrones — Landkreis Mainz-Bingen (Rheinland-Pfalz)

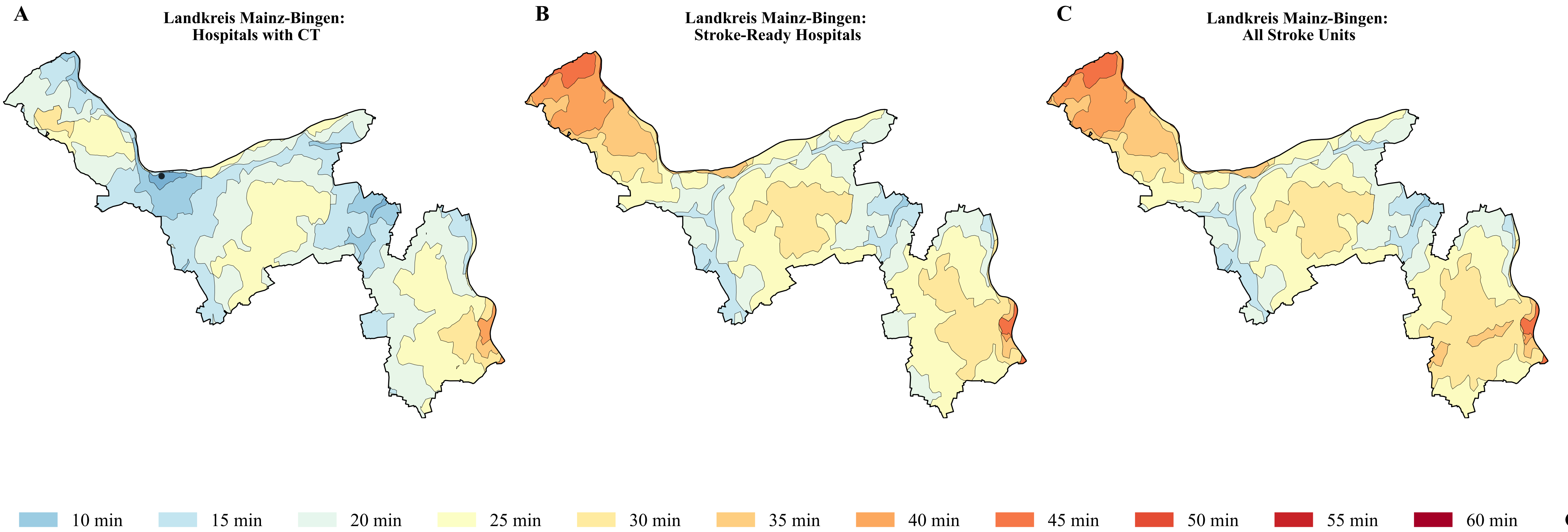

Isochrones — Landkreis Heidenheim (Baden-Württemberg)

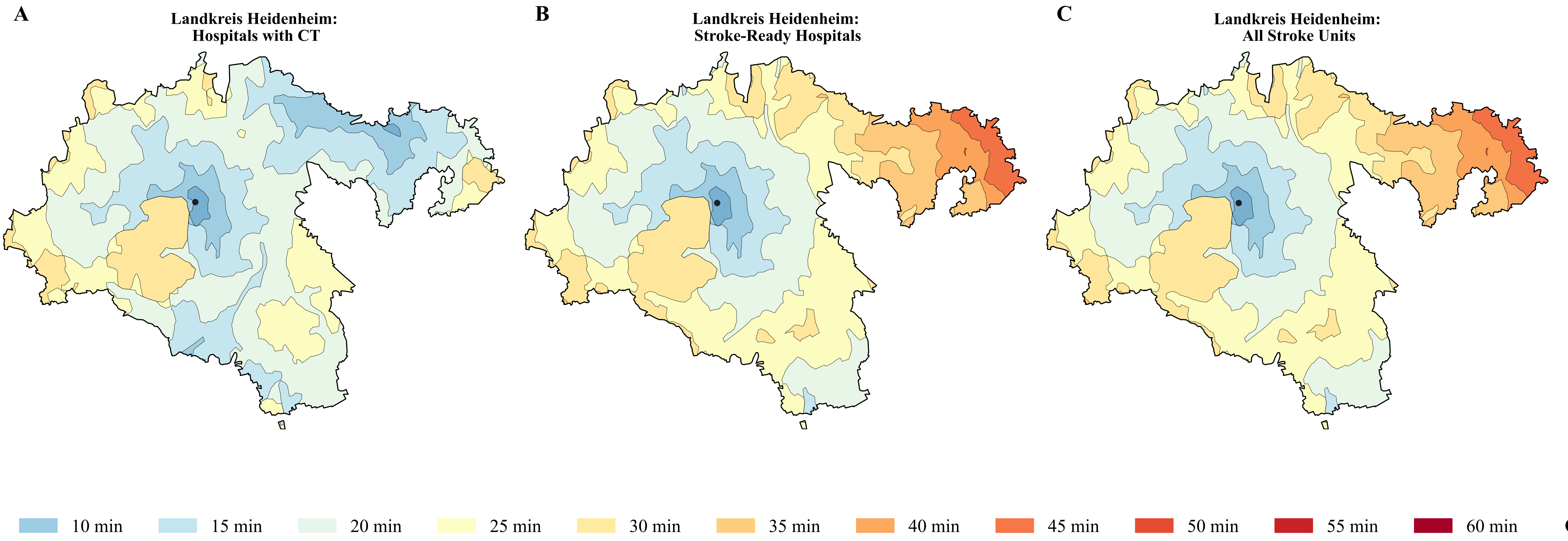

Isochrones — Stadtkreis Pforzheim (Baden-Württemberg)

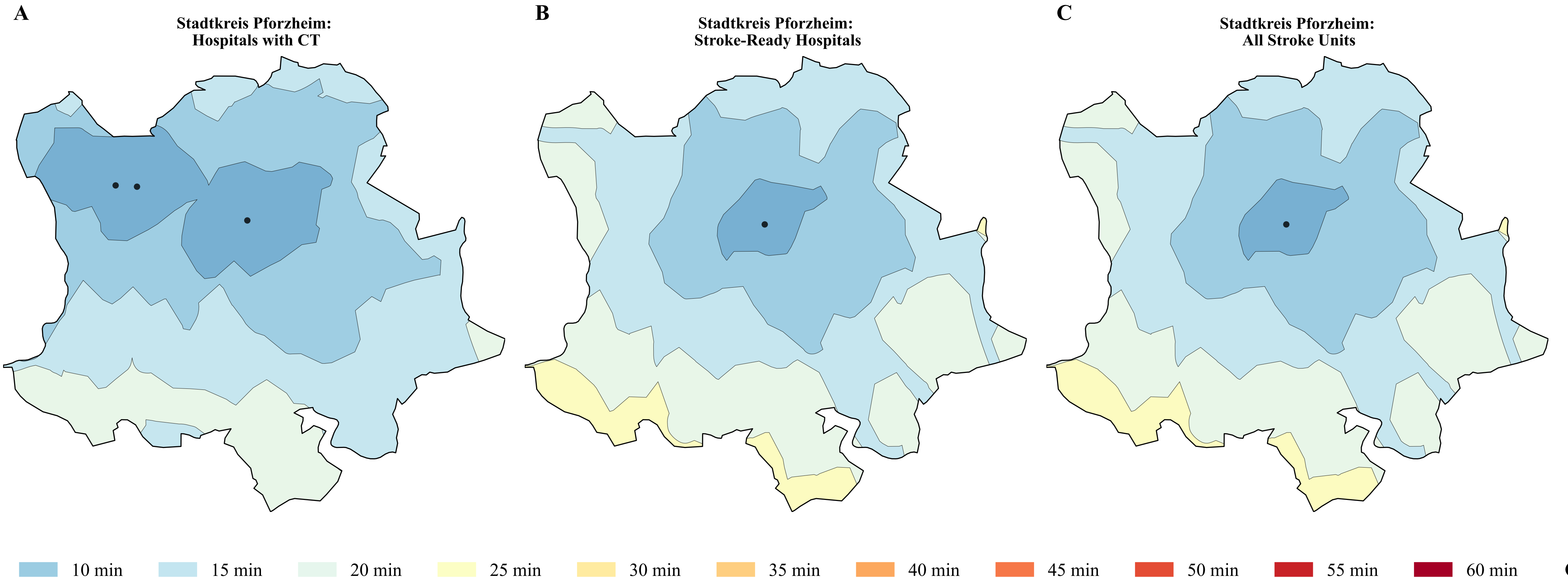

613 / 5833

Isochrones — Landkreis Tübingen (Baden-Württemberg)

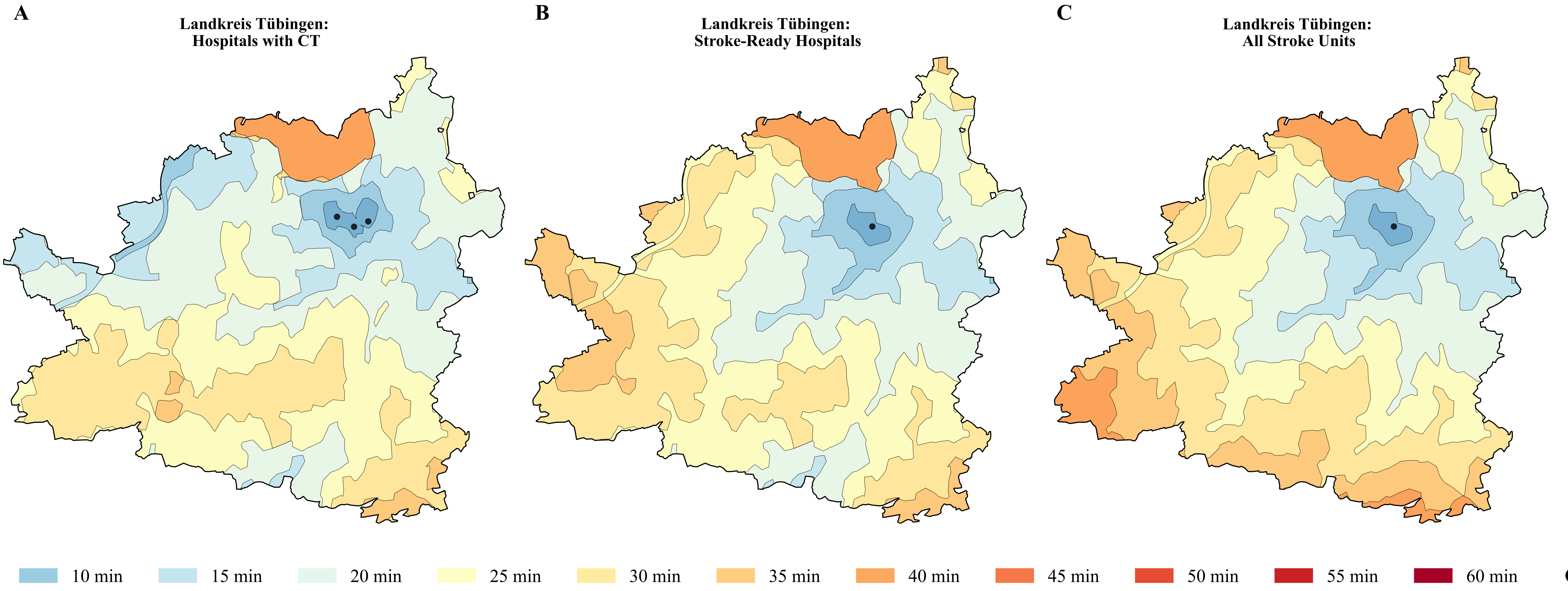

Isochrones — Landkreis Bodenseekreis (Baden-Württemberg)

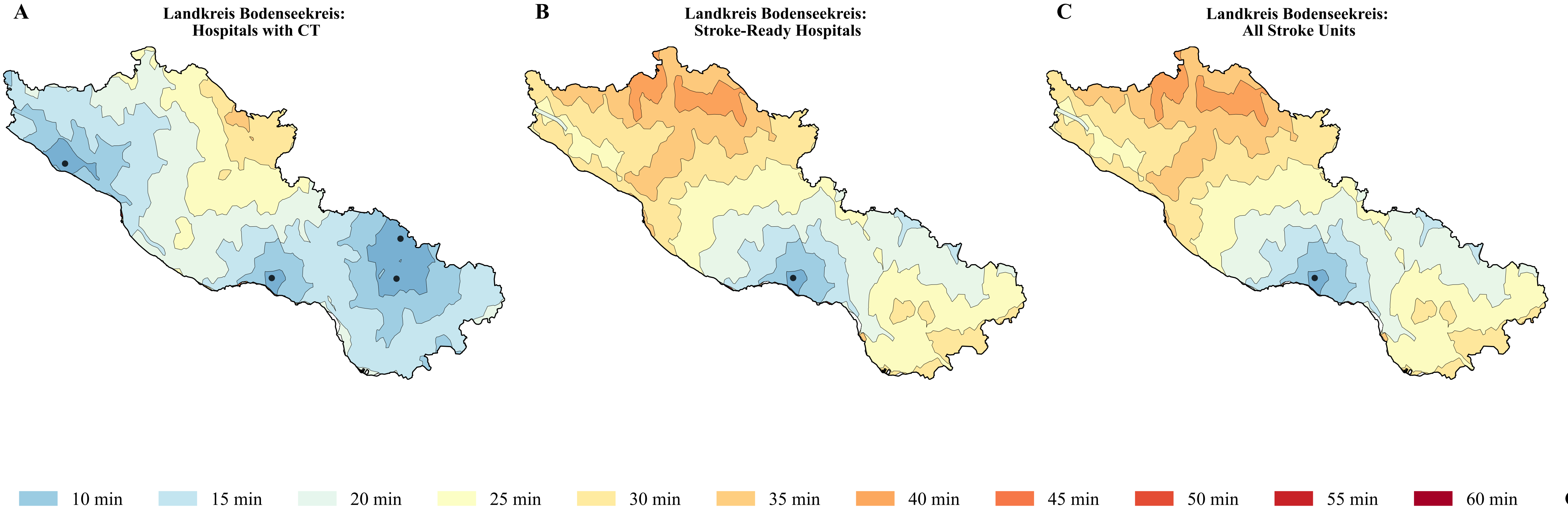

Isochrones — Landkreis Miesbach (Bayern)

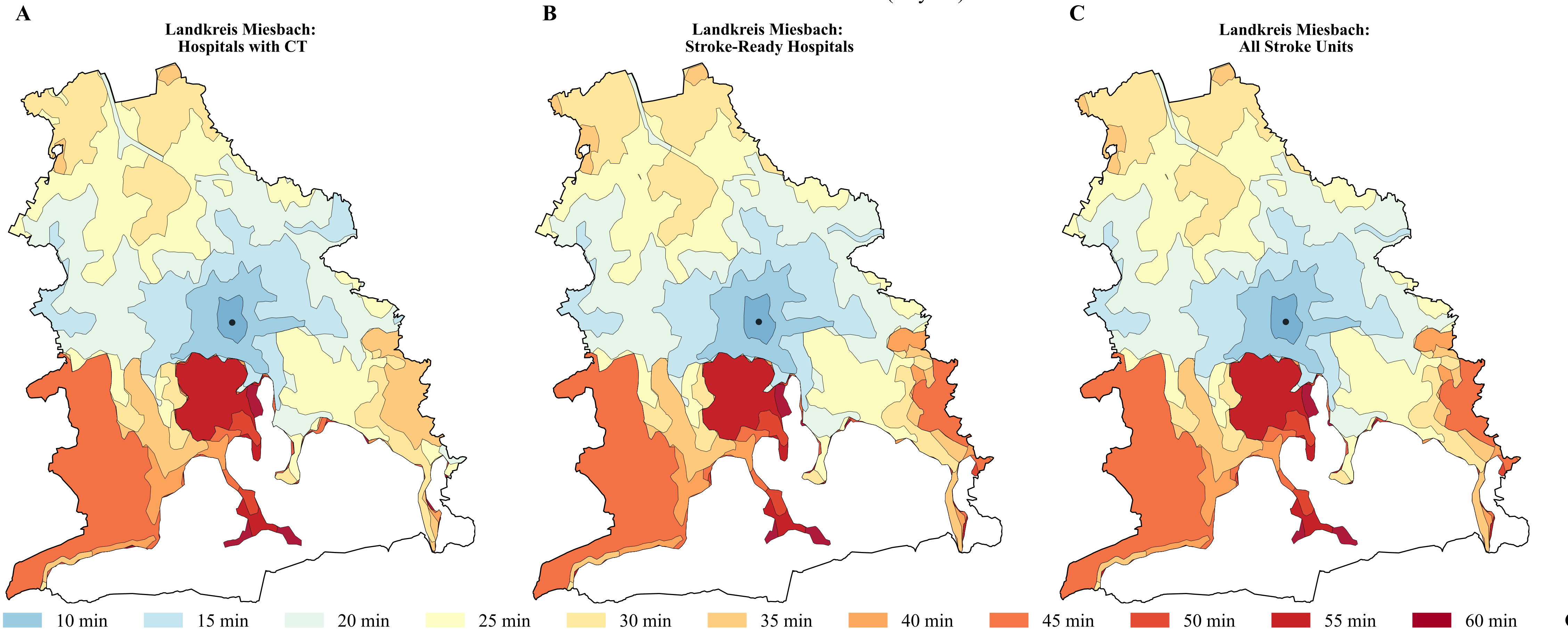

Isochrones — Landkreis Mühldorf a.Inn (Bayern)

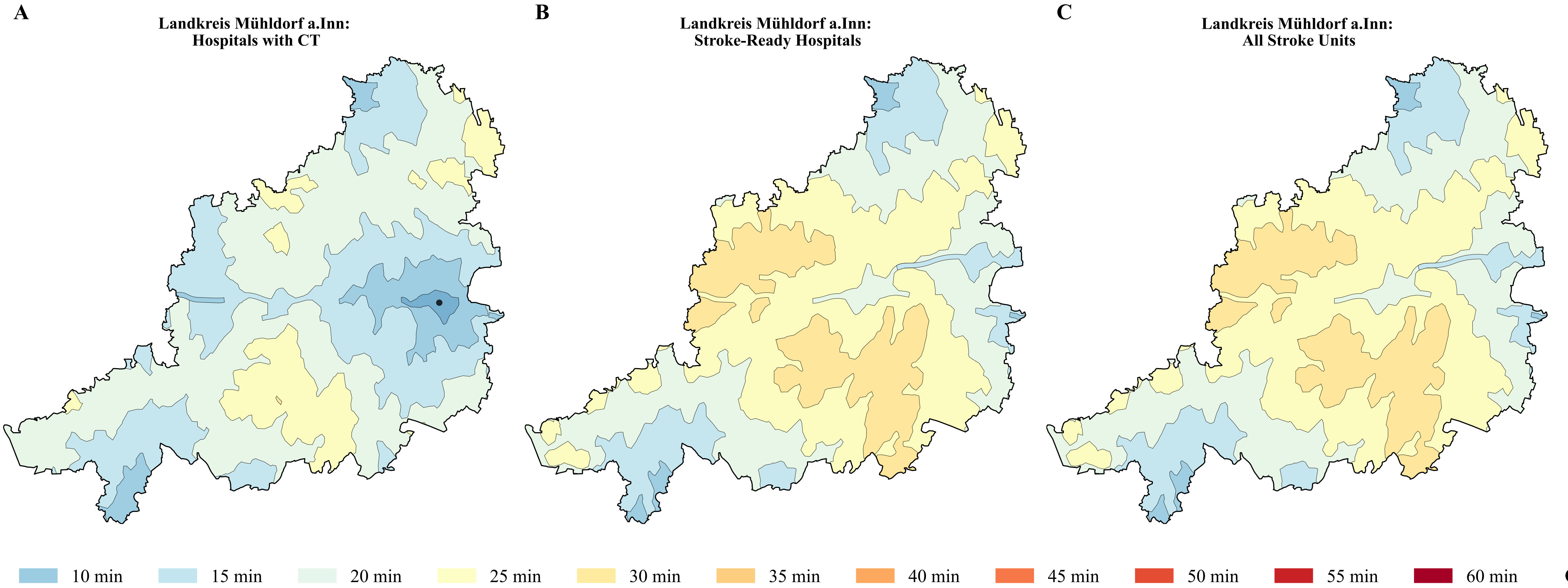

Isochrones — Landkreis Neuburg-Schrobenhausen (Bayern)

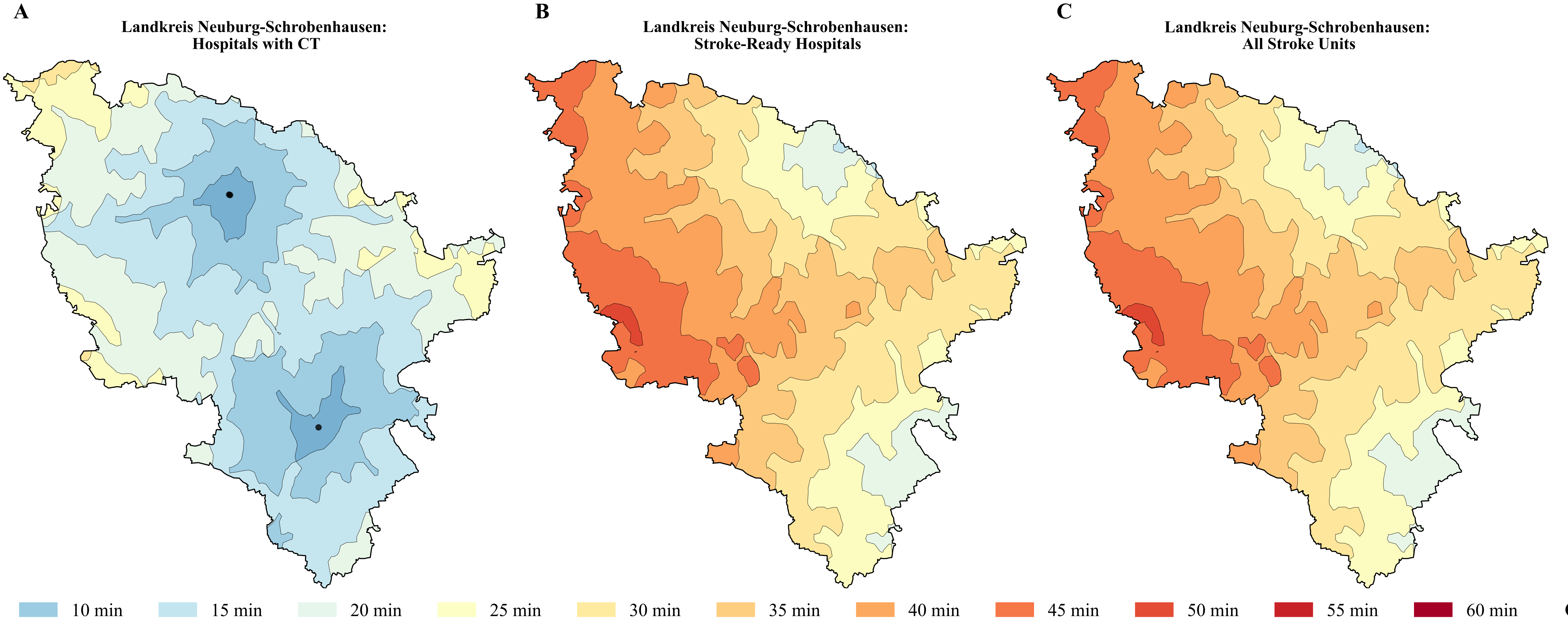

Isochrones — Landkreis Starnberg (Bayern)

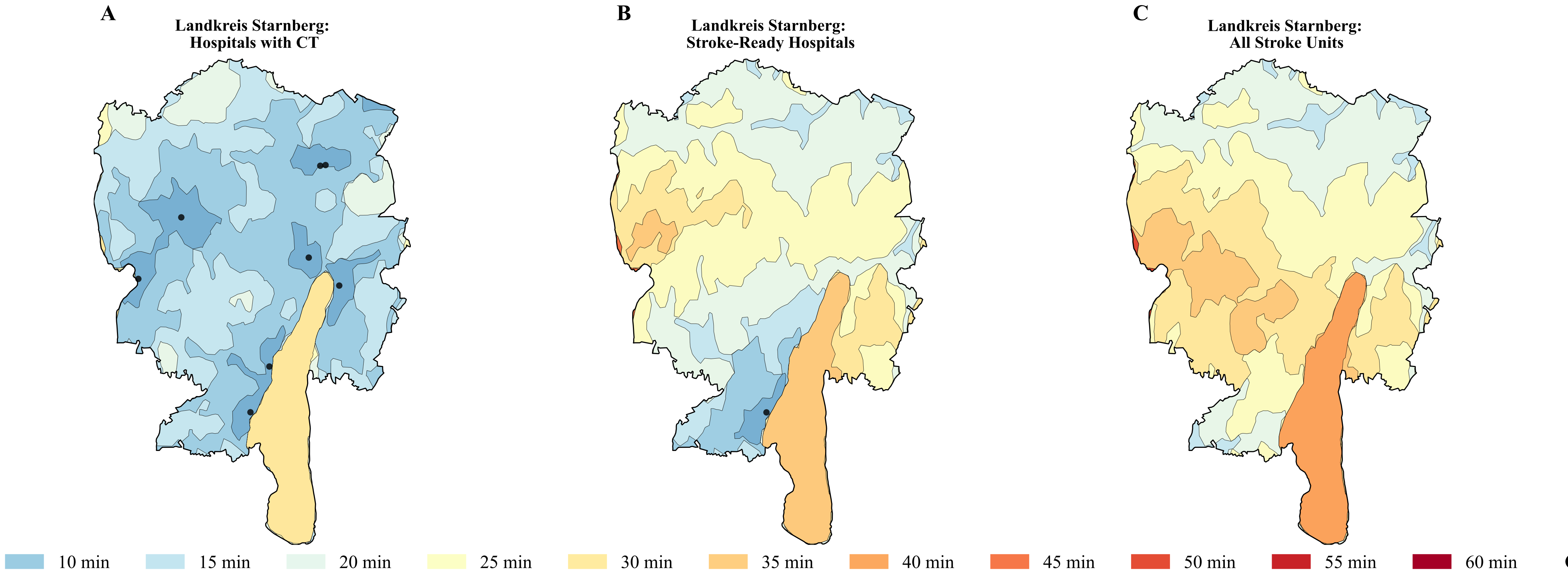

Isochrones — Landkreis Fürth (Bayern)

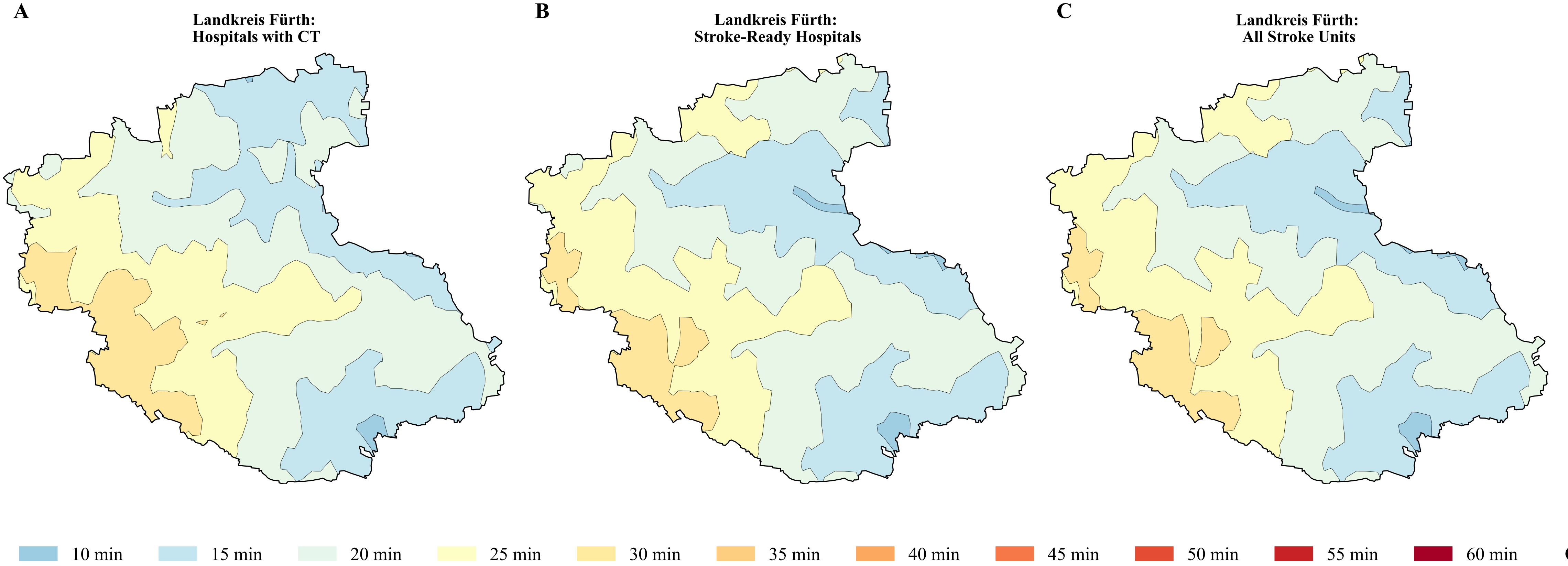

Isochrones — Landkreis Kitzingen (Bayern)

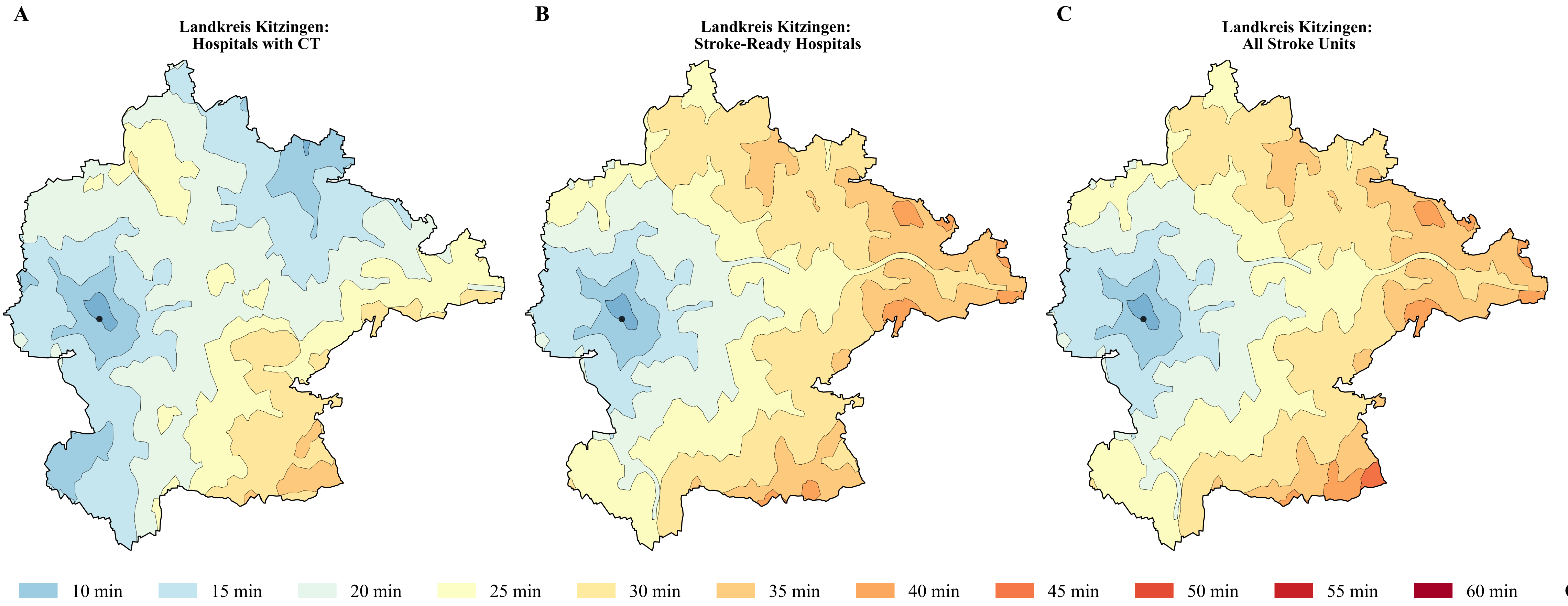

Isochrones — Landkreis Günzburg (Bayern)

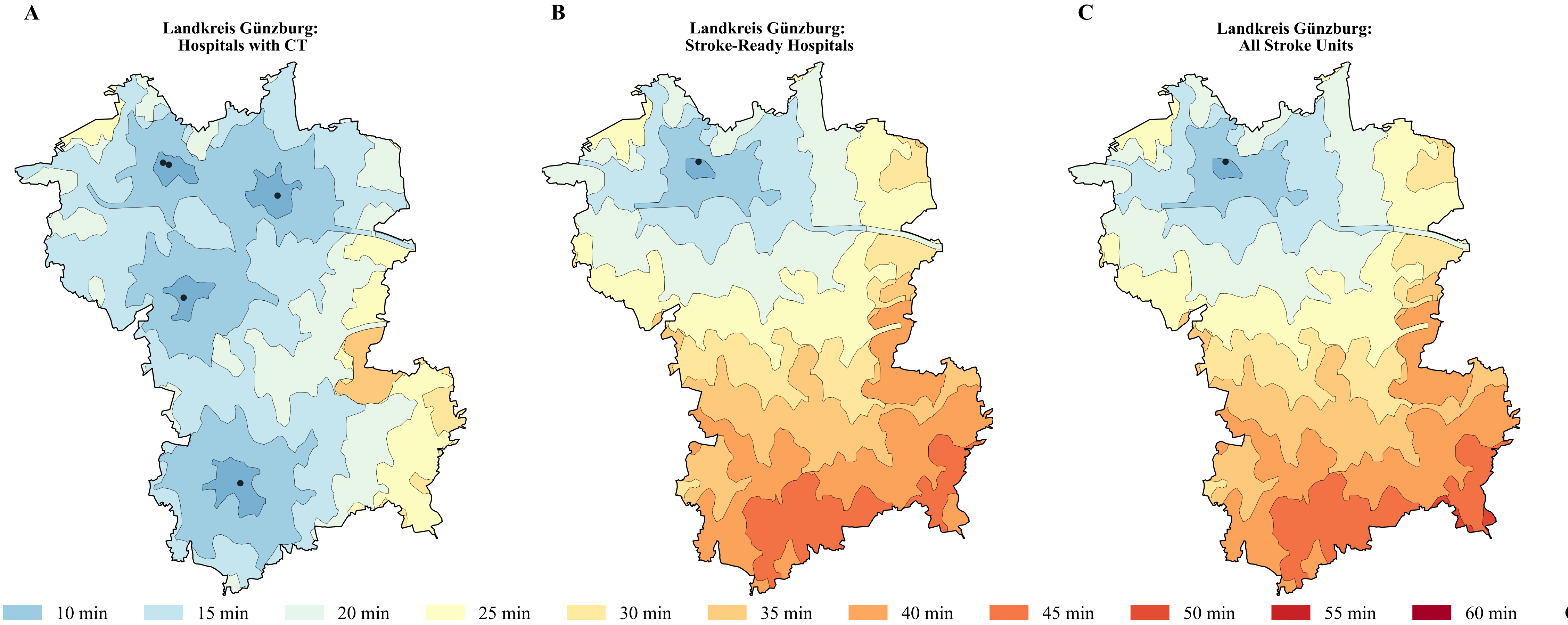

Isochrones — Landkreis Havelland (Brandenburg)

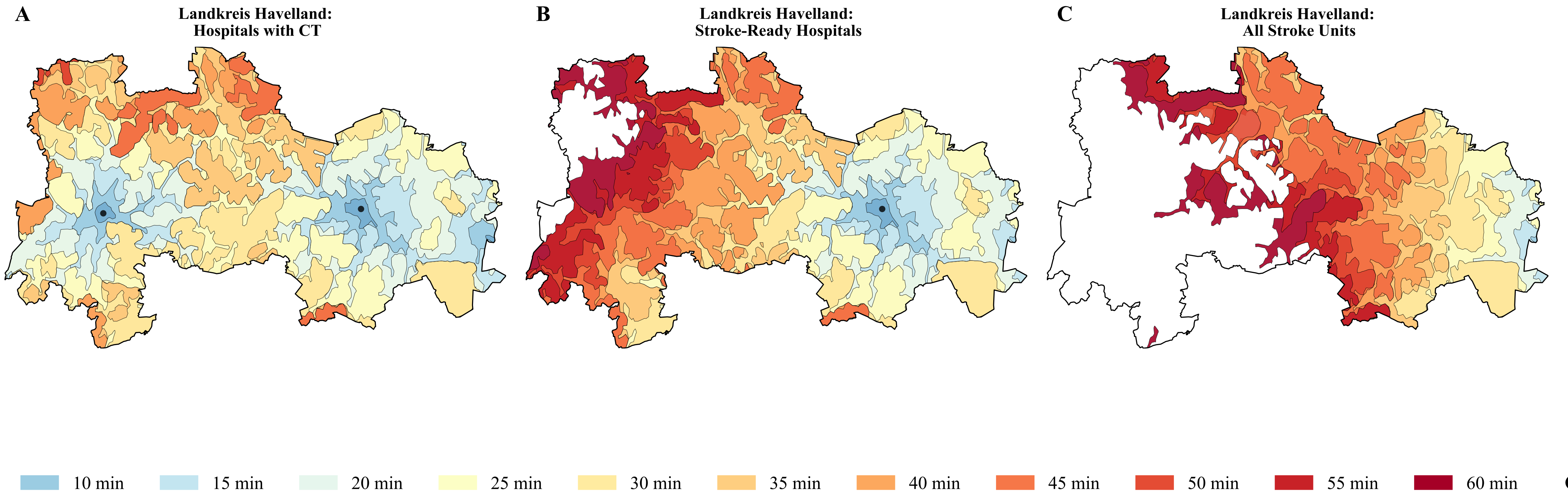

Isochrones — Landkreis Teltow-Fläming (Brandenburg)

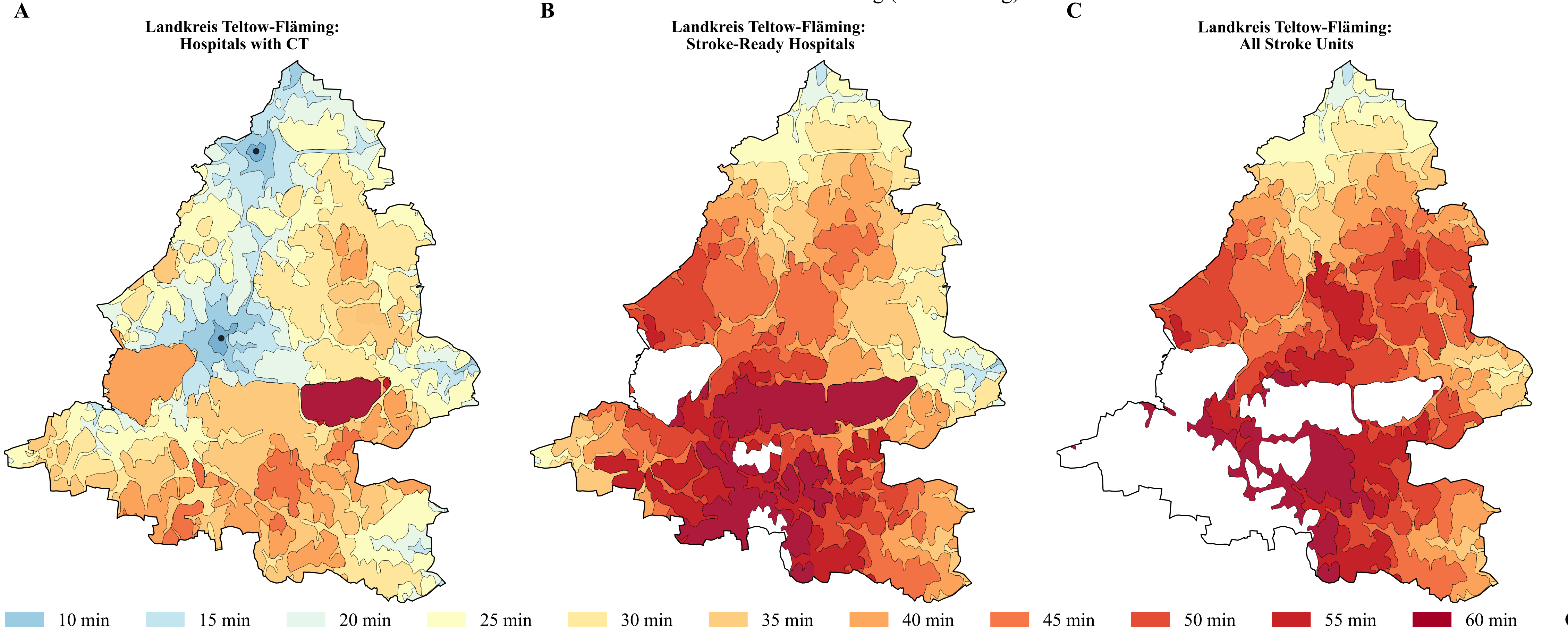

Isochrones — Landkreis Börde (Sachsen-Anhalt)

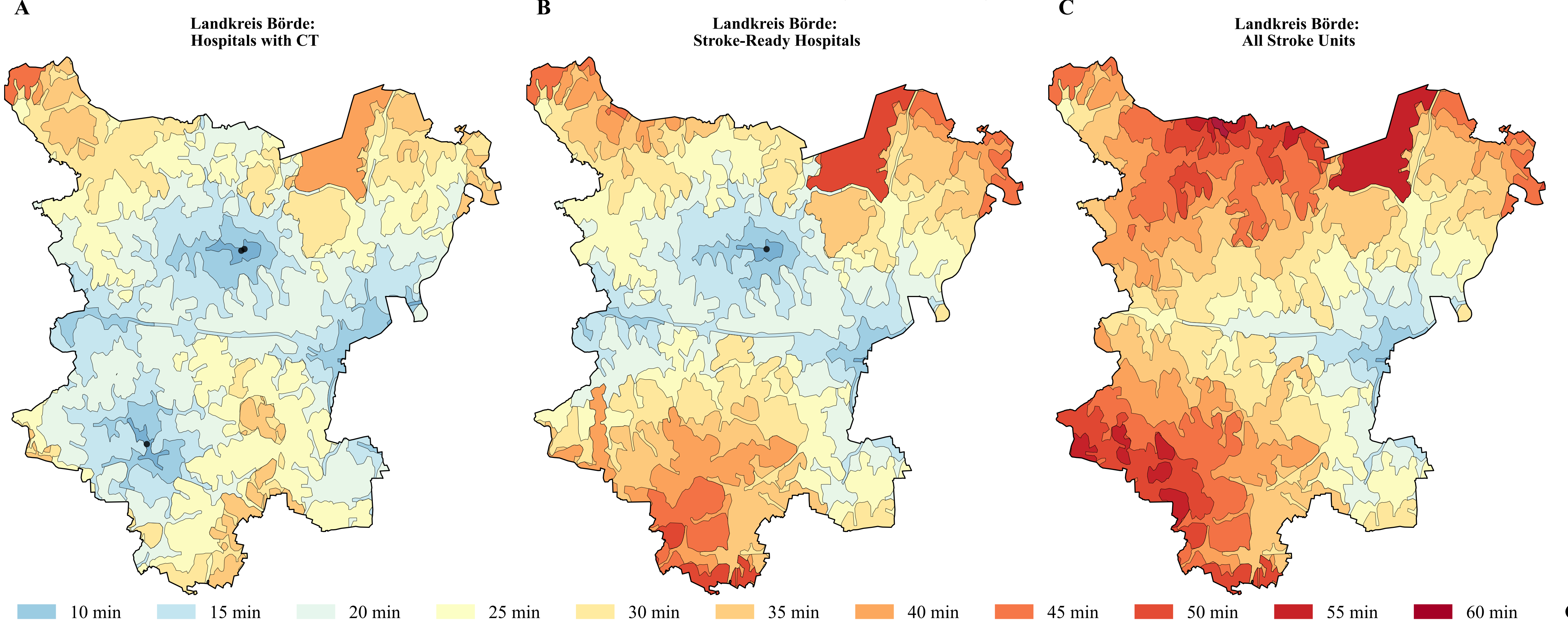

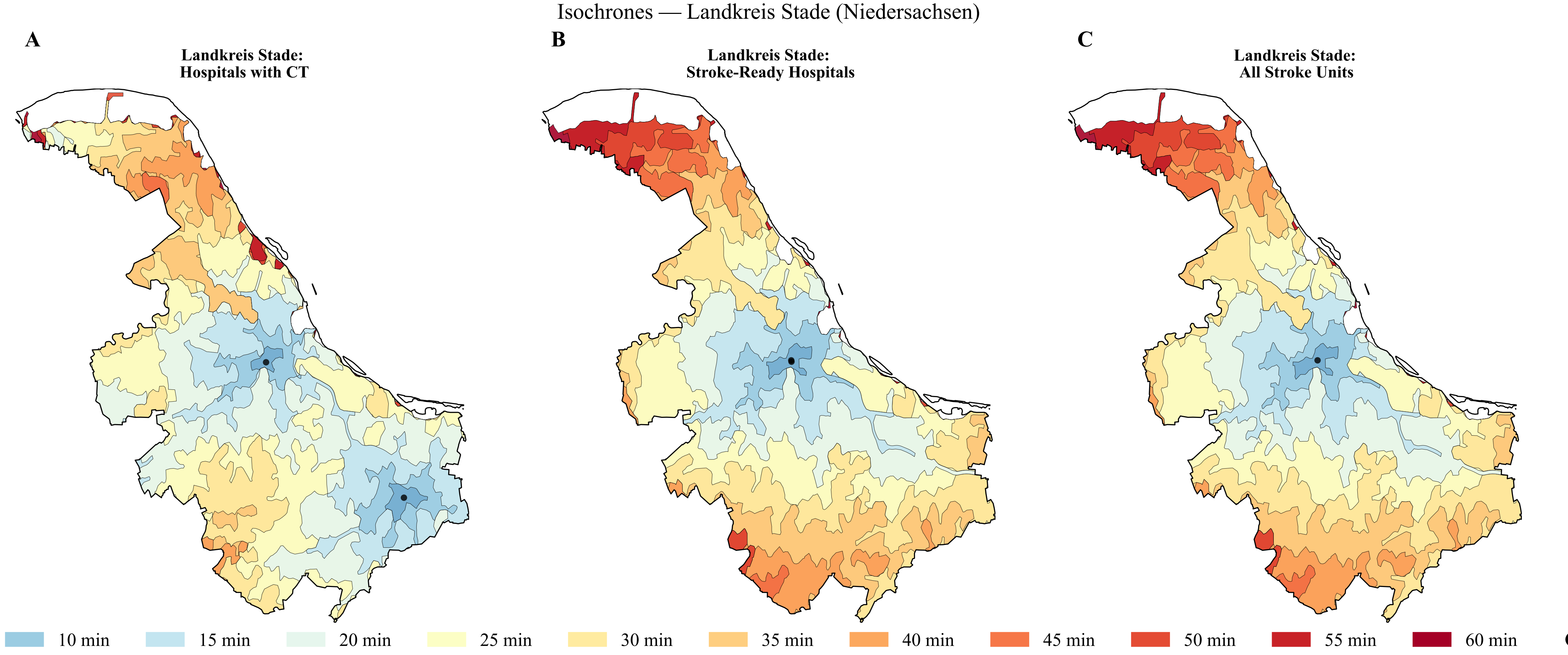

Isochrones — Landkreis Stade (Niedersachsen)
A
Landkreis Stade:
Hospitals with CT
B
Landkreis Stade:
Stroke-Ready Hospitals
C
Landkreis Stade:
All Stroke Units
10 min
15 min
20 min
25 min
30 min
35 min
40 min
45 min
50 min
55 min
60 min

Isochrones — Kreisfreie Stadt Oldenburg (Oldb) (Niedersachsen)

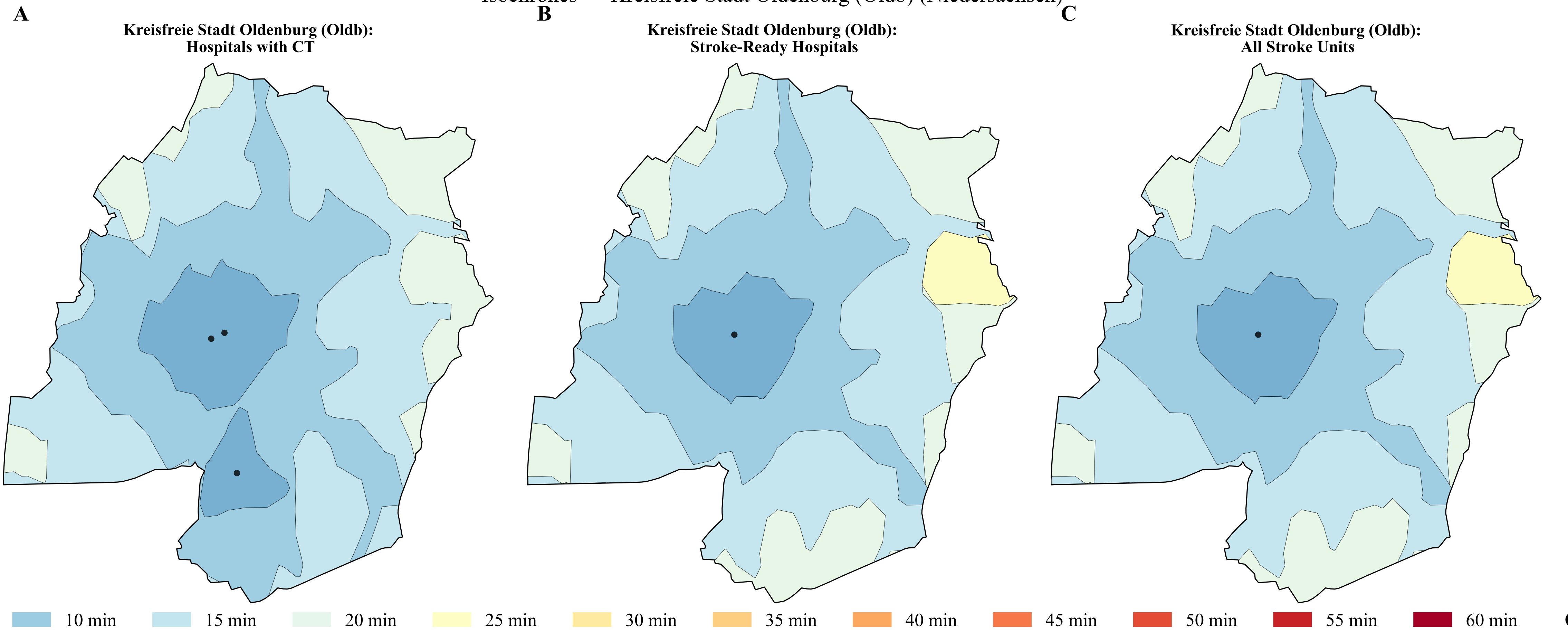

Isochrones — Landkreis Cloppenburg (Niedersachsen)

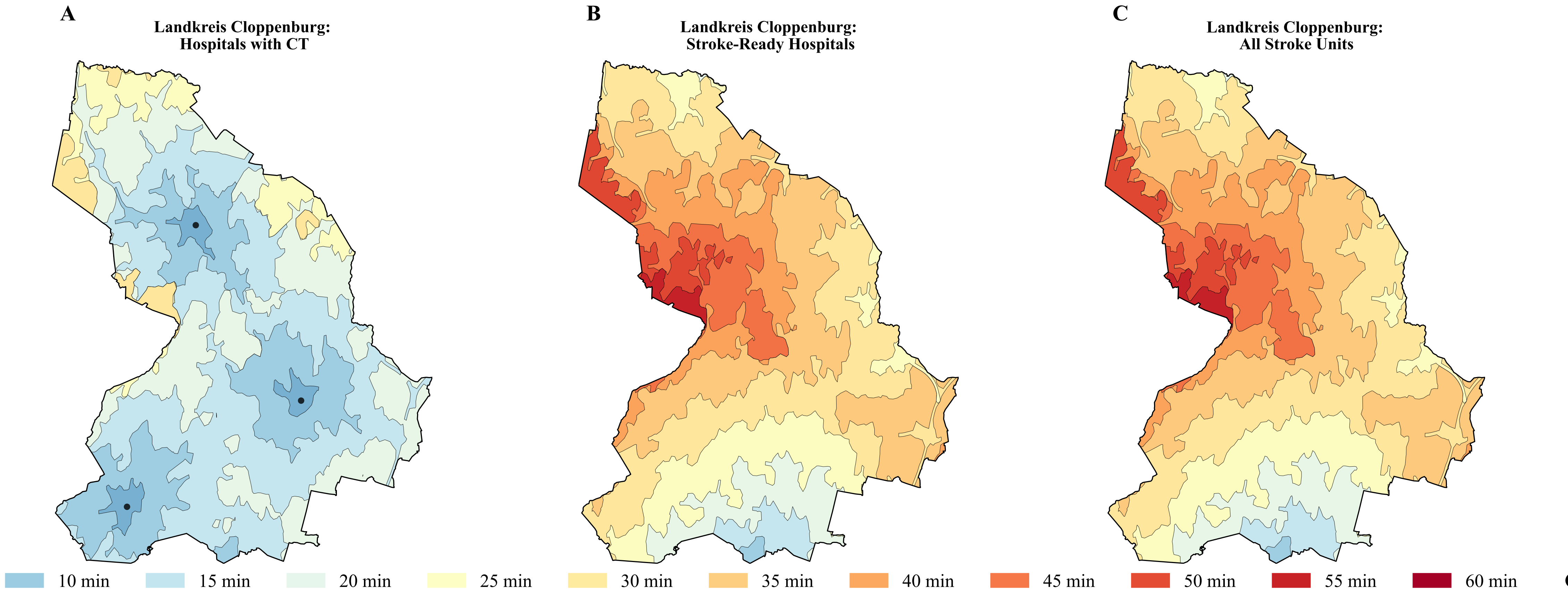

Isochrones — Kreisfreie Stadt Wuppertal (Nordrhein-Westfalen)

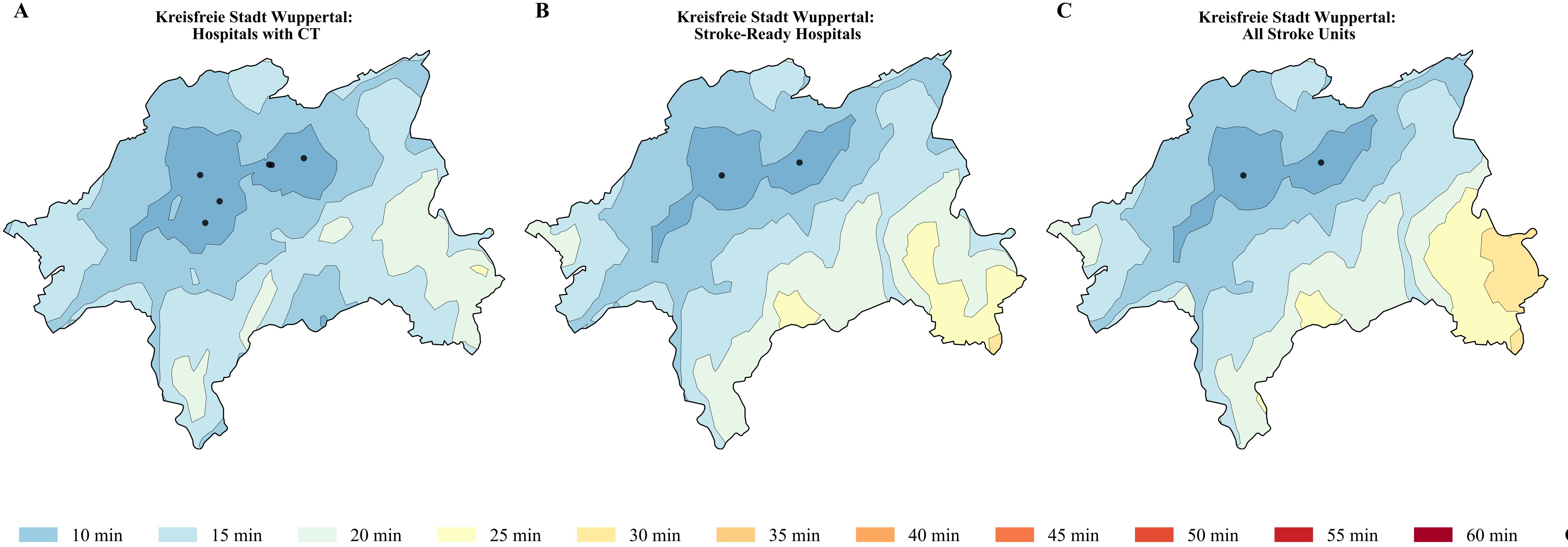

Isochrones — Landkreis Odenwaldkreis (Hessen)

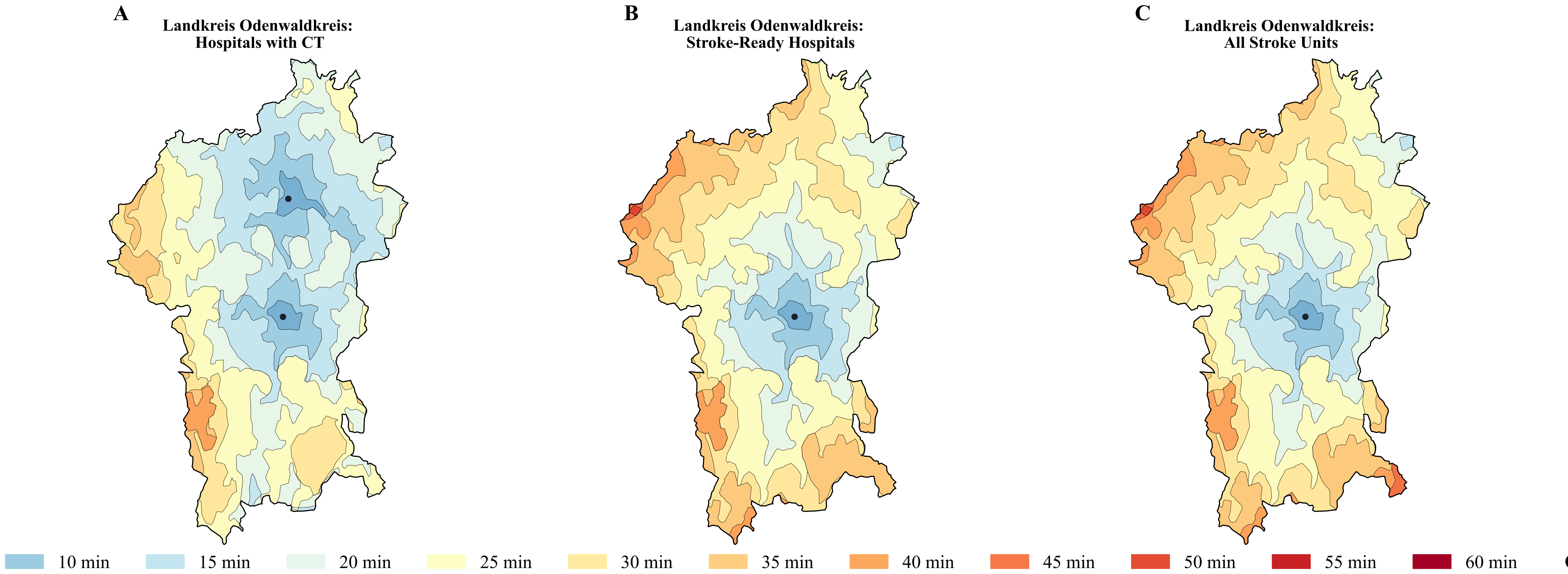

Isochrones — Landkreis Karlsruhe (Baden-Württemberg)

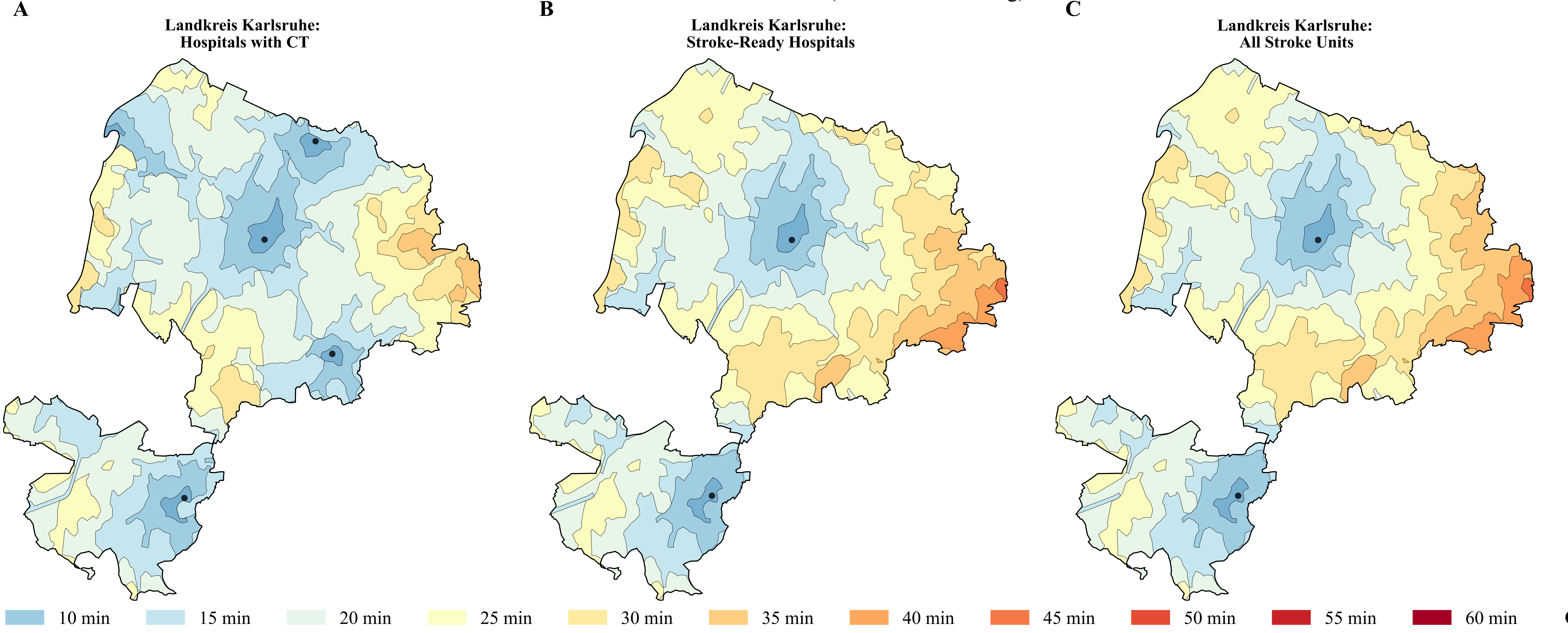

Isochrones — Kreisfreie Stadt Landshut (Bayern)

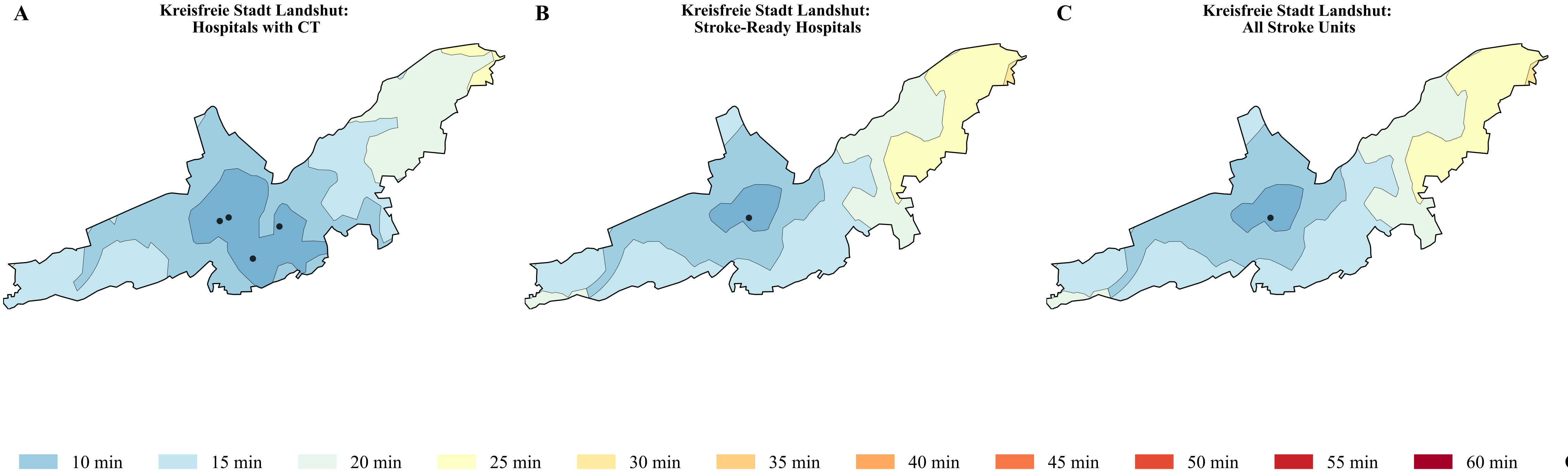

Isochrones — Kreisfreie Stadt Erlangen (Bayern)

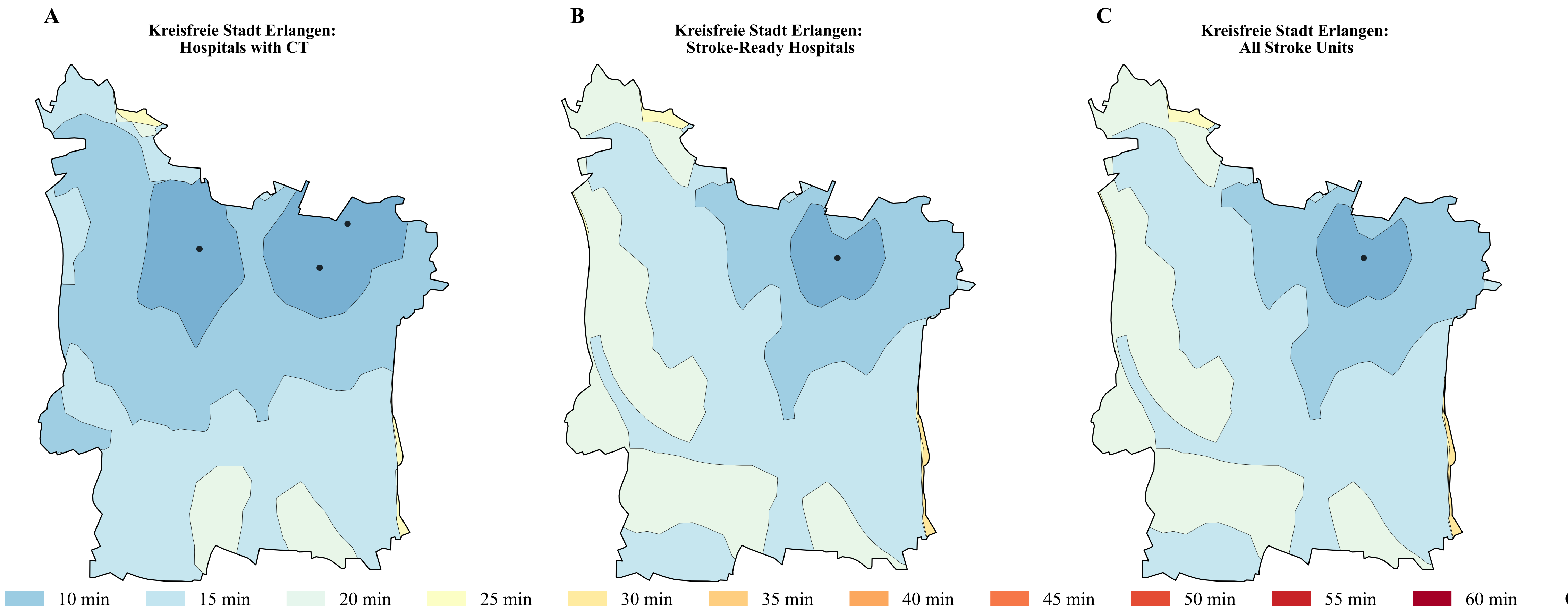

Isochrones — Landkreis Haßberge (Bayern)

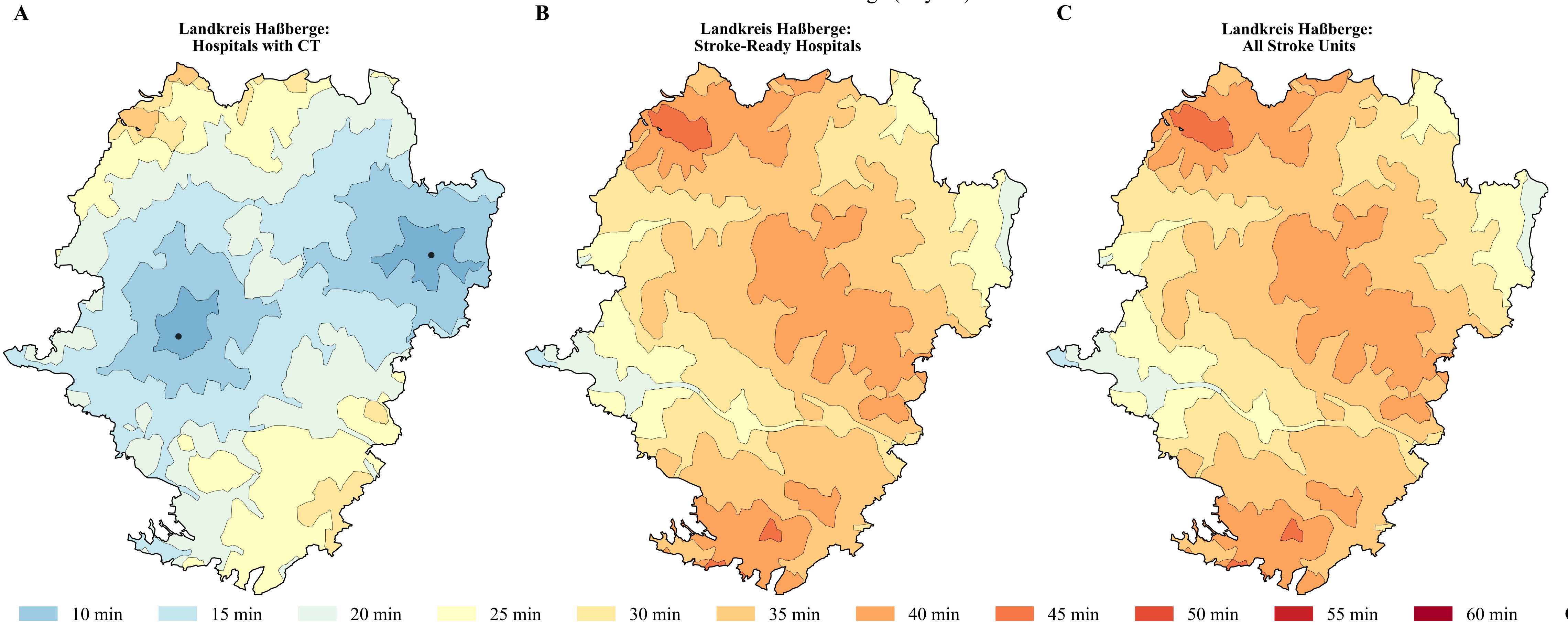

Isochrones — Landkreis Aichach-Friedberg (Bayern)

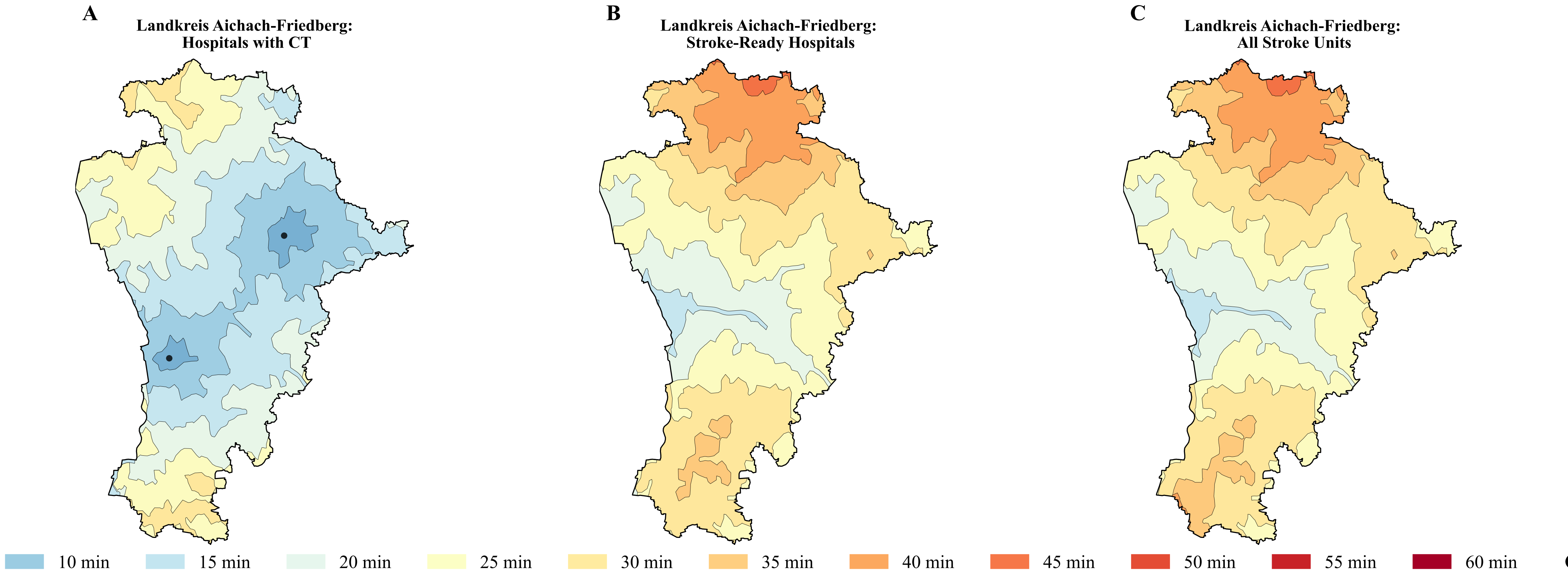

Isochrones — Landkreis Dillingen a.d.Donau (Bayern)

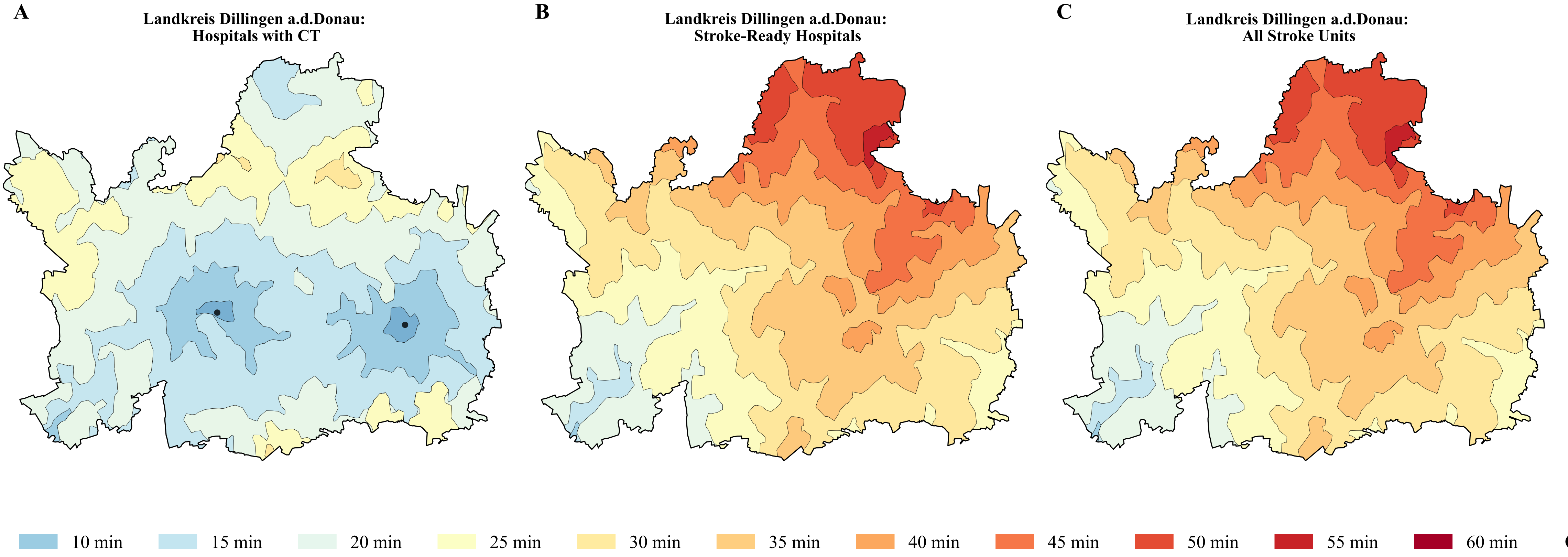

Isochrones — Landkreis Oberspreewald-Lausitz (Brandenburg)

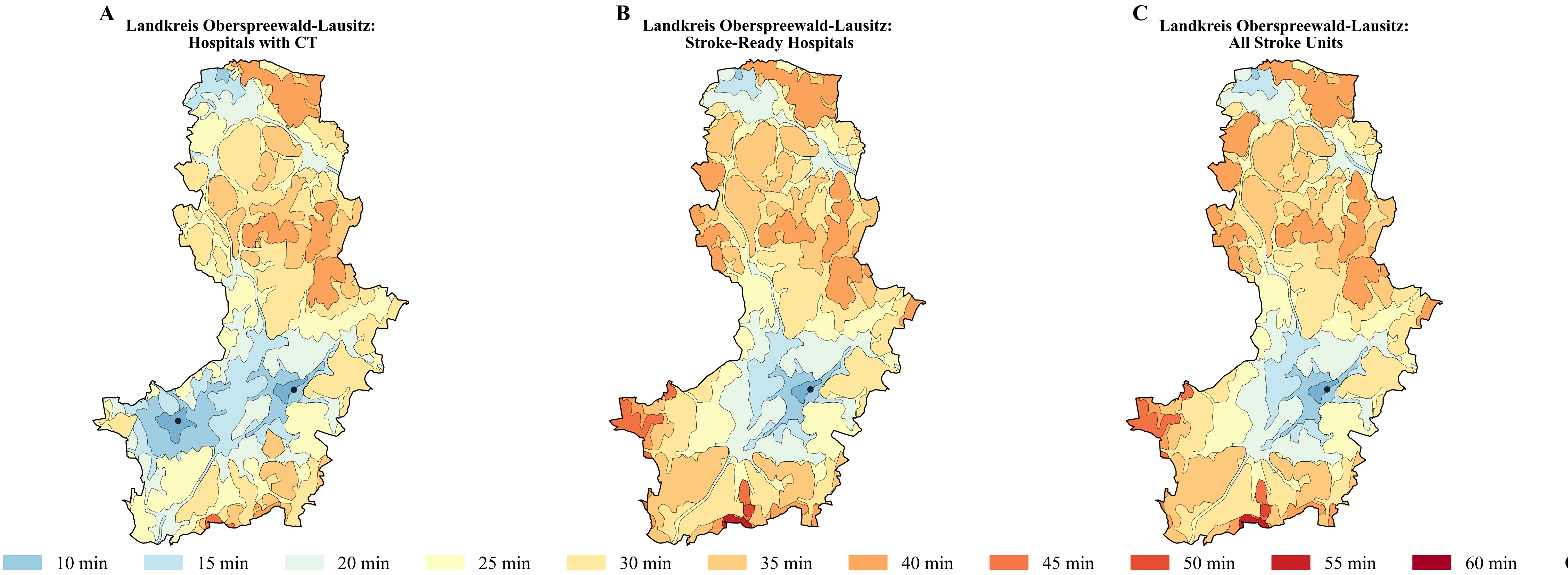

637 / 5833

Isochrones — Landkreis Bautzen (Sachsen)

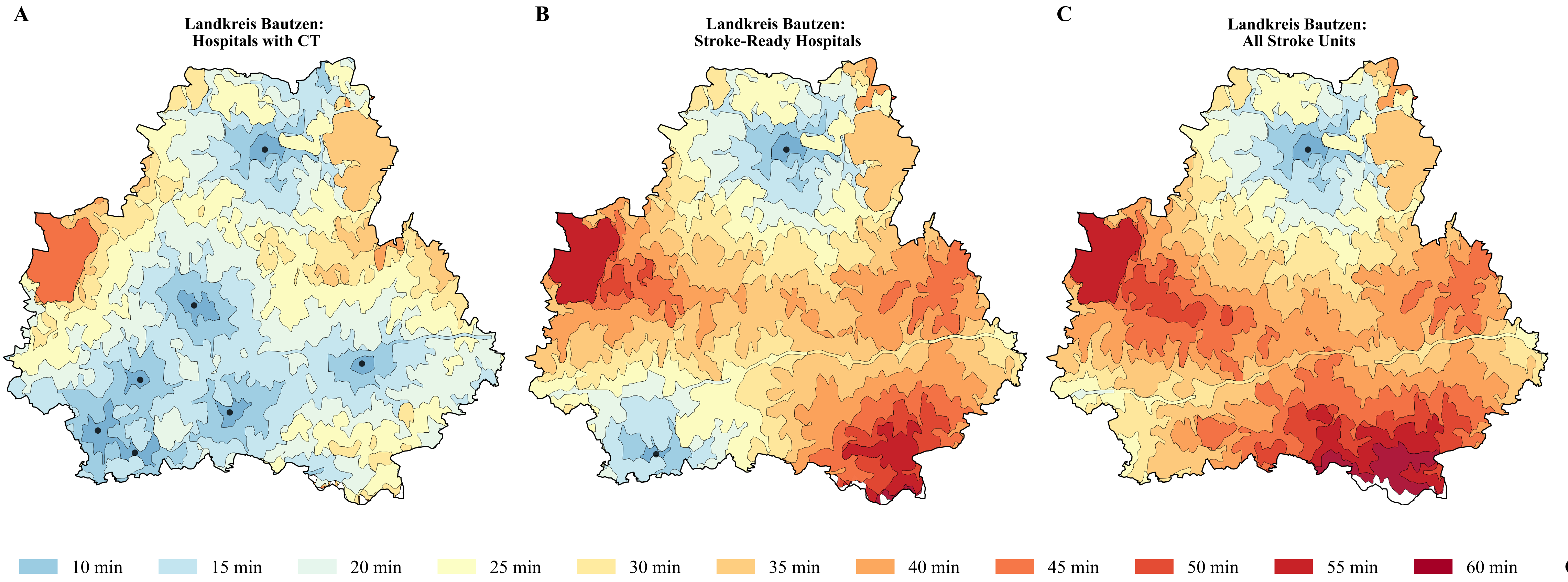

Isochrones — Kreisfreie Stadt Dessau-Roßlau (Sachsen-Anhalt)

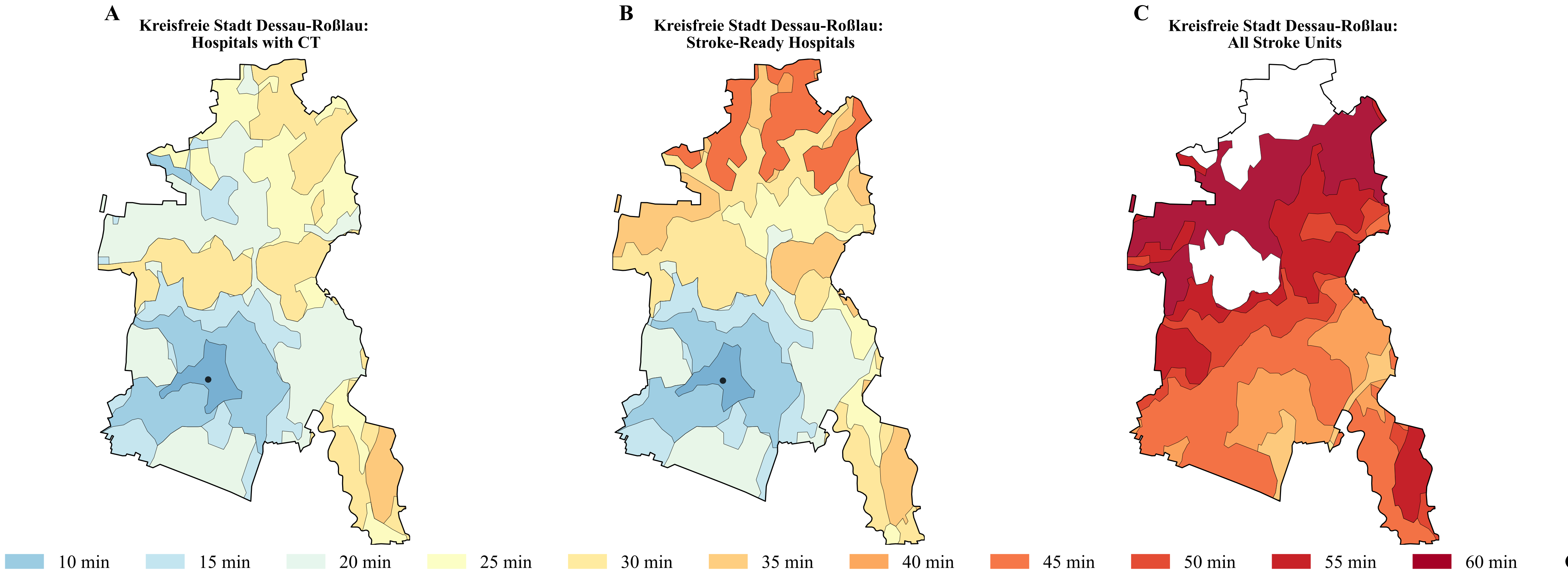

Isochrones — Landkreis Lüneburg (Niedersachsen)

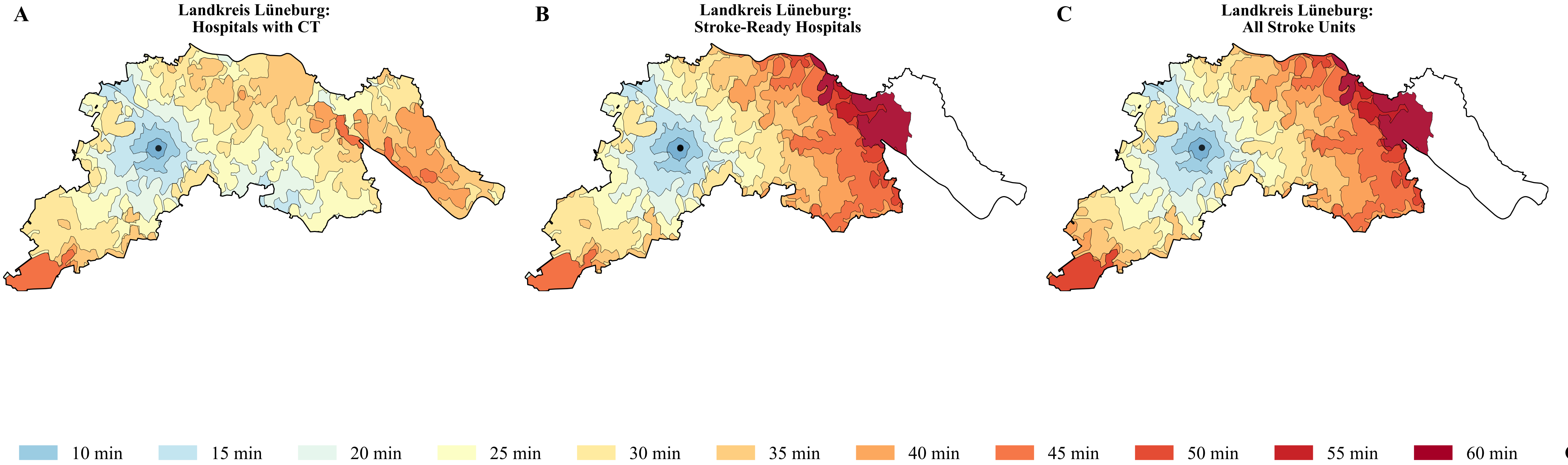

Isochrones — Kreisfreie Stadt Bremerhaven (Bremen)

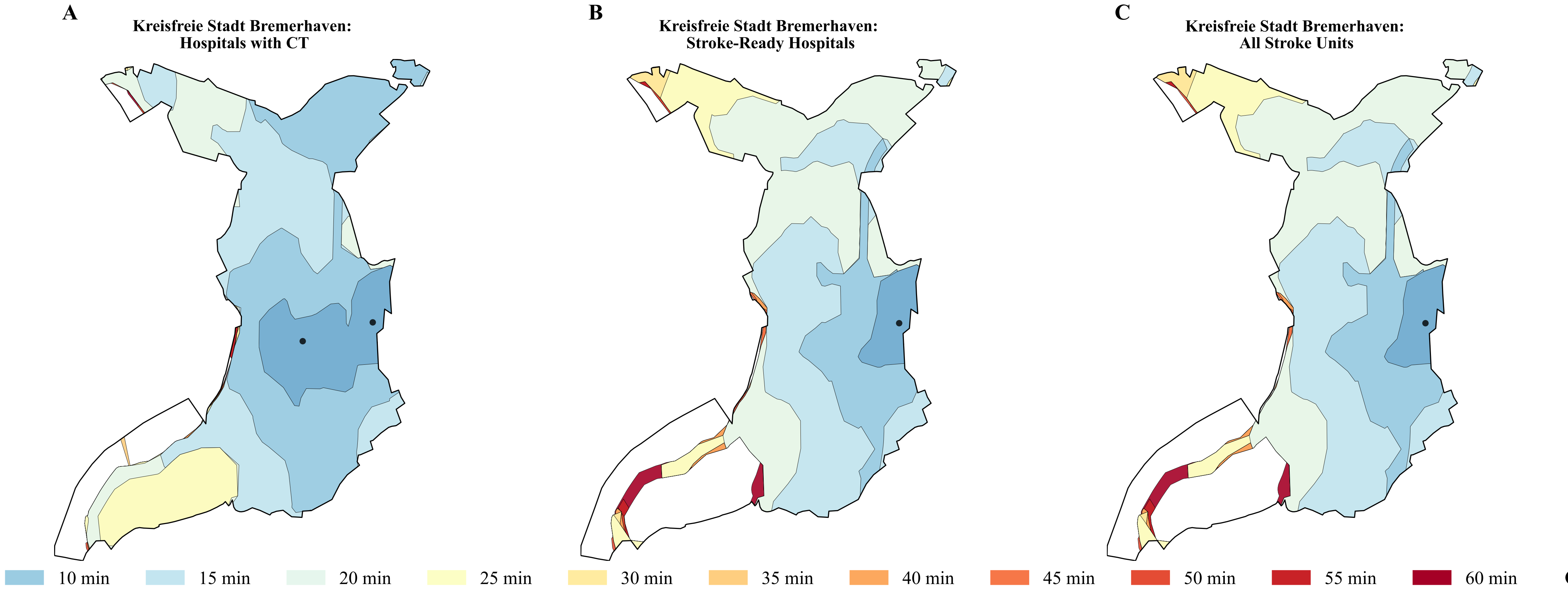

Isochrones — Kreisfreie Stadt Koblenz (Rheinland-Pfalz)

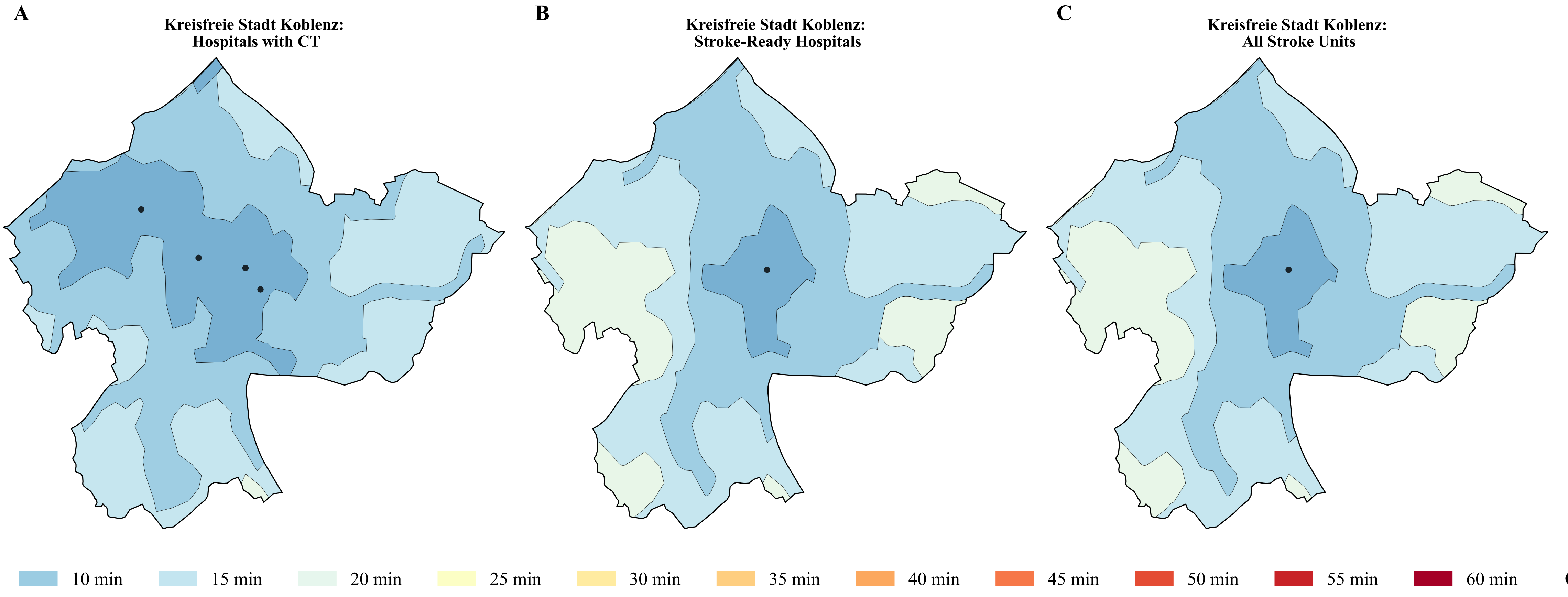

Isochrones — Kreisfreie Stadt Pirmasens (Rheinland-Pfalz)

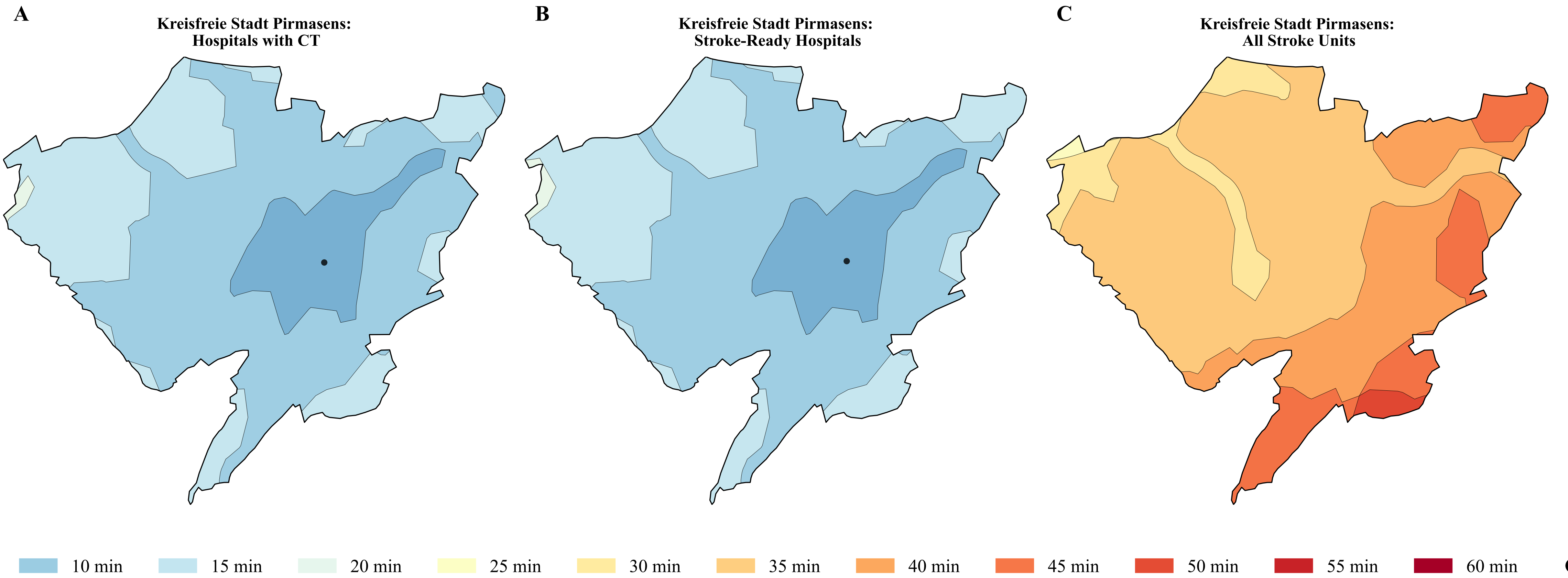

643 / 5833

Isochrones — Landkreis Donnersbergkreis (Rheinland-Pfalz)

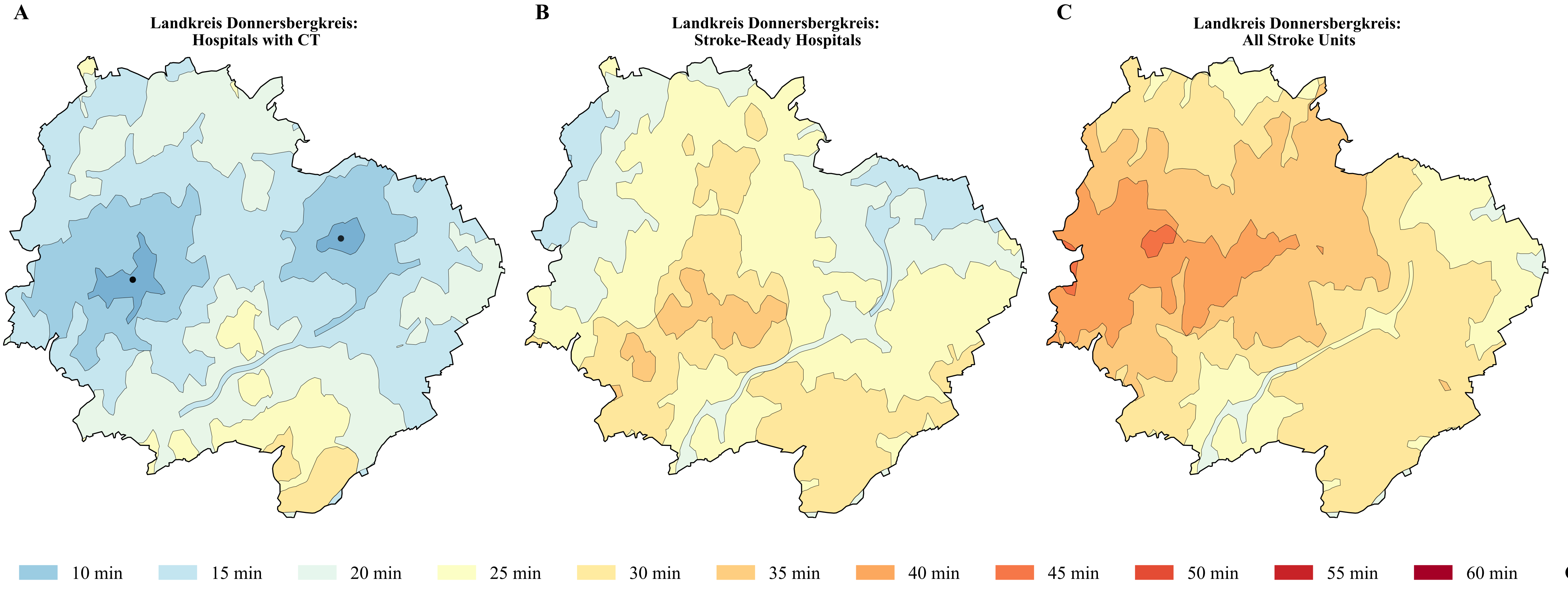

Isochrones — Stadtkreis Heidelberg (Baden-Württemberg)

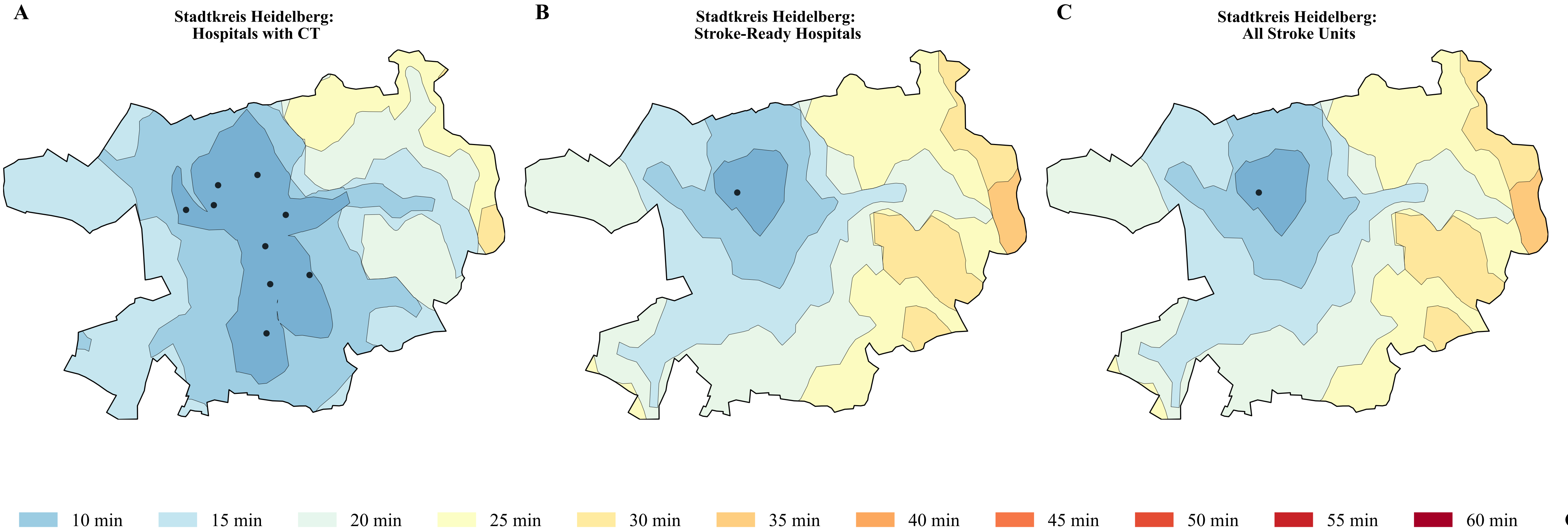

Isochrones — Kreisfreie Stadt Straubing (Bayern)

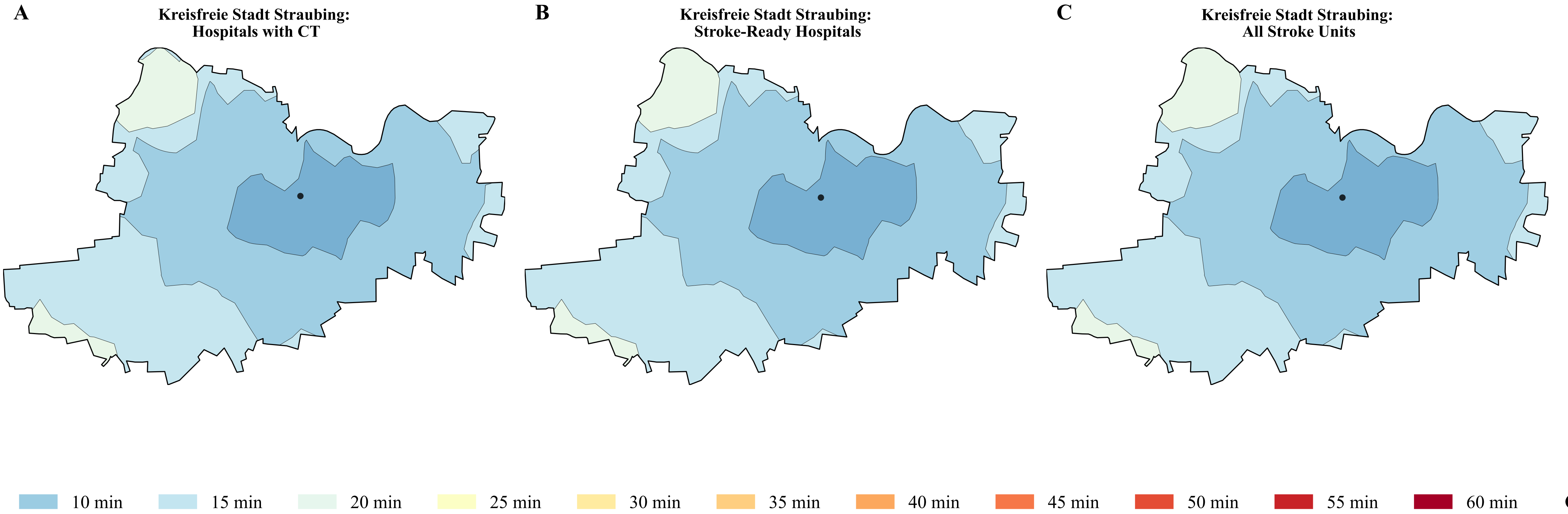

Isochrones — Landkreis Straubing-Bogen (Bayern)

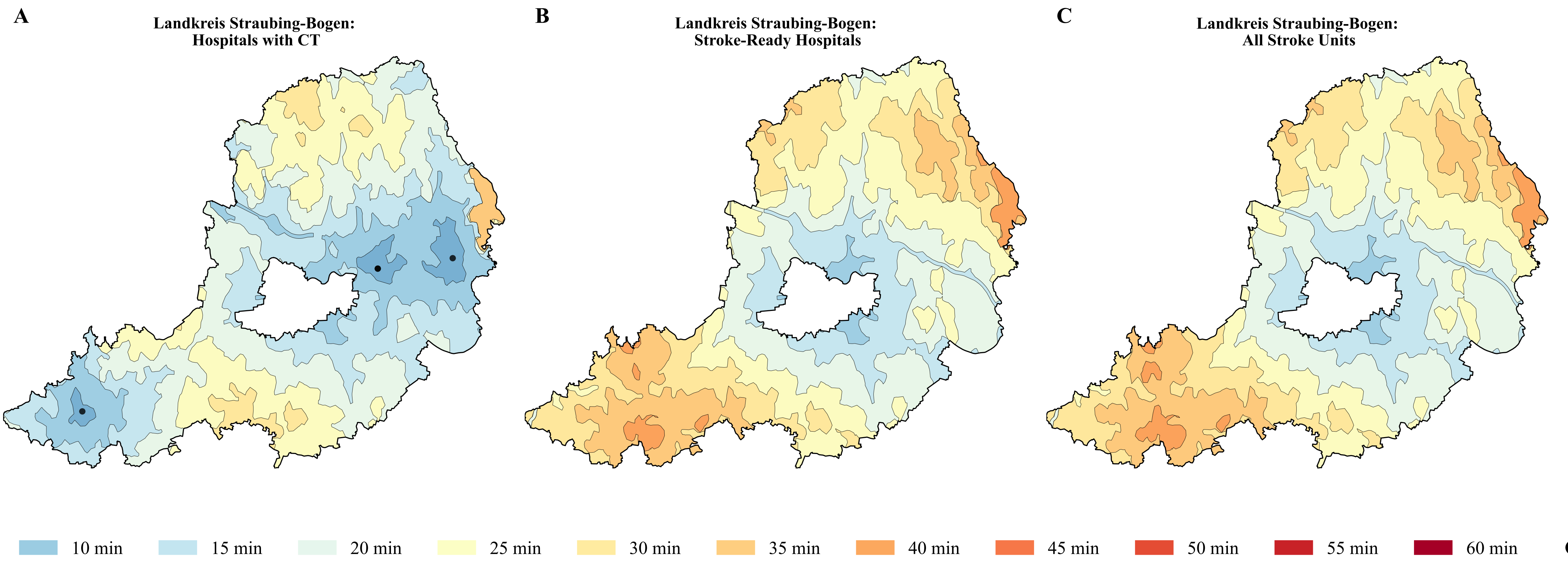

Isochrones — Landkreis Neumarkt i.d.OPf. (Bayern)

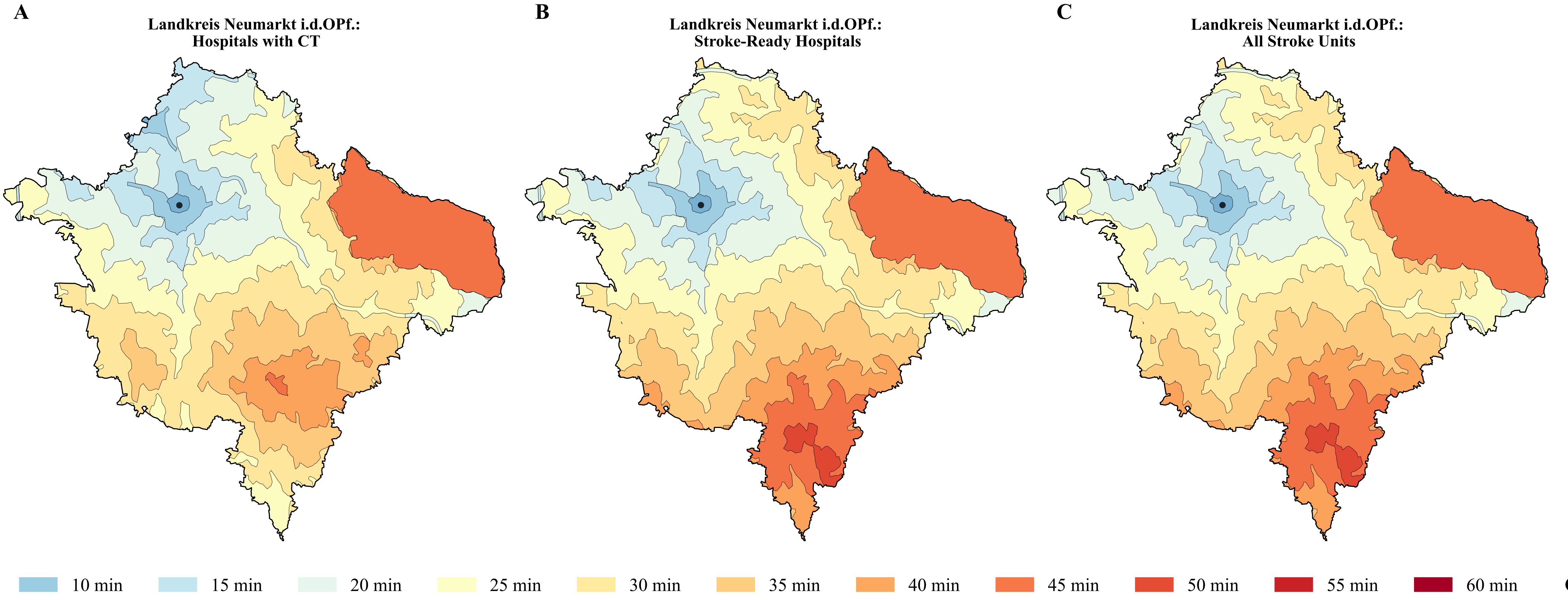

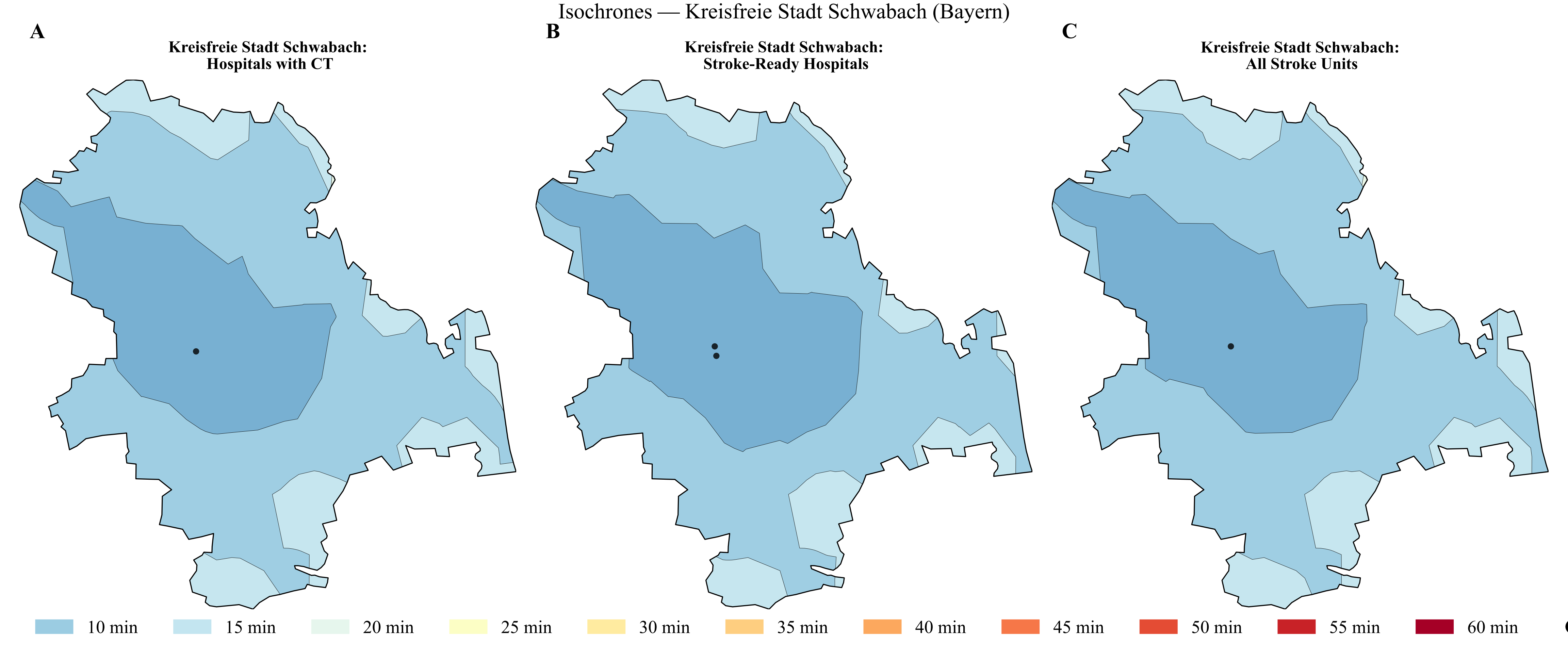
Isochrones — Kreisfreie Stadt Schwabach (Bayern)
A
Kreisfreie Stadt Schwabach:
Hospitals with CT
B
Kreisfreie Stadt Schwabach:
Stroke-Ready Hospitals
C
Kreisfreie Stadt Schwabach:
All Stroke Units
10 min
15 min
20 min
25 min
30 min
35 min
40 min
45 min
50 min
55 min
60 min

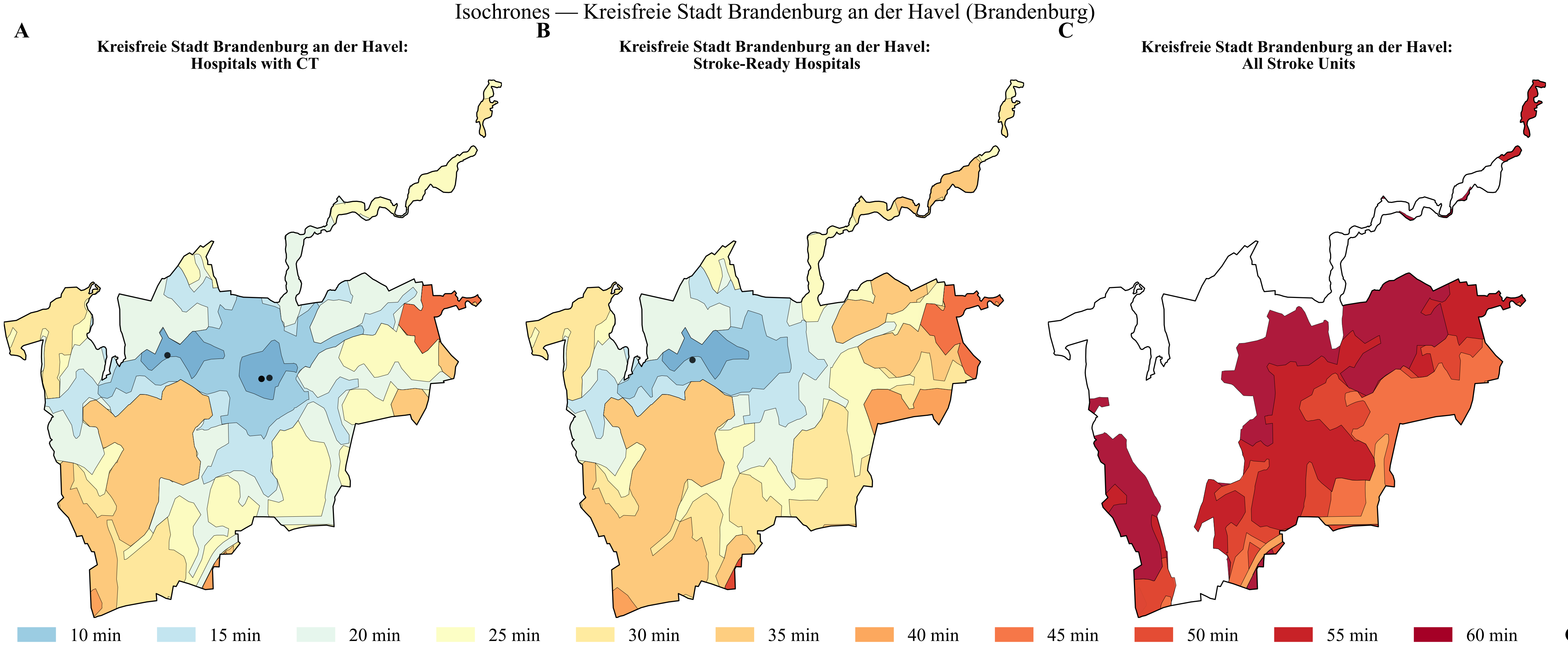
Isochrones — Kreisfreie Stadt Brandenburg an der Havel (Brandenburg)
A
Kreisfreie Stadt Brandenburg an der Havel:
Hospitals with CT
B
Kreisfreie Stadt Brandenburg an der Havel:
Stroke-Ready Hospitals
C
Kreisfreie Stadt Brandenburg an der Havel:
All Stroke Units
10 min
15 min
20 min
25 min
30 min
35 min
40 min
45 min
50 min
55 min
60 min

Isochrones — Landkreis Ludwigslust-Parchim (Mecklenburg-Vorpommern)

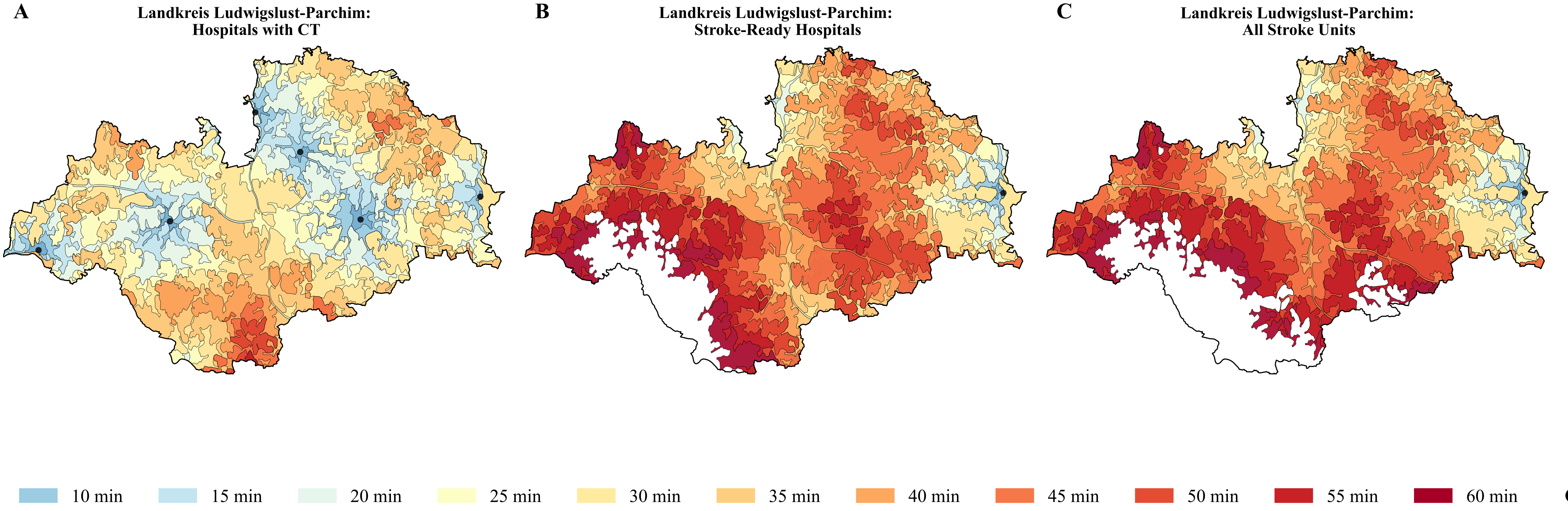

Isochrones — Landkreis Saalfeld-Rudolstadt (Thüringen)

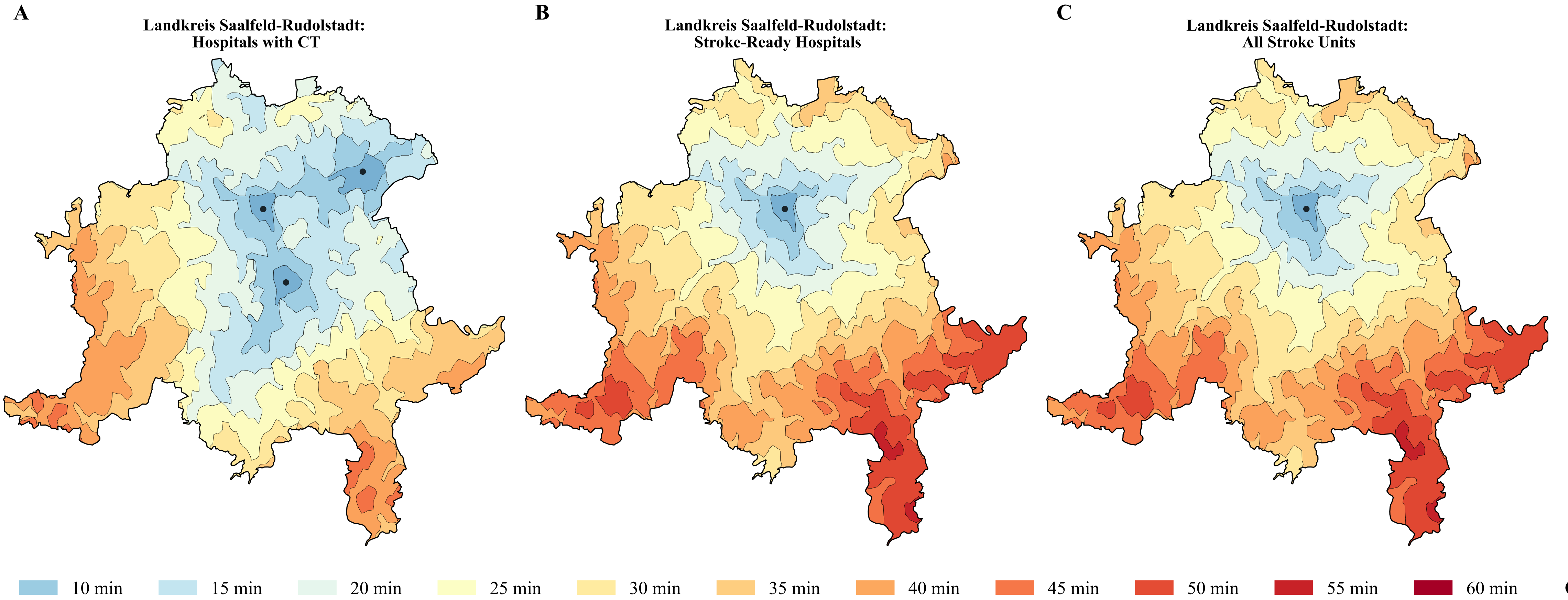

Isochrones — Kreis Ostholstein (Schleswig-Holstein)

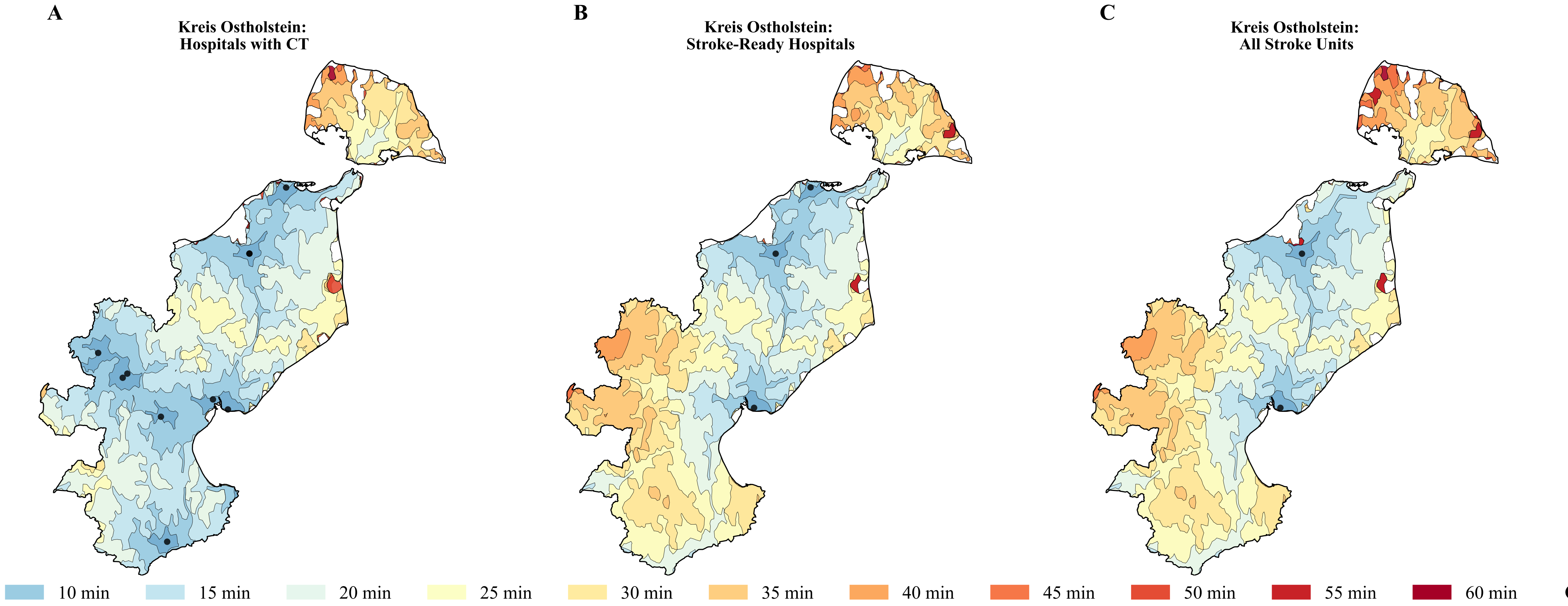

Isochrones — Kreis Rendsburg-Eckernförde (Schleswig-Holstein)

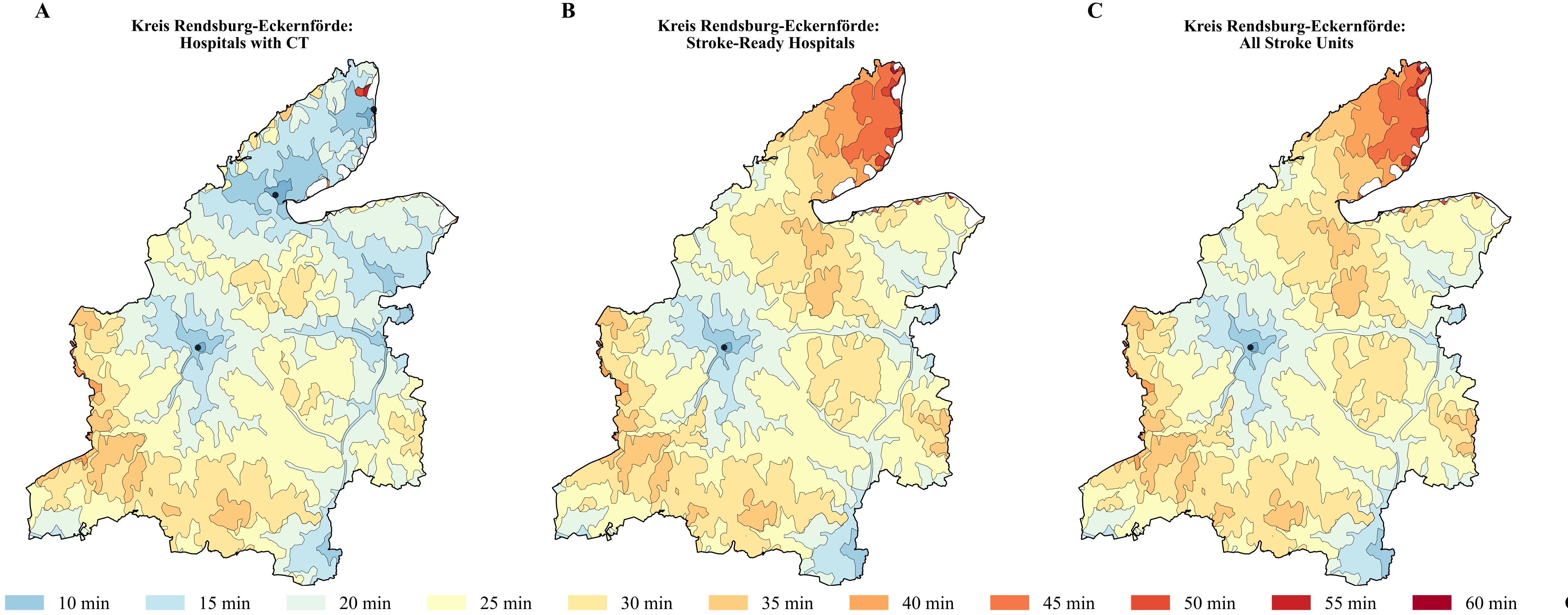

Isochrones — Landkreis Ostalbkreis (Baden-Württemberg)

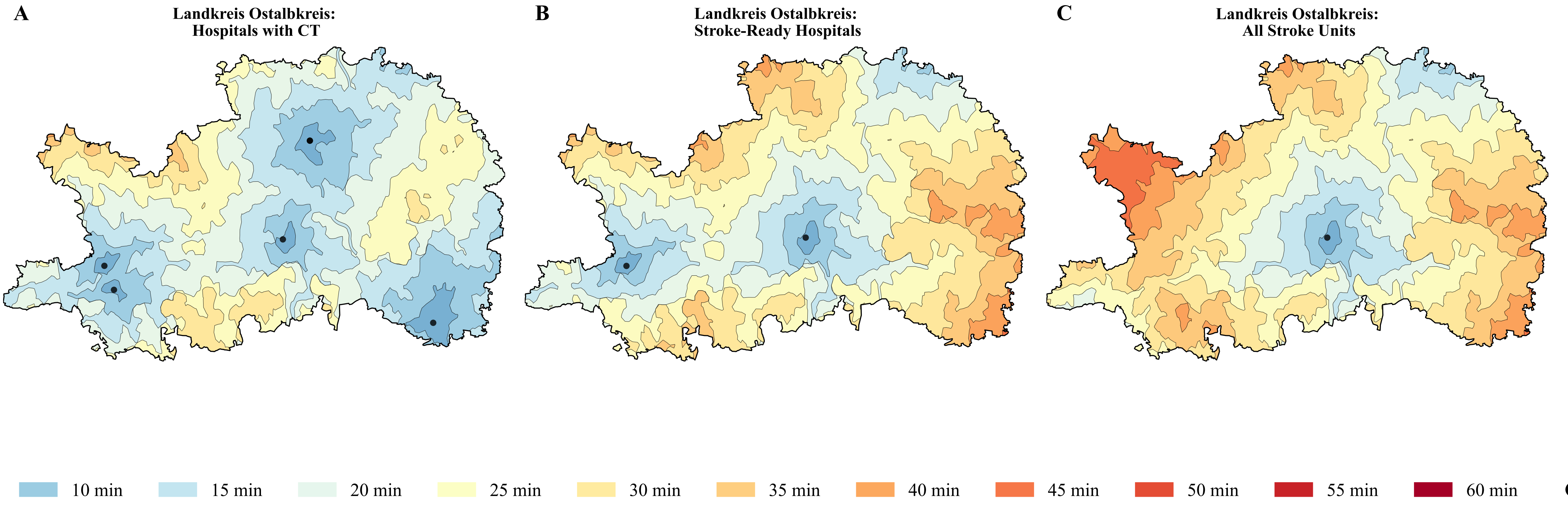

Isochrones — Landkreis Emmendingen (Baden-Württemberg)

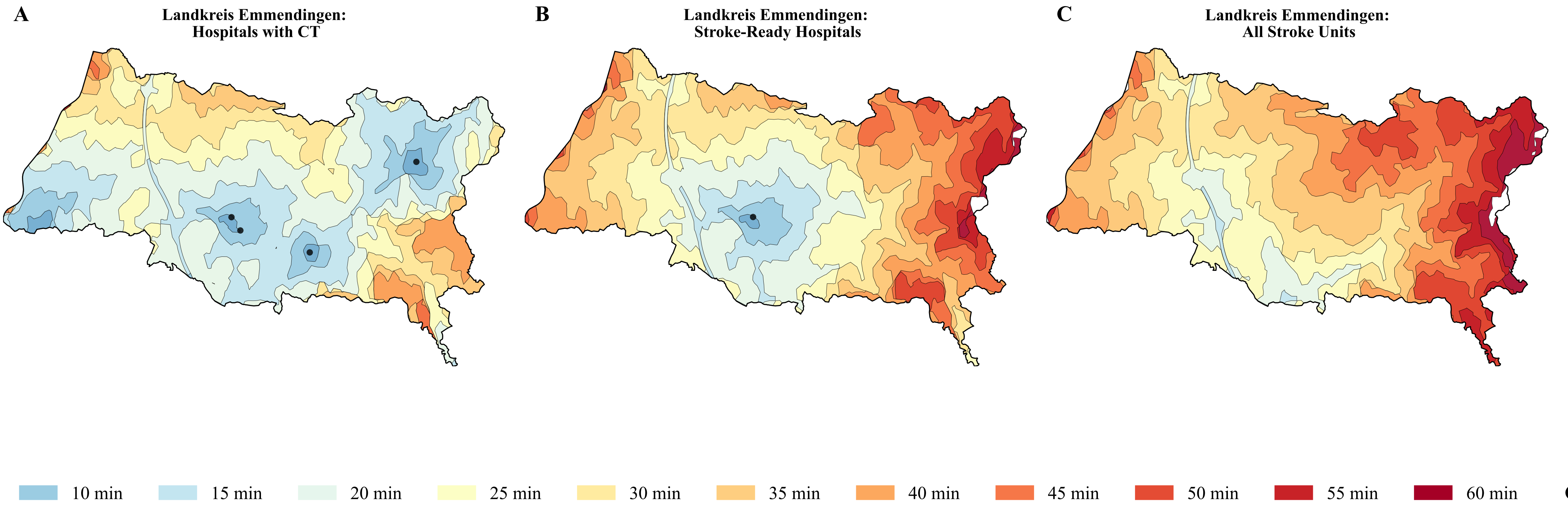

Isochrones — Kreisfreie Stadt München (Bayern)

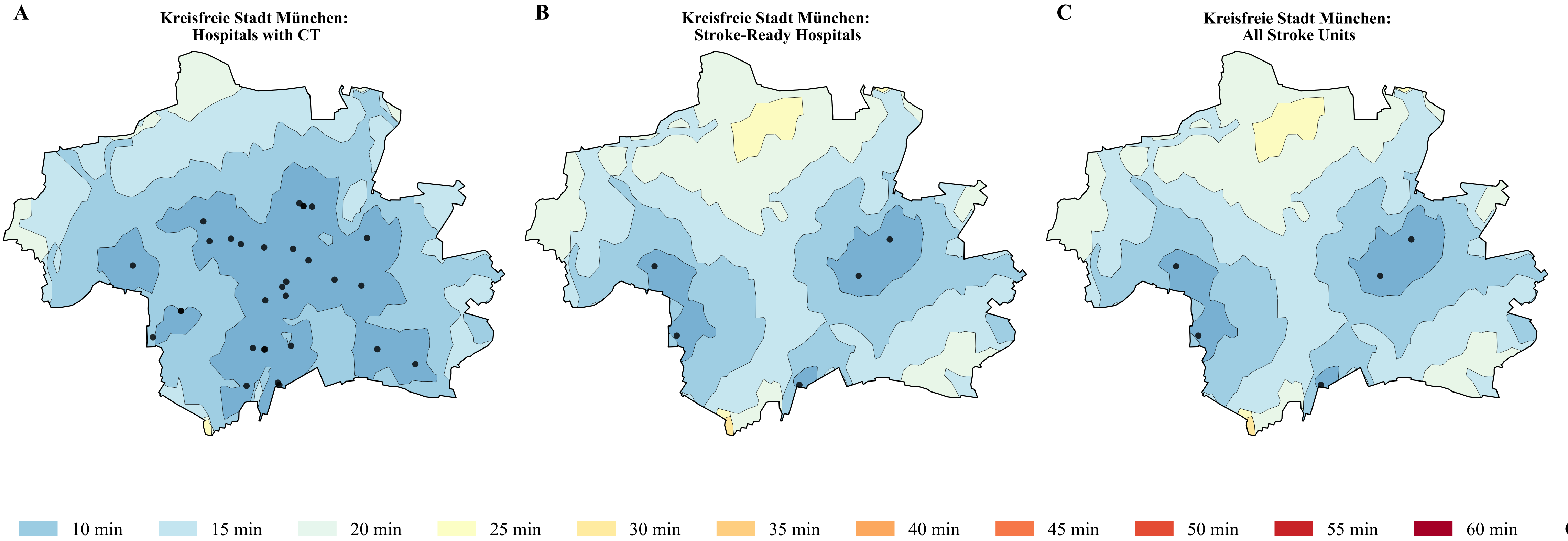

Isochrones — Landkreis Bayreuth (Bayern)

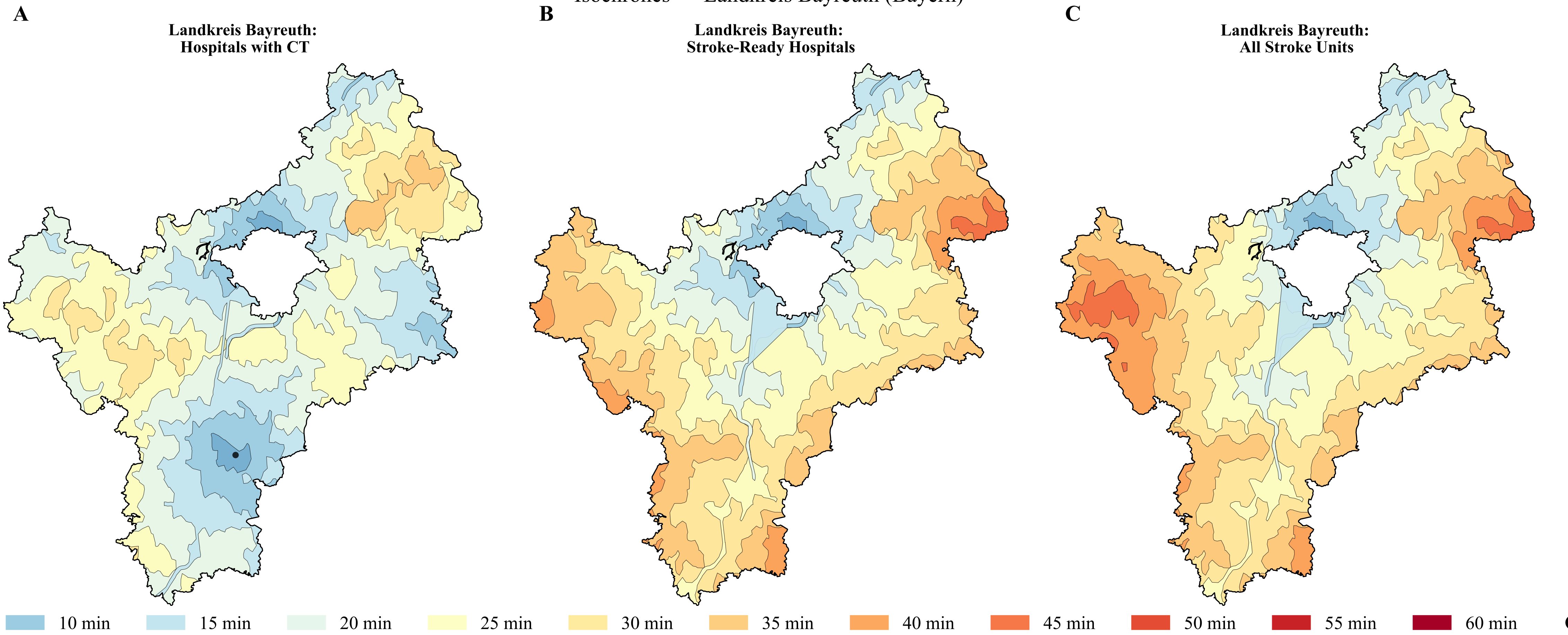

Isochrones — Landkreis Kronach (Bayern)

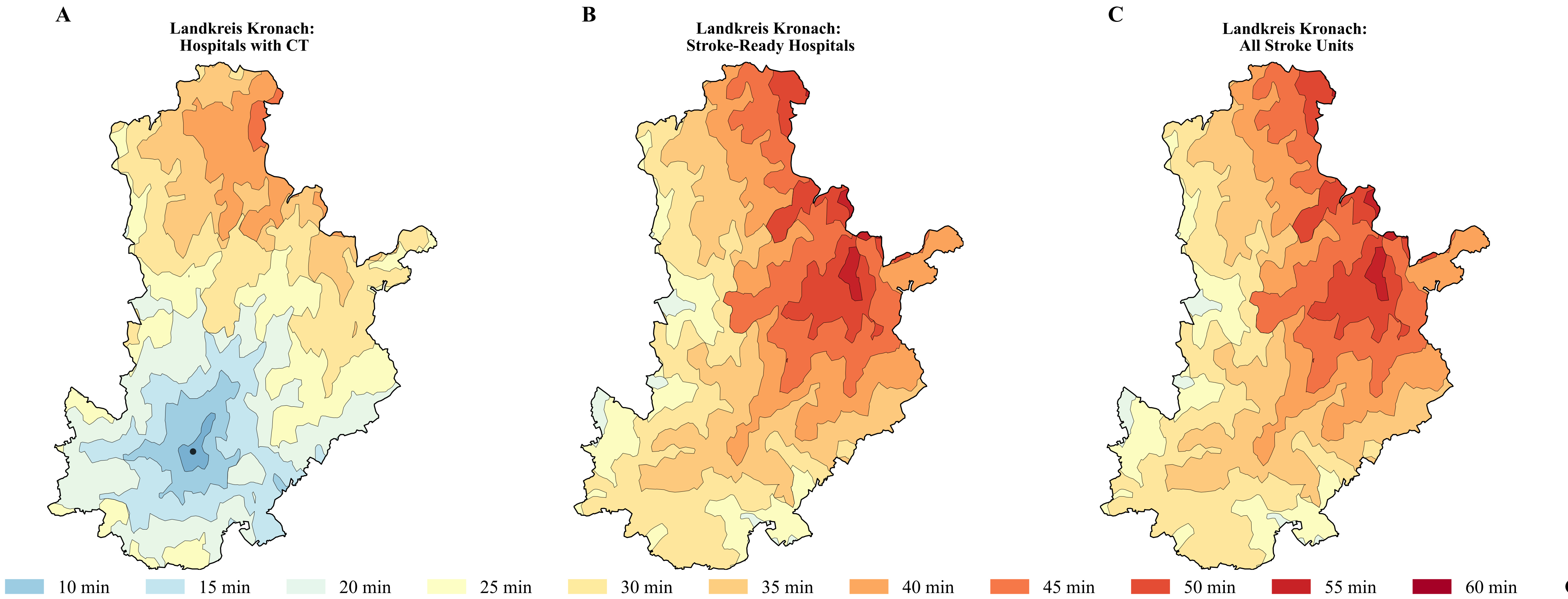

Isochrones — Landkreis Aschaffenburg (Bayern)

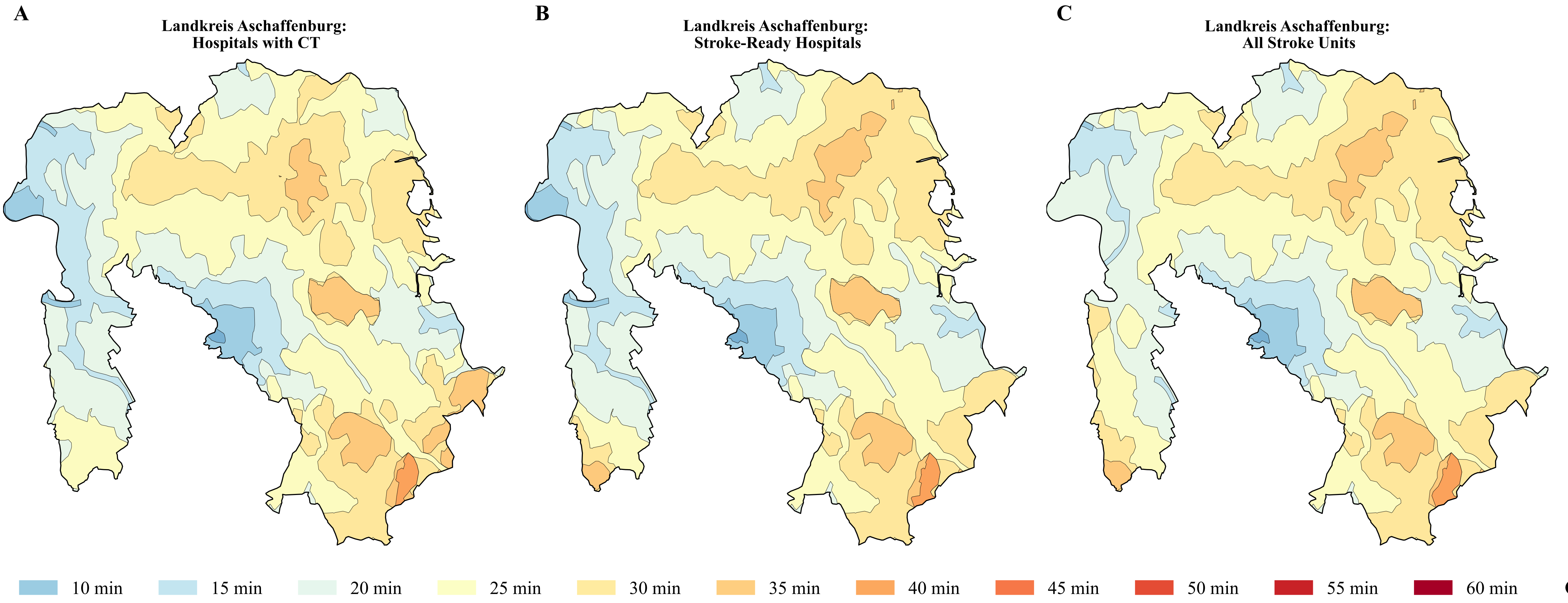

Isochrones — Landkreis Saalekreis (Sachsen-Anhalt)

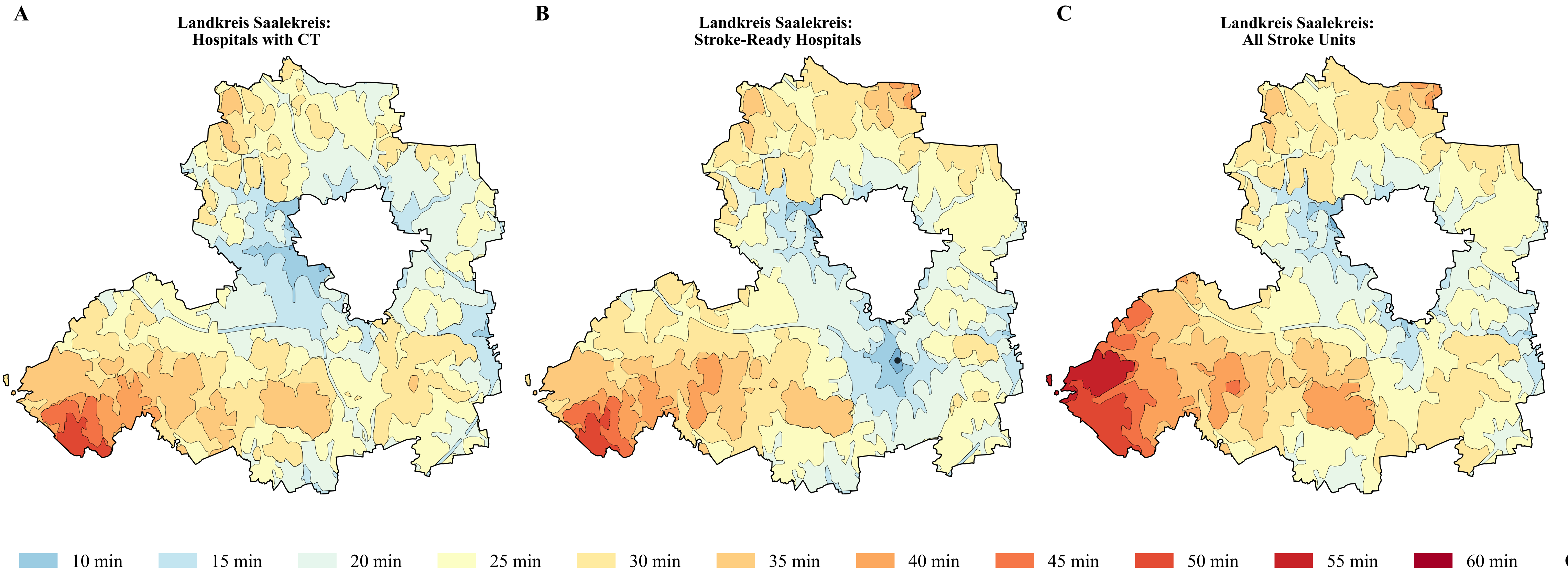

Isochrones — Kreisfreie Stadt Wolfsburg (Niedersachsen)

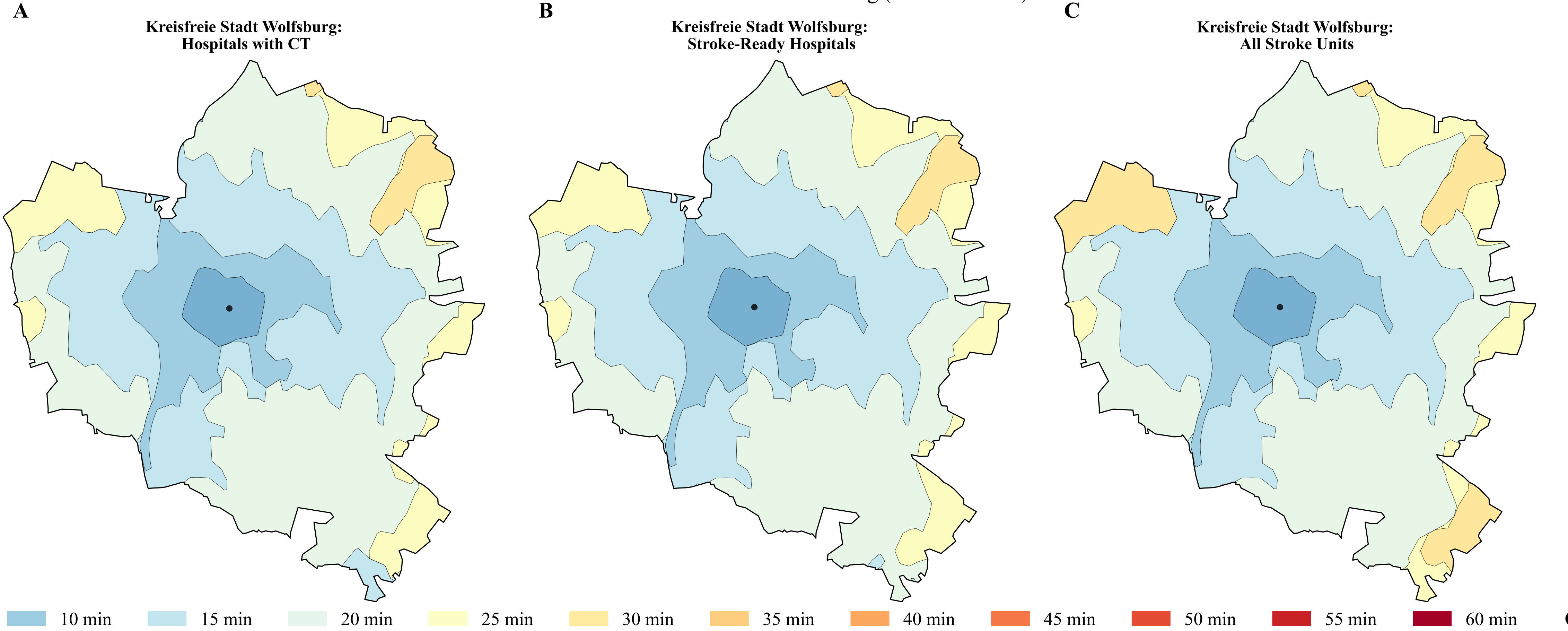

662 / 5833

Isochrones — Landkreis Wolfenbüttel (Niedersachsen)

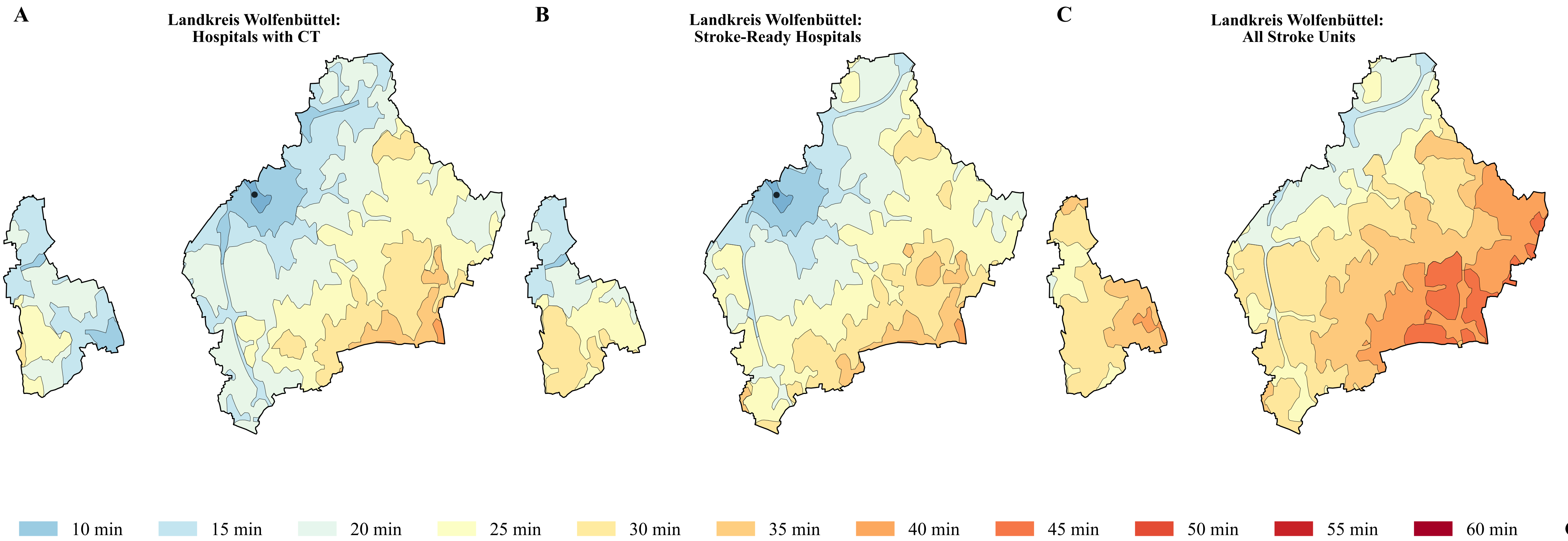

Isochrones — Landkreis Ammerland (Niedersachsen)

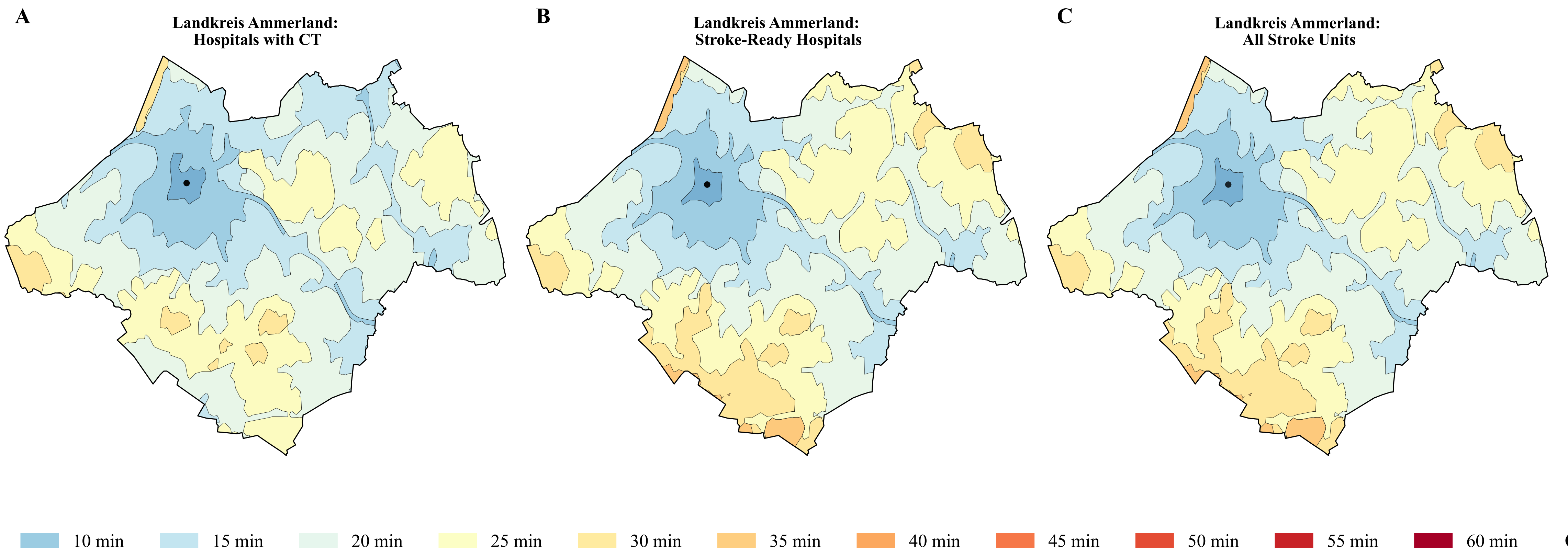

Isochrones — Kreis Rhein-Kreis Neuss (Nordrhein-Westfalen)

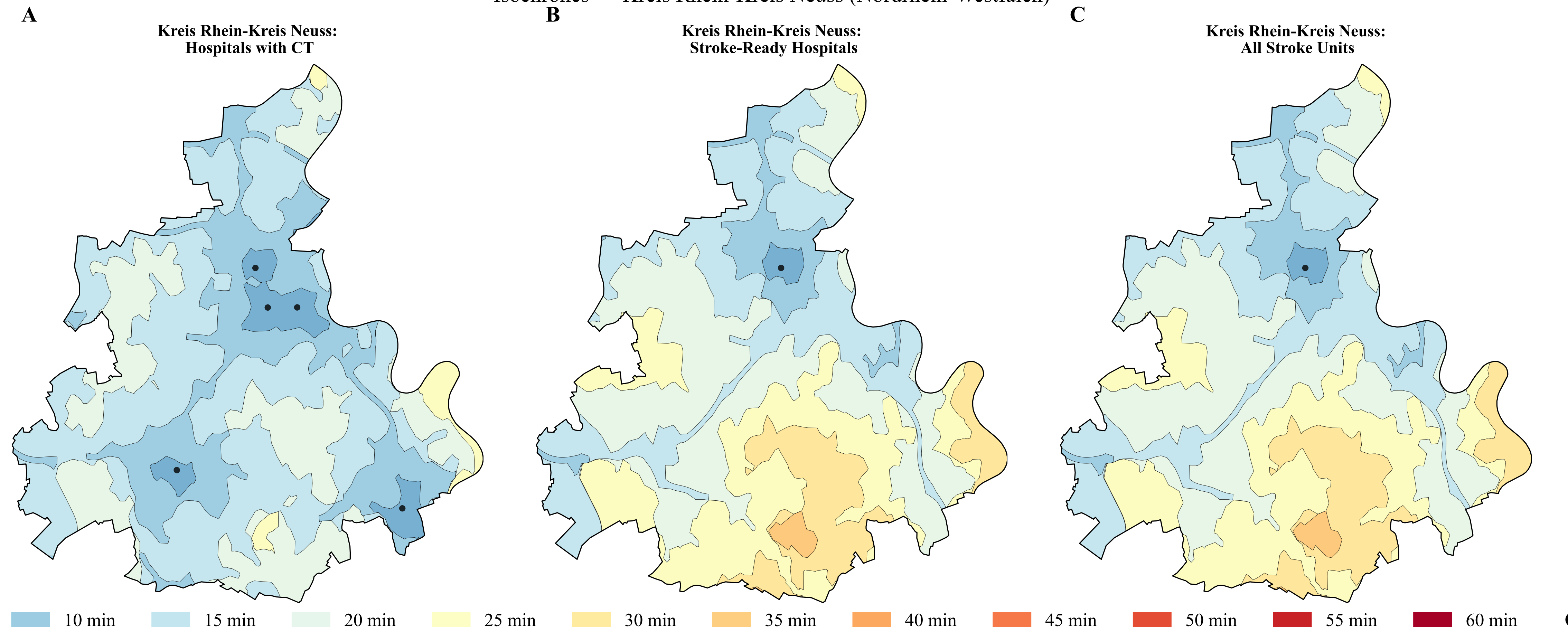

Isochrones — Kreis Lippe (Nordrhein-Westfalen)

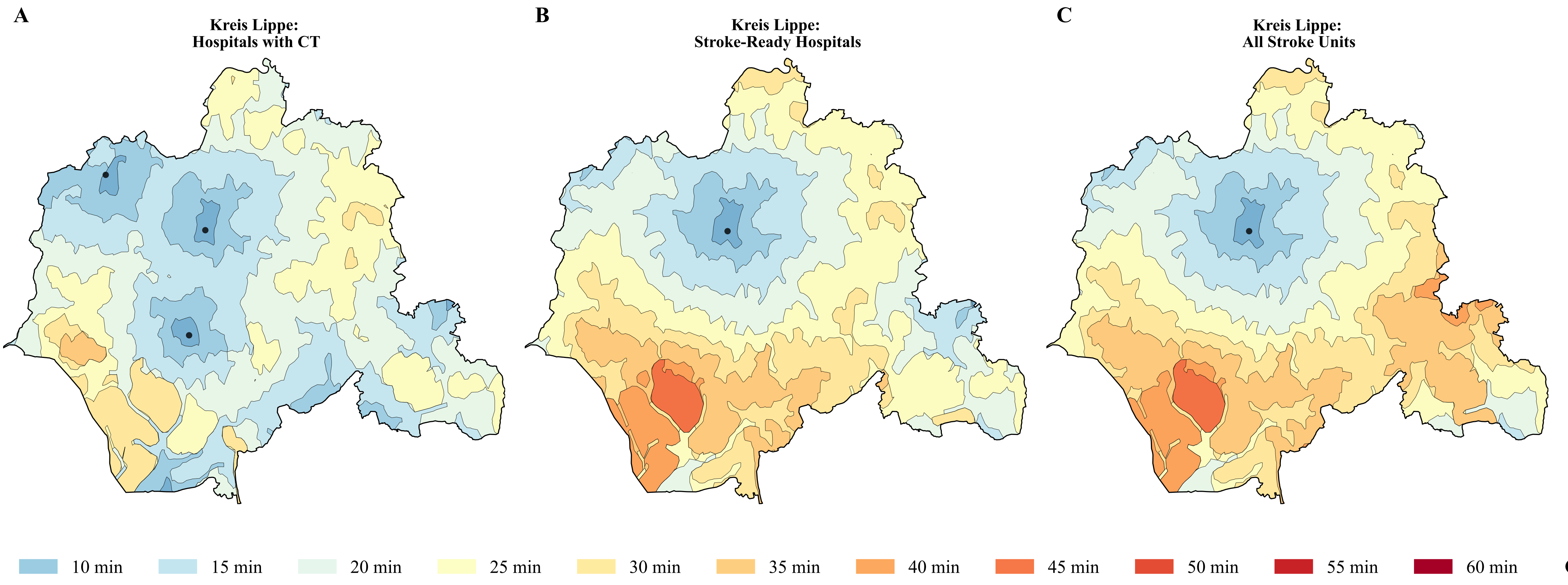

Isochrones — Kreisfreie Stadt Hagen (Nordrhein-Westfalen)

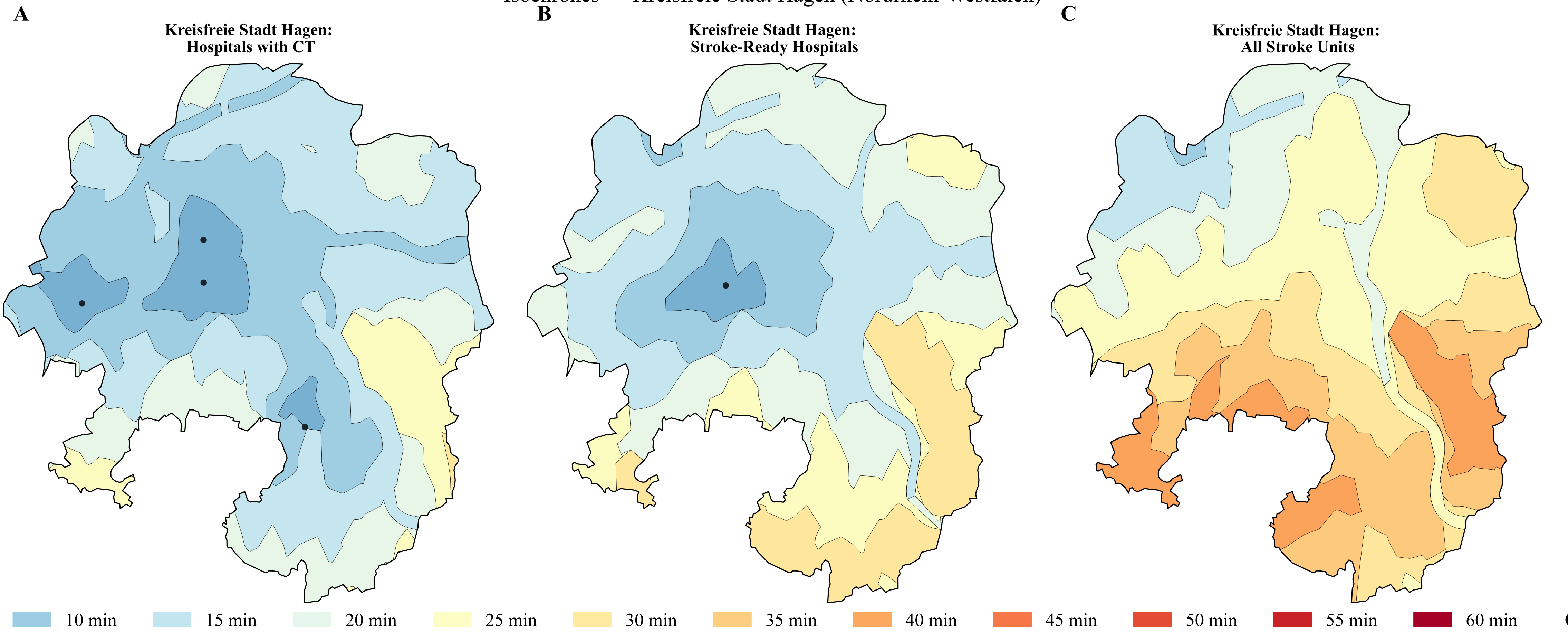

Isochrones — Landkreis Alzey-Worms (Rheinland-Pfalz)

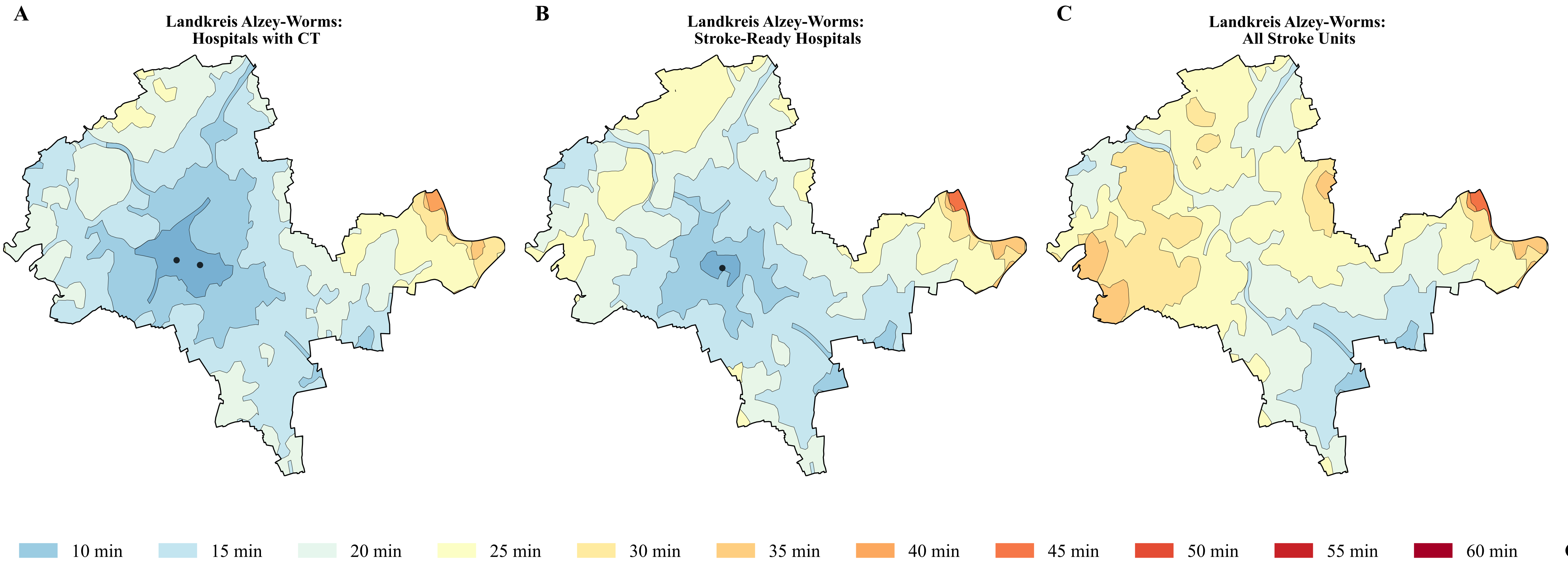

Isochrones — Landkreis Germersheim (Rheinland-Pfalz)

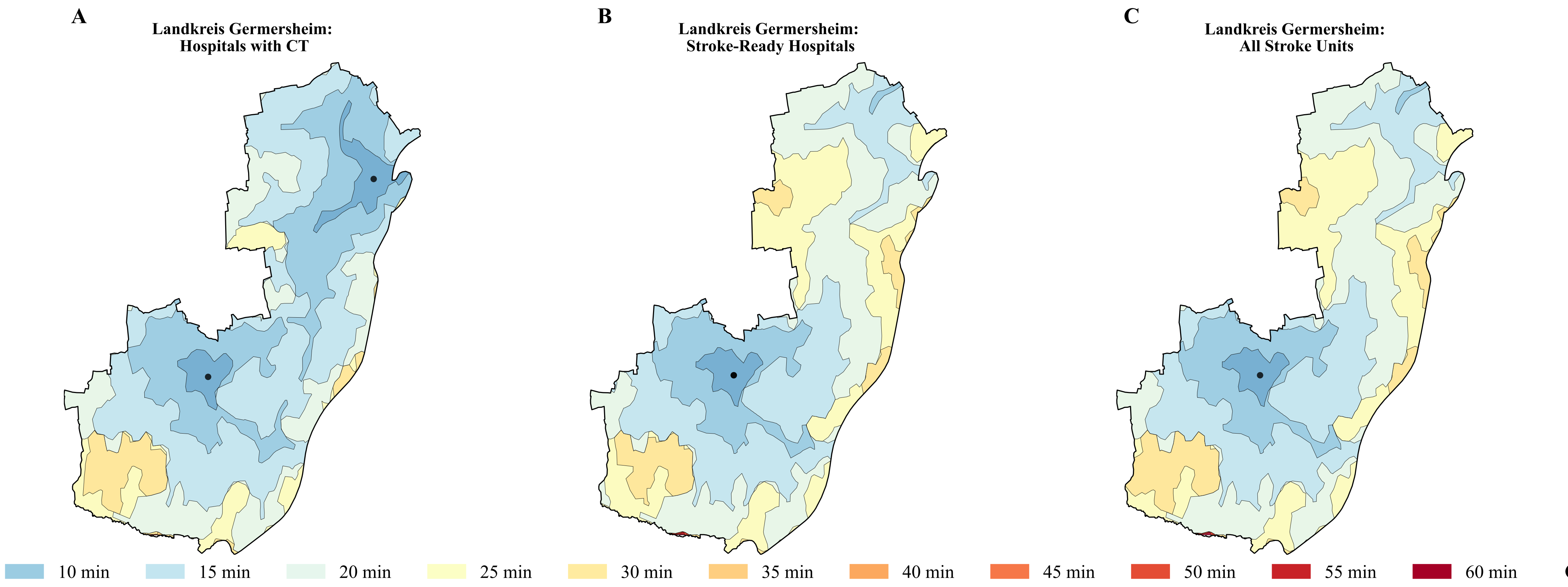

Isochrones — Landkreis Neckar-Odenwald-Kreis (Baden-Württemberg)

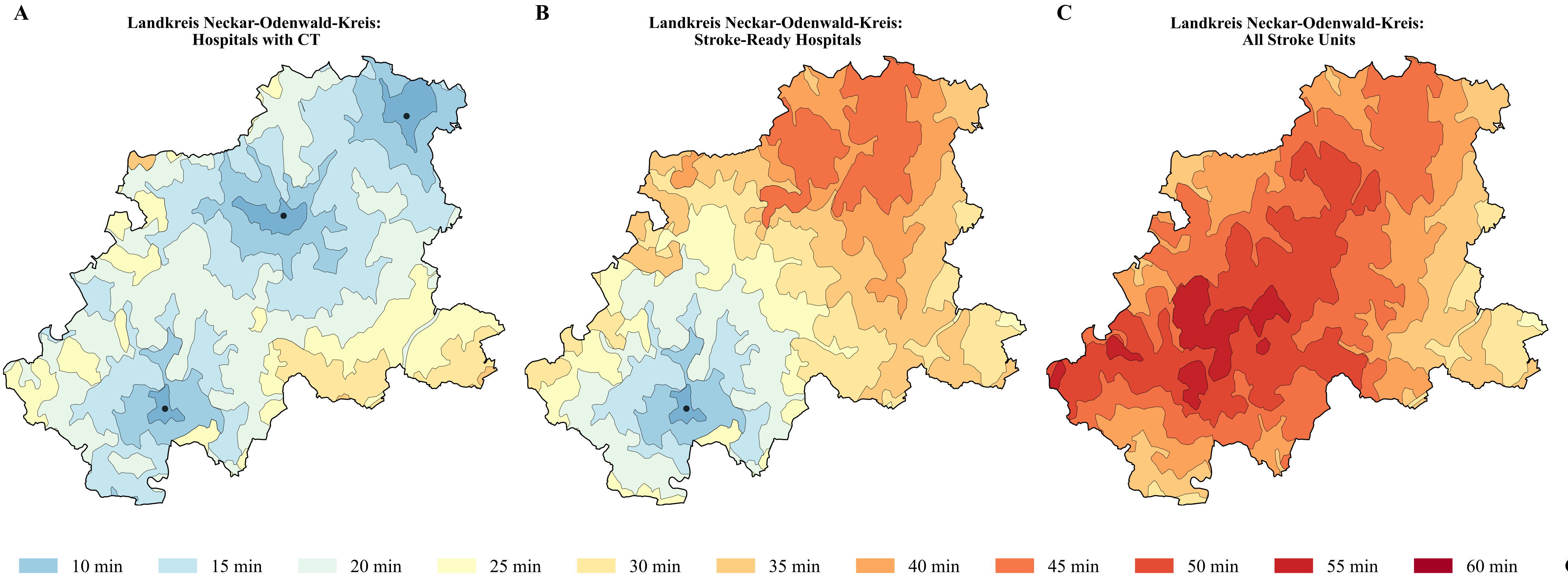

Isochrones — Landkreis Enzkreis (Baden-Württemberg)

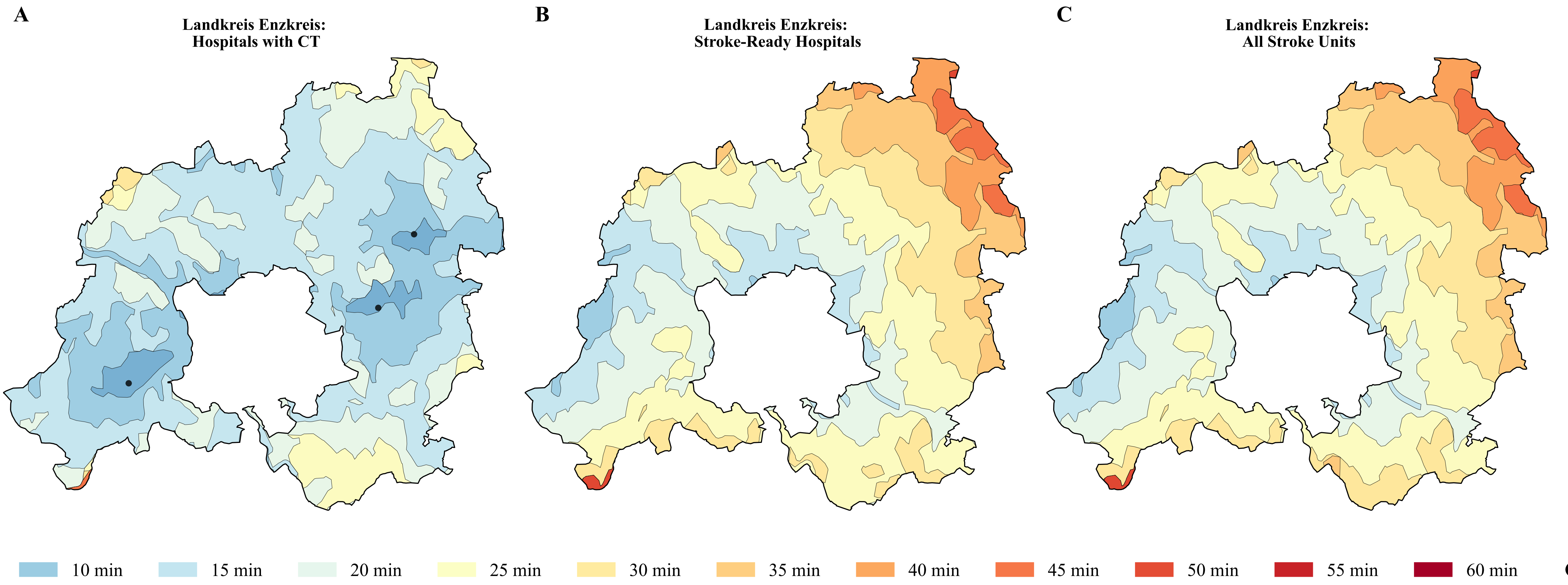

Isochrones — Landkreis Berchtesgadener Land (Bayern)

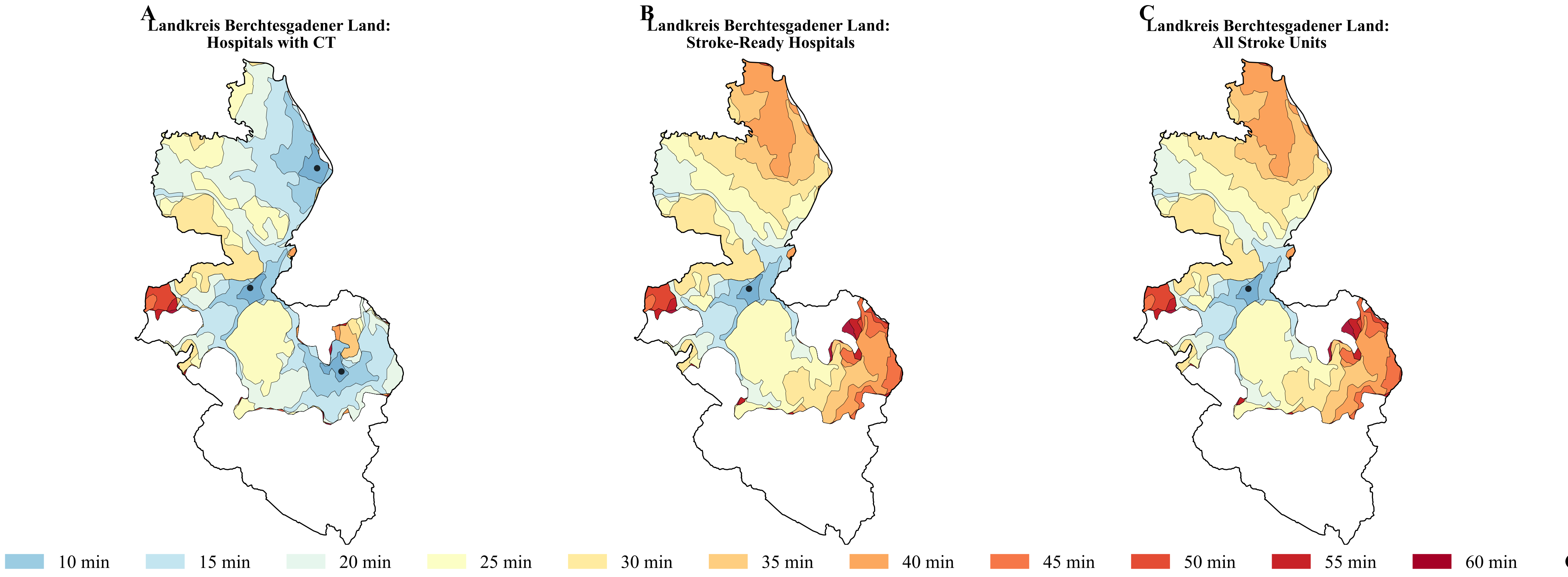

Isochrones — Kreisfreie Stadt Hof (Bayern)

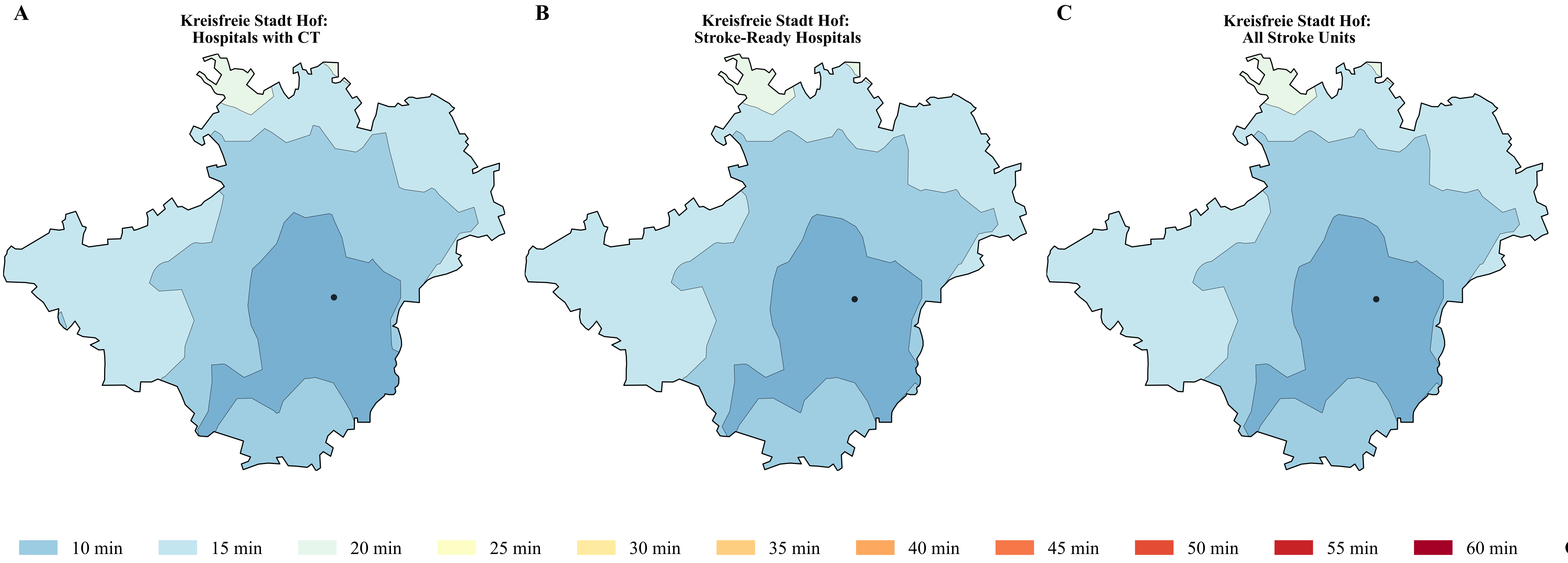

Isochrones — Landkreis Kulmbach (Bayern)

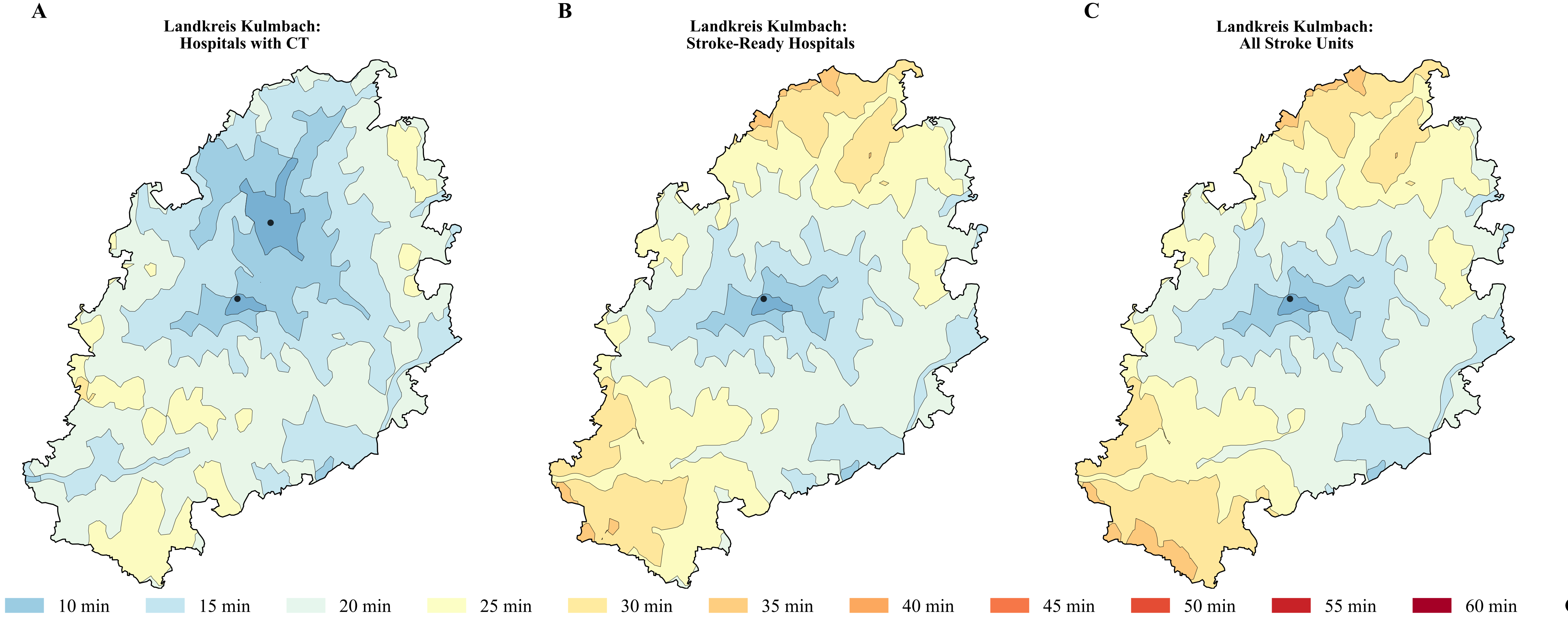

Isochrones — Kreisfreie Stadt Memmingen (Bayern)

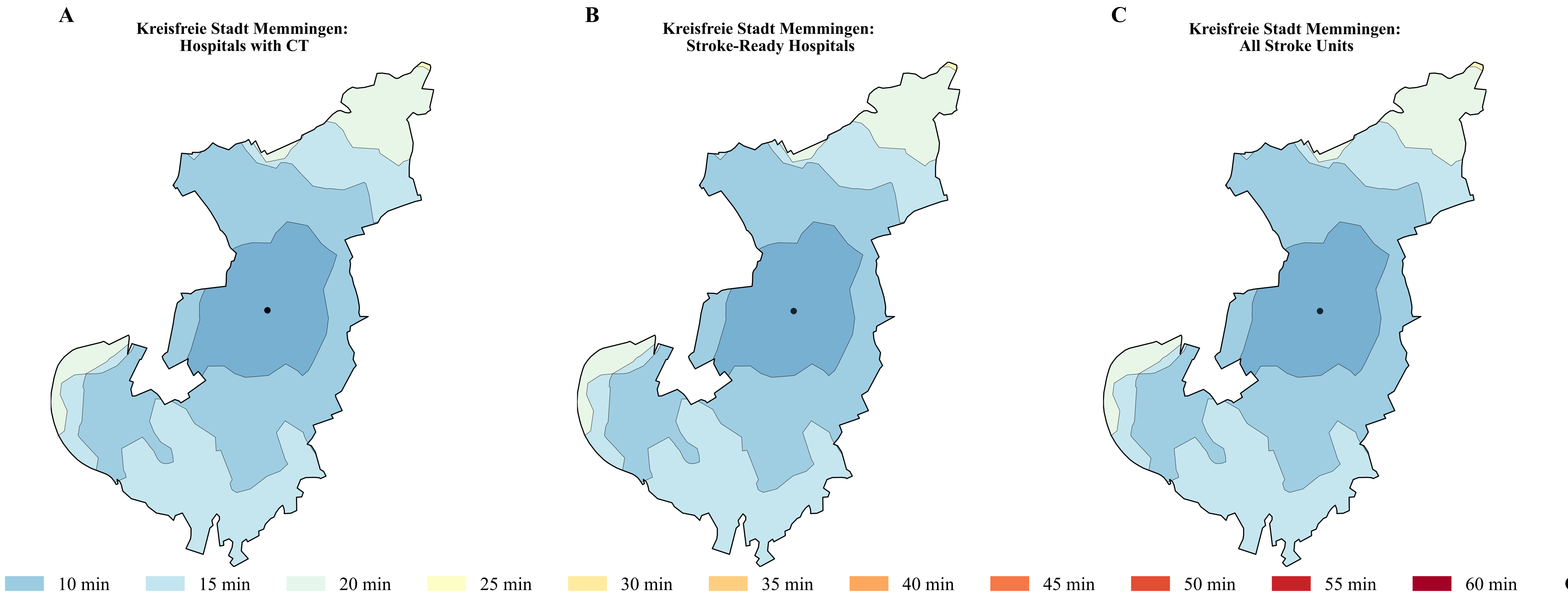

Isochrones — Landkreis Augsburg (Bayern)

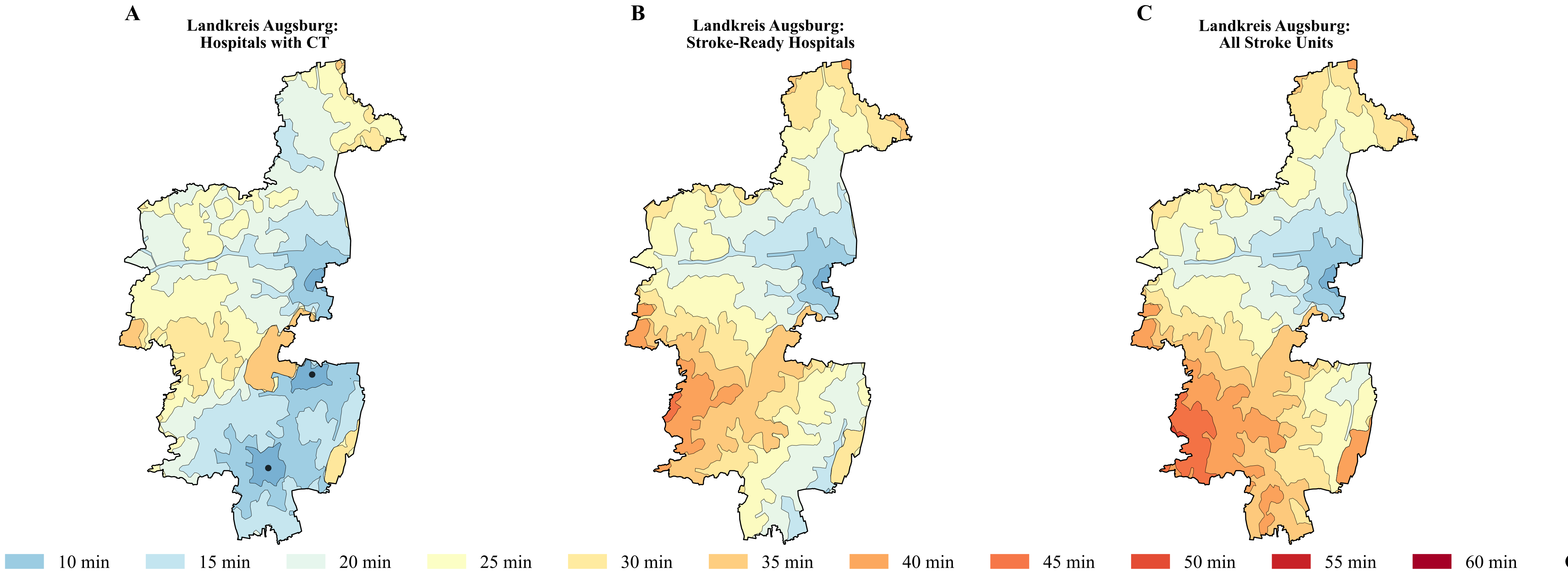

Isochrones — Landkreis Vorpommern-Rügen (Mecklenburg-Vorpommern)

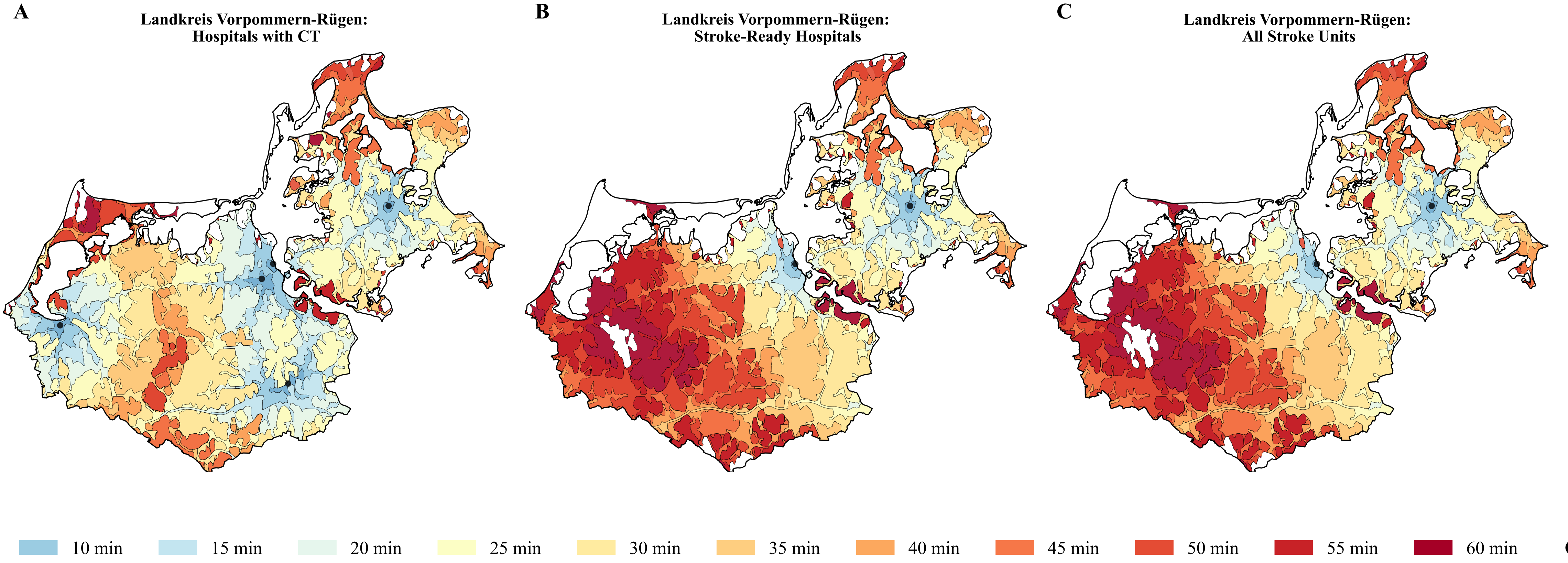

Isochrones — Landkreis Zwickau (Sachsen)

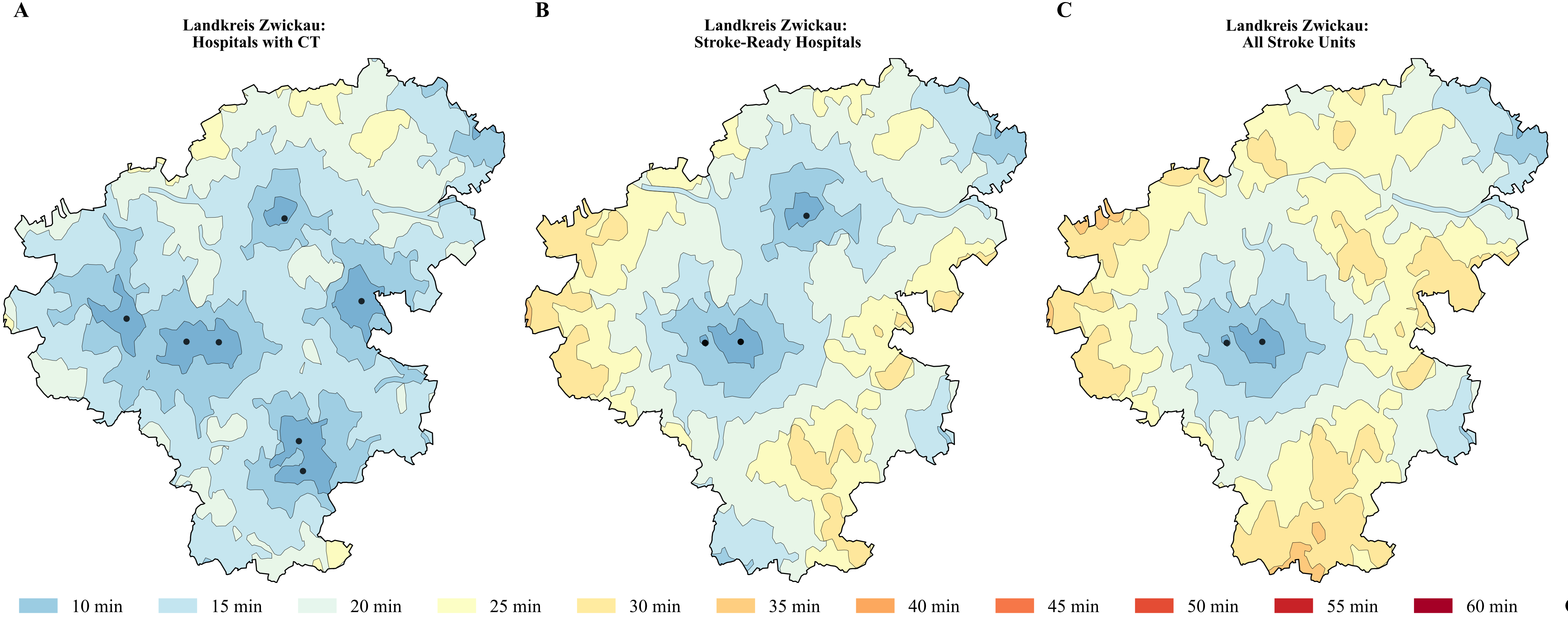

Isochrones — Landkreis Görlitz (Sachsen)

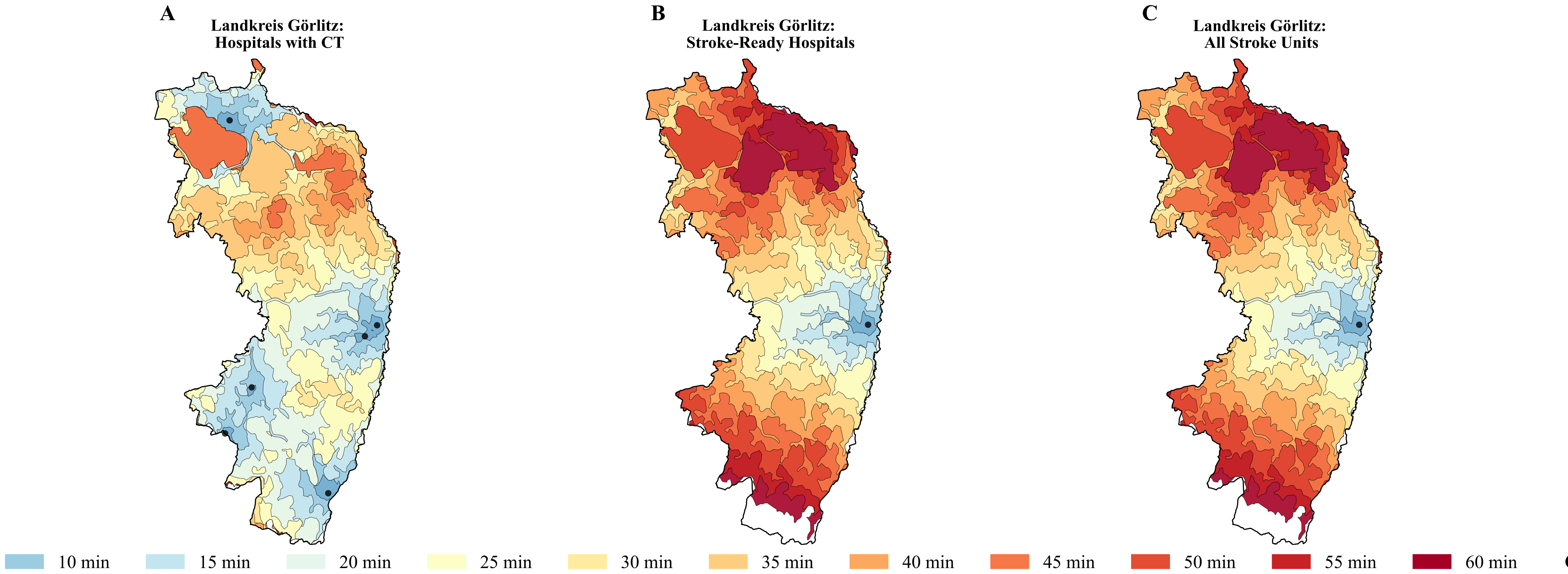

Isochrones — Landkreis Sonneberg (Thüringen)

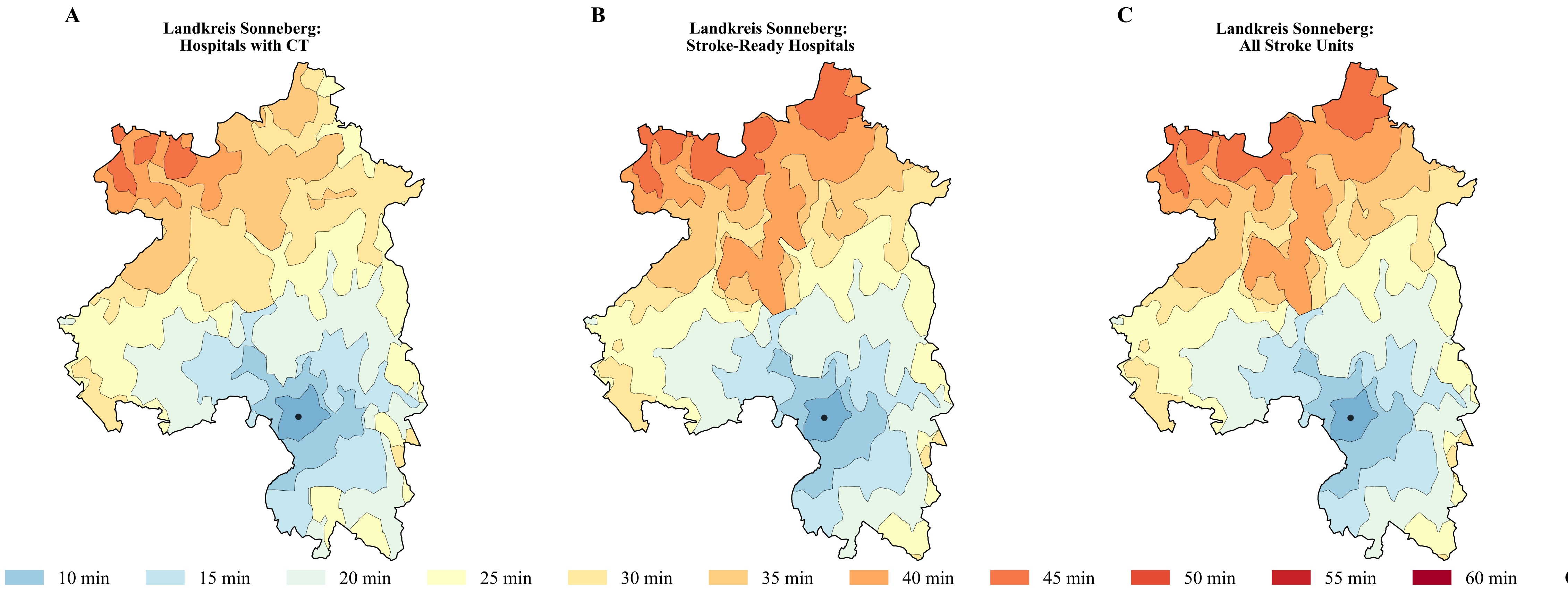

Isochrones — Kreisfreie Stadt Köln (Nordrhein-Westfalen)

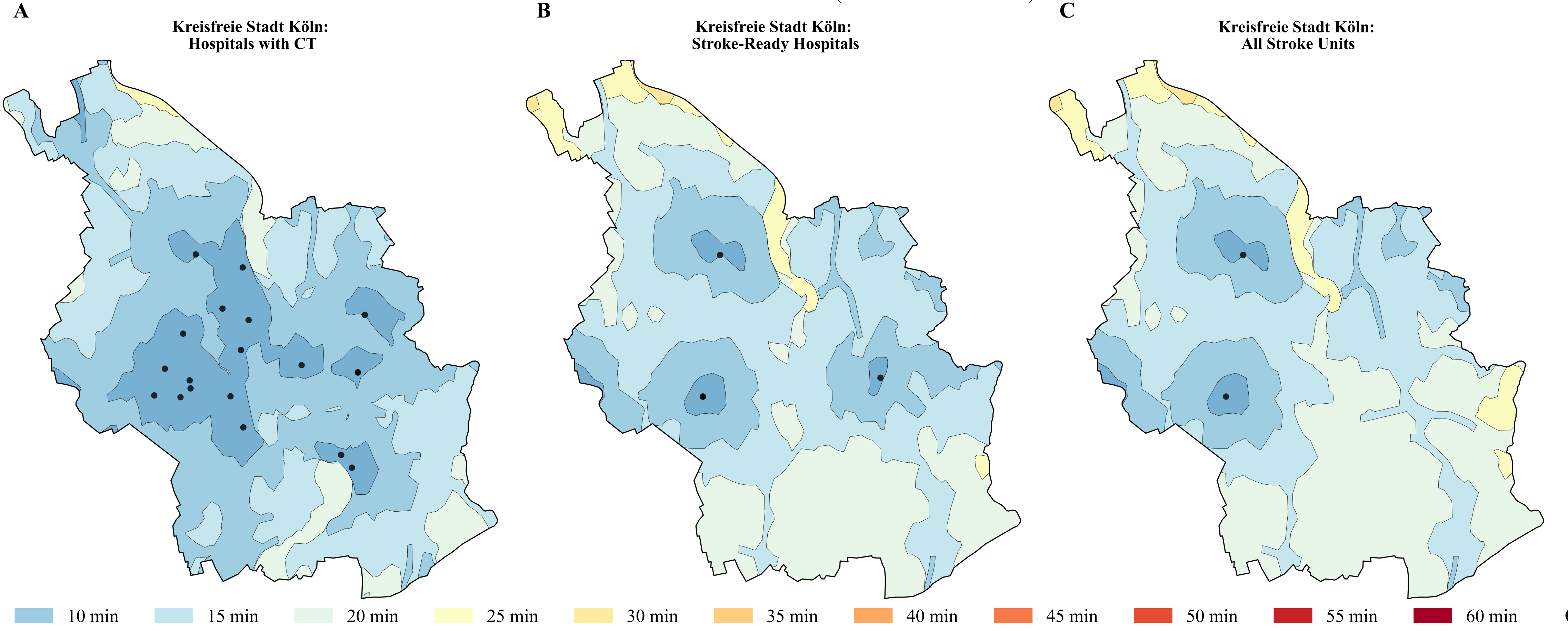

Isochrones — Kreis Heinsberg (Nordrhein-Westfalen)

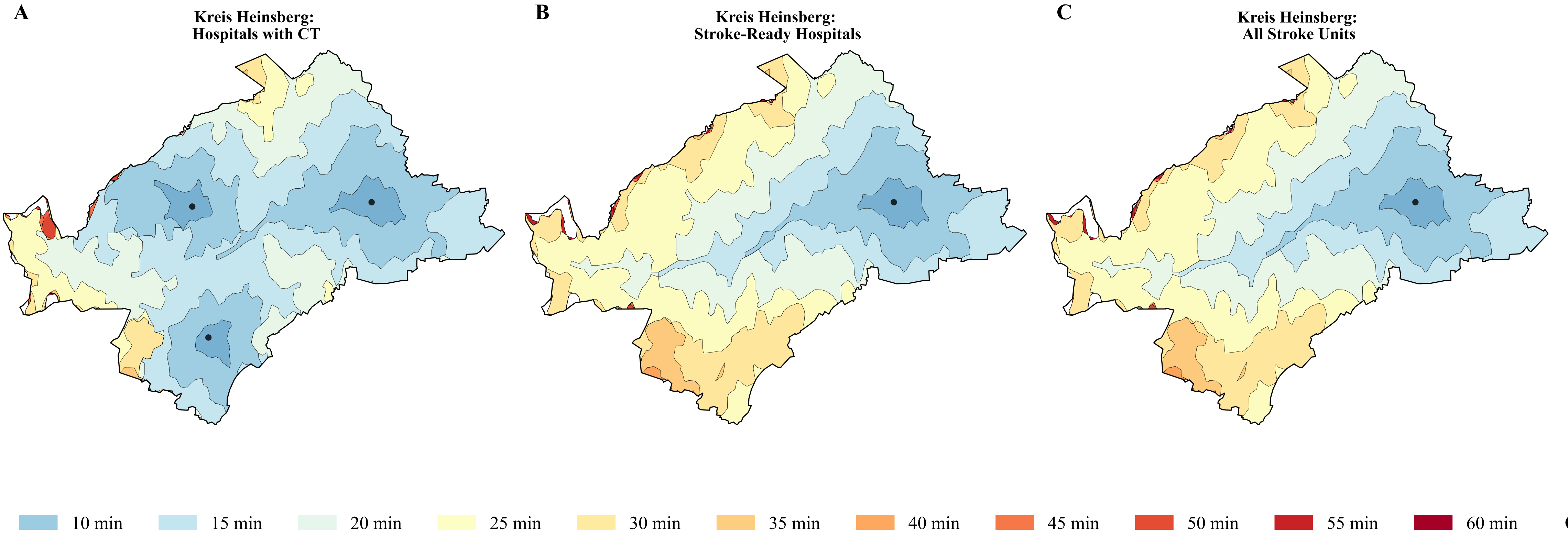

Isochrones — Landkreis Bergstraße (Hessen)

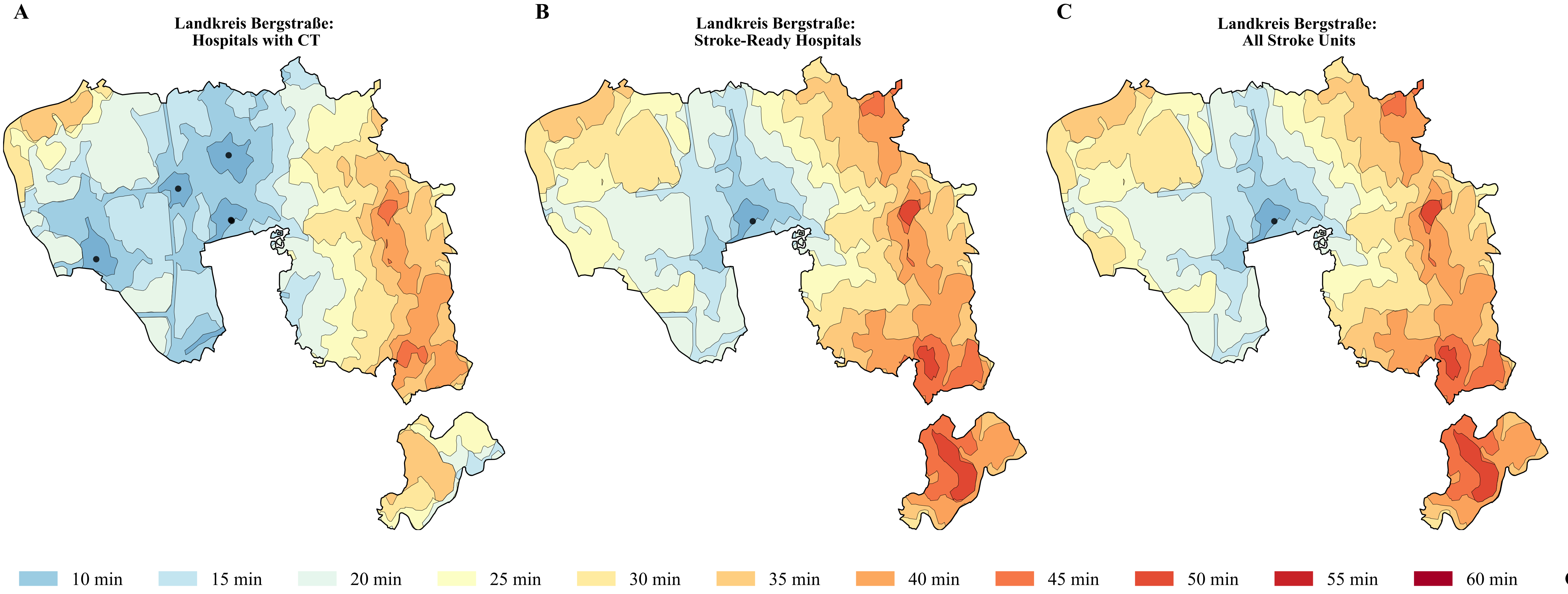

Isochrones — Landkreis Wetteraukreis (Hessen)

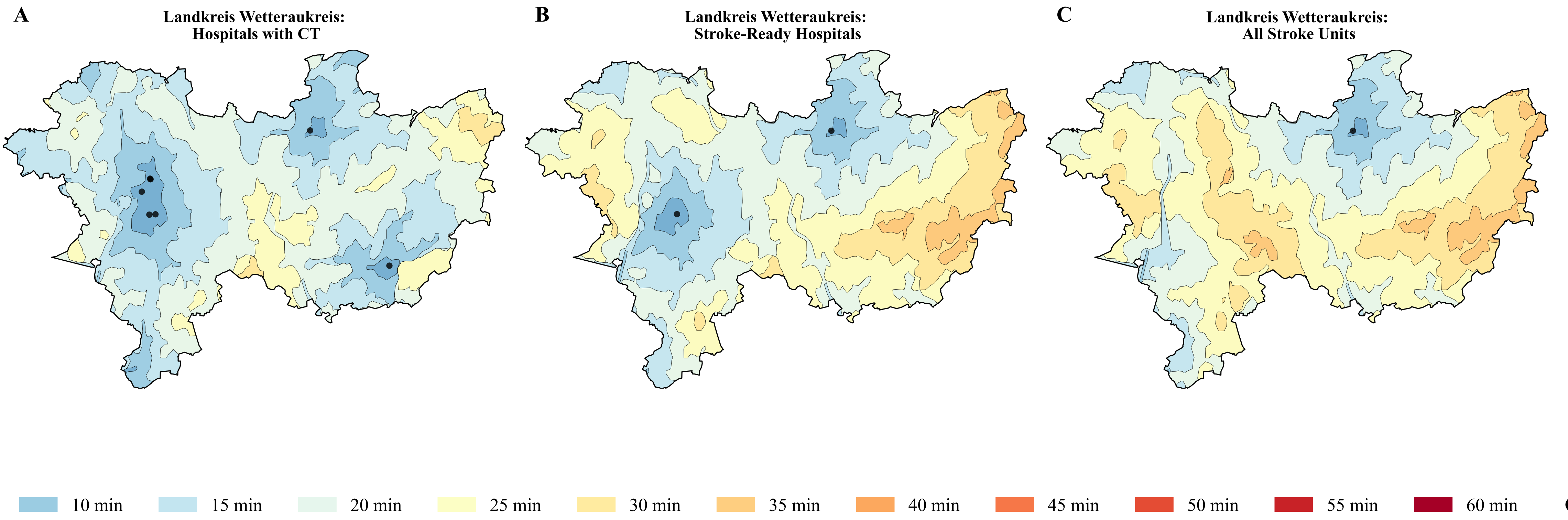

Isochrones — Kreisfreie Stadt Landau in der Pfalz (Rheinland-Pfalz)

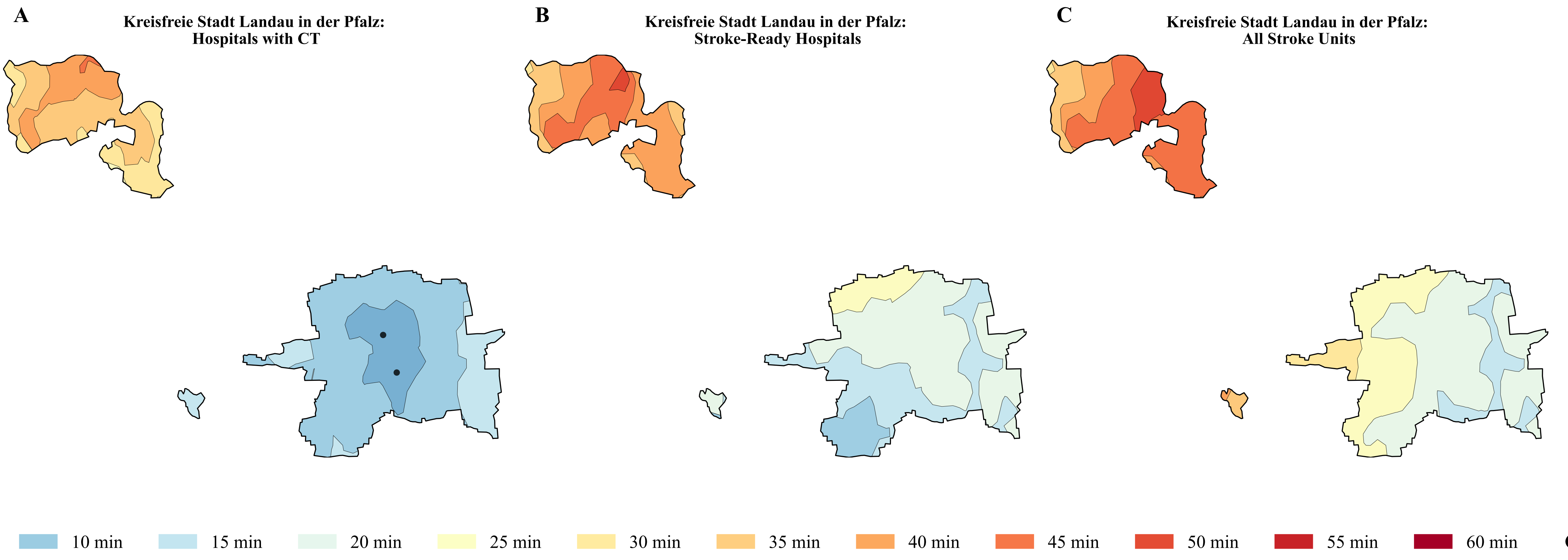

Isochrones — Stadtkreis Stuttgart (Baden-Württemberg)

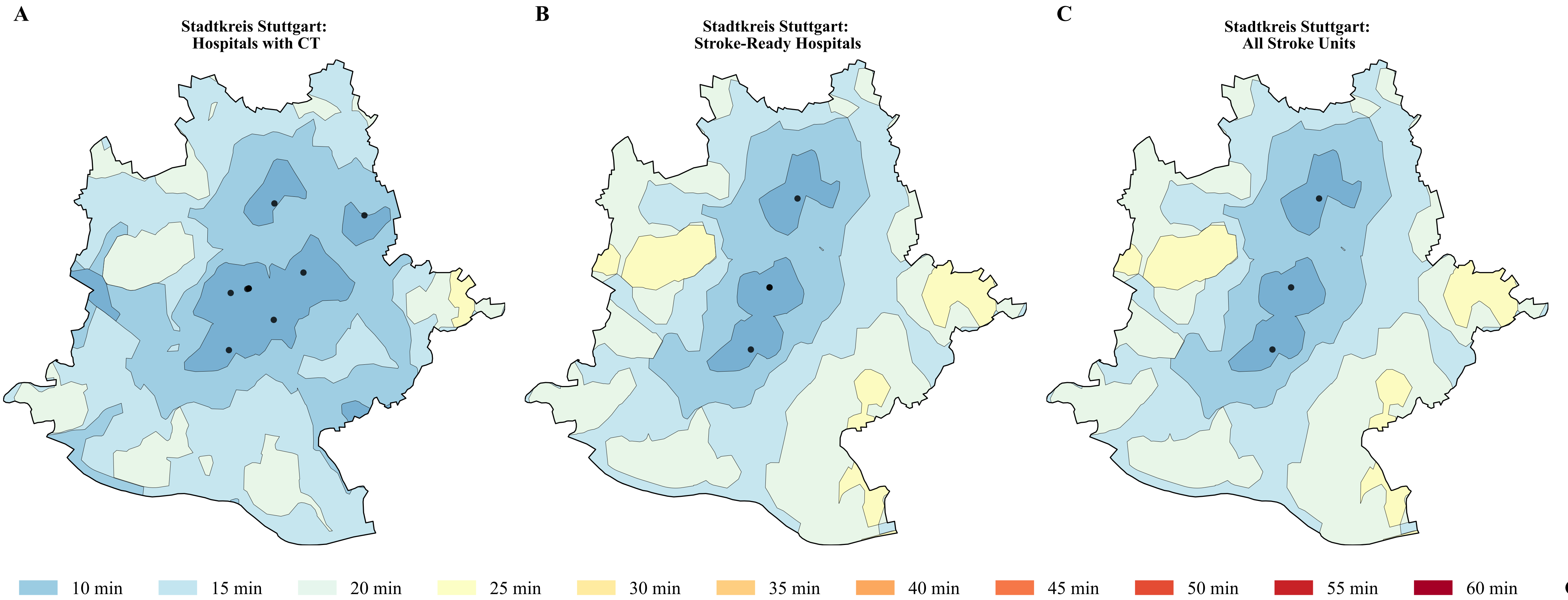

Isochrones — Landkreis Konstanz (Baden-Württemberg)

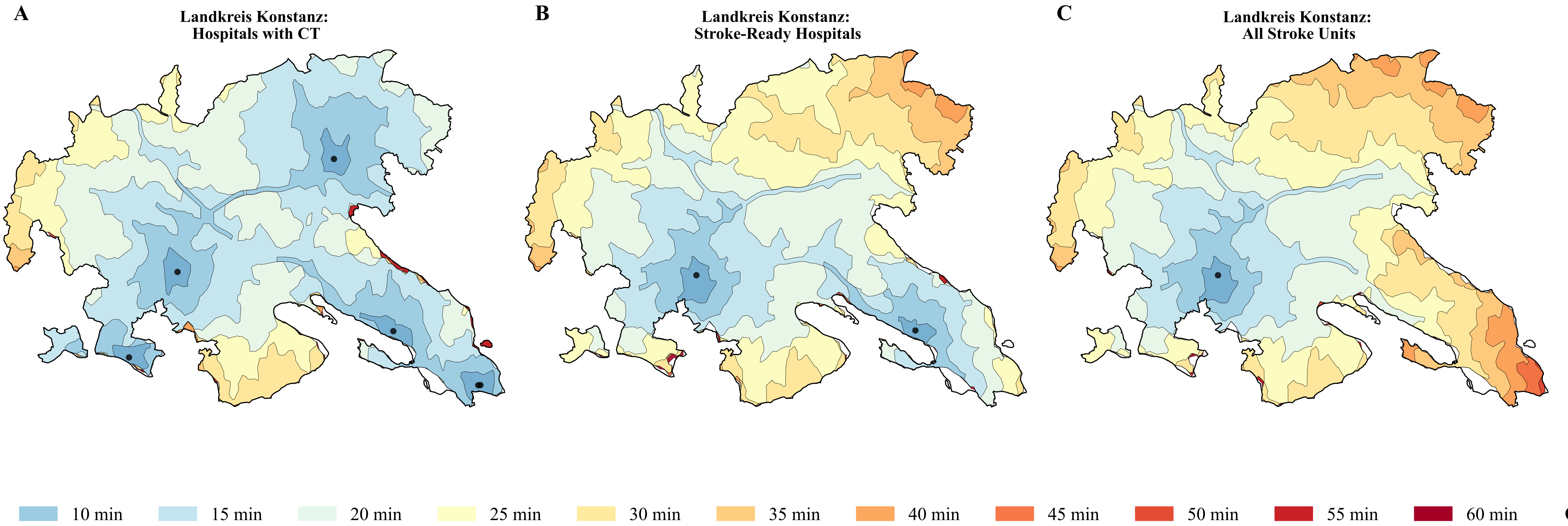

Isochrones — Landkreis Sigmaringen (Baden-Württemberg)

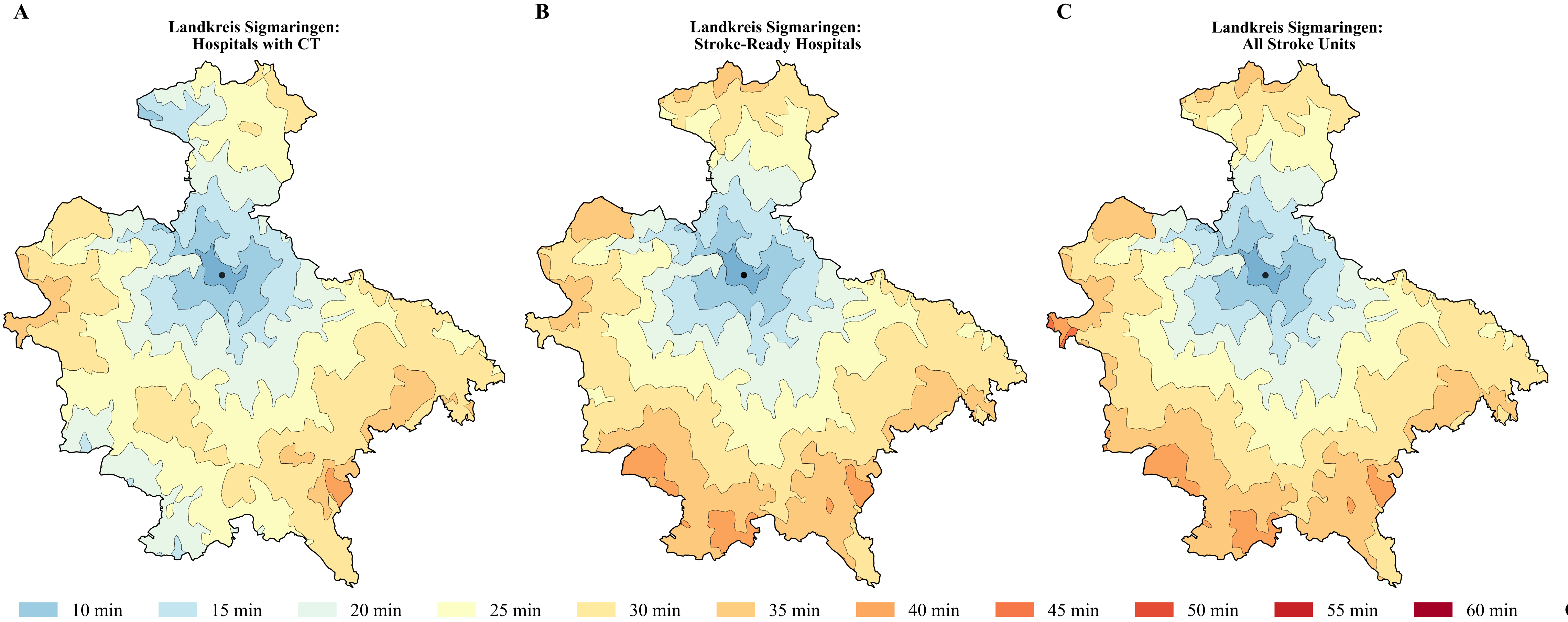

Isochrones — Landkreis Kelheim (Bayern)

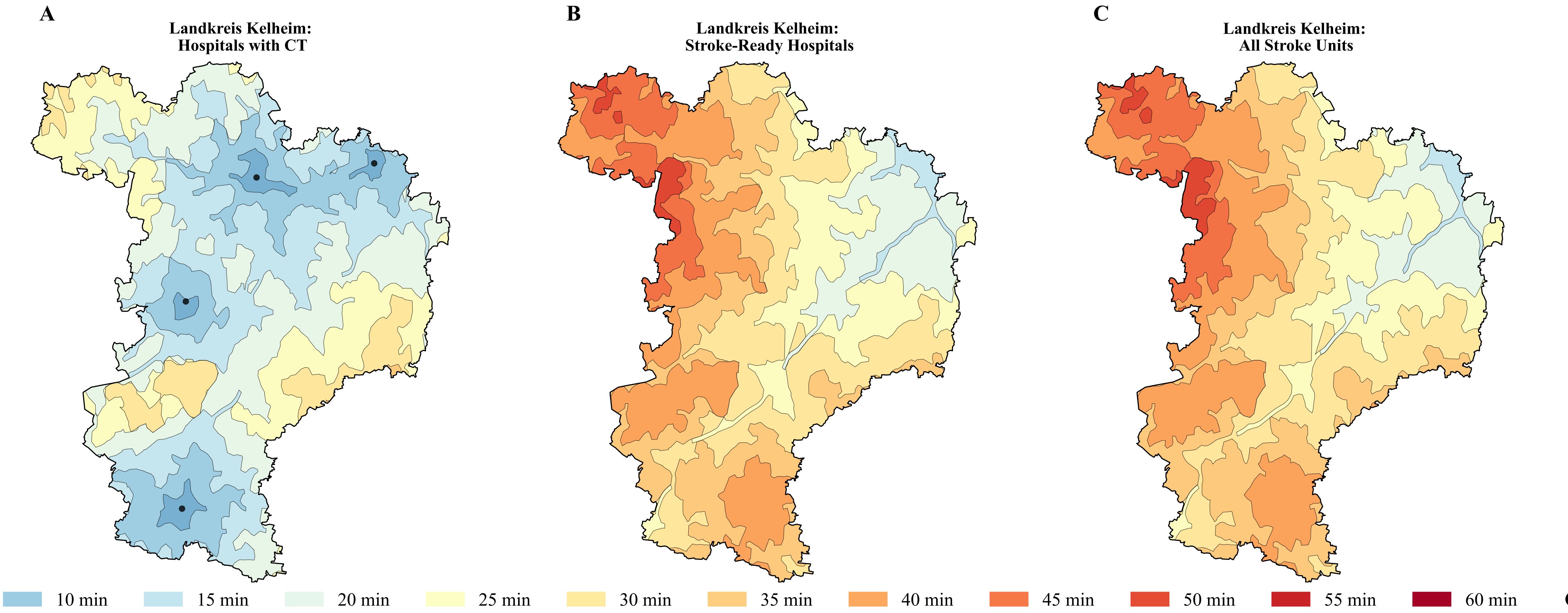

Isochrones — Kreisfreie Stadt Schweinfurt (Bayern)

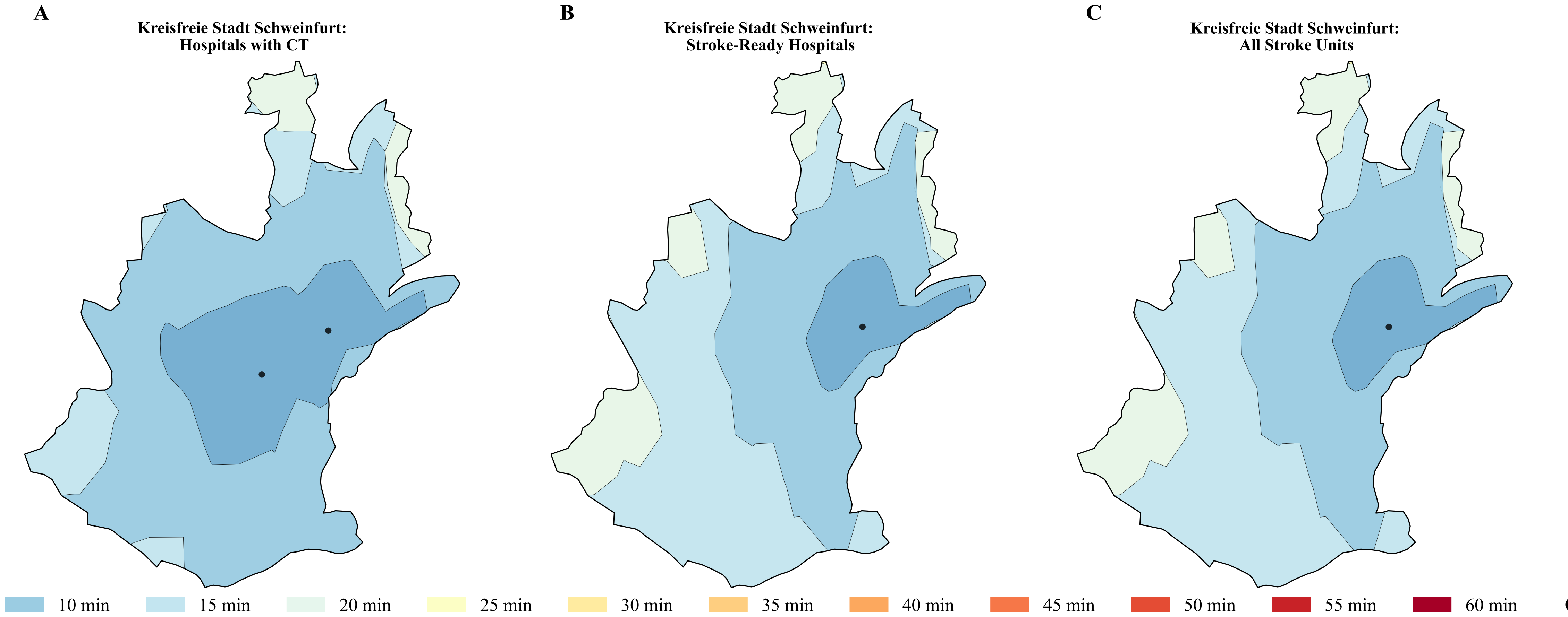

Isochrones — Landkreis Neu-Ulm (Bayern)

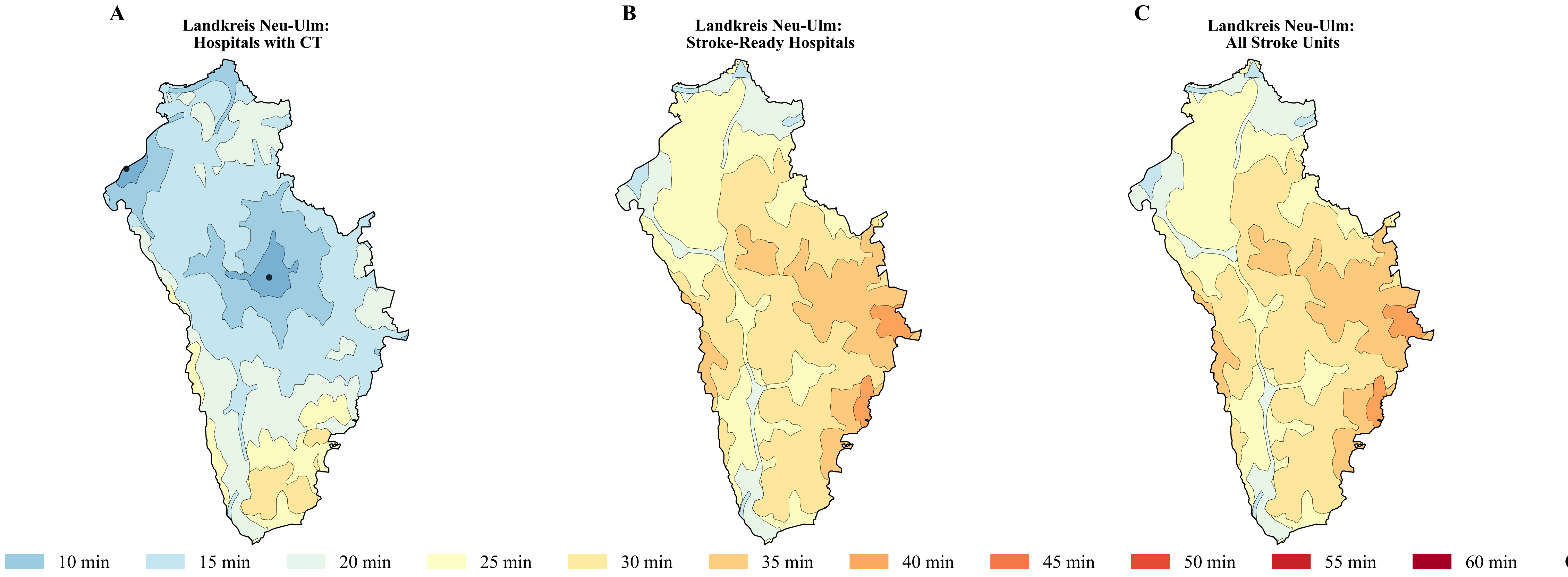

Isochrones — Landkreis Oberhavel (Brandenburg)

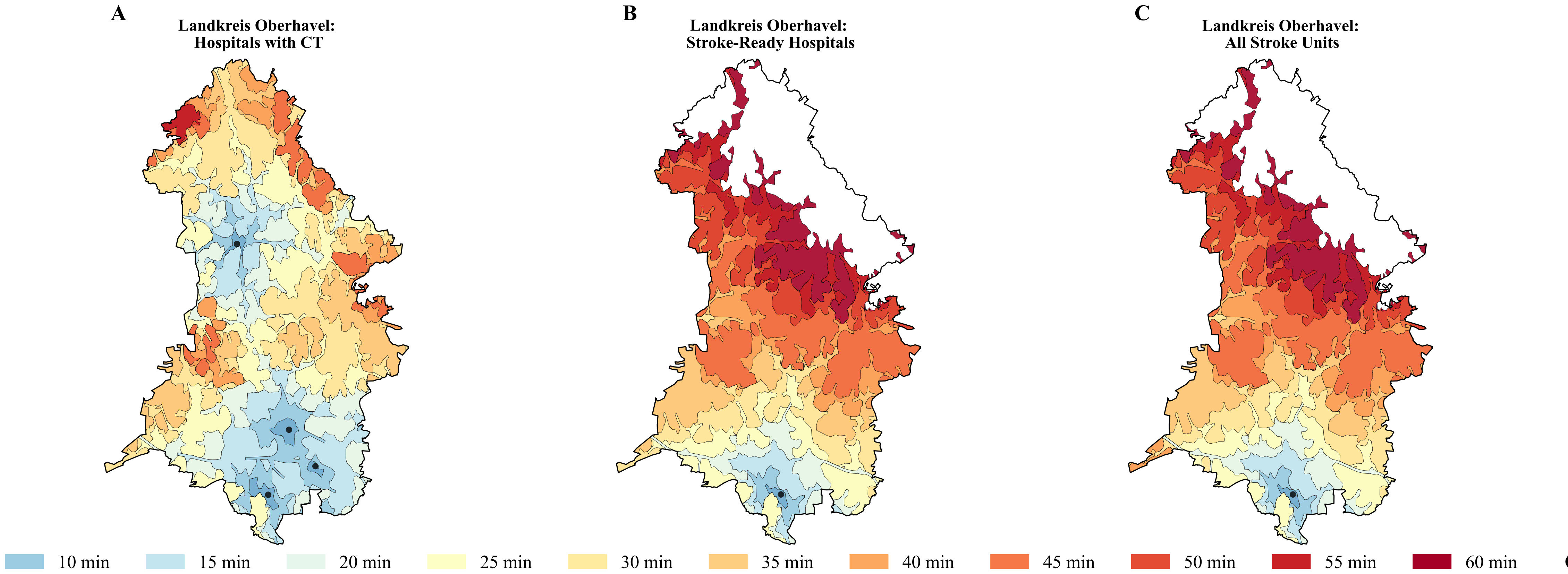

Isochrones — Landkreis Spree-Neiße (Brandenburg)

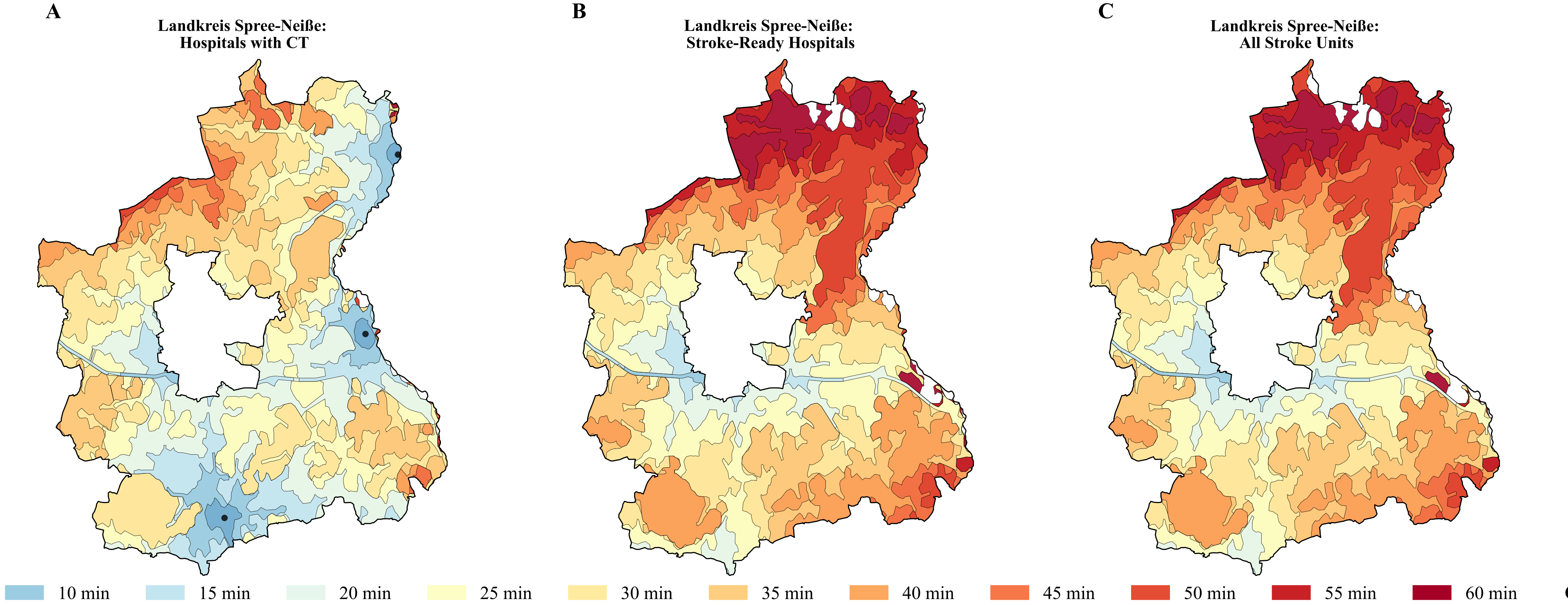

Isochrones — Landkreis Ilm-Kreis (Thüringen)

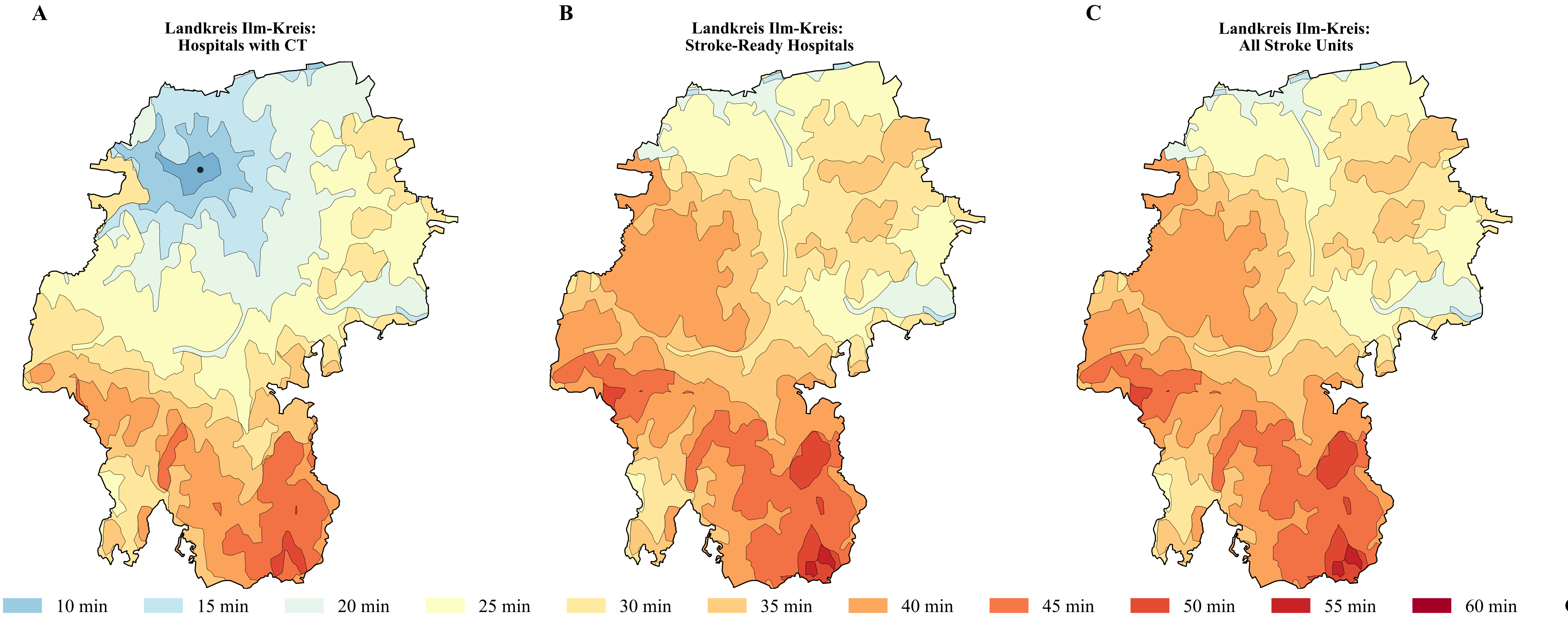

Isochrones — Landkreis Greiz (Thüringen)

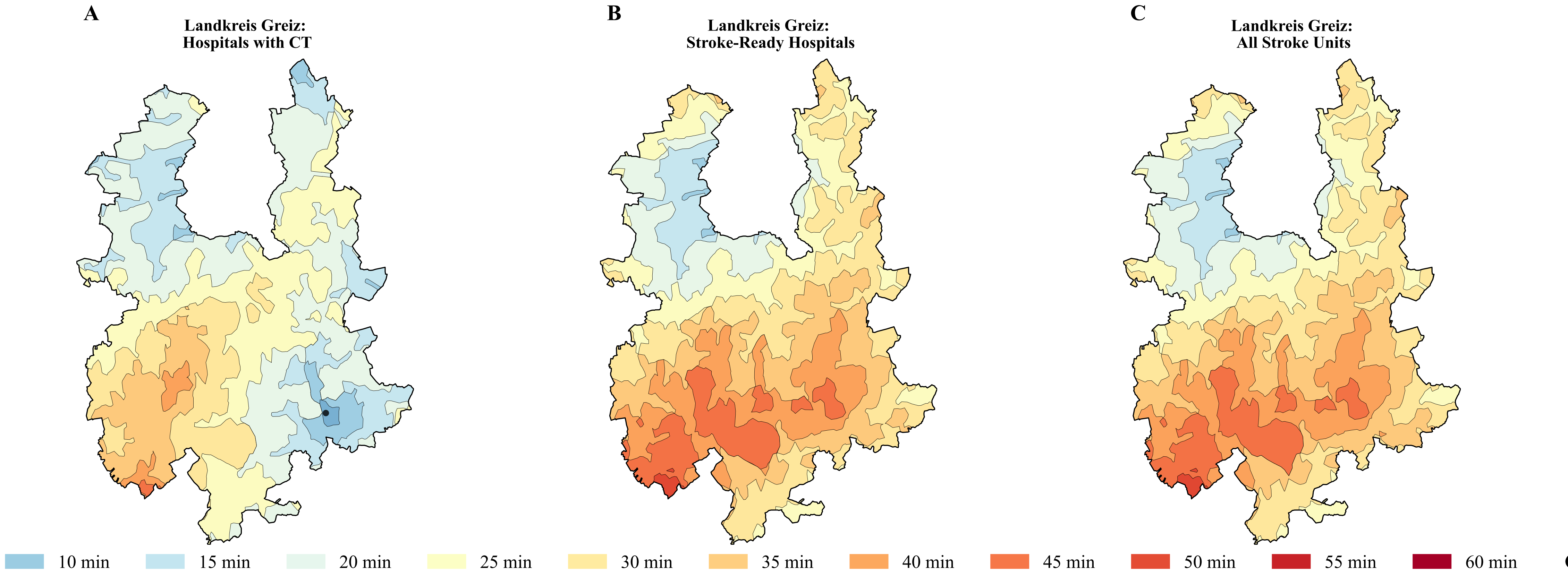

Isochrones — Kreisfreie Stadt Bottrop (Nordrhein-Westfalen)

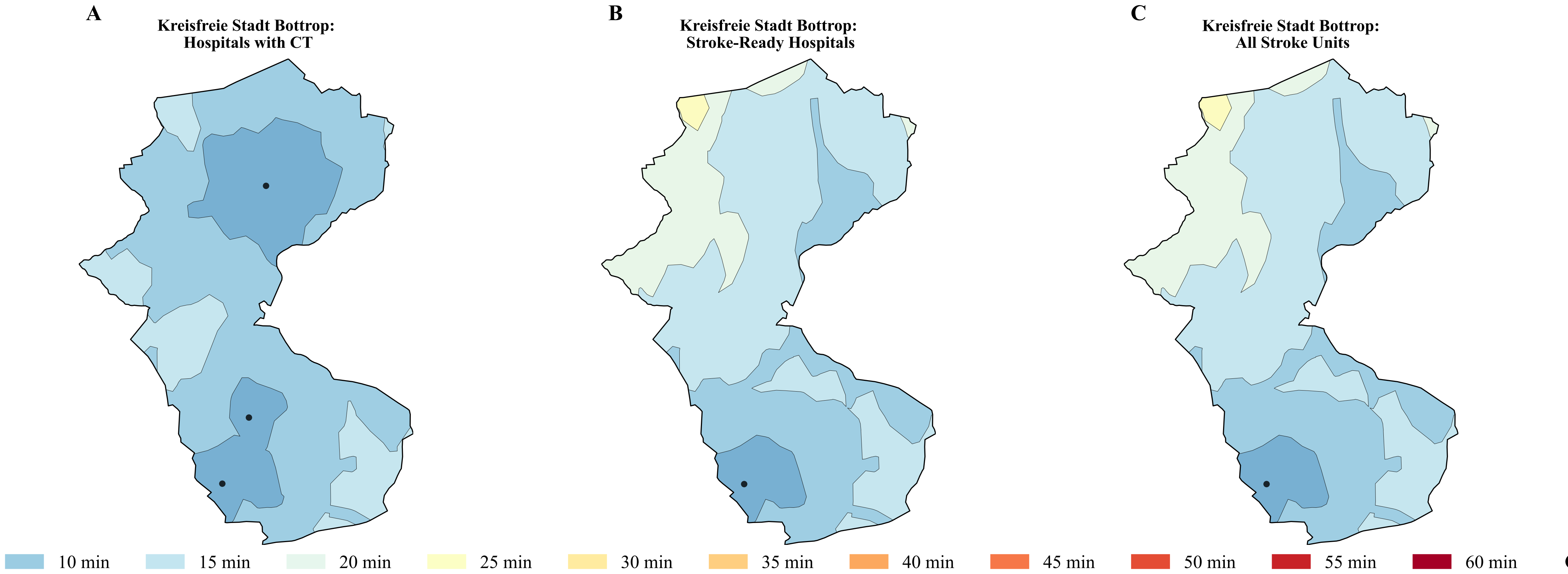

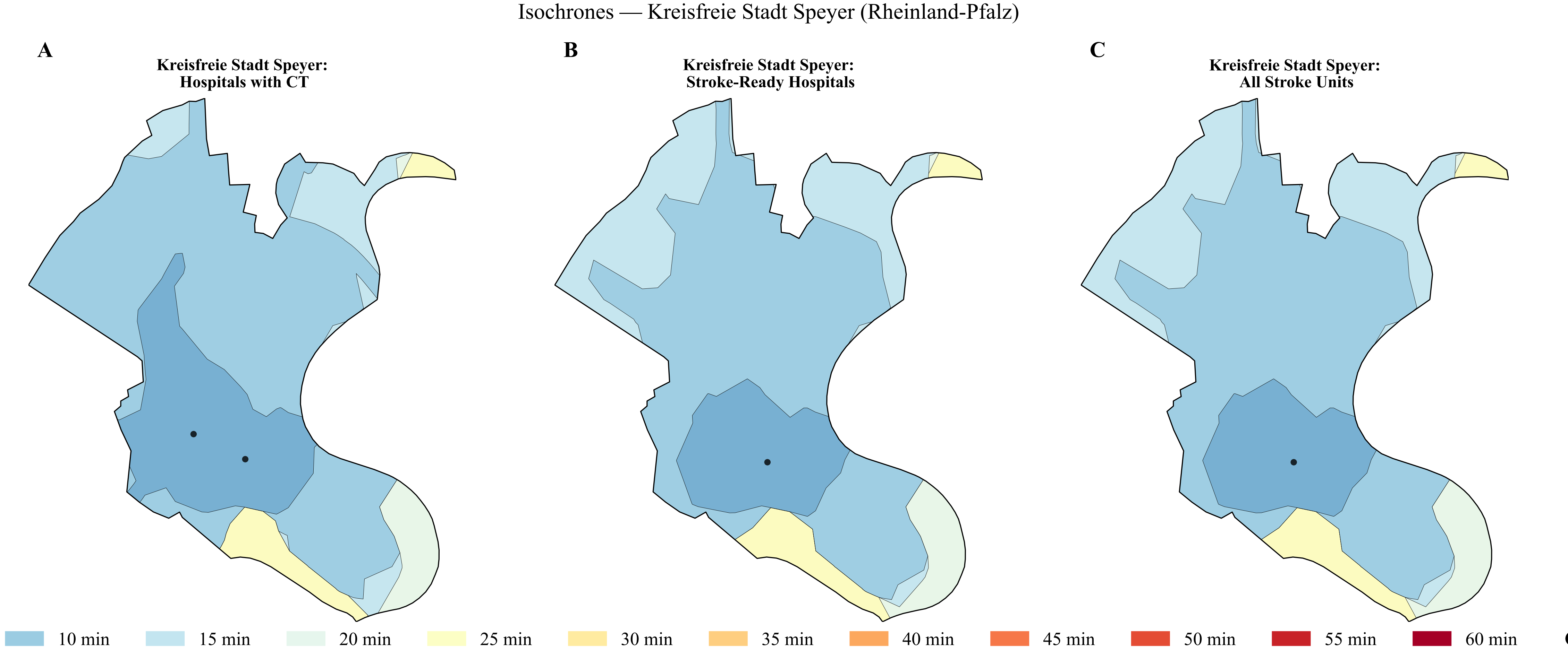

Isochrones — Kreisfreie Stadt Speyer (Rheinland-Pfalz)
A
Kreisfreie Stadt Speyer:
Hospitals with CT
B
Kreisfreie Stadt Speyer:
Stroke-Ready Hospitals
C
Kreisfreie Stadt Speyer:
All Stroke Units
10 min
15 min
20 min
25 min
30 min
35 min
40 min
45 min
50 min
55 min
60 min

Isochrones — Stadtkreis Mannheim (Baden-Württemberg)

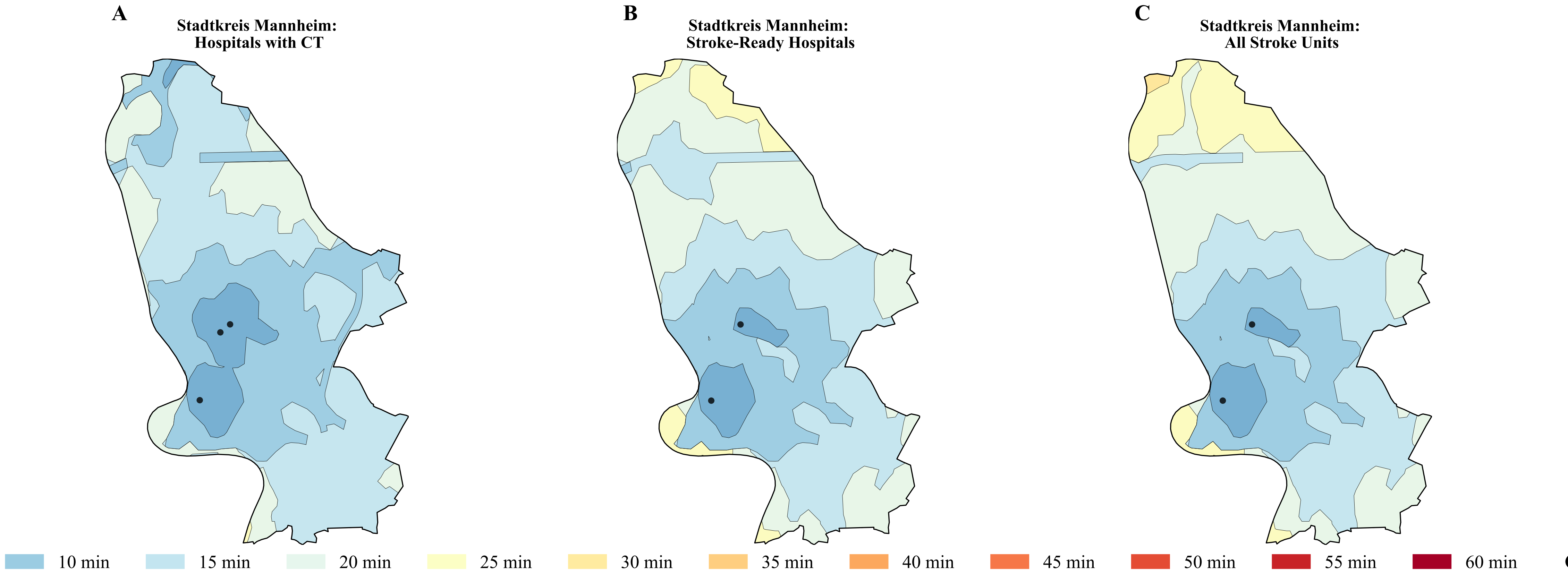

Isochrones — Landkreis Tuttlingen (Baden-Württemberg)

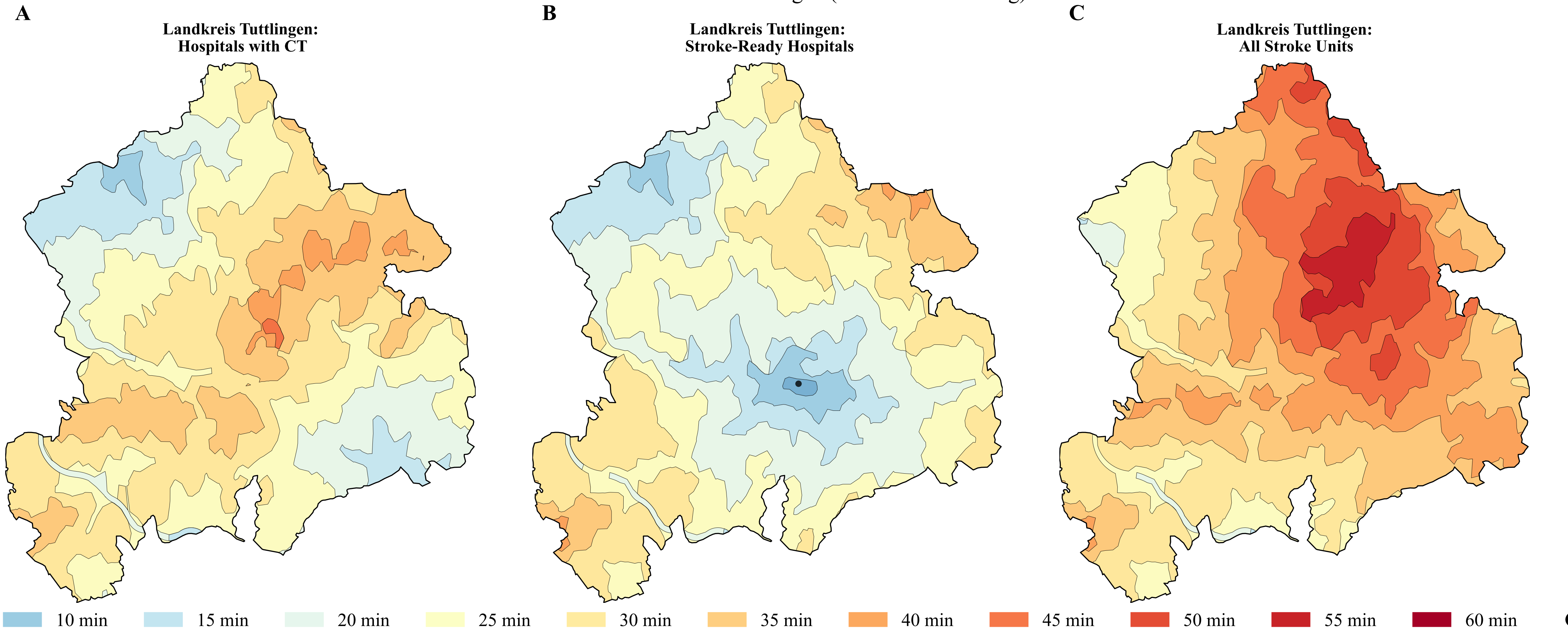

Isochrones — Landkreis Zollernalbkreis (Baden-Württemberg)

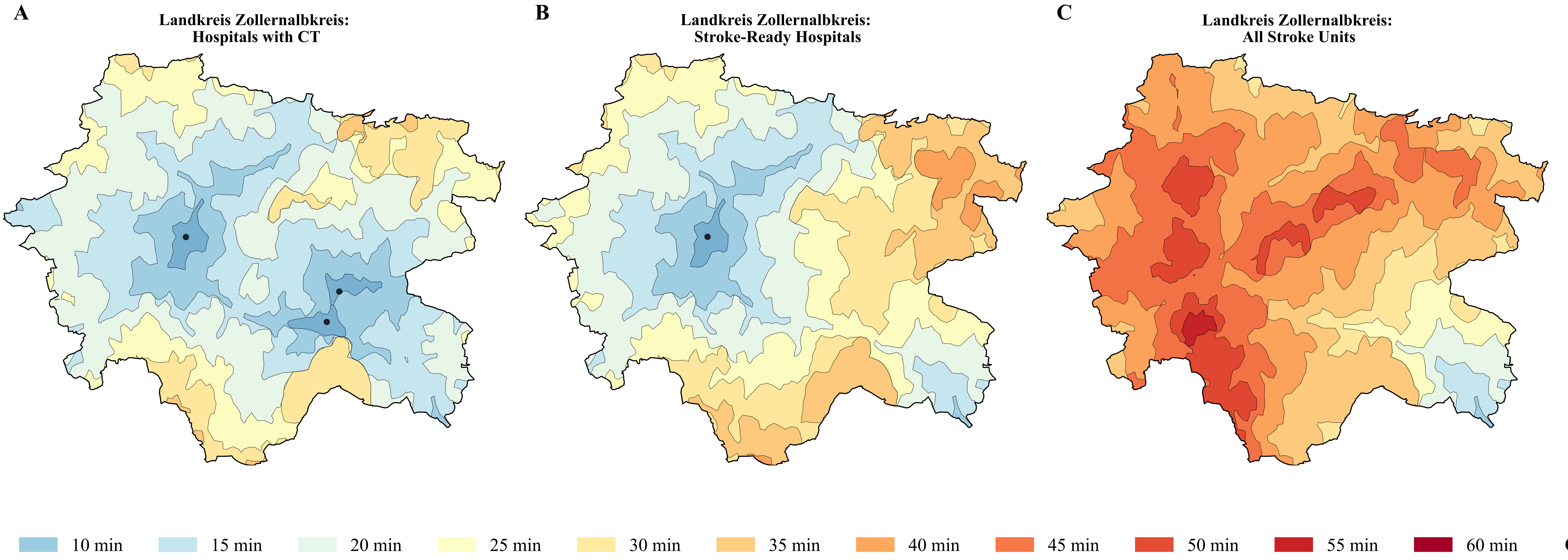

Isochrones — Landkreis Eichstätt (Bayern)

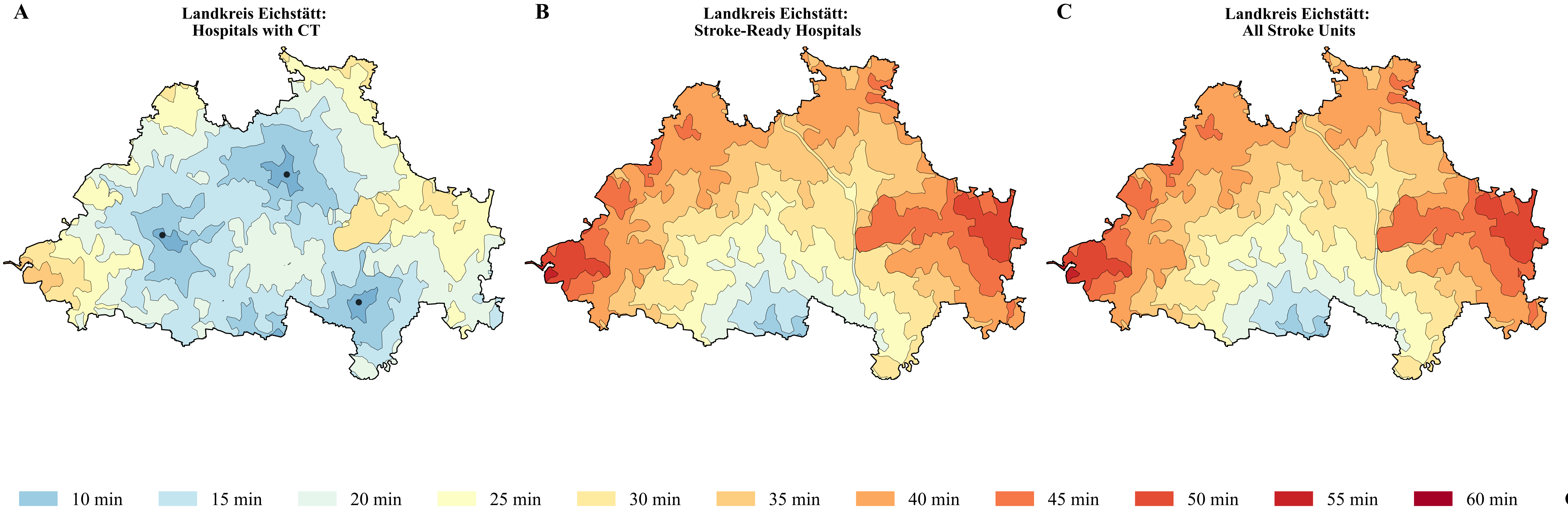

Isochrones — Landkreis Freyung-Grafenau (Bayern)

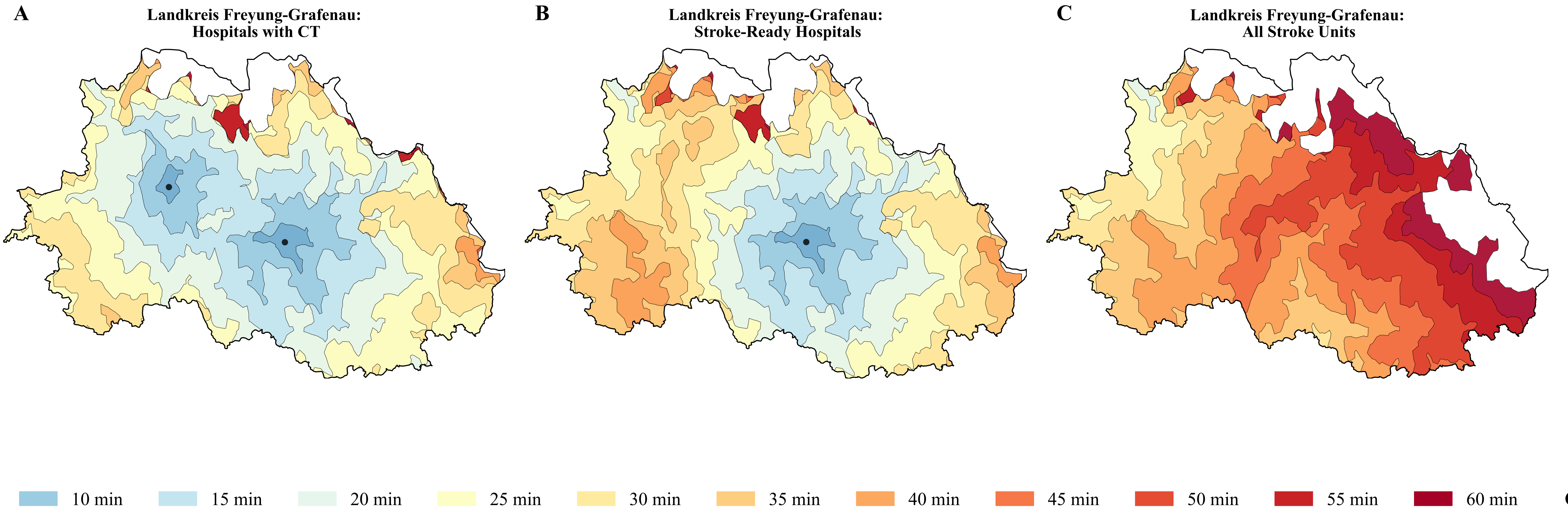

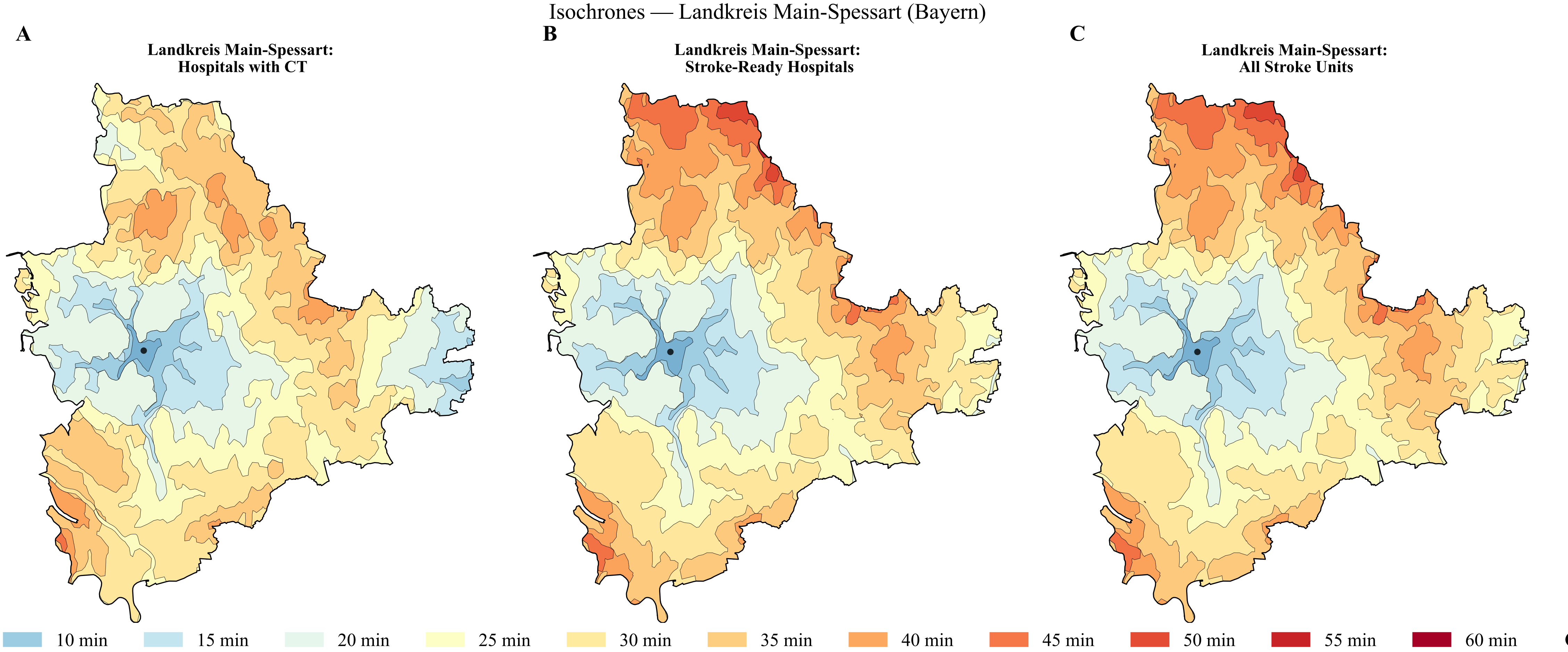
Isochrones — Landkreis Main-Spessart (Bayern)
A
Landkreis Main-Spessart:
Hospitals with CT
B
Landkreis Main-Spessart:
Stroke-Ready Hospitals
C
Landkreis Main-Spessart:
All Stroke Units
10 min
15 min
20 min
25 min
30 min
35 min
40 min
45 min
50 min
55 min
60 min

Isochrones — Kreisfreie Stadt Potsdam (Brandenburg)

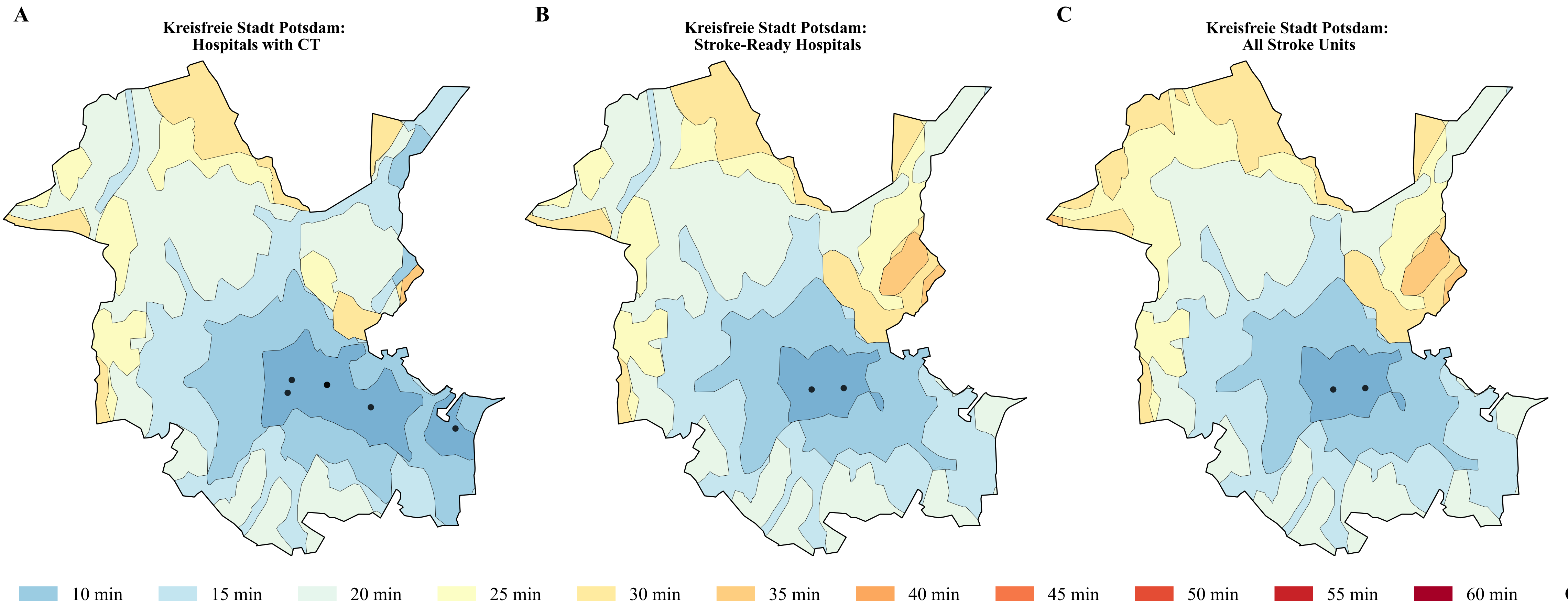

Isochrones — Landkreis Nordsachsen (Sachsen)

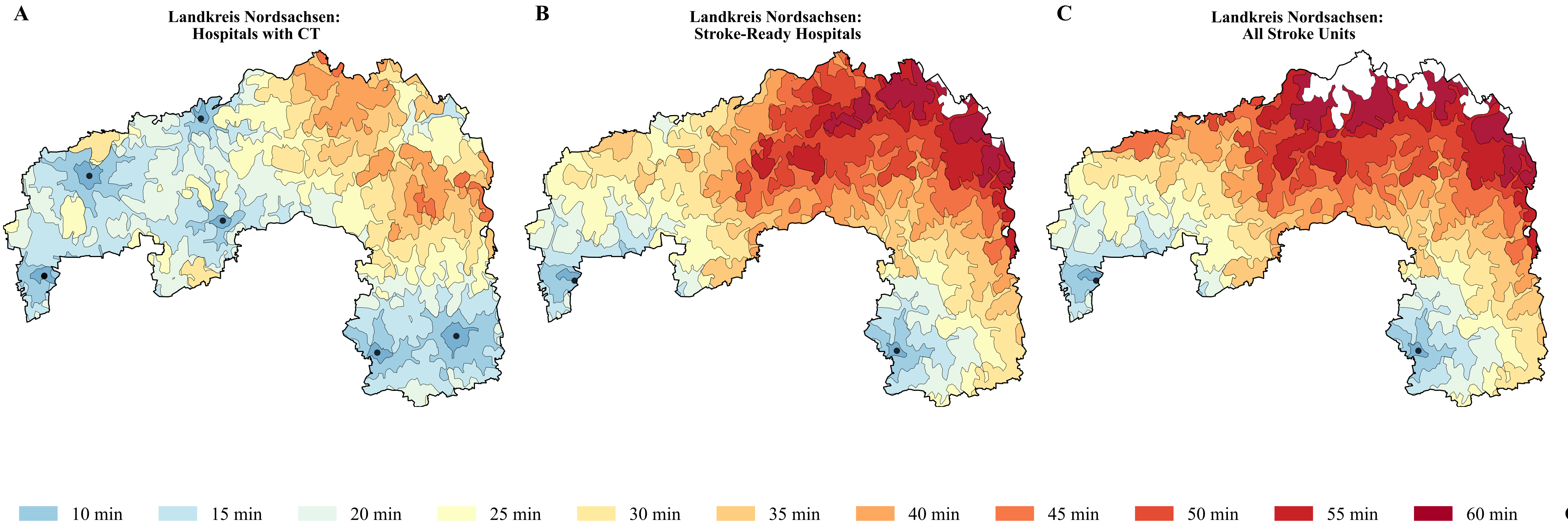

Isochrones — Landkreis Cuxhaven (Niedersachsen)

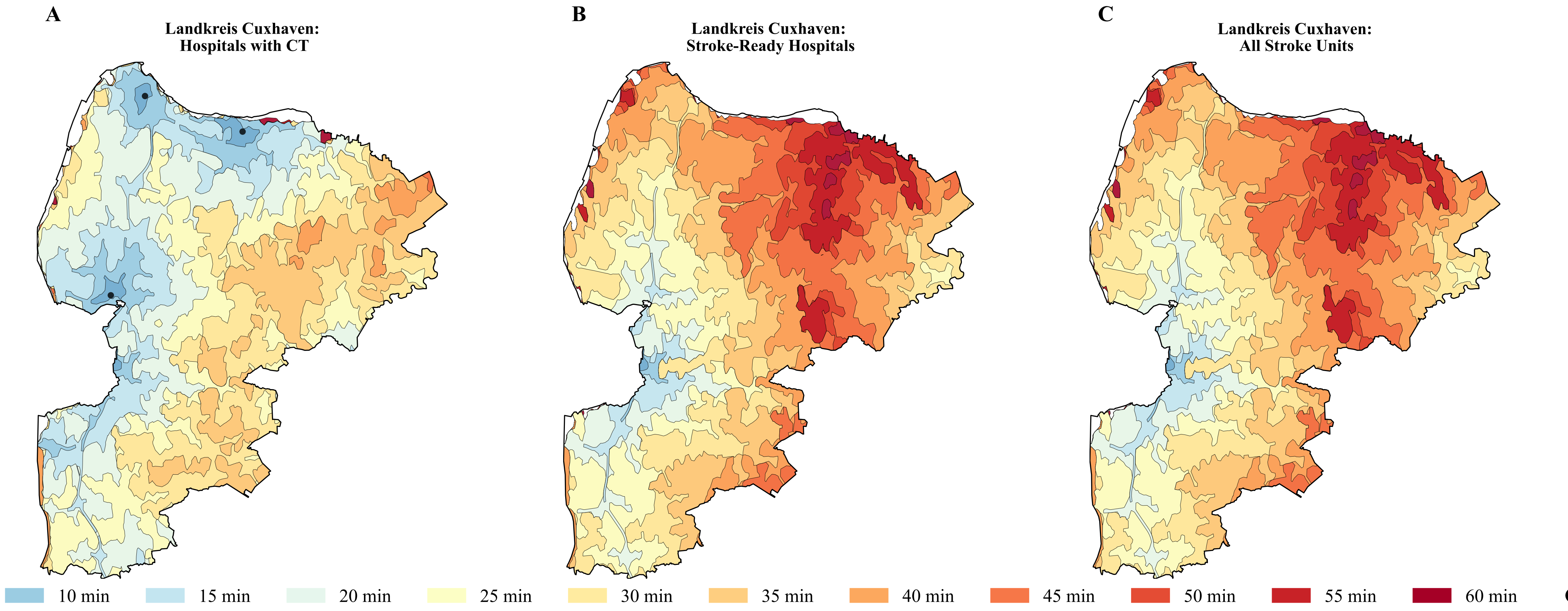

Isochrones — Kreisfreie Stadt Bremen (Bremen)

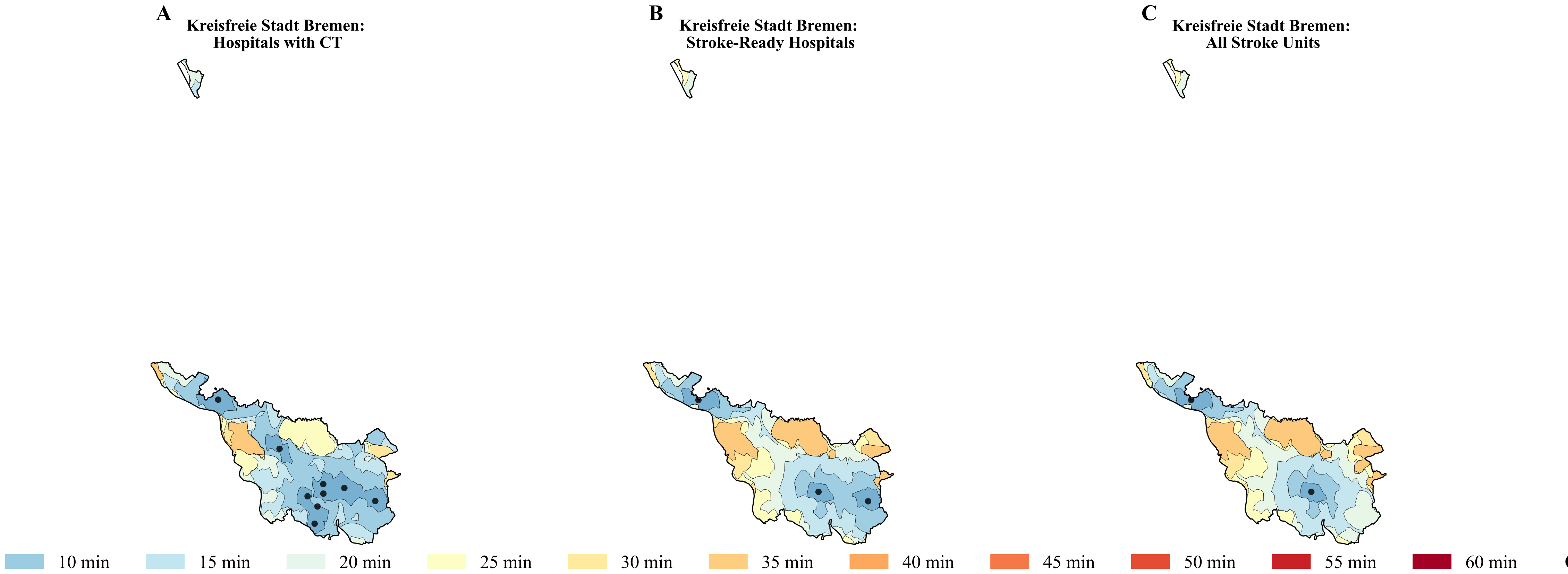

Isochrones — Landkreis Groß-Gerau (Hessen)

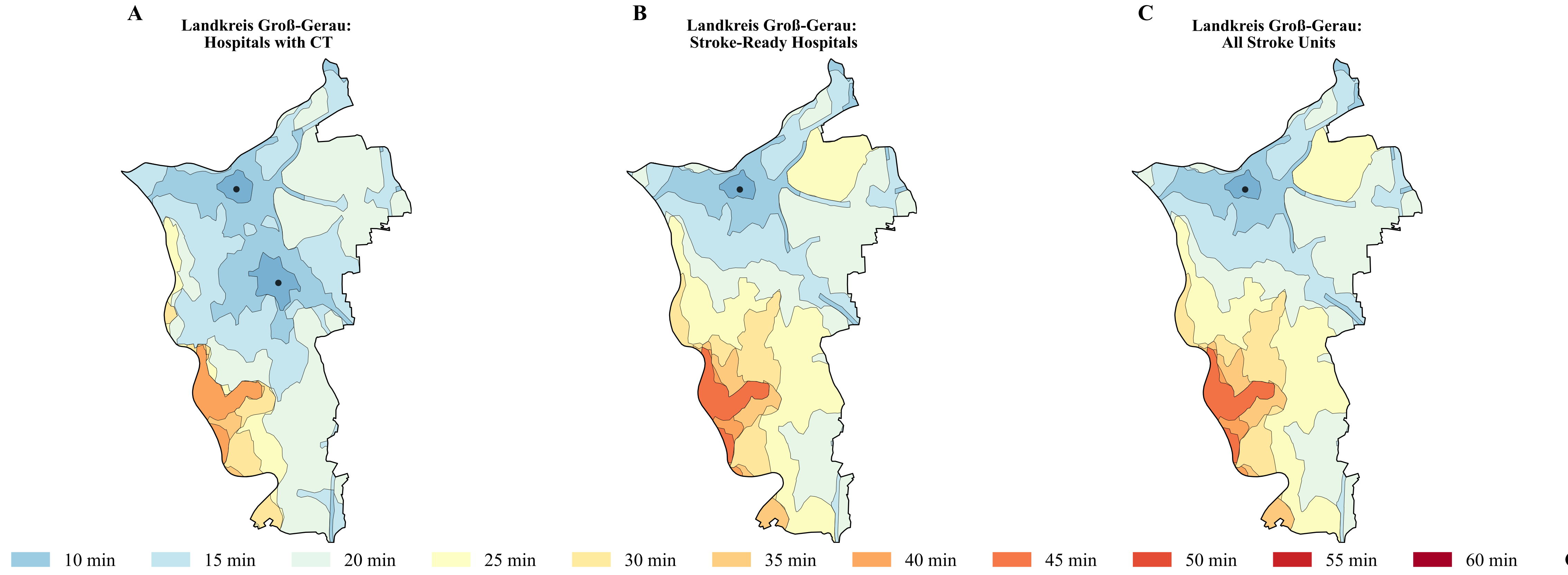

Isochrones — Landkreis Limburg-Weilburg (Hessen)

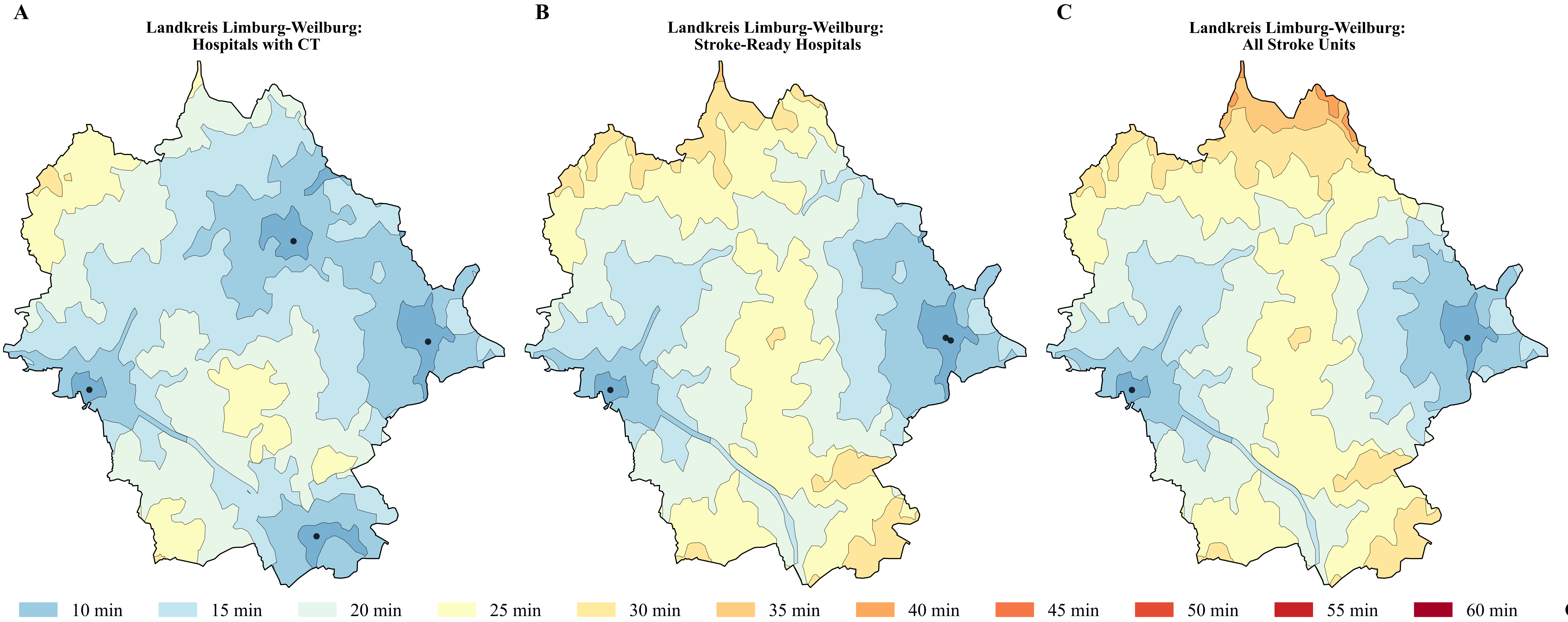

Isochrones — Landkreis Waldeck-Frankenberg (Hessen)

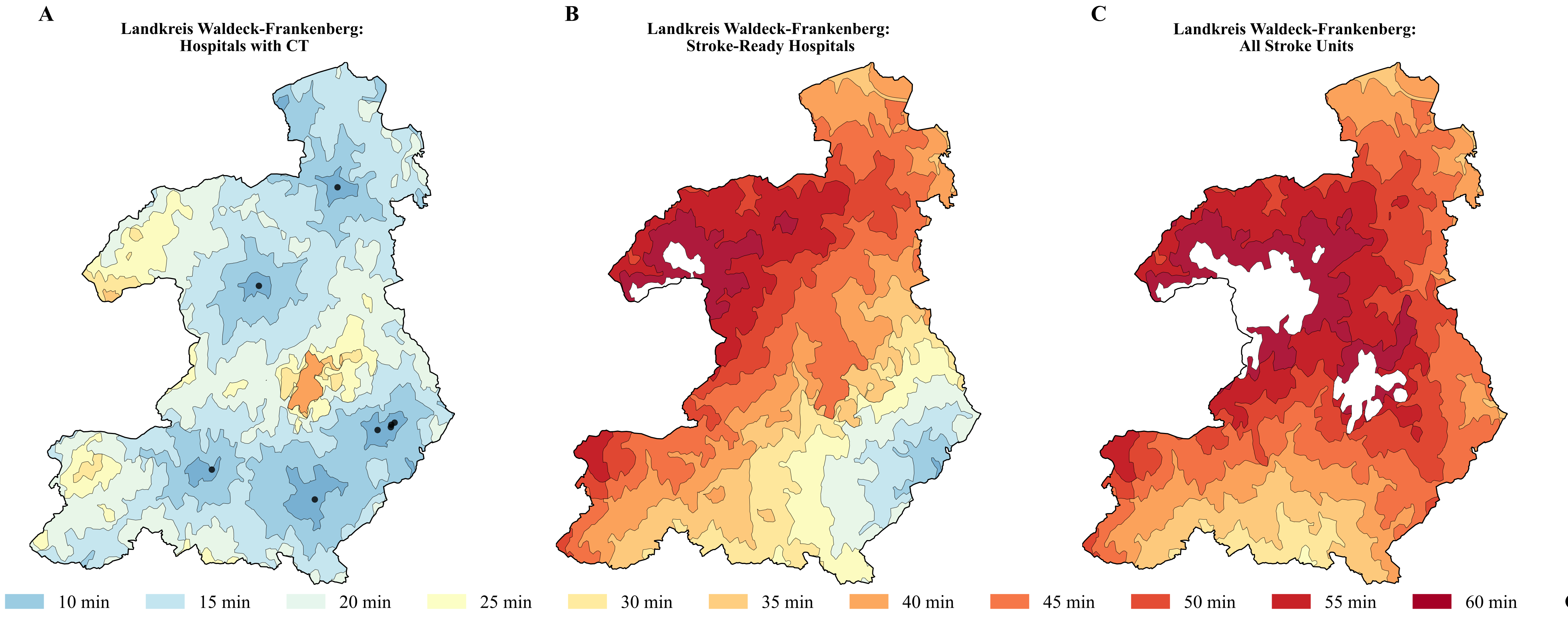

Isochrones — Landkreis Trier-Saarburg (Rheinland-Pfalz)

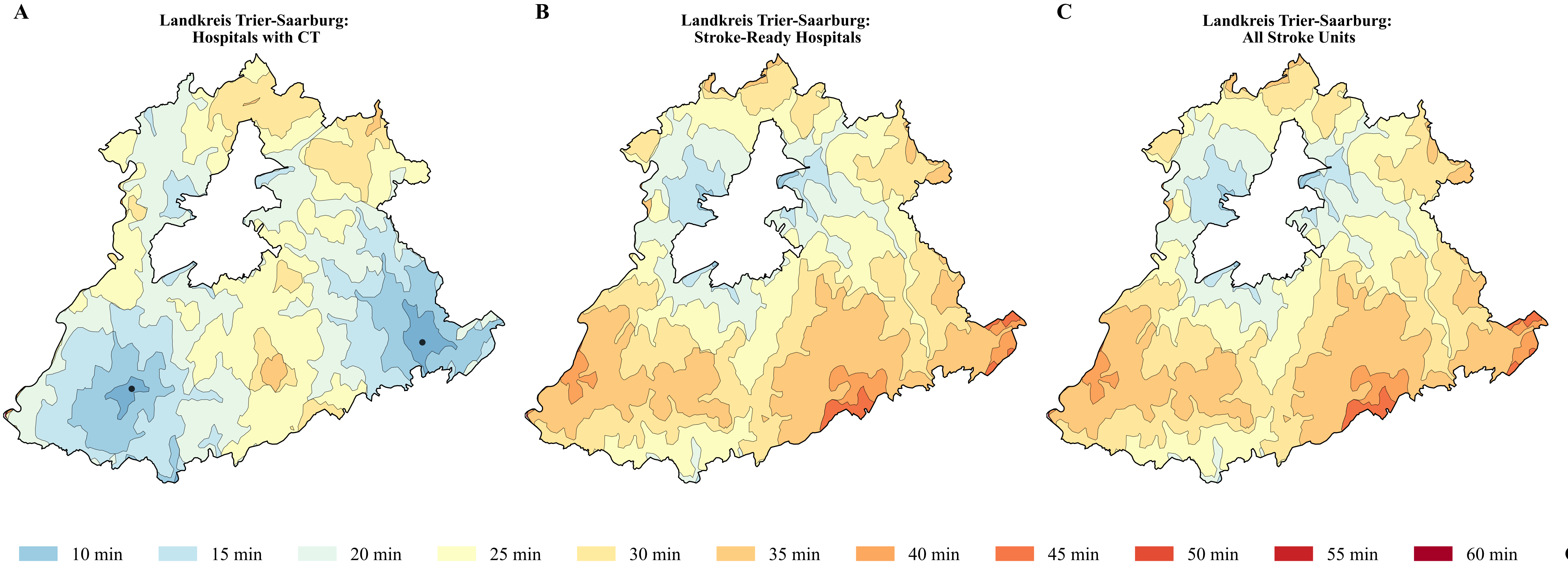

Isochrones — Landkreis Reutlingen (Baden-Württemberg)

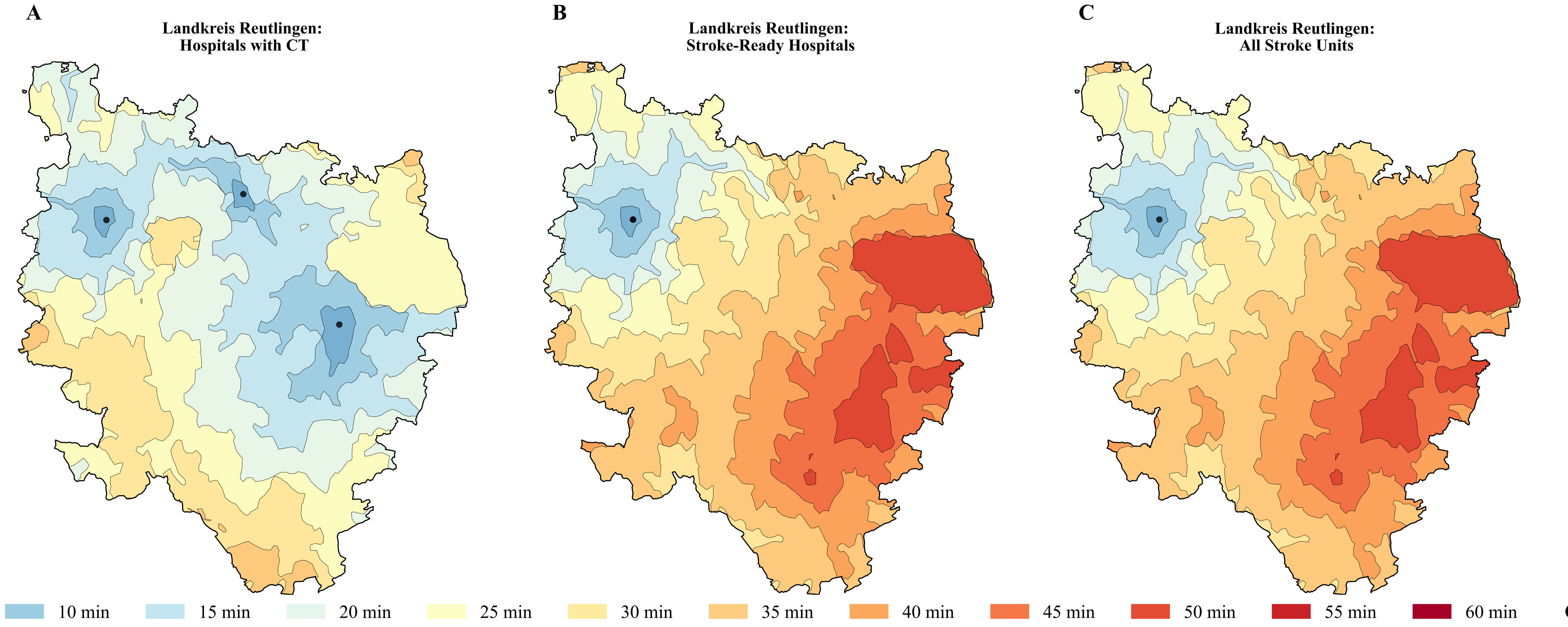

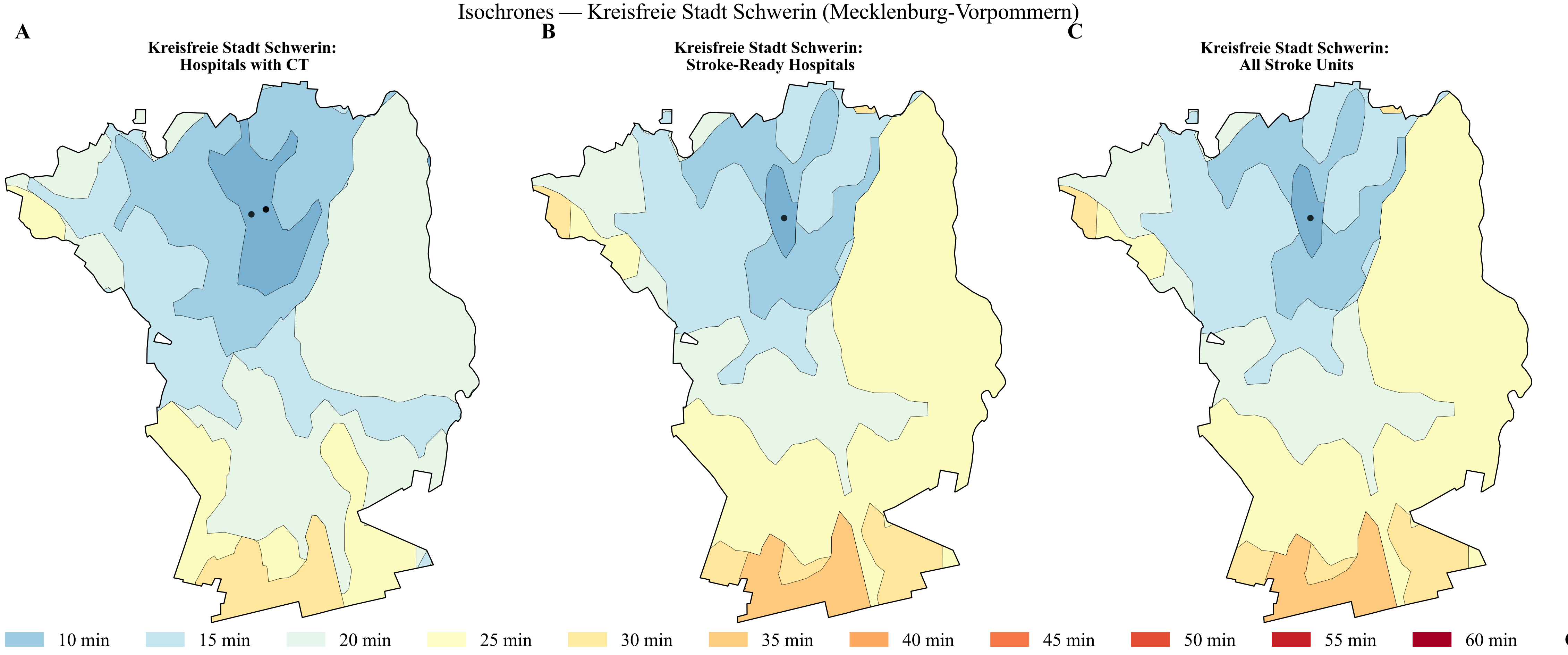
Isochrones — Kreisfreie Stadt Schwerin (Mecklenburg-Vorpommern)
A
Kreisfreie Stadt Schwerin:
Hospitals with CT
B
Kreisfreie Stadt Schwerin:
Stroke-Ready Hospitals
C
Kreisfreie Stadt Schwerin:
All Stroke Units
10 min
15 min
20 min
25 min
30 min
35 min
40 min
45 min
50 min
55 min
60 min

Isochrones — Kreisfreie Stadt Hamburg (Hamburg)

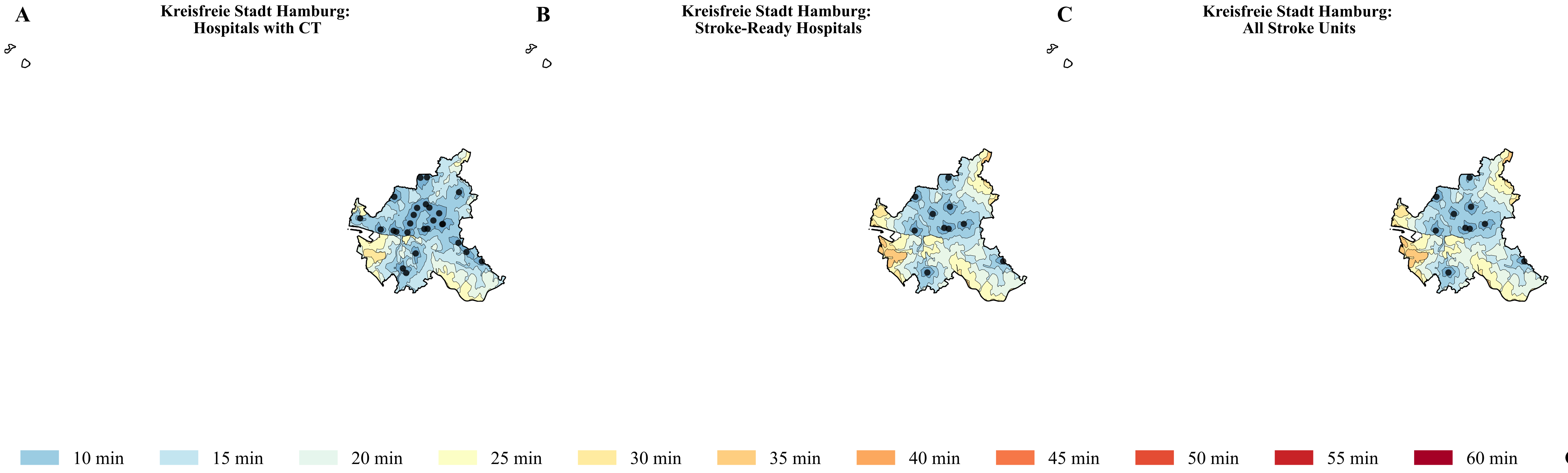

Isochrones — Kreisfreie Stadt Braunschweig (Niedersachsen)

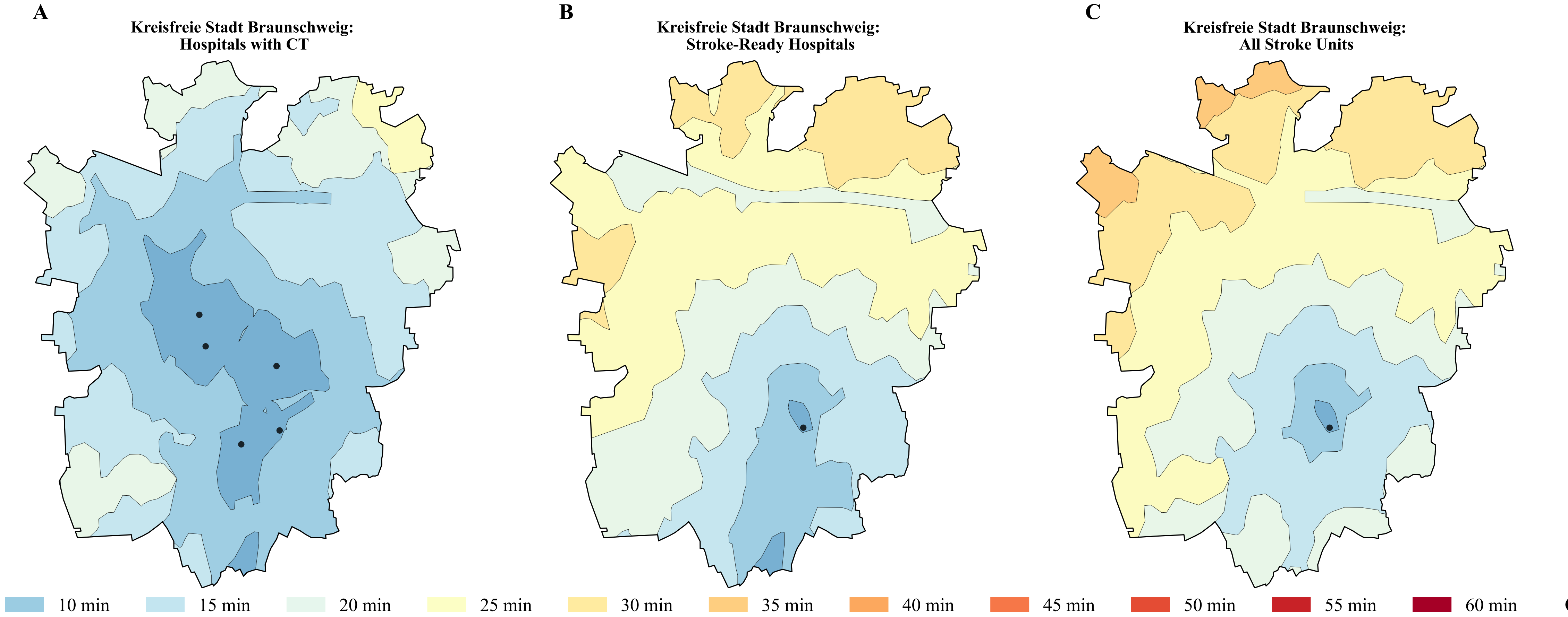

Isochrones — Landkreis Leer (Niedersachsen)

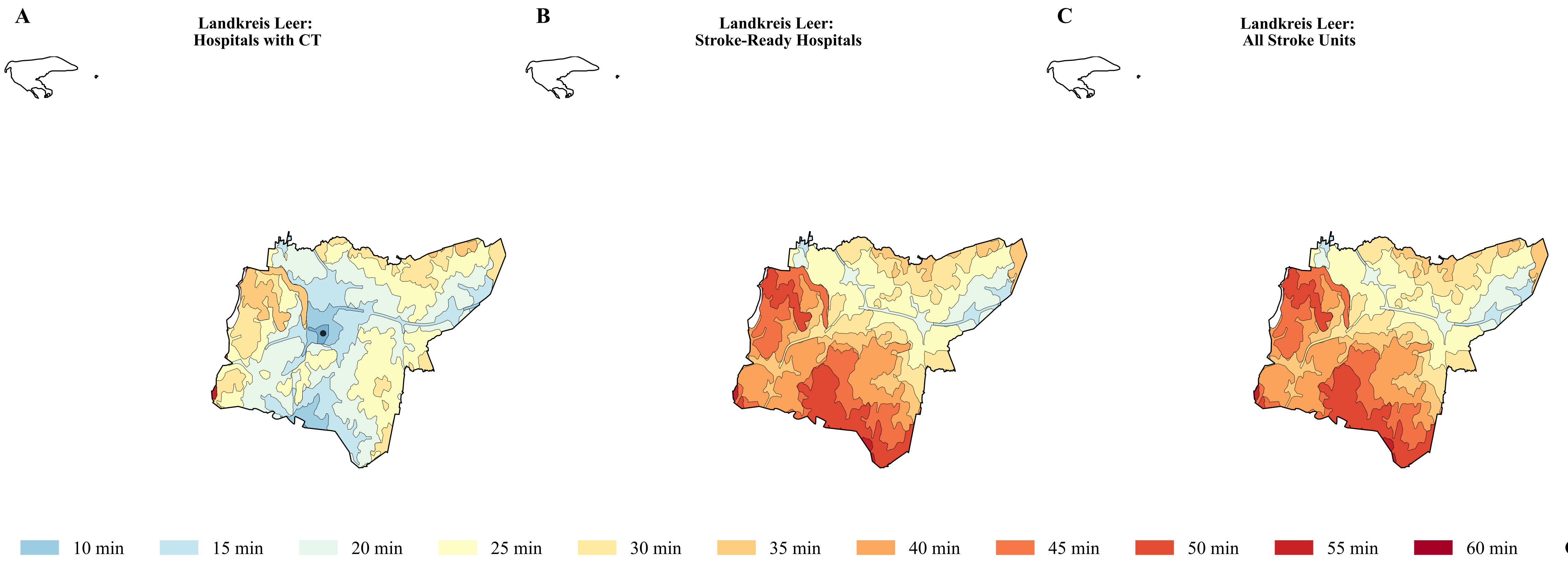

Isochrones — Kreisfreie Stadt Duisburg (Nordrhein-Westfalen)

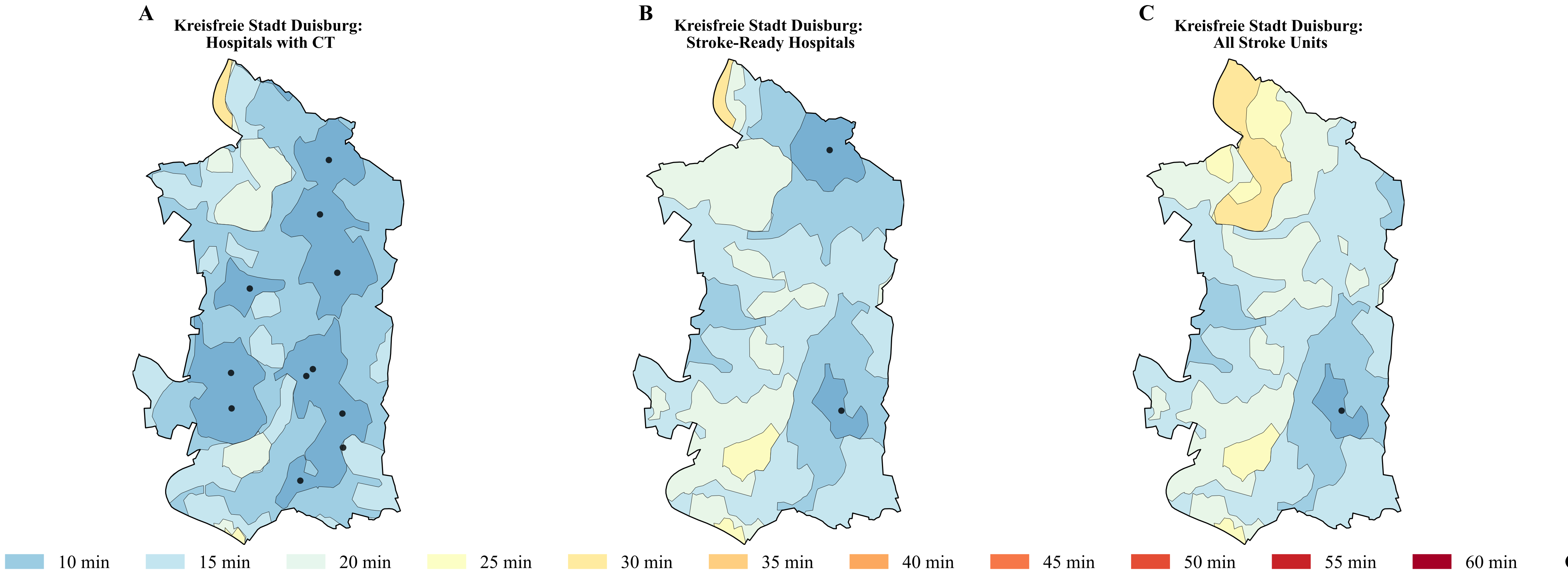

Isochrones — Kreis Siegen-Wittgenstein (Nordrhein-Westfalen)

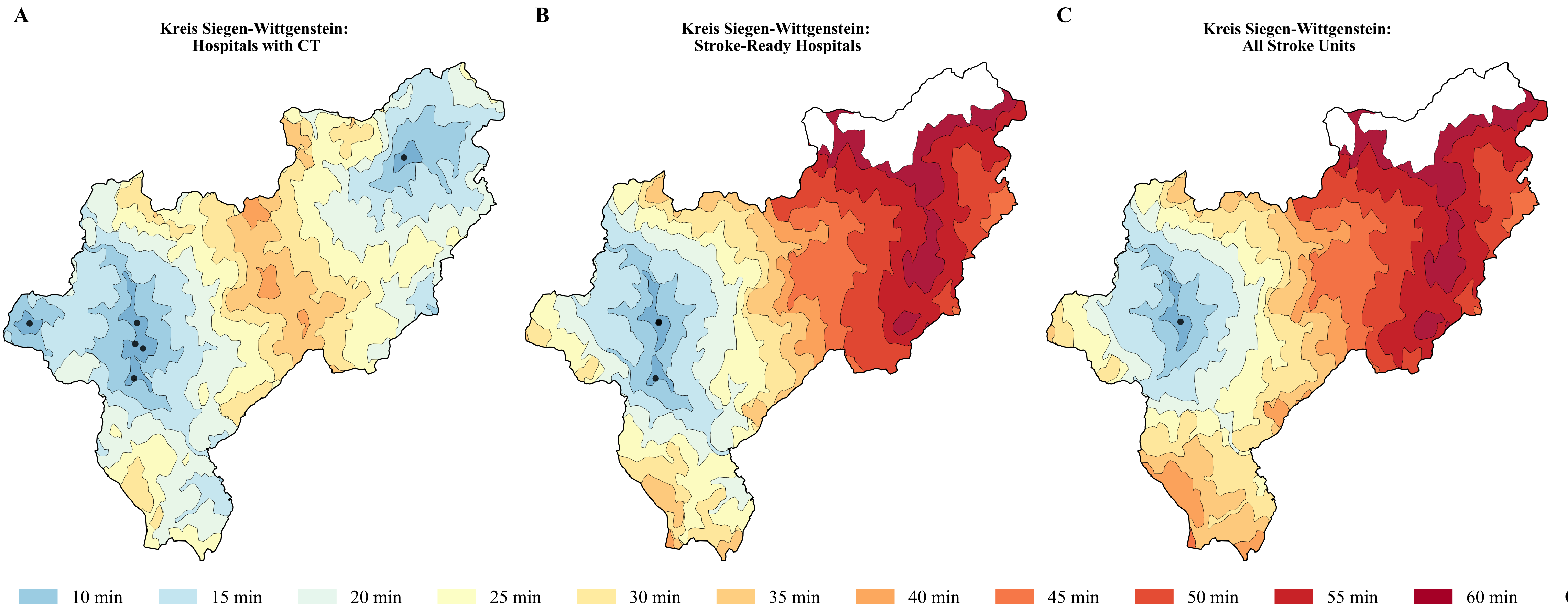

Isochrones — Landkreis Schwarzwald-Baar-Kreis (Baden-Württemberg)

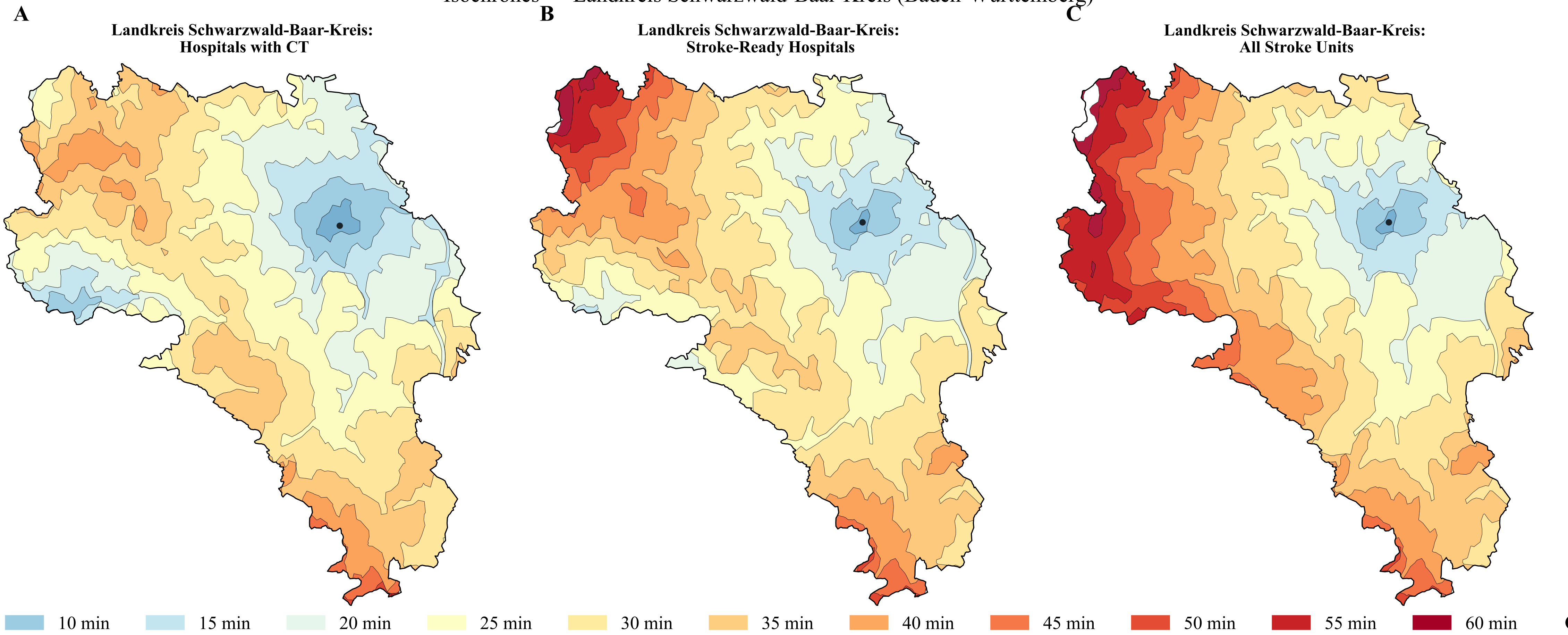

Isochrones — Kreisfreie Stadt Amberg (Bayern)

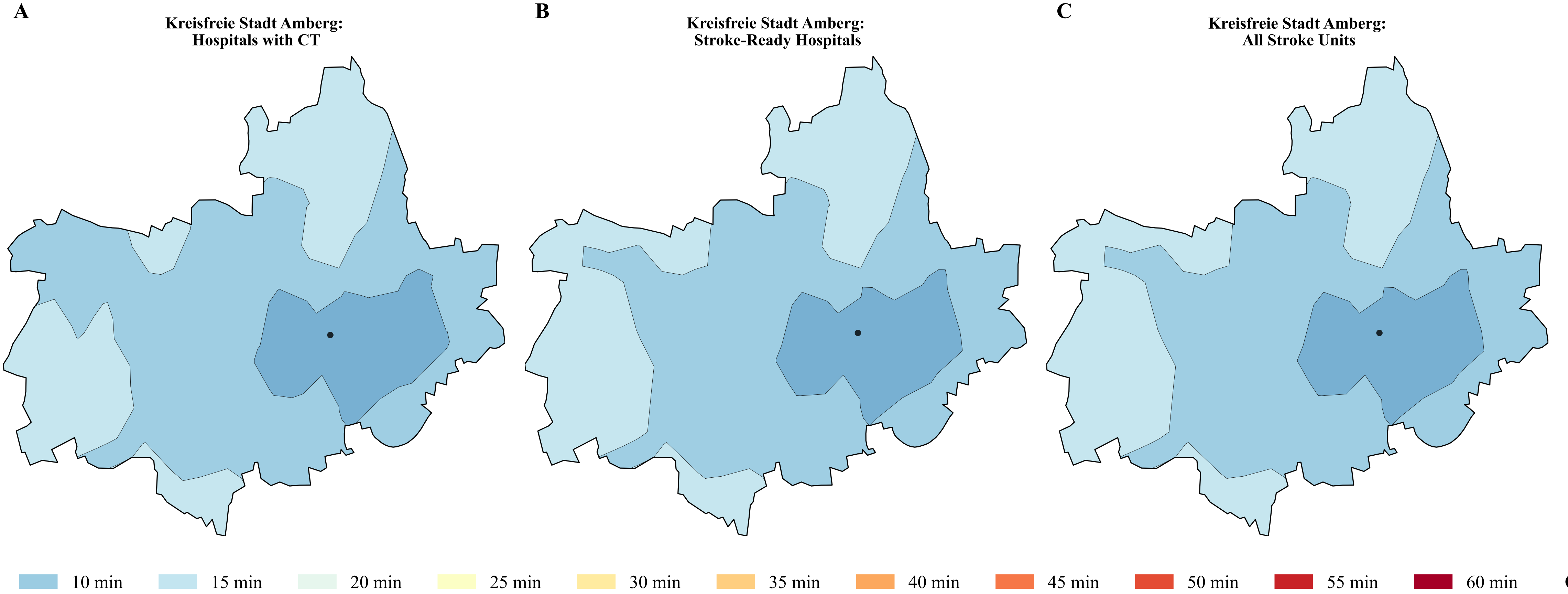

Isochrones — Kreisfreie Stadt Bayreuth (Bayern)

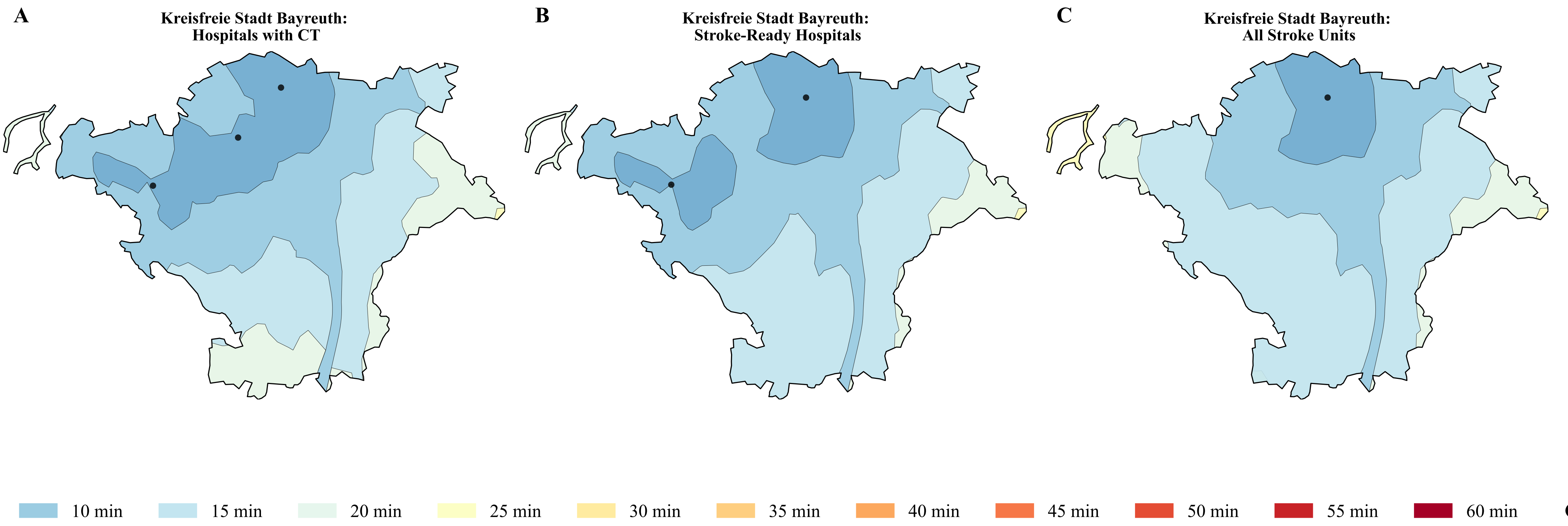

Isochrones — Landkreis Nürnberger Land (Bayern)

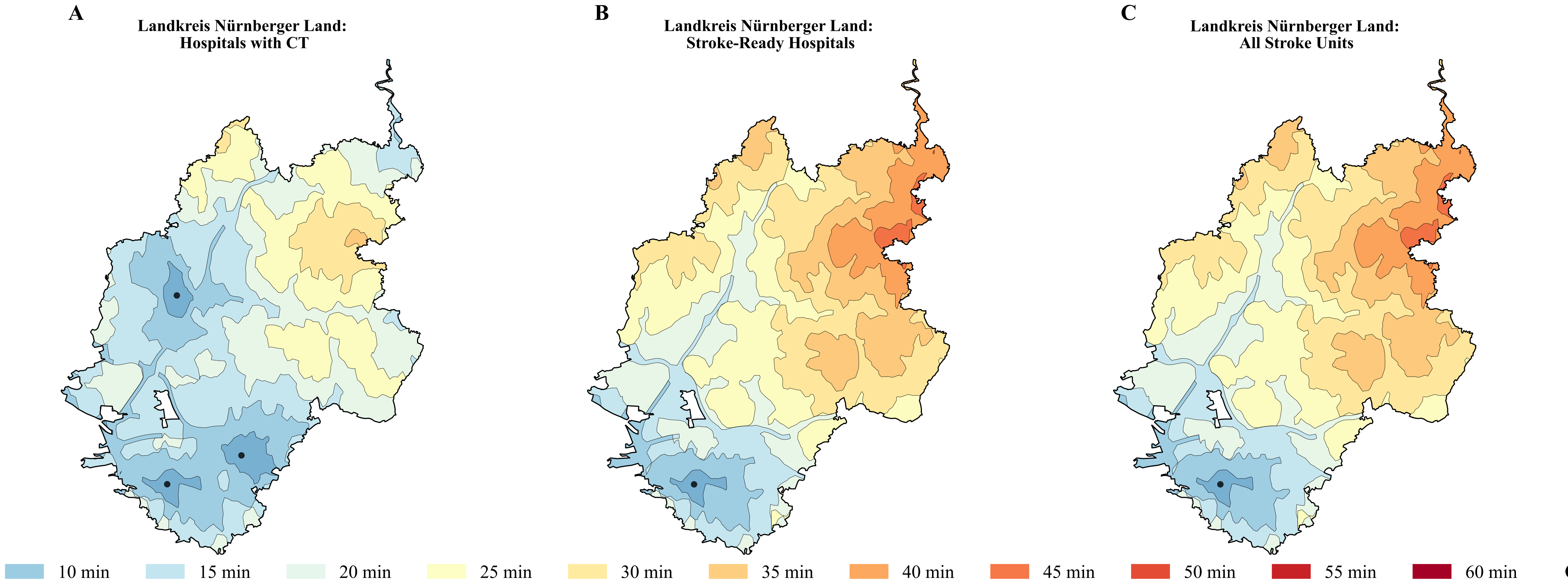

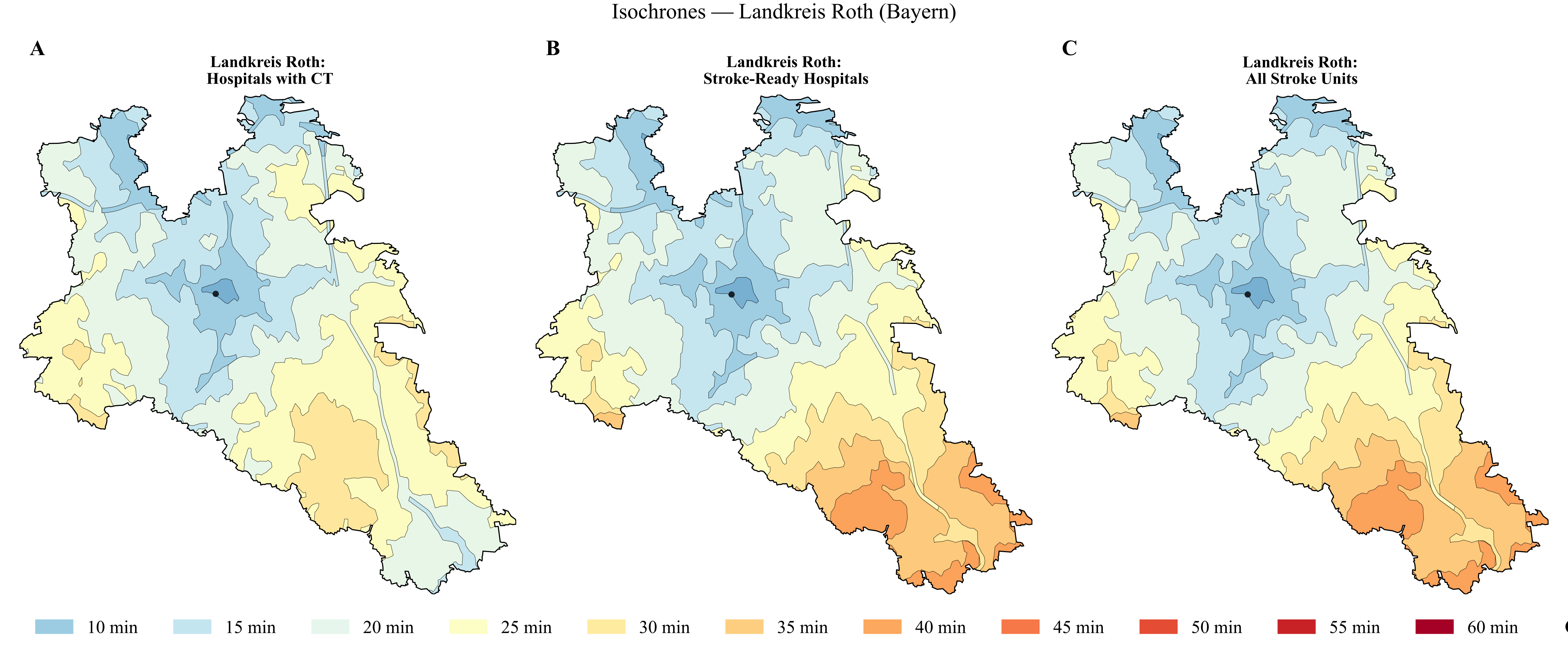
Isochrones — Landkreis Roth (Bayern)
A
Landkreis Roth:
Hospitals with CT
B
Landkreis Roth:
Stroke-Ready Hospitals
C
Landkreis Roth:
All Stroke Units
10 min
15 min
20 min
25 min
30 min
35 min
40 min
45 min
50 min
55 min
60 min

Isochrones — Kreisfreie Stadt Aschaffenburg (Bayern)

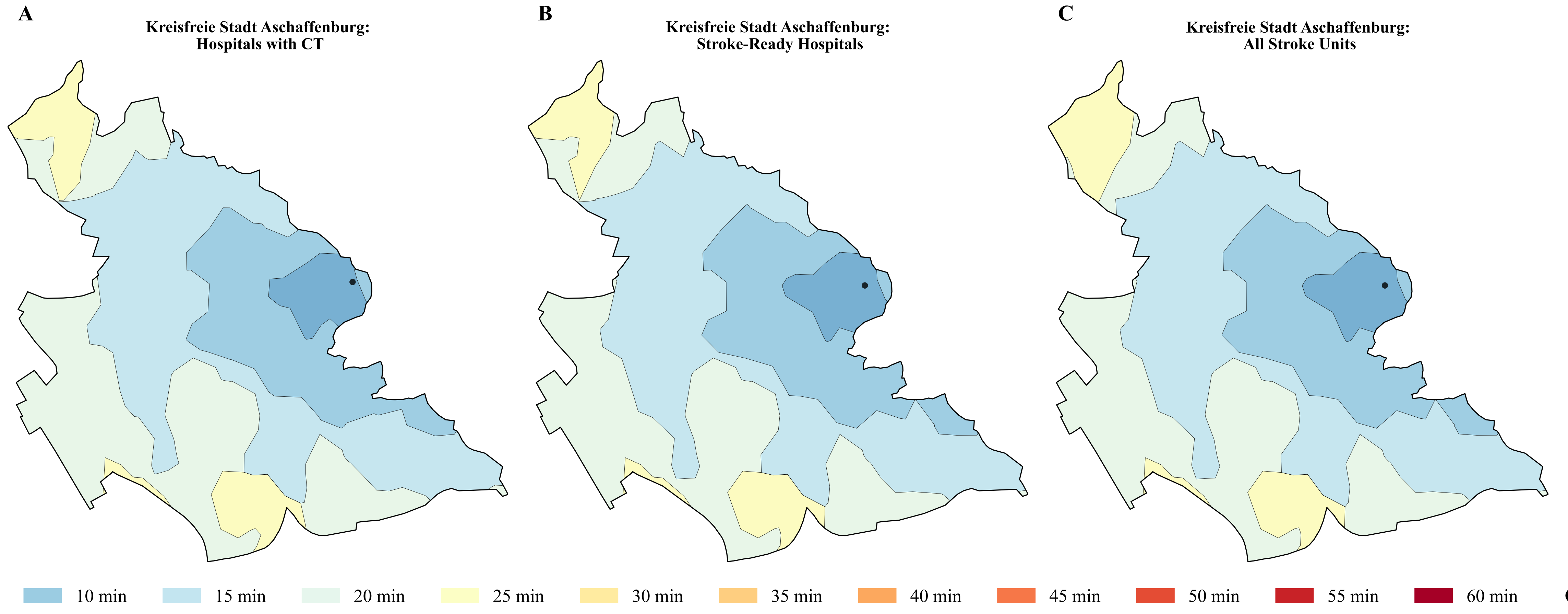

Isochrones — Landkreis Merzig-Wadern (Saarland)

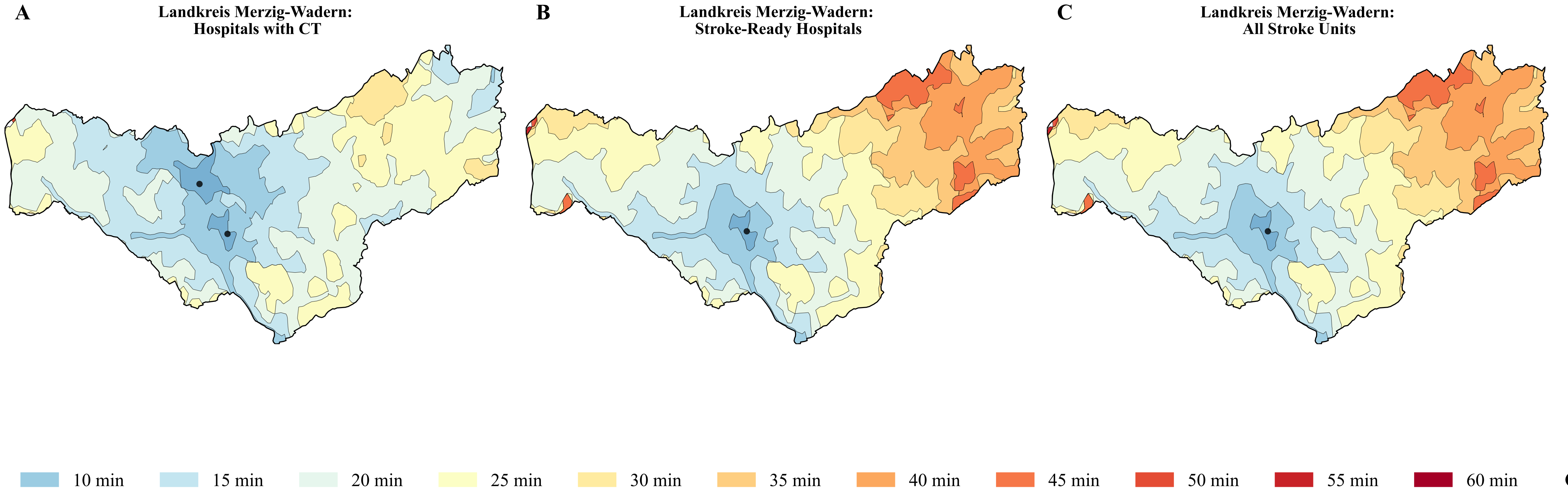

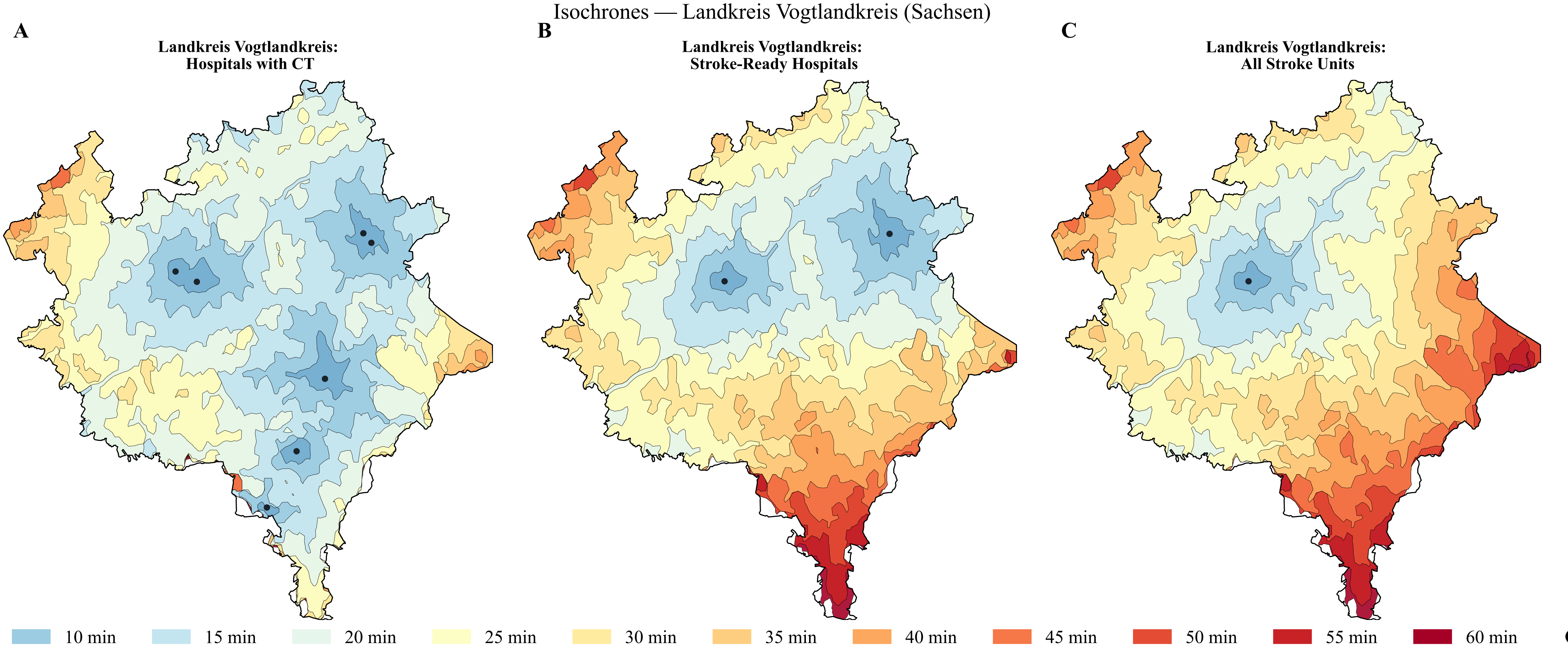
Isochrones — Landkreis Vogtlandkreis (Sachsen)
A
Landkreis Vogtlandkreis:
Hospitals with CT
B
Landkreis Vogtlandkreis:
Stroke-Ready Hospitals
C
Landkreis Vogtlandkreis:
All Stroke Units
10 min
15 min
20 min
25 min
30 min
35 min
40 min
45 min
50 min
55 min
60 min

Isochrones — Landkreis Altmarkkreis Salzwedel (Sachsen-Anhalt)

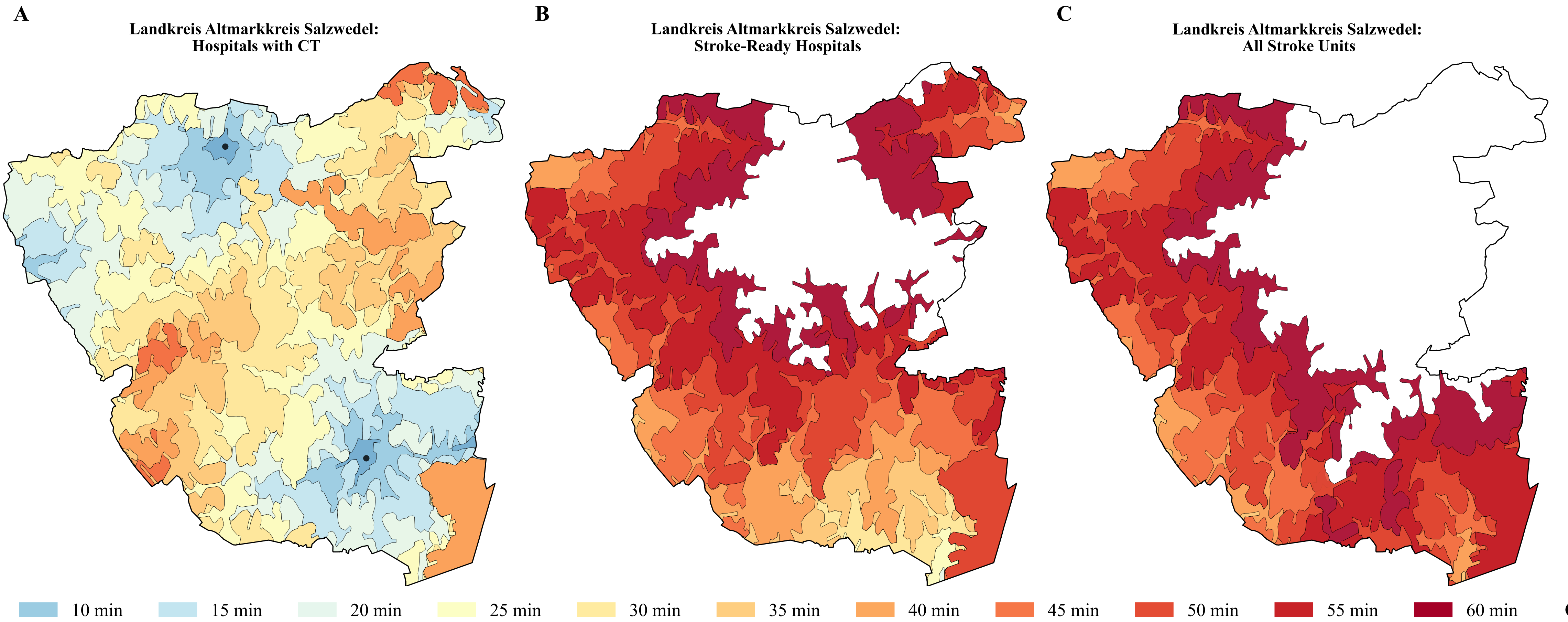

Isochrones — Kreis Segeberg (Schleswig-Holstein)

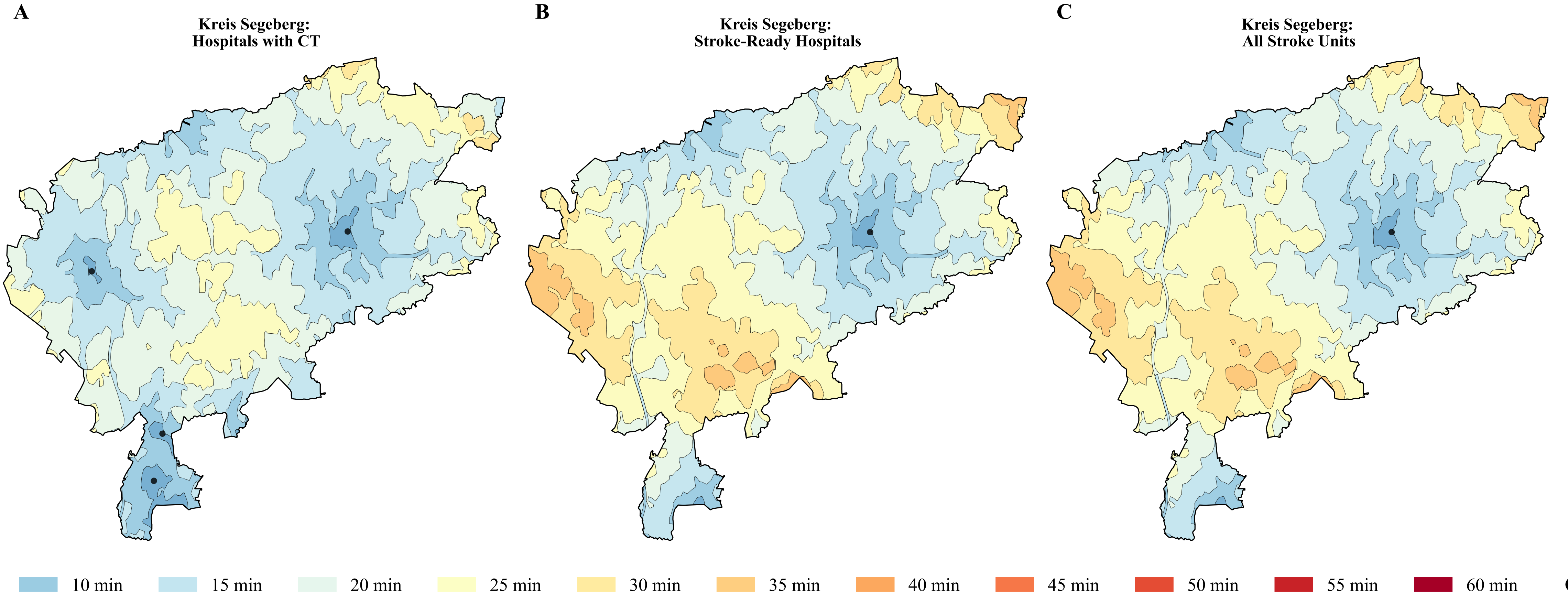

Isochrones — Landkreis Osnabrück (Niedersachsen)

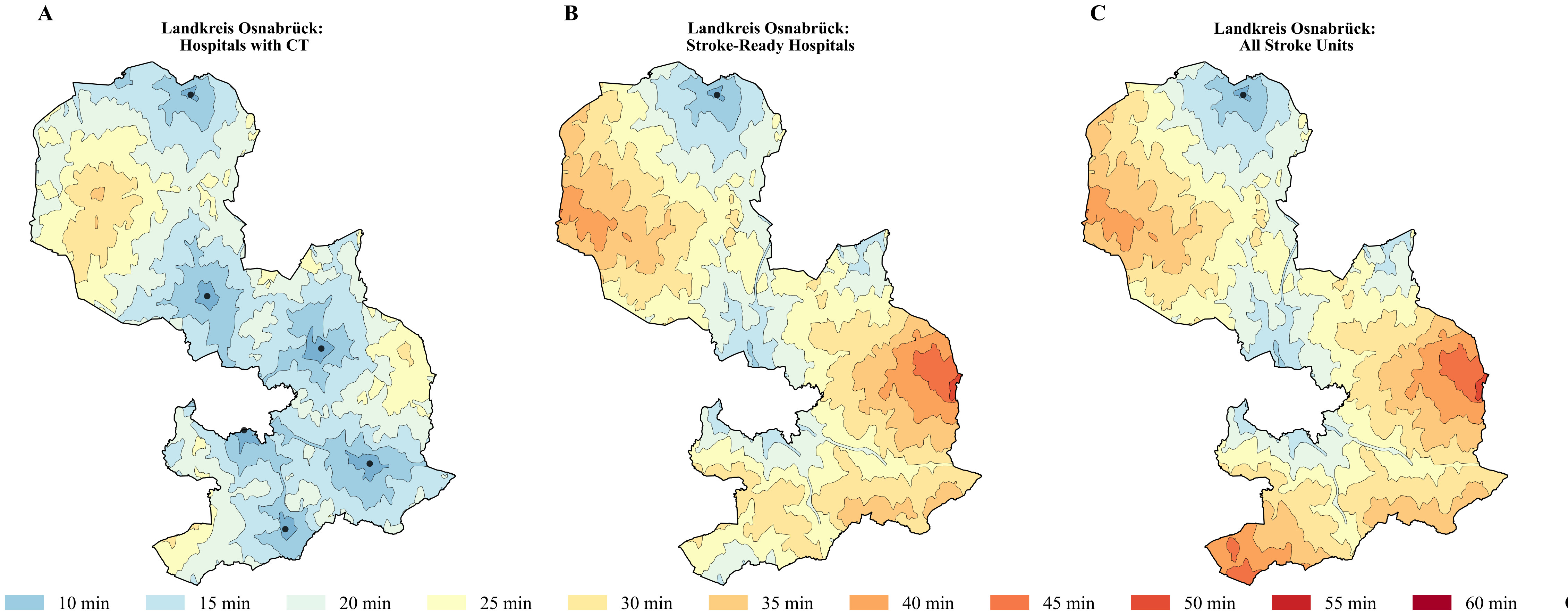

Isochrones — Kreisfreie Stadt Oberhausen (Nordrhein-Westfalen)

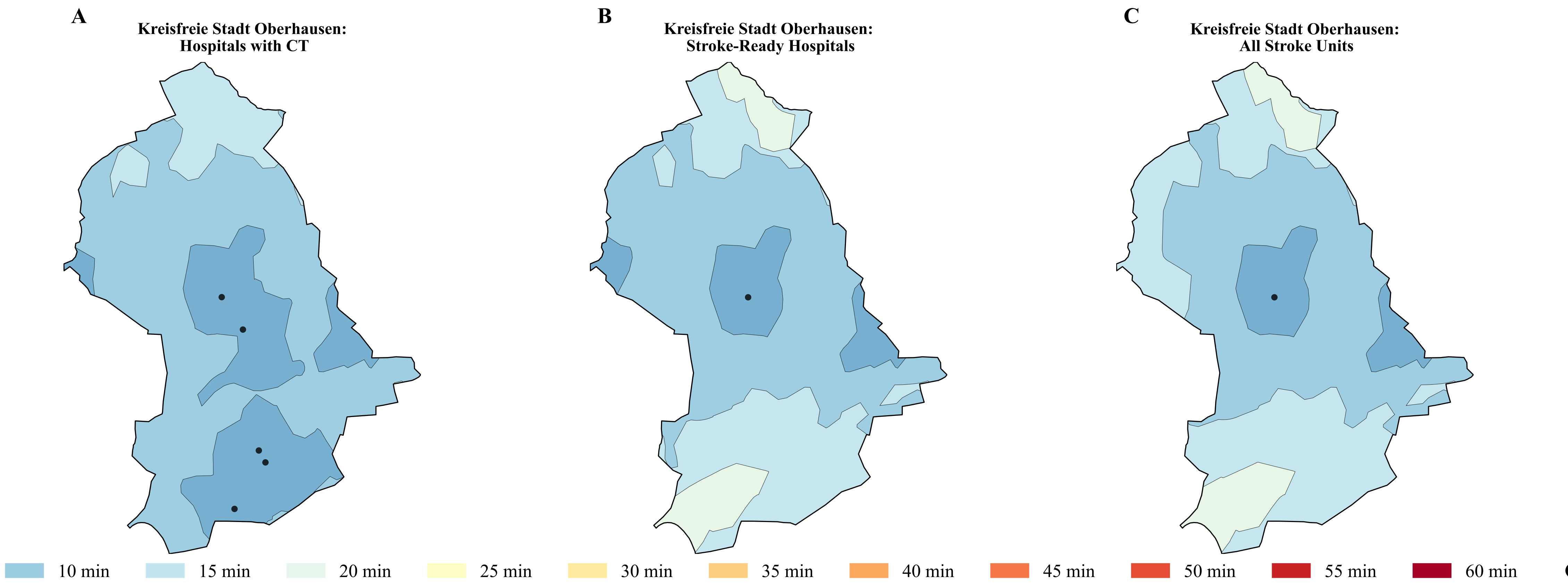

Isochrones — Kreisfreie Stadt Remscheid (Nordrhein-Westfalen)

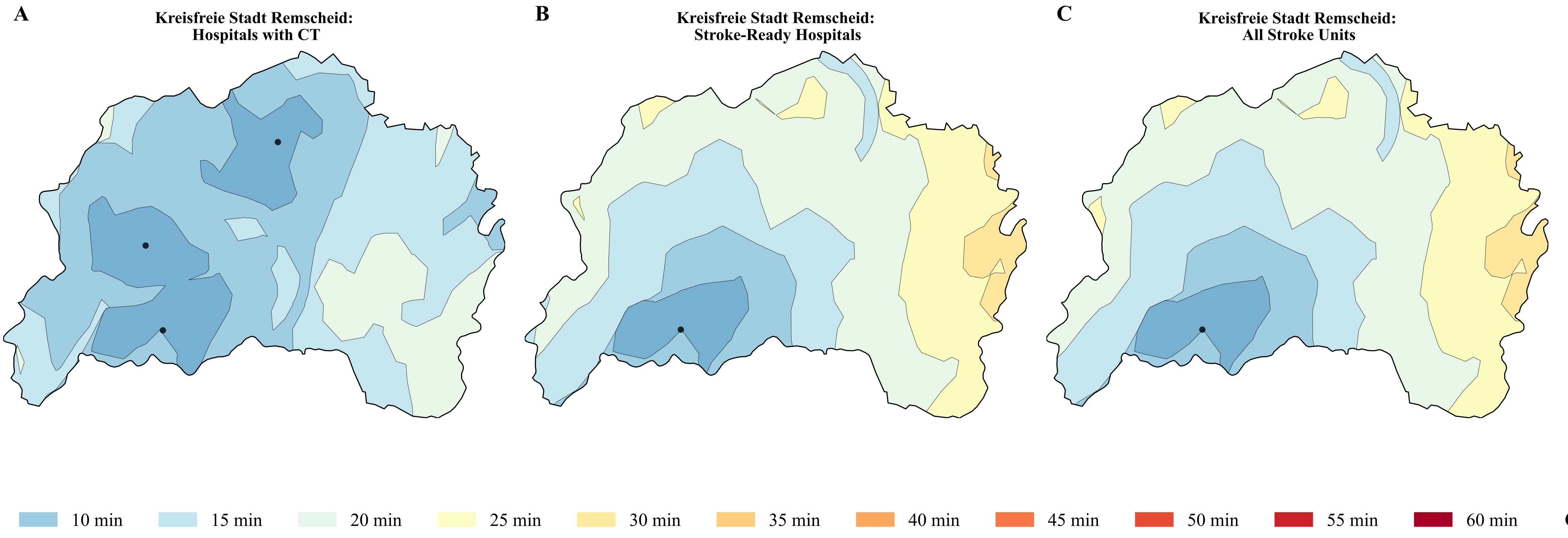

Isochrones — Kreis Mettmann (Nordrhein-Westfalen)

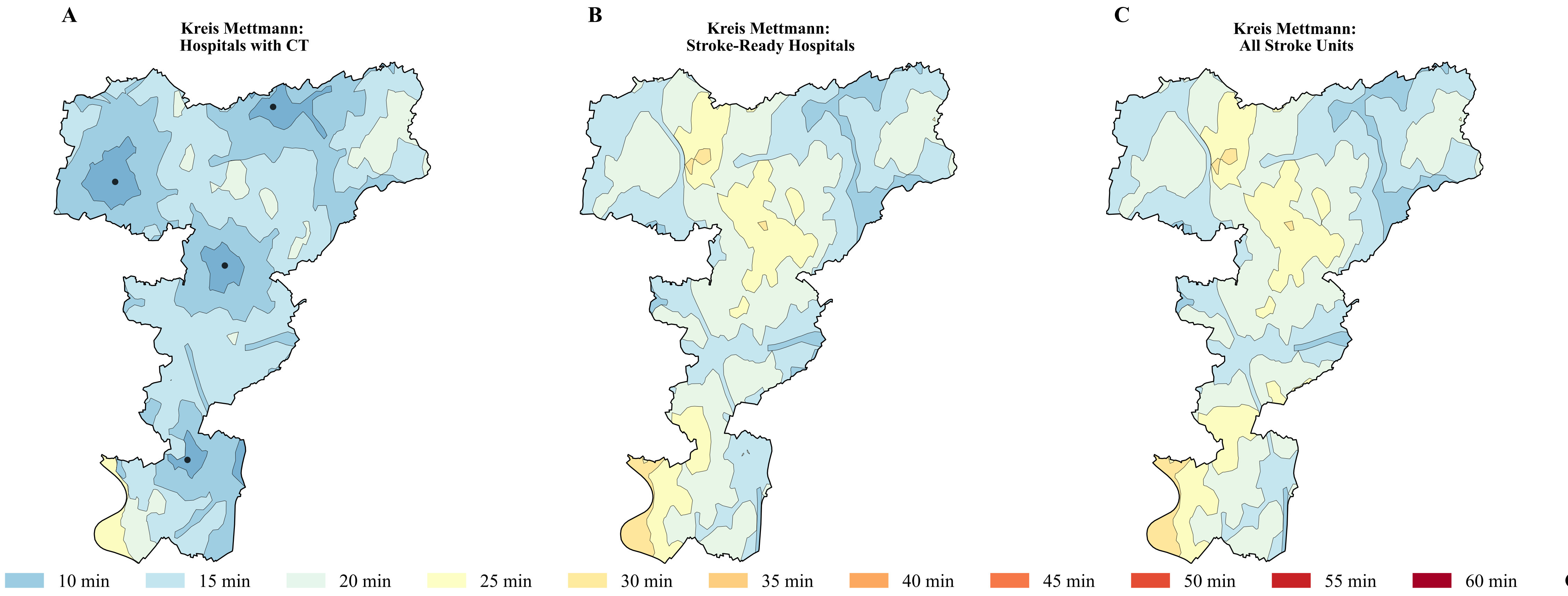

Isochrones — Kreis Rhein-Sieg-Kreis (Nordrhein-Westfalen)

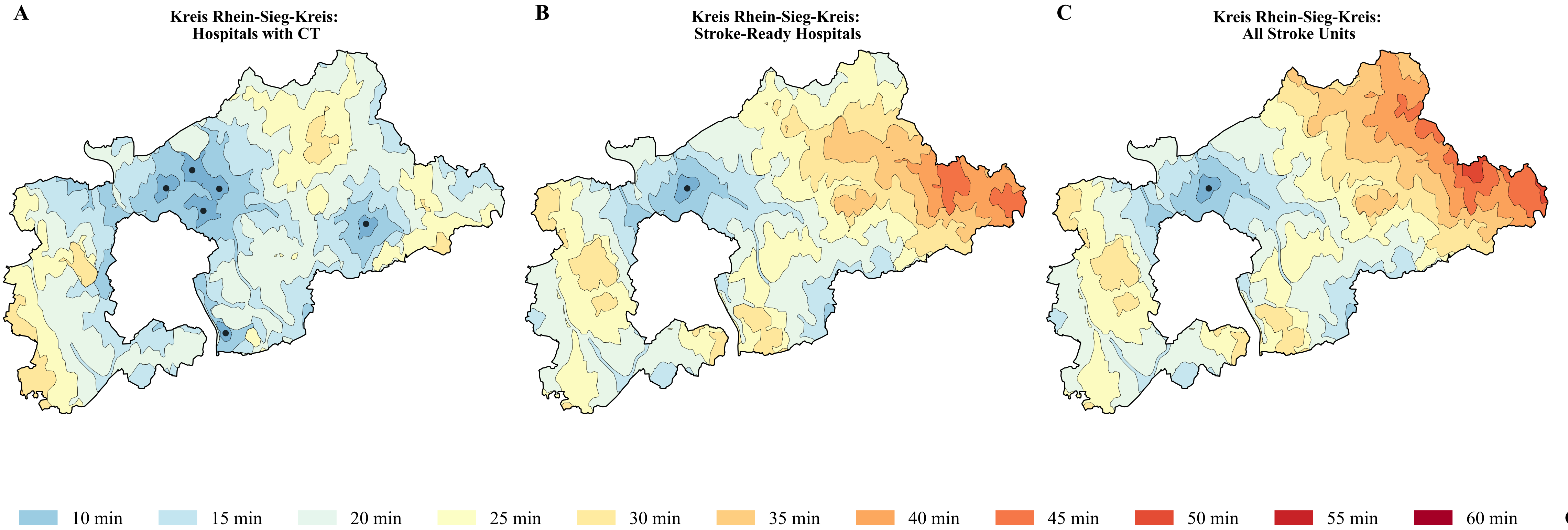

Isochrones — Kreis Borken (Nordrhein-Westfalen)

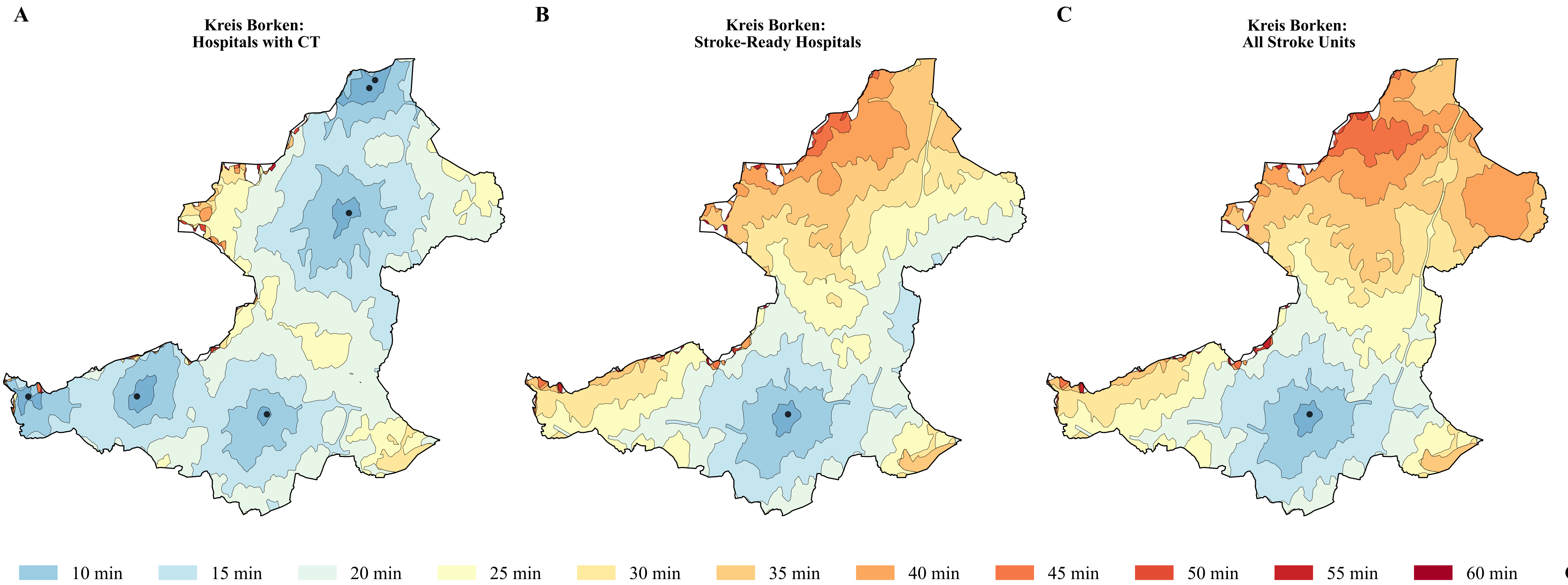

Isochrones — Landkreis Vulkaneifel (Rheinland-Pfalz)

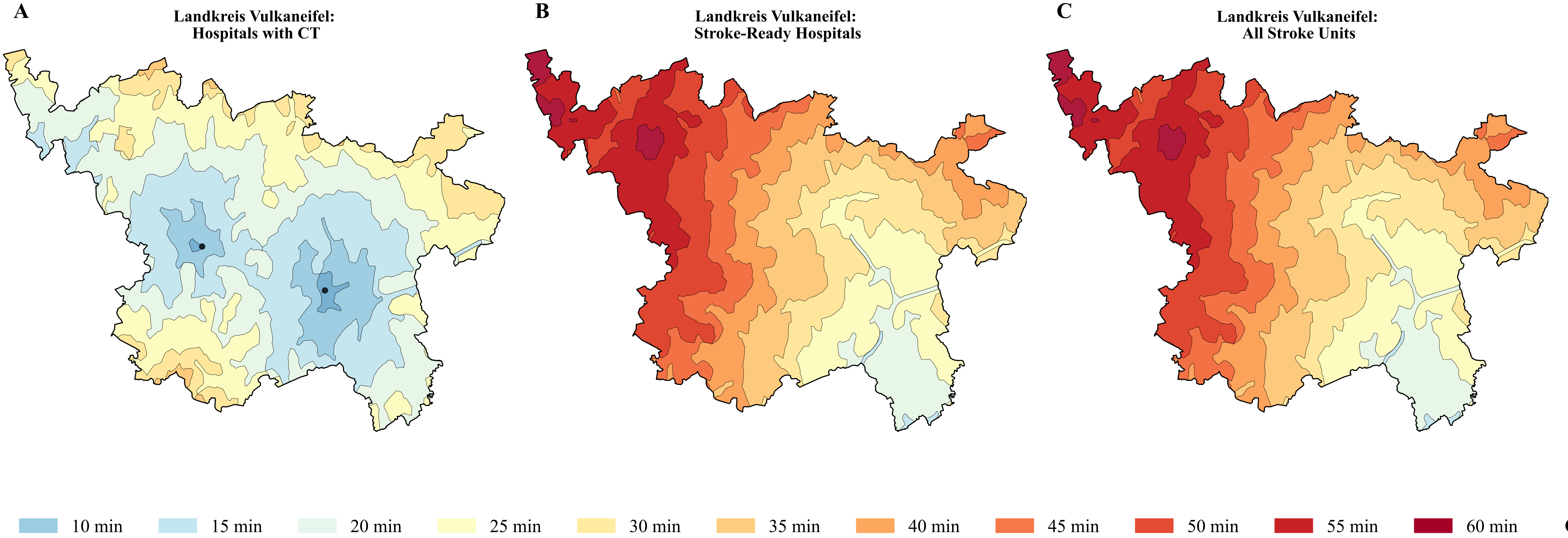

Isochrones — Landkreis Kaiserslautern (Rheinland-Pfalz)

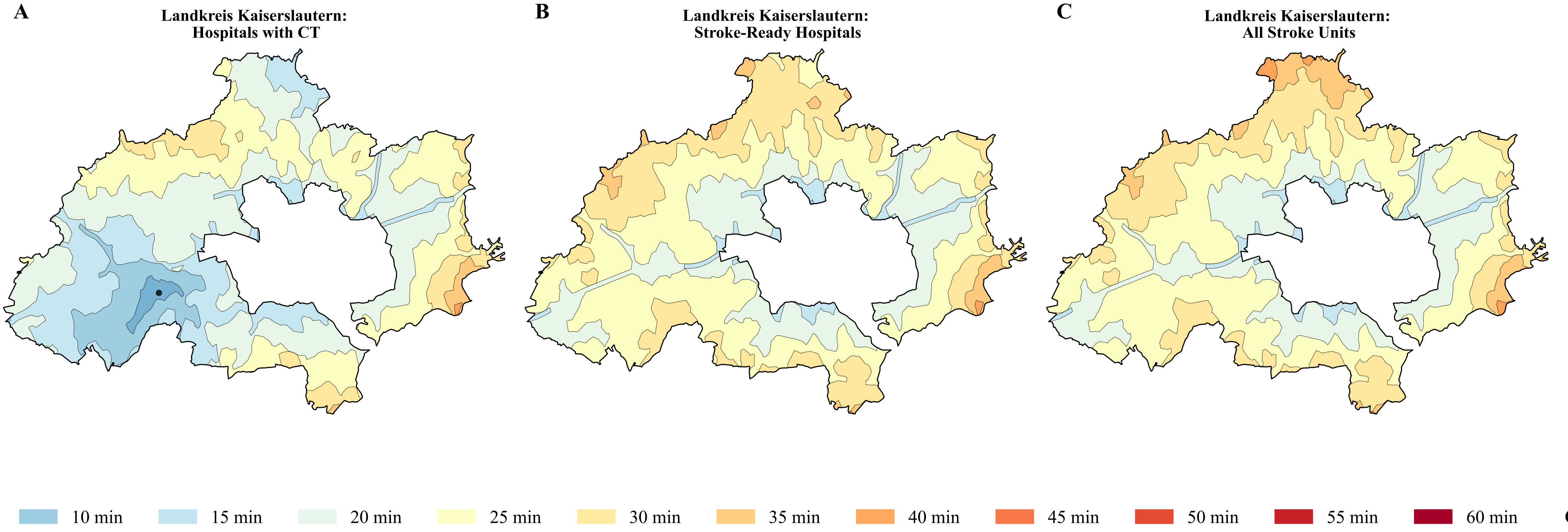

Isochrones — Landkreis Calw (Baden-Württemberg)

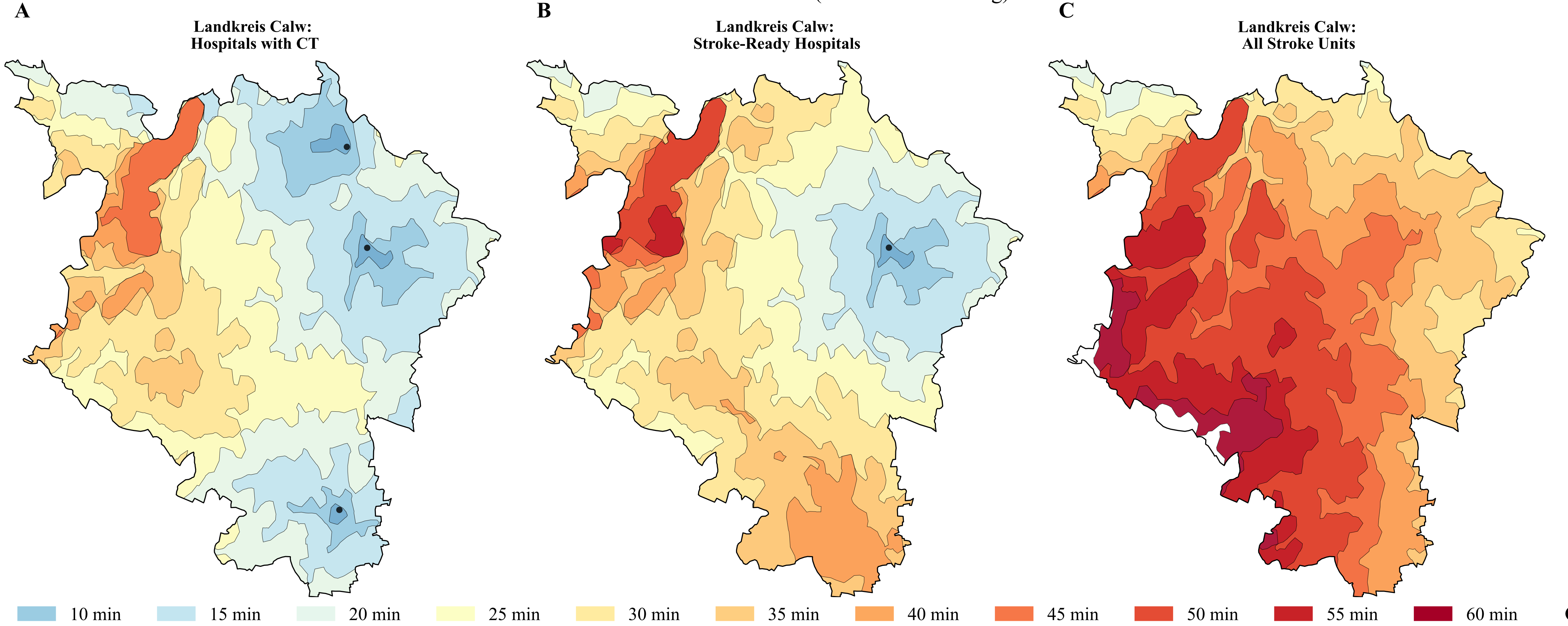

Isochrones — Kreisfreie Stadt Berlin (Berlin)

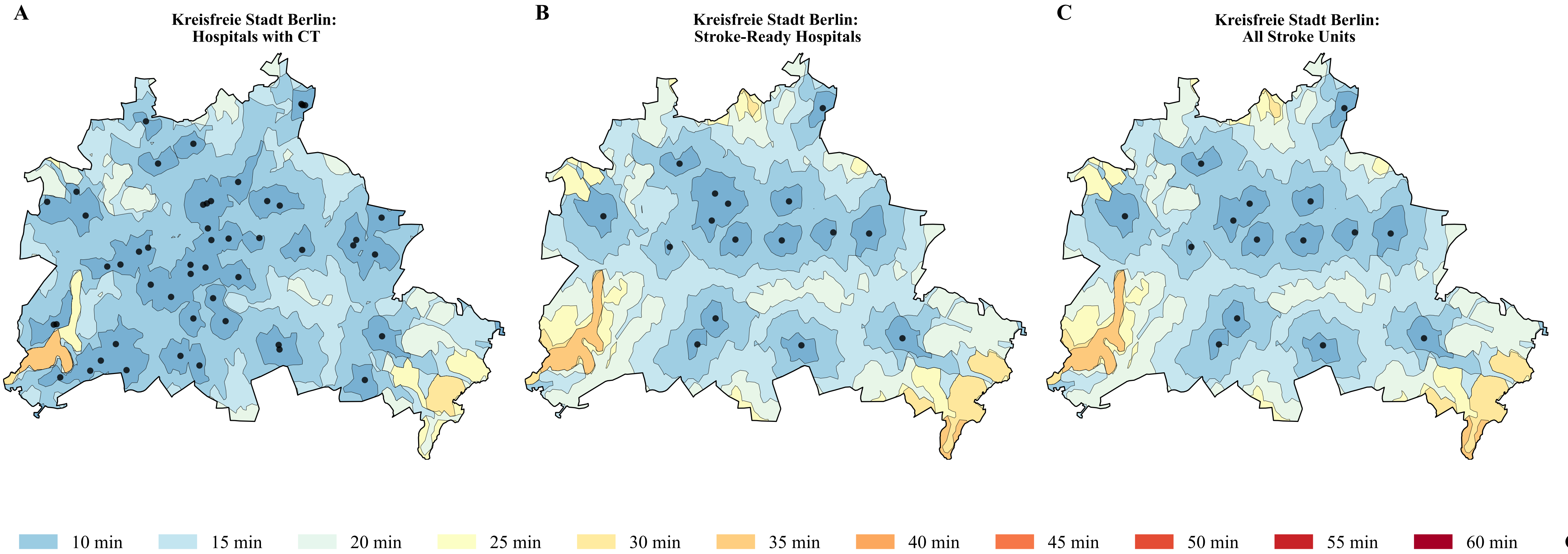

Isochrones — Kreis Stormarn (Schleswig-Holstein)

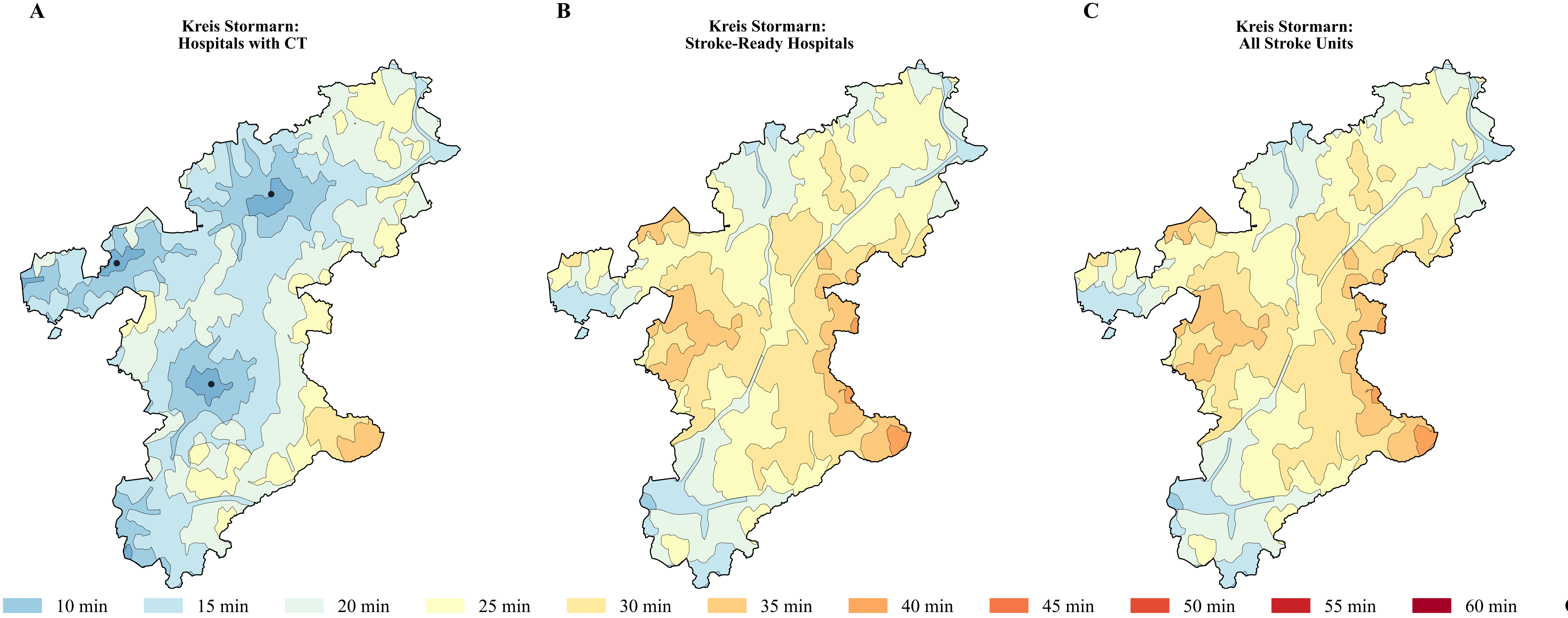

Isochrones — Landkreis Hildesheim (Niedersachsen)

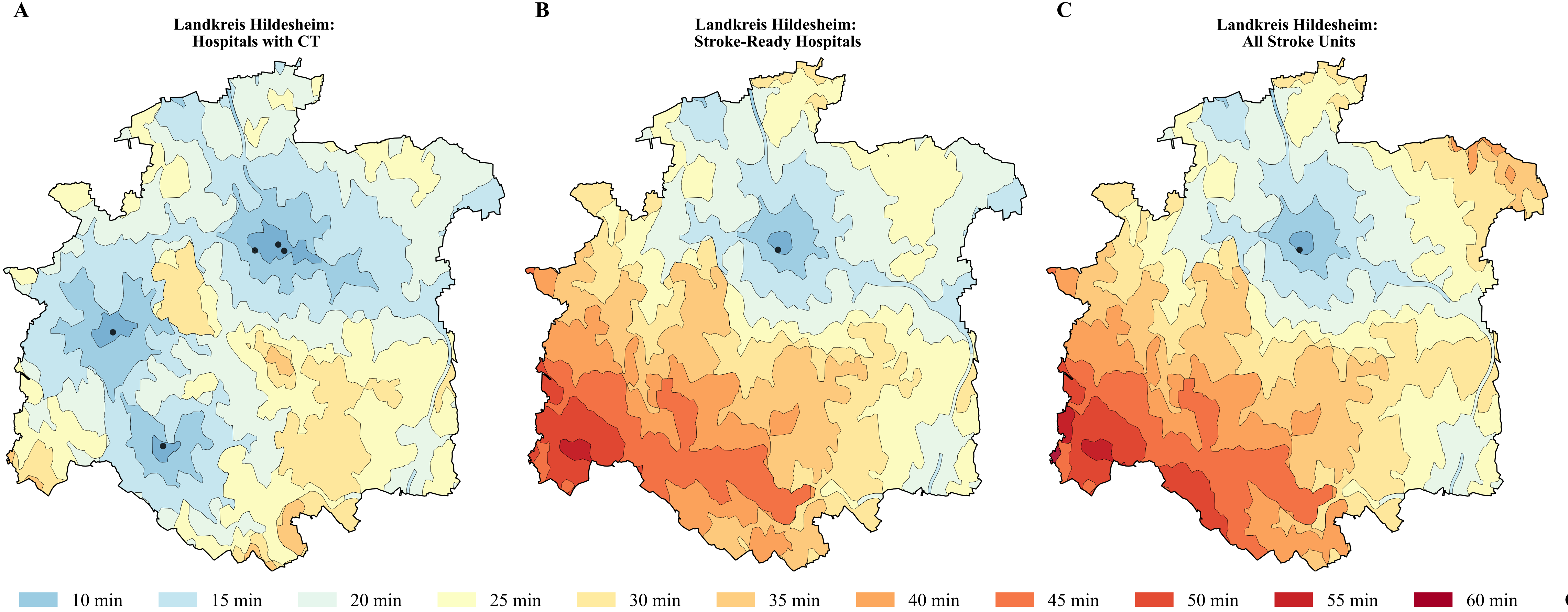

Isochrones — Kreis Viersen (Nordrhein-Westfalen)

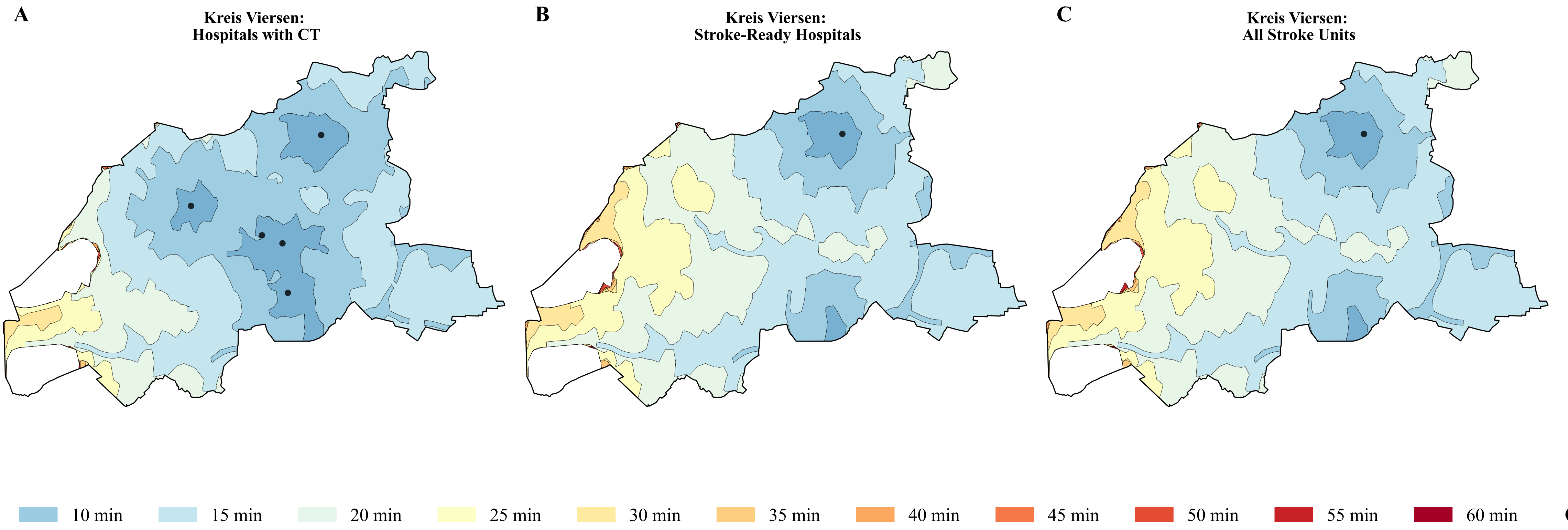

Isochrones — Kreis Rheinisch-Bergischer Kreis (Nordrhein-Westfalen)

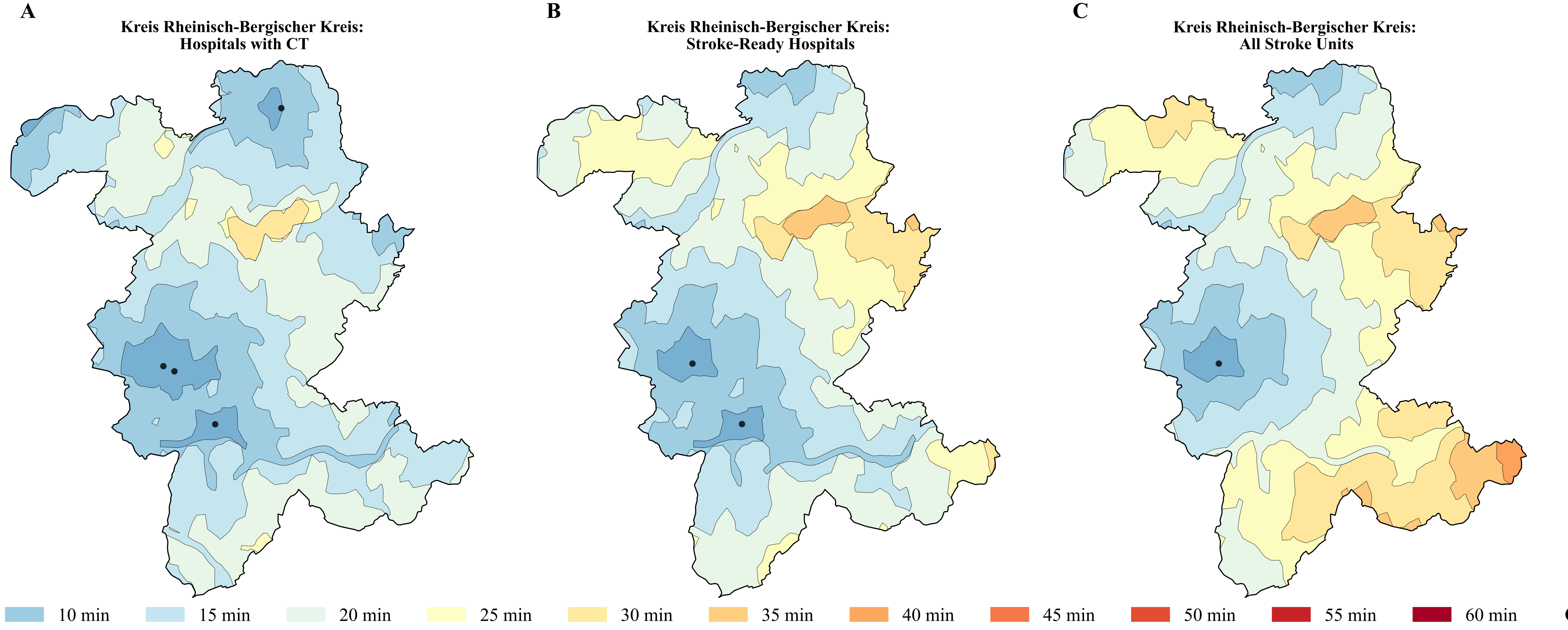

Isochrones — Kreisfreie Stadt Zweibrücken (Rheinland-Pfalz)

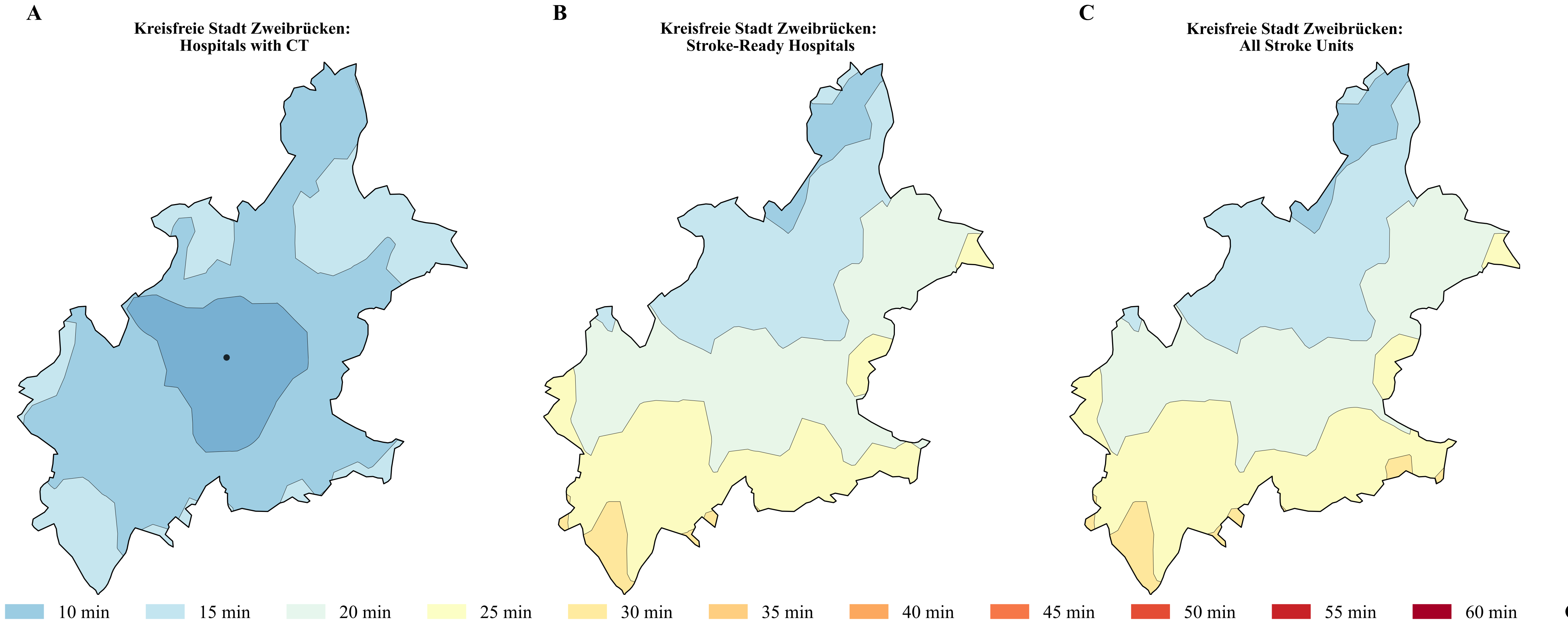

Isochrones — Landkreis Rhein-Pfalz-Kreis (Rheinland-Pfalz)

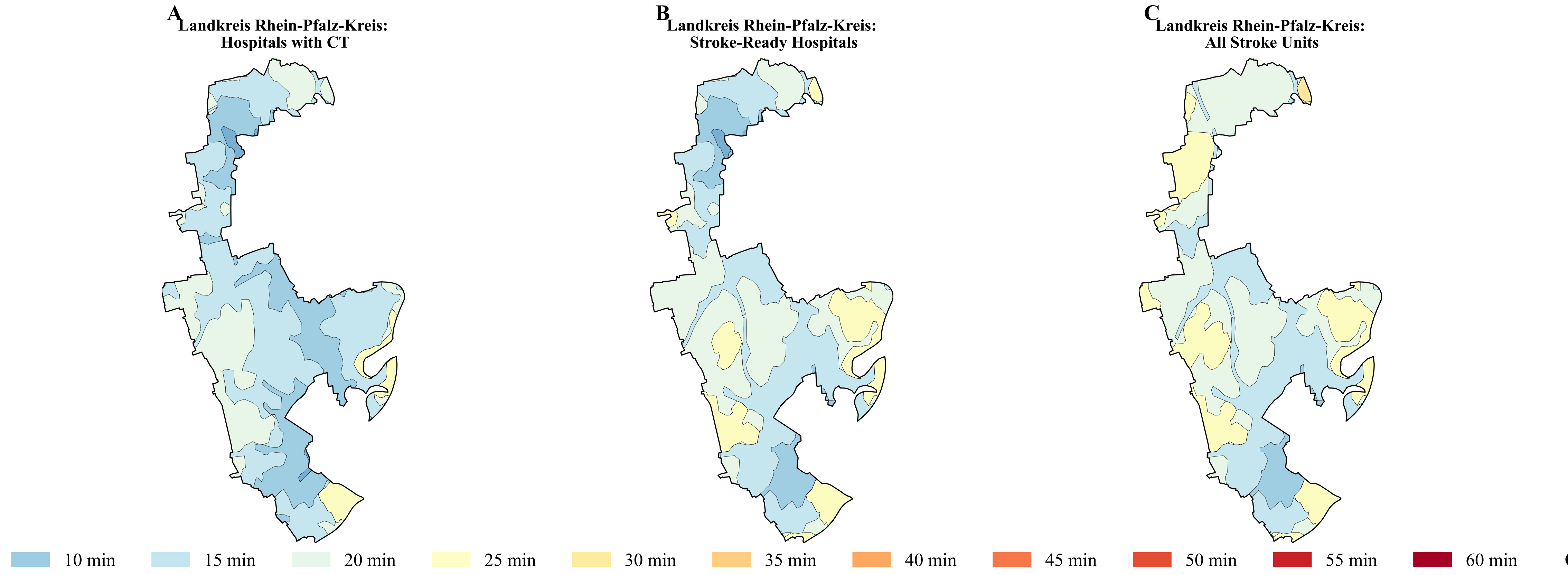

Isochrones — Landkreis Altötting (Bayern)

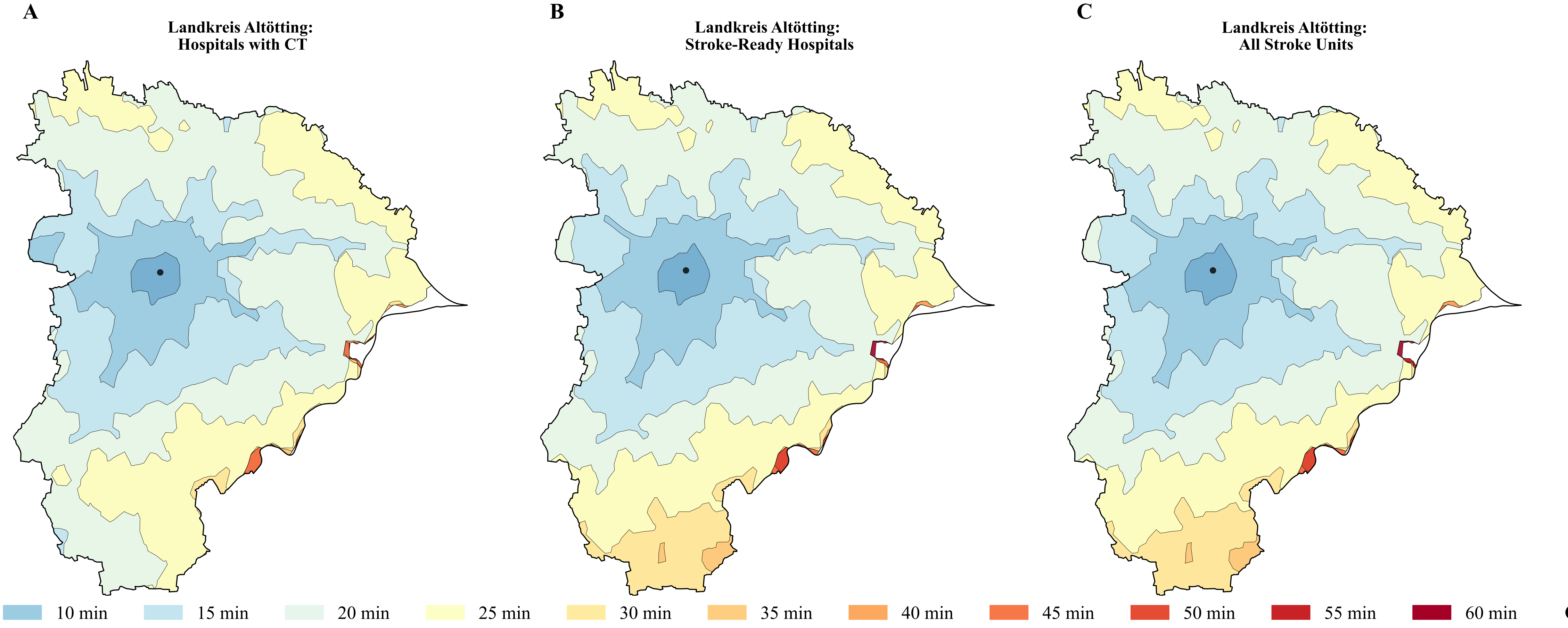

Isochrones — Landkreis Pfaffenhofen a.d.Ilm (Bayern)

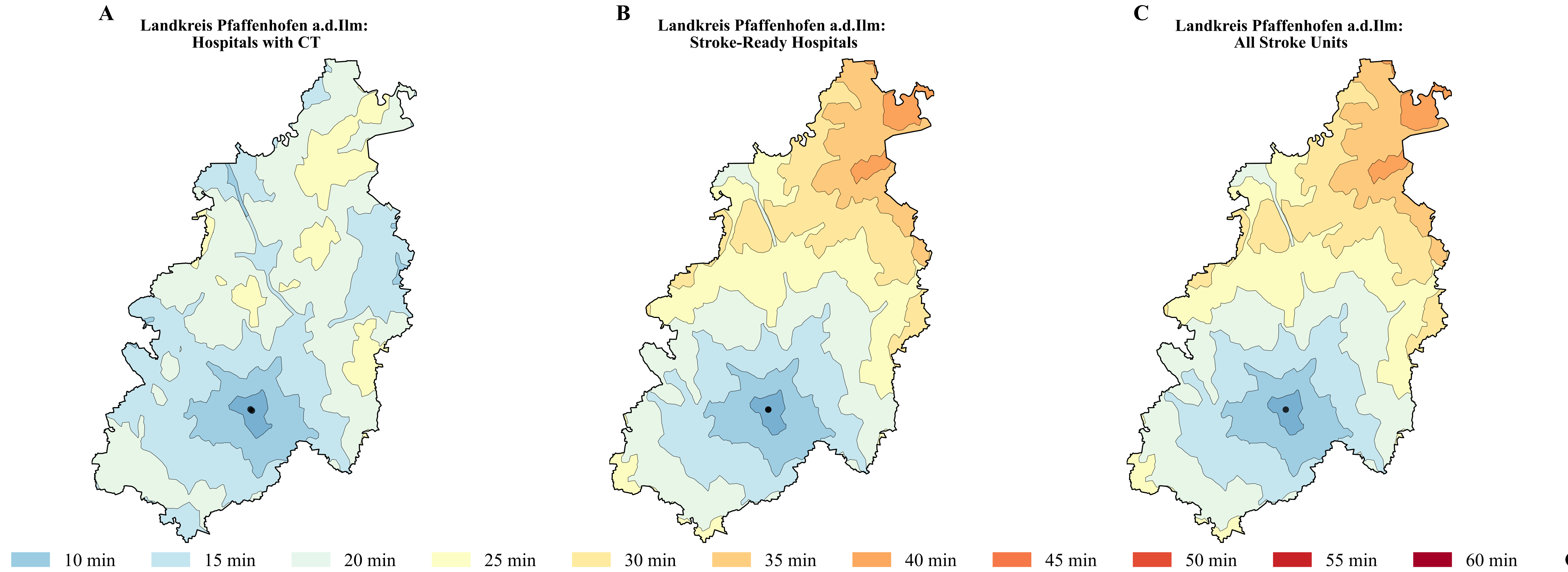

Isochrones — Kreisfreie Stadt Bamberg (Bayern)

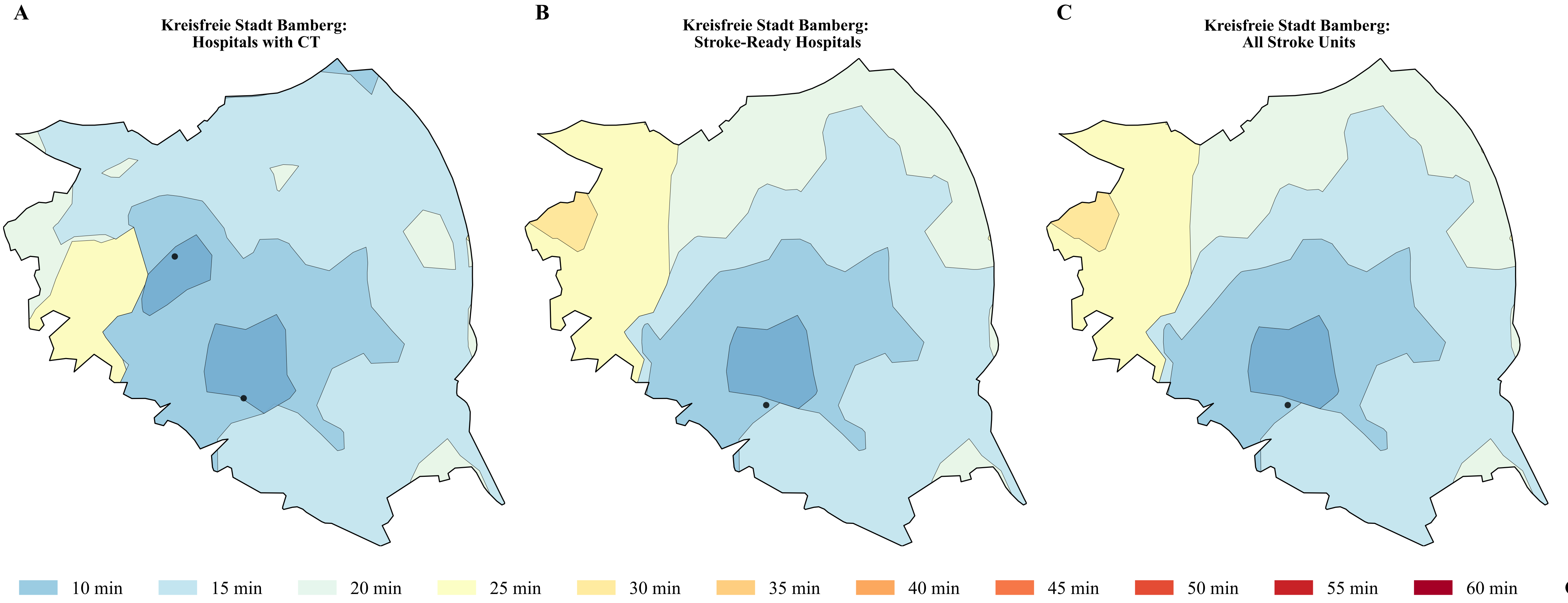

747 / 5833

Isochrones — Landkreis Hof (Bayern)

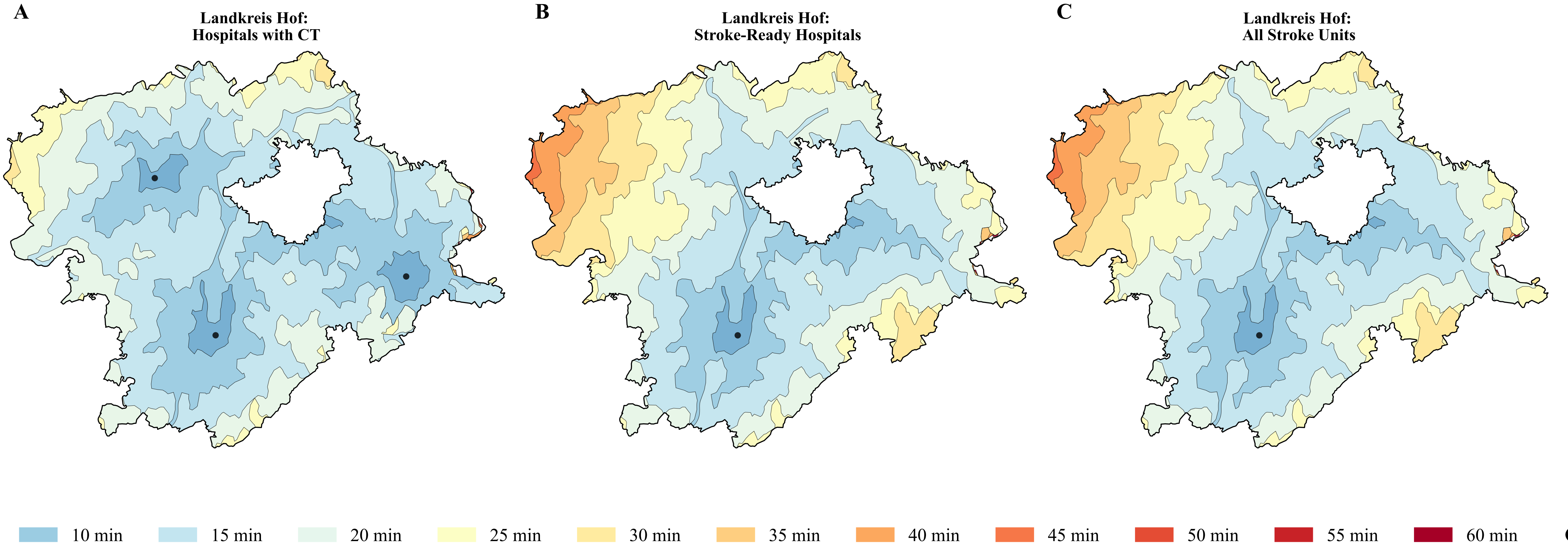

Isochrones — Landkreis Miltenberg (Bayern)

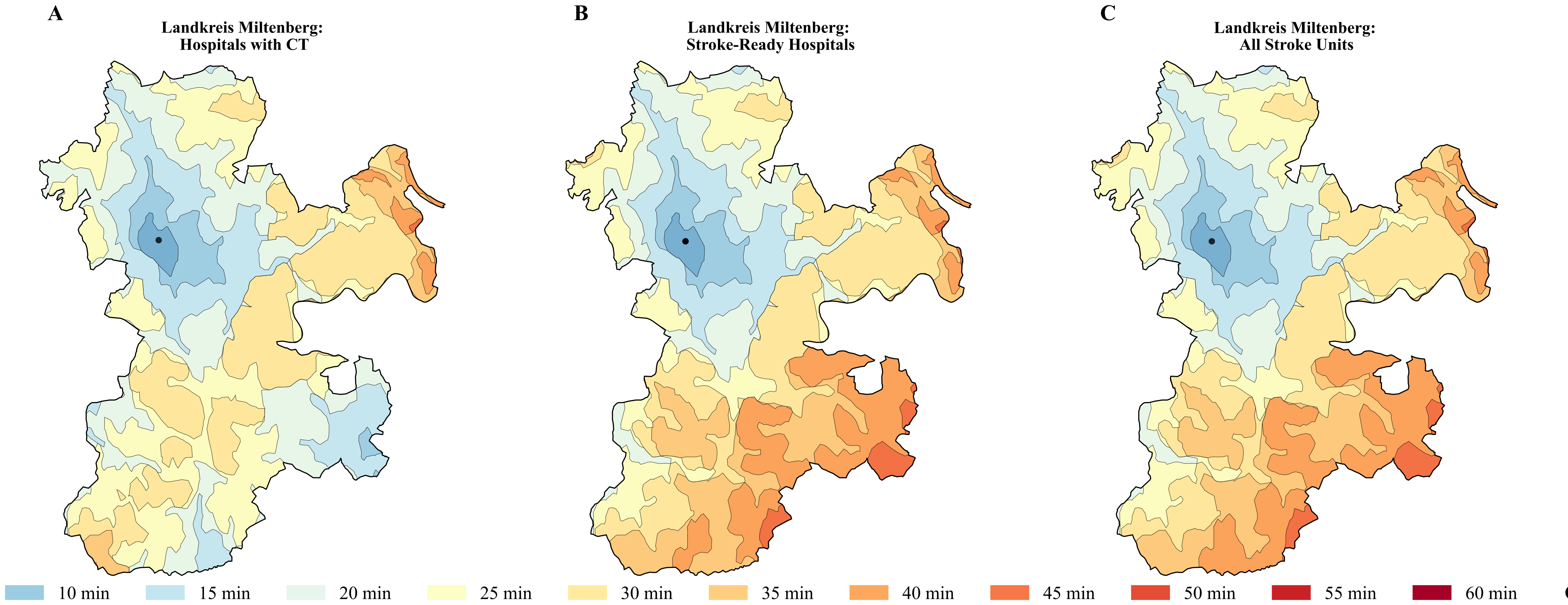

Isochrones — Kreisfreie Stadt Augsburg (Bayern)

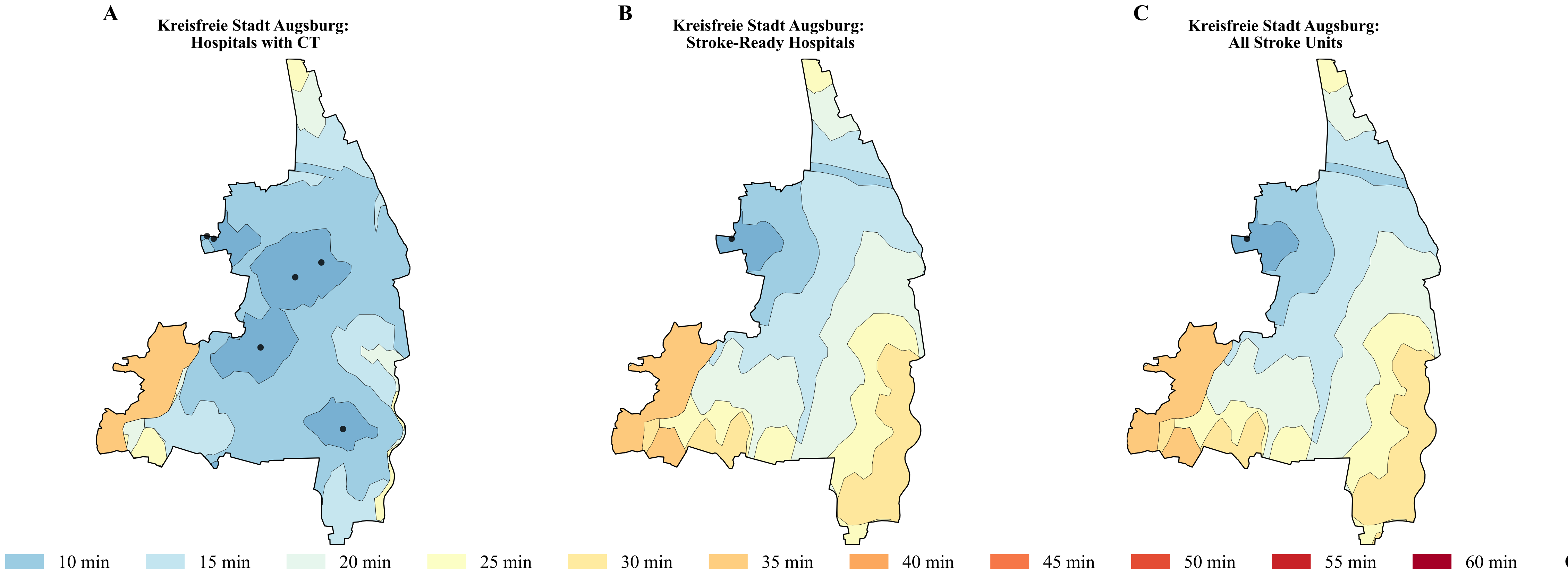

Isochrones — Landkreis Barnim (Brandenburg)

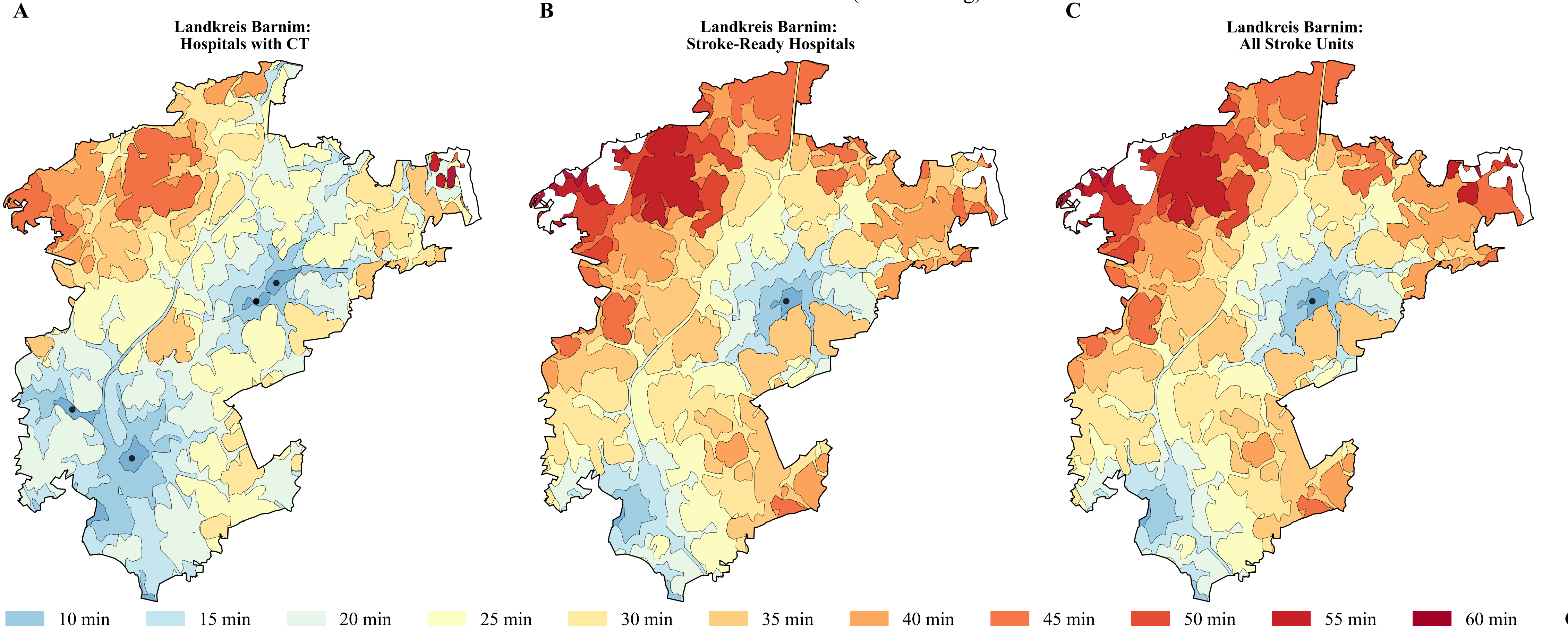

Isochrones — Landkreis Meißen (Sachsen)

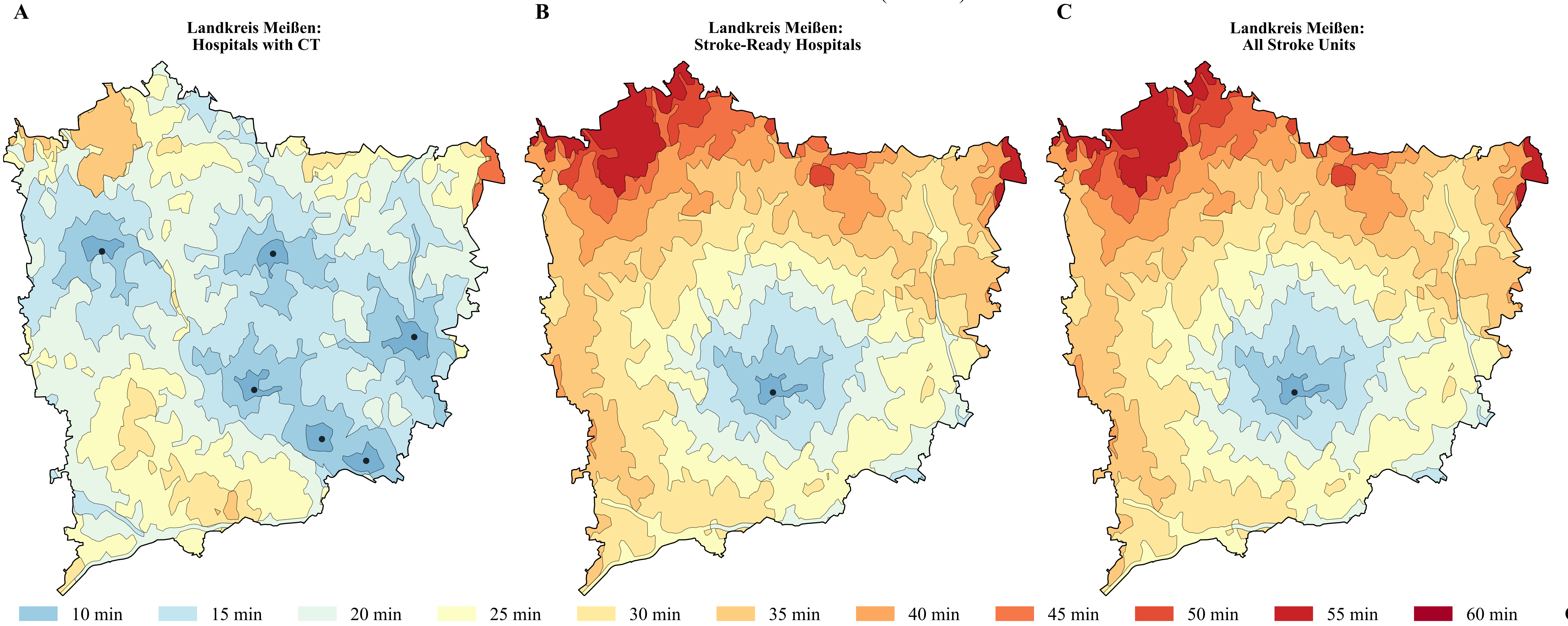

Isochrones — Kreisfreie Stadt Suhl (Thüringen)

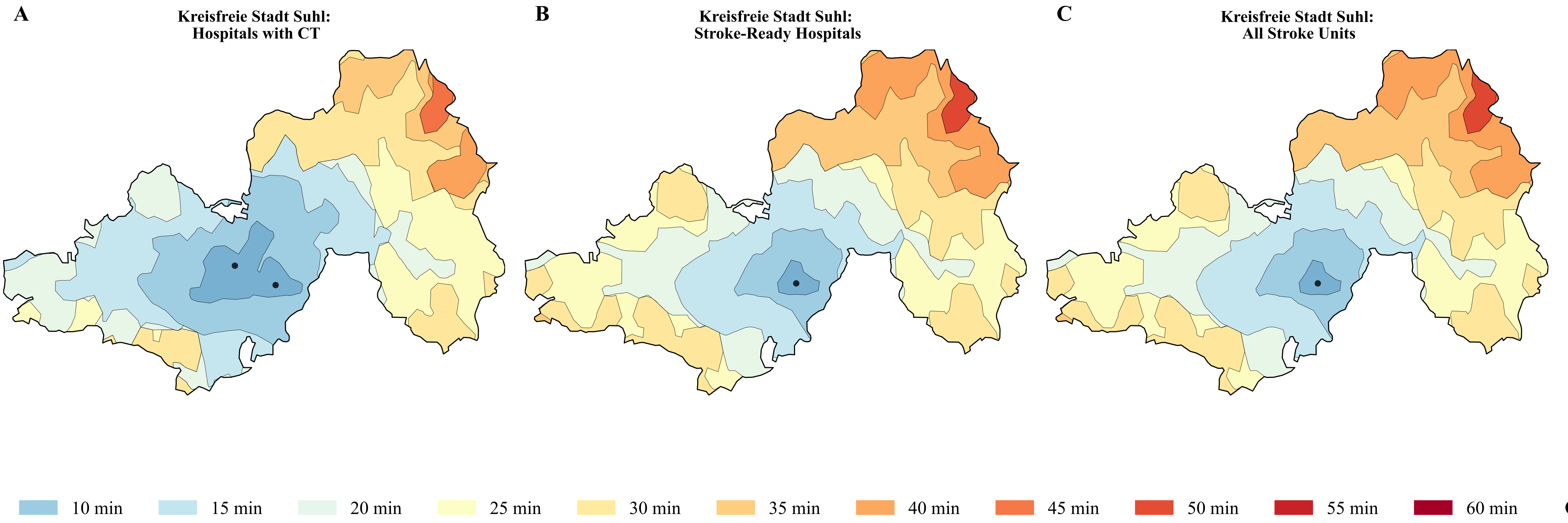

Isochrones — Landkreis Eichsfeld (Thüringen)

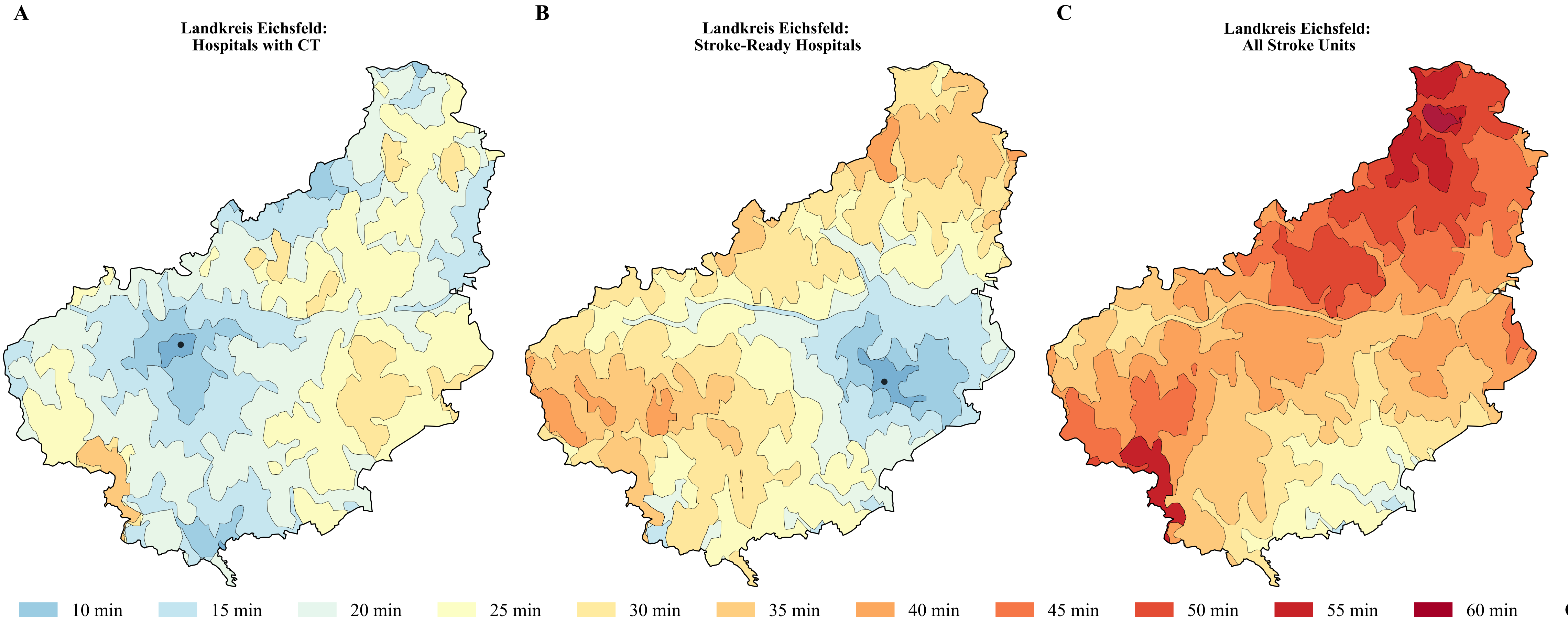

Isochrones — Landkreis Göttingen (Niedersachsen)

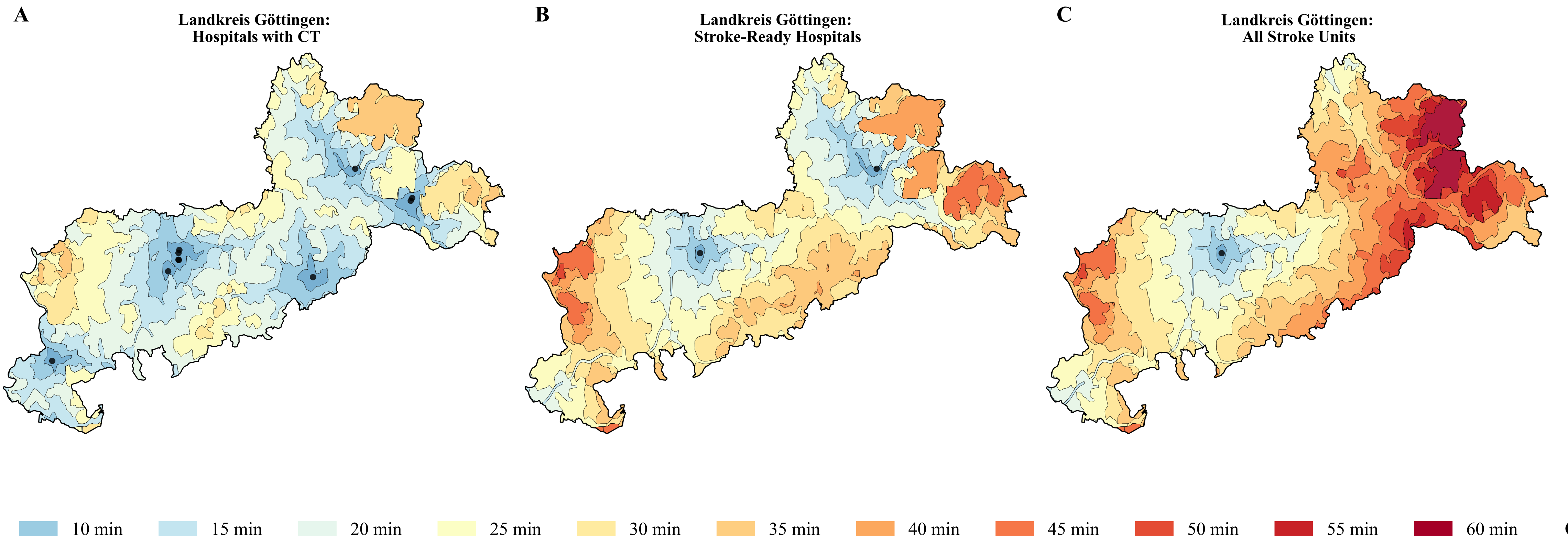

Isochrones — Landkreis Schaumburg (Niedersachsen)

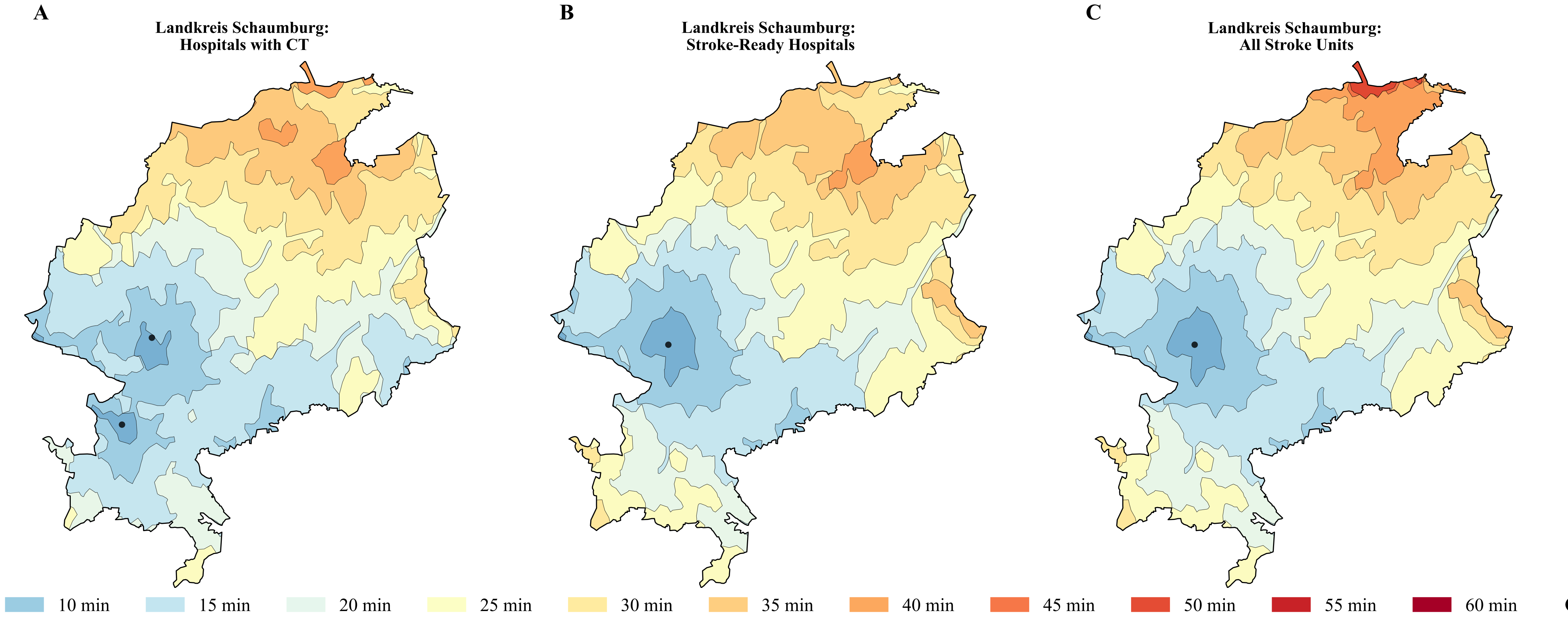

Isochrones — Landkreis Aurich (Niedersachsen)

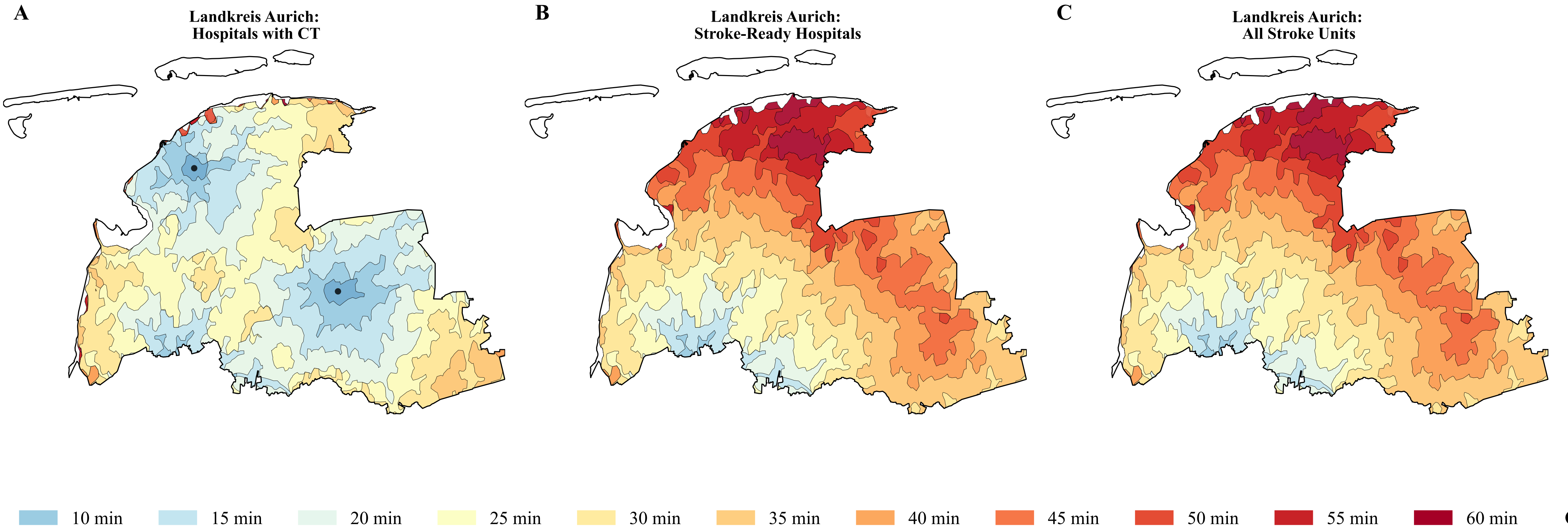

Isochrones — Landkreis Friesland (Niedersachsen)

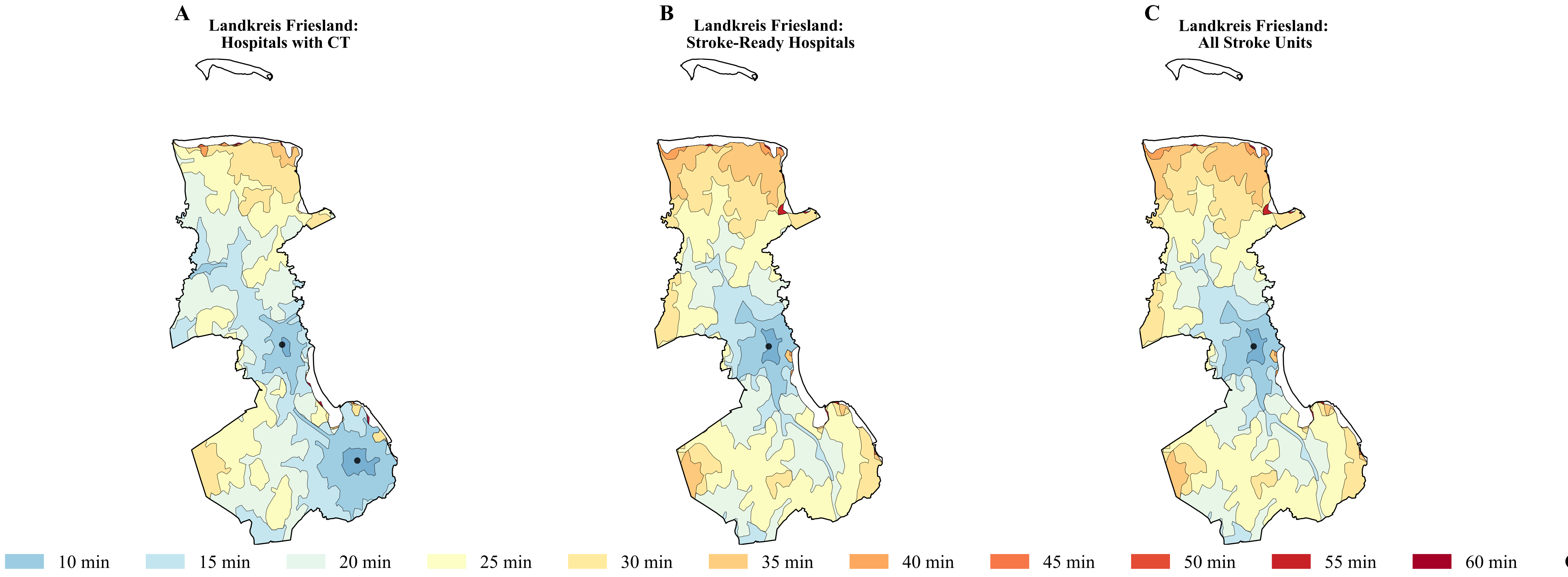

Isochrones — Landkreis Wesermarsch (Niedersachsen)

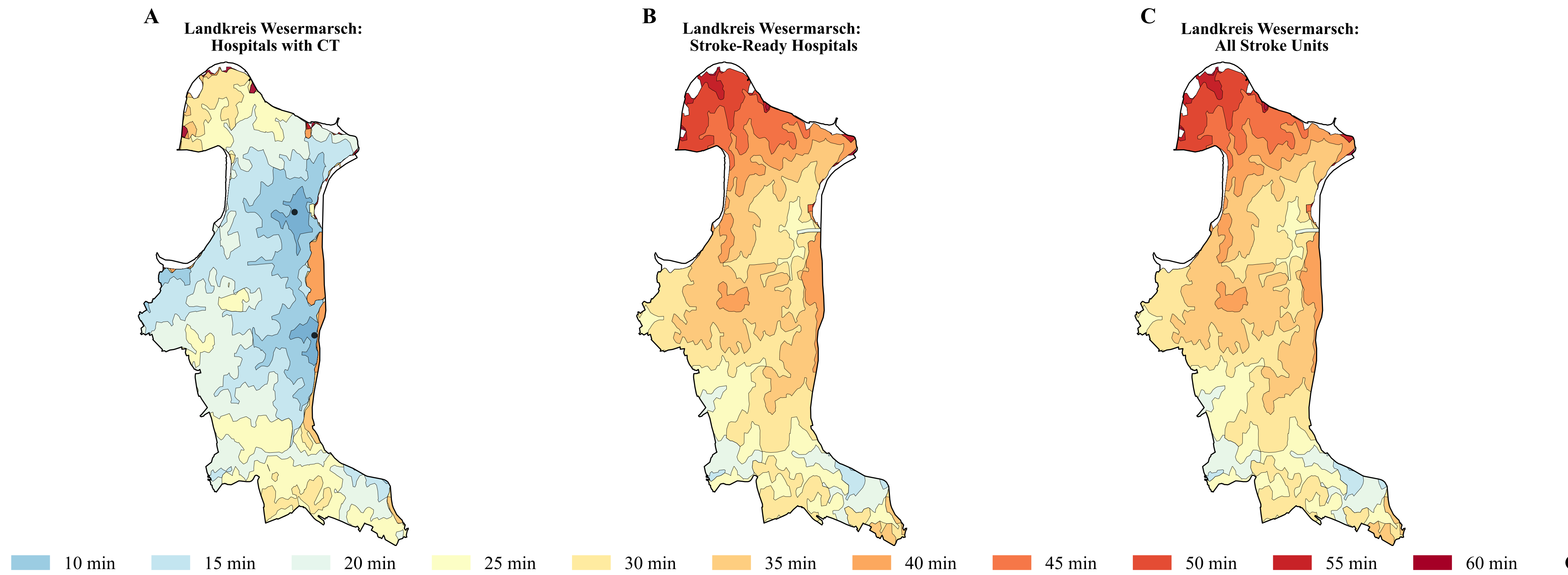

Isochrones — Landkreis Main-Kinzig-Kreis (Hessen)

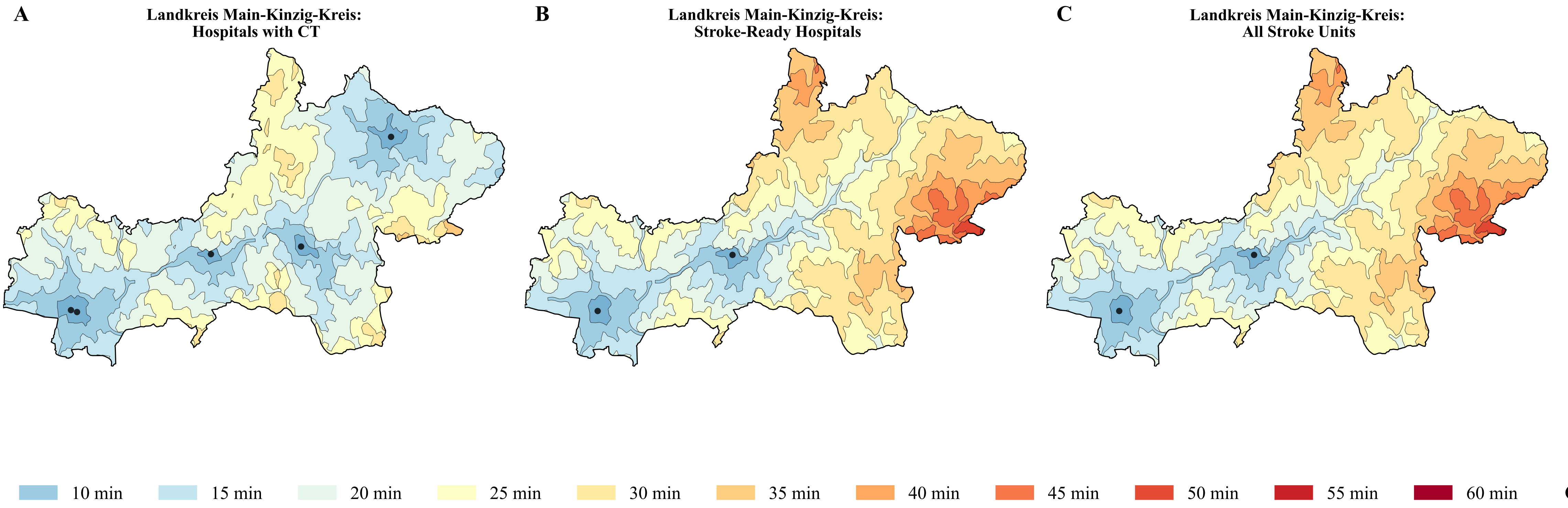

Isochrones — Landkreis Vogelsbergkreis (Hessen)

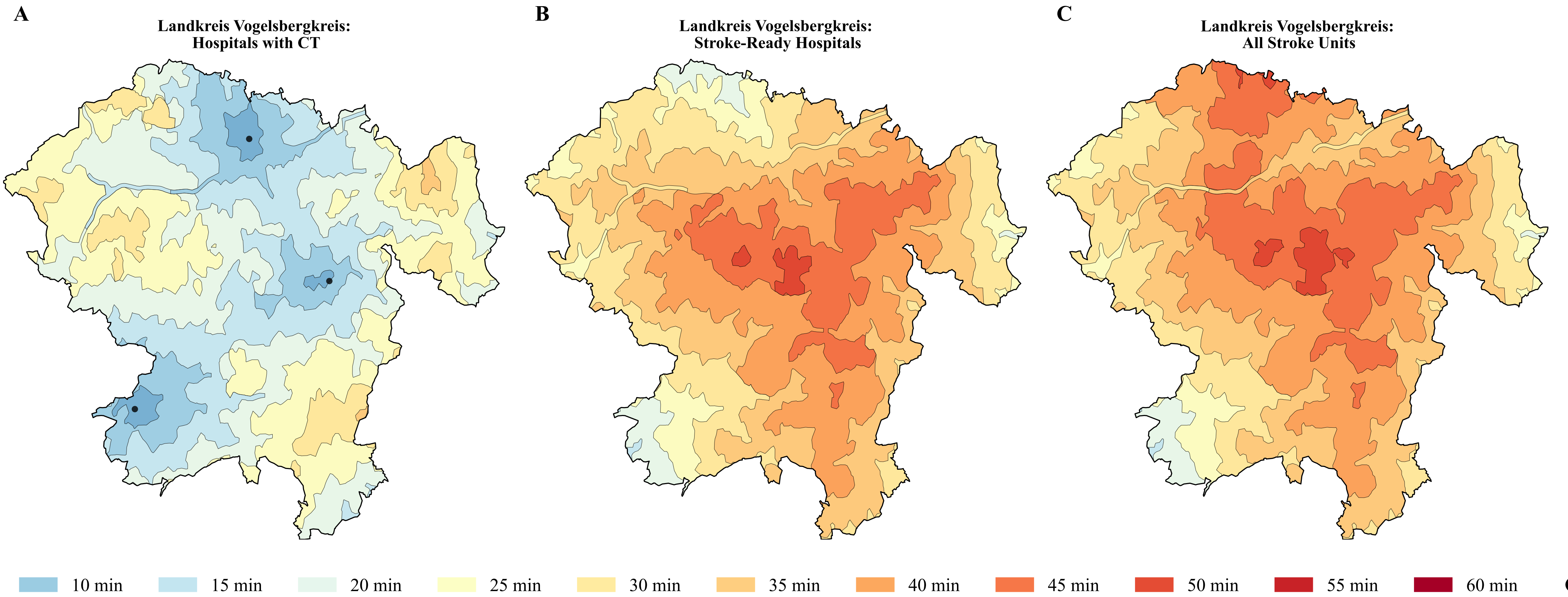

Isochrones — Landkreis Neuwied (Rheinland-Pfalz)

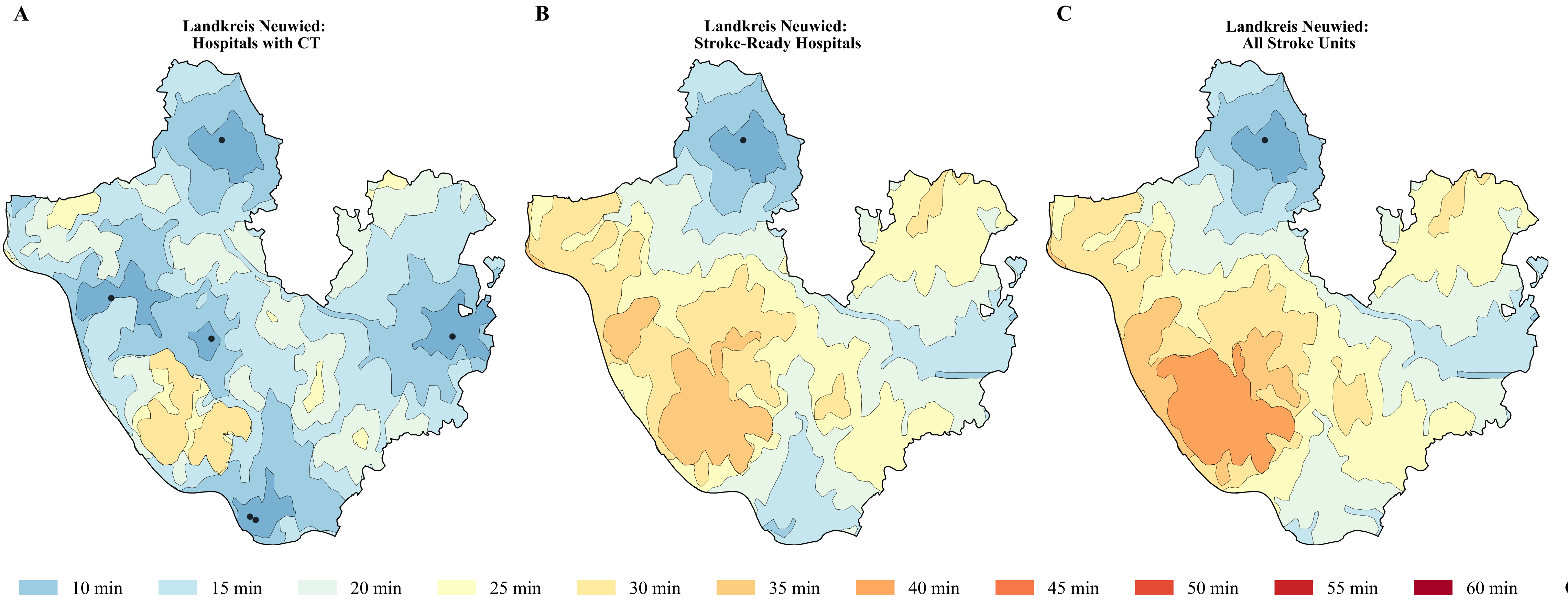

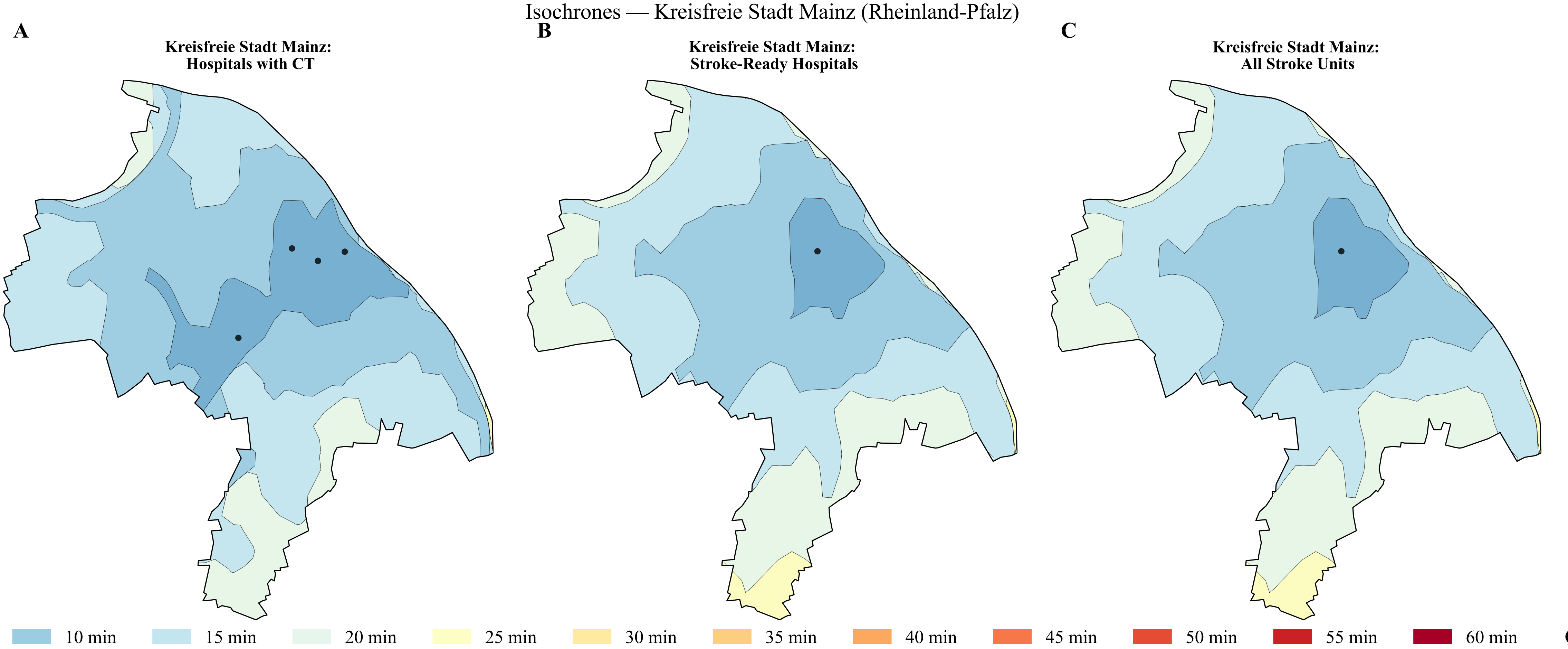
Isochrones — Kreisfreie Stadt Mainz (Rheinland-Pfalz)
A
Kreisfreie Stadt Mainz:
Hospitals with CT
B
Kreisfreie Stadt Mainz:
Stroke-Ready Hospitals
C
Kreisfreie Stadt Mainz:
All Stroke Units
10 min
15 min
20 min
25 min
30 min
35 min
40 min
45 min
50 min
55 min
60 min

Isochrones — Landkreis Hohenlohekreis (Baden-Württemberg)

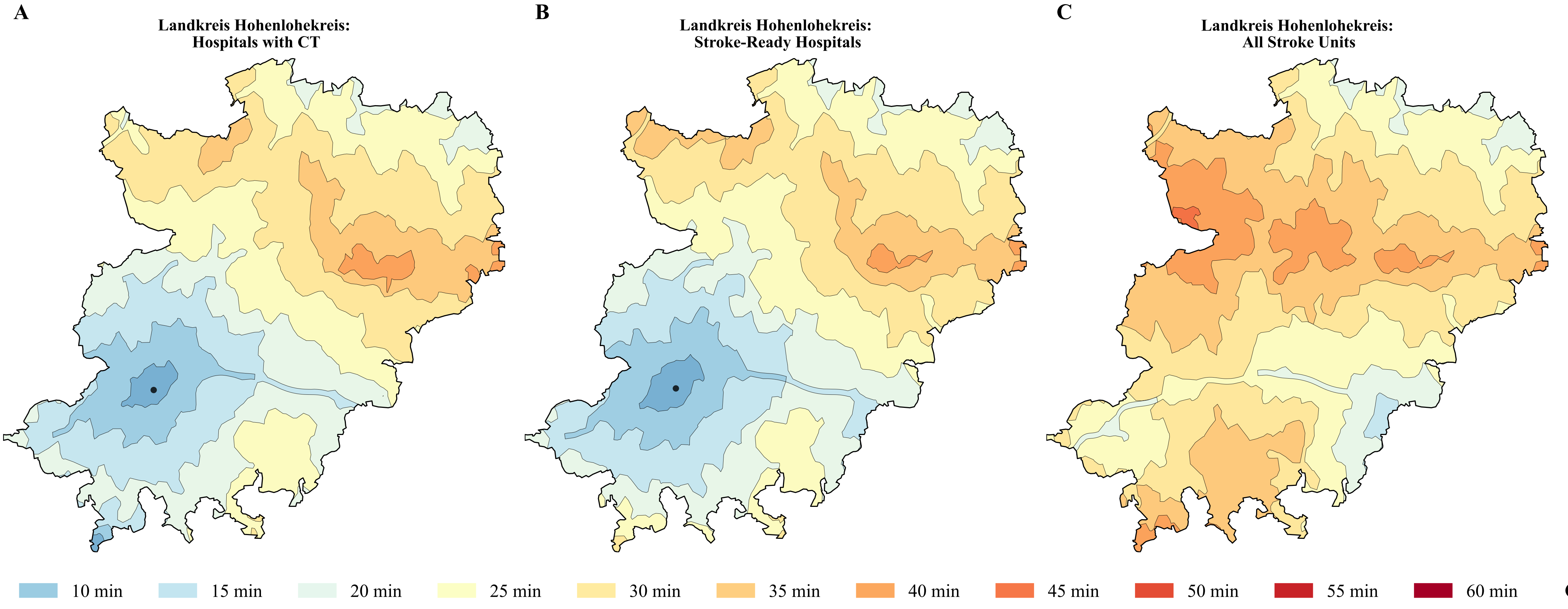

Isochrones — Landkreis Ortenaukreis (Baden-Württemberg)

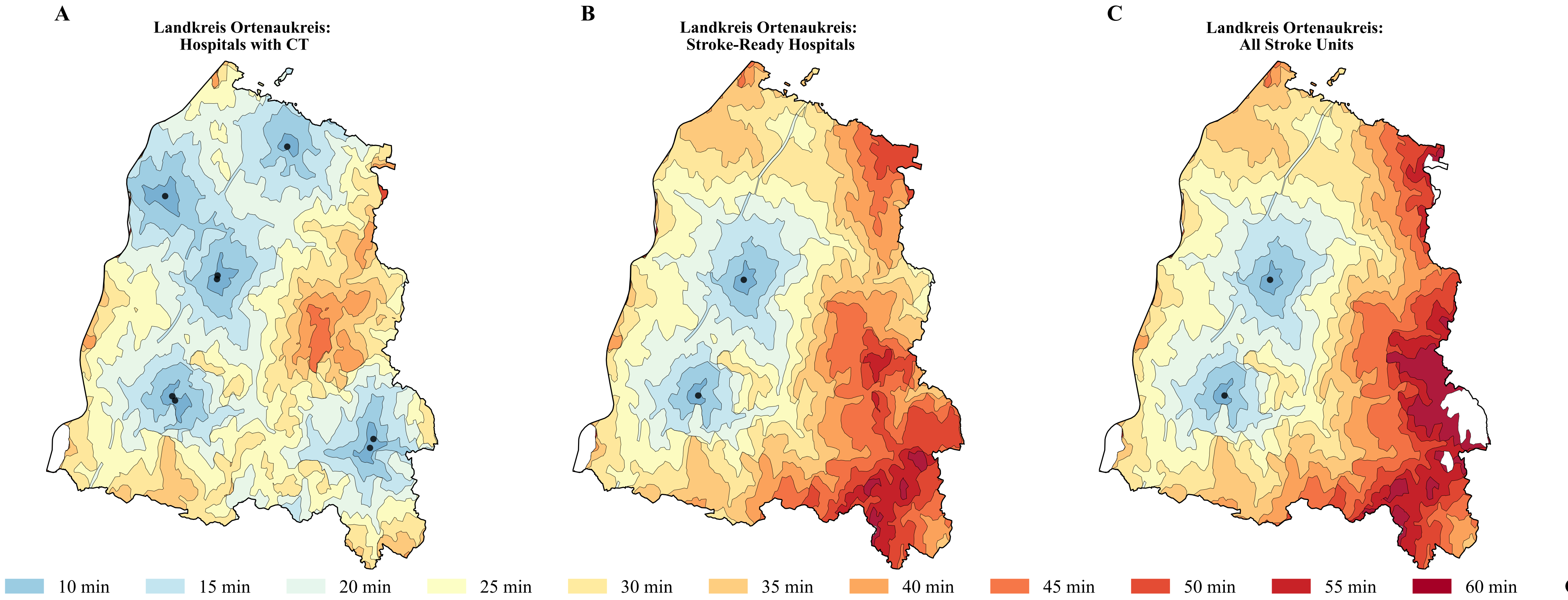

Isochrones — Landkreis Neustadt a.d.Aisch-Bad Windsheim (Bayern)

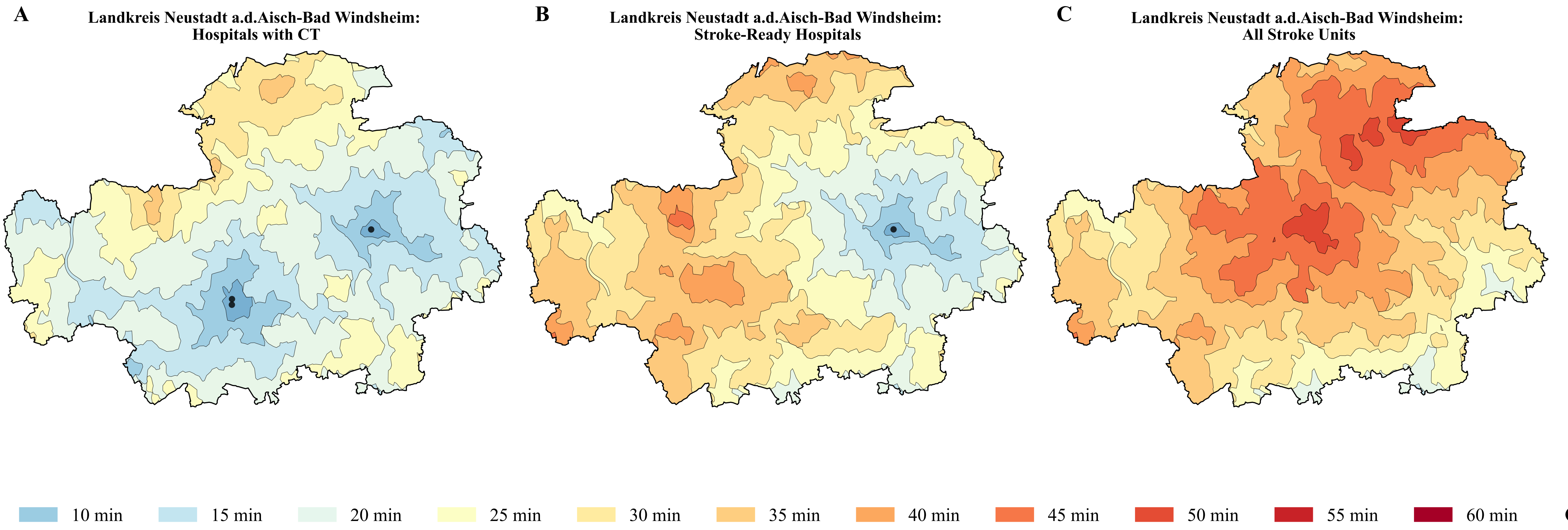

Isochrones — Landkreis Goslar (Niedersachsen)

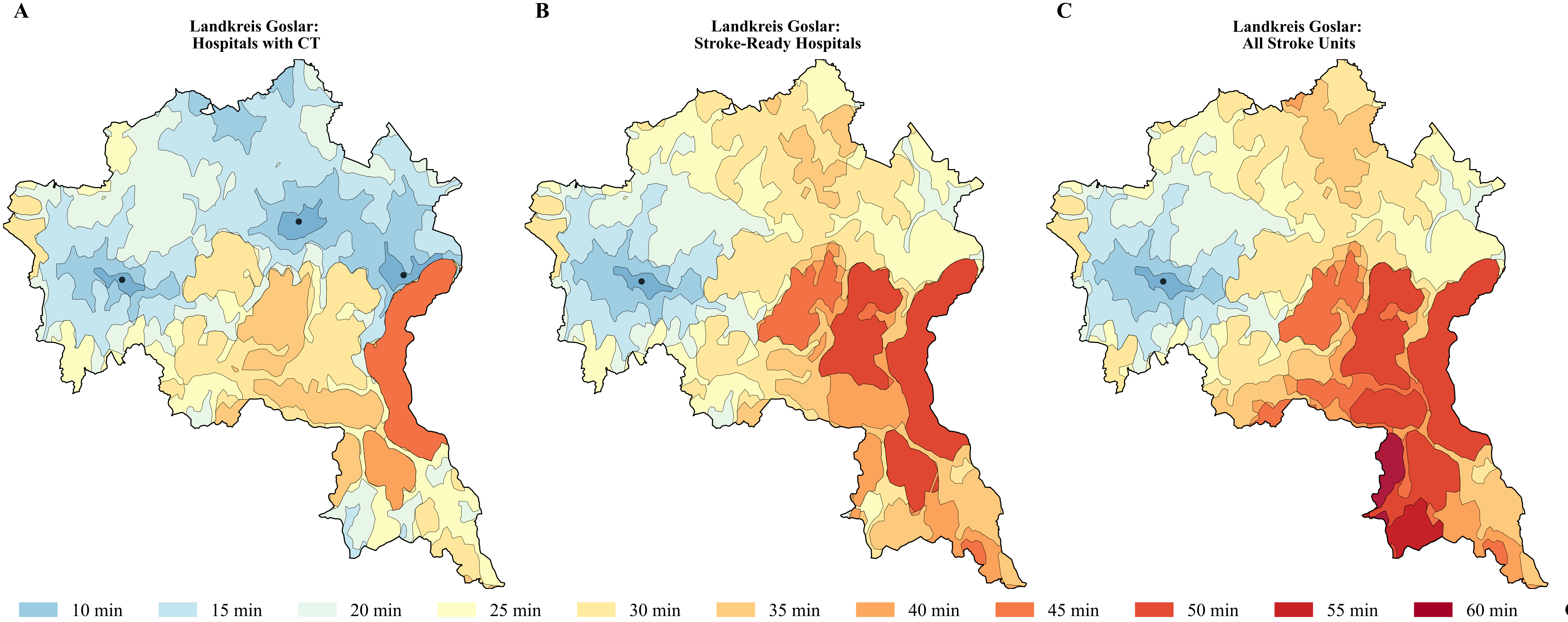

Isochrones — Landkreis Region Hannover (Niedersachsen)

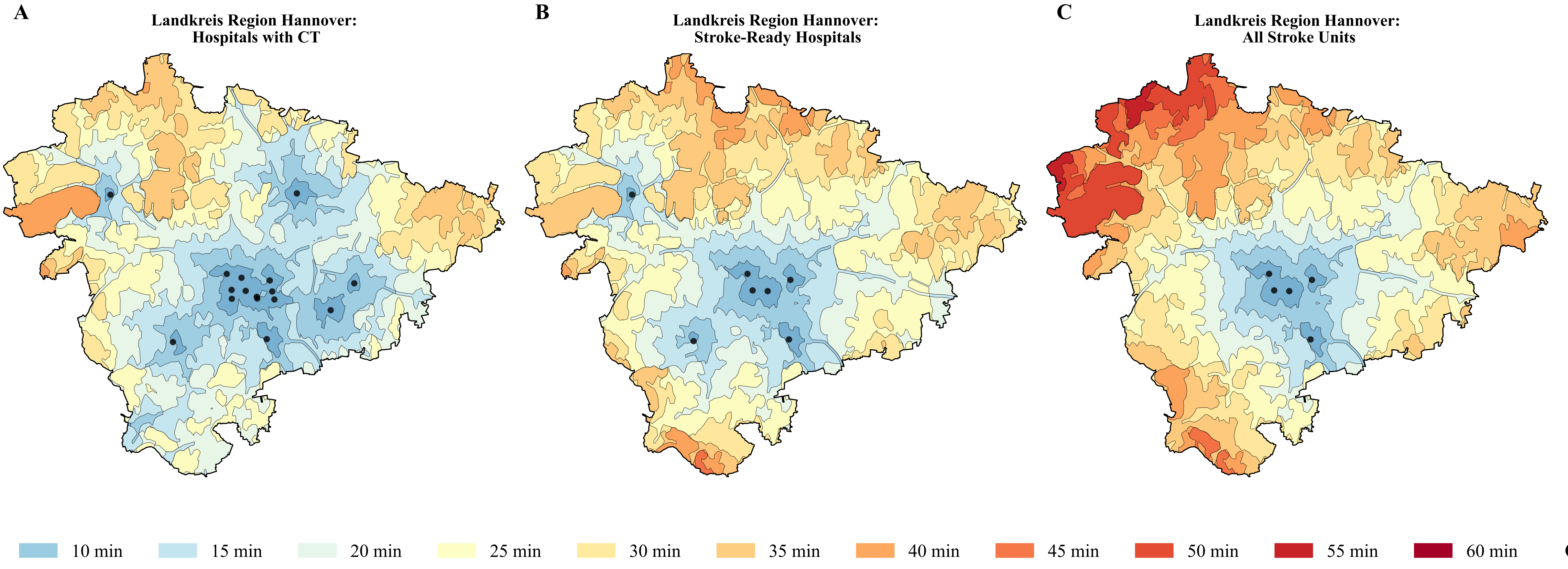

Isochrones — Landkreis Vechta (Niedersachsen)

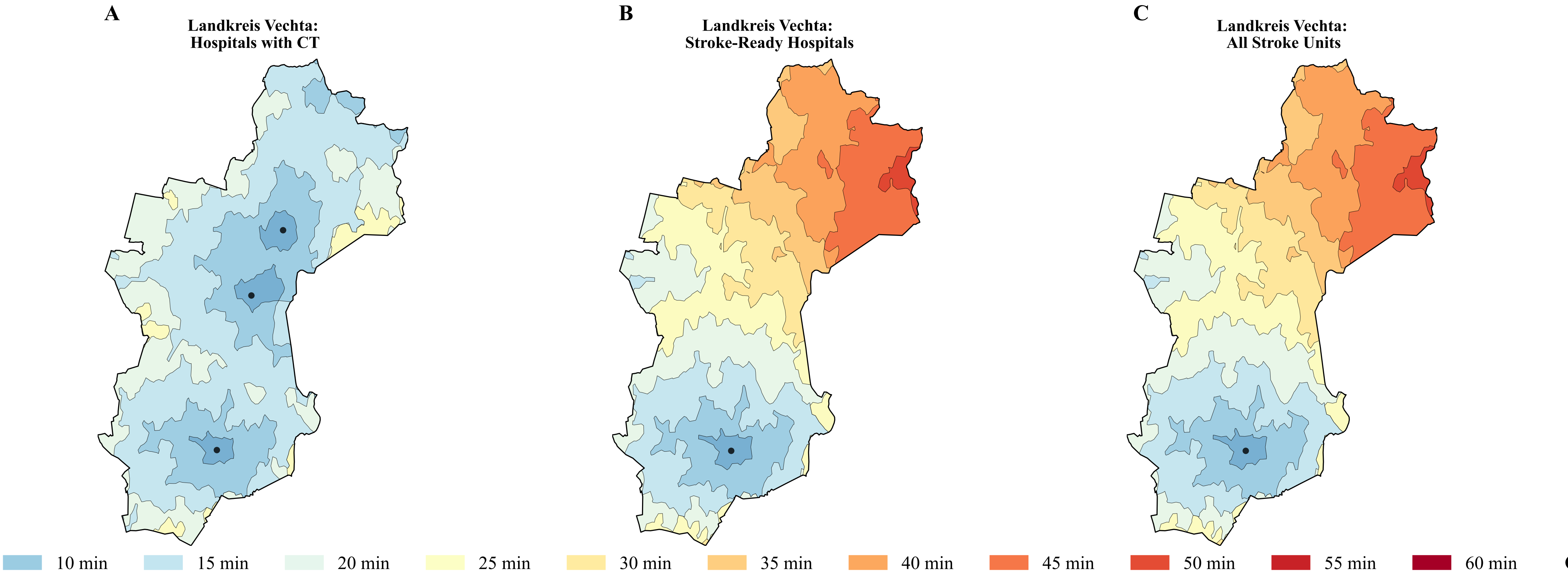

Isochrones — Kreis Städteregion Aachen (Nordrhein-Westfalen)

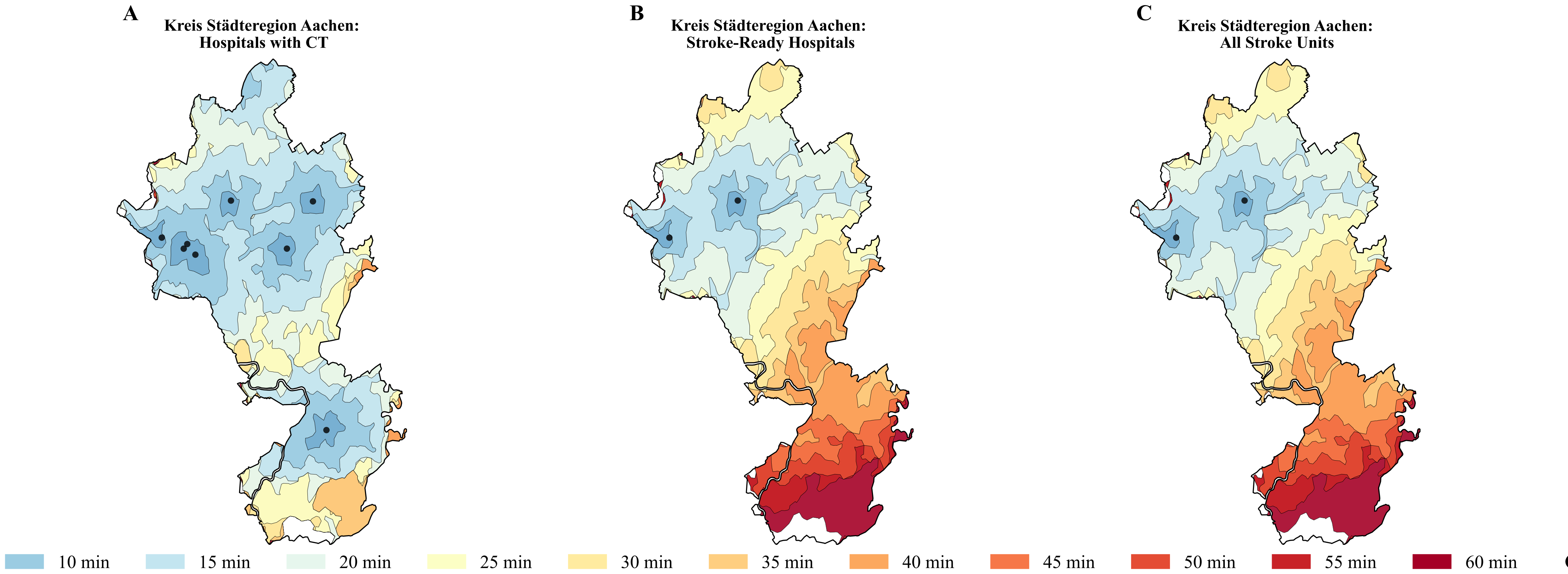

Isochrones — Kreis Steinfurt (Nordrhein-Westfalen)

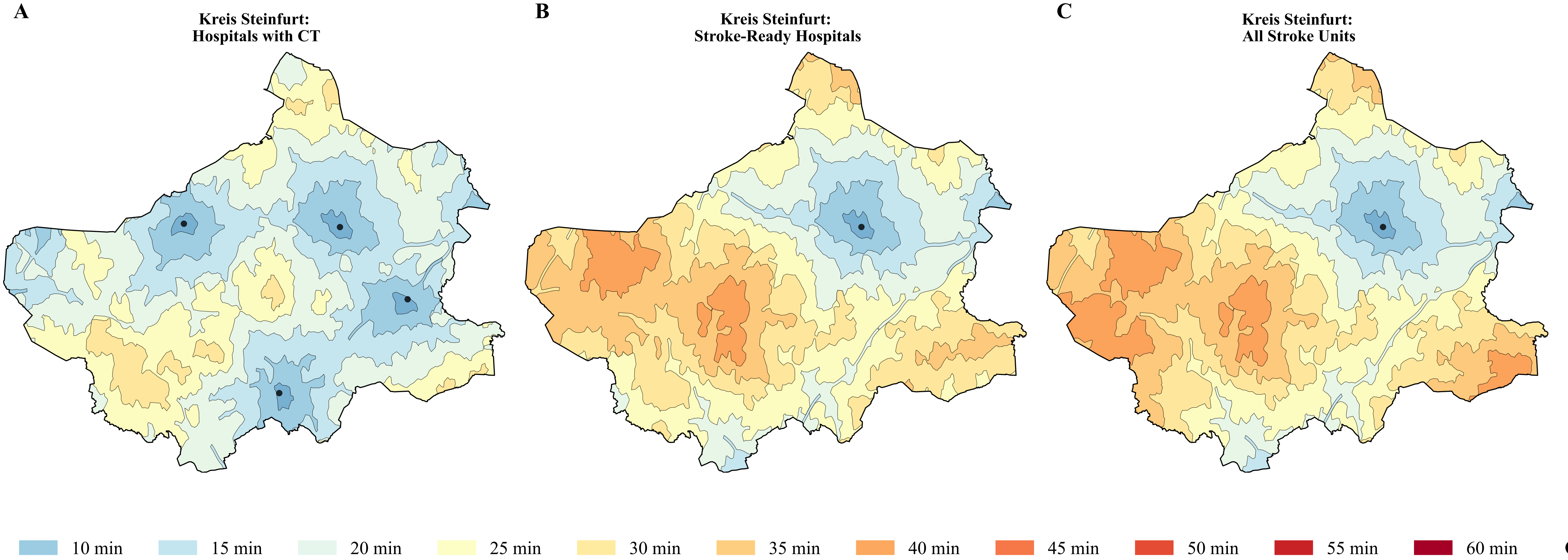

Isochrones — Kreis Gütersloh (Nordrhein-Westfalen)

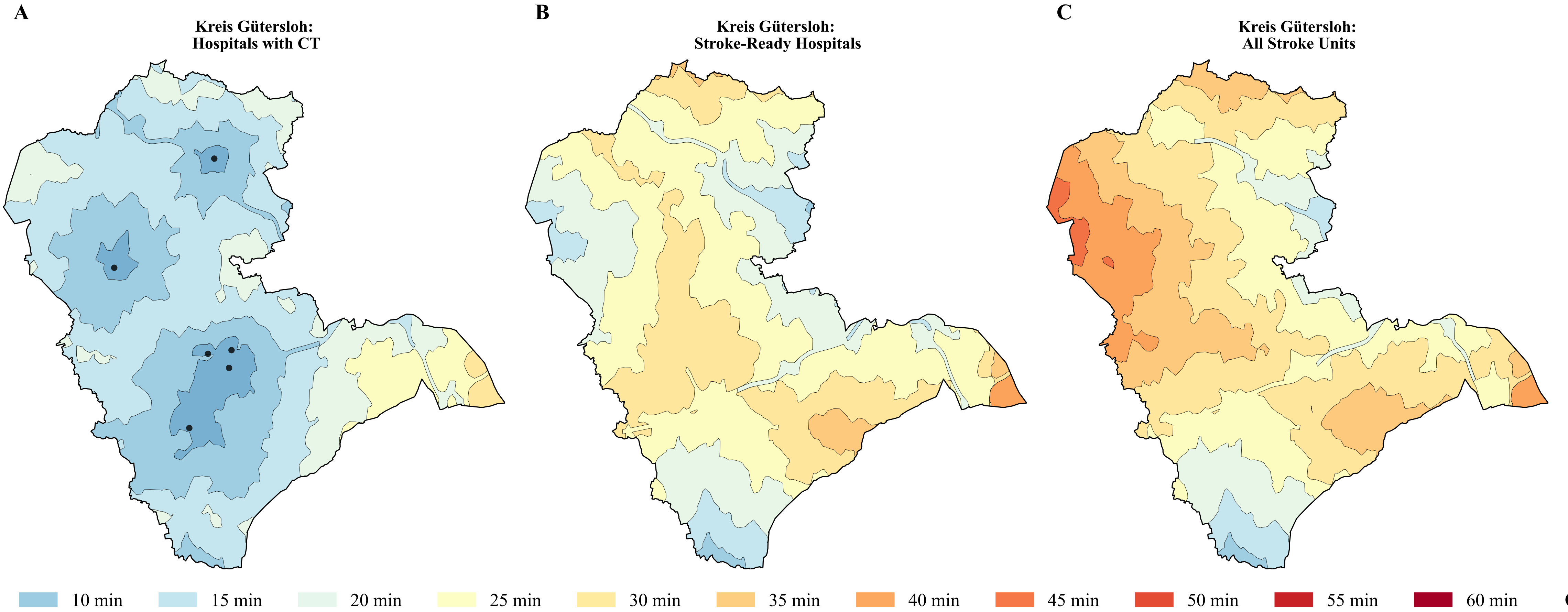

Isochrones — Kreis Unna (Nordrhein-Westfalen)

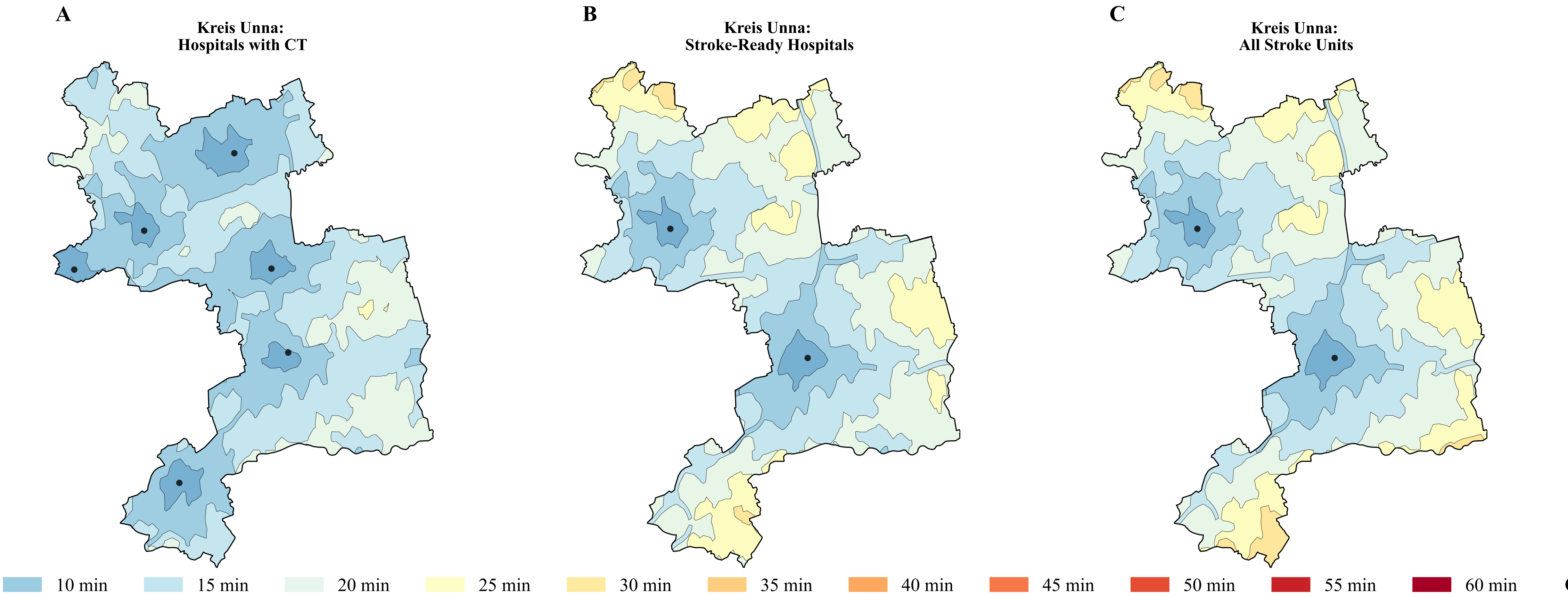

Isochrones — Landkreis Darmstadt-Dieburg (Hessen)

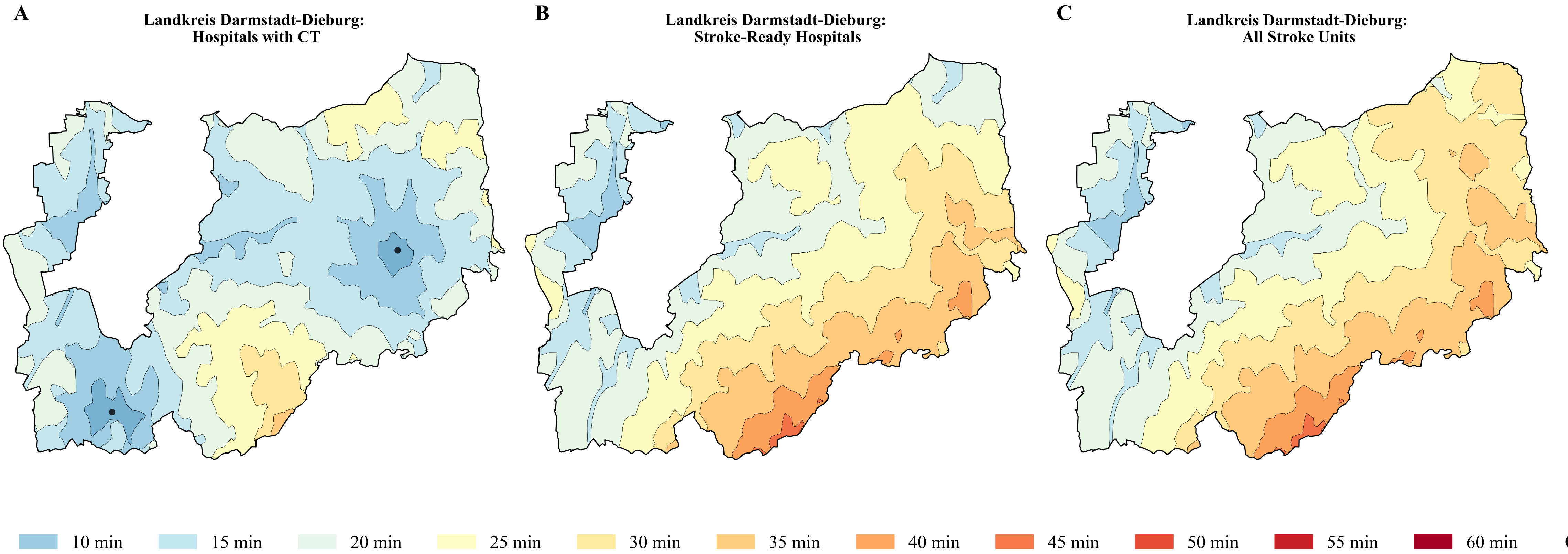

Isochrones — Landkreis Gießen (Hessen)

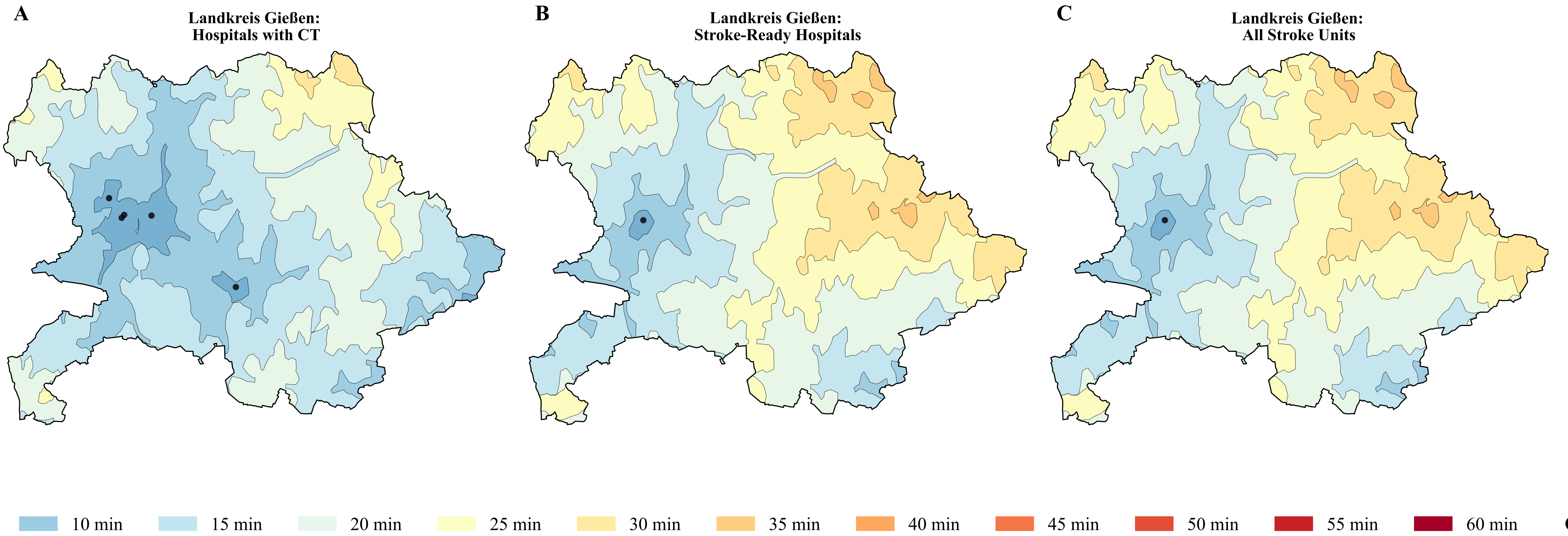

Isochrones — Kreisfreie Stadt Neustadt an der Weinstraße (Rheinland-Pfalz)

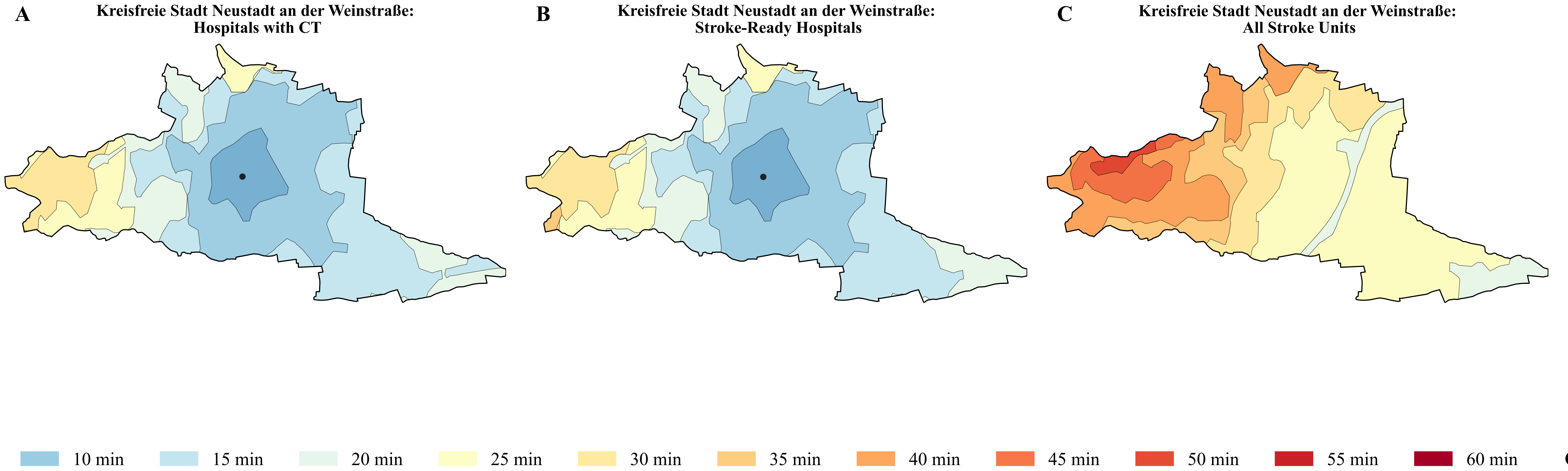

776 / 5833

Isochrones — Landkreis Main-Tauber-Kreis (Baden-Württemberg)

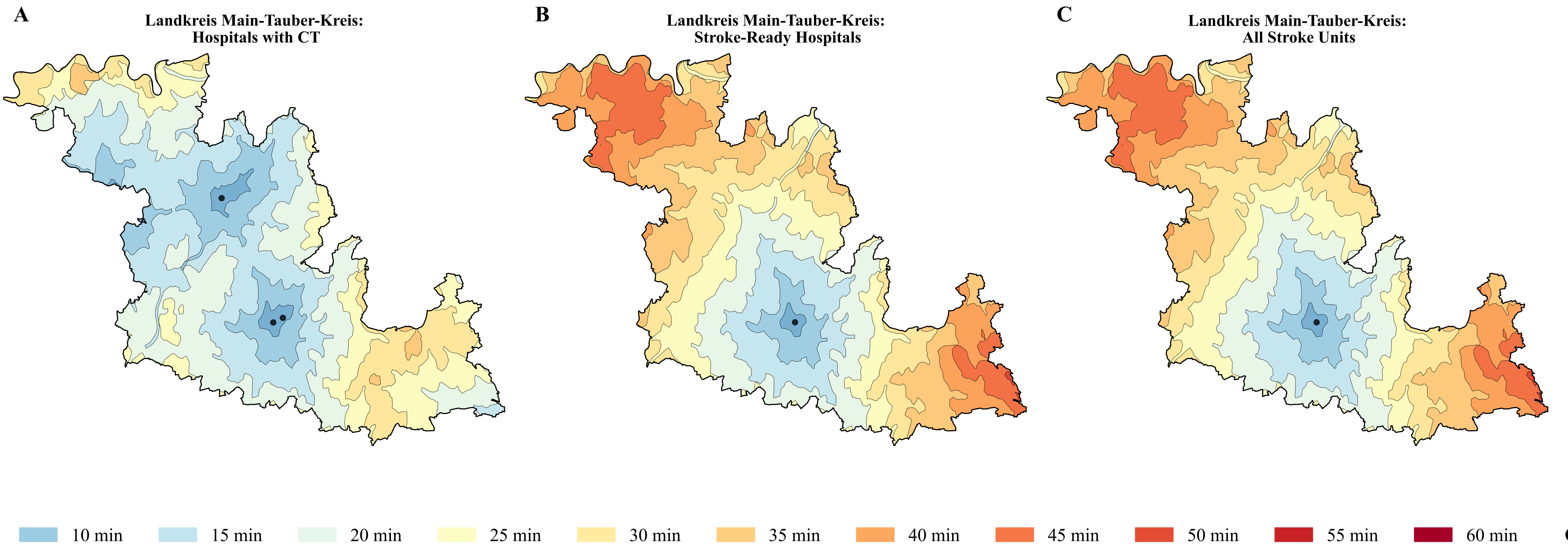

Isochrones — Landkreis Freudenstadt (Baden-Württemberg)

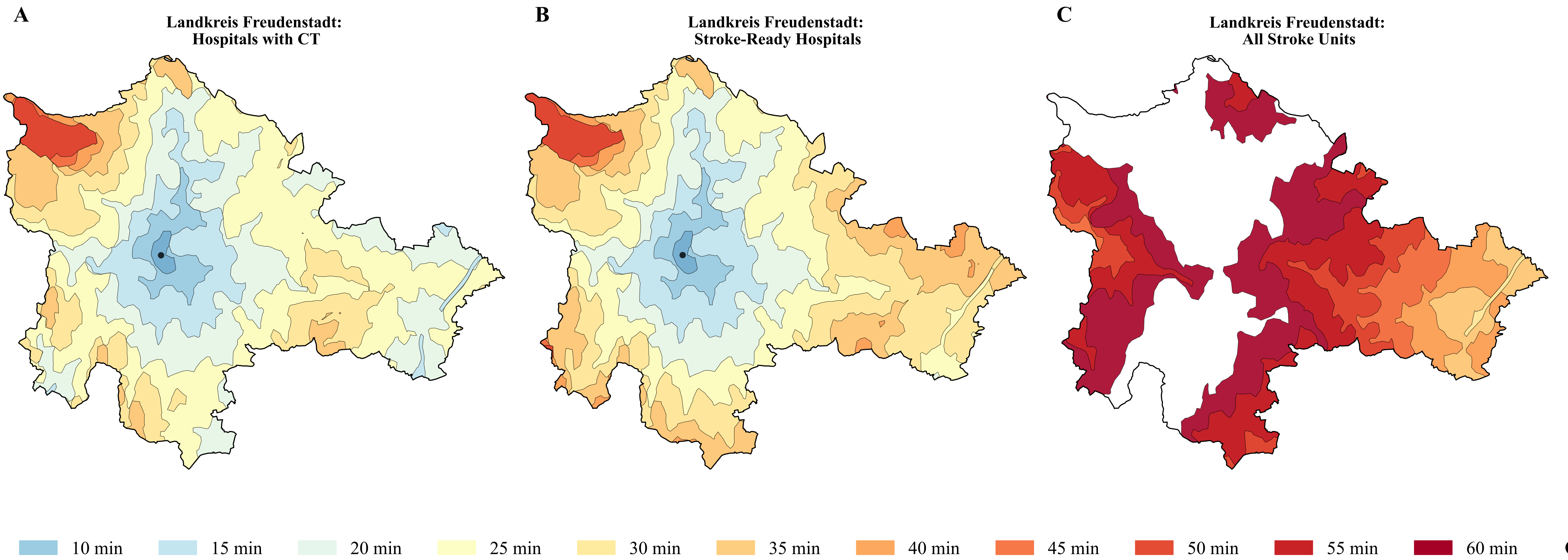

Isochrones — Landkreis Rosenheim (Bayern)

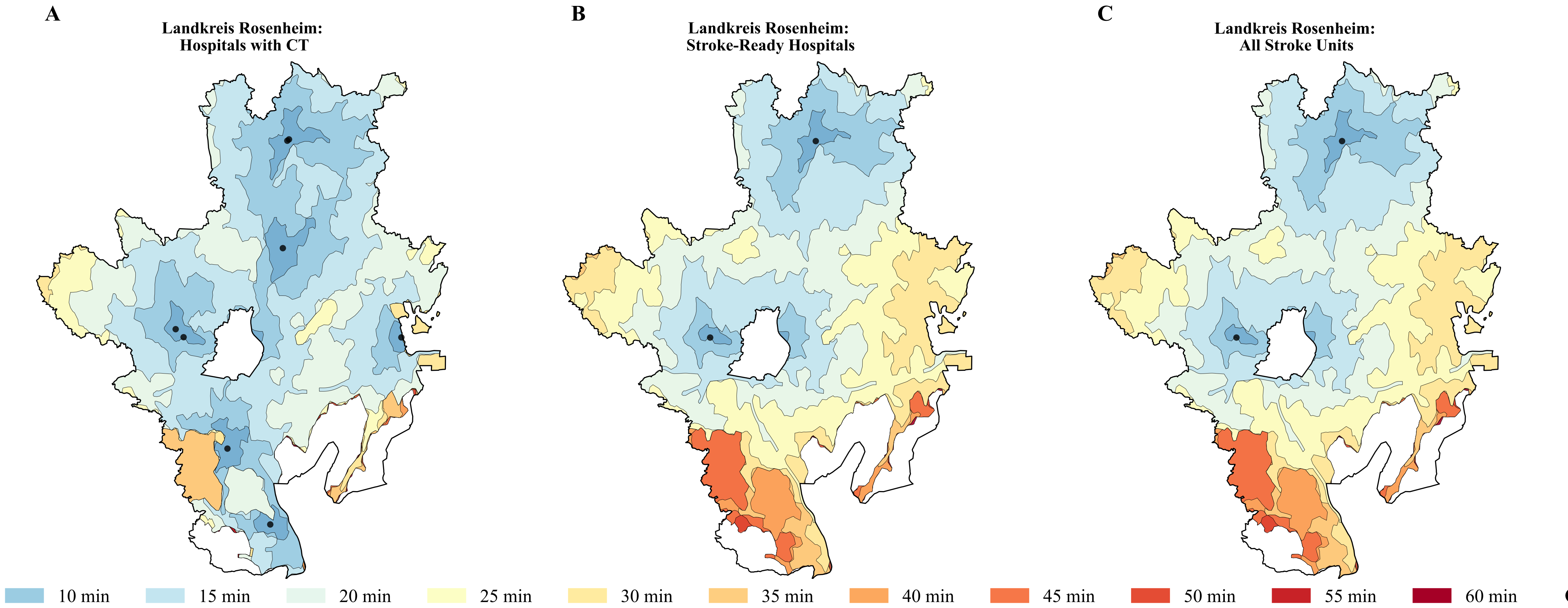

Isochrones — Kreisfreie Stadt Passau (Bayern)

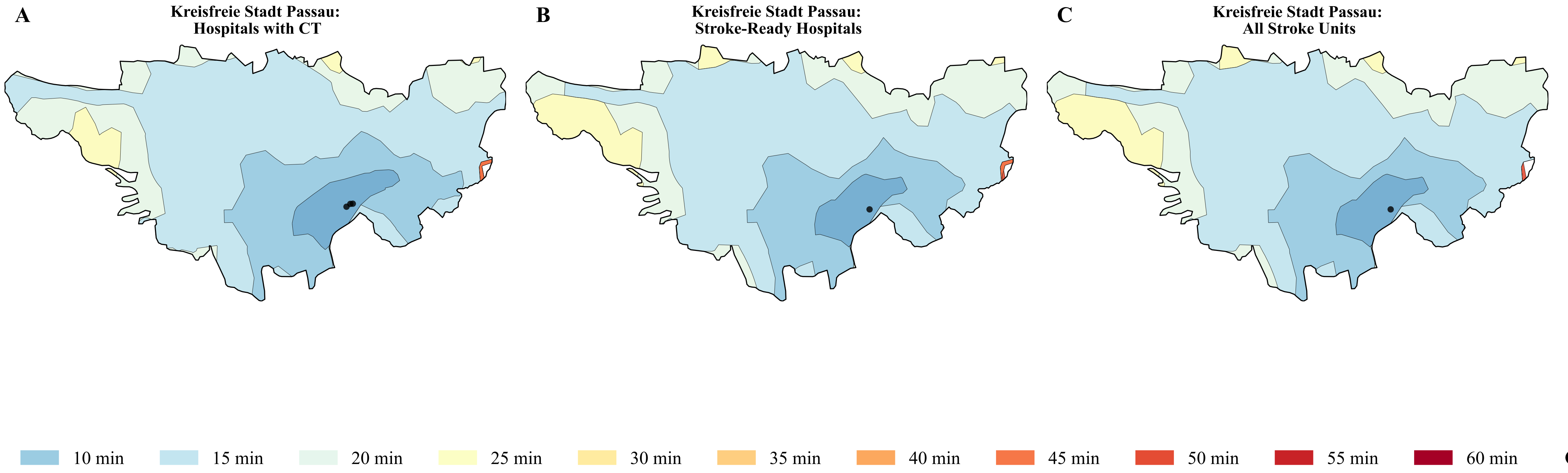

Isochrones — Landkreis Coburg (Bayern)

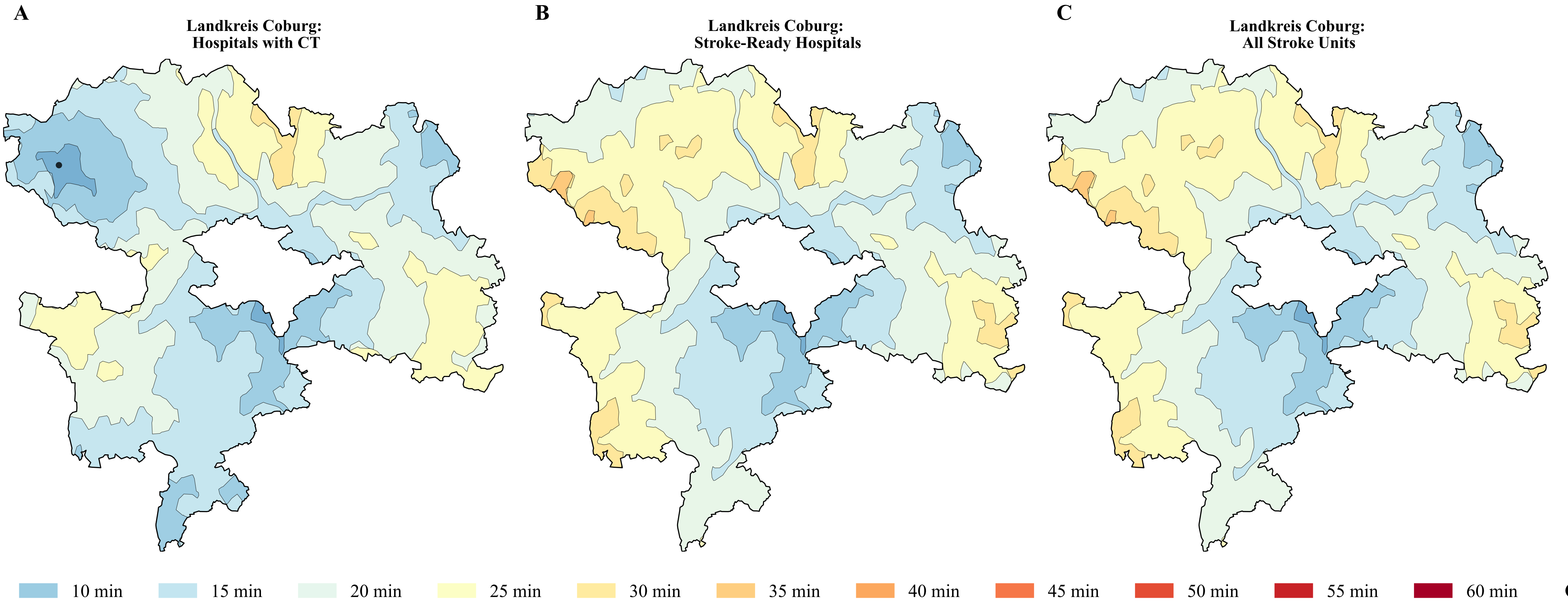

Isochrones — Landkreis Ostallgäu (Bayern)

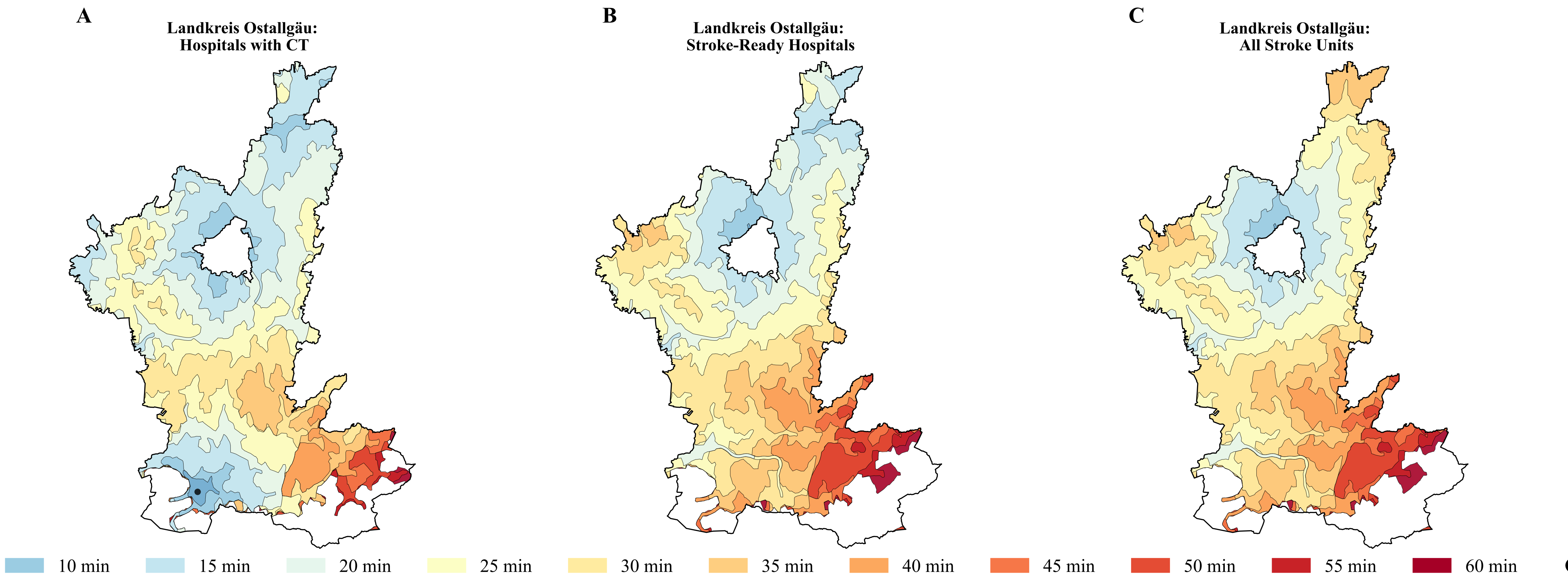

Isochrones — Landkreis Saarpfalz-Kreis (Saarland)

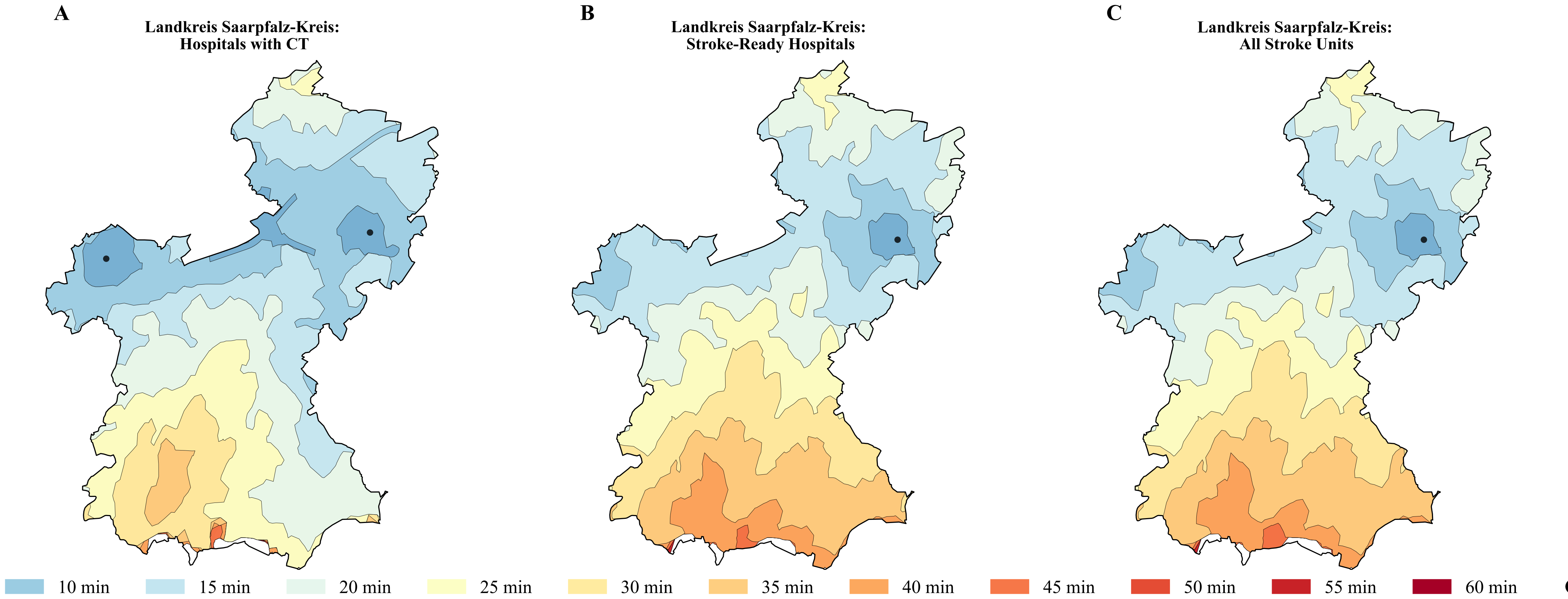

Isochrones — Landkreis Nordhausen (Thüringen)

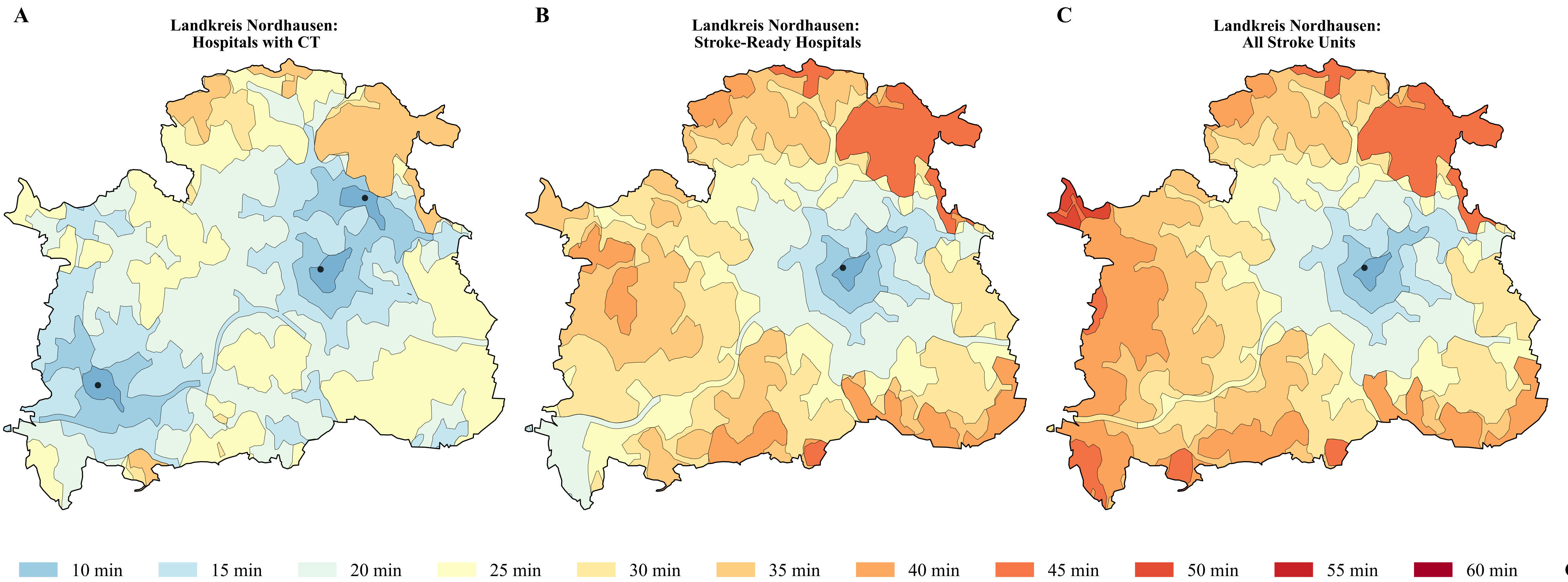

Isochrones — Landkreis Uelzen (Niedersachsen)

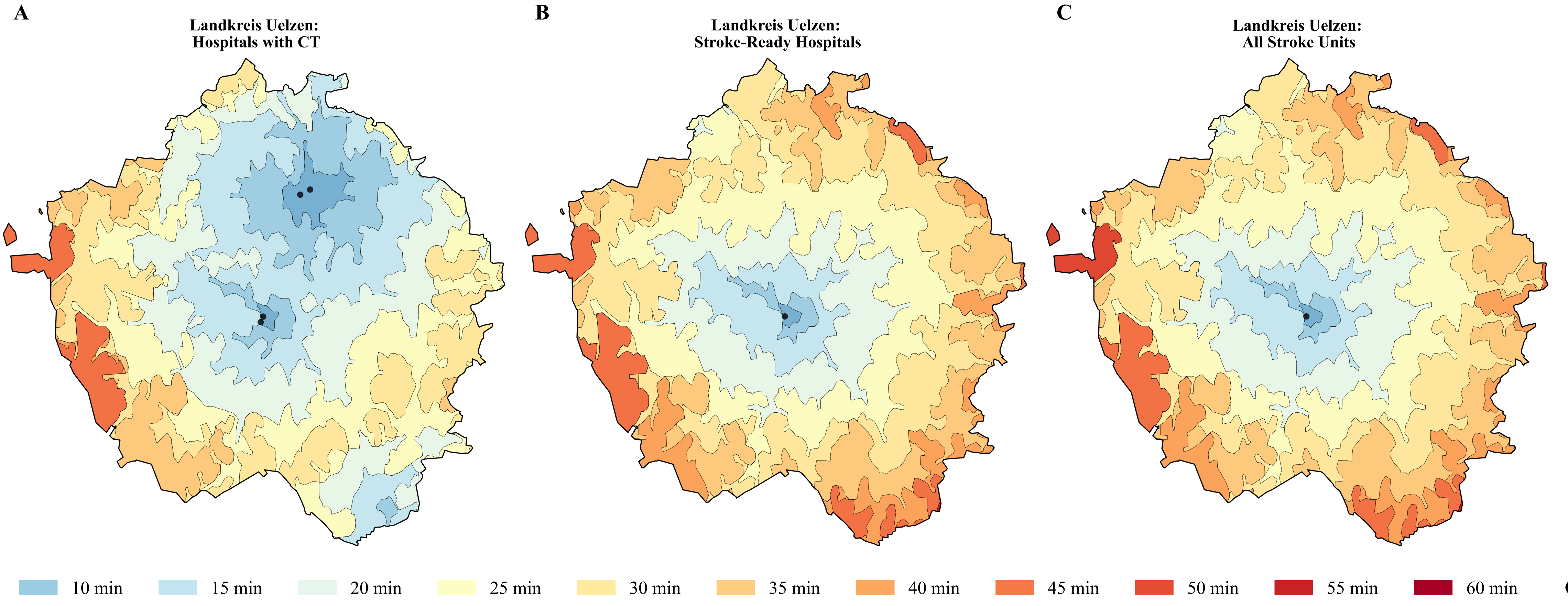

Isochrones — Kreisfreie Stadt Mülheim an der Ruhr (Nordrhein-Westfalen)

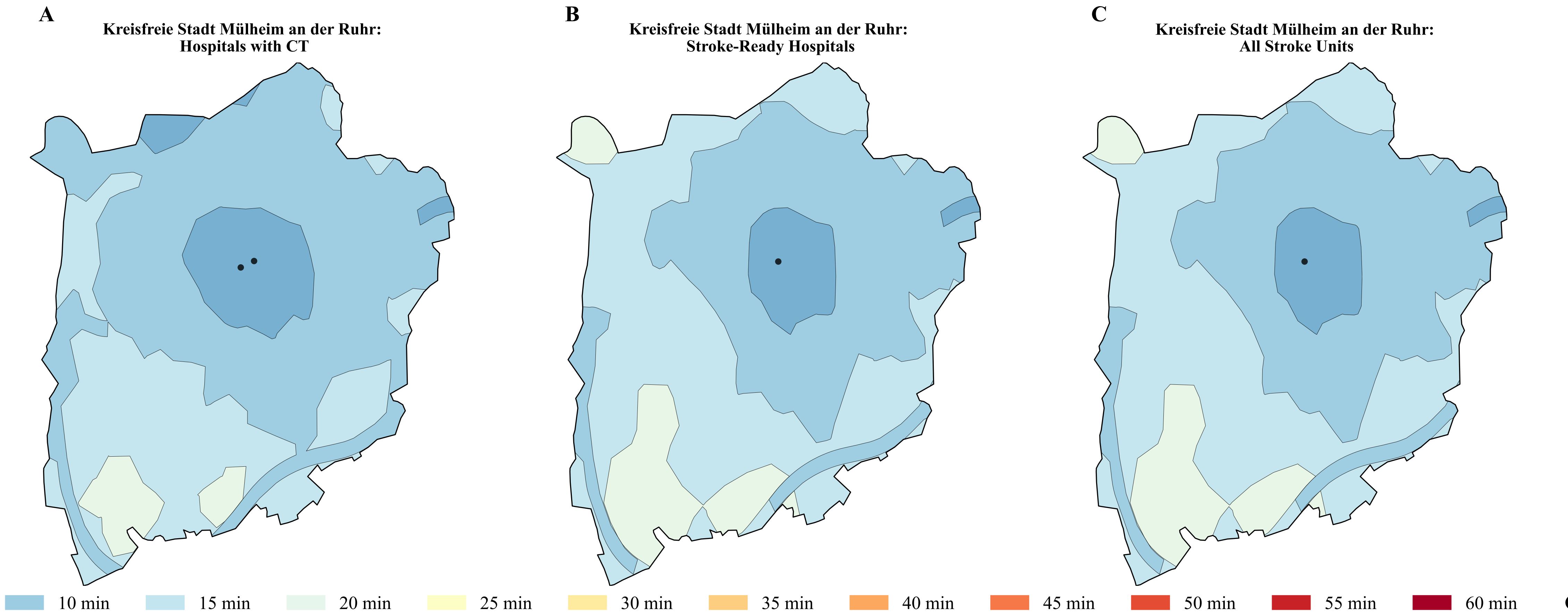

Isochrones — Kreisfreie Stadt Bonn (Nordrhein-Westfalen)

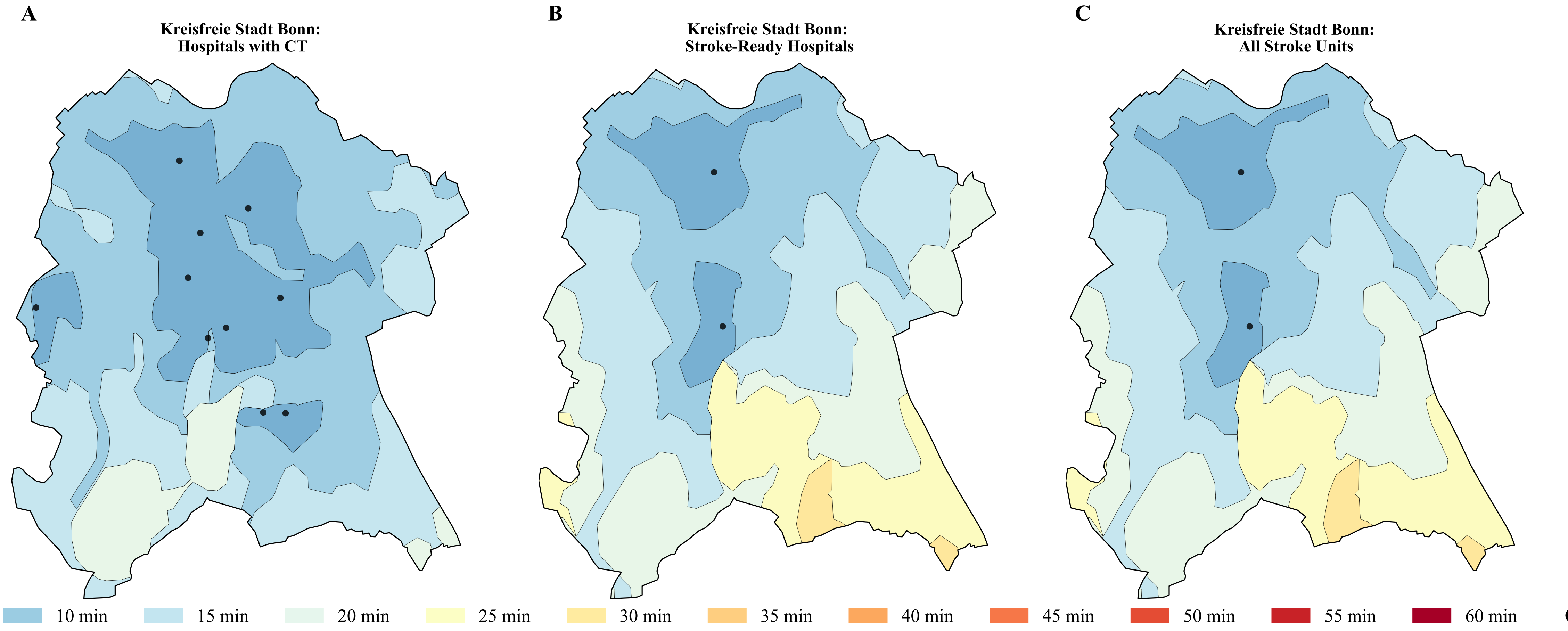

Isochrones — Landkreis Birkenfeld (Rheinland-Pfalz)

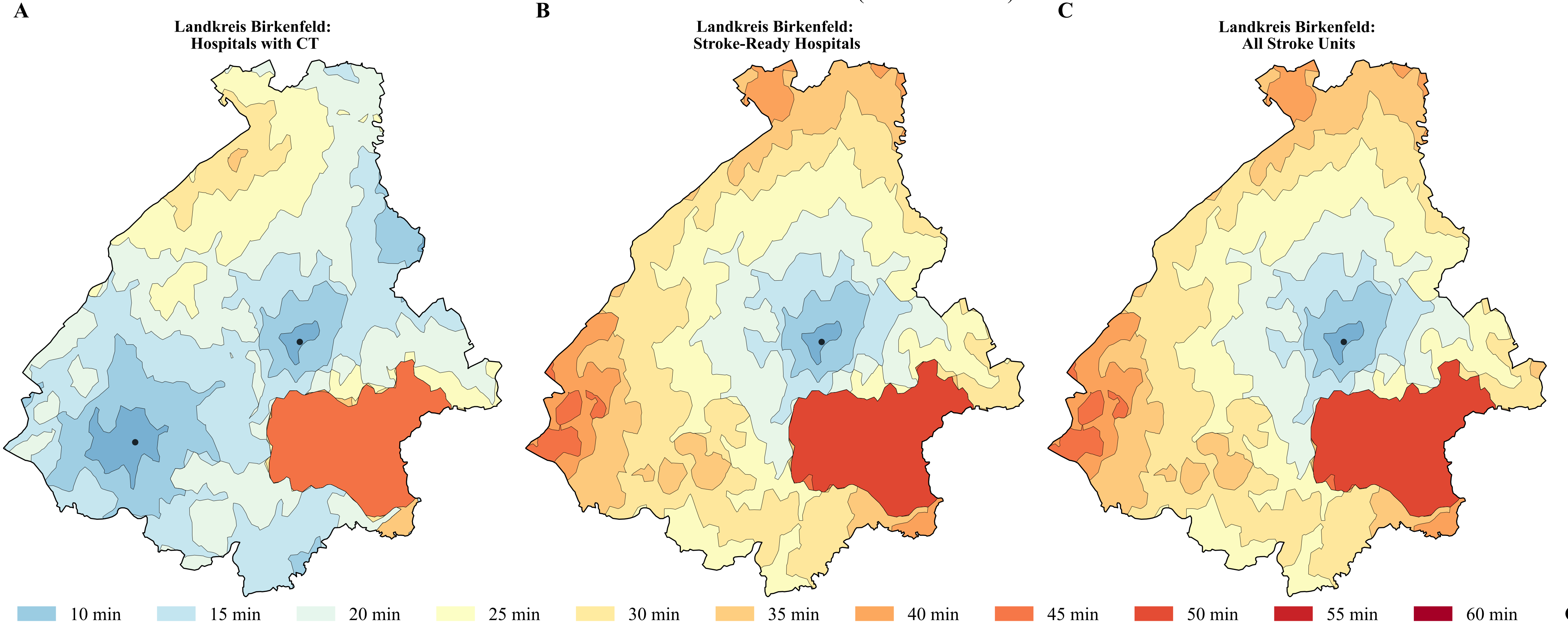

Isochrones — Landkreis Rems-Murr-Kreis (Baden-Württemberg)

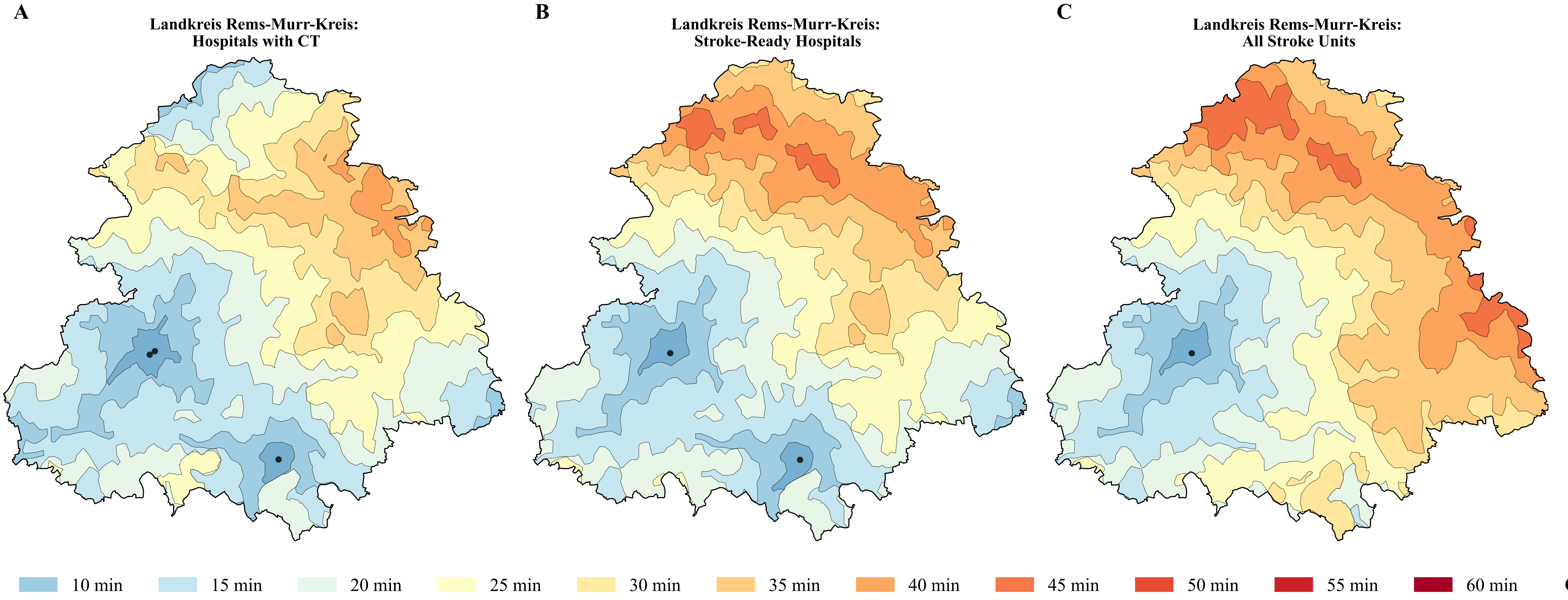

Isochrones — Landkreis Schwäbisch Hall (Baden-Württemberg)

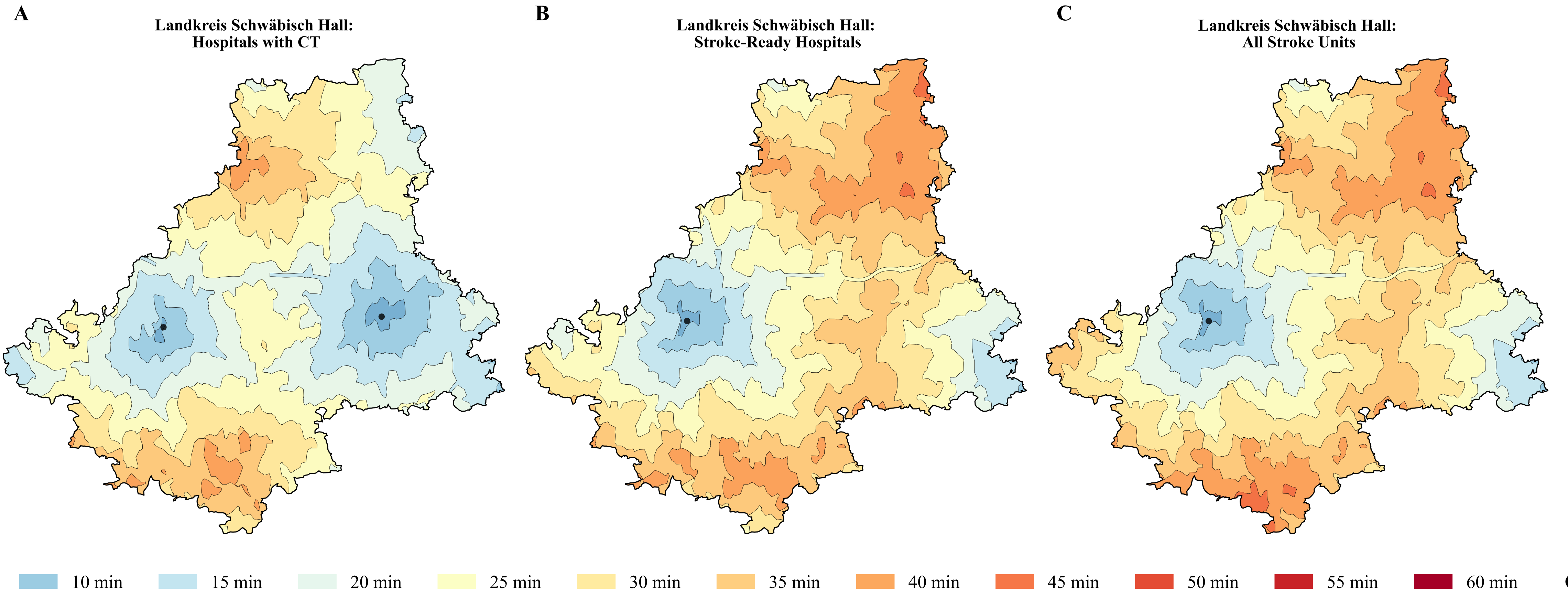

Isochrones — Landkreis Rottweil (Baden-Württemberg)

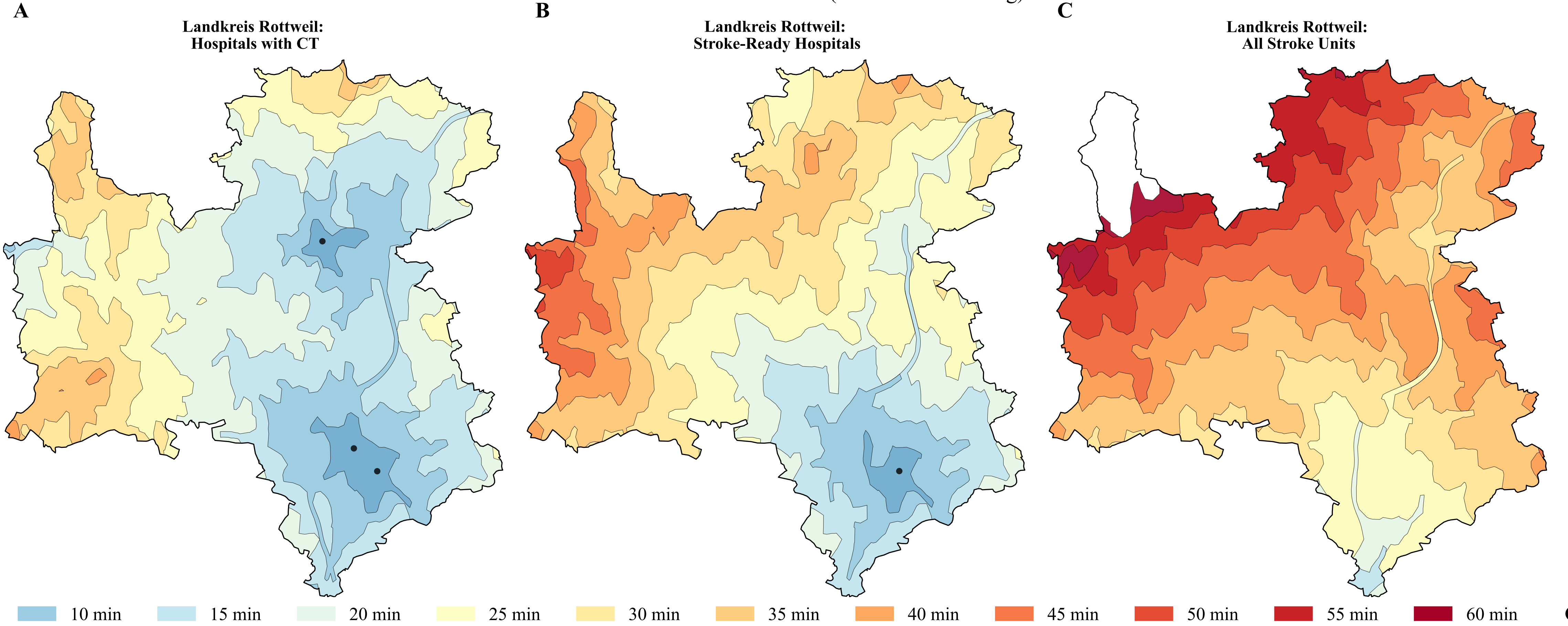

Isochrones — Landkreis Amberg-Sulzbach (Bayern)

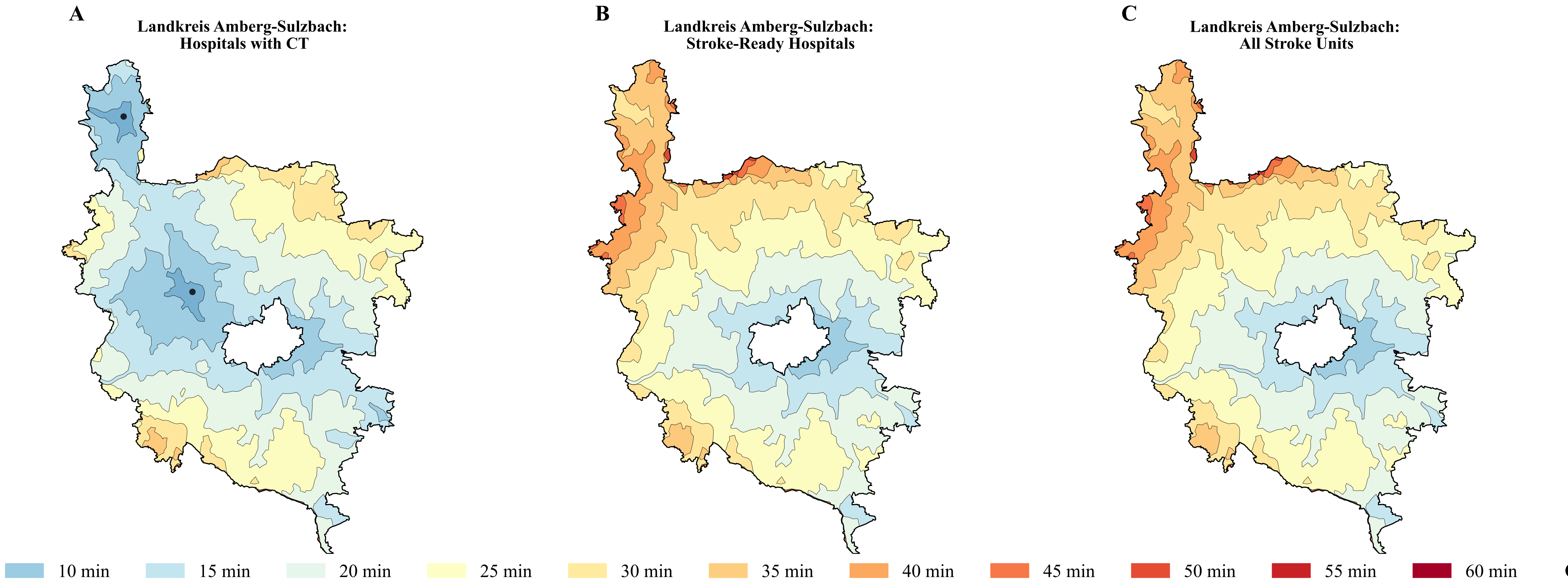

Isochrones — Kreisfreie Stadt Kempten (Allgäu) (Bayern)

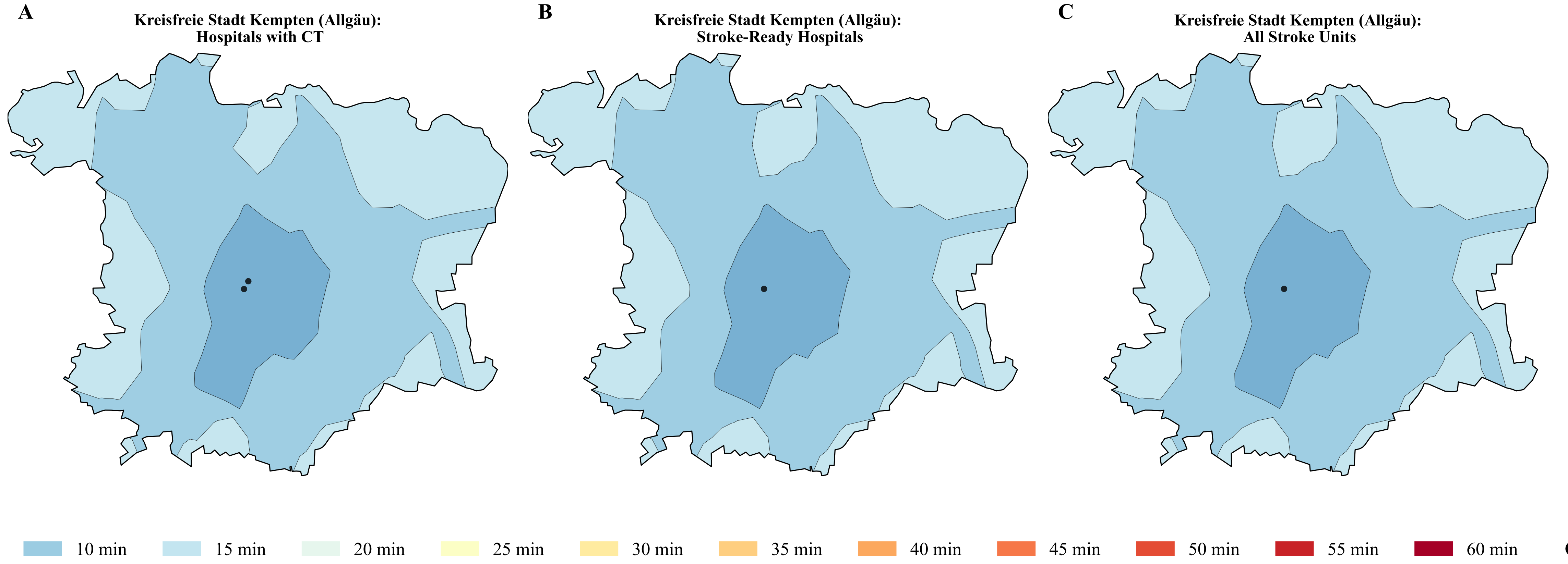

Isochrones — Landkreis Mecklenburgische Seenplatte (Mecklenburg-Vorpommern)

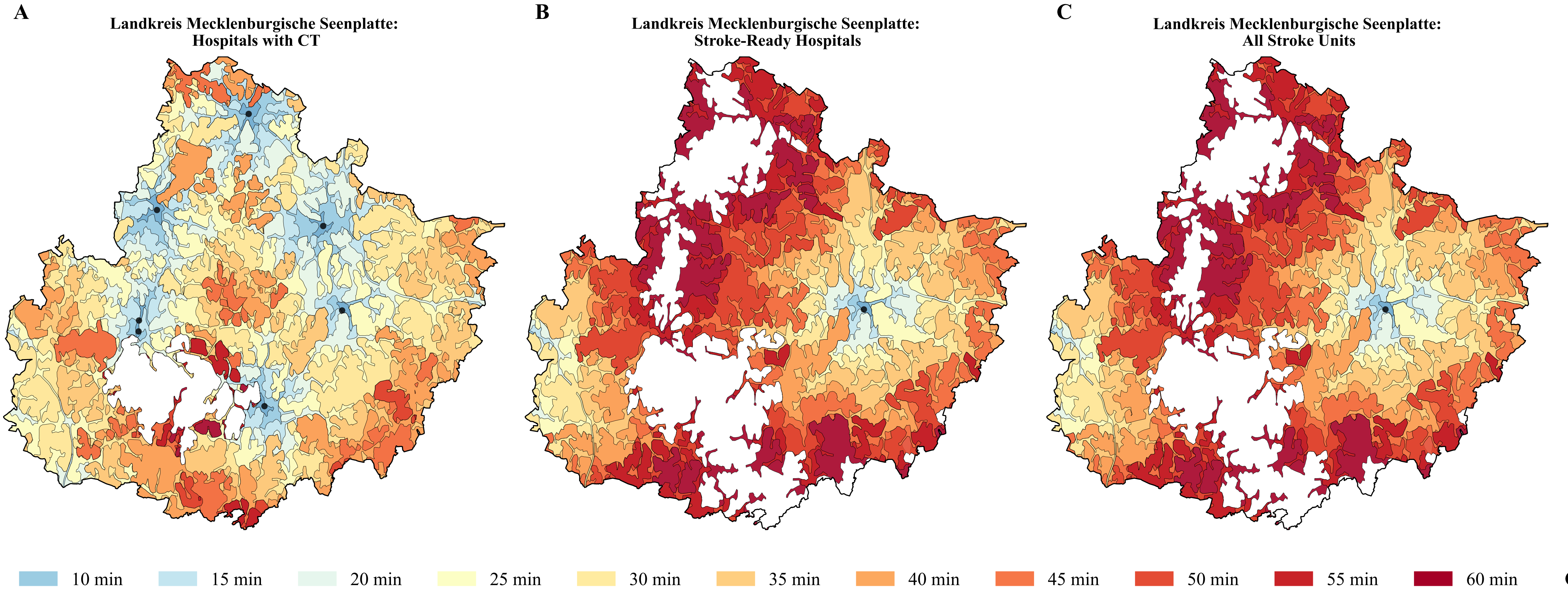

Isochrones — Kreisfreie Stadt Chemnitz (Sachsen)

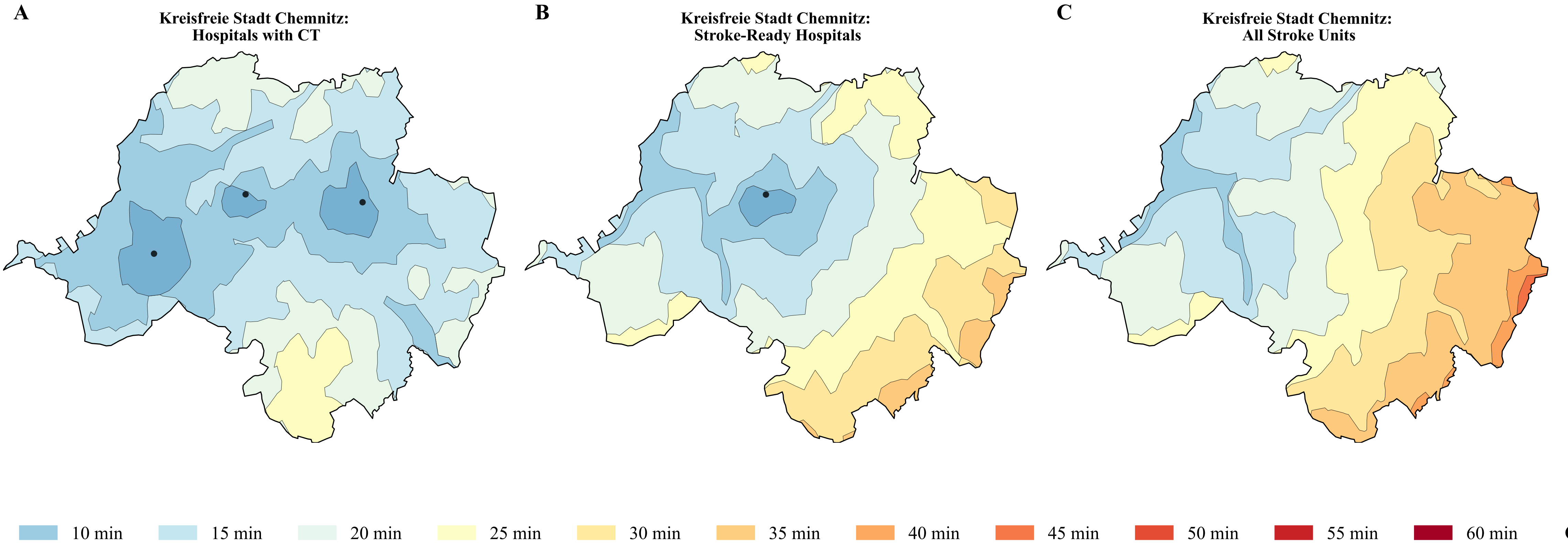

Isochrones — Landkreis Erzgebirgskreis (Sachsen)

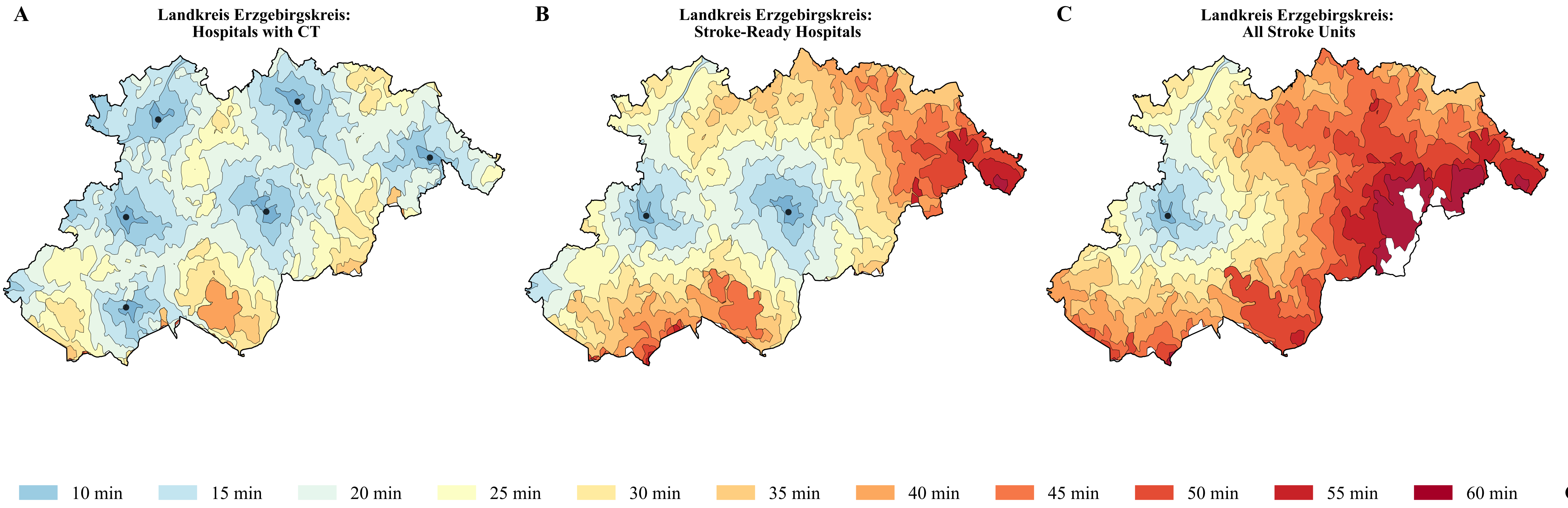

Isochrones — Kreisfreie Stadt Dresden (Sachsen)

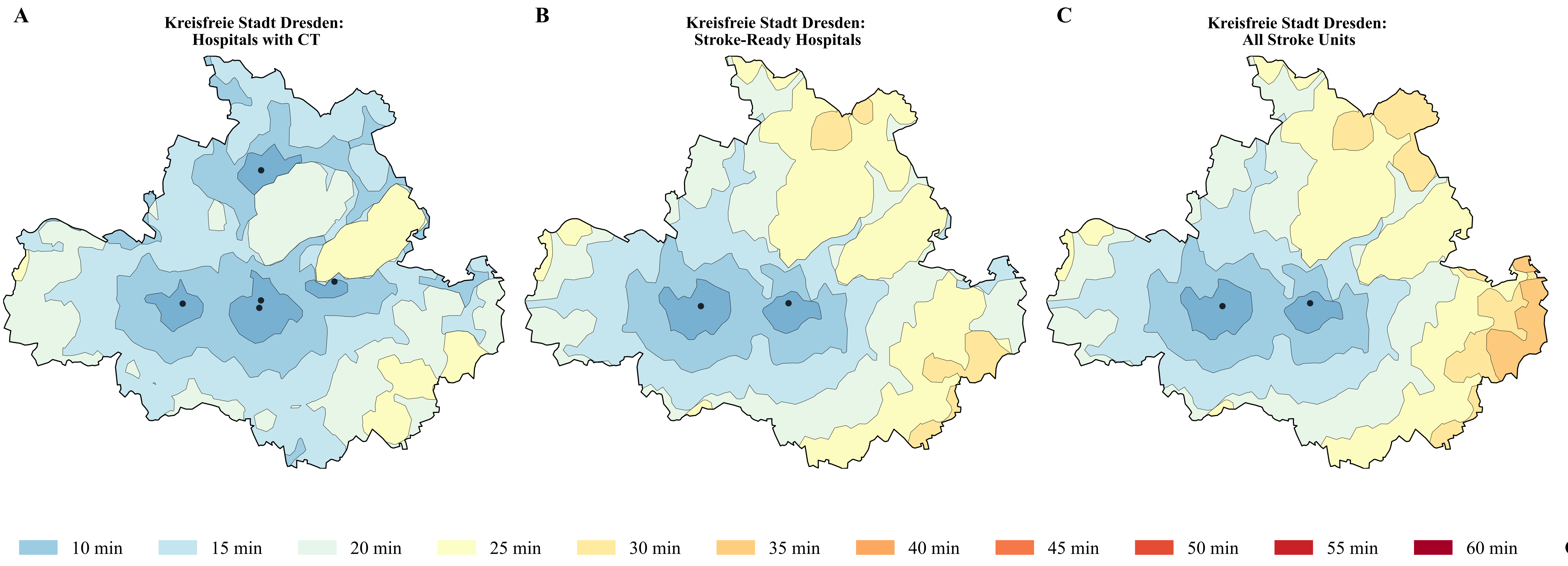

Isochrones — Landkreis Hildburghausen (Thüringen)

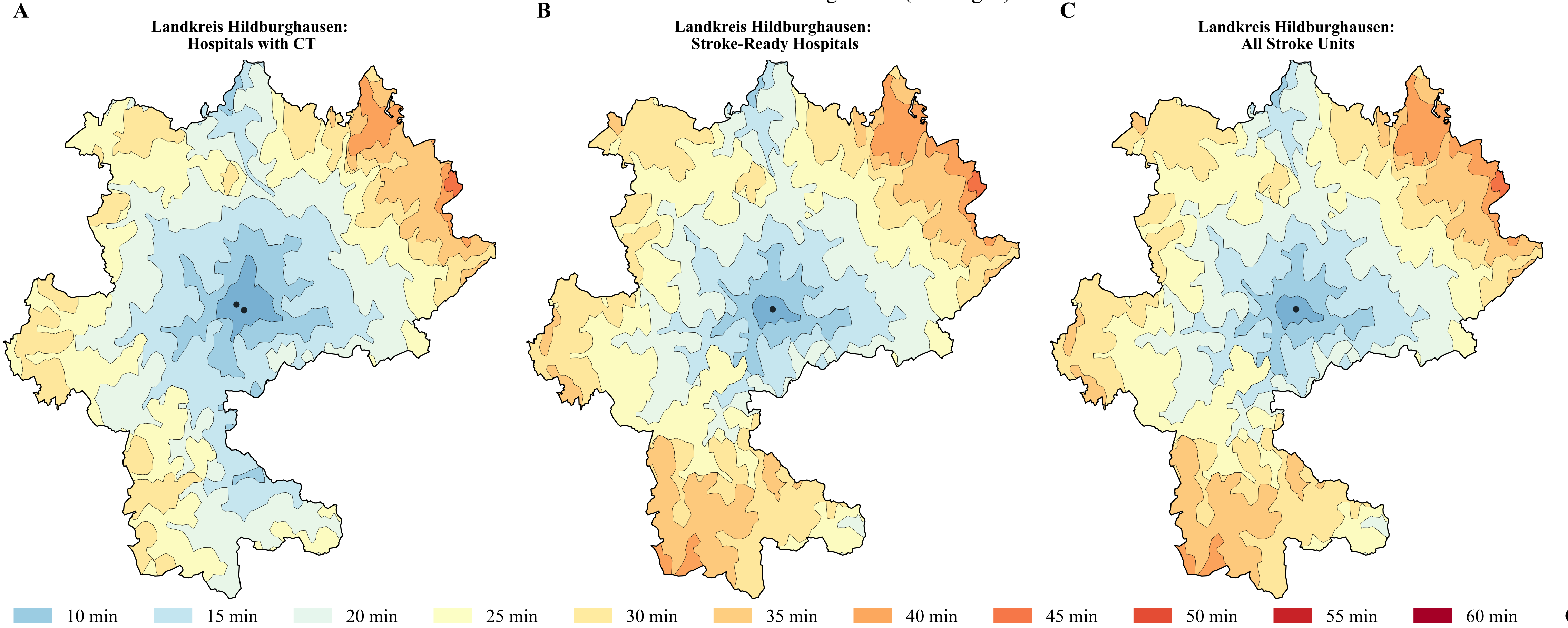

Isochrones — Kreis Herzogtum Lauenburg (Schleswig-Holstein)

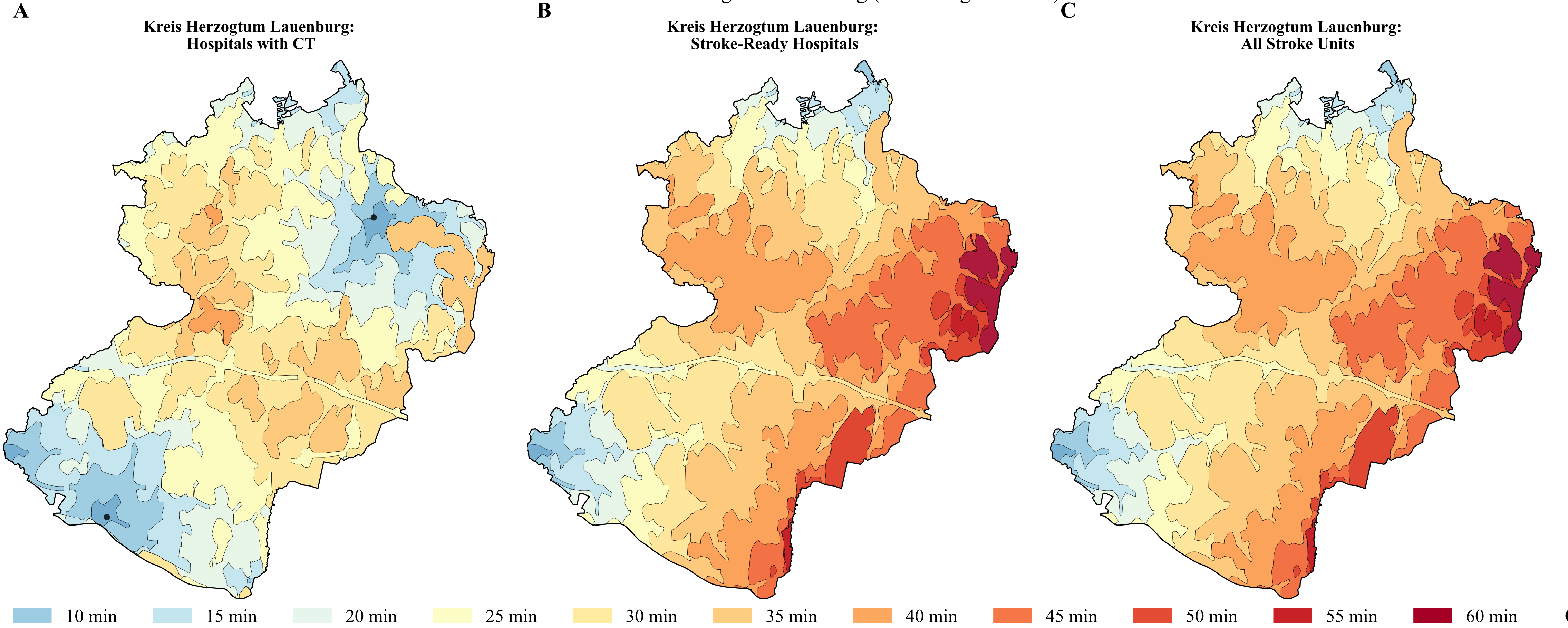

Isochrones — Landkreis Oldenburg (Niedersachsen)

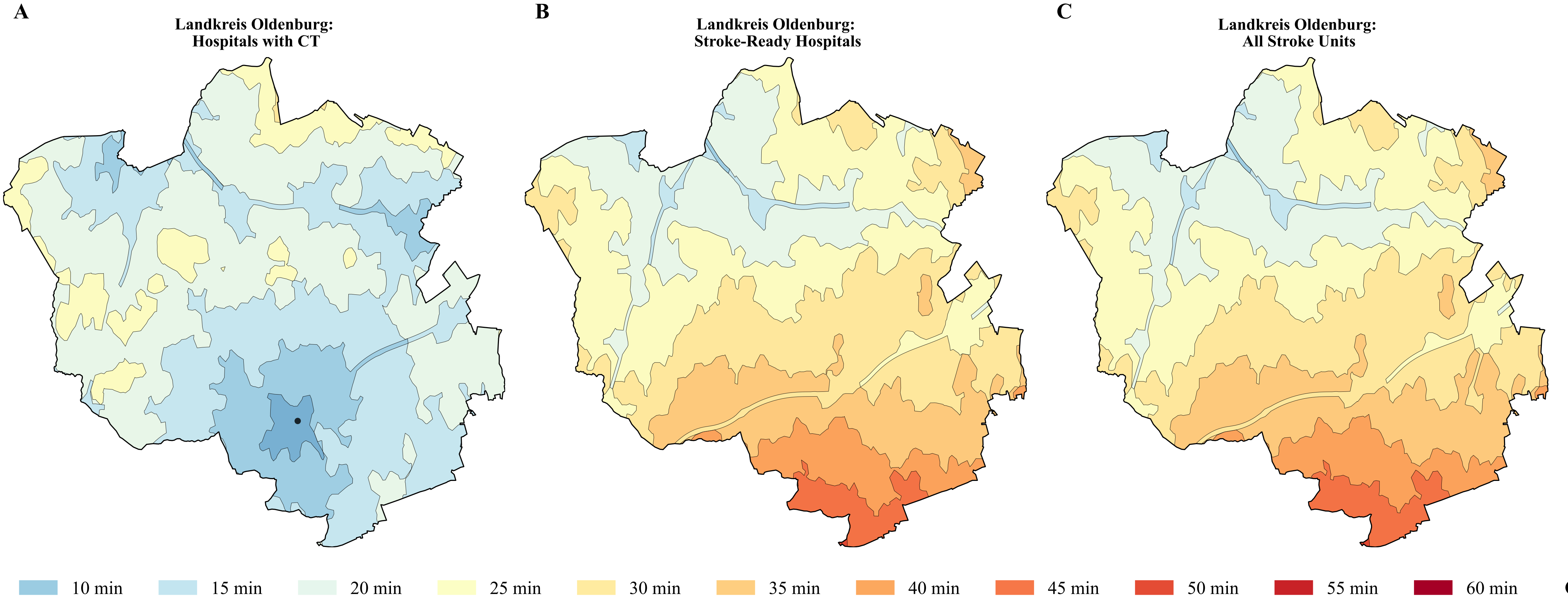

Isochrones — Kreisfreie Stadt Krefeld (Nordrhein-Westfalen)

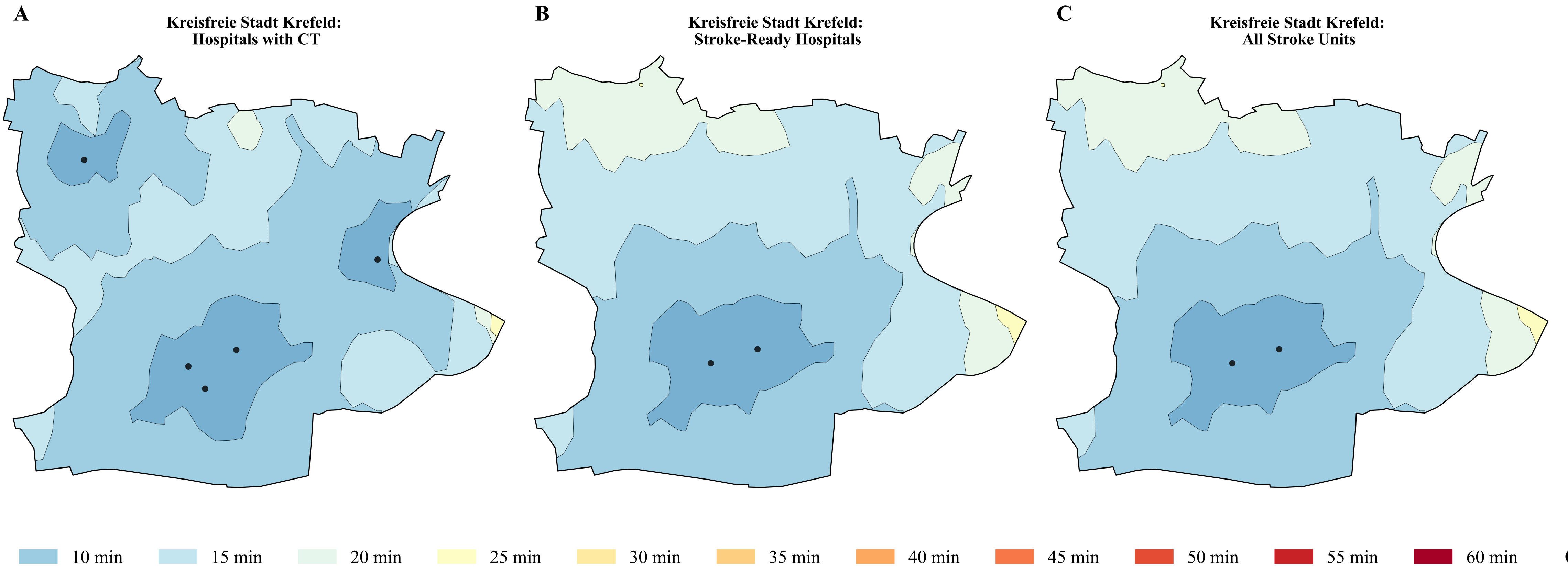

Isochrones — Kreis Oberbergischer Kreis (Nordrhein-Westfalen)

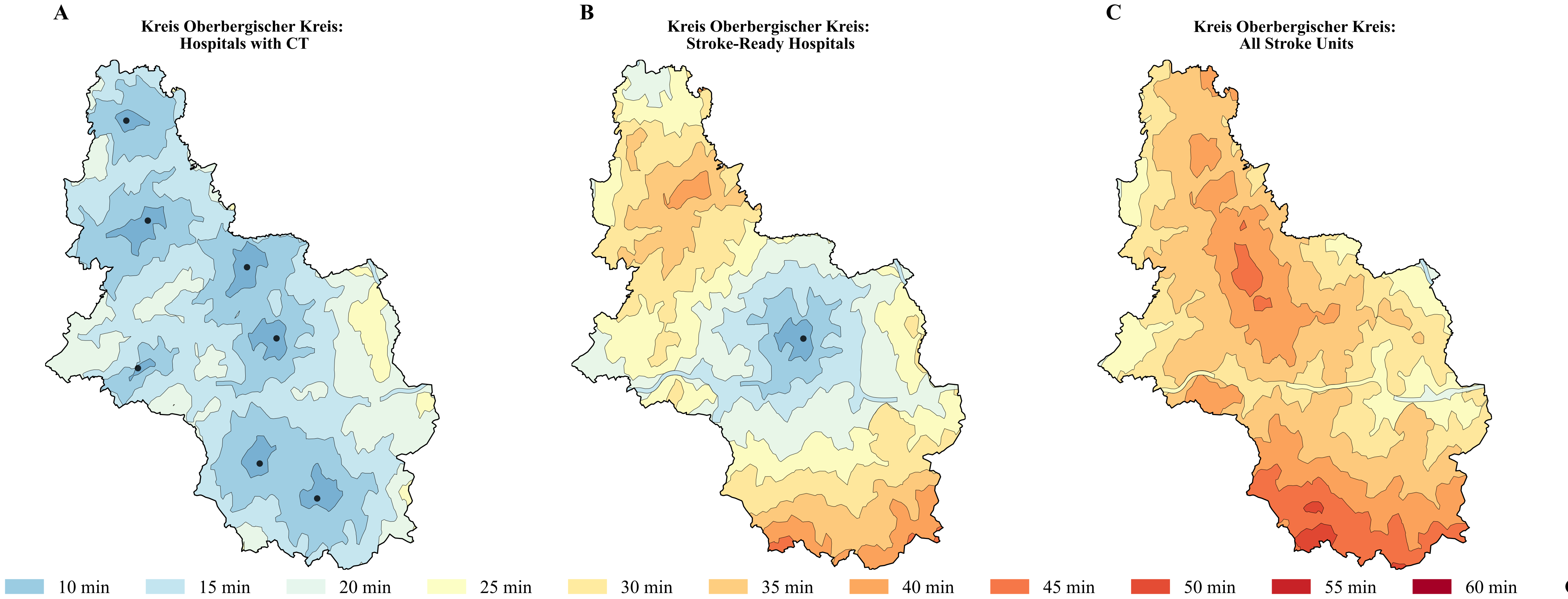

Isochrones — Landkreis Kusel (Rheinland-Pfalz)

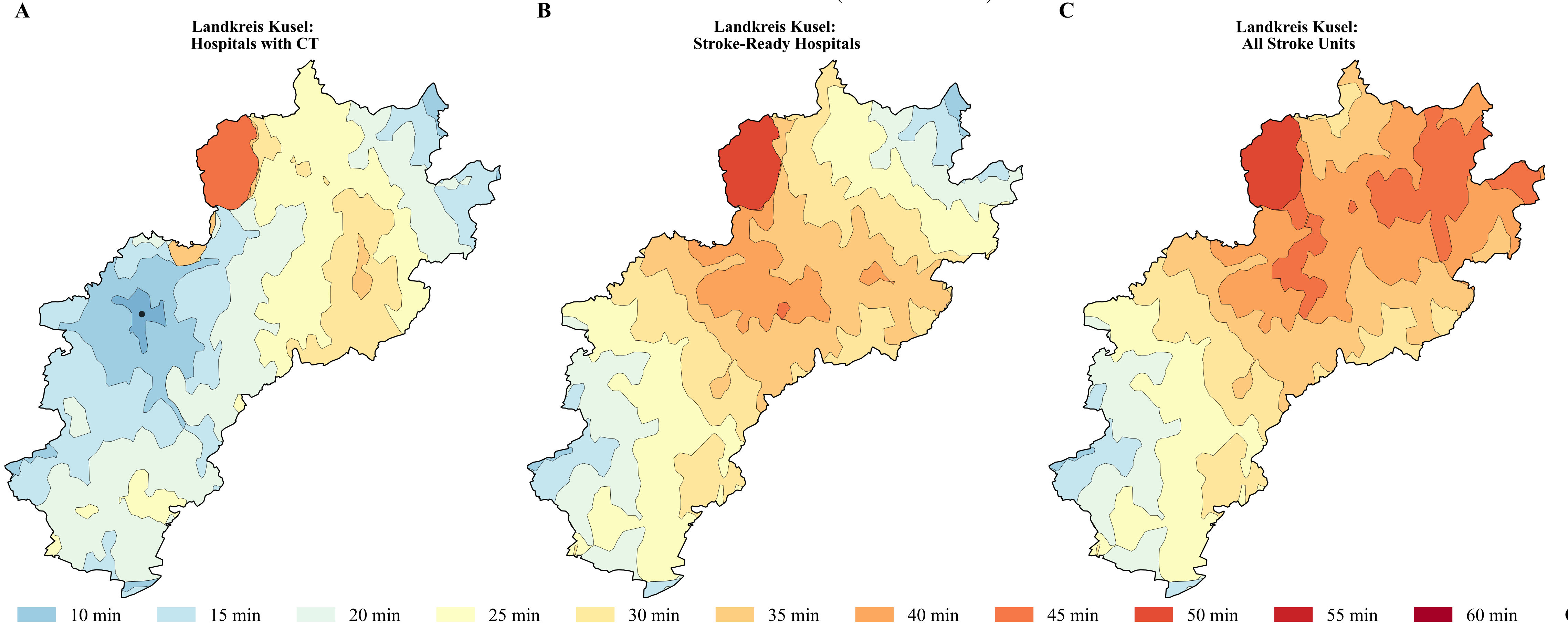

Isochrones — Landkreis Fürstenfeldbruck (Bayern)

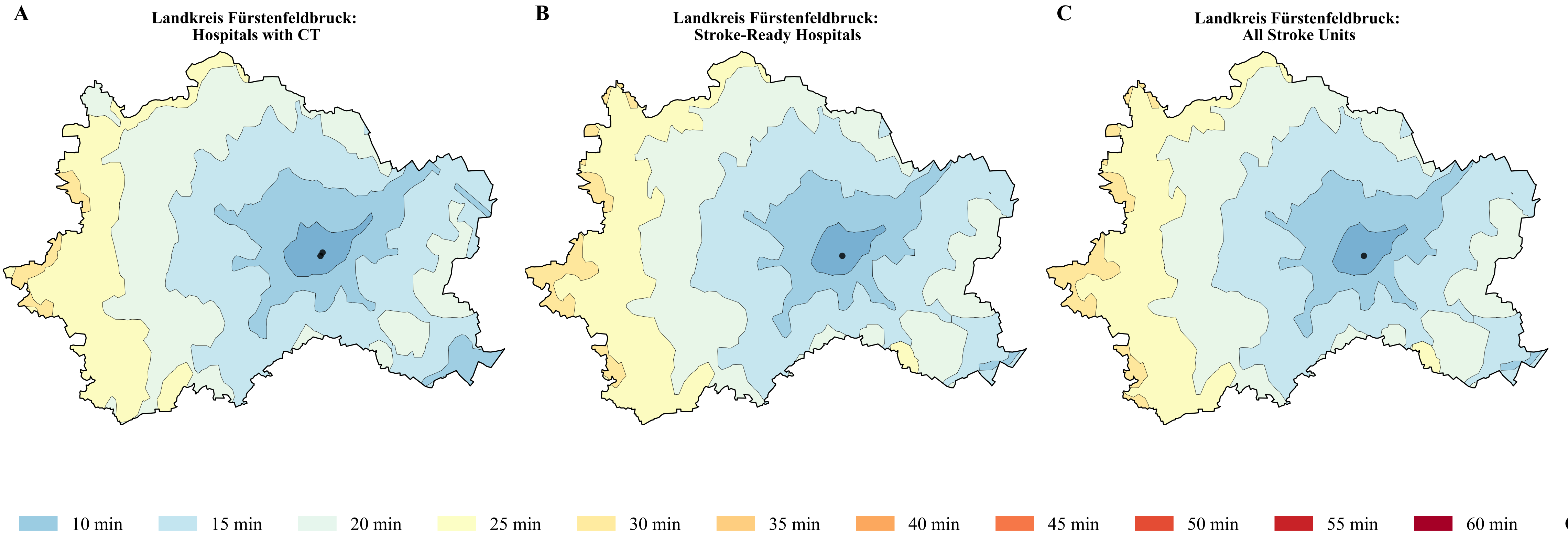

Isochrones — Landkreis Weilheim-Schongau (Bayern)

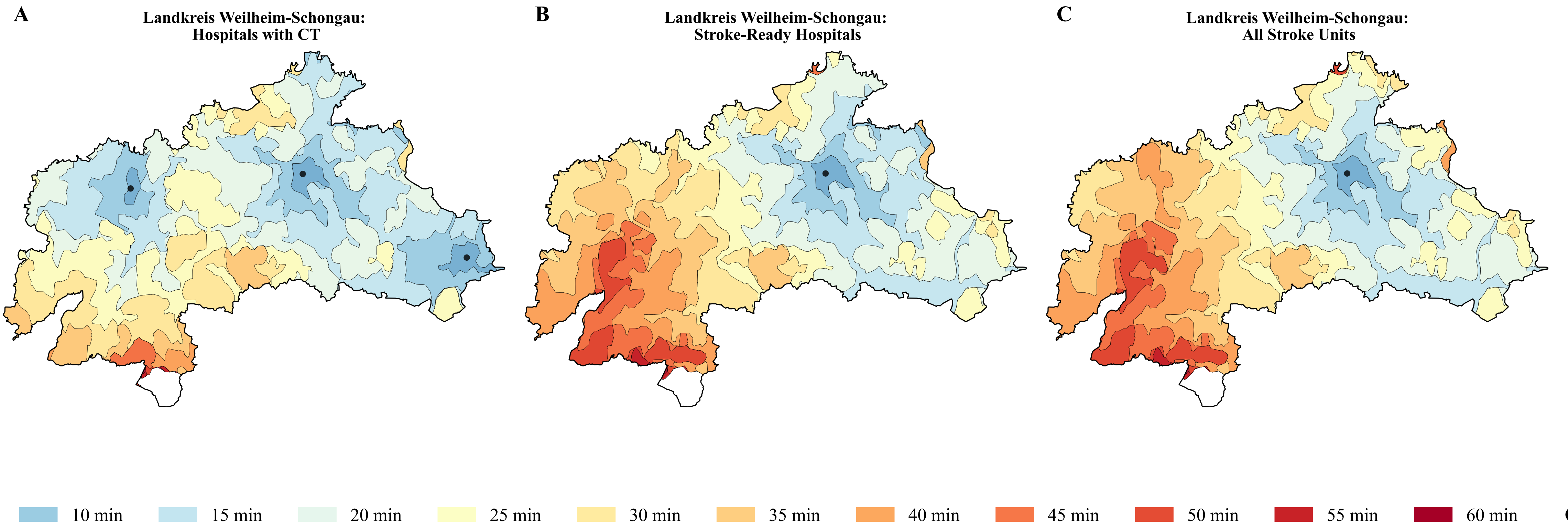

Isochrones — Landkreis Neustadt a.d.Waldnaab (Bayern)

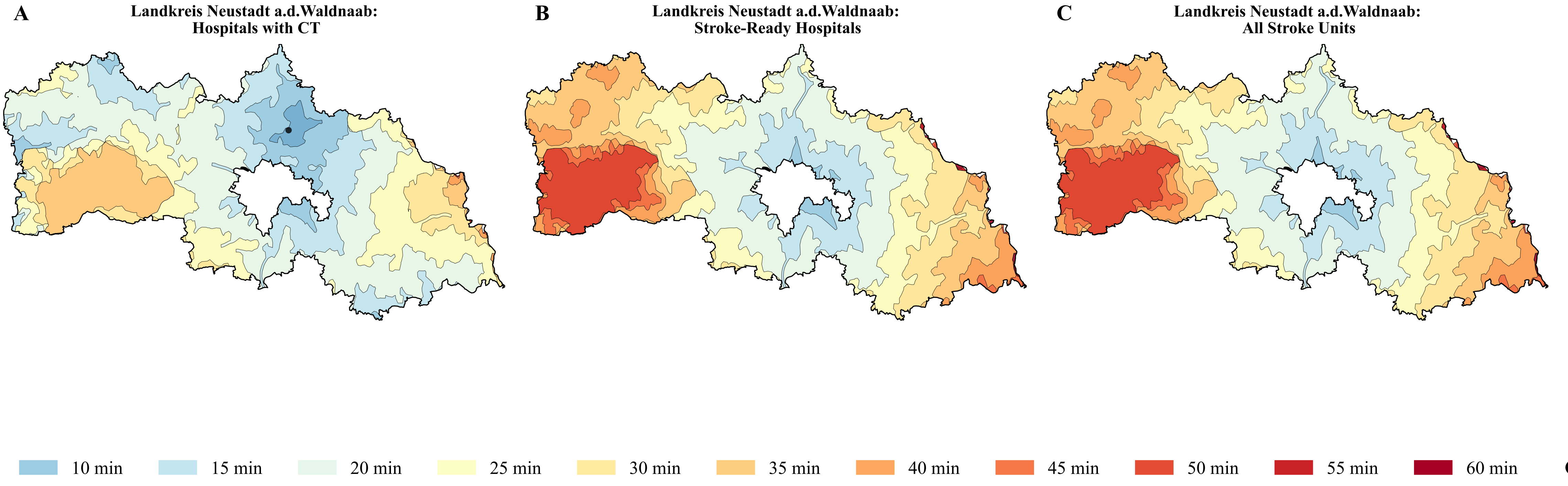

Isochrones — Landkreis Würzburg (Bayern)

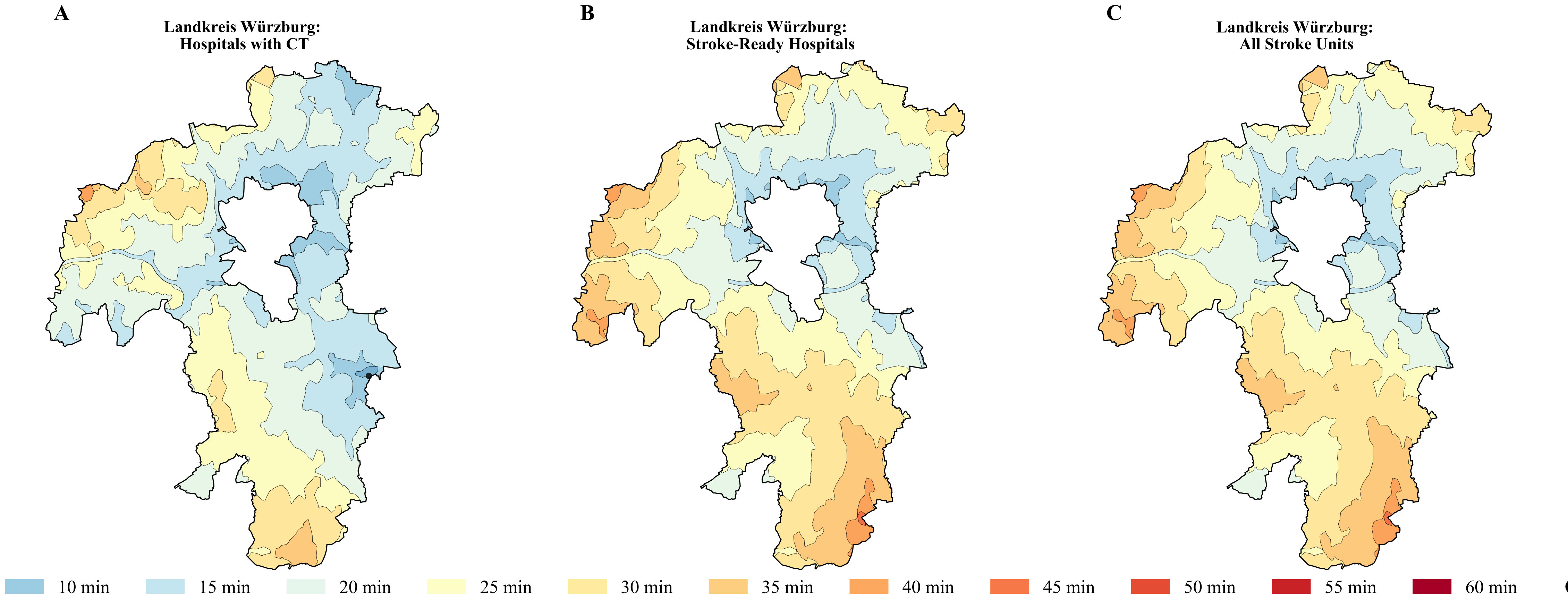

Isochrones — Landkreis Elbe-Elster (Brandenburg)

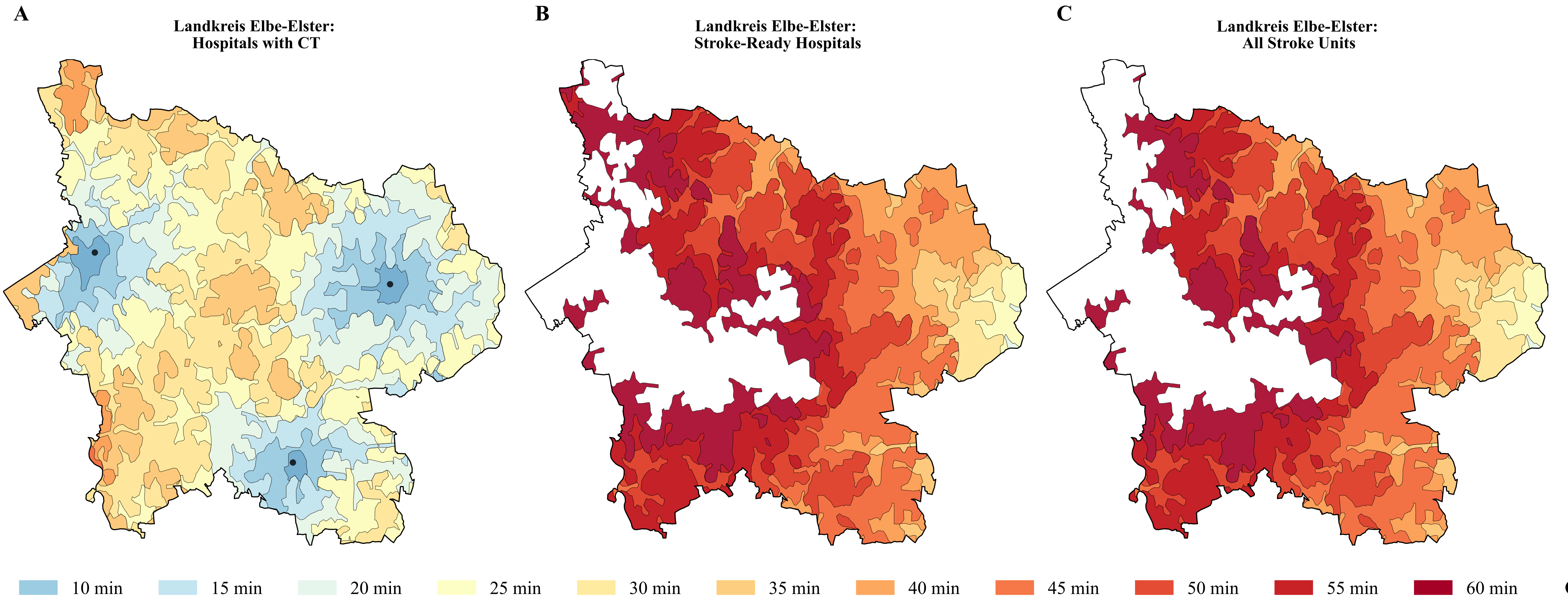

Isochrones — Landkreis Osterholz (Niedersachsen)

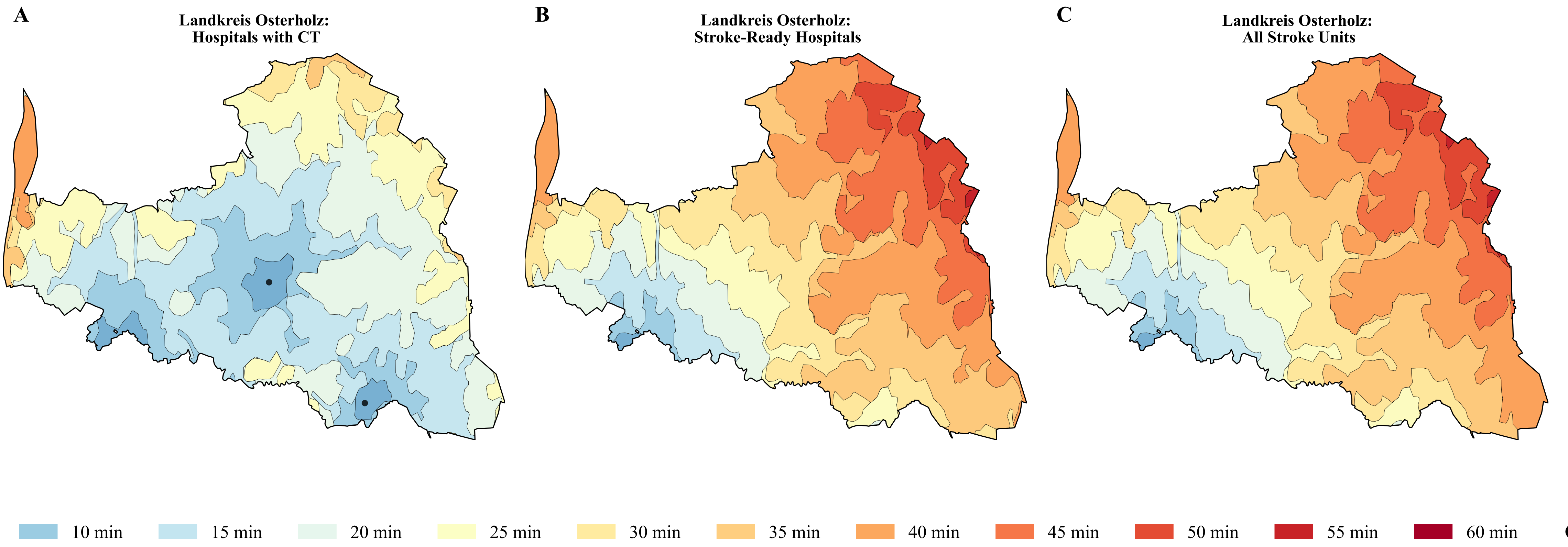

Isochrones — Landkreis Heidekreis (Niedersachsen)

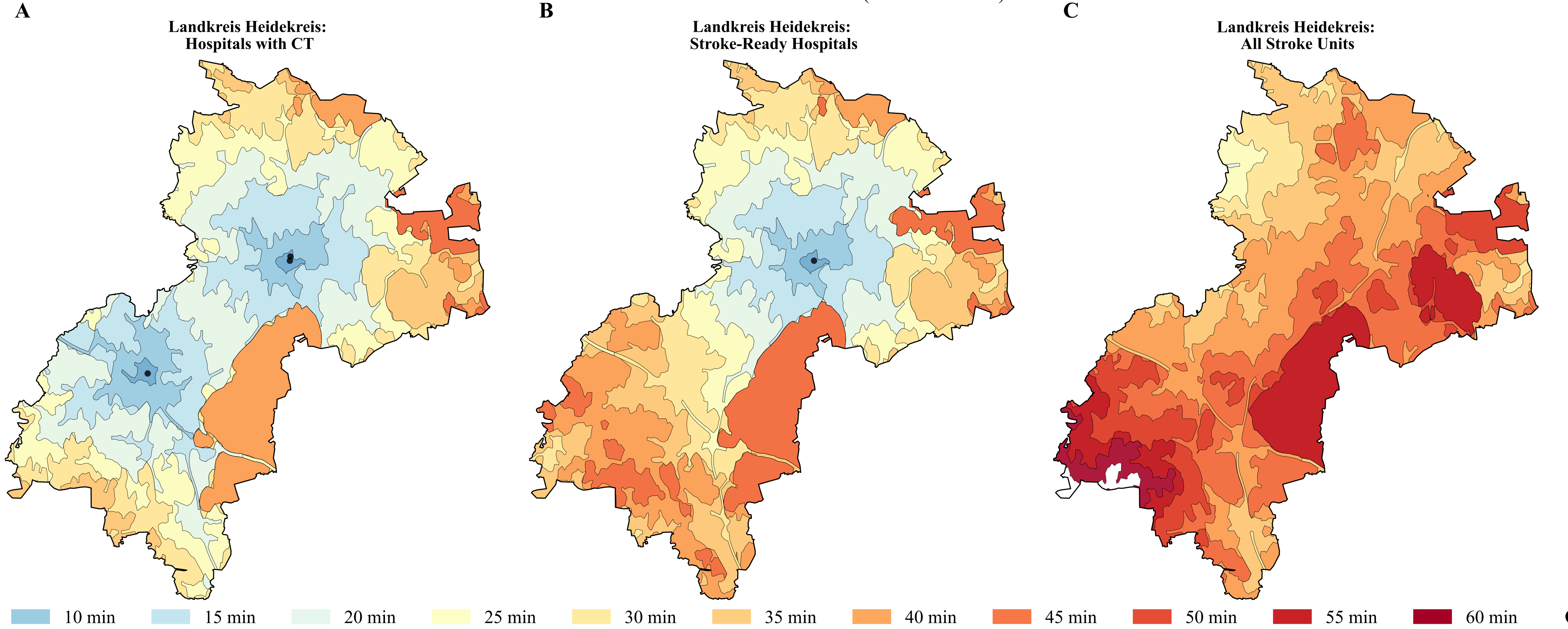

Isochrones — Kreisfreie Stadt Solingen (Nordrhein-Westfalen)

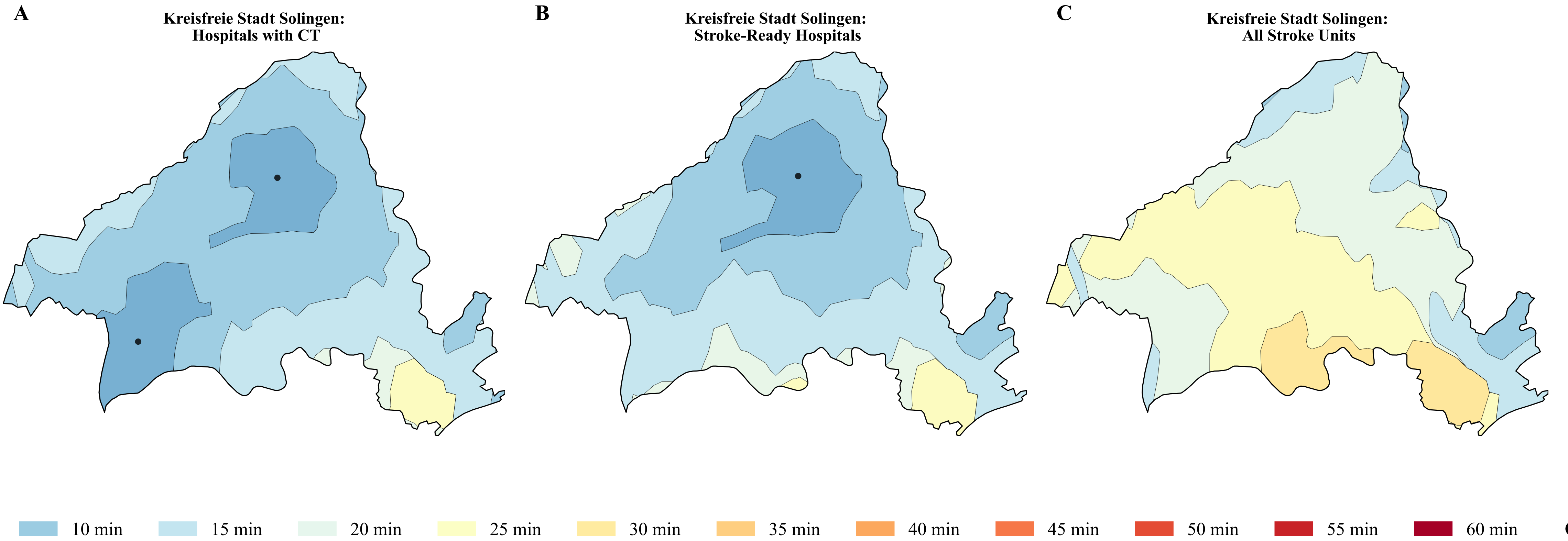

Isochrones — Landkreis Bad Kreuznach (Rheinland-Pfalz)

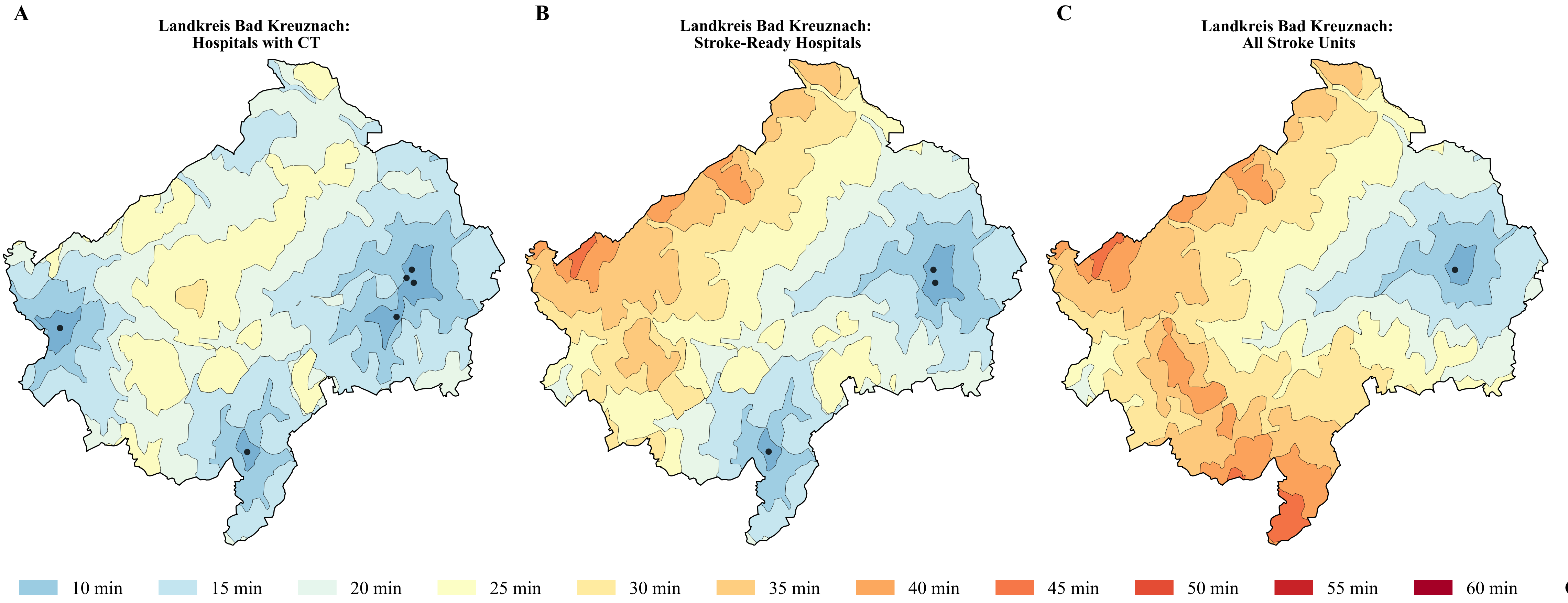

Isochrones — Landkreis Südwestpfalz (Rheinland-Pfalz)

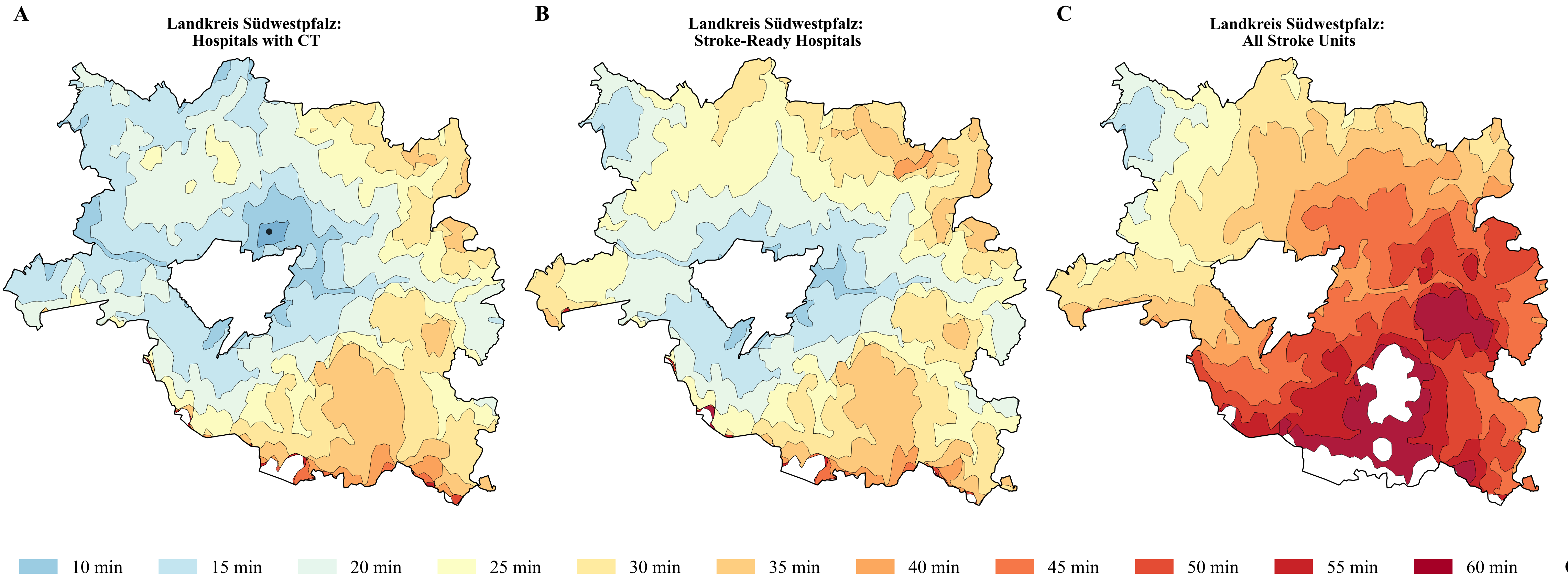

Isochrones — Landkreis Ludwigsburg (Baden-Württemberg)

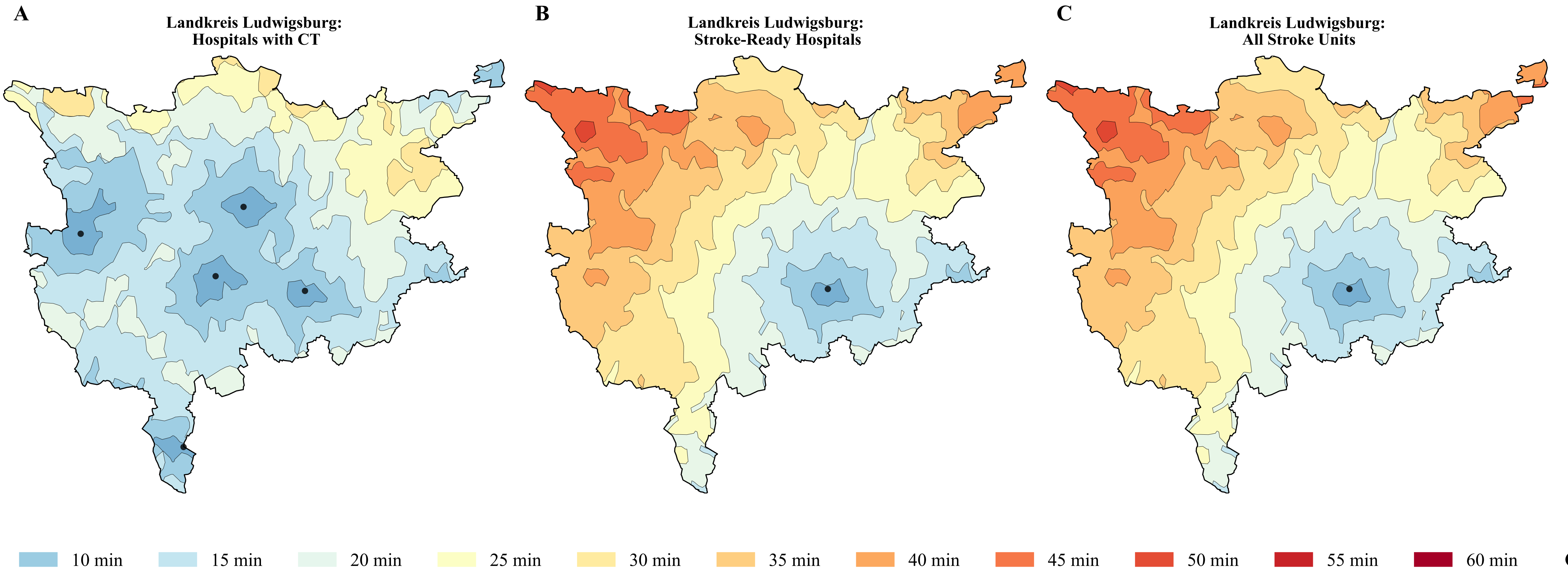

Isochrones — Landkreis Lörrach (Baden-Württemberg)

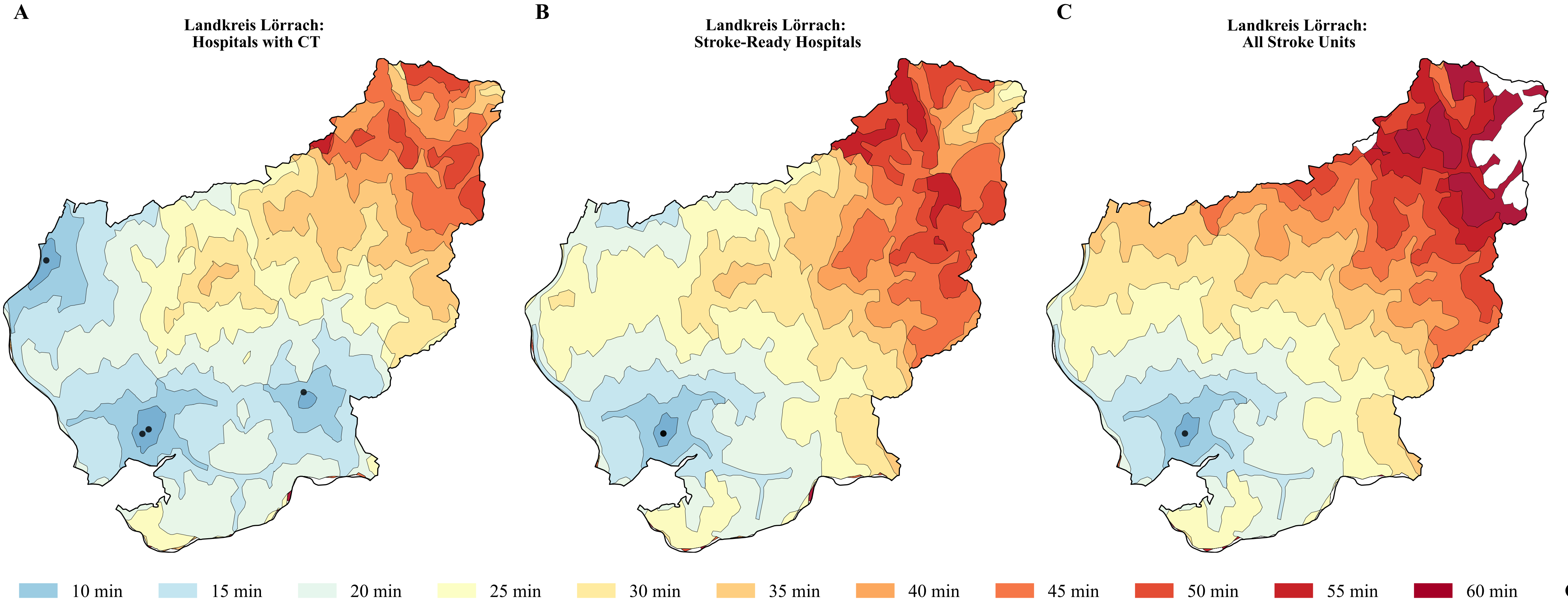

Isochrones — Landkreis Alb-Donau-Kreis (Baden-Württemberg)

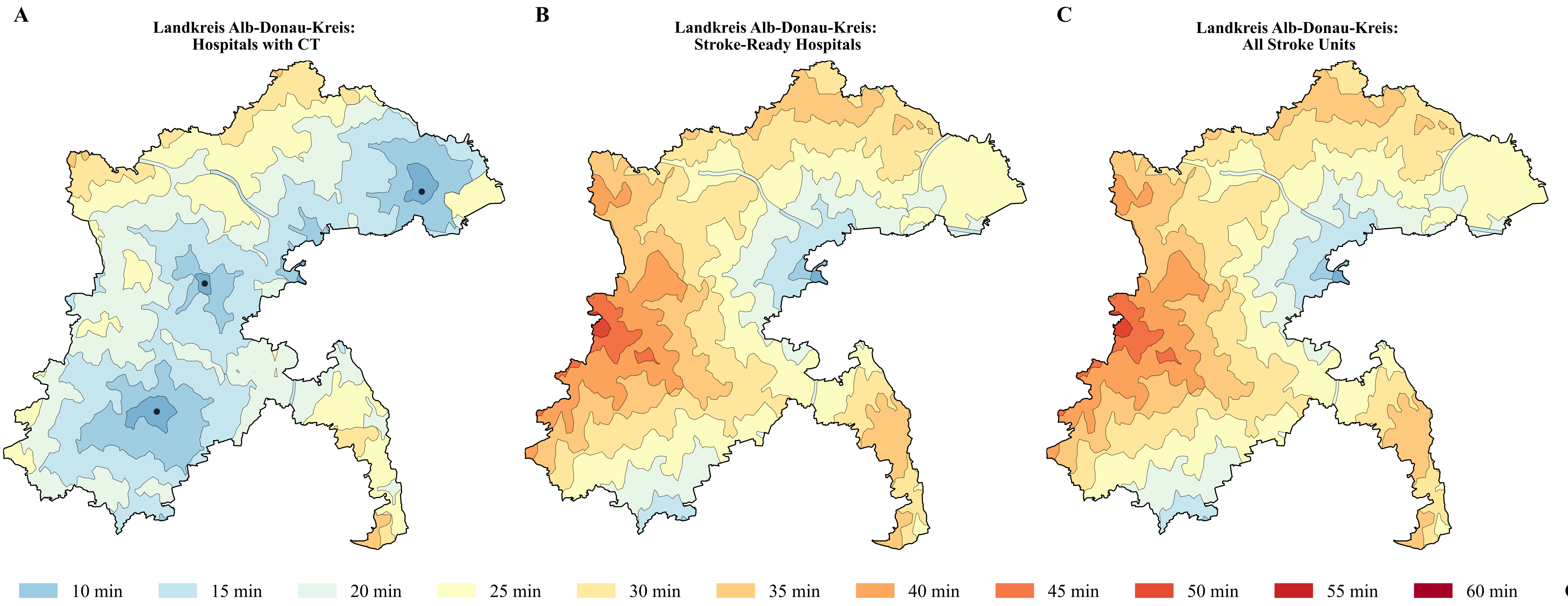

Isochrones — Landkreis Biberach (Baden-Württemberg)

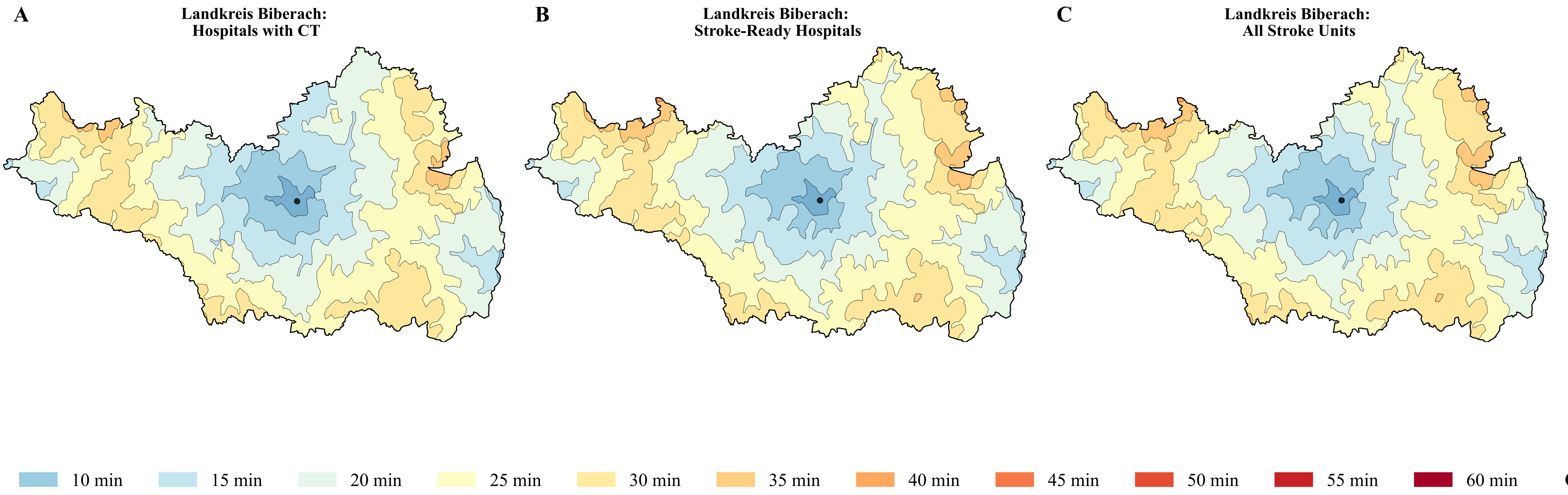

Isochrones — Kreisfreie Stadt Fürth (Bayern)

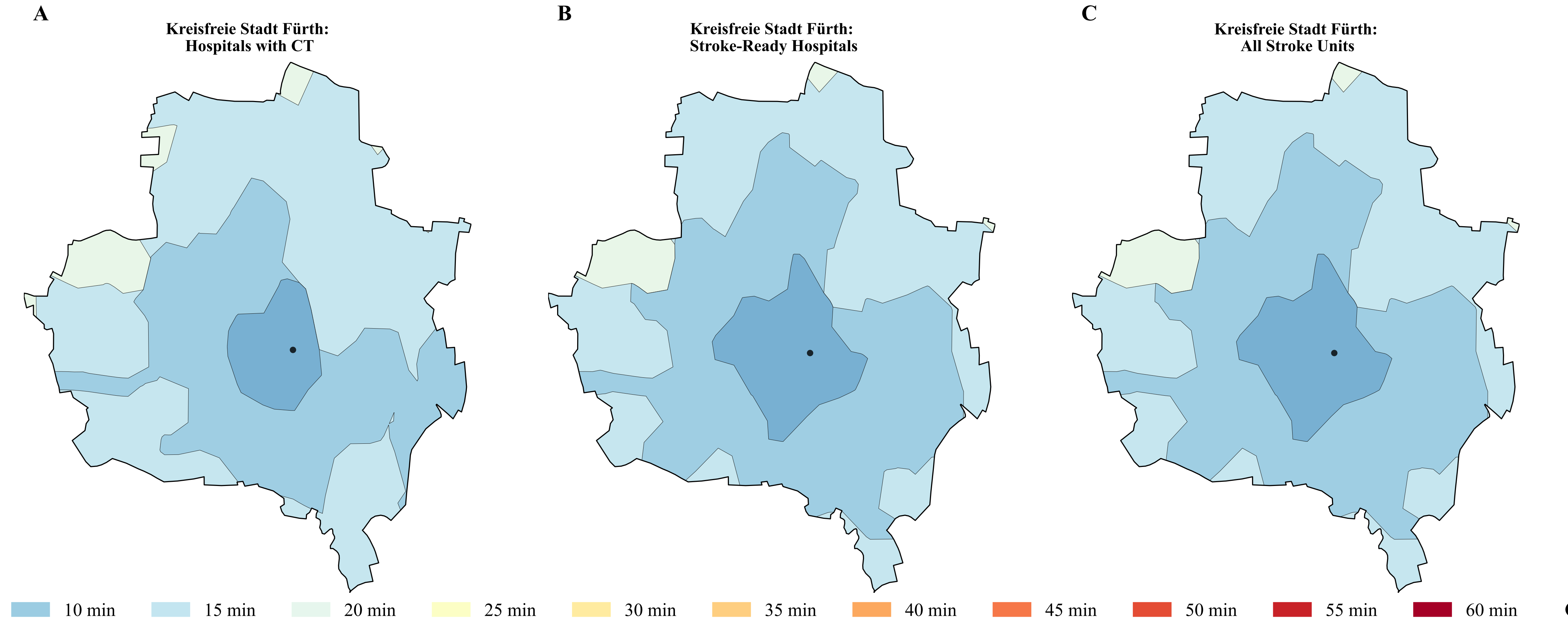

Isochrones — Kreisfreie Stadt Halle (Saale) (Sachsen-Anhalt)

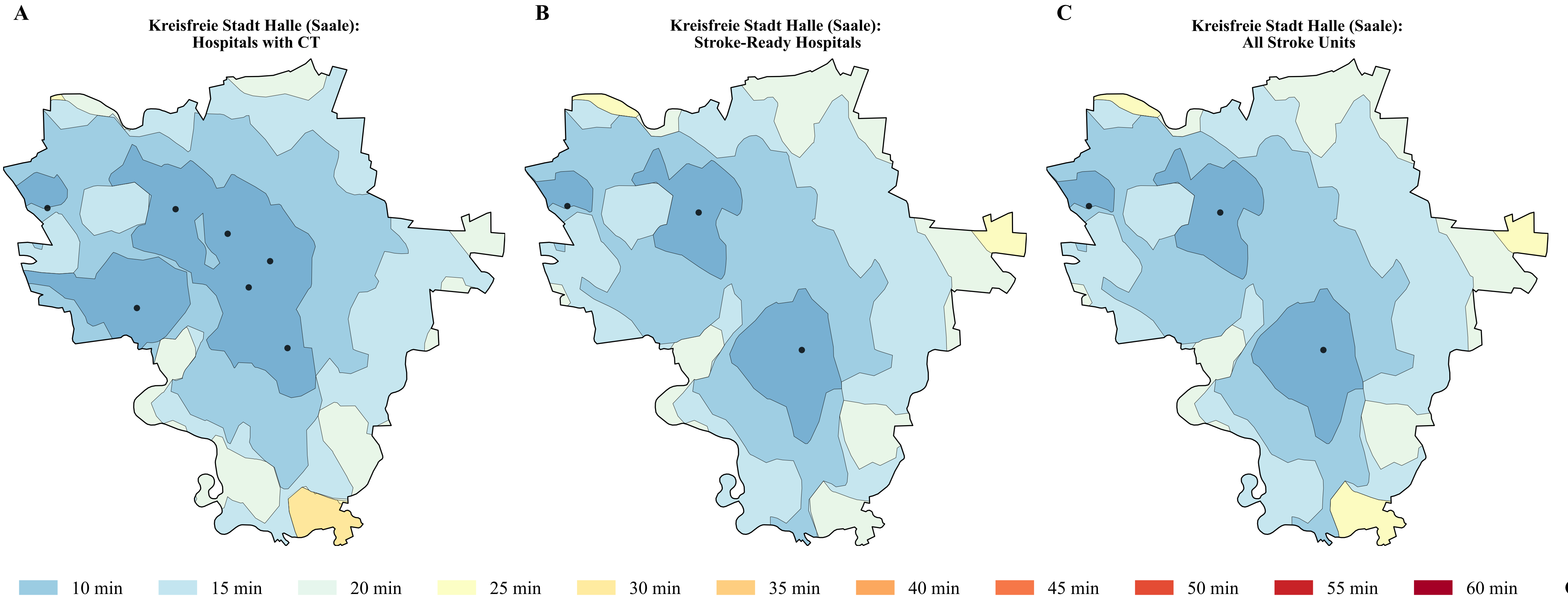

Isochrones — Landkreis Wittenberg (Sachsen-Anhalt)

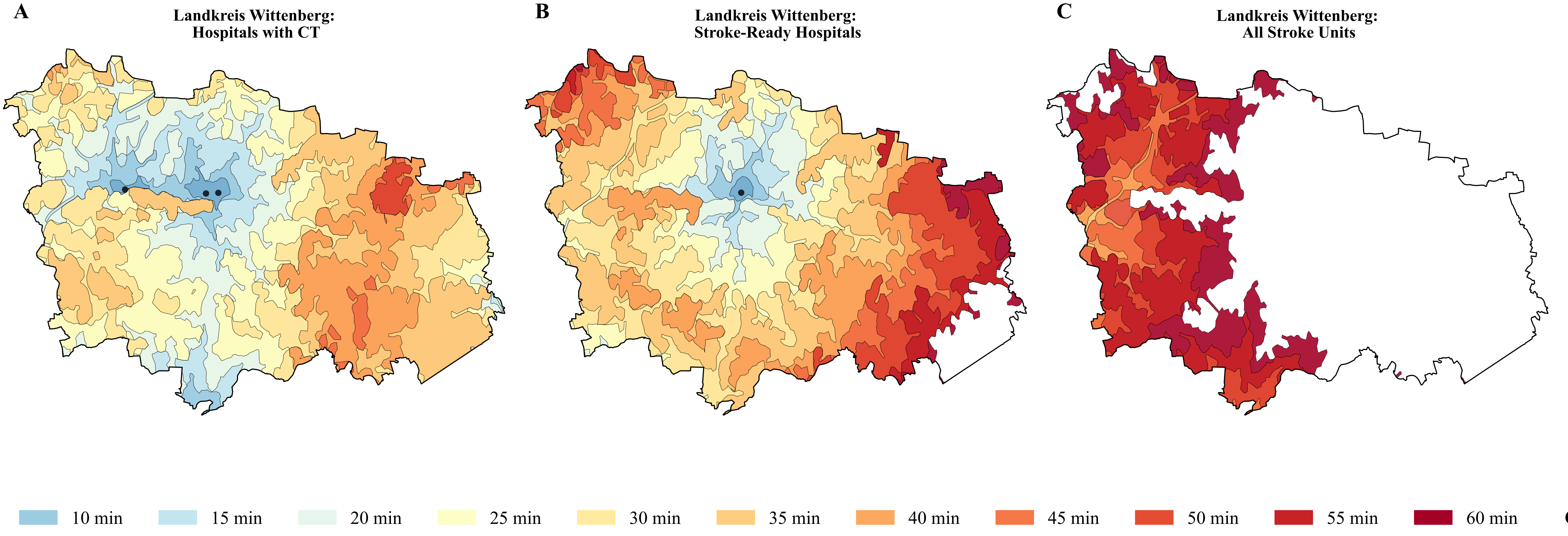

Isochrones — Landkreis Saale-Holzland-Kreis (Thüringen)

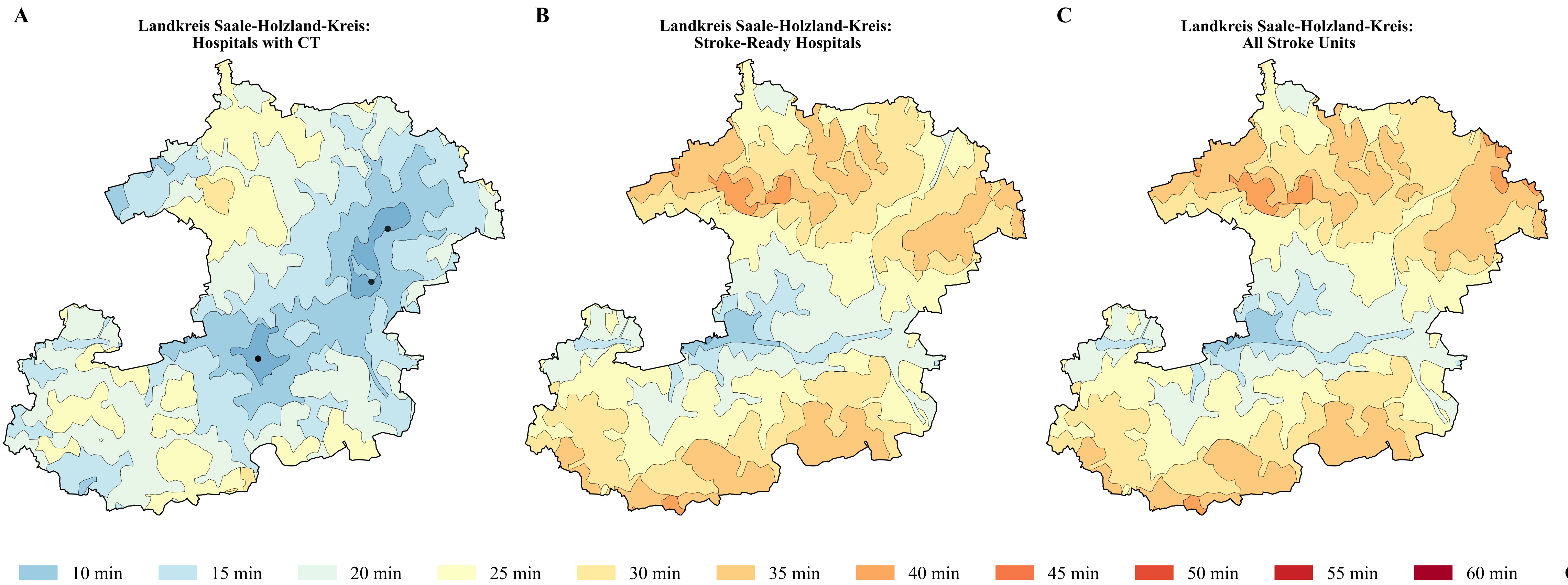

Isochrones — Kreisfreie Stadt Salzgitter (Niedersachsen)

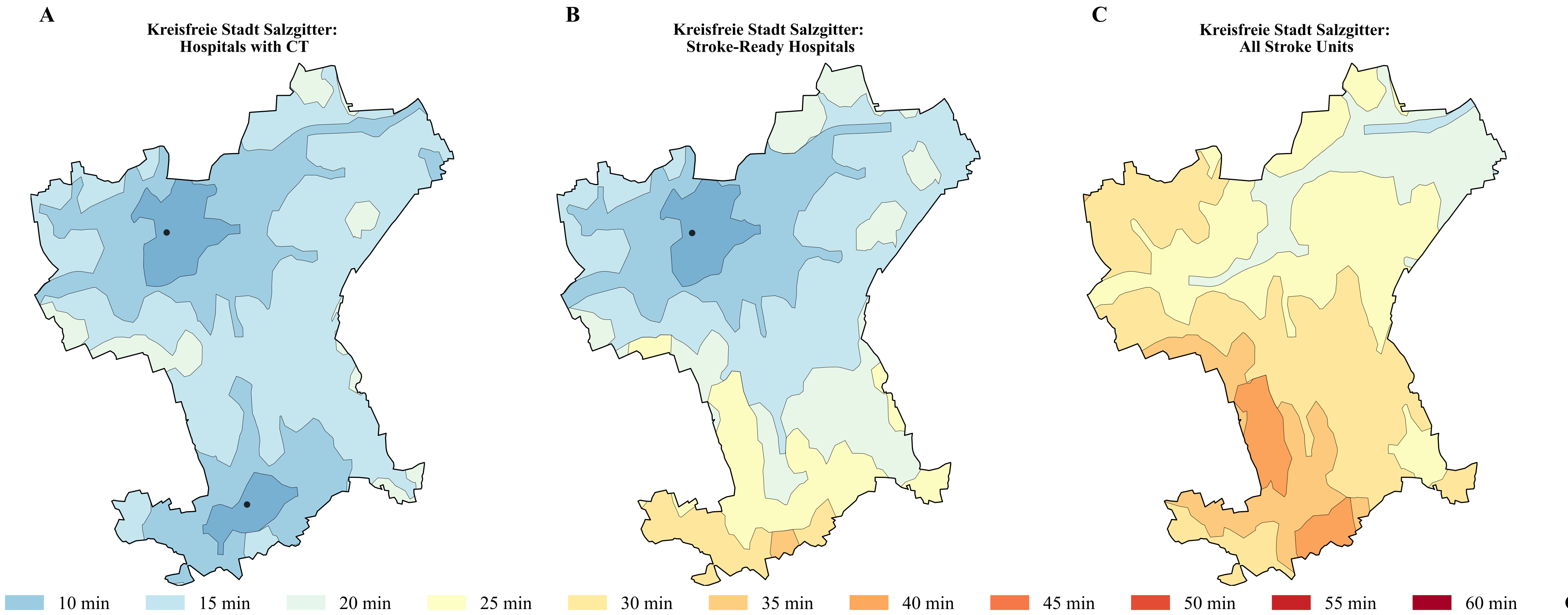

Isochrones — Kreis Wesel (Nordrhein-Westfalen)

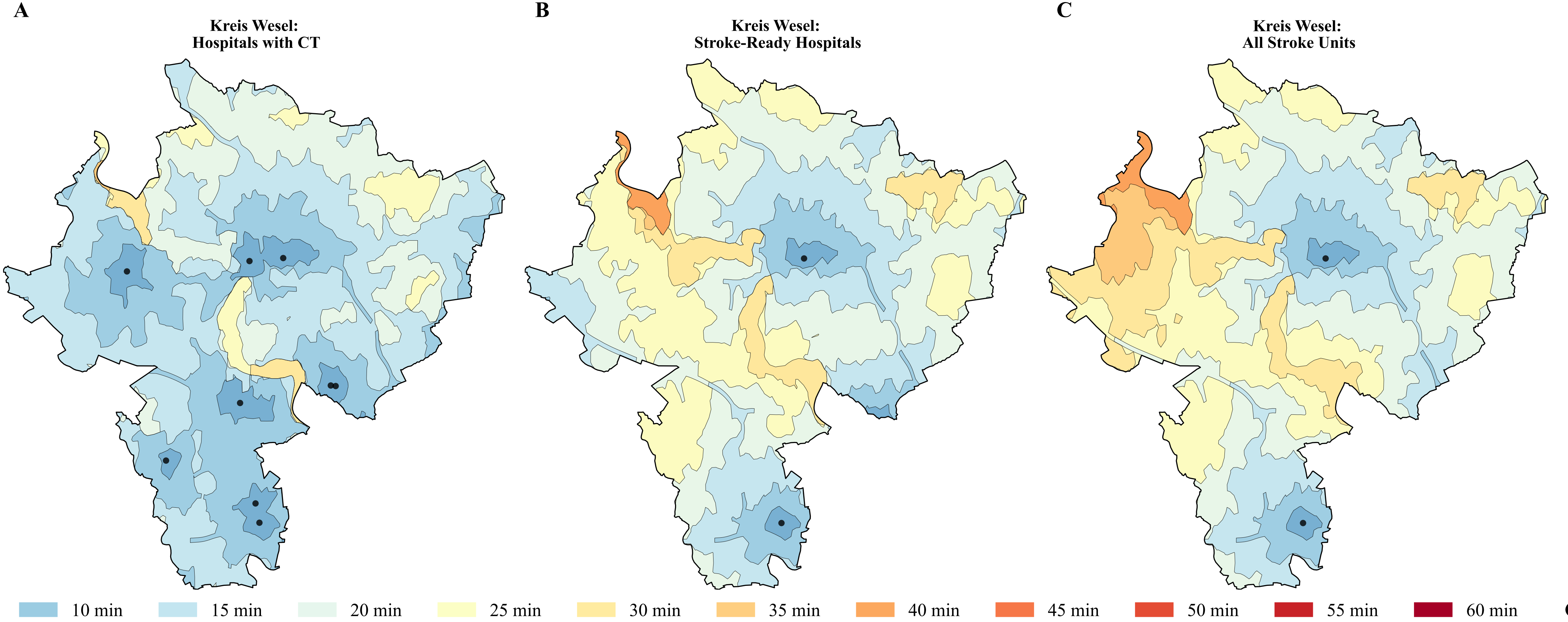

Isochrones — Kreis Rhein-Erft-Kreis (Nordrhein-Westfalen)

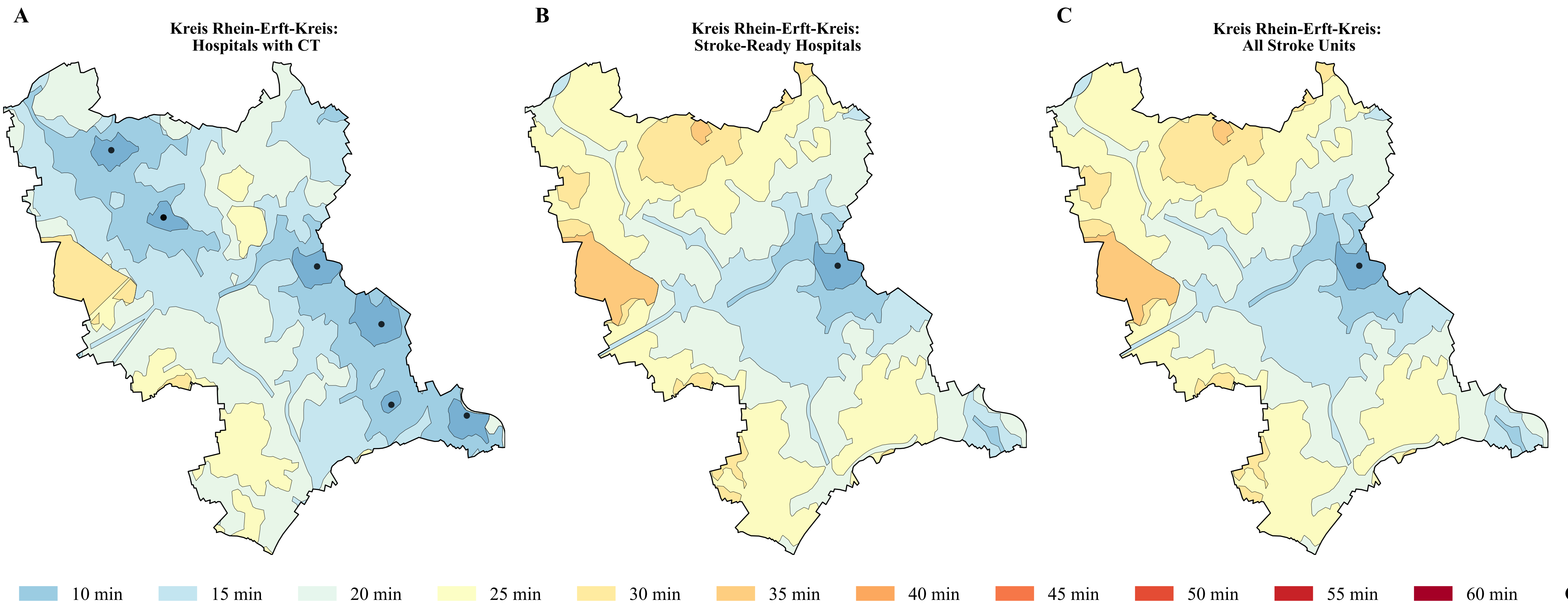

Isochrones — Kreisfreie Stadt Frankfurt am Main (Hessen)

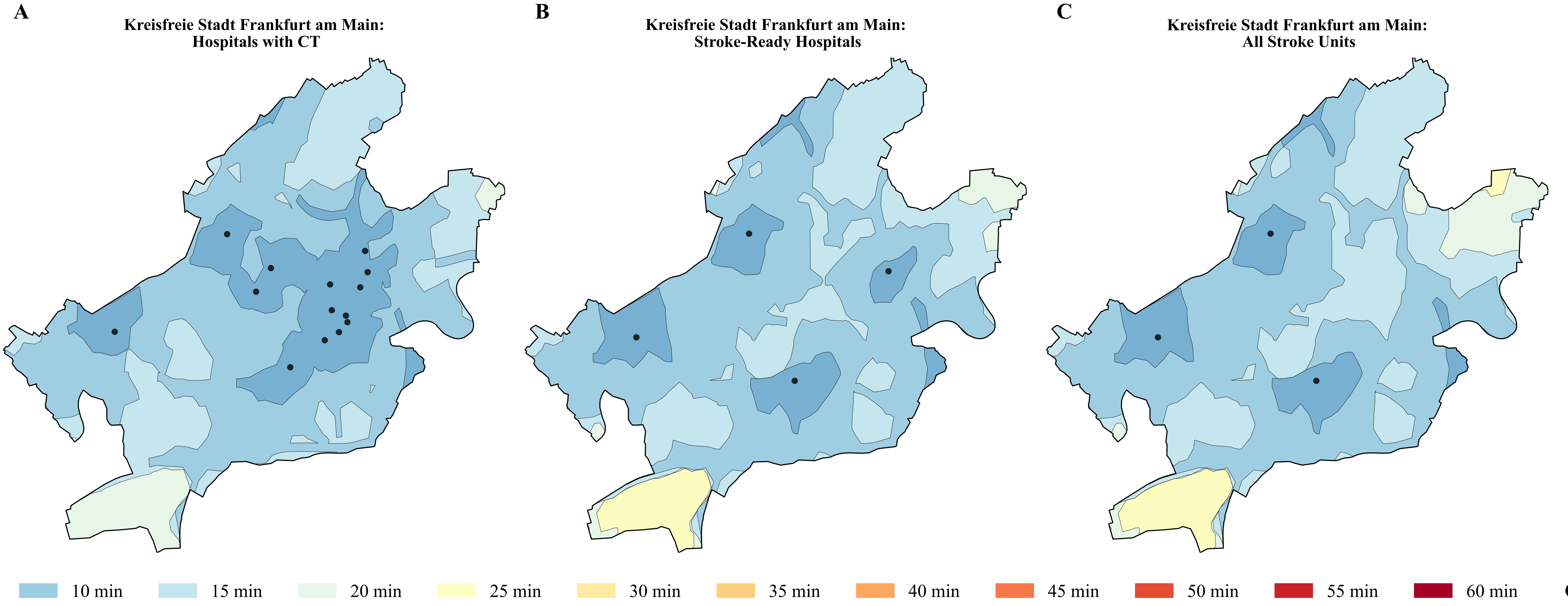

Isochrones — Landkreis Südliche Weinstraße (Rheinland-Pfalz)

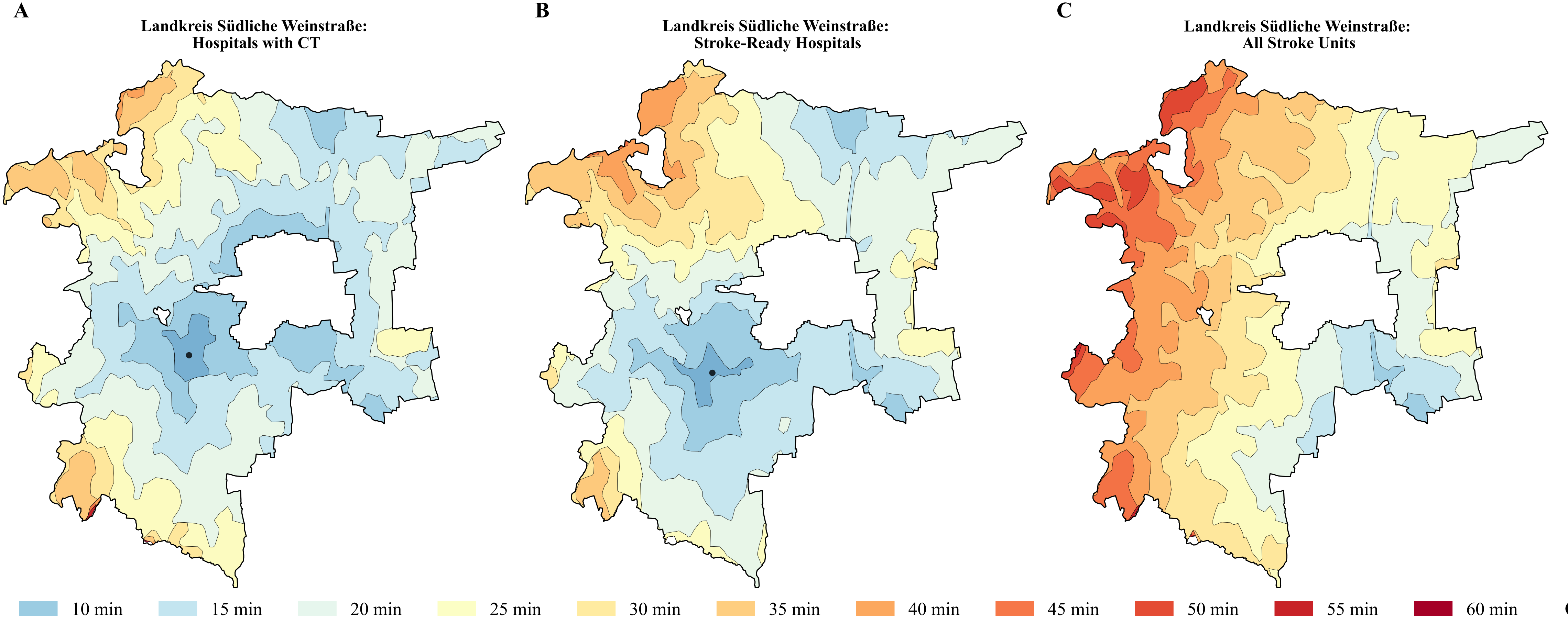

Isochrones — Stadtkreis Karlsruhe (Baden-Württemberg)

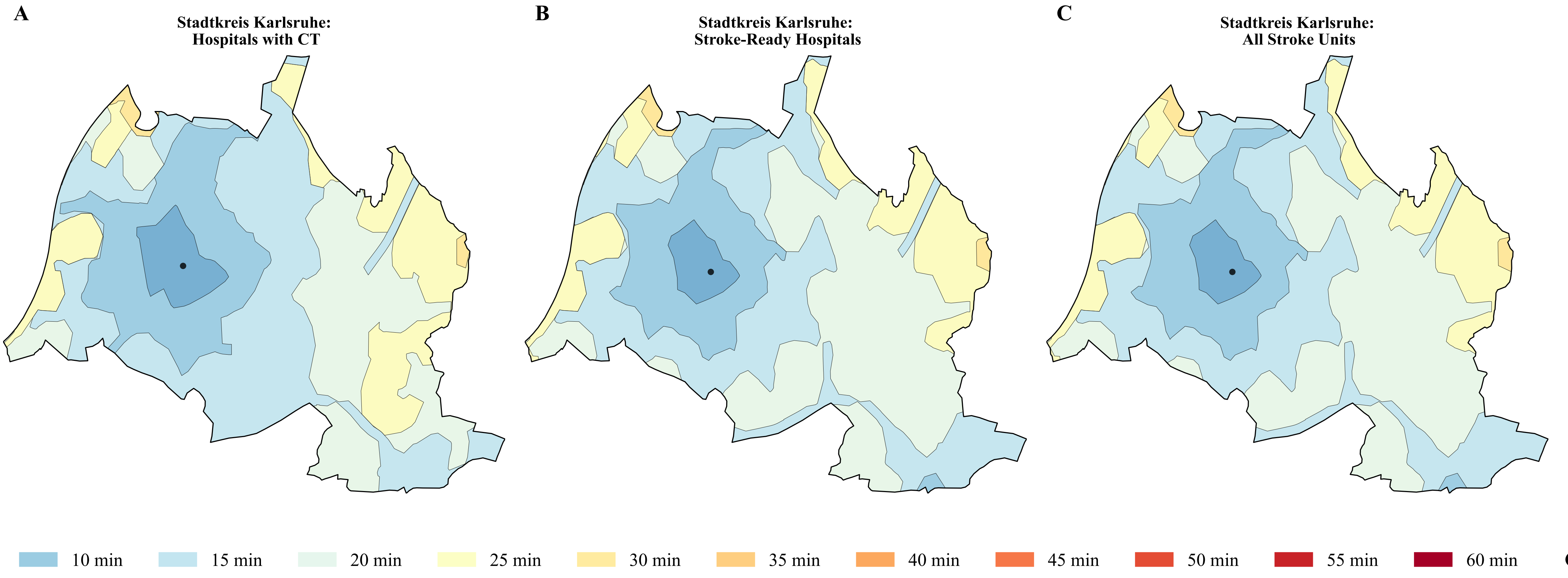

Isochrones — Landkreis Rhein-Neckar-Kreis (Baden-Württemberg)

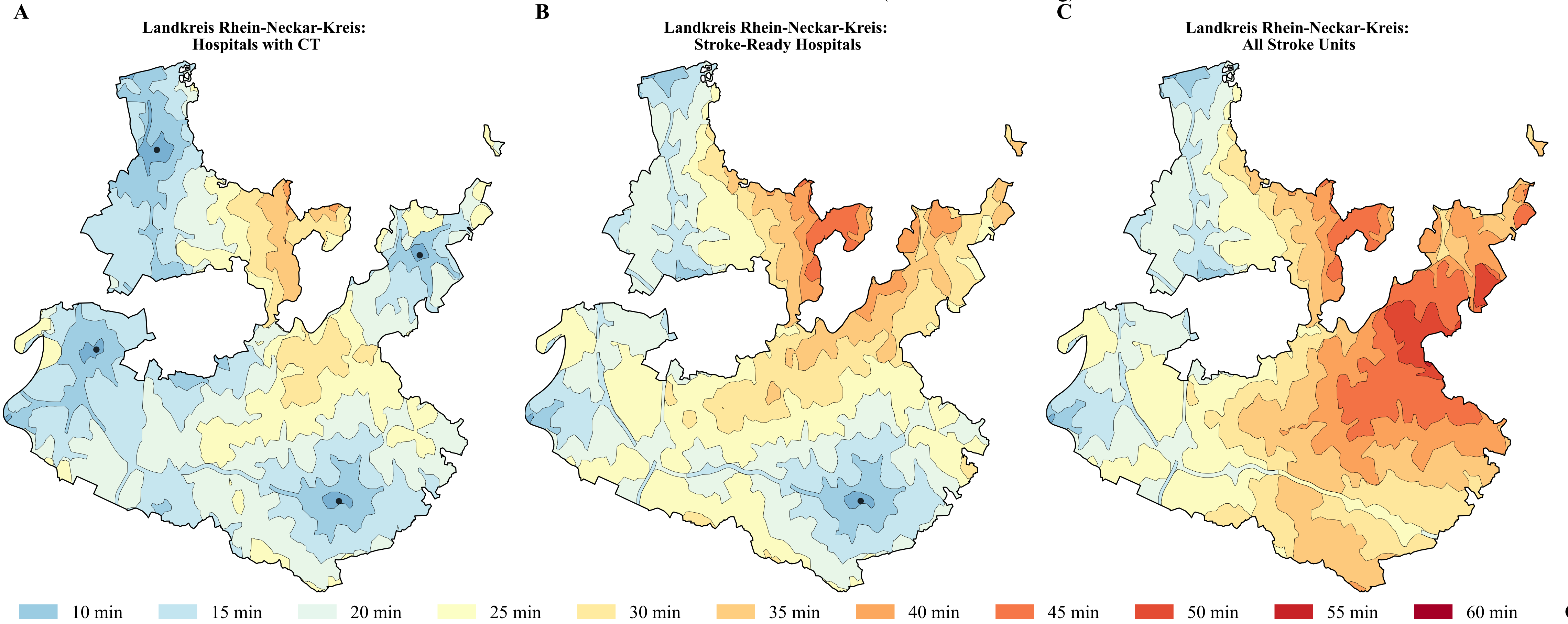

Isochrones — Landkreis München (Bayern)

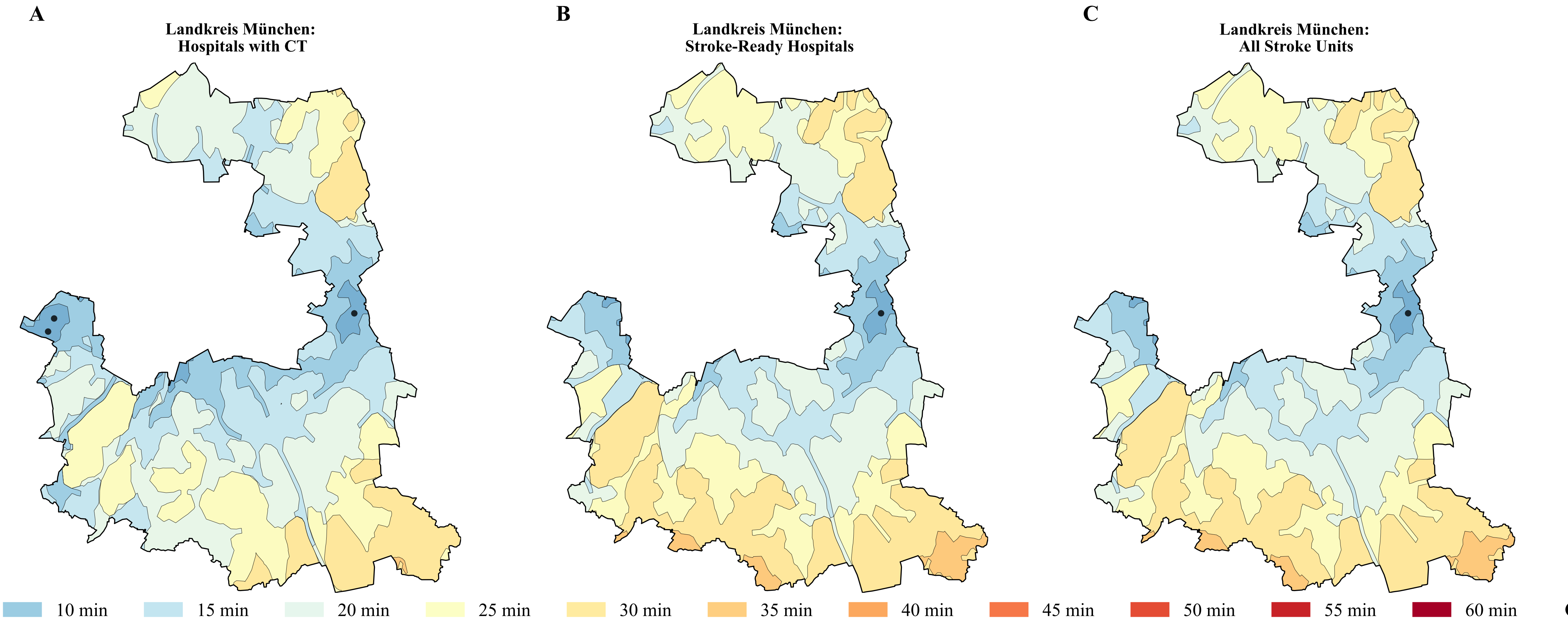

Isochrones — Landkreis Dingolfing-Landau (Bayern)

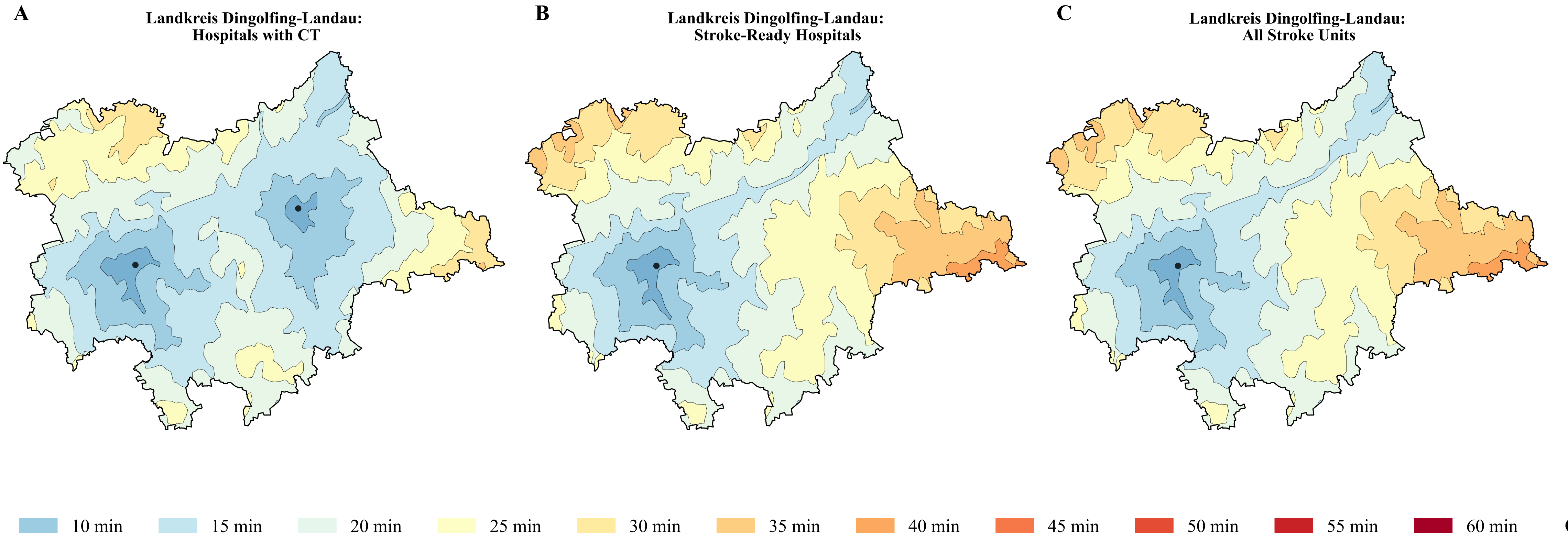

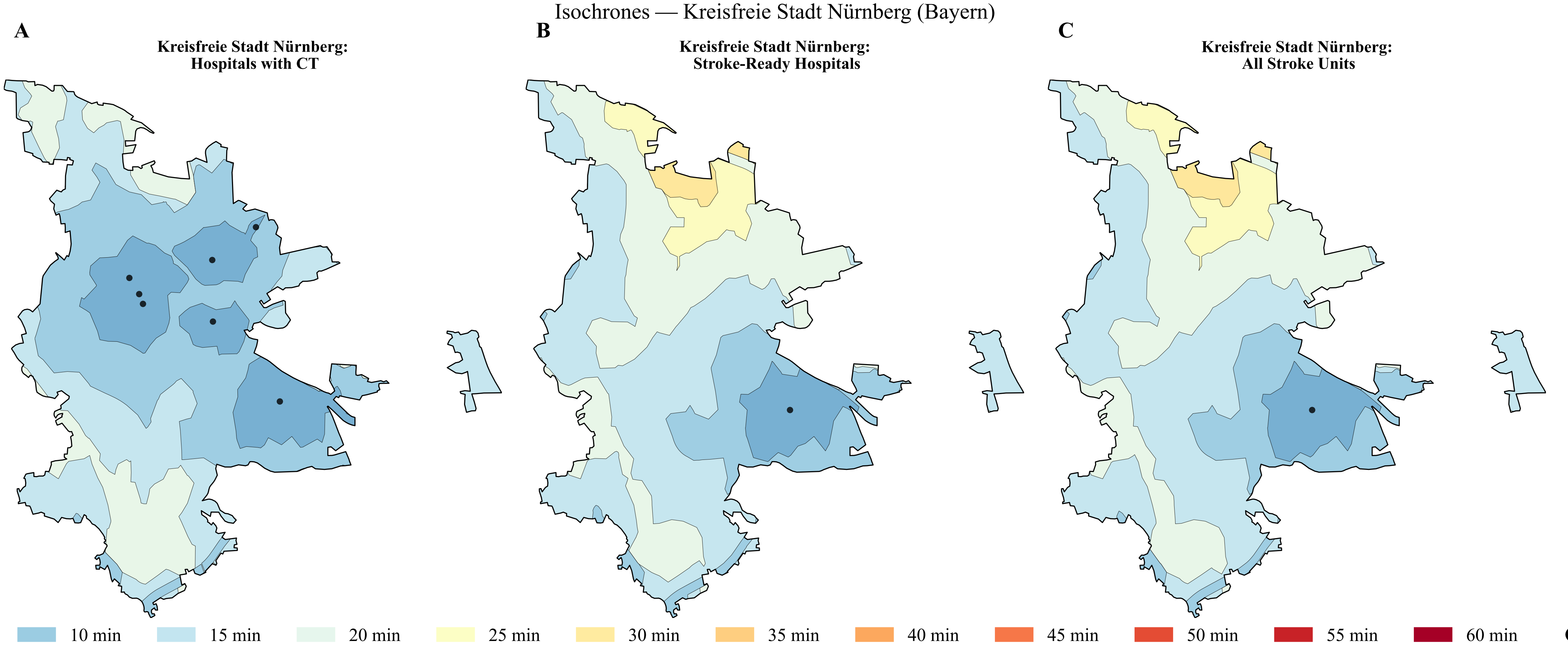
Isochrones — Kreisfreie Stadt Nürnberg (Bayern)
A
Kreisfreie Stadt Nürnberg:
Hospitals with CT
B
Kreisfreie Stadt Nürnberg:
Stroke-Ready Hospitals
C
Kreisfreie Stadt Nürnberg:
All Stroke Units
10 min
15 min
20 min
25 min
30 min
35 min
40 min
45 min
50 min
55 min
60 min

Isochrones — Kreisfreie Stadt Cottbus (Brandenburg)

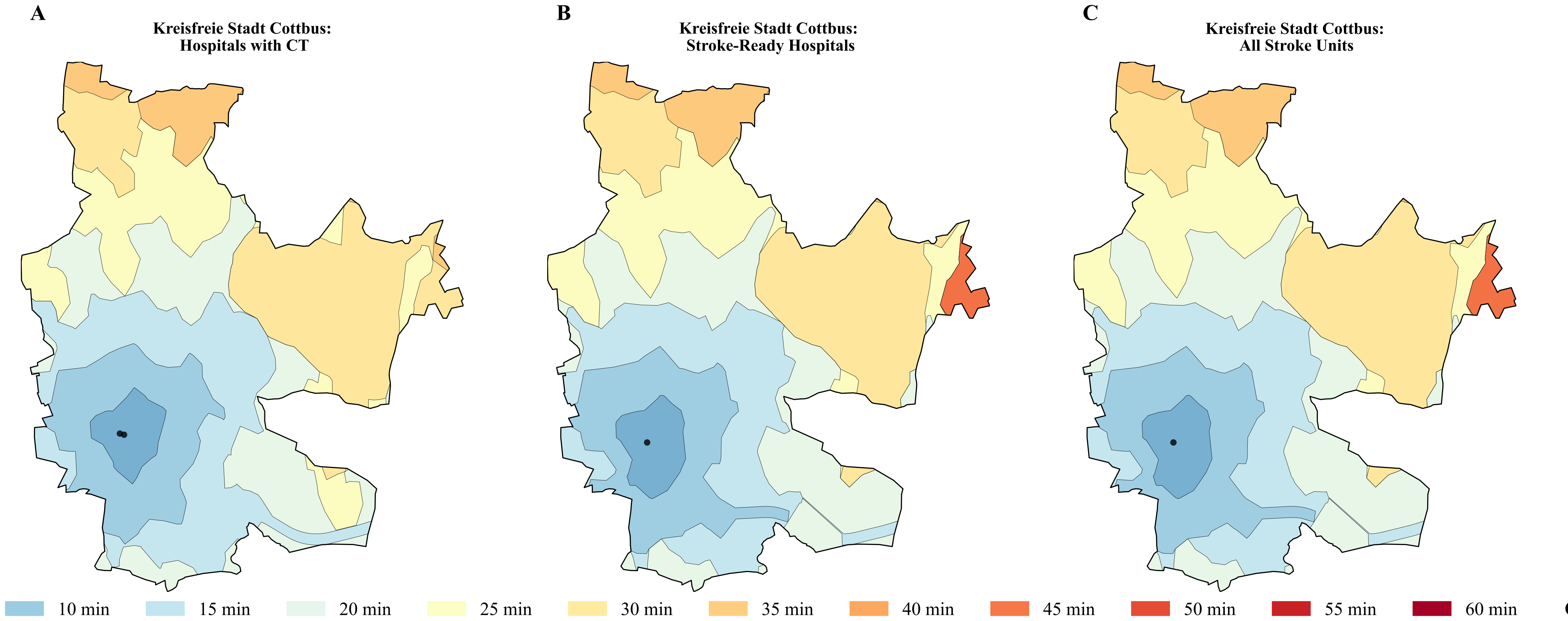

Isochrones — Landkreis Salzlandkreis (Sachsen-Anhalt)

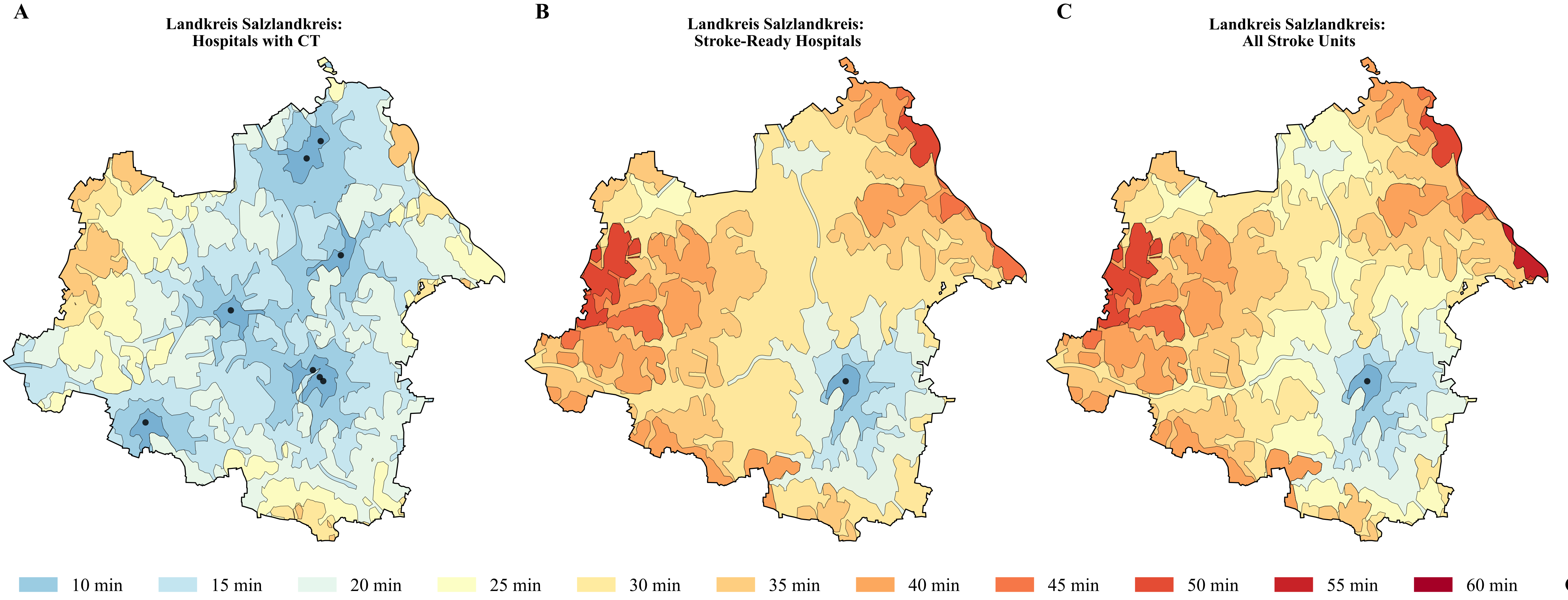

Isochrones — Landkreis Sömmerda (Thüringen)

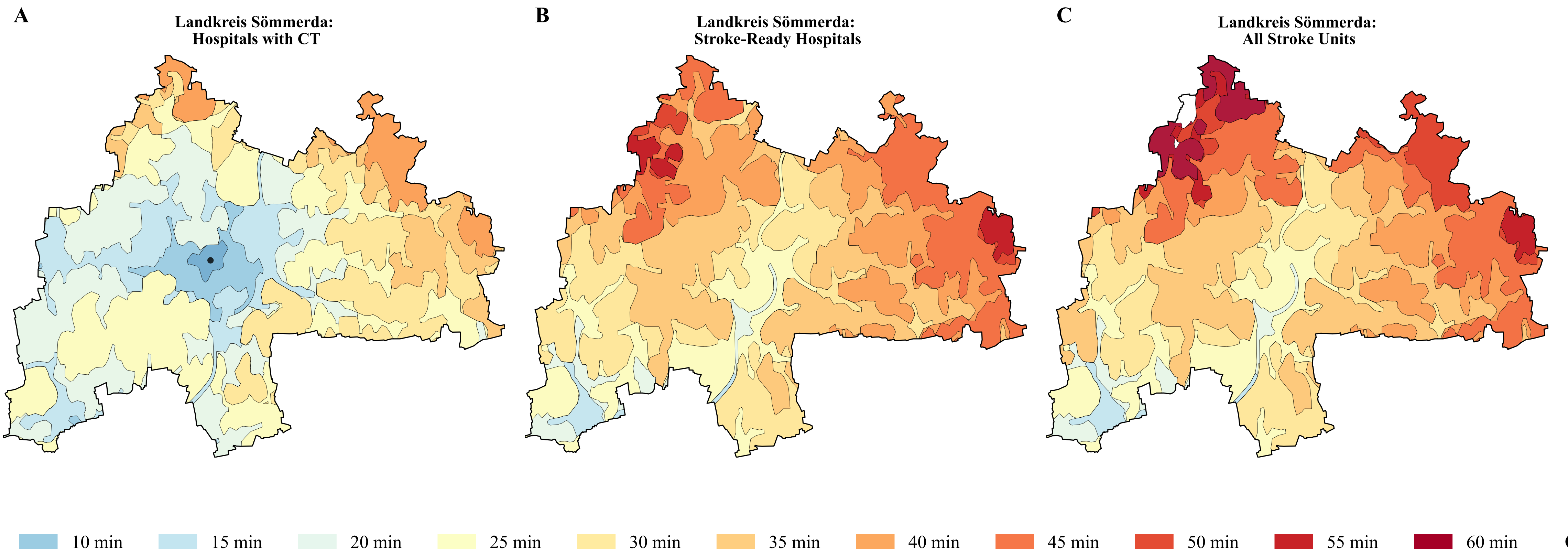

Isochrones — Landkreis Saale-Orla-Kreis (Thüringen)

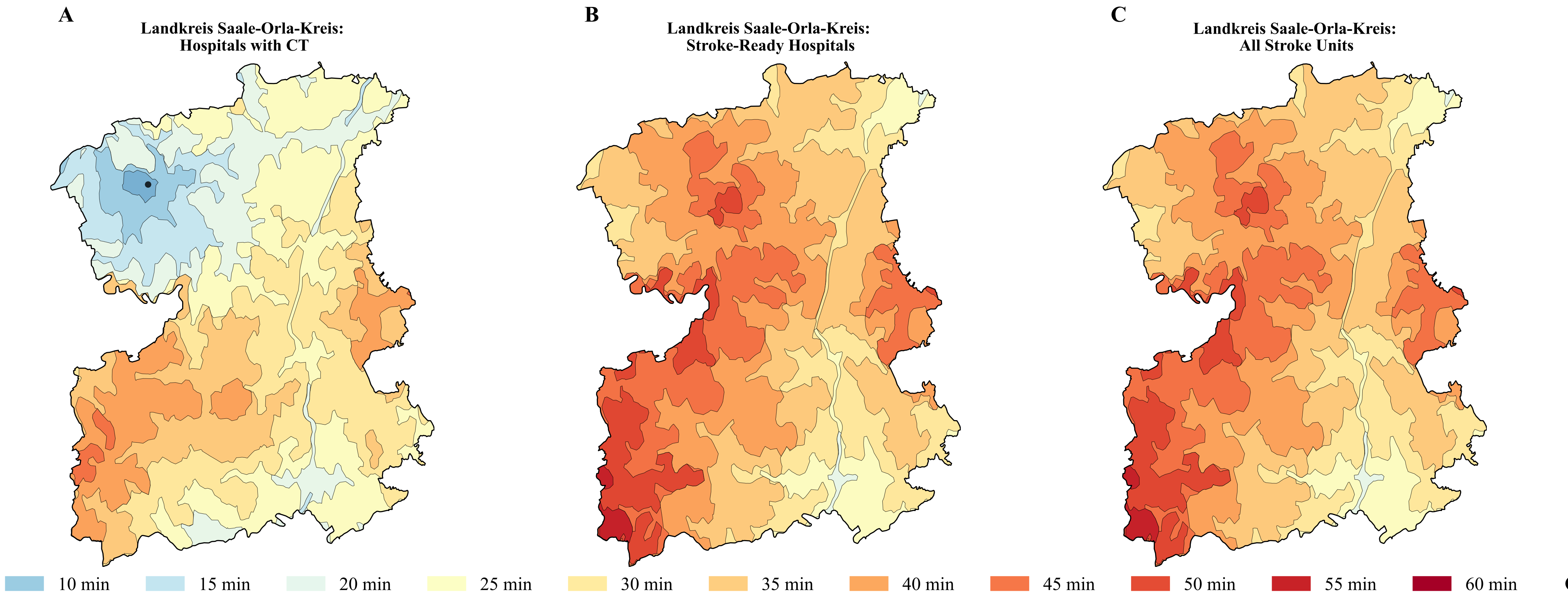

Supplement S7: Rurality/Urbanity Analysis of Resulting isochrones

Facility type	Time isochrone (min)	Total area (km²)	Urban Area (km²)	% German Urban Area Covered	Rural Area (km²)	% German Rural Area Covered	Other Area (km²)	% German Other Area Covered	Area Urban (% isochrone area)	Area Rural (% isochrone area)	Area Other (% isochrone area)
CT Hospitals	15	130 696	27 637	78·8	103 043	32·1	16	1.2	21·1	78·8	0·0
CT Hospitals	30	320 046	34 892	99·4	284 712	89·5	442	33.4	10 9	89·0	0·1
CT Hospitals	45	352 069	35 130	100	316 190	98·4	749	56.5	10·0	89·8	0·2
CT Hospitals	60	354 669	35 148	100	318 433	99·2	1 088	82.1	9·9	89·8	0·3
Stroke-Ready Hospitals	15	52 364	17 224	49·1	35 135	14·6	5	0.4	32·9	67·1	0·0
Stroke-Ready Hospitals	30	226 106	32 311	92·2	193 531	63·2	264	19.9	14·3	85·6	0·1
Stroke-Ready Hospitals	45	320 313	34 684	98·8	285 012	89·6	617	46.6	10·8	89·0	0·2
Stroke-Ready Hospitals	60	347 795	35 076	99·9	311 941	97·2	778	58.7%	10·1	89·7	0·2
Stroke Units	15	41 372	15 088	43·0	26 280	11·6	4	0.3	36·5	63·5	0·0
Stroke Units	30	195 465	30 573	87·1	164 643	54·6	249	18.8	15·6	84·2	0·1
Stroke Units	45	301 211	34 214	97·5	266 443	84·2	554	41.8	11·4	88·5	0·2
Stroke Units	60	338 703	34 922	99·5	303 014	94·7	767	57.9	10·3	89·5	0·2

Germany baseline areas:
Total area in dataset: 357,686 km² - Urban area ≈ 35,094 km² (9.8%) - Rural area ≈ 321,267 km² (89.8%) - Other area ≈ 1,325 km² (0.4%)

Supplemental Table S8: Rurality/Urbanity Analysis of benefit Areas (Normal Speed, Compared to Certified Stroke Units)

Benefit category	Area of Benefit Area Urban (%)	Area of Benefit Area Rural (%)	Area of Benefit Area Other or Uninhabited (%)
High (30+ min)	6·4	93·4	0·1
Likely irrelevant (<10 min)	13·5	86·3	0·2
Low (10-20 min)	8·3	91·5	0·2
Medium (20-30 min)	5·9	93·9	0·1
Neither reachable within 60 min	2·9	84·5	12·7
Only CT reachable within 60 min	1·2	98·0	0·8

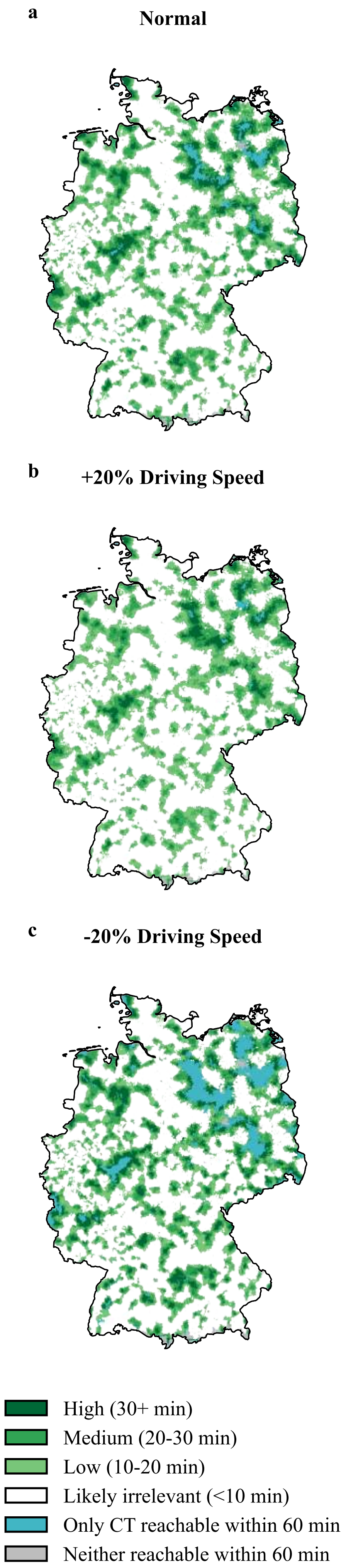
a
Normal
b
+20% Driving Speed
c
-20% Driving Speed
High (30+ min)
Medium (20-30 min)
Low (10-20 min)
Likely irrelevant (<10 min)
Only CT reachable within 60 min
Neither reachable within 60 min

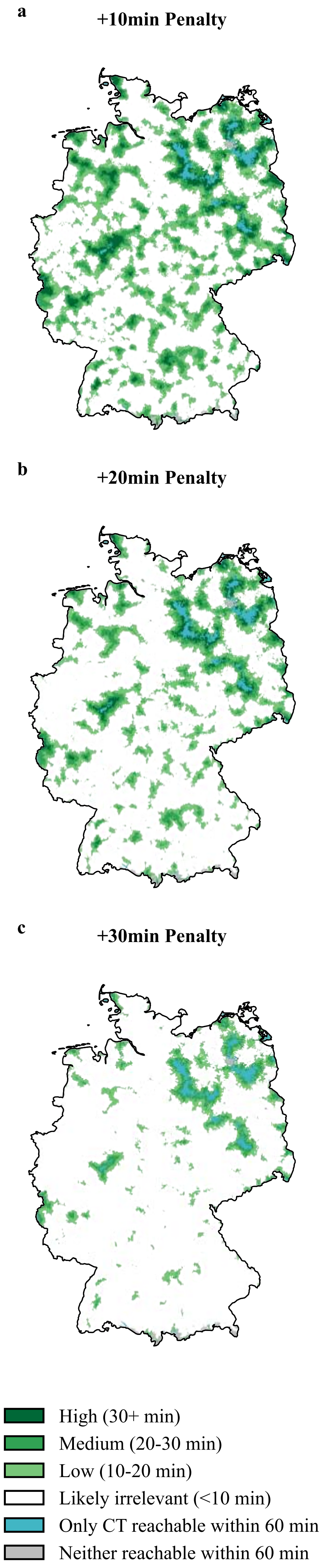
a
+10min Penalty
b
+20min Penalty
c
+30min Penalty
High (30+ min)
Medium (20-30 min)
Low (10-20 min)
Likely irrelevant (<10 min)
Only CT reachable within 60 min
Neither reachable within 60 min

Population and Area Coverage in Berlin (Stroke-Ready Hospitals) — Extended (+10min Penalty)

Category	Grid Cells	Area (%)	Population	Population (%)
High (30+ min)	0	0.0	0	0.0
Medium (20-30 min)	1	0.1	514	0.0
Low (10-20 min)	56	4.8	29176	0.8
Only CT reachable within 60 min	0	0.0	0	0.0
Neither reachable within 60 min	0	0.0	0	0.0
Likely irrelevant (<10 min)	1119	95.2	3583736	99.2

Population and Area Coverage in Baden-Württemberg (Stroke-Ready Hospitals) — Extended (+10min Penalty)

Category	Grid Cells	Area (%)	Population	Population (%)
High (30+ min)	640	1.5	41809	0.4
Medium (20-30 min)	3035	7.0	345259	3.2
Low (10-20 min)	8696	20.0	1655056	15.3
Only CT reachable within 60 min	43	0.1	2181	0.0
Neither reachable within 60 min	33	0.1	1470	0.0
Likely irrelevant (<10 min)	30931	71.3	8787370	81.1

Population and Area Coverage in Bremen (Stroke-Ready Hospitals) — Extended (+10min Penalty)

Category	Grid Cells	Area (%)	Population	Population (%)
High (30+ min)	0	0.0	0	0.0
Medium (20-30 min)	4	1.0	103	0.0
Low (10-20 min)	71	17.7	17295	3.3
Only CT reachable within 60 min	0	0.0	0	0.0
Neither reachable within 60 min	0	0.0	0	0.0
Likely irrelevant (<10 min)	327	81.3	506505	96.7

Population and Area Coverage in Hamburg (Stroke-Ready Hospitals) — Extended (+10min Penalty)

Category	Grid Cells	Area (%)	Population	Population (%)
High (30+ min)	0	0.0	0	0.0
Medium (20-30 min)	0	0.0	0	0.0
Low (10-20 min)	92	8.8	75373	4.1
Only CT reachable within 60 min	0	0.0	0	0.0
Neither reachable within 60 min	10	1.0	6092	0.3
Likely irrelevant (<10 min)	942	90.2	1761117	95.6

Population and Area Coverage in Brandenburg (Stroke-Ready Hospitals) — Extended (+10min Penalty)

Category	Grid Cells	Area (%)	Population	Population (%)
High (30+ min)	4105	10.6	203493	8.3
Medium (20-30 min)	6196	15.9	198884	8.1
Low (10-20 min)	9643	24.8	513382	21.0
Only CT reachable within 60 min	2529	6.5	43532	1.8
Neither reachable within 60 min	180	0.5	3399	0.1
Likely irrelevant (<10 min)	16204	41.7	1479292	60.6

Population and Area Coverage in Bayern (Stroke-Ready Hospitals) — Extended (+10min Penalty)

Category	Grid Cells	Area (%)	Population	Population (%)
High (30+ min)	676	0.8	44253	0.3
Medium (20-30 min)	8092	9.4	672226	5.1
Low (10-20 min)	21020	24.5	2092091	15.8
Only CT reachable within 60 min	78	0.1	2188	0.0
Neither reachable within 60 min	2186	2.6	15198	0.1
Likely irrelevant (<10 min)	53641	62.6	10445174	78.7

Population and Area Coverage in Hessen (Stroke-Ready Hospitals) — Extended (+10min Penalty)

Category	Grid Cells	Area (%)	Population	Population (%)
High (30+ min)	1194	4.5	91369	1.5
Medium (20-30 min)	3016	11.3	240916	3.9
Low (10-20 min)	7388	27.6	799670	12.8
Only CT reachable within 60 min	37	0.1	677	0.0
Neither reachable within 60 min	0	0.0	0	0.0
Likely irrelevant (<10 min)	15113	56.5	5121778	81.9

Population and Area Coverage in Mecklenburg-Vorpommern (Stroke-Ready Hospitals) — Extended (+10min Penalty)

Category	Grid Cells	Area (%)	Population	Population (%)
High (30+ min)	3331	10.7	165269	11.3
Medium (20-30 min)	5528	17.7	162339	11.1
Low (10-20 min)	7110	22.8	229848	15.7
Only CT reachable within 60 min	2481	8.0	46235	3.2
Neither reachable within 60 min	976	3.1	13806	0.9
Likely irrelevant (<10 min)	11755	37.7	846498	57.8

Population and Area Coverage in Saarland (Stroke-Ready Hospitals) — Extended (+10min Penalty)

Category	Grid Cells	Area (%)	Population	Population (%)
High (30+ min)	1	0.0	0	0.0
Medium (20-30 min)	152	4.7	12608	1.3
Low (10-20 min)	572	17.8	74423	7.7
Only CT reachable within 60 min	1	0.0	64	0.0
Neither reachable within 60 min	17	0.5	1958	0.2
Likely irrelevant (<10 min)	2477	76.9	881282	90.8

Population and Area Coverage in Rheinland-Pfalz (Stroke-Ready Hospitals) — Extended (+10min Penalty)

Category	Grid Cells	Area (%)	Population	Population (%)
High (30+ min)	1370	5.5	84404	2.0
Medium (20-30 min)	3244	13.0	251679	6.0
Low (10-20 min)	6241	25.1	661092	15.9
Only CT reachable within 60 min	130	0.5	1700	0.0
Neither reachable within 60 min	29	0.1	1114	0.0
Likely irrelevant (<10 min)	13880	55.8	3164296	76.0

Population and Area Coverage in Nordrhein-Westfalen (Stroke-Ready Hospitals) — Extended (+10min Penalty)

Category	Grid Cells	Area (%)	Population	Population (%)
High (30+ min)	1956	4.4	201321	1.1
Medium (20-30 min)	3235	7.3	378839	2.1
Low (10-20 min)	10112	22.9	2153452	11.7
Only CT reachable within 60 min	271	0.6	3238	0.0
Neither reachable within 60 min	116	0.3	8865	0.0
Likely irrelevant (<10 min)	28481	64.5	15621200	85.1

Population and Area Coverage in Niedersachsen (Stroke-Ready Hospitals) — Extended (+10min Penalty)

Category	Grid Cells	Area (%)	Population	Population (%)
High (30+ min)	2645	4.2	213060	2.7
Medium (20-30 min)	8187	13.0	628853	7.8
Low (10-20 min)	14419	22.8	1525232	19.0
Only CT reachable within 60 min	719	1.1	9806	0.1
Neither reachable within 60 min	353	0.6	17274	0.2
Likely irrelevant (<10 min)	36847	58.3	5645637	70.2

Population and Area Coverage in Sachsen-Anhalt (Stroke-Ready Hospitals) — Extended (+10min Penalty)

Category	Grid Cells	Area (%)	Population	Population (%)
High (30+ min)	2574	9.6	105819	5.0
Medium (20-30 min)	4755	17.7	236312	11.2
Low (10-20 min)	6322	23.5	384385	18.3
Only CT reachable within 60 min	1546	5.7	19488	0.9
Neither reachable within 60 min	0	0.0	0	0.0
Likely irrelevant (<10 min)	11739	43.6	1357210	64.5

Population and Area Coverage in Sachsen (Stroke-Ready Hospitals) — Extended (+10min Penalty)

Category	Grid Cells	Area (%)	Population	Population (%)
High (30+ min)	1230	5.3	154755	3.8
Medium (20-30 min)	3988	17.2	390371	9.6
Low (10-20 min)	6142	26.4	659150	16.2
Only CT reachable within 60 min	157	0.7	6222	0.2
Neither reachable within 60 min	102	0.4	656	0.0
Likely irrelevant (<10 min)	11633	50.0	2868351	70.3

Population and Area Coverage in Schleswig-Holstein (Stroke-Ready Hospitals) — Extended (+10min Penalty)

Category	Grid Cells	Area (%)	Population	Population (%)
High (30+ min)	754	3.6	49933	1.8
Medium (20-30 min)	1406	6.8	123275	4.5
Low (10-20 min)	3986	19.3	481250	17.5
Only CT reachable within 60 min	232	1.1	27767	1.0
Neither reachable within 60 min	336	1.6	11988	0.4
Likely irrelevant (<10 min)	13965	67.5	2048062	74.7

Population and Area Coverage in Thüringen (Stroke-Ready Hospitals) — Extended (+10min Penalty)

Category	Grid Cells	Area (%)	Population	Population (%)
High (30+ min)	118	0.6	8087	0.4
Medium (20-30 min)	1431	6.9	111314	5.5
Low (10-20 min)	6126	29.4	385312	19.0
Only CT reachable within 60 min	0	0.0	0	0.0
Neither reachable within 60 min	0	0.0	0	0.0
Likely irrelevant (<10 min)	13144	63.1	1525461	75.1

Population and Area Coverage in Berlin (Stroke Units) — Regular (+10min Penalty)

Category	Grid Cells	Area (%)	Population	Population (%)
High (30+ min)	0	0.0	0	0.0
Medium (20-30 min)	2	0.2	806	0.0
Low (10-20 min)	55	4.7	28884	0.8
Only CT reachable within 60 min	0	0.0	0	0.0
Neither reachable within 60 min	0	0.0	0	0.0
Likely irrelevant (<10 min)	1119	95.2	3583736	99.2

Population and Area Coverage in Baden-Württemberg (Stroke Units) — Regular (+10min Penalty)

Category	Grid Cells	Area (%)	Population	Population (%)
High (30+ min)	2546	5.9	309502	2.9
Medium (20-30 min)	6197	14.3	811790	7.5
Low (10-20 min)	10875	25.1	2128298	19.6
Only CT reachable within 60 min	1362	3.1	101057	0.9
Neither reachable within 60 min	33	0.1	1470	0.0
Likely irrelevant (<10 min)	22365	51.6	7481028	69.1

Population and Area Coverage in Bremen (Stroke Units) — Regular (+10min Penalty)

Category	Grid Cells	Area (%)	Population	Population (%)
High (30+ min)	0	0.0	0	0.0
Medium (20-30 min)	9	2.2	328	0.1
Low (10-20 min)	74	18.4	17755	3.4
Only CT reachable within 60 min	0	0.0	0	0.0
Neither reachable within 60 min	0	0.0	0	0.0
Likely irrelevant (<10 min)	319	79.4	505820	96.5

Population and Area Coverage in Hamburg (Stroke Units) — Regular (+10min Penalty)

Category	Grid Cells	Area (%)	Population	Population (%)
High (30+ min)	0	0.0	0	0.0
Medium (20-30 min)	0	0.0	0	0.0
Low (10-20 min)	92	8.8	75373	4.1
Only CT reachable within 60 min	0	0.0	0	0.0
Neither reachable within 60 min	10	1.0	6092	0.3
Likely irrelevant (<10 min)	942	90.2	1761117	95.6

Population and Area Coverage in Brandenburg (Stroke Units) — Regular (+10min Penalty)

Category	Grid Cells	Area (%)	Population	Population (%)
High (30+ min)	5730	14.7	308425	12.6
Medium (20-30 min)	6951	17.9	251403	10.3
Low (10-20 min)	9485	24.4	553304	22.7
Only CT reachable within 60 min	6658	17.1	175853	7.2
Neither reachable within 60 min	180	0.5	3399	0.1
Likely irrelevant (<10 min)	9853	25.4	1149598	47.1

Population and Area Coverage in Bayern (Stroke Units) — Regular (+10min Penalty)

Category	Grid Cells	Area (%)	Population	Population (%)
High (30+ min)	1214	1.4	75296	0.6
Medium (20-30 min)	10104	11.8	854975	6.4
Low (10-20 min)	22122	25.8	2273112	17.1
Only CT reachable within 60 min	159	0.2	2872	0.0
Neither reachable within 60 min	2186	2.6	15198	0.1
Likely irrelevant (<10 min)	49908	58.2	10049677	75.7

Population and Area Coverage in Hessen (Stroke Units) — Regular (+10min Penalty)

Category	Grid Cells	Area (%)	Population	Population (%)
High (30+ min)	2525	9.4	184500	2.9
Medium (20-30 min)	4379	16.4	388447	6.2
Low (10-20 min)	6922	25.9	861825	13.8
Only CT reachable within 60 min	185	0.7	6027	0.1
Neither reachable within 60 min	0	0.0	0	0.0
Likely irrelevant (<10 min)	12737	47.6	4813611	77.0

Population and Area Coverage in Mecklenburg-Vorpommern (Stroke Units) — Regular (+10min Penalty)

Category	Grid Cells	Area (%)	Population	Population (%)
High (30+ min)	3410	10.9	164913	11.3
Medium (20-30 min)	5551	17.8	165098	11.3
Low (10-20 min)	6961	22.3	243014	16.6
Only CT reachable within 60 min	3131	10.0	54174	3.7
Neither reachable within 60 min	977	3.1	13820	0.9
Likely irrelevant (<10 min)	11151	35.8	822976	56.2

Population and Area Coverage in Saarland (Stroke Units) — Regular (+10min Penalty)

Category	Grid Cells	Area (%)	Population	Population (%)
High (30+ min)	1	0.0	0	0.0
Medium (20-30 min)	154	4.8	12874	1.3
Low (10-20 min)	570	17.7	74158	7.6
Only CT reachable within 60 min	1	0.0	64	0.0
Neither reachable within 60 min	17	0.5	1958	0.2
Likely irrelevant (<10 min)	2477	76.9	881281	90.8

Population and Area Coverage in Rheinland-Pfalz (Stroke Units) — Regular (+10min Penalty)

Category	Grid Cells	Area (%)	Population	Population (%)
High (30+ min)	1698	6.8	112306	2.7
Medium (20-30 min)	4426	17.8	410371	9.9
Low (10-20 min)	7812	31.4	957321	23.0
Only CT reachable within 60 min	172	0.7	2166	0.1
Neither reachable within 60 min	30	0.1	1114	0.0
Likely irrelevant (<10 min)	10756	43.2	2681007	64.4

Population and Area Coverage in Nordrhein-Westfalen (Stroke Units) — Regular (+10min Penalty)

Category	Grid Cells	Area (%)	Population	Population (%)
High (30+ min)	2438	5.5	283059	1.5
Medium (20-30 min)	5435	12.3	820578	4.5
Low (10-20 min)	11851	26.8	2840119	15.5
Only CT reachable within 60 min	340	0.8	7778	0.0
Neither reachable within 60 min	116	0.3	8865	0.0
Likely irrelevant (<10 min)	23991	54.3	14406516	78.4

Population and Area Coverage in Niedersachsen (Stroke Units) — Regular (+10min Penalty)

Category	Grid Cells	Area (%)	Population	Population (%)
High (30+ min)	4827	7.6	393465	4.9
Medium (20-30 min)	11108	17.6	935173	11.6
Low (10-20 min)	17513	27.7	1985236	24.7
Only CT reachable within 60 min	1333	2.1	24361	0.3
Neither reachable within 60 min	354	0.6	17333	0.2
Likely irrelevant (<10 min)	28035	44.4	4684294	58.3

Population and Area Coverage in Sachsen-Anhalt (Stroke Units) — Regular (+10min Penalty)

Category	Grid Cells	Area (%)	Population	Population (%)
High (30+ min)	4035	15.0	274982	13.1
Medium (20-30 min)	4882	18.1	355102	16.9
Low (10-20 min)	6015	22.3	391360	18.6
Only CT reachable within 60 min	5810	21.6	139112	6.6
Neither reachable within 60 min	0	0.0	0	0.0
Likely irrelevant (<10 min)	6194	23.0	942658	44.8

Population and Area Coverage in Sachsen (Stroke Units) — Regular (+10min Penalty)

Category	Grid Cells	Area (%)	Population	Population (%)
High (30+ min)	2263	9.7	246830	6.1
Medium (20-30 min)	4642	20.0	497455	12.2
Low (10-20 min)	6045	26.0	811202	19.9
Only CT reachable within 60 min	441	1.9	19215	0.5
Neither reachable within 60 min	104	0.4	655	0.0
Likely irrelevant (<10 min)	9757	42.0	2504148	61.4

Population and Area Coverage in Schleswig-Holstein (Stroke Units) — Regular (+10min Penalty)

Category	Grid Cells	Area (%)	Population	Population (%)
High (30+ min)	765	3.7	50134	1.8
Medium (20-30 min)	1414	6.8	123712	4.5
Low (10-20 min)	4013	19.4	481612	17.6
Only CT reachable within 60 min	236	1.1	27810	1.0
Neither reachable within 60 min	343	1.7	12125	0.4
Likely irrelevant (<10 min)	13908	67.3	2046882	74.6

Population and Area Coverage in Thüringen (Stroke Units) — Regular (+10min Penalty)

Category	Grid Cells	Area (%)	Population	Population (%)
High (30+ min)	559	2.7	23669	1.2
Medium (20-30 min)	2757	13.2	203622	10.0
Low (10-20 min)	6251	30.0	394398	19.4
Only CT reachable within 60 min	16	0.1	6	0.0
Neither reachable within 60 min	0	0.0	0	0.0
Likely irrelevant (<10 min)	11236	54.0	1408479	69.4

Population and Area Coverage in Berlin (Stroke-Ready Hospitals) — Extended (+20% Speed (Emergency))

Category	Grid Cells	Area (%)	Population	Population (%)
High (30+ min)	0	0.0	0	0.0
Medium (20-30 min)	3	0.3	5339	0.1
Low (10-20 min)	144	12.2	309317	8.6
Only CT reachable within 60 min	0	0.0	0	0.0
Neither reachable within 60 min	0	0.0	0	0.0
Likely irrelevant (<10 min)	1029	87.5	3298770	91.3

Population and Area Coverage in Baden-Württemberg (Stroke-Ready Hospitals) — Extended (+20% Speed (Emergency))

Category	Grid Cells	Area (%)	Population	Population (%)
High (30+ min)	276	0.6	43327	0.4
Medium (20-30 min)	2521	5.8	395087	3.6
Low (10-20 min)	8733	20.1	1785431	16.5
Only CT reachable within 60 min	3	0.0	245	0.0
Neither reachable within 60 min	33	0.1	1479	0.0
Likely irrelevant (<10 min)	31812	73.3	8607576	79.5

Population and Area Coverage in Bremen (Stroke-Ready Hospitals) — Extended (+20% Speed (Emergency))

Category	Grid Cells	Area (%)	Population	Population (%)
High (30+ min)	0	0.0	0	0.0
Medium (20-30 min)	2	0.5	1316	0.3
Low (10-20 min)	60	14.9	69999	13.4
Only CT reachable within 60 min	0	0.0	0	0.0
Neither reachable within 60 min	0	0.0	0	0.0
Likely irrelevant (<10 min)	340	84.6	452588	86.4

Population and Area Coverage in Hamburg (Stroke-Ready Hospitals) — Extended (+20% Speed (Emergency))

Category	Grid Cells	Area (%)	Population	Population (%)
High (30+ min)	0	0.0	0	0.0
Medium (20-30 min)	0	0.0	0	0.0
Low (10-20 min)	127	12.2	152986	8.3
Only CT reachable within 60 min	0	0.0	0	0.0
Neither reachable within 60 min	10	1.0	6092	0.3
Likely irrelevant (<10 min)	907	86.9	1683504	91.4

Population and Area Coverage in Brandenburg (Stroke-Ready Hospitals) — Extended (+20% Speed (Emergency))

Category	Grid Cells	Area (%)	Population	Population (%)
High (30+ min)	3314	8.5	197529	8.1
Medium (20-30 min)	6574	16.9	249723	10.2
Low (10-20 min)	10893	28.0	563596	23.1
Only CT reachable within 60 min	411	1.1	2663	0.1
Neither reachable within 60 min	172	0.4	3319	0.1
Likely irrelevant (<10 min)	17493	45.0	1425152	58.4

Population and Area Coverage in Bayern (Stroke-Ready Hospitals) — Extended (+20% Speed (Emergency))

Category	Grid Cells	Area (%)	Population	Population (%)
High (30+ min)	185	0.2	44410	0.3
Medium (20-30 min)	6135	7.2	727320	5.5
Low (10-20 min)	21840	25.5	2762356	20.8
Only CT reachable within 60 min	50	0.1	3597	0.0
Neither reachable within 60 min	2163	2.5	13796	0.1
Likely irrelevant (<10 min)	55320	64.6	9719651	73.2

Population and Area Coverage in Hessen (Stroke-Ready Hospitals) — Extended (+20% Speed (Emergency))

Category	Grid Cells	Area (%)	Population	Population (%)
High (30+ min)	753	2.8	75673	1.2
Medium (20-30 min)	2608	9.8	249411	4.0
Low (10-20 min)	7889	29.5	952325	15.2
Only CT reachable within 60 min	0	0.0	0	0.0
Neither reachable within 60 min	0	0.0	0	0.0
Likely irrelevant (<10 min)	15498	57.9	4977001	79.6

Population and Area Coverage in Mecklenburg-Vorpommern (Stroke-Ready Hospitals) — Extended (+20% Speed (Emergency))

Category	Grid Cells	Area (%)	Population	Population (%)
High (30+ min)	2909	9.3	162247	11.1
Medium (20-30 min)	5411	17.4	166648	11.4
Low (10-20 min)	8532	27.4	234479	16.0
Only CT reachable within 60 min	986	3.2	16600	1.1
Neither reachable within 60 min	495	1.6	10401	0.7
Likely irrelevant (<10 min)	12848	41.2	873620	59.7

Population and Area Coverage in Saarland (Stroke-Ready Hospitals) — Extended (+20% Speed (Emergency))

Category	Grid Cells	Area (%)	Population	Population (%)
High (30+ min)	1	0.0	0	0.0
Medium (20-30 min)	83	2.6	10084	1.0
Low (10-20 min)	548	17.0	76672	7.9
Only CT reachable within 60 min	1	0.0	102	0.0
Neither reachable within 60 min	16	0.5	1976	0.2
Likely irrelevant (<10 min)	2571	79.8	881501	90.8

Population and Area Coverage in Rheinland-Pfalz (Stroke-Ready Hospitals) — Extended (+20% Speed (Emergency))

Category	Grid Cells	Area (%)	Population	Population (%)
High (30+ min)	707	2.8	50900	1.2
Medium (20-30 min)	3071	12.3	299592	7.2
Low (10-20 min)	6602	26.5	784262	18.8
Only CT reachable within 60 min	3	0.0	2	0.0
Neither reachable within 60 min	27	0.1	1114	0.0
Likely irrelevant (<10 min)	14484	58.2	3028415	72.7

Population and Area Coverage in Nordrhein-Westfalen (Stroke-Ready Hospitals) — Extended (+20% Speed (Emergency))

Category	Grid Cells	Area (%)	Population	Population (%)
High (30+ min)	1318	3.0	160504	0.9
Medium (20-30 min)	3230	7.3	490731	2.7
Low (10-20 min)	11092	25.1	3586975	19.5
Only CT reachable within 60 min	25	0.1	670	0.0
Neither reachable within 60 min	118	0.3	8358	0.0
Likely irrelevant (<10 min)	28388	64.3	14119677	76.9

Population and Area Coverage in Niedersachsen (Stroke-Ready Hospitals) — Extended (+20% Speed (Emergency))

Category	Grid Cells	Area (%)	Population	Population (%)
High (30+ min)	1609	2.5	157631	2.0
Medium (20-30 min)	6863	10.9	710765	8.8
Low (10-20 min)	15932	25.2	1676918	20.9
Only CT reachable within 60 min	153	0.2	3942	0.0
Neither reachable within 60 min	343	0.5	16681	0.2
Likely irrelevant (<10 min)	38270	60.6	5473925	68.1

Population and Area Coverage in Sachsen-Anhalt (Stroke-Ready Hospitals) — Extended (+20% Speed (Emergency))

Category	Grid Cells	Area (%)	Population	Population (%)
High (30+ min)	2190	8.1	95123	4.5
Medium (20-30 min)	4702	17.5	277669	13.2
Low (10-20 min)	7602	28.2	379193	18.0
Only CT reachable within 60 min	172	0.6	668	0.0
Neither reachable within 60 min	0	0.0	0	0.0
Likely irrelevant (<10 min)	12270	45.6	1350561	64.2

Population and Area Coverage in Sachsen (Stroke-Ready Hospitals) — Extended (+20% Speed (Emergency))

Category	Grid Cells	Area (%)	Population	Population (%)
High (30+ min)	704	3.0	120902	3.0
Medium (20-30 min)	3390	14.6	408865	10.0
Low (10-20 min)	6856	29.5	837305	20.5
Only CT reachable within 60 min	30	0.1	833	0.0
Neither reachable within 60 min	96	0.4	598	0.0
Likely irrelevant (<10 min)	12176	52.4	2711002	66.5

Population and Area Coverage in Schleswig-Holstein (Stroke-Ready Hospitals) — Extended (+20% Speed (Emergency))

Category	Grid Cells	Area (%)	Population	Population (%)
High (30+ min)	521	2.5	48018	1.8
Medium (20-30 min)	1244	6.0	131939	4.8
Low (10-20 min)	3911	18.9	548761	20.0
Only CT reachable within 60 min	184	0.9	24956	0.9
Neither reachable within 60 min	331	1.6	11554	0.4
Likely irrelevant (<10 min)	14488	70.1	1977047	72.1

Population and Area Coverage in Thüringen (Stroke-Ready Hospitals) — Extended (+20% Speed (Emergency))

Category	Grid Cells	Area (%)	Population	Population (%)
High (30+ min)	33	0.2	12088	0.6
Medium (20-30 min)	977	4.7	131676	6.5
Low (10-20 min)	5758	27.7	402118	19.8
Only CT reachable within 60 min	0	0.0	0	0.0
Neither reachable within 60 min	0	0.0	0	0.0
Likely irrelevant (<10 min)	14051	67.5	1484292	73.1

Population and Area Coverage in Berlin (Stroke Units) — Regular (+20% Speed (Emergency))

Category	Grid Cells	Area (%)	Population	Population (%)
High (30+ min)	0	0.0	0	0.0
Medium (20-30 min)	3	0.3	5339	0.1
Low (10-20 min)	145	12.3	309609	8.6
Only CT reachable within 60 min	0	0.0	0	0.0
Neither reachable within 60 min	0	0.0	0	0.0
Likely irrelevant (<10 min)	1028	87.4	3298478	91.3

Population and Area Coverage in Baden-Württemberg (Stroke Units) — Regular (+20% Speed (Emergency))

Category	Grid Cells	Area (%)	Population	Population (%)
High (30+ min)	2025	4.7	299427	2.8
Medium (20-30 min)	5639	13.0	869696	8.0
Low (10-20 min)	12168	28.1	2304799	21.3
Only CT reachable within 60 min	237	0.5	19641	0.2
Neither reachable within 60 min	33	0.1	1479	0.0
Likely irrelevant (<10 min)	23276	53.7	7338103	67.7

Population and Area Coverage in Bremen (Stroke Units) — Regular (+20% Speed (Emergency))

Category	Grid Cells	Area (%)	Population	Population (%)
High (30+ min)	0	0.0	0	0.0
Medium (20-30 min)	2	0.5	1316	0.3
Low (10-20 min)	82	20.4	103106	19.7
Only CT reachable within 60 min	0	0.0	0	0.0
Neither reachable within 60 min	0	0.0	0	0.0
Likely irrelevant (<10 min)	318	79.1	419481	80.1

Population and Area Coverage in Hamburg (Stroke Units) — Regular (+20% Speed (Emergency))

Category	Grid Cells	Area (%)	Population	Population (%)
High (30+ min)	0	0.0	0	0.0
Medium (20-30 min)	0	0.0	0	0.0
Low (10-20 min)	127	12.2	152986	8.3
Only CT reachable within 60 min	0	0.0	0	0.0
Neither reachable within 60 min	10	1.0	6092	0.3
Likely irrelevant (<10 min)	907	86.9	1683504	91.4

Population and Area Coverage in Brandenburg (Stroke Units) — Regular (+20% Speed (Emergency))

Category	Grid Cells	Area (%)	Population	Population (%)
High (30+ min)	6581	16.9	380946	15.6
Medium (20-30 min)	8523	21.9	310869	12.7
Low (10-20 min)	10969	28.2	593792	24.3
Only CT reachable within 60 min	1578	4.1	36688	1.5
Neither reachable within 60 min	173	0.4	3327	0.1
Likely irrelevant (<10 min)	11033	28.4	1116360	45.7

Population and Area Coverage in Bayern (Stroke Units) — Regular (+20% Speed (Emergency))

Category	Grid Cells	Area (%)	Population	Population (%)
High (30+ min)	539	0.6	65280	0.5
Medium (20-30 min)	7863	9.2	924144	7.0
Low (10-20 min)	23281	27.2	2909162	21.9
Only CT reachable within 60 min	53	0.1	3597	0.0
Neither reachable within 60 min	2164	2.5	13844	0.1
Likely irrelevant (<10 min)	51793	60.4	9355103	70.5

Population and Area Coverage in Hessen (Stroke Units) — Regular (+20% Speed (Emergency))

Category	Grid Cells	Area (%)	Population	Population (%)
High (30+ min)	1576	5.9	157497	2.5
Medium (20-30 min)	4501	16.8	399581	6.4
Low (10-20 min)	7519	28.1	1063990	17.0
Only CT reachable within 60 min	0	0.0	0	0.0
Neither reachable within 60 min	0	0.0	0	0.0
Likely irrelevant (<10 min)	13152	49.2	4633342	74.1

Population and Area Coverage in Mecklenburg-Vorpommern (Stroke Units) — Regular (+20% Speed (Emergency))

Category	Grid Cells	Area (%)	Population	Population (%)
High (30+ min)	3087	9.9	165049	11.3
Medium (20-30 min)	5673	18.2	170801	11.7
Low (10-20 min)	8518	27.3	245194	16.7
Only CT reachable within 60 min	1185	3.8	22074	1.5
Neither reachable within 60 min	496	1.6	10409	0.7
Likely irrelevant (<10 min)	12222	39.2	850468	58.1

Population and Area Coverage in Saarland (Stroke Units) — Regular (+20% Speed (Emergency))

Category	Grid Cells	Area (%)	Population	Population (%)
High (30+ min)	2	0.1	9	0.0
Medium (20-30 min)	83	2.6	10084	1.0
Low (10-20 min)	548	17.0	76663	7.9
Only CT reachable within 60 min	1	0.0	102	0.0
Neither reachable within 60 min	16	0.5	1976	0.2
Likely irrelevant (<10 min)	2570	79.8	881501	90.8

Population and Area Coverage in Rheinland-Pfalz (Stroke Units) — Regular (+20% Speed (Emergency))

Category	Grid Cells	Area (%)	Population	Population (%)
High (30+ min)	744	3.0	60278	1.4
Medium (20-30 min)	4193	16.8	463264	11.1
Low (10-20 min)	8524	34.2	1120357	26.9
Only CT reachable within 60 min	3	0.0	2	0.0
Neither reachable within 60 min	27	0.1	1114	0.0
Likely irrelevant (<10 min)	11403	45.8	2519270	60.5

Population and Area Coverage in Nordrhein-Westfalen (Stroke Units) — Regular (+20% Speed (Emergency))

Category	Grid Cells	Area (%)	Population	Population (%)
High (30+ min)	1683	3.8	247456	1.3
Medium (20-30 min)	5133	11.6	931144	5.1
Low (10-20 min)	13122	29.7	4473212	24.4
Only CT reachable within 60 min	26	0.1	672	0.0
Neither reachable within 60 min	118	0.3	8355	0.0
Likely irrelevant (<10 min)	24089	54.5	12706076	69.2

Population and Area Coverage in Niedersachsen (Stroke Units) — Regular (+20% Speed (Emergency))

Category	Grid Cells	Area (%)	Population	Population (%)
High (30+ min)	3120	4.9	304322	3.8
Medium (20-30 min)	9878	15.6	1010865	12.6
Low (10-20 min)	19739	31.2	2164616	26.9
Only CT reachable within 60 min	426	0.7	6387	0.1
Neither reachable within 60 min	344	0.5	16740	0.2
Likely irrelevant (<10 min)	29663	47.0	4536932	56.4

Population and Area Coverage in Sachsen-Anhalt (Stroke Units) — Regular (+20% Speed (Emergency))

Category	Grid Cells	Area (%)	Population	Population (%)
High (30+ min)	4878	18.1	284256	13.5
Medium (20-30 min)	4844	18.0	407587	19.4
Low (10-20 min)	7577	28.1	421683	20.0
Only CT reachable within 60 min	2939	10.9	57850	2.8
Neither reachable within 60 min	0	0.0	0	0.0
Likely irrelevant (<10 min)	6698	24.9	931838	44.3

Population and Area Coverage in Sachsen (Stroke Units) — Regular (+20% Speed (Emergency))

Category	Grid Cells	Area (%)	Population	Population (%)
High (30+ min)	1424	6.1	209364	5.1
Medium (20-30 min)	4518	19.4	554324	13.6
Low (10-20 min)	6787	29.2	925275	22.7
Only CT reachable within 60 min	110	0.5	3990	0.1
Neither reachable within 60 min	96	0.4	598	0.0
Likely irrelevant (<10 min)	10317	44.4	2385954	58.5

Population and Area Coverage in Schleswig-Holstein (Stroke Units) — Regular (+20% Speed (Emergency))

Category	Grid Cells	Area (%)	Population	Population (%)
High (30+ min)	529	2.6	47306	1.7
Medium (20-30 min)	1246	6.0	131905	4.8
Low (10-20 min)	3970	19.2	554455	20.2
Only CT reachable within 60 min	196	0.9	26732	1.0
Neither reachable within 60 min	332	1.6	11608	0.4
Likely irrelevant (<10 min)	14406	69.7	1970269	71.8

Population and Area Coverage in Thüringen (Stroke Units) — Regular (+20% Speed (Emergency))

Category	Grid Cells	Area (%)	Population	Population (%)
High (30+ min)	154	0.7	19291	1.0
Medium (20-30 min)	2185	10.5	205633	10.1
Low (10-20 min)	6487	31.2	437904	21.6
Only CT reachable within 60 min	0	0.0	0	0.0
Neither reachable within 60 min	0	0.0	0	0.0
Likely irrelevant (<10 min)	11993	57.6	1367346	67.4

Population and Area Coverage in Berlin (Stroke-Ready Hospitals) — Extended (+20min Penalty)

Category	Grid Cells	Area (%)	Population	Population (%)
High (30+ min)	0	0.0	0	0.0
Medium (20-30 min)	0	0.0	0	0.0
Low (10-20 min)	11	0.9	1257	0.0
Only CT reachable within 60 min	0	0.0	0	0.0
Neither reachable within 60 min	0	0.0	0	0.0
Likely irrelevant (<10 min)	1165	99.1	3612169	100.0

Population and Area Coverage in Baden-Württemberg (Stroke-Ready Hospitals) — Extended (+20min Penalty)

Category	Grid Cells	Area (%)	Population	Population (%)
High (30+ min)	93	0.2	2248	0.0
Medium (20-30 min)	1244	2.9	62391	0.6
Low (10-20 min)	5700	13.1	609395	5.6
Only CT reachable within 60 min	43	0.1	2189	0.0
Neither reachable within 60 min	33	0.1	1474	0.0
Likely irrelevant (<10 min)	36265	83.6	10155448	93.7

Population and Area Coverage in Bremen (Stroke-Ready Hospitals) — Extended (+20min Penalty)

Category	Grid Cells	Area (%)	Population	Population (%)
High (30+ min)	0	0.0	0	0.0
Medium (20-30 min)	0	0.0	0	0.0
Low (10-20 min)	42	10.4	1075	0.2
Only CT reachable within 60 min	0	0.0	0	0.0
Neither reachable within 60 min	0	0.0	0	0.0
Likely irrelevant (<10 min)	360	89.6	522828	99.8

Population and Area Coverage in Hamburg (Stroke-Ready Hospitals) — Extended (+20min Penalty)

Category	Grid Cells	Area (%)	Population	Population (%)
High (30+ min)	0	0.0	0	0.0
Medium (20-30 min)	0	0.0	0	0.0
Low (10-20 min)	13	1.2	1190	0.1
Only CT reachable within 60 min	0	0.0	0	0.0
Neither reachable within 60 min	10	1.0	6092	0.3
Likely irrelevant (<10 min)	1021	97.8	1835300	99.6

Population and Area Coverage in Brandenburg (Stroke-Ready Hospitals) — Extended (+20min Penalty)

Category	Grid Cells	Area (%)	Population	Population (%)
High (30+ min)	2292	5.9	79270	3.2
Medium (20-30 min)	5822	15.0	184446	7.6
Low (10-20 min)	9099	23.4	273975	11.2
Only CT reachable within 60 min	2529	6.5	43532	1.8
Neither reachable within 60 min	180	0.5	3399	0.1
Likely irrelevant (<10 min)	18935	48.7	1857360	76.1

Population and Area Coverage in Bayern (Stroke-Ready Hospitals) — Extended (+20min Penalty)

Category	Grid Cells	Area (%)	Population	Population (%)
High (30+ min)	46	0.1	1725	0.0
Medium (20-30 min)	2573	3.0	149995	1.1
Low (10-20 min)	13459	15.7	913302	6.9
Only CT reachable within 60 min	78	0.1	2189	0.0
Neither reachable within 60 min	2186	2.6	15199	0.1
Likely irrelevant (<10 min)	67351	78.6	12188720	91.8

Population and Area Coverage in Hessen (Stroke-Ready Hospitals) — Extended (+20min Penalty)

Category	Grid Cells	Area (%)	Population	Population (%)
High (30+ min)	383	1.4	14258	0.2
Medium (20-30 min)	1494	5.6	105358	1.7
Low (10-20 min)	4886	18.3	349316	5.6
Only CT reachable within 60 min	37	0.1	677	0.0
Neither reachable within 60 min	0	0.0	0	0.0
Likely irrelevant (<10 min)	19948	74.6	5784801	92.5

Population and Area Coverage in Mecklenburg-Vorpommern (Stroke-Ready Hospitals) — Extended (+20min Penalty)

Category	Grid Cells	Area (%)	Population	Population (%)
High (30+ min)	2255	7.2	85802	5.9
Medium (20-30 min)	4889	15.7	145341	9.9
Low (10-20 min)	7293	23.4	212524	14.5
Only CT reachable within 60 min	2481	8.0	46235	3.2
Neither reachable within 60 min	976	3.1	13806	0.9
Likely irrelevant (<10 min)	13287	42.6	960287	65.6

Population and Area Coverage in Saarland (Stroke-Ready Hospitals) — Extended (+20min Penalty)

Category	Grid Cells	Area (%)	Population	Population (%)
High (30+ min)	0	0.0	0	0.0
Medium (20-30 min)	26	0.8	144	0.0
Low (10-20 min)	366	11.4	30959	3.2
Only CT reachable within 60 min	1	0.0	64	0.0
Neither reachable within 60 min	17	0.5	1958	0.2
Likely irrelevant (<10 min)	2810	87.3	937210	96.6

Population and Area Coverage in Rheinland-Pfalz (Stroke-Ready Hospitals) — Extended (+20min Penalty)

Category	Grid Cells	Area (%)	Population	Population (%)
High (30+ min)	424	1.7	14792	0.4
Medium (20-30 min)	2208	8.9	126034	3.0
Low (10-20 min)	4603	18.5	343667	8.3
Only CT reachable within 60 min	130	0.5	1700	0.0
Neither reachable within 60 min	29	0.1	1114	0.0
Likely irrelevant (<10 min)	17500	70.3	3676978	88.3

Population and Area Coverage in Nordrhein-Westfalen (Stroke-Ready Hospitals) — Extended (+20min Penalty)

Category	Grid Cells	Area (%)	Population	Population (%)
High (30+ min)	936	2.1	59772	0.3
Medium (20-30 min)	1906	4.3	180070	1.0
Low (10-20 min)	4584	10.4	502007	2.7
Only CT reachable within 60 min	271	0.6	3238	0.0
Neither reachable within 60 min	116	0.3	8871	0.0
Likely irrelevant (<10 min)	36358	82.3	17612957	95.9

Population and Area Coverage in Niedersachsen (Stroke-Ready Hospitals) — Extended (+20min Penalty)

Category	Grid Cells	Area (%)	Population	Population (%)
High (30+ min)	811	1.3	32862	0.4
Medium (20-30 min)	4999	7.9	296260	3.7
Low (10-20 min)	11491	18.2	812452	10.1
Only CT reachable within 60 min	719	1.1	9806	0.1
Neither reachable within 60 min	353	0.6	17276	0.2
Likely irrelevant (<10 min)	44797	70.9	6871206	85.5

Population and Area Coverage in Sachsen-Anhalt (Stroke-Ready Hospitals) — Extended (+20min Penalty)

Category	Grid Cells	Area (%)	Population	Population (%)
High (30+ min)	1709	6.3	62270	3.0
Medium (20-30 min)	3602	13.4	88973	4.2
Low (10-20 min)	6069	22.5	292512	13.9
Only CT reachable within 60 min	1546	5.7	19488	0.9
Neither reachable within 60 min	0	0.0	0	0.0
Likely irrelevant (<10 min)	14010	52.0	1639971	78.0

Population and Area Coverage in Sachsen (Stroke-Ready Hospitals) — Extended (+20min Penalty)

Category	Grid Cells	Area (%)	Population	Population (%)
High (30+ min)	511	2.2	52465	1.3
Medium (20-30 min)	2209	9.5	174023	4.3
Low (10-20 min)	5267	22.7	433290	10.6
Only CT reachable within 60 min	157	0.7	6222	0.2
Neither reachable within 60 min	102	0.4	656	0.0
Likely irrelevant (<10 min)	15006	64.5	3412849	83.7

Population and Area Coverage in Schleswig-Holstein (Stroke-Ready Hospitals) — Extended (+20min Penalty)

Category	Grid Cells	Area (%)	Population	Population (%)
High (30+ min)	301	1.5	8323	0.3
Medium (20-30 min)	861	4.2	60151	2.2
Low (10-20 min)	2798	13.5	196489	7.2
Only CT reachable within 60 min	232	1.1	27767	1.0
Neither reachable within 60 min	336	1.6	11983	0.4
Likely irrelevant (<10 min)	16151	78.1	2437562	88.9

Population and Area Coverage in Thüringen (Stroke-Ready Hospitals) — Extended (+20min Penalty)

Category	Grid Cells	Area (%)	Population	Population (%)
High (30+ min)	13	0.1	5	0.0
Medium (20-30 min)	472	2.3	13910	0.7
Low (10-20 min)	4419	21.2	234319	11.5
Only CT reachable within 60 min	0	0.0	0	0.0
Neither reachable within 60 min	0	0.0	0	0.0
Likely irrelevant (<10 min)	15915	76.4	1781940	87.8

Population and Area Coverage in Berlin (Stroke Units) — Regular (+20min Penalty)

Category	Grid Cells	Area (%)	Population	Population (%)
High (30+ min)	0	0.0	0	0.0
Medium (20-30 min)	0	0.0	0	0.0
Low (10-20 min)	12	1.0	1549	0.0
Only CT reachable within 60 min	0	0.0	0	0.0
Neither reachable within 60 min	0	0.0	0	0.0
Likely irrelevant (<10 min)	1164	99.0	3611877	100.0

Population and Area Coverage in Baden-Württemberg (Stroke Units) — Regular (+20min Penalty)

Category	Grid Cells	Area (%)	Population	Population (%)
High (30+ min)	1001	2.3	101794	0.9
Medium (20-30 min)	3981	9.2	359186	3.3
Low (10-20 min)	8936	20.6	1186013	10.9
Only CT reachable within 60 min	1362	3.1	101048	0.9
Neither reachable within 60 min	33	0.1	1474	0.0
Likely irrelevant (<10 min)	28065	64.7	9083630	83.9

Population and Area Coverage in Bremen (Stroke Units) — Regular (+20min Penalty)

Category	Grid Cells	Area (%)	Population	Population (%)
High (30+ min)	0	0.0	0	0.0
Medium (20-30 min)	0	0.0	0	0.0
Low (10-20 min)	48	11.9	1326	0.3
Only CT reachable within 60 min	0	0.0	0	0.0
Neither reachable within 60 min	0	0.0	0	0.0
Likely irrelevant (<10 min)	354	88.1	522577	99.7

Population and Area Coverage in Hamburg (Stroke Units) — Regular (+20min Penalty)

Category	Grid Cells	Area (%)	Population	Population (%)
High (30+ min)	0	0.0	0	0.0
Medium (20-30 min)	0	0.0	0	0.0
Low (10-20 min)	13	1.2	1190	0.1
Only CT reachable within 60 min	0	0.0	0	0.0
Neither reachable within 60 min	10	1.0	6092	0.3
Likely irrelevant (<10 min)	1021	97.8	1835300	99.6

Population and Area Coverage in Brandenburg (Stroke Units) — Regular (+20min Penalty)

Category	Grid Cells	Area (%)	Population	Population (%)
High (30+ min)	3691	9.5	183779	7.5
Medium (20-30 min)	6785	17.5	202198	8.3
Low (10-20 min)	9059	23.3	333825	13.7
Only CT reachable within 60 min	6658	17.1	175853	7.2
Neither reachable within 60 min	180	0.5	3399	0.1
Likely irrelevant (<10 min)	12484	32.1	1542928	63.2

Population and Area Coverage in Bayern (Stroke Units) — Regular (+20min Penalty)

Category	Grid Cells	Area (%)	Population	Population (%)
High (30+ min)	230	0.3	9712	0.1
Medium (20-30 min)	3511	4.1	197929	1.5
Low (10-20 min)	15607	18.2	1118370	8.4
Only CT reachable within 60 min	159	0.2	2851	0.0
Neither reachable within 60 min	2186	2.6	15199	0.1
Likely irrelevant (<10 min)	64000	74.7	11927069	89.9

Population and Area Coverage in Hessen (Stroke Units) — Regular (+20min Penalty)

Category	Grid Cells	Area (%)	Population	Population (%)
High (30+ min)	729	2.7	46046	0.7
Medium (20-30 min)	3014	11.3	193787	3.1
Low (10-20 min)	5796	21.7	488536	7.8
Only CT reachable within 60 min	185	0.7	6027	0.1
Neither reachable within 60 min	0	0.0	0	0.0
Likely irrelevant (<10 min)	17024	63.6	5520014	88.3

Population and Area Coverage in Mecklenburg-Vorpommern (Stroke Units) — Regular (+20min Penalty)

Category	Grid Cells	Area (%)	Population	Population (%)
High (30+ min)	2343	7.5	85287	5.8
Medium (20-30 min)	4903	15.7	148291	10.1
Low (10-20 min)	7143	22.9	225328	15.4
Only CT reachable within 60 min	3131	10.0	54174	3.7
Neither reachable within 60 min	977	3.1	13819	0.9
Likely irrelevant (<10 min)	12684	40.7	937096	64.0

Population and Area Coverage in Saarland (Stroke Units) — Regular (+20min Penalty)

Category	Grid Cells	Area (%)	Population	Population (%)
High (30+ min)	0	0.0	0	0.0
Medium (20-30 min)	27	0.8	154	0.0
Low (10-20 min)	368	11.4	31206	3.2
Only CT reachable within 60 min	1	0.0	64	0.0
Neither reachable within 60 min	17	0.5	1958	0.2
Likely irrelevant (<10 min)	2807	87.2	936953	96.6

Population and Area Coverage in Rheinland-Pfalz (Stroke Units) — Regular (+20min Penalty)

Category	Grid Cells	Area (%)	Population	Population (%)
High (30+ min)	528	2.1	21904	0.5
Medium (20-30 min)	2833	11.4	171707	4.1
Low (10-20 min)	6060	24.3	506027	12.2
Only CT reachable within 60 min	172	0.7	2166	0.1
Neither reachable within 60 min	30	0.1	1114	0.0
Likely irrelevant (<10 min)	15271	61.3	3461367	83.1

Population and Area Coverage in Nordrhein-Westfalen (Stroke Units) — Regular (+20min Penalty)

Category	Grid Cells	Area (%)	Population	Population (%)
High (30+ min)	973	2.2	62710	0.3
Medium (20-30 min)	2431	5.5	264207	1.4
Low (10-20 min)	7608	17.2	1051197	5.7
Only CT reachable within 60 min	340	0.8	7778	0.0
Neither reachable within 60 min	116	0.3	8871	0.0
Likely irrelevant (<10 min)	32703	74.0	16972152	92.4

Population and Area Coverage in Niedersachsen (Stroke Units) — Regular (+20min Penalty)

Category	Grid Cells	Area (%)	Population	Population (%)
High (30+ min)	1948	3.1	93691	1.2
Medium (20-30 min)	7624	12.1	490468	6.1
Low (10-20 min)	14998	23.7	1189351	14.8
Only CT reachable within 60 min	1333	2.1	24361	0.3
Neither reachable within 60 min	354	0.6	17335	0.2
Likely irrelevant (<10 min)	36913	58.4	6224656	77.4

Population and Area Coverage in Sachsen-Anhalt (Stroke Units) — Regular (+20min Penalty)

Category	Grid Cells	Area (%)	Population	Population (%)
High (30+ min)	2649	9.8	125398	6.0
Medium (20-30 min)	4117	15.3	213634	10.2
Low (10-20 min)	6058	22.5	402134	19.1
Only CT reachable within 60 min	5810	21.6	139112	6.6
Neither reachable within 60 min	0	0.0	0	0.0
Likely irrelevant (<10 min)	8302	30.8	1222936	58.1

Population and Area Coverage in Sachsen (Stroke Units) — Regular (+20min Penalty)

Category	Grid Cells	Area (%)	Population	Population (%)
High (30+ min)	1003	4.3	84723	2.1
Medium (20-30 min)	2933	12.6	248159	6.1
Low (10-20 min)	5739	24.7	539436	13.2
Only CT reachable within 60 min	441	1.9	19215	0.5
Neither reachable within 60 min	104	0.4	655	0.0
Likely irrelevant (<10 min)	13032	56.0	3187317	78.1

Population and Area Coverage in Schleswig-Holstein (Stroke Units) — Regular (+20min Penalty)

Category	Grid Cells	Area (%)	Population	Population (%)
High (30+ min)	302	1.5	8424	0.3
Medium (20-30 min)	882	4.3	60464	2.2
Low (10-20 min)	2838	13.7	198280	7.2
Only CT reachable within 60 min	236	1.1	27810	1.0
Neither reachable within 60 min	343	1.7	12120	0.4
Likely irrelevant (<10 min)	16078	77.8	2435177	88.8

Population and Area Coverage in Thüringen (Stroke Units) — Regular (+20min Penalty)

Category	Grid Cells	Area (%)	Population	Population (%)
High (30+ min)	141	0.7	1968	0.1
Medium (20-30 min)	1567	7.5	62162	3.1
Low (10-20 min)	5503	26.4	327384	16.1
Only CT reachable within 60 min	16	0.1	6	0.0
Neither reachable within 60 min	0	0.0	0	0.0
Likely irrelevant (<10 min)	13592	65.3	1638654	80.7

Population and Area Coverage in Berlin (Stroke-Ready Hospitals) — Extended (+30min Penalty)

Category	Grid Cells	Area (%)	Population	Population (%)
High (30+ min)	0	0.0	0	0.0
Medium (20-30 min)	0	0.0	0	0.0
Low (10-20 min)	0	0.0	0	0.0
Only CT reachable within 60 min	0	0.0	0	0.0
Neither reachable within 60 min	0	0.0	0	0.0
Likely irrelevant (<10 min)	1176	100.0	3613426	100.0

Population and Area Coverage in Baden-Württemberg (Stroke-Ready Hospitals) — Extended (+30min Penalty)

Category	Grid Cells	Area (%)	Population	Population (%)
High (30+ min)	0	0.0	0	0.0
Medium (20-30 min)	138	0.3	2731	0.0
Low (10-20 min)	1734	4.0	76464	0.7
Only CT reachable within 60 min	43	0.1	2189	0.0
Neither reachable within 60 min	33	0.1	1470	0.0
Likely irrelevant (<10 min)	41430	95.5	10750291	99.2

Population and Area Coverage in Bremen (Stroke-Ready Hospitals) — Extended (+30min Penalty)

Category	Grid Cells	Area (%)	Population	Population (%)
High (30+ min)	0	0.0	0	0.0
Medium (20-30 min)	0	0.0	0	0.0
Low (10-20 min)	0	0.0	0	0.0
Only CT reachable within 60 min	0	0.0	0	0.0
Neither reachable within 60 min	0	0.0	0	0.0
Likely irrelevant (<10 min)	402	100.0	523903	100.0

Population and Area Coverage in Hamburg (Stroke-Ready Hospitals) — Extended (+30min Penalty)

Category	Grid Cells	Area (%)	Population	Population (%)
High (30+ min)	0	0.0	0	0.0
Medium (20-30 min)	0	0.0	0	0.0
Low (10-20 min)	0	0.0	0	0.0
Only CT reachable within 60 min	0	0.0	0	0.0
Neither reachable within 60 min	10	1.0	6092	0.3
Likely irrelevant (<10 min)	1034	99.0	1836490	99.7

Population and Area Coverage in Brandenburg (Stroke-Ready Hospitals) — Extended (+30min Penalty)

Category	Grid Cells	Area (%)	Population	Population (%)
High (30+ min)	0	0.0	0	0.0
Medium (20-30 min)	3672	9.5	93495	3.8
Low (10-20 min)	7538	19.4	199635	8.2
Only CT reachable within 60 min	2529	6.5	43532	1.8
Neither reachable within 60 min	180	0.5	3399	0.1
Likely irrelevant (<10 min)	24938	64.2	2101921	86.1

Population and Area Coverage in Bayern (Stroke-Ready Hospitals) — Extended (+30min Penalty)

Category	Grid Cells	Area (%)	Population	Population (%)
High (30+ min)	0	0.0	0	0.0
Medium (20-30 min)	183	0.2	5112	0.0
Low (10-20 min)	3533	4.1	178265	1.3
Only CT reachable within 60 min	78	0.1	2189	0.0
Neither reachable within 60 min	2186	2.6	15197	0.1
Likely irrelevant (<10 min)	79713	93.0	13070367	98.5

Population and Area Coverage in Hessen (Stroke-Ready Hospitals) — Extended (+30min Penalty)

Category	Grid Cells	Area (%)	Population	Population (%)
High (30+ min)	0	0.0	0	0.0
Medium (20-30 min)	395	1.5	14433	0.2
Low (10-20 min)	1615	6.0	105930	1.7
Only CT reachable within 60 min	37	0.1	677	0.0
Neither reachable within 60 min	0	0.0	0	0.0
Likely irrelevant (<10 min)	24701	92.3	6133370	98.1

Population and Area Coverage in Mecklenburg-Vorpommern (Stroke-Ready Hospitals) — Extended (+30min Penalty)

Category	Grid Cells	Area (%)	Population	Population (%)
High (30+ min)	0	0.0	0	0.0
Medium (20-30 min)	3480	11.2	102134	7.0
Low (10-20 min)	6538	21.0	163743	11.2
Only CT reachable within 60 min	2481	8.0	46227	3.2
Neither reachable within 60 min	976	3.1	13809	0.9
Likely irrelevant (<10 min)	17706	56.8	1138082	77.7

Population and Area Coverage in Saarland (Stroke-Ready Hospitals) — Extended (+30min Penalty)

Category	Grid Cells	Area (%)	Population	Population (%)
High (30+ min)	0	0.0	0	0.0
Medium (20-30 min)	0	0.0	0	0.0
Low (10-20 min)	41	1.3	177	0.0
Only CT reachable within 60 min	1	0.0	64	0.0
Neither reachable within 60 min	17	0.5	1958	0.2
Likely irrelevant (<10 min)	3161	98.2	968136	99.8

Population and Area Coverage in Rheinland-Pfalz (Stroke-Ready Hospitals) — Extended (+30min Penalty)

Category	Grid Cells	Area (%)	Population	Population (%)
High (30+ min)	0	0.0	0	0.0
Medium (20-30 min)	749	3.0	24212	0.6
Low (10-20 min)	2238	9.0	126319	3.0
Only CT reachable within 60 min	130	0.5	1700	0.0
Neither reachable within 60 min	29	0.1	1114	0.0
Likely irrelevant (<10 min)	21748	87.4	4010940	96.3

Population and Area Coverage in Nordrhein-Westfalen (Stroke-Ready Hospitals) — Extended (+30min Penalty)

Category	Grid Cells	Area (%)	Population	Population (%)
High (30+ min)	0	0.0	0	0.0
Medium (20-30 min)	1142	2.6	63928	0.3
Low (10-20 min)	1974	4.5	182376	1.0
Only CT reachable within 60 min	271	0.6	3238	0.0
Neither reachable within 60 min	116	0.3	8865	0.0
Likely irrelevant (<10 min)	40668	92.1	18108508	98.6

Population and Area Coverage in Niedersachsen (Stroke-Ready Hospitals) — Extended (+30min Penalty)

Category	Grid Cells	Area (%)	Population	Population (%)
High (30+ min)	0	0.0	0	0.0
Medium (20-30 min)	1208	1.9	39873	0.5
Low (10-20 min)	6100	9.7	319172	4.0
Only CT reachable within 60 min	719	1.1	9812	0.1
Neither reachable within 60 min	353	0.6	17283	0.2
Likely irrelevant (<10 min)	54790	86.7	7653722	95.2

Population and Area Coverage in Sachsen-Anhalt (Stroke-Ready Hospitals) — Extended (+30min Penalty)

Category	Grid Cells	Area (%)	Population	Population (%)
High (30+ min)	0	0.0	0	0.0
Medium (20-30 min)	2660	9.9	74300	3.5
Low (10-20 min)	4488	16.7	107731	5.1
Only CT reachable within 60 min	1546	5.7	19488	0.9
Neither reachable within 60 min	0	0.0	0	0.0
Likely irrelevant (<10 min)	18242	67.7	1901695	90.4

Population and Area Coverage in Sachsen (Stroke-Ready Hospitals) — Extended (+30min Penalty)

Category	Grid Cells	Area (%)	Population	Population (%)
High (30+ min)	0	0.0	0	0.0
Medium (20-30 min)	986	4.2	60736	1.5
Low (10-20 min)	2567	11.0	189315	4.6
Only CT reachable within 60 min	157	0.7	6222	0.2
Neither reachable within 60 min	102	0.4	655	0.0
Likely irrelevant (<10 min)	19440	83.6	3822577	93.7

Population and Area Coverage in Schleswig-Holstein (Stroke-Ready Hospitals) — Extended (+30min Penalty)

Category	Grid Cells	Area (%)	Population	Population (%)
High (30+ min)	0	0.0	0	0.0
Medium (20-30 min)	362	1.8	8792	0.3
Low (10-20 min)	1073	5.2	66802	2.4
Only CT reachable within 60 min	232	1.1	27767	1.0
Neither reachable within 60 min	336	1.6	11986	0.4
Likely irrelevant (<10 min)	18676	90.3	2626928	95.8

Population and Area Coverage in Thüringen (Stroke-Ready Hospitals) — Extended (+30min Penalty)

Category	Grid Cells	Area (%)	Population	Population (%)
High (30+ min)	0	0.0	0	0.0
Medium (20-30 min)	39	0.2	36	0.0
Low (10-20 min)	1241	6.0	34188	1.7
Only CT reachable within 60 min	0	0.0	0	0.0
Neither reachable within 60 min	0	0.0	0	0.0
Likely irrelevant (<10 min)	19539	93.9	1995950	98.3

Population and Area Coverage in Berlin (Stroke Units) — Regular (+30min Penalty)

Category	Grid Cells	Area (%)	Population	Population (%)
High (30+ min)	0	0.0	0	0.0
Medium (20-30 min)	0	0.0	0	0.0
Low (10-20 min)	0	0.0	0	0.0
Only CT reachable within 60 min	0	0.0	0	0.0
Neither reachable within 60 min	0	0.0	0	0.0
Likely irrelevant (<10 min)	1176	100.0	3613426	100.0

Population and Area Coverage in Baden-Württemberg (Stroke Units) — Regular (+30min Penalty)

Category	Grid Cells	Area (%)	Population	Population (%)
High (30+ min)	0	0.0	0	0.0
Medium (20-30 min)	1547	3.6	117870	1.1
Low (10-20 min)	4646	10.7	404952	3.7
Only CT reachable within 60 min	1362	3.1	101047	0.9
Neither reachable within 60 min	33	0.1	1470	0.0
Likely irrelevant (<10 min)	35790	82.5	10207806	94.2

Population and Area Coverage in Bremen (Stroke Units) — Regular (+30min Penalty)

Category	Grid Cells	Area (%)	Population	Population (%)
High (30+ min)	0	0.0	0	0.0
Medium (20-30 min)	0	0.0	0	0.0
Low (10-20 min)	0	0.0	0	0.0
Only CT reachable within 60 min	0	0.0	0	0.0
Neither reachable within 60 min	0	0.0	0	0.0
Likely irrelevant (<10 min)	402	100.0	523903	100.0

Population and Area Coverage in Hamburg (Stroke Units) — Regular (+30min Penalty)

Category	Grid Cells	Area (%)	Population	Population (%)
High (30+ min)	0	0.0	0	0.0
Medium (20-30 min)	0	0.0	0	0.0
Low (10-20 min)	0	0.0	0	0.0
Only CT reachable within 60 min	0	0.0	0	0.0
Neither reachable within 60 min	10	1.0	6092	0.3
Likely irrelevant (<10 min)	1034	99.0	1836490	99.7

Population and Area Coverage in Brandenburg (Stroke Units) — Regular (+30min Penalty)

Category	Grid Cells	Area (%)	Population	Population (%)
High (30+ min)	0	0.0	0	0.0
Medium (20-30 min)	5326	13.7	200417	8.2
Low (10-20 min)	8277	21.3	227424	9.3
Only CT reachable within 60 min	6658	17.1	175853	7.2
Neither reachable within 60 min	180	0.5	3399	0.1
Likely irrelevant (<10 min)	18416	47.4	1834889	75.1

Population and Area Coverage in Bayern (Stroke Units) — Regular (+30min Penalty)

Category	Grid Cells	Area (%)	Population	Population (%)
High (30+ min)	0	0.0	0	0.0
Medium (20-30 min)	380	0.4	14052	0.1
Low (10-20 min)	4470	5.2	224801	1.7
Only CT reachable within 60 min	159	0.2	2851	0.0
Neither reachable within 60 min	2186	2.6	15197	0.1
Likely irrelevant (<10 min)	78498	91.6	13014229	98.1

Population and Area Coverage in Hessen (Stroke Units) — Regular (+30min Penalty)

Category	Grid Cells	Area (%)	Population	Population (%)
High (30+ min)	0	0.0	0	0.0
Medium (20-30 min)	782	2.9	46667	0.7
Low (10-20 min)	3136	11.7	194621	3.1
Only CT reachable within 60 min	185	0.7	6027	0.1
Neither reachable within 60 min	0	0.0	0	0.0
Likely irrelevant (<10 min)	22645	84.7	6007095	96.0

Population and Area Coverage in Mecklenburg-Vorpommern (Stroke Units) — Regular (+30min Penalty)

Category	Grid Cells	Area (%)	Population	Population (%)
High (30+ min)	0	0.0	0	0.0
Medium (20-30 min)	3710	11.9	104446	7.1
Low (10-20 min)	6362	20.4	179422	12.3
Only CT reachable within 60 min	3131	10.0	54166	3.7
Neither reachable within 60 min	977	3.1	13823	0.9
Likely irrelevant (<10 min)	17001	54.5	1112138	76.0

Population and Area Coverage in Saarland (Stroke Units) — Regular (+30min Penalty)

Category	Grid Cells	Area (%)	Population	Population (%)
High (30+ min)	0	0.0	0	0.0
Medium (20-30 min)	0	0.0	0	0.0
Low (10-20 min)	42	1.3	186	0.0
Only CT reachable within 60 min	1	0.0	64	0.0
Neither reachable within 60 min	17	0.5	1958	0.2
Likely irrelevant (<10 min)	3160	98.1	968127	99.8

Population and Area Coverage in Rheinland-Pfalz (Stroke Units) — Regular (+30min Penalty)

Category	Grid Cells	Area (%)	Population	Population (%)
High (30+ min)	0	0.0	0	0.0
Medium (20-30 min)	926	3.7	33423	0.8
Low (10-20 min)	2991	12.0	172455	4.1
Only CT reachable within 60 min	172	0.7	2166	0.1
Neither reachable within 60 min	30	0.1	1114	0.0
Likely irrelevant (<10 min)	20775	83.5	3955127	95.0

Population and Area Coverage in Nordrhein-Westfalen (Stroke Units) — Regular (+30min Penalty)

Category	Grid Cells	Area (%)	Population	Population (%)
High (30+ min)	0	0.0	0	0.0
Medium (20-30 min)	1180	2.7	66942	0.4
Low (10-20 min)	2500	5.7	266396	1.5
Only CT reachable within 60 min	340	0.8	7778	0.0
Neither reachable within 60 min	116	0.3	8867	0.0
Likely irrelevant (<10 min)	40035	90.6	18016932	98.1

Population and Area Coverage in Niedersachsen (Stroke Units) — Regular (+30min Penalty)

Category	Grid Cells	Area (%)	Population	Population (%)
High (30+ min)	0	0.0	0	0.0
Medium (20-30 min)	2777	4.4	113900	1.4
Low (10-20 min)	9136	14.5	526948	6.6
Only CT reachable within 60 min	1333	2.1	24368	0.3
Neither reachable within 60 min	354	0.6	17342	0.2
Likely irrelevant (<10 min)	49570	78.5	7357304	91.5

Population and Area Coverage in Sachsen-Anhalt (Stroke Units) — Regular (+30min Penalty)

Category	Grid Cells	Area (%)	Population	Population (%)
High (30+ min)	0	0.0	0	0.0
Medium (20-30 min)	3500	13.0	140517	6.7
Low (10-20 min)	4964	18.4	225373	10.7
Only CT reachable within 60 min	5810	21.6	139112	6.6
Neither reachable within 60 min	0	0.0	0	0.0
Likely irrelevant (<10 min)	12662	47.0	1598212	76.0

Population and Area Coverage in Sachsen (Stroke Units) — Regular (+30min Penalty)

Category	Grid Cells	Area (%)	Population	Population (%)
High (30+ min)	0	0.0	0	0.0
Medium (20-30 min)	1431	6.2	94643	2.3
Low (10-20 min)	3357	14.4	261356	6.4
Only CT reachable within 60 min	441	1.9	19215	0.5
Neither reachable within 60 min	104	0.4	655	0.0
Likely irrelevant (<10 min)	17919	77.1	3703636	90.8

Population and Area Coverage in Schleswig-Holstein (Stroke Units) — Regular (+30min Penalty)

Category	Grid Cells	Area (%)	Population	Population (%)
High (30+ min)	0	0.0	0	0.0
Medium (20-30 min)	369	1.8	8966	0.3
Low (10-20 min)	1094	5.3	67158	2.4
Only CT reachable within 60 min	236	1.1	27810	1.0
Neither reachable within 60 min	343	1.7	12123	0.4
Likely irrelevant (<10 min)	18637	90.1	2626218	95.8

Population and Area Coverage in Thüringen (Stroke Units) — Regular (+30min Penalty)

Category	Grid Cells	Area (%)	Population	Population (%)
High (30+ min)	0	0.0	0	0.0
Medium (20-30 min)	318	1.5	6243	0.3
Low (10-20 min)	2462	11.8	84969	4.2
Only CT reachable within 60 min	16	0.1	6	0.0
Neither reachable within 60 min	0	0.0	0	0.0
Likely irrelevant (<10 min)	18023	86.6	1938956	95.5

Population and Area Coverage in Berlin (Stroke-Ready Hospitals) — Extended (-20% Speed (Traffic))

Category	Grid Cells	Area (%)	Population	Population (%)
High (30+ min)	0	0.0	0	0.0
Medium (20-30 min)	32	2.7	42255	1.2
Low (10-20 min)	268	22.8	762330	21.1
Only CT reachable within 60 min	0	0.0	0	0.0
Neither reachable within 60 min	0	0.0	0	0.0
Likely irrelevant (<10 min)	876	74.5	2808841	77.7

Population and Area Coverage in Baden-Württemberg (Stroke-Ready Hospitals) — Extended (-20% Speed (Traffic))

Category	Grid Cells	Area (%)	Population	Population (%)
High (30+ min)	2075	4.8	350271	3.2
Medium (20-30 min)	4557	10.5	891431	8.2
Low (10-20 min)	7812	18.0	1794171	16.6
Only CT reachable within 60 min	572	1.3	9043	0.1
Neither reachable within 60 min	65	0.1	1649	0.0
Likely irrelevant (<10 min)	28297	65.2	7786580	71.9

Population and Area Coverage in Bremen (Stroke-Ready Hospitals) — Extended (-20% Speed (Traffic))

Category	Grid Cells	Area (%)	Population	Population (%)
High (30+ min)	1	0.2	992	0.2
Medium (20-30 min)	21	5.2	17507	3.3
Low (10-20 min)	108	26.9	139389	26.6
Only CT reachable within 60 min	0	0.0	0	0.0
Neither reachable within 60 min	0	0.0	0	0.0
Likely irrelevant (<10 min)	272	67.7	366015	69.9

Population and Area Coverage in Hamburg (Stroke-Ready Hospitals) — Extended (-20% Speed (Traffic))

Category	Grid Cells	Area (%)	Population	Population (%)
High (30+ min)	0	0.0	0	0.0
Medium (20-30 min)	37	3.5	54561	3.0
Low (10-20 min)	191	18.3	295867	16.1
Only CT reachable within 60 min	0	0.0	0	0.0
Neither reachable within 60 min	10	1.0	6111	0.3
Likely irrelevant (<10 min)	806	77.2	1486043	80.7

Population and Area Coverage in Brandenburg (Stroke-Ready Hospitals) — Extended (-20% Speed (Traffic))

Category	Grid Cells	Area (%)	Population	Population (%)
High (30+ min)	3457	8.9	255057	10.4
Medium (20-30 min)	5229	13.5	315027	12.9
Low (10-20 min)	7174	18.5	484374	19.8
Only CT reachable within 60 min	7907	20.3	169720	7.0
Neither reachable within 60 min	392	1.0	4460	0.2
Likely irrelevant (<10 min)	14698	37.8	1213344	49.7

Population and Area Coverage in Bayern (Stroke-Ready Hospitals) — Extended (-20% Speed (Traffic))

Category	Grid Cells	Area (%)	Population	Population (%)
High (30+ min)	4993	5.8	658493	5.0
Medium (20-30 min)	11971	14.0	1263226	9.5
Low (10-20 min)	18352	21.4	2927369	22.1
Only CT reachable within 60 min	428	0.5	12360	0.1
Neither reachable within 60 min	2271	2.7	17056	0.1
Likely irrelevant (<10 min)	47678	55.6	8392626	63.2

Population and Area Coverage in Hessen (Stroke-Ready Hospitals) — Extended (-20% Speed (Traffic))

Category	Grid Cells	Area (%)	Population	Population (%)
High (30+ min)	2424	9.1	245514	3.9
Medium (20-30 min)	4150	15.5	454543	7.3
Low (10-20 min)	7024	26.3	1218978	19.5
Only CT reachable within 60 min	569	2.1	33103	0.5
Neither reachable within 60 min	0	0.0	0	0.0
Likely irrelevant (<10 min)	12581	47.0	4302272	68.8

Population and Area Coverage in Mecklenburg-Vorpommern (Stroke-Ready Hospitals) — Extended (-20% Speed (Traffic))

Category	Grid Cells	Area (%)	Population	Population (%)
High (30+ min)	2681	8.6	171802	11.7
Medium (20-30 min)	4184	13.4	121051	8.3
Low (10-20 min)	5349	17.2	216457	14.8
Only CT reachable within 60 min	7293	23.4	170874	11.7
Neither reachable within 60 min	1443	4.6	21553	1.5
Likely irrelevant (<10 min)	10231	32.8	762258	52.1

Population and Area Coverage in Saarland (Stroke-Ready Hospitals) — Extended (-20% Speed (Traffic))

Category	Grid Cells	Area (%)	Population	Population (%)
High (30+ min)	64	2.0	7243	0.7
Medium (20-30 min)	265	8.2	34345	3.5
Low (10-20 min)	627	19.5	119900	12.4
Only CT reachable within 60 min	0	0.0	1	0.0
Neither reachable within 60 min	16	0.5	1943	0.2
Likely irrelevant (<10 min)	2248	69.8	806903	83.2

Population and Area Coverage in Rheinland-Pfalz (Stroke-Ready Hospitals) — Extended (-20% Speed (Traffic))

Category	Grid Cells	Area (%)	Population	Population (%)
High (30+ min)	2342	9.4	272657	6.5
Medium (20-30 min)	3311	13.3	355195	8.5
Low (10-20 min)	6029	24.2	853888	20.5
Only CT reachable within 60 min	1233	5.0	43867	1.1
Neither reachable within 60 min	34	0.1	1232	0.0
Likely irrelevant (<10 min)	11945	48.0	2637446	63.3

Population and Area Coverage in Nordrhein-Westfalen (Stroke-Ready Hospitals) — Extended (-20% Speed (Traffic))

Category	Grid Cells	Area (%)	Population	Population (%)
High (30+ min)	2574	5.8	491274	2.7
Medium (20-30 min)	5280	12.0	1370329	7.5
Low (10-20 min)	11428	25.9	4660381	25.4
Only CT reachable within 60 min	1740	3.9	88455	0.5
Neither reachable within 60 min	127	0.3	8668	0.0
Likely irrelevant (<10 min)	23022	52.1	11747808	64.0

Population and Area Coverage in Niedersachsen (Stroke-Ready Hospitals) — Extended (-20% Speed (Traffic))

Category	Grid Cells	Area (%)	Population	Population (%)
High (30+ min)	5377	8.5	675336	8.4
Medium (20-30 min)	9509	15.1	991492	12.3
Low (10-20 min)	11617	18.4	1428755	17.8
Only CT reachable within 60 min	2491	3.9	80583	1.0
Neither reachable within 60 min	368	0.6	17924	0.2
Likely irrelevant (<10 min)	33808	53.5	4845772	60.3

Population and Area Coverage in Sachsen-Anhalt (Stroke-Ready Hospitals) — Extended (-20% Speed (Traffic))

Category	Grid Cells	Area (%)	Population	Population (%)
High (30+ min)	2406	8.9	249029	11.8
Medium (20-30 min)	3913	14.5	240605	11.4
Low (10-20 min)	4622	17.2	273489	13.0
Only CT reachable within 60 min	5199	19.3	119578	5.7
Neither reachable within 60 min	259	1.0	1046	0.0
Likely irrelevant (<10 min)	10537	39.1	1219467	58.0

Population and Area Coverage in Sachsen (Stroke-Ready Hospitals) — Extended (-20% Speed (Traffic))

Category	Grid Cells	Area (%)	Population	Population (%)
High (30+ min)	2410	10.4	388490	9.5
Medium (20-30 min)	4134	17.8	457868	11.2
Low (10-20 min)	4723	20.3	717669	17.6
Only CT reachable within 60 min	1641	7.1	95650	2.3
Neither reachable within 60 min	106	0.5	768	0.0
Likely irrelevant (<10 min)	10238	44.0	2419060	59.3

Population and Area Coverage in Schleswig-Holstein (Stroke-Ready Hospitals) — Extended (-20% Speed (Traffic))

Category	Grid Cells	Area (%)	Population	Population (%)
High (30+ min)	1133	5.5	141531	5.2
Medium (20-30 min)	1897	9.2	294552	10.7
Low (10-20 min)	3762	18.2	516452	18.8
Only CT reachable within 60 min	683	3.3	42299	1.5
Neither reachable within 60 min	360	1.7	12495	0.5
Likely irrelevant (<10 min)	12844	62.1	1734946	63.3

Population and Area Coverage in Thüringen (Stroke-Ready Hospitals) — Extended (-20% Speed (Traffic))

Category	Grid Cells	Area (%)	Population	Population (%)
High (30+ min)	779	3.7	127166	6.3
Medium (20-30 min)	2309	11.1	166709	8.2
Low (10-20 min)	5890	28.3	417641	20.6
Only CT reachable within 60 min	185	0.9	1760	0.1
Neither reachable within 60 min	0	0.0	0	0.0
Likely irrelevant (<10 min)	11656	56.0	1316898	64.9

Population and Area Coverage in Berlin (Stroke Units) — Regular (-20% Speed (Traffic))

Category	Grid Cells	Area (%)	Population	Population (%)
High (30+ min)	0	0.0	0	0.0
Medium (20-30 min)	32	2.7	42255	1.2
Low (10-20 min)	272	23.1	770346	21.3
Only CT reachable within 60 min	0	0.0	0	0.0
Neither reachable within 60 min	0	0.0	0	0.0
Likely irrelevant (<10 min)	872	74.1	2800825	77.5

Population and Area Coverage in Baden-Württemberg (Stroke Units) — Regular (-20% Speed (Traffic))

Category	Grid Cells	Area (%)	Population	Population (%)
High (30+ min)	3940	9.1	785155	7.2
Medium (20-30 min)	7250	16.7	1312385	12.1
Low (10-20 min)	8304	19.1	1946733	18.0
Only CT reachable within 60 min	3866	8.9	272160	2.5
Neither reachable within 60 min	68	0.2	1668	0.0
Likely irrelevant (<10 min)	19950	46.0	6515044	60.1

Population and Area Coverage in Bremen (Stroke Units) — Regular (-20% Speed (Traffic))

Category	Grid Cells	Area (%)	Population	Population (%)
High (30+ min)	1	0.2	992	0.2
Medium (20-30 min)	25	6.2	17590	3.4
Low (10-20 min)	191	47.5	206064	39.3
Only CT reachable within 60 min	0	0.0	0	0.0
Neither reachable within 60 min	0	0.0	0	0.0
Likely irrelevant (<10 min)	185	46.0	299257	57.1

Population and Area Coverage in Hamburg (Stroke Units) — Regular (-20% Speed (Traffic))

Category	Grid Cells	Area (%)	Population	Population (%)
High (30+ min)	0	0.0	0	0.0
Medium (20-30 min)	37	3.5	54561	3.0
Low (10-20 min)	191	18.3	295867	16.1
Only CT reachable within 60 min	0	0.0	0	0.0
Neither reachable within 60 min	10	1.0	6111	0.3
Likely irrelevant (<10 min)	806	77.2	1486043	80.7

Population and Area Coverage in Brandenburg (Stroke Units) — Regular (-20% Speed (Traffic))

Category	Grid Cells	Area (%)	Population	Population (%)
High (30+ min)	3717	9.6	270149	11.1
Medium (20-30 min)	5599	14.4	354952	14.5
Low (10-20 min)	6436	16.6	451631	18.5
Only CT reachable within 60 min	13902	35.8	422374	17.3
Neither reachable within 60 min	396	1.0	4485	0.2
Likely irrelevant (<10 min)	8807	22.7	938391	38.4

Population and Area Coverage in Bayern (Stroke Units) — Regular (-20% Speed (Traffic))

Category	Grid Cells	Area (%)	Population	Population (%)
High (30+ min)	6540	7.6	837548	6.3
Medium (20-30 min)	13357	15.6	1377967	10.4
Low (10-20 min)	18531	21.6	2991780	22.5
Only CT reachable within 60 min	795	0.9	25112	0.2
Neither reachable within 60 min	2271	2.7	17059	0.1
Likely irrelevant (<10 min)	44199	51.6	8021664	60.4

Population and Area Coverage in Hessen (Stroke Units) — Regular (-20% Speed (Traffic))

Category	Grid Cells	Area (%)	Population	Population (%)
High (30+ min)	4378	16.4	438533	7.0
Medium (20-30 min)	4236	15.8	520000	8.3
Low (10-20 min)	6295	23.5	1308931	20.9
Only CT reachable within 60 min	1312	4.9	72897	1.2
Neither reachable within 60 min	0	0.0	0	0.0
Likely irrelevant (<10 min)	10527	39.4	3914049	62.6

Population and Area Coverage in Mecklenburg-Vorpommern (Stroke Units) — Regular (-20% Speed (Traffic))

Category	Grid Cells	Area (%)	Population	Population (%)
High (30+ min)	2638	8.5	171272	11.7
Medium (20-30 min)	4207	13.5	124566	8.5
Low (10-20 min)	5131	16.5	233472	15.9
Only CT reachable within 60 min	8145	26.1	184711	12.6
Neither reachable within 60 min	1444	4.6	22243	1.5
Likely irrelevant (<10 min)	9616	30.8	727731	49.7

Population and Area Coverage in Saarland (Stroke Units) — Regular (-20% Speed (Traffic))

Category	Grid Cells	Area (%)	Population	Population (%)
High (30+ min)	64	2.0	7243	0.7
Medium (20-30 min)	268	8.3	34422	3.5
Low (10-20 min)	623	19.3	119813	12.3
Only CT reachable within 60 min	2	0.1	10	0.0
Neither reachable within 60 min	16	0.5	1943	0.2
Likely irrelevant (<10 min)	2247	69.8	806904	83.2

Population and Area Coverage in Rheinland-Pfalz (Stroke Units) — Regular (-20% Speed (Traffic))

Category	Grid Cells	Area (%)	Population	Population (%)
High (30+ min)	3144	12.6	413897	9.9
Medium (20-30 min)	4560	18.3	574481	13.8
Low (10-20 min)	6661	26.8	973551	23.4
Only CT reachable within 60 min	1541	6.2	57066	1.4
Neither reachable within 60 min	34	0.1	1232	0.0
Likely irrelevant (<10 min)	8954	36.0	2144058	51.5

Population and Area Coverage in Nordrhein-Westfalen (Stroke Units) — Regular (-20% Speed (Traffic))

Category	Grid Cells	Area (%)	Population	Population (%)
High (30+ min)	4474	10.1	963463	5.2
Medium (20-30 min)	7016	15.9	1873645	10.2
Low (10-20 min)	11893	26.9	5184256	28.2
Only CT reachable within 60 min	1858	4.2	99187	0.5
Neither reachable within 60 min	127	0.3	8667	0.0
Likely irrelevant (<10 min)	18803	42.6	10237697	55.7

Population and Area Coverage in Niedersachsen (Stroke Units) — Regular (-20% Speed (Traffic))

Category	Grid Cells	Area (%)	Population	Population (%)
High (30+ min)	7325	11.6	976677	12.1
Medium (20-30 min)	11872	18.8	1329569	16.5
Low (10-20 min)	13310	21.1	1695091	21.1
Only CT reachable within 60 min	5677	9.0	196127	2.4
Neither reachable within 60 min	368	0.6	17924	0.2
Likely irrelevant (<10 min)	24618	39.0	3824474	47.6

Population and Area Coverage in Sachsen-Anhalt (Stroke Units) — Regular (-20% Speed (Traffic))

Category	Grid Cells	Area (%)	Population	Population (%)
High (30+ min)	2847	10.6	414597	19.7
Medium (20-30 min)	4115	15.3	302370	14.4
Low (10-20 min)	4336	16.1	271135	12.9
Only CT reachable within 60 min	10179	37.8	316356	15.0
Neither reachable within 60 min	260	1.0	1045	0.0
Likely irrelevant (<10 min)	5199	19.3	797711	37.9

Population and Area Coverage in Sachsen (Stroke Units) — Regular (-20% Speed (Traffic))

Category	Grid Cells	Area (%)	Population	Population (%)
High (30+ min)	3456	14.9	539182	13.2
Medium (20-30 min)	4190	18.0	579409	14.2
Low (10-20 min)	4488	19.3	717931	17.6
Only CT reachable within 60 min	2512	10.8	153790	3.8
Neither reachable within 60 min	106	0.5	768	0.0
Likely irrelevant (<10 min)	8500	36.6	2088425	51.2

Population and Area Coverage in Schleswig-Holstein (Stroke Units) — Regular (-20% Speed (Traffic))

Category	Grid Cells	Area (%)	Population	Population (%)
High (30+ min)	1139	5.5	141629	5.2
Medium (20-30 min)	1922	9.3	295992	10.8
Low (10-20 min)	3793	18.3	522991	19.1
Only CT reachable within 60 min	701	3.4	42599	1.6
Neither reachable within 60 min	367	1.8	12643	0.5
Likely irrelevant (<10 min)	12757	61.7	1726421	63.0

Population and Area Coverage in Thüringen (Stroke Units) — Regular (-20% Speed (Traffic))

Category	Grid Cells	Area (%)	Population	Population (%)
High (30+ min)	1701	8.2	197696	9.7
Medium (20-30 min)	3039	14.6	200839	9.9
Low (10-20 min)	5566	26.7	404822	19.9
Only CT reachable within 60 min	652	3.1	17162	0.8
Neither reachable within 60 min	0	0.0	0	0.0
Likely irrelevant (<10 min)	9861	47.4	1209655	59.6

Population and Area Coverage in Berlin (Stroke-Ready Hospitals) — Extended (Normal Speed)

Category	Grid Cells	Area (%)	Population	Population (%)
High (30+ min)	1	0.1	514	0.0
Medium (20-30 min)	8	0.7	11042	0.3
Low (10-20 min)	242	20.6	643773	17.8
Only CT reachable within 60 min	0	0.0	0	0.0
Neither reachable within 60 min	0	0.0	0	0.0
Likely irrelevant (<10 min)	925	78.7	2958097	81.9

Population and Area Coverage in Baden-Württemberg (Stroke-Ready Hospitals) — Extended (Normal Speed)

Category	Grid Cells	Area (%)	Population	Population (%)
High (30+ min)	936	2.2	116705	1.1
Medium (20-30 min)	3572	8.2	645316	6.0
Low (10-20 min)	8634	19.9	1846660	17.0
Only CT reachable within 60 min	43	0.1	2181	0.0
Neither reachable within 60 min	33	0.1	1470	0.0
Likely irrelevant (<10 min)	30160	69.5	8220813	75.9

Population and Area Coverage in Bremen (Stroke-Ready Hospitals) — Extended (Normal Speed)

Category	Grid Cells	Area (%)	Population	Population (%)
High (30+ min)	0	0.0	0	0.0
Medium (20-30 min)	12	3.0	4542	0.9
Low (10-20 min)	113	28.1	109245	20.9
Only CT reachable within 60 min	0	0.0	0	0.0
Neither reachable within 60 min	0	0.0	0	0.0
Likely irrelevant (<10 min)	277	68.9	410116	78.3

Population and Area Coverage in Hamburg (Stroke-Ready Hospitals) — Extended (Normal Speed)

Category	Grid Cells	Area (%)	Population	Population (%)
High (30+ min)	0	0.0	0	0.0
Medium (20-30 min)	13	1.2	21004	1.1
Low (10-20 min)	159	15.2	213721	11.6
Only CT reachable within 60 min	0	0.0	0	0.0
Neither reachable within 60 min	10	1.0	6092	0.3
Likely irrelevant (<10 min)	862	82.6	1601765	86.9

Population and Area Coverage in Brandenburg (Stroke-Ready Hospitals) — Extended (Normal Speed)

Category	Grid Cells	Area (%)	Population	Population (%)
High (30+ min)	4406	11.3	260011	10.6
Medium (20-30 min)	6275	16.1	287939	11.8
Low (10-20 min)	9559	24.6	528370	21.6
Only CT reachable within 60 min	2529	6.5	43533	1.8
Neither reachable within 60 min	180	0.5	3399	0.1
Likely irrelevant (<10 min)	15908	40.9	1318730	54.0

Population and Area Coverage in Bayern (Stroke-Ready Hospitals) — Extended (Normal Speed)

Category	Grid Cells	Area (%)	Population	Population (%)
High (30+ min)	1268	1.5	208993	1.6
Medium (20-30 min)	9597	11.2	1035285	7.8
Low (10-20 min)	20907	24.4	2984116	22.5
Only CT reachable within 60 min	78	0.1	2188	0.0
Neither reachable within 60 min	2186	2.6	15199	0.1
Likely irrelevant (<10 min)	51657	60.3	9025349	68.0

Population and Area Coverage in Hessen (Stroke-Ready Hospitals) — Extended (Normal Speed)

Category	Grid Cells	Area (%)	Population	Population (%)
High (30+ min)	1438	5.4	141136	2.3
Medium (20-30 min)	3440	12.9	359958	5.8
Low (10-20 min)	7699	28.8	1086857	17.4
Only CT reachable within 60 min	37	0.1	677	0.0
Neither reachable within 60 min	0	0.0	0	0.0
Likely irrelevant (<10 min)	14134	52.8	4665782	74.6

Population and Area Coverage in Mecklenburg-Vorpommern (Stroke-Ready Hospitals) — Extended (Normal Speed)

Category	Grid Cells	Area (%)	Population	Population (%)
High (30+ min)	3562	11.4	211026	14.4
Medium (20-30 min)	5527	17.7	154395	10.5
Low (10-20 min)	6992	22.4	219220	15.0
Only CT reachable within 60 min	2481	8.0	46235	3.2
Neither reachable within 60 min	976	3.1	13806	0.9
Likely irrelevant (<10 min)	11643	37.3	819313	56.0

Population and Area Coverage in Saarland (Stroke-Ready Hospitals) — Extended (Normal Speed)

Category	Grid Cells	Area (%)	Population	Population (%)
High (30+ min)	4	0.1	325	0.0
Medium (20-30 min)	195	6.1	24200	2.5
Low (10-20 min)	597	18.5	91936	9.5
Only CT reachable within 60 min	1	0.0	64	0.0
Neither reachable within 60 min	17	0.5	1958	0.2
Likely irrelevant (<10 min)	2406	74.7	851852	87.8

Population and Area Coverage in Rheinland-Pfalz (Stroke-Ready Hospitals) — Extended (Normal Speed)

Category	Grid Cells	Area (%)	Population	Population (%)
High (30+ min)	1601	6.4	124856	3.0
Medium (20-30 min)	3560	14.3	360892	8.7
Low (10-20 min)	6312	25.4	831737	20.0
Only CT reachable within 60 min	130	0.5	1700	0.0
Neither reachable within 60 min	29	0.1	1114	0.0
Likely irrelevant (<10 min)	13262	53.3	2843986	68.3

Population and Area Coverage in Nordrhein-Westfalen (Stroke-Ready Hospitals) — Extended (Normal Speed)

Category	Grid Cells	Area (%)	Population	Population (%)
High (30+ min)	2317	5.2	317543	1.7
Medium (20-30 min)	4007	9.1	827777	4.5
Low (10-20 min)	11789	26.7	4157622	22.6
Only CT reachable within 60 min	271	0.6	3238	0.0
Neither reachable within 60 min	116	0.3	8872	0.0
Likely irrelevant (<10 min)	25671	58.1	13051863	71.1

Population and Area Coverage in Niedersachsen (Stroke-Ready Hospitals) — Extended (Normal Speed)

Category	Grid Cells	Area (%)	Population	Population (%)
High (30+ min)	3211	5.1	367633	4.6
Medium (20-30 min)	9059	14.3	915132	11.4
Low (10-20 min)	13745	21.8	1542219	19.2
Only CT reachable within 60 min	719	1.1	9815	0.1
Neither reachable within 60 min	353	0.6	17274	0.2
Likely irrelevant (<10 min)	36083	57.1	5187789	64.5

Population and Area Coverage in Sachsen-Anhalt (Stroke-Ready Hospitals) — Extended (Normal Speed)

Category	Grid Cells	Area (%)	Population	Population (%)
High (30+ min)	2832	10.5	174515	8.3
Medium (20-30 min)	4848	18.0	306348	14.6
Low (10-20 min)	6183	23.0	297707	14.2
Only CT reachable within 60 min	1546	5.7	19488	0.9
Neither reachable within 60 min	0	0.0	0	0.0
Likely irrelevant (<10 min)	11527	42.8	1305156	62.1

Population and Area Coverage in Sachsen (Stroke-Ready Hospitals) — Extended (Normal Speed)

Category	Grid Cells	Area (%)	Population	Population (%)
High (30+ min)	1509	6.5	258188	6.3
Medium (20-30 min)	4329	18.6	445550	10.9
Low (10-20 min)	5955	25.6	786210	19.3
Only CT reachable within 60 min	157	0.7	6222	0.2
Neither reachable within 60 min	102	0.4	656	0.0
Likely irrelevant (<10 min)	11200	48.2	2582679	63.3

Population and Area Coverage in Schleswig-Holstein (Stroke-Ready Hospitals) — Extended (Normal Speed)

Category	Grid Cells	Area (%)	Population	Population (%)
High (30+ min)	850	4.1	81419	3.0
Medium (20-30 min)	1613	7.8	221237	8.1
Low (10-20 min)	3925	19.0	511826	18.7
Only CT reachable within 60 min	232	1.1	27767	1.0
Neither reachable within 60 min	336	1.6	11988	0.4
Likely irrelevant (<10 min)	13723	66.4	1888038	68.8

Population and Area Coverage in Thüringen (Stroke-Ready Hospitals) — Extended (Normal Speed)

Category	Grid Cells	Area (%)	Population	Population (%)
High (30+ min)	199	1.0	50194	2.5
Medium (20-30 min)	1637	7.9	151954	7.5
Low (10-20 min)	6185	29.7	408082	20.1
Only CT reachable within 60 min	0	0.0	0	0.0
Neither reachable within 60 min	0	0.0	0	0.0
Likely irrelevant (<10 min)	12798	61.5	1419944	69.9

Population and Area Coverage in Berlin (Stroke Units) — Regular (Normal Speed)

Category	Grid Cells	Area (%)	Population	Population (%)
High (30+ min)	1	0.1	514	0.0
Medium (20-30 min)	9	0.8	11334	0.3
Low (10-20 min)	242	20.6	645709	17.9
Only CT reachable within 60 min	0	0.0	0	0.0
Neither reachable within 60 min	0	0.0	0	0.0
Likely irrelevant (<10 min)	924	78.6	2955869	81.8

Population and Area Coverage in Baden-Württemberg (Stroke Units) — Regular (Normal Speed)

Category	Grid Cells	Area (%)	Population	Population (%)
High (30+ min)	2973	6.9	470081	4.3
Medium (20-30 min)	6826	15.7	1146667	10.6
Low (10-20 min)	10494	24.2	2163589	20.0
Only CT reachable within 60 min	1362	3.1	101057	0.9
Neither reachable within 60 min	33	0.1	1470	0.0
Likely irrelevant (<10 min)	21690	50.0	6950281	64.2

Population and Area Coverage in Bremen (Stroke Units) — Regular (Normal Speed)

Category	Grid Cells	Area (%)	Population	Population (%)
High (30+ min)	0	0.0	0	0.0
Medium (20-30 min)	17	4.2	4767	0.9
Low (10-20 min)	153	38.1	174858	33.4
Only CT reachable within 60 min	0	0.0	0	0.0
Neither reachable within 60 min	0	0.0	0	0.0
Likely irrelevant (<10 min)	232	57.7	344278	65.7

Population and Area Coverage in Hamburg (Stroke Units) — Regular (Normal Speed)

Category	Grid Cells	Area (%)	Population	Population (%)
High (30+ min)	0	0.0	0	0.0
Medium (20-30 min)	13	1.2	21004	1.1
Low (10-20 min)	159	15.2	213721	11.6
Only CT reachable within 60 min	0	0.0	0	0.0
Neither reachable within 60 min	10	1.0	6092	0.3
Likely irrelevant (<10 min)	862	82.6	1601765	86.9

Population and Area Coverage in Brandenburg (Stroke Units) — Regular (Normal Speed)

Category	Grid Cells	Area (%)	Population	Population (%)
High (30+ min)	6048	15.6	375541	15.4
Medium (20-30 min)	7013	18.0	332850	13.6
Low (10-20 min)	9349	24.1	533889	21.9
Only CT reachable within 60 min	6658	17.1	175855	7.2
Neither reachable within 60 min	180	0.5	3399	0.1
Likely irrelevant (<10 min)	9609	24.7	1020448	41.8

Population and Area Coverage in Bayern (Stroke Units) — Regular (Normal Speed)

Category	Grid Cells	Area (%)	Population	Population (%)
High (30+ min)	2035	2.4	275240	2.1
Medium (20-30 min)	11481	13.4	1234440	9.3
Low (10-20 min)	21797	25.4	3083180	23.2
Only CT reachable within 60 min	159	0.2	2872	0.0
Neither reachable within 60 min	2186	2.6	15199	0.1
Likely irrelevant (<10 min)	48035	56.1	8660199	65.3

Population and Area Coverage in Hessen (Stroke Units) — Regular (Normal Speed)

Category	Grid Cells	Area (%)	Population	Population (%)
High (30+ min)	3096	11.6	299988	4.8
Medium (20-30 min)	4410	16.5	464075	7.4
Low (10-20 min)	7164	26.8	1230482	19.7
Only CT reachable within 60 min	185	0.7	6027	0.1
Neither reachable within 60 min	0	0.0	0	0.0
Likely irrelevant (<10 min)	11893	44.5	4253838	68.0

Population and Area Coverage in Mecklenburg-Vorpommern (Stroke Units) — Regular (Normal Speed)

Category	Grid Cells	Area (%)	Population	Population (%)
High (30+ min)	3642	11.7	210683	14.4
Medium (20-30 min)	5549	17.8	157141	10.7
Low (10-20 min)	6855	22.0	237095	16.2
Only CT reachable within 60 min	3131	10.0	54174	3.7
Neither reachable within 60 min	977	3.1	13820	0.9
Likely irrelevant (<10 min)	11027	35.4	791082	54.0

Population and Area Coverage in Saarland (Stroke Units) — Regular (Normal Speed)

Category	Grid Cells	Area (%)	Population	Population (%)
High (30+ min)	4	0.1	325	0.0
Medium (20-30 min)	197	6.1	24466	2.5
Low (10-20 min)	595	18.5	91670	9.4
Only CT reachable within 60 min	1	0.0	64	0.0
Neither reachable within 60 min	17	0.5	1958	0.2
Likely irrelevant (<10 min)	2406	74.7	851852	87.8

Population and Area Coverage in Rheinland-Pfalz (Stroke Units) — Regular (Normal Speed)

Category	Grid Cells	Area (%)	Population	Population (%)
High (30+ min)	2093	8.4	204376	4.9
Medium (20-30 min)	4690	18.8	537388	12.9
Low (10-20 min)	7763	31.2	1072504	25.8
Only CT reachable within 60 min	172	0.7	2166	0.1
Neither reachable within 60 min	30	0.1	1114	0.0
Likely irrelevant (<10 min)	10146	40.8	2346737	56.4

Population and Area Coverage in Nordrhein-Westfalen (Stroke Units) — Regular (Normal Speed)

Category	Grid Cells	Area (%)	Population	Population (%)
High (30+ min)	3364	7.6	580743	3.2
Medium (20-30 min)	5875	13.3	1271089	6.9
Low (10-20 min)	13102	29.7	4902560	26.7
Only CT reachable within 60 min	340	0.8	7778	0.0
Neither reachable within 60 min	116	0.3	8872	0.0
Likely irrelevant (<10 min)	21374	48.4	11595873	63.1

Population and Area Coverage in Niedersachsen (Stroke Units) — Regular (Normal Speed)

Category	Grid Cells	Area (%)	Population	Population (%)
High (30+ min)	5558	8.8	636610	7.9
Medium (20-30 min)	11913	18.9	1191450	14.8
Low (10-20 min)	16805	26.6	1963741	24.4
Only CT reachable within 60 min	1333	2.1	24371	0.3
Neither reachable within 60 min	354	0.6	17333	0.2
Likely irrelevant (<10 min)	27207	43.1	4206357	52.3

Population and Area Coverage in Sachsen-Anhalt (Stroke Units) — Regular (Normal Speed)

Category	Grid Cells	Area (%)	Population	Population (%)
High (30+ min)	4273	15.9	382458	18.2
Medium (20-30 min)	4940	18.3	380142	18.1
Low (10-20 min)	5941	22.1	310477	14.8
Only CT reachable within 60 min	5810	21.6	139112	6.6
Neither reachable within 60 min	0	0.0	0	0.0
Likely irrelevant (<10 min)	5972	22.2	891025	42.4

Population and Area Coverage in Sachsen (Stroke Units) — Regular (Normal Speed)

Category	Grid Cells	Area (%)	Population	Population (%)
High (30+ min)	2655	11.4	391828	9.6
Medium (20-30 min)	4939	21.2	587919	14.4
Low (10-20 min)	5688	24.5	824178	20.2
Only CT reachable within 60 min	441	1.9	19215	0.5
Neither reachable within 60 min	104	0.4	655	0.0
Likely irrelevant (<10 min)	9425	40.5	2255710	55.3

Population and Area Coverage in Schleswig-Holstein (Stroke Units) — Regular (Normal Speed)

Category	Grid Cells	Area (%)	Population	Population (%)
High (30+ min)	860	4.2	81613	3.0
Medium (20-30 min)	1622	7.8	221681	8.1
Low (10-20 min)	3964	19.2	518387	18.9
Only CT reachable within 60 min	236	1.1	27810	1.0
Neither reachable within 60 min	343	1.7	12125	0.4
Likely irrelevant (<10 min)	13654	66.0	1880659	68.6

Population and Area Coverage in Thüringen (Stroke Units) — Regular (Normal Speed)

Category	Grid Cells	Area (%)	Population	Population (%)
High (30+ min)	729	3.5	89177	4.4
Medium (20-30 min)	2827	13.6	211925	10.4
Low (10-20 min)	6317	30.3	421170	20.7
Only CT reachable within 60 min	16	0.1	6	0.0
Neither reachable within 60 min	0	0.0	0	0.0
Likely irrelevant (<10 min)	10930	52.5	1307896	64.4

Population and Area Coverage in Kreisfreie Stadt Neumünster (Stroke-Ready Hospitals) (Schleswig-Holstein) — Extended (+10min Penalty)

Category	Grid Cells	Area (%)	Population	Population (%)
High (30+ min)	0	0.0	0	0.0
Medium (20-30 min)	0	0.0	0	0.0
Low (10-20 min)	0	0.0	0	0.0
Only CT reachable within 60 min	0	0.0	0	0.0
Neither reachable within 60 min	0	0.0	0	0.0
Likely irrelevant (<10 min)	100	100.0	85923	100.0

Population and Area Coverage in Kreisfreie Stadt Osnabrück (Stroke-Ready Hospitals) (Niedersachsen) — Extended (+10min Penalty)

Category	Grid Cells	Area (%)	Population	Population (%)
High (30+ min)	0	0.0	0	0.0
Medium (20-30 min)	0	0.0	0	0.0
Low (10-20 min)	5	3.2	1979	1.3
Only CT reachable within 60 min	0	0.0	0	0.0
Neither reachable within 60 min	0	0.0	0	0.0
Likely irrelevant (<10 min)	150	96.8	152462	98.7

Population and Area Coverage in Kreisfreie Stadt Mönchengladbach (Stroke-Ready Hospitals) (Nordrhein-Westfalen) — Extended (+10min Penalty)

Category	Grid Cells	Area (%)	Population	Population (%)
High (30+ min)	0	0.0	0	0.0
Medium (20-30 min)	0	0.0	0	0.0
Low (10-20 min)	9	4.1	8837	3.2
Only CT reachable within 60 min	0	0.0	0	0.0
Neither reachable within 60 min	0	0.0	0	0.0
Likely irrelevant (<10 min)	211	95.9	265284	96.8

Population and Area Coverage in Kreis Coesfeld (Stroke-Ready Hospitals) (Nordrhein-Westfalen) — Extended (+10min Penalty)

Category	Grid Cells	Area (%)	Population	Population (%)
High (30+ min)	0	0.0	0	0.0
Medium (20-30 min)	6	0.4	136	0.1
Low (10-20 min)	301	20.7	47449	22.0
Only CT reachable within 60 min	0	0.0	0	0.0
Neither reachable within 60 min	0	0.0	0	0.0
Likely irrelevant (<10 min)	1147	78.9	168351	78.0

Population and Area Coverage in Kreis Paderborn (Stroke-Ready Hospitals) (Nordrhein-Westfalen) — Extended (+10min Penalty)

Category	Grid Cells	Area (%)	Population	Population (%)
High (30+ min)	28	1.7	10	0.0
Medium (20-30 min)	25	1.5	621	0.2
Low (10-20 min)	711	43.7	69972	21.2
Only CT reachable within 60 min	0	0.0	0	0.0
Neither reachable within 60 min	0	0.0	0	0.0
Likely irrelevant (<10 min)	862	53.0	259918	78.6

Population and Area Coverage in Kreisfreie Stadt Kassel (Stroke-Ready Hospitals) (Hessen) — Extended (+10min Penalty)

Category	Grid Cells	Area (%)	Population	Population (%)
High (30+ min)	0	0.0	0	0.0
Medium (20-30 min)	0	0.0	0	0.0
Low (10-20 min)	23	17.3	467	0.2
Only CT reachable within 60 min	0	0.0	0	0.0
Neither reachable within 60 min	0	0.0	0	0.0
Likely irrelevant (<10 min)	110	82.7	205252	99.8

Population and Area Coverage in Landkreis Hersfeld-Rotenburg (Stroke-Ready Hospitals) (Hessen) — Extended (+10min Penalty)

Category	Grid Cells	Area (%)	Population	Population (%)
High (30+ min)	10	0.7	66	0.1
Medium (20-30 min)	110	7.9	9914	8.2
Low (10-20 min)	266	19.0	22162	18.3
Only CT reachable within 60 min	0	0.0	0	0.0
Neither reachable within 60 min	0	0.0	0	0.0
Likely irrelevant (<10 min)	1015	72.4	88747	73.4

Population and Area Coverage in Landkreis Schwalm-Eder-Kreis (Stroke-Ready Hospitals) (Hessen) — Extended (+10min Penalty)

Category	Grid Cells	Area (%)	Population	Population (%)
High (30+ min)	7	0.4	130	0.1
Medium (20-30 min)	131	6.6	10182	5.6
Low (10-20 min)	779	39.4	62077	34.1
Only CT reachable within 60 min	0	0.0	0	0.0
Neither reachable within 60 min	0	0.0	0	0.0
Likely irrelevant (<10 min)	1062	53.7	109438	60.2

Population and Area Coverage in Landkreis Westerwaldkreis (Stroke-Ready Hospitals) (Rheinland-Pfalz) — Extended (+10min Penalty)

Category	Grid Cells	Area (%)	Population	Population (%)
High (30+ min)	0	0.0	0	0.0
Medium (20-30 min)	64	5.1	12163	5.9
Low (10-20 min)	389	31.0	48279	23.5
Only CT reachable within 60 min	0	0.0	0	0.0
Neither reachable within 60 min	0	0.0	0	0.0
Likely irrelevant (<10 min)	802	63.9	145382	70.6

Population and Area Coverage in Kreisfreie Stadt Frankenthal (Pfalz) (Stroke-Ready Hospitals) (Rheinland-Pfalz) — Extended (+10min Penalty)

Category	Grid Cells	Area (%)	Population	Population (%)
High (30+ min)	0	0.0	0	0.0
Medium (20-30 min)	0	0.0	0	0.0
Low (10-20 min)	0	0.0	0	0.0
Only CT reachable within 60 min	0	0.0	0	0.0
Neither reachable within 60 min	0	0.0	0	0.0
Likely irrelevant (<10 min)	54	100.0	45433	100.0

Population and Area Coverage in Landkreis Böblingen (Stroke-Ready Hospitals) (Baden-Württemberg) — Extended (+10min Penalty)

Category	Grid Cells	Area (%)	Population	Population (%)
High (30+ min)	0	0.0	0	0.0
Medium (20-30 min)	30	4.0	11128	3.0
Low (10-20 min)	305	40.7	141486	38.0
Only CT reachable within 60 min	0	0.0	0	0.0
Neither reachable within 60 min	0	0.0	0	0.0
Likely irrelevant (<10 min)	414	55.3	219735	59.0

Population and Area Coverage in Landkreis Cham (Stroke-Ready Hospitals) (Bayern) — Extended (+10min Penalty)

Category	Grid Cells	Area (%)	Population	Population (%)
High (30+ min)	1	0.1	7	0.0
Medium (20-30 min)	83	4.7	5172	4.1
Low (10-20 min)	525	29.7	32755	25.8
Only CT reachable within 60 min	0	0.0	0	0.0
Neither reachable within 60 min	0	0.0	0	0.0
Likely irrelevant (<10 min)	1158	65.5	88935	70.1

Population and Area Coverage in Landkreis Saarlouis (Stroke-Ready Hospitals) (Saarland) — Extended (+10min Penalty)

Category	Grid Cells	Area (%)	Population	Population (%)
High (30+ min)	0	0.0	0	0.0
Medium (20-30 min)	26	4.6	5040	2.7
Low (10-20 min)	112	19.9	28918	15.4
Only CT reachable within 60 min	1	0.2	320	0.2
Neither reachable within 60 min	2	0.4	41	0.0
Likely irrelevant (<10 min)	423	75.0	152890	81.7

Population and Area Coverage in Landkreis Anhalt-Bitterfeld (Stroke-Ready Hospitals) (Sachsen-Anhalt) — Extended (+10min Penalty)

Category	Grid Cells	Area (%)	Population	Population (%)
High (30+ min)	244	12.8	4484	3.0
Medium (20-30 min)	321	16.8	16802	11.4
Low (10-20 min)	410	21.5	37514	25.4
Only CT reachable within 60 min	20	1.0	0	0.0
Neither reachable within 60 min	0	0.0	0	0.0
Likely irrelevant (<10 min)	916	47.9	88697	60.1

Population and Area Coverage in Landkreis Jerichower Land (Stroke-Ready Hospitals) (Sachsen-Anhalt) — Extended (+10min Penalty)

Category	Grid Cells	Area (%)	Population	Population (%)
High (30+ min)	246	11.8	5907	7.3
Medium (20-30 min)	616	29.5	20337	25.3
Low (10-20 min)	844	40.4	46100	57.3
Only CT reachable within 60 min	154	7.4	916	1.1
Neither reachable within 60 min	0	0.0	0	0.0
Likely irrelevant (<10 min)	228	10.9	7239	9.0

Population and Area Coverage in Landkreis Kyffhäuserkreis (Stroke-Ready Hospitals) (Thüringen) — Extended (+10min Penalty)

Category	Grid Cells	Area (%)	Population	Population (%)
High (30+ min)	30	2.2	1383	2.0
Medium (20-30 min)	303	22.7	18359	26.3
Low (10-20 min)	345	25.8	14203	20.4
Only CT reachable within 60 min	0	0.0	0	0.0
Neither reachable within 60 min	0	0.0	0	0.0
Likely irrelevant (<10 min)	659	49.3	35747	51.3

Population and Area Coverage in Landkreis Peine (Stroke-Ready Hospitals) (Niedersachsen) — Extended (+10min Penalty)

Category	Grid Cells	Area (%)	Population	Population (%)
High (30+ min)	0	0.0	0	0.0
Medium (20-30 min)	0	0.0	0	0.0
Low (10-20 min)	28	3.9	4170	3.2
Only CT reachable within 60 min	0	0.0	0	0.0
Neither reachable within 60 min	0	0.0	0	0.0
Likely irrelevant (<10 min)	684	96.1	126547	96.8

Population and Area Coverage in Kreisfreie Stadt Leverkusen (Stroke-Ready Hospitals) (Nordrhein-Westfalen) — Extended (+10min Penalty)

Category	Grid Cells	Area (%)	Population	Population (%)
High (30+ min)	0	0.0	0	0.0
Medium (20-30 min)	0	0.0	0	0.0
Low (10-20 min)	5	4.9	1786	1.1
Only CT reachable within 60 min	0	0.0	0	0.0
Neither reachable within 60 min	0	0.0	0	0.0
Likely irrelevant (<10 min)	97	95.1	158884	98.9

Population and Area Coverage in Kreis Ennepe-Ruhr-Kreis (Stroke-Ready Hospitals) (Nordrhein-Westfalen) — Extended (+10min Penalty)

Category	Grid Cells	Area (%)	Population	Population (%)
High (30+ min)	0	0.0	0	0.0
Medium (20-30 min)	1	0.2	8	0.0
Low (10-20 min)	41	7.6	6536	2.0
Only CT reachable within 60 min	0	0.0	0	0.0
Neither reachable within 60 min	0	0.0	0	0.0
Likely irrelevant (<10 min)	494	92.2	326117	98.0

Population and Area Coverage in Kreis Märkischer Kreis (Stroke-Ready Hospitals) (Nordrhein-Westfalen) — Extended (+10min Penalty)

Category	Grid Cells	Area (%)	Population	Population (%)
High (30+ min)	14	1.0	580	0.1
Medium (20-30 min)	126	9.3	29213	7.0
Low (10-20 min)	427	31.5	93038	22.4
Only CT reachable within 60 min	0	0.0	0	0.0
Neither reachable within 60 min	0	0.0	0	0.0
Likely irrelevant (<10 min)	790	58.2	292563	70.4

Population and Area Coverage in Landkreis Ahrweiler (Stroke-Ready Hospitals) (Rheinland-Pfalz) — Extended (+10min Penalty)

Category	Grid Cells	Area (%)	Population	Population (%)
High (30+ min)	0	0.0	0	0.0
Medium (20-30 min)	0	0.0	0	0.0
Low (10-20 min)	217	21.9	30237	23.5
Only CT reachable within 60 min	0	0.0	0	0.0
Neither reachable within 60 min	0	0.0	0	0.0
Likely irrelevant (<10 min)	776	78.1	98650	76.5

Population and Area Coverage in Stadtkreis Baden-Baden (Stroke-Ready Hospitals) (Baden-Württemberg) — Extended (+10min Penalty)

Category	Grid Cells	Area (%)	Population	Population (%)
High (30+ min)	2	1.1	1	0.0
Medium (20-30 min)	75	41.9	7431	11.8
Low (10-20 min)	52	29.1	35484	56.4
Only CT reachable within 60 min	0	0.0	0	0.0
Neither reachable within 60 min	0	0.0	0	0.0
Likely irrelevant (<10 min)	50	27.9	20025	31.8

Population and Area Coverage in Landkreis Waldshut (Stroke-Ready Hospitals) (Baden-Württemberg) — Extended (+10min Penalty)

Category	Grid Cells	Area (%)	Population	Population (%)
High (30+ min)	0	0.0	0	0.0
Medium (20-30 min)	5	0.4	1898	1.3
Low (10-20 min)	77	6.0	14458	10.2
Only CT reachable within 60 min	0	0.0	0	0.0
Neither reachable within 60 min	2	0.2	204	0.1
Likely irrelevant (<10 min)	1196	93.4	124989	88.3

Population and Area Coverage in Landkreis Bad Tölz-Wolfratshausen (Stroke-Ready Hospitals) (Bayern) — Extended (+10min Penalty)

Category	Grid Cells	Area (%)	Population	Population (%)
High (30+ min)	0	0.0	0	0.0
Medium (20-30 min)	13	1.0	919	0.7
Low (10-20 min)	291	22.1	50782	40.3
Only CT reachable within 60 min	1	0.1	5	0.0
Neither reachable within 60 min	109	8.3	176	0.1
Likely irrelevant (<10 min)	905	68.6	74275	58.9

Population and Area Coverage in Landkreis Passau (Stroke-Ready Hospitals) (Bayern) — Extended (+10min Penalty)

Category	Grid Cells	Area (%)	Population	Population (%)
High (30+ min)	48	2.7	2798	1.5
Medium (20-30 min)	178	9.9	14102	7.4
Low (10-20 min)	404	22.4	40008	21.1
Only CT reachable within 60 min	1	0.1	113	0.1
Neither reachable within 60 min	10	0.6	139	0.1
Likely irrelevant (<10 min)	1159	64.4	132553	69.9

Population and Area Coverage in Landkreis Lichtenfels (Stroke-Ready Hospitals) (Bayern) — Extended (+10min Penalty)

Category	Grid Cells	Area (%)	Population	Population (%)
High (30+ min)	0	0.0	0	0.0
Medium (20-30 min)	0	0.0	0	0.0
Low (10-20 min)	65	9.9	1754	2.5
Only CT reachable within 60 min	0	0.0	0	0.0
Neither reachable within 60 min	0	0.0	0	0.0
Likely irrelevant (<10 min)	590	90.1	67443	97.5

Population and Area Coverage in Landkreis Wunsiedel i.Fichtelgebirge (Stroke-Ready Hospitals) (Bayern) — Extended (+10min Penalty)

Category	Grid Cells	Area (%)	Population	Population (%)
High (30+ min)	6	0.8	15	0.0
Medium (20-30 min)	128	17.9	24761	33.5
Low (10-20 min)	392	54.9	29272	39.6
Only CT reachable within 60 min	0	0.0	0	0.0
Neither reachable within 60 min	0	0.0	0	0.0
Likely irrelevant (<10 min)	188	26.3	19888	26.9

Population and Area Coverage in Landkreis Ansbach (Stroke-Ready Hospitals) (Bayern) — Extended (+10min Penalty)

Category	Grid Cells	Area (%)	Population	Population (%)
High (30+ min)	18	0.7	575	0.3
Medium (20-30 min)	167	6.9	17135	9.3
Low (10-20 min)	315	13.0	18351	9.9
Only CT reachable within 60 min	0	0.0	0	0.0
Neither reachable within 60 min	0	0.0	0	0.0
Likely irrelevant (<10 min)	1932	79.4	148815	80.5

Population and Area Coverage in Kreisfreie Stadt Würzburg (Stroke-Ready Hospitals) (Bayern) — Extended (+10min Penalty)

Category	Grid Cells	Area (%)	Population	Population (%)
High (30+ min)	0	0.0	0	0.0
Medium (20-30 min)	0	0.0	0	0.0
Low (10-20 min)	0	0.0	0	0.0
Only CT reachable within 60 min	0	0.0	0	0.0
Neither reachable within 60 min	0	0.0	0	0.0
Likely irrelevant (<10 min)	109	100.0	128110	100.0

Population and Area Coverage in Landkreis Oder-Spree (Stroke-Ready Hospitals) (Brandenburg) — Extended (+10min Penalty)

Category	Grid Cells	Area (%)	Population	Population (%)
High (30+ min)	28	0.9	2149	1.2
Medium (20-30 min)	322	10.8	11635	6.8
Low (10-20 min)	1078	36.3	52686	30.6
Only CT reachable within 60 min	39	1.3	198	0.1
Neither reachable within 60 min	7	0.2	49	0.0
Likely irrelevant (<10 min)	1495	50.4	105213	61.2

Population and Area Coverage in Landkreis Potsdam-Mittelmark (Stroke-Ready Hospitals) (Brandenburg) — Extended (+10min Penalty)

Category	Grid Cells	Area (%)	Population	Population (%)
High (30+ min)	432	12.7	15586	7.7
Medium (20-30 min)	851	25.0	29951	14.8
Low (10-20 min)	1055	31.0	42065	20.7
Only CT reachable within 60 min	204	6.0	723	0.4
Neither reachable within 60 min	0	0.0	0	0.0
Likely irrelevant (<10 min)	865	25.4	114673	56.5

Population and Area Coverage in Landkreis Uckermark (Stroke-Ready Hospitals) (Brandenburg) — Extended (+10min Penalty)

Category	Grid Cells	Area (%)	Population	Population (%)
High (30+ min)	756	18.3	27134	23.5
Medium (20-30 min)	889	21.6	11964	10.3
Low (10-20 min)	716	17.4	17331	15.0
Only CT reachable within 60 min	808	19.6	16472	14.2
Neither reachable within 60 min	85	2.1	1901	1.6
Likely irrelevant (<10 min)	867	21.0	40856	35.3

Population and Area Coverage in Landkreis Sächsische Schweiz-Osterzgebirge (Stroke-Ready Hospitals) (Sachsen) — Extended (+10min Penalty)

Category	Grid Cells	Area (%)	Population	Population (%)
High (30+ min)	209	10.1	14549	6.4
Medium (20-30 min)	579	27.9	63697	27.9
Low (10-20 min)	640	30.9	88891	39.0
Only CT reachable within 60 min	55	2.7	452	0.2
Neither reachable within 60 min	40	1.9	173	0.1
Likely irrelevant (<10 min)	549	26.5	60397	26.5

Population and Area Coverage in Kreisfreie Stadt Leipzig (Stroke-Ready Hospitals) (Sachsen) — Extended (+10min Penalty)

Category	Grid Cells	Area (%)	Population	Population (%)
High (30+ min)	0	0.0	0	0.0
Medium (20-30 min)	0	0.0	0	0.0
Low (10-20 min)	22	5.7	18160	2.9
Only CT reachable within 60 min	0	0.0	0	0.0
Neither reachable within 60 min	0	0.0	0	0.0
Likely irrelevant (<10 min)	362	94.3	601166	97.1

Population and Area Coverage in Kreisfreie Stadt Jena (Stroke-Ready Hospitals) (Thüringen) — Extended (+10min Penalty)

Category	Grid Cells	Area (%)	Population	Population (%)
High (30+ min)	0	0.0	0	0.0
Medium (20-30 min)	2	1.3	0	0.0
Low (10-20 min)	24	16.1	3222	3.2
Only CT reachable within 60 min	0	0.0	0	0.0
Neither reachable within 60 min	0	0.0	0	0.0
Likely irrelevant (<10 min)	123	82.6	97024	96.8

Population and Area Coverage in Landkreis Gotha (Stroke-Ready Hospitals) (Thüringen) — Extended (+10min Penalty)

Category	Grid Cells	Area (%)	Population	Population (%)
High (30+ min)	0	0.0	0	0.0
Medium (20-30 min)	7	0.6	42	0.0
Low (10-20 min)	252	21.0	10314	8.4
Only CT reachable within 60 min	0	0.0	0	0.0
Neither reachable within 60 min	0	0.0	0	0.0
Likely irrelevant (<10 min)	940	78.4	111920	91.5

Population and Area Coverage in Kreis Pinneberg (Stroke-Ready Hospitals) (Schleswig-Holstein) — Extended (+10min Penalty)

Category	Grid Cells	Area (%)	Population	Population (%)
High (30+ min)	0	0.0	0	0.0
Medium (20-30 min)	1	0.1	21	0.0
Low (10-20 min)	117	13.8	38325	12.8
Only CT reachable within 60 min	1	0.1	14	0.0
Neither reachable within 60 min	19	2.2	123	0.0
Likely irrelevant (<10 min)	712	83.8	262014	87.2

Population and Area Coverage in Kreis Schleswig-Flensburg (Stroke-Ready Hospitals) (Schleswig-Holstein) — Extended (+10min Penalty)

Category	Grid Cells	Area (%)	Population	Population (%)
High (30+ min)	0	0.0	0	0.0
Medium (20-30 min)	27	1.0	3724	1.9
Low (10-20 min)	136	5.0	10352	5.3
Only CT reachable within 60 min	4	0.1	38	0.0
Neither reachable within 60 min	8	0.3	231	0.1
Likely irrelevant (<10 min)	2522	93.5	182448	92.7

Population and Area Coverage in Kreis Steinburg (Stroke-Ready Hospitals) (Schleswig-Holstein) — Extended (+10min Penalty)

Category	Grid Cells	Area (%)	Population	Population (%)
High (30+ min)	0	0.0	0	0.0
Medium (20-30 min)	1	0.1	43	0.0
Low (10-20 min)	172	12.4	11484	8.7
Only CT reachable within 60 min	0	0.0	0	0.0
Neither reachable within 60 min	9	0.7	432	0.3
Likely irrelevant (<10 min)	1200	86.8	120774	91.0

Population and Area Coverage in Landkreis Verden (Stroke-Ready Hospitals) (Niedersachsen) — Extended (+10min Penalty)

Category	Grid Cells	Area (%)	Population	Population (%)
High (30+ min)	0	0.0	0	0.0
Medium (20-30 min)	129	12.3	9775	7.4
Low (10-20 min)	399	37.9	43767	33.0
Only CT reachable within 60 min	0	0.0	0	0.0
Neither reachable within 60 min	0	0.0	0	0.0
Likely irrelevant (<10 min)	524	49.8	79286	59.7

Population and Area Coverage in Kreisfreie Stadt Delmenhorst (Stroke-Ready Hospitals) (Niedersachsen) — Extended (+10min Penalty)

Category	Grid Cells	Area (%)	Population	Population (%)
High (30+ min)	0	0.0	0	0.0
Medium (20-30 min)	7	8.4	12322	16.8
Low (10-20 min)	67	80.7	60635	82.6
Only CT reachable within 60 min	0	0.0	0	0.0
Neither reachable within 60 min	0	0.0	0	0.0
Likely irrelevant (<10 min)	9	10.8	438	0.6

Population and Area Coverage in Kreis Kleve (Stroke-Ready Hospitals) (Nordrhein-Westfalen) — Extended (+10min Penalty)

Category	Grid Cells	Area (%)	Population	Population (%)
High (30+ min)	1	0.1	16	0.0
Medium (20-30 min)	24	1.5	2735	0.9
Low (10-20 min)	282	18.0	54276	17.9
Only CT reachable within 60 min	8	0.5	273	0.1
Neither reachable within 60 min	14	0.9	127	0.0
Likely irrelevant (<10 min)	1242	79.1	245772	81.1

Population and Area Coverage in Kreis Herford (Stroke-Ready Hospitals) (Nordrhein-Westfalen) — Extended (+10min Penalty)

Category	Grid Cells	Area (%)	Population	Population (%)
High (30+ min)	0	0.0	0	0.0
Medium (20-30 min)	32	5.4	3785	1.5
Low (10-20 min)	248	41.7	102043	40.8
Only CT reachable within 60 min	0	0.0	0	0.0
Neither reachable within 60 min	0	0.0	0	0.0
Likely irrelevant (<10 min)	315	52.9	144467	57.7

Population and Area Coverage in Kreisfreie Stadt Dortmund (Stroke-Ready Hospitals) (Nordrhein-Westfalen) — Extended (+10min Penalty)

Category	Grid Cells	Area (%)	Population	Population (%)
High (30+ min)	0	0.0	0	0.0
Medium (20-30 min)	0	0.0	0	0.0
Low (10-20 min)	0	0.0	0	0.0
Only CT reachable within 60 min	0	0.0	0	0.0
Neither reachable within 60 min	0	0.0	0	0.0
Likely irrelevant (<10 min)	363	100.0	611483	100.0

Population and Area Coverage in Kreisfreie Stadt Wiesbaden (Stroke-Ready Hospitals) (Hessen) — Extended (+10min Penalty)

Category	Grid Cells	Area (%)	Population	Population (%)
High (30+ min)	0	0.0	0	0.0
Medium (20-30 min)	0	0.0	0	0.0
Low (10-20 min)	1	0.4	0	0.0
Only CT reachable within 60 min	0	0.0	0	0.0
Neither reachable within 60 min	0	0.0	0	0.0
Likely irrelevant (<10 min)	251	99.6	277503	100.0

Population and Area Coverage in Landkreis Hochtaunuskreis (Stroke-Ready Hospitals) (Hessen) — Extended (+10min Penalty)

Category	Grid Cells	Area (%)	Population	Population (%)
High (30+ min)	0	0.0	0	0.0
Medium (20-30 min)	1	0.2	28	0.0
Low (10-20 min)	163	26.9	28392	12.2
Only CT reachable within 60 min	0	0.0	0	0.0
Neither reachable within 60 min	0	0.0	0	0.0
Likely irrelevant (<10 min)	442	72.9	204100	87.8

Population and Area Coverage in Landkreis Kassel (Stroke-Ready Hospitals) (Hessen) — Extended (+10min Penalty)

Category	Grid Cells	Area (%)	Population	Population (%)
High (30+ min)	50	3.0	2676	1.1
Medium (20-30 min)	504	30.2	34669	14.6
Low (10-20 min)	755	45.2	77096	32.5
Only CT reachable within 60 min	0	0.0	0	0.0
Neither reachable within 60 min	0	0.0	0	0.0
Likely irrelevant (<10 min)	361	21.6	122559	51.7

Population and Area Coverage in Landkreis Cochem-Zell (Stroke-Ready Hospitals) (Rheinland-Pfalz) — Extended (+10min Penalty)

Category	Grid Cells	Area (%)	Population	Population (%)
High (30+ min)	218	25.0	14277	22.2
Medium (20-30 min)	238	27.3	19783	30.7
Low (10-20 min)	225	25.8	18876	29.3
Only CT reachable within 60 min	5	0.6	34	0.1
Neither reachable within 60 min	0	0.0	0	0.0
Likely irrelevant (<10 min)	185	21.2	11386	17.7

Population and Area Coverage in Landkreis Rhein-Lahn-Kreis (Stroke-Ready Hospitals) (Rheinland-Pfalz) — Extended (+10min Penalty)

Category	Grid Cells	Area (%)	Population	Population (%)
High (30+ min)	131	13.3	8442	6.8
Medium (20-30 min)	208	21.1	17215	13.8
Low (10-20 min)	235	23.8	20999	16.8
Only CT reachable within 60 min	0	0.0	0	0.0
Neither reachable within 60 min	0	0.0	0	0.0
Likely irrelevant (<10 min)	412	41.8	78018	62.6

Population and Area Coverage in Stadtkreis Heilbronn (Stroke-Ready Hospitals) (Baden-Württemberg) — Extended (+10min Penalty)

Category	Grid Cells	Area (%)	Population	Population (%)
High (30+ min)	0	0.0	0	0.0
Medium (20-30 min)	0	0.0	0	0.0
Low (10-20 min)	0	0.0	8	0.0
Only CT reachable within 60 min	0	0.0	0	0.0
Neither reachable within 60 min	0	0.0	0	0.0
Likely irrelevant (<10 min)	121	100.0	123078	100.0

Population and Area Coverage in Landkreis Schwandorf (Stroke-Ready Hospitals) (Bayern) — Extended (+10min Penalty)

Category	Grid Cells	Area (%)	Population	Population (%)
High (30+ min)	6	0.3	521	0.4
Medium (20-30 min)	276	15.5	13006	8.8
Low (10-20 min)	455	25.5	21763	14.7
Only CT reachable within 60 min	0	0.0	0	0.0
Neither reachable within 60 min	0	0.0	0	0.0
Likely irrelevant (<10 min)	1049	58.7	113244	76.2

Population and Area Coverage in Landkreis Unterallgäu (Stroke-Ready Hospitals) (Bayern) — Extended (+10min Penalty)

Category	Grid Cells	Area (%)	Population	Population (%)
High (30+ min)	7	0.5	101	0.1
Medium (20-30 min)	139	9.4	7292	5.1
Low (10-20 min)	542	36.5	42875	30.1
Only CT reachable within 60 min	0	0.0	0	0.0
Neither reachable within 60 min	0	0.0	0	0.0
Likely irrelevant (<10 min)	798	53.7	92004	64.7

Population and Area Coverage in Kreisfreie Stadt Frankfurt (Oder) (Stroke-Ready Hospitals) (Brandenburg) — Extended (+10min Penalty)

Category	Grid Cells	Area (%)	Population	Population (%)
High (30+ min)	0	0.0	0	0.0
Medium (20-30 min)	1	0.5	0	0.0
Low (10-20 min)	7	3.6	61	0.1
Only CT reachable within 60 min	4	2.0	464	0.8
Neither reachable within 60 min	15	7.7	524	0.9
Likely irrelevant (<10 min)	169	86.2	55067	98.1

Population and Area Coverage in Landkreis Dahme-Spreewald (Stroke-Ready Hospitals) (Brandenburg) — Extended (+10min Penalty)

Category	Grid Cells	Area (%)	Population	Population (%)
High (30+ min)	0	0.0	0	0.0
Medium (20-30 min)	76	2.6	5743	3.6
Low (10-20 min)	848	28.6	77444	48.9
Only CT reachable within 60 min	0	0.0	0	0.0
Neither reachable within 60 min	0	0.0	0	0.0
Likely irrelevant (<10 min)	2045	68.9	75282	47.5

Population and Area Coverage in Landkreis Leipzig (Stroke-Ready Hospitals) (Sachsen) — Extended (+10min Penalty)

Category	Grid Cells	Area (%)	Population	Population (%)
High (30+ min)	1	0.0	5	0.0
Medium (20-30 min)	199	9.4	15422	6.0
Low (10-20 min)	617	29.0	80199	31.4
Only CT reachable within 60 min	0	0.0	0	0.0
Neither reachable within 60 min	0	0.0	0	0.0
Likely irrelevant (<10 min)	1307	61.5	159799	62.6

Population and Area Coverage in Kreisfreie Stadt Magdeburg (Stroke-Ready Hospitals) (Sachsen-Anhalt) — Extended (+10min Penalty)

Category	Grid Cells	Area (%)	Population	Population (%)
High (30+ min)	0	0.0	0	0.0
Medium (20-30 min)	4	1.5	820	0.3
Low (10-20 min)	53	20.2	6982	2.8
Only CT reachable within 60 min	0	0.0	0	0.0
Neither reachable within 60 min	0	0.0	0	0.0
Likely irrelevant (<10 min)	206	78.3	241498	96.9

Population and Area Coverage in Landkreis Wartburgkreis (Stroke-Ready Hospitals) (Thüringen) — Extended (+10min Penalty)

Category	Grid Cells	Area (%)	Population	Population (%)
High (30+ min)	0	0.0	0	0.0
Medium (20-30 min)	20	1.1	451	0.3
Low (10-20 min)	164	9.4	11889	8.6
Only CT reachable within 60 min	0	0.0	0	0.0
Neither reachable within 60 min	0	0.0	0	0.0
Likely irrelevant (<10 min)	1567	89.5	125735	91.1

Population and Area Coverage in Landkreis Unstrut-Hainich-Kreis (Stroke-Ready Hospitals) (Thüringen) — Extended (+10min Penalty)

Category	Grid Cells	Area (%)	Population	Population (%)
High (30+ min)	0	0.0	0	0.0
Medium (20-30 min)	37	3.1	501	0.6
Low (10-20 min)	312	26.2	13579	15.0
Only CT reachable within 60 min	0	0.0	0	0.0
Neither reachable within 60 min	0	0.0	0	0.0
Likely irrelevant (<10 min)	840	70.6	76189	84.4

Population and Area Coverage in Kreisfreie Stadt Bielefeld (Stroke-Ready Hospitals) (Nordrhein-Westfalen) — Extended (+10min Penalty)

Category	Grid Cells	Area (%)	Population	Population (%)
High (30+ min)	0	0.0	0	0.0
Medium (20-30 min)	0	0.0	0	0.0
Low (10-20 min)	22	6.5	17332	5.0
Only CT reachable within 60 min	0	0.0	0	0.0
Neither reachable within 60 min	0	0.0	0	0.0
Likely irrelevant (<10 min)	314	93.5	332290	95.0

Population and Area Coverage in Kreisfreie Stadt Bochum (Stroke-Ready Hospitals) (Nordrhein-Westfalen) — Extended (+10min Penalty)

Category	Grid Cells	Area (%)	Population	Population (%)
High (30+ min)	0	0.0	0	0.0
Medium (20-30 min)	0	0.0	0	0.0
Low (10-20 min)	0	0.0	0	0.0
Only CT reachable within 60 min	0	0.0	0	0.0
Neither reachable within 60 min	0	0.0	0	0.0
Likely irrelevant (<10 min)	185	100.0	386703	100.0

Population and Area Coverage in Landkreis Fulda (Stroke-Ready Hospitals) (Hessen) — Extended (+10min Penalty)

Category	Grid Cells	Area (%)	Population	Population (%)
High (30+ min)	0	0.0	0	0.0
Medium (20-30 min)	4	0.2	24	0.0
Low (10-20 min)	423	24.2	39391	17.9
Only CT reachable within 60 min	0	0.0	0	0.0
Neither reachable within 60 min	0	0.0	0	0.0
Likely irrelevant (<10 min)	1324	75.6	180844	82.1

Population and Area Coverage in Landkreis Rastatt (Stroke-Ready Hospitals) (Baden-Württemberg) — Extended (+10min Penalty)

Category	Grid Cells	Area (%)	Population	Population (%)
High (30+ min)	15	1.7	685	0.3
Medium (20-30 min)	184	20.5	35935	15.8
Low (10-20 min)	249	27.7	60195	26.5
Only CT reachable within 60 min	0	0.0	11	0.0
Neither reachable within 60 min	2	0.2	31	0.0
Likely irrelevant (<10 min)	448	49.9	130269	57.4

Population and Area Coverage in Landkreis Breisgau-Hochschwarzwald (Stroke-Ready Hospitals) (Baden-Württemberg) — Extended (+10min Penalty)

Category	Grid Cells	Area (%)	Population	Population (%)
High (30+ min)	6	0.4	24	0.0
Medium (20-30 min)	55	3.3	6191	2.5
Low (10-20 min)	249	15.2	32606	13.2
Only CT reachable within 60 min	0	0.0	0	0.0
Neither reachable within 60 min	0	0.0	3	0.0
Likely irrelevant (<10 min)	1333	81.1	207565	84.2

Population and Area Coverage in Landkreis Ebersberg (Stroke-Ready Hospitals) (Bayern) — Extended (+10min Penalty)

Category	Grid Cells	Area (%)	Population	Population (%)
High (30+ min)	0	0.0	0	0.0
Medium (20-30 min)	0	0.0	0	0.0
Low (10-20 min)	1	0.2	3	0.0
Only CT reachable within 60 min	0	0.0	0	0.0
Neither reachable within 60 min	0	0.0	0	0.0
Likely irrelevant (<10 min)	663	99.8	137982	100.0

Population and Area Coverage in Landkreis Rottal-Inn (Stroke-Ready Hospitals) (Bayern) — Extended (+10min Penalty)

Category	Grid Cells	Area (%)	Population	Population (%)
High (30+ min)	0	0.0	0	0.0
Medium (20-30 min)	1	0.1	35	0.0
Low (10-20 min)	359	23.3	23424	19.3
Only CT reachable within 60 min	0	0.0	0	0.0
Neither reachable within 60 min	2	0.1	63	0.1
Likely irrelevant (<10 min)	1177	76.5	98146	80.7

Population and Area Coverage in Landkreis Bamberg (Stroke-Ready Hospitals) (Bayern) — Extended (+10min Penalty)

Category	Grid Cells	Area (%)	Population	Population (%)
High (30+ min)	0	0.0	0	0.0
Medium (20-30 min)	268	18.3	9550	6.3
Low (10-20 min)	691	47.2	68493	45.4
Only CT reachable within 60 min	0	0.0	0	0.0
Neither reachable within 60 min	0	0.0	0	0.0
Likely irrelevant (<10 min)	506	34.5	72755	48.2

Population and Area Coverage in Landkreis Schweinfurt (Stroke-Ready Hospitals) (Bayern) — Extended (+10min Penalty)

Category	Grid Cells	Area (%)	Population	Population (%)
High (30+ min)	0	0.0	0	0.0
Medium (20-30 min)	49	4.6	489	0.4
Low (10-20 min)	380	35.9	32908	28.1
Only CT reachable within 60 min	0	0.0	0	0.0
Neither reachable within 60 min	0	0.0	0	0.0
Likely irrelevant (<10 min)	630	59.5	83552	71.4

Population and Area Coverage in Landkreis St. Wendel (Stroke-Ready Hospitals) (Saarland) — Extended (+10min Penalty)

Category	Grid Cells	Area (%)	Population	Population (%)
High (30+ min)	0	0.0	0	0.0
Medium (20-30 min)	53	8.9	2772	3.3
Low (10-20 min)	134	22.4	13717	16.1
Only CT reachable within 60 min	0	0.0	0	0.0
Neither reachable within 60 min	0	0.0	0	0.0
Likely irrelevant (<10 min)	411	68.7	68797	80.7

Population and Area Coverage in Landkreis Harz (Stroke-Ready Hospitals) (Sachsen-Anhalt) — Extended (+10min Penalty)

Category	Grid Cells	Area (%)	Population	Population (%)
High (30+ min)	33	1.2	1407	0.7
Medium (20-30 min)	766	27.9	64307	32.1
Low (10-20 min)	1063	38.7	70743	35.4
Only CT reachable within 60 min	0	0.0	0	0.0
Neither reachable within 60 min	0	0.0	0	0.0
Likely irrelevant (<10 min)	886	32.2	63645	31.8

Population and Area Coverage in Kreisfreie Stadt Gera (Stroke-Ready Hospitals) (Thüringen) — Extended (+10min Penalty)

Category	Grid Cells	Area (%)	Population	Population (%)
High (30+ min)	0	0.0	0	0.0
Medium (20-30 min)	5	2.6	316	0.3
Low (10-20 min)	44	23.0	3986	3.6
Only CT reachable within 60 min	0	0.0	0	0.0
Neither reachable within 60 min	0	0.0	0	0.0
Likely irrelevant (<10 min)	142	74.3	105355	96.1

Population and Area Coverage in Kreisfreie Stadt Weimar (Stroke-Ready Hospitals) (Thüringen) — Extended (+10min Penalty)

Category	Grid Cells	Area (%)	Population	Population (%)
High (30+ min)	0	0.0	0	0.0
Medium (20-30 min)	0	0.0	0	0.0
Low (10-20 min)	0	0.0	0	0.0
Only CT reachable within 60 min	0	0.0	0	0.0
Neither reachable within 60 min	0	0.0	0	0.0
Likely irrelevant (<10 min)	109	100.0	57698	100.0

Population and Area Coverage in Landkreis Holzminden (Stroke-Ready Hospitals) (Niedersachsen) — Extended (+10min Penalty)

Category	Grid Cells	Area (%)	Population	Population (%)
High (30+ min)	8	0.9	178	0.2
Medium (20-30 min)	41	4.5	6472	9.0
Low (10-20 min)	164	18.1	5250	7.3
Only CT reachable within 60 min	0	0.0	0	0.0
Neither reachable within 60 min	0	0.0	0	0.0
Likely irrelevant (<10 min)	691	76.4	59888	83.4

Population and Area Coverage in Landkreis Grafschaft Bentheim (Stroke-Ready Hospitals) (Niedersachsen) — Extended (+10min Penalty)

Category	Grid Cells	Area (%)	Population	Population (%)
High (30+ min)	0	0.0	0	0.0
Medium (20-30 min)	9	0.7	427	0.3
Low (10-20 min)	185	14.5	25189	18.4
Only CT reachable within 60 min	6	0.5	451	0.3
Neither reachable within 60 min	21	1.6	442	0.3
Likely irrelevant (<10 min)	1053	82.7	110167	80.6

Population and Area Coverage in Kreis Höxter (Stroke-Ready Hospitals) (Nordrhein-Westfalen) — Extended (+10min Penalty)

Category	Grid Cells	Area (%)	Population	Population (%)
High (30+ min)	0	0.0	0	0.0
Medium (20-30 min)	242	15.5	24541	17.2
Low (10-20 min)	414	26.5	38306	26.8
Only CT reachable within 60 min	0	0.0	0	0.0
Neither reachable within 60 min	0	0.0	0	0.0
Likely irrelevant (<10 min)	907	58.0	79944	56.0

Population and Area Coverage in Landkreis Marburg-Biedenkopf (Stroke-Ready Hospitals) (Hessen) — Extended (+10min Penalty)

Category	Grid Cells	Area (%)	Population	Population (%)
High (30+ min)	134	8.4	19438	8.0
Medium (20-30 min)	157	9.8	18777	7.7
Low (10-20 min)	362	22.6	36667	15.1
Only CT reachable within 60 min	0	0.0	0	0.0
Neither reachable within 60 min	0	0.0	0	0.0
Likely irrelevant (<10 min)	947	59.2	168480	69.2

Population and Area Coverage in Landkreis Rhein-Hunsrück-Kreis (Stroke-Ready Hospitals) (Rheinland-Pfalz) — Extended (+10min Penalty)

Category	Grid Cells	Area (%)	Population	Population (%)
High (30+ min)	399	32.1	28942	27.6
Medium (20-30 min)	594	47.7	47639	45.4
Low (10-20 min)	242	19.5	28045	26.7
Only CT reachable within 60 min	2	0.2	0	0.0
Neither reachable within 60 min	0	0.0	0	0.0
Likely irrelevant (<10 min)	7	0.6	336	0.3

Population and Area Coverage in Landkreis Eifelkreis Bitburg-Prüm (Stroke-Ready Hospitals) (Rheinland-Pfalz) — Extended (+10min Penalty)

Category	Grid Cells	Area (%)	Population	Population (%)
High (30+ min)	309	15.2	11543	11.5
Medium (20-30 min)	905	44.4	42913	42.8
Low (10-20 min)	592	29.1	38958	38.8
Only CT reachable within 60 min	100	4.9	1324	1.3
Neither reachable within 60 min	2	0.1	127	0.1
Likely irrelevant (<10 min)	128	6.3	5471	5.5

Population and Area Coverage in Landkreis Erding (Stroke-Ready Hospitals) (Bayern) — Extended (+10min Penalty)

Category	Grid Cells	Area (%)	Population	Population (%)
High (30+ min)	0	0.0	0	0.0
Medium (20-30 min)	1	0.1	60	0.0
Low (10-20 min)	428	40.6	44434	32.7
Only CT reachable within 60 min	0	0.0	0	0.0
Neither reachable within 60 min	0	0.0	0	0.0
Likely irrelevant (<10 min)	626	59.3	91237	67.2

Population and Area Coverage in Landkreis Deggendorf (Stroke-Ready Hospitals) (Bayern) — Extended (+10min Penalty)

Category	Grid Cells	Area (%)	Population	Population (%)
High (30+ min)	0	0.0	0	0.0
Medium (20-30 min)	2	0.2	45	0.0
Low (10-20 min)	131	12.4	12863	10.8
Only CT reachable within 60 min	0	0.0	0	0.0
Neither reachable within 60 min	0	0.0	0	0.0
Likely irrelevant (<10 min)	924	87.4	106542	89.2

Population and Area Coverage in Landkreis Weißenburg-Gunzenhausen (Stroke-Ready Hospitals) (Bayern) — Extended (+10min Penalty)

Category	Grid Cells	Area (%)	Population	Population (%)
High (30+ min)	3	0.3	3	0.0
Medium (20-30 min)	201	16.9	11966	12.5
Low (10-20 min)	452	38.1	39874	41.8
Only CT reachable within 60 min	0	0.0	0	0.0
Neither reachable within 60 min	0	0.0	0	0.0
Likely irrelevant (<10 min)	531	44.7	43568	45.7

Population and Area Coverage in Landkreis Lindau (Bodensee) (Stroke-Ready Hospitals) (Bayern) — Extended (+10min Penalty)

Category	Grid Cells	Area (%)	Population	Population (%)
High (30+ min)	2	0.5	1008	1.2
Medium (20-30 min)	212	55.8	60675	74.7
Low (10-20 min)	126	33.2	16141	19.9
Only CT reachable within 60 min	5	1.3	230	0.3
Neither reachable within 60 min	10	2.6	682	0.8
Likely irrelevant (<10 min)	25	6.6	2454	3.0

Population and Area Coverage in Kreisfreie Stadt Rostock (Stroke-Ready Hospitals) (Mecklenburg-Vorpommern) — Extended (+10min Penalty)

Category	Grid Cells	Area (%)	Population	Population (%)
High (30+ min)	0	0.0	0	0.0
Medium (20-30 min)	11	4.9	15	0.0
Low (10-20 min)	96	42.7	27573	14.8
Only CT reachable within 60 min	1	0.4	33	0.0
Neither reachable within 60 min	5	2.2	576	0.3
Likely irrelevant (<10 min)	112	49.8	158208	84.9

Population and Area Coverage in Landkreis Rostock (Stroke-Ready Hospitals) (Mecklenburg-Vorpommern) — Extended (+10min Penalty)

Category	Grid Cells	Area (%)	Population	Population (%)
High (30+ min)	291	6.2	9950	5.0
Medium (20-30 min)	775	16.6	33323	16.8
Low (10-20 min)	1293	27.6	56698	28.6
Only CT reachable within 60 min	65	1.4	531	0.3
Neither reachable within 60 min	30	0.6	586	0.3
Likely irrelevant (<10 min)	2223	47.5	97285	49.0

Population and Area Coverage in Landkreis Vorpommern-Greifswald (Stroke-Ready Hospitals) (Mecklenburg-Vorpommern) — Extended (+10min Penalty)

Category	Grid Cells	Area (%)	Population	Population (%)
High (30+ min)	410	8.1	25671	11.6
Medium (20-30 min)	1195	23.7	43124	19.4
Low (10-20 min)	1079	21.4	26833	12.1
Only CT reachable within 60 min	474	9.4	16882	7.6
Neither reachable within 60 min	139	2.8	3468	1.6
Likely irrelevant (<10 min)	1742	34.6	105917	47.7

Population and Area Coverage in Landkreis Mittelsachsen (Stroke-Ready Hospitals) (Sachsen) — Extended (+10min Penalty)

Category	Grid Cells	Area (%)	Population	Population (%)
High (30+ min)	13	0.5	611	0.2
Medium (20-30 min)	569	21.0	65588	22.3
Low (10-20 min)	643	23.7	64691	22.0
Only CT reachable within 60 min	2	0.1	0	0.0
Neither reachable within 60 min	0	0.0	0	0.0
Likely irrelevant (<10 min)	1483	54.7	163522	55.5

Population and Area Coverage in Landkreis Schmalkalden-Meiningen (Stroke-Ready Hospitals) (Thüringen) — Extended (+10min Penalty)

Category	Grid Cells	Area (%)	Population	Population (%)
High (30+ min)	43	2.7	3283	2.8
Medium (20-30 min)	146	9.2	23761	20.5
Low (10-20 min)	354	22.3	26399	22.8
Only CT reachable within 60 min	0	0.0	0	0.0
Neither reachable within 60 min	0	0.0	0	0.0
Likely irrelevant (<10 min)	1042	65.7	62356	53.8

Population and Area Coverage in Kreisfreie Stadt Lübeck (Stroke-Ready Hospitals) (Schleswig-Holstein) — Extended (+10min Penalty)

Category	Grid Cells	Area (%)	Population	Population (%)
High (30+ min)	0	0.0	0	0.0
Medium (20-30 min)	7	2.4	1779	0.8
Low (10-20 min)	70	24.2	23917	11.0
Only CT reachable within 60 min	2	0.7	2030	0.9
Neither reachable within 60 min	2	0.7	159	0.1
Likely irrelevant (<10 min)	208	72.0	190043	87.2

Population and Area Coverage in Kreis Nordfriesland (Stroke-Ready Hospitals) (Schleswig-Holstein) — Extended (+10min Penalty)

Category	Grid Cells	Area (%)	Population	Population (%)
High (30+ min)	627	23.8	43839	27.3
Medium (20-30 min)	576	21.9	45940	28.6
Low (10-20 min)	479	18.2	19922	12.4
Only CT reachable within 60 min	217	8.2	23066	14.4
Neither reachable within 60 min	150	5.7	4915	3.1
Likely irrelevant (<10 min)	584	22.2	23008	14.3

Population and Area Coverage in Landkreis Northeim (Stroke-Ready Hospitals) (Niedersachsen) — Extended (+10min Penalty)

Category	Grid Cells	Area (%)	Population	Population (%)
High (30+ min)	15	0.9	421	0.3
Medium (20-30 min)	101	6.1	13115	9.8
Low (10-20 min)	297	18.0	12545	9.4
Only CT reachable within 60 min	0	0.0	0	0.0
Neither reachable within 60 min	0	0.0	0	0.0
Likely irrelevant (<10 min)	1238	75.0	108031	80.6

Population and Area Coverage in Kreisfreie Stadt Wilhelmshaven (Stroke-Ready Hospitals) (Niedersachsen) — Extended (+10min Penalty)

Category	Grid Cells	Area (%)	Population	Population (%)
High (30+ min)	0	0.0	0	0.0
Medium (20-30 min)	0	0.0	0	0.0
Low (10-20 min)	7	5.8	6343	9.1
Only CT reachable within 60 min	0	0.0	0	0.0
Neither reachable within 60 min	3	2.5	693	1.0
Likely irrelevant (<10 min)	111	91.7	62492	89.9

Population and Area Coverage in Kreisfreie Stadt Essen (Stroke-Ready Hospitals) (Nordrhein-Westfalen) — Extended (+10min Penalty)

Category	Grid Cells	Area (%)	Population	Population (%)
High (30+ min)	0	0.0	0	0.0
Medium (20-30 min)	0	0.0	0	0.0
Low (10-20 min)	4	1.4	2784	0.5
Only CT reachable within 60 min	0	0.0	0	0.0
Neither reachable within 60 min	0	0.0	0	0.0
Likely irrelevant (<10 min)	272	98.6	593892	99.5

Population and Area Coverage in Kreisfreie Stadt Münster (Stroke-Ready Hospitals) (Nordrhein-Westfalen) — Extended (+10min Penalty)

Category	Grid Cells	Area (%)	Population	Population (%)
High (30+ min)	0	0.0	0	0.0
Medium (20-30 min)	0	0.0	0	0.0
Low (10-20 min)	43	10.8	13416	3.7
Only CT reachable within 60 min	0	0.0	0	0.0
Neither reachable within 60 min	0	0.0	0	0.0
Likely irrelevant (<10 min)	356	89.2	351415	96.3

Population and Area Coverage in Kreis Recklinghausen (Stroke-Ready Hospitals) (Nordrhein-Westfalen) — Extended (+10min Penalty)

Category	Grid Cells	Area (%)	Population	Population (%)
High (30+ min)	0	0.0	0	0.0
Medium (20-30 min)	0	0.0	0	0.0
Low (10-20 min)	117	11.9	35036	5.7
Only CT reachable within 60 min	0	0.0	0	0.0
Neither reachable within 60 min	0	0.0	0	0.0
Likely irrelevant (<10 min)	865	88.1	581377	94.3

Population and Area Coverage in Landkreis Offenbach (Stroke-Ready Hospitals) (Hessen) — Extended (+10min Penalty)

Category	Grid Cells	Area (%)	Population	Population (%)
High (30+ min)	0	0.0	0	0.0
Medium (20-30 min)	0	0.0	0	0.0
Low (10-20 min)	0	0.0	0	0.0
Only CT reachable within 60 min	0	0.0	0	0.0
Neither reachable within 60 min	0	0.0	0	0.0
Likely irrelevant (<10 min)	446	100.0	341311	100.0

Population and Area Coverage in Kreisfreie Stadt Trier (Stroke-Ready Hospitals) (Rheinland-Pfalz) — Extended (+10min Penalty)

Category	Grid Cells	Area (%)	Population	Population (%)
High (30+ min)	0	0.0	0	0.0
Medium (20-30 min)	0	0.0	0	0.0
Low (10-20 min)	0	0.0	0	0.0
Only CT reachable within 60 min	0	0.0	0	0.0
Neither reachable within 60 min	0	0.0	0	0.0
Likely irrelevant (<10 min)	146	100.0	128525	100.0

Population and Area Coverage in Landkreis Bernkastel-Wittlich (Stroke-Ready Hospitals) (Rheinland-Pfalz) — Extended (+10min Penalty)

Category	Grid Cells	Area (%)	Population	Population (%)
High (30+ min)	2	0.1	2	0.0
Medium (20-30 min)	93	6.4	2937	2.6
Low (10-20 min)	511	35.0	40401	35.2
Only CT reachable within 60 min	0	0.0	0	0.0
Neither reachable within 60 min	0	0.0	0	0.0
Likely irrelevant (<10 min)	855	58.5	71331	62.2

Population and Area Coverage in Kreisfreie Stadt Worms (Stroke-Ready Hospitals) (Rheinland-Pfalz) — Extended (+10min Penalty)

Category	Grid Cells	Area (%)	Population	Population (%)
High (30+ min)	0	0.0	0	0.0
Medium (20-30 min)	0	0.0	0	0.0
Low (10-20 min)	1	0.7	0	0.0
Only CT reachable within 60 min	0	0.0	0	0.0
Neither reachable within 60 min	0	0.0	0	0.0
Likely irrelevant (<10 min)	133	99.3	81874	100.0

Population and Area Coverage in Landkreis Heilbronn (Stroke-Ready Hospitals) (Baden-Württemberg) — Extended (+10min Penalty)

Category	Grid Cells	Area (%)	Population	Population (%)
High (30+ min)	0	0.0	0	0.0
Medium (20-30 min)	63	4.6	3005	0.9
Low (10-20 min)	268	19.7	74597	22.5
Only CT reachable within 60 min	0	0.0	0	0.0
Neither reachable within 60 min	0	0.0	0	0.0
Likely irrelevant (<10 min)	1030	75.7	253802	76.6

Population and Area Coverage in Kreisfreie Stadt Ingolstadt (Stroke-Ready Hospitals) (Bayern) — Extended (+10min Penalty)

Category	Grid Cells	Area (%)	Population	Population (%)
High (30+ min)	0	0.0	0	0.0
Medium (20-30 min)	0	0.0	0	0.0
Low (10-20 min)	5	3.0	865	0.6
Only CT reachable within 60 min	0	0.0	0	0.0
Neither reachable within 60 min	0	0.0	0	0.0
Likely irrelevant (<10 min)	160	97.0	134354	99.4

Population and Area Coverage in Landkreis Forchheim (Stroke-Ready Hospitals) (Bayern) — Extended (+10min Penalty)

Category	Grid Cells	Area (%)	Population	Population (%)
High (30+ min)	0	0.0	0	0.0
Medium (20-30 min)	70	8.7	3253	2.7
Low (10-20 min)	305	37.9	26020	22.0
Only CT reachable within 60 min	0	0.0	0	0.0
Neither reachable within 60 min	0	0.0	0	0.0
Likely irrelevant (<10 min)	430	53.4	89138	75.3

Population and Area Coverage in Landkreis Erlangen-Höchstadt (Stroke-Ready Hospitals) (Bayern) — Extended (+10min Penalty)

Category	Grid Cells	Area (%)	Population	Population (%)
High (30+ min)	0	0.0	0	0.0
Medium (20-30 min)	77	11.0	10438	7.6
Low (10-20 min)	354	50.7	66310	48.3
Only CT reachable within 60 min	0	0.0	0	0.0
Neither reachable within 60 min	0	0.0	0	0.0
Likely irrelevant (<10 min)	267	38.3	60651	44.1

Population and Area Coverage in Landkreis Mansfeld-Südharz (Stroke-Ready Hospitals) (Sachsen-Anhalt) — Extended (+10min Penalty)

Category	Grid Cells	Area (%)	Population	Population (%)
High (30+ min)	11	0.6	94	0.1
Medium (20-30 min)	230	12.2	21349	16.7
Low (10-20 min)	459	24.3	44146	34.5
Only CT reachable within 60 min	0	0.0	0	0.0
Neither reachable within 60 min	0	0.0	0	0.0
Likely irrelevant (<10 min)	1190	63.0	62348	48.7

Population and Area Coverage in Kreis Minden-Lübbecke (Stroke-Ready Hospitals) (Nordrhein-Westfalen) — Extended (+10min Penalty)

Category	Grid Cells	Area (%)	Population	Population (%)
High (30+ min)	147	9.7	18338	5.9
Medium (20-30 min)	386	25.4	59410	19.2
Low (10-20 min)	212	13.9	20892	6.8
Only CT reachable within 60 min	0	0.0	0	0.0
Neither reachable within 60 min	0	0.0	0	0.0
Likely irrelevant (<10 min)	775	51.0	210833	68.1

Population and Area Coverage in Kreisfreie Stadt Hamm (Stroke-Ready Hospitals) (Nordrhein-Westfalen) — Extended (+10min Penalty)

Category	Grid Cells	Area (%)	Population	Population (%)
High (30+ min)	0	0.0	0	0.0
Medium (20-30 min)	0	0.0	0	0.0
Low (10-20 min)	13	4.4	3619	2.1
Only CT reachable within 60 min	0	0.0	0	0.0
Neither reachable within 60 min	0	0.0	0	0.0
Likely irrelevant (<10 min)	280	95.6	171781	97.9

Population and Area Coverage in Landkreis Main-Taunus-Kreis (Stroke-Ready Hospitals) (Hessen) — Extended (+10min Penalty)

Category	Grid Cells	Area (%)	Population	Population (%)
High (30+ min)	0	0.0	0	0.0
Medium (20-30 min)	0	0.0	0	0.0
Low (10-20 min)	25	8.9	5580	2.4
Only CT reachable within 60 min	0	0.0	0	0.0
Neither reachable within 60 min	0	0.0	0	0.0
Likely irrelevant (<10 min)	256	91.1	225853	97.6

Population and Area Coverage in Kreisfreie Stadt Ludwigshafen am Rhein (Stroke-Ready Hospitals) (Rheinland-Pfalz) — Extended (+10min Penalty)

Category	Grid Cells	Area (%)	Population	Population (%)
High (30+ min)	0	0.0	0	0.0
Medium (20-30 min)	0	0.0	0	0.0
Low (10-20 min)	2	2.0	175	0.1
Only CT reachable within 60 min	0	0.0	0	0.0
Neither reachable within 60 min	0	0.0	0	0.0
Likely irrelevant (<10 min)	97	98.0	163026	99.9

Population and Area Coverage in Landkreis Esslingen (Stroke-Ready Hospitals) (Baden-Württemberg) — Extended (+10min Penalty)

Category	Grid Cells	Area (%)	Population	Population (%)
High (30+ min)	0	0.0	0	0.0
Medium (20-30 min)	3	0.4	127	0.0
Low (10-20 min)	137	17.8	75375	14.6
Only CT reachable within 60 min	0	0.0	0	0.0
Neither reachable within 60 min	0	0.0	0	0.0
Likely irrelevant (<10 min)	631	81.8	441657	85.4

Population and Area Coverage in Landkreis Dachau (Stroke-Ready Hospitals) (Bayern) — Extended (+10min Penalty)

Category	Grid Cells	Area (%)	Population	Population (%)
High (30+ min)	0	0.0	0	0.0
Medium (20-30 min)	4	0.6	87	0.1
Low (10-20 min)	210	29.8	22135	14.7
Only CT reachable within 60 min	0	0.0	0	0.0
Neither reachable within 60 min	0	0.0	0	0.0
Likely irrelevant (<10 min)	490	69.6	127909	85.2

Population and Area Coverage in Landkreis Bad Kissingen (Stroke-Ready Hospitals) (Bayern) — Extended (+10min Penalty)

Category	Grid Cells	Area (%)	Population	Population (%)
High (30+ min)	7	0.5	77	0.1
Medium (20-30 min)	400	27.9	36596	34.3
Low (10-20 min)	637	44.5	49601	46.5
Only CT reachable within 60 min	0	0.0	0	0.0
Neither reachable within 60 min	0	0.0	0	0.0
Likely irrelevant (<10 min)	389	27.1	20351	19.1

Population and Area Coverage in Kreisfreie Stadt Erfurt (Stroke-Ready Hospitals) (Thüringen) — Extended (+10min Penalty)

Category	Grid Cells	Area (%)	Population	Population (%)
High (30+ min)	0	0.0	0	0.0
Medium (20-30 min)	0	0.0	0	0.0
Low (10-20 min)	33	9.6	4727	1.9
Only CT reachable within 60 min	0	0.0	0	0.0
Neither reachable within 60 min	0	0.0	0	0.0
Likely irrelevant (<10 min)	309	90.4	242495	98.1

Population and Area Coverage in Kreisfreie Stadt Flensburg (Stroke-Ready Hospitals) (Schleswig-Holstein) — Extended (+10min Penalty)

Category	Grid Cells	Area (%)	Population	Population (%)
High (30+ min)	0	0.0	0	0.0
Medium (20-30 min)	0	0.0	0	0.0
Low (10-20 min)	0	0.0	0	0.0
Only CT reachable within 60 min	0	0.0	0	0.0
Neither reachable within 60 min	0	0.0	0	0.0
Likely irrelevant (<10 min)	62	100.0	80819	100.0

Population and Area Coverage in Kreis Dithmarschen (Stroke-Ready Hospitals) (Schleswig-Holstein) — Extended (+10min Penalty)

Category	Grid Cells	Area (%)	Population	Population (%)
High (30+ min)	0	0.0	0	0.0
Medium (20-30 min)	76	4.1	6106	4.6
Low (10-20 min)	269	14.5	24196	18.1
Only CT reachable within 60 min	11	0.6	1168	0.9
Neither reachable within 60 min	32	1.7	320	0.2
Likely irrelevant (<10 min)	1461	79.0	101828	76.2

Population and Area Coverage in Landkreis Hameln-Pyrmont (Stroke-Ready Hospitals) (Niedersachsen) — Extended (+10min Penalty)

Category	Grid Cells	Area (%)	Population	Population (%)
High (30+ min)	1	0.1	119	0.1
Medium (20-30 min)	203	19.4	16666	11.3
Low (10-20 min)	297	28.3	52668	35.7
Only CT reachable within 60 min	0	0.0	0	0.0
Neither reachable within 60 min	0	0.0	0	0.0
Likely irrelevant (<10 min)	547	52.2	77948	52.9

Population and Area Coverage in Landkreis Celle (Stroke-Ready Hospitals) (Niedersachsen) — Extended (+10min Penalty)

Category	Grid Cells	Area (%)	Population	Population (%)
High (30+ min)	0	0.0	0	0.0
Medium (20-30 min)	0	0.0	0	0.0
Low (10-20 min)	85	4.1	1537	0.9
Only CT reachable within 60 min	0	0.0	0	0.0
Neither reachable within 60 min	0	0.0	0	0.0
Likely irrelevant (<10 min)	1982	95.9	174755	99.1

Population and Area Coverage in Landkreis Harburg (Stroke-Ready Hospitals) (Niedersachsen) — Extended (+10min Penalty)

Category	Grid Cells	Area (%)	Population	Population (%)
High (30+ min)	0	0.0	0	0.0
Medium (20-30 min)	4	0.2	483	0.2
Low (10-20 min)	61	3.6	17557	7.2
Only CT reachable within 60 min	0	0.0	0	0.0
Neither reachable within 60 min	0	0.0	0	0.0
Likely irrelevant (<10 min)	1624	96.2	225324	92.6

Population and Area Coverage in Kreisfreie Stadt Düsseldorf (Stroke-Ready Hospitals) (Nordrhein-Westfalen) — Extended (+10min Penalty)

Category	Grid Cells	Area (%)	Population	Population (%)
High (30+ min)	0	0.0	0	0.0
Medium (20-30 min)	0	0.0	0	0.0
Low (10-20 min)	12	4.3	11937	1.8
Only CT reachable within 60 min	0	0.0	0	0.0
Neither reachable within 60 min	0	0.0	0	0.0
Likely irrelevant (<10 min)	267	95.7	664487	98.2

Population and Area Coverage in Kreis Düren (Stroke-Ready Hospitals) (Nordrhein-Westfalen) — Extended (+10min Penalty)

Category	Grid Cells	Area (%)	Population	Population (%)
High (30+ min)	2	0.2	0	0.0
Medium (20-30 min)	23	1.9	100	0.0
Low (10-20 min)	314	26.2	58448	22.8
Only CT reachable within 60 min	0	0.0	0	0.0
Neither reachable within 60 min	0	0.0	0	0.0
Likely irrelevant (<10 min)	861	71.8	197711	77.2

Population and Area Coverage in Kreis Soest (Stroke-Ready Hospitals) (Nordrhein-Westfalen) — Extended (+10min Penalty)

Category	Grid Cells	Area (%)	Population	Population (%)
High (30+ min)	0	0.0	0	0.0
Medium (20-30 min)	40	2.3	149	0.1
Low (10-20 min)	416	24.1	42254	14.3
Only CT reachable within 60 min	0	0.0	0	0.0
Neither reachable within 60 min	0	0.0	0	0.0
Likely irrelevant (<10 min)	1268	73.5	252742	85.6

Population and Area Coverage in Kreisfreie Stadt Darmstadt (Stroke-Ready Hospitals) (Hessen) — Extended (+10min Penalty)

Category	Grid Cells	Area (%)	Population	Population (%)
High (30+ min)	0	0.0	0	0.0
Medium (20-30 min)	0	0.0	0	0.0
Low (10-20 min)	0	0.0	0	0.0
Only CT reachable within 60 min	0	0.0	0	0.0
Neither reachable within 60 min	0	0.0	0	0.0
Likely irrelevant (<10 min)	154	100.0	151599	100.0

Population and Area Coverage in Kreisfreie Stadt Offenbach am Main (Stroke-Ready Hospitals) (Hessen) — Extended (+10min Penalty)

Category	Grid Cells	Area (%)	Population	Population (%)
High (30+ min)	0	0.0	0	0.0
Medium (20-30 min)	0	0.0	0	0.0
Low (10-20 min)	0	0.0	0	0.0
Only CT reachable within 60 min	0	0.0	0	0.0
Neither reachable within 60 min	0	0.0	0	0.0
Likely irrelevant (<10 min)	56	100.0	123120	100.0

Population and Area Coverage in Landkreis Werra-Meißner-Kreis (Stroke-Ready Hospitals) (Hessen) — Extended (+10min Penalty)

Category	Grid Cells	Area (%)	Population	Population (%)
High (30+ min)	3	0.2	52	0.1
Medium (20-30 min)	206	15.6	17105	17.0
Low (10-20 min)	279	21.2	19306	19.1
Only CT reachable within 60 min	0	0.0	0	0.0
Neither reachable within 60 min	0	0.0	0	0.0
Likely irrelevant (<10 min)	830	63.0	64385	63.8

Population and Area Coverage in Landkreis Mayen-Koblenz (Stroke-Ready Hospitals) (Rheinland-Pfalz) — Extended (+10min Penalty)

Category	Grid Cells	Area (%)	Population	Population (%)
High (30+ min)	0	0.0	0	0.0
Medium (20-30 min)	119	11.5	9929	4.6
Low (10-20 min)	320	30.9	36473	16.8
Only CT reachable within 60 min	0	0.0	0	0.0
Neither reachable within 60 min	0	0.0	0	0.0
Likely irrelevant (<10 min)	595	57.5	170638	78.6

Population and Area Coverage in Landkreis Bad Dürkheim (Stroke-Ready Hospitals) (Rheinland-Pfalz) — Extended (+10min Penalty)

Category	Grid Cells	Area (%)	Population	Population (%)
High (30+ min)	0	0.0	0	0.0
Medium (20-30 min)	15	2.1	381	0.3
Low (10-20 min)	162	22.2	35869	26.7
Only CT reachable within 60 min	0	0.0	0	0.0
Neither reachable within 60 min	0	0.0	0	0.0
Likely irrelevant (<10 min)	554	75.8	98188	73.0

Population and Area Coverage in Landkreis Landsberg am Lech (Stroke-Ready Hospitals) (Bayern) — Extended (+10min Penalty)

Category	Grid Cells	Area (%)	Population	Population (%)
High (30+ min)	0	0.0	0	0.0
Medium (20-30 min)	0	0.0	0	0.0
Low (10-20 min)	99	10.3	2707	2.2
Only CT reachable within 60 min	0	0.0	0	0.0
Neither reachable within 60 min	0	0.0	0	0.0
Likely irrelevant (<10 min)	863	89.7	118223	97.8

Population and Area Coverage in Landkreis Donau-Ries (Stroke-Ready Hospitals) (Bayern) — Extended (+10min Penalty)

Category	Grid Cells	Area (%)	Population	Population (%)
High (30+ min)	331	21.1	20649	15.5
Medium (20-30 min)	878	56.1	84506	63.4
Low (10-20 min)	286	18.3	25605	19.2
Only CT reachable within 60 min	0	0.0	0	0.0
Neither reachable within 60 min	0	0.0	0	0.0
Likely irrelevant (<10 min)	71	4.5	2564	1.9

Population and Area Coverage in Landkreis Märkisch-Oderland (Stroke-Ready Hospitals) (Brandenburg) — Extended (+10min Penalty)

Category	Grid Cells	Area (%)	Population	Population (%)
High (30+ min)	734	25.7	18702	10.2
Medium (20-30 min)	708	24.8	38240	20.8
Low (10-20 min)	595	20.8	37125	20.2
Only CT reachable within 60 min	61	2.1	620	0.3
Neither reachable within 60 min	76	2.7	610	0.3
Likely irrelevant (<10 min)	681	23.9	88808	48.2

Population and Area Coverage in Landkreis Prignitz (Stroke-Ready Hospitals) (Brandenburg) — Extended (+10min Penalty)

Category	Grid Cells	Area (%)	Population	Population (%)
High (30+ min)	9	0.3	197	0.3
Medium (20-30 min)	355	12.4	4501	6.1
Low (10-20 min)	725	25.3	16630	22.4
Only CT reachable within 60 min	41	1.4	235	0.3
Neither reachable within 60 min	0	0.0	0	0.0
Likely irrelevant (<10 min)	1741	60.6	52805	71.0

Population and Area Coverage in Landkreis Burgenlandkreis (Stroke-Ready Hospitals) (Sachsen-Anhalt) — Extended (+10min Penalty)

Category	Grid Cells	Area (%)	Population	Population (%)
High (30+ min)	0	0.0	0	0.0
Medium (20-30 min)	124	6.8	15377	9.3
Low (10-20 min)	286	15.7	26460	16.0
Only CT reachable within 60 min	0	0.0	0	0.0
Neither reachable within 60 min	0	0.0	0	0.0
Likely irrelevant (<10 min)	1409	77.5	123283	74.7

Population and Area Coverage in Landkreis Stendal (Stroke-Ready Hospitals) (Sachsen-Anhalt) — Extended (+10min Penalty)

Category	Grid Cells	Area (%)	Population	Population (%)
High (30+ min)	1049	32.4	62698	60.6
Medium (20-30 min)	917	28.4	16579	16.0
Low (10-20 min)	454	14.0	12535	12.1
Only CT reachable within 60 min	566	17.5	5489	5.3
Neither reachable within 60 min	0	0.0	0	0.0
Likely irrelevant (<10 min)	248	7.7	6206	6.0

Population and Area Coverage in Landkreis Weimarer Land (Stroke-Ready Hospitals) (Thüringen) — Extended (+10min Penalty)

Category	Grid Cells	Area (%)	Population	Population (%)
High (30+ min)	0	0.0	0	0.0
Medium (20-30 min)	157	15.3	15529	21.0
Low (10-20 min)	217	21.1	17530	23.8
Only CT reachable within 60 min	0	0.0	0	0.0
Neither reachable within 60 min	0	0.0	0	0.0
Likely irrelevant (<10 min)	653	63.6	40716	55.2

Population and Area Coverage in Kreisfreie Stadt Kiel (Stroke-Ready Hospitals) (Schleswig-Holstein) — Extended (+10min Penalty)

Category	Grid Cells	Area (%)	Population	Population (%)
High (30+ min)	1	0.6	221	0.1
Medium (20-30 min)	0	0.0	0	0.0
Low (10-20 min)	1	0.6	346	0.1
Only CT reachable within 60 min	0	0.0	0	0.0
Neither reachable within 60 min	0	0.0	1672	0.7
Likely irrelevant (<10 min)	153	98.7	254080	99.1

Population and Area Coverage in Landkreis Gifhorn (Stroke-Ready Hospitals) (Niedersachsen) — Extended (+10min Penalty)

Category	Grid Cells	Area (%)	Population	Population (%)
High (30+ min)	75	3.6	3163	1.8
Medium (20-30 min)	197	9.4	9730	5.7
Low (10-20 min)	338	16.2	14475	8.4
Only CT reachable within 60 min	0	0.0	0	0.0
Neither reachable within 60 min	0	0.0	0	0.0
Likely irrelevant (<10 min)	1480	70.8	144834	84.1

Population and Area Coverage in Landkreis Helmstedt (Stroke-Ready Hospitals) (Niedersachsen) — Extended (+10min Penalty)

Category	Grid Cells	Area (%)	Population	Population (%)
High (30+ min)	0	0.0	0	0.0
Medium (20-30 min)	0	0.0	0	0.0
Low (10-20 min)	1	0.1	0	0.0
Only CT reachable within 60 min	0	0.0	0	0.0
Neither reachable within 60 min	0	0.0	0	0.0
Likely irrelevant (<10 min)	894	99.9	89413	100.0

Population and Area Coverage in Landkreis Lüchow-Dannenberg (Stroke-Ready Hospitals) (Niedersachsen) — Extended (+10min Penalty)

Category	Grid Cells	Area (%)	Population	Population (%)
High (30+ min)	561	34.2	30512	62.5
Medium (20-30 min)	384	23.4	7800	16.0
Low (10-20 min)	270	16.5	5970	12.2
Only CT reachable within 60 min	394	24.0	4193	8.6
Neither reachable within 60 min	0	0.0	0	0.0
Likely irrelevant (<10 min)	32	2.0	366	0.7

Population and Area Coverage in Landkreis Rotenburg (Wümme) (Stroke-Ready Hospitals) (Niedersachsen) — Extended (+10min Penalty)

Category	Grid Cells	Area (%)	Population	Population (%)
High (30+ min)	174	6.2	10742	6.6
Medium (20-30 min)	562	20.1	25207	15.4
Low (10-20 min)	470	16.8	26891	16.4
Only CT reachable within 60 min	0	0.0	0	0.0
Neither reachable within 60 min	0	0.0	0	0.0
Likely irrelevant (<10 min)	1588	56.8	101134	61.7

Population and Area Coverage in Kreis Euskirchen (Stroke-Ready Hospitals) (Nordrhein-Westfalen) — Extended (+10min Penalty)

Category	Grid Cells	Area (%)	Population	Population (%)
High (30+ min)	192	12.1	15092	8.0
Medium (20-30 min)	289	18.3	16961	9.0
Low (10-20 min)	450	28.4	45136	24.0
Only CT reachable within 60 min	22	1.4	44	0.0
Neither reachable within 60 min	0	0.0	2	0.0
Likely irrelevant (<10 min)	630	39.8	111002	59.0

Population and Area Coverage in Kreis Olpe (Stroke-Ready Hospitals) (Nordrhein-Westfalen) — Extended (+10min Penalty)

Category	Grid Cells	Area (%)	Population	Population (%)
High (30+ min)	258	28.2	48349	35.7
Medium (20-30 min)	246	26.9	26215	19.4
Low (10-20 min)	266	29.1	36318	26.8
Only CT reachable within 60 min	0	0.0	0	0.0
Neither reachable within 60 min	0	0.0	0	0.0
Likely irrelevant (<10 min)	145	15.8	24536	18.1

Population and Area Coverage in Landkreis Lahn-Dill-Kreis (Stroke-Ready Hospitals) (Hessen) — Extended (+10min Penalty)

Category	Grid Cells	Area (%)	Population	Population (%)
High (30+ min)	0	0.0	0	0.0
Medium (20-30 min)	220	16.1	25616	10.0
Low (10-20 min)	466	34.2	81038	31.7
Only CT reachable within 60 min	0	0.0	0	0.0
Neither reachable within 60 min	0	0.0	0	0.0
Likely irrelevant (<10 min)	678	49.7	149068	58.3

Population and Area Coverage in Landkreis Ravensburg (Stroke-Ready Hospitals) (Baden-Württemberg) — Extended (+10min Penalty)

Category	Grid Cells	Area (%)	Population	Population (%)
High (30+ min)	0	0.0	0	0.0
Medium (20-30 min)	124	6.3	14031	5.1
Low (10-20 min)	357	18.2	48818	17.6
Only CT reachable within 60 min	0	0.0	0	0.0
Neither reachable within 60 min	0	0.0	0	0.0
Likely irrelevant (<10 min)	1479	75.5	213919	77.3

Population and Area Coverage in Kreisfreie Stadt Rosenheim (Stroke-Ready Hospitals) (Bayern) — Extended (+10min Penalty)

Category	Grid Cells	Area (%)	Population	Population (%)
High (30+ min)	0	0.0	0	0.0
Medium (20-30 min)	0	0.0	0	0.0
Low (10-20 min)	0	0.0	0	0.0
Only CT reachable within 60 min	0	0.0	0	0.0
Neither reachable within 60 min	0	0.0	0	0.0
Likely irrelevant (<10 min)	47	100.0	62621	100.0

Population and Area Coverage in Landkreis Freising (Stroke-Ready Hospitals) (Bayern) — Extended (+10min Penalty)

Category	Grid Cells	Area (%)	Population	Population (%)
High (30+ min)	0	0.0	0	0.0
Medium (20-30 min)	31	3.2	2725	1.6
Low (10-20 min)	77	7.9	7735	4.4
Only CT reachable within 60 min	0	0.0	0	0.0
Neither reachable within 60 min	0	0.0	0	0.0
Likely irrelevant (<10 min)	866	88.9	164466	94.0

Population and Area Coverage in Landkreis Garmisch-Partenkirchen (Stroke-Ready Hospitals) (Bayern) — Extended (+10min Penalty)

Category	Grid Cells	Area (%)	Population	Population (%)
High (30+ min)	6	0.5	776	0.9
Medium (20-30 min)	117	9.8	16972	19.5
Low (10-20 min)	26	2.2	21652	24.9
Only CT reachable within 60 min	9	0.8	108	0.1
Neither reachable within 60 min	429	36.0	1071	1.2
Likely irrelevant (<10 min)	606	50.8	46338	53.3

Population and Area Coverage in Landkreis Regensburg (Stroke-Ready Hospitals) (Bayern) — Extended (+10min Penalty)

Category	Grid Cells	Area (%)	Population	Population (%)
High (30+ min)	0	0.0	0	0.0
Medium (20-30 min)	8	0.5	750	0.4
Low (10-20 min)	425	24.7	20401	10.5
Only CT reachable within 60 min	0	0.0	0	0.0
Neither reachable within 60 min	0	0.0	0	0.0
Likely irrelevant (<10 min)	1286	74.8	172560	89.1

Population and Area Coverage in Landkreis Tirschenreuth (Stroke-Ready Hospitals) (Bayern) — Extended (+10min Penalty)

Category	Grid Cells	Area (%)	Population	Population (%)
High (30+ min)	39	3.0	772	1.0
Medium (20-30 min)	507	39.4	25375	34.1
Low (10-20 min)	626	48.6	41803	56.1
Only CT reachable within 60 min	3	0.2	0	0.0
Neither reachable within 60 min	3	0.2	0	0.0
Likely irrelevant (<10 min)	110	8.5	6501	8.7

Population and Area Coverage in Kreisfreie Stadt Kaufbeuren (Stroke-Ready Hospitals) (Bayern) — Extended (+10min Penalty)

Category	Grid Cells	Area (%)	Population	Population (%)
High (30+ min)	0	0.0	0	0.0
Medium (20-30 min)	0	0.0	0	0.0
Low (10-20 min)	0	0.0	0	0.0
Only CT reachable within 60 min	0	0.0	0	0.0
Neither reachable within 60 min	0	0.0	0	0.0
Likely irrelevant (<10 min)	50	100.0	42940	100.0

Population and Area Coverage in Landkreis Oberallgäu (Stroke-Ready Hospitals) (Bayern) — Extended (+10min Penalty)

Category	Grid Cells	Area (%)	Population	Population (%)
High (30+ min)	38	2.1	8583	5.5
Medium (20-30 min)	187	10.5	26207	16.9
Low (10-20 min)	254	14.3	41174	26.5
Only CT reachable within 60 min	13	0.7	311	0.2
Neither reachable within 60 min	539	30.3	4154	2.7
Likely irrelevant (<10 min)	745	41.9	75083	48.3

Population and Area Coverage in Landkreis Regionalverband Saarbrücken (Stroke-Ready Hospitals) (Saarland) — Extended (+10min Penalty)

Category	Grid Cells	Area (%)	Population	Population (%)
High (30+ min)	0	0.0	0	0.0
Medium (20-30 min)	1	0.2	72	0.0
Low (10-20 min)	13	2.7	4573	1.4
Only CT reachable within 60 min	1	0.2	8	0.0
Neither reachable within 60 min	4	0.8	286	0.1
Likely irrelevant (<10 min)	469	96.1	326951	98.5

Population and Area Coverage in Landkreis Altenburger Land (Stroke-Ready Hospitals) (Thüringen) — Extended (+10min Penalty)

Category	Grid Cells	Area (%)	Population	Population (%)
High (30+ min)	0	0.0	0	0.0
Medium (20-30 min)	7	1.0	1125	1.3
Low (10-20 min)	69	9.5	8096	9.6
Only CT reachable within 60 min	0	0.0	0	0.0
Neither reachable within 60 min	0	0.0	0	0.0
Likely irrelevant (<10 min)	650	89.5	74859	89.0

Population and Area Coverage in Landkreis Diepholz (Stroke-Ready Hospitals) (Niedersachsen) — Extended (+10min Penalty)

Category	Grid Cells	Area (%)	Population	Population (%)
High (30+ min)	534	20.2	28704	13.6
Medium (20-30 min)	1117	42.3	56094	26.6
Low (10-20 min)	684	25.9	71689	33.9
Only CT reachable within 60 min	0	0.0	0	0.0
Neither reachable within 60 min	0	0.0	0	0.0
Likely irrelevant (<10 min)	306	11.6	54683	25.9

Population and Area Coverage in Kreisfreie Stadt Emden (Stroke-Ready Hospitals) (Niedersachsen) — Extended (+10min Penalty)

Category	Grid Cells	Area (%)	Population	Population (%)
High (30+ min)	0	0.0	0	0.0
Medium (20-30 min)	0	0.0	0	0.0
Low (10-20 min)	1	0.8	922	1.9
Only CT reachable within 60 min	0	0.0	0	0.0
Neither reachable within 60 min	0	0.0	0	0.0
Likely irrelevant (<10 min)	131	99.2	48199	98.1

Population and Area Coverage in Kreisfreie Stadt Gelsenkirchen (Stroke-Ready Hospitals) (Nordrhein-Westfalen) — Extended (+10min Penalty)

Category	Grid Cells	Area (%)	Population	Population (%)
High (30+ min)	0	0.0	0	0.0
Medium (20-30 min)	0	0.0	0	0.0
Low (10-20 min)	0	0.0	0	0.0
Only CT reachable within 60 min	0	0.0	0	0.0
Neither reachable within 60 min	0	0.0	0	0.0
Likely irrelevant (<10 min)	134	100.0	279785	100.0

Population and Area Coverage in Kreis Warendorf (Stroke-Ready Hospitals) (Nordrhein-Westfalen) — Extended (+10min Penalty)

Category	Grid Cells	Area (%)	Population	Population (%)
High (30+ min)	0	0.0	0	0.0
Medium (20-30 min)	0	0.0	0	0.0
Low (10-20 min)	410	23.9	67629	24.9
Only CT reachable within 60 min	0	0.0	0	0.0
Neither reachable within 60 min	0	0.0	0	0.0
Likely irrelevant (<10 min)	1308	76.1	204441	75.1

Population and Area Coverage in Stadtkreis Ulm (Stroke-Ready Hospitals) (Baden-Württemberg) — Extended (+10min Penalty)

Category	Grid Cells	Area (%)	Population	Population (%)
High (30+ min)	0	0.0	0	0.0
Medium (20-30 min)	0	0.0	0	0.0
Low (10-20 min)	1	0.7	1113	0.9
Only CT reachable within 60 min	0	0.0	0	0.0
Neither reachable within 60 min	0	0.0	0	0.0
Likely irrelevant (<10 min)	142	99.3	119001	99.1

Population and Area Coverage in Landkreis Traunstein (Stroke-Ready Hospitals) (Bayern) — Extended (+10min Penalty)

Category	Grid Cells	Area (%)	Population	Population (%)
High (30+ min)	0	0.0	0	0.0
Medium (20-30 min)	19	1.0	1343	0.8
Low (10-20 min)	275	15.1	30173	17.2
Only CT reachable within 60 min	1	0.1	4	0.0
Neither reachable within 60 min	131	7.2	1464	0.8
Likely irrelevant (<10 min)	1391	76.6	142631	81.2

Population and Area Coverage in Kreisfreie Stadt Regensburg (Stroke-Ready Hospitals) (Bayern) — Extended (+10min Penalty)

Category	Grid Cells	Area (%)	Population	Population (%)
High (30+ min)	0	0.0	0	0.0
Medium (20-30 min)	0	0.0	0	0.0
Low (10-20 min)	0	0.0	1	0.0
Only CT reachable within 60 min	0	0.0	0	0.0
Neither reachable within 60 min	0	0.0	0	0.0
Likely irrelevant (<10 min)	97	100.0	170061	100.0

Population and Area Coverage in Kreisfreie Stadt Weiden i.d.OPf. (Stroke-Ready Hospitals) (Bayern) — Extended (+10min Penalty)

Category	Grid Cells	Area (%)	Population	Population (%)
High (30+ min)	0	0.0	0	0.0
Medium (20-30 min)	0	0.0	0	0.0
Low (10-20 min)	0	0.0	0	0.0
Only CT reachable within 60 min	0	0.0	0	0.0
Neither reachable within 60 min	0	0.0	0	0.0
Likely irrelevant (<10 min)	86	100.0	43316	100.0

Population and Area Coverage in Kreisfreie Stadt Ansbach (Stroke-Ready Hospitals) (Bayern) — Extended (+10min Penalty)

Category	Grid Cells	Area (%)	Population	Population (%)
High (30+ min)	0	0.0	0	0.0
Medium (20-30 min)	0	0.0	0	0.0
Low (10-20 min)	0	0.0	0	0.0
Only CT reachable within 60 min	0	0.0	0	0.0
Neither reachable within 60 min	0	0.0	0	0.0
Likely irrelevant (<10 min)	123	100.0	41201	100.0

Population and Area Coverage in Landkreis Rhön-Grabfeld (Stroke-Ready Hospitals) (Bayern) — Extended (+10min Penalty)

Category	Grid Cells	Area (%)	Population	Population (%)
High (30+ min)	0	0.0	0	0.0
Medium (20-30 min)	1	0.1	13	0.0
Low (10-20 min)	42	3.3	1909	2.3
Only CT reachable within 60 min	0	0.0	0	0.0
Neither reachable within 60 min	0	0.0	0	0.0
Likely irrelevant (<10 min)	1245	96.7	80256	97.7

Population and Area Coverage in Landkreis Ostprignitz-Ruppin (Stroke-Ready Hospitals) (Brandenburg) — Extended (+10min Penalty)

Category	Grid Cells	Area (%)	Population	Population (%)
High (30+ min)	343	10.1	6309	6.7
Medium (20-30 min)	650	19.2	23866	25.2
Low (10-20 min)	761	22.5	14757	15.6
Only CT reachable within 60 min	26	0.8	269	0.3
Neither reachable within 60 min	0	0.0	0	0.0
Likely irrelevant (<10 min)	1603	47.4	49509	52.3

Population and Area Coverage in Kreis Plön (Stroke-Ready Hospitals) (Schleswig-Holstein) — Extended (+10min Penalty)

Category	Grid Cells	Area (%)	Population	Population (%)
High (30+ min)	8	0.5	92	0.1
Medium (20-30 min)	163	11.0	13012	10.3
Low (10-20 min)	526	35.3	33778	26.7
Only CT reachable within 60 min	2	0.1	179	0.1
Neither reachable within 60 min	17	1.1	1426	1.1
Likely irrelevant (<10 min)	772	51.9	77866	61.6

Population and Area Coverage in Landkreis Nienburg (Weser) (Stroke-Ready Hospitals) (Niedersachsen) — Extended (+10min Penalty)

Category	Grid Cells	Area (%)	Population	Population (%)
High (30+ min)	2	0.1	29	0.0
Medium (20-30 min)	143	7.7	4150	3.4
Low (10-20 min)	340	18.2	15003	12.4
Only CT reachable within 60 min	0	0.0	0	0.0
Neither reachable within 60 min	0	0.0	0	0.0
Likely irrelevant (<10 min)	1380	74.0	102237	84.2

Population and Area Coverage in Landkreis Emsland (Stroke-Ready Hospitals) (Niedersachsen) — Extended (+10min Penalty)

Category	Grid Cells	Area (%)	Population	Population (%)
High (30+ min)	205	5.3	37519	11.6
Medium (20-30 min)	615	16.0	37569	11.7
Low (10-20 min)	1176	30.6	77498	24.0
Only CT reachable within 60 min	7	0.2	111	0.0
Neither reachable within 60 min	20	0.5	353	0.1
Likely irrelevant (<10 min)	1816	47.3	169194	52.5

Population and Area Coverage in Landkreis Wittmund (Stroke-Ready Hospitals) (Niedersachsen) — Extended (+10min Penalty)

Category	Grid Cells	Area (%)	Population	Population (%)
High (30+ min)	2	0.2	169	0.3
Medium (20-30 min)	296	33.3	18247	32.1
Low (10-20 min)	341	38.4	26323	46.3
Only CT reachable within 60 min	2	0.2	16	0.0
Neither reachable within 60 min	50	5.6	2637	4.6
Likely irrelevant (<10 min)	197	22.2	9480	16.7

Population and Area Coverage in Kreis Hochsauerlandkreis (Stroke-Ready Hospitals) (Nordrhein-Westfalen) — Extended (+10min Penalty)

Category	Grid Cells	Area (%)	Population	Population (%)
High (30+ min)	752	29.9	65883	25.1
Medium (20-30 min)	760	30.2	49936	19.0
Low (10-20 min)	474	18.9	53466	20.4
Only CT reachable within 60 min	122	4.9	1382	0.5
Neither reachable within 60 min	0	0.0	0	0.0
Likely irrelevant (<10 min)	406	16.1	91702	35.0

Population and Area Coverage in Landkreis Rheingau-Taunus-Kreis (Stroke-Ready Hospitals) (Hessen) — Extended (+10min Penalty)

Category	Grid Cells	Area (%)	Population	Population (%)
High (30+ min)	87	8.5	3187	1.7
Medium (20-30 min)	253	24.8	23485	12.8
Low (10-20 min)	485	47.6	90247	49.2
Only CT reachable within 60 min	0	0.0	0	0.0
Neither reachable within 60 min	0	0.0	0	0.0
Likely irrelevant (<10 min)	194	19.0	66336	36.2

Population and Area Coverage in Landkreis Göppingen (Stroke-Ready Hospitals) (Baden-Württemberg) — Extended (+10min Penalty)

Category	Grid Cells	Area (%)	Population	Population (%)
High (30+ min)	0	0.0	0	0.0
Medium (20-30 min)	0	0.0	0	0.0
Low (10-20 min)	7	0.9	88	0.0
Only CT reachable within 60 min	0	0.0	0	0.0
Neither reachable within 60 min	0	0.0	0	0.0
Likely irrelevant (<10 min)	774	99.1	249908	100.0

Population and Area Coverage in Stadtkreis Freiburg im Breisgau (Stroke-Ready Hospitals) (Baden-Württemberg) — Extended (+10min Penalty)

Category	Grid Cells	Area (%)	Population	Population (%)
High (30+ min)	0	0.0	0	0.0
Medium (20-30 min)	2	1.1	235	0.1
Low (10-20 min)	35	18.7	4049	1.6
Only CT reachable within 60 min	0	0.0	0	0.0
Neither reachable within 60 min	0	0.0	0	0.0
Likely irrelevant (<10 min)	150	80.2	254155	98.3

Population and Area Coverage in Landkreis Landshut (Stroke-Ready Hospitals) (Bayern) — Extended (+10min Penalty)

Category	Grid Cells	Area (%)	Population	Population (%)
High (30+ min)	0	0.0	0	0.0
Medium (20-30 min)	17	1.0	505	0.3
Low (10-20 min)	226	13.8	18711	12.0
Only CT reachable within 60 min	0	0.0	0	0.0
Neither reachable within 60 min	0	0.0	0	0.0
Likely irrelevant (<10 min)	1398	85.2	136474	87.7

Population and Area Coverage in Landkreis Regen (Stroke-Ready Hospitals) (Bayern) — Extended (+10min Penalty)

Category	Grid Cells	Area (%)	Population	Population (%)
High (30+ min)	1	0.1	0	0.0
Medium (20-30 min)	130	11.4	10154	13.1
Low (10-20 min)	160	14.0	10984	14.2
Only CT reachable within 60 min	0	0.0	0	0.0
Neither reachable within 60 min	22	1.9	23	0.0
Likely irrelevant (<10 min)	827	72.5	56366	72.7

Population and Area Coverage in Kreisfreie Stadt Coburg (Stroke-Ready Hospitals) (Bayern) — Extended (+10min Penalty)

Category	Grid Cells	Area (%)	Population	Population (%)
High (30+ min)	0	0.0	0	0.0
Medium (20-30 min)	0	0.0	0	0.0
Low (10-20 min)	0	0.0	0	0.0
Only CT reachable within 60 min	0	0.0	0	0.0
Neither reachable within 60 min	0	0.0	0	0.0
Likely irrelevant (<10 min)	57	100.0	41384	100.0

Population and Area Coverage in Landkreis Neunkirchen (Stroke-Ready Hospitals) (Saarland) — Extended (+10min Penalty)

Category	Grid Cells	Area (%)	Population	Population (%)
High (30+ min)	0	0.0	0	0.0
Medium (20-30 min)	0	0.0	0	0.0
Low (10-20 min)	15	4.8	5397	4.2
Only CT reachable within 60 min	0	0.0	0	0.0
Neither reachable within 60 min	0	0.0	0	0.0
Likely irrelevant (<10 min)	297	95.2	121946	95.8

Population and Area Coverage in Landkreis Nordwestmecklenburg (Stroke-Ready Hospitals) (Mecklenburg-Vorpommern) — Extended (+10min Penalty)

Category	Grid Cells	Area (%)	Population	Population (%)
High (30+ min)	11	0.4	139	0.1
Medium (20-30 min)	354	12.2	7626	5.3
Low (10-20 min)	774	26.7	32578	22.6
Only CT reachable within 60 min	13	0.4	412	0.3
Neither reachable within 60 min	54	1.9	2508	1.7
Likely irrelevant (<10 min)	1698	58.5	100733	70.0

Population and Area Coverage in Kreisfreie Stadt Herne (Stroke-Ready Hospitals) (Nordrhein-Westfalen) — Extended (+10min Penalty)

Category	Grid Cells	Area (%)	Population	Population (%)
High (30+ min)	0	0.0	0	0.0
Medium (20-30 min)	0	0.0	0	0.0
Low (10-20 min)	0	0.0	0	0.0
Only CT reachable within 60 min	0	0.0	0	0.0
Neither reachable within 60 min	0	0.0	0	0.0
Likely irrelevant (<10 min)	69	100.0	165244	100.0

Population and Area Coverage in Landkreis Altenkirchen (Westerwald) (Stroke-Ready Hospitals) (Rheinland-Pfalz) — Extended (+10min Penalty)

Category	Grid Cells	Area (%)	Population	Population (%)
High (30+ min)	2	0.2	119	0.1
Medium (20-30 min)	284	34.8	50311	37.7
Low (10-20 min)	268	32.9	47608	35.7
Only CT reachable within 60 min	0	0.0	0	0.0
Neither reachable within 60 min	0	0.0	0	0.0
Likely irrelevant (<10 min)	261	32.0	35371	26.5

Population and Area Coverage in Kreisfreie Stadt Kaiserslautern (Stroke-Ready Hospitals) (Rheinland-Pfalz) — Extended (+10min Penalty)

Category	Grid Cells	Area (%)	Population	Population (%)
High (30+ min)	0	0.0	0	0.0
Medium (20-30 min)	0	0.0	0	0.0
Low (10-20 min)	0	0.0	0	0.0
Only CT reachable within 60 min	0	0.0	0	0.0
Neither reachable within 60 min	0	0.0	0	0.0
Likely irrelevant (<10 min)	174	100.0	110813	100.0

Population and Area Coverage in Landkreis Mainz-Bingen (Stroke-Ready Hospitals) (Rheinland-Pfalz) — Extended (+10min Penalty)

Category	Grid Cells	Area (%)	Population	Population (%)
High (30+ min)	11	1.4	881	0.4
Medium (20-30 min)	40	5.3	5303	2.5
Low (10-20 min)	111	14.6	19288	9.2
Only CT reachable within 60 min	0	0.0	0	0.0
Neither reachable within 60 min	0	0.0	0	0.0
Likely irrelevant (<10 min)	599	78.7	184747	87.9

Population and Area Coverage in Landkreis Heidenheim (Stroke-Ready Hospitals) (Baden-Württemberg) — Extended (+10min Penalty)

Category	Grid Cells	Area (%)	Population	Population (%)
High (30+ min)	5	0.7	258	0.2
Medium (20-30 min)	66	8.7	2406	1.9
Low (10-20 min)	126	16.6	9098	7.1
Only CT reachable within 60 min	0	0.0	0	0.0
Neither reachable within 60 min	0	0.0	0	0.0
Likely irrelevant (<10 min)	562	74.0	116227	90.8

Population and Area Coverage in Stadtkreis Pforzheim (Stroke-Ready Hospitals) (Baden-Württemberg) — Extended (+10min Penalty)

Category	Grid Cells	Area (%)	Population	Population (%)
High (30+ min)	0	0.0	0	0.0
Medium (20-30 min)	0	0.0	0	0.0
Low (10-20 min)	1	0.8	5	0.0
Only CT reachable within 60 min	0	0.0	0	0.0
Neither reachable within 60 min	0	0.0	0	0.0
Likely irrelevant (<10 min)	119	99.2	124957	100.0

Population and Area Coverage in Landkreis Tübingen (Stroke-Ready Hospitals) (Baden-Württemberg) — Extended (+10min Penalty)

Category	Grid Cells	Area (%)	Population	Population (%)
High (30+ min)	0	0.0	0	0.0
Medium (20-30 min)	9	1.4	1560	0.7
Low (10-20 min)	69	11.0	14009	6.4
Only CT reachable within 60 min	0	0.0	0	0.0
Neither reachable within 60 min	0	0.0	0	0.0
Likely irrelevant (<10 min)	550	87.6	202969	92.9

Population and Area Coverage in Landkreis Bodenseekreis (Stroke-Ready Hospitals) (Baden-Württemberg) — Extended (+10min Penalty)

Category	Grid Cells	Area (%)	Population	Population (%)
High (30+ min)	0	0.0	0	0.0
Medium (20-30 min)	65	8.1	5078	2.3
Low (10-20 min)	347	43.3	92315	41.8
Only CT reachable within 60 min	0	0.0	635	0.3
Neither reachable within 60 min	0	0.0	1958	0.9
Likely irrelevant (<10 min)	390	48.6	121088	54.8

Population and Area Coverage in Landkreis Miesbach (Stroke-Ready Hospitals) (Bayern) — Extended (+10min Penalty)

Category	Grid Cells	Area (%)	Population	Population (%)
High (30+ min)	0	0.0	0	0.0
Medium (20-30 min)	2	0.2	63	0.1
Low (10-20 min)	17	1.6	295	0.3
Only CT reachable within 60 min	2	0.2	1141	1.2
Neither reachable within 60 min	246	23.7	1166	1.2
Likely irrelevant (<10 min)	770	74.3	96068	97.3

Population and Area Coverage in Landkreis Mühldorf a.Inn (Stroke-Ready Hospitals) (Bayern) — Extended (+10min Penalty)

Category	Grid Cells	Area (%)	Population	Population (%)
High (30+ min)	0	0.0	0	0.0
Medium (20-30 min)	0	0.0	0	0.0
Low (10-20 min)	265	27.3	35563	32.1
Only CT reachable within 60 min	0	0.0	0	0.0
Neither reachable within 60 min	0	0.0	0	0.0
Likely irrelevant (<10 min)	705	72.7	75073	67.9

Population and Area Coverage in Landkreis Neuburg-Schrobenhausen (Stroke-Ready Hospitals) (Bayern) — Extended (+10min Penalty)

Category	Grid Cells	Area (%)	Population	Population (%)
High (30+ min)	13	1.4	1564	1.6
Medium (20-30 min)	393	43.5	22879	24.0
Low (10-20 min)	398	44.0	58487	61.3
Only CT reachable within 60 min	0	0.0	0	0.0
Neither reachable within 60 min	0	0.0	0	0.0
Likely irrelevant (<10 min)	100	11.1	12553	13.1

Population and Area Coverage in Landkreis Starnberg (Stroke-Ready Hospitals) (Bayern) — Extended (+10min Penalty)

Category	Grid Cells	Area (%)	Population	Population (%)
High (30+ min)	0	0.0	0	0.0
Medium (20-30 min)	8	1.4	183	0.1
Low (10-20 min)	220	37.5	43706	32.5
Only CT reachable within 60 min	0	0.0	0	0.0
Neither reachable within 60 min	0	0.0	0	0.0
Likely irrelevant (<10 min)	358	61.1	90648	67.4

Population and Area Coverage in Landkreis Fürth (Stroke-Ready Hospitals) (Bayern) — Extended (+10min Penalty)

Category	Grid Cells	Area (%)	Population	Population (%)
High (30+ min)	0	0.0	0	0.0
Medium (20-30 min)	0	0.0	0	0.0
Low (10-20 min)	3	0.8	386	0.3
Only CT reachable within 60 min	0	0.0	0	0.0
Neither reachable within 60 min	0	0.0	0	0.0
Likely irrelevant (<10 min)	379	99.2	118319	99.7

Population and Area Coverage in Landkreis Kitzingen (Stroke-Ready Hospitals) (Bayern) — Extended (+10min Penalty)

Category	Grid Cells	Area (%)	Population	Population (%)
High (30+ min)	0	0.0	0	0.0
Medium (20-30 min)	32	3.8	551	0.6
Low (10-20 min)	265	31.4	23379	25.6
Only CT reachable within 60 min	0	0.0	0	0.0
Neither reachable within 60 min	0	0.0	0	0.0
Likely irrelevant (<10 min)	547	64.8	67499	73.8

Population and Area Coverage in Landkreis Günzburg (Stroke-Ready Hospitals) (Bayern) — Extended (+10min Penalty)

Category	Grid Cells	Area (%)	Population	Population (%)
High (30+ min)	45	4.9	3205	2.6
Medium (20-30 min)	198	21.5	25711	20.5
Low (10-20 min)	264	28.6	20124	16.1
Only CT reachable within 60 min	0	0.0	0	0.0
Neither reachable within 60 min	0	0.0	0	0.0
Likely irrelevant (<10 min)	416	45.1	76079	60.8

Population and Area Coverage in Landkreis Havelland (Stroke-Ready Hospitals) (Brandenburg) — Extended (+10min Penalty)

Category	Grid Cells	Area (%)	Population	Population (%)
High (30+ min)	264	11.6	26224	17.3
Medium (20-30 min)	201	8.8	6613	4.4
Low (10-20 min)	289	12.7	12042	8.0
Only CT reachable within 60 min	156	6.8	985	0.7
Neither reachable within 60 min	0	0.0	0	0.0
Likely irrelevant (<10 min)	1372	60.1	105549	69.7

Population and Area Coverage in Landkreis Teltow-Fläming (Stroke-Ready Hospitals) (Brandenburg) — Extended (+10min Penalty)

Category	Grid Cells	Area (%)	Population	Population (%)
High (30+ min)	265	9.6	29469	18.8
Medium (20-30 min)	594	21.5	13562	8.6
Low (10-20 min)	995	36.0	55715	35.5
Only CT reachable within 60 min	140	5.1	694	0.4
Neither reachable within 60 min	0	0.0	0	0.0
Likely irrelevant (<10 min)	771	27.9	57630	36.7

Population and Area Coverage in Landkreis Börde (Stroke-Ready Hospitals) (Sachsen-Anhalt) — Extended (+10min Penalty)

Category	Grid Cells	Area (%)	Population	Population (%)
High (30+ min)	5	0.2	452	0.3
Medium (20-30 min)	281	9.0	17702	11.4
Low (10-20 min)	582	18.6	16689	10.7
Only CT reachable within 60 min	0	0.0	0	0.0
Neither reachable within 60 min	0	0.0	0	0.0
Likely irrelevant (<10 min)	2255	72.2	120540	77.6

Population and Area Coverage in Landkreis Stade (Stroke-Ready Hospitals) (Niedersachsen) — Extended (+10min Penalty)

Category	Grid Cells	Area (%)	Population	Population (%)
High (30+ min)	20	1.2	608	0.3
Medium (20-30 min)	113	6.9	5762	2.9
Low (10-20 min)	342	20.8	61573	31.1
Only CT reachable within 60 min	10	0.6	135	0.1
Neither reachable within 60 min	100	6.1	4948	2.5
Likely irrelevant (<10 min)	1056	64.4	125140	63.1

Population and Area Coverage in Kreisfreie Stadt Oldenburg (Oldb) (Stroke-Ready Hospitals) (Niedersachsen) — Extended (+10min Penalty)

Category	Grid Cells	Area (%)	Population	Population (%)
High (30+ min)	0	0.0	0	0.0
Medium (20-30 min)	0	0.0	0	0.0
Low (10-20 min)	0	0.0	0	0.0
Only CT reachable within 60 min	0	0.0	0	0.0
Neither reachable within 60 min	0	0.0	0	0.0
Likely irrelevant (<10 min)	137	100.0	159948	100.0

Population and Area Coverage in Landkreis Cloppenburg (Stroke-Ready Hospitals) (Niedersachsen) — Extended (+10min Penalty)

Category	Grid Cells	Area (%)	Population	Population (%)
High (30+ min)	144	7.6	7287	4.4
Medium (20-30 min)	553	29.1	47586	29.0
Low (10-20 min)	886	46.6	87175	53.1
Only CT reachable within 60 min	0	0.0	0	0.0
Neither reachable within 60 min	0	0.0	0	0.0
Likely irrelevant (<10 min)	319	16.8	22028	13.4

Population and Area Coverage in Kreisfreie Stadt Wuppertal (Stroke-Ready Hospitals) (Nordrhein-Westfalen) — Extended (+10min Penalty)

Category	Grid Cells	Area (%)	Population	Population (%)
High (30+ min)	0	0.0	0	0.0
Medium (20-30 min)	0	0.0	0	0.0
Low (10-20 min)	7	3.3	325	0.1
Only CT reachable within 60 min	0	0.0	0	0.0
Neither reachable within 60 min	0	0.0	0	0.0
Likely irrelevant (<10 min)	206	96.7	358748	99.9

Population and Area Coverage in Landkreis Odenwaldkreis (Stroke-Ready Hospitals) (Hessen) — Extended (+10min Penalty)

Category	Grid Cells	Area (%)	Population	Population (%)
High (30+ min)	0	0.0	0	0.0
Medium (20-30 min)	8	1.0	946	1.0
Low (10-20 min)	229	29.5	32816	33.5
Only CT reachable within 60 min	0	0.0	0	0.0
Neither reachable within 60 min	0	0.0	0	0.0
Likely irrelevant (<10 min)	539	69.5	64128	65.5

Population and Area Coverage in Landkreis Karlsruhe (Stroke-Ready Hospitals) (Baden-Württemberg) — Extended (+10min Penalty)

Category	Grid Cells	Area (%)	Population	Population (%)
High (30+ min)	0	0.0	0	0.0
Medium (20-30 min)	16	1.2	1166	0.3
Low (10-20 min)	206	15.4	58340	13.4
Only CT reachable within 60 min	0	0.0	0	0.0
Neither reachable within 60 min	0	0.0	0	0.0
Likely irrelevant (<10 min)	1113	83.4	376329	86.3

Population and Area Coverage in Kreisfreie Stadt Landshut (Stroke-Ready Hospitals) (Bayern) — Extended (+10min Penalty)

Category	Grid Cells	Area (%)	Population	Population (%)
High (30+ min)	0	0.0	0	0.0
Medium (20-30 min)	0	0.0	0	0.0
Low (10-20 min)	1	1.2	1	0.0
Only CT reachable within 60 min	0	0.0	0	0.0
Neither reachable within 60 min	0	0.0	0	0.0
Likely irrelevant (<10 min)	80	98.8	69057	100.0

Population and Area Coverage in Kreisfreie Stadt Erlangen (Stroke-Ready Hospitals) (Bayern) — Extended (+10min Penalty)

Category	Grid Cells	Area (%)	Population	Population (%)
High (30+ min)	0	0.0	0	0.0
Medium (20-30 min)	0	0.0	0	0.0
Low (10-20 min)	0	0.0	0	0.0
Only CT reachable within 60 min	0	0.0	0	0.0
Neither reachable within 60 min	0	0.0	0	0.0
Likely irrelevant (<10 min)	96	100.0	126629	100.0

Population and Area Coverage in Landkreis Haßberge (Stroke-Ready Hospitals) (Bayern) — Extended (+10min Penalty)

Category	Grid Cells	Area (%)	Population	Population (%)
High (30+ min)	0	0.0	0	0.0
Medium (20-30 min)	387	32.2	17084	19.6
Low (10-20 min)	703	58.6	63751	73.1
Only CT reachable within 60 min	0	0.0	0	0.0
Neither reachable within 60 min	0	0.0	0	0.0
Likely irrelevant (<10 min)	110	9.2	6331	7.3

Population and Area Coverage in Landkreis Aichach-Friedberg (Stroke-Ready Hospitals) (Bayern) — Extended (+10min Penalty)

Category	Grid Cells	Area (%)	Population	Population (%)
High (30+ min)	0	0.0	0	0.0
Medium (20-30 min)	103	10.9	8269	6.2
Low (10-20 min)	478	50.6	76107	57.0
Only CT reachable within 60 min	0	0.0	0	0.0
Neither reachable within 60 min	0	0.0	0	0.0
Likely irrelevant (<10 min)	364	38.5	49035	36.8

Population and Area Coverage in Landkreis Dillingen a.d.Donau (Stroke-Ready Hospitals) (Bayern) — Extended (+10min Penalty)

Category	Grid Cells	Area (%)	Population	Population (%)
High (30+ min)	65	6.7	3275	3.4
Medium (20-30 min)	282	29.1	22611	23.3
Low (10-20 min)	408	42.1	50804	52.3
Only CT reachable within 60 min	0	0.0	0	0.0
Neither reachable within 60 min	0	0.0	0	0.0
Likely irrelevant (<10 min)	215	22.2	20408	21.0

Population and Area Coverage in Landkreis Oberspreewald-Lausitz (Stroke-Ready Hospitals) (Brandenburg) — Extended (+10min Penalty)

Category	Grid Cells	Area (%)	Population	Population (%)
High (30+ min)	1	0.1	0	0.0
Medium (20-30 min)	33	2.1	1494	1.4
Low (10-20 min)	279	17.7	20290	18.7
Only CT reachable within 60 min	0	0.0	0	0.0
Neither reachable within 60 min	0	0.0	0	0.0
Likely irrelevant (<10 min)	1267	80.2	86674	79.9

Population and Area Coverage in Landkreis Bautzen (Stroke-Ready Hospitals) (Sachsen) — Extended (+10min Penalty)

Category	Grid Cells	Area (%)	Population	Population (%)
High (30+ min)	124	4.0	18095	6.3
Medium (20-30 min)	736	23.9	92818	32.2
Low (10-20 min)	1007	32.7	65112	22.6
Only CT reachable within 60 min	2	0.1	120	0.0
Neither reachable within 60 min	5	0.2	89	0.0
Likely irrelevant (<10 min)	1208	39.2	111789	38.8

Population and Area Coverage in Kreisfreie Stadt Dessau-Roßlau (Stroke-Ready Hospitals) (Sachsen-Anhalt) — Extended (+10min Penalty)

Category	Grid Cells	Area (%)	Population	Population (%)
High (30+ min)	1	0.3	0	0.0
Medium (20-30 min)	17	5.3	105	0.1
Low (10-20 min)	64	20.0	2626	3.1
Only CT reachable within 60 min	0	0.0	0	0.0
Neither reachable within 60 min	0	0.0	0	0.0
Likely irrelevant (<10 min)	238	74.4	81382	96.8

Population and Area Coverage in Landkreis Lüneburg (Stroke-Ready Hospitals) (Niedersachsen) — Extended (+10min Penalty)

Category	Grid Cells	Area (%)	Population	Population (%)
High (30+ min)	45	2.5	583	0.3
Medium (20-30 min)	149	8.4	4297	2.4
Low (10-20 min)	335	18.8	16627	9.3
Only CT reachable within 60 min	246	13.8	3805	2.1
Neither reachable within 60 min	0	0.0	0	0.0
Likely irrelevant (<10 min)	1005	56.5	152956	85.8

Population and Area Coverage in Kreisfreie Stadt Bremerhaven (Stroke-Ready Hospitals) (Bremen) — Extended (+10min Penalty)

Category	Grid Cells	Area (%)	Population	Population (%)
High (30+ min)	2	2.0	73	0.1
Medium (20-30 min)	0	0.0	0	0.0
Low (10-20 min)	3	3.1	579	0.5
Only CT reachable within 60 min	10	10.2	278	0.2
Neither reachable within 60 min	4	4.1	10	0.0
Likely irrelevant (<10 min)	79	80.6	113476	99.2

Population and Area Coverage in Kreisfreie Stadt Koblenz (Stroke-Ready Hospitals) (Rheinland-Pfalz) — Extended (+10min Penalty)

Category	Grid Cells	Area (%)	Population	Population (%)
High (30+ min)	0	0.0	0	0.0
Medium (20-30 min)	0	0.0	0	0.0
Low (10-20 min)	0	0.0	0	0.0
Only CT reachable within 60 min	0	0.0	0	0.0
Neither reachable within 60 min	0	0.0	0	0.0
Likely irrelevant (<10 min)	136	100.0	125857	100.0

Population and Area Coverage in Kreisfreie Stadt Pirmasens (Stroke-Ready Hospitals) (Rheinland-Pfalz) — Extended (+10min Penalty)

Category	Grid Cells	Area (%)	Population	Population (%)
High (30+ min)	0	0.0	0	0.0
Medium (20-30 min)	0	0.0	0	0.0
Low (10-20 min)	0	0.0	0	0.0
Only CT reachable within 60 min	0	0.0	0	0.0
Neither reachable within 60 min	0	0.0	0	0.0
Likely irrelevant (<10 min)	76	100.0	37262	100.0

Population and Area Coverage in Landkreis Donnersbergkreis (Stroke-Ready Hospitals) (Rheinland-Pfalz) — Extended (+10min Penalty)

Category	Grid Cells	Area (%)	Population	Population (%)
High (30+ min)	0	0.0	0	0.0
Medium (20-30 min)	21	2.6	440	0.6
Low (10-20 min)	353	43.7	37302	48.1
Only CT reachable within 60 min	0	0.0	0	0.0
Neither reachable within 60 min	0	0.0	0	0.0
Likely irrelevant (<10 min)	433	53.7	39775	51.3

Population and Area Coverage in Stadtkreis Heidelberg (Stroke-Ready Hospitals) (Baden-Württemberg) — Extended (+10min Penalty)

Category	Grid Cells	Area (%)	Population	Population (%)
High (30+ min)	0	0.0	0	0.0
Medium (20-30 min)	0	0.0	0	0.0
Low (10-20 min)	27	20.1	4026	2.6
Only CT reachable within 60 min	0	0.0	0	0.0
Neither reachable within 60 min	0	0.0	0	0.0
Likely irrelevant (<10 min)	107	79.9	150340	97.4

Population and Area Coverage in Kreisfreie Stadt Straubing (Stroke-Ready Hospitals) (Bayern) — Extended (+10min Penalty)

Category	Grid Cells	Area (%)	Population	Population (%)
High (30+ min)	0	0.0	0	0.0
Medium (20-30 min)	0	0.0	0	0.0
Low (10-20 min)	0	0.0	0	0.0
Only CT reachable within 60 min	0	0.0	0	0.0
Neither reachable within 60 min	0	0.0	0	0.0
Likely irrelevant (<10 min)	85	100.0	46942	100.0

Population and Area Coverage in Landkreis Straubing-Bogen (Stroke-Ready Hospitals) (Bayern) — Extended (+10min Penalty)

Category	Grid Cells	Area (%)	Population	Population (%)
High (30+ min)	0	0.0	0	0.0
Medium (20-30 min)	106	7.2	7457	7.4
Low (10-20 min)	365	24.9	16061	16.0
Only CT reachable within 60 min	0	0.0	0	0.0
Neither reachable within 60 min	0	0.0	0	0.0
Likely irrelevant (<10 min)	995	67.9	77004	76.6

Population and Area Coverage in Landkreis Neumarkt i.d.OPf. (Stroke-Ready Hospitals) (Bayern) — Extended (+10min Penalty)

Category	Grid Cells	Area (%)	Population	Population (%)
High (30+ min)	0	0.0	0	0.0
Medium (20-30 min)	12	0.7	1311	1.0
Low (10-20 min)	153	9.2	7396	5.6
Only CT reachable within 60 min	0	0.0	0	0.0
Neither reachable within 60 min	0	0.0	0	0.0
Likely irrelevant (<10 min)	1499	90.1	123760	93.4

Population and Area Coverage in Kreisfreie Stadt Schwabach (Stroke-Ready Hospitals) (Bayern) — Extended (+10min Penalty)

Category	Grid Cells	Area (%)	Population	Population (%)
High (30+ min)	0	0.0	0	0.0
Medium (20-30 min)	0	0.0	0	0.0
Low (10-20 min)	0	0.0	0	0.0
Only CT reachable within 60 min	0	0.0	0	0.0
Neither reachable within 60 min	0	0.0	0	0.0
Likely irrelevant (<10 min)	52	100.0	42132	100.0

Population and Area Coverage in Kreisfreie Stadt Brandenburg an der Havel (Stroke-Ready Hospitals) (Brandenburg) — Extended (+10min Penalty)

Category	Grid Cells	Area (%)	Population	Population (%)
High (30+ min)	0	0.0	0	0.0
Medium (20-30 min)	1	0.3	1	0.0
Low (10-20 min)	58	19.1	3429	5.0
Only CT reachable within 60 min	0	0.0	0	0.0
Neither reachable within 60 min	0	0.0	0	0.0
Likely irrelevant (<10 min)	245	80.6	65662	95.0

Population and Area Coverage in Landkreis Ludwigslust-Parchim (Stroke-Ready Hospitals) (Mecklenburg-Vorpommern) — Extended (+10min Penalty)

Category	Grid Cells	Area (%)	Population	Population (%)
High (30+ min)	1029	15.9	46279	23.6
Medium (20-30 min)	1488	23.1	40484	20.7
Low (10-20 min)	1530	23.7	33283	17.0
Only CT reachable within 60 min	586	9.1	9928	5.1
Neither reachable within 60 min	0	0.0	0	0.0
Likely irrelevant (<10 min)	1822	28.2	66042	33.7

Population and Area Coverage in Landkreis Saalfeld-Rudolstadt (Stroke-Ready Hospitals) (Thüringen) — Extended (+10min Penalty)

Category	Grid Cells	Area (%)	Population	Population (%)
High (30+ min)	0	0.0	0	0.0
Medium (20-30 min)	17	1.3	444	0.5
Low (10-20 min)	633	49.3	23928	24.7
Only CT reachable within 60 min	0	0.0	0	0.0
Neither reachable within 60 min	0	0.0	0	0.0
Likely irrelevant (<10 min)	633	49.3	72448	74.8

Population and Area Coverage in Kreis Ostholstein (Stroke-Ready Hospitals) (Schleswig-Holstein) — Extended (+10min Penalty)

Category	Grid Cells	Area (%)	Population	Population (%)
High (30+ min)	0	0.0	7	0.0
Medium (20-30 min)	137	7.4	11689	6.0
Low (10-20 min)	412	22.1	48773	24.8
Only CT reachable within 60 min	7	0.4	417	0.2
Neither reachable within 60 min	73	3.9	2950	1.5
Likely irrelevant (<10 min)	1232	66.2	132565	67.5

Population and Area Coverage in Kreis Rendsburg-Eckernförde (Stroke-Ready Hospitals) (Schleswig-Holstein) — Extended (+10min Penalty)

Category	Grid Cells	Area (%)	Population	Population (%)
High (30+ min)	60	2.0	2640	1.0
Medium (20-30 min)	140	4.7	5294	1.9
Low (10-20 min)	316	10.6	36786	13.4
Only CT reachable within 60 min	2	0.1	1871	0.7
Neither reachable within 60 min	35	1.2	1603	0.6
Likely irrelevant (<10 min)	2435	81.5	226358	82.4

Population and Area Coverage in Landkreis Ostalbkreis (Stroke-Ready Hospitals) (Baden-Württemberg) — Extended (+10min Penalty)

Category	Grid Cells	Area (%)	Population	Population (%)
High (30+ min)	0	0.0	0	0.0
Medium (20-30 min)	114	6.1	8777	2.8
Low (10-20 min)	464	25.0	48494	15.6
Only CT reachable within 60 min	0	0.0	0	0.0
Neither reachable within 60 min	0	0.0	0	0.0
Likely irrelevant (<10 min)	1277	68.8	254060	81.6

Population and Area Coverage in Landkreis Emmendingen (Stroke-Ready Hospitals) (Baden-Württemberg) — Extended (+10min Penalty)

Category	Grid Cells	Area (%)	Population	Population (%)
High (30+ min)	70	8.6	1732	1.1
Medium (20-30 min)	106	13.0	16460	10.2
Low (10-20 min)	205	25.1	36307	22.4
Only CT reachable within 60 min	4	0.5	1	0.0
Neither reachable within 60 min	0	0.0	5	0.0
Likely irrelevant (<10 min)	433	52.9	107379	66.3

Population and Area Coverage in Kreisfreie Stadt München (Stroke-Ready Hospitals) (Bayern) — Extended (+10min Penalty)

Category	Grid Cells	Area (%)	Population	Population (%)
High (30+ min)	0	0.0	0	0.0
Medium (20-30 min)	0	0.0	0	0.0
Low (10-20 min)	12	3.2	28078	1.7
Only CT reachable within 60 min	0	0.0	0	0.0
Neither reachable within 60 min	0	0.0	0	0.0
Likely irrelevant (<10 min)	367	96.8	1643077	98.3

Population and Area Coverage in Landkreis Bayreuth (Stroke-Ready Hospitals) (Bayern) — Extended (+10min Penalty)

Category	Grid Cells	Area (%)	Population	Population (%)
High (30+ min)	0	0.0	0	0.0
Medium (20-30 min)	82	5.1	3050	2.8
Low (10-20 min)	573	35.9	37313	34.3
Only CT reachable within 60 min	0	0.0	0	0.0
Neither reachable within 60 min	0	0.0	0	0.0
Likely irrelevant (<10 min)	941	59.0	68547	62.9

Population and Area Coverage in Landkreis Kronach (Stroke-Ready Hospitals) (Bayern) — Extended (+10min Penalty)

Category	Grid Cells	Area (%)	Population	Population (%)
High (30+ min)	3	0.4	13	0.0
Medium (20-30 min)	158	19.5	18373	25.8
Low (10-20 min)	386	47.5	34436	48.4
Only CT reachable within 60 min	0	0.0	0	0.0
Neither reachable within 60 min	0	0.0	0	0.0
Likely irrelevant (<10 min)	265	32.6	18257	25.7

Population and Area Coverage in Landkreis Aschaffenburg (Stroke-Ready Hospitals) (Bayern) — Extended (+10min Penalty)

Category	Grid Cells	Area (%)	Population	Population (%)
High (30+ min)	0	0.0	0	0.0
Medium (20-30 min)	0	0.0	0	0.0
Low (10-20 min)	15	1.7	1902	1.1
Only CT reachable within 60 min	0	0.0	0	0.0
Neither reachable within 60 min	0	0.0	0	0.0
Likely irrelevant (<10 min)	859	98.3	177947	98.9

Population and Area Coverage in Landkreis Saalekreis (Stroke-Ready Hospitals) (Sachsen-Anhalt) — Extended (+10min Penalty)

Category	Grid Cells	Area (%)	Population	Population (%)
High (30+ min)	0	0.0	0	0.0
Medium (20-30 min)	3	0.2	93	0.1
Low (10-20 min)	46	2.5	4987	3.0
Only CT reachable within 60 min	0	0.0	0	0.0
Neither reachable within 60 min	0	0.0	0	0.0
Likely irrelevant (<10 min)	1819	97.4	163855	97.0

Population and Area Coverage in Kreisfreie Stadt Wolfsburg (Stroke-Ready Hospitals) (Niedersachsen) — Extended (+10min Penalty)

Category	Grid Cells	Area (%)	Population	Population (%)
High (30+ min)	0	0.0	0	0.0
Medium (20-30 min)	0	0.0	0	0.0
Low (10-20 min)	0	0.0	0	0.0
Only CT reachable within 60 min	0	0.0	0	0.0
Neither reachable within 60 min	0	0.0	0	0.0
Likely irrelevant (<10 min)	274	100.0	122066	100.0

Population and Area Coverage in Landkreis Wolfenbüttel (Stroke-Ready Hospitals) (Niedersachsen) — Extended (+10min Penalty)

Category	Grid Cells	Area (%)	Population	Population (%)
High (30+ min)	0	0.0	0	0.0
Medium (20-30 min)	0	0.0	0	0.0
Low (10-20 min)	65	6.8	4134	3.5
Only CT reachable within 60 min	0	0.0	0	0.0
Neither reachable within 60 min	0	0.0	0	0.0
Likely irrelevant (<10 min)	892	93.2	115660	96.5

Population and Area Coverage in Landkreis Ammerland (Stroke-Ready Hospitals) (Niedersachsen) — Extended (+10min Penalty)

Category	Grid Cells	Area (%)	Population	Population (%)
High (30+ min)	0	0.0	0	0.0
Medium (20-30 min)	0	0.0	0	0.0
Low (10-20 min)	70	7.2	5381	4.4
Only CT reachable within 60 min	0	0.0	0	0.0
Neither reachable within 60 min	0	0.0	0	0.0
Likely irrelevant (<10 min)	909	92.8	116081	95.6

Population and Area Coverage in Kreis Rhein-Kreis Neuss (Stroke-Ready Hospitals) (Nordrhein-Westfalen) — Extended (+10min Penalty)

Category	Grid Cells	Area (%)	Population	Population (%)
High (30+ min)	0	0.0	0	0.0
Medium (20-30 min)	0	0.0	0	0.0
Low (10-20 min)	181	24.6	56028	11.9
Only CT reachable within 60 min	0	0.0	0	0.0
Neither reachable within 60 min	0	0.0	0	0.0
Likely irrelevant (<10 min)	555	75.4	414816	88.1

Population and Area Coverage in Kreis Lippe (Stroke-Ready Hospitals) (Nordrhein-Westfalen) — Extended (+10min Penalty)

Category	Grid Cells	Area (%)	Population	Population (%)
High (30+ min)	0	0.0	0	0.0
Medium (20-30 min)	50	3.1	10347	3.0
Low (10-20 min)	418	25.6	99002	28.7
Only CT reachable within 60 min	0	0.0	0	0.0
Neither reachable within 60 min	0	0.0	0	0.0
Likely irrelevant (<10 min)	1165	71.3	235810	68.3

Population and Area Coverage in Kreisfreie Stadt Hagen (Stroke-Ready Hospitals) (Nordrhein-Westfalen) — Extended (+10min Penalty)

Category	Grid Cells	Area (%)	Population	Population (%)
High (30+ min)	0	0.0	0	0.0
Medium (20-30 min)	0	0.0	0	0.0
Low (10-20 min)	28	13.7	3120	1.6
Only CT reachable within 60 min	0	0.0	0	0.0
Neither reachable within 60 min	0	0.0	0	0.0
Likely irrelevant (<10 min)	176	86.3	196539	98.4

Population and Area Coverage in Landkreis Alzey-Worms (Stroke-Ready Hospitals) (Rheinland-Pfalz) — Extended (+10min Penalty)

Category	Grid Cells	Area (%)	Population	Population (%)
High (30+ min)	0	0.0	0	0.0
Medium (20-30 min)	0	0.0	0	0.0
Low (10-20 min)	5	0.7	1077	0.8
Only CT reachable within 60 min	0	0.0	0	0.0
Neither reachable within 60 min	0	0.0	0	0.0
Likely irrelevant (<10 min)	732	99.3	128317	99.2

Population and Area Coverage in Landkreis Germersheim (Stroke-Ready Hospitals) (Rheinland-Pfalz) — Extended (+10min Penalty)

Category	Grid Cells	Area (%)	Population	Population (%)
High (30+ min)	0	0.0	0	0.0
Medium (20-30 min)	0	0.0	0	0.0
Low (10-20 min)	50	8.8	7320	5.7
Only CT reachable within 60 min	0	0.0	0	0.0
Neither reachable within 60 min	0	0.0	0	0.0
Likely irrelevant (<10 min)	520	91.2	121402	94.3

Population and Area Coverage in Landkreis Neckar-Odenwald-Kreis (Stroke-Ready Hospitals) (Baden-Württemberg) — Extended (+10min Penalty)

Category	Grid Cells	Area (%)	Population	Population (%)
High (30+ min)	118	8.4	8562	6.0
Medium (20-30 min)	323	23.1	29237	20.4
Low (10-20 min)	323	23.1	21395	14.9
Only CT reachable within 60 min	0	0.0	0	0.0
Neither reachable within 60 min	0	0.0	0	0.0
Likely irrelevant (<10 min)	633	45.3	84176	58.7

Population and Area Coverage in Landkreis Enzkreis (Stroke-Ready Hospitals) (Baden-Württemberg) — Extended (+10min Penalty)

Category	Grid Cells	Area (%)	Population	Population (%)
High (30+ min)	5	0.7	5	0.0
Medium (20-30 min)	76	10.7	12613	6.4
Low (10-20 min)	272	38.4	67416	34.4
Only CT reachable within 60 min	0	0.0	0	0.0
Neither reachable within 60 min	0	0.0	0	0.0
Likely irrelevant (<10 min)	355	50.1	115902	59.2

Population and Area Coverage in Landkreis Berchtesgadener Land (Stroke-Ready Hospitals) (Bayern) — Extended (+10min Penalty)

Category	Grid Cells	Area (%)	Population	Population (%)
High (30+ min)	6	0.6	248	0.3
Medium (20-30 min)	120	12.9	20695	22.1
Low (10-20 min)	114	12.3	29620	31.6
Only CT reachable within 60 min	5	0.5	87	0.1
Neither reachable within 60 min	326	35.1	1727	1.8
Likely irrelevant (<10 min)	358	38.5	41258	44.1

Population and Area Coverage in Kreisfreie Stadt Hof (Stroke-Ready Hospitals) (Bayern) — Extended (+10min Penalty)

Category	Grid Cells	Area (%)	Population	Population (%)
High (30+ min)	0	0.0	0	0.0
Medium (20-30 min)	0	0.0	0	0.0
Low (10-20 min)	0	0.0	0	0.0
Only CT reachable within 60 min	0	0.0	0	0.0
Neither reachable within 60 min	0	0.0	0	0.0
Likely irrelevant (<10 min)	75	100.0	44749	100.0

Population and Area Coverage in Landkreis Kulmbach (Stroke-Ready Hospitals) (Bayern) — Extended (+10min Penalty)

Category	Grid Cells	Area (%)	Population	Population (%)
High (30+ min)	0	0.0	0	0.0
Medium (20-30 min)	2	0.2	3	0.0
Low (10-20 min)	170	20.5	4450	5.9
Only CT reachable within 60 min	0	0.0	0	0.0
Neither reachable within 60 min	0	0.0	0	0.0
Likely irrelevant (<10 min)	656	79.2	71225	94.1

Population and Area Coverage in Kreisfreie Stadt Memmingen (Stroke-Ready Hospitals) (Bayern) — Extended (+10min Penalty)

Category	Grid Cells	Area (%)	Population	Population (%)
High (30+ min)	0	0.0	0	0.0
Medium (20-30 min)	0	0.0	0	0.0
Low (10-20 min)	0	0.0	0	0.0
Only CT reachable within 60 min	0	0.0	0	0.0
Neither reachable within 60 min	0	0.0	0	0.0
Likely irrelevant (<10 min)	84	100.0	43302	100.0

Population and Area Coverage in Landkreis Augsburg (Stroke-Ready Hospitals) (Bayern) — Extended (+10min Penalty)

Category	Grid Cells	Area (%)	Population	Population (%)
High (30+ min)	0	0.0	0	0.0
Medium (20-30 min)	92	7.1	4558	1.8
Low (10-20 min)	293	22.6	53325	21.5
Only CT reachable within 60 min	0	0.0	0	0.0
Neither reachable within 60 min	0	0.0	0	0.0
Likely irrelevant (<10 min)	914	70.4	190382	76.7

Population and Area Coverage in Landkreis Vorpommern-Rügen (Stroke-Ready Hospitals) (Mecklenburg-Vorpommern) — Extended (+10min Penalty)

Category	Grid Cells	Area (%)	Population	Population (%)
High (30+ min)	413	9.9	21254	10.7
Medium (20-30 min)	673	16.2	18984	9.5
Low (10-20 min)	1068	25.7	28460	14.3
Only CT reachable within 60 min	231	5.5	6550	3.3
Neither reachable within 60 min	274	6.6	5801	2.9
Likely irrelevant (<10 min)	1504	36.1	118220	59.3

Population and Area Coverage in Landkreis Zwickau (Stroke-Ready Hospitals) (Sachsen) — Extended (+10min Penalty)

Category	Grid Cells	Area (%)	Population	Population (%)
High (30+ min)	0	0.0	0	0.0
Medium (20-30 min)	0	0.0	0	0.0
Low (10-20 min)	279	22.9	40718	12.8
Only CT reachable within 60 min	0	0.0	0	0.0
Neither reachable within 60 min	0	0.0	0	0.0
Likely irrelevant (<10 min)	942	77.1	276499	87.2

Population and Area Coverage in Landkreis Görlitz (Stroke-Ready Hospitals) (Sachsen) — Extended (+10min Penalty)

Category	Grid Cells	Area (%)	Population	Population (%)
High (30+ min)	425	16.4	89577	40.9
Medium (20-30 min)	489	18.9	24581	11.2
Low (10-20 min)	370	14.3	20261	9.3
Only CT reachable within 60 min	74	2.9	4473	2.0
Neither reachable within 60 min	12	0.5	216	0.1
Likely irrelevant (<10 min)	1217	47.0	79853	36.5

Population and Area Coverage in Landkreis Sonneberg (Stroke-Ready Hospitals) (Thüringen) — Extended (+10min Penalty)

Category	Grid Cells	Area (%)	Population	Population (%)
High (30+ min)	0	0.0	0	0.0
Medium (20-30 min)	0	0.0	23	0.0
Low (10-20 min)	59	10.0	2330	4.3
Only CT reachable within 60 min	0	0.0	0	0.0
Neither reachable within 60 min	0	0.0	0	0.0
Likely irrelevant (<10 min)	530	90.0	51315	95.6

Population and Area Coverage in Kreisfreie Stadt Köln (Stroke-Ready Hospitals) (Nordrhein-Westfalen) — Extended (+10min Penalty)

Category	Grid Cells	Area (%)	Population	Population (%)
High (30+ min)	0	0.0	0	0.0
Medium (20-30 min)	0	0.0	0	0.0
Low (10-20 min)	13	2.5	22929	1.9
Only CT reachable within 60 min	0	0.0	0	0.0
Neither reachable within 60 min	0	0.0	0	0.0
Likely irrelevant (<10 min)	514	97.5	1192255	98.1

Population and Area Coverage in Kreis Heinsberg (Stroke-Ready Hospitals) (Nordrhein-Westfalen) — Extended (+10min Penalty)

Category	Grid Cells	Area (%)	Population	Population (%)
High (30+ min)	1	0.1	1	0.0
Medium (20-30 min)	4	0.5	746	0.3
Low (10-20 min)	202	26.0	70332	28.3
Only CT reachable within 60 min	2	0.3	33	0.0
Neither reachable within 60 min	2	0.3	330	0.1
Likely irrelevant (<10 min)	567	72.9	176771	71.2

Population and Area Coverage in Landkreis Bergstraße (Stroke-Ready Hospitals) (Hessen) — Extended (+10min Penalty)

Category	Grid Cells	Area (%)	Population	Population (%)
High (30+ min)	0	0.0	0	0.0
Medium (20-30 min)	20	2.2	3974	1.5
Low (10-20 min)	246	27.4	53098	20.0
Only CT reachable within 60 min	0	0.0	0	0.0
Neither reachable within 60 min	0	0.0	0	0.0
Likely irrelevant (<10 min)	631	70.3	208985	78.5

Population and Area Coverage in Landkreis Wetteraukreis (Stroke-Ready Hospitals) (Hessen) — Extended (+10min Penalty)

Category	Grid Cells	Area (%)	Population	Population (%)
High (30+ min)	0	0.0	0	0.0
Medium (20-30 min)	24	1.7	1744	0.6
Low (10-20 min)	233	16.7	30146	10.1
Only CT reachable within 60 min	0	0.0	0	0.0
Neither reachable within 60 min	0	0.0	0	0.0
Likely irrelevant (<10 min)	1138	81.6	266973	89.3

Population and Area Coverage in Kreisfreie Stadt Landau in der Pfalz (Stroke-Ready Hospitals) (Rheinland-Pfalz) — Extended (+10min Penalty)

Category	Grid Cells	Area (%)	Population	Population (%)
High (30+ min)	0	0.0	0	0.0
Medium (20-30 min)	0	0.0	0	0.0
Low (10-20 min)	15	14.7	196	0.4
Only CT reachable within 60 min	0	0.0	0	0.0
Neither reachable within 60 min	0	0.0	0	0.0
Likely irrelevant (<10 min)	87	85.3	52400	99.6

Population and Area Coverage in Stadtkreis Stuttgart (Stroke-Ready Hospitals) (Baden-Württemberg) — Extended (+10min Penalty)

Category	Grid Cells	Area (%)	Population	Population (%)
High (30+ min)	0	0.0	0	0.0
Medium (20-30 min)	0	0.0	0	0.0
Low (10-20 min)	5	2.0	4503	0.7
Only CT reachable within 60 min	0	0.0	0	0.0
Neither reachable within 60 min	0	0.0	0	0.0
Likely irrelevant (<10 min)	250	98.0	635443	99.3

Population and Area Coverage in Landkreis Konstanz (Stroke-Ready Hospitals) (Baden-Württemberg) — Extended (+10min Penalty)

Category	Grid Cells	Area (%)	Population	Population (%)
High (30+ min)	0	0.0	1055	0.4
Medium (20-30 min)	38	4.0	2321	0.9
Low (10-20 min)	172	18.1	32412	12.7
Only CT reachable within 60 min	3	0.3	1906	0.7
Neither reachable within 60 min	7	0.7	1161	0.5
Likely irrelevant (<10 min)	731	76.9	217298	84.8

Population and Area Coverage in Landkreis Sigmaringen (Stroke-Ready Hospitals) (Baden-Württemberg) — Extended (+10min Penalty)

Category	Grid Cells	Area (%)	Population	Population (%)
High (30+ min)	0	0.0	0	0.0
Medium (20-30 min)	28	1.9	719	0.6
Low (10-20 min)	167	11.5	12783	9.9
Only CT reachable within 60 min	0	0.0	0	0.0
Neither reachable within 60 min	0	0.0	0	0.0
Likely irrelevant (<10 min)	1254	86.5	115156	89.5

Population and Area Coverage in Landkreis Kelheim (Stroke-Ready Hospitals) (Bayern) — Extended (+10min Penalty)

Category	Grid Cells	Area (%)	Population	Population (%)
High (30+ min)	6	0.5	12	0.0
Medium (20-30 min)	443	34.2	37327	31.3
Low (10-20 min)	477	36.8	49664	41.7
Only CT reachable within 60 min	0	0.0	0	0.0
Neither reachable within 60 min	0	0.0	0	0.0
Likely irrelevant (<10 min)	371	28.6	32144	27.0

Population and Area Coverage in Kreisfreie Stadt Schweinfurt (Stroke-Ready Hospitals) (Bayern) — Extended (+10min Penalty)

Category	Grid Cells	Area (%)	Population	Population (%)
High (30+ min)	0	0.0	0	0.0
Medium (20-30 min)	0	0.0	0	0.0
Low (10-20 min)	0	0.0	0	0.0
Only CT reachable within 60 min	0	0.0	0	0.0
Neither reachable within 60 min	0	0.0	0	0.0
Likely irrelevant (<10 min)	46	100.0	53903	100.0

Population and Area Coverage in Landkreis Neu-Ulm (Stroke-Ready Hospitals) (Bayern) — Extended (+10min Penalty)

Category	Grid Cells	Area (%)	Population	Population (%)
High (30+ min)	0	0.0	0	0.0
Medium (20-30 min)	77	12.3	3144	1.8
Low (10-20 min)	366	58.7	104045	60.1
Only CT reachable within 60 min	0	0.0	0	0.0
Neither reachable within 60 min	0	0.0	0	0.0
Likely irrelevant (<10 min)	181	29.0	66038	38.1

Population and Area Coverage in Landkreis Oberhavel (Stroke-Ready Hospitals) (Brandenburg) — Extended (+10min Penalty)

Category	Grid Cells	Area (%)	Population	Population (%)
High (30+ min)	361	15.0	19729	9.8
Medium (20-30 min)	338	14.0	8923	4.4
Low (10-20 min)	651	27.0	64462	32.1
Only CT reachable within 60 min	510	21.1	7248	3.6
Neither reachable within 60 min	0	0.0	0	0.0
Likely irrelevant (<10 min)	554	22.9	100254	50.0

Population and Area Coverage in Landkreis Spree-Neiße (Stroke-Ready Hospitals) (Brandenburg) — Extended (+10min Penalty)

Category	Grid Cells	Area (%)	Population	Population (%)
High (30+ min)	148	7.1	15383	15.3
Medium (20-30 min)	303	14.5	5425	5.4
Low (10-20 min)	544	26.1	33564	33.4
Only CT reachable within 60 min	20	1.0	64	0.1
Neither reachable within 60 min	0	0.0	0	0.0
Likely irrelevant (<10 min)	1073	51.4	45961	45.8

Population and Area Coverage in Landkreis Ilm-Kreis (Stroke-Ready Hospitals) (Thüringen) — Extended (+10min Penalty)

Category	Grid Cells	Area (%)	Population	Population (%)
High (30+ min)	0	0.0	0	0.0
Medium (20-30 min)	53	5.2	2214	2.3
Low (10-20 min)	437	42.6	56636	58.7
Only CT reachable within 60 min	0	0.0	0	0.0
Neither reachable within 60 min	0	0.0	0	0.0
Likely irrelevant (<10 min)	536	52.2	37651	39.0

Population and Area Coverage in Landkreis Greiz (Stroke-Ready Hospitals) (Thüringen) — Extended (+10min Penalty)

Category	Grid Cells	Area (%)	Population	Population (%)
High (30+ min)	1	0.1	8	0.0
Medium (20-30 min)	112	10.4	19514	21.3
Low (10-20 min)	358	33.1	24772	27.0
Only CT reachable within 60 min	0	0.0	0	0.0
Neither reachable within 60 min	0	0.0	0	0.0
Likely irrelevant (<10 min)	609	56.4	47512	51.8

Population and Area Coverage in Kreisfreie Stadt Bottrop (Stroke-Ready Hospitals) (Nordrhein-Westfalen) — Extended (+10min Penalty)

Category	Grid Cells	Area (%)	Population	Population (%)
High (30+ min)	0	0.0	0	0.0
Medium (20-30 min)	0	0.0	0	0.0
Low (10-20 min)	1	0.8	0	0.0
Only CT reachable within 60 min	0	0.0	0	0.0
Neither reachable within 60 min	0	0.0	0	0.0
Likely irrelevant (<10 min)	132	99.2	116967	100.0

Population and Area Coverage in Kreisfreie Stadt Speyer (Stroke-Ready Hospitals) (Rheinland-Pfalz) — Extended (+10min Penalty)

Category	Grid Cells	Area (%)	Population	Population (%)
High (30+ min)	0	0.0	0	0.0
Medium (20-30 min)	0	0.0	0	0.0
Low (10-20 min)	0	0.0	0	0.0
Only CT reachable within 60 min	0	0.0	0	0.0
Neither reachable within 60 min	0	0.0	0	0.0
Likely irrelevant (<10 min)	52	100.0	51373	100.0

Population and Area Coverage in Stadtkreis Mannheim (Stroke-Ready Hospitals) (Baden-Württemberg) — Extended (+10min Penalty)

Category	Grid Cells	Area (%)	Population	Population (%)
High (30+ min)	0	0.0	0	0.0
Medium (20-30 min)	0	0.0	0	0.0
Low (10-20 min)	5	2.8	117	0.0
Only CT reachable within 60 min	0	0.0	0	0.0
Neither reachable within 60 min	0	0.0	0	0.0
Likely irrelevant (<10 min)	173	97.2	346832	100.0

Population and Area Coverage in Landkreis Tuttlingen (Stroke-Ready Hospitals) (Baden-Württemberg) — Extended (+10min Penalty)

Category	Grid Cells	Area (%)	Population	Population (%)
High (30+ min)	0	0.0	0	0.0
Medium (20-30 min)	0	0.0	0	0.0
Low (10-20 min)	11	1.2	206	0.2
Only CT reachable within 60 min	0	0.0	0	0.0
Neither reachable within 60 min	0	0.0	0	0.0
Likely irrelevant (<10 min)	870	98.8	134878	99.8

Population and Area Coverage in Landkreis Zollernalbkreis (Stroke-Ready Hospitals) (Baden-Württemberg) — Extended (+10min Penalty)

Category	Grid Cells	Area (%)	Population	Population (%)
High (30+ min)	0	0.0	0	0.0
Medium (20-30 min)	17	1.5	1314	0.7
Low (10-20 min)	262	23.6	56954	30.4
Only CT reachable within 60 min	0	0.0	0	0.0
Neither reachable within 60 min	0	0.0	0	0.0
Likely irrelevant (<10 min)	831	74.9	129007	68.9

Population and Area Coverage in Landkreis Eichstätt (Stroke-Ready Hospitals) (Bayern) — Extended (+10min Penalty)

Category	Grid Cells	Area (%)	Population	Population (%)
High (30+ min)	6	0.4	553	0.4
Medium (20-30 min)	578	39.0	35396	27.1
Low (10-20 min)	695	46.9	54636	41.8
Only CT reachable within 60 min	0	0.0	0	0.0
Neither reachable within 60 min	0	0.0	0	0.0
Likely irrelevant (<10 min)	203	13.7	40032	30.6

Population and Area Coverage in Landkreis Freyung-Grafenau (Stroke-Ready Hospitals) (Bayern) — Extended (+10min Penalty)

Category	Grid Cells	Area (%)	Population	Population (%)
High (30+ min)	0	0.0	0	0.0
Medium (20-30 min)	14	1.2	408	0.5
Low (10-20 min)	237	20.9	22459	28.5
Only CT reachable within 60 min	1	0.1	0	0.0
Neither reachable within 60 min	51	4.5	35	0.0
Likely irrelevant (<10 min)	830	73.3	55828	70.9

Population and Area Coverage in Landkreis Main-Spessart (Stroke-Ready Hospitals) (Bayern) — Extended (+10min Penalty)

Category	Grid Cells	Area (%)	Population	Population (%)
High (30+ min)	0	0.0	0	0.0
Medium (20-30 min)	13	0.8	46	0.0
Low (10-20 min)	252	15.2	13709	10.4
Only CT reachable within 60 min	0	0.0	0	0.0
Neither reachable within 60 min	0	0.0	0	0.0
Likely irrelevant (<10 min)	1397	84.1	117700	89.5

Population and Area Coverage in Kreisfreie Stadt Potsdam (Stroke-Ready Hospitals) (Brandenburg) — Extended (+10min Penalty)

Category	Grid Cells	Area (%)	Population	Population (%)
High (30+ min)	0	0.0	0	0.0
Medium (20-30 min)	0	0.0	0	0.0
Low (10-20 min)	7	2.9	2862	1.4
Only CT reachable within 60 min	0	0.0	0	0.0
Neither reachable within 60 min	0	0.0	0	0.0
Likely irrelevant (<10 min)	237	97.1	202453	98.6

Population and Area Coverage in Landkreis Nordsachsen (Stroke-Ready Hospitals) (Sachsen) — Extended (+10min Penalty)

Category	Grid Cells	Area (%)	Population	Population (%)
High (30+ min)	156	6.0	4471	2.4
Medium (20-30 min)	600	23.0	40792	22.2
Low (10-20 min)	921	35.3	67421	36.6
Only CT reachable within 60 min	21	0.8	43	0.0
Neither reachable within 60 min	0	0.0	0	0.0
Likely irrelevant (<10 min)	913	35.0	71346	38.8

Population and Area Coverage in Landkreis Cuxhaven (Stroke-Ready Hospitals) (Niedersachsen) — Extended (+10min Penalty)

Category	Grid Cells	Area (%)	Population	Population (%)
High (30+ min)	206	7.4	18364	9.2
Medium (20-30 min)	389	14.0	47161	23.7
Low (10-20 min)	874	31.4	38806	19.5
Only CT reachable within 60 min	7	0.3	687	0.3
Neither reachable within 60 min	45	1.6	2606	1.3
Likely irrelevant (<10 min)	1259	45.3	91081	45.8

Population and Area Coverage in Kreisfreie Stadt Bremen (Stroke-Ready Hospitals) (Bremen) — Extended (+10min Penalty)

Category	Grid Cells	Area (%)	Population	Population (%)
High (30+ min)	0	0.0	0	0.0
Medium (20-30 min)	4	0.9	111	0.0
Low (10-20 min)	65	14.8	15086	2.6
Only CT reachable within 60 min	0	0.0	0	0.0
Neither reachable within 60 min	1	0.2	1881	0.3
Likely irrelevant (<10 min)	368	84.0	566134	97.1

Population and Area Coverage in Landkreis Groß-Gerau (Stroke-Ready Hospitals) (Hessen) — Extended (+10min Penalty)

Category	Grid Cells	Area (%)	Population	Population (%)
High (30+ min)	0	0.0	0	0.0
Medium (20-30 min)	0	0.0	0	0.0
Low (10-20 min)	88	15.6	19501	7.5
Only CT reachable within 60 min	0	0.0	0	0.0
Neither reachable within 60 min	0	0.0	0	0.0
Likely irrelevant (<10 min)	476	84.4	240092	92.5

Population and Area Coverage in Landkreis Limburg-Weilburg (Stroke-Ready Hospitals) (Hessen) — Extended (+10min Penalty)

Category	Grid Cells	Area (%)	Population	Population (%)
High (30+ min)	0	0.0	0	0.0
Medium (20-30 min)	0	0.0	0	0.0
Low (10-20 min)	177	19.0	21305	12.3
Only CT reachable within 60 min	0	0.0	0	0.0
Neither reachable within 60 min	0	0.0	0	0.0
Likely irrelevant (<10 min)	757	81.0	151532	87.7

Population and Area Coverage in Landkreis Waldeck-Frankenberg (Stroke-Ready Hospitals) (Hessen) — Extended (+10min Penalty)

Category	Grid Cells	Area (%)	Population	Population (%)
High (30+ min)	892	37.4	61986	39.2
Medium (20-30 min)	657	27.6	50183	31.8
Low (10-20 min)	458	19.2	20106	12.7
Only CT reachable within 60 min	22	0.9	141	0.1
Neither reachable within 60 min	0	0.0	0	0.0
Likely irrelevant (<10 min)	353	14.8	25587	16.2

Population and Area Coverage in Landkreis Trier-Saarburg (Stroke-Ready Hospitals) (Rheinland-Pfalz) — Extended (+10min Penalty)

Category	Grid Cells	Area (%)	Population	Population (%)
High (30+ min)	1	0.1	1	0.0
Medium (20-30 min)	205	14.9	17862	11.9
Low (10-20 min)	469	34.1	36667	24.4
Only CT reachable within 60 min	0	0.0	0	0.0
Neither reachable within 60 min	0	0.0	355	0.2
Likely irrelevant (<10 min)	699	50.9	95150	63.4

Population and Area Coverage in Landkreis Reutlingen (Stroke-Ready Hospitals) (Baden-Württemberg) — Extended (+10min Penalty)

Category	Grid Cells	Area (%)	Population	Population (%)
High (30+ min)	141	10.6	9519	3.2
Medium (20-30 min)	315	23.6	15422	5.2
Low (10-20 min)	347	26.0	38503	12.9
Only CT reachable within 60 min	0	0.0	0	0.0
Neither reachable within 60 min	0	0.0	0	0.0
Likely irrelevant (<10 min)	530	39.8	234267	78.7

Population and Area Coverage in Kreisfreie Stadt Schwerin (Stroke-Ready Hospitals) (Mecklenburg-Vorpommern) — Extended (+10min Penalty)

Category	Grid Cells	Area (%)	Population	Population (%)
High (30+ min)	0	0.0	0	0.0
Medium (20-30 min)	0	0.0	0	0.0
Low (10-20 min)	5	2.9	936	1.0
Only CT reachable within 60 min	0	0.0	0	0.0
Neither reachable within 60 min	0	0.0	0	0.0
Likely irrelevant (<10 min)	168	97.1	95836	99.0

Population and Area Coverage in Kreisfreie Stadt Hamburg (Stroke-Ready Hospitals) (Hamburg) — Extended (+10min Penalty)

Category	Grid Cells	Area (%)	Population	Population (%)
High (30+ min)	0	0.0	0	0.0
Medium (20-30 min)	0	0.0	0	0.0
Low (10-20 min)	81	8.1	70977	4.0
Only CT reachable within 60 min	0	0.0	0	0.0
Neither reachable within 60 min	6	0.6	6087	0.3
Likely irrelevant (<10 min)	910	91.3	1716404	95.7

Population and Area Coverage in Kreisfreie Stadt Braunschweig (Stroke-Ready Hospitals) (Niedersachsen) — Extended (+10min Penalty)

Category	Grid Cells	Area (%)	Population	Population (%)
High (30+ min)	0	0.0	0	0.0
Medium (20-30 min)	0	0.0	0	0.0
Low (10-20 min)	101	41.6	69410	28.1
Only CT reachable within 60 min	0	0.0	0	0.0
Neither reachable within 60 min	0	0.0	0	0.0
Likely irrelevant (<10 min)	142	58.4	177268	71.9

Population and Area Coverage in Landkreis Leer (Stroke-Ready Hospitals) (Niedersachsen) — Extended (+10min Penalty)

Category	Grid Cells	Area (%)	Population	Population (%)
High (30+ min)	121	8.5	15941	9.4
Medium (20-30 min)	251	17.7	30769	18.2
Low (10-20 min)	443	31.2	73927	43.8
Only CT reachable within 60 min	1	0.1	9	0.0
Neither reachable within 60 min	38	2.7	3609	2.1
Likely irrelevant (<10 min)	564	39.8	44583	26.4

Population and Area Coverage in Kreisfreie Stadt Duisburg (Stroke-Ready Hospitals) (Nordrhein-Westfalen) — Extended (+10min Penalty)

Category	Grid Cells	Area (%)	Population	Population (%)
High (30+ min)	0	0.0	0	0.0
Medium (20-30 min)	0	0.0	0	0.0
Low (10-20 min)	3	1.0	1205	0.2
Only CT reachable within 60 min	0	0.0	0	0.0
Neither reachable within 60 min	0	0.0	0	0.0
Likely irrelevant (<10 min)	294	99.0	551698	99.8

Population and Area Coverage in Kreis Siegen-Wittgenstein (Stroke-Ready Hospitals) (Nordrhein-Westfalen) — Extended (+10min Penalty)

Category	Grid Cells	Area (%)	Population	Population (%)
High (30+ min)	331	22.8	29524	10.1
Medium (20-30 min)	106	7.3	5186	1.8
Low (10-20 min)	155	10.7	16311	5.6
Only CT reachable within 60 min	105	7.2	540	0.2
Neither reachable within 60 min	0	0.0	0	0.0
Likely irrelevant (<10 min)	754	52.0	241758	82.4

Population and Area Coverage in Landkreis Schwarzwald-Baar-Kreis (Stroke-Ready Hospitals) (Baden-Württemberg) — Extended (+10min Penalty)

Category	Grid Cells	Area (%)	Population	Population (%)
High (30+ min)	11	0.9	78	0.0
Medium (20-30 min)	19	1.5	666	0.3
Low (10-20 min)	128	10.4	13410	6.5
Only CT reachable within 60 min	2	0.2	15	0.0
Neither reachable within 60 min	0	0.0	0	0.0
Likely irrelevant (<10 min)	1070	87.0	192819	93.2

Population and Area Coverage in Kreisfreie Stadt Amberg (Stroke-Ready Hospitals) (Bayern) — Extended (+10min Penalty)

Category	Grid Cells	Area (%)	Population	Population (%)
High (30+ min)	0	0.0	0	0.0
Medium (20-30 min)	0	0.0	0	0.0
Low (10-20 min)	0	0.0	0	0.0
Only CT reachable within 60 min	0	0.0	0	0.0
Neither reachable within 60 min	0	0.0	0	0.0
Likely irrelevant (<10 min)	61	100.0	42852	100.0

Population and Area Coverage in Kreisfreie Stadt Bayreuth (Stroke-Ready Hospitals) (Bayern) — Extended (+10min Penalty)

Category	Grid Cells	Area (%)	Population	Population (%)
High (30+ min)	0	0.0	0	0.0
Medium (20-30 min)	0	0.0	0	0.0
Low (10-20 min)	0	0.0	0	0.0
Only CT reachable within 60 min	0	0.0	0	0.0
Neither reachable within 60 min	0	0.0	0	0.0
Likely irrelevant (<10 min)	82	100.0	73464	100.0

Population and Area Coverage in Landkreis Nürnberger Land (Stroke-Ready Hospitals) (Bayern) — Extended (+10min Penalty)

Category	Grid Cells	Area (%)	Population	Population (%)
High (30+ min)	0	0.0	0	0.0
Medium (20-30 min)	32	3.2	3204	1.9
Low (10-20 min)	484	49.1	47922	28.2
Only CT reachable within 60 min	0	0.0	0	0.0
Neither reachable within 60 min	0	0.0	0	0.0
Likely irrelevant (<10 min)	470	47.7	118951	69.9

Population and Area Coverage in Landkreis Roth (Stroke-Ready Hospitals) (Bayern) — Extended (+10min Penalty)

Category	Grid Cells	Area (%)	Population	Population (%)
High (30+ min)	0	0.0	0	0.0
Medium (20-30 min)	15	1.4	171	0.1
Low (10-20 min)	134	12.1	7662	5.9
Only CT reachable within 60 min	0	0.0	0	0.0
Neither reachable within 60 min	0	0.0	0	0.0
Likely irrelevant (<10 min)	955	86.5	120944	93.9

Population and Area Coverage in Kreisfreie Stadt Aschaffenburg (Stroke-Ready Hospitals) (Bayern) — Extended (+10min Penalty)

Category	Grid Cells	Area (%)	Population	Population (%)
High (30+ min)	0	0.0	0	0.0
Medium (20-30 min)	0	0.0	0	0.0
Low (10-20 min)	0	0.0	0	0.0
Only CT reachable within 60 min	0	0.0	0	0.0
Neither reachable within 60 min	0	0.0	0	0.0
Likely irrelevant (<10 min)	78	100.0	70292	100.0

Population and Area Coverage in Landkreis Merzig-Wadern (Stroke-Ready Hospitals) (Saarland) — Extended (+10min Penalty)

Category	Grid Cells	Area (%)	Population	Population (%)
High (30+ min)	1	0.1	8	0.0
Medium (20-30 min)	48	6.9	4356	4.4
Low (10-20 min)	192	27.8	18494	18.5
Only CT reachable within 60 min	0	0.0	1	0.0
Neither reachable within 60 min	0	0.0	191	0.2
Likely irrelevant (<10 min)	450	65.1	76967	77.0

Population and Area Coverage in Landkreis Vogtlandkreis (Stroke-Ready Hospitals) (Sachsen) — Extended (+10min Penalty)

Category	Grid Cells	Area (%)	Population	Population (%)
High (30+ min)	72	4.1	4855	2.2
Medium (20-30 min)	146	8.3	13823	6.3
Low (10-20 min)	269	15.2	15183	7.0
Only CT reachable within 60 min	1	0.1	177	0.1
Neither reachable within 60 min	12	0.7	105	0.0
Likely irrelevant (<10 min)	1267	71.7	183906	84.3

Population and Area Coverage in Landkreis Altmarkkreis Salzwedel (Stroke-Ready Hospitals) (Sachsen-Anhalt) — Extended (+10min Penalty)

Category	Grid Cells	Area (%)	Population	Population (%)
High (30+ min)	974	31.8	30458	39.4
Medium (20-30 min)	838	27.4	26222	34.0
Low (10-20 min)	461	15.1	7532	9.8
Only CT reachable within 60 min	691	22.6	12440	16.1
Neither reachable within 60 min	0	0.0	0	0.0
Likely irrelevant (<10 min)	98	3.2	579	0.7

Population and Area Coverage in Kreis Segeberg (Stroke-Ready Hospitals) (Schleswig-Holstein) — Extended (+10min Penalty)

Category	Grid Cells	Area (%)	Population	Population (%)
High (30+ min)	0	0.0	0	0.0
Medium (20-30 min)	7	0.4	273	0.1
Low (10-20 min)	418	22.8	60135	22.0
Only CT reachable within 60 min	0	0.0	0	0.0
Neither reachable within 60 min	0	0.0	0	0.0
Likely irrelevant (<10 min)	1407	76.8	213503	77.9

Population and Area Coverage in Landkreis Osnabrück (Stroke-Ready Hospitals) (Niedersachsen) — Extended (+10min Penalty)

Category	Grid Cells	Area (%)	Population	Population (%)
High (30+ min)	1	0.0	226	0.1
Medium (20-30 min)	184	6.6	19324	5.5
Low (10-20 min)	1280	45.7	146834	41.6
Only CT reachable within 60 min	0	0.0	0	0.0
Neither reachable within 60 min	0	0.0	0	0.0
Likely irrelevant (<10 min)	1337	47.7	186773	52.9

Population and Area Coverage in Kreisfreie Stadt Oberhausen (Stroke-Ready Hospitals) (Nordrhein-Westfalen) — Extended (+10min Penalty)

Category	Grid Cells	Area (%)	Population	Population (%)
High (30+ min)	0	0.0	0	0.0
Medium (20-30 min)	0	0.0	0	0.0
Low (10-20 min)	0	0.0	0	0.0
Only CT reachable within 60 min	0	0.0	0	0.0
Neither reachable within 60 min	0	0.0	0	0.0
Likely irrelevant (<10 min)	101	100.0	215167	100.0

Population and Area Coverage in Kreisfreie Stadt Remscheid (Stroke-Ready Hospitals) (Nordrhein-Westfalen) — Extended (+10min Penalty)

Category	Grid Cells	Area (%)	Population	Population (%)
High (30+ min)	0	0.0	0	0.0
Medium (20-30 min)	0	0.0	0	0.0
Low (10-20 min)	15	15.5	10369	9.6
Only CT reachable within 60 min	0	0.0	0	0.0
Neither reachable within 60 min	0	0.0	0	0.0
Likely irrelevant (<10 min)	82	84.5	98121	90.4

Population and Area Coverage in Kreis Mettmann (Stroke-Ready Hospitals) (Nordrhein-Westfalen) — Extended (+10min Penalty)

Category	Grid Cells	Area (%)	Population	Population (%)
High (30+ min)	0	0.0	0	0.0
Medium (20-30 min)	0	0.0	0	0.0
Low (10-20 min)	66	12.6	49819	10.5
Only CT reachable within 60 min	0	0.0	0	0.0
Neither reachable within 60 min	0	0.0	0	0.0
Likely irrelevant (<10 min)	459	87.4	423907	89.5

Population and Area Coverage in Kreis Rhein-Sieg-Kreis (Stroke-Ready Hospitals) (Nordrhein-Westfalen) — Extended (+10min Penalty)

Category	Grid Cells	Area (%)	Population	Population (%)
High (30+ min)	0	0.0	0	0.0
Medium (20-30 min)	95	6.4	14703	2.5
Low (10-20 min)	304	20.6	71496	12.3
Only CT reachable within 60 min	0	0.0	0	0.0
Neither reachable within 60 min	0	0.0	0	0.0
Likely irrelevant (<10 min)	1075	72.9	497077	85.2

Population and Area Coverage in Kreis Borken (Stroke-Ready Hospitals) (Nordrhein-Westfalen) — Extended (+10min Penalty)

Category	Grid Cells	Area (%)	Population	Population (%)
High (30+ min)	9	0.5	431	0.1
Medium (20-30 min)	254	14.0	62449	17.0
Low (10-20 min)	505	27.8	118242	32.2
Only CT reachable within 60 min	10	0.5	185	0.1
Neither reachable within 60 min	15	0.8	153	0.0
Likely irrelevant (<10 min)	1026	56.4	185332	50.5

Population and Area Coverage in Landkreis Vulkaneifel (Stroke-Ready Hospitals) (Rheinland-Pfalz) — Extended (+10min Penalty)

Category	Grid Cells	Area (%)	Population	Population (%)
High (30+ min)	281	24.4	19552	31.1
Medium (20-30 min)	218	19.0	9587	15.3
Low (10-20 min)	506	44.0	27135	43.2
Only CT reachable within 60 min	0	0.0	0	0.0
Neither reachable within 60 min	0	0.0	0	0.0
Likely irrelevant (<10 min)	145	12.6	6581	10.5

Population and Area Coverage in Landkreis Kaiserslautern (Stroke-Ready Hospitals) (Rheinland-Pfalz) — Extended (+10min Penalty)

Category	Grid Cells	Area (%)	Population	Population (%)
High (30+ min)	0	0.0	0	0.0
Medium (20-30 min)	0	0.0	0	0.0
Low (10-20 min)	212	26.8	31881	29.8
Only CT reachable within 60 min	0	0.0	0	0.0
Neither reachable within 60 min	0	0.0	0	0.0
Likely irrelevant (<10 min)	579	73.2	75026	70.2

Population and Area Coverage in Landkreis Calw (Stroke-Ready Hospitals) (Baden-Württemberg) — Extended (+10min Penalty)

Category	Grid Cells	Area (%)	Population	Population (%)
High (30+ min)	0	0.0	0	0.0
Medium (20-30 min)	81	8.4	21786	14.4
Low (10-20 min)	208	21.5	35871	23.7
Only CT reachable within 60 min	0	0.0	0	0.0
Neither reachable within 60 min	0	0.0	0	0.0
Likely irrelevant (<10 min)	678	70.1	93972	62.0

Population and Area Coverage in Kreisfreie Stadt Berlin (Stroke-Ready Hospitals) (Berlin) — Extended (+10min Penalty)

Category	Grid Cells	Area (%)	Population	Population (%)
High (30+ min)	0	0.0	0	0.0
Medium (20-30 min)	1	0.1	514	0.0
Low (10-20 min)	63	5.3	39013	1.1
Only CT reachable within 60 min	0	0.0	0	0.0
Neither reachable within 60 min	0	0.0	0	0.0
Likely irrelevant (<10 min)	1120	94.6	3589171	98.9

Population and Area Coverage in Kreis Stormarn (Stroke-Ready Hospitals) (Schleswig-Holstein) — Extended (+10min Penalty)

Category	Grid Cells	Area (%)	Population	Population (%)
High (30+ min)	0	0.0	0	0.0
Medium (20-30 min)	18	1.7	7001	2.9
Low (10-20 min)	517	49.6	119190	48.9
Only CT reachable within 60 min	0	0.0	0	0.0
Neither reachable within 60 min	0	0.0	0	0.0
Likely irrelevant (<10 min)	507	48.7	117777	48.3

Population and Area Coverage in Landkreis Hildesheim (Stroke-Ready Hospitals) (Niedersachsen) — Extended (+10min Penalty)

Category	Grid Cells	Area (%)	Population	Population (%)
High (30+ min)	80	5.0	15467	5.3
Medium (20-30 min)	290	18.2	20861	7.1
Low (10-20 min)	232	14.6	28722	9.8
Only CT reachable within 60 min	0	0.0	0	0.0
Neither reachable within 60 min	0	0.0	0	0.0
Likely irrelevant (<10 min)	990	62.2	228665	77.9

Population and Area Coverage in Kreis Viersen (Stroke-Ready Hospitals) (Nordrhein-Westfalen) — Extended (+10min Penalty)

Category	Grid Cells	Area (%)	Population	Population (%)
High (30+ min)	0	0.0	0	0.0
Medium (20-30 min)	2	0.3	91	0.0
Low (10-20 min)	59	8.1	18283	6.2
Only CT reachable within 60 min	0	0.0	0	0.0
Neither reachable within 60 min	45	6.2	1588	0.5
Likely irrelevant (<10 min)	622	85.4	276082	93.3

Population and Area Coverage in Kreis Rheinisch-Bergischer Kreis (Stroke-Ready Hospitals) (Nordrhein-Westfalen) — Extended (+10min Penalty)

Category	Grid Cells	Area (%)	Population	Population (%)
High (30+ min)	0	0.0	0	0.0
Medium (20-30 min)	0	0.0	0	0.0
Low (10-20 min)	75	13.4	10265	3.4
Only CT reachable within 60 min	0	0.0	0	0.0
Neither reachable within 60 min	0	0.0	0	0.0
Likely irrelevant (<10 min)	486	86.6	291107	96.6

Population and Area Coverage in Kreisfreie Stadt Zweibrücken (Stroke-Ready Hospitals) (Rheinland-Pfalz) — Extended (+10min Penalty)

Category	Grid Cells	Area (%)	Population	Population (%)
High (30+ min)	0	0.0	0	0.0
Medium (20-30 min)	0	0.0	0	0.0
Low (10-20 min)	29	33.0	2569	7.9
Only CT reachable within 60 min	0	0.0	0	0.0
Neither reachable within 60 min	0	0.0	0	0.0
Likely irrelevant (<10 min)	59	67.0	30038	92.1

Population and Area Coverage in Landkreis Rhein-Pfalz-Kreis (Stroke-Ready Hospitals) (Rheinland-Pfalz) — Extended (+10min Penalty)

Category	Grid Cells	Area (%)	Population	Population (%)
High (30+ min)	0	0.0	0	0.0
Medium (20-30 min)	0	0.0	0	0.0
Low (10-20 min)	19	5.0	1324	0.9
Only CT reachable within 60 min	0	0.0	0	0.0
Neither reachable within 60 min	0	0.0	0	0.0
Likely irrelevant (<10 min)	362	95.0	151171	99.1

Population and Area Coverage in Landkreis Altötting (Stroke-Ready Hospitals) (Bayern) — Extended (+10min Penalty)

Category	Grid Cells	Area (%)	Population	Population (%)
High (30+ min)	0	0.0	0	0.0
Medium (20-30 min)	0	0.0	0	0.0
Low (10-20 min)	26	3.8	1610	1.5
Only CT reachable within 60 min	0	0.0	0	0.0
Neither reachable within 60 min	3	0.4	2068	1.9
Likely irrelevant (<10 min)	649	95.7	103617	96.6

Population and Area Coverage in Landkreis Pfaffenhofen a.d.Ilm (Stroke-Ready Hospitals) (Bayern) — Extended (+10min Penalty)

Category	Grid Cells	Area (%)	Population	Population (%)
High (30+ min)	0	0.0	0	0.0
Medium (20-30 min)	17	1.8	2440	2.0
Low (10-20 min)	265	28.7	41450	33.5
Only CT reachable within 60 min	0	0.0	0	0.0
Neither reachable within 60 min	0	0.0	0	0.0
Likely irrelevant (<10 min)	642	69.5	79985	64.6

Population and Area Coverage in Kreisfreie Stadt Bamberg (Stroke-Ready Hospitals) (Bayern) — Extended (+10min Penalty)

Category	Grid Cells	Area (%)	Population	Population (%)
High (30+ min)	0	0.0	0	0.0
Medium (20-30 min)	0	0.0	0	0.0
Low (10-20 min)	7	10.4	6005	7.7
Only CT reachable within 60 min	0	0.0	0	0.0
Neither reachable within 60 min	0	0.0	0	0.0
Likely irrelevant (<10 min)	60	89.6	71819	92.3

Population and Area Coverage in Landkreis Hof (Stroke-Ready Hospitals) (Bayern) — Extended (+10min Penalty)

Category	Grid Cells	Area (%)	Population	Population (%)
High (30+ min)	0	0.0	0	0.0
Medium (20-30 min)	1	0.1	30	0.0
Low (10-20 min)	237	21.6	18359	18.4
Only CT reachable within 60 min	0	0.0	0	0.0
Neither reachable within 60 min	0	0.0	0	0.0
Likely irrelevant (<10 min)	861	78.3	81308	81.6

Population and Area Coverage in Landkreis Miltenberg (Stroke-Ready Hospitals) (Bayern) — Extended (+10min Penalty)

Category	Grid Cells	Area (%)	Population	Population (%)
High (30+ min)	8	0.9	296	0.2
Medium (20-30 min)	70	7.8	3952	3.0
Low (10-20 min)	144	16.1	8503	6.4
Only CT reachable within 60 min	0	0.0	0	0.0
Neither reachable within 60 min	0	0.0	0	0.0
Likely irrelevant (<10 min)	674	75.2	120395	90.4

Population and Area Coverage in Kreisfreie Stadt Augsburg (Stroke-Ready Hospitals) (Bayern) — Extended (+10min Penalty)

Category	Grid Cells	Area (%)	Population	Population (%)
High (30+ min)	0	0.0	0	0.0
Medium (20-30 min)	0	0.0	0	0.0
Low (10-20 min)	49	27.2	11321	3.9
Only CT reachable within 60 min	0	0.0	0	0.0
Neither reachable within 60 min	0	0.0	0	0.0
Likely irrelevant (<10 min)	131	72.8	275368	96.1

Population and Area Coverage in Landkreis Barnim (Stroke-Ready Hospitals) (Brandenburg) — Extended (+10min Penalty)

Category	Grid Cells	Area (%)	Population	Population (%)
High (30+ min)	3	0.2	70	0.0
Medium (20-30 min)	152	7.7	3521	2.1
Low (10-20 min)	790	39.9	41643	24.4
Only CT reachable within 60 min	49	2.5	153	0.1
Neither reachable within 60 min	15	0.8	13	0.0
Likely irrelevant (<10 min)	970	49.0	125043	73.4

Population and Area Coverage in Landkreis Meißen (Stroke-Ready Hospitals) (Sachsen) — Extended (+10min Penalty)

Category	Grid Cells	Area (%)	Population	Population (%)
High (30+ min)	53	2.8	8020	3.5
Medium (20-30 min)	351	18.7	45319	19.8
Low (10-20 min)	765	40.8	69607	30.4
Only CT reachable within 60 min	0	0.0	0	0.0
Neither reachable within 60 min	0	0.0	0	0.0
Likely irrelevant (<10 min)	704	37.6	106124	46.3

Population and Area Coverage in Kreisfreie Stadt Suhl (Stroke-Ready Hospitals) (Thüringen) — Extended (+10min Penalty)

Category	Grid Cells	Area (%)	Population	Population (%)
High (30+ min)	0	0.0	0	0.0
Medium (20-30 min)	1	0.6	4	0.0
Low (10-20 min)	22	12.3	850	2.6
Only CT reachable within 60 min	0	0.0	0	0.0
Neither reachable within 60 min	0	0.0	0	0.0
Likely irrelevant (<10 min)	156	87.2	32454	97.4

Population and Area Coverage in Landkreis Eichsfeld (Stroke-Ready Hospitals) (Thüringen) — Extended (+10min Penalty)

Category	Grid Cells	Area (%)	Population	Population (%)
High (30+ min)	0	0.0	0	0.0
Medium (20-30 min)	75	5.8	1916	2.0
Low (10-20 min)	572	44.4	41986	44.3
Only CT reachable within 60 min	0	0.0	0	0.0
Neither reachable within 60 min	0	0.0	0	0.0
Likely irrelevant (<10 min)	641	49.8	50781	53.6

Population and Area Coverage in Landkreis Göttingen (Stroke-Ready Hospitals) (Niedersachsen) — Extended (+10min Penalty)

Category	Grid Cells	Area (%)	Population	Population (%)
High (30+ min)	0	0.0	0	0.0
Medium (20-30 min)	162	7.1	19740	5.9
Low (10-20 min)	714	31.3	61829	18.5
Only CT reachable within 60 min	0	0.0	0	0.0
Neither reachable within 60 min	0	0.0	0	0.0
Likely irrelevant (<10 min)	1404	61.6	253002	75.6

Population and Area Coverage in Landkreis Schaumburg (Stroke-Ready Hospitals) (Niedersachsen) — Extended (+10min Penalty)

Category	Grid Cells	Area (%)	Population	Population (%)
High (30+ min)	0	0.0	0	0.0
Medium (20-30 min)	0	0.0	0	0.0
Low (10-20 min)	46	5.2	3360	2.2
Only CT reachable within 60 min	0	0.0	0	0.0
Neither reachable within 60 min	0	0.0	0	0.0
Likely irrelevant (<10 min)	843	94.8	152482	97.8

Population and Area Coverage in Landkreis Aurich (Stroke-Ready Hospitals) (Niedersachsen) — Extended (+10min Penalty)

Category	Grid Cells	Area (%)	Population	Population (%)
High (30+ min)	282	16.7	34715	18.8
Medium (20-30 min)	457	27.0	68263	36.9
Low (10-20 min)	313	18.5	35229	19.0
Only CT reachable within 60 min	10	0.6	124	0.1
Neither reachable within 60 min	66	3.9	4909	2.7
Likely irrelevant (<10 min)	562	33.3	41828	22.6

Population and Area Coverage in Landkreis Friesland (Stroke-Ready Hospitals) (Niedersachsen) — Extended (+10min Penalty)

Category	Grid Cells	Area (%)	Population	Population (%)
High (30+ min)	3	0.4	419	0.4
Medium (20-30 min)	2	0.2	411	0.4
Low (10-20 min)	148	17.9	14405	14.8
Only CT reachable within 60 min	4	0.5	67	0.1
Neither reachable within 60 min	48	5.8	1966	2.0
Likely irrelevant (<10 min)	621	75.2	80165	82.3

Population and Area Coverage in Landkreis Wesermarsch (Stroke-Ready Hospitals) (Niedersachsen) — Extended (+10min Penalty)

Category	Grid Cells	Area (%)	Population	Population (%)
High (30+ min)	1	0.1	43	0.1
Medium (20-30 min)	386	35.2	34201	40.4
Low (10-20 min)	368	33.5	29211	34.5
Only CT reachable within 60 min	9	0.8	225	0.3
Neither reachable within 60 min	23	2.1	344	0.4
Likely irrelevant (<10 min)	310	28.3	20678	24.4

Population and Area Coverage in Landkreis Main-Kinzig-Kreis (Stroke-Ready Hospitals) (Hessen) — Extended (+10min Penalty)

Category	Grid Cells	Area (%)	Population	Population (%)
High (30+ min)	0	0.0	0	0.0
Medium (20-30 min)	117	6.6	8625	2.1
Low (10-20 min)	629	35.7	36689	9.0
Only CT reachable within 60 min	0	0.0	0	0.0
Neither reachable within 60 min	0	0.0	0	0.0
Likely irrelevant (<10 min)	1014	57.6	363870	88.9

Population and Area Coverage in Landkreis Vogelsbergkreis (Stroke-Ready Hospitals) (Hessen) — Extended (+10min Penalty)

Category	Grid Cells	Area (%)	Population	Population (%)
High (30+ min)	108	5.8	9123	8.5
Medium (20-30 min)	566	30.5	30367	28.3
Low (10-20 min)	817	44.0	45993	42.9
Only CT reachable within 60 min	0	0.0	0	0.0
Neither reachable within 60 min	0	0.0	0	0.0
Likely irrelevant (<10 min)	365	19.7	21841	20.4

Population and Area Coverage in Landkreis Neuwied (Stroke-Ready Hospitals) (Rheinland-Pfalz) — Extended (+10min Penalty)

Category	Grid Cells	Area (%)	Population	Population (%)
High (30+ min)	0	0.0	0	0.0
Medium (20-30 min)	17	2.1	1280	0.7
Low (10-20 min)	264	33.3	57029	30.8
Only CT reachable within 60 min	0	0.0	0	0.0
Neither reachable within 60 min	0	0.0	0	0.0
Likely irrelevant (<10 min)	512	64.6	127008	68.5

Population and Area Coverage in Kreisfreie Stadt Mainz (Stroke-Ready Hospitals) (Rheinland-Pfalz) — Extended (+10min Penalty)

Category	Grid Cells	Area (%)	Population	Population (%)
High (30+ min)	0	0.0	0	0.0
Medium (20-30 min)	0	0.0	0	0.0
Low (10-20 min)	1	0.8	9	0.0
Only CT reachable within 60 min	0	0.0	0	0.0
Neither reachable within 60 min	0	0.0	0	0.0
Likely irrelevant (<10 min)	120	99.2	214059	100.0

Population and Area Coverage in Landkreis Hohenlohekreis (Stroke-Ready Hospitals) (Baden-Württemberg) — Extended (+10min Penalty)

Category	Grid Cells	Area (%)	Population	Population (%)
High (30+ min)	0	0.0	0	0.0
Medium (20-30 min)	0	0.0	0	0.0
Low (10-20 min)	10	1.0	336	0.3
Only CT reachable within 60 min	0	0.0	0	0.0
Neither reachable within 60 min	0	0.0	0	0.0
Likely irrelevant (<10 min)	949	99.0	108990	99.7

Population and Area Coverage in Landkreis Ortenaukreis (Stroke-Ready Hospitals) (Baden-Württemberg) — Extended (+10min Penalty)

Category	Grid Cells	Area (%)	Population	Population (%)
High (30+ min)	193	8.6	16687	4.0
Medium (20-30 min)	249	11.1	28810	6.8
Low (10-20 min)	507	22.6	91599	21.8
Only CT reachable within 60 min	0	0.0	7	0.0
Neither reachable within 60 min	9	0.4	262	0.1
Likely irrelevant (<10 min)	1286	57.3	283335	67.3

Population and Area Coverage in Landkreis Neustadt a.d.Aisch-Bad Windsheim (Stroke-Ready Hospitals) (Bayern) — Extended (+10min Penalty)

Category	Grid Cells	Area (%)	Population	Population (%)
High (30+ min)	0	0.0	0	0.0
Medium (20-30 min)	124	7.8	10302	10.1
Low (10-20 min)	557	35.1	29388	29.0
Only CT reachable within 60 min	0	0.0	0	0.0
Neither reachable within 60 min	0	0.0	0	0.0
Likely irrelevant (<10 min)	905	57.1	61810	60.9

Population and Area Coverage in Landkreis Goslar (Stroke-Ready Hospitals) (Niedersachsen) — Extended (+10min Penalty)

Category	Grid Cells	Area (%)	Population	Population (%)
High (30+ min)	0	0.0	0	0.0
Medium (20-30 min)	114	9.1	4760	3.5
Low (10-20 min)	469	37.5	79738	58.3
Only CT reachable within 60 min	0	0.0	0	0.0
Neither reachable within 60 min	0	0.0	0	0.0
Likely irrelevant (<10 min)	669	53.4	52299	38.2

Population and Area Coverage in Landkreis Region Hannover (Stroke-Ready Hospitals) (Niedersachsen) — Extended (+10min Penalty)

Category	Grid Cells	Area (%)	Population	Population (%)
High (30+ min)	0	0.0	0	0.0
Medium (20-30 min)	27	0.9	222	0.0
Low (10-20 min)	607	20.1	87593	7.6
Only CT reachable within 60 min	0	0.0	0	0.0
Neither reachable within 60 min	0	0.0	0	0.0
Likely irrelevant (<10 min)	2391	79.0	1062939	92.4

Population and Area Coverage in Landkreis Vechta (Stroke-Ready Hospitals) (Niedersachsen) — Extended (+10min Penalty)

Category	Grid Cells	Area (%)	Population	Population (%)
High (30+ min)	81	7.5	7020	5.2
Medium (20-30 min)	246	22.8	40243	29.6
Low (10-20 min)	215	20.0	36259	26.7
Only CT reachable within 60 min	0	0.0	0	0.0
Neither reachable within 60 min	0	0.0	0	0.0
Likely irrelevant (<10 min)	535	49.7	52257	38.5

Population and Area Coverage in Kreis Städteregion Aachen (Stroke-Ready Hospitals) (Nordrhein-Westfalen) — Extended (+10min Penalty)

Category	Grid Cells	Area (%)	Population	Population (%)
High (30+ min)	133	14.9	18595	3.3
Medium (20-30 min)	116	13.0	10455	1.9
Low (10-20 min)	194	21.7	79764	14.4
Only CT reachable within 60 min	9	1.0	256	0.0
Neither reachable within 60 min	13	1.5	387	0.1
Likely irrelevant (<10 min)	427	47.9	445952	80.3

Population and Area Coverage in Kreis Steinfurt (Stroke-Ready Hospitals) (Nordrhein-Westfalen) — Extended (+10min Penalty)

Category	Grid Cells	Area (%)	Population	Population (%)
High (30+ min)	0	0.0	0	0.0
Medium (20-30 min)	152	6.4	22431	5.1
Low (10-20 min)	948	40.1	190916	43.8
Only CT reachable within 60 min	0	0.0	0	0.0
Neither reachable within 60 min	0	0.0	0	0.0
Likely irrelevant (<10 min)	1266	53.5	222560	51.1

Population and Area Coverage in Kreis Gütersloh (Stroke-Ready Hospitals) (Nordrhein-Westfalen) — Extended (+10min Penalty)

Category	Grid Cells	Area (%)	Population	Population (%)
High (30+ min)	0	0.0	0	0.0
Medium (20-30 min)	7	0.6	692	0.2
Low (10-20 min)	731	57.9	220338	62.1
Only CT reachable within 60 min	0	0.0	0	0.0
Neither reachable within 60 min	0	0.0	0	0.0
Likely irrelevant (<10 min)	524	41.5	133759	37.7

Population and Area Coverage in Kreis Unna (Stroke-Ready Hospitals) (Nordrhein-Westfalen) — Extended (+10min Penalty)

Category	Grid Cells	Area (%)	Population	Population (%)
High (30+ min)	0	0.0	0	0.0
Medium (20-30 min)	0	0.0	0	0.0
Low (10-20 min)	91	12.8	25145	6.5
Only CT reachable within 60 min	0	0.0	0	0.0
Neither reachable within 60 min	0	0.0	0	0.0
Likely irrelevant (<10 min)	620	87.2	362067	93.5

Population and Area Coverage in Landkreis Darmstadt-Dieburg (Stroke-Ready Hospitals) (Hessen) — Extended (+10min Penalty)

Category	Grid Cells	Area (%)	Population	Population (%)
High (30+ min)	0	0.0	0	0.0
Medium (20-30 min)	49	5.9	4405	1.5
Low (10-20 min)	289	35.0	60803	21.0
Only CT reachable within 60 min	0	0.0	0	0.0
Neither reachable within 60 min	0	0.0	0	0.0
Likely irrelevant (<10 min)	487	59.0	223758	77.4

Population and Area Coverage in Landkreis Gießen (Stroke-Ready Hospitals) (Hessen) — Extended (+10min Penalty)

Category	Grid Cells	Area (%)	Population	Population (%)
High (30+ min)	0	0.0	0	0.0
Medium (20-30 min)	0	0.0	0	0.0
Low (10-20 min)	222	20.5	23633	9.2
Only CT reachable within 60 min	0	0.0	0	0.0
Neither reachable within 60 min	0	0.0	0	0.0
Likely irrelevant (<10 min)	859	79.5	234365	90.8

Population and Area Coverage in Kreisfreie Stadt Neustadt an der Weinstraße (Stroke-Ready Hospitals) (Rheinland-Pfalz) — Extended (+10min Penalty)

Category	Grid Cells	Area (%)	Population	Population (%)
High (30+ min)	0	0.0	0	0.0
Medium (20-30 min)	0	0.0	0	0.0
Low (10-20 min)	0	0.0	0	0.0
Only CT reachable within 60 min	0	0.0	0	0.0
Neither reachable within 60 min	0	0.0	0	0.0
Likely irrelevant (<10 min)	147	100.0	53717	100.0

Population and Area Coverage in Landkreis Main-Tauber-Kreis (Stroke-Ready Hospitals) (Baden-Württemberg) — Extended (+10min Penalty)

Category	Grid Cells	Area (%)	Population	Population (%)
High (30+ min)	35	2.2	1094	0.8
Medium (20-30 min)	290	17.9	16547	12.6
Low (10-20 min)	486	30.0	36123	27.4
Only CT reachable within 60 min	0	0.0	0	0.0
Neither reachable within 60 min	0	0.0	0	0.0
Likely irrelevant (<10 min)	811	50.0	77855	59.2

Population and Area Coverage in Landkreis Freudenstadt (Stroke-Ready Hospitals) (Baden-Württemberg) — Extended (+10min Penalty)

Category	Grid Cells	Area (%)	Population	Population (%)
High (30+ min)	0	0.0	0	0.0
Medium (20-30 min)	7	0.7	627	0.5
Low (10-20 min)	155	14.7	19784	17.1
Only CT reachable within 60 min	0	0.0	0	0.0
Neither reachable within 60 min	0	0.0	0	0.0
Likely irrelevant (<10 min)	896	84.7	95496	82.4

Population and Area Coverage in Landkreis Rosenheim (Stroke-Ready Hospitals) (Bayern) — Extended (+10min Penalty)

Category	Grid Cells	Area (%)	Population	Population (%)
High (30+ min)	9	0.5	80	0.0
Medium (20-30 min)	69	4.0	3618	1.4
Low (10-20 min)	332	19.3	49893	19.8
Only CT reachable within 60 min	2	0.1	30	0.0
Neither reachable within 60 min	137	8.0	989	0.4
Likely irrelevant (<10 min)	1174	68.1	197137	78.3

Population and Area Coverage in Kreisfreie Stadt Passau (Stroke-Ready Hospitals) (Bayern) — Extended (+10min Penalty)

Category	Grid Cells	Area (%)	Population	Population (%)
High (30+ min)	0	0.0	0	0.0
Medium (20-30 min)	0	0.0	0	0.0
Low (10-20 min)	1	1.3	7	0.0
Only CT reachable within 60 min	0	0.0	0	0.0
Neither reachable within 60 min	0	0.0	0	0.0
Likely irrelevant (<10 min)	75	98.7	42444	100.0

Population and Area Coverage in Landkreis Coburg (Stroke-Ready Hospitals) (Bayern) — Extended (+10min Penalty)

Category	Grid Cells	Area (%)	Population	Population (%)
High (30+ min)	0	0.0	0	0.0
Medium (20-30 min)	3	0.4	0	0.0
Low (10-20 min)	102	13.6	3816	4.2
Only CT reachable within 60 min	0	0.0	0	0.0
Neither reachable within 60 min	0	0.0	0	0.0
Likely irrelevant (<10 min)	647	86.0	86070	95.8

Population and Area Coverage in Landkreis Ostallgäu (Stroke-Ready Hospitals) (Bayern) — Extended (+10min Penalty)

Category	Grid Cells	Area (%)	Population	Population (%)
High (30+ min)	3	0.2	79	0.1
Medium (20-30 min)	53	3.2	7959	5.7
Low (10-20 min)	346	20.7	38666	27.6
Only CT reachable within 60 min	35	2.1	96	0.1
Neither reachable within 60 min	158	9.4	1332	0.9
Likely irrelevant (<10 min)	1080	64.5	92203	65.7

Population and Area Coverage in Landkreis Saarpfalz-Kreis (Stroke-Ready Hospitals) (Saarland) — Extended (+10min Penalty)

Category	Grid Cells	Area (%)	Population	Population (%)
High (30+ min)	0	0.0	0	0.0
Medium (20-30 min)	0	0.0	0	0.0
Low (10-20 min)	88	17.4	5866	4.3
Only CT reachable within 60 min	0	0.0	0	0.0
Neither reachable within 60 min	4	0.8	0	0.0
Likely irrelevant (<10 min)	413	81.8	131178	95.7

Population and Area Coverage in Landkreis Nordhausen (Stroke-Ready Hospitals) (Thüringen) — Extended (+10min Penalty)

Category	Grid Cells	Area (%)	Population	Population (%)
High (30+ min)	0	0.0	0	0.0
Medium (20-30 min)	54	5.9	1235	1.6
Low (10-20 min)	428	46.7	17925	23.2
Only CT reachable within 60 min	0	0.0	0	0.0
Neither reachable within 60 min	0	0.0	0	0.0
Likely irrelevant (<10 min)	434	47.4	57943	75.2

Population and Area Coverage in Landkreis Uelzen (Stroke-Ready Hospitals) (Niedersachsen) — Extended (+10min Penalty)

Category	Grid Cells	Area (%)	Population	Population (%)
High (30+ min)	10	0.5	28	0.0
Medium (20-30 min)	131	6.7	1854	2.0
Low (10-20 min)	516	26.5	26196	28.1
Only CT reachable within 60 min	0	0.0	0	0.0
Neither reachable within 60 min	0	0.0	0	0.0
Likely irrelevant (<10 min)	1290	66.3	65035	69.8

Population and Area Coverage in Kreisfreie Stadt Mülheim an der Ruhr (Stroke-Ready Hospitals) (Nordrhein-Westfalen) — Extended (+10min Penalty)

Category	Grid Cells	Area (%)	Population	Population (%)
High (30+ min)	0	0.0	0	0.0
Medium (20-30 min)	0	0.0	0	0.0
Low (10-20 min)	0	0.0	0	0.0
Only CT reachable within 60 min	0	0.0	0	0.0
Neither reachable within 60 min	0	0.0	0	0.0
Likely irrelevant (<10 min)	113	100.0	180205	100.0

Population and Area Coverage in Kreisfreie Stadt Bonn (Stroke-Ready Hospitals) (Nordrhein-Westfalen) — Extended (+10min Penalty)

Category	Grid Cells	Area (%)	Population	Population (%)
High (30+ min)	0	0.0	0	0.0
Medium (20-30 min)	0	0.0	0	0.0
Low (10-20 min)	19	10.6	19001	5.8
Only CT reachable within 60 min	0	0.0	0	0.0
Neither reachable within 60 min	0	0.0	0	0.0
Likely irrelevant (<10 min)	161	89.4	306709	94.2

Population and Area Coverage in Landkreis Birkenfeld (Stroke-Ready Hospitals) (Rheinland-Pfalz) — Extended (+10min Penalty)

Category	Grid Cells	Area (%)	Population	Population (%)
High (30+ min)	7	0.7	0	0.0
Medium (20-30 min)	101	10.5	3798	4.6
Low (10-20 min)	340	35.2	23608	28.5
Only CT reachable within 60 min	0	0.0	0	0.0
Neither reachable within 60 min	0	0.0	0	0.0
Likely irrelevant (<10 min)	517	53.6	55343	66.9

Population and Area Coverage in Landkreis Rems-Murr-Kreis (Stroke-Ready Hospitals) (Baden-Württemberg) — Extended (+10min Penalty)

Category	Grid Cells	Area (%)	Population	Population (%)
High (30+ min)	4	0.4	274	0.1
Medium (20-30 min)	41	3.9	2623	0.6
Low (10-20 min)	80	7.6	12150	2.9
Only CT reachable within 60 min	0	0.0	0	0.0
Neither reachable within 60 min	0	0.0	0	0.0
Likely irrelevant (<10 min)	932	88.2	402772	96.4

Population and Area Coverage in Landkreis Schwäbisch Hall (Stroke-Ready Hospitals) (Baden-Württemberg) — Extended (+10min Penalty)

Category	Grid Cells	Area (%)	Population	Population (%)
High (30+ min)	0	0.0	88	0.0
Medium (20-30 min)	143	7.8	16018	8.4
Low (10-20 min)	482	26.4	48823	25.6
Only CT reachable within 60 min	0	0.0	0	0.0
Neither reachable within 60 min	0	0.0	0	0.0
Likely irrelevant (<10 min)	1200	65.8	125958	66.0

Population and Area Coverage in Landkreis Rottweil (Stroke-Ready Hospitals) (Baden-Württemberg) — Extended (+10min Penalty)

Category	Grid Cells	Area (%)	Population	Population (%)
High (30+ min)	11	1.2	489	0.4
Medium (20-30 min)	58	6.2	6044	4.4
Low (10-20 min)	275	29.2	37828	27.5
Only CT reachable within 60 min	0	0.0	0	0.0
Neither reachable within 60 min	0	0.0	0	0.0
Likely irrelevant (<10 min)	597	63.4	93195	67.8

Population and Area Coverage in Landkreis Amberg-Sulzbach (Stroke-Ready Hospitals) (Bayern) — Extended (+10min Penalty)

Category	Grid Cells	Area (%)	Population	Population (%)
High (30+ min)	0	0.0	21	0.0
Medium (20-30 min)	118	7.6	8253	7.7
Low (10-20 min)	339	21.7	19874	18.6
Only CT reachable within 60 min	0	0.0	0	0.0
Neither reachable within 60 min	0	0.0	0	0.0
Likely irrelevant (<10 min)	1102	70.7	78557	73.6

Population and Area Coverage in Kreisfreie Stadt Kempten (Allgäu) (Stroke-Ready Hospitals) (Bayern) — Extended (+10min Penalty)

Category	Grid Cells	Area (%)	Population	Population (%)
High (30+ min)	0	0.0	0	0.0
Medium (20-30 min)	0	0.0	0	0.0
Low (10-20 min)	0	0.0	0	0.0
Only CT reachable within 60 min	0	0.0	0	0.0
Neither reachable within 60 min	0	0.0	0	0.0
Likely irrelevant (<10 min)	77	100.0	68488	100.0

Population and Area Coverage in Kreisfreie Stadt Chemnitz (Stroke-Ready Hospitals) (Sachsen) — Extended (+10min Penalty)

Category	Grid Cells	Area (%)	Population	Population (%)
High (30+ min)	0	0.0	0	0.0
Medium (20-30 min)	1	0.4	230	0.1
Low (10-20 min)	73	26.4	21294	7.2
Only CT reachable within 60 min	0	0.0	0	0.0
Neither reachable within 60 min	0	0.0	0	0.0
Likely irrelevant (<10 min)	202	73.2	274647	92.7

Population and Area Coverage in Landkreis Mecklenburgische Seenplatte (Stroke-Ready Hospitals) (Mecklenburg-Vorpommern) — Extended (+10min Penalty)

Category	Grid Cells	Area (%)	Population	Population (%)
High (30+ min)	1181	15.8	62728	26.3
Medium (20-30 min)	1018	13.7	27737	11.6
Low (10-20 min)	1224	16.4	25160	10.5
Only CT reachable within 60 min	1138	15.3	12488	5.2
Neither reachable within 60 min	460	6.2	2134	0.9
Likely irrelevant (<10 min)	2432	32.6	108694	45.5

Population and Area Coverage in Landkreis Erzgebirgskreis (Stroke-Ready Hospitals) (Sachsen) — Extended (+10min Penalty)

Category	Grid Cells	Area (%)	Population	Population (%)
High (30+ min)	163	7.1	14902	4.6
Medium (20-30 min)	310	13.5	27398	8.4
Low (10-20 min)	521	22.8	81290	24.9
Only CT reachable within 60 min	4	0.2	103	0.0
Neither reachable within 60 min	3	0.1	160	0.0
Likely irrelevant (<10 min)	1289	56.3	202417	62.0

Population and Area Coverage in Kreisfreie Stadt Dresden (Stroke-Ready Hospitals) (Sachsen) — Extended (+10min Penalty)

Category	Grid Cells	Area (%)	Population	Population (%)
High (30+ min)	0	0.0	0	0.0
Medium (20-30 min)	0	0.0	0	0.0
Low (10-20 min)	54	12.9	27687	4.5
Only CT reachable within 60 min	0	0.0	0	0.0
Neither reachable within 60 min	0	0.0	0	0.0
Likely irrelevant (<10 min)	366	87.1	587719	95.5

Population and Area Coverage in Landkreis Hildburghausen (Stroke-Ready Hospitals) (Thüringen) — Extended (+10min Penalty)

Category	Grid Cells	Area (%)	Population	Population (%)
High (30+ min)	0	0.0	0	0.0
Medium (20-30 min)	8	0.7	65	0.1
Low (10-20 min)	133	11.2	2704	4.6
Only CT reachable within 60 min	0	0.0	0	0.0
Neither reachable within 60 min	0	0.0	0	0.0
Likely irrelevant (<10 min)	1047	88.1	56128	95.3

Population and Area Coverage in Kreis Herzogtum Lauenburg (Stroke-Ready Hospitals) (Schleswig-Holstein) — Extended (+10min Penalty)

Category	Grid Cells	Area (%)	Population	Population (%)
High (30+ min)	62	3.6	3701	1.9
Medium (20-30 min)	284	16.6	36454	18.7
Low (10-20 min)	597	34.9	62646	32.1
Only CT reachable within 60 min	0	0.0	0	0.0
Neither reachable within 60 min	0	0.0	0	0.0
Likely irrelevant (<10 min)	768	44.9	92552	47.4

Population and Area Coverage in Landkreis Oldenburg (Stroke-Ready Hospitals) (Niedersachsen) — Extended (+10min Penalty)

Category	Grid Cells	Area (%)	Population	Population (%)
High (30+ min)	40	2.8	1118	0.9
Medium (20-30 min)	264	18.5	25308	19.8
Low (10-20 min)	469	32.8	44450	34.8
Only CT reachable within 60 min	0	0.0	0	0.0
Neither reachable within 60 min	0	0.0	0	0.0
Likely irrelevant (<10 min)	655	45.9	56743	44.5

Population and Area Coverage in Kreisfreie Stadt Krefeld (Stroke-Ready Hospitals) (Nordrhein-Westfalen) — Extended (+10min Penalty)

Category	Grid Cells	Area (%)	Population	Population (%)
High (30+ min)	0	0.0	0	0.0
Medium (20-30 min)	0	0.0	0	0.0
Low (10-20 min)	0	0.0	0	0.0
Only CT reachable within 60 min	0	0.0	0	0.0
Neither reachable within 60 min	0	0.0	0	0.0
Likely irrelevant (<10 min)	181	100.0	241982	100.0

Population and Area Coverage in Kreis Oberbergischer Kreis (Stroke-Ready Hospitals) (Nordrhein-Westfalen) — Extended (+10min Penalty)

Category	Grid Cells	Area (%)	Population	Population (%)
High (30+ min)	0	0.0	0	0.0
Medium (20-30 min)	203	17.3	34714	12.9
Low (10-20 min)	424	36.1	85616	31.8
Only CT reachable within 60 min	0	0.0	0	0.0
Neither reachable within 60 min	0	0.0	0	0.0
Likely irrelevant (<10 min)	549	46.7	148538	55.2

Population and Area Coverage in Landkreis Kusel (Stroke-Ready Hospitals) (Rheinland-Pfalz) — Extended (+10min Penalty)

Category	Grid Cells	Area (%)	Population	Population (%)
High (30+ min)	0	0.0	0	0.0
Medium (20-30 min)	69	9.6	7676	10.5
Low (10-20 min)	236	32.9	23010	31.4
Only CT reachable within 60 min	0	0.0	0	0.0
Neither reachable within 60 min	0	0.0	0	0.0
Likely irrelevant (<10 min)	412	57.5	42500	58.1

Population and Area Coverage in Landkreis Fürstenfeldbruck (Stroke-Ready Hospitals) (Bayern) — Extended (+10min Penalty)

Category	Grid Cells	Area (%)	Population	Population (%)
High (30+ min)	0	0.0	0	0.0
Medium (20-30 min)	0	0.0	0	0.0
Low (10-20 min)	3	0.6	44	0.0
Only CT reachable within 60 min	0	0.0	0	0.0
Neither reachable within 60 min	0	0.0	0	0.0
Likely irrelevant (<10 min)	523	99.4	214894	100.0

Population and Area Coverage in Landkreis Weilheim-Schongau (Stroke-Ready Hospitals) (Bayern) — Extended (+10min Penalty)

Category	Grid Cells	Area (%)	Population	Population (%)
High (30+ min)	0	0.0	0	0.0
Medium (20-30 min)	117	10.1	15374	11.4
Low (10-20 min)	275	23.7	28574	21.2
Only CT reachable within 60 min	1	0.1	0	0.0
Neither reachable within 60 min	13	1.1	13	0.0
Likely irrelevant (<10 min)	754	65.0	91112	67.5

Population and Area Coverage in Landkreis Neustadt a.d.Waldnaab (Stroke-Ready Hospitals) (Bayern) — Extended (+10min Penalty)

Category	Grid Cells	Area (%)	Population	Population (%)
High (30+ min)	2	0.1	5	0.0
Medium (20-30 min)	194	11.3	4506	4.6
Low (10-20 min)	434	25.2	15433	15.8
Only CT reachable within 60 min	0	0.0	0	0.0
Neither reachable within 60 min	0	0.0	0	0.0
Likely irrelevant (<10 min)	1092	63.4	77475	79.5

Population and Area Coverage in Landkreis Würzburg (Stroke-Ready Hospitals) (Bayern) — Extended (+10min Penalty)

Category	Grid Cells	Area (%)	Population	Population (%)
High (30+ min)	0	0.0	0	0.0
Medium (20-30 min)	13	1.1	511	0.3
Low (10-20 min)	266	22.1	25781	15.6
Only CT reachable within 60 min	0	0.0	0	0.0
Neither reachable within 60 min	0	0.0	0	0.0
Likely irrelevant (<10 min)	926	76.8	138770	84.1

Population and Area Coverage in Landkreis Elbe-Elster (Stroke-Ready Hospitals) (Brandenburg) — Extended (+10min Penalty)

Category	Grid Cells	Area (%)	Population	Population (%)
High (30+ min)	798	32.3	43889	43.1
Medium (20-30 min)	788	31.9	33649	33.0
Low (10-20 min)	266	10.8	7184	7.0
Only CT reachable within 60 min	553	22.4	15857	15.6
Neither reachable within 60 min	0	0.0	0	0.0
Likely irrelevant (<10 min)	64	2.6	1360	1.3

Population and Area Coverage in Landkreis Osterholz (Stroke-Ready Hospitals) (Niedersachsen) — Extended (+10min Penalty)

Category	Grid Cells	Area (%)	Population	Population (%)
High (30+ min)	3	0.3	158	0.1
Medium (20-30 min)	409	46.5	29510	26.3
Low (10-20 min)	237	27.0	46943	41.9
Only CT reachable within 60 min	0	0.0	0	0.0
Neither reachable within 60 min	0	0.0	0	0.0
Likely irrelevant (<10 min)	230	26.2	35541	31.7

Population and Area Coverage in Landkreis Heidekreis (Stroke-Ready Hospitals) (Niedersachsen) — Extended (+10min Penalty)

Category	Grid Cells	Area (%)	Population	Population (%)
High (30+ min)	1	0.0	0	0.0
Medium (20-30 min)	286	11.4	8959	6.6
Low (10-20 min)	529	21.0	46568	34.3
Only CT reachable within 60 min	0	0.0	0	0.0
Neither reachable within 60 min	0	0.0	0	0.0
Likely irrelevant (<10 min)	1700	67.6	80317	59.1

Population and Area Coverage in Kreisfreie Stadt Solingen (Stroke-Ready Hospitals) (Nordrhein-Westfalen) — Extended (+10min Penalty)

Category	Grid Cells	Area (%)	Population	Population (%)
High (30+ min)	0	0.0	0	0.0
Medium (20-30 min)	0	0.0	0	0.0
Low (10-20 min)	0	0.0	7	0.0
Only CT reachable within 60 min	0	0.0	0	0.0
Neither reachable within 60 min	0	0.0	0	0.0
Likely irrelevant (<10 min)	115	100.0	158431	100.0

Population and Area Coverage in Landkreis Bad Kreuznach (Stroke-Ready Hospitals) (Rheinland-Pfalz) — Extended (+10min Penalty)

Category	Grid Cells	Area (%)	Population	Population (%)
High (30+ min)	0	0.0	0	0.0
Medium (20-30 min)	70	6.5	3856	2.4
Low (10-20 min)	259	24.2	20223	12.6
Only CT reachable within 60 min	0	0.0	0	0.0
Neither reachable within 60 min	0	0.0	0	0.0
Likely irrelevant (<10 min)	743	69.3	136090	85.0

Population and Area Coverage in Landkreis Südwestpfalz (Stroke-Ready Hospitals) (Rheinland-Pfalz) — Extended (+10min Penalty)

Category	Grid Cells	Area (%)	Population	Population (%)
High (30+ min)	0	0.0	0	0.0
Medium (20-30 min)	1	0.1	0	0.0
Low (10-20 min)	126	10.7	16092	14.3
Only CT reachable within 60 min	0	0.0	0	0.0
Neither reachable within 60 min	10	0.8	61	0.1
Likely irrelevant (<10 min)	1040	88.4	95988	85.6

Population and Area Coverage in Landkreis Ludwigsburg (Stroke-Ready Hospitals) (Baden-Württemberg) — Extended (+10min Penalty)

Category	Grid Cells	Area (%)	Population	Population (%)
High (30+ min)	17	2.0	1129	0.2
Medium (20-30 min)	186	22.0	57156	10.8
Low (10-20 min)	251	29.6	128953	24.4
Only CT reachable within 60 min	0	0.0	0	0.0
Neither reachable within 60 min	0	0.0	0	0.0
Likely irrelevant (<10 min)	393	46.4	340305	64.5

Population and Area Coverage in Landkreis Lörrach (Stroke-Ready Hospitals) (Baden-Württemberg) — Extended (+10min Penalty)

Category	Grid Cells	Area (%)	Population	Population (%)
High (30+ min)	0	0.0	0	0.0
Medium (20-30 min)	0	0.0	0	0.0
Low (10-20 min)	298	31.7	38275	17.7
Only CT reachable within 60 min	0	0.0	52	0.0
Neither reachable within 60 min	0	0.0	9	0.0
Likely irrelevant (<10 min)	641	68.3	178308	82.3

Population and Area Coverage in Landkreis Alb-Donau-Kreis (Stroke-Ready Hospitals) (Baden-Württemberg) — Extended (+10min Penalty)

Category	Grid Cells	Area (%)	Population	Population (%)
High (30+ min)	4	0.2	3	0.0
Medium (20-30 min)	218	13.1	10841	5.7
Low (10-20 min)	716	43.2	88732	46.4
Only CT reachable within 60 min	0	0.0	0	0.0
Neither reachable within 60 min	0	0.0	0	0.0
Likely irrelevant (<10 min)	720	43.4	91531	47.9

Population and Area Coverage in Landkreis Biberach (Stroke-Ready Hospitals) (Baden-Württemberg) — Extended (+10min Penalty)

Category	Grid Cells	Area (%)	Population	Population (%)
High (30+ min)	0	0.0	0	0.0
Medium (20-30 min)	0	0.0	0	0.0
Low (10-20 min)	30	1.8	2355	1.2
Only CT reachable within 60 min	0	0.0	0	0.0
Neither reachable within 60 min	0	0.0	0	0.0
Likely irrelevant (<10 min)	1674	98.2	190902	98.8

Population and Area Coverage in Kreisfreie Stadt Fürth (Stroke-Ready Hospitals) (Bayern) — Extended (+10min Penalty)

Category	Grid Cells	Area (%)	Population	Population (%)
High (30+ min)	0	0.0	0	0.0
Medium (20-30 min)	0	0.0	0	0.0
Low (10-20 min)	0	0.0	0	0.0
Only CT reachable within 60 min	0	0.0	0	0.0
Neither reachable within 60 min	0	0.0	0	0.0
Likely irrelevant (<10 min)	83	100.0	150301	100.0

Population and Area Coverage in Kreisfreie Stadt Halle (Saale) (Stroke-Ready Hospitals) (Sachsen-Anhalt) — Extended (+10min Penalty)

Category	Grid Cells	Area (%)	Population	Population (%)
High (30+ min)	0	0.0	0	0.0
Medium (20-30 min)	0	0.0	0	0.0
Low (10-20 min)	0	0.0	0	0.0
Only CT reachable within 60 min	0	0.0	0	0.0
Neither reachable within 60 min	0	0.0	0	0.0
Likely irrelevant (<10 min)	174	100.0	250172	100.0

Population and Area Coverage in Landkreis Wittenberg (Stroke-Ready Hospitals) (Sachsen-Anhalt) — Extended (+10min Penalty)

Category	Grid Cells	Area (%)	Population	Population (%)
High (30+ min)	23	0.9	111	0.1
Medium (20-30 min)	216	8.5	3631	3.2
Low (10-20 min)	723	28.5	22481	19.7
Only CT reachable within 60 min	112	4.4	482	0.4
Neither reachable within 60 min	0	0.0	0	0.0
Likely irrelevant (<10 min)	1463	57.7	87378	76.6

Population and Area Coverage in Landkreis Saale-Holzland-Kreis (Stroke-Ready Hospitals) (Thüringen) — Extended (+10min Penalty)

Category	Grid Cells	Area (%)	Population	Population (%)
High (30+ min)	0	0.0	0	0.0
Medium (20-30 min)	64	6.2	3215	4.2
Low (10-20 min)	527	50.8	36593	48.3
Only CT reachable within 60 min	0	0.0	0	0.0
Neither reachable within 60 min	0	0.0	0	0.0
Likely irrelevant (<10 min)	447	43.1	35894	47.4

Population and Area Coverage in Kreisfreie Stadt Salzgitter (Stroke-Ready Hospitals) (Niedersachsen) — Extended (+10min Penalty)

Category	Grid Cells	Area (%)	Population	Population (%)
High (30+ min)	0	0.0	0	0.0
Medium (20-30 min)	2	0.7	204	0.2
Low (10-20 min)	52	17.6	12518	12.5
Only CT reachable within 60 min	0	0.0	0	0.0
Neither reachable within 60 min	0	0.0	0	0.0
Likely irrelevant (<10 min)	241	81.7	87330	87.3

Population and Area Coverage in Kreis Wesel (Stroke-Ready Hospitals) (Nordrhein-Westfalen) — Extended (+10min Penalty)

Category	Grid Cells	Area (%)	Population	Population (%)
High (30+ min)	0	0.0	0	0.0
Medium (20-30 min)	1	0.1	59	0.0
Low (10-20 min)	275	20.3	53519	11.3
Only CT reachable within 60 min	0	0.0	0	0.0
Neither reachable within 60 min	0	0.0	0	0.0
Likely irrelevant (<10 min)	1077	79.6	421506	88.7

Population and Area Coverage in Kreis Rhein-Erft-Kreis (Stroke-Ready Hospitals) (Nordrhein-Westfalen) — Extended (+10min Penalty)

Category	Grid Cells	Area (%)	Population	Population (%)
High (30+ min)	0	0.0	0	0.0
Medium (20-30 min)	0	0.0	0	0.0
Low (10-20 min)	217	24.2	104586	23.0
Only CT reachable within 60 min	0	0.0	0	0.0
Neither reachable within 60 min	0	0.0	0	0.0
Likely irrelevant (<10 min)	681	75.8	349943	77.0

Population and Area Coverage in Kreisfreie Stadt Frankfurt am Main (Stroke-Ready Hospitals) (Hessen) — Extended (+10min Penalty)

Category	Grid Cells	Area (%)	Population	Population (%)
High (30+ min)	0	0.0	0	0.0
Medium (20-30 min)	0	0.0	0	0.0
Low (10-20 min)	0	0.0	0	0.0
Only CT reachable within 60 min	0	0.0	0	0.0
Neither reachable within 60 min	0	0.0	0	0.0
Likely irrelevant (<10 min)	310	100.0	833128	100.0

Population and Area Coverage in Landkreis Südliche Weinstraße (Stroke-Ready Hospitals) (Rheinland-Pfalz) — Extended (+10min Penalty)

Category	Grid Cells	Area (%)	Population	Population (%)
High (30+ min)	0	0.0	0	0.0
Medium (20-30 min)	0	0.0	0	0.0
Low (10-20 min)	81	10.2	10222	9.1
Only CT reachable within 60 min	1	0.1	1	0.0
Neither reachable within 60 min	0	0.0	0	0.0
Likely irrelevant (<10 min)	712	89.7	101637	90.9

Population and Area Coverage in Stadtkreis Karlsruhe (Stroke-Ready Hospitals) (Baden-Württemberg) — Extended (+10min Penalty)

Category	Grid Cells	Area (%)	Population	Population (%)
High (30+ min)	0	0.0	0	0.0
Medium (20-30 min)	0	0.0	0	0.0
Low (10-20 min)	0	0.0	0	0.0
Only CT reachable within 60 min	0	0.0	0	0.0
Neither reachable within 60 min	0	0.0	0	0.0
Likely irrelevant (<10 min)	214	100.0	336385	100.0

Population and Area Coverage in Landkreis Rhein-Neckar-Kreis (Stroke-Ready Hospitals) (Baden-Württemberg) — Extended (+10min Penalty)

Category	Grid Cells	Area (%)	Population	Population (%)
High (30+ min)	0	0.0	0	0.0
Medium (20-30 min)	30	2.3	8042	1.5
Low (10-20 min)	307	23.4	125616	23.2
Only CT reachable within 60 min	0	0.0	0	0.0
Neither reachable within 60 min	0	0.0	0	0.0
Likely irrelevant (<10 min)	977	74.4	407362	75.3

Population and Area Coverage in Landkreis München (Stroke-Ready Hospitals) (Bayern) — Extended (+10min Penalty)

Category	Grid Cells	Area (%)	Population	Population (%)
High (30+ min)	0	0.0	0	0.0
Medium (20-30 min)	1	0.1	0	0.0
Low (10-20 min)	41	5.1	8469	2.5
Only CT reachable within 60 min	0	0.0	0	0.0
Neither reachable within 60 min	0	0.0	0	0.0
Likely irrelevant (<10 min)	761	94.8	335856	97.5

Population and Area Coverage in Landkreis Dingolfing-Landau (Stroke-Ready Hospitals) (Bayern) — Extended (+10min Penalty)

Category	Grid Cells	Area (%)	Population	Population (%)
High (30+ min)	0	0.0	0	0.0
Medium (20-30 min)	10	0.9	325	0.3
Low (10-20 min)	264	24.5	20327	21.5
Only CT reachable within 60 min	0	0.0	0	0.0
Neither reachable within 60 min	0	0.0	0	0.0
Likely irrelevant (<10 min)	803	74.6	73812	78.1

Population and Area Coverage in Kreisfreie Stadt Nürnberg (Stroke-Ready Hospitals) (Bayern) — Extended (+10min Penalty)

Category	Grid Cells	Area (%)	Population	Population (%)
High (30+ min)	0	0.0	0	0.0
Medium (20-30 min)	0	0.0	0	0.0
Low (10-20 min)	19	8.2	20105	3.9
Only CT reachable within 60 min	0	0.0	0	0.0
Neither reachable within 60 min	0	0.0	0	0.0
Likely irrelevant (<10 min)	213	91.8	498087	96.1

Population and Area Coverage in Kreisfreie Stadt Cottbus (Stroke-Ready Hospitals) (Brandenburg) — Extended (+10min Penalty)

Category	Grid Cells	Area (%)	Population	Population (%)
High (30+ min)	0	0.0	0	0.0
Medium (20-30 min)	0	0.0	0	0.0
Low (10-20 min)	5	2.4	143	0.1
Only CT reachable within 60 min	0	0.0	0	0.0
Neither reachable within 60 min	0	0.0	0	0.0
Likely irrelevant (<10 min)	207	97.6	104361	99.9

Population and Area Coverage in Landkreis Salzlandkreis (Stroke-Ready Hospitals) (Sachsen-Anhalt) — Extended (+10min Penalty)

Category	Grid Cells	Area (%)	Population	Population (%)
High (30+ min)	4	0.2	192	0.1
Medium (20-30 min)	390	20.9	32703	18.5
Low (10-20 min)	874	46.8	85197	48.3
Only CT reachable within 60 min	0	0.0	0	0.0
Neither reachable within 60 min	0	0.0	0	0.0
Likely irrelevant (<10 min)	600	32.1	58225	33.0

Population and Area Coverage in Landkreis Sömmerda (Stroke-Ready Hospitals) (Thüringen) — Extended (+10min Penalty)

Category	Grid Cells	Area (%)	Population	Population (%)
High (30+ min)	10	1.0	5	0.0
Medium (20-30 min)	127	12.2	5376	8.4
Low (10-20 min)	589	56.4	42514	66.8
Only CT reachable within 60 min	0	0.0	0	0.0
Neither reachable within 60 min	0	0.0	0	0.0
Likely irrelevant (<10 min)	318	30.5	15781	24.8

Population and Area Coverage in Landkreis Saale-Orla-Kreis (Stroke-Ready Hospitals) (Thüringen) — Extended (+10min Penalty)

Category	Grid Cells	Area (%)	Population	Population (%)
High (30+ min)	34	2.3	3406	4.5
Medium (20-30 min)	251	17.1	18417	24.5
Low (10-20 min)	519	35.4	21140	28.1
Only CT reachable within 60 min	0	0.0	0	0.0
Neither reachable within 60 min	0	0.0	0	0.0
Likely irrelevant (<10 min)	661	45.1	32343	42.9

Population and Area Coverage in Kreisfreie Stadt Neumünster (Stroke Units) (Schleswig-Holstein) — Regular (+10min Penalty)

Category	Grid Cells	Area (%)	Population	Population (%)
High (30+ min)	0	0.0	0	0.0
Medium (20-30 min)	0	0.0	0	0.0
Low (10-20 min)	0	0.0	0	0.0
Only CT reachable within 60 min	0	0.0	0	0.0
Neither reachable within 60 min	0	0.0	0	0.0
Likely irrelevant (<10 min)	100	100.0	85923	100.0

Population and Area Coverage in Kreisfreie Stadt Osnabrück (Stroke Units) (Niedersachsen) — Regular (+10min Penalty)

Category	Grid Cells	Area (%)	Population	Population (%)
High (30+ min)	0	0.0	0	0.0
Medium (20-30 min)	0	0.0	0	0.0
Low (10-20 min)	5	3.2	1979	1.3
Only CT reachable within 60 min	0	0.0	0	0.0
Neither reachable within 60 min	0	0.0	0	0.0
Likely irrelevant (<10 min)	150	96.8	152462	98.7

Population and Area Coverage in Kreisfreie Stadt Mönchengladbach (Stroke Units) (Nordrhein-Westfalen) — Regular (+10min Penalty)

Category	Grid Cells	Area (%)	Population	Population (%)
High (30+ min)	0	0.0	0	0.0
Medium (20-30 min)	0	0.0	0	0.0
Low (10-20 min)	9	4.1	8837	3.2
Only CT reachable within 60 min	0	0.0	0	0.0
Neither reachable within 60 min	0	0.0	0	0.0
Likely irrelevant (<10 min)	211	95.9	265284	96.8

Population and Area Coverage in Kreis Coesfeld (Stroke Units) (Nordrhein-Westfalen) — Regular (+10min Penalty)

Category	Grid Cells	Area (%)	Population	Population (%)
High (30+ min)	0	0.0	0	0.0
Medium (20-30 min)	76	5.2	5376	2.5
Low (10-20 min)	591	40.6	100194	46.4
Only CT reachable within 60 min	0	0.0	0	0.0
Neither reachable within 60 min	0	0.0	0	0.0
Likely irrelevant (<10 min)	787	54.1	110366	51.1

Population and Area Coverage in Kreis Paderborn (Stroke Units) (Nordrhein-Westfalen) — Regular (+10min Penalty)

Category	Grid Cells	Area (%)	Population	Population (%)
High (30+ min)	28	1.7	10	0.0
Medium (20-30 min)	34	2.1	672	0.2
Low (10-20 min)	714	43.9	70541	21.3
Only CT reachable within 60 min	0	0.0	0	0.0
Neither reachable within 60 min	0	0.0	0	0.0
Likely irrelevant (<10 min)	850	52.3	259298	78.5

Population and Area Coverage in Kreisfreie Stadt Kassel (Stroke Units) (Hessen) — Regular (+10min Penalty)

Category	Grid Cells	Area (%)	Population	Population (%)
High (30+ min)	0	0.0	0	0.0
Medium (20-30 min)	0	0.0	0	0.0
Low (10-20 min)	23	17.3	467	0.2
Only CT reachable within 60 min	0	0.0	0	0.0
Neither reachable within 60 min	0	0.0	0	0.0
Likely irrelevant (<10 min)	110	82.7	205252	99.8

Population and Area Coverage in Landkreis Hersfeld-Rotenburg (Stroke Units) (Hessen) — Regular (+10min Penalty)

Category	Grid Cells	Area (%)	Population	Population (%)
High (30+ min)	10	0.7	66	0.1
Medium (20-30 min)	123	8.8	10139	8.4
Low (10-20 min)	270	19.3	23053	19.1
Only CT reachable within 60 min	0	0.0	0	0.0
Neither reachable within 60 min	0	0.0	0	0.0
Likely irrelevant (<10 min)	998	71.2	87631	72.5

Population and Area Coverage in Landkreis Schwalm-Eder-Kreis (Stroke Units) (Hessen) — Regular (+10min Penalty)

Category	Grid Cells	Area (%)	Population	Population (%)
High (30+ min)	414	20.9	33746	18.6
Medium (20-30 min)	769	38.9	65169	35.8
Low (10-20 min)	463	23.4	52339	28.8
Only CT reachable within 60 min	0	0.0	0	0.0
Neither reachable within 60 min	0	0.0	0	0.0
Likely irrelevant (<10 min)	333	16.8	30573	16.8

Population and Area Coverage in Landkreis Westerwaldkreis (Stroke Units) (Rheinland-Pfalz) — Regular (+10min Penalty)

Category	Grid Cells	Area (%)	Population	Population (%)
High (30+ min)	1	0.1	8	0.0
Medium (20-30 min)	92	7.3	14664	7.1
Low (10-20 min)	386	30.8	47872	23.3
Only CT reachable within 60 min	0	0.0	0	0.0
Neither reachable within 60 min	0	0.0	0	0.0
Likely irrelevant (<10 min)	776	61.8	143280	69.6

Population and Area Coverage in Kreisfreie Stadt Frankenthal (Pfalz) (Stroke Units) (Rheinland-Pfalz) — Regular (+10min Penalty)

Category	Grid Cells	Area (%)	Population	Population (%)
High (30+ min)	0	0.0	0	0.0
Medium (20-30 min)	0	0.0	0	0.0
Low (10-20 min)	0	0.0	17	0.0
Only CT reachable within 60 min	0	0.0	0	0.0
Neither reachable within 60 min	0	0.0	0	0.0
Likely irrelevant (<10 min)	54	100.0	45416	100.0

Population and Area Coverage in Landkreis Böblingen (Stroke Units) (Baden-Württemberg) — Regular (+10min Penalty)

Category	Grid Cells	Area (%)	Population	Population (%)
High (30+ min)	0	0.0	0	0.0
Medium (20-30 min)	59	7.9	18234	4.9
Low (10-20 min)	327	43.7	150060	40.3
Only CT reachable within 60 min	0	0.0	0	0.0
Neither reachable within 60 min	0	0.0	0	0.0
Likely irrelevant (<10 min)	363	48.5	204055	54.8

Population and Area Coverage in Landkreis Cham (Stroke Units) (Bayern) — Regular (+10min Penalty)

Category	Grid Cells	Area (%)	Population	Population (%)
High (30+ min)	1	0.1	7	0.0
Medium (20-30 min)	84	4.8	5191	4.1
Low (10-20 min)	524	29.7	32736	25.8
Only CT reachable within 60 min	0	0.0	0	0.0
Neither reachable within 60 min	0	0.0	0	0.0
Likely irrelevant (<10 min)	1158	65.5	88935	70.1

Population and Area Coverage in Landkreis Saarlouis (Stroke Units) (Saarland) — Regular (+10min Penalty)

Category	Grid Cells	Area (%)	Population	Population (%)
High (30+ min)	0	0.0	0	0.0
Medium (20-30 min)	26	4.6	5040	2.7
Low (10-20 min)	112	19.9	28918	15.4
Only CT reachable within 60 min	1	0.2	320	0.2
Neither reachable within 60 min	2	0.4	41	0.0
Likely irrelevant (<10 min)	423	75.0	152890	81.7

Population and Area Coverage in Landkreis Anhalt-Bitterfeld (Stroke Units) (Sachsen-Anhalt) — Regular (+10min Penalty)

Category	Grid Cells	Area (%)	Population	Population (%)
High (30+ min)	612	32.0	29171	19.8
Medium (20-30 min)	438	22.9	55419	37.6
Low (10-20 min)	501	26.2	50589	34.3
Only CT reachable within 60 min	184	9.6	1112	0.8
Neither reachable within 60 min	0	0.0	0	0.0
Likely irrelevant (<10 min)	176	9.2	11206	7.6

Population and Area Coverage in Landkreis Jerichower Land (Stroke Units) (Sachsen-Anhalt) — Regular (+10min Penalty)

Category	Grid Cells	Area (%)	Population	Population (%)
High (30+ min)	191	9.1	15135	18.8
Medium (20-30 min)	578	27.7	18861	23.4
Low (10-20 min)	772	37.0	36490	45.3
Only CT reachable within 60 min	498	23.9	6136	7.6
Neither reachable within 60 min	0	0.0	0	0.0
Likely irrelevant (<10 min)	49	2.3	3877	4.8

Population and Area Coverage in Landkreis Kyffhäuserkreis (Stroke Units) (Thüringen) — Regular (+10min Penalty)

Category	Grid Cells	Area (%)	Population	Population (%)
High (30+ min)	184	13.8	4192	6.0
Medium (20-30 min)	694	51.9	45876	65.8
Low (10-20 min)	440	32.9	19598	28.1
Only CT reachable within 60 min	9	0.7	4	0.0
Neither reachable within 60 min	0	0.0	0	0.0
Likely irrelevant (<10 min)	10	0.7	22	0.0

Population and Area Coverage in Landkreis Peine (Stroke Units) (Niedersachsen) — Regular (+10min Penalty)

Category	Grid Cells	Area (%)	Population	Population (%)
High (30+ min)	9	1.3	401	0.3
Medium (20-30 min)	184	25.8	32311	24.7
Low (10-20 min)	463	65.0	92324	70.6
Only CT reachable within 60 min	0	0.0	0	0.0
Neither reachable within 60 min	0	0.0	0	0.0
Likely irrelevant (<10 min)	56	7.9	5681	4.3

Population and Area Coverage in Kreisfreie Stadt Leverkusen (Stroke Units) (Nordrhein-Westfalen) — Regular (+10min Penalty)

Category	Grid Cells	Area (%)	Population	Population (%)
High (30+ min)	0	0.0	0	0.0
Medium (20-30 min)	0	0.0	0	0.0
Low (10-20 min)	5	4.9	1786	1.1
Only CT reachable within 60 min	0	0.0	0	0.0
Neither reachable within 60 min	0	0.0	0	0.0
Likely irrelevant (<10 min)	97	95.1	158884	98.9

Population and Area Coverage in Kreis Ennepe-Ruhr-Kreis (Stroke Units) (Nordrhein-Westfalen) — Regular (+10min Penalty)

Category	Grid Cells	Area (%)	Population	Population (%)
High (30+ min)	0	0.0	0	0.0
Medium (20-30 min)	53	9.9	10548	3.2
Low (10-20 min)	114	21.3	42914	12.9
Only CT reachable within 60 min	0	0.0	0	0.0
Neither reachable within 60 min	0	0.0	0	0.0
Likely irrelevant (<10 min)	369	68.8	279199	83.9

Population and Area Coverage in Kreis Märkischer Kreis (Stroke Units) (Nordrhein-Westfalen) — Regular (+10min Penalty)

Category	Grid Cells	Area (%)	Population	Population (%)
High (30+ min)	18	1.3	585	0.1
Medium (20-30 min)	231	17.0	80274	19.3
Low (10-20 min)	523	38.5	175100	42.2
Only CT reachable within 60 min	0	0.0	0	0.0
Neither reachable within 60 min	0	0.0	0	0.0
Likely irrelevant (<10 min)	585	43.1	159435	38.4

Population and Area Coverage in Landkreis Ahrweiler (Stroke Units) (Rheinland-Pfalz) — Regular (+10min Penalty)

Category	Grid Cells	Area (%)	Population	Population (%)
High (30+ min)	0	0.0	0	0.0
Medium (20-30 min)	3	0.3	1867	1.4
Low (10-20 min)	243	24.5	35085	27.2
Only CT reachable within 60 min	0	0.0	0	0.0
Neither reachable within 60 min	0	0.0	0	0.0
Likely irrelevant (<10 min)	747	75.2	91935	71.3

Population and Area Coverage in Stadtkreis Baden-Baden (Stroke Units) (Baden-Württemberg) — Regular (+10min Penalty)

Category	Grid Cells	Area (%)	Population	Population (%)
High (30+ min)	2	1.1	1	0.0
Medium (20-30 min)	75	41.9	7431	11.8
Low (10-20 min)	52	29.1	35484	56.4
Only CT reachable within 60 min	0	0.0	0	0.0
Neither reachable within 60 min	0	0.0	0	0.0
Likely irrelevant (<10 min)	50	27.9	20025	31.8

Population and Area Coverage in Landkreis Waldshut (Stroke Units) (Baden-Württemberg) — Regular (+10min Penalty)

Category	Grid Cells	Area (%)	Population	Population (%)
High (30+ min)	102	8.0	20779	14.7
Medium (20-30 min)	205	16.0	12603	8.9
Low (10-20 min)	254	19.8	30932	21.9
Only CT reachable within 60 min	639	49.9	69358	49.0
Neither reachable within 60 min	2	0.2	204	0.1
Likely irrelevant (<10 min)	78	6.1	7673	5.4

Population and Area Coverage in Landkreis Bad Tölz-Wolfratshausen (Stroke Units) (Bayern) — Regular (+10min Penalty)

Category	Grid Cells	Area (%)	Population	Population (%)
High (30+ min)	1	0.1	213	0.2
Medium (20-30 min)	12	0.9	705	0.6
Low (10-20 min)	291	22.1	51035	40.5
Only CT reachable within 60 min	1	0.1	5	0.0
Neither reachable within 60 min	109	8.3	176	0.1
Likely irrelevant (<10 min)	905	68.6	74023	58.7

Population and Area Coverage in Landkreis Passau (Stroke Units) (Bayern) — Regular (+10min Penalty)

Category	Grid Cells	Area (%)	Population	Population (%)
High (30+ min)	120	6.7	6127	3.2
Medium (20-30 min)	471	26.2	42463	22.4
Low (10-20 min)	566	31.4	68316	36.0
Only CT reachable within 60 min	2	0.1	131	0.1
Neither reachable within 60 min	10	0.6	139	0.1
Likely irrelevant (<10 min)	631	35.1	72537	38.2

Population and Area Coverage in Landkreis Lichtenfels (Stroke Units) (Bayern) — Regular (+10min Penalty)

Category	Grid Cells	Area (%)	Population	Population (%)
High (30+ min)	0	0.0	0	0.0
Medium (20-30 min)	0	0.0	0	0.0
Low (10-20 min)	65	9.9	1754	2.5
Only CT reachable within 60 min	0	0.0	0	0.0
Neither reachable within 60 min	0	0.0	0	0.0
Likely irrelevant (<10 min)	590	90.1	67443	97.5

Population and Area Coverage in Landkreis Wunsiedel i.Fichtelgebirge (Stroke Units) (Bayern) — Regular (+10min Penalty)

Category	Grid Cells	Area (%)	Population	Population (%)
High (30+ min)	6	0.8	15	0.0
Medium (20-30 min)	128	17.9	24761	33.5
Low (10-20 min)	392	54.9	29272	39.6
Only CT reachable within 60 min	0	0.0	0	0.0
Neither reachable within 60 min	0	0.0	0	0.0
Likely irrelevant (<10 min)	188	26.3	19888	26.9

Population and Area Coverage in Landkreis Ansbach (Stroke Units) (Bayern) — Regular (+10min Penalty)

Category	Grid Cells	Area (%)	Population	Population (%)
High (30+ min)	18	0.7	575	0.3
Medium (20-30 min)	167	6.9	17135	9.3
Low (10-20 min)	315	13.0	18351	9.9
Only CT reachable within 60 min	0	0.0	0	0.0
Neither reachable within 60 min	0	0.0	0	0.0
Likely irrelevant (<10 min)	1932	79.4	148815	80.5

Population and Area Coverage in Kreisfreie Stadt Würzburg (Stroke Units) (Bayern) — Regular (+10min Penalty)

Category	Grid Cells	Area (%)	Population	Population (%)
High (30+ min)	0	0.0	0	0.0
Medium (20-30 min)	0	0.0	0	0.0
Low (10-20 min)	0	0.0	0	0.0
Only CT reachable within 60 min	0	0.0	0	0.0
Neither reachable within 60 min	0	0.0	0	0.0
Likely irrelevant (<10 min)	109	100.0	128110	100.0

Population and Area Coverage in Landkreis Oder-Spree (Stroke Units) (Brandenburg) — Regular (+10min Penalty)

Category	Grid Cells	Area (%)	Population	Population (%)
High (30+ min)	31	1.0	2167	1.3
Medium (20-30 min)	674	22.7	21103	12.3
Low (10-20 min)	1379	46.4	88815	51.7
Only CT reachable within 60 min	40	1.3	207	0.1
Neither reachable within 60 min	7	0.2	49	0.0
Likely irrelevant (<10 min)	838	28.2	59589	34.7

Population and Area Coverage in Landkreis Potsdam-Mittelmark (Stroke Units) (Brandenburg) — Regular (+10min Penalty)

Category	Grid Cells	Area (%)	Population	Population (%)
High (30+ min)	867	25.4	28879	14.2
Medium (20-30 min)	876	25.7	32681	16.1
Low (10-20 min)	753	22.1	36210	17.8
Only CT reachable within 60 min	621	18.2	10098	5.0
Neither reachable within 60 min	0	0.0	0	0.0
Likely irrelevant (<10 min)	290	8.5	95130	46.9

Population and Area Coverage in Landkreis Uckermark (Stroke Units) (Brandenburg) — Regular (+10min Penalty)

Category	Grid Cells	Area (%)	Population	Population (%)
High (30+ min)	1170	28.4	52748	45.6
Medium (20-30 min)	937	22.7	20240	17.5
Low (10-20 min)	332	8.1	4349	3.8
Only CT reachable within 60 min	1549	37.6	36120	31.2
Neither reachable within 60 min	85	2.1	1901	1.6
Likely irrelevant (<10 min)	48	1.2	300	0.3

Population and Area Coverage in Landkreis Sächsische Schweiz-Osterzgebirge (Stroke Units) (Sachsen) — Regular (+10min Penalty)

Category	Grid Cells	Area (%)	Population	Population (%)
High (30+ min)	343	16.6	27278	12.0
Medium (20-30 min)	573	27.7	69712	30.6
Low (10-20 min)	574	27.7	73964	32.4
Only CT reachable within 60 min	202	9.7	11916	5.2
Neither reachable within 60 min	41	2.0	172	0.1
Likely irrelevant (<10 min)	339	16.4	45117	19.8

Population and Area Coverage in Kreisfreie Stadt Leipzig (Stroke Units) (Sachsen) — Regular (+10min Penalty)

Category	Grid Cells	Area (%)	Population	Population (%)
High (30+ min)	0	0.0	0	0.0
Medium (20-30 min)	0	0.0	0	0.0
Low (10-20 min)	22	5.7	18160	2.9
Only CT reachable within 60 min	0	0.0	0	0.0
Neither reachable within 60 min	0	0.0	0	0.0
Likely irrelevant (<10 min)	362	94.3	601166	97.1

Population and Area Coverage in Kreisfreie Stadt Jena (Stroke Units) (Thüringen) — Regular (+10min Penalty)

Category	Grid Cells	Area (%)	Population	Population (%)
High (30+ min)	0	0.0	0	0.0
Medium (20-30 min)	2	1.3	0	0.0
Low (10-20 min)	24	16.1	3222	3.2
Only CT reachable within 60 min	0	0.0	0	0.0
Neither reachable within 60 min	0	0.0	0	0.0
Likely irrelevant (<10 min)	123	82.6	97024	96.8

Population and Area Coverage in Landkreis Gotha (Stroke Units) (Thüringen) — Regular (+10min Penalty)

Category	Grid Cells	Area (%)	Population	Population (%)
High (30+ min)	0	0.0	0	0.0
Medium (20-30 min)	18	1.5	161	0.1
Low (10-20 min)	310	25.9	13996	11.4
Only CT reachable within 60 min	0	0.0	0	0.0
Neither reachable within 60 min	0	0.0	0	0.0
Likely irrelevant (<10 min)	871	72.6	108119	88.4

Population and Area Coverage in Kreis Pinneberg (Stroke Units) (Schleswig-Holstein) — Regular (+10min Penalty)

Category	Grid Cells	Area (%)	Population	Population (%)
High (30+ min)	0	0.0	0	0.0
Medium (20-30 min)	1	0.1	21	0.0
Low (10-20 min)	117	13.8	38325	12.8
Only CT reachable within 60 min	1	0.1	14	0.0
Neither reachable within 60 min	19	2.2	123	0.0
Likely irrelevant (<10 min)	712	83.8	262014	87.2

Population and Area Coverage in Kreis Schleswig-Flensburg (Stroke Units) (Schleswig-Holstein) — Regular (+10min Penalty)

Category	Grid Cells	Area (%)	Population	Population (%)
High (30+ min)	0	0.0	0	0.0
Medium (20-30 min)	27	1.0	3724	1.9
Low (10-20 min)	136	5.0	10352	5.3
Only CT reachable within 60 min	4	0.1	38	0.0
Neither reachable within 60 min	8	0.3	231	0.1
Likely irrelevant (<10 min)	2522	93.5	182448	92.7

Population and Area Coverage in Kreis Steinburg (Stroke Units) (Schleswig-Holstein) — Regular (+10min Penalty)

Category	Grid Cells	Area (%)	Population	Population (%)
High (30+ min)	0	0.0	0	0.0
Medium (20-30 min)	1	0.1	43	0.0
Low (10-20 min)	172	12.4	11484	8.7
Only CT reachable within 60 min	0	0.0	0	0.0
Neither reachable within 60 min	9	0.7	432	0.3
Likely irrelevant (<10 min)	1200	86.8	120774	91.0

Population and Area Coverage in Landkreis Verden (Stroke Units) (Niedersachsen) — Regular (+10min Penalty)

Category	Grid Cells	Area (%)	Population	Population (%)
High (30+ min)	147	14.0	9855	7.4
Medium (20-30 min)	209	19.9	33409	25.2
Low (10-20 min)	525	49.9	71159	53.6
Only CT reachable within 60 min	0	0.0	0	0.0
Neither reachable within 60 min	0	0.0	0	0.0
Likely irrelevant (<10 min)	171	16.3	18405	13.9

Population and Area Coverage in Kreisfreie Stadt Delmenhorst (Stroke Units) (Niedersachsen) — Regular (+10min Penalty)

Category	Grid Cells	Area (%)	Population	Population (%)
High (30+ min)	0	0.0	0	0.0
Medium (20-30 min)	7	8.4	12322	16.8
Low (10-20 min)	67	80.7	60635	82.6
Only CT reachable within 60 min	0	0.0	0	0.0
Neither reachable within 60 min	0	0.0	0	0.0
Likely irrelevant (<10 min)	9	10.8	438	0.6

Population and Area Coverage in Kreis Kleve (Stroke Units) (Nordrhein-Westfalen) — Regular (+10min Penalty)

Category	Grid Cells	Area (%)	Population	Population (%)
High (30+ min)	301	19.2	73138	24.1
Medium (20-30 min)	545	34.7	103010	34.0
Low (10-20 min)	422	26.9	81427	26.9
Only CT reachable within 60 min	9	0.6	280	0.1
Neither reachable within 60 min	14	0.9	127	0.0
Likely irrelevant (<10 min)	280	17.8	45217	14.9

Population and Area Coverage in Kreis Herford (Stroke Units) (Nordrhein-Westfalen) — Regular (+10min Penalty)

Category	Grid Cells	Area (%)	Population	Population (%)
High (30+ min)	0	0.0	0	0.0
Medium (20-30 min)	38	6.4	5649	2.3
Low (10-20 min)	248	41.7	100617	40.2
Only CT reachable within 60 min	0	0.0	0	0.0
Neither reachable within 60 min	0	0.0	0	0.0
Likely irrelevant (<10 min)	309	51.9	144029	57.5

Population and Area Coverage in Kreisfreie Stadt Dortmund (Stroke Units) (Nordrhein-Westfalen) — Regular (+10min Penalty)

Category	Grid Cells	Area (%)	Population	Population (%)
High (30+ min)	0	0.0	0	0.0
Medium (20-30 min)	0	0.0	0	0.0
Low (10-20 min)	0	0.0	0	0.0
Only CT reachable within 60 min	0	0.0	0	0.0
Neither reachable within 60 min	0	0.0	0	0.0
Likely irrelevant (<10 min)	363	100.0	611483	100.0

Population and Area Coverage in Kreisfreie Stadt Wiesbaden (Stroke Units) (Hessen) — Regular (+10min Penalty)

Category	Grid Cells	Area (%)	Population	Population (%)
High (30+ min)	0	0.0	0	0.0
Medium (20-30 min)	0	0.0	0	0.0
Low (10-20 min)	1	0.4	0	0.0
Only CT reachable within 60 min	0	0.0	0	0.0
Neither reachable within 60 min	0	0.0	0	0.0
Likely irrelevant (<10 min)	251	99.6	277503	100.0

Population and Area Coverage in Landkreis Hochtaunuskreis (Stroke Units) (Hessen) — Regular (+10min Penalty)

Category	Grid Cells	Area (%)	Population	Population (%)
High (30+ min)	0	0.0	0	0.0
Medium (20-30 min)	1	0.2	28	0.0
Low (10-20 min)	182	30.0	33317	14.3
Only CT reachable within 60 min	0	0.0	0	0.0
Neither reachable within 60 min	0	0.0	0	0.0
Likely irrelevant (<10 min)	423	69.8	199175	85.7

Population and Area Coverage in Landkreis Kassel (Stroke Units) (Hessen) — Regular (+10min Penalty)

Category	Grid Cells	Area (%)	Population	Population (%)
High (30+ min)	72	4.3	3183	1.3
Medium (20-30 min)	551	33.0	37080	15.6
Low (10-20 min)	686	41.1	74178	31.3
Only CT reachable within 60 min	0	0.0	0	0.0
Neither reachable within 60 min	0	0.0	0	0.0
Likely irrelevant (<10 min)	361	21.6	122559	51.7

Population and Area Coverage in Landkreis Cochem-Zell (Stroke Units) (Rheinland-Pfalz) — Regular (+10min Penalty)

Category	Grid Cells	Area (%)	Population	Population (%)
High (30+ min)	218	25.0	14277	22.2
Medium (20-30 min)	238	27.3	19783	30.7
Low (10-20 min)	225	25.8	18876	29.3
Only CT reachable within 60 min	5	0.6	34	0.1
Neither reachable within 60 min	0	0.0	0	0.0
Likely irrelevant (<10 min)	185	21.2	11386	17.7

Population and Area Coverage in Landkreis Rhein-Lahn-Kreis (Stroke Units) (Rheinland-Pfalz) — Regular (+10min Penalty)

Category	Grid Cells	Area (%)	Population	Population (%)
High (30+ min)	131	13.3	8442	6.8
Medium (20-30 min)	208	21.1	17215	13.8
Low (10-20 min)	235	23.8	20999	16.8
Only CT reachable within 60 min	0	0.0	0	0.0
Neither reachable within 60 min	0	0.0	0	0.0
Likely irrelevant (<10 min)	412	41.8	78018	62.6

Population and Area Coverage in Stadtkreis Heilbronn (Stroke Units) (Baden-Württemberg) — Regular (+10min Penalty)

Category	Grid Cells	Area (%)	Population	Population (%)
High (30+ min)	0	0.0	0	0.0
Medium (20-30 min)	0	0.0	0	0.0
Low (10-20 min)	0	0.0	9	0.0
Only CT reachable within 60 min	0	0.0	0	0.0
Neither reachable within 60 min	0	0.0	0	0.0
Likely irrelevant (<10 min)	121	100.0	123077	100.0

Population and Area Coverage in Landkreis Schwandorf (Stroke Units) (Bayern) — Regular (+10min Penalty)

Category	Grid Cells	Area (%)	Population	Population (%)
High (30+ min)	6	0.3	521	0.4
Medium (20-30 min)	276	15.5	13006	8.8
Low (10-20 min)	455	25.5	21763	14.7
Only CT reachable within 60 min	0	0.0	0	0.0
Neither reachable within 60 min	0	0.0	0	0.0
Likely irrelevant (<10 min)	1049	58.7	113244	76.2

Population and Area Coverage in Landkreis Unterallgäu (Stroke Units) (Bayern) — Regular (+10min Penalty)

Category	Grid Cells	Area (%)	Population	Population (%)
High (30+ min)	9	0.6	107	0.1
Medium (20-30 min)	231	15.5	12019	8.4
Low (10-20 min)	536	36.1	49591	34.9
Only CT reachable within 60 min	0	0.0	0	0.0
Neither reachable within 60 min	0	0.0	0	0.0
Likely irrelevant (<10 min)	710	47.8	80555	56.6

Population and Area Coverage in Kreisfreie Stadt Frankfurt (Oder) (Stroke Units) (Brandenburg) — Regular (+10min Penalty)

Category	Grid Cells	Area (%)	Population	Population (%)
High (30+ min)	0	0.0	0	0.0
Medium (20-30 min)	0	0.0	0	0.0
Low (10-20 min)	7	3.6	61	0.1
Only CT reachable within 60 min	5	2.6	464	0.8
Neither reachable within 60 min	15	7.7	524	0.9
Likely irrelevant (<10 min)	169	86.2	55067	98.1

Population and Area Coverage in Landkreis Dahme-Spreewald (Stroke Units) (Brandenburg) — Regular (+10min Penalty)

Category	Grid Cells	Area (%)	Population	Population (%)
High (30+ min)	7	0.2	308	0.2
Medium (20-30 min)	251	8.5	27511	17.4
Low (10-20 min)	1431	48.2	85554	54.0
Only CT reachable within 60 min	0	0.0	0	0.0
Neither reachable within 60 min	0	0.0	0	0.0
Likely irrelevant (<10 min)	1280	43.1	45096	28.5

Population and Area Coverage in Landkreis Leipzig (Stroke Units) (Sachsen) — Regular (+10min Penalty)

Category	Grid Cells	Area (%)	Population	Population (%)
High (30+ min)	1	0.0	5	0.0
Medium (20-30 min)	204	9.6	16075	6.3
Low (10-20 min)	640	30.1	80280	31.4
Only CT reachable within 60 min	0	0.0	0	0.0
Neither reachable within 60 min	0	0.0	0	0.0
Likely irrelevant (<10 min)	1279	60.2	159065	62.3

Population and Area Coverage in Kreisfreie Stadt Magdeburg (Stroke Units) (Sachsen-Anhalt) — Regular (+10min Penalty)

Category	Grid Cells	Area (%)	Population	Population (%)
High (30+ min)	0	0.0	0	0.0
Medium (20-30 min)	4	1.5	820	0.3
Low (10-20 min)	53	20.2	6982	2.8
Only CT reachable within 60 min	0	0.0	0	0.0
Neither reachable within 60 min	0	0.0	0	0.0
Likely irrelevant (<10 min)	206	78.3	241498	96.9

Population and Area Coverage in Landkreis Wartburgkreis (Stroke Units) (Thüringen) — Regular (+10min Penalty)

Category	Grid Cells	Area (%)	Population	Population (%)
High (30+ min)	0	0.0	0	0.0
Medium (20-30 min)	28	1.6	451	0.3
Low (10-20 min)	202	11.5	13237	9.6
Only CT reachable within 60 min	0	0.0	0	0.0
Neither reachable within 60 min	0	0.0	0	0.0
Likely irrelevant (<10 min)	1521	86.9	124387	90.1

Population and Area Coverage in Landkreis Unstrut-Hainich-Kreis (Stroke Units) (Thüringen) — Regular (+10min Penalty)

Category	Grid Cells	Area (%)	Population	Population (%)
High (30+ min)	114	9.6	2418	2.7
Medium (20-30 min)	264	22.2	18750	20.8
Low (10-20 min)	468	39.4	26209	29.0
Only CT reachable within 60 min	0	0.0	0	0.0
Neither reachable within 60 min	0	0.0	0	0.0
Likely irrelevant (<10 min)	343	28.8	42892	47.5

Population and Area Coverage in Kreisfreie Stadt Bielefeld (Stroke Units) (Nordrhein-Westfalen) — Regular (+10min Penalty)

Category	Grid Cells	Area (%)	Population	Population (%)
High (30+ min)	0	0.0	0	0.0
Medium (20-30 min)	0	0.0	0	0.0
Low (10-20 min)	63	18.8	44964	12.9
Only CT reachable within 60 min	0	0.0	0	0.0
Neither reachable within 60 min	0	0.0	0	0.0
Likely irrelevant (<10 min)	273	81.2	304658	87.1

Population and Area Coverage in Kreisfreie Stadt Bochum (Stroke Units) (Nordrhein-Westfalen) — Regular (+10min Penalty)

Category	Grid Cells	Area (%)	Population	Population (%)
High (30+ min)	0	0.0	0	0.0
Medium (20-30 min)	0	0.0	0	0.0
Low (10-20 min)	0	0.0	0	0.0
Only CT reachable within 60 min	0	0.0	0	0.0
Neither reachable within 60 min	0	0.0	0	0.0
Likely irrelevant (<10 min)	185	100.0	386703	100.0

Population and Area Coverage in Landkreis Fulda (Stroke Units) (Hessen) — Regular (+10min Penalty)

Category	Grid Cells	Area (%)	Population	Population (%)
High (30+ min)	0	0.0	0	0.0
Medium (20-30 min)	4	0.2	24	0.0
Low (10-20 min)	423	24.2	39391	17.9
Only CT reachable within 60 min	0	0.0	0	0.0
Neither reachable within 60 min	0	0.0	0	0.0
Likely irrelevant (<10 min)	1324	75.6	180844	82.1

Population and Area Coverage in Landkreis Rastatt (Stroke Units) (Baden-Württemberg) — Regular (+10min Penalty)

Category	Grid Cells	Area (%)	Population	Population (%)
High (30+ min)	18	2.0	807	0.4
Medium (20-30 min)	267	29.7	38274	16.9
Low (10-20 min)	180	20.0	57937	25.5
Only CT reachable within 60 min	59	6.6	113	0.0
Neither reachable within 60 min	2	0.2	31	0.0
Likely irrelevant (<10 min)	372	41.4	129964	57.2

Population and Area Coverage in Landkreis Breisgau-Hochschwarzwald (Stroke Units) (Baden-Württemberg) — Regular (+10min Penalty)

Category	Grid Cells	Area (%)	Population	Population (%)
High (30+ min)	254	15.5	19433	7.9
Medium (20-30 min)	392	23.9	54667	22.2
Low (10-20 min)	443	27.0	65865	26.7
Only CT reachable within 60 min	119	7.2	1279	0.5
Neither reachable within 60 min	0	0.0	3	0.0
Likely irrelevant (<10 min)	435	26.5	105142	42.7

Population and Area Coverage in Landkreis Ebersberg (Stroke Units) (Bayern) — Regular (+10min Penalty)

Category	Grid Cells	Area (%)	Population	Population (%)
High (30+ min)	0	0.0	0	0.0
Medium (20-30 min)	0	0.0	0	0.0
Low (10-20 min)	1	0.2	3	0.0
Only CT reachable within 60 min	0	0.0	0	0.0
Neither reachable within 60 min	0	0.0	0	0.0
Likely irrelevant (<10 min)	663	99.8	137982	100.0

Population and Area Coverage in Landkreis Rottal-Inn (Stroke Units) (Bayern) — Regular (+10min Penalty)

Category	Grid Cells	Area (%)	Population	Population (%)
High (30+ min)	2	0.1	10	0.0
Medium (20-30 min)	135	8.8	8772	7.2
Low (10-20 min)	533	34.6	39495	32.5
Only CT reachable within 60 min	0	0.0	0	0.0
Neither reachable within 60 min	2	0.1	63	0.1
Likely irrelevant (<10 min)	867	56.3	73328	60.3

Population and Area Coverage in Landkreis Bamberg (Stroke Units) (Bayern) — Regular (+10min Penalty)

Category	Grid Cells	Area (%)	Population	Population (%)
High (30+ min)	0	0.0	0	0.0
Medium (20-30 min)	276	18.8	10196	6.8
Low (10-20 min)	683	46.6	67847	45.0
Only CT reachable within 60 min	0	0.0	0	0.0
Neither reachable within 60 min	0	0.0	0	0.0
Likely irrelevant (<10 min)	506	34.5	72755	48.2

Population and Area Coverage in Landkreis Schweinfurt (Stroke Units) (Bayern) — Regular (+10min Penalty)

Category	Grid Cells	Area (%)	Population	Population (%)
High (30+ min)	0	0.0	0	0.0
Medium (20-30 min)	49	4.6	489	0.4
Low (10-20 min)	380	35.9	32908	28.1
Only CT reachable within 60 min	0	0.0	0	0.0
Neither reachable within 60 min	0	0.0	0	0.0
Likely irrelevant (<10 min)	630	59.5	83552	71.4

Population and Area Coverage in Landkreis St. Wendel (Stroke Units) (Saarland) — Regular (+10min Penalty)

Category	Grid Cells	Area (%)	Population	Population (%)
High (30+ min)	0	0.0	0	0.0
Medium (20-30 min)	53	8.9	2772	3.3
Low (10-20 min)	134	22.4	13717	16.1
Only CT reachable within 60 min	0	0.0	0	0.0
Neither reachable within 60 min	0	0.0	0	0.0
Likely irrelevant (<10 min)	411	68.7	68797	80.7

Population and Area Coverage in Landkreis Harz (Stroke Units) (Sachsen-Anhalt) — Regular (+10min Penalty)

Category	Grid Cells	Area (%)	Population	Population (%)
High (30+ min)	50	1.8	2409	1.2
Medium (20-30 min)	882	32.1	68189	34.1
Low (10-20 min)	999	36.4	66327	33.1
Only CT reachable within 60 min	0	0.0	0	0.0
Neither reachable within 60 min	0	0.0	0	0.0
Likely irrelevant (<10 min)	817	29.7	63177	31.6

Population and Area Coverage in Kreisfreie Stadt Gera (Stroke Units) (Thüringen) — Regular (+10min Penalty)

Category	Grid Cells	Area (%)	Population	Population (%)
High (30+ min)	0	0.0	0	0.0
Medium (20-30 min)	5	2.6	316	0.3
Low (10-20 min)	44	23.0	3986	3.6
Only CT reachable within 60 min	0	0.0	0	0.0
Neither reachable within 60 min	0	0.0	0	0.0
Likely irrelevant (<10 min)	142	74.3	105355	96.1

Population and Area Coverage in Kreisfreie Stadt Weimar (Stroke Units) (Thüringen) — Regular (+10min Penalty)

Category	Grid Cells	Area (%)	Population	Population (%)
High (30+ min)	0	0.0	0	0.0
Medium (20-30 min)	0	0.0	0	0.0
Low (10-20 min)	0	0.0	0	0.0
Only CT reachable within 60 min	0	0.0	0	0.0
Neither reachable within 60 min	0	0.0	0	0.0
Likely irrelevant (<10 min)	109	100.0	57698	100.0

Population and Area Coverage in Landkreis Holzminden (Stroke Units) (Niedersachsen) — Regular (+10min Penalty)

Category	Grid Cells	Area (%)	Population	Population (%)
High (30+ min)	43	4.8	4873	6.8
Medium (20-30 min)	120	13.3	5234	7.3
Low (10-20 min)	159	17.6	7206	10.0
Only CT reachable within 60 min	0	0.0	0	0.0
Neither reachable within 60 min	0	0.0	0	0.0
Likely irrelevant (<10 min)	582	64.4	54475	75.9

Population and Area Coverage in Landkreis Grafschaft Bentheim (Stroke Units) (Niedersachsen) — Regular (+10min Penalty)

Category	Grid Cells	Area (%)	Population	Population (%)
High (30+ min)	0	0.0	0	0.0
Medium (20-30 min)	9	0.7	427	0.3
Low (10-20 min)	185	14.5	25189	18.4
Only CT reachable within 60 min	6	0.5	451	0.3
Neither reachable within 60 min	22	1.7	501	0.4
Likely irrelevant (<10 min)	1052	82.6	110108	80.6

Population and Area Coverage in Kreis Höxter (Stroke Units) (Nordrhein-Westfalen) — Regular (+10min Penalty)

Category	Grid Cells	Area (%)	Population	Population (%)
High (30+ min)	22	1.4	863	0.6
Medium (20-30 min)	391	25.0	34615	24.2
Low (10-20 min)	603	38.6	52837	37.0
Only CT reachable within 60 min	0	0.0	0	0.0
Neither reachable within 60 min	0	0.0	0	0.0
Likely irrelevant (<10 min)	547	35.0	54476	38.2

Population and Area Coverage in Landkreis Marburg-Biedenkopf (Stroke Units) (Hessen) — Regular (+10min Penalty)

Category	Grid Cells	Area (%)	Population	Population (%)
High (30+ min)	154	9.6	20810	8.6
Medium (20-30 min)	194	12.1	26017	10.7
Low (10-20 min)	435	27.2	41762	17.2
Only CT reachable within 60 min	0	0.0	0	0.0
Neither reachable within 60 min	0	0.0	0	0.0
Likely irrelevant (<10 min)	817	51.1	154773	63.6

Population and Area Coverage in Landkreis Rhein-Hunsrück-Kreis (Stroke Units) (Rheinland-Pfalz) — Regular (+10min Penalty)

Category	Grid Cells	Area (%)	Population	Population (%)
High (30+ min)	399	32.1	28942	27.6
Medium (20-30 min)	594	47.7	47639	45.4
Low (10-20 min)	242	19.5	28045	26.7
Only CT reachable within 60 min	2	0.2	0	0.0
Neither reachable within 60 min	0	0.0	0	0.0
Likely irrelevant (<10 min)	7	0.6	336	0.3

Population and Area Coverage in Landkreis Eifelkreis Bitburg-Prüm (Stroke Units) (Rheinland-Pfalz) — Regular (+10min Penalty)

Category	Grid Cells	Area (%)	Population	Population (%)
High (30+ min)	309	15.2	11543	11.5
Medium (20-30 min)	905	44.4	42913	42.8
Low (10-20 min)	592	29.1	38958	38.8
Only CT reachable within 60 min	100	4.9	1324	1.3
Neither reachable within 60 min	2	0.1	127	0.1
Likely irrelevant (<10 min)	128	6.3	5471	5.5

Population and Area Coverage in Landkreis Erding (Stroke Units) (Bayern) — Regular (+10min Penalty)

Category	Grid Cells	Area (%)	Population	Population (%)
High (30+ min)	0	0.0	0	0.0
Medium (20-30 min)	1	0.1	60	0.0
Low (10-20 min)	428	40.6	44434	32.7
Only CT reachable within 60 min	0	0.0	0	0.0
Neither reachable within 60 min	0	0.0	0	0.0
Likely irrelevant (<10 min)	626	59.3	91237	67.2

Population and Area Coverage in Landkreis Deggendorf (Stroke Units) (Bayern) — Regular (+10min Penalty)

Category	Grid Cells	Area (%)	Population	Population (%)
High (30+ min)	0	0.0	0	0.0
Medium (20-30 min)	2	0.2	45	0.0
Low (10-20 min)	131	12.4	12863	10.8
Only CT reachable within 60 min	0	0.0	0	0.0
Neither reachable within 60 min	0	0.0	0	0.0
Likely irrelevant (<10 min)	924	87.4	106542	89.2

Population and Area Coverage in Landkreis Weißenburg-Gunzenhausen (Stroke Units) (Bayern) — Regular (+10min Penalty)

Category	Grid Cells	Area (%)	Population	Population (%)
High (30+ min)	3	0.3	3	0.0
Medium (20-30 min)	201	16.9	11966	12.5
Low (10-20 min)	452	38.1	39874	41.8
Only CT reachable within 60 min	0	0.0	0	0.0
Neither reachable within 60 min	0	0.0	0	0.0
Likely irrelevant (<10 min)	531	44.7	43568	45.7

Population and Area Coverage in Landkreis Lindau (Bodensee) (Stroke Units) (Bayern) — Regular (+10min Penalty)

Category	Grid Cells	Area (%)	Population	Population (%)
High (30+ min)	2	0.5	1008	1.2
Medium (20-30 min)	212	55.8	60675	74.7
Low (10-20 min)	126	33.2	16141	19.9
Only CT reachable within 60 min	5	1.3	230	0.3
Neither reachable within 60 min	10	2.6	682	0.8
Likely irrelevant (<10 min)	25	6.6	2454	3.0

Population and Area Coverage in Kreisfreie Stadt Rostock (Stroke Units) (Mecklenburg-Vorpommern) — Regular (+10min Penalty)

Category	Grid Cells	Area (%)	Population	Population (%)
High (30+ min)	0	0.0	0	0.0
Medium (20-30 min)	15	6.7	15	0.0
Low (10-20 min)	93	41.3	29488	15.8
Only CT reachable within 60 min	1	0.4	33	0.0
Neither reachable within 60 min	5	2.2	576	0.3
Likely irrelevant (<10 min)	111	49.3	156293	83.8

Population and Area Coverage in Landkreis Rostock (Stroke Units) (Mecklenburg-Vorpommern) — Regular (+10min Penalty)

Category	Grid Cells	Area (%)	Population	Population (%)
High (30+ min)	292	6.2	9944	5.0
Medium (20-30 min)	782	16.7	33918	17.1
Low (10-20 min)	1318	28.2	57053	28.8
Only CT reachable within 60 min	69	1.5	694	0.3
Neither reachable within 60 min	30	0.6	586	0.3
Likely irrelevant (<10 min)	2186	46.7	96178	48.5

Population and Area Coverage in Landkreis Vorpommern-Greifswald (Stroke Units) (Mecklenburg-Vorpommern) — Regular (+10min Penalty)

Category	Grid Cells	Area (%)	Population	Population (%)
High (30+ min)	402	8.0	25261	11.4
Medium (20-30 min)	1262	25.0	45103	20.3
Low (10-20 min)	940	18.7	24393	11.0
Only CT reachable within 60 min	624	12.4	18356	8.3
Neither reachable within 60 min	139	2.8	3468	1.6
Likely irrelevant (<10 min)	1672	33.2	105314	47.5

Population and Area Coverage in Landkreis Mittelsachsen (Stroke Units) (Sachsen) — Regular (+10min Penalty)

Category	Grid Cells	Area (%)	Population	Population (%)
High (30+ min)	20	0.7	690	0.2
Medium (20-30 min)	598	22.1	70702	24.0
Low (10-20 min)	618	22.8	61791	21.0
Only CT reachable within 60 min	2	0.1	0	0.0
Neither reachable within 60 min	0	0.0	0	0.0
Likely irrelevant (<10 min)	1472	54.3	161229	54.8

Population and Area Coverage in Landkreis Schmalkalden-Meiningen (Stroke Units) (Thüringen) — Regular (+10min Penalty)

Category	Grid Cells	Area (%)	Population	Population (%)
High (30+ min)	43	2.7	3283	2.8
Medium (20-30 min)	146	9.2	23761	20.5
Low (10-20 min)	354	22.3	26399	22.8
Only CT reachable within 60 min	0	0.0	0	0.0
Neither reachable within 60 min	0	0.0	0	0.0
Likely irrelevant (<10 min)	1042	65.7	62356	53.8

Population and Area Coverage in Kreisfreie Stadt Lübeck (Stroke Units) (Schleswig-Holstein) — Regular (+10min Penalty)

Category	Grid Cells	Area (%)	Population	Population (%)
High (30+ min)	0	0.0	0	0.0
Medium (20-30 min)	7	2.4	1779	0.8
Low (10-20 min)	70	24.2	23917	11.0
Only CT reachable within 60 min	2	0.7	2030	0.9
Neither reachable within 60 min	2	0.7	159	0.1
Likely irrelevant (<10 min)	208	72.0	190043	87.2

Population and Area Coverage in Kreis Nordfriesland (Stroke Units) (Schleswig-Holstein) — Regular (+10min Penalty)

Category	Grid Cells	Area (%)	Population	Population (%)
High (30+ min)	638	24.2	43958	27.4
Medium (20-30 min)	568	21.6	45879	28.6
Low (10-20 min)	499	19.0	20180	12.6
Only CT reachable within 60 min	218	8.3	23096	14.4
Neither reachable within 60 min	151	5.7	4918	3.1
Likely irrelevant (<10 min)	559	21.2	22659	14.1

Population and Area Coverage in Landkreis Northeim (Stroke Units) (Niedersachsen) — Regular (+10min Penalty)

Category	Grid Cells	Area (%)	Population	Population (%)
High (30+ min)	71	4.3	4589	3.4
Medium (20-30 min)	413	25.0	41591	31.0
Low (10-20 min)	707	42.8	60437	45.1
Only CT reachable within 60 min	0	0.0	0	0.0
Neither reachable within 60 min	0	0.0	0	0.0
Likely irrelevant (<10 min)	460	27.9	27495	20.5

Population and Area Coverage in Kreisfreie Stadt Wilhelmshaven (Stroke Units) (Niedersachsen) — Regular (+10min Penalty)

Category	Grid Cells	Area (%)	Population	Population (%)
High (30+ min)	0	0.0	0	0.0
Medium (20-30 min)	0	0.0	0	0.0
Low (10-20 min)	7	5.8	6343	9.1
Only CT reachable within 60 min	0	0.0	0	0.0
Neither reachable within 60 min	3	2.5	693	1.0
Likely irrelevant (<10 min)	111	91.7	62492	89.9

Population and Area Coverage in Kreisfreie Stadt Essen (Stroke Units) (Nordrhein-Westfalen) — Regular (+10min Penalty)

Category	Grid Cells	Area (%)	Population	Population (%)
High (30+ min)	0	0.0	0	0.0
Medium (20-30 min)	0	0.0	0	0.0
Low (10-20 min)	4	1.4	2784	0.5
Only CT reachable within 60 min	0	0.0	0	0.0
Neither reachable within 60 min	0	0.0	0	0.0
Likely irrelevant (<10 min)	272	98.6	593892	99.5

Population and Area Coverage in Kreisfreie Stadt Münster (Stroke Units) (Nordrhein-Westfalen) — Regular (+10min Penalty)

Category	Grid Cells	Area (%)	Population	Population (%)
High (30+ min)	0	0.0	0	0.0
Medium (20-30 min)	0	0.0	0	0.0
Low (10-20 min)	43	10.8	13416	3.7
Only CT reachable within 60 min	0	0.0	0	0.0
Neither reachable within 60 min	0	0.0	0	0.0
Likely irrelevant (<10 min)	356	89.2	351415	96.3

Population and Area Coverage in Kreis Recklinghausen (Stroke Units) (Nordrhein-Westfalen) — Regular (+10min Penalty)

Category	Grid Cells	Area (%)	Population	Population (%)
High (30+ min)	0	0.0	0	0.0
Medium (20-30 min)	0	0.0	0	0.0
Low (10-20 min)	118	12.0	35059	5.7
Only CT reachable within 60 min	0	0.0	0	0.0
Neither reachable within 60 min	0	0.0	0	0.0
Likely irrelevant (<10 min)	864	88.0	581354	94.3

Population and Area Coverage in Landkreis Offenbach (Stroke Units) (Hessen) — Regular (+10min Penalty)

Category	Grid Cells	Area (%)	Population	Population (%)
High (30+ min)	0	0.0	0	0.0
Medium (20-30 min)	0	0.0	0	0.0
Low (10-20 min)	8	1.8	27	0.0
Only CT reachable within 60 min	0	0.0	0	0.0
Neither reachable within 60 min	0	0.0	0	0.0
Likely irrelevant (<10 min)	438	98.2	341284	100.0

Population and Area Coverage in Kreisfreie Stadt Trier (Stroke Units) (Rheinland-Pfalz) — Regular (+10min Penalty)

Category	Grid Cells	Area (%)	Population	Population (%)
High (30+ min)	0	0.0	0	0.0
Medium (20-30 min)	0	0.0	0	0.0
Low (10-20 min)	0	0.0	0	0.0
Only CT reachable within 60 min	0	0.0	0	0.0
Neither reachable within 60 min	0	0.0	0	0.0
Likely irrelevant (<10 min)	146	100.0	128525	100.0

Population and Area Coverage in Landkreis Bernkastel-Wittlich (Stroke Units) (Rheinland-Pfalz) — Regular (+10min Penalty)

Category	Grid Cells	Area (%)	Population	Population (%)
High (30+ min)	2	0.1	2	0.0
Medium (20-30 min)	93	6.4	2937	2.6
Low (10-20 min)	511	35.0	40401	35.2
Only CT reachable within 60 min	0	0.0	0	0.0
Neither reachable within 60 min	0	0.0	0	0.0
Likely irrelevant (<10 min)	855	58.5	71331	62.2

Population and Area Coverage in Kreisfreie Stadt Worms (Stroke Units) (Rheinland-Pfalz) — Regular (+10min Penalty)

Category	Grid Cells	Area (%)	Population	Population (%)
High (30+ min)	0	0.0	0	0.0
Medium (20-30 min)	0	0.0	0	0.0
Low (10-20 min)	1	0.7	19	0.0
Only CT reachable within 60 min	0	0.0	0	0.0
Neither reachable within 60 min	0	0.0	0	0.0
Likely irrelevant (<10 min)	133	99.3	81855	100.0

Population and Area Coverage in Landkreis Heilbronn (Stroke Units) (Baden-Württemberg) — Regular (+10min Penalty)

Category	Grid Cells	Area (%)	Population	Population (%)
High (30+ min)	3	0.2	5	0.0
Medium (20-30 min)	152	11.2	12293	3.7
Low (10-20 min)	571	42.0	116508	35.2
Only CT reachable within 60 min	0	0.0	0	0.0
Neither reachable within 60 min	0	0.0	0	0.0
Likely irrelevant (<10 min)	635	46.7	202598	61.1

Population and Area Coverage in Kreisfreie Stadt Ingolstadt (Stroke Units) (Bayern) — Regular (+10min Penalty)

Category	Grid Cells	Area (%)	Population	Population (%)
High (30+ min)	0	0.0	0	0.0
Medium (20-30 min)	0	0.0	0	0.0
Low (10-20 min)	5	3.0	865	0.6
Only CT reachable within 60 min	0	0.0	0	0.0
Neither reachable within 60 min	0	0.0	0	0.0
Likely irrelevant (<10 min)	160	97.0	134354	99.4

Population and Area Coverage in Landkreis Forchheim (Stroke Units) (Bayern) — Regular (+10min Penalty)

Category	Grid Cells	Area (%)	Population	Population (%)
High (30+ min)	0	0.0	0	0.0
Medium (20-30 min)	74	9.2	3451	2.9
Low (10-20 min)	301	37.4	25823	21.8
Only CT reachable within 60 min	0	0.0	0	0.0
Neither reachable within 60 min	0	0.0	0	0.0
Likely irrelevant (<10 min)	430	53.4	89137	75.3

Population and Area Coverage in Landkreis Erlangen-Höchstadt (Stroke Units) (Bayern) — Regular (+10min Penalty)

Category	Grid Cells	Area (%)	Population	Population (%)
High (30+ min)	8	1.1	175	0.1
Medium (20-30 min)	156	22.3	16526	12.0
Low (10-20 min)	304	43.6	64142	46.7
Only CT reachable within 60 min	0	0.0	0	0.0
Neither reachable within 60 min	0	0.0	0	0.0
Likely irrelevant (<10 min)	230	33.0	56556	41.2

Population and Area Coverage in Landkreis Mansfeld-Südharz (Stroke Units) (Sachsen-Anhalt) — Regular (+10min Penalty)

Category	Grid Cells	Area (%)	Population	Population (%)
High (30+ min)	453	24.0	27883	21.8
Medium (20-30 min)	647	34.2	69758	54.5
Low (10-20 min)	451	23.9	18229	14.2
Only CT reachable within 60 min	24	1.3	113	0.1
Neither reachable within 60 min	0	0.0	0	0.0
Likely irrelevant (<10 min)	315	16.7	11954	9.3

Population and Area Coverage in Kreis Minden-Lübbecke (Stroke Units) (Nordrhein-Westfalen) — Regular (+10min Penalty)

Category	Grid Cells	Area (%)	Population	Population (%)
High (30+ min)	147	9.7	18338	5.9
Medium (20-30 min)	386	25.4	59410	19.2
Low (10-20 min)	215	14.1	20892	6.8
Only CT reachable within 60 min	0	0.0	0	0.0
Neither reachable within 60 min	0	0.0	0	0.0
Likely irrelevant (<10 min)	772	50.8	210833	68.1

Population and Area Coverage in Kreisfreie Stadt Hamm (Stroke Units) (Nordrhein-Westfalen) — Regular (+10min Penalty)

Category	Grid Cells	Area (%)	Population	Population (%)
High (30+ min)	0	0.0	0	0.0
Medium (20-30 min)	0	0.0	0	0.0
Low (10-20 min)	13	4.4	3619	2.1
Only CT reachable within 60 min	0	0.0	0	0.0
Neither reachable within 60 min	0	0.0	0	0.0
Likely irrelevant (<10 min)	280	95.6	171781	97.9

Population and Area Coverage in Landkreis Main-Taunus-Kreis (Stroke Units) (Hessen) — Regular (+10min Penalty)

Category	Grid Cells	Area (%)	Population	Population (%)
High (30+ min)	0	0.0	0	0.0
Medium (20-30 min)	0	0.0	0	0.0
Low (10-20 min)	25	8.9	5580	2.4
Only CT reachable within 60 min	0	0.0	0	0.0
Neither reachable within 60 min	0	0.0	0	0.0
Likely irrelevant (<10 min)	256	91.1	225853	97.6

Population and Area Coverage in Kreisfreie Stadt Ludwigshafen am Rhein (Stroke Units) (Rheinland-Pfalz) — Regular (+10min Penalty)

Category	Grid Cells	Area (%)	Population	Population (%)
High (30+ min)	0	0.0	0	0.0
Medium (20-30 min)	0	0.0	0	0.0
Low (10-20 min)	2	2.0	175	0.1
Only CT reachable within 60 min	0	0.0	0	0.0
Neither reachable within 60 min	0	0.0	0	0.0
Likely irrelevant (<10 min)	97	98.0	163026	99.9

Population and Area Coverage in Landkreis Esslingen (Stroke Units) (Baden-Württemberg) — Regular (+10min Penalty)

Category	Grid Cells	Area (%)	Population	Population (%)
High (30+ min)	0	0.0	0	0.0
Medium (20-30 min)	3	0.4	127	0.0
Low (10-20 min)	146	18.9	77372	15.0
Only CT reachable within 60 min	0	0.0	0	0.0
Neither reachable within 60 min	0	0.0	0	0.0
Likely irrelevant (<10 min)	622	80.7	439660	85.0

Population and Area Coverage in Landkreis Dachau (Stroke Units) (Bayern) — Regular (+10min Penalty)

Category	Grid Cells	Area (%)	Population	Population (%)
High (30+ min)	0	0.0	0	0.0
Medium (20-30 min)	4	0.6	87	0.1
Low (10-20 min)	222	31.5	24600	16.4
Only CT reachable within 60 min	0	0.0	0	0.0
Neither reachable within 60 min	0	0.0	0	0.0
Likely irrelevant (<10 min)	478	67.9	125444	83.6

Population and Area Coverage in Landkreis Bad Kissingen (Stroke Units) (Bayern) — Regular (+10min Penalty)

Category	Grid Cells	Area (%)	Population	Population (%)
High (30+ min)	7	0.5	77	0.1
Medium (20-30 min)	400	27.9	36596	34.3
Low (10-20 min)	637	44.5	49601	46.5
Only CT reachable within 60 min	0	0.0	0	0.0
Neither reachable within 60 min	0	0.0	0	0.0
Likely irrelevant (<10 min)	389	27.1	20351	19.1

Population and Area Coverage in Kreisfreie Stadt Erfurt (Stroke Units) (Thüringen) — Regular (+10min Penalty)

Category	Grid Cells	Area (%)	Population	Population (%)
High (30+ min)	0	0.0	0	0.0
Medium (20-30 min)	0	0.0	0	0.0
Low (10-20 min)	33	9.6	4727	1.9
Only CT reachable within 60 min	0	0.0	0	0.0
Neither reachable within 60 min	0	0.0	0	0.0
Likely irrelevant (<10 min)	309	90.4	242495	98.1

Population and Area Coverage in Kreisfreie Stadt Flensburg (Stroke Units) (Schleswig-Holstein) — Regular (+10min Penalty)

Category	Grid Cells	Area (%)	Population	Population (%)
High (30+ min)	0	0.0	0	0.0
Medium (20-30 min)	0	0.0	0	0.0
Low (10-20 min)	0	0.0	0	0.0
Only CT reachable within 60 min	0	0.0	0	0.0
Neither reachable within 60 min	0	0.0	0	0.0
Likely irrelevant (<10 min)	62	100.0	80819	100.0

Population and Area Coverage in Kreis Dithmarschen (Stroke Units) (Schleswig-Holstein) — Regular (+10min Penalty)

Category	Grid Cells	Area (%)	Population	Population (%)
High (30+ min)	0	0.0	0	0.0
Medium (20-30 min)	86	4.7	6521	4.9
Low (10-20 min)	280	15.1	24537	18.4
Only CT reachable within 60 min	11	0.6	1168	0.9
Neither reachable within 60 min	33	1.8	446	0.3
Likely irrelevant (<10 min)	1439	77.8	100946	75.5

Population and Area Coverage in Landkreis Hameln-Pyrmont (Stroke Units) (Niedersachsen) — Regular (+10min Penalty)

Category	Grid Cells	Area (%)	Population	Population (%)
High (30+ min)	29	2.8	3932	2.7
Medium (20-30 min)	416	39.7	42255	28.7
Low (10-20 min)	305	29.1	50787	34.5
Only CT reachable within 60 min	0	0.0	0	0.0
Neither reachable within 60 min	0	0.0	0	0.0
Likely irrelevant (<10 min)	298	28.4	50427	34.2

Population and Area Coverage in Landkreis Celle (Stroke Units) (Niedersachsen) — Regular (+10min Penalty)

Category	Grid Cells	Area (%)	Population	Population (%)
High (30+ min)	1	0.0	1	0.0
Medium (20-30 min)	15	0.7	213	0.1
Low (10-20 min)	303	14.7	8450	4.8
Only CT reachable within 60 min	0	0.0	0	0.0
Neither reachable within 60 min	0	0.0	0	0.0
Likely irrelevant (<10 min)	1748	84.6	167628	95.1

Population and Area Coverage in Landkreis Harburg (Stroke Units) (Niedersachsen) — Regular (+10min Penalty)

Category	Grid Cells	Area (%)	Population	Population (%)
High (30+ min)	0	0.0	0	0.0
Medium (20-30 min)	4	0.2	483	0.2
Low (10-20 min)	65	3.8	17563	7.2
Only CT reachable within 60 min	0	0.0	0	0.0
Neither reachable within 60 min	0	0.0	0	0.0
Likely irrelevant (<10 min)	1620	95.9	225318	92.6

Population and Area Coverage in Kreisfreie Stadt Düsseldorf (Stroke Units) (Nordrhein-Westfalen) — Regular (+10min Penalty)

Category	Grid Cells	Area (%)	Population	Population (%)
High (30+ min)	0	0.0	0	0.0
Medium (20-30 min)	0	0.0	0	0.0
Low (10-20 min)	12	4.3	11937	1.8
Only CT reachable within 60 min	0	0.0	0	0.0
Neither reachable within 60 min	0	0.0	0	0.0
Likely irrelevant (<10 min)	267	95.7	664487	98.2

Population and Area Coverage in Kreis Düren (Stroke Units) (Nordrhein-Westfalen) — Regular (+10min Penalty)

Category	Grid Cells	Area (%)	Population	Population (%)
High (30+ min)	2	0.2	0	0.0
Medium (20-30 min)	23	1.9	100	0.0
Low (10-20 min)	314	26.2	58448	22.8
Only CT reachable within 60 min	0	0.0	0	0.0
Neither reachable within 60 min	0	0.0	0	0.0
Likely irrelevant (<10 min)	861	71.8	197711	77.2

Population and Area Coverage in Kreis Soest (Stroke Units) (Nordrhein-Westfalen) — Regular (+10min Penalty)

Category	Grid Cells	Area (%)	Population	Population (%)
High (30+ min)	0	0.0	0	0.0
Medium (20-30 min)	40	2.3	149	0.1
Low (10-20 min)	424	24.6	45245	15.3
Only CT reachable within 60 min	0	0.0	0	0.0
Neither reachable within 60 min	0	0.0	0	0.0
Likely irrelevant (<10 min)	1260	73.1	249751	84.6

Population and Area Coverage in Kreisfreie Stadt Darmstadt (Stroke Units) (Hessen) — Regular (+10min Penalty)

Category	Grid Cells	Area (%)	Population	Population (%)
High (30+ min)	0	0.0	0	0.0
Medium (20-30 min)	0	0.0	0	0.0
Low (10-20 min)	0	0.0	0	0.0
Only CT reachable within 60 min	0	0.0	0	0.0
Neither reachable within 60 min	0	0.0	0	0.0
Likely irrelevant (<10 min)	154	100.0	151599	100.0

Population and Area Coverage in Kreisfreie Stadt Offenbach am Main (Stroke Units) (Hessen) — Regular (+10min Penalty)

Category	Grid Cells	Area (%)	Population	Population (%)
High (30+ min)	0	0.0	0	0.0
Medium (20-30 min)	0	0.0	0	0.0
Low (10-20 min)	0	0.0	0	0.0
Only CT reachable within 60 min	0	0.0	0	0.0
Neither reachable within 60 min	0	0.0	0	0.0
Likely irrelevant (<10 min)	56	100.0	123120	100.0

Population and Area Coverage in Landkreis Werra-Meißner-Kreis (Stroke Units) (Hessen) — Regular (+10min Penalty)

Category	Grid Cells	Area (%)	Population	Population (%)
High (30+ min)	219	16.6	16925	16.8
Medium (20-30 min)	615	46.7	60018	59.5
Low (10-20 min)	284	21.5	14496	14.4
Only CT reachable within 60 min	0	0.0	0	0.0
Neither reachable within 60 min	0	0.0	0	0.0
Likely irrelevant (<10 min)	200	15.2	9409	9.3

Population and Area Coverage in Landkreis Mayen-Koblenz (Stroke Units) (Rheinland-Pfalz) — Regular (+10min Penalty)

Category	Grid Cells	Area (%)	Population	Population (%)
High (30+ min)	0	0.0	0	0.0
Medium (20-30 min)	141	13.6	20001	9.2
Low (10-20 min)	394	38.1	51425	23.7
Only CT reachable within 60 min	0	0.0	0	0.0
Neither reachable within 60 min	0	0.0	0	0.0
Likely irrelevant (<10 min)	499	48.3	145614	67.1

Population and Area Coverage in Landkreis Bad Dürkheim (Stroke Units) (Rheinland-Pfalz) — Regular (+10min Penalty)

Category	Grid Cells	Area (%)	Population	Population (%)
High (30+ min)	0	0.0	0	0.0
Medium (20-30 min)	83	11.4	7670	5.7
Low (10-20 min)	383	52.4	95564	71.1
Only CT reachable within 60 min	0	0.0	0	0.0
Neither reachable within 60 min	0	0.0	0	0.0
Likely irrelevant (<10 min)	265	36.3	31204	23.2

Population and Area Coverage in Landkreis Landsberg am Lech (Stroke Units) (Bayern) — Regular (+10min Penalty)

Category	Grid Cells	Area (%)	Population	Population (%)
High (30+ min)	0	0.0	0	0.0
Medium (20-30 min)	294	30.6	36261	30.0
Low (10-20 min)	472	49.1	67736	56.0
Only CT reachable within 60 min	0	0.0	0	0.0
Neither reachable within 60 min	0	0.0	0	0.0
Likely irrelevant (<10 min)	196	20.4	16933	14.0

Population and Area Coverage in Landkreis Donau-Ries (Stroke Units) (Bayern) — Regular (+10min Penalty)

Category	Grid Cells	Area (%)	Population	Population (%)
High (30+ min)	331	21.1	20649	15.5
Medium (20-30 min)	878	56.1	84506	63.4
Low (10-20 min)	286	18.3	25605	19.2
Only CT reachable within 60 min	0	0.0	0	0.0
Neither reachable within 60 min	0	0.0	0	0.0
Likely irrelevant (<10 min)	71	4.5	2564	1.9

Population and Area Coverage in Landkreis Märkisch-Oderland (Stroke Units) (Brandenburg) — Regular (+10min Penalty)

Category	Grid Cells	Area (%)	Population	Population (%)
High (30+ min)	736	25.8	18637	10.1
Medium (20-30 min)	709	24.8	38410	20.9
Low (10-20 min)	589	20.6	36955	20.1
Only CT reachable within 60 min	66	2.3	827	0.4
Neither reachable within 60 min	76	2.7	610	0.3
Likely irrelevant (<10 min)	679	23.8	88666	48.2

Population and Area Coverage in Landkreis Prignitz (Stroke Units) (Brandenburg) — Regular (+10min Penalty)

Category	Grid Cells	Area (%)	Population	Population (%)
High (30+ min)	641	22.3	20754	27.9
Medium (20-30 min)	443	15.4	6224	8.4
Low (10-20 min)	180	6.3	1712	2.3
Only CT reachable within 60 min	1519	52.9	43592	58.6
Neither reachable within 60 min	0	0.0	0	0.0
Likely irrelevant (<10 min)	88	3.1	2086	2.8

Population and Area Coverage in Landkreis Burgenlandkreis (Stroke Units) (Sachsen-Anhalt) — Regular (+10min Penalty)

Category	Grid Cells	Area (%)	Population	Population (%)
High (30+ min)	9	0.5	1171	0.7
Medium (20-30 min)	232	12.8	36155	21.9
Low (10-20 min)	645	35.5	59460	36.0
Only CT reachable within 60 min	0	0.0	0	0.0
Neither reachable within 60 min	0	0.0	0	0.0
Likely irrelevant (<10 min)	933	51.3	68334	41.4

Population and Area Coverage in Landkreis Stendal (Stroke Units) (Sachsen-Anhalt) — Regular (+10min Penalty)

Category	Grid Cells	Area (%)	Population	Population (%)
High (30+ min)	592	18.3	50335	48.6
Medium (20-30 min)	222	6.9	2742	2.6
Low (10-20 min)	194	6.0	7056	6.8
Only CT reachable within 60 min	2222	68.7	43347	41.9
Neither reachable within 60 min	0	0.0	0	0.0
Likely irrelevant (<10 min)	4	0.1	27	0.0

Population and Area Coverage in Landkreis Weimarer Land (Stroke Units) (Thüringen) — Regular (+10min Penalty)

Category	Grid Cells	Area (%)	Population	Population (%)
High (30+ min)	0	0.0	0	0.0
Medium (20-30 min)	157	15.3	15529	21.0
Low (10-20 min)	217	21.1	17530	23.8
Only CT reachable within 60 min	0	0.0	0	0.0
Neither reachable within 60 min	0	0.0	0	0.0
Likely irrelevant (<10 min)	653	63.6	40716	55.2

Population and Area Coverage in Kreisfreie Stadt Kiel (Stroke Units) (Schleswig-Holstein) — Regular (+10min Penalty)

Category	Grid Cells	Area (%)	Population	Population (%)
High (30+ min)	1	0.6	221	0.1
Medium (20-30 min)	0	0.0	0	0.0
Low (10-20 min)	1	0.6	346	0.1
Only CT reachable within 60 min	0	0.0	0	0.0
Neither reachable within 60 min	0	0.0	1672	0.7
Likely irrelevant (<10 min)	153	98.7	254080	99.1

Population and Area Coverage in Landkreis Gifhorn (Stroke Units) (Niedersachsen) — Regular (+10min Penalty)

Category	Grid Cells	Area (%)	Population	Population (%)
High (30+ min)	250	12.0	15158	8.8
Medium (20-30 min)	475	22.7	41505	24.1
Low (10-20 min)	775	37.1	73463	42.7
Only CT reachable within 60 min	0	0.0	0	0.0
Neither reachable within 60 min	0	0.0	0	0.0
Likely irrelevant (<10 min)	590	28.2	42076	24.4

Population and Area Coverage in Landkreis Helmstedt (Stroke Units) (Niedersachsen) — Regular (+10min Penalty)

Category	Grid Cells	Area (%)	Population	Population (%)
High (30+ min)	1	0.1	2455	2.7
Medium (20-30 min)	233	26.0	18049	20.2
Low (10-20 min)	311	34.7	36903	41.3
Only CT reachable within 60 min	0	0.0	0	0.0
Neither reachable within 60 min	0	0.0	0	0.0
Likely irrelevant (<10 min)	350	39.1	32006	35.8

Population and Area Coverage in Landkreis Lüchow-Dannenberg (Stroke Units) (Niedersachsen) — Regular (+10min Penalty)

Category	Grid Cells	Area (%)	Population	Population (%)
High (30+ min)	509	31.0	29186	59.8
Medium (20-30 min)	317	19.3	6748	13.8
Low (10-20 min)	210	12.8	4514	9.2
Only CT reachable within 60 min	576	35.1	8175	16.7
Neither reachable within 60 min	0	0.0	0	0.0
Likely irrelevant (<10 min)	29	1.8	218	0.4

Population and Area Coverage in Landkreis Rotenburg (Wümme) (Stroke Units) (Niedersachsen) — Regular (+10min Penalty)

Category	Grid Cells	Area (%)	Population	Population (%)
High (30+ min)	175	6.3	10841	6.6
Medium (20-30 min)	605	21.7	29755	18.1
Low (10-20 min)	531	19.0	28455	17.4
Only CT reachable within 60 min	0	0.0	0	0.0
Neither reachable within 60 min	0	0.0	0	0.0
Likely irrelevant (<10 min)	1483	53.1	94923	57.9

Population and Area Coverage in Kreis Euskirchen (Stroke Units) (Nordrhein-Westfalen) — Regular (+10min Penalty)

Category	Grid Cells	Area (%)	Population	Population (%)
High (30+ min)	192	12.1	15092	8.0
Medium (20-30 min)	289	18.3	16961	9.0
Low (10-20 min)	450	28.4	45136	24.0
Only CT reachable within 60 min	22	1.4	44	0.0
Neither reachable within 60 min	0	0.0	2	0.0
Likely irrelevant (<10 min)	630	39.8	111002	59.0

Population and Area Coverage in Kreis Olpe (Stroke Units) (Nordrhein-Westfalen) — Regular (+10min Penalty)

Category	Grid Cells	Area (%)	Population	Population (%)
High (30+ min)	258	28.2	48349	35.7
Medium (20-30 min)	246	26.9	26215	19.4
Low (10-20 min)	275	30.1	37794	27.9
Only CT reachable within 60 min	0	0.0	0	0.0
Neither reachable within 60 min	0	0.0	0	0.0
Likely irrelevant (<10 min)	136	14.9	23060	17.0

Population and Area Coverage in Landkreis Lahn-Dill-Kreis (Stroke Units) (Hessen) — Regular (+10min Penalty)

Category	Grid Cells	Area (%)	Population	Population (%)
High (30+ min)	36	2.6	5119	2.0
Medium (20-30 min)	331	24.3	49667	19.4
Low (10-20 min)	424	31.1	65656	25.7
Only CT reachable within 60 min	0	0.0	0	0.0
Neither reachable within 60 min	0	0.0	0	0.0
Likely irrelevant (<10 min)	573	42.0	135280	52.9

Population and Area Coverage in Landkreis Ravensburg (Stroke Units) (Baden-Württemberg) — Regular (+10min Penalty)

Category	Grid Cells	Area (%)	Population	Population (%)
High (30+ min)	0	0.0	0	0.0
Medium (20-30 min)	124	6.3	14031	5.1
Low (10-20 min)	357	18.2	48818	17.6
Only CT reachable within 60 min	0	0.0	0	0.0
Neither reachable within 60 min	0	0.0	0	0.0
Likely irrelevant (<10 min)	1479	75.5	213919	77.3

Population and Area Coverage in Kreisfreie Stadt Rosenheim (Stroke Units) (Bayern) — Regular (+10min Penalty)

Category	Grid Cells	Area (%)	Population	Population (%)
High (30+ min)	0	0.0	0	0.0
Medium (20-30 min)	0	0.0	0	0.0
Low (10-20 min)	0	0.0	0	0.0
Only CT reachable within 60 min	0	0.0	0	0.0
Neither reachable within 60 min	0	0.0	0	0.0
Likely irrelevant (<10 min)	47	100.0	62621	100.0

Population and Area Coverage in Landkreis Freising (Stroke Units) (Bayern) — Regular (+10min Penalty)

Category	Grid Cells	Area (%)	Population	Population (%)
High (30+ min)	0	0.0	0	0.0
Medium (20-30 min)	31	3.2	2725	1.6
Low (10-20 min)	77	7.9	7735	4.4
Only CT reachable within 60 min	0	0.0	0	0.0
Neither reachable within 60 min	0	0.0	0	0.0
Likely irrelevant (<10 min)	866	88.9	164466	94.0

Population and Area Coverage in Landkreis Garmisch-Partenkirchen (Stroke Units) (Bayern) — Regular (+10min Penalty)

Category	Grid Cells	Area (%)	Population	Population (%)
High (30+ min)	6	0.5	776	0.9
Medium (20-30 min)	117	9.8	16972	19.5
Low (10-20 min)	26	2.2	21652	24.9
Only CT reachable within 60 min	10	0.8	402	0.5
Neither reachable within 60 min	429	36.0	1071	1.2
Likely irrelevant (<10 min)	605	50.7	46044	53.0

Population and Area Coverage in Landkreis Regensburg (Stroke Units) (Bayern) — Regular (+10min Penalty)

Category	Grid Cells	Area (%)	Population	Population (%)
High (30+ min)	0	0.0	0	0.0
Medium (20-30 min)	11	0.6	1022	0.5
Low (10-20 min)	461	26.8	20471	10.6
Only CT reachable within 60 min	0	0.0	0	0.0
Neither reachable within 60 min	0	0.0	0	0.0
Likely irrelevant (<10 min)	1247	72.5	172218	88.9

Population and Area Coverage in Landkreis Tirschenreuth (Stroke Units) (Bayern) — Regular (+10min Penalty)

Category	Grid Cells	Area (%)	Population	Population (%)
High (30+ min)	39	3.0	772	1.0
Medium (20-30 min)	507	39.4	25375	34.1
Low (10-20 min)	626	48.6	41803	56.1
Only CT reachable within 60 min	3	0.2	0	0.0
Neither reachable within 60 min	3	0.2	0	0.0
Likely irrelevant (<10 min)	110	8.5	6501	8.7

Population and Area Coverage in Kreisfreie Stadt Kaufbeuren (Stroke Units) (Bayern) — Regular (+10min Penalty)

Category	Grid Cells	Area (%)	Population	Population (%)
High (30+ min)	0	0.0	0	0.0
Medium (20-30 min)	0	0.0	0	0.0
Low (10-20 min)	0	0.0	0	0.0
Only CT reachable within 60 min	0	0.0	0	0.0
Neither reachable within 60 min	0	0.0	0	0.0
Likely irrelevant (<10 min)	50	100.0	42940	100.0

Population and Area Coverage in Landkreis Oberallgäu (Stroke Units) (Bayern) — Regular (+10min Penalty)

Category	Grid Cells	Area (%)	Population	Population (%)
High (30+ min)	38	2.1	8583	5.5
Medium (20-30 min)	187	10.5	26207	16.9
Low (10-20 min)	254	14.3	41174	26.5
Only CT reachable within 60 min	13	0.7	311	0.2
Neither reachable within 60 min	539	30.3	4154	2.7
Likely irrelevant (<10 min)	745	41.9	75083	48.3

Population and Area Coverage in Landkreis Regionalverband Saarbrücken (Stroke Units) (Saarland) — Regular (+10min Penalty)

Category	Grid Cells	Area (%)	Population	Population (%)
High (30+ min)	0	0.0	0	0.0
Medium (20-30 min)	1	0.2	72	0.0
Low (10-20 min)	13	2.7	4573	1.4
Only CT reachable within 60 min	1	0.2	8	0.0
Neither reachable within 60 min	4	0.8	286	0.1
Likely irrelevant (<10 min)	469	96.1	326951	98.5

Population and Area Coverage in Landkreis Altenburger Land (Stroke Units) (Thüringen) — Regular (+10min Penalty)

Category	Grid Cells	Area (%)	Population	Population (%)
High (30+ min)	0	0.0	0	0.0
Medium (20-30 min)	8	1.1	1125	1.3
Low (10-20 min)	86	11.8	9355	11.1
Only CT reachable within 60 min	0	0.0	0	0.0
Neither reachable within 60 min	0	0.0	0	0.0
Likely irrelevant (<10 min)	632	87.1	73600	87.5

Population and Area Coverage in Landkreis Diepholz (Stroke Units) (Niedersachsen) — Regular (+10min Penalty)

Category	Grid Cells	Area (%)	Population	Population (%)
High (30+ min)	886	33.5	51819	24.5
Medium (20-30 min)	941	35.6	47159	22.3
Low (10-20 min)	493	18.7	62495	29.6
Only CT reachable within 60 min	94	3.6	3198	1.5
Neither reachable within 60 min	0	0.0	0	0.0
Likely irrelevant (<10 min)	227	8.6	46499	22.0

Population and Area Coverage in Kreisfreie Stadt Emden (Stroke Units) (Niedersachsen) — Regular (+10min Penalty)

Category	Grid Cells	Area (%)	Population	Population (%)
High (30+ min)	0	0.0	0	0.0
Medium (20-30 min)	0	0.0	0	0.0
Low (10-20 min)	1	0.8	922	1.9
Only CT reachable within 60 min	0	0.0	0	0.0
Neither reachable within 60 min	0	0.0	0	0.0
Likely irrelevant (<10 min)	131	99.2	48199	98.1

Population and Area Coverage in Kreisfreie Stadt Gelsenkirchen (Stroke Units) (Nordrhein-Westfalen) — Regular (+10min Penalty)

Category	Grid Cells	Area (%)	Population	Population (%)
High (30+ min)	0	0.0	0	0.0
Medium (20-30 min)	0	0.0	0	0.0
Low (10-20 min)	0	0.0	0	0.0
Only CT reachable within 60 min	0	0.0	0	0.0
Neither reachable within 60 min	0	0.0	0	0.0
Likely irrelevant (<10 min)	134	100.0	279785	100.0

Population and Area Coverage in Kreis Warendorf (Stroke Units) (Nordrhein-Westfalen) — Regular (+10min Penalty)

Category	Grid Cells	Area (%)	Population	Population (%)
High (30+ min)	46	2.7	1532	0.6
Medium (20-30 min)	301	17.5	51210	18.8
Low (10-20 min)	611	35.6	92752	34.1
Only CT reachable within 60 min	0	0.0	0	0.0
Neither reachable within 60 min	0	0.0	0	0.0
Likely irrelevant (<10 min)	760	44.2	126576	46.5

Population and Area Coverage in Stadtkreis Ulm (Stroke Units) (Baden-Württemberg) — Regular (+10min Penalty)

Category	Grid Cells	Area (%)	Population	Population (%)
High (30+ min)	0	0.0	0	0.0
Medium (20-30 min)	0	0.0	0	0.0
Low (10-20 min)	1	0.7	1113	0.9
Only CT reachable within 60 min	0	0.0	0	0.0
Neither reachable within 60 min	0	0.0	0	0.0
Likely irrelevant (<10 min)	142	99.3	119001	99.1

Population and Area Coverage in Landkreis Traunstein (Stroke Units) (Bayern) — Regular (+10min Penalty)

Category	Grid Cells	Area (%)	Population	Population (%)
High (30+ min)	0	0.0	0	0.0
Medium (20-30 min)	19	1.0	1343	0.8
Low (10-20 min)	275	15.1	30173	17.2
Only CT reachable within 60 min	1	0.1	4	0.0
Neither reachable within 60 min	131	7.2	1464	0.8
Likely irrelevant (<10 min)	1391	76.6	142631	81.2

Population and Area Coverage in Kreisfreie Stadt Regensburg (Stroke Units) (Bayern) — Regular (+10min Penalty)

Category	Grid Cells	Area (%)	Population	Population (%)
High (30+ min)	0	0.0	0	0.0
Medium (20-30 min)	0	0.0	0	0.0
Low (10-20 min)	0	0.0	1	0.0
Only CT reachable within 60 min	0	0.0	0	0.0
Neither reachable within 60 min	0	0.0	0	0.0
Likely irrelevant (<10 min)	97	100.0	170061	100.0

Population and Area Coverage in Kreisfreie Stadt Weiden i.d.OPf. (Stroke Units) (Bayern) — Regular (+10min Penalty)

Category	Grid Cells	Area (%)	Population	Population (%)
High (30+ min)	0	0.0	0	0.0
Medium (20-30 min)	0	0.0	0	0.0
Low (10-20 min)	0	0.0	0	0.0
Only CT reachable within 60 min	0	0.0	0	0.0
Neither reachable within 60 min	0	0.0	0	0.0
Likely irrelevant (<10 min)	86	100.0	43316	100.0

Population and Area Coverage in Kreisfreie Stadt Ansbach (Stroke Units) (Bayern) — Regular (+10min Penalty)

Category	Grid Cells	Area (%)	Population	Population (%)
High (30+ min)	0	0.0	0	0.0
Medium (20-30 min)	0	0.0	0	0.0
Low (10-20 min)	0	0.0	0	0.0
Only CT reachable within 60 min	0	0.0	0	0.0
Neither reachable within 60 min	0	0.0	0	0.0
Likely irrelevant (<10 min)	123	100.0	41201	100.0

Population and Area Coverage in Landkreis Rhön-Grabfeld (Stroke Units) (Bayern) — Regular (+10min Penalty)

Category	Grid Cells	Area (%)	Population	Population (%)
High (30+ min)	0	0.0	0	0.0
Medium (20-30 min)	1	0.1	13	0.0
Low (10-20 min)	42	3.3	1909	2.3
Only CT reachable within 60 min	0	0.0	0	0.0
Neither reachable within 60 min	0	0.0	0	0.0
Likely irrelevant (<10 min)	1245	96.7	80256	97.7

Population and Area Coverage in Landkreis Ostprignitz-Ruppin (Stroke Units) (Brandenburg) — Regular (+10min Penalty)

Category	Grid Cells	Area (%)	Population	Population (%)
High (30+ min)	350	10.3	9952	10.5
Medium (20-30 min)	578	17.1	19485	20.6
Low (10-20 min)	765	22.6	14942	15.8
Only CT reachable within 60 min	135	4.0	1246	1.3
Neither reachable within 60 min	0	0.0	0	0.0
Likely irrelevant (<10 min)	1555	46.0	49085	51.8

Population and Area Coverage in Kreis Plön (Stroke Units) (Schleswig-Holstein) — Regular (+10min Penalty)

Category	Grid Cells	Area (%)	Population	Population (%)
High (30+ min)	8	0.5	92	0.1
Medium (20-30 min)	163	11.0	13012	10.3
Low (10-20 min)	526	35.3	33778	26.7
Only CT reachable within 60 min	2	0.1	179	0.1
Neither reachable within 60 min	17	1.1	1426	1.1
Likely irrelevant (<10 min)	772	51.9	77866	61.6

Population and Area Coverage in Landkreis Nienburg (Weser) (Stroke Units) (Niedersachsen) — Regular (+10min Penalty)

Category	Grid Cells	Area (%)	Population	Population (%)
High (30+ min)	594	31.8	61563	50.7
Medium (20-30 min)	494	26.5	24806	20.4
Low (10-20 min)	283	15.2	16737	13.8
Only CT reachable within 60 min	293	15.7	7306	6.0
Neither reachable within 60 min	0	0.0	0	0.0
Likely irrelevant (<10 min)	201	10.8	11007	9.1

Population and Area Coverage in Landkreis Emsland (Stroke Units) (Niedersachsen) — Regular (+10min Penalty)

Category	Grid Cells	Area (%)	Population	Population (%)
High (30+ min)	205	5.3	37519	11.6
Medium (20-30 min)	615	16.0	37569	11.7
Low (10-20 min)	1176	30.6	77498	24.0
Only CT reachable within 60 min	7	0.2	111	0.0
Neither reachable within 60 min	20	0.5	353	0.1
Likely irrelevant (<10 min)	1816	47.3	169194	52.5

Population and Area Coverage in Landkreis Wittmund (Stroke Units) (Niedersachsen) — Regular (+10min Penalty)

Category	Grid Cells	Area (%)	Population	Population (%)
High (30+ min)	2	0.2	169	0.3
Medium (20-30 min)	296	33.3	18247	32.1
Low (10-20 min)	341	38.4	26323	46.3
Only CT reachable within 60 min	2	0.2	16	0.0
Neither reachable within 60 min	50	5.6	2637	4.6
Likely irrelevant (<10 min)	197	22.2	9480	16.7

Population and Area Coverage in Kreis Hochsauerlandkreis (Stroke Units) (Nordrhein-Westfalen) — Regular (+10min Penalty)

Category	Grid Cells	Area (%)	Population	Population (%)
High (30+ min)	721	28.7	62378	23.8
Medium (20-30 min)	744	29.6	49337	18.8
Low (10-20 min)	474	18.9	53466	20.4
Only CT reachable within 60 min	169	6.7	5486	2.1
Neither reachable within 60 min	0	0.0	0	0.0
Likely irrelevant (<10 min)	406	16.1	91702	35.0

Population and Area Coverage in Landkreis Rheingau-Taunus-Kreis (Stroke Units) (Hessen) — Regular (+10min Penalty)

Category	Grid Cells	Area (%)	Population	Population (%)
High (30+ min)	87	8.5	3187	1.7
Medium (20-30 min)	259	25.4	23928	13.1
Low (10-20 min)	479	47.0	89805	49.0
Only CT reachable within 60 min	0	0.0	0	0.0
Neither reachable within 60 min	0	0.0	0	0.0
Likely irrelevant (<10 min)	194	19.0	66335	36.2

Population and Area Coverage in Landkreis Göppingen (Stroke Units) (Baden-Württemberg) — Regular (+10min Penalty)

Category	Grid Cells	Area (%)	Population	Population (%)
High (30+ min)	0	0.0	0	0.0
Medium (20-30 min)	0	0.0	0	0.0
Low (10-20 min)	7	0.9	164	0.1
Only CT reachable within 60 min	0	0.0	0	0.0
Neither reachable within 60 min	0	0.0	0	0.0
Likely irrelevant (<10 min)	774	99.1	249832	99.9

Population and Area Coverage in Stadtkreis Freiburg im Breisgau (Stroke Units) (Baden-Württemberg) — Regular (+10min Penalty)

Category	Grid Cells	Area (%)	Population	Population (%)
High (30+ min)	0	0.0	0	0.0
Medium (20-30 min)	2	1.1	235	0.1
Low (10-20 min)	35	18.7	4049	1.6
Only CT reachable within 60 min	0	0.0	0	0.0
Neither reachable within 60 min	0	0.0	0	0.0
Likely irrelevant (<10 min)	150	80.2	254155	98.3

Population and Area Coverage in Landkreis Landshut (Stroke Units) (Bayern) — Regular (+10min Penalty)

Category	Grid Cells	Area (%)	Population	Population (%)
High (30+ min)	0	0.0	0	0.0
Medium (20-30 min)	17	1.0	505	0.3
Low (10-20 min)	245	14.9	19375	12.4
Only CT reachable within 60 min	0	0.0	0	0.0
Neither reachable within 60 min	0	0.0	0	0.0
Likely irrelevant (<10 min)	1379	84.0	135810	87.2

Population and Area Coverage in Landkreis Regen (Stroke Units) (Bayern) — Regular (+10min Penalty)

Category	Grid Cells	Area (%)	Population	Population (%)
High (30+ min)	0	0.0	0	0.0
Medium (20-30 min)	130	11.4	10154	13.1
Low (10-20 min)	160	14.0	10984	14.2
Only CT reachable within 60 min	2	0.2	0	0.0
Neither reachable within 60 min	22	1.9	23	0.0
Likely irrelevant (<10 min)	826	72.5	56366	72.7

Population and Area Coverage in Kreisfreie Stadt Coburg (Stroke Units) (Bayern) — Regular (+10min Penalty)

Category	Grid Cells	Area (%)	Population	Population (%)
High (30+ min)	0	0.0	0	0.0
Medium (20-30 min)	0	0.0	0	0.0
Low (10-20 min)	0	0.0	0	0.0
Only CT reachable within 60 min	0	0.0	0	0.0
Neither reachable within 60 min	0	0.0	0	0.0
Likely irrelevant (<10 min)	57	100.0	41384	100.0

Population and Area Coverage in Landkreis Neunkirchen (Stroke Units) (Saarland) — Regular (+10min Penalty)

Category	Grid Cells	Area (%)	Population	Population (%)
High (30+ min)	0	0.0	0	0.0
Medium (20-30 min)	0	0.0	0	0.0
Low (10-20 min)	15	4.8	5397	4.2
Only CT reachable within 60 min	0	0.0	0	0.0
Neither reachable within 60 min	0	0.0	0	0.0
Likely irrelevant (<10 min)	297	95.2	121946	95.8

Population and Area Coverage in Landkreis Nordwestmecklenburg (Stroke Units) (Mecklenburg-Vorpommern) — Regular (+10min Penalty)

Category	Grid Cells	Area (%)	Population	Population (%)
High (30+ min)	11	0.4	139	0.1
Medium (20-30 min)	354	12.2	7626	5.3
Low (10-20 min)	773	26.6	32549	22.6
Only CT reachable within 60 min	16	0.6	504	0.4
Neither reachable within 60 min	55	1.9	2522	1.8
Likely irrelevant (<10 min)	1695	58.4	100656	69.9

Population and Area Coverage in Kreisfreie Stadt Herne (Stroke Units) (Nordrhein-Westfalen) — Regular (+10min Penalty)

Category	Grid Cells	Area (%)	Population	Population (%)
High (30+ min)	0	0.0	0	0.0
Medium (20-30 min)	0	0.0	0	0.0
Low (10-20 min)	0	0.0	0	0.0
Only CT reachable within 60 min	0	0.0	0	0.0
Neither reachable within 60 min	0	0.0	0	0.0
Likely irrelevant (<10 min)	69	100.0	165244	100.0

Population and Area Coverage in Landkreis Altenkirchen (Westerwald) (Stroke Units) (Rheinland-Pfalz) — Regular (+10min Penalty)

Category	Grid Cells	Area (%)	Population	Population (%)
High (30+ min)	25	3.1	2829	2.1
Medium (20-30 min)	320	39.3	57265	42.9
Low (10-20 min)	221	27.1	38386	28.8
Only CT reachable within 60 min	0	0.0	0	0.0
Neither reachable within 60 min	0	0.0	0	0.0
Likely irrelevant (<10 min)	249	30.6	34929	26.2

Population and Area Coverage in Kreisfreie Stadt Kaiserslautern (Stroke Units) (Rheinland-Pfalz) — Regular (+10min Penalty)

Category	Grid Cells	Area (%)	Population	Population (%)
High (30+ min)	0	0.0	0	0.0
Medium (20-30 min)	0	0.0	0	0.0
Low (10-20 min)	0	0.0	0	0.0
Only CT reachable within 60 min	0	0.0	0	0.0
Neither reachable within 60 min	0	0.0	0	0.0
Likely irrelevant (<10 min)	174	100.0	110813	100.0

Population and Area Coverage in Landkreis Mainz-Bingen (Stroke Units) (Rheinland-Pfalz) — Regular (+10min Penalty)

Category	Grid Cells	Area (%)	Population	Population (%)
High (30+ min)	11	1.4	881	0.4
Medium (20-30 min)	40	5.3	5303	2.5
Low (10-20 min)	143	18.8	23755	11.3
Only CT reachable within 60 min	0	0.0	0	0.0
Neither reachable within 60 min	0	0.0	0	0.0
Likely irrelevant (<10 min)	567	74.5	180280	85.8

Population and Area Coverage in Landkreis Heidenheim (Stroke Units) (Baden-Württemberg) — Regular (+10min Penalty)

Category	Grid Cells	Area (%)	Population	Population (%)
High (30+ min)	5	0.7	258	0.2
Medium (20-30 min)	66	8.7	2406	1.9
Low (10-20 min)	126	16.6	9098	7.1
Only CT reachable within 60 min	0	0.0	0	0.0
Neither reachable within 60 min	0	0.0	0	0.0
Likely irrelevant (<10 min)	562	74.0	116227	90.8

Population and Area Coverage in Stadtkreis Pforzheim (Stroke Units) (Baden-Württemberg) — Regular (+10min Penalty)

Category	Grid Cells	Area (%)	Population	Population (%)
High (30+ min)	0	0.0	0	0.0
Medium (20-30 min)	0	0.0	0	0.0
Low (10-20 min)	1	0.8	5	0.0
Only CT reachable within 60 min	0	0.0	0	0.0
Neither reachable within 60 min	0	0.0	0	0.0
Likely irrelevant (<10 min)	119	99.2	124957	100.0

Population and Area Coverage in Landkreis Tübingen (Stroke Units) (Baden-Württemberg) — Regular (+10min Penalty)

Category	Grid Cells	Area (%)	Population	Population (%)
High (30+ min)	0	0.0	0	0.0
Medium (20-30 min)	9	1.4	2600	1.2
Low (10-20 min)	118	18.8	26925	12.3
Only CT reachable within 60 min	0	0.0	0	0.0
Neither reachable within 60 min	0	0.0	0	0.0
Likely irrelevant (<10 min)	501	79.8	189013	86.5

Population and Area Coverage in Landkreis Bodenseekreis (Stroke Units) (Baden-Württemberg) — Regular (+10min Penalty)

Category	Grid Cells	Area (%)	Population	Population (%)
High (30+ min)	1	0.1	5	0.0
Medium (20-30 min)	64	8.0	5072	2.3
Low (10-20 min)	347	43.3	92315	41.8
Only CT reachable within 60 min	0	0.0	635	0.3
Neither reachable within 60 min	0	0.0	1958	0.9
Likely irrelevant (<10 min)	390	48.6	121089	54.8

Population and Area Coverage in Landkreis Miesbach (Stroke Units) (Bayern) — Regular (+10min Penalty)

Category	Grid Cells	Area (%)	Population	Population (%)
High (30+ min)	0	0.0	0	0.0
Medium (20-30 min)	2	0.2	63	0.1
Low (10-20 min)	17	1.6	295	0.3
Only CT reachable within 60 min	2	0.2	1141	1.2
Neither reachable within 60 min	246	23.7	1166	1.2
Likely irrelevant (<10 min)	770	74.3	96068	97.3

Population and Area Coverage in Landkreis Mühldorf a.Inn (Stroke Units) (Bayern) — Regular (+10min Penalty)

Category	Grid Cells	Area (%)	Population	Population (%)
High (30+ min)	0	0.0	0	0.0
Medium (20-30 min)	0	0.0	0	0.0
Low (10-20 min)	265	27.3	35563	32.1
Only CT reachable within 60 min	0	0.0	0	0.0
Neither reachable within 60 min	0	0.0	0	0.0
Likely irrelevant (<10 min)	705	72.7	75073	67.9

Population and Area Coverage in Landkreis Neuburg-Schrobenhausen (Stroke Units) (Bayern) — Regular (+10min Penalty)

Category	Grid Cells	Area (%)	Population	Population (%)
High (30+ min)	13	1.4	1564	1.6
Medium (20-30 min)	393	43.5	22879	24.0
Low (10-20 min)	398	44.0	58487	61.3
Only CT reachable within 60 min	0	0.0	0	0.0
Neither reachable within 60 min	0	0.0	0	0.0
Likely irrelevant (<10 min)	100	11.1	12553	13.1

Population and Area Coverage in Landkreis Starnberg (Stroke Units) (Bayern) — Regular (+10min Penalty)

Category	Grid Cells	Area (%)	Population	Population (%)
High (30+ min)	0	0.0	0	0.0
Medium (20-30 min)	56	9.6	7496	5.6
Low (10-20 min)	345	58.9	68869	51.2
Only CT reachable within 60 min	0	0.0	0	0.0
Neither reachable within 60 min	0	0.0	0	0.0
Likely irrelevant (<10 min)	185	31.6	58172	43.2

Population and Area Coverage in Landkreis Fürth (Stroke Units) (Bayern) — Regular (+10min Penalty)

Category	Grid Cells	Area (%)	Population	Population (%)
High (30+ min)	0	0.0	0	0.0
Medium (20-30 min)	0	0.0	0	0.0
Low (10-20 min)	3	0.8	386	0.3
Only CT reachable within 60 min	0	0.0	0	0.0
Neither reachable within 60 min	0	0.0	0	0.0
Likely irrelevant (<10 min)	379	99.2	118319	99.7

Population and Area Coverage in Landkreis Kitzingen (Stroke Units) (Bayern) — Regular (+10min Penalty)

Category	Grid Cells	Area (%)	Population	Population (%)
High (30+ min)	0	0.0	0	0.0
Medium (20-30 min)	32	3.8	551	0.6
Low (10-20 min)	267	31.6	23379	25.6
Only CT reachable within 60 min	0	0.0	0	0.0
Neither reachable within 60 min	0	0.0	0	0.0
Likely irrelevant (<10 min)	545	64.6	67499	73.8

Population and Area Coverage in Landkreis Günzburg (Stroke Units) (Bayern) — Regular (+10min Penalty)

Category	Grid Cells	Area (%)	Population	Population (%)
High (30+ min)	45	4.9	3205	2.6
Medium (20-30 min)	203	22.0	25996	20.8
Low (10-20 min)	259	28.1	19840	15.9
Only CT reachable within 60 min	0	0.0	0	0.0
Neither reachable within 60 min	0	0.0	0	0.0
Likely irrelevant (<10 min)	416	45.1	76078	60.8

Population and Area Coverage in Landkreis Havelland (Stroke Units) (Brandenburg) — Regular (+10min Penalty)

Category	Grid Cells	Area (%)	Population	Population (%)
High (30+ min)	226	9.9	6415	4.2
Medium (20-30 min)	361	15.8	18919	12.5
Low (10-20 min)	398	17.4	28846	19.1
Only CT reachable within 60 min	898	39.4	40675	26.9
Neither reachable within 60 min	0	0.0	0	0.0
Likely irrelevant (<10 min)	399	17.5	56558	37.4

Population and Area Coverage in Landkreis Teltow-Fläming (Stroke Units) (Brandenburg) — Regular (+10min Penalty)

Category	Grid Cells	Area (%)	Population	Population (%)
High (30+ min)	308	11.1	34357	21.9
Medium (20-30 min)	513	18.6	11934	7.6
Low (10-20 min)	1136	41.1	72232	46.0
Only CT reachable within 60 min	653	23.6	8220	5.2
Neither reachable within 60 min	0	0.0	0	0.0
Likely irrelevant (<10 min)	155	5.6	30327	19.3

Population and Area Coverage in Landkreis Börde (Stroke Units) (Sachsen-Anhalt) — Regular (+10min Penalty)

Category	Grid Cells	Area (%)	Population	Population (%)
High (30+ min)	273	8.7	13107	8.4
Medium (20-30 min)	707	22.6	28745	18.5
Low (10-20 min)	937	30.0	38885	25.0
Only CT reachable within 60 min	0	0.0	0	0.0
Neither reachable within 60 min	0	0.0	0	0.0
Likely irrelevant (<10 min)	1206	38.6	74646	48.0

Population and Area Coverage in Landkreis Stade (Stroke Units) (Niedersachsen) — Regular (+10min Penalty)

Category	Grid Cells	Area (%)	Population	Population (%)
High (30+ min)	20	1.2	608	0.3
Medium (20-30 min)	113	6.9	5762	2.9
Low (10-20 min)	342	20.8	61573	31.1
Only CT reachable within 60 min	10	0.6	135	0.1
Neither reachable within 60 min	100	6.1	4948	2.5
Likely irrelevant (<10 min)	1056	64.4	125140	63.1

Population and Area Coverage in Kreisfreie Stadt Oldenburg (Oldb) (Stroke Units) (Niedersachsen) — Regular (+10min Penalty)

Category	Grid Cells	Area (%)	Population	Population (%)
High (30+ min)	0	0.0	0	0.0
Medium (20-30 min)	0	0.0	0	0.0
Low (10-20 min)	0	0.0	0	0.0
Only CT reachable within 60 min	0	0.0	0	0.0
Neither reachable within 60 min	0	0.0	0	0.0
Likely irrelevant (<10 min)	137	100.0	159948	100.0

Population and Area Coverage in Landkreis Cloppenburg (Stroke Units) (Niedersachsen) — Regular (+10min Penalty)

Category	Grid Cells	Area (%)	Population	Population (%)
High (30+ min)	144	7.6	7287	4.4
Medium (20-30 min)	553	29.1	47586	29.0
Low (10-20 min)	886	46.6	87175	53.1
Only CT reachable within 60 min	0	0.0	0	0.0
Neither reachable within 60 min	0	0.0	0	0.0
Likely irrelevant (<10 min)	319	16.8	22028	13.4

Population and Area Coverage in Kreisfreie Stadt Wuppertal (Stroke Units) (Nordrhein-Westfalen) — Regular (+10min Penalty)

Category	Grid Cells	Area (%)	Population	Population (%)
High (30+ min)	0	0.0	0	0.0
Medium (20-30 min)	0	0.0	0	0.0
Low (10-20 min)	16	7.5	2582	0.7
Only CT reachable within 60 min	0	0.0	0	0.0
Neither reachable within 60 min	0	0.0	0	0.0
Likely irrelevant (<10 min)	197	92.5	356491	99.3

Population and Area Coverage in Landkreis Odenwaldkreis (Stroke Units) (Hessen) — Regular (+10min Penalty)

Category	Grid Cells	Area (%)	Population	Population (%)
High (30+ min)	0	0.0	0	0.0
Medium (20-30 min)	11	1.4	951	1.0
Low (10-20 min)	226	29.1	32811	33.5
Only CT reachable within 60 min	0	0.0	0	0.0
Neither reachable within 60 min	0	0.0	0	0.0
Likely irrelevant (<10 min)	539	69.5	64128	65.5

Population and Area Coverage in Landkreis Karlsruhe (Stroke Units) (Baden-Württemberg) — Regular (+10min Penalty)

Category	Grid Cells	Area (%)	Population	Population (%)
High (30+ min)	0	0.0	0	0.0
Medium (20-30 min)	16	1.2	1166	0.3
Low (10-20 min)	219	16.4	62884	14.4
Only CT reachable within 60 min	0	0.0	0	0.0
Neither reachable within 60 min	0	0.0	0	0.0
Likely irrelevant (<10 min)	1100	82.4	371785	85.3

Population and Area Coverage in Kreisfreie Stadt Landshut (Stroke Units) (Bayern) — Regular (+10min Penalty)

Category	Grid Cells	Area (%)	Population	Population (%)
High (30+ min)	0	0.0	0	0.0
Medium (20-30 min)	0	0.0	0	0.0
Low (10-20 min)	1	1.2	1	0.0
Only CT reachable within 60 min	0	0.0	0	0.0
Neither reachable within 60 min	0	0.0	0	0.0
Likely irrelevant (<10 min)	80	98.8	69057	100.0

Population and Area Coverage in Kreisfreie Stadt Erlangen (Stroke Units) (Bayern) — Regular (+10min Penalty)

Category	Grid Cells	Area (%)	Population	Population (%)
High (30+ min)	0	0.0	0	0.0
Medium (20-30 min)	0	0.0	0	0.0
Low (10-20 min)	0	0.0	0	0.0
Only CT reachable within 60 min	0	0.0	0	0.0
Neither reachable within 60 min	0	0.0	0	0.0
Likely irrelevant (<10 min)	96	100.0	126629	100.0

Population and Area Coverage in Landkreis Haßberge (Stroke Units) (Bayern) — Regular (+10min Penalty)

Category	Grid Cells	Area (%)	Population	Population (%)
High (30+ min)	0	0.0	0	0.0
Medium (20-30 min)	387	32.2	17084	19.6
Low (10-20 min)	703	58.6	63751	73.1
Only CT reachable within 60 min	0	0.0	0	0.0
Neither reachable within 60 min	0	0.0	0	0.0
Likely irrelevant (<10 min)	110	9.2	6331	7.3

Population and Area Coverage in Landkreis Aichach-Friedberg (Stroke Units) (Bayern) — Regular (+10min Penalty)

Category	Grid Cells	Area (%)	Population	Population (%)
High (30+ min)	0	0.0	0	0.0
Medium (20-30 min)	103	10.9	8269	6.2
Low (10-20 min)	491	52.0	76646	57.5
Only CT reachable within 60 min	0	0.0	0	0.0
Neither reachable within 60 min	0	0.0	0	0.0
Likely irrelevant (<10 min)	351	37.1	48496	36.4

Population and Area Coverage in Landkreis Dillingen a.d.Donau (Stroke Units) (Bayern) — Regular (+10min Penalty)

Category	Grid Cells	Area (%)	Population	Population (%)
High (30+ min)	65	6.7	3275	3.4
Medium (20-30 min)	282	29.1	22611	23.3
Low (10-20 min)	408	42.1	50804	52.3
Only CT reachable within 60 min	0	0.0	0	0.0
Neither reachable within 60 min	0	0.0	0	0.0
Likely irrelevant (<10 min)	215	22.2	20408	21.0

Population and Area Coverage in Landkreis Oberspreewald-Lausitz (Stroke Units) (Brandenburg) — Regular (+10min Penalty)

Category	Grid Cells	Area (%)	Population	Population (%)
High (30+ min)	1	0.1	0	0.0
Medium (20-30 min)	34	2.2	1501	1.4
Low (10-20 min)	278	17.6	20282	18.7
Only CT reachable within 60 min	0	0.0	0	0.0
Neither reachable within 60 min	0	0.0	0	0.0
Likely irrelevant (<10 min)	1267	80.2	86675	79.9

Population and Area Coverage in Landkreis Bautzen (Stroke Units) (Sachsen) — Regular (+10min Penalty)

Category	Grid Cells	Area (%)	Population	Population (%)
High (30+ min)	412	13.4	48854	17.0
Medium (20-30 min)	953	30.9	111363	38.7
Low (10-20 min)	734	23.8	55558	19.3
Only CT reachable within 60 min	7	0.2	735	0.3
Neither reachable within 60 min	6	0.2	89	0.0
Likely irrelevant (<10 min)	970	31.5	71424	24.8

Population and Area Coverage in Kreisfreie Stadt Dessau-Roßlau (Stroke Units) (Sachsen-Anhalt) — Regular (+10min Penalty)

Category	Grid Cells	Area (%)	Population	Population (%)
High (30+ min)	159	49.7	61147	72.7
Medium (20-30 min)	96	30.0	21211	25.2
Low (10-20 min)	21	6.6	1101	1.3
Only CT reachable within 60 min	44	13.8	653	0.8
Neither reachable within 60 min	0	0.0	0	0.0
Likely irrelevant (<10 min)	0	0.0	1	0.0

Population and Area Coverage in Landkreis Lüneburg (Stroke Units) (Niedersachsen) — Regular (+10min Penalty)

Category	Grid Cells	Area (%)	Population	Population (%)
High (30+ min)	45	2.5	583	0.3
Medium (20-30 min)	153	8.6	4421	2.5
Low (10-20 min)	346	19.4	16619	9.3
Only CT reachable within 60 min	246	13.8	3805	2.1
Neither reachable within 60 min	0	0.0	0	0.0
Likely irrelevant (<10 min)	990	55.6	152840	85.7

Population and Area Coverage in Kreisfreie Stadt Bremerhaven (Stroke Units) (Bremen) — Regular (+10min Penalty)

Category	Grid Cells	Area (%)	Population	Population (%)
High (30+ min)	2	2.0	73	0.1
Medium (20-30 min)	0	0.0	0	0.0
Low (10-20 min)	3	3.1	579	0.5
Only CT reachable within 60 min	10	10.2	278	0.2
Neither reachable within 60 min	4	4.1	10	0.0
Likely irrelevant (<10 min)	79	80.6	113476	99.2

Population and Area Coverage in Kreisfreie Stadt Koblenz (Stroke Units) (Rheinland-Pfalz) — Regular (+10min Penalty)

Category	Grid Cells	Area (%)	Population	Population (%)
High (30+ min)	0	0.0	0	0.0
Medium (20-30 min)	0	0.0	0	0.0
Low (10-20 min)	0	0.0	0	0.0
Only CT reachable within 60 min	0	0.0	0	0.0
Neither reachable within 60 min	0	0.0	0	0.0
Likely irrelevant (<10 min)	136	100.0	125857	100.0

Population and Area Coverage in Kreisfreie Stadt Pirmasens (Stroke Units) (Rheinland-Pfalz) — Regular (+10min Penalty)

Category	Grid Cells	Area (%)	Population	Population (%)
High (30+ min)	13	17.1	332	0.9
Medium (20-30 min)	57	75.0	35003	93.9
Low (10-20 min)	6	7.9	1926	5.2
Only CT reachable within 60 min	0	0.0	0	0.0
Neither reachable within 60 min	0	0.0	0	0.0
Likely irrelevant (<10 min)	0	0.0	1	0.0

Population and Area Coverage in Landkreis Donnersbergkreis (Stroke Units) (Rheinland-Pfalz) — Regular (+10min Penalty)

Category	Grid Cells	Area (%)	Population	Population (%)
High (30+ min)	4	0.5	0	0.0
Medium (20-30 min)	269	33.3	15169	19.6
Low (10-20 min)	395	48.9	52261	67.4
Only CT reachable within 60 min	0	0.0	0	0.0
Neither reachable within 60 min	0	0.0	0	0.0
Likely irrelevant (<10 min)	139	17.2	10087	13.0

Population and Area Coverage in Stadtkreis Heidelberg (Stroke Units) (Baden-Württemberg) — Regular (+10min Penalty)

Category	Grid Cells	Area (%)	Population	Population (%)
High (30+ min)	0	0.0	0	0.0
Medium (20-30 min)	0	0.0	0	0.0
Low (10-20 min)	27	20.1	4026	2.6
Only CT reachable within 60 min	0	0.0	0	0.0
Neither reachable within 60 min	0	0.0	0	0.0
Likely irrelevant (<10 min)	107	79.9	150340	97.4

Population and Area Coverage in Kreisfreie Stadt Straubing (Stroke Units) (Bayern) — Regular (+10min Penalty)

Category	Grid Cells	Area (%)	Population	Population (%)
High (30+ min)	0	0.0	0	0.0
Medium (20-30 min)	0	0.0	0	0.0
Low (10-20 min)	0	0.0	0	0.0
Only CT reachable within 60 min	0	0.0	0	0.0
Neither reachable within 60 min	0	0.0	0	0.0
Likely irrelevant (<10 min)	85	100.0	46942	100.0

Population and Area Coverage in Landkreis Straubing-Bogen (Stroke Units) (Bayern) — Regular (+10min Penalty)

Category	Grid Cells	Area (%)	Population	Population (%)
High (30+ min)	0	0.0	0	0.0
Medium (20-30 min)	114	7.8	7982	7.9
Low (10-20 min)	358	24.4	15594	15.5
Only CT reachable within 60 min	0	0.0	0	0.0
Neither reachable within 60 min	0	0.0	0	0.0
Likely irrelevant (<10 min)	994	67.8	76946	76.5

Population and Area Coverage in Landkreis Neumarkt i.d.OPf. (Stroke Units) (Bayern) — Regular (+10min Penalty)

Category	Grid Cells	Area (%)	Population	Population (%)
High (30+ min)	0	0.0	0	0.0
Medium (20-30 min)	12	0.7	1311	1.0
Low (10-20 min)	153	9.2	7396	5.6
Only CT reachable within 60 min	0	0.0	0	0.0
Neither reachable within 60 min	0	0.0	0	0.0
Likely irrelevant (<10 min)	1499	90.1	123760	93.4

Population and Area Coverage in Kreisfreie Stadt Schwabach (Stroke Units) (Bayern) — Regular (+10min Penalty)

Category	Grid Cells	Area (%)	Population	Population (%)
High (30+ min)	0	0.0	0	0.0
Medium (20-30 min)	0	0.0	0	0.0
Low (10-20 min)	0	0.0	0	0.0
Only CT reachable within 60 min	0	0.0	0	0.0
Neither reachable within 60 min	0	0.0	0	0.0
Likely irrelevant (<10 min)	52	100.0	42132	100.0

Population and Area Coverage in Kreisfreie Stadt Brandenburg an der Havel (Stroke Units) (Brandenburg) — Regular (+10min Penalty)

Category	Grid Cells	Area (%)	Population	Population (%)
High (30+ min)	118	38.8	56238	81.4
Medium (20-30 min)	47	15.5	2037	2.9
Low (10-20 min)	13	4.3	97	0.1
Only CT reachable within 60 min	126	41.4	10720	15.5
Neither reachable within 60 min	0	0.0	0	0.0
Likely irrelevant (<10 min)	0	0.0	0	0.0

Population and Area Coverage in Landkreis Ludwigslust-Parchim (Stroke Units) (Mecklenburg-Vorpommern) — Regular (+10min Penalty)

Category	Grid Cells	Area (%)	Population	Population (%)
High (30+ min)	1126	17.4	46704	23.8
Medium (20-30 min)	1424	22.1	40490	20.7
Low (10-20 min)	1506	23.3	46661	23.8
Only CT reachable within 60 min	1041	16.1	15717	8.0
Neither reachable within 60 min	0	0.0	0	0.0
Likely irrelevant (<10 min)	1358	21.0	46444	23.7

Population and Area Coverage in Landkreis Saalfeld-Rudolstadt (Stroke Units) (Thüringen) — Regular (+10min Penalty)

Category	Grid Cells	Area (%)	Population	Population (%)
High (30+ min)	0	0.0	0	0.0
Medium (20-30 min)	17	1.3	444	0.5
Low (10-20 min)	633	49.3	23928	24.7
Only CT reachable within 60 min	0	0.0	0	0.0
Neither reachable within 60 min	0	0.0	0	0.0
Likely irrelevant (<10 min)	633	49.3	72448	74.8

Population and Area Coverage in Kreis Ostholstein (Stroke Units) (Schleswig-Holstein) — Regular (+10min Penalty)

Category	Grid Cells	Area (%)	Population	Population (%)
High (30+ min)	1	0.1	98	0.0
Medium (20-30 min)	143	7.7	11763	6.0
Low (10-20 min)	414	22.2	48777	24.8
Only CT reachable within 60 min	10	0.5	430	0.2
Neither reachable within 60 min	78	4.2	2957	1.5
Likely irrelevant (<10 min)	1215	65.3	132376	67.4

Population and Area Coverage in Kreis Rendsburg-Eckernförde (Stroke Units) (Schleswig-Holstein) — Regular (+10min Penalty)

Category	Grid Cells	Area (%)	Population	Population (%)
High (30+ min)	60	2.0	2640	1.0
Medium (20-30 min)	140	4.7	5294	1.9
Low (10-20 min)	316	10.6	36786	13.4
Only CT reachable within 60 min	2	0.1	1871	0.7
Neither reachable within 60 min	35	1.2	1603	0.6
Likely irrelevant (<10 min)	2435	81.5	226358	82.4

Population and Area Coverage in Landkreis Ostalbkreis (Stroke Units) (Baden-Württemberg) — Regular (+10min Penalty)

Category	Grid Cells	Area (%)	Population	Population (%)
High (30+ min)	1	0.1	55	0.0
Medium (20-30 min)	229	12.3	42957	13.8
Low (10-20 min)	660	35.6	111826	35.9
Only CT reachable within 60 min	0	0.0	0	0.0
Neither reachable within 60 min	0	0.0	0	0.0
Likely irrelevant (<10 min)	965	52.0	156493	50.3

Population and Area Coverage in Landkreis Emmendingen (Stroke Units) (Baden-Württemberg) — Regular (+10min Penalty)

Category	Grid Cells	Area (%)	Population	Population (%)
High (30+ min)	112	13.7	7399	4.6
Medium (20-30 min)	154	18.8	15460	9.6
Low (10-20 min)	302	36.9	73941	45.7
Only CT reachable within 60 min	5	0.6	7	0.0
Neither reachable within 60 min	0	0.0	5	0.0
Likely irrelevant (<10 min)	245	30.0	65072	40.2

Population and Area Coverage in Kreisfreie Stadt München (Stroke Units) (Bayern) — Regular (+10min Penalty)

Category	Grid Cells	Area (%)	Population	Population (%)
High (30+ min)	0	0.0	0	0.0
Medium (20-30 min)	0	0.0	0	0.0
Low (10-20 min)	12	3.2	28078	1.7
Only CT reachable within 60 min	0	0.0	0	0.0
Neither reachable within 60 min	0	0.0	0	0.0
Likely irrelevant (<10 min)	367	96.8	1643077	98.3

Population and Area Coverage in Landkreis Bayreuth (Stroke Units) (Bayern) — Regular (+10min Penalty)

Category	Grid Cells	Area (%)	Population	Population (%)
High (30+ min)	0	0.0	0	0.0
Medium (20-30 min)	123	7.7	5203	4.8
Low (10-20 min)	672	42.1	43027	39.5
Only CT reachable within 60 min	0	0.0	0	0.0
Neither reachable within 60 min	0	0.0	0	0.0
Likely irrelevant (<10 min)	801	50.2	60680	55.7

Population and Area Coverage in Landkreis Kronach (Stroke Units) (Bayern) — Regular (+10min Penalty)

Category	Grid Cells	Area (%)	Population	Population (%)
High (30+ min)	3	0.4	13	0.0
Medium (20-30 min)	158	19.5	18373	25.8
Low (10-20 min)	386	47.5	34436	48.4
Only CT reachable within 60 min	0	0.0	0	0.0
Neither reachable within 60 min	0	0.0	0	0.0
Likely irrelevant (<10 min)	265	32.6	18257	25.7

Population and Area Coverage in Landkreis Aschaffenburg (Stroke Units) (Bayern) — Regular (+10min Penalty)

Category	Grid Cells	Area (%)	Population	Population (%)
High (30+ min)	0	0.0	0	0.0
Medium (20-30 min)	0	0.0	0	0.0
Low (10-20 min)	22	2.5	3219	1.8
Only CT reachable within 60 min	0	0.0	0	0.0
Neither reachable within 60 min	0	0.0	0	0.0
Likely irrelevant (<10 min)	852	97.5	176630	98.2

Population and Area Coverage in Landkreis Saalekreis (Stroke Units) (Sachsen-Anhalt) — Regular (+10min Penalty)

Category	Grid Cells	Area (%)	Population	Population (%)
High (30+ min)	0	0.0	0	0.0
Medium (20-30 min)	45	2.4	277	0.2
Low (10-20 min)	179	9.6	10372	6.1
Only CT reachable within 60 min	0	0.0	0	0.0
Neither reachable within 60 min	0	0.0	0	0.0
Likely irrelevant (<10 min)	1644	88.0	158286	93.7

Population and Area Coverage in Kreisfreie Stadt Wolfsburg (Stroke Units) (Niedersachsen) — Regular (+10min Penalty)

Category	Grid Cells	Area (%)	Population	Population (%)
High (30+ min)	0	0.0	0	0.0
Medium (20-30 min)	0	0.0	0	0.0
Low (10-20 min)	1	0.4	2	0.0
Only CT reachable within 60 min	0	0.0	0	0.0
Neither reachable within 60 min	0	0.0	0	0.0
Likely irrelevant (<10 min)	273	99.6	122064	100.0

Population and Area Coverage in Landkreis Wolfenbüttel (Stroke Units) (Niedersachsen) — Regular (+10min Penalty)

Category	Grid Cells	Area (%)	Population	Population (%)
High (30+ min)	0	0.0	0	0.0
Medium (20-30 min)	54	5.6	2377	2.0
Low (10-20 min)	559	58.4	62031	51.8
Only CT reachable within 60 min	0	0.0	0	0.0
Neither reachable within 60 min	0	0.0	0	0.0
Likely irrelevant (<10 min)	344	35.9	55386	46.2

Population and Area Coverage in Landkreis Ammerland (Stroke Units) (Niedersachsen) — Regular (+10min Penalty)

Category	Grid Cells	Area (%)	Population	Population (%)
High (30+ min)	0	0.0	0	0.0
Medium (20-30 min)	0	0.0	0	0.0
Low (10-20 min)	70	7.2	5381	4.4
Only CT reachable within 60 min	0	0.0	0	0.0
Neither reachable within 60 min	0	0.0	0	0.0
Likely irrelevant (<10 min)	909	92.8	116081	95.6

Population and Area Coverage in Kreis Rhein-Kreis Neuss (Stroke Units) (Nordrhein-Westfalen) — Regular (+10min Penalty)

Category	Grid Cells	Area (%)	Population	Population (%)
High (30+ min)	0	0.0	0	0.0
Medium (20-30 min)	0	0.0	0	0.0
Low (10-20 min)	181	24.6	56028	11.9
Only CT reachable within 60 min	0	0.0	0	0.0
Neither reachable within 60 min	0	0.0	0	0.0
Likely irrelevant (<10 min)	555	75.4	414816	88.1

Population and Area Coverage in Kreis Lippe (Stroke Units) (Nordrhein-Westfalen) — Regular (+10min Penalty)

Category	Grid Cells	Area (%)	Population	Population (%)
High (30+ min)	0	0.0	0	0.0
Medium (20-30 min)	91	5.6	15545	4.5
Low (10-20 min)	543	33.3	118609	34.4
Only CT reachable within 60 min	0	0.0	0	0.0
Neither reachable within 60 min	0	0.0	0	0.0
Likely irrelevant (<10 min)	999	61.2	211005	61.1

Population and Area Coverage in Kreisfreie Stadt Hagen (Stroke Units) (Nordrhein-Westfalen) — Regular (+10min Penalty)

Category	Grid Cells	Area (%)	Population	Population (%)
High (30+ min)	0	0.0	0	0.0
Medium (20-30 min)	32	15.7	3118	1.6
Low (10-20 min)	124	60.8	135872	68.1
Only CT reachable within 60 min	0	0.0	0	0.0
Neither reachable within 60 min	0	0.0	0	0.0
Likely irrelevant (<10 min)	48	23.5	60669	30.4

Population and Area Coverage in Landkreis Alzey-Worms (Stroke Units) (Rheinland-Pfalz) — Regular (+10min Penalty)

Category	Grid Cells	Area (%)	Population	Population (%)
High (30+ min)	0	0.0	0	0.0
Medium (20-30 min)	6	0.8	6	0.0
Low (10-20 min)	287	38.9	45789	35.4
Only CT reachable within 60 min	0	0.0	0	0.0
Neither reachable within 60 min	0	0.0	0	0.0
Likely irrelevant (<10 min)	444	60.2	83599	64.6

Population and Area Coverage in Landkreis Germersheim (Stroke Units) (Rheinland-Pfalz) — Regular (+10min Penalty)

Category	Grid Cells	Area (%)	Population	Population (%)
High (30+ min)	0	0.0	0	0.0
Medium (20-30 min)	0	0.0	0	0.0
Low (10-20 min)	50	8.8	7320	5.7
Only CT reachable within 60 min	0	0.0	0	0.0
Neither reachable within 60 min	0	0.0	0	0.0
Likely irrelevant (<10 min)	520	91.2	121402	94.3

Population and Area Coverage in Landkreis Neckar-Odenwald-Kreis (Stroke Units) (Baden-Württemberg) — Regular (+10min Penalty)

Category	Grid Cells	Area (%)	Population	Population (%)
High (30+ min)	617	44.2	80269	56.0
Medium (20-30 min)	543	38.9	46066	32.1
Low (10-20 min)	161	11.5	14156	9.9
Only CT reachable within 60 min	0	0.0	0	0.0
Neither reachable within 60 min	0	0.0	0	0.0
Likely irrelevant (<10 min)	76	5.4	2879	2.0

Population and Area Coverage in Landkreis Enzkreis (Stroke Units) (Baden-Württemberg) — Regular (+10min Penalty)

Category	Grid Cells	Area (%)	Population	Population (%)
High (30+ min)	5	0.7	5	0.0
Medium (20-30 min)	76	10.7	12613	6.4
Low (10-20 min)	275	38.8	68203	34.8
Only CT reachable within 60 min	0	0.0	0	0.0
Neither reachable within 60 min	0	0.0	0	0.0
Likely irrelevant (<10 min)	352	49.7	115115	58.8

Population and Area Coverage in Landkreis Berchtesgadener Land (Stroke Units) (Bayern) — Regular (+10min Penalty)

Category	Grid Cells	Area (%)	Population	Population (%)
High (30+ min)	6	0.6	248	0.3
Medium (20-30 min)	120	12.9	20695	22.1
Low (10-20 min)	114	12.3	29620	31.6
Only CT reachable within 60 min	5	0.5	87	0.1
Neither reachable within 60 min	326	35.1	1727	1.8
Likely irrelevant (<10 min)	358	38.5	41258	44.1

Population and Area Coverage in Kreisfreie Stadt Hof (Stroke Units) (Bayern) — Regular (+10min Penalty)

Category	Grid Cells	Area (%)	Population	Population (%)
High (30+ min)	0	0.0	0	0.0
Medium (20-30 min)	0	0.0	0	0.0
Low (10-20 min)	0	0.0	0	0.0
Only CT reachable within 60 min	0	0.0	0	0.0
Neither reachable within 60 min	0	0.0	0	0.0
Likely irrelevant (<10 min)	75	100.0	44749	100.0

Population and Area Coverage in Landkreis Kulmbach (Stroke Units) (Bayern) — Regular (+10min Penalty)

Category	Grid Cells	Area (%)	Population	Population (%)
High (30+ min)	0	0.0	0	0.0
Medium (20-30 min)	2	0.2	3	0.0
Low (10-20 min)	184	22.2	4737	6.3
Only CT reachable within 60 min	0	0.0	0	0.0
Neither reachable within 60 min	0	0.0	0	0.0
Likely irrelevant (<10 min)	642	77.5	70938	93.7

Population and Area Coverage in Kreisfreie Stadt Memmingen (Stroke Units) (Bayern) — Regular (+10min Penalty)

Category	Grid Cells	Area (%)	Population	Population (%)
High (30+ min)	0	0.0	0	0.0
Medium (20-30 min)	0	0.0	0	0.0
Low (10-20 min)	0	0.0	0	0.0
Only CT reachable within 60 min	0	0.0	0	0.0
Neither reachable within 60 min	0	0.0	0	0.0
Likely irrelevant (<10 min)	84	100.0	43302	100.0

Population and Area Coverage in Landkreis Augsburg (Stroke Units) (Bayern) — Regular (+10min Penalty)

Category	Grid Cells	Area (%)	Population	Population (%)
High (30+ min)	7	0.5	373	0.2
Medium (20-30 min)	218	16.8	13534	5.5
Low (10-20 min)	289	22.2	72568	29.2
Only CT reachable within 60 min	0	0.0	0	0.0
Neither reachable within 60 min	0	0.0	0	0.0
Likely irrelevant (<10 min)	785	60.4	161790	65.2

Population and Area Coverage in Landkreis Vorpommern-Rügen (Stroke Units) (Mecklenburg-Vorpommern) — Regular (+10min Penalty)

Category	Grid Cells	Area (%)	Population	Population (%)
High (30+ min)	401	9.6	21052	10.6
Medium (20-30 min)	673	16.2	18996	9.5
Low (10-20 min)	1068	25.7	28447	14.3
Only CT reachable within 60 min	244	5.9	6752	3.4
Neither reachable within 60 min	274	6.6	5801	2.9
Likely irrelevant (<10 min)	1503	36.1	118221	59.3

Population and Area Coverage in Landkreis Zwickau (Stroke Units) (Sachsen) — Regular (+10min Penalty)

Category	Grid Cells	Area (%)	Population	Population (%)
High (30+ min)	0	0.0	0	0.0
Medium (20-30 min)	5	0.4	258	0.1
Low (10-20 min)	457	37.4	80863	25.5
Only CT reachable within 60 min	0	0.0	0	0.0
Neither reachable within 60 min	0	0.0	0	0.0
Likely irrelevant (<10 min)	759	62.2	236096	74.4

Population and Area Coverage in Landkreis Görlitz (Stroke Units) (Sachsen) — Regular (+10min Penalty)

Category	Grid Cells	Area (%)	Population	Population (%)
High (30+ min)	425	16.4	89577	40.9
Medium (20-30 min)	489	18.9	24581	11.2
Low (10-20 min)	370	14.3	20261	9.3
Only CT reachable within 60 min	74	2.9	4473	2.0
Neither reachable within 60 min	12	0.5	216	0.1
Likely irrelevant (<10 min)	1217	47.0	79853	36.5

Population and Area Coverage in Landkreis Sonneberg (Stroke Units) (Thüringen) — Regular (+10min Penalty)

Category	Grid Cells	Area (%)	Population	Population (%)
High (30+ min)	0	0.0	0	0.0
Medium (20-30 min)	0	0.0	23	0.0
Low (10-20 min)	59	10.0	2330	4.3
Only CT reachable within 60 min	0	0.0	0	0.0
Neither reachable within 60 min	0	0.0	0	0.0
Likely irrelevant (<10 min)	530	90.0	51315	95.6

Population and Area Coverage in Kreisfreie Stadt Köln (Stroke Units) (Nordrhein-Westfalen) — Regular (+10min Penalty)

Category	Grid Cells	Area (%)	Population	Population (%)
High (30+ min)	0	0.0	0	0.0
Medium (20-30 min)	0	0.0	0	0.0
Low (10-20 min)	20	3.8	22990	1.9
Only CT reachable within 60 min	0	0.0	0	0.0
Neither reachable within 60 min	0	0.0	0	0.0
Likely irrelevant (<10 min)	507	96.2	1192194	98.1

Population and Area Coverage in Kreis Heinsberg (Stroke Units) (Nordrhein-Westfalen) — Regular (+10min Penalty)

Category	Grid Cells	Area (%)	Population	Population (%)
High (30+ min)	1	0.1	1	0.0
Medium (20-30 min)	4	0.5	746	0.3
Low (10-20 min)	202	26.0	70332	28.3
Only CT reachable within 60 min	2	0.3	33	0.0
Neither reachable within 60 min	2	0.3	330	0.1
Likely irrelevant (<10 min)	567	72.9	176771	71.2

Population and Area Coverage in Landkreis Bergstraße (Stroke Units) (Hessen) — Regular (+10min Penalty)

Category	Grid Cells	Area (%)	Population	Population (%)
High (30+ min)	1	0.1	179	0.1
Medium (20-30 min)	20	2.2	3902	1.5
Low (10-20 min)	256	28.5	60085	22.6
Only CT reachable within 60 min	0	0.0	0	0.0
Neither reachable within 60 min	0	0.0	0	0.0
Likely irrelevant (<10 min)	620	69.1	201891	75.9

Population and Area Coverage in Landkreis Wetteraukreis (Stroke Units) (Hessen) — Regular (+10min Penalty)

Category	Grid Cells	Area (%)	Population	Population (%)
High (30+ min)	0	0.0	0	0.0
Medium (20-30 min)	28	2.0	1964	0.7
Low (10-20 min)	446	32.0	108823	36.4
Only CT reachable within 60 min	0	0.0	0	0.0
Neither reachable within 60 min	0	0.0	0	0.0
Likely irrelevant (<10 min)	921	66.0	188076	62.9

Population and Area Coverage in Kreisfreie Stadt Landau in der Pfalz (Stroke Units) (Rheinland-Pfalz) — Regular (+10min Penalty)

Category	Grid Cells	Area (%)	Population	Population (%)
High (30+ min)	0	0.0	0	0.0
Medium (20-30 min)	0	0.0	0	0.0
Low (10-20 min)	41	40.2	6860	13.0
Only CT reachable within 60 min	0	0.0	0	0.0
Neither reachable within 60 min	0	0.0	0	0.0
Likely irrelevant (<10 min)	61	59.8	45736	87.0

Population and Area Coverage in Stadtkreis Stuttgart (Stroke Units) (Baden-Württemberg) — Regular (+10min Penalty)

Category	Grid Cells	Area (%)	Population	Population (%)
High (30+ min)	0	0.0	0	0.0
Medium (20-30 min)	0	0.0	0	0.0
Low (10-20 min)	5	2.0	4503	0.7
Only CT reachable within 60 min	0	0.0	0	0.0
Neither reachable within 60 min	0	0.0	0	0.0
Likely irrelevant (<10 min)	250	98.0	635443	99.3

Population and Area Coverage in Landkreis Konstanz (Stroke Units) (Baden-Württemberg) — Regular (+10min Penalty)

Category	Grid Cells	Area (%)	Population	Population (%)
High (30+ min)	6	0.6	13648	5.3
Medium (20-30 min)	110	11.6	47495	18.5
Low (10-20 min)	221	23.2	34863	13.6
Only CT reachable within 60 min	3	0.3	1916	0.7
Neither reachable within 60 min	7	0.7	1161	0.5
Likely irrelevant (<10 min)	604	63.5	157070	61.3

Population and Area Coverage in Landkreis Sigmaringen (Stroke Units) (Baden-Württemberg) — Regular (+10min Penalty)

Category	Grid Cells	Area (%)	Population	Population (%)
High (30+ min)	0	0.0	0	0.0
Medium (20-30 min)	30	2.1	947	0.7
Low (10-20 min)	172	11.9	12623	9.8
Only CT reachable within 60 min	0	0.0	0	0.0
Neither reachable within 60 min	0	0.0	0	0.0
Likely irrelevant (<10 min)	1247	86.1	115088	89.5

Population and Area Coverage in Landkreis Kelheim (Stroke Units) (Bayern) — Regular (+10min Penalty)

Category	Grid Cells	Area (%)	Population	Population (%)
High (30+ min)	11	0.8	104	0.1
Medium (20-30 min)	465	35.9	40066	33.6
Low (10-20 min)	487	37.5	48901	41.0
Only CT reachable within 60 min	0	0.0	0	0.0
Neither reachable within 60 min	0	0.0	0	0.0
Likely irrelevant (<10 min)	334	25.8	30076	25.2

Population and Area Coverage in Kreisfreie Stadt Schweinfurt (Stroke Units) (Bayern) — Regular (+10min Penalty)

Category	Grid Cells	Area (%)	Population	Population (%)
High (30+ min)	0	0.0	0	0.0
Medium (20-30 min)	0	0.0	0	0.0
Low (10-20 min)	0	0.0	0	0.0
Only CT reachable within 60 min	0	0.0	0	0.0
Neither reachable within 60 min	0	0.0	0	0.0
Likely irrelevant (<10 min)	46	100.0	53903	100.0

Population and Area Coverage in Landkreis Neu-Ulm (Stroke Units) (Bayern) — Regular (+10min Penalty)

Category	Grid Cells	Area (%)	Population	Population (%)
High (30+ min)	0	0.0	0	0.0
Medium (20-30 min)	77	12.3	3144	1.8
Low (10-20 min)	366	58.7	104045	60.1
Only CT reachable within 60 min	0	0.0	0	0.0
Neither reachable within 60 min	0	0.0	0	0.0
Likely irrelevant (<10 min)	181	29.0	66038	38.1

Population and Area Coverage in Landkreis Oberhavel (Stroke Units) (Brandenburg) — Regular (+10min Penalty)

Category	Grid Cells	Area (%)	Population	Population (%)
High (30+ min)	361	15.0	19729	9.8
Medium (20-30 min)	338	14.0	8923	4.4
Low (10-20 min)	657	27.2	64486	32.1
Only CT reachable within 60 min	510	21.1	7248	3.6
Neither reachable within 60 min	0	0.0	0	0.0
Likely irrelevant (<10 min)	548	22.7	100230	50.0

Population and Area Coverage in Landkreis Spree-Neiße (Stroke Units) (Brandenburg) — Regular (+10min Penalty)

Category	Grid Cells	Area (%)	Population	Population (%)
High (30+ min)	148	7.1	15383	15.3
Medium (20-30 min)	303	14.5	5425	5.4
Low (10-20 min)	544	26.1	33564	33.4
Only CT reachable within 60 min	20	1.0	64	0.1
Neither reachable within 60 min	0	0.0	0	0.0
Likely irrelevant (<10 min)	1073	51.4	45961	45.8

Population and Area Coverage in Landkreis Ilm-Kreis (Stroke Units) (Thüringen) — Regular (+10min Penalty)

Category	Grid Cells	Area (%)	Population	Population (%)
High (30+ min)	0	0.0	0	0.0
Medium (20-30 min)	53	5.2	2214	2.3
Low (10-20 min)	437	42.6	56636	58.7
Only CT reachable within 60 min	0	0.0	0	0.0
Neither reachable within 60 min	0	0.0	0	0.0
Likely irrelevant (<10 min)	536	52.2	37651	39.0

Population and Area Coverage in Landkreis Greiz (Stroke Units) (Thüringen) — Regular (+10min Penalty)

Category	Grid Cells	Area (%)	Population	Population (%)
High (30+ min)	1	0.1	8	0.0
Medium (20-30 min)	114	10.6	19828	21.6
Low (10-20 min)	357	33.1	24522	26.7
Only CT reachable within 60 min	0	0.0	0	0.0
Neither reachable within 60 min	0	0.0	0	0.0
Likely irrelevant (<10 min)	608	56.3	47448	51.7

Population and Area Coverage in Kreisfreie Stadt Bottrop (Stroke Units) (Nordrhein-Westfalen) — Regular (+10min Penalty)

Category	Grid Cells	Area (%)	Population	Population (%)
High (30+ min)	0	0.0	0	0.0
Medium (20-30 min)	0	0.0	0	0.0
Low (10-20 min)	1	0.8	0	0.0
Only CT reachable within 60 min	0	0.0	0	0.0
Neither reachable within 60 min	0	0.0	0	0.0
Likely irrelevant (<10 min)	132	99.2	116967	100.0

Population and Area Coverage in Kreisfreie Stadt Speyer (Stroke Units) (Rheinland-Pfalz) — Regular (+10min Penalty)

Category	Grid Cells	Area (%)	Population	Population (%)
High (30+ min)	0	0.0	0	0.0
Medium (20-30 min)	0	0.0	0	0.0
Low (10-20 min)	0	0.0	0	0.0
Only CT reachable within 60 min	0	0.0	0	0.0
Neither reachable within 60 min	0	0.0	0	0.0
Likely irrelevant (<10 min)	52	100.0	51373	100.0

Population and Area Coverage in Stadtkreis Mannheim (Stroke Units) (Baden-Württemberg) — Regular (+10min Penalty)

Category	Grid Cells	Area (%)	Population	Population (%)
High (30+ min)	0	0.0	0	0.0
Medium (20-30 min)	0	0.0	0	0.0
Low (10-20 min)	14	7.9	3228	0.9
Only CT reachable within 60 min	0	0.0	0	0.0
Neither reachable within 60 min	0	0.0	0	0.0
Likely irrelevant (<10 min)	164	92.1	343721	99.1

Population and Area Coverage in Landkreis Tuttlingen (Stroke Units) (Baden-Württemberg) — Regular (+10min Penalty)

Category	Grid Cells	Area (%)	Population	Population (%)
High (30+ min)	0	0.0	0	0.0
Medium (20-30 min)	175	19.9	25230	18.7
Low (10-20 min)	323	36.7	58445	43.3
Only CT reachable within 60 min	0	0.0	0	0.0
Neither reachable within 60 min	0	0.0	0	0.0
Likely irrelevant (<10 min)	383	43.5	51409	38.1

Population and Area Coverage in Landkreis Zollernalbkreis (Stroke Units) (Baden-Württemberg) — Regular (+10min Penalty)

Category	Grid Cells	Area (%)	Population	Population (%)
High (30+ min)	160	14.4	27295	14.6
Medium (20-30 min)	546	49.2	107566	57.4
Low (10-20 min)	278	25.0	40136	21.4
Only CT reachable within 60 min	0	0.0	0	0.0
Neither reachable within 60 min	0	0.0	0	0.0
Likely irrelevant (<10 min)	126	11.4	12278	6.6

Population and Area Coverage in Landkreis Eichstätt (Stroke Units) (Bayern) — Regular (+10min Penalty)

Category	Grid Cells	Area (%)	Population	Population (%)
High (30+ min)	7	0.5	560	0.4
Medium (20-30 min)	577	38.9	35389	27.1
Low (10-20 min)	695	46.9	54636	41.8
Only CT reachable within 60 min	0	0.0	0	0.0
Neither reachable within 60 min	0	0.0	0	0.0
Likely irrelevant (<10 min)	203	13.7	40032	30.6

Population and Area Coverage in Landkreis Freyung-Grafenau (Stroke Units) (Bayern) — Regular (+10min Penalty)

Category	Grid Cells	Area (%)	Population	Population (%)
High (30+ min)	373	32.9	25582	32.5
Medium (20-30 min)	313	27.6	30961	39.3
Low (10-20 min)	180	15.9	14975	19.0
Only CT reachable within 60 min	78	6.9	371	0.5
Neither reachable within 60 min	51	4.5	35	0.0
Likely irrelevant (<10 min)	138	12.2	6806	8.6

Population and Area Coverage in Landkreis Main-Spessart (Stroke Units) (Bayern) — Regular (+10min Penalty)

Category	Grid Cells	Area (%)	Population	Population (%)
High (30+ min)	0	0.0	0	0.0
Medium (20-30 min)	13	0.8	46	0.0
Low (10-20 min)	252	15.2	13709	10.4
Only CT reachable within 60 min	0	0.0	0	0.0
Neither reachable within 60 min	0	0.0	0	0.0
Likely irrelevant (<10 min)	1397	84.1	117700	89.5

Population and Area Coverage in Kreisfreie Stadt Potsdam (Stroke Units) (Brandenburg) — Regular (+10min Penalty)

Category	Grid Cells	Area (%)	Population	Population (%)
High (30+ min)	0	0.0	0	0.0
Medium (20-30 min)	0	0.0	0	0.0
Low (10-20 min)	11	4.5	2983	1.5
Only CT reachable within 60 min	0	0.0	0	0.0
Neither reachable within 60 min	0	0.0	0	0.0
Likely irrelevant (<10 min)	233	95.5	202332	98.5

Population and Area Coverage in Landkreis Nordsachsen (Stroke Units) (Sachsen) — Regular (+10min Penalty)

Category	Grid Cells	Area (%)	Population	Population (%)
High (30+ min)	327	12.5	12161	6.6
Medium (20-30 min)	540	20.7	42917	23.3
Low (10-20 min)	774	29.6	60087	32.6
Only CT reachable within 60 min	124	4.7	1294	0.7
Neither reachable within 60 min	0	0.0	0	0.0
Likely irrelevant (<10 min)	846	32.4	67614	36.7

Population and Area Coverage in Landkreis Cuxhaven (Stroke Units) (Niedersachsen) — Regular (+10min Penalty)

Category	Grid Cells	Area (%)	Population	Population (%)
High (30+ min)	206	7.4	18364	9.2
Medium (20-30 min)	389	14.0	47161	23.7
Low (10-20 min)	874	31.4	38806	19.5
Only CT reachable within 60 min	7	0.3	687	0.3
Neither reachable within 60 min	45	1.6	2606	1.3
Likely irrelevant (<10 min)	1259	45.3	91081	45.8

Population and Area Coverage in Kreisfreie Stadt Bremen (Stroke Units) (Bremen) — Regular (+10min Penalty)

Category	Grid Cells	Area (%)	Population	Population (%)
High (30+ min)	0	0.0	0	0.0
Medium (20-30 min)	9	2.1	336	0.1
Low (10-20 min)	68	15.5	15409	2.6
Only CT reachable within 60 min	0	0.0	0	0.0
Neither reachable within 60 min	1	0.2	1881	0.3
Likely irrelevant (<10 min)	360	82.2	565586	97.0

Population and Area Coverage in Landkreis Groß-Gerau (Stroke Units) (Hessen) — Regular (+10min Penalty)

Category	Grid Cells	Area (%)	Population	Population (%)
High (30+ min)	0	0.0	0	0.0
Medium (20-30 min)	0	0.0	0	0.0
Low (10-20 min)	88	15.6	19501	7.5
Only CT reachable within 60 min	0	0.0	0	0.0
Neither reachable within 60 min	0	0.0	0	0.0
Likely irrelevant (<10 min)	476	84.4	240092	92.5

Population and Area Coverage in Landkreis Limburg-Weilburg (Stroke Units) (Hessen) — Regular (+10min Penalty)

Category	Grid Cells	Area (%)	Population	Population (%)
High (30+ min)	0	0.0	0	0.0
Medium (20-30 min)	7	0.7	49	0.0
Low (10-20 min)	222	23.8	28762	16.6
Only CT reachable within 60 min	0	0.0	0	0.0
Neither reachable within 60 min	0	0.0	0	0.0
Likely irrelevant (<10 min)	705	75.5	144026	83.3

Population and Area Coverage in Landkreis Waldeck-Frankenberg (Stroke Units) (Hessen) — Regular (+10min Penalty)

Category	Grid Cells	Area (%)	Population	Population (%)
High (30+ min)	1405	59.0	93178	59.0
Medium (20-30 min)	668	28.0	52876	33.5
Low (10-20 min)	114	4.8	5948	3.8
Only CT reachable within 60 min	187	7.9	5993	3.8
Neither reachable within 60 min	0	0.0	0	0.0
Likely irrelevant (<10 min)	8	0.3	8	0.0

Population and Area Coverage in Landkreis Trier-Saarburg (Stroke Units) (Rheinland-Pfalz) — Regular (+10min Penalty)

Category	Grid Cells	Area (%)	Population	Population (%)
High (30+ min)	1	0.1	1	0.0
Medium (20-30 min)	205	14.9	17862	11.9
Low (10-20 min)	469	34.1	36667	24.4
Only CT reachable within 60 min	0	0.0	0	0.0
Neither reachable within 60 min	0	0.0	355	0.2
Likely irrelevant (<10 min)	699	50.9	95150	63.4

Population and Area Coverage in Landkreis Reutlingen (Stroke Units) (Baden-Württemberg) — Regular (+10min Penalty)

Category	Grid Cells	Area (%)	Population	Population (%)
High (30+ min)	141	10.6	9519	3.2
Medium (20-30 min)	316	23.7	15428	5.2
Low (10-20 min)	346	26.0	38496	12.9
Only CT reachable within 60 min	0	0.0	0	0.0
Neither reachable within 60 min	0	0.0	0	0.0
Likely irrelevant (<10 min)	530	39.8	234268	78.7

Population and Area Coverage in Kreisfreie Stadt Schwerin (Stroke Units) (Mecklenburg-Vorpommern) — Regular (+10min Penalty)

Category	Grid Cells	Area (%)	Population	Population (%)
High (30+ min)	0	0.0	0	0.0
Medium (20-30 min)	0	0.0	0	0.0
Low (10-20 min)	5	2.9	936	1.0
Only CT reachable within 60 min	0	0.0	0	0.0
Neither reachable within 60 min	0	0.0	0	0.0
Likely irrelevant (<10 min)	168	97.1	95836	99.0

Population and Area Coverage in Kreisfreie Stadt Hamburg (Stroke Units) (Hamburg) — Regular (+10min Penalty)

Category	Grid Cells	Area (%)	Population	Population (%)
High (30+ min)	0	0.0	0	0.0
Medium (20-30 min)	0	0.0	0	0.0
Low (10-20 min)	81	8.1	70977	4.0
Only CT reachable within 60 min	0	0.0	0	0.0
Neither reachable within 60 min	6	0.6	6087	0.3
Likely irrelevant (<10 min)	910	91.3	1716404	95.7

Population and Area Coverage in Kreisfreie Stadt Braunschweig (Stroke Units) (Niedersachsen) — Regular (+10min Penalty)

Category	Grid Cells	Area (%)	Population	Population (%)
High (30+ min)	0	0.0	0	0.0
Medium (20-30 min)	0	0.0	0	0.0
Low (10-20 min)	112	46.1	76448	31.0
Only CT reachable within 60 min	0	0.0	0	0.0
Neither reachable within 60 min	0	0.0	0	0.0
Likely irrelevant (<10 min)	131	53.9	170230	69.0

Population and Area Coverage in Landkreis Leer (Stroke Units) (Niedersachsen) — Regular (+10min Penalty)

Category	Grid Cells	Area (%)	Population	Population (%)
High (30+ min)	121	8.5	15941	9.4
Medium (20-30 min)	251	17.7	30769	18.2
Low (10-20 min)	443	31.2	73927	43.8
Only CT reachable within 60 min	1	0.1	9	0.0
Neither reachable within 60 min	38	2.7	3609	2.1
Likely irrelevant (<10 min)	564	39.8	44583	26.4

Population and Area Coverage in Kreisfreie Stadt Duisburg (Stroke Units) (Nordrhein-Westfalen) — Regular (+10min Penalty)

Category	Grid Cells	Area (%)	Population	Population (%)
High (30+ min)	0	0.0	0	0.0
Medium (20-30 min)	0	0.0	0	0.0
Low (10-20 min)	27	9.1	28889	5.2
Only CT reachable within 60 min	0	0.0	0	0.0
Neither reachable within 60 min	0	0.0	0	0.0
Likely irrelevant (<10 min)	270	90.9	524014	94.8

Population and Area Coverage in Kreis Siegen-Wittgenstein (Stroke Units) (Nordrhein-Westfalen) — Regular (+10min Penalty)

Category	Grid Cells	Area (%)	Population	Population (%)
High (30+ min)	338	23.3	29606	10.1
Medium (20-30 min)	102	7.0	5948	2.0
Low (10-20 min)	302	20.8	45740	15.6
Only CT reachable within 60 min	107	7.4	544	0.2
Neither reachable within 60 min	0	0.0	0	0.0
Likely irrelevant (<10 min)	602	41.5	211481	72.1

Population and Area Coverage in Landkreis Schwarzwald-Baar-Kreis (Stroke Units) (Baden-Württemberg) — Regular (+10min Penalty)

Category	Grid Cells	Area (%)	Population	Population (%)
High (30+ min)	61	5.0	2936	1.4
Medium (20-30 min)	73	5.9	7615	3.7
Low (10-20 min)	120	9.8	7159	3.5
Only CT reachable within 60 min	7	0.6	35	0.0
Neither reachable within 60 min	0	0.0	0	0.0
Likely irrelevant (<10 min)	969	78.8	189243	91.4

Population and Area Coverage in Kreisfreie Stadt Amberg (Stroke Units) (Bayern) — Regular (+10min Penalty)

Category	Grid Cells	Area (%)	Population	Population (%)
High (30+ min)	0	0.0	0	0.0
Medium (20-30 min)	0	0.0	0	0.0
Low (10-20 min)	0	0.0	0	0.0
Only CT reachable within 60 min	0	0.0	0	0.0
Neither reachable within 60 min	0	0.0	0	0.0
Likely irrelevant (<10 min)	61	100.0	42852	100.0

Population and Area Coverage in Kreisfreie Stadt Bayreuth (Stroke Units) (Bayern) — Regular (+10min Penalty)

Category	Grid Cells	Area (%)	Population	Population (%)
High (30+ min)	0	0.0	0	0.0
Medium (20-30 min)	0	0.0	0	0.0
Low (10-20 min)	0	0.0	0	0.0
Only CT reachable within 60 min	0	0.0	0	0.0
Neither reachable within 60 min	0	0.0	0	0.0
Likely irrelevant (<10 min)	82	100.0	73464	100.0

Population and Area Coverage in Landkreis Nürnberger Land (Stroke Units) (Bayern) — Regular (+10min Penalty)

Category	Grid Cells	Area (%)	Population	Population (%)
High (30+ min)	0	0.0	0	0.0
Medium (20-30 min)	32	3.2	3204	1.9
Low (10-20 min)	484	49.1	47922	28.2
Only CT reachable within 60 min	0	0.0	0	0.0
Neither reachable within 60 min	0	0.0	0	0.0
Likely irrelevant (<10 min)	470	47.7	118951	69.9

Population and Area Coverage in Landkreis Roth (Stroke Units) (Bayern) — Regular (+10min Penalty)

Category	Grid Cells	Area (%)	Population	Population (%)
High (30+ min)	0	0.0	0	0.0
Medium (20-30 min)	15	1.4	171	0.1
Low (10-20 min)	134	12.1	7662	5.9
Only CT reachable within 60 min	0	0.0	0	0.0
Neither reachable within 60 min	0	0.0	0	0.0
Likely irrelevant (<10 min)	955	86.5	120944	93.9

Population and Area Coverage in Kreisfreie Stadt Aschaffenburg (Stroke Units) (Bayern) — Regular (+10min Penalty)

Category	Grid Cells	Area (%)	Population	Population (%)
High (30+ min)	0	0.0	0	0.0
Medium (20-30 min)	0	0.0	0	0.0
Low (10-20 min)	0	0.0	0	0.0
Only CT reachable within 60 min	0	0.0	0	0.0
Neither reachable within 60 min	0	0.0	0	0.0
Likely irrelevant (<10 min)	78	100.0	70292	100.0

Population and Area Coverage in Landkreis Merzig-Wadern (Stroke Units) (Saarland) — Regular (+10min Penalty)

Category	Grid Cells	Area (%)	Population	Population (%)
High (30+ min)	1	0.1	8	0.0
Medium (20-30 min)	48	6.9	4356	4.4
Low (10-20 min)	192	27.8	18494	18.5
Only CT reachable within 60 min	0	0.0	1	0.0
Neither reachable within 60 min	0	0.0	191	0.2
Likely irrelevant (<10 min)	450	65.1	76967	77.0

Population and Area Coverage in Landkreis Vogtlandkreis (Stroke Units) (Sachsen) — Regular (+10min Penalty)

Category	Grid Cells	Area (%)	Population	Population (%)
High (30+ min)	84	4.8	5232	2.4
Medium (20-30 min)	337	19.1	30061	13.8
Low (10-20 min)	382	21.6	54271	24.9
Only CT reachable within 60 min	3	0.2	209	0.1
Neither reachable within 60 min	12	0.7	105	0.0
Likely irrelevant (<10 min)	949	53.7	128171	58.8

Population and Area Coverage in Landkreis Altmarkkreis Salzwedel (Stroke Units) (Sachsen-Anhalt) — Regular (+10min Penalty)

Category	Grid Cells	Area (%)	Population	Population (%)
High (30+ min)	1055	34.5	38748	50.2
Medium (20-30 min)	402	13.1	11005	14.2
Low (10-20 min)	313	10.2	5117	6.6
Only CT reachable within 60 min	1233	40.3	22203	28.7
Neither reachable within 60 min	0	0.0	0	0.0
Likely irrelevant (<10 min)	59	1.9	158	0.2

Population and Area Coverage in Kreis Segeberg (Stroke Units) (Schleswig-Holstein) — Regular (+10min Penalty)

Category	Grid Cells	Area (%)	Population	Population (%)
High (30+ min)	0	0.0	0	0.0
Medium (20-30 min)	7	0.4	273	0.1
Low (10-20 min)	418	22.8	60135	22.0
Only CT reachable within 60 min	0	0.0	0	0.0
Neither reachable within 60 min	0	0.0	0	0.0
Likely irrelevant (<10 min)	1407	76.8	213503	77.9

Population and Area Coverage in Landkreis Osnabrück (Stroke Units) (Niedersachsen) — Regular (+10min Penalty)

Category	Grid Cells	Area (%)	Population	Population (%)
High (30+ min)	2	0.1	236	0.1
Medium (20-30 min)	249	8.9	24521	6.9
Low (10-20 min)	1314	46.9	149866	42.4
Only CT reachable within 60 min	0	0.0	0	0.0
Neither reachable within 60 min	0	0.0	0	0.0
Likely irrelevant (<10 min)	1237	44.1	178534	50.6

Population and Area Coverage in Kreisfreie Stadt Oberhausen (Stroke Units) (Nordrhein-Westfalen) — Regular (+10min Penalty)

Category	Grid Cells	Area (%)	Population	Population (%)
High (30+ min)	0	0.0	0	0.0
Medium (20-30 min)	0	0.0	0	0.0
Low (10-20 min)	0	0.0	0	0.0
Only CT reachable within 60 min	0	0.0	0	0.0
Neither reachable within 60 min	0	0.0	0	0.0
Likely irrelevant (<10 min)	101	100.0	215167	100.0

Population and Area Coverage in Kreisfreie Stadt Remscheid (Stroke Units) (Nordrhein-Westfalen) — Regular (+10min Penalty)

Category	Grid Cells	Area (%)	Population	Population (%)
High (30+ min)	0	0.0	0	0.0
Medium (20-30 min)	0	0.0	0	0.0
Low (10-20 min)	15	15.5	10369	9.6
Only CT reachable within 60 min	0	0.0	0	0.0
Neither reachable within 60 min	0	0.0	0	0.0
Likely irrelevant (<10 min)	82	84.5	98121	90.4

Population and Area Coverage in Kreis Mettmann (Stroke Units) (Nordrhein-Westfalen) — Regular (+10min Penalty)

Category	Grid Cells	Area (%)	Population	Population (%)
High (30+ min)	0	0.0	0	0.0
Medium (20-30 min)	0	0.0	0	0.0
Low (10-20 min)	72	13.7	67185	14.2
Only CT reachable within 60 min	0	0.0	0	0.0
Neither reachable within 60 min	0	0.0	0	0.0
Likely irrelevant (<10 min)	453	86.3	406541	85.8

Population and Area Coverage in Kreis Rhein-Sieg-Kreis (Stroke Units) (Nordrhein-Westfalen) — Regular (+10min Penalty)

Category	Grid Cells	Area (%)	Population	Population (%)
High (30+ min)	27	1.8	2751	0.5
Medium (20-30 min)	119	8.1	18446	3.2
Low (10-20 min)	356	24.2	83801	14.4
Only CT reachable within 60 min	0	0.0	0	0.0
Neither reachable within 60 min	0	0.0	0	0.0
Likely irrelevant (<10 min)	972	65.9	478278	82.0

Population and Area Coverage in Kreis Borken (Stroke Units) (Nordrhein-Westfalen) — Regular (+10min Penalty)

Category	Grid Cells	Area (%)	Population	Population (%)
High (30+ min)	46	2.5	1858	0.5
Medium (20-30 min)	400	22.0	83787	22.8
Low (10-20 min)	495	27.2	110799	30.2
Only CT reachable within 60 min	10	0.5	185	0.1
Neither reachable within 60 min	15	0.8	153	0.0
Likely irrelevant (<10 min)	853	46.9	170010	46.4

Population and Area Coverage in Landkreis Vulkaneifel (Stroke Units) (Rheinland-Pfalz) — Regular (+10min Penalty)

Category	Grid Cells	Area (%)	Population	Population (%)
High (30+ min)	281	24.4	19552	31.1
Medium (20-30 min)	218	19.0	9587	15.3
Low (10-20 min)	506	44.0	27135	43.2
Only CT reachable within 60 min	0	0.0	0	0.0
Neither reachable within 60 min	0	0.0	0	0.0
Likely irrelevant (<10 min)	145	12.6	6581	10.5

Population and Area Coverage in Landkreis Kaiserslautern (Stroke Units) (Rheinland-Pfalz) — Regular (+10min Penalty)

Category	Grid Cells	Area (%)	Population	Population (%)
High (30+ min)	0	0.0	0	0.0
Medium (20-30 min)	9	1.1	553	0.5
Low (10-20 min)	205	25.9	31353	29.3
Only CT reachable within 60 min	0	0.0	0	0.0
Neither reachable within 60 min	0	0.0	0	0.0
Likely irrelevant (<10 min)	577	72.9	75001	70.2

Population and Area Coverage in Landkreis Calw (Stroke Units) (Baden-Württemberg) — Regular (+10min Penalty)

Category	Grid Cells	Area (%)	Population	Population (%)
High (30+ min)	163	16.9	31967	21.1
Medium (20-30 min)	469	48.5	75482	49.8
Low (10-20 min)	247	25.5	34661	22.9
Only CT reachable within 60 min	9	0.9	164	0.1
Neither reachable within 60 min	0	0.0	0	0.0
Likely irrelevant (<10 min)	79	8.2	9355	6.2

Population and Area Coverage in Kreisfreie Stadt Berlin (Stroke Units) (Berlin) — Regular (+10min Penalty)

Category	Grid Cells	Area (%)	Population	Population (%)
High (30+ min)	0	0.0	0	0.0
Medium (20-30 min)	1	0.1	514	0.0
Low (10-20 min)	63	5.3	39013	1.1
Only CT reachable within 60 min	0	0.0	0	0.0
Neither reachable within 60 min	0	0.0	0	0.0
Likely irrelevant (<10 min)	1120	94.6	3589171	98.9

Population and Area Coverage in Kreis Stormarn (Stroke Units) (Schleswig-Holstein) — Regular (+10min Penalty)

Category	Grid Cells	Area (%)	Population	Population (%)
High (30+ min)	0	0.0	0	0.0
Medium (20-30 min)	18	1.7	7001	2.9
Low (10-20 min)	517	49.6	119190	48.9
Only CT reachable within 60 min	0	0.0	0	0.0
Neither reachable within 60 min	0	0.0	0	0.0
Likely irrelevant (<10 min)	507	48.7	117777	48.3

Population and Area Coverage in Landkreis Hildesheim (Stroke Units) (Niedersachsen) — Regular (+10min Penalty)

Category	Grid Cells	Area (%)	Population	Population (%)
High (30+ min)	98	6.2	16475	5.6
Medium (20-30 min)	296	18.6	23836	8.1
Low (10-20 min)	272	17.1	32002	10.9
Only CT reachable within 60 min	0	0.0	0	0.0
Neither reachable within 60 min	0	0.0	0	0.0
Likely irrelevant (<10 min)	926	58.2	221402	75.4

Population and Area Coverage in Kreis Viersen (Stroke Units) (Nordrhein-Westfalen) — Regular (+10min Penalty)

Category	Grid Cells	Area (%)	Population	Population (%)
High (30+ min)	0	0.0	0	0.0
Medium (20-30 min)	2	0.3	91	0.0
Low (10-20 min)	59	8.1	18283	6.2
Only CT reachable within 60 min	0	0.0	0	0.0
Neither reachable within 60 min	45	6.2	1588	0.5
Likely irrelevant (<10 min)	622	85.4	276082	93.3

Population and Area Coverage in Kreis Rheinisch-Bergischer Kreis (Stroke Units) (Nordrhein-Westfalen) — Regular (+10min Penalty)

Category	Grid Cells	Area (%)	Population	Population (%)
High (30+ min)	0	0.0	0	0.0
Medium (20-30 min)	13	2.3	1319	0.4
Low (10-20 min)	182	32.4	45985	15.3
Only CT reachable within 60 min	0	0.0	0	0.0
Neither reachable within 60 min	0	0.0	0	0.0
Likely irrelevant (<10 min)	366	65.2	254068	84.3

Population and Area Coverage in Kreisfreie Stadt Zweibrücken (Stroke Units) (Rheinland-Pfalz) — Regular (+10min Penalty)

Category	Grid Cells	Area (%)	Population	Population (%)
High (30+ min)	0	0.0	0	0.0
Medium (20-30 min)	0	0.0	0	0.0
Low (10-20 min)	31	35.2	3273	10.0
Only CT reachable within 60 min	0	0.0	0	0.0
Neither reachable within 60 min	0	0.0	0	0.0
Likely irrelevant (<10 min)	57	64.8	29334	90.0

Population and Area Coverage in Landkreis Rhein-Pfalz-Kreis (Stroke Units) (Rheinland-Pfalz) — Regular (+10min Penalty)

Category	Grid Cells	Area (%)	Population	Population (%)
High (30+ min)	0	0.0	0	0.0
Medium (20-30 min)	0	0.0	0	0.0
Low (10-20 min)	42	11.0	9146	6.0
Only CT reachable within 60 min	0	0.0	0	0.0
Neither reachable within 60 min	0	0.0	0	0.0
Likely irrelevant (<10 min)	339	89.0	143349	94.0

Population and Area Coverage in Landkreis Altötting (Stroke Units) (Bayern) — Regular (+10min Penalty)

Category	Grid Cells	Area (%)	Population	Population (%)
High (30+ min)	0	0.0	0	0.0
Medium (20-30 min)	0	0.0	0	0.0
Low (10-20 min)	26	3.8	1610	1.5
Only CT reachable within 60 min	0	0.0	0	0.0
Neither reachable within 60 min	3	0.4	2068	1.9
Likely irrelevant (<10 min)	649	95.7	103617	96.6

Population and Area Coverage in Landkreis Pfaffenhofen a.d.Ilm (Stroke Units) (Bayern) — Regular (+10min Penalty)

Category	Grid Cells	Area (%)	Population	Population (%)
High (30+ min)	0	0.0	0	0.0
Medium (20-30 min)	17	1.8	2462	2.0
Low (10-20 min)	265	28.7	41428	33.4
Only CT reachable within 60 min	0	0.0	0	0.0
Neither reachable within 60 min	0	0.0	0	0.0
Likely irrelevant (<10 min)	642	69.5	79985	64.6

Population and Area Coverage in Kreisfreie Stadt Bamberg (Stroke Units) (Bayern) — Regular (+10min Penalty)

Category	Grid Cells	Area (%)	Population	Population (%)
High (30+ min)	0	0.0	0	0.0
Medium (20-30 min)	0	0.0	0	0.0
Low (10-20 min)	7	10.4	6005	7.7
Only CT reachable within 60 min	0	0.0	0	0.0
Neither reachable within 60 min	0	0.0	0	0.0
Likely irrelevant (<10 min)	60	89.6	71819	92.3

Population and Area Coverage in Landkreis Hof (Stroke Units) (Bayern) — Regular (+10min Penalty)

Category	Grid Cells	Area (%)	Population	Population (%)
High (30+ min)	0	0.0	0	0.0
Medium (20-30 min)	1	0.1	30	0.0
Low (10-20 min)	237	21.6	18359	18.4
Only CT reachable within 60 min	0	0.0	0	0.0
Neither reachable within 60 min	0	0.0	0	0.0
Likely irrelevant (<10 min)	861	78.3	81308	81.6

Population and Area Coverage in Landkreis Miltenberg (Stroke Units) (Bayern) — Regular (+10min Penalty)

Category	Grid Cells	Area (%)	Population	Population (%)
High (30+ min)	8	0.9	296	0.2
Medium (20-30 min)	74	8.3	4025	3.0
Low (10-20 min)	140	15.6	8429	6.3
Only CT reachable within 60 min	0	0.0	0	0.0
Neither reachable within 60 min	0	0.0	0	0.0
Likely irrelevant (<10 min)	674	75.2	120396	90.4

Population and Area Coverage in Kreisfreie Stadt Augsburg (Stroke Units) (Bayern) — Regular (+10min Penalty)

Category	Grid Cells	Area (%)	Population	Population (%)
High (30+ min)	0	0.0	0	0.0
Medium (20-30 min)	0	0.0	0	0.0
Low (10-20 min)	49	27.2	11321	3.9
Only CT reachable within 60 min	0	0.0	0	0.0
Neither reachable within 60 min	0	0.0	0	0.0
Likely irrelevant (<10 min)	131	72.8	275368	96.1

Population and Area Coverage in Landkreis Barnim (Stroke Units) (Brandenburg) — Regular (+10min Penalty)

Category	Grid Cells	Area (%)	Population	Population (%)
High (30+ min)	9	0.5	527	0.3
Medium (20-30 min)	169	8.5	4283	2.5
Low (10-20 min)	774	39.1	40683	23.9
Only CT reachable within 60 min	56	2.8	179	0.1
Neither reachable within 60 min	15	0.8	13	0.0
Likely irrelevant (<10 min)	956	48.3	124758	73.2

Population and Area Coverage in Landkreis Meißen (Stroke Units) (Sachsen) — Regular (+10min Penalty)

Category	Grid Cells	Area (%)	Population	Population (%)
High (30+ min)	53	2.8	8020	3.5
Medium (20-30 min)	351	18.7	45319	19.8
Low (10-20 min)	765	40.8	69607	30.4
Only CT reachable within 60 min	0	0.0	0	0.0
Neither reachable within 60 min	0	0.0	0	0.0
Likely irrelevant (<10 min)	704	37.6	106124	46.3

Population and Area Coverage in Kreisfreie Stadt Suhl (Stroke Units) (Thüringen) — Regular (+10min Penalty)

Category	Grid Cells	Area (%)	Population	Population (%)
High (30+ min)	0	0.0	0	0.0
Medium (20-30 min)	1	0.6	4	0.0
Low (10-20 min)	22	12.3	850	2.6
Only CT reachable within 60 min	0	0.0	0	0.0
Neither reachable within 60 min	0	0.0	0	0.0
Likely irrelevant (<10 min)	156	87.2	32454	97.4

Population and Area Coverage in Landkreis Eichsfeld (Stroke Units) (Thüringen) — Regular (+10min Penalty)

Category	Grid Cells	Area (%)	Population	Population (%)
High (30+ min)	162	12.6	10831	11.4
Medium (20-30 min)	566	43.9	37595	39.7
Low (10-20 min)	398	30.9	32716	34.6
Only CT reachable within 60 min	0	0.0	0	0.0
Neither reachable within 60 min	0	0.0	0	0.0
Likely irrelevant (<10 min)	162	12.6	13541	14.3

Population and Area Coverage in Landkreis Göttingen (Stroke Units) (Niedersachsen) — Regular (+10min Penalty)

Category	Grid Cells	Area (%)	Population	Population (%)
High (30+ min)	265	11.6	19607	5.9
Medium (20-30 min)	489	21.4	49022	14.7
Low (10-20 min)	674	29.6	67192	20.1
Only CT reachable within 60 min	0	0.0	0	0.0
Neither reachable within 60 min	0	0.0	0	0.0
Likely irrelevant (<10 min)	852	37.4	198750	59.4

Population and Area Coverage in Landkreis Schaumburg (Stroke Units) (Niedersachsen) — Regular (+10min Penalty)

Category	Grid Cells	Area (%)	Population	Population (%)
High (30+ min)	0	0.0	0	0.0
Medium (20-30 min)	1	0.1	16	0.0
Low (10-20 min)	68	7.6	8154	5.2
Only CT reachable within 60 min	0	0.0	0	0.0
Neither reachable within 60 min	0	0.0	0	0.0
Likely irrelevant (<10 min)	820	92.2	147672	94.8

Population and Area Coverage in Landkreis Aurich (Stroke Units) (Niedersachsen) — Regular (+10min Penalty)

Category	Grid Cells	Area (%)	Population	Population (%)
High (30+ min)	282	16.7	34715	18.8
Medium (20-30 min)	457	27.0	68263	36.9
Low (10-20 min)	313	18.5	35229	19.0
Only CT reachable within 60 min	10	0.6	124	0.1
Neither reachable within 60 min	66	3.9	4909	2.7
Likely irrelevant (<10 min)	562	33.3	41828	22.6

Population and Area Coverage in Landkreis Friesland (Stroke Units) (Niedersachsen) — Regular (+10min Penalty)

Category	Grid Cells	Area (%)	Population	Population (%)
High (30+ min)	3	0.4	419	0.4
Medium (20-30 min)	2	0.2	411	0.4
Low (10-20 min)	148	17.9	14405	14.8
Only CT reachable within 60 min	4	0.5	67	0.1
Neither reachable within 60 min	48	5.8	1966	2.0
Likely irrelevant (<10 min)	621	75.2	80165	82.3

Population and Area Coverage in Landkreis Wesermarsch (Stroke Units) (Niedersachsen) — Regular (+10min Penalty)

Category	Grid Cells	Area (%)	Population	Population (%)
High (30+ min)	1	0.1	43	0.1
Medium (20-30 min)	386	35.2	34201	40.4
Low (10-20 min)	368	33.5	29211	34.5
Only CT reachable within 60 min	9	0.8	225	0.3
Neither reachable within 60 min	23	2.1	344	0.4
Likely irrelevant (<10 min)	310	28.3	20678	24.4

Population and Area Coverage in Landkreis Main-Kinzig-Kreis (Stroke Units) (Hessen) — Regular (+10min Penalty)

Category	Grid Cells	Area (%)	Population	Population (%)
High (30+ min)	0	0.0	0	0.0
Medium (20-30 min)	117	6.6	8625	2.1
Low (10-20 min)	629	35.7	36695	9.0
Only CT reachable within 60 min	0	0.0	0	0.0
Neither reachable within 60 min	0	0.0	0	0.0
Likely irrelevant (<10 min)	1014	57.6	363864	88.9

Population and Area Coverage in Landkreis Vogelsbergkreis (Stroke Units) (Hessen) — Regular (+10min Penalty)

Category	Grid Cells	Area (%)	Population	Population (%)
High (30+ min)	211	11.4	13041	12.2
Medium (20-30 min)	633	34.1	42320	39.4
Low (10-20 min)	707	38.1	31415	29.3
Only CT reachable within 60 min	0	0.0	0	0.0
Neither reachable within 60 min	0	0.0	0	0.0
Likely irrelevant (<10 min)	305	16.4	20548	19.1

Population and Area Coverage in Landkreis Neuwied (Stroke Units) (Rheinland-Pfalz) — Regular (+10min Penalty)

Category	Grid Cells	Area (%)	Population	Population (%)
High (30+ min)	0	0.0	0	0.0
Medium (20-30 min)	50	6.3	12941	7.0
Low (10-20 min)	309	39.0	72266	39.0
Only CT reachable within 60 min	0	0.0	0	0.0
Neither reachable within 60 min	0	0.0	0	0.0
Likely irrelevant (<10 min)	434	54.7	100110	54.0

Population and Area Coverage in Kreisfreie Stadt Mainz (Stroke Units) (Rheinland-Pfalz) — Regular (+10min Penalty)

Category	Grid Cells	Area (%)	Population	Population (%)
High (30+ min)	0	0.0	0	0.0
Medium (20-30 min)	0	0.0	0	0.0
Low (10-20 min)	1	0.8	9	0.0
Only CT reachable within 60 min	0	0.0	0	0.0
Neither reachable within 60 min	0	0.0	0	0.0
Likely irrelevant (<10 min)	120	99.2	214059	100.0

Population and Area Coverage in Landkreis Hohenlohekreis (Stroke Units) (Baden-Württemberg) — Regular (+10min Penalty)

Category	Grid Cells	Area (%)	Population	Population (%)
High (30+ min)	0	0.0	0	0.0
Medium (20-30 min)	67	7.0	6760	6.2
Low (10-20 min)	350	36.5	55806	51.0
Only CT reachable within 60 min	0	0.0	0	0.0
Neither reachable within 60 min	0	0.0	0	0.0
Likely irrelevant (<10 min)	542	56.5	46760	42.8

Population and Area Coverage in Landkreis Ortenaukreis (Stroke Units) (Baden-Württemberg) — Regular (+10min Penalty)

Category	Grid Cells	Area (%)	Population	Population (%)
High (30+ min)	221	9.8	17961	4.3
Medium (20-30 min)	266	11.9	30002	7.1
Low (10-20 min)	499	22.2	91251	21.7
Only CT reachable within 60 min	35	1.6	296	0.1
Neither reachable within 60 min	9	0.4	262	0.1
Likely irrelevant (<10 min)	1214	54.1	280928	66.8

Population and Area Coverage in Landkreis Neustadt a.d.Aisch-Bad Windsheim (Stroke Units) (Bayern) — Regular (+10min Penalty)

Category	Grid Cells	Area (%)	Population	Population (%)
High (30+ min)	66	4.2	1199	1.2
Medium (20-30 min)	616	38.8	46530	45.8
Low (10-20 min)	670	42.2	40678	40.1
Only CT reachable within 60 min	0	0.0	0	0.0
Neither reachable within 60 min	0	0.0	0	0.0
Likely irrelevant (<10 min)	234	14.8	13093	12.9

Population and Area Coverage in Landkreis Goslar (Stroke Units) (Niedersachsen) — Regular (+10min Penalty)

Category	Grid Cells	Area (%)	Population	Population (%)
High (30+ min)	26	2.1	565	0.4
Medium (20-30 min)	164	13.1	7758	5.7
Low (10-20 min)	497	39.7	82446	60.3
Only CT reachable within 60 min	0	0.0	0	0.0
Neither reachable within 60 min	0	0.0	0	0.0
Likely irrelevant (<10 min)	565	45.1	46028	33.6

Population and Area Coverage in Landkreis Region Hannover (Stroke Units) (Niedersachsen) — Regular (+10min Penalty)

Category	Grid Cells	Area (%)	Population	Population (%)
High (30+ min)	1	0.0	27	0.0
Medium (20-30 min)	275	9.1	36419	3.2
Low (10-20 min)	1141	37.7	159469	13.9
Only CT reachable within 60 min	0	0.0	0	0.0
Neither reachable within 60 min	0	0.0	0	0.0
Likely irrelevant (<10 min)	1608	53.2	954839	83.0

Population and Area Coverage in Landkreis Vechta (Stroke Units) (Niedersachsen) — Regular (+10min Penalty)

Category	Grid Cells	Area (%)	Population	Population (%)
High (30+ min)	81	7.5	7020	5.2
Medium (20-30 min)	246	22.8	40243	29.6
Low (10-20 min)	215	20.0	36259	26.7
Only CT reachable within 60 min	0	0.0	0	0.0
Neither reachable within 60 min	0	0.0	0	0.0
Likely irrelevant (<10 min)	535	49.7	52257	38.5

Population and Area Coverage in Kreis Städteregion Aachen (Stroke Units) (Nordrhein-Westfalen) — Regular (+10min Penalty)

Category	Grid Cells	Area (%)	Population	Population (%)
High (30+ min)	133	14.9	18595	3.3
Medium (20-30 min)	116	13.0	10455	1.9
Low (10-20 min)	194	21.7	79764	14.4
Only CT reachable within 60 min	9	1.0	256	0.0
Neither reachable within 60 min	13	1.5	387	0.1
Likely irrelevant (<10 min)	427	47.9	445952	80.3

Population and Area Coverage in Kreis Steinfurt (Stroke Units) (Nordrhein-Westfalen) — Regular (+10min Penalty)

Category	Grid Cells	Area (%)	Population	Population (%)
High (30+ min)	0	0.0	0	0.0
Medium (20-30 min)	182	7.7	24374	5.6
Low (10-20 min)	1036	43.8	203348	46.6
Only CT reachable within 60 min	0	0.0	0	0.0
Neither reachable within 60 min	0	0.0	0	0.0
Likely irrelevant (<10 min)	1148	48.5	208185	47.8

Population and Area Coverage in Kreis Gütersloh (Stroke Units) (Nordrhein-Westfalen) — Regular (+10min Penalty)

Category	Grid Cells	Area (%)	Population	Population (%)
High (30+ min)	11	0.9	807	0.2
Medium (20-30 min)	269	21.3	49076	13.8
Low (10-20 min)	680	53.9	229361	64.6
Only CT reachable within 60 min	0	0.0	0	0.0
Neither reachable within 60 min	0	0.0	0	0.0
Likely irrelevant (<10 min)	302	23.9	75545	21.3

Population and Area Coverage in Kreis Unna (Stroke Units) (Nordrhein-Westfalen) — Regular (+10min Penalty)

Category	Grid Cells	Area (%)	Population	Population (%)
High (30+ min)	0	0.0	0	0.0
Medium (20-30 min)	0	0.0	0	0.0
Low (10-20 min)	103	14.5	31515	8.1
Only CT reachable within 60 min	0	0.0	0	0.0
Neither reachable within 60 min	0	0.0	0	0.0
Likely irrelevant (<10 min)	608	85.5	355697	91.9

Population and Area Coverage in Landkreis Darmstadt-Dieburg (Stroke Units) (Hessen) — Regular (+10min Penalty)

Category	Grid Cells	Area (%)	Population	Population (%)
High (30+ min)	0	0.0	0	0.0
Medium (20-30 min)	54	6.5	4548	1.6
Low (10-20 min)	334	40.5	81812	28.3
Only CT reachable within 60 min	0	0.0	0	0.0
Neither reachable within 60 min	0	0.0	0	0.0
Likely irrelevant (<10 min)	437	53.0	202606	70.1

Population and Area Coverage in Landkreis Gießen (Stroke Units) (Hessen) — Regular (+10min Penalty)

Category	Grid Cells	Area (%)	Population	Population (%)
High (30+ min)	0	0.0	0	0.0
Medium (20-30 min)	0	0.0	0	0.0
Low (10-20 min)	222	20.5	23633	9.2
Only CT reachable within 60 min	0	0.0	0	0.0
Neither reachable within 60 min	0	0.0	0	0.0
Likely irrelevant (<10 min)	859	79.5	234365	90.8

Population and Area Coverage in Kreisfreie Stadt Neustadt an der Weinstraße (Stroke Units) (Rheinland-Pfalz) — Regular (+10min Penalty)

Category	Grid Cells	Area (%)	Population	Population (%)
High (30+ min)	0	0.0	0	0.0
Medium (20-30 min)	32	21.8	1533	2.9
Low (10-20 min)	100	68.0	50695	94.4
Only CT reachable within 60 min	0	0.0	0	0.0
Neither reachable within 60 min	0	0.0	0	0.0
Likely irrelevant (<10 min)	15	10.2	1489	2.8

Population and Area Coverage in Landkreis Main-Tauber-Kreis (Stroke Units) (Baden-Württemberg) — Regular (+10min Penalty)

Category	Grid Cells	Area (%)	Population	Population (%)
High (30+ min)	35	2.2	1094	0.8
Medium (20-30 min)	290	17.9	16547	12.6
Low (10-20 min)	486	30.0	36123	27.4
Only CT reachable within 60 min	0	0.0	0	0.0
Neither reachable within 60 min	0	0.0	0	0.0
Likely irrelevant (<10 min)	811	50.0	77855	59.2

Population and Area Coverage in Landkreis Freudenstadt (Stroke Units) (Baden-Württemberg) — Regular (+10min Penalty)

Category	Grid Cells	Area (%)	Population	Population (%)
High (30+ min)	405	38.3	52050	44.9
Medium (20-30 min)	143	13.5	12157	10.5
Low (10-20 min)	128	12.1	23303	20.1
Only CT reachable within 60 min	382	36.1	28395	24.5
Neither reachable within 60 min	0	0.0	0	0.0
Likely irrelevant (<10 min)	0	0.0	2	0.0

Population and Area Coverage in Landkreis Rosenheim (Stroke Units) (Bayern) — Regular (+10min Penalty)

Category	Grid Cells	Area (%)	Population	Population (%)
High (30+ min)	9	0.5	80	0.0
Medium (20-30 min)	69	4.0	3618	1.4
Low (10-20 min)	332	19.3	49893	19.8
Only CT reachable within 60 min	2	0.1	30	0.0
Neither reachable within 60 min	137	8.0	989	0.4
Likely irrelevant (<10 min)	1174	68.1	197137	78.3

Population and Area Coverage in Kreisfreie Stadt Passau (Stroke Units) (Bayern) — Regular (+10min Penalty)

Category	Grid Cells	Area (%)	Population	Population (%)
High (30+ min)	0	0.0	0	0.0
Medium (20-30 min)	0	0.0	0	0.0
Low (10-20 min)	1	1.3	7	0.0
Only CT reachable within 60 min	0	0.0	0	0.0
Neither reachable within 60 min	0	0.0	0	0.0
Likely irrelevant (<10 min)	75	98.7	42444	100.0

Population and Area Coverage in Landkreis Coburg (Stroke Units) (Bayern) — Regular (+10min Penalty)

Category	Grid Cells	Area (%)	Population	Population (%)
High (30+ min)	0	0.0	0	0.0
Medium (20-30 min)	3	0.4	0	0.0
Low (10-20 min)	102	13.6	3816	4.2
Only CT reachable within 60 min	0	0.0	0	0.0
Neither reachable within 60 min	0	0.0	0	0.0
Likely irrelevant (<10 min)	647	86.0	86070	95.8

Population and Area Coverage in Landkreis Ostallgäu (Stroke Units) (Bayern) — Regular (+10min Penalty)

Category	Grid Cells	Area (%)	Population	Population (%)
High (30+ min)	3	0.2	79	0.1
Medium (20-30 min)	83	5.0	9272	6.6
Low (10-20 min)	445	26.6	54189	38.6
Only CT reachable within 60 min	35	2.1	96	0.1
Neither reachable within 60 min	158	9.4	1332	0.9
Likely irrelevant (<10 min)	951	56.8	75367	53.7

Population and Area Coverage in Landkreis Saarpfalz-Kreis (Stroke Units) (Saarland) — Regular (+10min Penalty)

Category	Grid Cells	Area (%)	Population	Population (%)
High (30+ min)	0	0.0	0	0.0
Medium (20-30 min)	2	0.4	265	0.2
Low (10-20 min)	86	17.0	5601	4.1
Only CT reachable within 60 min	0	0.0	0	0.0
Neither reachable within 60 min	4	0.8	0	0.0
Likely irrelevant (<10 min)	413	81.8	131178	95.7

Population and Area Coverage in Landkreis Nordhausen (Stroke Units) (Thüringen) — Regular (+10min Penalty)

Category	Grid Cells	Area (%)	Population	Population (%)
High (30+ min)	5	0.5	0	0.0
Medium (20-30 min)	155	16.9	7788	10.1
Low (10-20 min)	357	39.0	14162	18.4
Only CT reachable within 60 min	0	0.0	0	0.0
Neither reachable within 60 min	0	0.0	0	0.0
Likely irrelevant (<10 min)	399	43.6	55153	71.5

Population and Area Coverage in Landkreis Uelzen (Stroke Units) (Niedersachsen) — Regular (+10min Penalty)

Category	Grid Cells	Area (%)	Population	Population (%)
High (30+ min)	10	0.5	28	0.0
Medium (20-30 min)	132	6.8	1869	2.0
Low (10-20 min)	515	26.5	26181	28.1
Only CT reachable within 60 min	0	0.0	0	0.0
Neither reachable within 60 min	0	0.0	0	0.0
Likely irrelevant (<10 min)	1290	66.3	65035	69.8

Population and Area Coverage in Kreisfreie Stadt Mülheim an der Ruhr (Stroke Units) (Nordrhein-Westfalen) — Regular (+10min Penalty)

Category	Grid Cells	Area (%)	Population	Population (%)
High (30+ min)	0	0.0	0	0.0
Medium (20-30 min)	0	0.0	0	0.0
Low (10-20 min)	0	0.0	0	0.0
Only CT reachable within 60 min	0	0.0	0	0.0
Neither reachable within 60 min	0	0.0	0	0.0
Likely irrelevant (<10 min)	113	100.0	180205	100.0

Population and Area Coverage in Kreisfreie Stadt Bonn (Stroke Units) (Nordrhein-Westfalen) — Regular (+10min Penalty)

Category	Grid Cells	Area (%)	Population	Population (%)
High (30+ min)	0	0.0	0	0.0
Medium (20-30 min)	0	0.0	0	0.0
Low (10-20 min)	19	10.6	19001	5.8
Only CT reachable within 60 min	0	0.0	0	0.0
Neither reachable within 60 min	0	0.0	0	0.0
Likely irrelevant (<10 min)	161	89.4	306709	94.2

Population and Area Coverage in Landkreis Birkenfeld (Stroke Units) (Rheinland-Pfalz) — Regular (+10min Penalty)

Category	Grid Cells	Area (%)	Population	Population (%)
High (30+ min)	7	0.7	0	0.0
Medium (20-30 min)	101	10.5	3798	4.6
Low (10-20 min)	341	35.3	23649	28.6
Only CT reachable within 60 min	0	0.0	0	0.0
Neither reachable within 60 min	0	0.0	0	0.0
Likely irrelevant (<10 min)	516	53.5	55302	66.8

Population and Area Coverage in Landkreis Rems-Murr-Kreis (Stroke Units) (Baden-Württemberg) — Regular (+10min Penalty)

Category	Grid Cells	Area (%)	Population	Population (%)
High (30+ min)	16	1.5	1298	0.3
Medium (20-30 min)	62	5.9	5754	1.4
Low (10-20 min)	283	26.8	72613	17.4
Only CT reachable within 60 min	0	0.0	0	0.0
Neither reachable within 60 min	0	0.0	0	0.0
Likely irrelevant (<10 min)	696	65.8	338154	80.9

Population and Area Coverage in Landkreis Schwäbisch Hall (Stroke Units) (Baden-Württemberg) — Regular (+10min Penalty)

Category	Grid Cells	Area (%)	Population	Population (%)
High (30+ min)	0	0.0	88	0.0
Medium (20-30 min)	148	8.1	16472	8.6
Low (10-20 min)	510	27.9	50791	26.6
Only CT reachable within 60 min	0	0.0	0	0.0
Neither reachable within 60 min	0	0.0	0	0.0
Likely irrelevant (<10 min)	1167	63.9	123536	64.7

Population and Area Coverage in Landkreis Rottweil (Stroke Units) (Baden-Württemberg) — Regular (+10min Penalty)

Category	Grid Cells	Area (%)	Population	Population (%)
High (30+ min)	157	16.7	19280	14.0
Medium (20-30 min)	385	40.9	45796	33.3
Low (10-20 min)	293	31.1	63476	46.1
Only CT reachable within 60 min	36	3.8	403	0.3
Neither reachable within 60 min	0	0.0	0	0.0
Likely irrelevant (<10 min)	70	7.4	8601	6.3

Population and Area Coverage in Landkreis Amberg-Sulzbach (Stroke Units) (Bayern) — Regular (+10min Penalty)

Category	Grid Cells	Area (%)	Population	Population (%)
High (30+ min)	0	0.0	21	0.0
Medium (20-30 min)	118	7.6	8253	7.7
Low (10-20 min)	339	21.7	19874	18.6
Only CT reachable within 60 min	0	0.0	0	0.0
Neither reachable within 60 min	0	0.0	0	0.0
Likely irrelevant (<10 min)	1102	70.7	78557	73.6

Population and Area Coverage in Kreisfreie Stadt Kempten (Allgäu) (Stroke Units) (Bayern) — Regular (+10min Penalty)

Category	Grid Cells	Area (%)	Population	Population (%)
High (30+ min)	0	0.0	0	0.0
Medium (20-30 min)	0	0.0	0	0.0
Low (10-20 min)	0	0.0	0	0.0
Only CT reachable within 60 min	0	0.0	0	0.0
Neither reachable within 60 min	0	0.0	0	0.0
Likely irrelevant (<10 min)	77	100.0	68488	100.0

Population and Area Coverage in Kreisfreie Stadt Chemnitz (Stroke Units) (Sachsen) — Regular (+10min Penalty)

Category	Grid Cells	Area (%)	Population	Population (%)
High (30+ min)	0	0.0	0	0.0
Medium (20-30 min)	39	14.1	8553	2.9
Low (10-20 min)	83	30.1	127726	43.1
Only CT reachable within 60 min	0	0.0	0	0.0
Neither reachable within 60 min	0	0.0	0	0.0
Likely irrelevant (<10 min)	154	55.8	159892	54.0

Population and Area Coverage in Landkreis Mecklenburgische Seenplatte (Stroke Units) (Mecklenburg-Vorpommern) — Regular (+10min Penalty)

Category	Grid Cells	Area (%)	Population	Population (%)
High (30+ min)	1179	15.8	62551	26.2
Medium (20-30 min)	1018	13.7	27737	11.6
Low (10-20 min)	1224	16.4	25160	10.5
Only CT reachable within 60 min	1140	15.3	12665	5.3
Neither reachable within 60 min	460	6.2	2134	0.9
Likely irrelevant (<10 min)	2432	32.6	108694	45.5

Population and Area Coverage in Landkreis Erzgebirgskreis (Stroke Units) (Sachsen) — Regular (+10min Penalty)

Category	Grid Cells	Area (%)	Population	Population (%)
High (30+ min)	580	25.3	54717	16.8
Medium (20-30 min)	545	23.8	77011	23.6
Low (10-20 min)	604	26.4	78166	24.0
Only CT reachable within 60 min	41	1.8	741	0.2
Neither reachable within 60 min	3	0.1	159	0.0
Likely irrelevant (<10 min)	517	22.6	115476	35.4

Population and Area Coverage in Kreisfreie Stadt Dresden (Stroke Units) (Sachsen) — Regular (+10min Penalty)

Category	Grid Cells	Area (%)	Population	Population (%)
High (30+ min)	0	0.0	0	0.0
Medium (20-30 min)	1	0.2	19	0.0
Low (10-20 min)	78	18.6	32402	5.3
Only CT reachable within 60 min	0	0.0	0	0.0
Neither reachable within 60 min	0	0.0	0	0.0
Likely irrelevant (<10 min)	341	81.2	582985	94.7

Population and Area Coverage in Landkreis Hildburghausen (Stroke Units) (Thüringen) — Regular (+10min Penalty)

Category	Grid Cells	Area (%)	Population	Population (%)
High (30+ min)	0	0.0	0	0.0
Medium (20-30 min)	8	0.7	65	0.1
Low (10-20 min)	133	11.2	2704	4.6
Only CT reachable within 60 min	0	0.0	0	0.0
Neither reachable within 60 min	0	0.0	0	0.0
Likely irrelevant (<10 min)	1047	88.1	56128	95.3

Population and Area Coverage in Kreis Herzogtum Lauenburg (Stroke Units) (Schleswig-Holstein) — Regular (+10min Penalty)

Category	Grid Cells	Area (%)	Population	Population (%)
High (30+ min)	62	3.6	3701	1.9
Medium (20-30 min)	284	16.6	36454	18.7
Low (10-20 min)	597	34.9	62646	32.1
Only CT reachable within 60 min	0	0.0	0	0.0
Neither reachable within 60 min	0	0.0	0	0.0
Likely irrelevant (<10 min)	768	44.9	92552	47.4

Population and Area Coverage in Landkreis Oldenburg (Stroke Units) (Niedersachsen) — Regular (+10min Penalty)

Category	Grid Cells	Area (%)	Population	Population (%)
High (30+ min)	42	2.9	1157	0.9
Medium (20-30 min)	266	18.6	26727	20.9
Low (10-20 min)	469	32.8	43076	33.8
Only CT reachable within 60 min	0	0.0	0	0.0
Neither reachable within 60 min	0	0.0	0	0.0
Likely irrelevant (<10 min)	651	45.6	56659	44.4

Population and Area Coverage in Kreisfreie Stadt Krefeld (Stroke Units) (Nordrhein-Westfalen) — Regular (+10min Penalty)

Category	Grid Cells	Area (%)	Population	Population (%)
High (30+ min)	0	0.0	0	0.0
Medium (20-30 min)	0	0.0	0	0.0
Low (10-20 min)	0	0.0	0	0.0
Only CT reachable within 60 min	0	0.0	0	0.0
Neither reachable within 60 min	0	0.0	0	0.0
Likely irrelevant (<10 min)	181	100.0	241982	100.0

Population and Area Coverage in Kreis Oberbergischer Kreis (Stroke Units) (Nordrhein-Westfalen) — Regular (+10min Penalty)

Category	Grid Cells	Area (%)	Population	Population (%)
High (30+ min)	92	7.8	6041	2.2
Medium (20-30 min)	616	52.4	155686	57.9
Low (10-20 min)	369	31.4	94841	35.3
Only CT reachable within 60 min	0	0.0	0	0.0
Neither reachable within 60 min	0	0.0	0	0.0
Likely irrelevant (<10 min)	99	8.4	12300	4.6

Population and Area Coverage in Landkreis Kusel (Stroke Units) (Rheinland-Pfalz) — Regular (+10min Penalty)

Category	Grid Cells	Area (%)	Population	Population (%)
High (30+ min)	18	2.5	1106	1.5
Medium (20-30 min)	161	22.5	15255	20.8
Low (10-20 min)	296	41.3	26388	36.1
Only CT reachable within 60 min	0	0.0	0	0.0
Neither reachable within 60 min	0	0.0	0	0.0
Likely irrelevant (<10 min)	242	33.8	30437	41.6

Population and Area Coverage in Landkreis Fürstenfeldbruck (Stroke Units) (Bayern) — Regular (+10min Penalty)

Category	Grid Cells	Area (%)	Population	Population (%)
High (30+ min)	0	0.0	0	0.0
Medium (20-30 min)	0	0.0	0	0.0
Low (10-20 min)	3	0.6	44	0.0
Only CT reachable within 60 min	0	0.0	0	0.0
Neither reachable within 60 min	0	0.0	0	0.0
Likely irrelevant (<10 min)	523	99.4	214894	100.0

Population and Area Coverage in Landkreis Weilheim-Schongau (Stroke Units) (Bayern) — Regular (+10min Penalty)

Category	Grid Cells	Area (%)	Population	Population (%)
High (30+ min)	4	0.3	96	0.1
Medium (20-30 min)	148	12.8	22839	16.9
Low (10-20 min)	268	23.1	23070	17.1
Only CT reachable within 60 min	1	0.1	0	0.0
Neither reachable within 60 min	13	1.1	13	0.0
Likely irrelevant (<10 min)	726	62.6	89055	65.9

Population and Area Coverage in Landkreis Neustadt a.d.Waldnaab (Stroke Units) (Bayern) — Regular (+10min Penalty)

Category	Grid Cells	Area (%)	Population	Population (%)
High (30+ min)	2	0.1	5	0.0
Medium (20-30 min)	194	11.3	4506	4.6
Low (10-20 min)	434	25.2	15433	15.8
Only CT reachable within 60 min	0	0.0	0	0.0
Neither reachable within 60 min	0	0.0	0	0.0
Likely irrelevant (<10 min)	1092	63.4	77475	79.5

Population and Area Coverage in Landkreis Würzburg (Stroke Units) (Bayern) — Regular (+10min Penalty)

Category	Grid Cells	Area (%)	Population	Population (%)
High (30+ min)	0	0.0	0	0.0
Medium (20-30 min)	13	1.1	511	0.3
Low (10-20 min)	266	22.1	25781	15.6
Only CT reachable within 60 min	0	0.0	0	0.0
Neither reachable within 60 min	0	0.0	0	0.0
Likely irrelevant (<10 min)	926	76.8	138770	84.1

Population and Area Coverage in Landkreis Elbe-Elster (Stroke Units) (Brandenburg) — Regular (+10min Penalty)

Category	Grid Cells	Area (%)	Population	Population (%)
High (30+ min)	778	31.5	43369	42.5
Medium (20-30 min)	772	31.3	33501	32.9
Low (10-20 min)	266	10.8	7184	7.0
Only CT reachable within 60 min	589	23.9	16525	16.2
Neither reachable within 60 min	0	0.0	0	0.0
Likely irrelevant (<10 min)	64	2.6	1360	1.3

Population and Area Coverage in Landkreis Osterholz (Stroke Units) (Niedersachsen) — Regular (+10min Penalty)

Category	Grid Cells	Area (%)	Population	Population (%)
High (30+ min)	3	0.3	158	0.1
Medium (20-30 min)	420	47.8	30566	27.3
Low (10-20 min)	226	25.7	45887	40.9
Only CT reachable within 60 min	0	0.0	0	0.0
Neither reachable within 60 min	0	0.0	0	0.0
Likely irrelevant (<10 min)	230	26.2	35541	31.7

Population and Area Coverage in Landkreis Heidekreis (Stroke Units) (Niedersachsen) — Regular (+10min Penalty)

Category	Grid Cells	Area (%)	Population	Population (%)
High (30+ min)	517	20.5	38047	28.0
Medium (20-30 min)	857	34.1	44396	32.7
Low (10-20 min)	703	27.9	43165	31.8
Only CT reachable within 60 min	21	0.8	4	0.0
Neither reachable within 60 min	0	0.0	0	0.0
Likely irrelevant (<10 min)	418	16.6	10232	7.5

Population and Area Coverage in Kreisfreie Stadt Solingen (Stroke Units) (Nordrhein-Westfalen) — Regular (+10min Penalty)

Category	Grid Cells	Area (%)	Population	Population (%)
High (30+ min)	0	0.0	0	0.0
Medium (20-30 min)	0	0.0	0	0.0
Low (10-20 min)	46	40.0	74832	47.2
Only CT reachable within 60 min	0	0.0	0	0.0
Neither reachable within 60 min	0	0.0	0	0.0
Likely irrelevant (<10 min)	69	60.0	83606	52.8

Population and Area Coverage in Landkreis Bad Kreuznach (Stroke Units) (Rheinland-Pfalz) — Regular (+10min Penalty)

Category	Grid Cells	Area (%)	Population	Population (%)
High (30+ min)	11	1.0	641	0.4
Medium (20-30 min)	148	13.8	11430	7.1
Low (10-20 min)	380	35.4	29075	18.2
Only CT reachable within 60 min	0	0.0	0	0.0
Neither reachable within 60 min	0	0.0	0	0.0
Likely irrelevant (<10 min)	533	49.7	119023	74.3

Population and Area Coverage in Landkreis Südwestpfalz (Stroke Units) (Rheinland-Pfalz) — Regular (+10min Penalty)

Category	Grid Cells	Area (%)	Population	Population (%)
High (30+ min)	254	21.6	23132	20.6
Medium (20-30 min)	356	30.2	41206	36.7
Low (10-20 min)	372	31.6	35113	31.3
Only CT reachable within 60 min	45	3.8	585	0.5
Neither reachable within 60 min	10	0.8	61	0.1
Likely irrelevant (<10 min)	140	11.9	12044	10.7

Population and Area Coverage in Landkreis Ludwigsburg (Stroke Units) (Baden-Württemberg) — Regular (+10min Penalty)

Category	Grid Cells	Area (%)	Population	Population (%)
High (30+ min)	18	2.1	1140	0.2
Medium (20-30 min)	185	21.8	57146	10.8
Low (10-20 min)	251	29.6	128953	24.4
Only CT reachable within 60 min	0	0.0	0	0.0
Neither reachable within 60 min	0	0.0	0	0.0
Likely irrelevant (<10 min)	393	46.4	340304	64.5

Population and Area Coverage in Landkreis Lörrach (Stroke Units) (Baden-Württemberg) — Regular (+10min Penalty)

Category	Grid Cells	Area (%)	Population	Population (%)
High (30+ min)	1	0.1	1	0.0
Medium (20-30 min)	44	4.7	2879	1.3
Low (10-20 min)	452	48.1	50081	23.1
Only CT reachable within 60 min	37	3.9	1124	0.5
Neither reachable within 60 min	0	0.0	9	0.0
Likely irrelevant (<10 min)	405	43.1	162550	75.0

Population and Area Coverage in Landkreis Alb-Donau-Kreis (Stroke Units) (Baden-Württemberg) — Regular (+10min Penalty)

Category	Grid Cells	Area (%)	Population	Population (%)
High (30+ min)	4	0.2	3	0.0
Medium (20-30 min)	218	13.1	10841	5.7
Low (10-20 min)	716	43.2	88732	46.4
Only CT reachable within 60 min	0	0.0	0	0.0
Neither reachable within 60 min	0	0.0	0	0.0
Likely irrelevant (<10 min)	720	43.4	91531	47.9

Population and Area Coverage in Landkreis Biberach (Stroke Units) (Baden-Württemberg) — Regular (+10min Penalty)

Category	Grid Cells	Area (%)	Population	Population (%)
High (30+ min)	0	0.0	0	0.0
Medium (20-30 min)	0	0.0	0	0.0
Low (10-20 min)	30	1.8	2355	1.2
Only CT reachable within 60 min	0	0.0	0	0.0
Neither reachable within 60 min	0	0.0	0	0.0
Likely irrelevant (<10 min)	1674	98.2	190902	98.8

Population and Area Coverage in Kreisfreie Stadt Fürth (Stroke Units) (Bayern) — Regular (+10min Penalty)

Category	Grid Cells	Area (%)	Population	Population (%)
High (30+ min)	0	0.0	0	0.0
Medium (20-30 min)	0	0.0	0	0.0
Low (10-20 min)	0	0.0	0	0.0
Only CT reachable within 60 min	0	0.0	0	0.0
Neither reachable within 60 min	0	0.0	0	0.0
Likely irrelevant (<10 min)	83	100.0	150301	100.0

Population and Area Coverage in Kreisfreie Stadt Halle (Saale) (Stroke Units) (Sachsen-Anhalt) — Regular (+10min Penalty)

Category	Grid Cells	Area (%)	Population	Population (%)
High (30+ min)	0	0.0	0	0.0
Medium (20-30 min)	0	0.0	0	0.0
Low (10-20 min)	0	0.0	0	0.0
Only CT reachable within 60 min	0	0.0	0	0.0
Neither reachable within 60 min	0	0.0	0	0.0
Likely irrelevant (<10 min)	174	100.0	250172	100.0

Population and Area Coverage in Landkreis Wittenberg (Stroke Units) (Sachsen-Anhalt) — Regular (+10min Penalty)

Category	Grid Cells	Area (%)	Population	Population (%)
High (30+ min)	636	25.1	35155	30.8
Medium (20-30 min)	253	10.0	8791	7.7
Low (10-20 min)	59	2.3	4823	4.2
Only CT reachable within 60 min	1589	62.6	65311	57.2
Neither reachable within 60 min	0	0.0	0	0.0
Likely irrelevant (<10 min)	0	0.0	3	0.0

Population and Area Coverage in Landkreis Saale-Holzland-Kreis (Stroke Units) (Thüringen) — Regular (+10min Penalty)

Category	Grid Cells	Area (%)	Population	Population (%)
High (30+ min)	0	0.0	0	0.0
Medium (20-30 min)	90	8.7	4295	5.7
Low (10-20 min)	507	48.8	35767	47.2
Only CT reachable within 60 min	0	0.0	0	0.0
Neither reachable within 60 min	0	0.0	0	0.0
Likely irrelevant (<10 min)	441	42.5	35640	47.1

Population and Area Coverage in Kreisfreie Stadt Salzgitter (Stroke Units) (Niedersachsen) — Regular (+10min Penalty)

Category	Grid Cells	Area (%)	Population	Population (%)
High (30+ min)	0	0.0	0	0.0
Medium (20-30 min)	42	14.2	16150	16.1
Low (10-20 min)	205	69.5	64842	64.8
Only CT reachable within 60 min	0	0.0	0	0.0
Neither reachable within 60 min	0	0.0	0	0.0
Likely irrelevant (<10 min)	48	16.3	19060	19.1

Population and Area Coverage in Kreis Wesel (Stroke Units) (Nordrhein-Westfalen) — Regular (+10min Penalty)

Category	Grid Cells	Area (%)	Population	Population (%)
High (30+ min)	0	0.0	0	0.0
Medium (20-30 min)	53	3.9	6474	1.4
Low (10-20 min)	333	24.6	87604	18.4
Only CT reachable within 60 min	0	0.0	0	0.0
Neither reachable within 60 min	0	0.0	0	0.0
Likely irrelevant (<10 min)	967	71.5	381006	80.2

Population and Area Coverage in Kreis Rhein-Erft-Kreis (Stroke Units) (Nordrhein-Westfalen) — Regular (+10min Penalty)

Category	Grid Cells	Area (%)	Population	Population (%)
High (30+ min)	0	0.0	0	0.0
Medium (20-30 min)	0	0.0	0	0.0
Low (10-20 min)	217	24.2	104586	23.0
Only CT reachable within 60 min	0	0.0	0	0.0
Neither reachable within 60 min	0	0.0	0	0.0
Likely irrelevant (<10 min)	681	75.8	349943	77.0

Population and Area Coverage in Kreisfreie Stadt Frankfurt am Main (Stroke Units) (Hessen) — Regular (+10min Penalty)

Category	Grid Cells	Area (%)	Population	Population (%)
High (30+ min)	0	0.0	0	0.0
Medium (20-30 min)	0	0.0	0	0.0
Low (10-20 min)	1	0.3	0	0.0
Only CT reachable within 60 min	0	0.0	0	0.0
Neither reachable within 60 min	0	0.0	0	0.0
Likely irrelevant (<10 min)	309	99.7	833128	100.0

Population and Area Coverage in Landkreis Südliche Weinstraße (Stroke Units) (Rheinland-Pfalz) — Regular (+10min Penalty)

Category	Grid Cells	Area (%)	Population	Population (%)
High (30+ min)	1	0.1	0	0.0
Medium (20-30 min)	114	14.4	9687	8.7
Low (10-20 min)	405	51.0	58895	52.7
Only CT reachable within 60 min	1	0.1	1	0.0
Neither reachable within 60 min	0	0.0	0	0.0
Likely irrelevant (<10 min)	273	34.4	43277	38.7

Population and Area Coverage in Stadtkreis Karlsruhe (Stroke Units) (Baden-Württemberg) — Regular (+10min Penalty)

Category	Grid Cells	Area (%)	Population	Population (%)
High (30+ min)	0	0.0	0	0.0
Medium (20-30 min)	0	0.0	0	0.0
Low (10-20 min)	0	0.0	0	0.0
Only CT reachable within 60 min	0	0.0	0	0.0
Neither reachable within 60 min	0	0.0	0	0.0
Likely irrelevant (<10 min)	214	100.0	336385	100.0

Population and Area Coverage in Landkreis Rhein-Neckar-Kreis (Stroke Units) (Baden-Württemberg) — Regular (+10min Penalty)

Category	Grid Cells	Area (%)	Population	Population (%)
High (30+ min)	37	2.8	1929	0.4
Medium (20-30 min)	240	18.3	42443	7.8
Low (10-20 min)	530	40.3	189295	35.0
Only CT reachable within 60 min	0	0.0	0	0.0
Neither reachable within 60 min	0	0.0	0	0.0
Likely irrelevant (<10 min)	507	38.6	307353	56.8

Population and Area Coverage in Landkreis München (Stroke Units) (Bayern) — Regular (+10min Penalty)

Category	Grid Cells	Area (%)	Population	Population (%)
High (30+ min)	0	0.0	0	0.0
Medium (20-30 min)	1	0.1	0	0.0
Low (10-20 min)	41	5.1	8469	2.5
Only CT reachable within 60 min	0	0.0	0	0.0
Neither reachable within 60 min	0	0.0	0	0.0
Likely irrelevant (<10 min)	761	94.8	335856	97.5

Population and Area Coverage in Landkreis Dingolfing-Landau (Stroke Units) (Bayern) — Regular (+10min Penalty)

Category	Grid Cells	Area (%)	Population	Population (%)
High (30+ min)	0	0.0	0	0.0
Medium (20-30 min)	10	0.9	325	0.3
Low (10-20 min)	264	24.5	20327	21.5
Only CT reachable within 60 min	0	0.0	0	0.0
Neither reachable within 60 min	0	0.0	0	0.0
Likely irrelevant (<10 min)	803	74.6	73812	78.1

Population and Area Coverage in Kreisfreie Stadt Nürnberg (Stroke Units) (Bayern) — Regular (+10min Penalty)

Category	Grid Cells	Area (%)	Population	Population (%)
High (30+ min)	0	0.0	0	0.0
Medium (20-30 min)	0	0.0	0	0.0
Low (10-20 min)	19	8.2	20105	3.9
Only CT reachable within 60 min	0	0.0	0	0.0
Neither reachable within 60 min	0	0.0	0	0.0
Likely irrelevant (<10 min)	213	91.8	498087	96.1

Population and Area Coverage in Kreisfreie Stadt Cottbus (Stroke Units) (Brandenburg) — Regular (+10min Penalty)

Category	Grid Cells	Area (%)	Population	Population (%)
High (30+ min)	0	0.0	0	0.0
Medium (20-30 min)	0	0.0	0	0.0
Low (10-20 min)	5	2.4	143	0.1
Only CT reachable within 60 min	0	0.0	0	0.0
Neither reachable within 60 min	0	0.0	0	0.0
Likely irrelevant (<10 min)	207	97.6	104361	99.9

Population and Area Coverage in Landkreis Salzlandkreis (Stroke Units) (Sachsen-Anhalt) — Regular (+10min Penalty)

Category	Grid Cells	Area (%)	Population	Population (%)
High (30+ min)	14	0.7	194	0.1
Medium (20-30 min)	380	20.3	32719	18.6
Low (10-20 min)	876	46.9	85195	48.3
Only CT reachable within 60 min	0	0.0	0	0.0
Neither reachable within 60 min	0	0.0	0	0.0
Likely irrelevant (<10 min)	598	32.0	58209	33.0

Population and Area Coverage in Landkreis Sömmerda (Stroke Units) (Thüringen) — Regular (+10min Penalty)

Category	Grid Cells	Area (%)	Population	Population (%)
High (30+ min)	22	2.1	54	0.1
Medium (20-30 min)	175	16.8	8445	13.3
Low (10-20 min)	588	56.3	40119	63.0
Only CT reachable within 60 min	7	0.7	2	0.0
Neither reachable within 60 min	0	0.0	0	0.0
Likely irrelevant (<10 min)	252	24.1	15056	23.6

Population and Area Coverage in Landkreis Saale-Orla-Kreis (Stroke Units) (Thüringen) — Regular (+10min Penalty)

Category	Grid Cells	Area (%)	Population	Population (%)
High (30+ min)	34	2.3	3406	4.5
Medium (20-30 min)	251	17.1	18417	24.5
Low (10-20 min)	519	35.4	21140	28.1
Only CT reachable within 60 min	0	0.0	0	0.0
Neither reachable within 60 min	0	0.0	0	0.0
Likely irrelevant (<10 min)	661	45.1	32343	42.9

Population and Area Coverage in Kreisfreie Stadt Neumünster (Stroke-Ready Hospitals) (Schleswig-Holstein) — Extended (+20% Speed (Emergency))

Category	Grid Cells	Area (%)	Population	Population (%)
High (30+ min)	0	0.0	0	0.0
Medium (20-30 min)	0	0.0	0	0.0
Low (10-20 min)	0	0.0	0	0.0
Only CT reachable within 60 min	0	0.0	0	0.0
Neither reachable within 60 min	0	0.0	0	0.0
Likely irrelevant (<10 min)	100	100.0	85923	100.0

Population and Area Coverage in Kreisfreie Stadt Osnabrück (Stroke-Ready Hospitals) (Niedersachsen) — Extended (+20% Speed (Emergency))

Category	Grid Cells	Area (%)	Population	Population (%)
High (30+ min)	0	0.0	0	0.0
Medium (20-30 min)	0	0.0	0	0.0
Low (10-20 min)	21	13.5	19774	12.8
Only CT reachable within 60 min	0	0.0	0	0.0
Neither reachable within 60 min	0	0.0	0	0.0
Likely irrelevant (<10 min)	134	86.5	134667	87.2

Population and Area Coverage in Kreisfreie Stadt Mönchengladbach (Stroke-Ready Hospitals) (Nordrhein-Westfalen) — Extended (+20% Speed (Emergency))

Category	Grid Cells	Area (%)	Population	Population (%)
High (30+ min)	0	0.0	0	0.0
Medium (20-30 min)	0	0.0	0	0.0
Low (10-20 min)	20	9.1	44459	16.2
Only CT reachable within 60 min	0	0.0	0	0.0
Neither reachable within 60 min	0	0.0	0	0.0
Likely irrelevant (<10 min)	200	90.9	229662	83.8

Population and Area Coverage in Kreis Coesfeld (Stroke-Ready Hospitals) (Nordrhein-Westfalen) — Extended (+20% Speed (Emergency))

Category	Grid Cells	Area (%)	Population	Population (%)
High (30+ min)	0	0.0	0	0.0
Medium (20-30 min)	15	1.0	4665	2.2
Low (10-20 min)	319	21.9	43296	20.1
Only CT reachable within 60 min	0	0.0	0	0.0
Neither reachable within 60 min	0	0.0	0	0.0
Likely irrelevant (<10 min)	1120	77.0	167975	77.8

Population and Area Coverage in Kreis Paderborn (Stroke-Ready Hospitals) (Nordrhein-Westfalen) — Extended (+20% Speed (Emergency))

Category	Grid Cells	Area (%)	Population	Population (%)
High (30+ min)	0	0.0	0	0.0
Medium (20-30 min)	36	2.2	149	0.0
Low (10-20 min)	553	34.0	74468	22.5
Only CT reachable within 60 min	0	0.0	0	0.0
Neither reachable within 60 min	0	0.0	0	0.0
Likely irrelevant (<10 min)	1037	63.8	255904	77.4

Population and Area Coverage in Kreisfreie Stadt Kassel (Stroke-Ready Hospitals) (Hessen) — Extended (+20% Speed (Emergency))

Category	Grid Cells	Area (%)	Population	Population (%)
High (30+ min)	0	0.0	0	0.0
Medium (20-30 min)	0	0.0	0	0.0
Low (10-20 min)	54	40.6	36068	17.5
Only CT reachable within 60 min	0	0.0	0	0.0
Neither reachable within 60 min	0	0.0	0	0.0
Likely irrelevant (<10 min)	79	59.4	169651	82.5

Population and Area Coverage in Landkreis Hersfeld-Rotenburg (Stroke-Ready Hospitals) (Hessen) — Extended (+20% Speed (Emergency))

Category	Grid Cells	Area (%)	Population	Population (%)
High (30+ min)	0	0.0	0	0.0
Medium (20-30 min)	104	7.4	15682	13.0
Low (10-20 min)	248	17.7	14167	11.7
Only CT reachable within 60 min	0	0.0	0	0.0
Neither reachable within 60 min	0	0.0	0	0.0
Likely irrelevant (<10 min)	1049	74.9	91040	75.3

Population and Area Coverage in Landkreis Schwalm-Eder-Kreis (Stroke-Ready Hospitals) (Hessen) — Extended (+20% Speed (Emergency))

Category	Grid Cells	Area (%)	Population	Population (%)
High (30+ min)	1	0.1	0	0.0
Medium (20-30 min)	99	5.0	7144	3.9
Low (10-20 min)	753	38.0	63271	34.8
Only CT reachable within 60 min	0	0.0	0	0.0
Neither reachable within 60 min	0	0.0	0	0.0
Likely irrelevant (<10 min)	1126	56.9	111412	61.3

Population and Area Coverage in Landkreis Westerwaldkreis (Stroke-Ready Hospitals) (Rheinland-Pfalz) — Extended (+20% Speed (Emergency))

Category	Grid Cells	Area (%)	Population	Population (%)
High (30+ min)	0	0.0	0	0.0
Medium (20-30 min)	30	2.4	6477	3.1
Low (10-20 min)	375	29.9	49833	24.2
Only CT reachable within 60 min	0	0.0	0	0.0
Neither reachable within 60 min	0	0.0	0	0.0
Likely irrelevant (<10 min)	850	67.7	149514	72.6

Population and Area Coverage in Kreisfreie Stadt Frankenthal (Pfalz) (Stroke-Ready Hospitals) (Rheinland-Pfalz) — Extended (+20% Speed (Emergency))

Category	Grid Cells	Area (%)	Population	Population (%)
High (30+ min)	0	0.0	0	0.0
Medium (20-30 min)	0	0.0	0	0.0
Low (10-20 min)	0	0.0	0	0.0
Only CT reachable within 60 min	0	0.0	0	0.0
Neither reachable within 60 min	0	0.0	0	0.0
Likely irrelevant (<10 min)	54	100.0	45433	100.0

Population and Area Coverage in Landkreis Böblingen (Stroke-Ready Hospitals) (Baden-Württemberg) — Extended (+20% Speed (Emergency))

Category	Grid Cells	Area (%)	Population	Population (%)
High (30+ min)	0	0.0	0	0.0
Medium (20-30 min)	20	2.7	15411	4.1
Low (10-20 min)	298	39.8	151677	40.7
Only CT reachable within 60 min	0	0.0	0	0.0
Neither reachable within 60 min	0	0.0	0	0.0
Likely irrelevant (<10 min)	431	57.5	205261	55.1

Population and Area Coverage in Landkreis Cham (Stroke-Ready Hospitals) (Bayern) — Extended (+20% Speed (Emergency))

Category	Grid Cells	Area (%)	Population	Population (%)
High (30+ min)	0	0.0	0	0.0
Medium (20-30 min)	13	0.7	921	0.7
Low (10-20 min)	553	31.3	36954	29.1
Only CT reachable within 60 min	0	0.0	0	0.0
Neither reachable within 60 min	0	0.0	0	0.0
Likely irrelevant (<10 min)	1201	68.0	88994	70.1

Population and Area Coverage in Landkreis Saarlouis (Stroke-Ready Hospitals) (Saarland) — Extended (+20% Speed (Emergency))

Category	Grid Cells	Area (%)	Population	Population (%)
High (30+ min)	0	0.0	0	0.0
Medium (20-30 min)	28	5.0	7991	4.3
Low (10-20 min)	108	19.1	23913	12.8
Only CT reachable within 60 min	0	0.0	0	0.0
Neither reachable within 60 min	2	0.4	41	0.0
Likely irrelevant (<10 min)	426	75.5	155264	82.9

Population and Area Coverage in Landkreis Anhalt-Bitterfeld (Stroke-Ready Hospitals) (Sachsen-Anhalt) — Extended (+20% Speed (Emergency))

Category	Grid Cells	Area (%)	Population	Population (%)
High (30+ min)	77	4.0	3632	2.5
Medium (20-30 min)	369	19.3	30122	20.4
Low (10-20 min)	494	25.9	22221	15.1
Only CT reachable within 60 min	0	0.0	0	0.0
Neither reachable within 60 min	0	0.0	0	0.0
Likely irrelevant (<10 min)	971	50.8	91522	62.1

Population and Area Coverage in Landkreis Jerichower Land (Stroke-Ready Hospitals) (Sachsen-Anhalt) — Extended (+20% Speed (Emergency))

Category	Grid Cells	Area (%)	Population	Population (%)
High (30+ min)	103	4.9	2757	3.4
Medium (20-30 min)	571	27.3	25844	32.1
Low (10-20 min)	1178	56.4	46501	57.8
Only CT reachable within 60 min	0	0.0	0	0.0
Neither reachable within 60 min	0	0.0	0	0.0
Likely irrelevant (<10 min)	236	11.3	5397	6.7

Population and Area Coverage in Landkreis Kyffhäuserkreis (Stroke-Ready Hospitals) (Thüringen) — Extended (+20% Speed (Emergency))

Category	Grid Cells	Area (%)	Population	Population (%)
High (30+ min)	12	0.9	2572	3.7
Medium (20-30 min)	190	14.2	13102	18.8
Low (10-20 min)	428	32.0	16090	23.1
Only CT reachable within 60 min	0	0.0	0	0.0
Neither reachable within 60 min	0	0.0	0	0.0
Likely irrelevant (<10 min)	707	52.9	37928	54.4

Population and Area Coverage in Landkreis Peine (Stroke-Ready Hospitals) (Niedersachsen) — Extended (+20% Speed (Emergency))

Category	Grid Cells	Area (%)	Population	Population (%)
High (30+ min)	0	0.0	0	0.0
Medium (20-30 min)	0	0.0	0	0.0
Low (10-20 min)	22	3.1	3573	2.7
Only CT reachable within 60 min	0	0.0	0	0.0
Neither reachable within 60 min	0	0.0	0	0.0
Likely irrelevant (<10 min)	690	96.9	127144	97.3

Population and Area Coverage in Kreisfreie Stadt Leverkusen (Stroke-Ready Hospitals) (Nordrhein-Westfalen) — Extended (+20% Speed (Emergency))

Category	Grid Cells	Area (%)	Population	Population (%)
High (30+ min)	0	0.0	0	0.0
Medium (20-30 min)	0	0.0	0	0.0
Low (10-20 min)	9	8.8	8139	5.1
Only CT reachable within 60 min	0	0.0	0	0.0
Neither reachable within 60 min	0	0.0	0	0.0
Likely irrelevant (<10 min)	93	91.2	152531	94.9

Population and Area Coverage in Kreis Ennepe-Ruhr-Kreis (Stroke-Ready Hospitals) (Nordrhein-Westfalen) — Extended (+20% Speed (Emergency))

Category	Grid Cells	Area (%)	Population	Population (%)
High (30+ min)	0	0.0	0	0.0
Medium (20-30 min)	0	0.0	0	0.0
Low (10-20 min)	39	7.3	21133	6.4
Only CT reachable within 60 min	0	0.0	0	0.0
Neither reachable within 60 min	0	0.0	0	0.0
Likely irrelevant (<10 min)	497	92.7	311528	93.6

Population and Area Coverage in Kreis Märkischer Kreis (Stroke-Ready Hospitals) (Nordrhein-Westfalen) — Extended (+20% Speed (Emergency))

Category	Grid Cells	Area (%)	Population	Population (%)
High (30+ min)	5	0.4	197	0.0
Medium (20-30 min)	100	7.4	24420	5.9
Low (10-20 min)	438	32.3	134542	32.4
Only CT reachable within 60 min	0	0.0	0	0.0
Neither reachable within 60 min	0	0.0	0	0.0
Likely irrelevant (<10 min)	814	60.0	256235	61.7

Population and Area Coverage in Landkreis Ahrweiler (Stroke-Ready Hospitals) (Rheinland-Pfalz) — Extended (+20% Speed (Emergency))

Category	Grid Cells	Area (%)	Population	Population (%)
High (30+ min)	0	0.0	0	0.0
Medium (20-30 min)	2	0.2	308	0.2
Low (10-20 min)	179	18.0	31428	24.4
Only CT reachable within 60 min	0	0.0	0	0.0
Neither reachable within 60 min	0	0.0	0	0.0
Likely irrelevant (<10 min)	812	81.8	97151	75.4

Population and Area Coverage in Stadtkreis Baden-Baden (Stroke-Ready Hospitals) (Baden-Württemberg) — Extended (+20% Speed (Emergency))

Category	Grid Cells	Area (%)	Population	Population (%)
High (30+ min)	0	0.0	0	0.0
Medium (20-30 min)	55	30.7	16078	25.5
Low (10-20 min)	90	50.3	33586	53.4
Only CT reachable within 60 min	0	0.0	0	0.0
Neither reachable within 60 min	0	0.0	0	0.0
Likely irrelevant (<10 min)	34	19.0	13277	21.1

Population and Area Coverage in Landkreis Waldshut (Stroke-Ready Hospitals) (Baden-Württemberg) — Extended (+20% Speed (Emergency))

Category	Grid Cells	Area (%)	Population	Population (%)
High (30+ min)	0	0.0	0	0.0
Medium (20-30 min)	1	0.1	2	0.0
Low (10-20 min)	70	5.5	15871	11.2
Only CT reachable within 60 min	0	0.0	0	0.0
Neither reachable within 60 min	2	0.2	204	0.1
Likely irrelevant (<10 min)	1207	94.3	125472	88.6

Population and Area Coverage in Landkreis Bad Tölz-Wolfratshausen (Stroke-Ready Hospitals) (Bayern) — Extended (+20% Speed (Emergency))

Category	Grid Cells	Area (%)	Population	Population (%)
High (30+ min)	0	0.0	0	0.0
Medium (20-30 min)	25	1.9	16903	13.4
Low (10-20 min)	235	17.8	33243	26.4
Only CT reachable within 60 min	0	0.0	0	0.0
Neither reachable within 60 min	109	8.3	176	0.1
Likely irrelevant (<10 min)	950	72.0	75835	60.1

Population and Area Coverage in Landkreis Passau (Stroke-Ready Hospitals) (Bayern) — Extended (+20% Speed (Emergency))

Category	Grid Cells	Area (%)	Population	Population (%)
High (30+ min)	26	1.4	2227	1.2
Medium (20-30 min)	102	5.7	8508	4.5
Low (10-20 min)	456	25.3	48028	25.3
Only CT reachable within 60 min	2	0.1	150	0.1
Neither reachable within 60 min	8	0.4	95	0.1
Likely irrelevant (<10 min)	1206	67.0	130705	68.9

Population and Area Coverage in Landkreis Lichtenfels (Stroke-Ready Hospitals) (Bayern) — Extended (+20% Speed (Emergency))

Category	Grid Cells	Area (%)	Population	Population (%)
High (30+ min)	0	0.0	0	0.0
Medium (20-30 min)	0	0.0	0	0.0
Low (10-20 min)	79	12.1	6825	9.9
Only CT reachable within 60 min	0	0.0	0	0.0
Neither reachable within 60 min	0	0.0	0	0.0
Likely irrelevant (<10 min)	576	87.9	62372	90.1

Population and Area Coverage in Landkreis Wunsiedel i.Fichtelgebirge (Stroke-Ready Hospitals) (Bayern) — Extended (+20% Speed (Emergency))

Category	Grid Cells	Area (%)	Population	Population (%)
High (30+ min)	2	0.3	159	0.2
Medium (20-30 min)	79	11.1	16135	21.8
Low (10-20 min)	474	66.4	47767	64.6
Only CT reachable within 60 min	0	0.0	0	0.0
Neither reachable within 60 min	0	0.0	0	0.0
Likely irrelevant (<10 min)	159	22.3	9875	13.4

Population and Area Coverage in Landkreis Ansbach (Stroke-Ready Hospitals) (Bayern) — Extended (+20% Speed (Emergency))

Category	Grid Cells	Area (%)	Population	Population (%)
High (30+ min)	0	0.0	0	0.0
Medium (20-30 min)	144	5.9	16724	9.0
Low (10-20 min)	302	12.4	12763	6.9
Only CT reachable within 60 min	0	0.0	0	0.0
Neither reachable within 60 min	0	0.0	0	0.0
Likely irrelevant (<10 min)	1986	81.7	155389	84.1

Population and Area Coverage in Kreisfreie Stadt Würzburg (Stroke-Ready Hospitals) (Bayern) — Extended (+20% Speed (Emergency))

Category	Grid Cells	Area (%)	Population	Population (%)
High (30+ min)	0	0.0	0	0.0
Medium (20-30 min)	0	0.0	0	0.0
Low (10-20 min)	0	0.0	0	0.0
Only CT reachable within 60 min	0	0.0	0	0.0
Neither reachable within 60 min	0	0.0	0	0.0
Likely irrelevant (<10 min)	109	100.0	128110	100.0

Population and Area Coverage in Landkreis Oder-Spree (Stroke-Ready Hospitals) (Brandenburg) — Extended (+20% Speed (Emergency))

Category	Grid Cells	Area (%)	Population	Population (%)
High (30+ min)	18	0.6	312	0.2
Medium (20-30 min)	228	7.7	21873	12.7
Low (10-20 min)	959	32.3	59222	34.4
Only CT reachable within 60 min	7	0.2	40	0.0
Neither reachable within 60 min	6	0.2	49	0.0
Likely irrelevant (<10 min)	1751	59.0	90434	52.6

Population and Area Coverage in Landkreis Potsdam-Mittelmark (Stroke-Ready Hospitals) (Brandenburg) — Extended (+20% Speed (Emergency))

Category	Grid Cells	Area (%)	Population	Population (%)
High (30+ min)	330	9.7	13123	6.5
Medium (20-30 min)	683	20.0	24457	12.0
Low (10-20 min)	1375	40.4	68610	33.8
Only CT reachable within 60 min	0	0.0	0	0.0
Neither reachable within 60 min	0	0.0	0	0.0
Likely irrelevant (<10 min)	1019	29.9	96808	47.7

Population and Area Coverage in Landkreis Uckermark (Stroke-Ready Hospitals) (Brandenburg) — Extended (+20% Speed (Emergency))

Category	Grid Cells	Area (%)	Population	Population (%)
High (30+ min)	848	20.6	36680	31.7
Medium (20-30 min)	1030	25.0	19073	16.5
Low (10-20 min)	1038	25.2	15618	13.5
Only CT reachable within 60 min	229	5.6	1818	1.6
Neither reachable within 60 min	84	2.0	1946	1.7
Likely irrelevant (<10 min)	892	21.6	40523	35.0

Population and Area Coverage in Landkreis Sächsische Schweiz-Osterzgebirge (Stroke-Ready Hospitals) (Sachsen) — Extended (+20% Speed (Emergency))

Category	Grid Cells	Area (%)	Population	Population (%)
High (30+ min)	127	6.1	17930	7.9
Medium (20-30 min)	557	26.9	58580	25.7
Low (10-20 min)	718	34.7	89658	39.3
Only CT reachable within 60 min	8	0.4	4	0.0
Neither reachable within 60 min	37	1.8	172	0.1
Likely irrelevant (<10 min)	625	30.2	61815	27.1

Population and Area Coverage in Kreisfreie Stadt Leipzig (Stroke-Ready Hospitals) (Sachsen) — Extended (+20% Speed (Emergency))

Category	Grid Cells	Area (%)	Population	Population (%)
High (30+ min)	0	0.0	0	0.0
Medium (20-30 min)	0	0.0	0	0.0
Low (10-20 min)	39	10.2	60433	9.8
Only CT reachable within 60 min	0	0.0	0	0.0
Neither reachable within 60 min	0	0.0	0	0.0
Likely irrelevant (<10 min)	345	89.8	558893	90.2

Population and Area Coverage in Kreisfreie Stadt Jena (Stroke-Ready Hospitals) (Thüringen) — Extended (+20% Speed (Emergency))

Category	Grid Cells	Area (%)	Population	Population (%)
High (30+ min)	0	0.0	0	0.0
Medium (20-30 min)	4	2.7	129	0.1
Low (10-20 min)	16	10.7	2883	2.9
Only CT reachable within 60 min	0	0.0	0	0.0
Neither reachable within 60 min	0	0.0	0	0.0
Likely irrelevant (<10 min)	129	86.6	97234	97.0

Population and Area Coverage in Landkreis Gotha (Stroke-Ready Hospitals) (Thüringen) — Extended (+20% Speed (Emergency))

Category	Grid Cells	Area (%)	Population	Population (%)
High (30+ min)	0	0.0	0	0.0
Medium (20-30 min)	2	0.2	0	0.0
Low (10-20 min)	191	15.9	17925	14.7
Only CT reachable within 60 min	0	0.0	0	0.0
Neither reachable within 60 min	0	0.0	0	0.0
Likely irrelevant (<10 min)	1006	83.9	104351	85.3

Population and Area Coverage in Kreis Pinneberg (Stroke-Ready Hospitals) (Schleswig-Holstein) — Extended (+20% Speed (Emergency))

Category	Grid Cells	Area (%)	Population	Population (%)
High (30+ min)	1	0.1	121	0.0
Medium (20-30 min)	1	0.1	18	0.0
Low (10-20 min)	112	13.2	64405	21.4
Only CT reachable within 60 min	3	0.4	35	0.0
Neither reachable within 60 min	19	2.2	123	0.0
Likely irrelevant (<10 min)	714	84.0	235795	78.5

Population and Area Coverage in Kreis Schleswig-Flensburg (Stroke-Ready Hospitals) (Schleswig-Holstein) — Extended (+20% Speed (Emergency))

Category	Grid Cells	Area (%)	Population	Population (%)
High (30+ min)	0	0.0	0	0.0
Medium (20-30 min)	19	0.7	1743	0.9
Low (10-20 min)	118	4.4	11786	6.0
Only CT reachable within 60 min	0	0.0	0	0.0
Neither reachable within 60 min	9	0.3	217	0.1
Likely irrelevant (<10 min)	2551	94.6	183047	93.0

Population and Area Coverage in Kreis Steinburg (Stroke-Ready Hospitals) (Schleswig-Holstein) — Extended (+20% Speed (Emergency))

Category	Grid Cells	Area (%)	Population	Population (%)
High (30+ min)	1	0.1	14	0.0
Medium (20-30 min)	0	0.0	0	0.0
Low (10-20 min)	127	9.2	5823	4.4
Only CT reachable within 60 min	0	0.0	0	0.0
Neither reachable within 60 min	8	0.6	417	0.3
Likely irrelevant (<10 min)	1246	90.2	126479	95.3

Population and Area Coverage in Landkreis Verden (Stroke-Ready Hospitals) (Niedersachsen) — Extended (+20% Speed (Emergency))

Category	Grid Cells	Area (%)	Population	Population (%)
High (30+ min)	0	0.0	0	0.0
Medium (20-30 min)	88	8.4	19463	14.7
Low (10-20 min)	374	35.6	41244	31.1
Only CT reachable within 60 min	0	0.0	0	0.0
Neither reachable within 60 min	0	0.0	0	0.0
Likely irrelevant (<10 min)	590	56.1	72121	54.3

Population and Area Coverage in Kreisfreie Stadt Delmenhorst (Stroke-Ready Hospitals) (Niedersachsen) — Extended (+20% Speed (Emergency))

Category	Grid Cells	Area (%)	Population	Population (%)
High (30+ min)	0	0.0	0	0.0
Medium (20-30 min)	5	6.0	9843	13.4
Low (10-20 min)	59	71.1	61799	84.2
Only CT reachable within 60 min	0	0.0	0	0.0
Neither reachable within 60 min	0	0.0	0	0.0
Likely irrelevant (<10 min)	19	22.9	1753	2.4

Population and Area Coverage in Kreis Kleve (Stroke-Ready Hospitals) (Nordrhein-Westfalen) — Extended (+20% Speed (Emergency))

Category	Grid Cells	Area (%)	Population	Population (%)
High (30+ min)	0	0.0	0	0.0
Medium (20-30 min)	15	1.0	4652	1.5
Low (10-20 min)	305	19.4	76157	25.1
Only CT reachable within 60 min	5	0.3	155	0.1
Neither reachable within 60 min	14	0.9	129	0.0
Likely irrelevant (<10 min)	1232	78.4	222106	73.3

Population and Area Coverage in Kreis Herford (Stroke-Ready Hospitals) (Nordrhein-Westfalen) — Extended (+20% Speed (Emergency))

Category	Grid Cells	Area (%)	Population	Population (%)
High (30+ min)	0	0.0	0	0.0
Medium (20-30 min)	22	3.7	15075	6.0
Low (10-20 min)	304	51.1	123538	49.4
Only CT reachable within 60 min	0	0.0	0	0.0
Neither reachable within 60 min	0	0.0	0	0.0
Likely irrelevant (<10 min)	269	45.2	111682	44.6

Population and Area Coverage in Kreisfreie Stadt Dortmund (Stroke-Ready Hospitals) (Nordrhein-Westfalen) — Extended (+20% Speed (Emergency))

Category	Grid Cells	Area (%)	Population	Population (%)
High (30+ min)	0	0.0	0	0.0
Medium (20-30 min)	0	0.0	0	0.0
Low (10-20 min)	41	11.3	56305	9.2
Only CT reachable within 60 min	0	0.0	0	0.0
Neither reachable within 60 min	0	0.0	0	0.0
Likely irrelevant (<10 min)	322	88.7	555178	90.8

Population and Area Coverage in Kreisfreie Stadt Wiesbaden (Stroke-Ready Hospitals) (Hessen) — Extended (+20% Speed (Emergency))

Category	Grid Cells	Area (%)	Population	Population (%)
High (30+ min)	0	0.0	0	0.0
Medium (20-30 min)	0	0.0	0	0.0
Low (10-20 min)	1	0.4	6	0.0
Only CT reachable within 60 min	0	0.0	0	0.0
Neither reachable within 60 min	0	0.0	0	0.0
Likely irrelevant (<10 min)	251	99.6	277497	100.0

Population and Area Coverage in Landkreis Hochtaunuskreis (Stroke-Ready Hospitals) (Hessen) — Extended (+20% Speed (Emergency))

Category	Grid Cells	Area (%)	Population	Population (%)
High (30+ min)	0	0.0	0	0.0
Medium (20-30 min)	0	0.0	0	0.0
Low (10-20 min)	200	33.0	48864	21.0
Only CT reachable within 60 min	0	0.0	0	0.0
Neither reachable within 60 min	0	0.0	0	0.0
Likely irrelevant (<10 min)	406	67.0	183656	79.0

Population and Area Coverage in Landkreis Kassel (Stroke-Ready Hospitals) (Hessen) — Extended (+20% Speed (Emergency))

Category	Grid Cells	Area (%)	Population	Population (%)
High (30+ min)	13	0.8	1065	0.4
Medium (20-30 min)	484	29.0	43328	18.3
Low (10-20 min)	829	49.6	80906	34.1
Only CT reachable within 60 min	0	0.0	0	0.0
Neither reachable within 60 min	0	0.0	0	0.0
Likely irrelevant (<10 min)	344	20.6	111701	47.1

Population and Area Coverage in Landkreis Cochem-Zell (Stroke-Ready Hospitals) (Rheinland-Pfalz) — Extended (+20% Speed (Emergency))

Category	Grid Cells	Area (%)	Population	Population (%)
High (30+ min)	63	7.2	6330	9.8
Medium (20-30 min)	347	39.8	25341	39.4
Low (10-20 min)	220	25.3	16673	25.9
Only CT reachable within 60 min	0	0.0	0	0.0
Neither reachable within 60 min	0	0.0	0	0.0
Likely irrelevant (<10 min)	241	27.7	16012	24.9

Population and Area Coverage in Landkreis Rhein-Lahn-Kreis (Stroke-Ready Hospitals) (Rheinland-Pfalz) — Extended (+20% Speed (Emergency))

Category	Grid Cells	Area (%)	Population	Population (%)
High (30+ min)	75	7.6	8885	7.1
Medium (20-30 min)	212	21.5	14969	12.0
Low (10-20 min)	244	24.7	25221	20.2
Only CT reachable within 60 min	0	0.0	0	0.0
Neither reachable within 60 min	0	0.0	0	0.0
Likely irrelevant (<10 min)	455	46.1	75599	60.6

Population and Area Coverage in Stadtkreis Heilbronn (Stroke-Ready Hospitals) (Baden-Württemberg) — Extended (+20% Speed (Emergency))

Category	Grid Cells	Area (%)	Population	Population (%)
High (30+ min)	0	0.0	0	0.0
Medium (20-30 min)	0	0.0	0	0.0
Low (10-20 min)	0	0.0	12	0.0
Only CT reachable within 60 min	0	0.0	0	0.0
Neither reachable within 60 min	0	0.0	0	0.0
Likely irrelevant (<10 min)	121	100.0	123074	100.0

Population and Area Coverage in Landkreis Schwandorf (Stroke-Ready Hospitals) (Bayern) — Extended (+20% Speed (Emergency))

Category	Grid Cells	Area (%)	Population	Population (%)
High (30+ min)	10	0.6	1269	0.9
Medium (20-30 min)	207	11.6	10730	7.2
Low (10-20 min)	486	27.2	27358	18.4
Only CT reachable within 60 min	0	0.0	0	0.0
Neither reachable within 60 min	0	0.0	0	0.0
Likely irrelevant (<10 min)	1083	60.6	109177	73.5

Population and Area Coverage in Landkreis Unterallgäu (Stroke-Ready Hospitals) (Bayern) — Extended (+20% Speed (Emergency))

Category	Grid Cells	Area (%)	Population	Population (%)
High (30+ min)	1	0.1	3	0.0
Medium (20-30 min)	72	4.8	2379	1.7
Low (10-20 min)	624	42.0	52771	37.1
Only CT reachable within 60 min	0	0.0	0	0.0
Neither reachable within 60 min	0	0.0	0	0.0
Likely irrelevant (<10 min)	789	53.1	87119	61.2

Population and Area Coverage in Kreisfreie Stadt Frankfurt (Oder) (Stroke-Ready Hospitals) (Brandenburg) — Extended (+20% Speed (Emergency))

Category	Grid Cells	Area (%)	Population	Population (%)
High (30+ min)	0	0.0	0	0.0
Medium (20-30 min)	1	0.5	187	0.3
Low (10-20 min)	14	7.1	14111	25.1
Only CT reachable within 60 min	2	1.0	65	0.1
Neither reachable within 60 min	13	6.6	445	0.8
Likely irrelevant (<10 min)	166	84.7	41308	73.6

Population and Area Coverage in Landkreis Dahme-Spreewald (Stroke-Ready Hospitals) (Brandenburg) — Extended (+20% Speed (Emergency))

Category	Grid Cells	Area (%)	Population	Population (%)
High (30+ min)	0	0.0	0	0.0
Medium (20-30 min)	35	1.2	4918	3.1
Low (10-20 min)	833	28.1	80940	51.1
Only CT reachable within 60 min	0	0.0	0	0.0
Neither reachable within 60 min	0	0.0	0	0.0
Likely irrelevant (<10 min)	2101	70.8	72611	45.8

Population and Area Coverage in Landkreis Leipzig (Stroke-Ready Hospitals) (Sachsen) — Extended (+20% Speed (Emergency))

Category	Grid Cells	Area (%)	Population	Population (%)
High (30+ min)	1	0.0	439	0.2
Medium (20-30 min)	181	8.5	35873	14.0
Low (10-20 min)	570	26.8	55036	21.5
Only CT reachable within 60 min	0	0.0	0	0.0
Neither reachable within 60 min	0	0.0	0	0.0
Likely irrelevant (<10 min)	1372	64.6	164077	64.2

Population and Area Coverage in Kreisfreie Stadt Magdeburg (Stroke-Ready Hospitals) (Sachsen-Anhalt) — Extended (+20% Speed (Emergency))

Category	Grid Cells	Area (%)	Population	Population (%)
High (30+ min)	0	0.0	0	0.0
Medium (20-30 min)	1	0.4	7	0.0
Low (10-20 min)	72	27.4	25757	10.3
Only CT reachable within 60 min	0	0.0	0	0.0
Neither reachable within 60 min	0	0.0	0	0.0
Likely irrelevant (<10 min)	190	72.2	223536	89.7

Population and Area Coverage in Landkreis Wartburgkreis (Stroke-Ready Hospitals) (Thüringen) — Extended (+20% Speed (Emergency))

Category	Grid Cells	Area (%)	Population	Population (%)
High (30+ min)	0	0.0	0	0.0
Medium (20-30 min)	19	1.1	1455	1.1
Low (10-20 min)	120	6.9	10769	7.8
Only CT reachable within 60 min	0	0.0	0	0.0
Neither reachable within 60 min	0	0.0	0	0.0
Likely irrelevant (<10 min)	1612	92.1	125851	91.1

Population and Area Coverage in Landkreis Unstrut-Hainich-Kreis (Stroke-Ready Hospitals) (Thüringen) — Extended (+20% Speed (Emergency))

Category	Grid Cells	Area (%)	Population	Population (%)
High (30+ min)	0	0.0	0	0.0
Medium (20-30 min)	19	1.6	597	0.7
Low (10-20 min)	307	25.8	33507	37.1
Only CT reachable within 60 min	0	0.0	0	0.0
Neither reachable within 60 min	0	0.0	0	0.0
Likely irrelevant (<10 min)	863	72.6	56165	62.2

Population and Area Coverage in Kreisfreie Stadt Bielefeld (Stroke-Ready Hospitals) (Nordrhein-Westfalen) — Extended (+20% Speed (Emergency))

Category	Grid Cells	Area (%)	Population	Population (%)
High (30+ min)	0	0.0	0	0.0
Medium (20-30 min)	0	0.0	0	0.0
Low (10-20 min)	23	6.8	22349	6.4
Only CT reachable within 60 min	0	0.0	0	0.0
Neither reachable within 60 min	0	0.0	0	0.0
Likely irrelevant (<10 min)	313	93.2	327273	93.6

Population and Area Coverage in Kreisfreie Stadt Bochum (Stroke-Ready Hospitals) (Nordrhein-Westfalen) — Extended (+20% Speed (Emergency))

Category	Grid Cells	Area (%)	Population	Population (%)
High (30+ min)	0	0.0	0	0.0
Medium (20-30 min)	0	0.0	0	0.0
Low (10-20 min)	6	3.2	17215	4.5
Only CT reachable within 60 min	0	0.0	0	0.0
Neither reachable within 60 min	0	0.0	0	0.0
Likely irrelevant (<10 min)	179	96.8	369488	95.5

Population and Area Coverage in Landkreis Fulda (Stroke-Ready Hospitals) (Hessen) — Extended (+20% Speed (Emergency))

Category	Grid Cells	Area (%)	Population	Population (%)
High (30+ min)	0	0.0	0	0.0
Medium (20-30 min)	2	0.1	2296	1.0
Low (10-20 min)	366	20.9	37483	17.0
Only CT reachable within 60 min	0	0.0	0	0.0
Neither reachable within 60 min	0	0.0	0	0.0
Likely irrelevant (<10 min)	1383	79.0	180480	81.9

Population and Area Coverage in Landkreis Rastatt (Stroke-Ready Hospitals) (Baden-Württemberg) — Extended (+20% Speed (Emergency))

Category	Grid Cells	Area (%)	Population	Population (%)
High (30+ min)	3	0.3	525	0.2
Medium (20-30 min)	164	18.3	40292	17.7
Low (10-20 min)	243	27.1	51986	22.9
Only CT reachable within 60 min	0	0.0	0	0.0
Neither reachable within 60 min	2	0.2	31	0.0
Likely irrelevant (<10 min)	486	54.1	134292	59.1

Population and Area Coverage in Landkreis Breisgau-Hochschwarzwald (Stroke-Ready Hospitals) (Baden-Württemberg) — Extended (+20% Speed (Emergency))

Category	Grid Cells	Area (%)	Population	Population (%)
High (30+ min)	2	0.1	29	0.0
Medium (20-30 min)	53	3.2	5899	2.4
Low (10-20 min)	215	13.1	24478	9.9
Only CT reachable within 60 min	1	0.1	6	0.0
Neither reachable within 60 min	0	0.0	3	0.0
Likely irrelevant (<10 min)	1372	83.5	215974	87.7

Population and Area Coverage in Landkreis Ebersberg (Stroke-Ready Hospitals) (Bayern) — Extended (+20% Speed (Emergency))

Category	Grid Cells	Area (%)	Population	Population (%)
High (30+ min)	0	0.0	0	0.0
Medium (20-30 min)	0	0.0	0	0.0
Low (10-20 min)	0	0.0	0	0.0
Only CT reachable within 60 min	0	0.0	0	0.0
Neither reachable within 60 min	0	0.0	0	0.0
Likely irrelevant (<10 min)	664	100.0	137985	100.0

Population and Area Coverage in Landkreis Rottal-Inn (Stroke-Ready Hospitals) (Bayern) — Extended (+20% Speed (Emergency))

Category	Grid Cells	Area (%)	Population	Population (%)
High (30+ min)	0	0.0	0	0.0
Medium (20-30 min)	2	0.1	119	0.1
Low (10-20 min)	307	19.9	24182	19.9
Only CT reachable within 60 min	0	0.0	0	0.0
Neither reachable within 60 min	2	0.1	63	0.1
Likely irrelevant (<10 min)	1228	79.8	97304	80.0

Population and Area Coverage in Landkreis Bamberg (Stroke-Ready Hospitals) (Bayern) — Extended (+20% Speed (Emergency))

Category	Grid Cells	Area (%)	Population	Population (%)
High (30+ min)	0	0.0	0	0.0
Medium (20-30 min)	155	10.6	9313	6.2
Low (10-20 min)	725	49.5	55541	36.8
Only CT reachable within 60 min	0	0.0	0	0.0
Neither reachable within 60 min	0	0.0	0	0.0
Likely irrelevant (<10 min)	585	39.9	85944	57.0

Population and Area Coverage in Landkreis Schweinfurt (Stroke-Ready Hospitals) (Bayern) — Extended (+20% Speed (Emergency))

Category	Grid Cells	Area (%)	Population	Population (%)
High (30+ min)	0	0.0	0	0.0
Medium (20-30 min)	22	2.1	1241	1.1
Low (10-20 min)	375	35.4	35460	30.3
Only CT reachable within 60 min	0	0.0	0	0.0
Neither reachable within 60 min	0	0.0	0	0.0
Likely irrelevant (<10 min)	662	62.5	80248	68.6

Population and Area Coverage in Landkreis St. Wendel (Stroke-Ready Hospitals) (Saarland) — Extended (+20% Speed (Emergency))

Category	Grid Cells	Area (%)	Population	Population (%)
High (30+ min)	0	0.0	0	0.0
Medium (20-30 min)	25	4.2	1435	1.7
Low (10-20 min)	125	20.9	11585	13.6
Only CT reachable within 60 min	0	0.0	0	0.0
Neither reachable within 60 min	0	0.0	0	0.0
Likely irrelevant (<10 min)	448	74.9	72266	84.7

Population and Area Coverage in Landkreis Harz (Stroke-Ready Hospitals) (Sachsen-Anhalt) — Extended (+20% Speed (Emergency))

Category	Grid Cells	Area (%)	Population	Population (%)
High (30+ min)	6	0.2	40	0.0
Medium (20-30 min)	370	13.5	55021	27.5
Low (10-20 min)	1379	50.2	79200	39.6
Only CT reachable within 60 min	0	0.0	0	0.0
Neither reachable within 60 min	0	0.0	0	0.0
Likely irrelevant (<10 min)	993	36.1	65841	32.9

Population and Area Coverage in Kreisfreie Stadt Gera (Stroke-Ready Hospitals) (Thüringen) — Extended (+20% Speed (Emergency))

Category	Grid Cells	Area (%)	Population	Population (%)
High (30+ min)	0	0.0	0	0.0
Medium (20-30 min)	0	0.0	0	0.0
Low (10-20 min)	46	24.1	4538	4.1
Only CT reachable within 60 min	0	0.0	0	0.0
Neither reachable within 60 min	0	0.0	0	0.0
Likely irrelevant (<10 min)	145	75.9	105119	95.9

Population and Area Coverage in Kreisfreie Stadt Weimar (Stroke-Ready Hospitals) (Thüringen) — Extended (+20% Speed (Emergency))

Category	Grid Cells	Area (%)	Population	Population (%)
High (30+ min)	0	0.0	0	0.0
Medium (20-30 min)	0	0.0	0	0.0
Low (10-20 min)	0	0.0	0	0.0
Only CT reachable within 60 min	0	0.0	0	0.0
Neither reachable within 60 min	0	0.0	0	0.0
Likely irrelevant (<10 min)	109	100.0	57698	100.0

Population and Area Coverage in Landkreis Holzminden (Stroke-Ready Hospitals) (Niedersachsen) — Extended (+20% Speed (Emergency))

Category	Grid Cells	Area (%)	Population	Population (%)
High (30+ min)	0	0.0	0	0.0
Medium (20-30 min)	31	3.4	4962	6.9
Low (10-20 min)	161	17.8	6437	9.0
Only CT reachable within 60 min	0	0.0	0	0.0
Neither reachable within 60 min	0	0.0	0	0.0
Likely irrelevant (<10 min)	712	78.8	60389	84.1

Population and Area Coverage in Landkreis Grafschaft Bentheim (Stroke-Ready Hospitals) (Niedersachsen) — Extended (+20% Speed (Emergency))

Category	Grid Cells	Area (%)	Population	Population (%)
High (30+ min)	3	0.2	398	0.3
Medium (20-30 min)	22	1.7	2575	1.9
Low (10-20 min)	183	14.4	26618	19.5
Only CT reachable within 60 min	3	0.2	14	0.0
Neither reachable within 60 min	18	1.4	549	0.4
Likely irrelevant (<10 min)	1045	82.0	106522	77.9

Population and Area Coverage in Kreis Höxter (Stroke-Ready Hospitals) (Nordrhein-Westfalen) — Extended (+20% Speed (Emergency))

Category	Grid Cells	Area (%)	Population	Population (%)
High (30+ min)	0	0.0	0	0.0
Medium (20-30 min)	171	10.9	24045	16.8
Low (10-20 min)	487	31.2	39714	27.8
Only CT reachable within 60 min	0	0.0	0	0.0
Neither reachable within 60 min	0	0.0	0	0.0
Likely irrelevant (<10 min)	905	57.9	79032	55.3

Population and Area Coverage in Landkreis Marburg-Biedenkopf (Stroke-Ready Hospitals) (Hessen) — Extended (+20% Speed (Emergency))

Category	Grid Cells	Area (%)	Population	Population (%)
High (30+ min)	91	5.7	17184	7.1
Medium (20-30 min)	149	9.3	16155	6.6
Low (10-20 min)	342	21.4	45523	18.7
Only CT reachable within 60 min	0	0.0	0	0.0
Neither reachable within 60 min	0	0.0	0	0.0
Likely irrelevant (<10 min)	1018	63.6	164500	67.6

Population and Area Coverage in Landkreis Rhein-Hunsrück-Kreis (Stroke-Ready Hospitals) (Rheinland-Pfalz) — Extended (+20% Speed (Emergency))

Category	Grid Cells	Area (%)	Population	Population (%)
High (30+ min)	214	17.2	19644	18.7
Medium (20-30 min)	639	51.4	54989	52.4
Low (10-20 min)	376	30.2	29747	28.3
Only CT reachable within 60 min	0	0.0	0	0.0
Neither reachable within 60 min	0	0.0	0	0.0
Likely irrelevant (<10 min)	15	1.2	582	0.6

Population and Area Coverage in Landkreis Eifelkreis Bitburg-Prüm (Stroke-Ready Hospitals) (Rheinland-Pfalz) — Extended (+20% Speed (Emergency))

Category	Grid Cells	Area (%)	Population	Population (%)
High (30+ min)	196	9.6	7338	7.3
Medium (20-30 min)	839	41.2	42583	42.4
Low (10-20 min)	837	41.1	41480	41.3
Only CT reachable within 60 min	0	0.0	0	0.0
Neither reachable within 60 min	1	0.0	127	0.1
Likely irrelevant (<10 min)	163	8.0	8808	8.8

Population and Area Coverage in Landkreis Erding (Stroke-Ready Hospitals) (Bayern) — Extended (+20% Speed (Emergency))

Category	Grid Cells	Area (%)	Population	Population (%)
High (30+ min)	0	0.0	0	0.0
Medium (20-30 min)	66	6.3	15304	11.3
Low (10-20 min)	362	34.3	29591	21.8
Only CT reachable within 60 min	0	0.0	0	0.0
Neither reachable within 60 min	0	0.0	0	0.0
Likely irrelevant (<10 min)	627	59.4	90836	66.9

Population and Area Coverage in Landkreis Deggendorf (Stroke-Ready Hospitals) (Bayern) — Extended (+20% Speed (Emergency))

Category	Grid Cells	Area (%)	Population	Population (%)
High (30+ min)	0	0.0	0	0.0
Medium (20-30 min)	0	0.0	0	0.0
Low (10-20 min)	55	5.2	4039	3.4
Only CT reachable within 60 min	0	0.0	0	0.0
Neither reachable within 60 min	0	0.0	0	0.0
Likely irrelevant (<10 min)	1002	94.8	115411	96.6

Population and Area Coverage in Landkreis Weißenburg-Gunzenhausen (Stroke-Ready Hospitals) (Bayern) — Extended (+20% Speed (Emergency))

Category	Grid Cells	Area (%)	Population	Population (%)
High (30+ min)	0	0.0	0	0.0
Medium (20-30 min)	36	3.0	1145	1.2
Low (10-20 min)	571	48.1	49645	52.0
Only CT reachable within 60 min	0	0.0	0	0.0
Neither reachable within 60 min	0	0.0	0	0.0
Likely irrelevant (<10 min)	580	48.9	44621	46.8

Population and Area Coverage in Landkreis Lindau (Bodensee) (Stroke-Ready Hospitals) (Bayern) — Extended (+20% Speed (Emergency))

Category	Grid Cells	Area (%)	Population	Population (%)
High (30+ min)	1	0.3	1856	2.3
Medium (20-30 min)	129	33.9	45456	56.0
Low (10-20 min)	194	51.1	27491	33.9
Only CT reachable within 60 min	6	1.6	181	0.2
Neither reachable within 60 min	7	1.8	614	0.8
Likely irrelevant (<10 min)	43	11.3	5592	6.9

Population and Area Coverage in Kreisfreie Stadt Rostock (Stroke-Ready Hospitals) (Mecklenburg-Vorpommern) — Extended (+20% Speed (Emergency))

Category	Grid Cells	Area (%)	Population	Population (%)
High (30+ min)	0	0.0	0	0.0
Medium (20-30 min)	0	0.0	0	0.0
Low (10-20 min)	91	40.4	24464	13.1
Only CT reachable within 60 min	1	0.4	33	0.0
Neither reachable within 60 min	5	2.2	576	0.3
Likely irrelevant (<10 min)	128	56.9	161332	86.5

Population and Area Coverage in Landkreis Rostock (Stroke-Ready Hospitals) (Mecklenburg-Vorpommern) — Extended (+20% Speed (Emergency))

Category	Grid Cells	Area (%)	Population	Population (%)
High (30+ min)	179	3.8	9739	4.9
Medium (20-30 min)	610	13.0	28134	14.2
Low (10-20 min)	1422	30.4	53028	26.7
Only CT reachable within 60 min	1	0.0	252	0.1
Neither reachable within 60 min	30	0.6	535	0.3
Likely irrelevant (<10 min)	2435	52.1	106685	53.8

Population and Area Coverage in Landkreis Vorpommern-Greifswald (Stroke-Ready Hospitals) (Mecklenburg-Vorpommern) — Extended (+20% Speed (Emergency))

Category	Grid Cells	Area (%)	Population	Population (%)
High (30+ min)	310	6.2	26980	12.2
Medium (20-30 min)	1291	25.6	42518	19.2
Low (10-20 min)	1428	28.3	30953	13.9
Only CT reachable within 60 min	112	2.2	9810	4.4
Neither reachable within 60 min	133	2.6	3046	1.4
Likely irrelevant (<10 min)	1765	35.0	108588	48.9

Population and Area Coverage in Landkreis Mittelsachsen (Stroke-Ready Hospitals) (Sachsen) — Extended (+20% Speed (Emergency))

Category	Grid Cells	Area (%)	Population	Population (%)
High (30+ min)	3	0.1	1024	0.3
Medium (20-30 min)	351	13.0	53609	18.2
Low (10-20 min)	800	29.5	68600	23.3
Only CT reachable within 60 min	1	0.0	0	0.0
Neither reachable within 60 min	0	0.0	0	0.0
Likely irrelevant (<10 min)	1555	57.4	171179	58.1

Population and Area Coverage in Landkreis Schmalkalden-Meiningen (Stroke-Ready Hospitals) (Thüringen) — Extended (+20% Speed (Emergency))

Category	Grid Cells	Area (%)	Population	Population (%)
High (30+ min)	9	0.6	2137	1.8
Medium (20-30 min)	148	9.3	20943	18.1
Low (10-20 min)	344	21.7	26370	22.8
Only CT reachable within 60 min	0	0.0	0	0.0
Neither reachable within 60 min	0	0.0	0	0.0
Likely irrelevant (<10 min)	1084	68.4	66349	57.3

Population and Area Coverage in Kreisfreie Stadt Lübeck (Stroke-Ready Hospitals) (Schleswig-Holstein) — Extended (+20% Speed (Emergency))

Category	Grid Cells	Area (%)	Population	Population (%)
High (30+ min)	2	0.7	2030	0.9
Medium (20-30 min)	19	6.6	12388	5.7
Low (10-20 min)	69	23.9	25059	11.5
Only CT reachable within 60 min	0	0.0	0	0.0
Neither reachable within 60 min	2	0.7	159	0.1
Likely irrelevant (<10 min)	197	68.2	178292	81.8

Population and Area Coverage in Kreis Nordfriesland (Stroke-Ready Hospitals) (Schleswig-Holstein) — Extended (+20% Speed (Emergency))

Category	Grid Cells	Area (%)	Population	Population (%)
High (30+ min)	481	18.3	44099	27.4
Medium (20-30 min)	576	21.9	36252	22.6
Low (10-20 min)	577	21.9	27785	17.3
Only CT reachable within 60 min	172	6.5	22203	13.8
Neither reachable within 60 min	148	5.6	4474	2.8
Likely irrelevant (<10 min)	679	25.8	25877	16.1

Population and Area Coverage in Landkreis Northeim (Stroke-Ready Hospitals) (Niedersachsen) — Extended (+20% Speed (Emergency))

Category	Grid Cells	Area (%)	Population	Population (%)
High (30+ min)	5	0.3	16	0.0
Medium (20-30 min)	73	4.4	11276	8.4
Low (10-20 min)	310	18.8	23301	17.4
Only CT reachable within 60 min	0	0.0	0	0.0
Neither reachable within 60 min	0	0.0	0	0.0
Likely irrelevant (<10 min)	1263	76.5	99519	74.2

Population and Area Coverage in Kreisfreie Stadt Wilhelmshaven (Stroke-Ready Hospitals) (Niedersachsen) — Extended (+20% Speed (Emergency))

Category	Grid Cells	Area (%)	Population	Population (%)
High (30+ min)	0	0.0	0	0.0
Medium (20-30 min)	0	0.0	0	0.0
Low (10-20 min)	22	18.2	24522	35.3
Only CT reachable within 60 min	1	0.8	40	0.1
Neither reachable within 60 min	2	1.7	652	0.9
Likely irrelevant (<10 min)	96	79.3	44314	63.7

Population and Area Coverage in Kreisfreie Stadt Essen (Stroke-Ready Hospitals) (Nordrhein-Westfalen) — Extended (+20% Speed (Emergency))

Category	Grid Cells	Area (%)	Population	Population (%)
High (30+ min)	0	0.0	0	0.0
Medium (20-30 min)	0	0.0	0	0.0
Low (10-20 min)	35	12.7	49365	8.3
Only CT reachable within 60 min	0	0.0	0	0.0
Neither reachable within 60 min	0	0.0	0	0.0
Likely irrelevant (<10 min)	241	87.3	547311	91.7

Population and Area Coverage in Kreisfreie Stadt Münster (Stroke-Ready Hospitals) (Nordrhein-Westfalen) — Extended (+20% Speed (Emergency))

Category	Grid Cells	Area (%)	Population	Population (%)
High (30+ min)	0	0.0	0	0.0
Medium (20-30 min)	3	0.8	256	0.1
Low (10-20 min)	42	10.5	27149	7.4
Only CT reachable within 60 min	0	0.0	0	0.0
Neither reachable within 60 min	0	0.0	0	0.0
Likely irrelevant (<10 min)	354	88.7	337426	92.5

Population and Area Coverage in Kreis Recklinghausen (Stroke-Ready Hospitals) (Nordrhein-Westfalen) — Extended (+20% Speed (Emergency))

Category	Grid Cells	Area (%)	Population	Population (%)
High (30+ min)	0	0.0	0	0.0
Medium (20-30 min)	0	0.0	0	0.0
Low (10-20 min)	176	17.9	123301	20.0
Only CT reachable within 60 min	0	0.0	0	0.0
Neither reachable within 60 min	0	0.0	0	0.0
Likely irrelevant (<10 min)	806	82.1	493112	80.0

Population and Area Coverage in Landkreis Offenbach (Stroke-Ready Hospitals) (Hessen) — Extended (+20% Speed (Emergency))

Category	Grid Cells	Area (%)	Population	Population (%)
High (30+ min)	0	0.0	0	0.0
Medium (20-30 min)	0	0.0	0	0.0
Low (10-20 min)	0	0.0	0	0.0
Only CT reachable within 60 min	0	0.0	0	0.0
Neither reachable within 60 min	0	0.0	0	0.0
Likely irrelevant (<10 min)	446	100.0	341311	100.0

Population and Area Coverage in Kreisfreie Stadt Trier (Stroke-Ready Hospitals) (Rheinland-Pfalz) — Extended (+20% Speed (Emergency))

Category	Grid Cells	Area (%)	Population	Population (%)
High (30+ min)	0	0.0	0	0.0
Medium (20-30 min)	0	0.0	0	0.0
Low (10-20 min)	0	0.0	0	0.0
Only CT reachable within 60 min	0	0.0	0	0.0
Neither reachable within 60 min	0	0.0	0	0.0
Likely irrelevant (<10 min)	146	100.0	128525	100.0

Population and Area Coverage in Landkreis Bernkastel-Wittlich (Stroke-Ready Hospitals) (Rheinland-Pfalz) — Extended (+20% Speed (Emergency))

Category	Grid Cells	Area (%)	Population	Population (%)
High (30+ min)	0	0.0	0	0.0
Medium (20-30 min)	45	3.1	5013	4.4
Low (10-20 min)	481	32.9	32814	28.6
Only CT reachable within 60 min	0	0.0	0	0.0
Neither reachable within 60 min	0	0.0	0	0.0
Likely irrelevant (<10 min)	935	64.0	76844	67.0

Population and Area Coverage in Kreisfreie Stadt Worms (Stroke-Ready Hospitals) (Rheinland-Pfalz) — Extended (+20% Speed (Emergency))

Category	Grid Cells	Area (%)	Population	Population (%)
High (30+ min)	0	0.0	0	0.0
Medium (20-30 min)	0	0.0	0	0.0
Low (10-20 min)	0	0.0	4	0.0
Only CT reachable within 60 min	0	0.0	0	0.0
Neither reachable within 60 min	0	0.0	0	0.0
Likely irrelevant (<10 min)	134	100.0	81870	100.0

Population and Area Coverage in Landkreis Heilbronn (Stroke-Ready Hospitals) (Baden-Württemberg) — Extended (+20% Speed (Emergency))

Category	Grid Cells	Area (%)	Population	Population (%)
High (30+ min)	0	0.0	0	0.0
Medium (20-30 min)	53	3.9	3307	1.0
Low (10-20 min)	275	20.2	75140	22.7
Only CT reachable within 60 min	0	0.0	0	0.0
Neither reachable within 60 min	0	0.0	0	0.0
Likely irrelevant (<10 min)	1033	75.9	252957	76.3

Population and Area Coverage in Kreisfreie Stadt Ingolstadt (Stroke-Ready Hospitals) (Bayern) — Extended (+20% Speed (Emergency))

Category	Grid Cells	Area (%)	Population	Population (%)
High (30+ min)	0	0.0	0	0.0
Medium (20-30 min)	0	0.0	0	0.0
Low (10-20 min)	12	7.3	13106	9.7
Only CT reachable within 60 min	0	0.0	0	0.0
Neither reachable within 60 min	0	0.0	0	0.0
Likely irrelevant (<10 min)	153	92.7	122113	90.3

Population and Area Coverage in Landkreis Forchheim (Stroke-Ready Hospitals) (Bayern) — Extended (+20% Speed (Emergency))

Category	Grid Cells	Area (%)	Population	Population (%)
High (30+ min)	0	0.0	0	0.0
Medium (20-30 min)	23	2.9	964	0.8
Low (10-20 min)	317	39.4	27014	22.8
Only CT reachable within 60 min	0	0.0	0	0.0
Neither reachable within 60 min	0	0.0	0	0.0
Likely irrelevant (<10 min)	465	57.8	90433	76.4

Population and Area Coverage in Landkreis Erlangen-Höchstadt (Stroke-Ready Hospitals) (Bayern) — Extended (+20% Speed (Emergency))

Category	Grid Cells	Area (%)	Population	Population (%)
High (30+ min)	0	0.0	0	0.0
Medium (20-30 min)	76	10.9	14898	10.8
Low (10-20 min)	326	46.7	59091	43.0
Only CT reachable within 60 min	0	0.0	0	0.0
Neither reachable within 60 min	0	0.0	0	0.0
Likely irrelevant (<10 min)	296	42.4	63410	46.2

Population and Area Coverage in Landkreis Mansfeld-Südharz (Stroke-Ready Hospitals) (Sachsen-Anhalt) — Extended (+20% Speed (Emergency))

Category	Grid Cells	Area (%)	Population	Population (%)
High (30+ min)	6	0.3	487	0.4
Medium (20-30 min)	143	7.6	24776	19.4
Low (10-20 min)	460	24.3	35489	27.7
Only CT reachable within 60 min	0	0.0	0	0.0
Neither reachable within 60 min	0	0.0	0	0.0
Likely irrelevant (<10 min)	1281	67.8	67185	52.5

Population and Area Coverage in Kreis Minden-Lübbecke (Stroke-Ready Hospitals) (Nordrhein-Westfalen) — Extended (+20% Speed (Emergency))

Category	Grid Cells	Area (%)	Population	Population (%)
High (30+ min)	77	5.1	12784	4.1
Medium (20-30 min)	345	22.7	55464	17.9
Low (10-20 min)	316	20.8	57013	18.4
Only CT reachable within 60 min	0	0.0	0	0.0
Neither reachable within 60 min	0	0.0	0	0.0
Likely irrelevant (<10 min)	782	51.4	184212	59.5

Population and Area Coverage in Kreisfreie Stadt Hamm (Stroke-Ready Hospitals) (Nordrhein-Westfalen) — Extended (+20% Speed (Emergency))

Category	Grid Cells	Area (%)	Population	Population (%)
High (30+ min)	0	0.0	0	0.0
Medium (20-30 min)	0	0.0	0	0.0
Low (10-20 min)	10	3.4	3684	2.1
Only CT reachable within 60 min	0	0.0	0	0.0
Neither reachable within 60 min	0	0.0	0	0.0
Likely irrelevant (<10 min)	283	96.6	171716	97.9

Population and Area Coverage in Landkreis Main-Taunus-Kreis (Stroke-Ready Hospitals) (Hessen) — Extended (+20% Speed (Emergency))

Category	Grid Cells	Area (%)	Population	Population (%)
High (30+ min)	0	0.0	0	0.0
Medium (20-30 min)	0	0.0	0	0.0
Low (10-20 min)	20	7.1	3088	1.3
Only CT reachable within 60 min	0	0.0	0	0.0
Neither reachable within 60 min	0	0.0	0	0.0
Likely irrelevant (<10 min)	261	92.9	228345	98.7

Population and Area Coverage in Kreisfreie Stadt Ludwigshafen am Rhein (Stroke-Ready Hospitals) (Rheinland-Pfalz) — Extended (+20% Speed (Emergency))

Category	Grid Cells	Area (%)	Population	Population (%)
High (30+ min)	0	0.0	0	0.0
Medium (20-30 min)	0	0.0	0	0.0
Low (10-20 min)	9	9.1	9239	5.7
Only CT reachable within 60 min	0	0.0	0	0.0
Neither reachable within 60 min	0	0.0	0	0.0
Likely irrelevant (<10 min)	90	90.9	153962	94.3

Population and Area Coverage in Landkreis Esslingen (Stroke-Ready Hospitals) (Baden-Württemberg) — Extended (+20% Speed (Emergency))

Category	Grid Cells	Area (%)	Population	Population (%)
High (30+ min)	0	0.0	0	0.0
Medium (20-30 min)	3	0.4	54	0.0
Low (10-20 min)	154	20.0	101118	19.6
Only CT reachable within 60 min	0	0.0	0	0.0
Neither reachable within 60 min	0	0.0	0	0.0
Likely irrelevant (<10 min)	614	79.6	415987	80.4

Population and Area Coverage in Landkreis Dachau (Stroke-Ready Hospitals) (Bayern) — Extended (+20% Speed (Emergency))

Category	Grid Cells	Area (%)	Population	Population (%)
High (30+ min)	0	0.0	0	0.0
Medium (20-30 min)	0	0.0	0	0.0
Low (10-20 min)	215	30.5	25898	17.3
Only CT reachable within 60 min	0	0.0	0	0.0
Neither reachable within 60 min	0	0.0	0	0.0
Likely irrelevant (<10 min)	489	69.5	124233	82.7

Population and Area Coverage in Landkreis Bad Kissingen (Stroke-Ready Hospitals) (Bayern) — Extended (+20% Speed (Emergency))

Category	Grid Cells	Area (%)	Population	Population (%)
High (30+ min)	2	0.1	2337	2.2
Medium (20-30 min)	280	19.5	28550	26.8
Low (10-20 min)	704	49.1	48012	45.0
Only CT reachable within 60 min	0	0.0	0	0.0
Neither reachable within 60 min	0	0.0	0	0.0
Likely irrelevant (<10 min)	447	31.2	27726	26.0

Population and Area Coverage in Kreisfreie Stadt Erfurt (Stroke-Ready Hospitals) (Thüringen) — Extended (+20% Speed (Emergency))

Category	Grid Cells	Area (%)	Population	Population (%)
High (30+ min)	0	0.0	0	0.0
Medium (20-30 min)	0	0.0	0	0.0
Low (10-20 min)	29	8.5	23378	9.5
Only CT reachable within 60 min	0	0.0	0	0.0
Neither reachable within 60 min	0	0.0	0	0.0
Likely irrelevant (<10 min)	313	91.5	223844	90.5

Population and Area Coverage in Kreisfreie Stadt Flensburg (Stroke-Ready Hospitals) (Schleswig-Holstein) — Extended (+20% Speed (Emergency))

Category	Grid Cells	Area (%)	Population	Population (%)
High (30+ min)	0	0.0	0	0.0
Medium (20-30 min)	0	0.0	0	0.0
Low (10-20 min)	0	0.0	0	0.0
Only CT reachable within 60 min	0	0.0	0	0.0
Neither reachable within 60 min	0	0.0	0	0.0
Likely irrelevant (<10 min)	62	100.0	80819	100.0

Population and Area Coverage in Kreis Dithmarschen (Stroke-Ready Hospitals) (Schleswig-Holstein) — Extended (+20% Speed (Emergency))

Category	Grid Cells	Area (%)	Population	Population (%)
High (30+ min)	0	0.0	0	0.0
Medium (20-30 min)	34	1.8	6177	4.6
Low (10-20 min)	281	15.2	22844	17.1
Only CT reachable within 60 min	7	0.4	1106	0.8
Neither reachable within 60 min	37	2.0	509	0.4
Likely irrelevant (<10 min)	1490	80.6	102982	77.1

Population and Area Coverage in Landkreis Hameln-Pyrmont (Stroke-Ready Hospitals) (Niedersachsen) — Extended (+20% Speed (Emergency))

Category	Grid Cells	Area (%)	Population	Population (%)
High (30+ min)	1	0.1	140	0.1
Medium (20-30 min)	166	15.8	22715	15.4
Low (10-20 min)	312	29.8	65933	44.7
Only CT reachable within 60 min	0	0.0	0	0.0
Neither reachable within 60 min	0	0.0	0	0.0
Likely irrelevant (<10 min)	569	54.3	58613	39.8

Population and Area Coverage in Landkreis Celle (Stroke-Ready Hospitals) (Niedersachsen) — Extended (+20% Speed (Emergency))

Category	Grid Cells	Area (%)	Population	Population (%)
High (30+ min)	0	0.0	0	0.0
Medium (20-30 min)	0	0.0	0	0.0
Low (10-20 min)	75	3.6	1279	0.7
Only CT reachable within 60 min	0	0.0	0	0.0
Neither reachable within 60 min	0	0.0	0	0.0
Likely irrelevant (<10 min)	1992	96.4	175013	99.3

Population and Area Coverage in Landkreis Harburg (Stroke-Ready Hospitals) (Niedersachsen) — Extended (+20% Speed (Emergency))

Category	Grid Cells	Area (%)	Population	Population (%)
High (30+ min)	0	0.0	0	0.0
Medium (20-30 min)	0	0.0	0	0.0
Low (10-20 min)	55	3.3	17053	7.0
Only CT reachable within 60 min	0	0.0	0	0.0
Neither reachable within 60 min	0	0.0	0	0.0
Likely irrelevant (<10 min)	1634	96.7	226311	93.0

Population and Area Coverage in Kreisfreie Stadt Düsseldorf (Stroke-Ready Hospitals) (Nordrhein-Westfalen) — Extended (+20% Speed (Emergency))

Category	Grid Cells	Area (%)	Population	Population (%)
High (30+ min)	0	0.0	0	0.0
Medium (20-30 min)	0	0.0	0	0.0
Low (10-20 min)	24	8.6	44195	6.5
Only CT reachable within 60 min	0	0.0	0	0.0
Neither reachable within 60 min	0	0.0	0	0.0
Likely irrelevant (<10 min)	255	91.4	632229	93.5

Population and Area Coverage in Kreis Düren (Stroke-Ready Hospitals) (Nordrhein-Westfalen) — Extended (+20% Speed (Emergency))

Category	Grid Cells	Area (%)	Population	Population (%)
High (30+ min)	0	0.0	0	0.0
Medium (20-30 min)	19	1.6	7200	2.8
Low (10-20 min)	281	23.4	61519	24.0
Only CT reachable within 60 min	0	0.0	0	0.0
Neither reachable within 60 min	0	0.0	0	0.0
Likely irrelevant (<10 min)	900	75.0	187540	73.2

Population and Area Coverage in Kreis Soest (Stroke-Ready Hospitals) (Nordrhein-Westfalen) — Extended (+20% Speed (Emergency))

Category	Grid Cells	Area (%)	Population	Population (%)
High (30+ min)	0	0.0	0	0.0
Medium (20-30 min)	7	0.4	0	0.0
Low (10-20 min)	412	23.9	62117	21.0
Only CT reachable within 60 min	0	0.0	0	0.0
Neither reachable within 60 min	0	0.0	0	0.0
Likely irrelevant (<10 min)	1305	75.7	233028	79.0

Population and Area Coverage in Kreisfreie Stadt Darmstadt (Stroke-Ready Hospitals) (Hessen) — Extended (+20% Speed (Emergency))

Category	Grid Cells	Area (%)	Population	Population (%)
High (30+ min)	0	0.0	0	0.0
Medium (20-30 min)	0	0.0	0	0.0
Low (10-20 min)	2	1.3	7	0.0
Only CT reachable within 60 min	0	0.0	0	0.0
Neither reachable within 60 min	0	0.0	0	0.0
Likely irrelevant (<10 min)	152	98.7	151592	100.0

Population and Area Coverage in Kreisfreie Stadt Offenbach am Main (Stroke-Ready Hospitals) (Hessen) — Extended (+20% Speed (Emergency))

Category	Grid Cells	Area (%)	Population	Population (%)
High (30+ min)	0	0.0	0	0.0
Medium (20-30 min)	0	0.0	0	0.0
Low (10-20 min)	0	0.0	0	0.0
Only CT reachable within 60 min	0	0.0	0	0.0
Neither reachable within 60 min	0	0.0	0	0.0
Likely irrelevant (<10 min)	56	100.0	123120	100.0

Population and Area Coverage in Landkreis Werra-Meißner-Kreis (Stroke-Ready Hospitals) (Hessen) — Extended (+20% Speed (Emergency))

Category	Grid Cells	Area (%)	Population	Population (%)
High (30+ min)	0	0.0	0	0.0
Medium (20-30 min)	160	12.1	20456	20.3
Low (10-20 min)	302	22.9	15316	15.2
Only CT reachable within 60 min	0	0.0	0	0.0
Neither reachable within 60 min	0	0.0	0	0.0
Likely irrelevant (<10 min)	856	64.9	65076	64.5

Population and Area Coverage in Landkreis Mayen-Koblenz (Stroke-Ready Hospitals) (Rheinland-Pfalz) — Extended (+20% Speed (Emergency))

Category	Grid Cells	Area (%)	Population	Population (%)
High (30+ min)	0	0.0	0	0.0
Medium (20-30 min)	62	6.0	15795	7.3
Low (10-20 min)	343	33.2	51408	23.7
Only CT reachable within 60 min	0	0.0	0	0.0
Neither reachable within 60 min	0	0.0	0	0.0
Likely irrelevant (<10 min)	629	60.8	149837	69.0

Population and Area Coverage in Landkreis Bad Dürkheim (Stroke-Ready Hospitals) (Rheinland-Pfalz) — Extended (+20% Speed (Emergency))

Category	Grid Cells	Area (%)	Population	Population (%)
High (30+ min)	0	0.0	0	0.0
Medium (20-30 min)	10	1.4	3302	2.5
Low (10-20 min)	186	25.4	54726	40.7
Only CT reachable within 60 min	0	0.0	0	0.0
Neither reachable within 60 min	0	0.0	0	0.0
Likely irrelevant (<10 min)	535	73.2	76410	56.8

Population and Area Coverage in Landkreis Landsberg am Lech (Stroke-Ready Hospitals) (Bayern) — Extended (+20% Speed (Emergency))

Category	Grid Cells	Area (%)	Population	Population (%)
High (30+ min)	0	0.0	0	0.0
Medium (20-30 min)	0	0.0	4	0.0
Low (10-20 min)	89	9.3	2107	1.7
Only CT reachable within 60 min	0	0.0	0	0.0
Neither reachable within 60 min	0	0.0	0	0.0
Likely irrelevant (<10 min)	873	90.7	118819	98.3

Population and Area Coverage in Landkreis Donau-Ries (Stroke-Ready Hospitals) (Bayern) — Extended (+20% Speed (Emergency))

Category	Grid Cells	Area (%)	Population	Population (%)
High (30+ min)	72	4.6	20420	15.3
Medium (20-30 min)	945	60.3	75938	57.0
Low (10-20 min)	459	29.3	33814	25.4
Only CT reachable within 60 min	0	0.0	0	0.0
Neither reachable within 60 min	0	0.0	0	0.0
Likely irrelevant (<10 min)	90	5.7	3152	2.4

Population and Area Coverage in Landkreis Märkisch-Oderland (Stroke-Ready Hospitals) (Brandenburg) — Extended (+20% Speed (Emergency))

Category	Grid Cells	Area (%)	Population	Population (%)
High (30+ min)	403	14.1	15416	8.4
Medium (20-30 min)	947	33.2	41342	22.5
Low (10-20 min)	689	24.1	39657	21.5
Only CT reachable within 60 min	9	0.3	37	0.0
Neither reachable within 60 min	71	2.5	569	0.3
Likely irrelevant (<10 min)	736	25.8	87084	47.3

Population and Area Coverage in Landkreis Prignitz (Stroke-Ready Hospitals) (Brandenburg) — Extended (+20% Speed (Emergency))

Category	Grid Cells	Area (%)	Population	Population (%)
High (30+ min)	8	0.3	117	0.2
Medium (20-30 min)	179	6.2	5649	7.6
Low (10-20 min)	850	29.6	15028	20.2
Only CT reachable within 60 min	0	0.0	0	0.0
Neither reachable within 60 min	0	0.0	0	0.0
Likely irrelevant (<10 min)	1834	63.9	53574	72.0

Population and Area Coverage in Landkreis Burgenlandkreis (Stroke-Ready Hospitals) (Sachsen-Anhalt) — Extended (+20% Speed (Emergency))

Category	Grid Cells	Area (%)	Population	Population (%)
High (30+ min)	3	0.2	151	0.1
Medium (20-30 min)	92	5.1	14394	8.7
Low (10-20 min)	287	15.8	24890	15.1
Only CT reachable within 60 min	0	0.0	0	0.0
Neither reachable within 60 min	0	0.0	0	0.0
Likely irrelevant (<10 min)	1437	79.0	125685	76.1

Population and Area Coverage in Landkreis Stendal (Stroke-Ready Hospitals) (Sachsen-Anhalt) — Extended (+20% Speed (Emergency))

Category	Grid Cells	Area (%)	Population	Population (%)
High (30+ min)	911	28.2	53328	51.5
Medium (20-30 min)	1320	40.8	30233	29.2
Low (10-20 min)	666	20.6	12906	12.5
Only CT reachable within 60 min	88	2.7	565	0.5
Neither reachable within 60 min	0	0.0	0	0.0
Likely irrelevant (<10 min)	249	7.7	6475	6.3

Population and Area Coverage in Landkreis Weimarer Land (Stroke-Ready Hospitals) (Thüringen) — Extended (+20% Speed (Emergency))

Category	Grid Cells	Area (%)	Population	Population (%)
High (30+ min)	0	0.0	0	0.0
Medium (20-30 min)	100	9.7	20911	28.3
Low (10-20 min)	295	28.7	14079	19.1
Only CT reachable within 60 min	0	0.0	0	0.0
Neither reachable within 60 min	0	0.0	0	0.0
Likely irrelevant (<10 min)	632	61.5	38785	52.6

Population and Area Coverage in Kreisfreie Stadt Kiel (Stroke-Ready Hospitals) (Schleswig-Holstein) — Extended (+20% Speed (Emergency))

Category	Grid Cells	Area (%)	Population	Population (%)
High (30+ min)	0	0.0	0	0.0
Medium (20-30 min)	0	0.0	0	0.0
Low (10-20 min)	4	2.6	5203	2.0
Only CT reachable within 60 min	0	0.0	1571	0.6
Neither reachable within 60 min	0	0.0	98	0.0
Likely irrelevant (<10 min)	151	97.4	249447	97.3

Population and Area Coverage in Landkreis Gifhorn (Stroke-Ready Hospitals) (Niedersachsen) — Extended (+20% Speed (Emergency))

Category	Grid Cells	Area (%)	Population	Population (%)
High (30+ min)	53	2.5	5115	3.0
Medium (20-30 min)	158	7.6	4489	2.6
Low (10-20 min)	323	15.5	16070	9.3
Only CT reachable within 60 min	0	0.0	0	0.0
Neither reachable within 60 min	0	0.0	0	0.0
Likely irrelevant (<10 min)	1556	74.4	146528	85.1

Population and Area Coverage in Landkreis Helmstedt (Stroke-Ready Hospitals) (Niedersachsen) — Extended (+20% Speed (Emergency))

Category	Grid Cells	Area (%)	Population	Population (%)
High (30+ min)	0	0.0	0	0.0
Medium (20-30 min)	0	0.0	0	0.0
Low (10-20 min)	2	0.2	16	0.0
Only CT reachable within 60 min	0	0.0	0	0.0
Neither reachable within 60 min	0	0.0	0	0.0
Likely irrelevant (<10 min)	893	99.8	89397	100.0

Population and Area Coverage in Landkreis Lüchow-Dannenberg (Stroke-Ready Hospitals) (Niedersachsen) — Extended (+20% Speed (Emergency))

Category	Grid Cells	Area (%)	Population	Population (%)
High (30+ min)	614	37.4	23066	47.2
Medium (20-30 min)	500	30.5	16680	34.2
Low (10-20 min)	452	27.5	8248	16.9
Only CT reachable within 60 min	4	0.2	154	0.3
Neither reachable within 60 min	0	0.0	0	0.0
Likely irrelevant (<10 min)	71	4.3	693	1.4

Population and Area Coverage in Landkreis Rotenburg (Wümme) (Stroke-Ready Hospitals) (Niedersachsen) — Extended (+20% Speed (Emergency))

Category	Grid Cells	Area (%)	Population	Population (%)
High (30+ min)	6	0.2	13	0.0
Medium (20-30 min)	596	21.3	33881	20.7
Low (10-20 min)	560	20.0	24481	14.9
Only CT reachable within 60 min	0	0.0	0	0.0
Neither reachable within 60 min	0	0.0	0	0.0
Likely irrelevant (<10 min)	1632	58.4	105599	64.4

Population and Area Coverage in Kreis Euskirchen (Stroke-Ready Hospitals) (Nordrhein-Westfalen) — Extended (+20% Speed (Emergency))

Category	Grid Cells	Area (%)	Population	Population (%)
High (30+ min)	108	6.8	9499	5.0
Medium (20-30 min)	272	17.2	14009	7.4
Low (10-20 min)	530	33.5	55275	29.4
Only CT reachable within 60 min	0	0.0	0	0.0
Neither reachable within 60 min	0	0.0	2	0.0
Likely irrelevant (<10 min)	673	42.5	109452	58.1

Population and Area Coverage in Kreis Olpe (Stroke-Ready Hospitals) (Nordrhein-Westfalen) — Extended (+20% Speed (Emergency))

Category	Grid Cells	Area (%)	Population	Population (%)
High (30+ min)	144	15.7	38619	28.5
Medium (20-30 min)	304	33.2	33460	24.7
Low (10-20 min)	301	32.9	34417	25.4
Only CT reachable within 60 min	0	0.0	0	0.0
Neither reachable within 60 min	0	0.0	0	0.0
Likely irrelevant (<10 min)	166	18.1	28922	21.4

Population and Area Coverage in Landkreis Lahn-Dill-Kreis (Stroke-Ready Hospitals) (Hessen) — Extended (+20% Speed (Emergency))

Category	Grid Cells	Area (%)	Population	Population (%)
High (30+ min)	0	0.0	0	0.0
Medium (20-30 min)	110	8.1	18240	7.1
Low (10-20 min)	532	39.0	88205	34.5
Only CT reachable within 60 min	0	0.0	0	0.0
Neither reachable within 60 min	0	0.0	0	0.0
Likely irrelevant (<10 min)	722	52.9	149277	58.4

Population and Area Coverage in Landkreis Ravensburg (Stroke-Ready Hospitals) (Baden-Württemberg) — Extended (+20% Speed (Emergency))

Category	Grid Cells	Area (%)	Population	Population (%)
High (30+ min)	0	0.0	0	0.0
Medium (20-30 min)	90	4.6	23507	8.5
Low (10-20 min)	328	16.7	37950	13.7
Only CT reachable within 60 min	0	0.0	0	0.0
Neither reachable within 60 min	0	0.0	0	0.0
Likely irrelevant (<10 min)	1542	78.7	215311	77.8

Population and Area Coverage in Kreisfreie Stadt Rosenheim (Stroke-Ready Hospitals) (Bayern) — Extended (+20% Speed (Emergency))

Category	Grid Cells	Area (%)	Population	Population (%)
High (30+ min)	0	0.0	0	0.0
Medium (20-30 min)	0	0.0	0	0.0
Low (10-20 min)	0	0.0	0	0.0
Only CT reachable within 60 min	0	0.0	0	0.0
Neither reachable within 60 min	0	0.0	0	0.0
Likely irrelevant (<10 min)	47	100.0	62621	100.0

Population and Area Coverage in Landkreis Freising (Stroke-Ready Hospitals) (Bayern) — Extended (+20% Speed (Emergency))

Category	Grid Cells	Area (%)	Population	Population (%)
High (30+ min)	0	0.0	0	0.0
Medium (20-30 min)	16	1.6	1253	0.7
Low (10-20 min)	79	8.1	7144	4.1
Only CT reachable within 60 min	0	0.0	0	0.0
Neither reachable within 60 min	0	0.0	0	0.0
Likely irrelevant (<10 min)	879	90.2	166529	95.2

Population and Area Coverage in Landkreis Garmisch-Partenkirchen (Stroke-Ready Hospitals) (Bayern) — Extended (+20% Speed (Emergency))

Category	Grid Cells	Area (%)	Population	Population (%)
High (30+ min)	1	0.1	362	0.4
Medium (20-30 min)	114	9.6	26600	30.6
Low (10-20 min)	35	2.9	14963	17.2
Only CT reachable within 60 min	8	0.7	652	0.8
Neither reachable within 60 min	429	36.0	994	1.1
Likely irrelevant (<10 min)	606	50.8	43346	49.9

Population and Area Coverage in Landkreis Regensburg (Stroke-Ready Hospitals) (Bayern) — Extended (+20% Speed (Emergency))

Category	Grid Cells	Area (%)	Population	Population (%)
High (30+ min)	0	0.0	0	0.0
Medium (20-30 min)	0	0.0	0	0.0
Low (10-20 min)	424	24.7	31330	16.2
Only CT reachable within 60 min	0	0.0	0	0.0
Neither reachable within 60 min	0	0.0	0	0.0
Likely irrelevant (<10 min)	1295	75.3	162381	83.8

Population and Area Coverage in Landkreis Tirschenreuth (Stroke-Ready Hospitals) (Bayern) — Extended (+20% Speed (Emergency))

Category	Grid Cells	Area (%)	Population	Population (%)
High (30+ min)	2	0.2	151	0.2
Medium (20-30 min)	427	33.2	28245	37.9
Low (10-20 min)	693	53.8	37181	49.9
Only CT reachable within 60 min	0	0.0	0	0.0
Neither reachable within 60 min	2	0.2	0	0.0
Likely irrelevant (<10 min)	164	12.7	8874	11.9

Population and Area Coverage in Kreisfreie Stadt Kaufbeuren (Stroke-Ready Hospitals) (Bayern) — Extended (+20% Speed (Emergency))

Category	Grid Cells	Area (%)	Population	Population (%)
High (30+ min)	0	0.0	0	0.0
Medium (20-30 min)	0	0.0	0	0.0
Low (10-20 min)	0	0.0	0	0.0
Only CT reachable within 60 min	0	0.0	0	0.0
Neither reachable within 60 min	0	0.0	0	0.0
Likely irrelevant (<10 min)	50	100.0	42940	100.0

Population and Area Coverage in Landkreis Oberallgäu (Stroke-Ready Hospitals) (Bayern) — Extended (+20% Speed (Emergency))

Category	Grid Cells	Area (%)	Population	Population (%)
High (30+ min)	25	1.4	7477	4.8
Medium (20-30 min)	191	10.8	44945	28.9
Low (10-20 min)	238	13.4	22593	14.5
Only CT reachable within 60 min	14	0.8	438	0.3
Neither reachable within 60 min	536	30.2	4027	2.6
Likely irrelevant (<10 min)	772	43.5	76032	48.9

Population and Area Coverage in Landkreis Regionalverband Saarbrücken (Stroke-Ready Hospitals) (Saarland) — Extended (+20% Speed (Emergency))

Category	Grid Cells	Area (%)	Population	Population (%)
High (30+ min)	0	0.0	0	0.0
Medium (20-30 min)	0	0.0	0	0.0
Low (10-20 min)	8	1.6	10373	3.1
Only CT reachable within 60 min	1	0.2	136	0.0
Neither reachable within 60 min	3	0.6	158	0.0
Likely irrelevant (<10 min)	476	97.5	321223	96.8

Population and Area Coverage in Landkreis Altenburger Land (Stroke-Ready Hospitals) (Thüringen) — Extended (+20% Speed (Emergency))

Category	Grid Cells	Area (%)	Population	Population (%)
High (30+ min)	0	0.0	0	0.0
Medium (20-30 min)	7	1.0	1171	1.4
Low (10-20 min)	47	6.5	7216	8.6
Only CT reachable within 60 min	0	0.0	0	0.0
Neither reachable within 60 min	0	0.0	0	0.0
Likely irrelevant (<10 min)	672	92.6	75693	90.0

Population and Area Coverage in Landkreis Diepholz (Stroke-Ready Hospitals) (Niedersachsen) — Extended (+20% Speed (Emergency))

Category	Grid Cells	Area (%)	Population	Population (%)
High (30+ min)	217	8.2	16965	8.0
Medium (20-30 min)	1111	42.1	58829	27.9
Low (10-20 min)	925	35.0	75900	35.9
Only CT reachable within 60 min	0	0.0	0	0.0
Neither reachable within 60 min	0	0.0	0	0.0
Likely irrelevant (<10 min)	388	14.7	59476	28.2

Population and Area Coverage in Kreisfreie Stadt Emden (Stroke-Ready Hospitals) (Niedersachsen) — Extended (+20% Speed (Emergency))

Category	Grid Cells	Area (%)	Population	Population (%)
High (30+ min)	0	0.0	0	0.0
Medium (20-30 min)	3	2.3	2125	4.3
Low (10-20 min)	0	0.0	0	0.0
Only CT reachable within 60 min	0	0.0	0	0.0
Neither reachable within 60 min	0	0.0	0	0.0
Likely irrelevant (<10 min)	129	97.7	46996	95.7

Population and Area Coverage in Kreisfreie Stadt Gelsenkirchen (Stroke-Ready Hospitals) (Nordrhein-Westfalen) — Extended (+20% Speed (Emergency))

Category	Grid Cells	Area (%)	Population	Population (%)
High (30+ min)	0	0.0	0	0.0
Medium (20-30 min)	0	0.0	0	0.0
Low (10-20 min)	19	14.2	30735	11.0
Only CT reachable within 60 min	0	0.0	0	0.0
Neither reachable within 60 min	0	0.0	0	0.0
Likely irrelevant (<10 min)	115	85.8	249050	89.0

Population and Area Coverage in Kreis Warendorf (Stroke-Ready Hospitals) (Nordrhein-Westfalen) — Extended (+20% Speed (Emergency))

Category	Grid Cells	Area (%)	Population	Population (%)
High (30+ min)	0	0.0	0	0.0
Medium (20-30 min)	20	1.2	15130	5.6
Low (10-20 min)	377	21.9	56499	20.8
Only CT reachable within 60 min	0	0.0	0	0.0
Neither reachable within 60 min	0	0.0	0	0.0
Likely irrelevant (<10 min)	1321	76.9	200441	73.7

Population and Area Coverage in Stadtkreis Ulm (Stroke-Ready Hospitals) (Baden-Württemberg) — Extended (+20% Speed (Emergency))

Category	Grid Cells	Area (%)	Population	Population (%)
High (30+ min)	0	0.0	0	0.0
Medium (20-30 min)	0	0.0	0	0.0
Low (10-20 min)	7	4.9	14058	11.7
Only CT reachable within 60 min	0	0.0	0	0.0
Neither reachable within 60 min	0	0.0	0	0.0
Likely irrelevant (<10 min)	136	95.1	106056	88.3

Population and Area Coverage in Landkreis Traunstein (Stroke-Ready Hospitals) (Bayern) — Extended (+20% Speed (Emergency))

Category	Grid Cells	Area (%)	Population	Population (%)
High (30+ min)	0	0.0	0	0.0
Medium (20-30 min)	35	1.9	4780	2.7
Low (10-20 min)	214	11.8	22711	12.9
Only CT reachable within 60 min	3	0.2	637	0.4
Neither reachable within 60 min	127	7.0	719	0.4
Likely irrelevant (<10 min)	1438	79.1	146768	83.6

Population and Area Coverage in Kreisfreie Stadt Regensburg (Stroke-Ready Hospitals) (Bayern) — Extended (+20% Speed (Emergency))

Category	Grid Cells	Area (%)	Population	Population (%)
High (30+ min)	0	0.0	0	0.0
Medium (20-30 min)	0	0.0	0	0.0
Low (10-20 min)	0	0.0	174	0.1
Only CT reachable within 60 min	0	0.0	0	0.0
Neither reachable within 60 min	0	0.0	0	0.0
Likely irrelevant (<10 min)	97	100.0	169888	99.9

Population and Area Coverage in Kreisfreie Stadt Weiden i.d.OPf. (Stroke-Ready Hospitals) (Bayern) — Extended (+20% Speed (Emergency))

Category	Grid Cells	Area (%)	Population	Population (%)
High (30+ min)	0	0.0	0	0.0
Medium (20-30 min)	0	0.0	0	0.0
Low (10-20 min)	0	0.0	0	0.0
Only CT reachable within 60 min	0	0.0	0	0.0
Neither reachable within 60 min	0	0.0	0	0.0
Likely irrelevant (<10 min)	86	100.0	43316	100.0

Population and Area Coverage in Kreisfreie Stadt Ansbach (Stroke-Ready Hospitals) (Bayern) — Extended (+20% Speed (Emergency))

Category	Grid Cells	Area (%)	Population	Population (%)
High (30+ min)	0	0.0	0	0.0
Medium (20-30 min)	0	0.0	0	0.0
Low (10-20 min)	0	0.0	0	0.0
Only CT reachable within 60 min	0	0.0	0	0.0
Neither reachable within 60 min	0	0.0	0	0.0
Likely irrelevant (<10 min)	123	100.0	41201	100.0

Population and Area Coverage in Landkreis Rhön-Grabfeld (Stroke-Ready Hospitals) (Bayern) — Extended (+20% Speed (Emergency))

Category	Grid Cells	Area (%)	Population	Population (%)
High (30+ min)	0	0.0	0	0.0
Medium (20-30 min)	0	0.0	0	0.0
Low (10-20 min)	27	2.1	881	1.1
Only CT reachable within 60 min	0	0.0	0	0.0
Neither reachable within 60 min	0	0.0	0	0.0
Likely irrelevant (<10 min)	1261	97.9	81297	98.9

Population and Area Coverage in Landkreis Ostprignitz-Ruppin (Stroke-Ready Hospitals) (Brandenburg) — Extended (+20% Speed (Emergency))

Category	Grid Cells	Area (%)	Population	Population (%)
High (30+ min)	132	3.9	6769	7.1
Medium (20-30 min)	635	18.8	19320	20.4
Low (10-20 min)	793	23.4	15491	16.4
Only CT reachable within 60 min	0	0.0	0	0.0
Neither reachable within 60 min	0	0.0	0	0.0
Likely irrelevant (<10 min)	1823	53.9	53130	56.1

Population and Area Coverage in Kreis Plön (Stroke-Ready Hospitals) (Schleswig-Holstein) — Extended (+20% Speed (Emergency))

Category	Grid Cells	Area (%)	Population	Population (%)
High (30+ min)	0	0.0	0	0.0
Medium (20-30 min)	96	6.5	18824	14.9
Low (10-20 min)	500	33.6	26637	21.1
Only CT reachable within 60 min	1	0.1	31	0.0
Neither reachable within 60 min	22	1.5	1489	1.2
Likely irrelevant (<10 min)	869	58.4	79372	62.8

Population and Area Coverage in Landkreis Nienburg (Weser) (Stroke-Ready Hospitals) (Niedersachsen) — Extended (+20% Speed (Emergency))

Category	Grid Cells	Area (%)	Population	Population (%)
High (30+ min)	0	0.0	0	0.0
Medium (20-30 min)	61	3.3	1801	1.5
Low (10-20 min)	375	20.1	15711	12.9
Only CT reachable within 60 min	0	0.0	0	0.0
Neither reachable within 60 min	0	0.0	0	0.0
Likely irrelevant (<10 min)	1429	76.6	103907	85.6

Population and Area Coverage in Landkreis Emsland (Stroke-Ready Hospitals) (Niedersachsen) — Extended (+20% Speed (Emergency))

Category	Grid Cells	Area (%)	Population	Population (%)
High (30+ min)	87	2.3	29829	9.3
Medium (20-30 min)	434	11.3	29374	9.1
Low (10-20 min)	1454	37.9	118911	36.9
Only CT reachable within 60 min	8	0.2	159	0.0
Neither reachable within 60 min	21	0.5	331	0.1
Likely irrelevant (<10 min)	1835	47.8	143640	44.6

Population and Area Coverage in Landkreis Wittmund (Stroke-Ready Hospitals) (Niedersachsen) — Extended (+20% Speed (Emergency))

Category	Grid Cells	Area (%)	Population	Population (%)
High (30+ min)	0	0.0	0	0.0
Medium (20-30 min)	101	11.4	10422	18.3
Low (10-20 min)	532	59.9	34065	59.9
Only CT reachable within 60 min	1	0.1	9	0.0
Neither reachable within 60 min	50	5.6	2637	4.6
Likely irrelevant (<10 min)	204	23.0	9739	17.1

Population and Area Coverage in Kreis Hochsauerlandkreis (Stroke-Ready Hospitals) (Nordrhein-Westfalen) — Extended (+20% Speed (Emergency))

Category	Grid Cells	Area (%)	Population	Population (%)
High (30+ min)	477	19.0	46773	17.8
Medium (20-30 min)	961	38.2	72982	27.8
Low (10-20 min)	629	25.0	50716	19.3
Only CT reachable within 60 min	6	0.2	0	0.0
Neither reachable within 60 min	0	0.0	0	0.0
Likely irrelevant (<10 min)	441	17.5	91898	35.0

Population and Area Coverage in Landkreis Rheingau-Taunus-Kreis (Stroke-Ready Hospitals) (Hessen) — Extended (+20% Speed (Emergency))

Category	Grid Cells	Area (%)	Population	Population (%)
High (30+ min)	38	3.7	2207	1.2
Medium (20-30 min)	217	21.3	28797	15.7
Low (10-20 min)	555	54.5	85774	46.8
Only CT reachable within 60 min	0	0.0	0	0.0
Neither reachable within 60 min	0	0.0	0	0.0
Likely irrelevant (<10 min)	209	20.5	66477	36.3

Population and Area Coverage in Landkreis Göppingen (Stroke-Ready Hospitals) (Baden-Württemberg) — Extended (+20% Speed (Emergency))

Category	Grid Cells	Area (%)	Population	Population (%)
High (30+ min)	0	0.0	0	0.0
Medium (20-30 min)	0	0.0	0	0.0
Low (10-20 min)	2	0.3	76	0.0
Only CT reachable within 60 min	0	0.0	0	0.0
Neither reachable within 60 min	0	0.0	0	0.0
Likely irrelevant (<10 min)	779	99.7	249920	100.0

Population and Area Coverage in Stadtkreis Freiburg im Breisgau (Stroke-Ready Hospitals) (Baden-Württemberg) — Extended (+20% Speed (Emergency))

Category	Grid Cells	Area (%)	Population	Population (%)
High (30+ min)	0	0.0	0	0.0
Medium (20-30 min)	3	1.6	614	0.2
Low (10-20 min)	36	19.3	18884	7.3
Only CT reachable within 60 min	0	0.0	0	0.0
Neither reachable within 60 min	0	0.0	0	0.0
Likely irrelevant (<10 min)	148	79.1	238941	92.5

Population and Area Coverage in Landkreis Landshut (Stroke-Ready Hospitals) (Bayern) — Extended (+20% Speed (Emergency))

Category	Grid Cells	Area (%)	Population	Population (%)
High (30+ min)	0	0.0	0	0.0
Medium (20-30 min)	4	0.2	104	0.1
Low (10-20 min)	188	11.5	14422	9.3
Only CT reachable within 60 min	0	0.0	0	0.0
Neither reachable within 60 min	0	0.0	0	0.0
Likely irrelevant (<10 min)	1449	88.3	141164	90.7

Population and Area Coverage in Landkreis Regen (Stroke-Ready Hospitals) (Bayern) — Extended (+20% Speed (Emergency))

Category	Grid Cells	Area (%)	Population	Population (%)
High (30+ min)	0	0.0	0	0.0
Medium (20-30 min)	69	6.1	8848	11.4
Low (10-20 min)	194	17.0	9907	12.8
Only CT reachable within 60 min	2	0.2	4	0.0
Neither reachable within 60 min	24	2.1	24	0.0
Likely irrelevant (<10 min)	851	74.6	58744	75.8

Population and Area Coverage in Kreisfreie Stadt Coburg (Stroke-Ready Hospitals) (Bayern) — Extended (+20% Speed (Emergency))

Category	Grid Cells	Area (%)	Population	Population (%)
High (30+ min)	0	0.0	0	0.0
Medium (20-30 min)	0	0.0	0	0.0
Low (10-20 min)	0	0.0	0	0.0
Only CT reachable within 60 min	0	0.0	0	0.0
Neither reachable within 60 min	0	0.0	0	0.0
Likely irrelevant (<10 min)	57	100.0	41384	100.0

Population and Area Coverage in Landkreis Neunkirchen (Stroke-Ready Hospitals) (Saarland) — Extended (+20% Speed (Emergency))

Category	Grid Cells	Area (%)	Population	Population (%)
High (30+ min)	0	0.0	0	0.0
Medium (20-30 min)	0	0.0	0	0.0
Low (10-20 min)	12	3.8	2983	2.3
Only CT reachable within 60 min	0	0.0	0	0.0
Neither reachable within 60 min	0	0.0	0	0.0
Likely irrelevant (<10 min)	300	96.2	124360	97.7

Population and Area Coverage in Landkreis Nordwestmecklenburg (Stroke-Ready Hospitals) (Mecklenburg-Vorpommern) — Extended (+20% Speed (Emergency))

Category	Grid Cells	Area (%)	Population	Population (%)
High (30+ min)	13	0.4	364	0.3
Medium (20-30 min)	182	6.3	5399	3.7
Low (10-20 min)	856	29.5	29726	20.6
Only CT reachable within 60 min	10	0.3	416	0.3
Neither reachable within 60 min	51	1.8	1667	1.2
Likely irrelevant (<10 min)	1792	61.7	106424	73.9

Population and Area Coverage in Kreisfreie Stadt Herne (Stroke-Ready Hospitals) (Nordrhein-Westfalen) — Extended (+20% Speed (Emergency))

Category	Grid Cells	Area (%)	Population	Population (%)
High (30+ min)	0	0.0	0	0.0
Medium (20-30 min)	0	0.0	0	0.0
Low (10-20 min)	4	5.8	15534	9.4
Only CT reachable within 60 min	0	0.0	0	0.0
Neither reachable within 60 min	0	0.0	0	0.0
Likely irrelevant (<10 min)	65	94.2	149710	90.6

Population and Area Coverage in Landkreis Altenkirchen (Westerwald) (Stroke-Ready Hospitals) (Rheinland-Pfalz) — Extended (+20% Speed (Emergency))

Category	Grid Cells	Area (%)	Population	Population (%)
High (30+ min)	0	0.0	0	0.0
Medium (20-30 min)	166	20.4	41014	30.7
Low (10-20 min)	365	44.8	51215	38.4
Only CT reachable within 60 min	0	0.0	0	0.0
Neither reachable within 60 min	0	0.0	0	0.0
Likely irrelevant (<10 min)	284	34.8	41180	30.9

Population and Area Coverage in Kreisfreie Stadt Kaiserslautern (Stroke-Ready Hospitals) (Rheinland-Pfalz) — Extended (+20% Speed (Emergency))

Category	Grid Cells	Area (%)	Population	Population (%)
High (30+ min)	0	0.0	0	0.0
Medium (20-30 min)	0	0.0	0	0.0
Low (10-20 min)	0	0.0	0	0.0
Only CT reachable within 60 min	0	0.0	0	0.0
Neither reachable within 60 min	0	0.0	0	0.0
Likely irrelevant (<10 min)	174	100.0	110813	100.0

Population and Area Coverage in Landkreis Mainz-Bingen (Stroke-Ready Hospitals) (Rheinland-Pfalz) — Extended (+20% Speed (Emergency))

Category	Grid Cells	Area (%)	Population	Population (%)
High (30+ min)	1	0.1	257	0.1
Medium (20-30 min)	34	4.5	5013	2.4
Low (10-20 min)	107	14.1	22364	10.6
Only CT reachable within 60 min	0	0.0	0	0.0
Neither reachable within 60 min	0	0.0	0	0.0
Likely irrelevant (<10 min)	619	81.3	182585	86.9

Population and Area Coverage in Landkreis Heidenheim (Stroke-Ready Hospitals) (Baden-Württemberg) — Extended (+20% Speed (Emergency))

Category	Grid Cells	Area (%)	Population	Population (%)
High (30+ min)	1	0.1	12	0.0
Medium (20-30 min)	53	7.0	2149	1.7
Low (10-20 min)	109	14.4	6839	5.3
Only CT reachable within 60 min	0	0.0	0	0.0
Neither reachable within 60 min	0	0.0	0	0.0
Likely irrelevant (<10 min)	596	78.5	118989	93.0

Population and Area Coverage in Stadtkreis Pforzheim (Stroke-Ready Hospitals) (Baden-Württemberg) — Extended (+20% Speed (Emergency))

Category	Grid Cells	Area (%)	Population	Population (%)
High (30+ min)	0	0.0	0	0.0
Medium (20-30 min)	0	0.0	0	0.0
Low (10-20 min)	9	7.5	7539	6.0
Only CT reachable within 60 min	0	0.0	0	0.0
Neither reachable within 60 min	0	0.0	0	0.0
Likely irrelevant (<10 min)	111	92.5	117423	94.0

Population and Area Coverage in Landkreis Tübingen (Stroke-Ready Hospitals) (Baden-Württemberg) — Extended (+20% Speed (Emergency))

Category	Grid Cells	Area (%)	Population	Population (%)
High (30+ min)	0	0.0	0	0.0
Medium (20-30 min)	2	0.3	497	0.2
Low (10-20 min)	57	9.1	13174	6.0
Only CT reachable within 60 min	0	0.0	0	0.0
Neither reachable within 60 min	0	0.0	0	0.0
Likely irrelevant (<10 min)	569	90.6	204867	93.7

Population and Area Coverage in Landkreis Bodenseekreis (Stroke-Ready Hospitals) (Baden-Württemberg) — Extended (+20% Speed (Emergency))

Category	Grid Cells	Area (%)	Population	Population (%)
High (30+ min)	0	0.0	0	0.0
Medium (20-30 min)	17	2.1	13676	6.2
Low (10-20 min)	412	51.4	86268	39.0
Only CT reachable within 60 min	1	0.1	4	0.0
Neither reachable within 60 min	0	0.0	1958	0.9
Likely irrelevant (<10 min)	372	46.4	119168	53.9

Population and Area Coverage in Landkreis Miesbach (Stroke-Ready Hospitals) (Bayern) — Extended (+20% Speed (Emergency))

Category	Grid Cells	Area (%)	Population	Population (%)
High (30+ min)	0	0.0	0	0.0
Medium (20-30 min)	0	0.0	12	0.0
Low (10-20 min)	22	2.1	396	0.4
Only CT reachable within 60 min	5	0.5	1186	1.2
Neither reachable within 60 min	240	23.1	990	1.0
Likely irrelevant (<10 min)	770	74.3	96149	97.4

Population and Area Coverage in Landkreis Mühldorf a.Inn (Stroke-Ready Hospitals) (Bayern) — Extended (+20% Speed (Emergency))

Category	Grid Cells	Area (%)	Population	Population (%)
High (30+ min)	0	0.0	0	0.0
Medium (20-30 min)	0	0.0	0	0.0
Low (10-20 min)	222	22.9	46901	42.4
Only CT reachable within 60 min	0	0.0	0	0.0
Neither reachable within 60 min	0	0.0	0	0.0
Likely irrelevant (<10 min)	748	77.1	63735	57.6

Population and Area Coverage in Landkreis Neuburg-Schrobenhausen (Stroke-Ready Hospitals) (Bayern) — Extended (+20% Speed (Emergency))

Category	Grid Cells	Area (%)	Population	Population (%)
High (30+ min)	0	0.0	0	0.0
Medium (20-30 min)	339	37.5	36826	38.6
Low (10-20 min)	461	51.0	50857	53.3
Only CT reachable within 60 min	0	0.0	0	0.0
Neither reachable within 60 min	0	0.0	0	0.0
Likely irrelevant (<10 min)	104	11.5	7800	8.2

Population and Area Coverage in Landkreis Starnberg (Stroke-Ready Hospitals) (Bayern) — Extended (+20% Speed (Emergency))

Category	Grid Cells	Area (%)	Population	Population (%)
High (30+ min)	0	0.0	0	0.0
Medium (20-30 min)	35	6.0	11492	8.5
Low (10-20 min)	237	40.4	61349	45.6
Only CT reachable within 60 min	0	0.0	0	0.0
Neither reachable within 60 min	0	0.0	0	0.0
Likely irrelevant (<10 min)	314	53.6	61696	45.9

Population and Area Coverage in Landkreis Fürth (Stroke-Ready Hospitals) (Bayern) — Extended (+20% Speed (Emergency))

Category	Grid Cells	Area (%)	Population	Population (%)
High (30+ min)	0	0.0	0	0.0
Medium (20-30 min)	0	0.0	0	0.0
Low (10-20 min)	2	0.5	100	0.1
Only CT reachable within 60 min	0	0.0	0	0.0
Neither reachable within 60 min	0	0.0	0	0.0
Likely irrelevant (<10 min)	380	99.5	118605	99.9

Population and Area Coverage in Landkreis Kitzingen (Stroke-Ready Hospitals) (Bayern) — Extended (+20% Speed (Emergency))

Category	Grid Cells	Area (%)	Population	Population (%)
High (30+ min)	0	0.0	0	0.0
Medium (20-30 min)	12	1.4	161	0.2
Low (10-20 min)	233	27.6	20529	22.5
Only CT reachable within 60 min	0	0.0	0	0.0
Neither reachable within 60 min	0	0.0	0	0.0
Likely irrelevant (<10 min)	599	71.0	70739	77.4

Population and Area Coverage in Landkreis Günzburg (Stroke-Ready Hospitals) (Bayern) — Extended (+20% Speed (Emergency))

Category	Grid Cells	Area (%)	Population	Population (%)
High (30+ min)	24	2.6	7893	6.3
Medium (20-30 min)	156	16.9	14787	11.8
Low (10-20 min)	334	36.2	39828	31.8
Only CT reachable within 60 min	0	0.0	0	0.0
Neither reachable within 60 min	0	0.0	0	0.0
Likely irrelevant (<10 min)	409	44.3	62611	50.0

Population and Area Coverage in Landkreis Havelland (Stroke-Ready Hospitals) (Brandenburg) — Extended (+20% Speed (Emergency))

Category	Grid Cells	Area (%)	Population	Population (%)
High (30+ min)	232	10.2	21945	14.5
Medium (20-30 min)	282	12.4	10006	6.6
Low (10-20 min)	322	14.1	7496	5.0
Only CT reachable within 60 min	36	1.6	145	0.1
Neither reachable within 60 min	0	0.0	0	0.0
Likely irrelevant (<10 min)	1410	61.8	111821	73.9

Population and Area Coverage in Landkreis Teltow-Fläming (Stroke-Ready Hospitals) (Brandenburg) — Extended (+20% Speed (Emergency))

Category	Grid Cells	Area (%)	Population	Population (%)
High (30+ min)	110	4.0	21405	13.6
Medium (20-30 min)	591	21.4	24703	15.7
Low (10-20 min)	1255	45.4	50148	31.9
Only CT reachable within 60 min	0	0.0	0	0.0
Neither reachable within 60 min	0	0.0	0	0.0
Likely irrelevant (<10 min)	809	29.3	60814	38.7

Population and Area Coverage in Landkreis Börde (Stroke-Ready Hospitals) (Sachsen-Anhalt) — Extended (+20% Speed (Emergency))

Category	Grid Cells	Area (%)	Population	Population (%)
High (30+ min)	4	0.1	62	0.0
Medium (20-30 min)	166	5.3	15829	10.2
Low (10-20 min)	598	19.1	17141	11.0
Only CT reachable within 60 min	0	0.0	0	0.0
Neither reachable within 60 min	0	0.0	0	0.0
Likely irrelevant (<10 min)	2355	75.4	122351	78.7

Population and Area Coverage in Landkreis Stade (Stroke-Ready Hospitals) (Niedersachsen) — Extended (+20% Speed (Emergency))

Category	Grid Cells	Area (%)	Population	Population (%)
High (30+ min)	9	0.5	362	0.2
Medium (20-30 min)	71	4.3	17820	9.0
Low (10-20 min)	337	20.5	42465	21.4
Only CT reachable within 60 min	7	0.4	147	0.1
Neither reachable within 60 min	96	5.9	4777	2.4
Likely irrelevant (<10 min)	1121	68.3	132595	66.9

Population and Area Coverage in Kreisfreie Stadt Oldenburg (Oldb) (Stroke-Ready Hospitals) (Niedersachsen) — Extended (+20% Speed (Emergency))

Category	Grid Cells	Area (%)	Population	Population (%)
High (30+ min)	0	0.0	0	0.0
Medium (20-30 min)	0	0.0	0	0.0
Low (10-20 min)	4	2.9	6239	3.9
Only CT reachable within 60 min	0	0.0	0	0.0
Neither reachable within 60 min	0	0.0	0	0.0
Likely irrelevant (<10 min)	133	97.1	153709	96.1

Population and Area Coverage in Landkreis Cloppenburg (Stroke-Ready Hospitals) (Niedersachsen) — Extended (+20% Speed (Emergency))

Category	Grid Cells	Area (%)	Population	Population (%)
High (30+ min)	67	3.5	10599	6.5
Medium (20-30 min)	455	23.9	53197	32.4
Low (10-20 min)	970	51.0	73236	44.6
Only CT reachable within 60 min	0	0.0	0	0.0
Neither reachable within 60 min	0	0.0	0	0.0
Likely irrelevant (<10 min)	410	21.6	27044	16.5

Population and Area Coverage in Kreisfreie Stadt Wuppertal (Stroke-Ready Hospitals) (Nordrhein-Westfalen) — Extended (+20% Speed (Emergency))

Category	Grid Cells	Area (%)	Population	Population (%)
High (30+ min)	0	0.0	0	0.0
Medium (20-30 min)	0	0.0	0	0.0
Low (10-20 min)	6	2.8	13097	3.6
Only CT reachable within 60 min	0	0.0	0	0.0
Neither reachable within 60 min	0	0.0	0	0.0
Likely irrelevant (<10 min)	207	97.2	345976	96.4

Population and Area Coverage in Landkreis Odenwaldkreis (Stroke-Ready Hospitals) (Hessen) — Extended (+20% Speed (Emergency))

Category	Grid Cells	Area (%)	Population	Population (%)
High (30+ min)	0	0.0	0	0.0
Medium (20-30 min)	2	0.3	14	0.0
Low (10-20 min)	210	27.1	35042	35.8
Only CT reachable within 60 min	0	0.0	0	0.0
Neither reachable within 60 min	0	0.0	0	0.0
Likely irrelevant (<10 min)	564	72.7	62834	64.2

Population and Area Coverage in Landkreis Karlsruhe (Stroke-Ready Hospitals) (Baden-Württemberg) — Extended (+20% Speed (Emergency))

Category	Grid Cells	Area (%)	Population	Population (%)
High (30+ min)	0	0.0	0	0.0
Medium (20-30 min)	6	0.4	10310	2.4
Low (10-20 min)	188	14.1	51391	11.8
Only CT reachable within 60 min	0	0.0	0	0.0
Neither reachable within 60 min	0	0.0	0	0.0
Likely irrelevant (<10 min)	1141	85.5	374134	85.8

Population and Area Coverage in Kreisfreie Stadt Landshut (Stroke-Ready Hospitals) (Bayern) — Extended (+20% Speed (Emergency))

Category	Grid Cells	Area (%)	Population	Population (%)
High (30+ min)	0	0.0	0	0.0
Medium (20-30 min)	0	0.0	0	0.0
Low (10-20 min)	5	6.2	2218	3.2
Only CT reachable within 60 min	0	0.0	0	0.0
Neither reachable within 60 min	0	0.0	0	0.0
Likely irrelevant (<10 min)	76	93.8	66840	96.8

Population and Area Coverage in Kreisfreie Stadt Erlangen (Stroke-Ready Hospitals) (Bayern) — Extended (+20% Speed (Emergency))

Category	Grid Cells	Area (%)	Population	Population (%)
High (30+ min)	0	0.0	0	0.0
Medium (20-30 min)	0	0.0	0	0.0
Low (10-20 min)	3	3.1	5117	4.0
Only CT reachable within 60 min	0	0.0	0	0.0
Neither reachable within 60 min	0	0.0	0	0.0
Likely irrelevant (<10 min)	93	96.9	121512	96.0

Population and Area Coverage in Landkreis Haßberge (Stroke-Ready Hospitals) (Bayern) — Extended (+20% Speed (Emergency))

Category	Grid Cells	Area (%)	Population	Population (%)
High (30+ min)	0	0.0	0	0.0
Medium (20-30 min)	309	25.8	24910	28.6
Low (10-20 min)	729	60.8	52477	60.2
Only CT reachable within 60 min	0	0.0	0	0.0
Neither reachable within 60 min	0	0.0	0	0.0
Likely irrelevant (<10 min)	162	13.5	9779	11.2

Population and Area Coverage in Landkreis Aichach-Friedberg (Stroke-Ready Hospitals) (Bayern) — Extended (+20% Speed (Emergency))

Category	Grid Cells	Area (%)	Population	Population (%)
High (30+ min)	0	0.0	0	0.0
Medium (20-30 min)	78	8.3	14315	10.7
Low (10-20 min)	459	48.6	72920	54.7
Only CT reachable within 60 min	0	0.0	0	0.0
Neither reachable within 60 min	0	0.0	0	0.0
Likely irrelevant (<10 min)	408	43.2	46176	34.6

Population and Area Coverage in Landkreis Dillingen a.d.Donau (Stroke-Ready Hospitals) (Bayern) — Extended (+20% Speed (Emergency))

Category	Grid Cells	Area (%)	Population	Population (%)
High (30+ min)	11	1.1	689	0.7
Medium (20-30 min)	237	24.4	29448	30.3
Low (10-20 min)	461	47.5	43822	45.1
Only CT reachable within 60 min	0	0.0	0	0.0
Neither reachable within 60 min	0	0.0	0	0.0
Likely irrelevant (<10 min)	261	26.9	23139	23.8

Population and Area Coverage in Landkreis Oberspreewald-Lausitz (Stroke-Ready Hospitals) (Brandenburg) — Extended (+20% Speed (Emergency))

Category	Grid Cells	Area (%)	Population	Population (%)
High (30+ min)	1	0.1	0	0.0
Medium (20-30 min)	33	2.1	8556	7.9
Low (10-20 min)	255	16.1	13263	12.2
Only CT reachable within 60 min	0	0.0	0	0.0
Neither reachable within 60 min	0	0.0	0	0.0
Likely irrelevant (<10 min)	1291	81.7	86639	79.9

Population and Area Coverage in Landkreis Bautzen (Stroke-Ready Hospitals) (Sachsen) — Extended (+20% Speed (Emergency))

Category	Grid Cells	Area (%)	Population	Population (%)
High (30+ min)	11	0.4	4908	1.7
Medium (20-30 min)	549	17.8	89675	31.1
Low (10-20 min)	1161	37.7	87526	30.4
Only CT reachable within 60 min	3	0.1	121	0.0
Neither reachable within 60 min	5	0.2	89	0.0
Likely irrelevant (<10 min)	1353	43.9	105704	36.7

Population and Area Coverage in Kreisfreie Stadt Dessau-Roßlau (Stroke-Ready Hospitals) (Sachsen-Anhalt) — Extended (+20% Speed (Emergency))

Category	Grid Cells	Area (%)	Population	Population (%)
High (30+ min)	0	0.0	0	0.0
Medium (20-30 min)	22	6.9	99	0.1
Low (10-20 min)	50	15.6	2365	2.8
Only CT reachable within 60 min	0	0.0	0	0.0
Neither reachable within 60 min	0	0.0	0	0.0
Likely irrelevant (<10 min)	248	77.5	81649	97.1

Population and Area Coverage in Landkreis Lüneburg (Stroke-Ready Hospitals) (Niedersachsen) — Extended (+20% Speed (Emergency))

Category	Grid Cells	Area (%)	Population	Population (%)
High (30+ min)	99	5.6	2023	1.1
Medium (20-30 min)	137	7.7	2588	1.5
Low (10-20 min)	382	21.5	16842	9.4
Only CT reachable within 60 min	110	6.2	1316	0.7
Neither reachable within 60 min	0	0.0	0	0.0
Likely irrelevant (<10 min)	1052	59.1	155499	87.2

Population and Area Coverage in Kreisfreie Stadt Bremerhaven (Stroke-Ready Hospitals) (Bremen) — Extended (+20% Speed (Emergency))

Category	Grid Cells	Area (%)	Population	Population (%)
High (30+ min)	0	0.0	0	0.0
Medium (20-30 min)	0	0.0	0	0.0
Low (10-20 min)	17	17.3	6901	6.0
Only CT reachable within 60 min	1	1.0	1409	1.2
Neither reachable within 60 min	4	4.1	10	0.0
Likely irrelevant (<10 min)	76	77.6	106096	92.7

Population and Area Coverage in Kreisfreie Stadt Koblenz (Stroke-Ready Hospitals) (Rheinland-Pfalz) — Extended (+20% Speed (Emergency))

Category	Grid Cells	Area (%)	Population	Population (%)
High (30+ min)	0	0.0	0	0.0
Medium (20-30 min)	0	0.0	0	0.0
Low (10-20 min)	11	8.1	6546	5.2
Only CT reachable within 60 min	0	0.0	0	0.0
Neither reachable within 60 min	0	0.0	0	0.0
Likely irrelevant (<10 min)	125	91.9	119311	94.8

Population and Area Coverage in Kreisfreie Stadt Pirmasens (Stroke-Ready Hospitals) (Rheinland-Pfalz) — Extended (+20% Speed (Emergency))

Category	Grid Cells	Area (%)	Population	Population (%)
High (30+ min)	0	0.0	0	0.0
Medium (20-30 min)	0	0.0	0	0.0
Low (10-20 min)	0	0.0	0	0.0
Only CT reachable within 60 min	0	0.0	0	0.0
Neither reachable within 60 min	0	0.0	0	0.0
Likely irrelevant (<10 min)	76	100.0	37262	100.0

Population and Area Coverage in Landkreis Donnersbergkreis (Stroke-Ready Hospitals) (Rheinland-Pfalz) — Extended (+20% Speed (Emergency))

Category	Grid Cells	Area (%)	Population	Population (%)
High (30+ min)	0	0.0	0	0.0
Medium (20-30 min)	20	2.5	4734	6.1
Low (10-20 min)	315	39.0	29595	38.2
Only CT reachable within 60 min	0	0.0	0	0.0
Neither reachable within 60 min	0	0.0	0	0.0
Likely irrelevant (<10 min)	472	58.5	43188	55.7

Population and Area Coverage in Stadtkreis Heidelberg (Stroke-Ready Hospitals) (Baden-Württemberg) — Extended (+20% Speed (Emergency))

Category	Grid Cells	Area (%)	Population	Population (%)
High (30+ min)	0	0.0	0	0.0
Medium (20-30 min)	0	0.0	0	0.0
Low (10-20 min)	46	34.3	33954	22.0
Only CT reachable within 60 min	0	0.0	0	0.0
Neither reachable within 60 min	0	0.0	0	0.0
Likely irrelevant (<10 min)	88	65.7	120412	78.0

Population and Area Coverage in Kreisfreie Stadt Straubing (Stroke-Ready Hospitals) (Bayern) — Extended (+20% Speed (Emergency))

Category	Grid Cells	Area (%)	Population	Population (%)
High (30+ min)	0	0.0	0	0.0
Medium (20-30 min)	0	0.0	0	0.0
Low (10-20 min)	0	0.0	0	0.0
Only CT reachable within 60 min	0	0.0	0	0.0
Neither reachable within 60 min	0	0.0	0	0.0
Likely irrelevant (<10 min)	85	100.0	46942	100.0

Population and Area Coverage in Landkreis Straubing-Bogen (Stroke-Ready Hospitals) (Bayern) — Extended (+20% Speed (Emergency))

Category	Grid Cells	Area (%)	Population	Population (%)
High (30+ min)	0	0.0	0	0.0
Medium (20-30 min)	61	4.2	5772	5.7
Low (10-20 min)	476	32.5	31004	30.8
Only CT reachable within 60 min	0	0.0	0	0.0
Neither reachable within 60 min	0	0.0	0	0.0
Likely irrelevant (<10 min)	929	63.4	63746	63.4

Population and Area Coverage in Landkreis Neumarkt i.d.OPf. (Stroke-Ready Hospitals) (Bayern) — Extended (+20% Speed (Emergency))

Category	Grid Cells	Area (%)	Population	Population (%)
High (30+ min)	0	0.0	0	0.0
Medium (20-30 min)	0	0.0	0	0.0
Low (10-20 min)	133	8.0	6912	5.2
Only CT reachable within 60 min	0	0.0	0	0.0
Neither reachable within 60 min	0	0.0	0	0.0
Likely irrelevant (<10 min)	1531	92.0	125555	94.8

Population and Area Coverage in Kreisfreie Stadt Schwabach (Stroke-Ready Hospitals) (Bayern) — Extended (+20% Speed (Emergency))

Category	Grid Cells	Area (%)	Population	Population (%)
High (30+ min)	0	0.0	0	0.0
Medium (20-30 min)	0	0.0	0	0.0
Low (10-20 min)	0	0.0	0	0.0
Only CT reachable within 60 min	0	0.0	0	0.0
Neither reachable within 60 min	0	0.0	0	0.0
Likely irrelevant (<10 min)	52	100.0	42132	100.0

Population and Area Coverage in Kreisfreie Stadt Brandenburg an der Havel (Stroke-Ready Hospitals) (Brandenburg) — Extended (+20% Speed (Emergency))

Category	Grid Cells	Area (%)	Population	Population (%)
High (30+ min)	0	0.0	0	0.0
Medium (20-30 min)	0	0.0	0	0.0
Low (10-20 min)	51	16.8	5538	8.0
Only CT reachable within 60 min	0	0.0	0	0.0
Neither reachable within 60 min	0	0.0	0	0.0
Likely irrelevant (<10 min)	253	83.2	63554	92.0

Population and Area Coverage in Landkreis Ludwigslust-Parchim (Stroke-Ready Hospitals) (Mecklenburg-Vorpommern) — Extended (+20% Speed (Emergency))

Category	Grid Cells	Area (%)	Population	Population (%)
High (30+ min)	1001	15.5	51825	26.4
Medium (20-30 min)	1540	23.9	35120	17.9
Low (10-20 min)	1809	28.0	37113	18.9
Only CT reachable within 60 min	32	0.5	71	0.0
Neither reachable within 60 min	0	0.0	0	0.0
Likely irrelevant (<10 min)	2073	32.1	71887	36.7

Population and Area Coverage in Landkreis Saalfeld-Rudolstadt (Stroke-Ready Hospitals) (Thüringen) — Extended (+20% Speed (Emergency))

Category	Grid Cells	Area (%)	Population	Population (%)
High (30+ min)	0	0.0	0	0.0
Medium (20-30 min)	6	0.5	26	0.0
Low (10-20 min)	625	48.7	37071	38.3
Only CT reachable within 60 min	0	0.0	0	0.0
Neither reachable within 60 min	0	0.0	0	0.0
Likely irrelevant (<10 min)	652	50.8	59723	61.7

Population and Area Coverage in Kreis Ostholstein (Stroke-Ready Hospitals) (Schleswig-Holstein) — Extended (+20% Speed (Emergency))

Category	Grid Cells	Area (%)	Population	Population (%)
High (30+ min)	6	0.3	476	0.2
Medium (20-30 min)	123	6.6	19865	10.1
Low (10-20 min)	429	23.1	56987	29.0
Only CT reachable within 60 min	14	0.8	314	0.2
Neither reachable within 60 min	60	3.2	2612	1.3
Likely irrelevant (<10 min)	1229	66.0	116147	59.1

Population and Area Coverage in Kreis Rendsburg-Eckernförde (Stroke-Ready Hospitals) (Schleswig-Holstein) — Extended (+20% Speed (Emergency))

Category	Grid Cells	Area (%)	Population	Population (%)
High (30+ min)	33	1.1	1523	0.6
Medium (20-30 min)	147	4.9	13750	5.0
Low (10-20 min)	261	8.7	26032	9.5
Only CT reachable within 60 min	3	0.1	152	0.1
Neither reachable within 60 min	31	1.0	3263	1.2
Likely irrelevant (<10 min)	2513	84.1	229832	83.7

Population and Area Coverage in Landkreis Ostalbkreis (Stroke-Ready Hospitals) (Baden-Württemberg) — Extended (+20% Speed (Emergency))

Category	Grid Cells	Area (%)	Population	Population (%)
High (30+ min)	0	0.0	0	0.0
Medium (20-30 min)	114	6.1	12025	3.9
Low (10-20 min)	419	22.6	55645	17.9
Only CT reachable within 60 min	0	0.0	0	0.0
Neither reachable within 60 min	0	0.0	0	0.0
Likely irrelevant (<10 min)	1322	71.3	243661	78.3

Population and Area Coverage in Landkreis Emmendingen (Stroke-Ready Hospitals) (Baden-Württemberg) — Extended (+20% Speed (Emergency))

Category	Grid Cells	Area (%)	Population	Population (%)
High (30+ min)	31	3.8	2354	1.5
Medium (20-30 min)	120	14.7	13479	8.3
Low (10-20 min)	190	23.2	36064	22.3
Only CT reachable within 60 min	1	0.1	64	0.0
Neither reachable within 60 min	0	0.0	5	0.0
Likely irrelevant (<10 min)	476	58.2	109918	67.9

Population and Area Coverage in Kreisfreie Stadt München (Stroke-Ready Hospitals) (Bayern) — Extended (+20% Speed (Emergency))

Category	Grid Cells	Area (%)	Population	Population (%)
High (30+ min)	0	0.0	0	0.0
Medium (20-30 min)	0	0.0	0	0.0
Low (10-20 min)	62	16.4	323476	19.4
Only CT reachable within 60 min	0	0.0	0	0.0
Neither reachable within 60 min	0	0.0	0	0.0
Likely irrelevant (<10 min)	317	83.6	1347679	80.6

Population and Area Coverage in Landkreis Bayreuth (Stroke-Ready Hospitals) (Bayern) — Extended (+20% Speed (Emergency))

Category	Grid Cells	Area (%)	Population	Population (%)
High (30+ min)	0	0.0	0	0.0
Medium (20-30 min)	66	4.1	9992	9.2
Low (10-20 min)	514	32.2	24552	22.5
Only CT reachable within 60 min	0	0.0	0	0.0
Neither reachable within 60 min	0	0.0	0	0.0
Likely irrelevant (<10 min)	1016	63.7	74366	68.3

Population and Area Coverage in Landkreis Kronach (Stroke-Ready Hospitals) (Bayern) — Extended (+20% Speed (Emergency))

Category	Grid Cells	Area (%)	Population	Population (%)
High (30+ min)	1	0.1	143	0.2
Medium (20-30 min)	108	13.3	16991	23.9
Low (10-20 min)	384	47.3	29970	42.2
Only CT reachable within 60 min	0	0.0	0	0.0
Neither reachable within 60 min	0	0.0	0	0.0
Likely irrelevant (<10 min)	319	39.3	23975	33.7

Population and Area Coverage in Landkreis Aschaffenburg (Stroke-Ready Hospitals) (Bayern) — Extended (+20% Speed (Emergency))

Category	Grid Cells	Area (%)	Population	Population (%)
High (30+ min)	0	0.0	0	0.0
Medium (20-30 min)	0	0.0	0	0.0
Low (10-20 min)	8	0.9	778	0.4
Only CT reachable within 60 min	0	0.0	0	0.0
Neither reachable within 60 min	0	0.0	0	0.0
Likely irrelevant (<10 min)	866	99.1	179071	99.6

Population and Area Coverage in Landkreis Saalekreis (Stroke-Ready Hospitals) (Sachsen-Anhalt) — Extended (+20% Speed (Emergency))

Category	Grid Cells	Area (%)	Population	Population (%)
High (30+ min)	0	0.0	0	0.0
Medium (20-30 min)	2	0.1	75	0.0
Low (10-20 min)	24	1.3	1641	1.0
Only CT reachable within 60 min	0	0.0	0	0.0
Neither reachable within 60 min	0	0.0	0	0.0
Likely irrelevant (<10 min)	1842	98.6	167219	99.0

Population and Area Coverage in Kreisfreie Stadt Wolfsburg (Stroke-Ready Hospitals) (Niedersachsen) — Extended (+20% Speed (Emergency))

Category	Grid Cells	Area (%)	Population	Population (%)
High (30+ min)	0	0.0	0	0.0
Medium (20-30 min)	0	0.0	0	0.0
Low (10-20 min)	0	0.0	0	0.0
Only CT reachable within 60 min	0	0.0	0	0.0
Neither reachable within 60 min	0	0.0	0	0.0
Likely irrelevant (<10 min)	274	100.0	122066	100.0

Population and Area Coverage in Landkreis Wolfenbüttel (Stroke-Ready Hospitals) (Niedersachsen) — Extended (+20% Speed (Emergency))

Category	Grid Cells	Area (%)	Population	Population (%)
High (30+ min)	0	0.0	0	0.0
Medium (20-30 min)	0	0.0	0	0.0
Low (10-20 min)	42	4.4	3534	3.0
Only CT reachable within 60 min	0	0.0	0	0.0
Neither reachable within 60 min	0	0.0	0	0.0
Likely irrelevant (<10 min)	915	95.6	116260	97.0

Population and Area Coverage in Landkreis Ammerland (Stroke-Ready Hospitals) (Niedersachsen) — Extended (+20% Speed (Emergency))

Category	Grid Cells	Area (%)	Population	Population (%)
High (30+ min)	0	0.0	0	0.0
Medium (20-30 min)	0	0.0	0	0.0
Low (10-20 min)	30	3.1	1293	1.1
Only CT reachable within 60 min	0	0.0	0	0.0
Neither reachable within 60 min	0	0.0	0	0.0
Likely irrelevant (<10 min)	949	96.9	120169	98.9

Population and Area Coverage in Kreis Rhein-Kreis Neuss (Stroke-Ready Hospitals) (Nordrhein-Westfalen) — Extended (+20% Speed (Emergency))

Category	Grid Cells	Area (%)	Population	Population (%)
High (30+ min)	0	0.0	0	0.0
Medium (20-30 min)	0	0.0	0	0.0
Low (10-20 min)	188	25.5	95606	20.3
Only CT reachable within 60 min	0	0.0	0	0.0
Neither reachable within 60 min	0	0.0	0	0.0
Likely irrelevant (<10 min)	548	74.5	375238	79.7

Population and Area Coverage in Kreis Lippe (Stroke-Ready Hospitals) (Nordrhein-Westfalen) — Extended (+20% Speed (Emergency))

Category	Grid Cells	Area (%)	Population	Population (%)
High (30+ min)	0	0.0	0	0.0
Medium (20-30 min)	18	1.1	19467	5.6
Low (10-20 min)	414	25.4	104663	30.3
Only CT reachable within 60 min	0	0.0	0	0.0
Neither reachable within 60 min	0	0.0	0	0.0
Likely irrelevant (<10 min)	1201	73.5	221029	64.0

Population and Area Coverage in Kreisfreie Stadt Hagen (Stroke-Ready Hospitals) (Nordrhein-Westfalen) — Extended (+20% Speed (Emergency))

Category	Grid Cells	Area (%)	Population	Population (%)
High (30+ min)	0	0.0	0	0.0
Medium (20-30 min)	0	0.0	0	0.0
Low (10-20 min)	37	18.1	12587	6.3
Only CT reachable within 60 min	0	0.0	0	0.0
Neither reachable within 60 min	0	0.0	0	0.0
Likely irrelevant (<10 min)	167	81.9	187072	93.7

Population and Area Coverage in Landkreis Alzey-Worms (Stroke-Ready Hospitals) (Rheinland-Pfalz) — Extended (+20% Speed (Emergency))

Category	Grid Cells	Area (%)	Population	Population (%)
High (30+ min)	0	0.0	0	0.0
Medium (20-30 min)	0	0.0	0	0.0
Low (10-20 min)	2	0.3	528	0.4
Only CT reachable within 60 min	0	0.0	0	0.0
Neither reachable within 60 min	0	0.0	0	0.0
Likely irrelevant (<10 min)	735	99.7	128866	99.6

Population and Area Coverage in Landkreis Germersheim (Stroke-Ready Hospitals) (Rheinland-Pfalz) — Extended (+20% Speed (Emergency))

Category	Grid Cells	Area (%)	Population	Population (%)
High (30+ min)	0	0.0	0	0.0
Medium (20-30 min)	0	0.0	0	0.0
Low (10-20 min)	83	14.6	32164	25.0
Only CT reachable within 60 min	0	0.0	0	0.0
Neither reachable within 60 min	0	0.0	0	0.0
Likely irrelevant (<10 min)	487	85.4	96558	75.0

Population and Area Coverage in Landkreis Neckar-Odenwald-Kreis (Stroke-Ready Hospitals) (Baden-Württemberg) — Extended (+20% Speed (Emergency))

Category	Grid Cells	Area (%)	Population	Population (%)
High (30+ min)	50	3.6	7025	4.9
Medium (20-30 min)	338	24.2	28519	19.9
Low (10-20 min)	300	21.5	19367	13.5
Only CT reachable within 60 min	0	0.0	0	0.0
Neither reachable within 60 min	0	0.0	0	0.0
Likely irrelevant (<10 min)	709	50.8	88459	61.7

Population and Area Coverage in Landkreis Enzkreis (Stroke-Ready Hospitals) (Baden-Württemberg) — Extended (+20% Speed (Emergency))

Category	Grid Cells	Area (%)	Population	Population (%)
High (30+ min)	0	0.0	1	0.0
Medium (20-30 min)	60	8.5	14956	7.6
Low (10-20 min)	291	41.1	74784	38.2
Only CT reachable within 60 min	0	0.0	0	0.0
Neither reachable within 60 min	0	0.0	0	0.0
Likely irrelevant (<10 min)	357	50.4	106195	54.2

Population and Area Coverage in Landkreis Berchtesgadener Land (Stroke-Ready Hospitals) (Bayern) — Extended (+20% Speed (Emergency))

Category	Grid Cells	Area (%)	Population	Population (%)
High (30+ min)	0	0.0	0	0.0
Medium (20-30 min)	121	13.0	27571	29.4
Low (10-20 min)	122	13.1	22997	24.6
Only CT reachable within 60 min	3	0.3	58	0.1
Neither reachable within 60 min	326	35.1	1749	1.9
Likely irrelevant (<10 min)	357	38.4	41260	44.1

Population and Area Coverage in Kreisfreie Stadt Hof (Stroke-Ready Hospitals) (Bayern) — Extended (+20% Speed (Emergency))

Category	Grid Cells	Area (%)	Population	Population (%)
High (30+ min)	0	0.0	0	0.0
Medium (20-30 min)	0	0.0	0	0.0
Low (10-20 min)	0	0.0	0	0.0
Only CT reachable within 60 min	0	0.0	0	0.0
Neither reachable within 60 min	0	0.0	0	0.0
Likely irrelevant (<10 min)	75	100.0	44749	100.0

Population and Area Coverage in Landkreis Kulmbach (Stroke-Ready Hospitals) (Bayern) — Extended (+20% Speed (Emergency))

Category	Grid Cells	Area (%)	Population	Population (%)
High (30+ min)	0	0.0	0	0.0
Medium (20-30 min)	0	0.0	0	0.0
Low (10-20 min)	194	23.4	8053	10.6
Only CT reachable within 60 min	0	0.0	0	0.0
Neither reachable within 60 min	0	0.0	0	0.0
Likely irrelevant (<10 min)	634	76.6	67625	89.4

Population and Area Coverage in Kreisfreie Stadt Memmingen (Stroke-Ready Hospitals) (Bayern) — Extended (+20% Speed (Emergency))

Category	Grid Cells	Area (%)	Population	Population (%)
High (30+ min)	0	0.0	0	0.0
Medium (20-30 min)	0	0.0	0	0.0
Low (10-20 min)	0	0.0	0	0.0
Only CT reachable within 60 min	0	0.0	0	0.0
Neither reachable within 60 min	0	0.0	0	0.0
Likely irrelevant (<10 min)	84	100.0	43302	100.0

Population and Area Coverage in Landkreis Augsburg (Stroke-Ready Hospitals) (Bayern) — Extended (+20% Speed (Emergency))

Category	Grid Cells	Area (%)	Population	Population (%)
High (30+ min)	0	0.0	0	0.0
Medium (20-30 min)	39	3.0	3598	1.4
Low (10-20 min)	341	26.3	70609	28.4
Only CT reachable within 60 min	0	0.0	0	0.0
Neither reachable within 60 min	0	0.0	0	0.0
Likely irrelevant (<10 min)	919	70.7	174058	70.1

Population and Area Coverage in Landkreis Zwickau (Stroke-Ready Hospitals) (Sachsen) — Extended (+20% Speed (Emergency))

Category	Grid Cells	Area (%)	Population	Population (%)
High (30+ min)	0	0.0	0	0.0
Medium (20-30 min)	21	1.7	2946	0.9
Low (10-20 min)	317	26.0	62178	19.6
Only CT reachable within 60 min	0	0.0	0	0.0
Neither reachable within 60 min	0	0.0	0	0.0
Likely irrelevant (<10 min)	883	72.3	252093	79.5

Population and Area Coverage in Landkreis Vorpommern-Rügen (Stroke-Ready Hospitals) (Mecklenburg-Vorpommern) — Extended (+20% Speed (Emergency))

Category	Grid Cells	Area (%)	Population	Population (%)
High (30+ min)	337	8.1	19178	9.6
Medium (20-30 min)	472	11.3	16835	8.4
Low (10-20 min)	1208	29.0	35585	17.9
Only CT reachable within 60 min	125	3.0	2897	1.5
Neither reachable within 60 min	257	6.2	5653	2.8
Likely irrelevant (<10 min)	1764	42.4	119121	59.8

Population and Area Coverage in Landkreis Görlitz (Stroke-Ready Hospitals) (Sachsen) — Extended (+20% Speed (Emergency))

Category	Grid Cells	Area (%)	Population	Population (%)
High (30+ min)	364	14.1	80943	37.0
Medium (20-30 min)	503	19.4	32632	14.9
Low (10-20 min)	390	15.1	23456	10.7
Only CT reachable within 60 min	5	0.2	39	0.0
Neither reachable within 60 min	10	0.4	206	0.1
Likely irrelevant (<10 min)	1315	50.8	81685	37.3

Population and Area Coverage in Landkreis Sonneberg (Stroke-Ready Hospitals) (Thüringen) — Extended (+20% Speed (Emergency))

Category	Grid Cells	Area (%)	Population	Population (%)
High (30+ min)	0	0.0	0	0.0
Medium (20-30 min)	0	0.0	0	0.0
Low (10-20 min)	34	5.8	1902	3.5
Only CT reachable within 60 min	0	0.0	0	0.0
Neither reachable within 60 min	0	0.0	0	0.0
Likely irrelevant (<10 min)	555	94.2	51766	96.5

Population and Area Coverage in Kreisfreie Stadt Köln (Stroke-Ready Hospitals) (Nordrhein-Westfalen) — Extended (+20% Speed (Emergency))

Category	Grid Cells	Area (%)	Population	Population (%)
High (30+ min)	0	0.0	0	0.0
Medium (20-30 min)	0	0.0	0	0.0
Low (10-20 min)	79	15.0	256294	21.1
Only CT reachable within 60 min	0	0.0	0	0.0
Neither reachable within 60 min	0	0.0	0	0.0
Likely irrelevant (<10 min)	448	85.0	958890	78.9

Population and Area Coverage in Kreis Heinsberg (Stroke-Ready Hospitals) (Nordrhein-Westfalen) — Extended (+20% Speed (Emergency))

Category	Grid Cells	Area (%)	Population	Population (%)
High (30+ min)	0	0.0	410	0.2
Medium (20-30 min)	11	1.4	2893	1.2
Low (10-20 min)	205	26.3	79529	32.0
Only CT reachable within 60 min	1	0.1	68	0.0
Neither reachable within 60 min	2	0.3	263	0.1
Likely irrelevant (<10 min)	559	71.9	165050	66.5

Population and Area Coverage in Landkreis Bergstraße (Stroke-Ready Hospitals) (Hessen) — Extended (+20% Speed (Emergency))

Category	Grid Cells	Area (%)	Population	Population (%)
High (30+ min)	0	0.0	0	0.0
Medium (20-30 min)	10	1.1	2206	0.8
Low (10-20 min)	243	27.1	91709	34.5
Only CT reachable within 60 min	0	0.0	0	0.0
Neither reachable within 60 min	0	0.0	0	0.0
Likely irrelevant (<10 min)	644	71.8	172142	64.7

Population and Area Coverage in Landkreis Wetteraukreis (Stroke-Ready Hospitals) (Hessen) — Extended (+20% Speed (Emergency))

Category	Grid Cells	Area (%)	Population	Population (%)
High (30+ min)	0	0.0	0	0.0
Medium (20-30 min)	8	0.6	1572	0.5
Low (10-20 min)	221	15.8	31122	10.4
Only CT reachable within 60 min	0	0.0	0	0.0
Neither reachable within 60 min	0	0.0	0	0.0
Likely irrelevant (<10 min)	1166	83.6	266169	89.1

Population and Area Coverage in Kreisfreie Stadt Landau in der Pfalz (Stroke-Ready Hospitals) (Rheinland-Pfalz) — Extended (+20% Speed (Emergency))

Category	Grid Cells	Area (%)	Population	Population (%)
High (30+ min)	0	0.0	0	0.0
Medium (20-30 min)	0	0.0	0	0.0
Low (10-20 min)	34	33.3	37224	70.8
Only CT reachable within 60 min	0	0.0	0	0.0
Neither reachable within 60 min	0	0.0	0	0.0
Likely irrelevant (<10 min)	68	66.7	15372	29.2

Population and Area Coverage in Stadtkreis Stuttgart (Stroke-Ready Hospitals) (Baden-Württemberg) — Extended (+20% Speed (Emergency))

Category	Grid Cells	Area (%)	Population	Population (%)
High (30+ min)	0	0.0	0	0.0
Medium (20-30 min)	0	0.0	0	0.0
Low (10-20 min)	21	8.2	41113	6.4
Only CT reachable within 60 min	0	0.0	0	0.0
Neither reachable within 60 min	0	0.0	0	0.0
Likely irrelevant (<10 min)	234	91.8	598833	93.6

Population and Area Coverage in Landkreis Konstanz (Stroke-Ready Hospitals) (Baden-Württemberg) — Extended (+20% Speed (Emergency))

Category	Grid Cells	Area (%)	Population	Population (%)
High (30+ min)	0	0.0	1055	0.4
Medium (20-30 min)	21	2.2	3729	1.5
Low (10-20 min)	183	19.2	52420	20.5
Only CT reachable within 60 min	1	0.1	13	0.0
Neither reachable within 60 min	6	0.6	1147	0.4
Likely irrelevant (<10 min)	740	77.8	197789	77.2

Population and Area Coverage in Landkreis Sigmaringen (Stroke-Ready Hospitals) (Baden-Württemberg) — Extended (+20% Speed (Emergency))

Category	Grid Cells	Area (%)	Population	Population (%)
High (30+ min)	0	0.0	0	0.0
Medium (20-30 min)	6	0.4	110	0.1
Low (10-20 min)	162	11.2	10092	7.8
Only CT reachable within 60 min	0	0.0	0	0.0
Neither reachable within 60 min	0	0.0	0	0.0
Likely irrelevant (<10 min)	1281	88.4	118456	92.1

Population and Area Coverage in Landkreis Kelheim (Stroke-Ready Hospitals) (Bayern) — Extended (+20% Speed (Emergency))

Category	Grid Cells	Area (%)	Population	Population (%)
High (30+ min)	2	0.2	321	0.3
Medium (20-30 min)	373	28.8	42184	35.4
Low (10-20 min)	534	41.2	45237	38.0
Only CT reachable within 60 min	0	0.0	0	0.0
Neither reachable within 60 min	0	0.0	0	0.0
Likely irrelevant (<10 min)	388	29.9	31405	26.4

Population and Area Coverage in Kreisfreie Stadt Schweinfurt (Stroke-Ready Hospitals) (Bayern) — Extended (+20% Speed (Emergency))

Category	Grid Cells	Area (%)	Population	Population (%)
High (30+ min)	0	0.0	0	0.0
Medium (20-30 min)	0	0.0	0	0.0
Low (10-20 min)	0	0.0	0	0.0
Only CT reachable within 60 min	0	0.0	0	0.0
Neither reachable within 60 min	0	0.0	0	0.0
Likely irrelevant (<10 min)	46	100.0	53903	100.0

Population and Area Coverage in Landkreis Neu-Ulm (Stroke-Ready Hospitals) (Bayern) — Extended (+20% Speed (Emergency))

Category	Grid Cells	Area (%)	Population	Population (%)
High (30+ min)	0	0.0	0	0.0
Medium (20-30 min)	65	10.4	14981	8.6
Low (10-20 min)	346	55.4	104079	60.1
Only CT reachable within 60 min	0	0.0	0	0.0
Neither reachable within 60 min	0	0.0	0	0.0
Likely irrelevant (<10 min)	213	34.1	54167	31.3

Population and Area Coverage in Landkreis Oberhavel (Stroke-Ready Hospitals) (Brandenburg) — Extended (+20% Speed (Emergency))

Category	Grid Cells	Area (%)	Population	Population (%)
High (30+ min)	425	17.6	19839	9.9
Medium (20-30 min)	541	22.4	14220	7.1
Low (10-20 min)	752	31.2	71823	35.8
Only CT reachable within 60 min	108	4.5	473	0.2
Neither reachable within 60 min	0	0.0	0	0.0
Likely irrelevant (<10 min)	588	24.4	94261	47.0

Population and Area Coverage in Landkreis Spree-Neiße (Stroke-Ready Hospitals) (Brandenburg) — Extended (+20% Speed (Emergency))

Category	Grid Cells	Area (%)	Population	Population (%)
High (30+ min)	60	2.9	13933	13.9
Medium (20-30 min)	317	15.2	14923	14.9
Low (10-20 min)	531	25.4	24016	23.9
Only CT reachable within 60 min	2	0.1	0	0.0
Neither reachable within 60 min	0	0.0	0	0.0
Likely irrelevant (<10 min)	1178	56.4	47525	47.3

Population and Area Coverage in Landkreis Ilm-Kreis (Stroke-Ready Hospitals) (Thüringen) — Extended (+20% Speed (Emergency))

Category	Grid Cells	Area (%)	Population	Population (%)
High (30+ min)	0	0.0	0	0.0
Medium (20-30 min)	26	2.5	11622	12.0
Low (10-20 min)	342	33.3	40465	41.9
Only CT reachable within 60 min	0	0.0	0	0.0
Neither reachable within 60 min	0	0.0	0	0.0
Likely irrelevant (<10 min)	658	64.1	44414	46.0

Population and Area Coverage in Landkreis Greiz (Stroke-Ready Hospitals) (Thüringen) — Extended (+20% Speed (Emergency))

Category	Grid Cells	Area (%)	Population	Population (%)
High (30+ min)	3	0.3	212	0.2
Medium (20-30 min)	63	5.8	16836	18.3
Low (10-20 min)	347	32.1	25455	27.7
Only CT reachable within 60 min	0	0.0	0	0.0
Neither reachable within 60 min	0	0.0	0	0.0
Likely irrelevant (<10 min)	667	61.8	49303	53.7

Population and Area Coverage in Kreisfreie Stadt Bottrop (Stroke-Ready Hospitals) (Nordrhein-Westfalen) — Extended (+20% Speed (Emergency))

Category	Grid Cells	Area (%)	Population	Population (%)
High (30+ min)	0	0.0	0	0.0
Medium (20-30 min)	0	0.0	0	0.0
Low (10-20 min)	18	13.5	3544	3.0
Only CT reachable within 60 min	0	0.0	0	0.0
Neither reachable within 60 min	0	0.0	0	0.0
Likely irrelevant (<10 min)	115	86.5	113423	97.0

Population and Area Coverage in Kreisfreie Stadt Speyer (Stroke-Ready Hospitals) (Rheinland-Pfalz) — Extended (+20% Speed (Emergency))

Category	Grid Cells	Area (%)	Population	Population (%)
High (30+ min)	0	0.0	0	0.0
Medium (20-30 min)	0	0.0	0	0.0
Low (10-20 min)	0	0.0	0	0.0
Only CT reachable within 60 min	0	0.0	0	0.0
Neither reachable within 60 min	0	0.0	0	0.0
Likely irrelevant (<10 min)	52	100.0	51373	100.0

Population and Area Coverage in Stadtkreis Mannheim (Stroke-Ready Hospitals) (Baden-Württemberg) — Extended (+20% Speed (Emergency))

Category	Grid Cells	Area (%)	Population	Population (%)
High (30+ min)	0	0.0	0	0.0
Medium (20-30 min)	0	0.0	0	0.0
Low (10-20 min)	12	6.7	2432	0.7
Only CT reachable within 60 min	0	0.0	0	0.0
Neither reachable within 60 min	0	0.0	0	0.0
Likely irrelevant (<10 min)	166	93.3	344517	99.3

Population and Area Coverage in Landkreis Tuttlingen (Stroke-Ready Hospitals) (Baden-Württemberg) — Extended (+20% Speed (Emergency))

Category	Grid Cells	Area (%)	Population	Population (%)
High (30+ min)	0	0.0	0	0.0
Medium (20-30 min)	0	0.0	0	0.0
Low (10-20 min)	7	0.8	333	0.2
Only CT reachable within 60 min	0	0.0	0	0.0
Neither reachable within 60 min	0	0.0	0	0.0
Likely irrelevant (<10 min)	874	99.2	134751	99.8

Population and Area Coverage in Landkreis Zollernalbkreis (Stroke-Ready Hospitals) (Baden-Württemberg) — Extended (+20% Speed (Emergency))

Category	Grid Cells	Area (%)	Population	Population (%)
High (30+ min)	0	0.0	0	0.0
Medium (20-30 min)	19	1.7	8194	4.4
Low (10-20 min)	229	20.6	50028	26.7
Only CT reachable within 60 min	0	0.0	0	0.0
Neither reachable within 60 min	0	0.0	0	0.0
Likely irrelevant (<10 min)	862	77.7	129053	68.9

Population and Area Coverage in Landkreis Eichstätt (Stroke-Ready Hospitals) (Bayern) — Extended (+20% Speed (Emergency))

Category	Grid Cells	Area (%)	Population	Population (%)
High (30+ min)	0	0.0	0	0.0
Medium (20-30 min)	373	25.2	30154	23.1
Low (10-20 min)	880	59.4	62205	47.6
Only CT reachable within 60 min	0	0.0	0	0.0
Neither reachable within 60 min	0	0.0	0	0.0
Likely irrelevant (<10 min)	229	15.5	38258	29.3

Population and Area Coverage in Landkreis Freyung-Grafenau (Stroke-Ready Hospitals) (Bayern) — Extended (+20% Speed (Emergency))

Category	Grid Cells	Area (%)	Population	Population (%)
High (30+ min)	0	0.0	0	0.0
Medium (20-30 min)	15	1.3	3896	4.9
Low (10-20 min)	213	18.8	16148	20.5
Only CT reachable within 60 min	1	0.1	27	0.0
Neither reachable within 60 min	49	4.3	8	0.0
Likely irrelevant (<10 min)	855	75.5	58651	74.5

Population and Area Coverage in Landkreis Main-Spessart (Stroke-Ready Hospitals) (Bayern) — Extended (+20% Speed (Emergency))

Category	Grid Cells	Area (%)	Population	Population (%)
High (30+ min)	0	0.0	0	0.0
Medium (20-30 min)	5	0.3	0	0.0
Low (10-20 min)	204	12.3	10427	7.9
Only CT reachable within 60 min	0	0.0	0	0.0
Neither reachable within 60 min	0	0.0	0	0.0
Likely irrelevant (<10 min)	1453	87.4	121028	92.1

Population and Area Coverage in Kreisfreie Stadt Potsdam (Stroke-Ready Hospitals) (Brandenburg) — Extended (+20% Speed (Emergency))

Category	Grid Cells	Area (%)	Population	Population (%)
High (30+ min)	0	0.0	0	0.0
Medium (20-30 min)	0	0.0	0	0.0
Low (10-20 min)	13	5.3	15509	7.6
Only CT reachable within 60 min	0	0.0	0	0.0
Neither reachable within 60 min	0	0.0	0	0.0
Likely irrelevant (<10 min)	231	94.7	189806	92.4

Population and Area Coverage in Landkreis Nordsachsen (Stroke-Ready Hospitals) (Sachsen) — Extended (+20% Speed (Emergency))

Category	Grid Cells	Area (%)	Population	Population (%)
High (30+ min)	57	2.2	2406	1.3
Medium (20-30 min)	533	20.4	47384	25.7
Low (10-20 min)	993	38.0	66394	36.1
Only CT reachable within 60 min	9	0.3	11	0.0
Neither reachable within 60 min	0	0.0	0	0.0
Likely irrelevant (<10 min)	1019	39.0	67878	36.9

Population and Area Coverage in Landkreis Cuxhaven (Stroke-Ready Hospitals) (Niedersachsen) — Extended (+20% Speed (Emergency))

Category	Grid Cells	Area (%)	Population	Population (%)
High (30+ min)	124	4.5	10700	5.4
Medium (20-30 min)	348	12.5	50501	25.4
Low (10-20 min)	880	31.7	46942	23.6
Only CT reachable within 60 min	9	0.3	773	0.4
Neither reachable within 60 min	42	1.5	2418	1.2
Likely irrelevant (<10 min)	1377	49.5	87371	44.0

Population and Area Coverage in Kreisfreie Stadt Bremen (Stroke-Ready Hospitals) (Bremen) — Extended (+20% Speed (Emergency))

Category	Grid Cells	Area (%)	Population	Population (%)
High (30+ min)	0	0.0	0	0.0
Medium (20-30 min)	0	0.0	12	0.0
Low (10-20 min)	53	12.1	67844	11.6
Only CT reachable within 60 min	0	0.0	0	0.0
Neither reachable within 60 min	1	0.2	1881	0.3
Likely irrelevant (<10 min)	384	87.7	513475	88.0

Population and Area Coverage in Landkreis Groß-Gerau (Stroke-Ready Hospitals) (Hessen) — Extended (+20% Speed (Emergency))

Category	Grid Cells	Area (%)	Population	Population (%)
High (30+ min)	0	0.0	0	0.0
Medium (20-30 min)	1	0.2	824	0.3
Low (10-20 min)	95	16.8	31871	12.3
Only CT reachable within 60 min	0	0.0	0	0.0
Neither reachable within 60 min	0	0.0	0	0.0
Likely irrelevant (<10 min)	468	83.0	226898	87.4

Population and Area Coverage in Landkreis Limburg-Weilburg (Stroke-Ready Hospitals) (Hessen) — Extended (+20% Speed (Emergency))

Category	Grid Cells	Area (%)	Population	Population (%)
High (30+ min)	0	0.0	0	0.0
Medium (20-30 min)	5	0.5	80	0.0
Low (10-20 min)	170	18.2	24238	14.0
Only CT reachable within 60 min	0	0.0	0	0.0
Neither reachable within 60 min	0	0.0	0	0.0
Likely irrelevant (<10 min)	759	81.3	148519	85.9

Population and Area Coverage in Landkreis Waldeck-Frankenberg (Stroke-Ready Hospitals) (Hessen) — Extended (+20% Speed (Emergency))

Category	Grid Cells	Area (%)	Population	Population (%)
High (30+ min)	633	26.6	53835	34.1
Medium (20-30 min)	765	32.1	45520	28.8
Low (10-20 min)	677	28.4	46564	29.5
Only CT reachable within 60 min	0	0.0	0	0.0
Neither reachable within 60 min	0	0.0	0	0.0
Likely irrelevant (<10 min)	307	12.9	12084	7.6

Population and Area Coverage in Landkreis Trier-Saarburg (Stroke-Ready Hospitals) (Rheinland-Pfalz) — Extended (+20% Speed (Emergency))

Category	Grid Cells	Area (%)	Population	Population (%)
High (30+ min)	1	0.1	0	0.0
Medium (20-30 min)	142	10.3	19958	13.3
Low (10-20 min)	471	34.3	34223	22.8
Only CT reachable within 60 min	0	0.0	0	0.0
Neither reachable within 60 min	0	0.0	355	0.2
Likely irrelevant (<10 min)	760	55.3	95499	63.7

Population and Area Coverage in Landkreis Reutlingen (Stroke-Ready Hospitals) (Baden-Württemberg) — Extended (+20% Speed (Emergency))

Category	Grid Cells	Area (%)	Population	Population (%)
High (30+ min)	70	5.3	10133	3.4
Medium (20-30 min)	292	21.9	9778	3.3
Low (10-20 min)	373	28.0	41609	14.0
Only CT reachable within 60 min	0	0.0	0	0.0
Neither reachable within 60 min	0	0.0	0	0.0
Likely irrelevant (<10 min)	598	44.9	236191	79.3

Population and Area Coverage in Kreisfreie Stadt Schwerin (Stroke-Ready Hospitals) (Mecklenburg-Vorpommern) — Extended (+20% Speed (Emergency))

Category	Grid Cells	Area (%)	Population	Population (%)
High (30+ min)	0	0.0	0	0.0
Medium (20-30 min)	0	0.0	0	0.0
Low (10-20 min)	1	0.6	124	0.1
Only CT reachable within 60 min	0	0.0	0	0.0
Neither reachable within 60 min	0	0.0	0	0.0
Likely irrelevant (<10 min)	172	99.4	96648	99.9

Population and Area Coverage in Kreisfreie Stadt Hamburg (Stroke-Ready Hospitals) (Hamburg) — Extended (+20% Speed (Emergency))

Category	Grid Cells	Area (%)	Population	Population (%)
High (30+ min)	0	0.0	0	0.0
Medium (20-30 min)	0	0.0	0	0.0
Low (10-20 min)	112	11.2	149048	8.3
Only CT reachable within 60 min	0	0.0	0	0.0
Neither reachable within 60 min	6	0.6	6087	0.3
Likely irrelevant (<10 min)	879	88.2	1638333	91.3

Population and Area Coverage in Kreisfreie Stadt Braunschweig (Stroke-Ready Hospitals) (Niedersachsen) — Extended (+20% Speed (Emergency))

Category	Grid Cells	Area (%)	Population	Population (%)
High (30+ min)	0	0.0	0	0.0
Medium (20-30 min)	0	0.0	0	0.0
Low (10-20 min)	115	47.3	136891	55.5
Only CT reachable within 60 min	0	0.0	0	0.0
Neither reachable within 60 min	0	0.0	0	0.0
Likely irrelevant (<10 min)	128	52.7	109787	44.5

Population and Area Coverage in Landkreis Leer (Stroke-Ready Hospitals) (Niedersachsen) — Extended (+20% Speed (Emergency))

Category	Grid Cells	Area (%)	Population	Population (%)
High (30+ min)	60	4.2	9525	5.6
Medium (20-30 min)	236	16.6	39973	23.7
Low (10-20 min)	476	33.6	60988	36.1
Only CT reachable within 60 min	2	0.1	71	0.0
Neither reachable within 60 min	38	2.7	3553	2.1
Likely irrelevant (<10 min)	606	42.7	54728	32.4

Population and Area Coverage in Kreisfreie Stadt Duisburg (Stroke-Ready Hospitals) (Nordrhein-Westfalen) — Extended (+20% Speed (Emergency))

Category	Grid Cells	Area (%)	Population	Population (%)
High (30+ min)	0	0.0	0	0.0
Medium (20-30 min)	0	0.0	0	0.0
Low (10-20 min)	41	13.8	74204	13.4
Only CT reachable within 60 min	0	0.0	0	0.0
Neither reachable within 60 min	0	0.0	0	0.0
Likely irrelevant (<10 min)	256	86.2	478699	86.6

Population and Area Coverage in Kreis Siegen-Wittgenstein (Stroke-Ready Hospitals) (Nordrhein-Westfalen) — Extended (+20% Speed (Emergency))

Category	Grid Cells	Area (%)	Population	Population (%)
High (30+ min)	328	22.6	25221	8.6
Medium (20-30 min)	187	12.9	8487	2.9
Low (10-20 min)	135	9.3	15720	5.4
Only CT reachable within 60 min	11	0.8	5	0.0
Neither reachable within 60 min	0	0.0	0	0.0
Likely irrelevant (<10 min)	790	54.4	243886	83.1

Population and Area Coverage in Landkreis Schwarzwald-Baar-Kreis (Stroke-Ready Hospitals) (Baden-Württemberg) — Extended (+20% Speed (Emergency))

Category	Grid Cells	Area (%)	Population	Population (%)
High (30+ min)	5	0.4	32	0.0
Medium (20-30 min)	22	1.8	579	0.3
Low (10-20 min)	94	7.6	11494	5.6
Only CT reachable within 60 min	0	0.0	0	0.0
Neither reachable within 60 min	0	0.0	0	0.0
Likely irrelevant (<10 min)	1109	90.2	194883	94.2

Population and Area Coverage in Kreisfreie Stadt Amberg (Stroke-Ready Hospitals) (Bayern) — Extended (+20% Speed (Emergency))

Category	Grid Cells	Area (%)	Population	Population (%)
High (30+ min)	0	0.0	0	0.0
Medium (20-30 min)	0	0.0	0	0.0
Low (10-20 min)	0	0.0	0	0.0
Only CT reachable within 60 min	0	0.0	0	0.0
Neither reachable within 60 min	0	0.0	0	0.0
Likely irrelevant (<10 min)	61	100.0	42852	100.0

Population and Area Coverage in Kreisfreie Stadt Bayreuth (Stroke-Ready Hospitals) (Bayern) — Extended (+20% Speed (Emergency))

Category	Grid Cells	Area (%)	Population	Population (%)
High (30+ min)	0	0.0	0	0.0
Medium (20-30 min)	0	0.0	0	0.0
Low (10-20 min)	0	0.0	0	0.0
Only CT reachable within 60 min	0	0.0	0	0.0
Neither reachable within 60 min	0	0.0	0	0.0
Likely irrelevant (<10 min)	82	100.0	73464	100.0

Population and Area Coverage in Landkreis Nürnberger Land (Stroke-Ready Hospitals) (Bayern) — Extended (+20% Speed (Emergency))

Category	Grid Cells	Area (%)	Population	Population (%)
High (30+ min)	0	0.0	0	0.0
Medium (20-30 min)	18	1.8	2094	1.2
Low (10-20 min)	376	38.1	73165	43.0
Only CT reachable within 60 min	0	0.0	0	0.0
Neither reachable within 60 min	0	0.0	0	0.0
Likely irrelevant (<10 min)	592	60.0	94818	55.8

Population and Area Coverage in Landkreis Roth (Stroke-Ready Hospitals) (Bayern) — Extended (+20% Speed (Emergency))

Category	Grid Cells	Area (%)	Population	Population (%)
High (30+ min)	0	0.0	0	0.0
Medium (20-30 min)	2	0.2	2	0.0
Low (10-20 min)	131	11.9	6862	5.3
Only CT reachable within 60 min	0	0.0	0	0.0
Neither reachable within 60 min	0	0.0	0	0.0
Likely irrelevant (<10 min)	971	88.0	121913	94.7

Population and Area Coverage in Kreisfreie Stadt Aschaffenburg (Stroke-Ready Hospitals) (Bayern) — Extended (+20% Speed (Emergency))

Category	Grid Cells	Area (%)	Population	Population (%)
High (30+ min)	0	0.0	0	0.0
Medium (20-30 min)	0	0.0	0	0.0
Low (10-20 min)	0	0.0	0	0.0
Only CT reachable within 60 min	0	0.0	0	0.0
Neither reachable within 60 min	0	0.0	0	0.0
Likely irrelevant (<10 min)	78	100.0	70292	100.0

Population and Area Coverage in Landkreis Merzig-Wadern (Stroke-Ready Hospitals) (Saarland) — Extended (+20% Speed (Emergency))

Category	Grid Cells	Area (%)	Population	Population (%)
High (30+ min)	0	0.0	0	0.0
Medium (20-30 min)	14	2.0	289	0.3
Low (10-20 min)	182	26.3	21319	21.3
Only CT reachable within 60 min	0	0.0	1	0.0
Neither reachable within 60 min	0	0.0	191	0.2
Likely irrelevant (<10 min)	495	71.6	78217	78.2

Population and Area Coverage in Landkreis Vogtlandkreis (Stroke-Ready Hospitals) (Sachsen) — Extended (+20% Speed (Emergency))

Category	Grid Cells	Area (%)	Population	Population (%)
High (30+ min)	46	2.6	3825	1.8
Medium (20-30 min)	143	8.1	13705	6.3
Low (10-20 min)	286	16.2	17019	7.8
Only CT reachable within 60 min	1	0.1	88	0.0
Neither reachable within 60 min	12	0.7	105	0.0
Likely irrelevant (<10 min)	1279	72.4	183307	84.1

Population and Area Coverage in Landkreis Altmarkkreis Salzwedel (Stroke-Ready Hospitals) (Sachsen-Anhalt) — Extended (+20% Speed (Emergency))

Category	Grid Cells	Area (%)	Population	Population (%)
High (30+ min)	1044	34.1	33705	43.6
Medium (20-30 min)	1241	40.5	31632	41.0
Low (10-20 min)	655	21.4	10012	13.0
Only CT reachable within 60 min	0	0.0	0	0.0
Neither reachable within 60 min	0	0.0	0	0.0
Likely irrelevant (<10 min)	122	4.0	1882	2.4

Population and Area Coverage in Kreis Segeberg (Stroke-Ready Hospitals) (Schleswig-Holstein) — Extended (+20% Speed (Emergency))

Category	Grid Cells	Area (%)	Population	Population (%)
High (30+ min)	0	0.0	0	0.0
Medium (20-30 min)	4	0.2	300	0.1
Low (10-20 min)	385	21.0	81457	29.7
Only CT reachable within 60 min	0	0.0	0	0.0
Neither reachable within 60 min	0	0.0	0	0.0
Likely irrelevant (<10 min)	1443	78.8	192154	70.2

Population and Area Coverage in Landkreis Osnabrück (Stroke-Ready Hospitals) (Niedersachsen) — Extended (+20% Speed (Emergency))

Category	Grid Cells	Area (%)	Population	Population (%)
High (30+ min)	0	0.0	0	0.0
Medium (20-30 min)	94	3.4	18418	5.2
Low (10-20 min)	1199	42.8	166662	47.2
Only CT reachable within 60 min	0	0.0	0	0.0
Neither reachable within 60 min	0	0.0	0	0.0
Likely irrelevant (<10 min)	1509	53.9	168077	47.6

Population and Area Coverage in Kreisfreie Stadt Oberhausen (Stroke-Ready Hospitals) (Nordrhein-Westfalen) — Extended (+20% Speed (Emergency))

Category	Grid Cells	Area (%)	Population	Population (%)
High (30+ min)	0	0.0	0	0.0
Medium (20-30 min)	0	0.0	0	0.0
Low (10-20 min)	14	13.9	57172	26.6
Only CT reachable within 60 min	0	0.0	0	0.0
Neither reachable within 60 min	0	0.0	0	0.0
Likely irrelevant (<10 min)	87	86.1	157995	73.4

Population and Area Coverage in Kreisfreie Stadt Remscheid (Stroke-Ready Hospitals) (Nordrhein-Westfalen) — Extended (+20% Speed (Emergency))

Category	Grid Cells	Area (%)	Population	Population (%)
High (30+ min)	0	0.0	0	0.0
Medium (20-30 min)	0	0.0	0	0.0
Low (10-20 min)	30	30.9	27405	25.3
Only CT reachable within 60 min	0	0.0	0	0.0
Neither reachable within 60 min	0	0.0	0	0.0
Likely irrelevant (<10 min)	67	69.1	81085	74.7

Population and Area Coverage in Kreis Mettmann (Stroke-Ready Hospitals) (Nordrhein-Westfalen) — Extended (+20% Speed (Emergency))

Category	Grid Cells	Area (%)	Population	Population (%)
High (30+ min)	0	0.0	0	0.0
Medium (20-30 min)	0	0.0	0	0.0
Low (10-20 min)	105	20.0	125071	26.4
Only CT reachable within 60 min	0	0.0	0	0.0
Neither reachable within 60 min	0	0.0	0	0.0
Likely irrelevant (<10 min)	420	80.0	348655	73.6

Population and Area Coverage in Kreis Rhein-Sieg-Kreis (Stroke-Ready Hospitals) (Nordrhein-Westfalen) — Extended (+20% Speed (Emergency))

Category	Grid Cells	Area (%)	Population	Population (%)
High (30+ min)	0	0.0	0	0.0
Medium (20-30 min)	48	3.3	9458	1.6
Low (10-20 min)	334	22.7	128992	22.1
Only CT reachable within 60 min	0	0.0	0	0.0
Neither reachable within 60 min	0	0.0	0	0.0
Likely irrelevant (<10 min)	1092	74.1	444826	76.3

Population and Area Coverage in Kreis Borken (Stroke-Ready Hospitals) (Nordrhein-Westfalen) — Extended (+20% Speed (Emergency))

Category	Grid Cells	Area (%)	Population	Population (%)
High (30+ min)	17	0.9	7989	2.2
Medium (20-30 min)	235	12.9	70100	19.1
Low (10-20 min)	464	25.5	101559	27.7
Only CT reachable within 60 min	2	0.1	75	0.0
Neither reachable within 60 min	16	0.9	147	0.0
Likely irrelevant (<10 min)	1085	59.6	186922	51.0

Population and Area Coverage in Landkreis Vulkaneifel (Stroke-Ready Hospitals) (Rheinland-Pfalz) — Extended (+20% Speed (Emergency))

Category	Grid Cells	Area (%)	Population	Population (%)
High (30+ min)	145	12.6	7926	12.6
Medium (20-30 min)	275	23.9	22248	35.4
Low (10-20 min)	554	48.2	24896	39.6
Only CT reachable within 60 min	0	0.0	0	0.0
Neither reachable within 60 min	0	0.0	0	0.0
Likely irrelevant (<10 min)	176	15.3	7785	12.4

Population and Area Coverage in Landkreis Kaiserslautern (Stroke-Ready Hospitals) (Rheinland-Pfalz) — Extended (+20% Speed (Emergency))

Category	Grid Cells	Area (%)	Population	Population (%)
High (30+ min)	0	0.0	0	0.0
Medium (20-30 min)	4	0.5	517	0.5
Low (10-20 min)	174	22.0	27381	25.6
Only CT reachable within 60 min	0	0.0	0	0.0
Neither reachable within 60 min	0	0.0	0	0.0
Likely irrelevant (<10 min)	613	77.5	79009	73.9

Population and Area Coverage in Landkreis Calw (Stroke-Ready Hospitals) (Baden-Württemberg) — Extended (+20% Speed (Emergency))

Category	Grid Cells	Area (%)	Population	Population (%)
High (30+ min)	1	0.1	906	0.6
Medium (20-30 min)	53	5.5	14918	9.8
Low (10-20 min)	233	24.1	38590	25.5
Only CT reachable within 60 min	0	0.0	0	0.0
Neither reachable within 60 min	0	0.0	0	0.0
Likely irrelevant (<10 min)	680	70.3	97215	64.1

Population and Area Coverage in Kreisfreie Stadt Berlin (Stroke-Ready Hospitals) (Berlin) — Extended (+20% Speed (Emergency))

Category	Grid Cells	Area (%)	Population	Population (%)
High (30+ min)	0	0.0	0	0.0
Medium (20-30 min)	2	0.2	5567	0.2
Low (10-20 min)	152	12.8	323555	8.9
Only CT reachable within 60 min	0	0.0	0	0.0
Neither reachable within 60 min	0	0.0	0	0.0
Likely irrelevant (<10 min)	1030	87.0	3299576	90.9

Population and Area Coverage in Kreis Stormarn (Stroke-Ready Hospitals) (Schleswig-Holstein) — Extended (+20% Speed (Emergency))

Category	Grid Cells	Area (%)	Population	Population (%)
High (30+ min)	0	0.0	0	0.0
Medium (20-30 min)	9	0.9	5230	2.1
Low (10-20 min)	512	49.1	124500	51.0
Only CT reachable within 60 min	0	0.0	0	0.0
Neither reachable within 60 min	0	0.0	0	0.0
Likely irrelevant (<10 min)	521	50.0	114238	46.8

Population and Area Coverage in Landkreis Hildesheim (Stroke-Ready Hospitals) (Niedersachsen) — Extended (+20% Speed (Emergency))

Category	Grid Cells	Area (%)	Population	Population (%)
High (30+ min)	24	1.5	13985	4.8
Medium (20-30 min)	290	18.2	23431	8.0
Low (10-20 min)	254	16.0	24643	8.4
Only CT reachable within 60 min	0	0.0	0	0.0
Neither reachable within 60 min	0	0.0	0	0.0
Likely irrelevant (<10 min)	1024	64.3	231656	78.9

Population and Area Coverage in Kreis Viersen (Stroke-Ready Hospitals) (Nordrhein-Westfalen) — Extended (+20% Speed (Emergency))

Category	Grid Cells	Area (%)	Population	Population (%)
High (30+ min)	0	0.0	0	0.0
Medium (20-30 min)	1	0.1	2	0.0
Low (10-20 min)	122	16.8	53711	18.1
Only CT reachable within 60 min	0	0.0	0	0.0
Neither reachable within 60 min	45	6.2	1588	0.5
Likely irrelevant (<10 min)	560	76.9	240743	81.3

Population and Area Coverage in Kreis Rheinisch-Bergischer Kreis (Stroke-Ready Hospitals) (Nordrhein-Westfalen) — Extended (+20% Speed (Emergency))

Category	Grid Cells	Area (%)	Population	Population (%)
High (30+ min)	0	0.0	0	0.0
Medium (20-30 min)	0	0.0	0	0.0
Low (10-20 min)	61	10.9	15630	5.2
Only CT reachable within 60 min	0	0.0	0	0.0
Neither reachable within 60 min	0	0.0	0	0.0
Likely irrelevant (<10 min)	500	89.1	285742	94.8

Population and Area Coverage in Kreisfreie Stadt Zweibrücken (Stroke-Ready Hospitals) (Rheinland-Pfalz) — Extended (+20% Speed (Emergency))

Category	Grid Cells	Area (%)	Population	Population (%)
High (30+ min)	0	0.0	0	0.0
Medium (20-30 min)	0	0.0	0	0.0
Low (10-20 min)	58	65.9	29582	90.7
Only CT reachable within 60 min	0	0.0	0	0.0
Neither reachable within 60 min	0	0.0	0	0.0
Likely irrelevant (<10 min)	30	34.1	3025	9.3

Population and Area Coverage in Landkreis Rhein-Pfalz-Kreis (Stroke-Ready Hospitals) (Rheinland-Pfalz) — Extended (+20% Speed (Emergency))

Category	Grid Cells	Area (%)	Population	Population (%)
High (30+ min)	0	0.0	0	0.0
Medium (20-30 min)	0	0.0	0	0.0
Low (10-20 min)	5	1.3	738	0.5
Only CT reachable within 60 min	0	0.0	0	0.0
Neither reachable within 60 min	0	0.0	0	0.0
Likely irrelevant (<10 min)	376	98.7	151757	99.5

Population and Area Coverage in Landkreis Altötting (Stroke-Ready Hospitals) (Bayern) — Extended (+20% Speed (Emergency))

Category	Grid Cells	Area (%)	Population	Population (%)
High (30+ min)	0	0.0	0	0.0
Medium (20-30 min)	0	0.0	0	0.0
Low (10-20 min)	17	2.5	690	0.6
Only CT reachable within 60 min	0	0.0	0	0.0
Neither reachable within 60 min	3	0.4	2068	1.9
Likely irrelevant (<10 min)	658	97.1	104537	97.4

Population and Area Coverage in Landkreis Pfaffenhofen a.d.Ilm (Stroke-Ready Hospitals) (Bayern) — Extended (+20% Speed (Emergency))

Category	Grid Cells	Area (%)	Population	Population (%)
High (30+ min)	0	0.0	0	0.0
Medium (20-30 min)	3	0.3	405	0.3
Low (10-20 min)	204	22.1	23807	19.2
Only CT reachable within 60 min	0	0.0	0	0.0
Neither reachable within 60 min	0	0.0	0	0.0
Likely irrelevant (<10 min)	717	77.6	99663	80.5

Population and Area Coverage in Kreisfreie Stadt Bamberg (Stroke-Ready Hospitals) (Bayern) — Extended (+20% Speed (Emergency))

Category	Grid Cells	Area (%)	Population	Population (%)
High (30+ min)	0	0.0	0	0.0
Medium (20-30 min)	0	0.0	0	0.0
Low (10-20 min)	4	6.0	8027	10.3
Only CT reachable within 60 min	0	0.0	0	0.0
Neither reachable within 60 min	0	0.0	0	0.0
Likely irrelevant (<10 min)	63	94.0	69797	89.7

Population and Area Coverage in Landkreis Hof (Stroke-Ready Hospitals) (Bayern) — Extended (+20% Speed (Emergency))

Category	Grid Cells	Area (%)	Population	Population (%)
High (30+ min)	0	0.0	0	0.0
Medium (20-30 min)	0	0.0	0	0.0
Low (10-20 min)	257	23.4	27007	27.1
Only CT reachable within 60 min	0	0.0	0	0.0
Neither reachable within 60 min	0	0.0	0	0.0
Likely irrelevant (<10 min)	842	76.6	72690	72.9

Population and Area Coverage in Landkreis Miltenberg (Stroke-Ready Hospitals) (Bayern) — Extended (+20% Speed (Emergency))

Category	Grid Cells	Area (%)	Population	Population (%)
High (30+ min)	1	0.1	30	0.0
Medium (20-30 min)	48	5.4	2399	1.8
Low (10-20 min)	138	15.4	8727	6.6
Only CT reachable within 60 min	0	0.0	0	0.0
Neither reachable within 60 min	0	0.0	0	0.0
Likely irrelevant (<10 min)	709	79.1	121990	91.6

Population and Area Coverage in Kreisfreie Stadt Augsburg (Stroke-Ready Hospitals) (Bayern) — Extended (+20% Speed (Emergency))

Category	Grid Cells	Area (%)	Population	Population (%)
High (30+ min)	0	0.0	0	0.0
Medium (20-30 min)	2	1.1	8	0.0
Low (10-20 min)	80	44.4	119316	41.6
Only CT reachable within 60 min	0	0.0	0	0.0
Neither reachable within 60 min	0	0.0	0	0.0
Likely irrelevant (<10 min)	98	54.4	167365	58.4

Population and Area Coverage in Landkreis Barnim (Stroke-Ready Hospitals) (Brandenburg) — Extended (+20% Speed (Emergency))

Category	Grid Cells	Area (%)	Population	Population (%)
High (30+ min)	2	0.1	47	0.0
Medium (20-30 min)	76	3.8	852	0.5
Low (10-20 min)	728	36.8	53109	31.2
Only CT reachable within 60 min	4	0.2	25	0.0
Neither reachable within 60 min	15	0.8	13	0.0
Likely irrelevant (<10 min)	1154	58.3	116397	68.3

Population and Area Coverage in Landkreis Meißen (Stroke-Ready Hospitals) (Sachsen) — Extended (+20% Speed (Emergency))

Category	Grid Cells	Area (%)	Population	Population (%)
High (30+ min)	0	0.0	0	0.0
Medium (20-30 min)	277	14.8	45527	19.9
Low (10-20 min)	881	47.0	97238	42.4
Only CT reachable within 60 min	0	0.0	0	0.0
Neither reachable within 60 min	0	0.0	0	0.0
Likely irrelevant (<10 min)	715	38.2	86305	37.7

Population and Area Coverage in Kreisfreie Stadt Suhl (Stroke-Ready Hospitals) (Thüringen) — Extended (+20% Speed (Emergency))

Category	Grid Cells	Area (%)	Population	Population (%)
High (30+ min)	0	0.0	0	0.0
Medium (20-30 min)	0	0.0	0	0.0
Low (10-20 min)	13	7.3	3893	11.7
Only CT reachable within 60 min	0	0.0	0	0.0
Neither reachable within 60 min	0	0.0	0	0.0
Likely irrelevant (<10 min)	166	92.7	29415	88.3

Population and Area Coverage in Landkreis Eichsfeld (Stroke-Ready Hospitals) (Thüringen) — Extended (+20% Speed (Emergency))

Category	Grid Cells	Area (%)	Population	Population (%)
High (30+ min)	0	0.0	0	0.0
Medium (20-30 min)	32	2.5	4820	5.1
Low (10-20 min)	528	41.0	35824	37.8
Only CT reachable within 60 min	0	0.0	0	0.0
Neither reachable within 60 min	0	0.0	0	0.0
Likely irrelevant (<10 min)	728	56.5	54039	57.1

Population and Area Coverage in Landkreis Göttingen (Stroke-Ready Hospitals) (Niedersachsen) — Extended (+20% Speed (Emergency))

Category	Grid Cells	Area (%)	Population	Population (%)
High (30+ min)	1	0.0	51	0.0
Medium (20-30 min)	131	5.7	28557	8.5
Low (10-20 min)	669	29.3	65801	19.7
Only CT reachable within 60 min	0	0.0	0	0.0
Neither reachable within 60 min	0	0.0	0	0.0
Likely irrelevant (<10 min)	1479	64.9	240162	71.8

Population and Area Coverage in Landkreis Schaumburg (Stroke-Ready Hospitals) (Niedersachsen) — Extended (+20% Speed (Emergency))

Category	Grid Cells	Area (%)	Population	Population (%)
High (30+ min)	0	0.0	0	0.0
Medium (20-30 min)	0	0.0	0	0.0
Low (10-20 min)	38	4.3	11705	7.5
Only CT reachable within 60 min	0	0.0	0	0.0
Neither reachable within 60 min	0	0.0	0	0.0
Likely irrelevant (<10 min)	851	95.7	144137	92.5

Population and Area Coverage in Landkreis Aurich (Stroke-Ready Hospitals) (Niedersachsen) — Extended (+20% Speed (Emergency))

Category	Grid Cells	Area (%)	Population	Population (%)
High (30+ min)	170	10.1	31029	16.8
Medium (20-30 min)	433	25.6	57597	31.1
Low (10-20 min)	408	24.1	43387	23.4
Only CT reachable within 60 min	8	0.5	65	0.0
Neither reachable within 60 min	64	3.8	4908	2.7
Likely irrelevant (<10 min)	607	35.9	48082	26.0

Population and Area Coverage in Landkreis Friesland (Stroke-Ready Hospitals) (Niedersachsen) — Extended (+20% Speed (Emergency))

Category	Grid Cells	Area (%)	Population	Population (%)
High (30+ min)	0	0.0	0	0.0
Medium (20-30 min)	6	0.7	188	0.2
Low (10-20 min)	142	17.2	21320	21.9
Only CT reachable within 60 min	6	0.7	302	0.3
Neither reachable within 60 min	44	5.3	1642	1.7
Likely irrelevant (<10 min)	628	76.0	73981	75.9

Population and Area Coverage in Landkreis Wesermarsch (Stroke-Ready Hospitals) (Niedersachsen) — Extended (+20% Speed (Emergency))

Category	Grid Cells	Area (%)	Population	Population (%)
High (30+ min)	0	0.0	0	0.0
Medium (20-30 min)	233	21.2	29161	34.4
Low (10-20 min)	506	46.1	33192	39.2
Only CT reachable within 60 min	6	0.5	149	0.2
Neither reachable within 60 min	24	2.2	344	0.4
Likely irrelevant (<10 min)	328	29.9	21856	25.8

Population and Area Coverage in Landkreis Main-Kinzig-Kreis (Stroke-Ready Hospitals) (Hessen) — Extended (+20% Speed (Emergency))

Category	Grid Cells	Area (%)	Population	Population (%)
High (30+ min)	0	0.0	0	0.0
Medium (20-30 min)	55	3.1	5499	1.3
Low (10-20 min)	620	35.2	44855	11.0
Only CT reachable within 60 min	0	0.0	0	0.0
Neither reachable within 60 min	0	0.0	0	0.0
Likely irrelevant (<10 min)	1085	61.6	358830	87.7

Population and Area Coverage in Landkreis Vogelsbergkreis (Stroke-Ready Hospitals) (Hessen) — Extended (+20% Speed (Emergency))

Category	Grid Cells	Area (%)	Population	Population (%)
High (30+ min)	40	2.2	5599	5.2
Medium (20-30 min)	430	23.2	30228	28.2
Low (10-20 min)	979	52.7	47198	44.0
Only CT reachable within 60 min	0	0.0	0	0.0
Neither reachable within 60 min	0	0.0	0	0.0
Likely irrelevant (<10 min)	407	21.9	24299	22.6

Population and Area Coverage in Landkreis Neuwied (Stroke-Ready Hospitals) (Rheinland-Pfalz) — Extended (+20% Speed (Emergency))

Category	Grid Cells	Area (%)	Population	Population (%)
High (30+ min)	0	0.0	0	0.0
Medium (20-30 min)	35	4.4	9893	5.3
Low (10-20 min)	252	31.8	67120	36.2
Only CT reachable within 60 min	0	0.0	0	0.0
Neither reachable within 60 min	0	0.0	0	0.0
Likely irrelevant (<10 min)	506	63.8	108304	58.4

Population and Area Coverage in Kreisfreie Stadt Mainz (Stroke-Ready Hospitals) (Rheinland-Pfalz) — Extended (+20% Speed (Emergency))

Category	Grid Cells	Area (%)	Population	Population (%)
High (30+ min)	0	0.0	0	0.0
Medium (20-30 min)	0	0.0	0	0.0
Low (10-20 min)	1	0.8	476	0.2
Only CT reachable within 60 min	0	0.0	0	0.0
Neither reachable within 60 min	0	0.0	0	0.0
Likely irrelevant (<10 min)	120	99.2	213592	99.8

Population and Area Coverage in Landkreis Hohenlohekreis (Stroke-Ready Hospitals) (Baden-Württemberg) — Extended (+20% Speed (Emergency))

Category	Grid Cells	Area (%)	Population	Population (%)
High (30+ min)	0	0.0	0	0.0
Medium (20-30 min)	1	0.1	0	0.0
Low (10-20 min)	5	0.5	297	0.3
Only CT reachable within 60 min	0	0.0	0	0.0
Neither reachable within 60 min	0	0.0	0	0.0
Likely irrelevant (<10 min)	953	99.4	109029	99.7

Population and Area Coverage in Landkreis Ortenaukreis (Stroke-Ready Hospitals) (Baden-Württemberg) — Extended (+20% Speed (Emergency))

Category	Grid Cells	Area (%)	Population	Population (%)
High (30+ min)	104	4.6	11042	2.6
Medium (20-30 min)	257	11.5	39949	9.5
Low (10-20 min)	553	24.6	85876	20.4
Only CT reachable within 60 min	1	0.0	1	0.0
Neither reachable within 60 min	9	0.4	270	0.1
Likely irrelevant (<10 min)	1320	58.8	283562	67.4

Population and Area Coverage in Landkreis Neustadt a.d.Aisch-Bad Windsheim (Stroke-Ready Hospitals) (Bayern) — Extended (+20% Speed (Emergency))

Category	Grid Cells	Area (%)	Population	Population (%)
High (30+ min)	0	0.0	0	0.0
Medium (20-30 min)	80	5.0	10838	10.7
Low (10-20 min)	493	31.1	22184	21.9
Only CT reachable within 60 min	0	0.0	0	0.0
Neither reachable within 60 min	0	0.0	0	0.0
Likely irrelevant (<10 min)	1013	63.9	68478	67.5

Population and Area Coverage in Landkreis Goslar (Stroke-Ready Hospitals) (Niedersachsen) — Extended (+20% Speed (Emergency))

Category	Grid Cells	Area (%)	Population	Population (%)
High (30+ min)	0	0.0	0	0.0
Medium (20-30 min)	36	2.9	18711	13.7
Low (10-20 min)	458	36.6	63993	46.8
Only CT reachable within 60 min	0	0.0	0	0.0
Neither reachable within 60 min	0	0.0	0	0.0
Likely irrelevant (<10 min)	758	60.5	54093	39.5

Population and Area Coverage in Landkreis Region Hannover (Stroke-Ready Hospitals) (Niedersachsen) — Extended (+20% Speed (Emergency))

Category	Grid Cells	Area (%)	Population	Population (%)
High (30+ min)	0	0.0	0	0.0
Medium (20-30 min)	17	0.6	1855	0.2
Low (10-20 min)	590	19.5	96328	8.4
Only CT reachable within 60 min	0	0.0	0	0.0
Neither reachable within 60 min	0	0.0	0	0.0
Likely irrelevant (<10 min)	2418	79.9	1052571	91.5

Population and Area Coverage in Landkreis Vechta (Stroke-Ready Hospitals) (Niedersachsen) — Extended (+20% Speed (Emergency))

Category	Grid Cells	Area (%)	Population	Population (%)
High (30+ min)	13	1.2	1585	1.2
Medium (20-30 min)	301	27.9	58948	43.4
Low (10-20 min)	215	20.0	20104	14.8
Only CT reachable within 60 min	0	0.0	0	0.0
Neither reachable within 60 min	0	0.0	0	0.0
Likely irrelevant (<10 min)	548	50.9	55142	40.6

Population and Area Coverage in Kreis Städteregion Aachen (Stroke-Ready Hospitals) (Nordrhein-Westfalen) — Extended (+20% Speed (Emergency))

Category	Grid Cells	Area (%)	Population	Population (%)
High (30+ min)	100	11.2	14454	2.6
Medium (20-30 min)	128	14.3	12435	2.2
Low (10-20 min)	241	27.0	167559	30.2
Only CT reachable within 60 min	0	0.0	60	0.0
Neither reachable within 60 min	13	1.5	337	0.1
Likely irrelevant (<10 min)	410	46.0	360564	64.9

Population and Area Coverage in Kreis Steinfurt (Stroke-Ready Hospitals) (Nordrhein-Westfalen) — Extended (+20% Speed (Emergency))

Category	Grid Cells	Area (%)	Population	Population (%)
High (30+ min)	0	0.0	0	0.0
Medium (20-30 min)	19	0.8	22329	5.1
Low (10-20 min)	996	42.1	194852	44.7
Only CT reachable within 60 min	0	0.0	0	0.0
Neither reachable within 60 min	0	0.0	0	0.0
Likely irrelevant (<10 min)	1351	57.1	218726	50.2

Population and Area Coverage in Kreis Gütersloh (Stroke-Ready Hospitals) (Nordrhein-Westfalen) — Extended (+20% Speed (Emergency))

Category	Grid Cells	Area (%)	Population	Population (%)
High (30+ min)	0	0.0	0	0.0
Medium (20-30 min)	38	3.0	29301	8.3
Low (10-20 min)	697	55.2	195418	55.1
Only CT reachable within 60 min	0	0.0	0	0.0
Neither reachable within 60 min	0	0.0	0	0.0
Likely irrelevant (<10 min)	527	41.8	130070	36.7

Population and Area Coverage in Kreis Unna (Stroke-Ready Hospitals) (Nordrhein-Westfalen) — Extended (+20% Speed (Emergency))

Category	Grid Cells	Area (%)	Population	Population (%)
High (30+ min)	0	0.0	0	0.0
Medium (20-30 min)	0	0.0	0	0.0
Low (10-20 min)	154	21.7	108115	27.9
Only CT reachable within 60 min	0	0.0	0	0.0
Neither reachable within 60 min	0	0.0	0	0.0
Likely irrelevant (<10 min)	557	78.3	279097	72.1

Population and Area Coverage in Landkreis Darmstadt-Dieburg (Stroke-Ready Hospitals) (Hessen) — Extended (+20% Speed (Emergency))

Category	Grid Cells	Area (%)	Population	Population (%)
High (30+ min)	0	0.0	0	0.0
Medium (20-30 min)	38	4.6	10538	3.6
Low (10-20 min)	286	34.7	68530	23.7
Only CT reachable within 60 min	0	0.0	0	0.0
Neither reachable within 60 min	0	0.0	0	0.0
Likely irrelevant (<10 min)	501	60.7	209898	72.6

Population and Area Coverage in Landkreis Gießen (Stroke-Ready Hospitals) (Hessen) — Extended (+20% Speed (Emergency))

Category	Grid Cells	Area (%)	Population	Population (%)
High (30+ min)	0	0.0	0	0.0
Medium (20-30 min)	0	0.0	0	0.0
Low (10-20 min)	201	18.6	21343	8.3
Only CT reachable within 60 min	0	0.0	0	0.0
Neither reachable within 60 min	0	0.0	0	0.0
Likely irrelevant (<10 min)	880	81.4	236655	91.7

Population and Area Coverage in Kreisfreie Stadt Neustadt an der Weinstraße (Stroke-Ready Hospitals) (Rheinland-Pfalz) — Extended (+20% Speed (Emergency))

Category	Grid Cells	Area (%)	Population	Population (%)
High (30+ min)	0	0.0	0	0.0
Medium (20-30 min)	0	0.0	0	0.0
Low (10-20 min)	0	0.0	0	0.0
Only CT reachable within 60 min	0	0.0	0	0.0
Neither reachable within 60 min	0	0.0	0	0.0
Likely irrelevant (<10 min)	147	100.0	53717	100.0

Population and Area Coverage in Landkreis Main-Tauber-Kreis (Stroke-Ready Hospitals) (Baden-Württemberg) — Extended (+20% Speed (Emergency))

Category	Grid Cells	Area (%)	Population	Population (%)
High (30+ min)	0	0.0	13	0.0
Medium (20-30 min)	221	13.6	13270	10.1
Low (10-20 min)	545	33.6	38545	29.3
Only CT reachable within 60 min	0	0.0	0	0.0
Neither reachable within 60 min	0	0.0	0	0.0
Likely irrelevant (<10 min)	856	52.8	79791	60.6

Population and Area Coverage in Landkreis Freudenstadt (Stroke-Ready Hospitals) (Baden-Württemberg) — Extended (+20% Speed (Emergency))

Category	Grid Cells	Area (%)	Population	Population (%)
High (30+ min)	0	0.0	0	0.0
Medium (20-30 min)	2	0.2	1	0.0
Low (10-20 min)	119	11.2	13349	11.5
Only CT reachable within 60 min	0	0.0	0	0.0
Neither reachable within 60 min	0	0.0	0	0.0
Likely irrelevant (<10 min)	937	88.6	102557	88.5

Population and Area Coverage in Landkreis Rosenheim (Stroke-Ready Hospitals) (Bayern) — Extended (+20% Speed (Emergency))

Category	Grid Cells	Area (%)	Population	Population (%)
High (30+ min)	4	0.2	91	0.0
Medium (20-30 min)	93	5.4	11201	4.4
Low (10-20 min)	323	18.7	48561	19.3
Only CT reachable within 60 min	0	0.0	0	0.0
Neither reachable within 60 min	135	7.8	1003	0.4
Likely irrelevant (<10 min)	1168	67.8	190891	75.8

Population and Area Coverage in Kreisfreie Stadt Passau (Stroke-Ready Hospitals) (Bayern) — Extended (+20% Speed (Emergency))

Category	Grid Cells	Area (%)	Population	Population (%)
High (30+ min)	0	0.0	0	0.0
Medium (20-30 min)	0	0.0	0	0.0
Low (10-20 min)	0	0.0	0	0.0
Only CT reachable within 60 min	0	0.0	0	0.0
Neither reachable within 60 min	0	0.0	0	0.0
Likely irrelevant (<10 min)	76	100.0	42451	100.0

Population and Area Coverage in Landkreis Coburg (Stroke-Ready Hospitals) (Bayern) — Extended (+20% Speed (Emergency))

Category	Grid Cells	Area (%)	Population	Population (%)
High (30+ min)	0	0.0	0	0.0
Medium (20-30 min)	2	0.3	8	0.0
Low (10-20 min)	104	13.8	6031	6.7
Only CT reachable within 60 min	0	0.0	0	0.0
Neither reachable within 60 min	0	0.0	0	0.0
Likely irrelevant (<10 min)	646	85.9	83847	93.3

Population and Area Coverage in Landkreis Ostallgäu (Stroke-Ready Hospitals) (Bayern) — Extended (+20% Speed (Emergency))

Category	Grid Cells	Area (%)	Population	Population (%)
High (30+ min)	0	0.0	0	0.0
Medium (20-30 min)	45	2.7	9057	6.5
Low (10-20 min)	327	19.5	33619	24.0
Only CT reachable within 60 min	8	0.5	277	0.2
Neither reachable within 60 min	153	9.1	1036	0.7
Likely irrelevant (<10 min)	1142	68.2	96346	68.7

Population and Area Coverage in Landkreis Saarpfalz-Kreis (Stroke-Ready Hospitals) (Saarland) — Extended (+20% Speed (Emergency))

Category	Grid Cells	Area (%)	Population	Population (%)
High (30+ min)	1	0.2	0	0.0
Medium (20-30 min)	0	0.0	0	0.0
Low (10-20 min)	87	17.2	7599	5.5
Only CT reachable within 60 min	0	0.0	0	0.0
Neither reachable within 60 min	4	0.8	0	0.0
Likely irrelevant (<10 min)	413	81.8	129445	94.5

Population and Area Coverage in Landkreis Nordhausen (Stroke-Ready Hospitals) (Thüringen) — Extended (+20% Speed (Emergency))

Category	Grid Cells	Area (%)	Population	Population (%)
High (30+ min)	0	0.0	0	0.0
Medium (20-30 min)	27	2.9	4196	5.4
Low (10-20 min)	369	40.3	15029	19.5
Only CT reachable within 60 min	0	0.0	0	0.0
Neither reachable within 60 min	0	0.0	0	0.0
Likely irrelevant (<10 min)	520	56.8	57878	75.1

Population and Area Coverage in Landkreis Uelzen (Stroke-Ready Hospitals) (Niedersachsen) — Extended (+20% Speed (Emergency))

Category	Grid Cells	Area (%)	Population	Population (%)
High (30+ min)	4	0.2	2	0.0
Medium (20-30 min)	90	4.6	2290	2.5
Low (10-20 min)	516	26.5	24452	26.3
Only CT reachable within 60 min	0	0.0	0	0.0
Neither reachable within 60 min	0	0.0	0	0.0
Likely irrelevant (<10 min)	1337	68.7	66369	71.3

Population and Area Coverage in Kreisfreie Stadt Mülheim an der Ruhr (Stroke-Ready Hospitals) (Nordrhein-Westfalen) — Extended (+20% Speed (Emergency))

Category	Grid Cells	Area (%)	Population	Population (%)
High (30+ min)	0	0.0	0	0.0
Medium (20-30 min)	0	0.0	0	0.0
Low (10-20 min)	1	0.9	2910	1.6
Only CT reachable within 60 min	0	0.0	0	0.0
Neither reachable within 60 min	0	0.0	0	0.0
Likely irrelevant (<10 min)	112	99.1	177295	98.4

Population and Area Coverage in Kreisfreie Stadt Bonn (Stroke-Ready Hospitals) (Nordrhein-Westfalen) — Extended (+20% Speed (Emergency))

Category	Grid Cells	Area (%)	Population	Population (%)
High (30+ min)	0	0.0	0	0.0
Medium (20-30 min)	0	0.0	0	0.0
Low (10-20 min)	34	18.9	64999	20.0
Only CT reachable within 60 min	0	0.0	0	0.0
Neither reachable within 60 min	0	0.0	0	0.0
Likely irrelevant (<10 min)	146	81.1	260711	80.0

Population and Area Coverage in Landkreis Birkenfeld (Stroke-Ready Hospitals) (Rheinland-Pfalz) — Extended (+20% Speed (Emergency))

Category	Grid Cells	Area (%)	Population	Population (%)
High (30+ min)	1	0.1	0	0.0
Medium (20-30 min)	102	10.6	10542	12.7
Low (10-20 min)	295	30.6	14394	17.4
Only CT reachable within 60 min	0	0.0	0	0.0
Neither reachable within 60 min	0	0.0	0	0.0
Likely irrelevant (<10 min)	567	58.8	57813	69.9

Population and Area Coverage in Landkreis Rems-Murr-Kreis (Stroke-Ready Hospitals) (Baden-Württemberg) — Extended (+20% Speed (Emergency))

Category	Grid Cells	Area (%)	Population	Population (%)
High (30+ min)	0	0.0	0	0.0
Medium (20-30 min)	31	2.9	1971	0.5
Low (10-20 min)	84	7.9	10151	2.4
Only CT reachable within 60 min	0	0.0	0	0.0
Neither reachable within 60 min	0	0.0	0	0.0
Likely irrelevant (<10 min)	942	89.1	405697	97.1

Population and Area Coverage in Landkreis Schwäbisch Hall (Stroke-Ready Hospitals) (Baden-Württemberg) — Extended (+20% Speed (Emergency))

Category	Grid Cells	Area (%)	Population	Population (%)
High (30+ min)	0	0.0	0	0.0
Medium (20-30 min)	82	4.5	26693	14.0
Low (10-20 min)	497	27.2	37468	19.6
Only CT reachable within 60 min	0	0.0	0	0.0
Neither reachable within 60 min	0	0.0	0	0.0
Likely irrelevant (<10 min)	1246	68.3	126726	66.4

Population and Area Coverage in Landkreis Rottweil (Stroke-Ready Hospitals) (Baden-Württemberg) — Extended (+20% Speed (Emergency))

Category	Grid Cells	Area (%)	Population	Population (%)
High (30+ min)	1	0.1	244	0.2
Medium (20-30 min)	37	3.9	5649	4.1
Low (10-20 min)	257	27.3	33993	24.7
Only CT reachable within 60 min	0	0.0	0	0.0
Neither reachable within 60 min	0	0.0	0	0.0
Likely irrelevant (<10 min)	646	68.7	97670	71.0

Population and Area Coverage in Landkreis Amberg-Sulzbach (Stroke-Ready Hospitals) (Bayern) — Extended (+20% Speed (Emergency))

Category	Grid Cells	Area (%)	Population	Population (%)
High (30+ min)	0	0.0	0	0.0
Medium (20-30 min)	100	6.4	8526	8.0
Low (10-20 min)	379	24.3	29440	27.6
Only CT reachable within 60 min	0	0.0	0	0.0
Neither reachable within 60 min	0	0.0	0	0.0
Likely irrelevant (<10 min)	1080	69.3	68739	64.4

Population and Area Coverage in Kreisfreie Stadt Kempten (Allgäu) (Stroke-Ready Hospitals) (Bayern) — Extended (+20% Speed (Emergency))

Category	Grid Cells	Area (%)	Population	Population (%)
High (30+ min)	0	0.0	0	0.0
Medium (20-30 min)	0	0.0	0	0.0
Low (10-20 min)	0	0.0	0	0.0
Only CT reachable within 60 min	0	0.0	0	0.0
Neither reachable within 60 min	0	0.0	0	0.0
Likely irrelevant (<10 min)	77	100.0	68488	100.0

Population and Area Coverage in Kreisfreie Stadt Chemnitz (Stroke-Ready Hospitals) (Sachsen) — Extended (+20% Speed (Emergency))

Category	Grid Cells	Area (%)	Population	Population (%)
High (30+ min)	0	0.0	0	0.0
Medium (20-30 min)	0	0.0	0	0.0
Low (10-20 min)	91	33.0	80281	27.1
Only CT reachable within 60 min	0	0.0	0	0.0
Neither reachable within 60 min	0	0.0	0	0.0
Likely irrelevant (<10 min)	185	67.0	215890	72.9

Population and Area Coverage in Landkreis Mecklenburgische Seenplatte (Stroke-Ready Hospitals) (Mecklenburg-Vorpommern) — Extended (+20% Speed (Emergency))

Category	Grid Cells	Area (%)	Population	Population (%)
High (30+ min)	1099	14.7	55440	23.2
Medium (20-30 min)	1297	17.4	42818	17.9
Low (10-20 min)	1677	22.5	24161	10.1
Only CT reachable within 60 min	721	9.7	3517	1.5
Neither reachable within 60 min	0	0.0	0	0.0
Likely irrelevant (<10 min)	2659	35.7	113005	47.3

Population and Area Coverage in Landkreis Erzgebirgskreis (Stroke-Ready Hospitals) (Sachsen) — Extended (+20% Speed (Emergency))

Category	Grid Cells	Area (%)	Population	Population (%)
High (30+ min)	95	4.1	9752	3.0
Medium (20-30 min)	263	11.5	28093	8.6
Low (10-20 min)	589	25.7	80479	24.7
Only CT reachable within 60 min	1	0.0	81	0.0
Neither reachable within 60 min	2	0.1	128	0.0
Likely irrelevant (<10 min)	1340	58.5	207737	63.7

Population and Area Coverage in Kreisfreie Stadt Dresden (Stroke-Ready Hospitals) (Sachsen) — Extended (+20% Speed (Emergency))

Category	Grid Cells	Area (%)	Population	Population (%)
High (30+ min)	0	0.0	0	0.0
Medium (20-30 min)	1	0.2	2	0.0
Low (10-20 min)	59	14.0	50048	8.1
Only CT reachable within 60 min	0	0.0	0	0.0
Neither reachable within 60 min	0	0.0	0	0.0
Likely irrelevant (<10 min)	360	85.7	565356	91.9

Population and Area Coverage in Landkreis Hildburghausen (Stroke-Ready Hospitals) (Thüringen) — Extended (+20% Speed (Emergency))

Category	Grid Cells	Area (%)	Population	Population (%)
High (30+ min)	0	0.0	0	0.0
Medium (20-30 min)	2	0.2	7	0.0
Low (10-20 min)	108	9.1	2074	3.5
Only CT reachable within 60 min	0	0.0	0	0.0
Neither reachable within 60 min	0	0.0	0	0.0
Likely irrelevant (<10 min)	1078	90.7	56816	96.5

Population and Area Coverage in Kreis Herzogtum Lauenburg (Stroke-Ready Hospitals) (Schleswig-Holstein) — Extended (+20% Speed (Emergency))

Category	Grid Cells	Area (%)	Population	Population (%)
High (30+ min)	1	0.1	541	0.3
Medium (20-30 min)	241	14.1	22284	11.4
Low (10-20 min)	599	35.0	85154	43.6
Only CT reachable within 60 min	0	0.0	0	0.0
Neither reachable within 60 min	0	0.0	0	0.0
Likely irrelevant (<10 min)	870	50.8	87374	44.7

Population and Area Coverage in Landkreis Oldenburg (Stroke-Ready Hospitals) (Niedersachsen) — Extended (+20% Speed (Emergency))

Category	Grid Cells	Area (%)	Population	Population (%)
High (30+ min)	18	1.3	1178	0.9
Medium (20-30 min)	216	15.1	21807	17.1
Low (10-20 min)	490	34.3	43061	33.7
Only CT reachable within 60 min	0	0.0	0	0.0
Neither reachable within 60 min	0	0.0	0	0.0
Likely irrelevant (<10 min)	704	49.3	61573	48.2

Population and Area Coverage in Kreisfreie Stadt Krefeld (Stroke-Ready Hospitals) (Nordrhein-Westfalen) — Extended (+20% Speed (Emergency))

Category	Grid Cells	Area (%)	Population	Population (%)
High (30+ min)	0	0.0	0	0.0
Medium (20-30 min)	0	0.0	0	0.0
Low (10-20 min)	17	9.4	23177	9.6
Only CT reachable within 60 min	0	0.0	0	0.0
Neither reachable within 60 min	0	0.0	0	0.0
Likely irrelevant (<10 min)	164	90.6	218805	90.4

Population and Area Coverage in Kreis Oberbergischer Kreis (Stroke-Ready Hospitals) (Nordrhein-Westfalen) — Extended (+20% Speed (Emergency))

Category	Grid Cells	Area (%)	Population	Population (%)
High (30+ min)	0	0.0	0	0.0
Medium (20-30 min)	198	16.8	40445	15.0
Low (10-20 min)	465	39.5	91947	34.2
Only CT reachable within 60 min	0	0.0	0	0.0
Neither reachable within 60 min	0	0.0	0	0.0
Likely irrelevant (<10 min)	513	43.6	136476	50.8

Population and Area Coverage in Landkreis Kusel (Stroke-Ready Hospitals) (Rheinland-Pfalz) — Extended (+20% Speed (Emergency))

Category	Grid Cells	Area (%)	Population	Population (%)
High (30+ min)	0	0.0	0	0.0
Medium (20-30 min)	57	7.9	10107	13.8
Low (10-20 min)	214	29.8	18894	25.8
Only CT reachable within 60 min	0	0.0	0	0.0
Neither reachable within 60 min	0	0.0	0	0.0
Likely irrelevant (<10 min)	446	62.2	44185	60.4

Population and Area Coverage in Landkreis Fürstenfeldbruck (Stroke-Ready Hospitals) (Bayern) — Extended (+20% Speed (Emergency))

Category	Grid Cells	Area (%)	Population	Population (%)
High (30+ min)	0	0.0	0	0.0
Medium (20-30 min)	0	0.0	0	0.0
Low (10-20 min)	1	0.2	0	0.0
Only CT reachable within 60 min	0	0.0	0	0.0
Neither reachable within 60 min	0	0.0	0	0.0
Likely irrelevant (<10 min)	525	99.8	214938	100.0

Population and Area Coverage in Landkreis Weilheim-Schongau (Stroke-Ready Hospitals) (Bayern) — Extended (+20% Speed (Emergency))

Category	Grid Cells	Area (%)	Population	Population (%)
High (30+ min)	0	0.0	0	0.0
Medium (20-30 min)	64	5.5	15106	11.2
Low (10-20 min)	328	28.3	42107	31.2
Only CT reachable within 60 min	0	0.0	0	0.0
Neither reachable within 60 min	13	1.1	13	0.0
Likely irrelevant (<10 min)	755	65.1	77847	57.6

Population and Area Coverage in Landkreis Neustadt a.d.Waldnaab (Stroke-Ready Hospitals) (Bayern) — Extended (+20% Speed (Emergency))

Category	Grid Cells	Area (%)	Population	Population (%)
High (30+ min)	0	0.0	0	0.0
Medium (20-30 min)	67	3.9	1833	1.9
Low (10-20 min)	539	31.3	21328	21.9
Only CT reachable within 60 min	0	0.0	0	0.0
Neither reachable within 60 min	0	0.0	0	0.0
Likely irrelevant (<10 min)	1116	64.8	74258	76.2

Population and Area Coverage in Landkreis Würzburg (Stroke-Ready Hospitals) (Bayern) — Extended (+20% Speed (Emergency))

Category	Grid Cells	Area (%)	Population	Population (%)
High (30+ min)	0	0.0	0	0.0
Medium (20-30 min)	0	0.0	0	0.0
Low (10-20 min)	235	19.5	26583	16.1
Only CT reachable within 60 min	0	0.0	0	0.0
Neither reachable within 60 min	0	0.0	0	0.0
Likely irrelevant (<10 min)	970	80.5	138479	83.9

Population and Area Coverage in Landkreis Elbe-Elster (Stroke-Ready Hospitals) (Brandenburg) — Extended (+20% Speed (Emergency))

Category	Grid Cells	Area (%)	Population	Population (%)
High (30+ min)	786	31.8	48892	48.0
Medium (20-30 min)	1067	43.2	37724	37.0
Low (10-20 min)	515	20.9	12853	12.6
Only CT reachable within 60 min	27	1.1	123	0.1
Neither reachable within 60 min	0	0.0	0	0.0
Likely irrelevant (<10 min)	74	3.0	2347	2.3

Population and Area Coverage in Landkreis Osterholz (Stroke-Ready Hospitals) (Niedersachsen) — Extended (+20% Speed (Emergency))

Category	Grid Cells	Area (%)	Population	Population (%)
High (30+ min)	0	0.0	0	0.0
Medium (20-30 min)	221	25.1	43601	38.9
Low (10-20 min)	428	48.7	36249	32.3
Only CT reachable within 60 min	0	0.0	0	0.0
Neither reachable within 60 min	0	0.0	0	0.0
Likely irrelevant (<10 min)	230	26.2	32302	28.8

Population and Area Coverage in Landkreis Heidekreis (Stroke-Ready Hospitals) (Niedersachsen) — Extended (+20% Speed (Emergency))

Category	Grid Cells	Area (%)	Population	Population (%)
High (30+ min)	0	0.0	0	0.0
Medium (20-30 min)	208	8.3	16508	12.2
Low (10-20 min)	613	24.4	32540	24.0
Only CT reachable within 60 min	0	0.0	0	0.0
Neither reachable within 60 min	0	0.0	0	0.0
Likely irrelevant (<10 min)	1695	67.4	86796	63.9

Population and Area Coverage in Kreisfreie Stadt Solingen (Stroke-Ready Hospitals) (Nordrhein-Westfalen) — Extended (+20% Speed (Emergency))

Category	Grid Cells	Area (%)	Population	Population (%)
High (30+ min)	0	0.0	0	0.0
Medium (20-30 min)	0	0.0	0	0.0
Low (10-20 min)	6	5.2	3249	2.1
Only CT reachable within 60 min	0	0.0	0	0.0
Neither reachable within 60 min	0	0.0	0	0.0
Likely irrelevant (<10 min)	109	94.8	155189	97.9

Population and Area Coverage in Landkreis Bad Kreuznach (Stroke-Ready Hospitals) (Rheinland-Pfalz) — Extended (+20% Speed (Emergency))

Category	Grid Cells	Area (%)	Population	Population (%)
High (30+ min)	0	0.0	0	0.0
Medium (20-30 min)	46	4.3	6899	4.3
Low (10-20 min)	244	22.8	18914	11.8
Only CT reachable within 60 min	0	0.0	0	0.0
Neither reachable within 60 min	0	0.0	0	0.0
Likely irrelevant (<10 min)	782	72.9	134356	83.9

Population and Area Coverage in Landkreis Südwestpfalz (Stroke-Ready Hospitals) (Rheinland-Pfalz) — Extended (+20% Speed (Emergency))

Category	Grid Cells	Area (%)	Population	Population (%)
High (30+ min)	0	0.0	0	0.0
Medium (20-30 min)	0	0.0	0	0.0
Low (10-20 min)	107	9.1	17257	15.4
Only CT reachable within 60 min	0	0.0	1	0.0
Neither reachable within 60 min	10	0.8	60	0.1
Likely irrelevant (<10 min)	1060	90.1	94823	84.6

Population and Area Coverage in Landkreis Ludwigsburg (Stroke-Ready Hospitals) (Baden-Württemberg) — Extended (+20% Speed (Emergency))

Category	Grid Cells	Area (%)	Population	Population (%)
High (30+ min)	8	0.9	9892	1.9
Medium (20-30 min)	136	16.1	34743	6.6
Low (10-20 min)	340	40.1	169586	32.1
Only CT reachable within 60 min	0	0.0	0	0.0
Neither reachable within 60 min	0	0.0	0	0.0
Likely irrelevant (<10 min)	363	42.9	313322	59.4

Population and Area Coverage in Landkreis Lörrach (Stroke-Ready Hospitals) (Baden-Württemberg) — Extended (+20% Speed (Emergency))

Category	Grid Cells	Area (%)	Population	Population (%)
High (30+ min)	0	0.0	0	0.0
Medium (20-30 min)	1	0.1	433	0.2
Low (10-20 min)	274	29.2	41109	19.0
Only CT reachable within 60 min	1	0.1	918	0.4
Neither reachable within 60 min	0	0.0	9	0.0
Likely irrelevant (<10 min)	663	70.6	174175	80.4

Population and Area Coverage in Landkreis Alb-Donau-Kreis (Stroke-Ready Hospitals) (Baden-Württemberg) — Extended (+20% Speed (Emergency))

Category	Grid Cells	Area (%)	Population	Population (%)
High (30+ min)	0	0.0	0	0.0
Medium (20-30 min)	153	9.2	22192	11.6
Low (10-20 min)	728	43.9	78335	41.0
Only CT reachable within 60 min	0	0.0	0	0.0
Neither reachable within 60 min	0	0.0	0	0.0
Likely irrelevant (<10 min)	777	46.9	90580	47.4

Population and Area Coverage in Landkreis Biberach (Stroke-Ready Hospitals) (Baden-Württemberg) — Extended (+20% Speed (Emergency))

Category	Grid Cells	Area (%)	Population	Population (%)
High (30+ min)	0	0.0	0	0.0
Medium (20-30 min)	0	0.0	0	0.0
Low (10-20 min)	10	0.6	284	0.1
Only CT reachable within 60 min	0	0.0	0	0.0
Neither reachable within 60 min	0	0.0	0	0.0
Likely irrelevant (<10 min)	1694	99.4	192973	99.9

Population and Area Coverage in Kreisfreie Stadt Fürth (Stroke-Ready Hospitals) (Bayern) — Extended (+20% Speed (Emergency))

Category	Grid Cells	Area (%)	Population	Population (%)
High (30+ min)	0	0.0	0	0.0
Medium (20-30 min)	0	0.0	0	0.0
Low (10-20 min)	0	0.0	0	0.0
Only CT reachable within 60 min	0	0.0	0	0.0
Neither reachable within 60 min	0	0.0	0	0.0
Likely irrelevant (<10 min)	83	100.0	150301	100.0

Population and Area Coverage in Kreisfreie Stadt Halle (Saale) (Stroke-Ready Hospitals) (Sachsen-Anhalt) — Extended (+20% Speed (Emergency))

Category	Grid Cells	Area (%)	Population	Population (%)
High (30+ min)	0	0.0	0	0.0
Medium (20-30 min)	0	0.0	0	0.0
Low (10-20 min)	0	0.0	329	0.1
Only CT reachable within 60 min	0	0.0	0	0.0
Neither reachable within 60 min	0	0.0	0	0.0
Likely irrelevant (<10 min)	174	100.0	249843	99.9

Population and Area Coverage in Landkreis Wittenberg (Stroke-Ready Hospitals) (Sachsen-Anhalt) — Extended (+20% Speed (Emergency))

Category	Grid Cells	Area (%)	Population	Population (%)
High (30+ min)	28	1.1	708	0.6
Medium (20-30 min)	160	6.3	4981	4.4
Low (10-20 min)	672	26.5	21256	18.6
Only CT reachable within 60 min	79	3.1	66	0.1
Neither reachable within 60 min	0	0.0	0	0.0
Likely irrelevant (<10 min)	1598	63.0	87072	76.3

Population and Area Coverage in Landkreis Saale-Holzland-Kreis (Stroke-Ready Hospitals) (Thüringen) — Extended (+20% Speed (Emergency))

Category	Grid Cells	Area (%)	Population	Population (%)
High (30+ min)	0	0.0	0	0.0
Medium (20-30 min)	45	4.3	9072	12.0
Low (10-20 min)	544	52.4	36972	48.8
Only CT reachable within 60 min	0	0.0	0	0.0
Neither reachable within 60 min	0	0.0	0	0.0
Likely irrelevant (<10 min)	449	43.3	29658	39.2

Population and Area Coverage in Kreisfreie Stadt Salzgitter (Stroke-Ready Hospitals) (Niedersachsen) — Extended (+20% Speed (Emergency))

Category	Grid Cells	Area (%)	Population	Population (%)
High (30+ min)	0	0.0	0	0.0
Medium (20-30 min)	5	1.7	568	0.6
Low (10-20 min)	55	18.6	15609	15.6
Only CT reachable within 60 min	0	0.0	0	0.0
Neither reachable within 60 min	0	0.0	0	0.0
Likely irrelevant (<10 min)	235	79.7	83875	83.8

Population and Area Coverage in Kreis Wesel (Stroke-Ready Hospitals) (Nordrhein-Westfalen) — Extended (+20% Speed (Emergency))

Category	Grid Cells	Area (%)	Population	Population (%)
High (30+ min)	0	0.0	0	0.0
Medium (20-30 min)	3	0.2	565	0.1
Low (10-20 min)	297	22.0	94785	20.0
Only CT reachable within 60 min	0	0.0	0	0.0
Neither reachable within 60 min	0	0.0	0	0.0
Likely irrelevant (<10 min)	1053	77.8	379734	79.9

Population and Area Coverage in Kreis Rhein-Erft-Kreis (Stroke-Ready Hospitals) (Nordrhein-Westfalen) — Extended (+20% Speed (Emergency))

Category	Grid Cells	Area (%)	Population	Population (%)
High (30+ min)	0	0.0	0	0.0
Medium (20-30 min)	7	0.8	1646	0.4
Low (10-20 min)	205	22.8	142442	31.3
Only CT reachable within 60 min	0	0.0	0	0.0
Neither reachable within 60 min	0	0.0	0	0.0
Likely irrelevant (<10 min)	686	76.4	310441	68.3

Population and Area Coverage in Kreisfreie Stadt Frankfurt am Main (Stroke-Ready Hospitals) (Hessen) — Extended (+20% Speed (Emergency))

Category	Grid Cells	Area (%)	Population	Population (%)
High (30+ min)	0	0.0	0	0.0
Medium (20-30 min)	0	0.0	0	0.0
Low (10-20 min)	0	0.0	0	0.0
Only CT reachable within 60 min	0	0.0	0	0.0
Neither reachable within 60 min	0	0.0	0	0.0
Likely irrelevant (<10 min)	310	100.0	833128	100.0

Population and Area Coverage in Landkreis Südliche Weinstraße (Stroke-Ready Hospitals) (Rheinland-Pfalz) — Extended (+20% Speed (Emergency))

Category	Grid Cells	Area (%)	Population	Population (%)
High (30+ min)	0	0.0	0	0.0
Medium (20-30 min)	0	0.0	0	0.0
Low (10-20 min)	68	8.6	8773	7.8
Only CT reachable within 60 min	0	0.0	0	0.0
Neither reachable within 60 min	0	0.0	0	0.0
Likely irrelevant (<10 min)	726	91.4	103087	92.2

Population and Area Coverage in Stadtkreis Karlsruhe (Stroke-Ready Hospitals) (Baden-Württemberg) — Extended (+20% Speed (Emergency))

Category	Grid Cells	Area (%)	Population	Population (%)
High (30+ min)	0	0.0	0	0.0
Medium (20-30 min)	0	0.0	0	0.0
Low (10-20 min)	0	0.0	0	0.0
Only CT reachable within 60 min	0	0.0	0	0.0
Neither reachable within 60 min	0	0.0	0	0.0
Likely irrelevant (<10 min)	214	100.0	336385	100.0

Population and Area Coverage in Landkreis Rhein-Neckar-Kreis (Stroke-Ready Hospitals) (Baden-Württemberg) — Extended (+20% Speed (Emergency))

Category	Grid Cells	Area (%)	Population	Population (%)
High (30+ min)	0	0.0	0	0.0
Medium (20-30 min)	20	1.5	12851	2.4
Low (10-20 min)	268	20.4	128684	23.8
Only CT reachable within 60 min	0	0.0	0	0.0
Neither reachable within 60 min	0	0.0	0	0.0
Likely irrelevant (<10 min)	1026	78.1	399485	73.8

Population and Area Coverage in Landkreis München (Stroke-Ready Hospitals) (Bayern) — Extended (+20% Speed (Emergency))

Category	Grid Cells	Area (%)	Population	Population (%)
High (30+ min)	0	0.0	0	0.0
Medium (20-30 min)	0	0.0	0	0.0
Low (10-20 min)	46	5.7	15789	4.6
Only CT reachable within 60 min	0	0.0	0	0.0
Neither reachable within 60 min	0	0.0	0	0.0
Likely irrelevant (<10 min)	757	94.3	328536	95.4

Population and Area Coverage in Landkreis Dingolfing-Landau (Stroke-Ready Hospitals) (Bayern) — Extended (+20% Speed (Emergency))

Category	Grid Cells	Area (%)	Population	Population (%)
High (30+ min)	0	0.0	0	0.0
Medium (20-30 min)	3	0.3	145	0.2
Low (10-20 min)	201	18.7	19292	20.4
Only CT reachable within 60 min	0	0.0	0	0.0
Neither reachable within 60 min	0	0.0	0	0.0
Likely irrelevant (<10 min)	873	81.1	75027	79.4

Population and Area Coverage in Kreisfreie Stadt Nürnberg (Stroke-Ready Hospitals) (Bayern) — Extended (+20% Speed (Emergency))

Category	Grid Cells	Area (%)	Population	Population (%)
High (30+ min)	0	0.0	0	0.0
Medium (20-30 min)	0	0.0	0	0.0
Low (10-20 min)	57	24.6	212054	40.9
Only CT reachable within 60 min	0	0.0	0	0.0
Neither reachable within 60 min	0	0.0	0	0.0
Likely irrelevant (<10 min)	175	75.4	306138	59.1

Population and Area Coverage in Kreisfreie Stadt Cottbus (Stroke-Ready Hospitals) (Brandenburg) — Extended (+20% Speed (Emergency))

Category	Grid Cells	Area (%)	Population	Population (%)
High (30+ min)	0	0.0	0	0.0
Medium (20-30 min)	0	0.0	0	0.0
Low (10-20 min)	5	2.4	105	0.1
Only CT reachable within 60 min	0	0.0	0	0.0
Neither reachable within 60 min	0	0.0	0	0.0
Likely irrelevant (<10 min)	207	97.6	104399	99.9

Population and Area Coverage in Landkreis Salzlandkreis (Stroke-Ready Hospitals) (Sachsen-Anhalt) — Extended (+20% Speed (Emergency))

Category	Grid Cells	Area (%)	Population	Population (%)
High (30+ min)	3	0.2	12	0.0
Medium (20-30 min)	235	12.6	44195	25.1
Low (10-20 min)	1063	56.9	79605	45.1
Only CT reachable within 60 min	0	0.0	0	0.0
Neither reachable within 60 min	0	0.0	0	0.0
Likely irrelevant (<10 min)	567	30.4	52505	29.8

Population and Area Coverage in Landkreis Sömmerda (Stroke-Ready Hospitals) (Thüringen) — Extended (+20% Speed (Emergency))

Category	Grid Cells	Area (%)	Population	Population (%)
High (30+ min)	0	0.0	0	0.0
Medium (20-30 min)	92	8.8	14959	23.5
Low (10-20 min)	506	48.5	28636	45.0
Only CT reachable within 60 min	0	0.0	0	0.0
Neither reachable within 60 min	0	0.0	0	0.0
Likely irrelevant (<10 min)	446	42.7	20081	31.5

Population and Area Coverage in Landkreis Saale-Orla-Kreis (Stroke-Ready Hospitals) (Thüringen) — Extended (+20% Speed (Emergency))

Category	Grid Cells	Area (%)	Population	Population (%)
High (30+ min)	9	0.6	7165	9.5
Medium (20-30 min)	203	13.9	13020	17.3
Low (10-20 min)	483	33.0	17464	23.2
Only CT reachable within 60 min	0	0.0	0	0.0
Neither reachable within 60 min	0	0.0	0	0.0
Likely irrelevant (<10 min)	770	52.6	37657	50.0

Population and Area Coverage in Kreisfreie Stadt Neumünster (Stroke Units) (Schleswig-Holstein) — Regular (+20% Speed (Emergency))

Category	Grid Cells	Area (%)	Population	Population (%)
High (30+ min)	0	0.0	0	0.0
Medium (20-30 min)	0	0.0	0	0.0
Low (10-20 min)	0	0.0	0	0.0
Only CT reachable within 60 min	0	0.0	0	0.0
Neither reachable within 60 min	0	0.0	0	0.0
Likely irrelevant (<10 min)	100	100.0	85923	100.0

Population and Area Coverage in Kreisfreie Stadt Osnabrück (Stroke Units) (Niedersachsen) — Regular (+20% Speed (Emergency))

Category	Grid Cells	Area (%)	Population	Population (%)
High (30+ min)	0	0.0	0	0.0
Medium (20-30 min)	0	0.0	0	0.0
Low (10-20 min)	21	13.5	19774	12.8
Only CT reachable within 60 min	0	0.0	0	0.0
Neither reachable within 60 min	0	0.0	0	0.0
Likely irrelevant (<10 min)	134	86.5	134667	87.2

Population and Area Coverage in Kreisfreie Stadt Mönchengladbach (Stroke Units) (Nordrhein-Westfalen) — Regular (+20% Speed (Emergency))

Category	Grid Cells	Area (%)	Population	Population (%)
High (30+ min)	0	0.0	0	0.0
Medium (20-30 min)	0	0.0	0	0.0
Low (10-20 min)	20	9.1	44459	16.2
Only CT reachable within 60 min	0	0.0	0	0.0
Neither reachable within 60 min	0	0.0	0	0.0
Likely irrelevant (<10 min)	200	90.9	229662	83.8

Population and Area Coverage in Kreis Coesfeld (Stroke Units) (Nordrhein-Westfalen) — Regular (+20% Speed (Emergency))

Category	Grid Cells	Area (%)	Population	Population (%)
High (30+ min)	0	0.0	0	0.0
Medium (20-30 min)	66	4.5	18407	8.5
Low (10-20 min)	582	40.0	81377	37.7
Only CT reachable within 60 min	0	0.0	0	0.0
Neither reachable within 60 min	0	0.0	0	0.0
Likely irrelevant (<10 min)	806	55.4	116152	53.8

Population and Area Coverage in Kreis Paderborn (Stroke Units) (Nordrhein-Westfalen) — Regular (+20% Speed (Emergency))

Category	Grid Cells	Area (%)	Population	Population (%)
High (30+ min)	0	0.0	0	0.0
Medium (20-30 min)	45	2.8	200	0.1
Low (10-20 min)	556	34.2	74908	22.7
Only CT reachable within 60 min	0	0.0	0	0.0
Neither reachable within 60 min	0	0.0	0	0.0
Likely irrelevant (<10 min)	1025	63.0	255413	77.3

Population and Area Coverage in Kreisfreie Stadt Kassel (Stroke Units) (Hessen) — Regular (+20% Speed (Emergency))

Category	Grid Cells	Area (%)	Population	Population (%)
High (30+ min)	0	0.0	0	0.0
Medium (20-30 min)	0	0.0	0	0.0
Low (10-20 min)	54	40.6	36068	17.5
Only CT reachable within 60 min	0	0.0	0	0.0
Neither reachable within 60 min	0	0.0	0	0.0
Likely irrelevant (<10 min)	79	59.4	169651	82.5

Population and Area Coverage in Landkreis Hersfeld-Rotenburg (Stroke Units) (Hessen) — Regular (+20% Speed (Emergency))

Category	Grid Cells	Area (%)	Population	Population (%)
High (30+ min)	0	0.0	0	0.0
Medium (20-30 min)	105	7.5	15734	13.0
Low (10-20 min)	255	18.2	15074	12.5
Only CT reachable within 60 min	0	0.0	0	0.0
Neither reachable within 60 min	0	0.0	0	0.0
Likely irrelevant (<10 min)	1041	74.3	90081	74.5

Population and Area Coverage in Landkreis Schwalm-Eder-Kreis (Stroke Units) (Hessen) — Regular (+20% Speed (Emergency))

Category	Grid Cells	Area (%)	Population	Population (%)
High (30+ min)	166	8.4	23877	13.1
Medium (20-30 min)	869	43.9	68726	37.8
Low (10-20 min)	551	27.8	46350	25.5
Only CT reachable within 60 min	0	0.0	0	0.0
Neither reachable within 60 min	0	0.0	0	0.0
Likely irrelevant (<10 min)	393	19.9	42874	23.6

Population and Area Coverage in Landkreis Westerwaldkreis (Stroke Units) (Rheinland-Pfalz) — Regular (+20% Speed (Emergency))

Category	Grid Cells	Area (%)	Population	Population (%)
High (30+ min)	0	0.0	0	0.0
Medium (20-30 min)	37	2.9	7644	3.7
Low (10-20 min)	406	32.4	51956	25.2
Only CT reachable within 60 min	0	0.0	0	0.0
Neither reachable within 60 min	0	0.0	0	0.0
Likely irrelevant (<10 min)	812	64.7	146224	71.0

Population and Area Coverage in Kreisfreie Stadt Frankenthal (Pfalz) (Stroke Units) (Rheinland-Pfalz) — Regular (+20% Speed (Emergency))

Category	Grid Cells	Area (%)	Population	Population (%)
High (30+ min)	0	0.0	0	0.0
Medium (20-30 min)	0	0.0	0	0.0
Low (10-20 min)	11	20.4	16304	35.9
Only CT reachable within 60 min	0	0.0	0	0.0
Neither reachable within 60 min	0	0.0	0	0.0
Likely irrelevant (<10 min)	43	79.6	29129	64.1

Population and Area Coverage in Landkreis Böblingen (Stroke Units) (Baden-Württemberg) — Regular (+20% Speed (Emergency))

Category	Grid Cells	Area (%)	Population	Population (%)
High (30+ min)	0	0.0	0	0.0
Medium (20-30 min)	28	3.7	15756	4.2
Low (10-20 min)	335	44.7	158086	42.5
Only CT reachable within 60 min	0	0.0	0	0.0
Neither reachable within 60 min	0	0.0	0	0.0
Likely irrelevant (<10 min)	386	51.5	198507	53.3

Population and Area Coverage in Landkreis Cham (Stroke Units) (Bayern) — Regular (+20% Speed (Emergency))

Category	Grid Cells	Area (%)	Population	Population (%)
High (30+ min)	0	0.0	0	0.0
Medium (20-30 min)	13	0.7	921	0.7
Low (10-20 min)	553	31.3	36954	29.1
Only CT reachable within 60 min	0	0.0	0	0.0
Neither reachable within 60 min	0	0.0	0	0.0
Likely irrelevant (<10 min)	1201	68.0	88994	70.1

Population and Area Coverage in Landkreis Saarlouis (Stroke Units) (Saarland) — Regular (+20% Speed (Emergency))

Category	Grid Cells	Area (%)	Population	Population (%)
High (30+ min)	0	0.0	0	0.0
Medium (20-30 min)	28	5.0	7991	4.3
Low (10-20 min)	108	19.1	23913	12.8
Only CT reachable within 60 min	0	0.0	0	0.0
Neither reachable within 60 min	2	0.4	41	0.0
Likely irrelevant (<10 min)	426	75.5	155264	82.9

Population and Area Coverage in Landkreis Anhalt-Bitterfeld (Stroke Units) (Sachsen-Anhalt) — Regular (+20% Speed (Emergency))

Category	Grid Cells	Area (%)	Population	Population (%)
High (30+ min)	533	27.9	22131	15.0
Medium (20-30 min)	538	28.2	64579	43.8
Low (10-20 min)	590	30.9	47458	32.2
Only CT reachable within 60 min	5	0.3	9	0.0
Neither reachable within 60 min	0	0.0	0	0.0
Likely irrelevant (<10 min)	245	12.8	13320	9.0

Population and Area Coverage in Landkreis Jerichower Land (Stroke Units) (Sachsen-Anhalt) — Regular (+20% Speed (Emergency))

Category	Grid Cells	Area (%)	Population	Population (%)
High (30+ min)	346	16.6	13301	16.5
Medium (20-30 min)	528	25.3	28793	35.8
Low (10-20 min)	1102	52.8	35864	44.6
Only CT reachable within 60 min	61	2.9	401	0.5
Neither reachable within 60 min	0	0.0	0	0.0
Likely irrelevant (<10 min)	51	2.4	2140	2.7

Population and Area Coverage in Landkreis Kyffhäuserkreis (Stroke Units) (Thüringen) — Regular (+20% Speed (Emergency))

Category	Grid Cells	Area (%)	Population	Population (%)
High (30+ min)	39	2.9	2867	4.1
Medium (20-30 min)	501	37.5	29976	43.0
Low (10-20 min)	783	58.6	35896	51.5
Only CT reachable within 60 min	0	0.0	0	0.0
Neither reachable within 60 min	0	0.0	0	0.0
Likely irrelevant (<10 min)	14	1.0	953	1.4

Population and Area Coverage in Landkreis Peine (Stroke Units) (Niedersachsen) — Regular (+20% Speed (Emergency))

Category	Grid Cells	Area (%)	Population	Population (%)
High (30+ min)	0	0.0	0	0.0
Medium (20-30 min)	123	17.3	37112	28.4
Low (10-20 min)	471	66.2	84290	64.5
Only CT reachable within 60 min	0	0.0	0	0.0
Neither reachable within 60 min	0	0.0	0	0.0
Likely irrelevant (<10 min)	118	16.6	9315	7.1

Population and Area Coverage in Kreisfreie Stadt Leverkusen (Stroke Units) (Nordrhein-Westfalen) — Regular (+20% Speed (Emergency))

Category	Grid Cells	Area (%)	Population	Population (%)
High (30+ min)	0	0.0	0	0.0
Medium (20-30 min)	0	0.0	0	0.0
Low (10-20 min)	9	8.8	8139	5.1
Only CT reachable within 60 min	0	0.0	0	0.0
Neither reachable within 60 min	0	0.0	0	0.0
Likely irrelevant (<10 min)	93	91.2	152531	94.9

Population and Area Coverage in Kreis Ennepe-Ruhr-Kreis (Stroke Units) (Nordrhein-Westfalen) — Regular (+20% Speed (Emergency))

Category	Grid Cells	Area (%)	Population	Population (%)
High (30+ min)	0	0.0	0	0.0
Medium (20-30 min)	21	3.9	2507	0.8
Low (10-20 min)	154	28.7	82261	24.7
Only CT reachable within 60 min	0	0.0	0	0.0
Neither reachable within 60 min	0	0.0	0	0.0
Likely irrelevant (<10 min)	361	67.4	247893	74.5

Population and Area Coverage in Kreis Märkischer Kreis (Stroke Units) (Nordrhein-Westfalen) — Regular (+20% Speed (Emergency))

Category	Grid Cells	Area (%)	Population	Population (%)
High (30+ min)	16	1.2	1337	0.3
Medium (20-30 min)	190	14.0	104622	25.2
Low (10-20 min)	519	38.2	145017	34.9
Only CT reachable within 60 min	0	0.0	0	0.0
Neither reachable within 60 min	0	0.0	0	0.0
Likely irrelevant (<10 min)	632	46.6	164418	39.6

Population and Area Coverage in Landkreis Ahrweiler (Stroke Units) (Rheinland-Pfalz) — Regular (+20% Speed (Emergency))

Category	Grid Cells	Area (%)	Population	Population (%)
High (30+ min)	0	0.0	0	0.0
Medium (20-30 min)	2	0.2	309	0.2
Low (10-20 min)	204	20.5	40281	31.3
Only CT reachable within 60 min	0	0.0	0	0.0
Neither reachable within 60 min	0	0.0	0	0.0
Likely irrelevant (<10 min)	787	79.3	88297	68.5

Population and Area Coverage in Stadtkreis Baden-Baden (Stroke Units) (Baden-Württemberg) — Regular (+20% Speed (Emergency))

Category	Grid Cells	Area (%)	Population	Population (%)
High (30+ min)	0	0.0	0	0.0
Medium (20-30 min)	56	31.3	16078	25.5
Low (10-20 min)	89	49.7	33586	53.4
Only CT reachable within 60 min	0	0.0	0	0.0
Neither reachable within 60 min	0	0.0	0	0.0
Likely irrelevant (<10 min)	34	19.0	13277	21.1

Population and Area Coverage in Landkreis Waldshut (Stroke Units) (Baden-Württemberg) — Regular (+20% Speed (Emergency))

Category	Grid Cells	Area (%)	Population	Population (%)
High (30+ min)	398	31.1	57624	40.7
Medium (20-30 min)	259	20.2	18858	13.3
Low (10-20 min)	362	28.3	38187	27.0
Only CT reachable within 60 min	167	13.0	18912	13.4
Neither reachable within 60 min	2	0.2	204	0.1
Likely irrelevant (<10 min)	92	7.2	7764	5.5

Population and Area Coverage in Landkreis Bad Tölz-Wolfratshausen (Stroke Units) (Bayern) — Regular (+20% Speed (Emergency))

Category	Grid Cells	Area (%)	Population	Population (%)
High (30+ min)	0	0.0	0	0.0
Medium (20-30 min)	27	2.0	16953	13.4
Low (10-20 min)	233	17.7	33500	26.6
Only CT reachable within 60 min	0	0.0	0	0.0
Neither reachable within 60 min	109	8.3	176	0.1
Likely irrelevant (<10 min)	950	72.0	75528	59.9

Population and Area Coverage in Landkreis Passau (Stroke Units) (Bayern) — Regular (+20% Speed (Emergency))

Category	Grid Cells	Area (%)	Population	Population (%)
High (30+ min)	70	3.9	4208	2.2
Medium (20-30 min)	401	22.3	35487	18.7
Low (10-20 min)	596	33.1	66019	34.8
Only CT reachable within 60 min	2	0.1	150	0.1
Neither reachable within 60 min	8	0.4	95	0.1
Likely irrelevant (<10 min)	723	40.2	83754	44.1

Population and Area Coverage in Landkreis Lichtenfels (Stroke Units) (Bayern) — Regular (+20% Speed (Emergency))

Category	Grid Cells	Area (%)	Population	Population (%)
High (30+ min)	0	0.0	0	0.0
Medium (20-30 min)	0	0.0	0	0.0
Low (10-20 min)	79	12.1	6825	9.9
Only CT reachable within 60 min	0	0.0	0	0.0
Neither reachable within 60 min	0	0.0	0	0.0
Likely irrelevant (<10 min)	576	87.9	62372	90.1

Population and Area Coverage in Landkreis Wunsiedel i.Fichtelgebirge (Stroke Units) (Bayern) — Regular (+20% Speed (Emergency))

Category	Grid Cells	Area (%)	Population	Population (%)
High (30+ min)	2	0.3	159	0.2
Medium (20-30 min)	79	11.1	16135	21.8
Low (10-20 min)	474	66.4	47767	64.6
Only CT reachable within 60 min	0	0.0	0	0.0
Neither reachable within 60 min	0	0.0	0	0.0
Likely irrelevant (<10 min)	159	22.3	9875	13.4

Population and Area Coverage in Landkreis Ansbach (Stroke Units) (Bayern) — Regular (+20% Speed (Emergency))

Category	Grid Cells	Area (%)	Population	Population (%)
High (30+ min)	0	0.0	0	0.0
Medium (20-30 min)	144	5.9	16724	9.0
Low (10-20 min)	302	12.4	12763	6.9
Only CT reachable within 60 min	0	0.0	0	0.0
Neither reachable within 60 min	0	0.0	0	0.0
Likely irrelevant (<10 min)	1986	81.7	155389	84.1

Population and Area Coverage in Kreisfreie Stadt Würzburg (Stroke Units) (Bayern) — Regular (+20% Speed (Emergency))

Category	Grid Cells	Area (%)	Population	Population (%)
High (30+ min)	0	0.0	0	0.0
Medium (20-30 min)	0	0.0	0	0.0
Low (10-20 min)	0	0.0	0	0.0
Only CT reachable within 60 min	0	0.0	0	0.0
Neither reachable within 60 min	0	0.0	0	0.0
Likely irrelevant (<10 min)	109	100.0	128110	100.0

Population and Area Coverage in Landkreis Oder-Spree (Stroke Units) (Brandenburg) — Regular (+20% Speed (Emergency))

Category	Grid Cells	Area (%)	Population	Population (%)
High (30+ min)	18	0.6	312	0.2
Medium (20-30 min)	397	13.4	31137	18.1
Low (10-20 min)	1421	47.9	83906	48.8
Only CT reachable within 60 min	8	0.3	40	0.0
Neither reachable within 60 min	6	0.2	49	0.0
Likely irrelevant (<10 min)	1119	37.7	56486	32.9

Population and Area Coverage in Landkreis Potsdam-Mittelmark (Stroke Units) (Brandenburg) — Regular (+20% Speed (Emergency))

Category	Grid Cells	Area (%)	Population	Population (%)
High (30+ min)	957	28.1	32212	15.9
Medium (20-30 min)	912	26.8	26814	13.2
Low (10-20 min)	1172	34.4	69888	34.4
Only CT reachable within 60 min	44	1.3	271	0.1
Neither reachable within 60 min	0	0.0	0	0.0
Likely irrelevant (<10 min)	322	9.5	73813	36.4

Population and Area Coverage in Landkreis Uckermark (Stroke Units) (Brandenburg) — Regular (+20% Speed (Emergency))

Category	Grid Cells	Area (%)	Population	Population (%)
High (30+ min)	1505	36.5	76595	66.2
Medium (20-30 min)	1546	37.5	26716	23.1
Low (10-20 min)	683	16.6	7924	6.9
Only CT reachable within 60 min	269	6.5	2114	1.8
Neither reachable within 60 min	84	2.0	1946	1.7
Likely irrelevant (<10 min)	34	0.8	363	0.3

Population and Area Coverage in Landkreis Sächsische Schweiz-Osterzgebirge (Stroke Units) (Sachsen) — Regular (+20% Speed (Emergency))

Category	Grid Cells	Area (%)	Population	Population (%)
High (30+ min)	305	14.7	36083	15.8
Medium (20-30 min)	580	28.0	61795	27.1
Low (10-20 min)	663	32.0	81777	35.8
Only CT reachable within 60 min	83	4.0	3147	1.4
Neither reachable within 60 min	37	1.8	172	0.1
Likely irrelevant (<10 min)	404	19.5	45185	19.8

Population and Area Coverage in Kreisfreie Stadt Leipzig (Stroke Units) (Sachsen) — Regular (+20% Speed (Emergency))

Category	Grid Cells	Area (%)	Population	Population (%)
High (30+ min)	0	0.0	0	0.0
Medium (20-30 min)	0	0.0	0	0.0
Low (10-20 min)	39	10.2	60433	9.8
Only CT reachable within 60 min	0	0.0	0	0.0
Neither reachable within 60 min	0	0.0	0	0.0
Likely irrelevant (<10 min)	345	89.8	558893	90.2

Population and Area Coverage in Kreisfreie Stadt Jena (Stroke Units) (Thüringen) — Regular (+20% Speed (Emergency))

Category	Grid Cells	Area (%)	Population	Population (%)
High (30+ min)	0	0.0	0	0.0
Medium (20-30 min)	4	2.7	129	0.1
Low (10-20 min)	16	10.7	2883	2.9
Only CT reachable within 60 min	0	0.0	0	0.0
Neither reachable within 60 min	0	0.0	0	0.0
Likely irrelevant (<10 min)	129	86.6	97234	97.0

Population and Area Coverage in Landkreis Gotha (Stroke Units) (Thüringen) — Regular (+20% Speed (Emergency))

Category	Grid Cells	Area (%)	Population	Population (%)
High (30+ min)	0	0.0	0	0.0
Medium (20-30 min)	6	0.5	39	0.0
Low (10-20 min)	248	20.7	20764	17.0
Only CT reachable within 60 min	0	0.0	0	0.0
Neither reachable within 60 min	0	0.0	0	0.0
Likely irrelevant (<10 min)	945	78.8	101473	83.0

Population and Area Coverage in Kreis Pinneberg (Stroke Units) (Schleswig-Holstein) — Regular (+20% Speed (Emergency))

Category	Grid Cells	Area (%)	Population	Population (%)
High (30+ min)	0	0.0	0	0.0
Medium (20-30 min)	1	0.1	18	0.0
Low (10-20 min)	112	13.2	64405	21.4
Only CT reachable within 60 min	4	0.5	157	0.1
Neither reachable within 60 min	19	2.2	123	0.0
Likely irrelevant (<10 min)	714	84.0	235794	78.5

Population and Area Coverage in Kreis Schleswig-Flensburg (Stroke Units) (Schleswig-Holstein) — Regular (+20% Speed (Emergency))

Category	Grid Cells	Area (%)	Population	Population (%)
High (30+ min)	0	0.0	0	0.0
Medium (20-30 min)	19	0.7	1743	0.9
Low (10-20 min)	118	4.4	11786	6.0
Only CT reachable within 60 min	0	0.0	0	0.0
Neither reachable within 60 min	9	0.3	217	0.1
Likely irrelevant (<10 min)	2551	94.6	183047	93.0

Population and Area Coverage in Kreis Steinburg (Stroke Units) (Schleswig-Holstein) — Regular (+20% Speed (Emergency))

Category	Grid Cells	Area (%)	Population	Population (%)
High (30+ min)	1	0.1	14	0.0
Medium (20-30 min)	0	0.0	0	0.0
Low (10-20 min)	127	9.2	5823	4.4
Only CT reachable within 60 min	0	0.0	0	0.0
Neither reachable within 60 min	8	0.6	417	0.3
Likely irrelevant (<10 min)	1246	90.2	126479	95.3

Population and Area Coverage in Landkreis Verden (Stroke Units) (Niedersachsen) — Regular (+20% Speed (Emergency))

Category	Grid Cells	Area (%)	Population	Population (%)
High (30+ min)	8	0.8	614	0.5
Medium (20-30 min)	289	27.5	39630	29.8
Low (10-20 min)	508	48.3	73055	55.0
Only CT reachable within 60 min	0	0.0	0	0.0
Neither reachable within 60 min	0	0.0	0	0.0
Likely irrelevant (<10 min)	247	23.5	19529	14.7

Population and Area Coverage in Kreisfreie Stadt Delmenhorst (Stroke Units) (Niedersachsen) — Regular (+20% Speed (Emergency))

Category	Grid Cells	Area (%)	Population	Population (%)
High (30+ min)	0	0.0	0	0.0
Medium (20-30 min)	5	6.0	9843	13.4
Low (10-20 min)	59	71.1	61799	84.2
Only CT reachable within 60 min	0	0.0	0	0.0
Neither reachable within 60 min	0	0.0	0	0.0
Likely irrelevant (<10 min)	19	22.9	1753	2.4

Population and Area Coverage in Kreis Kleve (Stroke Units) (Nordrhein-Westfalen) — Regular (+20% Speed (Emergency))

Category	Grid Cells	Area (%)	Population	Population (%)
High (30+ min)	217	13.8	66246	21.8
Medium (20-30 min)	574	36.5	118146	39.0
Low (10-20 min)	414	26.4	67871	22.4
Only CT reachable within 60 min	5	0.3	154	0.1
Neither reachable within 60 min	14	0.9	127	0.0
Likely irrelevant (<10 min)	347	22.1	50655	16.7

Population and Area Coverage in Kreis Herford (Stroke Units) (Nordrhein-Westfalen) — Regular (+20% Speed (Emergency))

Category	Grid Cells	Area (%)	Population	Population (%)
High (30+ min)	0	0.0	0	0.0
Medium (20-30 min)	22	3.7	15075	6.0
Low (10-20 min)	314	52.8	124026	49.6
Only CT reachable within 60 min	0	0.0	0	0.0
Neither reachable within 60 min	0	0.0	0	0.0
Likely irrelevant (<10 min)	259	43.5	111194	44.4

Population and Area Coverage in Kreisfreie Stadt Dortmund (Stroke Units) (Nordrhein-Westfalen) — Regular (+20% Speed (Emergency))

Category	Grid Cells	Area (%)	Population	Population (%)
High (30+ min)	0	0.0	0	0.0
Medium (20-30 min)	0	0.0	0	0.0
Low (10-20 min)	42	11.6	56509	9.2
Only CT reachable within 60 min	0	0.0	0	0.0
Neither reachable within 60 min	0	0.0	0	0.0
Likely irrelevant (<10 min)	321	88.4	554974	90.8

Population and Area Coverage in Kreisfreie Stadt Wiesbaden (Stroke Units) (Hessen) — Regular (+20% Speed (Emergency))

Category	Grid Cells	Area (%)	Population	Population (%)
High (30+ min)	0	0.0	0	0.0
Medium (20-30 min)	0	0.0	0	0.0
Low (10-20 min)	1	0.4	6	0.0
Only CT reachable within 60 min	0	0.0	0	0.0
Neither reachable within 60 min	0	0.0	0	0.0
Likely irrelevant (<10 min)	251	99.6	277497	100.0

Population and Area Coverage in Landkreis Hochtaunuskreis (Stroke Units) (Hessen) — Regular (+20% Speed (Emergency))

Category	Grid Cells	Area (%)	Population	Population (%)
High (30+ min)	0	0.0	0	0.0
Medium (20-30 min)	0	0.0	0	0.0
Low (10-20 min)	217	35.8	50425	21.7
Only CT reachable within 60 min	0	0.0	0	0.0
Neither reachable within 60 min	0	0.0	0	0.0
Likely irrelevant (<10 min)	389	64.2	182095	78.3

Population and Area Coverage in Landkreis Kassel (Stroke Units) (Hessen) — Regular (+20% Speed (Emergency))

Category	Grid Cells	Area (%)	Population	Population (%)
High (30+ min)	23	1.4	2253	1.0
Medium (20-30 min)	539	32.3	43928	18.5
Low (10-20 min)	765	45.8	79119	33.4
Only CT reachable within 60 min	0	0.0	0	0.0
Neither reachable within 60 min	0	0.0	0	0.0
Likely irrelevant (<10 min)	343	20.5	111700	47.1

Population and Area Coverage in Landkreis Cochem-Zell (Stroke Units) (Rheinland-Pfalz) — Regular (+20% Speed (Emergency))

Category	Grid Cells	Area (%)	Population	Population (%)
High (30+ min)	63	7.2	6330	9.8
Medium (20-30 min)	348	40.0	25354	39.4
Low (10-20 min)	219	25.1	16660	25.9
Only CT reachable within 60 min	0	0.0	0	0.0
Neither reachable within 60 min	0	0.0	0	0.0
Likely irrelevant (<10 min)	241	27.7	16012	24.9

Population and Area Coverage in Landkreis Rhein-Lahn-Kreis (Stroke Units) (Rheinland-Pfalz) — Regular (+20% Speed (Emergency))

Category	Grid Cells	Area (%)	Population	Population (%)
High (30+ min)	75	7.6	8885	7.1
Medium (20-30 min)	212	21.5	14969	12.0
Low (10-20 min)	244	24.7	25221	20.2
Only CT reachable within 60 min	0	0.0	0	0.0
Neither reachable within 60 min	0	0.0	0	0.0
Likely irrelevant (<10 min)	455	46.1	75599	60.6

Population and Area Coverage in Stadtkreis Heilbronn (Stroke Units) (Baden-Württemberg) — Regular (+20% Speed (Emergency))

Category	Grid Cells	Area (%)	Population	Population (%)
High (30+ min)	0	0.0	0	0.0
Medium (20-30 min)	0	0.0	0	0.0
Low (10-20 min)	0	0.0	12	0.0
Only CT reachable within 60 min	0	0.0	0	0.0
Neither reachable within 60 min	0	0.0	0	0.0
Likely irrelevant (<10 min)	121	100.0	123074	100.0

Population and Area Coverage in Landkreis Schwandorf (Stroke Units) (Bayern) — Regular (+20% Speed (Emergency))

Category	Grid Cells	Area (%)	Population	Population (%)
High (30+ min)	10	0.6	1269	0.9
Medium (20-30 min)	207	11.6	10730	7.2
Low (10-20 min)	486	27.2	27358	18.4
Only CT reachable within 60 min	0	0.0	0	0.0
Neither reachable within 60 min	0	0.0	0	0.0
Likely irrelevant (<10 min)	1083	60.6	109177	73.5

Population and Area Coverage in Landkreis Unterallgäu (Stroke Units) (Bayern) — Regular (+20% Speed (Emergency))

Category	Grid Cells	Area (%)	Population	Population (%)
High (30+ min)	1	0.1	3	0.0
Medium (20-30 min)	126	8.5	8407	5.9
Low (10-20 min)	667	44.9	60216	42.3
Only CT reachable within 60 min	0	0.0	0	0.0
Neither reachable within 60 min	0	0.0	0	0.0
Likely irrelevant (<10 min)	692	46.6	73646	51.8

Population and Area Coverage in Kreisfreie Stadt Frankfurt (Oder) (Stroke Units) (Brandenburg) — Regular (+20% Speed (Emergency))

Category	Grid Cells	Area (%)	Population	Population (%)
High (30+ min)	1	0.5	187	0.3
Medium (20-30 min)	1	0.5	48	0.1
Low (10-20 min)	14	7.1	14111	25.1
Only CT reachable within 60 min	2	1.0	66	0.1
Neither reachable within 60 min	13	6.6	445	0.8
Likely irrelevant (<10 min)	165	84.2	41259	73.5

Population and Area Coverage in Landkreis Dahme-Spreewald (Stroke Units) (Brandenburg) — Regular (+20% Speed (Emergency))

Category	Grid Cells	Area (%)	Population	Population (%)
High (30+ min)	0	0.0	0	0.0
Medium (20-30 min)	127	4.3	29093	18.4
Low (10-20 min)	1458	49.1	82677	52.2
Only CT reachable within 60 min	0	0.0	0	0.0
Neither reachable within 60 min	0	0.0	0	0.0
Likely irrelevant (<10 min)	1384	46.6	46699	29.5

Population and Area Coverage in Landkreis Leipzig (Stroke Units) (Sachsen) — Regular (+20% Speed (Emergency))

Category	Grid Cells	Area (%)	Population	Population (%)
High (30+ min)	1	0.0	439	0.2
Medium (20-30 min)	181	8.5	35873	14.0
Low (10-20 min)	590	27.8	55575	21.8
Only CT reachable within 60 min	0	0.0	0	0.0
Neither reachable within 60 min	0	0.0	0	0.0
Likely irrelevant (<10 min)	1352	63.7	163538	64.0

Population and Area Coverage in Kreisfreie Stadt Magdeburg (Stroke Units) (Sachsen-Anhalt) — Regular (+20% Speed (Emergency))

Category	Grid Cells	Area (%)	Population	Population (%)
High (30+ min)	0	0.0	0	0.0
Medium (20-30 min)	1	0.4	7	0.0
Low (10-20 min)	72	27.4	25757	10.3
Only CT reachable within 60 min	0	0.0	0	0.0
Neither reachable within 60 min	0	0.0	0	0.0
Likely irrelevant (<10 min)	190	72.2	223536	89.7

Population and Area Coverage in Landkreis Wartburgkreis (Stroke Units) (Thüringen) — Regular (+20% Speed (Emergency))

Category	Grid Cells	Area (%)	Population	Population (%)
High (30+ min)	0	0.0	0	0.0
Medium (20-30 min)	24	1.4	1455	1.1
Low (10-20 min)	160	9.1	12231	8.9
Only CT reachable within 60 min	0	0.0	0	0.0
Neither reachable within 60 min	0	0.0	0	0.0
Likely irrelevant (<10 min)	1567	89.5	124389	90.1

Population and Area Coverage in Landkreis Unstrut-Hainich-Kreis (Stroke Units) (Thüringen) — Regular (+20% Speed (Emergency))

Category	Grid Cells	Area (%)	Population	Population (%)
High (30+ min)	36	3.0	692	0.8
Medium (20-30 min)	303	25.5	21842	24.2
Low (10-20 min)	506	42.6	42633	47.2
Only CT reachable within 60 min	0	0.0	0	0.0
Neither reachable within 60 min	0	0.0	0	0.0
Likely irrelevant (<10 min)	344	28.9	25102	27.8

Population and Area Coverage in Kreisfreie Stadt Bielefeld (Stroke Units) (Nordrhein-Westfalen) — Regular (+20% Speed (Emergency))

Category	Grid Cells	Area (%)	Population	Population (%)
High (30+ min)	0	0.0	0	0.0
Medium (20-30 min)	0	0.0	0	0.0
Low (10-20 min)	91	27.1	86465	24.7
Only CT reachable within 60 min	0	0.0	0	0.0
Neither reachable within 60 min	0	0.0	0	0.0
Likely irrelevant (<10 min)	245	72.9	263157	75.3

Population and Area Coverage in Kreisfreie Stadt Bochum (Stroke Units) (Nordrhein-Westfalen) — Regular (+20% Speed (Emergency))

Category	Grid Cells	Area (%)	Population	Population (%)
High (30+ min)	0	0.0	0	0.0
Medium (20-30 min)	0	0.0	0	0.0
Low (10-20 min)	7	3.8	18717	4.8
Only CT reachable within 60 min	0	0.0	0	0.0
Neither reachable within 60 min	0	0.0	0	0.0
Likely irrelevant (<10 min)	178	96.2	367986	95.2

Population and Area Coverage in Landkreis Fulda (Stroke Units) (Hessen) — Regular (+20% Speed (Emergency))

Category	Grid Cells	Area (%)	Population	Population (%)
High (30+ min)	0	0.0	0	0.0
Medium (20-30 min)	2	0.1	2296	1.0
Low (10-20 min)	366	20.9	37483	17.0
Only CT reachable within 60 min	0	0.0	0	0.0
Neither reachable within 60 min	0	0.0	0	0.0
Likely irrelevant (<10 min)	1383	79.0	180480	81.9

Population and Area Coverage in Landkreis Rastatt (Stroke Units) (Baden-Württemberg) — Regular (+20% Speed (Emergency))

Category	Grid Cells	Area (%)	Population	Population (%)
High (30+ min)	12	1.3	544	0.2
Medium (20-30 min)	314	35.0	43959	19.4
Low (10-20 min)	162	18.0	48630	21.4
Only CT reachable within 60 min	1	0.1	1	0.0
Neither reachable within 60 min	2	0.2	31	0.0
Likely irrelevant (<10 min)	407	45.3	133961	59.0

Population and Area Coverage in Landkreis Breisgau-Hochschwarzwald (Stroke Units) (Baden-Württemberg) — Regular (+20% Speed (Emergency))

Category	Grid Cells	Area (%)	Population	Population (%)
High (30+ min)	123	7.5	7727	3.1
Medium (20-30 min)	501	30.5	59210	24.0
Low (10-20 min)	522	31.8	63287	25.7
Only CT reachable within 60 min	1	0.1	15	0.0
Neither reachable within 60 min	0	0.0	3	0.0
Likely irrelevant (<10 min)	496	30.2	116147	47.1

Population and Area Coverage in Landkreis Ebersberg (Stroke Units) (Bayern) — Regular (+20% Speed (Emergency))

Category	Grid Cells	Area (%)	Population	Population (%)
High (30+ min)	0	0.0	0	0.0
Medium (20-30 min)	0	0.0	0	0.0
Low (10-20 min)	0	0.0	0	0.0
Only CT reachable within 60 min	0	0.0	0	0.0
Neither reachable within 60 min	0	0.0	0	0.0
Likely irrelevant (<10 min)	664	100.0	137985	100.0

Population and Area Coverage in Landkreis Rottal-Inn (Stroke Units) (Bayern) — Regular (+20% Speed (Emergency))

Category	Grid Cells	Area (%)	Population	Population (%)
High (30+ min)	0	0.0	0	0.0
Medium (20-30 min)	86	5.6	5604	4.6
Low (10-20 min)	555	36.1	43715	35.9
Only CT reachable within 60 min	0	0.0	0	0.0
Neither reachable within 60 min	2	0.1	63	0.1
Likely irrelevant (<10 min)	896	58.2	72286	59.4

Population and Area Coverage in Landkreis Bamberg (Stroke Units) (Bayern) — Regular (+20% Speed (Emergency))

Category	Grid Cells	Area (%)	Population	Population (%)
High (30+ min)	0	0.0	0	0.0
Medium (20-30 min)	166	11.3	10831	7.2
Low (10-20 min)	714	48.7	54024	35.8
Only CT reachable within 60 min	0	0.0	0	0.0
Neither reachable within 60 min	0	0.0	0	0.0
Likely irrelevant (<10 min)	585	39.9	85943	57.0

Population and Area Coverage in Landkreis Schweinfurt (Stroke Units) (Bayern) — Regular (+20% Speed (Emergency))

Category	Grid Cells	Area (%)	Population	Population (%)
High (30+ min)	0	0.0	0	0.0
Medium (20-30 min)	22	2.1	1241	1.1
Low (10-20 min)	375	35.4	35460	30.3
Only CT reachable within 60 min	0	0.0	0	0.0
Neither reachable within 60 min	0	0.0	0	0.0
Likely irrelevant (<10 min)	662	62.5	80248	68.6

Population and Area Coverage in Landkreis St. Wendel (Stroke Units) (Saarland) — Regular (+20% Speed (Emergency))

Category	Grid Cells	Area (%)	Population	Population (%)
High (30+ min)	0	0.0	0	0.0
Medium (20-30 min)	25	4.2	1435	1.7
Low (10-20 min)	125	20.9	11585	13.6
Only CT reachable within 60 min	0	0.0	0	0.0
Neither reachable within 60 min	0	0.0	0	0.0
Likely irrelevant (<10 min)	448	74.9	72266	84.7

Population and Area Coverage in Landkreis Harz (Stroke Units) (Sachsen-Anhalt) — Regular (+20% Speed (Emergency))

Category	Grid Cells	Area (%)	Population	Population (%)
High (30+ min)	7	0.3	62	0.0
Medium (20-30 min)	434	15.8	61079	30.5
Low (10-20 min)	1383	50.3	76630	38.3
Only CT reachable within 60 min	0	0.0	0	0.0
Neither reachable within 60 min	0	0.0	0	0.0
Likely irrelevant (<10 min)	924	33.6	62331	31.1

Population and Area Coverage in Kreisfreie Stadt Gera (Stroke Units) (Thüringen) — Regular (+20% Speed (Emergency))

Category	Grid Cells	Area (%)	Population	Population (%)
High (30+ min)	0	0.0	0	0.0
Medium (20-30 min)	0	0.0	0	0.0
Low (10-20 min)	46	24.1	4538	4.1
Only CT reachable within 60 min	0	0.0	0	0.0
Neither reachable within 60 min	0	0.0	0	0.0
Likely irrelevant (<10 min)	145	75.9	105119	95.9

Population and Area Coverage in Kreisfreie Stadt Weimar (Stroke Units) (Thüringen) — Regular (+20% Speed (Emergency))

Category	Grid Cells	Area (%)	Population	Population (%)
High (30+ min)	0	0.0	0	0.0
Medium (20-30 min)	0	0.0	0	0.0
Low (10-20 min)	0	0.0	0	0.0
Only CT reachable within 60 min	0	0.0	0	0.0
Neither reachable within 60 min	0	0.0	0	0.0
Likely irrelevant (<10 min)	109	100.0	57698	100.0

Population and Area Coverage in Landkreis Holzminden (Stroke Units) (Niedersachsen) — Regular (+20% Speed (Emergency))

Category	Grid Cells	Area (%)	Population	Population (%)
High (30+ min)	1	0.1	790	1.1
Medium (20-30 min)	103	11.4	7368	10.3
Low (10-20 min)	179	19.8	7577	10.6
Only CT reachable within 60 min	0	0.0	0	0.0
Neither reachable within 60 min	0	0.0	0	0.0
Likely irrelevant (<10 min)	621	68.7	56053	78.1

Population and Area Coverage in Landkreis Grafschaft Bentheim (Stroke Units) (Niedersachsen) — Regular (+20% Speed (Emergency))

Category	Grid Cells	Area (%)	Population	Population (%)
High (30+ min)	2	0.2	368	0.3
Medium (20-30 min)	21	1.6	2529	1.9
Low (10-20 min)	183	14.4	26618	19.5
Only CT reachable within 60 min	5	0.4	89	0.1
Neither reachable within 60 min	19	1.5	612	0.4
Likely irrelevant (<10 min)	1044	81.9	106460	77.9

Population and Area Coverage in Kreis Höxter (Stroke Units) (Nordrhein-Westfalen) — Regular (+20% Speed (Emergency))

Category	Grid Cells	Area (%)	Population	Population (%)
High (30+ min)	9	0.6	872	0.6
Medium (20-30 min)	320	20.5	37202	26.1
Low (10-20 min)	666	42.6	49465	34.6
Only CT reachable within 60 min	0	0.0	0	0.0
Neither reachable within 60 min	0	0.0	0	0.0
Likely irrelevant (<10 min)	568	36.3	55252	38.7

Population and Area Coverage in Landkreis Marburg-Biedenkopf (Stroke Units) (Hessen) — Regular (+20% Speed (Emergency))

Category	Grid Cells	Area (%)	Population	Population (%)
High (30+ min)	99	6.2	17584	7.2
Medium (20-30 min)	184	11.5	21093	8.7
Low (10-20 min)	412	25.8	50857	20.9
Only CT reachable within 60 min	0	0.0	0	0.0
Neither reachable within 60 min	0	0.0	0	0.0
Likely irrelevant (<10 min)	905	56.6	153828	63.2

Population and Area Coverage in Landkreis Rhein-Hunsrück-Kreis (Stroke Units) (Rheinland-Pfalz) — Regular (+20% Speed (Emergency))

Category	Grid Cells	Area (%)	Population	Population (%)
High (30+ min)	214	17.2	19644	18.7
Medium (20-30 min)	639	51.4	54989	52.4
Low (10-20 min)	377	30.3	29747	28.3
Only CT reachable within 60 min	0	0.0	0	0.0
Neither reachable within 60 min	0	0.0	0	0.0
Likely irrelevant (<10 min)	14	1.1	582	0.6

Population and Area Coverage in Landkreis Eifelkreis Bitburg-Prüm (Stroke Units) (Rheinland-Pfalz) — Regular (+20% Speed (Emergency))

Category	Grid Cells	Area (%)	Population	Population (%)
High (30+ min)	196	9.6	7338	7.3
Medium (20-30 min)	839	41.2	42583	42.4
Low (10-20 min)	837	41.1	41480	41.3
Only CT reachable within 60 min	0	0.0	0	0.0
Neither reachable within 60 min	1	0.0	127	0.1
Likely irrelevant (<10 min)	163	8.0	8808	8.8

Population and Area Coverage in Landkreis Erding (Stroke Units) (Bayern) — Regular (+20% Speed (Emergency))

Category	Grid Cells	Area (%)	Population	Population (%)
High (30+ min)	0	0.0	0	0.0
Medium (20-30 min)	66	6.3	15304	11.3
Low (10-20 min)	362	34.3	29591	21.8
Only CT reachable within 60 min	0	0.0	0	0.0
Neither reachable within 60 min	0	0.0	0	0.0
Likely irrelevant (<10 min)	627	59.4	90836	66.9

Population and Area Coverage in Landkreis Deggendorf (Stroke Units) (Bayern) — Regular (+20% Speed (Emergency))

Category	Grid Cells	Area (%)	Population	Population (%)
High (30+ min)	0	0.0	0	0.0
Medium (20-30 min)	0	0.0	0	0.0
Low (10-20 min)	55	5.2	4039	3.4
Only CT reachable within 60 min	0	0.0	0	0.0
Neither reachable within 60 min	0	0.0	0	0.0
Likely irrelevant (<10 min)	1002	94.8	115411	96.6

Population and Area Coverage in Landkreis Weißenburg-Gunzenhausen (Stroke Units) (Bayern) — Regular (+20% Speed (Emergency))

Category	Grid Cells	Area (%)	Population	Population (%)
High (30+ min)	0	0.0	0	0.0
Medium (20-30 min)	36	3.0	1145	1.2
Low (10-20 min)	571	48.1	49645	52.0
Only CT reachable within 60 min	0	0.0	0	0.0
Neither reachable within 60 min	0	0.0	0	0.0
Likely irrelevant (<10 min)	580	48.9	44621	46.8

Population and Area Coverage in Landkreis Lindau (Bodensee) (Stroke Units) (Bayern) — Regular (+20% Speed (Emergency))

Category	Grid Cells	Area (%)	Population	Population (%)
High (30+ min)	1	0.3	1856	2.3
Medium (20-30 min)	129	33.9	45456	56.0
Low (10-20 min)	194	51.1	27491	33.9
Only CT reachable within 60 min	6	1.6	181	0.2
Neither reachable within 60 min	7	1.8	614	0.8
Likely irrelevant (<10 min)	43	11.3	5592	6.9

Population and Area Coverage in Kreisfreie Stadt Rostock (Stroke Units) (Mecklenburg-Vorpommern) — Regular (+20% Speed (Emergency))

Category	Grid Cells	Area (%)	Population	Population (%)
High (30+ min)	0	0.0	0	0.0
Medium (20-30 min)	0	0.0	0	0.0
Low (10-20 min)	94	41.8	27376	14.7
Only CT reachable within 60 min	2	0.9	1948	1.0
Neither reachable within 60 min	5	2.2	576	0.3
Likely irrelevant (<10 min)	124	55.1	156505	84.0

Population and Area Coverage in Landkreis Rostock (Stroke Units) (Mecklenburg-Vorpommern) — Regular (+20% Speed (Emergency))

Category	Grid Cells	Area (%)	Population	Population (%)
High (30+ min)	180	3.8	9753	4.9
Medium (20-30 min)	626	13.4	28239	14.2
Low (10-20 min)	1463	31.3	53929	27.2
Only CT reachable within 60 min	2	0.0	434	0.2
Neither reachable within 60 min	31	0.7	542	0.3
Likely irrelevant (<10 min)	2375	50.8	105476	53.2

Population and Area Coverage in Landkreis Vorpommern-Greifswald (Stroke Units) (Mecklenburg-Vorpommern) — Regular (+20% Speed (Emergency))

Category	Grid Cells	Area (%)	Population	Population (%)
High (30+ min)	347	6.9	27260	12.3
Medium (20-30 min)	1379	27.4	44200	19.9
Low (10-20 min)	1366	27.1	29977	13.5
Only CT reachable within 60 min	137	2.7	10066	4.5
Neither reachable within 60 min	133	2.6	3046	1.4
Likely irrelevant (<10 min)	1677	33.3	107346	48.4

Population and Area Coverage in Landkreis Mittelsachsen (Stroke Units) (Sachsen) — Regular (+20% Speed (Emergency))

Category	Grid Cells	Area (%)	Population	Population (%)
High (30+ min)	3	0.1	1024	0.3
Medium (20-30 min)	368	13.6	56292	19.1
Low (10-20 min)	804	29.7	68644	23.3
Only CT reachable within 60 min	1	0.0	0	0.0
Neither reachable within 60 min	0	0.0	0	0.0
Likely irrelevant (<10 min)	1534	56.6	168452	57.2

Population and Area Coverage in Landkreis Schmalkalden-Meiningen (Stroke Units) (Thüringen) — Regular (+20% Speed (Emergency))

Category	Grid Cells	Area (%)	Population	Population (%)
High (30+ min)	9	0.6	2137	1.8
Medium (20-30 min)	148	9.3	20943	18.1
Low (10-20 min)	344	21.7	26370	22.8
Only CT reachable within 60 min	0	0.0	0	0.0
Neither reachable within 60 min	0	0.0	0	0.0
Likely irrelevant (<10 min)	1084	68.4	66349	57.3

Population and Area Coverage in Kreisfreie Stadt Lübeck (Stroke Units) (Schleswig-Holstein) — Regular (+20% Speed (Emergency))

Category	Grid Cells	Area (%)	Population	Population (%)
High (30+ min)	1	0.3	957	0.4
Medium (20-30 min)	19	6.6	12388	5.7
Low (10-20 min)	69	23.9	25062	11.5
Only CT reachable within 60 min	1	0.3	1072	0.5
Neither reachable within 60 min	2	0.7	159	0.1
Likely irrelevant (<10 min)	197	68.2	178290	81.8

Population and Area Coverage in Kreis Nordfriesland (Stroke Units) (Schleswig-Holstein) — Regular (+20% Speed (Emergency))

Category	Grid Cells	Area (%)	Population	Population (%)
High (30+ min)	488	18.5	44193	27.5
Medium (20-30 min)	576	21.9	36177	22.5
Low (10-20 min)	587	22.3	27981	17.4
Only CT reachable within 60 min	173	6.6	22207	13.8
Neither reachable within 60 min	150	5.7	4529	2.8
Likely irrelevant (<10 min)	659	25.0	25603	15.9

Population and Area Coverage in Landkreis Northeim (Stroke Units) (Niedersachsen) — Regular (+20% Speed (Emergency))

Category	Grid Cells	Area (%)	Population	Population (%)
High (30+ min)	21	1.3	9553	7.1
Medium (20-30 min)	305	18.5	33390	24.9
Low (10-20 min)	805	48.8	56403	42.1
Only CT reachable within 60 min	0	0.0	0	0.0
Neither reachable within 60 min	0	0.0	0	0.0
Likely irrelevant (<10 min)	520	31.5	34766	25.9

Population and Area Coverage in Kreisfreie Stadt Wilhelmshaven (Stroke Units) (Niedersachsen) — Regular (+20% Speed (Emergency))

Category	Grid Cells	Area (%)	Population	Population (%)
High (30+ min)	0	0.0	0	0.0
Medium (20-30 min)	0	0.0	0	0.0
Low (10-20 min)	22	18.2	24522	35.3
Only CT reachable within 60 min	1	0.8	40	0.1
Neither reachable within 60 min	2	1.7	652	0.9
Likely irrelevant (<10 min)	96	79.3	44314	63.7

Population and Area Coverage in Kreisfreie Stadt Essen (Stroke Units) (Nordrhein-Westfalen) — Regular (+20% Speed (Emergency))

Category	Grid Cells	Area (%)	Population	Population (%)
High (30+ min)	0	0.0	0	0.0
Medium (20-30 min)	0	0.0	0	0.0
Low (10-20 min)	35	12.7	49365	8.3
Only CT reachable within 60 min	0	0.0	0	0.0
Neither reachable within 60 min	0	0.0	0	0.0
Likely irrelevant (<10 min)	241	87.3	547311	91.7

Population and Area Coverage in Kreisfreie Stadt Münster (Stroke Units) (Nordrhein-Westfalen) — Regular (+20% Speed (Emergency))

Category	Grid Cells	Area (%)	Population	Population (%)
High (30+ min)	0	0.0	0	0.0
Medium (20-30 min)	3	0.8	256	0.1
Low (10-20 min)	42	10.5	27149	7.4
Only CT reachable within 60 min	0	0.0	0	0.0
Neither reachable within 60 min	0	0.0	0	0.0
Likely irrelevant (<10 min)	354	88.7	337426	92.5

Population and Area Coverage in Kreis Recklinghausen (Stroke Units) (Nordrhein-Westfalen) — Regular (+20% Speed (Emergency))

Category	Grid Cells	Area (%)	Population	Population (%)
High (30+ min)	0	0.0	0	0.0
Medium (20-30 min)	0	0.0	0	0.0
Low (10-20 min)	176	17.9	123301	20.0
Only CT reachable within 60 min	0	0.0	0	0.0
Neither reachable within 60 min	0	0.0	0	0.0
Likely irrelevant (<10 min)	806	82.1	493112	80.0

Population and Area Coverage in Landkreis Offenbach (Stroke Units) (Hessen) — Regular (+20% Speed (Emergency))

Category	Grid Cells	Area (%)	Population	Population (%)
High (30+ min)	0	0.0	0	0.0
Medium (20-30 min)	0	0.0	0	0.0
Low (10-20 min)	37	8.3	18212	5.3
Only CT reachable within 60 min	0	0.0	0	0.0
Neither reachable within 60 min	0	0.0	0	0.0
Likely irrelevant (<10 min)	409	91.7	323099	94.7

Population and Area Coverage in Kreisfreie Stadt Trier (Stroke Units) (Rheinland-Pfalz) — Regular (+20% Speed (Emergency))

Category	Grid Cells	Area (%)	Population	Population (%)
High (30+ min)	0	0.0	0	0.0
Medium (20-30 min)	0	0.0	0	0.0
Low (10-20 min)	0	0.0	0	0.0
Only CT reachable within 60 min	0	0.0	0	0.0
Neither reachable within 60 min	0	0.0	0	0.0
Likely irrelevant (<10 min)	146	100.0	128525	100.0

Population and Area Coverage in Landkreis Bernkastel-Wittlich (Stroke Units) (Rheinland-Pfalz) — Regular (+20% Speed (Emergency))

Category	Grid Cells	Area (%)	Population	Population (%)
High (30+ min)	0	0.0	0	0.0
Medium (20-30 min)	45	3.1	5013	4.4
Low (10-20 min)	481	32.9	32814	28.6
Only CT reachable within 60 min	0	0.0	0	0.0
Neither reachable within 60 min	0	0.0	0	0.0
Likely irrelevant (<10 min)	935	64.0	76844	67.0

Population and Area Coverage in Kreisfreie Stadt Worms (Stroke Units) (Rheinland-Pfalz) — Regular (+20% Speed (Emergency))

Category	Grid Cells	Area (%)	Population	Population (%)
High (30+ min)	0	0.0	0	0.0
Medium (20-30 min)	0	0.0	0	0.0
Low (10-20 min)	0	0.0	19	0.0
Only CT reachable within 60 min	0	0.0	0	0.0
Neither reachable within 60 min	0	0.0	0	0.0
Likely irrelevant (<10 min)	134	100.0	81855	100.0

Population and Area Coverage in Landkreis Heilbronn (Stroke Units) (Baden-Württemberg) — Regular (+20% Speed (Emergency))

Category	Grid Cells	Area (%)	Population	Population (%)
High (30+ min)	14	1.0	703	0.2
Medium (20-30 min)	79	5.8	8280	2.5
Low (10-20 min)	583	42.8	120792	36.4
Only CT reachable within 60 min	0	0.0	0	0.0
Neither reachable within 60 min	0	0.0	0	0.0
Likely irrelevant (<10 min)	685	50.3	201629	60.8

Population and Area Coverage in Kreisfreie Stadt Ingolstadt (Stroke Units) (Bayern) — Regular (+20% Speed (Emergency))

Category	Grid Cells	Area (%)	Population	Population (%)
High (30+ min)	0	0.0	0	0.0
Medium (20-30 min)	0	0.0	0	0.0
Low (10-20 min)	12	7.3	13106	9.7
Only CT reachable within 60 min	0	0.0	0	0.0
Neither reachable within 60 min	0	0.0	0	0.0
Likely irrelevant (<10 min)	153	92.7	122113	90.3

Population and Area Coverage in Landkreis Forchheim (Stroke Units) (Bayern) — Regular (+20% Speed (Emergency))

Category	Grid Cells	Area (%)	Population	Population (%)
High (30+ min)	0	0.0	0	0.0
Medium (20-30 min)	26	3.2	1037	0.9
Low (10-20 min)	314	39.0	26941	22.8
Only CT reachable within 60 min	0	0.0	0	0.0
Neither reachable within 60 min	0	0.0	0	0.0
Likely irrelevant (<10 min)	465	57.8	90433	76.4

Population and Area Coverage in Landkreis Erlangen-Höchstadt (Stroke Units) (Bayern) — Regular (+20% Speed (Emergency))

Category	Grid Cells	Area (%)	Population	Population (%)
High (30+ min)	3	0.4	521	0.4
Medium (20-30 min)	146	20.9	18709	13.6
Low (10-20 min)	295	42.3	58073	42.3
Only CT reachable within 60 min	0	0.0	0	0.0
Neither reachable within 60 min	0	0.0	0	0.0
Likely irrelevant (<10 min)	254	36.4	60096	43.7

Population and Area Coverage in Landkreis Mansfeld-Südharz (Stroke Units) (Sachsen-Anhalt) — Regular (+20% Speed (Emergency))

Category	Grid Cells	Area (%)	Population	Population (%)
High (30+ min)	190	10.1	26890	21.0
Medium (20-30 min)	765	40.5	65393	51.1
Low (10-20 min)	540	28.6	20628	16.1
Only CT reachable within 60 min	0	0.0	0	0.0
Neither reachable within 60 min	0	0.0	0	0.0
Likely irrelevant (<10 min)	395	20.9	15026	11.7

Population and Area Coverage in Kreis Minden-Lübbecke (Stroke Units) (Nordrhein-Westfalen) — Regular (+20% Speed (Emergency))

Category	Grid Cells	Area (%)	Population	Population (%)
High (30+ min)	77	5.1	12784	4.1
Medium (20-30 min)	345	22.7	55464	17.9
Low (10-20 min)	316	20.8	57013	18.4
Only CT reachable within 60 min	0	0.0	0	0.0
Neither reachable within 60 min	0	0.0	0	0.0
Likely irrelevant (<10 min)	782	51.4	184212	59.5

Population and Area Coverage in Kreisfreie Stadt Hamm (Stroke Units) (Nordrhein-Westfalen) — Regular (+20% Speed (Emergency))

Category	Grid Cells	Area (%)	Population	Population (%)
High (30+ min)	0	0.0	0	0.0
Medium (20-30 min)	0	0.0	0	0.0
Low (10-20 min)	10	3.4	3684	2.1
Only CT reachable within 60 min	0	0.0	0	0.0
Neither reachable within 60 min	0	0.0	0	0.0
Likely irrelevant (<10 min)	283	96.6	171716	97.9

Population and Area Coverage in Landkreis Main-Taunus-Kreis (Stroke Units) (Hessen) — Regular (+20% Speed (Emergency))

Category	Grid Cells	Area (%)	Population	Population (%)
High (30+ min)	0	0.0	0	0.0
Medium (20-30 min)	0	0.0	0	0.0
Low (10-20 min)	20	7.1	3088	1.3
Only CT reachable within 60 min	0	0.0	0	0.0
Neither reachable within 60 min	0	0.0	0	0.0
Likely irrelevant (<10 min)	261	92.9	228345	98.7

Population and Area Coverage in Kreisfreie Stadt Ludwigshafen am Rhein (Stroke Units) (Rheinland-Pfalz) — Regular (+20% Speed (Emergency))

Category	Grid Cells	Area (%)	Population	Population (%)
High (30+ min)	0	0.0	0	0.0
Medium (20-30 min)	0	0.0	0	0.0
Low (10-20 min)	9	9.1	9239	5.7
Only CT reachable within 60 min	0	0.0	0	0.0
Neither reachable within 60 min	0	0.0	0	0.0
Likely irrelevant (<10 min)	90	90.9	153962	94.3

Population and Area Coverage in Landkreis Esslingen (Stroke Units) (Baden-Württemberg) — Regular (+20% Speed (Emergency))

Category	Grid Cells	Area (%)	Population	Population (%)
High (30+ min)	0	0.0	0	0.0
Medium (20-30 min)	3	0.4	54	0.0
Low (10-20 min)	161	20.9	102243	19.8
Only CT reachable within 60 min	0	0.0	0	0.0
Neither reachable within 60 min	0	0.0	0	0.0
Likely irrelevant (<10 min)	607	78.7	414862	80.2

Population and Area Coverage in Landkreis Dachau (Stroke Units) (Bayern) — Regular (+20% Speed (Emergency))

Category	Grid Cells	Area (%)	Population	Population (%)
High (30+ min)	0	0.0	0	0.0
Medium (20-30 min)	0	0.0	0	0.0
Low (10-20 min)	225	32.0	27230	18.1
Only CT reachable within 60 min	0	0.0	0	0.0
Neither reachable within 60 min	0	0.0	0	0.0
Likely irrelevant (<10 min)	479	68.0	122901	81.9

Population and Area Coverage in Landkreis Bad Kissingen (Stroke Units) (Bayern) — Regular (+20% Speed (Emergency))

Category	Grid Cells	Area (%)	Population	Population (%)
High (30+ min)	2	0.1	2337	2.2
Medium (20-30 min)	280	19.5	28550	26.8
Low (10-20 min)	704	49.1	48012	45.0
Only CT reachable within 60 min	0	0.0	0	0.0
Neither reachable within 60 min	0	0.0	0	0.0
Likely irrelevant (<10 min)	447	31.2	27726	26.0

Population and Area Coverage in Kreisfreie Stadt Erfurt (Stroke Units) (Thüringen) — Regular (+20% Speed (Emergency))

Category	Grid Cells	Area (%)	Population	Population (%)
High (30+ min)	0	0.0	0	0.0
Medium (20-30 min)	0	0.0	0	0.0
Low (10-20 min)	29	8.5	23378	9.5
Only CT reachable within 60 min	0	0.0	0	0.0
Neither reachable within 60 min	0	0.0	0	0.0
Likely irrelevant (<10 min)	313	91.5	223844	90.5

Population and Area Coverage in Kreisfreie Stadt Flensburg (Stroke Units) (Schleswig-Holstein) — Regular (+20% Speed (Emergency))

Category	Grid Cells	Area (%)	Population	Population (%)
High (30+ min)	0	0.0	0	0.0
Medium (20-30 min)	0	0.0	0	0.0
Low (10-20 min)	0	0.0	0	0.0
Only CT reachable within 60 min	0	0.0	0	0.0
Neither reachable within 60 min	0	0.0	0	0.0
Likely irrelevant (<10 min)	62	100.0	80819	100.0

Population and Area Coverage in Kreis Dithmarschen (Stroke Units) (Schleswig-Holstein) — Regular (+20% Speed (Emergency))

Category	Grid Cells	Area (%)	Population	Population (%)
High (30+ min)	0	0.0	0	0.0
Medium (20-30 min)	34	1.8	6177	4.6
Low (10-20 min)	300	16.2	23480	17.6
Only CT reachable within 60 min	7	0.4	1106	0.8
Neither reachable within 60 min	37	2.0	509	0.4
Likely irrelevant (<10 min)	1471	79.6	102346	76.6

Population and Area Coverage in Landkreis Hameln-Pyrmont (Stroke Units) (Niedersachsen) — Regular (+20% Speed (Emergency))

Category	Grid Cells	Area (%)	Population	Population (%)
High (30+ min)	9	0.9	5238	3.6
Medium (20-30 min)	279	26.6	39620	26.9
Low (10-20 min)	453	43.2	74513	50.6
Only CT reachable within 60 min	0	0.0	0	0.0
Neither reachable within 60 min	0	0.0	0	0.0
Likely irrelevant (<10 min)	307	29.3	28030	19.0

Population and Area Coverage in Landkreis Celle (Stroke Units) (Niedersachsen) — Regular (+20% Speed (Emergency))

Category	Grid Cells	Area (%)	Population	Population (%)
High (30+ min)	0	0.0	0	0.0
Medium (20-30 min)	3	0.1	52	0.0
Low (10-20 min)	277	13.4	5409	3.1
Only CT reachable within 60 min	0	0.0	0	0.0
Neither reachable within 60 min	0	0.0	0	0.0
Likely irrelevant (<10 min)	1787	86.5	170831	96.9

Population and Area Coverage in Landkreis Harburg (Stroke Units) (Niedersachsen) — Regular (+20% Speed (Emergency))

Category	Grid Cells	Area (%)	Population	Population (%)
High (30+ min)	0	0.0	0	0.0
Medium (20-30 min)	0	0.0	0	0.0
Low (10-20 min)	55	3.3	17053	7.0
Only CT reachable within 60 min	0	0.0	0	0.0
Neither reachable within 60 min	0	0.0	0	0.0
Likely irrelevant (<10 min)	1634	96.7	226311	93.0

Population and Area Coverage in Kreisfreie Stadt Düsseldorf (Stroke Units) (Nordrhein-Westfalen) — Regular (+20% Speed (Emergency))

Category	Grid Cells	Area (%)	Population	Population (%)
High (30+ min)	0	0.0	0	0.0
Medium (20-30 min)	0	0.0	0	0.0
Low (10-20 min)	24	8.6	44195	6.5
Only CT reachable within 60 min	0	0.0	0	0.0
Neither reachable within 60 min	0	0.0	0	0.0
Likely irrelevant (<10 min)	255	91.4	632229	93.5

Population and Area Coverage in Kreis Düren (Stroke Units) (Nordrhein-Westfalen) — Regular (+20% Speed (Emergency))

Category	Grid Cells	Area (%)	Population	Population (%)
High (30+ min)	0	0.0	0	0.0
Medium (20-30 min)	19	1.6	7200	2.8
Low (10-20 min)	281	23.4	61519	24.0
Only CT reachable within 60 min	0	0.0	0	0.0
Neither reachable within 60 min	0	0.0	0	0.0
Likely irrelevant (<10 min)	900	75.0	187540	73.2

Population and Area Coverage in Kreis Soest (Stroke Units) (Nordrhein-Westfalen) — Regular (+20% Speed (Emergency))

Category	Grid Cells	Area (%)	Population	Population (%)
High (30+ min)	0	0.0	0	0.0
Medium (20-30 min)	7	0.4	0	0.0
Low (10-20 min)	415	24.1	63131	21.4
Only CT reachable within 60 min	0	0.0	0	0.0
Neither reachable within 60 min	0	0.0	0	0.0
Likely irrelevant (<10 min)	1302	75.5	232014	78.6

Population and Area Coverage in Kreisfreie Stadt Darmstadt (Stroke Units) (Hessen) — Regular (+20% Speed (Emergency))

Category	Grid Cells	Area (%)	Population	Population (%)
High (30+ min)	0	0.0	0	0.0
Medium (20-30 min)	0	0.0	0	0.0
Low (10-20 min)	2	1.3	7	0.0
Only CT reachable within 60 min	0	0.0	0	0.0
Neither reachable within 60 min	0	0.0	0	0.0
Likely irrelevant (<10 min)	152	98.7	151592	100.0

Population and Area Coverage in Kreisfreie Stadt Offenbach am Main (Stroke Units) (Hessen) — Regular (+20% Speed (Emergency))

Category	Grid Cells	Area (%)	Population	Population (%)
High (30+ min)	0	0.0	0	0.0
Medium (20-30 min)	0	0.0	0	0.0
Low (10-20 min)	0	0.0	0	0.0
Only CT reachable within 60 min	0	0.0	0	0.0
Neither reachable within 60 min	0	0.0	0	0.0
Likely irrelevant (<10 min)	56	100.0	123120	100.0

Population and Area Coverage in Landkreis Werra-Meißner-Kreis (Stroke Units) (Hessen) — Regular (+20% Speed (Emergency))

Category	Grid Cells	Area (%)	Population	Population (%)
High (30+ min)	53	4.0	16444	16.3
Medium (20-30 min)	674	51.1	58427	57.9
Low (10-20 min)	373	28.3	16141	16.0
Only CT reachable within 60 min	0	0.0	0	0.0
Neither reachable within 60 min	0	0.0	0	0.0
Likely irrelevant (<10 min)	218	16.5	9836	9.8

Population and Area Coverage in Landkreis Mayen-Koblenz (Stroke Units) (Rheinland-Pfalz) — Regular (+20% Speed (Emergency))

Category	Grid Cells	Area (%)	Population	Population (%)
High (30+ min)	0	0.0	0	0.0
Medium (20-30 min)	87	8.4	19686	9.1
Low (10-20 min)	429	41.5	93127	42.9
Only CT reachable within 60 min	0	0.0	0	0.0
Neither reachable within 60 min	0	0.0	0	0.0
Likely irrelevant (<10 min)	518	50.1	104227	48.0

Population and Area Coverage in Landkreis Bad Dürkheim (Stroke Units) (Rheinland-Pfalz) — Regular (+20% Speed (Emergency))

Category	Grid Cells	Area (%)	Population	Population (%)
High (30+ min)	0	0.0	0	0.0
Medium (20-30 min)	44	6.0	16406	12.2
Low (10-20 min)	370	50.6	76367	56.8
Only CT reachable within 60 min	0	0.0	0	0.0
Neither reachable within 60 min	0	0.0	0	0.0
Likely irrelevant (<10 min)	317	43.4	41665	31.0

Population and Area Coverage in Landkreis Landsberg am Lech (Stroke Units) (Bayern) — Regular (+20% Speed (Emergency))

Category	Grid Cells	Area (%)	Population	Population (%)
High (30+ min)	0	0.0	0	0.0
Medium (20-30 min)	128	13.3	40896	33.8
Low (10-20 min)	578	60.1	56125	46.4
Only CT reachable within 60 min	0	0.0	0	0.0
Neither reachable within 60 min	0	0.0	0	0.0
Likely irrelevant (<10 min)	256	26.6	23909	19.8

Population and Area Coverage in Landkreis Donau-Ries (Stroke Units) (Bayern) — Regular (+20% Speed (Emergency))

Category	Grid Cells	Area (%)	Population	Population (%)
High (30+ min)	72	4.6	20420	15.3
Medium (20-30 min)	945	60.3	75938	57.0
Low (10-20 min)	459	29.3	33814	25.4
Only CT reachable within 60 min	0	0.0	0	0.0
Neither reachable within 60 min	0	0.0	0	0.0
Likely irrelevant (<10 min)	90	5.7	3152	2.4

Population and Area Coverage in Landkreis Märkisch-Oderland (Stroke Units) (Brandenburg) — Regular (+20% Speed (Emergency))

Category	Grid Cells	Area (%)	Population	Population (%)
High (30+ min)	405	14.2	15481	8.4
Medium (20-30 min)	982	34.4	41556	22.6
Low (10-20 min)	652	22.8	39378	21.4
Only CT reachable within 60 min	9	0.3	37	0.0
Neither reachable within 60 min	72	2.5	577	0.3
Likely irrelevant (<10 min)	735	25.7	87076	47.3

Population and Area Coverage in Landkreis Prignitz (Stroke Units) (Brandenburg) — Regular (+20% Speed (Emergency))

Category	Grid Cells	Area (%)	Population	Population (%)
High (30+ min)	1189	41.4	36554	49.2
Medium (20-30 min)	622	21.7	9375	12.6
Low (10-20 min)	272	9.5	4019	5.4
Only CT reachable within 60 min	676	23.5	22390	30.1
Neither reachable within 60 min	0	0.0	0	0.0
Likely irrelevant (<10 min)	112	3.9	2030	2.7

Population and Area Coverage in Landkreis Burgenlandkreis (Stroke Units) (Sachsen-Anhalt) — Regular (+20% Speed (Emergency))

Category	Grid Cells	Area (%)	Population	Population (%)
High (30+ min)	3	0.2	151	0.1
Medium (20-30 min)	155	8.5	34741	21.0
Low (10-20 min)	630	34.6	60892	36.9
Only CT reachable within 60 min	0	0.0	0	0.0
Neither reachable within 60 min	0	0.0	0	0.0
Likely irrelevant (<10 min)	1031	56.7	69336	42.0

Population and Area Coverage in Landkreis Stendal (Stroke Units) (Sachsen-Anhalt) — Regular (+20% Speed (Emergency))

Category	Grid Cells	Area (%)	Population	Population (%)
High (30+ min)	1152	35.6	61139	59.1
Medium (20-30 min)	317	9.8	8054	7.8
Low (10-20 min)	243	7.5	7774	7.5
Only CT reachable within 60 min	1497	46.3	26294	25.4
Neither reachable within 60 min	0	0.0	0	0.0
Likely irrelevant (<10 min)	25	0.8	246	0.2

Population and Area Coverage in Landkreis Weimarer Land (Stroke Units) (Thüringen) — Regular (+20% Speed (Emergency))

Category	Grid Cells	Area (%)	Population	Population (%)
High (30+ min)	0	0.0	0	0.0
Medium (20-30 min)	100	9.7	20911	28.3
Low (10-20 min)	295	28.7	14079	19.1
Only CT reachable within 60 min	0	0.0	0	0.0
Neither reachable within 60 min	0	0.0	0	0.0
Likely irrelevant (<10 min)	632	61.5	38785	52.6

Population and Area Coverage in Kreisfreie Stadt Kiel (Stroke Units) (Schleswig-Holstein) — Regular (+20% Speed (Emergency))

Category	Grid Cells	Area (%)	Population	Population (%)
High (30+ min)	0	0.0	0	0.0
Medium (20-30 min)	0	0.0	0	0.0
Low (10-20 min)	4	2.6	5203	2.0
Only CT reachable within 60 min	0	0.0	1571	0.6
Neither reachable within 60 min	0	0.0	98	0.0
Likely irrelevant (<10 min)	151	97.4	249447	97.3

Population and Area Coverage in Landkreis Gifhorn (Stroke Units) (Niedersachsen) — Regular (+20% Speed (Emergency))

Category	Grid Cells	Area (%)	Population	Population (%)
High (30+ min)	103	4.9	7460	4.3
Medium (20-30 min)	535	25.6	39572	23.0
Low (10-20 min)	798	38.2	81624	47.4
Only CT reachable within 60 min	0	0.0	0	0.0
Neither reachable within 60 min	0	0.0	0	0.0
Likely irrelevant (<10 min)	654	31.3	43546	25.3

Population and Area Coverage in Landkreis Helmstedt (Stroke Units) (Niedersachsen) — Regular (+20% Speed (Emergency))

Category	Grid Cells	Area (%)	Population	Population (%)
High (30+ min)	0	0.0	0	0.0
Medium (20-30 min)	198	22.1	38483	43.0
Low (10-20 min)	341	38.1	21253	23.8
Only CT reachable within 60 min	0	0.0	0	0.0
Neither reachable within 60 min	0	0.0	0	0.0
Likely irrelevant (<10 min)	356	39.8	29677	33.2

Population and Area Coverage in Landkreis Lüchow-Dannenberg (Stroke Units) (Niedersachsen) — Regular (+20% Speed (Emergency))

Category	Grid Cells	Area (%)	Population	Population (%)
High (30+ min)	685	41.7	24750	50.7
Medium (20-30 min)	356	21.7	14729	30.2
Low (10-20 min)	339	20.7	6606	13.5
Only CT reachable within 60 min	209	12.7	2352	4.8
Neither reachable within 60 min	0	0.0	0	0.0
Likely irrelevant (<10 min)	52	3.2	404	0.8

Population and Area Coverage in Landkreis Rotenburg (Wümme) (Stroke Units) (Niedersachsen) — Regular (+20% Speed (Emergency))

Category	Grid Cells	Area (%)	Population	Population (%)
High (30+ min)	6	0.2	13	0.0
Medium (20-30 min)	630	22.5	36739	22.4
Low (10-20 min)	607	21.7	26496	16.2
Only CT reachable within 60 min	0	0.0	0	0.0
Neither reachable within 60 min	0	0.0	0	0.0
Likely irrelevant (<10 min)	1551	55.5	100726	61.4

Population and Area Coverage in Kreis Euskirchen (Stroke Units) (Nordrhein-Westfalen) — Regular (+20% Speed (Emergency))

Category	Grid Cells	Area (%)	Population	Population (%)
High (30+ min)	108	6.8	9499	5.0
Medium (20-30 min)	272	17.2	14009	7.4
Low (10-20 min)	530	33.5	55275	29.4
Only CT reachable within 60 min	0	0.0	0	0.0
Neither reachable within 60 min	0	0.0	2	0.0
Likely irrelevant (<10 min)	673	42.5	109452	58.1

Population and Area Coverage in Kreis Olpe (Stroke Units) (Nordrhein-Westfalen) — Regular (+20% Speed (Emergency))

Category	Grid Cells	Area (%)	Population	Population (%)
High (30+ min)	144	15.7	38619	28.5
Medium (20-30 min)	306	33.4	33555	24.8
Low (10-20 min)	307	33.6	35483	26.2
Only CT reachable within 60 min	0	0.0	0	0.0
Neither reachable within 60 min	0	0.0	0	0.0
Likely irrelevant (<10 min)	158	17.3	27761	20.5

Population and Area Coverage in Landkreis Lahn-Dill-Kreis (Stroke Units) (Hessen) — Regular (+20% Speed (Emergency))

Category	Grid Cells	Area (%)	Population	Population (%)
High (30+ min)	0	0.0	0	0.0
Medium (20-30 min)	287	21.0	47253	18.5
Low (10-20 min)	524	38.4	88638	34.7
Only CT reachable within 60 min	0	0.0	0	0.0
Neither reachable within 60 min	0	0.0	0	0.0
Likely irrelevant (<10 min)	553	40.5	119831	46.9

Population and Area Coverage in Landkreis Ravensburg (Stroke Units) (Baden-Württemberg) — Regular (+20% Speed (Emergency))

Category	Grid Cells	Area (%)	Population	Population (%)
High (30+ min)	0	0.0	0	0.0
Medium (20-30 min)	90	4.6	23507	8.5
Low (10-20 min)	328	16.7	37950	13.7
Only CT reachable within 60 min	0	0.0	0	0.0
Neither reachable within 60 min	0	0.0	0	0.0
Likely irrelevant (<10 min)	1542	78.7	215311	77.8

Population and Area Coverage in Kreisfreie Stadt Rosenheim (Stroke Units) (Bayern) — Regular (+20% Speed (Emergency))

Category	Grid Cells	Area (%)	Population	Population (%)
High (30+ min)	0	0.0	0	0.0
Medium (20-30 min)	0	0.0	0	0.0
Low (10-20 min)	0	0.0	0	0.0
Only CT reachable within 60 min	0	0.0	0	0.0
Neither reachable within 60 min	0	0.0	0	0.0
Likely irrelevant (<10 min)	47	100.0	62621	100.0

Population and Area Coverage in Landkreis Freising (Stroke Units) (Bayern) — Regular (+20% Speed (Emergency))

Category	Grid Cells	Area (%)	Population	Population (%)
High (30+ min)	0	0.0	0	0.0
Medium (20-30 min)	16	1.6	1253	0.7
Low (10-20 min)	79	8.1	7144	4.1
Only CT reachable within 60 min	0	0.0	0	0.0
Neither reachable within 60 min	0	0.0	0	0.0
Likely irrelevant (<10 min)	879	90.2	166529	95.2

Population and Area Coverage in Landkreis Garmisch-Partenkirchen (Stroke Units) (Bayern) — Regular (+20% Speed (Emergency))

Category	Grid Cells	Area (%)	Population	Population (%)
High (30+ min)	1	0.1	362	0.4
Medium (20-30 min)	114	9.6	26600	30.6
Low (10-20 min)	35	2.9	14963	17.2
Only CT reachable within 60 min	8	0.7	652	0.8
Neither reachable within 60 min	430	36.0	1041	1.2
Likely irrelevant (<10 min)	605	50.7	43299	49.8

Population and Area Coverage in Landkreis Regensburg (Stroke Units) (Bayern) — Regular (+20% Speed (Emergency))

Category	Grid Cells	Area (%)	Population	Population (%)
High (30+ min)	0	0.0	0	0.0
Medium (20-30 min)	1	0.1	1	0.0
Low (10-20 min)	443	25.8	32299	16.7
Only CT reachable within 60 min	0	0.0	0	0.0
Neither reachable within 60 min	0	0.0	0	0.0
Likely irrelevant (<10 min)	1275	74.2	161411	83.3

Population and Area Coverage in Landkreis Tirschenreuth (Stroke Units) (Bayern) — Regular (+20% Speed (Emergency))

Category	Grid Cells	Area (%)	Population	Population (%)
High (30+ min)	2	0.2	151	0.2
Medium (20-30 min)	427	33.2	28245	37.9
Low (10-20 min)	693	53.8	37181	49.9
Only CT reachable within 60 min	0	0.0	0	0.0
Neither reachable within 60 min	2	0.2	0	0.0
Likely irrelevant (<10 min)	164	12.7	8874	11.9

Population and Area Coverage in Kreisfreie Stadt Kaufbeuren (Stroke Units) (Bayern) — Regular (+20% Speed (Emergency))

Category	Grid Cells	Area (%)	Population	Population (%)
High (30+ min)	0	0.0	0	0.0
Medium (20-30 min)	0	0.0	0	0.0
Low (10-20 min)	0	0.0	0	0.0
Only CT reachable within 60 min	0	0.0	0	0.0
Neither reachable within 60 min	0	0.0	0	0.0
Likely irrelevant (<10 min)	50	100.0	42940	100.0

Population and Area Coverage in Landkreis Oberallgäu (Stroke Units) (Bayern) — Regular (+20% Speed (Emergency))

Category	Grid Cells	Area (%)	Population	Population (%)
High (30+ min)	25	1.4	7477	4.8
Medium (20-30 min)	191	10.8	44945	28.9
Low (10-20 min)	238	13.4	22593	14.5
Only CT reachable within 60 min	14	0.8	438	0.3
Neither reachable within 60 min	536	30.2	4027	2.6
Likely irrelevant (<10 min)	772	43.5	76032	48.9

Population and Area Coverage in Landkreis Regionalverband Saarbrücken (Stroke Units) (Saarland) — Regular (+20% Speed (Emergency))

Category	Grid Cells	Area (%)	Population	Population (%)
High (30+ min)	0	0.0	0	0.0
Medium (20-30 min)	0	0.0	0	0.0
Low (10-20 min)	8	1.6	10373	3.1
Only CT reachable within 60 min	1	0.2	136	0.0
Neither reachable within 60 min	3	0.6	158	0.0
Likely irrelevant (<10 min)	476	97.5	321223	96.8

Population and Area Coverage in Landkreis Altenburger Land (Stroke Units) (Thüringen) — Regular (+20% Speed (Emergency))

Category	Grid Cells	Area (%)	Population	Population (%)
High (30+ min)	0	0.0	0	0.0
Medium (20-30 min)	7	1.0	1171	1.4
Low (10-20 min)	69	9.5	8215	9.8
Only CT reachable within 60 min	0	0.0	0	0.0
Neither reachable within 60 min	0	0.0	0	0.0
Likely irrelevant (<10 min)	650	89.5	74694	88.8

Population and Area Coverage in Landkreis Diepholz (Stroke Units) (Niedersachsen) — Regular (+20% Speed (Emergency))

Category	Grid Cells	Area (%)	Population	Population (%)
High (30+ min)	638	24.2	34302	16.2
Medium (20-30 min)	988	37.4	60284	28.5
Low (10-20 min)	738	27.9	63456	30.0
Only CT reachable within 60 min	0	0.0	0	0.0
Neither reachable within 60 min	0	0.0	0	0.0
Likely irrelevant (<10 min)	277	10.5	53128	25.2

Population and Area Coverage in Kreisfreie Stadt Emden (Stroke Units) (Niedersachsen) — Regular (+20% Speed (Emergency))

Category	Grid Cells	Area (%)	Population	Population (%)
High (30+ min)	0	0.0	0	0.0
Medium (20-30 min)	3	2.3	2125	4.3
Low (10-20 min)	0	0.0	0	0.0
Only CT reachable within 60 min	0	0.0	0	0.0
Neither reachable within 60 min	0	0.0	0	0.0
Likely irrelevant (<10 min)	129	97.7	46996	95.7

Population and Area Coverage in Kreisfreie Stadt Gelsenkirchen (Stroke Units) (Nordrhein-Westfalen) — Regular (+20% Speed (Emergency))

Category	Grid Cells	Area (%)	Population	Population (%)
High (30+ min)	0	0.0	0	0.0
Medium (20-30 min)	0	0.0	0	0.0
Low (10-20 min)	19	14.2	30735	11.0
Only CT reachable within 60 min	0	0.0	0	0.0
Neither reachable within 60 min	0	0.0	0	0.0
Likely irrelevant (<10 min)	115	85.8	249050	89.0

Population and Area Coverage in Kreis Warendorf (Stroke Units) (Nordrhein-Westfalen) — Regular (+20% Speed (Emergency))

Category	Grid Cells	Area (%)	Population	Population (%)
High (30+ min)	1	0.1	51	0.0
Medium (20-30 min)	290	16.9	53695	19.7
Low (10-20 min)	640	37.3	97964	36.0
Only CT reachable within 60 min	0	0.0	0	0.0
Neither reachable within 60 min	0	0.0	0	0.0
Likely irrelevant (<10 min)	787	45.8	120360	44.2

Population and Area Coverage in Stadtkreis Ulm (Stroke Units) (Baden-Württemberg) — Regular (+20% Speed (Emergency))

Category	Grid Cells	Area (%)	Population	Population (%)
High (30+ min)	0	0.0	0	0.0
Medium (20-30 min)	0	0.0	0	0.0
Low (10-20 min)	7	4.9	14058	11.7
Only CT reachable within 60 min	0	0.0	0	0.0
Neither reachable within 60 min	0	0.0	0	0.0
Likely irrelevant (<10 min)	136	95.1	106056	88.3

Population and Area Coverage in Landkreis Traunstein (Stroke Units) (Bayern) — Regular (+20% Speed (Emergency))

Category	Grid Cells	Area (%)	Population	Population (%)
High (30+ min)	0	0.0	0	0.0
Medium (20-30 min)	35	1.9	4780	2.7
Low (10-20 min)	214	11.8	22711	12.9
Only CT reachable within 60 min	3	0.2	637	0.4
Neither reachable within 60 min	127	7.0	719	0.4
Likely irrelevant (<10 min)	1438	79.1	146768	83.6

Population and Area Coverage in Kreisfreie Stadt Regensburg (Stroke Units) (Bayern) — Regular (+20% Speed (Emergency))

Category	Grid Cells	Area (%)	Population	Population (%)
High (30+ min)	0	0.0	0	0.0
Medium (20-30 min)	0	0.0	0	0.0
Low (10-20 min)	0	0.0	174	0.1
Only CT reachable within 60 min	0	0.0	0	0.0
Neither reachable within 60 min	0	0.0	0	0.0
Likely irrelevant (<10 min)	97	100.0	169888	99.9

Population and Area Coverage in Kreisfreie Stadt Weiden i.d.OPf. (Stroke Units) (Bayern) — Regular (+20% Speed (Emergency))

Category	Grid Cells	Area (%)	Population	Population (%)
High (30+ min)	0	0.0	0	0.0
Medium (20-30 min)	0	0.0	0	0.0
Low (10-20 min)	0	0.0	0	0.0
Only CT reachable within 60 min	0	0.0	0	0.0
Neither reachable within 60 min	0	0.0	0	0.0
Likely irrelevant (<10 min)	86	100.0	43316	100.0

Population and Area Coverage in Kreisfreie Stadt Ansbach (Stroke Units) (Bayern) — Regular (+20% Speed (Emergency))

Category	Grid Cells	Area (%)	Population	Population (%)
High (30+ min)	0	0.0	0	0.0
Medium (20-30 min)	0	0.0	0	0.0
Low (10-20 min)	0	0.0	0	0.0
Only CT reachable within 60 min	0	0.0	0	0.0
Neither reachable within 60 min	0	0.0	0	0.0
Likely irrelevant (<10 min)	123	100.0	41201	100.0

Population and Area Coverage in Landkreis Rhön-Grabfeld (Stroke Units) (Bayern) — Regular (+20% Speed (Emergency))

Category	Grid Cells	Area (%)	Population	Population (%)
High (30+ min)	0	0.0	0	0.0
Medium (20-30 min)	0	0.0	0	0.0
Low (10-20 min)	27	2.1	881	1.1
Only CT reachable within 60 min	0	0.0	0	0.0
Neither reachable within 60 min	0	0.0	0	0.0
Likely irrelevant (<10 min)	1261	97.9	81297	98.9

Population and Area Coverage in Landkreis Ostprignitz-Ruppin (Stroke Units) (Brandenburg) — Regular (+20% Speed (Emergency))

Category	Grid Cells	Area (%)	Population	Population (%)
High (30+ min)	253	7.5	9448	10.0
Medium (20-30 min)	592	17.5	17642	18.6
Low (10-20 min)	751	22.2	14694	15.5
Only CT reachable within 60 min	32	0.9	152	0.2
Neither reachable within 60 min	0	0.0	0	0.0
Likely irrelevant (<10 min)	1755	51.9	52774	55.7

Population and Area Coverage in Kreis Plön (Stroke Units) (Schleswig-Holstein) — Regular (+20% Speed (Emergency))

Category	Grid Cells	Area (%)	Population	Population (%)
High (30+ min)	0	0.0	0	0.0
Medium (20-30 min)	96	6.5	18824	14.9
Low (10-20 min)	512	34.4	26813	21.2
Only CT reachable within 60 min	1	0.1	31	0.0
Neither reachable within 60 min	22	1.5	1489	1.2
Likely irrelevant (<10 min)	857	57.6	79196	62.7

Population and Area Coverage in Landkreis Nienburg (Weser) (Stroke Units) (Niedersachsen) — Regular (+20% Speed (Emergency))

Category	Grid Cells	Area (%)	Population	Population (%)
High (30+ min)	580	31.1	55146	45.4
Medium (20-30 min)	683	36.6	32359	26.7
Low (10-20 min)	360	19.3	21716	17.9
Only CT reachable within 60 min	0	0.0	0	0.0
Neither reachable within 60 min	0	0.0	0	0.0
Likely irrelevant (<10 min)	242	13.0	12198	10.0

Population and Area Coverage in Landkreis Emsland (Stroke Units) (Niedersachsen) — Regular (+20% Speed (Emergency))

Category	Grid Cells	Area (%)	Population	Population (%)
High (30+ min)	87	2.3	29829	9.3
Medium (20-30 min)	434	11.3	29374	9.1
Low (10-20 min)	1454	37.9	118911	36.9
Only CT reachable within 60 min	8	0.2	159	0.0
Neither reachable within 60 min	21	0.5	331	0.1
Likely irrelevant (<10 min)	1835	47.8	143640	44.6

Population and Area Coverage in Landkreis Wittmund (Stroke Units) (Niedersachsen) — Regular (+20% Speed (Emergency))

Category	Grid Cells	Area (%)	Population	Population (%)
High (30+ min)	0	0.0	0	0.0
Medium (20-30 min)	101	11.4	10422	18.3
Low (10-20 min)	532	59.9	34065	59.9
Only CT reachable within 60 min	1	0.1	9	0.0
Neither reachable within 60 min	50	5.6	2637	4.6
Likely irrelevant (<10 min)	204	23.0	9739	17.1

Population and Area Coverage in Kreis Hochsauerlandkreis (Stroke Units) (Nordrhein-Westfalen) — Regular (+20% Speed (Emergency))

Category	Grid Cells	Area (%)	Population	Population (%)
High (30+ min)	551	21.9	52276	19.9
Medium (20-30 min)	887	35.3	67479	25.7
Low (10-20 min)	629	25.0	50716	19.3
Only CT reachable within 60 min	6	0.2	0	0.0
Neither reachable within 60 min	0	0.0	0	0.0
Likely irrelevant (<10 min)	441	17.5	91898	35.0

Population and Area Coverage in Landkreis Rheingau-Taunus-Kreis (Stroke Units) (Hessen) — Regular (+20% Speed (Emergency))

Category	Grid Cells	Area (%)	Population	Population (%)
High (30+ min)	38	3.7	2207	1.2
Medium (20-30 min)	220	21.6	29139	15.9
Low (10-20 min)	552	54.2	85432	46.6
Only CT reachable within 60 min	0	0.0	0	0.0
Neither reachable within 60 min	0	0.0	0	0.0
Likely irrelevant (<10 min)	209	20.5	66477	36.3

Population and Area Coverage in Landkreis Göppingen (Stroke Units) (Baden-Württemberg) — Regular (+20% Speed (Emergency))

Category	Grid Cells	Area (%)	Population	Population (%)
High (30+ min)	0	0.0	0	0.0
Medium (20-30 min)	0	0.0	0	0.0
Low (10-20 min)	6	0.8	380	0.2
Only CT reachable within 60 min	0	0.0	0	0.0
Neither reachable within 60 min	0	0.0	0	0.0
Likely irrelevant (<10 min)	775	99.2	249616	99.8

Population and Area Coverage in Stadtkreis Freiburg im Breisgau (Stroke Units) (Baden-Württemberg) — Regular (+20% Speed (Emergency))

Category	Grid Cells	Area (%)	Population	Population (%)
High (30+ min)	0	0.0	0	0.0
Medium (20-30 min)	3	1.6	614	0.2
Low (10-20 min)	36	19.3	18884	7.3
Only CT reachable within 60 min	0	0.0	0	0.0
Neither reachable within 60 min	0	0.0	0	0.0
Likely irrelevant (<10 min)	148	79.1	238941	92.5

Population and Area Coverage in Landkreis Landshut (Stroke Units) (Bayern) — Regular (+20% Speed (Emergency))

Category	Grid Cells	Area (%)	Population	Population (%)
High (30+ min)	0	0.0	0	0.0
Medium (20-30 min)	4	0.2	104	0.1
Low (10-20 min)	199	12.1	14920	9.6
Only CT reachable within 60 min	0	0.0	0	0.0
Neither reachable within 60 min	0	0.0	0	0.0
Likely irrelevant (<10 min)	1438	87.6	140666	90.4

Population and Area Coverage in Landkreis Regen (Stroke Units) (Bayern) — Regular (+20% Speed (Emergency))

Category	Grid Cells	Area (%)	Population	Population (%)
High (30+ min)	0	0.0	0	0.0
Medium (20-30 min)	69	6.1	8848	11.4
Low (10-20 min)	194	17.0	9907	12.8
Only CT reachable within 60 min	2	0.2	4	0.0
Neither reachable within 60 min	24	2.1	24	0.0
Likely irrelevant (<10 min)	851	74.6	58744	75.8

Population and Area Coverage in Kreisfreie Stadt Coburg (Stroke Units) (Bayern) — Regular (+20% Speed (Emergency))

Category	Grid Cells	Area (%)	Population	Population (%)
High (30+ min)	0	0.0	0	0.0
Medium (20-30 min)	0	0.0	0	0.0
Low (10-20 min)	0	0.0	0	0.0
Only CT reachable within 60 min	0	0.0	0	0.0
Neither reachable within 60 min	0	0.0	0	0.0
Likely irrelevant (<10 min)	57	100.0	41384	100.0

Population and Area Coverage in Landkreis Neunkirchen (Stroke Units) (Saarland) — Regular (+20% Speed (Emergency))

Category	Grid Cells	Area (%)	Population	Population (%)
High (30+ min)	0	0.0	0	0.0
Medium (20-30 min)	0	0.0	0	0.0
Low (10-20 min)	12	3.8	2983	2.3
Only CT reachable within 60 min	0	0.0	0	0.0
Neither reachable within 60 min	0	0.0	0	0.0
Likely irrelevant (<10 min)	300	96.2	124360	97.7

Population and Area Coverage in Landkreis Nordwestmecklenburg (Stroke Units) (Mecklenburg-Vorpommern) — Regular (+20% Speed (Emergency))

Category	Grid Cells	Area (%)	Population	Population (%)
High (30+ min)	12	0.4	364	0.3
Medium (20-30 min)	182	6.3	5399	3.7
Low (10-20 min)	855	29.4	29719	20.6
Only CT reachable within 60 min	15	0.5	1188	0.8
Neither reachable within 60 min	51	1.8	1667	1.2
Likely irrelevant (<10 min)	1789	61.6	105659	73.4

Population and Area Coverage in Kreisfreie Stadt Herne (Stroke Units) (Nordrhein-Westfalen) — Regular (+20% Speed (Emergency))

Category	Grid Cells	Area (%)	Population	Population (%)
High (30+ min)	0	0.0	0	0.0
Medium (20-30 min)	0	0.0	0	0.0
Low (10-20 min)	4	5.8	15534	9.4
Only CT reachable within 60 min	0	0.0	0	0.0
Neither reachable within 60 min	0	0.0	0	0.0
Likely irrelevant (<10 min)	65	94.2	149710	90.6

Population and Area Coverage in Landkreis Altenkirchen (Westerwald) (Stroke Units) (Rheinland-Pfalz) — Regular (+20% Speed (Emergency))

Category	Grid Cells	Area (%)	Population	Population (%)
High (30+ min)	0	0.0	0	0.0
Medium (20-30 min)	194	23.8	42710	32.0
Low (10-20 min)	360	44.2	55506	41.6
Only CT reachable within 60 min	0	0.0	0	0.0
Neither reachable within 60 min	0	0.0	0	0.0
Likely irrelevant (<10 min)	261	32.0	35193	26.4

Population and Area Coverage in Kreisfreie Stadt Kaiserslautern (Stroke Units) (Rheinland-Pfalz) — Regular (+20% Speed (Emergency))

Category	Grid Cells	Area (%)	Population	Population (%)
High (30+ min)	0	0.0	0	0.0
Medium (20-30 min)	0	0.0	0	0.0
Low (10-20 min)	0	0.0	0	0.0
Only CT reachable within 60 min	0	0.0	0	0.0
Neither reachable within 60 min	0	0.0	0	0.0
Likely irrelevant (<10 min)	174	100.0	110813	100.0

Population and Area Coverage in Landkreis Mainz-Bingen (Stroke Units) (Rheinland-Pfalz) — Regular (+20% Speed (Emergency))

Category	Grid Cells	Area (%)	Population	Population (%)
High (30+ min)	1	0.1	257	0.1
Medium (20-30 min)	34	4.5	5013	2.4
Low (10-20 min)	128	16.8	23855	11.3
Only CT reachable within 60 min	0	0.0	0	0.0
Neither reachable within 60 min	0	0.0	0	0.0
Likely irrelevant (<10 min)	598	78.6	181094	86.1

Population and Area Coverage in Landkreis Heidenheim (Stroke Units) (Baden-Württemberg) — Regular (+20% Speed (Emergency))

Category	Grid Cells	Area (%)	Population	Population (%)
High (30+ min)	1	0.1	12	0.0
Medium (20-30 min)	53	7.0	2149	1.7
Low (10-20 min)	109	14.4	6839	5.3
Only CT reachable within 60 min	0	0.0	0	0.0
Neither reachable within 60 min	0	0.0	0	0.0
Likely irrelevant (<10 min)	596	78.5	118989	93.0

Population and Area Coverage in Stadtkreis Pforzheim (Stroke Units) (Baden-Württemberg) — Regular (+20% Speed (Emergency))

Category	Grid Cells	Area (%)	Population	Population (%)
High (30+ min)	0	0.0	0	0.0
Medium (20-30 min)	0	0.0	0	0.0
Low (10-20 min)	9	7.5	7539	6.0
Only CT reachable within 60 min	0	0.0	0	0.0
Neither reachable within 60 min	0	0.0	0	0.0
Likely irrelevant (<10 min)	111	92.5	117423	94.0

Population and Area Coverage in Landkreis Tübingen (Stroke Units) (Baden-Württemberg) — Regular (+20% Speed (Emergency))

Category	Grid Cells	Area (%)	Population	Population (%)
High (30+ min)	0	0.0	0	0.0
Medium (20-30 min)	2	0.3	497	0.2
Low (10-20 min)	90	14.3	21418	9.8
Only CT reachable within 60 min	0	0.0	0	0.0
Neither reachable within 60 min	0	0.0	0	0.0
Likely irrelevant (<10 min)	536	85.4	196623	90.0

Population and Area Coverage in Landkreis Bodenseekreis (Stroke Units) (Baden-Württemberg) — Regular (+20% Speed (Emergency))

Category	Grid Cells	Area (%)	Population	Population (%)
High (30+ min)	0	0.0	0	0.0
Medium (20-30 min)	17	2.1	13676	6.2
Low (10-20 min)	412	51.4	86268	39.0
Only CT reachable within 60 min	1	0.1	4	0.0
Neither reachable within 60 min	0	0.0	1958	0.9
Likely irrelevant (<10 min)	372	46.4	119168	53.9

Population and Area Coverage in Landkreis Miesbach (Stroke Units) (Bayern) — Regular (+20% Speed (Emergency))

Category	Grid Cells	Area (%)	Population	Population (%)
High (30+ min)	0	0.0	0	0.0
Medium (20-30 min)	0	0.0	12	0.0
Low (10-20 min)	22	2.1	396	0.4
Only CT reachable within 60 min	5	0.5	1186	1.2
Neither reachable within 60 min	240	23.1	990	1.0
Likely irrelevant (<10 min)	770	74.3	96149	97.4

Population and Area Coverage in Landkreis Mühldorf a.Inn (Stroke Units) (Bayern) — Regular (+20% Speed (Emergency))

Category	Grid Cells	Area (%)	Population	Population (%)
High (30+ min)	0	0.0	0	0.0
Medium (20-30 min)	0	0.0	0	0.0
Low (10-20 min)	222	22.9	46901	42.4
Only CT reachable within 60 min	0	0.0	0	0.0
Neither reachable within 60 min	0	0.0	0	0.0
Likely irrelevant (<10 min)	748	77.1	63735	57.6

Population and Area Coverage in Landkreis Neuburg-Schrobenhausen (Stroke Units) (Bayern) — Regular (+20% Speed (Emergency))

Category	Grid Cells	Area (%)	Population	Population (%)
High (30+ min)	0	0.0	0	0.0
Medium (20-30 min)	339	37.5	36826	38.6
Low (10-20 min)	461	51.0	50857	53.3
Only CT reachable within 60 min	0	0.0	0	0.0
Neither reachable within 60 min	0	0.0	0	0.0
Likely irrelevant (<10 min)	104	11.5	7800	8.2

Population and Area Coverage in Landkreis Starnberg (Stroke Units) (Bayern) — Regular (+20% Speed (Emergency))

Category	Grid Cells	Area (%)	Population	Population (%)
High (30+ min)	1	0.2	260	0.2
Medium (20-30 min)	71	12.1	25995	19.3
Low (10-20 min)	371	63.3	68960	51.3
Only CT reachable within 60 min	0	0.0	0	0.0
Neither reachable within 60 min	0	0.0	0	0.0
Likely irrelevant (<10 min)	143	24.4	39322	29.2

Population and Area Coverage in Landkreis Fürth (Stroke Units) (Bayern) — Regular (+20% Speed (Emergency))

Category	Grid Cells	Area (%)	Population	Population (%)
High (30+ min)	0	0.0	0	0.0
Medium (20-30 min)	0	0.0	0	0.0
Low (10-20 min)	2	0.5	100	0.1
Only CT reachable within 60 min	0	0.0	0	0.0
Neither reachable within 60 min	0	0.0	0	0.0
Likely irrelevant (<10 min)	380	99.5	118605	99.9

Population and Area Coverage in Landkreis Kitzingen (Stroke Units) (Bayern) — Regular (+20% Speed (Emergency))

Category	Grid Cells	Area (%)	Population	Population (%)
High (30+ min)	0	0.0	0	0.0
Medium (20-30 min)	12	1.4	161	0.2
Low (10-20 min)	234	27.7	20529	22.5
Only CT reachable within 60 min	0	0.0	0	0.0
Neither reachable within 60 min	0	0.0	0	0.0
Likely irrelevant (<10 min)	598	70.9	70739	77.4

Population and Area Coverage in Landkreis Günzburg (Stroke Units) (Bayern) — Regular (+20% Speed (Emergency))

Category	Grid Cells	Area (%)	Population	Population (%)
High (30+ min)	24	2.6	7893	6.3
Medium (20-30 min)	160	17.3	15060	12.0
Low (10-20 min)	330	35.8	39555	31.6
Only CT reachable within 60 min	0	0.0	0	0.0
Neither reachable within 60 min	0	0.0	0	0.0
Likely irrelevant (<10 min)	409	44.3	62611	50.0

Population and Area Coverage in Landkreis Havelland (Stroke Units) (Brandenburg) — Regular (+20% Speed (Emergency))

Category	Grid Cells	Area (%)	Population	Population (%)
High (30+ min)	474	20.8	31808	21.0
Medium (20-30 min)	584	25.6	21864	14.4
Low (10-20 min)	408	17.9	20606	13.6
Only CT reachable within 60 min	340	14.9	10169	6.7
Neither reachable within 60 min	0	0.0	0	0.0
Likely irrelevant (<10 min)	476	20.9	66966	44.2

Population and Area Coverage in Landkreis Teltow-Fläming (Stroke Units) (Brandenburg) — Regular (+20% Speed (Emergency))

Category	Grid Cells	Area (%)	Population	Population (%)
High (30+ min)	335	12.1	31839	20.3
Medium (20-30 min)	697	25.2	23104	14.7
Low (10-20 min)	1416	51.2	66000	42.0
Only CT reachable within 60 min	92	3.3	516	0.3
Neither reachable within 60 min	0	0.0	0	0.0
Likely irrelevant (<10 min)	225	8.1	35611	22.7

Population and Area Coverage in Landkreis Börde (Stroke Units) (Sachsen-Anhalt) — Regular (+20% Speed (Emergency))

Category	Grid Cells	Area (%)	Population	Population (%)
High (30+ min)	103	3.3	4818	3.1
Medium (20-30 min)	561	18.0	30959	19.9
Low (10-20 min)	1160	37.1	42788	27.5
Only CT reachable within 60 min	0	0.0	0	0.0
Neither reachable within 60 min	0	0.0	0	0.0
Likely irrelevant (<10 min)	1299	41.6	76818	49.4

Population and Area Coverage in Landkreis Stade (Stroke Units) (Niedersachsen) — Regular (+20% Speed (Emergency))

Category	Grid Cells	Area (%)	Population	Population (%)
High (30+ min)	11	0.7	550	0.3
Medium (20-30 min)	71	4.3	17820	9.0
Low (10-20 min)	337	20.5	42465	21.4
Only CT reachable within 60 min	7	0.4	147	0.1
Neither reachable within 60 min	96	5.9	4777	2.4
Likely irrelevant (<10 min)	1119	68.2	132407	66.8

Population and Area Coverage in Kreisfreie Stadt Oldenburg (Oldb) (Stroke Units) (Niedersachsen) — Regular (+20% Speed (Emergency))

Category	Grid Cells	Area (%)	Population	Population (%)
High (30+ min)	0	0.0	0	0.0
Medium (20-30 min)	0	0.0	0	0.0
Low (10-20 min)	4	2.9	6239	3.9
Only CT reachable within 60 min	0	0.0	0	0.0
Neither reachable within 60 min	0	0.0	0	0.0
Likely irrelevant (<10 min)	133	97.1	153709	96.1

Population and Area Coverage in Landkreis Cloppenburg (Stroke Units) (Niedersachsen) — Regular (+20% Speed (Emergency))

Category	Grid Cells	Area (%)	Population	Population (%)
High (30+ min)	67	3.5	10599	6.5
Medium (20-30 min)	455	23.9	53197	32.4
Low (10-20 min)	970	51.0	73236	44.6
Only CT reachable within 60 min	0	0.0	0	0.0
Neither reachable within 60 min	0	0.0	0	0.0
Likely irrelevant (<10 min)	410	21.6	27044	16.5

Population and Area Coverage in Kreisfreie Stadt Wuppertal (Stroke Units) (Nordrhein-Westfalen) — Regular (+20% Speed (Emergency))

Category	Grid Cells	Area (%)	Population	Population (%)
High (30+ min)	0	0.0	0	0.0
Medium (20-30 min)	0	0.0	0	0.0
Low (10-20 min)	16	7.5	15423	4.3
Only CT reachable within 60 min	0	0.0	0	0.0
Neither reachable within 60 min	0	0.0	0	0.0
Likely irrelevant (<10 min)	197	92.5	343650	95.7

Population and Area Coverage in Landkreis Odenwaldkreis (Stroke Units) (Hessen) — Regular (+20% Speed (Emergency))

Category	Grid Cells	Area (%)	Population	Population (%)
High (30+ min)	0	0.0	0	0.0
Medium (20-30 min)	2	0.3	14	0.0
Low (10-20 min)	210	27.1	35042	35.8
Only CT reachable within 60 min	0	0.0	0	0.0
Neither reachable within 60 min	0	0.0	0	0.0
Likely irrelevant (<10 min)	564	72.7	62834	64.2

Population and Area Coverage in Landkreis Karlsruhe (Stroke Units) (Baden-Württemberg) — Regular (+20% Speed (Emergency))

Category	Grid Cells	Area (%)	Population	Population (%)
High (30+ min)	0	0.0	0	0.0
Medium (20-30 min)	6	0.4	10310	2.4
Low (10-20 min)	193	14.5	51737	11.9
Only CT reachable within 60 min	0	0.0	0	0.0
Neither reachable within 60 min	0	0.0	0	0.0
Likely irrelevant (<10 min)	1136	85.1	373788	85.8

Population and Area Coverage in Kreisfreie Stadt Landshut (Stroke Units) (Bayern) — Regular (+20% Speed (Emergency))

Category	Grid Cells	Area (%)	Population	Population (%)
High (30+ min)	0	0.0	0	0.0
Medium (20-30 min)	0	0.0	0	0.0
Low (10-20 min)	5	6.2	2218	3.2
Only CT reachable within 60 min	0	0.0	0	0.0
Neither reachable within 60 min	0	0.0	0	0.0
Likely irrelevant (<10 min)	76	93.8	66840	96.8

Population and Area Coverage in Kreisfreie Stadt Erlangen (Stroke Units) (Bayern) — Regular (+20% Speed (Emergency))

Category	Grid Cells	Area (%)	Population	Population (%)
High (30+ min)	0	0.0	0	0.0
Medium (20-30 min)	0	0.0	0	0.0
Low (10-20 min)	3	3.1	5117	4.0
Only CT reachable within 60 min	0	0.0	0	0.0
Neither reachable within 60 min	0	0.0	0	0.0
Likely irrelevant (<10 min)	93	96.9	121512	96.0

Population and Area Coverage in Landkreis Haßberge (Stroke Units) (Bayern) — Regular (+20% Speed (Emergency))

Category	Grid Cells	Area (%)	Population	Population (%)
High (30+ min)	0	0.0	0	0.0
Medium (20-30 min)	309	25.8	24910	28.6
Low (10-20 min)	729	60.8	52477	60.2
Only CT reachable within 60 min	0	0.0	0	0.0
Neither reachable within 60 min	0	0.0	0	0.0
Likely irrelevant (<10 min)	162	13.5	9779	11.2

Population and Area Coverage in Landkreis Aichach-Friedberg (Stroke Units) (Bayern) — Regular (+20% Speed (Emergency))

Category	Grid Cells	Area (%)	Population	Population (%)
High (30+ min)	0	0.0	0	0.0
Medium (20-30 min)	78	8.3	14315	10.7
Low (10-20 min)	468	49.5	73283	54.9
Only CT reachable within 60 min	0	0.0	0	0.0
Neither reachable within 60 min	0	0.0	0	0.0
Likely irrelevant (<10 min)	399	42.2	45813	34.3

Population and Area Coverage in Landkreis Dillingen a.d.Donau (Stroke Units) (Bayern) — Regular (+20% Speed (Emergency))

Category	Grid Cells	Area (%)	Population	Population (%)
High (30+ min)	11	1.1	689	0.7
Medium (20-30 min)	237	24.4	29448	30.3
Low (10-20 min)	461	47.5	43822	45.1
Only CT reachable within 60 min	0	0.0	0	0.0
Neither reachable within 60 min	0	0.0	0	0.0
Likely irrelevant (<10 min)	261	26.9	23139	23.8

Population and Area Coverage in Landkreis Oberspreewald-Lausitz (Stroke Units) (Brandenburg) — Regular (+20% Speed (Emergency))

Category	Grid Cells	Area (%)	Population	Population (%)
High (30+ min)	1	0.1	0	0.0
Medium (20-30 min)	34	2.2	8556	7.9
Low (10-20 min)	255	16.1	13271	12.2
Only CT reachable within 60 min	0	0.0	0	0.0
Neither reachable within 60 min	0	0.0	0	0.0
Likely irrelevant (<10 min)	1290	81.6	86631	79.9

Population and Area Coverage in Landkreis Bautzen (Stroke Units) (Sachsen) — Regular (+20% Speed (Emergency))

Category	Grid Cells	Area (%)	Population	Population (%)
High (30+ min)	184	6.0	31811	11.0
Medium (20-30 min)	918	29.8	126099	43.8
Low (10-20 min)	849	27.5	50684	17.6
Only CT reachable within 60 min	3	0.1	121	0.0
Neither reachable within 60 min	5	0.2	89	0.0
Likely irrelevant (<10 min)	1123	36.4	79219	27.5

Population and Area Coverage in Kreisfreie Stadt Dessau-Roßlau (Stroke Units) (Sachsen-Anhalt) — Regular (+20% Speed (Emergency))

Category	Grid Cells	Area (%)	Population	Population (%)
High (30+ min)	153	47.8	44655	53.1
Medium (20-30 min)	119	37.2	36304	43.2
Low (10-20 min)	48	15.0	3153	3.7
Only CT reachable within 60 min	0	0.0	0	0.0
Neither reachable within 60 min	0	0.0	0	0.0
Likely irrelevant (<10 min)	0	0.0	1	0.0

Population and Area Coverage in Landkreis Lüneburg (Stroke Units) (Niedersachsen) — Regular (+20% Speed (Emergency))

Category	Grid Cells	Area (%)	Population	Population (%)
High (30+ min)	78	4.4	1923	1.1
Medium (20-30 min)	138	7.8	2626	1.5
Low (10-20 min)	401	22.5	17435	9.8
Only CT reachable within 60 min	133	7.5	1417	0.8
Neither reachable within 60 min	0	0.0	0	0.0
Likely irrelevant (<10 min)	1030	57.9	154867	86.9

Population and Area Coverage in Kreisfreie Stadt Bremerhaven (Stroke Units) (Bremen) — Regular (+20% Speed (Emergency))

Category	Grid Cells	Area (%)	Population	Population (%)
High (30+ min)	0	0.0	0	0.0
Medium (20-30 min)	0	0.0	0	0.0
Low (10-20 min)	17	17.3	6901	6.0
Only CT reachable within 60 min	1	1.0	1409	1.2
Neither reachable within 60 min	4	4.1	10	0.0
Likely irrelevant (<10 min)	76	77.6	106096	92.7

Population and Area Coverage in Kreisfreie Stadt Koblenz (Stroke Units) (Rheinland-Pfalz) — Regular (+20% Speed (Emergency))

Category	Grid Cells	Area (%)	Population	Population (%)
High (30+ min)	0	0.0	0	0.0
Medium (20-30 min)	0	0.0	0	0.0
Low (10-20 min)	11	8.1	6546	5.2
Only CT reachable within 60 min	0	0.0	0	0.0
Neither reachable within 60 min	0	0.0	0	0.0
Likely irrelevant (<10 min)	125	91.9	119311	94.8

Population and Area Coverage in Kreisfreie Stadt Pirmasens (Stroke Units) (Rheinland-Pfalz) — Regular (+20% Speed (Emergency))

Category	Grid Cells	Area (%)	Population	Population (%)
High (30+ min)	5	6.6	1019	2.7
Medium (20-30 min)	62	81.6	34204	91.8
Low (10-20 min)	9	11.8	2038	5.5
Only CT reachable within 60 min	0	0.0	0	0.0
Neither reachable within 60 min	0	0.0	0	0.0
Likely irrelevant (<10 min)	0	0.0	1	0.0

Population and Area Coverage in Landkreis Donnersbergkreis (Stroke Units) (Rheinland-Pfalz) — Regular (+20% Speed (Emergency))

Category	Grid Cells	Area (%)	Population	Population (%)
High (30+ min)	9	1.1	1292	1.7
Medium (20-30 min)	202	25.0	18558	23.9
Low (10-20 min)	407	50.4	42751	55.2
Only CT reachable within 60 min	0	0.0	0	0.0
Neither reachable within 60 min	0	0.0	0	0.0
Likely irrelevant (<10 min)	189	23.4	14916	19.2

Population and Area Coverage in Stadtkreis Heidelberg (Stroke Units) (Baden-Württemberg) — Regular (+20% Speed (Emergency))

Category	Grid Cells	Area (%)	Population	Population (%)
High (30+ min)	0	0.0	0	0.0
Medium (20-30 min)	0	0.0	0	0.0
Low (10-20 min)	46	34.3	33954	22.0
Only CT reachable within 60 min	0	0.0	0	0.0
Neither reachable within 60 min	0	0.0	0	0.0
Likely irrelevant (<10 min)	88	65.7	120412	78.0

Population and Area Coverage in Kreisfreie Stadt Straubing (Stroke Units) (Bayern) — Regular (+20% Speed (Emergency))

Category	Grid Cells	Area (%)	Population	Population (%)
High (30+ min)	0	0.0	0	0.0
Medium (20-30 min)	0	0.0	0	0.0
Low (10-20 min)	0	0.0	0	0.0
Only CT reachable within 60 min	0	0.0	0	0.0
Neither reachable within 60 min	0	0.0	0	0.0
Likely irrelevant (<10 min)	85	100.0	46942	100.0

Population and Area Coverage in Landkreis Straubing-Bogen (Stroke Units) (Bayern) — Regular (+20% Speed (Emergency))

Category	Grid Cells	Area (%)	Population	Population (%)
High (30+ min)	0	0.0	0	0.0
Medium (20-30 min)	65	4.4	5805	5.8
Low (10-20 min)	472	32.2	30971	30.8
Only CT reachable within 60 min	0	0.0	0	0.0
Neither reachable within 60 min	0	0.0	0	0.0
Likely irrelevant (<10 min)	929	63.4	63746	63.4

Population and Area Coverage in Landkreis Neumarkt i.d.OPf. (Stroke Units) (Bayern) — Regular (+20% Speed (Emergency))

Category	Grid Cells	Area (%)	Population	Population (%)
High (30+ min)	0	0.0	0	0.0
Medium (20-30 min)	0	0.0	0	0.0
Low (10-20 min)	133	8.0	6912	5.2
Only CT reachable within 60 min	0	0.0	0	0.0
Neither reachable within 60 min	0	0.0	0	0.0
Likely irrelevant (<10 min)	1531	92.0	125555	94.8

Population and Area Coverage in Kreisfreie Stadt Schwabach (Stroke Units) (Bayern) — Regular (+20% Speed (Emergency))

Category	Grid Cells	Area (%)	Population	Population (%)
High (30+ min)	0	0.0	0	0.0
Medium (20-30 min)	0	0.0	0	0.0
Low (10-20 min)	0	0.0	0	0.0
Only CT reachable within 60 min	0	0.0	0	0.0
Neither reachable within 60 min	0	0.0	0	0.0
Likely irrelevant (<10 min)	52	100.0	42132	100.0

Population and Area Coverage in Kreisfreie Stadt Brandenburg an der Havel (Stroke Units) (Brandenburg) — Regular (+20% Speed (Emergency))

Category	Grid Cells	Area (%)	Population	Population (%)
High (30+ min)	207	68.1	64462	93.3
Medium (20-30 min)	70	23.0	4041	5.8
Low (10-20 min)	27	8.9	587	0.8
Only CT reachable within 60 min	0	0.0	1	0.0
Neither reachable within 60 min	0	0.0	0	0.0
Likely irrelevant (<10 min)	0	0.0	1	0.0

Population and Area Coverage in Landkreis Ludwigslust-Parchim (Stroke Units) (Mecklenburg-Vorpommern) — Regular (+20% Speed (Emergency))

Category	Grid Cells	Area (%)	Population	Population (%)
High (30+ min)	1130	17.5	54680	27.9
Medium (20-30 min)	1674	25.9	37045	18.9
Low (10-20 min)	1813	28.1	45290	23.1
Only CT reachable within 60 min	196	3.0	1924	1.0
Neither reachable within 60 min	0	0.0	0	0.0
Likely irrelevant (<10 min)	1642	25.4	57077	29.1

Population and Area Coverage in Landkreis Saalfeld-Rudolstadt (Stroke Units) (Thüringen) — Regular (+20% Speed (Emergency))

Category	Grid Cells	Area (%)	Population	Population (%)
High (30+ min)	0	0.0	0	0.0
Medium (20-30 min)	6	0.5	26	0.0
Low (10-20 min)	625	48.7	37071	38.3
Only CT reachable within 60 min	0	0.0	0	0.0
Neither reachable within 60 min	0	0.0	0	0.0
Likely irrelevant (<10 min)	652	50.8	59723	61.7

Population and Area Coverage in Kreis Ostholstein (Stroke Units) (Schleswig-Holstein) — Regular (+20% Speed (Emergency))

Category	Grid Cells	Area (%)	Population	Population (%)
High (30+ min)	9	0.5	603	0.3
Medium (20-30 min)	124	6.7	19897	10.1
Low (10-20 min)	448	24.1	61580	31.4
Only CT reachable within 60 min	22	1.2	1108	0.6
Neither reachable within 60 min	60	3.2	2612	1.3
Likely irrelevant (<10 min)	1198	64.4	110601	56.3

Population and Area Coverage in Kreis Rendsburg-Eckernförde (Stroke Units) (Schleswig-Holstein) — Regular (+20% Speed (Emergency))

Category	Grid Cells	Area (%)	Population	Population (%)
High (30+ min)	33	1.1	1523	0.6
Medium (20-30 min)	147	4.9	13750	5.0
Low (10-20 min)	261	8.7	26032	9.5
Only CT reachable within 60 min	3	0.1	152	0.1
Neither reachable within 60 min	31	1.0	3263	1.2
Likely irrelevant (<10 min)	2513	84.1	229832	83.7

Population and Area Coverage in Landkreis Ostalbkreis (Stroke Units) (Baden-Württemberg) — Regular (+20% Speed (Emergency))

Category	Grid Cells	Area (%)	Population	Population (%)
High (30+ min)	0	0.0	0	0.0
Medium (20-30 min)	202	10.9	59955	19.3
Low (10-20 min)	618	33.3	90395	29.0
Only CT reachable within 60 min	0	0.0	0	0.0
Neither reachable within 60 min	0	0.0	0	0.0
Likely irrelevant (<10 min)	1035	55.8	160981	51.7

Population and Area Coverage in Landkreis Emmendingen (Stroke Units) (Baden-Württemberg) — Regular (+20% Speed (Emergency))

Category	Grid Cells	Area (%)	Population	Population (%)
High (30+ min)	82	10.0	7657	4.7
Medium (20-30 min)	142	17.4	17626	10.9
Low (10-20 min)	320	39.1	67972	42.0
Only CT reachable within 60 min	1	0.1	64	0.0
Neither reachable within 60 min	0	0.0	5	0.0
Likely irrelevant (<10 min)	273	33.4	68560	42.4

Population and Area Coverage in Kreisfreie Stadt München (Stroke Units) (Bayern) — Regular (+20% Speed (Emergency))

Category	Grid Cells	Area (%)	Population	Population (%)
High (30+ min)	0	0.0	0	0.0
Medium (20-30 min)	0	0.0	0	0.0
Low (10-20 min)	62	16.4	323476	19.4
Only CT reachable within 60 min	0	0.0	0	0.0
Neither reachable within 60 min	0	0.0	0	0.0
Likely irrelevant (<10 min)	317	83.6	1347679	80.6

Population and Area Coverage in Landkreis Bayreuth (Stroke Units) (Bayern) — Regular (+20% Speed (Emergency))

Category	Grid Cells	Area (%)	Population	Population (%)
High (30+ min)	0	0.0	0	0.0
Medium (20-30 min)	68	4.3	9999	9.2
Low (10-20 min)	632	39.6	33300	30.6
Only CT reachable within 60 min	0	0.0	0	0.0
Neither reachable within 60 min	0	0.0	0	0.0
Likely irrelevant (<10 min)	896	56.1	65611	60.2

Population and Area Coverage in Landkreis Kronach (Stroke Units) (Bayern) — Regular (+20% Speed (Emergency))

Category	Grid Cells	Area (%)	Population	Population (%)
High (30+ min)	1	0.1	143	0.2
Medium (20-30 min)	108	13.3	16991	23.9
Low (10-20 min)	384	47.3	29970	42.2
Only CT reachable within 60 min	0	0.0	0	0.0
Neither reachable within 60 min	0	0.0	0	0.0
Likely irrelevant (<10 min)	319	39.3	23975	33.7

Population and Area Coverage in Landkreis Aschaffenburg (Stroke Units) (Bayern) — Regular (+20% Speed (Emergency))

Category	Grid Cells	Area (%)	Population	Population (%)
High (30+ min)	0	0.0	0	0.0
Medium (20-30 min)	0	0.0	0	0.0
Low (10-20 min)	10	1.1	988	0.5
Only CT reachable within 60 min	0	0.0	0	0.0
Neither reachable within 60 min	0	0.0	0	0.0
Likely irrelevant (<10 min)	864	98.9	178861	99.5

Population and Area Coverage in Landkreis Saalekreis (Stroke Units) (Sachsen-Anhalt) — Regular (+20% Speed (Emergency))

Category	Grid Cells	Area (%)	Population	Population (%)
High (30+ min)	0	0.0	0	0.0
Medium (20-30 min)	6	0.3	91	0.1
Low (10-20 min)	140	7.5	4636	2.7
Only CT reachable within 60 min	0	0.0	0	0.0
Neither reachable within 60 min	0	0.0	0	0.0
Likely irrelevant (<10 min)	1722	92.2	164208	97.2

Population and Area Coverage in Kreisfreie Stadt Wolfsburg (Stroke Units) (Niedersachsen) — Regular (+20% Speed (Emergency))

Category	Grid Cells	Area (%)	Population	Population (%)
High (30+ min)	0	0.0	0	0.0
Medium (20-30 min)	0	0.0	0	0.0
Low (10-20 min)	0	0.0	0	0.0
Only CT reachable within 60 min	0	0.0	0	0.0
Neither reachable within 60 min	0	0.0	0	0.0
Likely irrelevant (<10 min)	274	100.0	122066	100.0

Population and Area Coverage in Landkreis Wolfenbüttel (Stroke Units) (Niedersachsen) — Regular (+20% Speed (Emergency))

Category	Grid Cells	Area (%)	Population	Population (%)
High (30+ min)	0	0.0	0	0.0
Medium (20-30 min)	29	3.0	1594	1.3
Low (10-20 min)	451	47.1	77157	64.4
Only CT reachable within 60 min	0	0.0	0	0.0
Neither reachable within 60 min	0	0.0	0	0.0
Likely irrelevant (<10 min)	477	49.8	41043	34.3

Population and Area Coverage in Landkreis Ammerland (Stroke Units) (Niedersachsen) — Regular (+20% Speed (Emergency))

Category	Grid Cells	Area (%)	Population	Population (%)
High (30+ min)	0	0.0	0	0.0
Medium (20-30 min)	0	0.0	0	0.0
Low (10-20 min)	30	3.1	1293	1.1
Only CT reachable within 60 min	0	0.0	0	0.0
Neither reachable within 60 min	0	0.0	0	0.0
Likely irrelevant (<10 min)	949	96.9	120169	98.9

Population and Area Coverage in Kreis Rhein-Kreis Neuss (Stroke Units) (Nordrhein-Westfalen) — Regular (+20% Speed (Emergency))

Category	Grid Cells	Area (%)	Population	Population (%)
High (30+ min)	0	0.0	0	0.0
Medium (20-30 min)	0	0.0	0	0.0
Low (10-20 min)	188	25.5	95606	20.3
Only CT reachable within 60 min	0	0.0	0	0.0
Neither reachable within 60 min	0	0.0	0	0.0
Likely irrelevant (<10 min)	548	74.5	375238	79.7

Population and Area Coverage in Kreis Lippe (Stroke Units) (Nordrhein-Westfalen) — Regular (+20% Speed (Emergency))

Category	Grid Cells	Area (%)	Population	Population (%)
High (30+ min)	0	0.0	0	0.0
Medium (20-30 min)	35	2.1	23805	6.9
Low (10-20 min)	534	32.7	118514	34.3
Only CT reachable within 60 min	0	0.0	0	0.0
Neither reachable within 60 min	0	0.0	0	0.0
Likely irrelevant (<10 min)	1064	65.2	202840	58.8

Population and Area Coverage in Kreisfreie Stadt Hagen (Stroke Units) (Nordrhein-Westfalen) — Regular (+20% Speed (Emergency))

Category	Grid Cells	Area (%)	Population	Population (%)
High (30+ min)	0	0.0	0	0.0
Medium (20-30 min)	20	9.8	13581	6.8
Low (10-20 min)	144	70.6	149674	75.0
Only CT reachable within 60 min	0	0.0	0	0.0
Neither reachable within 60 min	0	0.0	0	0.0
Likely irrelevant (<10 min)	40	19.6	36404	18.2

Population and Area Coverage in Landkreis Alzey-Worms (Stroke Units) (Rheinland-Pfalz) — Regular (+20% Speed (Emergency))

Category	Grid Cells	Area (%)	Population	Population (%)
High (30+ min)	0	0.0	0	0.0
Medium (20-30 min)	3	0.4	190	0.1
Low (10-20 min)	313	42.5	48998	37.9
Only CT reachable within 60 min	0	0.0	0	0.0
Neither reachable within 60 min	0	0.0	0	0.0
Likely irrelevant (<10 min)	421	57.1	80206	62.0

Population and Area Coverage in Landkreis Germersheim (Stroke Units) (Rheinland-Pfalz) — Regular (+20% Speed (Emergency))

Category	Grid Cells	Area (%)	Population	Population (%)
High (30+ min)	0	0.0	0	0.0
Medium (20-30 min)	0	0.0	0	0.0
Low (10-20 min)	83	14.6	32164	25.0
Only CT reachable within 60 min	0	0.0	0	0.0
Neither reachable within 60 min	0	0.0	0	0.0
Likely irrelevant (<10 min)	487	85.4	96558	75.0

Population and Area Coverage in Landkreis Neckar-Odenwald-Kreis (Stroke Units) (Baden-Württemberg) — Regular (+20% Speed (Emergency))

Category	Grid Cells	Area (%)	Population	Population (%)
High (30+ min)	345	24.7	51686	36.1
Medium (20-30 min)	682	48.8	63381	44.2
Low (10-20 min)	268	19.2	24741	17.3
Only CT reachable within 60 min	0	0.0	0	0.0
Neither reachable within 60 min	0	0.0	0	0.0
Likely irrelevant (<10 min)	102	7.3	3562	2.5

Population and Area Coverage in Landkreis Enzkreis (Stroke Units) (Baden-Württemberg) — Regular (+20% Speed (Emergency))

Category	Grid Cells	Area (%)	Population	Population (%)
High (30+ min)	0	0.0	1	0.0
Medium (20-30 min)	60	8.5	14956	7.6
Low (10-20 min)	293	41.4	74815	38.2
Only CT reachable within 60 min	0	0.0	0	0.0
Neither reachable within 60 min	0	0.0	0	0.0
Likely irrelevant (<10 min)	355	50.1	106164	54.2

Population and Area Coverage in Landkreis Berchtesgadener Land (Stroke Units) (Bayern) — Regular (+20% Speed (Emergency))

Category	Grid Cells	Area (%)	Population	Population (%)
High (30+ min)	0	0.0	0	0.0
Medium (20-30 min)	121	13.0	27571	29.4
Low (10-20 min)	122	13.1	22997	24.6
Only CT reachable within 60 min	3	0.3	58	0.1
Neither reachable within 60 min	326	35.1	1749	1.9
Likely irrelevant (<10 min)	357	38.4	41260	44.1

Population and Area Coverage in Kreisfreie Stadt Hof (Stroke Units) (Bayern) — Regular (+20% Speed (Emergency))

Category	Grid Cells	Area (%)	Population	Population (%)
High (30+ min)	0	0.0	0	0.0
Medium (20-30 min)	0	0.0	0	0.0
Low (10-20 min)	0	0.0	0	0.0
Only CT reachable within 60 min	0	0.0	0	0.0
Neither reachable within 60 min	0	0.0	0	0.0
Likely irrelevant (<10 min)	75	100.0	44749	100.0

Population and Area Coverage in Landkreis Kulmbach (Stroke Units) (Bayern) — Regular (+20% Speed (Emergency))

Category	Grid Cells	Area (%)	Population	Population (%)
High (30+ min)	0	0.0	0	0.0
Medium (20-30 min)	0	0.0	0	0.0
Low (10-20 min)	202	24.4	8149	10.8
Only CT reachable within 60 min	0	0.0	0	0.0
Neither reachable within 60 min	0	0.0	0	0.0
Likely irrelevant (<10 min)	626	75.6	67529	89.2

Population and Area Coverage in Kreisfreie Stadt Memmingen (Stroke Units) (Bayern) — Regular (+20% Speed (Emergency))

Category	Grid Cells	Area (%)	Population	Population (%)
High (30+ min)	0	0.0	0	0.0
Medium (20-30 min)	0	0.0	0	0.0
Low (10-20 min)	0	0.0	0	0.0
Only CT reachable within 60 min	0	0.0	0	0.0
Neither reachable within 60 min	0	0.0	0	0.0
Likely irrelevant (<10 min)	84	100.0	43302	100.0

Population and Area Coverage in Landkreis Augsburg (Stroke Units) (Bayern) — Regular (+20% Speed (Emergency))

Category	Grid Cells	Area (%)	Population	Population (%)
High (30+ min)	0	0.0	0	0.0
Medium (20-30 min)	180	13.9	24957	10.1
Low (10-20 min)	286	22.0	68653	27.7
Only CT reachable within 60 min	0	0.0	0	0.0
Neither reachable within 60 min	0	0.0	0	0.0
Likely irrelevant (<10 min)	833	64.1	154655	62.3

Population and Area Coverage in Landkreis Zwickau (Stroke Units) (Sachsen) — Regular (+20% Speed (Emergency))

Category	Grid Cells	Area (%)	Population	Population (%)
High (30+ min)	0	0.0	0	0.0
Medium (20-30 min)	28	2.3	5351	1.7
Low (10-20 min)	460	37.7	90408	28.5
Only CT reachable within 60 min	0	0.0	0	0.0
Neither reachable within 60 min	0	0.0	0	0.0
Likely irrelevant (<10 min)	733	60.0	221458	69.8

Population and Area Coverage in Landkreis Vorpommern-Rügen (Stroke Units) (Mecklenburg-Vorpommern) — Regular (+20% Speed (Emergency))

Category	Grid Cells	Area (%)	Population	Population (%)
High (30+ min)	338	8.1	19169	9.6
Medium (20-30 min)	479	11.5	16933	8.5
Low (10-20 min)	1202	28.9	35457	17.8
Only CT reachable within 60 min	126	3.0	2935	1.5
Neither reachable within 60 min	257	6.2	5653	2.8
Likely irrelevant (<10 min)	1761	42.3	119122	59.8

Population and Area Coverage in Landkreis Görlitz (Stroke Units) (Sachsen) — Regular (+20% Speed (Emergency))

Category	Grid Cells	Area (%)	Population	Population (%)
High (30+ min)	364	14.1	80943	37.0
Medium (20-30 min)	503	19.4	32630	14.9
Low (10-20 min)	390	15.1	23456	10.7
Only CT reachable within 60 min	5	0.2	39	0.0
Neither reachable within 60 min	10	0.4	206	0.1
Likely irrelevant (<10 min)	1315	50.8	81687	37.3

Population and Area Coverage in Landkreis Sonneberg (Stroke Units) (Thüringen) — Regular (+20% Speed (Emergency))

Category	Grid Cells	Area (%)	Population	Population (%)
High (30+ min)	0	0.0	0	0.0
Medium (20-30 min)	0	0.0	0	0.0
Low (10-20 min)	34	5.8	1902	3.5
Only CT reachable within 60 min	0	0.0	0	0.0
Neither reachable within 60 min	0	0.0	0	0.0
Likely irrelevant (<10 min)	555	94.2	51766	96.5

Population and Area Coverage in Kreisfreie Stadt Köln (Stroke Units) (Nordrhein-Westfalen) — Regular (+20% Speed (Emergency))

Category	Grid Cells	Area (%)	Population	Population (%)
High (30+ min)	0	0.0	0	0.0
Medium (20-30 min)	0	0.0	0	0.0
Low (10-20 min)	96	18.2	304415	25.1
Only CT reachable within 60 min	0	0.0	0	0.0
Neither reachable within 60 min	0	0.0	0	0.0
Likely irrelevant (<10 min)	431	81.8	910769	74.9

Population and Area Coverage in Kreis Heinsberg (Stroke Units) (Nordrhein-Westfalen) — Regular (+20% Speed (Emergency))

Category	Grid Cells	Area (%)	Population	Population (%)
High (30+ min)	0	0.0	410	0.2
Medium (20-30 min)	11	1.4	2893	1.2
Low (10-20 min)	205	26.3	79529	32.0
Only CT reachable within 60 min	1	0.1	68	0.0
Neither reachable within 60 min	2	0.3	263	0.1
Likely irrelevant (<10 min)	559	71.9	165050	66.5

Population and Area Coverage in Landkreis Bergstraße (Stroke Units) (Hessen) — Regular (+20% Speed (Emergency))

Category	Grid Cells	Area (%)	Population	Population (%)
High (30+ min)	0	0.0	0	0.0
Medium (20-30 min)	10	1.1	2206	0.8
Low (10-20 min)	244	27.2	91710	34.5
Only CT reachable within 60 min	0	0.0	0	0.0
Neither reachable within 60 min	0	0.0	0	0.0
Likely irrelevant (<10 min)	643	71.7	172141	64.7

Population and Area Coverage in Landkreis Wetteraukreis (Stroke Units) (Hessen) — Regular (+20% Speed (Emergency))

Category	Grid Cells	Area (%)	Population	Population (%)
High (30+ min)	0	0.0	0	0.0
Medium (20-30 min)	13	0.9	5972	2.0
Low (10-20 min)	425	30.5	103856	34.8
Only CT reachable within 60 min	0	0.0	0	0.0
Neither reachable within 60 min	0	0.0	0	0.0
Likely irrelevant (<10 min)	957	68.6	189035	63.3

Population and Area Coverage in Kreisfreie Stadt Landau in der Pfalz (Stroke Units) (Rheinland-Pfalz) — Regular (+20% Speed (Emergency))

Category	Grid Cells	Area (%)	Population	Population (%)
High (30+ min)	0	0.0	0	0.0
Medium (20-30 min)	0	0.0	0	0.0
Low (10-20 min)	63	61.8	40541	77.1
Only CT reachable within 60 min	0	0.0	0	0.0
Neither reachable within 60 min	0	0.0	0	0.0
Likely irrelevant (<10 min)	39	38.2	12055	22.9

Population and Area Coverage in Stadtkreis Stuttgart (Stroke Units) (Baden-Württemberg) — Regular (+20% Speed (Emergency))

Category	Grid Cells	Area (%)	Population	Population (%)
High (30+ min)	0	0.0	0	0.0
Medium (20-30 min)	0	0.0	0	0.0
Low (10-20 min)	21	8.2	41113	6.4
Only CT reachable within 60 min	0	0.0	0	0.0
Neither reachable within 60 min	0	0.0	0	0.0
Likely irrelevant (<10 min)	234	91.8	598833	93.6

Population and Area Coverage in Landkreis Konstanz (Stroke Units) (Baden-Württemberg) — Regular (+20% Speed (Emergency))

Category	Grid Cells	Area (%)	Population	Population (%)
High (30+ min)	8	0.8	25977	10.1
Medium (20-30 min)	56	5.9	36935	14.4
Low (10-20 min)	268	28.2	40391	15.8
Only CT reachable within 60 min	2	0.2	17	0.0
Neither reachable within 60 min	6	0.6	1163	0.5
Likely irrelevant (<10 min)	611	64.2	151670	59.2

Population and Area Coverage in Landkreis Sigmaringen (Stroke Units) (Baden-Württemberg) — Regular (+20% Speed (Emergency))

Category	Grid Cells	Area (%)	Population	Population (%)
High (30+ min)	0	0.0	0	0.0
Medium (20-30 min)	6	0.4	110	0.1
Low (10-20 min)	169	11.7	10286	8.0
Only CT reachable within 60 min	0	0.0	0	0.0
Neither reachable within 60 min	0	0.0	0	0.0
Likely irrelevant (<10 min)	1274	87.9	118262	91.9

Population and Area Coverage in Landkreis Kelheim (Stroke Units) (Bayern) — Regular (+20% Speed (Emergency))

Category	Grid Cells	Area (%)	Population	Population (%)
High (30+ min)	3	0.2	383	0.3
Medium (20-30 min)	416	32.1	46596	39.1
Low (10-20 min)	515	39.7	47273	39.7
Only CT reachable within 60 min	0	0.0	0	0.0
Neither reachable within 60 min	0	0.0	0	0.0
Likely irrelevant (<10 min)	363	28.0	24895	20.9

Population and Area Coverage in Kreisfreie Stadt Schweinfurt (Stroke Units) (Bayern) — Regular (+20% Speed (Emergency))

Category	Grid Cells	Area (%)	Population	Population (%)
High (30+ min)	0	0.0	0	0.0
Medium (20-30 min)	0	0.0	0	0.0
Low (10-20 min)	0	0.0	0	0.0
Only CT reachable within 60 min	0	0.0	0	0.0
Neither reachable within 60 min	0	0.0	0	0.0
Likely irrelevant (<10 min)	46	100.0	53903	100.0

Population and Area Coverage in Landkreis Neu-Ulm (Stroke Units) (Bayern) — Regular (+20% Speed (Emergency))

Category	Grid Cells	Area (%)	Population	Population (%)
High (30+ min)	0	0.0	0	0.0
Medium (20-30 min)	65	10.4	14981	8.6
Low (10-20 min)	346	55.4	104079	60.1
Only CT reachable within 60 min	0	0.0	0	0.0
Neither reachable within 60 min	0	0.0	0	0.0
Likely irrelevant (<10 min)	213	34.1	54167	31.3

Population and Area Coverage in Landkreis Oberhavel (Stroke Units) (Brandenburg) — Regular (+20% Speed (Emergency))

Category	Grid Cells	Area (%)	Population	Population (%)
High (30+ min)	425	17.6	19839	9.9
Medium (20-30 min)	541	22.4	14220	7.1
Low (10-20 min)	754	31.2	71838	35.8
Only CT reachable within 60 min	108	4.5	473	0.2
Neither reachable within 60 min	0	0.0	0	0.0
Likely irrelevant (<10 min)	586	24.3	94246	47.0

Population and Area Coverage in Landkreis Spree-Neiße (Stroke Units) (Brandenburg) — Regular (+20% Speed (Emergency))

Category	Grid Cells	Area (%)	Population	Population (%)
High (30+ min)	60	2.9	13933	13.9
Medium (20-30 min)	317	15.2	14923	14.9
Low (10-20 min)	531	25.4	24016	23.9
Only CT reachable within 60 min	2	0.1	0	0.0
Neither reachable within 60 min	0	0.0	0	0.0
Likely irrelevant (<10 min)	1178	56.4	47525	47.3

Population and Area Coverage in Landkreis Ilm-Kreis (Stroke Units) (Thüringen) — Regular (+20% Speed (Emergency))

Category	Grid Cells	Area (%)	Population	Population (%)
High (30+ min)	0	0.0	0	0.0
Medium (20-30 min)	26	2.5	11622	12.0
Low (10-20 min)	342	33.3	40465	41.9
Only CT reachable within 60 min	0	0.0	0	0.0
Neither reachable within 60 min	0	0.0	0	0.0
Likely irrelevant (<10 min)	658	64.1	44414	46.0

Population and Area Coverage in Landkreis Greiz (Stroke Units) (Thüringen) — Regular (+20% Speed (Emergency))

Category	Grid Cells	Area (%)	Population	Population (%)
High (30+ min)	3	0.3	212	0.2
Medium (20-30 min)	65	6.0	17144	18.7
Low (10-20 min)	346	32.0	25211	27.5
Only CT reachable within 60 min	0	0.0	0	0.0
Neither reachable within 60 min	0	0.0	0	0.0
Likely irrelevant (<10 min)	666	61.7	49239	53.6

Population and Area Coverage in Kreisfreie Stadt Bottrop (Stroke Units) (Nordrhein-Westfalen) — Regular (+20% Speed (Emergency))

Category	Grid Cells	Area (%)	Population	Population (%)
High (30+ min)	0	0.0	0	0.0
Medium (20-30 min)	0	0.0	0	0.0
Low (10-20 min)	18	13.5	3544	3.0
Only CT reachable within 60 min	0	0.0	0	0.0
Neither reachable within 60 min	0	0.0	0	0.0
Likely irrelevant (<10 min)	115	86.5	113423	97.0

Population and Area Coverage in Kreisfreie Stadt Speyer (Stroke Units) (Rheinland-Pfalz) — Regular (+20% Speed (Emergency))

Category	Grid Cells	Area (%)	Population	Population (%)
High (30+ min)	0	0.0	0	0.0
Medium (20-30 min)	0	0.0	0	0.0
Low (10-20 min)	0	0.0	0	0.0
Only CT reachable within 60 min	0	0.0	0	0.0
Neither reachable within 60 min	0	0.0	0	0.0
Likely irrelevant (<10 min)	52	100.0	51373	100.0

Population and Area Coverage in Stadtkreis Mannheim (Stroke Units) (Baden-Württemberg) — Regular (+20% Speed (Emergency))

Category	Grid Cells	Area (%)	Population	Population (%)
High (30+ min)	0	0.0	0	0.0
Medium (20-30 min)	0	0.0	0	0.0
Low (10-20 min)	16	9.0	4149	1.2
Only CT reachable within 60 min	0	0.0	0	0.0
Neither reachable within 60 min	0	0.0	0	0.0
Likely irrelevant (<10 min)	162	91.0	342800	98.8

Population and Area Coverage in Landkreis Tuttlingen (Stroke Units) (Baden-Württemberg) — Regular (+20% Speed (Emergency))

Category	Grid Cells	Area (%)	Population	Population (%)
High (30+ min)	0	0.0	0	0.0
Medium (20-30 min)	21	2.4	1627	1.2
Low (10-20 min)	437	49.6	78577	58.2
Only CT reachable within 60 min	0	0.0	0	0.0
Neither reachable within 60 min	0	0.0	0	0.0
Likely irrelevant (<10 min)	423	48.0	54880	40.6

Population and Area Coverage in Landkreis Zollernalbkreis (Stroke Units) (Baden-Württemberg) — Regular (+20% Speed (Emergency))

Category	Grid Cells	Area (%)	Population	Population (%)
High (30+ min)	67	6.0	24302	13.0
Medium (20-30 min)	449	40.5	94497	50.5
Low (10-20 min)	452	40.7	56337	30.1
Only CT reachable within 60 min	0	0.0	0	0.0
Neither reachable within 60 min	0	0.0	0	0.0
Likely irrelevant (<10 min)	142	12.8	12139	6.5

Population and Area Coverage in Landkreis Eichstätt (Stroke Units) (Bayern) — Regular (+20% Speed (Emergency))

Category	Grid Cells	Area (%)	Population	Population (%)
High (30+ min)	0	0.0	0	0.0
Medium (20-30 min)	375	25.3	30157	23.1
Low (10-20 min)	878	59.2	62203	47.6
Only CT reachable within 60 min	0	0.0	0	0.0
Neither reachable within 60 min	0	0.0	0	0.0
Likely irrelevant (<10 min)	229	15.5	38257	29.3

Population and Area Coverage in Landkreis Freyung-Grafenau (Stroke Units) (Bayern) — Regular (+20% Speed (Emergency))

Category	Grid Cells	Area (%)	Population	Population (%)
High (30+ min)	302	26.7	18036	22.9
Medium (20-30 min)	388	34.2	34096	43.3
Low (10-20 min)	228	20.1	17134	21.8
Only CT reachable within 60 min	3	0.3	27	0.0
Neither reachable within 60 min	49	4.3	8	0.0
Likely irrelevant (<10 min)	163	14.4	9429	12.0

Population and Area Coverage in Landkreis Main-Spessart (Stroke Units) (Bayern) — Regular (+20% Speed (Emergency))

Category	Grid Cells	Area (%)	Population	Population (%)
High (30+ min)	0	0.0	0	0.0
Medium (20-30 min)	5	0.3	0	0.0
Low (10-20 min)	204	12.3	10427	7.9
Only CT reachable within 60 min	0	0.0	0	0.0
Neither reachable within 60 min	0	0.0	0	0.0
Likely irrelevant (<10 min)	1453	87.4	121028	92.1

Population and Area Coverage in Kreisfreie Stadt Potsdam (Stroke Units) (Brandenburg) — Regular (+20% Speed (Emergency))

Category	Grid Cells	Area (%)	Population	Population (%)
High (30+ min)	0	0.0	0	0.0
Medium (20-30 min)	0	0.0	0	0.0
Low (10-20 min)	17	7.0	15795	7.7
Only CT reachable within 60 min	0	0.0	0	0.0
Neither reachable within 60 min	0	0.0	0	0.0
Likely irrelevant (<10 min)	227	93.0	189520	92.3

Population and Area Coverage in Landkreis Nordsachsen (Stroke Units) (Sachsen) — Regular (+20% Speed (Emergency))

Category	Grid Cells	Area (%)	Population	Population (%)
High (30+ min)	186	7.1	10869	5.9
Medium (20-30 min)	613	23.5	45496	24.7
Low (10-20 min)	869	33.3	62645	34.0
Only CT reachable within 60 min	12	0.5	12	0.0
Neither reachable within 60 min	0	0.0	0	0.0
Likely irrelevant (<10 min)	931	35.7	65051	35.3

Population and Area Coverage in Landkreis Cuxhaven (Stroke Units) (Niedersachsen) — Regular (+20% Speed (Emergency))

Category	Grid Cells	Area (%)	Population	Population (%)
High (30+ min)	124	4.5	10700	5.4
Medium (20-30 min)	348	12.5	50501	25.4
Low (10-20 min)	881	31.7	46949	23.6
Only CT reachable within 60 min	9	0.3	773	0.4
Neither reachable within 60 min	42	1.5	2418	1.2
Likely irrelevant (<10 min)	1376	49.5	87364	44.0

Population and Area Coverage in Kreisfreie Stadt Bremen (Stroke Units) (Bremen) — Regular (+20% Speed (Emergency))

Category	Grid Cells	Area (%)	Population	Population (%)
High (30+ min)	0	0.0	0	0.0
Medium (20-30 min)	0	0.0	12	0.0
Low (10-20 min)	75	17.1	100535	17.2
Only CT reachable within 60 min	0	0.0	0	0.0
Neither reachable within 60 min	1	0.2	1881	0.3
Likely irrelevant (<10 min)	362	82.6	480784	82.4

Population and Area Coverage in Landkreis Groß-Gerau (Stroke Units) (Hessen) — Regular (+20% Speed (Emergency))

Category	Grid Cells	Area (%)	Population	Population (%)
High (30+ min)	0	0.0	0	0.0
Medium (20-30 min)	1	0.2	824	0.3
Low (10-20 min)	95	16.8	31871	12.3
Only CT reachable within 60 min	0	0.0	0	0.0
Neither reachable within 60 min	0	0.0	0	0.0
Likely irrelevant (<10 min)	468	83.0	226898	87.4

Population and Area Coverage in Landkreis Limburg-Weilburg (Stroke Units) (Hessen) — Regular (+20% Speed (Emergency))

Category	Grid Cells	Area (%)	Population	Population (%)
High (30+ min)	0	0.0	0	0.0
Medium (20-30 min)	7	0.7	135	0.1
Low (10-20 min)	225	24.1	31529	18.2
Only CT reachable within 60 min	0	0.0	0	0.0
Neither reachable within 60 min	0	0.0	0	0.0
Likely irrelevant (<10 min)	702	75.2	141173	81.7

Population and Area Coverage in Landkreis Waldeck-Frankenberg (Stroke Units) (Hessen) — Regular (+20% Speed (Emergency))

Category	Grid Cells	Area (%)	Population	Population (%)
High (30+ min)	1163	48.8	90143	57.1
Medium (20-30 min)	985	41.4	52572	33.3
Low (10-20 min)	218	9.2	15281	9.7
Only CT reachable within 60 min	0	0.0	0	0.0
Neither reachable within 60 min	0	0.0	0	0.0
Likely irrelevant (<10 min)	16	0.7	7	0.0

Population and Area Coverage in Landkreis Trier-Saarburg (Stroke Units) (Rheinland-Pfalz) — Regular (+20% Speed (Emergency))

Category	Grid Cells	Area (%)	Population	Population (%)
High (30+ min)	1	0.1	0	0.0
Medium (20-30 min)	142	10.3	19958	13.3
Low (10-20 min)	471	34.3	34223	22.8
Only CT reachable within 60 min	0	0.0	0	0.0
Neither reachable within 60 min	0	0.0	355	0.2
Likely irrelevant (<10 min)	760	55.3	95499	63.7

Population and Area Coverage in Landkreis Reutlingen (Stroke Units) (Baden-Württemberg) — Regular (+20% Speed (Emergency))

Category	Grid Cells	Area (%)	Population	Population (%)
High (30+ min)	70	5.3	10133	3.4
Medium (20-30 min)	292	21.9	9778	3.3
Low (10-20 min)	373	28.0	41609	14.0
Only CT reachable within 60 min	0	0.0	0	0.0
Neither reachable within 60 min	0	0.0	0	0.0
Likely irrelevant (<10 min)	598	44.9	236191	79.3

Population and Area Coverage in Kreisfreie Stadt Schwerin (Stroke Units) (Mecklenburg-Vorpommern) — Regular (+20% Speed (Emergency))

Category	Grid Cells	Area (%)	Population	Population (%)
High (30+ min)	0	0.0	0	0.0
Medium (20-30 min)	0	0.0	0	0.0
Low (10-20 min)	1	0.6	124	0.1
Only CT reachable within 60 min	0	0.0	0	0.0
Neither reachable within 60 min	0	0.0	0	0.0
Likely irrelevant (<10 min)	172	99.4	96648	99.9

Population and Area Coverage in Kreisfreie Stadt Hamburg (Stroke Units) (Hamburg) — Regular (+20% Speed (Emergency))

Category	Grid Cells	Area (%)	Population	Population (%)
High (30+ min)	0	0.0	0	0.0
Medium (20-30 min)	0	0.0	0	0.0
Low (10-20 min)	112	11.2	149048	8.3
Only CT reachable within 60 min	0	0.0	0	0.0
Neither reachable within 60 min	6	0.6	6087	0.3
Likely irrelevant (<10 min)	879	88.2	1638333	91.3

Population and Area Coverage in Kreisfreie Stadt Braunschweig (Stroke Units) (Niedersachsen) — Regular (+20% Speed (Emergency))

Category	Grid Cells	Area (%)	Population	Population (%)
High (30+ min)	0	0.0	0	0.0
Medium (20-30 min)	0	0.0	0	0.0
Low (10-20 min)	124	51.0	145440	59.0
Only CT reachable within 60 min	0	0.0	0	0.0
Neither reachable within 60 min	0	0.0	0	0.0
Likely irrelevant (<10 min)	119	49.0	101238	41.0

Population and Area Coverage in Landkreis Leer (Stroke Units) (Niedersachsen) — Regular (+20% Speed (Emergency))

Category	Grid Cells	Area (%)	Population	Population (%)
High (30+ min)	60	4.2	9525	5.6
Medium (20-30 min)	236	16.6	39973	23.7
Low (10-20 min)	476	33.6	60988	36.1
Only CT reachable within 60 min	2	0.1	71	0.0
Neither reachable within 60 min	38	2.7	3553	2.1
Likely irrelevant (<10 min)	606	42.7	54728	32.4

Population and Area Coverage in Kreisfreie Stadt Duisburg (Stroke Units) (Nordrhein-Westfalen) — Regular (+20% Speed (Emergency))

Category	Grid Cells	Area (%)	Population	Population (%)
High (30+ min)	0	0.0	0	0.0
Medium (20-30 min)	0	0.0	0	0.0
Low (10-20 min)	108	36.4	225974	40.9
Only CT reachable within 60 min	0	0.0	0	0.0
Neither reachable within 60 min	0	0.0	0	0.0
Likely irrelevant (<10 min)	189	63.6	326929	59.1

Population and Area Coverage in Kreis Siegen-Wittgenstein (Stroke Units) (Nordrhein-Westfalen) — Regular (+20% Speed (Emergency))

Category	Grid Cells	Area (%)	Population	Population (%)
High (30+ min)	331	22.8	25233	8.6
Medium (20-30 min)	188	13.0	9246	3.2
Low (10-20 min)	271	18.7	42215	14.4
Only CT reachable within 60 min	12	0.8	9	0.0
Neither reachable within 60 min	0	0.0	0	0.0
Likely irrelevant (<10 min)	649	44.7	216616	73.8

Population and Area Coverage in Landkreis Schwarzwald-Baar-Kreis (Stroke Units) (Baden-Württemberg) — Regular (+20% Speed (Emergency))

Category	Grid Cells	Area (%)	Population	Population (%)
High (30+ min)	35	2.8	1025	0.5
Medium (20-30 min)	77	6.3	7346	3.5
Low (10-20 min)	127	10.3	9376	4.5
Only CT reachable within 60 min	0	0.0	0	0.0
Neither reachable within 60 min	0	0.0	0	0.0
Likely irrelevant (<10 min)	991	80.6	189241	91.4

Population and Area Coverage in Kreisfreie Stadt Amberg (Stroke Units) (Bayern) — Regular (+20% Speed (Emergency))

Category	Grid Cells	Area (%)	Population	Population (%)
High (30+ min)	0	0.0	0	0.0
Medium (20-30 min)	0	0.0	0	0.0
Low (10-20 min)	0	0.0	0	0.0
Only CT reachable within 60 min	0	0.0	0	0.0
Neither reachable within 60 min	0	0.0	0	0.0
Likely irrelevant (<10 min)	61	100.0	42852	100.0

Population and Area Coverage in Kreisfreie Stadt Bayreuth (Stroke Units) (Bayern) — Regular (+20% Speed (Emergency))

Category	Grid Cells	Area (%)	Population	Population (%)
High (30+ min)	0	0.0	0	0.0
Medium (20-30 min)	0	0.0	0	0.0
Low (10-20 min)	2	2.4	993	1.4
Only CT reachable within 60 min	0	0.0	0	0.0
Neither reachable within 60 min	0	0.0	0	0.0
Likely irrelevant (<10 min)	80	97.6	72471	98.6

Population and Area Coverage in Landkreis Nürnberger Land (Stroke Units) (Bayern) — Regular (+20% Speed (Emergency))

Category	Grid Cells	Area (%)	Population	Population (%)
High (30+ min)	0	0.0	0	0.0
Medium (20-30 min)	18	1.8	2094	1.2
Low (10-20 min)	376	38.1	73165	43.0
Only CT reachable within 60 min	0	0.0	0	0.0
Neither reachable within 60 min	0	0.0	0	0.0
Likely irrelevant (<10 min)	592	60.0	94818	55.8

Population and Area Coverage in Landkreis Roth (Stroke Units) (Bayern) — Regular (+20% Speed (Emergency))

Category	Grid Cells	Area (%)	Population	Population (%)
High (30+ min)	0	0.0	0	0.0
Medium (20-30 min)	2	0.2	2	0.0
Low (10-20 min)	131	11.9	6862	5.3
Only CT reachable within 60 min	0	0.0	0	0.0
Neither reachable within 60 min	0	0.0	0	0.0
Likely irrelevant (<10 min)	971	88.0	121913	94.7

Population and Area Coverage in Kreisfreie Stadt Aschaffenburg (Stroke Units) (Bayern) — Regular (+20% Speed (Emergency))

Category	Grid Cells	Area (%)	Population	Population (%)
High (30+ min)	0	0.0	0	0.0
Medium (20-30 min)	0	0.0	0	0.0
Low (10-20 min)	0	0.0	0	0.0
Only CT reachable within 60 min	0	0.0	0	0.0
Neither reachable within 60 min	0	0.0	0	0.0
Likely irrelevant (<10 min)	78	100.0	70292	100.0

Population and Area Coverage in Landkreis Merzig-Wadern (Stroke Units) (Saarland) — Regular (+20% Speed (Emergency))

Category	Grid Cells	Area (%)	Population	Population (%)
High (30+ min)	0	0.0	0	0.0
Medium (20-30 min)	14	2.0	289	0.3
Low (10-20 min)	182	26.3	21319	21.3
Only CT reachable within 60 min	0	0.0	1	0.0
Neither reachable within 60 min	0	0.0	191	0.2
Likely irrelevant (<10 min)	495	71.6	78217	78.2

Population and Area Coverage in Landkreis Vogtlandkreis (Stroke Units) (Sachsen) — Regular (+20% Speed (Emergency))

Category	Grid Cells	Area (%)	Population	Population (%)
High (30+ min)	48	2.7	3941	1.8
Medium (20-30 min)	332	18.8	39171	18.0
Low (10-20 min)	385	21.8	43670	20.0
Only CT reachable within 60 min	2	0.1	93	0.0
Neither reachable within 60 min	12	0.7	105	0.0
Likely irrelevant (<10 min)	988	55.9	131069	60.1

Population and Area Coverage in Landkreis Altmarkkreis Salzwedel (Stroke Units) (Sachsen-Anhalt) — Regular (+20% Speed (Emergency))

Category	Grid Cells	Area (%)	Population	Population (%)
High (30+ min)	1316	43.0	44565	57.7
Medium (20-30 min)	656	21.4	15864	20.5
Low (10-20 min)	450	14.7	6357	8.2
Only CT reachable within 60 min	580	18.9	9182	11.9
Neither reachable within 60 min	0	0.0	0	0.0
Likely irrelevant (<10 min)	60	2.0	1263	1.6

Population and Area Coverage in Kreis Segeberg (Stroke Units) (Schleswig-Holstein) — Regular (+20% Speed (Emergency))

Category	Grid Cells	Area (%)	Population	Population (%)
High (30+ min)	0	0.0	0	0.0
Medium (20-30 min)	4	0.2	300	0.1
Low (10-20 min)	385	21.0	81457	29.7
Only CT reachable within 60 min	0	0.0	0	0.0
Neither reachable within 60 min	0	0.0	0	0.0
Likely irrelevant (<10 min)	1443	78.8	192154	70.2

Population and Area Coverage in Landkreis Osnabrück (Stroke Units) (Niedersachsen) — Regular (+20% Speed (Emergency))

Category	Grid Cells	Area (%)	Population	Population (%)
High (30+ min)	0	0.0	0	0.0
Medium (20-30 min)	114	4.1	19238	5.4
Low (10-20 min)	1298	46.3	175675	49.7
Only CT reachable within 60 min	0	0.0	0	0.0
Neither reachable within 60 min	0	0.0	0	0.0
Likely irrelevant (<10 min)	1390	49.6	158244	44.8

Population and Area Coverage in Kreisfreie Stadt Oberhausen (Stroke Units) (Nordrhein-Westfalen) — Regular (+20% Speed (Emergency))

Category	Grid Cells	Area (%)	Population	Population (%)
High (30+ min)	0	0.0	0	0.0
Medium (20-30 min)	0	0.0	0	0.0
Low (10-20 min)	14	13.9	58460	27.2
Only CT reachable within 60 min	0	0.0	0	0.0
Neither reachable within 60 min	0	0.0	0	0.0
Likely irrelevant (<10 min)	87	86.1	156707	72.8

Population and Area Coverage in Kreisfreie Stadt Remscheid (Stroke Units) (Nordrhein-Westfalen) — Regular (+20% Speed (Emergency))

Category	Grid Cells	Area (%)	Population	Population (%)
High (30+ min)	0	0.0	0	0.0
Medium (20-30 min)	0	0.0	0	0.0
Low (10-20 min)	30	30.9	27405	25.3
Only CT reachable within 60 min	0	0.0	0	0.0
Neither reachable within 60 min	0	0.0	0	0.0
Likely irrelevant (<10 min)	67	69.1	81085	74.7

Population and Area Coverage in Kreis Mettmann (Stroke Units) (Nordrhein-Westfalen) — Regular (+20% Speed (Emergency))

Category	Grid Cells	Area (%)	Population	Population (%)
High (30+ min)	0	0.0	0	0.0
Medium (20-30 min)	0	0.0	0	0.0
Low (10-20 min)	109	20.8	134373	28.4
Only CT reachable within 60 min	0	0.0	0	0.0
Neither reachable within 60 min	0	0.0	0	0.0
Likely irrelevant (<10 min)	416	79.2	339353	71.6

Population and Area Coverage in Kreis Rhein-Sieg-Kreis (Stroke Units) (Nordrhein-Westfalen) — Regular (+20% Speed (Emergency))

Category	Grid Cells	Area (%)	Population	Population (%)
High (30+ min)	2	0.1	205	0.0
Medium (20-30 min)	89	6.0	14677	2.5
Low (10-20 min)	386	26.2	138042	23.7
Only CT reachable within 60 min	0	0.0	0	0.0
Neither reachable within 60 min	0	0.0	0	0.0
Likely irrelevant (<10 min)	997	67.6	430352	73.8

Population and Area Coverage in Kreis Borken (Stroke Units) (Nordrhein-Westfalen) — Regular (+20% Speed (Emergency))

Category	Grid Cells	Area (%)	Population	Population (%)
High (30+ min)	18	1.0	8024	2.2
Medium (20-30 min)	344	18.9	79287	21.6
Low (10-20 min)	540	29.7	109181	29.8
Only CT reachable within 60 min	2	0.1	81	0.0
Neither reachable within 60 min	16	0.9	147	0.0
Likely irrelevant (<10 min)	899	49.4	170072	46.4

Population and Area Coverage in Landkreis Vulkaneifel (Stroke Units) (Rheinland-Pfalz) — Regular (+20% Speed (Emergency))

Category	Grid Cells	Area (%)	Population	Population (%)
High (30+ min)	145	12.6	7926	12.6
Medium (20-30 min)	275	23.9	22248	35.4
Low (10-20 min)	554	48.2	24896	39.6
Only CT reachable within 60 min	0	0.0	0	0.0
Neither reachable within 60 min	0	0.0	0	0.0
Likely irrelevant (<10 min)	176	15.3	7785	12.4

Population and Area Coverage in Landkreis Kaiserslautern (Stroke Units) (Rheinland-Pfalz) — Regular (+20% Speed (Emergency))

Category	Grid Cells	Area (%)	Population	Population (%)
High (30+ min)	0	0.0	0	0.0
Medium (20-30 min)	5	0.6	580	0.5
Low (10-20 min)	180	22.8	27553	25.8
Only CT reachable within 60 min	0	0.0	0	0.0
Neither reachable within 60 min	0	0.0	0	0.0
Likely irrelevant (<10 min)	606	76.6	78774	73.7

Population and Area Coverage in Landkreis Calw (Stroke Units) (Baden-Württemberg) — Regular (+20% Speed (Emergency))

Category	Grid Cells	Area (%)	Population	Population (%)
High (30+ min)	56	5.8	17437	11.5
Medium (20-30 min)	459	47.5	74872	49.4
Low (10-20 min)	381	39.4	49861	32.9
Only CT reachable within 60 min	0	0.0	0	0.0
Neither reachable within 60 min	0	0.0	0	0.0
Likely irrelevant (<10 min)	71	7.3	9459	6.2

Population and Area Coverage in Kreisfreie Stadt Berlin (Stroke Units) (Berlin) — Regular (+20% Speed (Emergency))

Category	Grid Cells	Area (%)	Population	Population (%)
High (30+ min)	0	0.0	0	0.0
Medium (20-30 min)	2	0.2	5567	0.2
Low (10-20 min)	152	12.8	323555	8.9
Only CT reachable within 60 min	0	0.0	0	0.0
Neither reachable within 60 min	0	0.0	0	0.0
Likely irrelevant (<10 min)	1030	87.0	3299576	90.9

Population and Area Coverage in Kreis Stormarn (Stroke Units) (Schleswig-Holstein) — Regular (+20% Speed (Emergency))

Category	Grid Cells	Area (%)	Population	Population (%)
High (30+ min)	0	0.0	0	0.0
Medium (20-30 min)	9	0.9	5230	2.1
Low (10-20 min)	512	49.1	124500	51.0
Only CT reachable within 60 min	0	0.0	0	0.0
Neither reachable within 60 min	0	0.0	0	0.0
Likely irrelevant (<10 min)	521	50.0	114238	46.8

Population and Area Coverage in Landkreis Hildesheim (Stroke Units) (Niedersachsen) — Regular (+20% Speed (Emergency))

Category	Grid Cells	Area (%)	Population	Population (%)
High (30+ min)	35	2.2	14951	5.1
Medium (20-30 min)	293	18.4	23988	8.2
Low (10-20 min)	298	18.7	28152	9.6
Only CT reachable within 60 min	0	0.0	0	0.0
Neither reachable within 60 min	0	0.0	0	0.0
Likely irrelevant (<10 min)	966	60.7	226624	77.2

Population and Area Coverage in Kreis Viersen (Stroke Units) (Nordrhein-Westfalen) — Regular (+20% Speed (Emergency))

Category	Grid Cells	Area (%)	Population	Population (%)
High (30+ min)	0	0.0	0	0.0
Medium (20-30 min)	1	0.1	2	0.0
Low (10-20 min)	122	16.8	53711	18.1
Only CT reachable within 60 min	0	0.0	0	0.0
Neither reachable within 60 min	45	6.2	1588	0.5
Likely irrelevant (<10 min)	560	76.9	240743	81.3

Population and Area Coverage in Kreis Rheinisch-Bergischer Kreis (Stroke Units) (Nordrhein-Westfalen) — Regular (+20% Speed (Emergency))

Category	Grid Cells	Area (%)	Population	Population (%)
High (30+ min)	0	0.0	0	0.0
Medium (20-30 min)	7	1.2	282	0.1
Low (10-20 min)	183	32.6	64927	21.5
Only CT reachable within 60 min	0	0.0	0	0.0
Neither reachable within 60 min	0	0.0	0	0.0
Likely irrelevant (<10 min)	371	66.1	236163	78.4

Population and Area Coverage in Kreisfreie Stadt Zweibrücken (Stroke Units) (Rheinland-Pfalz) — Regular (+20% Speed (Emergency))

Category	Grid Cells	Area (%)	Population	Population (%)
High (30+ min)	0	0.0	0	0.0
Medium (20-30 min)	0	0.0	0	0.0
Low (10-20 min)	59	67.0	30002	92.0
Only CT reachable within 60 min	0	0.0	0	0.0
Neither reachable within 60 min	0	0.0	0	0.0
Likely irrelevant (<10 min)	29	33.0	2605	8.0

Population and Area Coverage in Landkreis Rhein-Pfalz-Kreis (Stroke Units) (Rheinland-Pfalz) — Regular (+20% Speed (Emergency))

Category	Grid Cells	Area (%)	Population	Population (%)
High (30+ min)	0	0.0	0	0.0
Medium (20-30 min)	0	0.0	0	0.0
Low (10-20 min)	29	7.6	13830	9.1
Only CT reachable within 60 min	0	0.0	0	0.0
Neither reachable within 60 min	0	0.0	0	0.0
Likely irrelevant (<10 min)	352	92.4	138665	90.9

Population and Area Coverage in Landkreis Altötting (Stroke Units) (Bayern) — Regular (+20% Speed (Emergency))

Category	Grid Cells	Area (%)	Population	Population (%)
High (30+ min)	0	0.0	0	0.0
Medium (20-30 min)	0	0.0	0	0.0
Low (10-20 min)	17	2.5	690	0.6
Only CT reachable within 60 min	0	0.0	0	0.0
Neither reachable within 60 min	3	0.4	2068	1.9
Likely irrelevant (<10 min)	658	97.1	104537	97.4

Population and Area Coverage in Landkreis Pfaffenhofen a.d.Ilm (Stroke Units) (Bayern) — Regular (+20% Speed (Emergency))

Category	Grid Cells	Area (%)	Population	Population (%)
High (30+ min)	0	0.0	0	0.0
Medium (20-30 min)	5	0.5	1541	1.2
Low (10-20 min)	202	21.9	22671	18.3
Only CT reachable within 60 min	0	0.0	0	0.0
Neither reachable within 60 min	0	0.0	0	0.0
Likely irrelevant (<10 min)	717	77.6	99663	80.5

Population and Area Coverage in Kreisfreie Stadt Bamberg (Stroke Units) (Bayern) — Regular (+20% Speed (Emergency))

Category	Grid Cells	Area (%)	Population	Population (%)
High (30+ min)	0	0.0	0	0.0
Medium (20-30 min)	0	0.0	0	0.0
Low (10-20 min)	4	6.0	8027	10.3
Only CT reachable within 60 min	0	0.0	0	0.0
Neither reachable within 60 min	0	0.0	0	0.0
Likely irrelevant (<10 min)	63	94.0	69797	89.7

Population and Area Coverage in Landkreis Hof (Stroke Units) (Bayern) — Regular (+20% Speed (Emergency))

Category	Grid Cells	Area (%)	Population	Population (%)
High (30+ min)	0	0.0	0	0.0
Medium (20-30 min)	0	0.0	0	0.0
Low (10-20 min)	257	23.4	27007	27.1
Only CT reachable within 60 min	0	0.0	0	0.0
Neither reachable within 60 min	0	0.0	0	0.0
Likely irrelevant (<10 min)	842	76.6	72690	72.9

Population and Area Coverage in Landkreis Miltenberg (Stroke Units) (Bayern) — Regular (+20% Speed (Emergency))

Category	Grid Cells	Area (%)	Population	Population (%)
High (30+ min)	1	0.1	31	0.0
Medium (20-30 min)	52	5.8	2473	1.9
Low (10-20 min)	134	15.0	8653	6.5
Only CT reachable within 60 min	0	0.0	0	0.0
Neither reachable within 60 min	0	0.0	0	0.0
Likely irrelevant (<10 min)	709	79.1	121989	91.6

Population and Area Coverage in Kreisfreie Stadt Augsburg (Stroke Units) (Bayern) — Regular (+20% Speed (Emergency))

Category	Grid Cells	Area (%)	Population	Population (%)
High (30+ min)	0	0.0	0	0.0
Medium (20-30 min)	2	1.1	8	0.0
Low (10-20 min)	80	44.4	119316	41.6
Only CT reachable within 60 min	0	0.0	0	0.0
Neither reachable within 60 min	0	0.0	0	0.0
Likely irrelevant (<10 min)	98	54.4	167365	58.4

Population and Area Coverage in Landkreis Barnim (Stroke Units) (Brandenburg) — Regular (+20% Speed (Emergency))

Category	Grid Cells	Area (%)	Population	Population (%)
High (30+ min)	5	0.3	56	0.0
Medium (20-30 min)	100	5.1	2447	1.4
Low (10-20 min)	709	35.8	51506	30.2
Only CT reachable within 60 min	9	0.5	45	0.0
Neither reachable within 60 min	15	0.8	13	0.0
Likely irrelevant (<10 min)	1141	57.7	116376	68.3

Population and Area Coverage in Landkreis Meißen (Stroke Units) (Sachsen) — Regular (+20% Speed (Emergency))

Category	Grid Cells	Area (%)	Population	Population (%)
High (30+ min)	0	0.0	0	0.0
Medium (20-30 min)	277	14.8	45532	19.9
Low (10-20 min)	881	47.0	97233	42.4
Only CT reachable within 60 min	0	0.0	0	0.0
Neither reachable within 60 min	0	0.0	0	0.0
Likely irrelevant (<10 min)	715	38.2	86305	37.7

Population and Area Coverage in Kreisfreie Stadt Suhl (Stroke Units) (Thüringen) — Regular (+20% Speed (Emergency))

Category	Grid Cells	Area (%)	Population	Population (%)
High (30+ min)	0	0.0	0	0.0
Medium (20-30 min)	0	0.0	0	0.0
Low (10-20 min)	13	7.3	3893	11.7
Only CT reachable within 60 min	0	0.0	0	0.0
Neither reachable within 60 min	0	0.0	0	0.0
Likely irrelevant (<10 min)	166	92.7	29415	88.3

Population and Area Coverage in Landkreis Eichsfeld (Stroke Units) (Thüringen) — Regular (+20% Speed (Emergency))

Category	Grid Cells	Area (%)	Population	Population (%)
High (30+ min)	65	5.0	6820	7.2
Medium (20-30 min)	473	36.7	32619	34.5
Low (10-20 min)	559	43.4	40397	42.7
Only CT reachable within 60 min	0	0.0	0	0.0
Neither reachable within 60 min	0	0.0	0	0.0
Likely irrelevant (<10 min)	191	14.8	14847	15.7

Population and Area Coverage in Landkreis Göttingen (Stroke Units) (Niedersachsen) — Regular (+20% Speed (Emergency))

Category	Grid Cells	Area (%)	Population	Population (%)
High (30+ min)	172	7.5	21909	6.5
Medium (20-30 min)	473	20.7	47566	14.2
Low (10-20 min)	730	32.0	76266	22.8
Only CT reachable within 60 min	0	0.0	0	0.0
Neither reachable within 60 min	0	0.0	0	0.0
Likely irrelevant (<10 min)	905	39.7	188830	56.4

Population and Area Coverage in Landkreis Schaumburg (Stroke Units) (Niedersachsen) — Regular (+20% Speed (Emergency))

Category	Grid Cells	Area (%)	Population	Population (%)
High (30+ min)	0	0.0	0	0.0
Medium (20-30 min)	1	0.1	248	0.2
Low (10-20 min)	60	6.7	16081	10.3
Only CT reachable within 60 min	0	0.0	0	0.0
Neither reachable within 60 min	0	0.0	0	0.0
Likely irrelevant (<10 min)	828	93.1	139513	89.5

Population and Area Coverage in Landkreis Aurich (Stroke Units) (Niedersachsen) — Regular (+20% Speed (Emergency))

Category	Grid Cells	Area (%)	Population	Population (%)
High (30+ min)	170	10.1	31029	16.8
Medium (20-30 min)	433	25.6	57597	31.1
Low (10-20 min)	408	24.1	43387	23.4
Only CT reachable within 60 min	8	0.5	65	0.0
Neither reachable within 60 min	64	3.8	4908	2.7
Likely irrelevant (<10 min)	607	35.9	48082	26.0

Population and Area Coverage in Landkreis Friesland (Stroke Units) (Niedersachsen) — Regular (+20% Speed (Emergency))

Category	Grid Cells	Area (%)	Population	Population (%)
High (30+ min)	0	0.0	0	0.0
Medium (20-30 min)	6	0.7	188	0.2
Low (10-20 min)	142	17.2	21320	21.9
Only CT reachable within 60 min	6	0.7	302	0.3
Neither reachable within 60 min	44	5.3	1642	1.7
Likely irrelevant (<10 min)	628	76.0	73981	75.9

Population and Area Coverage in Landkreis Wesermarsch (Stroke Units) (Niedersachsen) — Regular (+20% Speed (Emergency))

Category	Grid Cells	Area (%)	Population	Population (%)
High (30+ min)	0	0.0	0	0.0
Medium (20-30 min)	233	21.2	29161	34.4
Low (10-20 min)	506	46.1	33192	39.2
Only CT reachable within 60 min	6	0.5	149	0.2
Neither reachable within 60 min	24	2.2	344	0.4
Likely irrelevant (<10 min)	328	29.9	21856	25.8

Population and Area Coverage in Landkreis Main-Kinzig-Kreis (Stroke Units) (Hessen) — Regular (+20% Speed (Emergency))

Category	Grid Cells	Area (%)	Population	Population (%)
High (30+ min)	0	0.0	0	0.0
Medium (20-30 min)	55	3.1	5499	1.3
Low (10-20 min)	620	35.2	44855	11.0
Only CT reachable within 60 min	0	0.0	0	0.0
Neither reachable within 60 min	0	0.0	0	0.0
Likely irrelevant (<10 min)	1085	61.6	358830	87.7

Population and Area Coverage in Landkreis Vogelsbergkreis (Stroke Units) (Hessen) — Regular (+20% Speed (Emergency))

Category	Grid Cells	Area (%)	Population	Population (%)
High (30+ min)	104	5.6	9293	8.7
Medium (20-30 min)	531	28.6	34090	31.8
Low (10-20 min)	868	46.8	41138	38.3
Only CT reachable within 60 min	0	0.0	0	0.0
Neither reachable within 60 min	0	0.0	0	0.0
Likely irrelevant (<10 min)	353	19.0	22803	21.2

Population and Area Coverage in Landkreis Neuwied (Stroke Units) (Rheinland-Pfalz) — Regular (+20% Speed (Emergency))

Category	Grid Cells	Area (%)	Population	Population (%)
High (30+ min)	0	0.0	0	0.0
Medium (20-30 min)	52	6.6	13451	7.3
Low (10-20 min)	350	44.1	115493	62.3
Only CT reachable within 60 min	0	0.0	0	0.0
Neither reachable within 60 min	0	0.0	0	0.0
Likely irrelevant (<10 min)	391	49.3	56373	30.4

Population and Area Coverage in Kreisfreie Stadt Mainz (Stroke Units) (Rheinland-Pfalz) — Regular (+20% Speed (Emergency))

Category	Grid Cells	Area (%)	Population	Population (%)
High (30+ min)	0	0.0	0	0.0
Medium (20-30 min)	0	0.0	0	0.0
Low (10-20 min)	1	0.8	476	0.2
Only CT reachable within 60 min	0	0.0	0	0.0
Neither reachable within 60 min	0	0.0	0	0.0
Likely irrelevant (<10 min)	120	99.2	213592	99.8

Population and Area Coverage in Landkreis Hohenlohekreis (Stroke Units) (Baden-Württemberg) — Regular (+20% Speed (Emergency))

Category	Grid Cells	Area (%)	Population	Population (%)
High (30+ min)	1	0.1	0	0.0
Medium (20-30 min)	34	3.5	14759	13.5
Low (10-20 min)	350	36.5	42465	38.8
Only CT reachable within 60 min	0	0.0	0	0.0
Neither reachable within 60 min	0	0.0	0	0.0
Likely irrelevant (<10 min)	574	59.9	52102	47.7

Population and Area Coverage in Landkreis Ortenaukreis (Stroke Units) (Baden-Württemberg) — Regular (+20% Speed (Emergency))

Category	Grid Cells	Area (%)	Population	Population (%)
High (30+ min)	183	8.2	12797	3.0
Medium (20-30 min)	249	11.1	39380	9.4
Low (10-20 min)	554	24.7	86828	20.6
Only CT reachable within 60 min	2	0.1	26	0.0
Neither reachable within 60 min	9	0.4	270	0.1
Likely irrelevant (<10 min)	1247	55.6	281399	66.9

Population and Area Coverage in Landkreis Neustadt a.d.Aisch-Bad Windsheim (Stroke Units) (Bayern) — Regular (+20% Speed (Emergency))

Category	Grid Cells	Area (%)	Population	Population (%)
High (30+ min)	3	0.2	16	0.0
Medium (20-30 min)	513	32.3	44848	44.2
Low (10-20 min)	727	45.8	38299	37.7
Only CT reachable within 60 min	0	0.0	0	0.0
Neither reachable within 60 min	0	0.0	0	0.0
Likely irrelevant (<10 min)	343	21.6	18337	18.1

Population and Area Coverage in Landkreis Goslar (Stroke Units) (Niedersachsen) — Regular (+20% Speed (Emergency))

Category	Grid Cells	Area (%)	Population	Population (%)
High (30+ min)	4	0.3	54	0.0
Medium (20-30 min)	93	7.4	20981	15.3
Low (10-20 min)	499	39.9	66049	48.3
Only CT reachable within 60 min	0	0.0	0	0.0
Neither reachable within 60 min	0	0.0	0	0.0
Likely irrelevant (<10 min)	656	52.4	49713	36.3

Population and Area Coverage in Landkreis Region Hannover (Stroke Units) (Niedersachsen) — Regular (+20% Speed (Emergency))

Category	Grid Cells	Area (%)	Population	Population (%)
High (30+ min)	0	0.0	0	0.0
Medium (20-30 min)	99	3.3	25267	2.2
Low (10-20 min)	1281	42.3	180101	15.7
Only CT reachable within 60 min	0	0.0	0	0.0
Neither reachable within 60 min	0	0.0	0	0.0
Likely irrelevant (<10 min)	1645	54.4	945386	82.2

Population and Area Coverage in Landkreis Vechta (Stroke Units) (Niedersachsen) — Regular (+20% Speed (Emergency))

Category	Grid Cells	Area (%)	Population	Population (%)
High (30+ min)	13	1.2	1585	1.2
Medium (20-30 min)	301	27.9	58948	43.4
Low (10-20 min)	215	20.0	20104	14.8
Only CT reachable within 60 min	0	0.0	0	0.0
Neither reachable within 60 min	0	0.0	0	0.0
Likely irrelevant (<10 min)	548	50.9	55142	40.6

Population and Area Coverage in Kreis Städteregion Aachen (Stroke Units) (Nordrhein-Westfalen) — Regular (+20% Speed (Emergency))

Category	Grid Cells	Area (%)	Population	Population (%)
High (30+ min)	100	11.2	14454	2.6
Medium (20-30 min)	128	14.3	12435	2.2
Low (10-20 min)	241	27.0	167559	30.2
Only CT reachable within 60 min	0	0.0	60	0.0
Neither reachable within 60 min	13	1.5	337	0.1
Likely irrelevant (<10 min)	410	46.0	360564	64.9

Population and Area Coverage in Kreis Steinfurt (Stroke Units) (Nordrhein-Westfalen) — Regular (+20% Speed (Emergency))

Category	Grid Cells	Area (%)	Population	Population (%)
High (30+ min)	0	0.0	0	0.0
Medium (20-30 min)	33	1.4	23688	5.4
Low (10-20 min)	1095	46.3	206991	47.5
Only CT reachable within 60 min	0	0.0	0	0.0
Neither reachable within 60 min	0	0.0	0	0.0
Likely irrelevant (<10 min)	1238	52.3	205228	47.1

Population and Area Coverage in Kreis Gütersloh (Stroke Units) (Nordrhein-Westfalen) — Regular (+20% Speed (Emergency))

Category	Grid Cells	Area (%)	Population	Population (%)
High (30+ min)	5	0.4	2125	0.6
Medium (20-30 min)	241	19.1	66933	18.9
Low (10-20 min)	703	55.7	207161	58.4
Only CT reachable within 60 min	0	0.0	0	0.0
Neither reachable within 60 min	0	0.0	0	0.0
Likely irrelevant (<10 min)	313	24.8	78570	22.1

Population and Area Coverage in Kreis Unna (Stroke Units) (Nordrhein-Westfalen) — Regular (+20% Speed (Emergency))

Category	Grid Cells	Area (%)	Population	Population (%)
High (30+ min)	0	0.0	0	0.0
Medium (20-30 min)	0	0.0	0	0.0
Low (10-20 min)	166	23.3	114065	29.5
Only CT reachable within 60 min	0	0.0	0	0.0
Neither reachable within 60 min	0	0.0	0	0.0
Likely irrelevant (<10 min)	545	76.7	273147	70.5

Population and Area Coverage in Landkreis Darmstadt-Dieburg (Stroke Units) (Hessen) — Regular (+20% Speed (Emergency))

Category	Grid Cells	Area (%)	Population	Population (%)
High (30+ min)	0	0.0	0	0.0
Medium (20-30 min)	38	4.6	10538	3.6
Low (10-20 min)	302	36.6	75249	26.0
Only CT reachable within 60 min	0	0.0	0	0.0
Neither reachable within 60 min	0	0.0	0	0.0
Likely irrelevant (<10 min)	485	58.8	203179	70.3

Population and Area Coverage in Landkreis Gießen (Stroke Units) (Hessen) — Regular (+20% Speed (Emergency))

Category	Grid Cells	Area (%)	Population	Population (%)
High (30+ min)	0	0.0	0	0.0
Medium (20-30 min)	0	0.0	0	0.0
Low (10-20 min)	201	18.6	21343	8.3
Only CT reachable within 60 min	0	0.0	0	0.0
Neither reachable within 60 min	0	0.0	0	0.0
Likely irrelevant (<10 min)	880	81.4	236655	91.7

Population and Area Coverage in Kreisfreie Stadt Neustadt an der Weinstraße (Stroke Units) (Rheinland-Pfalz) — Regular (+20% Speed (Emergency))

Category	Grid Cells	Area (%)	Population	Population (%)
High (30+ min)	0	0.0	0	0.0
Medium (20-30 min)	29	19.7	27558	51.3
Low (10-20 min)	101	68.7	23603	43.9
Only CT reachable within 60 min	0	0.0	0	0.0
Neither reachable within 60 min	0	0.0	0	0.0
Likely irrelevant (<10 min)	17	11.6	2556	4.8

Population and Area Coverage in Landkreis Main-Tauber-Kreis (Stroke Units) (Baden-Württemberg) — Regular (+20% Speed (Emergency))

Category	Grid Cells	Area (%)	Population	Population (%)
High (30+ min)	0	0.0	13	0.0
Medium (20-30 min)	221	13.6	13270	10.1
Low (10-20 min)	545	33.6	38545	29.3
Only CT reachable within 60 min	0	0.0	0	0.0
Neither reachable within 60 min	0	0.0	0	0.0
Likely irrelevant (<10 min)	856	52.8	79791	60.6

Population and Area Coverage in Landkreis Freudenstadt (Stroke Units) (Baden-Württemberg) — Regular (+20% Speed (Emergency))

Category	Grid Cells	Area (%)	Population	Population (%)
High (30+ min)	543	51.3	62780	54.2
Medium (20-30 min)	255	24.1	22056	19.0
Low (10-20 min)	194	18.3	30477	26.3
Only CT reachable within 60 min	64	6.0	530	0.5
Neither reachable within 60 min	0	0.0	0	0.0
Likely irrelevant (<10 min)	2	0.2	64	0.1

Population and Area Coverage in Landkreis Rosenheim (Stroke Units) (Bayern) — Regular (+20% Speed (Emergency))

Category	Grid Cells	Area (%)	Population	Population (%)
High (30+ min)	4	0.2	91	0.0
Medium (20-30 min)	93	5.4	11201	4.4
Low (10-20 min)	323	18.7	48561	19.3
Only CT reachable within 60 min	0	0.0	0	0.0
Neither reachable within 60 min	135	7.8	1003	0.4
Likely irrelevant (<10 min)	1168	67.8	190891	75.8

Population and Area Coverage in Kreisfreie Stadt Passau (Stroke Units) (Bayern) — Regular (+20% Speed (Emergency))

Category	Grid Cells	Area (%)	Population	Population (%)
High (30+ min)	0	0.0	0	0.0
Medium (20-30 min)	0	0.0	0	0.0
Low (10-20 min)	0	0.0	0	0.0
Only CT reachable within 60 min	0	0.0	0	0.0
Neither reachable within 60 min	0	0.0	0	0.0
Likely irrelevant (<10 min)	76	100.0	42451	100.0

Population and Area Coverage in Landkreis Coburg (Stroke Units) (Bayern) — Regular (+20% Speed (Emergency))

Category	Grid Cells	Area (%)	Population	Population (%)
High (30+ min)	0	0.0	0	0.0
Medium (20-30 min)	2	0.3	8	0.0
Low (10-20 min)	104	13.8	6031	6.7
Only CT reachable within 60 min	0	0.0	0	0.0
Neither reachable within 60 min	0	0.0	0	0.0
Likely irrelevant (<10 min)	646	85.9	83847	93.3

Population and Area Coverage in Landkreis Ostallgäu (Stroke Units) (Bayern) — Regular (+20% Speed (Emergency))

Category	Grid Cells	Area (%)	Population	Population (%)
High (30+ min)	0	0.0	0	0.0
Medium (20-30 min)	51	3.0	9426	6.7
Low (10-20 min)	437	26.1	50816	36.2
Only CT reachable within 60 min	9	0.5	277	0.2
Neither reachable within 60 min	153	9.1	1036	0.7
Likely irrelevant (<10 min)	1025	61.2	78780	56.1

Population and Area Coverage in Landkreis Saarpfalz-Kreis (Stroke Units) (Saarland) — Regular (+20% Speed (Emergency))

Category	Grid Cells	Area (%)	Population	Population (%)
High (30+ min)	2	0.4	9	0.0
Medium (20-30 min)	0	0.0	0	0.0
Low (10-20 min)	87	17.2	7590	5.5
Only CT reachable within 60 min	0	0.0	0	0.0
Neither reachable within 60 min	4	0.8	0	0.0
Likely irrelevant (<10 min)	412	81.6	129445	94.5

Population and Area Coverage in Landkreis Nordhausen (Stroke Units) (Thüringen) — Regular (+20% Speed (Emergency))

Category	Grid Cells	Area (%)	Population	Population (%)
High (30+ min)	0	0.0	0	0.0
Medium (20-30 min)	101	11.0	8177	10.6
Low (10-20 min)	329	35.9	13725	17.8
Only CT reachable within 60 min	0	0.0	0	0.0
Neither reachable within 60 min	0	0.0	0	0.0
Likely irrelevant (<10 min)	486	53.1	55201	71.6

Population and Area Coverage in Landkreis Uelzen (Stroke Units) (Niedersachsen) — Regular (+20% Speed (Emergency))

Category	Grid Cells	Area (%)	Population	Population (%)
High (30+ min)	4	0.2	2	0.0
Medium (20-30 min)	91	4.7	2305	2.5
Low (10-20 min)	515	26.5	24437	26.2
Only CT reachable within 60 min	0	0.0	0	0.0
Neither reachable within 60 min	0	0.0	0	0.0
Likely irrelevant (<10 min)	1337	68.7	66369	71.3

Population and Area Coverage in Kreisfreie Stadt Mülheim an der Ruhr (Stroke Units) (Nordrhein-Westfalen) — Regular (+20% Speed (Emergency))

Category	Grid Cells	Area (%)	Population	Population (%)
High (30+ min)	0	0.0	0	0.0
Medium (20-30 min)	0	0.0	0	0.0
Low (10-20 min)	1	0.9	2910	1.6
Only CT reachable within 60 min	0	0.0	0	0.0
Neither reachable within 60 min	0	0.0	0	0.0
Likely irrelevant (<10 min)	112	99.1	177295	98.4

Population and Area Coverage in Kreisfreie Stadt Bonn (Stroke Units) (Nordrhein-Westfalen) — Regular (+20% Speed (Emergency))

Category	Grid Cells	Area (%)	Population	Population (%)
High (30+ min)	0	0.0	0	0.0
Medium (20-30 min)	0	0.0	0	0.0
Low (10-20 min)	34	18.9	64999	20.0
Only CT reachable within 60 min	0	0.0	0	0.0
Neither reachable within 60 min	0	0.0	0	0.0
Likely irrelevant (<10 min)	146	81.1	260711	80.0

Population and Area Coverage in Landkreis Birkenfeld (Stroke Units) (Rheinland-Pfalz) — Regular (+20% Speed (Emergency))

Category	Grid Cells	Area (%)	Population	Population (%)
High (30+ min)	1	0.1	0	0.0
Medium (20-30 min)	102	10.6	10542	12.7
Low (10-20 min)	296	30.7	14435	17.4
Only CT reachable within 60 min	0	0.0	0	0.0
Neither reachable within 60 min	0	0.0	0	0.0
Likely irrelevant (<10 min)	566	58.7	57772	69.8

Population and Area Coverage in Landkreis Rems-Murr-Kreis (Stroke Units) (Baden-Württemberg) — Regular (+20% Speed (Emergency))

Category	Grid Cells	Area (%)	Population	Population (%)
High (30+ min)	5	0.5	464	0.1
Medium (20-30 min)	48	4.5	4678	1.1
Low (10-20 min)	276	26.1	67918	16.3
Only CT reachable within 60 min	0	0.0	0	0.0
Neither reachable within 60 min	0	0.0	0	0.0
Likely irrelevant (<10 min)	728	68.9	344759	82.5

Population and Area Coverage in Landkreis Schwäbisch Hall (Stroke Units) (Baden-Württemberg) — Regular (+20% Speed (Emergency))

Category	Grid Cells	Area (%)	Population	Population (%)
High (30+ min)	0	0.0	0	0.0
Medium (20-30 min)	82	4.5	26693	14.0
Low (10-20 min)	521	28.5	39687	20.8
Only CT reachable within 60 min	0	0.0	0	0.0
Neither reachable within 60 min	0	0.0	0	0.0
Likely irrelevant (<10 min)	1222	67.0	124507	65.2

Population and Area Coverage in Landkreis Rottweil (Stroke Units) (Baden-Württemberg) — Regular (+20% Speed (Emergency))

Category	Grid Cells	Area (%)	Population	Population (%)
High (30+ min)	76	8.1	9945	7.2
Medium (20-30 min)	360	38.3	55041	40.0
Low (10-20 min)	419	44.5	62991	45.8
Only CT reachable within 60 min	3	0.3	12	0.0
Neither reachable within 60 min	0	0.0	0	0.0
Likely irrelevant (<10 min)	83	8.8	9567	7.0

Population and Area Coverage in Landkreis Amberg-Sulzbach (Stroke Units) (Bayern) — Regular (+20% Speed (Emergency))

Category	Grid Cells	Area (%)	Population	Population (%)
High (30+ min)	0	0.0	0	0.0
Medium (20-30 min)	100	6.4	8526	8.0
Low (10-20 min)	379	24.3	29440	27.6
Only CT reachable within 60 min	0	0.0	0	0.0
Neither reachable within 60 min	0	0.0	0	0.0
Likely irrelevant (<10 min)	1080	69.3	68739	64.4

Population and Area Coverage in Kreisfreie Stadt Kempten (Allgäu) (Stroke Units) (Bayern) — Regular (+20% Speed (Emergency))

Category	Grid Cells	Area (%)	Population	Population (%)
High (30+ min)	0	0.0	0	0.0
Medium (20-30 min)	0	0.0	0	0.0
Low (10-20 min)	0	0.0	0	0.0
Only CT reachable within 60 min	0	0.0	0	0.0
Neither reachable within 60 min	0	0.0	0	0.0
Likely irrelevant (<10 min)	77	100.0	68488	100.0

Population and Area Coverage in Kreisfreie Stadt Chemnitz (Stroke Units) (Sachsen) — Regular (+20% Speed (Emergency))

Category	Grid Cells	Area (%)	Population	Population (%)
High (30+ min)	0	0.0	0	0.0
Medium (20-30 min)	34	12.3	32577	11.0
Low (10-20 min)	104	37.7	148134	50.0
Only CT reachable within 60 min	0	0.0	0	0.0
Neither reachable within 60 min	0	0.0	0	0.0
Likely irrelevant (<10 min)	138	50.0	115460	39.0

Population and Area Coverage in Landkreis Mecklenburgische Seenplatte (Stroke Units) (Mecklenburg-Vorpommern) — Regular (+20% Speed (Emergency))

Category	Grid Cells	Area (%)	Population	Population (%)
High (30+ min)	1096	14.7	54998	23.0
Medium (20-30 min)	1296	17.4	42818	17.9
Low (10-20 min)	1676	22.5	23981	10.0
Only CT reachable within 60 min	726	9.7	4139	1.7
Neither reachable within 60 min	0	0.0	0	0.0
Likely irrelevant (<10 min)	2659	35.7	113005	47.3

Population and Area Coverage in Landkreis Erzgebirgskreis (Stroke Units) (Sachsen) — Regular (+20% Speed (Emergency))

Category	Grid Cells	Area (%)	Population	Population (%)
High (30+ min)	335	14.6	44529	13.6
Medium (20-30 min)	670	29.3	73096	22.4
Low (10-20 min)	714	31.2	88103	27.0
Only CT reachable within 60 min	6	0.3	133	0.0
Neither reachable within 60 min	2	0.1	128	0.0
Likely irrelevant (<10 min)	563	24.6	120281	36.9

Population and Area Coverage in Kreisfreie Stadt Dresden (Stroke Units) (Sachsen) — Regular (+20% Speed (Emergency))

Category	Grid Cells	Area (%)	Population	Population (%)
High (30+ min)	0	0.0	0	0.0
Medium (20-30 min)	2	0.5	21	0.0
Low (10-20 min)	83	19.8	55542	9.0
Only CT reachable within 60 min	0	0.0	0	0.0
Neither reachable within 60 min	0	0.0	0	0.0
Likely irrelevant (<10 min)	335	79.8	559843	91.0

Population and Area Coverage in Landkreis Hildburghausen (Stroke Units) (Thüringen) — Regular (+20% Speed (Emergency))

Category	Grid Cells	Area (%)	Population	Population (%)
High (30+ min)	0	0.0	0	0.0
Medium (20-30 min)	2	0.2	7	0.0
Low (10-20 min)	108	9.1	2074	3.5
Only CT reachable within 60 min	0	0.0	0	0.0
Neither reachable within 60 min	0	0.0	0	0.0
Likely irrelevant (<10 min)	1078	90.7	56816	96.5

Population and Area Coverage in Kreis Herzogtum Lauenburg (Stroke Units) (Schleswig-Holstein) — Regular (+20% Speed (Emergency))

Category	Grid Cells	Area (%)	Population	Population (%)
High (30+ min)	1	0.1	541	0.3
Medium (20-30 min)	241	14.1	22284	11.4
Low (10-20 min)	599	35.0	85154	43.6
Only CT reachable within 60 min	0	0.0	0	0.0
Neither reachable within 60 min	0	0.0	0	0.0
Likely irrelevant (<10 min)	870	50.8	87374	44.7

Population and Area Coverage in Landkreis Oldenburg (Stroke Units) (Niedersachsen) — Regular (+20% Speed (Emergency))

Category	Grid Cells	Area (%)	Population	Population (%)
High (30+ min)	20	1.4	1933	1.5
Medium (20-30 min)	219	15.3	21092	16.5
Low (10-20 min)	486	34.0	43026	33.7
Only CT reachable within 60 min	0	0.0	0	0.0
Neither reachable within 60 min	0	0.0	0	0.0
Likely irrelevant (<10 min)	703	49.2	61568	48.2

Population and Area Coverage in Kreisfreie Stadt Krefeld (Stroke Units) (Nordrhein-Westfalen) — Regular (+20% Speed (Emergency))

Category	Grid Cells	Area (%)	Population	Population (%)
High (30+ min)	0	0.0	0	0.0
Medium (20-30 min)	0	0.0	0	0.0
Low (10-20 min)	17	9.4	23177	9.6
Only CT reachable within 60 min	0	0.0	0	0.0
Neither reachable within 60 min	0	0.0	0	0.0
Likely irrelevant (<10 min)	164	90.6	218805	90.4

Population and Area Coverage in Kreis Oberbergischer Kreis (Stroke Units) (Nordrhein-Westfalen) — Regular (+20% Speed (Emergency))

Category	Grid Cells	Area (%)	Population	Population (%)
High (30+ min)	43	3.7	11051	4.1
Medium (20-30 min)	578	49.1	138507	51.5
Low (10-20 min)	425	36.1	103744	38.6
Only CT reachable within 60 min	0	0.0	0	0.0
Neither reachable within 60 min	0	0.0	0	0.0
Likely irrelevant (<10 min)	130	11.1	15566	5.8

Population and Area Coverage in Landkreis Kusel (Stroke Units) (Rheinland-Pfalz) — Regular (+20% Speed (Emergency))

Category	Grid Cells	Area (%)	Population	Population (%)
High (30+ min)	0	0.0	0	0.0
Medium (20-30 min)	129	18.0	14926	20.4
Low (10-20 min)	314	43.8	26409	36.1
Only CT reachable within 60 min	0	0.0	0	0.0
Neither reachable within 60 min	0	0.0	0	0.0
Likely irrelevant (<10 min)	274	38.2	31851	43.5

Population and Area Coverage in Landkreis Fürstenfeldbruck (Stroke Units) (Bayern) — Regular (+20% Speed (Emergency))

Category	Grid Cells	Area (%)	Population	Population (%)
High (30+ min)	0	0.0	0	0.0
Medium (20-30 min)	0	0.0	0	0.0
Low (10-20 min)	1	0.2	0	0.0
Only CT reachable within 60 min	0	0.0	0	0.0
Neither reachable within 60 min	0	0.0	0	0.0
Likely irrelevant (<10 min)	525	99.8	214938	100.0

Population and Area Coverage in Landkreis Weilheim-Schongau (Stroke Units) (Bayern) — Regular (+20% Speed (Emergency))

Category	Grid Cells	Area (%)	Population	Population (%)
High (30+ min)	0	0.0	0	0.0
Medium (20-30 min)	92	7.9	20751	15.4
Low (10-20 min)	312	26.9	38717	28.7
Only CT reachable within 60 min	0	0.0	0	0.0
Neither reachable within 60 min	13	1.1	13	0.0
Likely irrelevant (<10 min)	743	64.1	75592	56.0

Population and Area Coverage in Landkreis Neustadt a.d.Waldnaab (Stroke Units) (Bayern) — Regular (+20% Speed (Emergency))

Category	Grid Cells	Area (%)	Population	Population (%)
High (30+ min)	0	0.0	0	0.0
Medium (20-30 min)	67	3.9	1833	1.9
Low (10-20 min)	539	31.3	21328	21.9
Only CT reachable within 60 min	0	0.0	0	0.0
Neither reachable within 60 min	0	0.0	0	0.0
Likely irrelevant (<10 min)	1116	64.8	74258	76.2

Population and Area Coverage in Landkreis Würzburg (Stroke Units) (Bayern) — Regular (+20% Speed (Emergency))

Category	Grid Cells	Area (%)	Population	Population (%)
High (30+ min)	0	0.0	0	0.0
Medium (20-30 min)	0	0.0	0	0.0
Low (10-20 min)	235	19.5	26583	16.1
Only CT reachable within 60 min	0	0.0	0	0.0
Neither reachable within 60 min	0	0.0	0	0.0
Likely irrelevant (<10 min)	970	80.5	138479	83.9

Population and Area Coverage in Landkreis Elbe-Elster (Stroke Units) (Brandenburg) — Regular (+20% Speed (Emergency))

Category	Grid Cells	Area (%)	Population	Population (%)
High (30+ min)	802	32.5	49204	48.3
Medium (20-30 min)	1059	42.9	37494	36.8
Low (10-20 min)	506	20.5	12767	12.5
Only CT reachable within 60 min	28	1.1	128	0.1
Neither reachable within 60 min	0	0.0	0	0.0
Likely irrelevant (<10 min)	74	3.0	2346	2.3

Population and Area Coverage in Landkreis Osterholz (Stroke Units) (Niedersachsen) — Regular (+20% Speed (Emergency))

Category	Grid Cells	Area (%)	Population	Population (%)
High (30+ min)	0	0.0	0	0.0
Medium (20-30 min)	236	26.8	44512	39.7
Low (10-20 min)	413	47.0	35338	31.5
Only CT reachable within 60 min	0	0.0	0	0.0
Neither reachable within 60 min	0	0.0	0	0.0
Likely irrelevant (<10 min)	230	26.2	32302	28.8

Population and Area Coverage in Landkreis Heidekreis (Stroke Units) (Niedersachsen) — Regular (+20% Speed (Emergency))

Category	Grid Cells	Area (%)	Population	Population (%)
High (30+ min)	186	7.4	29259	21.5
Medium (20-30 min)	913	36.3	45915	33.8
Low (10-20 min)	991	39.4	48425	35.6
Only CT reachable within 60 min	0	0.0	0	0.0
Neither reachable within 60 min	0	0.0	0	0.0
Likely irrelevant (<10 min)	426	16.9	12245	9.0

Population and Area Coverage in Kreisfreie Stadt Solingen (Stroke Units) (Nordrhein-Westfalen) — Regular (+20% Speed (Emergency))

Category	Grid Cells	Area (%)	Population	Population (%)
High (30+ min)	0	0.0	0	0.0
Medium (20-30 min)	0	0.0	0	0.0
Low (10-20 min)	76	66.1	141929	89.6
Only CT reachable within 60 min	0	0.0	0	0.0
Neither reachable within 60 min	0	0.0	0	0.0
Likely irrelevant (<10 min)	39	33.9	16509	10.4

Population and Area Coverage in Landkreis Bad Kreuznach (Stroke Units) (Rheinland-Pfalz) — Regular (+20% Speed (Emergency))

Category	Grid Cells	Area (%)	Population	Population (%)
High (30+ min)	3	0.3	574	0.4
Medium (20-30 min)	105	9.8	12102	7.6
Low (10-20 min)	389	36.3	30836	19.3
Only CT reachable within 60 min	0	0.0	0	0.0
Neither reachable within 60 min	0	0.0	0	0.0
Likely irrelevant (<10 min)	575	53.6	116657	72.8

Population and Area Coverage in Landkreis Südwestpfalz (Stroke Units) (Rheinland-Pfalz) — Regular (+20% Speed (Emergency))

Category	Grid Cells	Area (%)	Population	Population (%)
High (30+ min)	20	1.7	6491	5.8
Medium (20-30 min)	513	43.6	44867	40.0
Low (10-20 min)	453	38.5	45067	40.2
Only CT reachable within 60 min	0	0.0	5	0.0
Neither reachable within 60 min	10	0.8	60	0.1
Likely irrelevant (<10 min)	181	15.4	15651	14.0

Population and Area Coverage in Landkreis Ludwigsburg (Stroke Units) (Baden-Württemberg) — Regular (+20% Speed (Emergency))

Category	Grid Cells	Area (%)	Population	Population (%)
High (30+ min)	9	1.1	9894	1.9
Medium (20-30 min)	135	15.9	34741	6.6
Low (10-20 min)	340	40.1	169586	32.1
Only CT reachable within 60 min	0	0.0	0	0.0
Neither reachable within 60 min	0	0.0	0	0.0
Likely irrelevant (<10 min)	363	42.9	313322	59.4

Population and Area Coverage in Landkreis Lörrach (Stroke Units) (Baden-Württemberg) — Regular (+20% Speed (Emergency))

Category	Grid Cells	Area (%)	Population	Population (%)
High (30+ min)	0	0.0	0	0.0
Medium (20-30 min)	37	3.9	4390	2.0
Low (10-20 min)	459	48.9	51707	23.9
Only CT reachable within 60 min	1	0.1	971	0.4
Neither reachable within 60 min	0	0.0	9	0.0
Likely irrelevant (<10 min)	442	47.1	159567	73.7

Population and Area Coverage in Landkreis Alb-Donau-Kreis (Stroke Units) (Baden-Württemberg) — Regular (+20% Speed (Emergency))

Category	Grid Cells	Area (%)	Population	Population (%)
High (30+ min)	0	0.0	0	0.0
Medium (20-30 min)	153	9.2	22192	11.6
Low (10-20 min)	728	43.9	78335	41.0
Only CT reachable within 60 min	0	0.0	0	0.0
Neither reachable within 60 min	0	0.0	0	0.0
Likely irrelevant (<10 min)	777	46.9	90580	47.4

Population and Area Coverage in Landkreis Biberach (Stroke Units) (Baden-Württemberg) — Regular (+20% Speed (Emergency))

Category	Grid Cells	Area (%)	Population	Population (%)
High (30+ min)	0	0.0	0	0.0
Medium (20-30 min)	0	0.0	0	0.0
Low (10-20 min)	10	0.6	284	0.1
Only CT reachable within 60 min	0	0.0	0	0.0
Neither reachable within 60 min	0	0.0	0	0.0
Likely irrelevant (<10 min)	1694	99.4	192973	99.9

Population and Area Coverage in Kreisfreie Stadt Fürth (Stroke Units) (Bayern) — Regular (+20% Speed (Emergency))

Category	Grid Cells	Area (%)	Population	Population (%)
High (30+ min)	0	0.0	0	0.0
Medium (20-30 min)	0	0.0	0	0.0
Low (10-20 min)	0	0.0	0	0.0
Only CT reachable within 60 min	0	0.0	0	0.0
Neither reachable within 60 min	0	0.0	0	0.0
Likely irrelevant (<10 min)	83	100.0	150301	100.0

Population and Area Coverage in Kreisfreie Stadt Halle (Saale) (Stroke Units) (Sachsen-Anhalt) — Regular (+20% Speed (Emergency))

Category	Grid Cells	Area (%)	Population	Population (%)
High (30+ min)	0	0.0	0	0.0
Medium (20-30 min)	0	0.0	0	0.0
Low (10-20 min)	0	0.0	329	0.1
Only CT reachable within 60 min	0	0.0	0	0.0
Neither reachable within 60 min	0	0.0	0	0.0
Likely irrelevant (<10 min)	174	100.0	249843	99.9

Population and Area Coverage in Landkreis Wittenberg (Stroke Units) (Sachsen-Anhalt) — Regular (+20% Speed (Emergency))

Category	Grid Cells	Area (%)	Population	Population (%)
High (30+ min)	1025	40.4	65612	57.5
Medium (20-30 min)	536	21.1	17426	15.3
Low (10-20 min)	163	6.4	8961	7.9
Only CT reachable within 60 min	813	32.0	22082	19.4
Neither reachable within 60 min	0	0.0	0	0.0
Likely irrelevant (<10 min)	0	0.0	2	0.0

Population and Area Coverage in Landkreis Saale-Holzland-Kreis (Stroke Units) (Thüringen) — Regular (+20% Speed (Emergency))

Category	Grid Cells	Area (%)	Population	Population (%)
High (30+ min)	0	0.0	0	0.0
Medium (20-30 min)	67	6.5	9609	12.7
Low (10-20 min)	529	51.0	36510	48.2
Only CT reachable within 60 min	0	0.0	0	0.0
Neither reachable within 60 min	0	0.0	0	0.0
Likely irrelevant (<10 min)	442	42.6	29583	39.1

Population and Area Coverage in Kreisfreie Stadt Salzgitter (Stroke Units) (Niedersachsen) — Regular (+20% Speed (Emergency))

Category	Grid Cells	Area (%)	Population	Population (%)
High (30+ min)	1	0.3	822	0.8
Medium (20-30 min)	48	16.3	17881	17.9
Low (10-20 min)	190	64.4	55046	55.0
Only CT reachable within 60 min	0	0.0	0	0.0
Neither reachable within 60 min	0	0.0	0	0.0
Likely irrelevant (<10 min)	56	19.0	26303	26.3

Population and Area Coverage in Kreis Wesel (Stroke Units) (Nordrhein-Westfalen) — Regular (+20% Speed (Emergency))

Category	Grid Cells	Area (%)	Population	Population (%)
High (30+ min)	0	0.0	0	0.0
Medium (20-30 min)	58	4.3	15628	3.3
Low (10-20 min)	345	25.5	122966	25.9
Only CT reachable within 60 min	0	0.0	0	0.0
Neither reachable within 60 min	0	0.0	0	0.0
Likely irrelevant (<10 min)	950	70.2	336490	70.8

Population and Area Coverage in Kreis Rhein-Erft-Kreis (Stroke Units) (Nordrhein-Westfalen) — Regular (+20% Speed (Emergency))

Category	Grid Cells	Area (%)	Population	Population (%)
High (30+ min)	0	0.0	0	0.0
Medium (20-30 min)	7	0.8	1646	0.4
Low (10-20 min)	205	22.8	142442	31.3
Only CT reachable within 60 min	0	0.0	0	0.0
Neither reachable within 60 min	0	0.0	0	0.0
Likely irrelevant (<10 min)	686	76.4	310441	68.3

Population and Area Coverage in Kreisfreie Stadt Frankfurt am Main (Stroke Units) (Hessen) — Regular (+20% Speed (Emergency))

Category	Grid Cells	Area (%)	Population	Population (%)
High (30+ min)	0	0.0	0	0.0
Medium (20-30 min)	0	0.0	0	0.0
Low (10-20 min)	10	3.2	54512	6.5
Only CT reachable within 60 min	0	0.0	0	0.0
Neither reachable within 60 min	0	0.0	0	0.0
Likely irrelevant (<10 min)	300	96.8	778616	93.5

Population and Area Coverage in Landkreis Südliche Weinstraße (Stroke Units) (Rheinland-Pfalz) — Regular (+20% Speed (Emergency))

Category	Grid Cells	Area (%)	Population	Population (%)
High (30+ min)	1	0.1	1	0.0
Medium (20-30 min)	92	11.6	9984	8.9
Low (10-20 min)	373	47.0	47848	42.8
Only CT reachable within 60 min	0	0.0	0	0.0
Neither reachable within 60 min	0	0.0	0	0.0
Likely irrelevant (<10 min)	328	41.3	54027	48.3

Population and Area Coverage in Stadtkreis Karlsruhe (Stroke Units) (Baden-Württemberg) — Regular (+20% Speed (Emergency))

Category	Grid Cells	Area (%)	Population	Population (%)
High (30+ min)	0	0.0	0	0.0
Medium (20-30 min)	0	0.0	0	0.0
Low (10-20 min)	0	0.0	0	0.0
Only CT reachable within 60 min	0	0.0	0	0.0
Neither reachable within 60 min	0	0.0	0	0.0
Likely irrelevant (<10 min)	214	100.0	336385	100.0

Population and Area Coverage in Landkreis Rhein-Neckar-Kreis (Stroke Units) (Baden-Württemberg) — Regular (+20% Speed (Emergency))

Category	Grid Cells	Area (%)	Population	Population (%)
High (30+ min)	1	0.1	106	0.0
Medium (20-30 min)	191	14.5	39061	7.2
Low (10-20 min)	586	44.6	212643	39.3
Only CT reachable within 60 min	0	0.0	0	0.0
Neither reachable within 60 min	0	0.0	0	0.0
Likely irrelevant (<10 min)	536	40.8	289210	53.5

Population and Area Coverage in Landkreis München (Stroke Units) (Bayern) — Regular (+20% Speed (Emergency))

Category	Grid Cells	Area (%)	Population	Population (%)
High (30+ min)	0	0.0	0	0.0
Medium (20-30 min)	0	0.0	0	0.0
Low (10-20 min)	46	5.7	15789	4.6
Only CT reachable within 60 min	0	0.0	0	0.0
Neither reachable within 60 min	0	0.0	0	0.0
Likely irrelevant (<10 min)	757	94.3	328536	95.4

Population and Area Coverage in Landkreis Dingolfing-Landau (Stroke Units) (Bayern) — Regular (+20% Speed (Emergency))

Category	Grid Cells	Area (%)	Population	Population (%)
High (30+ min)	0	0.0	0	0.0
Medium (20-30 min)	3	0.3	145	0.2
Low (10-20 min)	201	18.7	19292	20.4
Only CT reachable within 60 min	0	0.0	0	0.0
Neither reachable within 60 min	0	0.0	0	0.0
Likely irrelevant (<10 min)	873	81.1	75027	79.4

Population and Area Coverage in Kreisfreie Stadt Nürnberg (Stroke Units) (Bayern) — Regular (+20% Speed (Emergency))

Category	Grid Cells	Area (%)	Population	Population (%)
High (30+ min)	0	0.0	0	0.0
Medium (20-30 min)	0	0.0	0	0.0
Low (10-20 min)	57	24.6	212054	40.9
Only CT reachable within 60 min	0	0.0	0	0.0
Neither reachable within 60 min	0	0.0	0	0.0
Likely irrelevant (<10 min)	175	75.4	306138	59.1

Population and Area Coverage in Kreisfreie Stadt Cottbus (Stroke Units) (Brandenburg) — Regular (+20% Speed (Emergency))

Category	Grid Cells	Area (%)	Population	Population (%)
High (30+ min)	0	0.0	0	0.0
Medium (20-30 min)	0	0.0	0	0.0
Low (10-20 min)	5	2.4	105	0.1
Only CT reachable within 60 min	0	0.0	0	0.0
Neither reachable within 60 min	0	0.0	0	0.0
Likely irrelevant (<10 min)	207	97.6	104399	99.9

Population and Area Coverage in Landkreis Salzlandkreis (Stroke Units) (Sachsen-Anhalt) — Regular (+20% Speed (Emergency))

Category	Grid Cells	Area (%)	Population	Population (%)
High (30+ min)	20	1.1	20	0.0
Medium (20-30 min)	241	12.9	44202	25.1
Low (10-20 min)	1044	55.9	79607	45.1
Only CT reachable within 60 min	0	0.0	0	0.0
Neither reachable within 60 min	0	0.0	0	0.0
Likely irrelevant (<10 min)	563	30.1	52488	29.8

Population and Area Coverage in Landkreis Sömmerda (Stroke Units) (Thüringen) — Regular (+20% Speed (Emergency))

Category	Grid Cells	Area (%)	Population	Population (%)
High (30+ min)	0	0.0	0	0.0
Medium (20-30 min)	137	13.1	18273	28.7
Low (10-20 min)	569	54.5	26535	41.7
Only CT reachable within 60 min	0	0.0	0	0.0
Neither reachable within 60 min	0	0.0	0	0.0
Likely irrelevant (<10 min)	338	32.4	18868	29.6

Population and Area Coverage in Landkreis Saale-Orla-Kreis (Stroke Units) (Thüringen) — Regular (+20% Speed (Emergency))

Category	Grid Cells	Area (%)	Population	Population (%)
High (30+ min)	9	0.6	7165	9.5
Medium (20-30 min)	203	13.9	13020	17.3
Low (10-20 min)	483	33.0	17464	23.2
Only CT reachable within 60 min	0	0.0	0	0.0
Neither reachable within 60 min	0	0.0	0	0.0
Likely irrelevant (<10 min)	770	52.6	37657	50.0

Population and Area Coverage in Kreisfreie Stadt Neumünster (Stroke-Ready Hospitals) (Schleswig-Holstein) — Extended (+20min Penalty)

Category	Grid Cells	Area (%)	Population	Population (%)
High (30+ min)	0	0.0	0	0.0
Medium (20-30 min)	0	0.0	0	0.0
Low (10-20 min)	0	0.0	0	0.0
Only CT reachable within 60 min	0	0.0	0	0.0
Neither reachable within 60 min	0	0.0	0	0.0
Likely irrelevant (<10 min)	100	100.0	85923	100.0

Population and Area Coverage in Kreisfreie Stadt Osnabrück (Stroke-Ready Hospitals) (Niedersachsen) — Extended (+20min Penalty)

Category	Grid Cells	Area (%)	Population	Population (%)
High (30+ min)	0	0.0	0	0.0
Medium (20-30 min)	0	0.0	0	0.0
Low (10-20 min)	0	0.0	0	0.0
Only CT reachable within 60 min	0	0.0	0	0.0
Neither reachable within 60 min	0	0.0	0	0.0
Likely irrelevant (<10 min)	155	100.0	154441	100.0

Population and Area Coverage in Kreisfreie Stadt Mönchengladbach (Stroke-Ready Hospitals) (Nordrhein-Westfalen) — Extended (+20min Penalty)

Category	Grid Cells	Area (%)	Population	Population (%)
High (30+ min)	0	0.0	0	0.0
Medium (20-30 min)	0	0.0	0	0.0
Low (10-20 min)	0	0.0	0	0.0
Only CT reachable within 60 min	0	0.0	0	0.0
Neither reachable within 60 min	0	0.0	0	0.0
Likely irrelevant (<10 min)	220	100.0	274121	100.0

Population and Area Coverage in Kreis Coesfeld (Stroke-Ready Hospitals) (Nordrhein-Westfalen) — Extended (+20min Penalty)

Category	Grid Cells	Area (%)	Population	Population (%)
High (30+ min)	0	0.0	0	0.0
Medium (20-30 min)	0	0.0	0	0.0
Low (10-20 min)	6	0.4	136	0.1
Only CT reachable within 60 min	0	0.0	0	0.0
Neither reachable within 60 min	0	0.0	0	0.0
Likely irrelevant (<10 min)	1448	99.6	215800	99.9

Population and Area Coverage in Kreis Paderborn (Stroke-Ready Hospitals) (Nordrhein-Westfalen) — Extended (+20min Penalty)

Category	Grid Cells	Area (%)	Population	Population (%)
High (30+ min)	0	0.0	0	0.0
Medium (20-30 min)	28	1.7	10	0.0
Low (10-20 min)	199	12.2	5730	1.7
Only CT reachable within 60 min	0	0.0	0	0.0
Neither reachable within 60 min	0	0.0	0	0.0
Likely irrelevant (<10 min)	1399	86.0	324781	98.3

Population and Area Coverage in Kreisfreie Stadt Kassel (Stroke-Ready Hospitals) (Hessen) — Extended (+20min Penalty)

Category	Grid Cells	Area (%)	Population	Population (%)
High (30+ min)	0	0.0	0	0.0
Medium (20-30 min)	0	0.0	0	0.0
Low (10-20 min)	0	0.0	0	0.0
Only CT reachable within 60 min	0	0.0	0	0.0
Neither reachable within 60 min	0	0.0	0	0.0
Likely irrelevant (<10 min)	133	100.0	205719	100.0

Population and Area Coverage in Landkreis Hersfeld-Rotenburg (Stroke-Ready Hospitals) (Hessen) — Extended (+20min Penalty)

Category	Grid Cells	Area (%)	Population	Population (%)
High (30+ min)	0	0.0	0	0.0
Medium (20-30 min)	40	2.9	290	0.2
Low (10-20 min)	205	14.6	12709	10.5
Only CT reachable within 60 min	0	0.0	0	0.0
Neither reachable within 60 min	0	0.0	0	0.0
Likely irrelevant (<10 min)	1156	82.5	107890	89.2

Population and Area Coverage in Landkreis Schwalm-Eder-Kreis (Stroke-Ready Hospitals) (Hessen) — Extended (+20min Penalty)

Category	Grid Cells	Area (%)	Population	Population (%)
High (30+ min)	0	0.0	0	0.0
Medium (20-30 min)	75	3.8	4759	2.6
Low (10-20 min)	393	19.9	23088	12.7
Only CT reachable within 60 min	0	0.0	0	0.0
Neither reachable within 60 min	0	0.0	0	0.0
Likely irrelevant (<10 min)	1511	76.4	153980	84.7

Population and Area Coverage in Landkreis Westerwaldkreis (Stroke-Ready Hospitals) (Rheinland-Pfalz) — Extended (+20min Penalty)

Category	Grid Cells	Area (%)	Population	Population (%)
High (30+ min)	0	0.0	0	0.0
Medium (20-30 min)	0	0.0	0	0.0
Low (10-20 min)	223	17.8	29036	14.1
Only CT reachable within 60 min	0	0.0	0	0.0
Neither reachable within 60 min	0	0.0	0	0.0
Likely irrelevant (<10 min)	1032	82.2	176788	85.9

Population and Area Coverage in Kreisfreie Stadt Frankenthal (Pfalz) (Stroke-Ready Hospitals) (Rheinland-Pfalz) — Extended (+20min Penalty)

Category	Grid Cells	Area (%)	Population	Population (%)
High (30+ min)	0	0.0	0	0.0
Medium (20-30 min)	0	0.0	0	0.0
Low (10-20 min)	0	0.0	0	0.0
Only CT reachable within 60 min	0	0.0	0	0.0
Neither reachable within 60 min	0	0.0	0	0.0
Likely irrelevant (<10 min)	54	100.0	45433	100.0

Population and Area Coverage in Landkreis Böblingen (Stroke-Ready Hospitals) (Baden-Württemberg) — Extended (+20min Penalty)

Category	Grid Cells	Area (%)	Population	Population (%)
High (30+ min)	0	0.0	0	0.0
Medium (20-30 min)	0	0.0	0	0.0
Low (10-20 min)	57	7.6	12239	3.3
Only CT reachable within 60 min	0	0.0	0	0.0
Neither reachable within 60 min	0	0.0	0	0.0
Likely irrelevant (<10 min)	692	92.4	360110	96.7

Population and Area Coverage in Landkreis Cham (Stroke-Ready Hospitals) (Bayern) — Extended (+20min Penalty)

Category	Grid Cells	Area (%)	Population	Population (%)
High (30+ min)	0	0.0	0	0.0
Medium (20-30 min)	39	2.2	1700	1.3
Low (10-20 min)	272	15.4	13638	10.7
Only CT reachable within 60 min	0	0.0	0	0.0
Neither reachable within 60 min	0	0.0	0	0.0
Likely irrelevant (<10 min)	1456	82.4	111531	87.9

Population and Area Coverage in Landkreis Saarlouis (Stroke-Ready Hospitals) (Saarland) — Extended (+20min Penalty)

Category	Grid Cells	Area (%)	Population	Population (%)
High (30+ min)	0	0.0	0	0.0
Medium (20-30 min)	1	0.2	3	0.0
Low (10-20 min)	42	7.4	5639	3.0
Only CT reachable within 60 min	1	0.2	320	0.2
Neither reachable within 60 min	2	0.4	41	0.0
Likely irrelevant (<10 min)	518	91.8	181206	96.8

Population and Area Coverage in Landkreis Anhalt-Bitterfeld (Stroke-Ready Hospitals) (Sachsen-Anhalt) — Extended (+20min Penalty)

Category	Grid Cells	Area (%)	Population	Population (%)
High (30+ min)	74	3.9	240	0.2
Medium (20-30 min)	373	19.5	6302	4.3
Low (10-20 min)	241	12.6	16718	11.3
Only CT reachable within 60 min	20	1.0	0	0.0
Neither reachable within 60 min	0	0.0	0	0.0
Likely irrelevant (<10 min)	1203	63.0	124237	84.2

Population and Area Coverage in Landkreis Jerichower Land (Stroke-Ready Hospitals) (Sachsen-Anhalt) — Extended (+20min Penalty)

Category	Grid Cells	Area (%)	Population	Population (%)
High (30+ min)	126	6.0	1465	1.8
Medium (20-30 min)	559	26.8	13353	16.6
Low (10-20 min)	808	38.7	30292	37.6
Only CT reachable within 60 min	154	7.4	916	1.1
Neither reachable within 60 min	0	0.0	0	0.0
Likely irrelevant (<10 min)	441	21.1	34473	42.8

Population and Area Coverage in Landkreis Kyffhäuserkreis (Stroke-Ready Hospitals) (Thüringen) — Extended (+20min Penalty)

Category	Grid Cells	Area (%)	Population	Population (%)
High (30+ min)	3	0.2	0	0.0
Medium (20-30 min)	191	14.3	4024	5.8
Low (10-20 min)	433	32.4	26395	37.9
Only CT reachable within 60 min	0	0.0	0	0.0
Neither reachable within 60 min	0	0.0	0	0.0
Likely irrelevant (<10 min)	710	53.1	39273	56.4

Population and Area Coverage in Landkreis Peine (Stroke-Ready Hospitals) (Niedersachsen) — Extended (+20min Penalty)

Category	Grid Cells	Area (%)	Population	Population (%)
High (30+ min)	0	0.0	0	0.0
Medium (20-30 min)	0	0.0	0	0.0
Low (10-20 min)	0	0.0	0	0.0
Only CT reachable within 60 min	0	0.0	0	0.0
Neither reachable within 60 min	0	0.0	0	0.0
Likely irrelevant (<10 min)	712	100.0	130717	100.0

Population and Area Coverage in Kreisfreie Stadt Leverkusen (Stroke-Ready Hospitals) (Nordrhein-Westfalen) — Extended (+20min Penalty)

Category	Grid Cells	Area (%)	Population	Population (%)
High (30+ min)	0	0.0	0	0.0
Medium (20-30 min)	0	0.0	0	0.0
Low (10-20 min)	0	0.0	0	0.0
Only CT reachable within 60 min	0	0.0	0	0.0
Neither reachable within 60 min	0	0.0	0	0.0
Likely irrelevant (<10 min)	102	100.0	160670	100.0

Population and Area Coverage in Kreis Ennepe-Ruhr-Kreis (Stroke-Ready Hospitals) (Nordrhein-Westfalen) — Extended (+20min Penalty)

Category	Grid Cells	Area (%)	Population	Population (%)
High (30+ min)	0	0.0	0	0.0
Medium (20-30 min)	0	0.0	0	0.0
Low (10-20 min)	20	3.7	3739	1.1
Only CT reachable within 60 min	0	0.0	0	0.0
Neither reachable within 60 min	0	0.0	0	0.0
Likely irrelevant (<10 min)	516	96.3	328922	98.9

Population and Area Coverage in Kreis Märkischer Kreis (Stroke-Ready Hospitals) (Nordrhein-Westfalen) — Extended (+20min Penalty)

Category	Grid Cells	Area (%)	Population	Population (%)
High (30+ min)	0	0.0	15	0.0
Medium (20-30 min)	17	1.3	669	0.2
Low (10-20 min)	203	15.0	31985	7.7
Only CT reachable within 60 min	0	0.0	0	0.0
Neither reachable within 60 min	0	0.0	0	0.0
Likely irrelevant (<10 min)	1137	83.8	382725	92.1

Population and Area Coverage in Landkreis Ahrweiler (Stroke-Ready Hospitals) (Rheinland-Pfalz) — Extended (+20min Penalty)

Category	Grid Cells	Area (%)	Population	Population (%)
High (30+ min)	0	0.0	0	0.0
Medium (20-30 min)	0	0.0	0	0.0
Low (10-20 min)	169	17.0	6104	4.7
Only CT reachable within 60 min	0	0.0	0	0.0
Neither reachable within 60 min	0	0.0	0	0.0
Likely irrelevant (<10 min)	824	83.0	122783	95.3

Population and Area Coverage in Stadtkreis Baden-Baden (Stroke-Ready Hospitals) (Baden-Württemberg) — Extended (+20min Penalty)

Category	Grid Cells	Area (%)	Population	Population (%)
High (30+ min)	0	0.0	0	0.0
Medium (20-30 min)	39	21.8	27	0.0
Low (10-20 min)	43	24.0	7756	12.3
Only CT reachable within 60 min	0	0.0	0	0.0
Neither reachable within 60 min	0	0.0	0	0.0
Likely irrelevant (<10 min)	97	54.2	55158	87.6

Population and Area Coverage in Landkreis Waldshut (Stroke-Ready Hospitals) (Baden-Württemberg) — Extended (+20min Penalty)

Category	Grid Cells	Area (%)	Population	Population (%)
High (30+ min)	0	0.0	0	0.0
Medium (20-30 min)	0	0.0	0	0.0
Low (10-20 min)	75	5.9	13047	9.2
Only CT reachable within 60 min	0	0.0	0	0.0
Neither reachable within 60 min	2	0.2	204	0.1
Likely irrelevant (<10 min)	1203	94.0	128298	90.6

Population and Area Coverage in Landkreis Bad Tölz-Wolfratshausen (Stroke-Ready Hospitals) (Bayern) — Extended (+20min Penalty)

Category	Grid Cells	Area (%)	Population	Population (%)
High (30+ min)	0	0.0	0	0.0
Medium (20-30 min)	4	0.3	34	0.0
Low (10-20 min)	53	4.0	1894	1.5
Only CT reachable within 60 min	1	0.1	5	0.0
Neither reachable within 60 min	109	8.3	176	0.1
Likely irrelevant (<10 min)	1152	87.3	124048	98.3

Population and Area Coverage in Landkreis Passau (Stroke-Ready Hospitals) (Bayern) — Extended (+20min Penalty)

Category	Grid Cells	Area (%)	Population	Population (%)
High (30+ min)	0	0.0	0	0.0
Medium (20-30 min)	55	3.1	2901	1.5
Low (10-20 min)	323	17.9	26465	14.0
Only CT reachable within 60 min	1	0.1	113	0.1
Neither reachable within 60 min	10	0.6	139	0.1
Likely irrelevant (<10 min)	1411	78.4	160095	84.4

Population and Area Coverage in Landkreis Lichtenfels (Stroke-Ready Hospitals) (Bayern) — Extended (+20min Penalty)

Category	Grid Cells	Area (%)	Population	Population (%)
High (30+ min)	0	0.0	0	0.0
Medium (20-30 min)	0	0.0	0	0.0
Low (10-20 min)	0	0.0	0	0.0
Only CT reachable within 60 min	0	0.0	0	0.0
Neither reachable within 60 min	0	0.0	0	0.0
Likely irrelevant (<10 min)	655	100.0	69197	100.0

Population and Area Coverage in Landkreis Wunsiedel i.Fichtelgebirge (Stroke-Ready Hospitals) (Bayern) — Extended (+20min Penalty)

Category	Grid Cells	Area (%)	Population	Population (%)
High (30+ min)	0	0.0	0	0.0
Medium (20-30 min)	29	4.1	1581	2.1
Low (10-20 min)	274	38.4	30702	41.5
Only CT reachable within 60 min	0	0.0	0	0.0
Neither reachable within 60 min	0	0.0	0	0.0
Likely irrelevant (<10 min)	411	57.6	41653	56.3

Population and Area Coverage in Landkreis Ansbach (Stroke-Ready Hospitals) (Bayern) — Extended (+20min Penalty)

Category	Grid Cells	Area (%)	Population	Population (%)
High (30+ min)	0	0.0	0	0.0
Medium (20-30 min)	30	1.2	960	0.5
Low (10-20 min)	255	10.5	19092	10.3
Only CT reachable within 60 min	0	0.0	0	0.0
Neither reachable within 60 min	0	0.0	0	0.0
Likely irrelevant (<10 min)	2147	88.3	164824	89.2

Population and Area Coverage in Kreisfreie Stadt Würzburg (Stroke-Ready Hospitals) (Bayern) — Extended (+20min Penalty)

Category	Grid Cells	Area (%)	Population	Population (%)
High (30+ min)	0	0.0	0	0.0
Medium (20-30 min)	0	0.0	0	0.0
Low (10-20 min)	0	0.0	0	0.0
Only CT reachable within 60 min	0	0.0	0	0.0
Neither reachable within 60 min	0	0.0	0	0.0
Likely irrelevant (<10 min)	109	100.0	128110	100.0

Population and Area Coverage in Landkreis Oder-Spree (Stroke-Ready Hospitals) (Brandenburg) — Extended (+20min Penalty)

Category	Grid Cells	Area (%)	Population	Population (%)
High (30+ min)	12	0.4	561	0.3
Medium (20-30 min)	181	6.1	4237	2.5
Low (10-20 min)	768	25.9	13953	8.1
Only CT reachable within 60 min	39	1.3	198	0.1
Neither reachable within 60 min	7	0.2	49	0.0
Likely irrelevant (<10 min)	1962	66.1	152932	89.0

Population and Area Coverage in Landkreis Potsdam-Mittelmark (Stroke-Ready Hospitals) (Brandenburg) — Extended (+20min Penalty)

Category	Grid Cells	Area (%)	Population	Population (%)
High (30+ min)	176	5.2	3374	1.7
Medium (20-30 min)	717	21.0	18652	9.2
Low (10-20 min)	1161	34.1	40724	20.1
Only CT reachable within 60 min	204	6.0	723	0.4
Neither reachable within 60 min	0	0.0	0	0.0
Likely irrelevant (<10 min)	1149	33.7	139525	68.7

Population and Area Coverage in Landkreis Uckermark (Stroke-Ready Hospitals) (Brandenburg) — Extended (+20min Penalty)

Category	Grid Cells	Area (%)	Population	Population (%)
High (30+ min)	588	14.3	9330	8.1
Medium (20-30 min)	923	22.4	26844	23.2
Low (10-20 min)	754	18.3	10945	9.5
Only CT reachable within 60 min	808	19.6	16472	14.2
Neither reachable within 60 min	85	2.1	1901	1.6
Likely irrelevant (<10 min)	963	23.4	50166	43.4

Population and Area Coverage in Landkreis Sächsische Schweiz-Osterzgebirge (Stroke-Ready Hospitals) (Sachsen) — Extended (+20min Penalty)

Category	Grid Cells	Area (%)	Population	Population (%)
High (30+ min)	94	4.5	4459	2.0
Medium (20-30 min)	361	17.4	22663	9.9
Low (10-20 min)	724	34.9	67723	29.7
Only CT reachable within 60 min	55	2.7	452	0.2
Neither reachable within 60 min	40	1.9	173	0.1
Likely irrelevant (<10 min)	798	38.5	132689	58.2

Population and Area Coverage in Kreisfreie Stadt Leipzig (Stroke-Ready Hospitals) (Sachsen) — Extended (+20min Penalty)

Category	Grid Cells	Area (%)	Population	Population (%)
High (30+ min)	0	0.0	0	0.0
Medium (20-30 min)	0	0.0	0	0.0
Low (10-20 min)	0	0.0	0	0.0
Only CT reachable within 60 min	0	0.0	0	0.0
Neither reachable within 60 min	0	0.0	0	0.0
Likely irrelevant (<10 min)	384	100.0	619326	100.0

Population and Area Coverage in Kreisfreie Stadt Jena (Stroke-Ready Hospitals) (Thüringen) — Extended (+20min Penalty)

Category	Grid Cells	Area (%)	Population	Population (%)
High (30+ min)	0	0.0	0	0.0
Medium (20-30 min)	0	0.0	0	0.0
Low (10-20 min)	2	1.3	0	0.0
Only CT reachable within 60 min	0	0.0	0	0.0
Neither reachable within 60 min	0	0.0	0	0.0
Likely irrelevant (<10 min)	147	98.7	100246	100.0

Population and Area Coverage in Landkreis Gotha (Stroke-Ready Hospitals) (Thüringen) — Extended (+20min Penalty)

Category	Grid Cells	Area (%)	Population	Population (%)
High (30+ min)	0	0.0	0	0.0
Medium (20-30 min)	2	0.2	5	0.0
Low (10-20 min)	156	13.0	568	0.5
Only CT reachable within 60 min	0	0.0	0	0.0
Neither reachable within 60 min	0	0.0	0	0.0
Likely irrelevant (<10 min)	1041	86.8	121703	99.5

Population and Area Coverage in Kreis Pinneberg (Stroke-Ready Hospitals) (Schleswig-Holstein) — Extended (+20min Penalty)

Category	Grid Cells	Area (%)	Population	Population (%)
High (30+ min)	0	0.0	0	0.0
Medium (20-30 min)	1	0.1	21	0.0
Low (10-20 min)	20	2.4	984	0.3
Only CT reachable within 60 min	1	0.1	14	0.0
Neither reachable within 60 min	19	2.2	121	0.0
Likely irrelevant (<10 min)	809	95.2	299357	99.6

Population and Area Coverage in Kreis Schleswig-Flensburg (Stroke-Ready Hospitals) (Schleswig-Holstein) — Extended (+20min Penalty)

Category	Grid Cells	Area (%)	Population	Population (%)
High (30+ min)	0	0.0	0	0.0
Medium (20-30 min)	10	0.4	1011	0.5
Low (10-20 min)	152	5.6	13011	6.6
Only CT reachable within 60 min	4	0.1	38	0.0
Neither reachable within 60 min	8	0.3	231	0.1
Likely irrelevant (<10 min)	2523	93.5	182502	92.7

Population and Area Coverage in Kreis Steinburg (Stroke-Ready Hospitals) (Schleswig-Holstein) — Extended (+20min Penalty)

Category	Grid Cells	Area (%)	Population	Population (%)
High (30+ min)	0	0.0	0	0.0
Medium (20-30 min)	1	0.1	43	0.0
Low (10-20 min)	76	5.5	3678	2.8
Only CT reachable within 60 min	0	0.0	0	0.0
Neither reachable within 60 min	9	0.7	432	0.3
Likely irrelevant (<10 min)	1296	93.8	128580	96.9

Population and Area Coverage in Landkreis Verden (Stroke-Ready Hospitals) (Niedersachsen) — Extended (+20min Penalty)

Category	Grid Cells	Area (%)	Population	Population (%)
High (30+ min)	0	0.0	0	0.0
Medium (20-30 min)	33	3.1	812	0.6
Low (10-20 min)	235	22.3	14363	10.8
Only CT reachable within 60 min	0	0.0	0	0.0
Neither reachable within 60 min	0	0.0	0	0.0
Likely irrelevant (<10 min)	784	74.5	117653	88.6

Population and Area Coverage in Kreisfreie Stadt Delmenhorst (Stroke-Ready Hospitals) (Niedersachsen) — Extended (+20min Penalty)

Category	Grid Cells	Area (%)	Population	Population (%)
High (30+ min)	0	0.0	0	0.0
Medium (20-30 min)	0	0.0	0	0.0
Low (10-20 min)	10	12.0	12550	17.1
Only CT reachable within 60 min	0	0.0	0	0.0
Neither reachable within 60 min	0	0.0	0	0.0
Likely irrelevant (<10 min)	73	88.0	60845	82.9

Population and Area Coverage in Kreis Kleve (Stroke-Ready Hospitals) (Nordrhein-Westfalen) — Extended (+20min Penalty)

Category	Grid Cells	Area (%)	Population	Population (%)
High (30+ min)	1	0.1	16	0.0
Medium (20-30 min)	7	0.4	191	0.1
Low (10-20 min)	76	4.8	5030	1.7
Only CT reachable within 60 min	8	0.5	273	0.1
Neither reachable within 60 min	14	0.9	127	0.0
Likely irrelevant (<10 min)	1465	93.3	297562	98.1

Population and Area Coverage in Kreis Herford (Stroke-Ready Hospitals) (Nordrhein-Westfalen) — Extended (+20min Penalty)

Category	Grid Cells	Area (%)	Population	Population (%)
High (30+ min)	0	0.0	0	0.0
Medium (20-30 min)	0	0.0	0	0.0
Low (10-20 min)	53	8.9	7085	2.8
Only CT reachable within 60 min	0	0.0	0	0.0
Neither reachable within 60 min	0	0.0	0	0.0
Likely irrelevant (<10 min)	542	91.1	243210	97.2

Population and Area Coverage in Kreisfreie Stadt Dortmund (Stroke-Ready Hospitals) (Nordrhein-Westfalen) — Extended (+20min Penalty)

Category	Grid Cells	Area (%)	Population	Population (%)
High (30+ min)	0	0.0	0	0.0
Medium (20-30 min)	0	0.0	0	0.0
Low (10-20 min)	0	0.0	0	0.0
Only CT reachable within 60 min	0	0.0	0	0.0
Neither reachable within 60 min	0	0.0	0	0.0
Likely irrelevant (<10 min)	363	100.0	611483	100.0

Population and Area Coverage in Kreisfreie Stadt Wiesbaden (Stroke-Ready Hospitals) (Hessen) — Extended (+20min Penalty)

Category	Grid Cells	Area (%)	Population	Population (%)
High (30+ min)	0	0.0	0	0.0
Medium (20-30 min)	0	0.0	0	0.0
Low (10-20 min)	0	0.0	0	0.0
Only CT reachable within 60 min	0	0.0	0	0.0
Neither reachable within 60 min	0	0.0	0	0.0
Likely irrelevant (<10 min)	252	100.0	277503	100.0

Population and Area Coverage in Landkreis Hochtaunuskreis (Stroke-Ready Hospitals) (Hessen) — Extended (+20min Penalty)

Category	Grid Cells	Area (%)	Population	Population (%)
High (30+ min)	0	0.0	0	0.0
Medium (20-30 min)	0	0.0	0	0.0
Low (10-20 min)	4	0.7	118	0.1
Only CT reachable within 60 min	0	0.0	0	0.0
Neither reachable within 60 min	0	0.0	0	0.0
Likely irrelevant (<10 min)	602	99.3	232402	99.9

Population and Area Coverage in Landkreis Kassel (Stroke-Ready Hospitals) (Hessen) — Extended (+20min Penalty)

Category	Grid Cells	Area (%)	Population	Population (%)
High (30+ min)	0	0.0	0	0.0
Medium (20-30 min)	84	5.0	3101	1.3
Low (10-20 min)	677	40.5	44276	18.7
Only CT reachable within 60 min	0	0.0	0	0.0
Neither reachable within 60 min	0	0.0	0	0.0
Likely irrelevant (<10 min)	909	54.4	189623	80.0

Population and Area Coverage in Landkreis Cochem-Zell (Stroke-Ready Hospitals) (Rheinland-Pfalz) — Extended (+20min Penalty)

Category	Grid Cells	Area (%)	Population	Population (%)
High (30+ min)	69	7.9	2988	4.6
Medium (20-30 min)	286	32.8	17571	27.3
Low (10-20 min)	257	29.5	24060	37.4
Only CT reachable within 60 min	5	0.6	34	0.1
Neither reachable within 60 min	0	0.0	0	0.0
Likely irrelevant (<10 min)	254	29.2	19703	30.6

Population and Area Coverage in Landkreis Rhein-Lahn-Kreis (Stroke-Ready Hospitals) (Rheinland-Pfalz) — Extended (+20min Penalty)

Category	Grid Cells	Area (%)	Population	Population (%)
High (30+ min)	25	2.5	900	0.7
Medium (20-30 min)	127	12.9	8520	6.8
Low (10-20 min)	273	27.7	21930	17.6
Only CT reachable within 60 min	0	0.0	0	0.0
Neither reachable within 60 min	0	0.0	0	0.0
Likely irrelevant (<10 min)	561	56.9	93324	74.9

Population and Area Coverage in Stadtkreis Heilbronn (Stroke-Ready Hospitals) (Baden-Württemberg) — Extended (+20min Penalty)

Category	Grid Cells	Area (%)	Population	Population (%)
High (30+ min)	0	0.0	0	0.0
Medium (20-30 min)	0	0.0	0	0.0
Low (10-20 min)	0	0.0	0	0.0
Only CT reachable within 60 min	0	0.0	0	0.0
Neither reachable within 60 min	0	0.0	0	0.0
Likely irrelevant (<10 min)	121	100.0	123086	100.0

Population and Area Coverage in Landkreis Schwandorf (Stroke-Ready Hospitals) (Bayern) — Extended (+20min Penalty)

Category	Grid Cells	Area (%)	Population	Population (%)
High (30+ min)	0	0.0	0	0.0
Medium (20-30 min)	42	2.4	2375	1.6
Low (10-20 min)	299	16.7	12049	8.1
Only CT reachable within 60 min	0	0.0	0	0.0
Neither reachable within 60 min	0	0.0	0	0.0
Likely irrelevant (<10 min)	1445	80.9	134110	90.3

Population and Area Coverage in Landkreis Unterallgäu (Stroke-Ready Hospitals) (Bayern) — Extended (+20min Penalty)

Category	Grid Cells	Area (%)	Population	Population (%)
High (30+ min)	0	0.0	0	0.0
Medium (20-30 min)	25	1.7	998	0.7
Low (10-20 min)	298	20.1	15499	10.9
Only CT reachable within 60 min	0	0.0	0	0.0
Neither reachable within 60 min	0	0.0	0	0.0
Likely irrelevant (<10 min)	1163	78.3	125775	88.4

Population and Area Coverage in Kreisfreie Stadt Frankfurt (Oder) (Stroke-Ready Hospitals) (Brandenburg) — Extended (+20min Penalty)

Category	Grid Cells	Area (%)	Population	Population (%)
High (30+ min)	0	0.0	0	0.0
Medium (20-30 min)	1	0.5	0	0.0
Low (10-20 min)	0	0.0	0	0.0
Only CT reachable within 60 min	4	2.0	464	0.8
Neither reachable within 60 min	15	7.7	524	0.9
Likely irrelevant (<10 min)	176	89.8	55128	98.2

Population and Area Coverage in Landkreis Dahme-Spreewald (Stroke-Ready Hospitals) (Brandenburg) — Extended (+20min Penalty)

Category	Grid Cells	Area (%)	Population	Population (%)
High (30+ min)	0	0.0	0	0.0
Medium (20-30 min)	35	1.2	329	0.2
Low (10-20 min)	514	17.3	13278	8.4
Only CT reachable within 60 min	0	0.0	0	0.0
Neither reachable within 60 min	0	0.0	0	0.0
Likely irrelevant (<10 min)	2420	81.5	144862	91.4

Population and Area Coverage in Landkreis Leipzig (Stroke-Ready Hospitals) (Sachsen) — Extended (+20min Penalty)

Category	Grid Cells	Area (%)	Population	Population (%)
High (30+ min)	0	0.0	0	0.0
Medium (20-30 min)	3	0.1	93	0.0
Low (10-20 min)	294	13.8	21237	8.3
Only CT reachable within 60 min	0	0.0	0	0.0
Neither reachable within 60 min	0	0.0	0	0.0
Likely irrelevant (<10 min)	1827	86.0	234095	91.6

Population and Area Coverage in Kreisfreie Stadt Magdeburg (Stroke-Ready Hospitals) (Sachsen-Anhalt) — Extended (+20min Penalty)

Category	Grid Cells	Area (%)	Population	Population (%)
High (30+ min)	0	0.0	0	0.0
Medium (20-30 min)	0	0.0	0	0.0
Low (10-20 min)	32	12.2	1613	0.6
Only CT reachable within 60 min	0	0.0	0	0.0
Neither reachable within 60 min	0	0.0	0	0.0
Likely irrelevant (<10 min)	231	87.8	247687	99.4

Population and Area Coverage in Landkreis Wartburgkreis (Stroke-Ready Hospitals) (Thüringen) — Extended (+20min Penalty)

Category	Grid Cells	Area (%)	Population	Population (%)
High (30+ min)	0	0.0	0	0.0
Medium (20-30 min)	0	0.0	0	0.0
Low (10-20 min)	119	6.8	4283	3.1
Only CT reachable within 60 min	0	0.0	0	0.0
Neither reachable within 60 min	0	0.0	0	0.0
Likely irrelevant (<10 min)	1632	93.2	133792	96.9

Population and Area Coverage in Landkreis Unstrut-Hainich-Kreis (Stroke-Ready Hospitals) (Thüringen) — Extended (+20min Penalty)

Category	Grid Cells	Area (%)	Population	Population (%)
High (30+ min)	0	0.0	0	0.0
Medium (20-30 min)	10	0.8	65	0.1
Low (10-20 min)	122	10.3	1701	1.9
Only CT reachable within 60 min	0	0.0	0	0.0
Neither reachable within 60 min	0	0.0	0	0.0
Likely irrelevant (<10 min)	1057	88.9	88503	98.0

Population and Area Coverage in Kreisfreie Stadt Bielefeld (Stroke-Ready Hospitals) (Nordrhein-Westfalen) — Extended (+20min Penalty)

Category	Grid Cells	Area (%)	Population	Population (%)
High (30+ min)	0	0.0	0	0.0
Medium (20-30 min)	0	0.0	0	0.0
Low (10-20 min)	0	0.0	0	0.0
Only CT reachable within 60 min	0	0.0	0	0.0
Neither reachable within 60 min	0	0.0	0	0.0
Likely irrelevant (<10 min)	336	100.0	349622	100.0

Population and Area Coverage in Kreisfreie Stadt Bochum (Stroke-Ready Hospitals) (Nordrhein-Westfalen) — Extended (+20min Penalty)

Category	Grid Cells	Area (%)	Population	Population (%)
High (30+ min)	0	0.0	0	0.0
Medium (20-30 min)	0	0.0	0	0.0
Low (10-20 min)	0	0.0	0	0.0
Only CT reachable within 60 min	0	0.0	0	0.0
Neither reachable within 60 min	0	0.0	0	0.0
Likely irrelevant (<10 min)	185	100.0	386703	100.0

Population and Area Coverage in Landkreis Fulda (Stroke-Ready Hospitals) (Hessen) — Extended (+20min Penalty)

Category	Grid Cells	Area (%)	Population	Population (%)
High (30+ min)	0	0.0	0	0.0
Medium (20-30 min)	0	0.0	0	0.0
Low (10-20 min)	142	8.1	5410	2.5
Only CT reachable within 60 min	0	0.0	0	0.0
Neither reachable within 60 min	0	0.0	0	0.0
Likely irrelevant (<10 min)	1609	91.9	214849	97.5

Population and Area Coverage in Landkreis Rastatt (Stroke-Ready Hospitals) (Baden-Württemberg) — Extended (+20min Penalty)

Category	Grid Cells	Area (%)	Population	Population (%)
High (30+ min)	0	0.0	0	0.0
Medium (20-30 min)	101	11.2	4075	1.8
Low (10-20 min)	238	26.5	41452	18.3
Only CT reachable within 60 min	0	0.0	11	0.0
Neither reachable within 60 min	2	0.2	31	0.0
Likely irrelevant (<10 min)	557	62.0	181557	79.9

Population and Area Coverage in Landkreis Breisgau-Hochschwarzwald (Stroke-Ready Hospitals) (Baden-Württemberg) — Extended (+20min Penalty)

Category	Grid Cells	Area (%)	Population	Population (%)
High (30+ min)	3	0.2	19	0.0
Medium (20-30 min)	4	0.2	10	0.0
Low (10-20 min)	185	11.3	14157	5.7
Only CT reachable within 60 min	0	0.0	0	0.0
Neither reachable within 60 min	0	0.0	3	0.0
Likely irrelevant (<10 min)	1451	88.3	232200	94.2

Population and Area Coverage in Landkreis Ebersberg (Stroke-Ready Hospitals) (Bayern) — Extended (+20min Penalty)

Category	Grid Cells	Area (%)	Population	Population (%)
High (30+ min)	0	0.0	0	0.0
Medium (20-30 min)	0	0.0	0	0.0
Low (10-20 min)	0	0.0	0	0.0
Only CT reachable within 60 min	0	0.0	0	0.0
Neither reachable within 60 min	0	0.0	0	0.0
Likely irrelevant (<10 min)	664	100.0	137985	100.0

Population and Area Coverage in Landkreis Rottal-Inn (Stroke-Ready Hospitals) (Bayern) — Extended (+20min Penalty)

Category	Grid Cells	Area (%)	Population	Population (%)
High (30+ min)	0	0.0	0	0.0
Medium (20-30 min)	0	0.0	0	0.0
Low (10-20 min)	109	7.1	4715	3.9
Only CT reachable within 60 min	0	0.0	0	0.0
Neither reachable within 60 min	2	0.1	63	0.1
Likely irrelevant (<10 min)	1428	92.8	116890	96.1

Population and Area Coverage in Landkreis Bamberg (Stroke-Ready Hospitals) (Bayern) — Extended (+20min Penalty)

Category	Grid Cells	Area (%)	Population	Population (%)
High (30+ min)	0	0.0	0	0.0
Medium (20-30 min)	33	2.3	114	0.1
Low (10-20 min)	419	28.6	15192	10.1
Only CT reachable within 60 min	0	0.0	0	0.0
Neither reachable within 60 min	0	0.0	0	0.0
Likely irrelevant (<10 min)	1013	69.1	135492	89.8

Population and Area Coverage in Landkreis Schweinfurt (Stroke-Ready Hospitals) (Bayern) — Extended (+20min Penalty)

Category	Grid Cells	Area (%)	Population	Population (%)
High (30+ min)	0	0.0	0	0.0
Medium (20-30 min)	0	0.0	0	0.0
Low (10-20 min)	66	6.2	494	0.4
Only CT reachable within 60 min	0	0.0	0	0.0
Neither reachable within 60 min	0	0.0	0	0.0
Likely irrelevant (<10 min)	993	93.8	116455	99.6

Population and Area Coverage in Landkreis St. Wendel (Stroke-Ready Hospitals) (Saarland) — Extended (+20min Penalty)

Category	Grid Cells	Area (%)	Population	Population (%)
High (30+ min)	0	0.0	0	0.0
Medium (20-30 min)	9	1.5	24	0.0
Low (10-20 min)	83	13.9	4446	5.2
Only CT reachable within 60 min	0	0.0	0	0.0
Neither reachable within 60 min	0	0.0	0	0.0
Likely irrelevant (<10 min)	506	84.6	80816	94.8

Population and Area Coverage in Landkreis Harz (Stroke-Ready Hospitals) (Sachsen-Anhalt) — Extended (+20min Penalty)

Category	Grid Cells	Area (%)	Population	Population (%)
High (30+ min)	2	0.1	11	0.0
Medium (20-30 min)	452	16.4	8042	4.0
Low (10-20 min)	1077	39.2	78871	39.4
Only CT reachable within 60 min	0	0.0	0	0.0
Neither reachable within 60 min	0	0.0	0	0.0
Likely irrelevant (<10 min)	1217	44.3	113178	56.6

Population and Area Coverage in Kreisfreie Stadt Gera (Stroke-Ready Hospitals) (Thüringen) — Extended (+20min Penalty)

Category	Grid Cells	Area (%)	Population	Population (%)
High (30+ min)	0	0.0	0	0.0
Medium (20-30 min)	0	0.0	0	0.0
Low (10-20 min)	20	10.5	930	0.8
Only CT reachable within 60 min	0	0.0	0	0.0
Neither reachable within 60 min	0	0.0	0	0.0
Likely irrelevant (<10 min)	171	89.5	108727	99.2

Population and Area Coverage in Kreisfreie Stadt Weimar (Stroke-Ready Hospitals) (Thüringen) — Extended (+20min Penalty)

Category	Grid Cells	Area (%)	Population	Population (%)
High (30+ min)	0	0.0	0	0.0
Medium (20-30 min)	0	0.0	0	0.0
Low (10-20 min)	0	0.0	0	0.0
Only CT reachable within 60 min	0	0.0	0	0.0
Neither reachable within 60 min	0	0.0	0	0.0
Likely irrelevant (<10 min)	109	100.0	57698	100.0

Population and Area Coverage in Landkreis Holzminden (Stroke-Ready Hospitals) (Niedersachsen) — Extended (+20min Penalty)

Category	Grid Cells	Area (%)	Population	Population (%)
High (30+ min)	0	0.0	0	0.0
Medium (20-30 min)	40	4.4	2984	4.2
Low (10-20 min)	163	18.0	8468	11.8
Only CT reachable within 60 min	0	0.0	0	0.0
Neither reachable within 60 min	0	0.0	0	0.0
Likely irrelevant (<10 min)	701	77.5	60336	84.0

Population and Area Coverage in Landkreis Grafschaft Bentheim (Stroke-Ready Hospitals) (Niedersachsen) — Extended (+20min Penalty)

Category	Grid Cells	Area (%)	Population	Population (%)
High (30+ min)	0	0.0	0	0.0
Medium (20-30 min)	1	0.1	16	0.0
Low (10-20 min)	32	2.5	719	0.5
Only CT reachable within 60 min	6	0.5	451	0.3
Neither reachable within 60 min	21	1.6	442	0.3
Likely irrelevant (<10 min)	1214	95.3	135048	98.8

Population and Area Coverage in Kreis Höxter (Stroke-Ready Hospitals) (Nordrhein-Westfalen) — Extended (+20min Penalty)

Category	Grid Cells	Area (%)	Population	Population (%)
High (30+ min)	0	0.0	0	0.0
Medium (20-30 min)	1	0.1	0	0.0
Low (10-20 min)	343	21.9	26335	18.4
Only CT reachable within 60 min	0	0.0	0	0.0
Neither reachable within 60 min	0	0.0	0	0.0
Likely irrelevant (<10 min)	1219	78.0	116456	81.6

Population and Area Coverage in Landkreis Marburg-Biedenkopf (Stroke-Ready Hospitals) (Hessen) — Extended (+20min Penalty)

Category	Grid Cells	Area (%)	Population	Population (%)
High (30+ min)	27	1.7	314	0.1
Medium (20-30 min)	188	11.8	29630	12.2
Low (10-20 min)	205	12.8	22177	9.1
Only CT reachable within 60 min	0	0.0	0	0.0
Neither reachable within 60 min	0	0.0	0	0.0
Likely irrelevant (<10 min)	1180	73.8	191241	78.6

Population and Area Coverage in Landkreis Rhein-Hunsrück-Kreis (Stroke-Ready Hospitals) (Rheinland-Pfalz) — Extended (+20min Penalty)

Category	Grid Cells	Area (%)	Population	Population (%)
High (30+ min)	61	4.9	3751	3.6
Medium (20-30 min)	475	38.2	31241	29.8
Low (10-20 min)	624	50.2	54829	52.2
Only CT reachable within 60 min	2	0.2	0	0.0
Neither reachable within 60 min	0	0.0	0	0.0
Likely irrelevant (<10 min)	82	6.6	15141	14.4

Population and Area Coverage in Landkreis Eifelkreis Bitburg-Prüm (Stroke-Ready Hospitals) (Rheinland-Pfalz) — Extended (+20min Penalty)

Category	Grid Cells	Area (%)	Population	Population (%)
High (30+ min)	123	6.0	1867	1.9
Medium (20-30 min)	857	42.1	31942	31.8
Low (10-20 min)	628	30.8	41045	40.9
Only CT reachable within 60 min	100	4.9	1324	1.3
Neither reachable within 60 min	2	0.1	127	0.1
Likely irrelevant (<10 min)	326	16.0	24031	24.0

Population and Area Coverage in Landkreis Erding (Stroke-Ready Hospitals) (Bayern) — Extended (+20min Penalty)

Category	Grid Cells	Area (%)	Population	Population (%)
High (30+ min)	0	0.0	0	0.0
Medium (20-30 min)	0	0.0	0	0.0
Low (10-20 min)	1	0.1	60	0.0
Only CT reachable within 60 min	0	0.0	0	0.0
Neither reachable within 60 min	0	0.0	0	0.0
Likely irrelevant (<10 min)	1054	99.9	135671	100.0

Population and Area Coverage in Landkreis Deggendorf (Stroke-Ready Hospitals) (Bayern) — Extended (+20min Penalty)

Category	Grid Cells	Area (%)	Population	Population (%)
High (30+ min)	0	0.0	0	0.0
Medium (20-30 min)	0	0.0	0	0.0
Low (10-20 min)	37	3.5	1513	1.3
Only CT reachable within 60 min	0	0.0	0	0.0
Neither reachable within 60 min	0	0.0	0	0.0
Likely irrelevant (<10 min)	1020	96.5	117937	98.7

Population and Area Coverage in Landkreis Weißenburg-Gunzenhausen (Stroke-Ready Hospitals) (Bayern) — Extended (+20min Penalty)

Category	Grid Cells	Area (%)	Population	Population (%)
High (30+ min)	0	0.0	0	0.0
Medium (20-30 min)	66	5.6	3150	3.3
Low (10-20 min)	372	31.3	19678	20.6
Only CT reachable within 60 min	0	0.0	0	0.0
Neither reachable within 60 min	0	0.0	0	0.0
Likely irrelevant (<10 min)	749	63.1	72583	76.1

Population and Area Coverage in Landkreis Lindau (Bodensee) (Stroke-Ready Hospitals) (Bayern) — Extended (+20min Penalty)

Category	Grid Cells	Area (%)	Population	Population (%)
High (30+ min)	0	0.0	0	0.0
Medium (20-30 min)	105	27.6	25472	31.4
Low (10-20 min)	215	56.6	45528	56.1
Only CT reachable within 60 min	5	1.3	230	0.3
Neither reachable within 60 min	10	2.6	682	0.8
Likely irrelevant (<10 min)	45	11.8	9278	11.4

Population and Area Coverage in Kreisfreie Stadt Rostock (Stroke-Ready Hospitals) (Mecklenburg-Vorpommern) — Extended (+20min Penalty)

Category	Grid Cells	Area (%)	Population	Population (%)
High (30+ min)	0	0.0	0	0.0
Medium (20-30 min)	11	4.9	2	0.0
Low (10-20 min)	60	26.7	1222	0.7
Only CT reachable within 60 min	1	0.4	33	0.0
Neither reachable within 60 min	5	2.2	576	0.3
Likely irrelevant (<10 min)	148	65.8	184572	99.0

Population and Area Coverage in Landkreis Rostock (Stroke-Ready Hospitals) (Mecklenburg-Vorpommern) — Extended (+20min Penalty)

Category	Grid Cells	Area (%)	Population	Population (%)
High (30+ min)	160	3.4	2540	1.3
Medium (20-30 min)	480	10.3	22289	11.2
Low (10-20 min)	1387	29.7	41983	21.2
Only CT reachable within 60 min	65	1.4	531	0.3
Neither reachable within 60 min	30	0.6	586	0.3
Likely irrelevant (<10 min)	2555	54.6	130444	65.8

Population and Area Coverage in Landkreis Mittelsachsen (Stroke-Ready Hospitals) (Sachsen) — Extended (+20min Penalty)

Category	Grid Cells	Area (%)	Population	Population (%)
High (30+ min)	0	0.0	0	0.0
Medium (20-30 min)	179	6.6	12886	4.4
Low (10-20 min)	689	25.4	73211	24.9
Only CT reachable within 60 min	2	0.1	0	0.0
Neither reachable within 60 min	0	0.0	0	0.0
Likely irrelevant (<10 min)	1840	67.9	208315	70.8

Population and Area Coverage in Landkreis Vorpommern-Greifswald (Stroke-Ready Hospitals) (Mecklenburg-Vorpommern) — Extended (+20min Penalty)

Category	Grid Cells	Area (%)	Population	Population (%)
High (30+ min)	265	5.3	12404	5.6
Medium (20-30 min)	876	17.4	24808	11.2
Low (10-20 min)	1298	25.8	49088	22.1
Only CT reachable within 60 min	474	9.4	16882	7.6
Neither reachable within 60 min	139	2.8	3468	1.6
Likely irrelevant (<10 min)	1987	39.4	115245	51.9

Population and Area Coverage in Landkreis Schmalkalden-Meiningen (Stroke-Ready Hospitals) (Thüringen) — Extended (+20min Penalty)

Category	Grid Cells	Area (%)	Population	Population (%)
High (30+ min)	0	0.0	0	0.0
Medium (20-30 min)	87	5.5	3441	3.0
Low (10-20 min)	323	20.4	32583	28.1
Only CT reachable within 60 min	0	0.0	0	0.0
Neither reachable within 60 min	0	0.0	0	0.0
Likely irrelevant (<10 min)	1175	74.1	79775	68.9

Population and Area Coverage in Kreisfreie Stadt Lübeck (Stroke-Ready Hospitals) (Schleswig-Holstein) — Extended (+20min Penalty)

Category	Grid Cells	Area (%)	Population	Population (%)
High (30+ min)	0	0.0	0	0.0
Medium (20-30 min)	0	0.0	0	0.0
Low (10-20 min)	40	13.8	3604	1.7
Only CT reachable within 60 min	2	0.7	2030	0.9
Neither reachable within 60 min	2	0.7	159	0.1
Likely irrelevant (<10 min)	245	84.8	212135	97.3

Population and Area Coverage in Kreis Nordfriesland (Stroke-Ready Hospitals) (Schleswig-Holstein) — Extended (+20min Penalty)

Category	Grid Cells	Area (%)	Population	Population (%)
High (30+ min)	288	10.9	7910	4.9
Medium (20-30 min)	571	21.7	48194	30.0
Low (10-20 min)	794	30.2	52318	32.6
Only CT reachable within 60 min	217	8.2	23066	14.4
Neither reachable within 60 min	150	5.7	4915	3.1
Likely irrelevant (<10 min)	613	23.3	24287	15.1

Population and Area Coverage in Landkreis Northeim (Stroke-Ready Hospitals) (Niedersachsen) — Extended (+20min Penalty)

Category	Grid Cells	Area (%)	Population	Population (%)
High (30+ min)	0	0.0	0	0.0
Medium (20-30 min)	51	3.1	1160	0.9
Low (10-20 min)	226	13.7	15651	11.7
Only CT reachable within 60 min	0	0.0	0	0.0
Neither reachable within 60 min	0	0.0	0	0.0
Likely irrelevant (<10 min)	1374	83.2	117301	87.5

Population and Area Coverage in Kreisfreie Stadt Wilhelmshaven (Stroke-Ready Hospitals) (Niedersachsen) — Extended (+20min Penalty)

Category	Grid Cells	Area (%)	Population	Population (%)
High (30+ min)	0	0.0	0	0.0
Medium (20-30 min)	0	0.0	0	0.0
Low (10-20 min)	0	0.0	0	0.0
Only CT reachable within 60 min	0	0.0	0	0.0
Neither reachable within 60 min	3	2.5	693	1.0
Likely irrelevant (<10 min)	118	97.5	68835	99.0

Population and Area Coverage in Kreisfreie Stadt Essen (Stroke-Ready Hospitals) (Nordrhein-Westfalen) — Extended (+20min Penalty)

Category	Grid Cells	Area (%)	Population	Population (%)
High (30+ min)	0	0.0	0	0.0
Medium (20-30 min)	0	0.0	0	0.0
Low (10-20 min)	0	0.0	0	0.0
Only CT reachable within 60 min	0	0.0	0	0.0
Neither reachable within 60 min	0	0.0	0	0.0
Likely irrelevant (<10 min)	276	100.0	596676	100.0

Population and Area Coverage in Kreisfreie Stadt Münster (Stroke-Ready Hospitals) (Nordrhein-Westfalen) — Extended (+20min Penalty)

Category	Grid Cells	Area (%)	Population	Population (%)
High (30+ min)	0	0.0	0	0.0
Medium (20-30 min)	0	0.0	0	0.0
Low (10-20 min)	0	0.0	0	0.0
Only CT reachable within 60 min	0	0.0	0	0.0
Neither reachable within 60 min	0	0.0	0	0.0
Likely irrelevant (<10 min)	399	100.0	364831	100.0

Population and Area Coverage in Kreis Recklinghausen (Stroke-Ready Hospitals) (Nordrhein-Westfalen) — Extended (+20min Penalty)

Category	Grid Cells	Area (%)	Population	Population (%)
High (30+ min)	0	0.0	0	0.0
Medium (20-30 min)	0	0.0	0	0.0
Low (10-20 min)	0	0.0	0	0.0
Only CT reachable within 60 min	0	0.0	0	0.0
Neither reachable within 60 min	0	0.0	0	0.0
Likely irrelevant (<10 min)	982	100.0	616413	100.0

Population and Area Coverage in Landkreis Offenbach (Stroke-Ready Hospitals) (Hessen) — Extended (+20min Penalty)

Category	Grid Cells	Area (%)	Population	Population (%)
High (30+ min)	0	0.0	0	0.0
Medium (20-30 min)	0	0.0	0	0.0
Low (10-20 min)	0	0.0	0	0.0
Only CT reachable within 60 min	0	0.0	0	0.0
Neither reachable within 60 min	0	0.0	0	0.0
Likely irrelevant (<10 min)	446	100.0	341311	100.0

Population and Area Coverage in Kreisfreie Stadt Trier (Stroke-Ready Hospitals) (Rheinland-Pfalz) — Extended (+20min Penalty)

Category	Grid Cells	Area (%)	Population	Population (%)
High (30+ min)	0	0.0	0	0.0
Medium (20-30 min)	0	0.0	0	0.0
Low (10-20 min)	0	0.0	0	0.0
Only CT reachable within 60 min	0	0.0	0	0.0
Neither reachable within 60 min	0	0.0	0	0.0
Likely irrelevant (<10 min)	146	100.0	128525	100.0

Population and Area Coverage in Landkreis Bernkastel-Wittlich (Stroke-Ready Hospitals) (Rheinland-Pfalz) — Extended (+20min Penalty)

Category	Grid Cells	Area (%)	Population	Population (%)
High (30+ min)	0	0.0	0	0.0
Medium (20-30 min)	29	2.0	251	0.2
Low (10-20 min)	409	28.0	21138	18.4
Only CT reachable within 60 min	0	0.0	0	0.0
Neither reachable within 60 min	0	0.0	0	0.0
Likely irrelevant (<10 min)	1023	70.0	93282	81.3

Population and Area Coverage in Kreisfreie Stadt Worms (Stroke-Ready Hospitals) (Rheinland-Pfalz) — Extended (+20min Penalty)

Category	Grid Cells	Area (%)	Population	Population (%)
High (30+ min)	0	0.0	0	0.0
Medium (20-30 min)	0	0.0	0	0.0
Low (10-20 min)	1	0.7	0	0.0
Only CT reachable within 60 min	0	0.0	0	0.0
Neither reachable within 60 min	0	0.0	0	0.0
Likely irrelevant (<10 min)	133	99.3	81874	100.0

Population and Area Coverage in Landkreis Heilbronn (Stroke-Ready Hospitals) (Baden-Württemberg) — Extended (+20min Penalty)

Category	Grid Cells	Area (%)	Population	Population (%)
High (30+ min)	0	0.0	0	0.0
Medium (20-30 min)	13	1.0	387	0.1
Low (10-20 min)	107	7.9	16078	4.9
Only CT reachable within 60 min	0	0.0	0	0.0
Neither reachable within 60 min	0	0.0	0	0.0
Likely irrelevant (<10 min)	1241	91.2	314939	95.0

Population and Area Coverage in Kreisfreie Stadt Ingolstadt (Stroke-Ready Hospitals) (Bayern) — Extended (+20min Penalty)

Category	Grid Cells	Area (%)	Population	Population (%)
High (30+ min)	0	0.0	0	0.0
Medium (20-30 min)	0	0.0	0	0.0
Low (10-20 min)	1	0.6	0	0.0
Only CT reachable within 60 min	0	0.0	0	0.0
Neither reachable within 60 min	0	0.0	0	0.0
Likely irrelevant (<10 min)	164	99.4	135219	100.0

Population and Area Coverage in Landkreis Forchheim (Stroke-Ready Hospitals) (Bayern) — Extended (+20min Penalty)

Category	Grid Cells	Area (%)	Population	Population (%)
High (30+ min)	0	0.0	0	0.0
Medium (20-30 min)	0	0.0	0	0.0
Low (10-20 min)	219	27.2	15220	12.9
Only CT reachable within 60 min	0	0.0	0	0.0
Neither reachable within 60 min	0	0.0	0	0.0
Likely irrelevant (<10 min)	586	72.8	103191	87.1

Population and Area Coverage in Landkreis Erlangen-Höchstadt (Stroke-Ready Hospitals) (Bayern) — Extended (+20min Penalty)

Category	Grid Cells	Area (%)	Population	Population (%)
High (30+ min)	0	0.0	0	0.0
Medium (20-30 min)	0	0.0	0	0.0
Low (10-20 min)	88	12.6	10629	7.7
Only CT reachable within 60 min	0	0.0	0	0.0
Neither reachable within 60 min	0	0.0	0	0.0
Likely irrelevant (<10 min)	610	87.4	126770	92.3

Population and Area Coverage in Landkreis Mansfeld-Südharz (Stroke-Ready Hospitals) (Sachsen-Anhalt) — Extended (+20min Penalty)

Category	Grid Cells	Area (%)	Population	Population (%)
High (30+ min)	0	0.0	0	0.0
Medium (20-30 min)	34	1.8	439	0.3
Low (10-20 min)	446	23.6	24907	19.5
Only CT reachable within 60 min	0	0.0	0	0.0
Neither reachable within 60 min	0	0.0	0	0.0
Likely irrelevant (<10 min)	1410	74.6	102591	80.2

Population and Area Coverage in Kreis Minden-Lübbecke (Stroke-Ready Hospitals) (Nordrhein-Westfalen) — Extended (+20min Penalty)

Category	Grid Cells	Area (%)	Population	Population (%)
High (30+ min)	0	0.0	0	0.0
Medium (20-30 min)	190	12.5	25643	8.3
Low (10-20 min)	439	28.9	56174	18.2
Only CT reachable within 60 min	0	0.0	0	0.0
Neither reachable within 60 min	0	0.0	0	0.0
Likely irrelevant (<10 min)	891	58.6	227656	73.6

Population and Area Coverage in Kreisfreie Stadt Hamm (Stroke-Ready Hospitals) (Nordrhein-Westfalen) — Extended (+20min Penalty)

Category	Grid Cells	Area (%)	Population	Population (%)
High (30+ min)	0	0.0	0	0.0
Medium (20-30 min)	0	0.0	0	0.0
Low (10-20 min)	0	0.0	0	0.0
Only CT reachable within 60 min	0	0.0	0	0.0
Neither reachable within 60 min	0	0.0	0	0.0
Likely irrelevant (<10 min)	293	100.0	175400	100.0

Population and Area Coverage in Landkreis Main-Taunus-Kreis (Stroke-Ready Hospitals) (Hessen) — Extended (+20min Penalty)

Category	Grid Cells	Area (%)	Population	Population (%)
High (30+ min)	0	0.0	0	0.0
Medium (20-30 min)	0	0.0	0	0.0
Low (10-20 min)	1	0.4	0	0.0
Only CT reachable within 60 min	0	0.0	0	0.0
Neither reachable within 60 min	0	0.0	0	0.0
Likely irrelevant (<10 min)	280	99.6	231433	100.0

Population and Area Coverage in Kreisfreie Stadt Ludwigshafen am Rhein (Stroke-Ready Hospitals) (Rheinland-Pfalz) — Extended (+20min Penalty)

Category	Grid Cells	Area (%)	Population	Population (%)
High (30+ min)	0	0.0	0	0.0
Medium (20-30 min)	0	0.0	0	0.0
Low (10-20 min)	0	0.0	0	0.0
Only CT reachable within 60 min	0	0.0	0	0.0
Neither reachable within 60 min	0	0.0	0	0.0
Likely irrelevant (<10 min)	99	100.0	163201	100.0

Population and Area Coverage in Landkreis Esslingen (Stroke-Ready Hospitals) (Baden-Württemberg) — Extended (+20min Penalty)

Category	Grid Cells	Area (%)	Population	Population (%)
High (30+ min)	0	0.0	0	0.0
Medium (20-30 min)	0	0.0	0	0.0
Low (10-20 min)	26	3.4	689	0.1
Only CT reachable within 60 min	0	0.0	0	0.0
Neither reachable within 60 min	0	0.0	0	0.0
Likely irrelevant (<10 min)	745	96.6	516470	99.9

Population and Area Coverage in Landkreis Dachau (Stroke-Ready Hospitals) (Bayern) — Extended (+20min Penalty)

Category	Grid Cells	Area (%)	Population	Population (%)
High (30+ min)	0	0.0	0	0.0
Medium (20-30 min)	0	0.0	0	0.0
Low (10-20 min)	7	1.0	103	0.1
Only CT reachable within 60 min	0	0.0	0	0.0
Neither reachable within 60 min	0	0.0	0	0.0
Likely irrelevant (<10 min)	697	99.0	150028	99.9

Population and Area Coverage in Landkreis Bad Kissingen (Stroke-Ready Hospitals) (Bayern) — Extended (+20min Penalty)

Category	Grid Cells	Area (%)	Population	Population (%)
High (30+ min)	0	0.0	0	0.0
Medium (20-30 min)	116	8.1	1445	1.4
Low (10-20 min)	666	46.5	54691	51.3
Only CT reachable within 60 min	0	0.0	0	0.0
Neither reachable within 60 min	0	0.0	0	0.0
Likely irrelevant (<10 min)	651	45.4	50489	47.4

Population and Area Coverage in Kreisfreie Stadt Erfurt (Stroke-Ready Hospitals) (Thüringen) — Extended (+20min Penalty)

Category	Grid Cells	Area (%)	Population	Population (%)
High (30+ min)	0	0.0	0	0.0
Medium (20-30 min)	0	0.0	0	0.0
Low (10-20 min)	0	0.0	0	0.0
Only CT reachable within 60 min	0	0.0	0	0.0
Neither reachable within 60 min	0	0.0	0	0.0
Likely irrelevant (<10 min)	342	100.0	247222	100.0

Population and Area Coverage in Kreisfreie Stadt Flensburg (Stroke-Ready Hospitals) (Schleswig-Holstein) — Extended (+20min Penalty)

Category	Grid Cells	Area (%)	Population	Population (%)
High (30+ min)	0	0.0	0	0.0
Medium (20-30 min)	0	0.0	0	0.0
Low (10-20 min)	0	0.0	0	0.0
Only CT reachable within 60 min	0	0.0	0	0.0
Neither reachable within 60 min	0	0.0	0	0.0
Likely irrelevant (<10 min)	62	100.0	80819	100.0

Population and Area Coverage in Kreis Dithmarschen (Stroke-Ready Hospitals) (Schleswig-Holstein) — Extended (+20min Penalty)

Category	Grid Cells	Area (%)	Population	Population (%)
High (30+ min)	0	0.0	0	0.0
Medium (20-30 min)	5	0.3	96	0.1
Low (10-20 min)	234	12.7	17509	13.1
Only CT reachable within 60 min	11	0.6	1168	0.9
Neither reachable within 60 min	32	1.7	320	0.2
Likely irrelevant (<10 min)	1567	84.7	114525	85.7

Population and Area Coverage in Landkreis Hameln-Pyrmont (Stroke-Ready Hospitals) (Niedersachsen) — Extended (+20min Penalty)

Category	Grid Cells	Area (%)	Population	Population (%)
High (30+ min)	0	0.0	0	0.0
Medium (20-30 min)	60	5.7	2531	1.7
Low (10-20 min)	185	17.7	14656	9.9
Only CT reachable within 60 min	0	0.0	0	0.0
Neither reachable within 60 min	0	0.0	0	0.0
Likely irrelevant (<10 min)	803	76.6	130214	88.3

Population and Area Coverage in Landkreis Celle (Stroke-Ready Hospitals) (Niedersachsen) — Extended (+20min Penalty)

Category	Grid Cells	Area (%)	Population	Population (%)
High (30+ min)	0	0.0	0	0.0
Medium (20-30 min)	0	0.0	0	0.0
Low (10-20 min)	68	3.3	1403	0.8
Only CT reachable within 60 min	0	0.0	0	0.0
Neither reachable within 60 min	0	0.0	0	0.0
Likely irrelevant (<10 min)	1999	96.7	174889	99.2

Population and Area Coverage in Landkreis Harburg (Stroke-Ready Hospitals) (Niedersachsen) — Extended (+20min Penalty)

Category	Grid Cells	Area (%)	Population	Population (%)
High (30+ min)	0	0.0	0	0.0
Medium (20-30 min)	0	0.0	0	0.0
Low (10-20 min)	9	0.5	699	0.3
Only CT reachable within 60 min	0	0.0	0	0.0
Neither reachable within 60 min	0	0.0	0	0.0
Likely irrelevant (<10 min)	1680	99.5	242665	99.7

Population and Area Coverage in Kreisfreie Stadt Düsseldorf (Stroke-Ready Hospitals) (Nordrhein-Westfalen) — Extended (+20min Penalty)

Category	Grid Cells	Area (%)	Population	Population (%)
High (30+ min)	0	0.0	0	0.0
Medium (20-30 min)	0	0.0	0	0.0
Low (10-20 min)	0	0.0	0	0.0
Only CT reachable within 60 min	0	0.0	0	0.0
Neither reachable within 60 min	0	0.0	0	0.0
Likely irrelevant (<10 min)	279	100.0	676424	100.0

Population and Area Coverage in Kreis Düren (Stroke-Ready Hospitals) (Nordrhein-Westfalen) — Extended (+20min Penalty)

Category	Grid Cells	Area (%)	Population	Population (%)
High (30+ min)	0	0.0	0	0.0
Medium (20-30 min)	18	1.5	87	0.0
Low (10-20 min)	62	5.2	1995	0.8
Only CT reachable within 60 min	0	0.0	0	0.0
Neither reachable within 60 min	0	0.0	0	0.0
Likely irrelevant (<10 min)	1120	93.3	254177	99.2

Population and Area Coverage in Kreis Soest (Stroke-Ready Hospitals) (Nordrhein-Westfalen) — Extended (+20min Penalty)

Category	Grid Cells	Area (%)	Population	Population (%)
High (30+ min)	0	0.0	0	0.0
Medium (20-30 min)	29	1.7	22	0.0
Low (10-20 min)	134	7.8	16022	5.4
Only CT reachable within 60 min	0	0.0	0	0.0
Neither reachable within 60 min	0	0.0	0	0.0
Likely irrelevant (<10 min)	1561	90.5	279101	94.6

Population and Area Coverage in Kreisfreie Stadt Darmstadt (Stroke-Ready Hospitals) (Hessen) — Extended (+20min Penalty)

Category	Grid Cells	Area (%)	Population	Population (%)
High (30+ min)	0	0.0	0	0.0
Medium (20-30 min)	0	0.0	0	0.0
Low (10-20 min)	0	0.0	0	0.0
Only CT reachable within 60 min	0	0.0	0	0.0
Neither reachable within 60 min	0	0.0	0	0.0
Likely irrelevant (<10 min)	154	100.0	151599	100.0

Population and Area Coverage in Kreisfreie Stadt Offenbach am Main (Stroke-Ready Hospitals) (Hessen) — Extended (+20min Penalty)

Category	Grid Cells	Area (%)	Population	Population (%)
High (30+ min)	0	0.0	0	0.0
Medium (20-30 min)	0	0.0	0	0.0
Low (10-20 min)	0	0.0	0	0.0
Only CT reachable within 60 min	0	0.0	0	0.0
Neither reachable within 60 min	0	0.0	0	0.0
Likely irrelevant (<10 min)	56	100.0	123120	100.0

Population and Area Coverage in Landkreis Werra-Meißner-Kreis (Stroke-Ready Hospitals) (Hessen) — Extended (+20min Penalty)

Category	Grid Cells	Area (%)	Population	Population (%)
High (30+ min)	0	0.0	0	0.0
Medium (20-30 min)	30	2.3	61	0.1
Low (10-20 min)	291	22.1	21696	21.5
Only CT reachable within 60 min	0	0.0	0	0.0
Neither reachable within 60 min	0	0.0	0	0.0
Likely irrelevant (<10 min)	997	75.6	79091	78.4

Population and Area Coverage in Landkreis Mayen-Koblenz (Stroke-Ready Hospitals) (Rheinland-Pfalz) — Extended (+20min Penalty)

Category	Grid Cells	Area (%)	Population	Population (%)
High (30+ min)	0	0.0	0	0.0
Medium (20-30 min)	33	3.2	1171	0.5
Low (10-20 min)	248	24.0	15407	7.1
Only CT reachable within 60 min	0	0.0	0	0.0
Neither reachable within 60 min	0	0.0	0	0.0
Likely irrelevant (<10 min)	753	72.8	200462	92.4

Population and Area Coverage in Landkreis Bad Dürkheim (Stroke-Ready Hospitals) (Rheinland-Pfalz) — Extended (+20min Penalty)

Category	Grid Cells	Area (%)	Population	Population (%)
High (30+ min)	0	0.0	0	0.0
Medium (20-30 min)	0	0.0	0	0.0
Low (10-20 min)	44	6.0	735	0.5
Only CT reachable within 60 min	0	0.0	0	0.0
Neither reachable within 60 min	0	0.0	0	0.0
Likely irrelevant (<10 min)	687	94.0	133703	99.5

Population and Area Coverage in Landkreis Landsberg am Lech (Stroke-Ready Hospitals) (Bayern) — Extended (+20min Penalty)

Category	Grid Cells	Area (%)	Population	Population (%)
High (30+ min)	0	0.0	0	0.0
Medium (20-30 min)	0	0.0	0	0.0
Low (10-20 min)	59	6.1	933	0.8
Only CT reachable within 60 min	0	0.0	0	0.0
Neither reachable within 60 min	0	0.0	0	0.0
Likely irrelevant (<10 min)	903	93.9	119997	99.2

Population and Area Coverage in Landkreis Donau-Ries (Stroke-Ready Hospitals) (Bayern) — Extended (+20min Penalty)

Category	Grid Cells	Area (%)	Population	Population (%)
High (30+ min)	19	1.2	315	0.2
Medium (20-30 min)	761	48.6	44133	33.1
Low (10-20 min)	592	37.8	72487	54.4
Only CT reachable within 60 min	0	0.0	0	0.0
Neither reachable within 60 min	0	0.0	0	0.0
Likely irrelevant (<10 min)	194	12.4	16389	12.3

Population and Area Coverage in Landkreis Märkisch-Oderland (Stroke-Ready Hospitals) (Brandenburg) — Extended (+20min Penalty)

Category	Grid Cells	Area (%)	Population	Population (%)
High (30+ min)	329	11.5	6902	3.7
Medium (20-30 min)	695	24.3	16852	9.2
Low (10-20 min)	887	31.1	44526	24.2
Only CT reachable within 60 min	61	2.1	620	0.3
Neither reachable within 60 min	76	2.7	610	0.3
Likely irrelevant (<10 min)	807	28.3	114595	62.2

Population and Area Coverage in Landkreis Prignitz (Stroke-Ready Hospitals) (Brandenburg) — Extended (+20min Penalty)

Category	Grid Cells	Area (%)	Population	Population (%)
High (30+ min)	0	0.0	2	0.0
Medium (20-30 min)	154	5.4	979	1.3
Low (10-20 min)	713	24.8	8258	11.1
Only CT reachable within 60 min	41	1.4	235	0.3
Neither reachable within 60 min	0	0.0	0	0.0
Likely irrelevant (<10 min)	1963	68.4	64894	87.3

Population and Area Coverage in Landkreis Burgenlandkreis (Stroke-Ready Hospitals) (Sachsen-Anhalt) — Extended (+20min Penalty)

Category	Grid Cells	Area (%)	Population	Population (%)
High (30+ min)	0	0.0	0	0.0
Medium (20-30 min)	2	0.1	63	0.0
Low (10-20 min)	302	16.6	21529	13.0
Only CT reachable within 60 min	0	0.0	0	0.0
Neither reachable within 60 min	0	0.0	0	0.0
Likely irrelevant (<10 min)	1515	83.3	143528	86.9

Population and Area Coverage in Landkreis Stendal (Stroke-Ready Hospitals) (Sachsen-Anhalt) — Extended (+20min Penalty)

Category	Grid Cells	Area (%)	Population	Population (%)
High (30+ min)	813	25.1	37146	35.9
Medium (20-30 min)	933	28.8	35095	33.9
Low (10-20 min)	607	18.8	16314	15.8
Only CT reachable within 60 min	566	17.5	5489	5.3
Neither reachable within 60 min	0	0.0	0	0.0
Likely irrelevant (<10 min)	315	9.7	9463	9.1

Population and Area Coverage in Landkreis Weimarer Land (Stroke-Ready Hospitals) (Thüringen) — Extended (+20min Penalty)

Category	Grid Cells	Area (%)	Population	Population (%)
High (30+ min)	0	0.0	0	0.0
Medium (20-30 min)	12	1.2	336	0.5
Low (10-20 min)	225	21.9	18319	24.8
Only CT reachable within 60 min	0	0.0	0	0.0
Neither reachable within 60 min	0	0.0	0	0.0
Likely irrelevant (<10 min)	790	76.9	55120	74.7

Population and Area Coverage in Kreisfreie Stadt Kiel (Stroke-Ready Hospitals) (Schleswig-Holstein) — Extended (+20min Penalty)

Category	Grid Cells	Area (%)	Population	Population (%)
High (30+ min)	1	0.6	221	0.1
Medium (20-30 min)	0	0.0	0	0.0
Low (10-20 min)	0	0.0	0	0.0
Only CT reachable within 60 min	0	0.0	0	0.0
Neither reachable within 60 min	0	0.0	1670	0.7
Likely irrelevant (<10 min)	154	99.4	254428	99.3

Population and Area Coverage in Landkreis Gifhorn (Stroke-Ready Hospitals) (Niedersachsen) — Extended (+20min Penalty)

Category	Grid Cells	Area (%)	Population	Population (%)
High (30+ min)	4	0.2	0	0.0
Medium (20-30 min)	101	4.8	3381	2.0
Low (10-20 min)	446	21.3	15339	8.9
Only CT reachable within 60 min	0	0.0	0	0.0
Neither reachable within 60 min	0	0.0	0	0.0
Likely irrelevant (<10 min)	1539	73.6	153482	89.1

Population and Area Coverage in Landkreis Helmstedt (Stroke-Ready Hospitals) (Niedersachsen) — Extended (+20min Penalty)

Category	Grid Cells	Area (%)	Population	Population (%)
High (30+ min)	0	0.0	0	0.0
Medium (20-30 min)	0	0.0	0	0.0
Low (10-20 min)	0	0.0	0	0.0
Only CT reachable within 60 min	0	0.0	0	0.0
Neither reachable within 60 min	0	0.0	0	0.0
Likely irrelevant (<10 min)	895	100.0	89413	100.0

Population and Area Coverage in Landkreis Lüchow-Dannenberg (Stroke-Ready Hospitals) (Niedersachsen) — Extended (+20min Penalty)

Category	Grid Cells	Area (%)	Population	Population (%)
High (30+ min)	358	21.8	18641	38.2
Medium (20-30 min)	513	31.3	17958	36.8
Low (10-20 min)	344	21.0	7683	15.7
Only CT reachable within 60 min	394	24.0	4193	8.6
Neither reachable within 60 min	0	0.0	0	0.0
Likely irrelevant (<10 min)	32	2.0	366	0.7

Population and Area Coverage in Landkreis Rotenburg (Wümme) (Stroke-Ready Hospitals) (Niedersachsen) — Extended (+20min Penalty)

Category	Grid Cells	Area (%)	Population	Population (%)
High (30+ min)	52	1.9	1018	0.6
Medium (20-30 min)	472	16.9	19494	11.9
Low (10-20 min)	614	22.0	35701	21.8
Only CT reachable within 60 min	0	0.0	0	0.0
Neither reachable within 60 min	0	0.0	0	0.0
Likely irrelevant (<10 min)	1656	59.3	107761	65.7

Population and Area Coverage in Kreis Euskirchen (Stroke-Ready Hospitals) (Nordrhein-Westfalen) — Extended (+20min Penalty)

Category	Grid Cells	Area (%)	Population	Population (%)
High (30+ min)	99	6.3	3890	2.1
Medium (20-30 min)	283	17.9	16245	8.6
Low (10-20 min)	317	20.0	23453	12.5
Only CT reachable within 60 min	22	1.4	44	0.0
Neither reachable within 60 min	0	0.0	2	0.0
Likely irrelevant (<10 min)	862	54.5	144603	76.8

Population and Area Coverage in Kreis Olpe (Stroke-Ready Hospitals) (Nordrhein-Westfalen) — Extended (+20min Penalty)

Category	Grid Cells	Area (%)	Population	Population (%)
High (30+ min)	127	13.9	6592	4.9
Medium (20-30 min)	214	23.4	47518	35.1
Low (10-20 min)	252	27.5	22099	16.3
Only CT reachable within 60 min	0	0.0	0	0.0
Neither reachable within 60 min	0	0.0	0	0.0
Likely irrelevant (<10 min)	322	35.2	59209	43.7

Population and Area Coverage in Landkreis Lahn-Dill-Kreis (Stroke-Ready Hospitals) (Hessen) — Extended (+20min Penalty)

Category	Grid Cells	Area (%)	Population	Population (%)
High (30+ min)	0	0.0	0	0.0
Medium (20-30 min)	30	2.2	2076	0.8
Low (10-20 min)	413	30.3	39764	15.5
Only CT reachable within 60 min	0	0.0	0	0.0
Neither reachable within 60 min	0	0.0	0	0.0
Likely irrelevant (<10 min)	921	67.5	213882	83.6

Population and Area Coverage in Landkreis Ravensburg (Stroke-Ready Hospitals) (Baden-Württemberg) — Extended (+20min Penalty)

Category	Grid Cells	Area (%)	Population	Population (%)
High (30+ min)	0	0.0	0	0.0
Medium (20-30 min)	7	0.4	321	0.1
Low (10-20 min)	228	11.6	27090	9.8
Only CT reachable within 60 min	0	0.0	0	0.0
Neither reachable within 60 min	0	0.0	0	0.0
Likely irrelevant (<10 min)	1725	88.0	249357	90.1

Population and Area Coverage in Kreisfreie Stadt Rosenheim (Stroke-Ready Hospitals) (Bayern) — Extended (+20min Penalty)

Category	Grid Cells	Area (%)	Population	Population (%)
High (30+ min)	0	0.0	0	0.0
Medium (20-30 min)	0	0.0	0	0.0
Low (10-20 min)	0	0.0	0	0.0
Only CT reachable within 60 min	0	0.0	0	0.0
Neither reachable within 60 min	0	0.0	0	0.0
Likely irrelevant (<10 min)	47	100.0	62621	100.0

Population and Area Coverage in Landkreis Freising (Stroke-Ready Hospitals) (Bayern) — Extended (+20min Penalty)

Category	Grid Cells	Area (%)	Population	Population (%)
High (30+ min)	0	0.0	0	0.0
Medium (20-30 min)	0	0.0	0	0.0
Low (10-20 min)	67	6.9	4945	2.8
Only CT reachable within 60 min	0	0.0	0	0.0
Neither reachable within 60 min	0	0.0	0	0.0
Likely irrelevant (<10 min)	907	93.1	169981	97.2

Population and Area Coverage in Landkreis Garmisch-Partenkirchen (Stroke-Ready Hospitals) (Bayern) — Extended (+20min Penalty)

Category	Grid Cells	Area (%)	Population	Population (%)
High (30+ min)	2	0.2	563	0.6
Medium (20-30 min)	87	7.3	9955	11.5
Low (10-20 min)	42	3.5	7300	8.4
Only CT reachable within 60 min	9	0.8	108	0.1
Neither reachable within 60 min	429	36.0	1071	1.2
Likely irrelevant (<10 min)	624	52.3	67920	78.1

Population and Area Coverage in Landkreis Regensburg (Stroke-Ready Hospitals) (Bayern) — Extended (+20min Penalty)

Category	Grid Cells	Area (%)	Population	Population (%)
High (30+ min)	0	0.0	0	0.0
Medium (20-30 min)	3	0.2	0	0.0
Low (10-20 min)	101	5.9	4191	2.2
Only CT reachable within 60 min	0	0.0	0	0.0
Neither reachable within 60 min	0	0.0	0	0.0
Likely irrelevant (<10 min)	1615	93.9	189520	97.8

Population and Area Coverage in Landkreis Tirschenreuth (Stroke-Ready Hospitals) (Bayern) — Extended (+20min Penalty)

Category	Grid Cells	Area (%)	Population	Population (%)
High (30+ min)	0	0.0	0	0.0
Medium (20-30 min)	175	13.6	5265	7.1
Low (10-20 min)	591	45.9	31687	42.6
Only CT reachable within 60 min	3	0.2	0	0.0
Neither reachable within 60 min	3	0.2	0	0.0
Likely irrelevant (<10 min)	516	40.1	37499	50.4

Population and Area Coverage in Kreisfreie Stadt Kaufbeuren (Stroke-Ready Hospitals) (Bayern) — Extended (+20min Penalty)

Category	Grid Cells	Area (%)	Population	Population (%)
High (30+ min)	0	0.0	0	0.0
Medium (20-30 min)	0	0.0	0	0.0
Low (10-20 min)	0	0.0	0	0.0
Only CT reachable within 60 min	0	0.0	0	0.0
Neither reachable within 60 min	0	0.0	0	0.0
Likely irrelevant (<10 min)	50	100.0	42940	100.0

Population and Area Coverage in Landkreis Oberallgäu (Stroke-Ready Hospitals) (Bayern) — Extended (+20min Penalty)

Category	Grid Cells	Area (%)	Population	Population (%)
High (30+ min)	14	0.8	424	0.3
Medium (20-30 min)	110	6.2	15438	9.9
Low (10-20 min)	224	12.6	21896	14.1
Only CT reachable within 60 min	13	0.7	311	0.2
Neither reachable within 60 min	539	30.3	4155	2.7
Likely irrelevant (<10 min)	876	49.3	113288	72.8

Population and Area Coverage in Landkreis Regionalverband Saarbrücken (Stroke-Ready Hospitals) (Saarland) — Extended (+20min Penalty)

Category	Grid Cells	Area (%)	Population	Population (%)
High (30+ min)	0	0.0	0	0.0
Medium (20-30 min)	0	0.0	0	0.0
Low (10-20 min)	1	0.2	72	0.0
Only CT reachable within 60 min	1	0.2	8	0.0
Neither reachable within 60 min	4	0.8	286	0.1
Likely irrelevant (<10 min)	482	98.8	331524	99.9

Population and Area Coverage in Landkreis Altenburger Land (Stroke-Ready Hospitals) (Thüringen) — Extended (+20min Penalty)

Category	Grid Cells	Area (%)	Population	Population (%)
High (30+ min)	0	0.0	0	0.0
Medium (20-30 min)	0	0.0	0	0.0
Low (10-20 min)	22	3.0	1457	1.7
Only CT reachable within 60 min	0	0.0	0	0.0
Neither reachable within 60 min	0	0.0	0	0.0
Likely irrelevant (<10 min)	704	97.0	82623	98.3

Population and Area Coverage in Landkreis Diepholz (Stroke-Ready Hospitals) (Niedersachsen) — Extended (+20min Penalty)

Category	Grid Cells	Area (%)	Population	Population (%)
High (30+ min)	27	1.0	217	0.1
Medium (20-30 min)	908	34.4	38537	18.2
Low (10-20 min)	1070	40.5	74448	35.3
Only CT reachable within 60 min	0	0.0	0	0.0
Neither reachable within 60 min	0	0.0	0	0.0
Likely irrelevant (<10 min)	636	24.1	97968	46.4

Population and Area Coverage in Kreisfreie Stadt Emden (Stroke-Ready Hospitals) (Niedersachsen) — Extended (+20min Penalty)

Category	Grid Cells	Area (%)	Population	Population (%)
High (30+ min)	0	0.0	0	0.0
Medium (20-30 min)	0	0.0	0	0.0
Low (10-20 min)	1	0.8	922	1.9
Only CT reachable within 60 min	0	0.0	0	0.0
Neither reachable within 60 min	0	0.0	0	0.0
Likely irrelevant (<10 min)	131	99.2	48199	98.1

Population and Area Coverage in Kreisfreie Stadt Gelsenkirchen (Stroke-Ready Hospitals) (Nordrhein-Westfalen) — Extended (+20min Penalty)

Category	Grid Cells	Area (%)	Population	Population (%)
High (30+ min)	0	0.0	0	0.0
Medium (20-30 min)	0	0.0	0	0.0
Low (10-20 min)	0	0.0	0	0.0
Only CT reachable within 60 min	0	0.0	0	0.0
Neither reachable within 60 min	0	0.0	0	0.0
Likely irrelevant (<10 min)	134	100.0	279785	100.0

Population and Area Coverage in Kreis Warendorf (Stroke-Ready Hospitals) (Nordrhein-Westfalen) — Extended (+20min Penalty)

Category	Grid Cells	Area (%)	Population	Population (%)
High (30+ min)	0	0.0	0	0.0
Medium (20-30 min)	0	0.0	0	0.0
Low (10-20 min)	6	0.3	37	0.0
Only CT reachable within 60 min	0	0.0	0	0.0
Neither reachable within 60 min	0	0.0	0	0.0
Likely irrelevant (<10 min)	1712	99.7	272033	100.0

Population and Area Coverage in Stadtkreis Ulm (Stroke-Ready Hospitals) (Baden-Württemberg) — Extended (+20min Penalty)

Category	Grid Cells	Area (%)	Population	Population (%)
High (30+ min)	0	0.0	0	0.0
Medium (20-30 min)	0	0.0	0	0.0
Low (10-20 min)	0	0.0	0	0.0
Only CT reachable within 60 min	0	0.0	0	0.0
Neither reachable within 60 min	0	0.0	0	0.0
Likely irrelevant (<10 min)	143	100.0	120114	100.0

Population and Area Coverage in Landkreis Traunstein (Stroke-Ready Hospitals) (Bayern) — Extended (+20min Penalty)

Category	Grid Cells	Area (%)	Population	Population (%)
High (30+ min)	0	0.0	0	0.0
Medium (20-30 min)	0	0.0	0	0.0
Low (10-20 min)	59	3.2	4789	2.7
Only CT reachable within 60 min	1	0.1	4	0.0
Neither reachable within 60 min	131	7.2	1464	0.8
Likely irrelevant (<10 min)	1626	89.5	169358	96.4

Population and Area Coverage in Kreisfreie Stadt Regensburg (Stroke-Ready Hospitals) (Bayern) — Extended (+20min Penalty)

Category	Grid Cells	Area (%)	Population	Population (%)
High (30+ min)	0	0.0	0	0.0
Medium (20-30 min)	0	0.0	0	0.0
Low (10-20 min)	0	0.0	0	0.0
Only CT reachable within 60 min	0	0.0	0	0.0
Neither reachable within 60 min	0	0.0	0	0.0
Likely irrelevant (<10 min)	97	100.0	170062	100.0

Population and Area Coverage in Kreisfreie Stadt Weiden i.d.OPf. (Stroke-Ready Hospitals) (Bayern) — Extended (+20min Penalty)

Category	Grid Cells	Area (%)	Population	Population (%)
High (30+ min)	0	0.0	0	0.0
Medium (20-30 min)	0	0.0	0	0.0
Low (10-20 min)	0	0.0	0	0.0
Only CT reachable within 60 min	0	0.0	0	0.0
Neither reachable within 60 min	0	0.0	0	0.0
Likely irrelevant (<10 min)	86	100.0	43316	100.0

Population and Area Coverage in Kreisfreie Stadt Ansbach (Stroke-Ready Hospitals) (Bayern) — Extended (+20min Penalty)

Category	Grid Cells	Area (%)	Population	Population (%)
High (30+ min)	0	0.0	0	0.0
Medium (20-30 min)	0	0.0	0	0.0
Low (10-20 min)	0	0.0	0	0.0
Only CT reachable within 60 min	0	0.0	0	0.0
Neither reachable within 60 min	0	0.0	0	0.0
Likely irrelevant (<10 min)	123	100.0	41201	100.0

Population and Area Coverage in Landkreis Rhön-Grabfeld (Stroke-Ready Hospitals) (Bayern) — Extended (+20min Penalty)

Category	Grid Cells	Area (%)	Population	Population (%)
High (30+ min)	0	0.0	0	0.0
Medium (20-30 min)	1	0.1	13	0.0
Low (10-20 min)	38	3.0	1835	2.2
Only CT reachable within 60 min	0	0.0	0	0.0
Neither reachable within 60 min	0	0.0	0	0.0
Likely irrelevant (<10 min)	1249	97.0	80330	97.8

Population and Area Coverage in Landkreis Ostprignitz-Ruppin (Stroke-Ready Hospitals) (Brandenburg) — Extended (+20min Penalty)

Category	Grid Cells	Area (%)	Population	Population (%)
High (30+ min)	95	2.8	1110	1.2
Medium (20-30 min)	662	19.6	10748	11.3
Low (10-20 min)	933	27.6	30107	31.8
Only CT reachable within 60 min	26	0.8	269	0.3
Neither reachable within 60 min	0	0.0	0	0.0
Likely irrelevant (<10 min)	1667	49.3	52476	55.4

Population and Area Coverage in Kreis Plön (Stroke-Ready Hospitals) (Schleswig-Holstein) — Extended (+20min Penalty)

Category	Grid Cells	Area (%)	Population	Population (%)
High (30+ min)	0	0.0	0	0.0
Medium (20-30 min)	10	0.7	175	0.1
Low (10-20 min)	446	30.0	19499	15.4
Only CT reachable within 60 min	2	0.1	179	0.1
Neither reachable within 60 min	17	1.1	1426	1.1
Likely irrelevant (<10 min)	1013	68.1	105074	83.2

Population and Area Coverage in Landkreis Nienburg (Weser) (Stroke-Ready Hospitals) (Niedersachsen) — Extended (+20min Penalty)

Category	Grid Cells	Area (%)	Population	Population (%)
High (30+ min)	0	0.0	0	0.0
Medium (20-30 min)	102	5.5	2428	2.0
Low (10-20 min)	380	20.4	16699	13.8
Only CT reachable within 60 min	0	0.0	0	0.0
Neither reachable within 60 min	0	0.0	0	0.0
Likely irrelevant (<10 min)	1383	74.2	102292	84.2

Population and Area Coverage in Landkreis Emsland (Stroke-Ready Hospitals) (Niedersachsen) — Extended (+20min Penalty)

Category	Grid Cells	Area (%)	Population	Population (%)
High (30+ min)	48	1.3	2433	0.8
Medium (20-30 min)	465	12.1	50004	15.5
Low (10-20 min)	788	20.5	45564	14.1
Only CT reachable within 60 min	7	0.2	111	0.0
Neither reachable within 60 min	20	0.5	353	0.1
Likely irrelevant (<10 min)	2511	65.4	223779	69.4

Population and Area Coverage in Landkreis Wittmund (Stroke-Ready Hospitals) (Niedersachsen) — Extended (+20min Penalty)

Category	Grid Cells	Area (%)	Population	Population (%)
High (30+ min)	2	0.2	169	0.3
Medium (20-30 min)	162	18.2	11250	19.8
Low (10-20 min)	328	36.9	18395	32.3
Only CT reachable within 60 min	2	0.2	16	0.0
Neither reachable within 60 min	50	5.6	2637	4.6
Likely irrelevant (<10 min)	344	38.7	24405	42.9

Population and Area Coverage in Kreis Hochsauerlandkreis (Stroke-Ready Hospitals) (Nordrhein-Westfalen) — Extended (+20min Penalty)

Category	Grid Cells	Area (%)	Population	Population (%)
High (30+ min)	357	14.2	21585	8.2
Medium (20-30 min)	693	27.6	53769	20.5
Low (10-20 min)	694	27.6	52310	19.9
Only CT reachable within 60 min	122	4.9	1382	0.5
Neither reachable within 60 min	0	0.0	0	0.0
Likely irrelevant (<10 min)	648	25.8	133323	50.8

Population and Area Coverage in Landkreis Rheingau-Taunus-Kreis (Stroke-Ready Hospitals) (Hessen) — Extended (+20min Penalty)

Category	Grid Cells	Area (%)	Population	Population (%)
High (30+ min)	18	1.8	447	0.2
Medium (20-30 min)	130	12.8	4527	2.5
Low (10-20 min)	344	33.8	32411	17.7
Only CT reachable within 60 min	0	0.0	0	0.0
Neither reachable within 60 min	0	0.0	0	0.0
Likely irrelevant (<10 min)	527	51.7	145870	79.6

Population and Area Coverage in Landkreis Göppingen (Stroke-Ready Hospitals) (Baden-Württemberg) — Extended (+20min Penalty)

Category	Grid Cells	Area (%)	Population	Population (%)
High (30+ min)	0	0.0	0	0.0
Medium (20-30 min)	0	0.0	0	0.0
Low (10-20 min)	4	0.5	19	0.0
Only CT reachable within 60 min	0	0.0	0	0.0
Neither reachable within 60 min	0	0.0	0	0.0
Likely irrelevant (<10 min)	777	99.5	249977	100.0

Population and Area Coverage in Stadtkreis Freiburg im Breisgau (Stroke-Ready Hospitals) (Baden-Württemberg) — Extended (+20min Penalty)

Category	Grid Cells	Area (%)	Population	Population (%)
High (30+ min)	0	0.0	0	0.0
Medium (20-30 min)	0	0.0	0	0.0
Low (10-20 min)	24	12.8	2170	0.8
Only CT reachable within 60 min	0	0.0	0	0.0
Neither reachable within 60 min	0	0.0	0	0.0
Likely irrelevant (<10 min)	163	87.2	256269	99.2

Population and Area Coverage in Landkreis Landshut (Stroke-Ready Hospitals) (Bayern) — Extended (+20min Penalty)

Category	Grid Cells	Area (%)	Population	Population (%)
High (30+ min)	0	0.0	0	0.0
Medium (20-30 min)	0	0.0	0	0.0
Low (10-20 min)	90	5.5	3589	2.3
Only CT reachable within 60 min	0	0.0	0	0.0
Neither reachable within 60 min	0	0.0	0	0.0
Likely irrelevant (<10 min)	1551	94.5	152101	97.7

Population and Area Coverage in Landkreis Regen (Stroke-Ready Hospitals) (Bayern) — Extended (+20min Penalty)

Category	Grid Cells	Area (%)	Population	Population (%)
High (30+ min)	1	0.1	0	0.0
Medium (20-30 min)	3	0.3	0	0.0
Low (10-20 min)	202	17.7	12232	15.8
Only CT reachable within 60 min	0	0.0	0	0.0
Neither reachable within 60 min	22	1.9	23	0.0
Likely irrelevant (<10 min)	912	80.0	65272	84.2

Population and Area Coverage in Kreisfreie Stadt Coburg (Stroke-Ready Hospitals) (Bayern) — Extended (+20min Penalty)

Category	Grid Cells	Area (%)	Population	Population (%)
High (30+ min)	0	0.0	0	0.0
Medium (20-30 min)	0	0.0	0	0.0
Low (10-20 min)	0	0.0	0	0.0
Only CT reachable within 60 min	0	0.0	0	0.0
Neither reachable within 60 min	0	0.0	0	0.0
Likely irrelevant (<10 min)	57	100.0	41384	100.0

Population and Area Coverage in Landkreis Neunkirchen (Stroke-Ready Hospitals) (Saarland) — Extended (+20min Penalty)

Category	Grid Cells	Area (%)	Population	Population (%)
High (30+ min)	0	0.0	0	0.0
Medium (20-30 min)	0	0.0	0	0.0
Low (10-20 min)	0	0.0	0	0.0
Only CT reachable within 60 min	0	0.0	0	0.0
Neither reachable within 60 min	0	0.0	0	0.0
Likely irrelevant (<10 min)	312	100.0	127343	100.0

Population and Area Coverage in Landkreis Nordwestmecklenburg (Stroke-Ready Hospitals) (Mecklenburg-Vorpommern) — Extended (+20min Penalty)

Category	Grid Cells	Area (%)	Population	Population (%)
High (30+ min)	9	0.3	139	0.1
Medium (20-30 min)	161	5.5	2217	1.5
Low (10-20 min)	710	24.4	19356	13.4
Only CT reachable within 60 min	13	0.4	412	0.3
Neither reachable within 60 min	54	1.9	2508	1.7
Likely irrelevant (<10 min)	1957	67.4	119364	82.9

Population and Area Coverage in Kreisfreie Stadt Herne (Stroke-Ready Hospitals) (Nordrhein-Westfalen) — Extended (+20min Penalty)

Category	Grid Cells	Area (%)	Population	Population (%)
High (30+ min)	0	0.0	0	0.0
Medium (20-30 min)	0	0.0	0	0.0
Low (10-20 min)	0	0.0	0	0.0
Only CT reachable within 60 min	0	0.0	0	0.0
Neither reachable within 60 min	0	0.0	0	0.0
Likely irrelevant (<10 min)	69	100.0	165244	100.0

Population and Area Coverage in Landkreis Altenkirchen (Westerwald) (Stroke-Ready Hospitals) (Rheinland-Pfalz) — Extended (+20min Penalty)

Category	Grid Cells	Area (%)	Population	Population (%)
High (30+ min)	0	0.0	0	0.0
Medium (20-30 min)	104	12.8	16137	12.1
Low (10-20 min)	330	40.5	54721	41.0
Only CT reachable within 60 min	0	0.0	0	0.0
Neither reachable within 60 min	0	0.0	0	0.0
Likely irrelevant (<10 min)	381	46.7	62551	46.9

Population and Area Coverage in Kreisfreie Stadt Kaiserslautern (Stroke-Ready Hospitals) (Rheinland-Pfalz) — Extended (+20min Penalty)

Category	Grid Cells	Area (%)	Population	Population (%)
High (30+ min)	0	0.0	0	0.0
Medium (20-30 min)	0	0.0	0	0.0
Low (10-20 min)	0	0.0	0	0.0
Only CT reachable within 60 min	0	0.0	0	0.0
Neither reachable within 60 min	0	0.0	0	0.0
Likely irrelevant (<10 min)	174	100.0	110813	100.0

Population and Area Coverage in Landkreis Mainz-Bingen (Stroke-Ready Hospitals) (Rheinland-Pfalz) — Extended (+20min Penalty)

Category	Grid Cells	Area (%)	Population	Population (%)
High (30+ min)	0	0.0	0	0.0
Medium (20-30 min)	13	1.7	1040	0.5
Low (10-20 min)	85	11.2	5956	2.8
Only CT reachable within 60 min	0	0.0	0	0.0
Neither reachable within 60 min	0	0.0	0	0.0
Likely irrelevant (<10 min)	663	87.1	203223	96.7

Population and Area Coverage in Landkreis Heidenheim (Stroke-Ready Hospitals) (Baden-Württemberg) — Extended (+20min Penalty)

Category	Grid Cells	Area (%)	Population	Population (%)
High (30+ min)	0	0.0	0	0.0
Medium (20-30 min)	13	1.7	379	0.3
Low (10-20 min)	78	10.3	3149	2.5
Only CT reachable within 60 min	0	0.0	0	0.0
Neither reachable within 60 min	0	0.0	0	0.0
Likely irrelevant (<10 min)	668	88.0	124461	97.2

Population and Area Coverage in Stadtkreis Pforzheim (Stroke-Ready Hospitals) (Baden-Württemberg) — Extended (+20min Penalty)

Category	Grid Cells	Area (%)	Population	Population (%)
High (30+ min)	0	0.0	0	0.0
Medium (20-30 min)	0	0.0	0	0.0
Low (10-20 min)	0	0.0	0	0.0
Only CT reachable within 60 min	0	0.0	0	0.0
Neither reachable within 60 min	0	0.0	0	0.0
Likely irrelevant (<10 min)	120	100.0	124962	100.0

Population and Area Coverage in Landkreis Tübingen (Stroke-Ready Hospitals) (Baden-Württemberg) — Extended (+20min Penalty)

Category	Grid Cells	Area (%)	Population	Population (%)
High (30+ min)	0	0.0	0	0.0
Medium (20-30 min)	0	0.0	0	0.0
Low (10-20 min)	21	3.3	6045	2.8
Only CT reachable within 60 min	0	0.0	0	0.0
Neither reachable within 60 min	0	0.0	0	0.0
Likely irrelevant (<10 min)	607	96.7	212493	97.2

Population and Area Coverage in Landkreis Bodenseekreis (Stroke-Ready Hospitals) (Baden-Württemberg) — Extended (+20min Penalty)

Category	Grid Cells	Area (%)	Population	Population (%)
High (30+ min)	0	0.0	0	0.0
Medium (20-30 min)	0	0.0	0	0.0
Low (10-20 min)	150	18.7	14288	6.5
Only CT reachable within 60 min	0	0.0	635	0.3
Neither reachable within 60 min	0	0.0	1958	0.9
Likely irrelevant (<10 min)	652	81.3	204193	92.4

Population and Area Coverage in Landkreis Miesbach (Stroke-Ready Hospitals) (Bayern) — Extended (+20min Penalty)

Category	Grid Cells	Area (%)	Population	Population (%)
High (30+ min)	0	0.0	0	0.0
Medium (20-30 min)	1	0.1	27	0.0
Low (10-20 min)	18	1.7	331	0.3
Only CT reachable within 60 min	2	0.2	1141	1.2
Neither reachable within 60 min	246	23.7	1166	1.2
Likely irrelevant (<10 min)	770	74.3	96068	97.3

Population and Area Coverage in Landkreis Mühldorf a.Inn (Stroke-Ready Hospitals) (Bayern) — Extended (+20min Penalty)

Category	Grid Cells	Area (%)	Population	Population (%)
High (30+ min)	0	0.0	0	0.0
Medium (20-30 min)	0	0.0	0	0.0
Low (10-20 min)	0	0.0	0	0.0
Only CT reachable within 60 min	0	0.0	0	0.0
Neither reachable within 60 min	0	0.0	0	0.0
Likely irrelevant (<10 min)	970	100.0	110636	100.0

Population and Area Coverage in Landkreis Neuburg-Schrobenhausen (Stroke-Ready Hospitals) (Bayern) — Extended (+20min Penalty)

Category	Grid Cells	Area (%)	Population	Population (%)
High (30+ min)	0	0.0	0	0.0
Medium (20-30 min)	112	12.4	5955	6.2
Low (10-20 min)	368	40.7	21750	22.8
Only CT reachable within 60 min	0	0.0	0	0.0
Neither reachable within 60 min	0	0.0	0	0.0
Likely irrelevant (<10 min)	424	46.9	67778	71.0

Population and Area Coverage in Landkreis Starnberg (Stroke-Ready Hospitals) (Bayern) — Extended (+20min Penalty)

Category	Grid Cells	Area (%)	Population	Population (%)
High (30+ min)	0	0.0	0	0.0
Medium (20-30 min)	0	0.0	0	0.0
Low (10-20 min)	11	1.9	884	0.7
Only CT reachable within 60 min	0	0.0	0	0.0
Neither reachable within 60 min	0	0.0	0	0.0
Likely irrelevant (<10 min)	575	98.1	133653	99.3

Population and Area Coverage in Landkreis Fürth (Stroke-Ready Hospitals) (Bayern) — Extended (+20min Penalty)

Category	Grid Cells	Area (%)	Population	Population (%)
High (30+ min)	0	0.0	0	0.0
Medium (20-30 min)	0	0.0	0	0.0
Low (10-20 min)	0	0.0	0	0.0
Only CT reachable within 60 min	0	0.0	0	0.0
Neither reachable within 60 min	0	0.0	0	0.0
Likely irrelevant (<10 min)	382	100.0	118705	100.0

Population and Area Coverage in Landkreis Kitzingen (Stroke-Ready Hospitals) (Bayern) — Extended (+20min Penalty)

Category	Grid Cells	Area (%)	Population	Population (%)
High (30+ min)	0	0.0	0	0.0
Medium (20-30 min)	0	0.0	0	0.0
Low (10-20 min)	102	12.1	2255	2.5
Only CT reachable within 60 min	0	0.0	0	0.0
Neither reachable within 60 min	0	0.0	0	0.0
Likely irrelevant (<10 min)	742	87.9	89174	97.5

Population and Area Coverage in Landkreis Günzburg (Stroke-Ready Hospitals) (Bayern) — Extended (+20min Penalty)

Category	Grid Cells	Area (%)	Population	Population (%)
High (30+ min)	0	0.0	0	0.0
Medium (20-30 min)	68	7.4	3827	3.1
Low (10-20 min)	251	27.2	29077	23.2
Only CT reachable within 60 min	0	0.0	0	0.0
Neither reachable within 60 min	0	0.0	0	0.0
Likely irrelevant (<10 min)	604	65.4	92215	73.7

Population and Area Coverage in Landkreis Havelland (Stroke-Ready Hospitals) (Brandenburg) — Extended (+20min Penalty)

Category	Grid Cells	Area (%)	Population	Population (%)
High (30+ min)	180	7.9	4344	2.9
Medium (20-30 min)	225	9.9	24203	16.0
Low (10-20 min)	332	14.5	9193	6.1
Only CT reachable within 60 min	156	6.8	985	0.7
Neither reachable within 60 min	0	0.0	0	0.0
Likely irrelevant (<10 min)	1389	60.9	112688	74.4

Population and Area Coverage in Landkreis Teltow-Fläming (Stroke-Ready Hospitals) (Brandenburg) — Extended (+20min Penalty)

Category	Grid Cells	Area (%)	Population	Population (%)
High (30+ min)	82	3.0	6041	3.8
Medium (20-30 min)	705	25.5	33839	21.5
Low (10-20 min)	925	33.5	27326	17.4
Only CT reachable within 60 min	140	5.1	694	0.4
Neither reachable within 60 min	0	0.0	0	0.0
Likely irrelevant (<10 min)	913	33.0	89170	56.8

Population and Area Coverage in Landkreis Börde (Stroke-Ready Hospitals) (Sachsen-Anhalt) — Extended (+20min Penalty)

Category	Grid Cells	Area (%)	Population	Population (%)
High (30+ min)	0	0.0	0	0.0
Medium (20-30 min)	112	3.6	2344	1.5
Low (10-20 min)	574	18.4	25366	16.3
Only CT reachable within 60 min	0	0.0	0	0.0
Neither reachable within 60 min	0	0.0	0	0.0
Likely irrelevant (<10 min)	2437	78.0	127673	82.2

Population and Area Coverage in Landkreis Stade (Stroke-Ready Hospitals) (Niedersachsen) — Extended (+20min Penalty)

Category	Grid Cells	Area (%)	Population	Population (%)
High (30+ min)	20	1.2	608	0.3
Medium (20-30 min)	41	2.5	2318	1.2
Low (10-20 min)	250	15.2	11193	5.6
Only CT reachable within 60 min	10	0.6	135	0.1
Neither reachable within 60 min	100	6.1	4948	2.5
Likely irrelevant (<10 min)	1220	74.3	178964	90.3

Population and Area Coverage in Kreisfreie Stadt Oldenburg (Oldb) (Stroke-Ready Hospitals) (Niedersachsen) — Extended (+20min Penalty)

Category	Grid Cells	Area (%)	Population	Population (%)
High (30+ min)	0	0.0	0	0.0
Medium (20-30 min)	0	0.0	0	0.0
Low (10-20 min)	0	0.0	0	0.0
Only CT reachable within 60 min	0	0.0	0	0.0
Neither reachable within 60 min	0	0.0	0	0.0
Likely irrelevant (<10 min)	137	100.0	159948	100.0

Population and Area Coverage in Landkreis Cloppenburg (Stroke-Ready Hospitals) (Niedersachsen) — Extended (+20min Penalty)

Category	Grid Cells	Area (%)	Population	Population (%)
High (30+ min)	1	0.1	2	0.0
Medium (20-30 min)	234	12.3	8369	5.1
Low (10-20 min)	715	37.6	59398	36.2
Only CT reachable within 60 min	0	0.0	0	0.0
Neither reachable within 60 min	0	0.0	0	0.0
Likely irrelevant (<10 min)	952	50.1	96307	58.7

Population and Area Coverage in Kreisfreie Stadt Wuppertal (Stroke-Ready Hospitals) (Nordrhein-Westfalen) — Extended (+20min Penalty)

Category	Grid Cells	Area (%)	Population	Population (%)
High (30+ min)	0	0.0	0	0.0
Medium (20-30 min)	0	0.0	0	0.0
Low (10-20 min)	0	0.0	0	0.0
Only CT reachable within 60 min	0	0.0	0	0.0
Neither reachable within 60 min	0	0.0	0	0.0
Likely irrelevant (<10 min)	213	100.0	359073	100.0

Population and Area Coverage in Landkreis Odenwaldkreis (Stroke-Ready Hospitals) (Hessen) — Extended (+20min Penalty)

Category	Grid Cells	Area (%)	Population	Population (%)
High (30+ min)	0	0.0	0	0.0
Medium (20-30 min)	1	0.1	0	0.0
Low (10-20 min)	72	9.3	8461	8.6
Only CT reachable within 60 min	0	0.0	0	0.0
Neither reachable within 60 min	0	0.0	0	0.0
Likely irrelevant (<10 min)	703	90.6	89429	91.4

Population and Area Coverage in Landkreis Karlsruhe (Stroke-Ready Hospitals) (Baden-Württemberg) — Extended (+20min Penalty)

Category	Grid Cells	Area (%)	Population	Population (%)
High (30+ min)	0	0.0	0	0.0
Medium (20-30 min)	0	0.0	0	0.0
Low (10-20 min)	53	4.0	8919	2.0
Only CT reachable within 60 min	0	0.0	0	0.0
Neither reachable within 60 min	0	0.0	0	0.0
Likely irrelevant (<10 min)	1282	96.0	426916	98.0

Population and Area Coverage in Kreisfreie Stadt Landshut (Stroke-Ready Hospitals) (Bayern) — Extended (+20min Penalty)

Category	Grid Cells	Area (%)	Population	Population (%)
High (30+ min)	0	0.0	0	0.0
Medium (20-30 min)	0	0.0	0	0.0
Low (10-20 min)	0	0.0	0	0.0
Only CT reachable within 60 min	0	0.0	0	0.0
Neither reachable within 60 min	0	0.0	0	0.0
Likely irrelevant (<10 min)	81	100.0	69058	100.0

Population and Area Coverage in Kreisfreie Stadt Erlangen (Stroke-Ready Hospitals) (Bayern) — Extended (+20min Penalty)

Category	Grid Cells	Area (%)	Population	Population (%)
High (30+ min)	0	0.0	0	0.0
Medium (20-30 min)	0	0.0	0	0.0
Low (10-20 min)	0	0.0	0	0.0
Only CT reachable within 60 min	0	0.0	0	0.0
Neither reachable within 60 min	0	0.0	0	0.0
Likely irrelevant (<10 min)	96	100.0	126629	100.0

Population and Area Coverage in Landkreis Haßberge (Stroke-Ready Hospitals) (Bayern) — Extended (+20min Penalty)

Category	Grid Cells	Area (%)	Population	Population (%)
High (30+ min)	0	0.0	0	0.0
Medium (20-30 min)	3	0.2	1	0.0
Low (10-20 min)	726	60.5	31737	36.4
Only CT reachable within 60 min	0	0.0	0	0.0
Neither reachable within 60 min	0	0.0	0	0.0
Likely irrelevant (<10 min)	471	39.2	55428	63.6

Population and Area Coverage in Landkreis Aichach-Friedberg (Stroke-Ready Hospitals) (Bayern) — Extended (+20min Penalty)

Category	Grid Cells	Area (%)	Population	Population (%)
High (30+ min)	0	0.0	0	0.0
Medium (20-30 min)	8	0.8	309	0.2
Low (10-20 min)	192	20.3	21081	15.8
Only CT reachable within 60 min	0	0.0	0	0.0
Neither reachable within 60 min	0	0.0	0	0.0
Likely irrelevant (<10 min)	745	78.8	112021	84.0

Population and Area Coverage in Landkreis Dillingen a.d.Donau (Stroke-Ready Hospitals) (Bayern) — Extended (+20min Penalty)

Category	Grid Cells	Area (%)	Population	Population (%)
High (30+ min)	5	0.5	151	0.2
Medium (20-30 min)	145	14.9	5111	5.3
Low (10-20 min)	324	33.4	27867	28.7
Only CT reachable within 60 min	0	0.0	0	0.0
Neither reachable within 60 min	0	0.0	0	0.0
Likely irrelevant (<10 min)	496	51.1	63969	65.9

Population and Area Coverage in Landkreis Oberspreewald-Lausitz (Stroke-Ready Hospitals) (Brandenburg) — Extended (+20min Penalty)

Category	Grid Cells	Area (%)	Population	Population (%)
High (30+ min)	0	0.0	0	0.0
Medium (20-30 min)	15	0.9	132	0.1
Low (10-20 min)	194	12.3	4023	3.7
Only CT reachable within 60 min	0	0.0	0	0.0
Neither reachable within 60 min	0	0.0	0	0.0
Likely irrelevant (<10 min)	1371	86.8	104303	96.2

Population and Area Coverage in Landkreis Bautzen (Stroke-Ready Hospitals) (Sachsen) — Extended (+20min Penalty)

Category	Grid Cells	Area (%)	Population	Population (%)
High (30+ min)	46	1.5	7908	2.7
Medium (20-30 min)	279	9.1	25834	9.0
Low (10-20 min)	1144	37.1	97216	33.8
Only CT reachable within 60 min	2	0.1	120	0.0
Neither reachable within 60 min	5	0.2	89	0.0
Likely irrelevant (<10 min)	1606	52.1	156856	54.5

Population and Area Coverage in Kreisfreie Stadt Dessau-Roßlau (Stroke-Ready Hospitals) (Sachsen-Anhalt) — Extended (+20min Penalty)

Category	Grid Cells	Area (%)	Population	Population (%)
High (30+ min)	0	0.0	0	0.0
Medium (20-30 min)	17	5.3	94	0.1
Low (10-20 min)	42	13.1	472	0.6
Only CT reachable within 60 min	0	0.0	0	0.0
Neither reachable within 60 min	0	0.0	0	0.0
Likely irrelevant (<10 min)	261	81.6	83547	99.3

Population and Area Coverage in Landkreis Lüneburg (Stroke-Ready Hospitals) (Niedersachsen) — Extended (+20min Penalty)

Category	Grid Cells	Area (%)	Population	Population (%)
High (30+ min)	45	2.5	581	0.3
Medium (20-30 min)	108	6.1	2357	1.3
Low (10-20 min)	355	19.9	17897	10.0
Only CT reachable within 60 min	246	13.8	3805	2.1
Neither reachable within 60 min	0	0.0	0	0.0
Likely irrelevant (<10 min)	1026	57.6	153628	86.2

Population and Area Coverage in Kreisfreie Stadt Bremerhaven (Stroke-Ready Hospitals) (Bremen) — Extended (+20min Penalty)

Category	Grid Cells	Area (%)	Population	Population (%)
High (30+ min)	1	1.0	5	0.0
Medium (20-30 min)	1	1.0	67	0.1
Low (10-20 min)	0	0.0	0	0.0
Only CT reachable within 60 min	10	10.2	278	0.2
Neither reachable within 60 min	4	4.1	10	0.0
Likely irrelevant (<10 min)	82	83.7	114056	99.7

Population and Area Coverage in Kreisfreie Stadt Koblenz (Stroke-Ready Hospitals) (Rheinland-Pfalz) — Extended (+20min Penalty)

Category	Grid Cells	Area (%)	Population	Population (%)
High (30+ min)	0	0.0	0	0.0
Medium (20-30 min)	0	0.0	0	0.0
Low (10-20 min)	0	0.0	0	0.0
Only CT reachable within 60 min	0	0.0	0	0.0
Neither reachable within 60 min	0	0.0	0	0.0
Likely irrelevant (<10 min)	136	100.0	125857	100.0

Population and Area Coverage in Kreisfreie Stadt Pirmasens (Stroke-Ready Hospitals) (Rheinland-Pfalz) — Extended (+20min Penalty)

Category	Grid Cells	Area (%)	Population	Population (%)
High (30+ min)	0	0.0	0	0.0
Medium (20-30 min)	0	0.0	0	0.0
Low (10-20 min)	0	0.0	0	0.0
Only CT reachable within 60 min	0	0.0	0	0.0
Neither reachable within 60 min	0	0.0	0	0.0
Likely irrelevant (<10 min)	76	100.0	37262	100.0

Population and Area Coverage in Landkreis Donnersbergkreis (Stroke-Ready Hospitals) (Rheinland-Pfalz) — Extended (+20min Penalty)

Category	Grid Cells	Area (%)	Population	Population (%)
High (30+ min)	0	0.0	0	0.0
Medium (20-30 min)	0	0.0	0	0.0
Low (10-20 min)	35	4.3	494	0.6
Only CT reachable within 60 min	0	0.0	0	0.0
Neither reachable within 60 min	0	0.0	0	0.0
Likely irrelevant (<10 min)	772	95.7	77023	99.4

Population and Area Coverage in Stadtkreis Heidelberg (Stroke-Ready Hospitals) (Baden-Württemberg) — Extended (+20min Penalty)

Category	Grid Cells	Area (%)	Population	Population (%)
High (30+ min)	0	0.0	0	0.0
Medium (20-30 min)	0	0.0	0	0.0
Low (10-20 min)	2	1.5	15	0.0
Only CT reachable within 60 min	0	0.0	0	0.0
Neither reachable within 60 min	0	0.0	0	0.0
Likely irrelevant (<10 min)	132	98.5	154351	100.0

Population and Area Coverage in Kreisfreie Stadt Straubing (Stroke-Ready Hospitals) (Bayern) — Extended (+20min Penalty)

Category	Grid Cells	Area (%)	Population	Population (%)
High (30+ min)	0	0.0	0	0.0
Medium (20-30 min)	0	0.0	0	0.0
Low (10-20 min)	0	0.0	0	0.0
Only CT reachable within 60 min	0	0.0	0	0.0
Neither reachable within 60 min	0	0.0	0	0.0
Likely irrelevant (<10 min)	85	100.0	46942	100.0

Population and Area Coverage in Landkreis Straubing-Bogen (Stroke-Ready Hospitals) (Bayern) — Extended (+20min Penalty)

Category	Grid Cells	Area (%)	Population	Population (%)
High (30+ min)	0	0.0	0	0.0
Medium (20-30 min)	0	0.0	0	0.0
Low (10-20 min)	215	14.7	10066	10.0
Only CT reachable within 60 min	0	0.0	0	0.0
Neither reachable within 60 min	0	0.0	0	0.0
Likely irrelevant (<10 min)	1251	85.3	90456	90.0

Population and Area Coverage in Landkreis Neumarkt i.d.OPf. (Stroke-Ready Hospitals) (Bayern) — Extended (+20min Penalty)

Category	Grid Cells	Area (%)	Population	Population (%)
High (30+ min)	0	0.0	0	0.0
Medium (20-30 min)	12	0.7	1311	1.0
Low (10-20 min)	126	7.6	6444	4.9
Only CT reachable within 60 min	0	0.0	0	0.0
Neither reachable within 60 min	0	0.0	0	0.0
Likely irrelevant (<10 min)	1526	91.7	124712	94.1

Population and Area Coverage in Kreisfreie Stadt Schwabach (Stroke-Ready Hospitals) (Bayern) — Extended (+20min Penalty)

Category	Grid Cells	Area (%)	Population	Population (%)
High (30+ min)	0	0.0	0	0.0
Medium (20-30 min)	0	0.0	0	0.0
Low (10-20 min)	0	0.0	0	0.0
Only CT reachable within 60 min	0	0.0	0	0.0
Neither reachable within 60 min	0	0.0	0	0.0
Likely irrelevant (<10 min)	52	100.0	42132	100.0

Population and Area Coverage in Kreisfreie Stadt Brandenburg an der Havel (Stroke-Ready Hospitals) (Brandenburg) — Extended (+20min Penalty)

Category	Grid Cells	Area (%)	Population	Population (%)
High (30+ min)	0	0.0	0	0.0
Medium (20-30 min)	1	0.3	1	0.0
Low (10-20 min)	33	10.9	1319	1.9
Only CT reachable within 60 min	0	0.0	0	0.0
Neither reachable within 60 min	0	0.0	0	0.0
Likely irrelevant (<10 min)	270	88.8	67772	98.1

Population and Area Coverage in Landkreis Ludwigslust-Parchim (Stroke-Ready Hospitals) (Mecklenburg-Vorpommern) — Extended (+20min Penalty)

Category	Grid Cells	Area (%)	Population	Population (%)
High (30+ min)	587	9.1	19477	9.9
Medium (20-30 min)	1586	24.6	45704	23.3
Low (10-20 min)	1677	26.0	45653	23.3
Only CT reachable within 60 min	586	9.1	9928	5.1
Neither reachable within 60 min	0	0.0	0	0.0
Likely irrelevant (<10 min)	2019	31.3	75254	38.4

Population and Area Coverage in Landkreis Saalfeld-Rudolstadt (Stroke-Ready Hospitals) (Thüringen) — Extended (+20min Penalty)

Category	Grid Cells	Area (%)	Population	Population (%)
High (30+ min)	0	0.0	0	0.0
Medium (20-30 min)	3	0.2	84	0.1
Low (10-20 min)	402	31.3	11777	12.2
Only CT reachable within 60 min	0	0.0	0	0.0
Neither reachable within 60 min	0	0.0	0	0.0
Likely irrelevant (<10 min)	878	68.4	84959	87.7

Population and Area Coverage in Kreis Ostholstein (Stroke-Ready Hospitals) (Schleswig-Holstein) — Extended (+20min Penalty)

Category	Grid Cells	Area (%)	Population	Population (%)
High (30+ min)	0	0.0	0	0.0
Medium (20-30 min)	4	0.2	110	0.1
Low (10-20 min)	176	9.5	12922	6.6
Only CT reachable within 60 min	7	0.4	417	0.2
Neither reachable within 60 min	73	3.9	2950	1.5
Likely irrelevant (<10 min)	1601	86.0	180002	91.7

Population and Area Coverage in Kreis Rendsburg-Eckernförde (Stroke-Ready Hospitals) (Schleswig-Holstein) — Extended (+20min Penalty)

Category	Grid Cells	Area (%)	Population	Population (%)
High (30+ min)	2	0.1	138	0.1
Medium (20-30 min)	81	2.7	2972	1.1
Low (10-20 min)	154	5.2	5694	2.1
Only CT reachable within 60 min	2	0.1	1871	0.7
Neither reachable within 60 min	35	1.2	1603	0.6
Likely irrelevant (<10 min)	2714	90.8	262274	95.5

Population and Area Coverage in Landkreis Ostalbkreis (Stroke-Ready Hospitals) (Baden-Württemberg) — Extended (+20min Penalty)

Category	Grid Cells	Area (%)	Population	Population (%)
High (30+ min)	0	0.0	0	0.0
Medium (20-30 min)	0	0.0	0	0.0
Low (10-20 min)	247	13.3	18575	6.0
Only CT reachable within 60 min	0	0.0	0	0.0
Neither reachable within 60 min	0	0.0	0	0.0
Likely irrelevant (<10 min)	1608	86.7	292756	94.0

Population and Area Coverage in Landkreis Emmendingen (Stroke-Ready Hospitals) (Baden-Württemberg) — Extended (+20min Penalty)

Category	Grid Cells	Area (%)	Population	Population (%)
High (30+ min)	13	1.6	56	0.0
Medium (20-30 min)	73	8.9	1780	1.1
Low (10-20 min)	213	26.0	22483	13.9
Only CT reachable within 60 min	4	0.5	1	0.0
Neither reachable within 60 min	0	0.0	5	0.0
Likely irrelevant (<10 min)	515	63.0	137559	85.0

Population and Area Coverage in Kreisfreie Stadt München (Stroke-Ready Hospitals) (Bayern) — Extended (+20min Penalty)

Category	Grid Cells	Area (%)	Population	Population (%)
High (30+ min)	0	0.0	0	0.0
Medium (20-30 min)	0	0.0	0	0.0
Low (10-20 min)	0	0.0	0	0.0
Only CT reachable within 60 min	0	0.0	0	0.0
Neither reachable within 60 min	0	0.0	0	0.0
Likely irrelevant (<10 min)	379	100.0	1671155	100.0

Population and Area Coverage in Landkreis Bayreuth (Stroke-Ready Hospitals) (Bayern) — Extended (+20min Penalty)

Category	Grid Cells	Area (%)	Population	Population (%)
High (30+ min)	0	0.0	0	0.0
Medium (20-30 min)	11	0.7	839	0.8
Low (10-20 min)	307	19.2	10257	9.4
Only CT reachable within 60 min	0	0.0	0	0.0
Neither reachable within 60 min	0	0.0	0	0.0
Likely irrelevant (<10 min)	1278	80.1	97814	89.8

Population and Area Coverage in Landkreis Kronach (Stroke-Ready Hospitals) (Bayern) — Extended (+20min Penalty)

Category	Grid Cells	Area (%)	Population	Population (%)
High (30+ min)	0	0.0	0	0.0
Medium (20-30 min)	72	8.9	1455	2.0
Low (10-20 min)	327	40.3	27859	39.2
Only CT reachable within 60 min	0	0.0	0	0.0
Neither reachable within 60 min	0	0.0	0	0.0
Likely irrelevant (<10 min)	413	50.9	41765	58.8

Population and Area Coverage in Landkreis Aschaffenburg (Stroke-Ready Hospitals) (Bayern) — Extended (+20min Penalty)

Category	Grid Cells	Area (%)	Population	Population (%)
High (30+ min)	0	0.0	0	0.0
Medium (20-30 min)	0	0.0	0	0.0
Low (10-20 min)	7	0.8	12	0.0
Only CT reachable within 60 min	0	0.0	0	0.0
Neither reachable within 60 min	0	0.0	0	0.0
Likely irrelevant (<10 min)	867	99.2	179837	100.0

Population and Area Coverage in Landkreis Saalekreis (Stroke-Ready Hospitals) (Sachsen-Anhalt) — Extended (+20min Penalty)

Category	Grid Cells	Area (%)	Population	Population (%)
High (30+ min)	0	0.0	0	0.0
Medium (20-30 min)	0	0.0	0	0.0
Low (10-20 min)	23	1.2	886	0.5
Only CT reachable within 60 min	0	0.0	0	0.0
Neither reachable within 60 min	0	0.0	0	0.0
Likely irrelevant (<10 min)	1845	98.8	168049	99.5

Population and Area Coverage in Kreisfreie Stadt Wolfsburg (Stroke-Ready Hospitals) (Niedersachsen) — Extended (+20min Penalty)

Category	Grid Cells	Area (%)	Population	Population (%)
High (30+ min)	0	0.0	0	0.0
Medium (20-30 min)	0	0.0	0	0.0
Low (10-20 min)	0	0.0	0	0.0
Only CT reachable within 60 min	0	0.0	0	0.0
Neither reachable within 60 min	0	0.0	0	0.0
Likely irrelevant (<10 min)	274	100.0	122066	100.0

Population and Area Coverage in Landkreis Wolfenbüttel (Stroke-Ready Hospitals) (Niedersachsen) — Extended (+20min Penalty)

Category	Grid Cells	Area (%)	Population	Population (%)
High (30+ min)	0	0.0	0	0.0
Medium (20-30 min)	0	0.0	0	0.0
Low (10-20 min)	3	0.3	13	0.0
Only CT reachable within 60 min	0	0.0	0	0.0
Neither reachable within 60 min	0	0.0	0	0.0
Likely irrelevant (<10 min)	954	99.7	119781	100.0

Population and Area Coverage in Landkreis Ammerland (Stroke-Ready Hospitals) (Niedersachsen) — Extended (+20min Penalty)

Category	Grid Cells	Area (%)	Population	Population (%)
High (30+ min)	0	0.0	0	0.0
Medium (20-30 min)	0	0.0	0	0.0
Low (10-20 min)	10	1.0	342	0.3
Only CT reachable within 60 min	0	0.0	0	0.0
Neither reachable within 60 min	0	0.0	0	0.0
Likely irrelevant (<10 min)	969	99.0	121120	99.7

Population and Area Coverage in Kreis Rhein-Kreis Neuss (Stroke-Ready Hospitals) (Nordrhein-Westfalen) — Extended (+20min Penalty)

Category	Grid Cells	Area (%)	Population	Population (%)
High (30+ min)	0	0.0	0	0.0
Medium (20-30 min)	0	0.0	0	0.0
Low (10-20 min)	6	0.8	251	0.1
Only CT reachable within 60 min	0	0.0	0	0.0
Neither reachable within 60 min	0	0.0	0	0.0
Likely irrelevant (<10 min)	730	99.2	470593	99.9

Population and Area Coverage in Kreis Lippe (Stroke-Ready Hospitals) (Nordrhein-Westfalen) — Extended (+20min Penalty)

Category	Grid Cells	Area (%)	Population	Population (%)
High (30+ min)	0	0.0	0	0.0
Medium (20-30 min)	0	0.0	0	0.0
Low (10-20 min)	214	13.1	23673	6.9
Only CT reachable within 60 min	0	0.0	0	0.0
Neither reachable within 60 min	0	0.0	0	0.0
Likely irrelevant (<10 min)	1419	86.9	321486	93.1

Population and Area Coverage in Kreisfreie Stadt Hagen (Stroke-Ready Hospitals) (Nordrhein-Westfalen) — Extended (+20min Penalty)

Category	Grid Cells	Area (%)	Population	Population (%)
High (30+ min)	0	0.0	0	0.0
Medium (20-30 min)	0	0.0	0	0.0
Low (10-20 min)	0	0.0	0	0.0
Only CT reachable within 60 min	0	0.0	0	0.0
Neither reachable within 60 min	0	0.0	0	0.0
Likely irrelevant (<10 min)	204	100.0	199659	100.0

Population and Area Coverage in Landkreis Alzey-Worms (Stroke-Ready Hospitals) (Rheinland-Pfalz) — Extended (+20min Penalty)

Category	Grid Cells	Area (%)	Population	Population (%)
High (30+ min)	0	0.0	0	0.0
Medium (20-30 min)	0	0.0	0	0.0
Low (10-20 min)	0	0.0	160	0.1
Only CT reachable within 60 min	0	0.0	0	0.0
Neither reachable within 60 min	0	0.0	0	0.0
Likely irrelevant (<10 min)	737	100.0	129234	99.9

Population and Area Coverage in Landkreis Germersheim (Stroke-Ready Hospitals) (Rheinland-Pfalz) — Extended (+20min Penalty)

Category	Grid Cells	Area (%)	Population	Population (%)
High (30+ min)	0	0.0	0	0.0
Medium (20-30 min)	0	0.0	0	0.0
Low (10-20 min)	0	0.0	0	0.0
Only CT reachable within 60 min	0	0.0	0	0.0
Neither reachable within 60 min	0	0.0	0	0.0
Likely irrelevant (<10 min)	570	100.0	128722	100.0

Population and Area Coverage in Landkreis Neckar-Odenwald-Kreis (Stroke-Ready Hospitals) (Baden-Württemberg) — Extended (+20min Penalty)

Category	Grid Cells	Area (%)	Population	Population (%)
High (30+ min)	0	0.0	0	0.0
Medium (20-30 min)	156	11.2	8815	6.1
Low (10-20 min)	417	29.8	36544	25.5
Only CT reachable within 60 min	0	0.0	0	0.0
Neither reachable within 60 min	0	0.0	0	0.0
Likely irrelevant (<10 min)	824	59.0	98011	68.4

Population and Area Coverage in Landkreis Enzkreis (Stroke-Ready Hospitals) (Baden-Württemberg) — Extended (+20min Penalty)

Category	Grid Cells	Area (%)	Population	Population (%)
High (30+ min)	0	0.0	0	0.0
Medium (20-30 min)	19	2.7	457	0.2
Low (10-20 min)	98	13.8	16499	8.4
Only CT reachable within 60 min	0	0.0	0	0.0
Neither reachable within 60 min	0	0.0	0	0.0
Likely irrelevant (<10 min)	591	83.5	178980	91.3

Population and Area Coverage in Landkreis Berchtesgadener Land (Stroke-Ready Hospitals) (Bayern) — Extended (+20min Penalty)

Category	Grid Cells	Area (%)	Population	Population (%)
High (30+ min)	5	0.5	204	0.2
Medium (20-30 min)	11	1.2	541	0.6
Low (10-20 min)	131	14.1	21721	23.2
Only CT reachable within 60 min	5	0.5	87	0.1
Neither reachable within 60 min	326	35.1	1727	1.8
Likely irrelevant (<10 min)	451	48.5	69355	74.1

Population and Area Coverage in Kreisfreie Stadt Hof (Stroke-Ready Hospitals) (Bayern) — Extended (+20min Penalty)

Category	Grid Cells	Area (%)	Population	Population (%)
High (30+ min)	0	0.0	0	0.0
Medium (20-30 min)	0	0.0	0	0.0
Low (10-20 min)	0	0.0	0	0.0
Only CT reachable within 60 min	0	0.0	0	0.0
Neither reachable within 60 min	0	0.0	0	0.0
Likely irrelevant (<10 min)	75	100.0	44749	100.0

Population and Area Coverage in Landkreis Kulmbach (Stroke-Ready Hospitals) (Bayern) — Extended (+20min Penalty)

Category	Grid Cells	Area (%)	Population	Population (%)
High (30+ min)	0	0.0	0	0.0
Medium (20-30 min)	0	0.0	0	0.0
Low (10-20 min)	12	1.4	101	0.1
Only CT reachable within 60 min	0	0.0	0	0.0
Neither reachable within 60 min	0	0.0	0	0.0
Likely irrelevant (<10 min)	816	98.6	75577	99.9

Population and Area Coverage in Kreisfreie Stadt Memmingen (Stroke-Ready Hospitals) (Bayern) — Extended (+20min Penalty)

Category	Grid Cells	Area (%)	Population	Population (%)
High (30+ min)	0	0.0	0	0.0
Medium (20-30 min)	0	0.0	0	0.0
Low (10-20 min)	0	0.0	0	0.0
Only CT reachable within 60 min	0	0.0	0	0.0
Neither reachable within 60 min	0	0.0	0	0.0
Likely irrelevant (<10 min)	84	100.0	43302	100.0

Population and Area Coverage in Landkreis Augsburg (Stroke-Ready Hospitals) (Bayern) — Extended (+20min Penalty)

Category	Grid Cells	Area (%)	Population	Population (%)
High (30+ min)	0	0.0	0	0.0
Medium (20-30 min)	3	0.2	132	0.1
Low (10-20 min)	172	13.2	8943	3.6
Only CT reachable within 60 min	0	0.0	0	0.0
Neither reachable within 60 min	0	0.0	0	0.0
Likely irrelevant (<10 min)	1124	86.5	239190	96.3

Population and Area Coverage in Landkreis Vorpommern-Rügen (Stroke-Ready Hospitals) (Mecklenburg-Vorpommern) — Extended (+20min Penalty)

Category	Grid Cells	Area (%)	Population	Population (%)
High (30+ min)	340	8.2	14147	7.1
Medium (20-30 min)	634	15.2	14808	7.4
Low (10-20 min)	855	20.5	28658	14.4
Only CT reachable within 60 min	231	5.5	6550	3.3
Neither reachable within 60 min	274	6.6	5801	2.9
Likely irrelevant (<10 min)	1829	43.9	129305	64.9

Population and Area Coverage in Landkreis Zwickau (Stroke-Ready Hospitals) (Sachsen) — Extended (+20min Penalty)

Category	Grid Cells	Area (%)	Population	Population (%)
High (30+ min)	0	0.0	0	0.0
Medium (20-30 min)	0	0.0	0	0.0
Low (10-20 min)	2	0.2	23	0.0
Only CT reachable within 60 min	0	0.0	0	0.0
Neither reachable within 60 min	0	0.0	0	0.0
Likely irrelevant (<10 min)	1219	99.8	317194	100.0

Population and Area Coverage in Landkreis Görlitz (Stroke-Ready Hospitals) (Sachsen) — Extended (+20min Penalty)

Category	Grid Cells	Area (%)	Population	Population (%)
High (30+ min)	161	6.2	32390	14.8
Medium (20-30 min)	557	21.5	63762	29.1
Low (10-20 min)	516	19.9	26569	12.1
Only CT reachable within 60 min	74	2.9	4473	2.0
Neither reachable within 60 min	12	0.5	216	0.1
Likely irrelevant (<10 min)	1267	49.0	91551	41.8

Population and Area Coverage in Landkreis Sonneberg (Stroke-Ready Hospitals) (Thüringen) — Extended (+20min Penalty)

Category	Grid Cells	Area (%)	Population	Population (%)
High (30+ min)	0	0.0	0	0.0
Medium (20-30 min)	0	0.0	0	0.0
Low (10-20 min)	59	10.0	2347	4.4
Only CT reachable within 60 min	0	0.0	0	0.0
Neither reachable within 60 min	0	0.0	0	0.0
Likely irrelevant (<10 min)	530	90.0	51321	95.6

Population and Area Coverage in Kreisfreie Stadt Köln (Stroke-Ready Hospitals) (Nordrhein-Westfalen) — Extended (+20min Penalty)

Category	Grid Cells	Area (%)	Population	Population (%)
High (30+ min)	0	0.0	0	0.0
Medium (20-30 min)	0	0.0	0	0.0
Low (10-20 min)	0	0.0	0	0.0
Only CT reachable within 60 min	0	0.0	0	0.0
Neither reachable within 60 min	0	0.0	0	0.0
Likely irrelevant (<10 min)	527	100.0	1215184	100.0

Population and Area Coverage in Kreis Heinsberg (Stroke-Ready Hospitals) (Nordrhein-Westfalen) — Extended (+20min Penalty)

Category	Grid Cells	Area (%)	Population	Population (%)
High (30+ min)	1	0.1	1	0.0
Medium (20-30 min)	0	0.0	0	0.0
Low (10-20 min)	5	0.6	747	0.3
Only CT reachable within 60 min	2	0.3	33	0.0
Neither reachable within 60 min	2	0.3	330	0.1
Likely irrelevant (<10 min)	768	98.7	247102	99.6

Population and Area Coverage in Landkreis Bergstraße (Stroke-Ready Hospitals) (Hessen) — Extended (+20min Penalty)

Category	Grid Cells	Area (%)	Population	Population (%)
High (30+ min)	0	0.0	0	0.0
Medium (20-30 min)	8	0.9	493	0.2
Low (10-20 min)	131	14.6	20195	7.6
Only CT reachable within 60 min	0	0.0	0	0.0
Neither reachable within 60 min	0	0.0	0	0.0
Likely irrelevant (<10 min)	758	84.5	245369	92.2

Population and Area Coverage in Landkreis Wetteraukreis (Stroke-Ready Hospitals) (Hessen) — Extended (+20min Penalty)

Category	Grid Cells	Area (%)	Population	Population (%)
High (30+ min)	0	0.0	0	0.0
Medium (20-30 min)	0	0.0	0	0.0
Low (10-20 min)	50	3.6	2188	0.7
Only CT reachable within 60 min	0	0.0	0	0.0
Neither reachable within 60 min	0	0.0	0	0.0
Likely irrelevant (<10 min)	1345	96.4	296675	99.3

Population and Area Coverage in Kreisfreie Stadt Landau in der Pfalz (Stroke-Ready Hospitals) (Rheinland-Pfalz) — Extended (+20min Penalty)

Category	Grid Cells	Area (%)	Population	Population (%)
High (30+ min)	0	0.0	0	0.0
Medium (20-30 min)	0	0.0	0	0.0
Low (10-20 min)	9	8.8	27	0.1
Only CT reachable within 60 min	0	0.0	0	0.0
Neither reachable within 60 min	0	0.0	0	0.0
Likely irrelevant (<10 min)	93	91.2	52569	99.9

Population and Area Coverage in Stadtkreis Stuttgart (Stroke-Ready Hospitals) (Baden-Württemberg) — Extended (+20min Penalty)

Category	Grid Cells	Area (%)	Population	Population (%)
High (30+ min)	0	0.0	0	0.0
Medium (20-30 min)	0	0.0	0	0.0
Low (10-20 min)	0	0.0	0	0.0
Only CT reachable within 60 min	0	0.0	0	0.0
Neither reachable within 60 min	0	0.0	0	0.0
Likely irrelevant (<10 min)	255	100.0	639946	100.0

Population and Area Coverage in Landkreis Konstanz (Stroke-Ready Hospitals) (Baden-Württemberg) — Extended (+20min Penalty)

Category	Grid Cells	Area (%)	Population	Population (%)
High (30+ min)	0	0.0	0	0.0
Medium (20-30 min)	0	0.0	1055	0.4
Low (10-20 min)	48	5.0	2569	1.0
Only CT reachable within 60 min	3	0.3	1914	0.7
Neither reachable within 60 min	7	0.7	1161	0.5
Likely irrelevant (<10 min)	893	93.9	249454	97.4

Population and Area Coverage in Landkreis Sigmaringen (Stroke-Ready Hospitals) (Baden-Württemberg) — Extended (+20min Penalty)

Category	Grid Cells	Area (%)	Population	Population (%)
High (30+ min)	0	0.0	0	0.0
Medium (20-30 min)	0	0.0	0	0.0
Low (10-20 min)	145	10.0	6334	4.9
Only CT reachable within 60 min	0	0.0	0	0.0
Neither reachable within 60 min	0	0.0	0	0.0
Likely irrelevant (<10 min)	1304	90.0	122324	95.1

Population and Area Coverage in Landkreis Kelheim (Stroke-Ready Hospitals) (Bayern) — Extended (+20min Penalty)

Category	Grid Cells	Area (%)	Population	Population (%)
High (30+ min)	0	0.0	0	0.0
Medium (20-30 min)	109	8.4	3797	3.2
Low (10-20 min)	509	39.2	37930	31.8
Only CT reachable within 60 min	0	0.0	0	0.0
Neither reachable within 60 min	0	0.0	0	0.0
Likely irrelevant (<10 min)	679	52.4	77420	65.0

Population and Area Coverage in Kreisfreie Stadt Schweinfurt (Stroke-Ready Hospitals) (Bayern) — Extended (+20min Penalty)

Category	Grid Cells	Area (%)	Population	Population (%)
High (30+ min)	0	0.0	0	0.0
Medium (20-30 min)	0	0.0	0	0.0
Low (10-20 min)	0	0.0	0	0.0
Only CT reachable within 60 min	0	0.0	0	0.0
Neither reachable within 60 min	0	0.0	0	0.0
Likely irrelevant (<10 min)	46	100.0	53903	100.0

Population and Area Coverage in Landkreis Neu-Ulm (Stroke-Ready Hospitals) (Bayern) — Extended (+20min Penalty)

Category	Grid Cells	Area (%)	Population	Population (%)
High (30+ min)	0	0.0	0	0.0
Medium (20-30 min)	0	0.0	0	0.0
Low (10-20 min)	114	18.3	5370	3.1
Only CT reachable within 60 min	0	0.0	0	0.0
Neither reachable within 60 min	0	0.0	0	0.0
Likely irrelevant (<10 min)	510	81.7	167857	96.9

Population and Area Coverage in Landkreis Oberhavel (Stroke-Ready Hospitals) (Brandenburg) — Extended (+20min Penalty)

Category	Grid Cells	Area (%)	Population	Population (%)
High (30+ min)	257	10.6	13602	6.8
Medium (20-30 min)	366	15.2	10402	5.2
Low (10-20 min)	482	20.0	13543	6.8
Only CT reachable within 60 min	510	21.1	7248	3.6
Neither reachable within 60 min	0	0.0	0	0.0
Likely irrelevant (<10 min)	799	33.1	155821	77.7

Population and Area Coverage in Landkreis Spree-Neiße (Stroke-Ready Hospitals) (Brandenburg) — Extended (+20min Penalty)

Category	Grid Cells	Area (%)	Population	Population (%)
High (30+ min)	93	4.5	12851	12.8
Medium (20-30 min)	250	12.0	4486	4.5
Low (10-20 min)	456	21.8	10296	10.3
Only CT reachable within 60 min	20	1.0	64	0.1
Neither reachable within 60 min	0	0.0	0	0.0
Likely irrelevant (<10 min)	1269	60.8	72700	72.4

Population and Area Coverage in Landkreis Ilm-Kreis (Stroke-Ready Hospitals) (Thüringen) — Extended (+20min Penalty)

Category	Grid Cells	Area (%)	Population	Population (%)
High (30+ min)	0	0.0	0	0.0
Medium (20-30 min)	0	0.0	0	0.0
Low (10-20 min)	321	31.3	30744	31.9
Only CT reachable within 60 min	0	0.0	0	0.0
Neither reachable within 60 min	0	0.0	0	0.0
Likely irrelevant (<10 min)	705	68.7	65757	68.1

Population and Area Coverage in Landkreis Greiz (Stroke-Ready Hospitals) (Thüringen) — Extended (+20min Penalty)

Category	Grid Cells	Area (%)	Population	Population (%)
High (30+ min)	0	0.0	0	0.0
Medium (20-30 min)	16	1.5	425	0.5
Low (10-20 min)	324	30.0	34112	37.2
Only CT reachable within 60 min	0	0.0	0	0.0
Neither reachable within 60 min	0	0.0	0	0.0
Likely irrelevant (<10 min)	740	68.5	57269	62.4

Population and Area Coverage in Kreisfreie Stadt Bottrop (Stroke-Ready Hospitals) (Nordrhein-Westfalen) — Extended (+20min Penalty)

Category	Grid Cells	Area (%)	Population	Population (%)
High (30+ min)	0	0.0	0	0.0
Medium (20-30 min)	0	0.0	0	0.0
Low (10-20 min)	0	0.0	0	0.0
Only CT reachable within 60 min	0	0.0	0	0.0
Neither reachable within 60 min	0	0.0	0	0.0
Likely irrelevant (<10 min)	133	100.0	116967	100.0

Population and Area Coverage in Kreisfreie Stadt Speyer (Stroke-Ready Hospitals) (Rheinland-Pfalz) — Extended (+20min Penalty)

Category	Grid Cells	Area (%)	Population	Population (%)
High (30+ min)	0	0.0	0	0.0
Medium (20-30 min)	0	0.0	0	0.0
Low (10-20 min)	0	0.0	0	0.0
Only CT reachable within 60 min	0	0.0	0	0.0
Neither reachable within 60 min	0	0.0	0	0.0
Likely irrelevant (<10 min)	52	100.0	51373	100.0

Population and Area Coverage in Stadtkreis Mannheim (Stroke-Ready Hospitals) (Baden-Württemberg) — Extended (+20min Penalty)

Category	Grid Cells	Area (%)	Population	Population (%)
High (30+ min)	0	0.0	0	0.0
Medium (20-30 min)	0	0.0	0	0.0
Low (10-20 min)	0	0.0	0	0.0
Only CT reachable within 60 min	0	0.0	0	0.0
Neither reachable within 60 min	0	0.0	0	0.0
Likely irrelevant (<10 min)	178	100.0	346949	100.0

Population and Area Coverage in Landkreis Tuttlingen (Stroke-Ready Hospitals) (Baden-Württemberg) — Extended (+20min Penalty)

Category	Grid Cells	Area (%)	Population	Population (%)
High (30+ min)	0	0.0	0	0.0
Medium (20-30 min)	0	0.0	0	0.0
Low (10-20 min)	0	0.0	0	0.0
Only CT reachable within 60 min	0	0.0	0	0.0
Neither reachable within 60 min	0	0.0	0	0.0
Likely irrelevant (<10 min)	881	100.0	135084	100.0

Population and Area Coverage in Landkreis Zollernalbkreis (Stroke-Ready Hospitals) (Baden-Württemberg) — Extended (+20min Penalty)

Category	Grid Cells	Area (%)	Population	Population (%)
High (30+ min)	0	0.0	0	0.0
Medium (20-30 min)	0	0.0	0	0.0
Low (10-20 min)	89	8.0	3999	2.1
Only CT reachable within 60 min	0	0.0	0	0.0
Neither reachable within 60 min	0	0.0	0	0.0
Likely irrelevant (<10 min)	1021	92.0	183276	97.9

Population and Area Coverage in Landkreis Eichstätt (Stroke-Ready Hospitals) (Bayern) — Extended (+20min Penalty)

Category	Grid Cells	Area (%)	Population	Population (%)
High (30+ min)	0	0.0	0	0.0
Medium (20-30 min)	176	11.9	10168	7.8
Low (10-20 min)	710	47.9	35130	26.9
Only CT reachable within 60 min	0	0.0	0	0.0
Neither reachable within 60 min	0	0.0	0	0.0
Likely irrelevant (<10 min)	596	40.2	85319	65.3

Population and Area Coverage in Landkreis Freyung-Grafenau (Stroke-Ready Hospitals) (Bayern) — Extended (+20min Penalty)

Category	Grid Cells	Area (%)	Population	Population (%)
High (30+ min)	0	0.0	0	0.0
Medium (20-30 min)	2	0.2	0	0.0
Low (10-20 min)	110	9.7	4607	5.9
Only CT reachable within 60 min	1	0.1	0	0.0
Neither reachable within 60 min	51	4.5	35	0.0
Likely irrelevant (<10 min)	969	85.5	74088	94.1

Population and Area Coverage in Landkreis Main-Spessart (Stroke-Ready Hospitals) (Bayern) — Extended (+20min Penalty)

Category	Grid Cells	Area (%)	Population	Population (%)
High (30+ min)	0	0.0	0	0.0
Medium (20-30 min)	10	0.6	1	0.0
Low (10-20 min)	181	10.9	6758	5.1
Only CT reachable within 60 min	0	0.0	0	0.0
Neither reachable within 60 min	0	0.0	0	0.0
Likely irrelevant (<10 min)	1471	88.5	124696	94.9

Population and Area Coverage in Kreisfreie Stadt Potsdam (Stroke-Ready Hospitals) (Brandenburg) — Extended (+20min Penalty)

Category	Grid Cells	Area (%)	Population	Population (%)
High (30+ min)	0	0.0	0	0.0
Medium (20-30 min)	0	0.0	0	0.0
Low (10-20 min)	2	0.8	0	0.0
Only CT reachable within 60 min	0	0.0	0	0.0
Neither reachable within 60 min	0	0.0	0	0.0
Likely irrelevant (<10 min)	242	99.2	205315	100.0

Population and Area Coverage in Landkreis Nordsachsen (Stroke-Ready Hospitals) (Sachsen) — Extended (+20min Penalty)

Category	Grid Cells	Area (%)	Population	Population (%)
High (30+ min)	125	4.8	3757	2.0
Medium (20-30 min)	404	15.5	19286	10.5
Low (10-20 min)	695	26.6	40856	22.2
Only CT reachable within 60 min	21	0.8	43	0.0
Neither reachable within 60 min	0	0.0	0	0.0
Likely irrelevant (<10 min)	1366	52.3	120131	65.3

Population and Area Coverage in Landkreis Cuxhaven (Stroke-Ready Hospitals) (Niedersachsen) — Extended (+20min Penalty)

Category	Grid Cells	Area (%)	Population	Population (%)
High (30+ min)	92	3.3	4061	2.0
Medium (20-30 min)	321	11.5	20069	10.1
Low (10-20 min)	737	26.5	59033	29.7
Only CT reachable within 60 min	7	0.3	687	0.3
Neither reachable within 60 min	45	1.6	2609	1.3
Likely irrelevant (<10 min)	1578	56.8	112246	56.5

Population and Area Coverage in Kreisfreie Stadt Bremen (Stroke-Ready Hospitals) (Bremen) — Extended (+20min Penalty)

Category	Grid Cells	Area (%)	Population	Population (%)
High (30+ min)	0	0.0	0	0.0
Medium (20-30 min)	0	0.0	0	0.0
Low (10-20 min)	41	9.4	1087	0.2
Only CT reachable within 60 min	0	0.0	0	0.0
Neither reachable within 60 min	1	0.2	1881	0.3
Likely irrelevant (<10 min)	396	90.4	580244	99.5

Population and Area Coverage in Landkreis Groß-Gerau (Stroke-Ready Hospitals) (Hessen) — Extended (+20min Penalty)

Category	Grid Cells	Area (%)	Population	Population (%)
High (30+ min)	0	0.0	0	0.0
Medium (20-30 min)	0	0.0	0	0.0
Low (10-20 min)	6	1.1	32	0.0
Only CT reachable within 60 min	0	0.0	0	0.0
Neither reachable within 60 min	0	0.0	0	0.0
Likely irrelevant (<10 min)	558	98.9	259561	100.0

Population and Area Coverage in Landkreis Limburg-Weilburg (Stroke-Ready Hospitals) (Hessen) — Extended (+20min Penalty)

Category	Grid Cells	Area (%)	Population	Population (%)
High (30+ min)	0	0.0	0	0.0
Medium (20-30 min)	0	0.0	0	0.0
Low (10-20 min)	1	0.1	4	0.0
Only CT reachable within 60 min	0	0.0	0	0.0
Neither reachable within 60 min	0	0.0	0	0.0
Likely irrelevant (<10 min)	933	99.9	172833	100.0

Population and Area Coverage in Landkreis Waldeck-Frankenberg (Stroke-Ready Hospitals) (Hessen) — Extended (+20min Penalty)

Category	Grid Cells	Area (%)	Population	Population (%)
High (30+ min)	392	16.5	14155	9.0
Medium (20-30 min)	681	28.6	51770	32.8
Low (10-20 min)	613	25.7	51292	32.5
Only CT reachable within 60 min	22	0.9	141	0.1
Neither reachable within 60 min	0	0.0	0	0.0
Likely irrelevant (<10 min)	674	28.3	40645	25.7

Population and Area Coverage in Landkreis Trier-Saarburg (Stroke-Ready Hospitals) (Rheinland-Pfalz) — Extended (+20min Penalty)

Category	Grid Cells	Area (%)	Population	Population (%)
High (30+ min)	0	0.0	0	0.0
Medium (20-30 min)	9	0.7	49	0.0
Low (10-20 min)	370	26.9	25982	17.3
Only CT reachable within 60 min	0	0.0	0	0.0
Neither reachable within 60 min	0	0.0	355	0.2
Likely irrelevant (<10 min)	995	72.4	123649	82.4

Population and Area Coverage in Landkreis Reutlingen (Stroke-Ready Hospitals) (Baden-Württemberg) — Extended (+20min Penalty)

Category	Grid Cells	Area (%)	Population	Population (%)
High (30+ min)	0	0.0	0	0.0
Medium (20-30 min)	277	20.8	10607	3.6
Low (10-20 min)	421	31.6	30607	10.3
Only CT reachable within 60 min	0	0.0	0	0.0
Neither reachable within 60 min	0	0.0	0	0.0
Likely irrelevant (<10 min)	635	47.6	256497	86.2

Population and Area Coverage in Kreisfreie Stadt Schwerin (Stroke-Ready Hospitals) (Mecklenburg-Vorpommern) — Extended (+20min Penalty)

Category	Grid Cells	Area (%)	Population	Population (%)
High (30+ min)	0	0.0	0	0.0
Medium (20-30 min)	0	0.0	0	0.0
Low (10-20 min)	0	0.0	0	0.0
Only CT reachable within 60 min	0	0.0	0	0.0
Neither reachable within 60 min	0	0.0	0	0.0
Likely irrelevant (<10 min)	173	100.0	96772	100.0

Population and Area Coverage in Kreisfreie Stadt Hamburg (Stroke-Ready Hospitals) (Hamburg) — Extended (+20min Penalty)

Category	Grid Cells	Area (%)	Population	Population (%)
High (30+ min)	0	0.0	0	0.0
Medium (20-30 min)	0	0.0	0	0.0
Low (10-20 min)	16	1.6	994	0.1
Only CT reachable within 60 min	0	0.0	0	0.0
Neither reachable within 60 min	6	0.6	6087	0.3
Likely irrelevant (<10 min)	975	97.8	1786387	99.6

Population and Area Coverage in Kreisfreie Stadt Braunschweig (Stroke-Ready Hospitals) (Niedersachsen) — Extended (+20min Penalty)

Category	Grid Cells	Area (%)	Population	Population (%)
High (30+ min)	0	0.0	0	0.0
Medium (20-30 min)	0	0.0	0	0.0
Low (10-20 min)	0	0.0	0	0.0
Only CT reachable within 60 min	0	0.0	0	0.0
Neither reachable within 60 min	0	0.0	0	0.0
Likely irrelevant (<10 min)	243	100.0	246678	100.0

Population and Area Coverage in Landkreis Leer (Stroke-Ready Hospitals) (Niedersachsen) — Extended (+20min Penalty)

Category	Grid Cells	Area (%)	Population	Population (%)
High (30+ min)	3	0.2	26	0.0
Medium (20-30 min)	222	15.7	24426	14.5
Low (10-20 min)	473	33.4	59447	35.2
Only CT reachable within 60 min	1	0.1	9	0.0
Neither reachable within 60 min	38	2.7	3609	2.1
Likely irrelevant (<10 min)	681	48.0	81321	48.2

Population and Area Coverage in Kreisfreie Stadt Duisburg (Stroke-Ready Hospitals) (Nordrhein-Westfalen) — Extended (+20min Penalty)

Category	Grid Cells	Area (%)	Population	Population (%)
High (30+ min)	0	0.0	0	0.0
Medium (20-30 min)	0	0.0	0	0.0
Low (10-20 min)	0	0.0	0	0.0
Only CT reachable within 60 min	0	0.0	0	0.0
Neither reachable within 60 min	0	0.0	0	0.0
Likely irrelevant (<10 min)	297	100.0	552903	100.0

Population and Area Coverage in Kreis Siegen-Wittgenstein (Stroke-Ready Hospitals) (Nordrhein-Westfalen) — Extended (+20min Penalty)

Category	Grid Cells	Area (%)	Population	Population (%)
High (30+ min)	248	17.1	21661	7.4
Medium (20-30 min)	189	13.0	13028	4.4
Low (10-20 min)	98	6.8	5283	1.8
Only CT reachable within 60 min	105	7.2	540	0.2
Neither reachable within 60 min	0	0.0	0	0.0
Likely irrelevant (<10 min)	811	55.9	252807	86.2

Population and Area Coverage in Landkreis Schwarzwald-Baar-Kreis (Stroke-Ready Hospitals) (Baden-Württemberg) — Extended (+20min Penalty)

Category	Grid Cells	Area (%)	Population	Population (%)
High (30+ min)	11	0.9	78	0.0
Medium (20-30 min)	19	1.5	666	0.3
Low (10-20 min)	98	8.0	11569	5.6
Only CT reachable within 60 min	2	0.2	15	0.0
Neither reachable within 60 min	0	0.0	0	0.0
Likely irrelevant (<10 min)	1100	89.4	194660	94.0

Population and Area Coverage in Kreisfreie Stadt Amberg (Stroke-Ready Hospitals) (Bayern) — Extended (+20min Penalty)

Category	Grid Cells	Area (%)	Population	Population (%)
High (30+ min)	0	0.0	0	0.0
Medium (20-30 min)	0	0.0	0	0.0
Low (10-20 min)	0	0.0	0	0.0
Only CT reachable within 60 min	0	0.0	0	0.0
Neither reachable within 60 min	0	0.0	0	0.0
Likely irrelevant (<10 min)	61	100.0	42852	100.0

Population and Area Coverage in Kreisfreie Stadt Bayreuth (Stroke-Ready Hospitals) (Bayern) — Extended (+20min Penalty)

Category	Grid Cells	Area (%)	Population	Population (%)
High (30+ min)	0	0.0	0	0.0
Medium (20-30 min)	0	0.0	0	0.0
Low (10-20 min)	0	0.0	0	0.0
Only CT reachable within 60 min	0	0.0	0	0.0
Neither reachable within 60 min	0	0.0	0	0.0
Likely irrelevant (<10 min)	82	100.0	73464	100.0

Population and Area Coverage in Landkreis Nürnberger Land (Stroke-Ready Hospitals) (Bayern) — Extended (+20min Penalty)

Category	Grid Cells	Area (%)	Population	Population (%)
High (30+ min)	0	0.0	0	0.0
Medium (20-30 min)	3	0.3	32	0.0
Low (10-20 min)	208	21.1	12179	7.2
Only CT reachable within 60 min	0	0.0	0	0.0
Neither reachable within 60 min	0	0.0	0	0.0
Likely irrelevant (<10 min)	775	78.6	157866	92.8

Population and Area Coverage in Landkreis Roth (Stroke-Ready Hospitals) (Bayern) — Extended (+20min Penalty)

Category	Grid Cells	Area (%)	Population	Population (%)
High (30+ min)	0	0.0	0	0.0
Medium (20-30 min)	0	0.0	0	0.0
Low (10-20 min)	136	12.3	6378	5.0
Only CT reachable within 60 min	0	0.0	0	0.0
Neither reachable within 60 min	0	0.0	0	0.0
Likely irrelevant (<10 min)	968	87.7	122399	95.0

Population and Area Coverage in Kreisfreie Stadt Aschaffenburg (Stroke-Ready Hospitals) (Bayern) — Extended (+20min Penalty)

Category	Grid Cells	Area (%)	Population	Population (%)
High (30+ min)	0	0.0	0	0.0
Medium (20-30 min)	0	0.0	0	0.0
Low (10-20 min)	0	0.0	0	0.0
Only CT reachable within 60 min	0	0.0	0	0.0
Neither reachable within 60 min	0	0.0	0	0.0
Likely irrelevant (<10 min)	78	100.0	70292	100.0

Population and Area Coverage in Landkreis Merzig-Wadern (Stroke-Ready Hospitals) (Saarland) — Extended (+20min Penalty)

Category	Grid Cells	Area (%)	Population	Population (%)
High (30+ min)	1	0.1	8	0.0
Medium (20-30 min)	13	1.9	117	0.1
Low (10-20 min)	147	21.3	16848	16.8
Only CT reachable within 60 min	0	0.0	1	0.0
Neither reachable within 60 min	0	0.0	191	0.2
Likely irrelevant (<10 min)	530	76.7	82852	82.8

Population and Area Coverage in Landkreis Vogtlandkreis (Stroke-Ready Hospitals) (Sachsen) — Extended (+20min Penalty)

Category	Grid Cells	Area (%)	Population	Population (%)
High (30+ min)	16	0.9	604	0.3
Medium (20-30 min)	74	4.2	5135	2.4
Low (10-20 min)	208	11.8	16939	7.8
Only CT reachable within 60 min	1	0.1	177	0.1
Neither reachable within 60 min	12	0.7	105	0.0
Likely irrelevant (<10 min)	1456	82.4	195089	89.5

Population and Area Coverage in Landkreis Altmarkkreis Salzwedel (Stroke-Ready Hospitals) (Sachsen-Anhalt) — Extended (+20min Penalty)

Category	Grid Cells	Area (%)	Population	Population (%)
High (30+ min)	681	22.2	23242	30.1
Medium (20-30 min)	911	29.8	19923	25.8
Low (10-20 min)	618	20.2	19142	24.8
Only CT reachable within 60 min	691	22.6	12440	16.1
Neither reachable within 60 min	0	0.0	0	0.0
Likely irrelevant (<10 min)	161	5.3	2484	3.2

Population and Area Coverage in Kreis Segeberg (Stroke-Ready Hospitals) (Schleswig-Holstein) — Extended (+20min Penalty)

Category	Grid Cells	Area (%)	Population	Population (%)
High (30+ min)	0	0.0	0	0.0
Medium (20-30 min)	0	0.0	0	0.0
Low (10-20 min)	71	3.9	2142	0.8
Only CT reachable within 60 min	0	0.0	0	0.0
Neither reachable within 60 min	0	0.0	0	0.0
Likely irrelevant (<10 min)	1761	96.1	271769	99.2

Population and Area Coverage in Landkreis Osnabrück (Stroke-Ready Hospitals) (Niedersachsen) — Extended (+20min Penalty)

Category	Grid Cells	Area (%)	Population	Population (%)
High (30+ min)	0	0.0	0	0.0
Medium (20-30 min)	40	1.4	4964	1.4
Low (10-20 min)	442	15.8	30468	8.6
Only CT reachable within 60 min	0	0.0	0	0.0
Neither reachable within 60 min	0	0.0	0	0.0
Likely irrelevant (<10 min)	2320	82.8	317725	90.0

Population and Area Coverage in Kreisfreie Stadt Oberhausen (Stroke-Ready Hospitals) (Nordrhein-Westfalen) — Extended (+20min Penalty)

Category	Grid Cells	Area (%)	Population	Population (%)
High (30+ min)	0	0.0	0	0.0
Medium (20-30 min)	0	0.0	0	0.0
Low (10-20 min)	0	0.0	0	0.0
Only CT reachable within 60 min	0	0.0	0	0.0
Neither reachable within 60 min	0	0.0	0	0.0
Likely irrelevant (<10 min)	101	100.0	215167	100.0

Population and Area Coverage in Kreisfreie Stadt Remscheid (Stroke-Ready Hospitals) (Nordrhein-Westfalen) — Extended (+20min Penalty)

Category	Grid Cells	Area (%)	Population	Population (%)
High (30+ min)	0	0.0	0	0.0
Medium (20-30 min)	0	0.0	0	0.0
Low (10-20 min)	0	0.0	0	0.0
Only CT reachable within 60 min	0	0.0	0	0.0
Neither reachable within 60 min	0	0.0	0	0.0
Likely irrelevant (<10 min)	97	100.0	108490	100.0

Population and Area Coverage in Kreis Mettmann (Stroke-Ready Hospitals) (Nordrhein-Westfalen) — Extended (+20min Penalty)

Category	Grid Cells	Area (%)	Population	Population (%)
High (30+ min)	0	0.0	0	0.0
Medium (20-30 min)	0	0.0	0	0.0
Low (10-20 min)	0	0.0	0	0.0
Only CT reachable within 60 min	0	0.0	0	0.0
Neither reachable within 60 min	0	0.0	0	0.0
Likely irrelevant (<10 min)	525	100.0	473726	100.0

Population and Area Coverage in Kreis Rhein-Sieg-Kreis (Stroke-Ready Hospitals) (Nordrhein-Westfalen) — Extended (+20min Penalty)

Category	Grid Cells	Area (%)	Population	Population (%)
High (30+ min)	0	0.0	0	0.0
Medium (20-30 min)	31	2.1	3153	0.5
Low (10-20 min)	155	10.5	23630	4.1
Only CT reachable within 60 min	0	0.0	0	0.0
Neither reachable within 60 min	0	0.0	0	0.0
Likely irrelevant (<10 min)	1288	87.4	556493	95.4

Population and Area Coverage in Kreis Borken (Stroke-Ready Hospitals) (Nordrhein-Westfalen) — Extended (+20min Penalty)

Category	Grid Cells	Area (%)	Population	Population (%)
High (30+ min)	1	0.1	17	0.0
Medium (20-30 min)	15	0.8	889	0.2
Low (10-20 min)	345	19.0	66927	18.2
Only CT reachable within 60 min	10	0.5	185	0.1
Neither reachable within 60 min	15	0.8	153	0.0
Likely irrelevant (<10 min)	1433	78.8	298621	81.4

Population and Area Coverage in Landkreis Vulkaneifel (Stroke-Ready Hospitals) (Rheinland-Pfalz) — Extended (+20min Penalty)

Category	Grid Cells	Area (%)	Population	Population (%)
High (30+ min)	143	12.4	5268	8.4
Medium (20-30 min)	265	23.0	18657	29.7
Low (10-20 min)	354	30.8	14196	22.6
Only CT reachable within 60 min	0	0.0	0	0.0
Neither reachable within 60 min	0	0.0	0	0.0
Likely irrelevant (<10 min)	388	33.7	24734	39.4

Population and Area Coverage in Landkreis Kaiserslautern (Stroke-Ready Hospitals) (Rheinland-Pfalz) — Extended (+20min Penalty)

Category	Grid Cells	Area (%)	Population	Population (%)
High (30+ min)	0	0.0	0	0.0
Medium (20-30 min)	0	0.0	0	0.0
Low (10-20 min)	8	1.0	235	0.2
Only CT reachable within 60 min	0	0.0	0	0.0
Neither reachable within 60 min	0	0.0	0	0.0
Likely irrelevant (<10 min)	783	99.0	106672	99.8

Population and Area Coverage in Landkreis Calw (Stroke-Ready Hospitals) (Baden-Württemberg) — Extended (+20min Penalty)

Category	Grid Cells	Area (%)	Population	Population (%)
High (30+ min)	0	0.0	0	0.0
Medium (20-30 min)	0	0.0	0	0.0
Low (10-20 min)	178	18.4	36727	24.2
Only CT reachable within 60 min	0	0.0	0	0.0
Neither reachable within 60 min	0	0.0	0	0.0
Likely irrelevant (<10 min)	789	81.6	114902	75.8

Population and Area Coverage in Kreisfreie Stadt Berlin (Stroke-Ready Hospitals) (Berlin) — Extended (+20min Penalty)

Category	Grid Cells	Area (%)	Population	Population (%)
High (30+ min)	0	0.0	0	0.0
Medium (20-30 min)	0	0.0	0	0.0
Low (10-20 min)	13	1.1	1858	0.1
Only CT reachable within 60 min	0	0.0	0	0.0
Neither reachable within 60 min	0	0.0	0	0.0
Likely irrelevant (<10 min)	1171	98.9	3626840	99.9

Population and Area Coverage in Kreis Stormarn (Stroke-Ready Hospitals) (Schleswig-Holstein) — Extended (+20min Penalty)

Category	Grid Cells	Area (%)	Population	Population (%)
High (30+ min)	0	0.0	0	0.0
Medium (20-30 min)	0	0.0	0	0.0
Low (10-20 min)	74	7.1	11580	4.7
Only CT reachable within 60 min	0	0.0	0	0.0
Neither reachable within 60 min	0	0.0	0	0.0
Likely irrelevant (<10 min)	968	92.9	232388	95.3

Population and Area Coverage in Landkreis Hildesheim (Stroke-Ready Hospitals) (Niedersachsen) — Extended (+20min Penalty)

Category	Grid Cells	Area (%)	Population	Population (%)
High (30+ min)	2	0.1	12	0.0
Medium (20-30 min)	174	10.9	19414	6.6
Low (10-20 min)	304	19.1	25845	8.8
Only CT reachable within 60 min	0	0.0	0	0.0
Neither reachable within 60 min	0	0.0	0	0.0
Likely irrelevant (<10 min)	1112	69.8	248444	84.6

Population and Area Coverage in Kreis Viersen (Stroke-Ready Hospitals) (Nordrhein-Westfalen) — Extended (+20min Penalty)

Category	Grid Cells	Area (%)	Population	Population (%)
High (30+ min)	0	0.0	0	0.0
Medium (20-30 min)	1	0.1	10	0.0
Low (10-20 min)	2	0.3	83	0.0
Only CT reachable within 60 min	0	0.0	0	0.0
Neither reachable within 60 min	45	6.2	1588	0.5
Likely irrelevant (<10 min)	680	93.4	294363	99.4

Population and Area Coverage in Kreis Rheinisch-Bergischer Kreis (Stroke-Ready Hospitals) (Nordrhein-Westfalen) — Extended (+20min Penalty)

Category	Grid Cells	Area (%)	Population	Population (%)
High (30+ min)	0	0.0	0	0.0
Medium (20-30 min)	0	0.0	0	0.0
Low (10-20 min)	1	0.2	88	0.0
Only CT reachable within 60 min	0	0.0	0	0.0
Neither reachable within 60 min	0	0.0	0	0.0
Likely irrelevant (<10 min)	560	99.8	301284	100.0

Population and Area Coverage in Kreisfreie Stadt Zweibrücken (Stroke-Ready Hospitals) (Rheinland-Pfalz) — Extended (+20min Penalty)

Category	Grid Cells	Area (%)	Population	Population (%)
High (30+ min)	0	0.0	0	0.0
Medium (20-30 min)	0	0.0	0	0.0
Low (10-20 min)	0	0.0	0	0.0
Only CT reachable within 60 min	0	0.0	0	0.0
Neither reachable within 60 min	0	0.0	0	0.0
Likely irrelevant (<10 min)	88	100.0	32607	100.0

Population and Area Coverage in Landkreis Rhein-Pfalz-Kreis (Stroke-Ready Hospitals) (Rheinland-Pfalz) — Extended (+20min Penalty)

Category	Grid Cells	Area (%)	Population	Population (%)
High (30+ min)	0	0.0	0	0.0
Medium (20-30 min)	0	0.0	0	0.0
Low (10-20 min)	0	0.0	0	0.0
Only CT reachable within 60 min	0	0.0	0	0.0
Neither reachable within 60 min	0	0.0	0	0.0
Likely irrelevant (<10 min)	381	100.0	152495	100.0

Population and Area Coverage in Landkreis Altötting (Stroke-Ready Hospitals) (Bayern) — Extended (+20min Penalty)

Category	Grid Cells	Area (%)	Population	Population (%)
High (30+ min)	0	0.0	0	0.0
Medium (20-30 min)	0	0.0	0	0.0
Low (10-20 min)	3	0.4	96	0.1
Only CT reachable within 60 min	0	0.0	0	0.0
Neither reachable within 60 min	3	0.4	2068	1.9
Likely irrelevant (<10 min)	672	99.1	105131	98.0

Population and Area Coverage in Landkreis Pfaffenhofen a.d.Ilm (Stroke-Ready Hospitals) (Bayern) — Extended (+20min Penalty)

Category	Grid Cells	Area (%)	Population	Population (%)
High (30+ min)	0	0.0	0	0.0
Medium (20-30 min)	0	0.0	0	0.0
Low (10-20 min)	109	11.8	11924	9.6
Only CT reachable within 60 min	0	0.0	0	0.0
Neither reachable within 60 min	0	0.0	0	0.0
Likely irrelevant (<10 min)	815	88.2	111951	90.4

Population and Area Coverage in Kreisfreie Stadt Bamberg (Stroke-Ready Hospitals) (Bayern) — Extended (+20min Penalty)

Category	Grid Cells	Area (%)	Population	Population (%)
High (30+ min)	0	0.0	0	0.0
Medium (20-30 min)	0	0.0	0	0.0
Low (10-20 min)	0	0.0	0	0.0
Only CT reachable within 60 min	0	0.0	0	0.0
Neither reachable within 60 min	0	0.0	0	0.0
Likely irrelevant (<10 min)	67	100.0	77824	100.0

Population and Area Coverage in Landkreis Hof (Stroke-Ready Hospitals) (Bayern) — Extended (+20min Penalty)

Category	Grid Cells	Area (%)	Population	Population (%)
High (30+ min)	0	0.0	0	0.0
Medium (20-30 min)	0	0.0	0	0.0
Low (10-20 min)	77	7.0	4390	4.4
Only CT reachable within 60 min	0	0.0	0	0.0
Neither reachable within 60 min	0	0.0	0	0.0
Likely irrelevant (<10 min)	1022	93.0	95307	95.6

Population and Area Coverage in Landkreis Miltenberg (Stroke-Ready Hospitals) (Bayern) — Extended (+20min Penalty)

Category	Grid Cells	Area (%)	Population	Population (%)
High (30+ min)	0	0.0	0	0.0
Medium (20-30 min)	15	1.7	592	0.4
Low (10-20 min)	194	21.7	9207	6.9
Only CT reachable within 60 min	0	0.0	0	0.0
Neither reachable within 60 min	0	0.0	0	0.0
Likely irrelevant (<10 min)	687	76.7	123347	92.6

Population and Area Coverage in Kreisfreie Stadt Augsburg (Stroke-Ready Hospitals) (Bayern) — Extended (+20min Penalty)

Category	Grid Cells	Area (%)	Population	Population (%)
High (30+ min)	0	0.0	0	0.0
Medium (20-30 min)	0	0.0	0	0.0
Low (10-20 min)	3	1.7	5	0.0
Only CT reachable within 60 min	0	0.0	0	0.0
Neither reachable within 60 min	0	0.0	0	0.0
Likely irrelevant (<10 min)	177	98.3	286684	100.0

Population and Area Coverage in Landkreis Barnim (Stroke-Ready Hospitals) (Brandenburg) — Extended (+20min Penalty)

Category	Grid Cells	Area (%)	Population	Population (%)
High (30+ min)	1	0.1	3	0.0
Medium (20-30 min)	94	4.7	501	0.3
Low (10-20 min)	552	27.9	17771	10.4
Only CT reachable within 60 min	49	2.5	153	0.1
Neither reachable within 60 min	15	0.8	13	0.0
Likely irrelevant (<10 min)	1268	64.1	152002	89.2

Population and Area Coverage in Landkreis Meißen (Stroke-Ready Hospitals) (Sachsen) — Extended (+20min Penalty)

Category	Grid Cells	Area (%)	Population	Population (%)
High (30+ min)	13	0.7	421	0.2
Medium (20-30 min)	158	8.4	11225	4.9
Low (10-20 min)	562	30.0	51735	22.6
Only CT reachable within 60 min	0	0.0	0	0.0
Neither reachable within 60 min	0	0.0	0	0.0
Likely irrelevant (<10 min)	1140	60.9	165689	72.3

Population and Area Coverage in Kreisfreie Stadt Suhl (Stroke-Ready Hospitals) (Thüringen) — Extended (+20min Penalty)

Category	Grid Cells	Area (%)	Population	Population (%)
High (30+ min)	0	0.0	0	0.0
Medium (20-30 min)	0	0.0	0	0.0
Low (10-20 min)	3	1.7	8	0.0
Only CT reachable within 60 min	0	0.0	0	0.0
Neither reachable within 60 min	0	0.0	0	0.0
Likely irrelevant (<10 min)	176	98.3	33300	100.0

Population and Area Coverage in Landkreis Eichsfeld (Stroke-Ready Hospitals) (Thüringen) — Extended (+20min Penalty)

Category	Grid Cells	Area (%)	Population	Population (%)
High (30+ min)	0	0.0	0	0.0
Medium (20-30 min)	0	0.0	0	0.0
Low (10-20 min)	220	17.1	5920	6.3
Only CT reachable within 60 min	0	0.0	0	0.0
Neither reachable within 60 min	0	0.0	0	0.0
Likely irrelevant (<10 min)	1068	82.9	88763	93.7

Population and Area Coverage in Landkreis Göttingen (Stroke-Ready Hospitals) (Niedersachsen) — Extended (+20min Penalty)

Category	Grid Cells	Area (%)	Population	Population (%)
High (30+ min)	0	0.0	0	0.0
Medium (20-30 min)	14	0.6	92	0.0
Low (10-20 min)	469	20.6	26835	8.0
Only CT reachable within 60 min	0	0.0	0	0.0
Neither reachable within 60 min	0	0.0	0	0.0
Likely irrelevant (<10 min)	1797	78.8	307644	92.0

Population and Area Coverage in Landkreis Schaumburg (Stroke-Ready Hospitals) (Niedersachsen) — Extended (+20min Penalty)

Category	Grid Cells	Area (%)	Population	Population (%)
High (30+ min)	0	0.0	0	0.0
Medium (20-30 min)	0	0.0	0	0.0
Low (10-20 min)	2	0.2	0	0.0
Only CT reachable within 60 min	0	0.0	0	0.0
Neither reachable within 60 min	0	0.0	0	0.0
Likely irrelevant (<10 min)	887	99.8	155842	100.0

Population and Area Coverage in Landkreis Aurich (Stroke-Ready Hospitals) (Niedersachsen) — Extended (+20min Penalty)

Category	Grid Cells	Area (%)	Population	Population (%)
High (30+ min)	122	7.2	4049	2.2
Medium (20-30 min)	332	19.6	43916	23.7
Low (10-20 min)	489	28.9	71501	38.6
Only CT reachable within 60 min	10	0.6	124	0.1
Neither reachable within 60 min	66	3.9	4909	2.7
Likely irrelevant (<10 min)	671	39.7	60569	32.7

Population and Area Coverage in Landkreis Friesland (Stroke-Ready Hospitals) (Niedersachsen) — Extended (+20min Penalty)

Category	Grid Cells	Area (%)	Population	Population (%)
High (30+ min)	3	0.4	419	0.4
Medium (20-30 min)	1	0.1	244	0.3
Low (10-20 min)	17	2.1	541	0.6
Only CT reachable within 60 min	4	0.5	67	0.1
Neither reachable within 60 min	48	5.8	1966	2.0
Likely irrelevant (<10 min)	753	91.2	94196	96.7

Population and Area Coverage in Landkreis Wesermarsch (Stroke-Ready Hospitals) (Niedersachsen) — Extended (+20min Penalty)

Category	Grid Cells	Area (%)	Population	Population (%)
High (30+ min)	1	0.1	43	0.1
Medium (20-30 min)	119	10.8	4681	5.5
Low (10-20 min)	357	32.5	30755	36.3
Only CT reachable within 60 min	9	0.8	225	0.3
Neither reachable within 60 min	23	2.1	344	0.4
Likely irrelevant (<10 min)	588	53.6	48654	57.4

Population and Area Coverage in Landkreis Main-Kinzig-Kreis (Stroke-Ready Hospitals) (Hessen) — Extended (+20min Penalty)

Category	Grid Cells	Area (%)	Population	Population (%)
High (30+ min)	0	0.0	0	0.0
Medium (20-30 min)	31	1.8	1008	0.2
Low (10-20 min)	282	16.0	14120	3.5
Only CT reachable within 60 min	0	0.0	0	0.0
Neither reachable within 60 min	0	0.0	0	0.0
Likely irrelevant (<10 min)	1447	82.2	394056	96.3

Population and Area Coverage in Landkreis Vogelsbergkreis (Stroke-Ready Hospitals) (Hessen) — Extended (+20min Penalty)

Category	Grid Cells	Area (%)	Population	Population (%)
High (30+ min)	0	0.0	0	0.0
Medium (20-30 min)	258	13.9	12373	11.5
Low (10-20 min)	916	49.4	44337	41.3
Only CT reachable within 60 min	0	0.0	0	0.0
Neither reachable within 60 min	0	0.0	0	0.0
Likely irrelevant (<10 min)	682	36.7	50614	47.2

Population and Area Coverage in Landkreis Neuwied (Stroke-Ready Hospitals) (Rheinland-Pfalz) — Extended (+20min Penalty)

Category	Grid Cells	Area (%)	Population	Population (%)
High (30+ min)	0	0.0	0	0.0
Medium (20-30 min)	0	0.0	0	0.0
Low (10-20 min)	31	3.9	1898	1.0
Only CT reachable within 60 min	0	0.0	0	0.0
Neither reachable within 60 min	0	0.0	0	0.0
Likely irrelevant (<10 min)	762	96.1	183419	99.0

Population and Area Coverage in Kreisfreie Stadt Mainz (Stroke-Ready Hospitals) (Rheinland-Pfalz) — Extended (+20min Penalty)

Category	Grid Cells	Area (%)	Population	Population (%)
High (30+ min)	0	0.0	0	0.0
Medium (20-30 min)	0	0.0	0	0.0
Low (10-20 min)	0	0.0	0	0.0
Only CT reachable within 60 min	0	0.0	0	0.0
Neither reachable within 60 min	0	0.0	0	0.0
Likely irrelevant (<10 min)	121	100.0	214068	100.0

Population and Area Coverage in Landkreis Hohenlohekreis (Stroke-Ready Hospitals) (Baden-Württemberg) — Extended (+20min Penalty)

Category	Grid Cells	Area (%)	Population	Population (%)
High (30+ min)	0	0.0	0	0.0
Medium (20-30 min)	0	0.0	0	0.0
Low (10-20 min)	4	0.4	324	0.3
Only CT reachable within 60 min	0	0.0	0	0.0
Neither reachable within 60 min	0	0.0	0	0.0
Likely irrelevant (<10 min)	955	99.6	109002	99.7

Population and Area Coverage in Landkreis Ortenaukreis (Stroke-Ready Hospitals) (Baden-Württemberg) — Extended (+20min Penalty)

Category	Grid Cells	Area (%)	Population	Population (%)
High (30+ min)	64	2.9	2085	0.5
Medium (20-30 min)	269	12.0	21171	5.0
Low (10-20 min)	369	16.4	44437	10.6
Only CT reachable within 60 min	0	0.0	7	0.0
Neither reachable within 60 min	9	0.4	266	0.1
Likely irrelevant (<10 min)	1533	68.3	352734	83.8

Population and Area Coverage in Landkreis Neustadt a.d.Aisch-Bad Windsheim (Stroke-Ready Hospitals) (Bayern) — Extended (+20min Penalty)

Category	Grid Cells	Area (%)	Population	Population (%)
High (30+ min)	0	0.0	0	0.0
Medium (20-30 min)	0	0.0	0	0.0
Low (10-20 min)	351	22.1	18124	17.9
Only CT reachable within 60 min	0	0.0	0	0.0
Neither reachable within 60 min	0	0.0	0	0.0
Likely irrelevant (<10 min)	1235	77.9	83376	82.1

Population and Area Coverage in Landkreis Goslar (Stroke-Ready Hospitals) (Niedersachsen) — Extended (+20min Penalty)

Category	Grid Cells	Area (%)	Population	Population (%)
High (30+ min)	0	0.0	0	0.0
Medium (20-30 min)	65	5.2	428	0.3
Low (10-20 min)	214	17.1	9354	6.8
Only CT reachable within 60 min	0	0.0	0	0.0
Neither reachable within 60 min	0	0.0	0	0.0
Likely irrelevant (<10 min)	973	77.7	127015	92.8

Population and Area Coverage in Landkreis Region Hannover (Stroke-Ready Hospitals) (Niedersachsen) — Extended (+20min Penalty)

Category	Grid Cells	Area (%)	Population	Population (%)
High (30+ min)	0	0.0	0	0.0
Medium (20-30 min)	6	0.2	1	0.0
Low (10-20 min)	210	6.9	5185	0.5
Only CT reachable within 60 min	0	0.0	0	0.0
Neither reachable within 60 min	0	0.0	0	0.0
Likely irrelevant (<10 min)	2809	92.9	1145568	99.5

Population and Area Coverage in Landkreis Vechta (Stroke-Ready Hospitals) (Niedersachsen) — Extended (+20min Penalty)

Category	Grid Cells	Area (%)	Population	Population (%)
High (30+ min)	0	0.0	0	0.0
Medium (20-30 min)	129	12.0	8047	5.9
Low (10-20 min)	218	20.2	39960	29.4
Only CT reachable within 60 min	0	0.0	0	0.0
Neither reachable within 60 min	0	0.0	0	0.0
Likely irrelevant (<10 min)	730	67.8	87772	64.6

Population and Area Coverage in Kreis Städteregion Aachen (Stroke-Ready Hospitals) (Nordrhein-Westfalen) — Extended (+20min Penalty)

Category	Grid Cells	Area (%)	Population	Population (%)
High (30+ min)	57	6.4	5451	1.0
Medium (20-30 min)	112	12.6	13844	2.5
Low (10-20 min)	138	15.5	13071	2.4
Only CT reachable within 60 min	9	1.0	256	0.0
Neither reachable within 60 min	13	1.5	387	0.1
Likely irrelevant (<10 min)	563	63.1	522400	94.1

Population and Area Coverage in Kreis Steinfurt (Stroke-Ready Hospitals) (Nordrhein-Westfalen) — Extended (+20min Penalty)

Category	Grid Cells	Area (%)	Population	Population (%)
High (30+ min)	0	0.0	0	0.0
Medium (20-30 min)	0	0.0	0	0.0
Low (10-20 min)	518	21.9	76173	17.5
Only CT reachable within 60 min	0	0.0	0	0.0
Neither reachable within 60 min	0	0.0	0	0.0
Likely irrelevant (<10 min)	1848	78.1	359734	82.5

Population and Area Coverage in Kreis Gütersloh (Stroke-Ready Hospitals) (Nordrhein-Westfalen) — Extended (+20min Penalty)

Category	Grid Cells	Area (%)	Population	Population (%)
High (30+ min)	0	0.0	0	0.0
Medium (20-30 min)	0	0.0	0	0.0
Low (10-20 min)	33	2.6	1959	0.6
Only CT reachable within 60 min	0	0.0	0	0.0
Neither reachable within 60 min	0	0.0	0	0.0
Likely irrelevant (<10 min)	1229	97.4	352830	99.4

Population and Area Coverage in Kreis Unna (Stroke-Ready Hospitals) (Nordrhein-Westfalen) — Extended (+20min Penalty)

Category	Grid Cells	Area (%)	Population	Population (%)
High (30+ min)	0	0.0	0	0.0
Medium (20-30 min)	0	0.0	0	0.0
Low (10-20 min)	0	0.0	0	0.0
Only CT reachable within 60 min	0	0.0	0	0.0
Neither reachable within 60 min	0	0.0	0	0.0
Likely irrelevant (<10 min)	711	100.0	387212	100.0

Population and Area Coverage in Landkreis Darmstadt-Dieburg (Stroke-Ready Hospitals) (Hessen) — Extended (+20min Penalty)

Category	Grid Cells	Area (%)	Population	Population (%)
High (30+ min)	0	0.0	0	0.0
Medium (20-30 min)	0	0.0	3	0.0
Low (10-20 min)	133	16.1	10948	3.8
Only CT reachable within 60 min	0	0.0	0	0.0
Neither reachable within 60 min	0	0.0	0	0.0
Likely irrelevant (<10 min)	692	83.9	278015	96.2

Population and Area Coverage in Landkreis Gießen (Stroke-Ready Hospitals) (Hessen) — Extended (+20min Penalty)

Category	Grid Cells	Area (%)	Population	Population (%)
High (30+ min)	0	0.0	0	0.0
Medium (20-30 min)	0	0.0	0	0.0
Low (10-20 min)	12	1.1	434	0.2
Only CT reachable within 60 min	0	0.0	0	0.0
Neither reachable within 60 min	0	0.0	0	0.0
Likely irrelevant (<10 min)	1069	98.9	257564	99.8

Population and Area Coverage in Kreisfreie Stadt Neustadt an der Weinstraße (Stroke-Ready Hospitals) (Rheinland-Pfalz) — Extended (+20min Penalty)

Category	Grid Cells	Area (%)	Population	Population (%)
High (30+ min)	0	0.0	0	0.0
Medium (20-30 min)	0	0.0	0	0.0
Low (10-20 min)	0	0.0	0	0.0
Only CT reachable within 60 min	0	0.0	0	0.0
Neither reachable within 60 min	0	0.0	0	0.0
Likely irrelevant (<10 min)	147	100.0	53717	100.0

Population and Area Coverage in Landkreis Main-Tauber-Kreis (Stroke-Ready Hospitals) (Baden-Württemberg) — Extended (+20min Penalty)

Category	Grid Cells	Area (%)	Population	Population (%)
High (30+ min)	0	0.0	0	0.0
Medium (20-30 min)	110	6.8	7676	5.8
Low (10-20 min)	434	26.8	23961	18.2
Only CT reachable within 60 min	0	0.0	0	0.0
Neither reachable within 60 min	0	0.0	0	0.0
Likely irrelevant (<10 min)	1078	66.5	99982	76.0

Population and Area Coverage in Landkreis Freudenstadt (Stroke-Ready Hospitals) (Baden-Württemberg) — Extended (+20min Penalty)

Category	Grid Cells	Area (%)	Population	Population (%)
High (30+ min)	0	0.0	0	0.0
Medium (20-30 min)	0	0.0	0	0.0
Low (10-20 min)	112	10.6	12540	10.8
Only CT reachable within 60 min	0	0.0	0	0.0
Neither reachable within 60 min	0	0.0	0	0.0
Likely irrelevant (<10 min)	946	89.4	103367	89.2

Population and Area Coverage in Landkreis Rosenheim (Stroke-Ready Hospitals) (Bayern) — Extended (+20min Penalty)

Category	Grid Cells	Area (%)	Population	Population (%)
High (30+ min)	0	0.0	0	0.0
Medium (20-30 min)	13	0.8	121	0.0
Low (10-20 min)	123	7.1	4435	1.8
Only CT reachable within 60 min	2	0.1	30	0.0
Neither reachable within 60 min	137	8.0	989	0.4
Likely irrelevant (<10 min)	1448	84.0	246172	97.8

Population and Area Coverage in Kreisfreie Stadt Passau (Stroke-Ready Hospitals) (Bayern) — Extended (+20min Penalty)

Category	Grid Cells	Area (%)	Population	Population (%)
High (30+ min)	0	0.0	0	0.0
Medium (20-30 min)	0	0.0	0	0.0
Low (10-20 min)	0	0.0	0	0.0
Only CT reachable within 60 min	0	0.0	0	0.0
Neither reachable within 60 min	0	0.0	0	0.0
Likely irrelevant (<10 min)	76	100.0	42451	100.0

Population and Area Coverage in Landkreis Coburg (Stroke-Ready Hospitals) (Bayern) — Extended (+20min Penalty)

Category	Grid Cells	Area (%)	Population	Population (%)
High (30+ min)	0	0.0	0	0.0
Medium (20-30 min)	0	0.0	0	0.0
Low (10-20 min)	3	0.4	0	0.0
Only CT reachable within 60 min	0	0.0	0	0.0
Neither reachable within 60 min	0	0.0	0	0.0
Likely irrelevant (<10 min)	749	99.6	89886	100.0

Population and Area Coverage in Landkreis Ostallgäu (Stroke-Ready Hospitals) (Bayern) — Extended (+20min Penalty)

Category	Grid Cells	Area (%)	Population	Population (%)
High (30+ min)	1	0.1	61	0.0
Medium (20-30 min)	7	0.4	390	0.3
Low (10-20 min)	279	16.7	29287	20.9
Only CT reachable within 60 min	35	2.1	97	0.1
Neither reachable within 60 min	158	9.4	1332	0.9
Likely irrelevant (<10 min)	1195	71.3	109168	77.8

Population and Area Coverage in Landkreis Saarpfalz-Kreis (Stroke-Ready Hospitals) (Saarland) — Extended (+20min Penalty)

Category	Grid Cells	Area (%)	Population	Population (%)
High (30+ min)	0	0.0	0	0.0
Medium (20-30 min)	0	0.0	0	0.0
Low (10-20 min)	58	11.5	3395	2.5
Only CT reachable within 60 min	0	0.0	0	0.0
Neither reachable within 60 min	4	0.8	0	0.0
Likely irrelevant (<10 min)	443	87.7	133649	97.5

Population and Area Coverage in Landkreis Nordhausen (Stroke-Ready Hospitals) (Thüringen) — Extended (+20min Penalty)

Category	Grid Cells	Area (%)	Population	Population (%)
High (30+ min)	0	0.0	0	0.0
Medium (20-30 min)	3	0.3	0	0.0
Low (10-20 min)	285	31.1	3727	4.8
Only CT reachable within 60 min	0	0.0	0	0.0
Neither reachable within 60 min	0	0.0	0	0.0
Likely irrelevant (<10 min)	628	68.6	73376	95.2

Population and Area Coverage in Landkreis Uelzen (Stroke-Ready Hospitals) (Niedersachsen) — Extended (+20min Penalty)

Category	Grid Cells	Area (%)	Population	Population (%)
High (30+ min)	0	0.0	0	0.0
Medium (20-30 min)	37	1.9	50	0.1
Low (10-20 min)	261	13.4	4029	4.3
Only CT reachable within 60 min	0	0.0	0	0.0
Neither reachable within 60 min	0	0.0	0	0.0
Likely irrelevant (<10 min)	1649	84.7	89034	95.6

Population and Area Coverage in Kreisfreie Stadt Mülheim an der Ruhr (Stroke-Ready Hospitals) (Nordrhein-Westfalen) — Extended (+20min Penalty)

Category	Grid Cells	Area (%)	Population	Population (%)
High (30+ min)	0	0.0	0	0.0
Medium (20-30 min)	0	0.0	0	0.0
Low (10-20 min)	0	0.0	0	0.0
Only CT reachable within 60 min	0	0.0	0	0.0
Neither reachable within 60 min	0	0.0	0	0.0
Likely irrelevant (<10 min)	113	100.0	180205	100.0

Population and Area Coverage in Kreisfreie Stadt Bonn (Stroke-Ready Hospitals) (Nordrhein-Westfalen) — Extended (+20min Penalty)

Category	Grid Cells	Area (%)	Population	Population (%)
High (30+ min)	0	0.0	0	0.0
Medium (20-30 min)	0	0.0	0	0.0
Low (10-20 min)	0	0.0	0	0.0
Only CT reachable within 60 min	0	0.0	0	0.0
Neither reachable within 60 min	0	0.0	0	0.0
Likely irrelevant (<10 min)	180	100.0	325710	100.0

Population and Area Coverage in Landkreis Birkenfeld (Stroke-Ready Hospitals) (Rheinland-Pfalz) — Extended (+20min Penalty)

Category	Grid Cells	Area (%)	Population	Population (%)
High (30+ min)	0	0.0	0	0.0
Medium (20-30 min)	17	1.8	203	0.2
Low (10-20 min)	156	16.2	5395	6.5
Only CT reachable within 60 min	0	0.0	0	0.0
Neither reachable within 60 min	0	0.0	0	0.0
Likely irrelevant (<10 min)	792	82.1	77151	93.2

Population and Area Coverage in Landkreis Rems-Murr-Kreis (Stroke-Ready Hospitals) (Baden-Württemberg) — Extended (+20min Penalty)

Category	Grid Cells	Area (%)	Population	Population (%)
High (30+ min)	0	0.0	0	0.0
Medium (20-30 min)	11	1.0	573	0.1
Low (10-20 min)	109	10.3	14079	3.4
Only CT reachable within 60 min	0	0.0	0	0.0
Neither reachable within 60 min	0	0.0	0	0.0
Likely irrelevant (<10 min)	937	88.6	403167	96.5

Population and Area Coverage in Landkreis Schwäbisch Hall (Stroke-Ready Hospitals) (Baden-Württemberg) — Extended (+20min Penalty)

Category	Grid Cells	Area (%)	Population	Population (%)
High (30+ min)	0	0.0	0	0.0
Medium (20-30 min)	9	0.5	227	0.1
Low (10-20 min)	378	20.7	26034	13.6
Only CT reachable within 60 min	0	0.0	0	0.0
Neither reachable within 60 min	0	0.0	0	0.0
Likely irrelevant (<10 min)	1438	78.8	164626	86.2

Population and Area Coverage in Landkreis Rottweil (Stroke-Ready Hospitals) (Baden-Württemberg) — Extended (+20min Penalty)

Category	Grid Cells	Area (%)	Population	Population (%)
High (30+ min)	1	0.1	7	0.0
Medium (20-30 min)	32	3.4	1816	1.3
Low (10-20 min)	147	15.6	12359	9.0
Only CT reachable within 60 min	0	0.0	0	0.0
Neither reachable within 60 min	0	0.0	0	0.0
Likely irrelevant (<10 min)	761	80.9	123374	89.7

Population and Area Coverage in Landkreis Amberg-Sulzbach (Stroke-Ready Hospitals) (Bayern) — Extended (+20min Penalty)

Category	Grid Cells	Area (%)	Population	Population (%)
High (30+ min)	0	0.0	0	0.0
Medium (20-30 min)	6	0.4	103	0.1
Low (10-20 min)	194	12.4	12391	11.6
Only CT reachable within 60 min	0	0.0	0	0.0
Neither reachable within 60 min	0	0.0	0	0.0
Likely irrelevant (<10 min)	1359	87.2	94211	88.3

Population and Area Coverage in Kreisfreie Stadt Kempten (Allgäu) (Stroke-Ready Hospitals) (Bayern) — Extended (+20min Penalty)

Category	Grid Cells	Area (%)	Population	Population (%)
High (30+ min)	0	0.0	0	0.0
Medium (20-30 min)	0	0.0	0	0.0
Low (10-20 min)	0	0.0	0	0.0
Only CT reachable within 60 min	0	0.0	0	0.0
Neither reachable within 60 min	0	0.0	0	0.0
Likely irrelevant (<10 min)	77	100.0	68488	100.0

Population and Area Coverage in Landkreis Mecklenburgische Seenplatte (Stroke-Ready Hospitals) (Mecklenburg-Vorpommern) — Extended (+20min Penalty)

Category	Grid Cells	Area (%)	Population	Population (%)
High (30+ min)	903	12.1	37770	15.8
Medium (20-30 min)	1119	15.0	38935	16.3
Low (10-20 min)	1268	17.0	32222	13.5
Only CT reachable within 60 min	1138	15.3	12488	5.2
Neither reachable within 60 min	460	6.2	2134	0.9
Likely irrelevant (<10 min)	2565	34.4	115392	48.3

Population and Area Coverage in Kreisfreie Stadt Chemnitz (Stroke-Ready Hospitals) (Sachsen) — Extended (+20min Penalty)

Category	Grid Cells	Area (%)	Population	Population (%)
High (30+ min)	0	0.0	0	0.0
Medium (20-30 min)	0	0.0	0	0.0
Low (10-20 min)	6	2.2	758	0.3
Only CT reachable within 60 min	0	0.0	0	0.0
Neither reachable within 60 min	0	0.0	0	0.0
Likely irrelevant (<10 min)	270	97.8	295413	99.7

Population and Area Coverage in Landkreis Erzgebirgskreis (Stroke-Ready Hospitals) (Sachsen) — Extended (+20min Penalty)

Category	Grid Cells	Area (%)	Population	Population (%)
High (30+ min)	42	1.8	2336	0.7
Medium (20-30 min)	179	7.8	13765	4.2
Low (10-20 min)	450	19.7	36845	11.3
Only CT reachable within 60 min	4	0.2	103	0.0
Neither reachable within 60 min	3	0.1	160	0.0
Likely irrelevant (<10 min)	1612	70.4	273061	83.7

Population and Area Coverage in Kreisfreie Stadt Dresden (Stroke-Ready Hospitals) (Sachsen) — Extended (+20min Penalty)

Category	Grid Cells	Area (%)	Population	Population (%)
High (30+ min)	0	0.0	0	0.0
Medium (20-30 min)	0	0.0	0	0.0
Low (10-20 min)	0	0.0	0	0.0
Only CT reachable within 60 min	0	0.0	0	0.0
Neither reachable within 60 min	0	0.0	0	0.0
Likely irrelevant (<10 min)	420	100.0	615406	100.0

Population and Area Coverage in Landkreis Hildburghausen (Stroke-Ready Hospitals) (Thüringen) — Extended (+20min Penalty)

Category	Grid Cells	Area (%)	Population	Population (%)
High (30+ min)	0	0.0	0	0.0
Medium (20-30 min)	0	0.0	0	0.0
Low (10-20 min)	55	4.6	404	0.7
Only CT reachable within 60 min	0	0.0	0	0.0
Neither reachable within 60 min	0	0.0	0	0.0
Likely irrelevant (<10 min)	1133	95.4	58493	99.3

Population and Area Coverage in Kreis Herzogtum Lauenburg (Stroke-Ready Hospitals) (Schleswig-Holstein) — Extended (+20min Penalty)

Category	Grid Cells	Area (%)	Population	Population (%)
High (30+ min)	7	0.4	43	0.0
Medium (20-30 min)	201	11.7	9557	4.9
Low (10-20 min)	601	35.1	61571	31.5
Only CT reachable within 60 min	0	0.0	0	0.0
Neither reachable within 60 min	0	0.0	0	0.0
Likely irrelevant (<10 min)	902	52.7	124182	63.6

Population and Area Coverage in Landkreis Oldenburg (Stroke-Ready Hospitals) (Niedersachsen) — Extended (+20min Penalty)

Category	Grid Cells	Area (%)	Population	Population (%)
High (30+ min)	0	0.0	0	0.0
Medium (20-30 min)	48	3.4	1127	0.9
Low (10-20 min)	297	20.8	28465	22.3
Only CT reachable within 60 min	0	0.0	0	0.0
Neither reachable within 60 min	0	0.0	0	0.0
Likely irrelevant (<10 min)	1083	75.8	98027	76.8

Population and Area Coverage in Kreisfreie Stadt Krefeld (Stroke-Ready Hospitals) (Nordrhein-Westfalen) — Extended (+20min Penalty)

Category	Grid Cells	Area (%)	Population	Population (%)
High (30+ min)	0	0.0	0	0.0
Medium (20-30 min)	0	0.0	0	0.0
Low (10-20 min)	0	0.0	0	0.0
Only CT reachable within 60 min	0	0.0	0	0.0
Neither reachable within 60 min	0	0.0	0	0.0
Likely irrelevant (<10 min)	181	100.0	241982	100.0

Population and Area Coverage in Kreis Oberbergischer Kreis (Stroke-Ready Hospitals) (Nordrhein-Westfalen) — Extended (+20min Penalty)

Category	Grid Cells	Area (%)	Population	Population (%)
High (30+ min)	0	0.0	0	0.0
Medium (20-30 min)	2	0.2	114	0.0
Low (10-20 min)	214	18.2	35126	13.1
Only CT reachable within 60 min	0	0.0	0	0.0
Neither reachable within 60 min	0	0.0	0	0.0
Likely irrelevant (<10 min)	960	81.6	233628	86.9

Population and Area Coverage in Landkreis Kusel (Stroke-Ready Hospitals) (Rheinland-Pfalz) — Extended (+20min Penalty)

Category	Grid Cells	Area (%)	Population	Population (%)
High (30+ min)	0	0.0	0	0.0
Medium (20-30 min)	1	0.1	1	0.0
Low (10-20 min)	161	22.5	13195	18.0
Only CT reachable within 60 min	0	0.0	0	0.0
Neither reachable within 60 min	0	0.0	0	0.0
Likely irrelevant (<10 min)	555	77.4	59990	82.0

Population and Area Coverage in Landkreis Fürstenfeldbruck (Stroke-Ready Hospitals) (Bayern) — Extended (+20min Penalty)

Category	Grid Cells	Area (%)	Population	Population (%)
High (30+ min)	0	0.0	0	0.0
Medium (20-30 min)	0	0.0	0	0.0
Low (10-20 min)	0	0.0	0	0.0
Only CT reachable within 60 min	0	0.0	0	0.0
Neither reachable within 60 min	0	0.0	0	0.0
Likely irrelevant (<10 min)	526	100.0	214938	100.0

Population and Area Coverage in Landkreis Weilheim-Schongau (Stroke-Ready Hospitals) (Bayern) — Extended (+20min Penalty)

Category	Grid Cells	Area (%)	Population	Population (%)
High (30+ min)	0	0.0	0	0.0
Medium (20-30 min)	40	3.4	635	0.5
Low (10-20 min)	242	20.9	21930	16.2
Only CT reachable within 60 min	1	0.1	0	0.0
Neither reachable within 60 min	13	1.1	13	0.0
Likely irrelevant (<10 min)	864	74.5	112495	83.3

Population and Area Coverage in Landkreis Neustadt a.d.Waldnaab (Stroke-Ready Hospitals) (Bayern) — Extended (+20min Penalty)

Category	Grid Cells	Area (%)	Population	Population (%)
High (30+ min)	0	0.0	0	0.0
Medium (20-30 min)	72	4.2	387	0.4
Low (10-20 min)	403	23.4	11005	11.3
Only CT reachable within 60 min	0	0.0	0	0.0
Neither reachable within 60 min	0	0.0	0	0.0
Likely irrelevant (<10 min)	1247	72.4	86027	88.3

Population and Area Coverage in Landkreis Würzburg (Stroke-Ready Hospitals) (Bayern) — Extended (+20min Penalty)

Category	Grid Cells	Area (%)	Population	Population (%)
High (30+ min)	0	0.0	0	0.0
Medium (20-30 min)	0	0.0	0	0.0
Low (10-20 min)	100	8.3	6644	4.0
Only CT reachable within 60 min	0	0.0	0	0.0
Neither reachable within 60 min	0	0.0	0	0.0
Likely irrelevant (<10 min)	1105	91.7	158418	96.0

Population and Area Coverage in Landkreis Elbe-Elster (Stroke-Ready Hospitals) (Brandenburg) — Extended (+20min Penalty)

Category	Grid Cells	Area (%)	Population	Population (%)
High (30+ min)	500	20.3	21772	21.4
Medium (20-30 min)	881	35.7	33135	32.5
Low (10-20 min)	420	17.0	28419	27.9
Only CT reachable within 60 min	553	22.4	15857	15.6
Neither reachable within 60 min	0	0.0	0	0.0
Likely irrelevant (<10 min)	115	4.7	2756	2.7

Population and Area Coverage in Landkreis Osterholz (Stroke-Ready Hospitals) (Niedersachsen) — Extended (+20min Penalty)

Category	Grid Cells	Area (%)	Population	Population (%)
High (30+ min)	1	0.1	55	0.0
Medium (20-30 min)	157	17.9	5743	5.1
Low (10-20 min)	344	39.1	28583	25.5
Only CT reachable within 60 min	0	0.0	0	0.0
Neither reachable within 60 min	0	0.0	0	0.0
Likely irrelevant (<10 min)	377	42.9	77771	69.3

Population and Area Coverage in Landkreis Heidekreis (Stroke-Ready Hospitals) (Niedersachsen) — Extended (+20min Penalty)

Category	Grid Cells	Area (%)	Population	Population (%)
High (30+ min)	0	0.0	0	0.0
Medium (20-30 min)	52	2.1	483	0.4
Low (10-20 min)	558	22.2	26852	19.8
Only CT reachable within 60 min	0	0.0	0	0.0
Neither reachable within 60 min	0	0.0	0	0.0
Likely irrelevant (<10 min)	1906	75.8	108509	79.9

Population and Area Coverage in Kreisfreie Stadt Solingen (Stroke-Ready Hospitals) (Nordrhein-Westfalen) — Extended (+20min Penalty)

Category	Grid Cells	Area (%)	Population	Population (%)
High (30+ min)	0	0.0	0	0.0
Medium (20-30 min)	0	0.0	0	0.0
Low (10-20 min)	0	0.0	0	0.0
Only CT reachable within 60 min	0	0.0	0	0.0
Neither reachable within 60 min	0	0.0	0	0.0
Likely irrelevant (<10 min)	115	100.0	158438	100.0

Population and Area Coverage in Landkreis Bad Kreuznach (Stroke-Ready Hospitals) (Rheinland-Pfalz) — Extended (+20min Penalty)

Category	Grid Cells	Area (%)	Population	Population (%)
High (30+ min)	0	0.0	0	0.0
Medium (20-30 min)	4	0.4	0	0.0
Low (10-20 min)	163	15.2	5872	3.7
Only CT reachable within 60 min	0	0.0	0	0.0
Neither reachable within 60 min	0	0.0	0	0.0
Likely irrelevant (<10 min)	905	84.4	154297	96.3

Population and Area Coverage in Landkreis Südwestpfalz (Stroke-Ready Hospitals) (Rheinland-Pfalz) — Extended (+20min Penalty)

Category	Grid Cells	Area (%)	Population	Population (%)
High (30+ min)	0	0.0	0	0.0
Medium (20-30 min)	1	0.1	0	0.0
Low (10-20 min)	13	1.1	473	0.4
Only CT reachable within 60 min	0	0.0	0	0.0
Neither reachable within 60 min	10	0.8	61	0.1
Likely irrelevant (<10 min)	1153	98.0	111607	99.5

Population and Area Coverage in Landkreis Ludwigsburg (Stroke-Ready Hospitals) (Baden-Württemberg) — Extended (+20min Penalty)

Category	Grid Cells	Area (%)	Population	Population (%)
High (30+ min)	0	0.0	0	0.0
Medium (20-30 min)	46	5.4	1797	0.3
Low (10-20 min)	225	26.6	70913	13.4
Only CT reachable within 60 min	0	0.0	0	0.0
Neither reachable within 60 min	0	0.0	0	0.0
Likely irrelevant (<10 min)	576	68.0	454833	86.2

Population and Area Coverage in Landkreis Lörrach (Stroke-Ready Hospitals) (Baden-Württemberg) — Extended (+20min Penalty)

Category	Grid Cells	Area (%)	Population	Population (%)
High (30+ min)	0	0.0	0	0.0
Medium (20-30 min)	0	0.0	0	0.0
Low (10-20 min)	173	18.4	11507	5.3
Only CT reachable within 60 min	0	0.0	52	0.0
Neither reachable within 60 min	0	0.0	9	0.0
Likely irrelevant (<10 min)	766	81.6	205076	94.7

Population and Area Coverage in Landkreis Alb-Donau-Kreis (Stroke-Ready Hospitals) (Baden-Württemberg) — Extended (+20min Penalty)

Category	Grid Cells	Area (%)	Population	Population (%)
High (30+ min)	0	0.0	0	0.0
Medium (20-30 min)	36	2.2	440	0.2
Low (10-20 min)	373	22.5	24806	13.0
Only CT reachable within 60 min	0	0.0	0	0.0
Neither reachable within 60 min	0	0.0	0	0.0
Likely irrelevant (<10 min)	1249	75.3	165861	86.8

Population and Area Coverage in Landkreis Biberach (Stroke-Ready Hospitals) (Baden-Württemberg) — Extended (+20min Penalty)

Category	Grid Cells	Area (%)	Population	Population (%)
High (30+ min)	0	0.0	0	0.0
Medium (20-30 min)	0	0.0	0	0.0
Low (10-20 min)	11	0.6	585	0.3
Only CT reachable within 60 min	0	0.0	0	0.0
Neither reachable within 60 min	0	0.0	0	0.0
Likely irrelevant (<10 min)	1693	99.4	192672	99.7

Population and Area Coverage in Kreisfreie Stadt Fürth (Stroke-Ready Hospitals) (Bayern) — Extended (+20min Penalty)

Category	Grid Cells	Area (%)	Population	Population (%)
High (30+ min)	0	0.0	0	0.0
Medium (20-30 min)	0	0.0	0	0.0
Low (10-20 min)	0	0.0	0	0.0
Only CT reachable within 60 min	0	0.0	0	0.0
Neither reachable within 60 min	0	0.0	0	0.0
Likely irrelevant (<10 min)	83	100.0	150301	100.0

Population and Area Coverage in Kreisfreie Stadt Halle (Saale) (Stroke-Ready Hospitals) (Sachsen-Anhalt) — Extended (+20min Penalty)

Category	Grid Cells	Area (%)	Population	Population (%)
High (30+ min)	0	0.0	0	0.0
Medium (20-30 min)	0	0.0	0	0.0
Low (10-20 min)	0	0.0	0	0.0
Only CT reachable within 60 min	0	0.0	0	0.0
Neither reachable within 60 min	0	0.0	0	0.0
Likely irrelevant (<10 min)	174	100.0	250172	100.0

Population and Area Coverage in Landkreis Wittenberg (Stroke-Ready Hospitals) (Sachsen-Anhalt) — Extended (+20min Penalty)

Category	Grid Cells	Area (%)	Population	Population (%)
High (30+ min)	23	0.9	111	0.1
Medium (20-30 min)	147	5.8	2638	2.3
Low (10-20 min)	621	24.5	14857	13.0
Only CT reachable within 60 min	112	4.4	482	0.4
Neither reachable within 60 min	0	0.0	0	0.0
Likely irrelevant (<10 min)	1634	64.4	95995	84.1

Population and Area Coverage in Landkreis Saale-Holzland-Kreis (Stroke-Ready Hospitals) (Thüringen) — Extended (+20min Penalty)

Category	Grid Cells	Area (%)	Population	Population (%)
High (30+ min)	0	0.0	0	0.0
Medium (20-30 min)	0	0.0	0	0.0
Low (10-20 min)	212	20.4	5896	7.8
Only CT reachable within 60 min	0	0.0	0	0.0
Neither reachable within 60 min	0	0.0	0	0.0
Likely irrelevant (<10 min)	826	79.6	69806	92.2

Population and Area Coverage in Kreisfreie Stadt Salzgitter (Stroke-Ready Hospitals) (Niedersachsen) — Extended (+20min Penalty)

Category	Grid Cells	Area (%)	Population	Population (%)
High (30+ min)	0	0.0	0	0.0
Medium (20-30 min)	0	0.0	0	0.0
Low (10-20 min)	2	0.7	204	0.2
Only CT reachable within 60 min	0	0.0	0	0.0
Neither reachable within 60 min	0	0.0	0	0.0
Likely irrelevant (<10 min)	293	99.3	99848	99.8

Population and Area Coverage in Kreis Wesel (Stroke-Ready Hospitals) (Nordrhein-Westfalen) — Extended (+20min Penalty)

Category	Grid Cells	Area (%)	Population	Population (%)
High (30+ min)	0	0.0	0	0.0
Medium (20-30 min)	0	0.0	0	0.0
Low (10-20 min)	12	0.9	304	0.1
Only CT reachable within 60 min	0	0.0	0	0.0
Neither reachable within 60 min	0	0.0	0	0.0
Likely irrelevant (<10 min)	1341	99.1	474780	99.9

Population and Area Coverage in Kreis Rhein-Erft-Kreis (Stroke-Ready Hospitals) (Nordrhein-Westfalen) — Extended (+20min Penalty)

Category	Grid Cells	Area (%)	Population	Population (%)
High (30+ min)	0	0.0	0	0.0
Medium (20-30 min)	0	0.0	0	0.0
Low (10-20 min)	6	0.7	138	0.0
Only CT reachable within 60 min	0	0.0	0	0.0
Neither reachable within 60 min	0	0.0	0	0.0
Likely irrelevant (<10 min)	892	99.3	454391	100.0

Population and Area Coverage in Kreisfreie Stadt Frankfurt am Main (Stroke-Ready Hospitals) (Hessen) — Extended (+20min Penalty)

Category	Grid Cells	Area (%)	Population	Population (%)
High (30+ min)	0	0.0	0	0.0
Medium (20-30 min)	0	0.0	0	0.0
Low (10-20 min)	0	0.0	0	0.0
Only CT reachable within 60 min	0	0.0	0	0.0
Neither reachable within 60 min	0	0.0	0	0.0
Likely irrelevant (<10 min)	310	100.0	833128	100.0

Population and Area Coverage in Landkreis Südliche Weinstraße (Stroke-Ready Hospitals) (Rheinland-Pfalz) — Extended (+20min Penalty)

Category	Grid Cells	Area (%)	Population	Population (%)
High (30+ min)	0	0.0	0	0.0
Medium (20-30 min)	0	0.0	0	0.0
Low (10-20 min)	20	2.5	16	0.0
Only CT reachable within 60 min	1	0.1	1	0.0
Neither reachable within 60 min	0	0.0	0	0.0
Likely irrelevant (<10 min)	773	97.4	111843	100.0

Population and Area Coverage in Stadtkreis Karlsruhe (Stroke-Ready Hospitals) (Baden-Württemberg) — Extended (+20min Penalty)

Category	Grid Cells	Area (%)	Population	Population (%)
High (30+ min)	0	0.0	0	0.0
Medium (20-30 min)	0	0.0	0	0.0
Low (10-20 min)	0	0.0	0	0.0
Only CT reachable within 60 min	0	0.0	0	0.0
Neither reachable within 60 min	0	0.0	0	0.0
Likely irrelevant (<10 min)	214	100.0	336385	100.0

Population and Area Coverage in Landkreis Rhein-Neckar-Kreis (Stroke-Ready Hospitals) (Baden-Württemberg) — Extended (+20min Penalty)

Category	Grid Cells	Area (%)	Population	Population (%)
High (30+ min)	0	0.0	0	0.0
Medium (20-30 min)	2	0.2	0	0.0
Low (10-20 min)	111	8.4	17428	3.2
Only CT reachable within 60 min	0	0.0	0	0.0
Neither reachable within 60 min	0	0.0	0	0.0
Likely irrelevant (<10 min)	1201	91.4	523592	96.8

Population and Area Coverage in Landkreis München (Stroke-Ready Hospitals) (Bayern) — Extended (+20min Penalty)

Category	Grid Cells	Area (%)	Population	Population (%)
High (30+ min)	0	0.0	0	0.0
Medium (20-30 min)	0	0.0	0	0.0
Low (10-20 min)	9	1.1	97	0.0
Only CT reachable within 60 min	0	0.0	0	0.0
Neither reachable within 60 min	0	0.0	0	0.0
Likely irrelevant (<10 min)	794	98.9	344228	100.0

Population and Area Coverage in Landkreis Dingolfing-Landau (Stroke-Ready Hospitals) (Bayern) — Extended (+20min Penalty)

Category	Grid Cells	Area (%)	Population	Population (%)
High (30+ min)	0	0.0	0	0.0
Medium (20-30 min)	0	0.0	0	0.0
Low (10-20 min)	80	7.4	4622	4.9
Only CT reachable within 60 min	0	0.0	0	0.0
Neither reachable within 60 min	0	0.0	0	0.0
Likely irrelevant (<10 min)	997	92.6	89842	95.1

Population and Area Coverage in Kreisfreie Stadt Nürnberg (Stroke-Ready Hospitals) (Bayern) — Extended (+20min Penalty)

Category	Grid Cells	Area (%)	Population	Population (%)
High (30+ min)	0	0.0	0	0.0
Medium (20-30 min)	0	0.0	0	0.0
Low (10-20 min)	0	0.0	0	0.0
Only CT reachable within 60 min	0	0.0	0	0.0
Neither reachable within 60 min	0	0.0	0	0.0
Likely irrelevant (<10 min)	232	100.0	518192	100.0

Population and Area Coverage in Kreisfreie Stadt Cottbus (Stroke-Ready Hospitals) (Brandenburg) — Extended (+20min Penalty)

Category	Grid Cells	Area (%)	Population	Population (%)
High (30+ min)	0	0.0	0	0.0
Medium (20-30 min)	0	0.0	0	0.0
Low (10-20 min)	4	1.9	84	0.1
Only CT reachable within 60 min	0	0.0	0	0.0
Neither reachable within 60 min	0	0.0	0	0.0
Likely irrelevant (<10 min)	208	98.1	104420	99.9

Population and Area Coverage in Landkreis Salzlandkreis (Stroke-Ready Hospitals) (Sachsen-Anhalt) — Extended (+20min Penalty)

Category	Grid Cells	Area (%)	Population	Population (%)
High (30+ min)	0	0.0	0	0.0
Medium (20-30 min)	49	2.6	629	0.4
Low (10-20 min)	656	35.1	40954	23.2
Only CT reachable within 60 min	0	0.0	0	0.0
Neither reachable within 60 min	0	0.0	0	0.0
Likely irrelevant (<10 min)	1163	62.3	134734	76.4

Population and Area Coverage in Landkreis Sömmerda (Stroke-Ready Hospitals) (Thüringen) — Extended (+20min Penalty)

Category	Grid Cells	Area (%)	Population	Population (%)
High (30+ min)	10	1.0	5	0.0
Medium (20-30 min)	42	4.0	530	0.8
Low (10-20 min)	480	46.0	20113	31.6
Only CT reachable within 60 min	0	0.0	0	0.0
Neither reachable within 60 min	0	0.0	0	0.0
Likely irrelevant (<10 min)	512	49.0	43028	67.6

Population and Area Coverage in Landkreis Saale-Orla-Kreis (Stroke-Ready Hospitals) (Thüringen) — Extended (+20min Penalty)

Category	Grid Cells	Area (%)	Population	Population (%)
High (30+ min)	0	0.0	0	0.0
Medium (20-30 min)	106	7.2	4996	6.6
Low (10-20 min)	656	44.8	34732	46.1
Only CT reachable within 60 min	0	0.0	0	0.0
Neither reachable within 60 min	0	0.0	0	0.0
Likely irrelevant (<10 min)	703	48.0	35578	47.2

Population and Area Coverage in Kreisfreie Stadt Neumünster (Stroke Units) (Schleswig-Holstein) — Regular (+20min Penalty)

Category	Grid Cells	Area (%)	Population	Population (%)
High (30+ min)	0	0.0	0	0.0
Medium (20-30 min)	0	0.0	0	0.0
Low (10-20 min)	0	0.0	0	0.0
Only CT reachable within 60 min	0	0.0	0	0.0
Neither reachable within 60 min	0	0.0	0	0.0
Likely irrelevant (<10 min)	100	100.0	85923	100.0

Population and Area Coverage in Kreisfreie Stadt Osnabrück (Stroke Units) (Niedersachsen) — Regular (+20min Penalty)

Category	Grid Cells	Area (%)	Population	Population (%)
High (30+ min)	0	0.0	0	0.0
Medium (20-30 min)	0	0.0	0	0.0
Low (10-20 min)	0	0.0	0	0.0
Only CT reachable within 60 min	0	0.0	0	0.0
Neither reachable within 60 min	0	0.0	0	0.0
Likely irrelevant (<10 min)	155	100.0	154441	100.0

Population and Area Coverage in Kreisfreie Stadt Mönchengladbach (Stroke Units) (Nordrhein-Westfalen) — Regular (+20min Penalty)

Category	Grid Cells	Area (%)	Population	Population (%)
High (30+ min)	0	0.0	0	0.0
Medium (20-30 min)	0	0.0	0	0.0
Low (10-20 min)	0	0.0	0	0.0
Only CT reachable within 60 min	0	0.0	0	0.0
Neither reachable within 60 min	0	0.0	0	0.0
Likely irrelevant (<10 min)	220	100.0	274121	100.0

Population and Area Coverage in Kreis Coesfeld (Stroke Units) (Nordrhein-Westfalen) — Regular (+20min Penalty)

Category	Grid Cells	Area (%)	Population	Population (%)
High (30+ min)	0	0.0	0	0.0
Medium (20-30 min)	0	0.0	0	0.0
Low (10-20 min)	112	7.7	7747	3.6
Only CT reachable within 60 min	0	0.0	0	0.0
Neither reachable within 60 min	0	0.0	0	0.0
Likely irrelevant (<10 min)	1342	92.3	208189	96.4

Population and Area Coverage in Kreis Paderborn (Stroke Units) (Nordrhein-Westfalen) — Regular (+20min Penalty)

Category	Grid Cells	Area (%)	Population	Population (%)
High (30+ min)	0	0.0	0	0.0
Medium (20-30 min)	28	1.7	10	0.0
Low (10-20 min)	216	13.3	6139	1.9
Only CT reachable within 60 min	0	0.0	0	0.0
Neither reachable within 60 min	0	0.0	0	0.0
Likely irrelevant (<10 min)	1382	85.0	324372	98.1

Population and Area Coverage in Kreisfreie Stadt Kassel (Stroke Units) (Hessen) — Regular (+20min Penalty)

Category	Grid Cells	Area (%)	Population	Population (%)
High (30+ min)	0	0.0	0	0.0
Medium (20-30 min)	0	0.0	0	0.0
Low (10-20 min)	0	0.0	0	0.0
Only CT reachable within 60 min	0	0.0	0	0.0
Neither reachable within 60 min	0	0.0	0	0.0
Likely irrelevant (<10 min)	133	100.0	205719	100.0

Population and Area Coverage in Landkreis Hersfeld-Rotenburg (Stroke Units) (Hessen) — Regular (+20min Penalty)

Category	Grid Cells	Area (%)	Population	Population (%)
High (30+ min)	0	0.0	0	0.0
Medium (20-30 min)	47	3.4	379	0.3
Low (10-20 min)	215	15.3	13736	11.4
Only CT reachable within 60 min	0	0.0	0	0.0
Neither reachable within 60 min	0	0.0	0	0.0
Likely irrelevant (<10 min)	1139	81.3	106774	88.3

Population and Area Coverage in Landkreis Schwalm-Eder-Kreis (Stroke Units) (Hessen) — Regular (+20min Penalty)

Category	Grid Cells	Area (%)	Population	Population (%)
High (30+ min)	9	0.5	160	0.1
Medium (20-30 min)	657	33.2	48065	26.4
Low (10-20 min)	832	42.0	76286	42.0
Only CT reachable within 60 min	0	0.0	0	0.0
Neither reachable within 60 min	0	0.0	0	0.0
Likely irrelevant (<10 min)	481	24.3	57316	31.5

Population and Area Coverage in Landkreis Westerwaldkreis (Stroke Units) (Rheinland-Pfalz) — Regular (+20min Penalty)

Category	Grid Cells	Area (%)	Population	Population (%)
High (30+ min)	0	0.0	0	0.0
Medium (20-30 min)	14	1.1	79	0.0
Low (10-20 min)	245	19.5	32001	15.5
Only CT reachable within 60 min	0	0.0	0	0.0
Neither reachable within 60 min	0	0.0	0	0.0
Likely irrelevant (<10 min)	996	79.4	173744	84.4

Population and Area Coverage in Kreisfreie Stadt Frankenthal (Pfalz) (Stroke Units) (Rheinland-Pfalz) — Regular (+20min Penalty)

Category	Grid Cells	Area (%)	Population	Population (%)
High (30+ min)	0	0.0	0	0.0
Medium (20-30 min)	0	0.0	0	0.0
Low (10-20 min)	0	0.0	0	0.0
Only CT reachable within 60 min	0	0.0	0	0.0
Neither reachable within 60 min	0	0.0	0	0.0
Likely irrelevant (<10 min)	54	100.0	45433	100.0

Population and Area Coverage in Landkreis Böblingen (Stroke Units) (Baden-Württemberg) — Regular (+20min Penalty)

Category	Grid Cells	Area (%)	Population	Population (%)
High (30+ min)	0	0.0	0	0.0
Medium (20-30 min)	0	0.0	0	0.0
Low (10-20 min)	93	12.4	21190	5.7
Only CT reachable within 60 min	0	0.0	0	0.0
Neither reachable within 60 min	0	0.0	0	0.0
Likely irrelevant (<10 min)	656	87.6	351159	94.3

Population and Area Coverage in Landkreis Cham (Stroke Units) (Bayern) — Regular (+20min Penalty)

Category	Grid Cells	Area (%)	Population	Population (%)
High (30+ min)	0	0.0	0	0.0
Medium (20-30 min)	39	2.2	1700	1.3
Low (10-20 min)	272	15.4	13638	10.7
Only CT reachable within 60 min	0	0.0	0	0.0
Neither reachable within 60 min	0	0.0	0	0.0
Likely irrelevant (<10 min)	1456	82.4	111531	87.9

Population and Area Coverage in Landkreis Saarlouis (Stroke Units) (Saarland) — Regular (+20min Penalty)

Category	Grid Cells	Area (%)	Population	Population (%)
High (30+ min)	0	0.0	0	0.0
Medium (20-30 min)	1	0.2	3	0.0
Low (10-20 min)	42	7.4	5639	3.0
Only CT reachable within 60 min	1	0.2	320	0.2
Neither reachable within 60 min	2	0.4	41	0.0
Likely irrelevant (<10 min)	518	91.8	181206	96.8

Population and Area Coverage in Landkreis Anhalt-Bitterfeld (Stroke Units) (Sachsen-Anhalt) — Regular (+20min Penalty)

Category	Grid Cells	Area (%)	Population	Population (%)
High (30+ min)	399	20.9	6520	4.4
Medium (20-30 min)	371	19.4	27594	18.7
Low (10-20 min)	551	28.8	62474	42.4
Only CT reachable within 60 min	184	9.6	1112	0.8
Neither reachable within 60 min	0	0.0	0	0.0
Likely irrelevant (<10 min)	406	21.2	49797	33.8

Population and Area Coverage in Landkreis Jerichower Land (Stroke Units) (Sachsen-Anhalt) — Regular (+20min Penalty)

Category	Grid Cells	Area (%)	Population	Population (%)
High (30+ min)	142	6.8	13502	16.8
Medium (20-30 min)	466	22.3	10265	12.8
Low (10-20 min)	720	34.5	19485	24.2
Only CT reachable within 60 min	498	23.9	6136	7.6
Neither reachable within 60 min	0	0.0	0	0.0
Likely irrelevant (<10 min)	262	12.5	31111	38.6

Population and Area Coverage in Landkreis Kyffhäuserkreis (Stroke Units) (Thüringen) — Regular (+20min Penalty)

Category	Grid Cells	Area (%)	Population	Population (%)
High (30+ min)	80	6.0	690	1.0
Medium (20-30 min)	643	48.1	29605	42.5
Low (10-20 min)	574	42.9	36846	52.9
Only CT reachable within 60 min	9	0.7	4	0.0
Neither reachable within 60 min	0	0.0	0	0.0
Likely irrelevant (<10 min)	31	2.3	2547	3.7

Population and Area Coverage in Landkreis Peine (Stroke Units) (Niedersachsen) — Regular (+20min Penalty)

Category	Grid Cells	Area (%)	Population	Population (%)
High (30+ min)	0	0.0	0	0.0
Medium (20-30 min)	9	1.3	401	0.3
Low (10-20 min)	413	58.0	52236	40.0
Only CT reachable within 60 min	0	0.0	0	0.0
Neither reachable within 60 min	0	0.0	0	0.0
Likely irrelevant (<10 min)	290	40.7	78080	59.7

Population and Area Coverage in Kreisfreie Stadt Leverkusen (Stroke Units) (Nordrhein-Westfalen) — Regular (+20min Penalty)

Category	Grid Cells	Area (%)	Population	Population (%)
High (30+ min)	0	0.0	0	0.0
Medium (20-30 min)	0	0.0	0	0.0
Low (10-20 min)	0	0.0	0	0.0
Only CT reachable within 60 min	0	0.0	0	0.0
Neither reachable within 60 min	0	0.0	0	0.0
Likely irrelevant (<10 min)	102	100.0	160670	100.0

Population and Area Coverage in Kreis Ennepe-Ruhr-Kreis (Stroke Units) (Nordrhein-Westfalen) — Regular (+20min Penalty)

Category	Grid Cells	Area (%)	Population	Population (%)
High (30+ min)	0	0.0	0	0.0
Medium (20-30 min)	6	1.1	198	0.1
Low (10-20 min)	109	20.3	23001	6.9
Only CT reachable within 60 min	0	0.0	0	0.0
Neither reachable within 60 min	0	0.0	0	0.0
Likely irrelevant (<10 min)	421	78.5	309462	93.0

Population and Area Coverage in Kreis Märkischer Kreis (Stroke Units) (Nordrhein-Westfalen) — Regular (+20min Penalty)

Category	Grid Cells	Area (%)	Population	Population (%)
High (30+ min)	0	0.0	15	0.0
Medium (20-30 min)	21	1.5	674	0.2
Low (10-20 min)	332	24.5	86594	20.8
Only CT reachable within 60 min	0	0.0	0	0.0
Neither reachable within 60 min	0	0.0	0	0.0
Likely irrelevant (<10 min)	1004	74.0	328111	79.0

Population and Area Coverage in Landkreis Ahrweiler (Stroke Units) (Rheinland-Pfalz) — Regular (+20min Penalty)

Category	Grid Cells	Area (%)	Population	Population (%)
High (30+ min)	0	0.0	0	0.0
Medium (20-30 min)	0	0.0	0	0.0
Low (10-20 min)	173	17.4	9348	7.3
Only CT reachable within 60 min	0	0.0	0	0.0
Neither reachable within 60 min	0	0.0	0	0.0
Likely irrelevant (<10 min)	820	82.6	119539	92.7

Population and Area Coverage in Stadtkreis Baden-Baden (Stroke Units) (Baden-Württemberg) — Regular (+20min Penalty)

Category	Grid Cells	Area (%)	Population	Population (%)
High (30+ min)	0	0.0	0	0.0
Medium (20-30 min)	39	21.8	27	0.0
Low (10-20 min)	43	24.0	7756	12.3
Only CT reachable within 60 min	0	0.0	0	0.0
Neither reachable within 60 min	0	0.0	0	0.0
Likely irrelevant (<10 min)	97	54.2	55158	87.6

Population and Area Coverage in Landkreis Waldshut (Stroke Units) (Baden-Württemberg) — Regular (+20min Penalty)

Category	Grid Cells	Area (%)	Population	Population (%)
High (30+ min)	92	7.2	18408	13.0
Medium (20-30 min)	206	16.1	12912	9.1
Low (10-20 min)	257	20.1	29849	21.1
Only CT reachable within 60 min	639	49.9	69358	49.0
Neither reachable within 60 min	2	0.2	204	0.1
Likely irrelevant (<10 min)	84	6.6	10818	7.6

Population and Area Coverage in Landkreis Bad Tölz-Wolfratshausen (Stroke Units) (Bayern) — Regular (+20min Penalty)

Category	Grid Cells	Area (%)	Population	Population (%)
High (30+ min)	0	0.0	0	0.0
Medium (20-30 min)	5	0.4	248	0.2
Low (10-20 min)	52	3.9	1933	1.5
Only CT reachable within 60 min	1	0.1	5	0.0
Neither reachable within 60 min	109	8.3	176	0.1
Likely irrelevant (<10 min)	1152	87.3	123795	98.1

Population and Area Coverage in Landkreis Passau (Stroke Units) (Bayern) — Regular (+20min Penalty)

Category	Grid Cells	Area (%)	Population	Population (%)
High (30+ min)	30	1.7	1530	0.8
Medium (20-30 min)	144	8.0	7242	3.8
Low (10-20 min)	591	32.8	56264	29.7
Only CT reachable within 60 min	2	0.1	126	0.1
Neither reachable within 60 min	10	0.6	139	0.1
Likely irrelevant (<10 min)	1023	56.8	124412	65.6

Population and Area Coverage in Landkreis Lichtenfels (Stroke Units) (Bayern) — Regular (+20min Penalty)

Category	Grid Cells	Area (%)	Population	Population (%)
High (30+ min)	0	0.0	0	0.0
Medium (20-30 min)	0	0.0	0	0.0
Low (10-20 min)	0	0.0	0	0.0
Only CT reachable within 60 min	0	0.0	0	0.0
Neither reachable within 60 min	0	0.0	0	0.0
Likely irrelevant (<10 min)	655	100.0	69197	100.0

Population and Area Coverage in Landkreis Wunsiedel i.Fichtelgebirge (Stroke Units) (Bayern) — Regular (+20min Penalty)

Category	Grid Cells	Area (%)	Population	Population (%)
High (30+ min)	0	0.0	0	0.0
Medium (20-30 min)	29	4.1	1581	2.1
Low (10-20 min)	274	38.4	30702	41.5
Only CT reachable within 60 min	0	0.0	0	0.0
Neither reachable within 60 min	0	0.0	0	0.0
Likely irrelevant (<10 min)	411	57.6	41653	56.3

Population and Area Coverage in Landkreis Ansbach (Stroke Units) (Bayern) — Regular (+20min Penalty)

Category	Grid Cells	Area (%)	Population	Population (%)
High (30+ min)	0	0.0	0	0.0
Medium (20-30 min)	30	1.2	960	0.5
Low (10-20 min)	255	10.5	19092	10.3
Only CT reachable within 60 min	0	0.0	0	0.0
Neither reachable within 60 min	0	0.0	0	0.0
Likely irrelevant (<10 min)	2147	88.3	164824	89.2

Population and Area Coverage in Kreisfreie Stadt Würzburg (Stroke Units) (Bayern) — Regular (+20min Penalty)

Category	Grid Cells	Area (%)	Population	Population (%)
High (30+ min)	0	0.0	0	0.0
Medium (20-30 min)	0	0.0	0	0.0
Low (10-20 min)	0	0.0	0	0.0
Only CT reachable within 60 min	0	0.0	0	0.0
Neither reachable within 60 min	0	0.0	0	0.0
Likely irrelevant (<10 min)	109	100.0	128110	100.0

Population and Area Coverage in Landkreis Oder-Spree (Stroke Units) (Brandenburg) — Regular (+20min Penalty)

Category	Grid Cells	Area (%)	Population	Population (%)
High (30+ min)	13	0.4	561	0.3
Medium (20-30 min)	339	11.4	6986	4.1
Low (10-20 min)	1194	40.2	29457	17.1
Only CT reachable within 60 min	40	1.3	207	0.1
Neither reachable within 60 min	7	0.2	49	0.0
Likely irrelevant (<10 min)	1376	46.3	134670	78.3

Population and Area Coverage in Landkreis Potsdam-Mittelmark (Stroke Units) (Brandenburg) — Regular (+20min Penalty)

Category	Grid Cells	Area (%)	Population	Population (%)
High (30+ min)	666	19.5	18546	9.1
Medium (20-30 min)	766	22.5	21213	10.4
Low (10-20 min)	844	24.8	34805	17.1
Only CT reachable within 60 min	621	18.2	10098	5.0
Neither reachable within 60 min	0	0.0	0	0.0
Likely irrelevant (<10 min)	510	15.0	118336	58.3

Population and Area Coverage in Landkreis Uckermark (Stroke Units) (Brandenburg) — Regular (+20min Penalty)

Category	Grid Cells	Area (%)	Population	Population (%)
High (30+ min)	831	20.2	37211	32.2
Medium (20-30 min)	1206	29.3	27341	23.6
Low (10-20 min)	398	9.7	12767	11.0
Only CT reachable within 60 min	1549	37.6	36120	31.2
Neither reachable within 60 min	85	2.1	1901	1.6
Likely irrelevant (<10 min)	52	1.3	318	0.3

Population and Area Coverage in Landkreis Sächsische Schweiz-Osterzgebirge (Stroke Units) (Sachsen) — Regular (+20min Penalty)

Category	Grid Cells	Area (%)	Population	Population (%)
High (30+ min)	208	10.0	15184	6.7
Medium (20-30 min)	373	18.0	23290	10.2
Low (10-20 min)	721	34.8	76602	33.6
Only CT reachable within 60 min	202	9.7	11916	5.2
Neither reachable within 60 min	41	2.0	172	0.1
Likely irrelevant (<10 min)	527	25.4	100995	44.3

Population and Area Coverage in Kreisfreie Stadt Leipzig (Stroke Units) (Sachsen) — Regular (+20min Penalty)

Category	Grid Cells	Area (%)	Population	Population (%)
High (30+ min)	0	0.0	0	0.0
Medium (20-30 min)	0	0.0	0	0.0
Low (10-20 min)	0	0.0	0	0.0
Only CT reachable within 60 min	0	0.0	0	0.0
Neither reachable within 60 min	0	0.0	0	0.0
Likely irrelevant (<10 min)	384	100.0	619326	100.0

Population and Area Coverage in Kreisfreie Stadt Jena (Stroke Units) (Thüringen) — Regular (+20min Penalty)

Category	Grid Cells	Area (%)	Population	Population (%)
High (30+ min)	0	0.0	0	0.0
Medium (20-30 min)	0	0.0	0	0.0
Low (10-20 min)	2	1.3	0	0.0
Only CT reachable within 60 min	0	0.0	0	0.0
Neither reachable within 60 min	0	0.0	0	0.0
Likely irrelevant (<10 min)	147	98.7	100246	100.0

Population and Area Coverage in Landkreis Gotha (Stroke Units) (Thüringen) — Regular (+20min Penalty)

Category	Grid Cells	Area (%)	Population	Population (%)
High (30+ min)	0	0.0	0	0.0
Medium (20-30 min)	2	0.2	5	0.0
Low (10-20 min)	189	15.8	782	0.6
Only CT reachable within 60 min	0	0.0	0	0.0
Neither reachable within 60 min	0	0.0	0	0.0
Likely irrelevant (<10 min)	1008	84.1	121489	99.4

Population and Area Coverage in Kreis Pinneberg (Stroke Units) (Schleswig-Holstein) — Regular (+20min Penalty)

Category	Grid Cells	Area (%)	Population	Population (%)
High (30+ min)	0	0.0	0	0.0
Medium (20-30 min)	1	0.1	21	0.0
Low (10-20 min)	20	2.4	984	0.3
Only CT reachable within 60 min	1	0.1	14	0.0
Neither reachable within 60 min	19	2.2	121	0.0
Likely irrelevant (<10 min)	809	95.2	299357	99.6

Population and Area Coverage in Kreis Schleswig-Flensburg (Stroke Units) (Schleswig-Holstein) — Regular (+20min Penalty)

Category	Grid Cells	Area (%)	Population	Population (%)
High (30+ min)	0	0.0	0	0.0
Medium (20-30 min)	10	0.4	1011	0.5
Low (10-20 min)	152	5.6	13011	6.6
Only CT reachable within 60 min	4	0.1	38	0.0
Neither reachable within 60 min	8	0.3	231	0.1
Likely irrelevant (<10 min)	2523	93.5	182502	92.7

Population and Area Coverage in Kreis Steinburg (Stroke Units) (Schleswig-Holstein) — Regular (+20min Penalty)

Category	Grid Cells	Area (%)	Population	Population (%)
High (30+ min)	0	0.0	0	0.0
Medium (20-30 min)	1	0.1	43	0.0
Low (10-20 min)	76	5.5	3678	2.8
Only CT reachable within 60 min	0	0.0	0	0.0
Neither reachable within 60 min	9	0.7	432	0.3
Likely irrelevant (<10 min)	1296	93.8	128580	96.9

Population and Area Coverage in Landkreis Verden (Stroke Units) (Niedersachsen) — Regular (+20min Penalty)

Category	Grid Cells	Area (%)	Population	Population (%)
High (30+ min)	58	5.5	2511	1.9
Medium (20-30 min)	126	12.0	9325	7.0
Low (10-20 min)	412	39.2	45470	34.2
Only CT reachable within 60 min	0	0.0	0	0.0
Neither reachable within 60 min	0	0.0	0	0.0
Likely irrelevant (<10 min)	456	43.3	75522	56.9

Population and Area Coverage in Kreisfreie Stadt Delmenhorst (Stroke Units) (Niedersachsen) — Regular (+20min Penalty)

Category	Grid Cells	Area (%)	Population	Population (%)
High (30+ min)	0	0.0	0	0.0
Medium (20-30 min)	0	0.0	0	0.0
Low (10-20 min)	10	12.0	12550	17.1
Only CT reachable within 60 min	0	0.0	0	0.0
Neither reachable within 60 min	0	0.0	0	0.0
Likely irrelevant (<10 min)	73	88.0	60845	82.9

Population and Area Coverage in Kreis Kleve (Stroke Units) (Nordrhein-Westfalen) — Regular (+20min Penalty)

Category	Grid Cells	Area (%)	Population	Population (%)
High (30+ min)	72	4.6	7209	2.4
Medium (20-30 min)	287	18.3	68477	22.6
Low (10-20 min)	528	33.6	104421	34.4
Only CT reachable within 60 min	9	0.6	280	0.1
Neither reachable within 60 min	14	0.9	127	0.0
Likely irrelevant (<10 min)	661	42.1	122685	40.5

Population and Area Coverage in Kreis Herford (Stroke Units) (Nordrhein-Westfalen) — Regular (+20min Penalty)

Category	Grid Cells	Area (%)	Population	Population (%)
High (30+ min)	0	0.0	0	0.0
Medium (20-30 min)	0	0.0	0	0.0
Low (10-20 min)	71	11.9	9618	3.8
Only CT reachable within 60 min	0	0.0	0	0.0
Neither reachable within 60 min	0	0.0	0	0.0
Likely irrelevant (<10 min)	524	88.1	240677	96.2

Population and Area Coverage in Kreisfreie Stadt Dortmund (Stroke Units) (Nordrhein-Westfalen) — Regular (+20min Penalty)

Category	Grid Cells	Area (%)	Population	Population (%)
High (30+ min)	0	0.0	0	0.0
Medium (20-30 min)	0	0.0	0	0.0
Low (10-20 min)	0	0.0	0	0.0
Only CT reachable within 60 min	0	0.0	0	0.0
Neither reachable within 60 min	0	0.0	0	0.0
Likely irrelevant (<10 min)	363	100.0	611483	100.0

Population and Area Coverage in Kreisfreie Stadt Wiesbaden (Stroke Units) (Hessen) — Regular (+20min Penalty)

Category	Grid Cells	Area (%)	Population	Population (%)
High (30+ min)	0	0.0	0	0.0
Medium (20-30 min)	0	0.0	0	0.0
Low (10-20 min)	0	0.0	0	0.0
Only CT reachable within 60 min	0	0.0	0	0.0
Neither reachable within 60 min	0	0.0	0	0.0
Likely irrelevant (<10 min)	252	100.0	277503	100.0

Population and Area Coverage in Landkreis Hochtaunuskreis (Stroke Units) (Hessen) — Regular (+20min Penalty)

Category	Grid Cells	Area (%)	Population	Population (%)
High (30+ min)	0	0.0	0	0.0
Medium (20-30 min)	0	0.0	0	0.0
Low (10-20 min)	4	0.7	118	0.1
Only CT reachable within 60 min	0	0.0	0	0.0
Neither reachable within 60 min	0	0.0	0	0.0
Likely irrelevant (<10 min)	602	99.3	232402	99.9

Population and Area Coverage in Landkreis Kassel (Stroke Units) (Hessen) — Regular (+20min Penalty)

Category	Grid Cells	Area (%)	Population	Population (%)
High (30+ min)	0	0.0	0	0.0
Medium (20-30 min)	122	7.3	3691	1.6
Low (10-20 min)	700	41.9	46552	19.6
Only CT reachable within 60 min	0	0.0	0	0.0
Neither reachable within 60 min	0	0.0	0	0.0
Likely irrelevant (<10 min)	848	50.8	186757	78.8

Population and Area Coverage in Landkreis Cochem-Zell (Stroke Units) (Rheinland-Pfalz) — Regular (+20min Penalty)

Category	Grid Cells	Area (%)	Population	Population (%)
High (30+ min)	69	7.9	2988	4.6
Medium (20-30 min)	286	32.8	17571	27.3
Low (10-20 min)	257	29.5	24060	37.4
Only CT reachable within 60 min	5	0.6	34	0.1
Neither reachable within 60 min	0	0.0	0	0.0
Likely irrelevant (<10 min)	254	29.2	19703	30.6

Population and Area Coverage in Landkreis Rhein-Lahn-Kreis (Stroke Units) (Rheinland-Pfalz) — Regular (+20min Penalty)

Category	Grid Cells	Area (%)	Population	Population (%)
High (30+ min)	25	2.5	900	0.7
Medium (20-30 min)	127	12.9	8520	6.8
Low (10-20 min)	273	27.7	21930	17.6
Only CT reachable within 60 min	0	0.0	0	0.0
Neither reachable within 60 min	0	0.0	0	0.0
Likely irrelevant (<10 min)	561	56.9	93324	74.9

Population and Area Coverage in Stadtkreis Heilbronn (Stroke Units) (Baden-Württemberg) — Regular (+20min Penalty)

Category	Grid Cells	Area (%)	Population	Population (%)
High (30+ min)	0	0.0	0	0.0
Medium (20-30 min)	0	0.0	0	0.0
Low (10-20 min)	0	0.0	0	0.0
Only CT reachable within 60 min	0	0.0	0	0.0
Neither reachable within 60 min	0	0.0	0	0.0
Likely irrelevant (<10 min)	121	100.0	123086	100.0

Population and Area Coverage in Landkreis Schwandorf (Stroke Units) (Bayern) — Regular (+20min Penalty)

Category	Grid Cells	Area (%)	Population	Population (%)
High (30+ min)	0	0.0	0	0.0
Medium (20-30 min)	42	2.4	2375	1.6
Low (10-20 min)	299	16.7	12049	8.1
Only CT reachable within 60 min	0	0.0	0	0.0
Neither reachable within 60 min	0	0.0	0	0.0
Likely irrelevant (<10 min)	1445	80.9	134110	90.3

Population and Area Coverage in Landkreis Unterallgäu (Stroke Units) (Bayern) — Regular (+20min Penalty)

Category	Grid Cells	Area (%)	Population	Population (%)
High (30+ min)	0	0.0	0	0.0
Medium (20-30 min)	66	4.4	1961	1.4
Low (10-20 min)	336	22.6	18568	13.1
Only CT reachable within 60 min	0	0.0	0	0.0
Neither reachable within 60 min	0	0.0	0	0.0
Likely irrelevant (<10 min)	1084	72.9	121743	85.6

Population and Area Coverage in Kreisfreie Stadt Frankfurt (Oder) (Stroke Units) (Brandenburg) — Regular (+20min Penalty)

Category	Grid Cells	Area (%)	Population	Population (%)
High (30+ min)	0	0.0	0	0.0
Medium (20-30 min)	0	0.0	0	0.0
Low (10-20 min)	0	0.0	0	0.0
Only CT reachable within 60 min	5	2.6	464	0.8
Neither reachable within 60 min	15	7.7	524	0.9
Likely irrelevant (<10 min)	176	89.8	55128	98.2

Population and Area Coverage in Landkreis Dahme-Spreewald (Stroke Units) (Brandenburg) — Regular (+20min Penalty)

Category	Grid Cells	Area (%)	Population	Population (%)
High (30+ min)	2	0.1	22	0.0
Medium (20-30 min)	116	3.9	1399	0.9
Low (10-20 min)	1095	36.9	45683	28.8
Only CT reachable within 60 min	0	0.0	0	0.0
Neither reachable within 60 min	0	0.0	0	0.0
Likely irrelevant (<10 min)	1756	59.1	111365	70.3

Population and Area Coverage in Landkreis Leipzig (Stroke Units) (Sachsen) — Regular (+20min Penalty)

Category	Grid Cells	Area (%)	Population	Population (%)
High (30+ min)	0	0.0	0	0.0
Medium (20-30 min)	3	0.1	93	0.0
Low (10-20 min)	327	15.4	22184	8.7
Only CT reachable within 60 min	0	0.0	0	0.0
Neither reachable within 60 min	0	0.0	0	0.0
Likely irrelevant (<10 min)	1794	84.5	233148	91.3

Population and Area Coverage in Kreisfreie Stadt Magdeburg (Stroke Units) (Sachsen-Anhalt) — Regular (+20min Penalty)

Category	Grid Cells	Area (%)	Population	Population (%)
High (30+ min)	0	0.0	0	0.0
Medium (20-30 min)	0	0.0	0	0.0
Low (10-20 min)	32	12.2	1613	0.6
Only CT reachable within 60 min	0	0.0	0	0.0
Neither reachable within 60 min	0	0.0	0	0.0
Likely irrelevant (<10 min)	231	87.8	247687	99.4

Population and Area Coverage in Landkreis Wartburgkreis (Stroke Units) (Thüringen) — Regular (+20min Penalty)

Category	Grid Cells	Area (%)	Population	Population (%)
High (30+ min)	0	0.0	0	0.0
Medium (20-30 min)	0	0.0	0	0.0
Low (10-20 min)	147	8.4	5003	3.6
Only CT reachable within 60 min	0	0.0	0	0.0
Neither reachable within 60 min	0	0.0	0	0.0
Likely irrelevant (<10 min)	1604	91.6	133072	96.4

Population and Area Coverage in Landkreis Unstrut-Hainich-Kreis (Stroke Units) (Thüringen) — Regular (+20min Penalty)

Category	Grid Cells	Area (%)	Population	Population (%)
High (30+ min)	4	0.3	0	0.0
Medium (20-30 min)	172	14.5	3624	4.0
Low (10-20 min)	477	40.1	24918	27.6
Only CT reachable within 60 min	0	0.0	0	0.0
Neither reachable within 60 min	0	0.0	0	0.0
Likely irrelevant (<10 min)	536	45.1	61727	68.4

Population and Area Coverage in Kreisfreie Stadt Bielefeld (Stroke Units) (Nordrhein-Westfalen) — Regular (+20min Penalty)

Category	Grid Cells	Area (%)	Population	Population (%)
High (30+ min)	0	0.0	0	0.0
Medium (20-30 min)	0	0.0	0	0.0
Low (10-20 min)	0	0.0	0	0.0
Only CT reachable within 60 min	0	0.0	0	0.0
Neither reachable within 60 min	0	0.0	0	0.0
Likely irrelevant (<10 min)	336	100.0	349622	100.0

Population and Area Coverage in Kreisfreie Stadt Bochum (Stroke Units) (Nordrhein-Westfalen) — Regular (+20min Penalty)

Category	Grid Cells	Area (%)	Population	Population (%)
High (30+ min)	0	0.0	0	0.0
Medium (20-30 min)	0	0.0	0	0.0
Low (10-20 min)	0	0.0	0	0.0
Only CT reachable within 60 min	0	0.0	0	0.0
Neither reachable within 60 min	0	0.0	0	0.0
Likely irrelevant (<10 min)	185	100.0	386703	100.0

Population and Area Coverage in Landkreis Fulda (Stroke Units) (Hessen) — Regular (+20min Penalty)

Category	Grid Cells	Area (%)	Population	Population (%)
High (30+ min)	0	0.0	0	0.0
Medium (20-30 min)	0	0.0	0	0.0
Low (10-20 min)	142	8.1	5410	2.5
Only CT reachable within 60 min	0	0.0	0	0.0
Neither reachable within 60 min	0	0.0	0	0.0
Likely irrelevant (<10 min)	1609	91.9	214849	97.5

Population and Area Coverage in Landkreis Rastatt (Stroke Units) (Baden-Württemberg) — Regular (+20min Penalty)

Category	Grid Cells	Area (%)	Population	Population (%)
High (30+ min)	2	0.2	54	0.0
Medium (20-30 min)	185	20.6	6482	2.9
Low (10-20 min)	169	18.8	39193	17.3
Only CT reachable within 60 min	59	6.6	113	0.0
Neither reachable within 60 min	2	0.2	31	0.0
Likely irrelevant (<10 min)	481	53.6	181253	79.8

Population and Area Coverage in Landkreis Breisgau-Hochschwarzwald (Stroke Units) (Baden-Württemberg) — Regular (+20min Penalty)

Category	Grid Cells	Area (%)	Population	Population (%)
High (30+ min)	78	4.7	2987	1.2
Medium (20-30 min)	375	22.8	31133	12.6
Low (10-20 min)	510	31.0	70498	28.6
Only CT reachable within 60 min	119	7.2	1279	0.5
Neither reachable within 60 min	0	0.0	3	0.0
Likely irrelevant (<10 min)	561	34.1	140489	57.0

Population and Area Coverage in Landkreis Ebersberg (Stroke Units) (Bayern) — Regular (+20min Penalty)

Category	Grid Cells	Area (%)	Population	Population (%)
High (30+ min)	0	0.0	0	0.0
Medium (20-30 min)	0	0.0	0	0.0
Low (10-20 min)	0	0.0	0	0.0
Only CT reachable within 60 min	0	0.0	0	0.0
Neither reachable within 60 min	0	0.0	0	0.0
Likely irrelevant (<10 min)	664	100.0	137985	100.0

Population and Area Coverage in Landkreis Rottal-Inn (Stroke Units) (Bayern) — Regular (+20min Penalty)

Category	Grid Cells	Area (%)	Population	Population (%)
High (30+ min)	0	0.0	0	0.0
Medium (20-30 min)	8	0.5	219	0.2
Low (10-20 min)	364	23.7	19902	16.4
Only CT reachable within 60 min	0	0.0	0	0.0
Neither reachable within 60 min	2	0.1	63	0.1
Likely irrelevant (<10 min)	1165	75.7	101484	83.4

Population and Area Coverage in Landkreis Bamberg (Stroke Units) (Bayern) — Regular (+20min Penalty)

Category	Grid Cells	Area (%)	Population	Population (%)
High (30+ min)	0	0.0	0	0.0
Medium (20-30 min)	35	2.4	115	0.1
Low (10-20 min)	417	28.5	15191	10.1
Only CT reachable within 60 min	0	0.0	0	0.0
Neither reachable within 60 min	0	0.0	0	0.0
Likely irrelevant (<10 min)	1013	69.1	135492	89.8

Population and Area Coverage in Landkreis Schweinfurt (Stroke Units) (Bayern) — Regular (+20min Penalty)

Category	Grid Cells	Area (%)	Population	Population (%)
High (30+ min)	0	0.0	0	0.0
Medium (20-30 min)	0	0.0	0	0.0
Low (10-20 min)	66	6.2	494	0.4
Only CT reachable within 60 min	0	0.0	0	0.0
Neither reachable within 60 min	0	0.0	0	0.0
Likely irrelevant (<10 min)	993	93.8	116455	99.6

Population and Area Coverage in Landkreis St. Wendel (Stroke Units) (Saarland) — Regular (+20min Penalty)

Category	Grid Cells	Area (%)	Population	Population (%)
High (30+ min)	0	0.0	0	0.0
Medium (20-30 min)	9	1.5	24	0.0
Low (10-20 min)	83	13.9	4446	5.2
Only CT reachable within 60 min	0	0.0	0	0.0
Neither reachable within 60 min	0	0.0	0	0.0
Likely irrelevant (<10 min)	506	84.6	80816	94.8

Population and Area Coverage in Landkreis Harz (Stroke Units) (Sachsen-Anhalt) — Regular (+20min Penalty)

Category	Grid Cells	Area (%)	Population	Population (%)
High (30+ min)	10	0.4	788	0.4
Medium (20-30 min)	592	21.5	14401	7.2
Low (10-20 min)	998	36.3	72204	36.1
Only CT reachable within 60 min	0	0.0	0	0.0
Neither reachable within 60 min	0	0.0	0	0.0
Likely irrelevant (<10 min)	1148	41.8	112709	56.3

Population and Area Coverage in Kreisfreie Stadt Gera (Stroke Units) (Thüringen) — Regular (+20min Penalty)

Category	Grid Cells	Area (%)	Population	Population (%)
High (30+ min)	0	0.0	0	0.0
Medium (20-30 min)	0	0.0	0	0.0
Low (10-20 min)	20	10.5	930	0.8
Only CT reachable within 60 min	0	0.0	0	0.0
Neither reachable within 60 min	0	0.0	0	0.0
Likely irrelevant (<10 min)	171	89.5	108727	99.2

Population and Area Coverage in Kreisfreie Stadt Weimar (Stroke Units) (Thüringen) — Regular (+20min Penalty)

Category	Grid Cells	Area (%)	Population	Population (%)
High (30+ min)	0	0.0	0	0.0
Medium (20-30 min)	0	0.0	0	0.0
Low (10-20 min)	0	0.0	0	0.0
Only CT reachable within 60 min	0	0.0	0	0.0
Neither reachable within 60 min	0	0.0	0	0.0
Likely irrelevant (<10 min)	109	100.0	57698	100.0

Population and Area Coverage in Landkreis Holzminden (Stroke Units) (Niedersachsen) — Regular (+20min Penalty)

Category	Grid Cells	Area (%)	Population	Population (%)
High (30+ min)	13	1.4	413	0.6
Medium (20-30 min)	147	16.3	9354	13.0
Low (10-20 min)	146	16.2	7204	10.0
Only CT reachable within 60 min	0	0.0	0	0.0
Neither reachable within 60 min	0	0.0	0	0.0
Likely irrelevant (<10 min)	598	66.2	54817	76.4

Population and Area Coverage in Landkreis Grafschaft Bentheim (Stroke Units) (Niedersachsen) — Regular (+20min Penalty)

Category	Grid Cells	Area (%)	Population	Population (%)
High (30+ min)	0	0.0	0	0.0
Medium (20-30 min)	1	0.1	16	0.0
Low (10-20 min)	32	2.5	719	0.5
Only CT reachable within 60 min	6	0.5	451	0.3
Neither reachable within 60 min	22	1.7	501	0.4
Likely irrelevant (<10 min)	1213	95.2	134989	98.8

Population and Area Coverage in Kreis Höxter (Stroke Units) (Nordrhein-Westfalen) — Regular (+20min Penalty)

Category	Grid Cells	Area (%)	Population	Population (%)
High (30+ min)	0	0.0	0	0.0
Medium (20-30 min)	24	1.5	869	0.6
Low (10-20 min)	637	40.8	42083	29.5
Only CT reachable within 60 min	0	0.0	0	0.0
Neither reachable within 60 min	0	0.0	0	0.0
Likely irrelevant (<10 min)	902	57.7	99839	69.9

Population and Area Coverage in Landkreis Marburg-Biedenkopf (Stroke Units) (Hessen) — Regular (+20min Penalty)

Category	Grid Cells	Area (%)	Population	Population (%)
High (30+ min)	50	3.1	2231	0.9
Medium (20-30 min)	170	10.6	28531	11.7
Low (10-20 min)	306	19.1	30667	12.6
Only CT reachable within 60 min	0	0.0	0	0.0
Neither reachable within 60 min	0	0.0	0	0.0
Likely irrelevant (<10 min)	1074	67.1	181933	74.8

Population and Area Coverage in Landkreis Rhein-Hunsrück-Kreis (Stroke Units) (Rheinland-Pfalz) — Regular (+20min Penalty)

Category	Grid Cells	Area (%)	Population	Population (%)
High (30+ min)	61	4.9	3751	3.6
Medium (20-30 min)	475	38.2	31241	29.8
Low (10-20 min)	624	50.2	54829	52.2
Only CT reachable within 60 min	2	0.2	0	0.0
Neither reachable within 60 min	0	0.0	0	0.0
Likely irrelevant (<10 min)	82	6.6	15141	14.4

Population and Area Coverage in Landkreis Eifelkreis Bitburg-Prüm (Stroke Units) (Rheinland-Pfalz) — Regular (+20min Penalty)

Category	Grid Cells	Area (%)	Population	Population (%)
High (30+ min)	123	6.0	1867	1.9
Medium (20-30 min)	857	42.1	31942	31.8
Low (10-20 min)	628	30.8	41045	40.9
Only CT reachable within 60 min	100	4.9	1324	1.3
Neither reachable within 60 min	2	0.1	127	0.1
Likely irrelevant (<10 min)	326	16.0	24031	24.0

Population and Area Coverage in Landkreis Erding (Stroke Units) (Bayern) — Regular (+20min Penalty)

Category	Grid Cells	Area (%)	Population	Population (%)
High (30+ min)	0	0.0	0	0.0
Medium (20-30 min)	0	0.0	0	0.0
Low (10-20 min)	1	0.1	60	0.0
Only CT reachable within 60 min	0	0.0	0	0.0
Neither reachable within 60 min	0	0.0	0	0.0
Likely irrelevant (<10 min)	1054	99.9	135671	100.0

Population and Area Coverage in Landkreis Deggendorf (Stroke Units) (Bayern) — Regular (+20min Penalty)

Category	Grid Cells	Area (%)	Population	Population (%)
High (30+ min)	0	0.0	0	0.0
Medium (20-30 min)	0	0.0	0	0.0
Low (10-20 min)	37	3.5	1513	1.3
Only CT reachable within 60 min	0	0.0	0	0.0
Neither reachable within 60 min	0	0.0	0	0.0
Likely irrelevant (<10 min)	1020	96.5	117937	98.7

Population and Area Coverage in Landkreis Weißenburg-Gunzenhausen (Stroke Units) (Bayern) — Regular (+20min Penalty)

Category	Grid Cells	Area (%)	Population	Population (%)
High (30+ min)	0	0.0	0	0.0
Medium (20-30 min)	66	5.6	3150	3.3
Low (10-20 min)	372	31.3	19678	20.6
Only CT reachable within 60 min	0	0.0	0	0.0
Neither reachable within 60 min	0	0.0	0	0.0
Likely irrelevant (<10 min)	749	63.1	72583	76.1

Population and Area Coverage in Landkreis Lindau (Bodensee) (Stroke Units) (Bayern) — Regular (+20min Penalty)

Category	Grid Cells	Area (%)	Population	Population (%)
High (30+ min)	0	0.0	0	0.0
Medium (20-30 min)	105	27.6	25472	31.4
Low (10-20 min)	215	56.6	45528	56.1
Only CT reachable within 60 min	5	1.3	230	0.3
Neither reachable within 60 min	10	2.6	682	0.8
Likely irrelevant (<10 min)	45	11.8	9278	11.4

Population and Area Coverage in Kreisfreie Stadt Rostock (Stroke Units) (Mecklenburg-Vorpommern) — Regular (+20min Penalty)

Category	Grid Cells	Area (%)	Population	Population (%)
High (30+ min)	0	0.0	0	0.0
Medium (20-30 min)	15	6.7	2	0.0
Low (10-20 min)	57	25.3	3136	1.7
Only CT reachable within 60 min	1	0.4	33	0.0
Neither reachable within 60 min	5	2.2	576	0.3
Likely irrelevant (<10 min)	147	65.3	182658	98.0

Population and Area Coverage in Landkreis Rostock (Stroke Units) (Mecklenburg-Vorpommern) — Regular (+20min Penalty)

Category	Grid Cells	Area (%)	Population	Population (%)
High (30+ min)	165	3.5	2533	1.3
Medium (20-30 min)	481	10.3	22757	11.5
Low (10-20 min)	1413	30.2	42135	21.2
Only CT reachable within 60 min	69	1.5	694	0.3
Neither reachable within 60 min	30	0.6	586	0.3
Likely irrelevant (<10 min)	2519	53.9	129668	65.4

Population and Area Coverage in Landkreis Mittelsachsen (Stroke Units) (Sachsen) — Regular (+20min Penalty)

Category	Grid Cells	Area (%)	Population	Population (%)
High (30+ min)	0	0.0	0	0.0
Medium (20-30 min)	197	7.3	13768	4.7
Low (10-20 min)	705	26.0	80184	27.2
Only CT reachable within 60 min	2	0.1	0	0.0
Neither reachable within 60 min	0	0.0	0	0.0
Likely irrelevant (<10 min)	1806	66.6	200460	68.1

Population and Area Coverage in Landkreis Vorpommern-Greifswald (Stroke Units) (Mecklenburg-Vorpommern) — Regular (+20min Penalty)

Category	Grid Cells	Area (%)	Population	Population (%)
High (30+ min)	258	5.1	12003	5.4
Medium (20-30 min)	942	18.7	26778	12.1
Low (10-20 min)	1159	23.0	46648	21.0
Only CT reachable within 60 min	624	12.4	18356	8.3
Neither reachable within 60 min	139	2.8	3468	1.6
Likely irrelevant (<10 min)	1917	38.0	114642	51.7

Population and Area Coverage in Landkreis Schmalkalden-Meiningen (Stroke Units) (Thüringen) — Regular (+20min Penalty)

Category	Grid Cells	Area (%)	Population	Population (%)
High (30+ min)	0	0.0	0	0.0
Medium (20-30 min)	87	5.5	3441	3.0
Low (10-20 min)	323	20.4	32583	28.1
Only CT reachable within 60 min	0	0.0	0	0.0
Neither reachable within 60 min	0	0.0	0	0.0
Likely irrelevant (<10 min)	1175	74.1	79775	68.9

Population and Area Coverage in Kreisfreie Stadt Lübeck (Stroke Units) (Schleswig-Holstein) — Regular (+20min Penalty)

Category	Grid Cells	Area (%)	Population	Population (%)
High (30+ min)	0	0.0	0	0.0
Medium (20-30 min)	0	0.0	0	0.0
Low (10-20 min)	40	13.8	3604	1.7
Only CT reachable within 60 min	2	0.7	2030	0.9
Neither reachable within 60 min	2	0.7	159	0.1
Likely irrelevant (<10 min)	245	84.8	212135	97.3

Population and Area Coverage in Kreis Nordfriesland (Stroke Units) (Schleswig-Holstein) — Regular (+20min Penalty)

Category	Grid Cells	Area (%)	Population	Population (%)
High (30+ min)	289	11.0	7929	4.9
Medium (20-30 min)	579	22.0	48293	30.1
Low (10-20 min)	809	30.7	52565	32.7
Only CT reachable within 60 min	218	8.3	23096	14.4
Neither reachable within 60 min	151	5.7	4918	3.1
Likely irrelevant (<10 min)	587	22.3	23889	14.9

Population and Area Coverage in Landkreis Northeim (Stroke Units) (Niedersachsen) — Regular (+20min Penalty)

Category	Grid Cells	Area (%)	Population	Population (%)
High (30+ min)	1	0.1	2	0.0
Medium (20-30 min)	210	12.7	8732	6.5
Low (10-20 min)	661	40.0	52704	39.3
Only CT reachable within 60 min	0	0.0	0	0.0
Neither reachable within 60 min	0	0.0	0	0.0
Likely irrelevant (<10 min)	779	47.2	72674	54.2

Population and Area Coverage in Kreisfreie Stadt Wilhelmshaven (Stroke Units) (Niedersachsen) — Regular (+20min Penalty)

Category	Grid Cells	Area (%)	Population	Population (%)
High (30+ min)	0	0.0	0	0.0
Medium (20-30 min)	0	0.0	0	0.0
Low (10-20 min)	0	0.0	0	0.0
Only CT reachable within 60 min	0	0.0	0	0.0
Neither reachable within 60 min	3	2.5	693	1.0
Likely irrelevant (<10 min)	118	97.5	68835	99.0

Population and Area Coverage in Kreisfreie Stadt Essen (Stroke Units) (Nordrhein-Westfalen) — Regular (+20min Penalty)

Category	Grid Cells	Area (%)	Population	Population (%)
High (30+ min)	0	0.0	0	0.0
Medium (20-30 min)	0	0.0	0	0.0
Low (10-20 min)	0	0.0	0	0.0
Only CT reachable within 60 min	0	0.0	0	0.0
Neither reachable within 60 min	0	0.0	0	0.0
Likely irrelevant (<10 min)	276	100.0	596676	100.0

Population and Area Coverage in Kreisfreie Stadt Münster (Stroke Units) (Nordrhein-Westfalen) — Regular (+20min Penalty)

Category	Grid Cells	Area (%)	Population	Population (%)
High (30+ min)	0	0.0	0	0.0
Medium (20-30 min)	0	0.0	0	0.0
Low (10-20 min)	0	0.0	0	0.0
Only CT reachable within 60 min	0	0.0	0	0.0
Neither reachable within 60 min	0	0.0	0	0.0
Likely irrelevant (<10 min)	399	100.0	364831	100.0

Population and Area Coverage in Kreis Recklinghausen (Stroke Units) (Nordrhein-Westfalen) — Regular (+20min Penalty)

Category	Grid Cells	Area (%)	Population	Population (%)
High (30+ min)	0	0.0	0	0.0
Medium (20-30 min)	0	0.0	0	0.0
Low (10-20 min)	0	0.0	0	0.0
Only CT reachable within 60 min	0	0.0	0	0.0
Neither reachable within 60 min	0	0.0	0	0.0
Likely irrelevant (<10 min)	982	100.0	616413	100.0

Population and Area Coverage in Landkreis Offenbach (Stroke Units) (Hessen) — Regular (+20min Penalty)

Category	Grid Cells	Area (%)	Population	Population (%)
High (30+ min)	0	0.0	0	0.0
Medium (20-30 min)	0	0.0	0	0.0
Low (10-20 min)	0	0.0	0	0.0
Only CT reachable within 60 min	0	0.0	0	0.0
Neither reachable within 60 min	0	0.0	0	0.0
Likely irrelevant (<10 min)	446	100.0	341311	100.0

Population and Area Coverage in Kreisfreie Stadt Trier (Stroke Units) (Rheinland-Pfalz) — Regular (+20min Penalty)

Category	Grid Cells	Area (%)	Population	Population (%)
High (30+ min)	0	0.0	0	0.0
Medium (20-30 min)	0	0.0	0	0.0
Low (10-20 min)	0	0.0	0	0.0
Only CT reachable within 60 min	0	0.0	0	0.0
Neither reachable within 60 min	0	0.0	0	0.0
Likely irrelevant (<10 min)	146	100.0	128525	100.0

Population and Area Coverage in Landkreis Bernkastel-Wittlich (Stroke Units) (Rheinland-Pfalz) — Regular (+20min Penalty)

Category	Grid Cells	Area (%)	Population	Population (%)
High (30+ min)	0	0.0	0	0.0
Medium (20-30 min)	29	2.0	251	0.2
Low (10-20 min)	409	28.0	21138	18.4
Only CT reachable within 60 min	0	0.0	0	0.0
Neither reachable within 60 min	0	0.0	0	0.0
Likely irrelevant (<10 min)	1023	70.0	93282	81.3

Population and Area Coverage in Kreisfreie Stadt Worms (Stroke Units) (Rheinland-Pfalz) — Regular (+20min Penalty)

Category	Grid Cells	Area (%)	Population	Population (%)
High (30+ min)	0	0.0	0	0.0
Medium (20-30 min)	0	0.0	0	0.0
Low (10-20 min)	1	0.7	0	0.0
Only CT reachable within 60 min	0	0.0	0	0.0
Neither reachable within 60 min	0	0.0	0	0.0
Likely irrelevant (<10 min)	133	99.3	81874	100.0

Population and Area Coverage in Landkreis Heilbronn (Stroke Units) (Baden-Württemberg) — Regular (+20min Penalty)

Category	Grid Cells	Area (%)	Population	Population (%)
High (30+ min)	0	0.0	0	0.0
Medium (20-30 min)	53	3.9	1411	0.4
Low (10-20 min)	357	26.2	46460	14.0
Only CT reachable within 60 min	0	0.0	0	0.0
Neither reachable within 60 min	0	0.0	0	0.0
Likely irrelevant (<10 min)	951	69.9	283533	85.6

Population and Area Coverage in Kreisfreie Stadt Ingolstadt (Stroke Units) (Bayern) — Regular (+20min Penalty)

Category	Grid Cells	Area (%)	Population	Population (%)
High (30+ min)	0	0.0	0	0.0
Medium (20-30 min)	0	0.0	0	0.0
Low (10-20 min)	1	0.6	0	0.0
Only CT reachable within 60 min	0	0.0	0	0.0
Neither reachable within 60 min	0	0.0	0	0.0
Likely irrelevant (<10 min)	164	99.4	135219	100.0

Population and Area Coverage in Landkreis Forchheim (Stroke Units) (Bayern) — Regular (+20min Penalty)

Category	Grid Cells	Area (%)	Population	Population (%)
High (30+ min)	0	0.0	0	0.0
Medium (20-30 min)	1	0.1	0	0.0
Low (10-20 min)	218	27.1	15220	12.9
Only CT reachable within 60 min	0	0.0	0	0.0
Neither reachable within 60 min	0	0.0	0	0.0
Likely irrelevant (<10 min)	586	72.8	103191	87.1

Population and Area Coverage in Landkreis Erlangen-Höchstadt (Stroke Units) (Bayern) — Regular (+20min Penalty)

Category	Grid Cells	Area (%)	Population	Population (%)
High (30+ min)	0	0.0	0	0.0
Medium (20-30 min)	36	5.2	1245	0.9
Low (10-20 min)	143	20.5	15840	11.5
Only CT reachable within 60 min	0	0.0	0	0.0
Neither reachable within 60 min	0	0.0	0	0.0
Likely irrelevant (<10 min)	519	74.4	120314	87.6

Population and Area Coverage in Landkreis Mansfeld-Südharz (Stroke Units) (Sachsen-Anhalt) — Regular (+20min Penalty)

Category	Grid Cells	Area (%)	Population	Population (%)
High (30+ min)	152	8.0	3326	2.6
Medium (20-30 min)	575	30.4	29009	22.7
Low (10-20 min)	724	38.3	71457	55.9
Only CT reachable within 60 min	24	1.3	113	0.1
Neither reachable within 60 min	0	0.0	0	0.0
Likely irrelevant (<10 min)	415	22.0	24032	18.8

Population and Area Coverage in Kreis Minden-Lübbecke (Stroke Units) (Nordrhein-Westfalen) — Regular (+20min Penalty)

Category	Grid Cells	Area (%)	Population	Population (%)
High (30+ min)	0	0.0	0	0.0
Medium (20-30 min)	190	12.5	25643	8.3
Low (10-20 min)	442	29.1	56175	18.2
Only CT reachable within 60 min	0	0.0	0	0.0
Neither reachable within 60 min	0	0.0	0	0.0
Likely irrelevant (<10 min)	888	58.4	227655	73.6

Population and Area Coverage in Kreisfreie Stadt Hamm (Stroke Units) (Nordrhein-Westfalen) — Regular (+20min Penalty)

Category	Grid Cells	Area (%)	Population	Population (%)
High (30+ min)	0	0.0	0	0.0
Medium (20-30 min)	0	0.0	0	0.0
Low (10-20 min)	0	0.0	0	0.0
Only CT reachable within 60 min	0	0.0	0	0.0
Neither reachable within 60 min	0	0.0	0	0.0
Likely irrelevant (<10 min)	293	100.0	175400	100.0

Population and Area Coverage in Landkreis Main-Taunus-Kreis (Stroke Units) (Hessen) — Regular (+20min Penalty)

Category	Grid Cells	Area (%)	Population	Population (%)
High (30+ min)	0	0.0	0	0.0
Medium (20-30 min)	0	0.0	0	0.0
Low (10-20 min)	1	0.4	0	0.0
Only CT reachable within 60 min	0	0.0	0	0.0
Neither reachable within 60 min	0	0.0	0	0.0
Likely irrelevant (<10 min)	280	99.6	231433	100.0

Population and Area Coverage in Kreisfreie Stadt Ludwigshafen am Rhein (Stroke Units) (Rheinland-Pfalz) — Regular (+20min Penalty)

Category	Grid Cells	Area (%)	Population	Population (%)
High (30+ min)	0	0.0	0	0.0
Medium (20-30 min)	0	0.0	0	0.0
Low (10-20 min)	0	0.0	0	0.0
Only CT reachable within 60 min	0	0.0	0	0.0
Neither reachable within 60 min	0	0.0	0	0.0
Likely irrelevant (<10 min)	99	100.0	163201	100.0

Population and Area Coverage in Landkreis Esslingen (Stroke Units) (Baden-Württemberg) — Regular (+20min Penalty)

Category	Grid Cells	Area (%)	Population	Population (%)
High (30+ min)	0	0.0	0	0.0
Medium (20-30 min)	0	0.0	0	0.0
Low (10-20 min)	26	3.4	689	0.1
Only CT reachable within 60 min	0	0.0	0	0.0
Neither reachable within 60 min	0	0.0	0	0.0
Likely irrelevant (<10 min)	745	96.6	516470	99.9

Population and Area Coverage in Landkreis Dachau (Stroke Units) (Bayern) — Regular (+20min Penalty)

Category	Grid Cells	Area (%)	Population	Population (%)
High (30+ min)	0	0.0	0	0.0
Medium (20-30 min)	0	0.0	0	0.0
Low (10-20 min)	9	1.3	1262	0.8
Only CT reachable within 60 min	0	0.0	0	0.0
Neither reachable within 60 min	0	0.0	0	0.0
Likely irrelevant (<10 min)	695	98.7	148869	99.2

Population and Area Coverage in Landkreis Bad Kissingen (Stroke Units) (Bayern) — Regular (+20min Penalty)

Category	Grid Cells	Area (%)	Population	Population (%)
High (30+ min)	0	0.0	0	0.0
Medium (20-30 min)	116	8.1	1445	1.4
Low (10-20 min)	666	46.5	54691	51.3
Only CT reachable within 60 min	0	0.0	0	0.0
Neither reachable within 60 min	0	0.0	0	0.0
Likely irrelevant (<10 min)	651	45.4	50489	47.4

Population and Area Coverage in Kreisfreie Stadt Erfurt (Stroke Units) (Thüringen) — Regular (+20min Penalty)

Category	Grid Cells	Area (%)	Population	Population (%)
High (30+ min)	0	0.0	0	0.0
Medium (20-30 min)	0	0.0	0	0.0
Low (10-20 min)	0	0.0	0	0.0
Only CT reachable within 60 min	0	0.0	0	0.0
Neither reachable within 60 min	0	0.0	0	0.0
Likely irrelevant (<10 min)	342	100.0	247222	100.0

Population and Area Coverage in Kreisfreie Stadt Flensburg (Stroke Units) (Schleswig-Holstein) — Regular (+20min Penalty)

Category	Grid Cells	Area (%)	Population	Population (%)
High (30+ min)	0	0.0	0	0.0
Medium (20-30 min)	0	0.0	0	0.0
Low (10-20 min)	0	0.0	0	0.0
Only CT reachable within 60 min	0	0.0	0	0.0
Neither reachable within 60 min	0	0.0	0	0.0
Likely irrelevant (<10 min)	62	100.0	80819	100.0

Population and Area Coverage in Kreis Dithmarschen (Stroke Units) (Schleswig-Holstein) — Regular (+20min Penalty)

Category	Grid Cells	Area (%)	Population	Population (%)
High (30+ min)	0	0.0	0	0.0
Medium (20-30 min)	13	0.7	257	0.2
Low (10-20 min)	263	14.2	19259	14.4
Only CT reachable within 60 min	11	0.6	1168	0.9
Neither reachable within 60 min	33	1.8	446	0.3
Likely irrelevant (<10 min)	1529	82.7	112488	84.2

Population and Area Coverage in Landkreis Hameln-Pyrmont (Stroke Units) (Niedersachsen) — Regular (+20min Penalty)

Category	Grid Cells	Area (%)	Population	Population (%)
High (30+ min)	14	1.3	410	0.3
Medium (20-30 min)	89	8.5	5521	3.7
Low (10-20 min)	410	39.1	41955	28.5
Only CT reachable within 60 min	0	0.0	0	0.0
Neither reachable within 60 min	0	0.0	0	0.0
Likely irrelevant (<10 min)	535	51.0	99515	67.5

Population and Area Coverage in Landkreis Celle (Stroke Units) (Niedersachsen) — Regular (+20min Penalty)

Category	Grid Cells	Area (%)	Population	Population (%)
High (30+ min)	1	0.0	1	0.0
Medium (20-30 min)	12	0.6	58	0.0
Low (10-20 min)	289	14.0	8471	4.8
Only CT reachable within 60 min	0	0.0	0	0.0
Neither reachable within 60 min	0	0.0	0	0.0
Likely irrelevant (<10 min)	1765	85.4	167762	95.2

Population and Area Coverage in Landkreis Harburg (Stroke Units) (Niedersachsen) — Regular (+20min Penalty)

Category	Grid Cells	Area (%)	Population	Population (%)
High (30+ min)	0	0.0	0	0.0
Medium (20-30 min)	0	0.0	0	0.0
Low (10-20 min)	13	0.8	706	0.3
Only CT reachable within 60 min	0	0.0	0	0.0
Neither reachable within 60 min	0	0.0	0	0.0
Likely irrelevant (<10 min)	1676	99.2	242658	99.7

Population and Area Coverage in Kreisfreie Stadt Düsseldorf (Stroke Units) (Nordrhein-Westfalen) — Regular (+20min Penalty)

Category	Grid Cells	Area (%)	Population	Population (%)
High (30+ min)	0	0.0	0	0.0
Medium (20-30 min)	0	0.0	0	0.0
Low (10-20 min)	0	0.0	0	0.0
Only CT reachable within 60 min	0	0.0	0	0.0
Neither reachable within 60 min	0	0.0	0	0.0
Likely irrelevant (<10 min)	279	100.0	676424	100.0

Population and Area Coverage in Kreis Düren (Stroke Units) (Nordrhein-Westfalen) — Regular (+20min Penalty)

Category	Grid Cells	Area (%)	Population	Population (%)
High (30+ min)	0	0.0	0	0.0
Medium (20-30 min)	18	1.5	87	0.0
Low (10-20 min)	62	5.2	1995	0.8
Only CT reachable within 60 min	0	0.0	0	0.0
Neither reachable within 60 min	0	0.0	0	0.0
Likely irrelevant (<10 min)	1120	93.3	254177	99.2

Population and Area Coverage in Kreis Soest (Stroke Units) (Nordrhein-Westfalen) — Regular (+20min Penalty)

Category	Grid Cells	Area (%)	Population	Population (%)
High (30+ min)	0	0.0	0	0.0
Medium (20-30 min)	29	1.7	22	0.0
Low (10-20 min)	134	7.8	16022	5.4
Only CT reachable within 60 min	0	0.0	0	0.0
Neither reachable within 60 min	0	0.0	0	0.0
Likely irrelevant (<10 min)	1561	90.5	279101	94.6

Population and Area Coverage in Kreisfreie Stadt Darmstadt (Stroke Units) (Hessen) — Regular (+20min Penalty)

Category	Grid Cells	Area (%)	Population	Population (%)
High (30+ min)	0	0.0	0	0.0
Medium (20-30 min)	0	0.0	0	0.0
Low (10-20 min)	0	0.0	0	0.0
Only CT reachable within 60 min	0	0.0	0	0.0
Neither reachable within 60 min	0	0.0	0	0.0
Likely irrelevant (<10 min)	154	100.0	151599	100.0

Population and Area Coverage in Kreisfreie Stadt Offenbach am Main (Stroke Units) (Hessen) — Regular (+20min Penalty)

Category	Grid Cells	Area (%)	Population	Population (%)
High (30+ min)	0	0.0	0	0.0
Medium (20-30 min)	0	0.0	0	0.0
Low (10-20 min)	0	0.0	0	0.0
Only CT reachable within 60 min	0	0.0	0	0.0
Neither reachable within 60 min	0	0.0	0	0.0
Likely irrelevant (<10 min)	56	100.0	123120	100.0

Population and Area Coverage in Landkreis Werra-Meißner-Kreis (Stroke Units) (Hessen) — Regular (+20min Penalty)

Category	Grid Cells	Area (%)	Population	Population (%)
High (30+ min)	45	3.4	1702	1.7
Medium (20-30 min)	457	34.7	26879	26.7
Low (10-20 min)	511	38.8	58183	57.7
Only CT reachable within 60 min	0	0.0	0	0.0
Neither reachable within 60 min	0	0.0	0	0.0
Likely irrelevant (<10 min)	305	23.1	14084	14.0

Population and Area Coverage in Landkreis Mayen-Koblenz (Stroke Units) (Rheinland-Pfalz) — Regular (+20min Penalty)

Category	Grid Cells	Area (%)	Population	Population (%)
High (30+ min)	0	0.0	0	0.0
Medium (20-30 min)	50	4.8	1527	0.7
Low (10-20 min)	249	24.1	25242	11.6
Only CT reachable within 60 min	0	0.0	0	0.0
Neither reachable within 60 min	0	0.0	0	0.0
Likely irrelevant (<10 min)	735	71.1	190271	87.7

Population and Area Coverage in Landkreis Bad Dürkheim (Stroke Units) (Rheinland-Pfalz) — Regular (+20min Penalty)

Category	Grid Cells	Area (%)	Population	Population (%)
High (30+ min)	0	0.0	0	0.0
Medium (20-30 min)	27	3.7	2986	2.2
Low (10-20 min)	187	25.6	7965	5.9
Only CT reachable within 60 min	0	0.0	0	0.0
Neither reachable within 60 min	0	0.0	0	0.0
Likely irrelevant (<10 min)	517	70.7	123487	91.9

Population and Area Coverage in Landkreis Landsberg am Lech (Stroke Units) (Bayern) — Regular (+20min Penalty)

Category	Grid Cells	Area (%)	Population	Population (%)
High (30+ min)	0	0.0	0	0.0
Medium (20-30 min)	58	6.0	891	0.7
Low (10-20 min)	519	54.0	58609	48.5
Only CT reachable within 60 min	0	0.0	0	0.0
Neither reachable within 60 min	0	0.0	0	0.0
Likely irrelevant (<10 min)	385	40.0	61430	50.8

Population and Area Coverage in Landkreis Donau-Ries (Stroke Units) (Bayern) — Regular (+20min Penalty)

Category	Grid Cells	Area (%)	Population	Population (%)
High (30+ min)	19	1.2	315	0.2
Medium (20-30 min)	761	48.6	44133	33.1
Low (10-20 min)	592	37.8	72487	54.4
Only CT reachable within 60 min	0	0.0	0	0.0
Neither reachable within 60 min	0	0.0	0	0.0
Likely irrelevant (<10 min)	194	12.4	16389	12.3

Population and Area Coverage in Landkreis Märkisch-Oderland (Stroke Units) (Brandenburg) — Regular (+20min Penalty)

Category	Grid Cells	Area (%)	Population	Population (%)
High (30+ min)	328	11.5	7082	3.8
Medium (20-30 min)	701	24.6	16777	9.1
Low (10-20 min)	879	30.8	44356	24.1
Only CT reachable within 60 min	66	2.3	827	0.4
Neither reachable within 60 min	76	2.7	610	0.3
Likely irrelevant (<10 min)	805	28.2	114453	62.2

Population and Area Coverage in Landkreis Prignitz (Stroke Units) (Brandenburg) — Regular (+20min Penalty)

Category	Grid Cells	Area (%)	Population	Population (%)
High (30+ min)	472	16.4	9735	13.1
Medium (20-30 min)	560	19.5	15328	20.6
Low (10-20 min)	228	7.9	3539	4.8
Only CT reachable within 60 min	1519	52.9	43592	58.6
Neither reachable within 60 min	0	0.0	0	0.0
Likely irrelevant (<10 min)	92	3.2	2174	2.9

Population and Area Coverage in Landkreis Burgenlandkreis (Stroke Units) (Sachsen-Anhalt) — Regular (+20min Penalty)

Category	Grid Cells	Area (%)	Population	Population (%)
High (30+ min)	0	0.0	0	0.0
Medium (20-30 min)	34	1.9	2349	1.4
Low (10-20 min)	571	31.4	52700	31.9
Only CT reachable within 60 min	0	0.0	0	0.0
Neither reachable within 60 min	0	0.0	0	0.0
Likely irrelevant (<10 min)	1214	66.7	110071	66.7

Population and Area Coverage in Landkreis Stendal (Stroke Units) (Sachsen-Anhalt) — Regular (+20min Penalty)

Category	Grid Cells	Area (%)	Population	Population (%)
High (30+ min)	457	14.1	27700	26.8
Medium (20-30 min)	334	10.3	24549	23.7
Low (10-20 min)	215	6.6	7881	7.6
Only CT reachable within 60 min	2222	68.7	43347	41.9
Neither reachable within 60 min	0	0.0	0	0.0
Likely irrelevant (<10 min)	6	0.2	30	0.0

Population and Area Coverage in Landkreis Weimarer Land (Stroke Units) (Thüringen) — Regular (+20min Penalty)

Category	Grid Cells	Area (%)	Population	Population (%)
High (30+ min)	0	0.0	0	0.0
Medium (20-30 min)	12	1.2	336	0.5
Low (10-20 min)	225	21.9	18319	24.8
Only CT reachable within 60 min	0	0.0	0	0.0
Neither reachable within 60 min	0	0.0	0	0.0
Likely irrelevant (<10 min)	790	76.9	55120	74.7

Population and Area Coverage in Kreisfreie Stadt Kiel (Stroke Units) (Schleswig-Holstein) — Regular (+20min Penalty)

Category	Grid Cells	Area (%)	Population	Population (%)
High (30+ min)	1	0.6	221	0.1
Medium (20-30 min)	0	0.0	0	0.0
Low (10-20 min)	0	0.0	0	0.0
Only CT reachable within 60 min	0	0.0	0	0.0
Neither reachable within 60 min	0	0.0	1670	0.7
Likely irrelevant (<10 min)	154	99.4	254428	99.3

Population and Area Coverage in Landkreis Gifhorn (Stroke Units) (Niedersachsen) — Regular (+20min Penalty)

Category	Grid Cells	Area (%)	Population	Population (%)
High (30+ min)	45	2.2	505	0.3
Medium (20-30 min)	471	22.5	24835	14.4
Low (10-20 min)	870	41.6	60366	35.1
Only CT reachable within 60 min	0	0.0	0	0.0
Neither reachable within 60 min	0	0.0	0	0.0
Likely irrelevant (<10 min)	704	33.7	86496	50.2

Population and Area Coverage in Landkreis Helmstedt (Stroke Units) (Niedersachsen) — Regular (+20min Penalty)

Category	Grid Cells	Area (%)	Population	Population (%)
High (30+ min)	0	0.0	0	0.0
Medium (20-30 min)	83	9.3	8054	9.0
Low (10-20 min)	237	26.5	16202	18.1
Only CT reachable within 60 min	0	0.0	0	0.0
Neither reachable within 60 min	0	0.0	0	0.0
Likely irrelevant (<10 min)	575	64.2	65157	72.9

Population and Area Coverage in Landkreis Lüchow-Dannenberg (Stroke Units) (Niedersachsen) — Regular (+20min Penalty)

Category	Grid Cells	Area (%)	Population	Population (%)
High (30+ min)	306	18.6	17315	35.5
Medium (20-30 min)	446	27.2	16906	34.6
Low (10-20 min)	284	17.3	6228	12.8
Only CT reachable within 60 min	576	35.1	8175	16.7
Neither reachable within 60 min	0	0.0	0	0.0
Likely irrelevant (<10 min)	29	1.8	217	0.4

Population and Area Coverage in Landkreis Rotenburg (Wümme) (Stroke Units) (Niedersachsen) — Regular (+20min Penalty)

Category	Grid Cells	Area (%)	Population	Population (%)
High (30+ min)	53	1.9	1117	0.7
Medium (20-30 min)	511	18.3	23694	14.4
Low (10-20 min)	640	22.9	32215	19.6
Only CT reachable within 60 min	0	0.0	0	0.0
Neither reachable within 60 min	0	0.0	0	0.0
Likely irrelevant (<10 min)	1590	56.9	106948	65.2

Population and Area Coverage in Kreis Euskirchen (Stroke Units) (Nordrhein-Westfalen) — Regular (+20min Penalty)

Category	Grid Cells	Area (%)	Population	Population (%)
High (30+ min)	99	6.3	3890	2.1
Medium (20-30 min)	283	17.9	16245	8.6
Low (10-20 min)	317	20.0	23453	12.5
Only CT reachable within 60 min	22	1.4	44	0.0
Neither reachable within 60 min	0	0.0	2	0.0
Likely irrelevant (<10 min)	862	54.5	144603	76.8

Population and Area Coverage in Kreis Olpe (Stroke Units) (Nordrhein-Westfalen) — Regular (+20min Penalty)

Category	Grid Cells	Area (%)	Population	Population (%)
High (30+ min)	127	13.9	6592	4.9
Medium (20-30 min)	214	23.4	47518	35.1
Low (10-20 min)	252	27.5	22099	16.3
Only CT reachable within 60 min	0	0.0	0	0.0
Neither reachable within 60 min	0	0.0	0	0.0
Likely irrelevant (<10 min)	322	35.2	59209	43.7

Population and Area Coverage in Landkreis Lahn-Dill-Kreis (Stroke Units) (Hessen) — Regular (+20min Penalty)

Category	Grid Cells	Area (%)	Population	Population (%)
High (30+ min)	0	0.0	0	0.0
Medium (20-30 min)	127	9.3	13746	5.4
Low (10-20 min)	514	37.7	59719	23.4
Only CT reachable within 60 min	0	0.0	0	0.0
Neither reachable within 60 min	0	0.0	0	0.0
Likely irrelevant (<10 min)	723	53.0	182257	71.3

Population and Area Coverage in Landkreis Ravensburg (Stroke Units) (Baden-Württemberg) — Regular (+20min Penalty)

Category	Grid Cells	Area (%)	Population	Population (%)
High (30+ min)	0	0.0	0	0.0
Medium (20-30 min)	7	0.4	321	0.1
Low (10-20 min)	228	11.6	27090	9.8
Only CT reachable within 60 min	0	0.0	0	0.0
Neither reachable within 60 min	0	0.0	0	0.0
Likely irrelevant (<10 min)	1725	88.0	249357	90.1

Population and Area Coverage in Kreisfreie Stadt Rosenheim (Stroke Units) (Bayern) — Regular (+20min Penalty)

Category	Grid Cells	Area (%)	Population	Population (%)
High (30+ min)	0	0.0	0	0.0
Medium (20-30 min)	0	0.0	0	0.0
Low (10-20 min)	0	0.0	0	0.0
Only CT reachable within 60 min	0	0.0	0	0.0
Neither reachable within 60 min	0	0.0	0	0.0
Likely irrelevant (<10 min)	47	100.0	62621	100.0

Population and Area Coverage in Landkreis Freising (Stroke Units) (Bayern) — Regular (+20min Penalty)

Category	Grid Cells	Area (%)	Population	Population (%)
High (30+ min)	0	0.0	0	0.0
Medium (20-30 min)	0	0.0	0	0.0
Low (10-20 min)	67	6.9	4945	2.8
Only CT reachable within 60 min	0	0.0	0	0.0
Neither reachable within 60 min	0	0.0	0	0.0
Likely irrelevant (<10 min)	907	93.1	169981	97.2

Population and Area Coverage in Landkreis Garmisch-Partenkirchen (Stroke Units) (Bayern) — Regular (+20min Penalty)

Category	Grid Cells	Area (%)	Population	Population (%)
High (30+ min)	2	0.2	563	0.6
Medium (20-30 min)	87	7.3	9955	11.5
Low (10-20 min)	42	3.5	7300	8.4
Only CT reachable within 60 min	10	0.8	385	0.4
Neither reachable within 60 min	429	36.0	1071	1.2
Likely irrelevant (<10 min)	623	52.2	67643	77.8

Population and Area Coverage in Landkreis Regensburg (Stroke Units) (Bayern) — Regular (+20min Penalty)

Category	Grid Cells	Area (%)	Population	Population (%)
High (30+ min)	0	0.0	0	0.0
Medium (20-30 min)	3	0.2	0	0.0
Low (10-20 min)	141	8.2	5142	2.7
Only CT reachable within 60 min	0	0.0	0	0.0
Neither reachable within 60 min	0	0.0	0	0.0
Likely irrelevant (<10 min)	1575	91.6	188569	97.3

Population and Area Coverage in Landkreis Tirschenreuth (Stroke Units) (Bayern) — Regular (+20min Penalty)

Category	Grid Cells	Area (%)	Population	Population (%)
High (30+ min)	0	0.0	0	0.0
Medium (20-30 min)	175	13.6	5265	7.1
Low (10-20 min)	591	45.9	31687	42.6
Only CT reachable within 60 min	3	0.2	0	0.0
Neither reachable within 60 min	3	0.2	0	0.0
Likely irrelevant (<10 min)	516	40.1	37499	50.4

Population and Area Coverage in Kreisfreie Stadt Kaufbeuren (Stroke Units) (Bayern) — Regular (+20min Penalty)

Category	Grid Cells	Area (%)	Population	Population (%)
High (30+ min)	0	0.0	0	0.0
Medium (20-30 min)	0	0.0	0	0.0
Low (10-20 min)	0	0.0	0	0.0
Only CT reachable within 60 min	0	0.0	0	0.0
Neither reachable within 60 min	0	0.0	0	0.0
Likely irrelevant (<10 min)	50	100.0	42940	100.0

Population and Area Coverage in Landkreis Oberallgäu (Stroke Units) (Bayern) — Regular (+20min Penalty)

Category	Grid Cells	Area (%)	Population	Population (%)
High (30+ min)	14	0.8	424	0.3
Medium (20-30 min)	110	6.2	15438	9.9
Low (10-20 min)	224	12.6	21896	14.1
Only CT reachable within 60 min	13	0.7	311	0.2
Neither reachable within 60 min	539	30.3	4155	2.7
Likely irrelevant (<10 min)	876	49.3	113288	72.8

Population and Area Coverage in Landkreis Regionalverband Saarbrücken (Stroke Units) (Saarland) — Regular (+20min Penalty)

Category	Grid Cells	Area (%)	Population	Population (%)
High (30+ min)	0	0.0	0	0.0
Medium (20-30 min)	0	0.0	0	0.0
Low (10-20 min)	1	0.2	72	0.0
Only CT reachable within 60 min	1	0.2	8	0.0
Neither reachable within 60 min	4	0.8	286	0.1
Likely irrelevant (<10 min)	482	98.8	331524	99.9

Population and Area Coverage in Landkreis Altenburger Land (Stroke Units) (Thüringen) — Regular (+20min Penalty)

Category	Grid Cells	Area (%)	Population	Population (%)
High (30+ min)	0	0.0	0	0.0
Medium (20-30 min)	0	0.0	0	0.0
Low (10-20 min)	36	5.0	1896	2.3
Only CT reachable within 60 min	0	0.0	0	0.0
Neither reachable within 60 min	0	0.0	0	0.0
Likely irrelevant (<10 min)	690	95.0	82184	97.7

Population and Area Coverage in Landkreis Diepholz (Stroke Units) (Niedersachsen) — Regular (+20min Penalty)

Category	Grid Cells	Area (%)	Population	Population (%)
High (30+ min)	391	14.8	22909	10.8
Medium (20-30 min)	945	35.8	43445	20.6
Low (10-20 min)	674	25.5	49078	23.2
Only CT reachable within 60 min	94	3.6	3198	1.5
Neither reachable within 60 min	0	0.0	0	0.0
Likely irrelevant (<10 min)	537	20.3	92540	43.8

Population and Area Coverage in Kreisfreie Stadt Emden (Stroke Units) (Niedersachsen) — Regular (+20min Penalty)

Category	Grid Cells	Area (%)	Population	Population (%)
High (30+ min)	0	0.0	0	0.0
Medium (20-30 min)	0	0.0	0	0.0
Low (10-20 min)	1	0.8	922	1.9
Only CT reachable within 60 min	0	0.0	0	0.0
Neither reachable within 60 min	0	0.0	0	0.0
Likely irrelevant (<10 min)	131	99.2	48199	98.1

Population and Area Coverage in Kreisfreie Stadt Gelsenkirchen (Stroke Units) (Nordrhein-Westfalen) — Regular (+20min Penalty)

Category	Grid Cells	Area (%)	Population	Population (%)
High (30+ min)	0	0.0	0	0.0
Medium (20-30 min)	0	0.0	0	0.0
Low (10-20 min)	0	0.0	0	0.0
Only CT reachable within 60 min	0	0.0	0	0.0
Neither reachable within 60 min	0	0.0	0	0.0
Likely irrelevant (<10 min)	134	100.0	279785	100.0

Population and Area Coverage in Kreis Warendorf (Stroke Units) (Nordrhein-Westfalen) — Regular (+20min Penalty)

Category	Grid Cells	Area (%)	Population	Population (%)
High (30+ min)	0	0.0	0	0.0
Medium (20-30 min)	72	4.2	2472	0.9
Low (10-20 min)	401	23.3	57808	21.2
Only CT reachable within 60 min	0	0.0	0	0.0
Neither reachable within 60 min	0	0.0	0	0.0
Likely irrelevant (<10 min)	1245	72.5	211790	77.8

Population and Area Coverage in Stadtkreis Ulm (Stroke Units) (Baden-Württemberg) — Regular (+20min Penalty)

Category	Grid Cells	Area (%)	Population	Population (%)
High (30+ min)	0	0.0	0	0.0
Medium (20-30 min)	0	0.0	0	0.0
Low (10-20 min)	0	0.0	0	0.0
Only CT reachable within 60 min	0	0.0	0	0.0
Neither reachable within 60 min	0	0.0	0	0.0
Likely irrelevant (<10 min)	143	100.0	120114	100.0

Population and Area Coverage in Landkreis Traunstein (Stroke Units) (Bayern) — Regular (+20min Penalty)

Category	Grid Cells	Area (%)	Population	Population (%)
High (30+ min)	0	0.0	0	0.0
Medium (20-30 min)	0	0.0	0	0.0
Low (10-20 min)	59	3.2	4789	2.7
Only CT reachable within 60 min	1	0.1	4	0.0
Neither reachable within 60 min	131	7.2	1464	0.8
Likely irrelevant (<10 min)	1626	89.5	169358	96.4

Population and Area Coverage in Kreisfreie Stadt Regensburg (Stroke Units) (Bayern) — Regular (+20min Penalty)

Category	Grid Cells	Area (%)	Population	Population (%)
High (30+ min)	0	0.0	0	0.0
Medium (20-30 min)	0	0.0	0	0.0
Low (10-20 min)	0	0.0	0	0.0
Only CT reachable within 60 min	0	0.0	0	0.0
Neither reachable within 60 min	0	0.0	0	0.0
Likely irrelevant (<10 min)	97	100.0	170062	100.0

Population and Area Coverage in Kreisfreie Stadt Weiden i.d.OPf. (Stroke Units) (Bayern) — Regular (+20min Penalty)

Category	Grid Cells	Area (%)	Population	Population (%)
High (30+ min)	0	0.0	0	0.0
Medium (20-30 min)	0	0.0	0	0.0
Low (10-20 min)	0	0.0	0	0.0
Only CT reachable within 60 min	0	0.0	0	0.0
Neither reachable within 60 min	0	0.0	0	0.0
Likely irrelevant (<10 min)	86	100.0	43316	100.0

Population and Area Coverage in Kreisfreie Stadt Ansbach (Stroke Units) (Bayern) — Regular (+20min Penalty)

Category	Grid Cells	Area (%)	Population	Population (%)
High (30+ min)	0	0.0	0	0.0
Medium (20-30 min)	0	0.0	0	0.0
Low (10-20 min)	0	0.0	0	0.0
Only CT reachable within 60 min	0	0.0	0	0.0
Neither reachable within 60 min	0	0.0	0	0.0
Likely irrelevant (<10 min)	123	100.0	41201	100.0

Population and Area Coverage in Landkreis Rhön-Grabfeld (Stroke Units) (Bayern) — Regular (+20min Penalty)

Category	Grid Cells	Area (%)	Population	Population (%)
High (30+ min)	0	0.0	0	0.0
Medium (20-30 min)	1	0.1	13	0.0
Low (10-20 min)	38	3.0	1835	2.2
Only CT reachable within 60 min	0	0.0	0	0.0
Neither reachable within 60 min	0	0.0	0	0.0
Likely irrelevant (<10 min)	1249	97.0	80330	97.8

Population and Area Coverage in Landkreis Ostprignitz-Ruppin (Stroke Units) (Brandenburg) — Regular (+20min Penalty)

Category	Grid Cells	Area (%)	Population	Population (%)
High (30+ min)	134	4.0	2342	2.5
Medium (20-30 min)	597	17.6	12540	13.2
Low (10-20 min)	896	26.5	26519	28.0
Only CT reachable within 60 min	135	4.0	1246	1.3
Neither reachable within 60 min	0	0.0	0	0.0
Likely irrelevant (<10 min)	1621	47.9	52063	55.0

Population and Area Coverage in Kreis Plön (Stroke Units) (Schleswig-Holstein) — Regular (+20min Penalty)

Category	Grid Cells	Area (%)	Population	Population (%)
High (30+ min)	0	0.0	0	0.0
Medium (20-30 min)	10	0.7	175	0.1
Low (10-20 min)	446	30.0	19499	15.4
Only CT reachable within 60 min	2	0.1	179	0.1
Neither reachable within 60 min	17	1.1	1426	1.1
Likely irrelevant (<10 min)	1013	68.1	105074	83.2

Population and Area Coverage in Landkreis Nienburg (Weser) (Stroke Units) (Niedersachsen) — Regular (+20min Penalty)

Category	Grid Cells	Area (%)	Population	Population (%)
High (30+ min)	465	24.9	31180	25.7
Medium (20-30 min)	596	32.0	53837	44.3
Low (10-20 min)	310	16.6	18089	14.9
Only CT reachable within 60 min	293	15.7	7306	6.0
Neither reachable within 60 min	0	0.0	0	0.0
Likely irrelevant (<10 min)	201	10.8	11007	9.1

Population and Area Coverage in Landkreis Emsland (Stroke Units) (Niedersachsen) — Regular (+20min Penalty)

Category	Grid Cells	Area (%)	Population	Population (%)
High (30+ min)	48	1.3	2433	0.8
Medium (20-30 min)	465	12.1	50004	15.5
Low (10-20 min)	788	20.5	45564	14.1
Only CT reachable within 60 min	7	0.2	111	0.0
Neither reachable within 60 min	20	0.5	353	0.1
Likely irrelevant (<10 min)	2511	65.4	223779	69.4

Population and Area Coverage in Landkreis Wittmund (Stroke Units) (Niedersachsen) — Regular (+20min Penalty)

Category	Grid Cells	Area (%)	Population	Population (%)
High (30+ min)	2	0.2	169	0.3
Medium (20-30 min)	162	18.2	11250	19.8
Low (10-20 min)	328	36.9	18395	32.3
Only CT reachable within 60 min	2	0.2	16	0.0
Neither reachable within 60 min	50	5.6	2637	4.6
Likely irrelevant (<10 min)	344	38.7	24405	42.9

Population and Area Coverage in Kreis Hochsauerlandkreis (Stroke Units) (Nordrhein-Westfalen) — Regular (+20min Penalty)

Category	Grid Cells	Area (%)	Population	Population (%)
High (30+ min)	342	13.6	18385	7.0
Medium (20-30 min)	661	26.3	52865	20.1
Low (10-20 min)	694	27.6	52310	19.9
Only CT reachable within 60 min	169	6.7	5486	2.1
Neither reachable within 60 min	0	0.0	0	0.0
Likely irrelevant (<10 min)	648	25.8	133323	50.8

Population and Area Coverage in Landkreis Rheingau-Taunus-Kreis (Stroke Units) (Hessen) — Regular (+20min Penalty)

Category	Grid Cells	Area (%)	Population	Population (%)
High (30+ min)	18	1.8	447	0.2
Medium (20-30 min)	130	12.8	4527	2.5
Low (10-20 min)	350	34.3	32853	17.9
Only CT reachable within 60 min	0	0.0	0	0.0
Neither reachable within 60 min	0	0.0	0	0.0
Likely irrelevant (<10 min)	521	51.1	145428	79.4

Population and Area Coverage in Landkreis Göppingen (Stroke Units) (Baden-Württemberg) — Regular (+20min Penalty)

Category	Grid Cells	Area (%)	Population	Population (%)
High (30+ min)	0	0.0	0	0.0
Medium (20-30 min)	0	0.0	0	0.0
Low (10-20 min)	4	0.5	19	0.0
Only CT reachable within 60 min	0	0.0	0	0.0
Neither reachable within 60 min	0	0.0	0	0.0
Likely irrelevant (<10 min)	777	99.5	249977	100.0

Population and Area Coverage in Stadtkreis Freiburg im Breisgau (Stroke Units) (Baden-Württemberg) — Regular (+20min Penalty)

Category	Grid Cells	Area (%)	Population	Population (%)
High (30+ min)	0	0.0	0	0.0
Medium (20-30 min)	0	0.0	0	0.0
Low (10-20 min)	24	12.8	2170	0.8
Only CT reachable within 60 min	0	0.0	0	0.0
Neither reachable within 60 min	0	0.0	0	0.0
Likely irrelevant (<10 min)	163	87.2	256269	99.2

Population and Area Coverage in Landkreis Landshut (Stroke Units) (Bayern) — Regular (+20min Penalty)

Category	Grid Cells	Area (%)	Population	Population (%)
High (30+ min)	0	0.0	0	0.0
Medium (20-30 min)	0	0.0	0	0.0
Low (10-20 min)	106	6.5	4061	2.6
Only CT reachable within 60 min	0	0.0	0	0.0
Neither reachable within 60 min	0	0.0	0	0.0
Likely irrelevant (<10 min)	1535	93.5	151629	97.4

Population and Area Coverage in Landkreis Regen (Stroke Units) (Bayern) — Regular (+20min Penalty)

Category	Grid Cells	Area (%)	Population	Population (%)
High (30+ min)	0	0.0	0	0.0
Medium (20-30 min)	3	0.3	0	0.0
Low (10-20 min)	202	17.7	12232	15.8
Only CT reachable within 60 min	2	0.2	0	0.0
Neither reachable within 60 min	22	1.9	23	0.0
Likely irrelevant (<10 min)	911	79.9	65272	84.2

Population and Area Coverage in Kreisfreie Stadt Coburg (Stroke Units) (Bayern) — Regular (+20min Penalty)

Category	Grid Cells	Area (%)	Population	Population (%)
High (30+ min)	0	0.0	0	0.0
Medium (20-30 min)	0	0.0	0	0.0
Low (10-20 min)	0	0.0	0	0.0
Only CT reachable within 60 min	0	0.0	0	0.0
Neither reachable within 60 min	0	0.0	0	0.0
Likely irrelevant (<10 min)	57	100.0	41384	100.0

Population and Area Coverage in Landkreis Neunkirchen (Stroke Units) (Saarland) — Regular (+20min Penalty)

Category	Grid Cells	Area (%)	Population	Population (%)
High (30+ min)	0	0.0	0	0.0
Medium (20-30 min)	0	0.0	0	0.0
Low (10-20 min)	0	0.0	0	0.0
Only CT reachable within 60 min	0	0.0	0	0.0
Neither reachable within 60 min	0	0.0	0	0.0
Likely irrelevant (<10 min)	312	100.0	127343	100.0

Population and Area Coverage in Landkreis Nordwestmecklenburg (Stroke Units) (Mecklenburg-Vorpommern) — Regular (+20min Penalty)

Category	Grid Cells	Area (%)	Population	Population (%)
High (30+ min)	9	0.3	139	0.1
Medium (20-30 min)	161	5.5	2217	1.5
Low (10-20 min)	709	24.4	19327	13.4
Only CT reachable within 60 min	16	0.6	504	0.4
Neither reachable within 60 min	55	1.9	2522	1.8
Likely irrelevant (<10 min)	1954	67.3	119287	82.8

Population and Area Coverage in Kreisfreie Stadt Herne (Stroke Units) (Nordrhein-Westfalen) — Regular (+20min Penalty)

Category	Grid Cells	Area (%)	Population	Population (%)
High (30+ min)	0	0.0	0	0.0
Medium (20-30 min)	0	0.0	0	0.0
Low (10-20 min)	0	0.0	0	0.0
Only CT reachable within 60 min	0	0.0	0	0.0
Neither reachable within 60 min	0	0.0	0	0.0
Likely irrelevant (<10 min)	69	100.0	165244	100.0

Population and Area Coverage in Landkreis Altenkirchen (Westerwald) (Stroke Units) (Rheinland-Pfalz) — Regular (+20min Penalty)

Category	Grid Cells	Area (%)	Population	Population (%)
High (30+ min)	0	0.0	0	0.0
Medium (20-30 min)	146	17.9	22387	16.8
Low (10-20 min)	324	39.8	56333	42.2
Only CT reachable within 60 min	0	0.0	0	0.0
Neither reachable within 60 min	0	0.0	0	0.0
Likely irrelevant (<10 min)	345	42.3	54689	41.0

Population and Area Coverage in Kreisfreie Stadt Kaiserslautern (Stroke Units) (Rheinland-Pfalz) — Regular (+20min Penalty)

Category	Grid Cells	Area (%)	Population	Population (%)
High (30+ min)	0	0.0	0	0.0
Medium (20-30 min)	0	0.0	0	0.0
Low (10-20 min)	0	0.0	0	0.0
Only CT reachable within 60 min	0	0.0	0	0.0
Neither reachable within 60 min	0	0.0	0	0.0
Likely irrelevant (<10 min)	174	100.0	110813	100.0

Population and Area Coverage in Landkreis Mainz-Bingen (Stroke Units) (Rheinland-Pfalz) — Regular (+20min Penalty)

Category	Grid Cells	Area (%)	Population	Population (%)
High (30+ min)	0	0.0	0	0.0
Medium (20-30 min)	13	1.7	1040	0.5
Low (10-20 min)	92	12.1	6131	2.9
Only CT reachable within 60 min	0	0.0	0	0.0
Neither reachable within 60 min	0	0.0	0	0.0
Likely irrelevant (<10 min)	656	86.2	203048	96.6

Population and Area Coverage in Landkreis Heidenheim (Stroke Units) (Baden-Württemberg) — Regular (+20min Penalty)

Category	Grid Cells	Area (%)	Population	Population (%)
High (30+ min)	0	0.0	0	0.0
Medium (20-30 min)	13	1.7	379	0.3
Low (10-20 min)	78	10.3	3149	2.5
Only CT reachable within 60 min	0	0.0	0	0.0
Neither reachable within 60 min	0	0.0	0	0.0
Likely irrelevant (<10 min)	668	88.0	124461	97.2

Population and Area Coverage in Stadtkreis Pforzheim (Stroke Units) (Baden-Württemberg) — Regular (+20min Penalty)

Category	Grid Cells	Area (%)	Population	Population (%)
High (30+ min)	0	0.0	0	0.0
Medium (20-30 min)	0	0.0	0	0.0
Low (10-20 min)	0	0.0	0	0.0
Only CT reachable within 60 min	0	0.0	0	0.0
Neither reachable within 60 min	0	0.0	0	0.0
Likely irrelevant (<10 min)	120	100.0	124962	100.0

Population and Area Coverage in Landkreis Tübingen (Stroke Units) (Baden-Württemberg) — Regular (+20min Penalty)

Category	Grid Cells	Area (%)	Population	Population (%)
High (30+ min)	0	0.0	0	0.0
Medium (20-30 min)	0	0.0	0	0.0
Low (10-20 min)	58	9.2	13328	6.1
Only CT reachable within 60 min	0	0.0	0	0.0
Neither reachable within 60 min	0	0.0	0	0.0
Likely irrelevant (<10 min)	570	90.8	205210	93.9

Population and Area Coverage in Landkreis Bodenseekreis (Stroke Units) (Baden-Württemberg) — Regular (+20min Penalty)

Category	Grid Cells	Area (%)	Population	Population (%)
High (30+ min)	0	0.0	0	0.0
Medium (20-30 min)	1	0.1	5	0.0
Low (10-20 min)	149	18.6	14283	6.5
Only CT reachable within 60 min	0	0.0	655	0.3
Neither reachable within 60 min	0	0.0	1958	0.9
Likely irrelevant (<10 min)	652	81.3	204173	92.4

Population and Area Coverage in Landkreis Miesbach (Stroke Units) (Bayern) — Regular (+20min Penalty)

Category	Grid Cells	Area (%)	Population	Population (%)
High (30+ min)	0	0.0	0	0.0
Medium (20-30 min)	1	0.1	27	0.0
Low (10-20 min)	18	1.7	331	0.3
Only CT reachable within 60 min	2	0.2	1141	1.2
Neither reachable within 60 min	246	23.7	1166	1.2
Likely irrelevant (<10 min)	770	74.3	96068	97.3

Population and Area Coverage in Landkreis Mühldorf a.Inn (Stroke Units) (Bayern) — Regular (+20min Penalty)

Category	Grid Cells	Area (%)	Population	Population (%)
High (30+ min)	0	0.0	0	0.0
Medium (20-30 min)	0	0.0	0	0.0
Low (10-20 min)	0	0.0	0	0.0
Only CT reachable within 60 min	0	0.0	0	0.0
Neither reachable within 60 min	0	0.0	0	0.0
Likely irrelevant (<10 min)	970	100.0	110636	100.0

Population and Area Coverage in Landkreis Neuburg-Schrobenhausen (Stroke Units) (Bayern) — Regular (+20min Penalty)

Category	Grid Cells	Area (%)	Population	Population (%)
High (30+ min)	0	0.0	0	0.0
Medium (20-30 min)	112	12.4	5955	6.2
Low (10-20 min)	368	40.7	21750	22.8
Only CT reachable within 60 min	0	0.0	0	0.0
Neither reachable within 60 min	0	0.0	0	0.0
Likely irrelevant (<10 min)	424	46.9	67778	71.0

Population and Area Coverage in Landkreis Starnberg (Stroke Units) (Bayern) — Regular (+20min Penalty)

Category	Grid Cells	Area (%)	Population	Population (%)
High (30+ min)	0	0.0	0	0.0
Medium (20-30 min)	1	0.2	603	0.4
Low (10-20 min)	123	21.0	7918	5.9
Only CT reachable within 60 min	0	0.0	0	0.0
Neither reachable within 60 min	0	0.0	0	0.0
Likely irrelevant (<10 min)	462	78.8	126016	93.7

Population and Area Coverage in Landkreis Fürth (Stroke Units) (Bayern) — Regular (+20min Penalty)

Category	Grid Cells	Area (%)	Population	Population (%)
High (30+ min)	0	0.0	0	0.0
Medium (20-30 min)	0	0.0	0	0.0
Low (10-20 min)	0	0.0	0	0.0
Only CT reachable within 60 min	0	0.0	0	0.0
Neither reachable within 60 min	0	0.0	0	0.0
Likely irrelevant (<10 min)	382	100.0	118705	100.0

Population and Area Coverage in Landkreis Kitzingen (Stroke Units) (Bayern) — Regular (+20min Penalty)

Category	Grid Cells	Area (%)	Population	Population (%)
High (30+ min)	0	0.0	0	0.0
Medium (20-30 min)	0	0.0	0	0.0
Low (10-20 min)	104	12.3	2255	2.5
Only CT reachable within 60 min	0	0.0	0	0.0
Neither reachable within 60 min	0	0.0	0	0.0
Likely irrelevant (<10 min)	740	87.7	89174	97.5

Population and Area Coverage in Landkreis Günzburg (Stroke Units) (Bayern) — Regular (+20min Penalty)

Category	Grid Cells	Area (%)	Population	Population (%)
High (30+ min)	0	0.0	0	0.0
Medium (20-30 min)	73	7.9	4111	3.3
Low (10-20 min)	246	26.7	28793	23.0
Only CT reachable within 60 min	0	0.0	0	0.0
Neither reachable within 60 min	0	0.0	0	0.0
Likely irrelevant (<10 min)	604	65.4	92215	73.7

Population and Area Coverage in Landkreis Havelland (Stroke Units) (Brandenburg) — Regular (+20min Penalty)

Category	Grid Cells	Area (%)	Population	Population (%)
High (30+ min)	168	7.4	4773	3.2
Medium (20-30 min)	279	12.2	6159	4.1
Low (10-20 min)	422	18.5	20702	13.7
Only CT reachable within 60 min	898	39.4	40675	26.9
Neither reachable within 60 min	0	0.0	0	0.0
Likely irrelevant (<10 min)	515	22.6	79104	52.2

Population and Area Coverage in Landkreis Teltow-Fläming (Stroke Units) (Brandenburg) — Regular (+20min Penalty)

Category	Grid Cells	Area (%)	Population	Population (%)
High (30+ min)	144	5.2	11576	7.4
Medium (20-30 min)	621	22.5	32318	20.6
Low (10-20 min)	1024	37.0	42450	27.0
Only CT reachable within 60 min	653	23.6	8220	5.2
Neither reachable within 60 min	0	0.0	0	0.0
Likely irrelevant (<10 min)	323	11.7	62506	39.8

Population and Area Coverage in Landkreis Börde (Stroke Units) (Sachsen-Anhalt) — Regular (+20min Penalty)

Category	Grid Cells	Area (%)	Population	Population (%)
High (30+ min)	45	1.4	133	0.1
Medium (20-30 min)	584	18.7	20148	13.0
Low (10-20 min)	967	31.0	36694	23.6
Only CT reachable within 60 min	0	0.0	0	0.0
Neither reachable within 60 min	0	0.0	0	0.0
Likely irrelevant (<10 min)	1527	48.9	98408	63.3

Population and Area Coverage in Landkreis Stade (Stroke Units) (Niedersachsen) — Regular (+20min Penalty)

Category	Grid Cells	Area (%)	Population	Population (%)
High (30+ min)	20	1.2	608	0.3
Medium (20-30 min)	41	2.5	2318	1.2
Low (10-20 min)	250	15.2	11193	5.6
Only CT reachable within 60 min	10	0.6	135	0.1
Neither reachable within 60 min	100	6.1	4948	2.5
Likely irrelevant (<10 min)	1220	74.3	178964	90.3

Population and Area Coverage in Kreisfreie Stadt Oldenburg (Oldb) (Stroke Units) (Niedersachsen) — Regular (+20min Penalty)

Category	Grid Cells	Area (%)	Population	Population (%)
High (30+ min)	0	0.0	0	0.0
Medium (20-30 min)	0	0.0	0	0.0
Low (10-20 min)	0	0.0	0	0.0
Only CT reachable within 60 min	0	0.0	0	0.0
Neither reachable within 60 min	0	0.0	0	0.0
Likely irrelevant (<10 min)	137	100.0	159948	100.0

Population and Area Coverage in Landkreis Cloppenburg (Stroke Units) (Niedersachsen) — Regular (+20min Penalty)

Category	Grid Cells	Area (%)	Population	Population (%)
High (30+ min)	1	0.1	2	0.0
Medium (20-30 min)	234	12.3	8369	5.1
Low (10-20 min)	715	37.6	59398	36.2
Only CT reachable within 60 min	0	0.0	0	0.0
Neither reachable within 60 min	0	0.0	0	0.0
Likely irrelevant (<10 min)	952	50.1	96307	58.7

Population and Area Coverage in Kreisfreie Stadt Wuppertal (Stroke Units) (Nordrhein-Westfalen) — Regular (+20min Penalty)

Category	Grid Cells	Area (%)	Population	Population (%)
High (30+ min)	0	0.0	0	0.0
Medium (20-30 min)	0	0.0	0	0.0
Low (10-20 min)	0	0.0	0	0.0
Only CT reachable within 60 min	0	0.0	0	0.0
Neither reachable within 60 min	0	0.0	0	0.0
Likely irrelevant (<10 min)	213	100.0	359073	100.0

Population and Area Coverage in Landkreis Odenwaldkreis (Stroke Units) (Hessen) — Regular (+20min Penalty)

Category	Grid Cells	Area (%)	Population	Population (%)
High (30+ min)	0	0.0	0	0.0
Medium (20-30 min)	4	0.5	0	0.0
Low (10-20 min)	70	9.0	8495	8.7
Only CT reachable within 60 min	0	0.0	0	0.0
Neither reachable within 60 min	0	0.0	0	0.0
Likely irrelevant (<10 min)	702	90.5	89395	91.3

Population and Area Coverage in Landkreis Karlsruhe (Stroke Units) (Baden-Württemberg) — Regular (+20min Penalty)

Category	Grid Cells	Area (%)	Population	Population (%)
High (30+ min)	0	0.0	0	0.0
Medium (20-30 min)	0	0.0	0	0.0
Low (10-20 min)	63	4.7	13187	3.0
Only CT reachable within 60 min	0	0.0	0	0.0
Neither reachable within 60 min	0	0.0	0	0.0
Likely irrelevant (<10 min)	1272	95.3	422648	97.0

Population and Area Coverage in Kreisfreie Stadt Landshut (Stroke Units) (Bayern) — Regular (+20min Penalty)

Category	Grid Cells	Area (%)	Population	Population (%)
High (30+ min)	0	0.0	0	0.0
Medium (20-30 min)	0	0.0	0	0.0
Low (10-20 min)	0	0.0	0	0.0
Only CT reachable within 60 min	0	0.0	0	0.0
Neither reachable within 60 min	0	0.0	0	0.0
Likely irrelevant (<10 min)	81	100.0	69058	100.0

Population and Area Coverage in Kreisfreie Stadt Erlangen (Stroke Units) (Bayern) — Regular (+20min Penalty)

Category	Grid Cells	Area (%)	Population	Population (%)
High (30+ min)	0	0.0	0	0.0
Medium (20-30 min)	0	0.0	0	0.0
Low (10-20 min)	0	0.0	0	0.0
Only CT reachable within 60 min	0	0.0	0	0.0
Neither reachable within 60 min	0	0.0	0	0.0
Likely irrelevant (<10 min)	96	100.0	126629	100.0

Population and Area Coverage in Landkreis Haßberge (Stroke Units) (Bayern) — Regular (+20min Penalty)

Category	Grid Cells	Area (%)	Population	Population (%)
High (30+ min)	0	0.0	0	0.0
Medium (20-30 min)	3	0.2	1	0.0
Low (10-20 min)	726	60.5	31737	36.4
Only CT reachable within 60 min	0	0.0	0	0.0
Neither reachable within 60 min	0	0.0	0	0.0
Likely irrelevant (<10 min)	471	39.2	55428	63.6

Population and Area Coverage in Landkreis Aichach-Friedberg (Stroke Units) (Bayern) — Regular (+20min Penalty)

Category	Grid Cells	Area (%)	Population	Population (%)
High (30+ min)	0	0.0	0	0.0
Medium (20-30 min)	8	0.8	309	0.2
Low (10-20 min)	204	21.6	21596	16.2
Only CT reachable within 60 min	0	0.0	0	0.0
Neither reachable within 60 min	0	0.0	0	0.0
Likely irrelevant (<10 min)	733	77.6	111506	83.6

Population and Area Coverage in Landkreis Dillingen a.d.Donau (Stroke Units) (Bayern) — Regular (+20min Penalty)

Category	Grid Cells	Area (%)	Population	Population (%)
High (30+ min)	5	0.5	151	0.2
Medium (20-30 min)	145	14.9	5111	5.3
Low (10-20 min)	324	33.4	27867	28.7
Only CT reachable within 60 min	0	0.0	0	0.0
Neither reachable within 60 min	0	0.0	0	0.0
Likely irrelevant (<10 min)	496	51.1	63969	65.9

Population and Area Coverage in Landkreis Oberspreewald-Lausitz (Stroke Units) (Brandenburg) — Regular (+20min Penalty)

Category	Grid Cells	Area (%)	Population	Population (%)
High (30+ min)	0	0.0	0	0.0
Medium (20-30 min)	15	0.9	132	0.1
Low (10-20 min)	195	12.3	4030	3.7
Only CT reachable within 60 min	0	0.0	0	0.0
Neither reachable within 60 min	0	0.0	0	0.0
Likely irrelevant (<10 min)	1370	86.7	104296	96.2

Population and Area Coverage in Landkreis Bautzen (Stroke Units) (Sachsen) — Regular (+20min Penalty)

Category	Grid Cells	Area (%)	Population	Population (%)
High (30+ min)	146	4.7	20043	7.0
Medium (20-30 min)	523	17.0	43331	15.0
Low (10-20 min)	1195	38.8	111773	38.8
Only CT reachable within 60 min	7	0.2	735	0.3
Neither reachable within 60 min	6	0.2	89	0.0
Likely irrelevant (<10 min)	1205	39.1	112052	38.9

Population and Area Coverage in Kreisfreie Stadt Dessau-Roßlau (Stroke Units) (Sachsen-Anhalt) — Regular (+20min Penalty)

Category	Grid Cells	Area (%)	Population	Population (%)
High (30+ min)	108	33.8	14932	17.8
Medium (20-30 min)	110	34.4	51183	60.9
Low (10-20 min)	58	18.1	17344	20.6
Only CT reachable within 60 min	44	13.8	653	0.8
Neither reachable within 60 min	0	0.0	0	0.0
Likely irrelevant (<10 min)	0	0.0	1	0.0

Population and Area Coverage in Landkreis Lüneburg (Stroke Units) (Niedersachsen) — Regular (+20min Penalty)

Category	Grid Cells	Area (%)	Population	Population (%)
High (30+ min)	45	2.5	581	0.3
Medium (20-30 min)	112	6.3	2481	1.4
Low (10-20 min)	365	20.5	17930	10.1
Only CT reachable within 60 min	246	13.8	3805	2.1
Neither reachable within 60 min	0	0.0	0	0.0
Likely irrelevant (<10 min)	1012	56.9	153471	86.1

Population and Area Coverage in Kreisfreie Stadt Bremerhaven (Stroke Units) (Bremen) — Regular (+20min Penalty)

Category	Grid Cells	Area (%)	Population	Population (%)
High (30+ min)	2	2.0	73	0.1
Medium (20-30 min)	0	0.0	0	0.0
Low (10-20 min)	0	0.0	0	0.0
Only CT reachable within 60 min	10	10.2	278	0.2
Neither reachable within 60 min	4	4.1	10	0.0
Likely irrelevant (<10 min)	82	83.7	114055	99.7

Population and Area Coverage in Kreisfreie Stadt Koblenz (Stroke Units) (Rheinland-Pfalz) — Regular (+20min Penalty)

Category	Grid Cells	Area (%)	Population	Population (%)
High (30+ min)	0	0.0	0	0.0
Medium (20-30 min)	0	0.0	0	0.0
Low (10-20 min)	0	0.0	0	0.0
Only CT reachable within 60 min	0	0.0	0	0.0
Neither reachable within 60 min	0	0.0	0	0.0
Likely irrelevant (<10 min)	136	100.0	125857	100.0

Population and Area Coverage in Kreisfreie Stadt Pirmasens (Stroke Units) (Rheinland-Pfalz) — Regular (+20min Penalty)

Category	Grid Cells	Area (%)	Population	Population (%)
High (30+ min)	0	0.0	0	0.0
Medium (20-30 min)	13	17.1	332	0.9
Low (10-20 min)	57	75.0	35003	93.9
Only CT reachable within 60 min	0	0.0	0	0.0
Neither reachable within 60 min	0	0.0	0	0.0
Likely irrelevant (<10 min)	6	7.9	1927	5.2

Population and Area Coverage in Landkreis Donnersbergkreis (Stroke Units) (Rheinland-Pfalz) — Regular (+20min Penalty)

Category	Grid Cells	Area (%)	Population	Population (%)
High (30+ min)	0	0.0	0	0.0
Medium (20-30 min)	4	0.5	0	0.0
Low (10-20 min)	324	40.1	16023	20.7
Only CT reachable within 60 min	0	0.0	0	0.0
Neither reachable within 60 min	0	0.0	0	0.0
Likely irrelevant (<10 min)	479	59.4	61494	79.3

Population and Area Coverage in Stadtkreis Heidelberg (Stroke Units) (Baden-Württemberg) — Regular (+20min Penalty)

Category	Grid Cells	Area (%)	Population	Population (%)
High (30+ min)	0	0.0	0	0.0
Medium (20-30 min)	0	0.0	0	0.0
Low (10-20 min)	2	1.5	15	0.0
Only CT reachable within 60 min	0	0.0	0	0.0
Neither reachable within 60 min	0	0.0	0	0.0
Likely irrelevant (<10 min)	132	98.5	154351	100.0

Population and Area Coverage in Kreisfreie Stadt Straubing (Stroke Units) (Bayern) — Regular (+20min Penalty)

Category	Grid Cells	Area (%)	Population	Population (%)
High (30+ min)	0	0.0	0	0.0
Medium (20-30 min)	0	0.0	0	0.0
Low (10-20 min)	0	0.0	0	0.0
Only CT reachable within 60 min	0	0.0	0	0.0
Neither reachable within 60 min	0	0.0	0	0.0
Likely irrelevant (<10 min)	85	100.0	46942	100.0

Population and Area Coverage in Landkreis Straubing-Bogen (Stroke Units) (Bayern) — Regular (+20min Penalty)

Category	Grid Cells	Area (%)	Population	Population (%)
High (30+ min)	0	0.0	0	0.0
Medium (20-30 min)	0	0.0	0	0.0
Low (10-20 min)	222	15.1	10587	10.5
Only CT reachable within 60 min	0	0.0	0	0.0
Neither reachable within 60 min	0	0.0	0	0.0
Likely irrelevant (<10 min)	1244	84.9	89935	89.5

Population and Area Coverage in Landkreis Neumarkt i.d.OPf. (Stroke Units) (Bayern) — Regular (+20min Penalty)

Category	Grid Cells	Area (%)	Population	Population (%)
High (30+ min)	0	0.0	0	0.0
Medium (20-30 min)	12	0.7	1311	1.0
Low (10-20 min)	126	7.6	6444	4.9
Only CT reachable within 60 min	0	0.0	0	0.0
Neither reachable within 60 min	0	0.0	0	0.0
Likely irrelevant (<10 min)	1526	91.7	124712	94.1

Population and Area Coverage in Kreisfreie Stadt Schwabach (Stroke Units) (Bayern) — Regular (+20min Penalty)

Category	Grid Cells	Area (%)	Population	Population (%)
High (30+ min)	0	0.0	0	0.0
Medium (20-30 min)	0	0.0	0	0.0
Low (10-20 min)	0	0.0	0	0.0
Only CT reachable within 60 min	0	0.0	0	0.0
Neither reachable within 60 min	0	0.0	0	0.0
Likely irrelevant (<10 min)	52	100.0	42132	100.0

Population and Area Coverage in Kreisfreie Stadt Brandenburg an der Havel (Stroke Units) (Brandenburg) — Regular (+20min Penalty)

Category	Grid Cells	Area (%)	Population	Population (%)
High (30+ min)	102	33.6	44525	64.4
Medium (20-30 min)	55	18.1	13525	19.6
Low (10-20 min)	21	6.9	320	0.5
Only CT reachable within 60 min	126	41.4	10720	15.5
Neither reachable within 60 min	0	0.0	0	0.0
Likely irrelevant (<10 min)	0	0.0	2	0.0

Population and Area Coverage in Landkreis Ludwigslust-Parchim (Stroke Units) (Mecklenburg-Vorpommern) — Regular (+20min Penalty)

Category	Grid Cells	Area (%)	Population	Population (%)
High (30+ min)	685	10.6	19655	10.0
Medium (20-30 min)	1523	23.6	46117	23.5
Low (10-20 min)	1651	25.6	58872	30.0
Only CT reachable within 60 min	1041	16.1	15717	8.0
Neither reachable within 60 min	0	0.0	0	0.0
Likely irrelevant (<10 min)	1555	24.1	55655	28.4

Population and Area Coverage in Landkreis Saalfeld-Rudolstadt (Stroke Units) (Thüringen) — Regular (+20min Penalty)

Category	Grid Cells	Area (%)	Population	Population (%)
High (30+ min)	0	0.0	0	0.0
Medium (20-30 min)	3	0.2	84	0.1
Low (10-20 min)	402	31.3	11777	12.2
Only CT reachable within 60 min	0	0.0	0	0.0
Neither reachable within 60 min	0	0.0	0	0.0
Likely irrelevant (<10 min)	878	68.4	84959	87.7

Population and Area Coverage in Kreis Ostholstein (Stroke Units) (Schleswig-Holstein) — Regular (+20min Penalty)

Category	Grid Cells	Area (%)	Population	Population (%)
High (30+ min)	1	0.1	90	0.0
Medium (20-30 min)	8	0.4	152	0.1
Low (10-20 min)	179	9.6	12958	6.6
Only CT reachable within 60 min	10	0.5	430	0.2
Neither reachable within 60 min	78	4.2	2957	1.5
Likely irrelevant (<10 min)	1585	85.2	179814	91.6

Population and Area Coverage in Kreis Rendsburg-Eckernförde (Stroke Units) (Schleswig-Holstein) — Regular (+20min Penalty)

Category	Grid Cells	Area (%)	Population	Population (%)
High (30+ min)	2	0.1	138	0.1
Medium (20-30 min)	81	2.7	2972	1.1
Low (10-20 min)	154	5.2	5694	2.1
Only CT reachable within 60 min	2	0.1	1871	0.7
Neither reachable within 60 min	35	1.2	1603	0.6
Likely irrelevant (<10 min)	2714	90.8	262274	95.5

Population and Area Coverage in Landkreis Ostalbkreis (Stroke Units) (Baden-Württemberg) — Regular (+20min Penalty)

Category	Grid Cells	Area (%)	Population	Population (%)
High (30+ min)	0	0.0	0	0.0
Medium (20-30 min)	29	1.6	3681	1.2
Low (10-20 min)	428	23.1	54014	17.3
Only CT reachable within 60 min	0	0.0	0	0.0
Neither reachable within 60 min	0	0.0	0	0.0
Likely irrelevant (<10 min)	1398	75.4	253636	81.5

Population and Area Coverage in Landkreis Emmendingen (Stroke Units) (Baden-Württemberg) — Regular (+20min Penalty)

Category	Grid Cells	Area (%)	Population	Population (%)
High (30+ min)	45	5.5	833	0.5
Medium (20-30 min)	119	14.5	7563	4.7
Low (10-20 min)	286	35.0	24912	15.4
Only CT reachable within 60 min	5	0.6	7	0.0
Neither reachable within 60 min	0	0.0	5	0.0
Likely irrelevant (<10 min)	363	44.4	128564	79.4

Population and Area Coverage in Kreisfreie Stadt München (Stroke Units) (Bayern) — Regular (+20min Penalty)

Category	Grid Cells	Area (%)	Population	Population (%)
High (30+ min)	0	0.0	0	0.0
Medium (20-30 min)	0	0.0	0	0.0
Low (10-20 min)	0	0.0	0	0.0
Only CT reachable within 60 min	0	0.0	0	0.0
Neither reachable within 60 min	0	0.0	0	0.0
Likely irrelevant (<10 min)	379	100.0	1671155	100.0

Population and Area Coverage in Landkreis Bayreuth (Stroke Units) (Bayern) — Regular (+20min Penalty)

Category	Grid Cells	Area (%)	Population	Population (%)
High (30+ min)	0	0.0	0	0.0
Medium (20-30 min)	27	1.7	1287	1.2
Low (10-20 min)	389	24.4	16372	15.0
Only CT reachable within 60 min	0	0.0	0	0.0
Neither reachable within 60 min	0	0.0	0	0.0
Likely irrelevant (<10 min)	1180	73.9	91251	83.8

Population and Area Coverage in Landkreis Kronach (Stroke Units) (Bayern) — Regular (+20min Penalty)

Category	Grid Cells	Area (%)	Population	Population (%)
High (30+ min)	0	0.0	0	0.0
Medium (20-30 min)	72	8.9	1455	2.0
Low (10-20 min)	327	40.3	27859	39.2
Only CT reachable within 60 min	0	0.0	0	0.0
Neither reachable within 60 min	0	0.0	0	0.0
[truncated: 321,679 more chars]
